# Filamentous virus-like particles are present in coral dinoflagellates across genera and ocean basins

**DOI:** 10.1038/s41396-023-01526-6

**Published:** 2023-11-01

**Authors:** Lauren I. Howe-Kerr, Anna M. Knochel, Matthew D. Meyer, Jordan A. Sims, Carly E. Karrick, Carsten G. B. Grupstra, Alex J. Veglia, Andrew R. Thurber, Rebecca L. Vega Thurber, Adrienne M. S. Correa

**Affiliations:** 1 BioSciences Department, Rice University, Houston, TX USA; 2 Department of Biological Sciences, Florida International University, Miami, FL USA; 3 Shared Equipment Authority, Rice University, Houston, TX USA; 4 Environmental Science and Policy, George Mason University, Fairfax, VA USA; 5 Department of Biology, Boston University, Boston, MA USA; 6 Department of Biology, University of Puerto Rico, Mayagüez, PR USA; 7 Department of Microbiology, Oregon State University, Corvallis, OR USA; 8 College of Earth Ocean and Atmospheric Sciences, Oregon State University, Corvallis, OR USA; 9 Department of Environmental Science, Policy and Management, University of California, Berkeley, CA USA

**Keywords:** Environmental microbiology, Microbial ecology

## Abstract

Filamentous viruses are hypothesized to play a role in stony coral tissue loss disease (SCTLD) through infection of the endosymbiotic dinoflagellates (Family Symbiodiniaceae) of corals. To evaluate this hypothesis, it is critical to understand the global distribution of filamentous virus infections across the genetic diversity of Symbiodiniaceae hosts. Using transmission electron microscopy, we demonstrate that filamentous virus-like particles (VLPs) are present in over 60% of Symbiodiniaceae cells (genus *Cladocopium*) within Pacific corals (*Acropora hyacinthus*, *Porites c.f. lobata*); these VLPs are more prevalent in Symbiodiniaceae of in situ colonies experiencing heat stress. Symbiodiniaceae expelled from *A. hyacinthus* also contain filamentous VLPs, and these cells are more degraded than their *in hospite* counterparts. Similar to VLPs reported from SCTLD-affected Caribbean reefs, VLPs range from ~150 to 1500 nm in length and 16–37 nm in diameter and appear to constitute various stages in a replication cycle. Finally, we demonstrate that SCTLD-affected corals containing filamentous VLPs are dominated by diverse Symbiodiniaceae lineages from the genera *Breviolum, Cladocopium*, and *Durusdinium*. Although this study cannot definitively confirm or refute the role of filamentous VLPs in SCTLD, it demonstrates that filamentous VLPs are not solely observed in SCTLD-affected corals or reef regions, nor are they solely associated with corals dominated by members of a particular Symbiodiniaceae genus. We hypothesize that filamentous viruses are a widespread, common group that infects Symbiodiniaceae. Genomic characterization of these viruses and empirical tests of the impacts of filamentous virus infection on Symbiodiniaceae and coral colonies should be prioritized.

## Introduction

Stony coral tissue loss disease (SCTLD) is among the most persistent and widespread diseases of coral colonies reported to date. Infecting at least 22 of the 45 Caribbean stony coral species [1], SCTLD has driven significant losses of coral colonies throughout the Caribbean since 2014 [2]. SCTLD is presumed to be contagious and transmitted via water or direct contact evidenced by spatial modeling of disease spread [3–6], in situ observations [7–10], and ex situ transmission experiments [11–13]. Numerous studies have examined the role of bacteria in the etiology of SCTLD, and although some opportunistic bacterial groups have been implicated, a causative agent has yet to be identified [14–21]. Filamentous virus-like particles (VLPs) were recently documented within the endosymbiotic dinoflagellates (Family Symbiodiniaceae) associated with SCTLD-affected and apparently healthy coral colonies from the middle Florida Keys, using transmission electron microscopy (TEM) imaging [22]. The documented VLPs were morphologically similar to positive-sense, single-stranded RNA ( + ssRNA) viruses typically associated with plant disease (e.g., Flexiviridae [23], Potyviridae [24], and Closteroviridae [25]). Genomic evidence of filamentous viruses, specifically alphaflexiviruses, was subsequently detected in U.S. Virgin Island corals sampled during a SCTLD transmission experiment, in both SCTLD-exposed and unexposed colonies [26]. Furthermore, histological evidence indicates that a breakdown of the coral-Symbiodiniaceae partnership occurs in SCTLD-affected coral tissues [27], concomitantly with upregulation of antiviral immunity-related genes in Symbiodiniaceae [28]. Based on these observations, it has been hypothesized that viral infections of Symbiodiniaceae within coral tissues play a role in SCTLD.

Although our understanding of the coral reef virosphere has improved over the last two decades (reviewed in [29–32]), most environmental virology data for reefs originates from a limited number of geographic regions and host species [33, 34]. Furthermore, although filamentous viruses have been detected from coral reef environments ([22, 26, 35–42]; Supplementary Table 1 for comprehensive TEM-based list), they are not typically the focus of coral virology studies and have primarily been tangentially observed (but see [22, 34]). To date, most reports of filamentous VLPs or sequence similarities to filamentous +ssRNA viruses have been from Caribbean corals on SCTLD-affected reefs [22, 26] or from Symbiodiniaceae cultures (included in Supplementary Table 1). However, one study reported filamentous VLPs from Symbiodiniaceae within *Porites australiensis* exhibiting white patch syndrome (WPS) on the Great Barrier Reef (Australia, [41]). Additional TEM imaging of Symbiodiniaceae cells from Pacific stony corals will help clarify the geographic distribution and prevalence of filamentous virus infections across the Family Symbiodiniaceae; this work is critical to evaluating the hypothesis that filamentous viruses contribute to SCTLD signs.

Further development of our understanding of the host range of Symbiodiniaceae-infecting filamentous viruses may additionally reveal or explain patterns of SCTLD susceptibility or resistance, as well as aid in predicting which colonies may persist on reefs during pandemic and endemic phases of SCTLD infection. For example, Symbiodiniaceae genetic identity has been implicated in coral colony susceptibility to other diseases [43–45]. Differences in Symbiodiniaceae community diversity in diseased versus healthy *Siderastrea siderea* holobionts have also previously been reported from the Florida Keys (USA) and U.S. Virgin Islands in the context of dark spot syndrome [46]. Coral holobionts vary in their susceptibility to SCTLD infection [8, 9, 12, 47], and it has been hypothesized that Symbiodiniaceae genetic identity may play a role in this variation [28, 48]. For example, Symbiodiniaceae lineages within the genus *Breviolum* are hypothesized to be the most susceptible to SCTLD infection, followed by symbionts in the genera *Cladocopium* and *Durusdinium*, with symbionts in *Symbiodinium* predicted to be the least susceptible [49]. Yet, genetic characterization of Symbiodiniaceae communities has yet to be reported for any coral colonies from which filamentous VLPs have been documented via TEM (studies in Supplementary Table 1).

Here we aim to understand the prevalence of filamentous virus infections in Symbiodiniaceae and genetically characterize the Symbiodiniaceae lineages that filamentous VLPs are associated with. To do this we: (1) characterize putative filamentous virus infections in Symbiodiniaceae of Pacific stony corals, including healthy, bleached, and temperature stressed colonies using TEM imaging, and assess the dominant Symbiodiniaceae lineages within these colonies using molecular approaches, and (2) assess the diversity of Symbiodiniaceae within Caribbean coral colonies (SCTLD-affected and apparently healthy states) from which filamentous VLPs were previously reported by [22]. By generating baseline information on the distribution of filamentous VLPs within Symbiodiniaceae in Pacific coral holobionts and exploring the host range for Symbiodiniaceae-associated filamentous viruses, this study advances efforts to understand the role of viruses in SCTLD.

## Materials and methods

### Experimental design and sample collection

Coral-Symbiodiniaceae holobionts included in this study originated from reefs in the South Pacific (Mo’orea, French Polynesia) and Caribbean (Florida, USA). All samples were collected between March 2018 and August 2019.

#### Mo’orea, French Polynesia

The South Pacific coral holobionts in this study, *Acropora hyacinthus* and *Porites cf. lobata*, reside in part of a broad geographic area that has yet to be affected by SCTLD. Yet, a mass bleaching event impacted the reefs of Mo’orea from March to August 2019 [50], exposing all colonies on the reef to heat stress. The majority of *A. hyacinthus* colonies bleached during this period, while *P. cf. lobata* did not exhibit visible signs of bleaching [51]. We analyzed tissue biopsies collected from colonies in situ during the heat stress event, as well as during ambient conditions. These in situ samples were supplemented with biopsies collected during an aquaria-based heat stress experiment. Additionally, expelled Symbiodiniaceae, *ex hospite* cells that had recently exited *A. hyacinthus* colonies, were collected. In total, eight types of samples from South Pacific reefs are presented here: 1) *P. cf. lobata* tissues collected in situ during ambient conditions; 2) *P. cf. lobata* tissues collected in situ during heat stress; 3) *A. hyacinthus* tissues collected in situ during heat stress conditions; 4) *A. hyacinthus* tissues collected from an ambient (control) treatment in an aquaria-based experiment; 5) *A. hyacinthus* tissues collected during a heat stress treatment in an aquaria-based experiment; 6) expelled Symbiodiniaceae recently exited from *A. hyacinthus* colonies collected in situ during heat stress, 7) expelled Symbiodiniaceae recently exited from *A. hyacinthus* colonies in an ambient (control) treatment in an aquaria-based experiment, and 8) expelled Symbiodiniaceae recently exited from *A. hyacinthus* colonies during a heat stress treatment in an aquaria-based experiment. Symbiodiniaceae taxonomy and morphology in addition to visible signs of potential viral infection were examined and compared across these categories using Symbiodiniaceae marker gene amplicon sequencing and TEM imaging.

*Porites cf. lobata* colonies were tagged with unique identifiers on the fringe and forereef of Mo’orea’s north shore; their tissues were biopsied during ambient temperature conditions in March 2018 (TEM samples), August 2018 (amplicon sequencing samples), and again during elevated temperature conditions in late March 2019 (for both TEM and amplicon, *n* = 6 colonies, 12 samples in total for TEM and amplicon sequencing respectively, [51]). In March 2019, none of the sampled *P. cf. lobata* were visibly bleached. Based on thermistors placed on the reef bottom at 10 m around the island, anomalously high temperatures for Mo’orea (surpassing 29 °C) began in December 2018; corals sampled at the end of March had experienced 2 weeks in which water temperatures were +0.1°C above the maximum monthly mean [50]. Monitoring of these colonies over time (see [51]) suggest that this marine heatwave was a physiological stress for *P. cf. lobata* colonies, associated with partial mortality by October 2020. At each timepoint, biopsies were collected via SCUBA or snorkel using bone cutters and placed in individual whirl-paks and processed using standard methods [52]. Upon surfacing to the boat ( ~5–45 minutes after underwater collection), a small ( ~0.5 cm^2^) fragment for TEM was fixed in 2% paraformaldehyde in 3X phosphate buffered saline. Samples were placed in a cooler and then stored at 4 °C until further processing. An additional ~3–4 small fragments (1 cm^2^) of skeleton/tissue from each coral were preserved in DNA/RNA shield (ZymoResearch, Irvine, California, USA), vortexed for 25 min at full speed with 5 ceramic beads and 1.35 g garnet matrix (MP Biomedicals), and then frozen at –40 °C until further processing.

Tissue biopsies of bleaching *A. hyacinthus* colonies (*n* = 5) were collected on the forereef of the north shore of Mo’orea in July 2019 during the bleaching event (after >4 months of heat stress). Biopsies were preserved for TEM imaging and amplicon sequencing as described above for *P. cf. lobata*. Three of these *A. hyacinthus* colonies were paling but still visibly pigmented with Symbiodiniaceae when tissue biopsies were collected, whereas two were stark white but had living tissue (i.e., were bleached). All five colonies were confirmed to be dead in October 2020.

Biopsies of *A. hyacinthus* coral tissues were also collected from control and heat stress treatments during an aquaria-based experiment and similarly preserved for TEM and amplicon sequencing. Briefly, coral colonies (*n* = 4) were collected from the north shore forereef in May 2018 and fragmented into ten pieces. Half of the fragments were randomly distributed across three flow-through seawater tanks at ambient temperature ( ~29 °C) and half across three heated flow-through seawater tanks ( ~33 °C) at the Richard B. Gump Research Station (Mo’orea, French Polynesia). After experiencing 78 hours of treatment conditions, one sample each per coral colony ( ~genotype) was collected from the control condition and the heat treatment (*n* = 8 samples total). Fragments were not visibly bleached; visible bleaching was not expected given the short duration of the experiment, yet coral holobiont stress responses, as well as shifts in bacteria and virus activity could occur within this timeframe [53–55]. Symbiodiniaceae cells expelled from a subset of *A. hyacinthus* samples in both the aquaria-based experiment (*n* = 8) and on the reef (*n* = 2) were additionally collected and preserved for TEM imaging. Briefly, this involved placing a symbiont capture device we devised around a ~5 cm^2^ branch for 3–6 hours and subsequently concentrating the cells in the device via centrifugation (see Supplementary methods for more details).

#### Caribbean

The Caribbean holobionts analyzed in this study for Symbiodiniaceae diversity were originally collected in situ from the middle Florida Keys, including from SCTLD-affected reefs and reefs where SCTLD was apparently absent, generating the following sample types: 1) disease lesions from SCTLD-affected coral colonies; 2) apparently healthy coral tissue from the same SCTLD-affected coral colonies; and 3) tissue from apparently healthy colonies (*Pseudodiploria strigosa* only). In total, 16 samples, belonging to five coral species, were collected for Symbiodiniaceae diversity analyses. These five species included three that are considered moderately susceptible to SCTLD (*Orbicella faveolata, Montastrea cavernosa, Siderastrea siderea*) and two that are considered highly susceptible to SCTLD (*Pseudodiploria strigosa, Colpophyllia natans*) [1], though susceptibility may vary according to geographic area and/or other factors, e.g., [11]). These samples correspond to colonies TEM-imaged by [22]. Coral tissues were collected as described in [18]; briefly, coral tissues were collected on the coral surface using a 10-ml plastic blunt tip syringe. The slurry of coral mucus and tissue was then immediately transferred from the syringe to a 15-ml tube, flash frozen in a liquid nitrogen dewar, and stored at –80 °C. Frozen tissue slurries were later thawed, transferred to ZR BashingBead Lysis Tubes (containing 0.1 and 0.5 mm beads and DNA/RNA shield; Zymo Research), homogenized by vortexing for 25 min at full speed, and re-frozen at –80 °C until further processing.

### Transmission electron microscopy (TEM) imaging

Samples fixed for TEM were decalcified in 1.5% ascorbic acid, through daily replacement of the ascorbic acid solution for 7-14 days (depending on the size of the sample). After decalcification, samples were fixed overnight in Karnovsky’s fixative [56]. Samples were then post-fixed in 1% osmium tetroxide, dehydrated using a graded series of ethanol, embedded in epoxy resin and heat-polymerized. Semi-thin sections of 1 μm thickness were cut using an ultramicrotome, stained with toluidine blue and analyzed with a compound light microscope to identify the presence or absence of Symbiodiniaceae cells. If Symbiodiniaceae cells were absent, deeper semi-thin sections were cut. If Symbiodiniaceae cells were present, ultra-thin sections of 100 nm thickness were cut and then stained with saturated methanolic uranyl acetate and Reynold’s lead citrate for TEM imaging. Electron micrographs were collected using a JEOL JEM-1230 equipped with a Gatan CCD camera (images of *A. hyacinthus* Symbiodiniaceae from the aquaria experiment) or a JEOL JEM-1400Flash equipped with an AMT NanoSprint15 Mk-II sCMOS camera (all other images). For each sample, multiple sections were viewed and all visible Symbiodiniaceae cells and associated virus-like particles were imaged at a range of magnifications.

Evidence of possible viral infection was documented in TEM images of Symbiodiniaceae cells from colonies under ambient and heat stress conditions in both Pacific species of corals. Filamentous virus-like particles and putative early viral inclusions and intermediate to late viral infections were identified based on comparisons to [22]; representative images of VLPs and infection progression were selected to visually depict the findings. Whorled electron-dense substances, often with layers of visible filamentous VLPs or a striated texture, were considered indications of early viral inclusions, in addition to dense clusters of filamentous VLPs. Less dense clusters of filamentous VLPs in a cellular cavity, sometimes stacked or in a stellate cluster, were considered indications of intermediate viral infections. Dispersed VLPs in large cellular cavities were considered indicative of late viral infections. A positive filamentous viral detection was documented if a cell contained clear visible signs of any or all of these putative stages. Cross-sectional width and length of VLPs was measured from the highest magnification images from each coral species in ImageJ. In conjunction with indications of viral infection, the following Symbiodiniaceae cell characteristics were noted as either “present” or “absent”: 1) intact cell membrane, 2) distinct organelles (two or more organelles identifiable), 3) presence of large vacuoles, and 4) intact thylakoid membranes. Symbiodiniaceae cells with two or more signs of stress, as indicated by the categories above, were categorized as “degraded.” In total, 257 Symbiodiniaceae cells were imaged from 13 samples of *A. hyacinthus*, and 317 Symbiodiniaceae cells were imaged from 12 samples of *P. cf. lobata*. Additionally, 137 cells were imaged from 10 samples of Symbiodiniaceae cells that were expelled from *A. hyacinthus* fragments.

All statistical tests were conducted using R (version 4.2.2). Binomial logistic regressions were used to assess associations among heat stress (heat vs ambient), cell degradation (degraded vs not degraded), and presence of filamentous VLPs (present vs absent) using the glm() function in the stats package (v 4.2.2). Type II *p* values were calculated using an ANOVA in the car package (v 3.1.1; Anova command). Model residuals were visually checked by plotting the distribution of residuals and checking Q-Q plots.

### Characterization of Symbiodiniaceae genetic diversity

To characterize the Symbiodiniaceae present in all Pacific and Caribbean colonies, coral tissue slurries preserved in DNA/RNA Shield (ZymoResearch) were incubated with Proteinase K (20 mg ml^-1^) and DNA was then extracted following the ZymoBIOMICs DNA/RNA Kit (ZymoResearch) manufacturer’s instructions. For samples of *A. hyacinthus* and Florida coral species, PCR reactions were conducted to amplify the Internal Transcribed Spacer-2 (ITS-2) region (Sym_VAR_5.8SII and Sym_VAR_REV, [57]) of Symbiodiniaceae rDNA at Oregon State University’s Center for Quantitative Life Sciences (OSU CQLS, Corvallis, OR, USA) and sequenced on a PE300 run using the MiSeq System (Illumina, see Supplementary methods for additional sequencing details). For *P. cf. lobata* samples, the D1–D2 region of the large subunit (LSU) nuclear ribosomal RNA gene was amplified and sequenced on the same MiSeq System using the primers LSU1F_illu and LSU1R_illu ([58], as described in [51]). *Porites cf. lobata* frequently harbors *Cladocopium* C15 symbionts; the ITS-2 region does not typically distinguish ecologically relevant variants of *Cladocopium* C15 [59], so the LSU was targeted for these samples.

Symbiodiniaceae ITS-2 sequences from *A. hyacinthus* and Florida coral species [22] were processed with Symportal [60] to identify dominant ITS-2 profiles based on defining intragenomic variants (DIVs). Since Symportal is specific to the ITS-2, *P. cf. lobata* Symbiodiniaceae LSU sequences were analyzed with the DADA2 pipeline [61], which clusters and merges potentially erroneous ASVs (see [51]). A recent consensus paper on best practices to assess Symbiodiniaceae diversity [59] highlighted that accounting for differences in ITS-2 rDNA copy number across lineages representing distinct Symbiodiniaceae genera is crucial when examining the relative abundance of symbiont lineages within a sample. Since Florida coral samples contained lineages representing multiple Symbiodiniaceae genera, these samples were corrected for inter-generic copy number differences by dividing counts of Symbiodiniaceae DIV absolute abundances by the mean copy number estimate for that genus (i.e., 1,721 for *Symbiodinium*, 195 for *Breviolum*, 2,119 for *Cladocopium*, and 362 for *Durusdinium*, from [62]). In this study, one sample of *Montastrea cavernosa* contained *Gerakladium*. Since a copy number estimate is not yet available for this genus, the sample was excluded from downstream analyses of Symbiodiniaceae relative abundance. Symbiodiniaceae relative abundances in each sample without adjustments for copy number are provided in Supplementary Material.

## Results

### Filamentous VLPs are present in Symbiodiniaceae from apparently healthy and heat stressed Pacific coral colonies

**Fig. 1:**
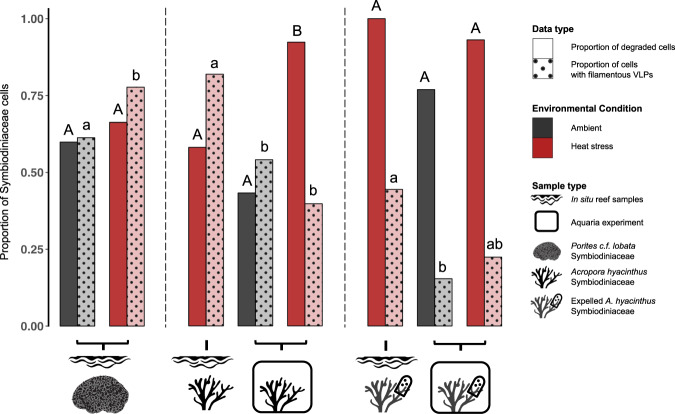
Proportions of Symbiodiniaceae cells per sample type that showed signs of degradation or contained filamentous virus-like particles (VLPs). Letters denote significant differences among groups within each facet (i.e., sample types within areas bounded by dashed lines were statistically compared using binomial logistic regressions and Tukey tests for multiple comparisons). Capital letters denote significant differences between proportions of degraded cells and lowercase letters signify significant differences between proportions of cells with filamentous VLPs. In addition to the statistical comparisons displayed in the graph, expelled *Acropora hyacinthus* Symbiodiniaceae cells were significantly more degraded than those *in hospite*.

**Fig. 2:**
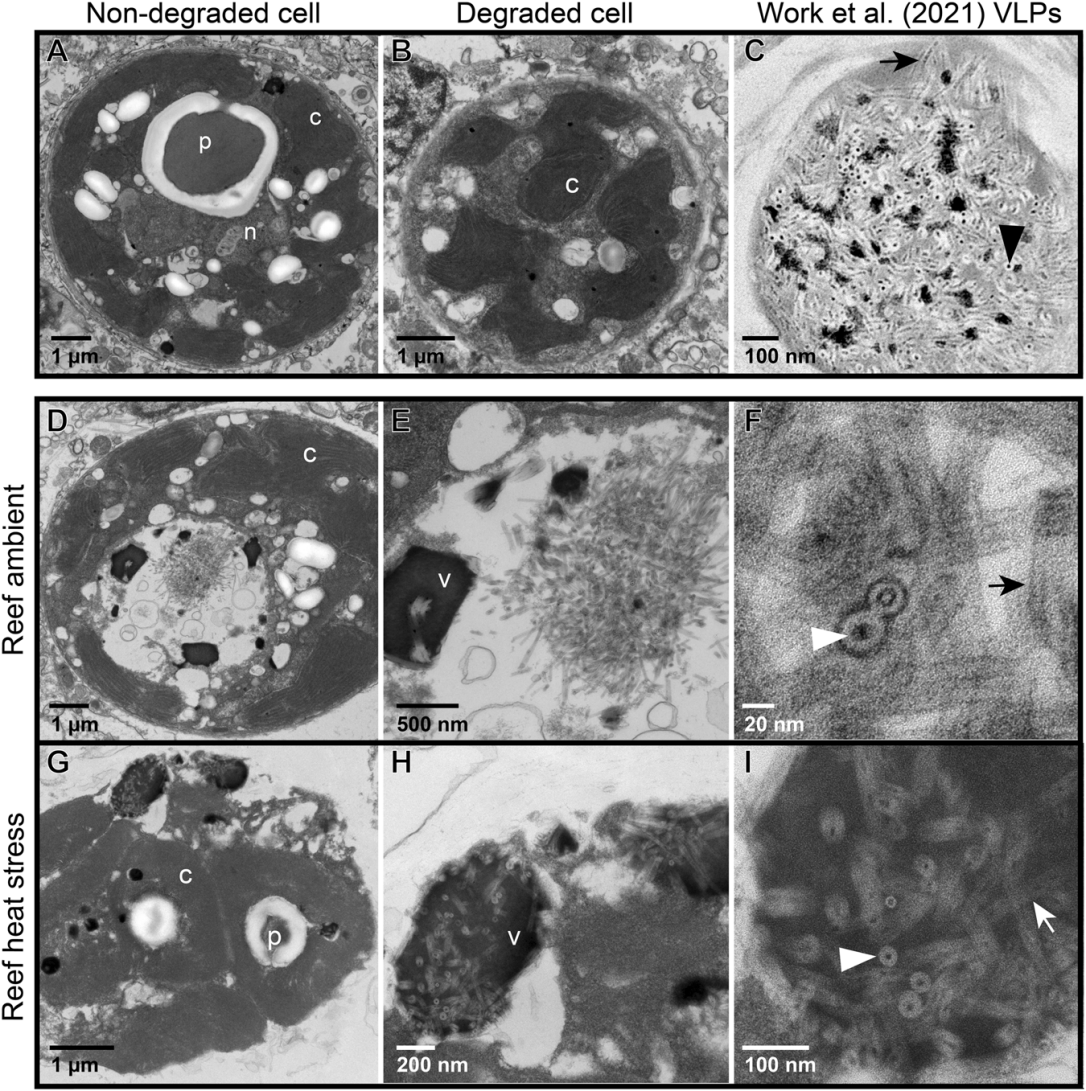
Representative transmission electron microscopy (TEM) images of Symbiodiniaceae from in situ colonies of the Pacific stony coral, *Porites cf. lobata*. **A** Apparently healthy cell; **B** degraded cell with no significant visible filamentous virus-like particles (VLPs); **C** representative Symbiodiniaceae cell reproduced from [22] containing filamentous VLPs from a stony coral tissue loss disease (SCTLD)-affected coral colony. **D**–**F** Symbiodiniaceae cell containing filamentous VLPs similar to those in [22], sampled from an apparently healthy *P. cf. lobata* colony. **E** is an inset of (**D**); (**F**) is an inset of (**E**). **G**–**I** Symbiodiniaceae cell containing filamentous VLPs similar to those in [22], sampled from a *P. cf. lobata* colony exposed to elevated temperatures. **H** is an inset of (**G**); **I** is an inset of (**H**). Arrows point to the sagittal length of a VLP; arrowheads point to the cross section of a VLP. p – pyrenoid body; n – nucleus; c – chloroplast; v – putative viroplasm.

**Fig. 3:**
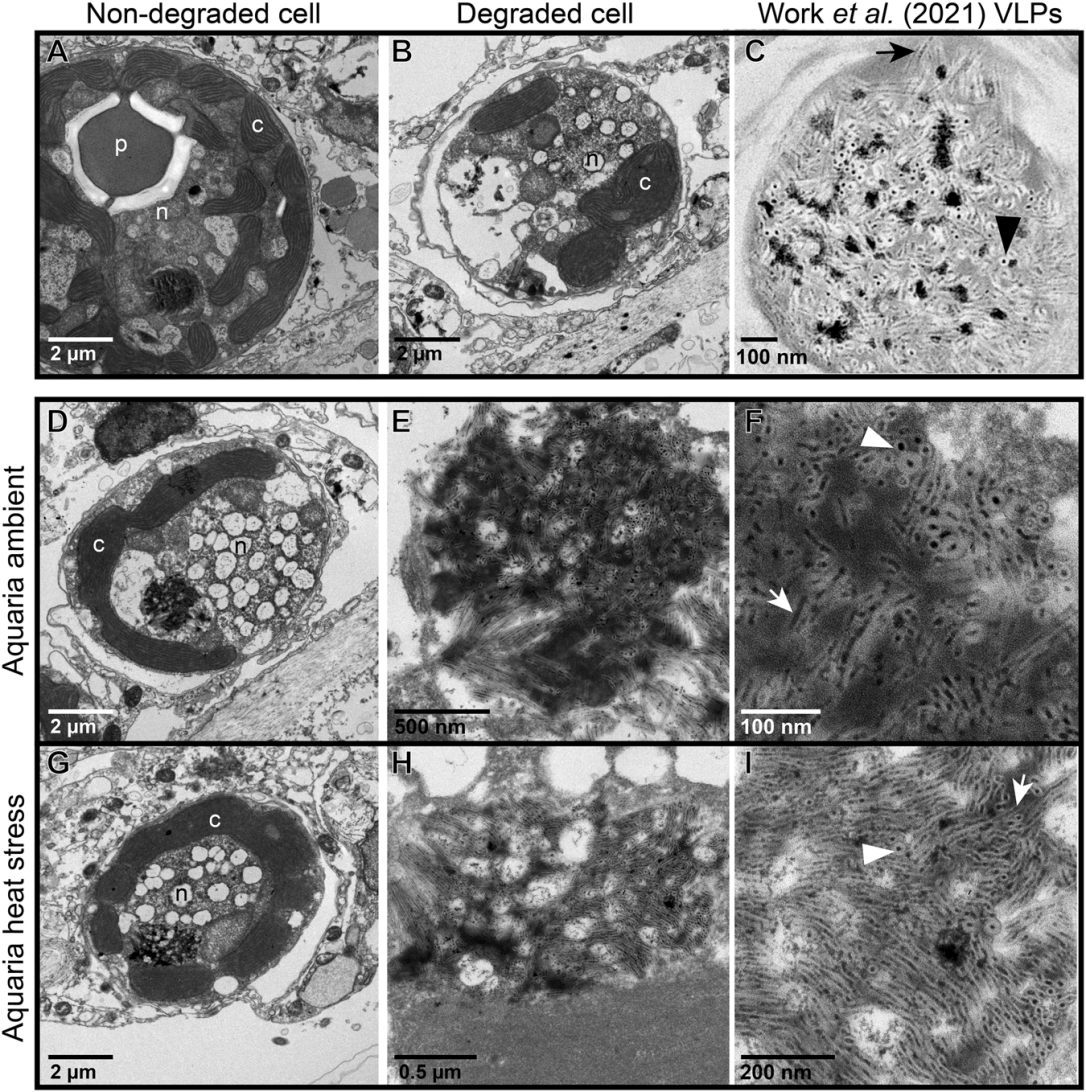
Representative transmission electron microscopy (TEM) images of Symbiodiniaceae from colonies of the Pacific stony coral, *Acropora hyacinthus*, in an aquaria-based experiment. **A** Apparently healthy cell; **B** degraded cell with no significant visible filamentous virus-like particles (VLPs); **C** representative Symbiodiniaceae cell reproduced from [22] containing filamentous VLPs from a stony coral tissue loss disease (SCTLD)-affected coral colony. **D**–**F** Symbiodiniaceae cell containing filamentous VLPs similar to those in [22], sampled from an apparently healthy *A. hyacinthus* colony. **E** is an inset of (**D**); **F** is an inset of (**E**). **G**–**I** Symbiodiniaceae cell containing filamentous VLPs similar to those in [22], sampled from a heat stressed *A. hyacinthus* colony. (H) is an inset of (**G**); **I** is an inset of (**H**). Arrows point to the sagittal length of a VLP; arrowheads point to the cross section of a VLP. P – pyrenoid body; n – nucleus; c – chloroplast.

**Fig. 4:**
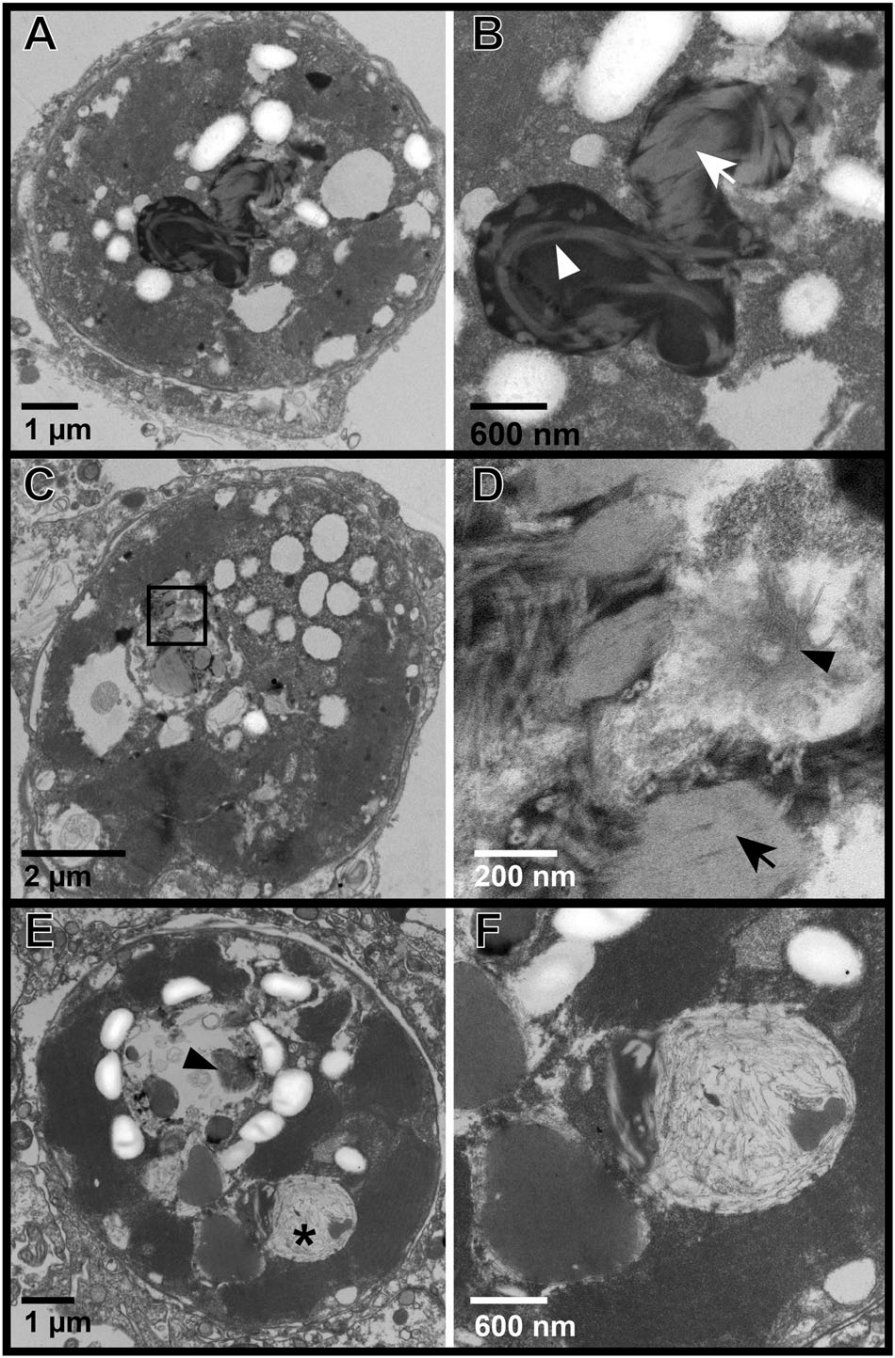
Representative transmission electron microscopy (TEM) images of Symbiodiniaceae from in situ colonies of the Pacific stony coral, *Porites cf. lobata*, showing progressive stages of a putative viral infection. **A**, **B** Putative early-stage viral inclusion: Symbiodiniaceae cell with whorled electron-dense viroplasm; (**B**) is an inset of (**A**); white arrowhead shows curved whorl, white arrow shows stacked parallel whorls. **C**, **D** Putative intermediate stage viral infection: filamentous VLPs in haphazard, stacked (black arrow), and stellate (black arrowhead) patterns within a Symbiodiniaceae cavity; (**D**) is an inset of the box in (**C**). **E**, **F** Putative late-stage infection: Symbiodiniaceae cell with coarse VLPs in large cavities; black arrowhead is a dense cluster of fine filamentous VLPs with visible cross sections; **F** is an inset of the cavity with coarse VLP (*) in (**E**). Putative stages follow those in [22].

**Fig. 5:**
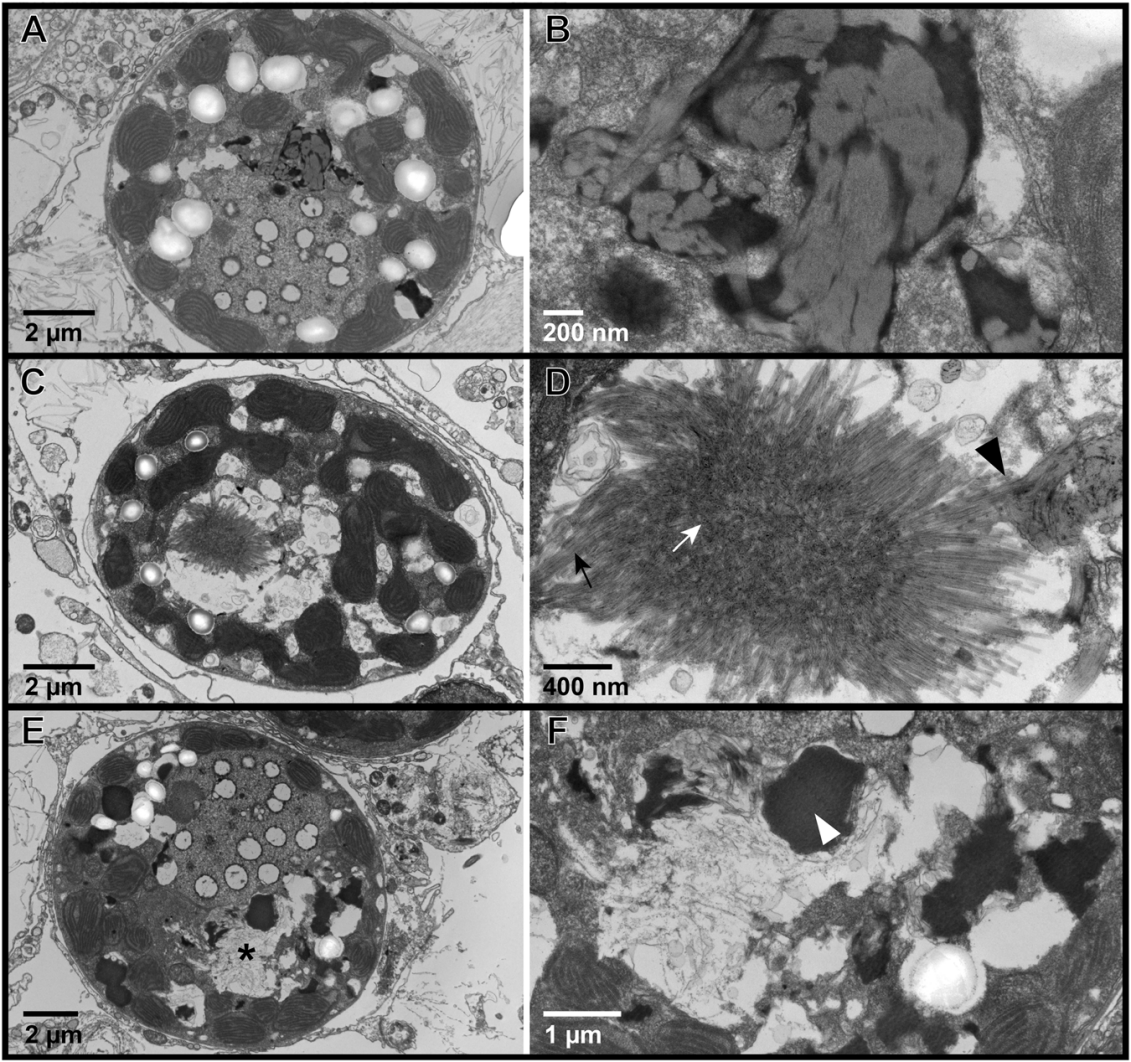
Representative transmission electron microscopy (TEM) images of Symbiodiniaceae from in situ colonies of the Pacific stony coral, *Acropora hyacinthus*, showing progressive stages of putative viral infection. **A**, **B** Putative early-stage viral inclusion: Symbiodiniaceae cell with whorled electron-dense viroplasm; (**B**) is an inset of (**A**). **C**, **D** Putative intermediate stage viral infection: filamentous VLPs in a stellate cluster within a Symbiodiniaceae cavity (arrows, black - sagittal length of a VLP; white - cross section), whorled electron dense material to the right of VLP cluster with VLPs extending out (black arrowhead); (**D**) is an inset of (**C**). **E**, **F** Putative late-stage infection: Symbiodiniaceae cell with coarse VLPs in large cavities with striated viroplasm (white arrowhead); (**F**) is an inset of (**E**). Putative stages follow those in [22].

**Fig. 6:**
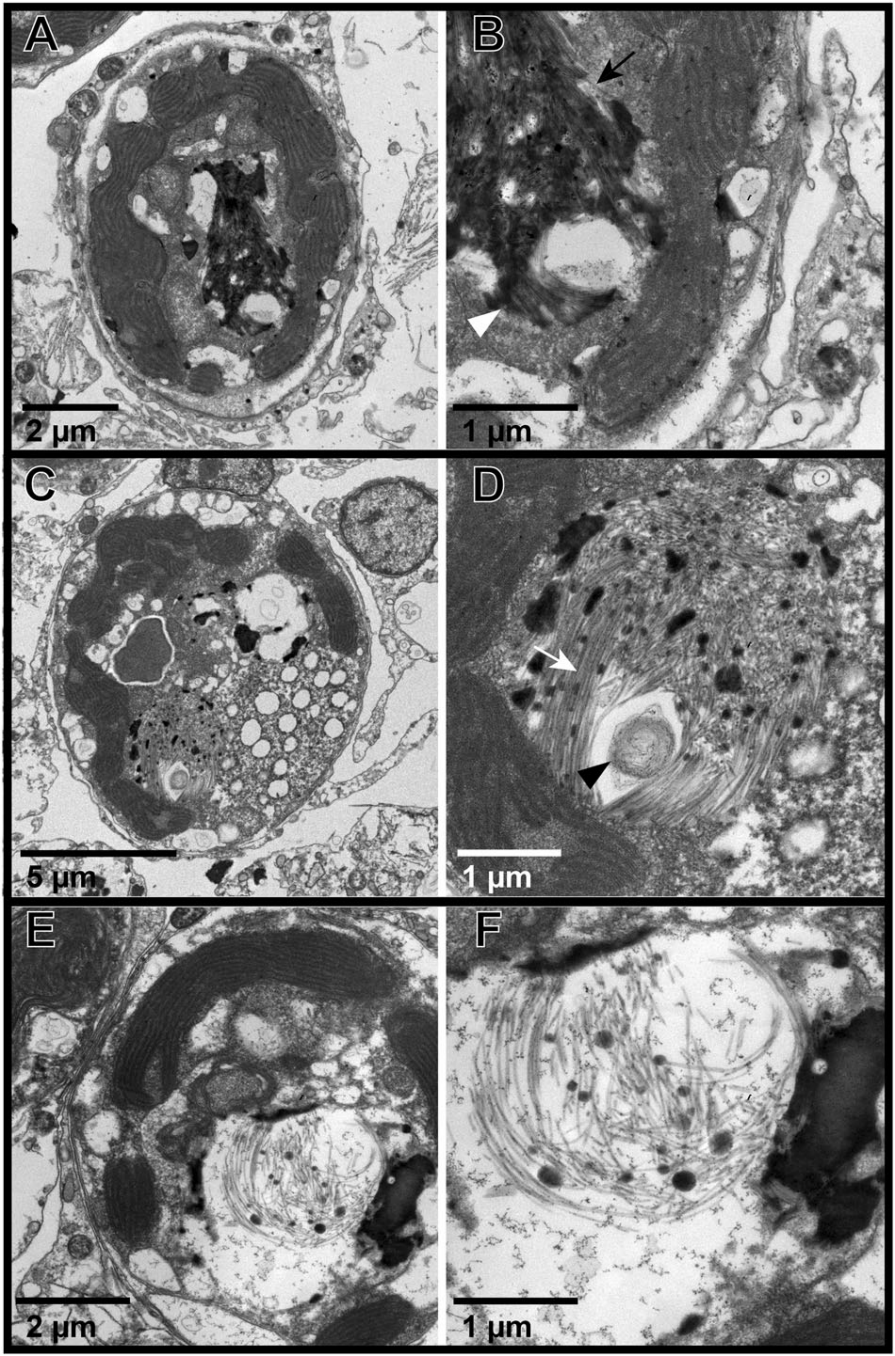
Representative transmission electron microscopy (TEM) images of Symbiodiniaceae from colonies of the Pacific stony coral, *Acropora hyacinthus*, in an aquaria-based experiment, showing progressive stages of putative viral infection. **A**, **B** Putative early-stage viral inclusion: Symbiodiniaceae cell with whorled electron-dense viroplasm and visible VLPs (black arrow - sagittal length of a VLP; white arrowhead - cross section); (**B**) is an inset of (**A**). **C**, **D** Putative intermediate stage viral infections: VLP in haphazard patterns within a Symbiodiniaceae cavity; (**D**) is an inset of (**C**). Note visible sagittal lengths (white arrow) and cross sections of VLPs, and circular cluster of thin filaments (black arrowhead). **E**, **F** Putative late-stage infection: Symbiodiniaceae cell with coarse VLPs in large cavities; (**F**) is an inset of (**E**). Putative stages follow those in [22].

Symbiodiniaceae containing filamentous VLPs were documented in all sample types of Pacific coral tissue biopsies and expelled Symbiodiniaceae samples in this study (Fig. 1). In coral biopsies from both coral species in ambient and heat stress conditions, we documented high magnification images of distinct VLP clusters, where both cross sections and sagittal lengths of the curved linear structures with a filamentous core were visible. Based on measurements from these high magnification images of distinct VLP clusters (i.e., F and I in Figs. 2 and 3), VLP widths ranged from 22 to 33 nm in *P. cf. lobata* and 16–37 nm in *A. hyacinthus*. Filamentous VLP length ranged from 189 to 865 nm in *P. cf. lobata* and 155–1500 nm in *A. hyacinthus*. Some VLPs, which were typically observed in haphazard swirls in cavities near viroplasm (such as those in Fig. 4F), were thinner and ranged from ~5 to 10 nm in width. These clusters of filamentous VLPs were found in cytoplasmic vacuoles in the cell, and occasionally within thylakoid membranes and/or in close proximity to the pyrenoid body. Clusters of stellate VLPs were occasionally observed (e.g., Figs. 4D and  5D), as well as other cluster patterns, including striated and parallel (Fig. 6C, D) and striated in a concentric circular pattern (Fig. 6E, F). Several severely degraded and lysed Symbiodiniaceae cells were found with large masses of distinct and densely clustered VLPs in a *P. cf. lobata* sample (e.g., Fig. 2G-I); however, most VLPs and electron dense viroplasm were more centrally located and surrounded by chloroplasts within Symbiodiniaceae cells.

**Fig. 7:**
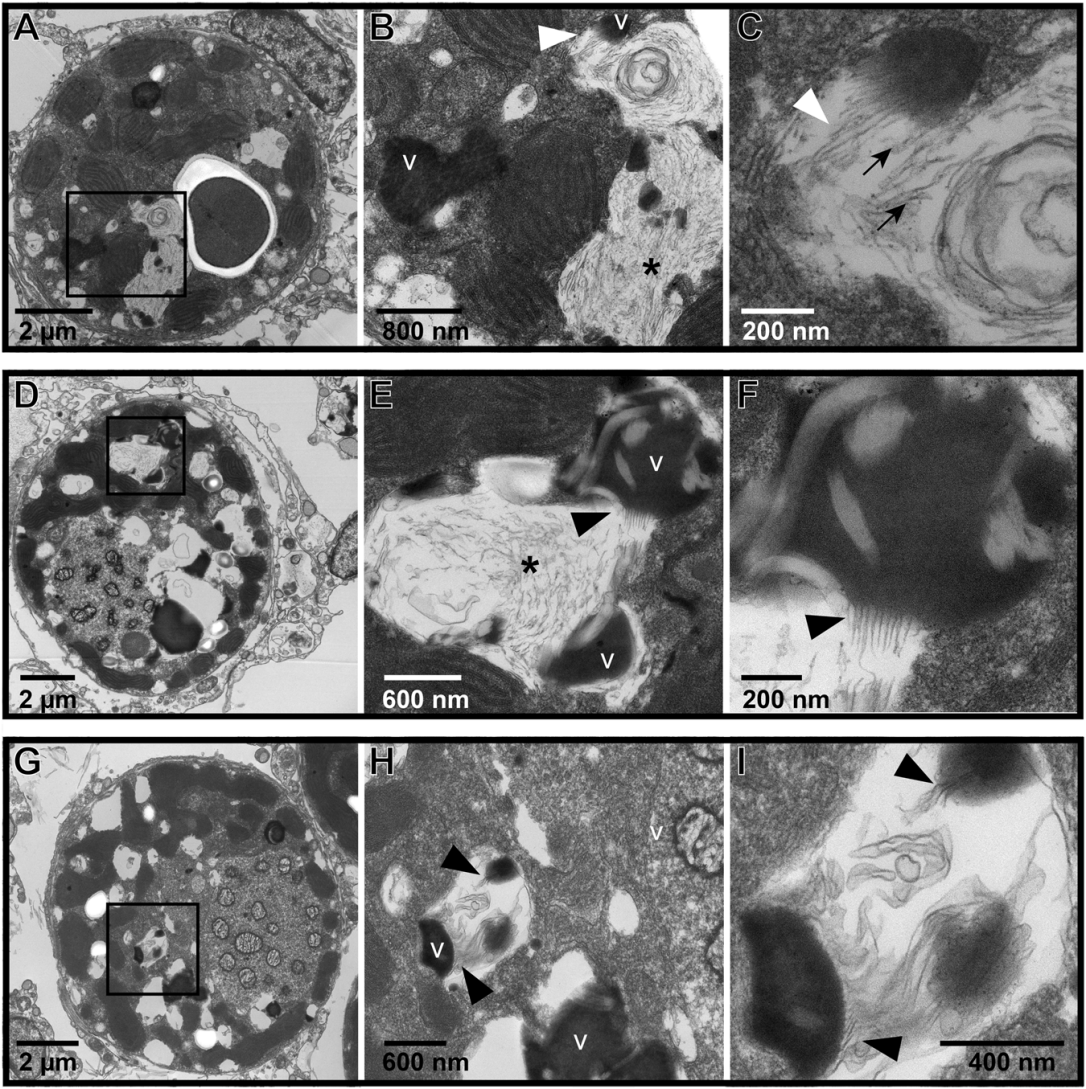
Transmission electron microscopy (TEM) images of putative virion formation in Symbiodiniaceae cells from bleaching colonies of the Pacific stony coral, *Acropora hyacinthus*, sampled during an in situ heat stress event. **A**–**C** Symbiodiniaceae cell with electron-dense viroplasm and putative extruding virions; (**B**) is a higher resolution of the boxed area from (**A**); (**C**) is an inset of (**B**). Double membranes are visible in (**C**) of some filaments (white arrowhead). Cross sections with electron-dense cores are indicated by black arrows. **D**–**F** and **G**–**I** show similar examples of a Symbiodiniaceae cell with putative exocytosis of virions into the cytoplasm. V indicates putative viroplasm; * indicates a cavity with coarse filamentous VLPs; black arrowheads highlight areas with putative virion exocytosis. The identity of these structures cannot be confirmed; other possibilities include viral nucleic acid strands being released for later assembly in the cytoplasm, host cell degradation of filamentous VLPs (visible in the electron-dense putative viroplasm) to recycle nucleic acids, or other non-viral intracellular structure.

Images of Symbiodiniaceae from *P. cf. lobata* and *A. hyacinthus* holobionts revealed putative viral infections with different morphologies (Figs. 4–6). These morphologies appeared comparable to those described in [22]: (1) putative early-stage viral inclusions where whorled, electron-dense viroplasm is visible in a Symbiodiniaceae cell cytoplasm; (2) putative intermediate stage viral infections where filamentous VLPs are arranged within a cytoplasmic cavity; and (3) putative late-stage infections where coarse filamentous VLPs are dispersed in large cytoplasmic cavities. We use the early to late-stage infection terminology here to draw parallels to the hypothesized viral infection progression outlined in [22] but note that stages of viral morphogenesis cannot be confirmed without time course sampling. Putative viroplasm found in association with distinct filamentous VLPs frequently contained the following morphological characteristics: striated or subtle fibril texture (e.g., Supplementary Fig. 1D, Supplementary Fig. 2D), clear-presenting filaments (e.g., Fig. 4A, B, Fig. 5A, B, Supplementary Fig. 1 E, F, Supplementary Fig. 2B), clear-cut cavities along the perimeter (Supplementary Fig. 1, Supplementary Fig. 2D) that were frequently associated with dense dots (e.g., Supplementary Fig. 1A–D), and/or thinner filamentous VLPs in the immediate proximity (e.g., Fig. 4E, F and Fig. 5E, F). These thin filamentous VLPs were different from the wider filamentous VLPs (like those shown in Figs. 2–3); these two VLP morphologies were often observed in conjunction with each other (i.e., Fig. 6C, D; thin concentric filaments in immediate proximity to a cluster of filamentous VLPs). Electron dense, sometimes whorled, material transitioning into defined putative virus-like filament structures was observed in several images (e.g., Figs. 4D,  5D and F). Putative ‘virion exocytosis’ was also documented in Symbiodiniaceae cells of bleaching *A. hyacinthus* on the reef, where parallel lines of thin filamentous VLPs were distinctly protruding from electron-dense putative viroplasm (Fig. 7).

Signs of putative filamentous viral infections (either putative electron dense viroplasm, clusters of distinct filamentous VLPs, or more dispersed, coarse filamentous VLPs) were widespread across all sample types. Collectively, 70% (223/317) of *P. cf. lobata* Symbiodiniaceae cells contained visible signs of putative filamentous viral infections. Sixty-one percent (in situ and aquaria experiment samples combined, 157/257) of *A. hyacinthus* Symbiodiniaceae cells contained visible signs of putative filamentous viral infections. *Acropora hyacinthus* samples generally had more Symbiodiniaceae cells with putative filamentous virions (like those seen in Figs. 2–3), while *P. cf. lobata* had more cells with putative viroplasm (as seen in Supplementary Figs. 1 and 2). However, both coral species contained Symbiodiniaceae cells displaying each of the morphologies/potential stages of viral infection. Twenty-four percent (33/137) of Symbiodiniaceae cells expelled from *A. hyacinthus* colonies contained visible signs of putative filamentous viruses (electron dense viroplasm and coarse filamentous VLPs, Supplementary Fig. 3); no expelled cells contained distinct VLP clusters (such as those pictured in Figs. 2 and 3).

Filamentous VLPs were observed in both heat-stress and ambient conditions in Symbiodiniaceae cells of *P. cf. lobata* (Fig. 2) and A. *hyacinthus* colonies (Fig. 3). *Porites cf. lobata* colonies sampled during ambient temperatures on the reef displayed signs of viral infection in 61.2% (87/142) of imaged Symbiodiniaceae cells. During the reef-wide heat stress event, when the tagged *P. cf. lobata* were resampled, the percentage with signs of viral infection increased significantly to 77.7% (136/175; binomial logistic regression; heat stress χ2 = 10.5, *p* < 0.01). For the heat stress aquaria-based experiment with *A. hyacinthus*, treatment did not drive a significant difference in number of symbiont cells with filamentous VLPs (heat stress χ2 = 3.13, *p* = 0.08); 54.1% of imaged Symbiodiniaceae cells from control temperature fragments displayed signs of viral infection (40/74) and 39.7% (31/78) in the heat-stressed fragments. However, in the five colonies of *A. hyacinthus* sampled during the reef bleaching event, 81.9% (86/105) of imaged Symbiodiniaceae cells displayed signs of viral infection; this was significantly more signs of viral infection than imaged from Symbiodiniaceae within the control colonies of the aquaria experiment (54.1%, 40/74; binomial logistic regression with Tukey test for multiple comparisons; *z* = −3.91, *p* < 0.01; Supplementary Table 1 for all pairwise comparisons). For expelled cells from *A. hyacinthus*, since image resolution and severe cell degradation may have limited detection of VLPs, VLP detections within expelled cells from control, heat-stress, and heat-stress on the reef were compared to one another but not to their *in hospite* counterparts. In the aquaria experiment, 15.4% (8/52) of cells expelled from control fragments contained signs of infection by filamentous viruses compared to 22.4% (13/58) from the experimental heat stressed fragments. While expelled samples from control and heat-stressed fragments did not significantly differ in terms of number of symbiont cells with filamentous VLPs, expelled samples from in situ bleaching *A. hyacinthus* had significantly more cells with filamentous VLPs (44.4%–12/27) than the expelled samples from aquaria control fragments (Supplementary Table 1 for pairwise comparisons).

### Additional putative viruses

**Fig. 8:**
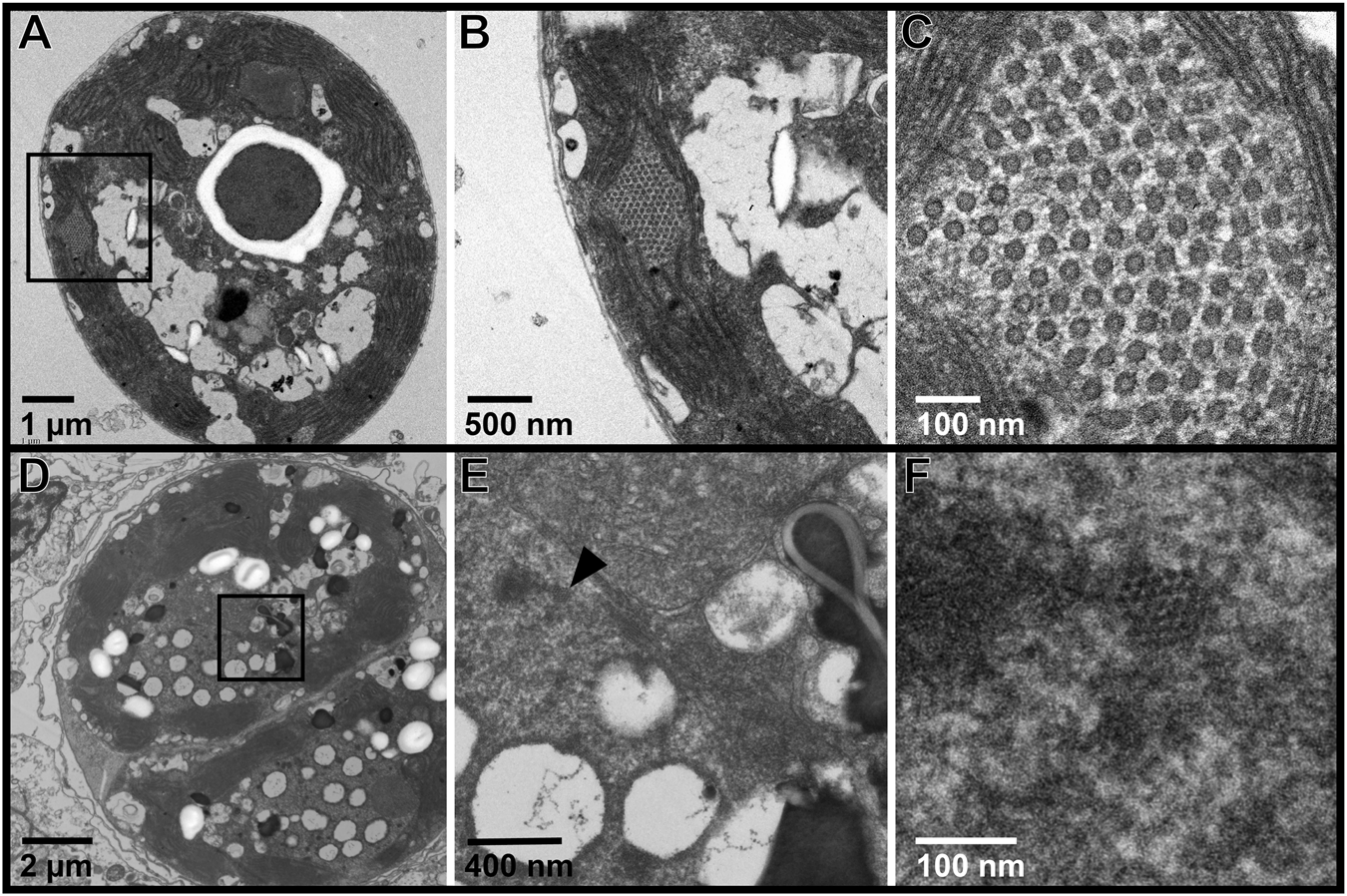
Crystalline arrays of virus-like particles (VLPs) were observed in two of the imaged Symbiodiniaceae cells. **A** An array of ~30 nm diameter VLPs was observed in a Symbiodiniaceae cell expelled from an *Acropora hyacinthus* coral fragment under ambient temperature conditions in an aquaria-based experiment. **B** Higher resolution of the boxed area from (**A**). **C** Inset of (**B**). **D** An array of ~10 nm VLPs was observed in the nucleus of an *in hospite* Symbiodiniaceae cell from an *A. hyacinthus* coral sampled on the forereef of Mo’orea, French Polynesia. **E** Higher resolution of the boxed area from (**D**). Black arrowhead indicates area shown in higher resolution in (**F**). **F** Inset of (**E**).

Filamentous VLPs were the most common and abundant morphology of putative virus documented in Symbiodiniaceae cells. However, additional VLP morphologies, including icosahedral non-enveloped VLPs that ranged between ~90 and 230 nm, were also observed within symbiont cells. Of note were two crystalline arrays; one was observed in between thylakoid membranes and the other in a nucleus. In the array between thylakoid membranes, the mean diameter of individual VLPs was 30.7 ± 1.3 nm (Fig. 8A–C). In a different Symbiodiniaceae cell, in the array within the nucleus (Fig. 8D–F), individual VLPs had a mean diameter of 10.7 ± 1.1 nm. Whorled, electron dense viroplasm (associated with filamentous VLPs) was evident in this symbiont cell as well (Fig. 8E).

### Symbiodiniaceae morphology

Apparently viable as well as degraded Symbiodiniaceae cells were observed in *P. cf. lobata* and *A. hyacinthus* coral species in ambient and in heat stress conditions. Apparently viable cells were most often observed in *A. hyacinthus* colonies in ambient temperature conditions (Fig. 1). In degraded Symbiodiniaceae cells, while some structures, including the nucleus and pyrenoid, were frequently deteriorated or entirely absent, chloroplasts remained visibly intact and were frequently the only identifiable organelle (across both coral holobionts, e.g., Figs. 2D and 6A). *A. hyacinthus* colonies in heat stress treatments of the aquaria-based experiment often had more severely degraded cells, including thylakoid membranes that appeared segmented and disintegrated (Supplementary Fig. 4).

In *P. cf. lobata* colonies sampled during ambient temperatures on the reef, 60% of cells (85/142) were scored as degraded; this increased to 66.3% (116/175) when the same colonies were sampled and imaged during the reef-wide heat stress event. This increase in degraded cells during the heat stress event was dependent on coral colony ( ~genotype), with some colonies driving this overall pattern (binomial logistic regression; colony χ2 = 15.12, *p* < 0.01; heat stress χ2 = 1.74, *p* = 0.19; colony*heat stress χ2 = 23.60, *p* < 0.01). Filamentous VLPs were more prevalent in non-degraded Symbiodiniaceae cells and also varied by coral colony (cell degradation χ2 = 8.95, *p* < 0.01; colony χ2 = 27.54, *p* < 0.01), with no interacting effects; this pattern was documented in addition to evidence that heat stress drove filamentous VLP prevalence (see first results section).

In the aquaria experiment with *A. hyacinthus*, heat-stressed coral fragments had significantly more degraded Symbiodiniaceae cells than control fragments (92.3% – 72/78 versus 43.2% – 32/74 cells; binomial logistic regression; χ2 = 42.24, *p* < 0.01; Fig. 1). Coral colony ( ~genotype) also significantly affected the number of degraded cells in a sample (χ2 = 8.46, *p* = 0.04). Filamentous VLPs (including putative viroplasm) were more frequently documented in non-degraded Symbiodiniaceae cells (χ2 = 9.17, *p* < 0.01) and there were no interacting effects among treatment, cell degradation, and coral colony on documentation of filamentous VLPs. In the five colonies of *A. hyacinthus* sampled during the reef bleaching event, 58% (61/105) of imaged Symbiodiniaceae were degraded; there was no significant association between cell degradation and documentation of filamentous VLPs in this set of samples (χ2 < 0.01, *p* = 0.98).

Symbiodiniaceae cells expelled from *A. hyacinthus* colonies were more degraded than their *in hospite* counterparts (χ2 = 25.61, *p* < 0.01; Fig. 1). These expelled cells, collected from all eight *A. hyacinthus* fragments from the aquaria-based heat stress experiment and from two colonies sampled during the bleaching event in situ, were severely degraded, often with extensive membrane blebbing (Supplementary. Fig. 3A, C, & G). While 93% (54/58) of Symbiodiniaceae cells expelled from heat stressed fragments in the aquarium experiment and 100% expelled from bleaching corals on the reef (27/27) were degraded relative to 77% (40/52) from control fragments, pairwise differences were not significant (Supplementary Table 2).

### Symbiodiniaceae genetic diversity

**Fig. 9:**
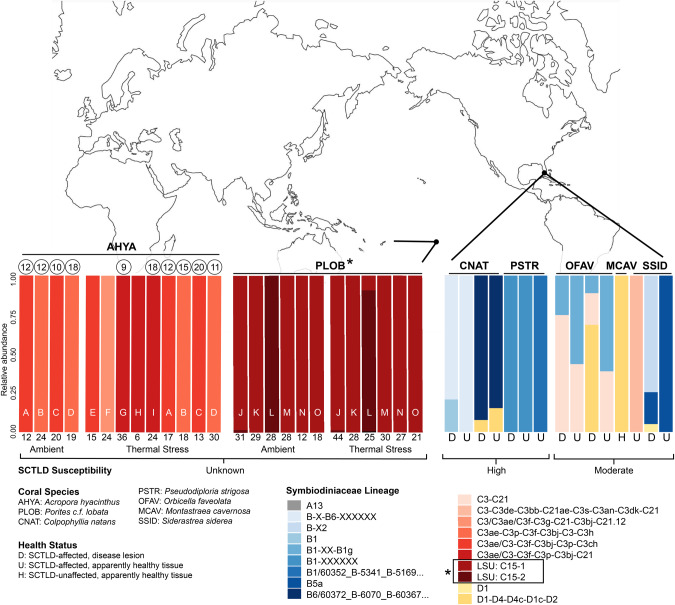
Summary of the dominant Symbiodiniaceae lineages detected from South Pacific and Caribbean coral colonies, from which filamentous virus-like particles (VLPs) were observed. Rightmost vertical bars correspond to Caribbean coral colonies from which filamentous VLPs were described by [22], whereas the leftmost and middle vertical bars correspond to South Pacific coral colonies from which filamentous VLPs are described in this study. Across the total dataset, colonies containing Symbiodiniaceae exhibiting filamentous VLPs were dominated by symbiont lineages within the genera *Breviolum*, *Cladocopium* or *Durusdinium*, or combinations thereof. Each stacked bar depicts the relative abundance of Symbiodiniaceae lineages within an individual sample. Lineages belonging to the same Symbiodiniaceae genus have different tones of the same color. *Acropora hyacinthus* and Caribbean Symbiodiniaceae are represented as Internal Transcribed Spacer-2 region (ITS-2) rDNA DIV profiles; *Porites cf. lobata* Symbiodiniaceae are curated D1-D2 large subunit (LSU) amplicon sequence variants (lineages within black box in color key). To account for differences in ITS-2 copy number variation among Symbiodiniaceae genera, relative abundances were adjusted based on [62] (see methods; versions of these graphs without adjustments for copy number are provided in Supplementary materials). Letters on South Pacific samples distinguish coral colonies ( ~genotypes); for *Acropora hyacinthus*, colonies A–D were used in the aquaria experiment whereas E–I refer to colonies sampled during bleaching on the reef. The number of Symbiodiniaceae cells imaged with transmission electron microscopy for each South Pacific sample is indicated immediately below each bar. Colonies from which expelled Symbiodiniaceae cells were collected have a circle above their bar; circles contain the number of expelled cells imaged from that colony. Map outline from www.freeworldmaps.net/world/pacific-centered/.

In Pacific coral holobionts, two lineages of *Cladocopium* C15 symbionts were detected within *P. cf. lobata* colonies based on D1-D2 of the LSU (Fig. 9, lineages in black box). Only one of the two lineages were detected from most samples (75%, 9/12); both lineages were detected from colony J during both timepoints, and both lineages were detected from colony L only at the 2019 time point (Fig. 9). *Cladocopium* C15-1 was the most commonly detected lineage, found in ~92% (11/12) of the samples examined. *A. hyacinthus* colonies were dominated by *Cladocopium* C3ae-C3 or C3/C3ae ITS-2 DIV profiles (Fig. 9). Colonies used in the aquaria experiment were dominated by one of two DIV profiles (colonies A-D, Fig. 9), whereas colonies sampled on the reef during bleaching (E-I) were dominated by one of three DIV profiles. In Caribbean coral samples (all of which contained filamentous VLPs; samples correspond to images in [22]) corrected for copy number variation (CNV) among Symbiodiniaceae genera, *C. natans, P. strigosa*, and *S. siderea* coral colonies tended to be dominated ( >50% relative abundance) by Symbiodiniaceae lineages in the genus *Breviolum*, but sometimes also contained symbiont lineages in the genus *Durusdinium* (Fig. 9, Supplementary Fig. 6). Eight *Breviolum* DIV profiles were represented across these samples, with one sample simultaneously containing two DIV profiles (leftmost bar of CNAT, Fig. 9). *M. cavernosa* was dominated by a Symbiodiniaceae lineage in the genus *Cladocopium* (C3-C3de DIV profile). *O. faveolata* colonies varied across samples with some dominated by *Breviolum*, *Cladocopium*, or *Durusdinium*, when CNV was accounted for. Without CNV correction, *O. faveolata* colonies appeared more consistently dominated by *Cladocopium* (Supplementary Figs. 7 & 8); for all other coral holobionts, Symbiodiniaceae dominance patterns were comparable with and without CNV correction. Overall, various Caribbean samples that contained filamentous VLPs appeared to harbor Symbiodiniaceae belonging to only one genus - either *Breviolum, Cladocopium*, or *Durusdinium* (Fig. 9).

## Discussion

Reef-associated virus research is accelerating due to the recent documentation of Symbiodiniaceae-associated filamentous VLPs on SCTLD-affected reefs. Multiple lines of evidence now associate both SCTLD-affected and apparently healthy corals with putative filamentous virus activity, based on: (1) the presence of filamentous VLPs in TEM images of Symbiodiniaceae cells from Caribbean corals (middle Florida Keys, U.S.A., [22]); and (2) draft genome assemblies similar to an alphaflexivirus (St. Thomas, U.S. Virgin Islands, [26]). Additional lines of inquiry have been prioritized to further clarify and test the potential role of viruses in SCTLD, including isolation, characterization, and culturing of viruses associated with SCTLD-affected and apparently healthy corals; visualization and quantification of viral infections spatiotemporally within SCTLD-affected corals (e.g., using approaches such as virus lineage-specific primers and quantitative PCR, or double stranded RNA immunofluorescence, [63]); and testing the extent to which Symbiodiniaceae lineages are differentially susceptible to SCTLD and/or viral infection [22, 26, 49]. Furthermore, characterizing the geographic and phylogenetic extent to which Symbiodiniaceae display evidence of filamentous VLPs will provide foundational information required to assess the potential role of viruses in SCTLD. By documenting extensive morphological evidence of filamentous VLPs in coral holobionts from the putatively SCTLD-free Pacific, this study establishes the critical baseline that filamentous viral infections are prevalent across ocean basins and Symbiodiniaceae genetic lineages.

### Filamentous VLPs are present in Symbiodiniaceae cells from stony corals in the Pacific

Filamentous VLPs are highly prevalent in the Symbiodiniaceae of dominant reef-building corals in the South Pacific, a putatively SCTLD-free reef region. We sampled coral colonies (*n* = 15 genotypes, represented by capital letters in Fig. 9) of two different species, examining *in hospite* and expelled Symbiodiniaceae in ambient and heat stress conditions from both experimental (aquaria) and in situ (reef) conditions. Filamentous VLPs were evident in all of these sample types and in 68% of the over 700 Symbiodiniaceae cells imaged with TEM. These images outline multiple viral morphologies which may represent a progression of viral infection (Figs. 4–7).

*Cladocopium* lineages of Symbiodiniaceae within *Porites cf. lobata* and *Acropora hyacinthus* contained distinct, filamentous VLPs in the cytoplasm that were comparable in size and morphology to those documented in [22] (see comparison Figs. 2–3). The VLPs in this study also share similar virion size and morphology to filamentous VLPs previously observed in Symbiodiniaceae and other symbiotic dinoflagellates, including those in [35, 37, 42, 64] (Supplementary Table 1). Similar to [22], we interpret these filamentous VLPs in Symbiodiniaceae on South Pacific reefs to be positive-sense single-stranded RNA viruses, as all known filamentous plant viruses are +ssRNA viruses [23]. Within the +ssRNA viruses, three families share similar morphology to the putative filamentous viruses described here: Flexiviridae [23], Potyviridae [24], and Closteroviridae [25]. Filamentous viruses of coral holobionts have been compared to all three of these families [32]. The length of some of the larger virions observed in this and earlier studies [22, 35, 37] is more consistent with Closteroviridae (650-2,220 nm [25]), than Flexiviridae (470-1,000 nm [23]) or Potyviridae (680-900 nm, [24]). Because virions were primarily overlapping and clustered, virion length was difficult to measure; purifying and then imaging this virus will help increase the accuracy of the length range. The width of observed filamentous VLPs from Symbiodiniaceae is generally larger (closer to ~30 nm, Supplementary Table 1) than what is reported for Flexiviridae (12-14 nm [23], Potyviridae (11-20 nm, [24]), or Closteroviridae ( ~12 nm [25]). Other morphological aspects of the putative viral infections in this study, such as the circular membranes/filament bodies and stellate clusters of filamentous VLPs, share similarity to the characteristic cylindrical inclusion bodies of several potyviruses [65, 66]. Without further phylogenomic information, the filamentous VLPs documented here could belong to any of these three +ssRNA viral families, another viral family (e.g., fungal dsRNA viruses, [67]), or may constitute their own unique clade.

Consistent patterns in TEM images, across sample types and coral species, are interpreted as putative stages of viral infection progression, providing further support that the filamentous VLPs observed here represent viral infection of Symbiodiniaceae. The prevalence of progressive stages of the maturation of putative viral particles corroborates the interpretation that these particles are not other miscellaneous isolated structures in the cell but active viral infections. For example, the electron-dense bodies (characterized here as putative viroplasm) were frequently observed in conjunction with VLPs (e.g., Fig. 6) and in cells where distinct VLPs were not evident. In past studies, comparable electron-dense bodies have been labeled as lipids [37], electron-dense droplets [64], apoptotic bodies [64], and viroplasm [22]. While the composition and identity of this material cannot be definitely determined from this dataset, the Symbiodiniaceae cells imaged in this study include numerous images that support the interpretation of most of this electron dense material as viroplasm of filamentous viruses. Many of these electron dense bodies contained visible filamentous particles within them (e.g., Fig. 2 G-I) or clear whorls or stacked filaments of comparable morphology to the filamentous VLPs (e.g., Figs. 4–5A-B). Clear “cavities” were frequently associated with whorled electron dense material (e.g., Supplementary Fig. 1E-F) or stippled electron-dense material (e.g., Supplementary Fig. 1C-D); an additional indicator of viroplasm (as opposed to lipid or other structure). Cavities in the putative viroplasm often had dense dots associated with them (visible in Supplementary Fig. 1B and D), which could represent an early stage of virion formation. We also documented apparent virus maturation: exocytosis of filamentous virions from some electron-dense bodies (Fig. 7). Given the dearth of information on comparable structures, particularly in Symbiodiniaceae, it is also possible that these filaments are not virions. For example, they (Fig. 7) could be viral nucleic acid strands being released for later assembly in the cytoplasm. Alternatively, host cells may be degrading the filamentous structures (visible in the electron-dense putative viroplasm) to recycle nucleic acids. Regardless, there are numerous co-occurrences of putative viroplasm and filamentous particles (both distinct clusters of filamentous VLPs and thinner coarse filamentous VLPs), indicating that these structures likely belong to the same viral lineage and represent different stages of infection. It is also possible that there are multiple types of filamentous viral infections co-occurring in the imaged Symbiodiniaceae cells or that some of the observed morphologies are non-viral. Genomic characterization of these putative viruses followed by spatially resolved virus quantification approaches are critical next steps in confirming their identity.

In addition to the widespread filamentous VLPs observed in the Symbiodiniaceae cells in this study, we document evidence of infection by different types of non-filamentous viruses. Icosahedral crystalline arrays – assemblies of virus capsids – were observed in between Symbiodiniaceae thylakoid membranes and within a symbiont nucleus (Fig. 8). The ~30 nm VLPs in Fig. 7A-C share similarity to a ssRNA virus that infects the free-living dinoflagellate, *Heterocapsa circularisquama*, which replicates in the cytoplasm and has a ~ 30 nm diameter icosahedral capsid [68]. These could be images of Symbiodiniaceae-infecting dinoRNAVs [51, 55, 69, 70]. Only one other study has reported crystalline arrays in Symbiodiniaceae cells; [42] documented 17-18 nm VLPs in the cytoplasm of Symbiodiniaceae cultures. These observations add to the diversity of reported viral morphologies from coral holobionts. Extensive genomic viral diversity has been documented from coral holobionts worldwide [33, 36, 71–74]; solving the capsid structures for core members of the coral virosphere and visualizing viral infections in vivo (e.g., [63]) is crucial for linking morphological and ‘Omics evidence of viruses, and better understanding viral impacts on holobionts.

### Filamentous VLPs are present in stony coral colonies dominated by lineages from diverse Symbiodiniaceae genera

To explore the host range of Symbiodiniaceae-associated filamentous viruses, we characterized symbiont diversity from Pacific and Caribbean corals from which filamentous VLPs had been previously documented using TEM imaging. We documented diverse lineages of Symbiodiniaceae, including members of *Breviolum*, *Cladocopium*, and/or *Durusdinium*, within these coral holobionts. The observation of consistent putative viral structures across multiple Symbiodiniaceae lineages, coral species, and geographic areas indicates that filamentous virus infections are likely common across the Family Symbiodiniaceae, and perhaps, other dinoflagellates as well. Additional lines of evidence (e.g., ‘Omics data, culture data) will be useful in further characterizing the host range of the putative filamentous viruses documented here.

Characterization of the Symbiodiniaceae genetic diversity of the Caribbean coral holobionts in this study contributes to our understanding of associations between SCTLD disease state, SCTLD susceptibility and Symbiodiniaceae identity. Past reports suggest colonies of coral species considered to be highly susceptible to SCTLD tended to be dominated by *Breviolum*, whereas coral species considered to be moderately susceptible to SCTLD were more frequently dominated by *Cladocopium* [49]. Additionally, [48] reported lower SCTLD infection in *P. strigosa* colonies harboring *Durusdinium*, relative to offshore corals harboring *Breviolum*. This could indicate that some symbiont lineages in *Breviolum* are more susceptible to SCTLD than some lineages in *Cladocopium* or *Durusdinium*. Our data provide some support (despite limited sample sizes) for these previously documented patterns. Our findings corroborate previous results in that, in this study, all samples from high susceptibility species, but only some samples from moderately susceptibility species, were dominated by *Breviolum* (Fig. 9; 100%, *n* = 7/7 vs 50%, *n* = 4/8). *Breviolum* was often documented in higher proportions in apparently healthy tissues from SCTLD-affected colonies, than in tissues sampled at the disease lesion of these same colonies (*O. faveolata, S. siderea* samples in Fig. 9); future studies should test whether there is differential mortality of Symbiodiniaceae lineages along disease lesions. Larger datasets are needed to test how generalizable these apparent relationships are among coral species, Symbiodiniaceae lineages, and/or SCTLD susceptibility across geographic locations or reef zones. Susceptibility to SCTLD may also be primarily driven by a factor other than Symbiodiniaceae identity, and the observed trends in Symbiodiniaceae dominance merely reflect specificity in the symbiotic associations formed by different coral species or populations.

Apparent Symbiodiniaceae dominance in coral fragments differed between CNV corrected and uncorrected data. The high copy number of *Cladocopium* obscured the prevalence of *Breviolum* and *Durusdinum* (particularly in *O. faveolata*, Supplementary Figs. 6 & 8), highlighting the importance of accounting for these differences when making interpretations based on symbiont composition [59]. The CNV-corrected data in this study is based on [62], which used Symbiodiniaceae lineages within each genus that differed from the specific lineages documented in our study. However, even if the corrections used here are not exact, they likely provide a more biologically realistic characterization than the uncorrected results (Supplementary Figs. 7 and 8), especially since inter-generic comparisons are being made [59, 75, 76]. It remains unknown how much variation in copy number exists within each Symbiodiniaceae genus, and efforts should be made to determine copy number estimates for the ITS-2 of additional lineages of Symbiodiniaceae per genus.

### Filamentous VLPs are more prevalent during reef bleaching conditions and are evident in Symbiodiniaceae cells expelled from corals

Elevated temperatures have been hypothesized to induce viral infections in coral Symbiodiniaceae and potentially contribute to coral bleaching ([30, 36, 69, 77], though evidence of this association for specific viral lineages remains limited (but see [51, 55]). The severe marine heatwave that occurred in Mo’orea (South Pacific study site) in 2019, provided an opportunity to investigate how the putative filamentous viruses documented here may react to changing environmental conditions. This is particularly important since marine heatwaves will continue to increase in frequency and severity as anthropogenic climate change progresses, and since all *A. hyacinthus* colonies in this study bleached and eventually died following the 2019 event, while *P. c.f. lobata* colonies survived. Of all sample types in this study (represented by sets of bars in Fig. 1), putative viral infections were most prevalent in the Symbiodiniaceae cells of colonies sampled during this heat wave. Specifically, bleaching *A. hyacinthus* colonies had the highest proportion of infected cells, followed closely by the heat-stressed *P. c.f. lobata* (Fig. 1). Aspects of this thermal stress event, whether it was the prolonged exposure, bleaching process, or other factor(s), appear to have been conducive to filamentous viral infection across multiple Symbiodiniaceae hosts and coral taxa.

In the aquaria-based experiment, heat-stressed *A. hyacinthus* fragments exhibited more degraded Symbiodiniaceae cells, but the number of putative viral infections detected did not differ by treatment. Overall, *A. hyacinthus* colonies in the aquaria-based experiment also had lower proportions of cells with putative viral infections than *A. hyacinthus* colonies on the reef. This is not surprising since colonies in the aquaria-based experiment were collected from the reef during ambient temperature conditions, and an intense heat stress ( + 4 °C above ambient) was applied without ramping. A reduced number of viral infections in the heat-stressed aquaria-based *A. hyacinthus* may be explained by degradation of Symbiodiniaceae cells at a rate too fast for viral infections to develop. Aspects of compromised Symbiodiniaceae cell integrity in the aquaria-based experiment, including the dissolution of thylakoid membranes, further support that the heat treatment was a more extreme stress than that experienced during the in situ bleaching event. Together, these results suggest that heat stress – up to a point – is conducive to filamentous virus infection, and species like *A. hyacinthus* that are more susceptible to bleaching may also exhibit more severe infections. When heat stress is severe (e.g., extremely high and/or rapid onset), deterioration of Symbiodiniaceae cells may outpace viral replication cycles, ultimately reducing observations of VLPs.

The presence of filamentous VLPs in Symbiodiniaceae cells expelled from *A. hyacinthus* colonies suggests a route by which viruses (especially late-stage infections) may exit coral colonies and enter environmental compartments on the reef (e.g., near reef water, sediments, other corals), potentially leading to infections of free-living Symbiodiniaceae or symbionts in nearby corals. When symbionts are lost at higher rates from their hosts during coral bleaching, viruses may become more prevalent in the water column (i.e., virus-mediated vortex of coral reef decline, [30]). Similar to past studies that show reduced photosynthetic capacity of expelled Symbiodiniaceae cells [78, 79], the expelled cells imaged here were severely degraded with indistinct thylakoid membranes and lysed and/or deformed cell walls (Supplementary Fig. 3). As these cells further degrade and disintegrate in the water column, filamentous viruses may be released.

### Towards understanding the roles of filamentous viruses in Symbiodiniaceae

Further work is needed to understand the consequences of infection by putative filamentous viruses to individual Symbiodiniaceae cells and the coral holobiont, during bleaching and disease progression. The VLPs in this study are morphologically similar to three filamentous +ssRNA virus families (Closteroviridae, Flexiviridae, and Potyviridae) which include well-described pathogens that infect flowering plants [80, 81]. However, given the divergence between dinoflagellates and angiosperms, it is likely that the putative filamentous viruses documented here are novel; their pathogenicity requires investigation. Similarly, previously described +ssRNA viruses that infect free-living dinoflagellates (HcRNAV) appear phylogenetically distinct relative to other +ssRNA viruses [82]. In this study, there was not a clear association between Symbiodiniaceae health (cell degradation) and infection by putative filamentous viruses. However, Symbiodiniaceae within heat stressed corals on the reef had the highest prevalence of filamentous VLPs, suggesting a possible association with bleaching.

SCTLD has decimated coral populations across the greater Caribbean (e.g., [2, 7, 10, 83, 84]) and there is growing concern about its potential to spread globally; it is therefore critical for coral reef management, conservation, and restoration initiatives to identify the causative agent(s) of this disease. If the causative agent of SCTLD is a novel pathogen unique to the disease, it should be documented strictly in areas where the disease is present. If filamentous +ssRNA viruses akin to those documented in this study and in [22] contribute to SCTLD signs, it would be expected that filamentous VLPs would be documented in at least higher frequency in SCTLD-susceptible hosts and geographic areas; this pattern was not detected in this study. Similarly, the prevalence of putative filamentous viruses in this study increased when colonies were heat stressed (Fig. 1), but heat stress has not been shown to exacerbate SCTLD [3, 9]. Alternatively, SCTLD could be caused by other non-viral members of the coral holobiont that become more pathogenic under certain contexts (e.g., in other systems [85]). In this case, viruses could cause secondary infections in SCTLD-affected colonies, as a result of physiological stress associated with SCTLD and/or thermal stress (as suggested with changes in bacterial communities in SCTLD [15], and as shown in other systems [86]). As global change continues to raise ocean temperatures and increase global connectivity, novel pathogens and disease are anticipated to spread [87, 88], requiring additional fundamental understanding of coral viruses.

## Conclusion

This study cannot confirm or deny the role of filamentous viruses in SCTLD. However, it does demonstrate that filamentous VLPs are not solely observed in SCTLD-affected or SCTLD-exposed corals or reef regions, nor are they solely associated with corals dominated by a particular Symbiodiniaceae genus (e.g., *Breviolum*). Filamentous VLPs are likely indicative of a widespread, common viral group that infects Symbiodiniaceae. Heat stress may exacerbate these infections, both locally within coral colonies and reef-wide through the release of infected Symbiodiniaceae cells into the water column. Expanded research efforts in coral reef virology are necessary to understand the contexts in which viral infections contribute to declines in coral health and to advance mitigation efforts of coral diseases like SCTLD.

### Supplementary information

Supplementary Materials

Supplementary TEM Images POR

Supplementary TEM Images ACR in situ

Supplementary TEM Images ACR aquaria experiment

Supplementary TEM Images expelled ACR symbionts aquaria

Supplementary TEM Images expelled ACR symbionts in situ

## Data Availability

The microscopy datasets analyzed for this study can be found as supplementary files. The sequence datasets generated and analyzed for this study can be found at the Sequence Read Archive under accession number PRJNA955224.
