# Supplementary material for: Filamentous virus-like particles are present in coral dinoflagellates across genera and ocean basins
Source: ISME J. 2023 Nov 1;17(12):2389–402. doi: 10.1038/s41396-023-01526-6 (PMC10689786; doi:10.1038/s41396-023-01526-6)
Supplement: Supplementary file 1 — Supplementary Materials [file 41396_2023_1526_MOESM1_ESM.docx]

**Filamentous virus-like particles are present in coral dinoflagellates across genera and ocean basins**

*Supplementary Materials*

Corresponding authors: Lauren I. Howe-Kerr, Adrienne M.S. Correa

Emails: [lihowekerr@gmail.com,](about:blank) [amsc@berkeley.edu](mailto:amsc@berkeley.edu)

[Supplementary Methods Text 2](#_Toc135255863)

[Supplementary Tables 4](#_Toc135255864)

[**Supplementary Table 1.** 4](#_Toc135255865)

[**Supplementary Table 2.** 6](#_Toc135255866)

[Supplementary Figures – TEM 7](#_Toc135255867)

[**Supplementary Figure 1.** 7](#_Toc135255868)

[**Supplementary Figure 2.** 8](#_Toc135255869)

[**Supplementary Figure 3.** 9](#_Toc135255870)

[**Supplementary Figure 4.** 10](#_Toc135255871)

[Supplementary Figures – Symbiont Capture Device 11](#_Toc135255872)

[**Supplementary Figure 5.** 11](#_Toc135255873)

[Supplementary Figures – Symbiodiniaceae Genetic Diversity 12](#_Toc135255874)

[**Supplementary Figure 6.** 12](#_Toc135255875)

[**Supplementary Figure 7.** 13](#_Toc135255876)

[**Supplementary Figure 8.** 13](#_Toc135255877)

[Supplementary Data File Descriptions 14](#_Toc135255878)

[References 15](#_Toc135255879)

## **Supplementary Methods Text**

Sampling of expelled Symbiodiniaceae cells

To sample Symbiodiniaceae cells expelled from *Acropora hyacinthus* corals, symbiont capture devices were designed. Capture devices were constructed using sterile 50 mL conical polyethylene tubes with screw-on snap cap lids (Celltreat 229427), nitrile gloves (size small or medium), and cable ties (Supplementary Figure 5). Scissors were sterilized and the finger of a nitrile glove was cut in two locations to produce a nitrile cylindrical sleeve (open on both sides). One end of this sleeve was stretched over thread on the outside of the 50 ml conical tube; the sleeve was secured either with a cable tie or by gently screwing the rim of the conical tube lid over the glove. The snap caps were then closed until deployment to prevent seawater contamination during transport.

For sampling in aquariums, capture devices were filled with seawater and deployed on *A. hyacinthus* fragments for three hours. The end of the nitrile sleeve was gently stretched over 3-4 branches, so that the tips of the branches extended several centimeters into the conical tube (Supplementary Figure 5A). After three hours, the capture devices were removed by taking the coral fragment out of the tank upside-down, gently pulling the nitrile glove off of the coral branch and closing the snap cap lid. Conical tubes were then centrifuged for 10 minutes at 4000 rpm to concentrate expelled Symbiodiniaceae cells. A small (~1 mm^2^) pellet was then visible; half of this pellet was placed in TEM fixative. For *in situ* sampling on the reef, the sampling process followed that of the aquaria sampling, but devices were left on *A. hyacinthus* branches for ~5 hours (Supplementary Figure 5B).

Symbiodiniaceae amplicon sequencing and analysis

To characterize the dominant Symbiodiniaceae lineages present in each *A. hyacinthus* colony, coral tissue slurries preserved in DNA/RNA shield were thawed, and a 300 μL aliquot used for DNA extractions. Slurries were incubated with Proteinase K (20 mg/ml) for two hours at 50 ℃ prior to extraction following the ZymoBIOMICs DNA/RNA Kit (ZymoResearch) manufacturer’s instructions. PCR reactions were conducted to amplify the Internal Transcribed Spacer-2 (ITS-2) region (using primers Sym_VAR_5.8SII and Sym_VAR_REV, [1]) of Symbiodiniaceae rDNA at Oregon State University’s Center for Quantitative Life Sciences (OSU CQLS, Corvallis, OR, USA). Each PCR reaction consisted of 5 μL of DNA at a concentration of 5 ng/μL, 2.5 μL of forward primer and MiSeq Adapter, 2.5 μL of reverse primer and MiSeq Adapter, 12.5 μL 2x KAPA HiFi HotStart ReadyMix, and 2.5 μL molecular grade water. Each reaction had a total volume of 25 μL. We used the following PCR cycle protocol: 95 °C for 3 min, then 15 cycles of 95 °C for 30 sec, 56 °C for 30 sec, and 72 °C for 30 sec, and then 72 °C for 4 min for the final step. Using Agencourt AMPure XP Magnetic Beads, we cleaned up the PCR reactions, then added Illumina indexing primers to 50 μL of the purified product. Then, we used this PCR-primer mixture to run a second round of PCR to incorporate unique barcodes in each sample. Each reaction contained 5 μL of purified PCR product from the first round, 5 μL Illumina Indexed Primer 1 (i5), 5 μL Illumina Indexed Primer 2 (i7), 25 μL 2x KAPA HiFi HotStart ReadyMix, and 10 μL molecular grade water, for a total volume of 50 μL. We used the following PCR cycle protocol: 95 °C for 3 min, then 20 cycles of 95 °C for 30 sec, 56 °C for 30 sec, and 72 °C for 30 sec, and then 72 °C for 4 min as the final step. Once again, we purified the resulting product using AgenCourt AMPure XP Magnetic Beads. We quantified these purified samples using qPCR, with the KAPA library quantification kit (Roche Sequencing Solutions, Pleasanton, CA). The samples were then normalized, pooled in equal molar amounts, and then sequenced using the Illumina MiSeq platform (PE300 run with 25% PhiX at the OSU CQLS).

## **Supplementary Tables**

**Supplementary Table 1.** Summary of studies in which filamentous virus-like particles (VLPs) have been reported from cnidarian tissues, dinoflagellates *in hospite*, cultured Symbiodiniaceae, or seawater in the vicinity of symbiotic cnidarians.

| **Sample type** | **Ocean Basin & Collection location** | **Notes (treatment condition, VLP location)** | **Size of VLP** | **Reference** |
| --- | --- | --- | --- | --- |
| Seawater | Pacific; Aquaria and Heron Island, Australia | Qualitatively observed more filamentous VLPs in seawater surrounding heat-shocked *Acropora formosa* | L: ~3 um | Davy et al. 2006 - [2] |
| Symbiodiniaceae culture | Pacific; Culture | Filamentous VLPs induced via UV exposure; VLPs in cytoplasm and periphery of Symbiodiniaceae | D: ~30 nm  L: 2-3 um | Lohr et al. 2007 - [3] |
| *Acropora muricota* and *Porites* spp. | Pacific; Heron Island, Australia | Filamentous VLPs in coral surface mucus layer | L: <100 - >500 nm | Davy & Patten 2007 - [4] |
| *Acropora muricota* | Pacific; Heron Island, Australia | Filamentous VLPs observed in epidermal coral tissue.  Filamentous VLPs only in healthy colonies and not in white syndrome-affected colonies | L: 220-2,000 nm | Patten et al. 2008 - [5] |
| Symbiodiniaceae Cultures | Caribbean; Cultures | Observed in *Symbiodinium* and *Cladocopium* cultures in control conditions and after UV exposure    In Symbiodiniaceae cytoplasm, cell periphery, and external to cell | L: 100-500 nm | Lawrence et al. 2014 - [6] |
| Symbiodiniaceae within *Porites lutea* | Pacific; Kaneohe Bay, Hawaii | In Symbiodiniaceae cytoplasm and cell periphery of both healthy and white-patch syndrome *Porites* colonies | L: ~50-100 nm | Lawrence et al. 2015 - [7] |
| *Acropora aspera* and *Acropora millepora* | Pacific; Heron Island, Australia | Coral tissue of bleaching *Acropora* | Not reported | Correa et al. 2016 - [8] |
| Symbiodiniaceae cultures | Pacific; Cultures from Magnetic Island, Australia | Untreated Symbiodiniaceae cytoplasm and nuclei; sometimes associated with damaged nuclei; not observed in UV or heat-treated Symbiodiniaceae | D: 20-30 nm L: 1000-2500 nm | Weynberg et al. 2017 - [9] |
| Symbiotic Dinoflagellate (Collodarian-associated) | Mediterranean Sea; Bay of Villefranchesur-  Mer, France | Collodarian-dinoflagellate cytoplasm (control and heat-treated) | Not reported | Villar et al. 2018 - [10] |
| Symbiodiniaceae within *Montastrea cavernosa, Siderastrea siderea*, *Colpophyllia natans, Orbicella faveolata, Pseudodiploria strigosa* | Caribbean; Florida | Cytoplasm of Symbiodiniaceae from various species of apparently healthy and SCTLD-affected corals | D: 15-20 nm L: 558-6,697 nm | Work et al 2021 - [11] |

**Supplementary Table 2.** Pairwise comparisons of proportions of *Acropora hyacinthus*-associated Symbiodiniaceae that are either degraded or containing filamentous virus-like particles (VLPs). Comparisons are among three categories: 1) aquaria control conditions, 2) aquaria heat-stress conditions, and 3) *in situ* reef heat stress conditions. After binomial logistic regressions were run, pairwise comparisons between the three sample categories were conducted using Tukey tests for multiple comparisons.

| **Symbiont sample type**  (*in hospite* from coral colonies vs expelled) | **Model**  (family= “binomial” for all) | **Pairwise comparison** | **Statistic**  (p values < 0.05 in bold) |
| --- | --- | --- | --- |
| *A. hyacinthus coral colonies* | glm(Degraded ~ Category) | aquaria control vs aquaria heat stress | *z =* -5.68, ***p <* 0.01** |
|  |  | aquaria control vs reef heat stress | *z =* -1.95, *p =* 0.12 |
|  |  | aquaria heat stress vs reef heat stress | *z =* 4.61, ***p <* 0.01** |
| *A. hyacinthus coral colonies* | glm(VLPs ~ Category) | aquaria control vs aquaria heat stress | *z =* 1.76, *p =* 0.18 |
|  |  | aquaria control vs reef heat stress | *z =* -3.91, ***p <* 0.01** |
|  |  | aquaria heat stress vs reef heat stress | *z =* -5.61, ***p <* 0.01** |
| *A. hyacinthus expelled cells* | glm(Degraded ~ Category) | aquaria control vs aquaria heat stress | *z =* -2.28, *p =* 0.06 |
|  |  | aquaria control vs reef heat stress | *z =* -0.01, *p =* 0.99 |
|  |  | aquaria heat stress vs reef heat stress | *z =* -0.01, *p =* 0.99 |
| *A. hyacinthus expelled cells* | glm(VLPs ~ Category) | aquaria control vs aquaria heat stress | *z =* -0.93, *p =* 0.62 |
|  |  | aquaria control vs reef heat stress | *z =* -2.72, ***p =* 0.02** |
|  |  | aquaria heat stress vs reef heat stress | *z =* -2.04, *p =* 0.10 |

##

## **Supplementary Figures – TEM**

**
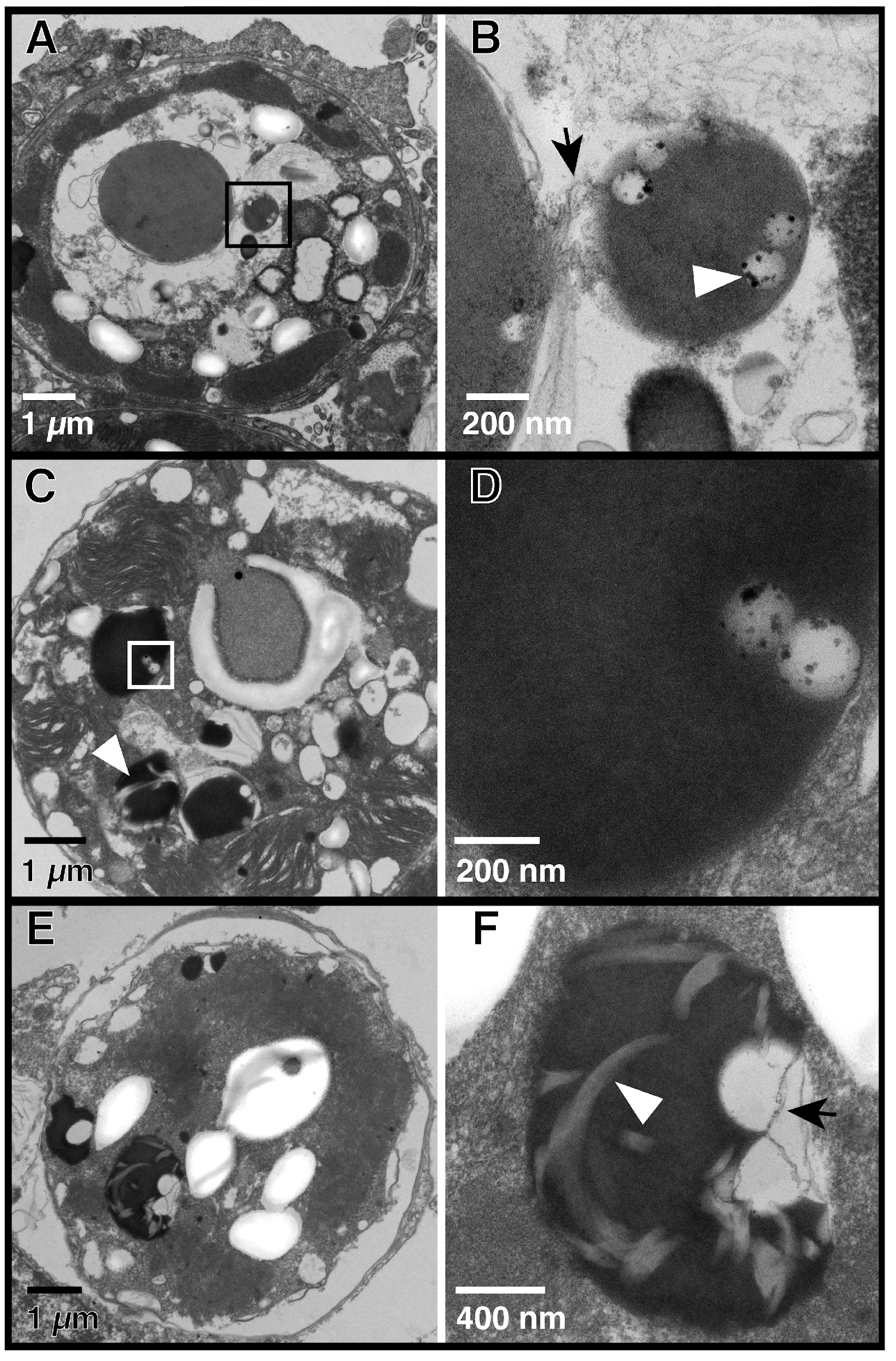
**

**Supplementary Figure 1. Representative transmission electron microscopy (TEM) images of Symbiodiniaceae from *in situ* colonies of the Pacific stony coral, *Porites c.f. lobata*, showing electron-dense putative viroplasm.** (A-B) large masses of putative viroplasm with clear-cut cavities containing electron dense particles (white arrowhead); (B) is an inset of (A); black arrow shows thin filaments separating from putative viroplasm. (C-D) additional cell with large masses of putative viroplasm, with clear cut cavities (box) and whorls (white arrowhead). (D) is an inset of (C). (E-F) whorled viroplasm (white arrowhead) with cavities and then filaments (black arrow); (F) is an inset of (E).

**
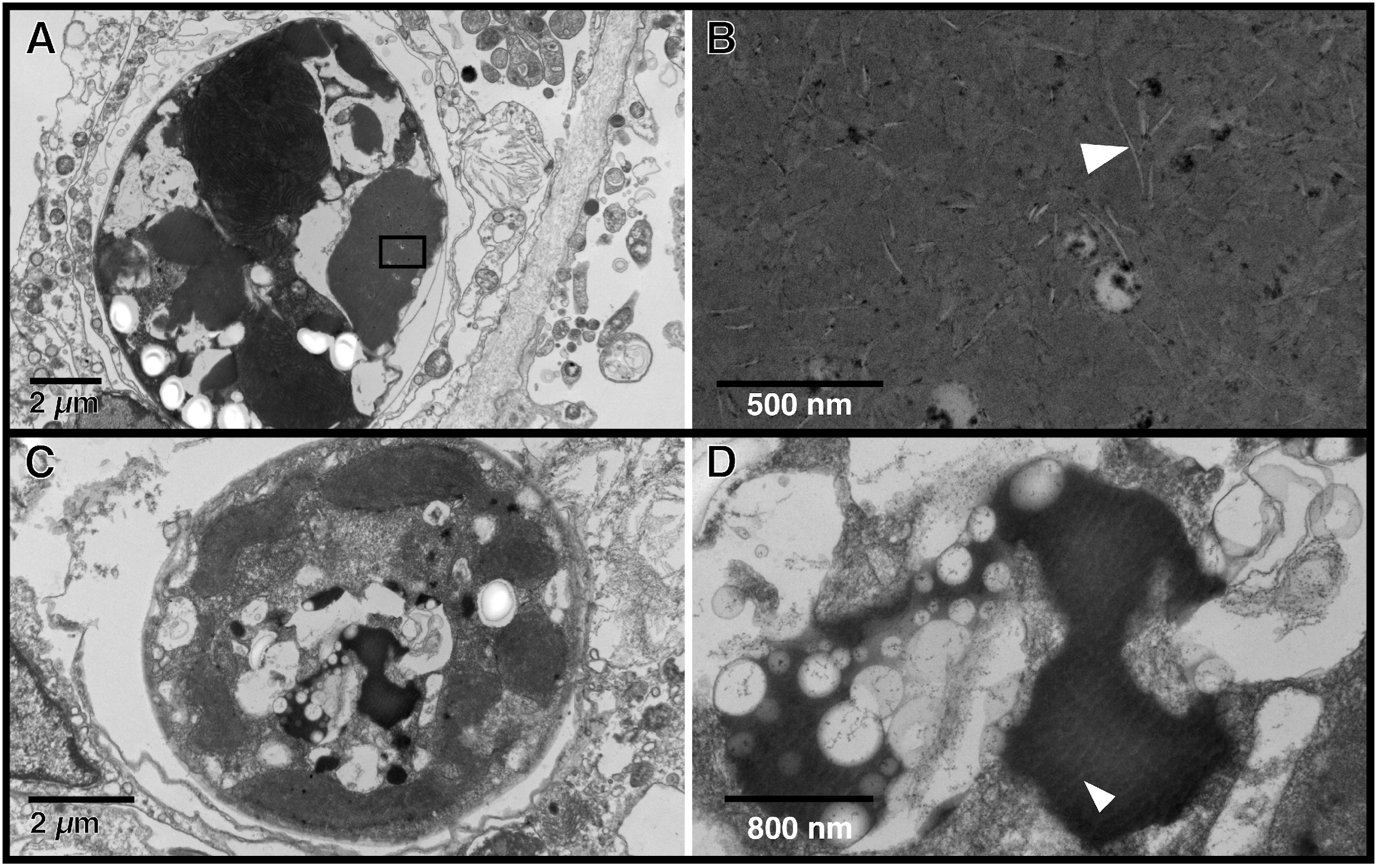
**

**Supplementary Figure 2.** **Representative transmission electron microscopy (TEM) images of Symbiodiniaceae from *in situ* colonies of the Pacific stony coral, *Acropora hyacinthus*, showing electron-dense putative viroplasm.** (A-B) electron-dense viroplasm containing visible filamentous particles (white arrowhead); (B) is an inset of (A). (C-D) election-dense viroplasm with ‘cavities’ and a striated texture (white arrowhead). (D) is an inset of (C).


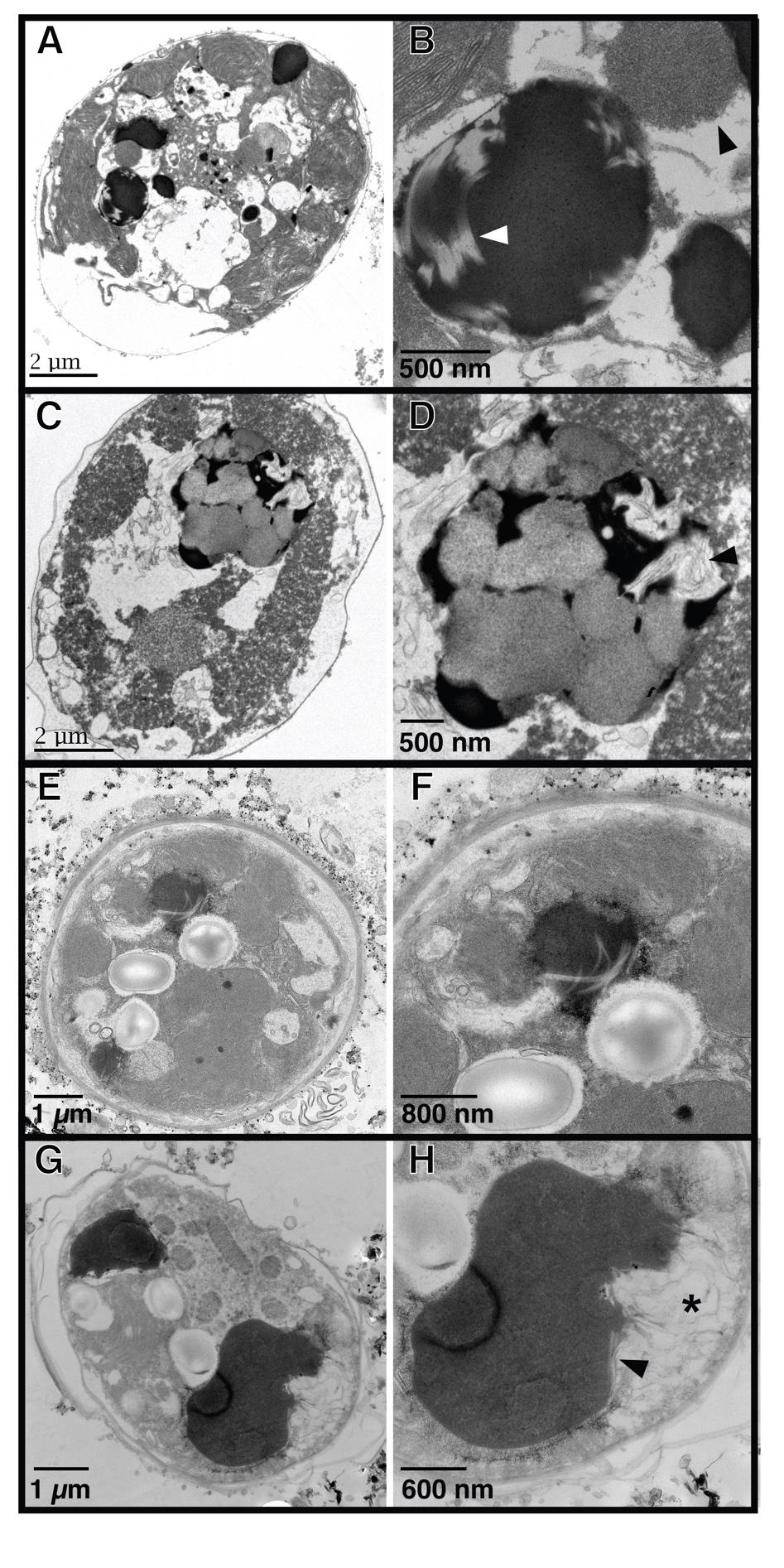


**Supplementary Figure 3. Representative transmission electron microscopy (TEM) images of expelled Symbiodiniaceae from colonies of the Pacific stony coral, *Acropora hyacinthus*, showing electron-dense putative viroplasm and coarse filamentous virus-like particles (VLPs).** (A-D) depict expelled cells sampled from colonies in an aquarium-based experiment while (E-H) depict expelled cells sampled from *in situ* colonies. (A-B) Whorled, electron-dense viroplasm (whorls indicated by white arrowhead) with a circular cluster of putative later-stage viroplasm (black arrowhead); (B) is an inset of (A). (C-D) large mass of election-dense viroplasm with coarse VLPs (black arrowhead) in a cell cavity. (D) is an inset of (C). (E-F) Whorled, electron-dense viroplasm in a Symbiodiniaceae cell expelled from a bleaching *A. hyacinthus* colony; (F) is an inset of (E). (G-H) large masses of election-dense viroplasm with separating filaments around its perimeter (black arrowhead) and coarse filamentous VLPs in the surrounding cell cavity (*).

**
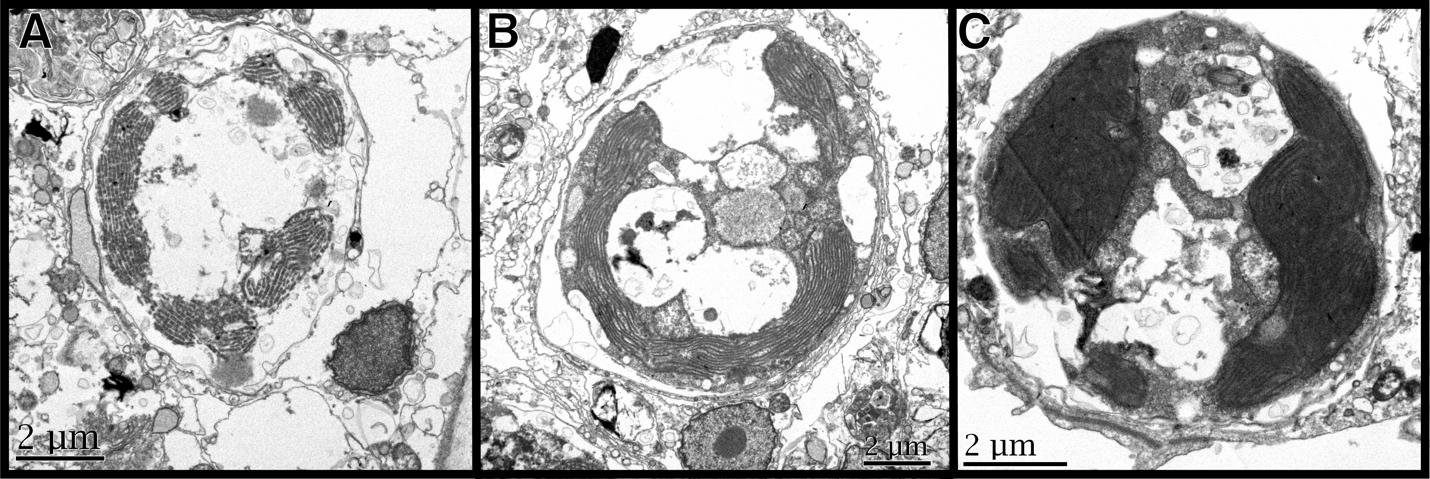
**

**Supplementary Figure 4. Degraded Symbiodiniaceae cells within heat-stressed fragments of *Acropora hyacinthus*, in an aquaria-based experiment.** After 78 hours of heat stress, *in hospite* Symbiodiniaceae cells were severely degraded, with chloroplasts frequently the only identifiable organelle (A-C); thylakoid membranes of some chloroplasts were separating (A-B) and appeared to be segmented (A). Others clumped together, making thylakoid membranes less distinct (C).

## **Supplementary Figures – Symbiont Capture Device**


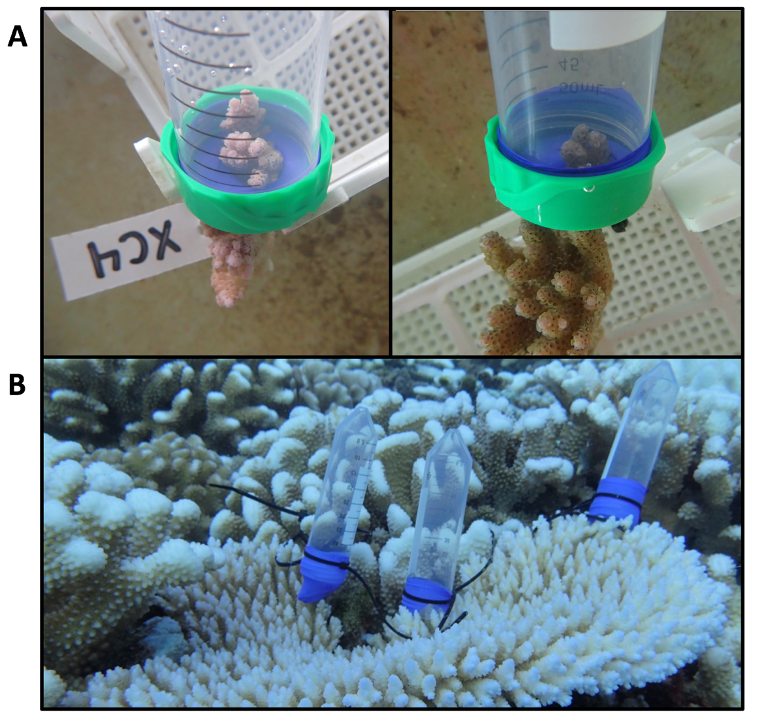


**Supplementary Figure 5. Representative images of the symbiont capture devices designed to capture expelled Symbiodiniaceae cells from *Acropora hyacinthus* coral colonies.** Devices were deployed in an aquaria-based experiment (A) and *in situ* on colonies on the north shore fore reef of Mo’orea, French Polynesia, South Pacific (B).

## **Supplementary Figures – Symbiodiniaceae Genetic Diversity**


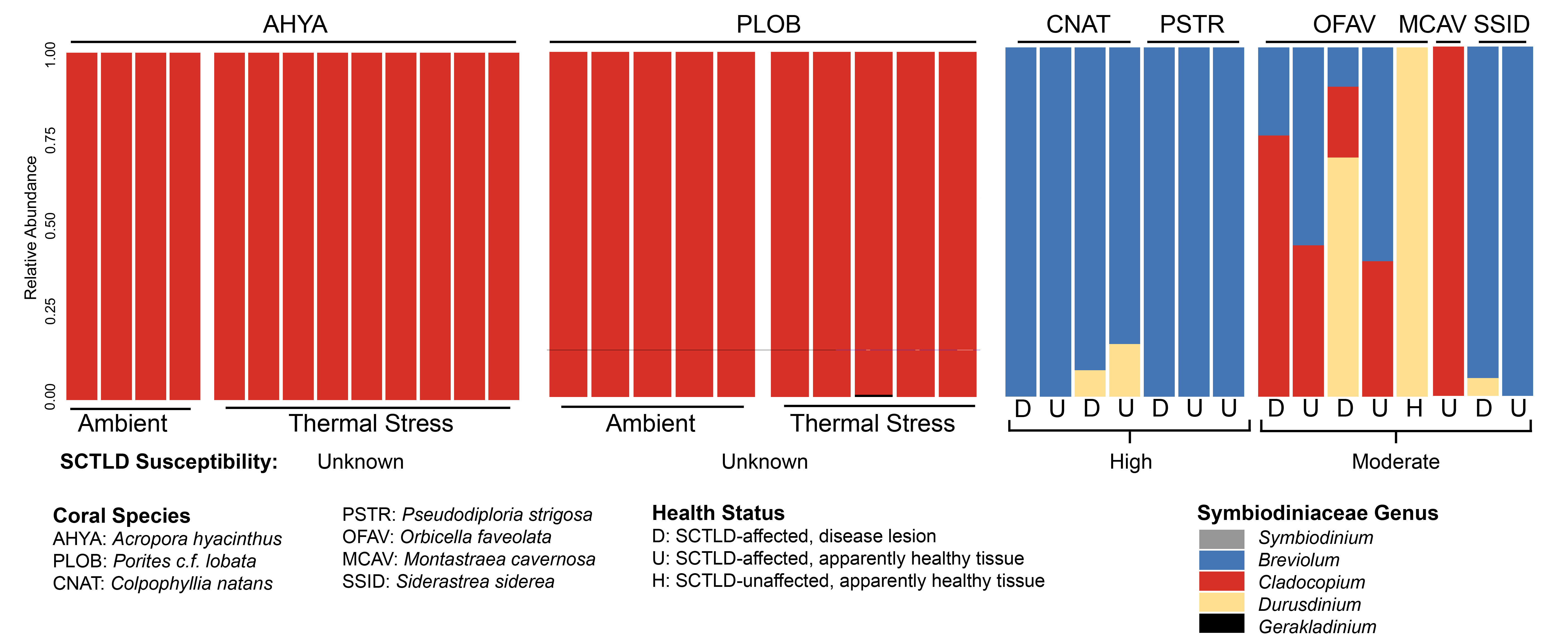


**Supplementary Figure 6. Summary of the dominant Symbiodiniaceae genera within coral colonies containing filamentous virus-like particles (VLPs), with copy number correction.** Each stacked bar depicts the relative abundance of Symbiodiniaceae genera within an individual sample. Lineages are based on Internal Transcribed Spacer-2 (ITS-2) region sequences, except for *Porites cf. lobata* which are sequences from the D1–D2 region of the large subunit (LSU) nuclear ribosomal RNA gene. To account for variation in copy number within the ITS-2 among Symbiodiniaceae genera, relative abundances were adjusted based on [12]. Caribbean coral samples correspond to a subset of samples from which filamentous VLPs were described by [11]. Across the total dataset, colonies containing Symbiodiniaceae exhibiting filamentous VLPs were dominated by symbionts in the genera *Breviolum*, *Cladocopium* or *Durusdinium*, or combinations thereof.


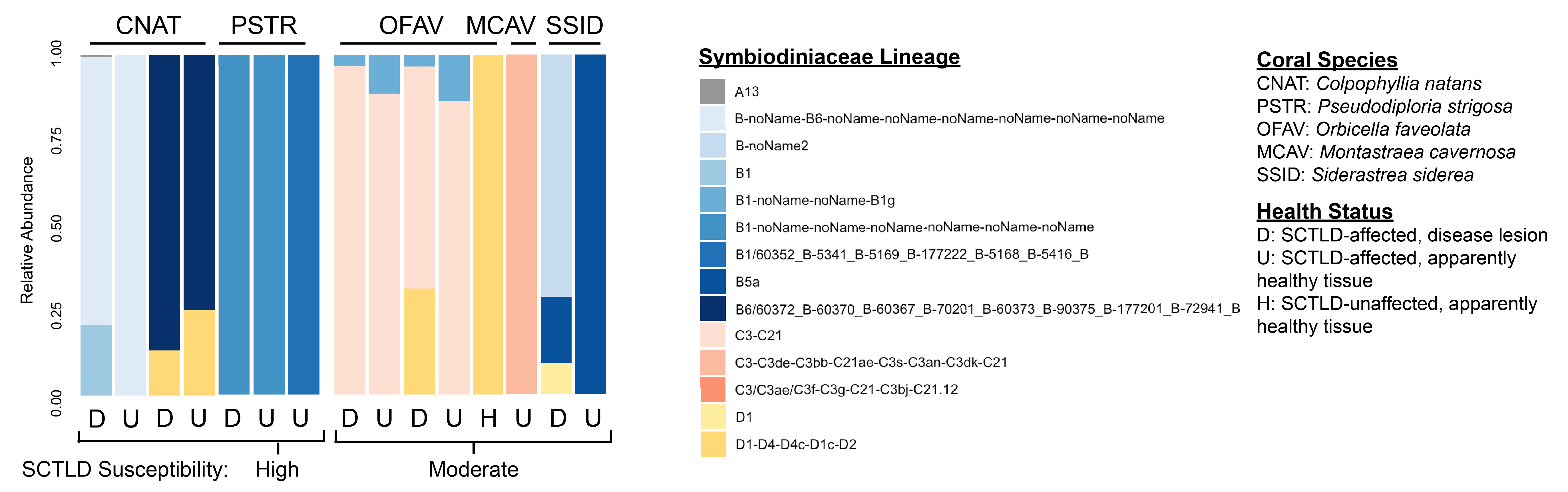


**Supplementary Figure 7. Summary of the dominant Symbiodiniaceae lineages from SCTLD-affected corals containing filamentous virus-like particles (VLPs).** Coral samples correspond to a subset of Caribbean samples from which filamentous VLPs were described by [11]. Lineages are based on Internal Transcribed Spacer-2 (ITS-2) region sequences, and lineages belonging to the same Symbiodiniaceae genus are depicted with different tones of the same color. Each stacked bar depicts the relative abundance of Symbiodiniaceae lineages within an individual sample. Note that unlike Figure 9 and Supplementary Figure 6, data are not corrected for copy number variation.

**
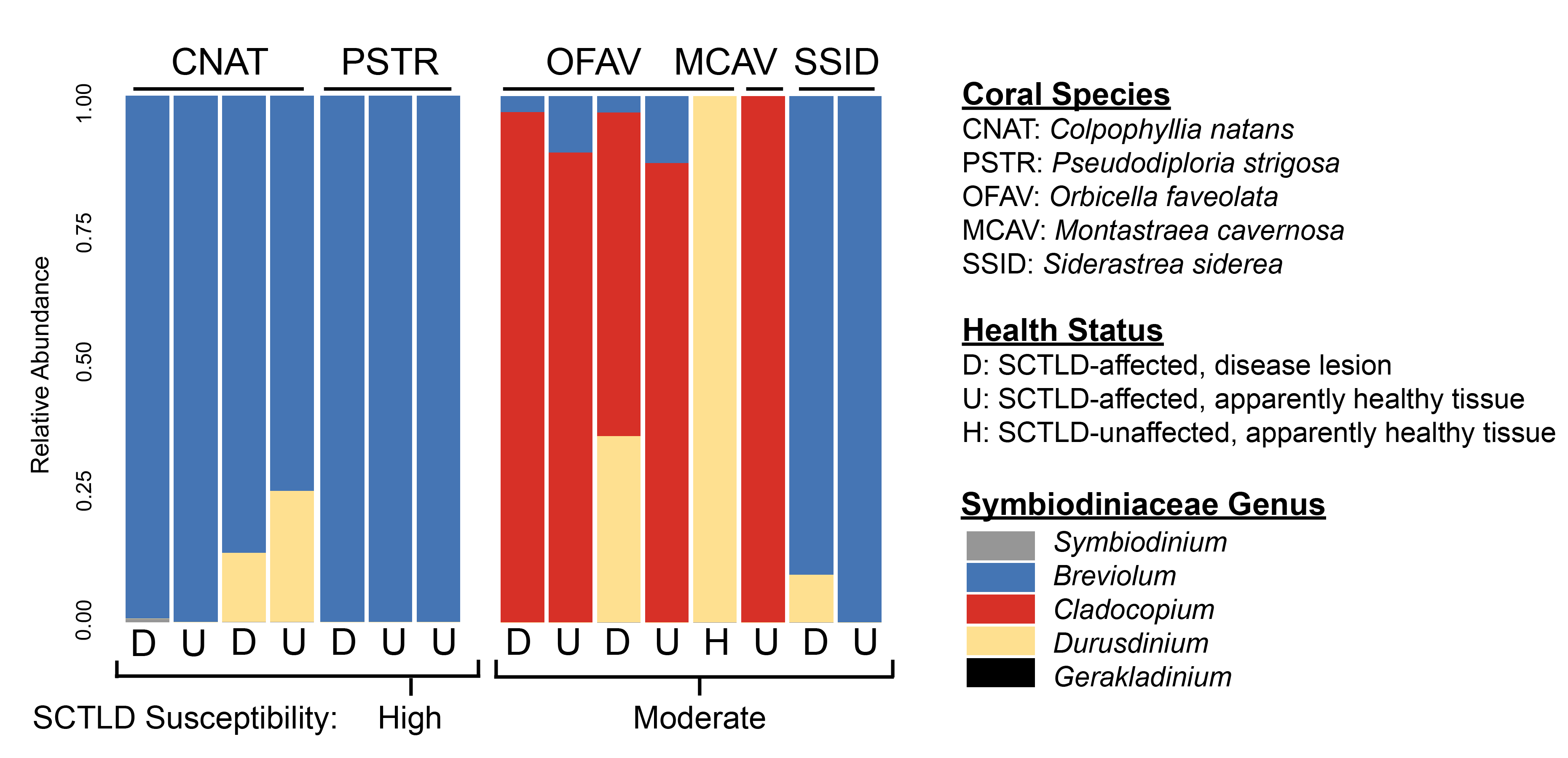
**

**Supplementary Figure 8. Summary of the dominant Symbiodiniaceae genera from SCTLD-affected corals containing filamentous virus-like particles (VLPs).** Coral samples correspond to a subset of Caribbean samples from which filamentous VLPs were described by [11]. Genus identification is based on Internal Transcribed Spacer-2 (ITS-2) region sequences. Each stacked bar depicts the relative abundance of Symbiodiniaceae genera within an individual sample. Note that unlike Figure 9 and Supplementary Figure 6, data are not corrected for copy number variation.

## **Supplementary Data File Descriptions**

**Supplementary Data File S1. (separate file - <Supplementary TEM Images** [**POR.pdf**](https://drive.google.com/file/d/1gYKPmTLRpxhN6qf0w7gdzlHPShMm3bX7/view?usp=share_link)**>)**

Symbiodiniaceae transmission electron microscopy images from *Porites cf. lobata in situ* sampling. Contains images of every cell imaged from the six colonies of *Porites cf. lobata* sampled on the north shore of Mo’orea, French Polynesia in March 2018 (ambient temperature conditions) and March 2019 (elevated temperature conditions). Unique colony identifiers are indicated by letters (J-O) which correspond to the colony labels in Figure 9.

**Supplementary Data File S2. (separate file - <Supplementary TEM Images ACR Aquaria.pdf>)**

Symbiodiniaceae transmission electron microscopy images from *Acropora hyacinthus* aquaria experiment. Contains images of every cell imaged from control and heat fragments of *Acropora hyacinthus.* The four coral genotypes are indicated by letters (A-D), and heat versus control conditions are indicated by “H” or “C”.

**Supplementary Data File S3. (separate file - <Supplementary TEM Images ACR in situ.pdf>)**

Symbiodiniaceae transmission electron microscopy images from *Acropora hyacinthus in situ* sampling. Contains images of every cell imaged from the six colonies of *Acropora hyacinthus* sampled on the north shore fore reef of Mo’orea, French Polynesia in July 2019 during a massive bleaching event. Unique colony identifiers are indicated by letters (E-I) which correspond to the colony labels in Figure 9.

**Supplementary Data File S4. (separate file - <Supplementary TEM Images Expelled ACR Aquaria.pdf>)**

Symbiodiniaceae transmission electron microscopy images from cells expelled from *Acropora hyacinthus* colonies during an aquaria experiment. Contains images of every cell imaged from the the eight samples of Symbiodiniaceae expelled from *A. hyacinthus* fragments in heat stress and control conditions during an aquaria-based experiment. The four coral genotypes from which expelled samples were collected are indicated by letters (A-D), and heat versus control conditions are indicated by “H” or “C”.

**Supplementary Data File S5. (separate file -** <**Supplementary TEM Images Expelled ACR in situ.pdf**>)

Symbiodiniaceae transmission electron microscopy images from cells expelled from *in situ* *Acropora hyacinthus* colonies. Contains images of every cell imaged from the two samples of Symbiodiniaceae expelled from *A. hyacinthus* on the reef during July 2019 during a massive bleaching event.

## **References**

1. Hume BCC, Ziegler M, Poulain J, Pochon X, Romac S, Boissin E, et al. An improved primer set and amplification protocol with increased specificity and sensitivity targeting the *Symbiodinium* ITS2 region. *PeerJ* 2018; **6**:e4816.

2. Davy SK, Burchett SG, Dale AL, Davies P, Davy JE, Muncke C, et al. Viruses: agents of coral disease? *Dis Aquat Organ* 2006; **69**:101–110.

3. Lohr J, Munn CB, Wilson WH. Characterization of a latent virus-like infection of symbiotic zooxanthellae. *Appl Environ Microbiol* 2007; **73**:2976–2981.

4. Davy JE, Patten NL. Morphological diversity of virus-like particles within the surface microlayer of scleractinian corals. *Aquatic Microbial Ecology* 2007; **47**:37–44.

5. Patten NL, Harrison PL, Mitchell JG. Prevalence of virus-like particles within a staghorn scleractinian coral (*Acropora muricata*) from the Great Barrier Reef. *Coral Reefs* 2008; **27**:569–580.

6. Lawrence SA, Wilson WH, Davy JE, Davy SK. Latent virus-like infections are present in a diverse range of *Symbiodinium* spp. (Dinophyta). *J Phycol* 2014; **50**:984–997.

7. Lawrence SA, Davy JE, Wilson WH, Hoegh-Guldberg O, Davy SK. *Porites* white patch syndrome: associated viruses and disease physiology. *Coral Reefs* 2015; **34**:249–257.

8. Correa AMS, Ainsworth TD, Rosales SM, Thurber AR, Butler CR, Vega Thurber RL. Viral outbreak in corals associated with an *in situ* bleaching event: Atypical herpes-like viruses and a new megavirus infecting *Symbiodinium*. *Front Microbiol* 2016; **7**:127.

9. Weynberg KD, Neave M, Clode PL, Voolstra CR, Brownlee C, Laffy P, et al. Prevalent and persistent viral infection in cultures of the coral algal endosymbiont *Symbiodinium.* *Coral Reefs* 2017; **36**:773–784.

10. Villar E, Dani V, Bigeard E, Linhart T, Mendez-Sandin M, Bachy C, et al. Symbiont chloroplasts remain active during bleaching-like response induced by thermal stress in *Collozoum pelagicum* (Collodaria, Retaria). *Front Mar Sci* 2018; **5**:387.

11. Work TM, Weatherby TM, Landsberg JH, Kiryu Y, Cook SM, Peters EC. Viral-like particles are associated with endosymbiont pathology in Florida corals affected by stony coral tissue loss disease. *Front Mar Sci* 2021; **8**:750658.

12. Saad OS, Lin X, Ng TY, Li L, Ang P, Lin S. Genome size, rDNA copy, and qPCR assays for Symbiodiniaceae. *Front Microbiol* 2020; **11**:847.
