# Supplementary material for: Filamentous virus-like particles are present in coral dinoflagellates across genera and ocean basins
Source: ISME J. 2023 Nov 1;17(12):2389–402. doi: 10.1038/s41396-023-01526-6 (PMC10689786; doi:10.1038/s41396-023-01526-6)
Supplement: Supplementary file 3 — Supplementary TEM Images ACR in situ [file 41396_2023_1526_MOESM3_ESM.pdf]

Filamentous virus-like particles are present in coral dinoflagellates across genera and ocean basins

Supplementary Data- *In situ Acropora hyacinthus* Symbiodiniaceae TEM images

ACR Colony E

ACR Colony F

ACR Colony G

ACR Colony H

ACR Colony I

ACR Colony E

Cell 1

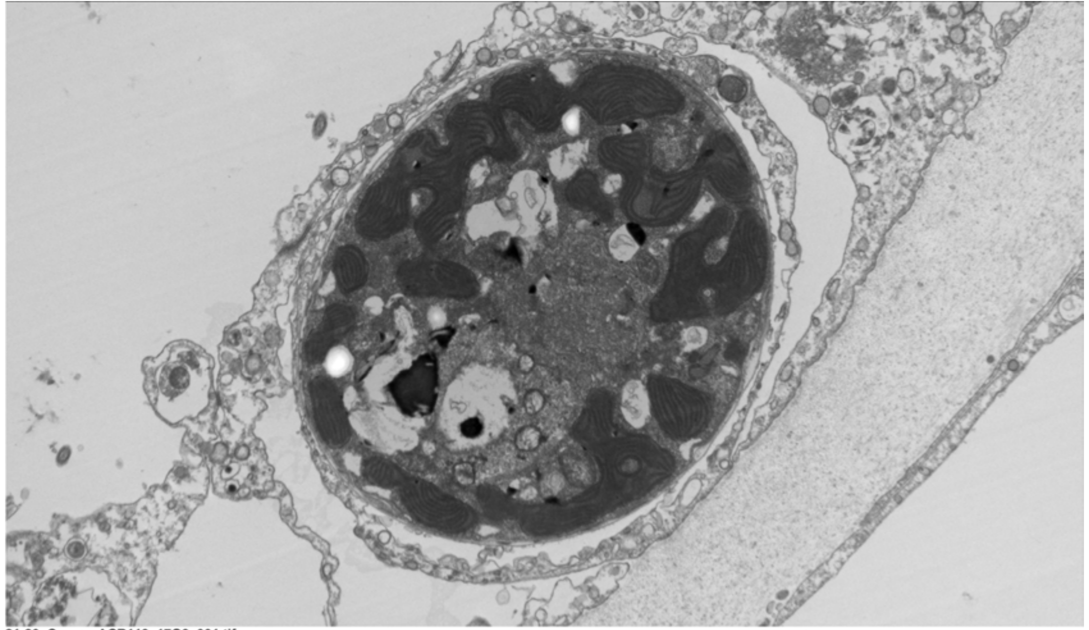

21-20_Correa_ACR113_17O3_001.tif
ACR 113 tissue
Biological Electron Microscopy Lab
Rice University - SEA
Microscopist: MD Meyer

2 µm
HV=80kV
Direct Mag: 1500 x

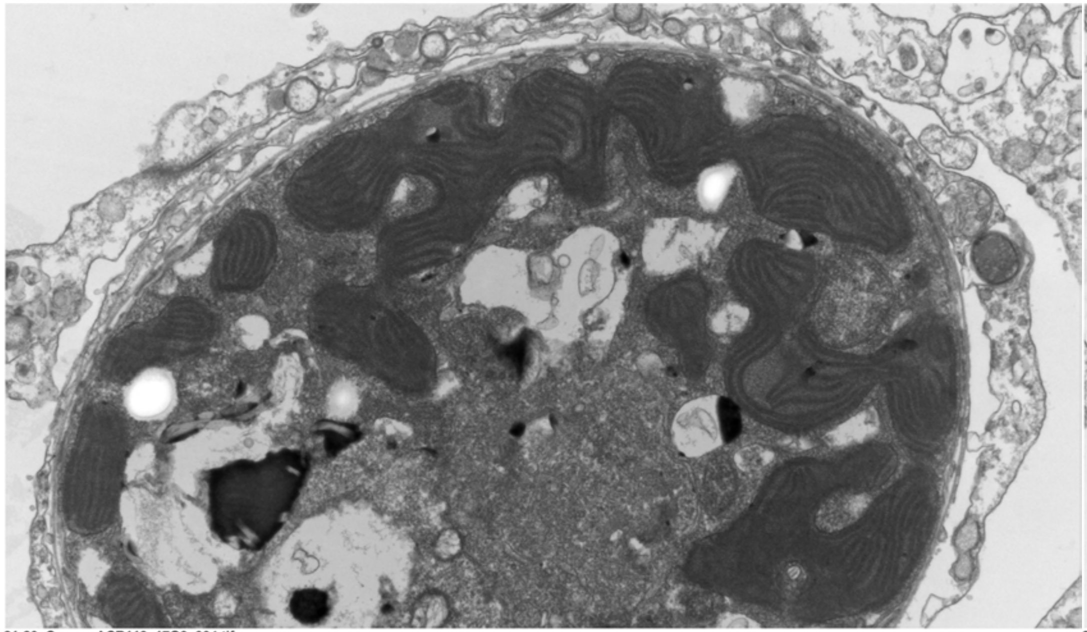

21-20_Correa_ACR113_17O3_004.tif
ACR 113 tissue
Biological Electron Microscopy Lab
Rice University - SEA
Microscopist: MD Meyer

1 µm
HV=80kV
Direct Mag: 3000 x

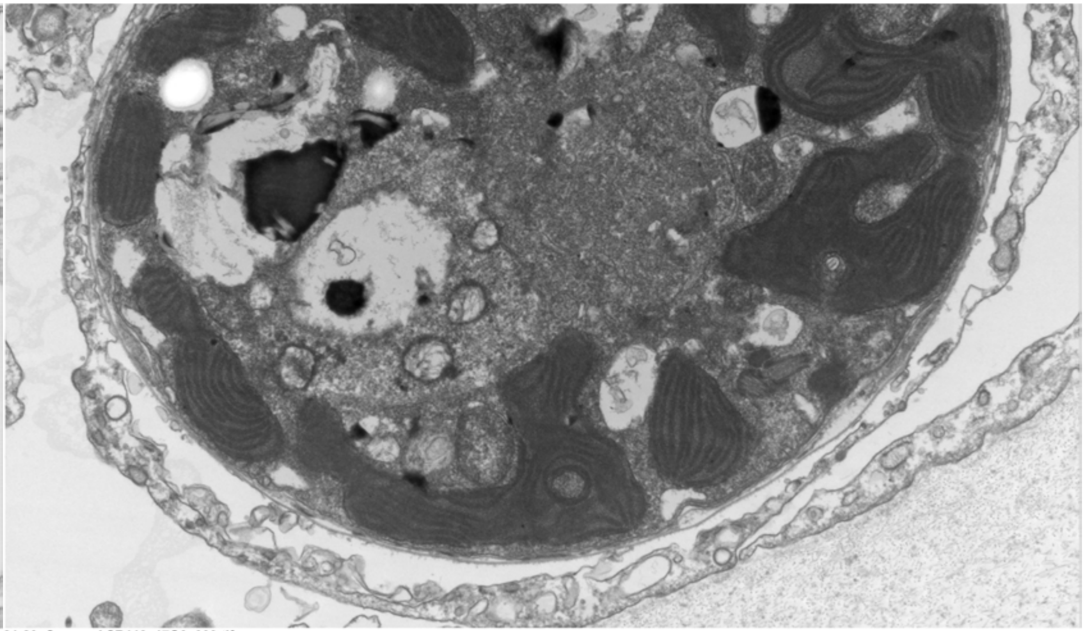

21-20_Correa_ACR113_17O3_003.tif
ACR 113 tissue
Biological Electron Microscopy Lab
Rice University - SEA
Microscopist: MD Meyer

1 µm
HV=80kV
Direct Mag: 3000 x

Cell 2

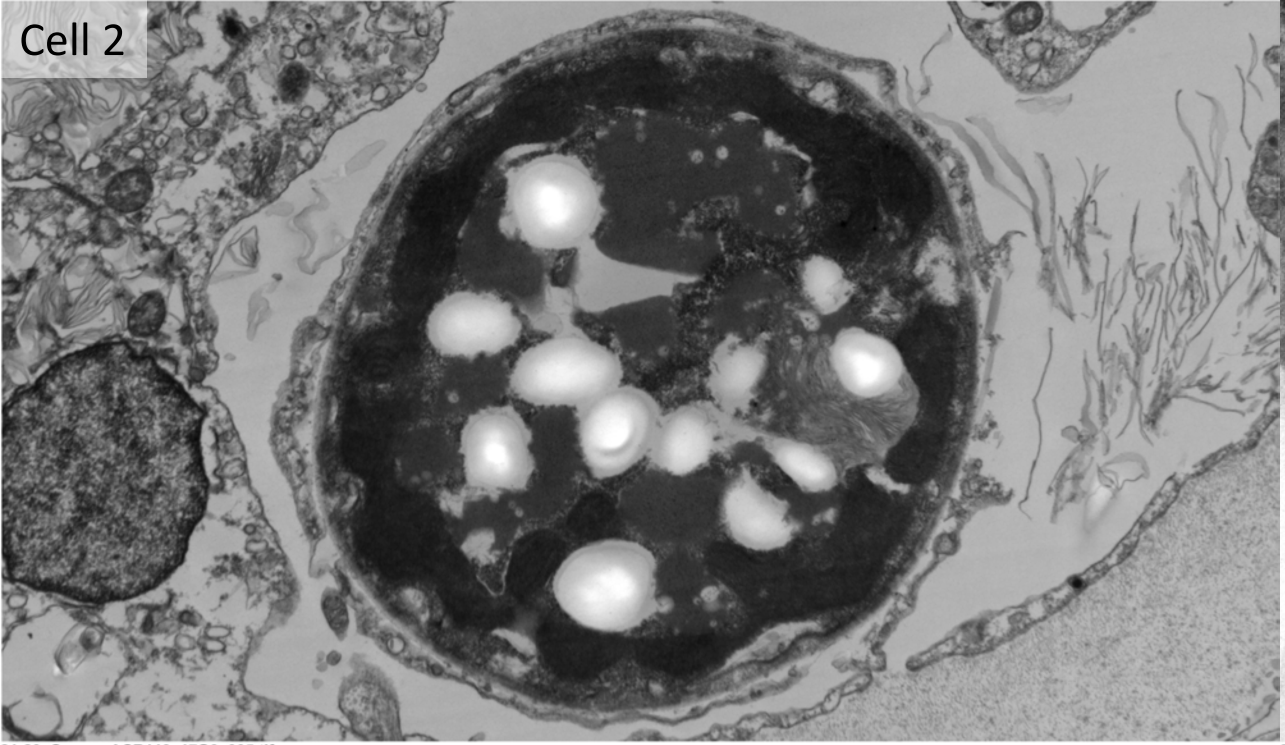

21-20_Correa_ACR113_17O3_005.tif
ACR 113 tissue
Biological Electron Microscopy Lab
Rice University - SEA
Microscopist: MD Meyer

2 µm
HV=80kV
Direct Mag: 2000 x

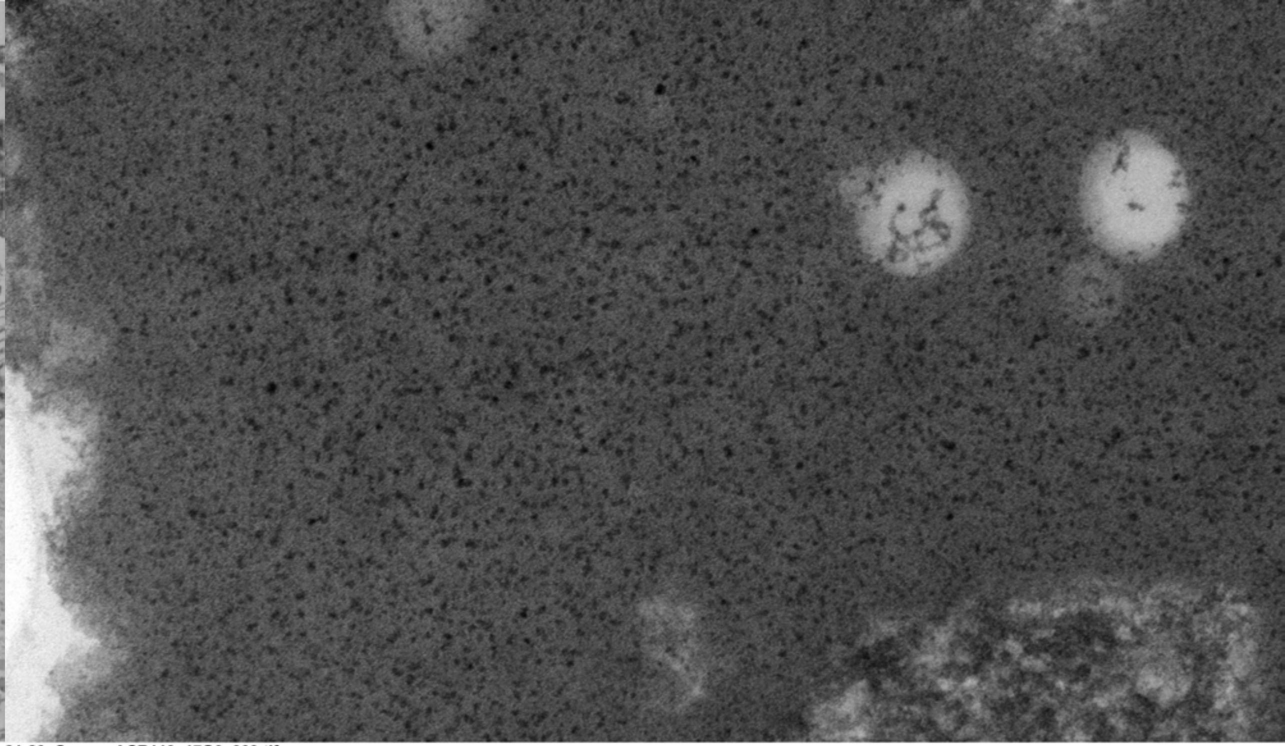

21-20_Correa_ACR113_17O3_009.tif
ACR 113 tissue
Biological Electron Microscopy Lab
Rice University - SEA
Microscopist: MD Meyer

200 nm
HV=80kV
Direct Mag: 20000 x

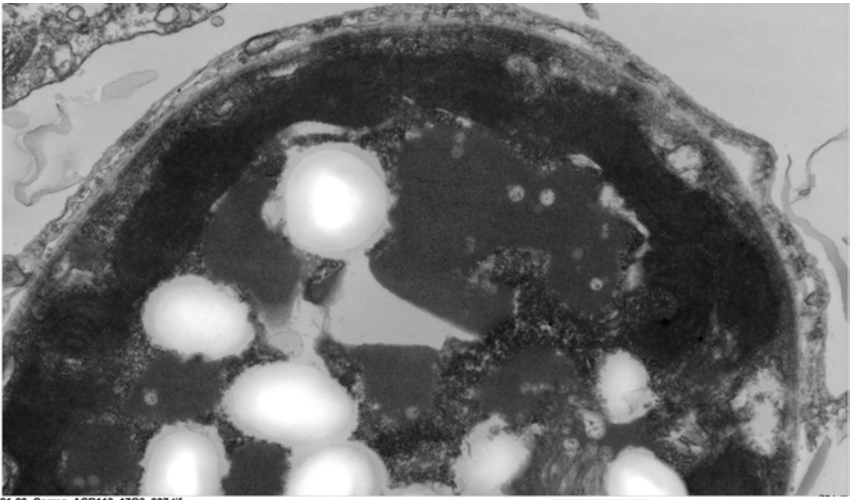

21-20_Correa_ACR113_17O3_007.tif
ACR 113 tissue
Biological Electron Microscopy Lab
Rice University - SEA
Microscopist: MD Meyer

1 µm
HV=80kV
Direct Mag: 4000 x

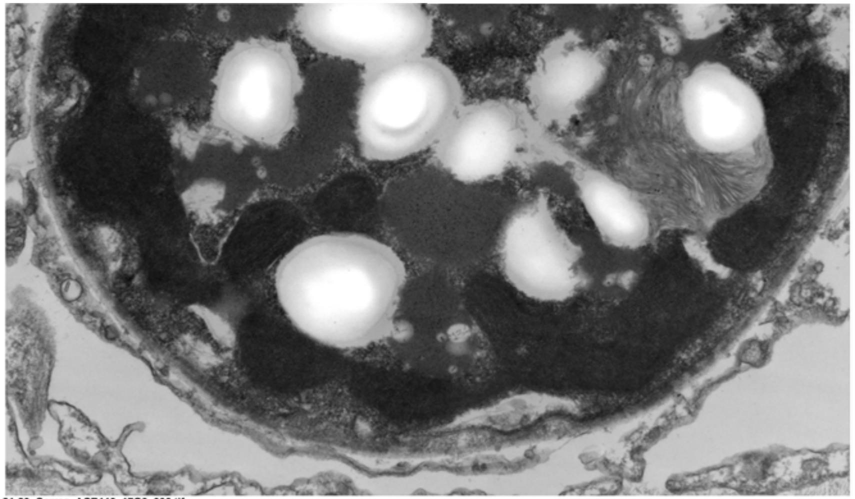

21-20_Correa_ACR113_17O3_006.tif
ACR 113 tissue
Biological Electron Microscopy Lab
Rice University - SEA
Microscopist: MD Meyer

1 µm
HV=80kV
Direct Mag: 4000 x

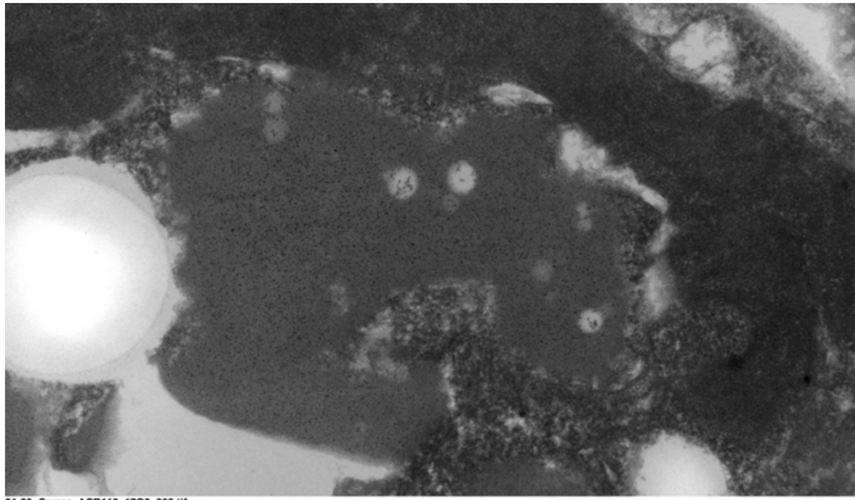

21-20_Correa_ACR113_17O3_008.tif
ACR 113 tissue
Biological Electron Microscopy Lab
Rice University - SEA
Microscopist: MD Meyer

500 nm
HV=80kV
Direct Mag: 8000 x

Cell 3

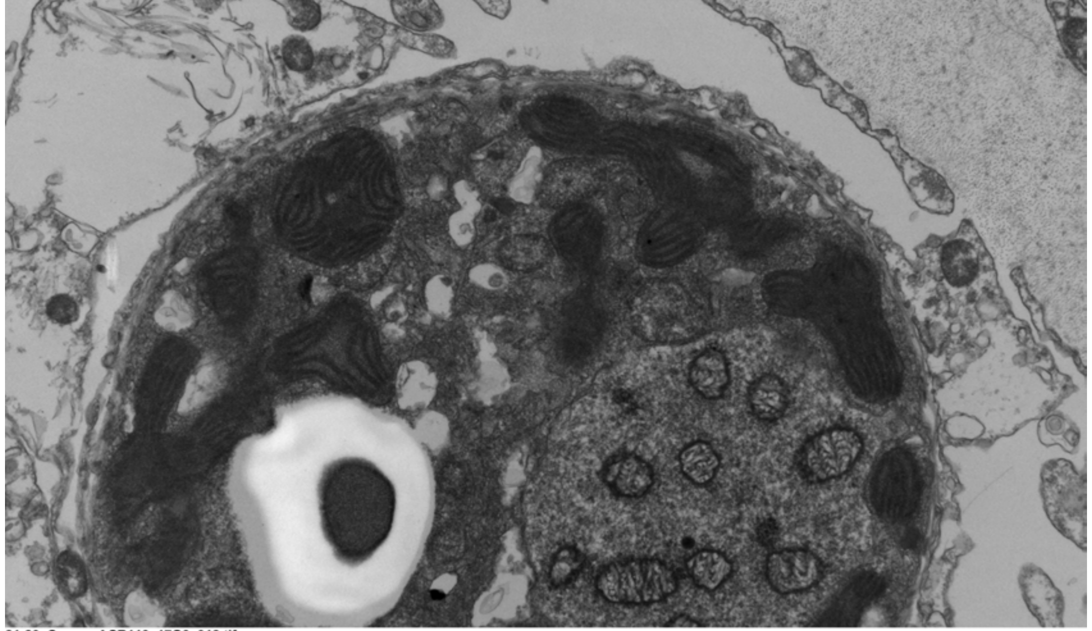

21-20_Correa_ACR113_17O3_012.tif
ACR 113 tissue
Biological Electron Microscopy Lab
Rice University - SEA
Microscopist: MD Meyer

1 μm
HV=80kV
Direct Mag: 3000 x

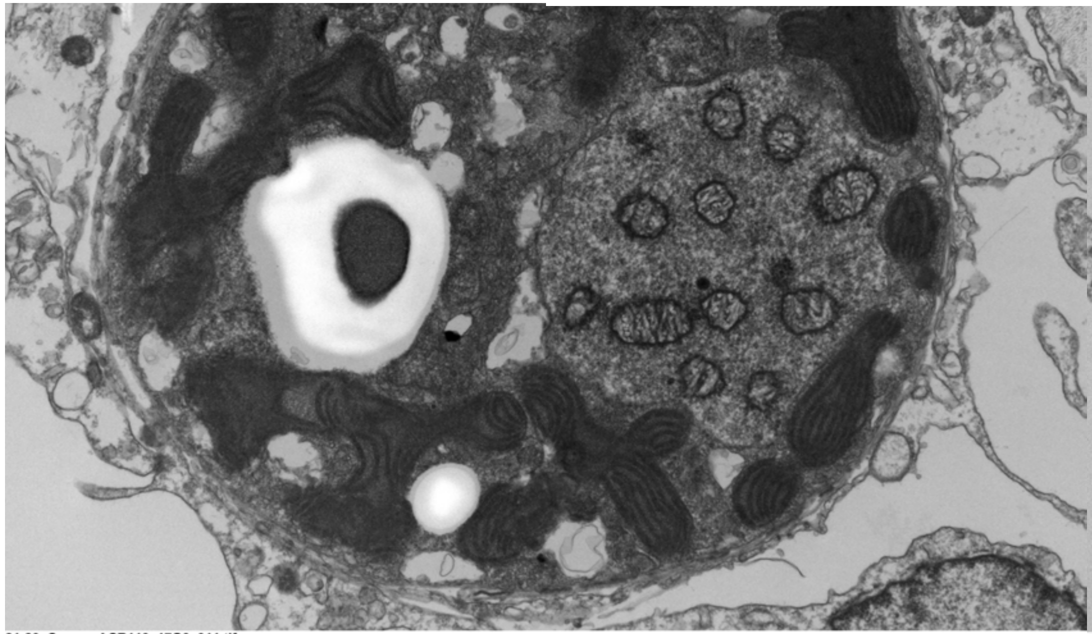

21-20_Correa_ACR113_17O3_011.tif
ACR 113 tissue
Biological Electron Microscopy Lab
Rice University - SEA
Microscopist: MD Meyer

1 μm
HV=80kV
Direct Mag: 3000 x

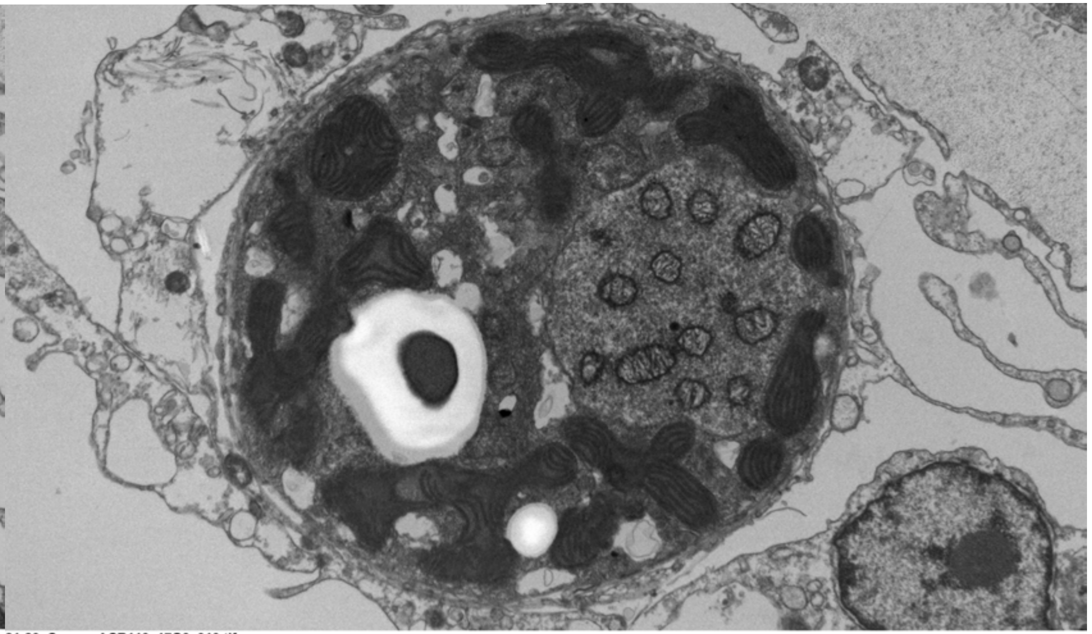

21-20_Correa_ACR113_17O3_010.tif
ACR 113 tissue
Biological Electron Microscopy Lab
Rice University - SEA
Microscopist: MD Meyer

2 μm
HV=80kV
Direct Mag: 2000 x

Cell 4

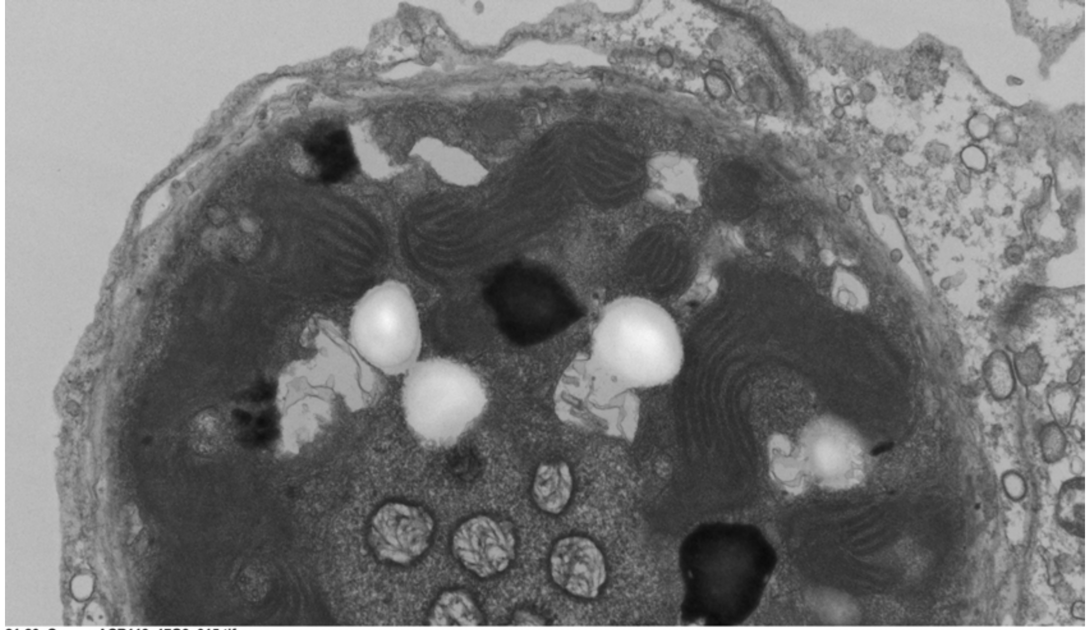

21-20_Correa_ACR113_17O3_015.tif
ACR 113 tissue
Biological Electron Microscopy Lab
Rice University - SEA
Microscopist: MD Meyer

1 µm
HV=80kV
Direct Mag: 4000 x

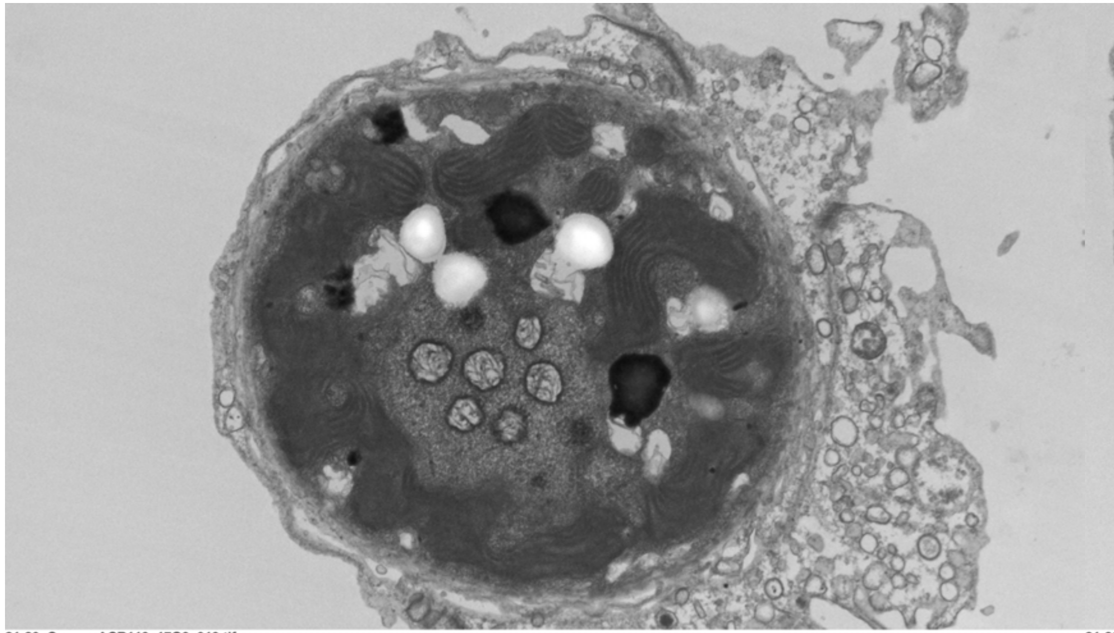

21-20_Correa_ACR113_17O3_013.tif
ACR 113 tissue
Biological Electron Microscopy Lab
Rice University - SEA
Microscopist: MD Meyer

1 µm
HV=80kV
Direct Mag: 2500 x

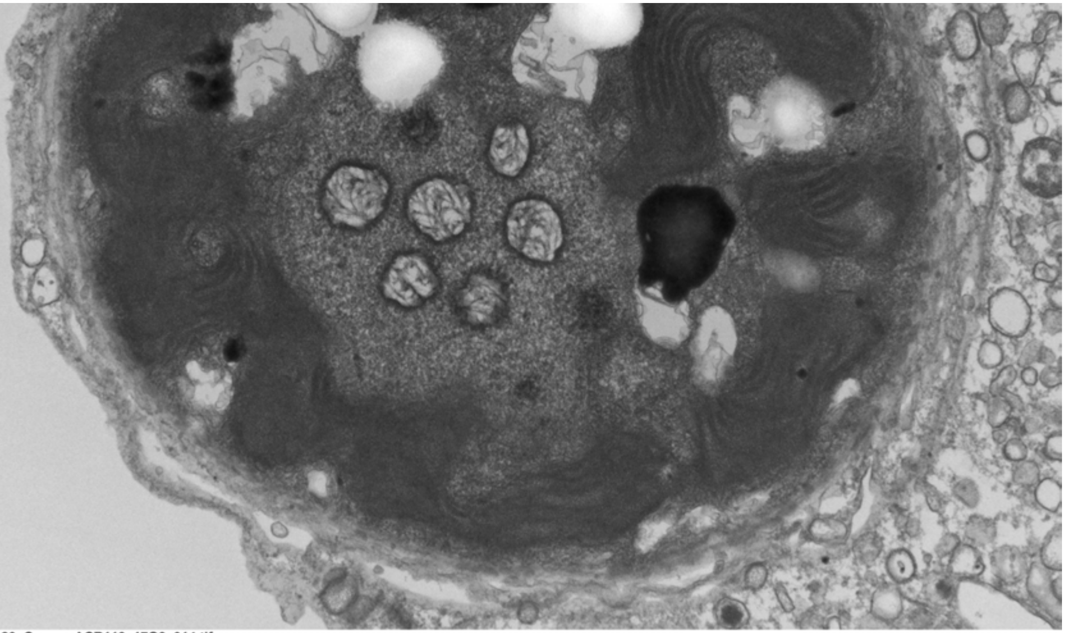

21-20_Correa_ACR113_17O3_014.tif
ACR 113 tissue
Biological Electron Microscopy Lab
Rice University - SEA
Microscopist: MD Meyer

1 µm
HV=80kV
Direct Mag: 4000 x

Cell 5

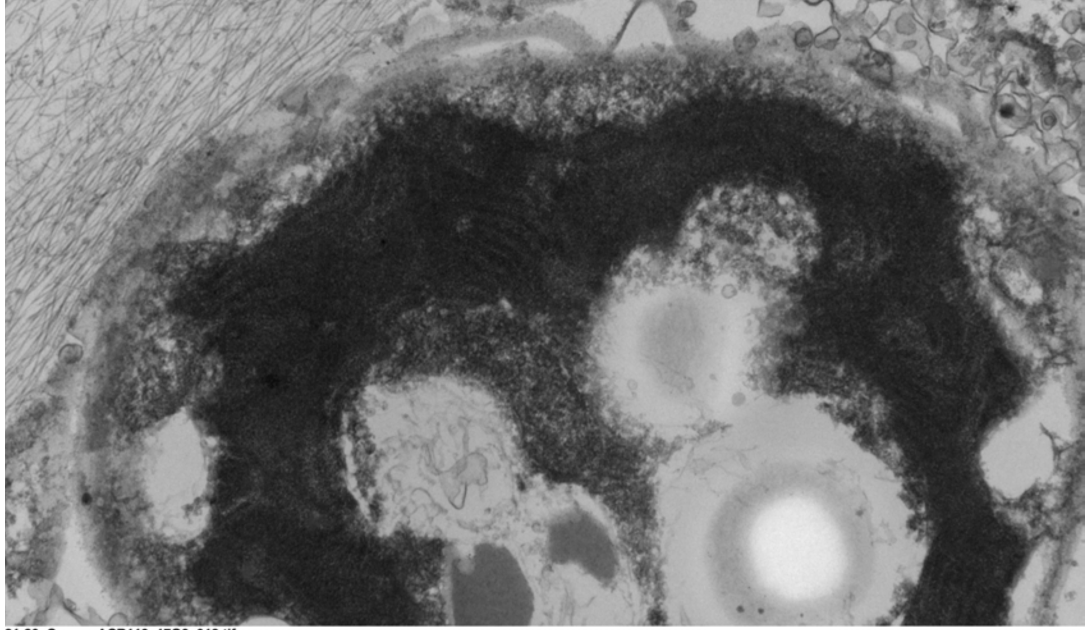

21-20_Correa_ACR113_17O3_018.tif
ACR 113 tissue
Biological Electron Microscopy Lab
Rice University - SEA
Microscopist: MD Meyer

800 nm
HV=80kV
Direct Mag: 5000 x

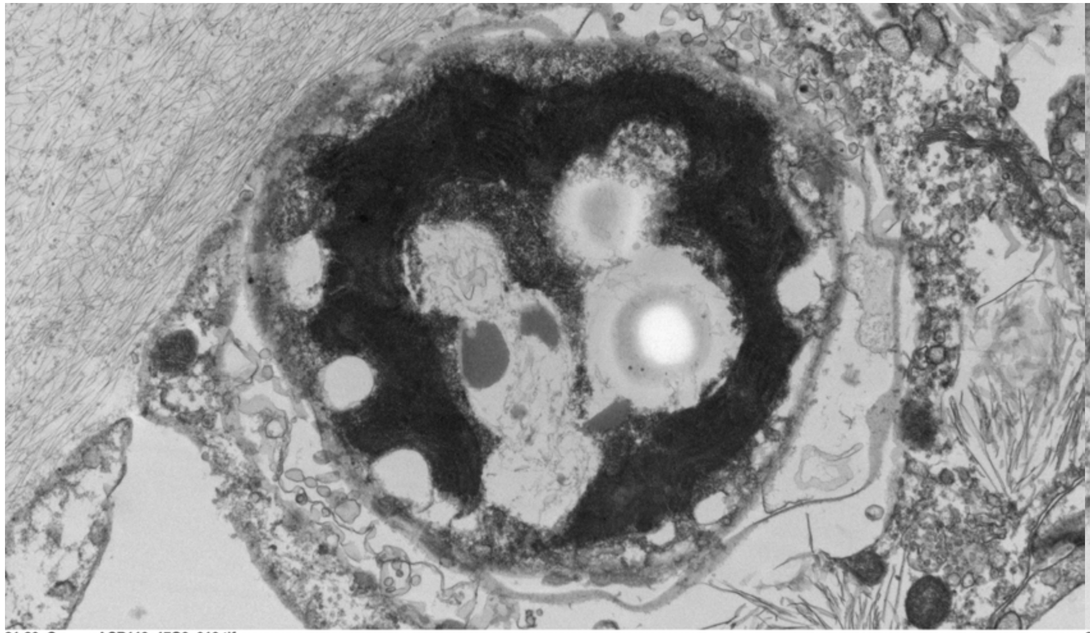

21-20_Correa_ACR113_17O3_016.tif
ACR 113 tissue
Biological Electron Microscopy Lab
Rice University - SEA
Microscopist: MD Meyer

1 µm
HV=80kV
Direct Mag: 3000 x

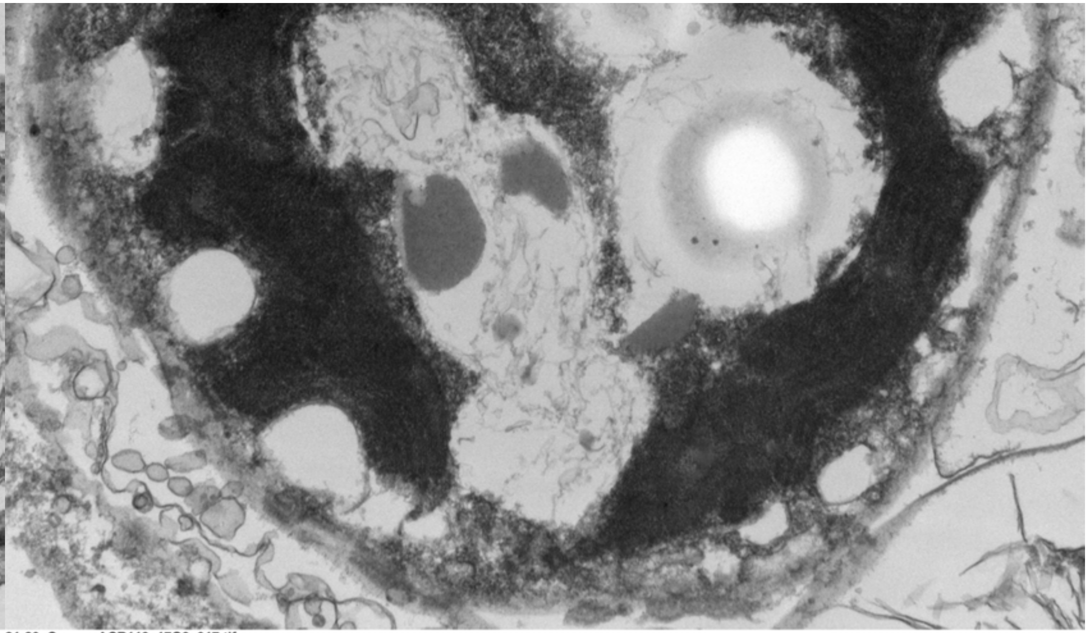

21-20_Correa_ACR113_17O3_017.tif
ACR 113 tissue
Biological Electron Microscopy Lab
Rice University - SEA
Microscopist: MD Meyer

800 nm
HV=80kV
Direct Mag: 5000 x

Cell 6

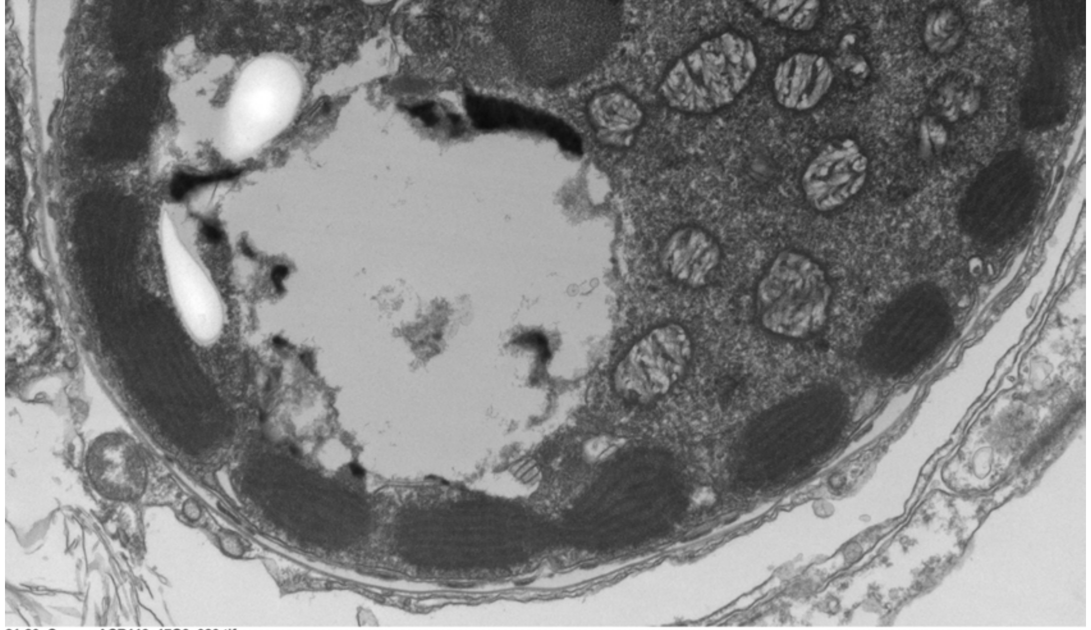

21-20_Correa_ACR113_17O3_020.tif
ACR 113 tissue
Biological Electron Microscopy Lab
Rice University - SEA
Microscopist: MD Meyer

1 µm
HV=80kV
Direct Mag: 4000 x

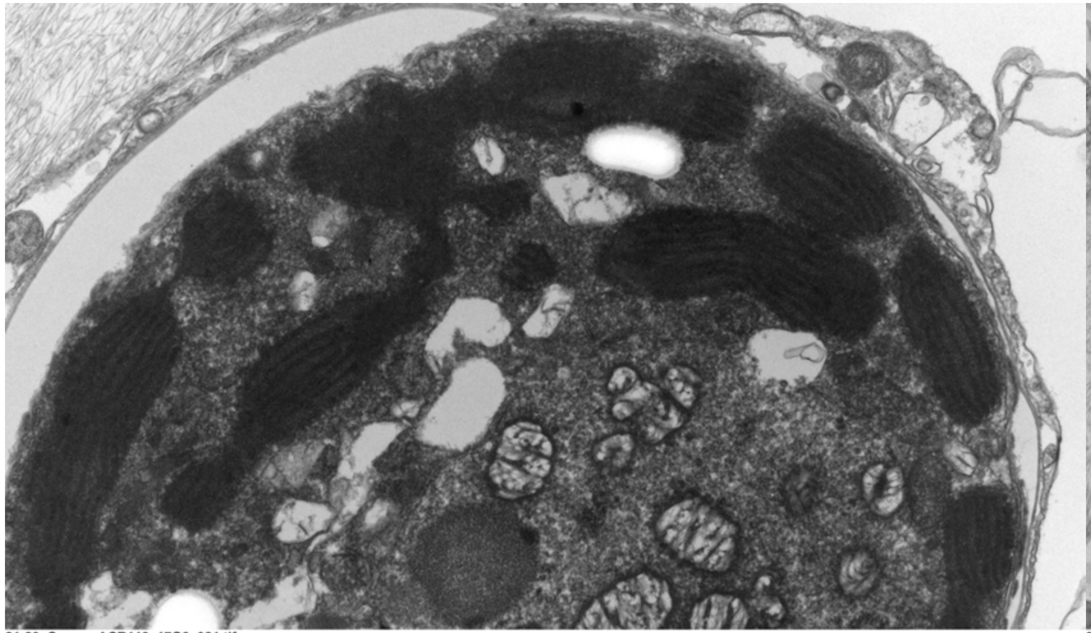

21-20_Correa_ACR113_17O3_021.tif
ACR 113 tissue
Biological Electron Microscopy Lab
Rice University - SEA
Microscopist: MD Meyer

1 µm
HV=80kV
Direct Mag: 4000 x

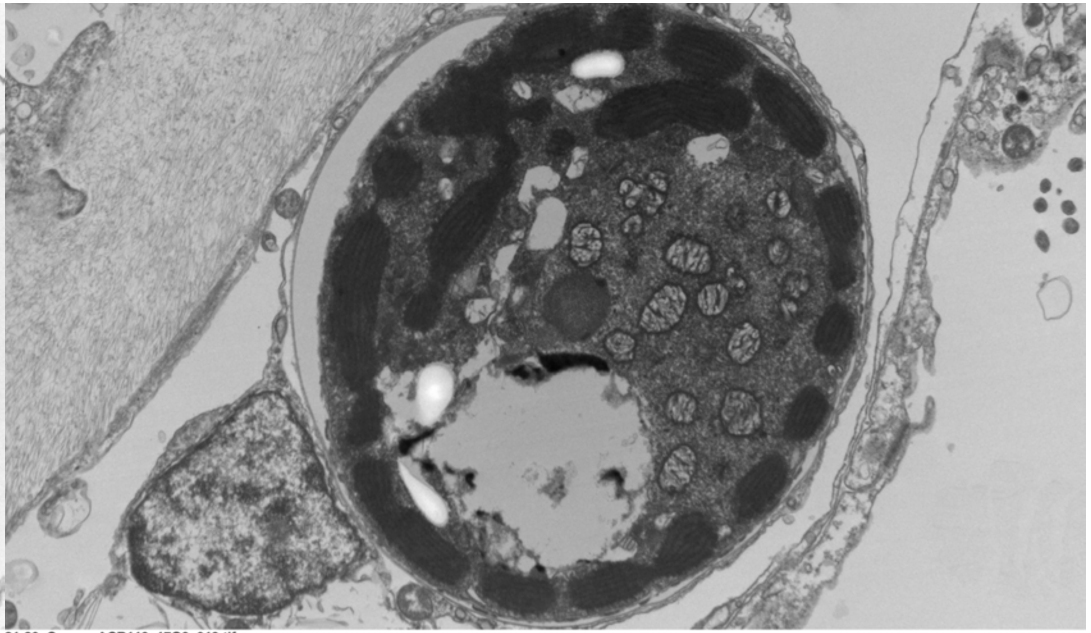

21-20_Correa_ACR113_17O3_019.tif
ACR 113 tissue
Biological Electron Microscopy Lab
Rice University - SEA
Microscopist: MD Meyer

2 µm
HV=80kV
Direct Mag: 2000 x

Cell 7

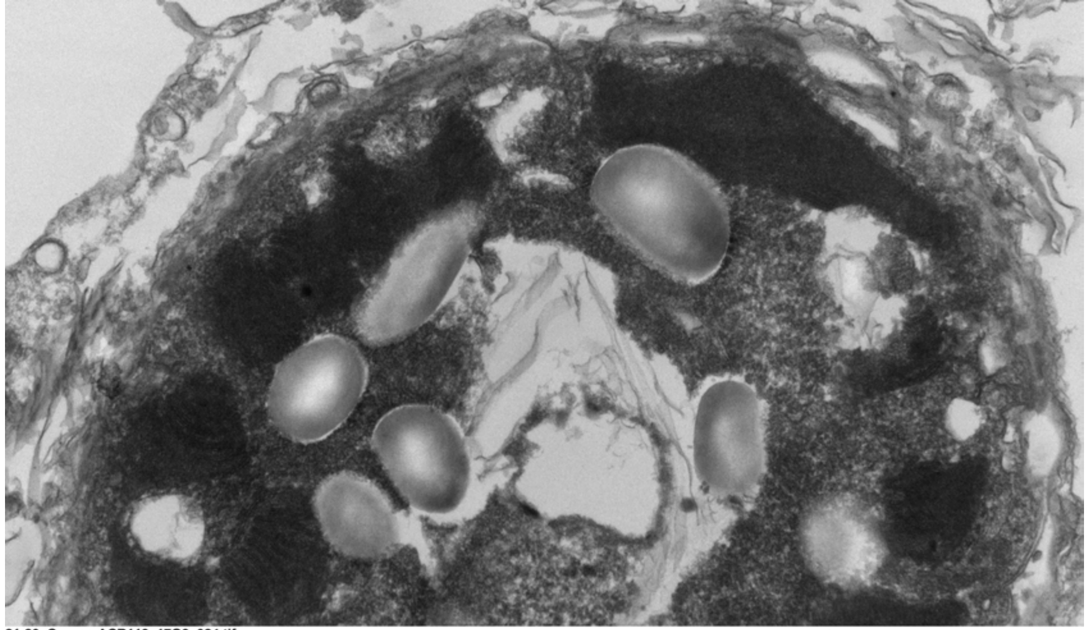

21-20_Correa_ACR113_17O3_024.tif
ACR 113 tissue
Biological Electron Microscopy Lab
Rice University - SEA
Microscopist: MD Meyer

800 nm
HV=80kV
Direct Mag: 5000 x

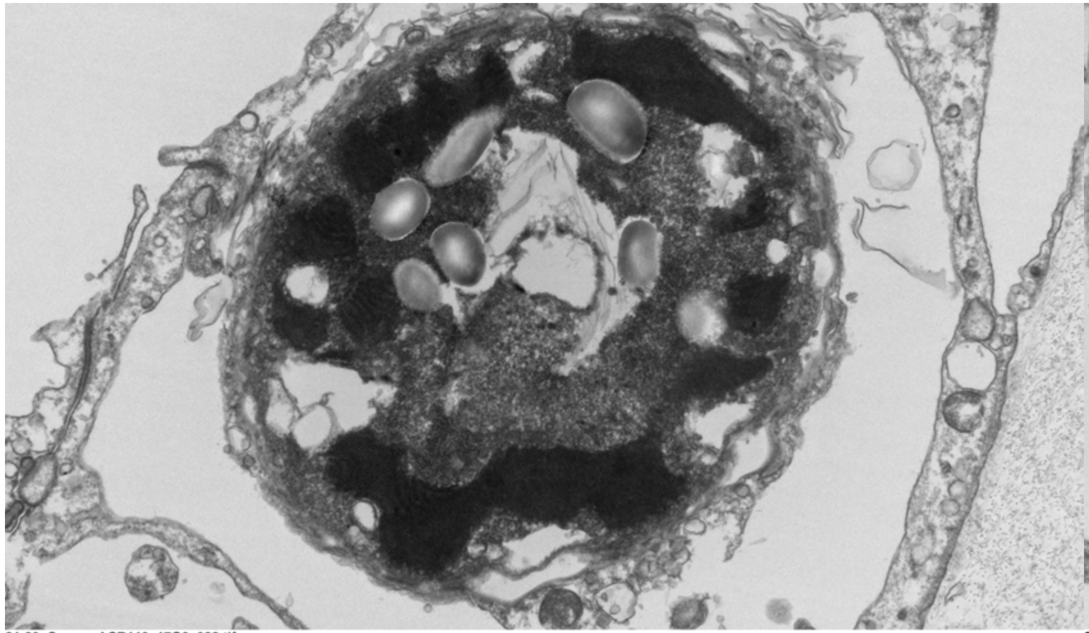

21-20_Correa_ACR113_17O3_022.tif
ACR 113 tissue
Biological Electron Microscopy Lab
Rice University - SEA
Microscopist: MD Meyer

1 μm
HV=80kV
Direct Mag: 3000 x

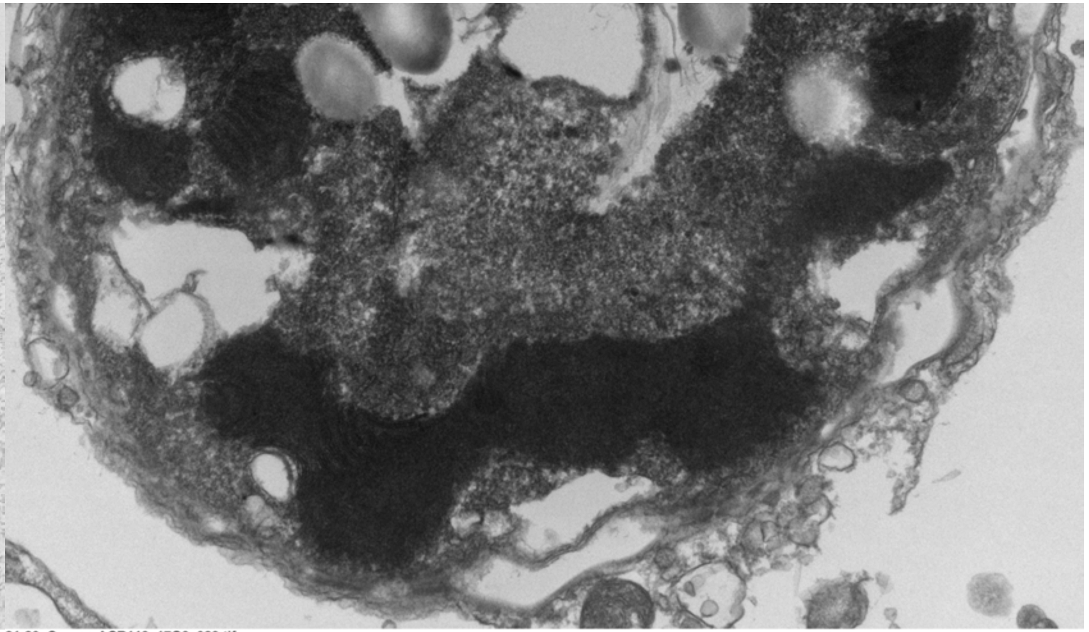

21-20_Correa_ACR113_17O3_023.tif
ACR 113 tissue
Biological Electron Microscopy Lab
Rice University - SEA
Microscopist: MD Meyer

800 nm
HV=80kV
Direct Mag: 5000 x

Cell 8

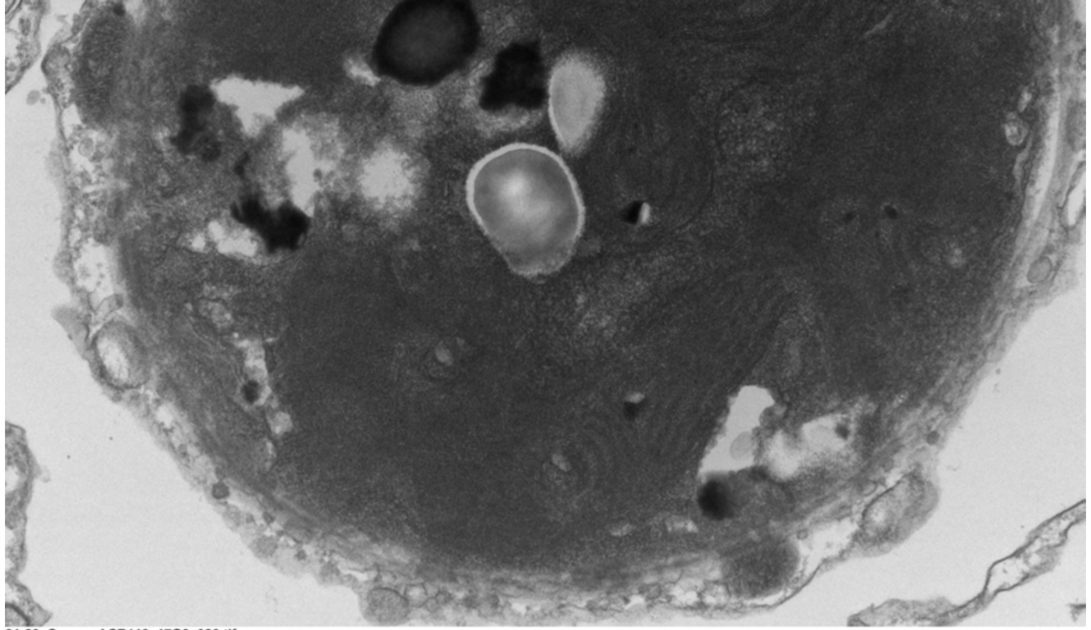

21-20_Correa_ACR113_17O3_026.tif
ACR 113 tissue
Biological Electron Microscopy Lab
Rice University - SEA
Microscopist: MD Meyer

800 nm
HV=80kV
Direct Mag: 5000 x

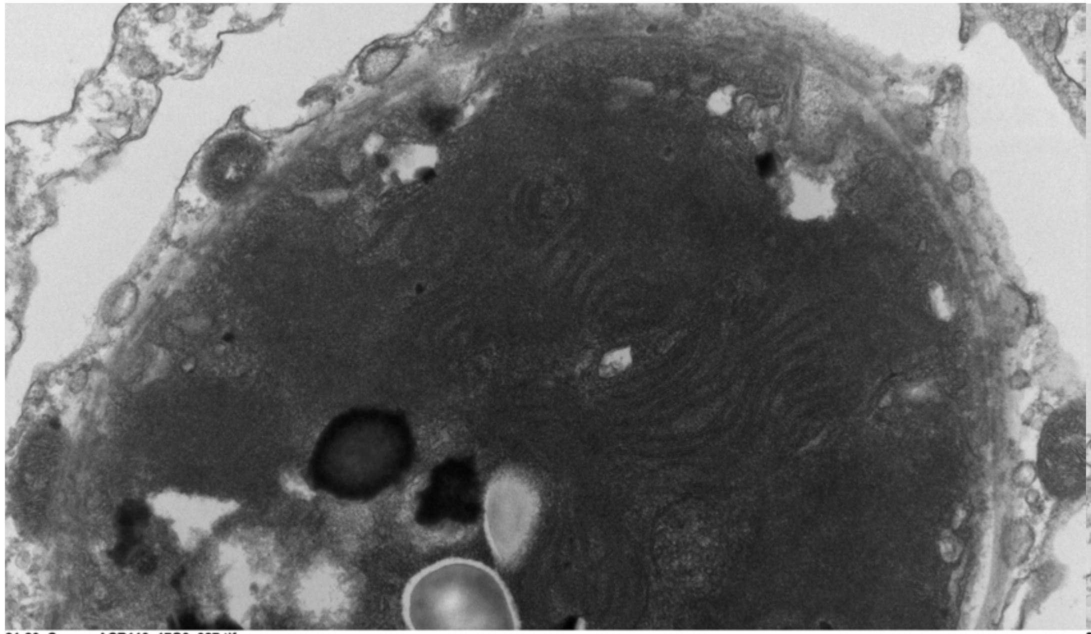

21-20_Correa_ACR113_17O3_027.tif
ACR 113 tissue
Biological Electron Microscopy Lab
Rice University - SEA
Microscopist: MD Meyer

800 nm
HV=80kV
Direct Mag: 5000 x

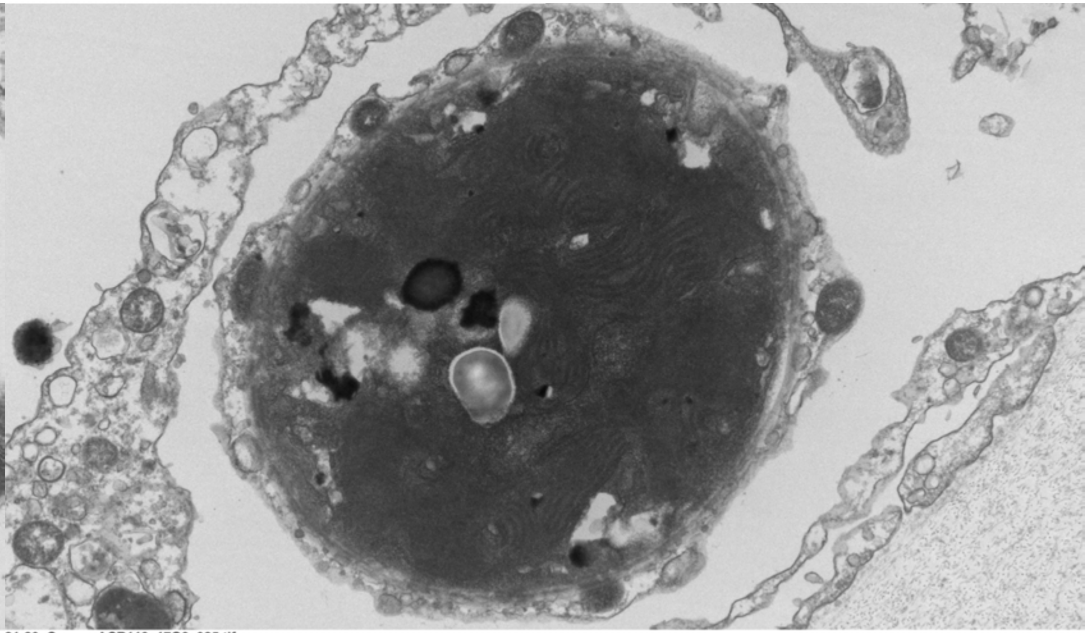

21-20_Correa_ACR113_17O3_025.tif
ACR 113 tissue
Biological Electron Microscopy Lab
Rice University - SEA
Microscopist: MD Meyer

1 μm
HV=80kV
Direct Mag: 3000 x

Cell 9

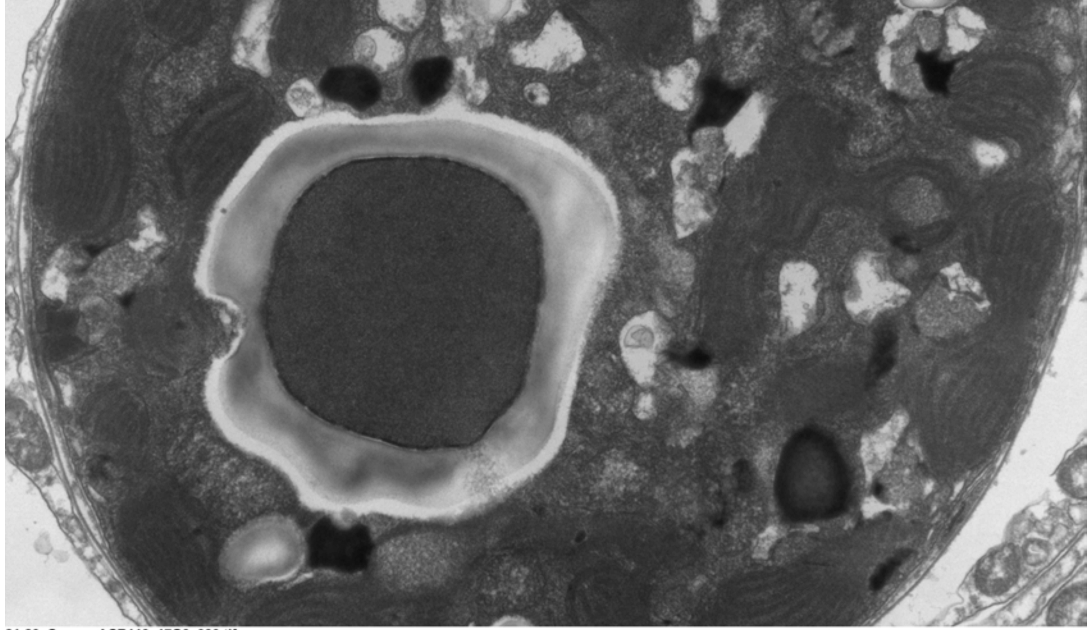

21-20_Correa_ACR113_17O3_030.tif
ACR 113 tissue
Biological Electron Microscopy Lab
Rice University - SEA
Microscopist: MD Meyer

1 µm
HV=80kV
Direct Mag: 4000 x

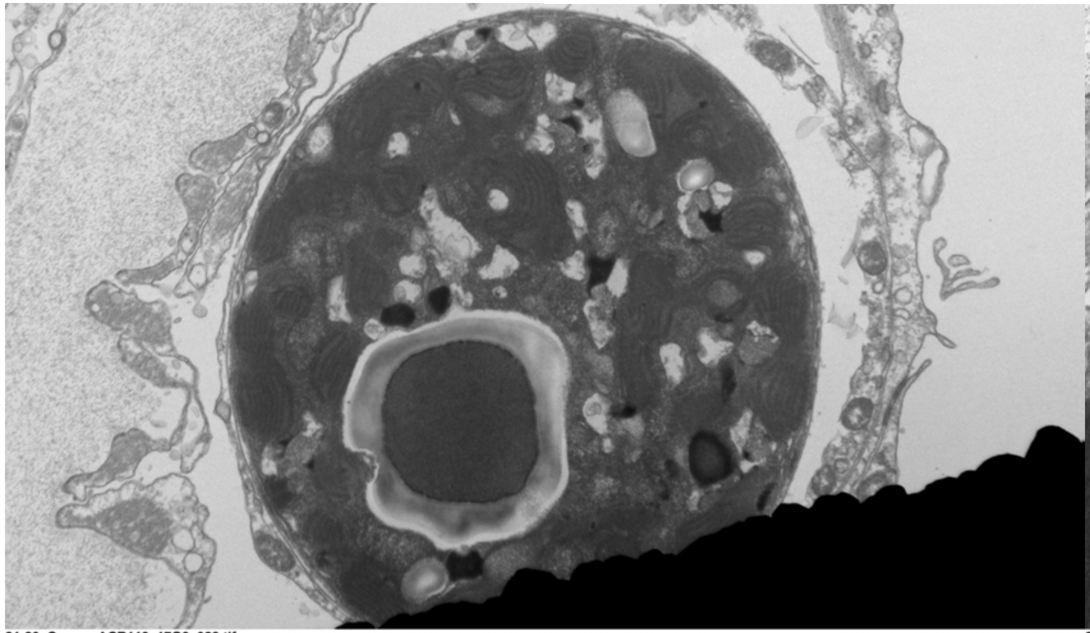

21-20_Correa_ACR113_17O3_028.tif
ACR 113 tissue
Biological Electron Microscopy Lab
Rice University - SEA
Microscopist: MD Meyer

2 µm
HV=80kV
Direct Mag: 2000 x

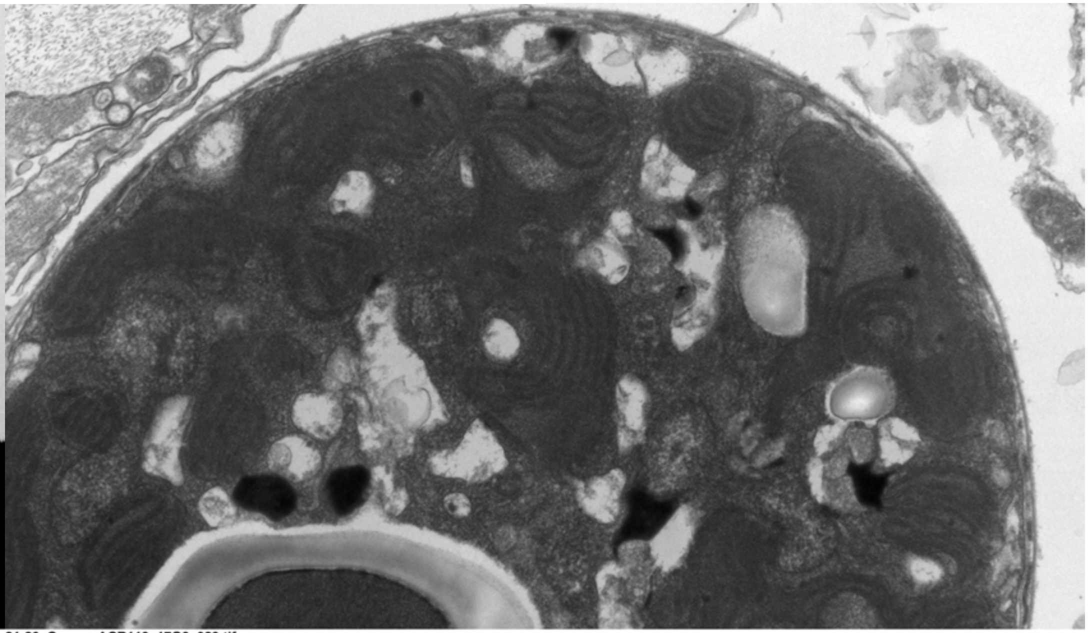

21-20_Correa_ACR113_17O3_029.tif
ACR 113 tissue
Biological Electron Microscopy Lab
Rice University - SEA
Microscopist: MD Meyer

1 µm
HV=80kV
Direct Mag: 4000 x

Cell 10

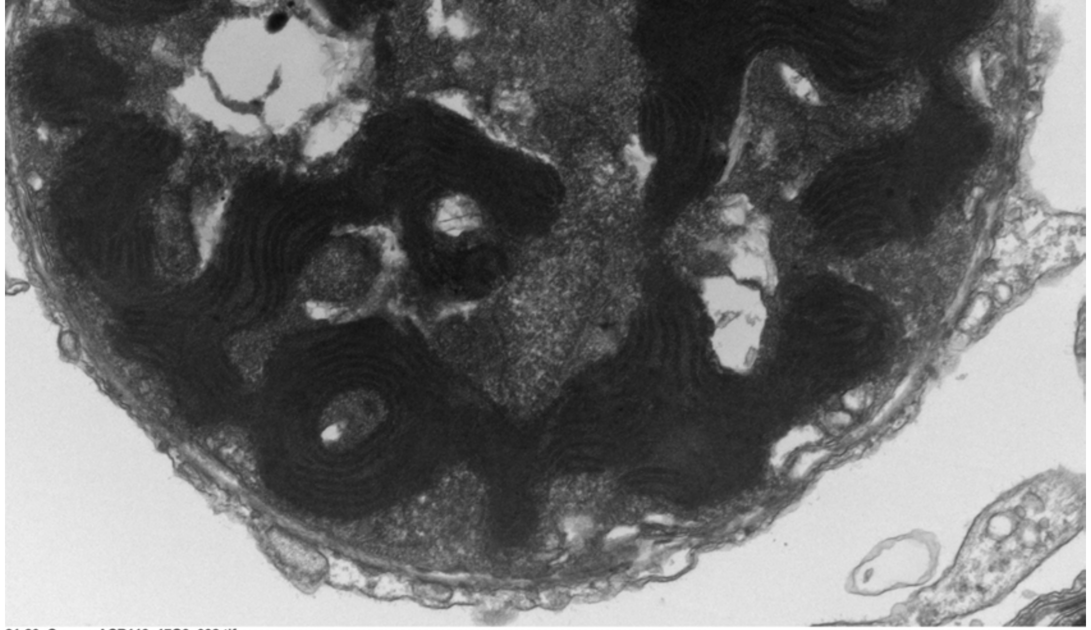

21-20_Correa_ACR113_17O3_032.tif
ACR 113 tissue
Biological Electron Microscopy Lab
Rice University - SEA
Microscopist: MD Meyer

1 µm
HV=80kV
Direct Mag: 4000 x

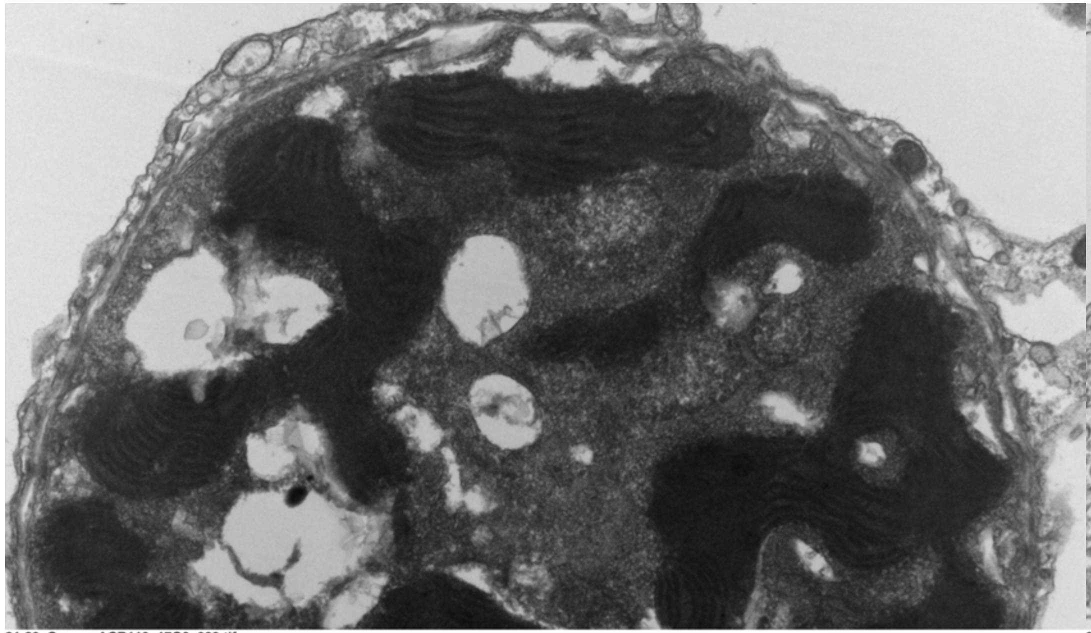

21-20_Correa_ACR113_17O3_033.tif
ACR 113 tissue
Biological Electron Microscopy Lab
Rice University - SEA
Microscopist: MD Meyer

1 µm
HV=80kV
Direct Mag: 4000 x

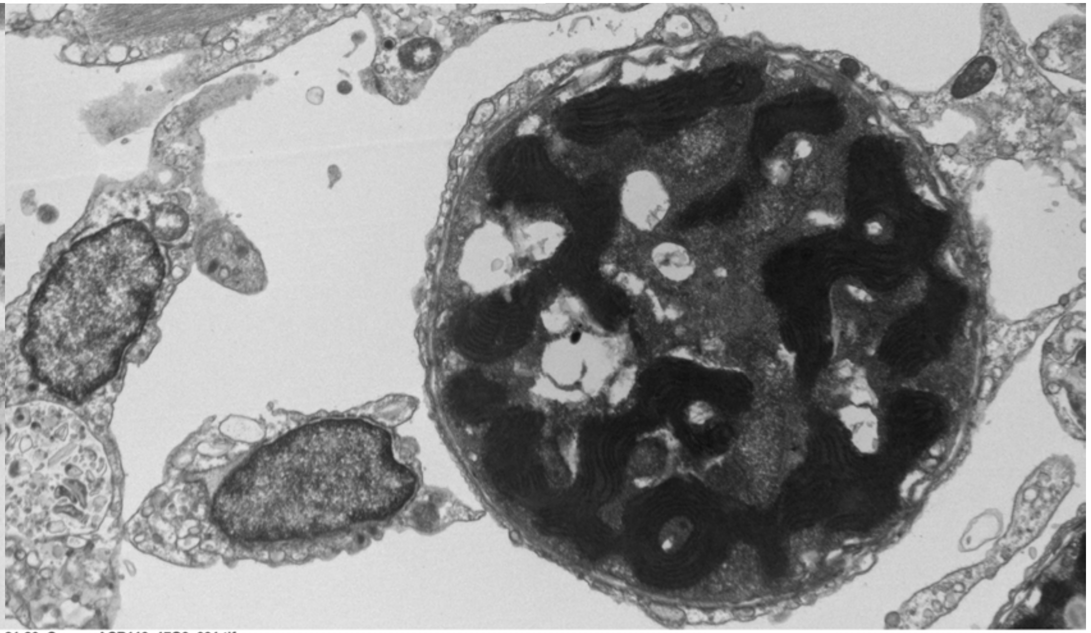

21-20_Correa_ACR113_17O3_031.tif
ACR 113 tissue
Biological Electron Microscopy Lab
Rice University - SEA
Microscopist: MD Meyer

2 µm
HV=80kV
Direct Mag: 2000 x

Cell 11

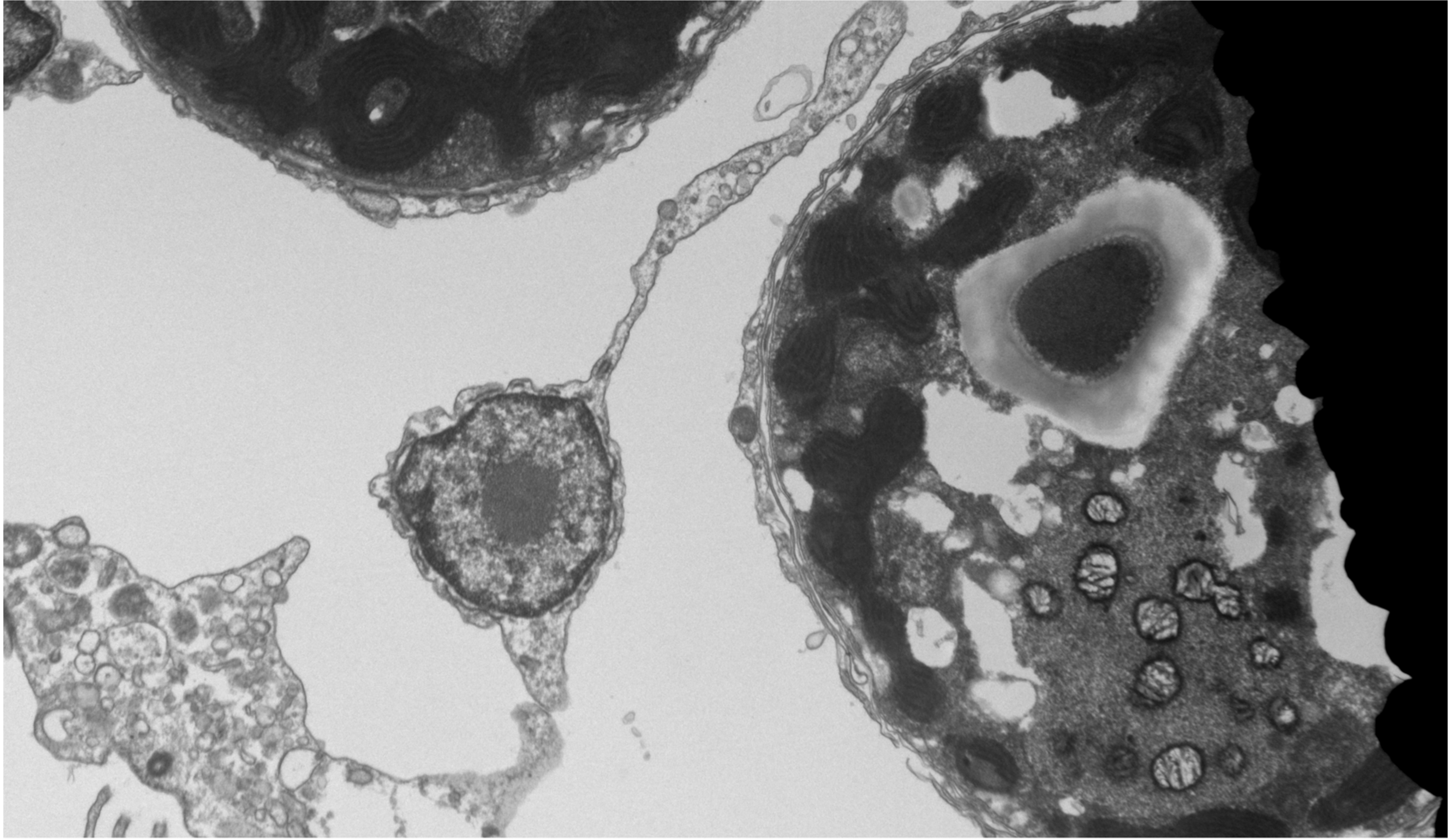

21-20_Correa_ACR113_17O3_034.tif
ACR 113 tissue
Biological Electron Microscopy Lab
Rice University - SEA
Microscopist: MD Meyer

2 µm
HV=80kV
Direct Mag: 2000 x

Cell 12

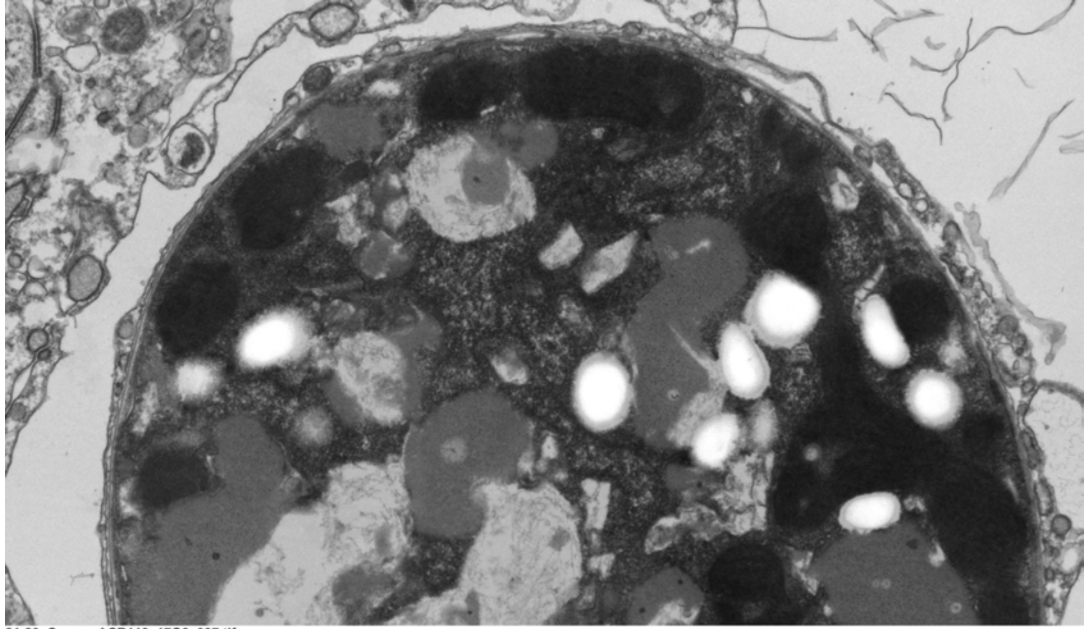

21-20_Correa_ACR113_17O3_037.tif
ACR 113 tissue
Biological Electron Microscopy Lab
Rice University - SEA
Microscopist: MD Meyer

1 µm
HV=80kV
Direct Mag: 3000 x

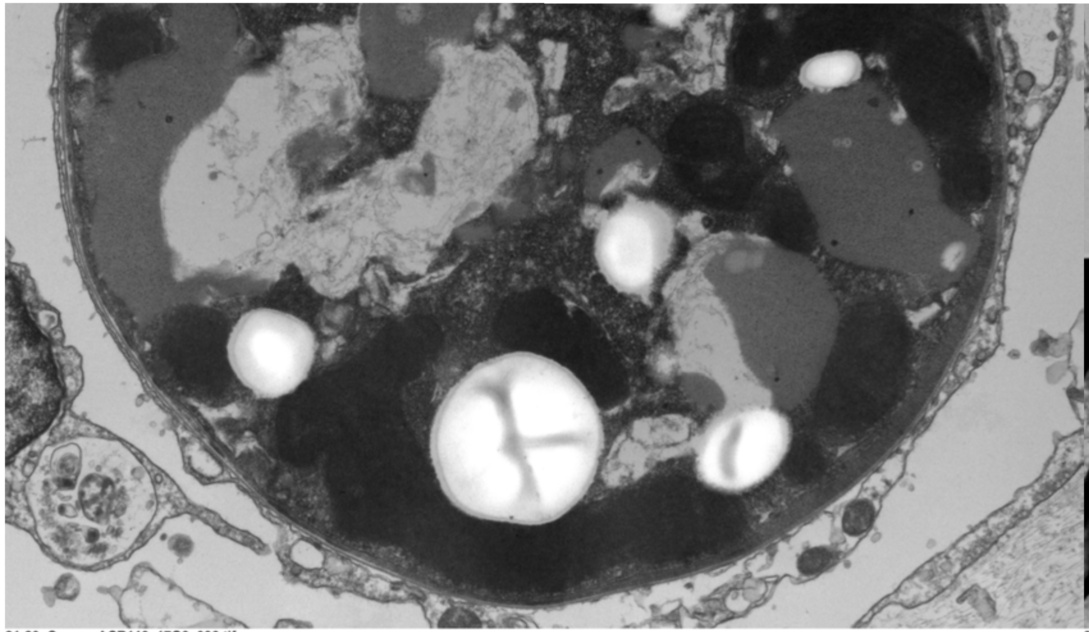

21-20_Correa_ACR113_17O3_036.tif
ACR 113 tissue
Biological Electron Microscopy Lab
Rice University - SEA
Microscopist: MD Meyer

1 µm
HV=80kV
Direct Mag: 3000 x

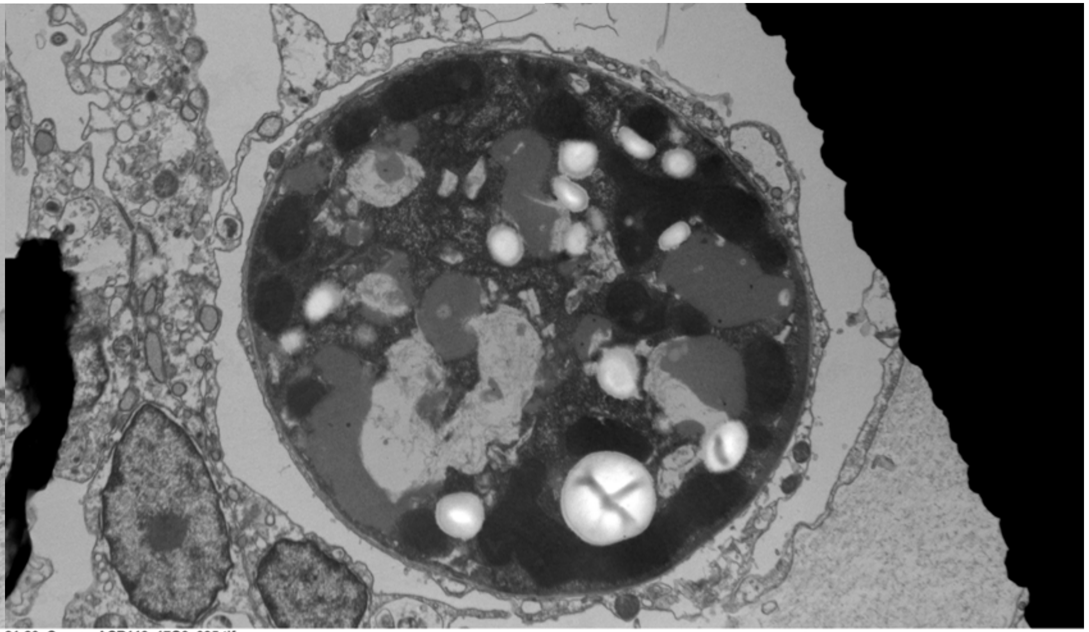

21-20_Correa_ACR113_17O3_035.tif
ACR 113 tissue
Biological Electron Microscopy Lab
Rice University - SEA
Microscopist: MD Meyer

2 µm
HV=80kV
Direct Mag: 1500 x

Cell 13

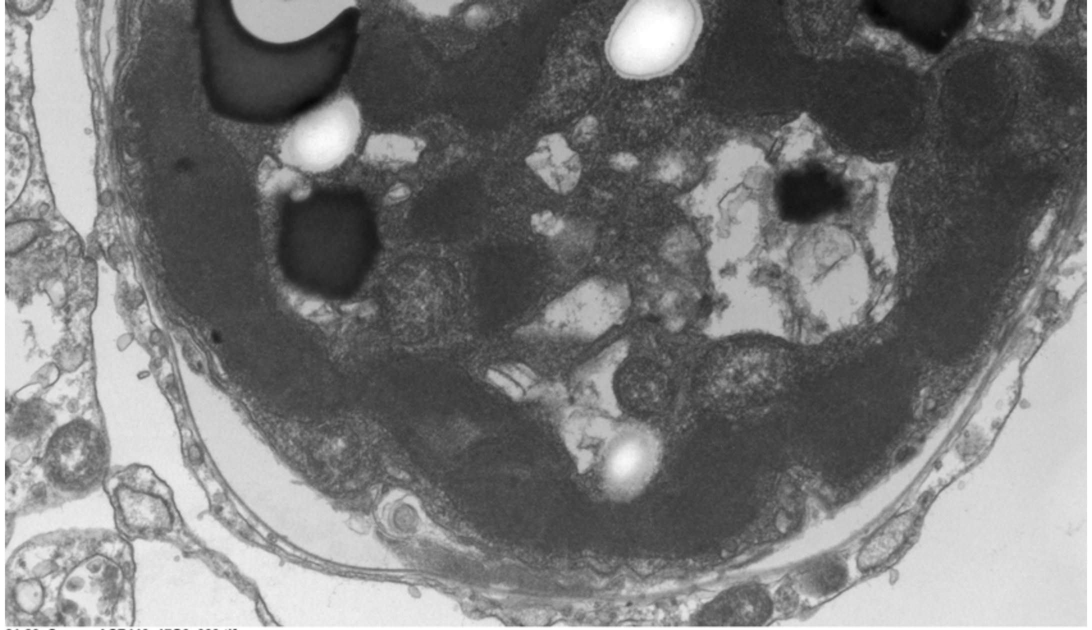

21-20_Correa_ACR113_17O3_039.tif
ACR 113 tissue
Biological Electron Microscopy Lab
Rice University - SEA
Microscopist: MD Meyer

1 μm
HV=80kV
Direct Mag: 4000 x

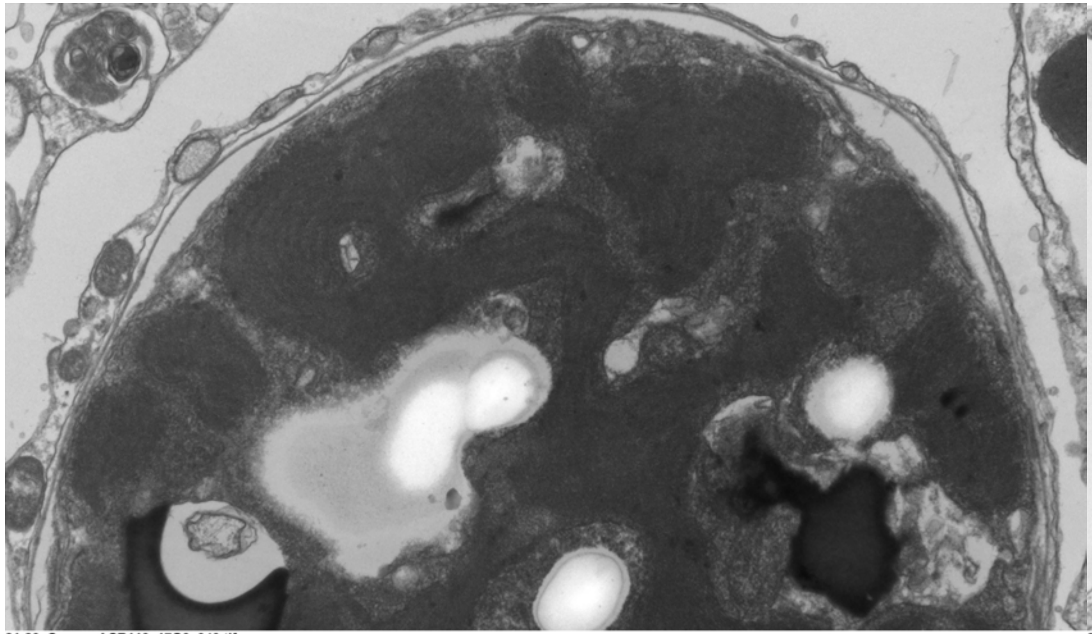

21-20_Correa_ACR113_17O3_040.tif
ACR 113 tissue
Biological Electron Microscopy Lab
Rice University - SEA
Microscopist: MD Meyer

1 μm
HV=80kV
Direct Mag: 4000 x

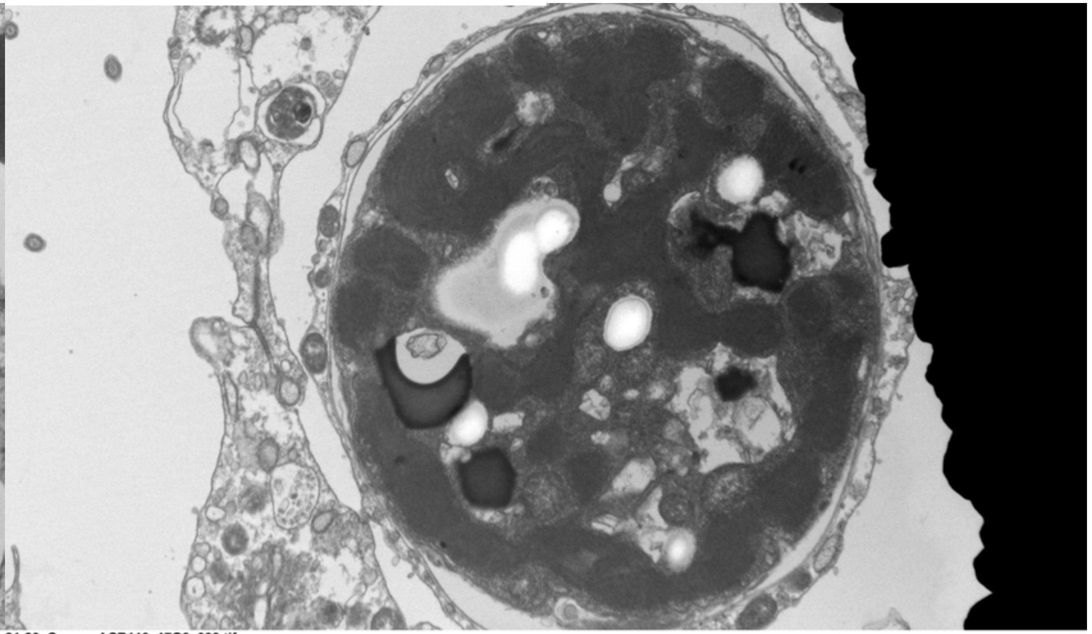

21-20_Correa_ACR113_17O3_038.tif
ACR 113 tissue
Biological Electron Microscopy Lab
Rice University - SEA
Microscopist: MD Meyer

2 μm
HV=80kV
Direct Mag: 2000 x

Cell 14

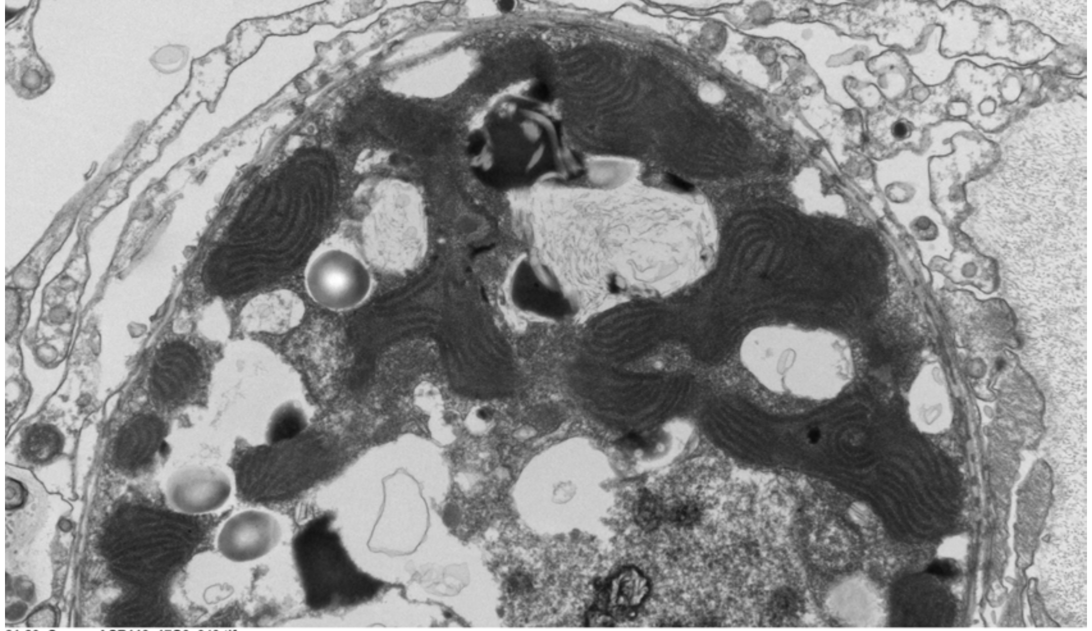

21-20_Correa_ACR113_17O3_043.tif
ACR 113 tissue
Biological Electron Microscopy Lab
Rice University - SEA
Microscopist: MD Meyer

1 µm
HV=80kV
Direct Mag: 3000 x

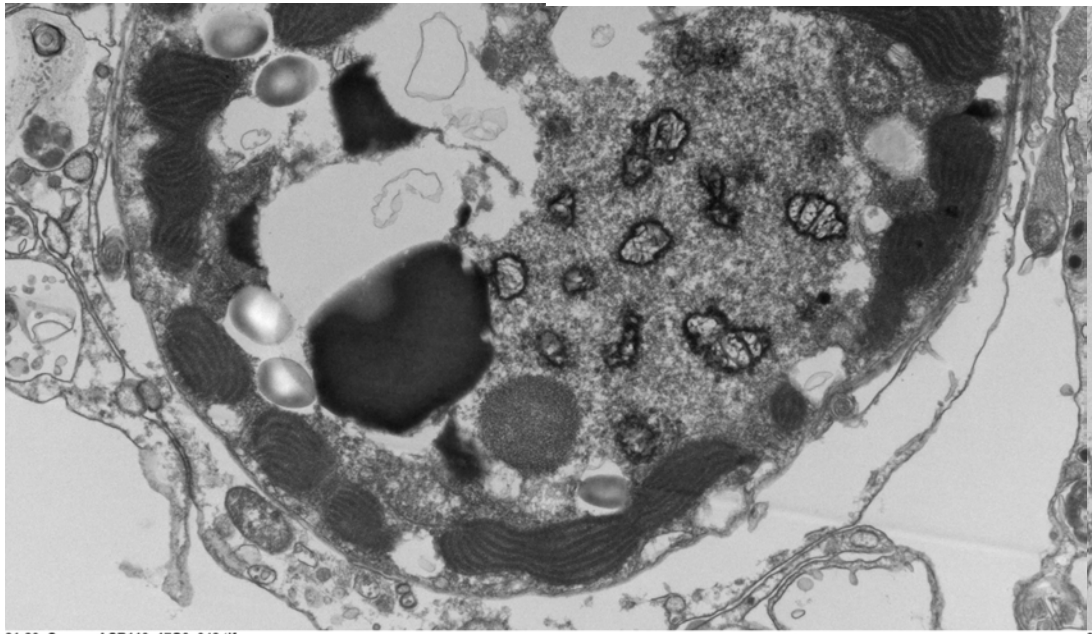

21-20_Correa_ACR113_17O3_042.tif
ACR 113 tissue
Biological Electron Microscopy Lab
Rice University - SEA
Microscopist: MD Meyer

1 µm
HV=80kV
Direct Mag: 3000 x

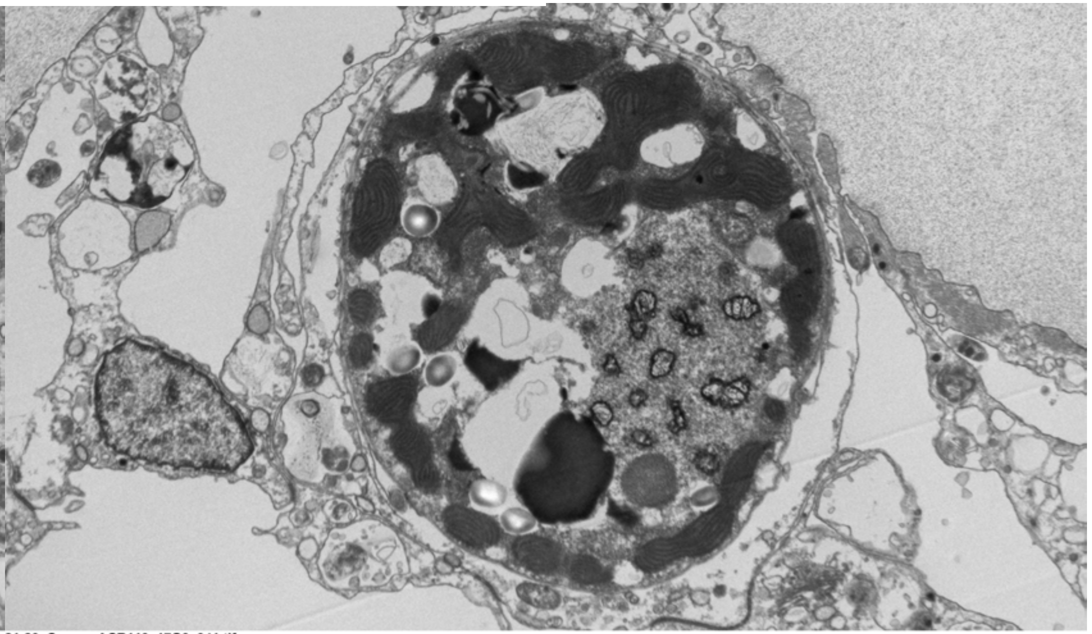

21-20_Correa_ACR113_17O3_041.tif
ACR 113 tissue
Biological Electron Microscopy Lab
Rice University - SEA
Microscopist: MD Meyer

2 µm
HV=80kV
Direct Mag: 1500 x

Cell 15

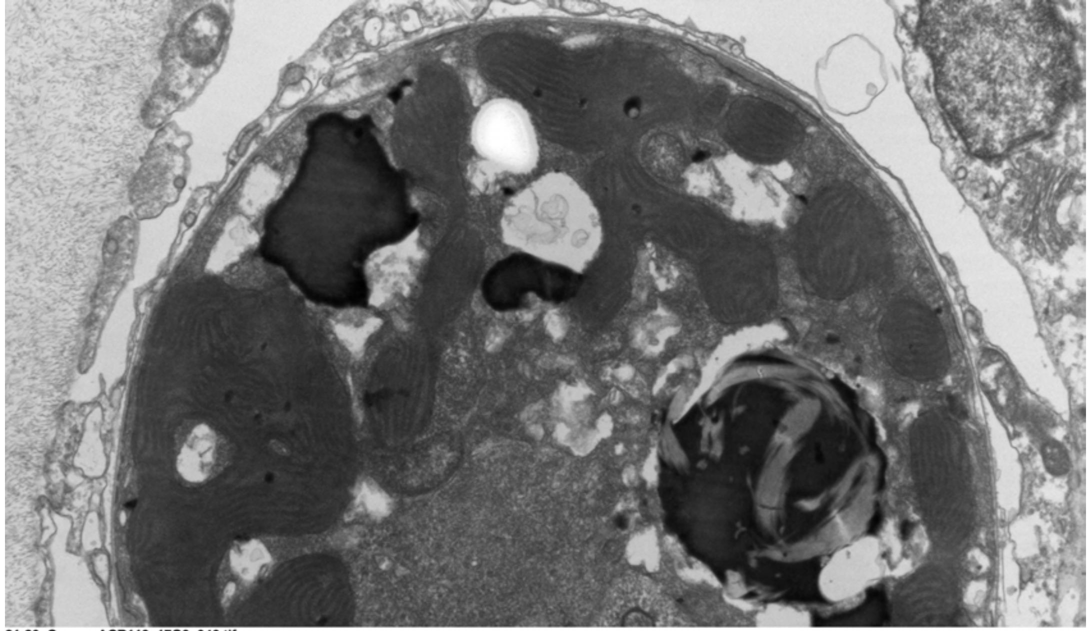

21-20_Correa_ACR113_17O3_046.tif
ACR 113 tissue
Biological Electron Microscopy Lab
Rice University - SEA
Microscopist: MD Meyer

1 µm
HV=80kV
Direct Mag: 3000 x

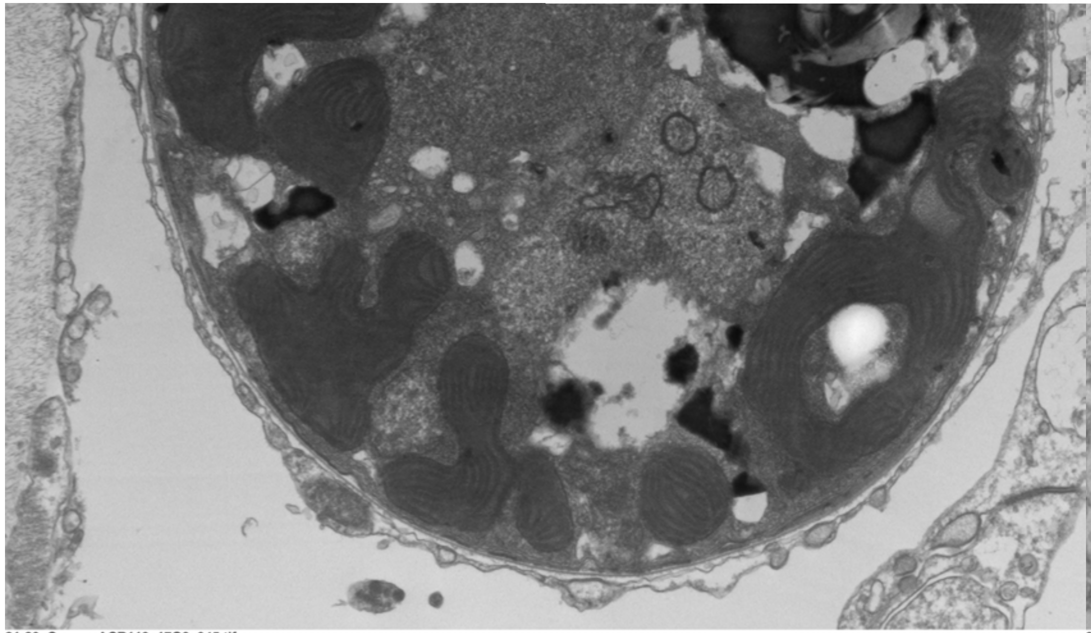

21-20_Correa_ACR113_17O3_045.tif
ACR 113 tissue
Biological Electron Microscopy Lab
Rice University - SEA
Microscopist: MD Meyer

1 µm
HV=80kV
Direct Mag: 3000 x

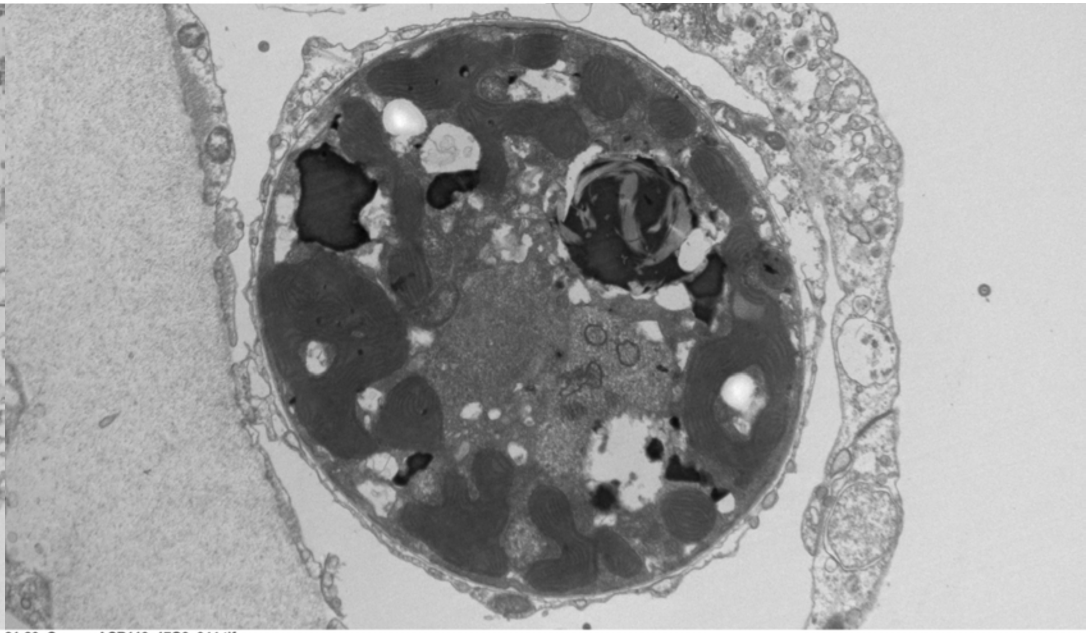

21-20_Correa_ACR113_17O3_044.tif
ACR 113 tissue
Biological Electron Microscopy Lab
Rice University - SEA
Microscopist: MD Meyer

2 µm
HV=80kV
Direct Mag: 1500 x

ACR Colony F

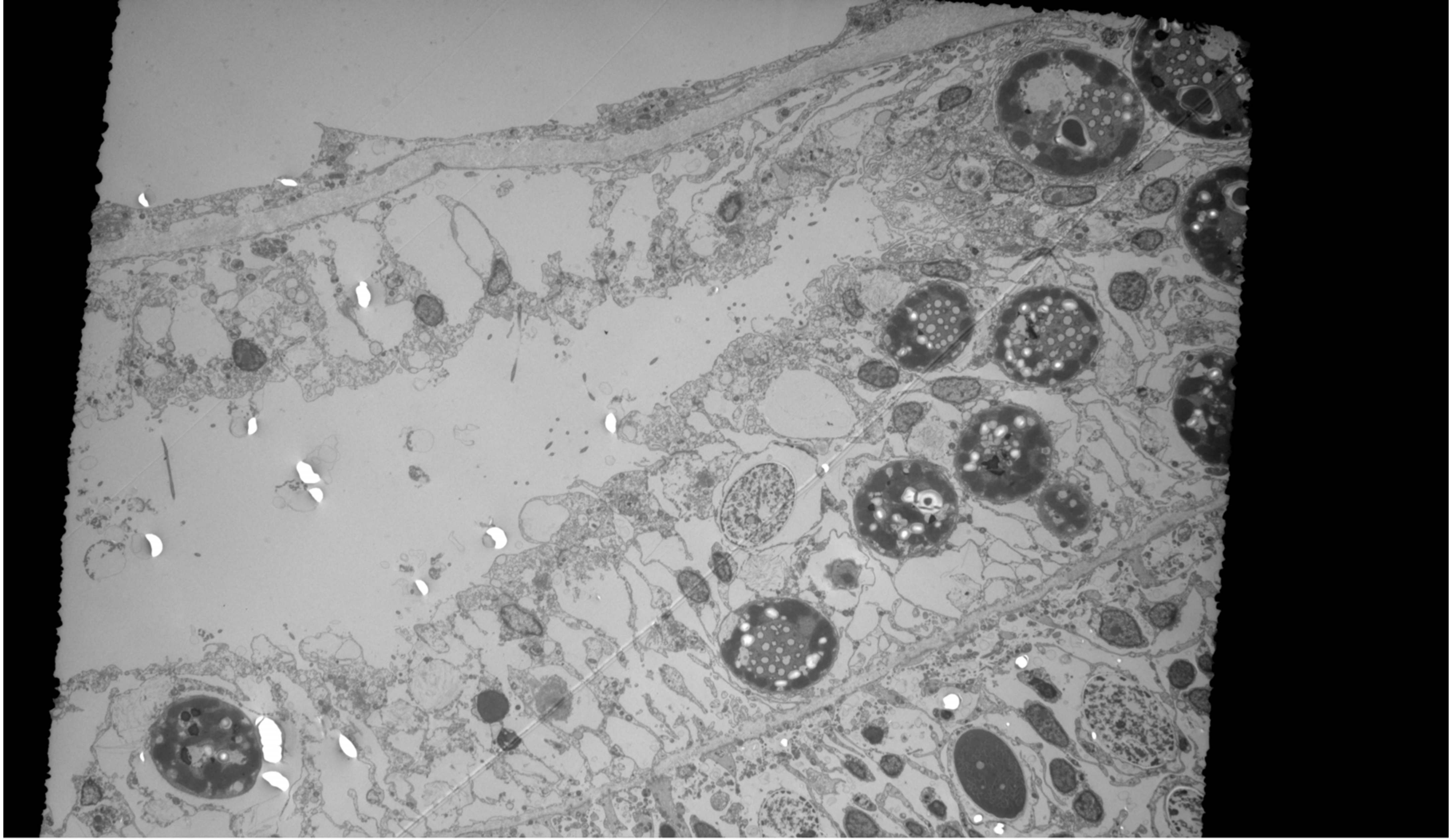

21-20_Correa_ACR116_17F3_001.tif
ARC 116
Biological Electron Microscopy Lab
Rice University - SEA
Microscopist: MD Meyer

10 µm
HV=80kV
Direct Mag: 300 x

Cell 1

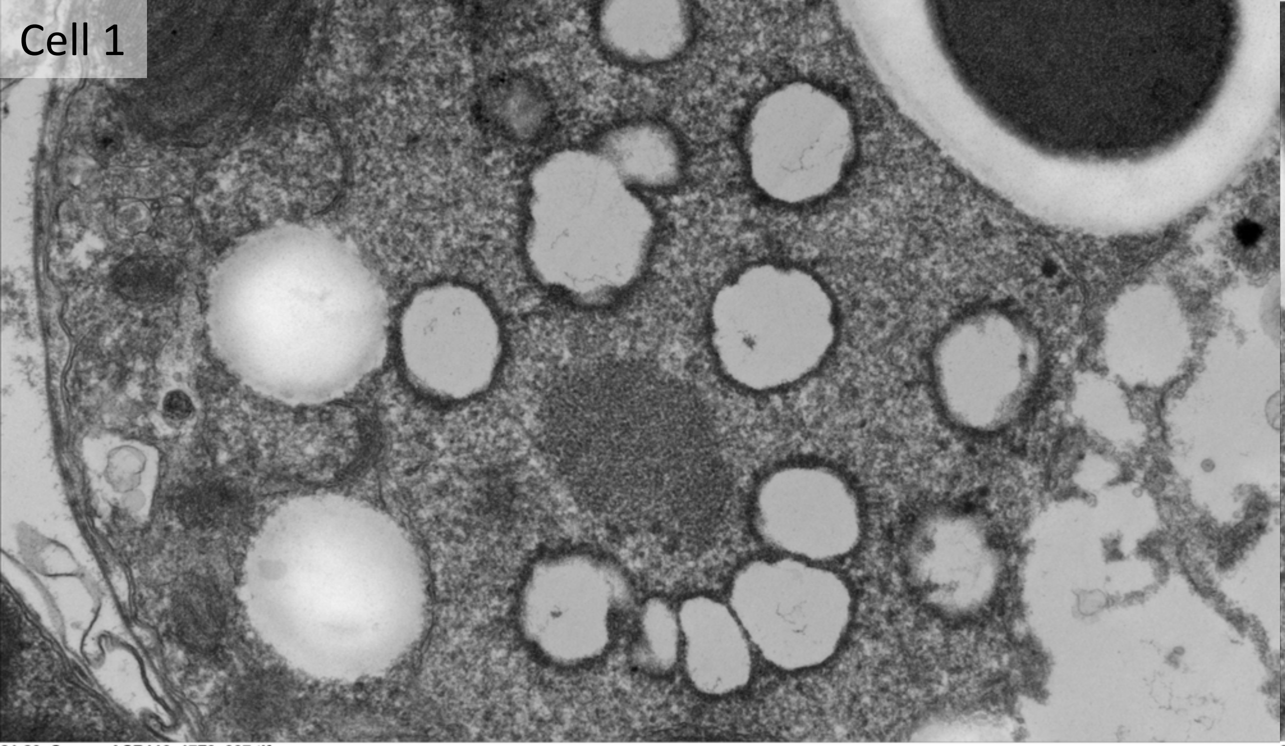

21-20_Correa_ACR116_17F3_007.tif
ARC 116
Biological Electron Microscopy Lab
Rice University - SEA
Microscopist: MD Meyer

800 nm
HV=80kV
Direct Mag: 5000 x

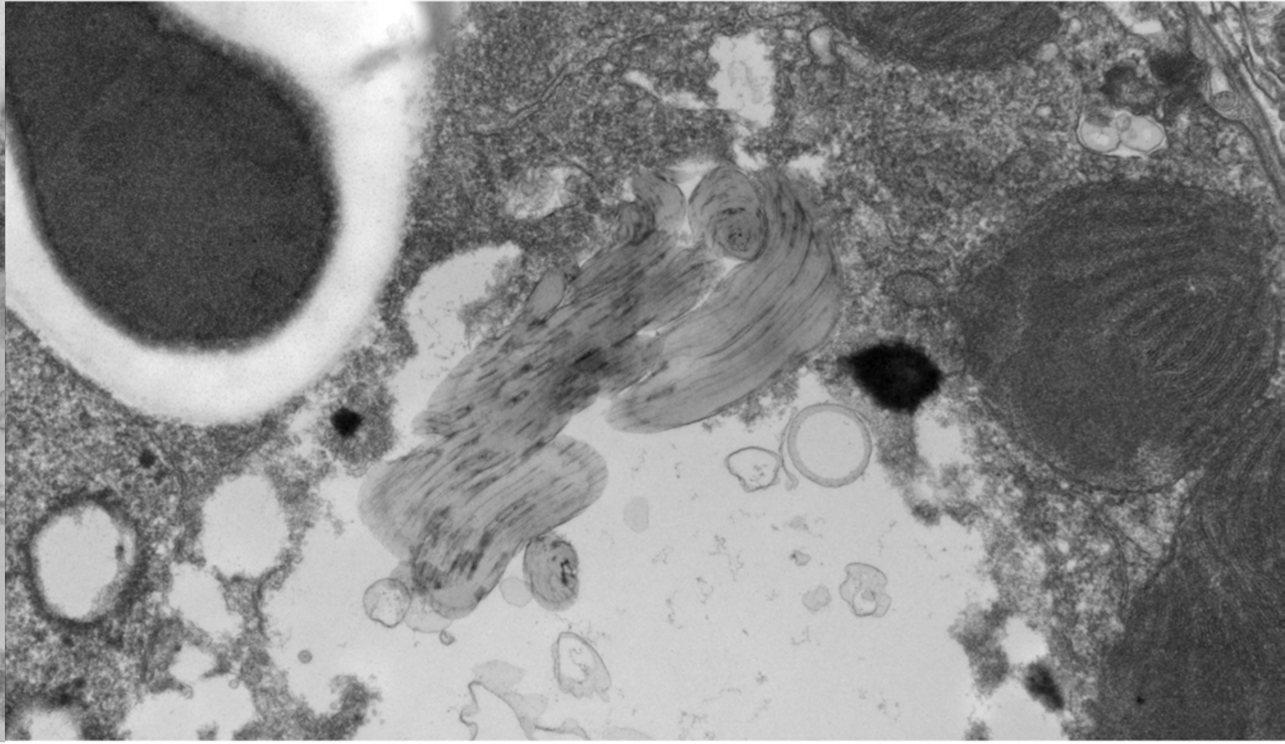

21-20_Correa_ACR116_17F3_005.tif
ARC 116
Biological Electron Microscopy Lab
Rice University - SEA
Microscopist: MD Meyer

800 nm
HV=80kV
Direct Mag: 5000 x

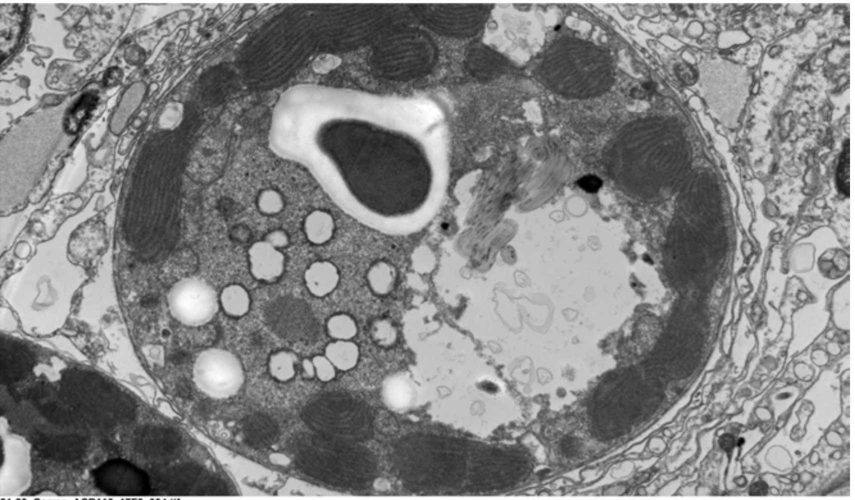

21-20_Correa_ACR116_17F3_004.tif
ARC 116
Biological Electron Microscopy Lab
Rice University - SEA
Microscopist: MD Meyer

2 µm
HV=80kV
Direct Mag: 2000 x

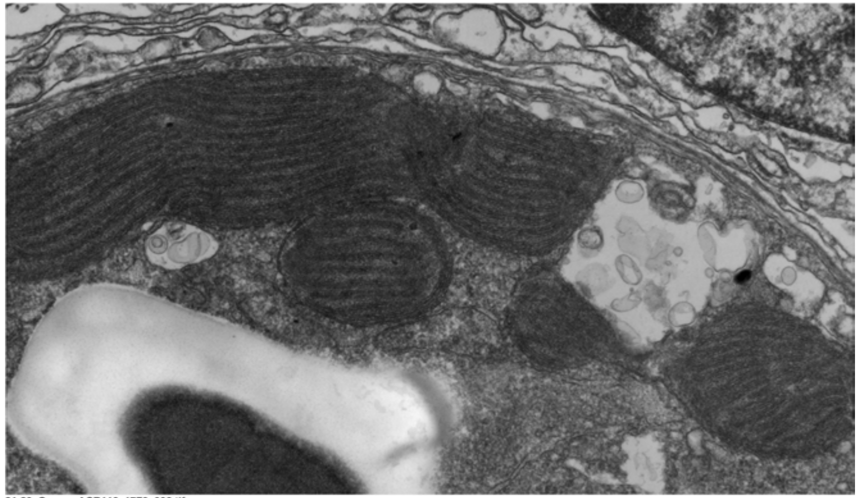

21-20_Correa_ACR116_17F3_006.tif
ARC 116
Biological Electron Microscopy Lab
Rice University - SEA
Microscopist: MD Meyer

800 nm
HV=80kV
Direct Mag: 5000 x

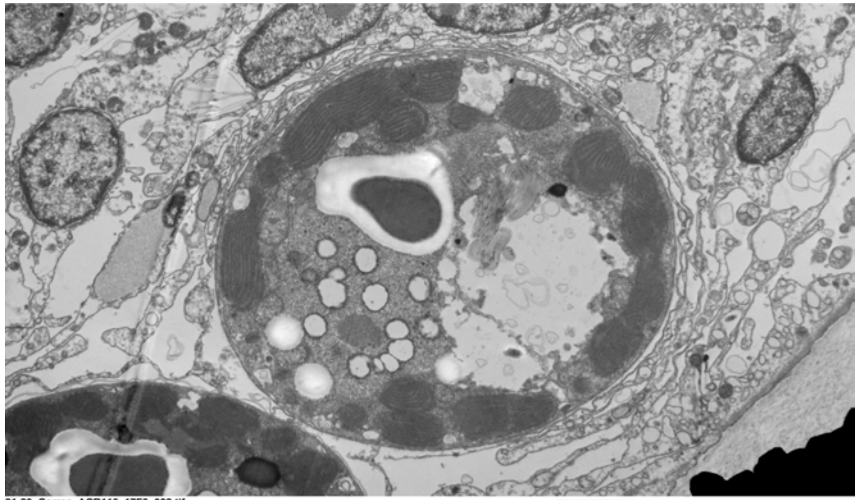

21-20_Correa_ACR116_17F3_002.tif
ARC 116
Biological Electron Microscopy Lab
Rice University - SEA
Microscopist: MD Meyer

2 µm
HV=80kV
Direct Mag: 1500 x

Cell 2

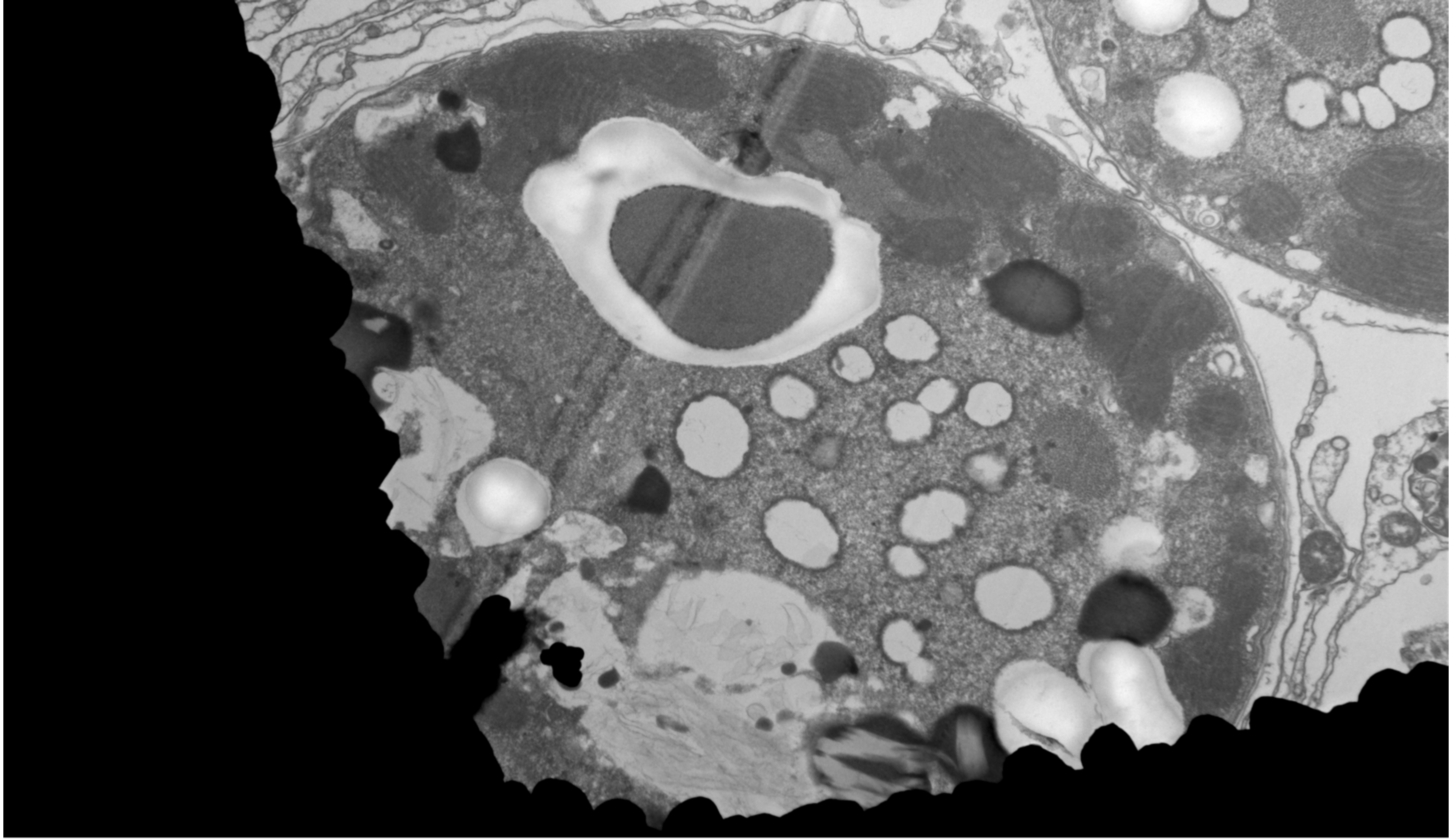

21-20_Correa_ACR116_17F3_008.tif
ARC 116
Biological Electron Microscopy Lab
Rice University - SEA
Microscopist: MD Meyer

HV=80kV
Direct Mag: 2000 x

Cell 3

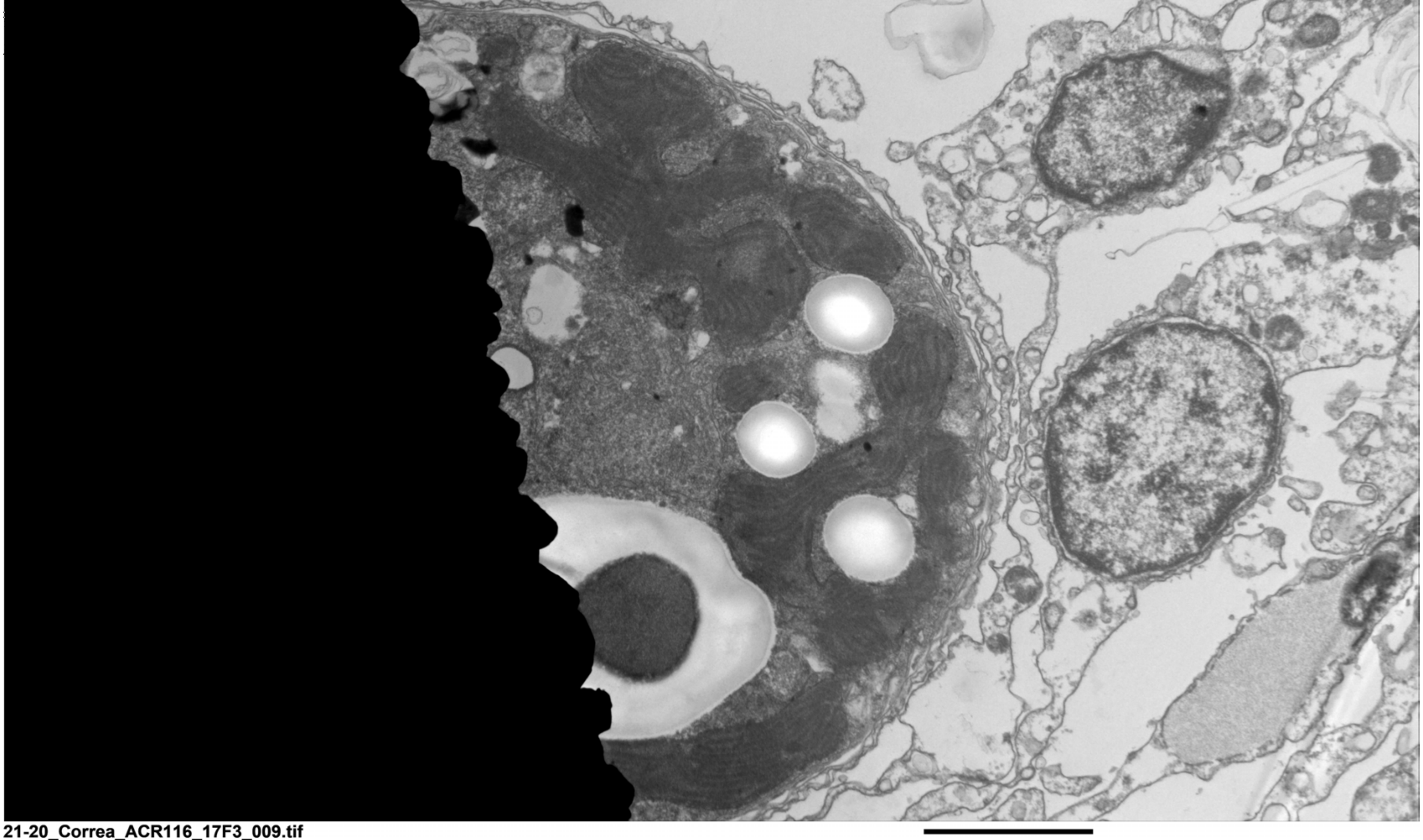

21-20_Correa_ACR116_17F3_009.tif
ARC 116
Biological Electron Microscopy Lab
Rice University - SEA
Microscopist: MD Meyer

2 µm
HV=80kV
Direct Mag: 2000 x

Cell 4

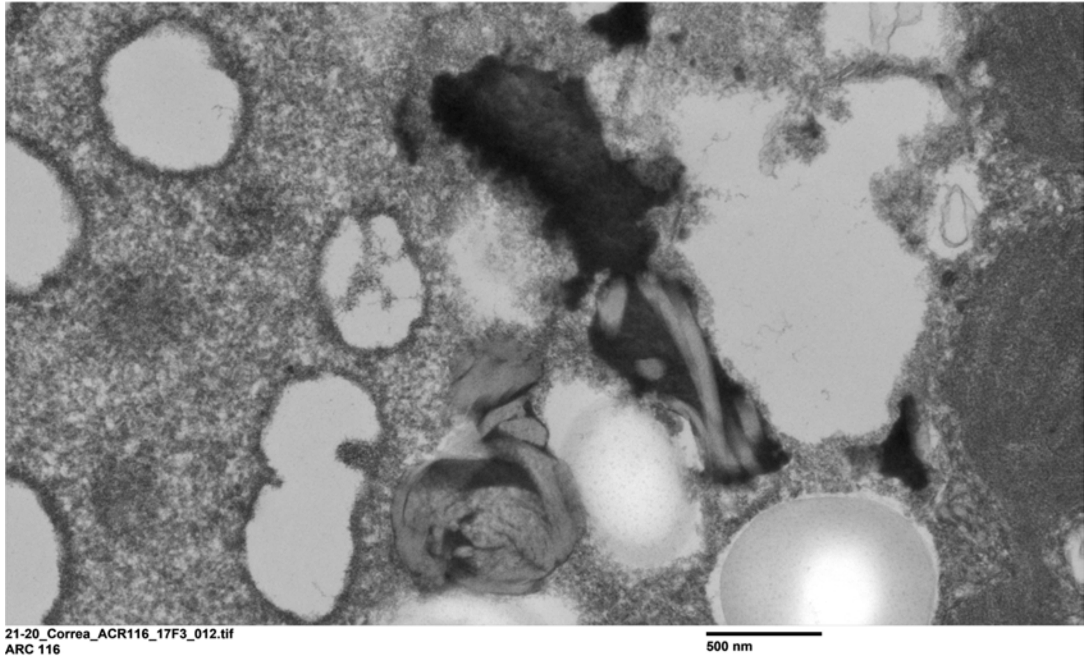

21-20_Correa_ACR116_17F3_012.tif
ARC 116
Biological Electron Microscopy Lab
Rice University - SEA
Microscopist: MD Meyer

HV=80kV
Direct Mag: 8000 x

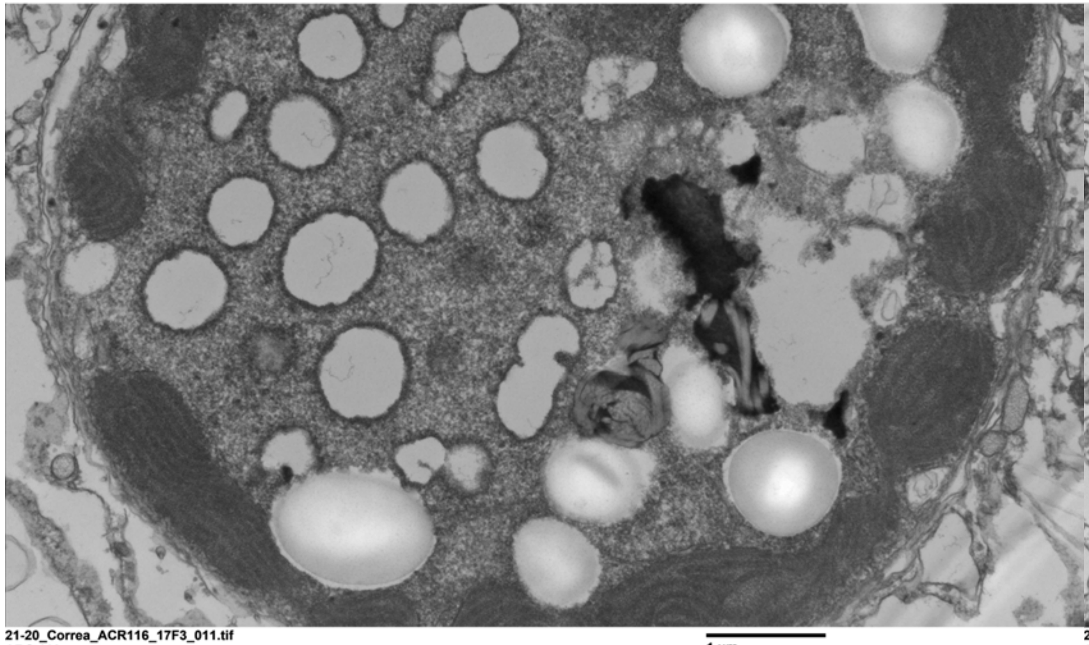

21-20_Correa_ACR116_17F3_011.tif
ARC 116
Biological Electron Microscopy Lab
Rice University - SEA
Microscopist: MD Meyer

HV=80kV
Direct Mag: 4000 x

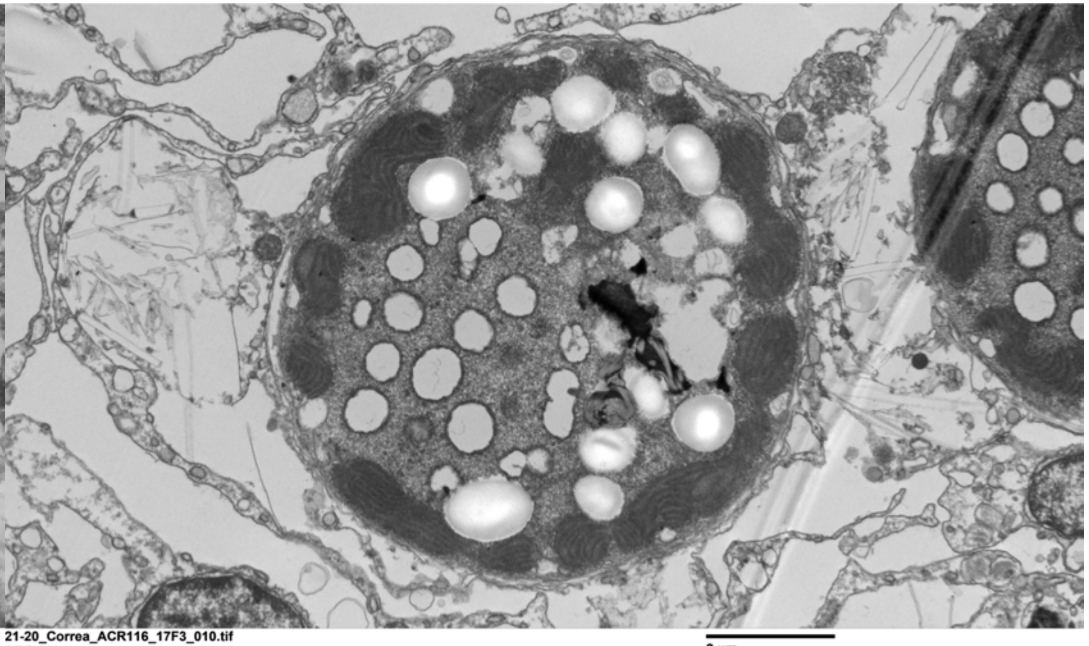

21-20_Correa_ACR116_17F3_010.tif
ARC 116
Biological Electron Microscopy Lab
Rice University - SEA
Microscopist: MD Meyer

HV=80kV
Direct Mag: 2000 x

Cell 5

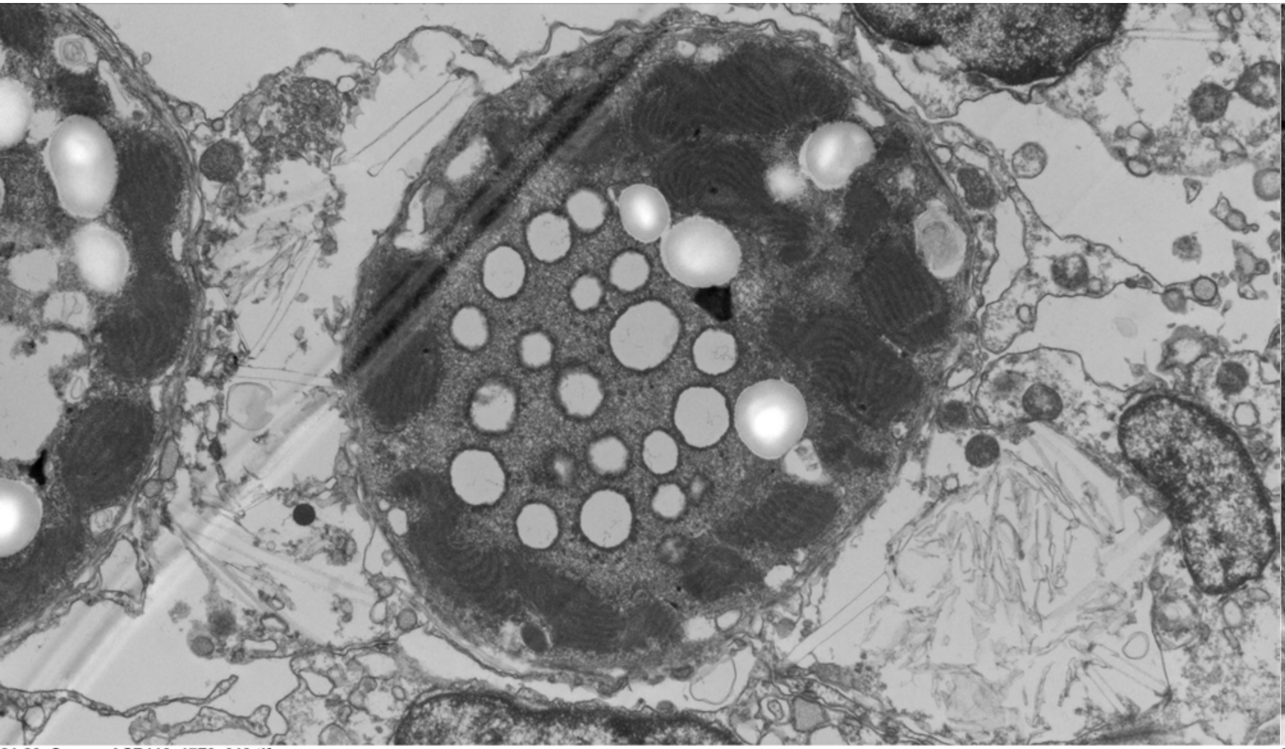

21-20_Correa_ACR116_17F3_013.tif
ARC 116
Biological Electron Microscopy Lab
Rice University - SEA
Microscopist: MD Meyer

1 µm
HV=80kV
Direct Mag: 2500 x

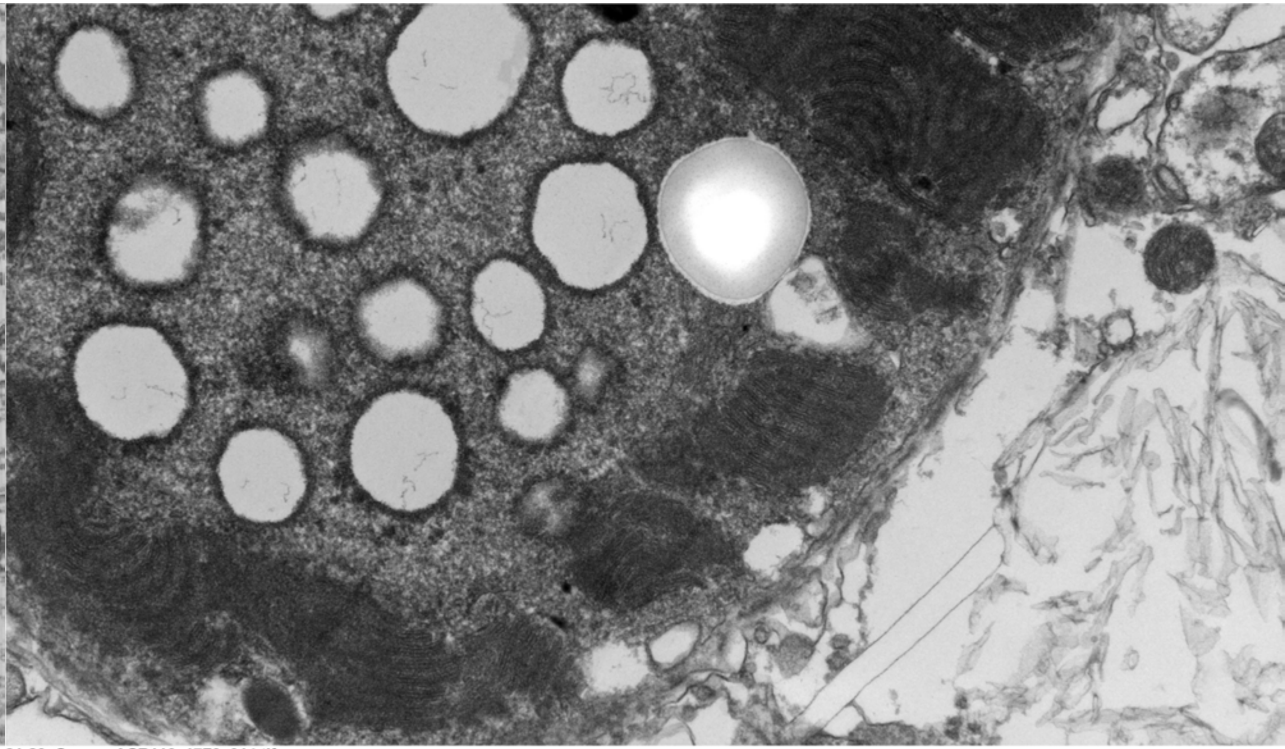

21-20_Correa_ACR116_17F3_014.tif
ARC 116
Biological Electron Microscopy Lab
Rice University - SEA
Microscopist: MD Meyer

800 nm
HV=80kV
Direct Mag: 5000 x

Cell 6

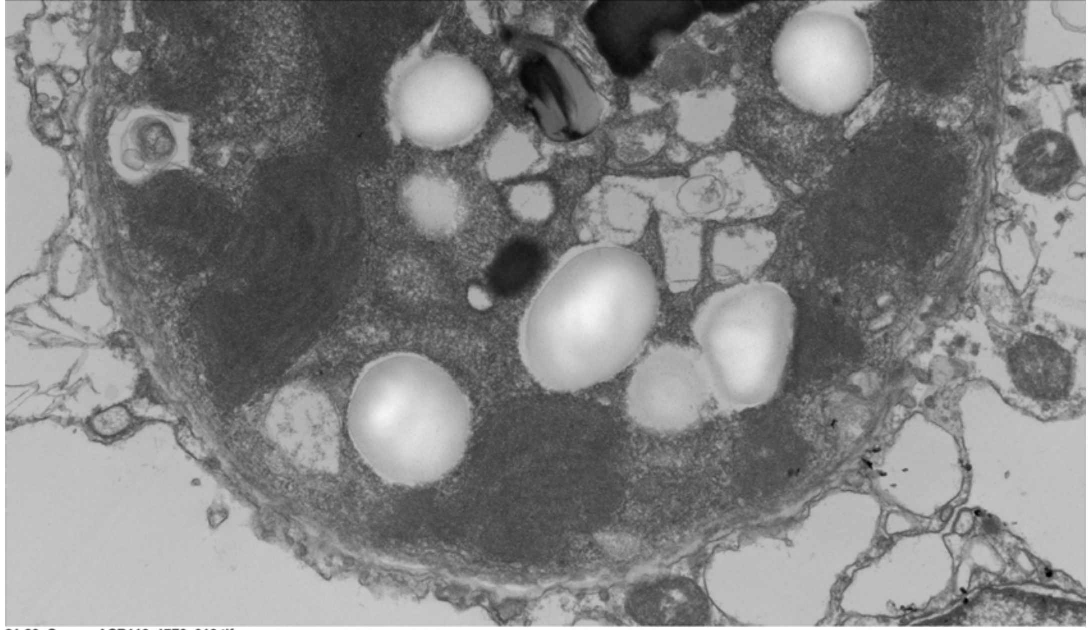

21-20_Correa_ACR116_17F3_016.tif
ARC 116
Biological Electron Microscopy Lab
Rice University - SEA
Microscopist: MD Meyer

1 µm
HV=80kV
Direct Mag: 4000 x

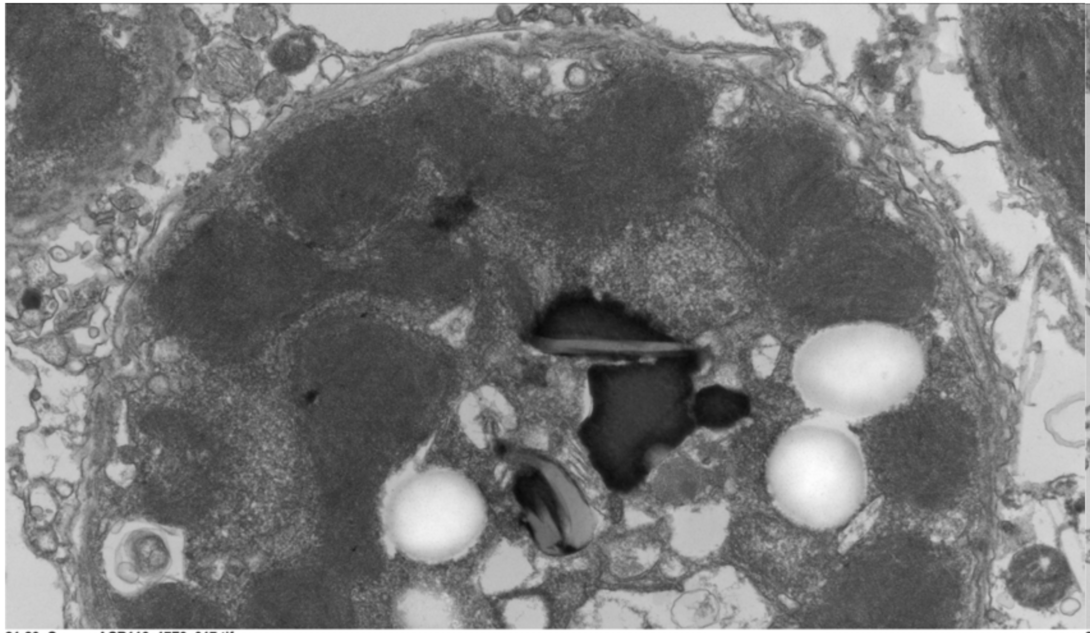

21-20_Correa_ACR116_17F3_017.tif
ARC 116
Biological Electron Microscopy Lab
Rice University - SEA
Microscopist: MD Meyer

1 µm
HV=80kV
Direct Mag: 4000 x

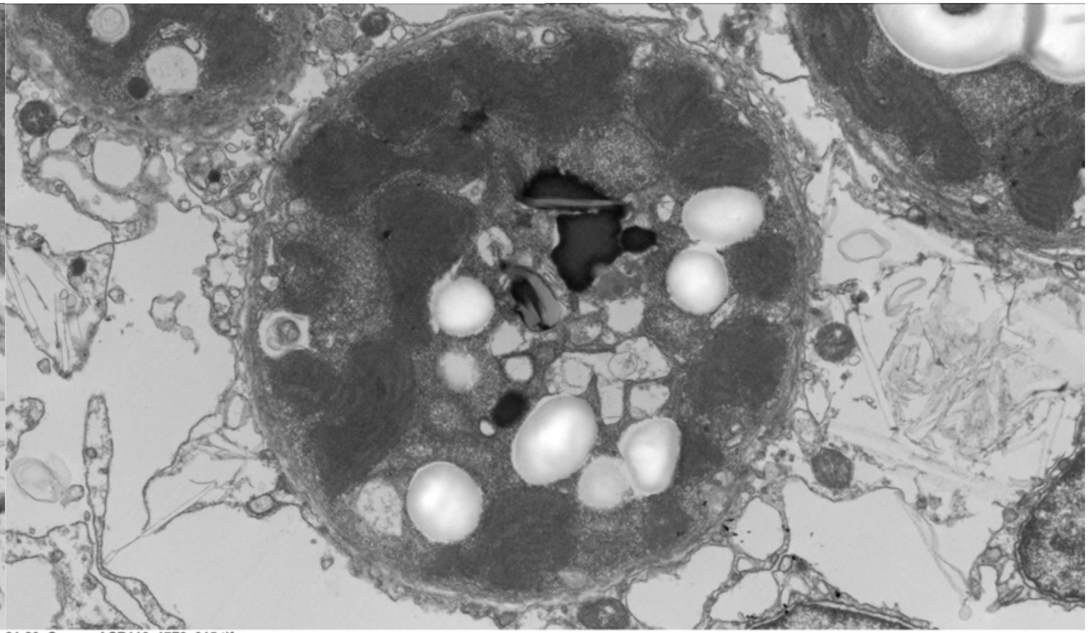

21-20_Correa_ACR116_17F3_015.tif
ARC 116
Biological Electron Microscopy Lab
Rice University - SEA
Microscopist: MD Meyer

1 µm
HV=80kV
Direct Mag: 2500 x

Cell 7

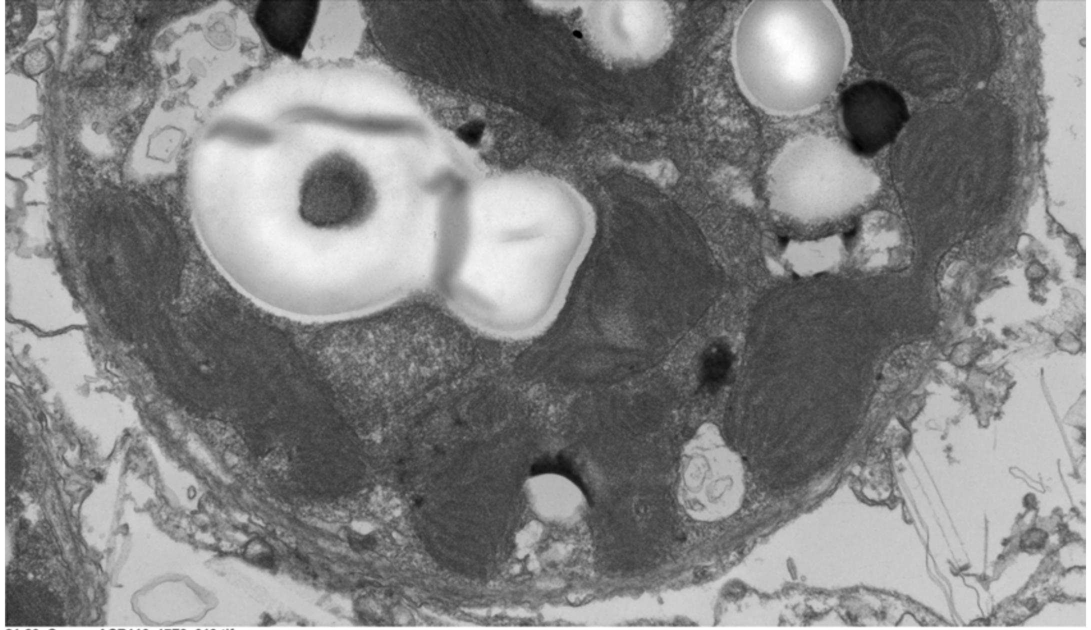

21-20_Correa_ACR116_17F3_019.tif
ARC 116
Biological Electron Microscopy Lab
Rice University - SEA
Microscopist: MD Meyer

1 µm
HV=80kV
Direct Mag: 4000 x

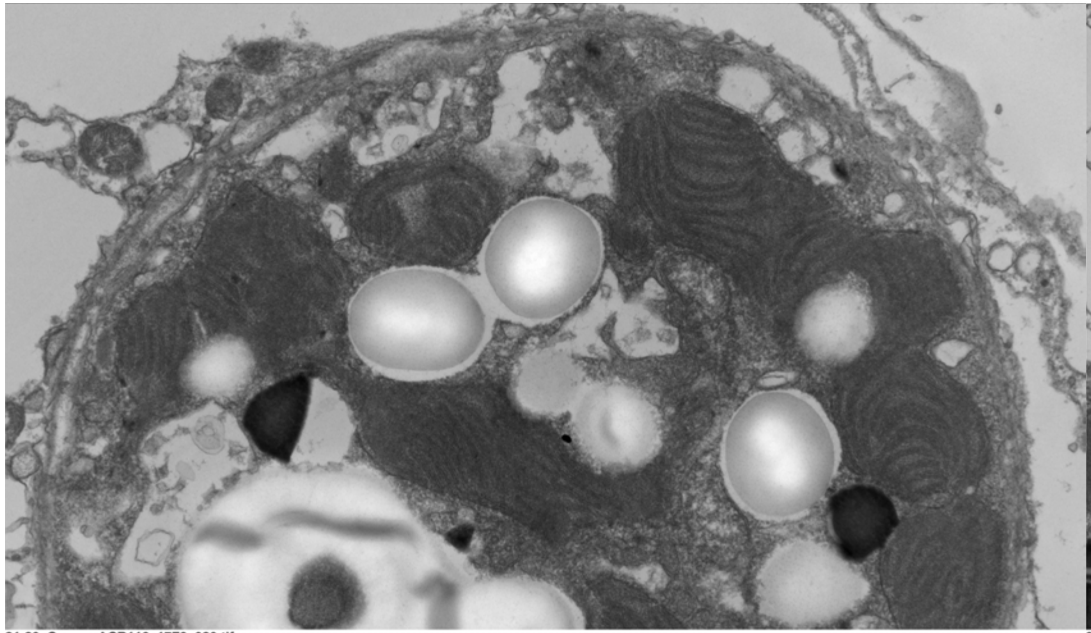

21-20_Correa_ACR116_17F3_020.tif
ARC 116
Biological Electron Microscopy Lab
Rice University - SEA
Microscopist: MD Meyer

1 µm
HV=80kV
Direct Mag: 4000 x

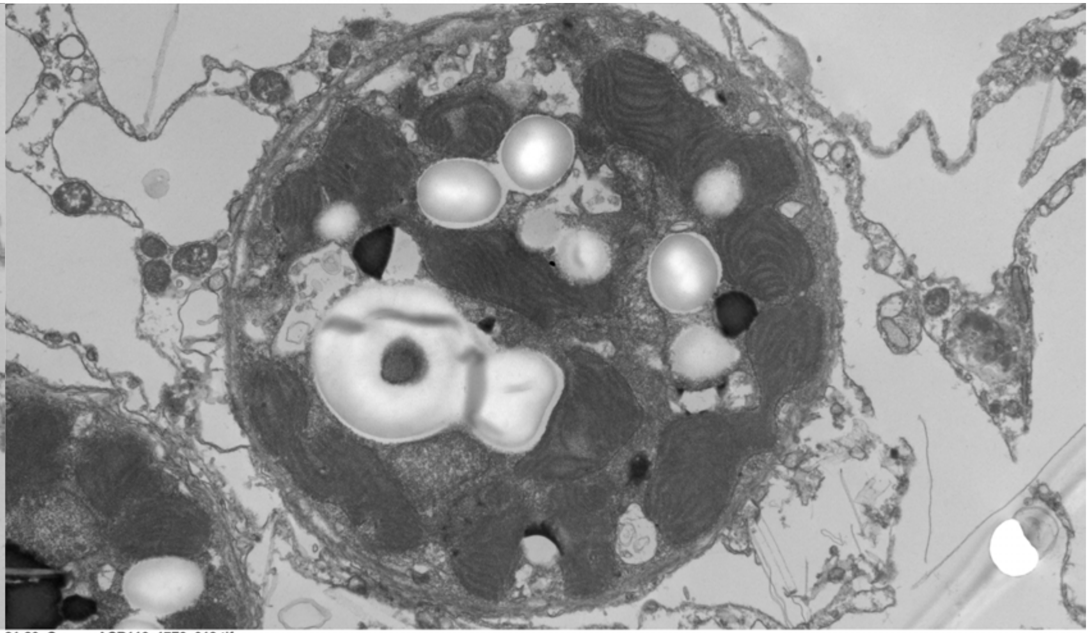

21-20_Correa_ACR116_17F3_018.tif
ARC 116
Biological Electron Microscopy Lab
Rice University - SEA
Microscopist: MD Meyer

1 µm
HV=80kV
Direct Mag: 2500 x

Cell 8

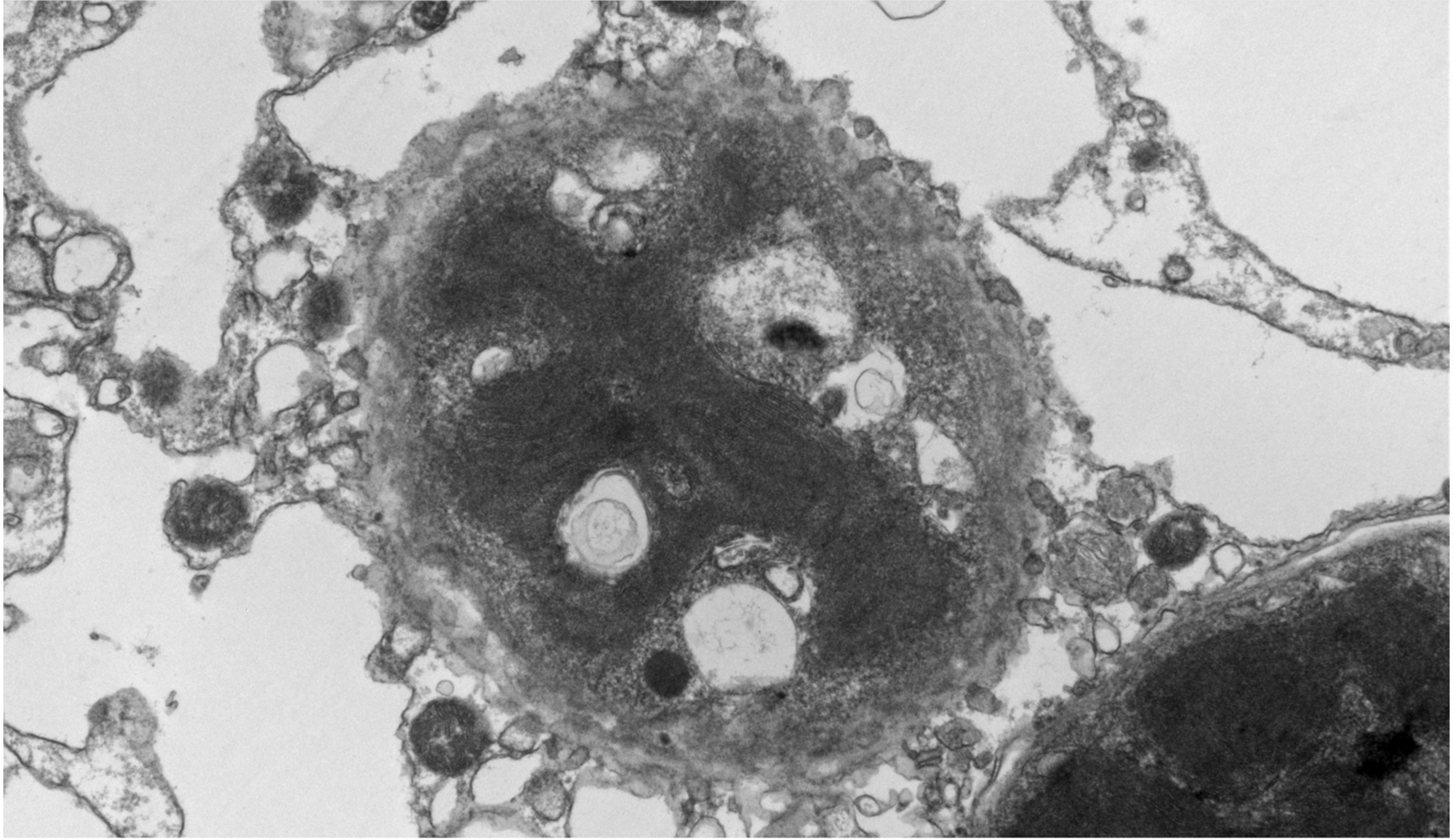

21-20_Correa_ACR116_17F3_021.tif
ARC 116
Biological Electron Microscopy Lab
Rice University - SEA
Microscopist: MD Meyer

1 µm
HV=80kV
Direct Mag: 4000 x

Cell 9

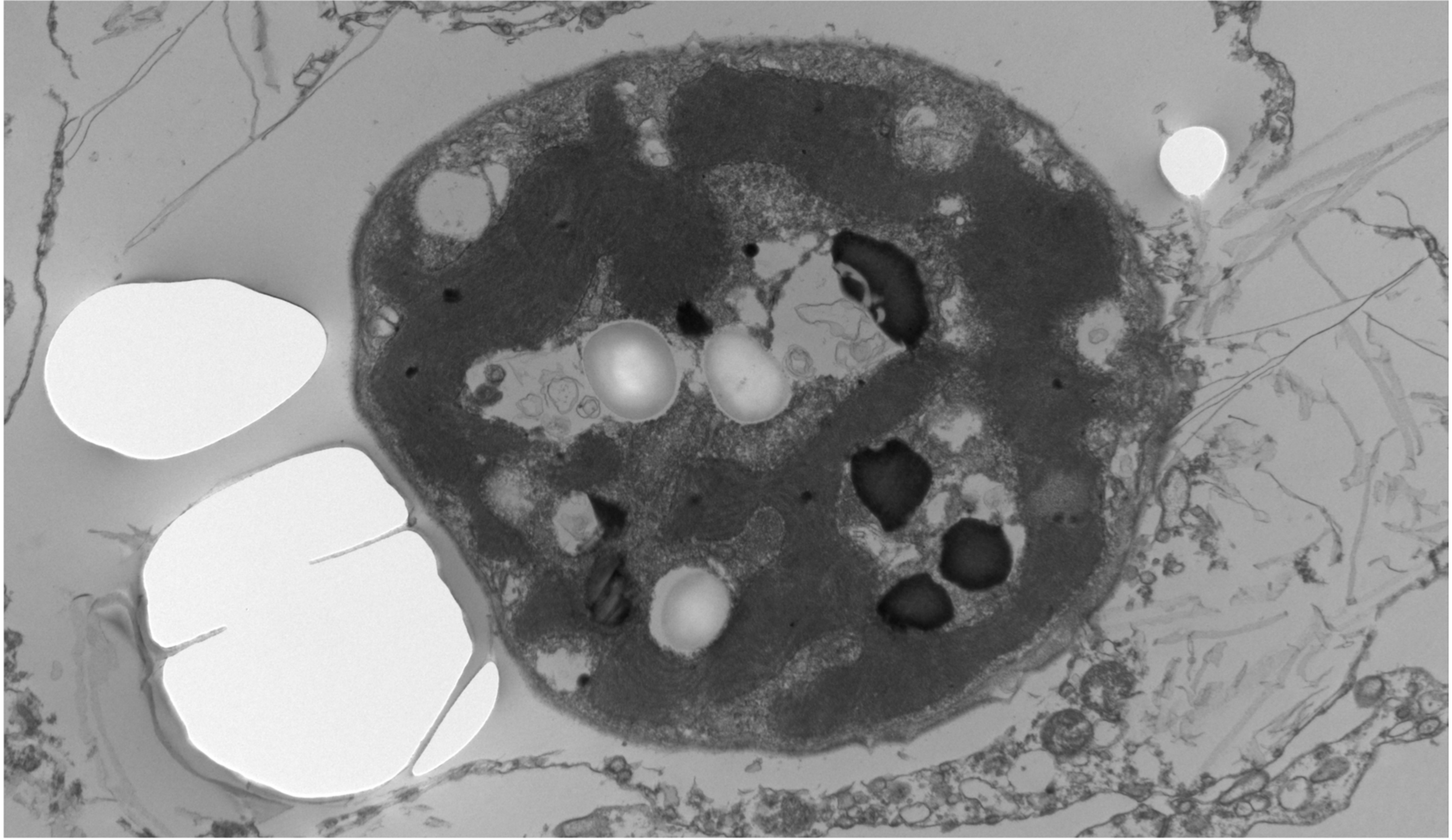

21-20_Correa_ACR116_17F3_022.tif
ARC 116
Biological Electron Microscopy Lab
Rice University - SEA
Microscopist: MD Meyer

2 µm
HV=80kV
Direct Mag: 2000 x

Cell 10

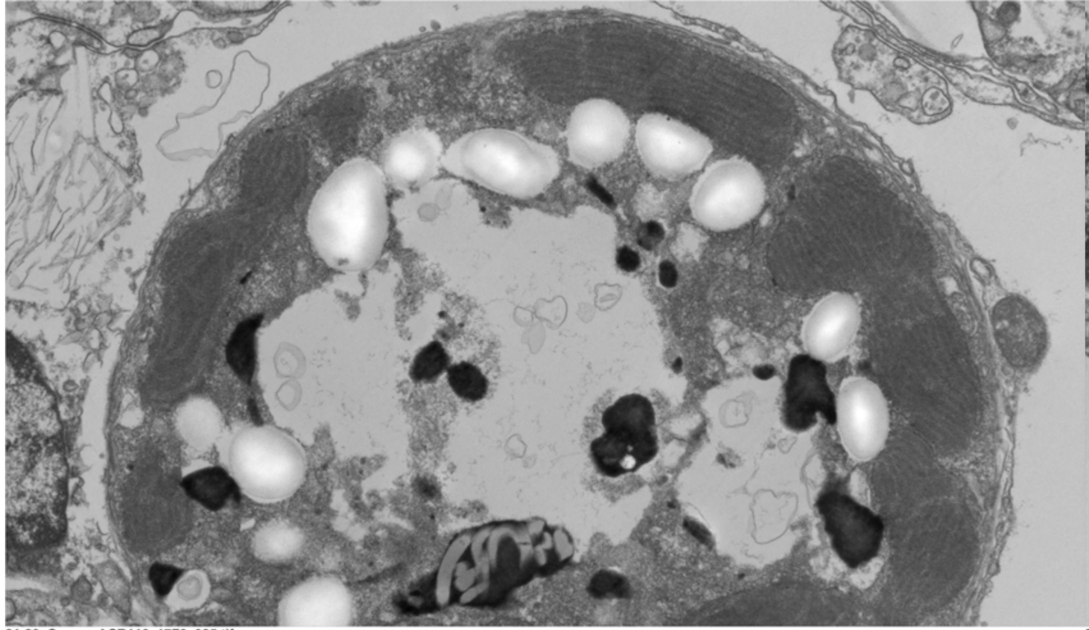

21-20_Correa_ACR116_17F3_025.tif
ARC 116
Biological Electron Microscopy Lab
Rice University - SEA
Microscopist: MD Meyer

1 µm
HV=80kV
Direct Mag: 3000 x

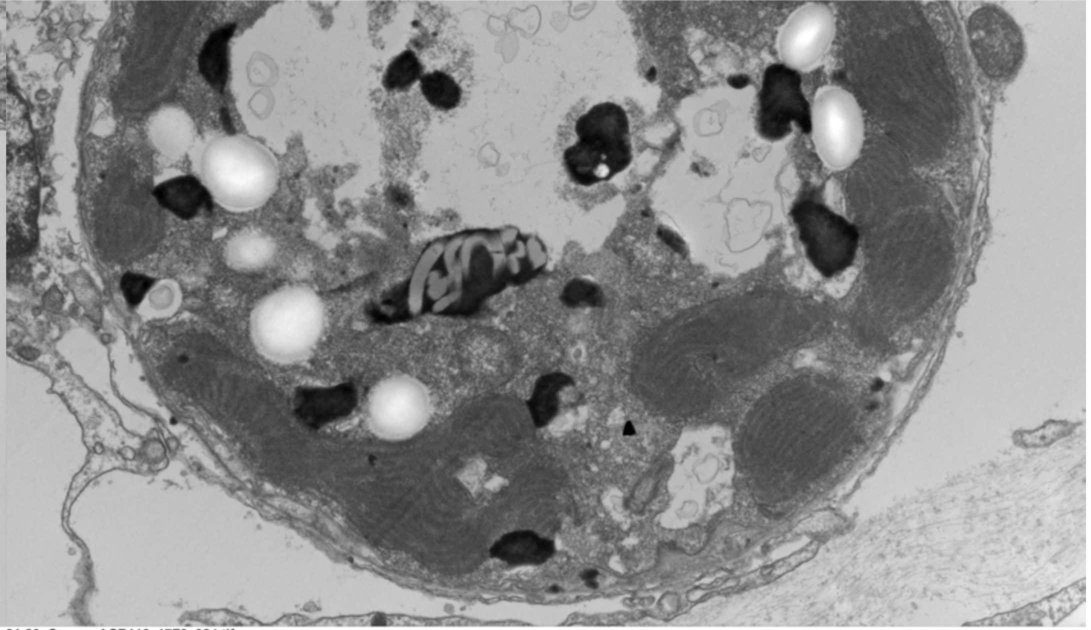

21-20_Correa_ACR116_17F3_024.tif
ARC 116
Biological Electron Microscopy Lab
Rice University - SEA
Microscopist: MD Meyer

1 µm
HV=80kV
Direct Mag: 3000 x

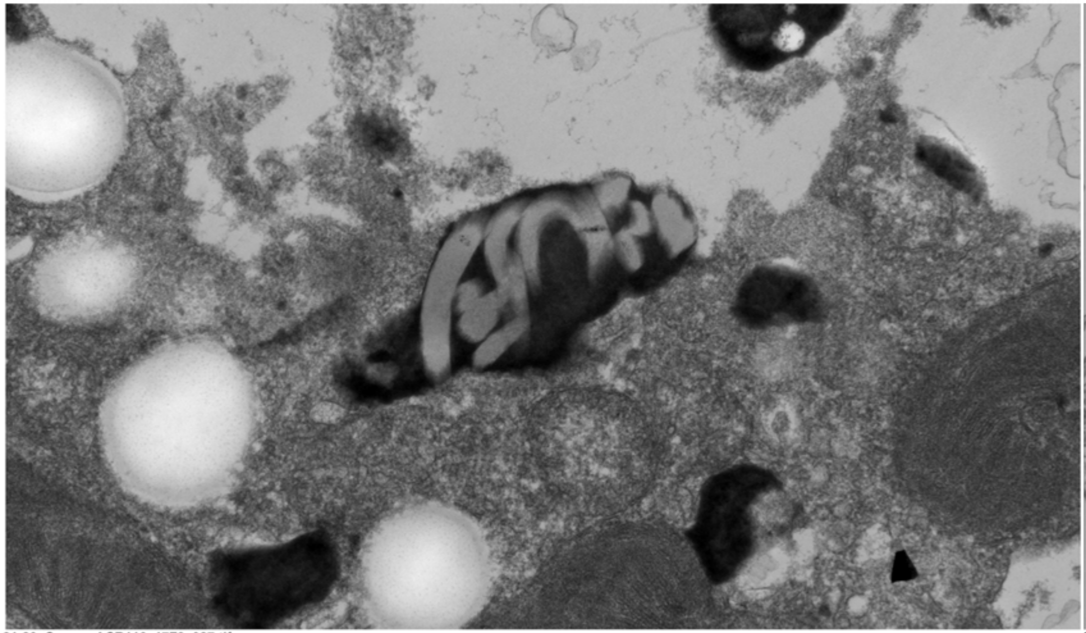

21-20_Correa_ACR116_17F3_027.tif
ARC 116
Biological Electron Microscopy Lab
Rice University - SEA
Microscopist: MD Meyer

600 nm
HV=80kV
Direct Mag: 6000 x

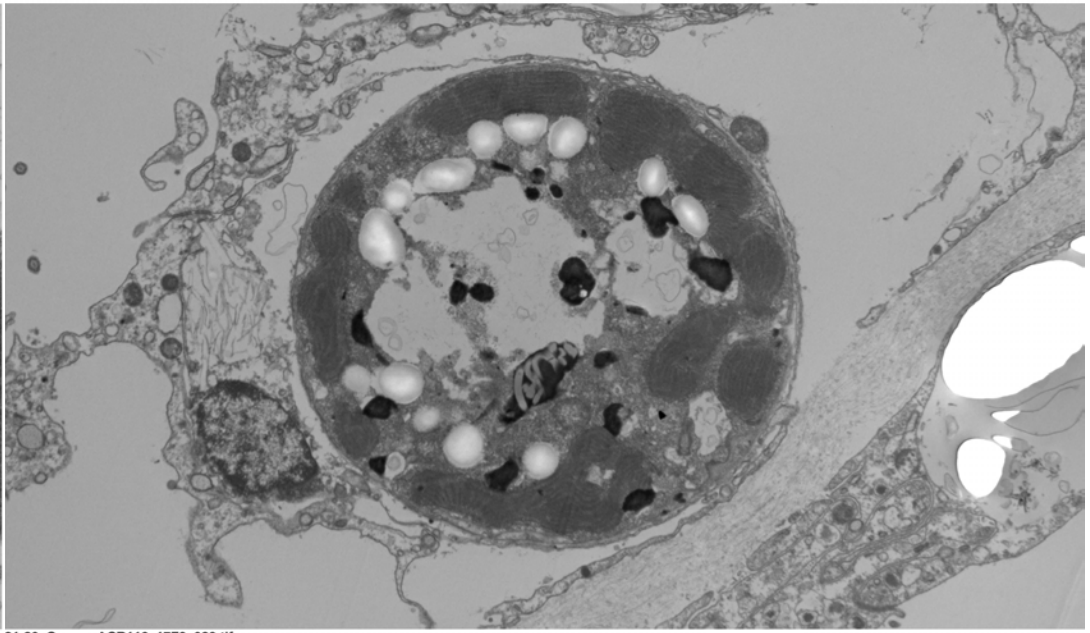

21-20_Correa_ACR116_17F3_023.tif
ARC 116
Biological Electron Microscopy Lab
Rice University - SEA
Microscopist: MD Meyer

2 µm
HV=80kV
Direct Mag: 1500 x

Cell 11

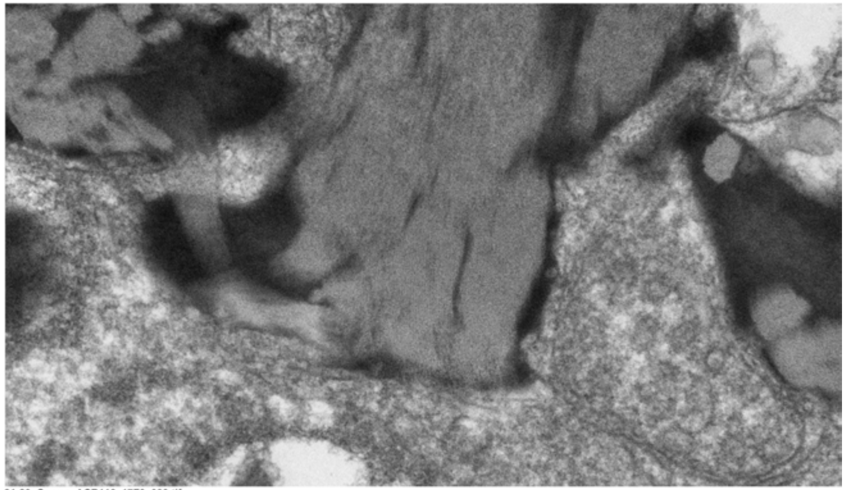

21-20_Correa_ACR116_17F3_033.tif
ARC 116
Biological Electron Microscopy Lab
Rice University - SEA
Microscopist: MD Meyer

200 nm
HV=80kV
Direct Mag: 20000 x

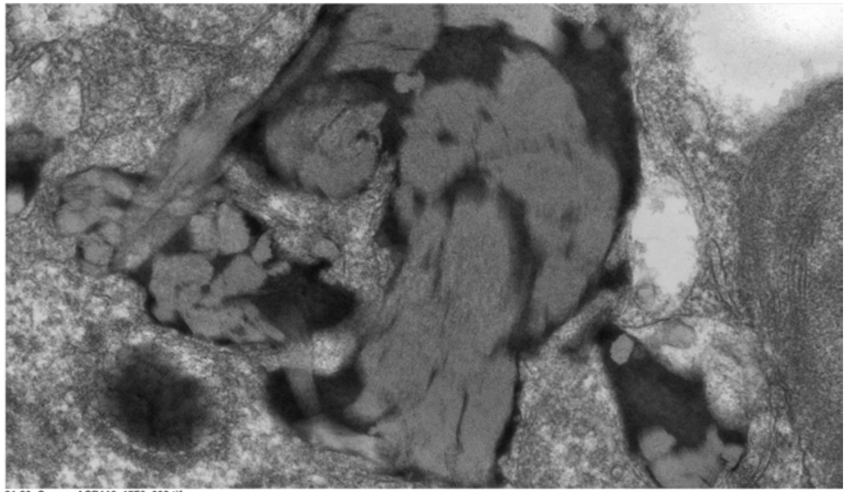

21-20_Correa_ACR116_17F3_032.tif
ARC 116
Biological Electron Microscopy Lab
Rice University - SEA
Microscopist: MD Meyer

200 nm
HV=80kV
Direct Mag: 12000 x

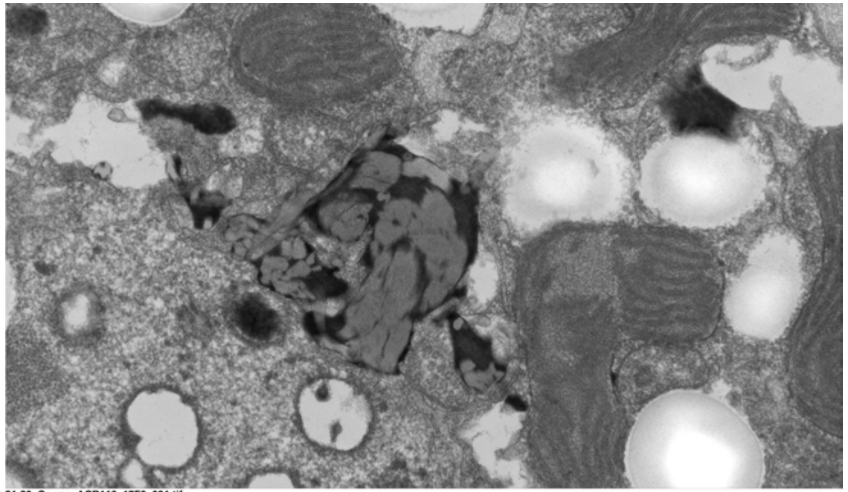

21-20_Correa_ACR116_17F3_031.tif
ARC 116
Biological Electron Microscopy Lab
Rice University - SEA
Microscopist: MD Meyer

800 nm
HV=80kV
Direct Mag: 5000 x

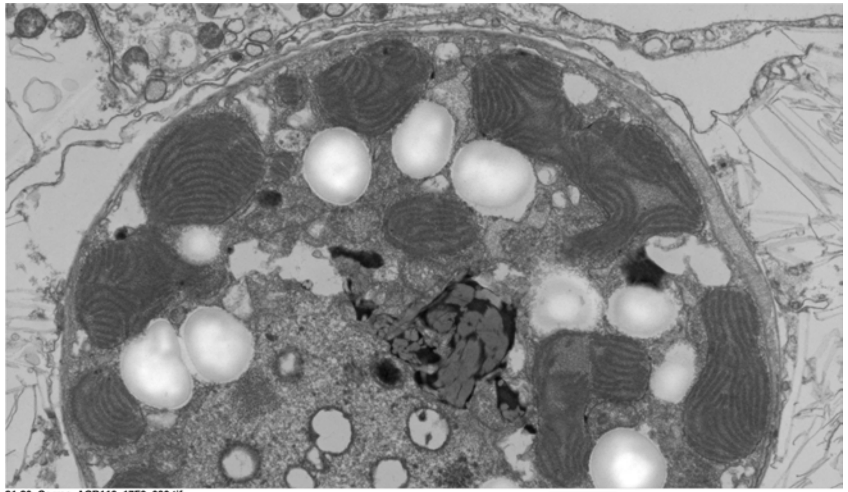

21-20_Correa_ACR116_17F3_030.tif
ARC 116
Biological Electron Microscopy Lab
Rice University - SEA
Microscopist: MD Meyer

1 μm
HV=80kV
Direct Mag: 3000 x

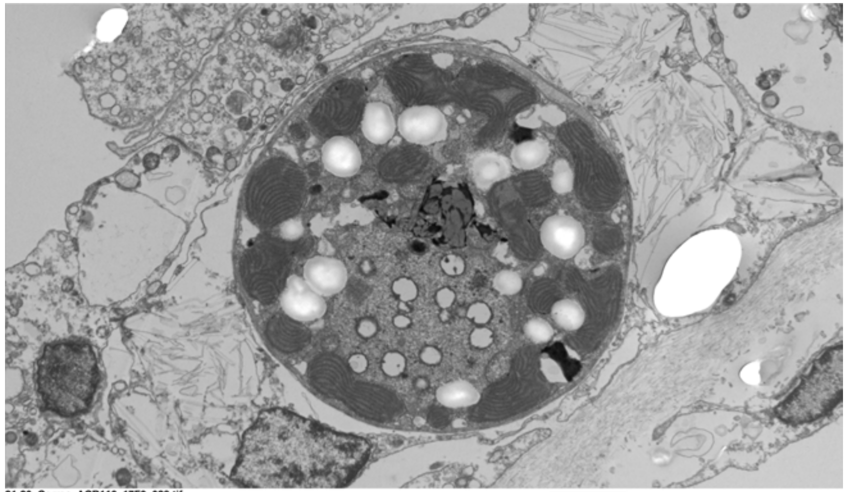

21-20_Correa_ACR116_17F3_028.tif
ARC 116
Biological Electron Microscopy Lab
Rice University - SEA
Microscopist: MD Meyer

2 μm
HV=80kV
Direct Mag: 1500 x

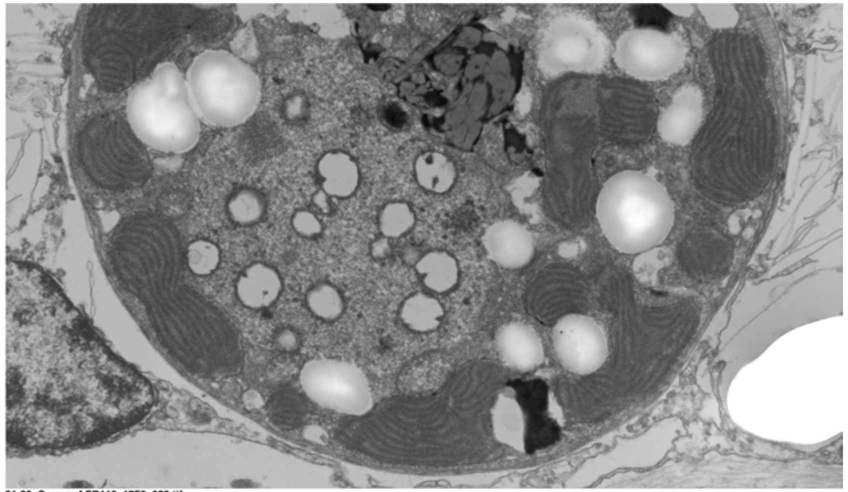

21-20_Correa_ACR116_17F3_029.tif
ARC 116
Biological Electron Microscopy Lab
Rice University - SEA
Microscopist: MD Meyer

1 μm
HV=80kV
Direct Mag: 3000 x

Cell 12

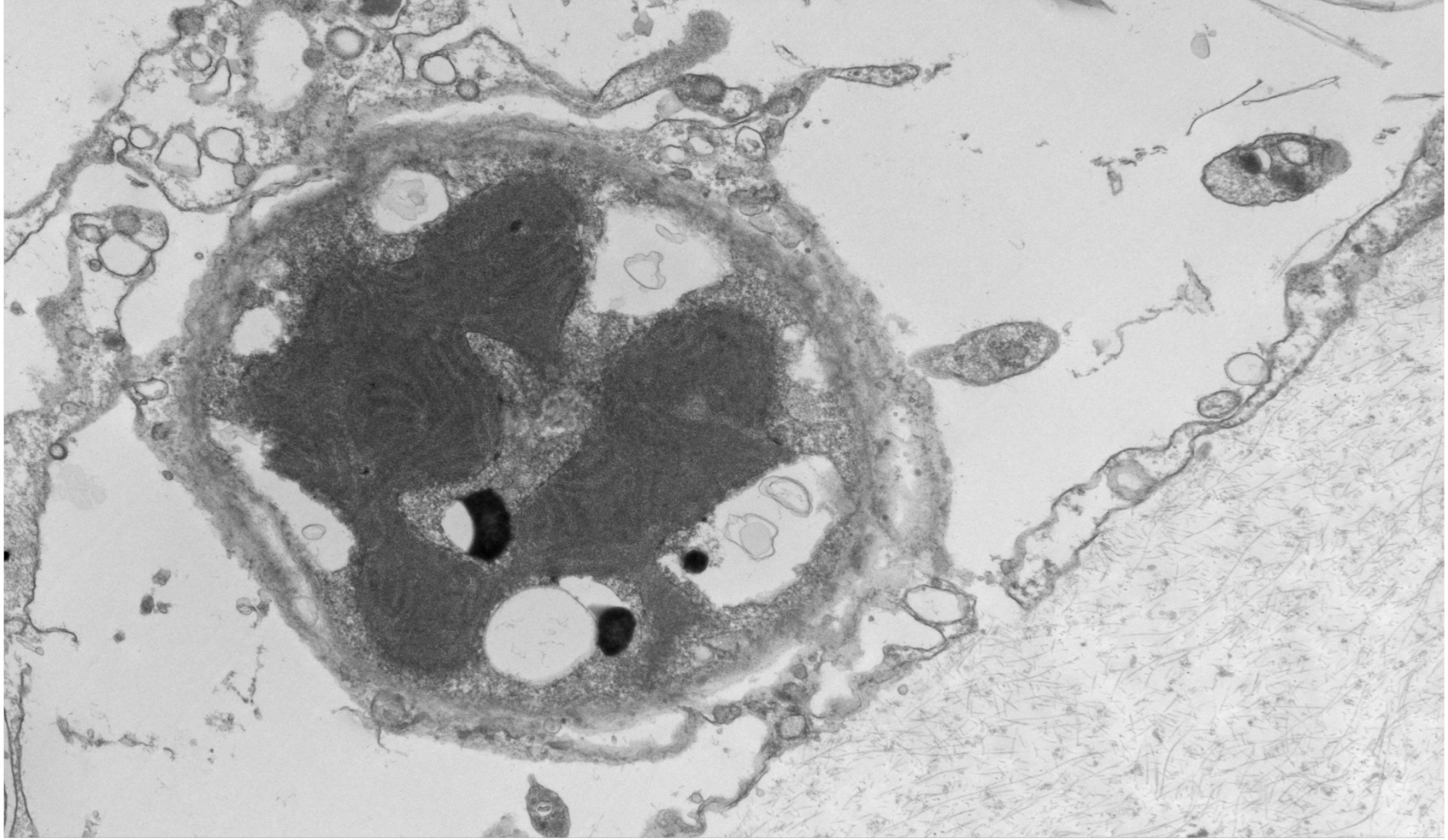

21-20_Correa_ACR116_17F3_034.tif
ARC 116
Biological Electron Microscopy Lab
Rice University - SEA
Microscopist: MD Meyer

1 µm
HV=80kV
Direct Mag: 3000 x

Cell 13

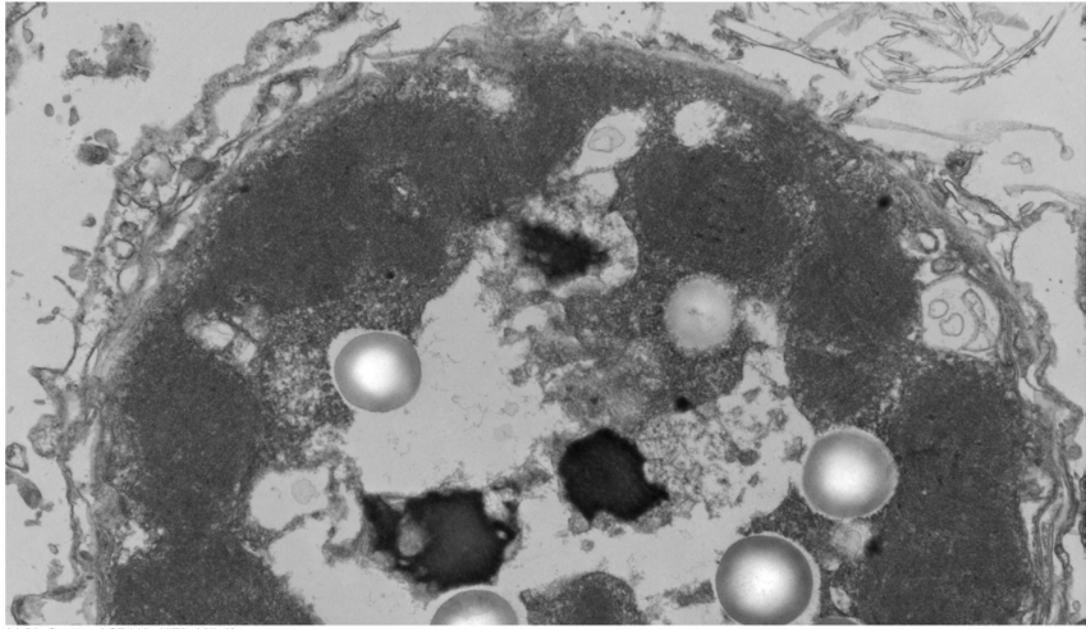

21-20_Correa_ACR116_17F3_037.tif
ARC 116
Biological Electron Microscopy Lab
Rice University - SEA
Microscopist: MD Meyer

1 µm
HV=80kV
Direct Mag: 4000 x

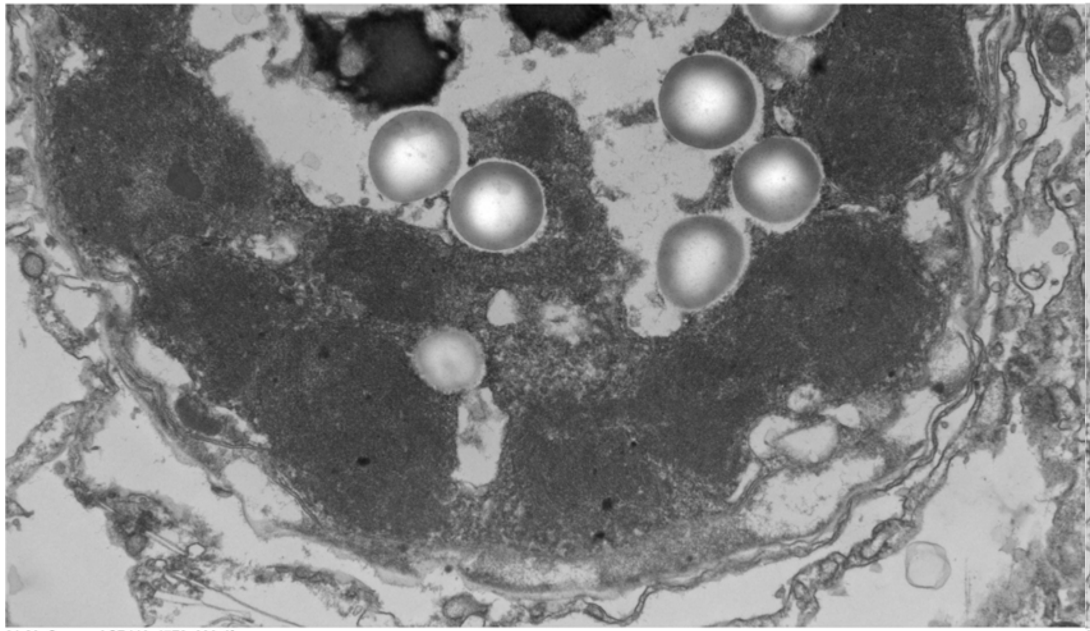

21-20_Correa_ACR116_17F3_036.tif
ARC 116
Biological Electron Microscopy Lab
Rice University - SEA
Microscopist: MD Meyer

1 µm
HV=80kV
Direct Mag: 4000 x

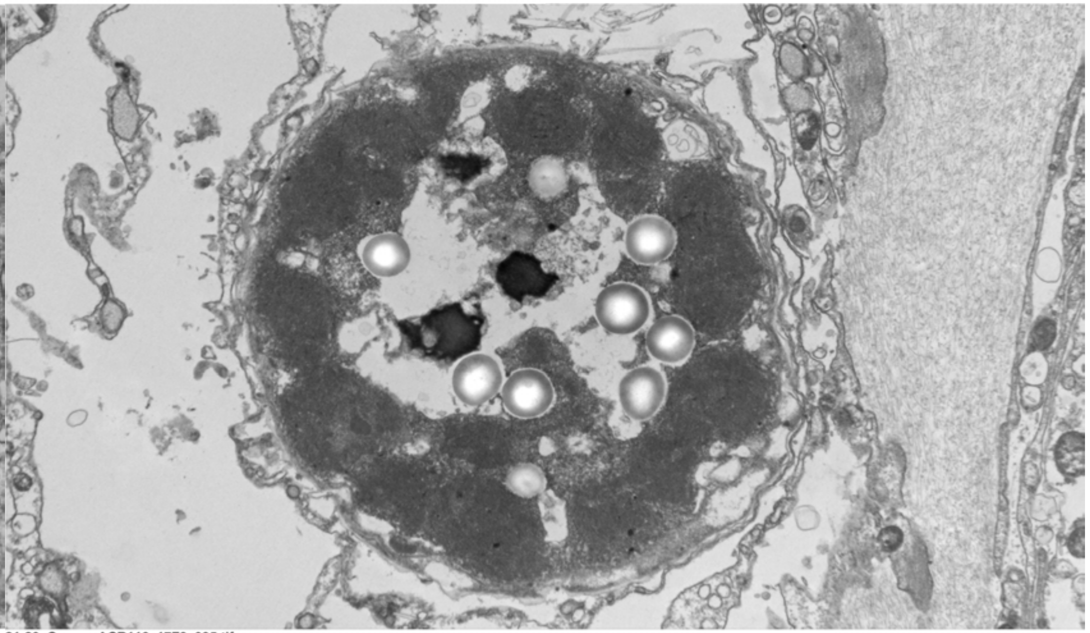

21-20_Correa_ACR116_17F3_035.tif
ARC 116
Biological Electron Microscopy Lab
Rice University - SEA
Microscopist: MD Meyer

2 µm
HV=80kV
Direct Mag: 2000 x

Cell 14

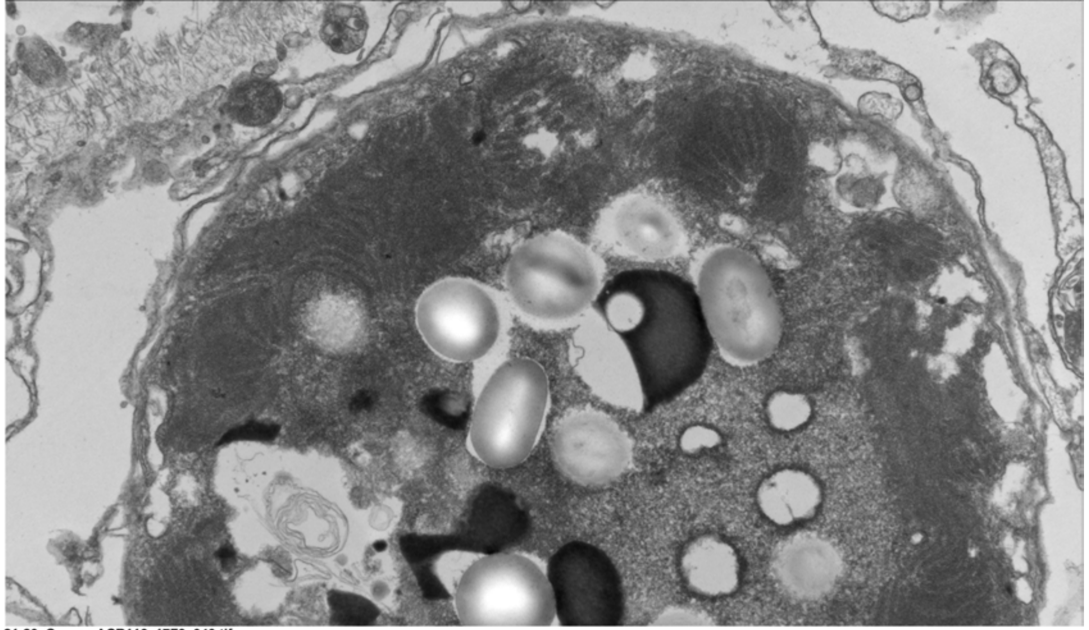

21-20_Correa_ACR116_17F3_040.tif
ARC 116
Biological Electron Microscopy Lab
Rice University - SEA
Microscopist: MD Meyer

1 µm
HV=80kV
Direct Mag: 4000 x

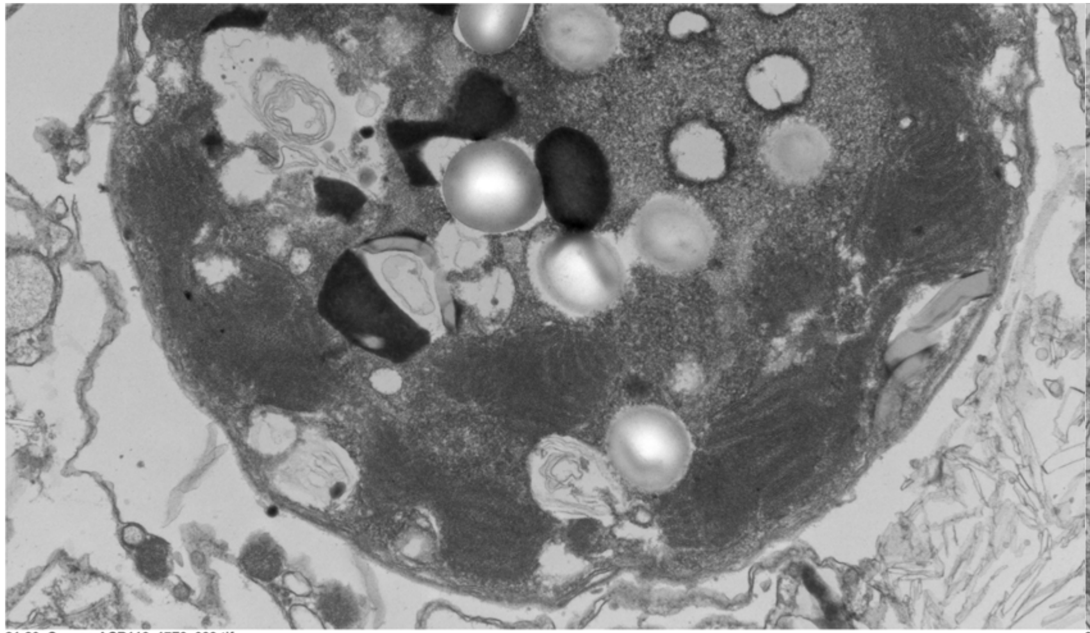

21-20_Correa_ACR116_17F3_039.tif
ARC 116
Biological Electron Microscopy Lab
Rice University - SEA
Microscopist: MD Meyer

1 µm
HV=80kV
Direct Mag: 4000 x

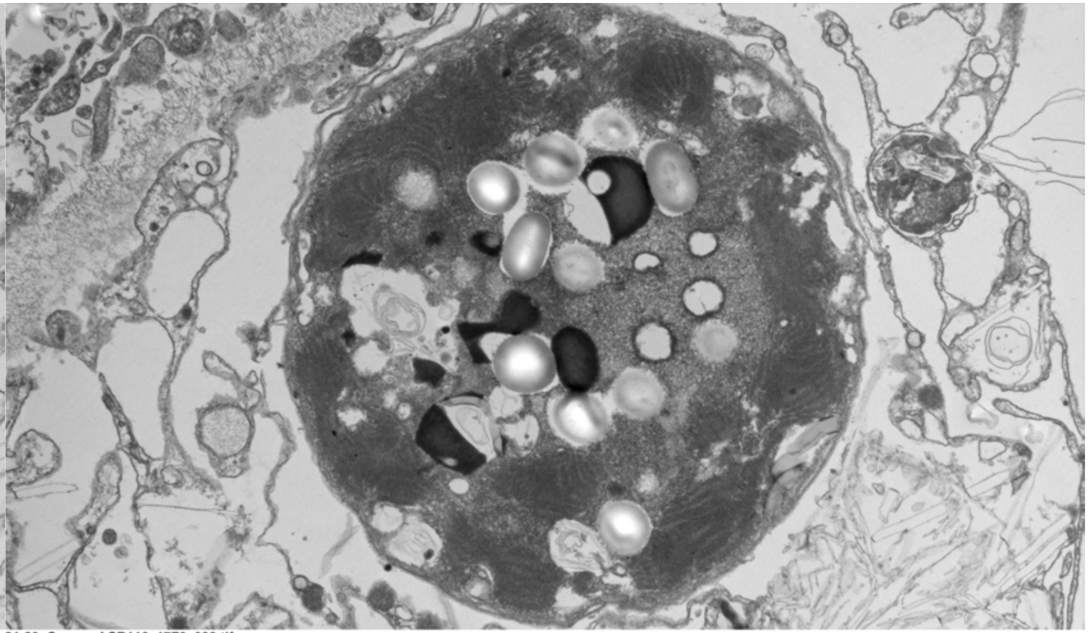

21-20_Correa_ACR116_17F3_038.tif
ARC 116
Biological Electron Microscopy Lab
Rice University - SEA
Microscopist: MD Meyer

1 µm
HV=80kV
Direct Mag: 2500 x

Cell 15

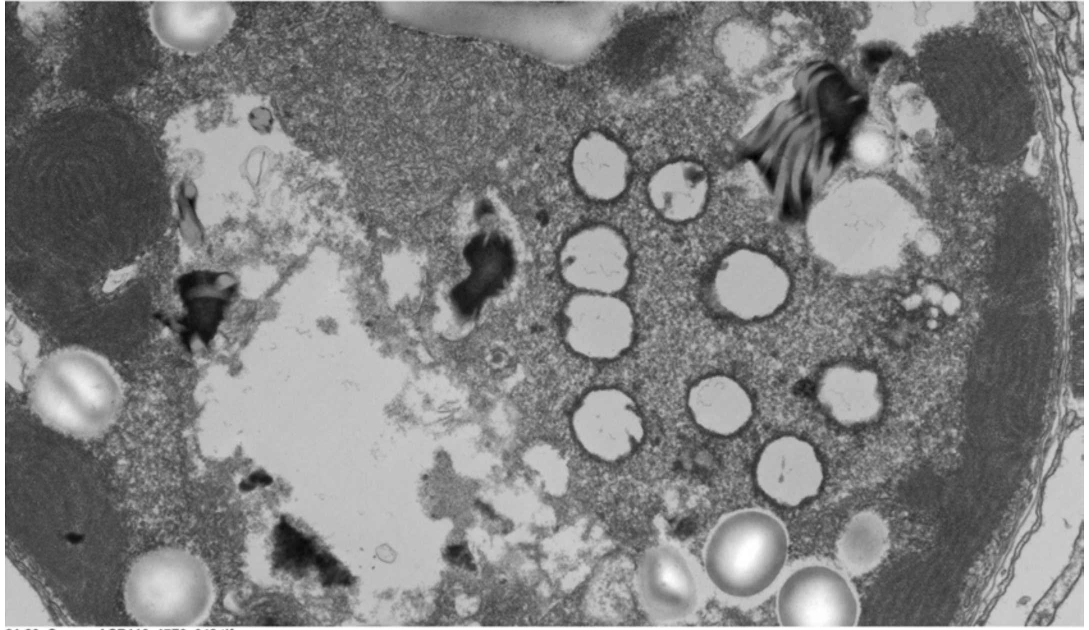

21-20_Correa_ACR116_17F3_042.tif
ARC 116
Biological Electron Microscopy Lab
Rice University - SEA
Microscopist: MD Meyer

1 µm
HV=80kV
Direct Mag: 4000 x

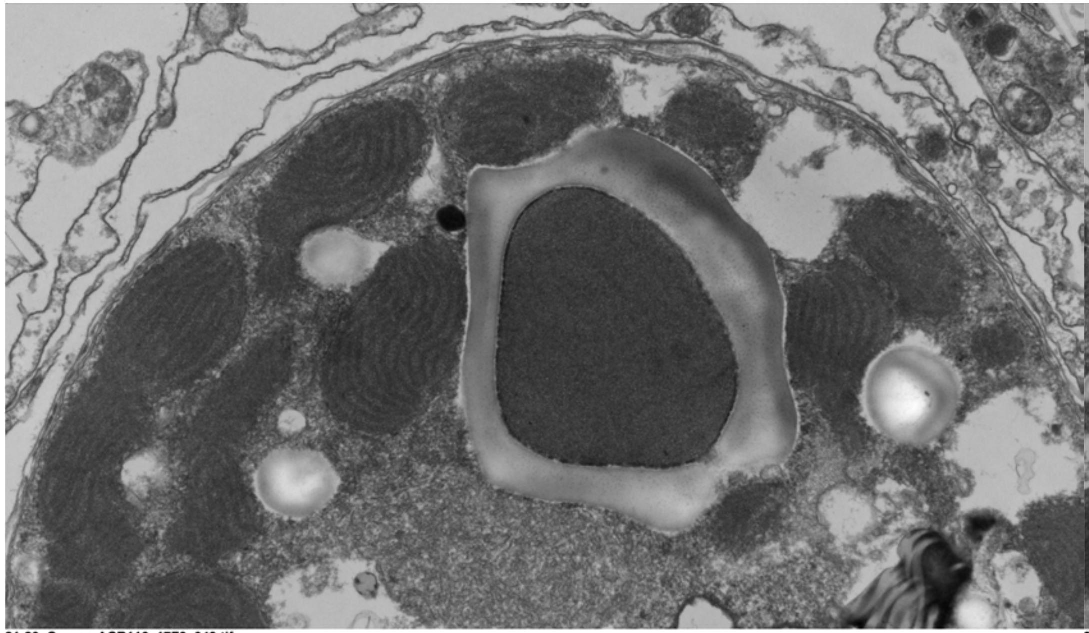

21-20_Correa_ACR116_17F3_043.tif
ARC 116
Biological Electron Microscopy Lab
Rice University - SEA
Microscopist: MD Meyer

1 µm
HV=80kV
Direct Mag: 4000 x

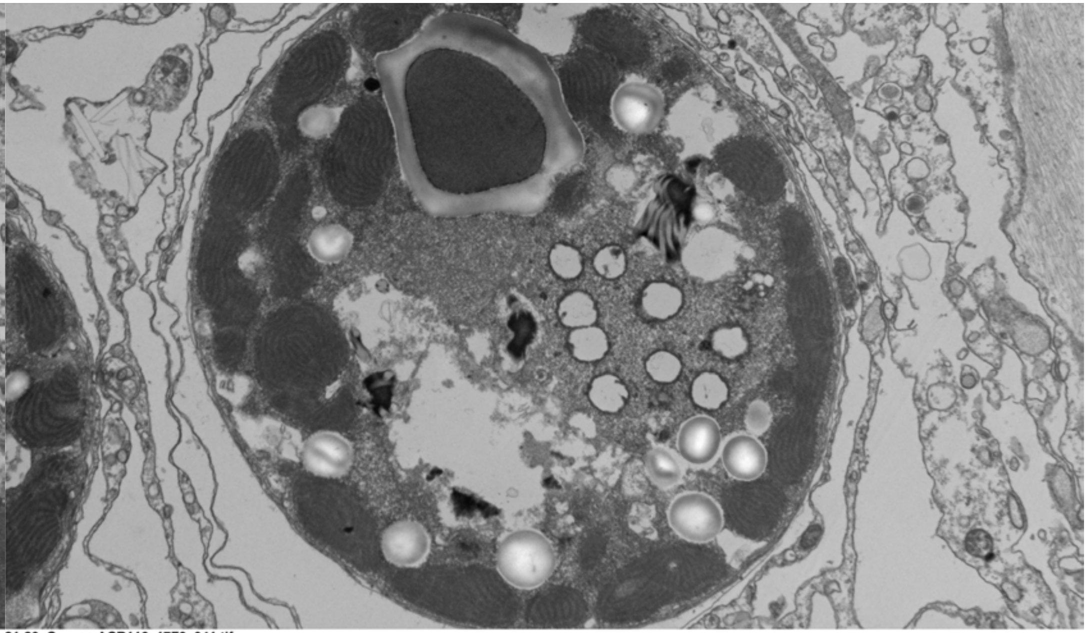

21-20_Correa_ACR116_17F3_041.tif
ARC 116
Biological Electron Microscopy Lab
Rice University - SEA
Microscopist: MD Meyer

2 µm
HV=80kV
Direct Mag: 2000 x

Cell 16

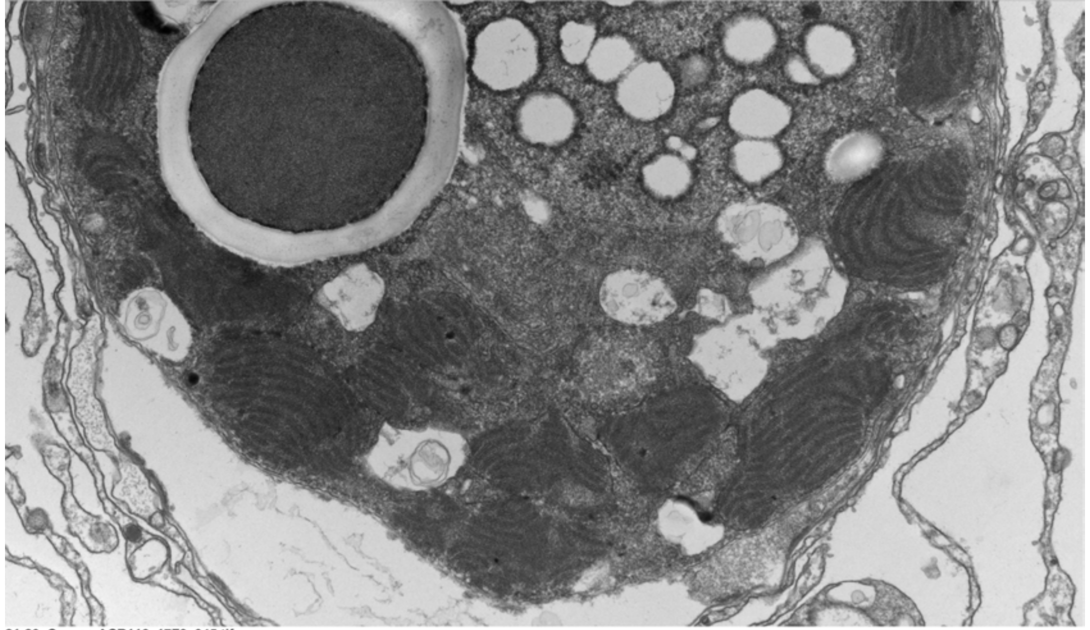

21-20_Correa_ACR116_17F3_045.tif
ARC 116
Biological Electron Microscopy Lab
Rice University - SEA
Microscopist: MD Meyer

1 µm
HV=80kV
Direct Mag: 4000 x

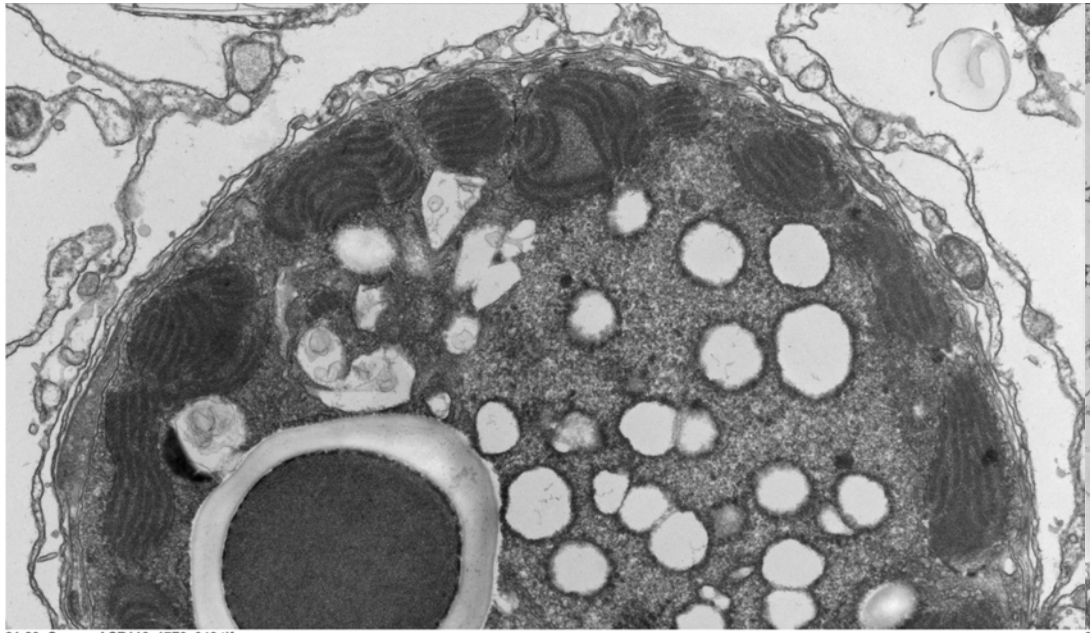

21-20_Correa_ACR116_17F3_046.tif
ARC 116
Biological Electron Microscopy Lab
Rice University - SEA
Microscopist: MD Meyer

1 µm
HV=80kV
Direct Mag: 4000 x

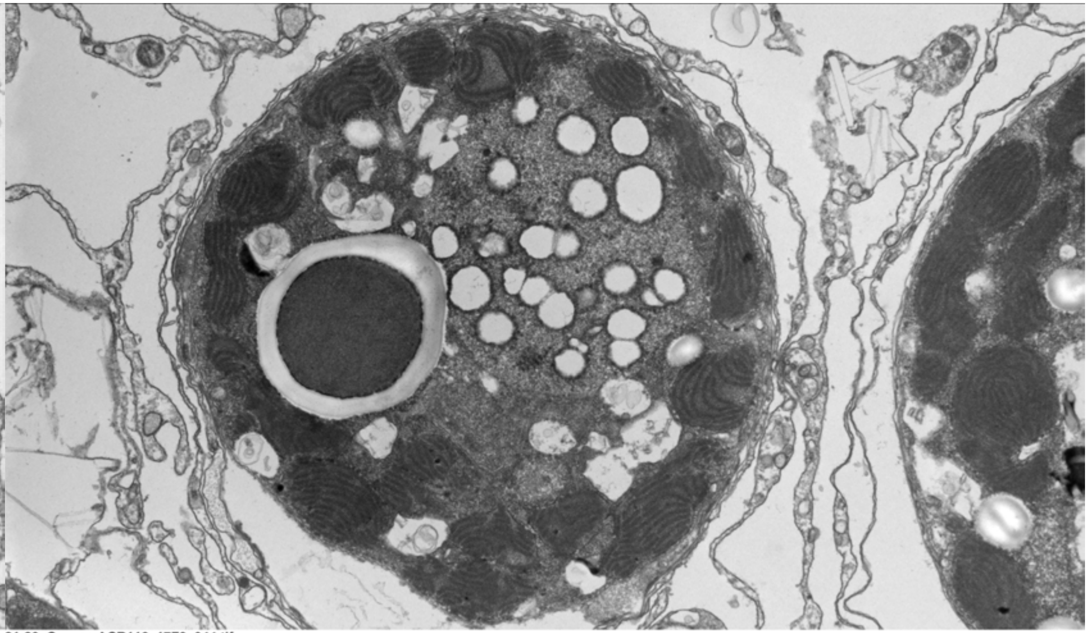

21-20_Correa_ACR116_17F3_044.tif
ARC 116
Biological Electron Microscopy Lab
Rice University - SEA
Microscopist: MD Meyer

1 µm
HV=80kV
Direct Mag: 2500 x

Cell 17

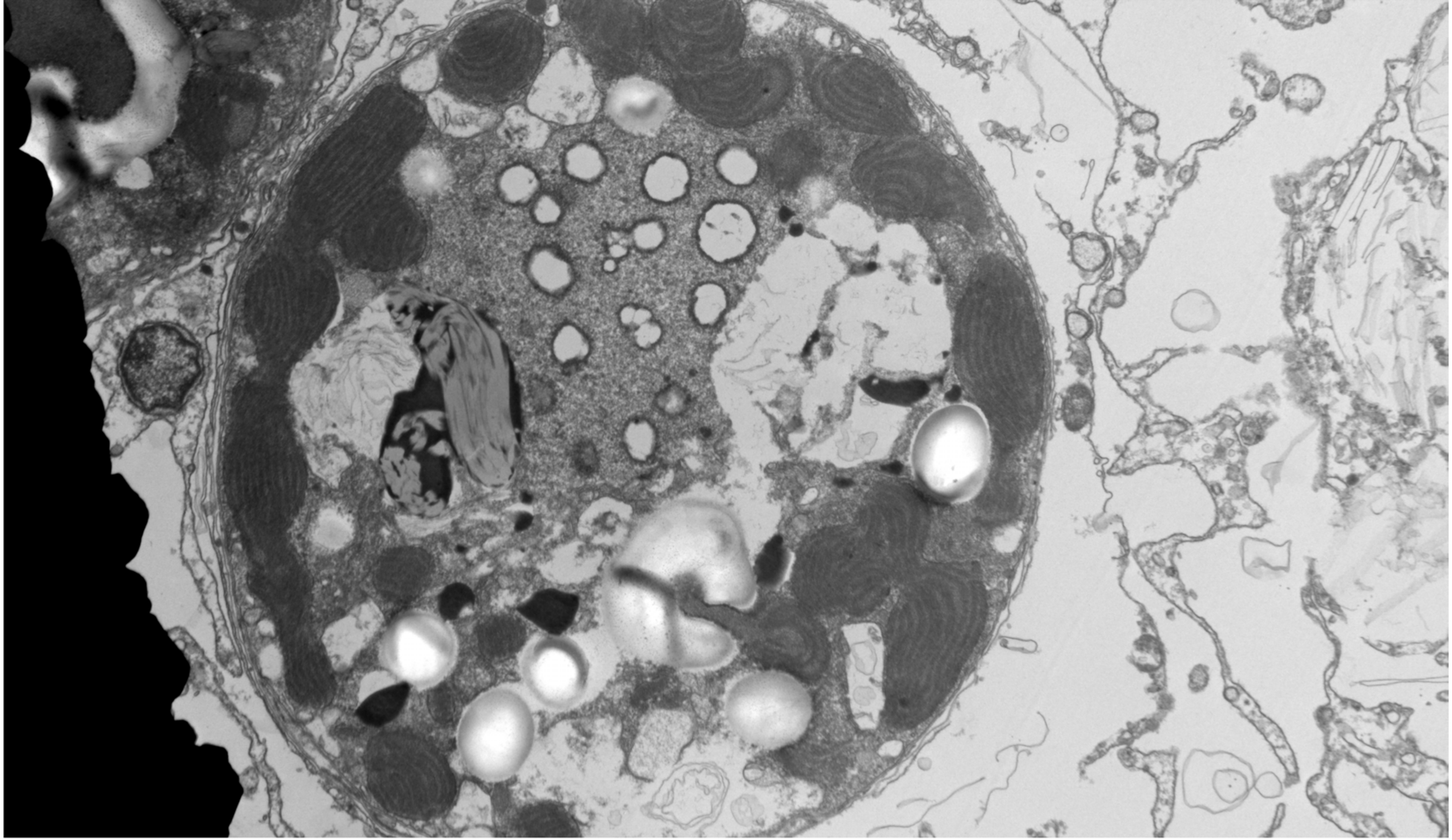

21-20_Correa_ACR116_17F3_047.tif
ARC 116
Biological Electron Microscopy Lab
Rice University - SEA
Microscopist: MD Meyer

HV=80kV
Direct Mag: 2000 x

Cell 18

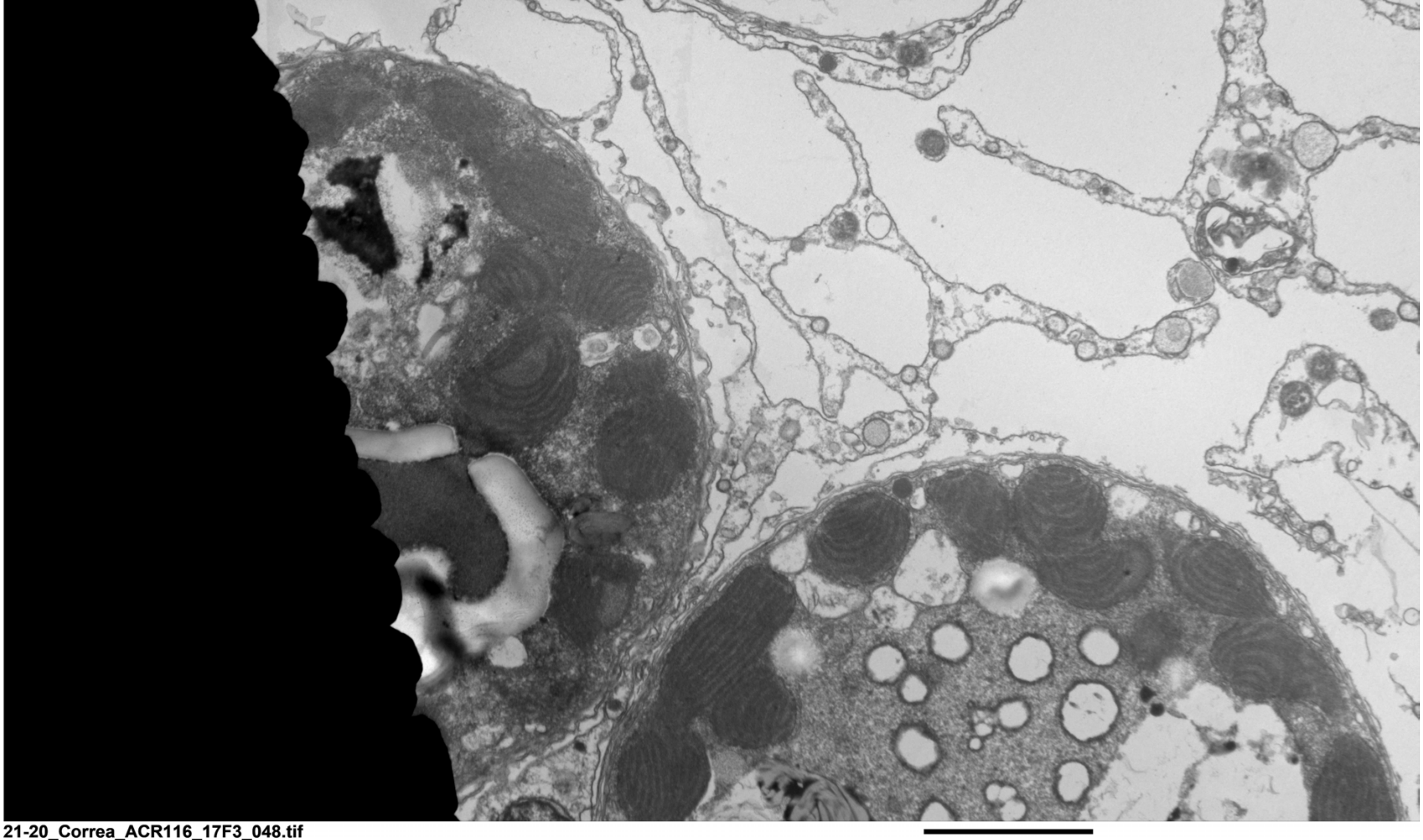

21-20_Correa_ACR116_17F3_048.tif
ARC 116
Biological Electron Microscopy Lab
Rice University - SEA
Microscopist: MD Meyer

2 µm
HV=80kV
Direct Mag: 2000 x

Cell 19

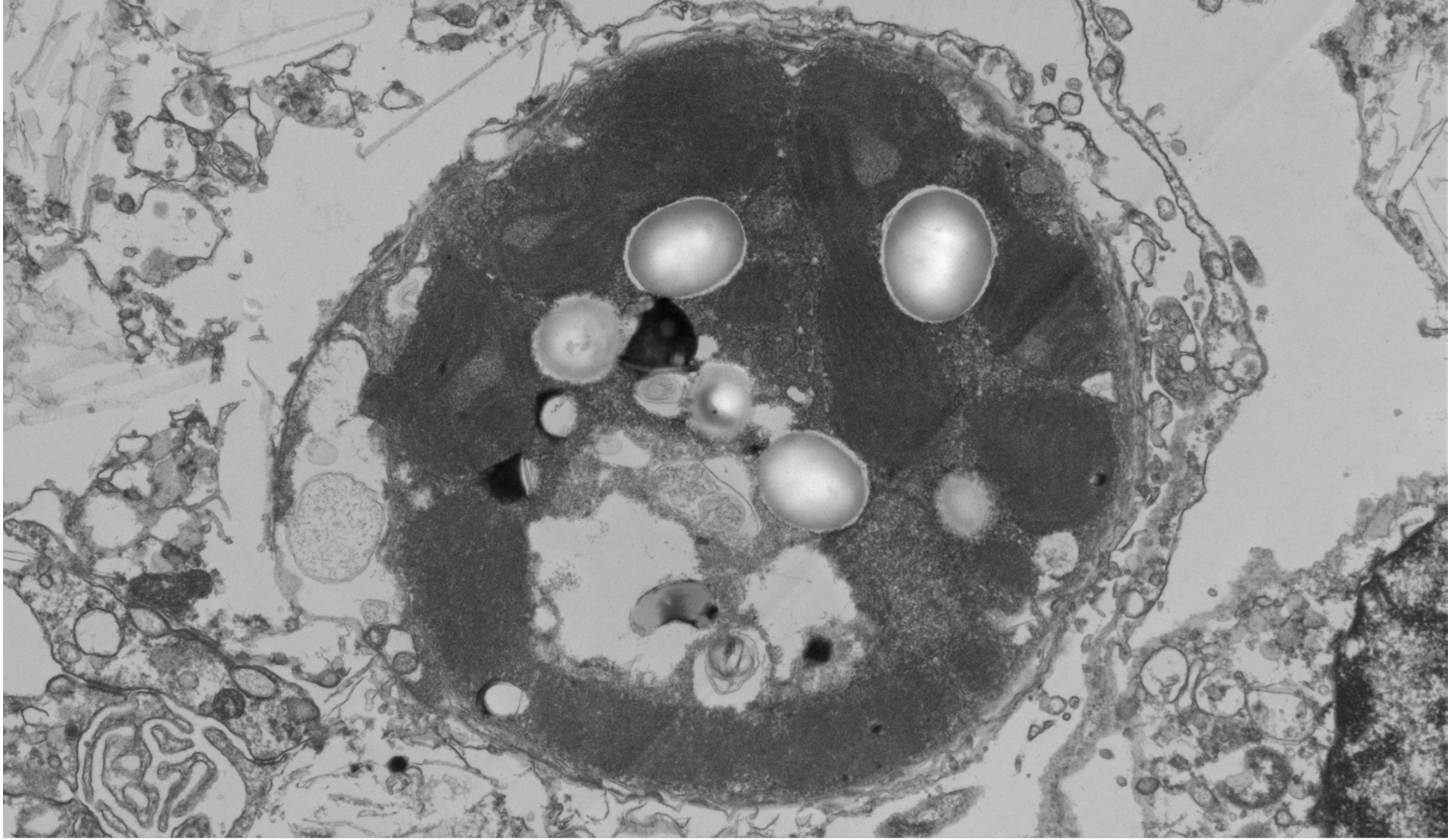

21-20_Correa_ACR116_17F3_050.tif
ARC 116
Biological Electron Microscopy Lab
Rice University - SEA
Microscopist: MD Meyer

HV=80kV
Direct Mag: 3000 x

Cell 20

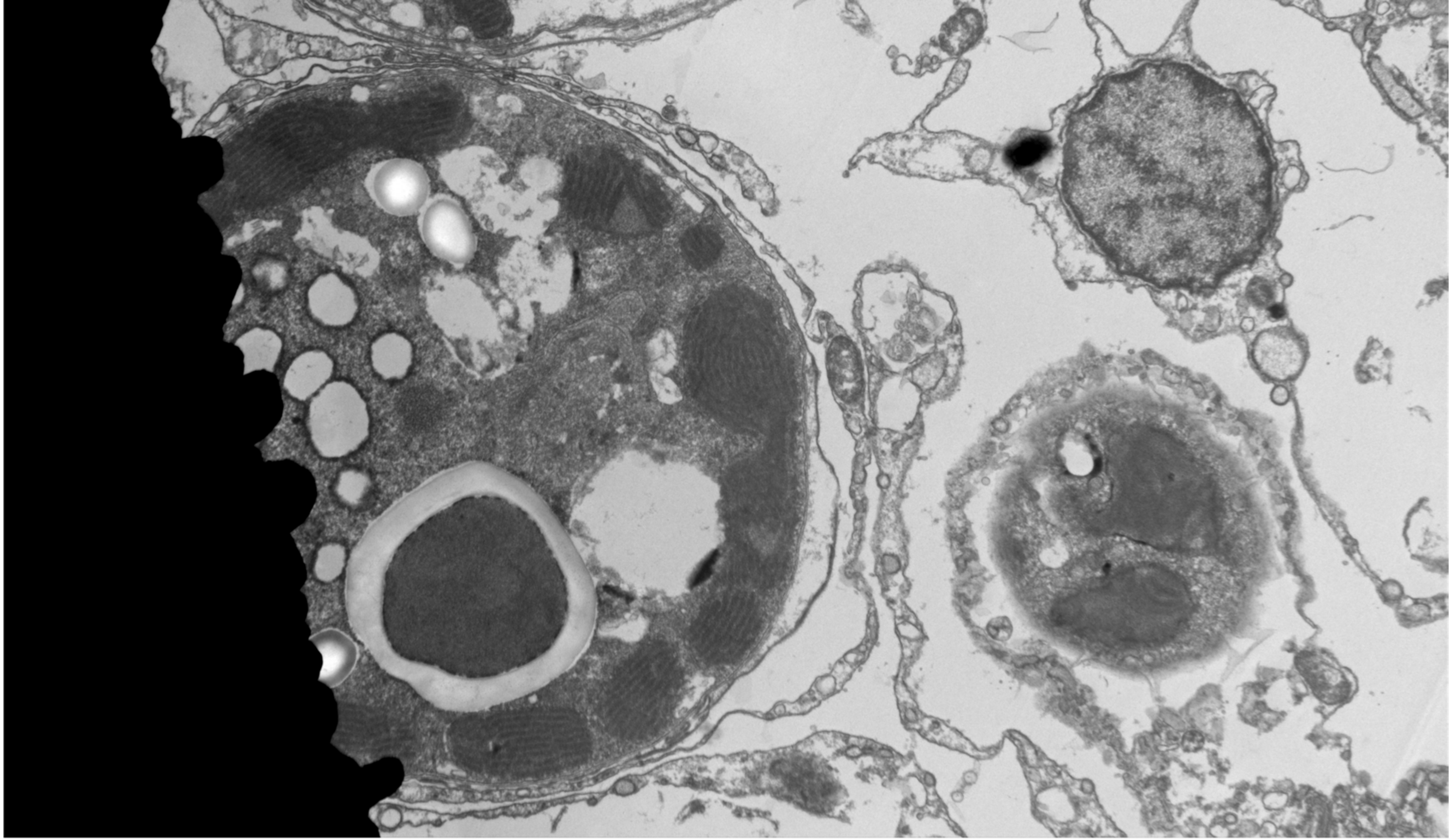

21-20_Correa_ACR116_17F3_051.tif
ARC 116
Biological Electron Microscopy Lab
Rice University - SEA
Microscopist: MD Meyer

2 µm
HV=80kV
Direct Mag: 2000 x

Cell 21

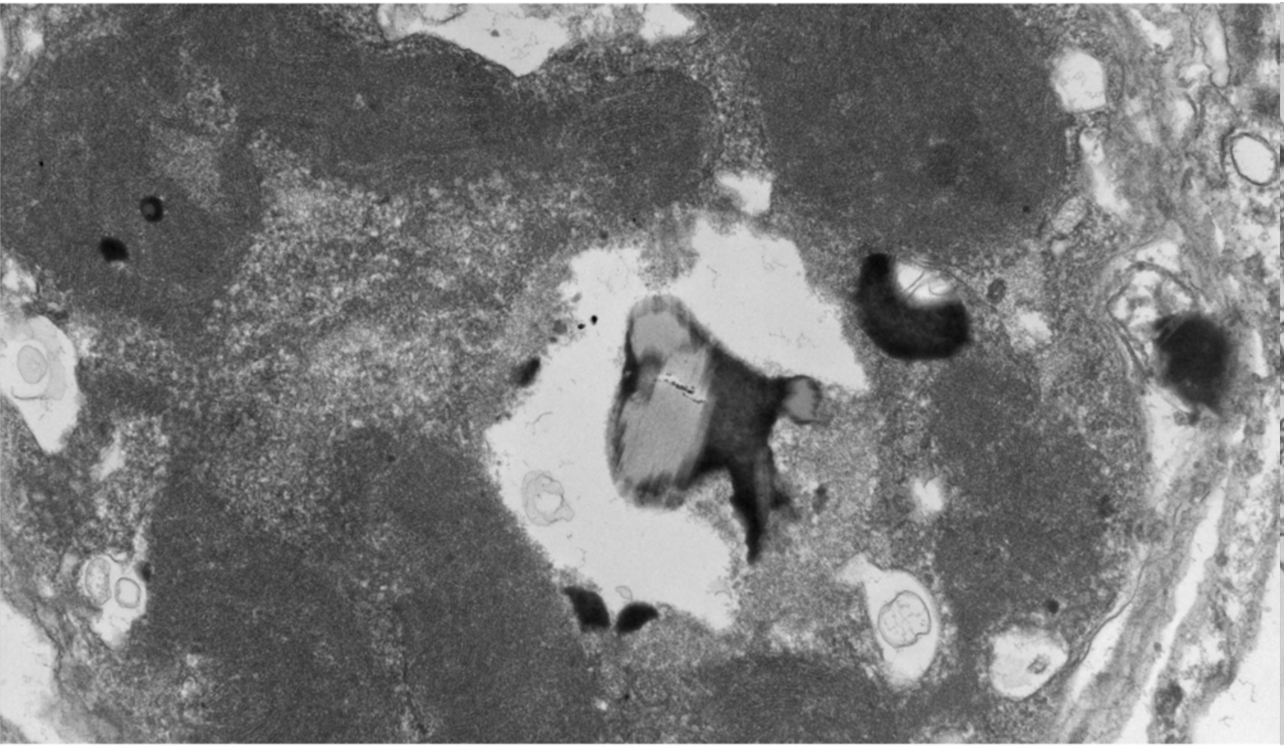

21-20_Correa_ACR116_17F3_053.tif
ARC 116
Biological Electron Microscopy Lab
Rice University - SEA
Microscopist: MD Meyer

800 nm
HV=80kV
Direct Mag: 5000 x

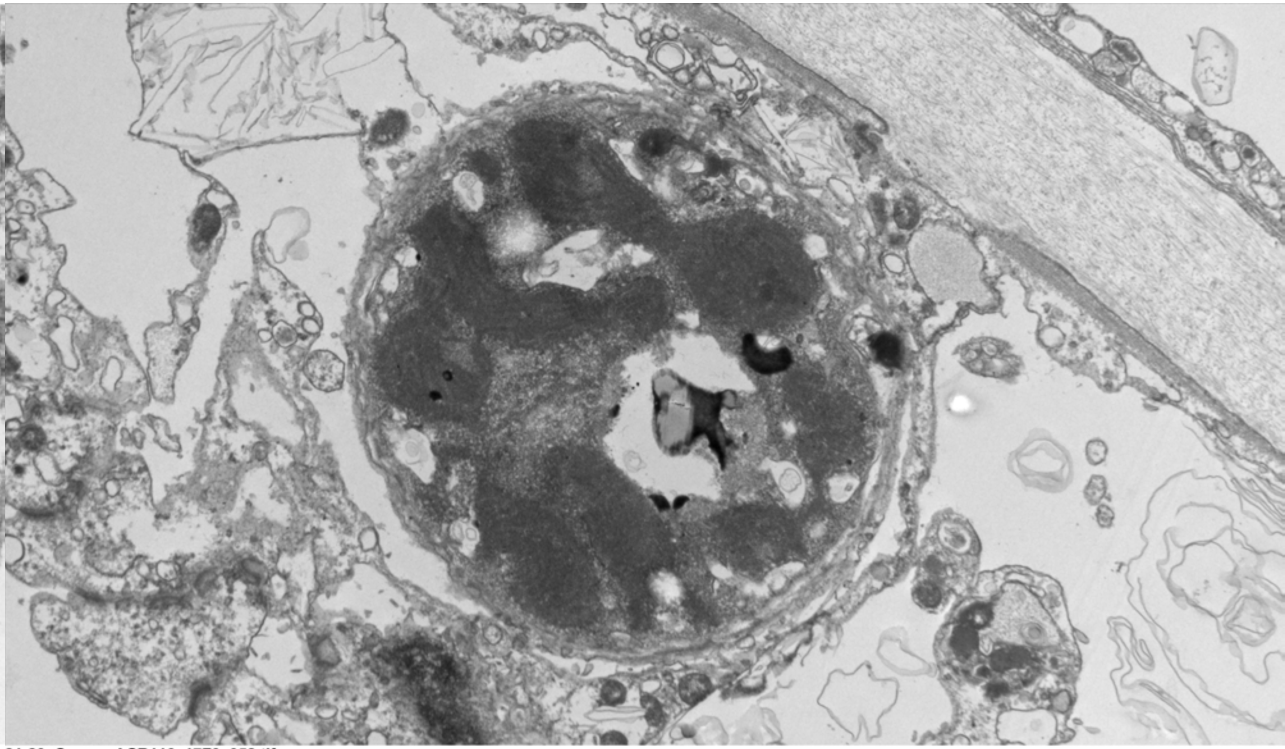

21-20_Correa_ACR116_17F3_052.tif
ARC 116
Biological Electron Microscopy Lab
Rice University - SEA
Microscopist: MD Meyer

2 µm
HV=80kV
Direct Mag: 2000 x

Cell 22

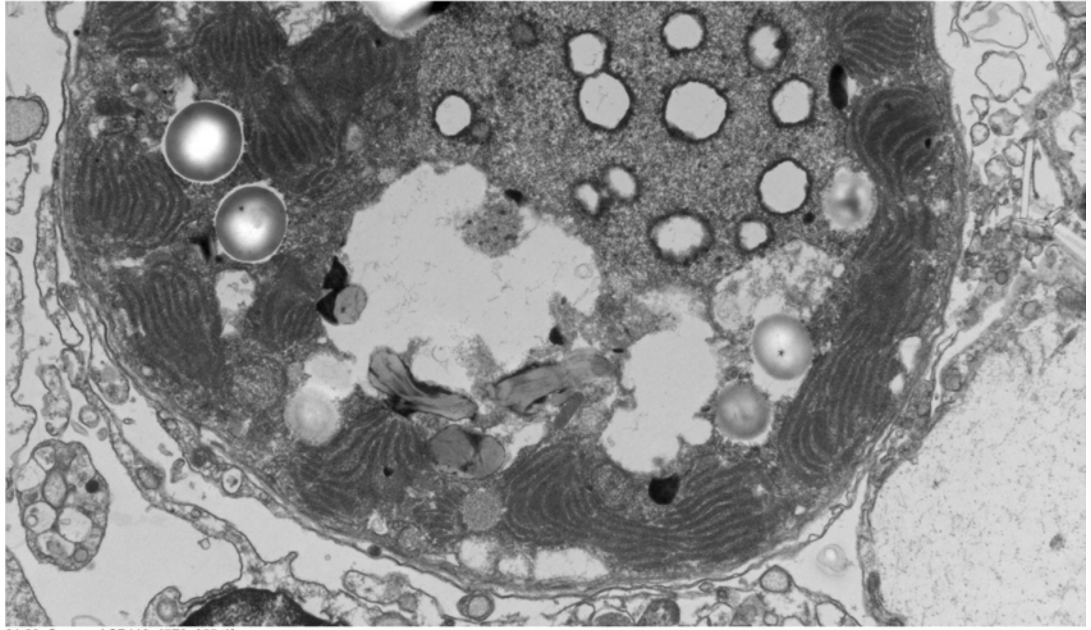

21-20_Correa_ACR116_17F3_055.tif
ARC 116
Biological Electron Microscopy Lab
Rice University - SEA
Microscopist: MD Meyer

1 µm
HV=80kV
Direct Mag: 3000 x

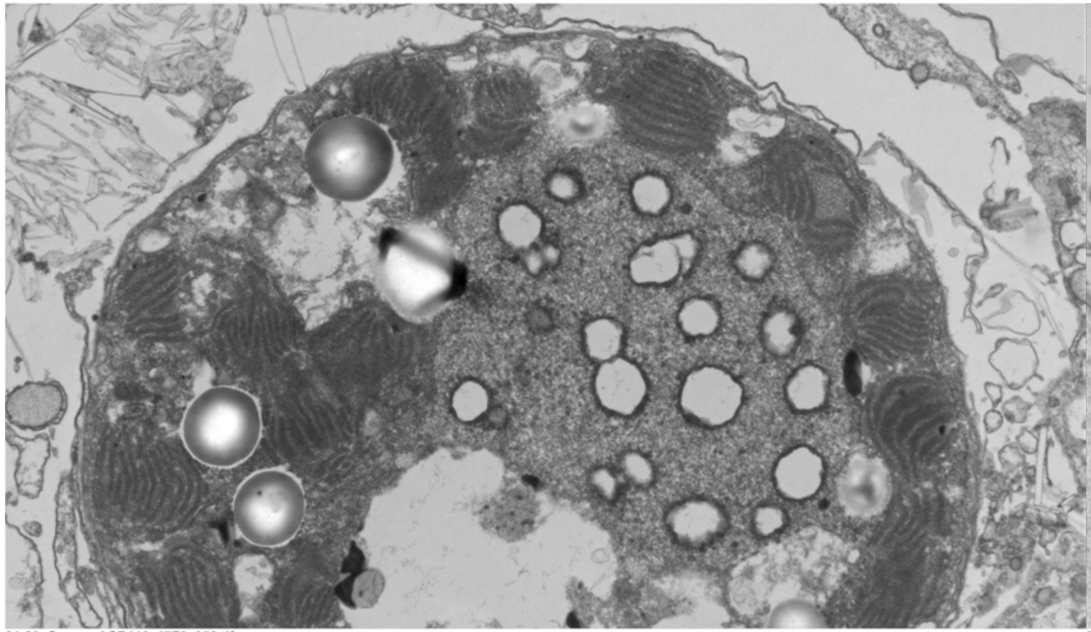

21-20_Correa_ACR116_17F3_056.tif
ARC 116
Biological Electron Microscopy Lab
Rice University - SEA
Microscopist: MD Meyer

1 µm
HV=80kV
Direct Mag: 3000 x

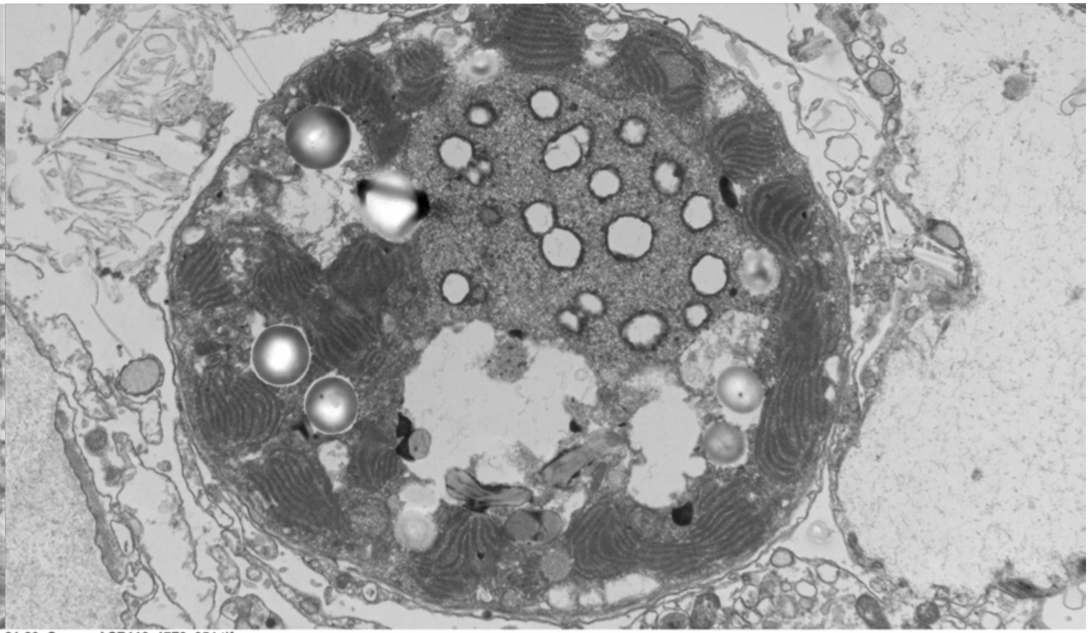

21-20_Correa_ACR116_17F3_054.tif
ARC 116
Biological Electron Microscopy Lab
Rice University - SEA
Microscopist: MD Meyer

2 µm
HV=80kV
Direct Mag: 2000 x

Cell 23

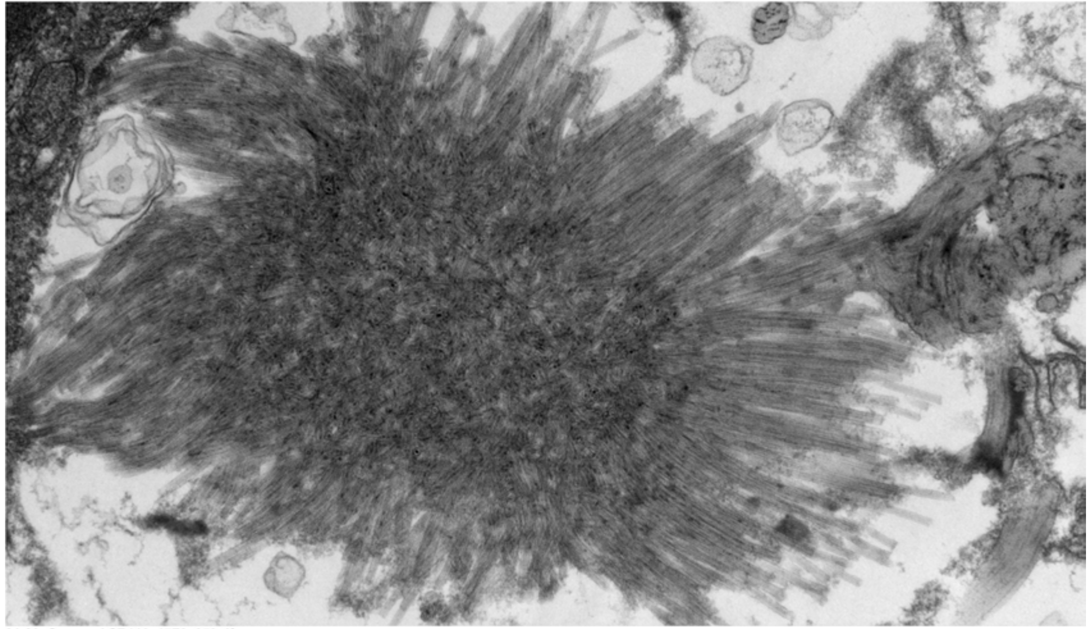

21-20_Correa_ACR116_17F3_059.tif
ARC 116
Biological Electron Microscopy Lab
Rice University - SEA
Microscopist: MD Meyer

400 nm
HV=80kV
Direct Mag: 10000 x

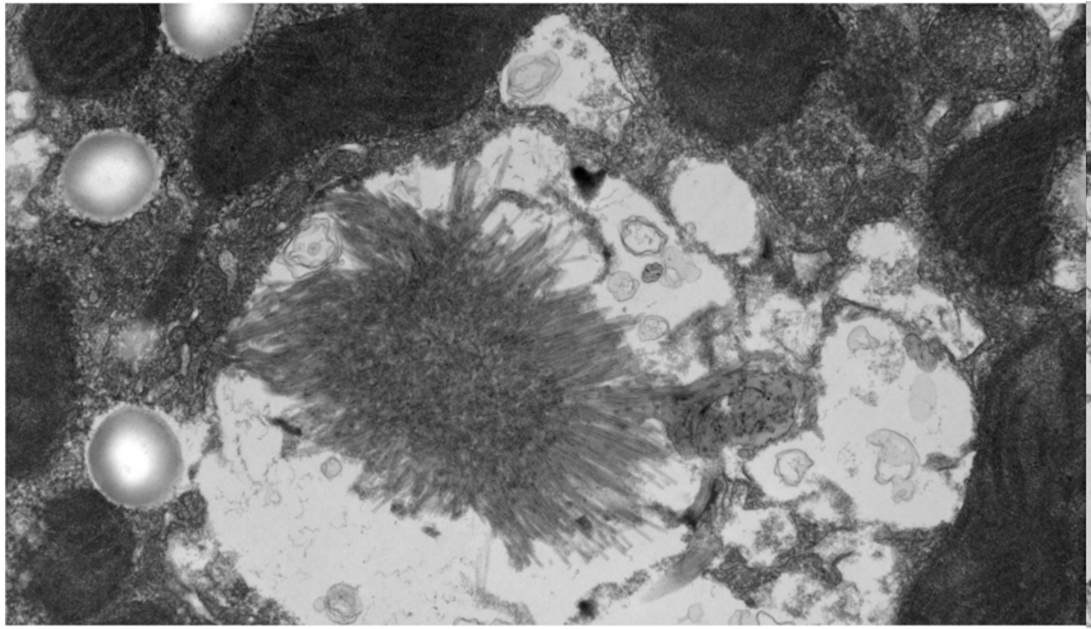

21-20_Correa_ACR116_17F3_058.tif
ARC 116
Biological Electron Microscopy Lab
Rice University - SEA
Microscopist: MD Meyer

800 nm
HV=80kV
Direct Mag: 5000 x

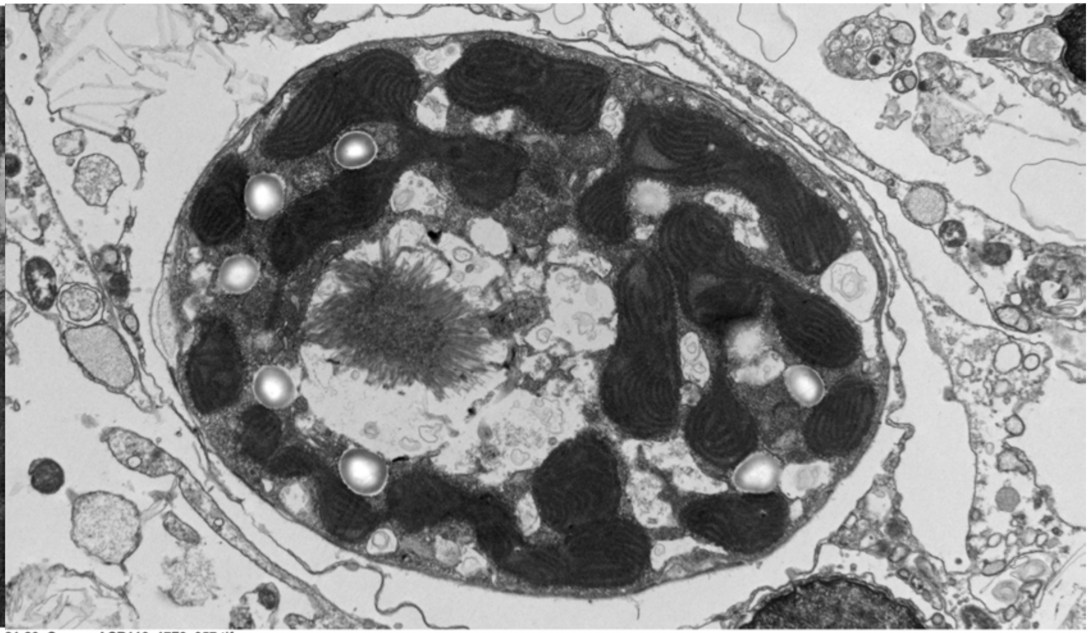

21-20_Correa_ACR116_17F3_057.tif
ARC 116
Biological Electron Microscopy Lab
Rice University - SEA
Microscopist: MD Meyer

2 µm
HV=80kV
Direct Mag: 2000 x

Cell 24

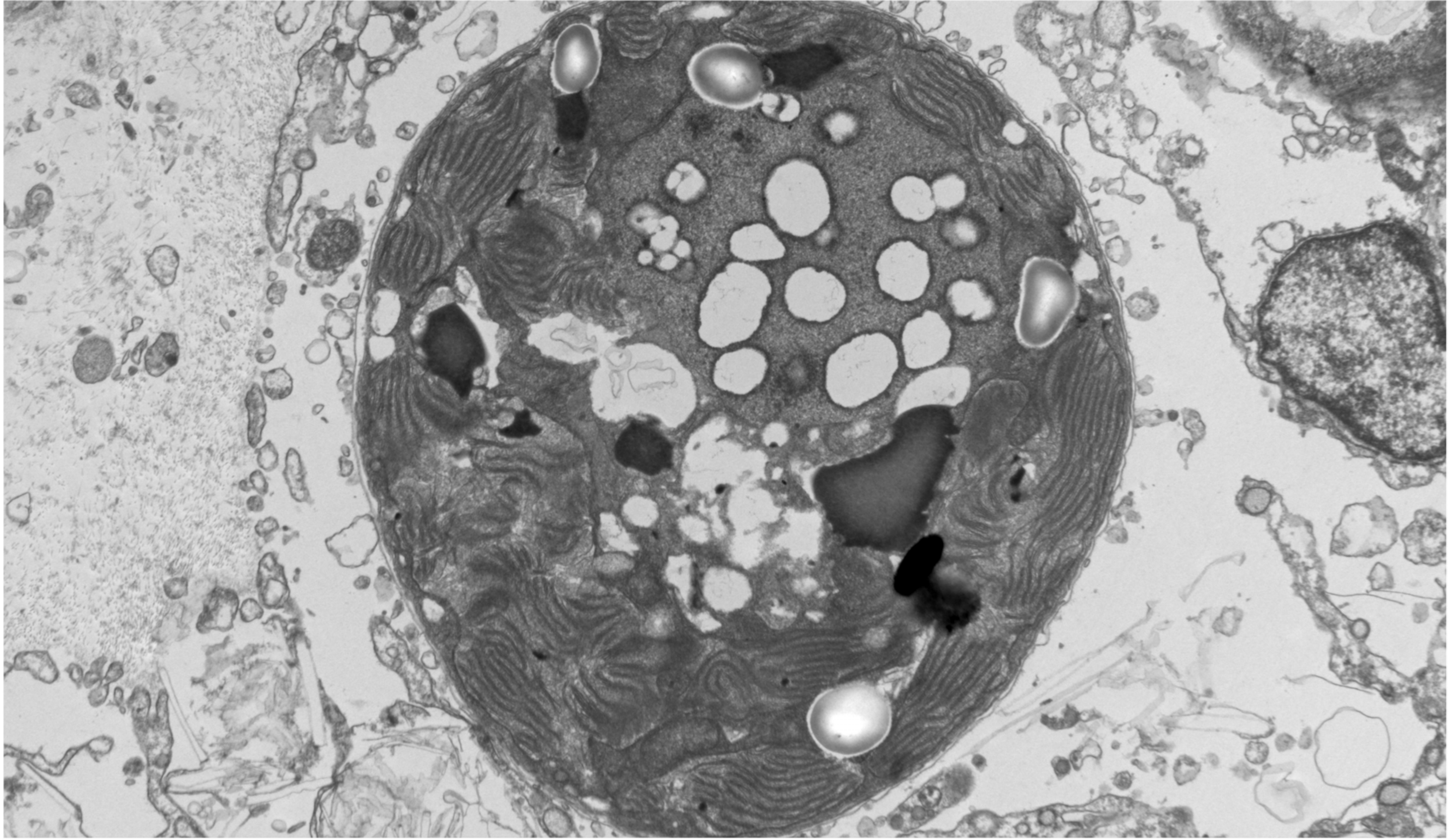

21-20_Correa_ACR116_17F3_060.tif
ARC 116
Biological Electron Microscopy Lab
Rice University - SEA
Microscopist: MD Meyer

2 µm
HV=80kV
Direct Mag: 2000 x

ACR Colony G

Cell 1

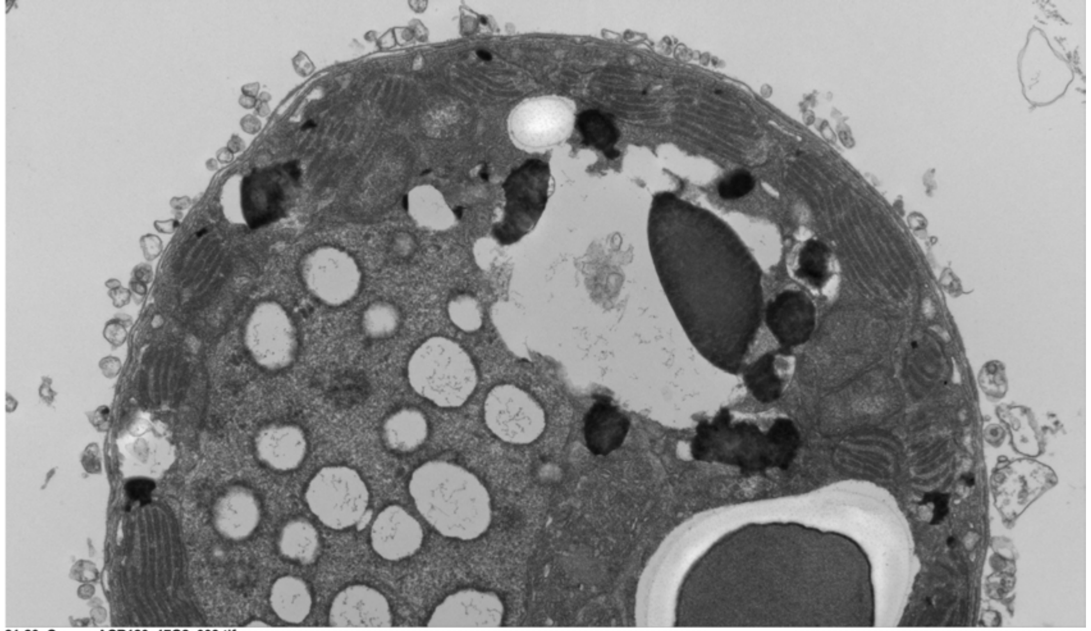

21-20_Correa_ACR120_17G2_003.tif
ACR 120
Biological Electron Microscopy Lab
Rice University - SEA
Microscopist: MD Meyer

1 µm
HV=80kV
Direct Mag: 3000 x

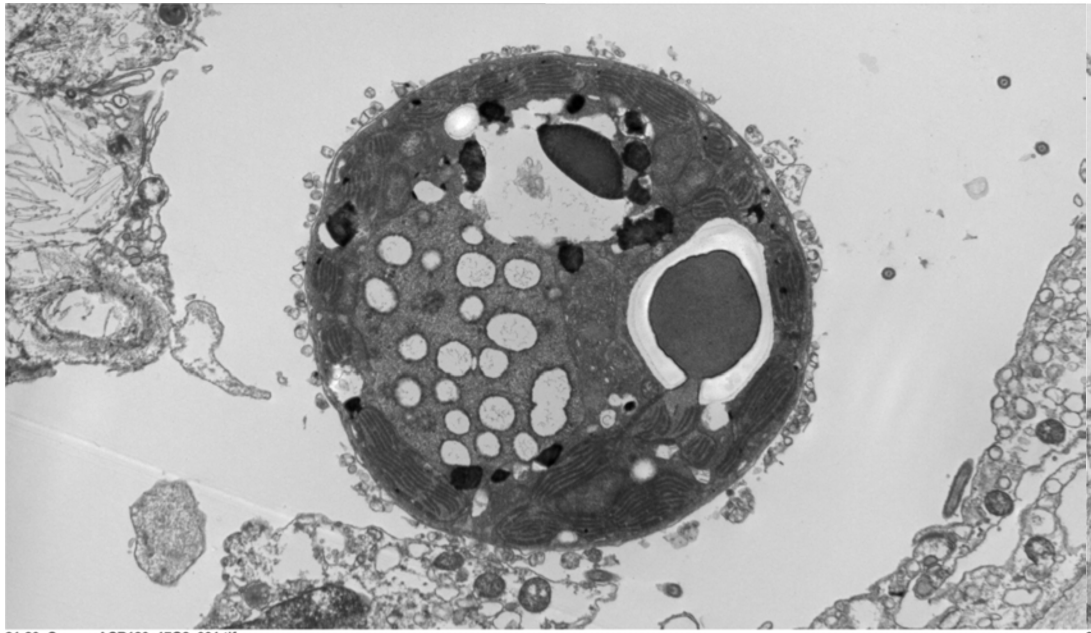

21-20_Correa_ACR120_17G2_001.tif
ACR 120
Biological Electron Microscopy Lab
Rice University - SEA
Microscopist: MD Meyer

2 µm
HV=80kV
Direct Mag: 1500 x

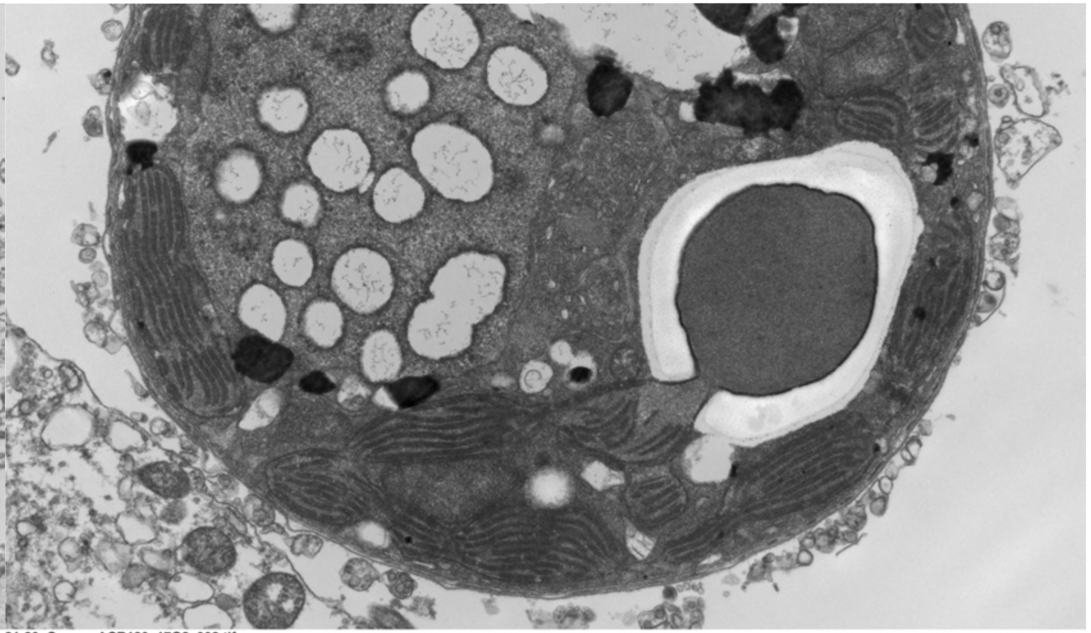

21-20_Correa_ACR120_17G2_002.tif
ACR 120
Biological Electron Microscopy Lab
Rice University - SEA
Microscopist: MD Meyer

1 µm
HV=80kV
Direct Mag: 3000 x

Cell 2

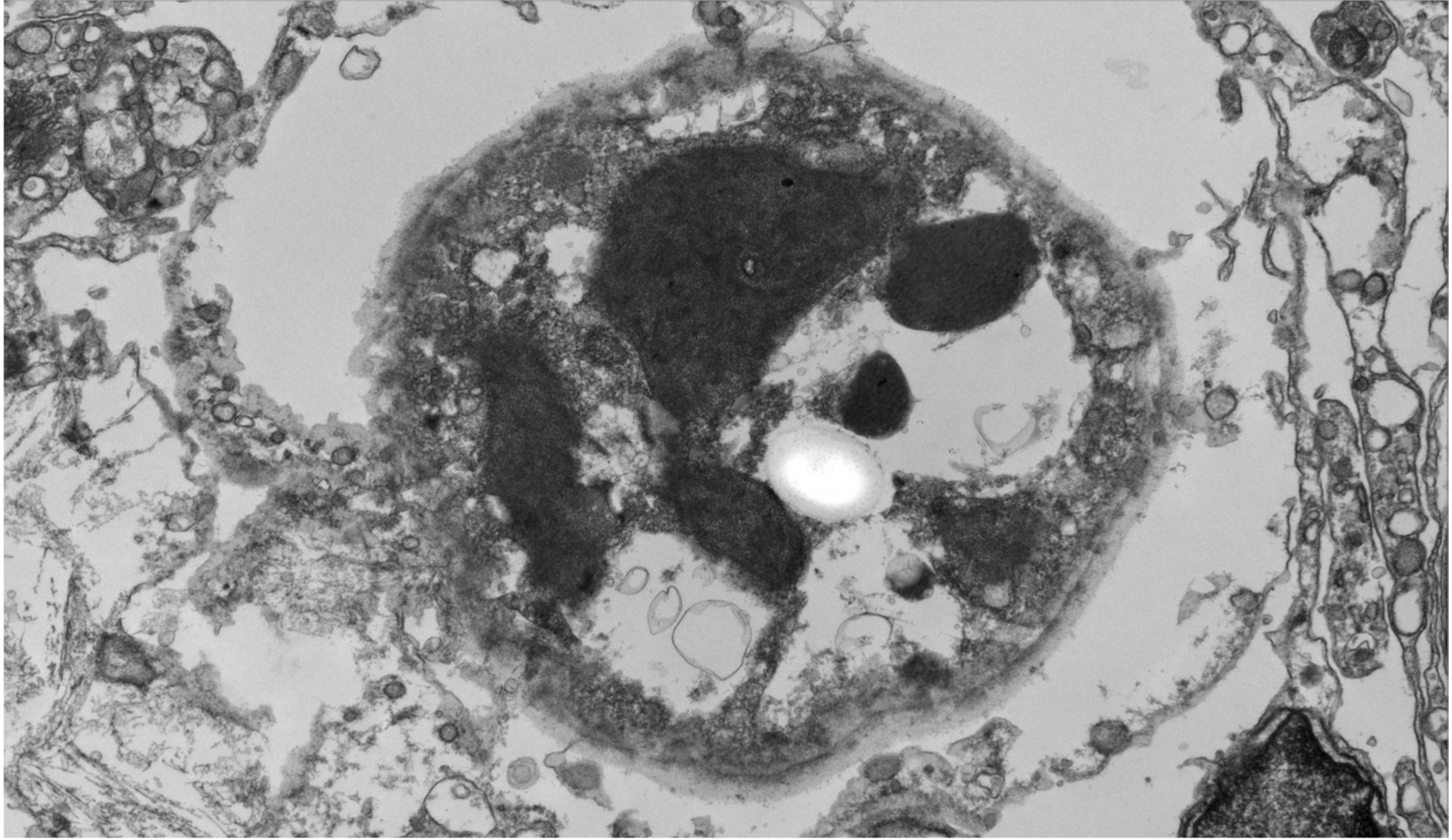

21-20_Correa_ACR120_17G2_004.tif
ACR 120
Biological Electron Microscopy Lab
Rice University - SEA
Microscopist: MD Meyer

1 µm
HV=80kV
Direct Mag: 3000 x

Cell 3

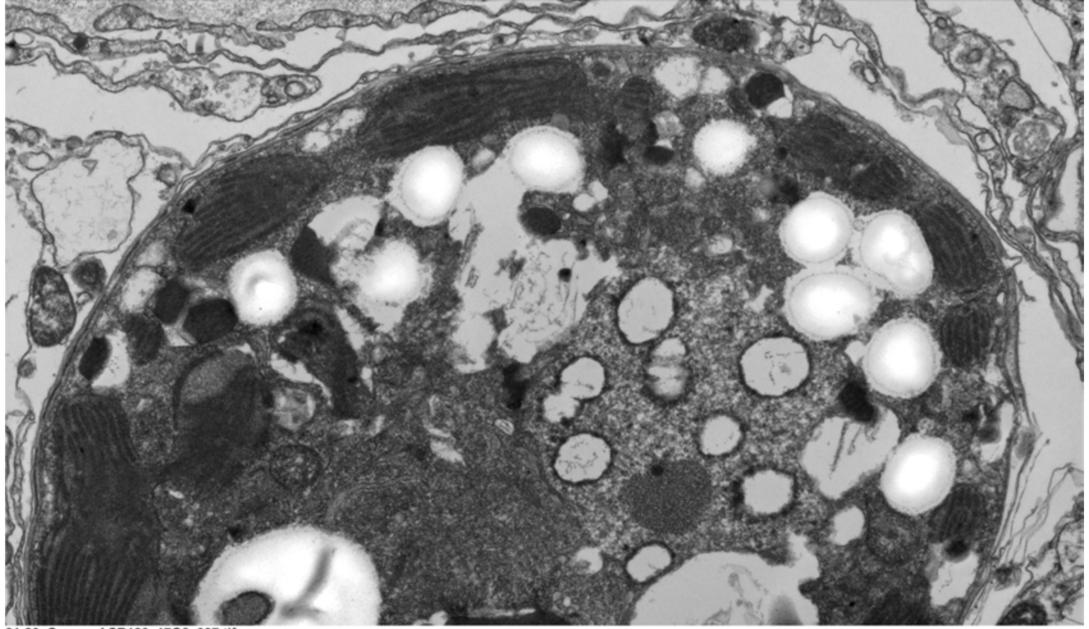

21-20_Correa_ACR120_17G2_007.tif
ACR 120
Biological Electron Microscopy Lab
Rice University - SEA
Microscopist: MD Meyer

1 µm
HV=80kV
Direct Mag: 3000 x

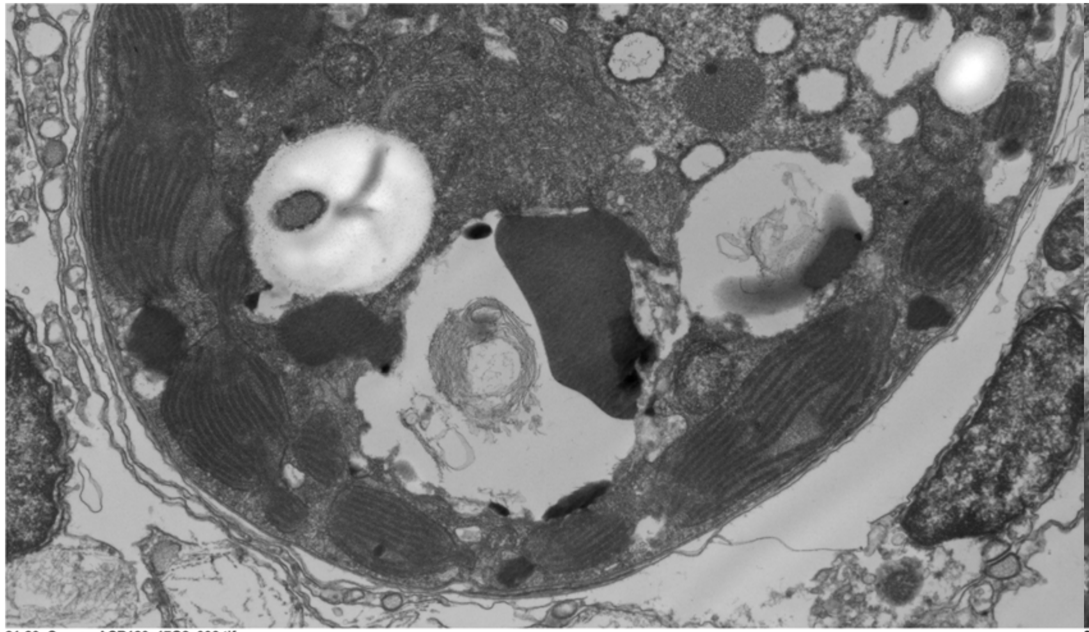

21-20_Correa_ACR120_17G2_006.tif
ACR 120
Biological Electron Microscopy Lab
Rice University - SEA
Microscopist: MD Meyer

1 µm
HV=80kV
Direct Mag: 3000 x

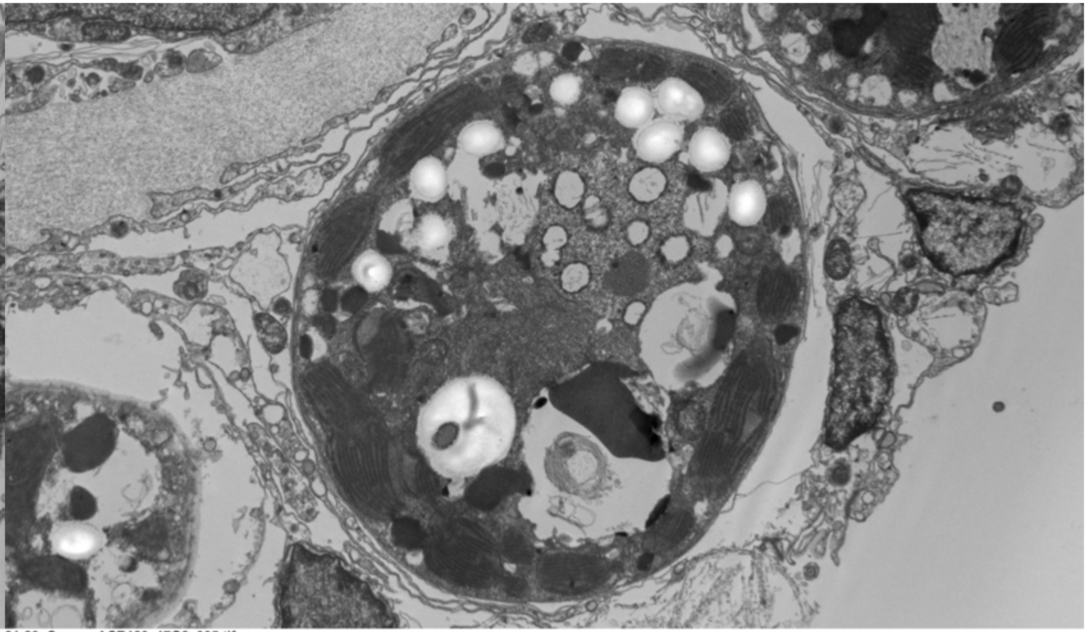

21-20_Correa_ACR120_17G2_005.tif
ACR 120
Biological Electron Microscopy Lab
Rice University - SEA
Microscopist: MD Meyer

2 µm
HV=80kV
Direct Mag: 1500 x

Cell 4

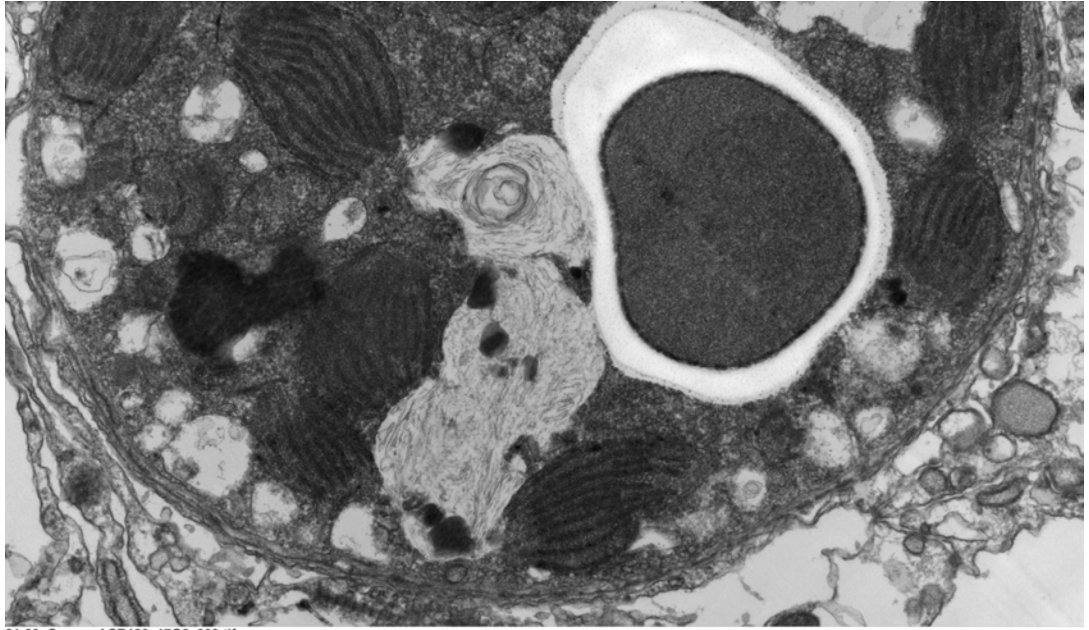

21-20_Correa_ACR120_17G2_009.tif
ACR 120
Biological Electron Microscopy Lab
Rice University - SEA
Microscopist: MD Meyer

1 µm
HV=80kV
Direct Mag: 4000 x

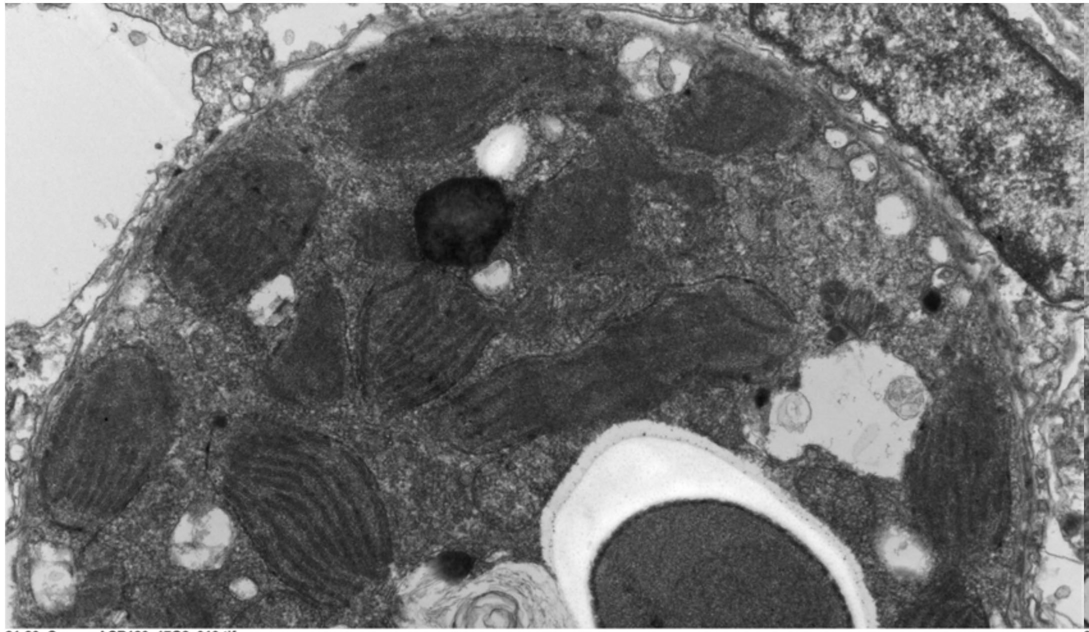

21-20_Correa_ACR120_17G2_010.tif
ACR 120
Biological Electron Microscopy Lab
Rice University - SEA
Microscopist: MD Meyer

1 µm
HV=80kV
Direct Mag: 4000 x

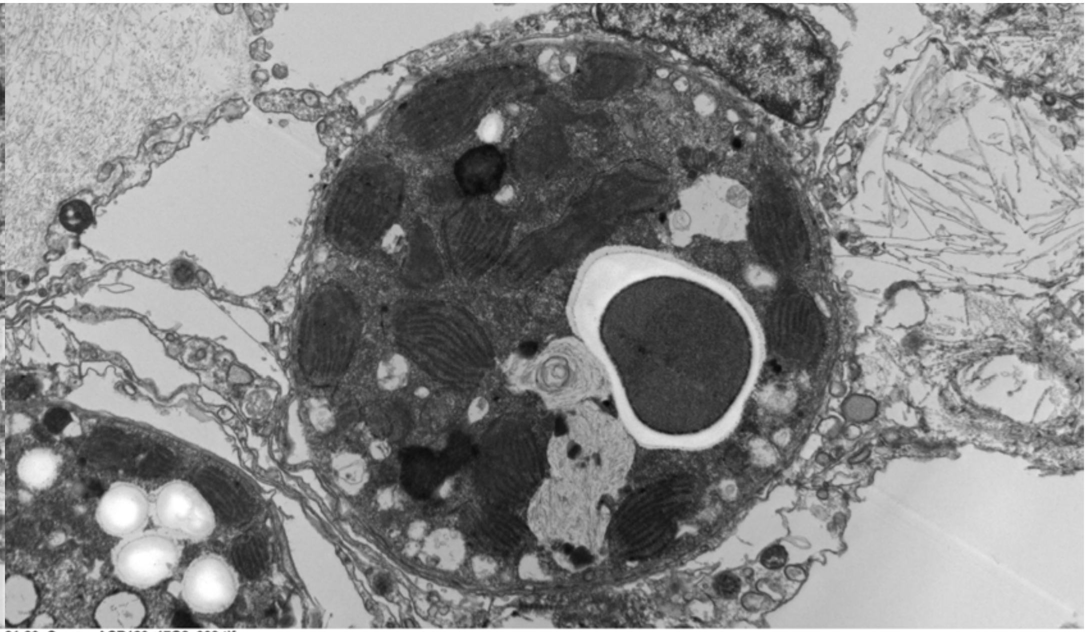

21-20_Correa_ACR120_17G2_008.tif
ACR 120
Biological Electron Microscopy Lab
Rice University - SEA
Microscopist: MD Meyer

2 µm
HV=80kV
Direct Mag: 2000 x

Cell 5

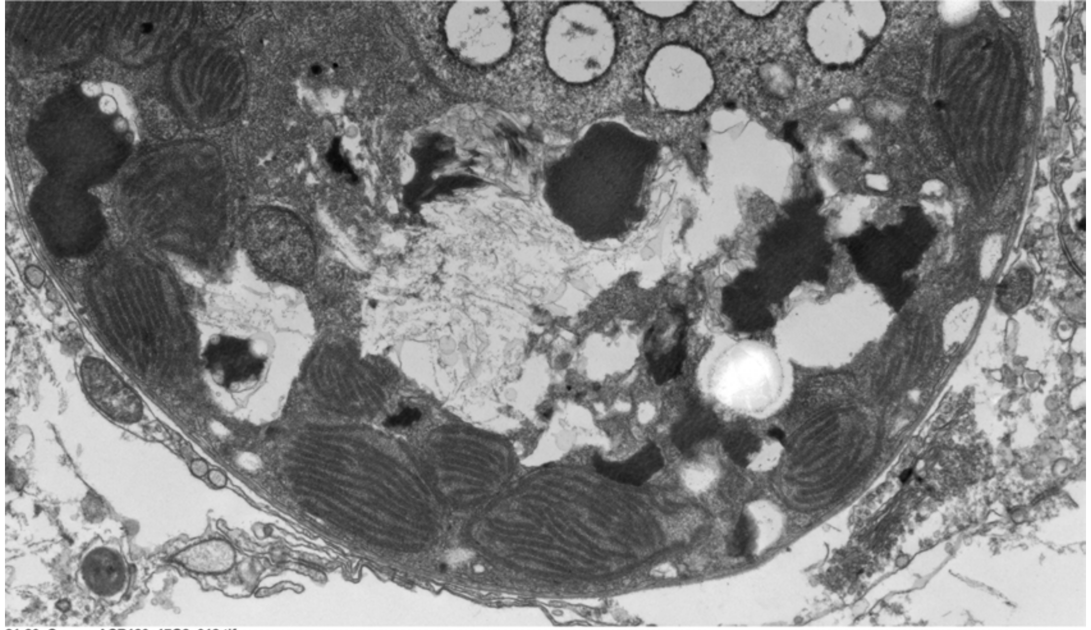

21-20_Correa_ACR120_17G2_012.tif
ACR 120
Biological Electron Microscopy Lab
Rice University - SEA
Microscopist: MD Meyer

1 µm
HV=80kV
Direct Mag: 3000 x

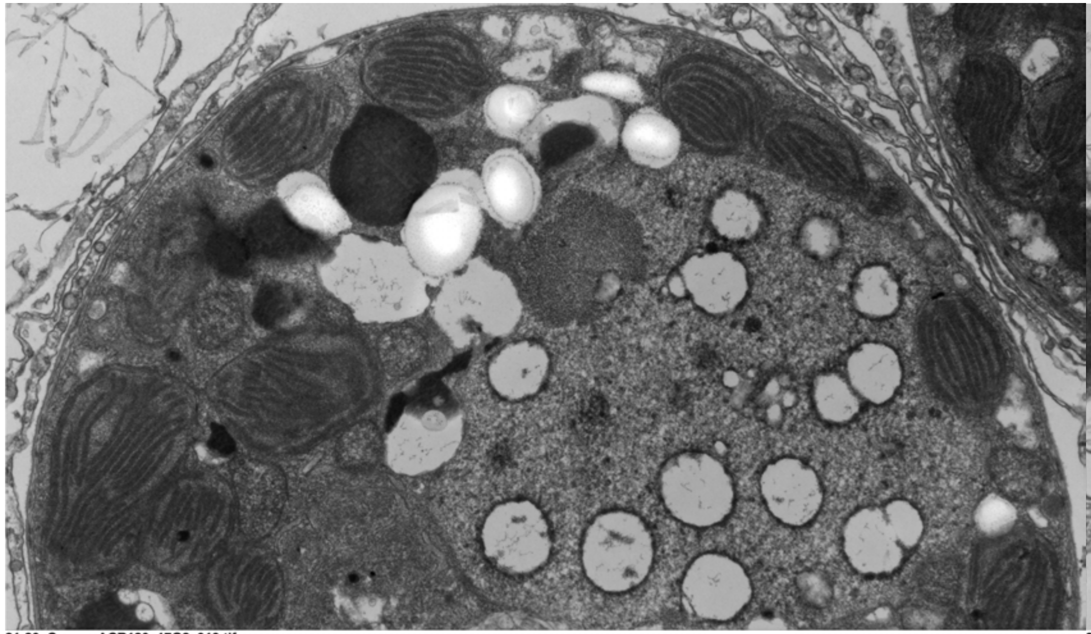

21-20_Correa_ACR120_17G2_013.tif
ACR 120
Biological Electron Microscopy Lab
Rice University - SEA
Microscopist: MD Meyer

1 µm
HV=80kV
Direct Mag: 3000 x

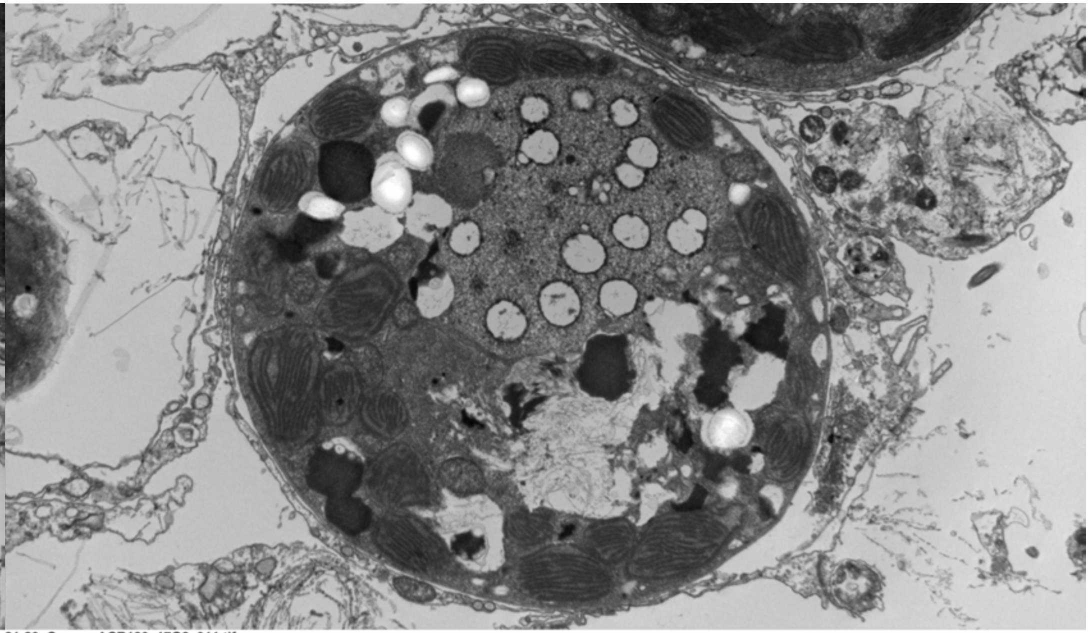

21-20_Correa_ACR120_17G2_011.tif
ACR 120
Biological Electron Microscopy Lab
Rice University - SEA
Microscopist: MD Meyer

2 µm
HV=80kV
Direct Mag: 1500 x

Cell 6

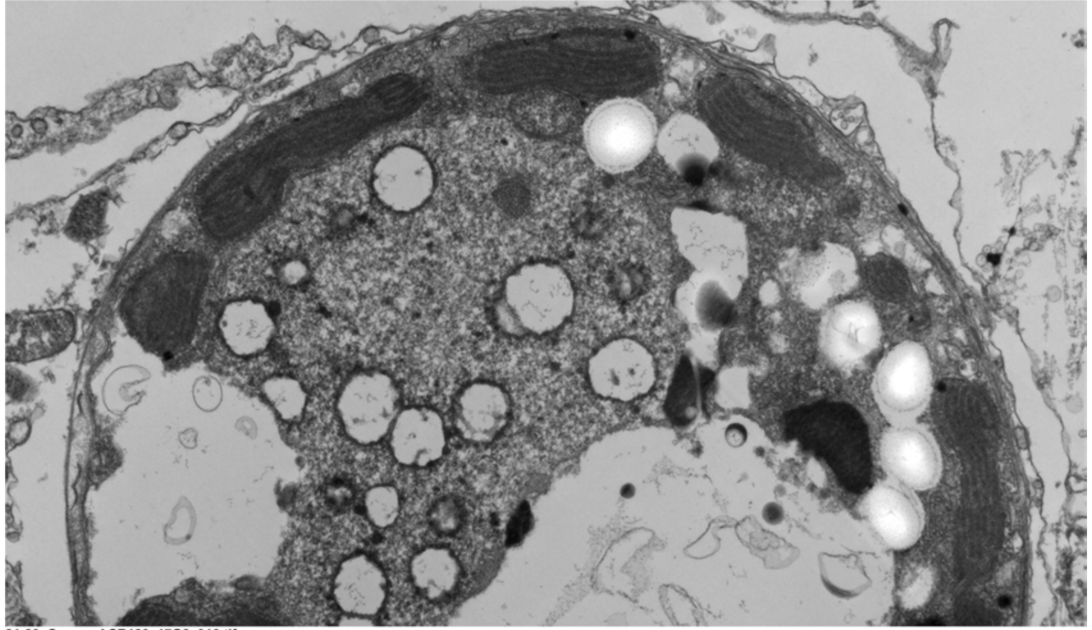

21-20_Correa_ACR120_17G2_016.tif
ACR 120
Biological Electron Microscopy Lab
Rice University - SEA
Microscopist: MD Meyer

1 µm
HV=80kV
Direct Mag: 3000 x

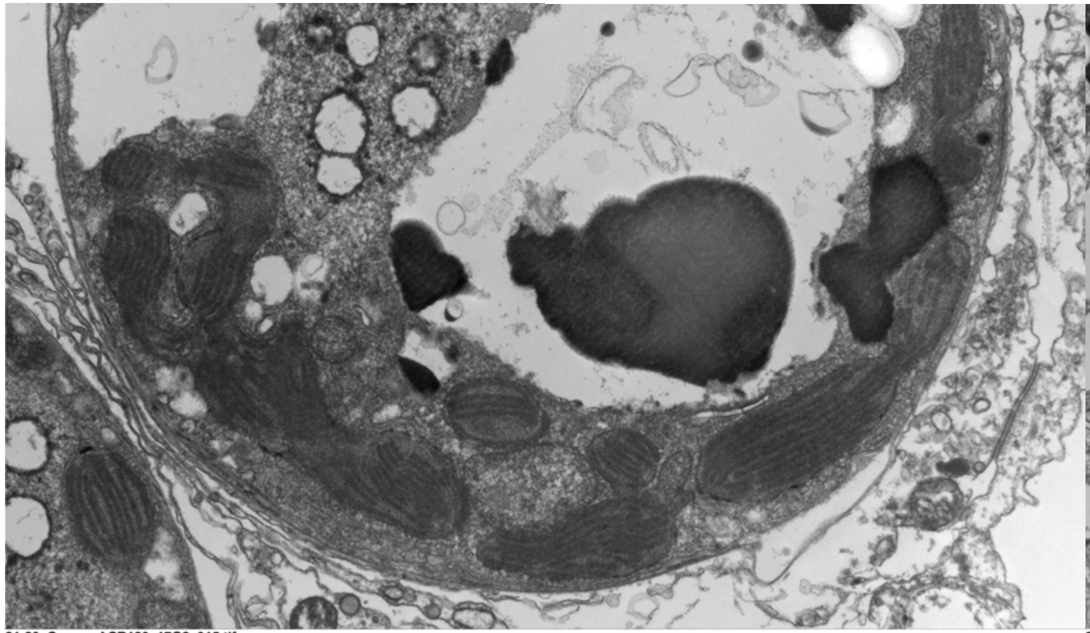

21-20_Correa_ACR120_17G2_015.tif
ACR 120
Biological Electron Microscopy Lab
Rice University - SEA
Microscopist: MD Meyer

1 µm
HV=80kV
Direct Mag: 3000 x

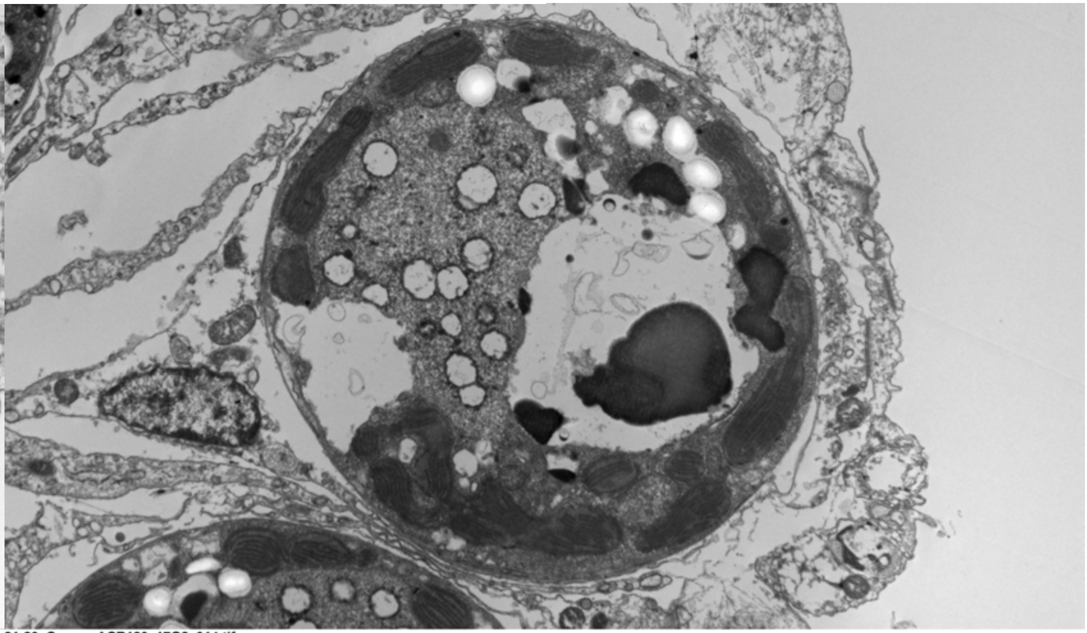

21-20_Correa_ACR120_17G2_014.tif
ACR 120
Biological Electron Microscopy Lab
Rice University - SEA
Microscopist: MD Meyer

2 µm
HV=80kV
Direct Mag: 1500 x

Cell 7

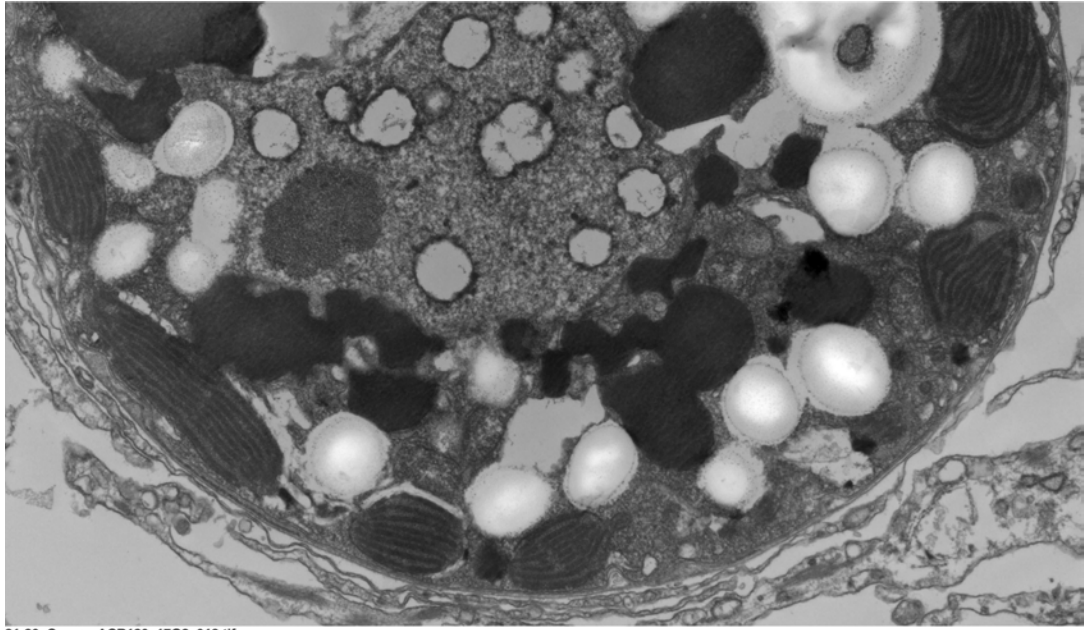

21-20_Correa_ACR120_17G2_018.tif
ACR 120
Biological Electron Microscopy Lab
Rice University - SEA
Microscopist: MD Meyer

1 µm
HV=80kV
Direct Mag: 3000 x

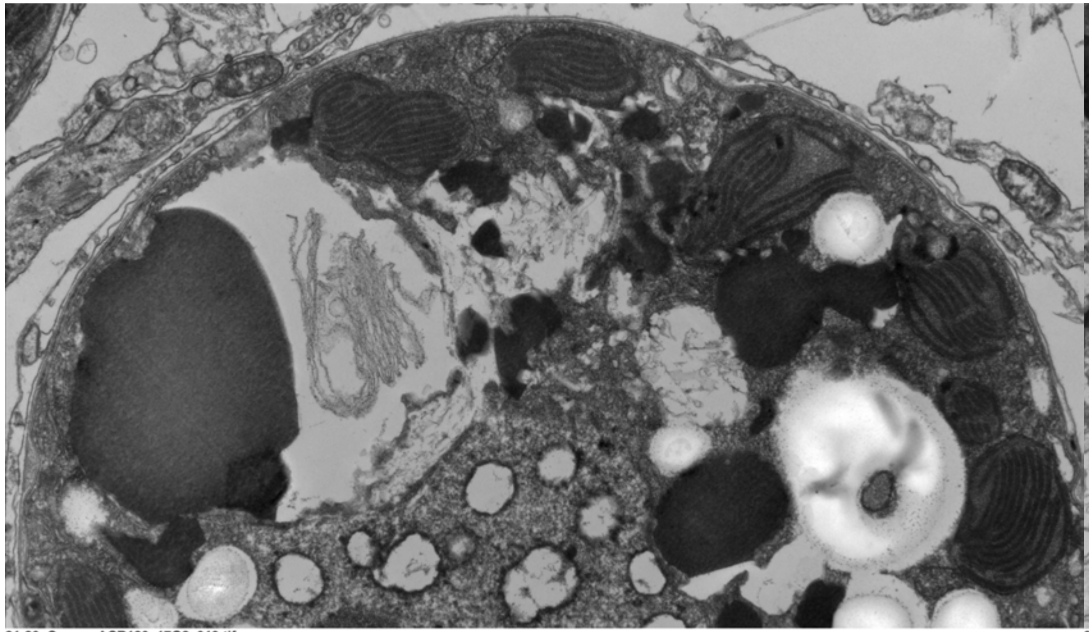

21-20_Correa_ACR120_17G2_019.tif
ACR 120
Biological Electron Microscopy Lab
Rice University - SEA
Microscopist: MD Meyer

1 µm
HV=80kV
Direct Mag: 3000 x

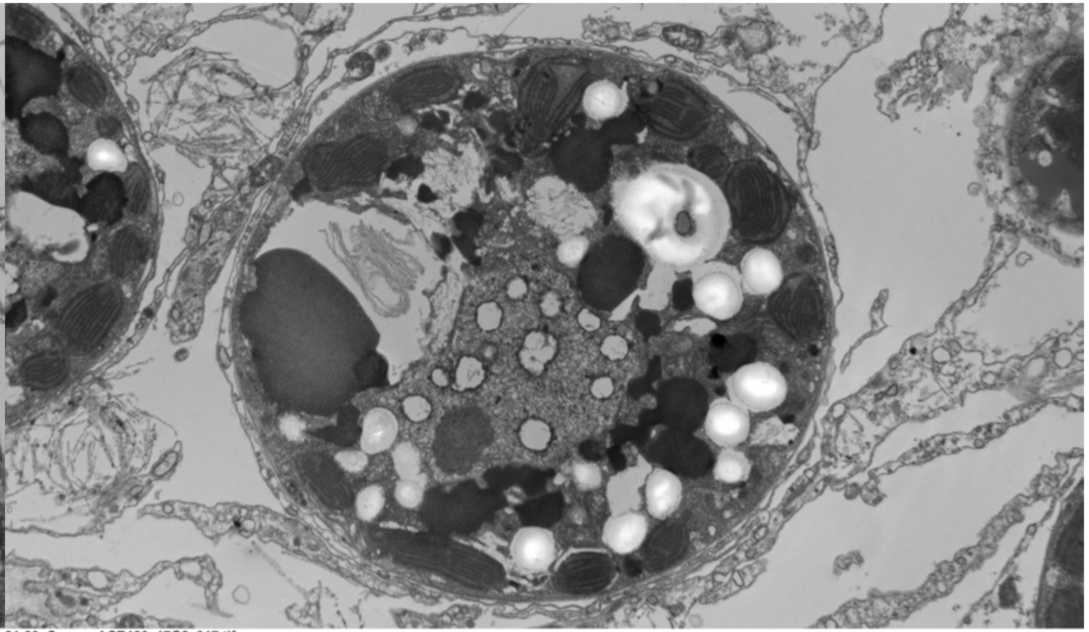

21-20_Correa_ACR120_17G2_017.tif
ACR 120
Biological Electron Microscopy Lab
Rice University - SEA
Microscopist: MD Meyer

2 µm
HV=80kV
Direct Mag: 1500 x

Cell 8

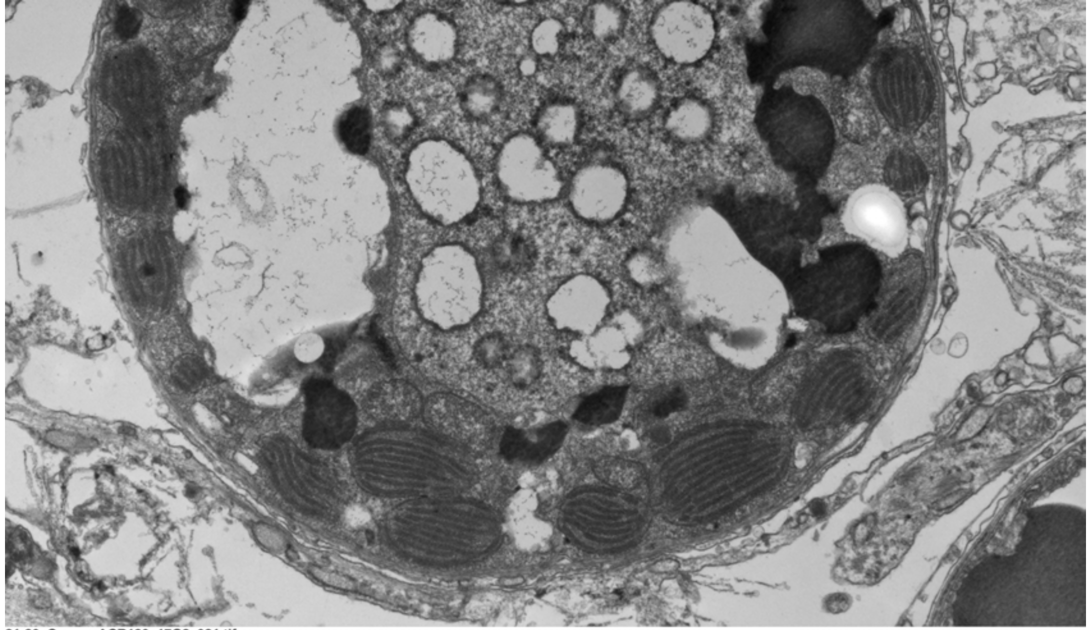

21-20_Correa_ACR120_17G2_021.tif
ACR 120
Biological Electron Microscopy Lab
Rice University - SEA
Microscopist: MD Meyer

1 µm
HV=80kV
Direct Mag: 3000 x

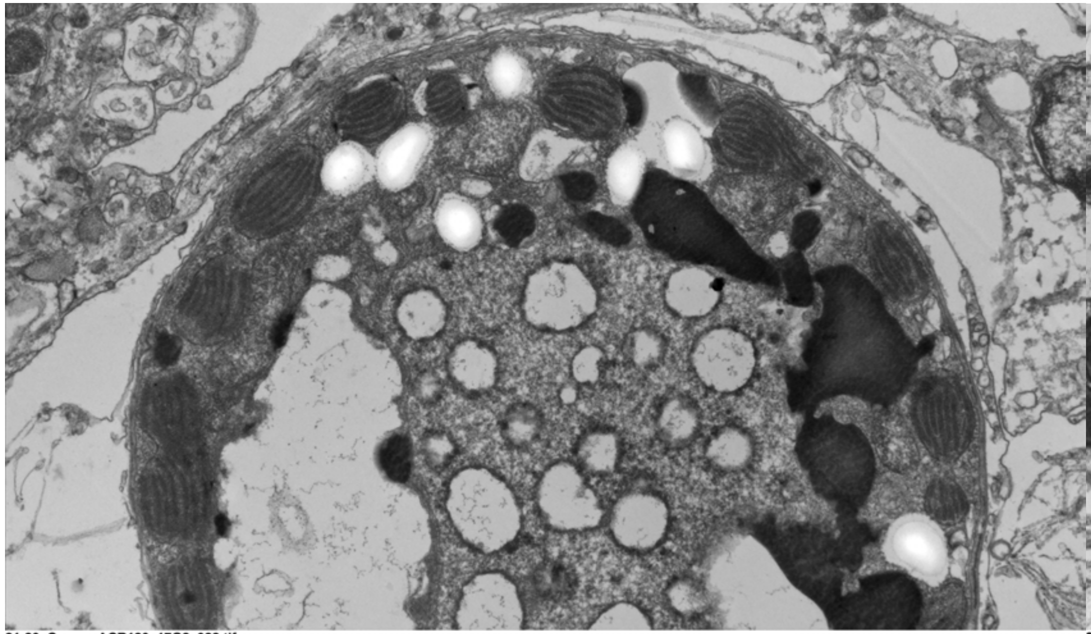

21-20_Correa_ACR120_17G2_022.tif
ACR 120
Biological Electron Microscopy Lab
Rice University - SEA
Microscopist: MD Meyer

1 µm
HV=80kV
Direct Mag: 3000 x

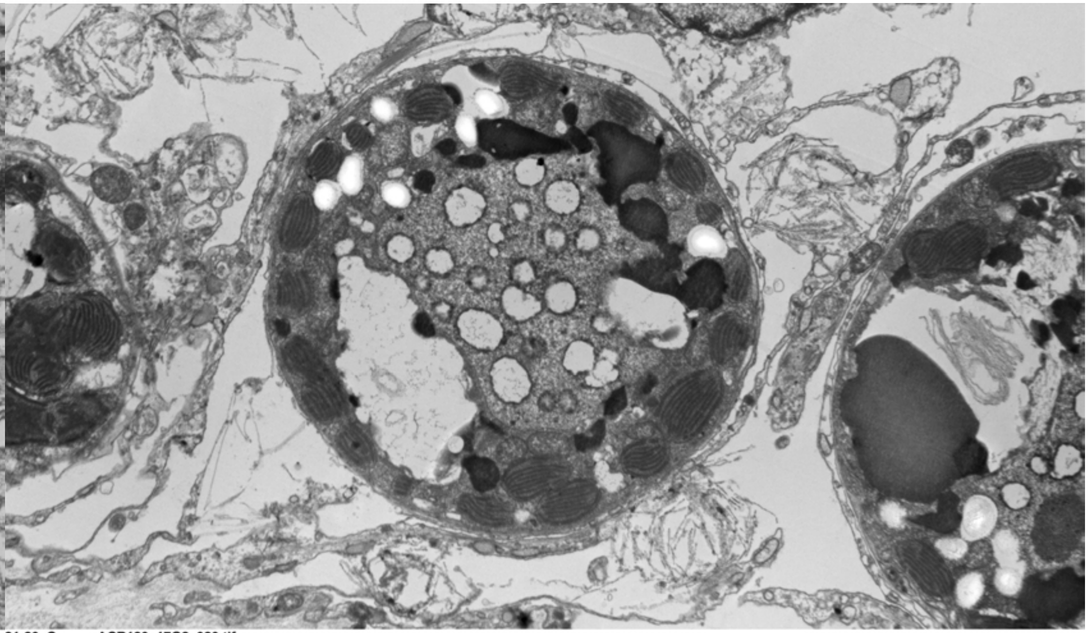

21-20_Correa_ACR120_17G2_020.tif
ACR 120
Biological Electron Microscopy Lab
Rice University - SEA
Microscopist: MD Meyer

2 µm
HV=80kV
Direct Mag: 1500 x

Cell 9

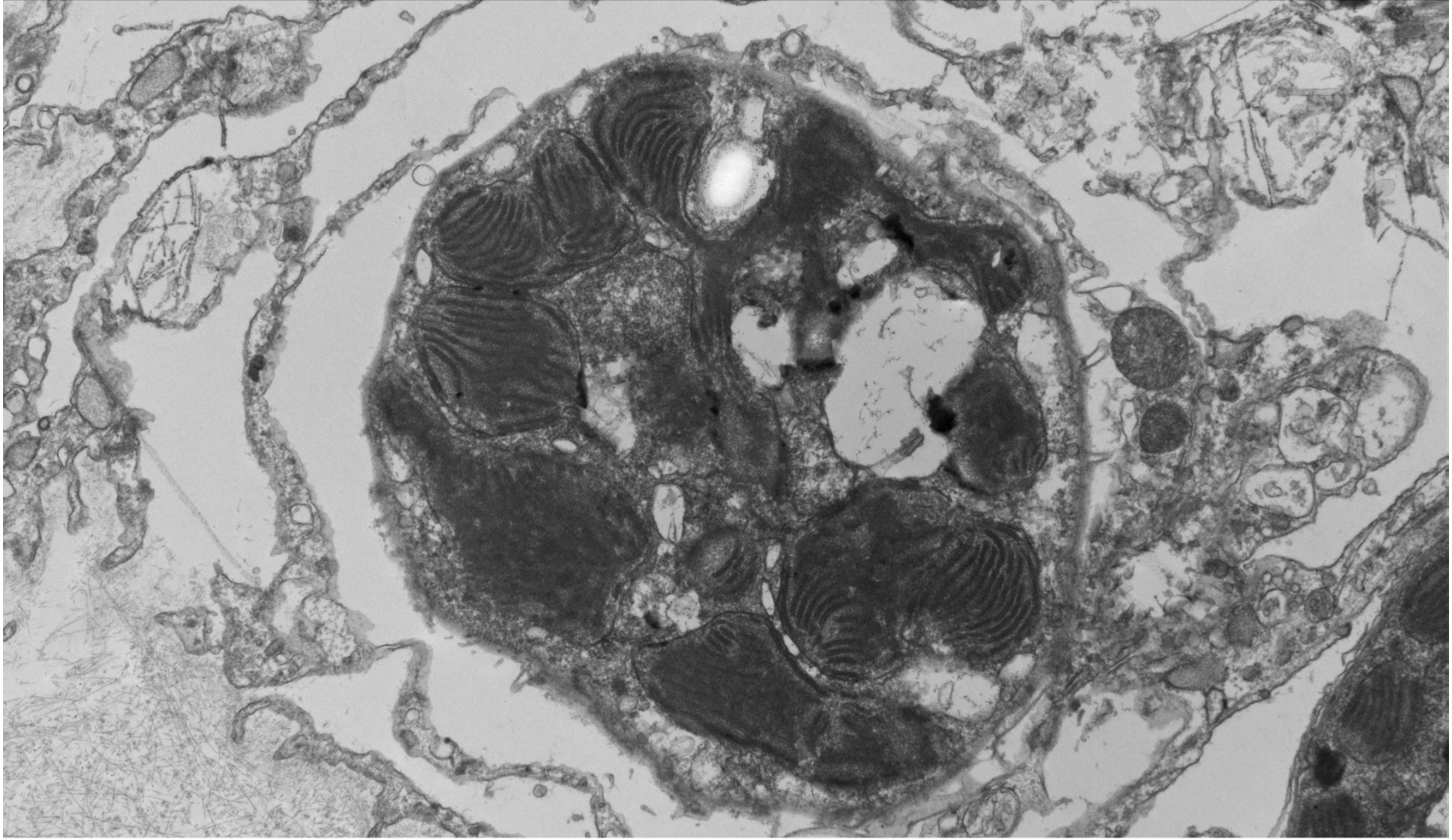

21-20_Correa_ACR120_17G2_023.tif
ACR 120
Biological Electron Microscopy Lab
Rice University - SEA
Microscopist: MD Meyer

1 µm
HV=80kV
Direct Mag: 2500 x

Cell 10

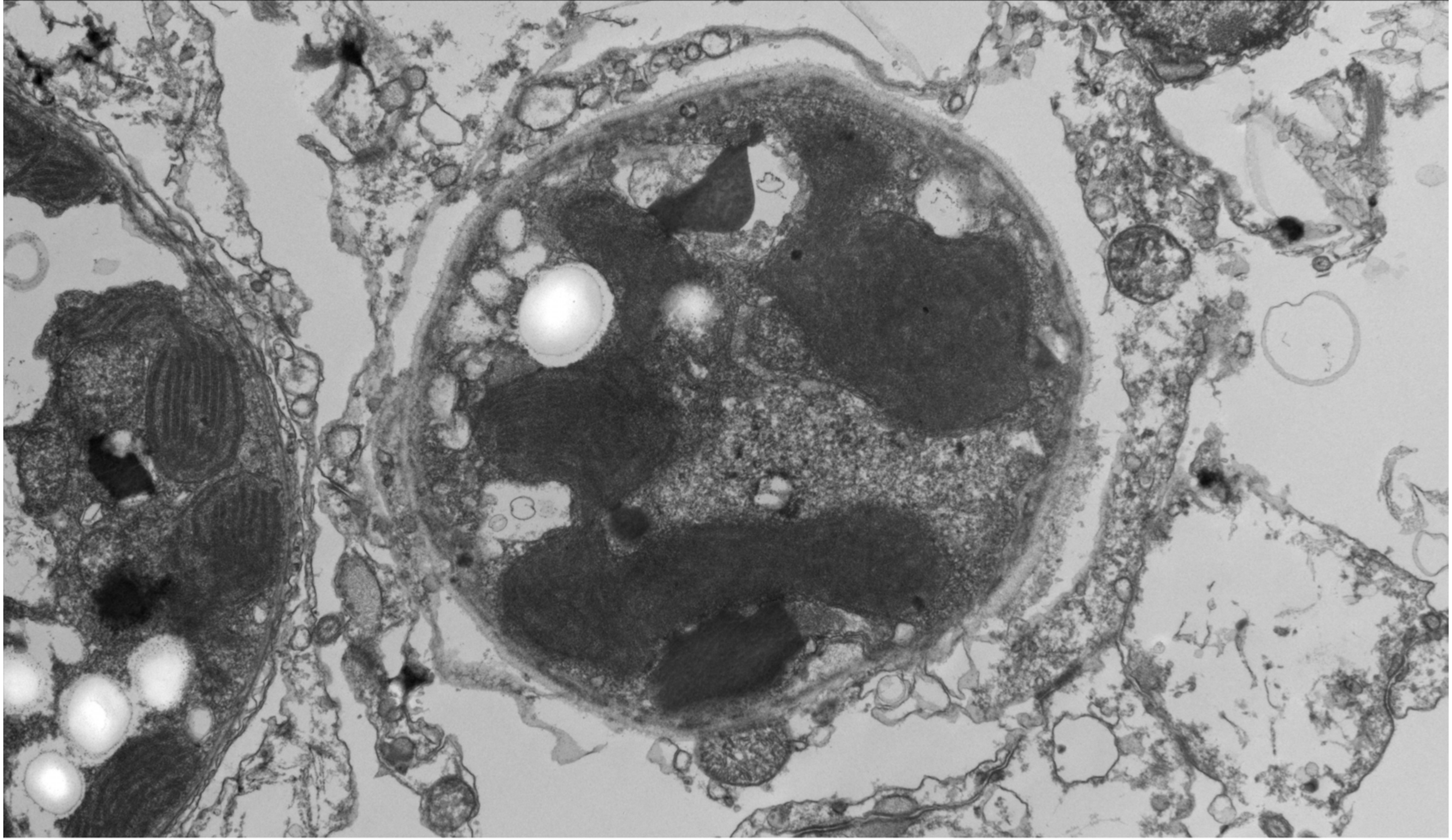

21-20_Correa_ACR120_17G2_024.tif
ACR 120
Biological Electron Microscopy Lab
Rice University - SEA
Microscopist: MD Meyer

1 µm
HV=80kV
Direct Mag: 2500 x

Cell 11

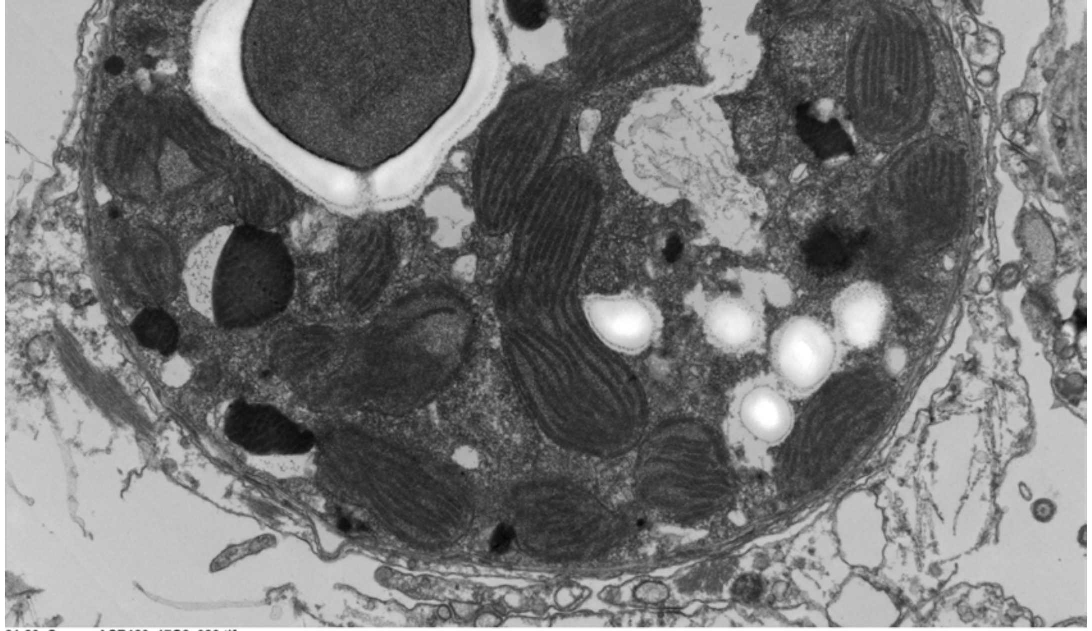

21-20_Correa_ACR120_17G2_026.tif
ACR 120
Biological Electron Microscopy Lab
Rice University - SEA
Microscopist: MD Meyer

1 µm
HV=80kV
Direct Mag: 3000 x

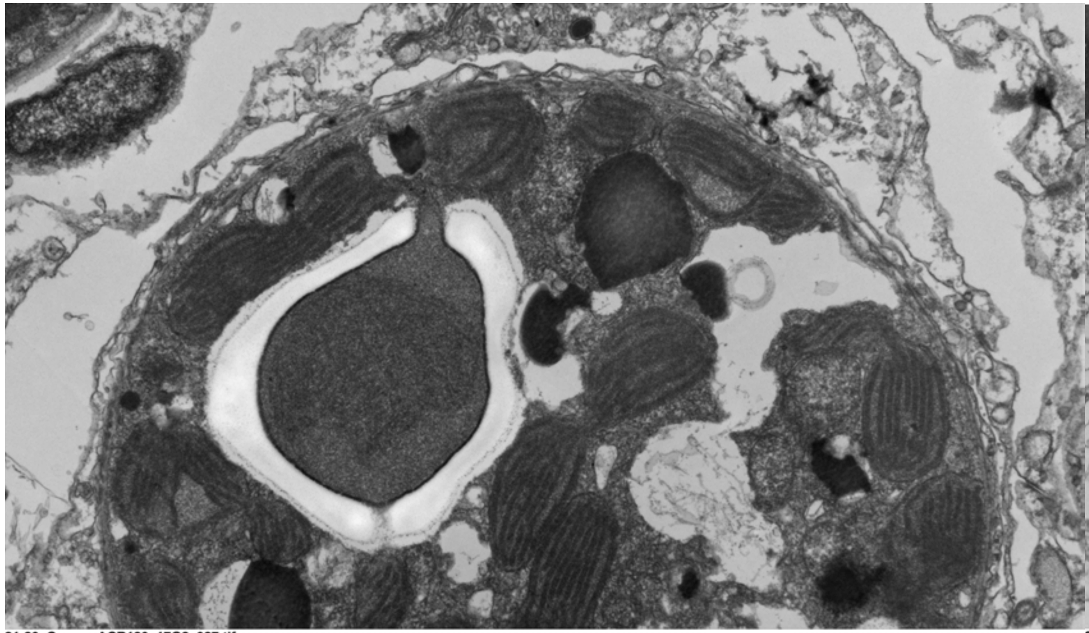

21-20_Correa_ACR120_17G2_027.tif
ACR 120
Biological Electron Microscopy Lab
Rice University - SEA
Microscopist: MD Meyer

1 µm
HV=80kV
Direct Mag: 3000 x

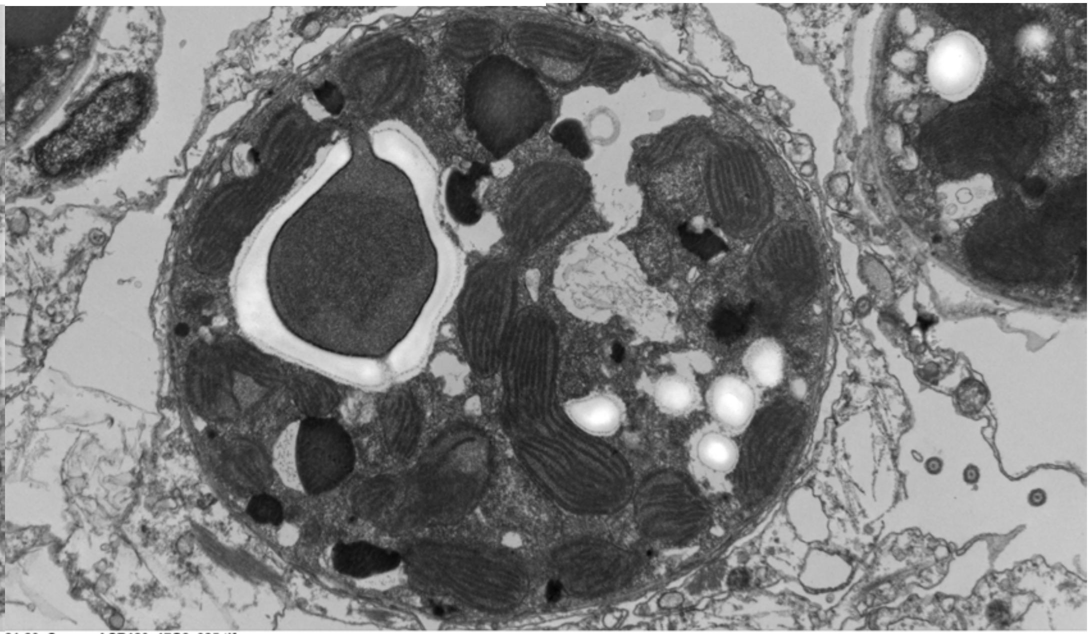

21-20_Correa_ACR120_17G2_025.tif
ACR 120
Biological Electron Microscopy Lab
Rice University - SEA
Microscopist: MD Meyer

2 µm
HV=80kV
Direct Mag: 2000 x

Cell 12

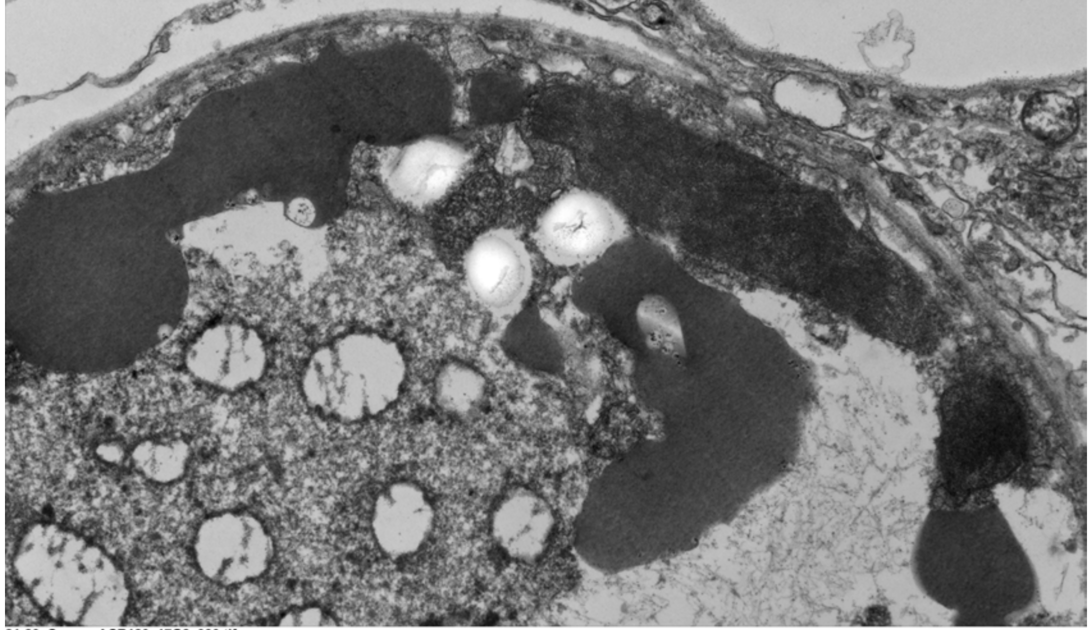

21-20_Correa_ACR120_17G2_030.tif
ACR 120
Biological Electron Microscopy Lab
Rice University - SEA
Microscopist: MD Meyer

1 µm
HV=80kV
Direct Mag: 4000 x

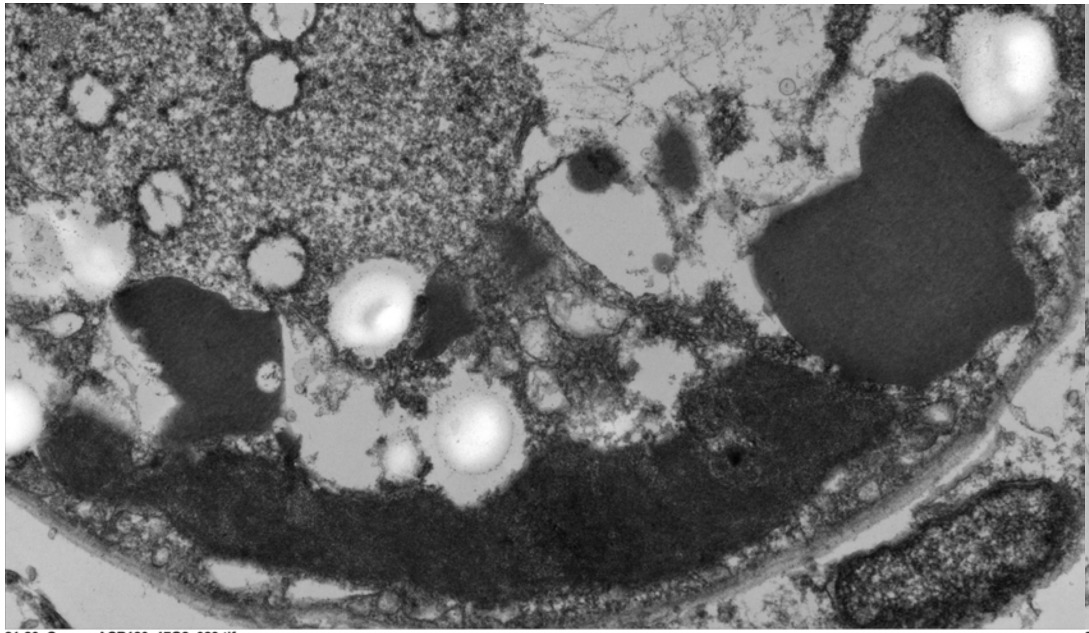

21-20_Correa_ACR120_17G2_029.tif
ACR 120
Biological Electron Microscopy Lab
Rice University - SEA
Microscopist: MD Meyer

1 µm
HV=80kV
Direct Mag: 4000 x

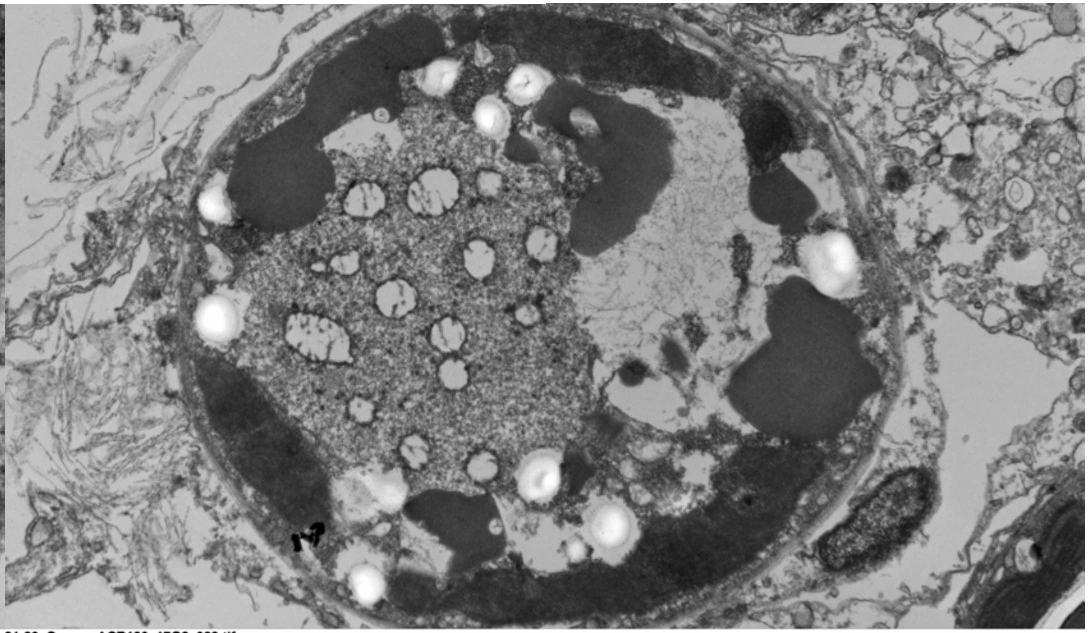

21-20_Correa_ACR120_17G2_028.tif
ACR 120
Biological Electron Microscopy Lab
Rice University - SEA
Microscopist: MD Meyer

2 µm
HV=80kV
Direct Mag: 2000 x

Cell 13

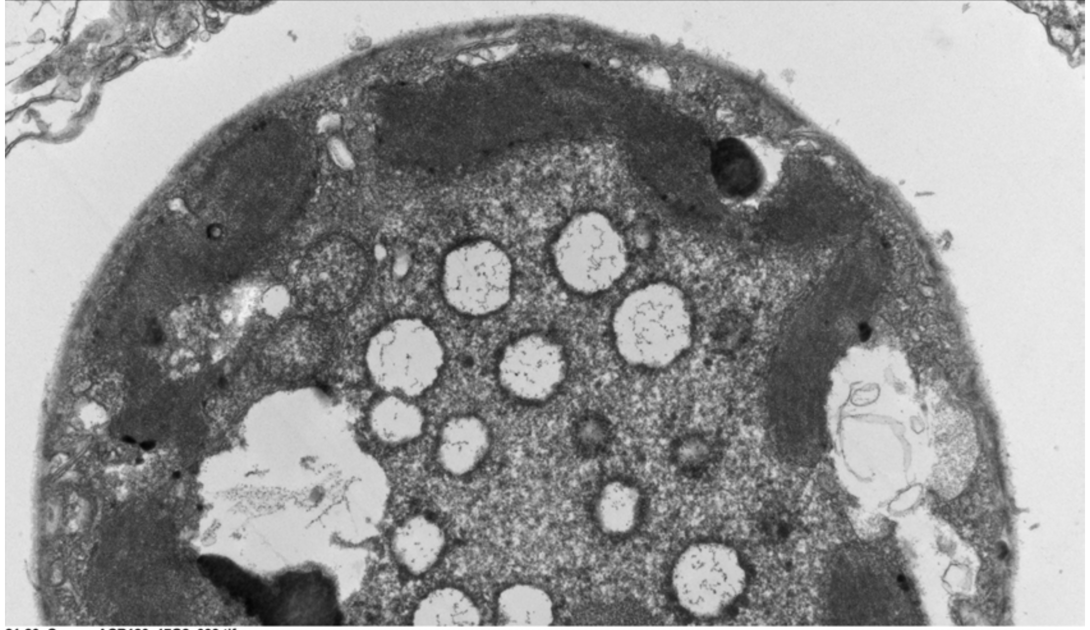

21-20_Correa_ACR120_17G2_033.tif
ACR 120
Biological Electron Microscopy Lab
Rice University - SEA
Microscopist: MD Meyer

1 µm
HV=80kV
Direct Mag: 4000 x

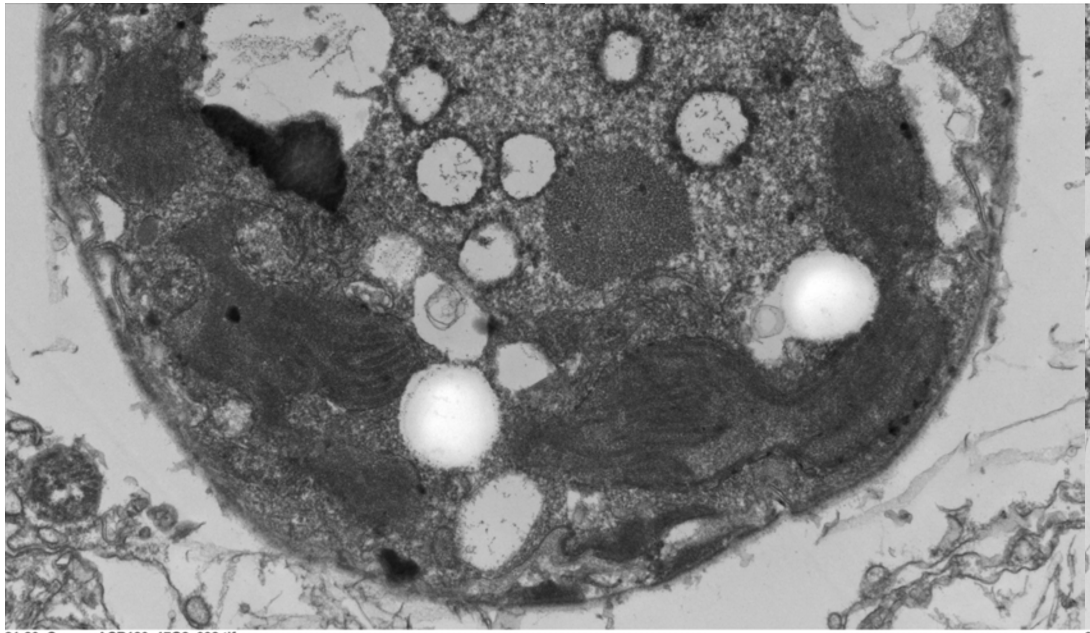

21-20_Correa_ACR120_17G2_032.tif
ACR 120
Biological Electron Microscopy Lab
Rice University - SEA
Microscopist: MD Meyer

1 µm
HV=80kV
Direct Mag: 4000 x

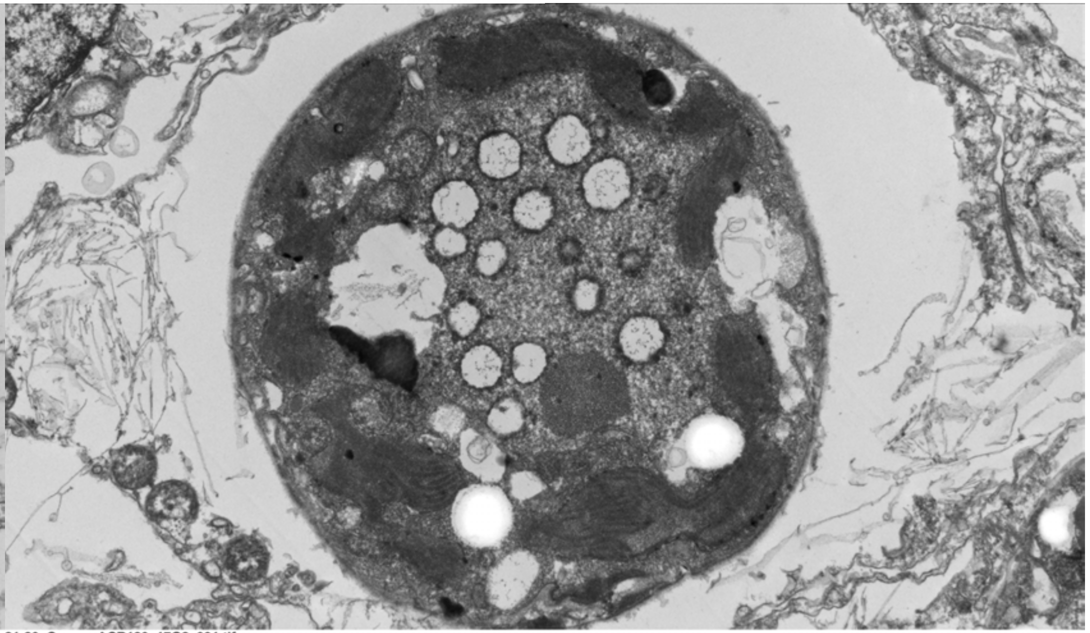

21-20_Correa_ACR120_17G2_031.tif
ACR 120
Biological Electron Microscopy Lab
Rice University - SEA
Microscopist: MD Meyer

1 µm
HV=80kV
Direct Mag: 2500 x

Cell 14

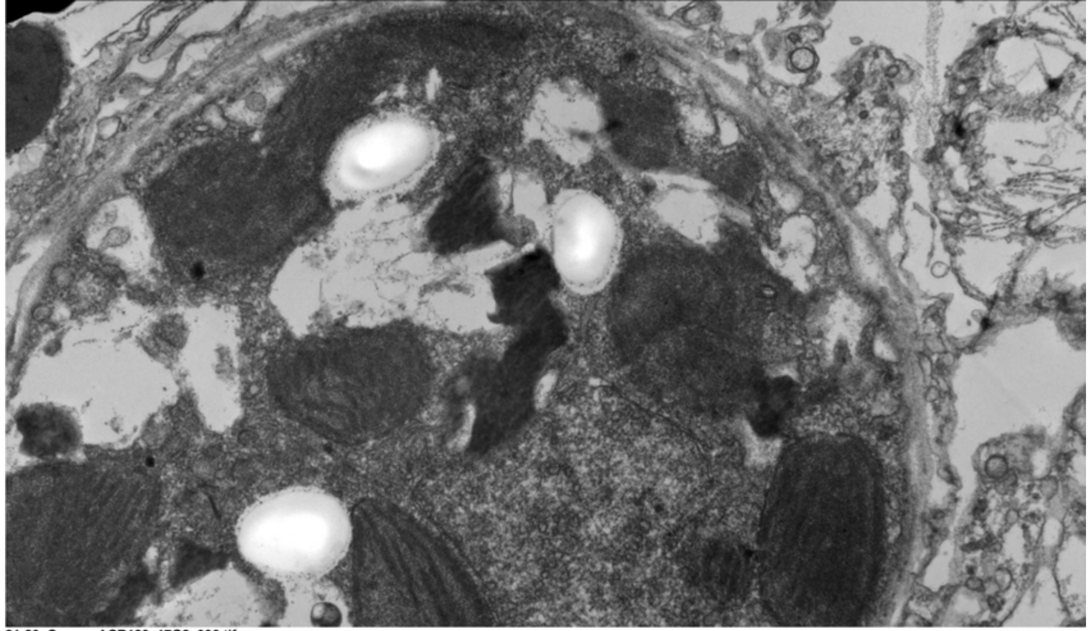

21-20_Correa_ACR120_17G2_036.tif
ACR 120
Biological Electron Microscopy Lab
Rice University - SEA
Microscopist: MD Meyer

1 µm
HV=80kV
Direct Mag: 4000 x

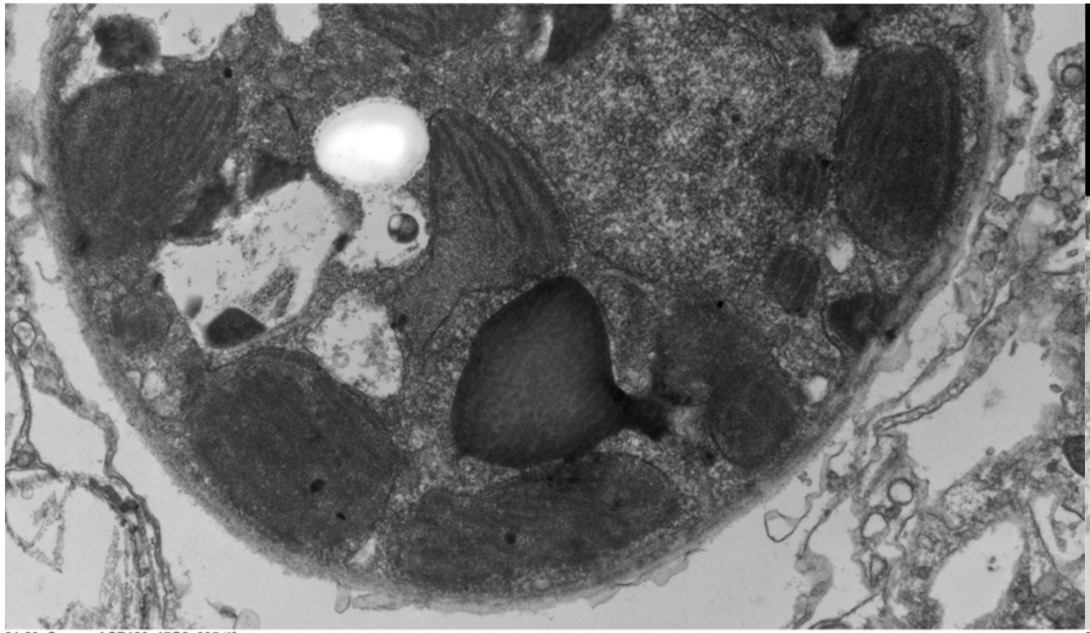

21-20_Correa_ACR120_17G2_035.tif
ACR 120
Biological Electron Microscopy Lab
Rice University - SEA
Microscopist: MD Meyer

1 µm
HV=80kV
Direct Mag: 4000 x

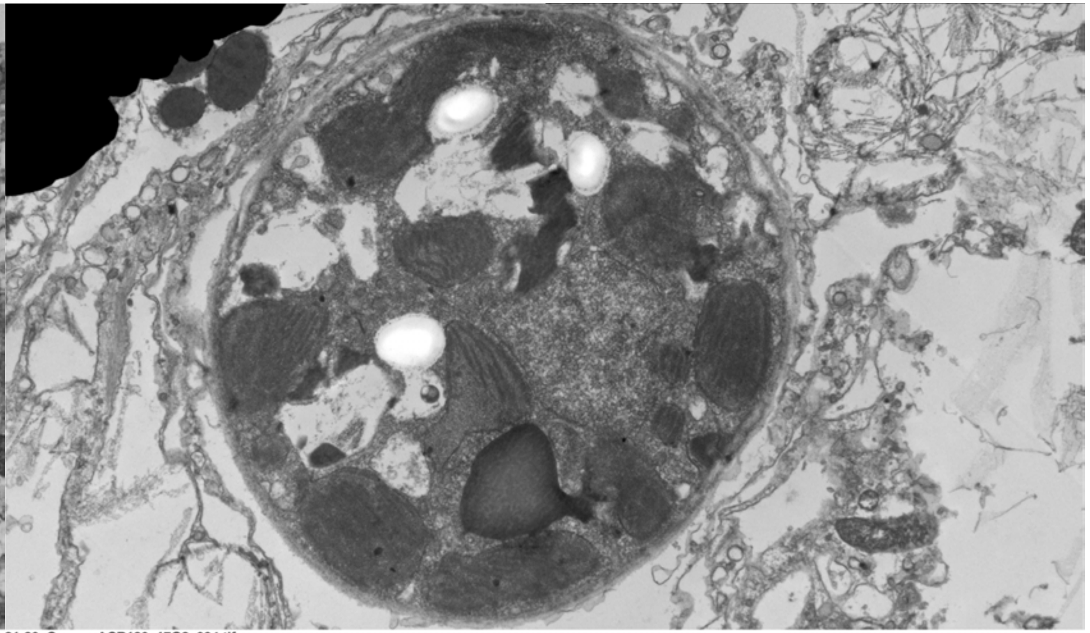

21-20_Correa_ACR120_17G2_034.tif
ACR 120
Biological Electron Microscopy Lab
Rice University - SEA
Microscopist: MD Meyer

1 µm
HV=80kV
Direct Mag: 2500 x

Cell 15

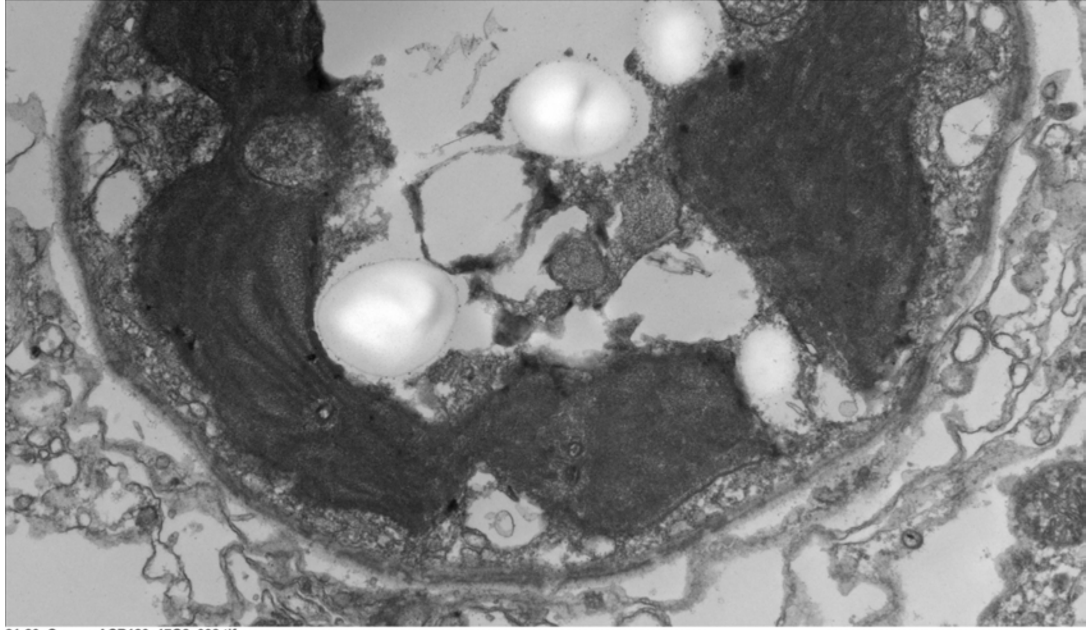

21-20_Correa_ACR120_17G2_038.tif
ACR 120
Biological Electron Microscopy Lab
Rice University - SEA
Microscopist: MD Meyer

1 µm
HV=80kV
Direct Mag: 4000 x

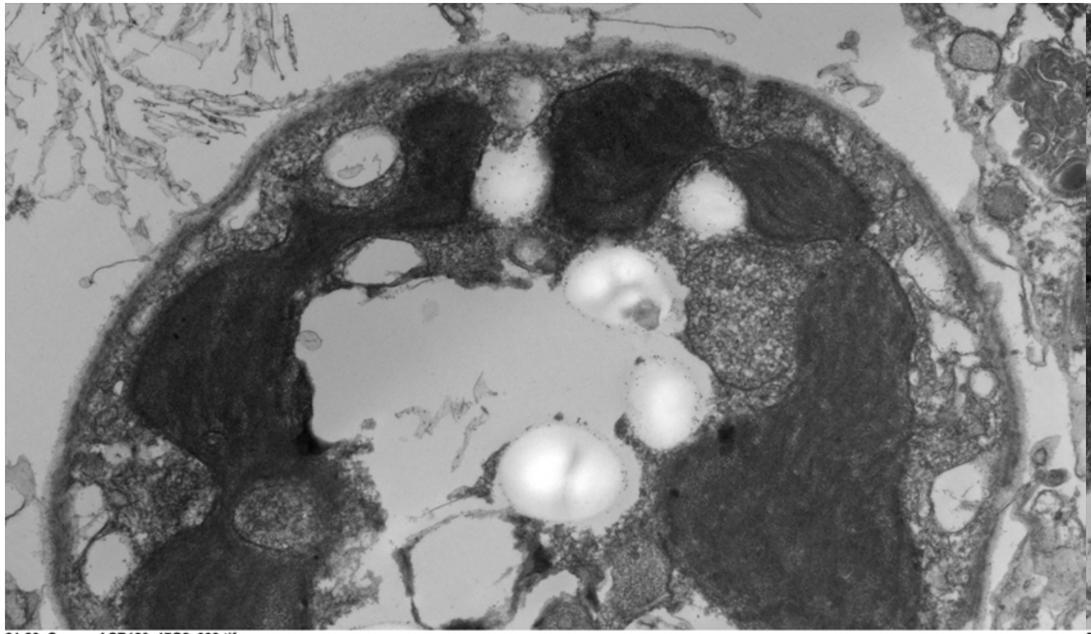

21-20_Correa_ACR120_17G2_039.tif
ACR 120
Biological Electron Microscopy Lab
Rice University - SEA
Microscopist: MD Meyer

1 µm
HV=80kV
Direct Mag: 4000 x

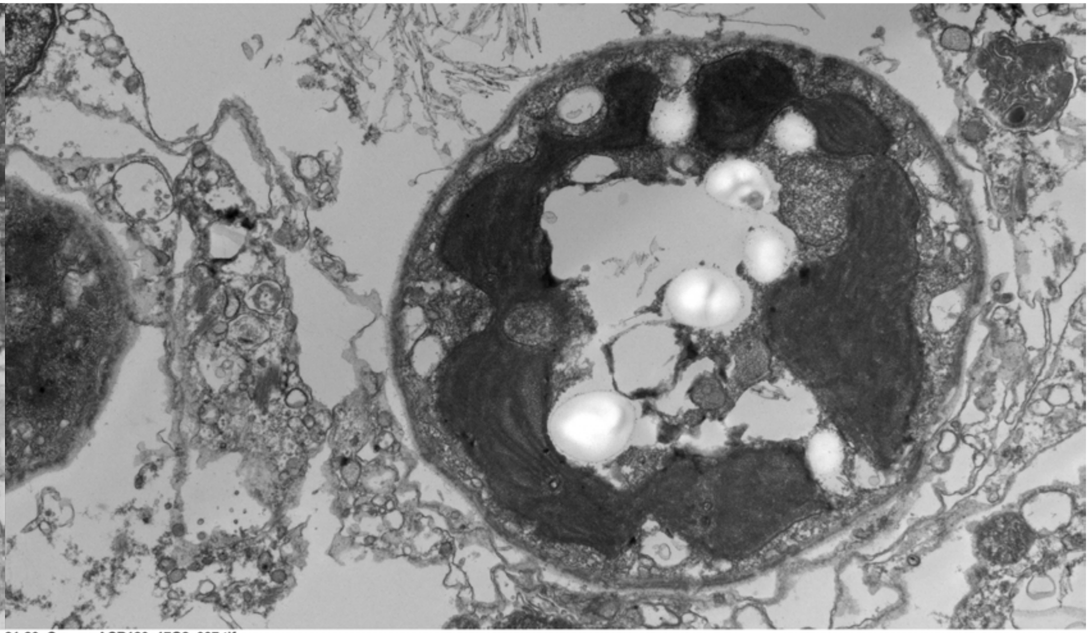

21-20_Correa_ACR120_17G2_037.tif
ACR 120
Biological Electron Microscopy Lab
Rice University - SEA
Microscopist: MD Meyer

1 µm
HV=80kV
Direct Mag: 2500 x

Cell 16

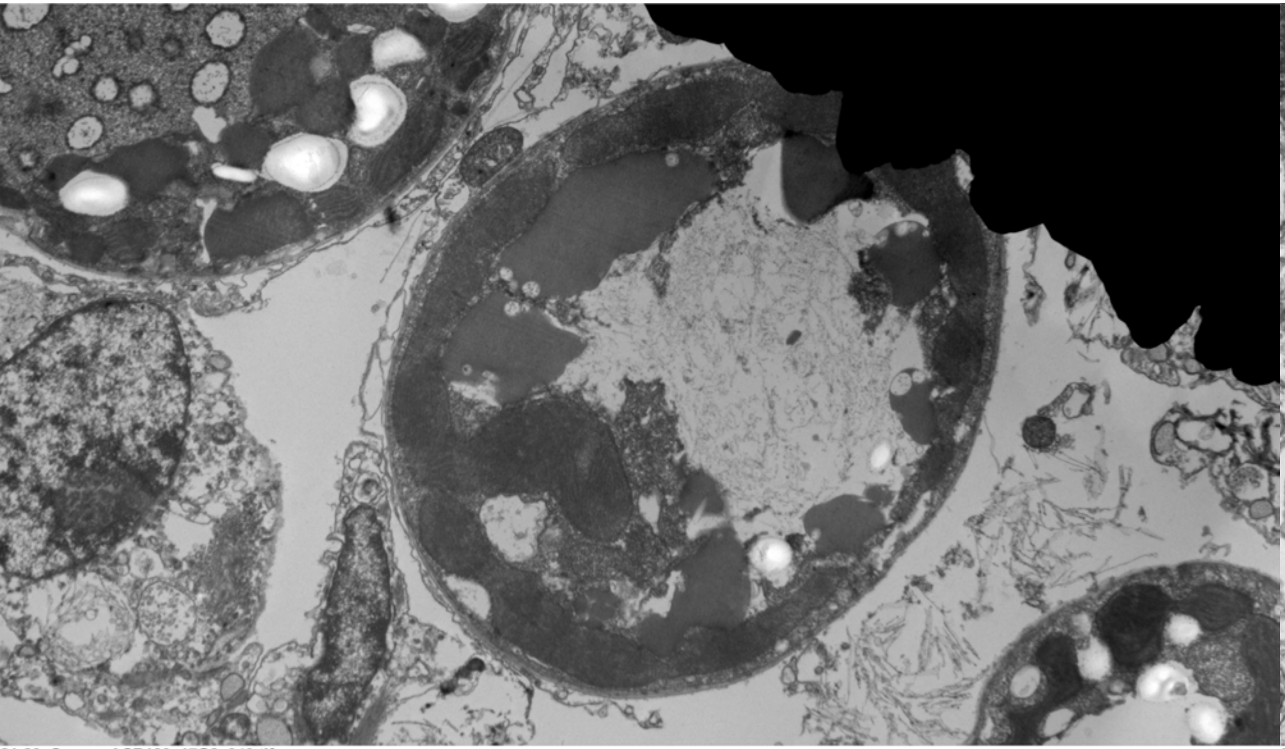

21-20_Correa_ACR120_17G2_040.tif
ACR 120
Biological Electron Microscopy Lab
Rice University - SEA
Microscopist: MD Meyer

2 µm
HV=80kV
Direct Mag: 1500 x

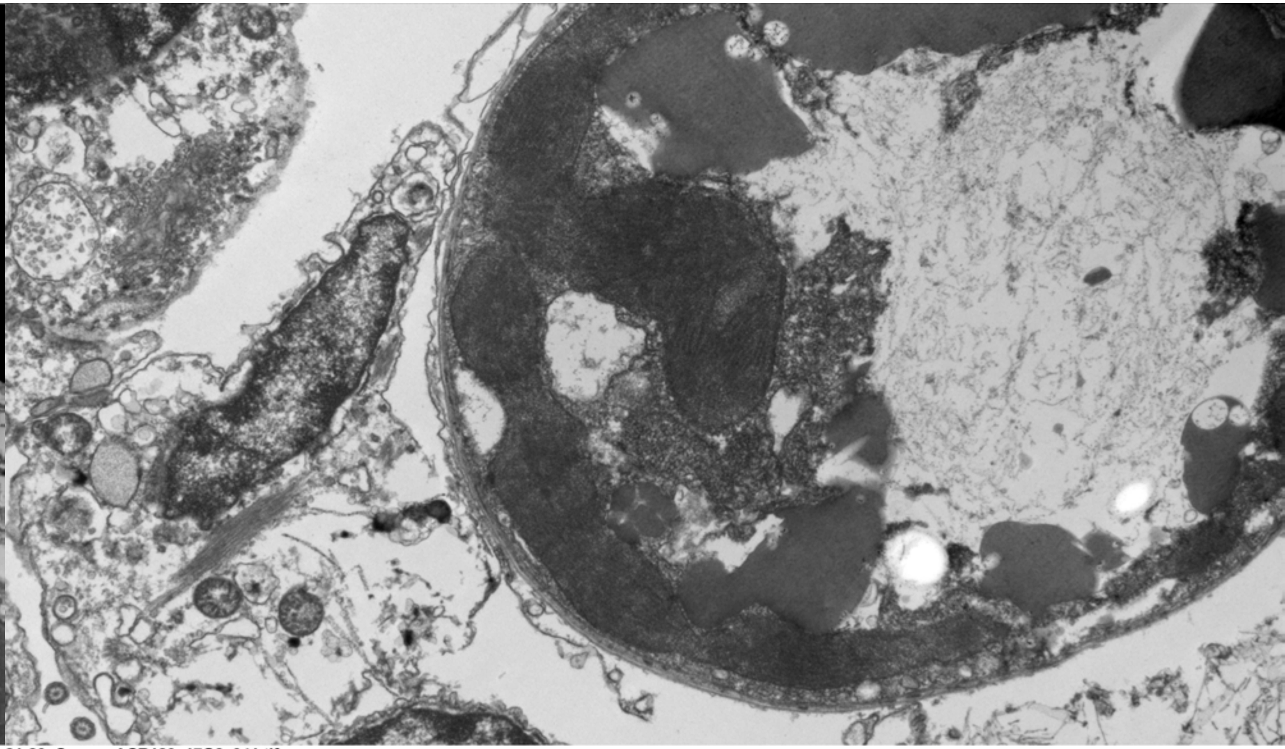

21-20_Correa_ACR120_17G2_041.tif
ACR 120
Biological Electron Microscopy Lab
Rice University - SEA
Microscopist: MD Meyer

1 µm
HV=80kV
Direct Mag: 2500 x

Cell 17

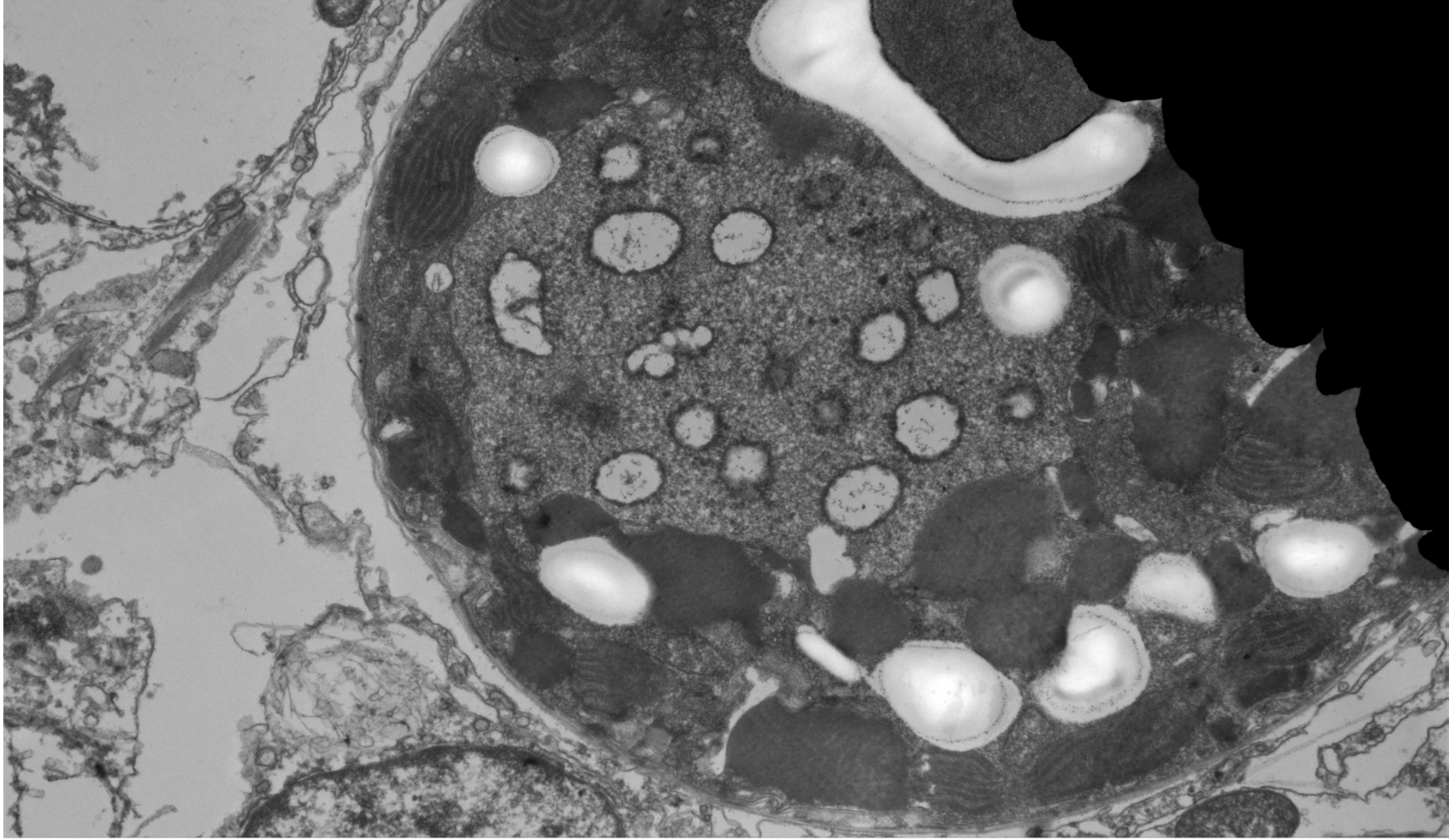

21-20_Correa_ACR120_17G2_042.tif
ACR 120
Biological Electron Microscopy Lab
Rice University - SEA
Microscopist: MD Meyer

1 μm
HV=80kV
Direct Mag: 2500 x

Cell 18

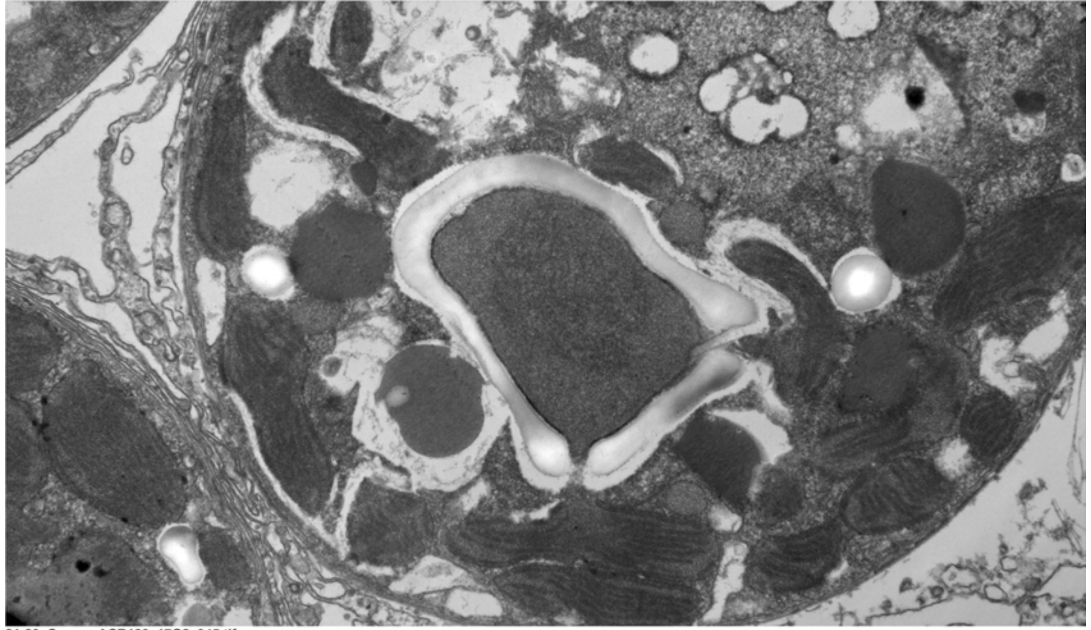

21-20_Correa_ACR120_17G2_045.tif
ACR 120
Biological Electron Microscopy Lab
Rice University - SEA
Microscopist: MD Meyer

1 µm
HV=80kV
Direct Mag: 3000 x

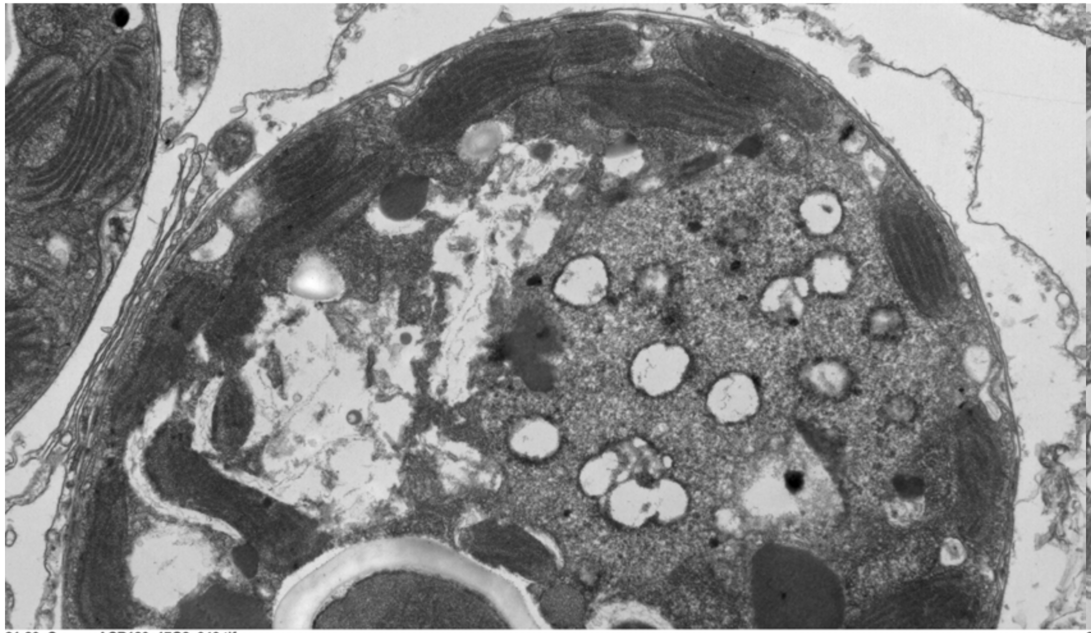

21-20_Correa_ACR120_17G2_046.tif
ACR 120
Biological Electron Microscopy Lab
Rice University - SEA
Microscopist: MD Meyer

1 µm
HV=80kV
Direct Mag: 3000 x

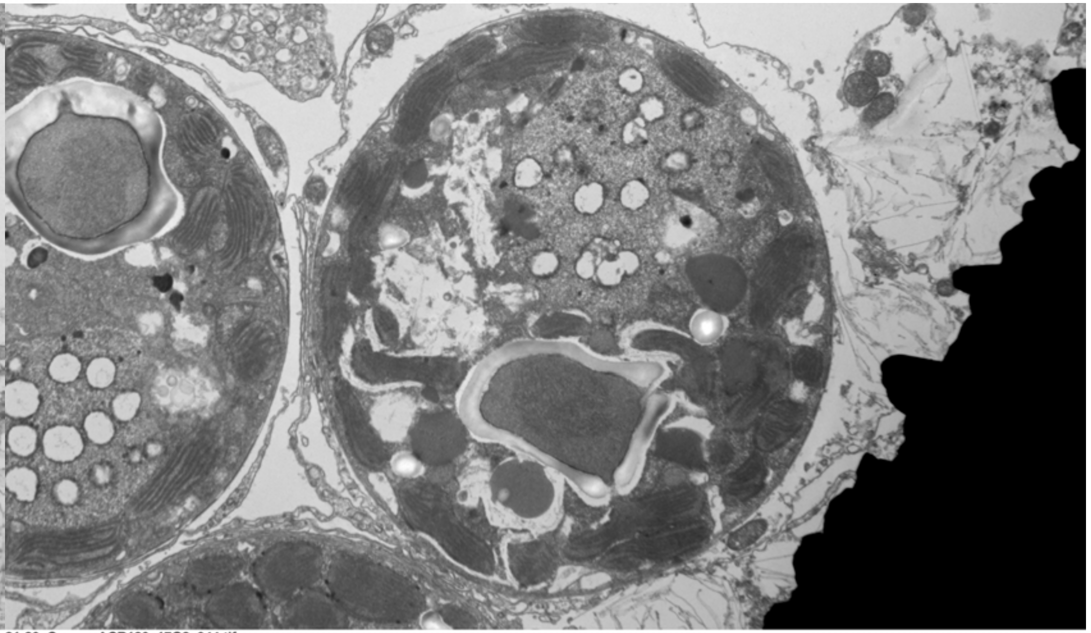

21-20_Correa_ACR120_17G2_044.tif
ACR 120
Biological Electron Microscopy Lab
Rice University - SEA
Microscopist: MD Meyer

2 µm
HV=80kV
Direct Mag: 1500 x

Cell 19

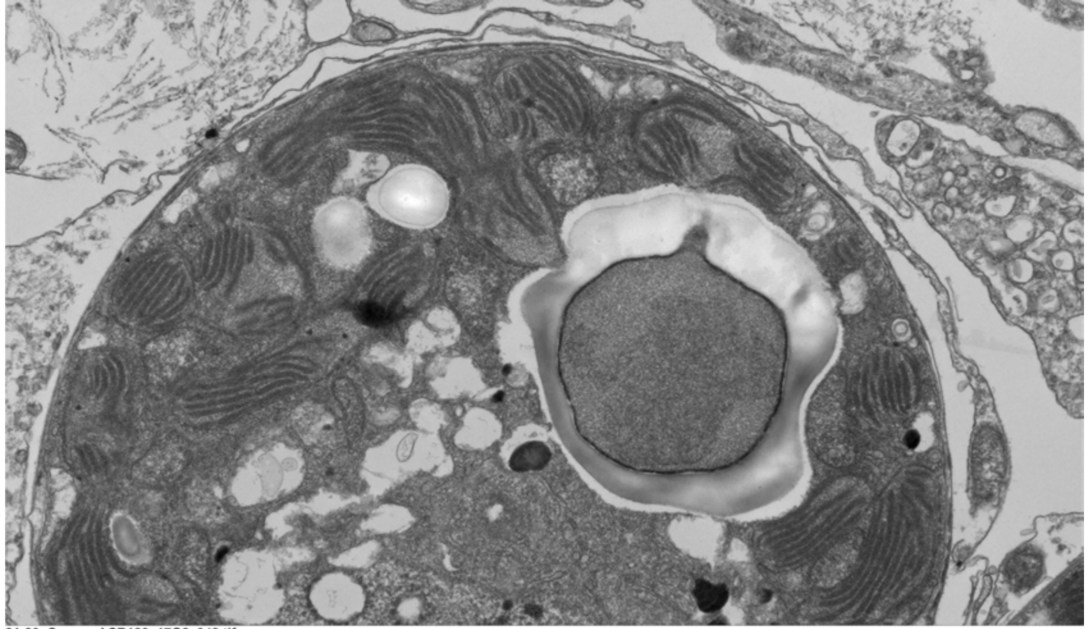

21-20_Correa_ACR120_17G2_048.tif
ACR 120
Biological Electron Microscopy Lab
Rice University - SEA
Microscopist: MD Meyer

1 μm
HV=80kV
Direct Mag: 3000 x

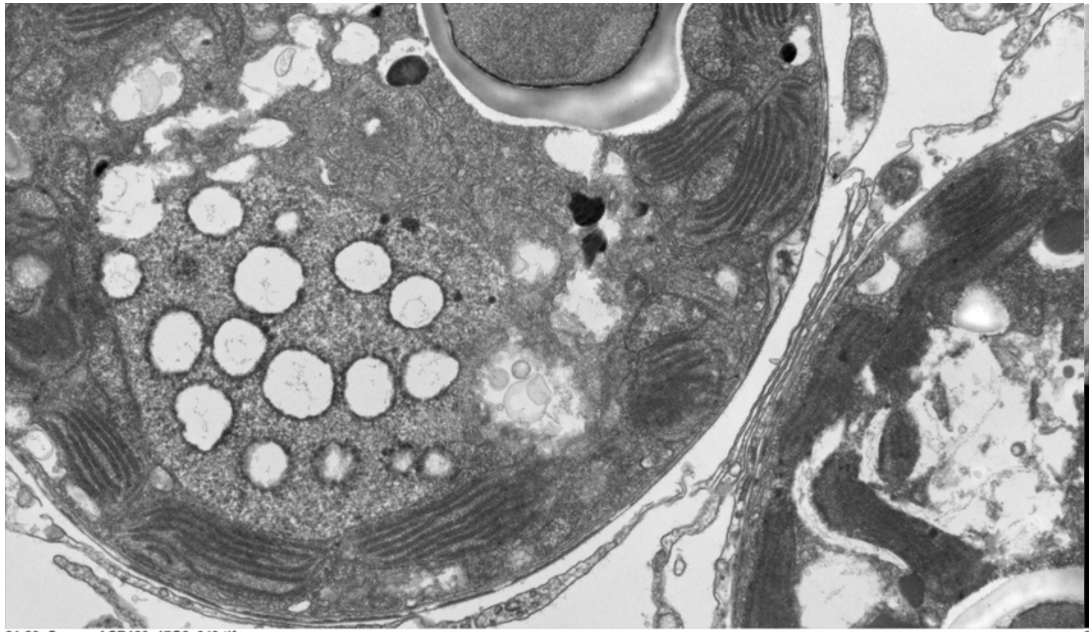

21-20_Correa_ACR120_17G2_049.tif
ACR 120
Biological Electron Microscopy Lab
Rice University - SEA
Microscopist: MD Meyer

1 μm
HV=80kV
Direct Mag: 3000 x

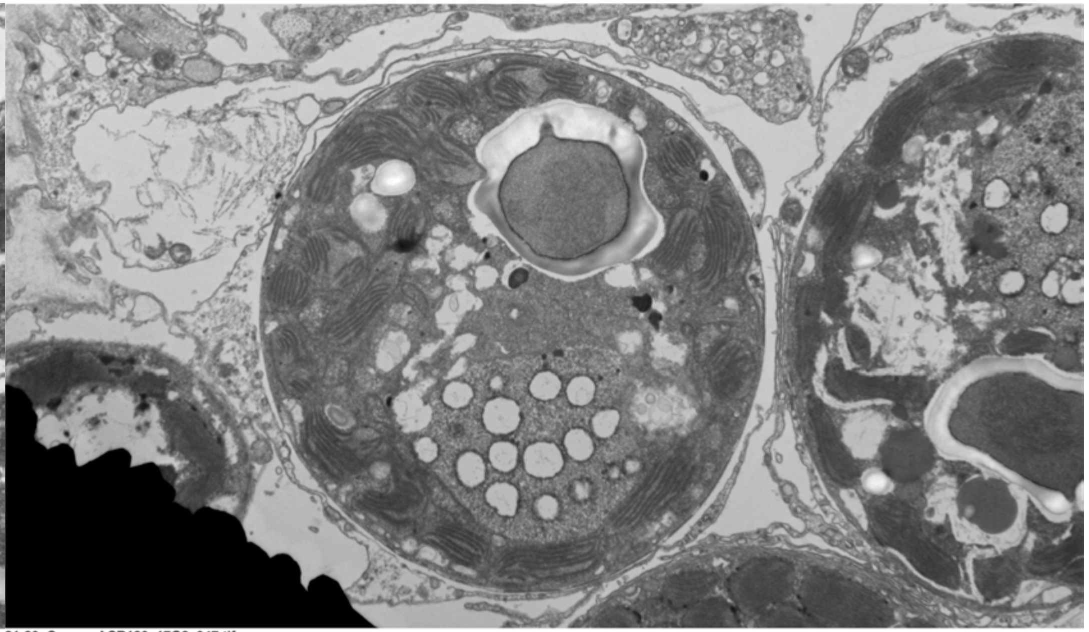

21-20_Correa_ACR120_17G2_047.tif
ACR 120
Biological Electron Microscopy Lab
Rice University - SEA
Microscopist: MD Meyer

2 μm
HV=80kV
Direct Mag: 1500 x

Cell 20

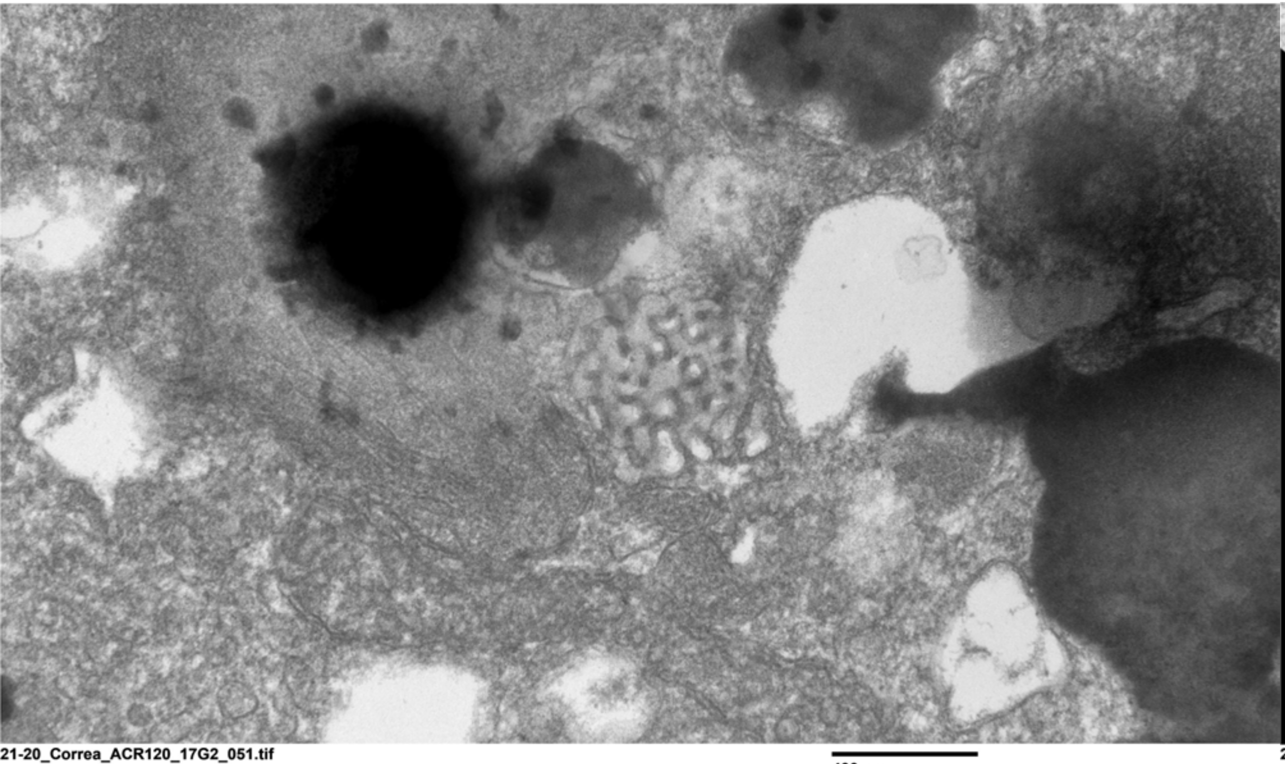

21-20_Correa_ACR120_17G2_051.tif
ACR 120
Biological Electron Microscopy Lab
Rice University - SEA
Microscopist: MD Meyer

HV=80kV
Direct Mag: 10000 x

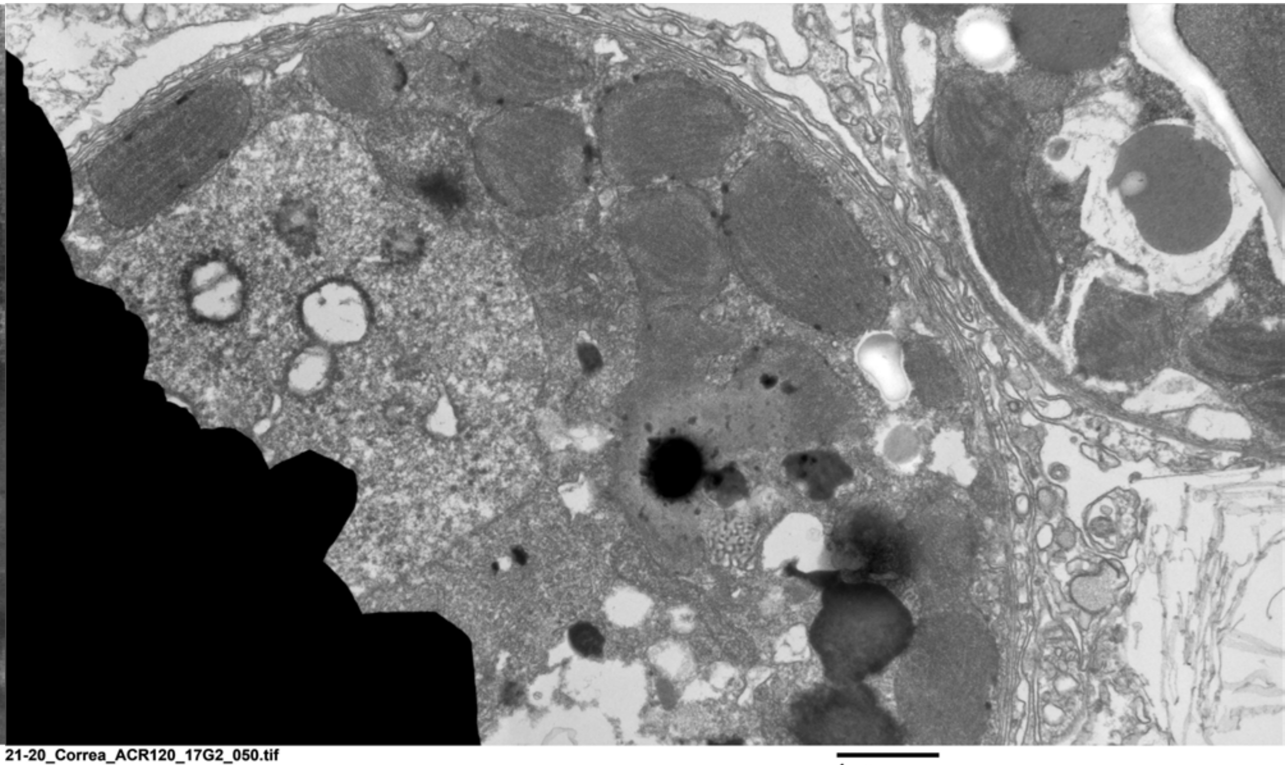

21-20_Correa_ACR120_17G2_050.tif
ACR 120
Biological Electron Microscopy Lab
Rice University - SEA
Microscopist: MD Meyer

HV=80kV
Direct Mag: 3000 x

Cell 21

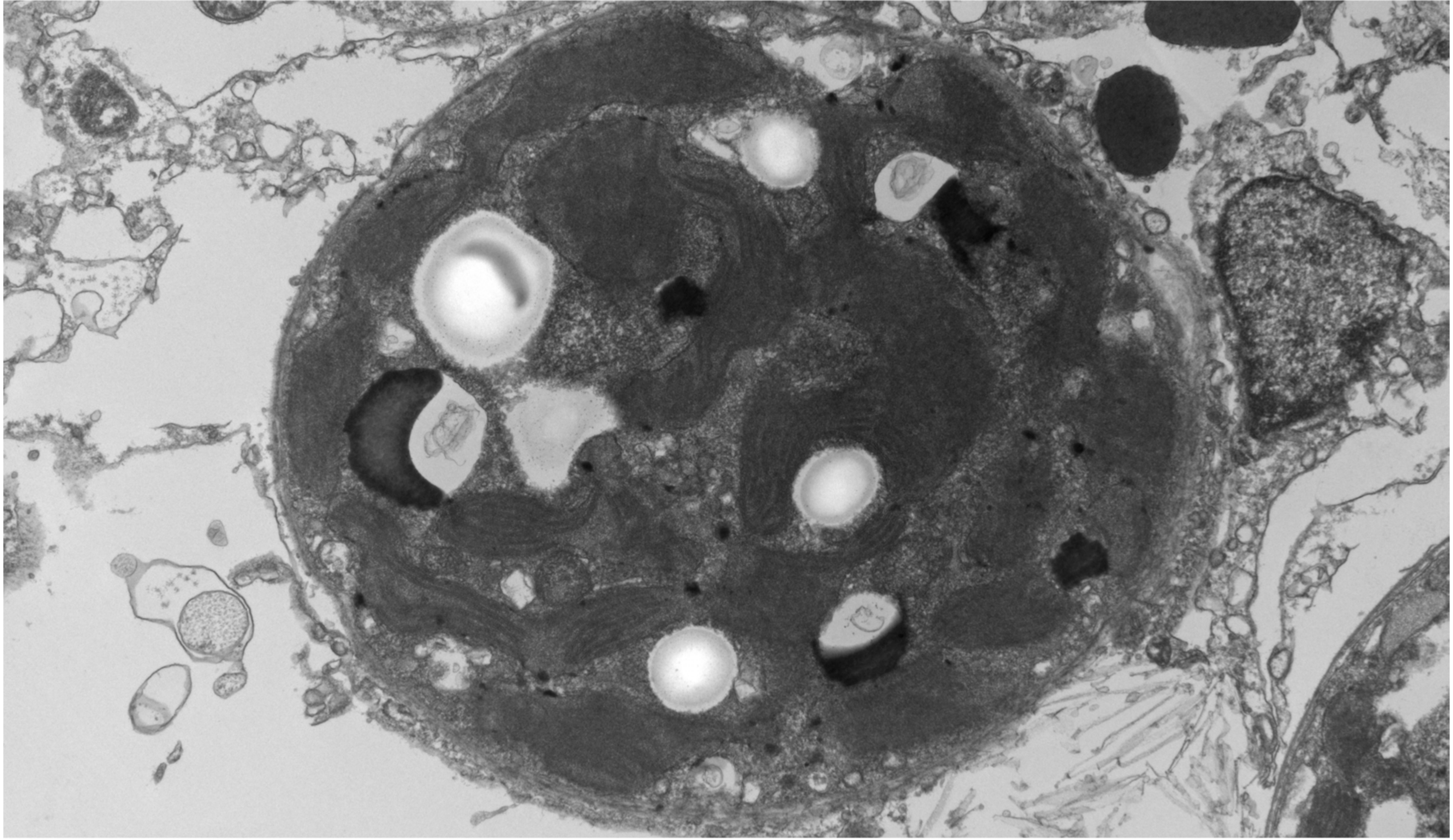

21-20_Correa_ACR120_17G2_052.tif
ACR 120
Biological Electron Microscopy Lab
Rice University - SEA
Microscopist: MD Meyer

1 µm
HV=80kV
Direct Mag: 2500 x

Cell 22

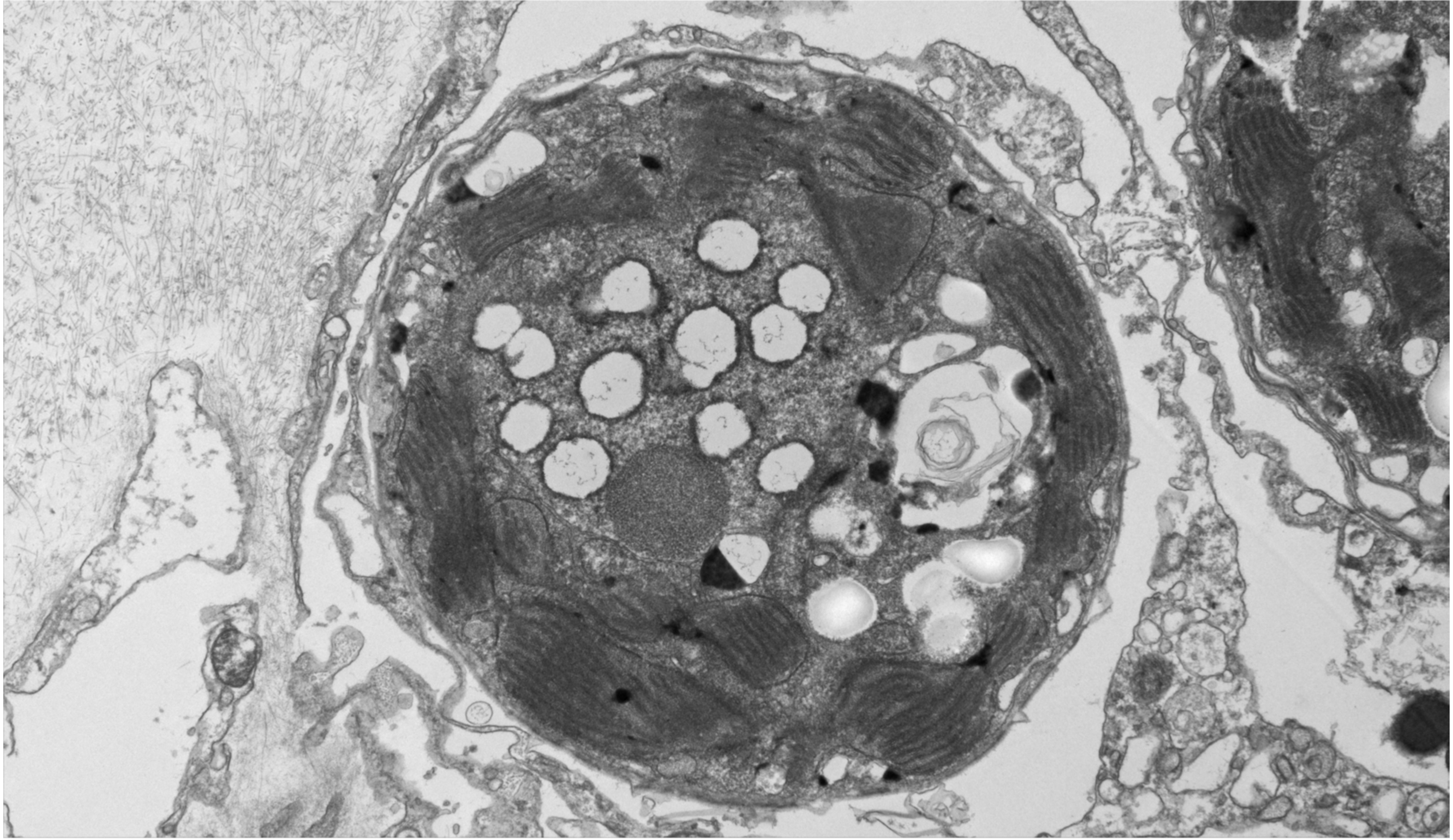

21-20_Correa_ACR120_17G2_053.tif
ACR 120
Biological Electron Microscopy Lab
Rice University - SEA
Microscopist: MD Meyer

1 µm
HV=80kV
Direct Mag: 2500 x

Cell 23

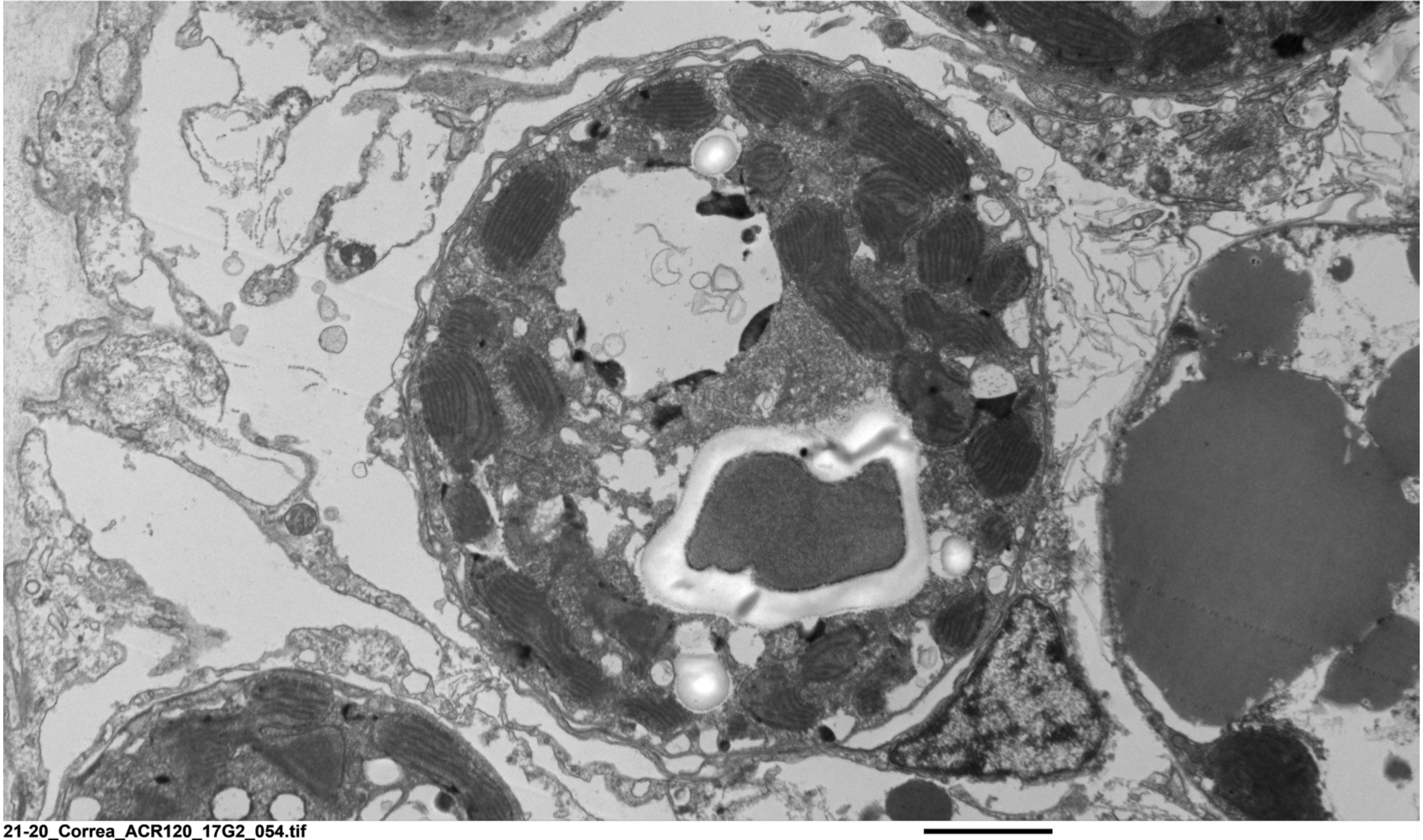

21-20_Correa_ACR120_17G2_054.tif
ACR 120
Biological Electron Microscopy Lab
Rice University - SEA
Microscopist: MD Meyer

HV=80kV
Direct Mag: 1500 x

Cell 24

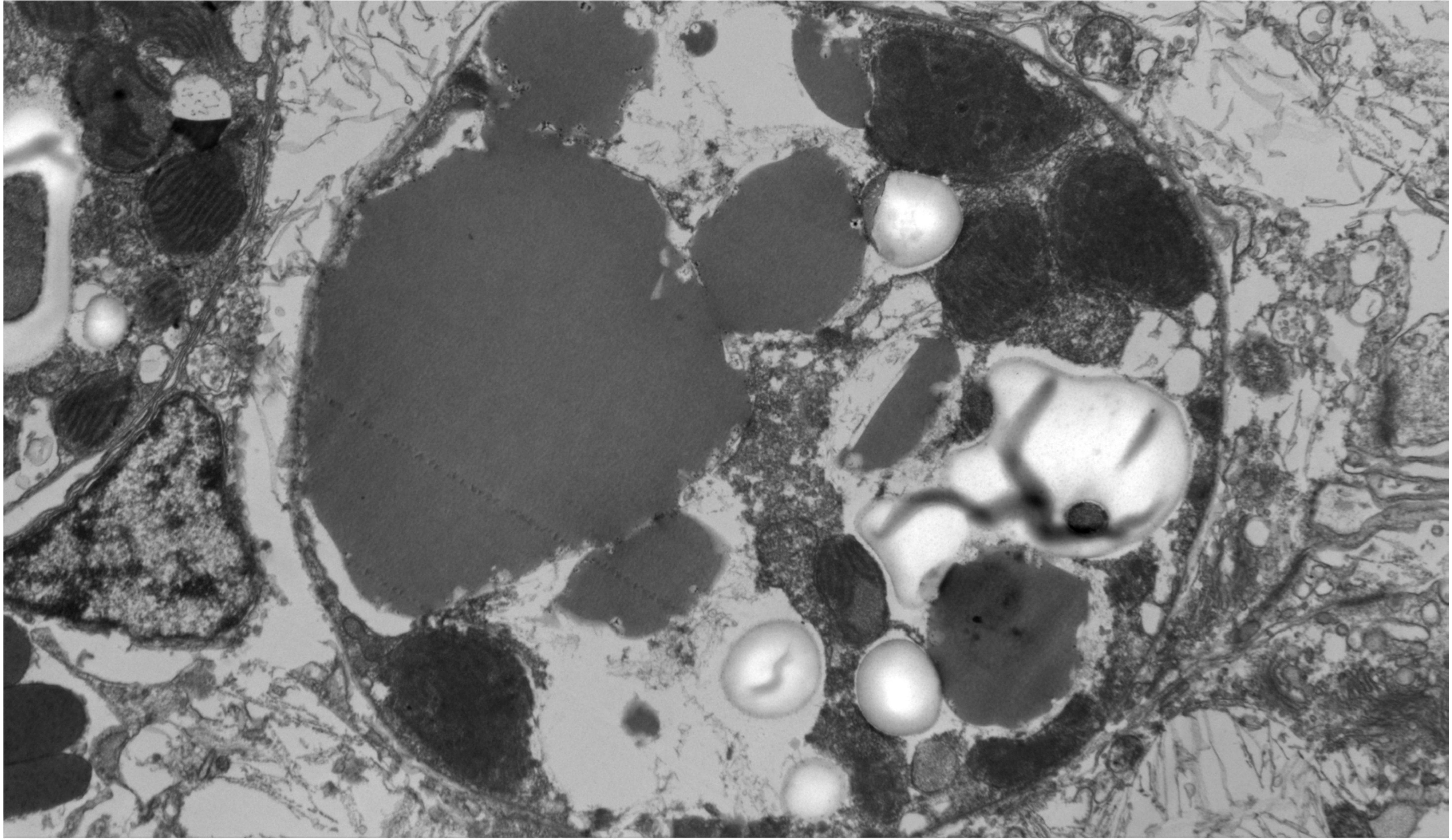

21-20_Correa_ACR120_17G2_055.tif
ACR 120
Biological Electron Microscopy Lab
Rice University - SEA
Microscopist: MD Meyer

2 μm
HV=80kV
Direct Mag: 2000 x

Cell 25

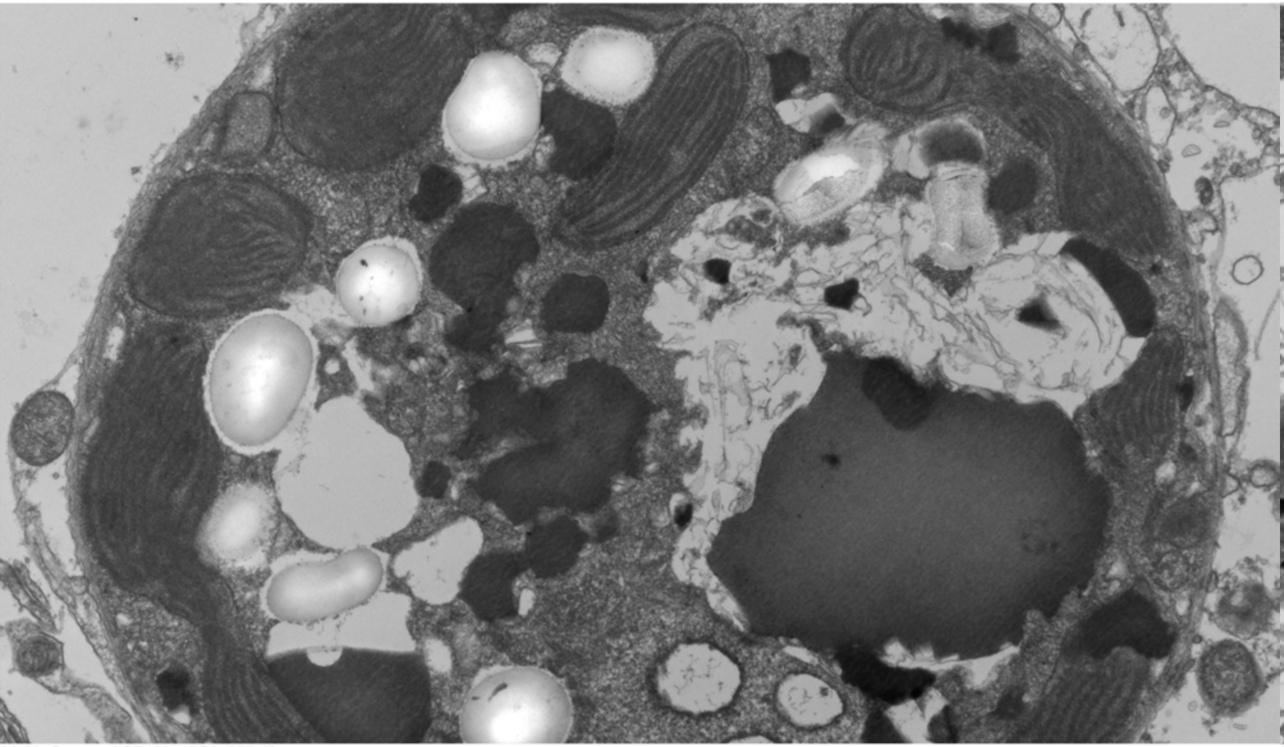

21-20_Correa_ACR120_17G2_057.tif
ACR 120
Biological Electron Microscopy Lab
Rice University - SEA
Microscopist: MD Meyer

1 µm
HV=80kV
Direct Mag: 3000 x

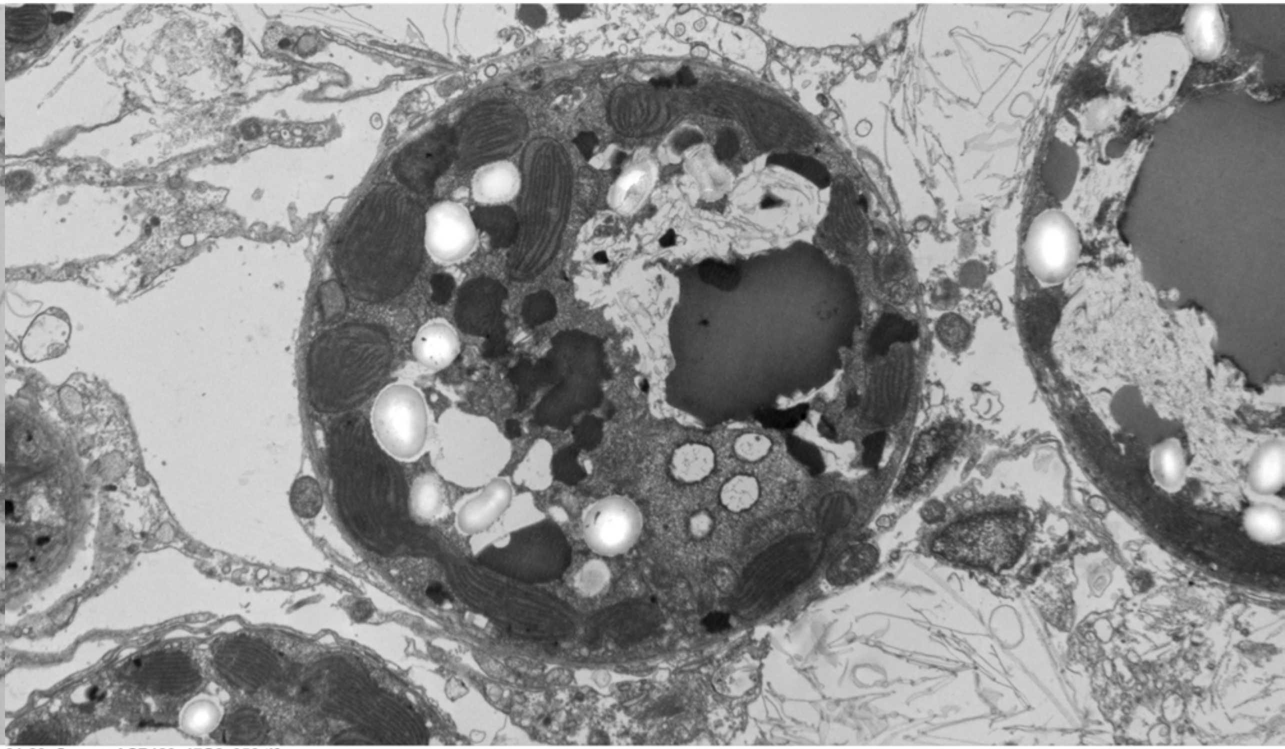

21-20_Correa_ACR120_17G2_056.tif
ACR 120
Biological Electron Microscopy Lab
Rice University - SEA
Microscopist: MD Meyer

2 µm
HV=80kV
Direct Mag: 1500 x

Cell 26

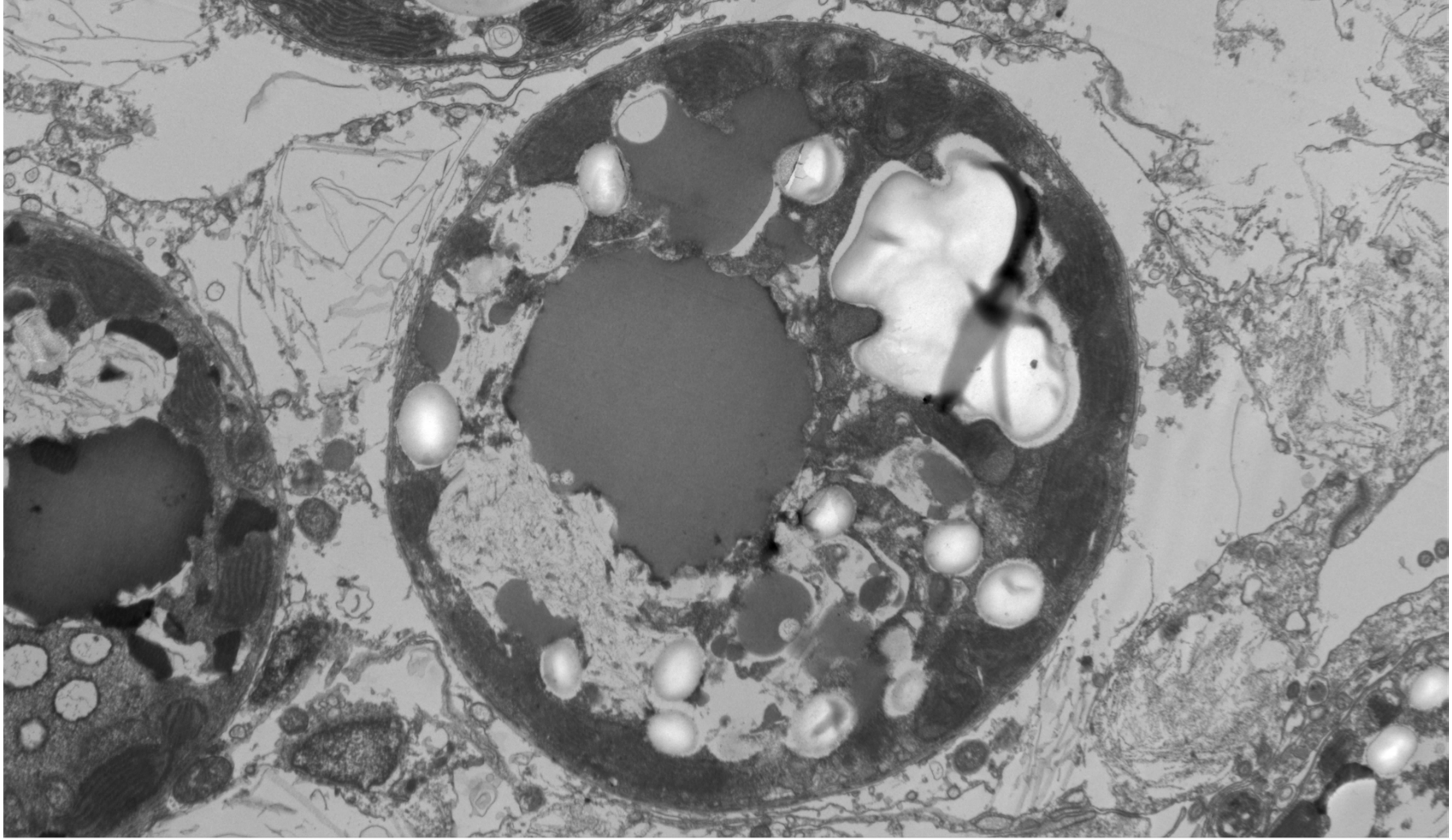

21-20_Correa_ACR120_17G2_058.tif
ACR 120
Biological Electron Microscopy Lab
Rice University - SEA
Microscopist: MD Meyer

2 µm
HV=80kV
Direct Mag: 1500 x

Cell 27 & 28

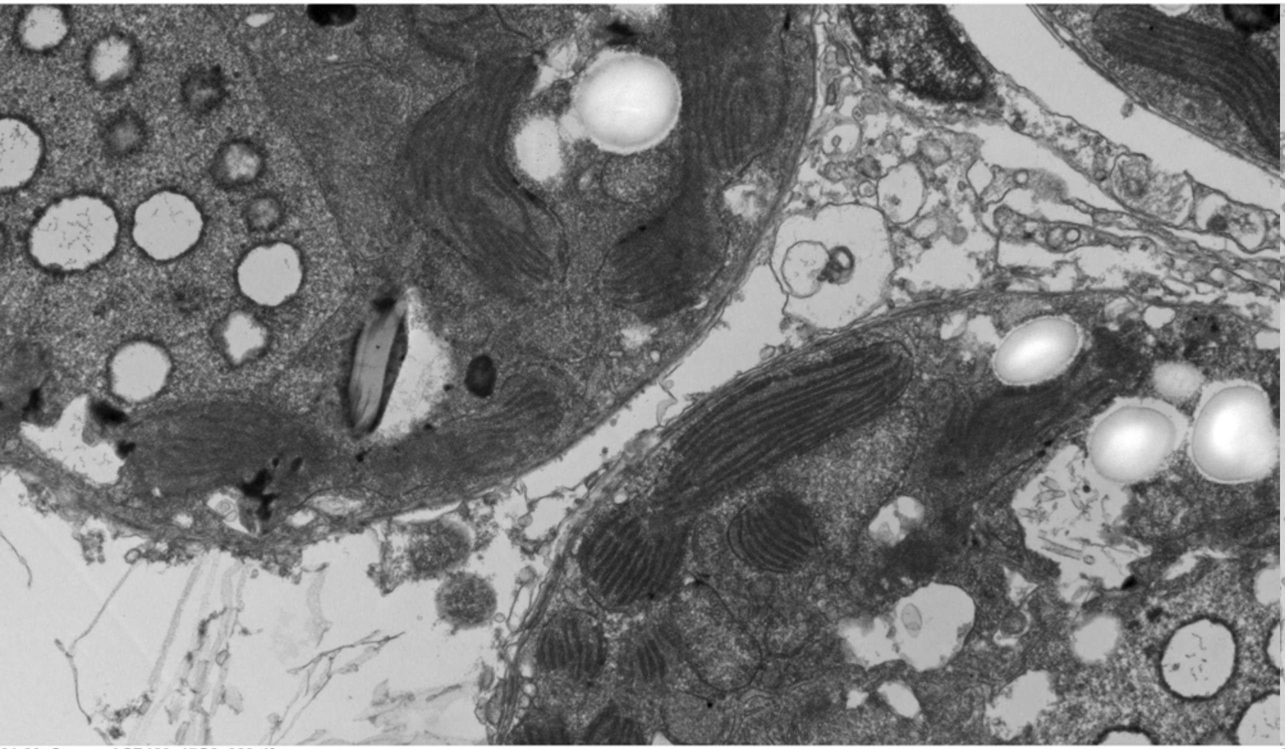

21-20_Correa_ACR120_17G2_060.tif
ACR 120
Biological Electron Microscopy Lab
Rice University - SEA
Microscopist: MD Meyer

1 μm
HV=80kV
Direct Mag: 3000 x

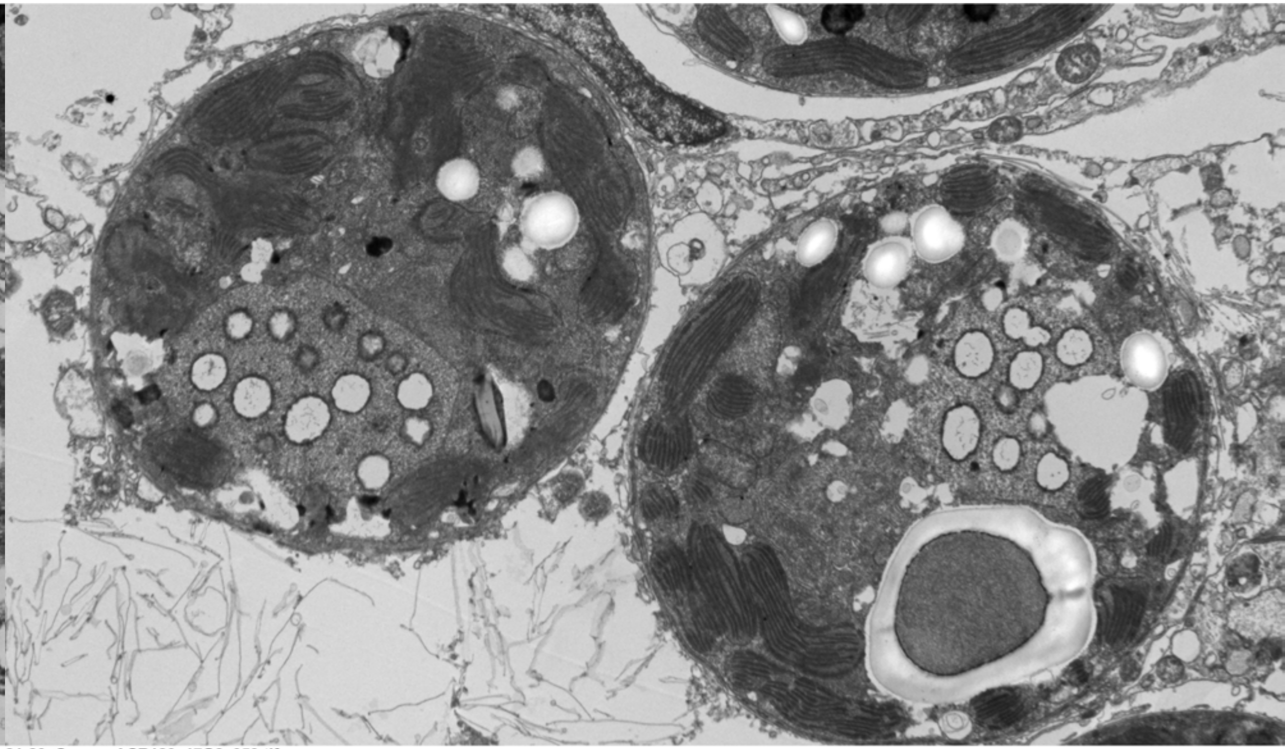

21-20_Correa_ACR120_17G2_059.tif
ACR 120
Biological Electron Microscopy Lab
Rice University - SEA
Microscopist: MD Meyer

2 μm
HV=80kV
Direct Mag: 1500 x

Cell 29

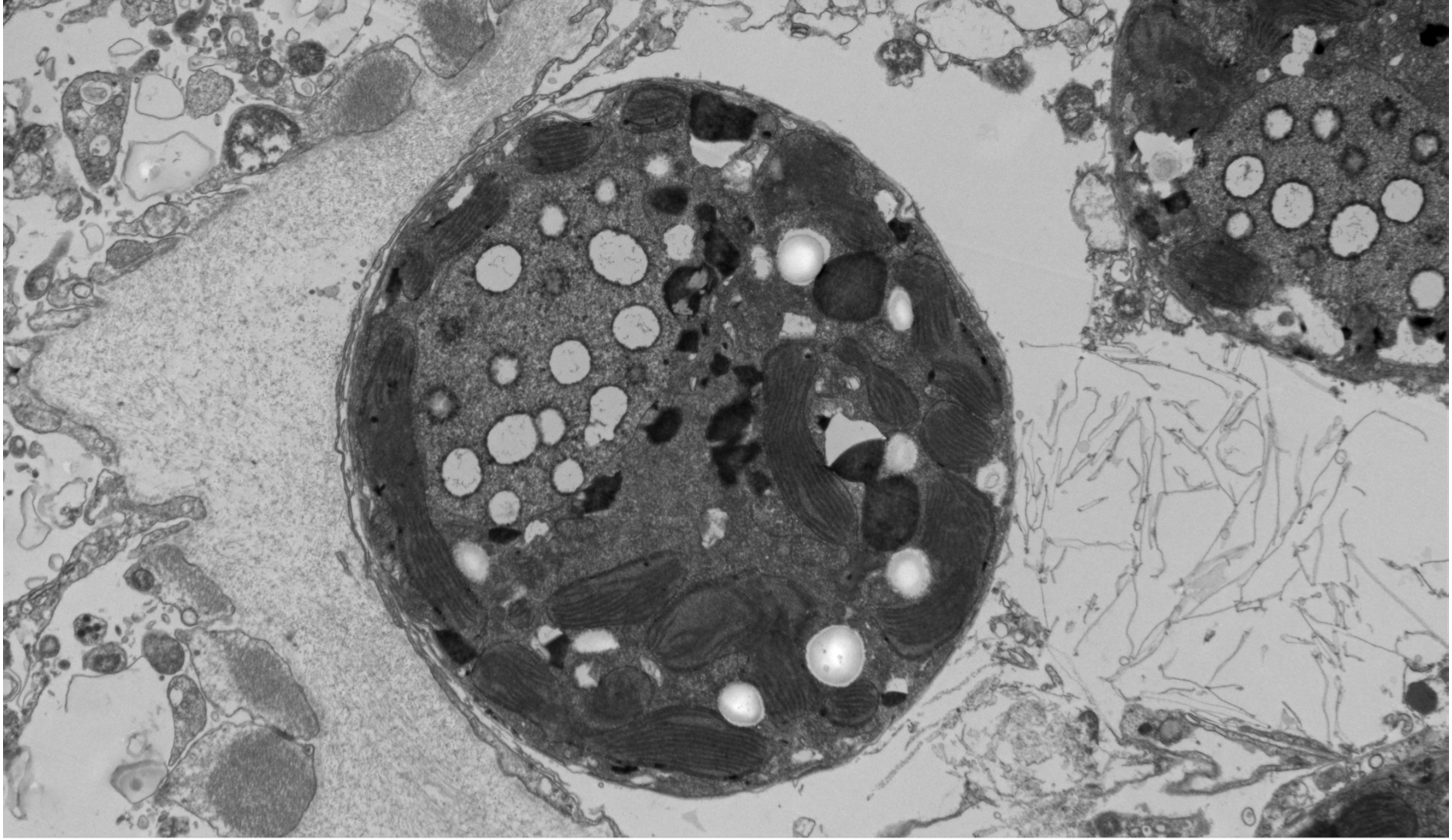

21-20_Correa_ACR120_17G2_061.tif
ACR 120
Biological Electron Microscopy Lab
Rice University - SEA
Microscopist: MD Meyer

2 µm
HV=80kV
Direct Mag: 1500 x

Cell 30

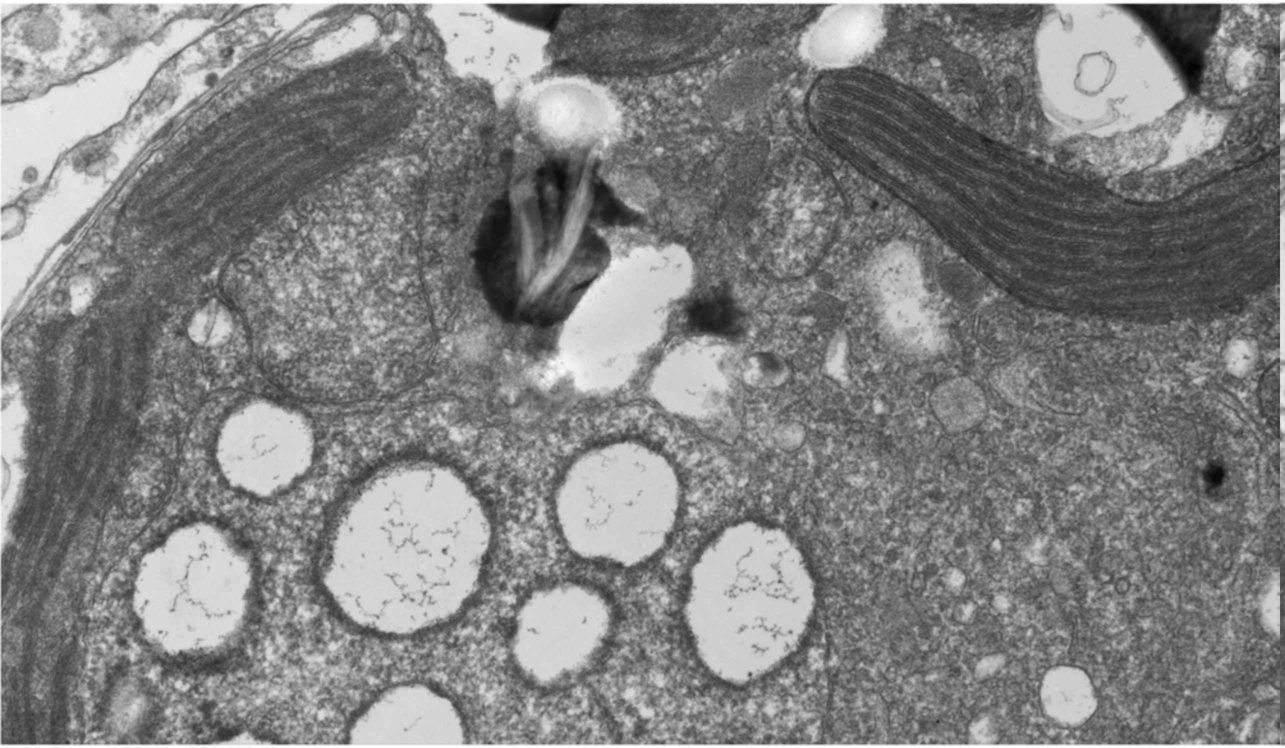

21-20_Correa_ACR120_17G2_063.tif
ACR 120
Biological Electron Microscopy Lab
Rice University - SEA
Microscopist: MD Meyer

800 nm
HV=80kV
Direct Mag: 5000 x

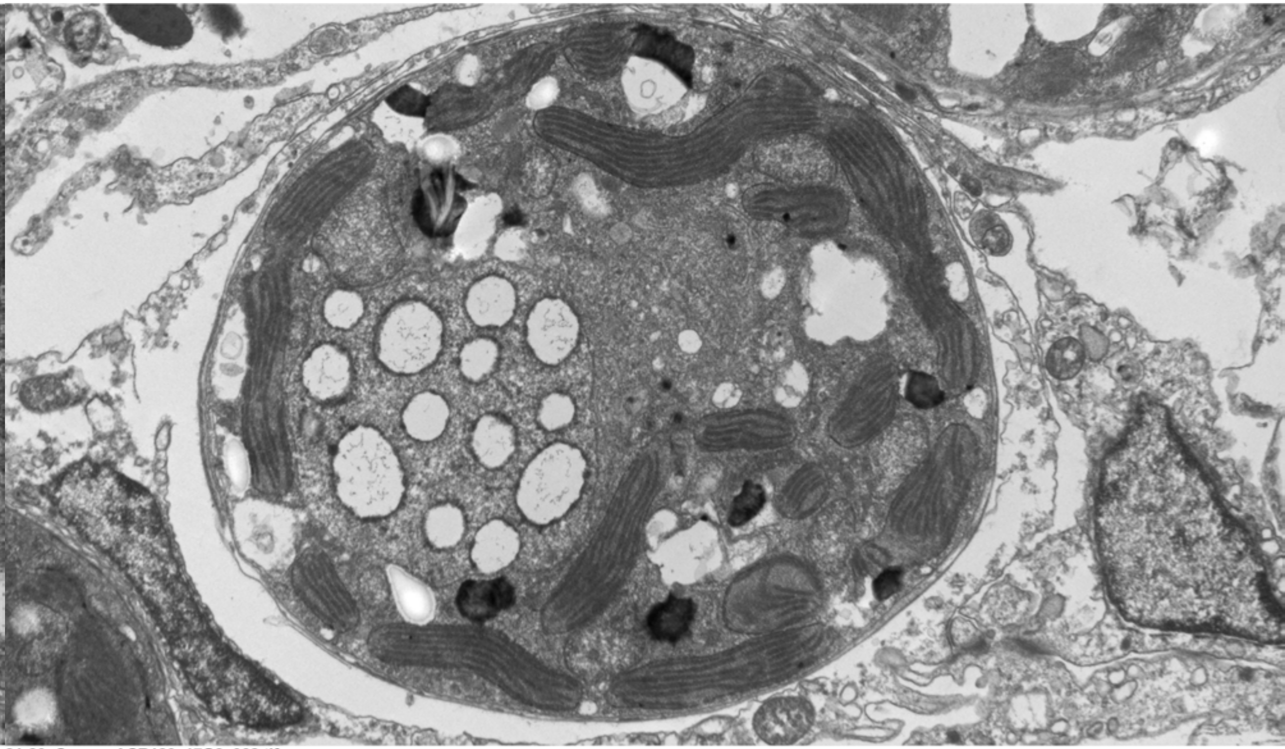

21-20_Correa_ACR120_17G2_062.tif
ACR 120
Biological Electron Microscopy Lab
Rice University - SEA
Microscopist: MD Meyer

2 μm
HV=80kV
Direct Mag: 2000 x

Cell 31 & 32

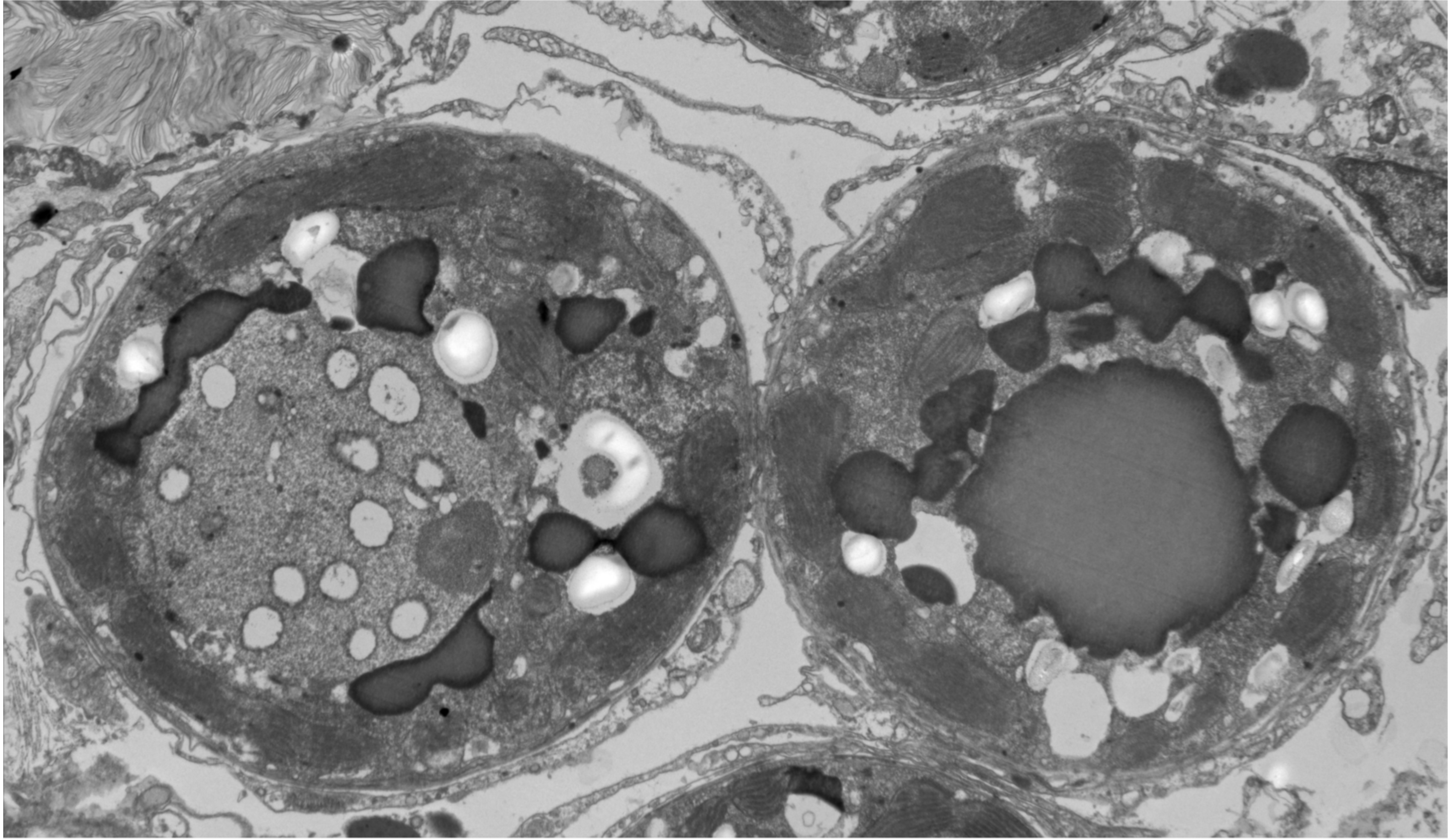

21-20_Correa_ACR120_17G2_064.tif
ACR 120
Biological Electron Microscopy Lab
Rice University - SEA
Microscopist: MD Meyer

2 µm
HV=80kV
Direct Mag: 1500 x

Cell 33 & 34

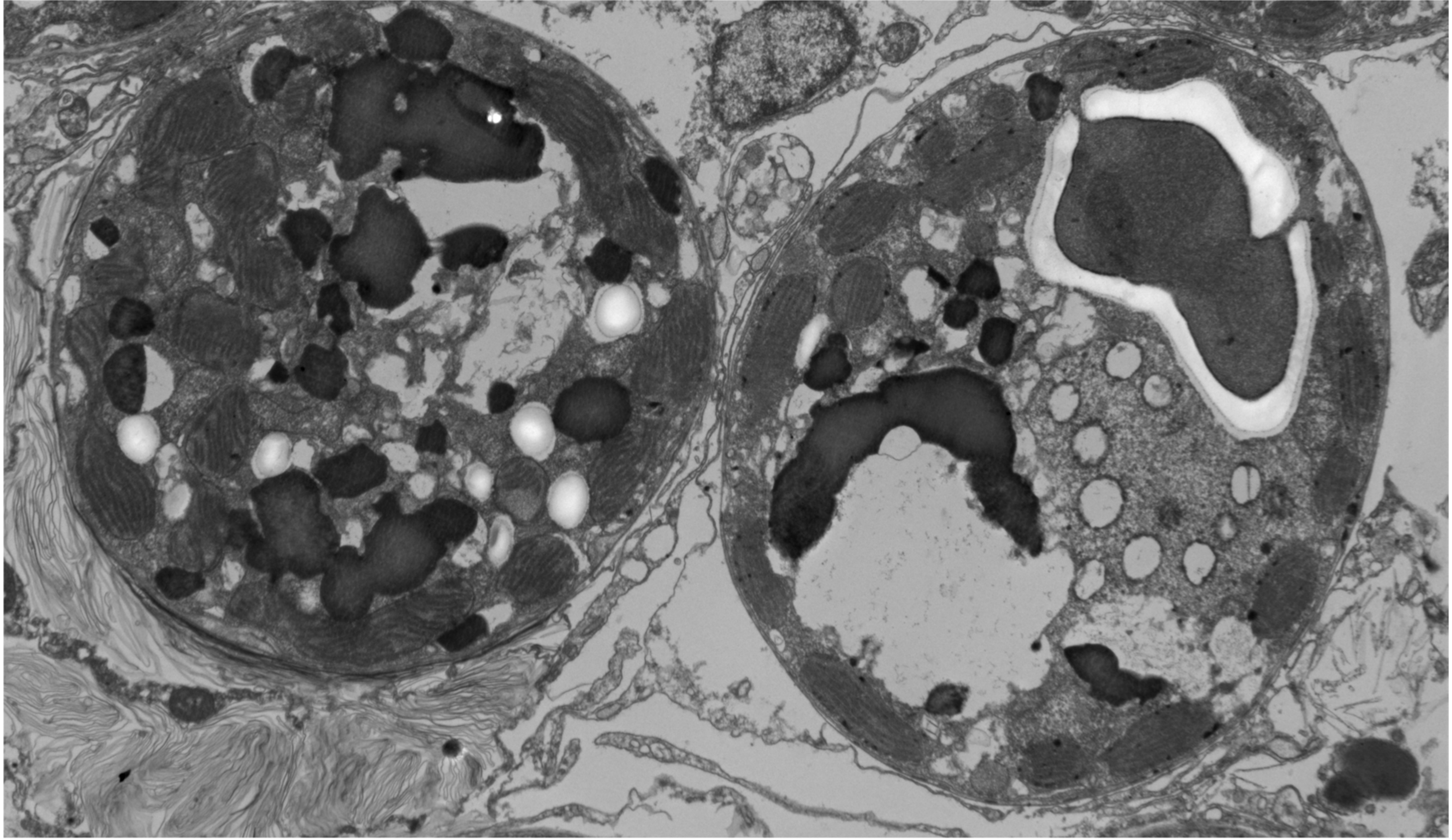

21-20_Correa_ACR120_17G2_065.tif
ACR 120
Biological Electron Microscopy Lab
Rice University - SEA
Microscopist: MD Meyer

2 µm
HV=80kV
Direct Mag: 1500 x

Cell 35

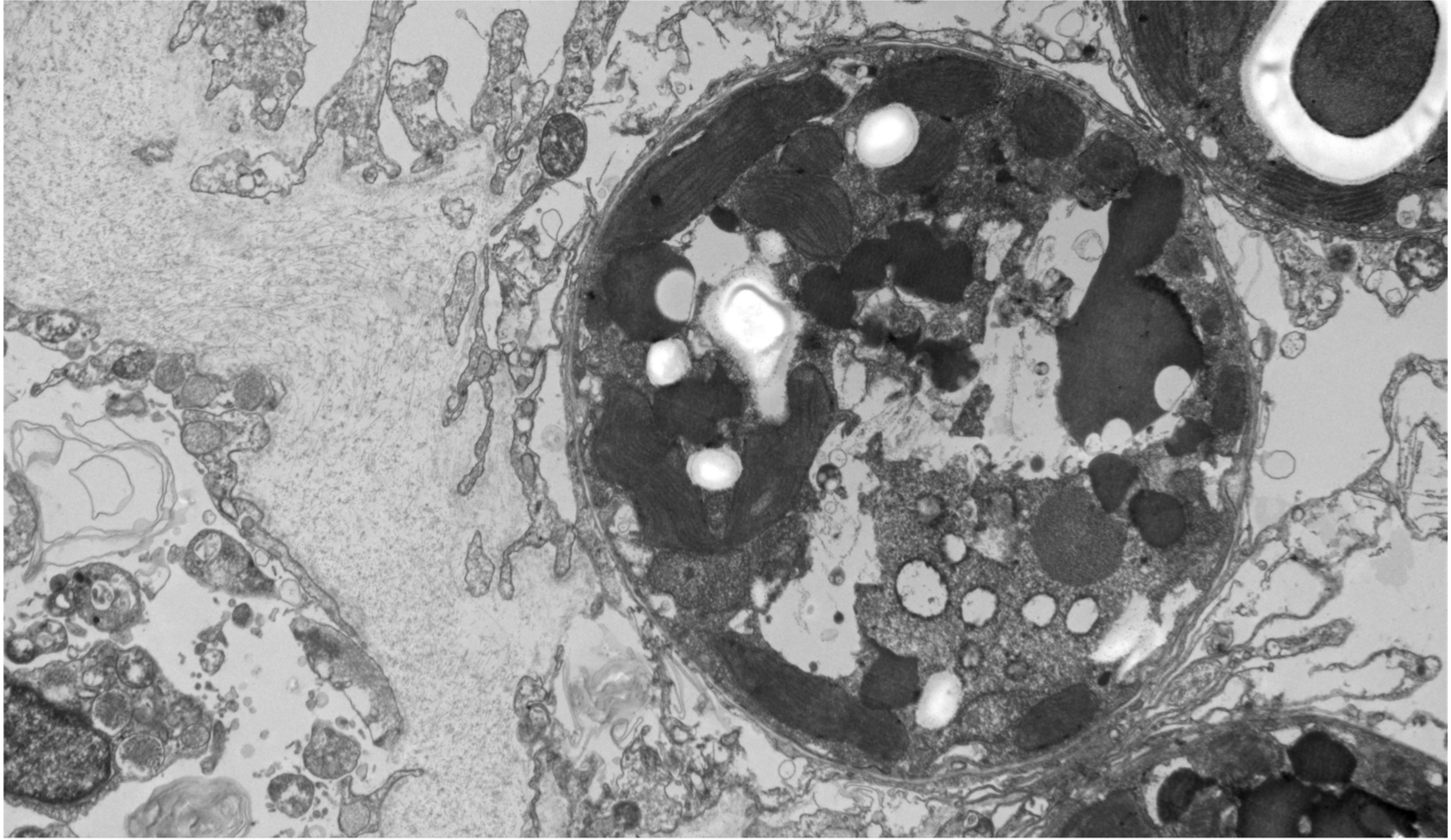

21-20_Correa_ACR120_17G2_066.tif
ACR 120
Biological Electron Microscopy Lab
Rice University - SEA
Microscopist: MD Meyer

2 µm
HV=80kV
Direct Mag: 1500 x

Cell 36

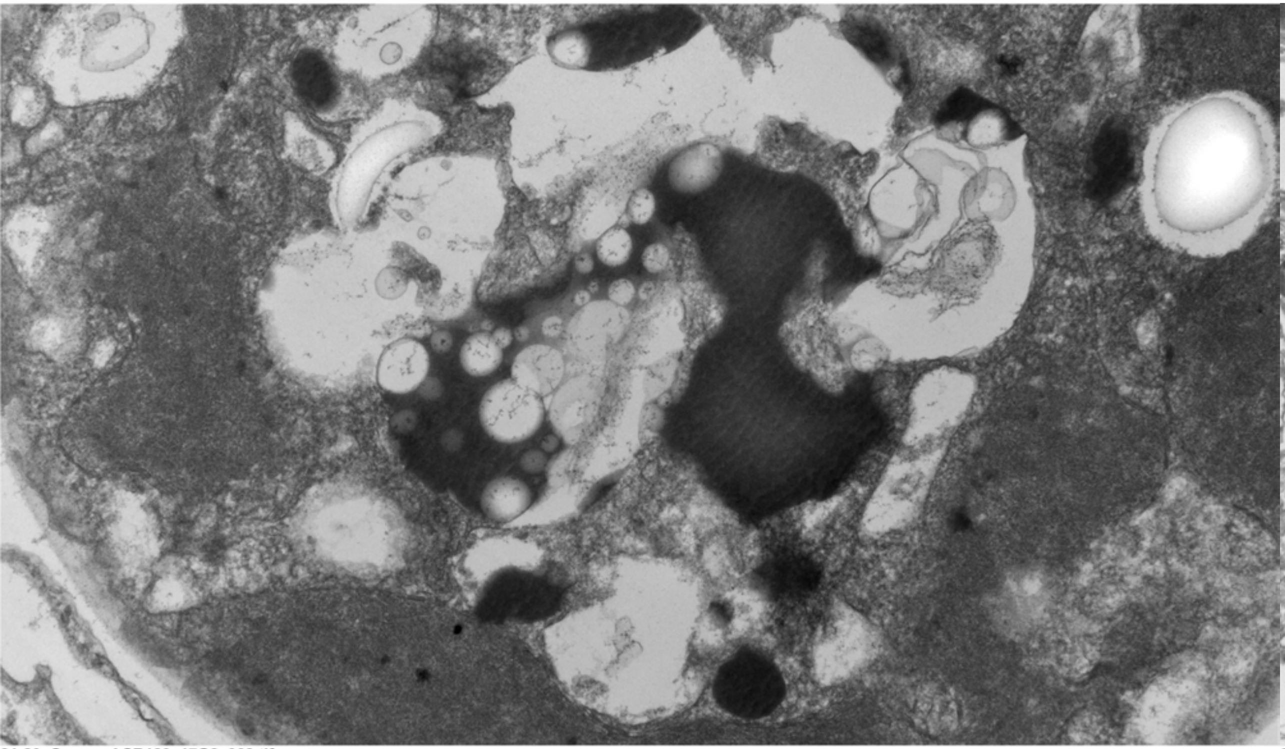

21-20_Correa_ACR120_17G2_068.tif
ACR 120
Biological Electron Microscopy Lab
Rice University - SEA
Microscopist: MD Meyer

800 nm
HV=80kV
Direct Mag: 5000 x

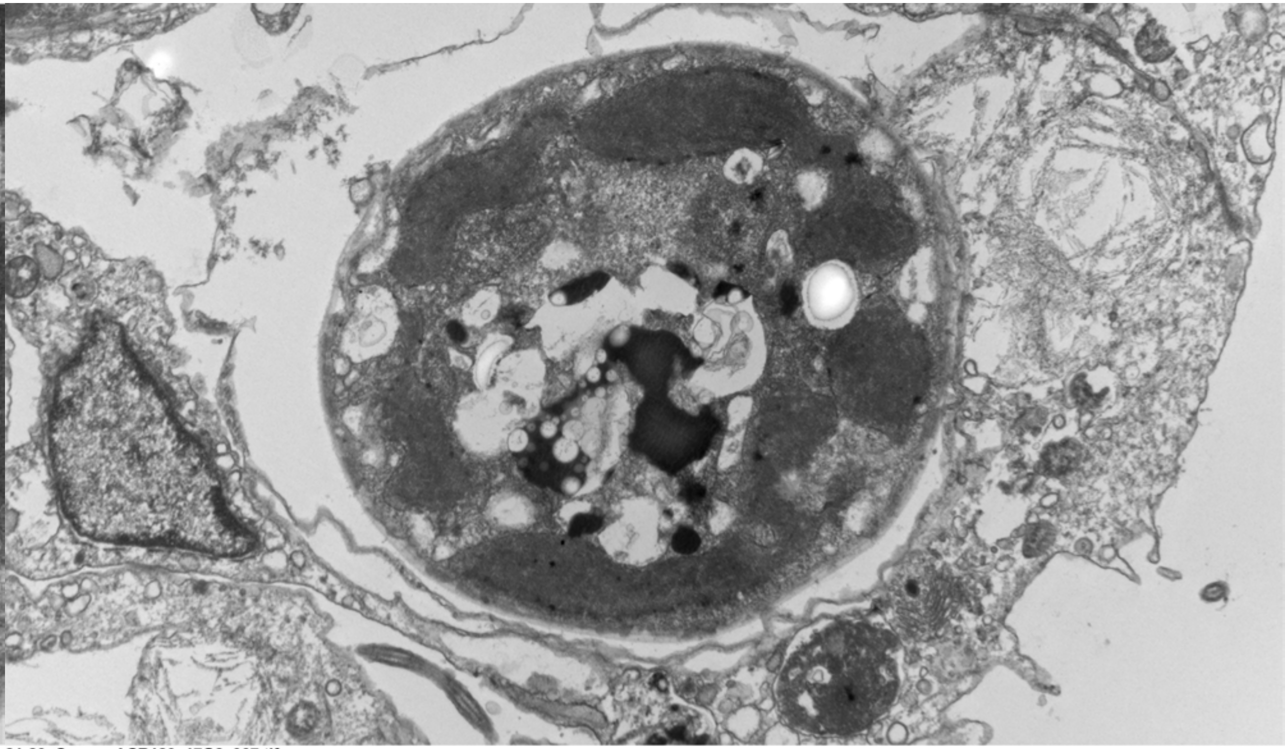

21-20_Correa_ACR120_17G2_067.tif
ACR 120
Biological Electron Microscopy Lab
Rice University - SEA
Microscopist: MD Meyer

2 µm
HV=80kV
Direct Mag: 2000 x

ACR Colony H

Cell 1

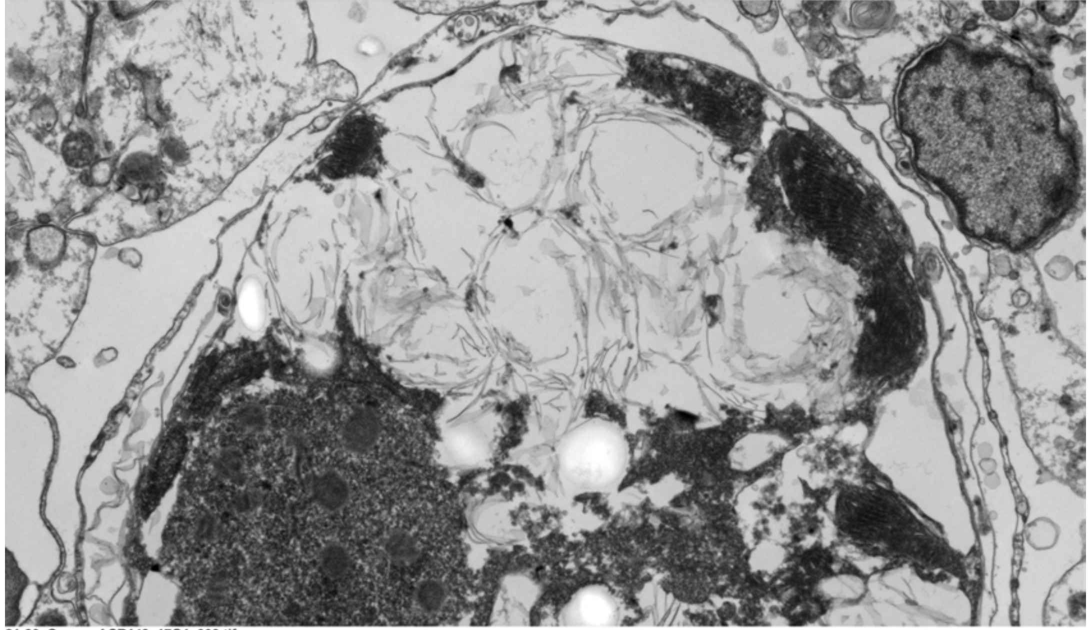

21-20_Correa_ACR143_17O1_003.tif
ACR143 tissue
Biological Electron Microscopy Lab
Rice University - SEA
Microscopist: MD Meyer

1 µm
HV=80kV
Direct Mag: 2500 x

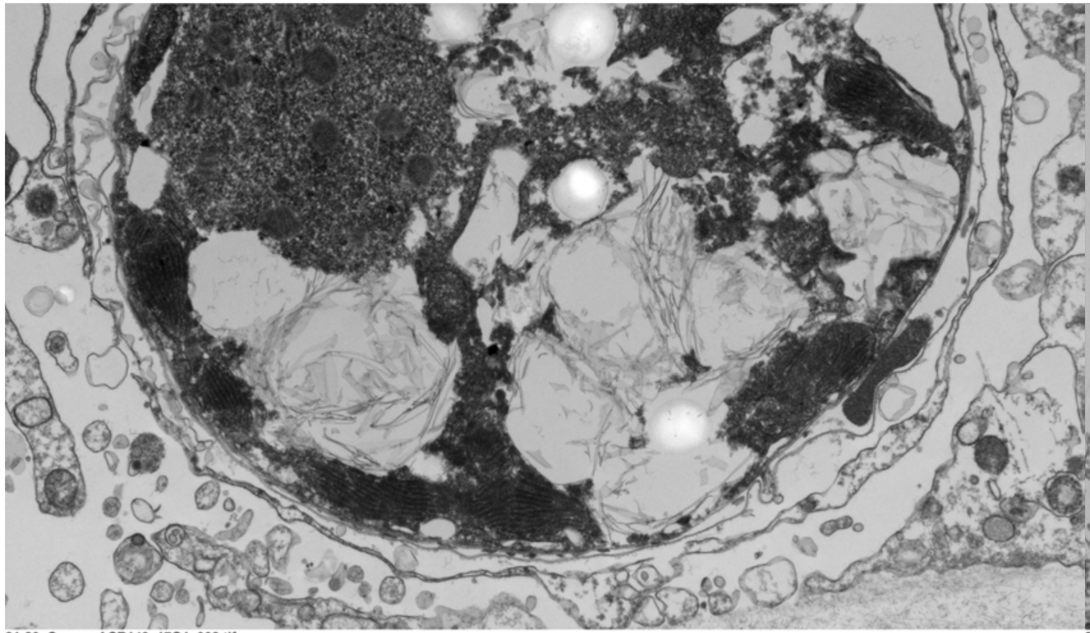

21-20_Correa_ACR143_17O1_002.tif
ACR143 tissue
Biological Electron Microscopy Lab
Rice University - SEA
Microscopist: MD Meyer

1 µm
HV=80kV
Direct Mag: 2500 x

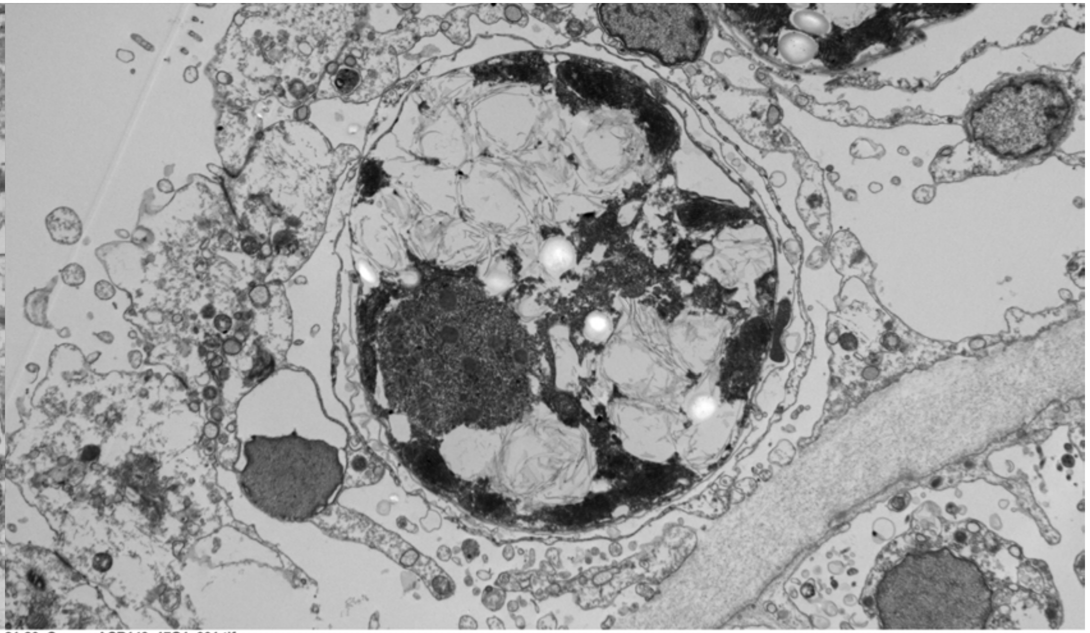

21-20_Correa_ACR143_17O1_001.tif
ACR143 tissue
Biological Electron Microscopy Lab
Rice University - SEA
Microscopist: MD Meyer

2 µm
HV=80kV
Direct Mag: 1200 x

Cell 2

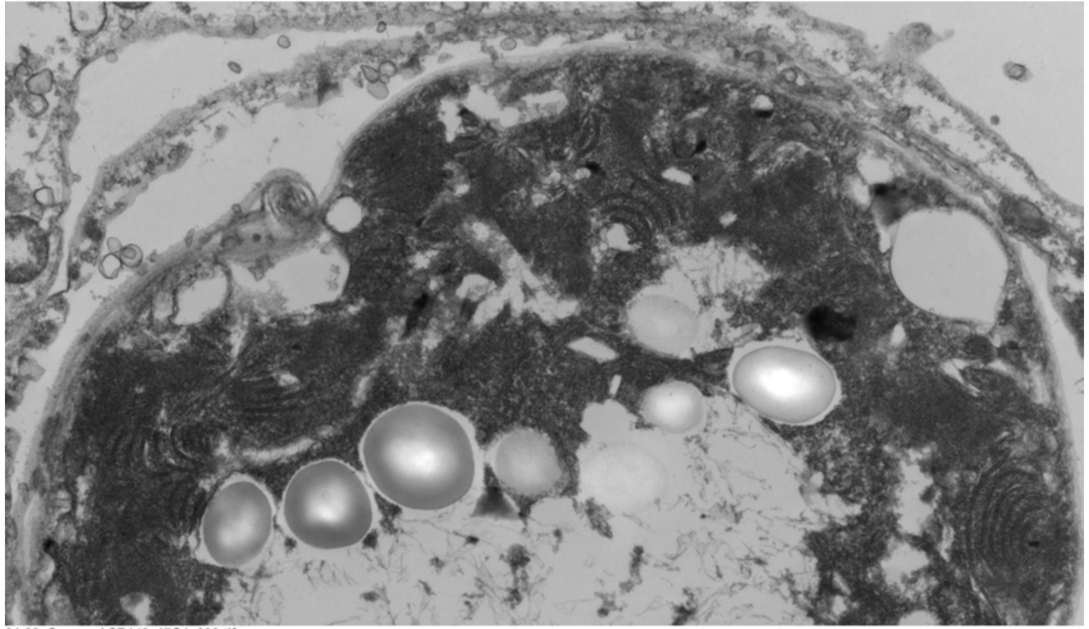

21-20_Correa_ACR143_17O1_006.tif
ACR143 tissue
Biological Electron Microscopy Lab
Rice University - SEA
Microscopist: MD Meyer

1 µm
HV=80kV
Direct Mag: 4000 x

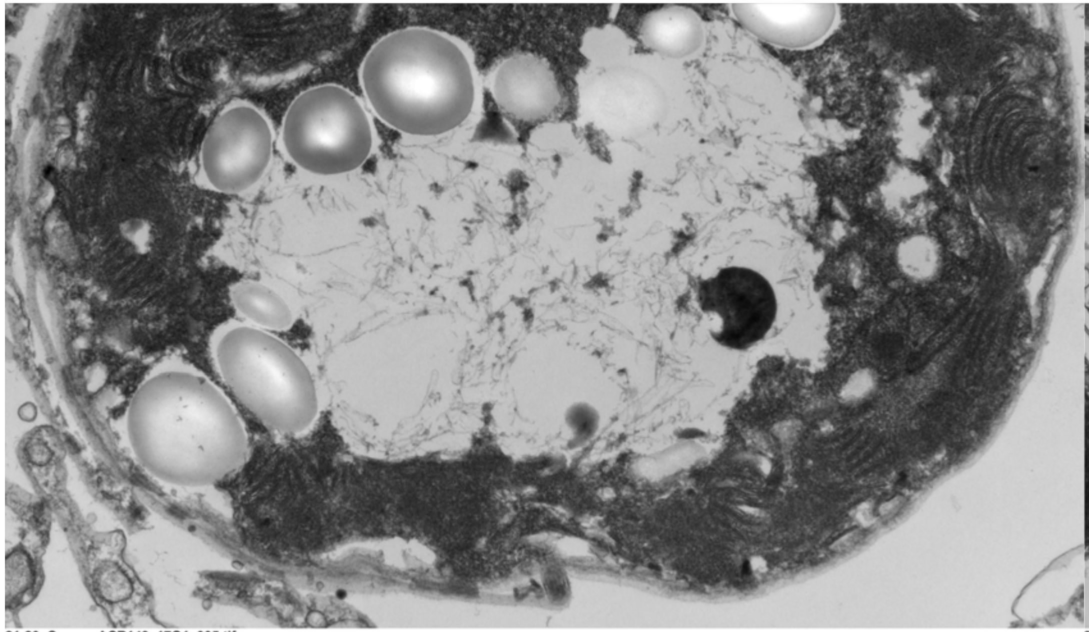

21-20_Correa_ACR143_17O1_005.tif
ACR143 tissue
Biological Electron Microscopy Lab
Rice University - SEA
Microscopist: MD Meyer

1 µm
HV=80kV
Direct Mag: 4000 x

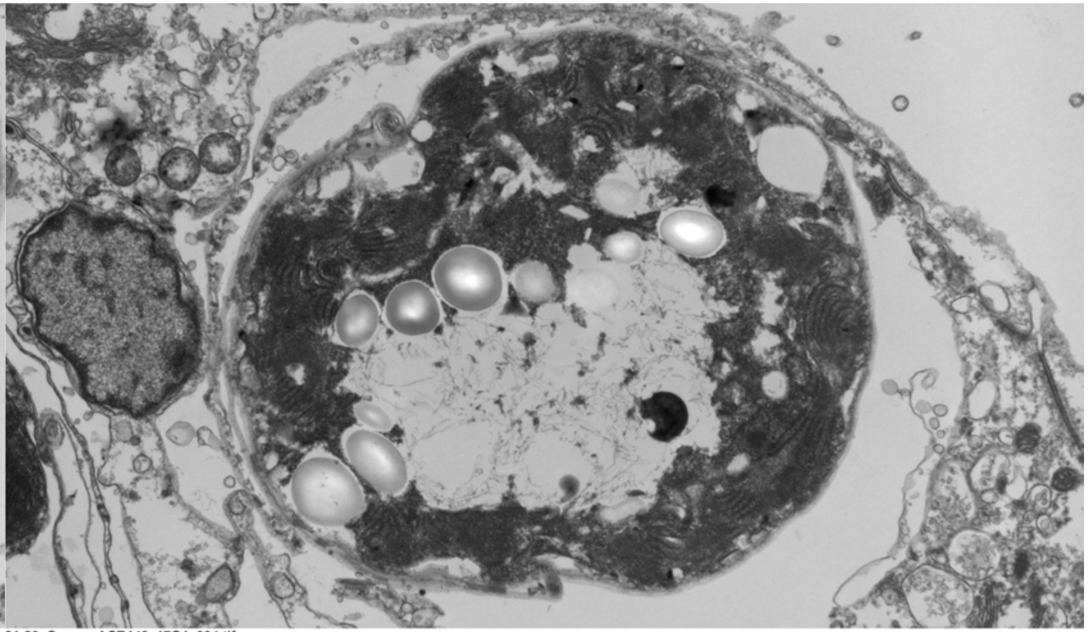

21-20_Correa_ACR143_17O1_004.tif
ACR143 tissue
Biological Electron Microscopy Lab
Rice University - SEA
Microscopist: MD Meyer

1 µm
HV=80kV
Direct Mag: 2500 x

Cell 3

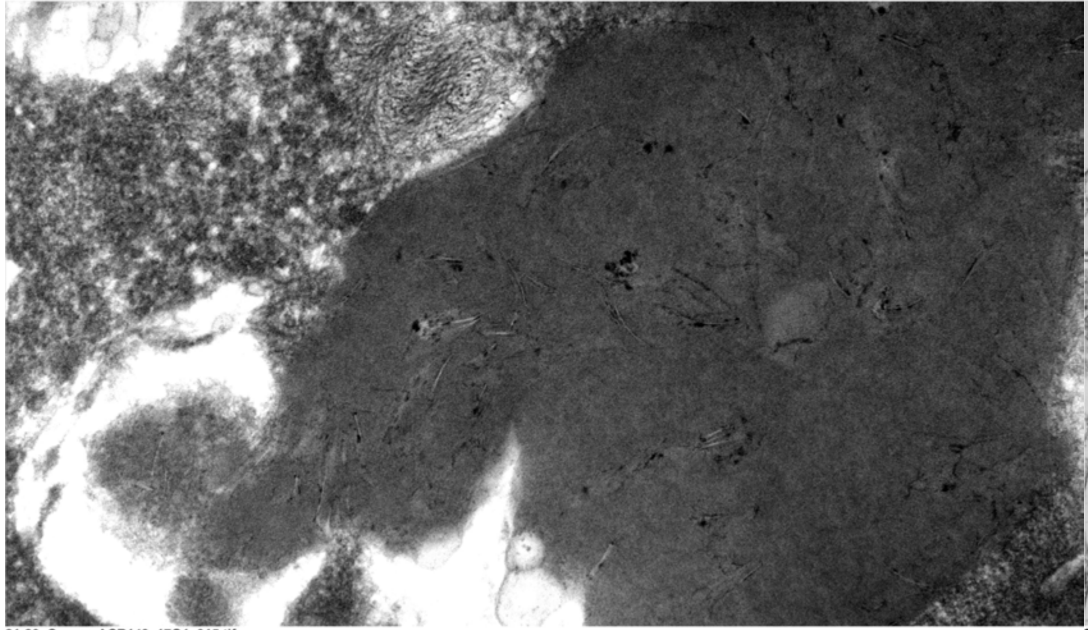

21-20_Correa_ACR143_17O1_015.tif
ACR143 tissue
Biological Electron Microscopy Lab
Rice University - SEA
Microscopist: MD Meyer

200 nm
HV=80kV
Direct Mag: 12000 x

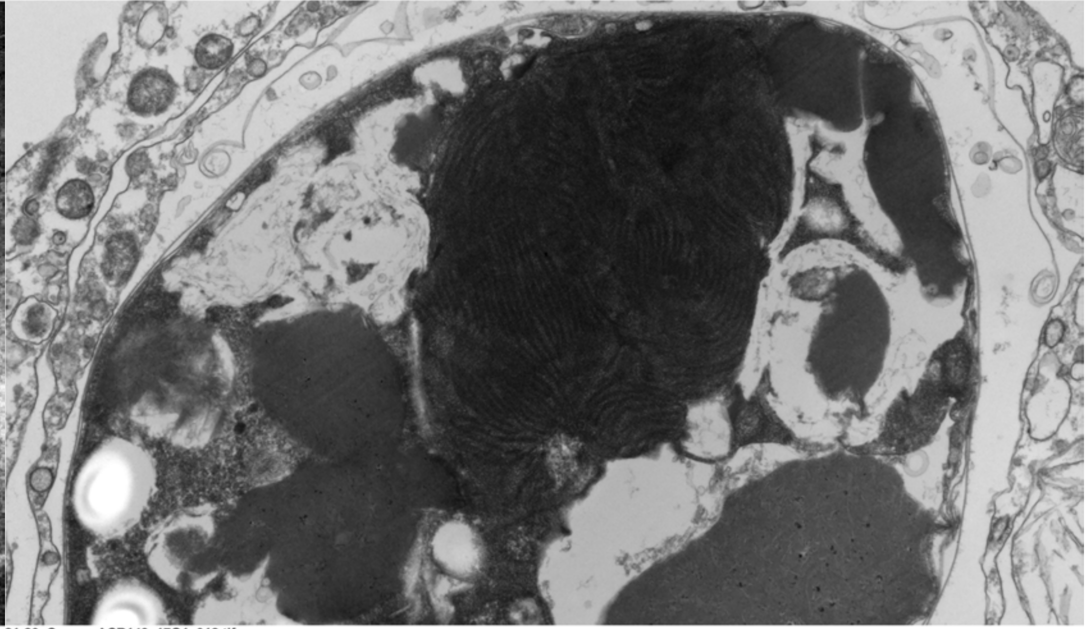

21-20_Correa_ACR143_17O1_012.tif
ACR143 tissue
Biological Electron Microscopy Lab
Rice University - SEA
Microscopist: MD Meyer

1 µm
HV=80kV
Direct Mag: 3000 x

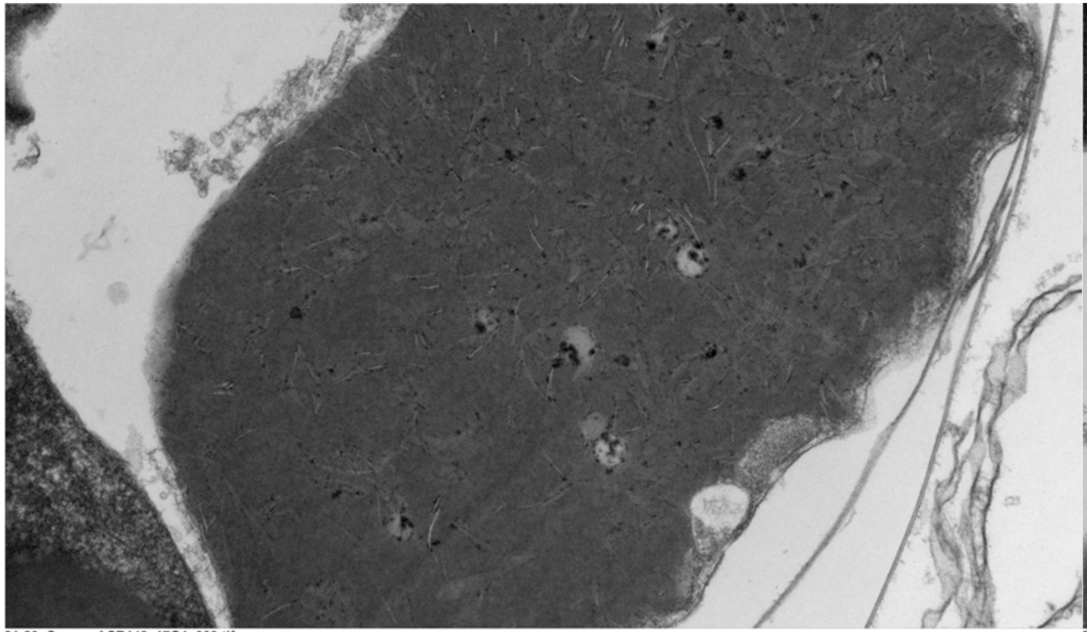

21-20_Correa_ACR143_17O1_009.tif
ACR143 tissue
Biological Electron Microscopy Lab
Rice University - SEA
Microscopist: MD Meyer

500 nm
HV=80kV
Direct Mag: 8000 x

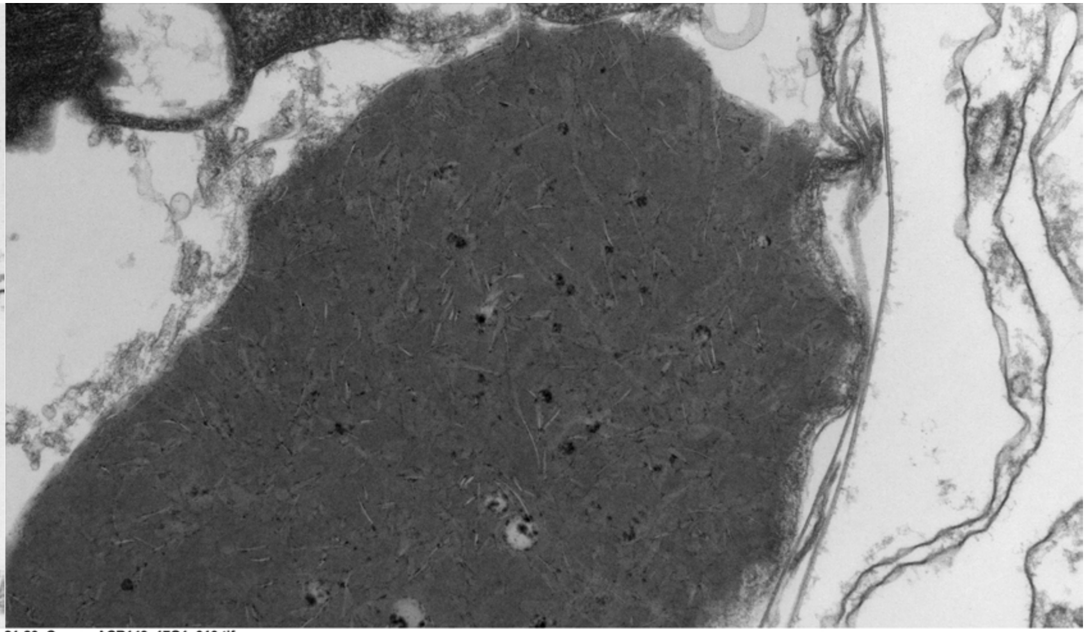

21-20_Correa_ACR143_17O1_010.tif
ACR143 tissue
Biological Electron Microscopy Lab
Rice University - SEA
Microscopist: MD Meyer

500 nm
HV=80kV
Direct Mag: 8000 x

Cell 3

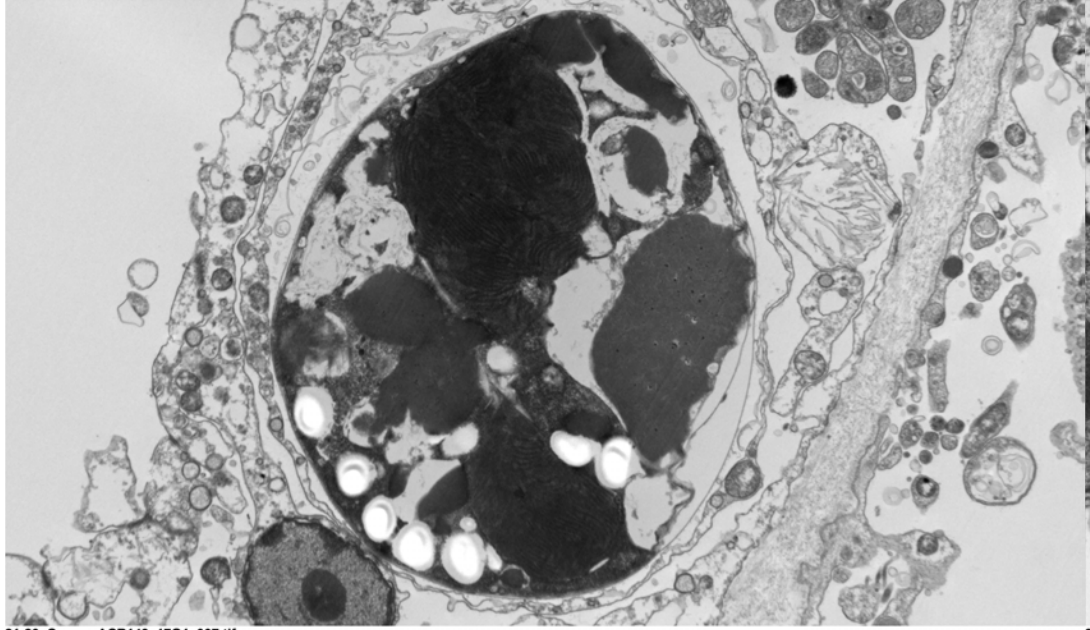

21-20_Correa_ACR143_17O1_007.tif
ACR143 tissue
Biological Electron Microscopy Lab
Rice University - SEA
Microscopist: MD Meyer

2 µm
HV=80kV
Direct Mag: 1500 x

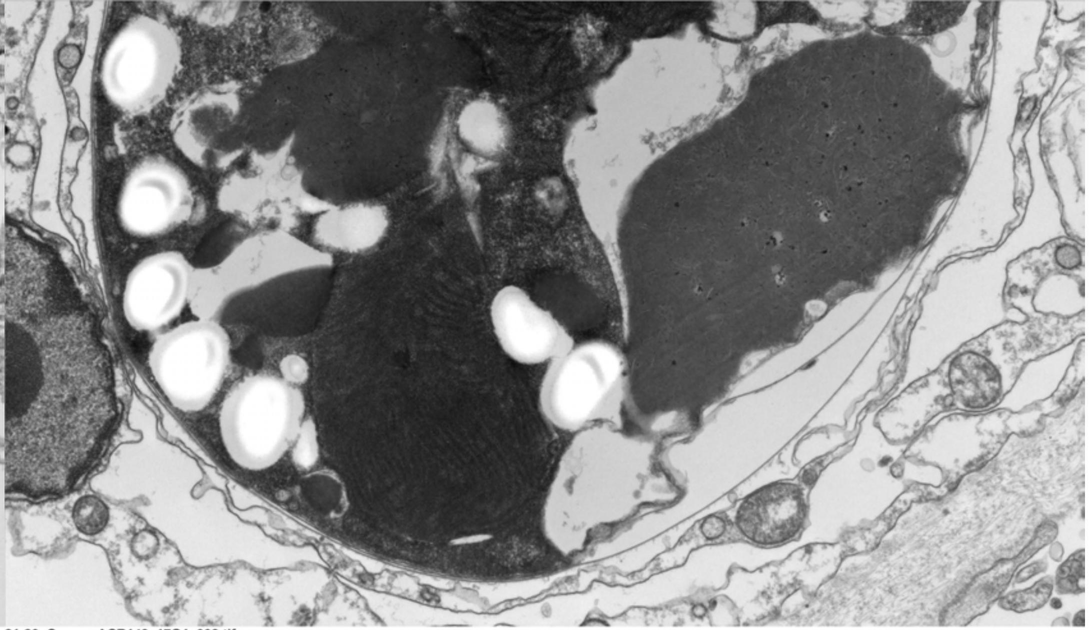

21-20_Correa_ACR143_17O1_008.tif
ACR143 tissue
Biological Electron Microscopy Lab
Rice University - SEA
Microscopist: MD Meyer

1 µm
HV=80kV
Direct Mag: 3000 x

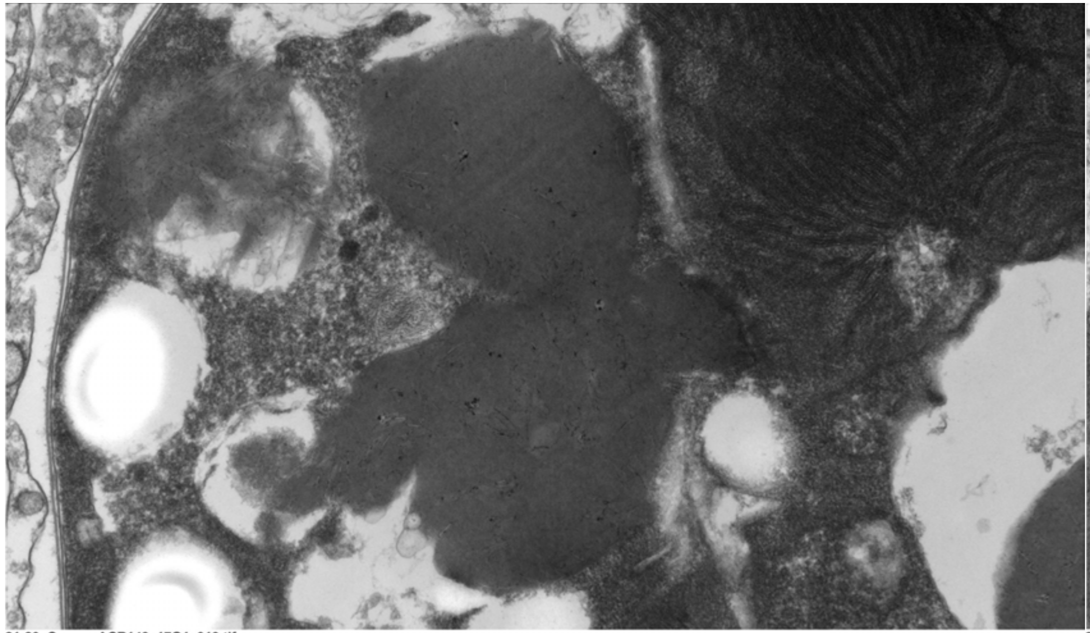

21-20_Correa_ACR143_17O1_013.tif
ACR143 tissue
Biological Electron Microscopy Lab
Rice University - SEA
Microscopist: MD Meyer

800 nm
HV=80kV
Direct Mag: 5000 x

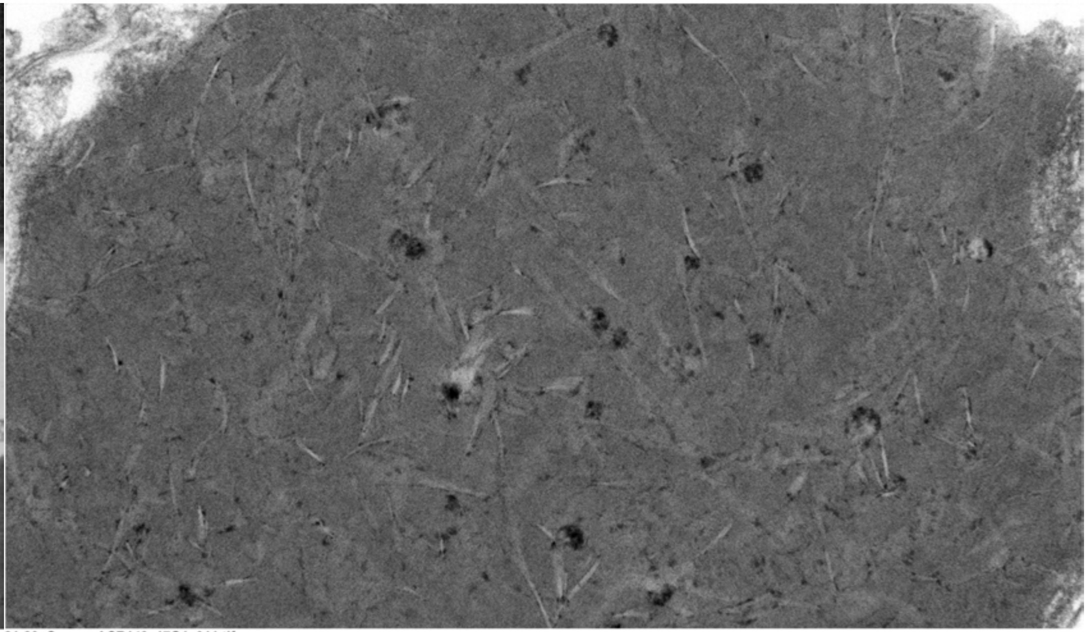

21-20_Correa_ACR143_17O1_011.tif
ACR143 tissue
Biological Electron Microscopy Lab
Rice University - SEA
Microscopist: MD Meyer

200 nm
HV=80kV
Direct Mag: 15000 x

Cell 4

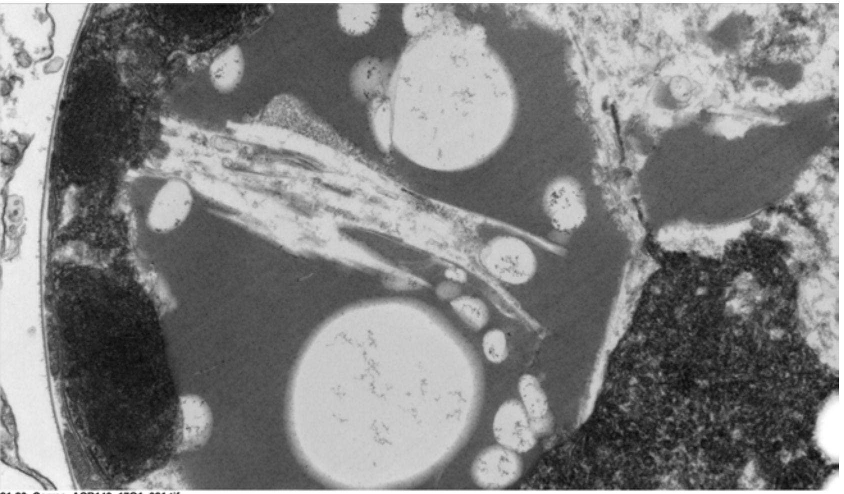

21-20_Correa_ACR143_17O1_021.tif
ACR143 tissue
Biological Electron Microscopy Lab
Rice University - SEA
Microscopist: MD Meyer
800 nm
HV=80kV
Direct Mag: 5000 x

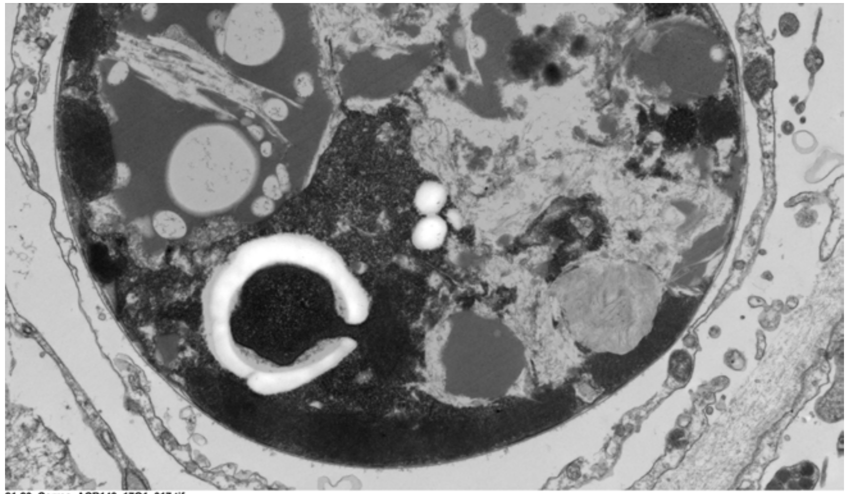

21-20_Correa_ACR143_17O1_017.tif
ACR143 tissue
Biological Electron Microscopy Lab
Rice University - SEA
Microscopist: MD Meyer
1 µm
HV=80kV
Direct Mag: 2500 x

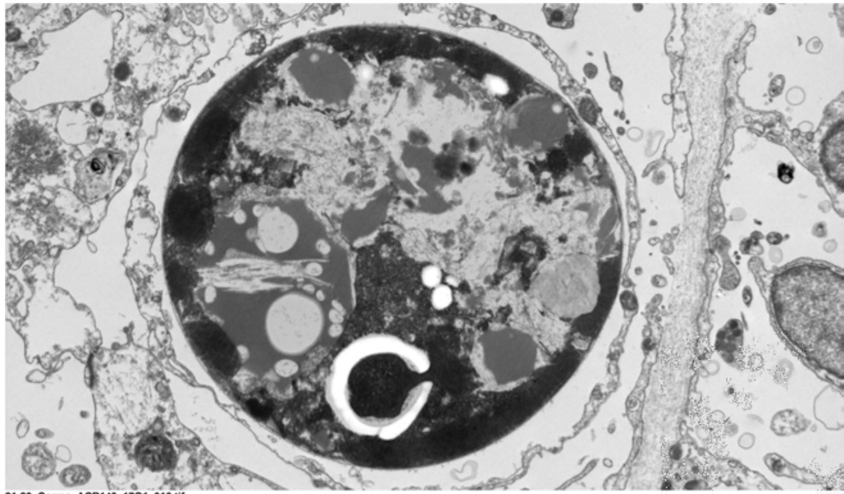

21-20_Correa_ACR143_17O1_016.tif
ACR143 tissue
Biological Electron Microscopy Lab
Rice University - SEA
Microscopist: MD Meyer
2 µm
HV=80kV
Direct Mag: 1500 x

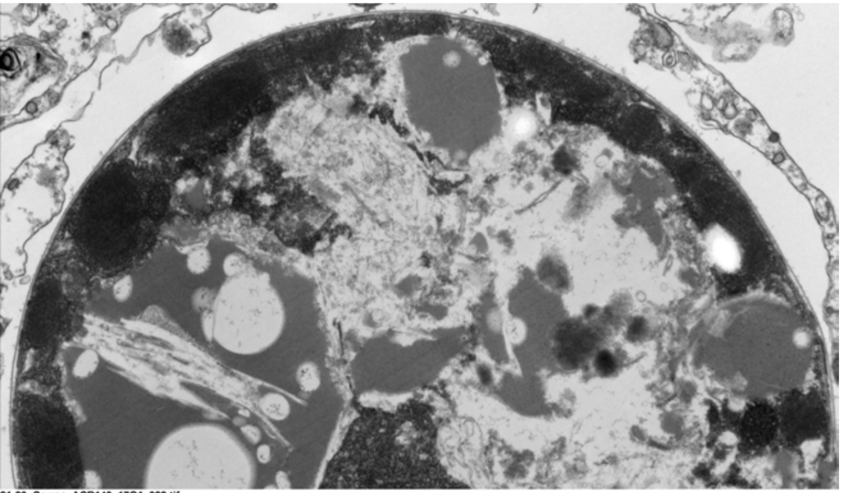

21-20_Correa_ACR143_17O1_022.tif
ACR143 tissue
Biological Electron Microscopy Lab
Rice University - SEA
Microscopist: MD Meyer
1 µm
HV=80kV
Direct Mag: 3000 x

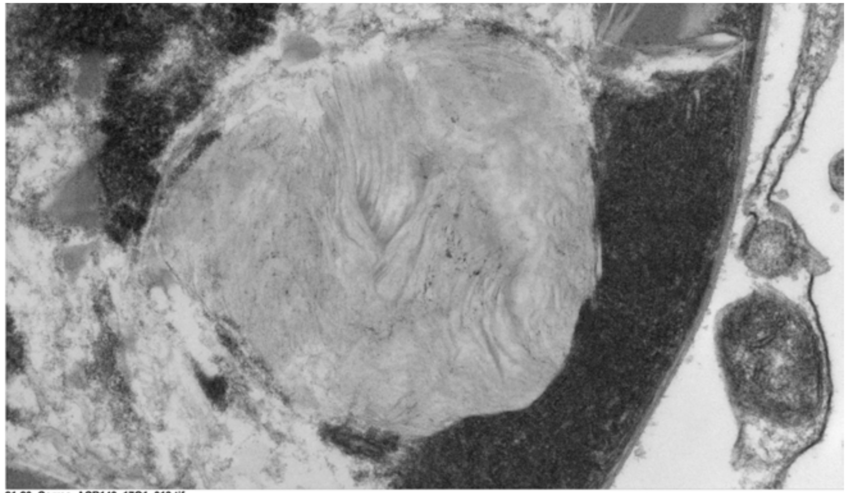

21-20_Correa_ACR143_17O1_019.tif
ACR143 tissue
Biological Electron Microscopy Lab
Rice University - SEA
Microscopist: MD Meyer
400 nm
HV=80kV
Direct Mag: 10000 x

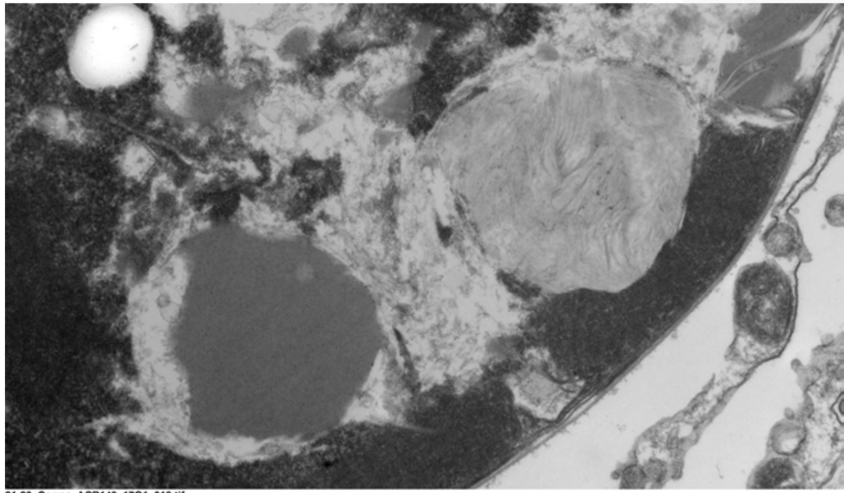

21-20_Correa_ACR143_17O1_018.tif
ACR143 tissue
Biological Electron Microscopy Lab
Rice University - SEA
Microscopist: MD Meyer
600 nm
HV=80kV
Direct Mag: 6000 x

Cell 5

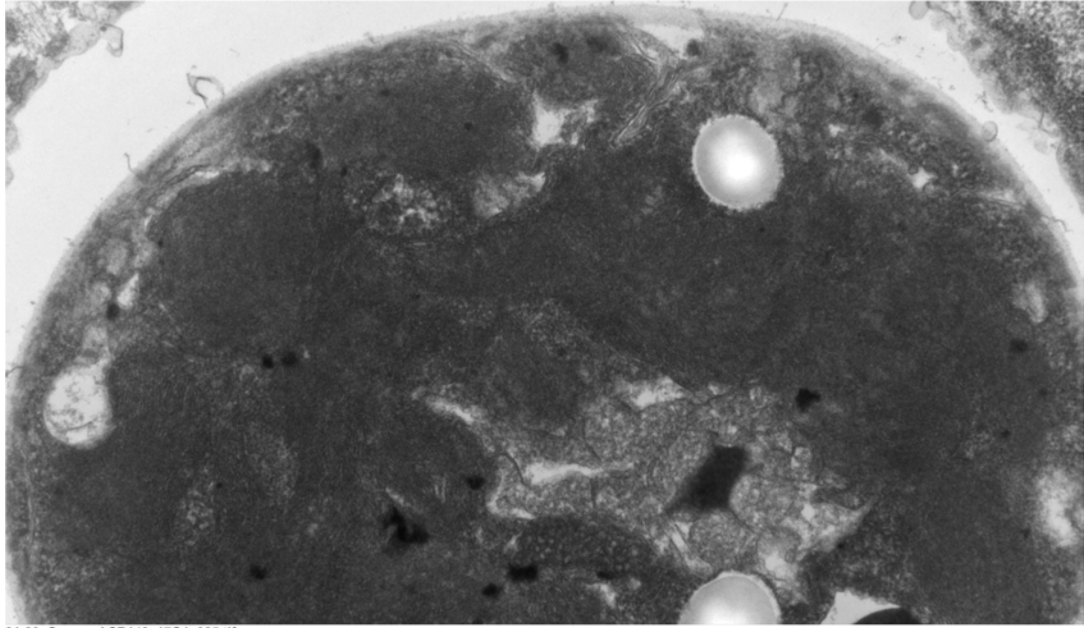

21-20_Correa_ACR143_17O1_025.tif
ACR143 tissue
Biological Electron Microscopy Lab
Rice University - SEA
Microscopist: MD Meyer

800 nm
HV=80kV
Direct Mag: 5000 x

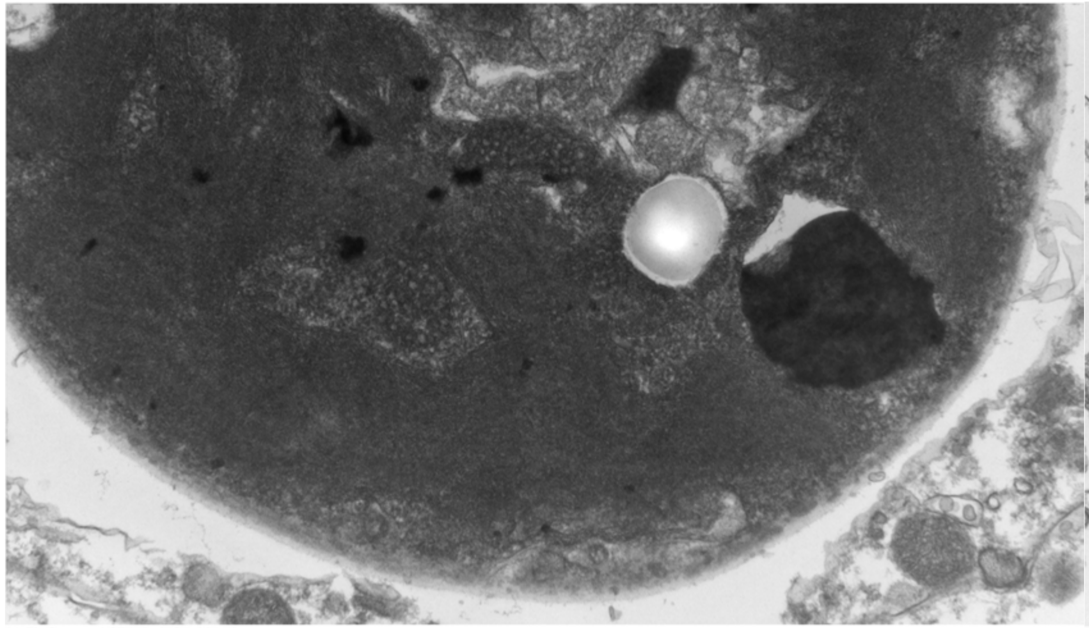

21-20_Correa_ACR143_17O1_024.tif
ACR143 tissue
Biological Electron Microscopy Lab
Rice University - SEA
Microscopist: MD Meyer

800 nm
HV=80kV
Direct Mag: 5000 x

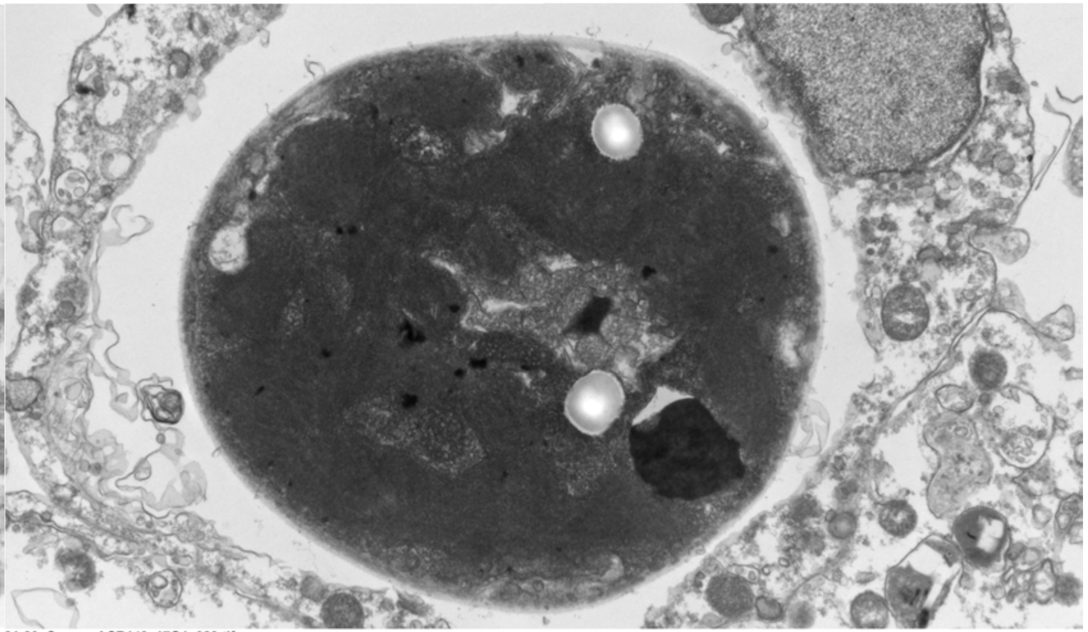

21-20_Correa_ACR143_17O1_023.tif
ACR143 tissue
Biological Electron Microscopy Lab
Rice University - SEA
Microscopist: MD Meyer

1 µm
HV=80kV
Direct Mag: 3000 x

Cell 6

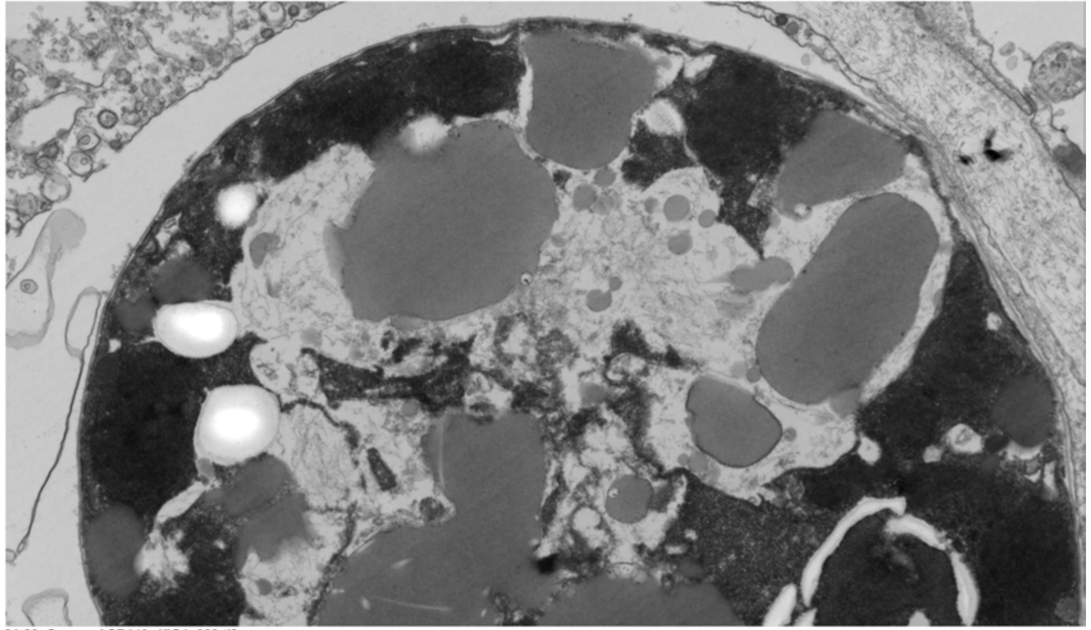

21-20_Correa_ACR143_17O1_028.tif
ACR143 tissue
Biological Electron Microscopy Lab
Rice University - SEA
Microscopist: MD Meyer

1 µm
HV=80kV
Direct Mag: 3000 x

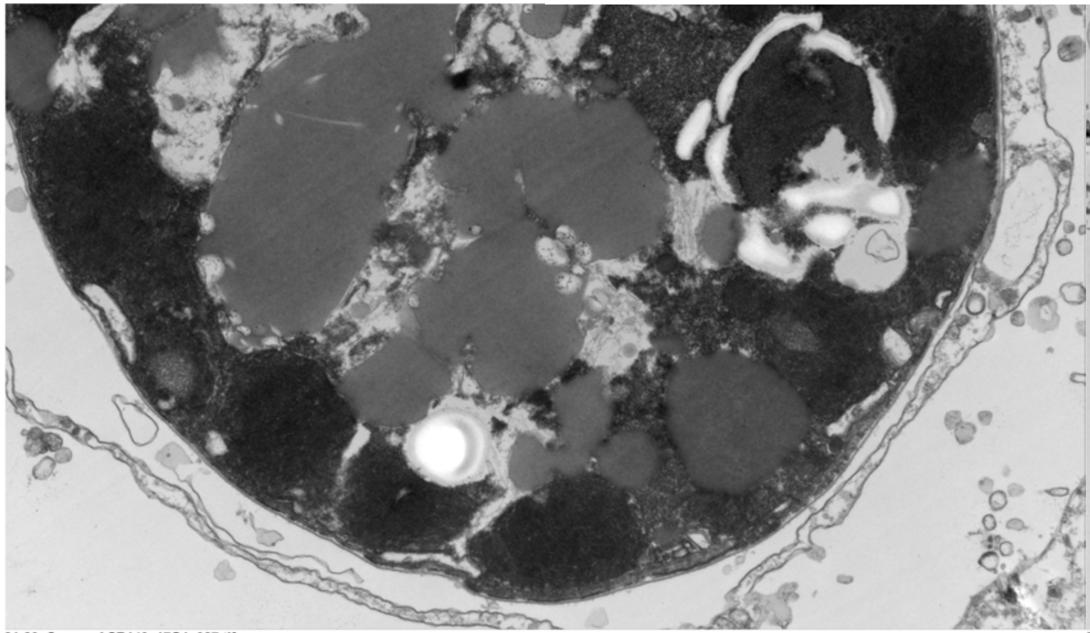

21-20_Correa_ACR143_17O1_027.tif
ACR143 tissue
Biological Electron Microscopy Lab
Rice University - SEA
Microscopist: MD Meyer

1 µm
HV=80kV
Direct Mag: 3000 x

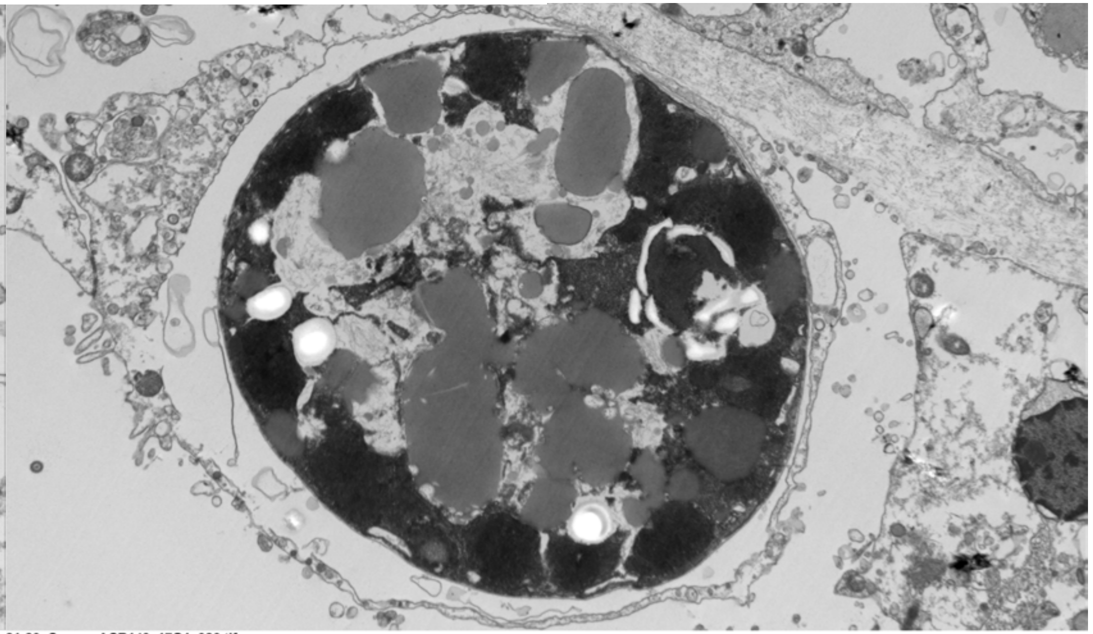

21-20_Correa_ACR143_17O1_026.tif
ACR143 tissue
Biological Electron Microscopy Lab
Rice University - SEA
Microscopist: MD Meyer

2 µm
HV=80kV
Direct Mag: 1500 x

ACR Colony I

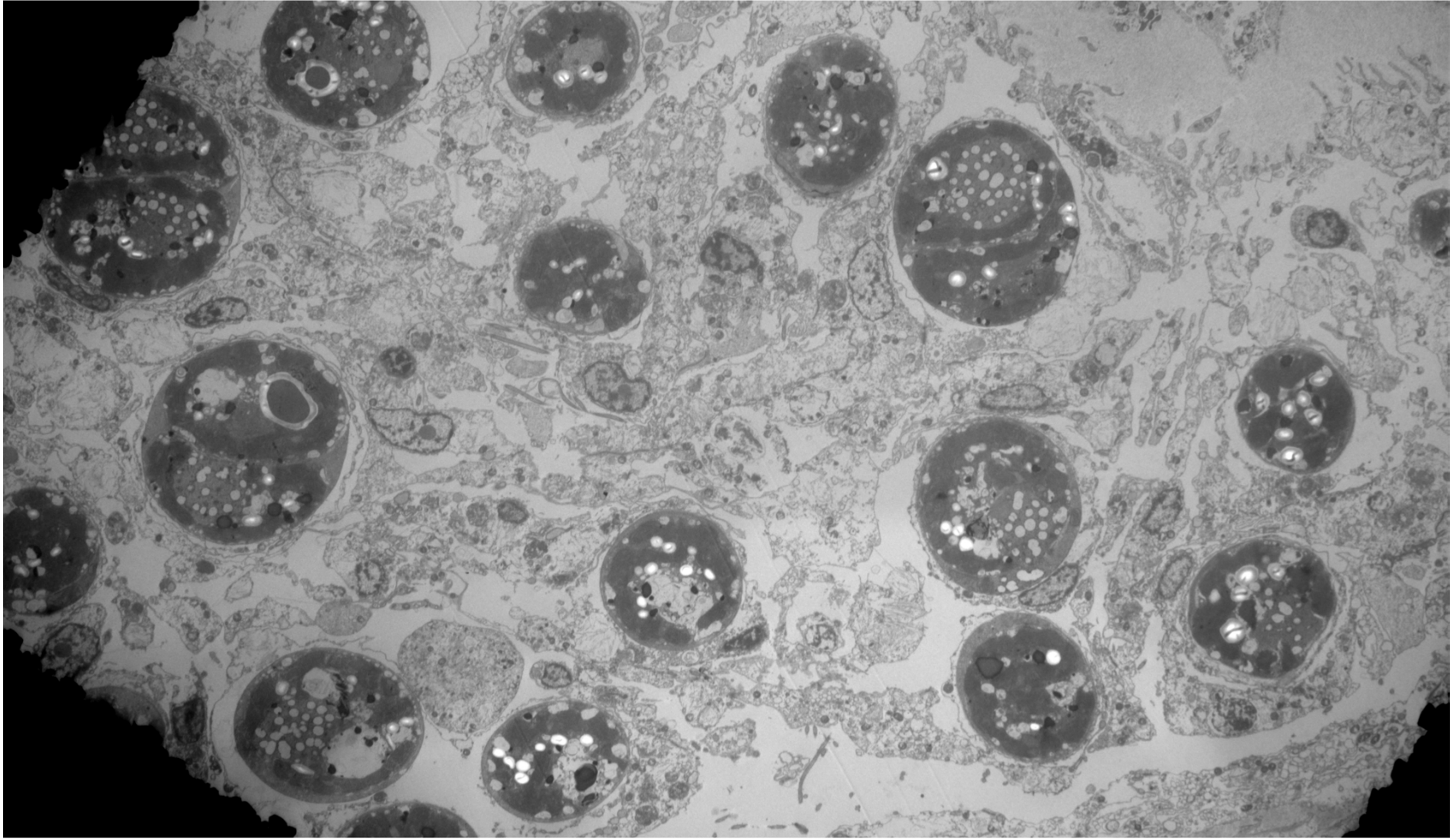

21-20_Correa_ACR158_17L1_001.tif
ACR 158
Biological Electron Microscopy Lab
Rice University - SEA
Microscopist: MD Meyer

10 µm
HV=80kV
Direct Mag: 400 x

Cell 1

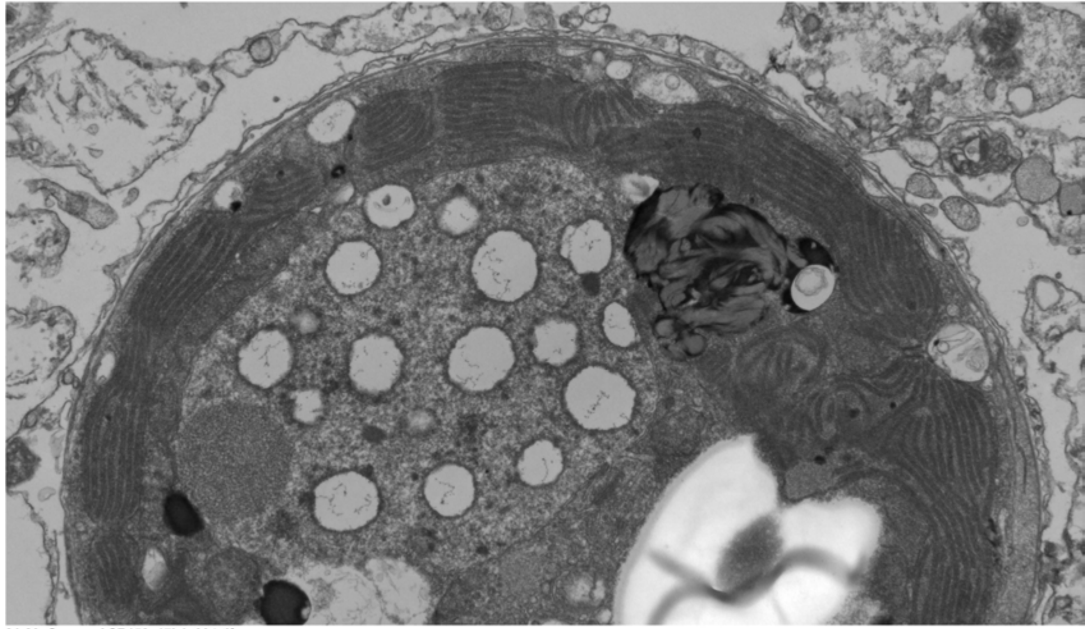

21-20_Correa_ACR158_17L1_004.tif
ACR 158
Biological Electron Microscopy Lab
Rice University - SEA
Microscopist: MD Meyer

1 µm
HV=80kV
Direct Mag: 3000 x

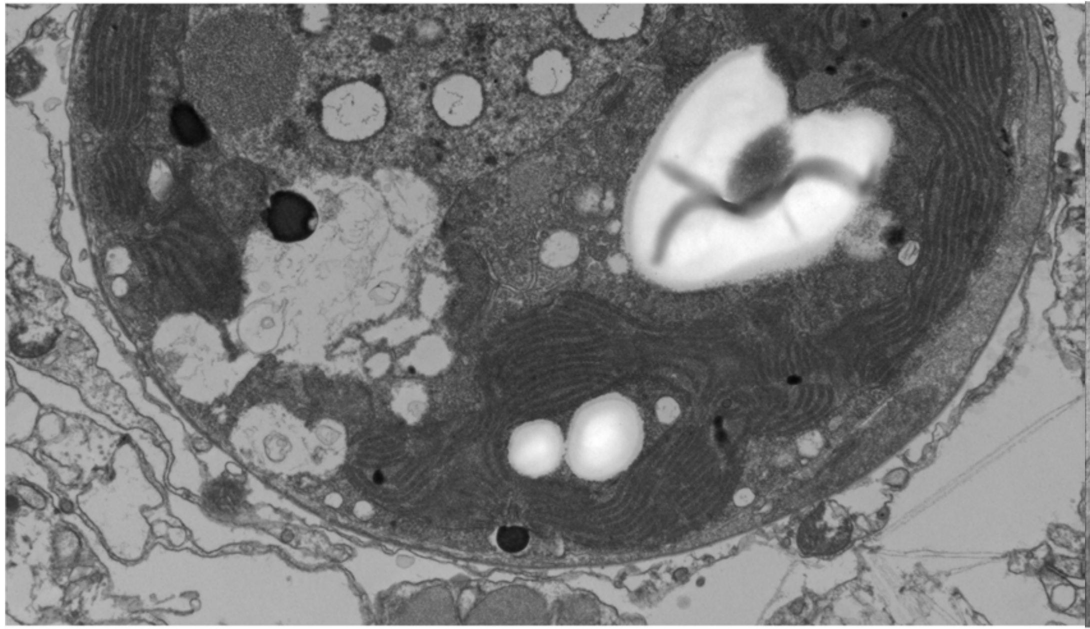

21-20_Correa_ACR158_17L1_003.tif
ACR 158
Biological Electron Microscopy Lab
Rice University - SEA
Microscopist: MD Meyer

1 µm
HV=80kV
Direct Mag: 3000 x

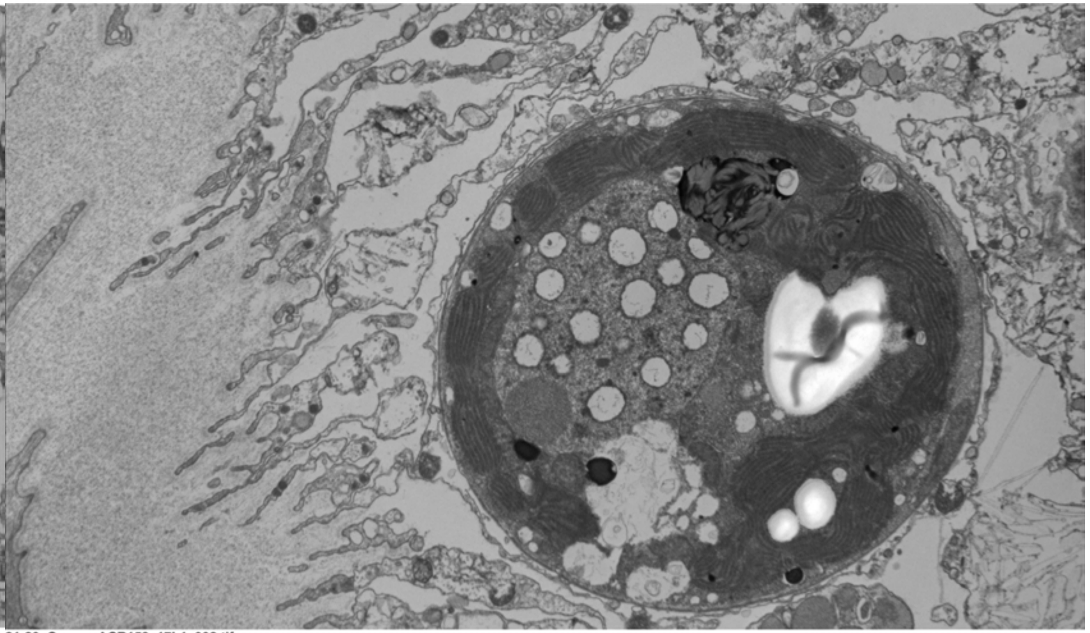

21-20_Correa_ACR158_17L1_002.tif
ACR 158
Biological Electron Microscopy Lab
Rice University - SEA
Microscopist: MD Meyer

2 µm
HV=80kV
Direct Mag: 1500 x

Cell 2

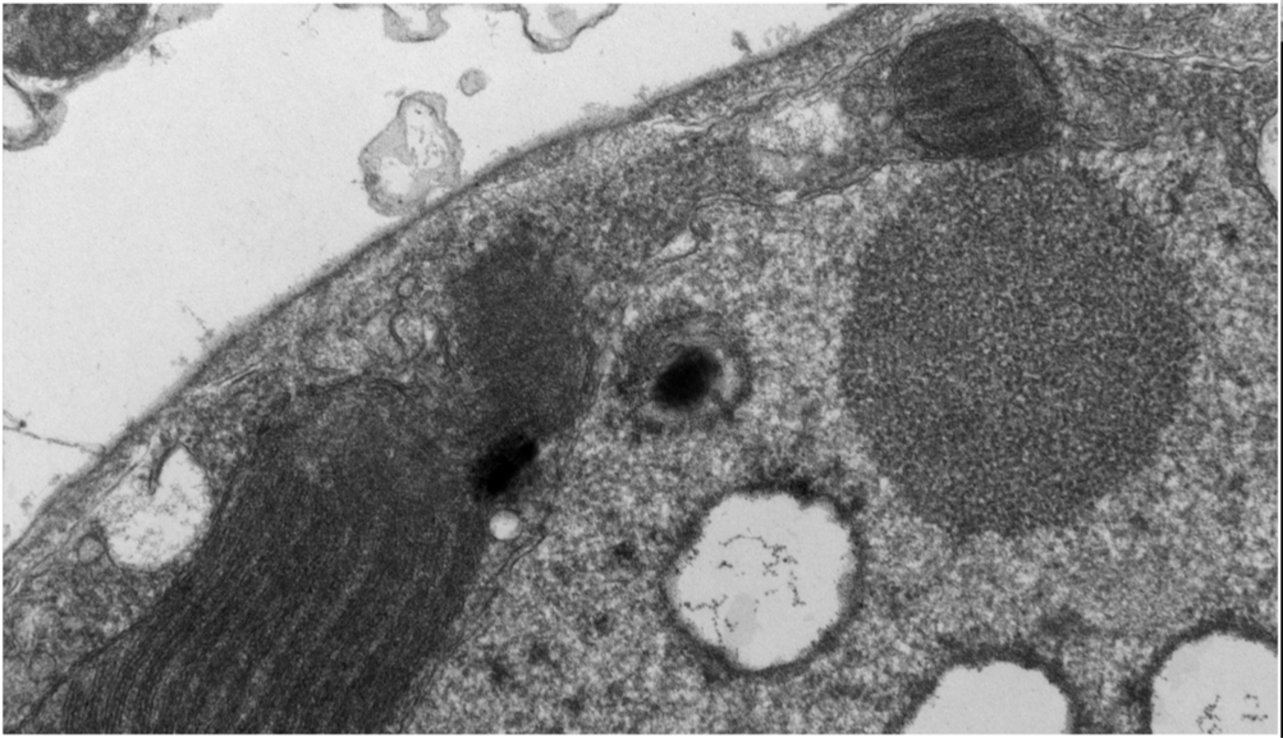

21-20_Correa_ACR158_17L1_006.tif
ACR 158
Biological Electron Microscopy Lab
Rice University - SEA
Microscopist: MD Meyer

500 nm
HV=80kV
Direct Mag: 8000 x

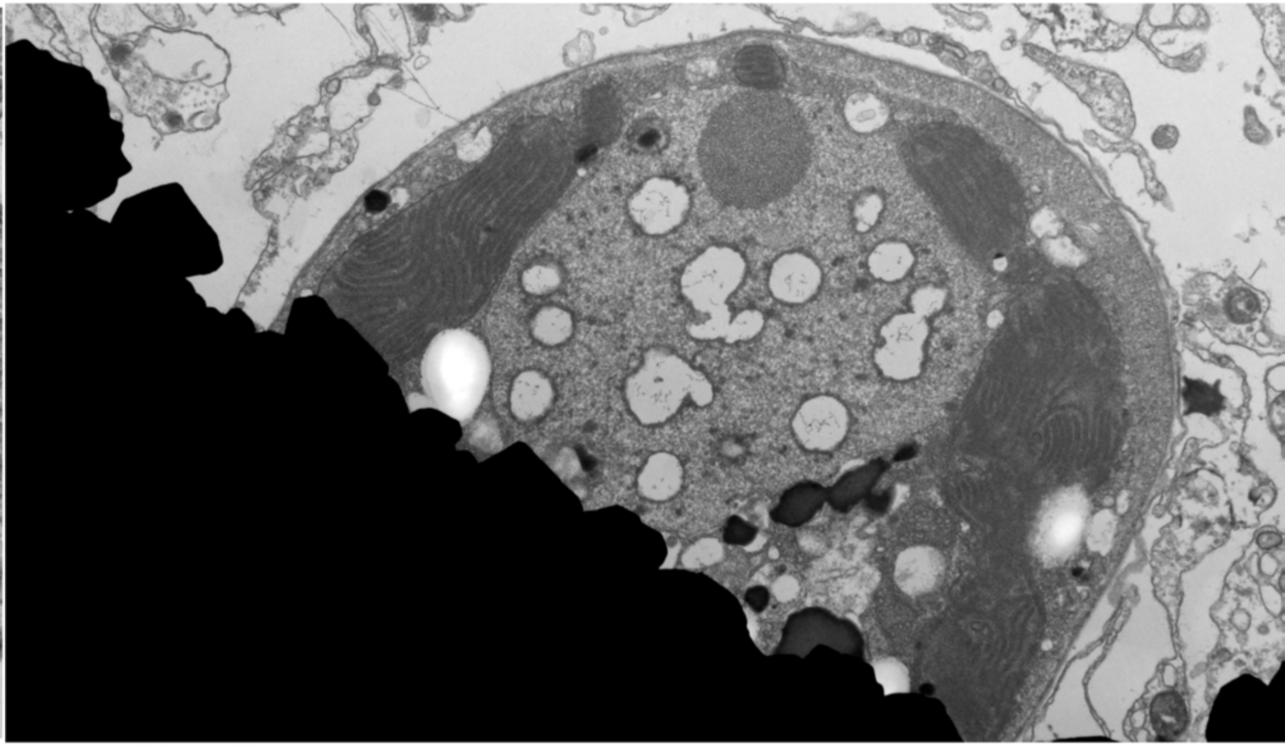

21-20_Correa_ACR158_17L1_005.tif
ACR 158
Biological Electron Microscopy Lab
Rice University - SEA
Microscopist: MD Meyer

1 µm
HV=80kV
Direct Mag: 2500 x

Cell 3

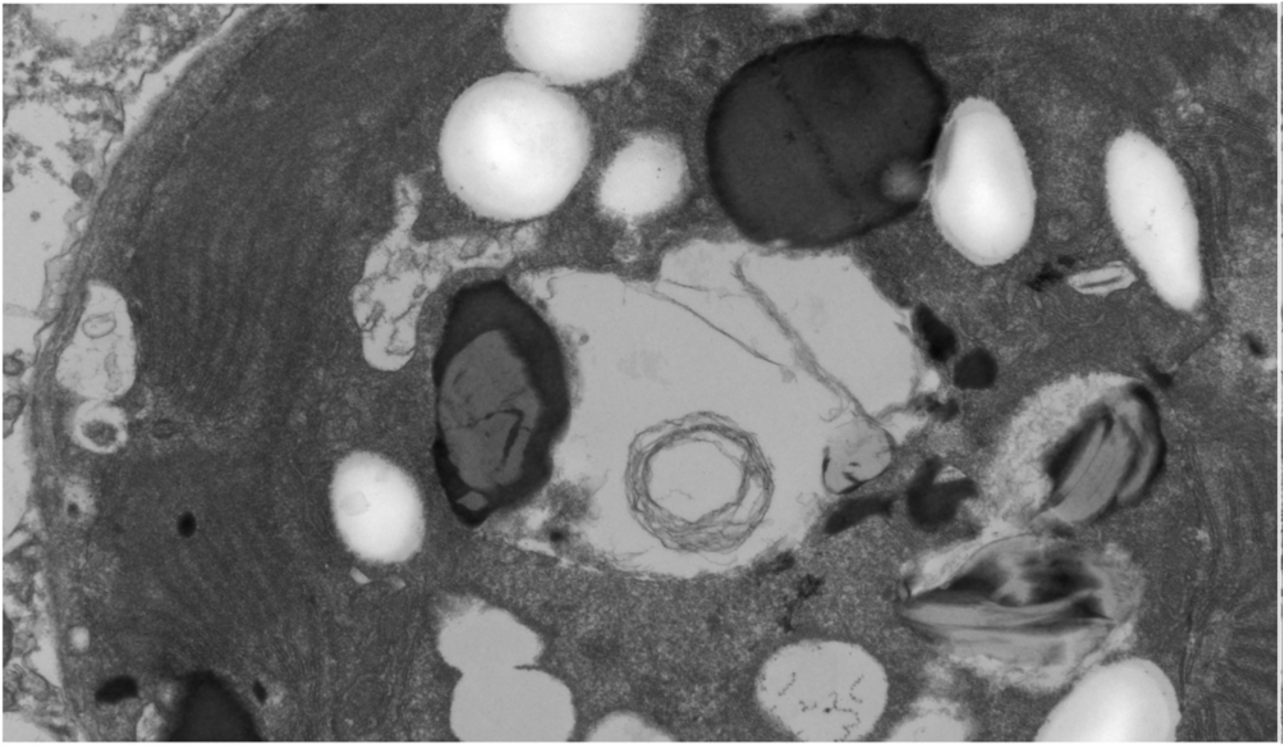

21-20_Correa_ACR158_17L1_008.tif
ACR 158
Biological Electron Microscopy Lab
Rice University - SEA
Microscopist: MD Meyer

800 nm
HV=80kV
Direct Mag: 5000 x

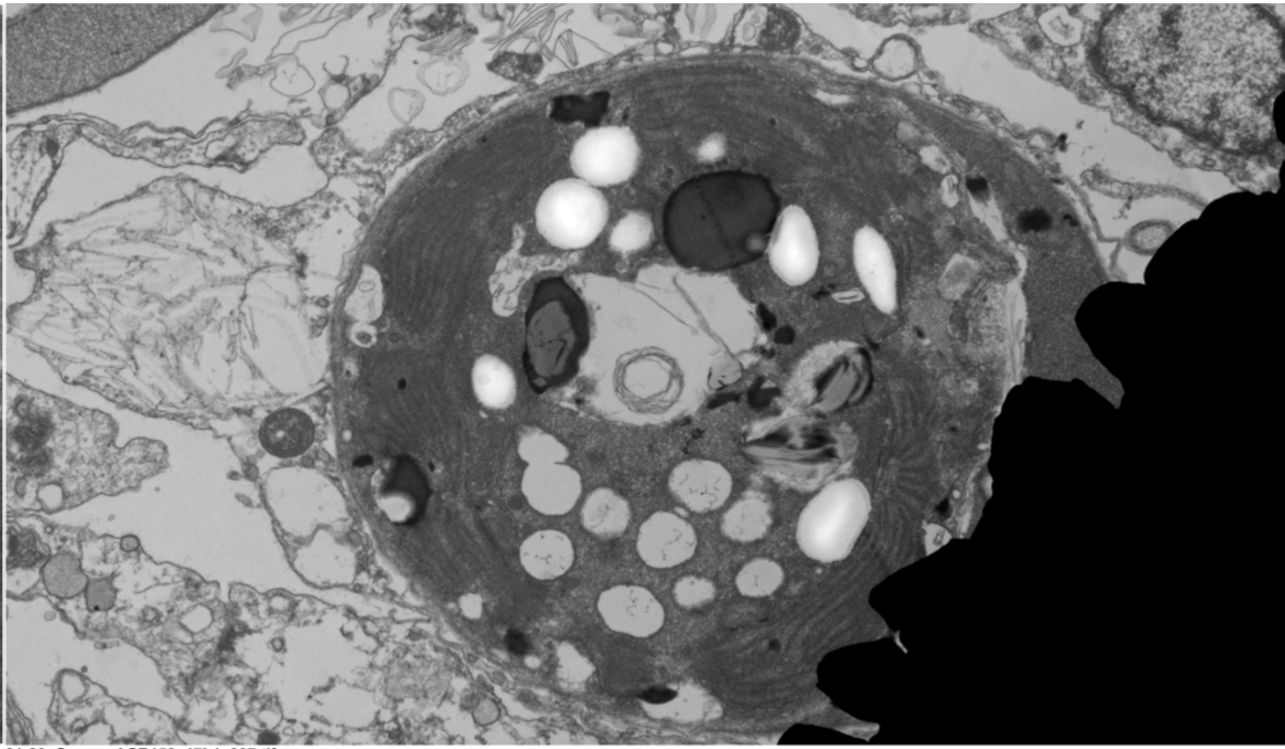

21-20_Correa_ACR158_17L1_007.tif
ACR 158
Biological Electron Microscopy Lab
Rice University - SEA
Microscopist: MD Meyer

1 μm
HV=80kV
Direct Mag: 2500 x

Cell 4

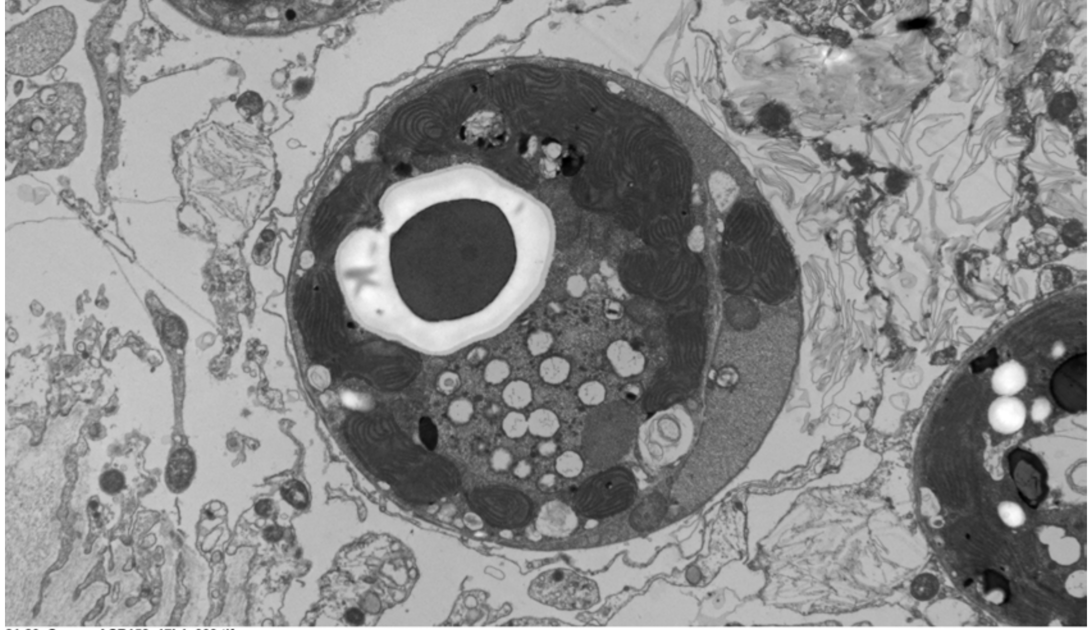

21-20_Correa_ACR158_17L1_009.tif
ACR 158
Biological Electron Microscopy Lab
Rice University - SEA
Microscopist: MD Meyer

2 µm
HV=80kV
Direct Mag: 1500 x

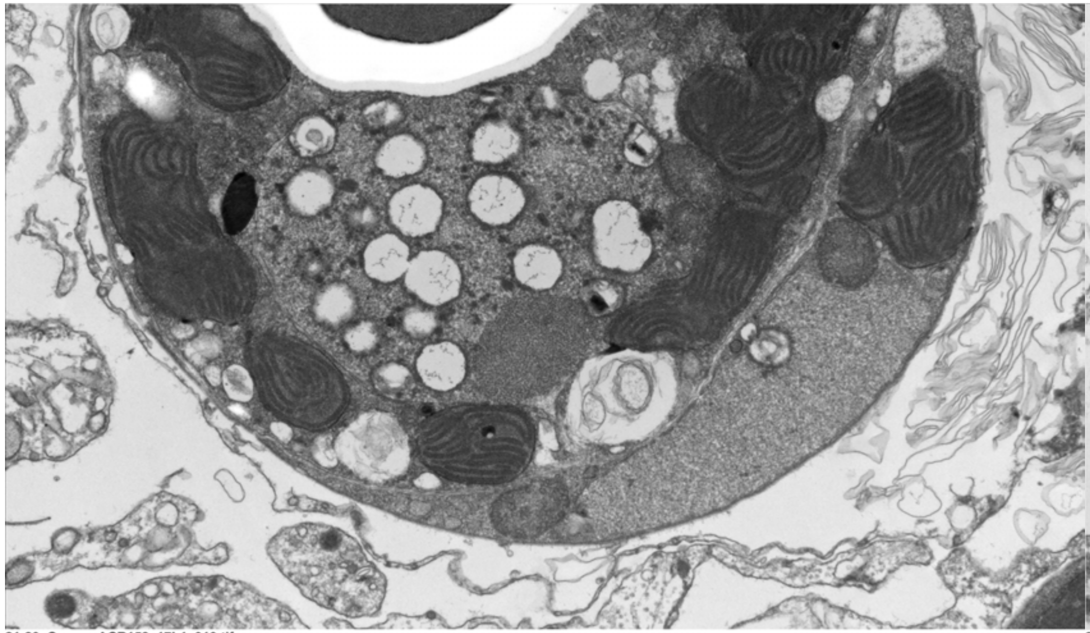

21-20_Correa_ACR158_17L1_010.tif
ACR 158
Biological Electron Microscopy Lab
Rice University - SEA
Microscopist: MD Meyer

1 µm
HV=80kV
Direct Mag: 3000 x

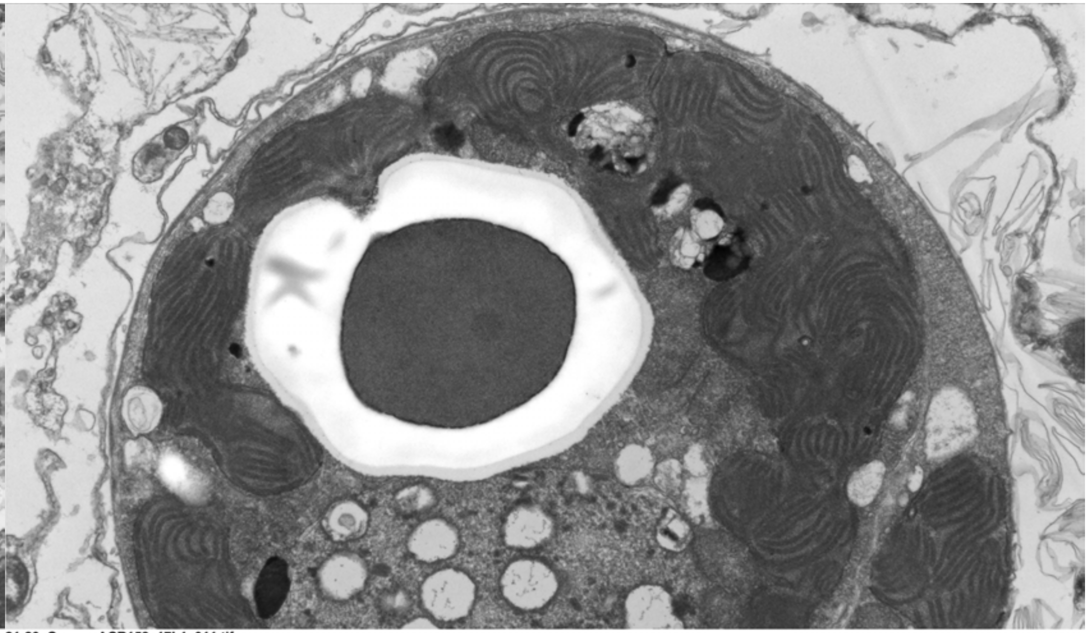

21-20_Correa_ACR158_17L1_011.tif
ACR 158
Biological Electron Microscopy Lab
Rice University - SEA
Microscopist: MD Meyer

1 µm
HV=80kV
Direct Mag: 3000 x

Cell 4

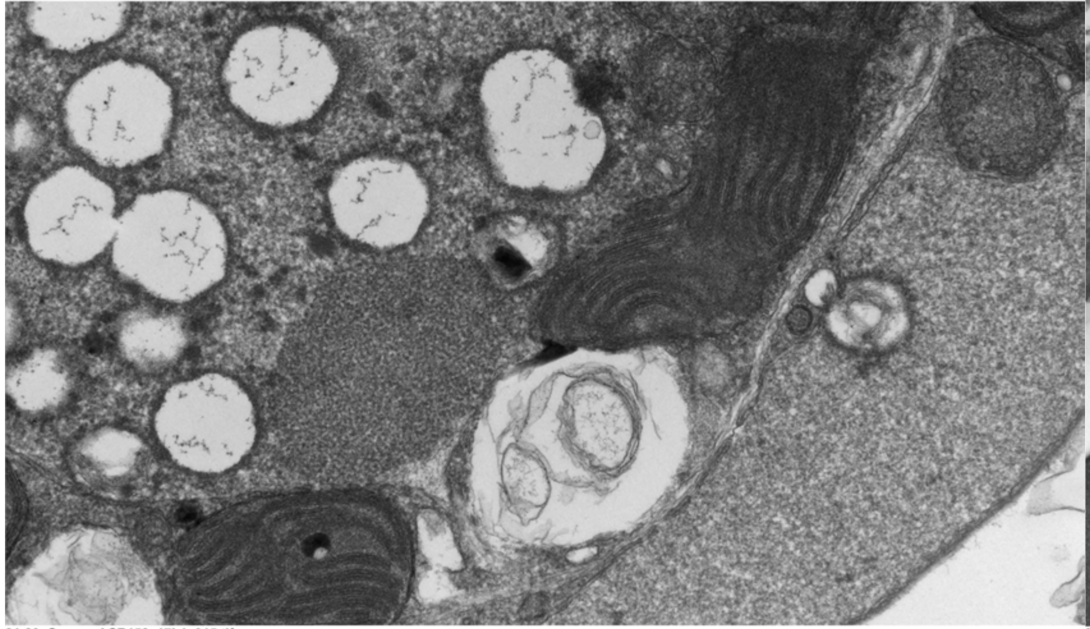

21-20_Correa_ACR158_17L1_015.tif
ACR 158
Biological Electron Microscopy Lab
Rice University - SEA
Microscopist: MD Meyer

600 nm
HV=80kV
Direct Mag: 6000 x

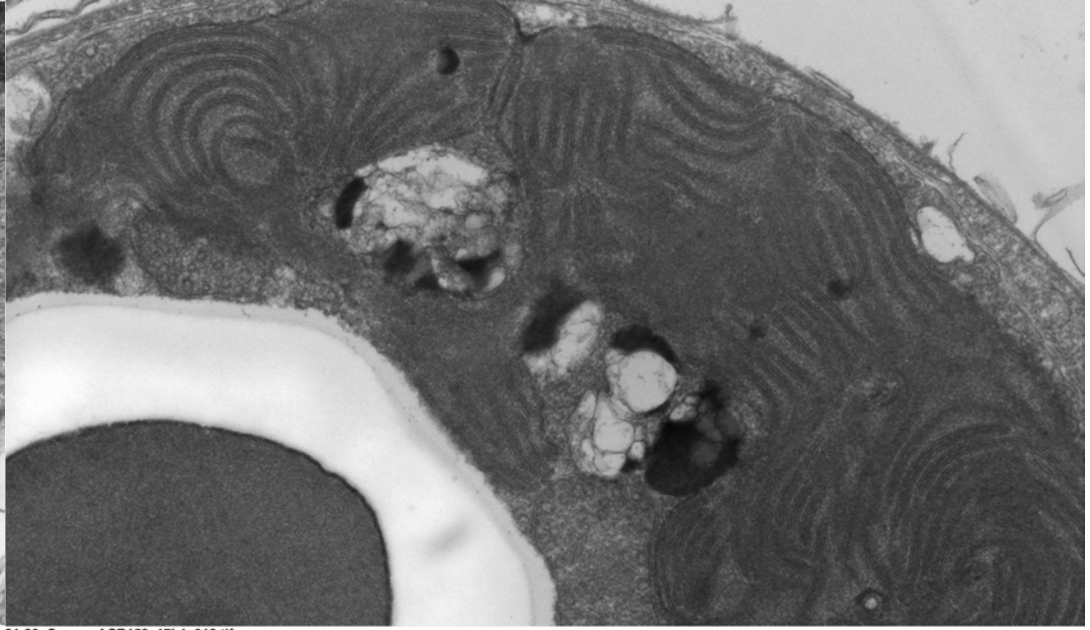

21-20_Correa_ACR158_17L1_012.tif
ACR 158
Biological Electron Microscopy Lab
Rice University - SEA
Microscopist: MD Meyer

600 nm
HV=80kV
Direct Mag: 6000 x

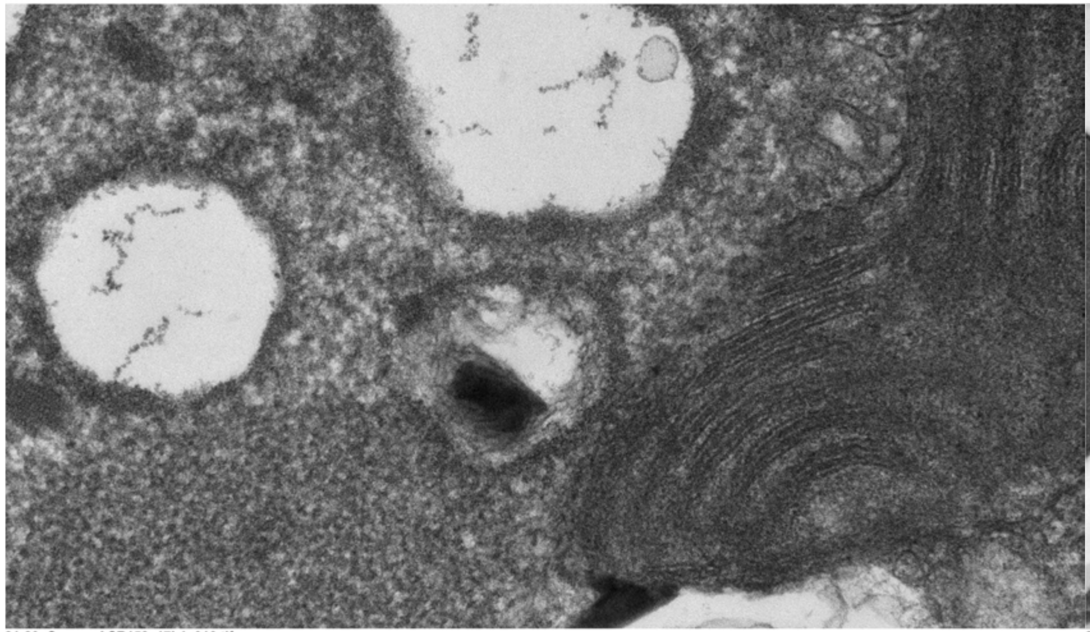

21-20_Correa_ACR158_17L1_016.tif
ACR 158
Biological Electron Microscopy Lab
Rice University - SEA
Microscopist: MD Meyer

200 nm
HV=80kV
Direct Mag: 15000 x

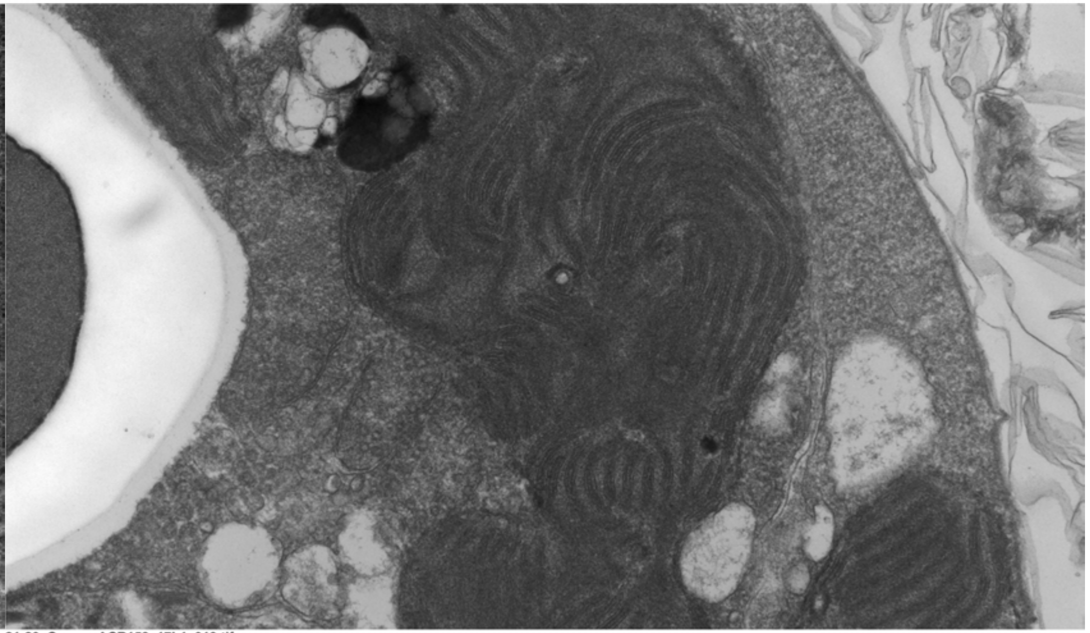

21-20_Correa_ACR158_17L1_013.tif
ACR 158
Biological Electron Microscopy Lab
Rice University - SEA
Microscopist: MD Meyer

600 nm
HV=80kV
Direct Mag: 6000 x

Cell 5

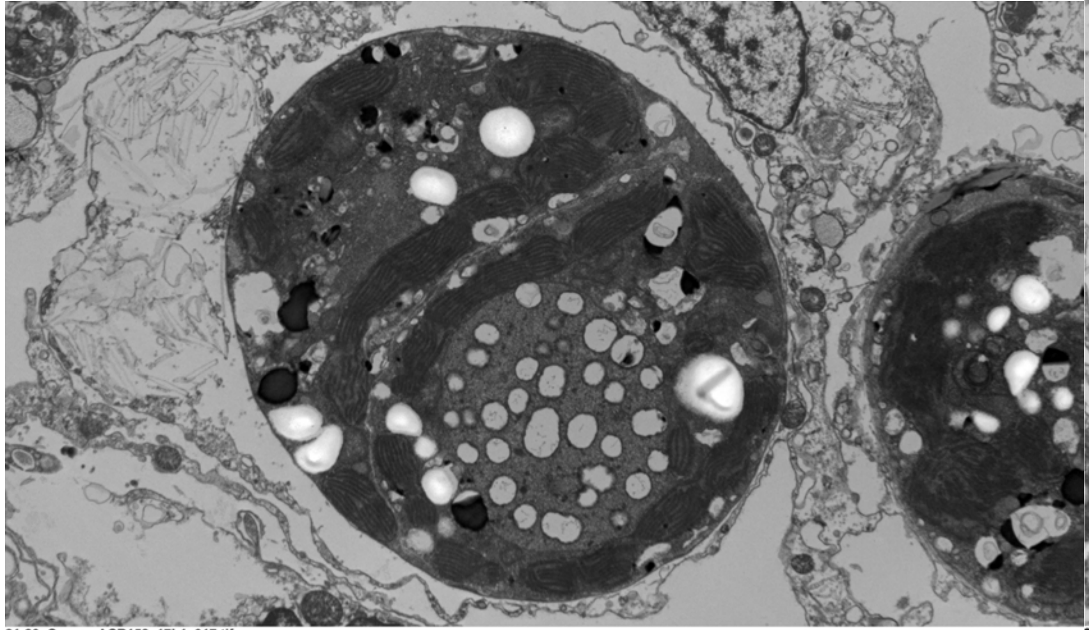

21-20_Correa_ACR158_17L1_017.tif
ACR 158
Biological Electron Microscopy Lab
Rice University - SEA
Microscopist: MD Meyer

2 µm
HV=80kV
Direct Mag: 1500 x

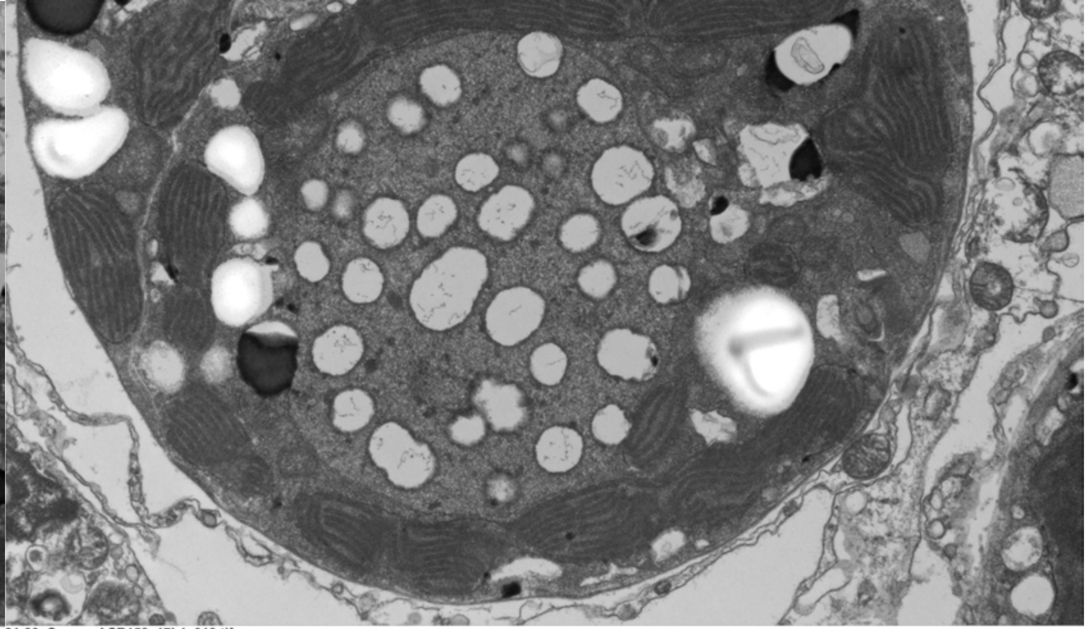

21-20_Correa_ACR158_17L1_018.tif
ACR 158
Biological Electron Microscopy Lab
Rice University - SEA
Microscopist: MD Meyer

1 µm
HV=80kV
Direct Mag: 3000 x

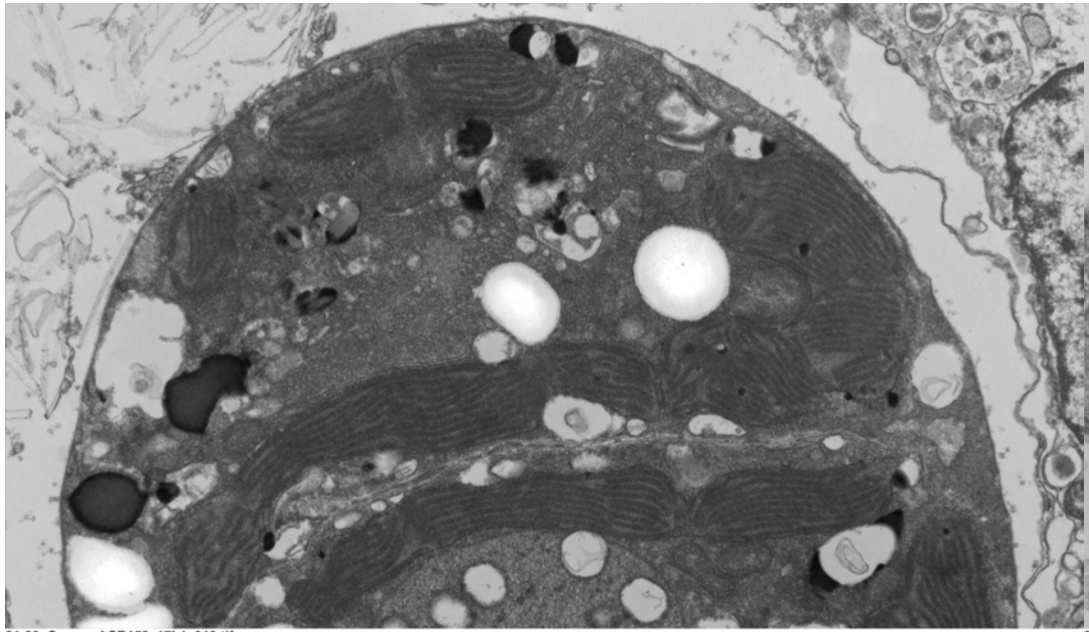

21-20_Correa_ACR158_17L1_019.tif
ACR 158
Biological Electron Microscopy Lab
Rice University - SEA
Microscopist: MD Meyer

1 µm
HV=80kV
Direct Mag: 3000 x

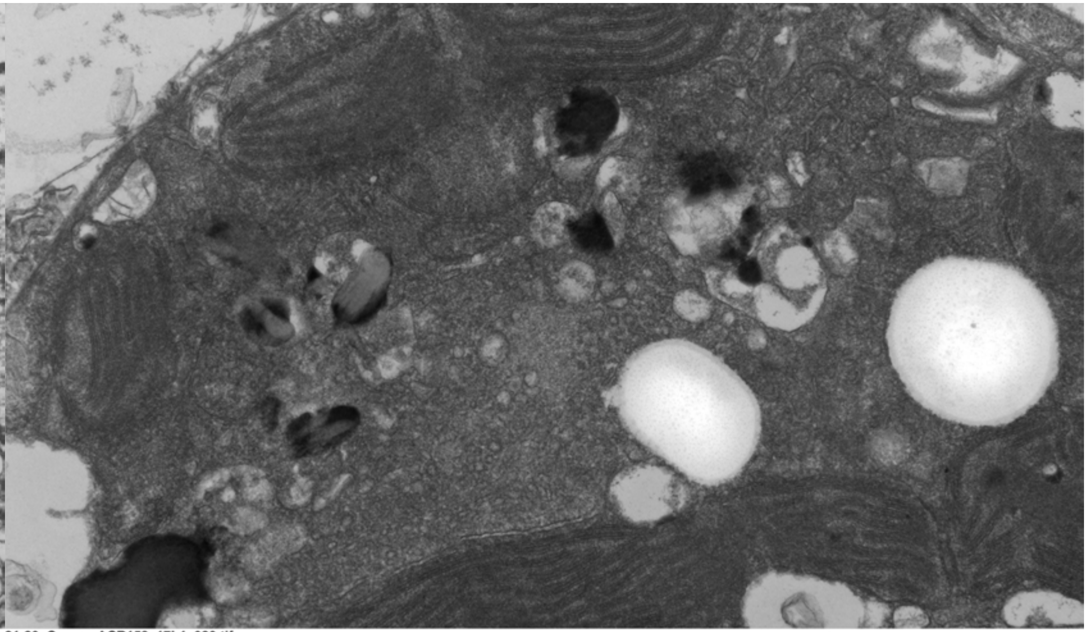

21-20_Correa_ACR158_17L1_020.tif
ACR 158
Biological Electron Microscopy Lab
Rice University - SEA
Microscopist: MD Meyer

800 nm
HV=80kV
Direct Mag: 5000 x

Cell 6

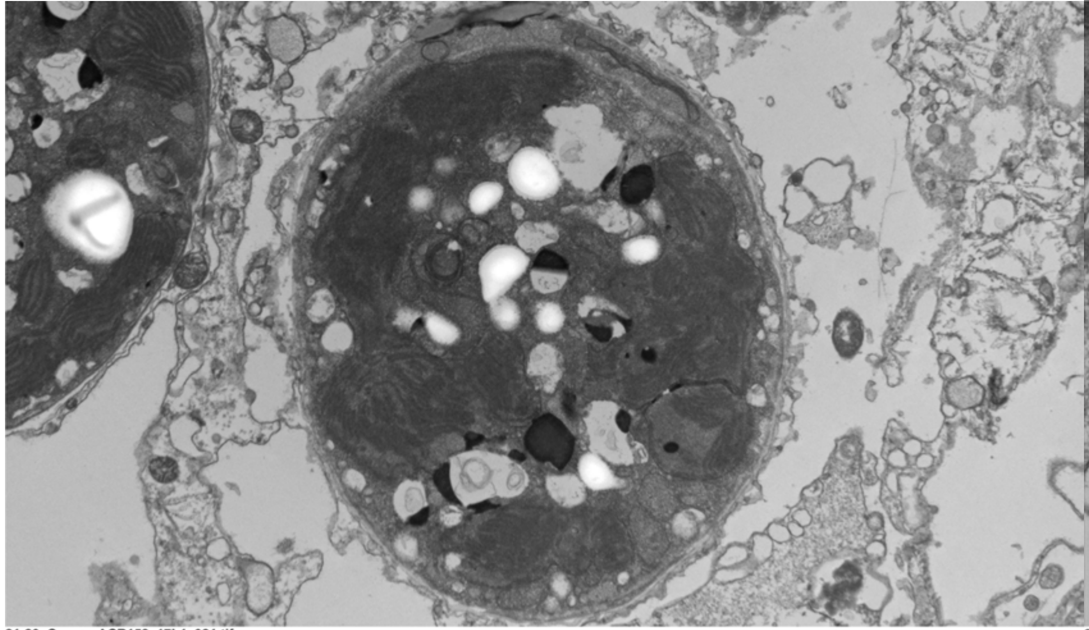

21-20_Correa_ACR158_17L1_021.tif
ACR 158
Biological Electron Microscopy Lab
Rice University - SEA
Microscopist: MD Meyer

2 µm
HV=80kV
Direct Mag: 2000 x

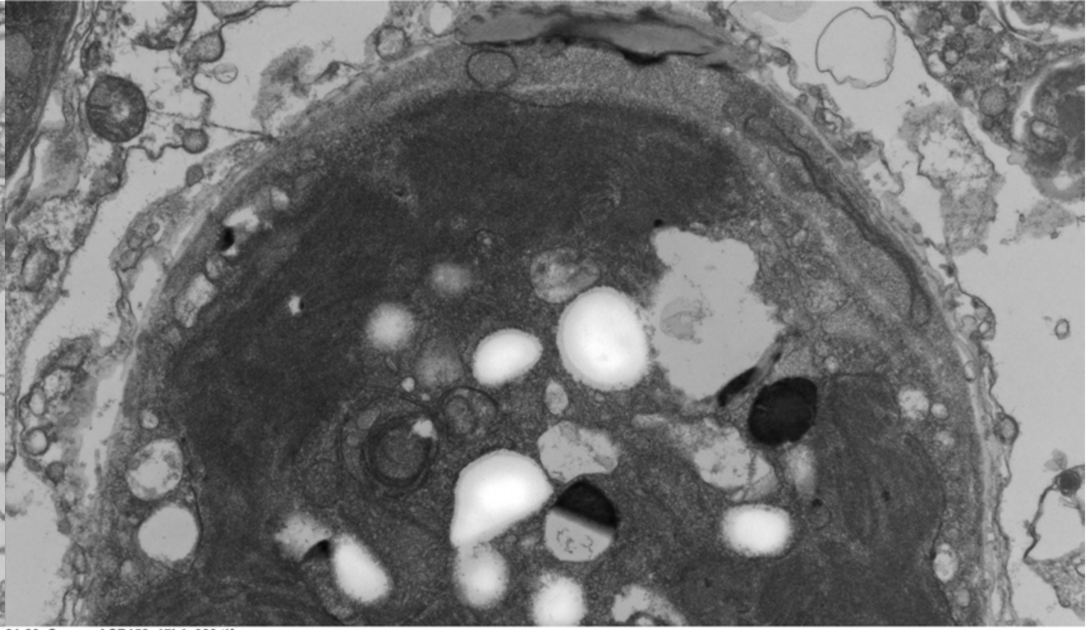

21-20_Correa_ACR158_17L1_023.tif
ACR 158
Biological Electron Microscopy Lab
Rice University - SEA
Microscopist: MD Meyer

1 µm
HV=80kV
Direct Mag: 4000 x

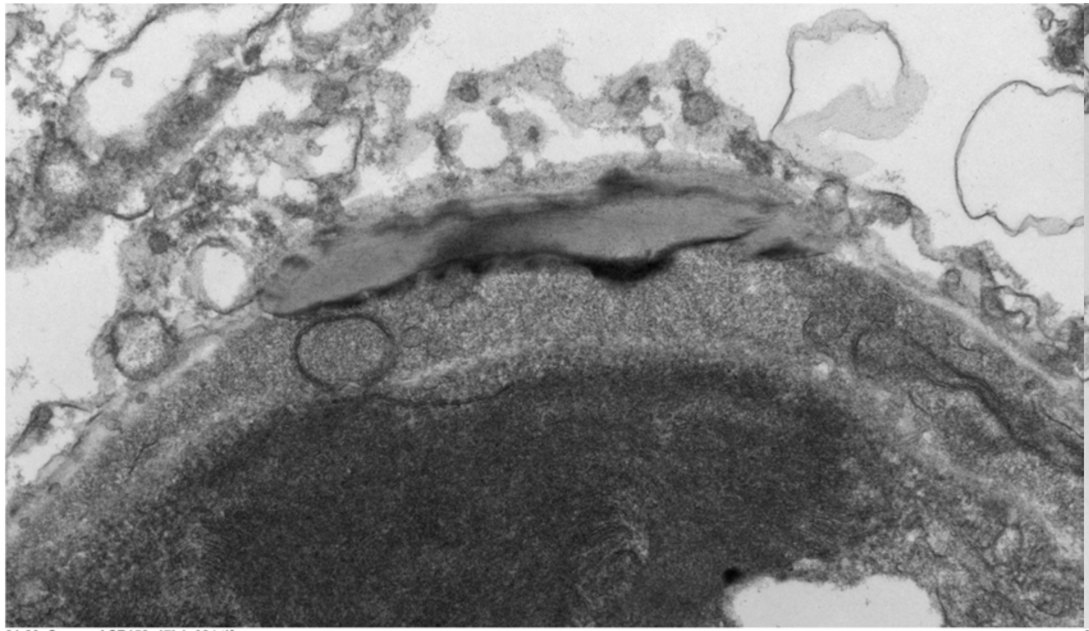

21-20_Correa_ACR158_17L1_024.tif
ACR 158
Biological Electron Microscopy Lab
Rice University - SEA
Microscopist: MD Meyer

500 nm
HV=80kV
Direct Mag: 8000 x

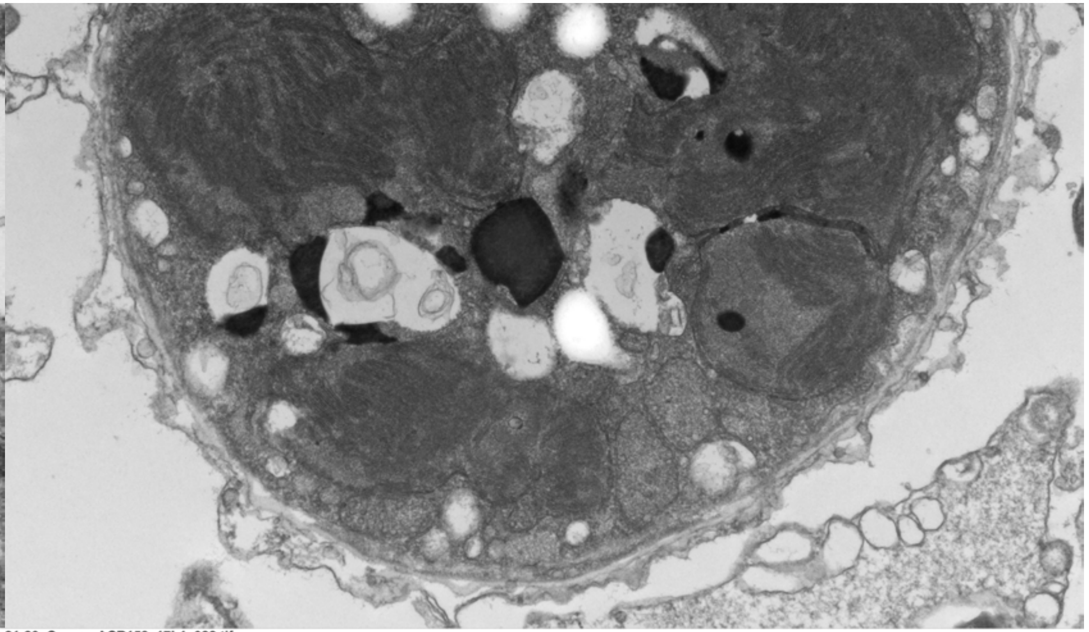

21-20_Correa_ACR158_17L1_022.tif
ACR 158
Biological Electron Microscopy Lab
Rice University - SEA
Microscopist: MD Meyer

1 µm
HV=80kV
Direct Mag: 4000 x

Cell 7

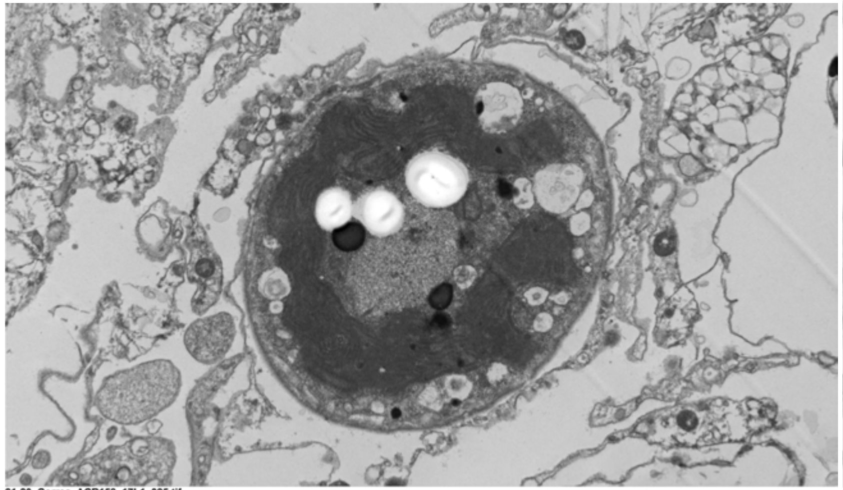

21-20_Correa_ACR158_17L1_025.tif
ACR 158
Biological Electron Microscopy Lab
Rice University - SEA
Microscopist: MD Meyer

2 µm
HV=80kV
Direct Mag: 2000 x

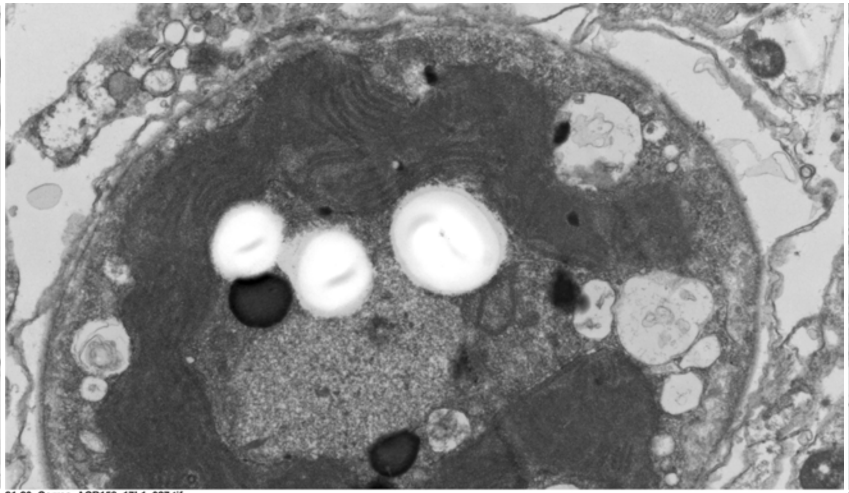

21-20_Correa_ACR158_17L1_027.tif
ACR 158
Biological Electron Microscopy Lab
Rice University - SEA
Microscopist: MD Meyer

1 µm
HV=80kV
Direct Mag: 4000 x

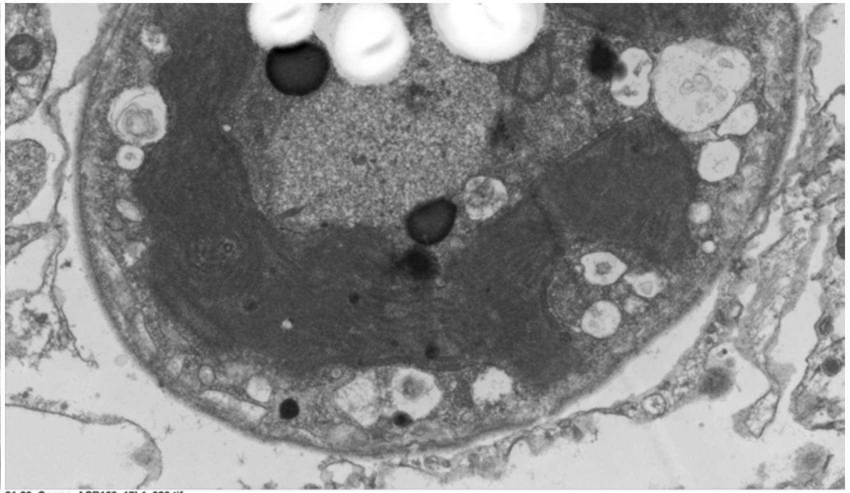

21-20_Correa_ACR158_17L1_026.tif
ACR 158
Biological Electron Microscopy Lab
Rice University - SEA
Microscopist: MD Meyer

1 µm
HV=80kV
Direct Mag: 4000 x

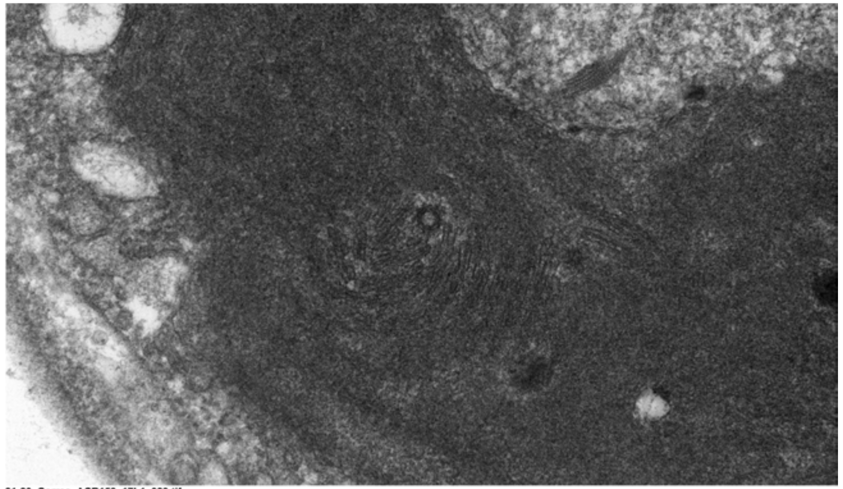

21-20_Correa_ACR158_17L1_029.tif
ACR 158
Biological Electron Microscopy Lab
Rice University - SEA
Microscopist: MD Meyer

200 nm
HV=80kV
Direct Mag: 12000 x

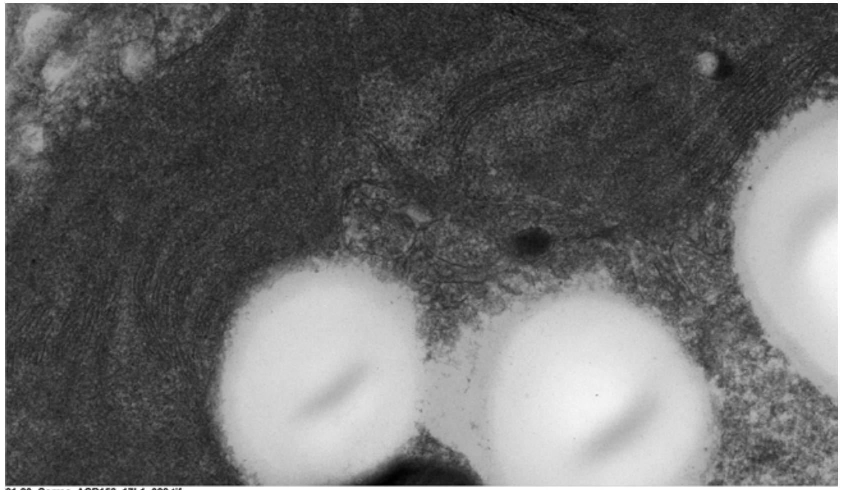

21-20_Correa_ACR158_17L1_028.tif
ACR 158
Biological Electron Microscopy Lab
Rice University - SEA
Microscopist: MD Meyer

200 nm
HV=80kV
Direct Mag: 12000 x

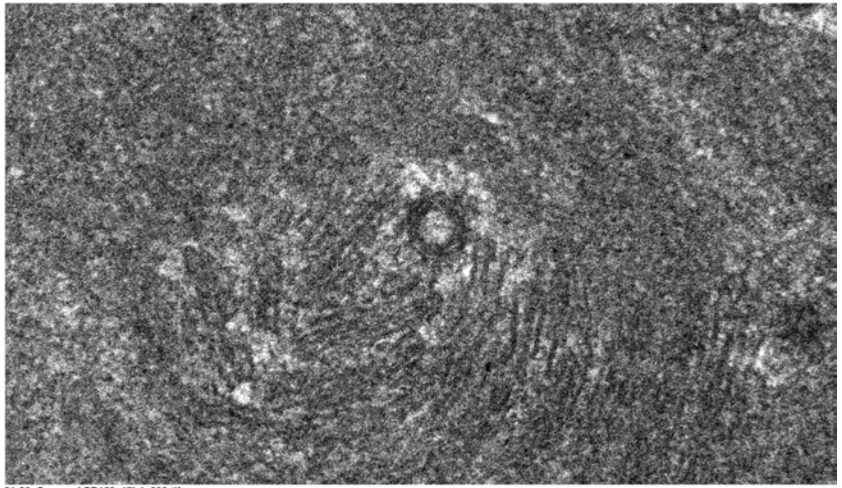

21-20_Correa_ACR158_17L1_030.tif
ACR 158
Biological Electron Microscopy Lab
Rice University - SEA
Microscopist: MD Meyer

100 nm
HV=80kV
Direct Mag: 30000 x

Cell 8

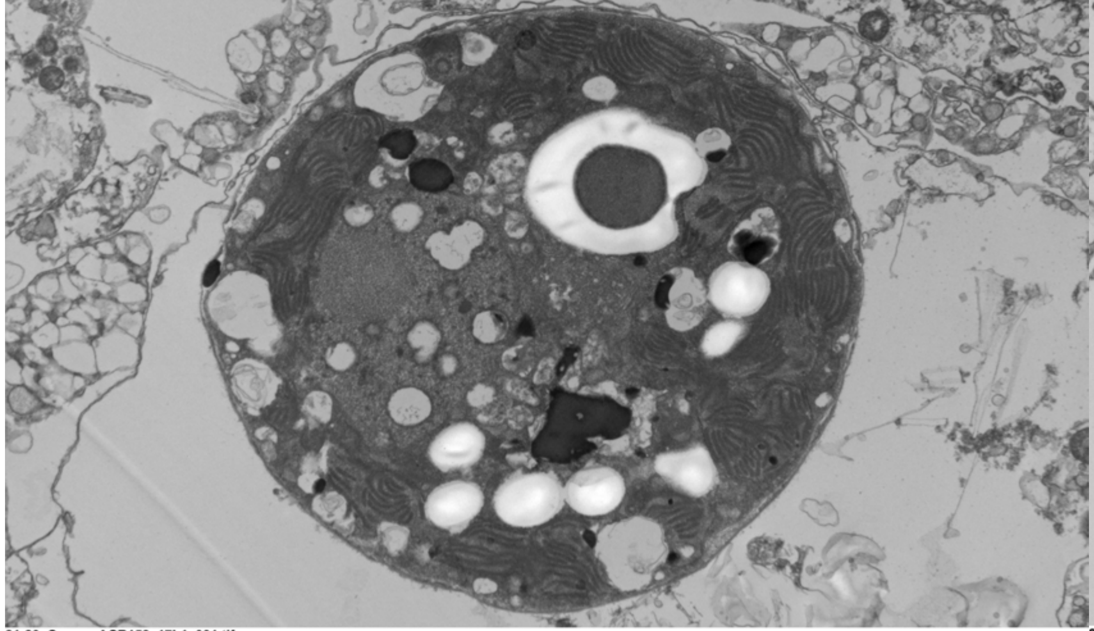

21-20_Correa_ACR158_17L1_031.tif
ACR 158
Biological Electron Microscopy Lab
Rice University - SEA
Microscopist: MD Meyer

2 µm
HV=80kV
Direct Mag: 2000 x

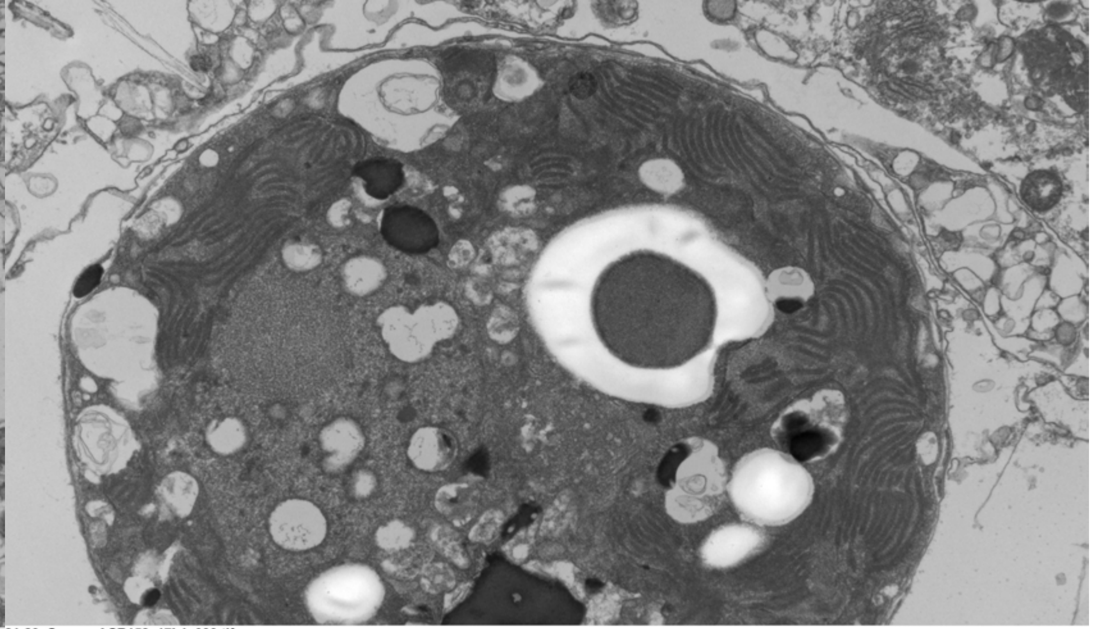

21-20_Correa_ACR158_17L1_033.tif
ACR 158
Biological Electron Microscopy Lab
Rice University - SEA
Microscopist: MD Meyer

1 µm
HV=80kV
Direct Mag: 3000 x

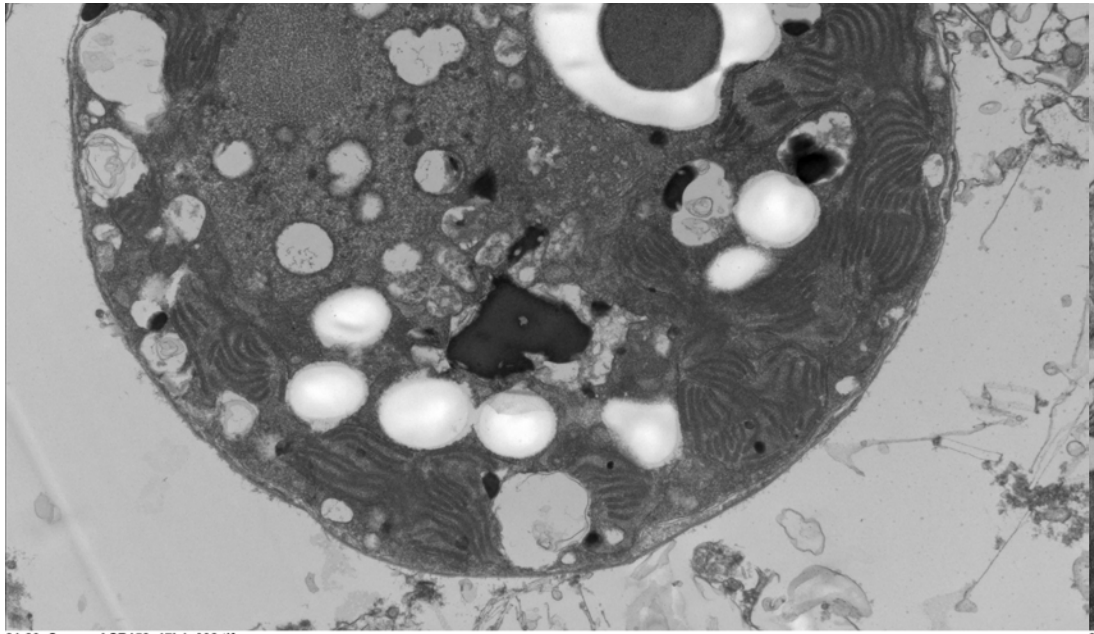

21-20_Correa_ACR158_17L1_032.tif
ACR 158
Biological Electron Microscopy Lab
Rice University - SEA
Microscopist: MD Meyer

1 µm
HV=80kV
Direct Mag: 3000 x

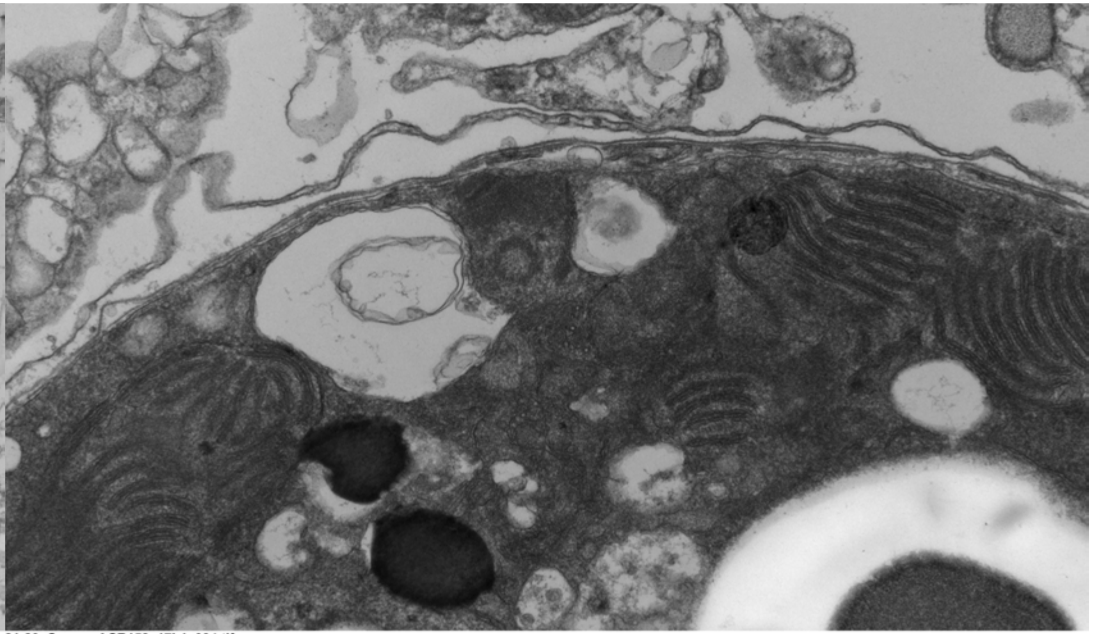

21-20_Correa_ACR158_17L1_034.tif
ACR 158
Biological Electron Microscopy Lab
Rice University - SEA
Microscopist: MD Meyer

600 nm
HV=80kV
Direct Mag: 6000 x

Cell 9

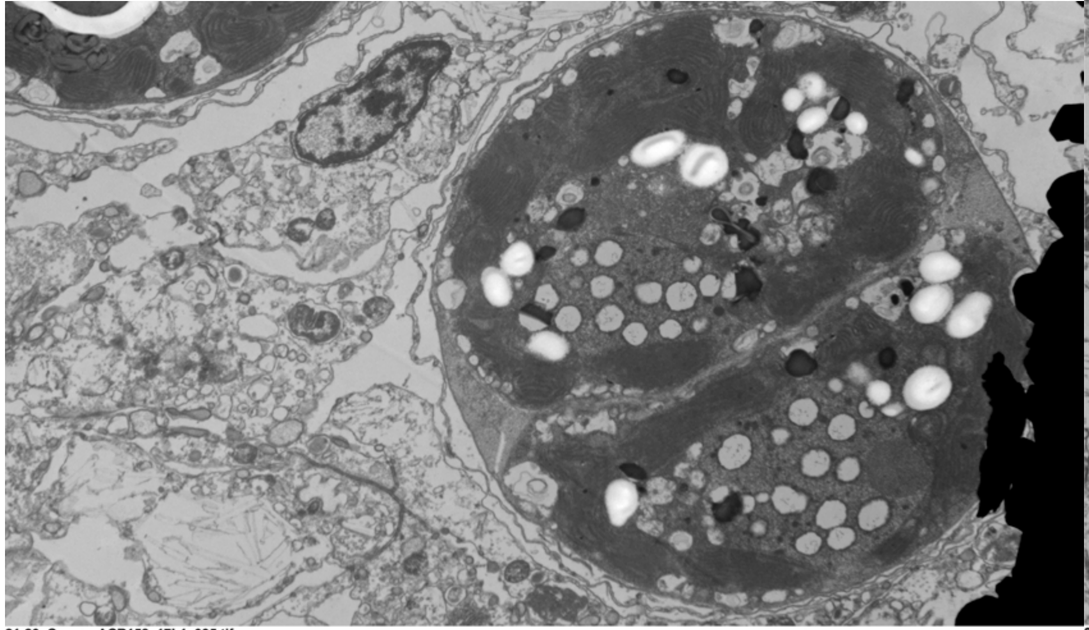

21-20_Correa_ACR158_17L1_035.tif
ACR 158
Biological Electron Microscopy Lab
Rice University - SEA
Microscopist: MD Meyer

2 µm
HV=80kV
Direct Mag: 1500 x

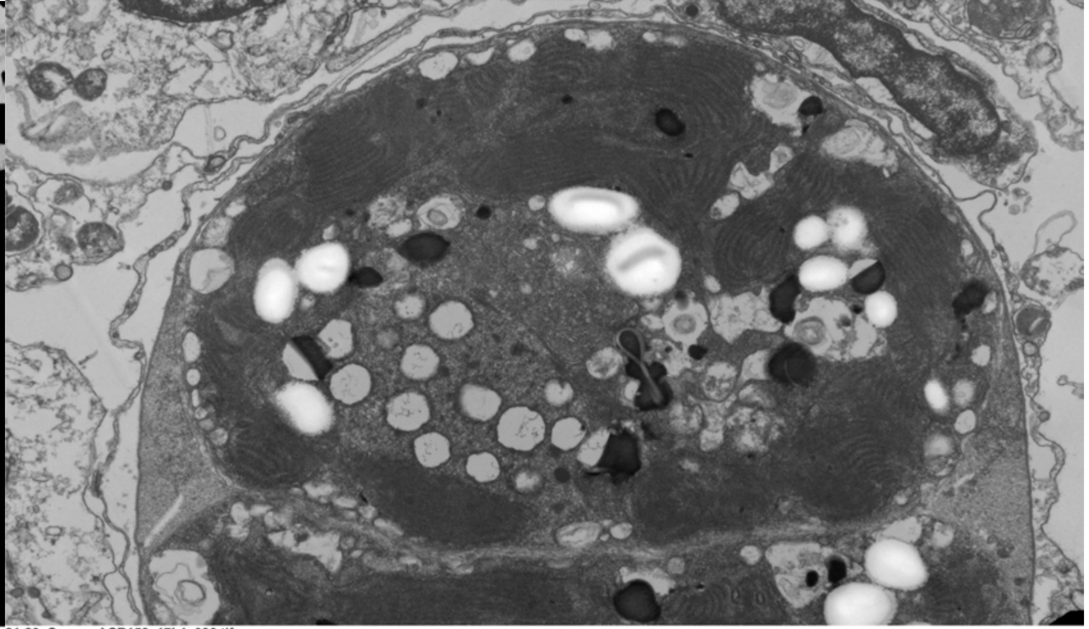

21-20_Correa_ACR158_17L1_036.tif
ACR 158
Biological Electron Microscopy Lab
Rice University - SEA
Microscopist: MD Meyer

1 µm
HV=80kV
Direct Mag: 2500 x

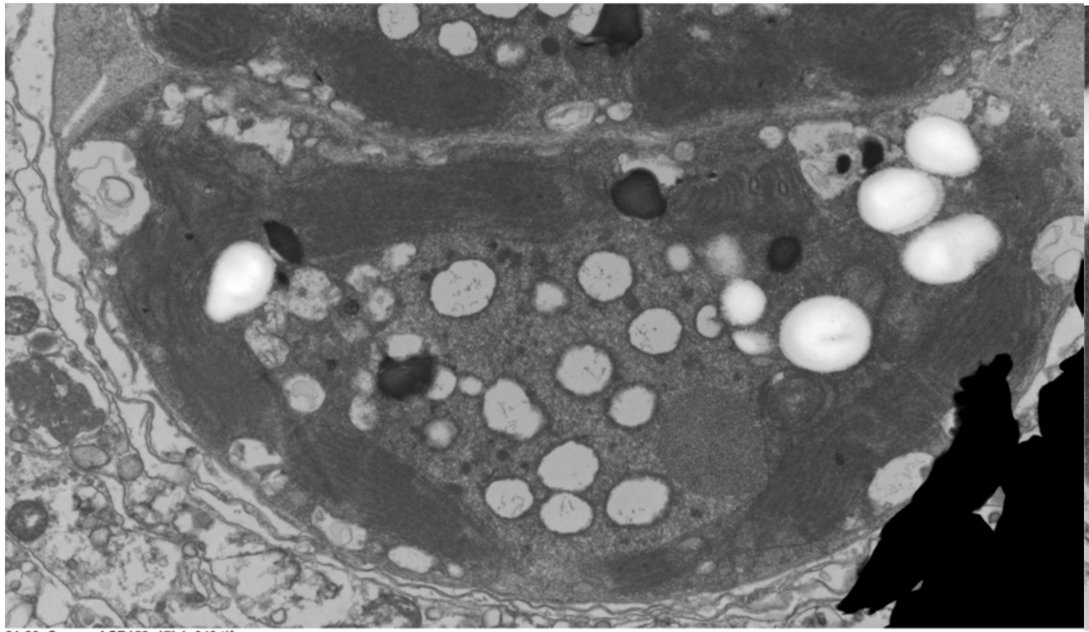

21-20_Correa_ACR158_17L1_043.tif
ACR 158
Biological Electron Microscopy Lab
Rice University - SEA
Microscopist: MD Meyer

1 µm
HV=80kV
Direct Mag: 3000 x

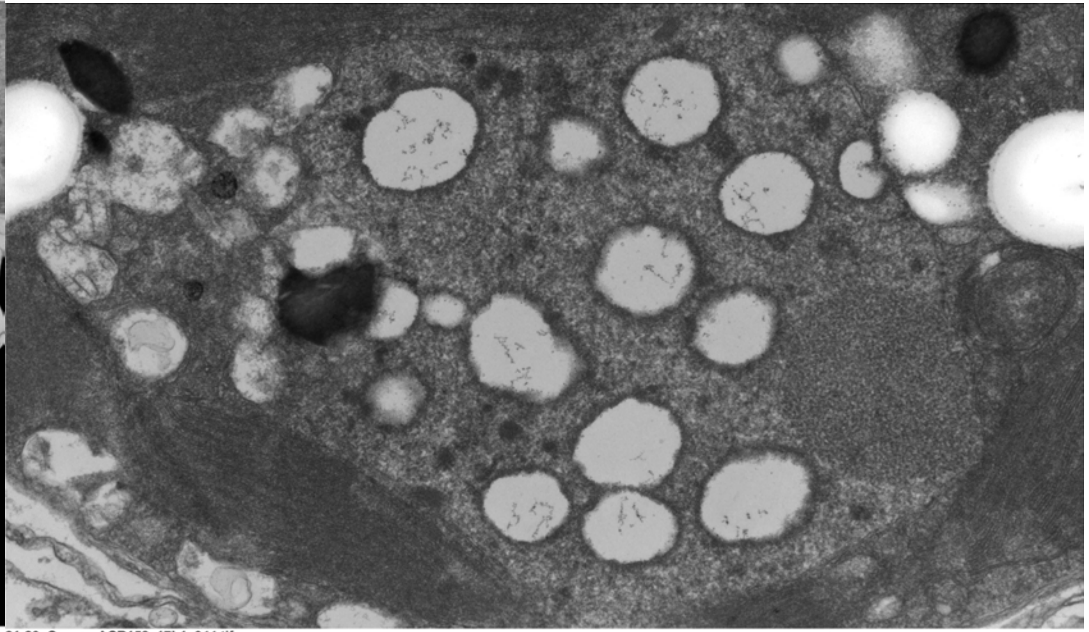

21-20_Correa_ACR158_17L1_044.tif
ACR 158
Biological Electron Microscopy Lab
Rice University - SEA
Microscopist: MD Meyer

800 nm
HV=80kV
Direct Mag: 5000 x

Cell 9

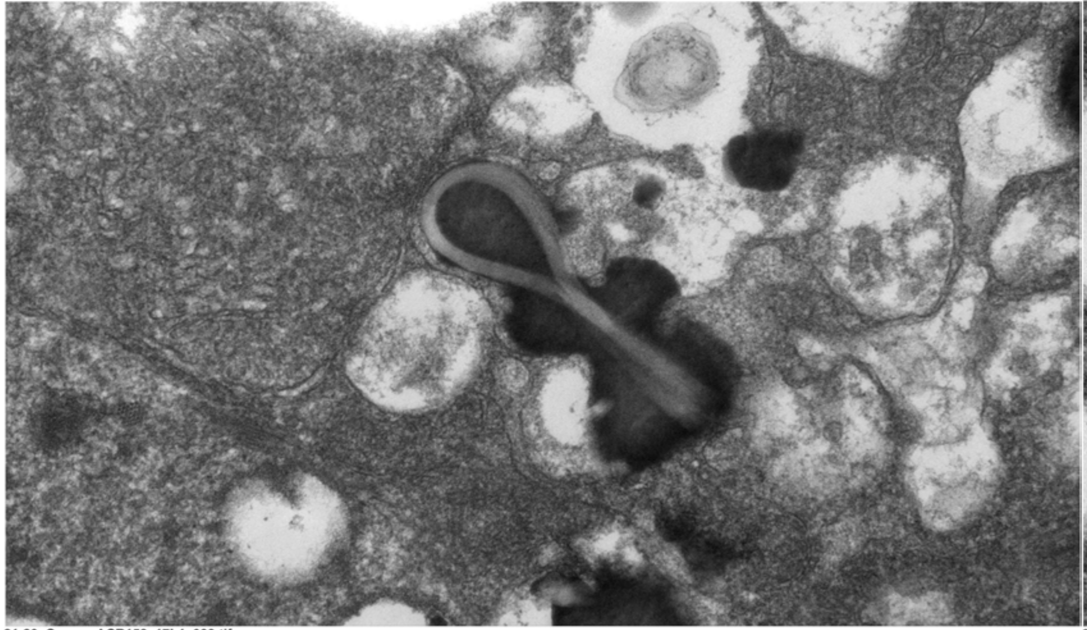

21-20_Correa_ACR158_17L1_039.tif
ACR 158
Biological Electron Microscopy Lab
Rice University - SEA
Microscopist: MD Meyer

400 nm
HV=80kV
Direct Mag: 10000 x

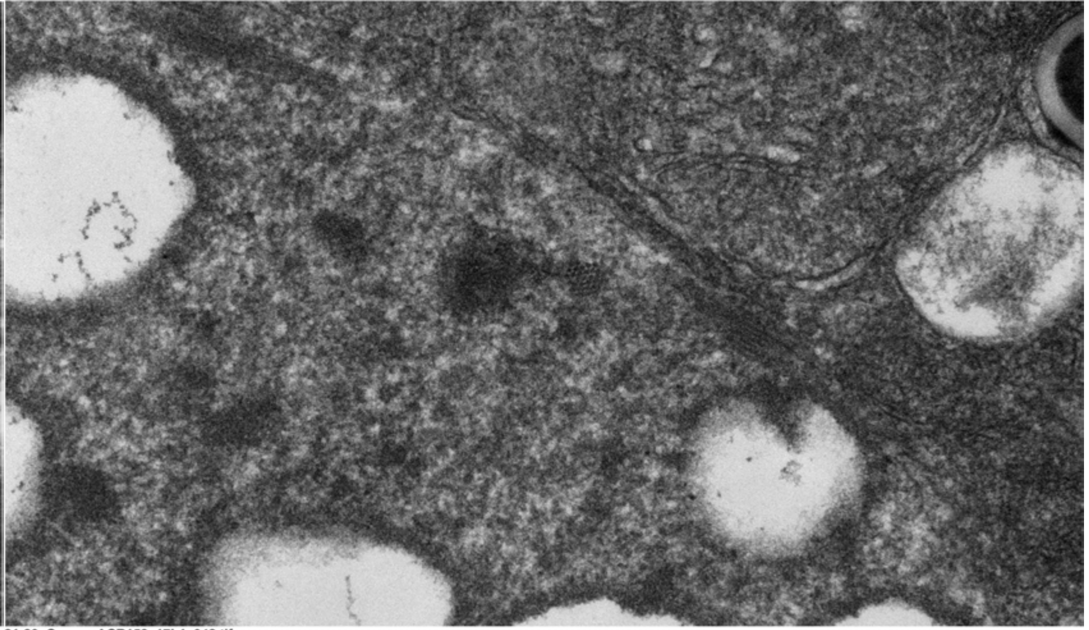

21-20_Correa_ACR158_17L1_042.tif
ACR 158
Biological Electron Microscopy Lab
Rice University - SEA
Microscopist: MD Meyer

200 nm
HV=80kV
Direct Mag: 15000 x

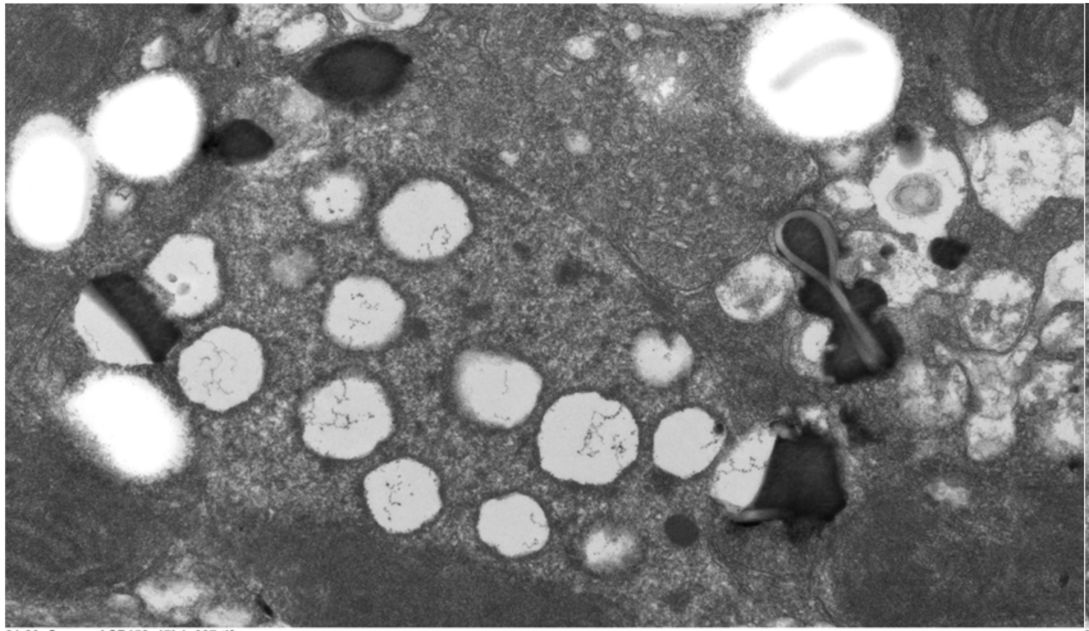

21-20_Correa_ACR158_17L1_037.tif
ACR 158
Biological Electron Microscopy Lab
Rice University - SEA
Microscopist: MD Meyer

800 nm
HV=80kV
Direct Mag: 5000 x

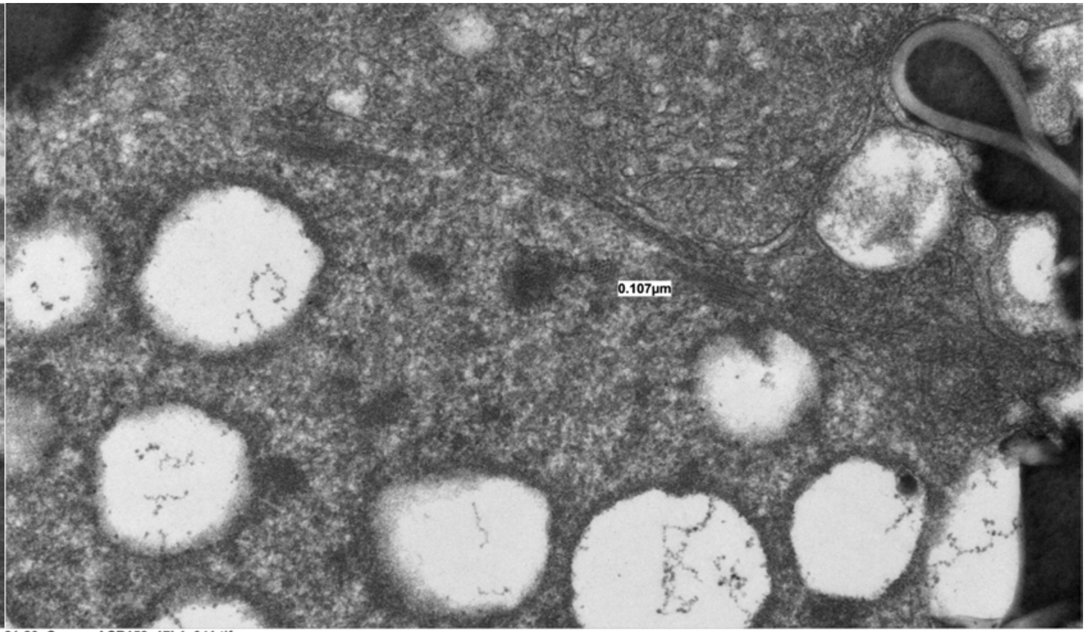

21-20_Correa_ACR158_17L1_041.tif
ACR 158
Biological Electron Microscopy Lab
Rice University - SEA
Microscopist: MD Meyer

400 nm
HV=80kV
Direct Mag: 10000 x

Cell 10

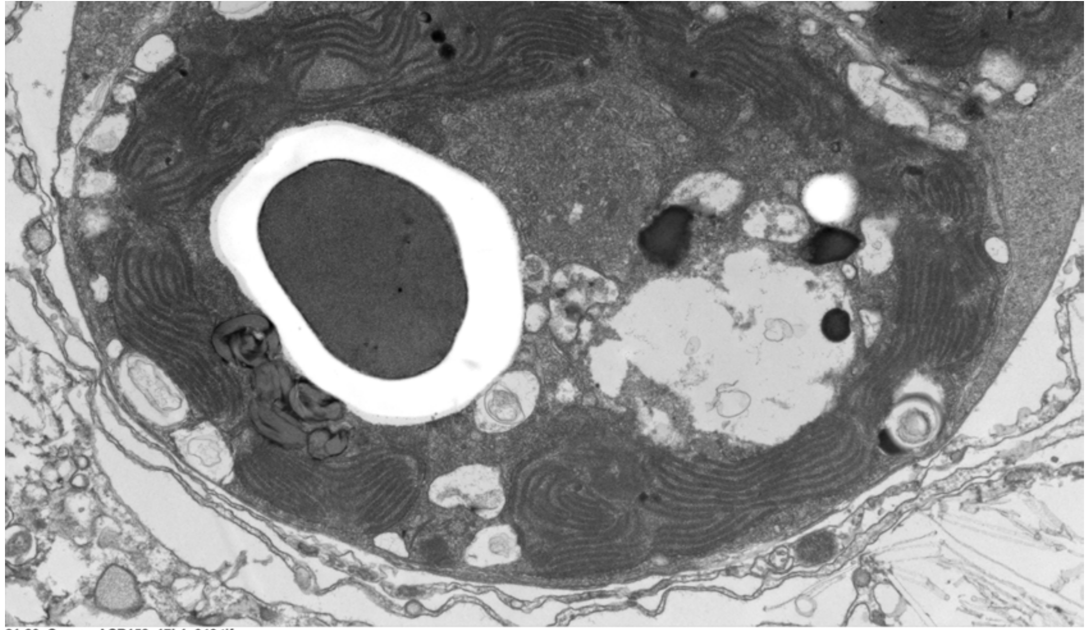

21-20_Correa_ACR158_17L1_046.tif
ACR 158
Biological Electron Microscopy Lab
Rice University - SEA
Microscopist: MD Meyer

1 µm
HV=80kV
Direct Mag: 3000 x

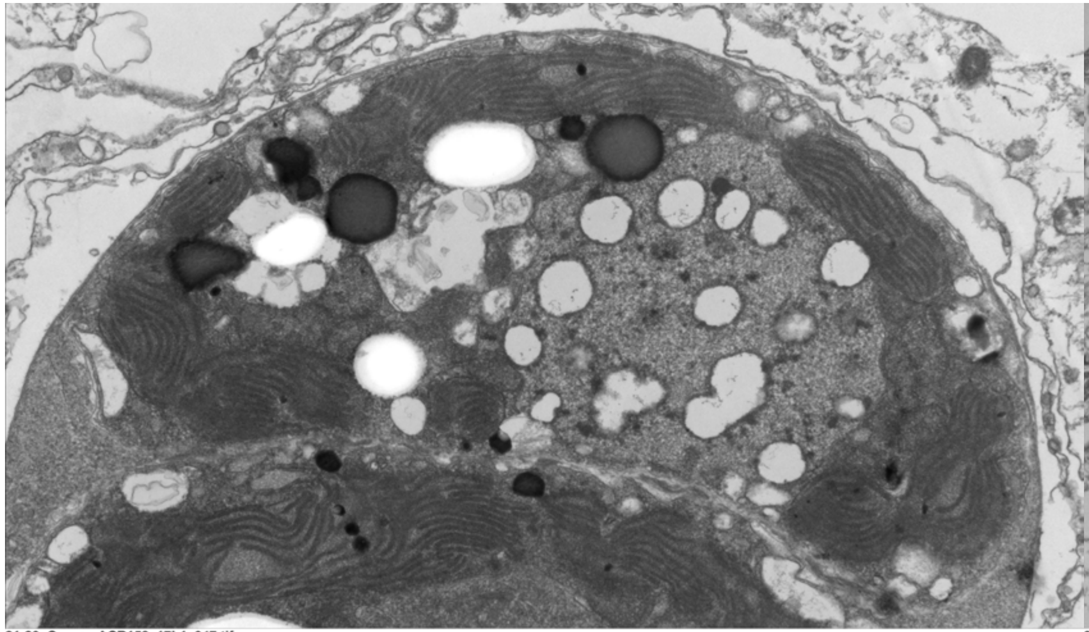

21-20_Correa_ACR158_17L1_047.tif
ACR 158
Biological Electron Microscopy Lab
Rice University - SEA
Microscopist: MD Meyer

1 µm
HV=80kV
Direct Mag: 3000 x

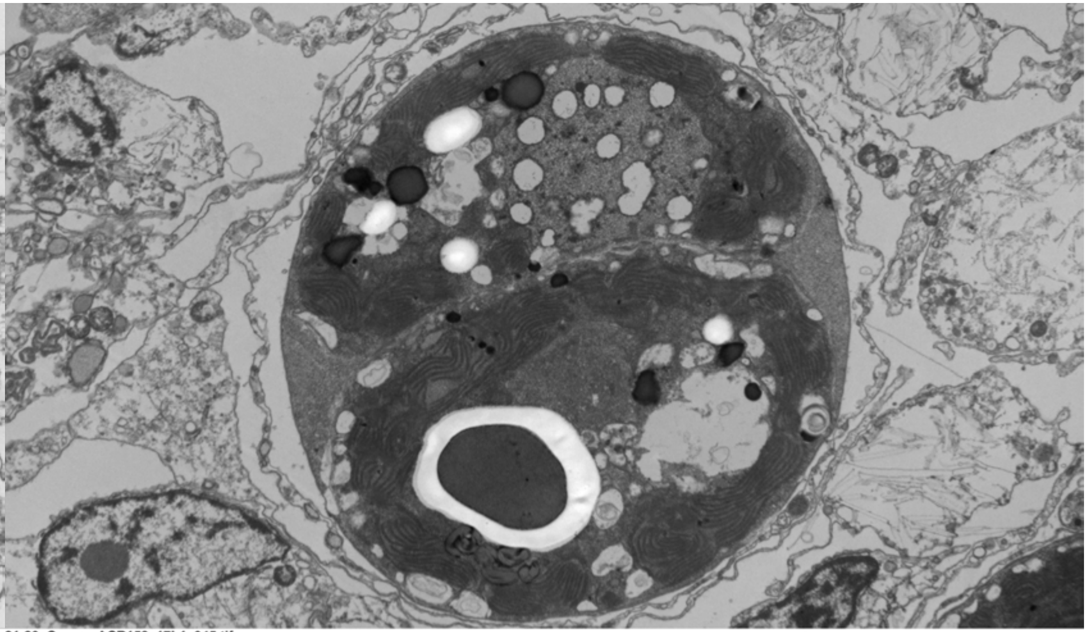

21-20_Correa_ACR158_17L1_045.tif
ACR 158
Biological Electron Microscopy Lab
Rice University - SEA
Microscopist: MD Meyer

2 µm
HV=80kV
Direct Mag: 1500 x

Cell 11

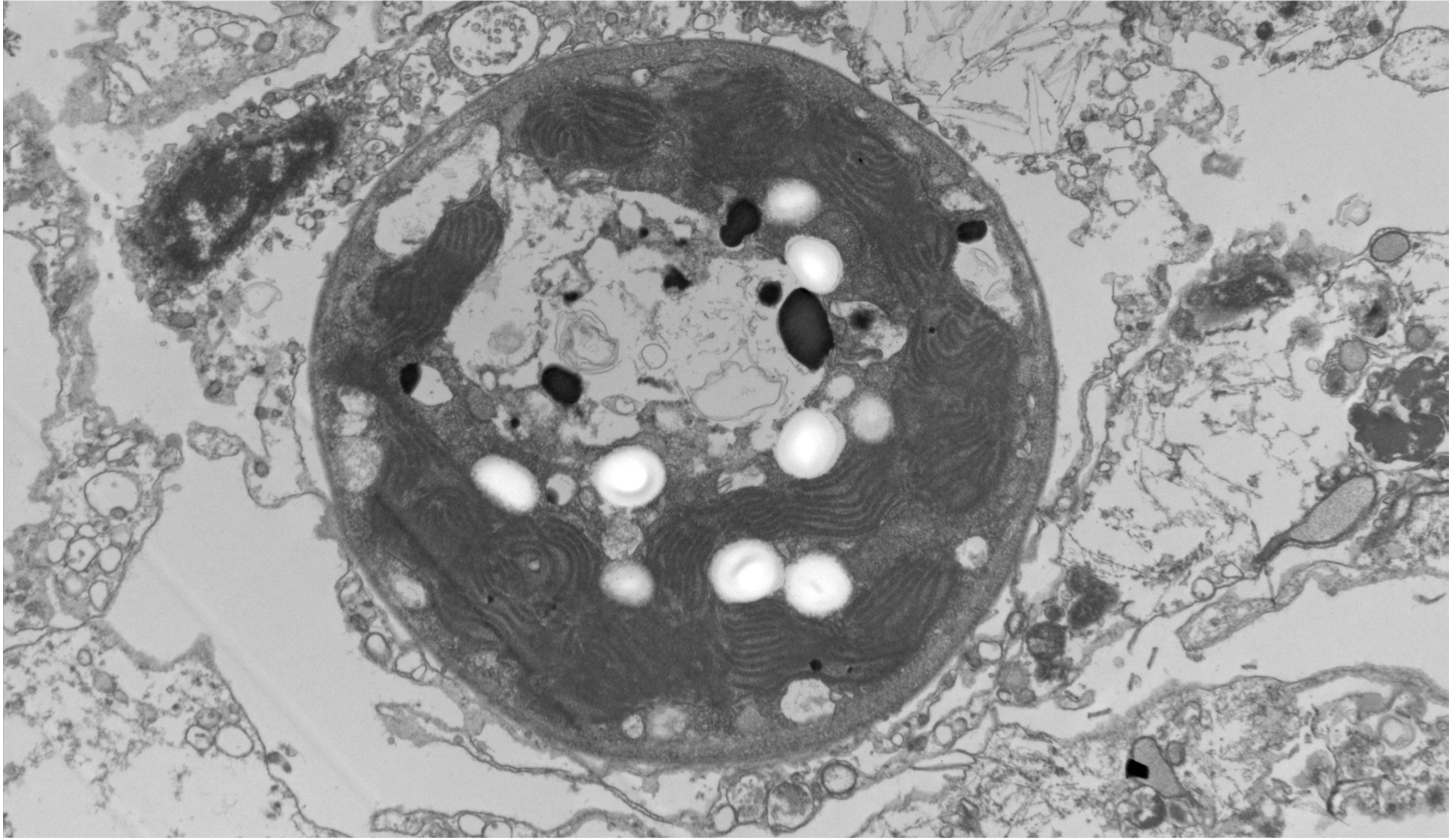

21-20_Correa_ACR158_17L1_048.tif
ACR 158
Biological Electron Microscopy Lab
Rice University - SEA
Microscopist: MD Meyer

2 μm
HV=80kV
Direct Mag: 2000 x

Cell 12

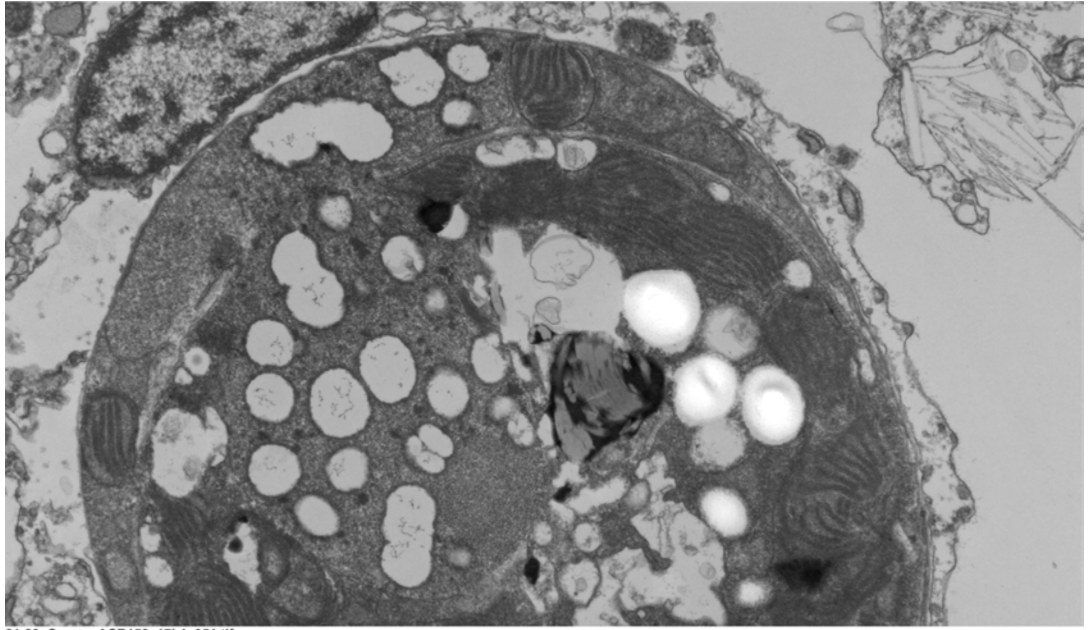

21-20_Correa_ACR158_17L1_051.tif
ACR 158
Biological Electron Microscopy Lab
Rice University - SEA
Microscopist: MD Meyer

1 µm
HV=80kV
Direct Mag: 3000 x

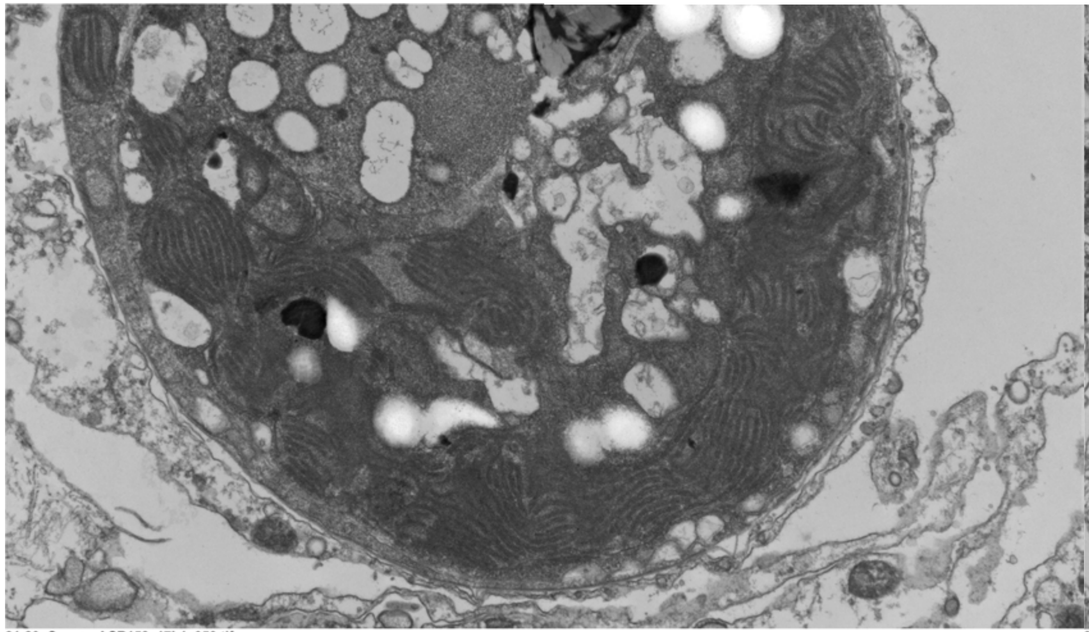

21-20_Correa_ACR158_17L1_050.tif
ACR 158
Biological Electron Microscopy Lab
Rice University - SEA
Microscopist: MD Meyer

1 µm
HV=80kV
Direct Mag: 3000 x

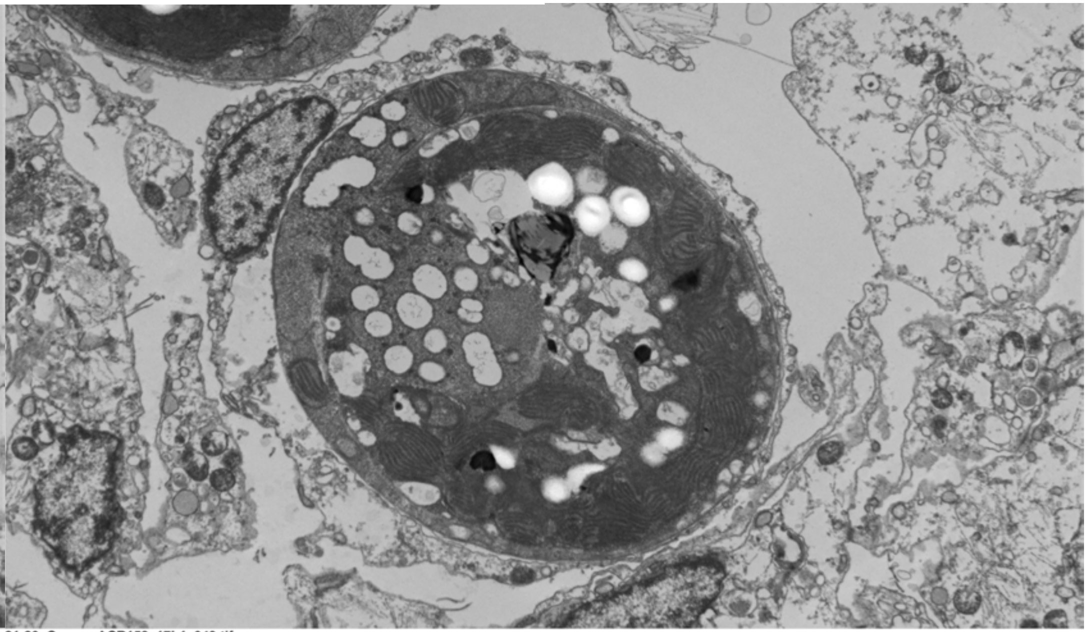

21-20_Correa_ACR158_17L1_049.tif
ACR 158
Biological Electron Microscopy Lab
Rice University - SEA
Microscopist: MD Meyer

2 µm
HV=80kV
Direct Mag: 1500 x

Cell 13

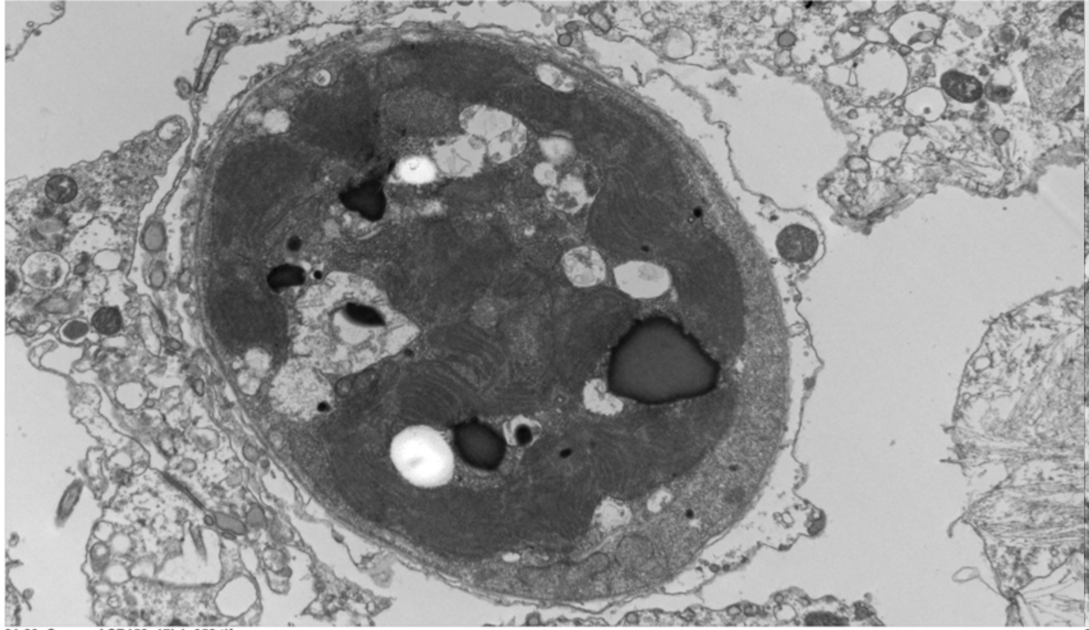

21-20_Correa_ACR158_17L1_052.tif
ACR 158
Biological Electron Microscopy Lab
Rice University - SEA
Microscopist: MD Meyer

2 µm
HV=80kV
Direct Mag: 2000 x

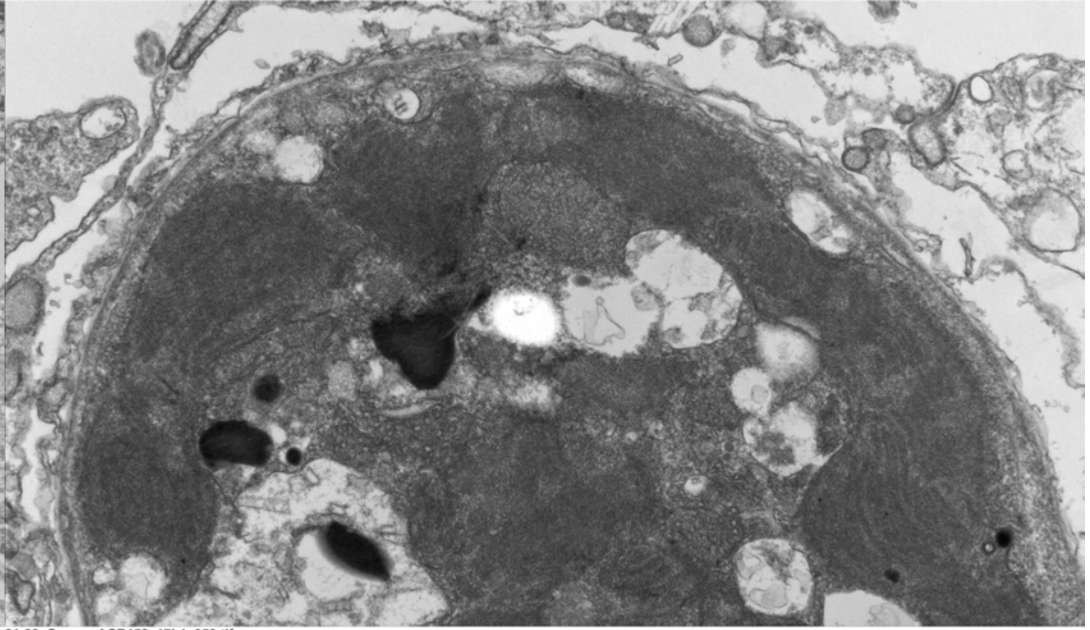

21-20_Correa_ACR158_17L1_053.tif
ACR 158
Biological Electron Microscopy Lab
Rice University - SEA
Microscopist: MD Meyer

1 µm
HV=80kV
Direct Mag: 4000 x

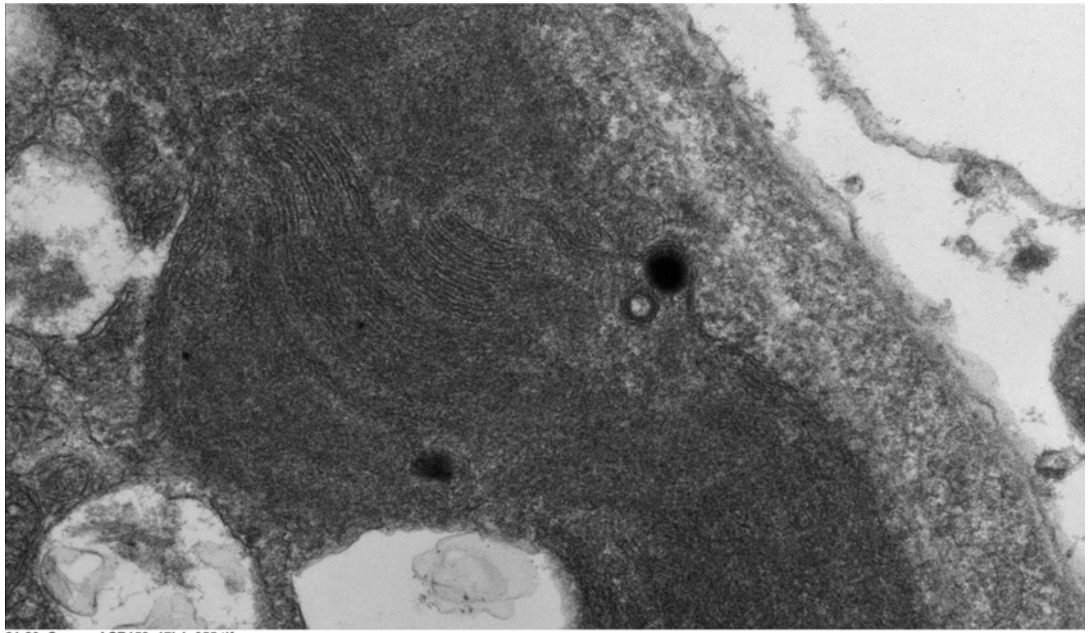

21-20_Correa_ACR158_17L1_055.tif
ACR 158
Biological Electron Microscopy Lab
Rice University - SEA
Microscopist: MD Meyer

400 nm
HV=80kV
Direct Mag: 10000 x

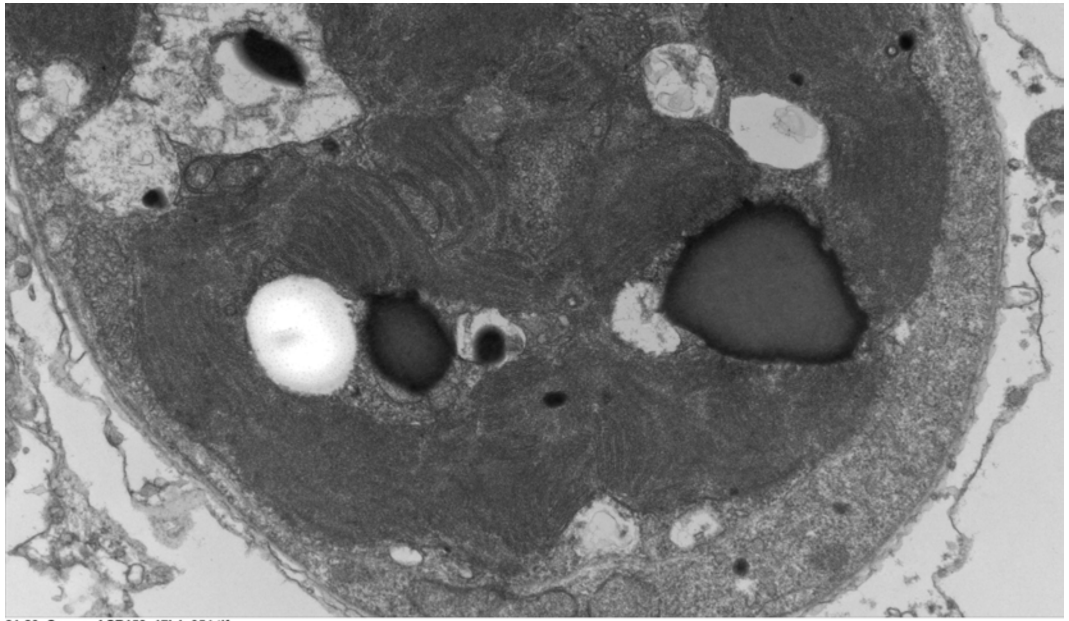

21-20_Correa_ACR158_17L1_054.tif
ACR 158
Biological Electron Microscopy Lab
Rice University - SEA
Microscopist: MD Meyer

1 µm
HV=80kV
Direct Mag: 4000 x

Cell 14

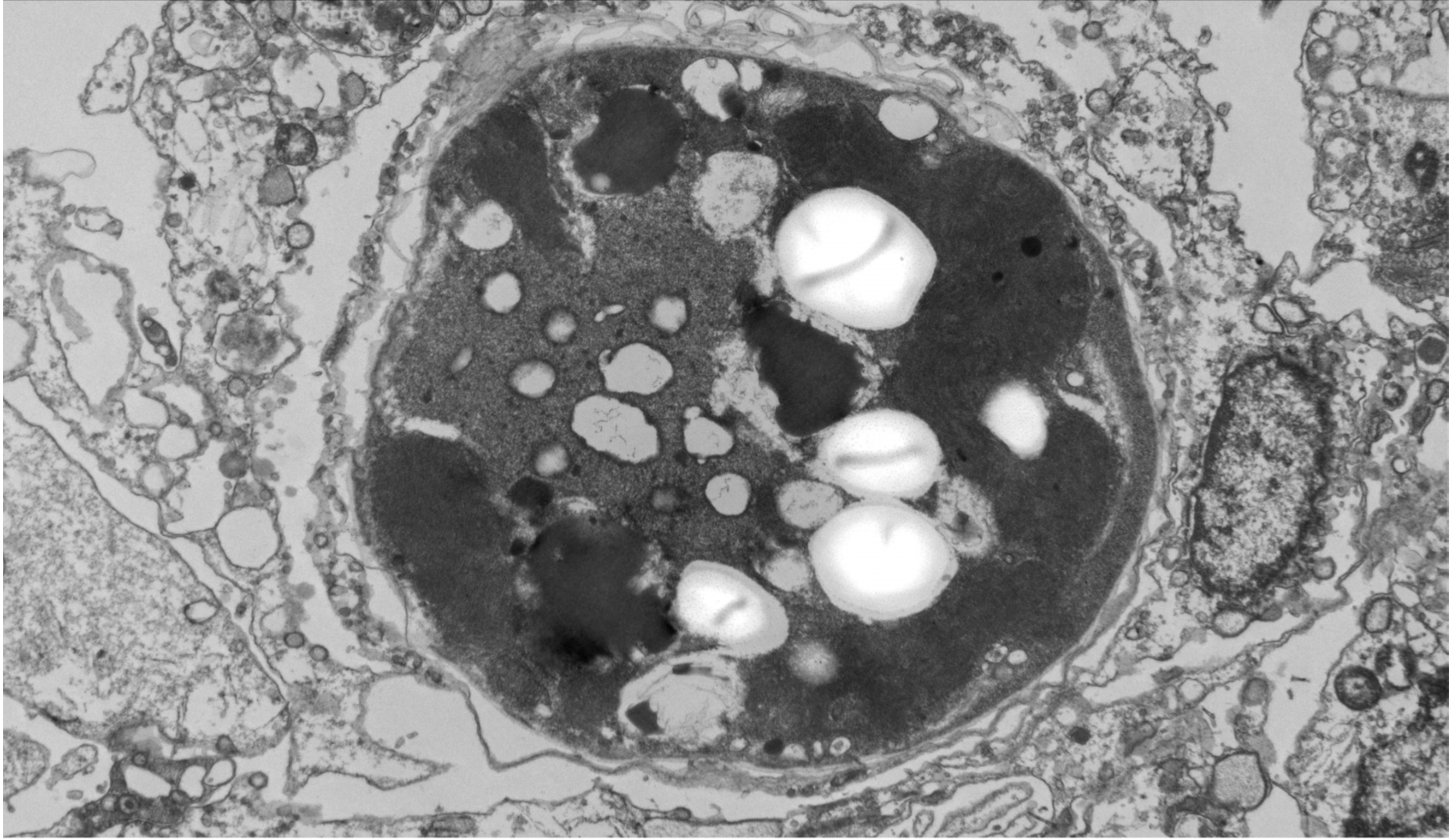

21-20_Correa_ACR158_17L1_056.tif
ACR 158
Biological Electron Microscopy Lab
Rice University - SEA
Microscopist: MD Meyer

2 µm
HV=80kV
Direct Mag: 2000 x

Cell 15

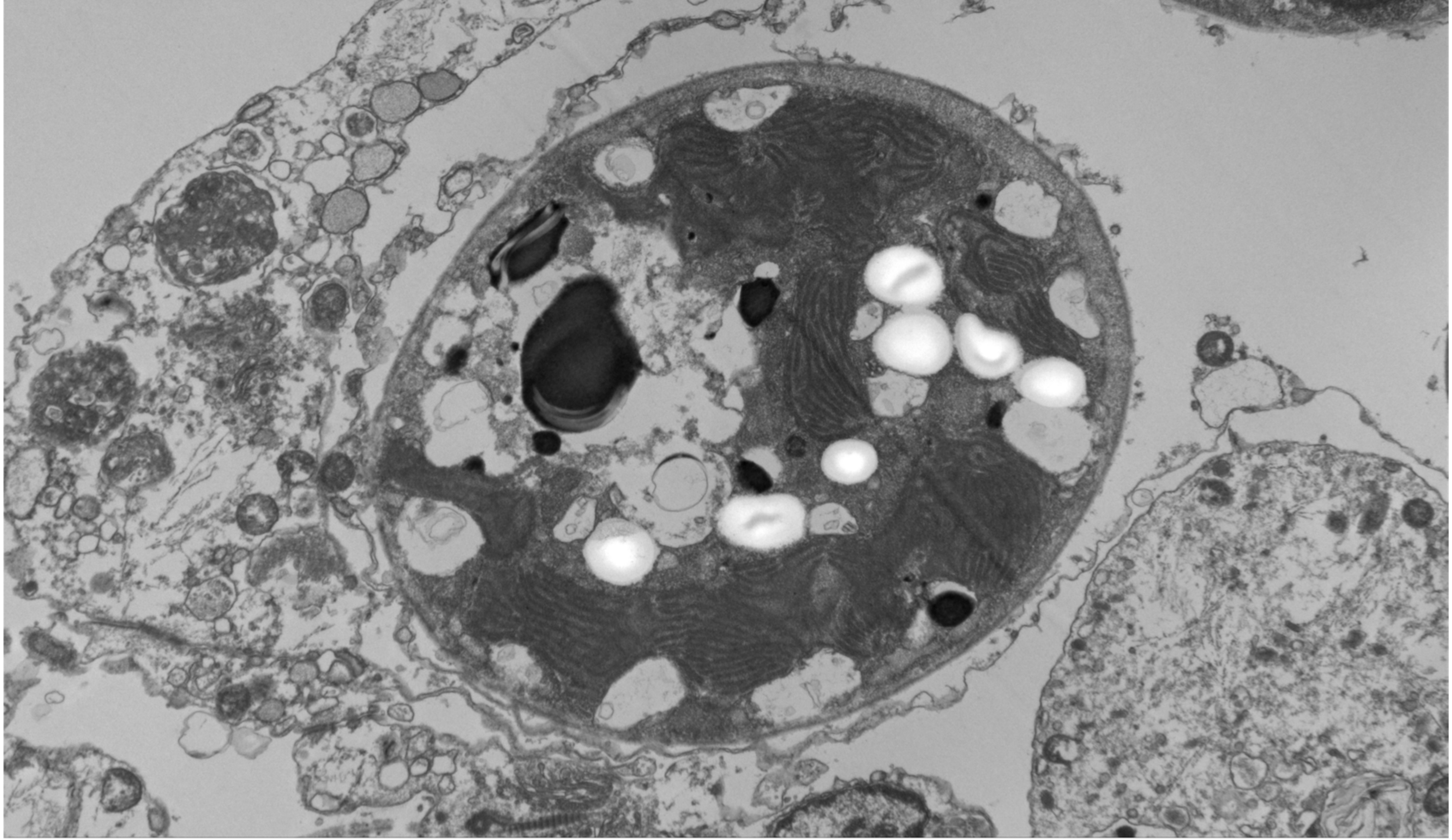

21-20_Correa_ACR158_17L1_057.tif
ACR 158
Biological Electron Microscopy Lab
Rice University - SEA
Microscopist: MD Meyer

2 µm
HV=80kV
Direct Mag: 2000 x

Cell 16

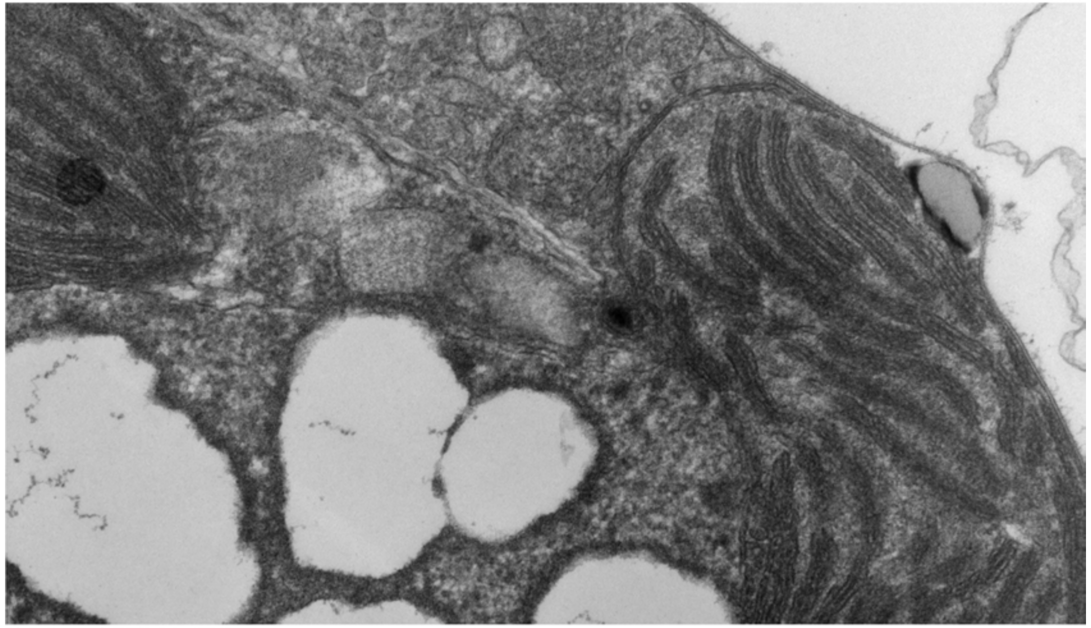

21-20_Correa_ACR158_17L1_060.tif
ACR 158
Biological Electron Microscopy Lab
Rice University - SEA
Microscopist: MD Meyer

400 nm
HV=80kV
Direct Mag: 10000 x

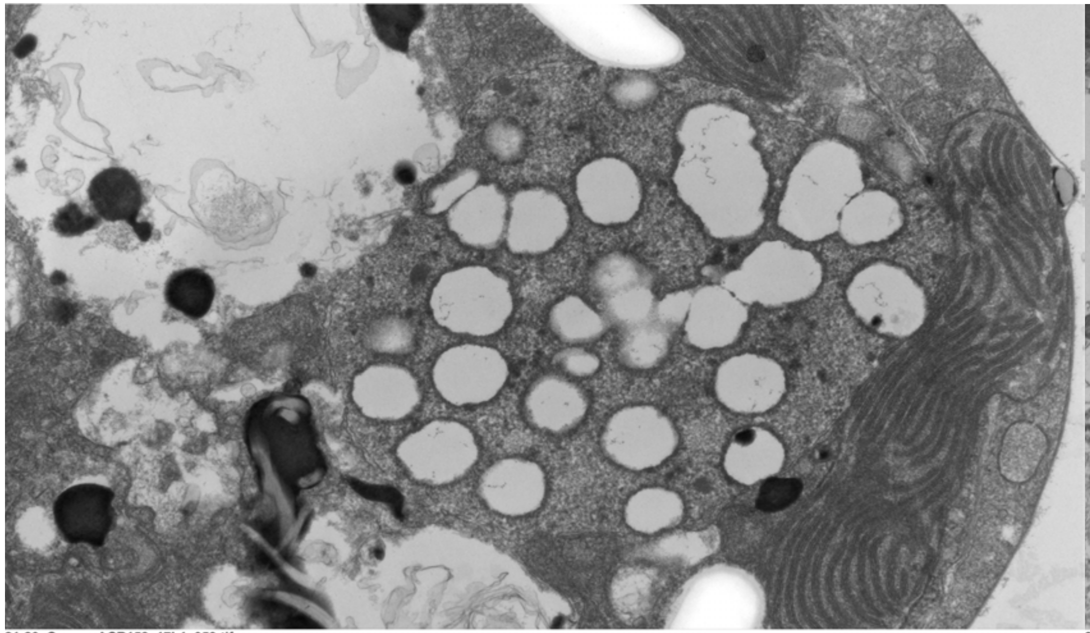

21-20_Correa_ACR158_17L1_059.tif
ACR 158
Biological Electron Microscopy Lab
Rice University - SEA
Microscopist: MD Meyer

1 µm
HV=80kV
Direct Mag: 4000 x

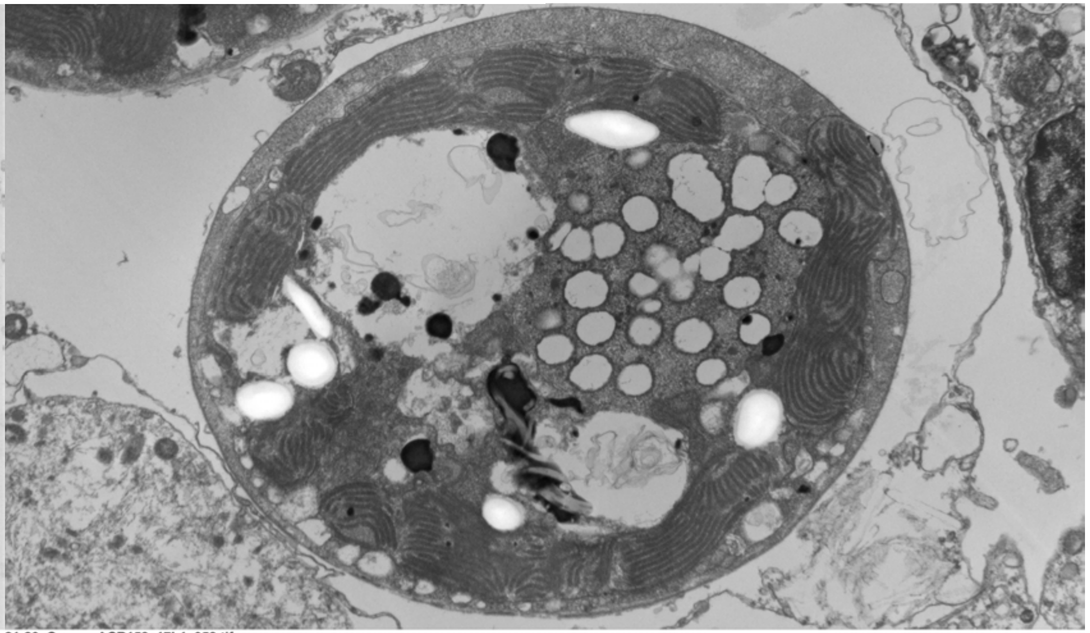

21-20_Correa_ACR158_17L1_058.tif
ACR 158
Biological Electron Microscopy Lab
Rice University - SEA
Microscopist: MD Meyer

2 µm
HV=80kV
Direct Mag: 2000 x

Cell 17

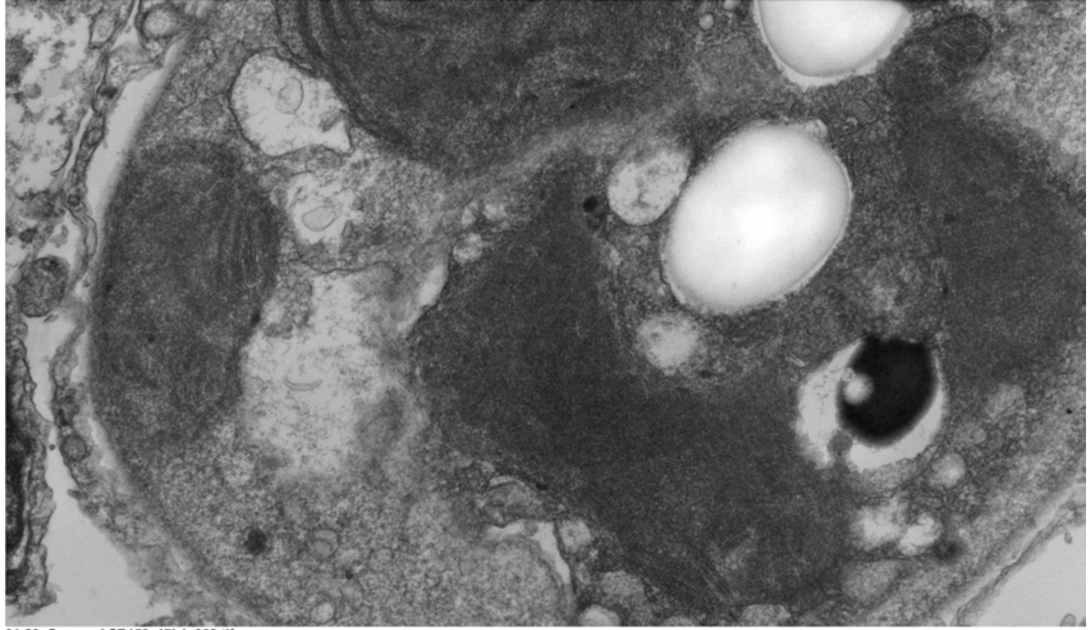

21-20_Correa_ACR158_17L1_062.tif
ACR 158
Biological Electron Microscopy Lab
Rice University - SEA
Microscopist: MD Meyer

800 nm
HV=80kV
Direct Mag: 5000 x

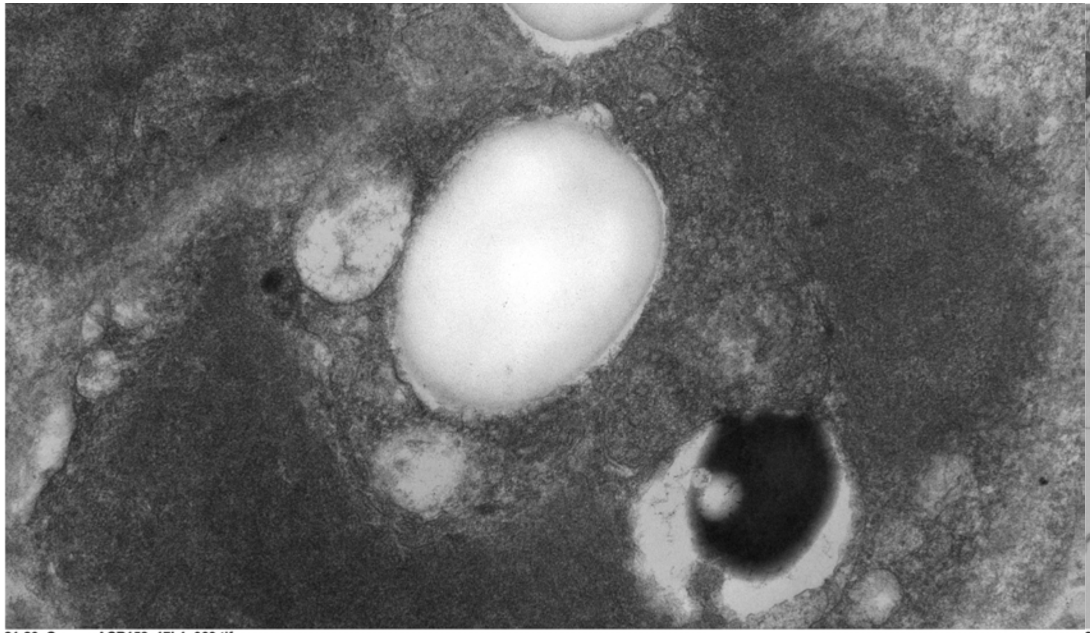

21-20_Correa_ACR158_17L1_063.tif
ACR 158
Biological Electron Microscopy Lab
Rice University - SEA
Microscopist: MD Meyer

500 nm
HV=80kV
Direct Mag: 8000 x

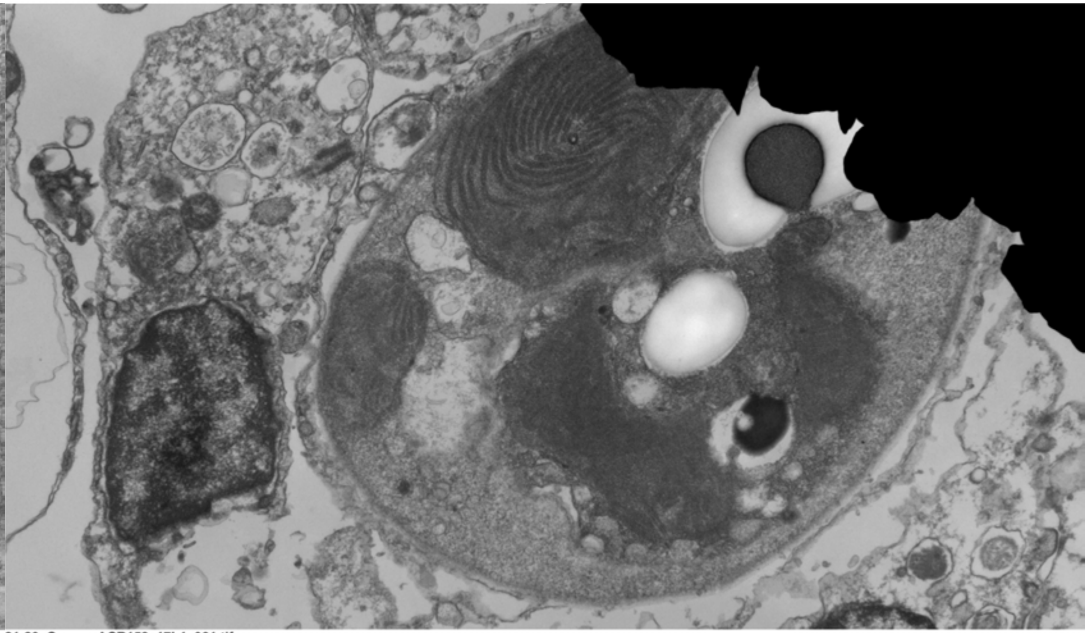

21-20_Correa_ACR158_17L1_061.tif
ACR 158
Biological Electron Microscopy Lab
Rice University - SEA
Microscopist: MD Meyer

1 µm
HV=80kV
Direct Mag: 3000 x

Cell 18

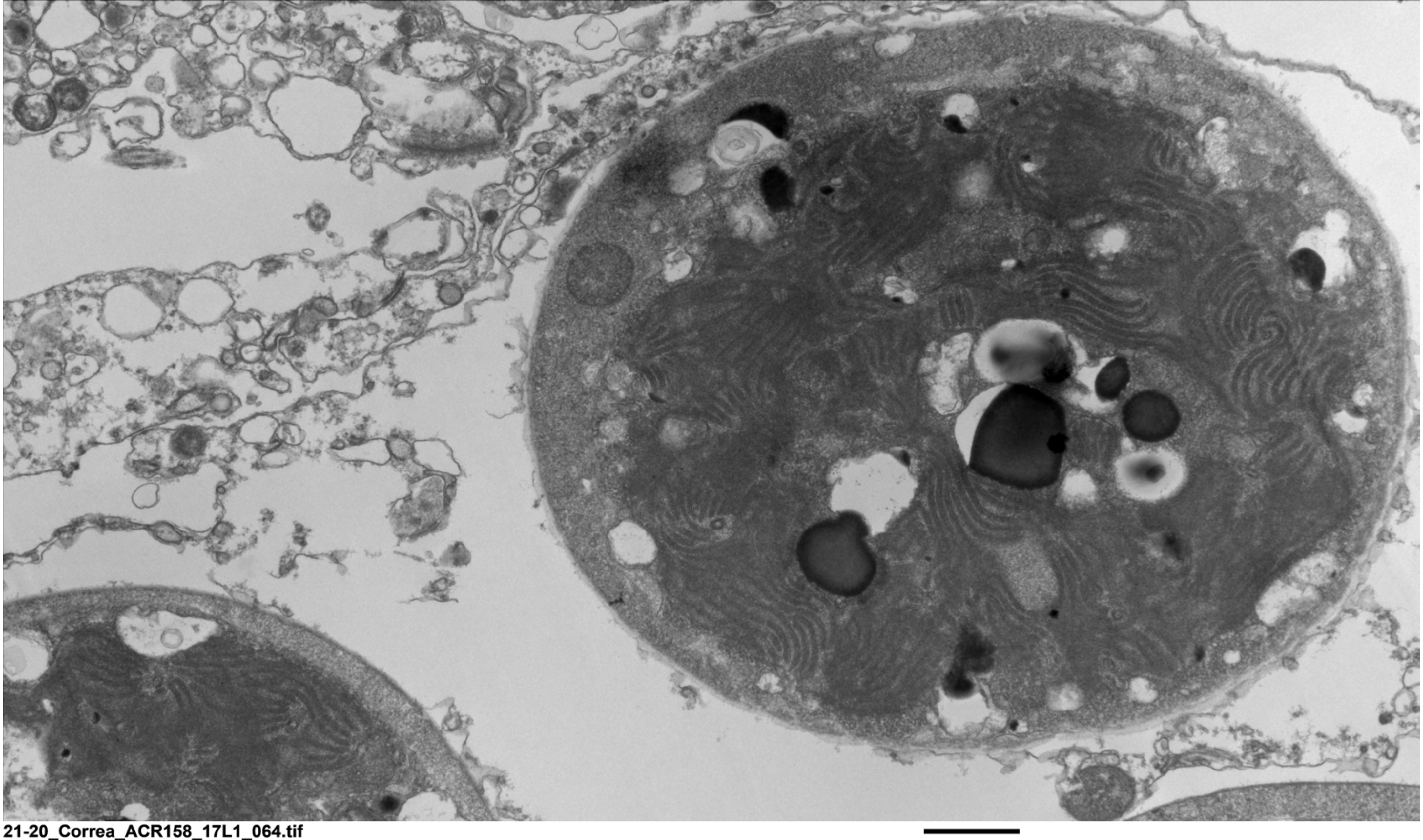

21-20_Correa_ACR158_17L1_064.tif
ACR 158
Biological Electron Microscopy Lab
Rice University - SEA
Microscopist: MD Meyer

1 μm
HV=80kV
Direct Mag: 2500 x

Cell 19

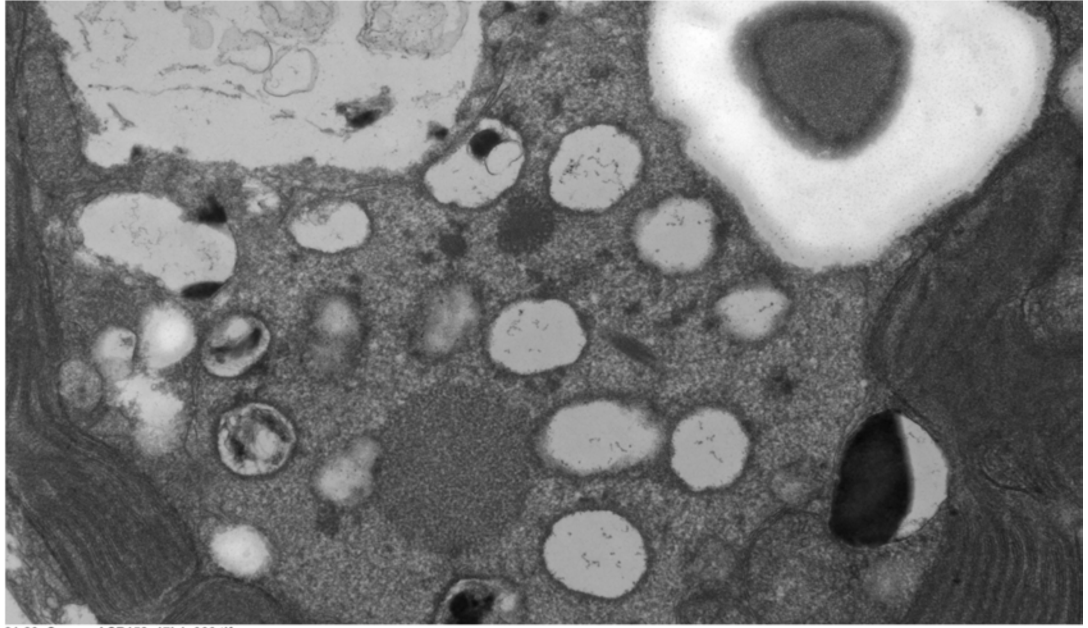

21-20_Correa_ACR158_17L1_066.tif
ACR 158
Biological Electron Microscopy Lab
Rice University - SEA
Microscopist: MD Meyer

800 nm
HV=80kV
Direct Mag: 5000 x

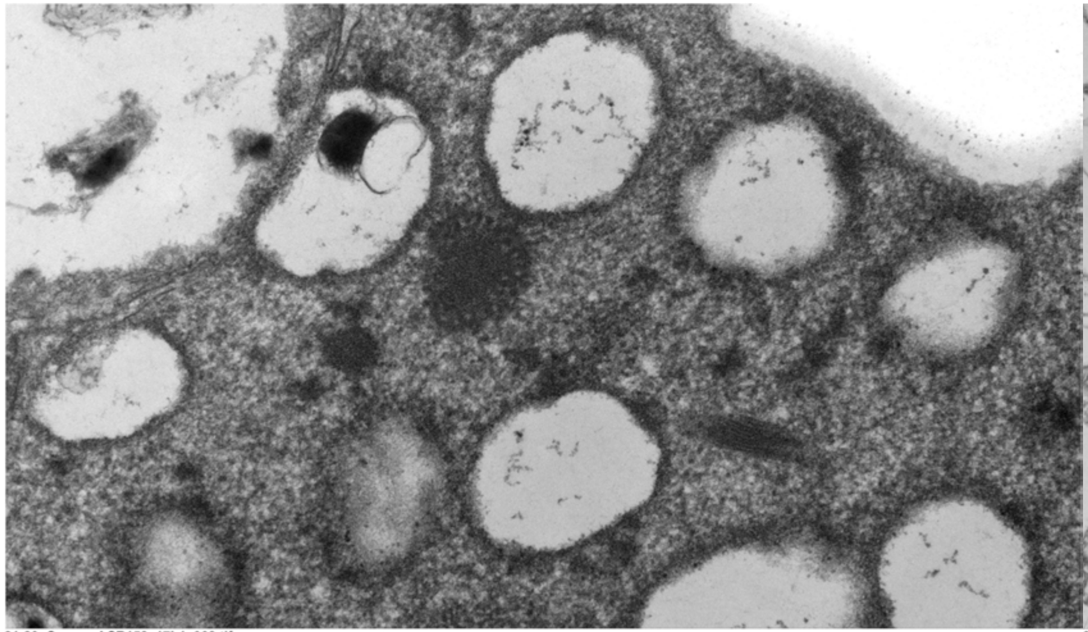

21-20_Correa_ACR158_17L1_068.tif
ACR 158
Biological Electron Microscopy Lab
Rice University - SEA
Microscopist: MD Meyer

400 nm
HV=80kV
Direct Mag: 10000 x

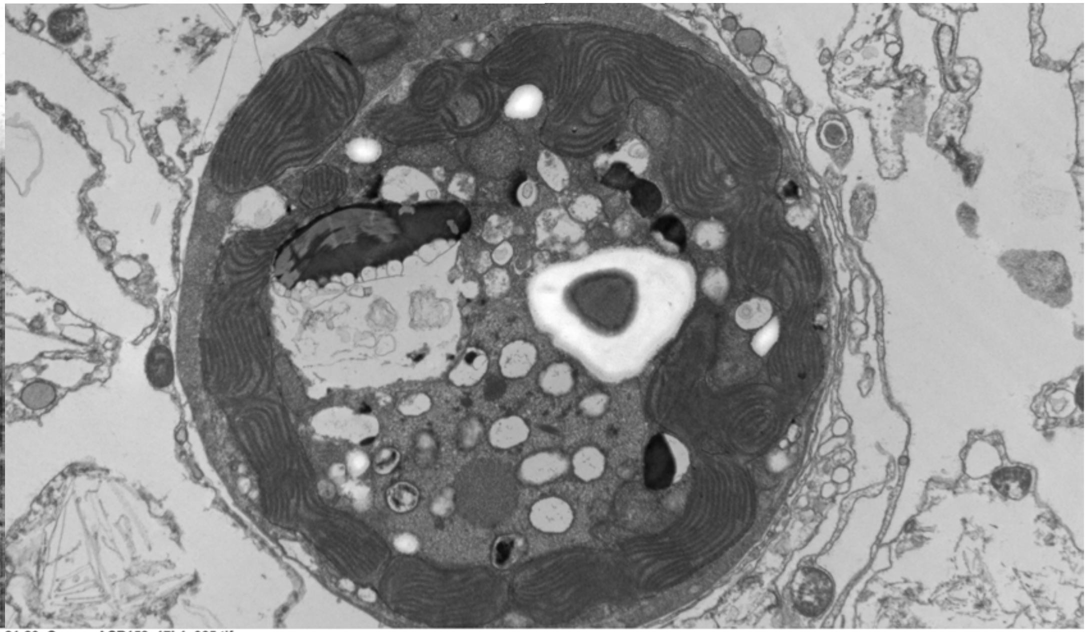

21-20_Correa_ACR158_17L1_065.tif
ACR 158
Biological Electron Microscopy Lab
Rice University - SEA
Microscopist: MD Meyer

2 µm
HV=80kV
Direct Mag: 2000 x

Cell 20

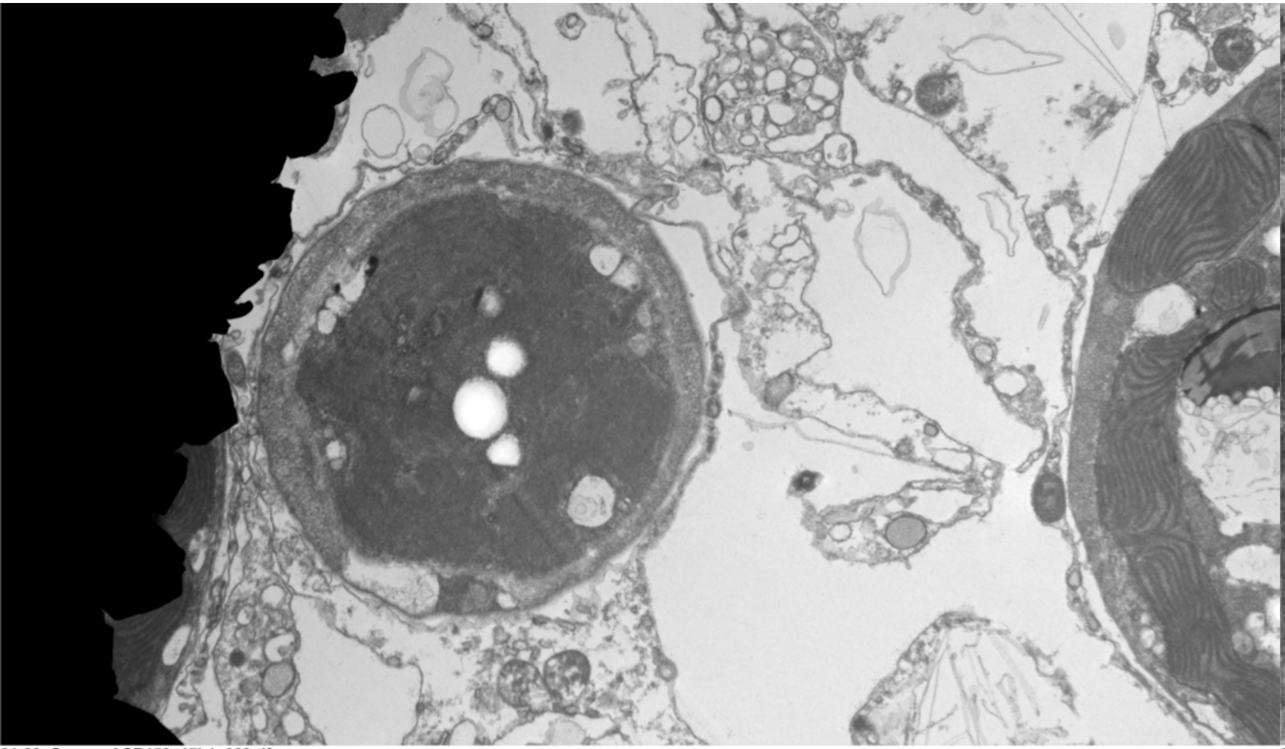

21-20_Correa_ACR158_17L1_069.tif
ACR 158
Biological Electron Microscopy Lab
Rice University - SEA
Microscopist: MD Meyer

2 µm
HV=80kV
Direct Mag: 2000 x

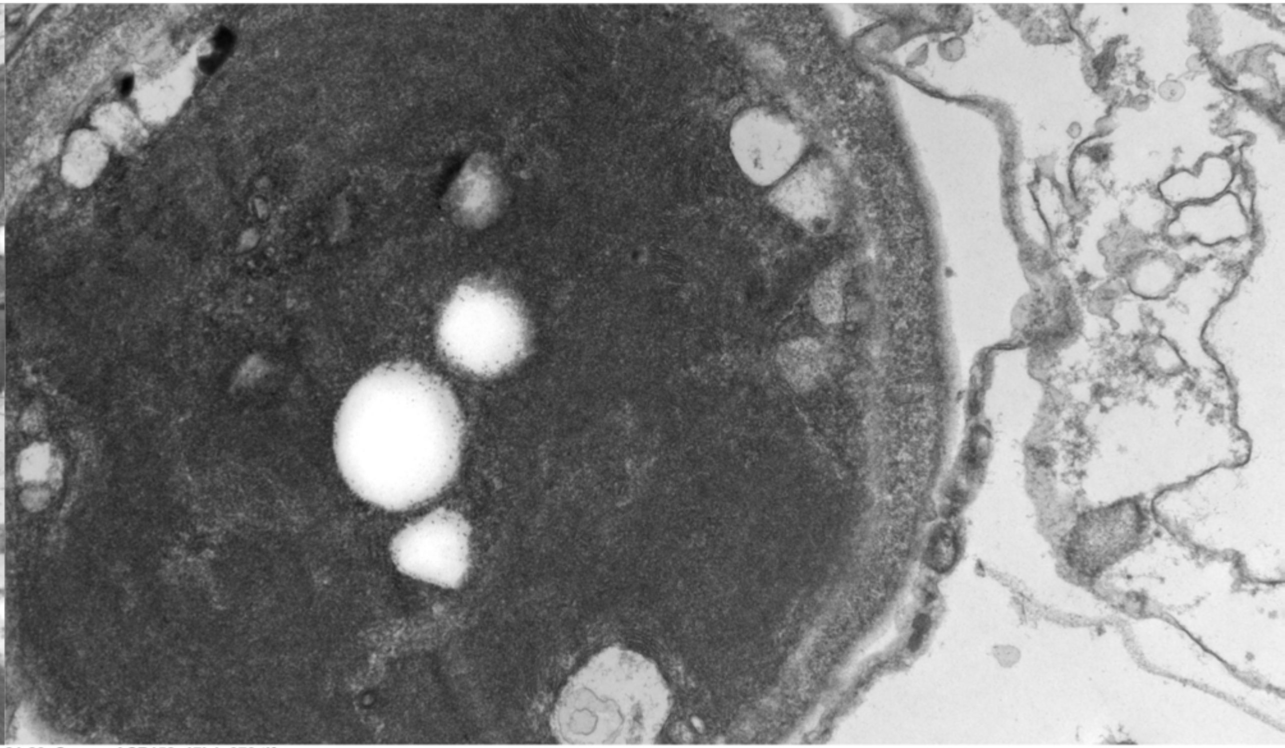

21-20_Correa_ACR158_17L1_070.tif
ACR 158
Biological Electron Microscopy Lab
Rice University - SEA
Microscopist: MD Meyer

800 nm
HV=80kV
Direct Mag: 5000 x

Cell 21

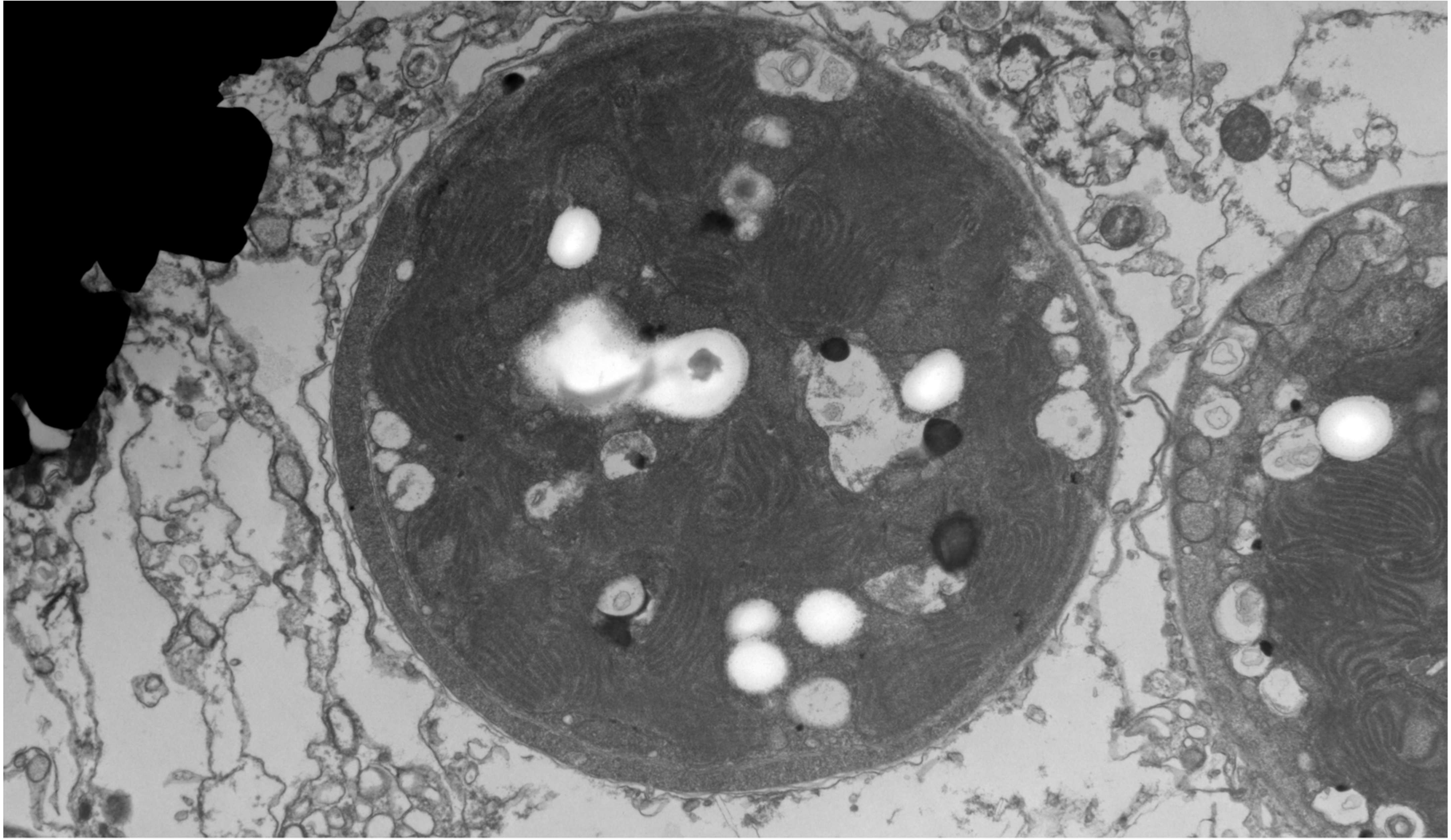

21-20_Correa_ACR158_17L1_071.tif
ACR 158
Biological Electron Microscopy Lab
Rice University - SEA
Microscopist: MD Meyer

1 µm
HV=80kV
Direct Mag: 2500 x

Cell 22

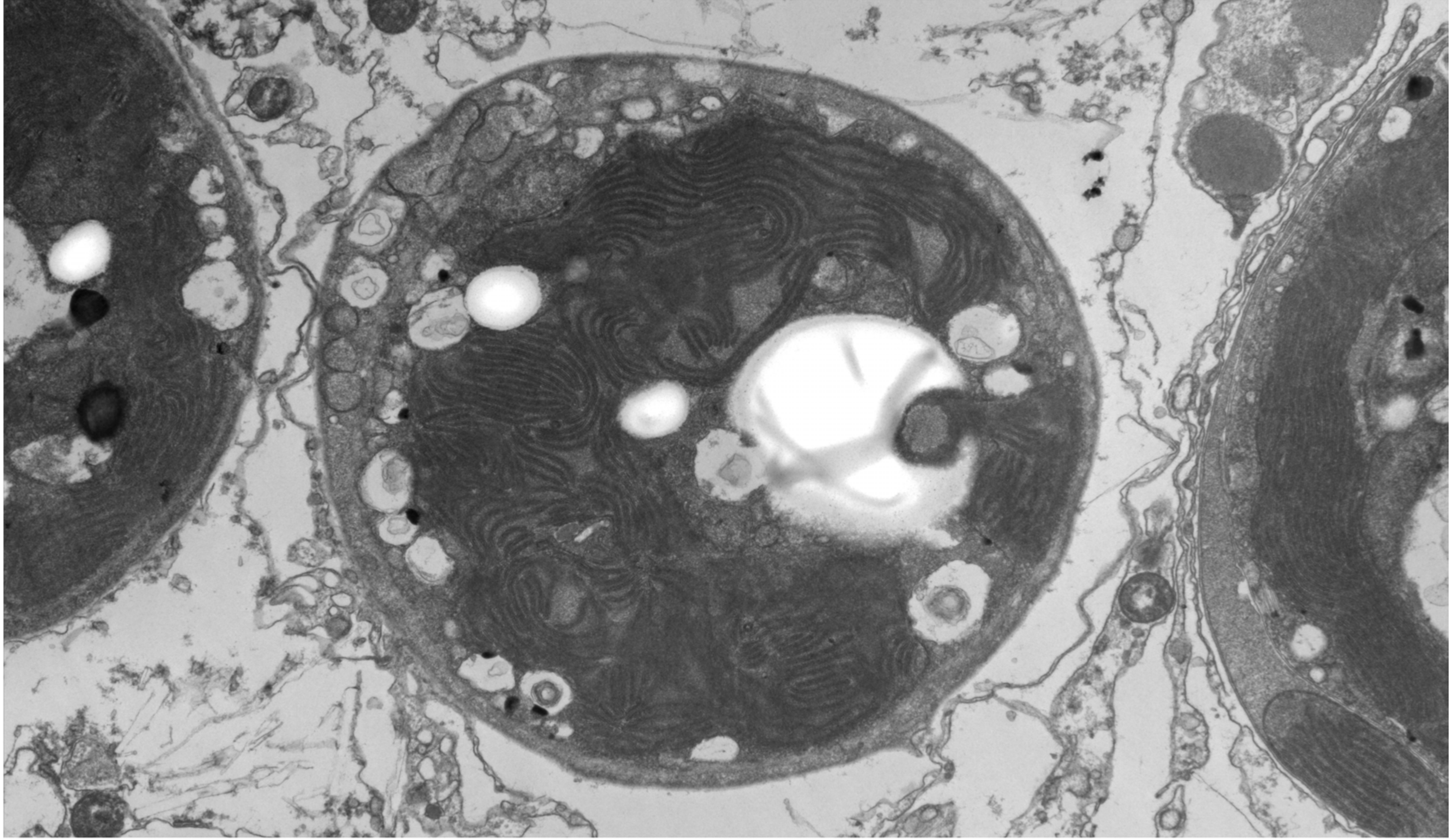

21-20_Correa_ACR158_17L1_072.tif
ACR 158
Biological Electron Microscopy Lab
Rice University - SEA
Microscopist: MD Meyer

1 µm
HV=80kV
Direct Mag: 2500 x

Cell 23

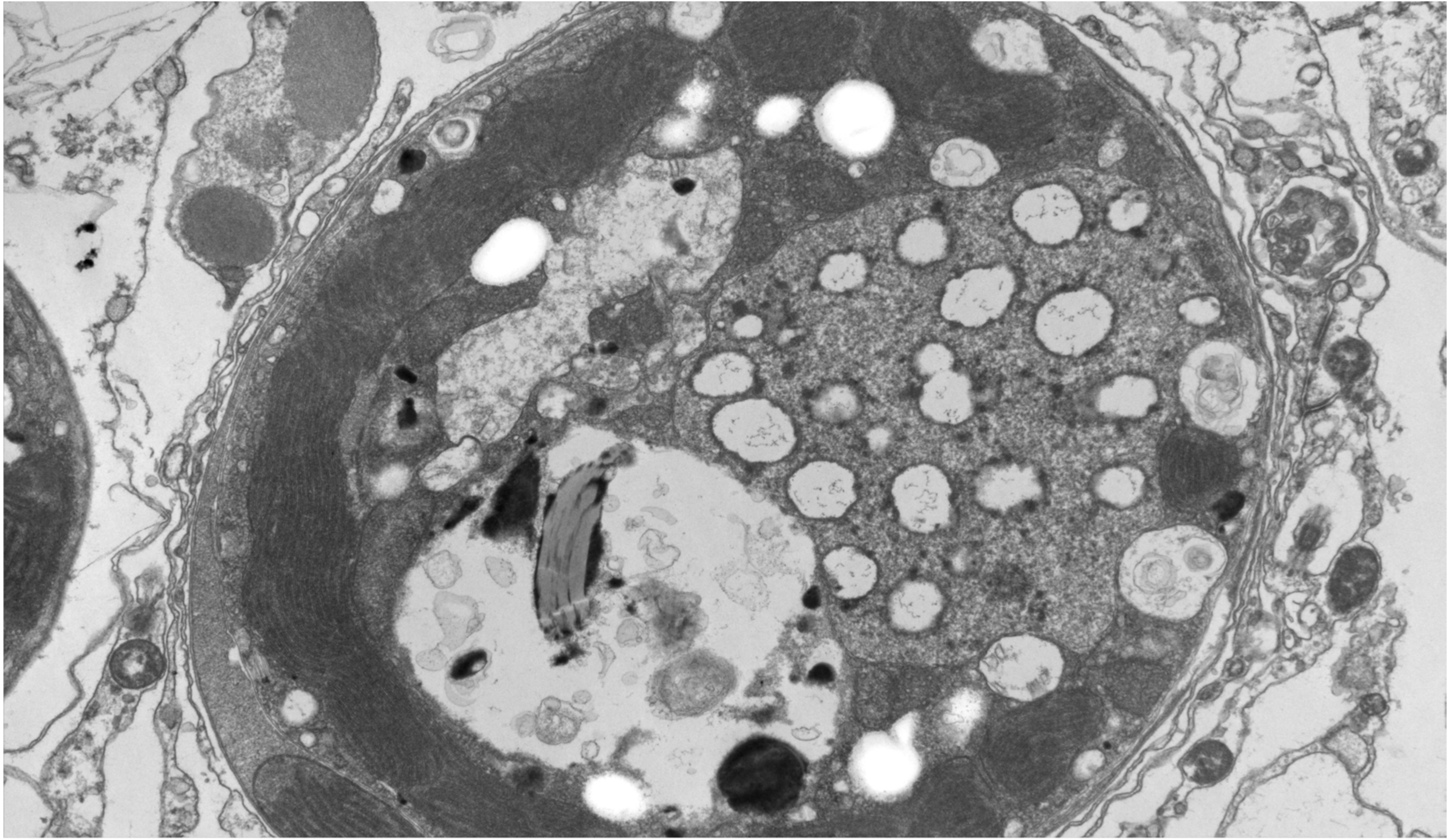

21-20_Correa_ACR158_17L1_073.tif
ACR 158
Biological Electron Microscopy Lab
Rice University - SEA
Microscopist: MD Meyer

1 µm
HV=80kV
Direct Mag: 2500 x

Cell 24

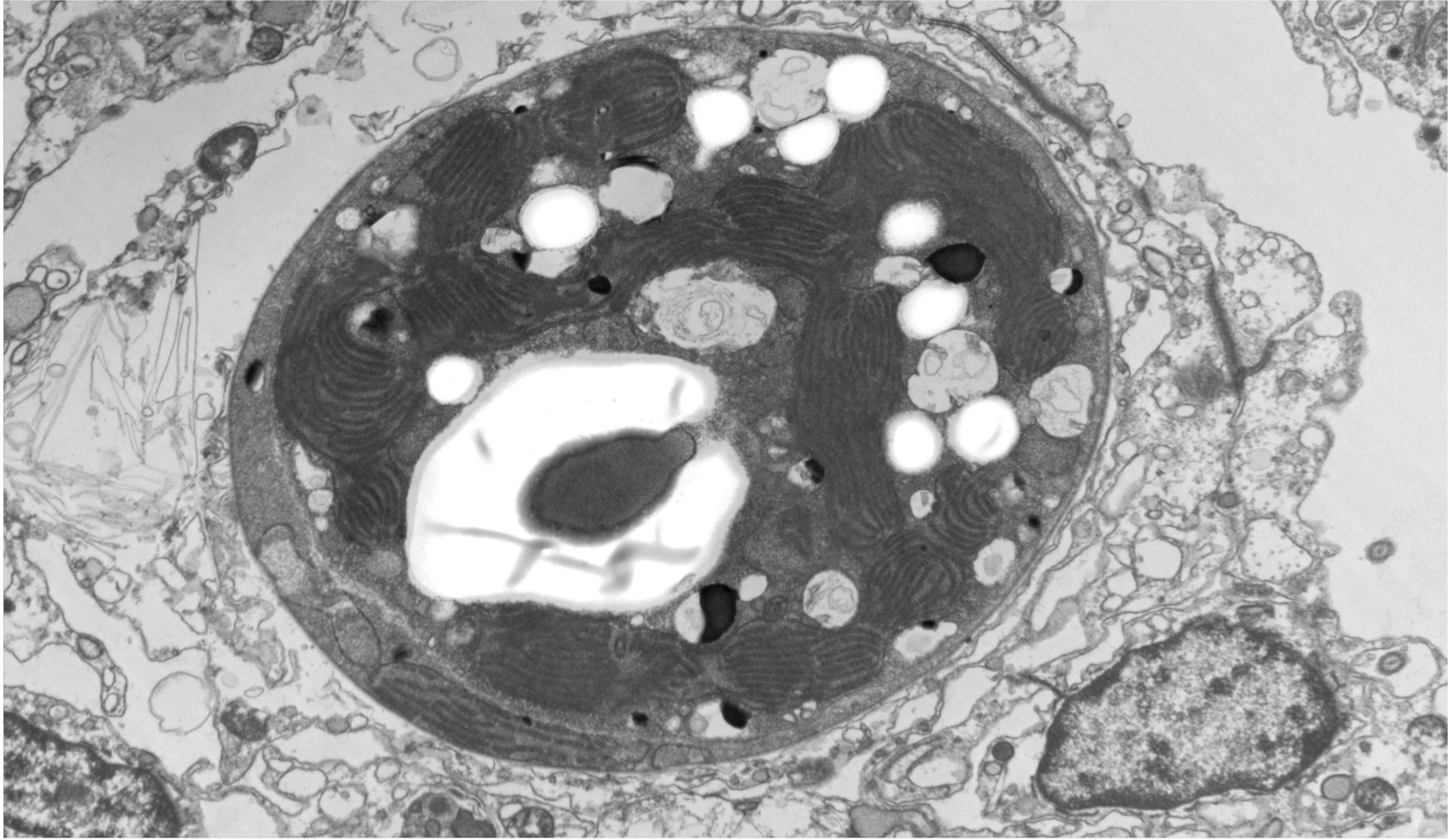

21-20_Correa_ACR158_17L1_074.tif
ACR 158
Biological Electron Microscopy Lab
Rice University - SEA
Microscopist: MD Meyer

2 µm
HV=80kV
Direct Mag: 2000 x
