# Supplementary material for: Filamentous virus-like particles are present in coral dinoflagellates across genera and ocean basins
Source: ISME J. 2023 Nov 1;17(12):2389–402. doi: 10.1038/s41396-023-01526-6 (PMC10689786; doi:10.1038/s41396-023-01526-6)
Supplement: Supplementary file 5 — Supplementary TEM Images expelled ACR symbionts aquaria [file 41396_2023_1526_MOESM5_ESM.pdf]

Filamentous virus-like particles are present in coral dinoflagellates across genera and ocean basins

Supplementary Data- Expelled Symbiodiniaceae from aquaria experiment *Acropora hyacinthus* TEM images

Expelled - ACR Colony A
- Ambient
- Heat

Expelled - ACR Colony B
- Ambient
- Heat

Expelled - ACR Colony C
- Ambient
- Heat

Expelled - ACR Colony D
- Ambient
- Heat

ACR Colony A Expelled- Ambient

Cell 1

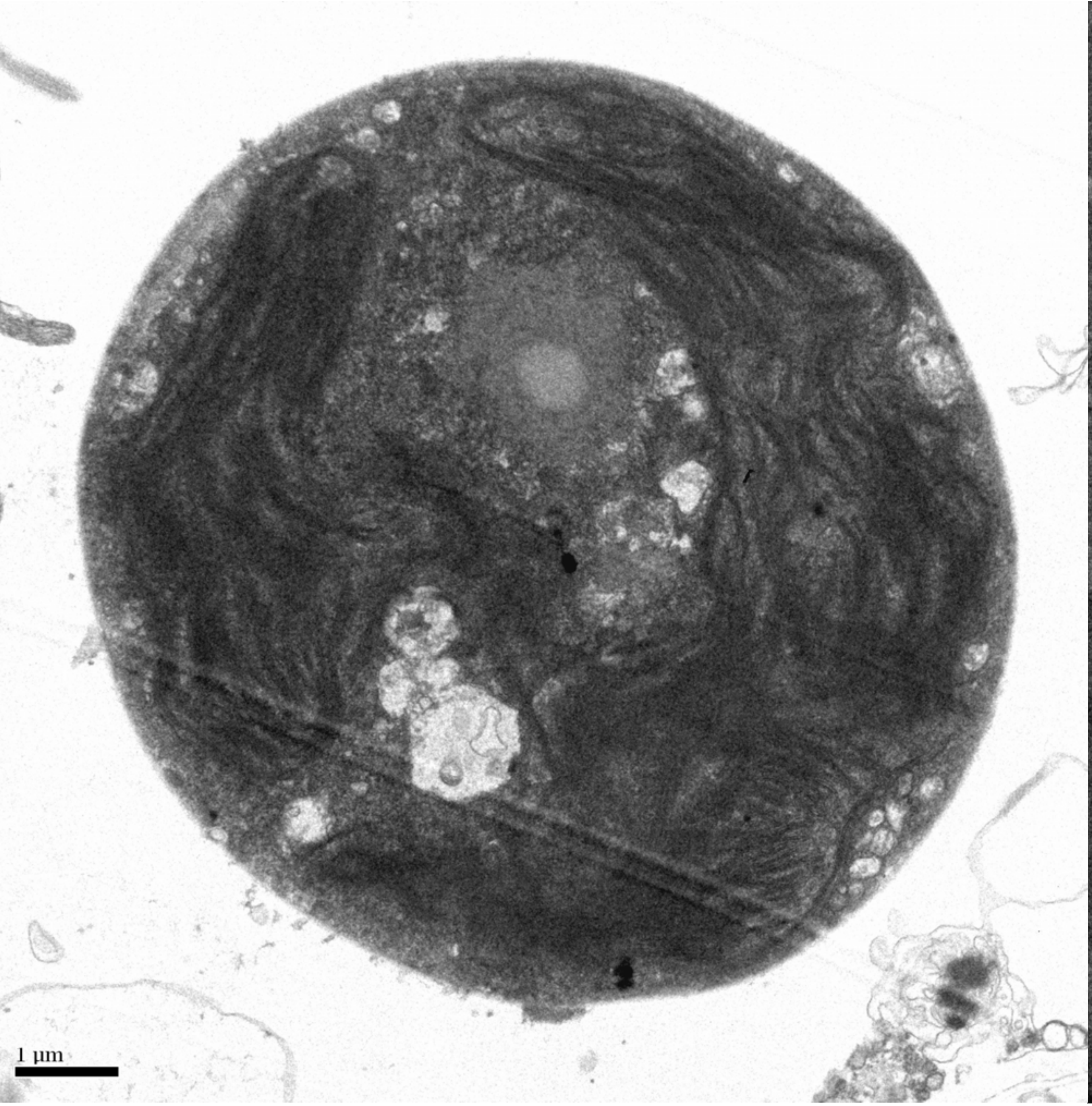

18-7_Correa_JC4_2GridC9_02

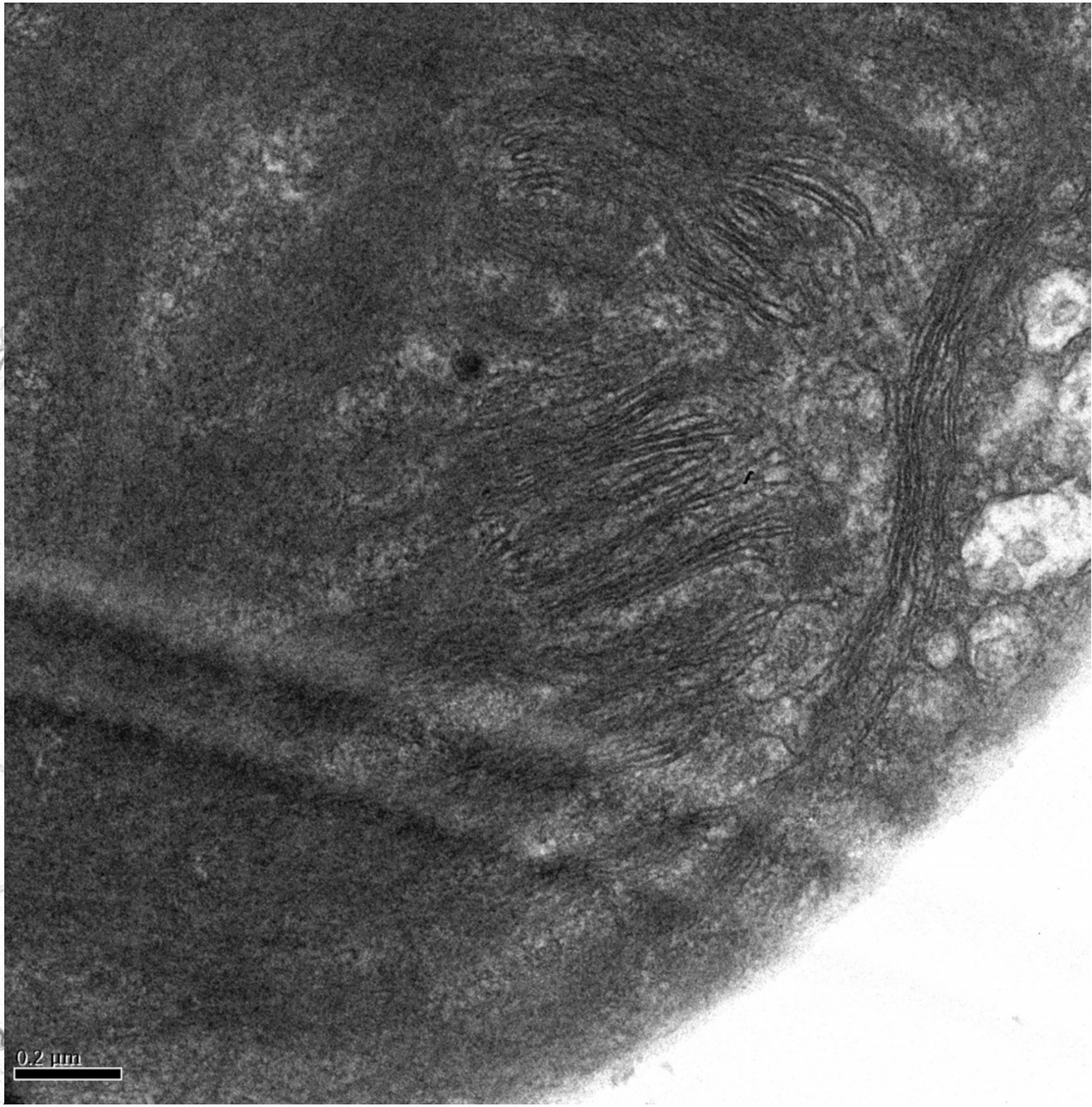

18-7_Correa_JC4_2GridC9_05

Cell 2

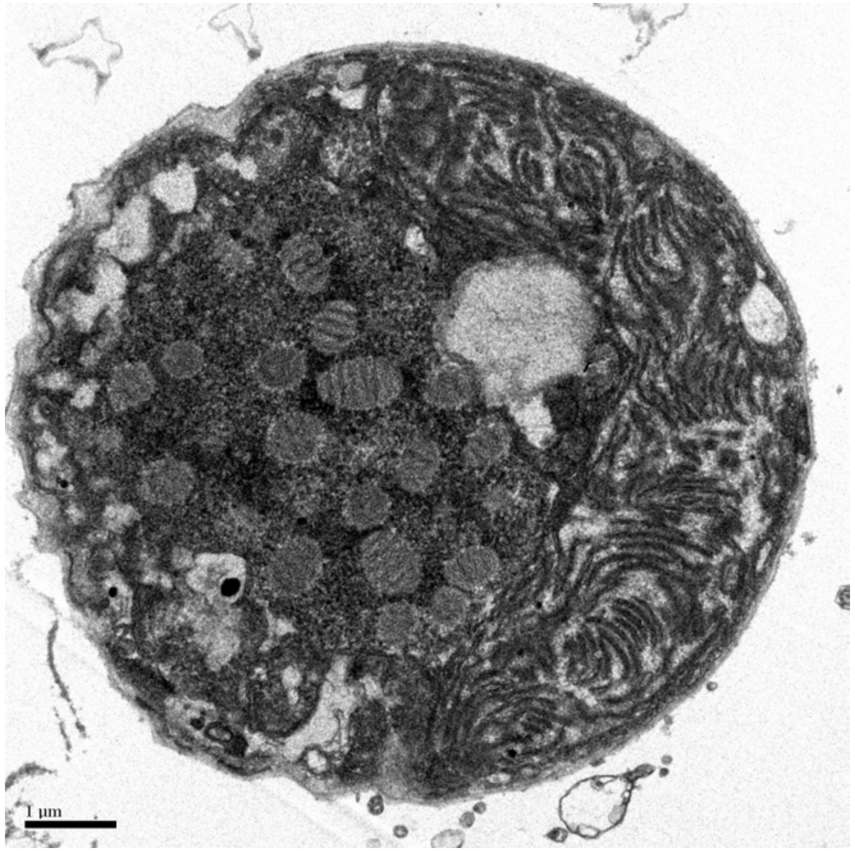

18-7_Correa_JC4_2GridC9_18

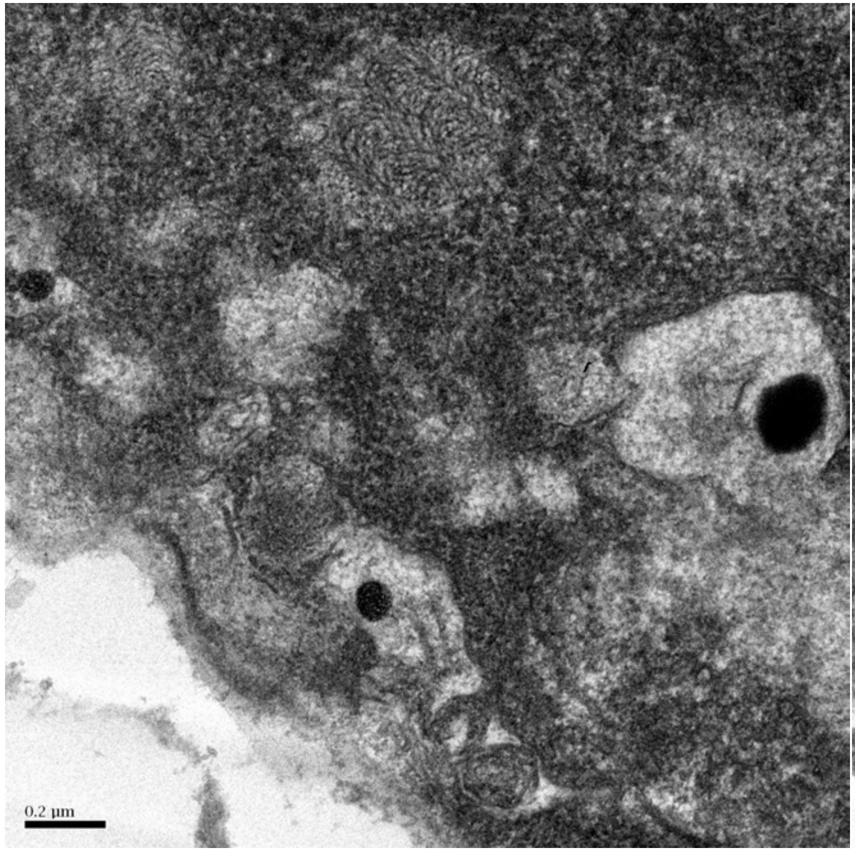

18-7_Correa_JC4_2GridC9_20

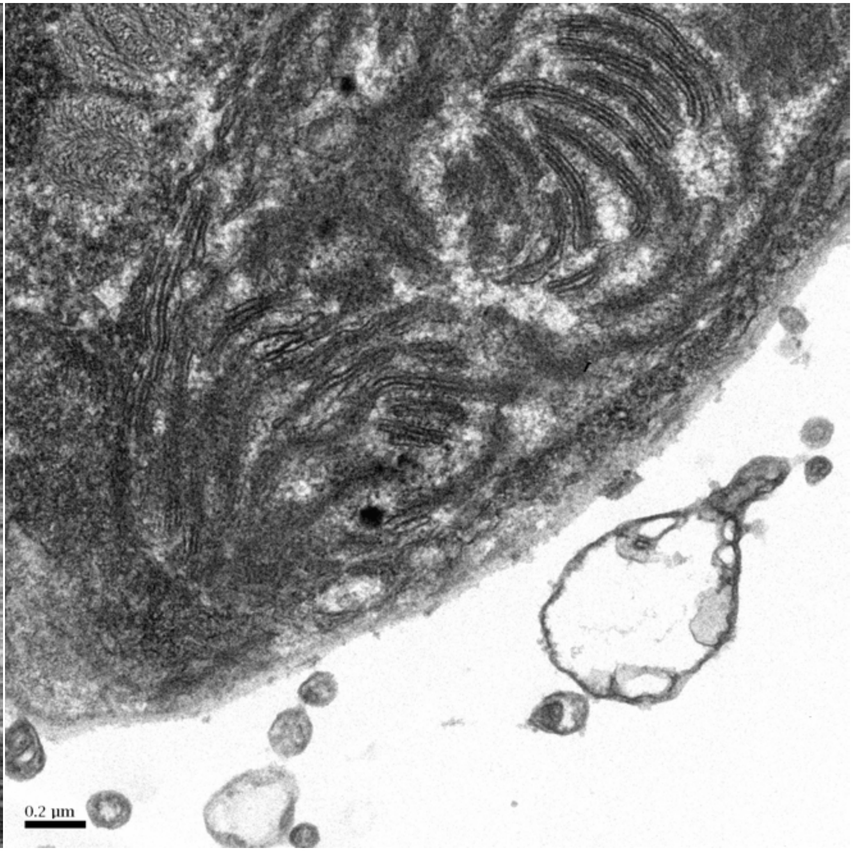

18-7_Correa_JC4_2GridC9_21

Cell 3

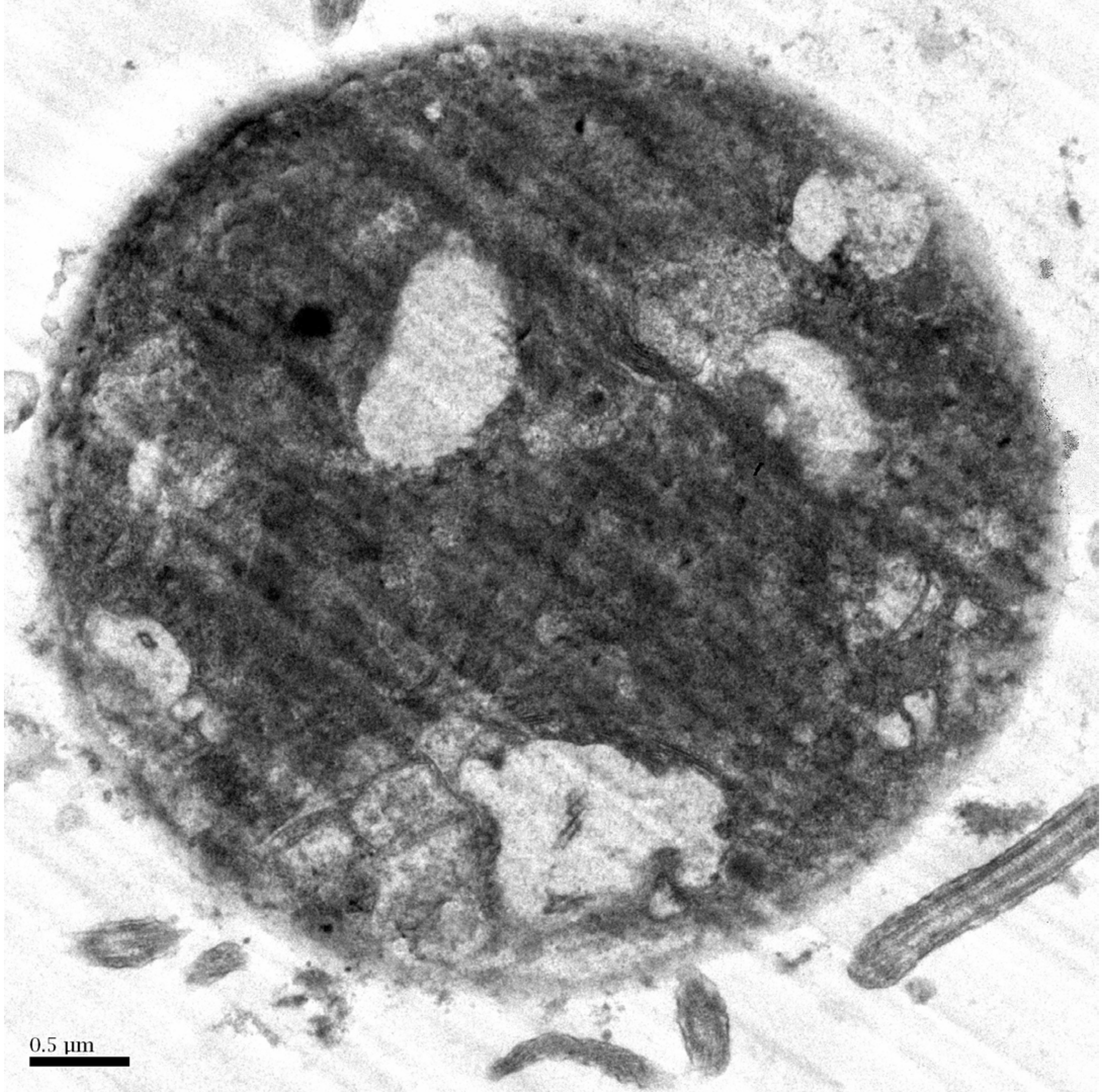

18-7_Correa_JC4_2GridC9_25

Cell 4

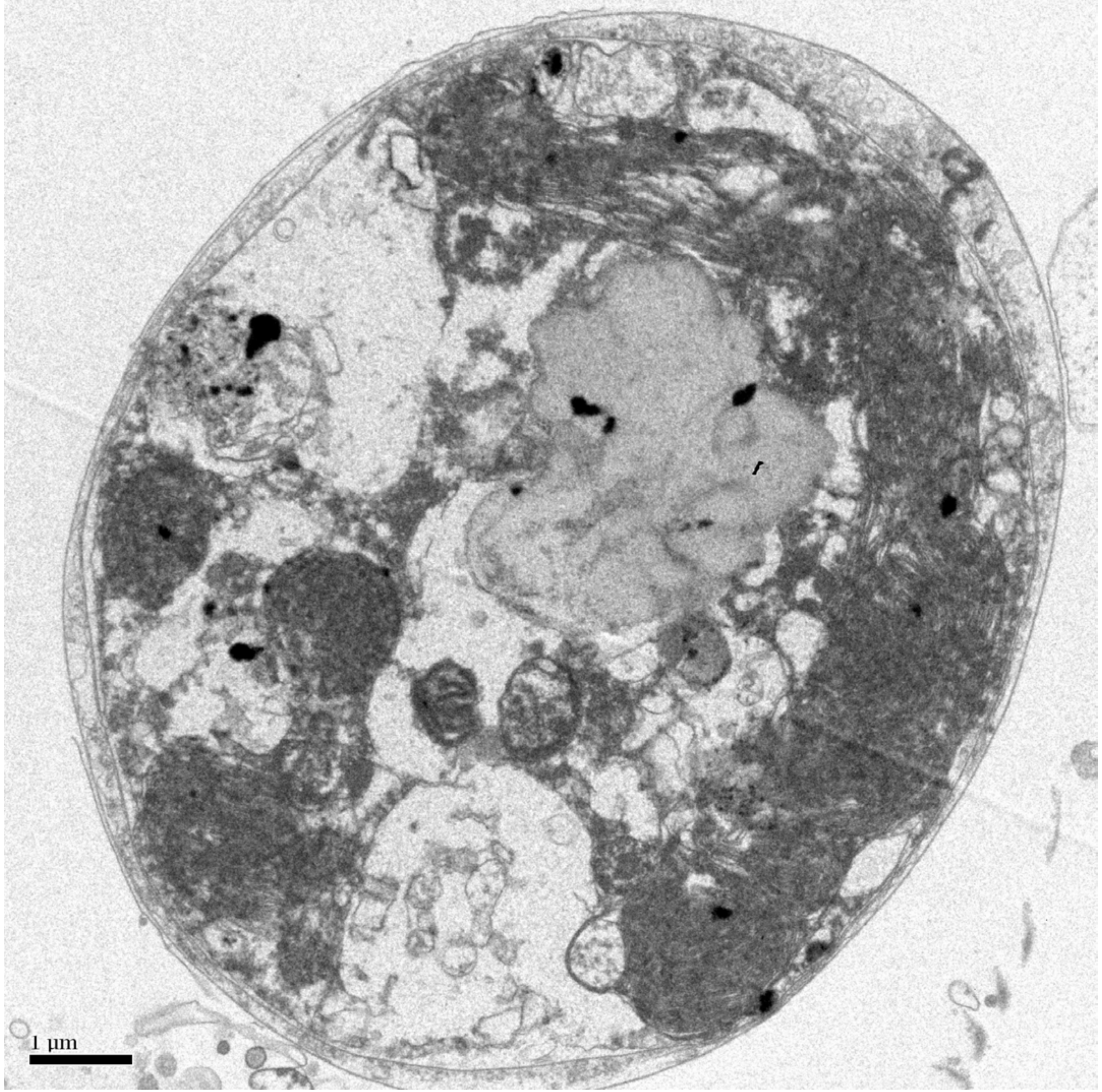

18-7_Correa_JC4_2GridC9_26

Cell 5

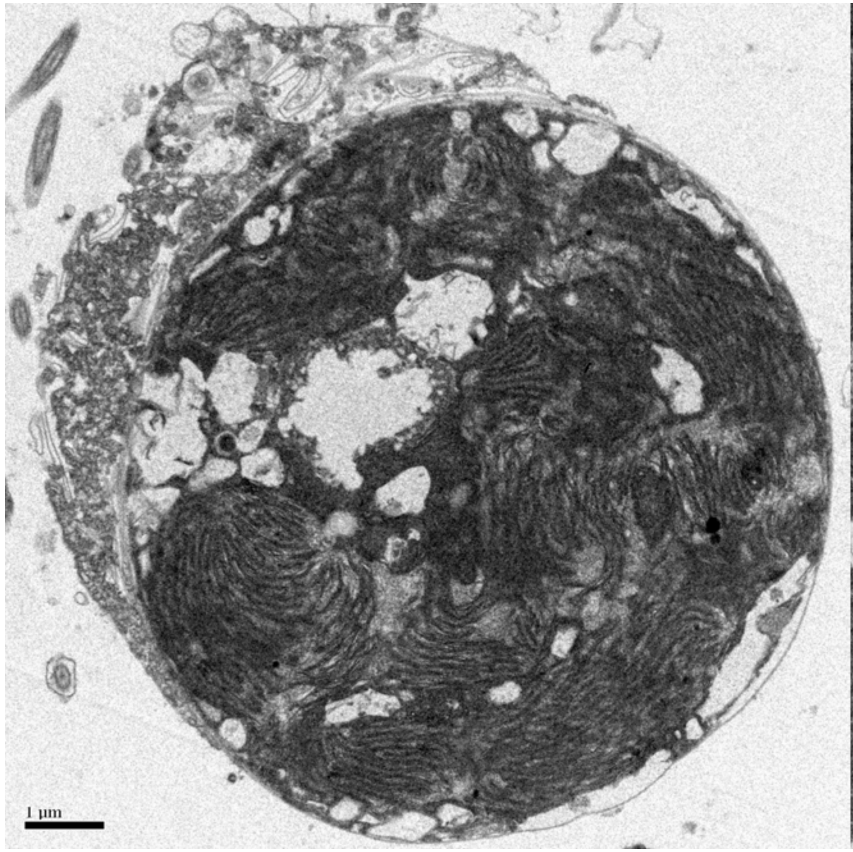

18-7_Correa_JC4_2GridC9_27

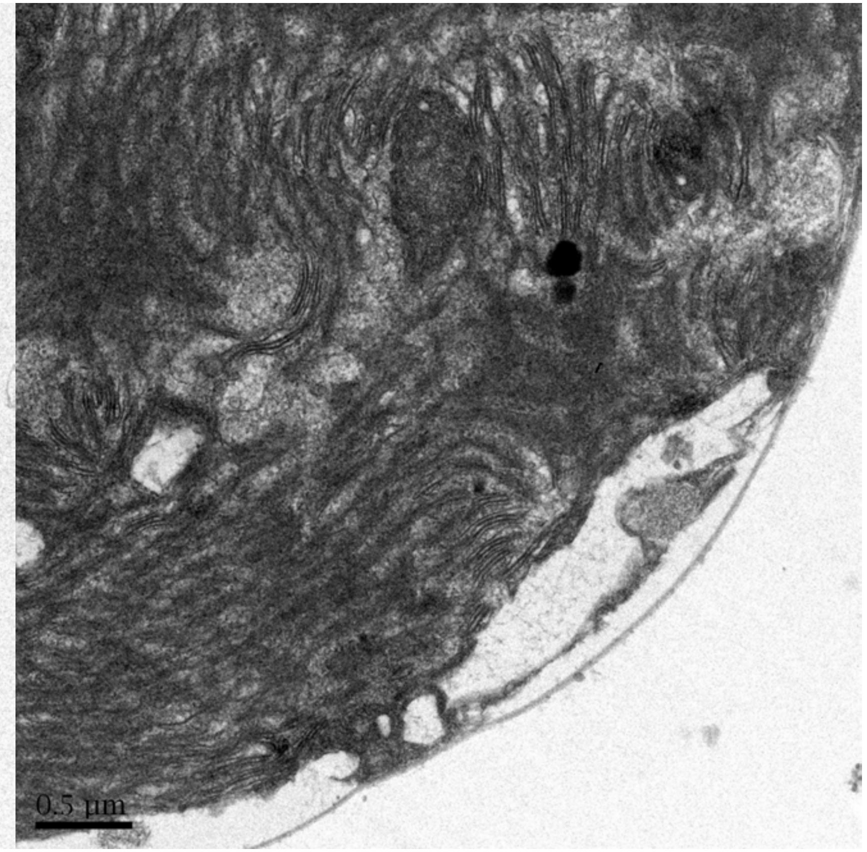

18-7_Correa_JC4_2GridC9_29

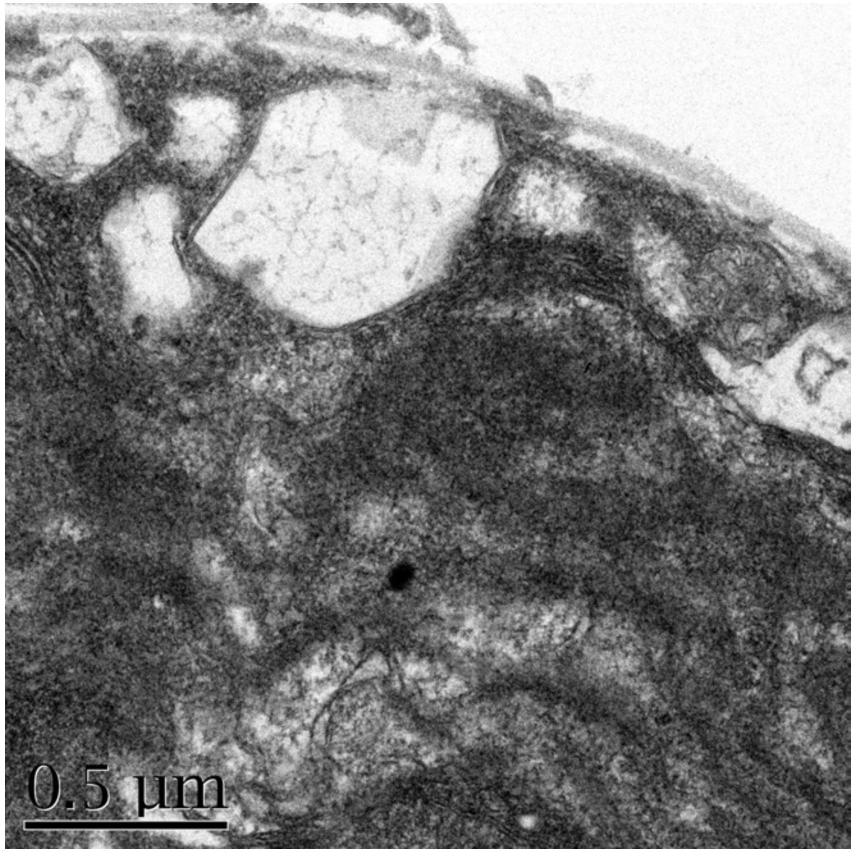

18-7_Correa_JC4_2GridC9_32

Cell 6

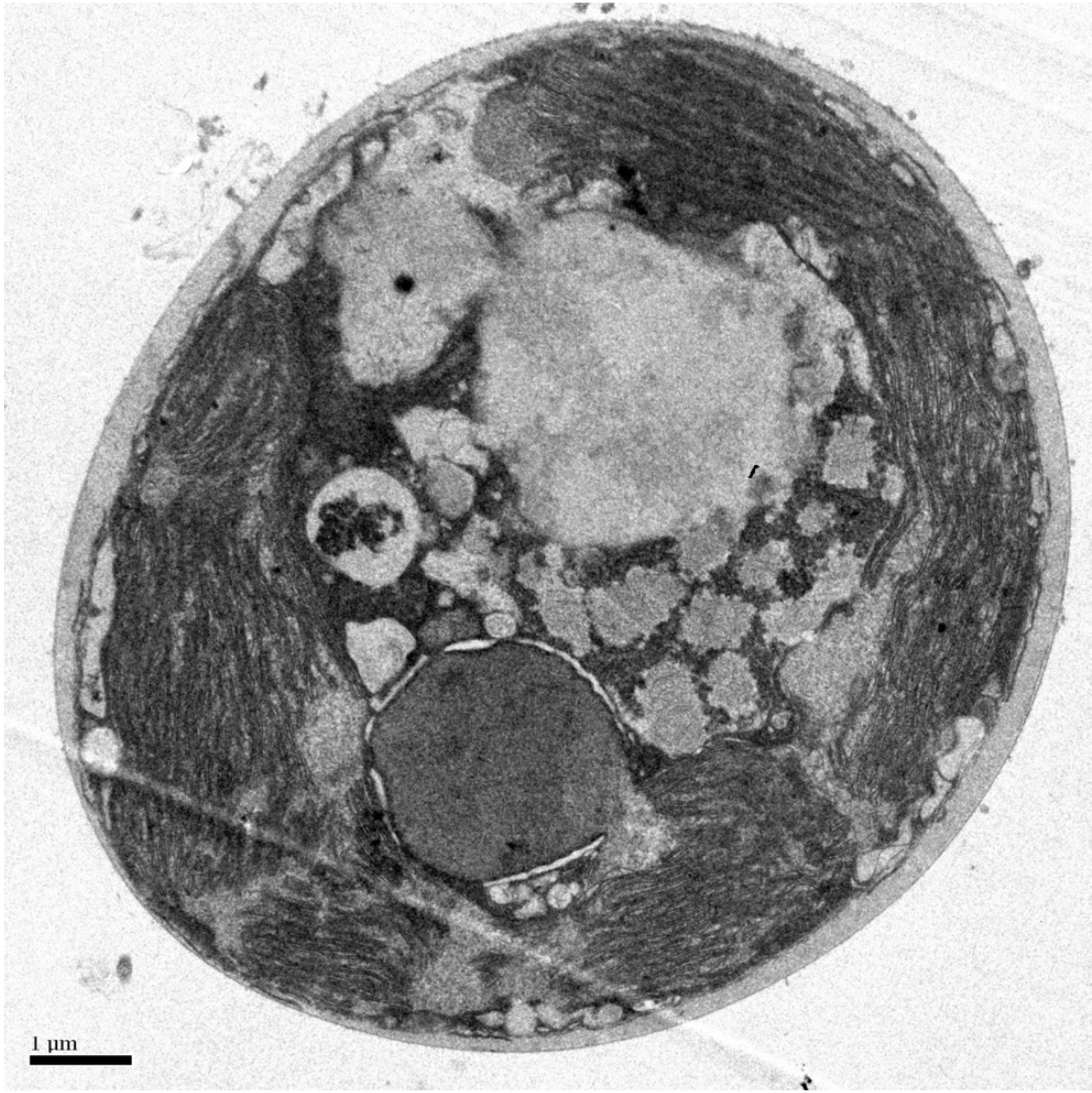

18-7_Correa_JC4_2GridC9_37

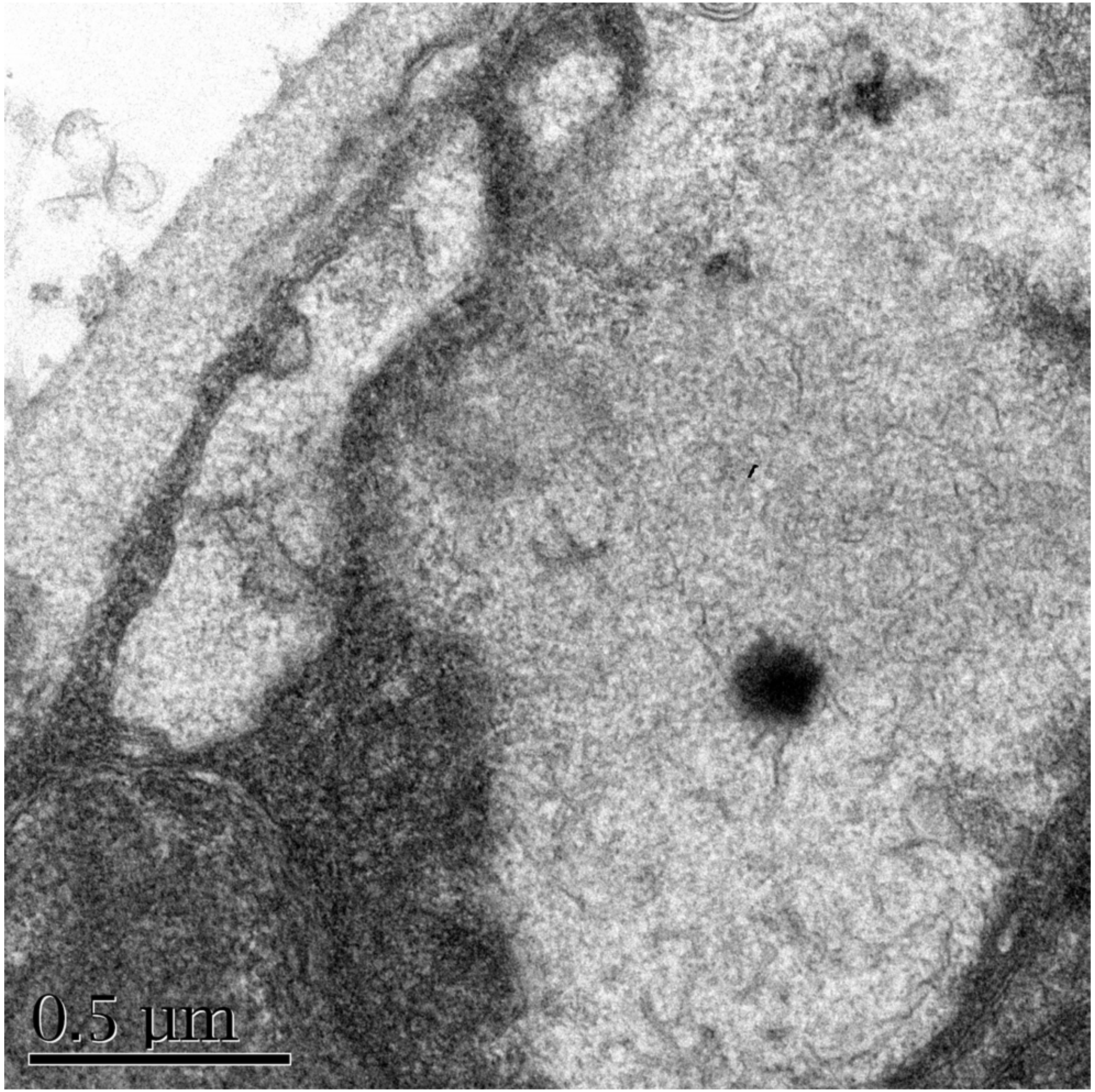

18-7_Correa_JC4_2GridC9_38

Cell 7

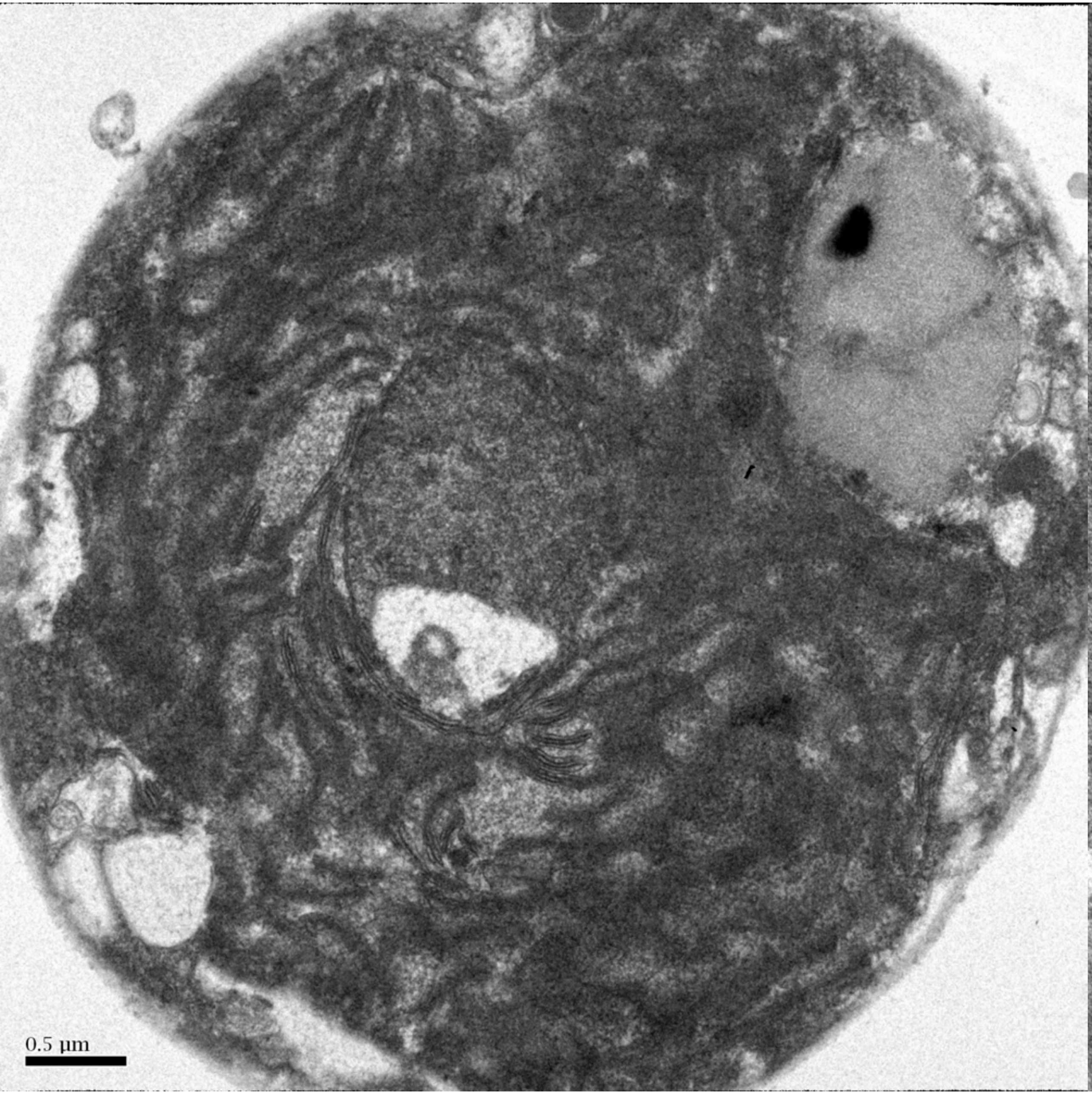

18-7_Correa_JC4_2C10_4

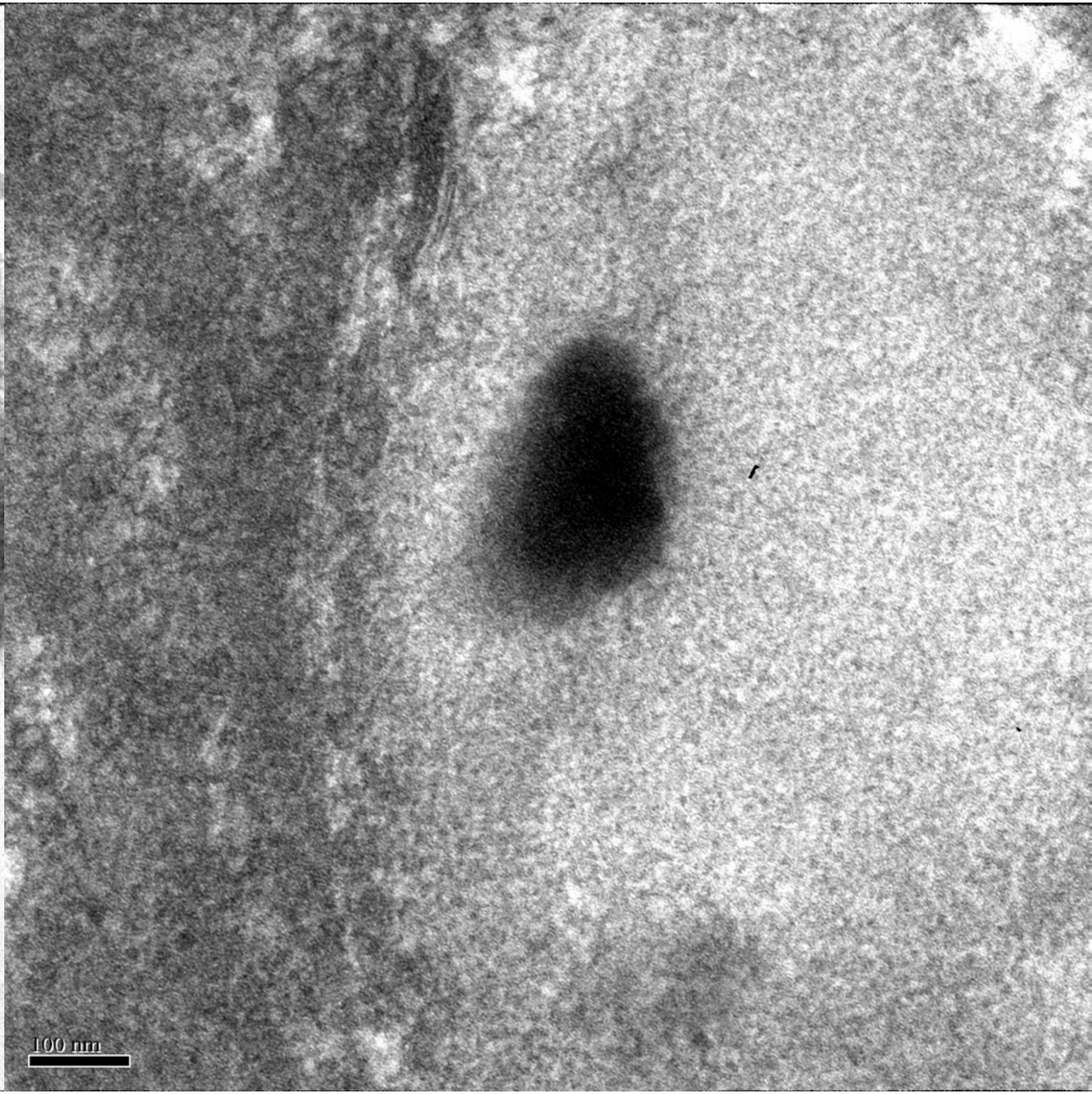

18-7_Correa_JC4_2C10_5

Cell 8

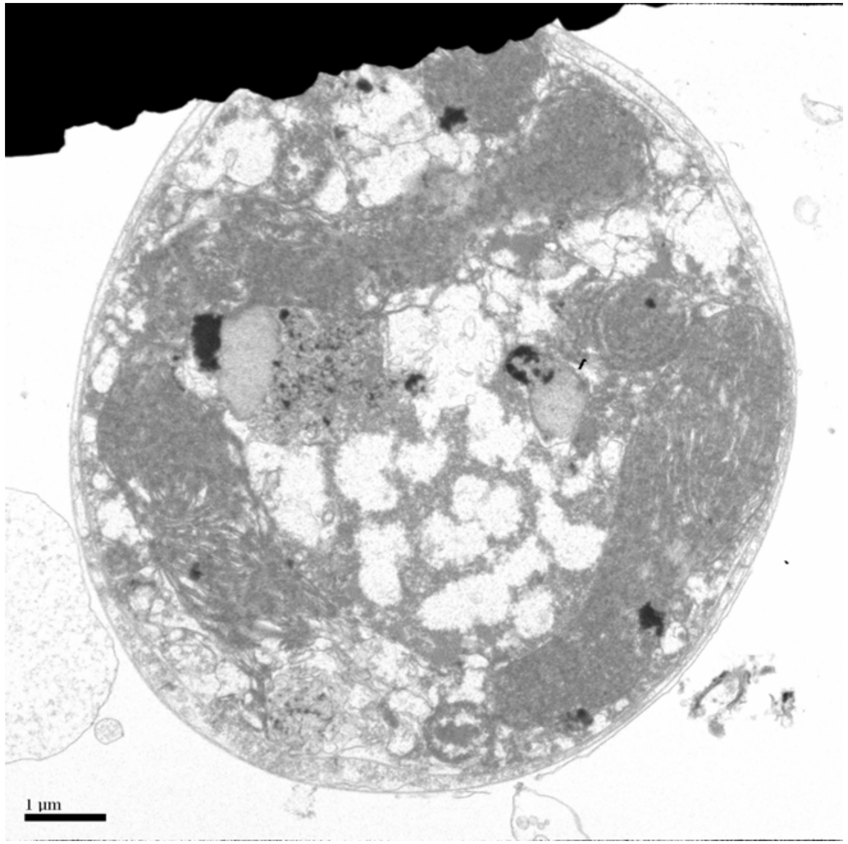

18-7_Correa_JC4_2C10_8

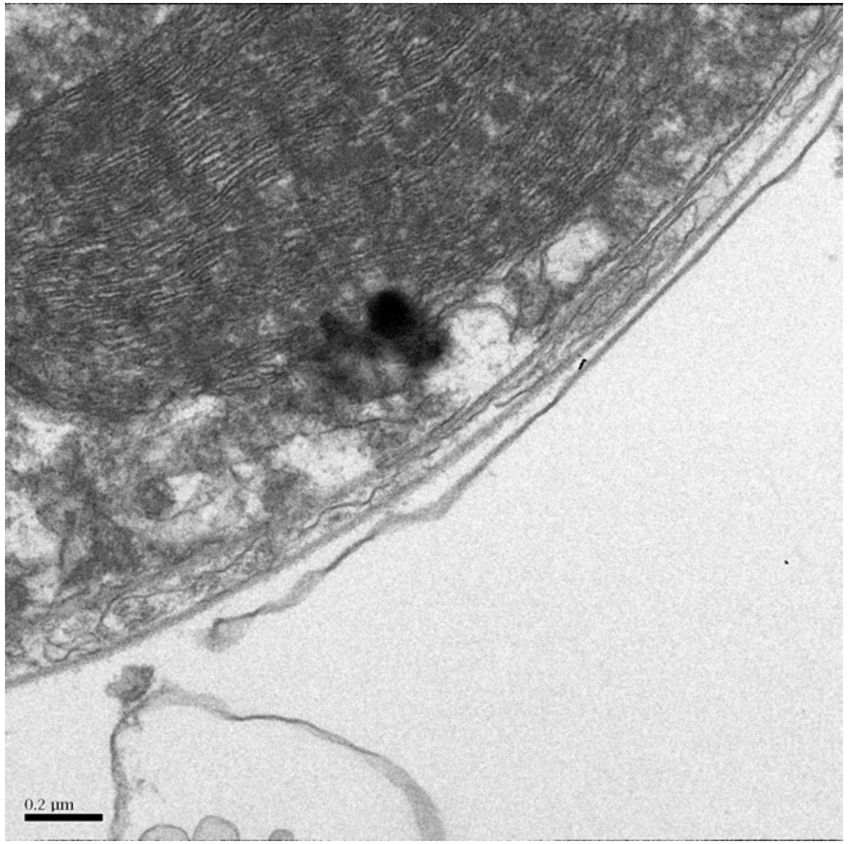

18-7_Correa_JC4_2C10_9

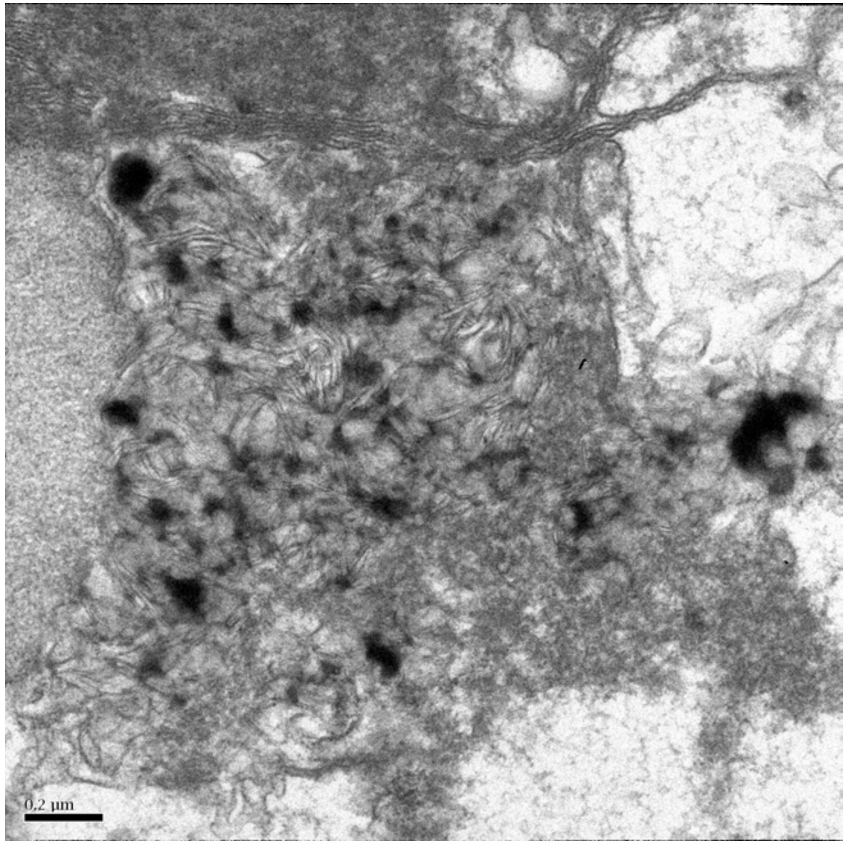

18-7_Correa_JC4_2C10_13

Cell 9

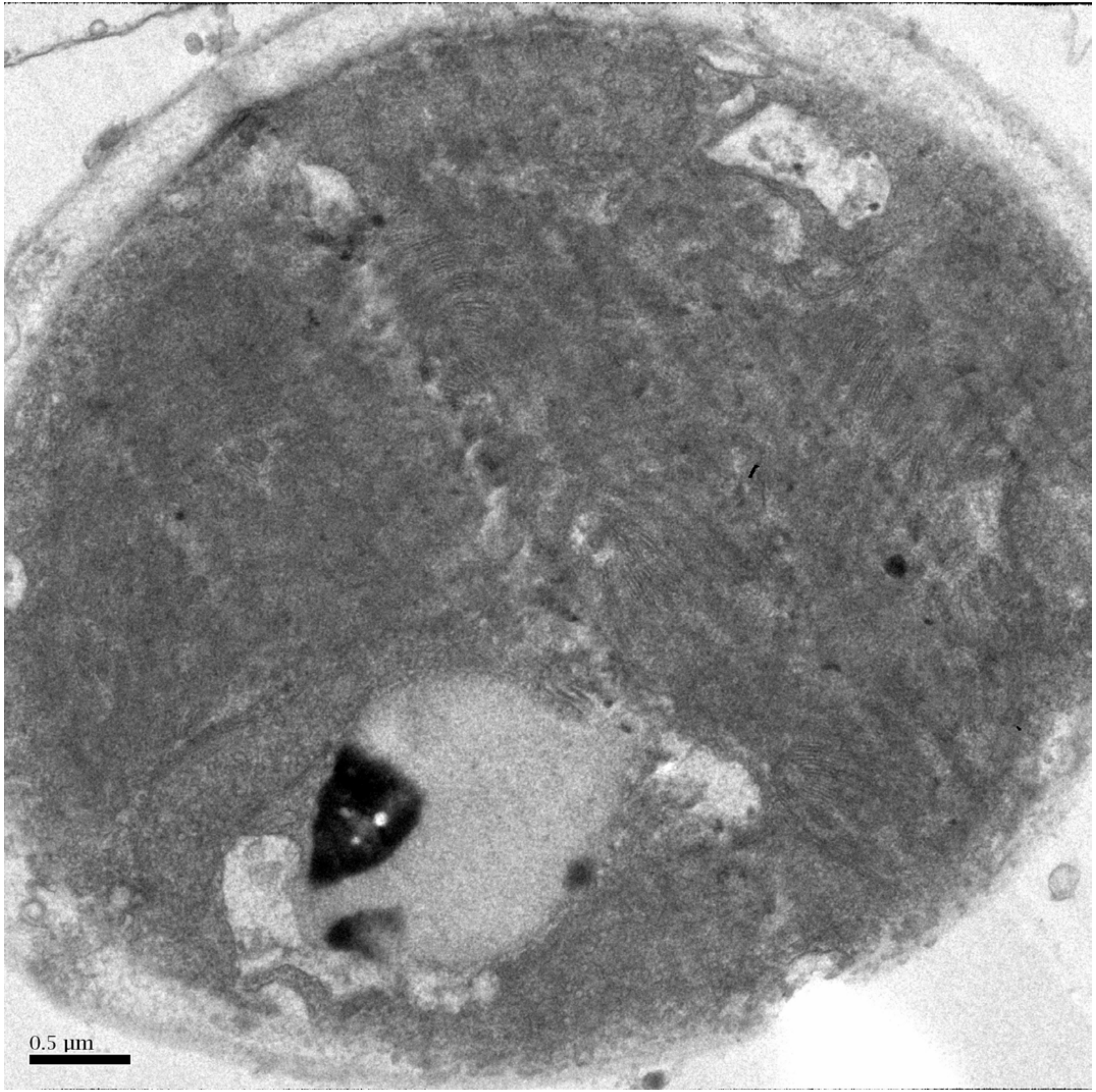

18-7_Correa_JC4_2C10_16

Cell 10

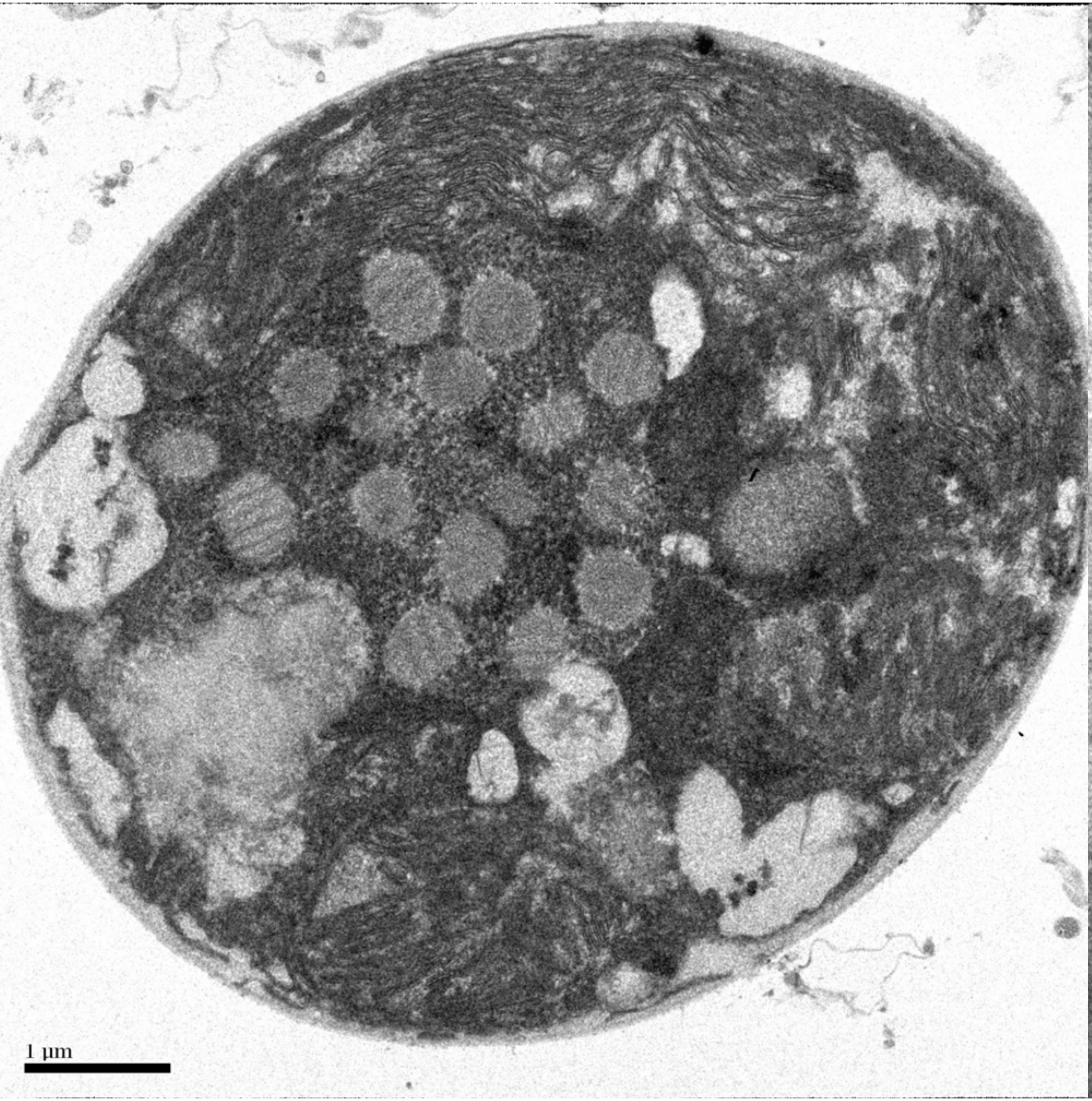

18-7_Correa_JC4_2C10_20

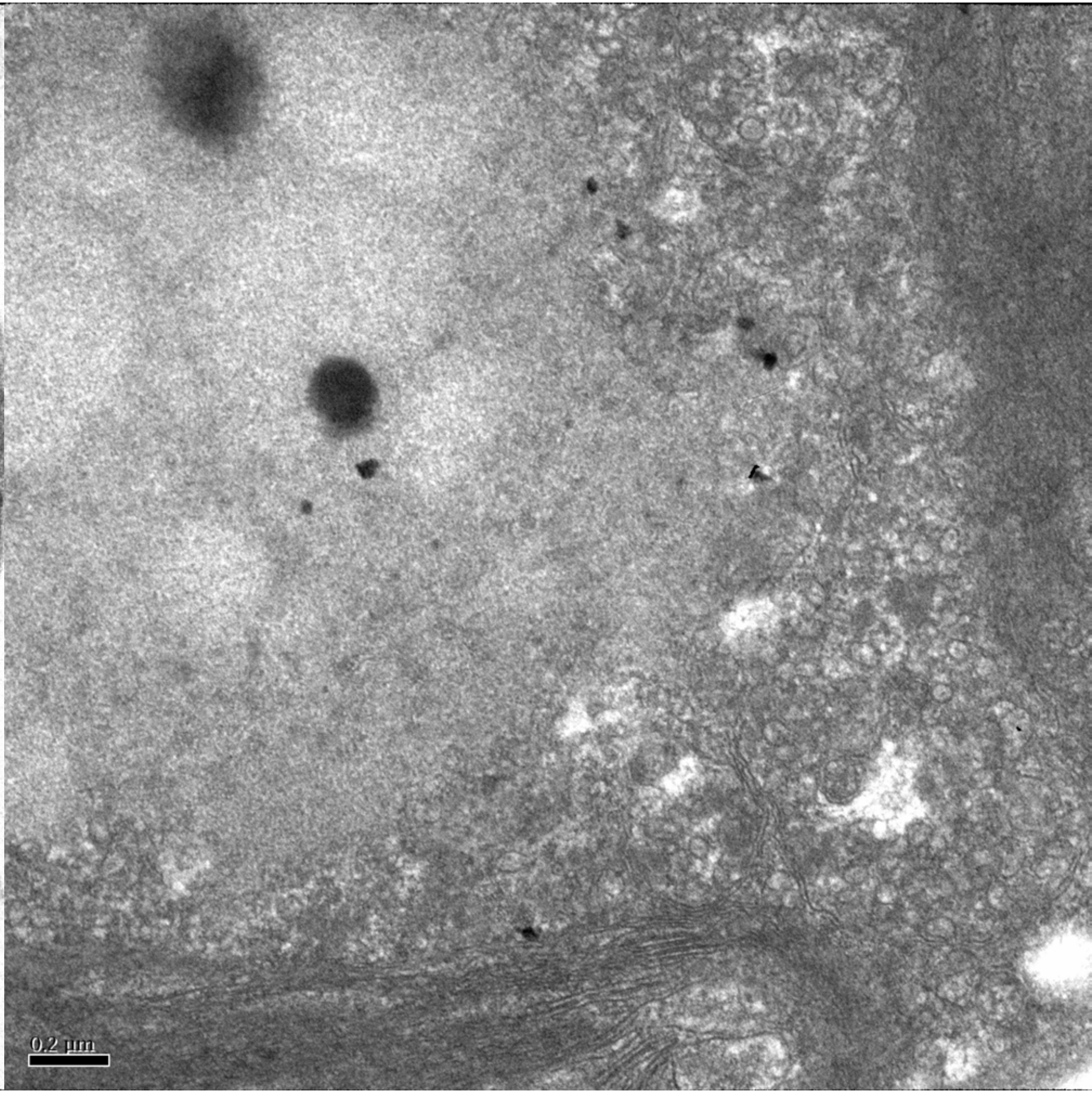

18-7_Correa_JC4_2C10_27

Cell 11

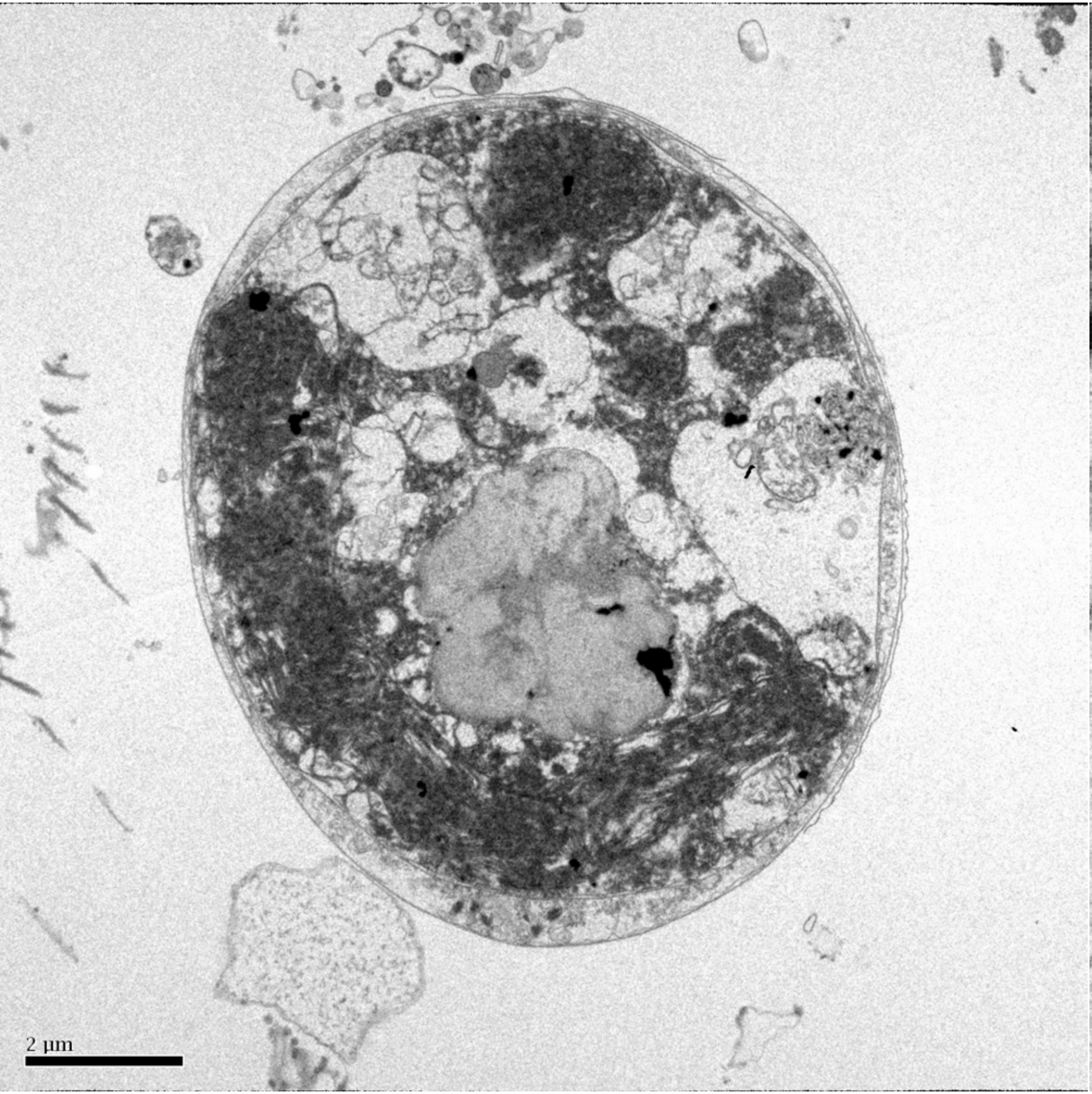

18-7_Correa_JC4_2C10_30

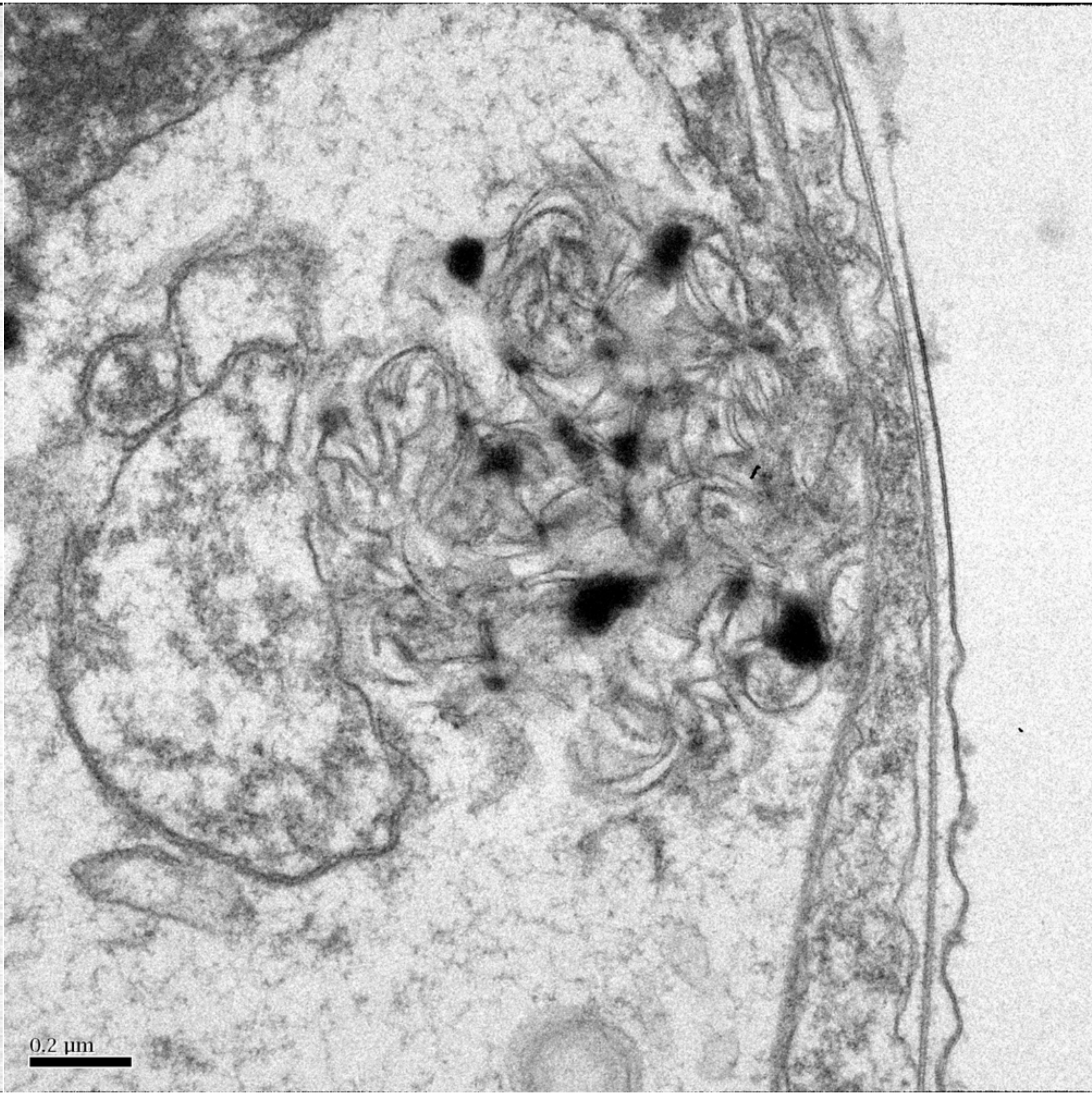

18-7_Correa_JC4_2C10_33

Cell 12

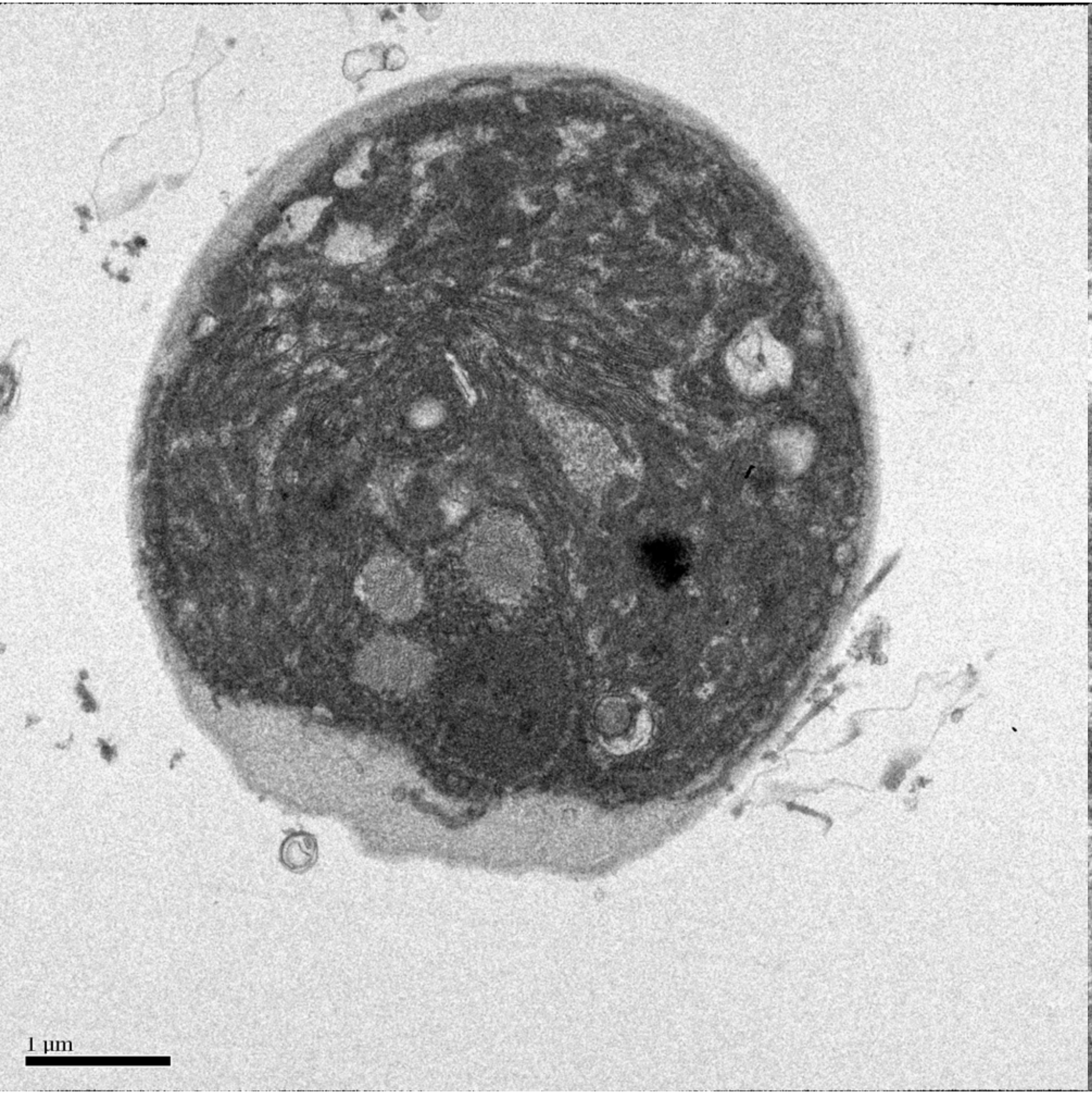

18-7_Correa_JC4_2C10_34

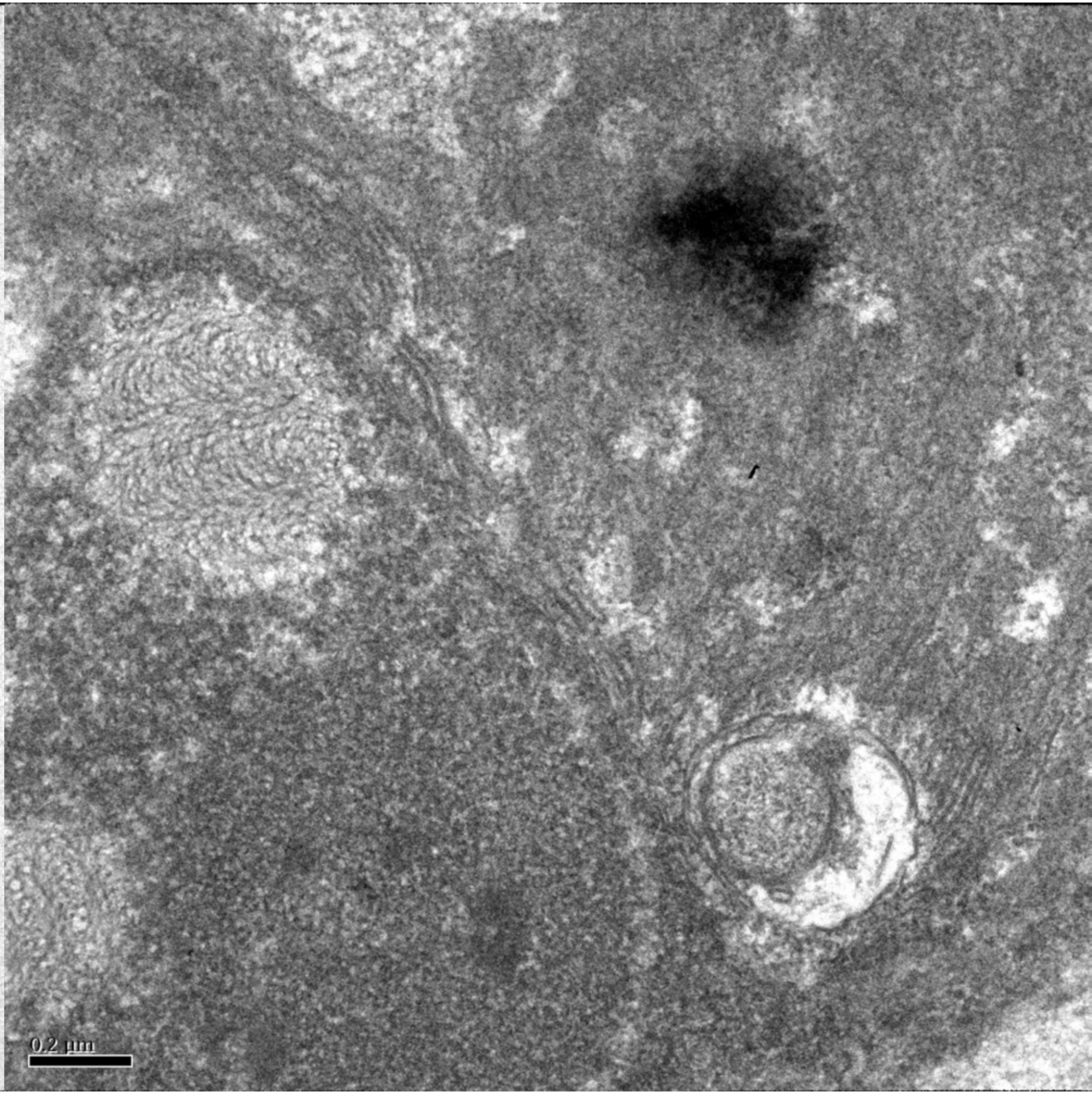

18-7_Correa_JC4_2C10_36

ACR Colony A Expelled- Heat

Cell 1

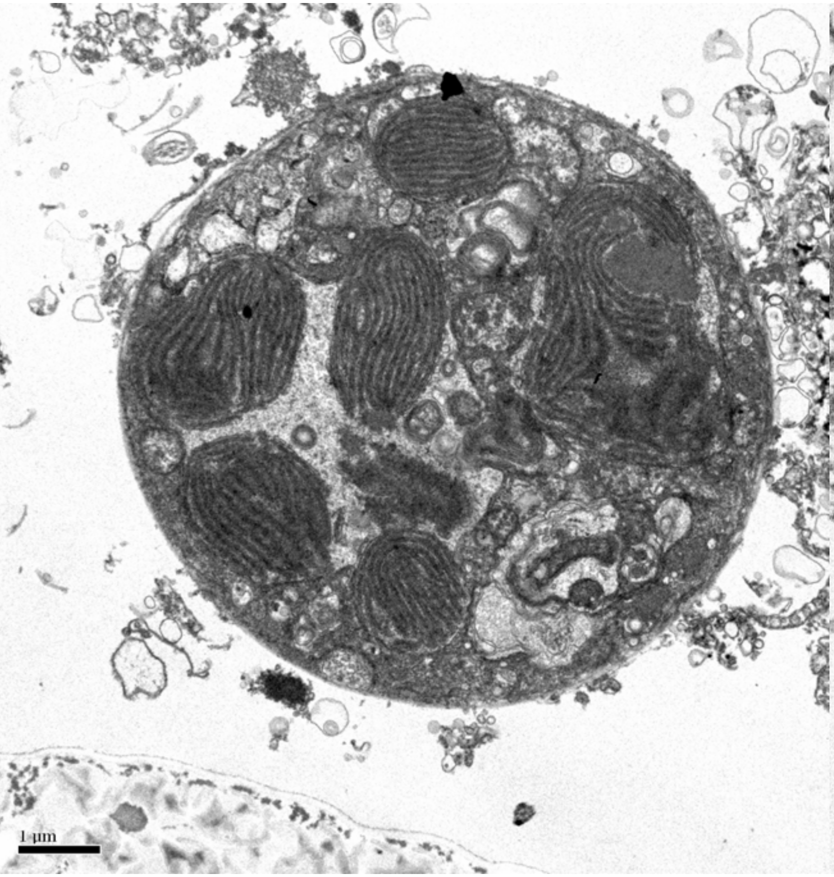

18-7_Correa_JH4_2GridD8_03

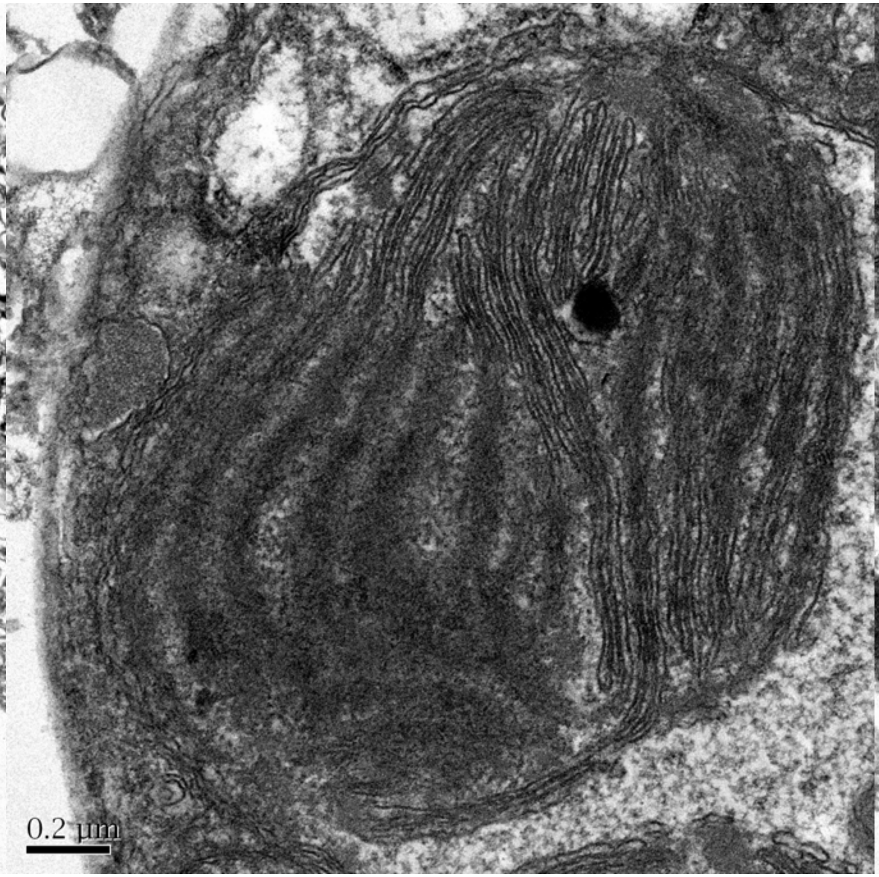

18-7_Correa_JH4_2GridD8_11

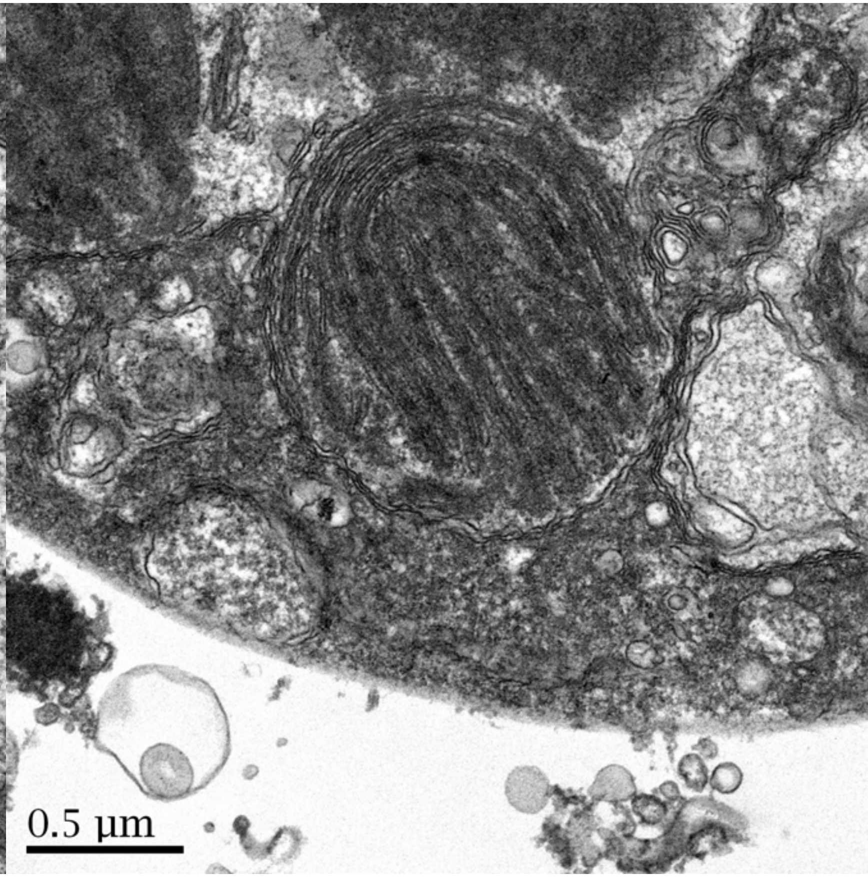

18-7_Correa_JH4_2GridD8_05

Cell 2

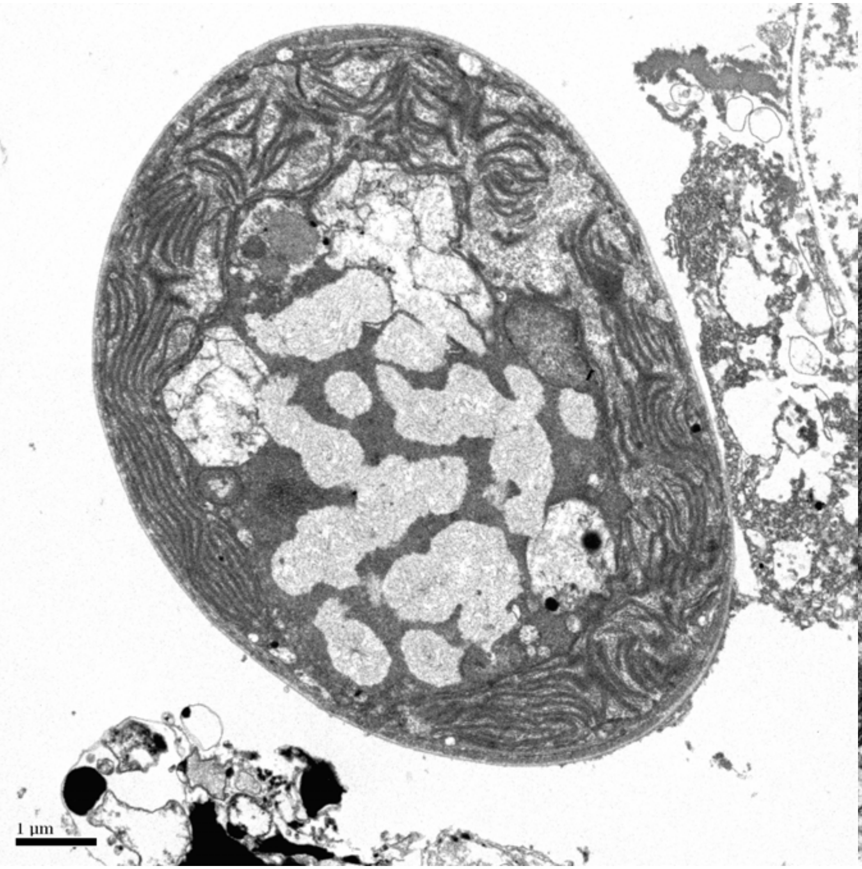

18-7_Correa_JH4_2GridD8_15

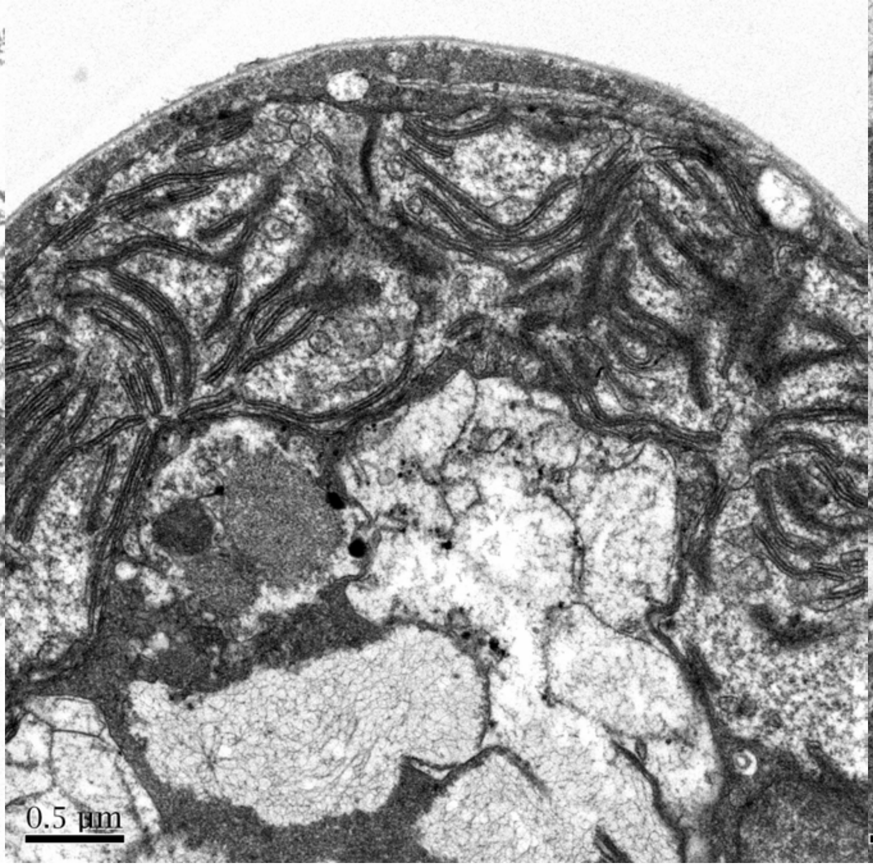

18-7_Correa_JH4_2GridD8_22

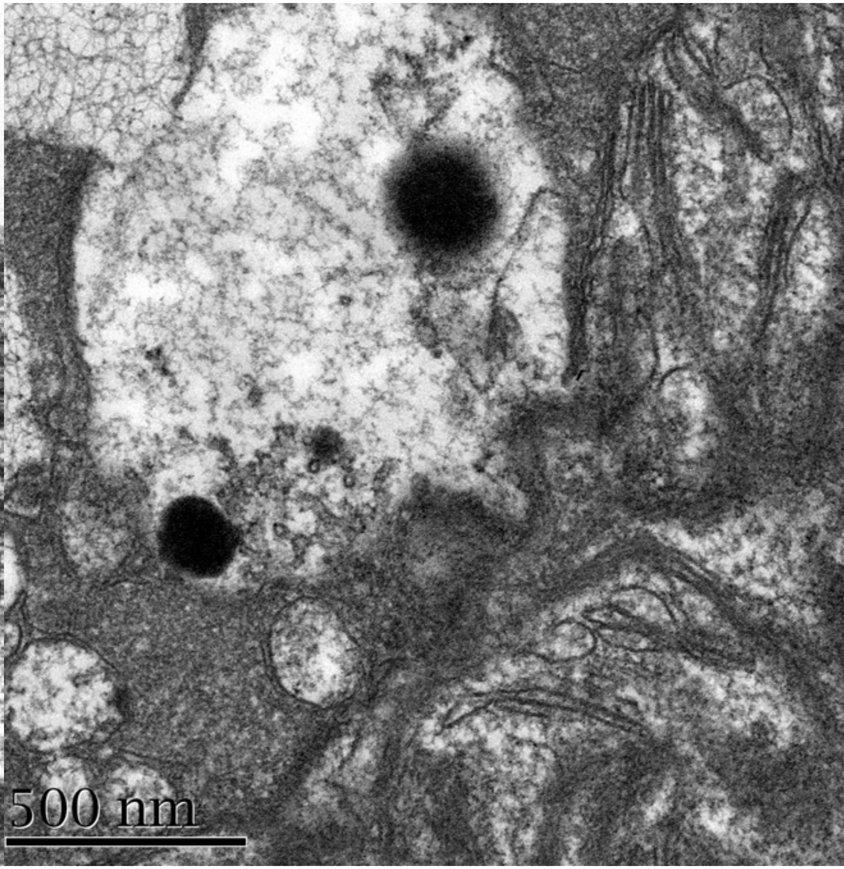

18-7_Correa_JH4_2GridD8_19

Cell 3

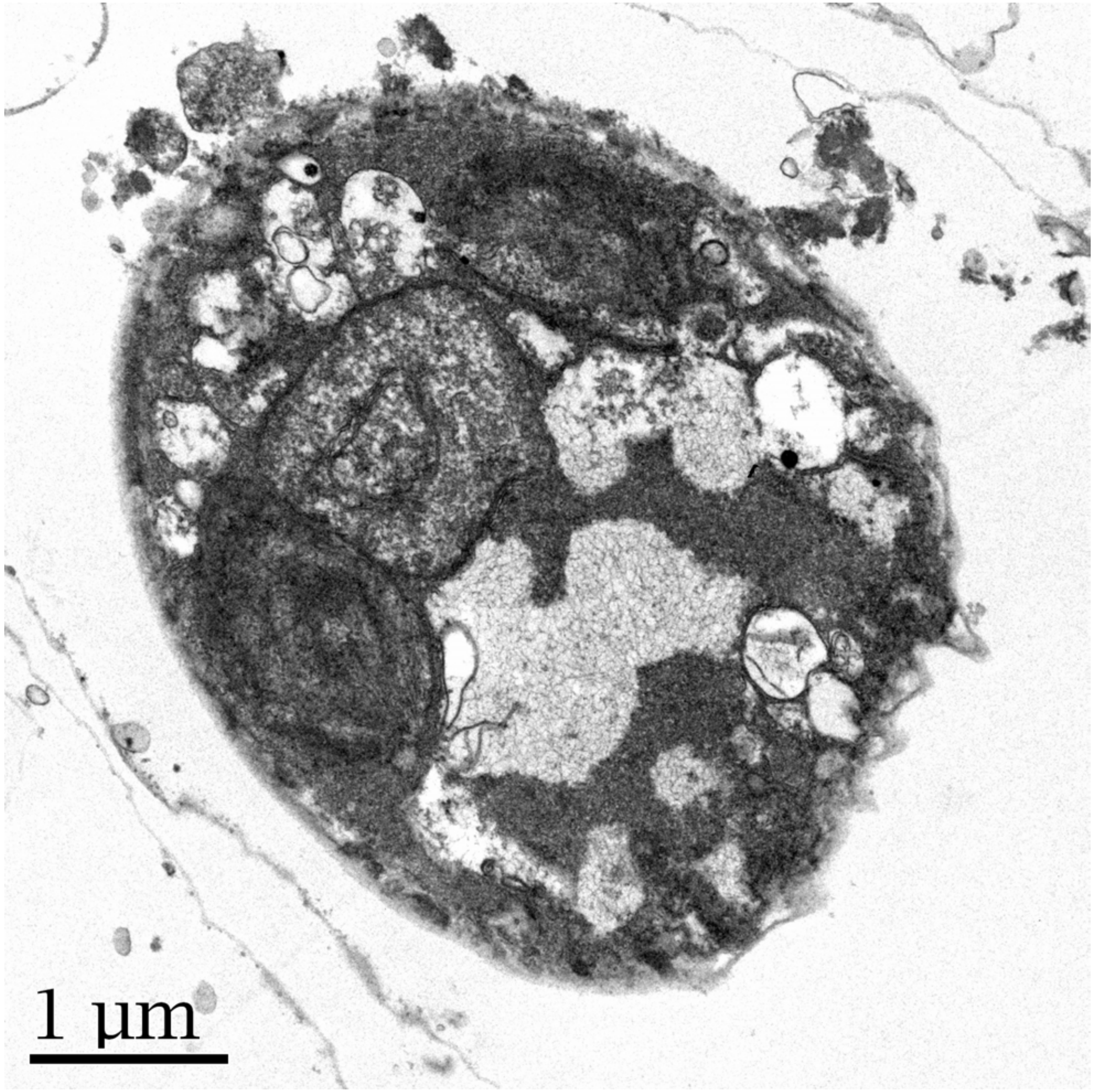

18-7_Correa_JH4_2GridD8_24

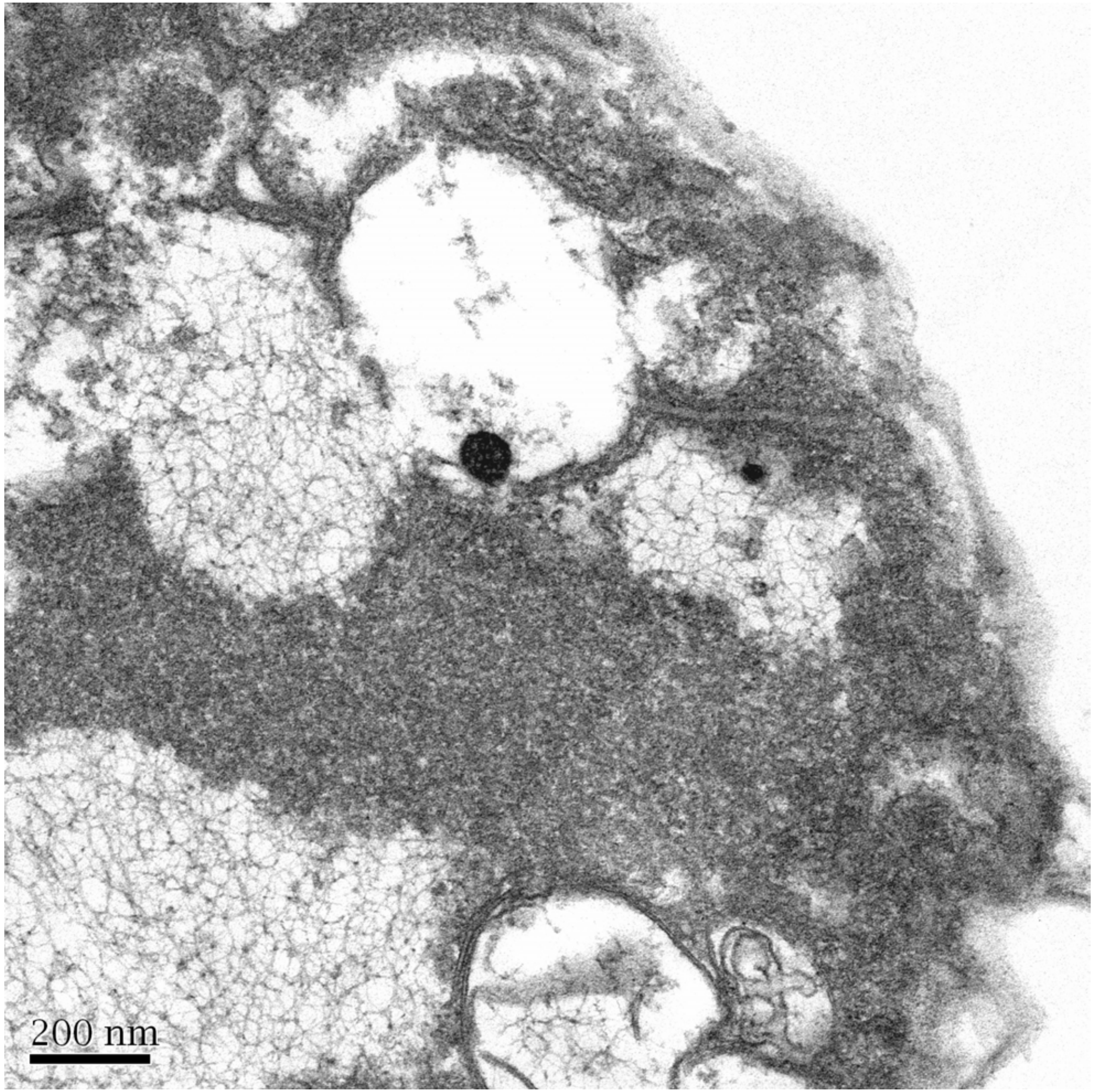

18-7_Correa_JH4_2GridD8_25

Cell 4

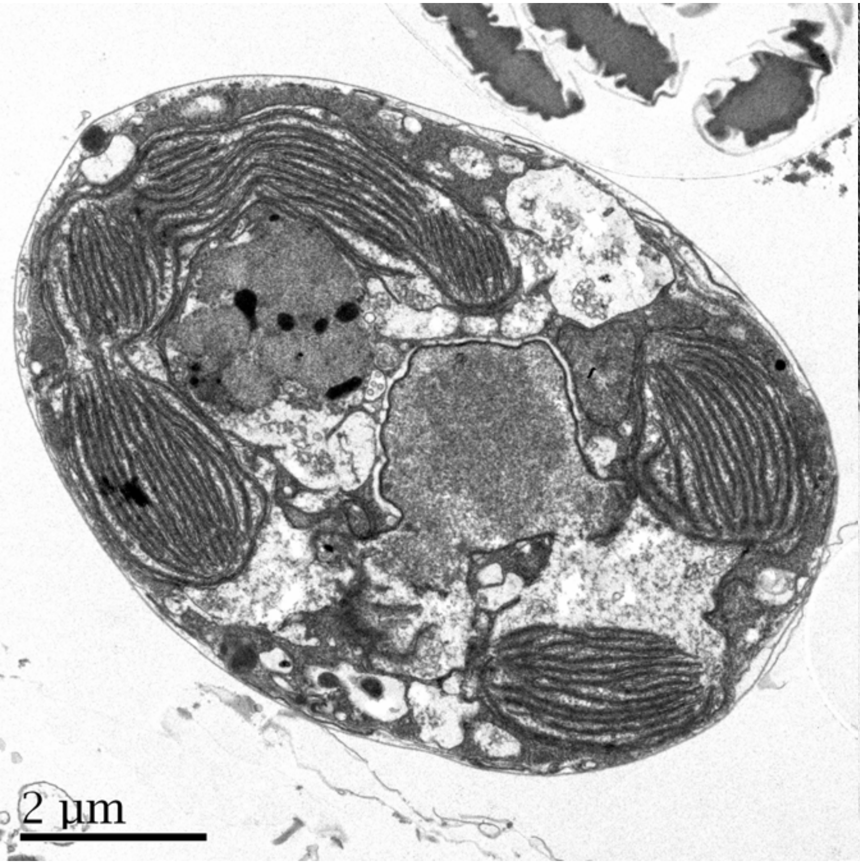

18-7_Correa_JH4_2GridD8_27

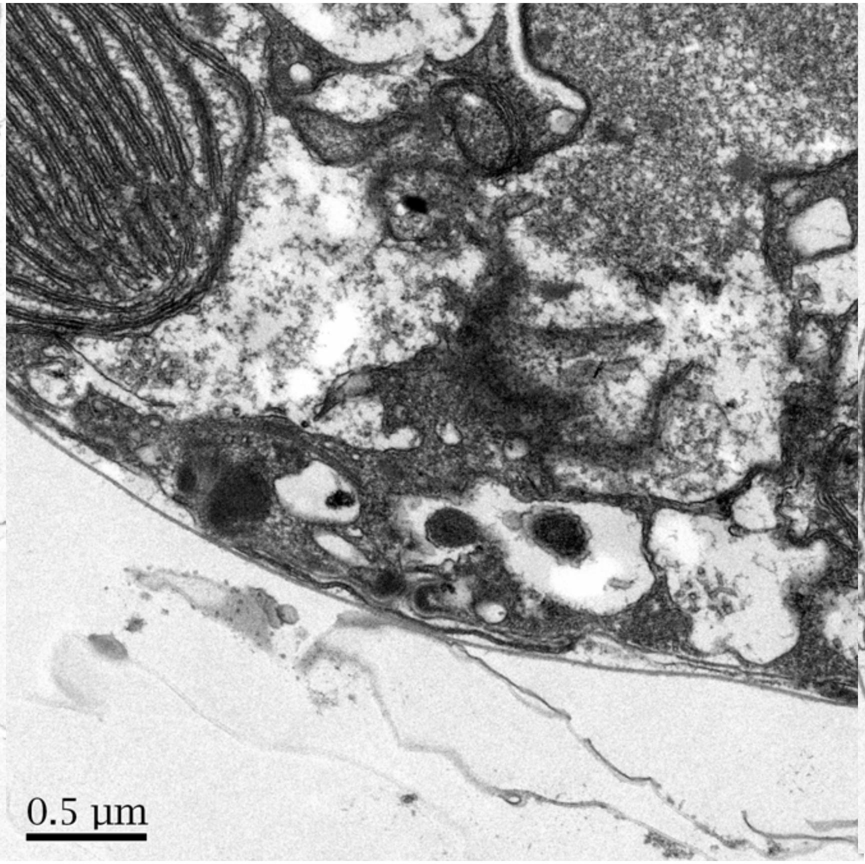

18-7_Correa_JH4_2GridD8_28

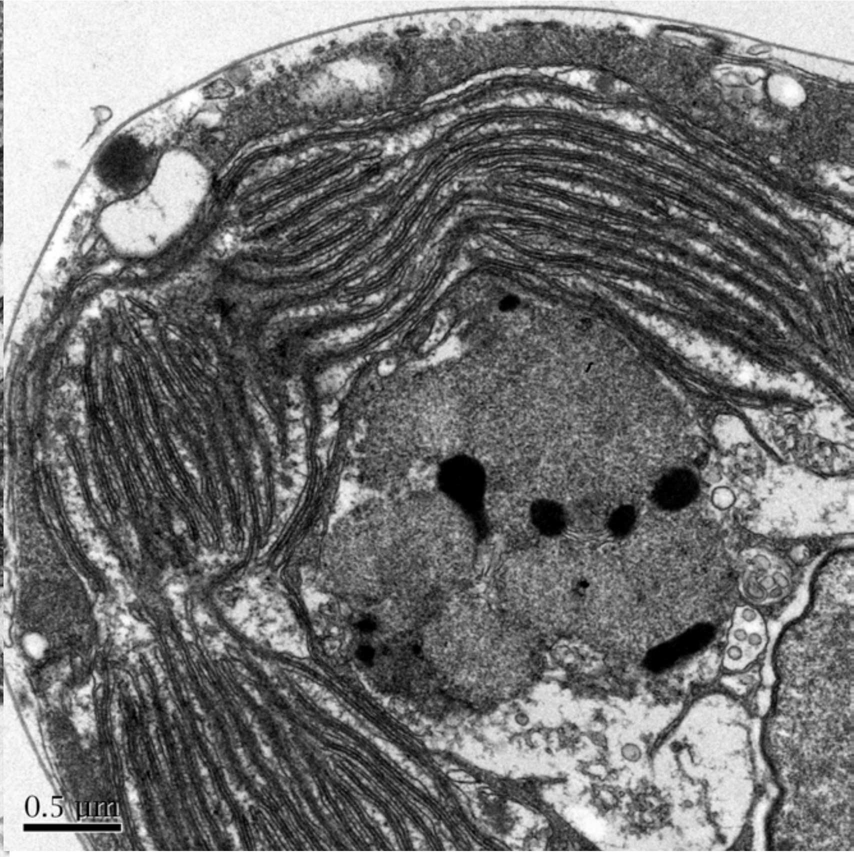

18-7_Correa_JH4_2GridD8_30

Cell 5

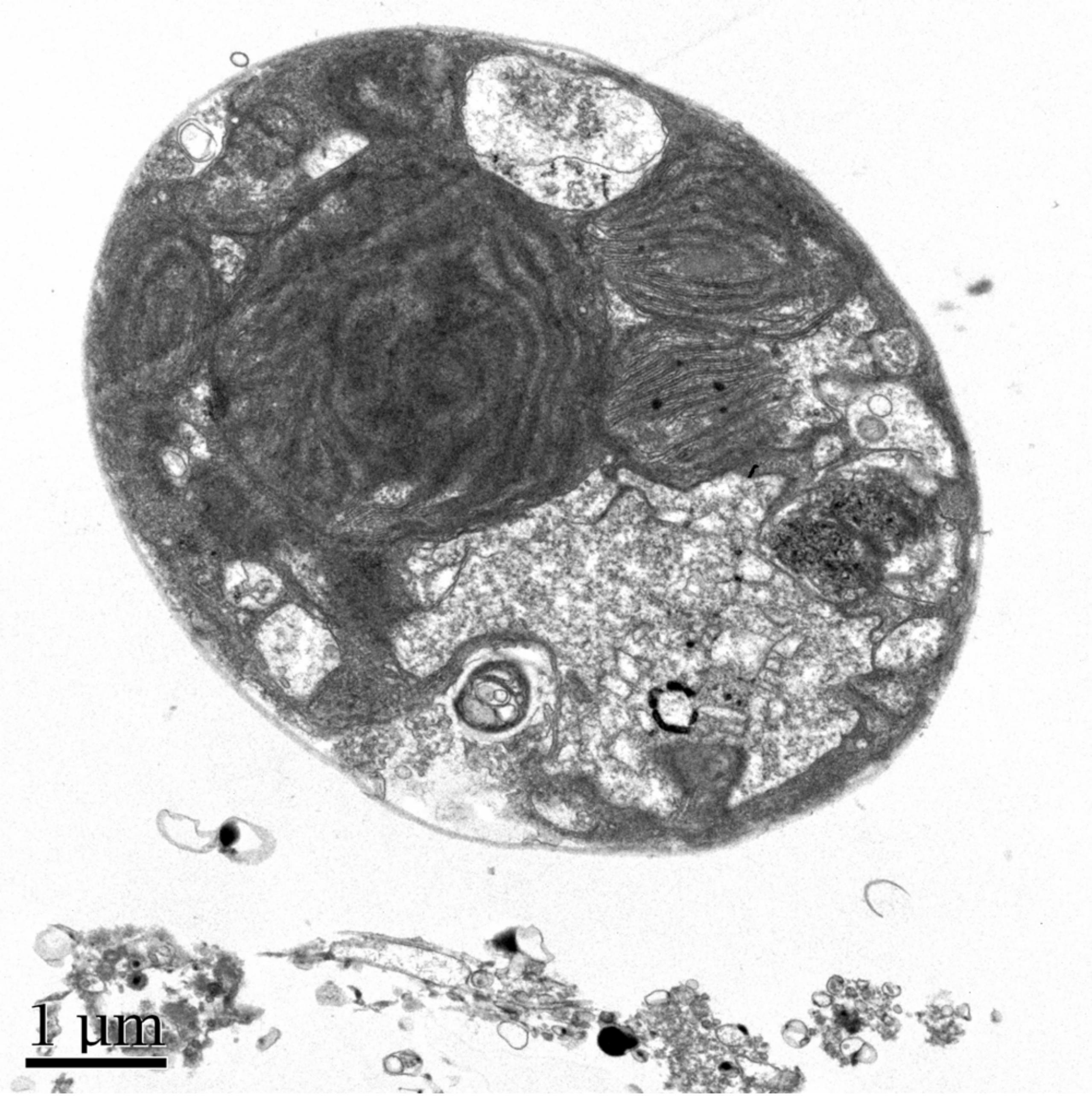

18-7_Correa_JH4_2GridD8_34

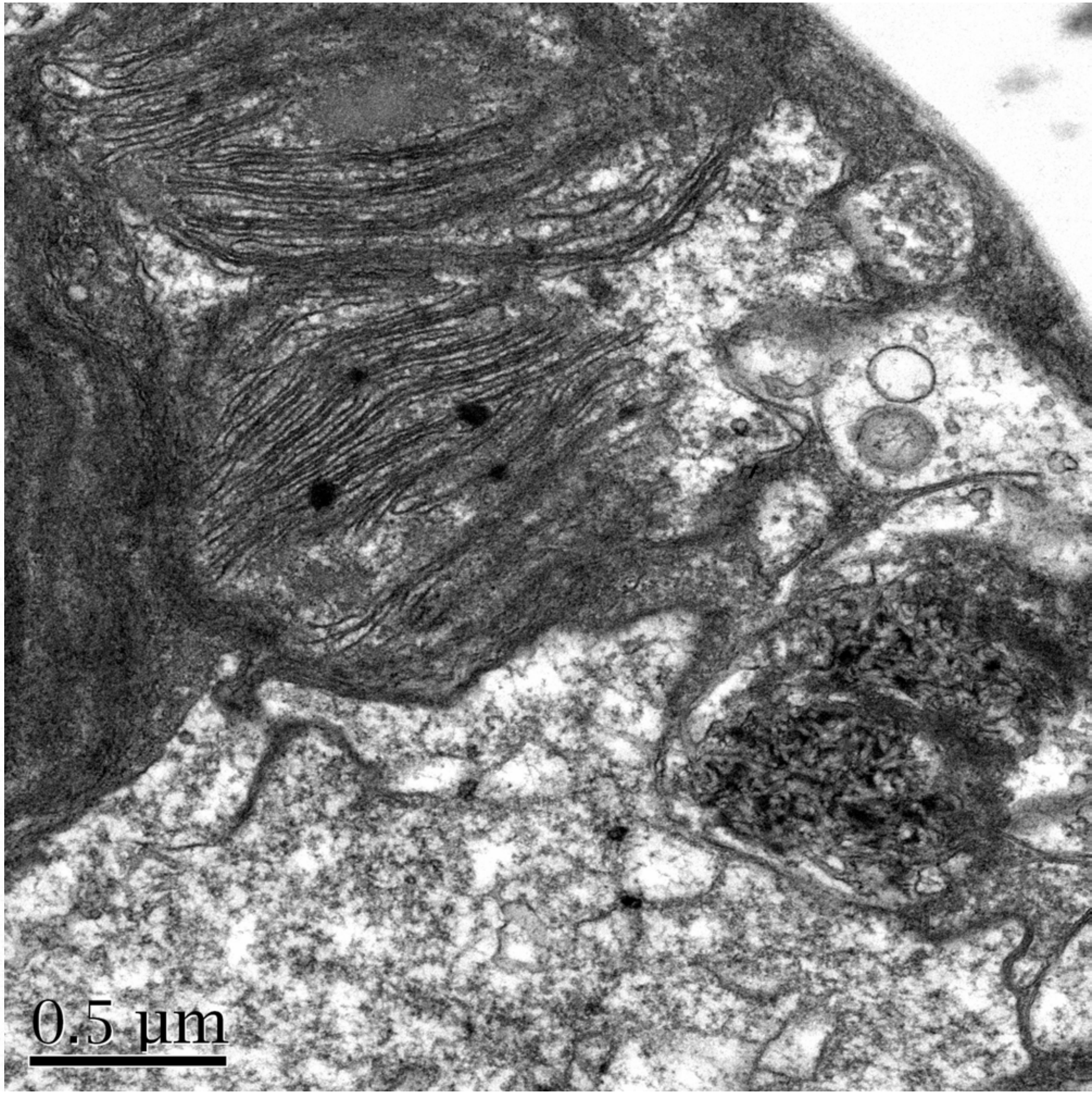

18-7_Correa_JH4_2GridD8_35

Cell 6

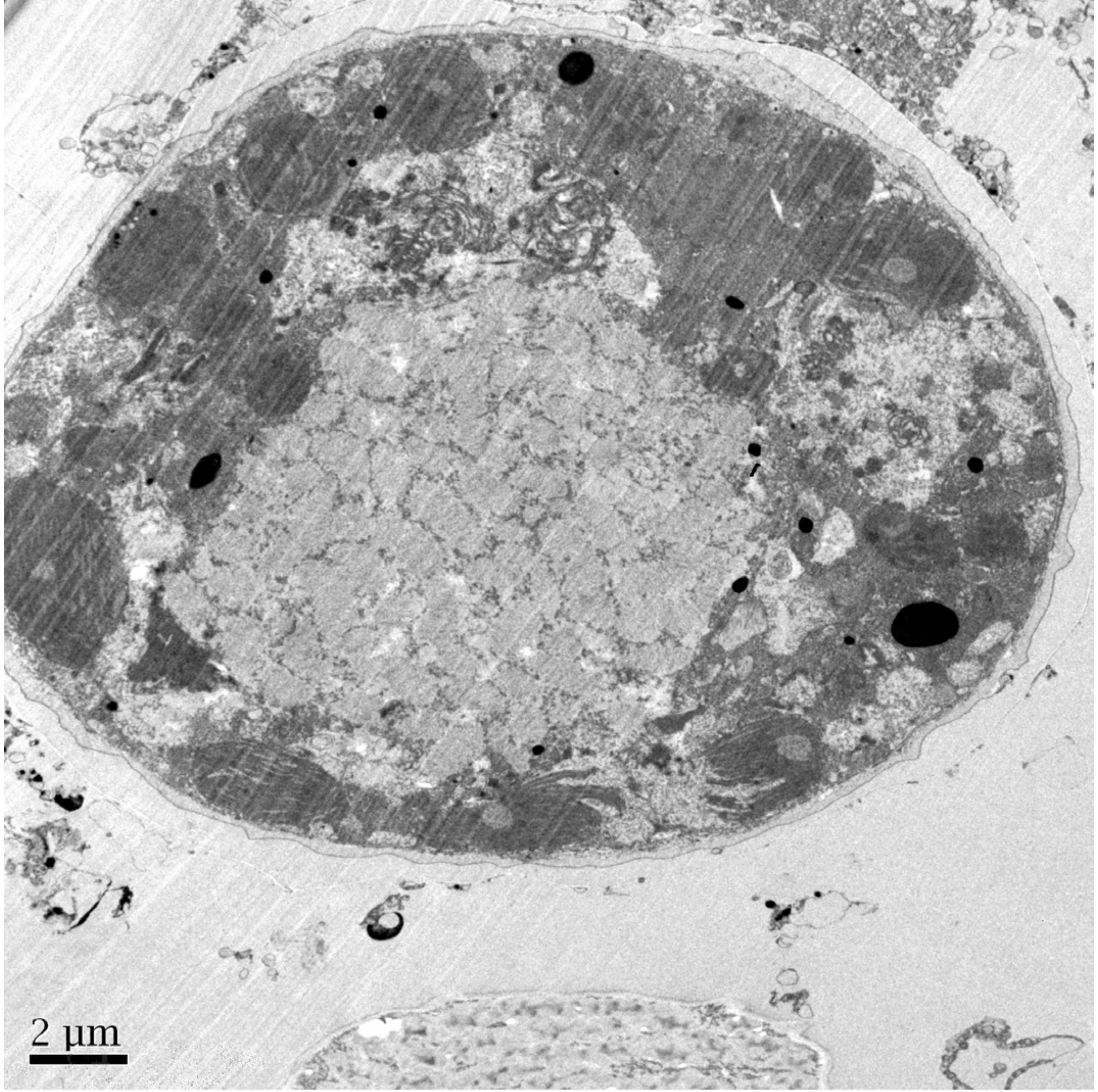

18-7_Correa_JH4_2GridD8_37

Cell 7

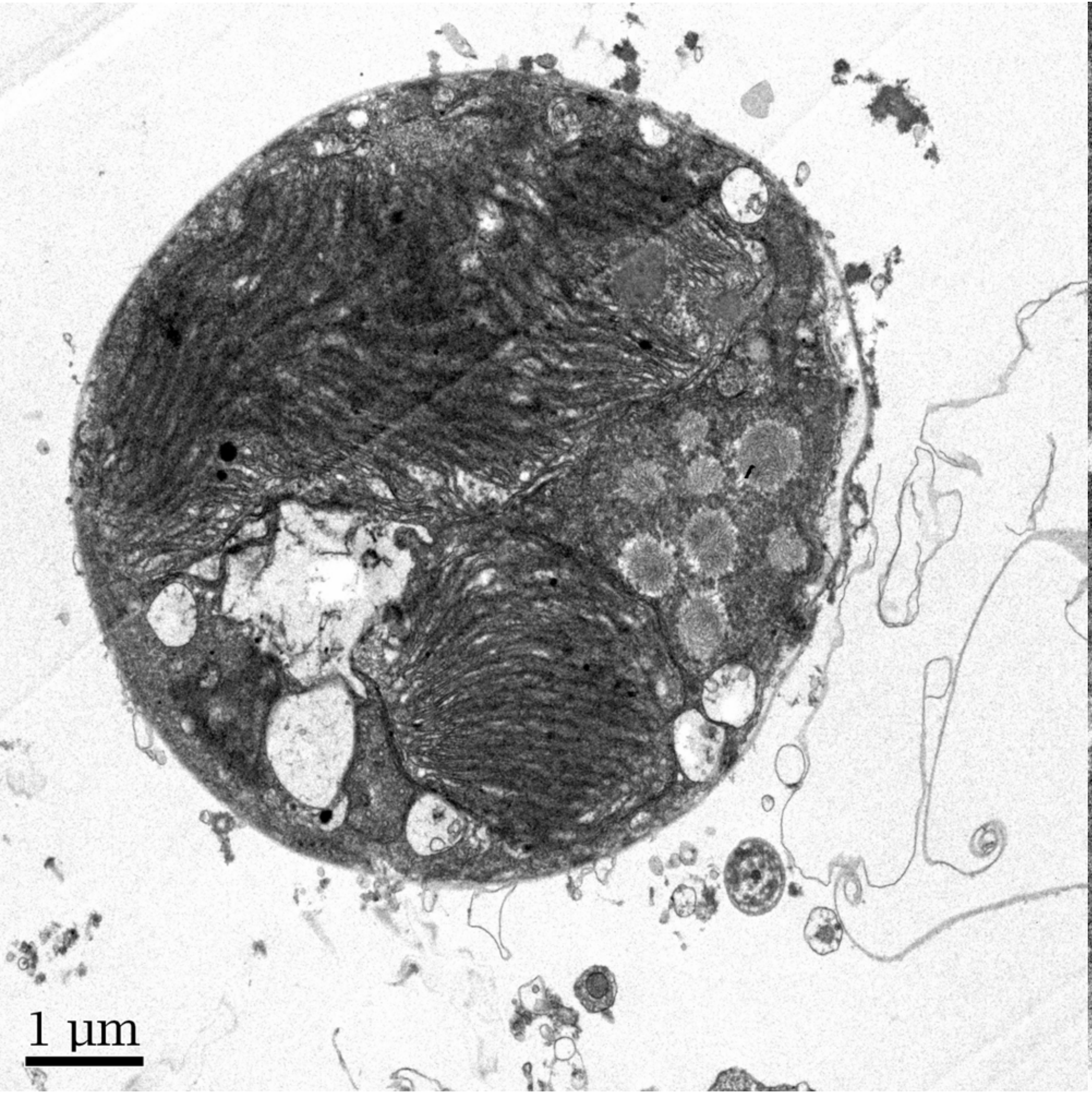

18-7_Correa_JH4_2GridD8_42

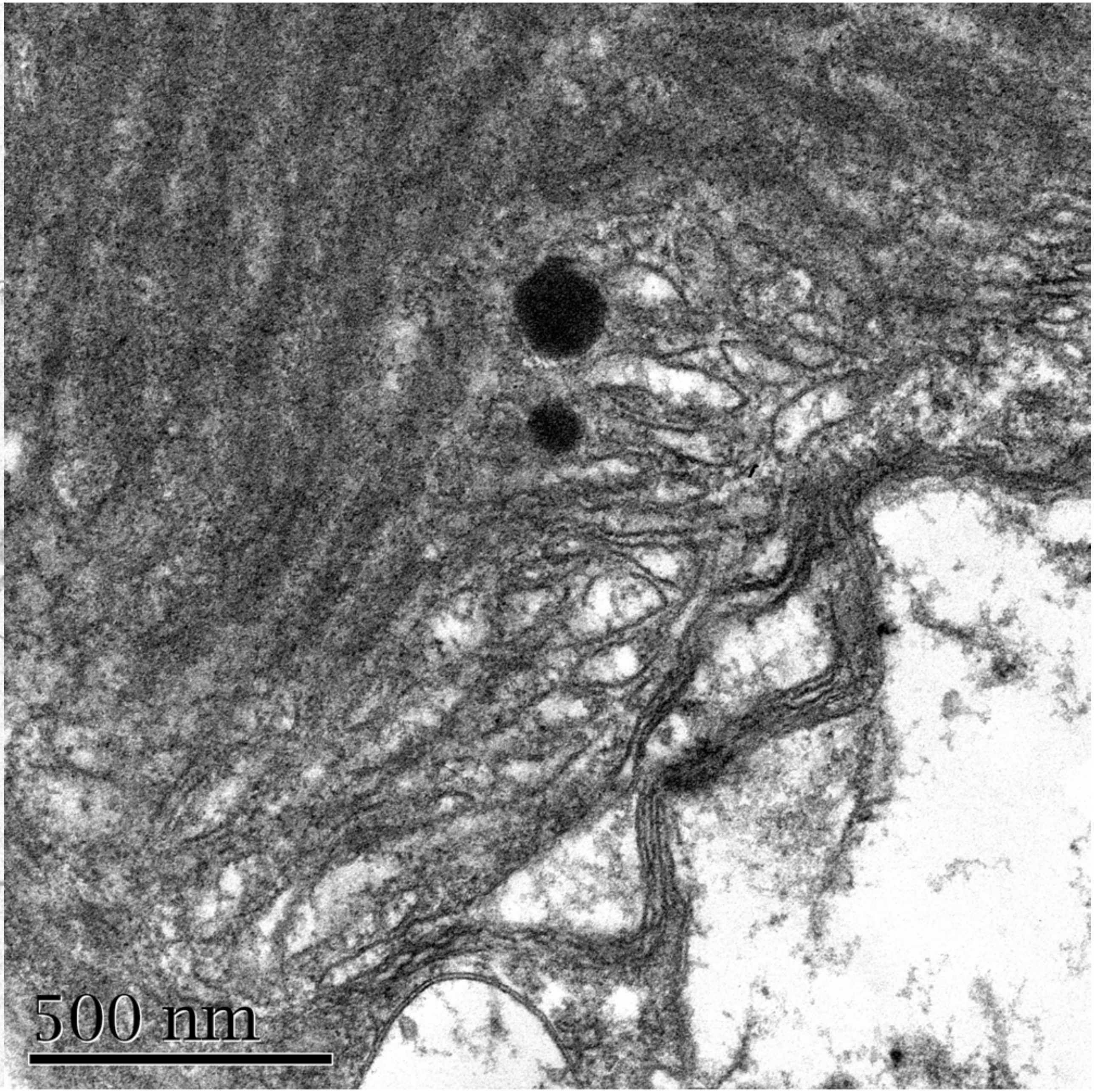

18-7_Correa_JH4_2GridD8_44

Cell 8

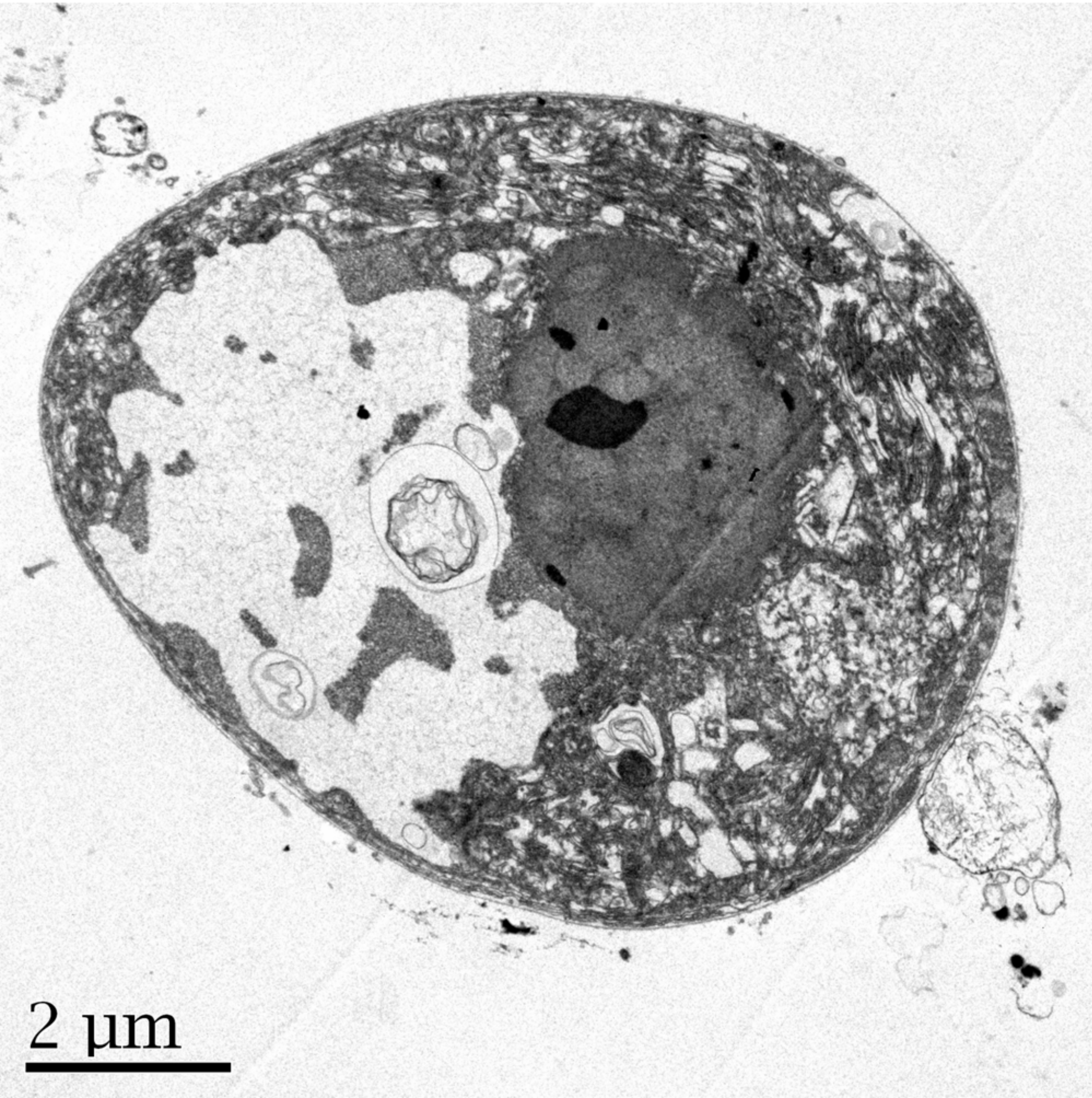

18-7_Correa_JH4_2GridD8_46

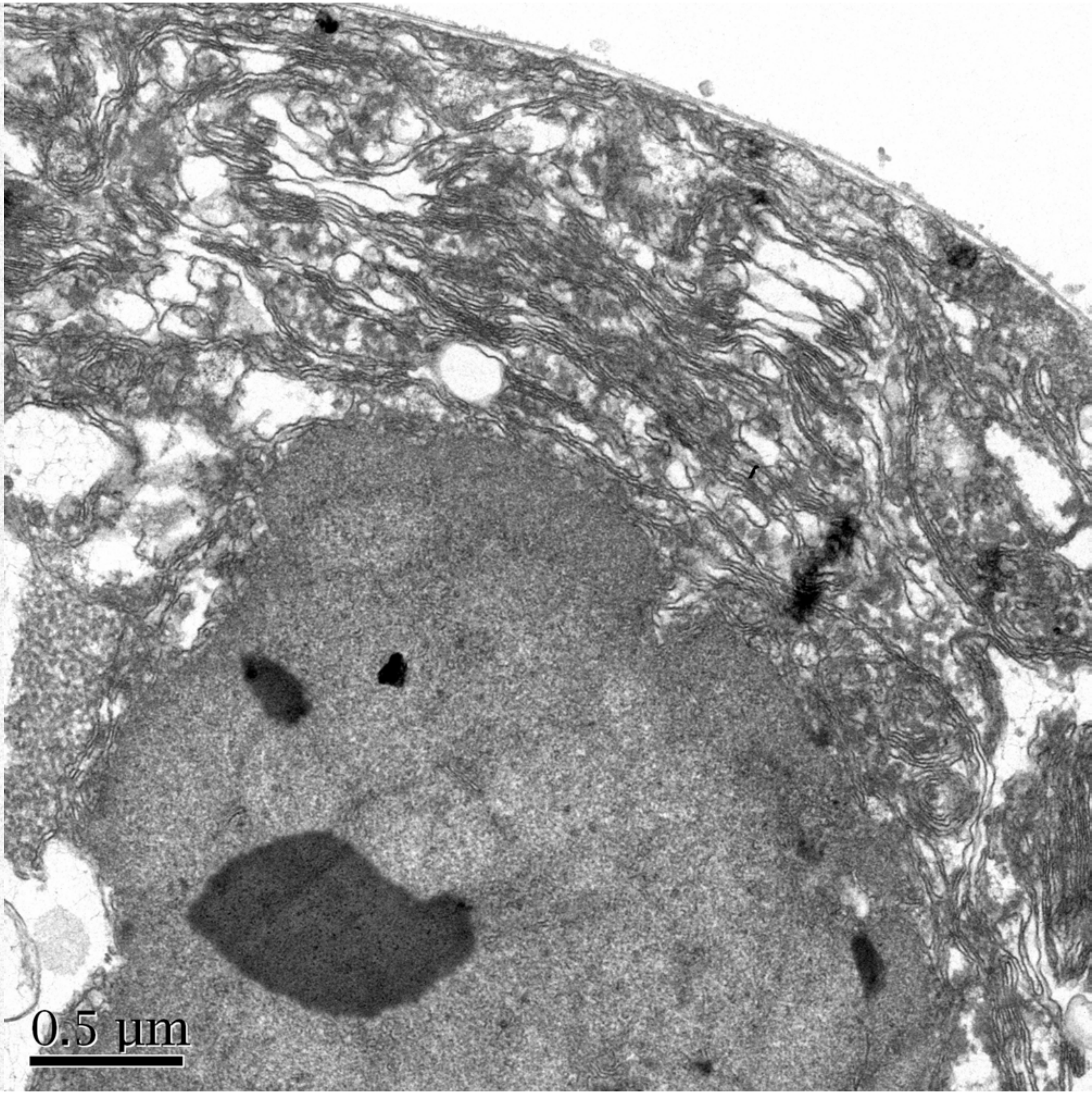

18-7_Correa_JH4_2GridD8_48

Cell 9

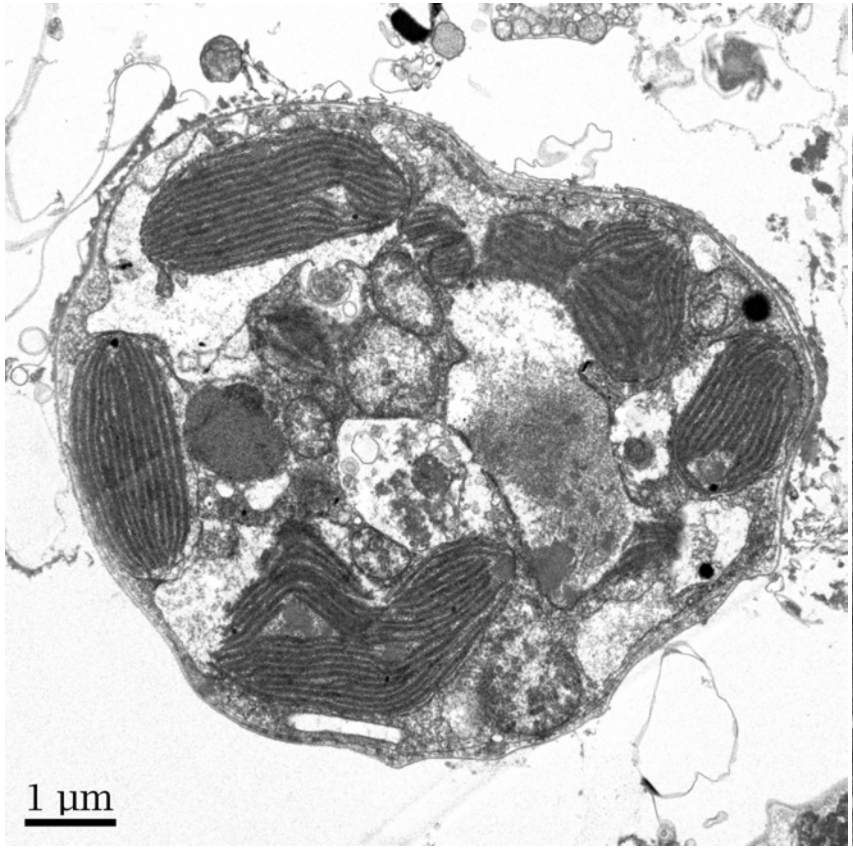

18-7_Correa_JH4_2GridD8_49

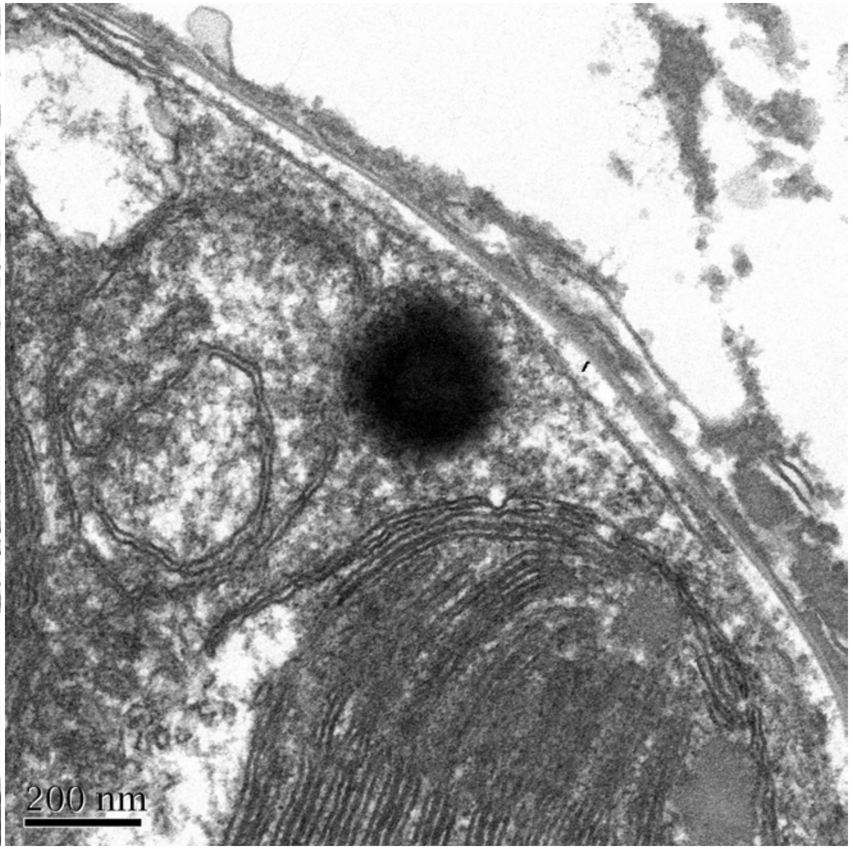

18-7_Correa_JH4_2GridD8_53

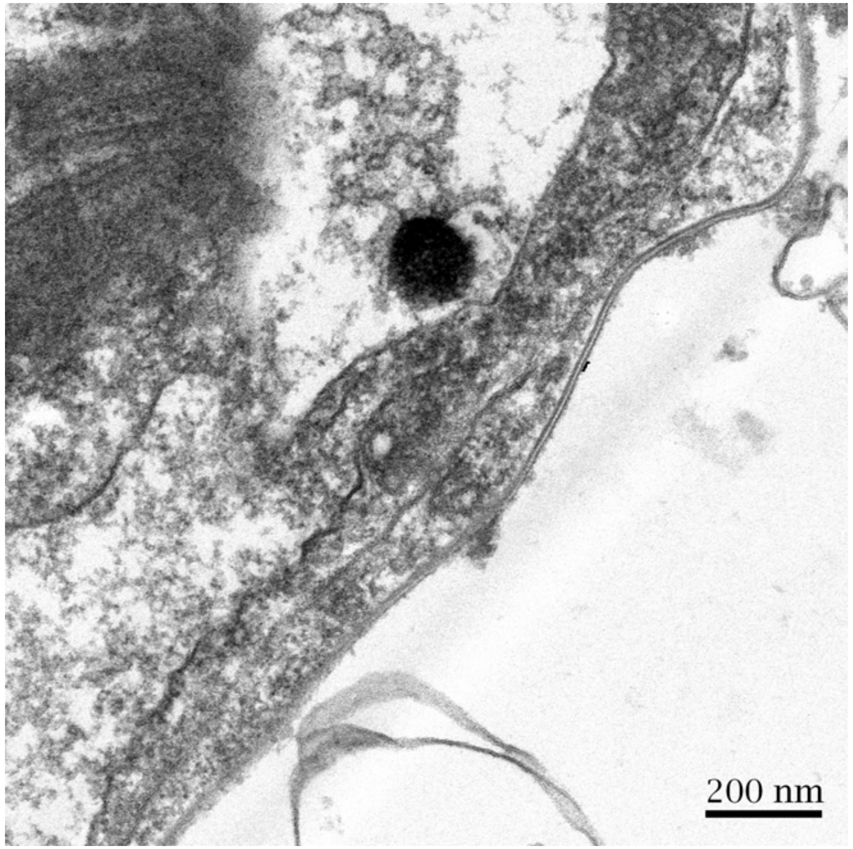

18-7_Correa_JH4_2GridD8_51

Cell 10

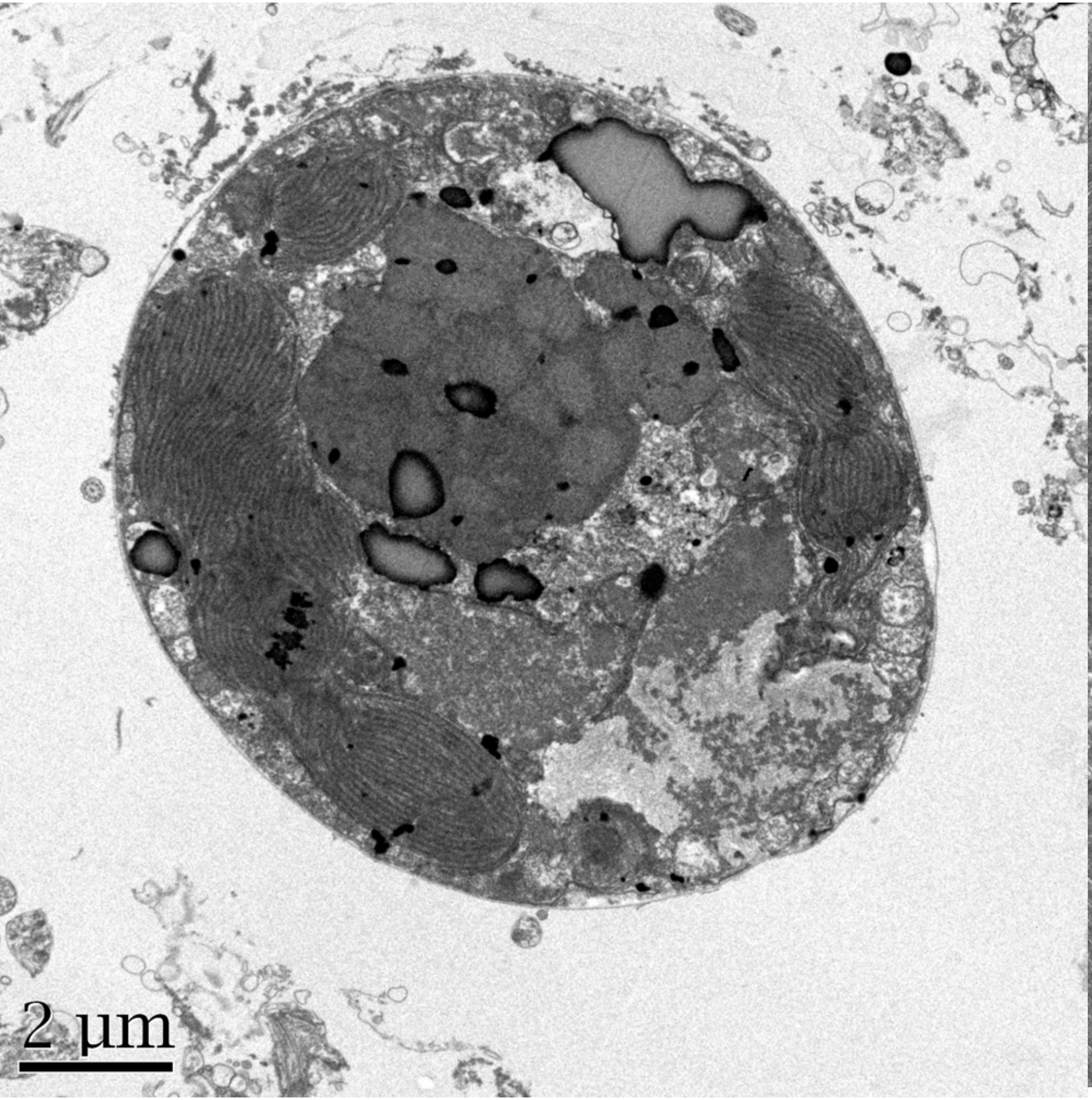

18-7_Correa_JH4_2GridD8_56

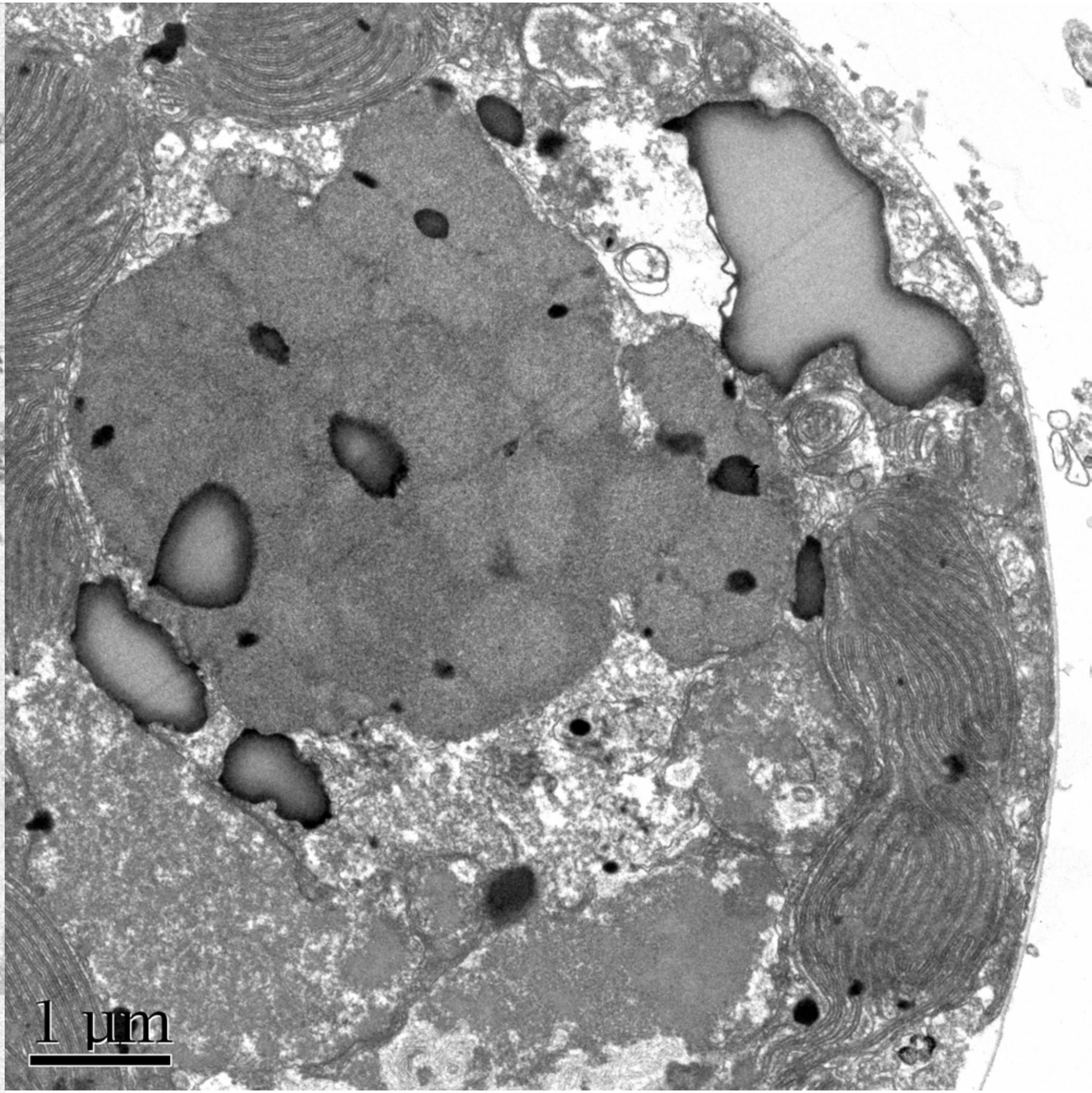

18-7_Correa_JH4_2GridD8_57

Cell 11

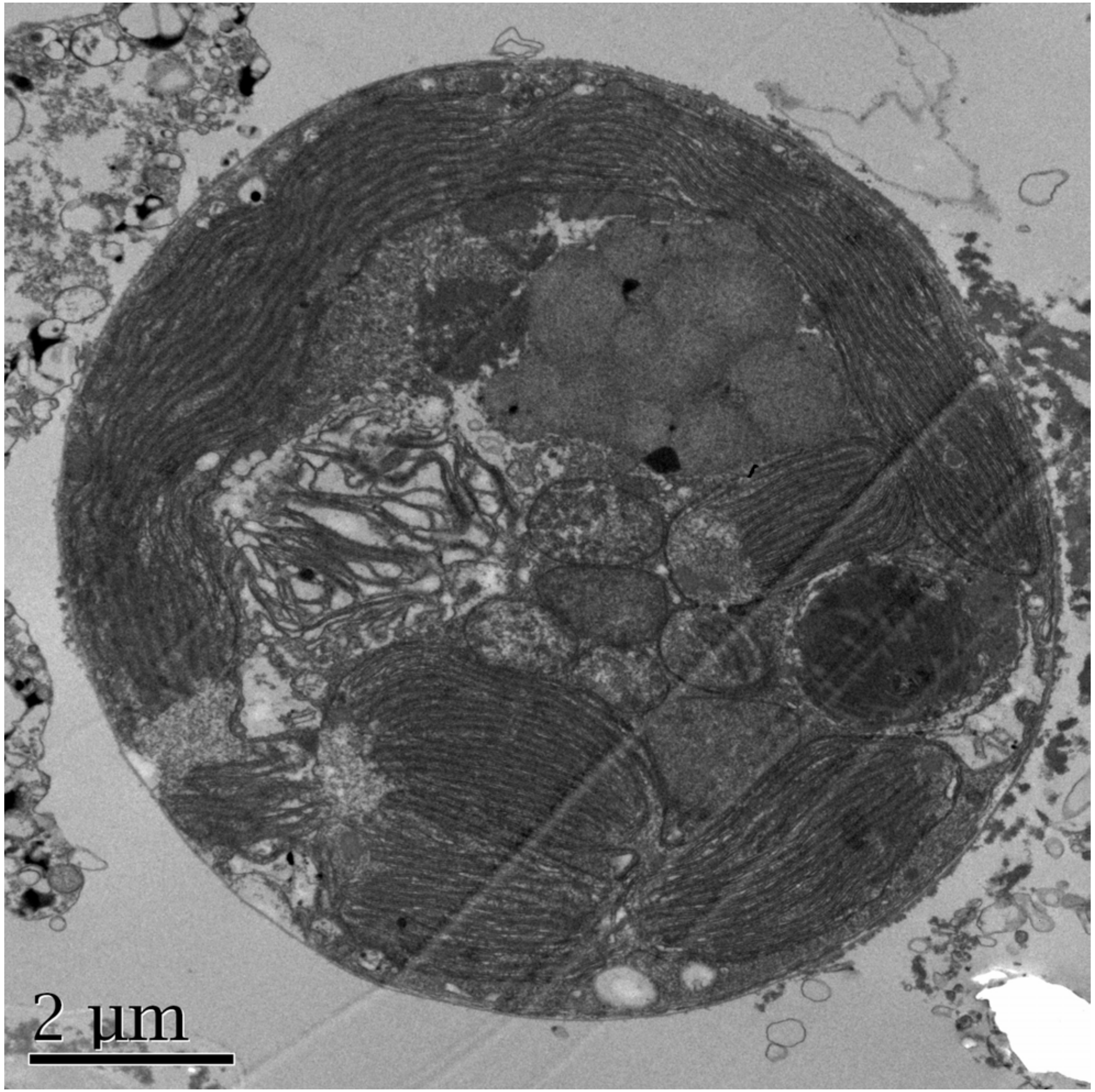

18-7_Correa_JH4_2GridD8_60

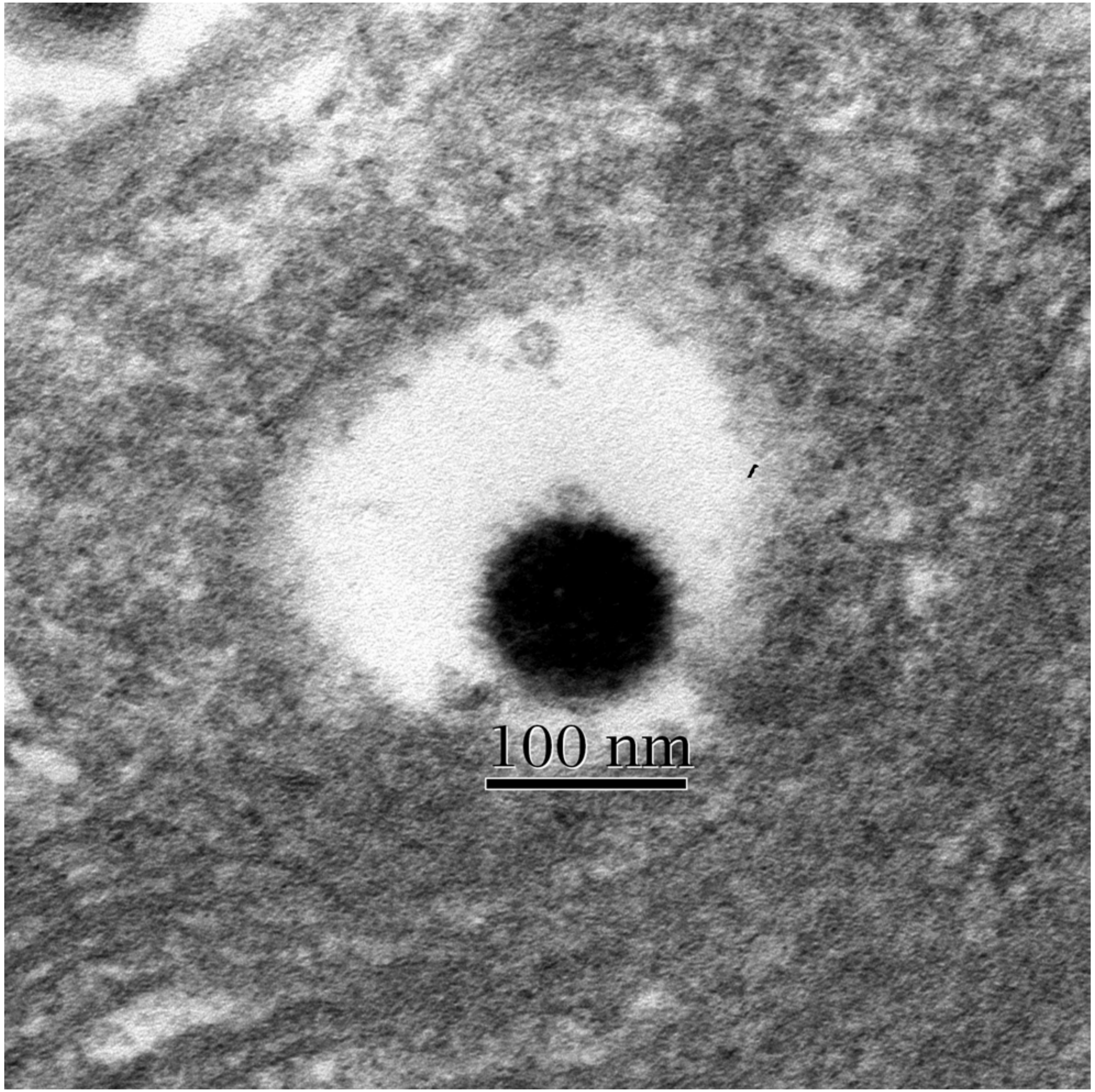

18-7_Correa_JH4_2GridD8_64

Cell 12

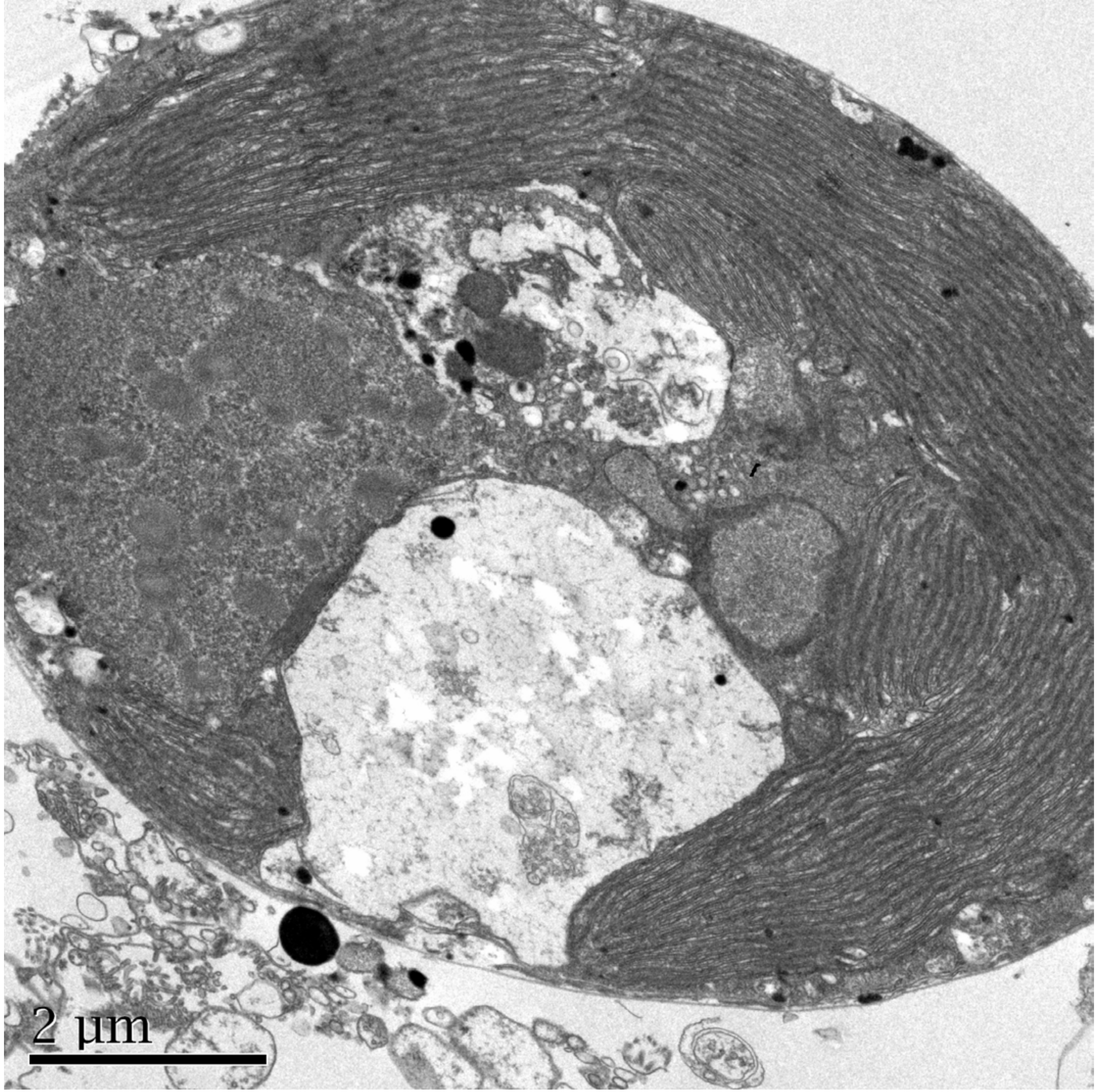

18-7_Correa_JH4_2GridD8_65

ACR Colony B Expelled- Ambient

Cell 1

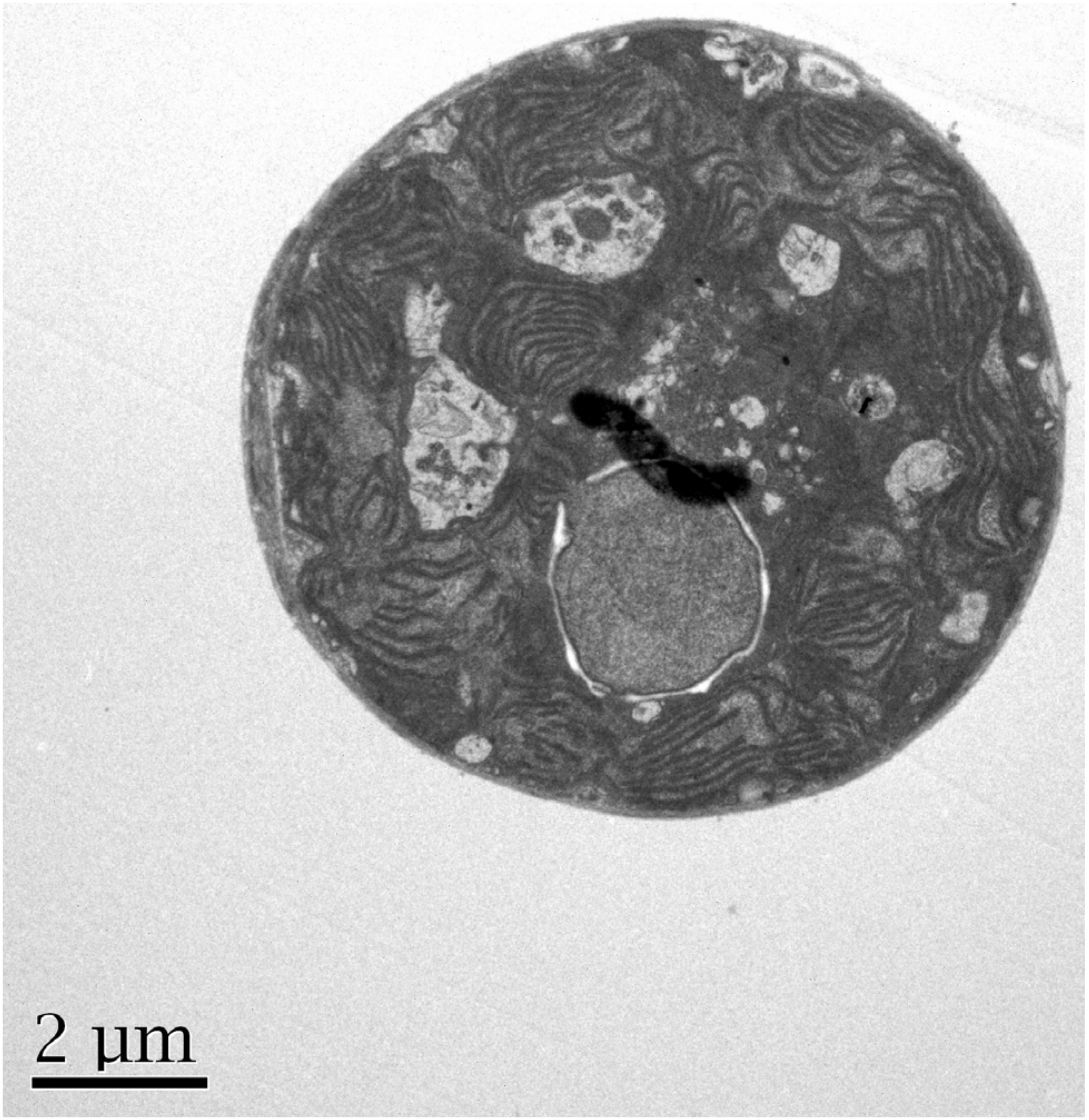

18-7_Correa_KC4_2GridD9_1

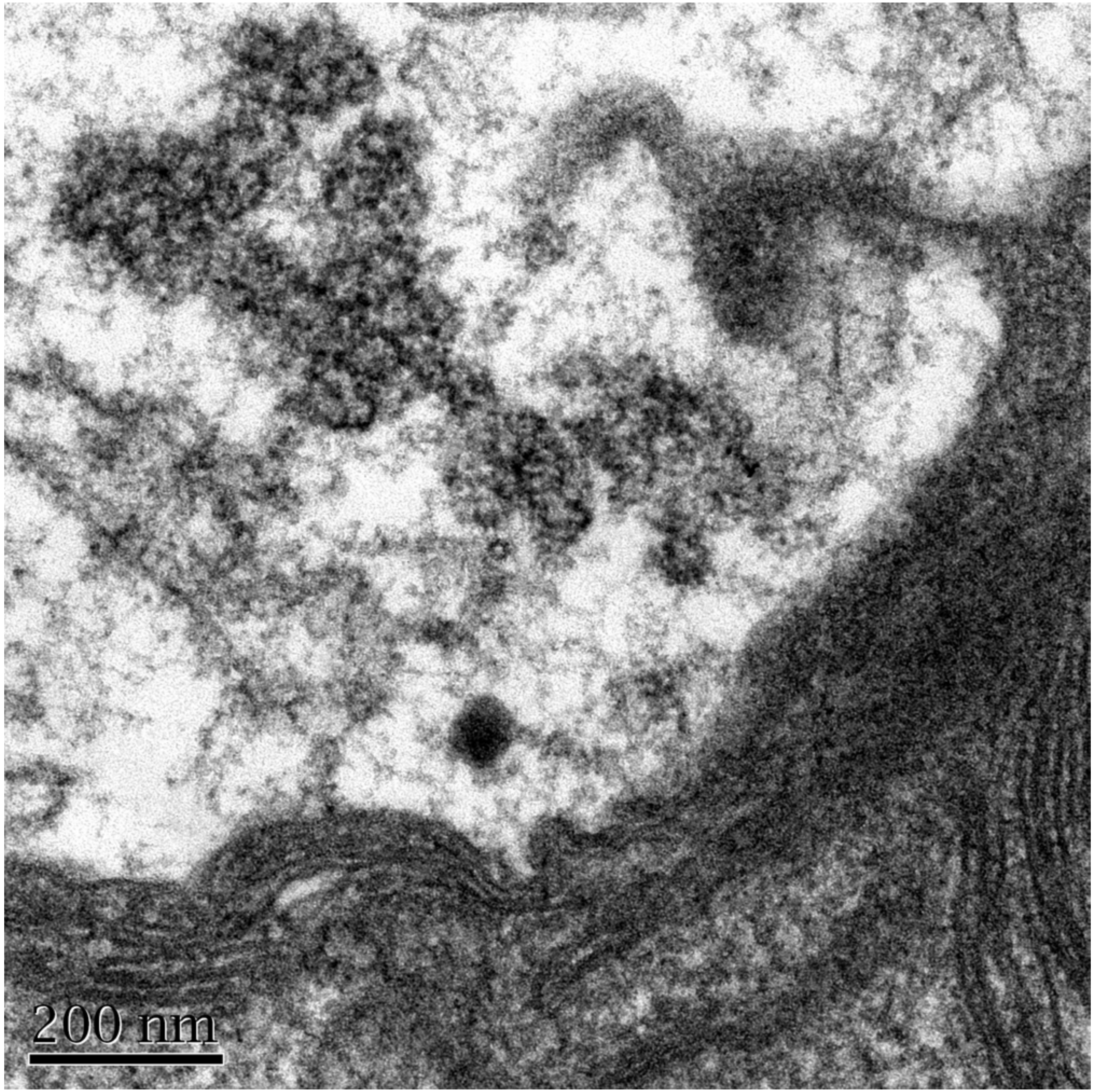

18-7_Correa_KC4_2GridD9_11

Cell 2

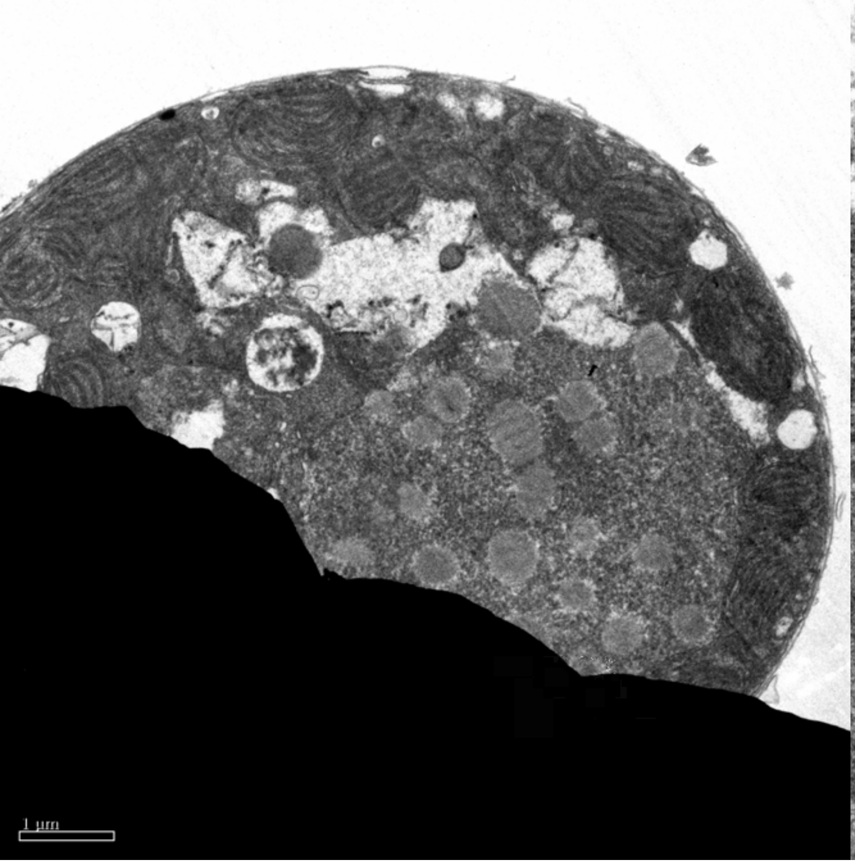

18-7_Correa_KC4_2GridD9_13

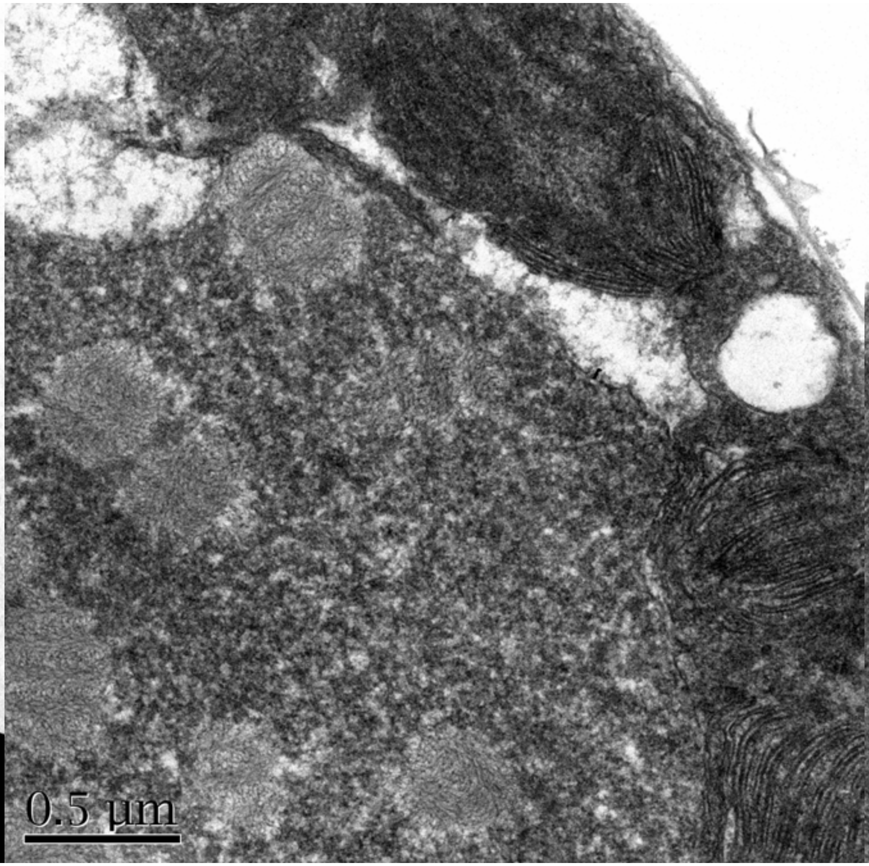

18-7_Correa_KC4_2GridD9_20

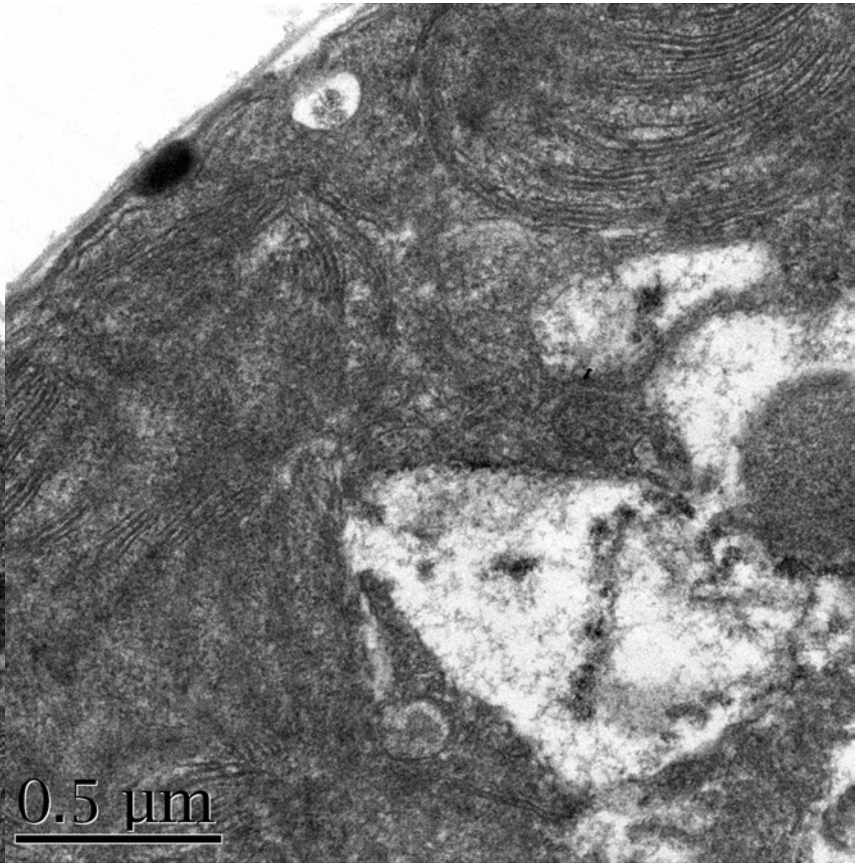

18-7_Correa_KC4_2GridD9_16

Cell 3

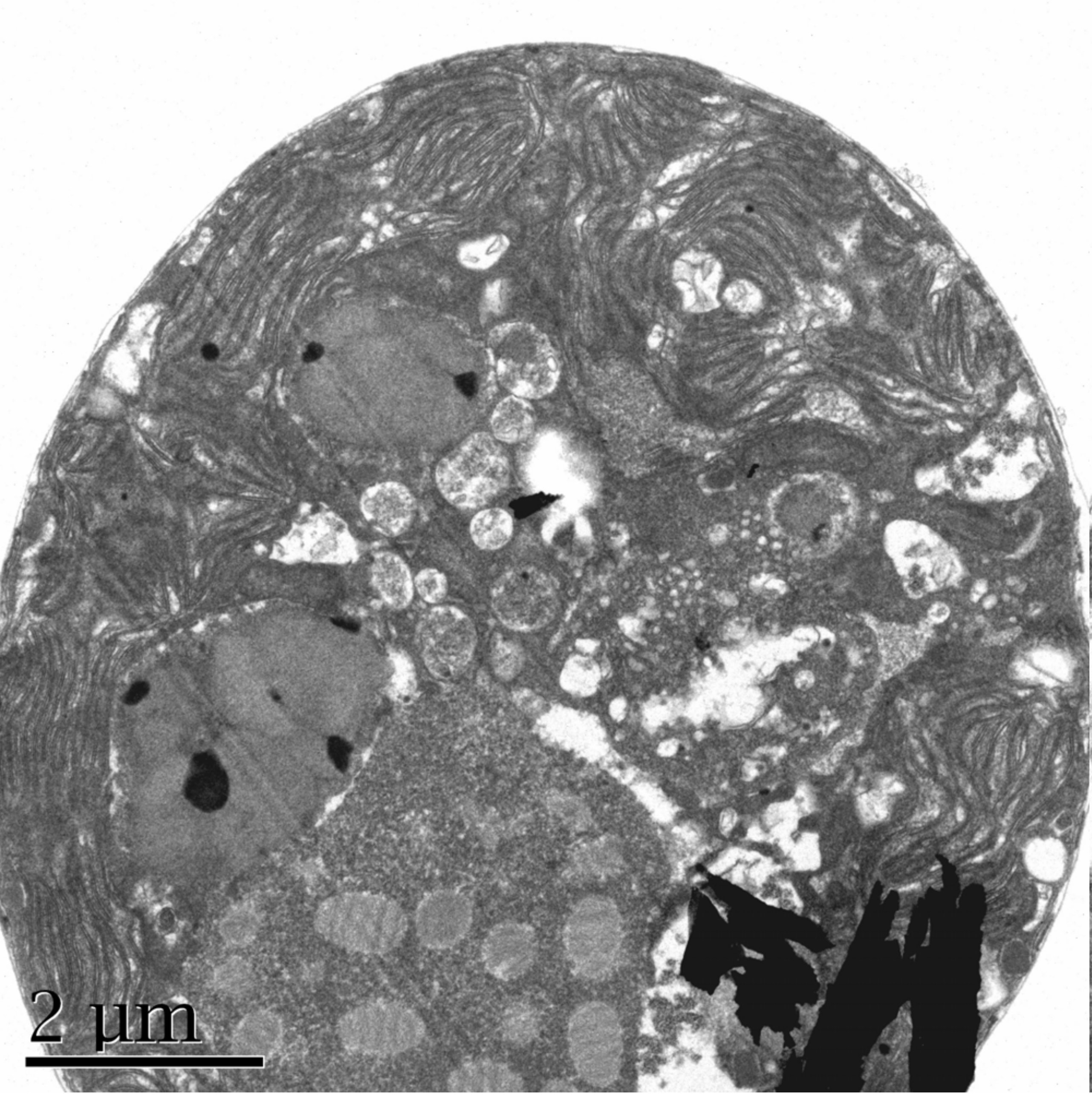

18-7_Correa_KC4_2GridD9_24

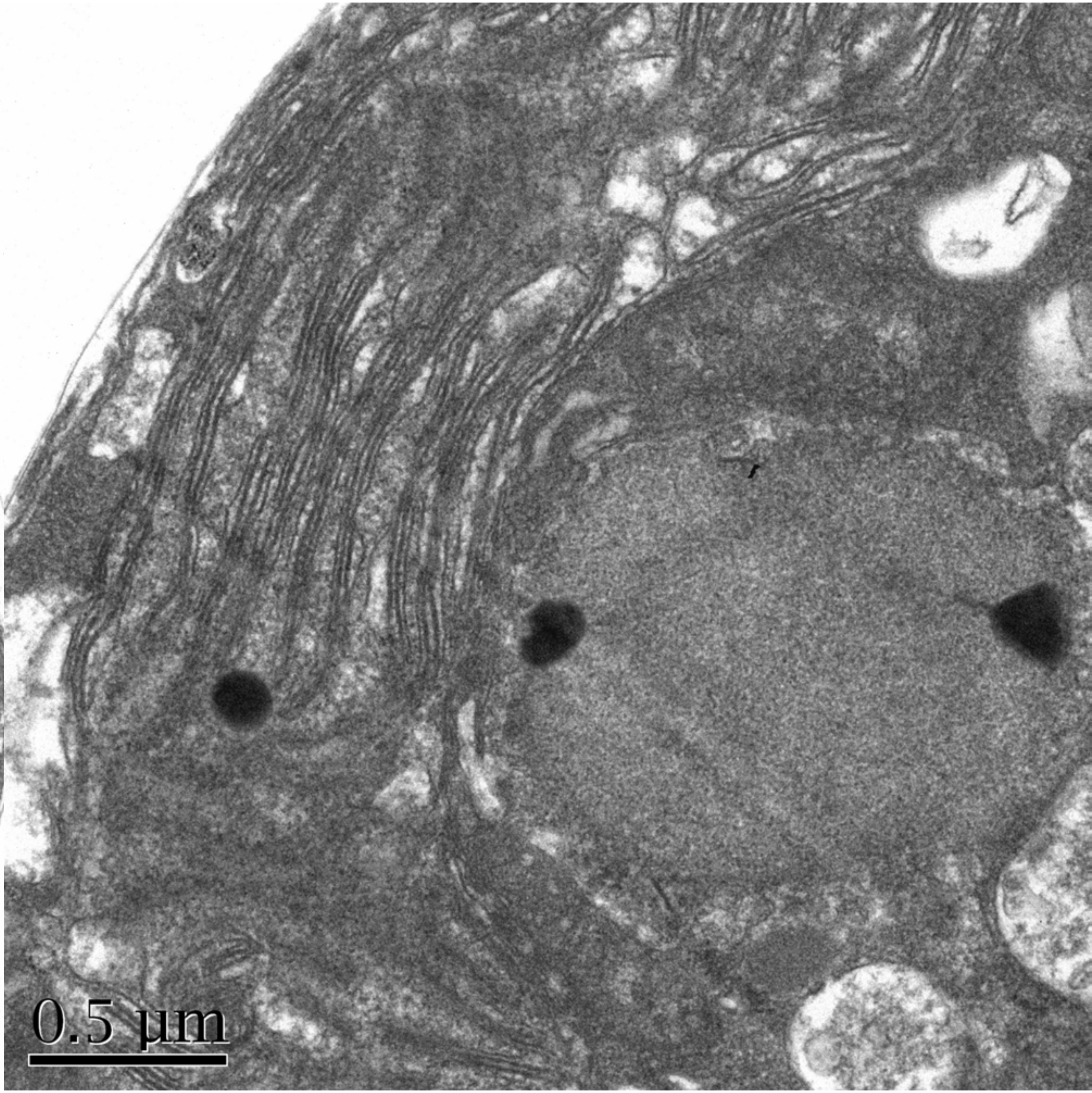

18-7_Correa_KC4_2GridD9_30

Cell 4

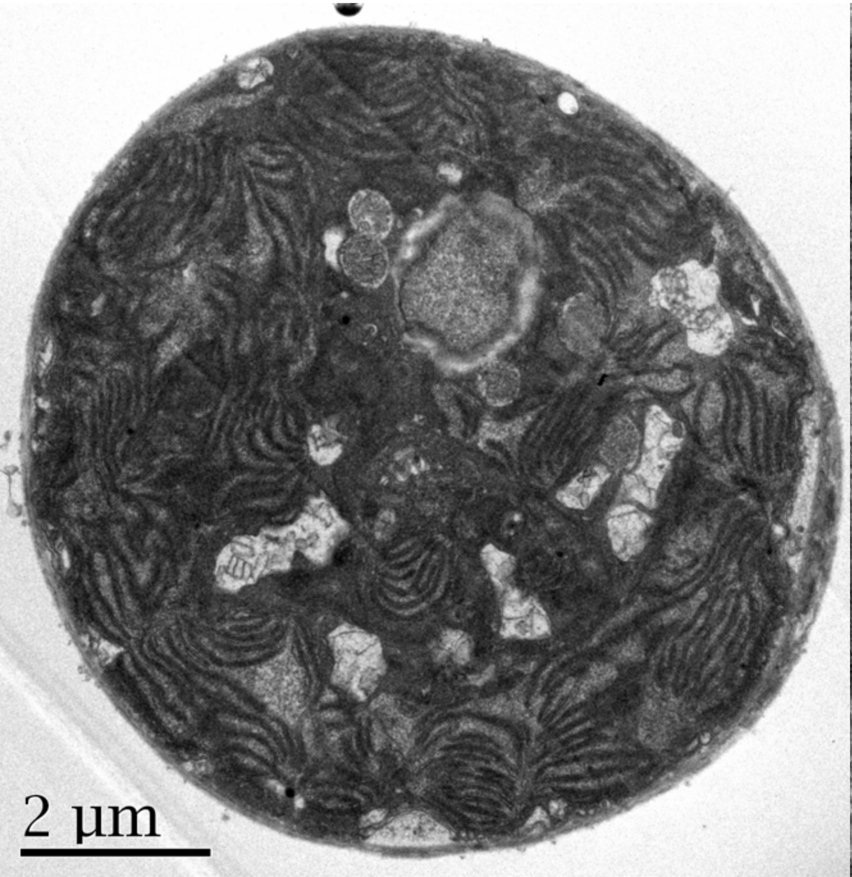

18-7_Correa_KC4_2GridD9_31

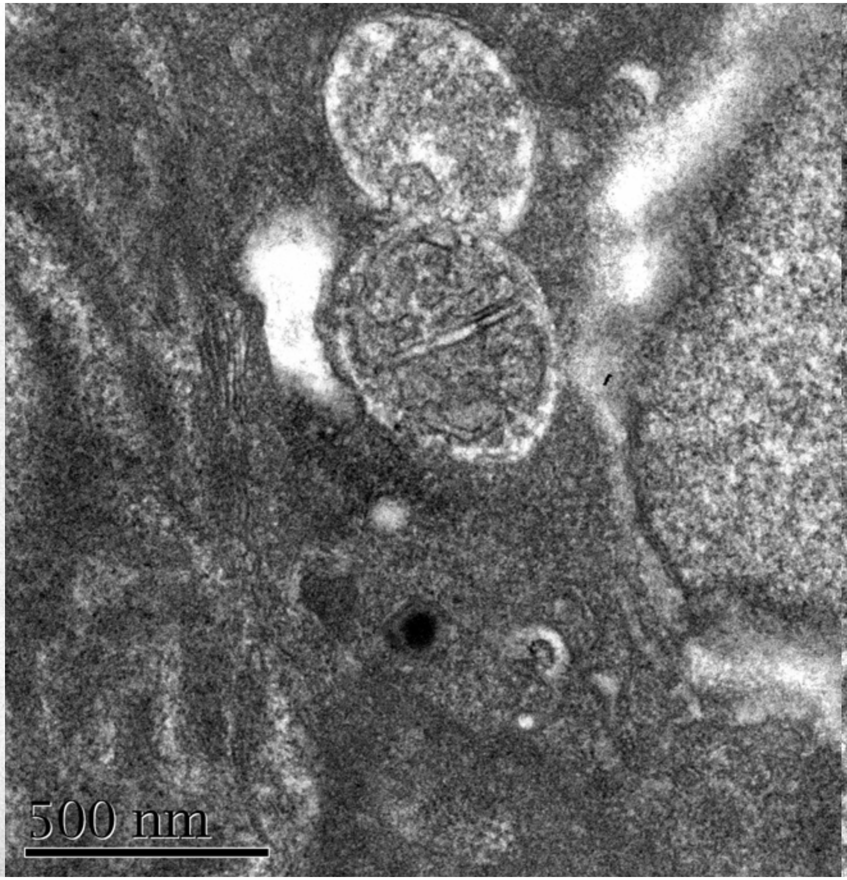

18-7_Correa_KC4_2GridD9_35

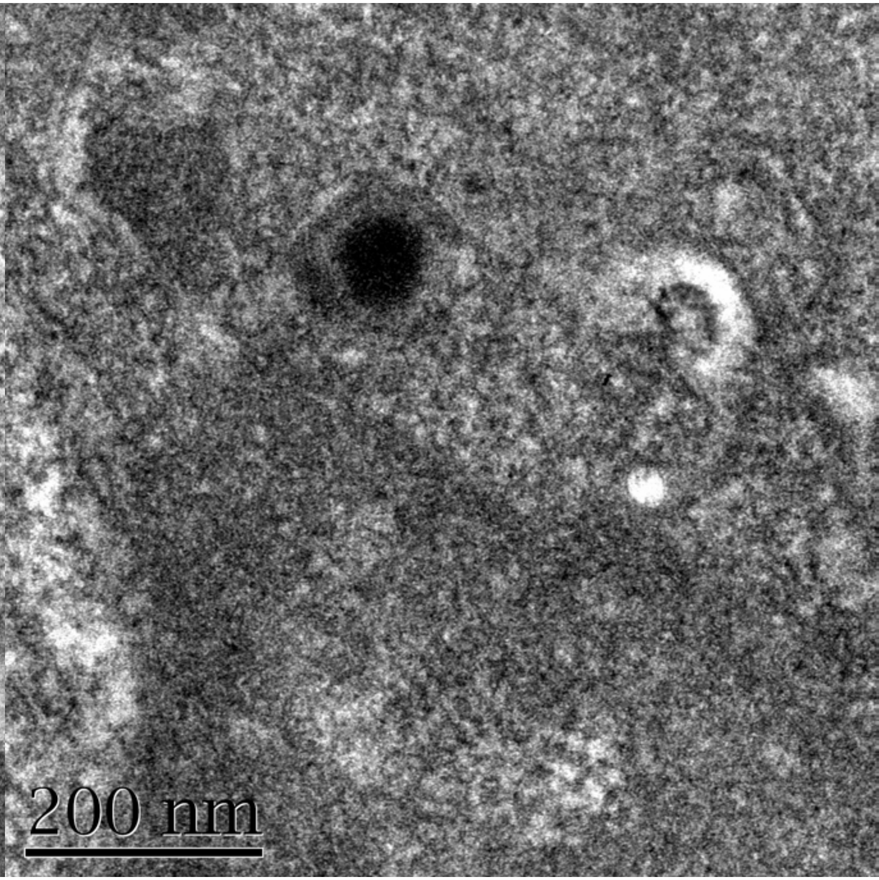

18-7_Correa_KC4_2GridD9_36

Cell 5

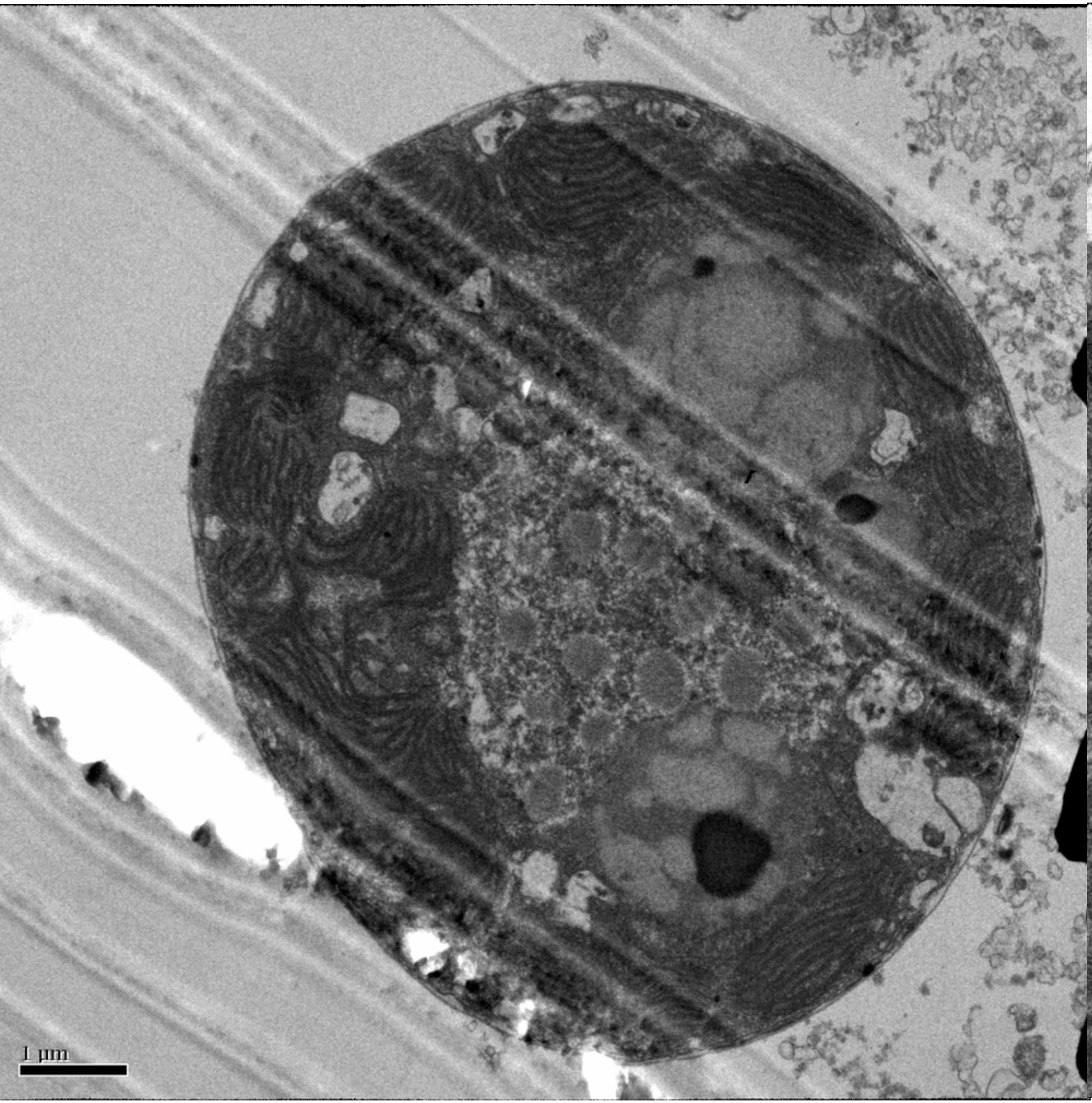

18-7_Correa_KC4_8T3_2

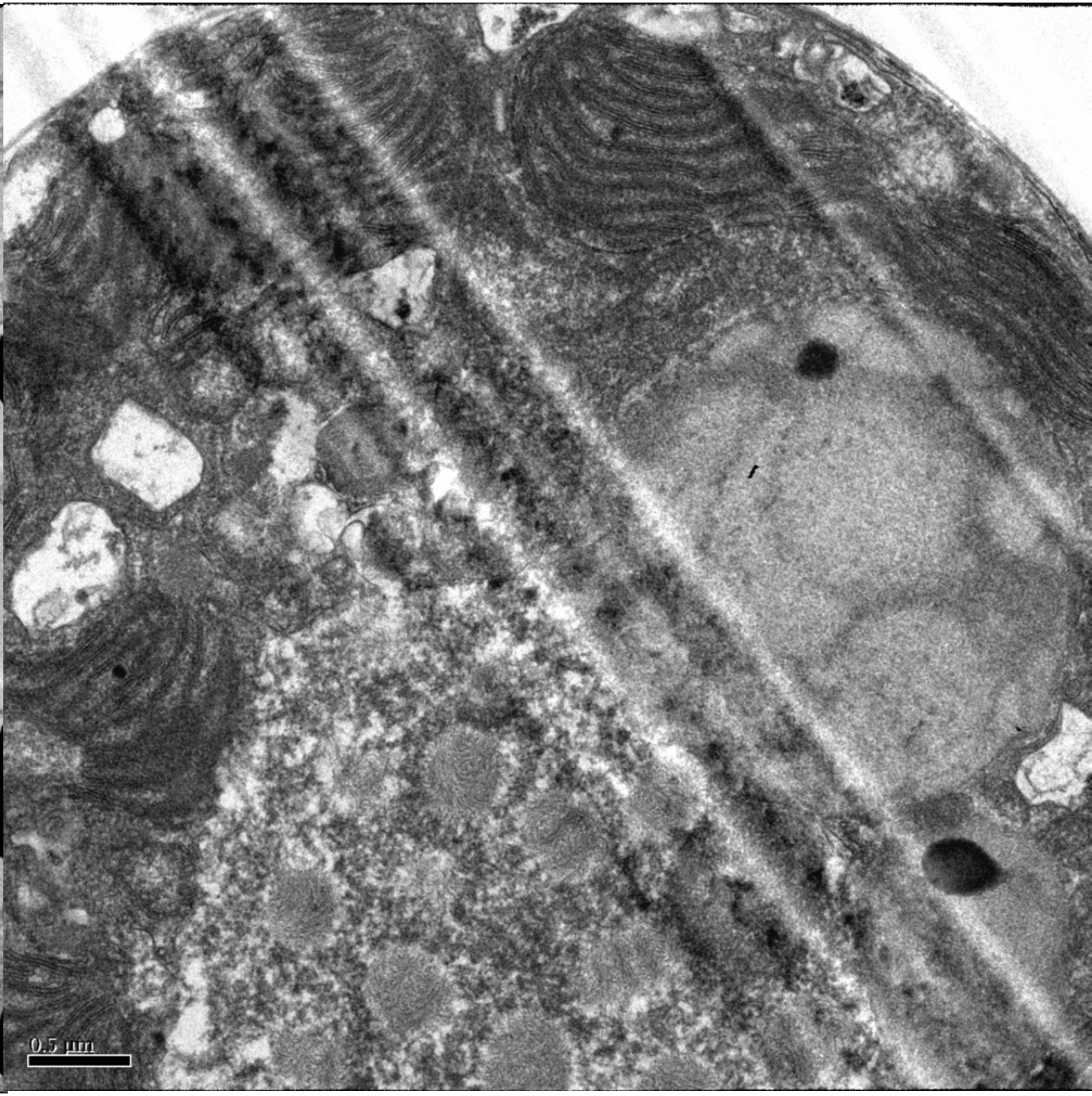

18-7_Correa_KC4_8T3_5

Cell 6

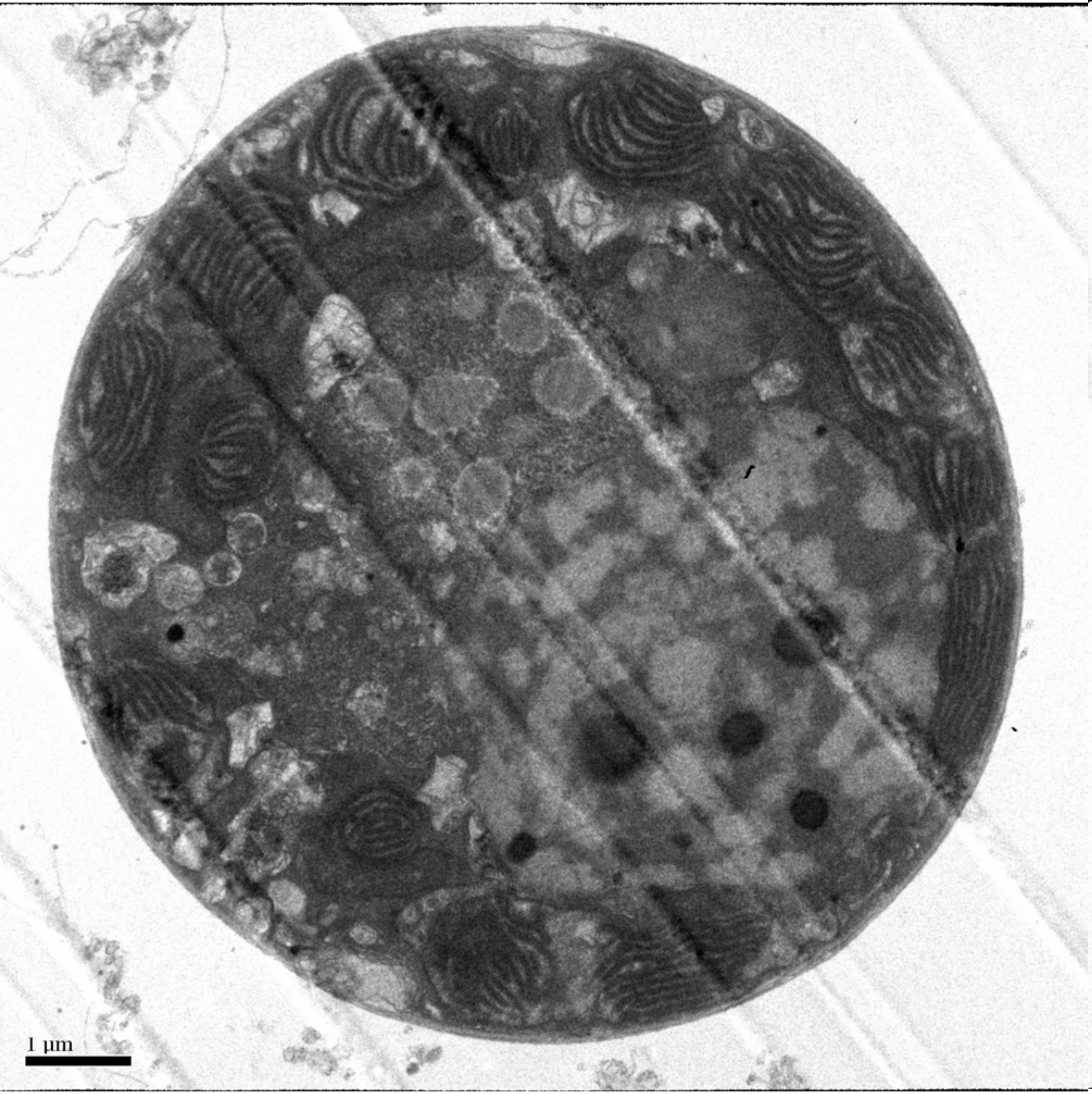

18-7_Correa_KC4_8T4_3

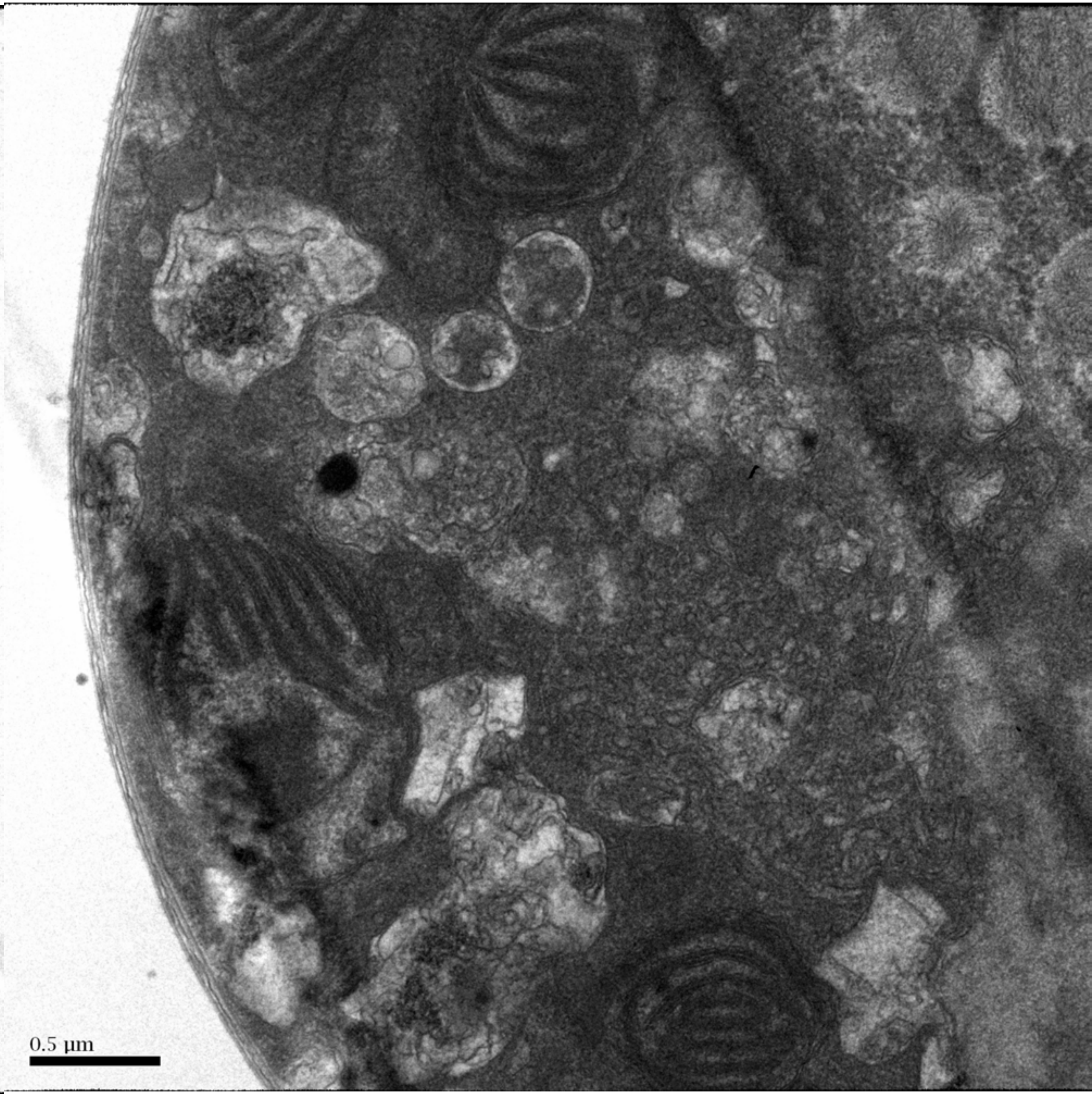

18-7_Correa_KC4_8T4_4

Cell 7

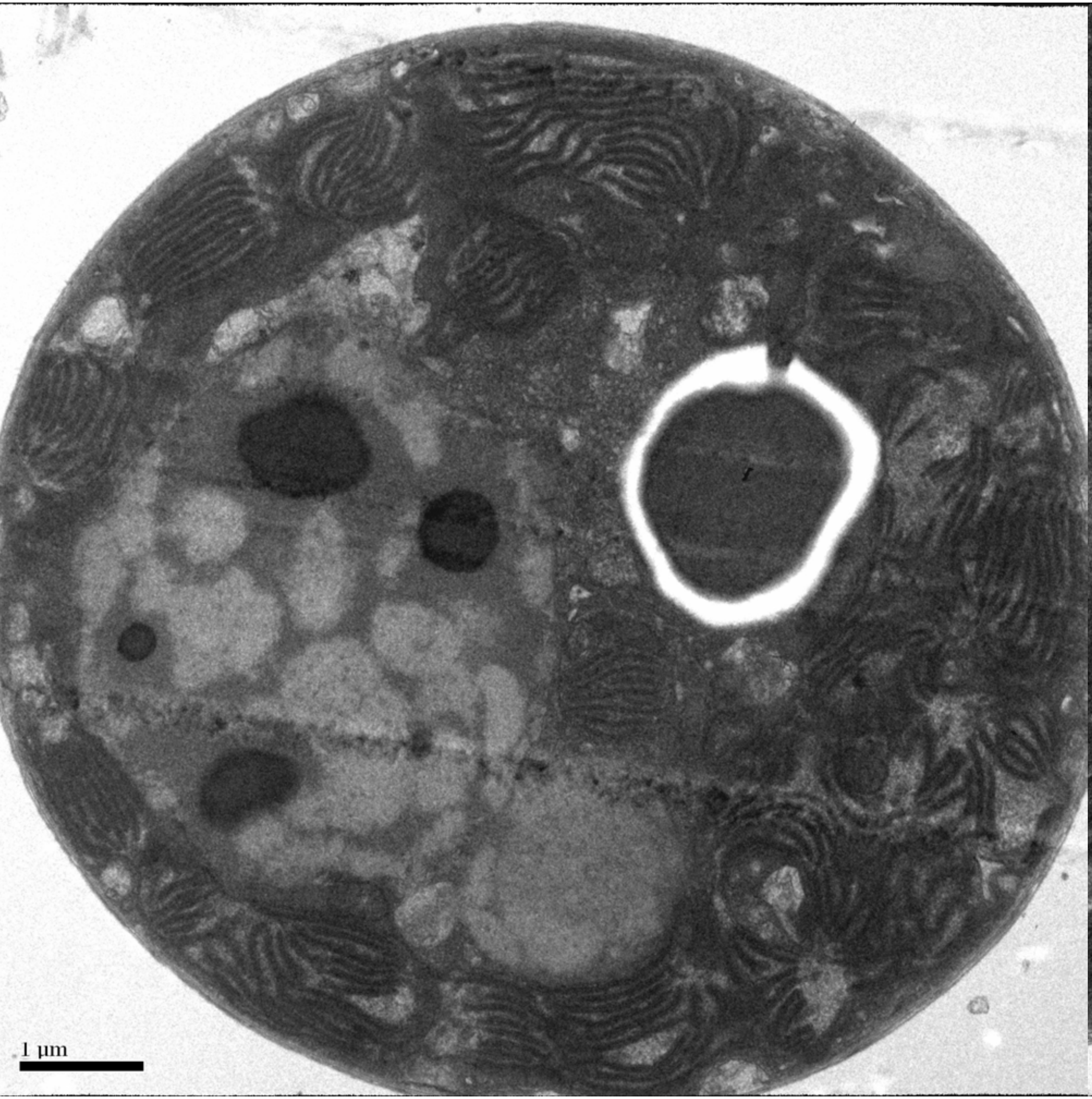

18-7 Correa KC4 8T4 11

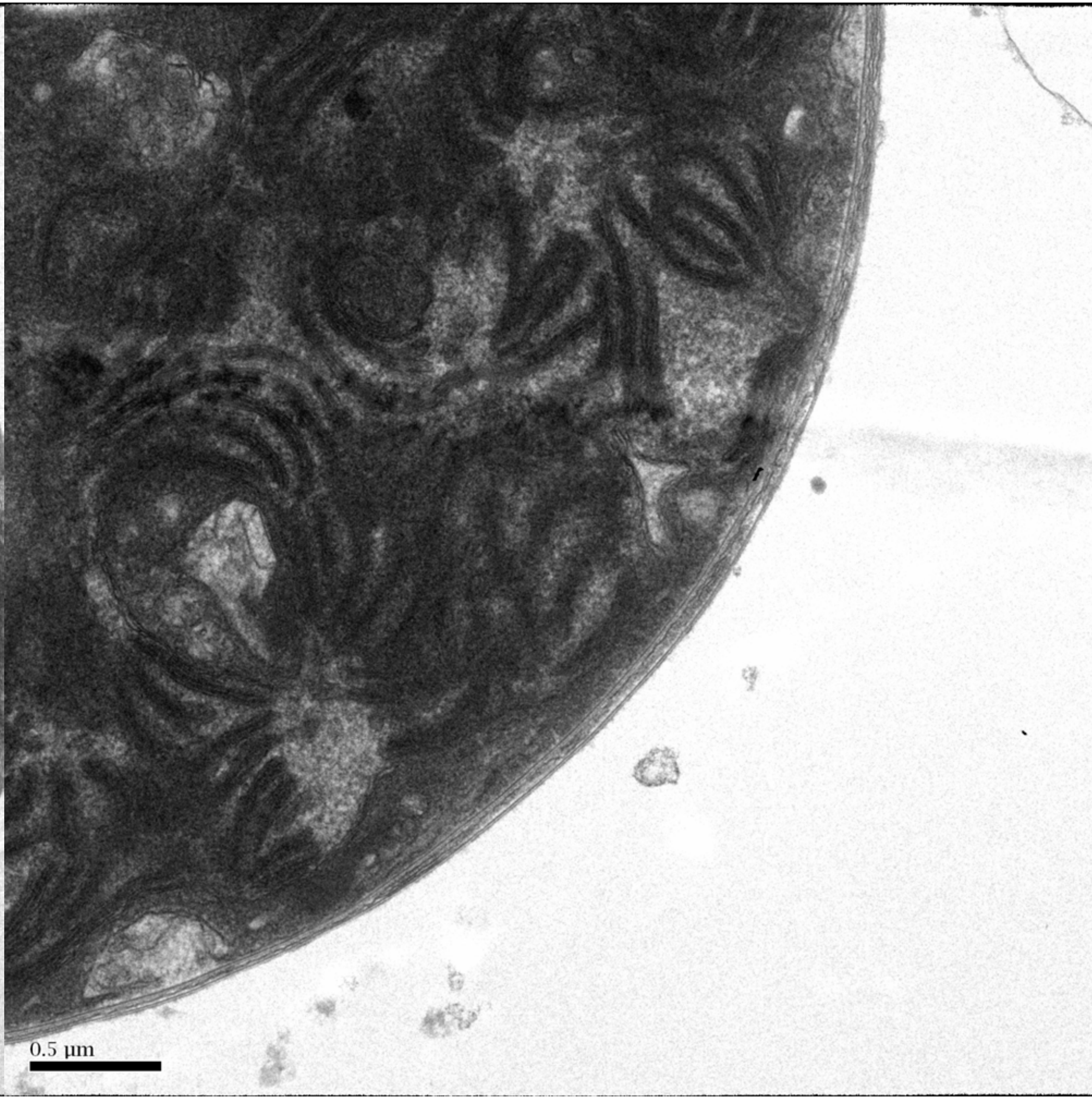

18-7 Correa KC4 8T4 13

Cell 8

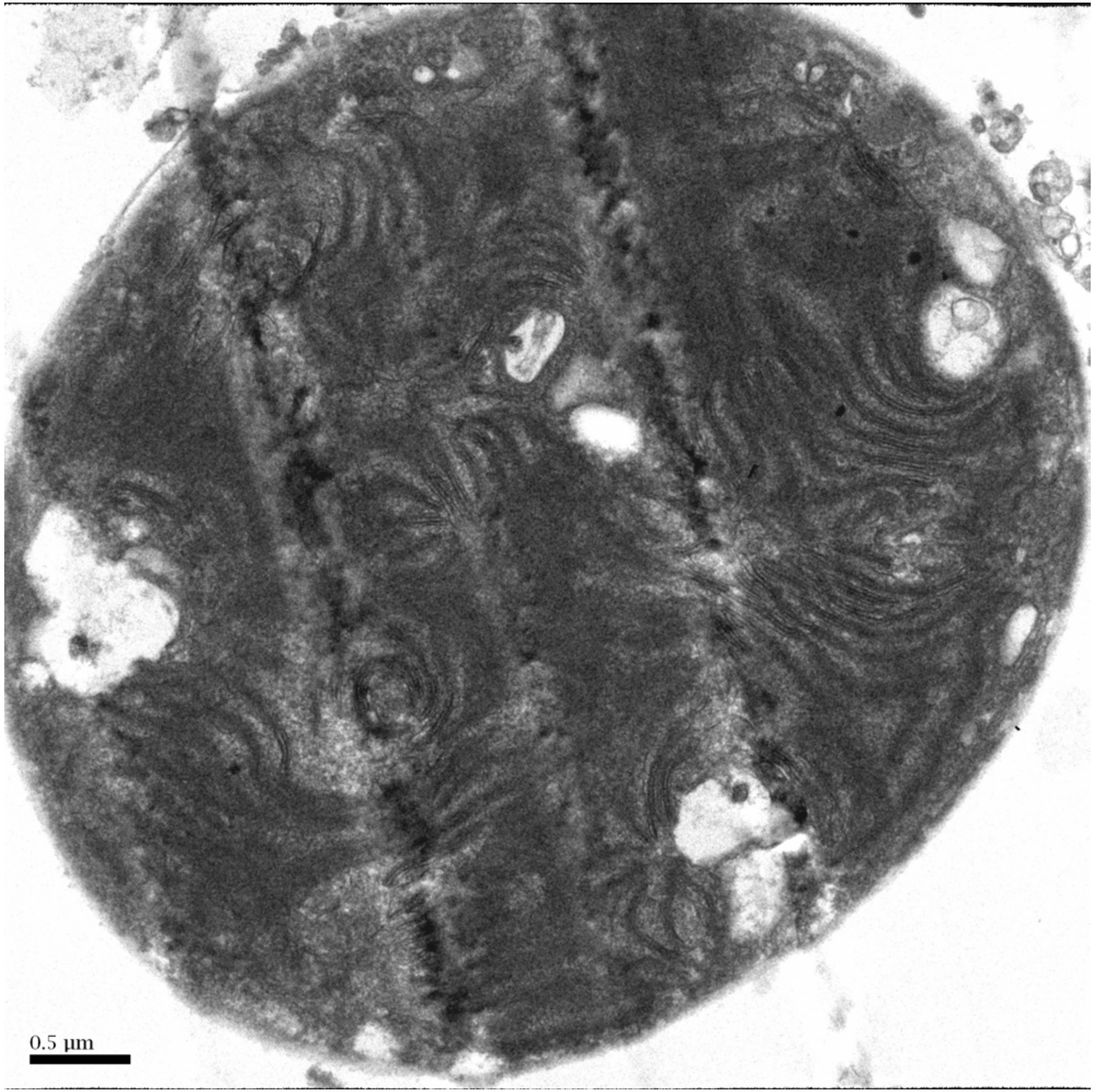

18-7_Correa_KC4_8U1_2

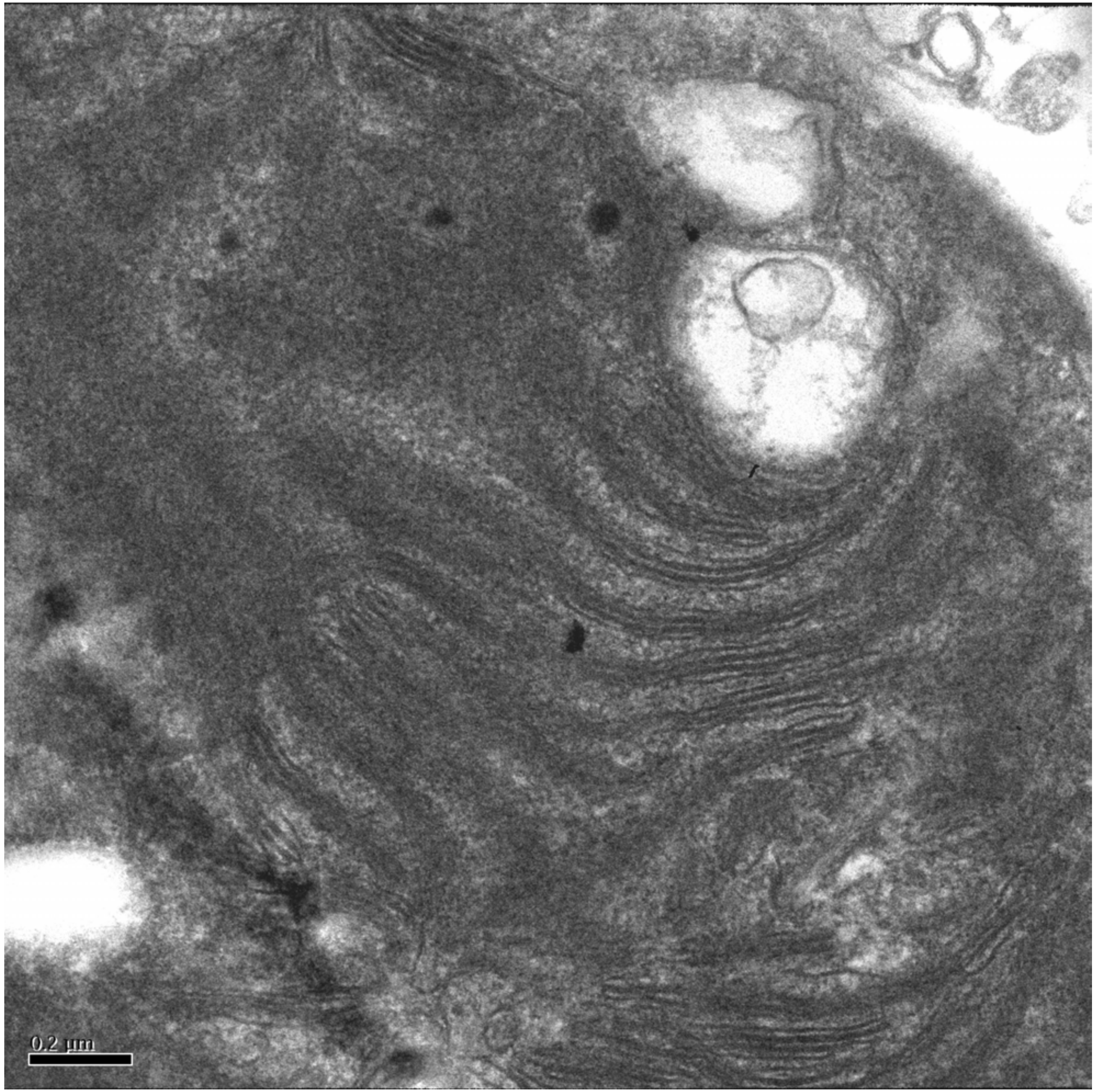

18-7_Correa_KC4_8U1_3

Cell 9

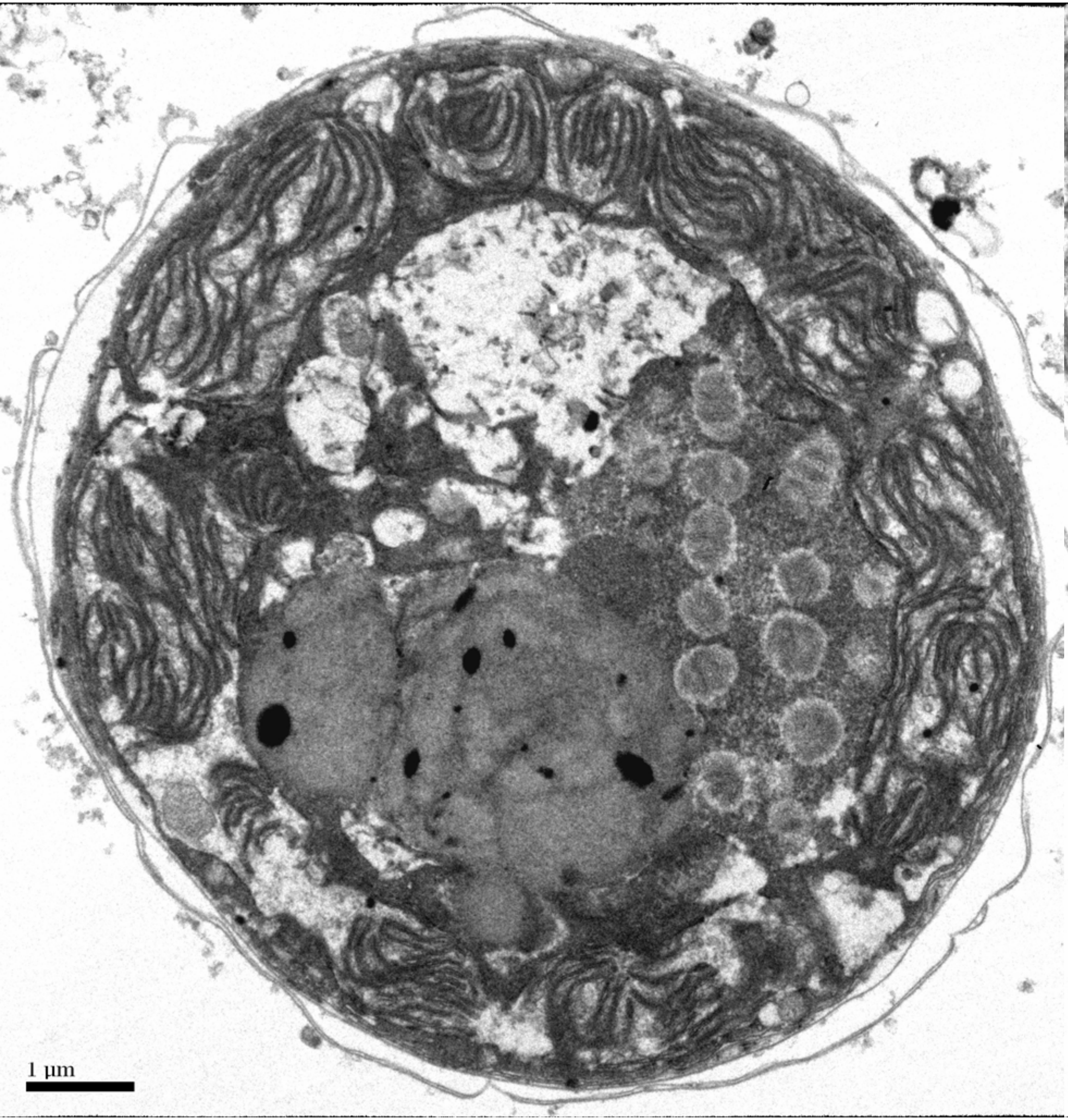

18-7_Correa_KC4_9A1_2

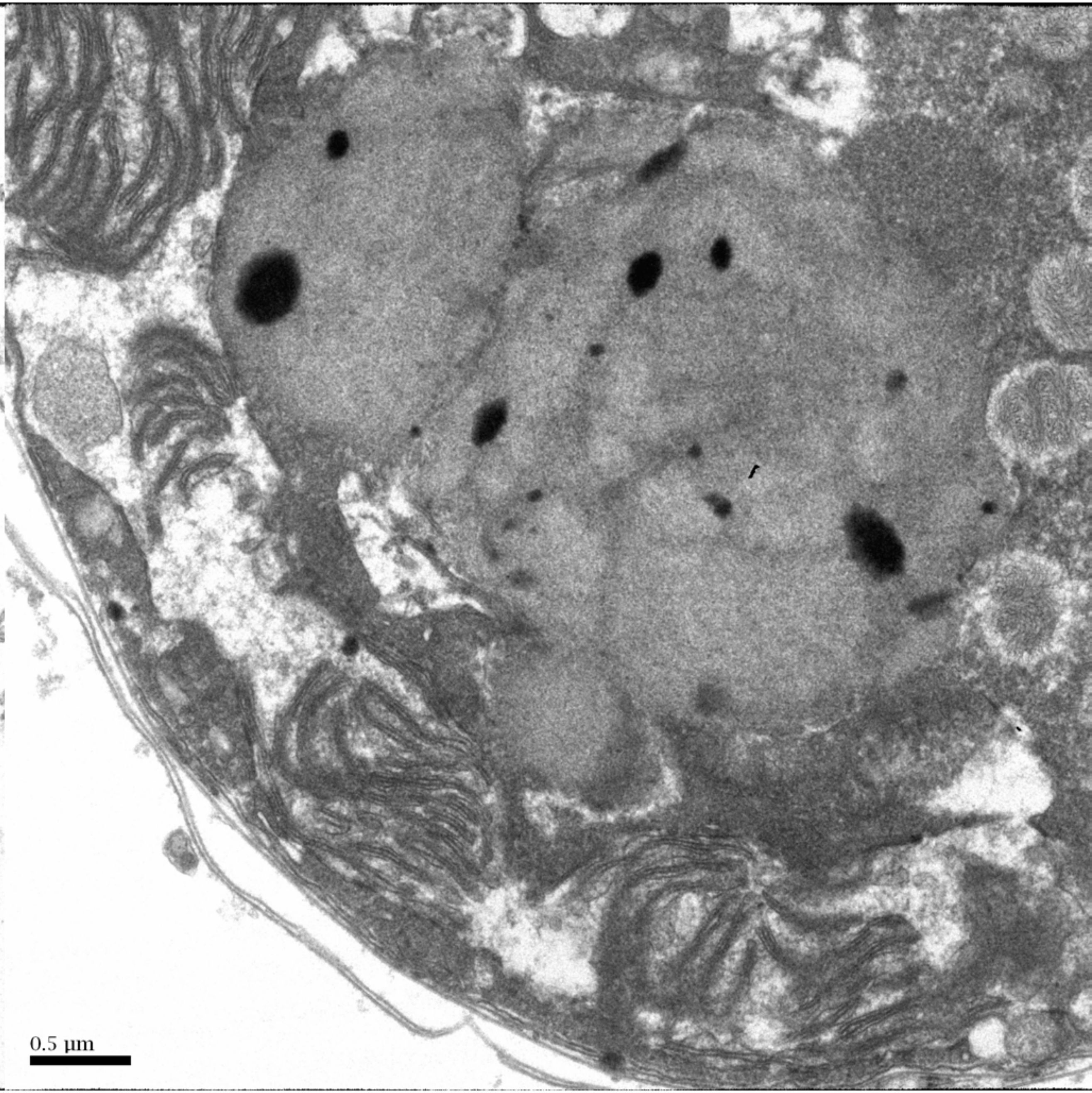

18-7_Correa_KC4_9A1_3

Cell 10

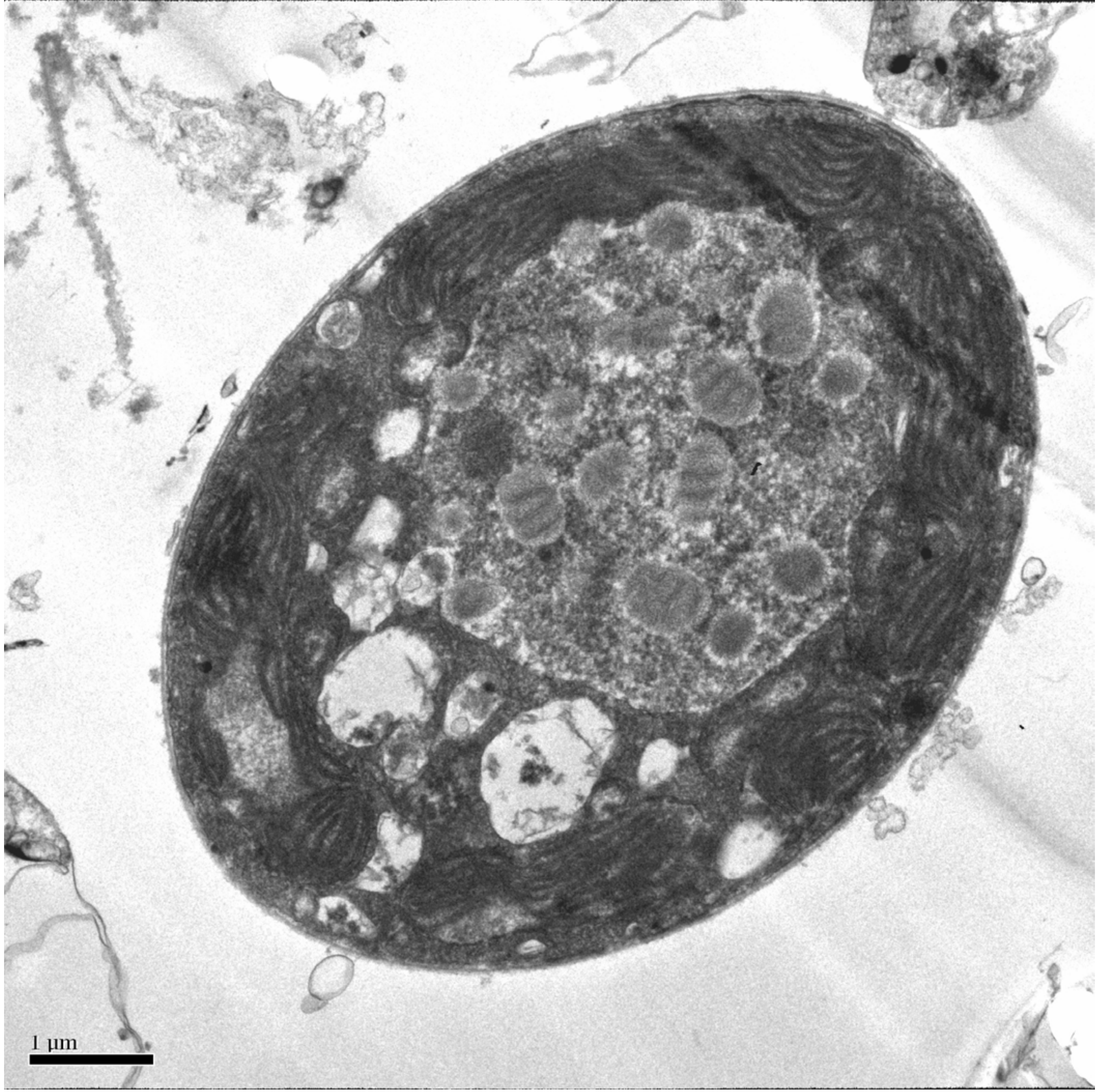

18-7_Correa_KC4_9A4_1

Cell 11

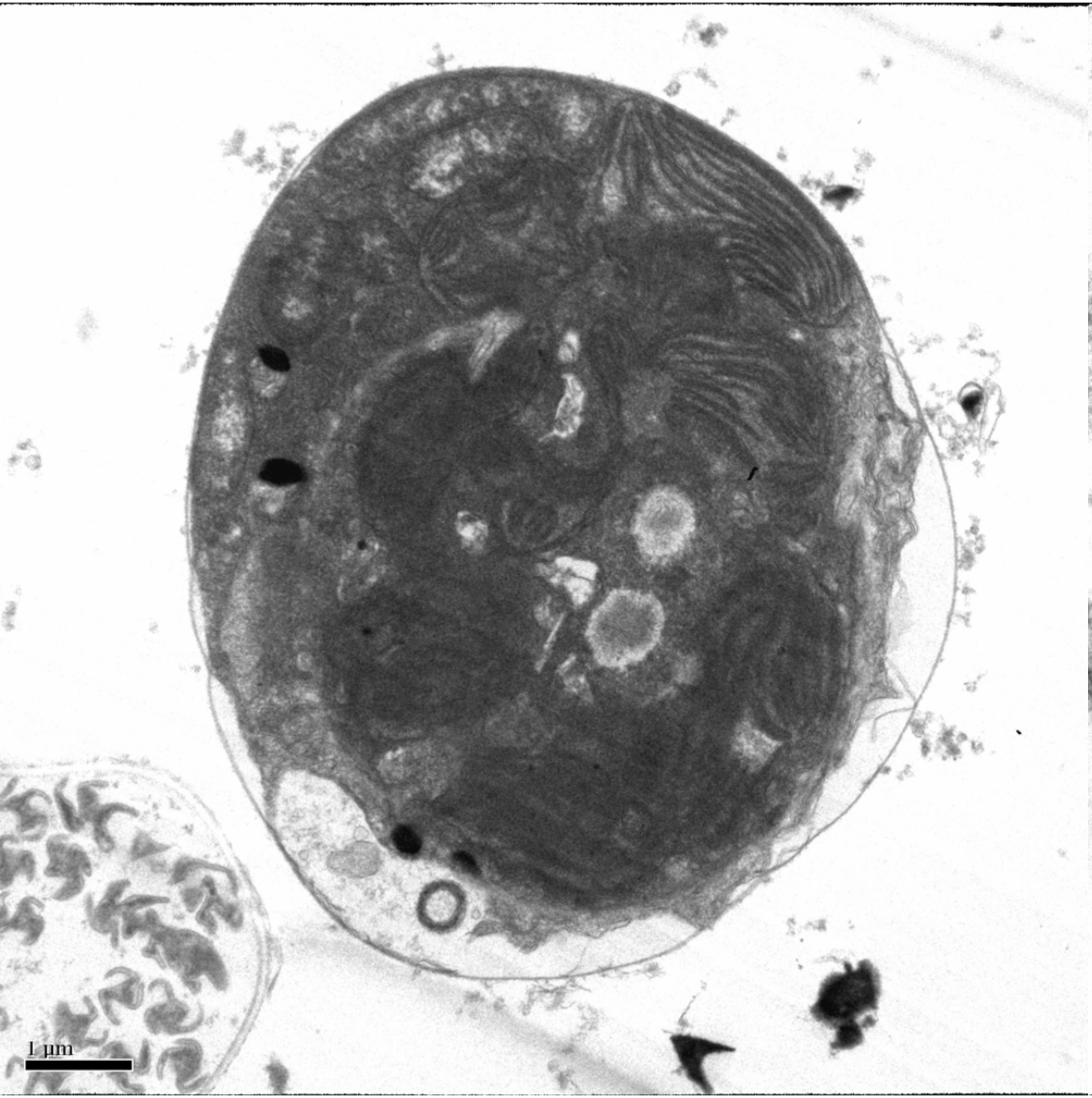

18-7_Correa_KC4_9A4_5

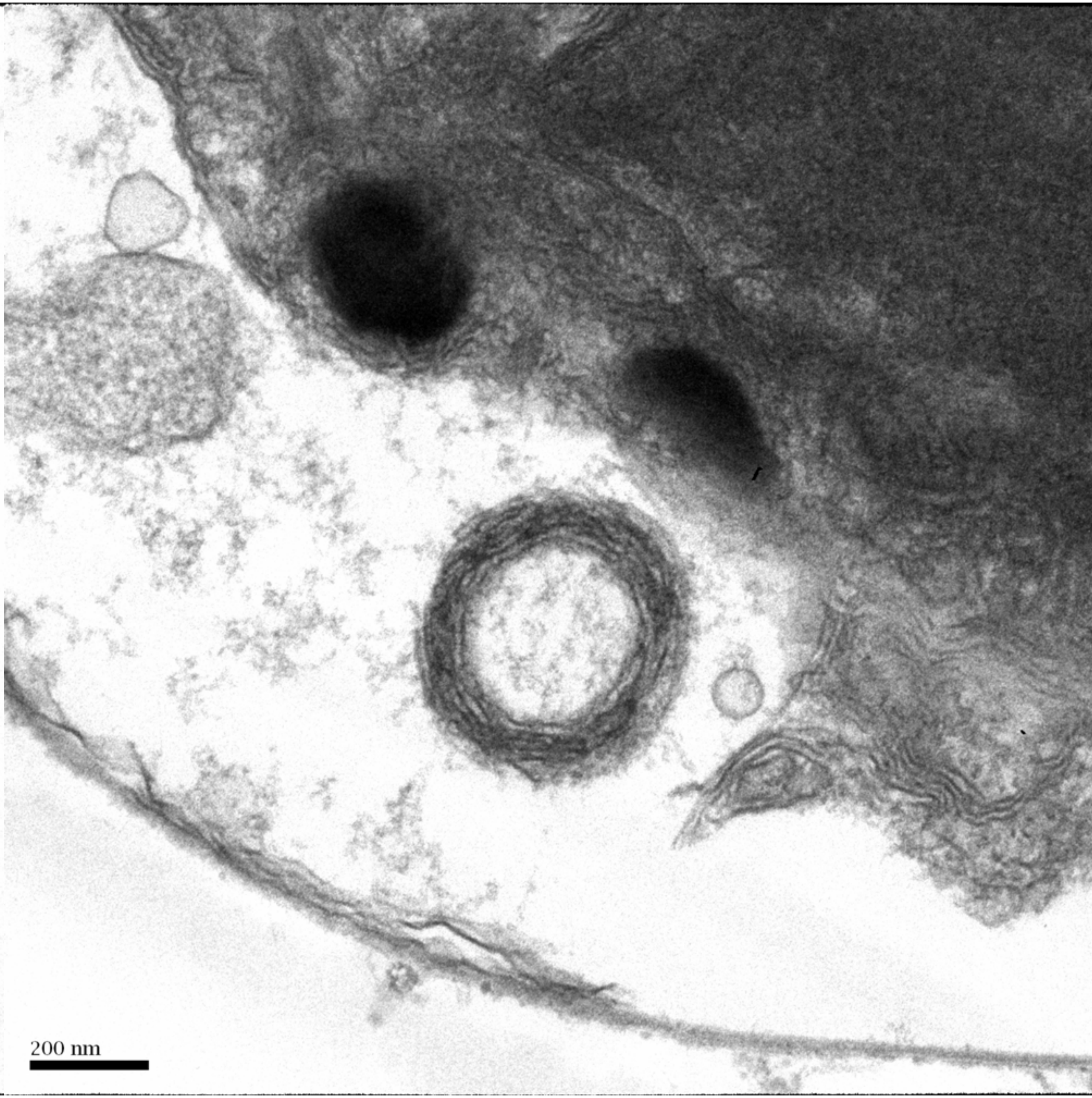

18-7_Correa_KC4_9A4_7

Cell 12

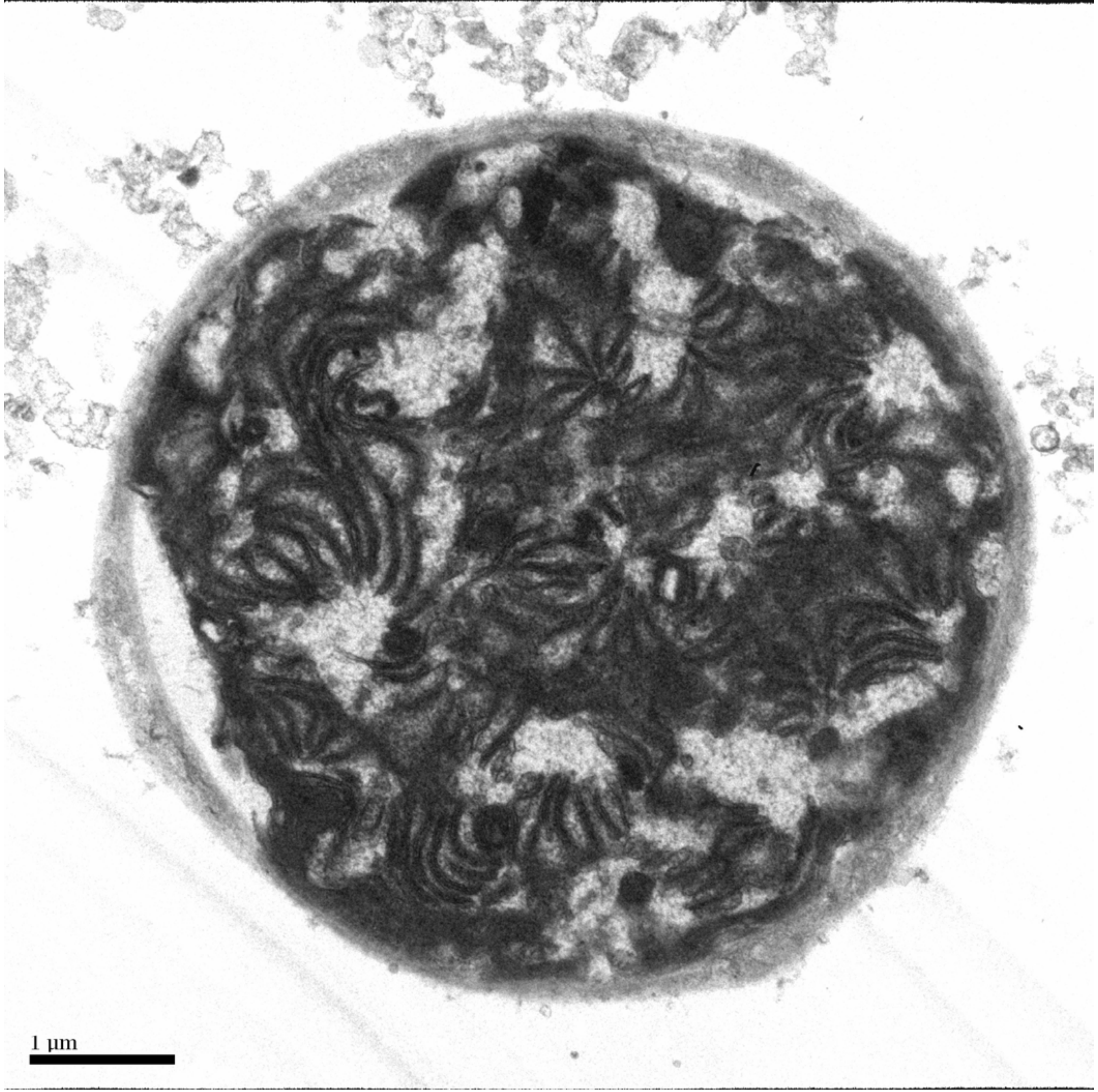

18-7_Correa_KC4_9A4_11

ACR Colony B Expelled- Heat

Cell 1

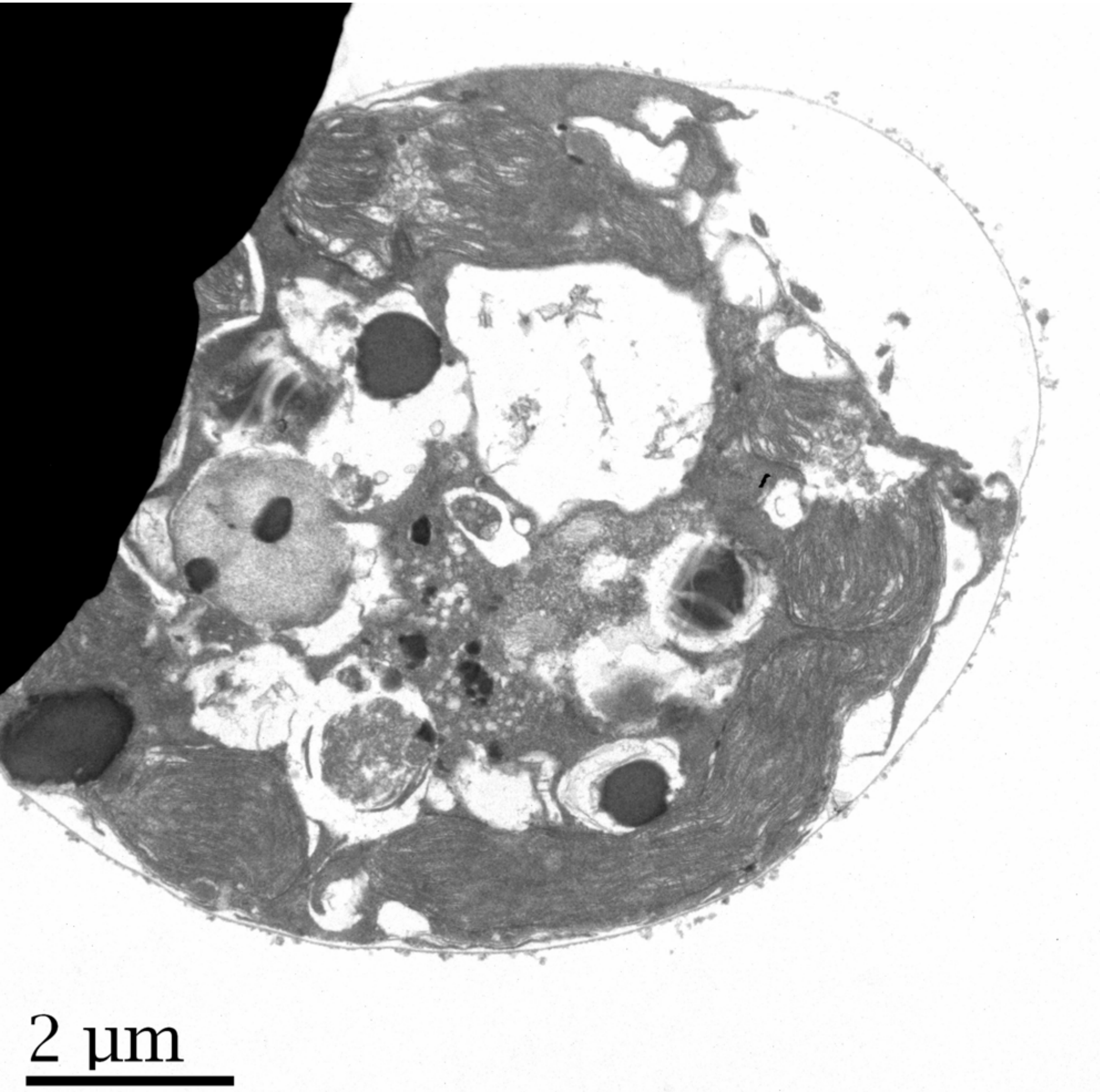

18-7_Correa_KH4_2GridE7_1

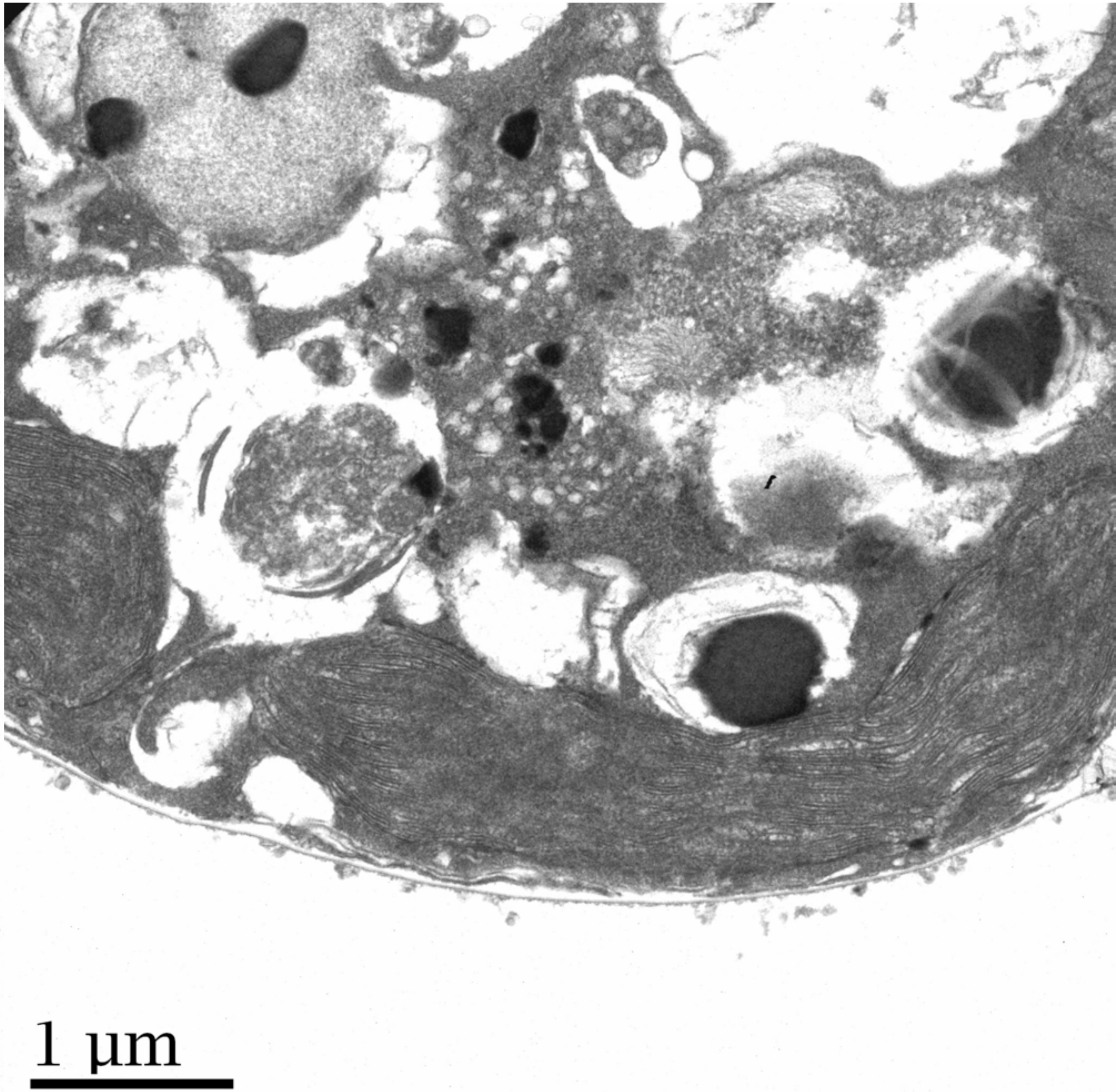

18-7_Correa_KH4_2GridE7_2

Cell 2

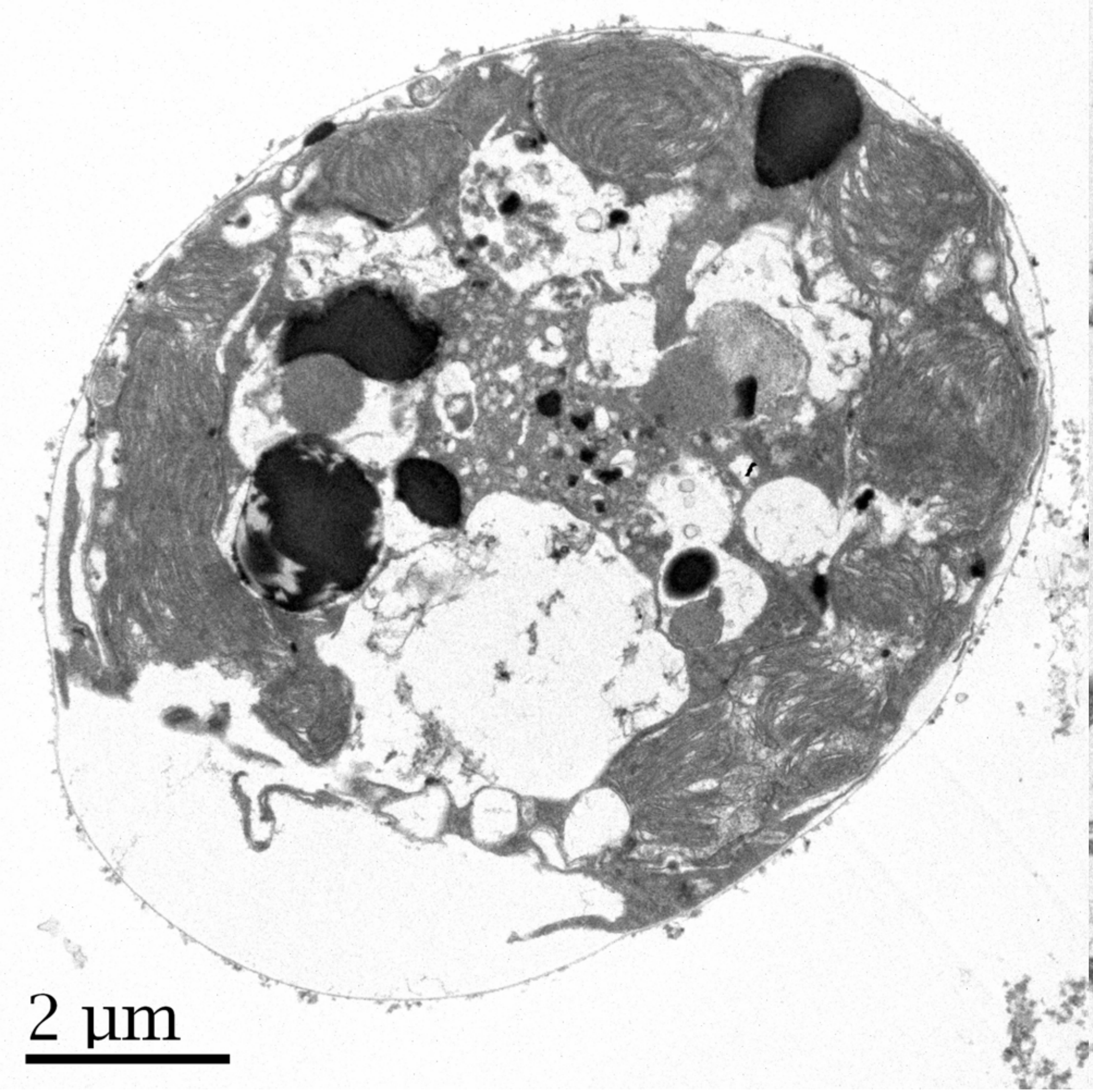

18-7_Correa_KH4_2GridE8_4

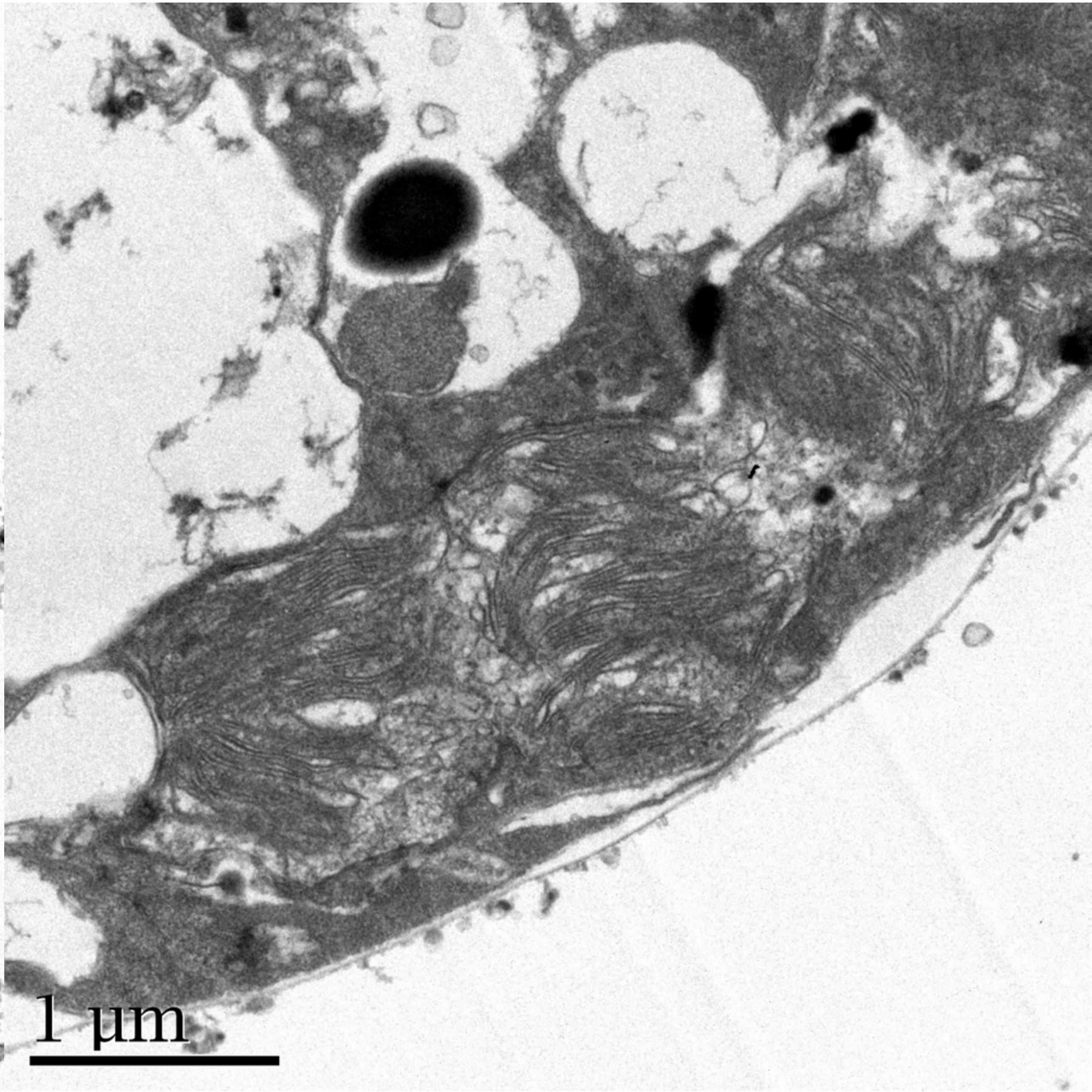

18-7_Correa_KH4_2GridE8_6

Cell 3

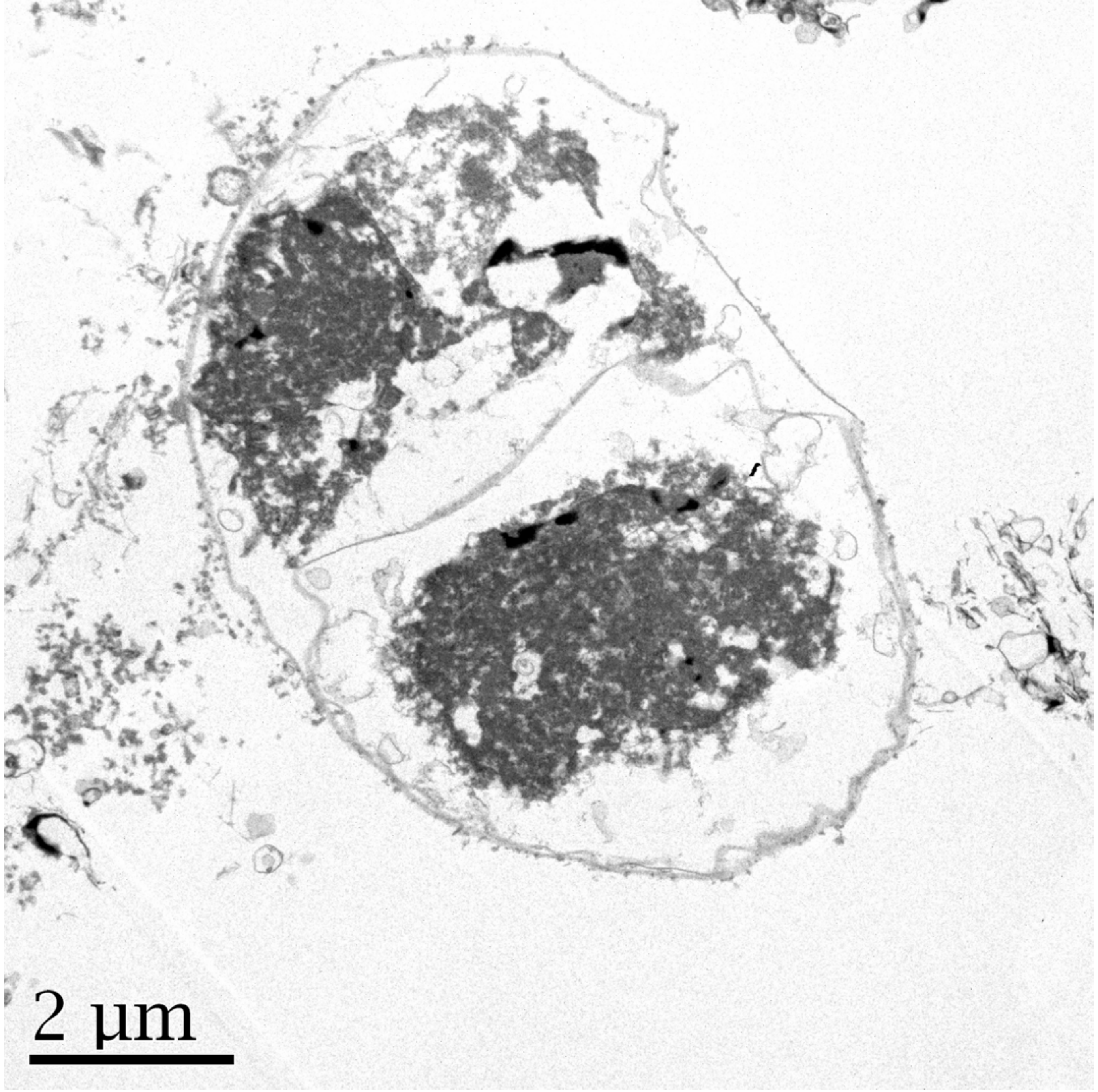

18-7_Correa_KH4_2GridE8_11

Cell 4

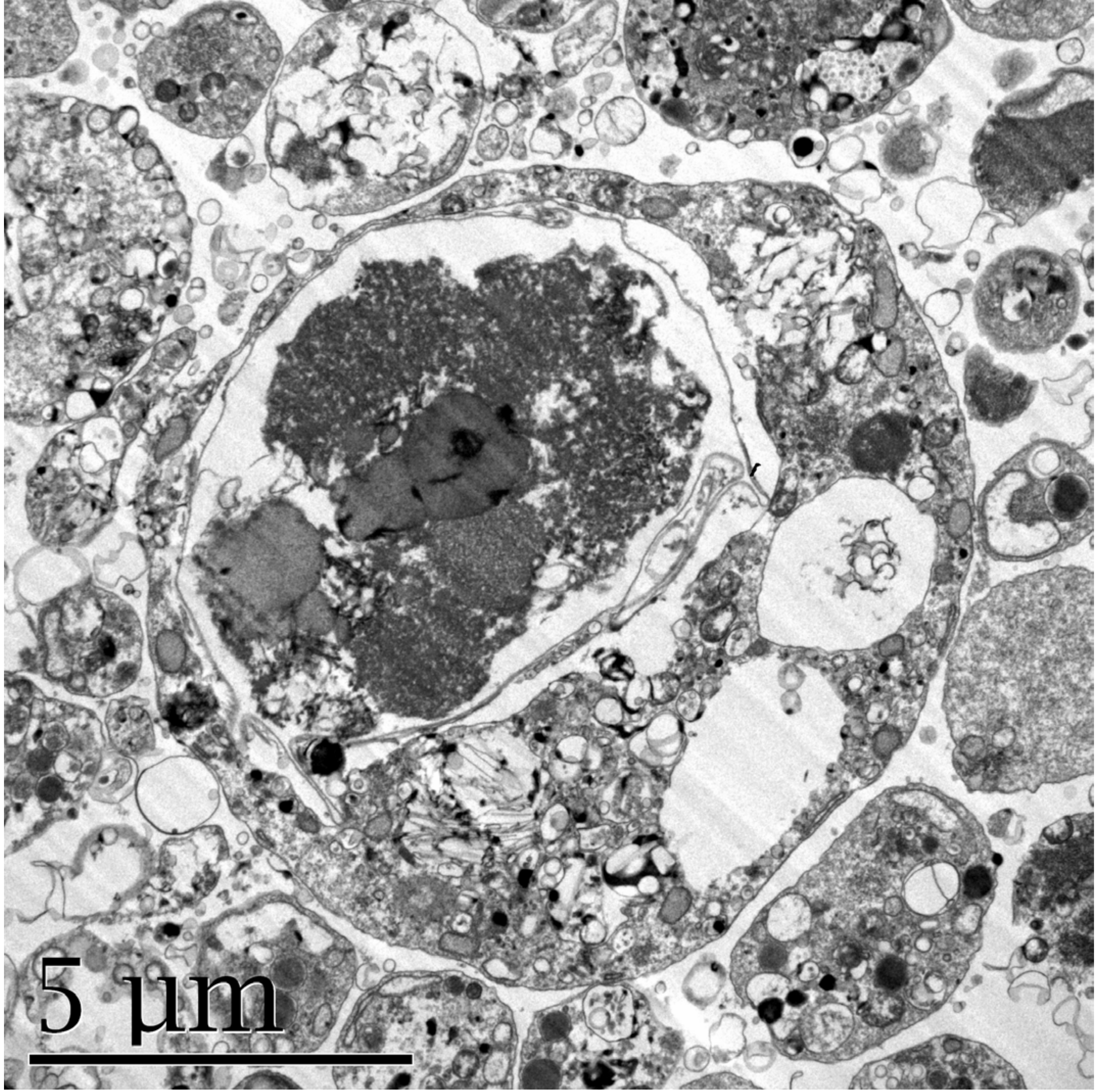

18-7_Correa_KH4_3GridA10_3

Cell 5

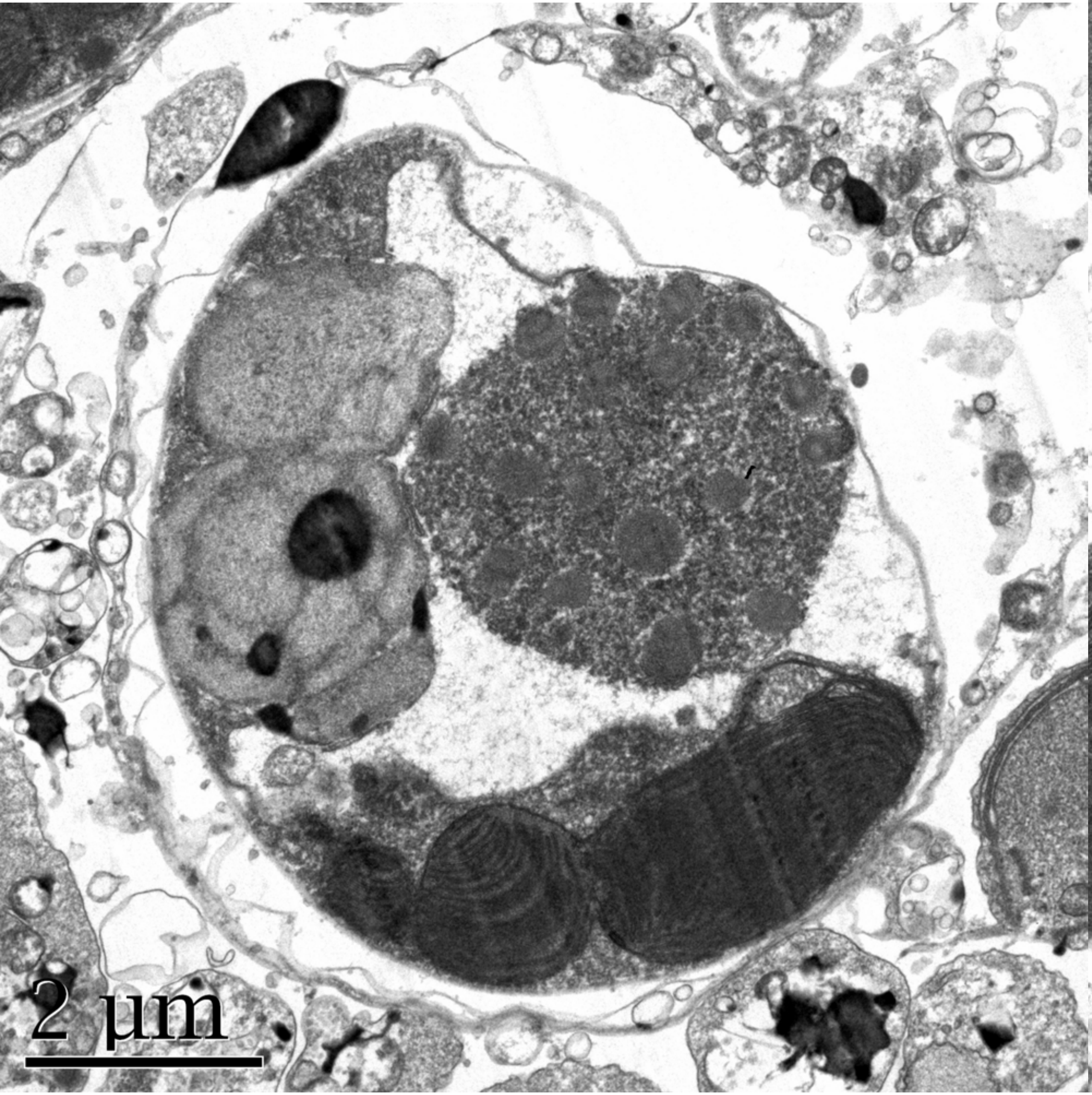

18-7_Correa_KH4_3GridA10_5

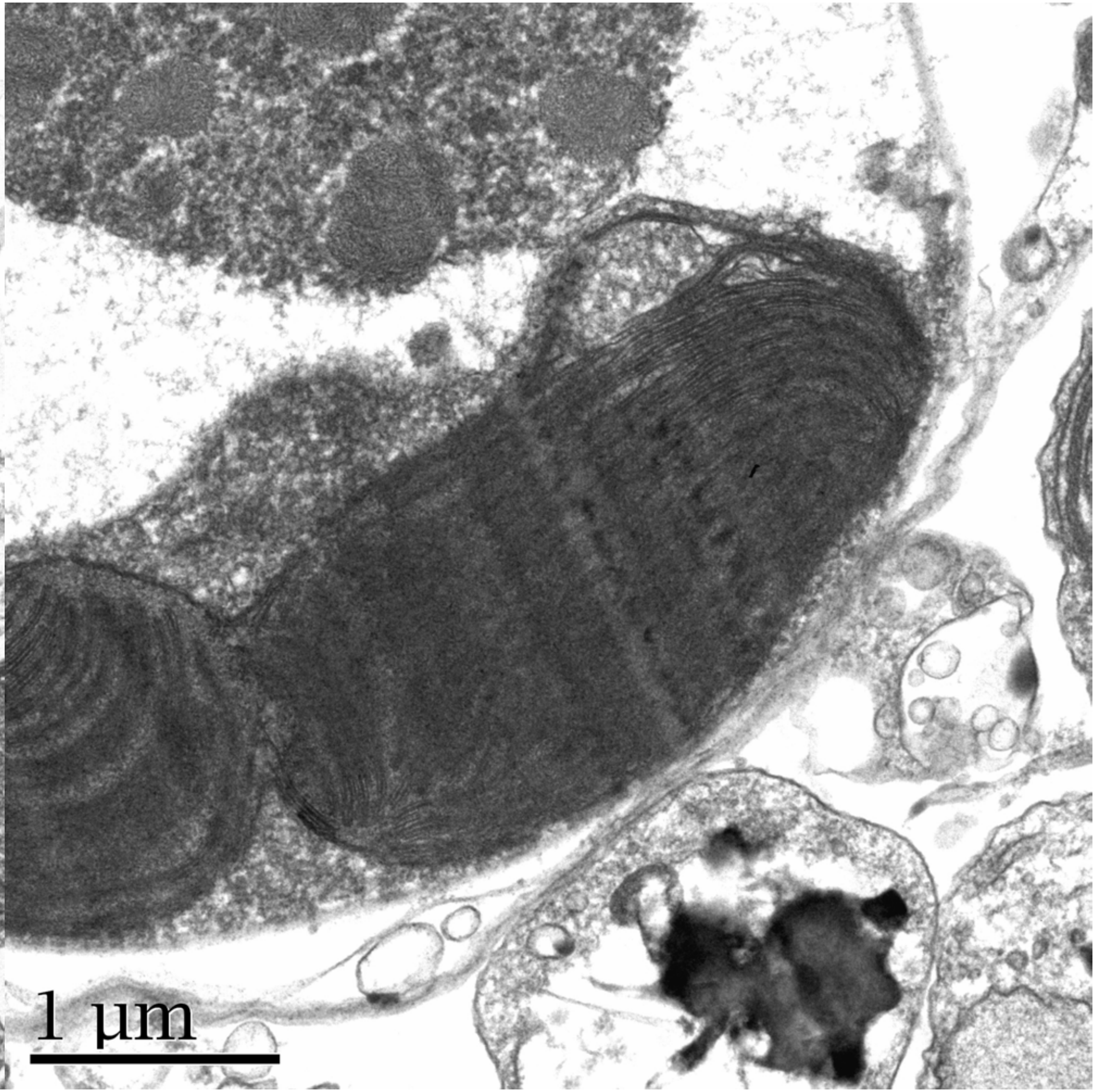

18-7_Correa_KH4_3GridA10_6

Cell 6

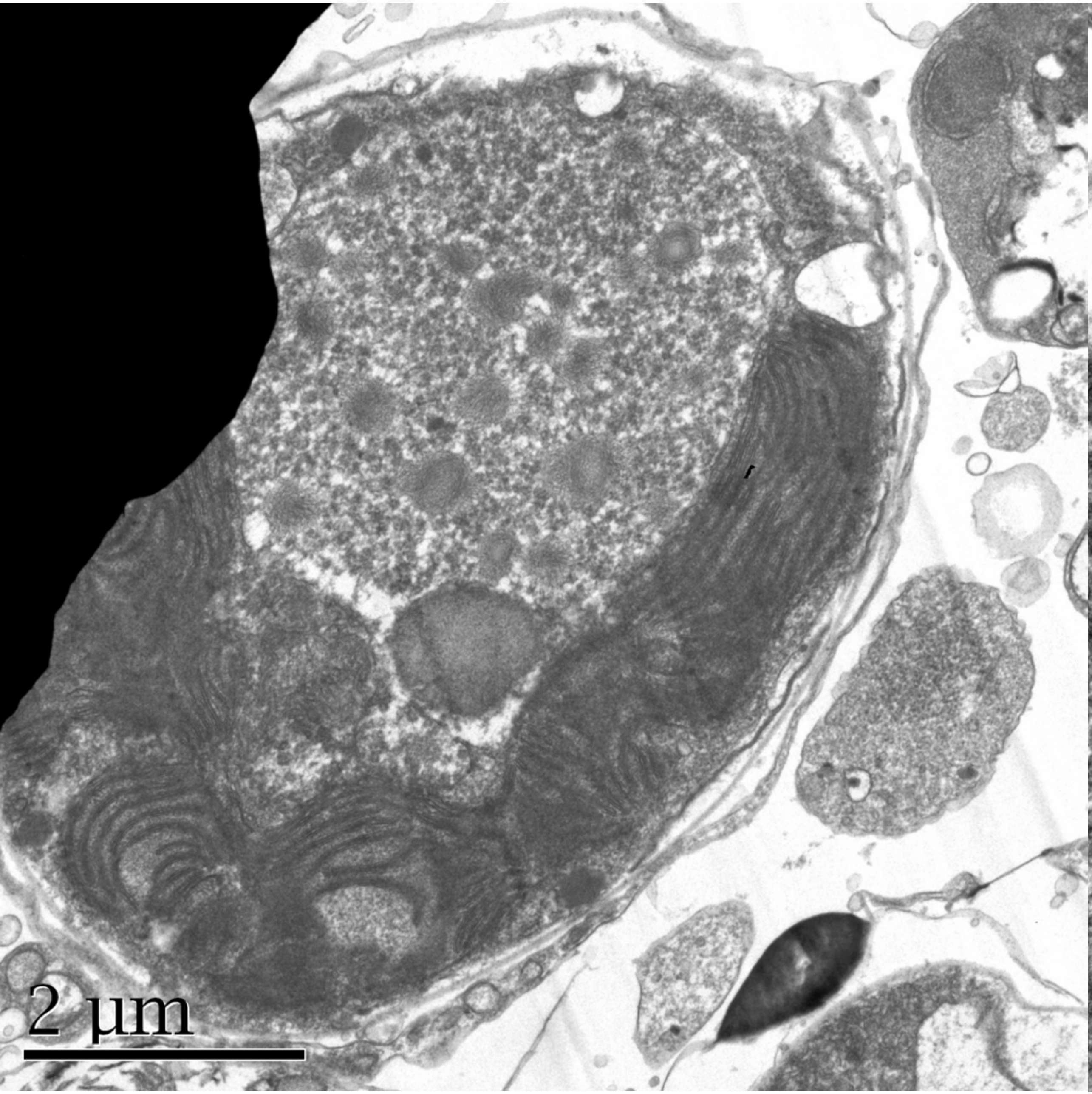

18-7_Correa_KH4_3GridA10_8

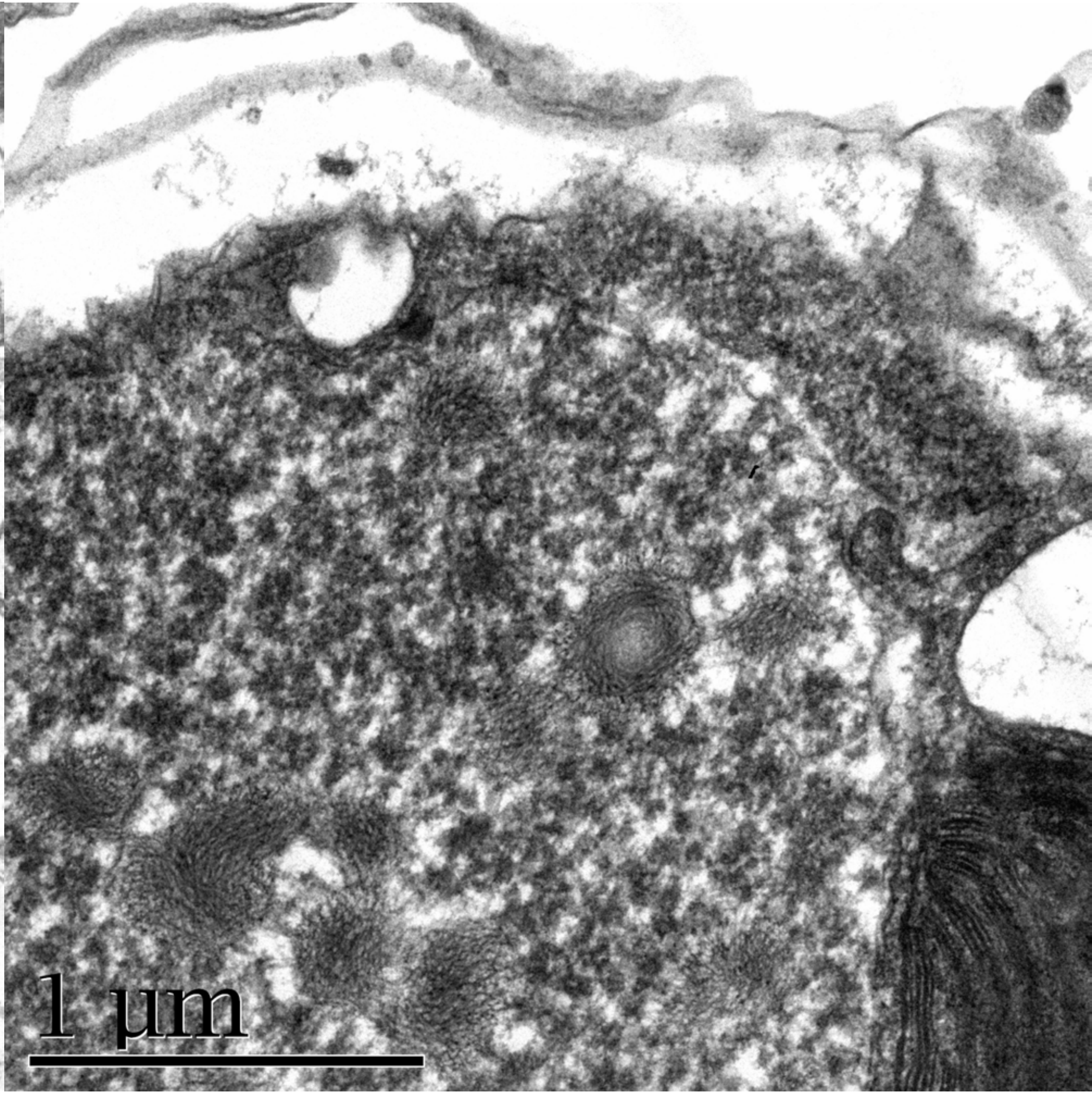

18-7_Correa_KH4_3GridA10_11

Cell 7

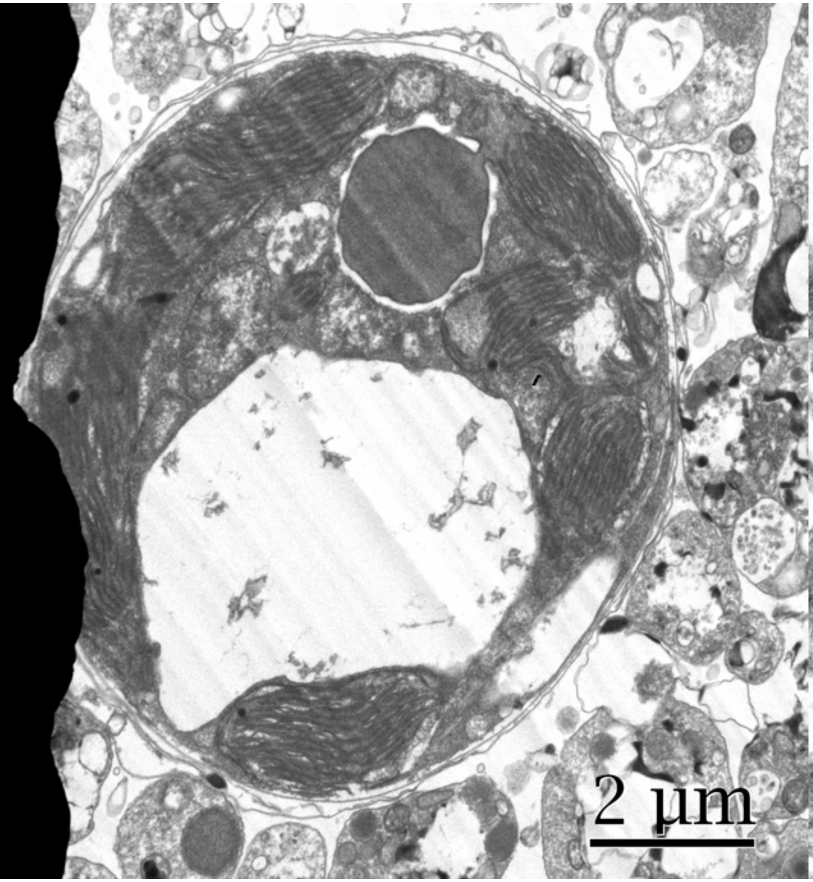

18-7_Correa_KH4_3GridA10_12

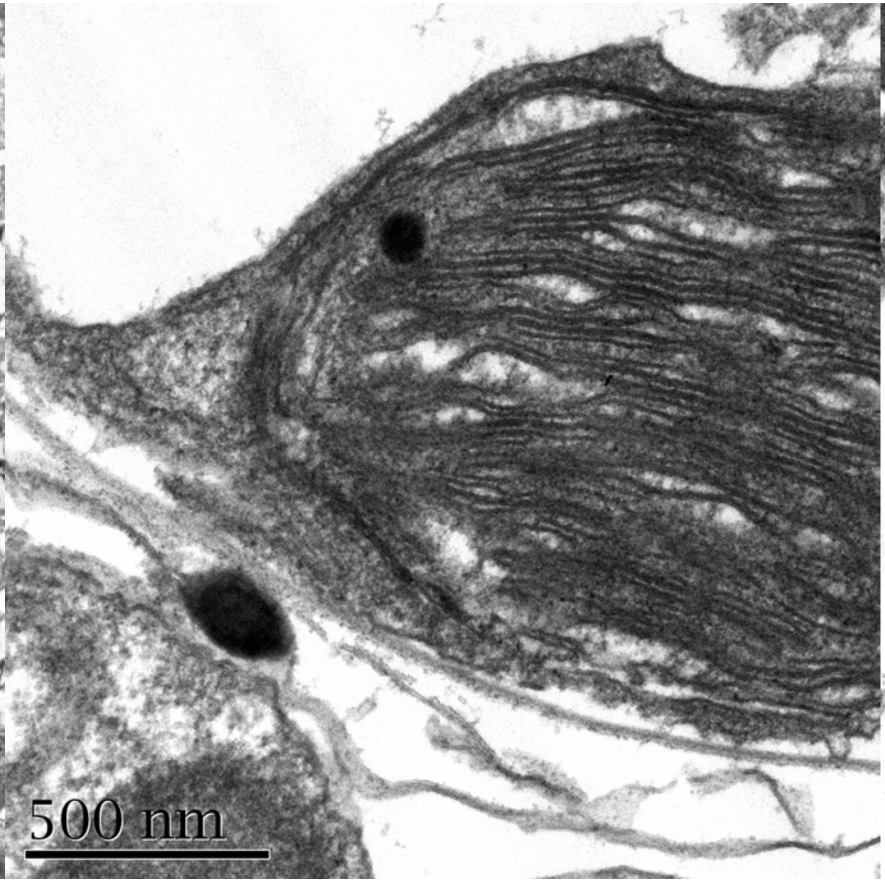

18-7_Correa_KH4_3GridA10_14

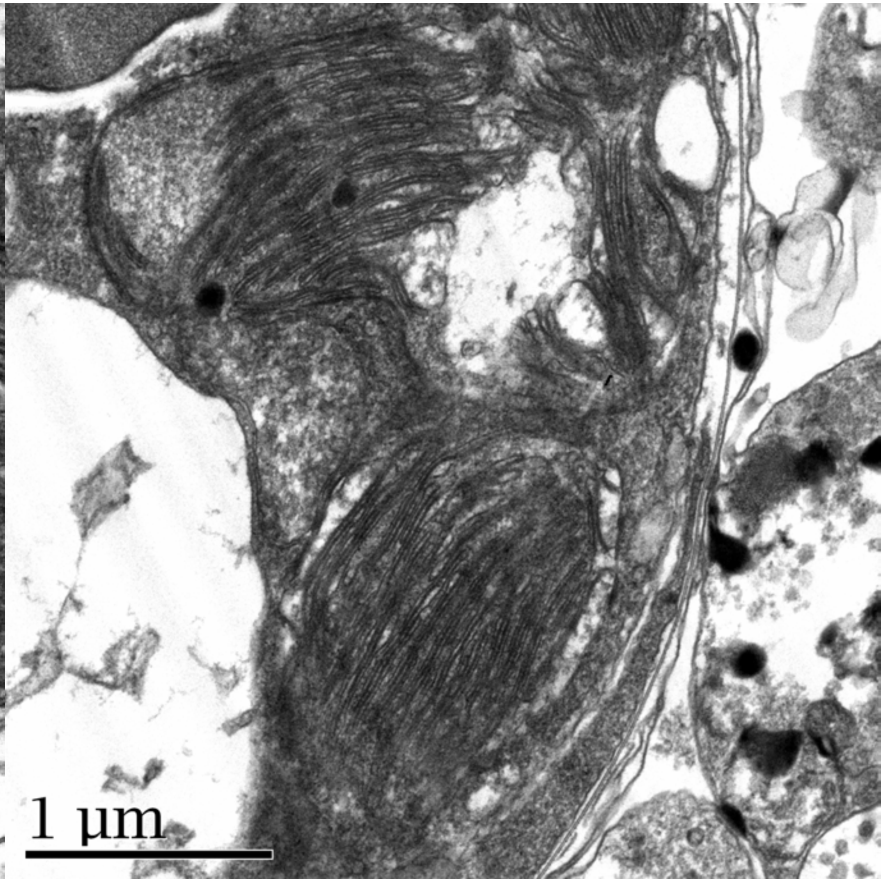

18-7_Correa_KH4_3GridA10_15

Cell 8

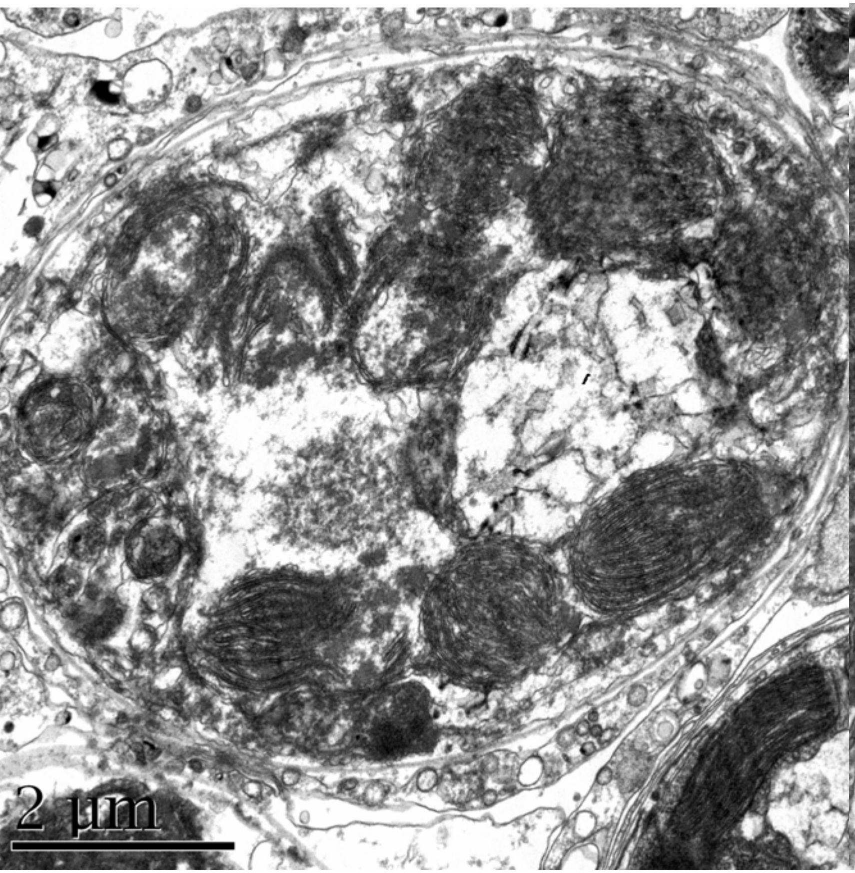

18-7_Correa_KH4_3GridA10_18

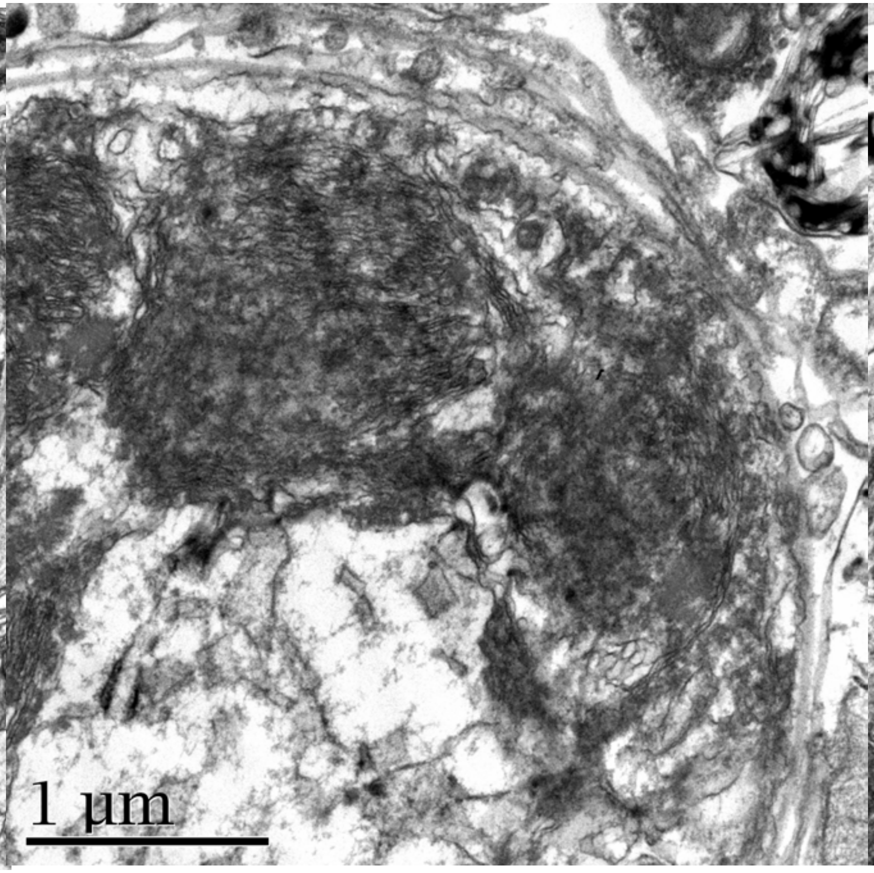

18-7_Correa_KH4_3GridA10_21

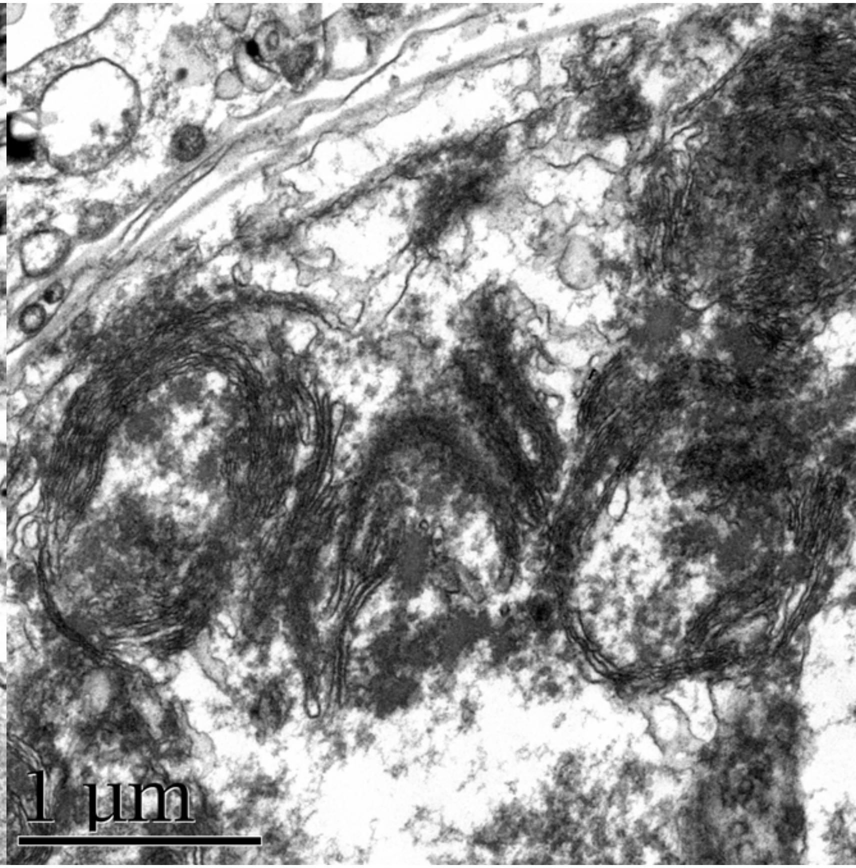

18-7_Correa_KH4_3GridA10_22

Cell 9

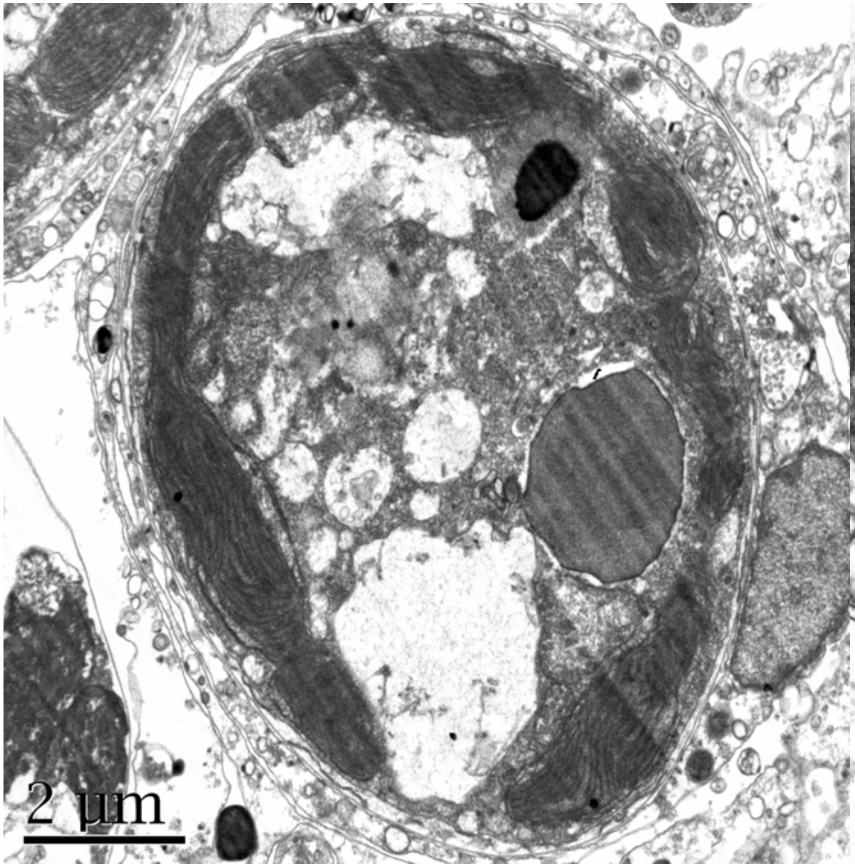

18-7_Correa_KH4_3GridA10_23

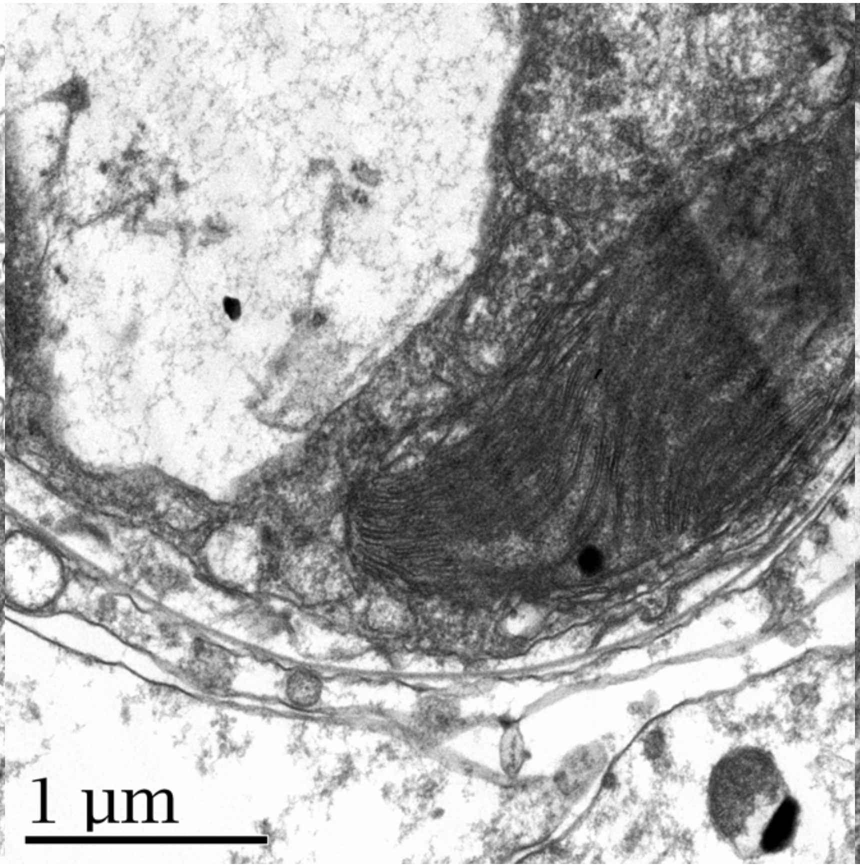

18-7_Correa_KH4_3GridA10_24

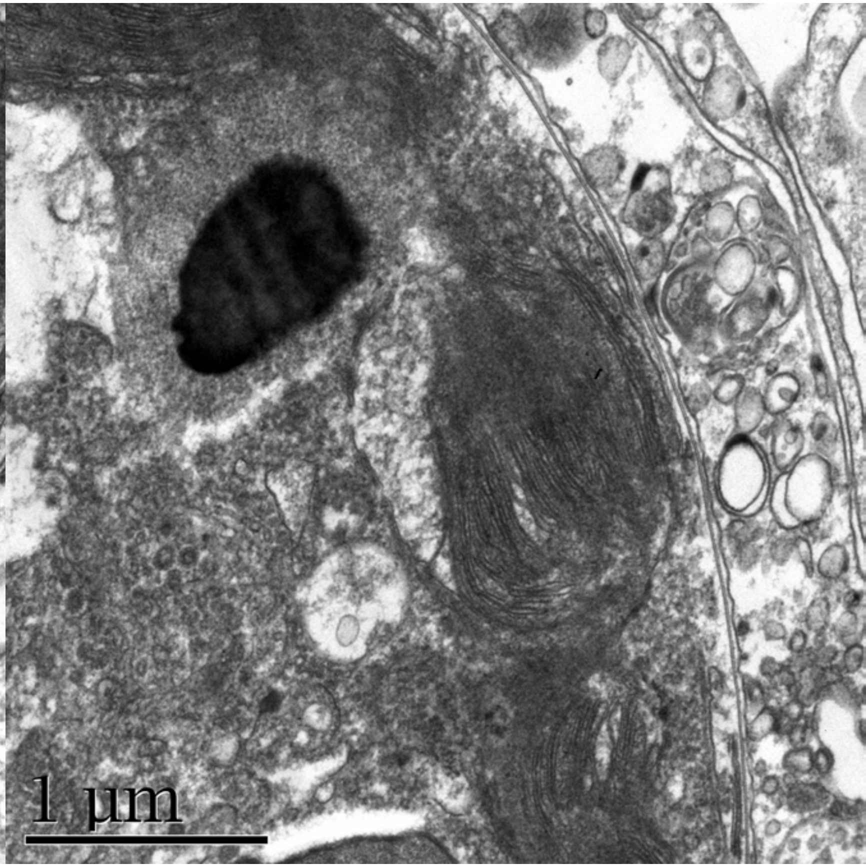

18-7_Correa_KH4_3GridA10_26

Cell 10

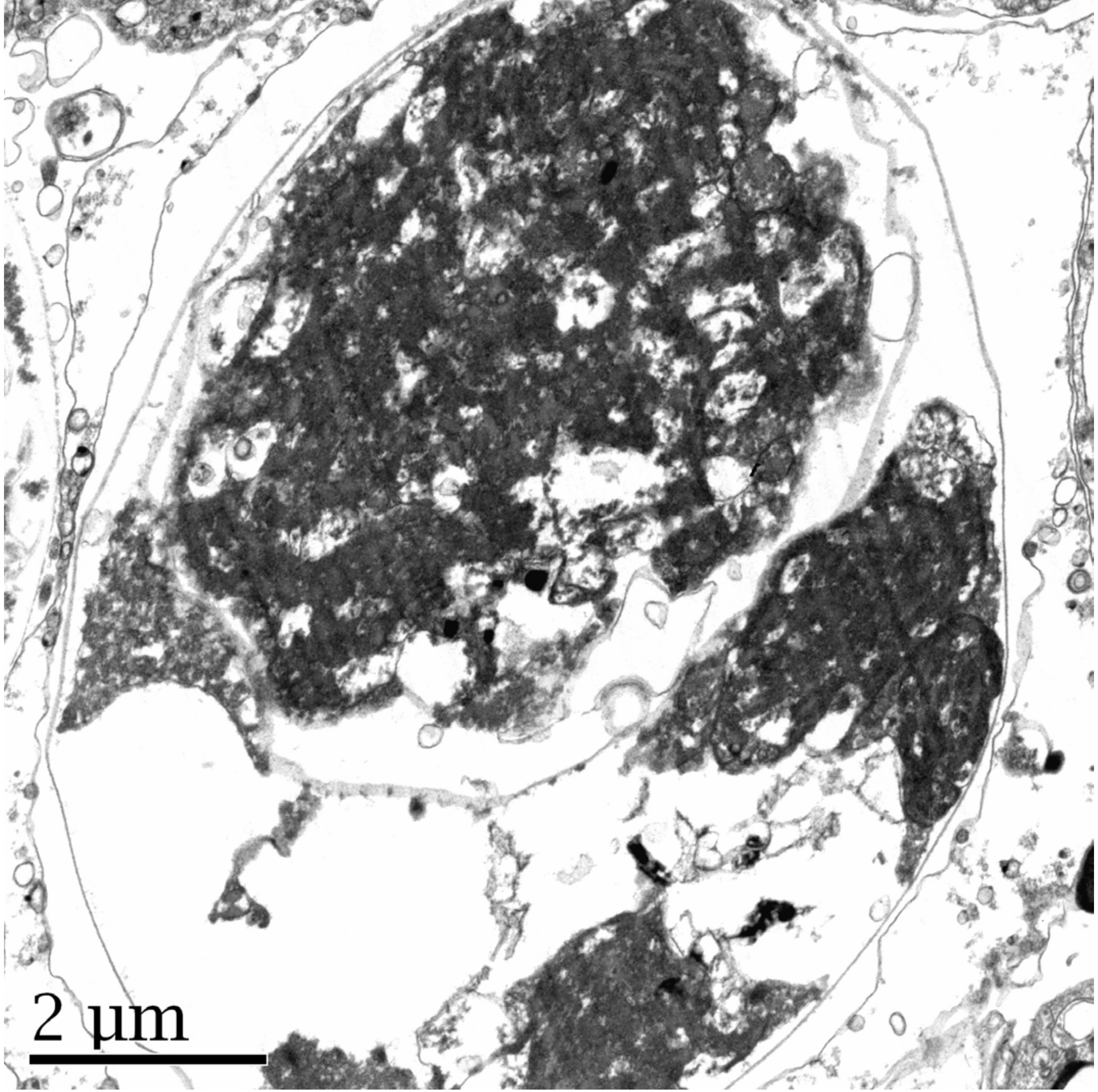

18-7_Correa_KH4_3GridA10_27

Cell 11

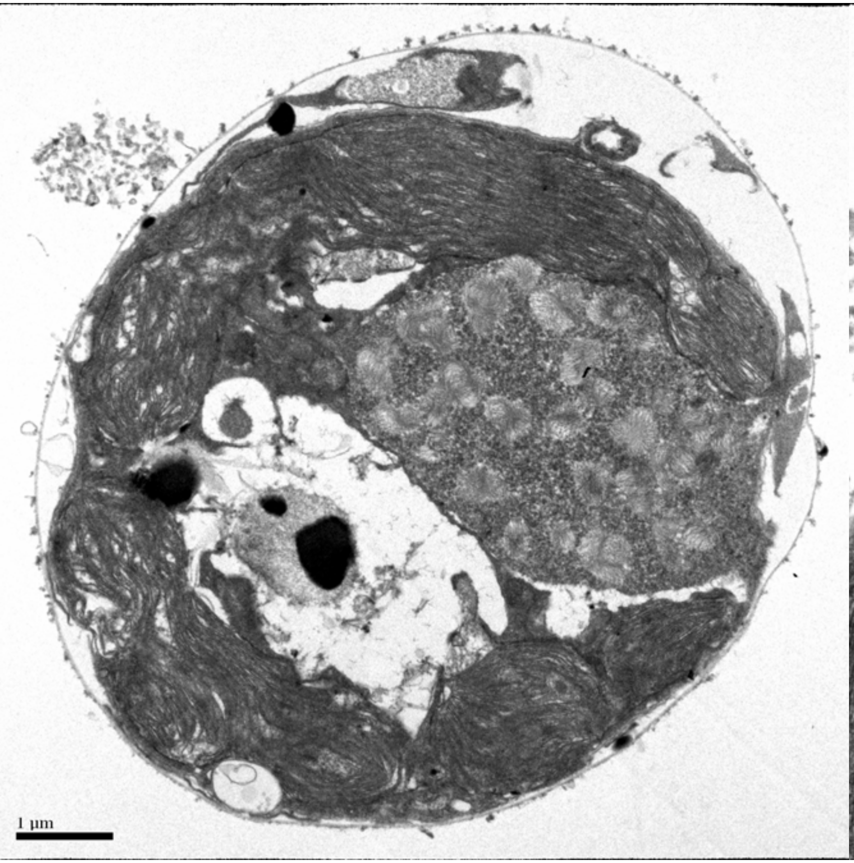

18-7_Correa_KH4_2E9_2

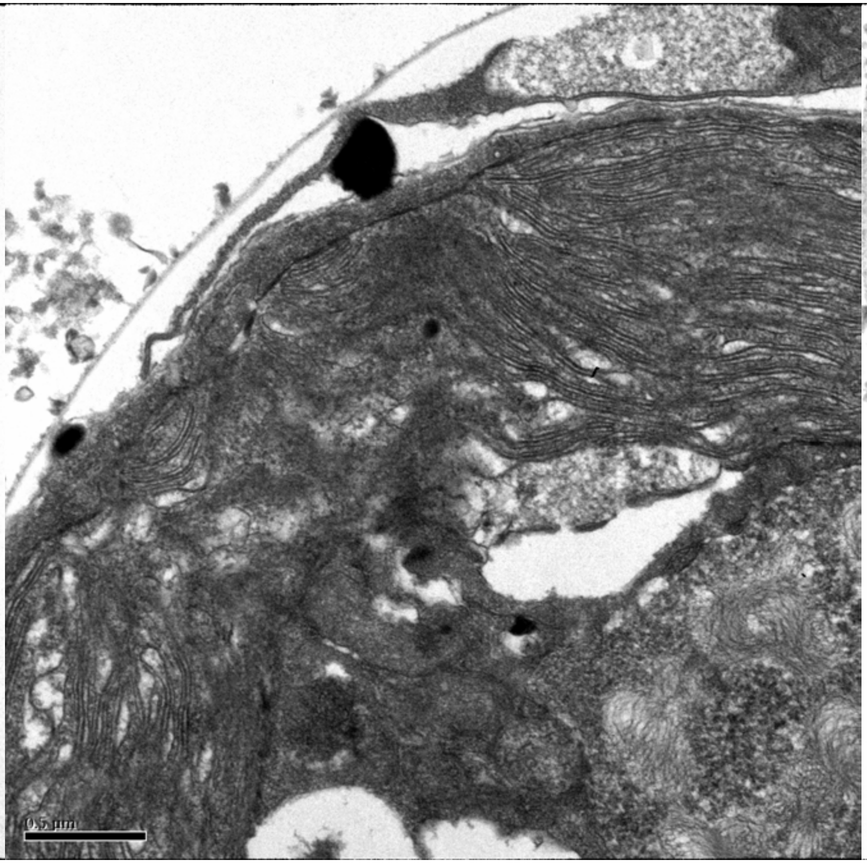

18-7_Correa_KH4_2E9_4

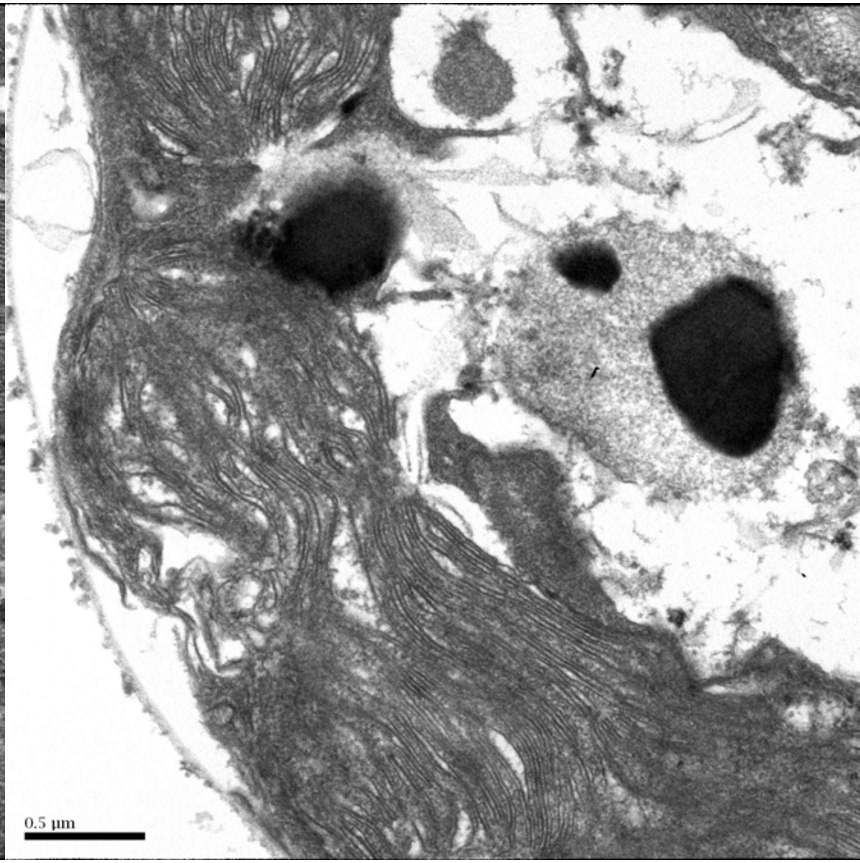

18-7_Correa_KH4_2E9_3

Cell 12

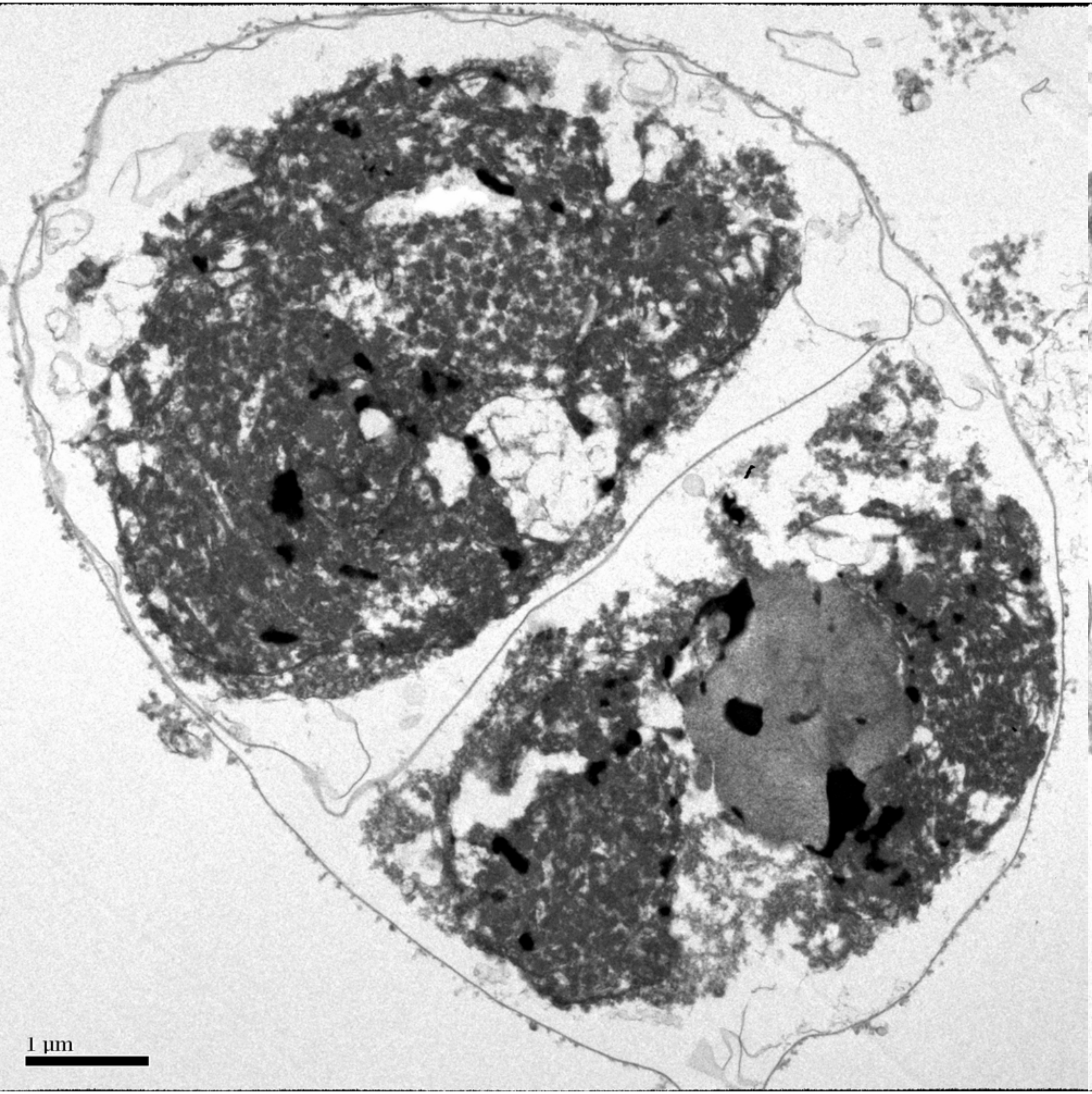

18-7_Correa_KH4_2E9_5

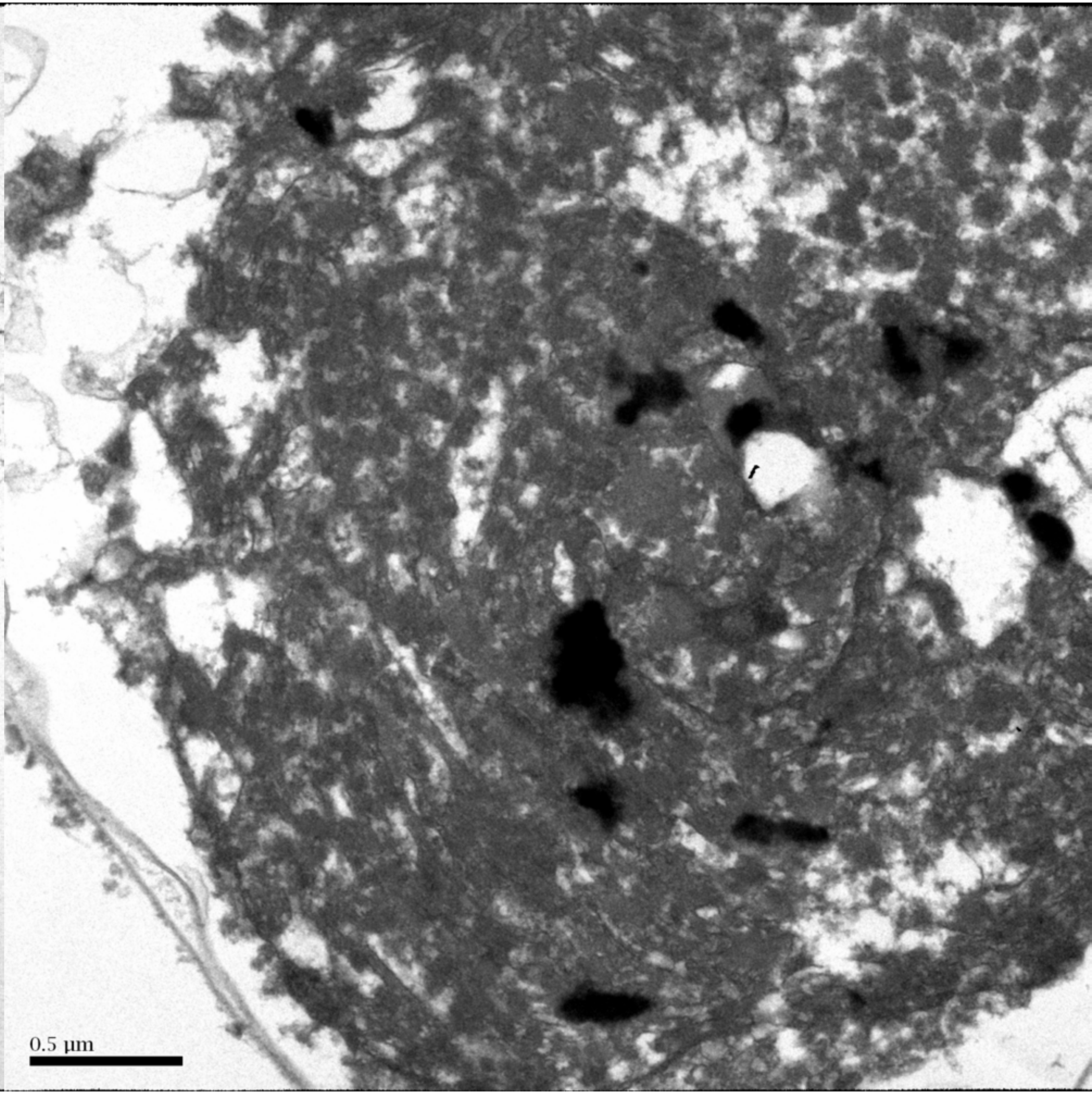

18-7_Correa_KH4_2E9_8

Cell 13

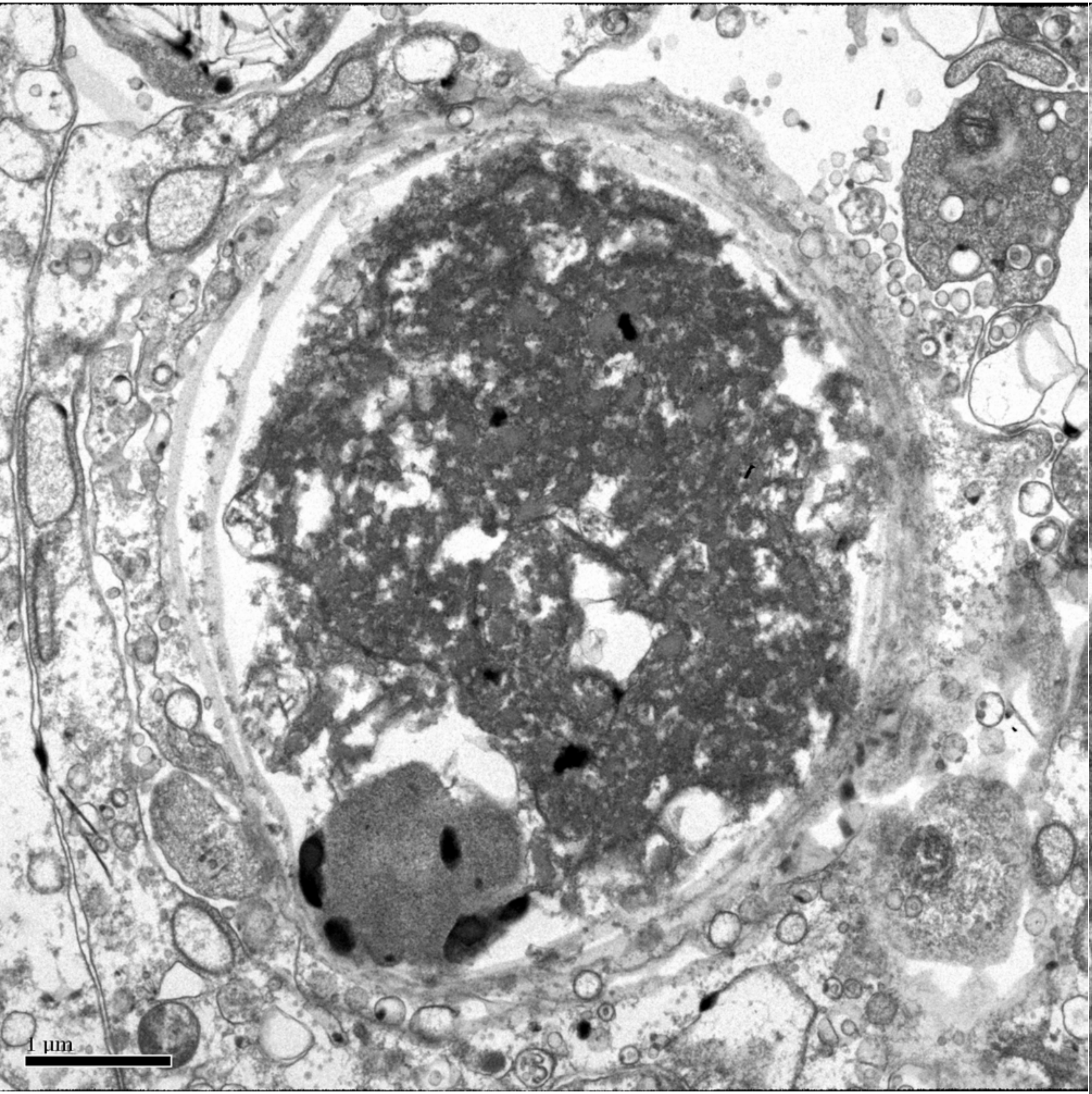

18-7_Correa_KH4_3B6_1

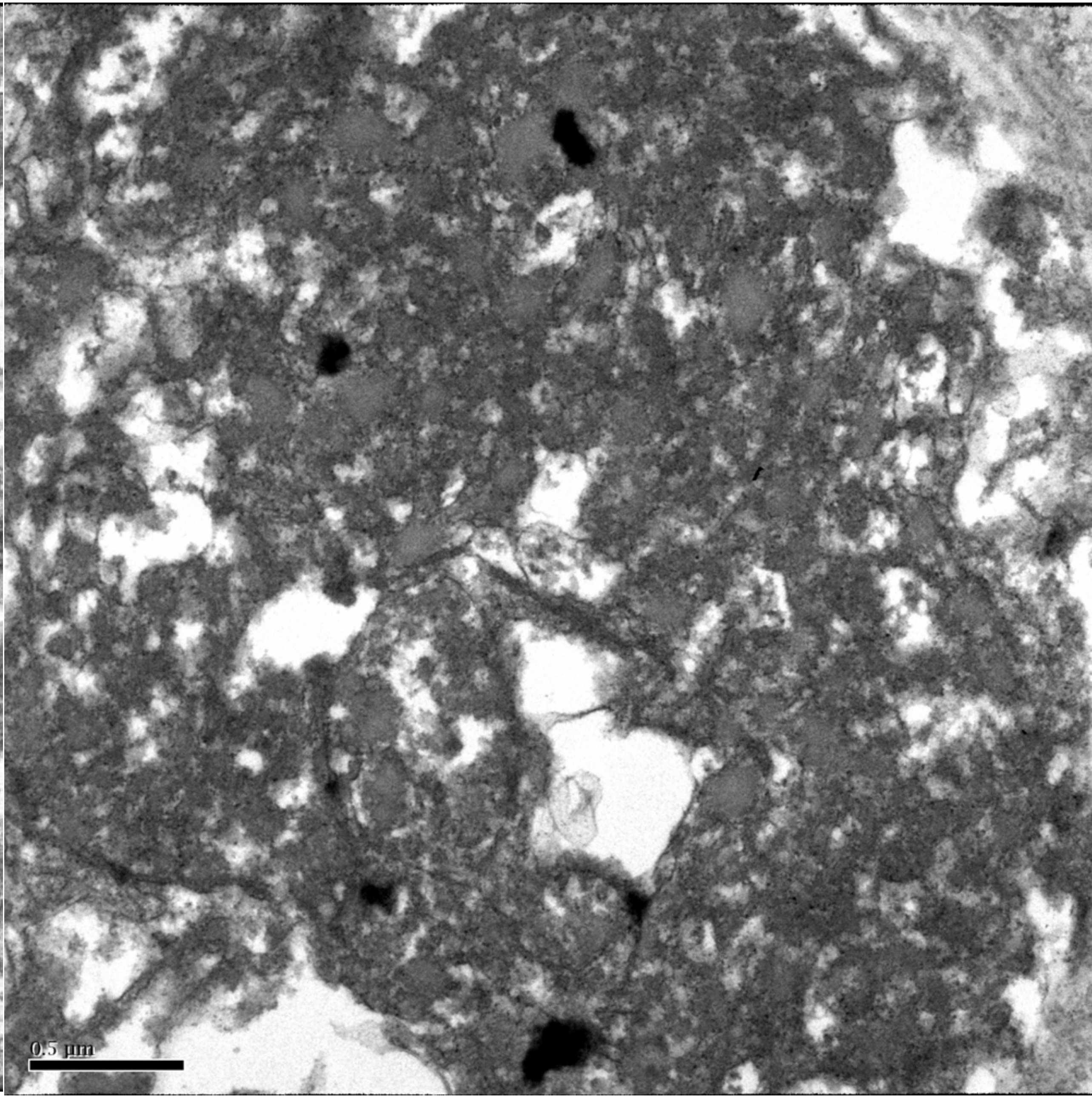

18-7_Correa_KH4_3B6_3

Cell 14

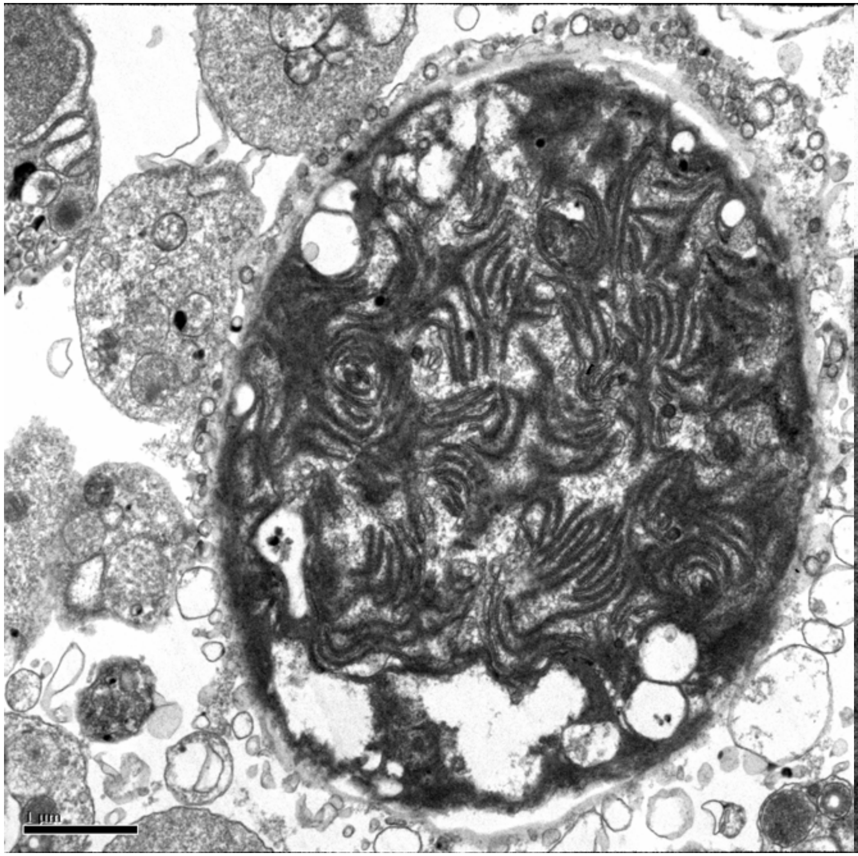

18-7_Correa_KH4_3B6_4

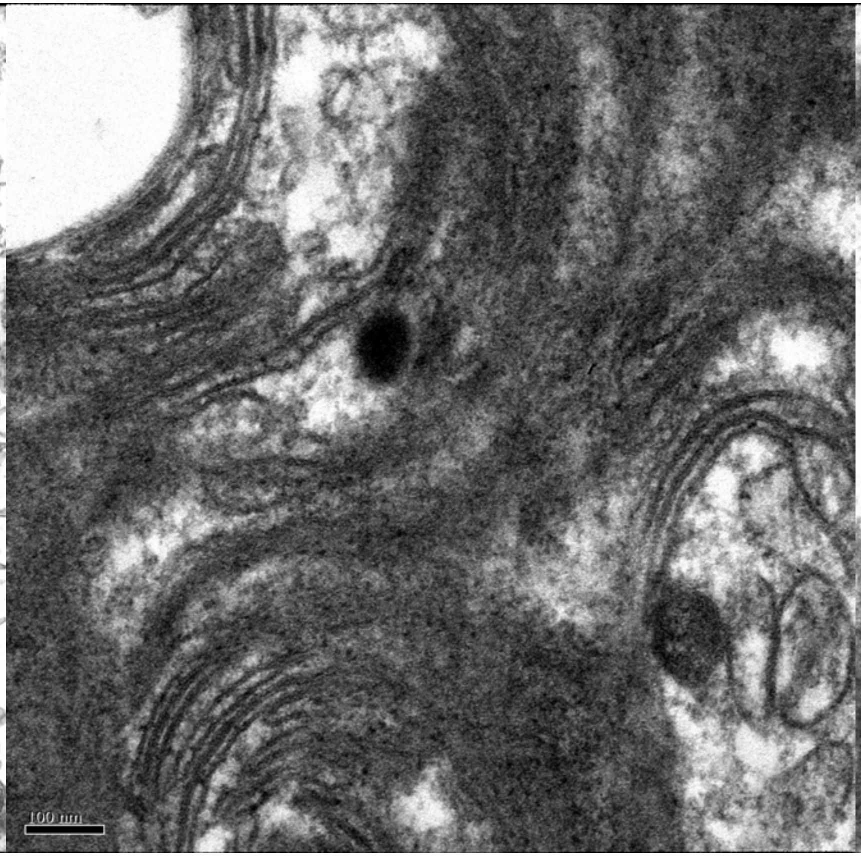

18-7_Correa_KH4_3B6_6

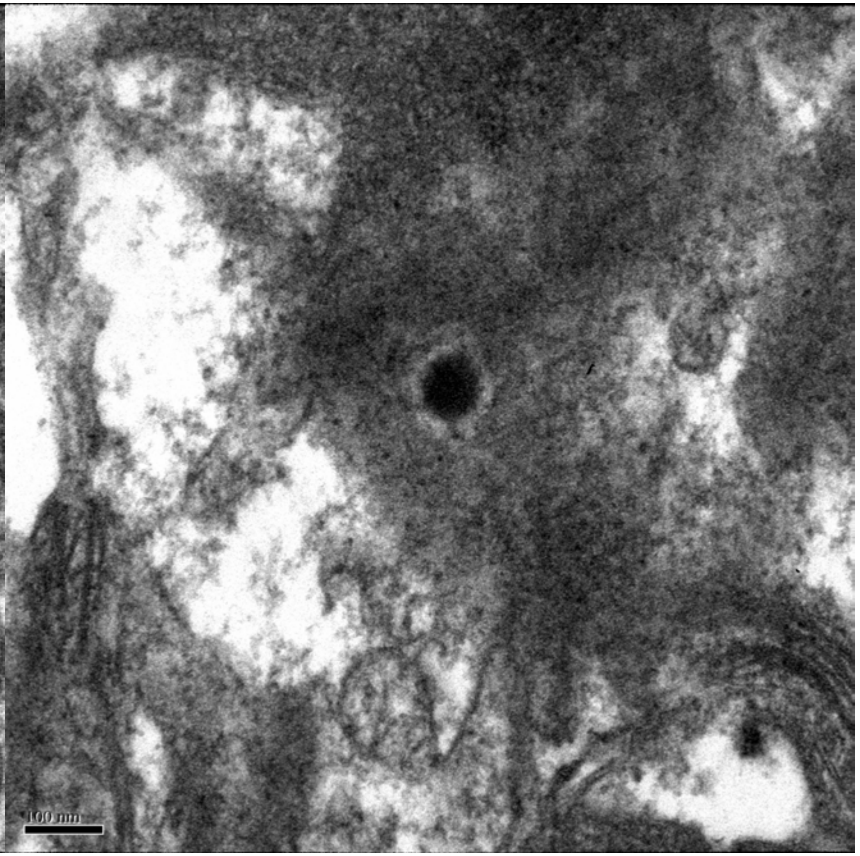

18-7_Correa_KH4_3B6_7

Cell 15

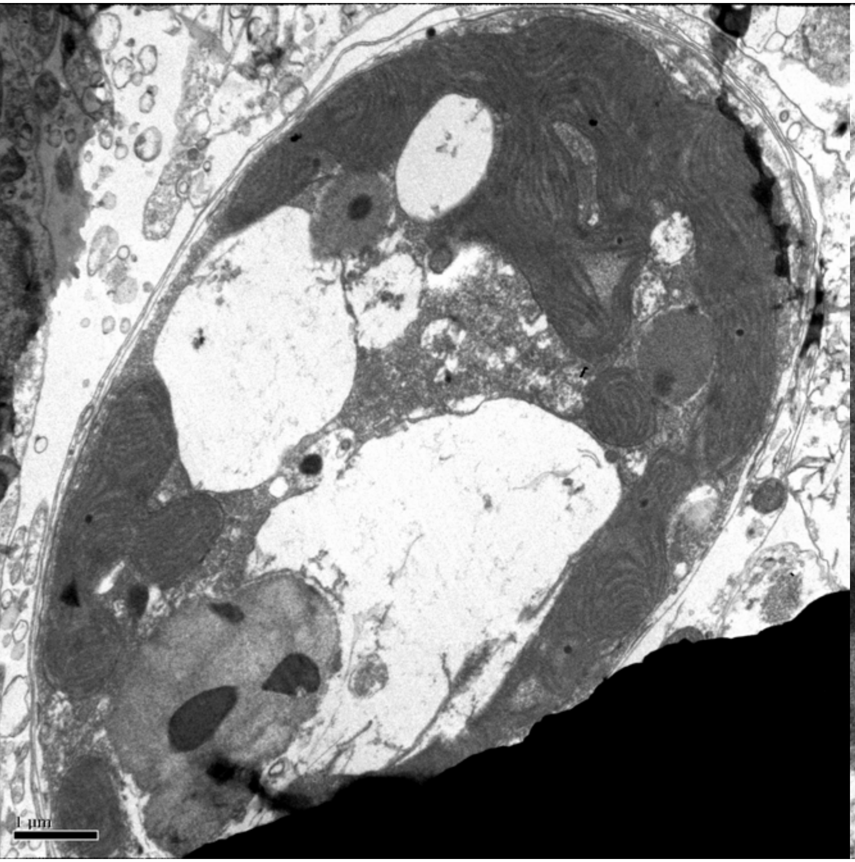

18-7_Correa_KH4_3B6_8

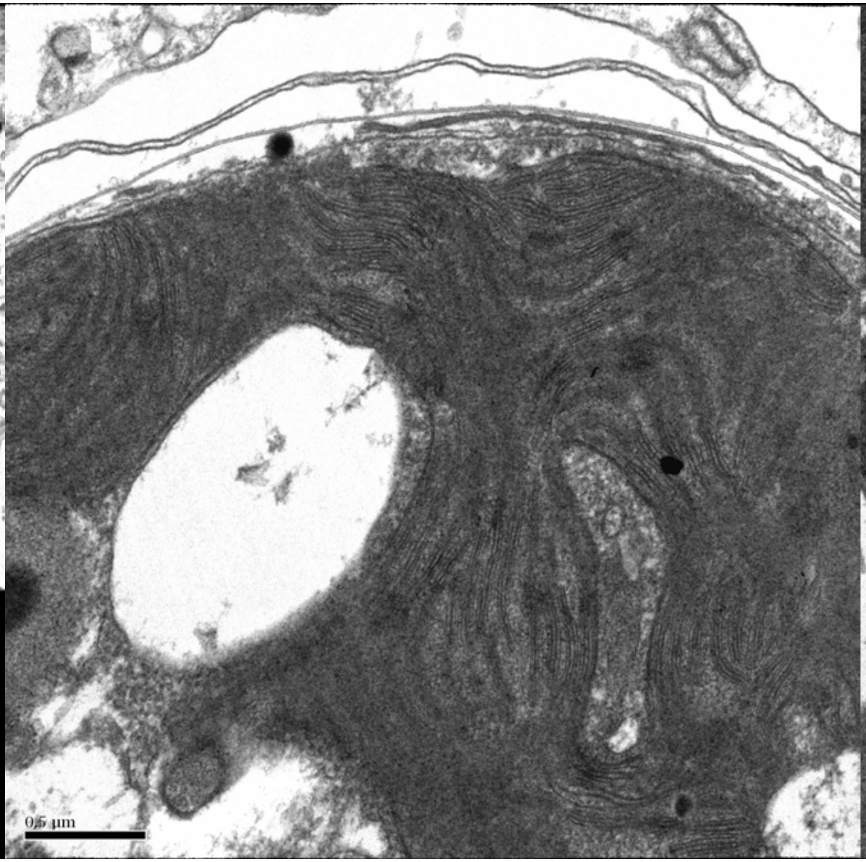

18-7_Correa_KH4_3B6_11

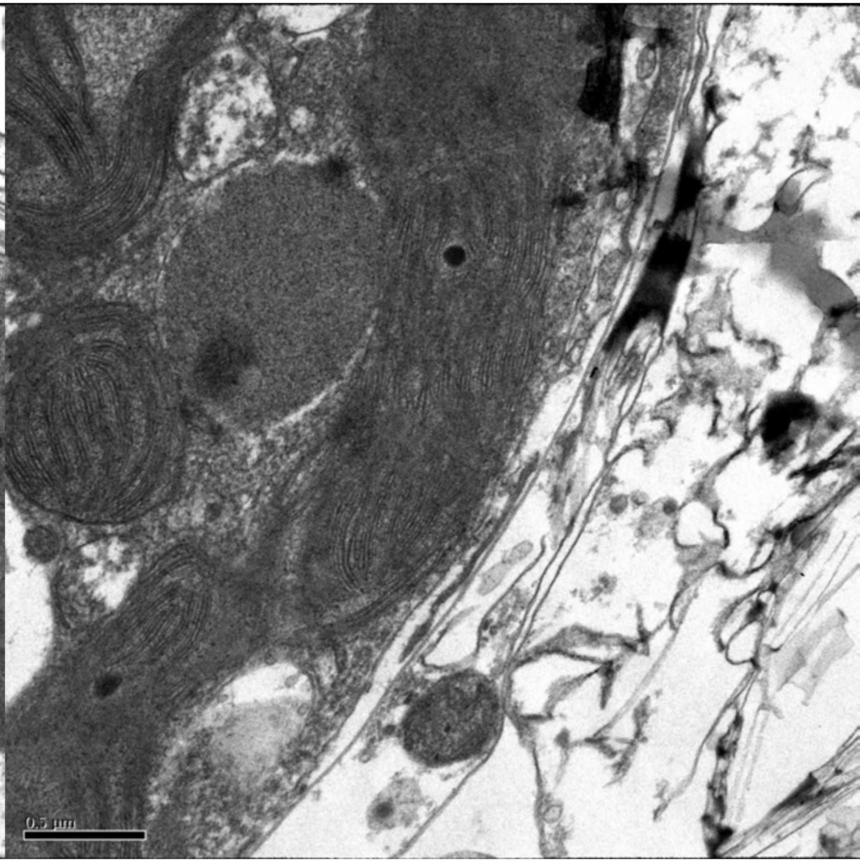

18-7_Correa_KH4_3B6_9

ACR Colony C Expelled- Ambient

Cell 1

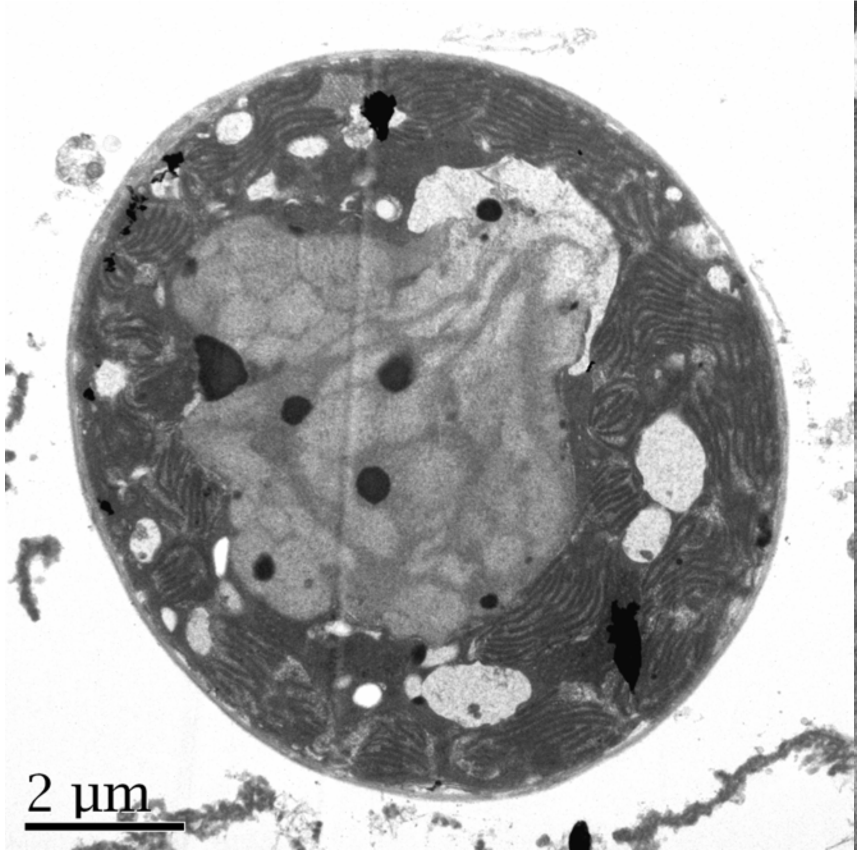

18-7_Correa_XC4_3GridB3_2

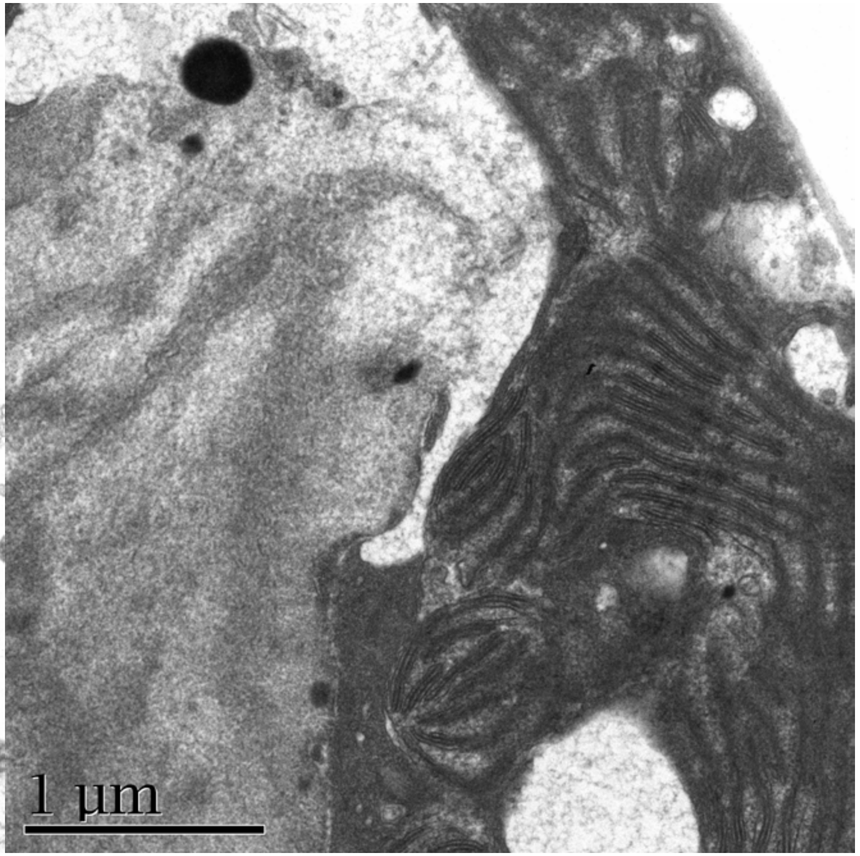

18-7_Correa_XC4_3GridB3_10

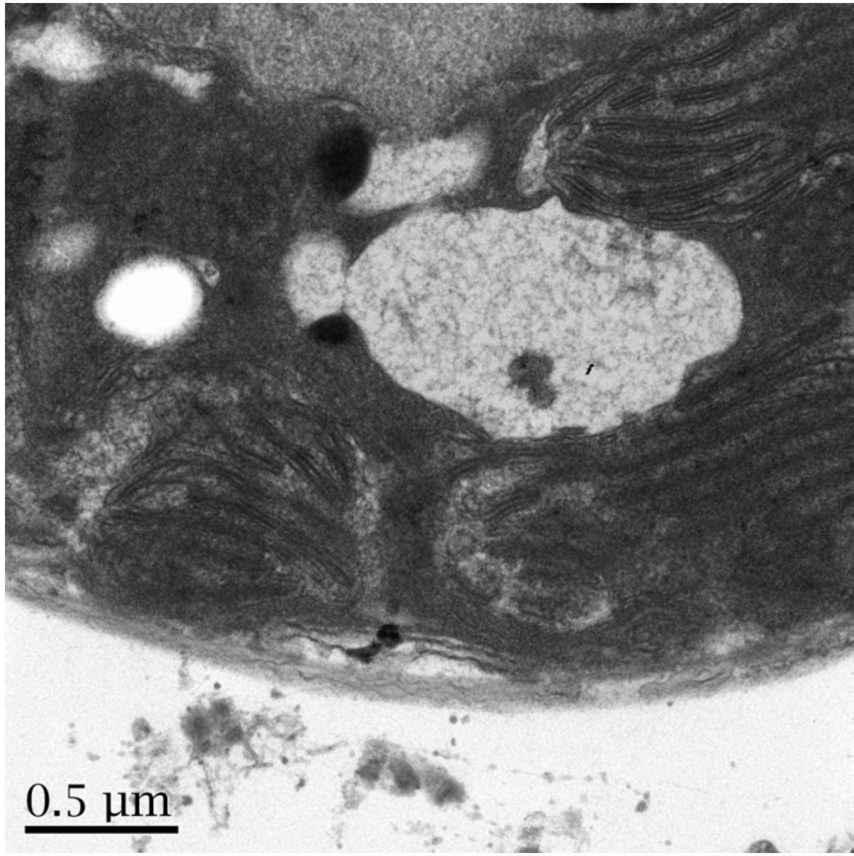

18-7_Correa_XC4_3GridB3_3

Cell 2

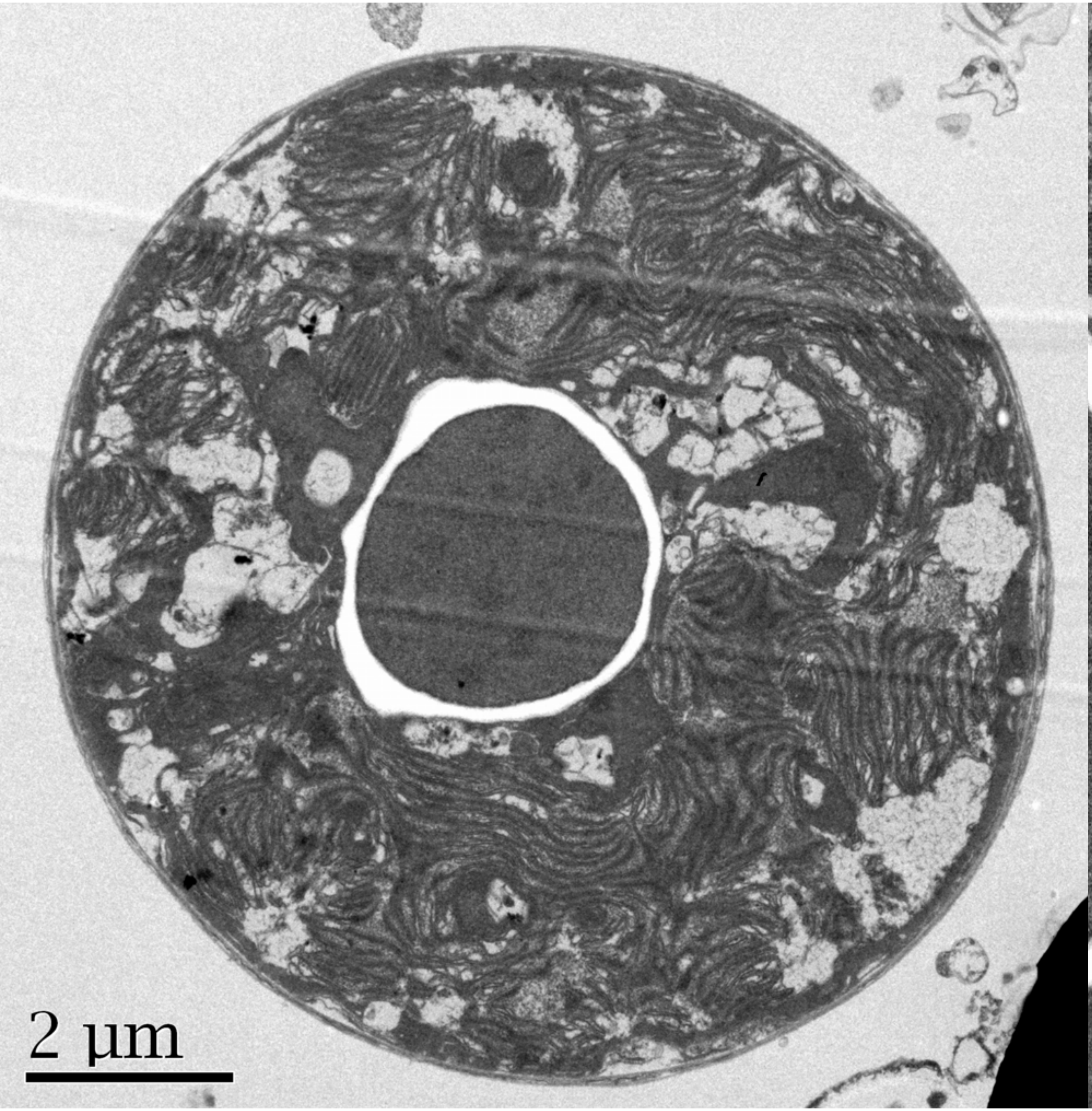

18-7_Correa_XC4_3GridB7_1

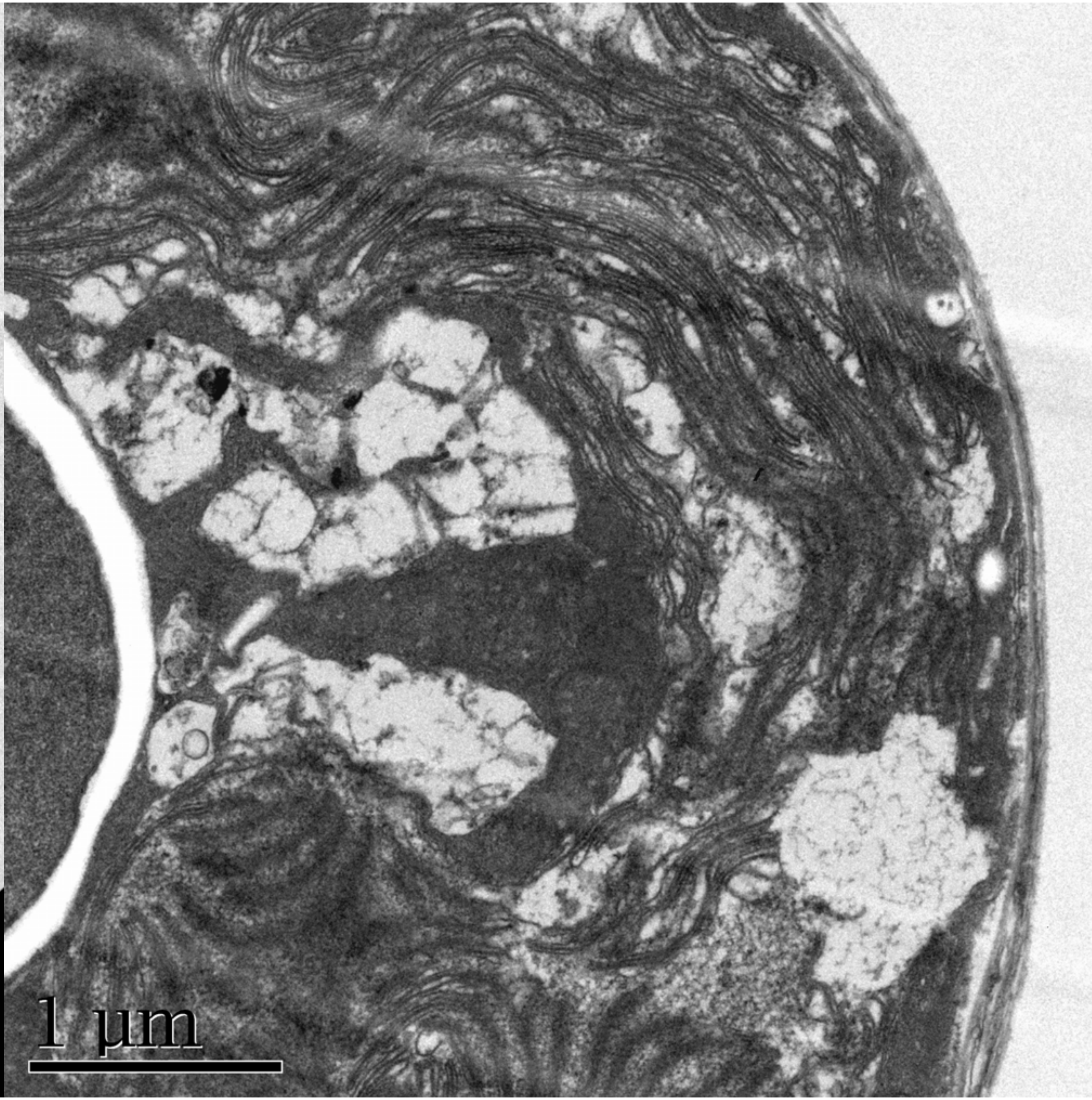

18-7_Correa_XC4_3GridB7_4

Cell 3

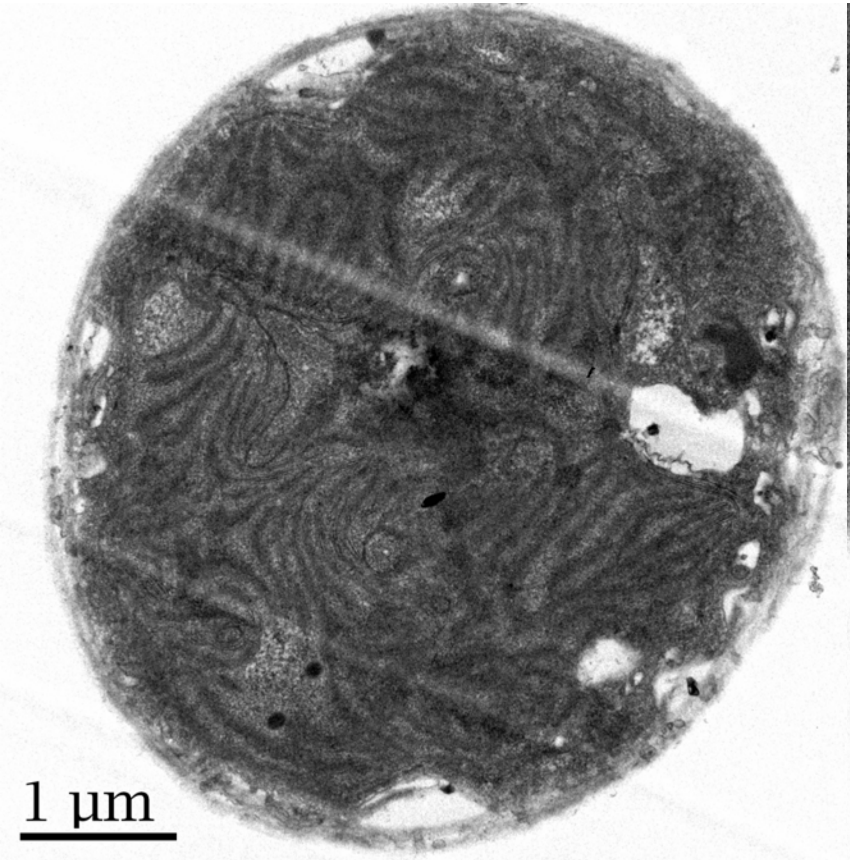

18-7_Correa_XC4_3GridB7_8

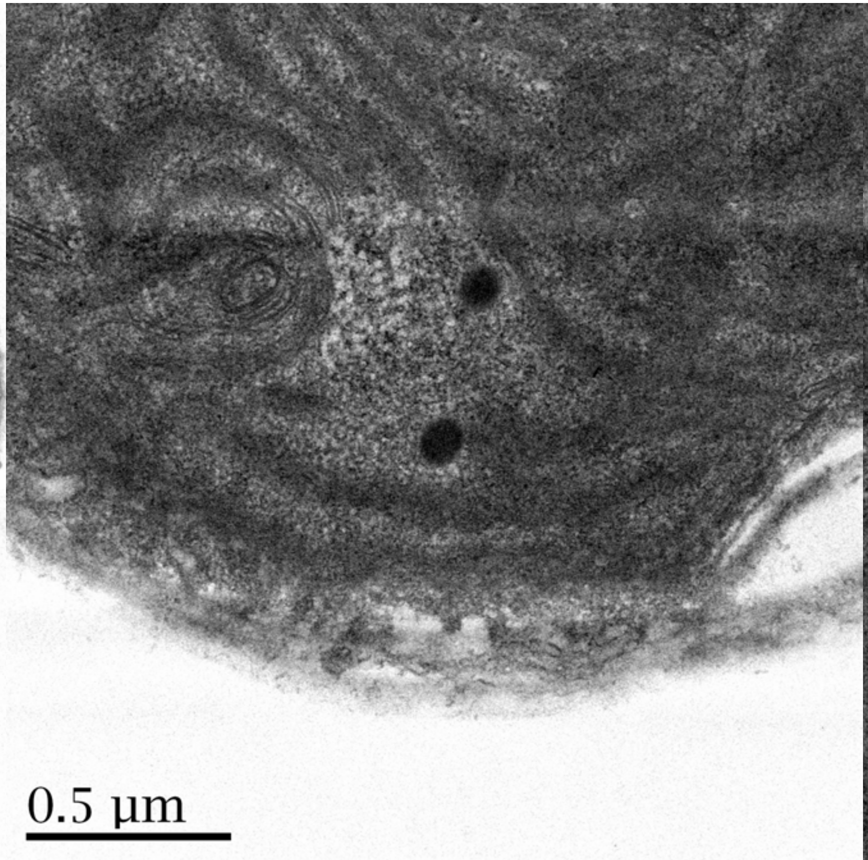

18-7_Correa_XC4_3GridB7_9

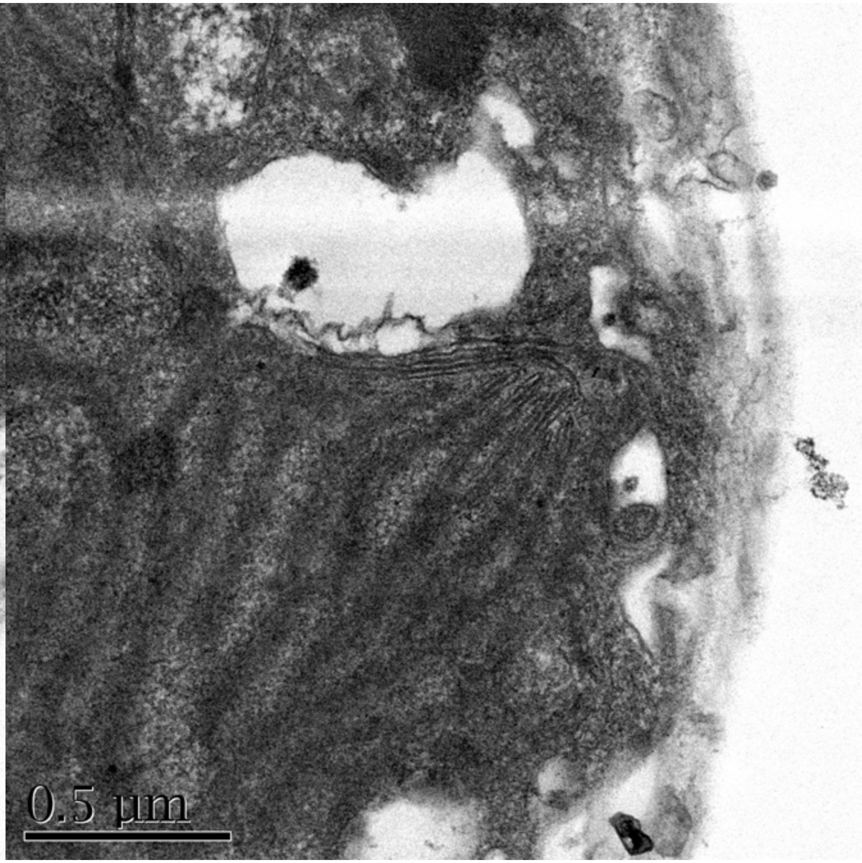

18-7_Correa_XC4_3GridB7_10

Cell 4

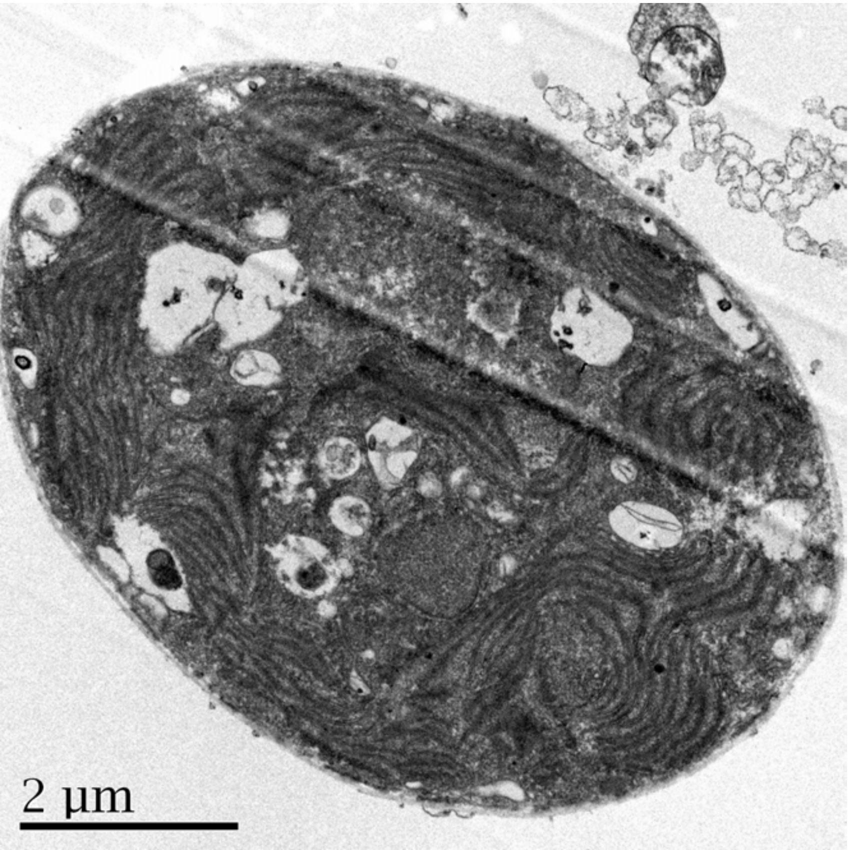

18-7_Correa_XC4_3GridB7_16

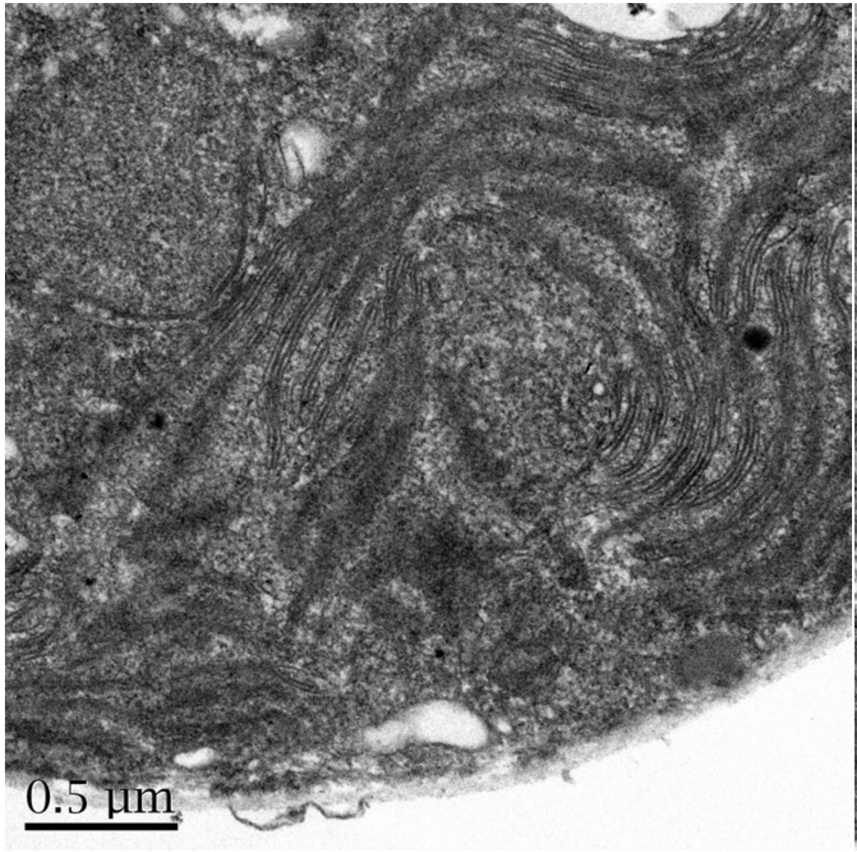

18-7_Correa_XC4_3GridB7_18

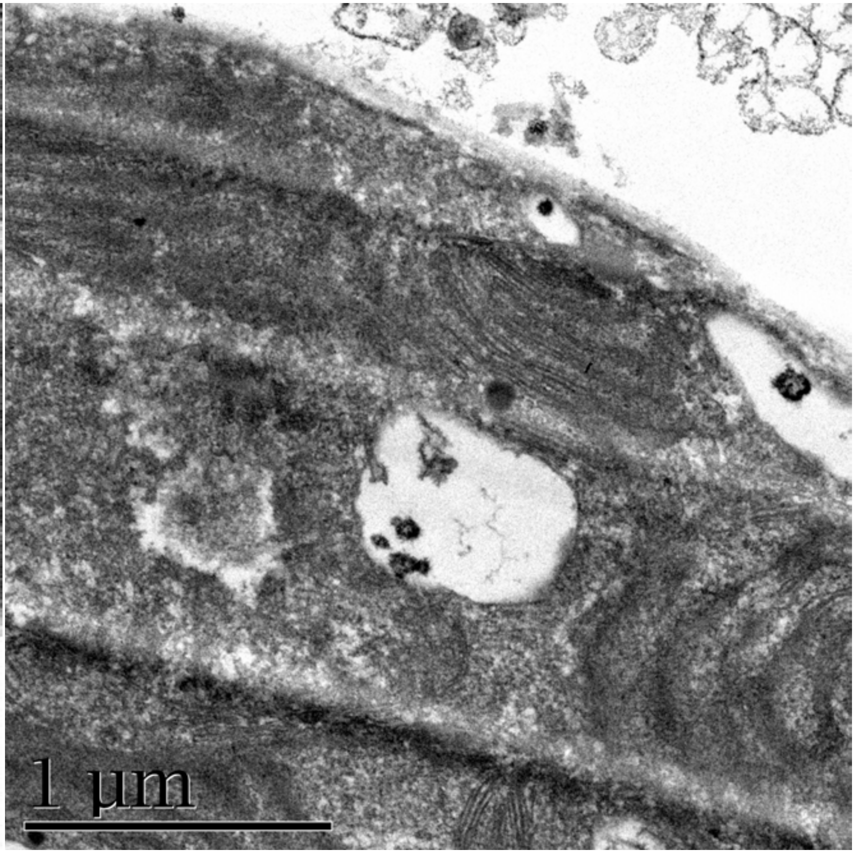

18-7_Correa_XC4_3GridB7_19

Cell 5

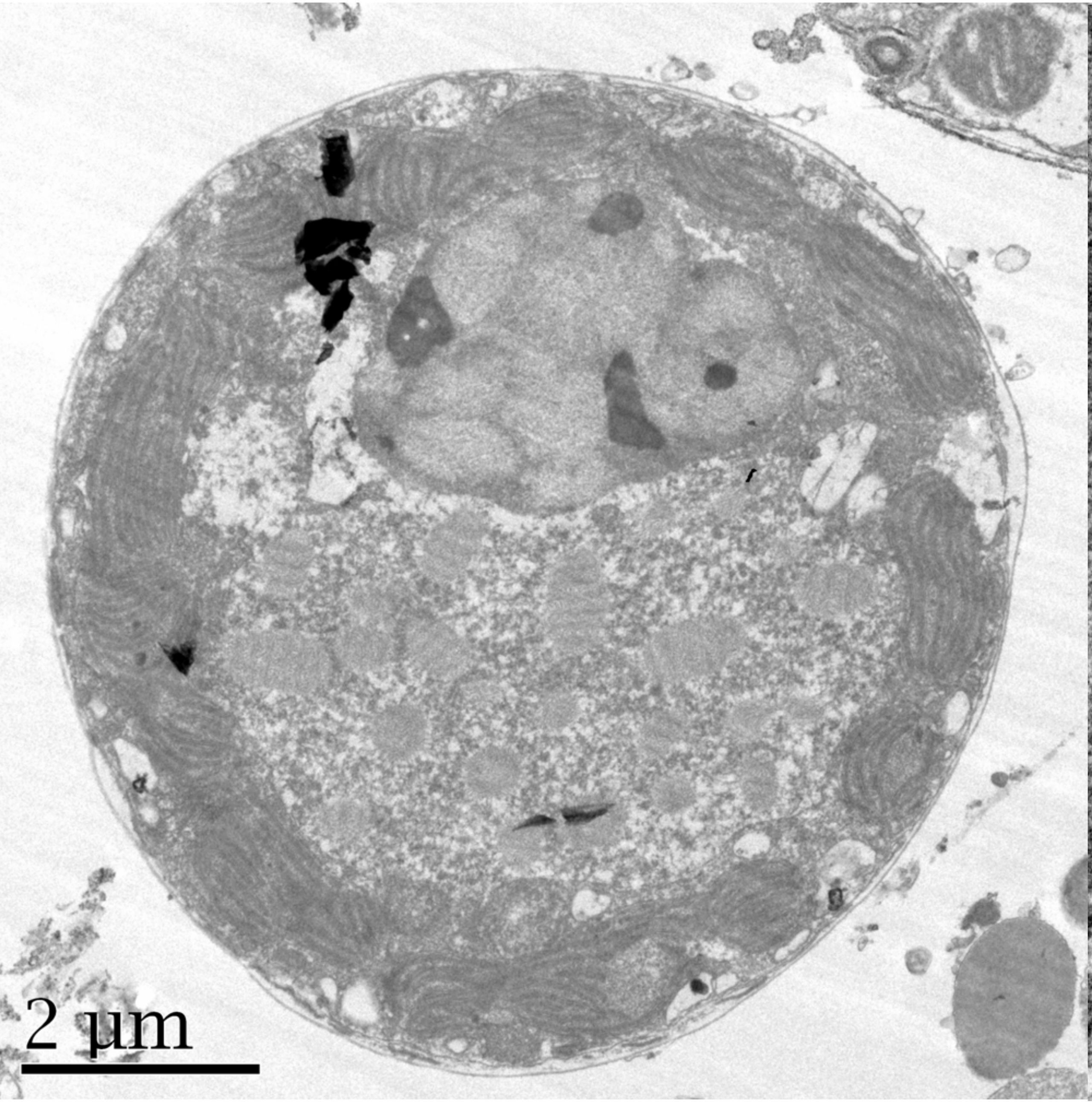

18-7_Correa_XC4_3GridB7_21

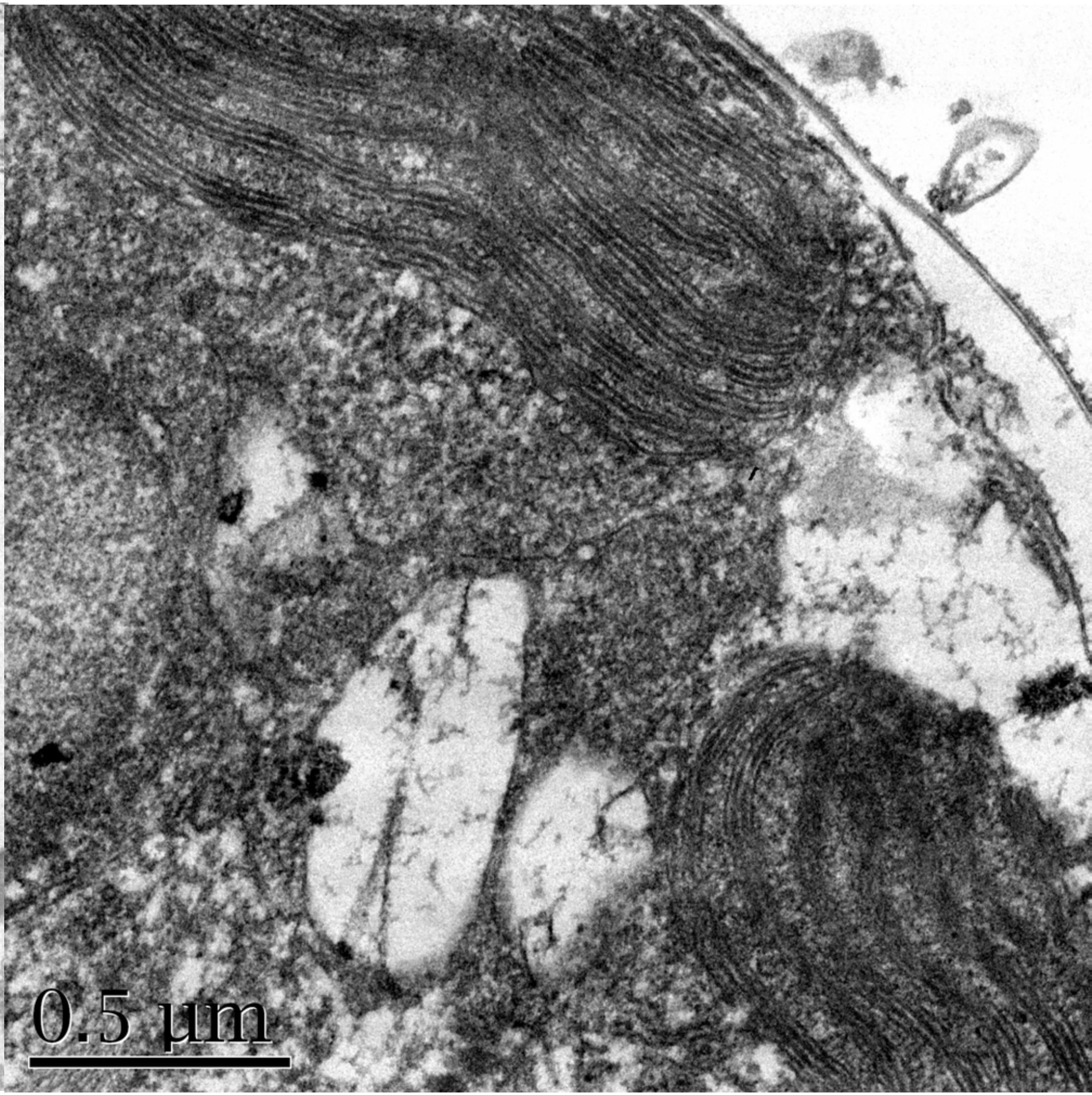

18-7_Correa_XC4_3GridB7_24

Cell 6

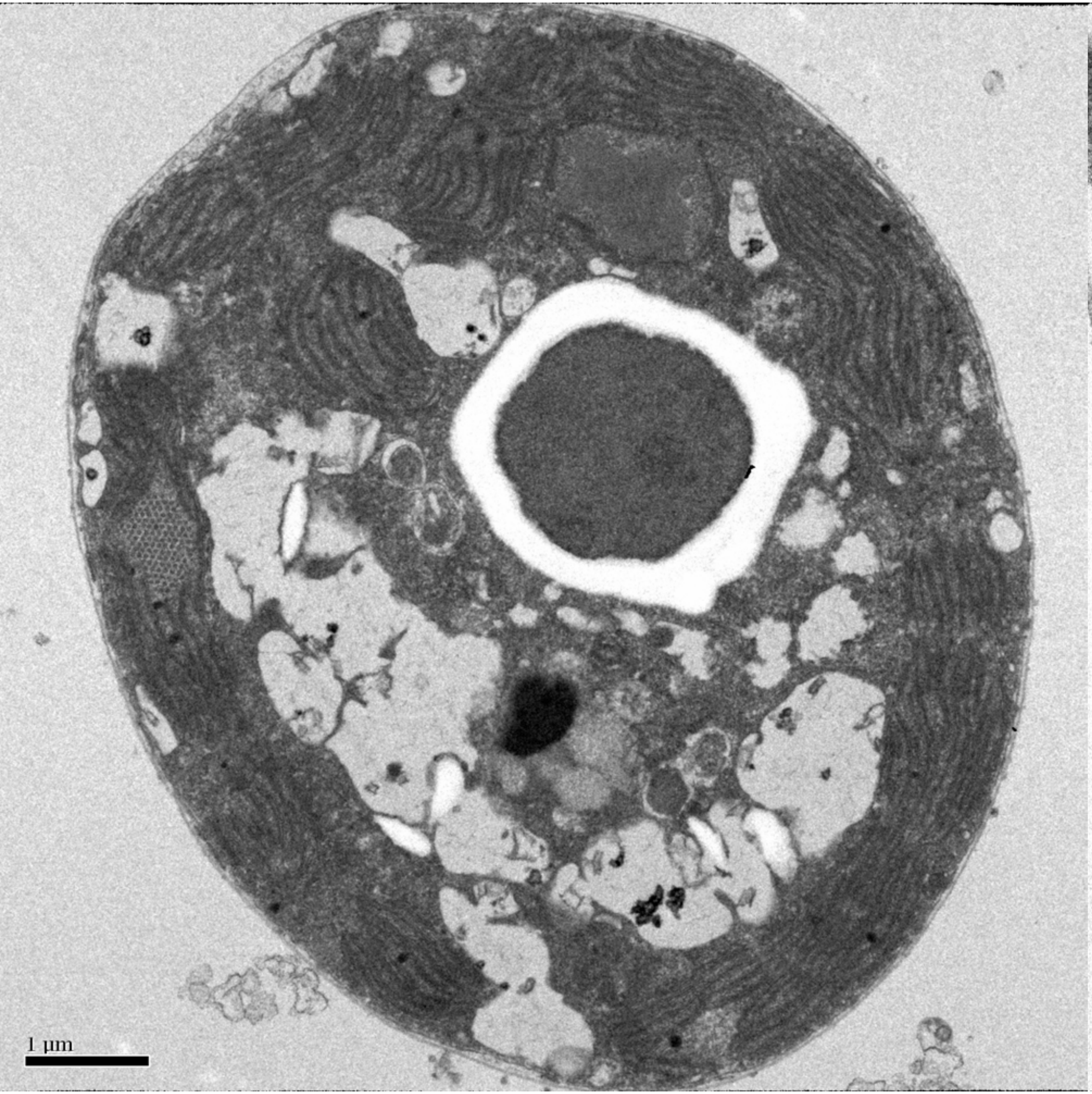

18-7_Correa_XC4_3B8_3

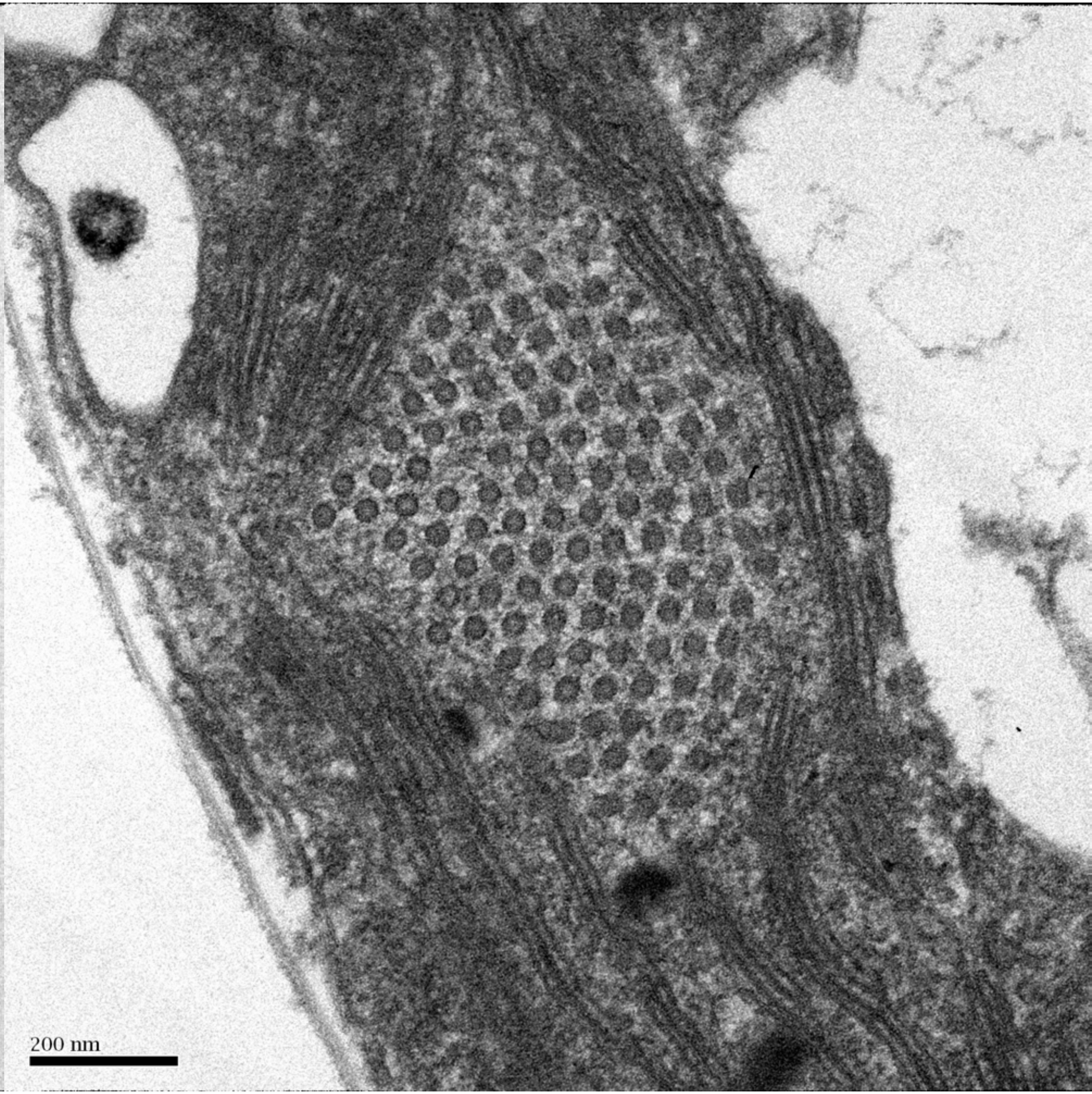

18-7_Correa_XC4_3B8_6

Cell 7

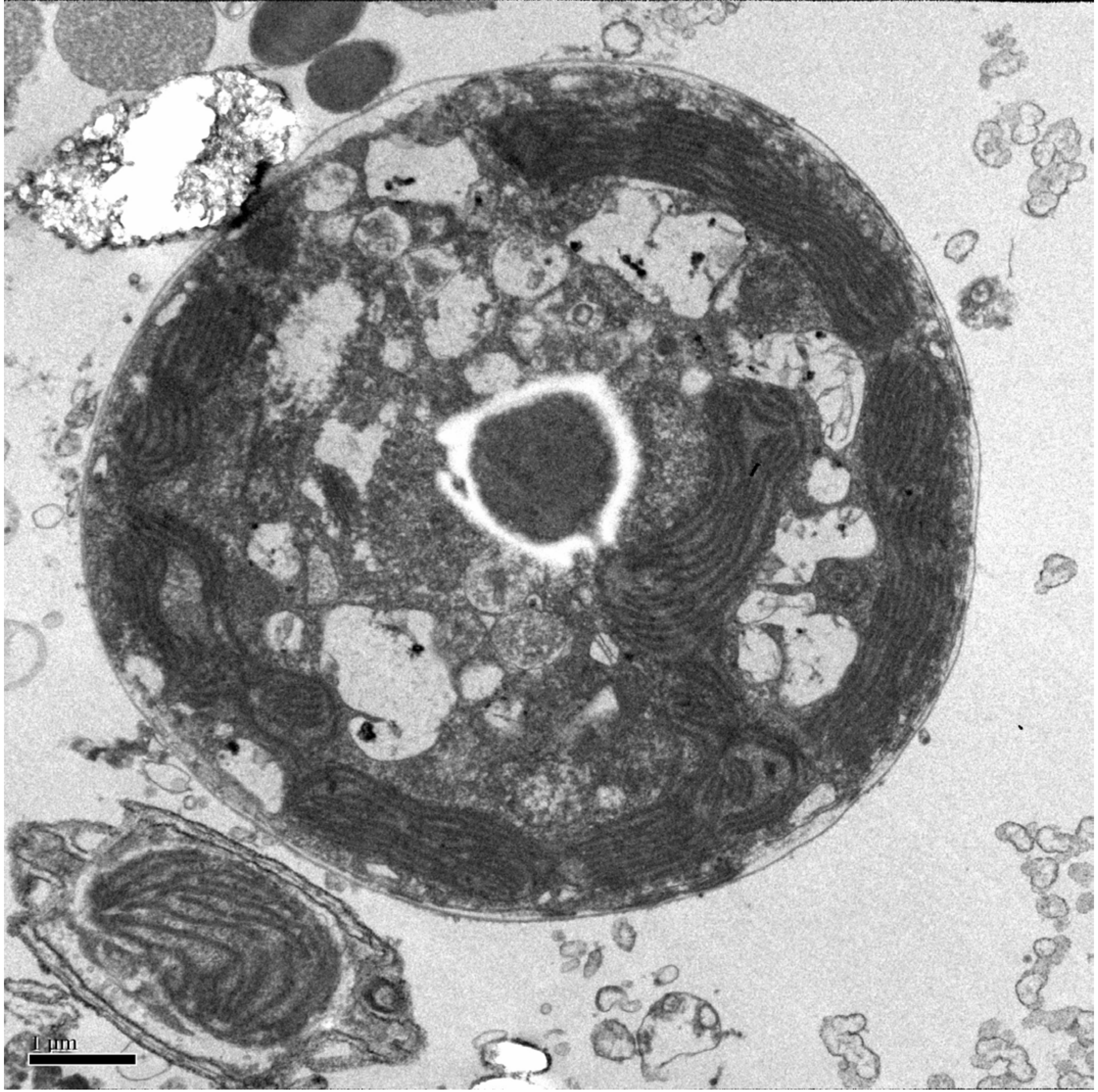

18-7_Correa_XC4_3B8_8

Cell 8

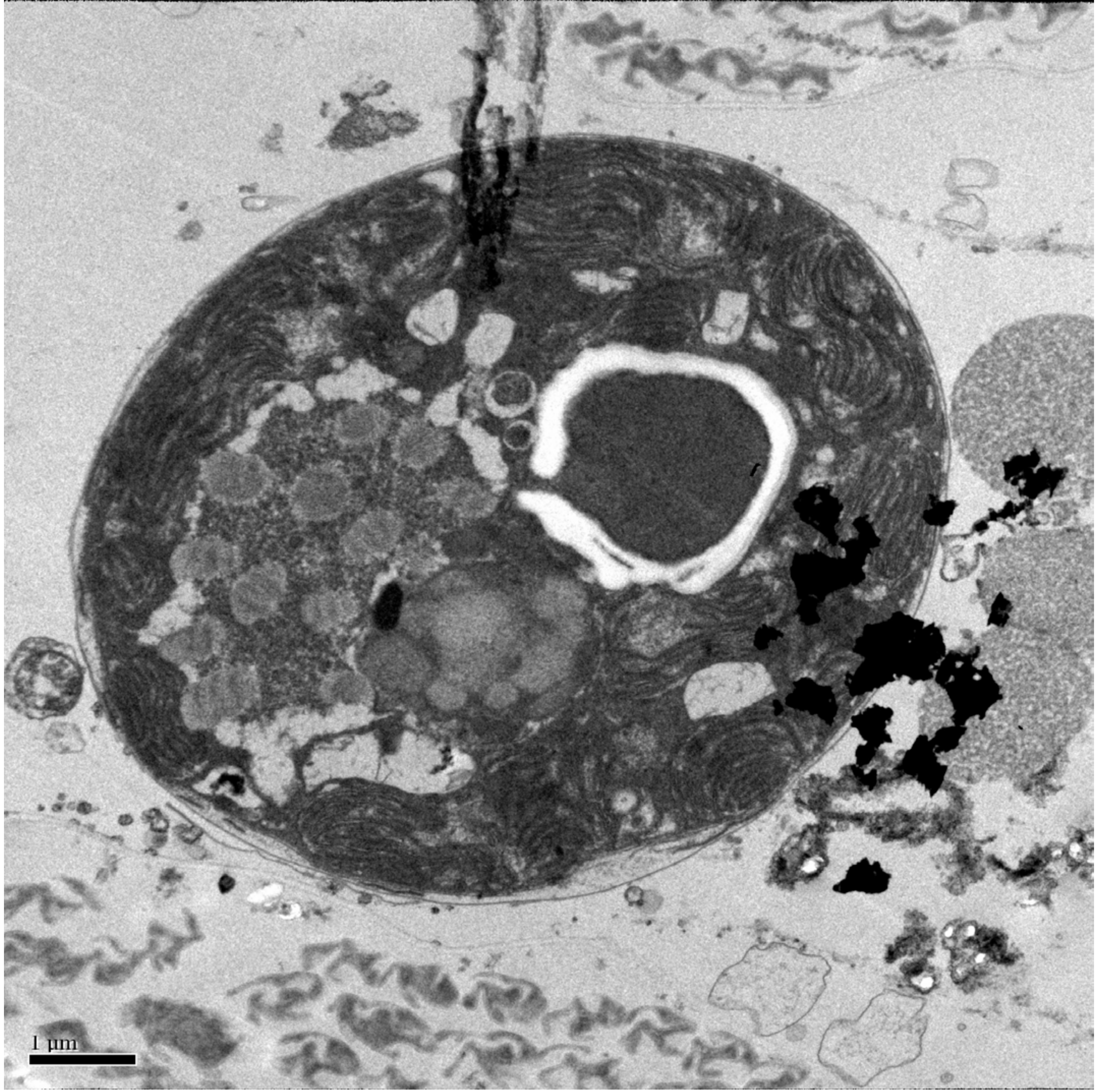

18-7_Correa_XC4_3B8_9

Cell 9

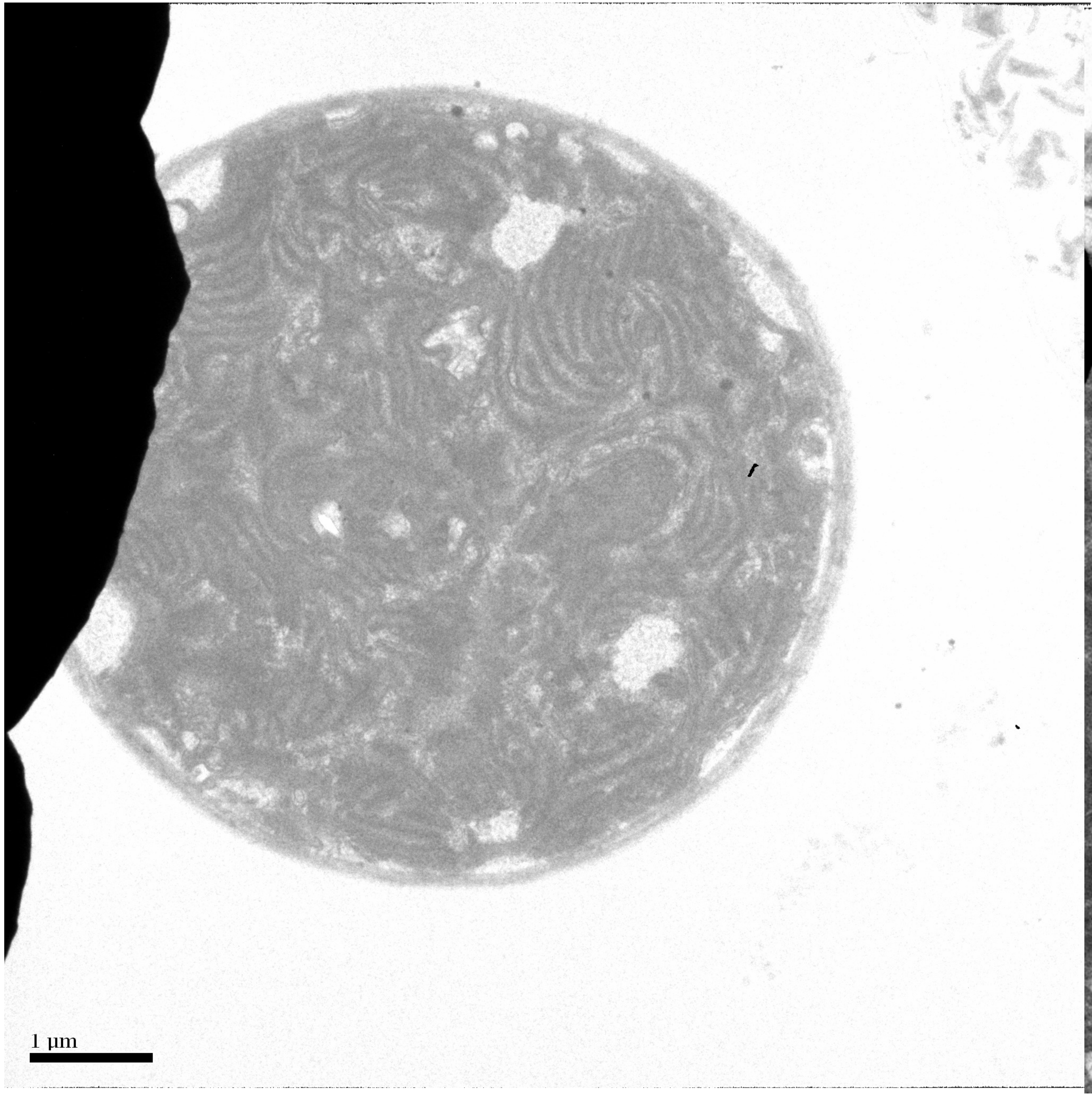

18-7_Correa_XC4_3B8_11

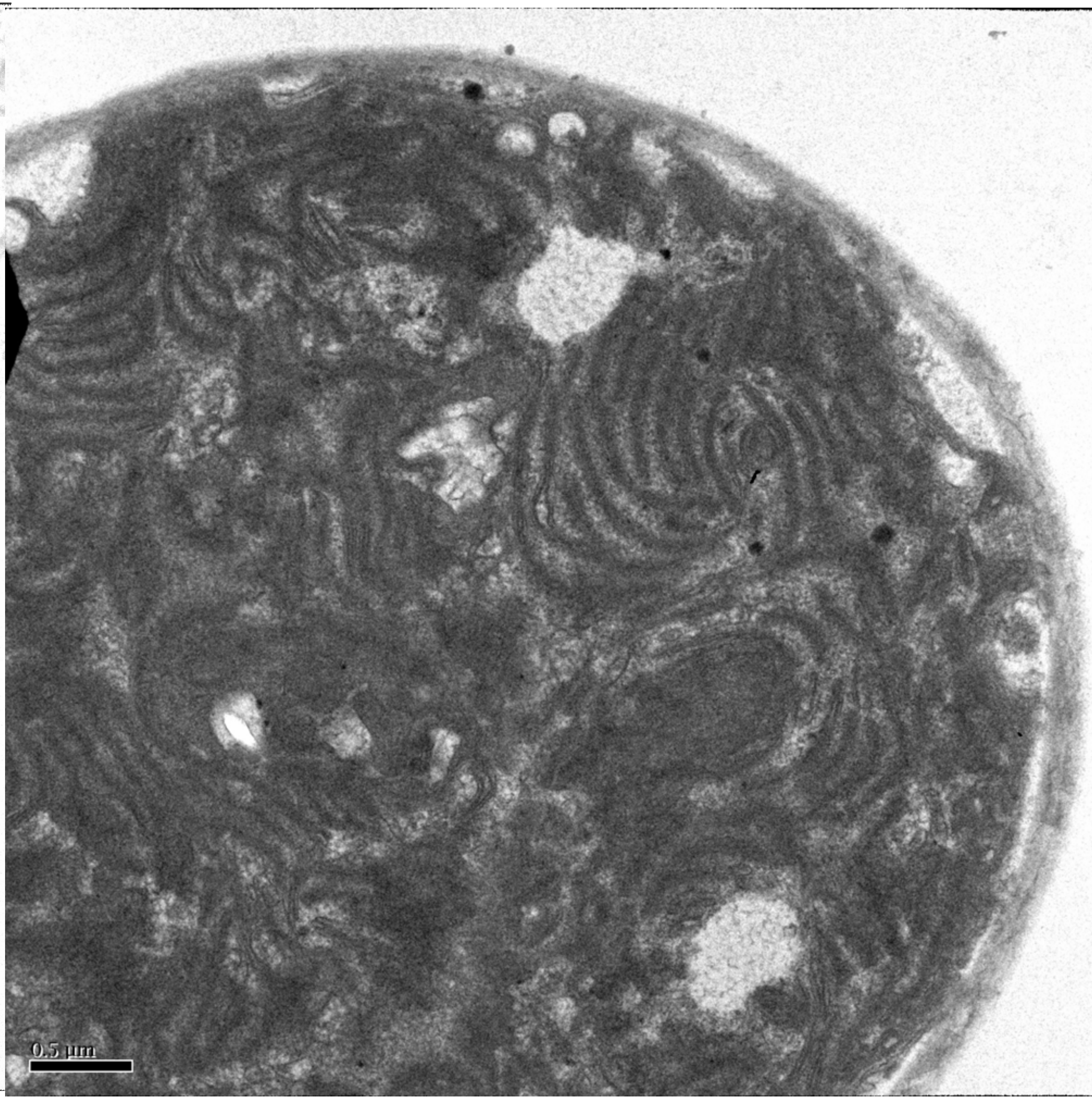

18-7_Correa_XC4_3B8_13

Cell 10

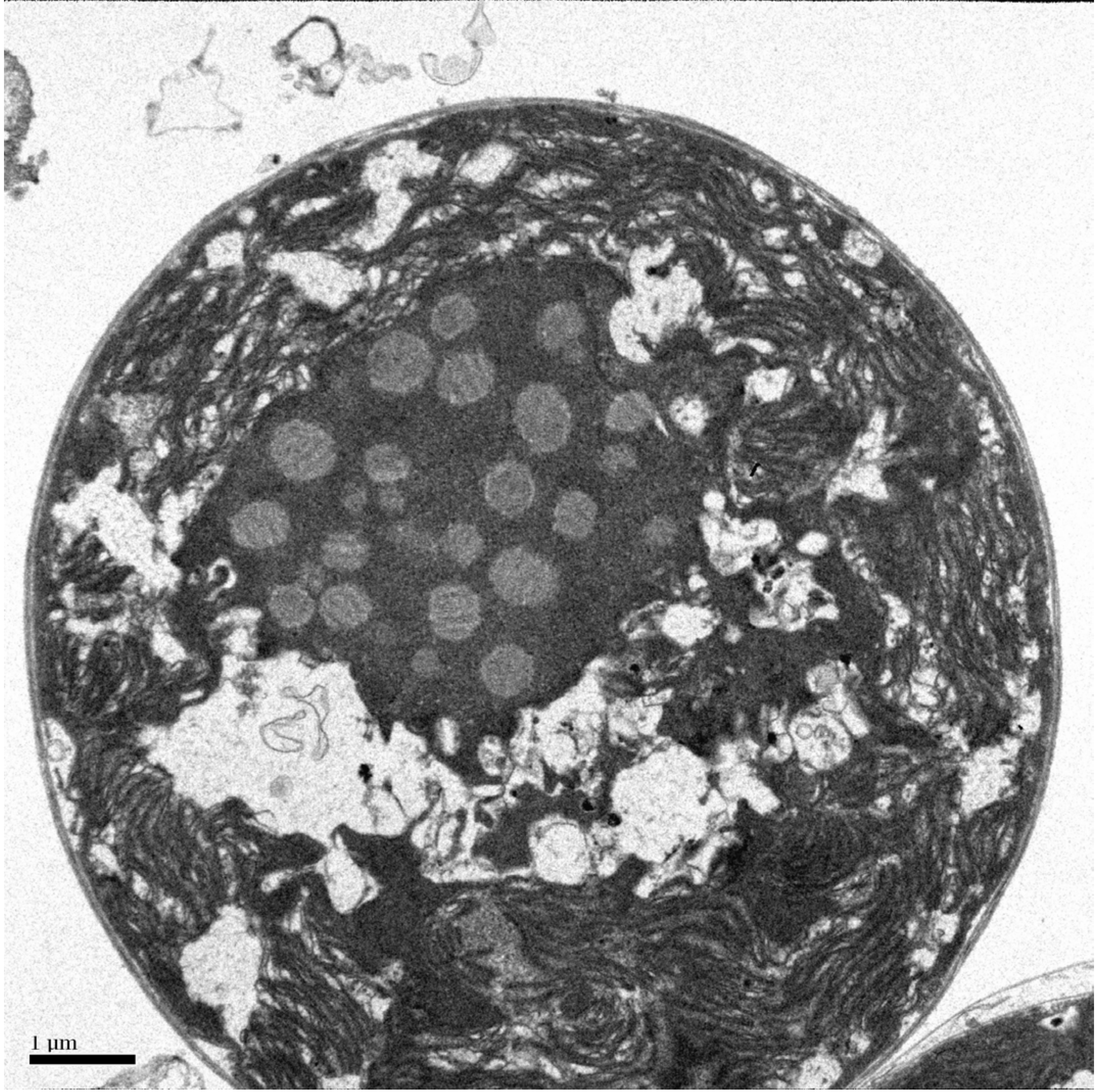

18-7_Correa_XC4_3B8_17

ACR Colony C Expelled- Heat

Cell 1

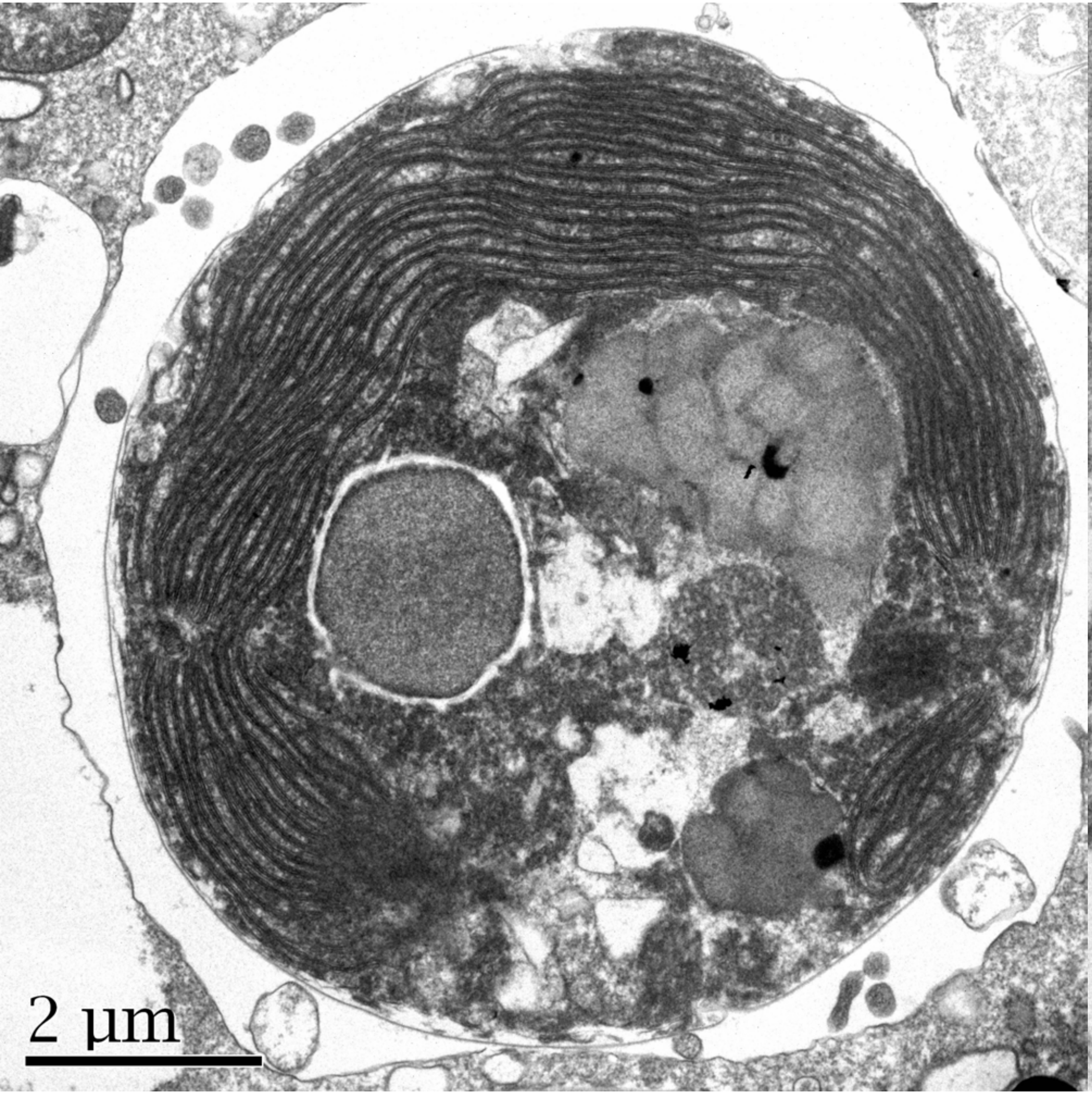

18-7_Correa_XH4_3GrridC1_4

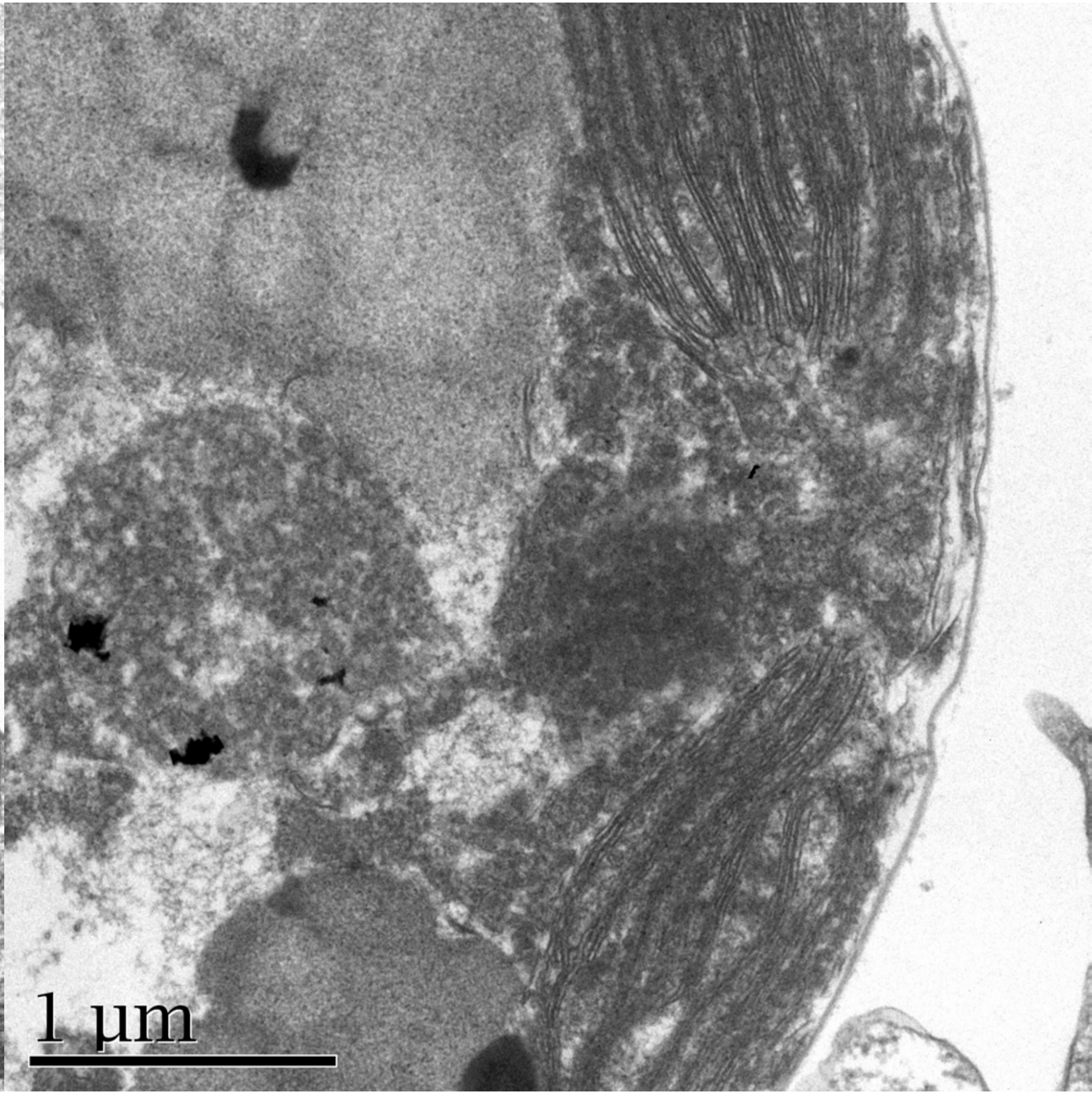

18-7_Correa_XH4_3GrridC1_6

Cell 2

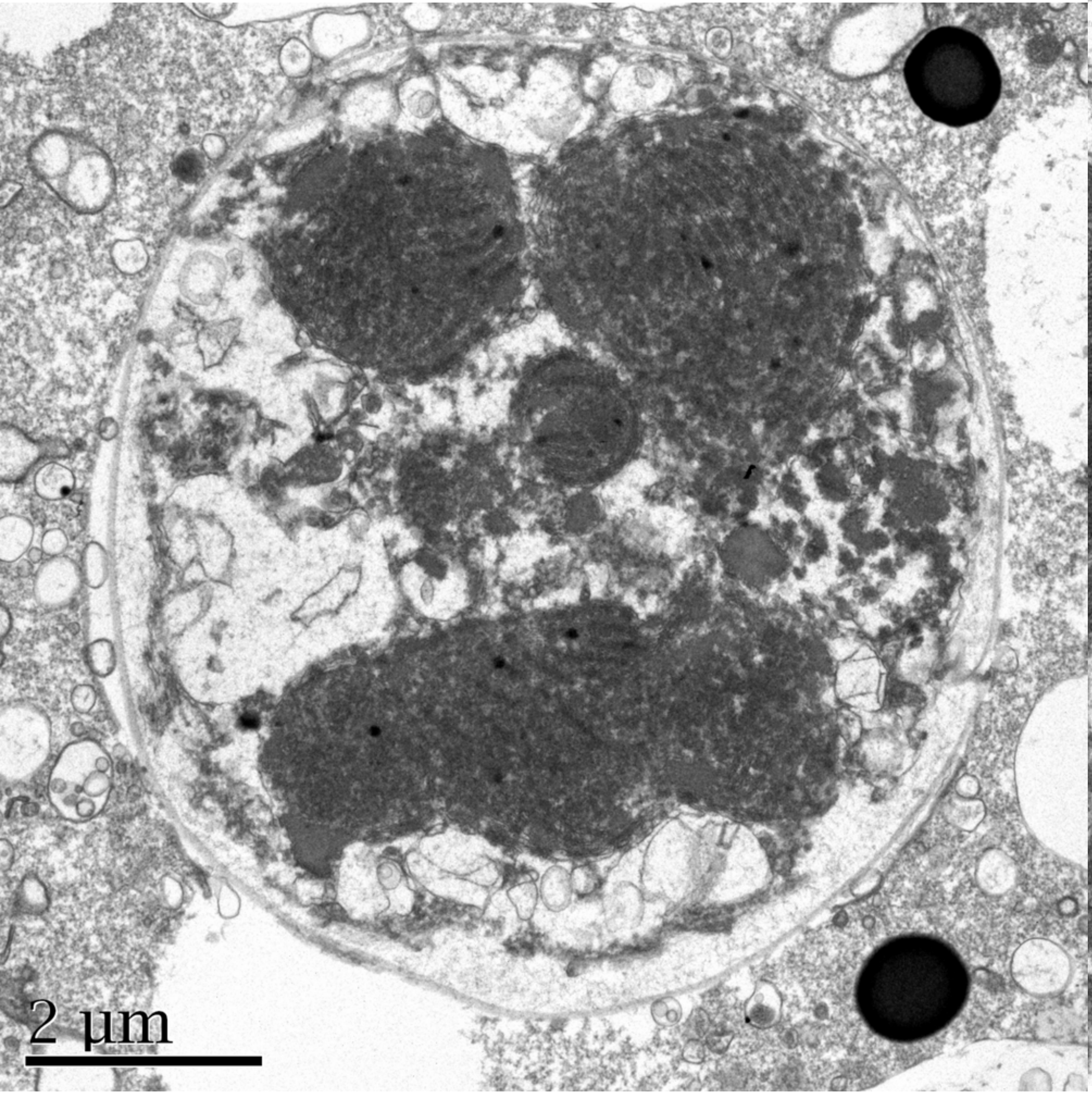

18-7_Correa_XH4_3GrridC1_12

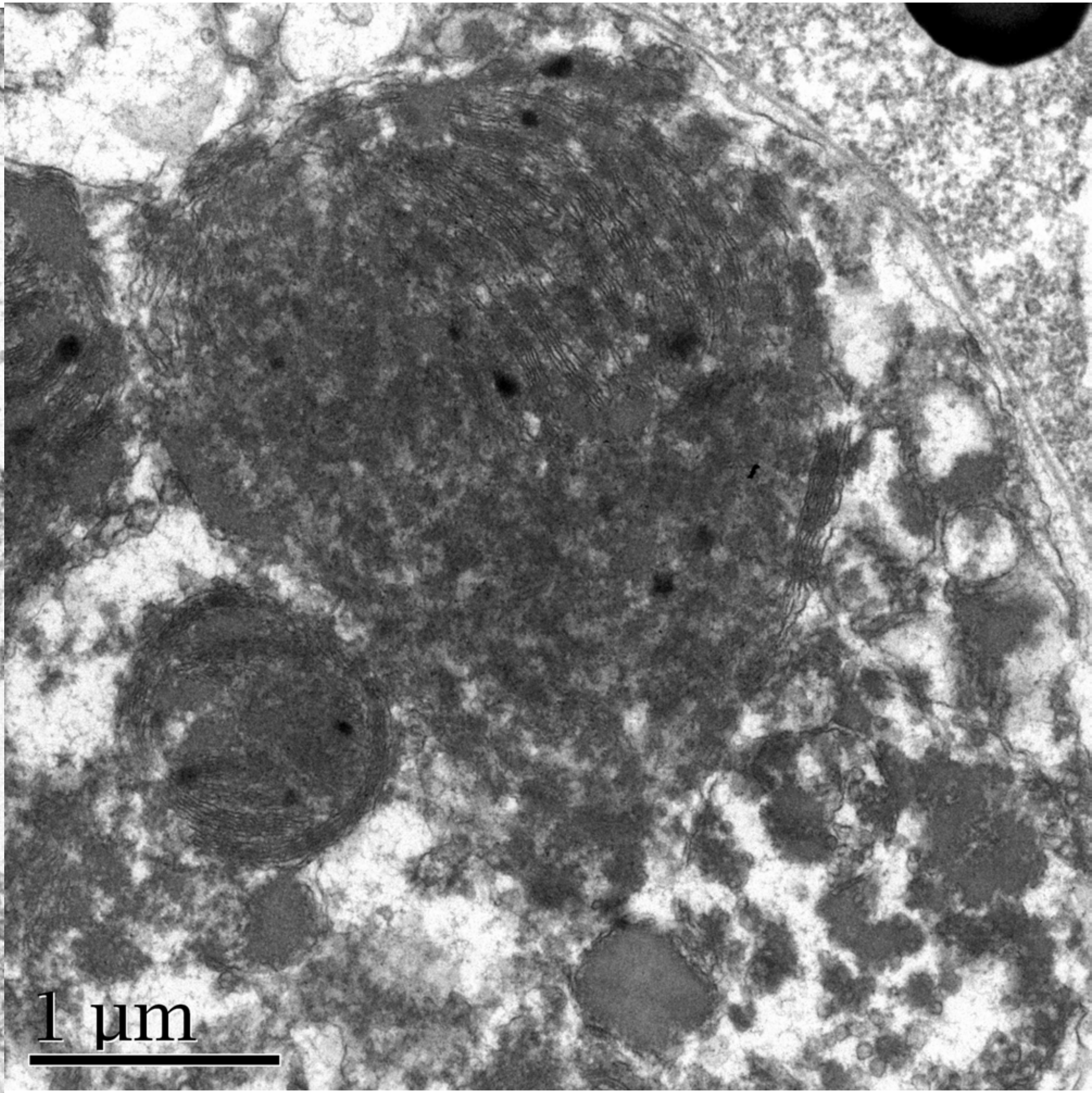

18-7_Correa_XH4_3GrridC1_17

Cell 3

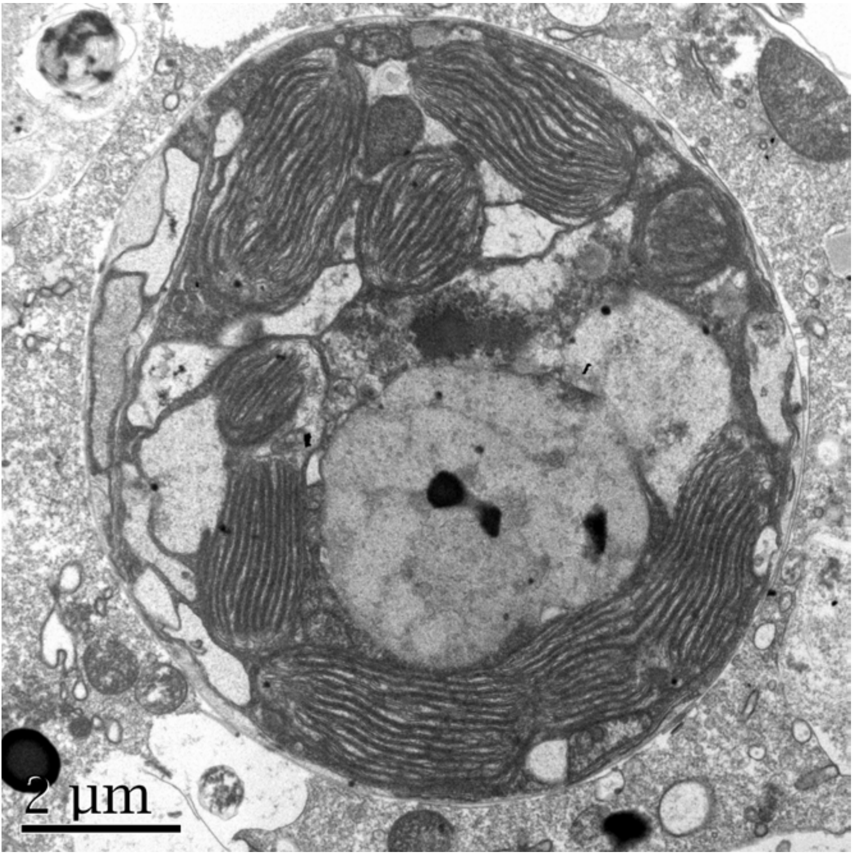

18-7_Correa_XH4_3GrridC1_21

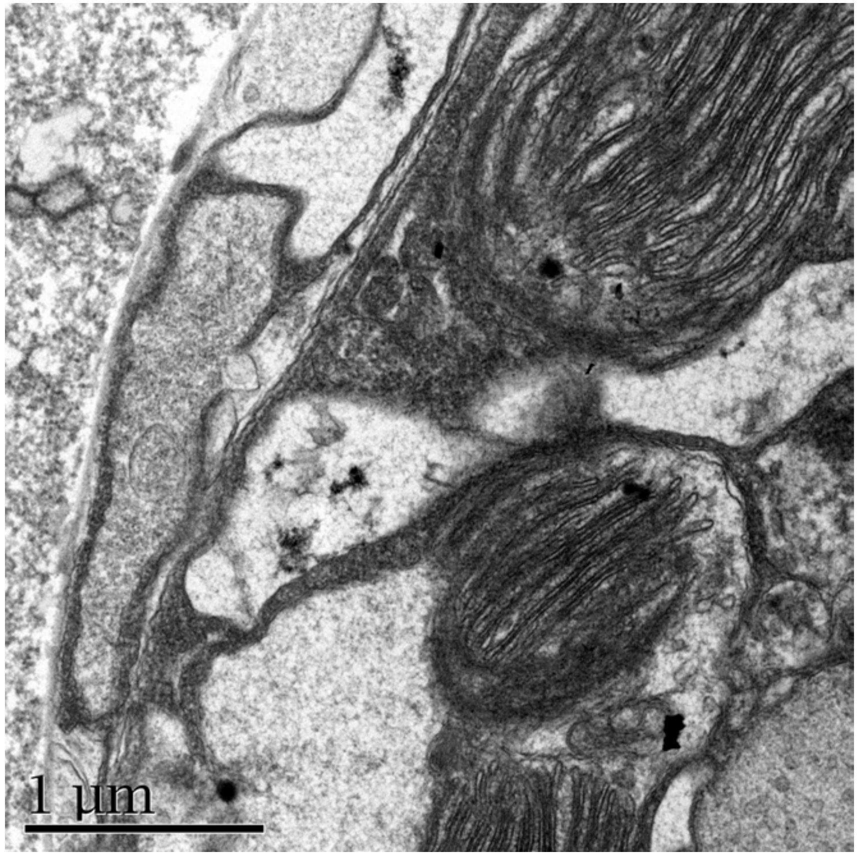

18-7_Correa_XH4_3GrridC1_26

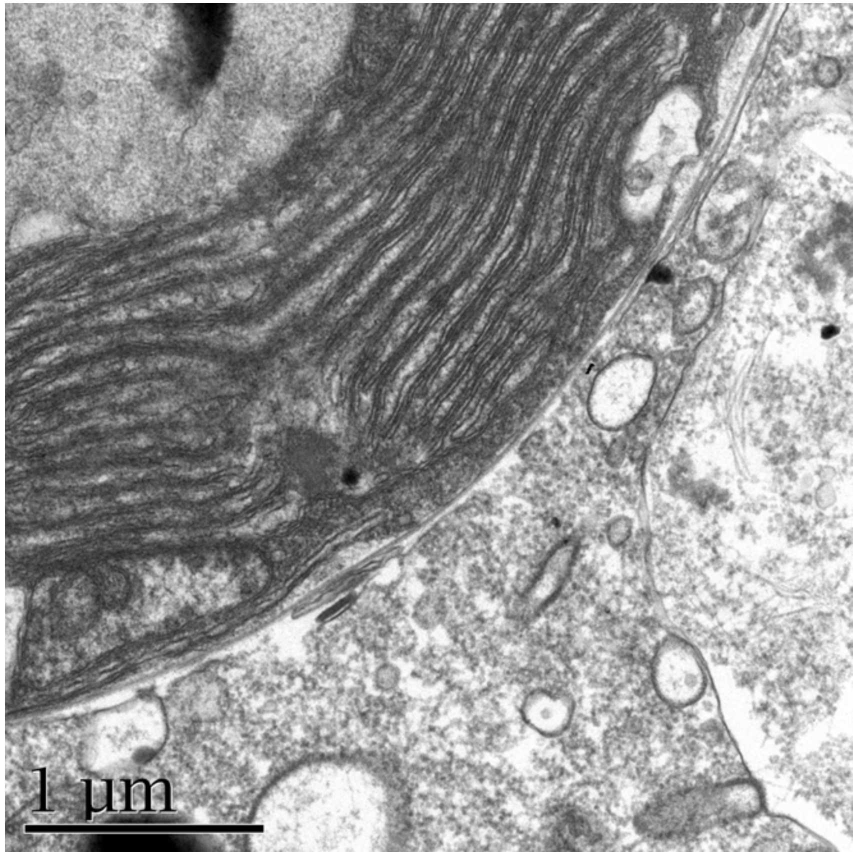

18-7_Correa_XH4_3GrridC1_22

Cell 4

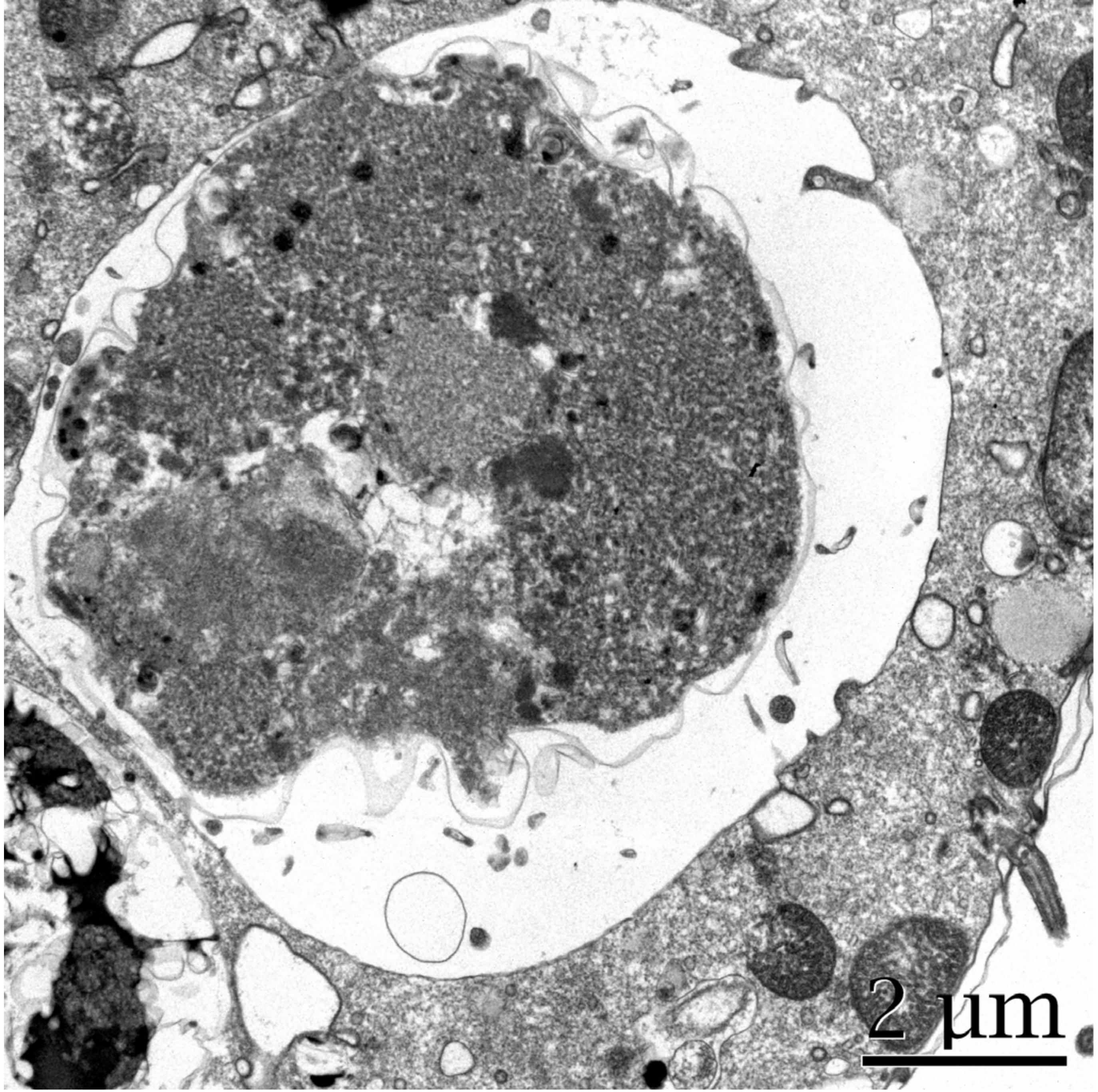

18-7_Correa_XH4_3GrridC1_28

Cell 5

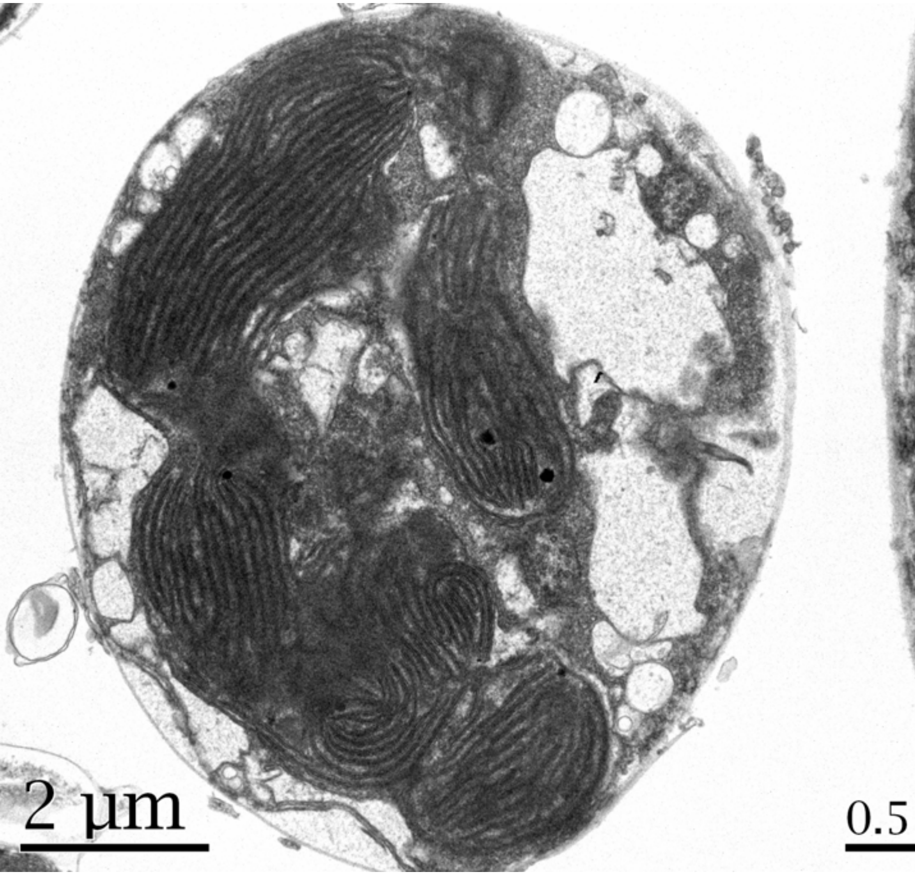

18-7_Correa_XH4_3GrridC1_32

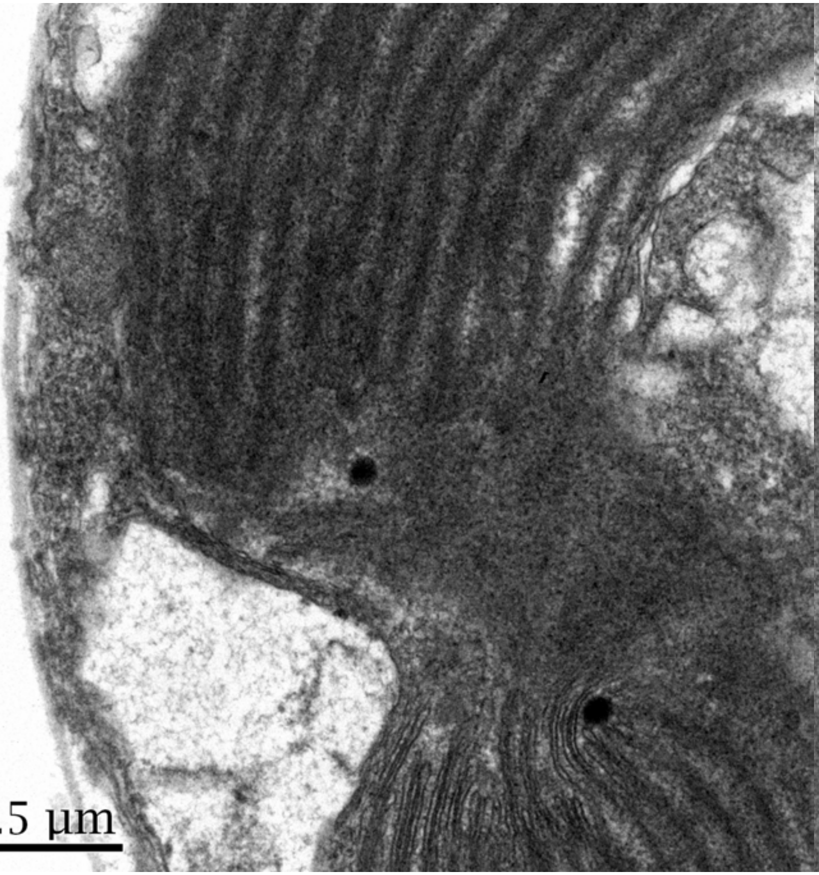

18-7_Correa_XH4_3GrridC1_35

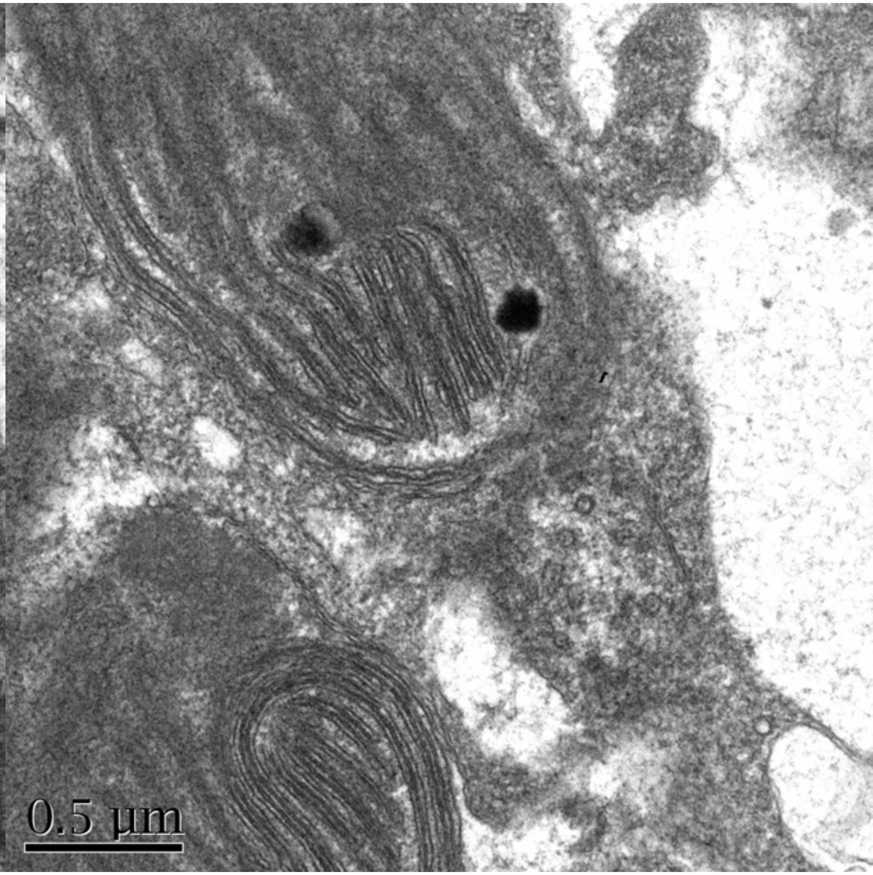

18-7_Correa_XH4_3GrridC1_34

Cell 6

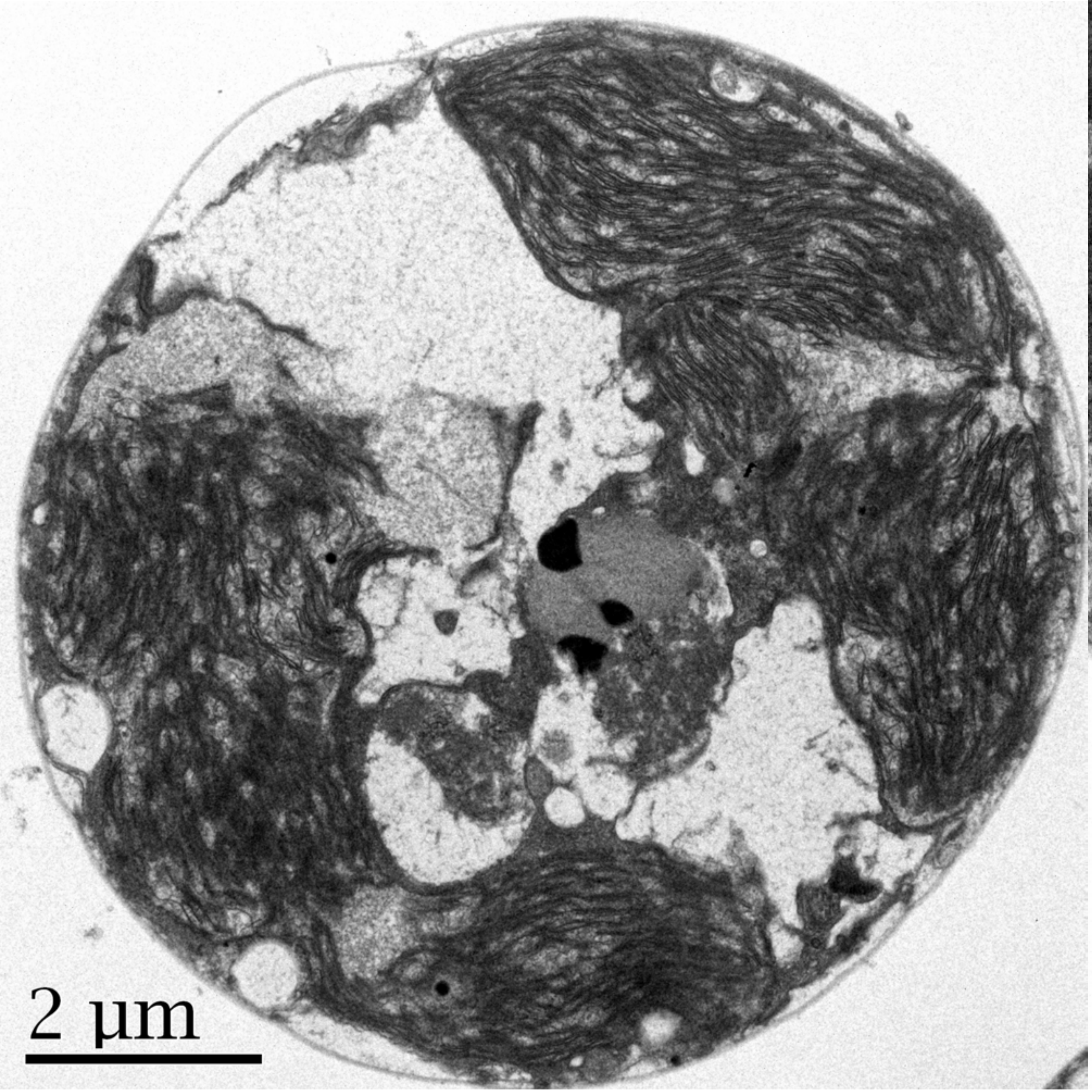

18-7_Correa_XH4_3GrridC1_36

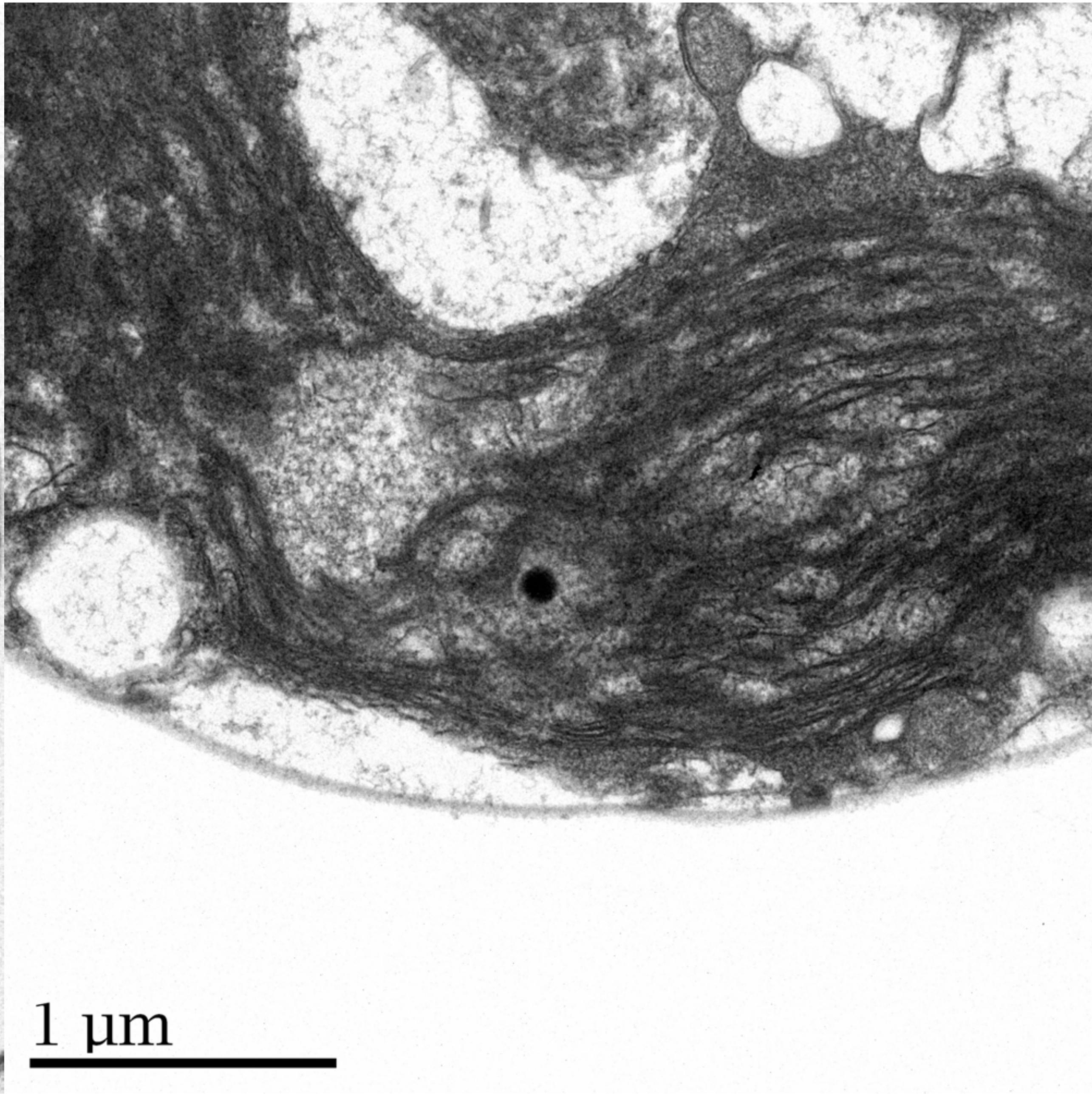

18-7_Correa_XH4_3GrridC1_37

Cell 7

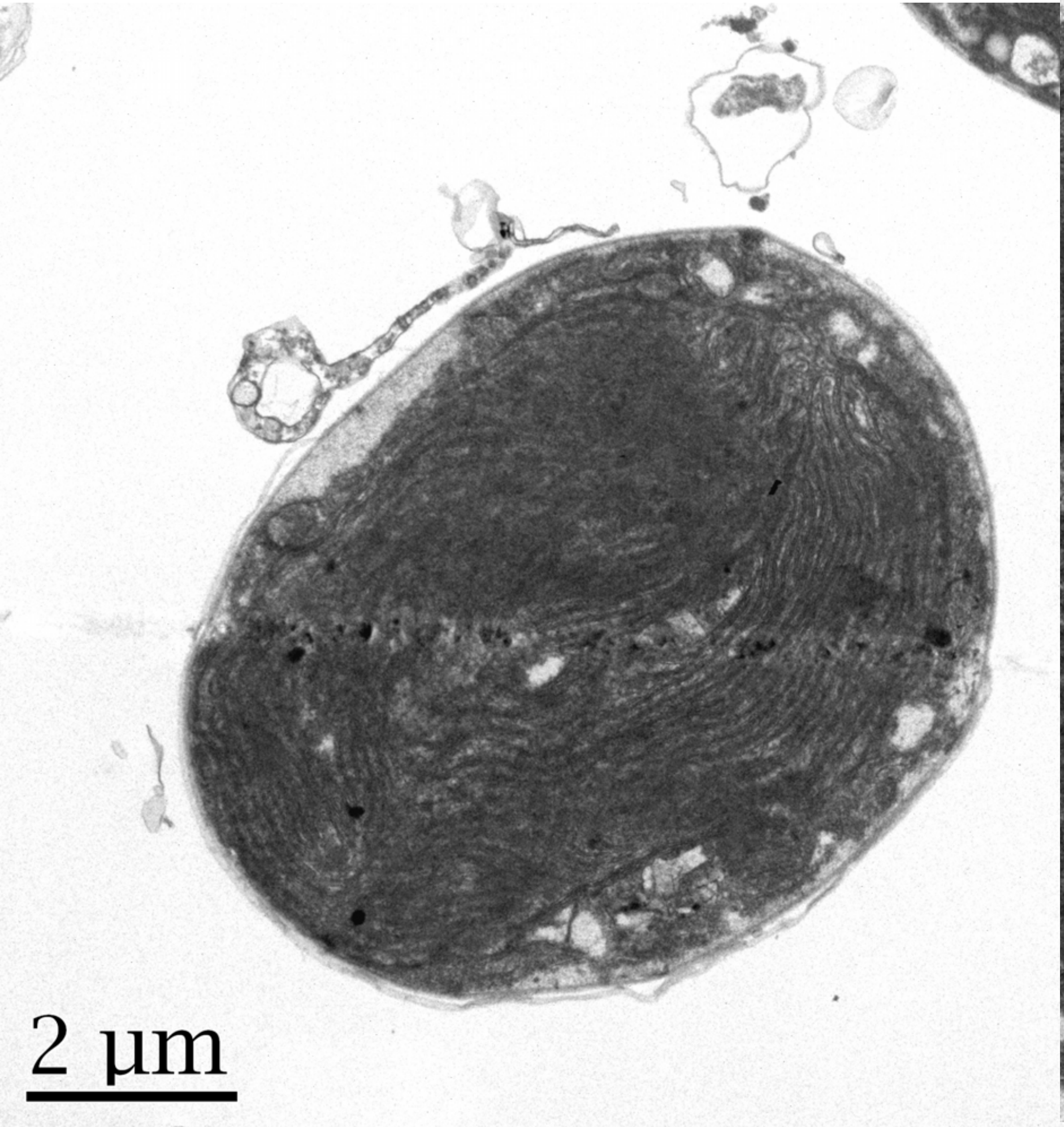

18-7_Correa_XH4_3GrridC1_39

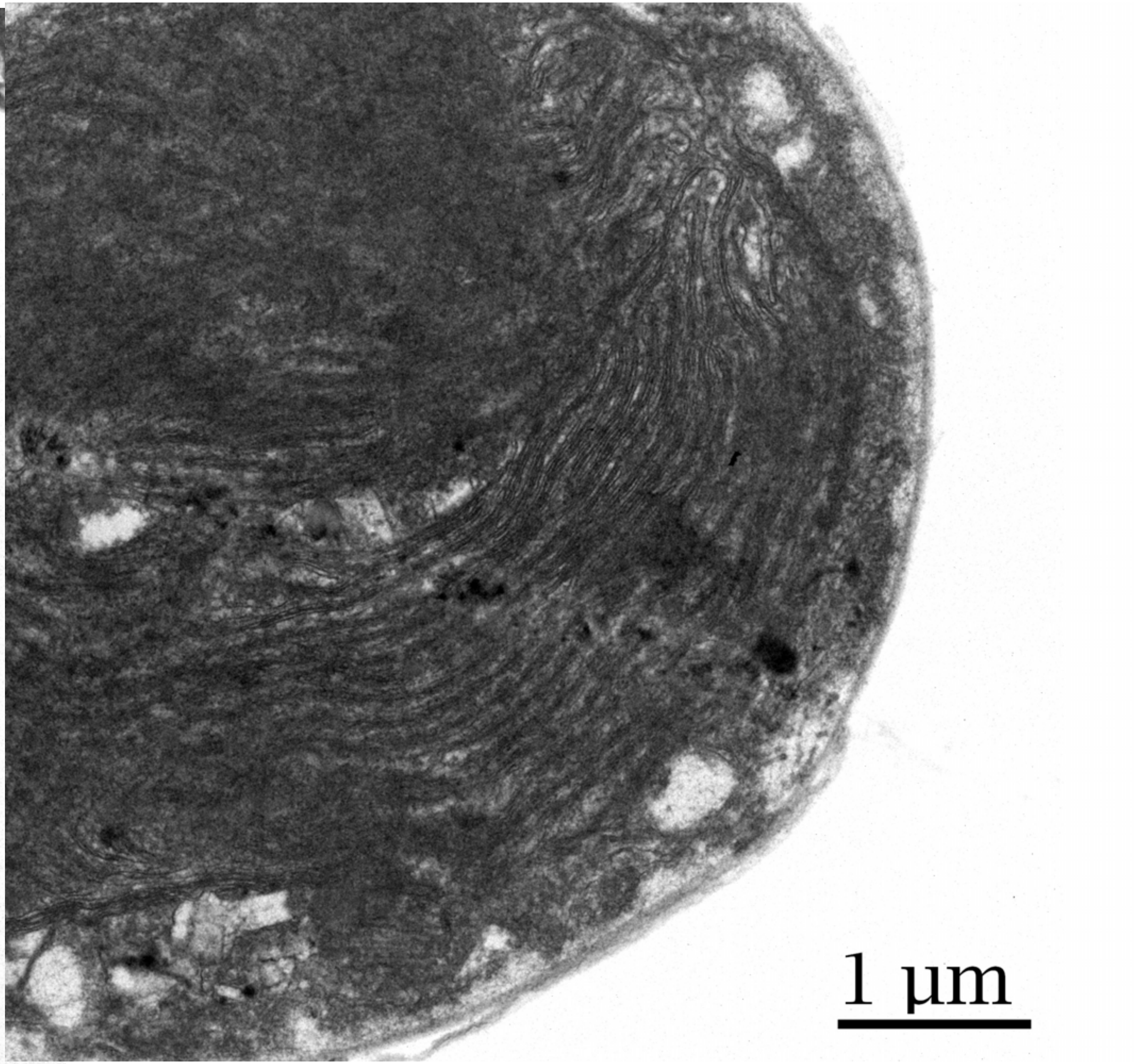

18-7_Correa_XH4_3GrridC1_41

Cell 8

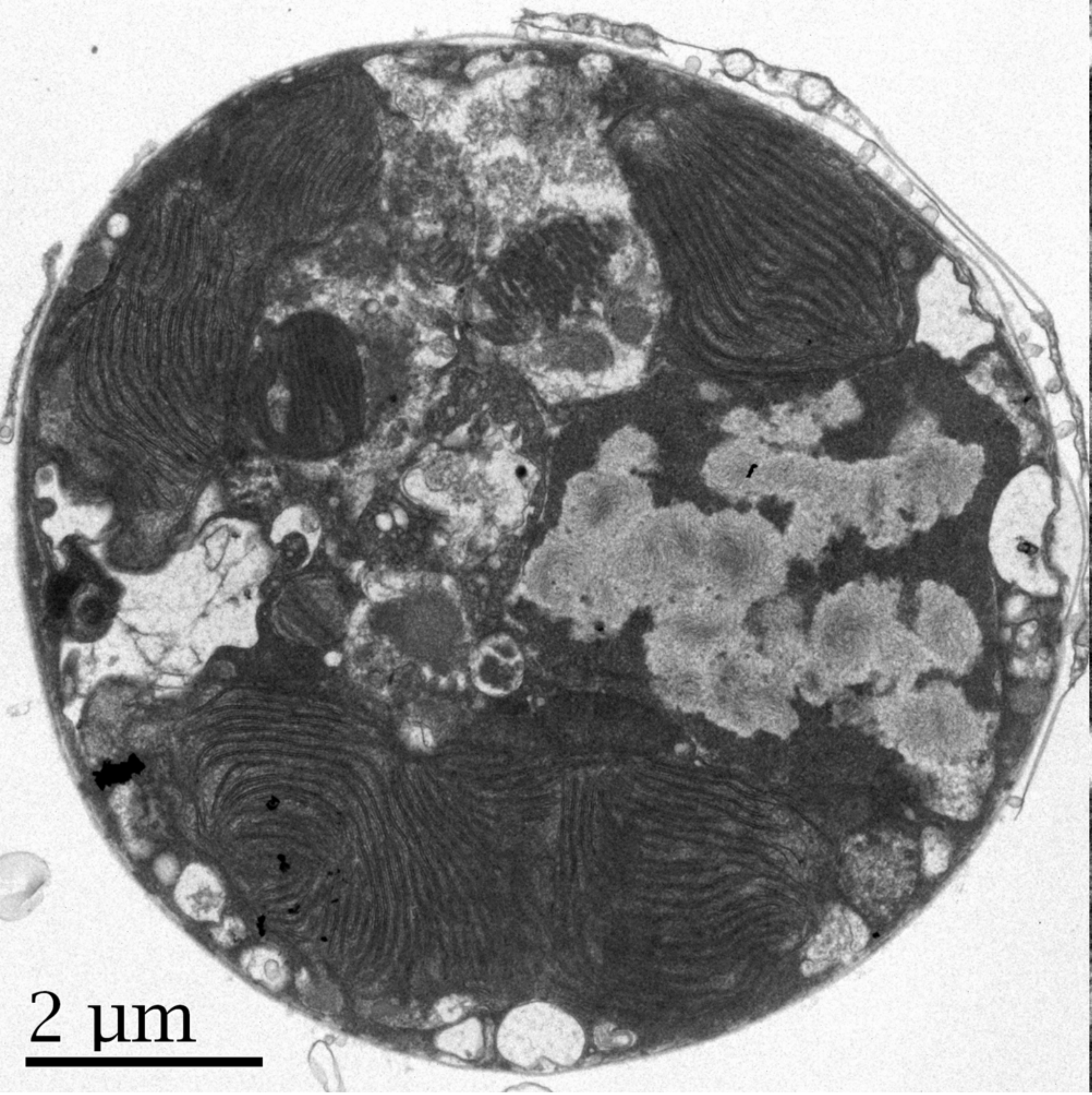

18-7_Correa_XH4_3GrridC1_42

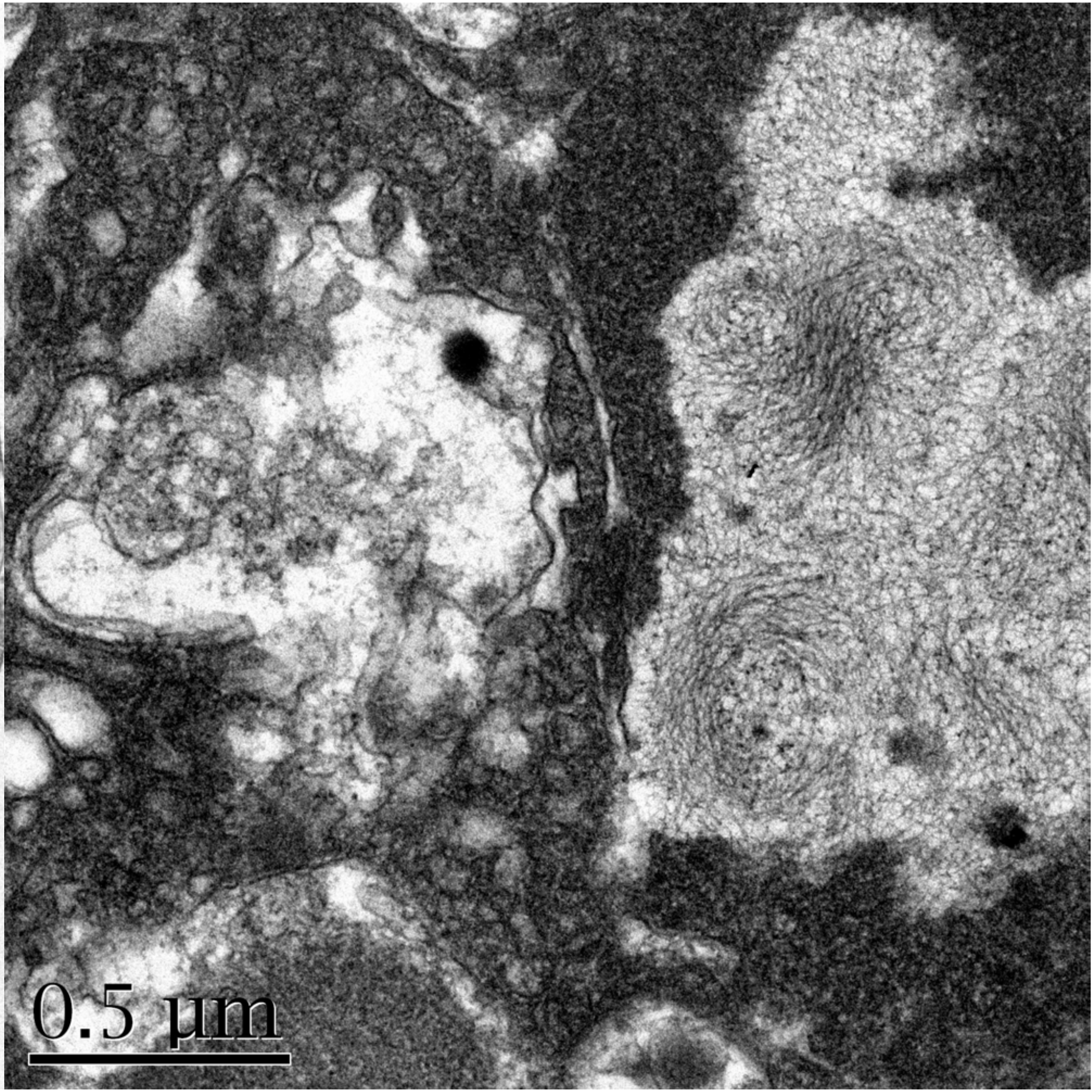

18-7_Correa_XH4_3GrridC1_43

Cell 9

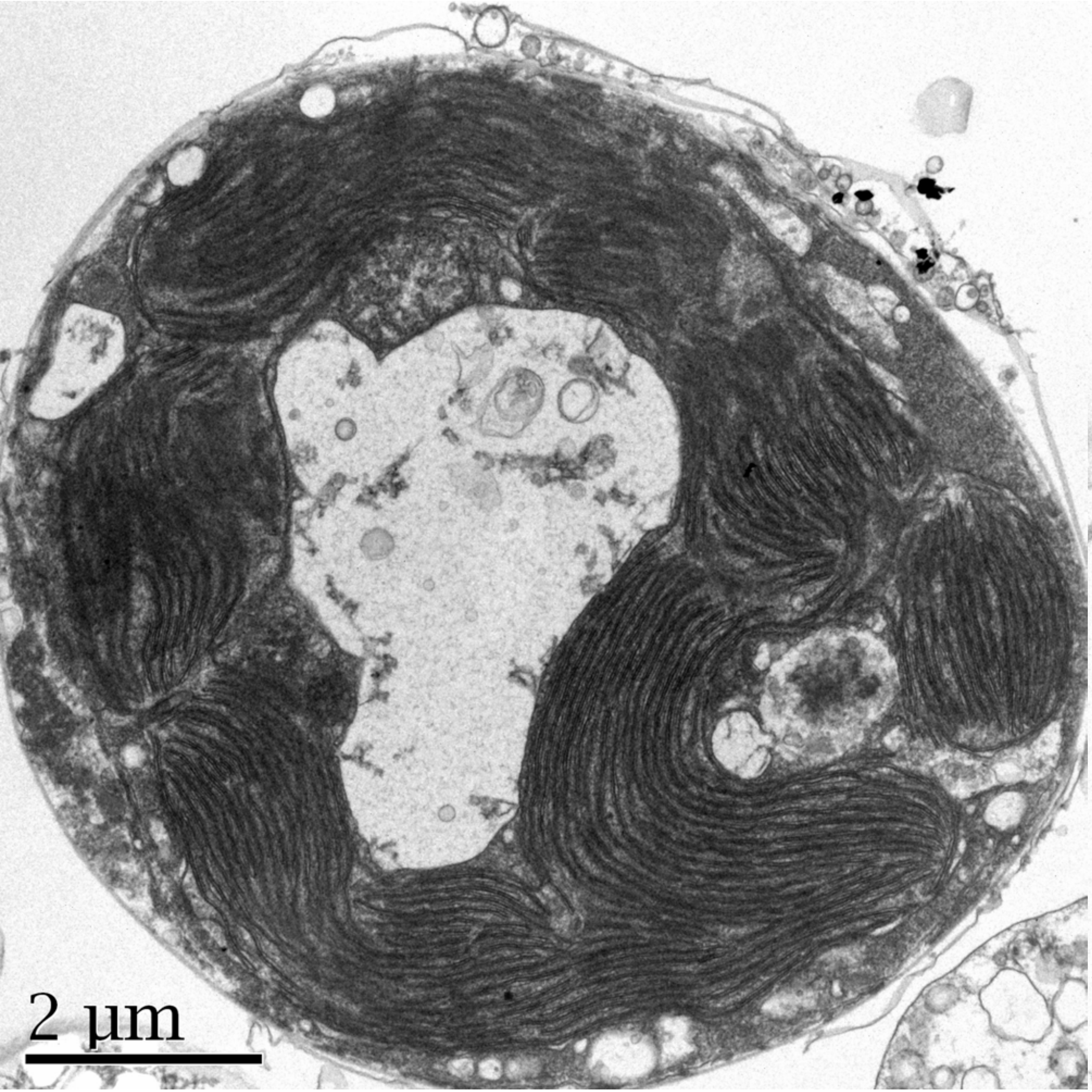

18-7_Correa_XH4_3GrridC1_46

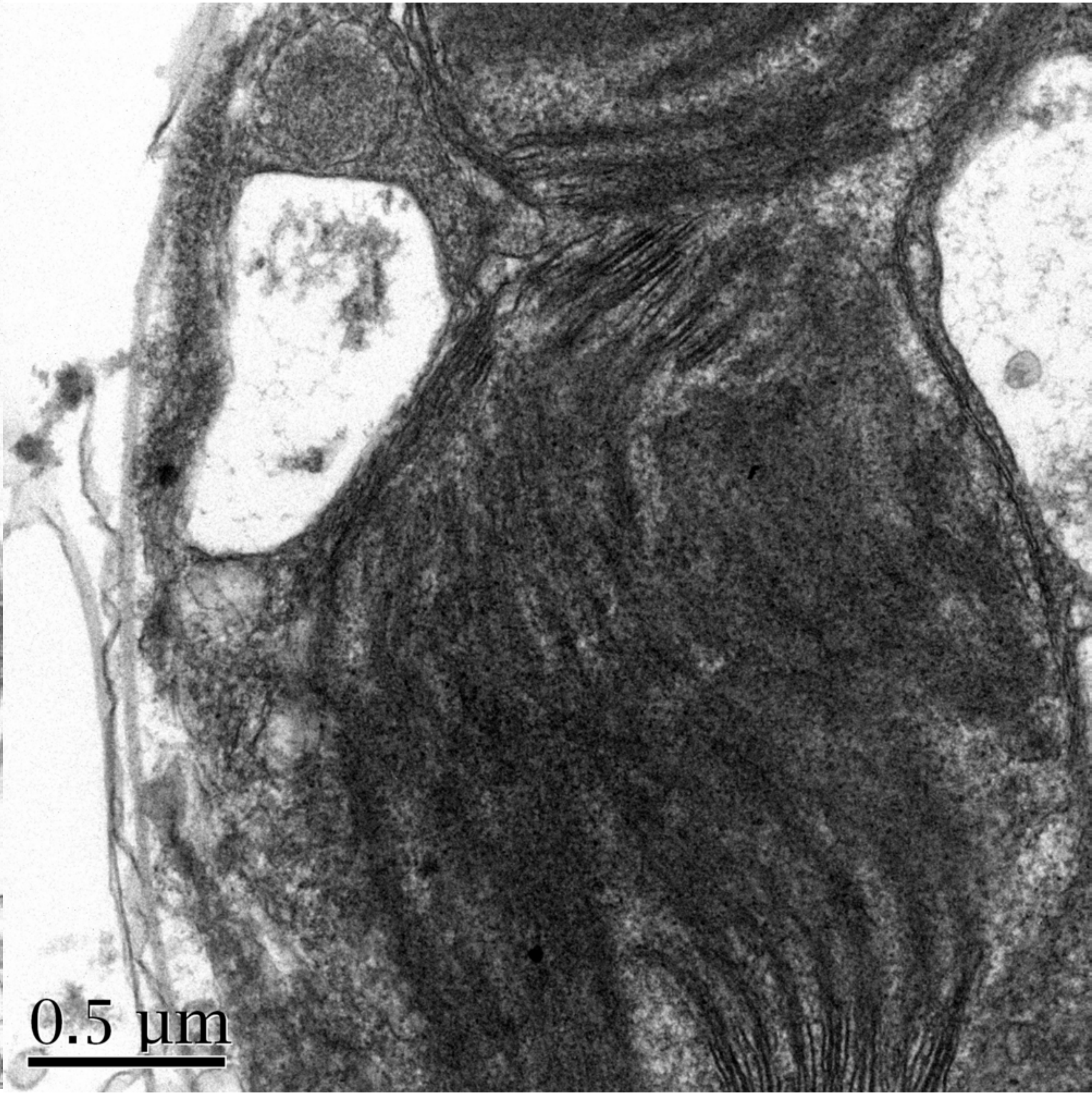

18-7_Correa_XH4_3GrridC1_48

Cell 10

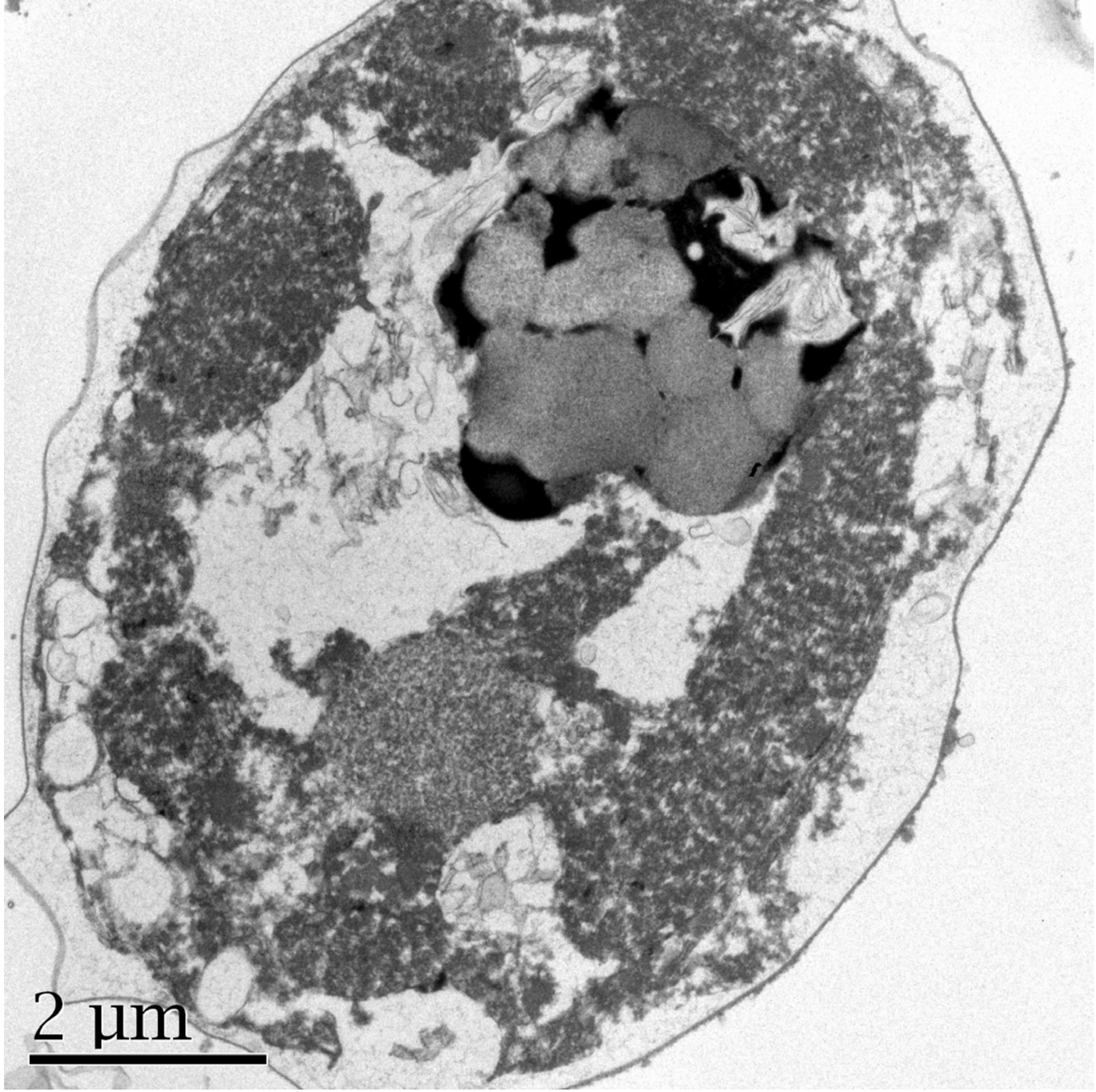

18-7_Correa_XH4_3GrridC1_50

Cell 11

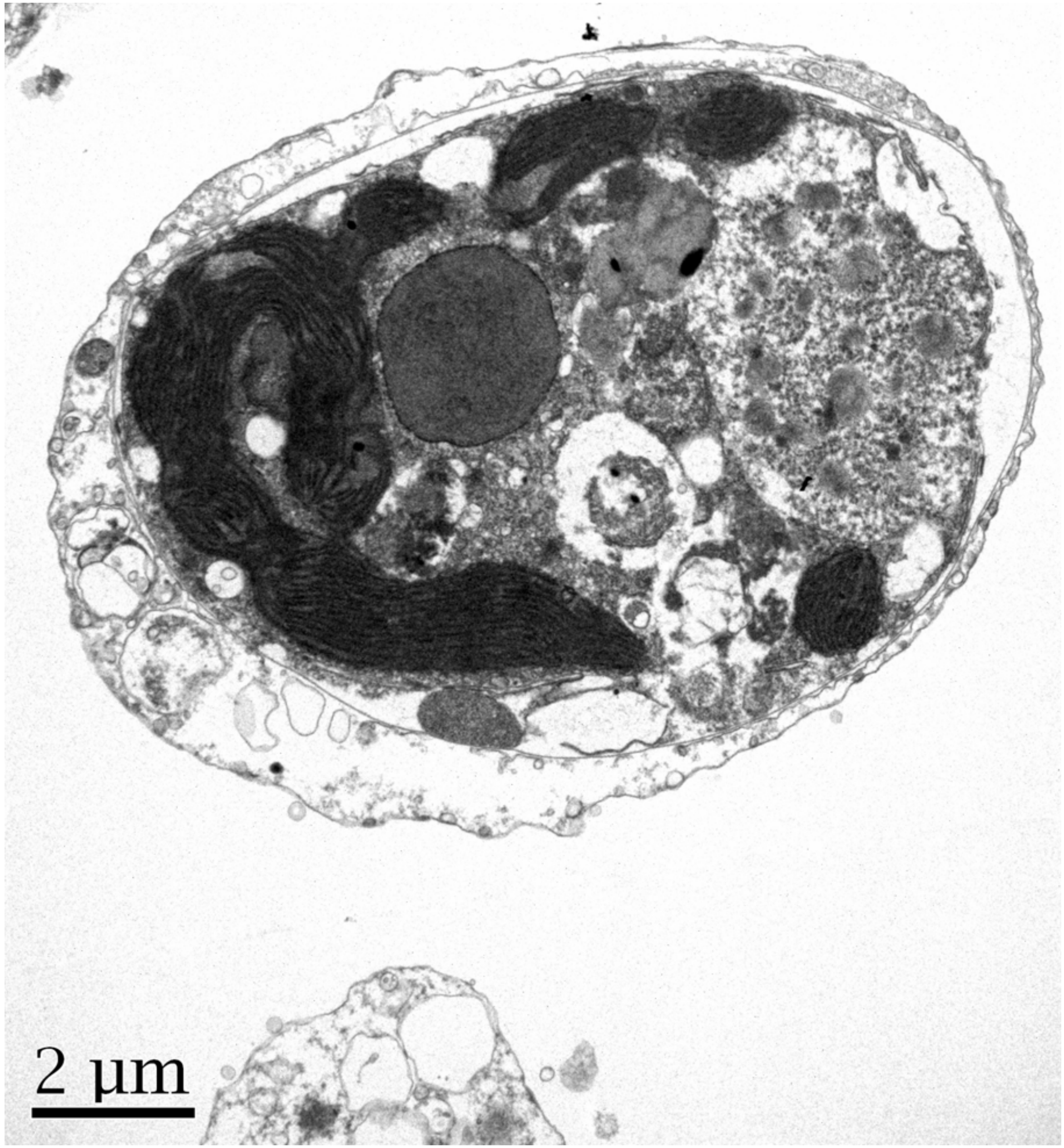

18-7_Correa_XH4_3GrridC1_51

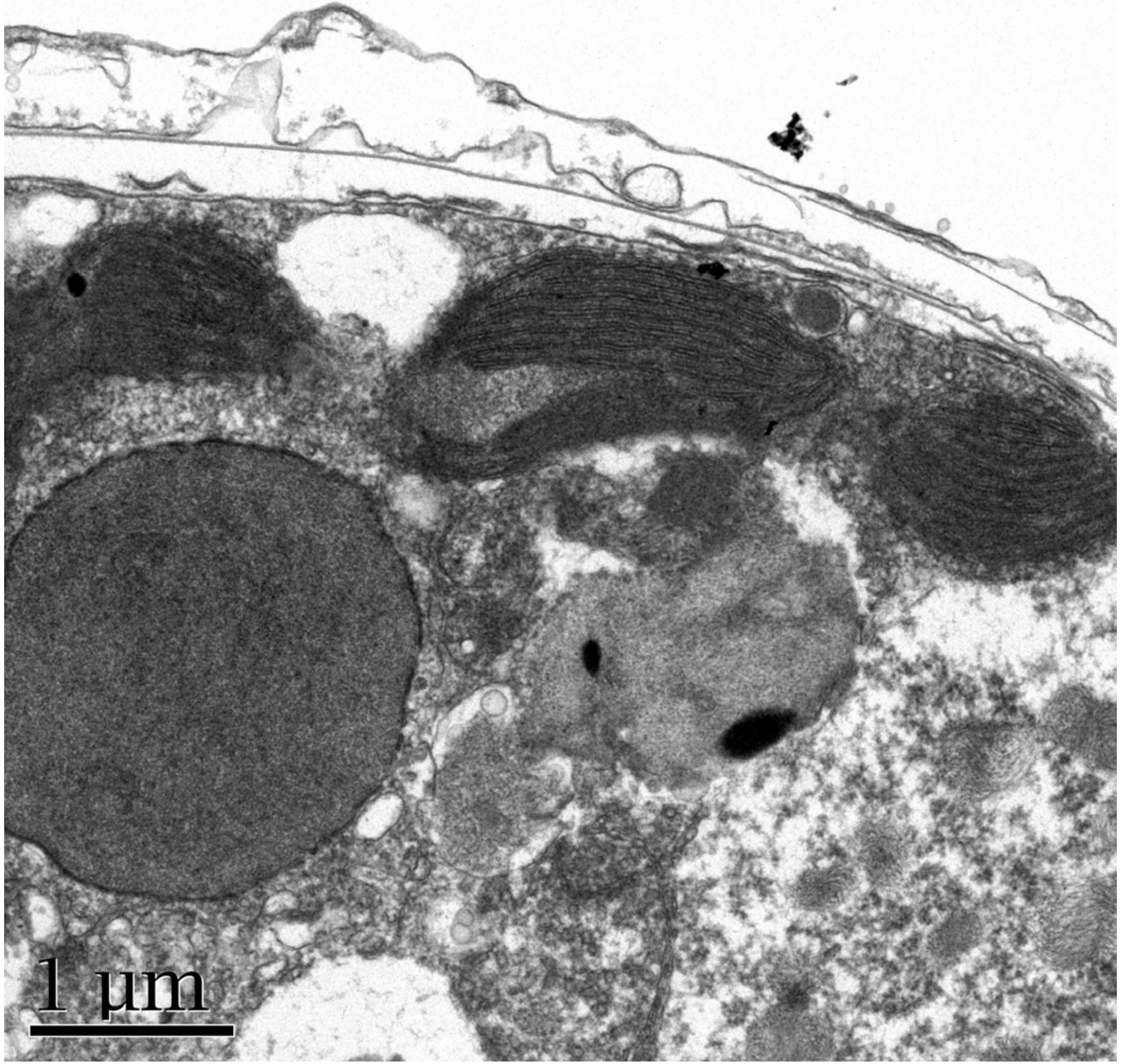

18-7_Correa_XH4_3GrridC1_54

Cell 12

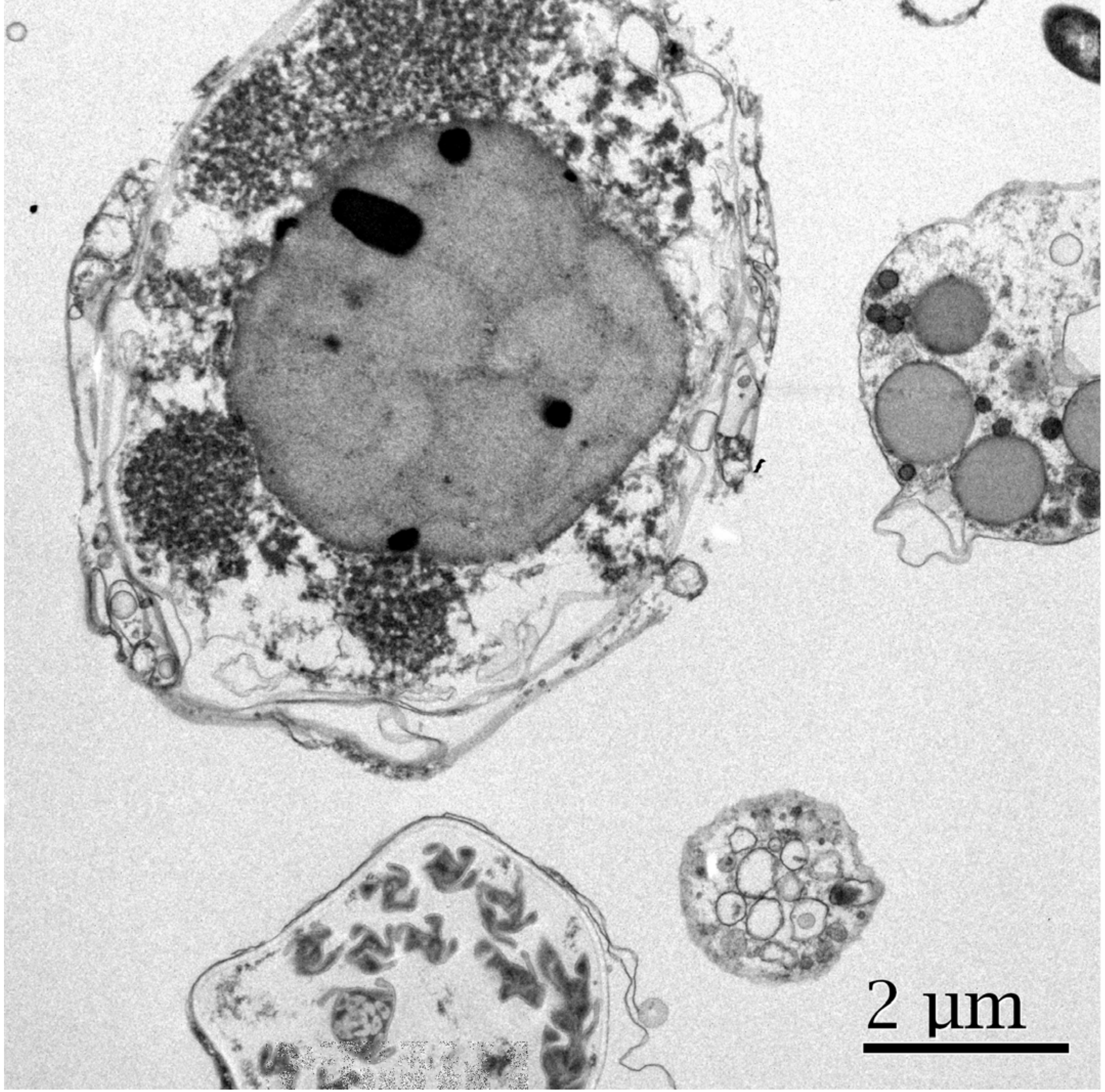

18-7_Correa_XH4_3GrridC1_56

Cell 13

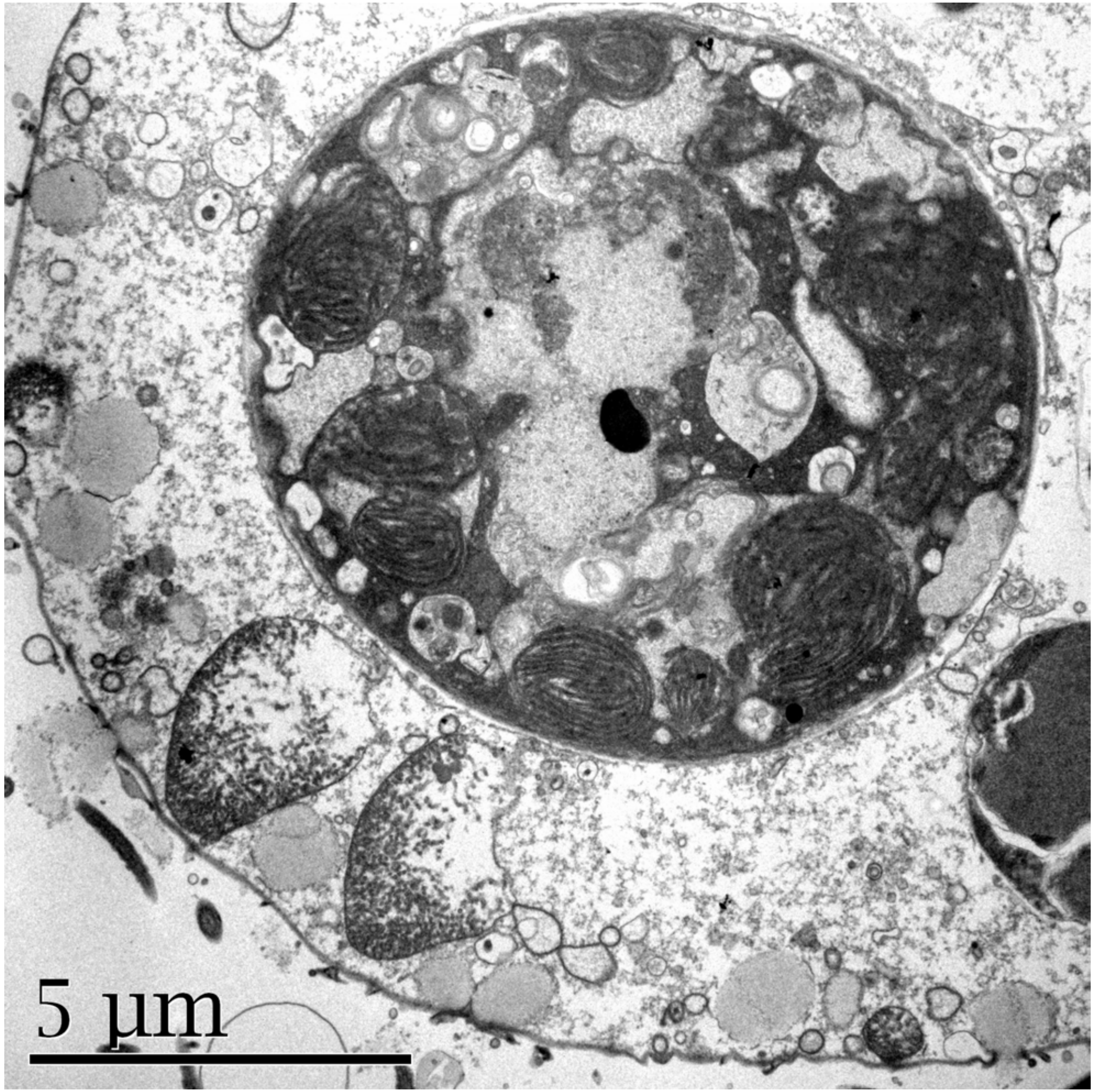

18-7_Correa_XH4_3GrridC1_58

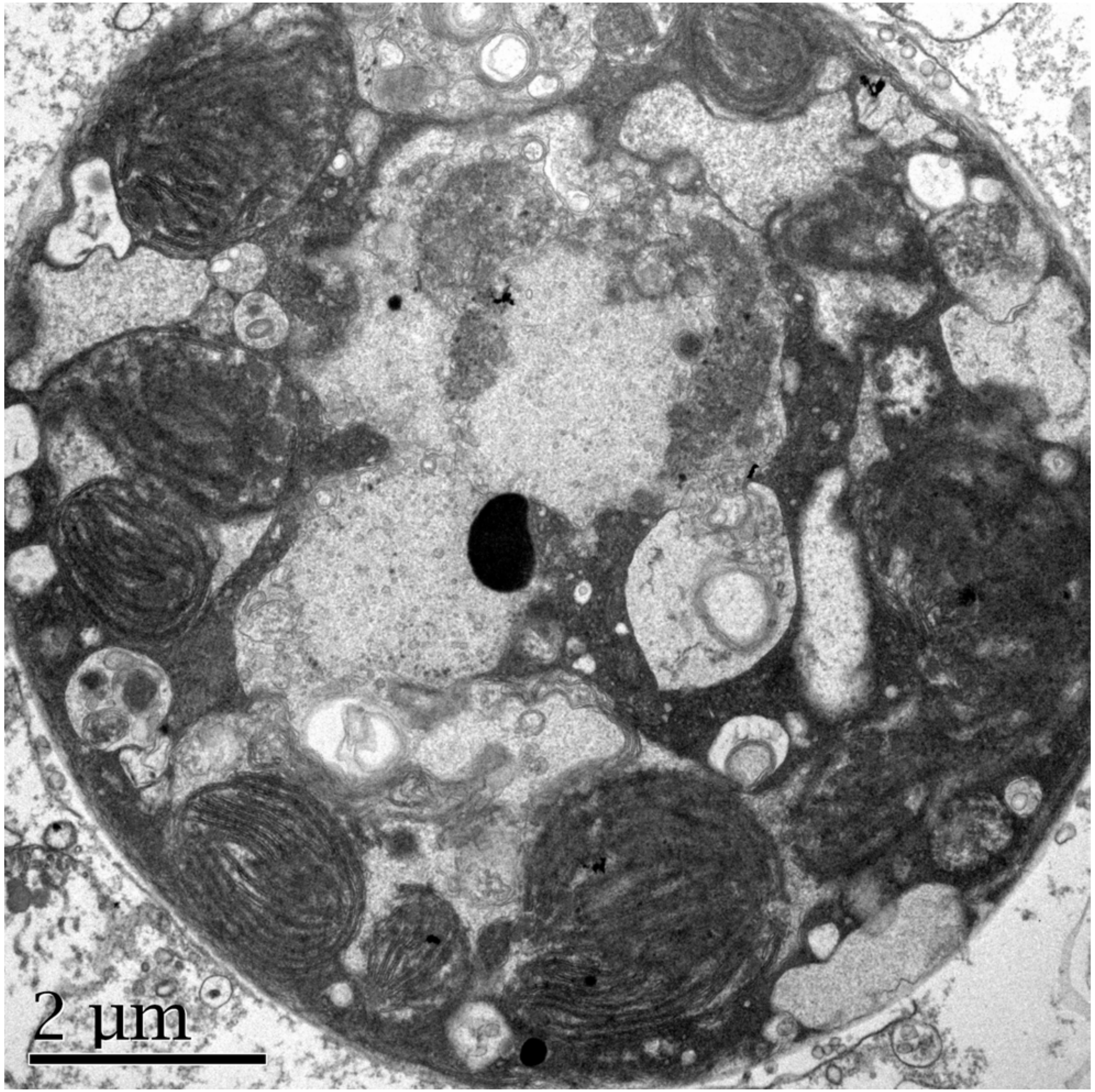

18-7_Correa_XH4_3GrridC1_59

Cell 14

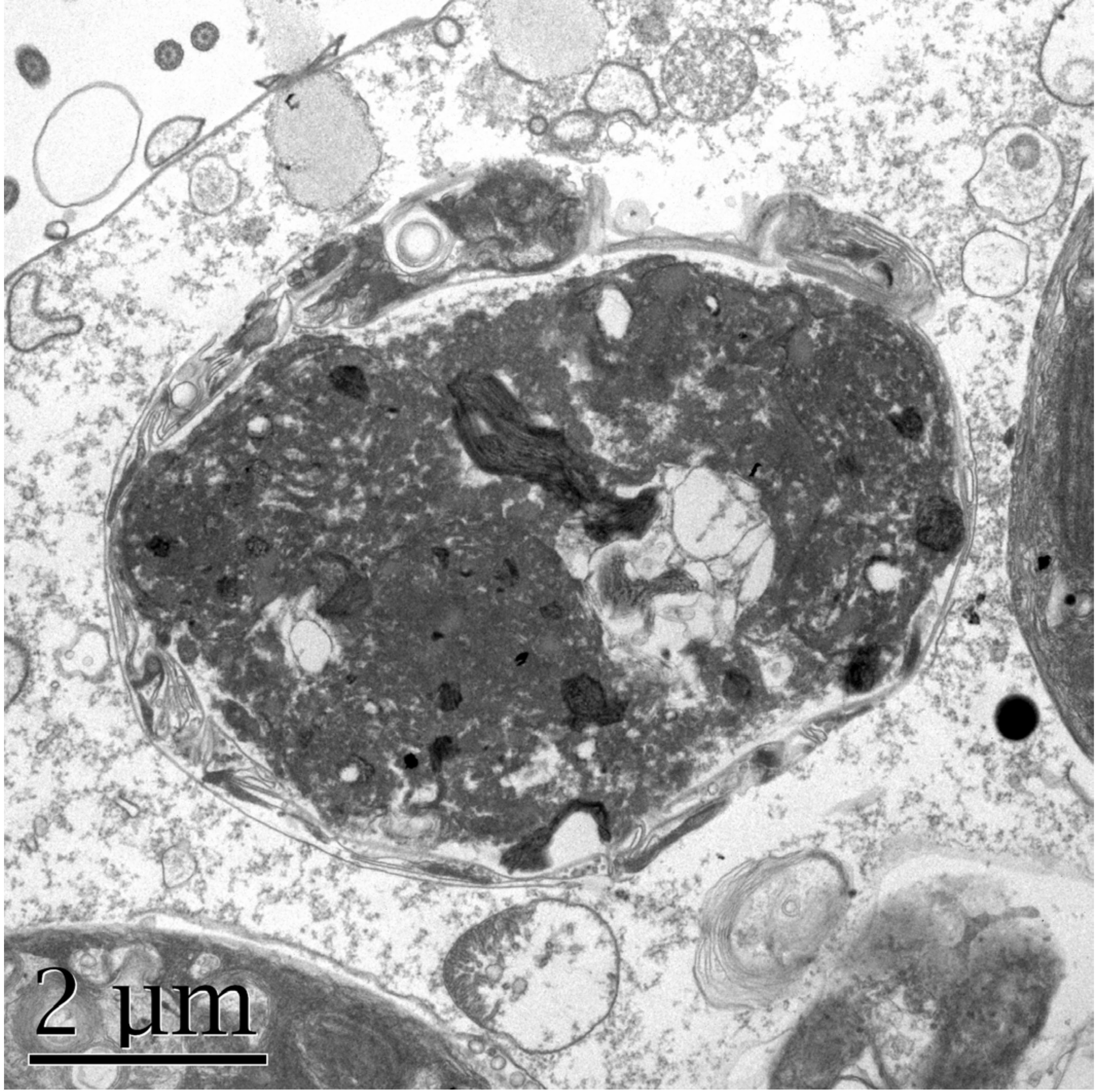

18-7_Correa_XH4_3GrridC1_60

Cell 15

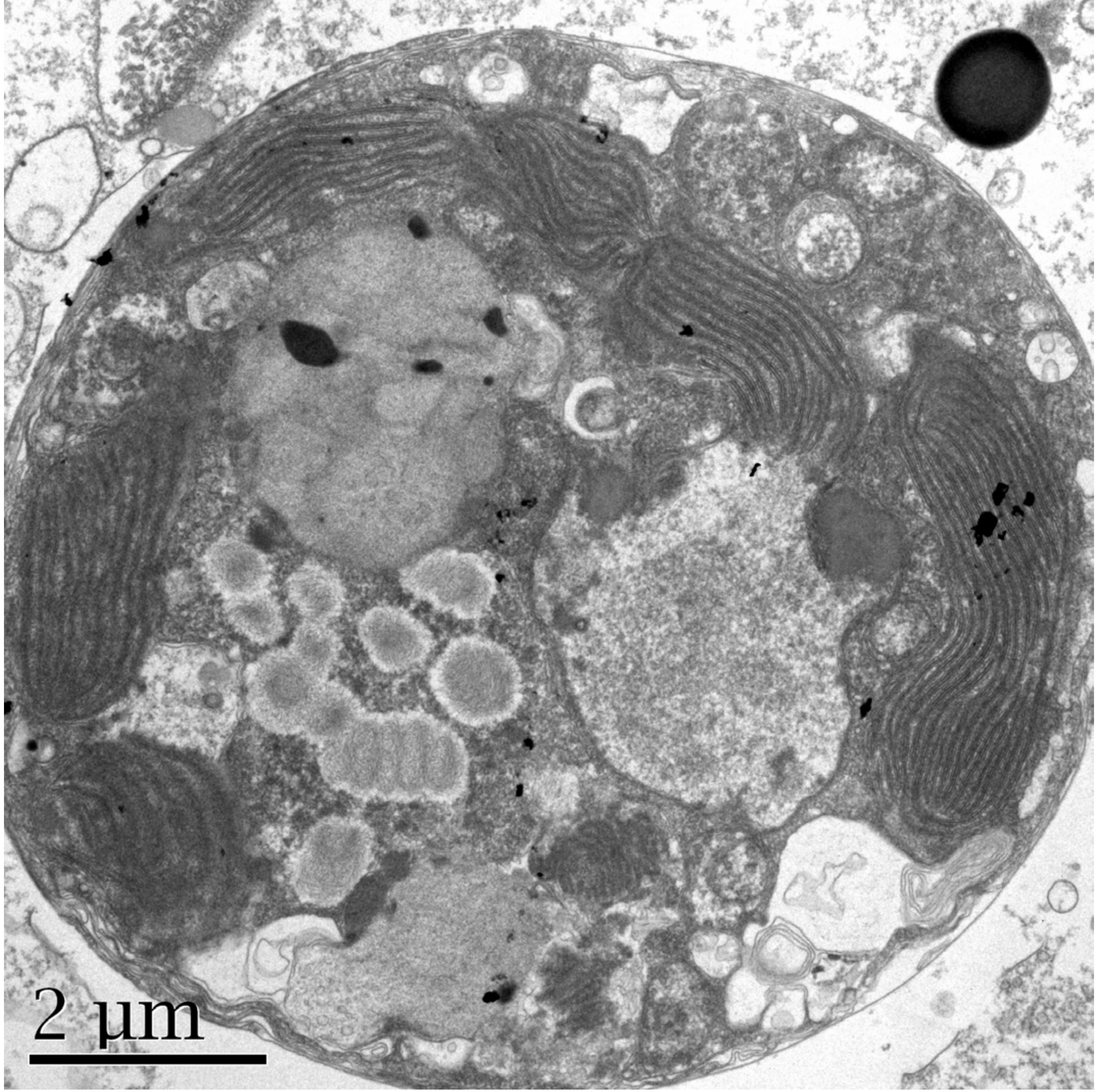

18-7_Correa_XH4_3GrridC1_61

Cell 16

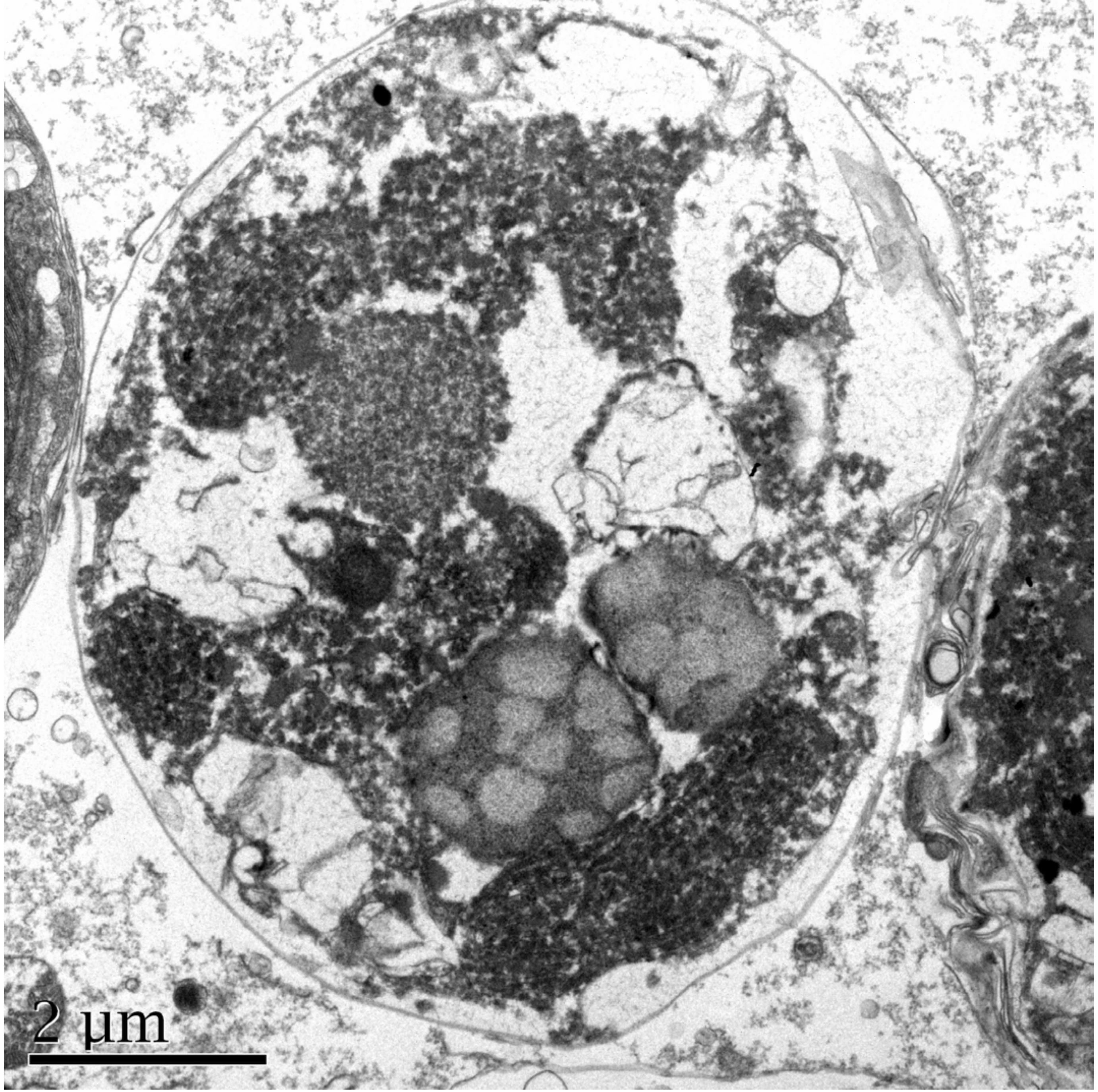

18-7_Correa_XH4_3GrridC1_62

Cell 17

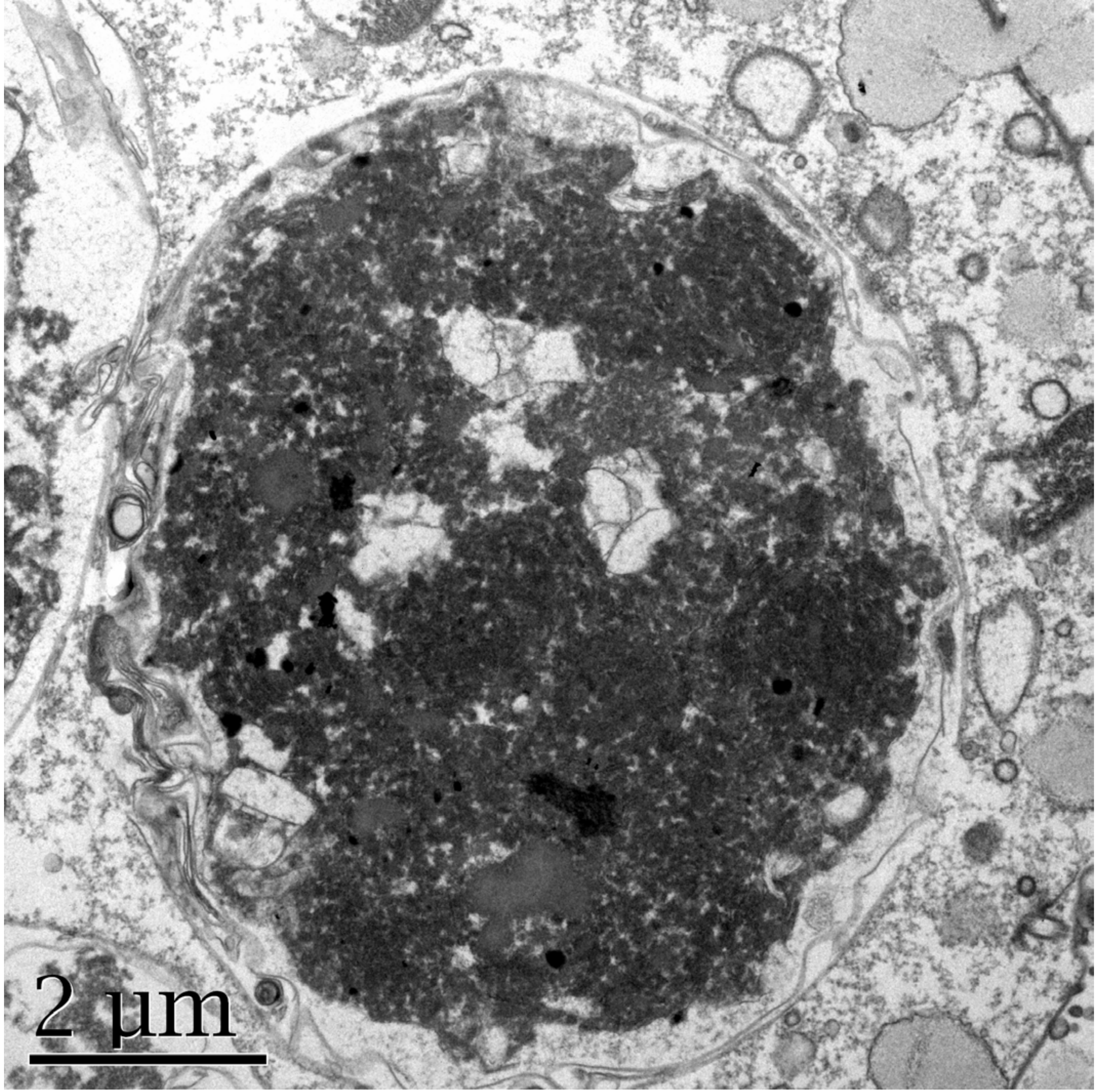

18-7_Correa_XH4_3GrridC1_63

Cell 18

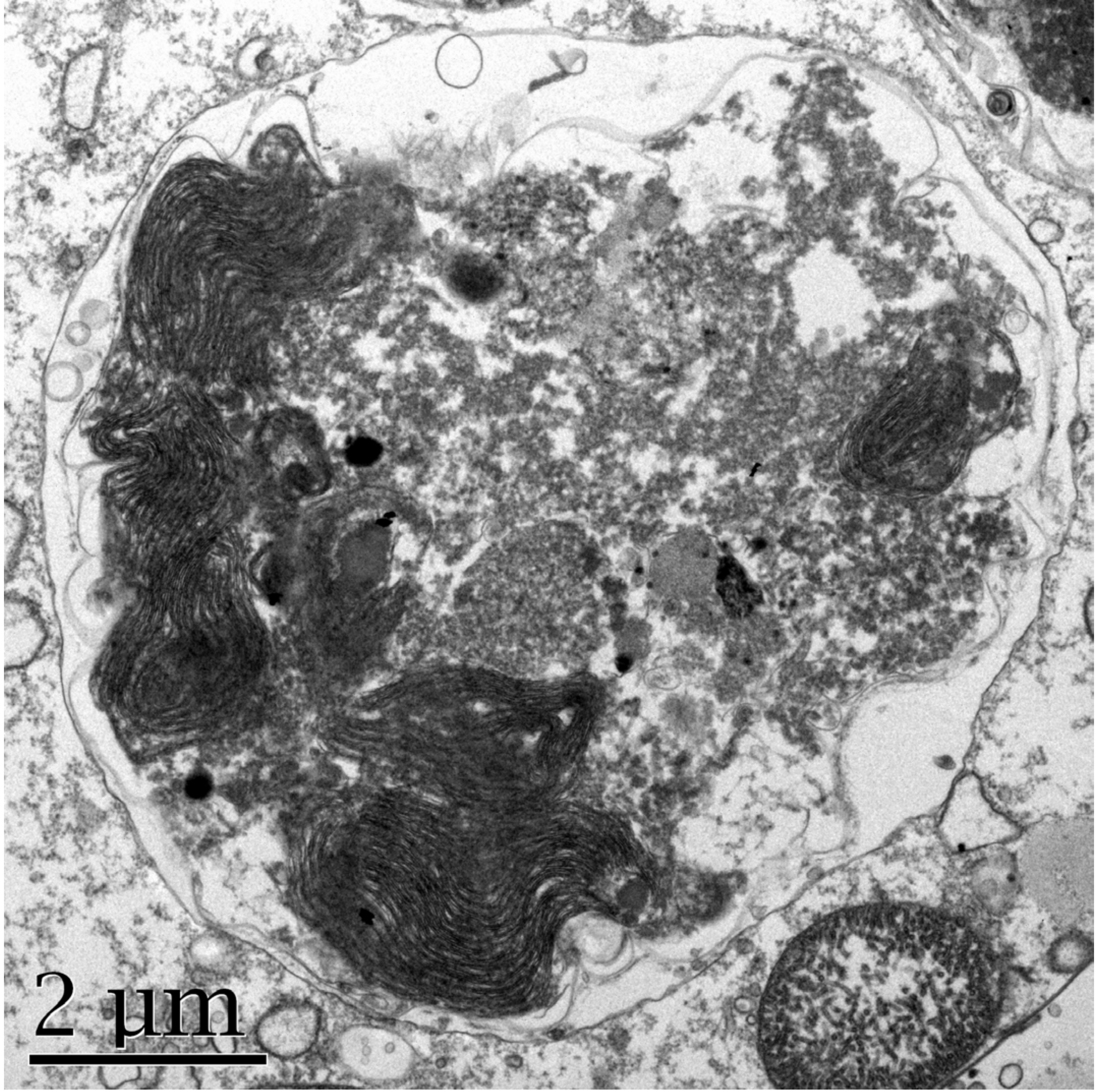

18-7_Correa_XH4_3GrridC1_64

Cell 19

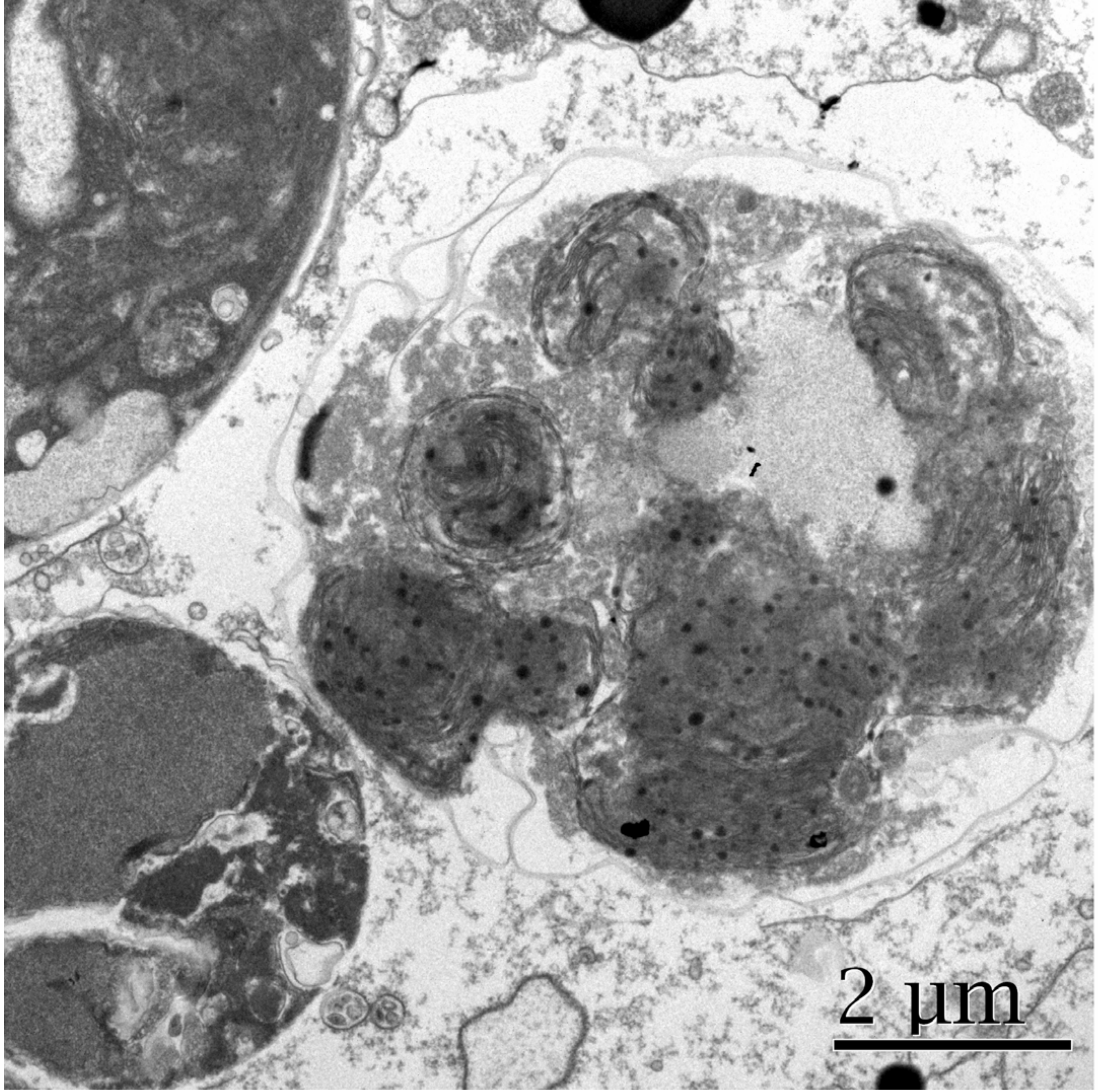

18-7_Correa_XH4_3GrridC1_65

Cell 20

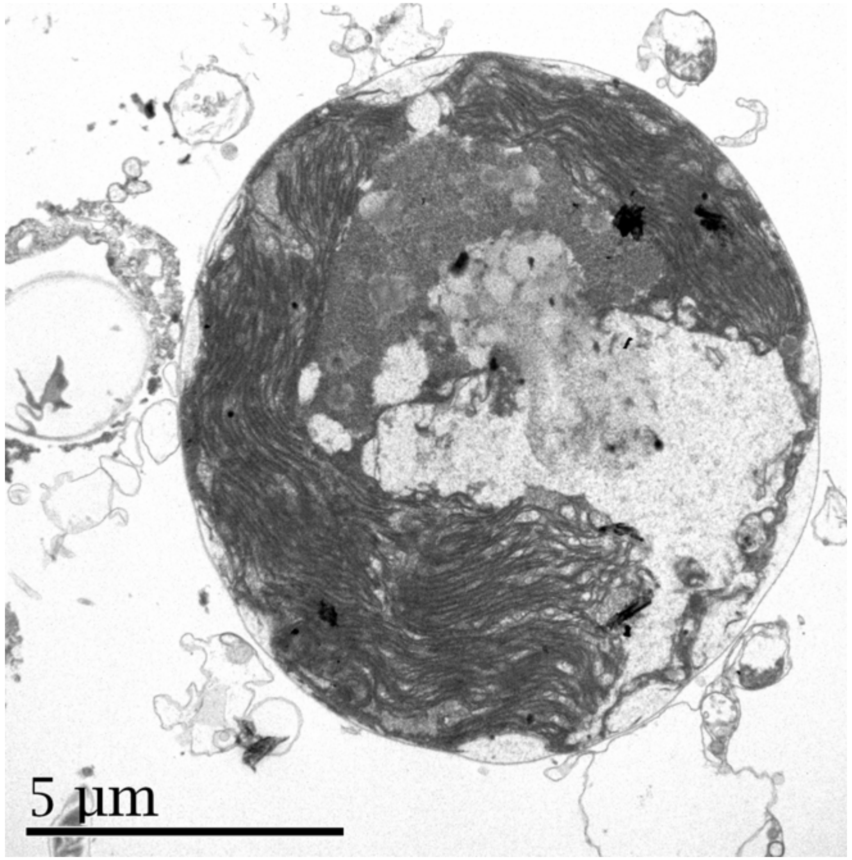

18-7_Correa_XH4_3GrridC1_67

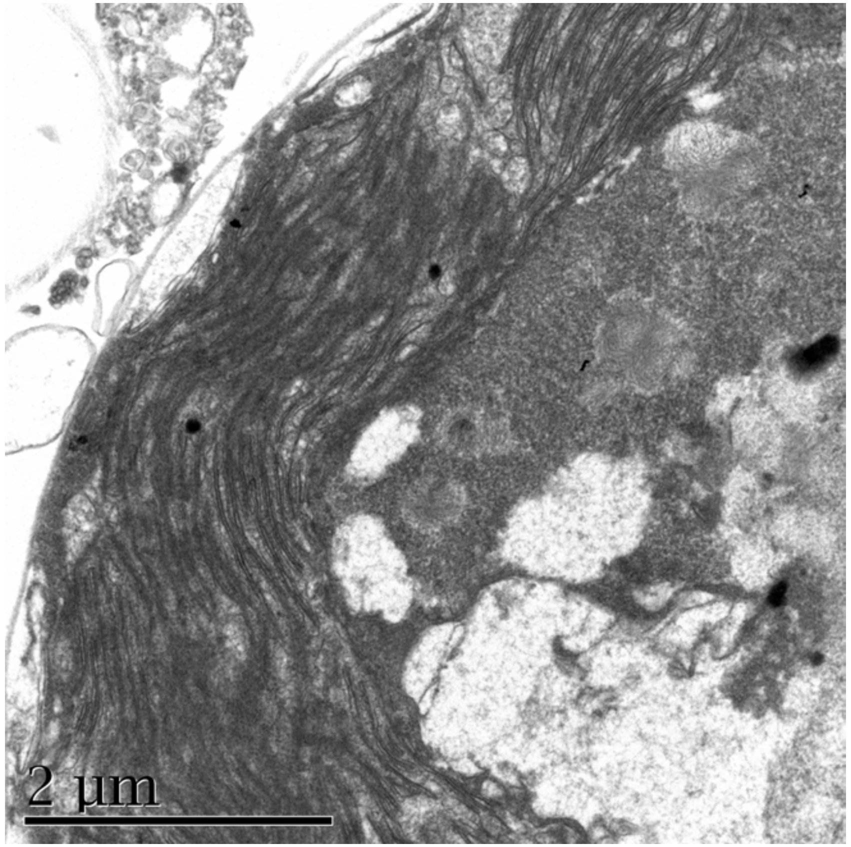

18-7_Correa_XH4_3GrridC1_68

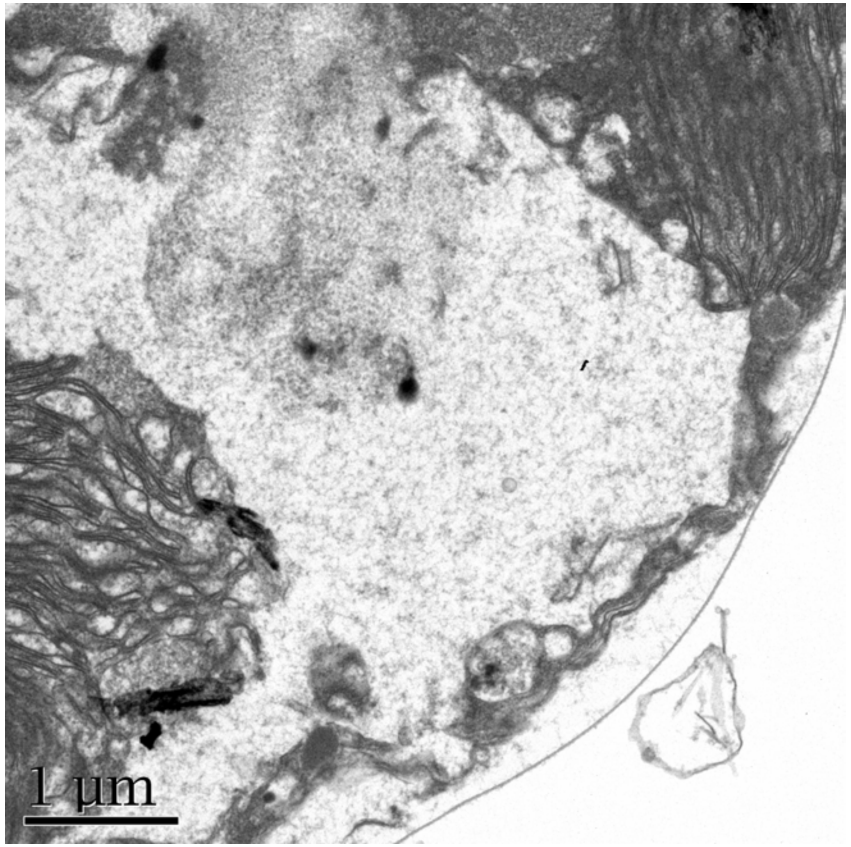

18-7_Correa_XH4_3GrridC1_70

ACR Colony D Expelled- Ambient

Cell 1

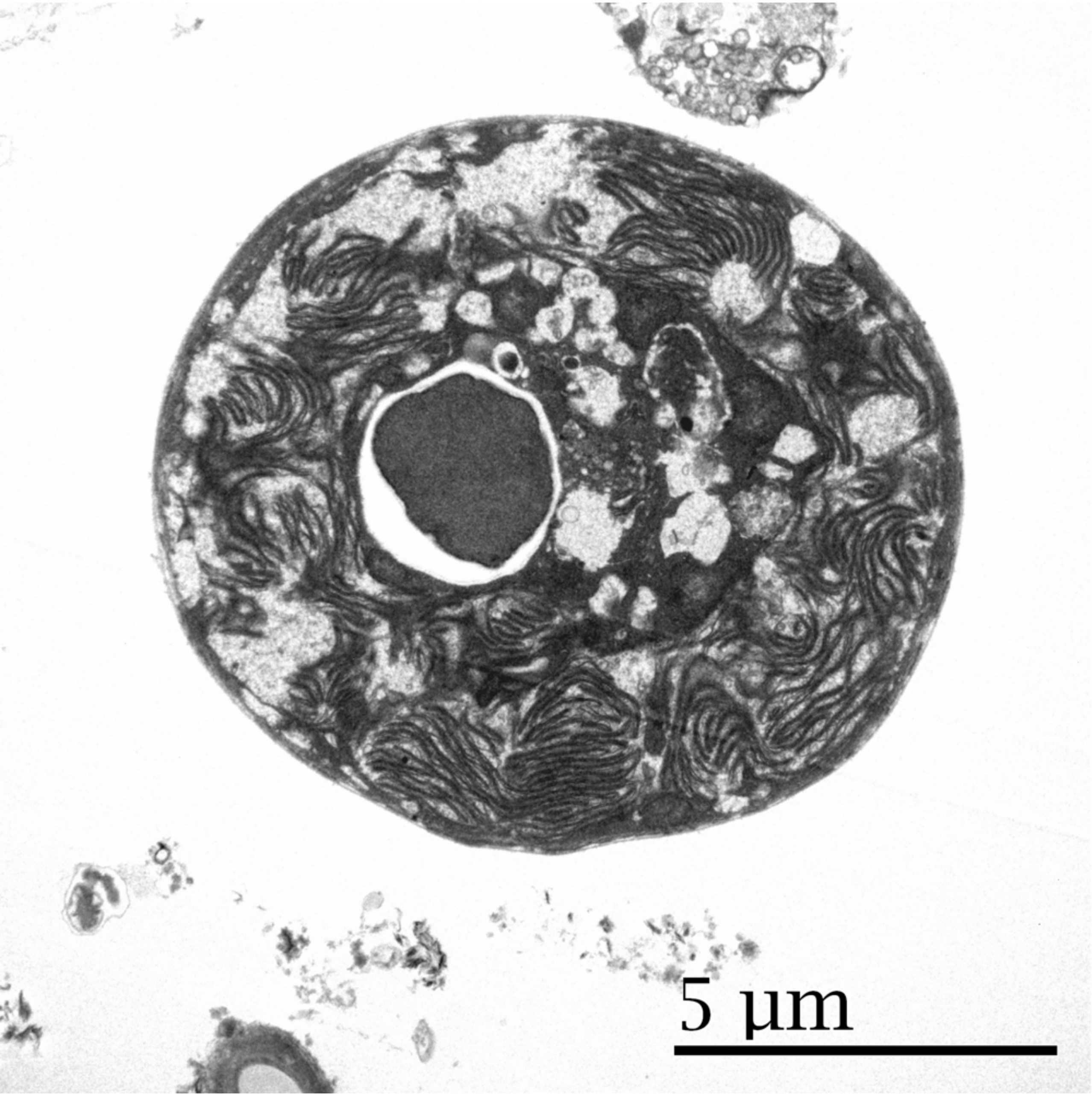

18-7_Correa_YC4_3GridC4_3

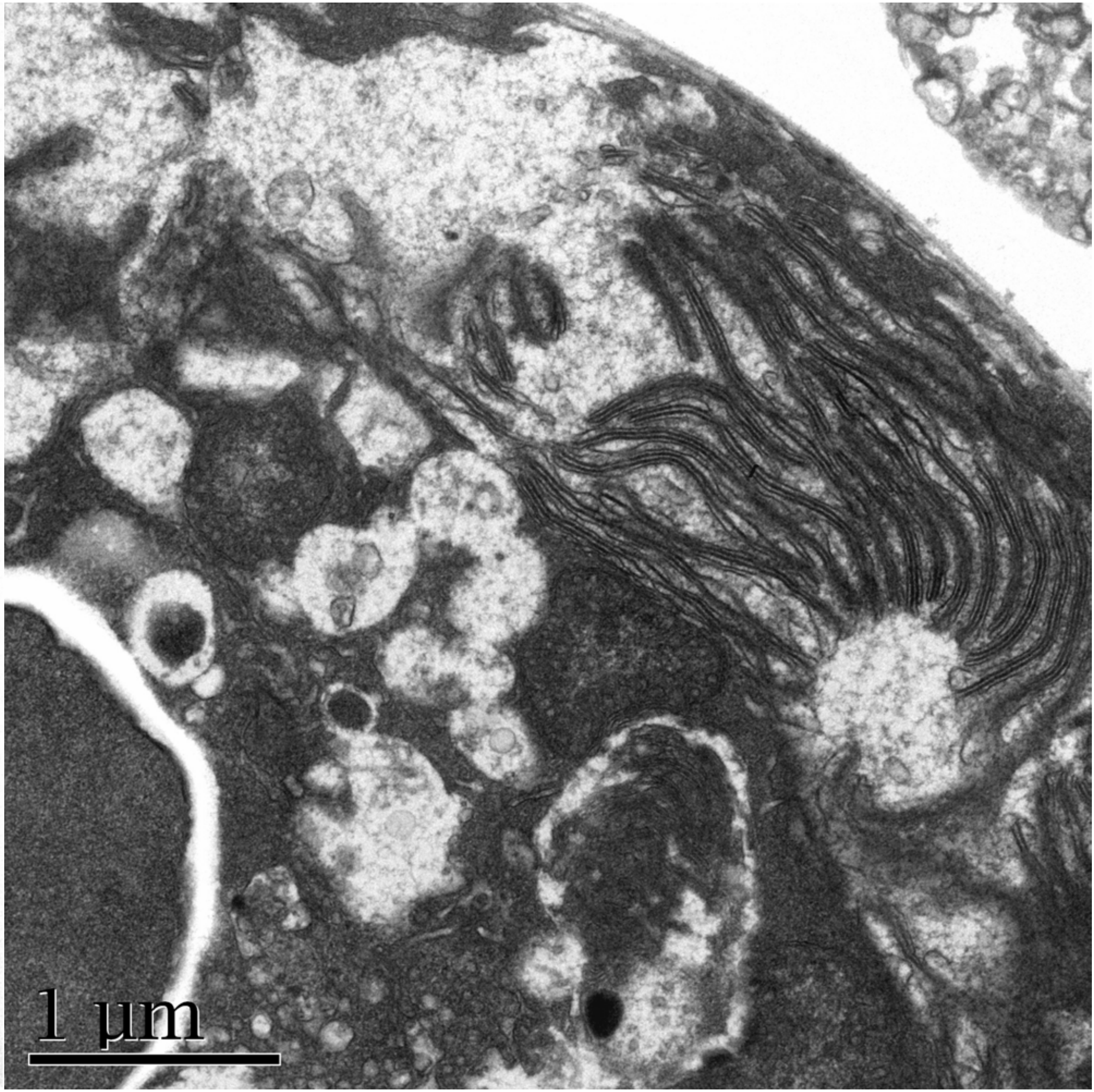

18-7_Correa_YC4_3GridC4_8

Cell 2

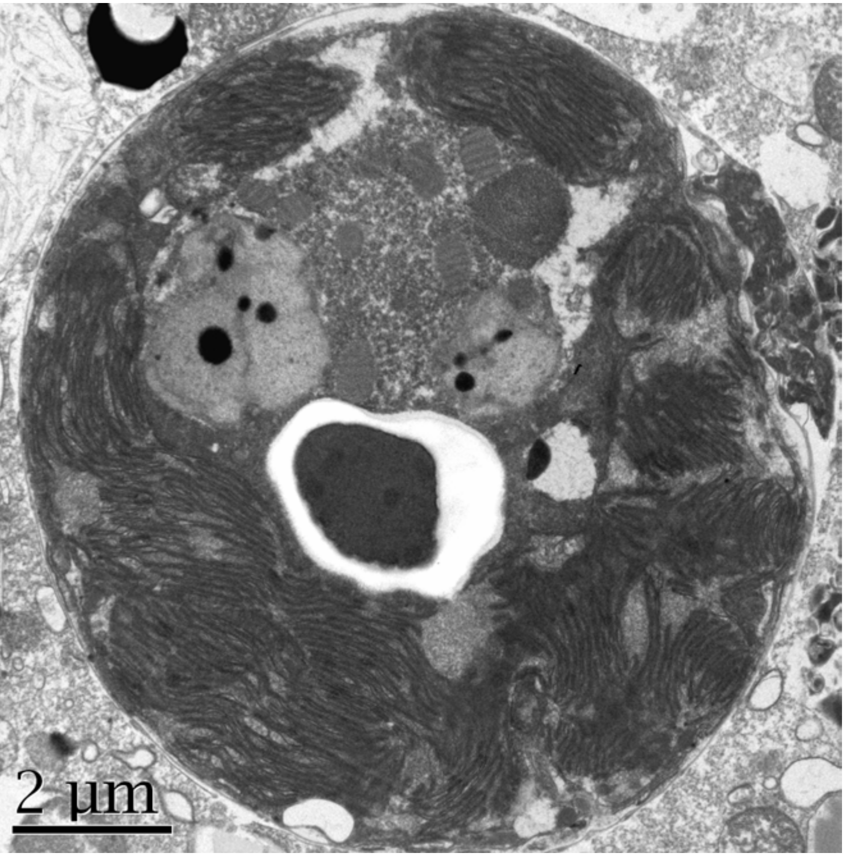

18-7_Correa_YC4_3GridC4_25

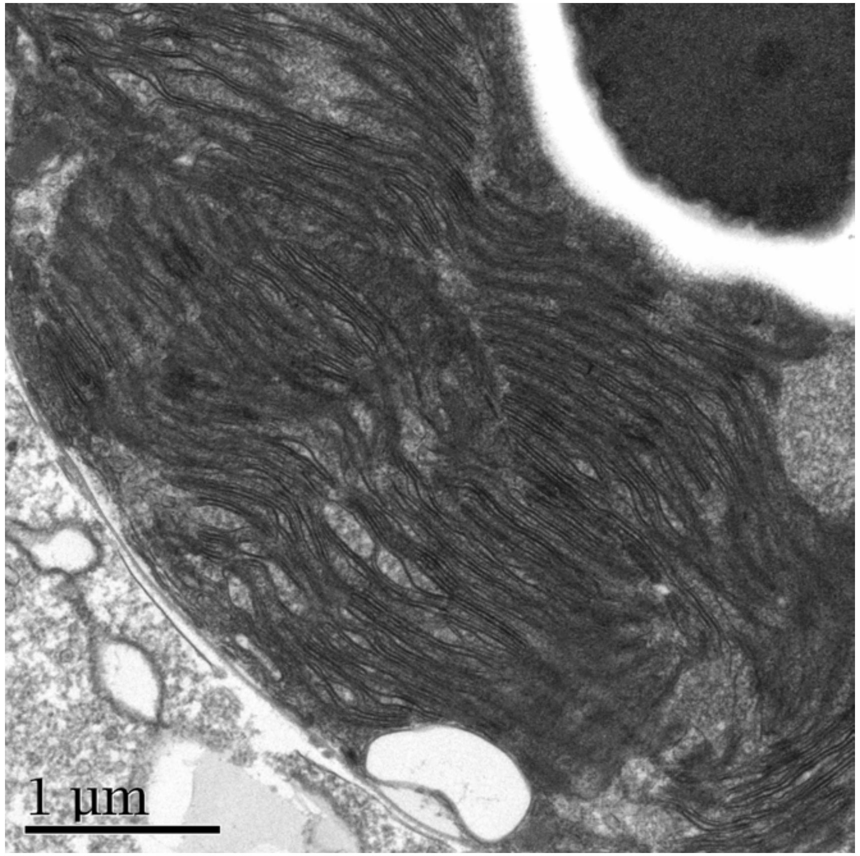

18-7_Correa_YC4_3GridC4_26

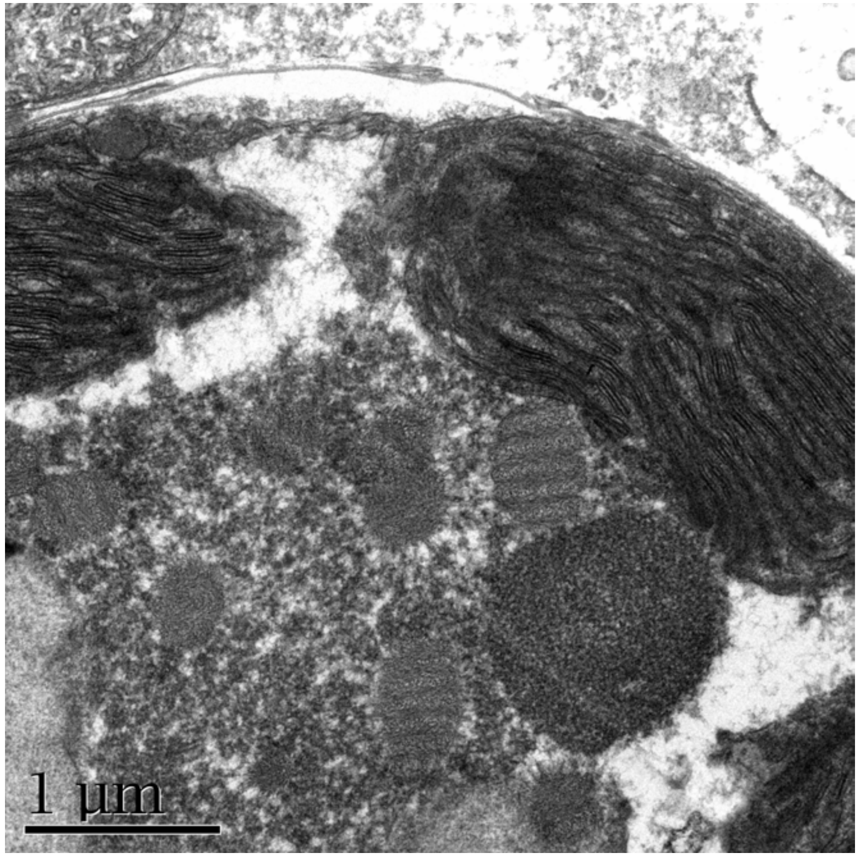

18-7_Correa_YC4_3GridC4_30

Cell 3

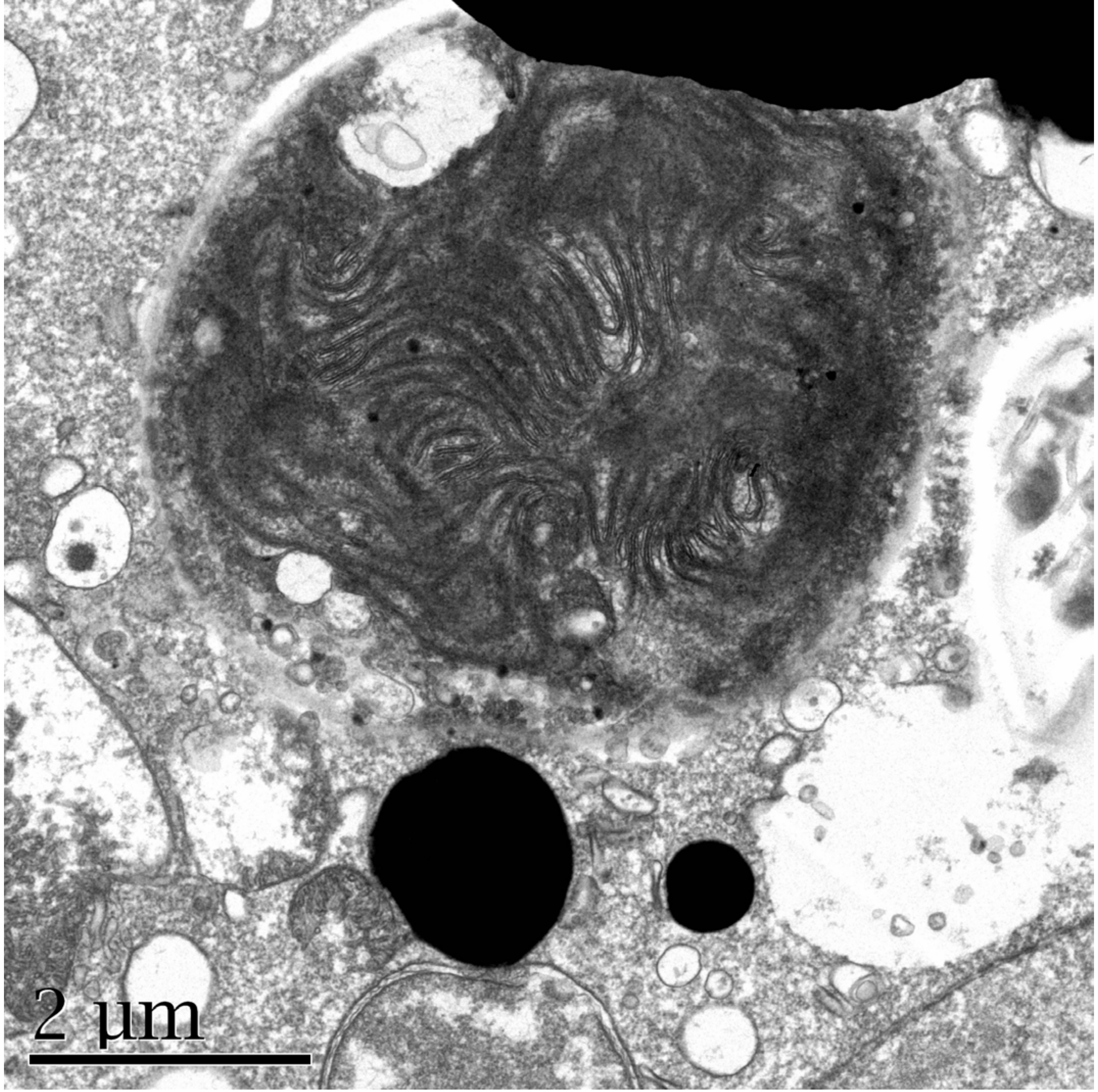

18-7_Correa_YC4_3GridC4_32

Cell 4

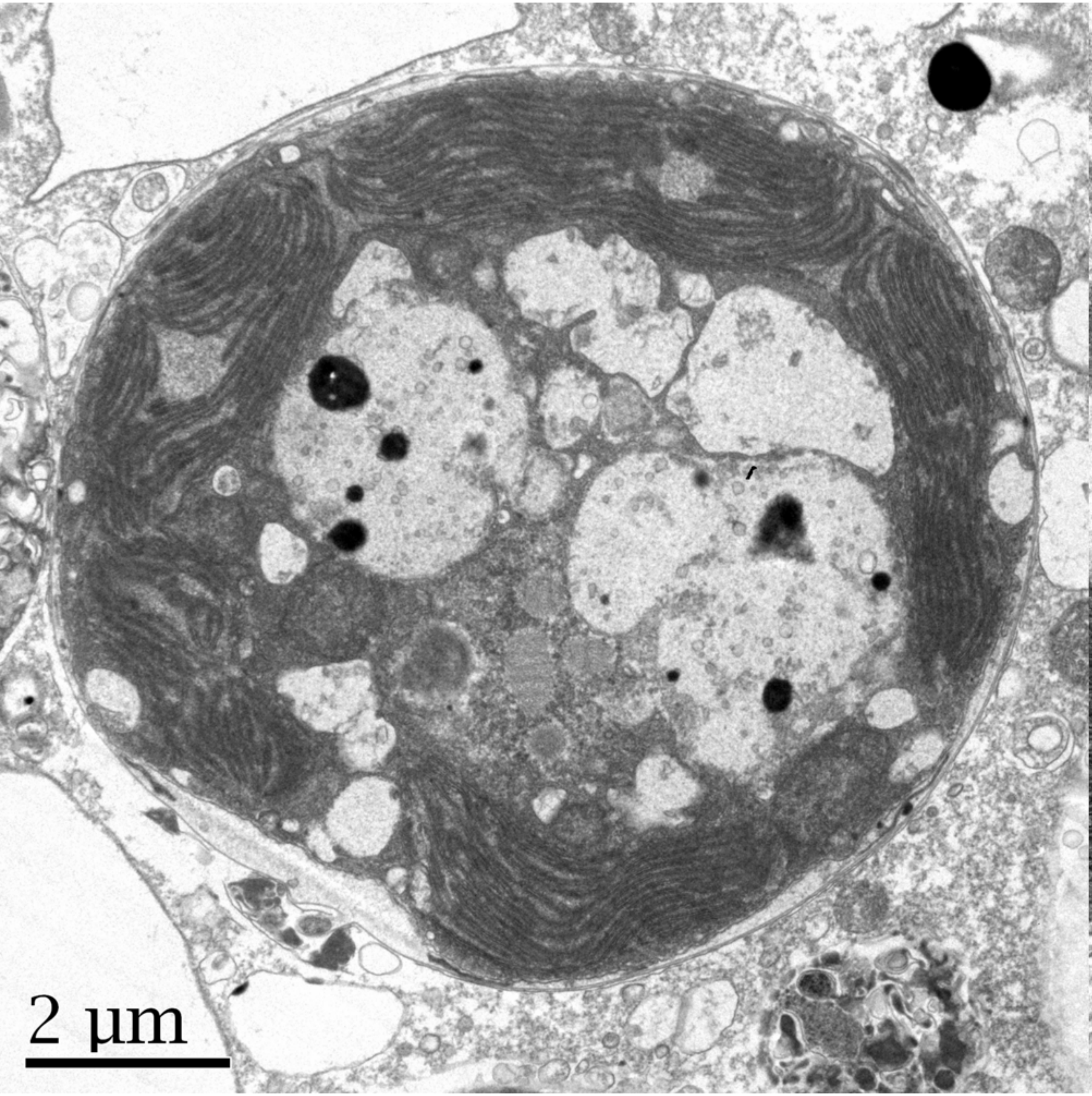

18-7_Correa_YC4_3GridC4_38

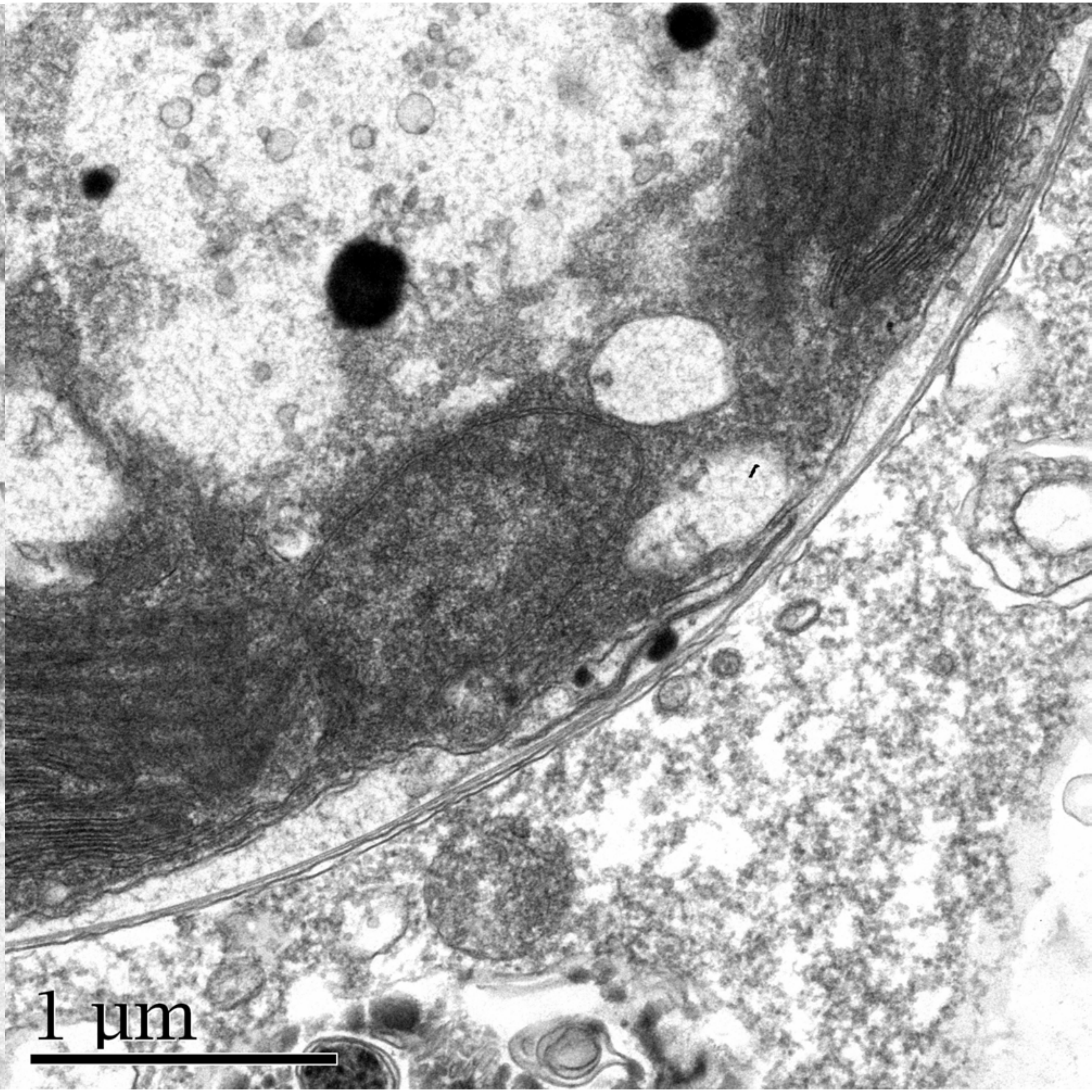

18-7_Correa_YC4_3GridC4_39

Cell 5

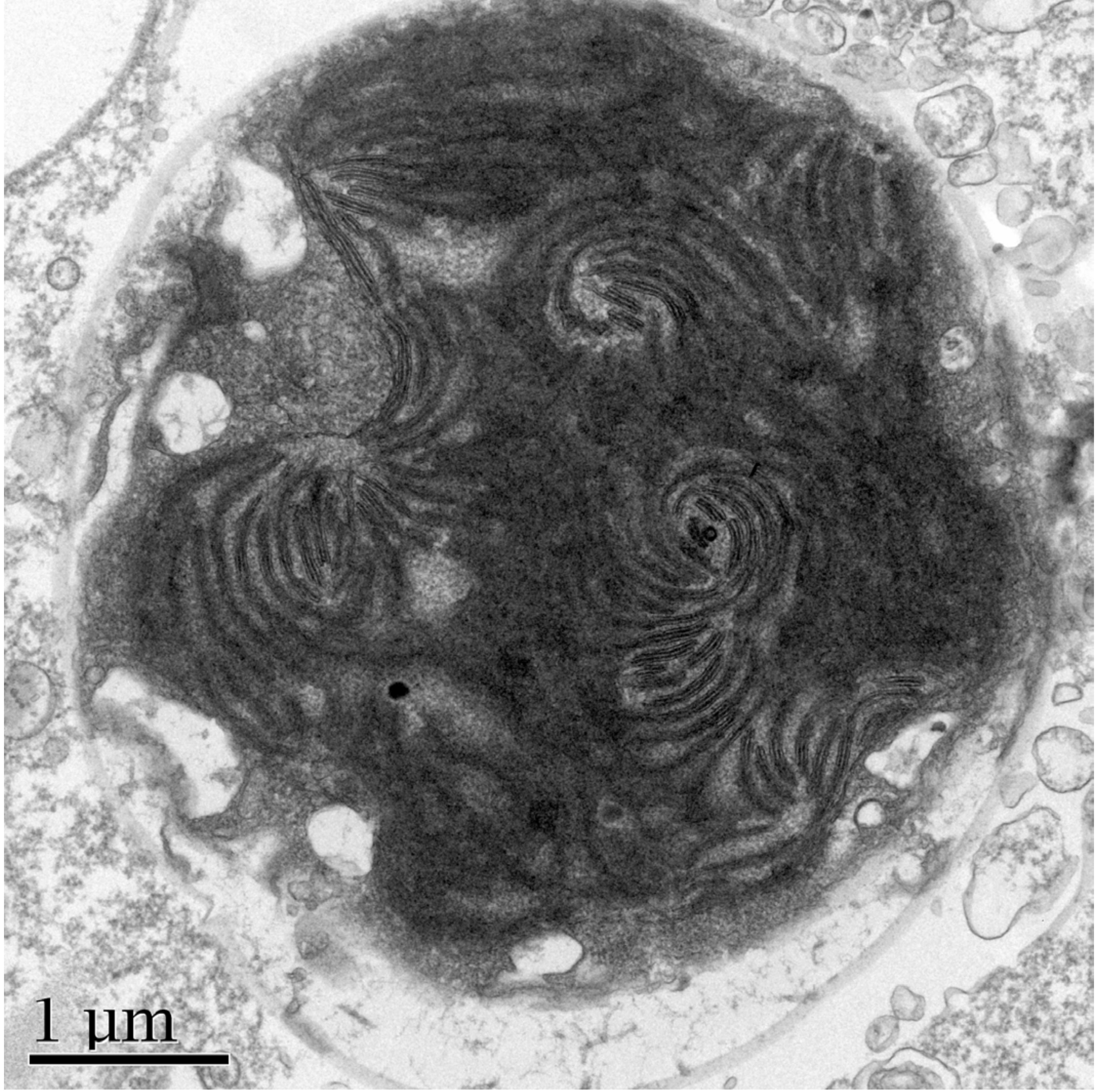

18-7_Correa_YC4_3GridC4_42

Cell 6

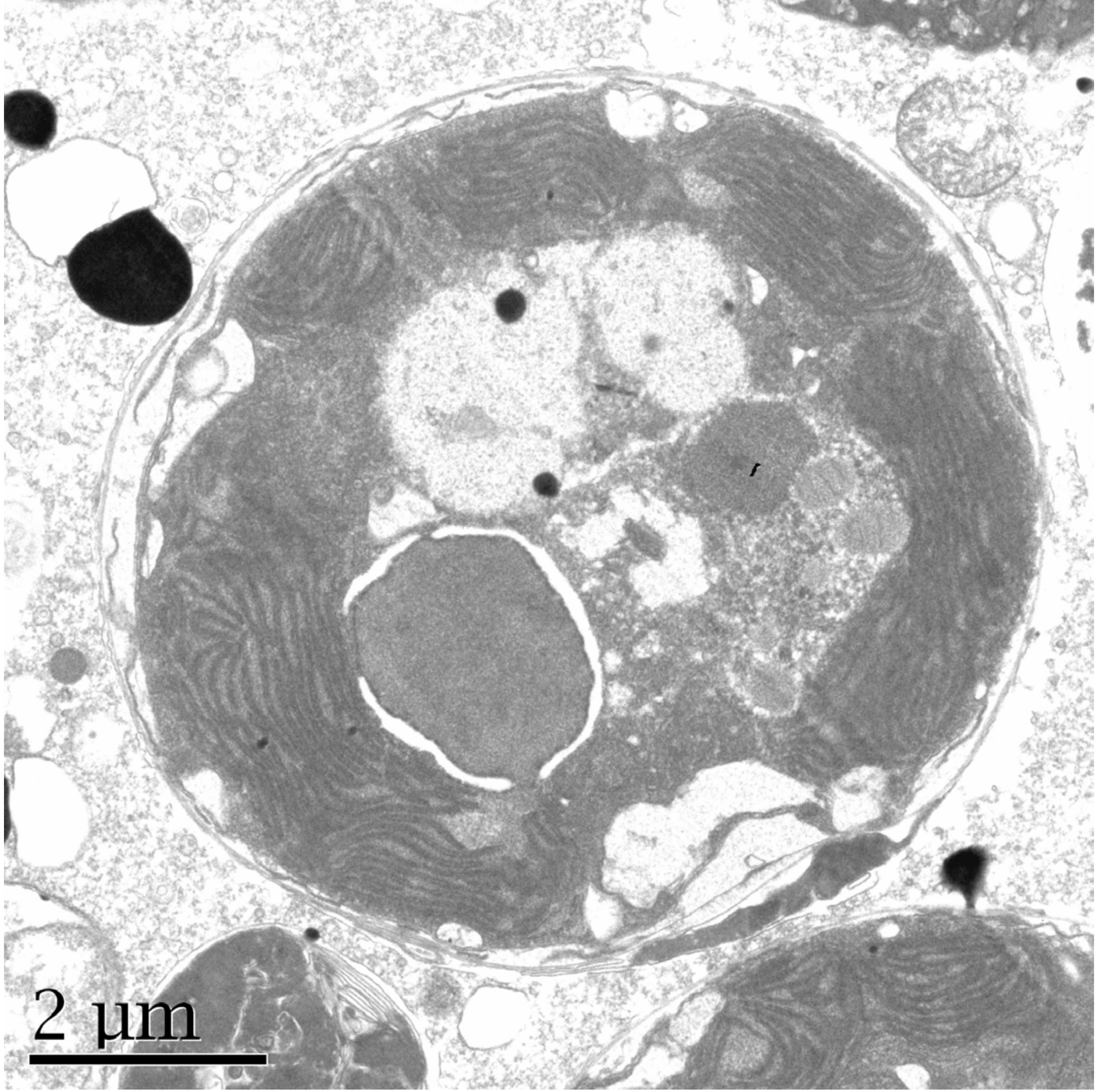

18-7_Correa_YC4_3GridC4_44

Cell 7

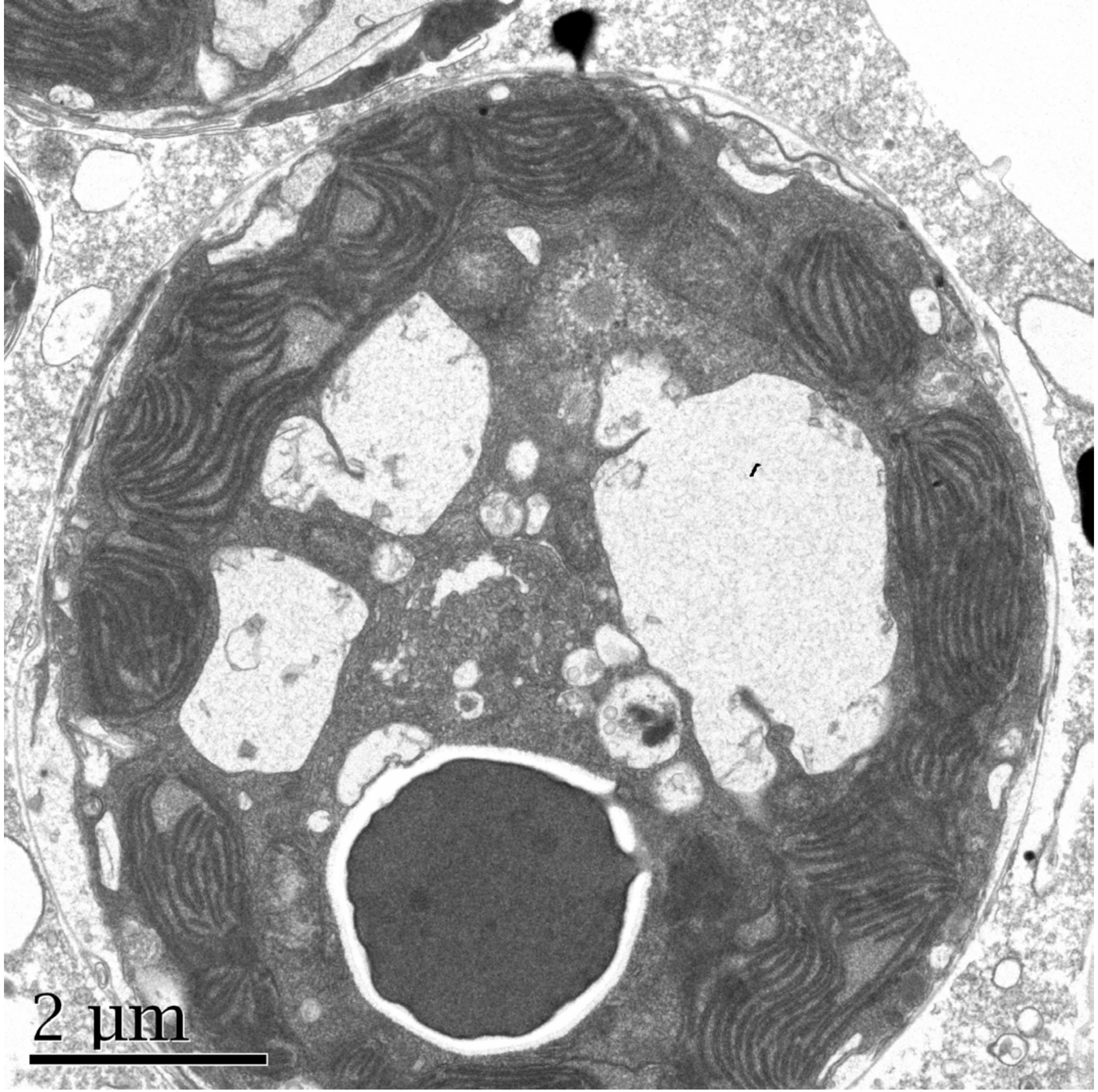

18-7_Correa_YC4_3GridC4_45

Cell 8

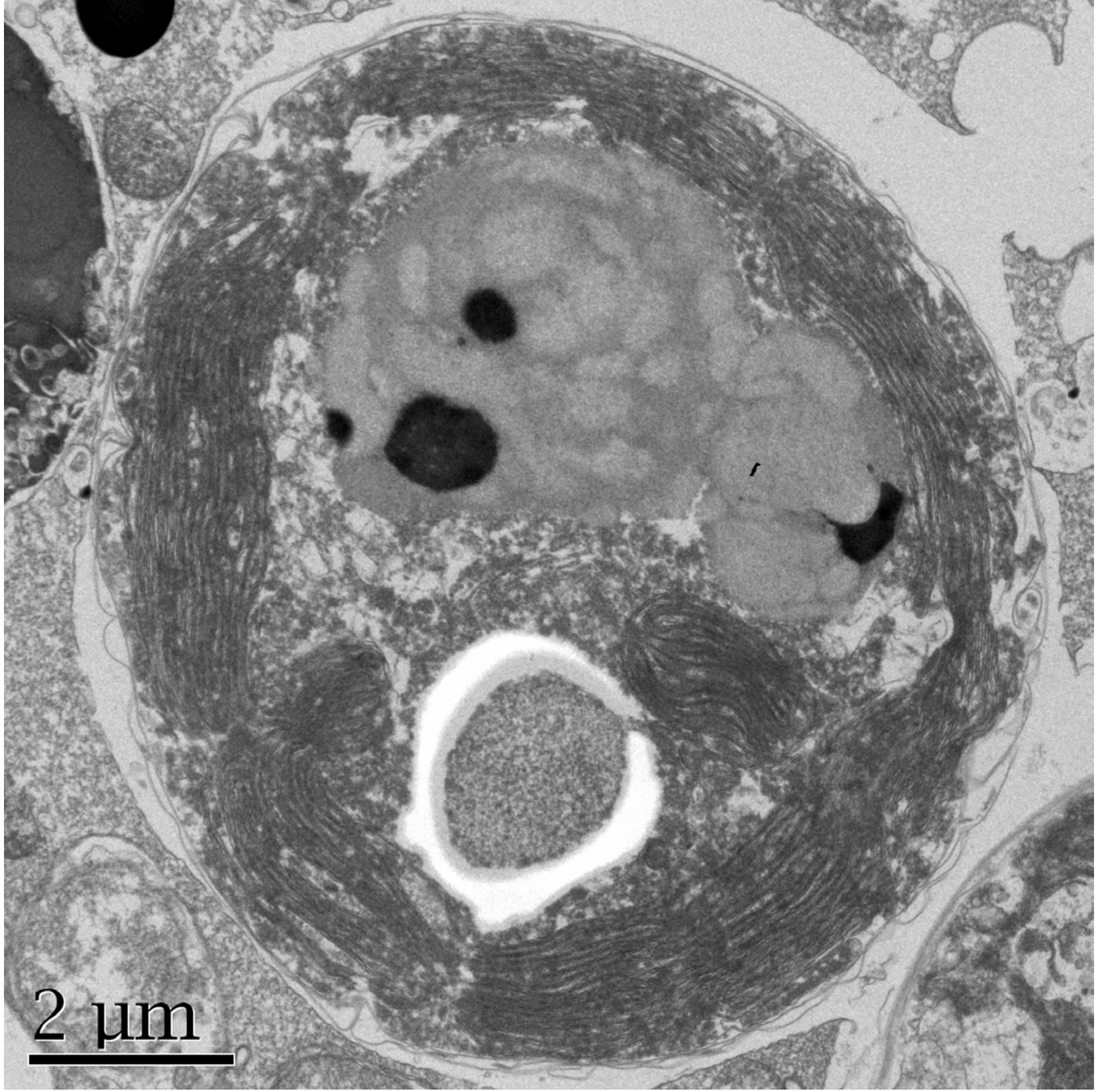

18-7_Correa_YC4_3GridC4_49

Cell 9

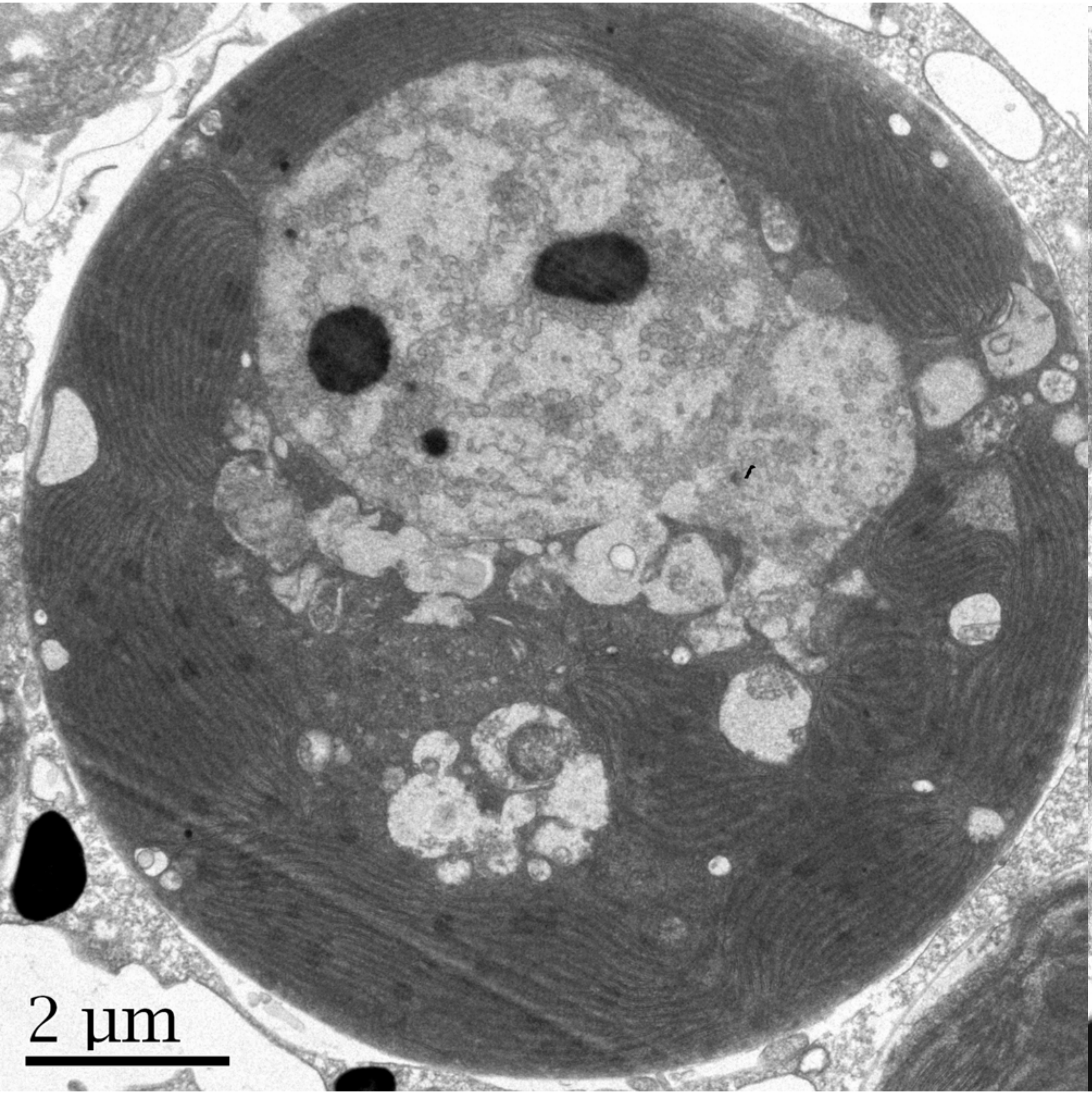

18-7_Correa_YC4_3GridC4_50

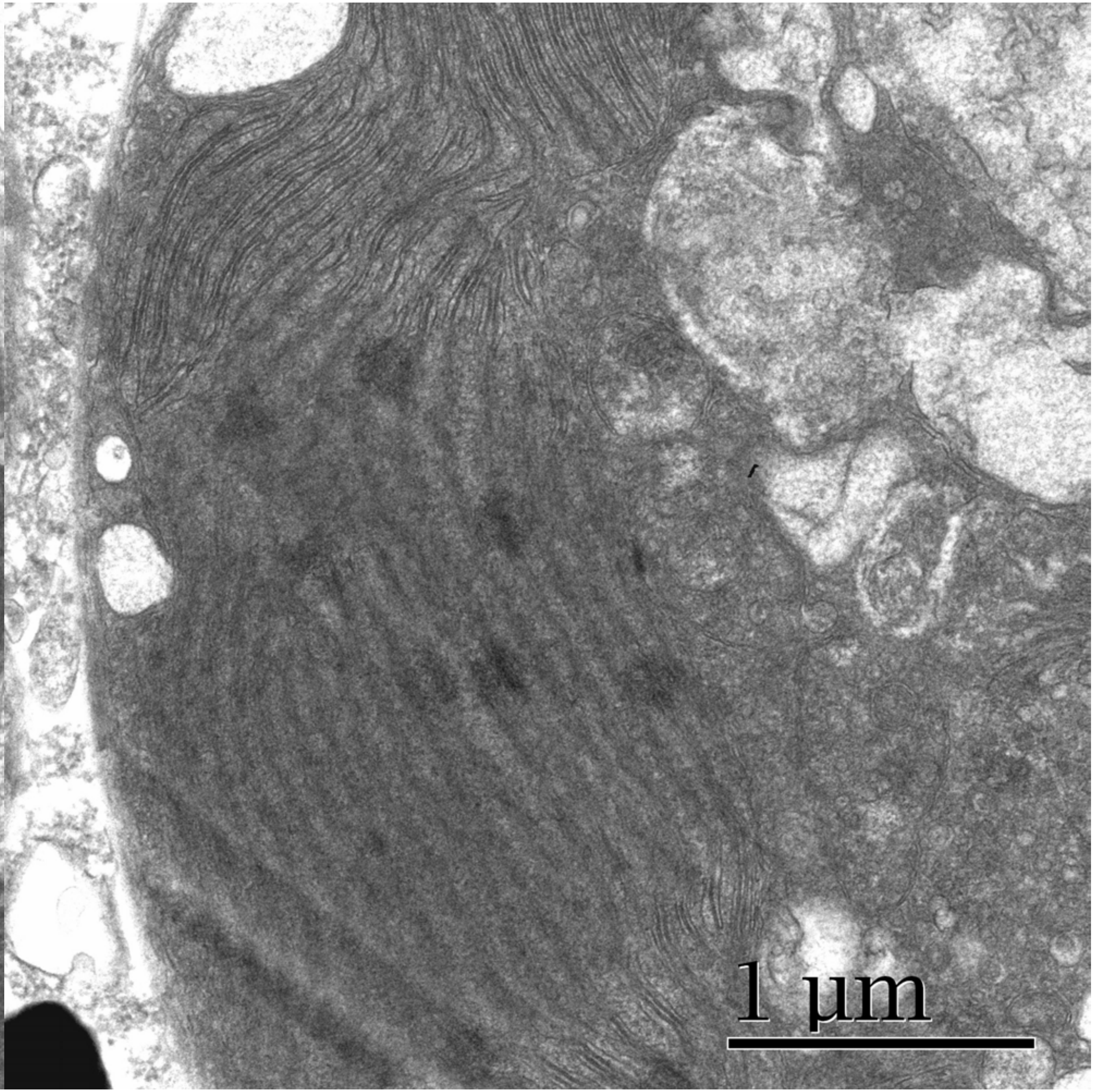

18-7_Correa_YC4_3GridC4_51

Cell 10

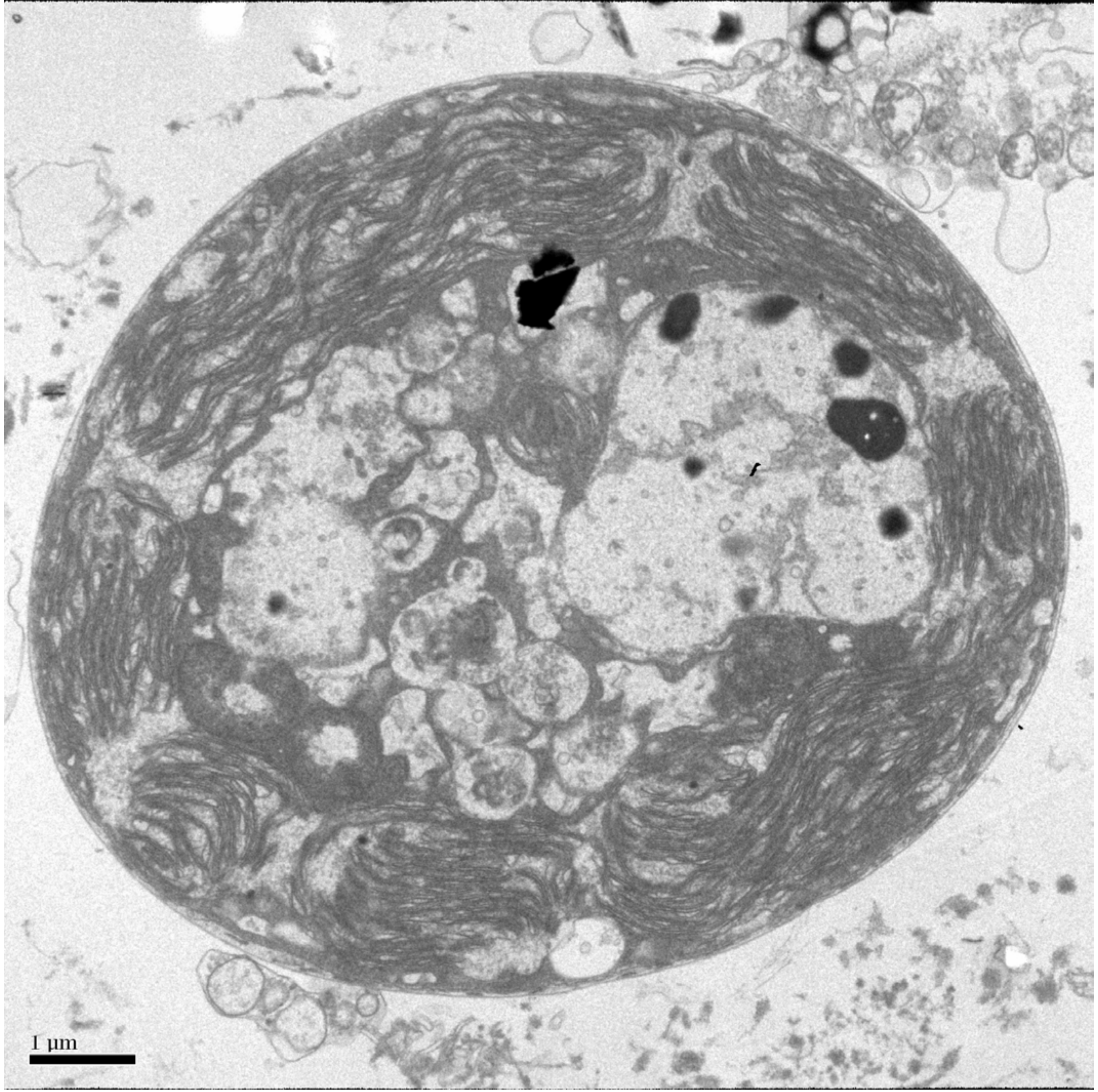

18-7_Correa_YC4_3C5_1

Cell 11

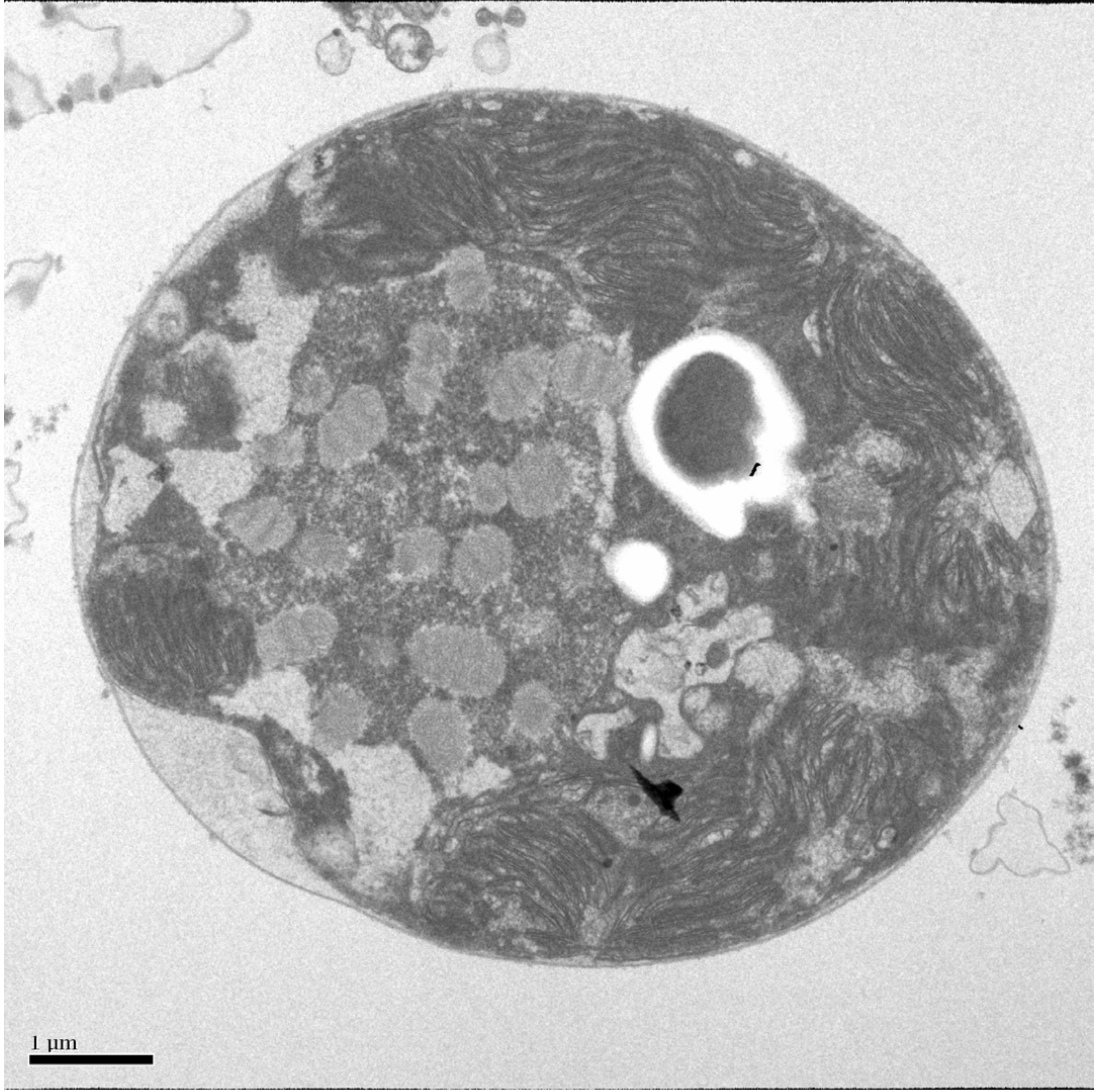

18-7_Correa_YC4_3C5_3

Cell 12

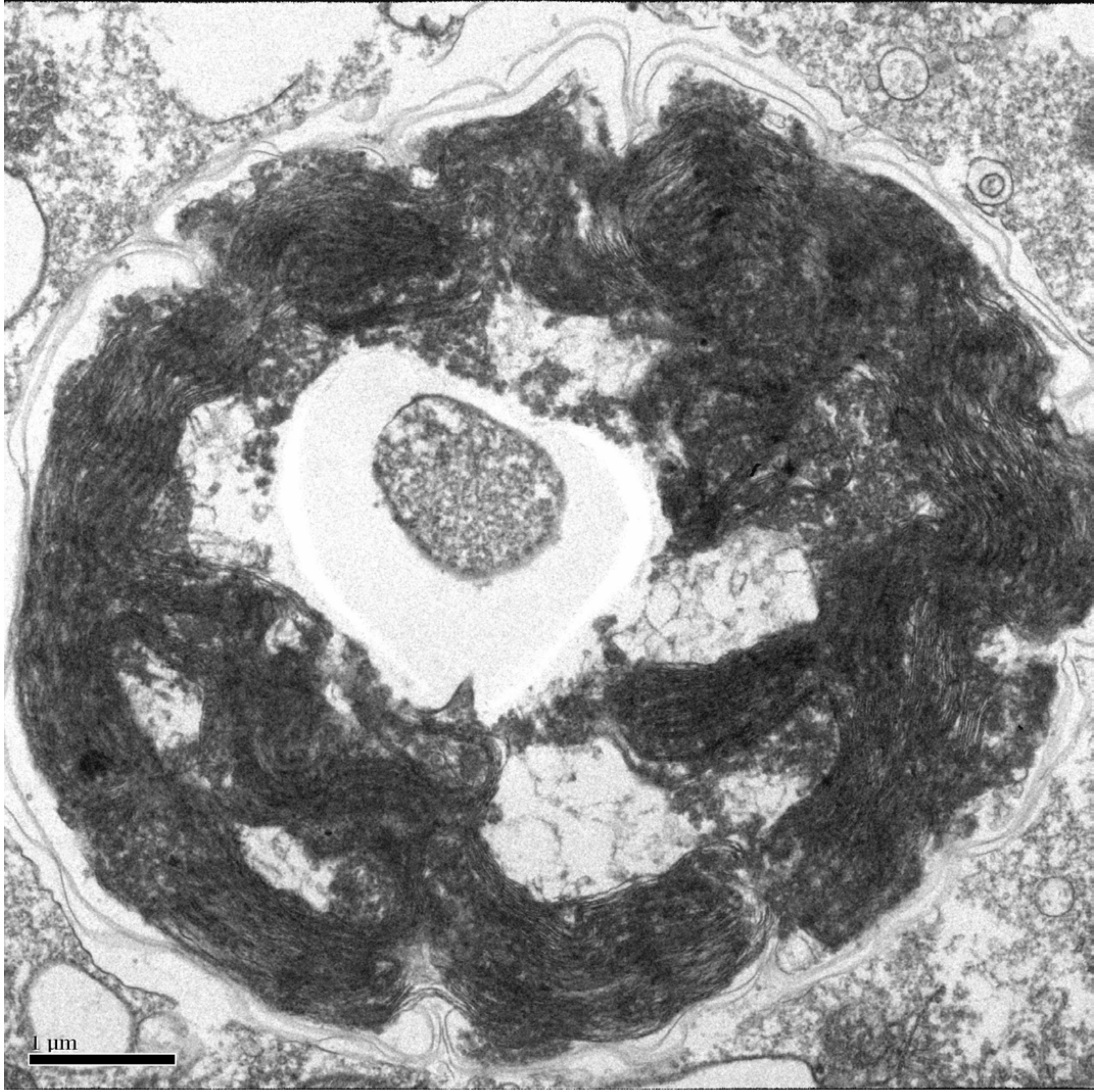

18-7_Correa_YC4_3C5_7

Cell 13

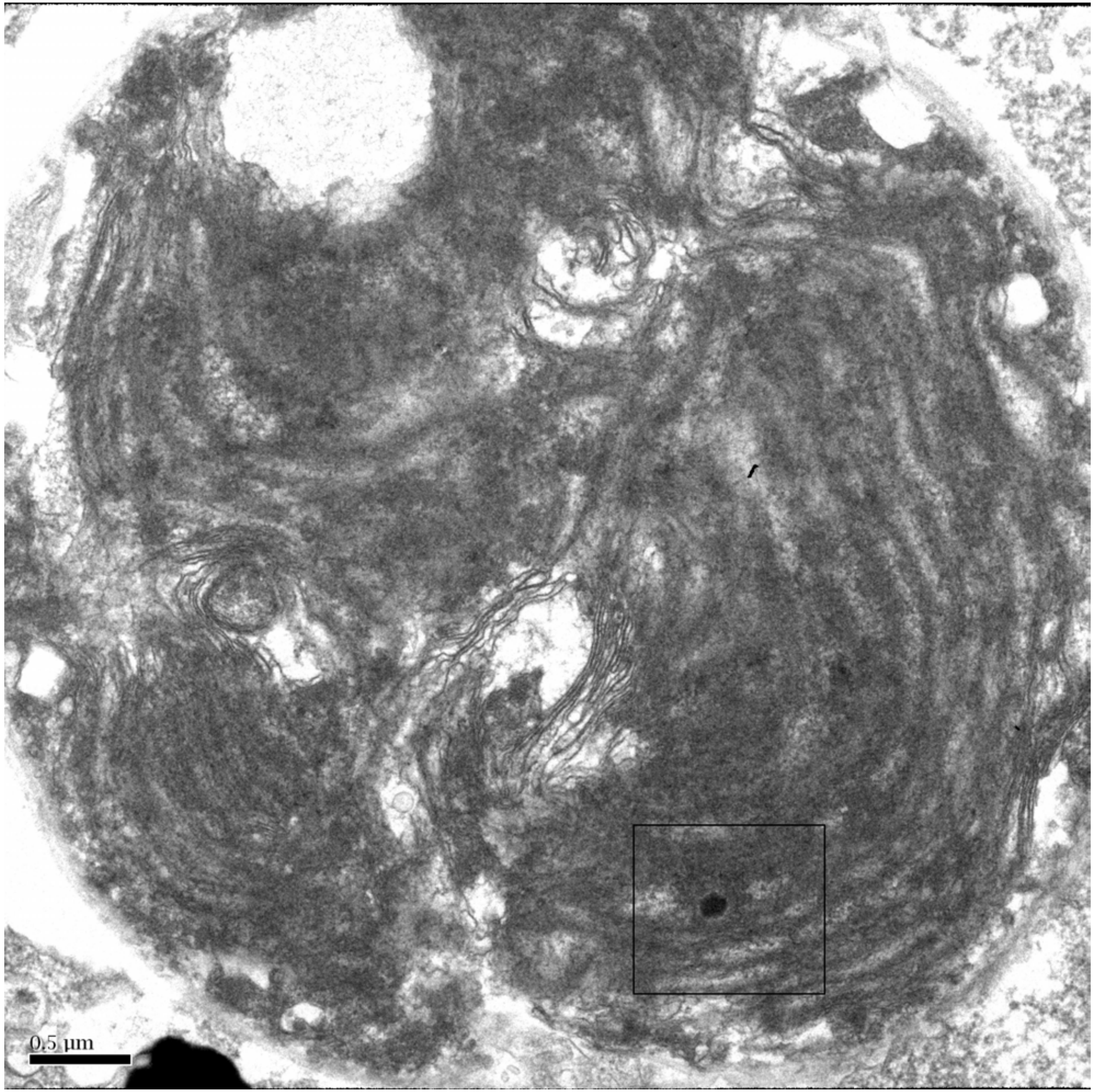

18-7_Correa_YC4_3C5_9

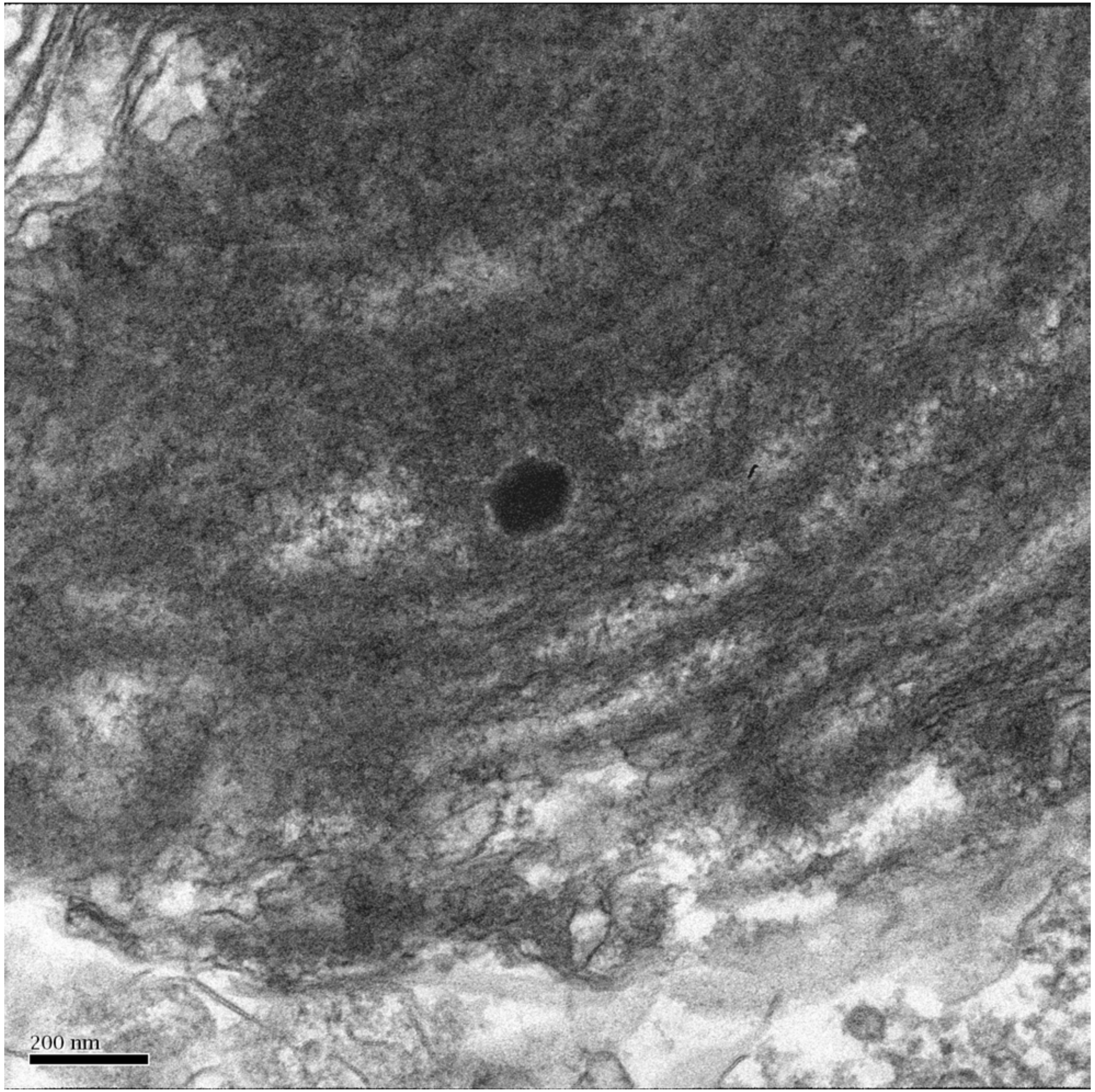

18-7_Correa_YC4_3C5_11

Cell 14

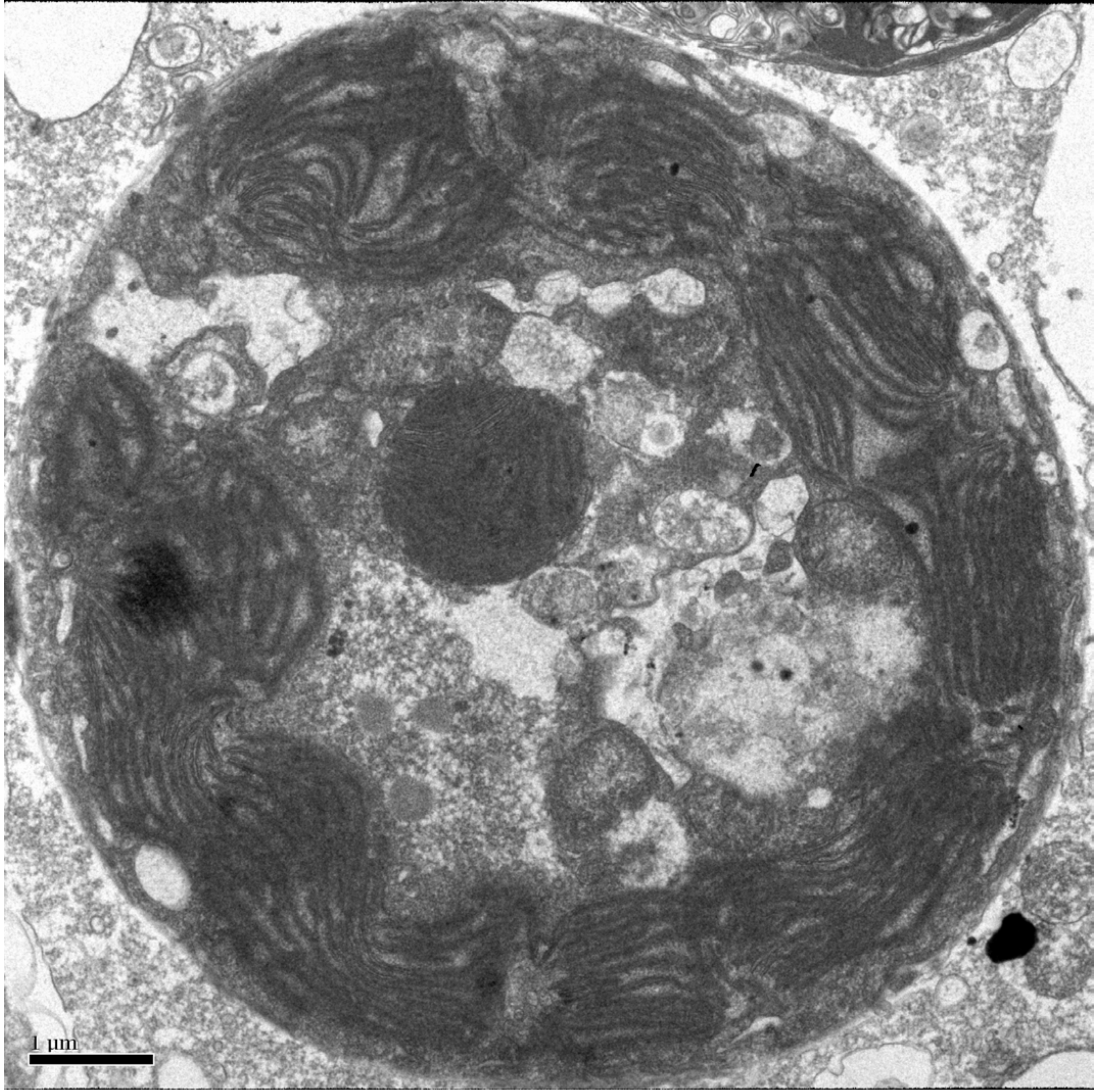

18-7_Correa_YC4_3C5_13

Cell 15

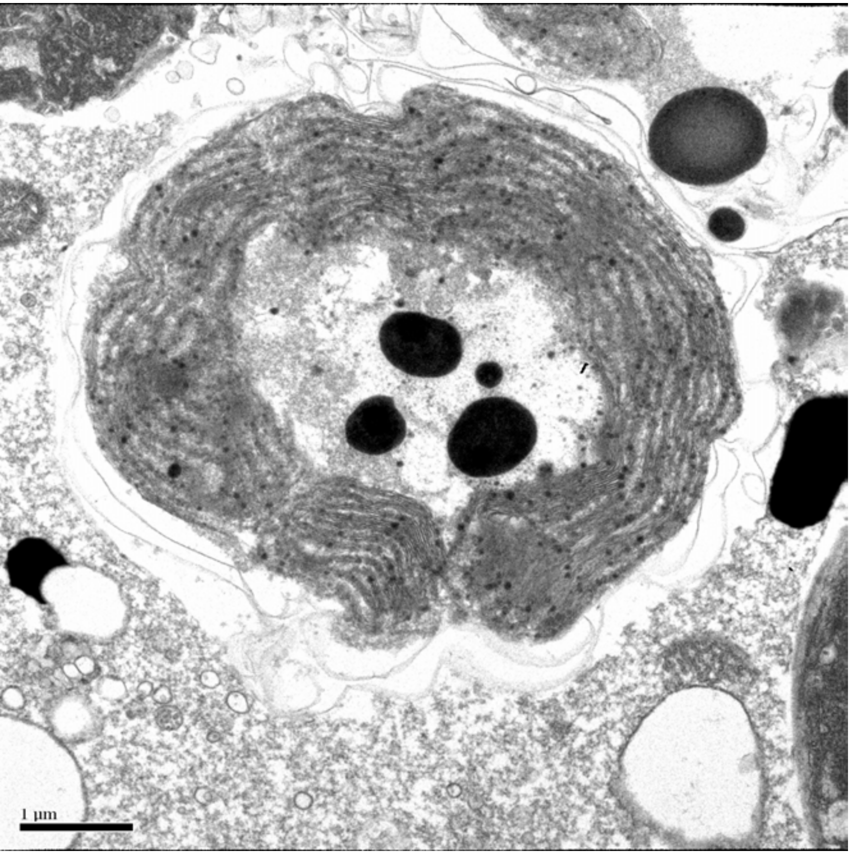

18-7_Correa_YC4_3C5_16

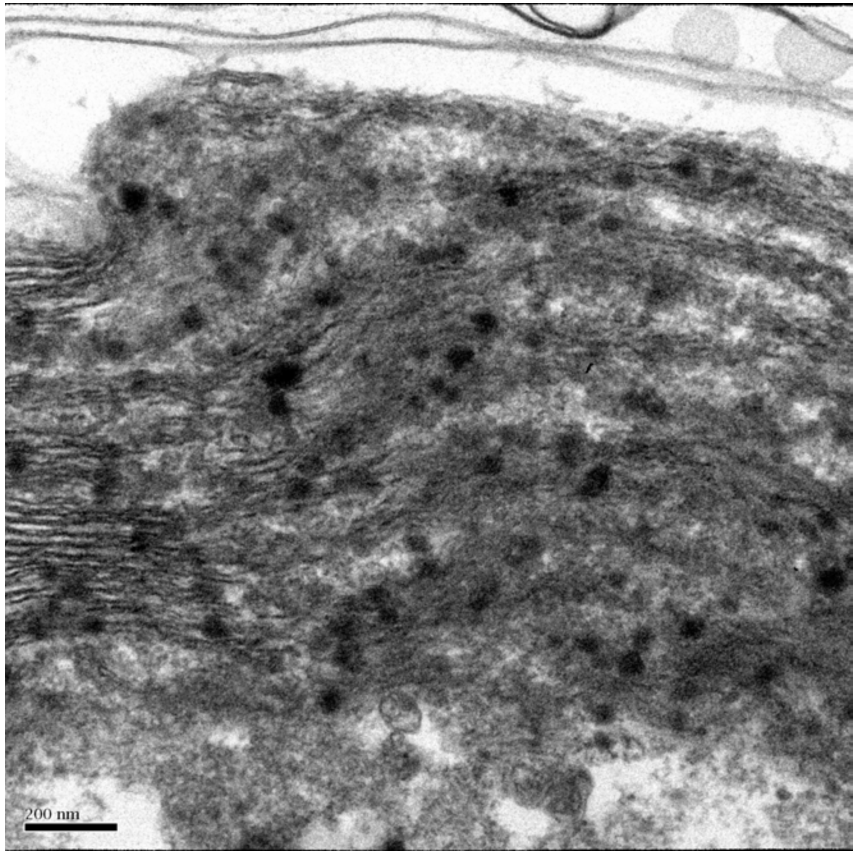

18-7_Correa_YC4_3C5_19

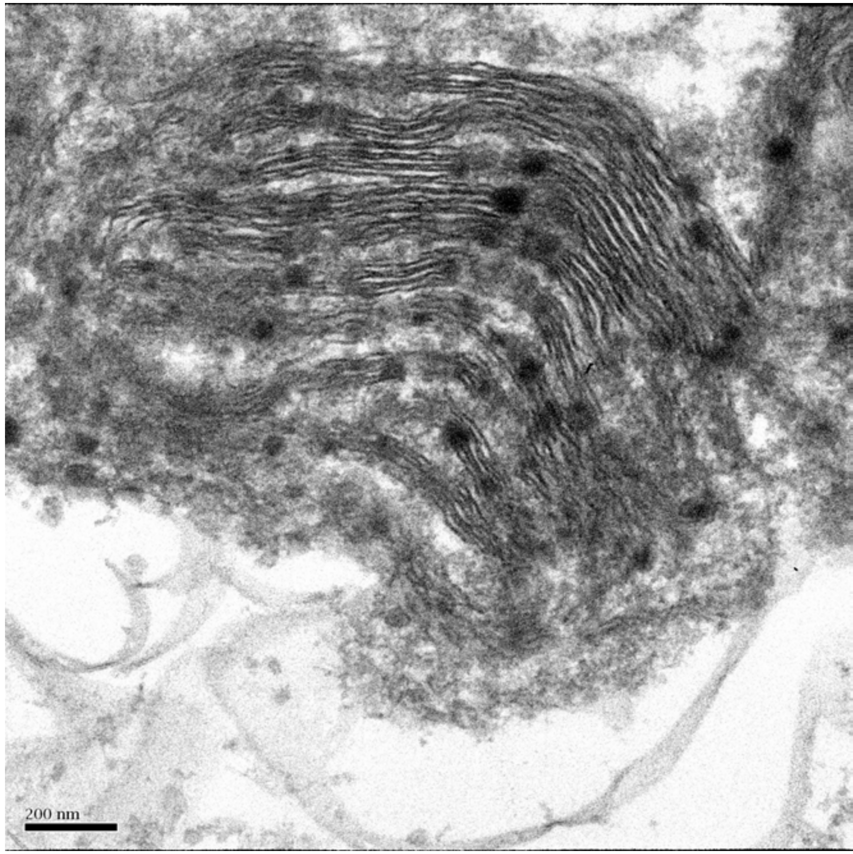

18-7_Correa_YC4_3C5_17

Cell 16

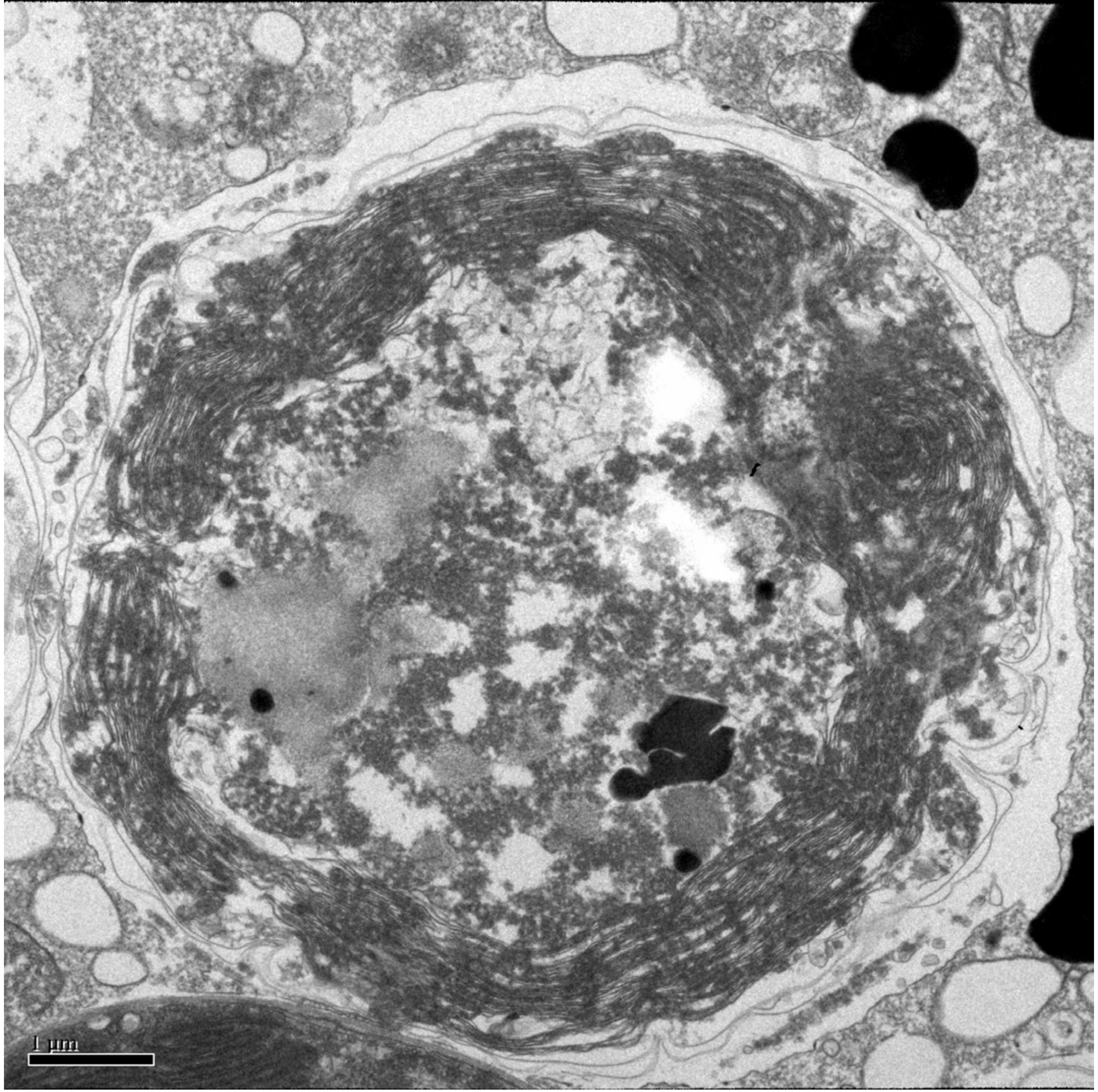

18-7_Correa_YC4_3C5_20

Cell 17

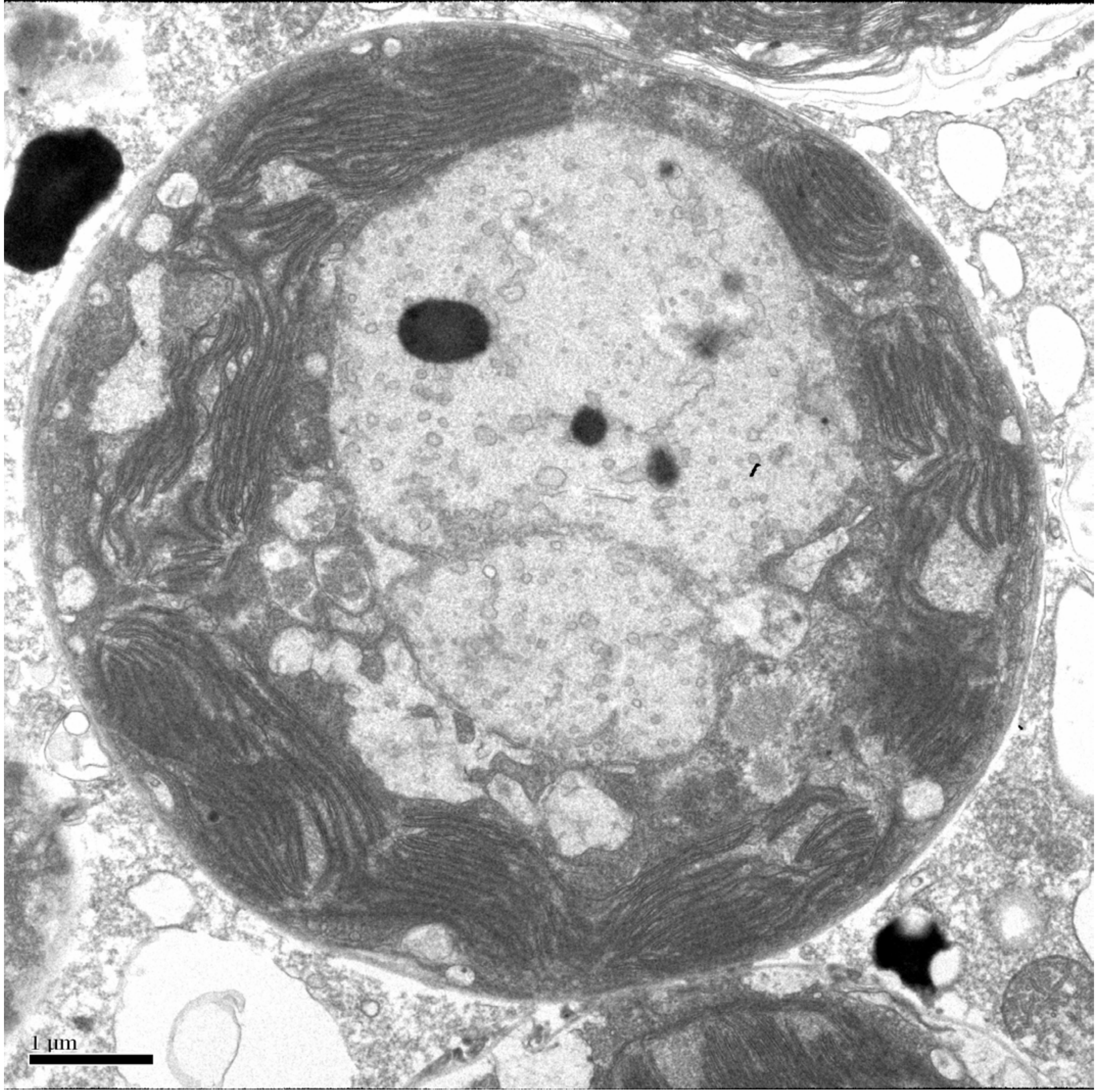

18-7_Correa_YC4_3C5_21

Cell 18

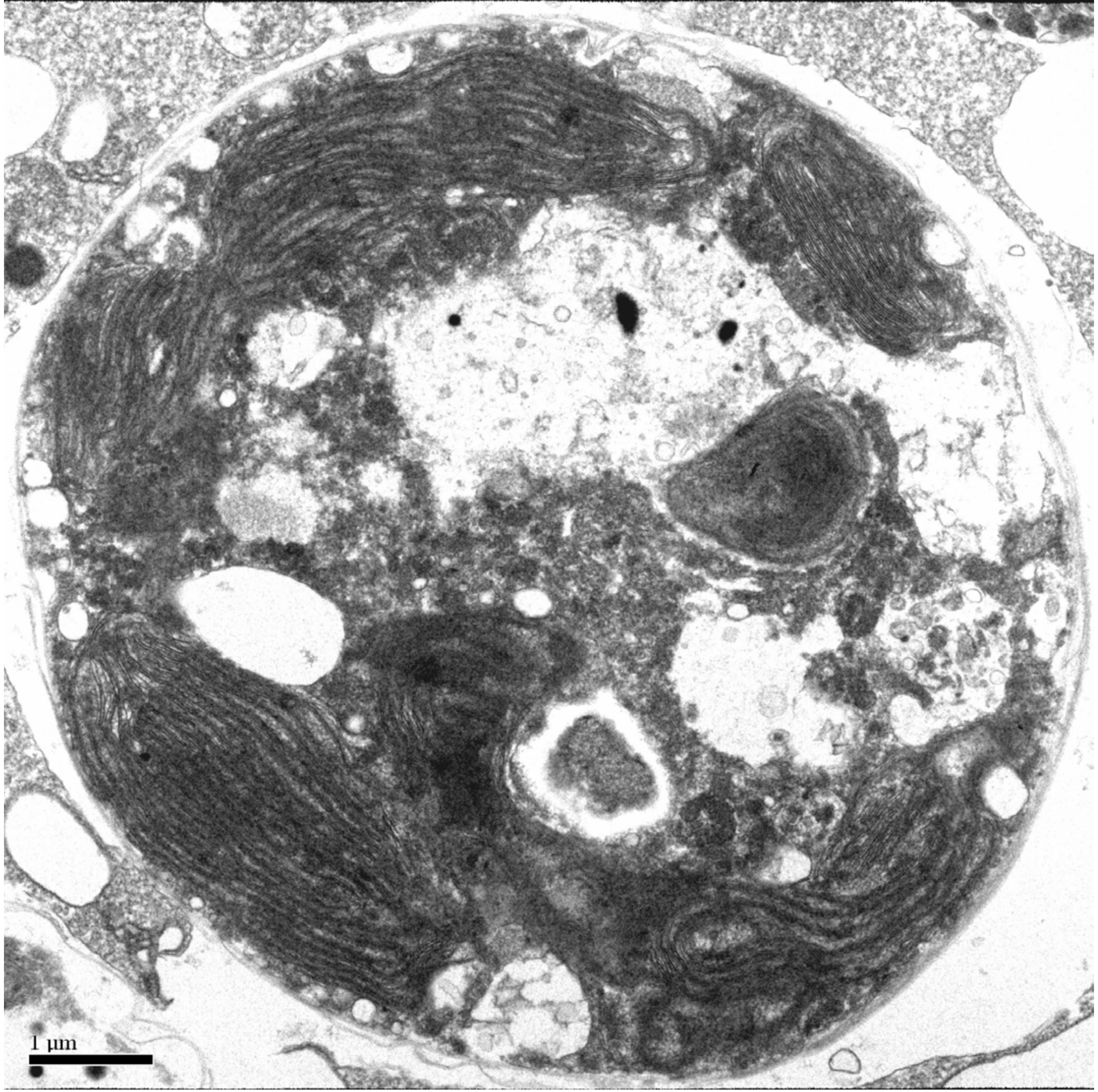

18-7_Correa_YC4_3C5_23

ACR Colony D Expelled- Heat

Cell 1

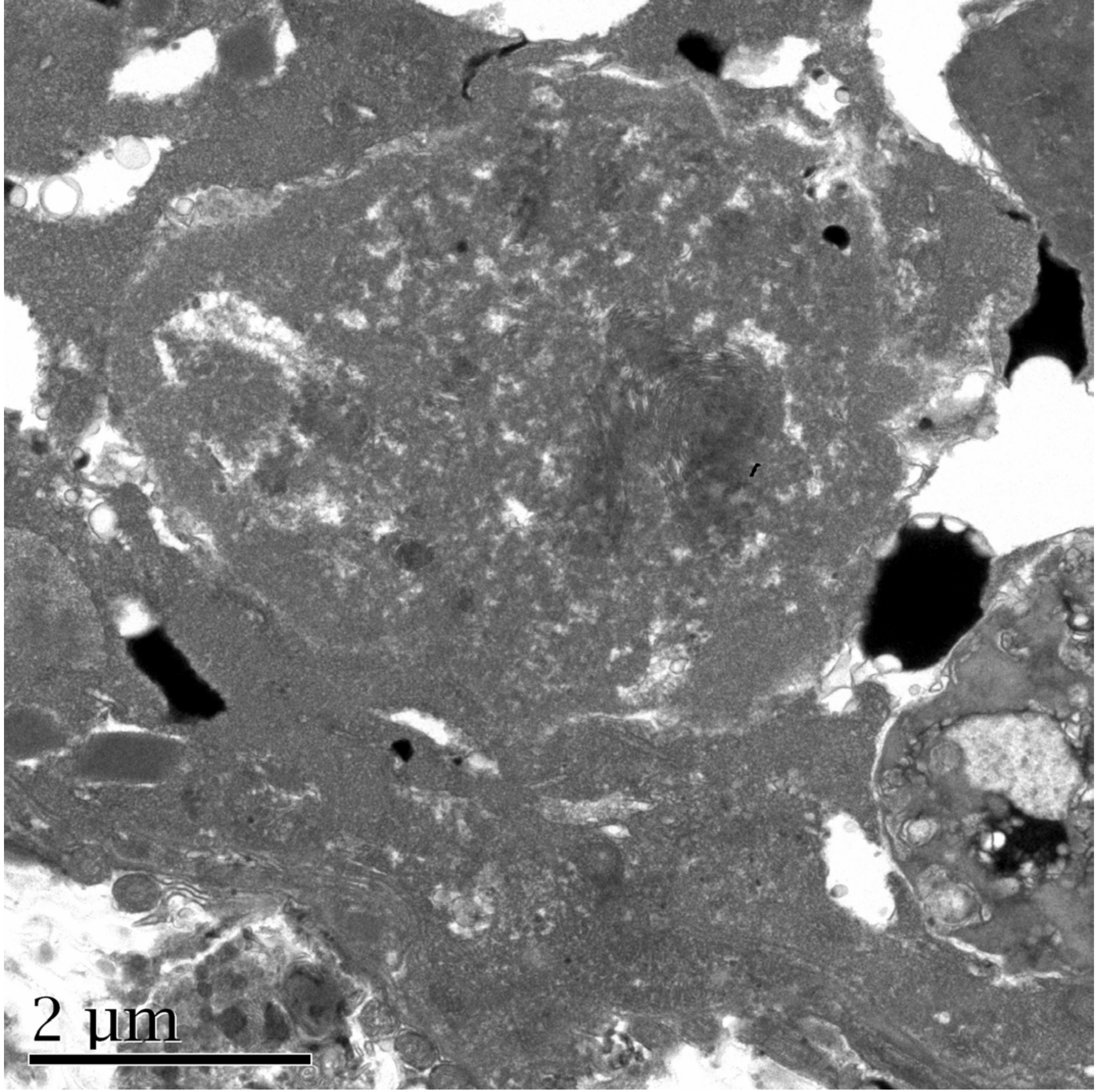

18-7_Correa_YH4_3GridD4_7

Cell 2

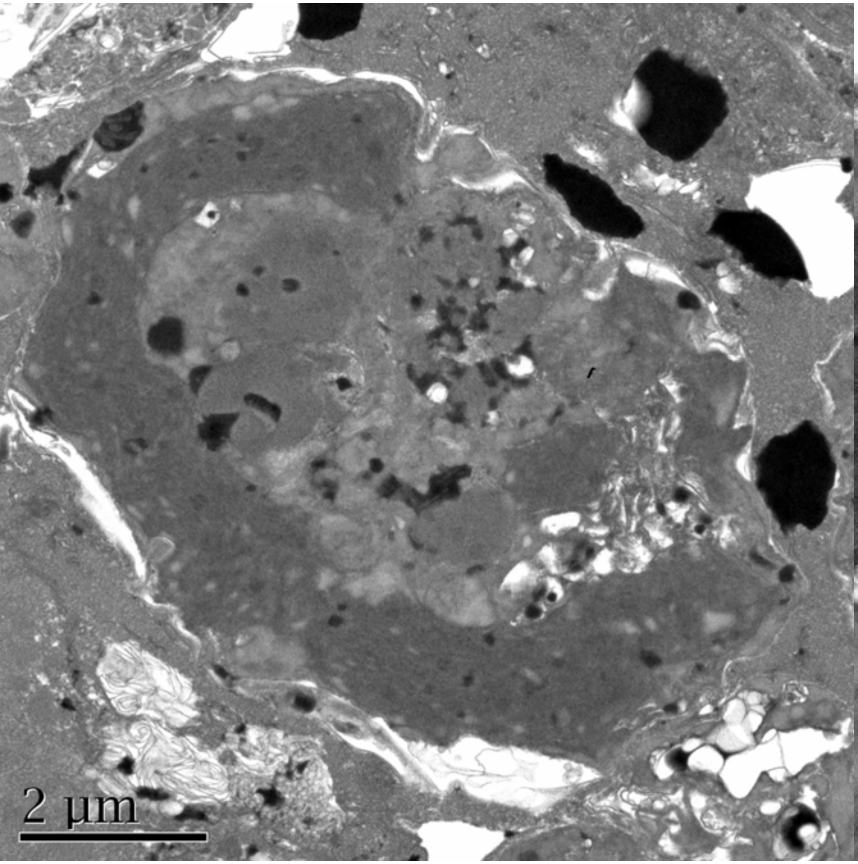

18-7_Correa_YH4_3GridD4_11

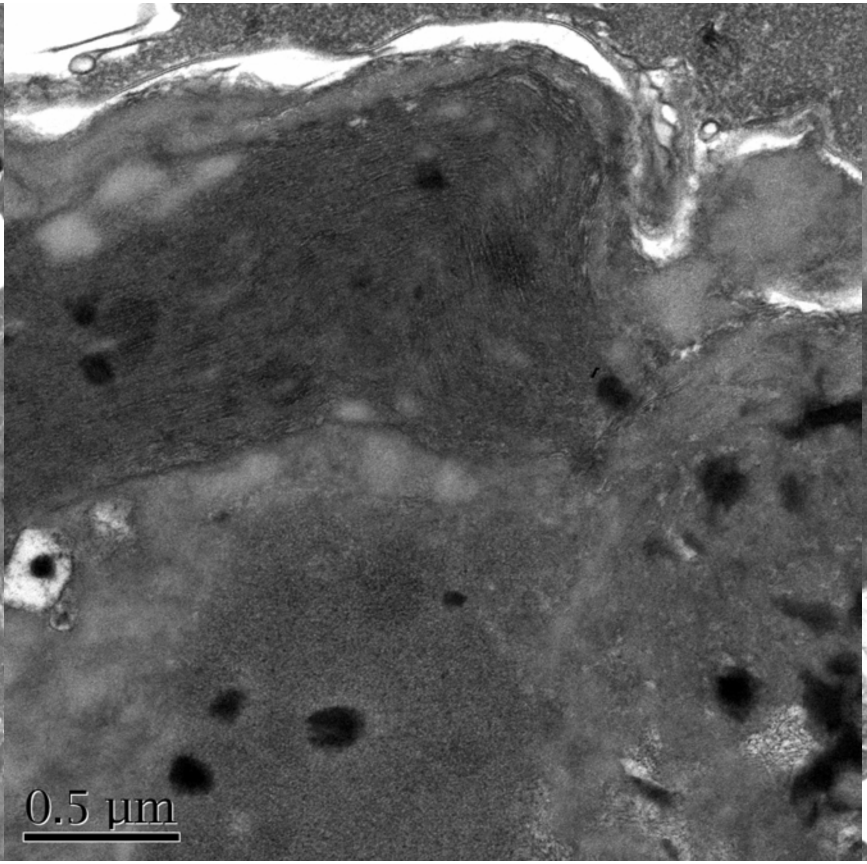

18-7_Correa_YH4_3GridD4_30

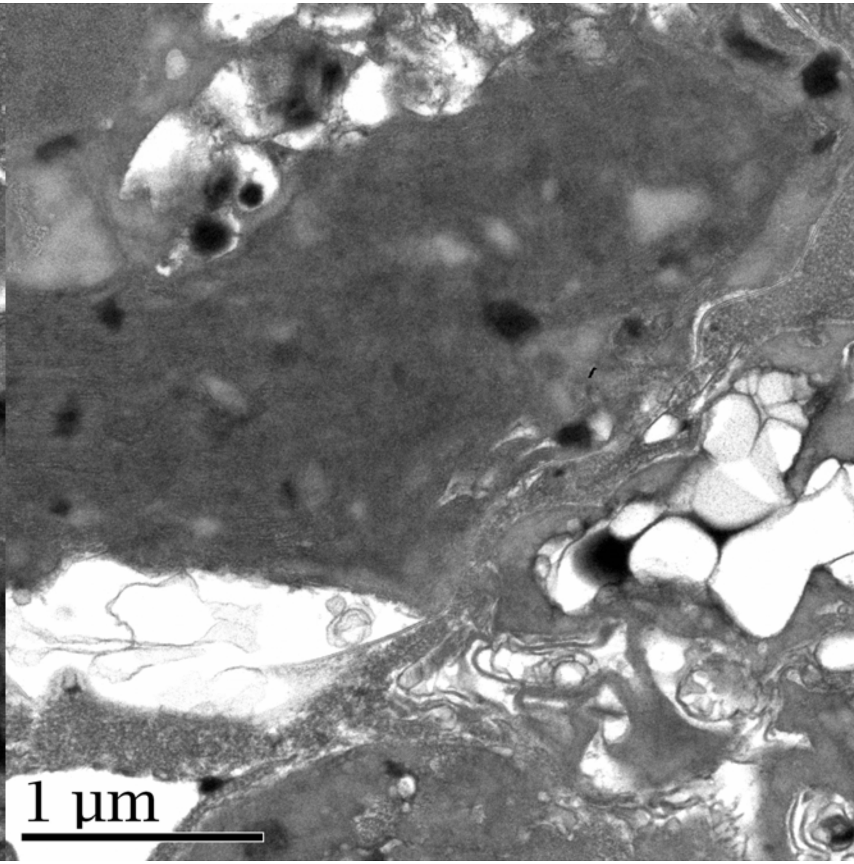

18-7_Correa_YH4_3GridD4_12

Cell 3

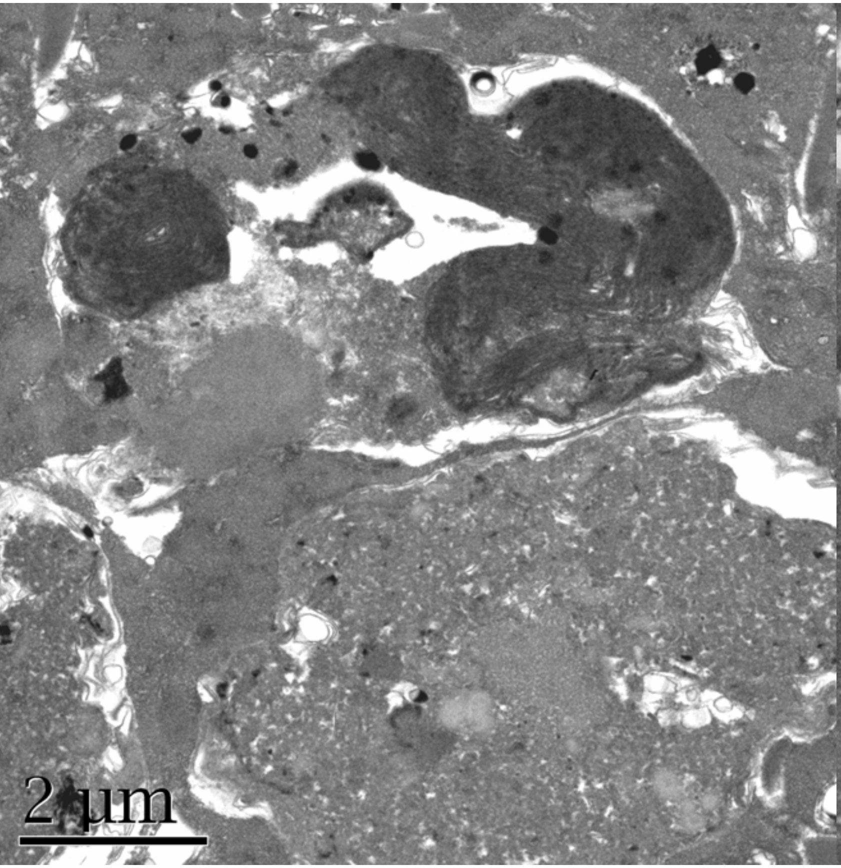

18-7_Correa_YH4_3GridD4_22

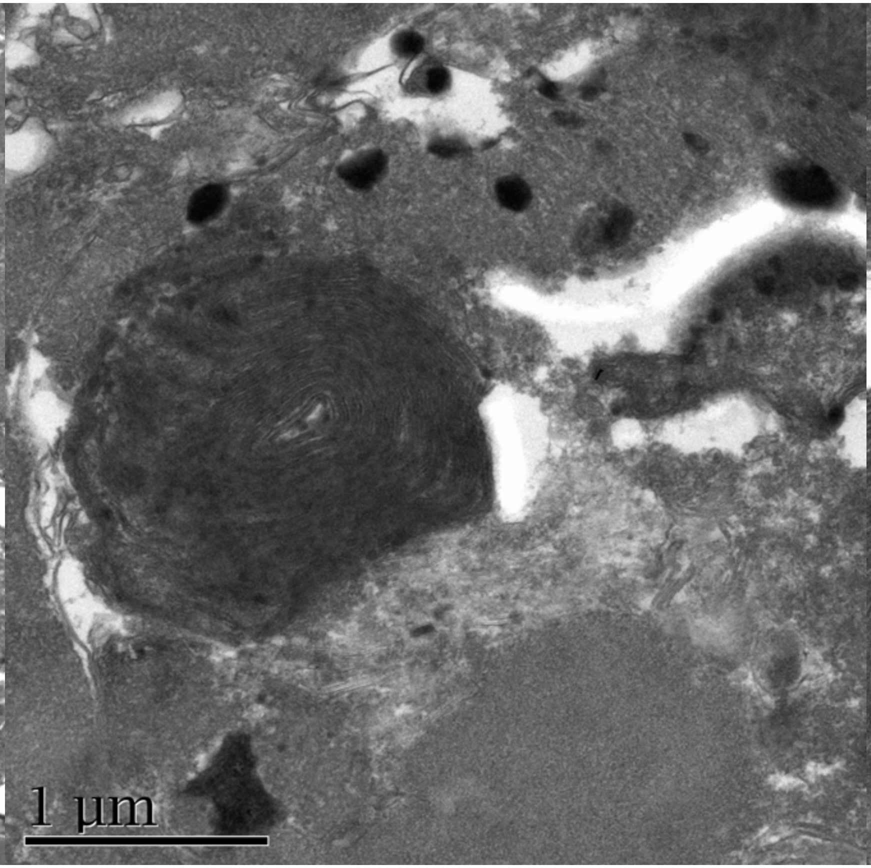

18-7_Correa_YH4_3GridD4_25

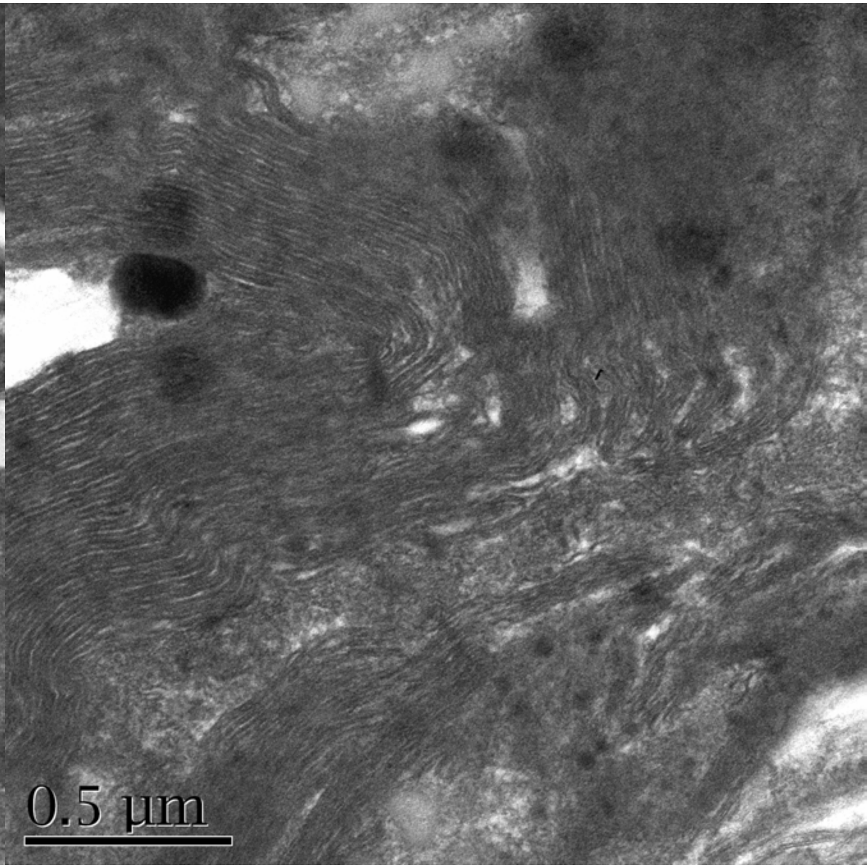

18-7_Correa_YH4_3GridD4_24

Cell 4

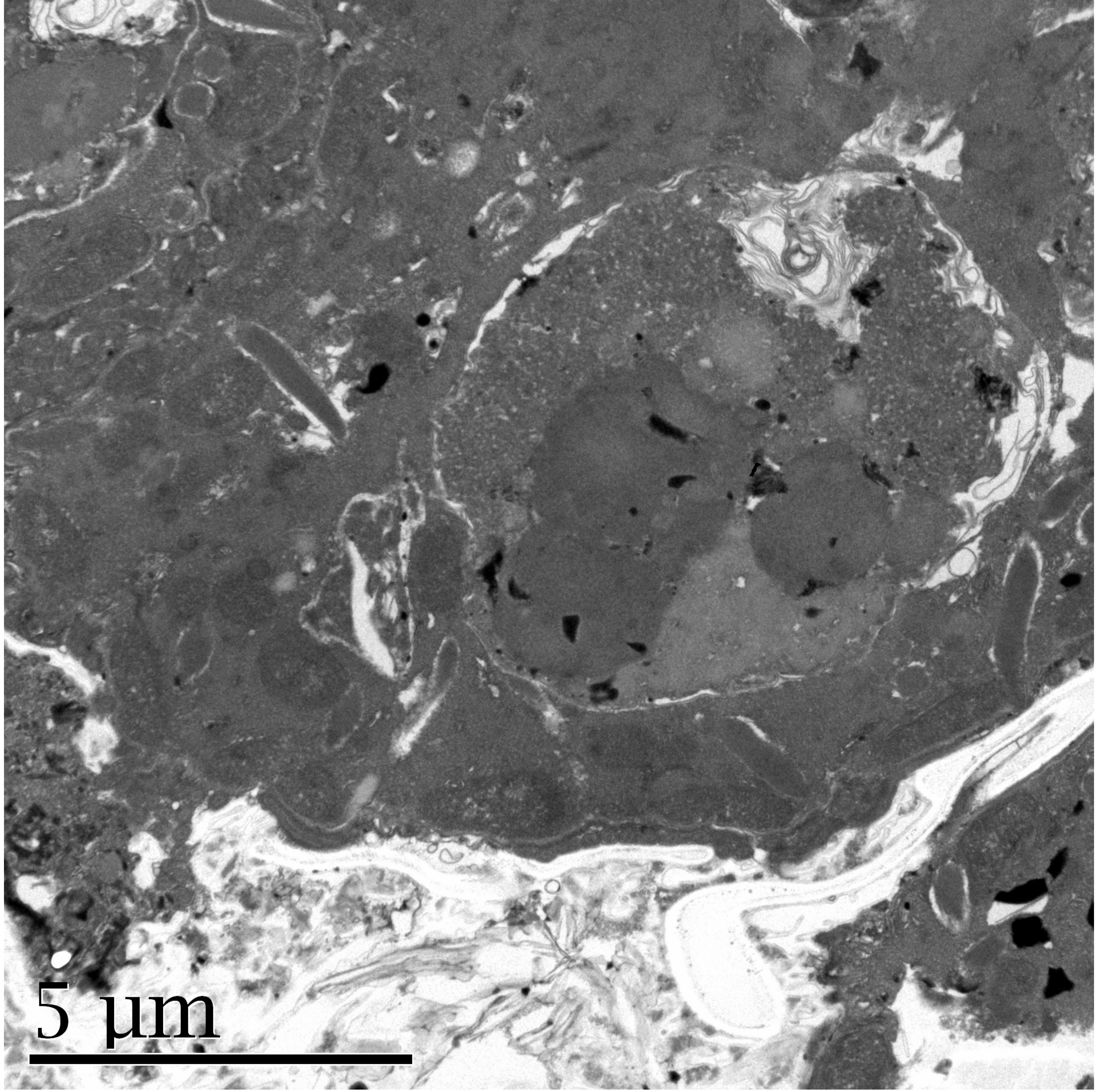

18-7_Correa_YH4_3GridD4_26

Cell 5

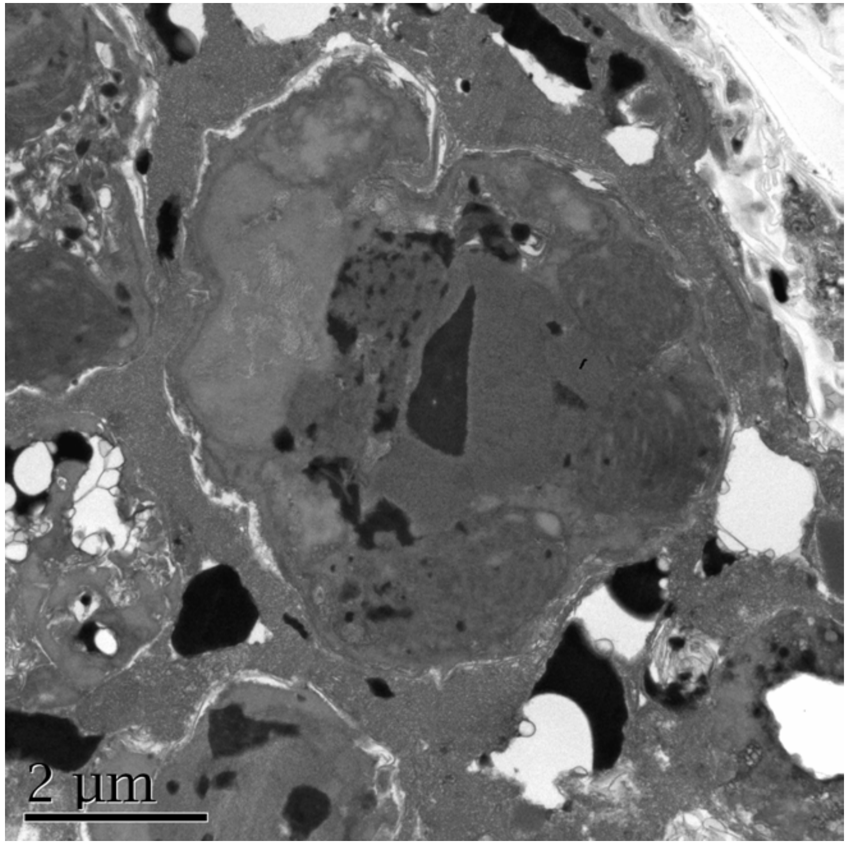

18-7_Correa_YH4_3GridD4_38

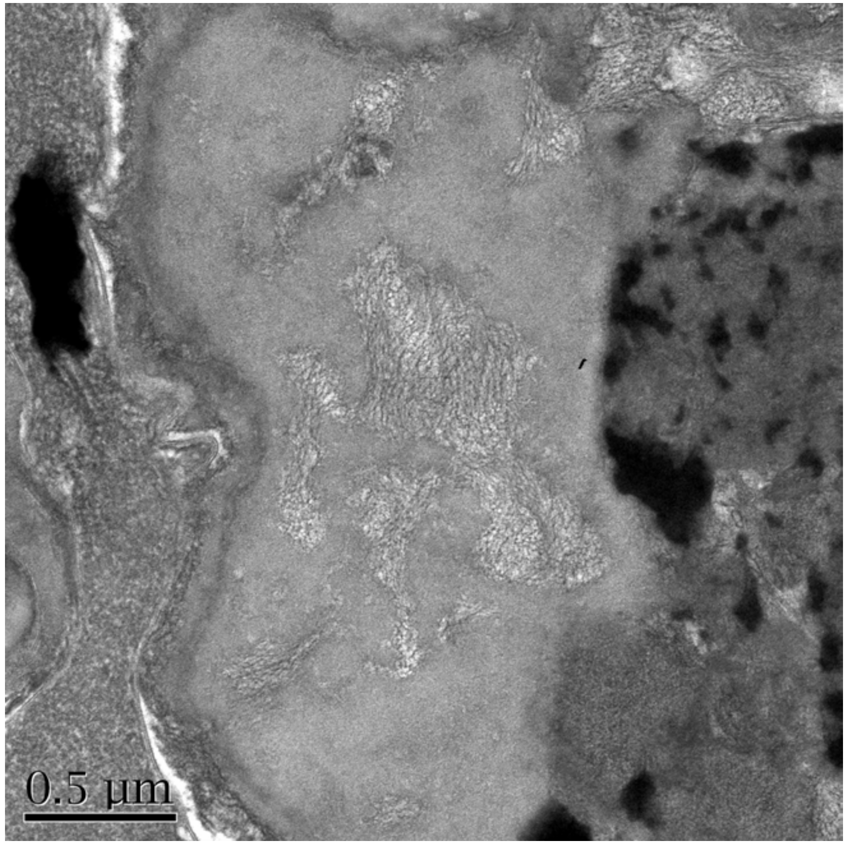

18-7_Correa_YH4_3GridD4_43

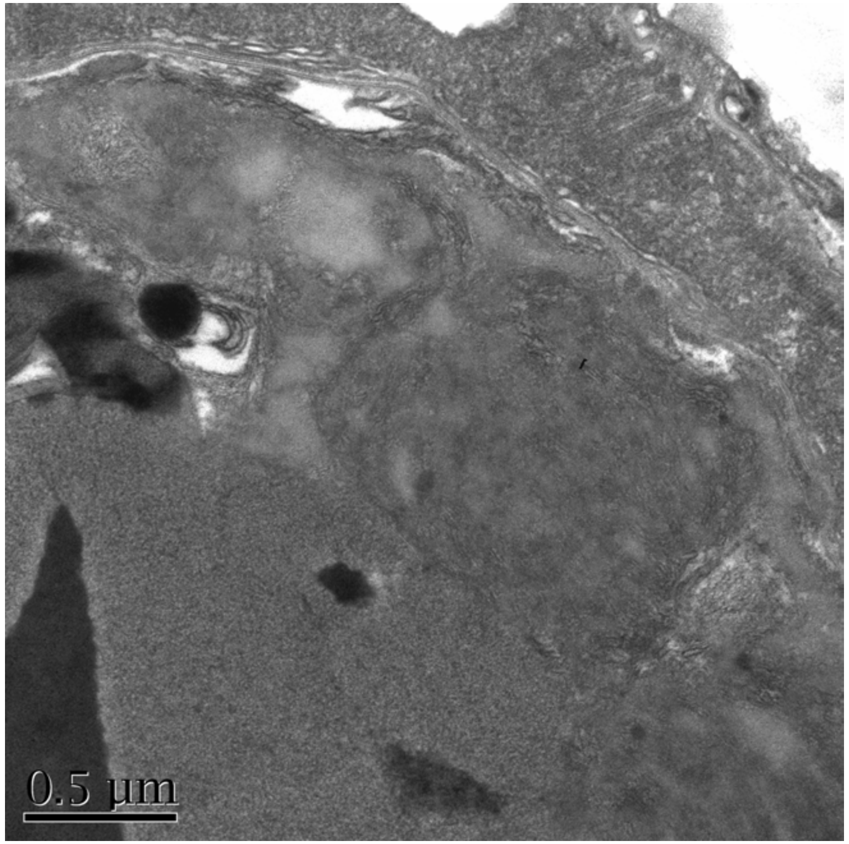

18-7_Correa_YH4_3GridD4_41

Cell 6

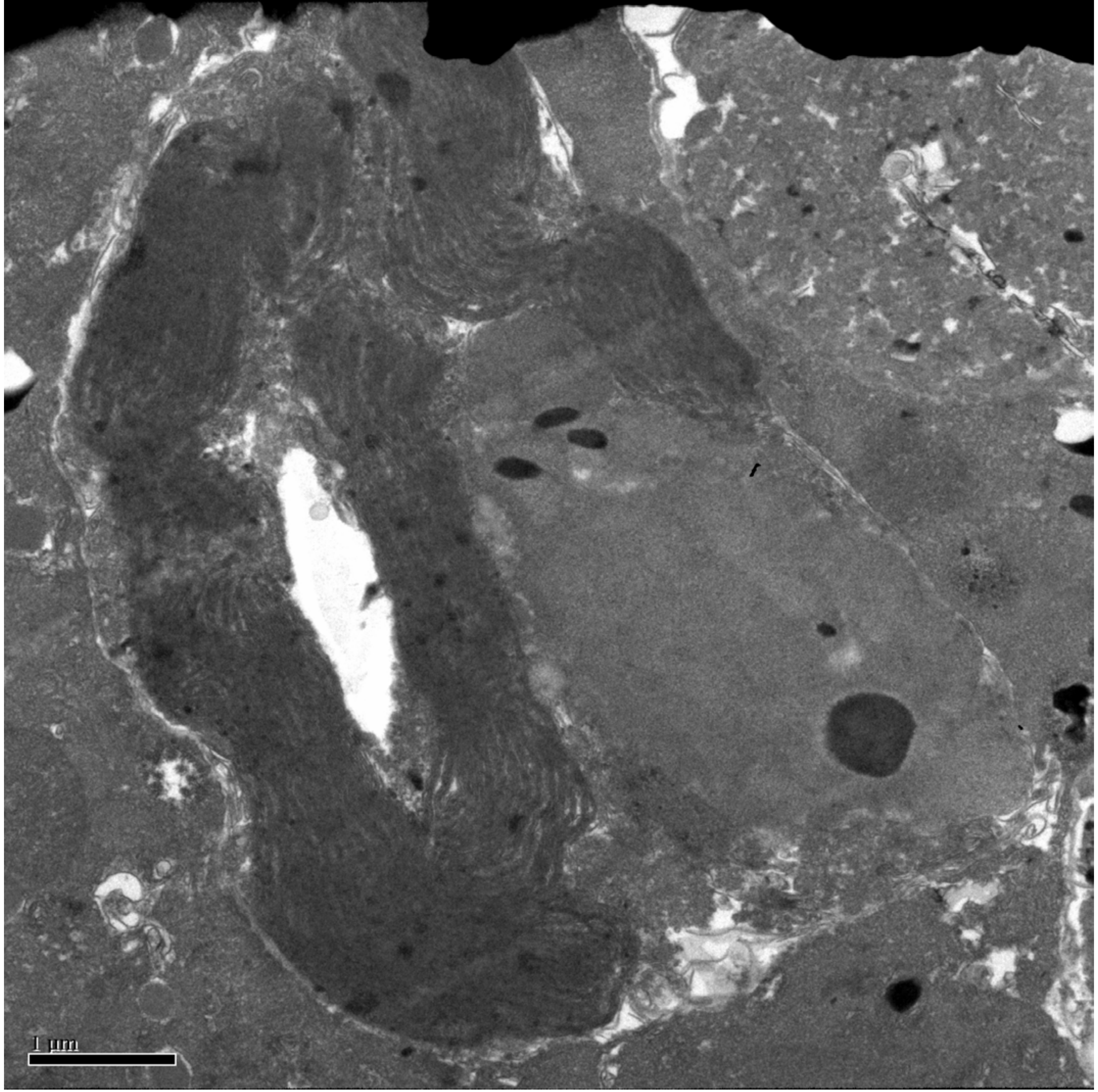

18-7_Correa_YH4_3D5_1

Cell 7

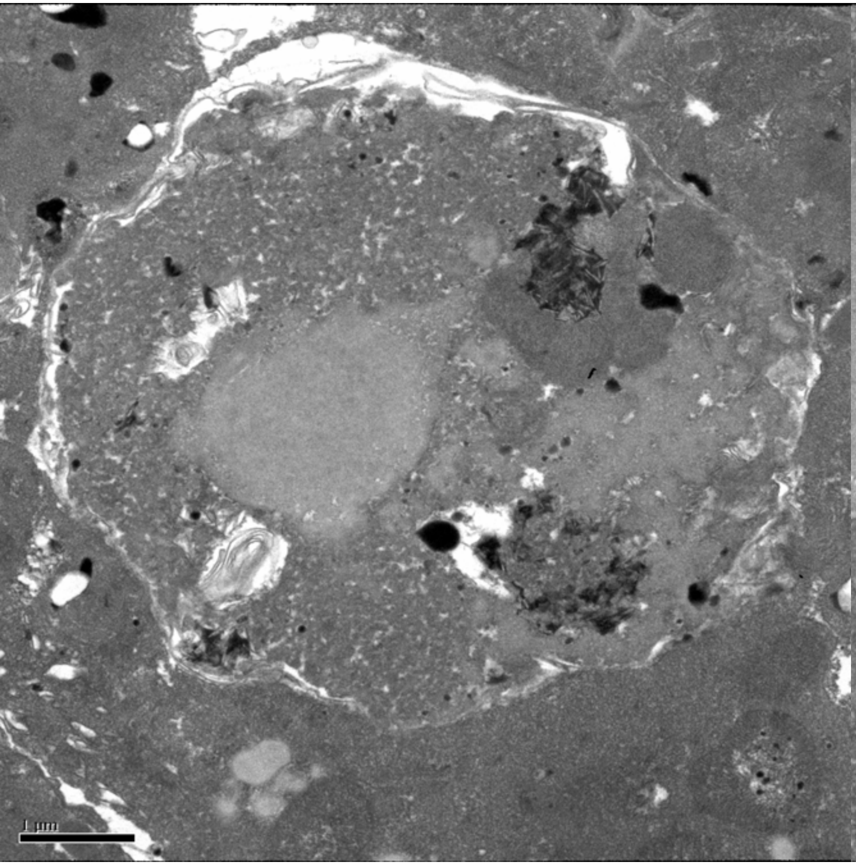

18-7_Correa_YH4_3D5_5

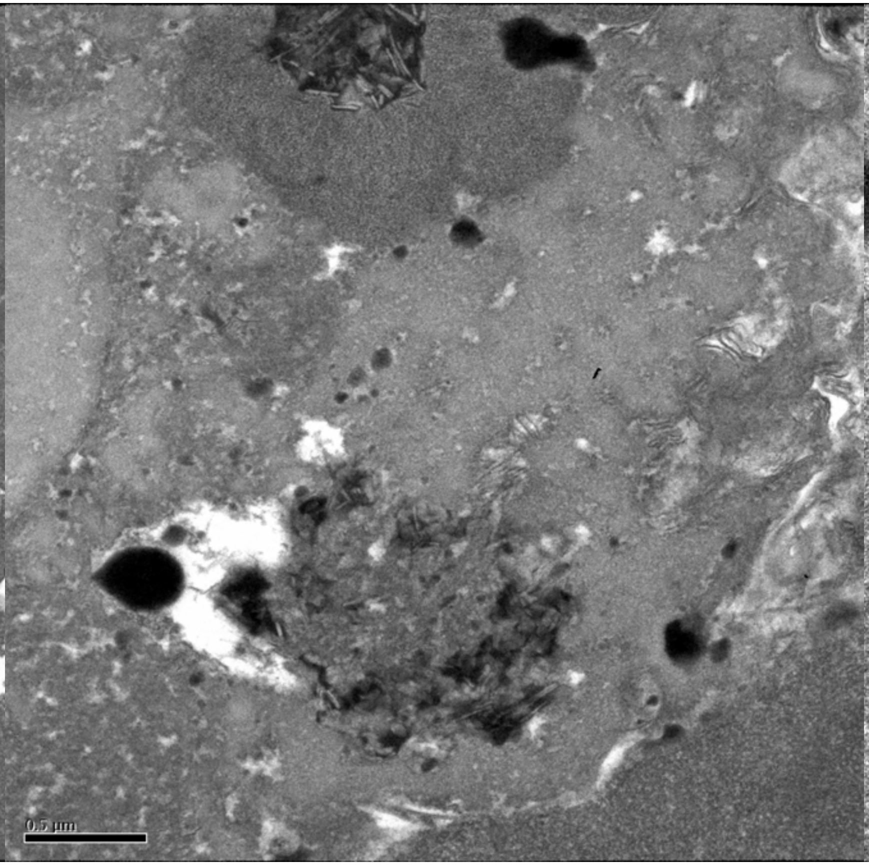

18-7_Correa_YH4_3D5_6

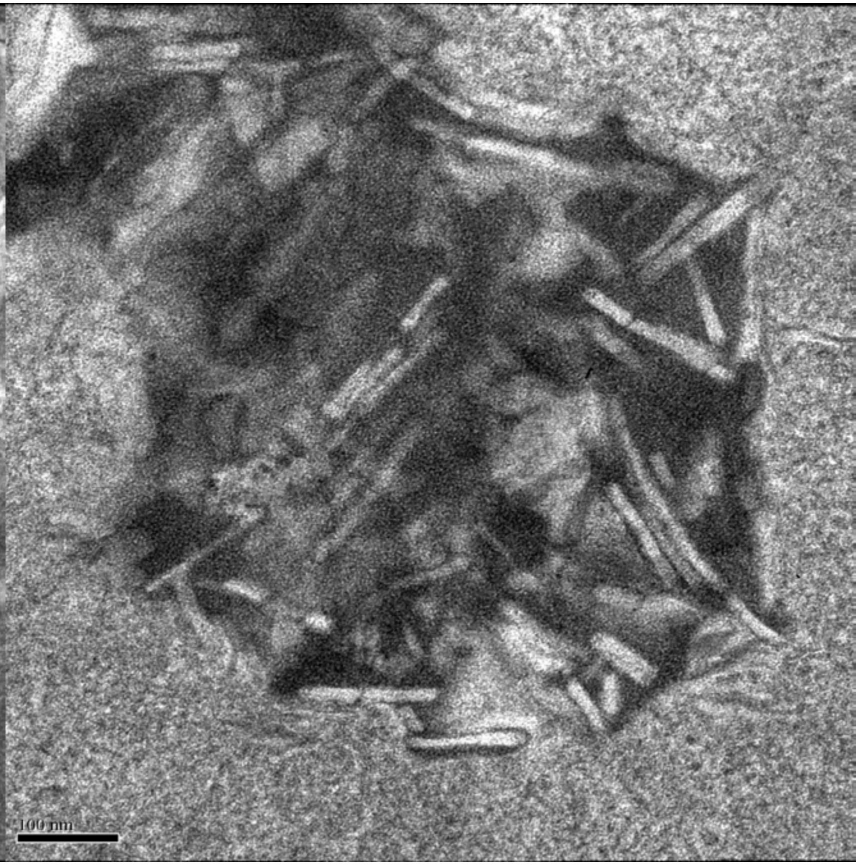

18-7_Correa_YH4_3D5_7

Cell 8

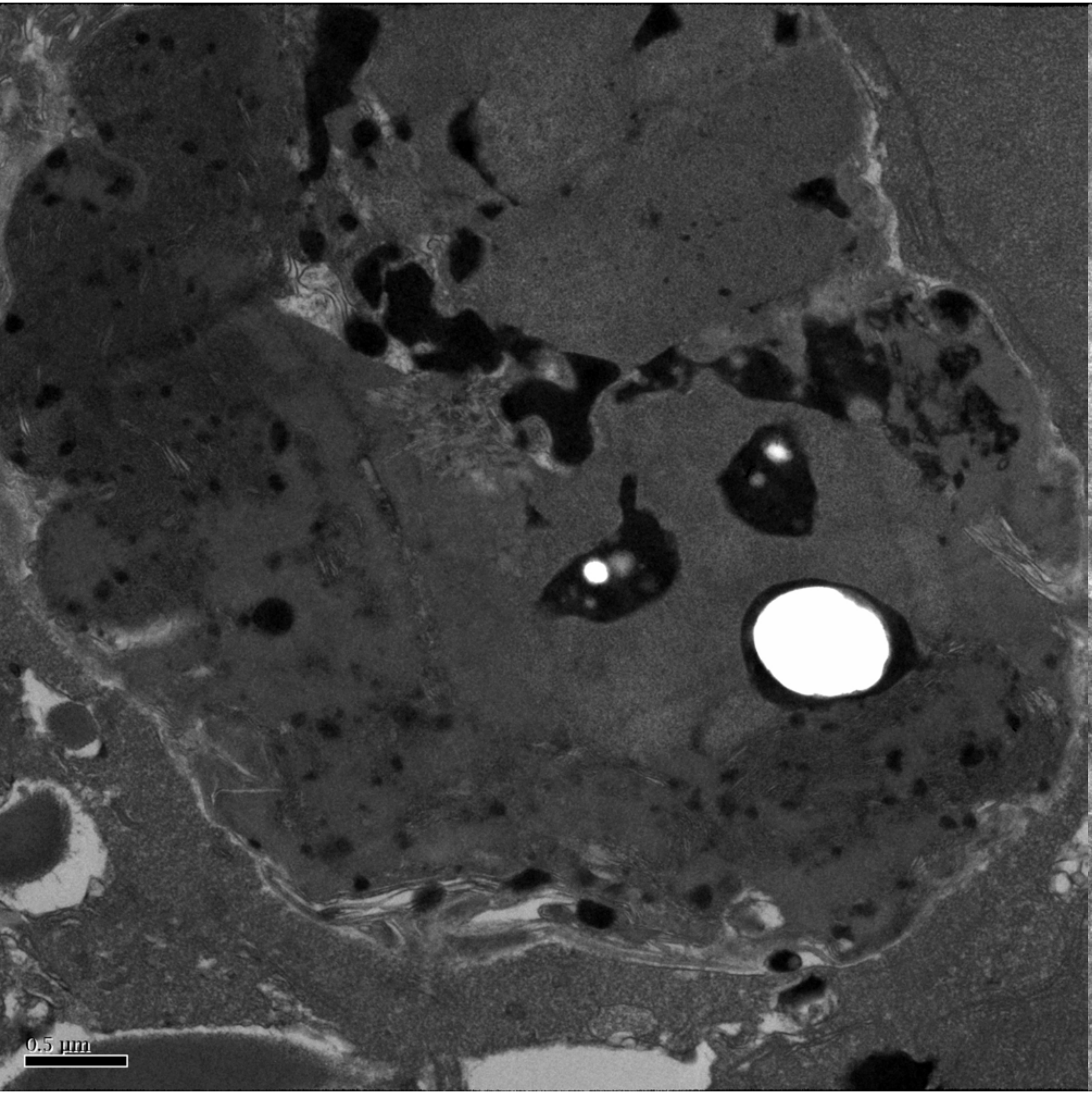

18-7_Correa_YH4_3D5_9

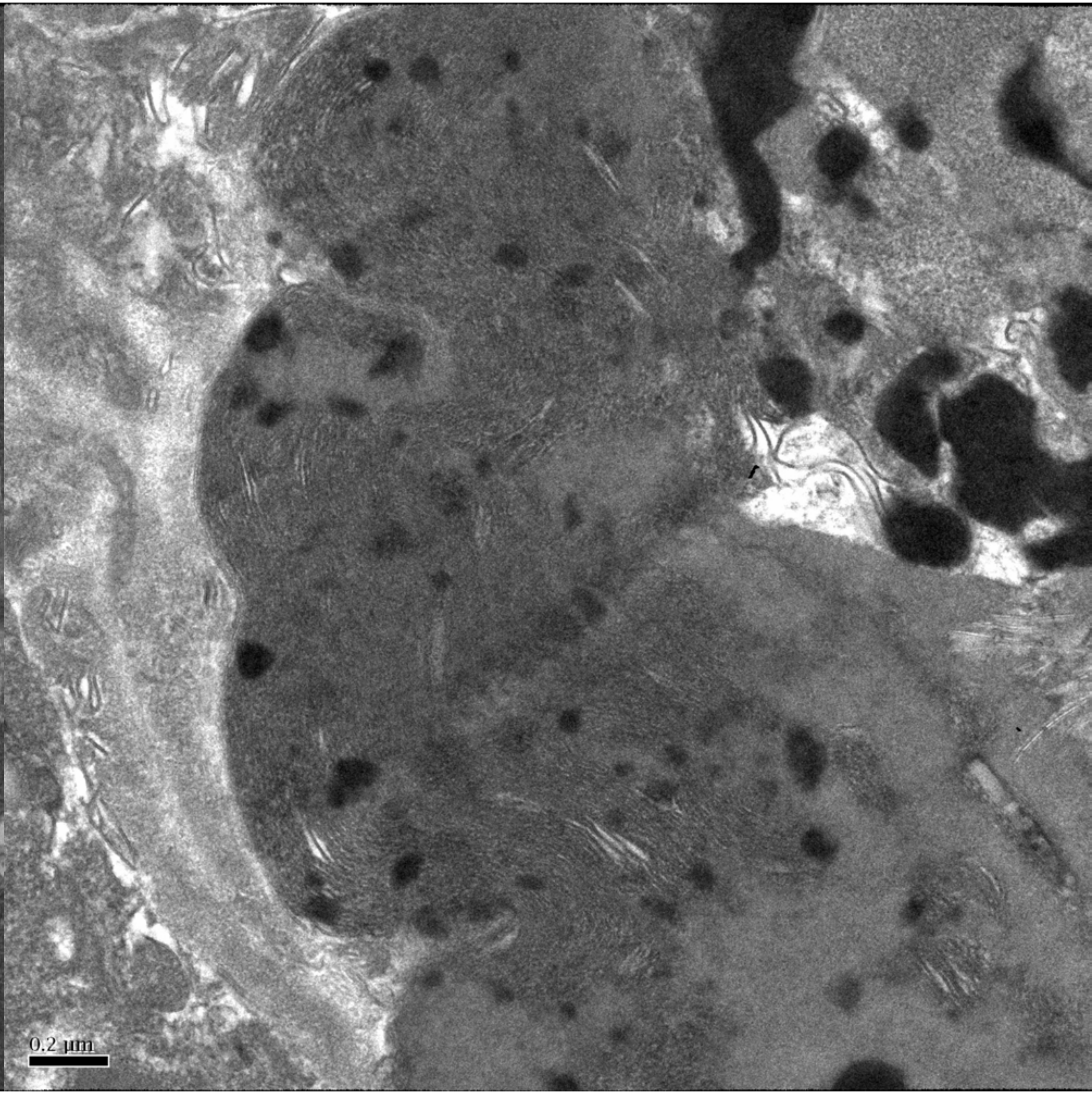

18-7_Correa_YH4_3D5_10

Cell 9

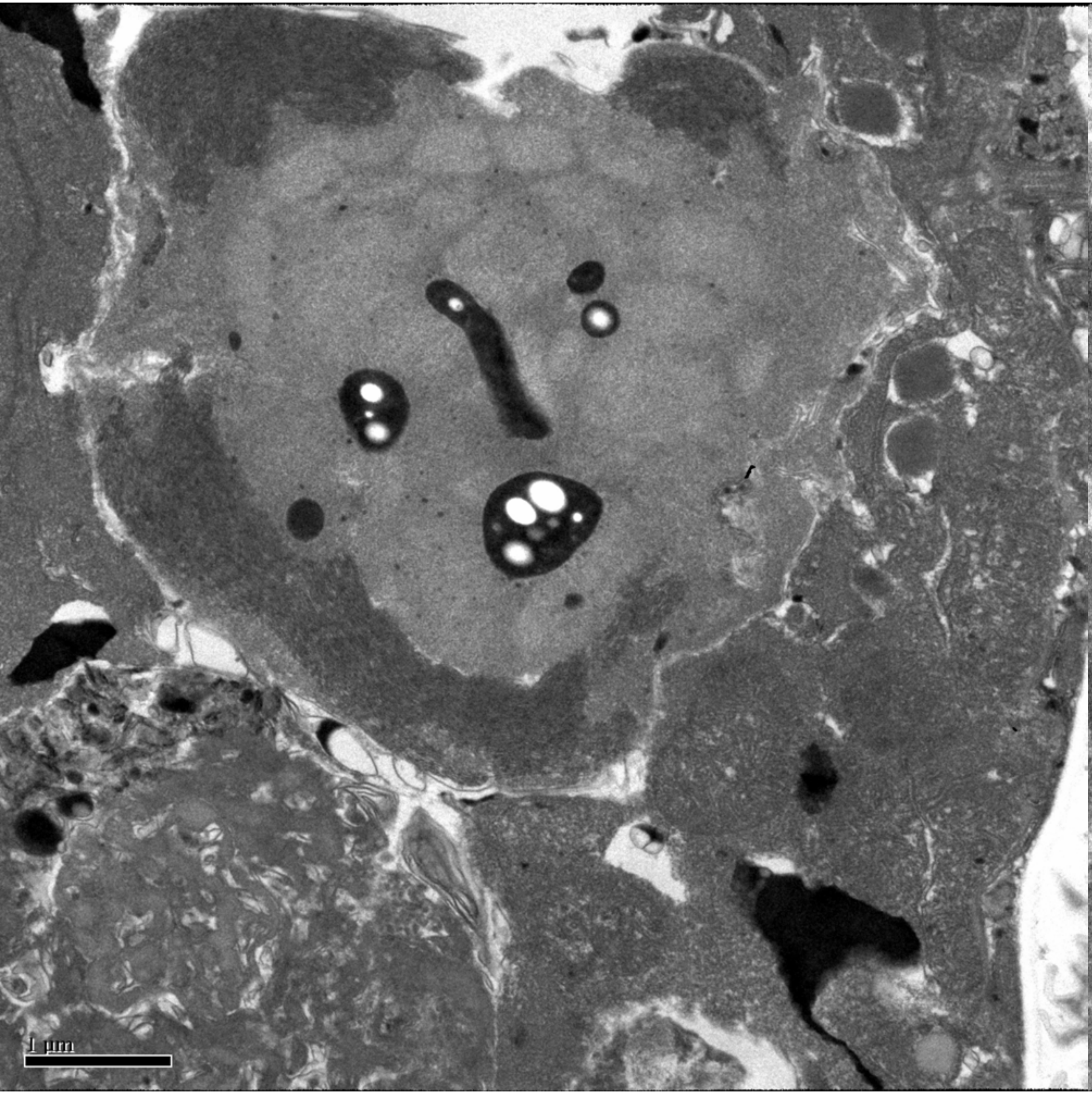

18-7_Correa_YH4_3D5_14

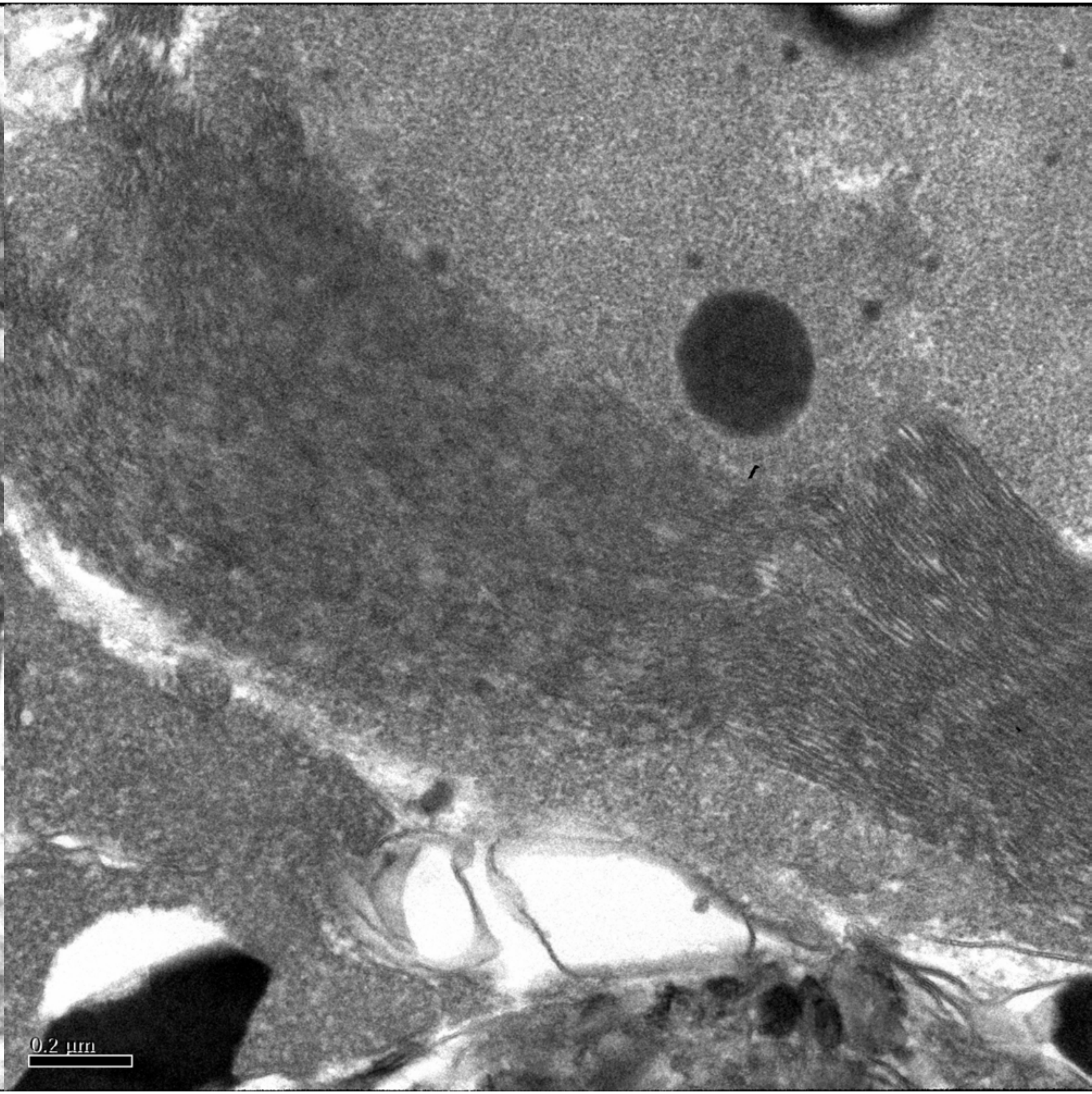

18-7_Correa_YH4_3D5_17

Cell 10

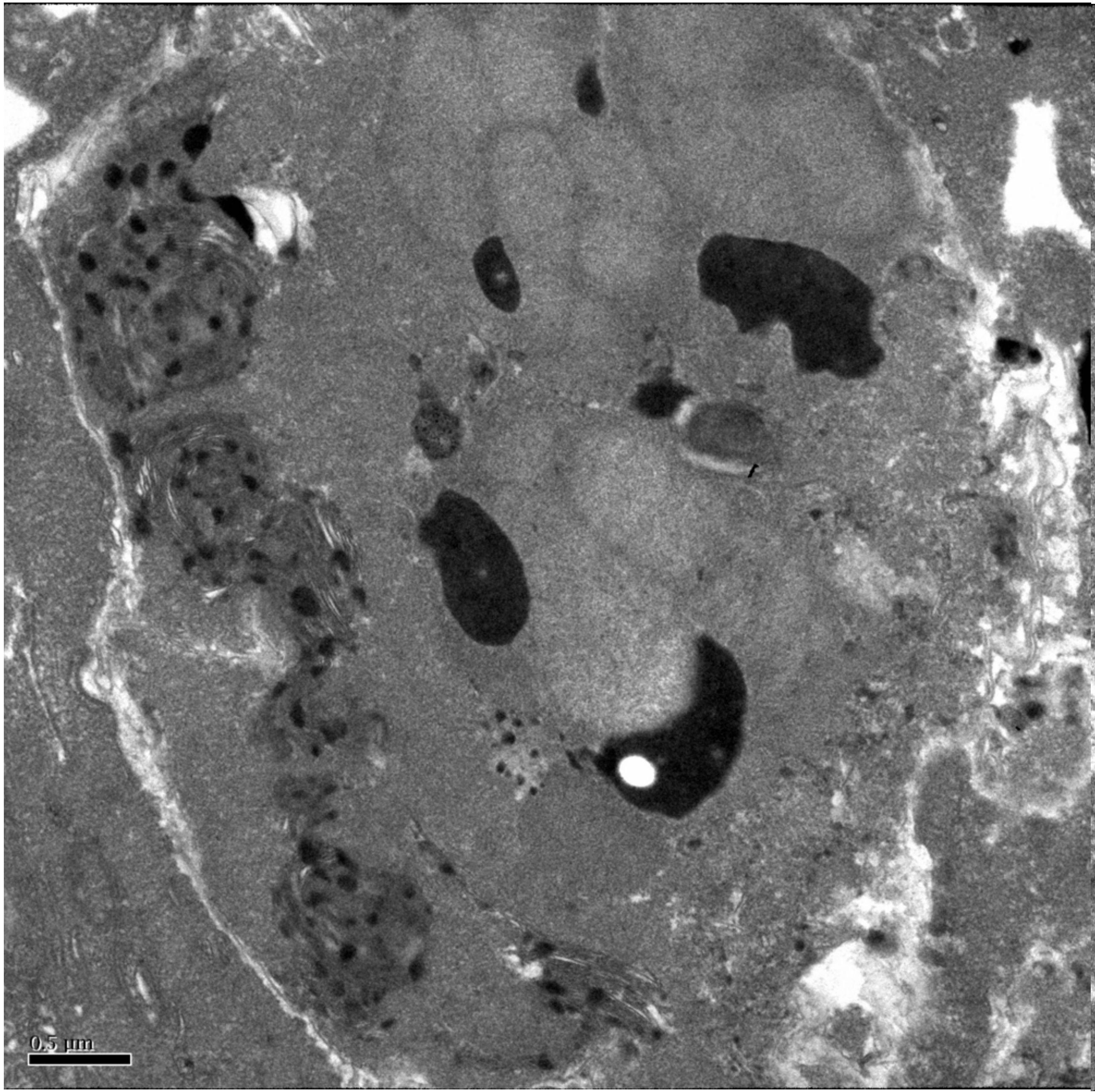

18-7_Correa_YH4_3D5_21

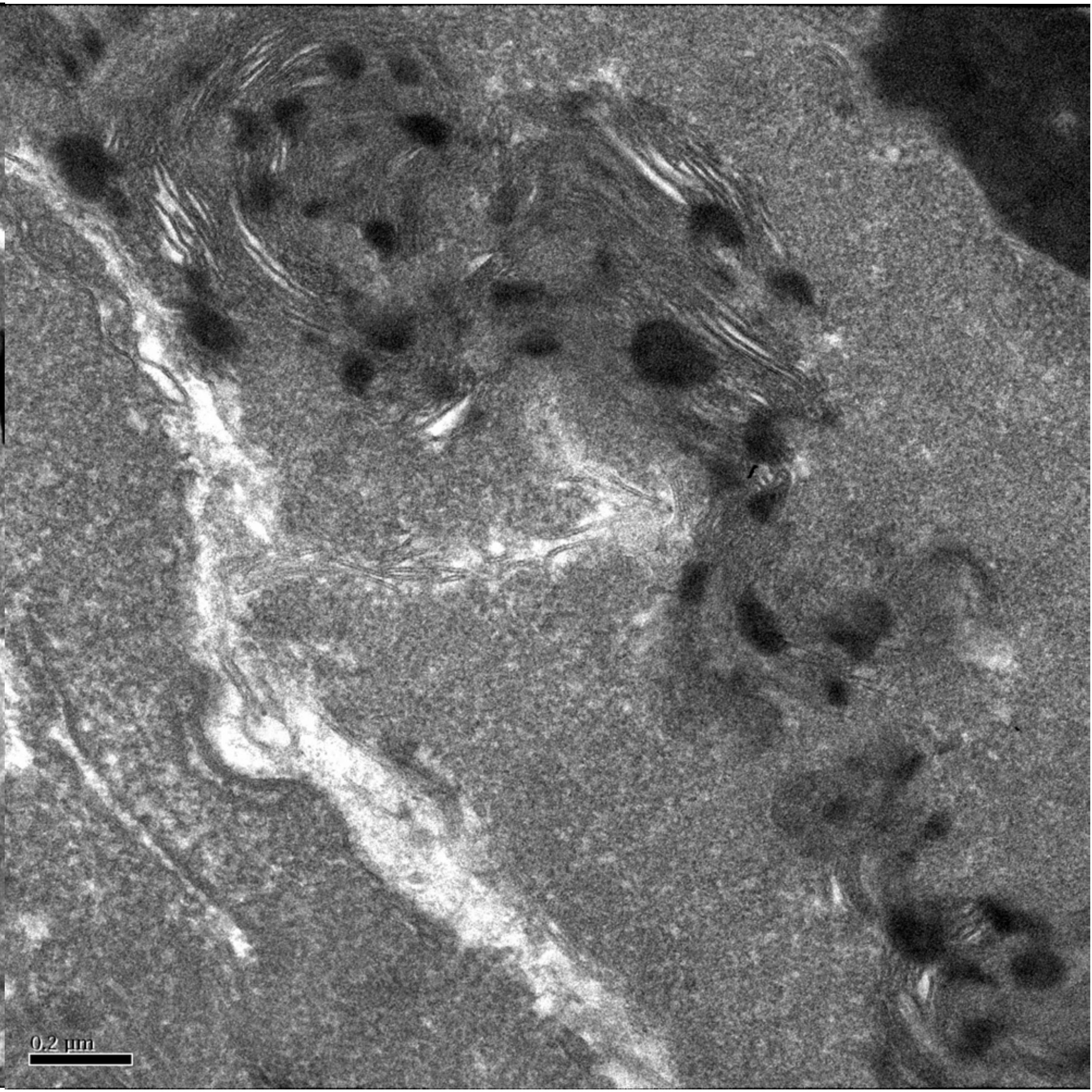

18-7_Correa_YH4_3D5_23

Cell 11

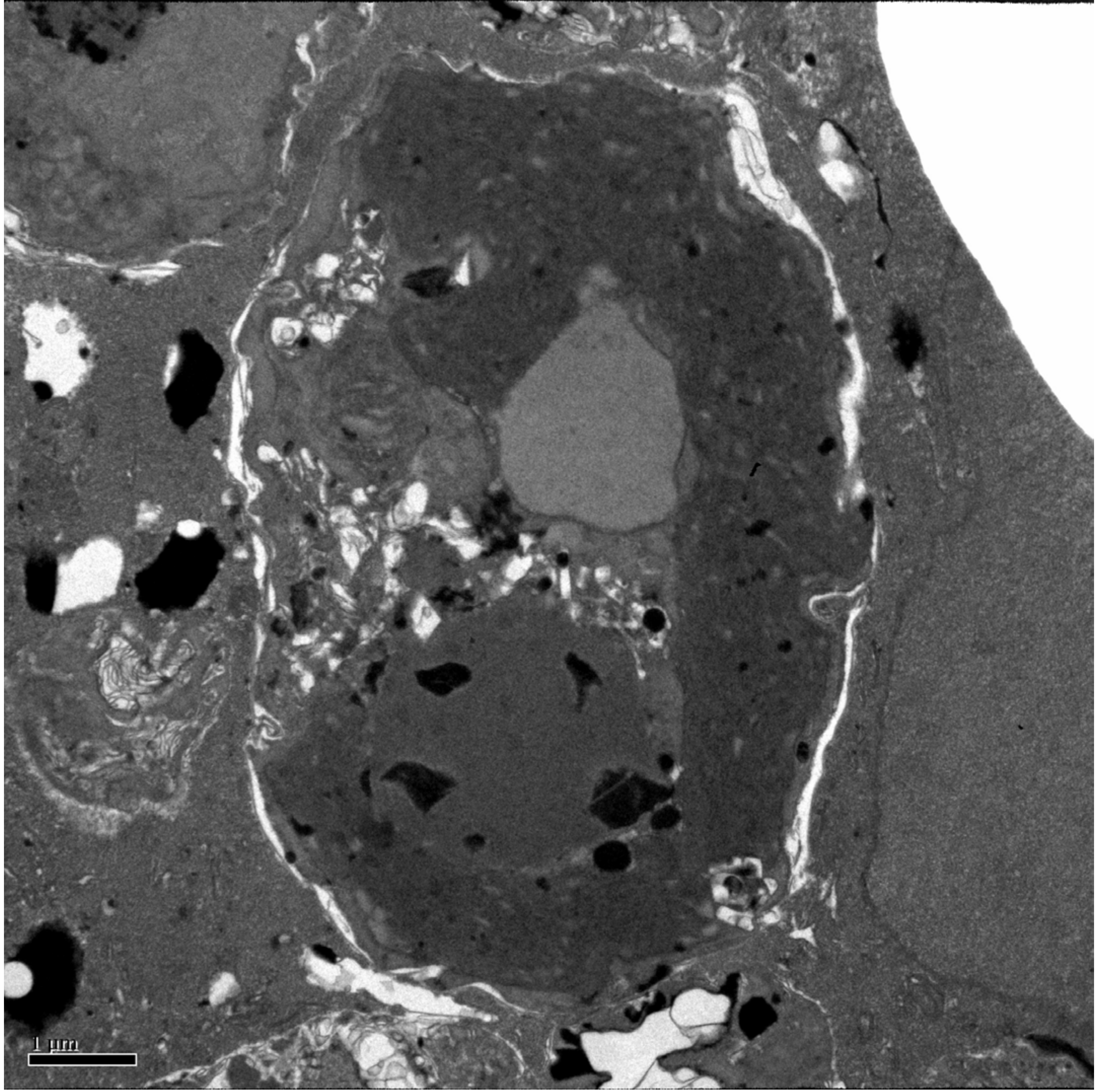

18-7_Correa_YH4_3D5_24
