# Supplementary material for: Filamentous virus-like particles are present in coral dinoflagellates across genera and ocean basins
Source: ISME J. 2023 Nov 1;17(12):2389–402. doi: 10.1038/s41396-023-01526-6 (PMC10689786; doi:10.1038/s41396-023-01526-6)
Supplement: Supplementary file 6 — Supplementary TEM Images expelled ACR symbionts in situ [file 41396_2023_1526_MOESM6_ESM.pdf]

Filamentous virus-like particles are present in coral dinoflagellates across genera and ocean basins

Supplementary Data- Expelled Symbiodiniaceae from *in situ Acropora hyacinthus* TEM images

Expelled- ACR Colony G

Expelled- ACR Colony I

Expelled- ACR Colony G

Cell 1

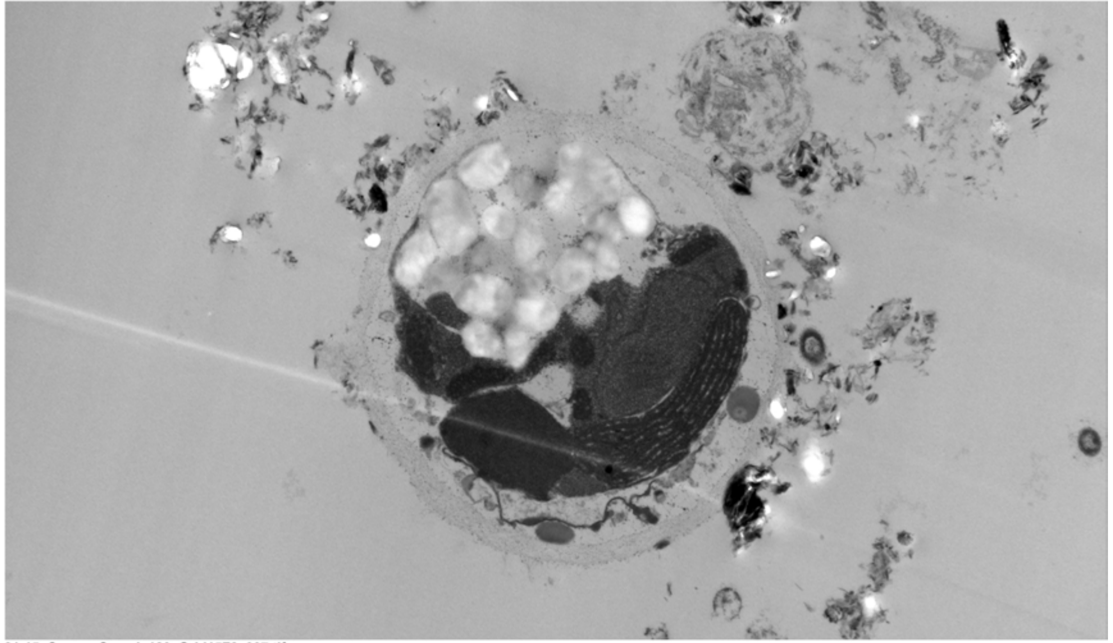

21-15_Correa_Sample120_Grid15F2_007.tif
1 µm
Cal: 0.002919 µm/pix
HV=80kV
Microscopist: Meyer
Direct Mag: 2500 x

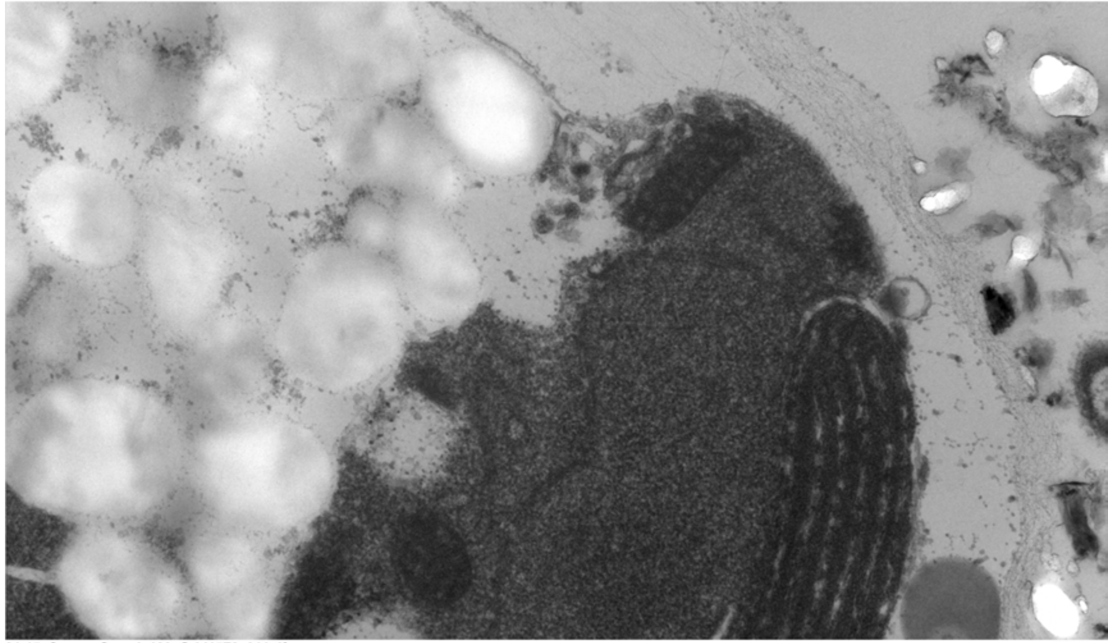

21-15_Correa_Sample120_Grid15F2_011.tif
500 nm
Cal: 0.000921 µm/pix
HV=80kV
Microscopist: Meyer
Direct Mag: 8000 x

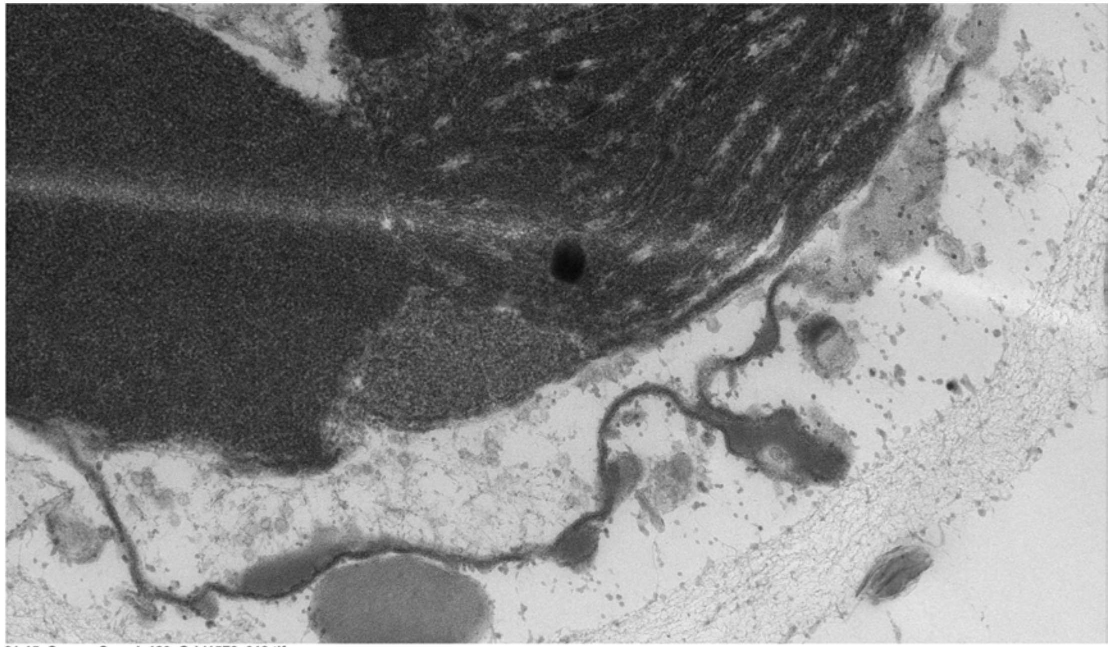

21-15_Correa_Sample120_Grid15F2_012.tif
200 nm
Cal: 0.000596 µm/pix
HV=80kV
Microscopist: Meyer
Direct Mag: 12000 x

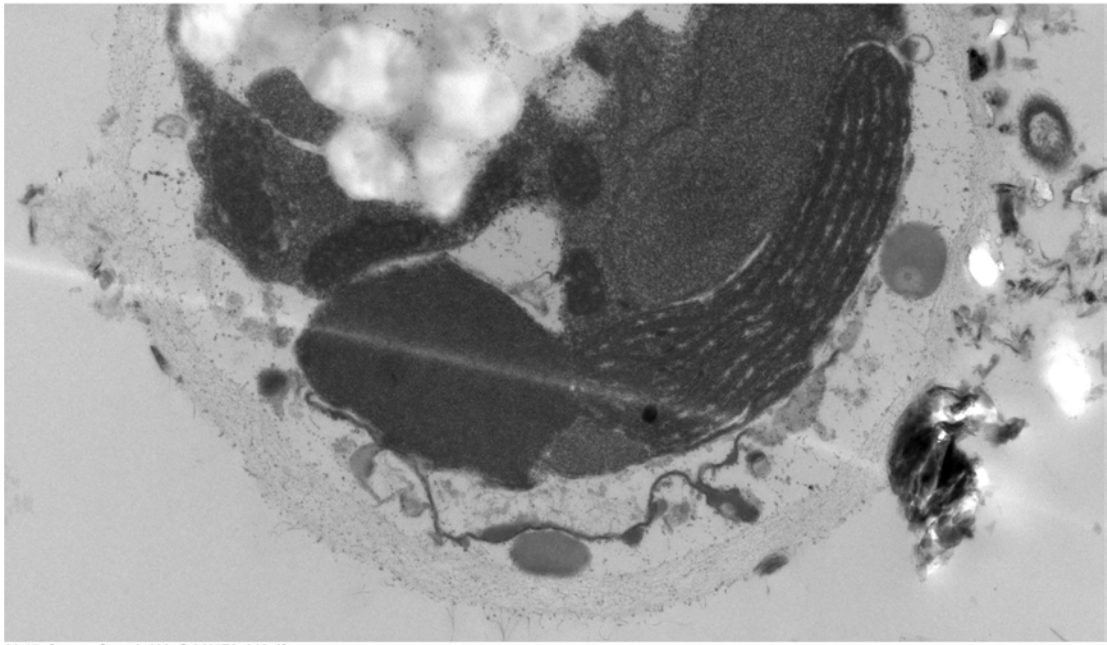

21-15_Correa_Sample120_Grid15F2_010.tif
800 nm
Cal: 0.001381 µm/pix
HV=80kV
Microscopist: Meyer
Direct Mag: 5000 x

Cell 2

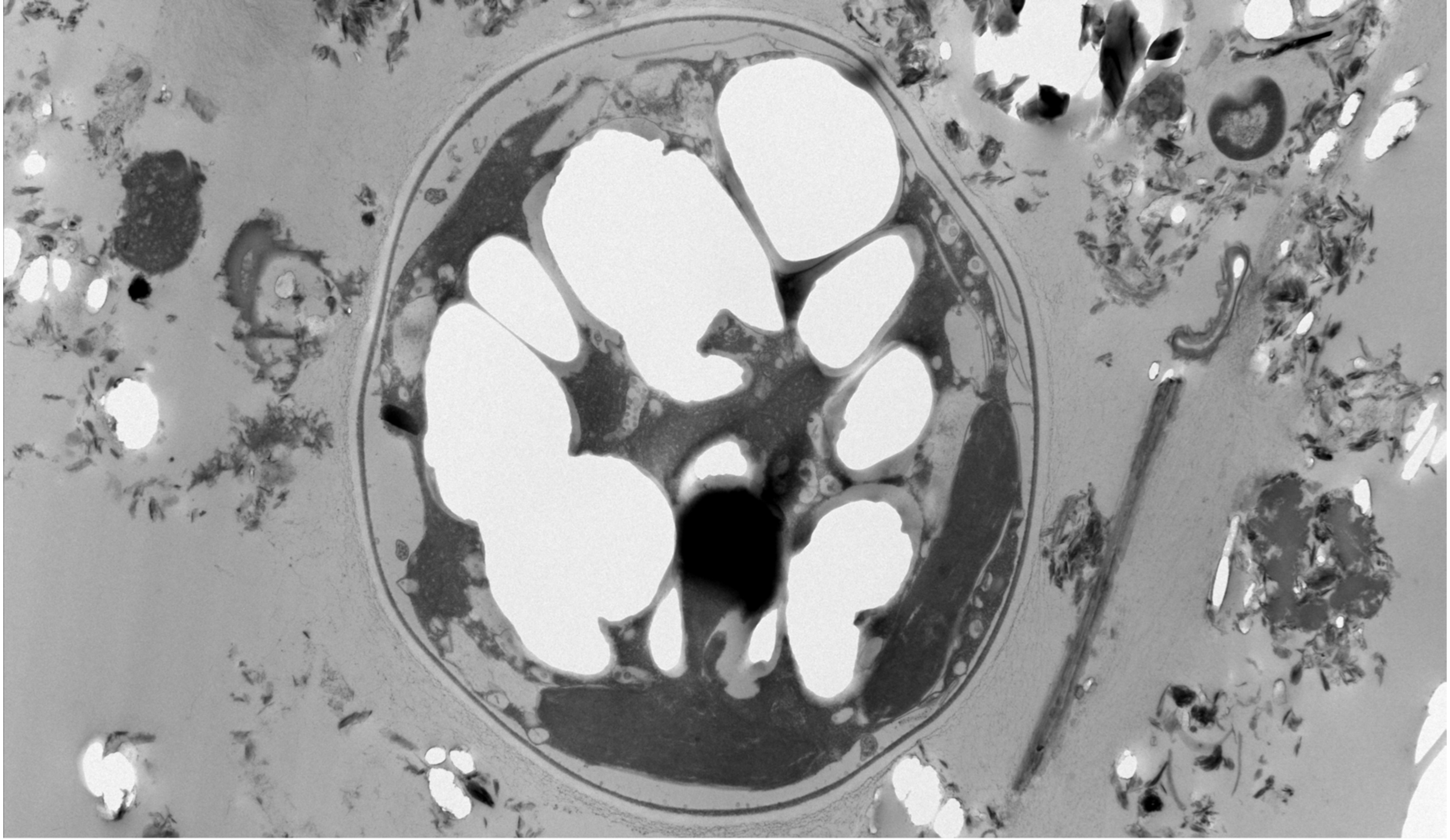

21-15_Correa_Sample120_Grid15F2_013.tif

Cal: 0.002471 µm/pix
Microscopist: Meyer

1 µm
HV=80kV
Direct Mag: 3000 x

Cell 3

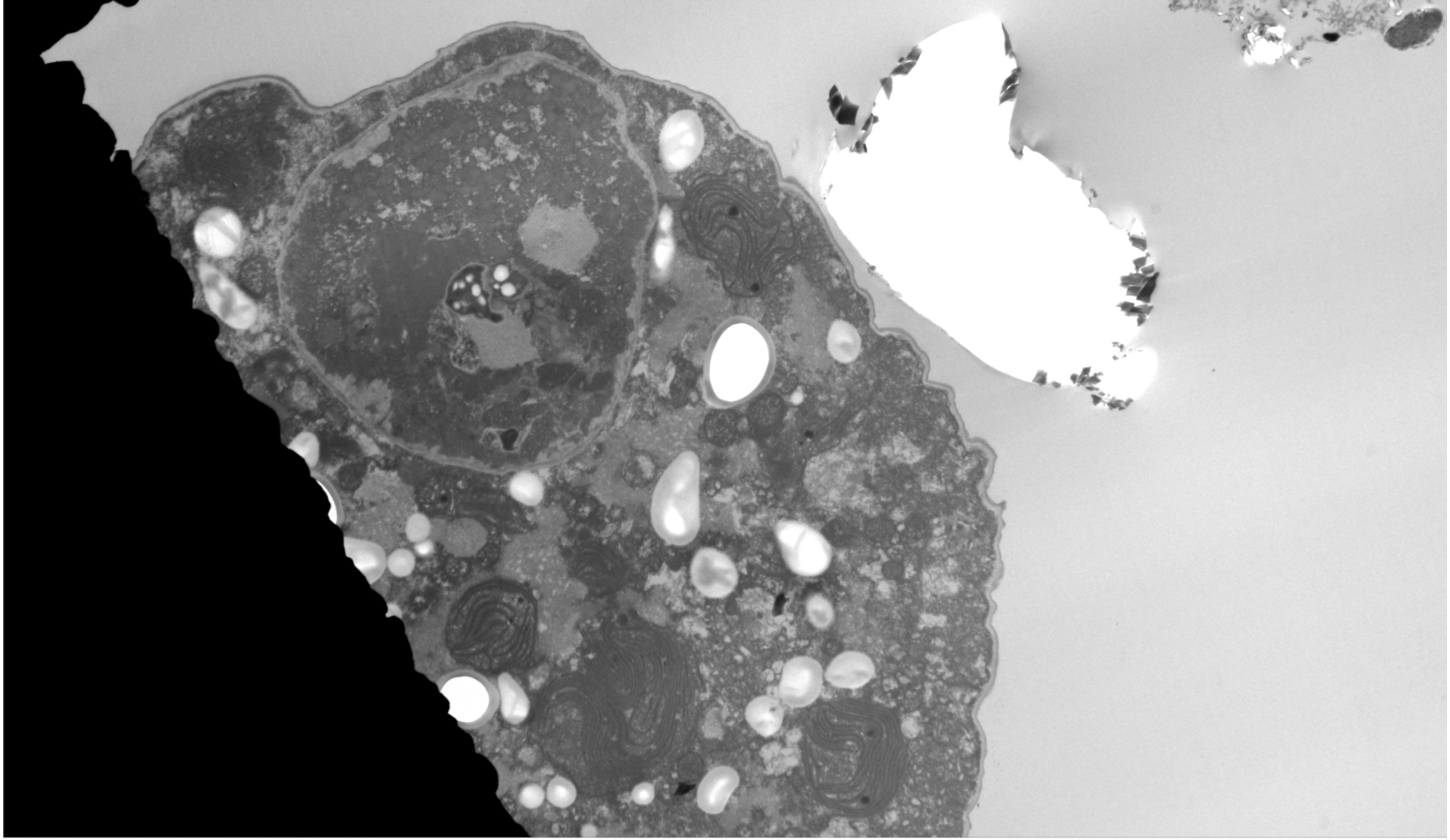

21-15_Correa_Sample120_Grid15F2_015.tif

Cal: 0.007156 µm/pix
Microscopist: Meyer

4 µm
HV=80kV
Direct Mag: 1000 x

Cells
4-5

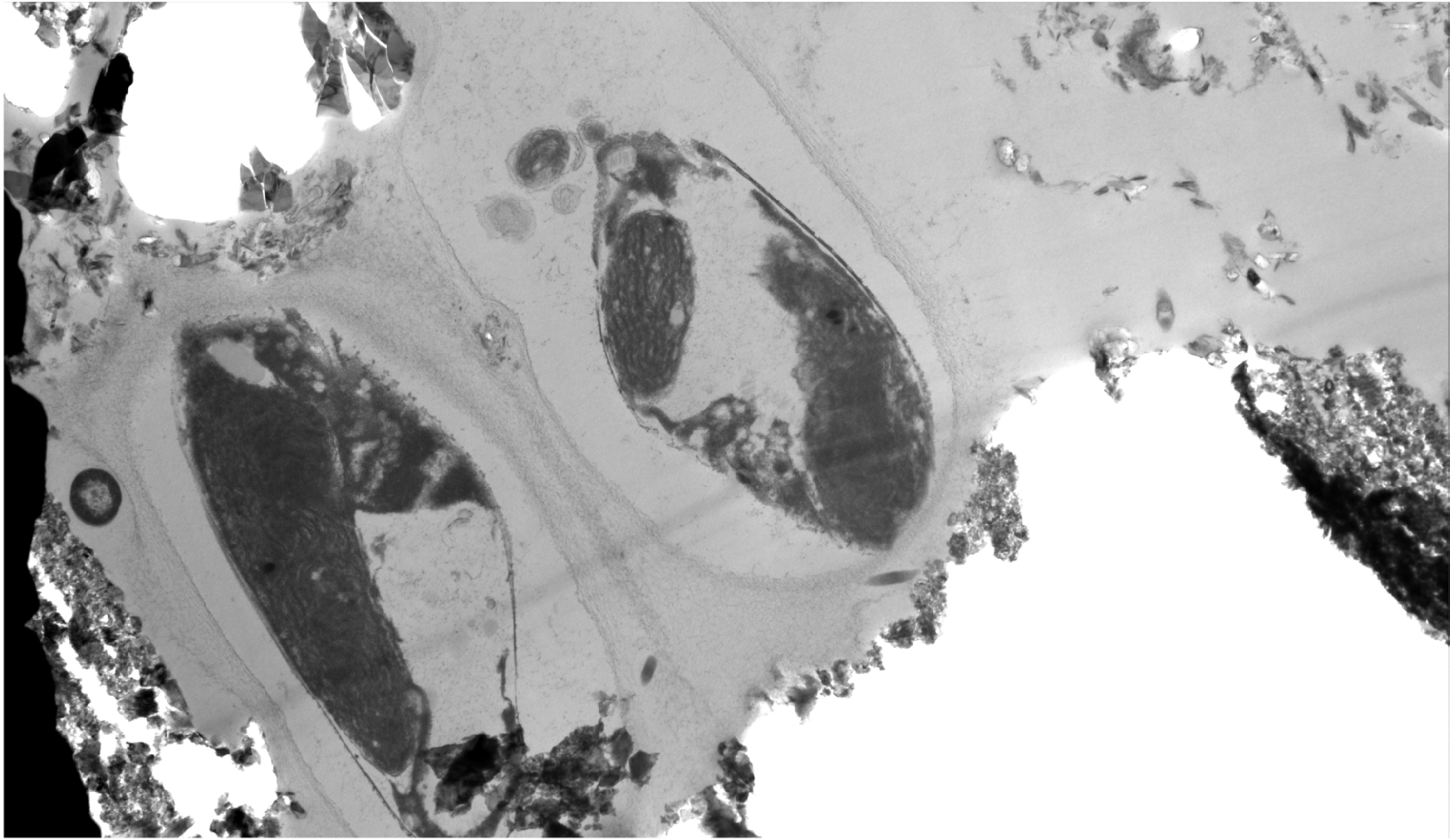

21-15_Correa_Sample120_Grid15F2_016.tif

Cal: 0.002919 µm/pix
Microscopist: Meyer

1 µm
HV=80kV
Direct Mag: 2500 x

Cell 6

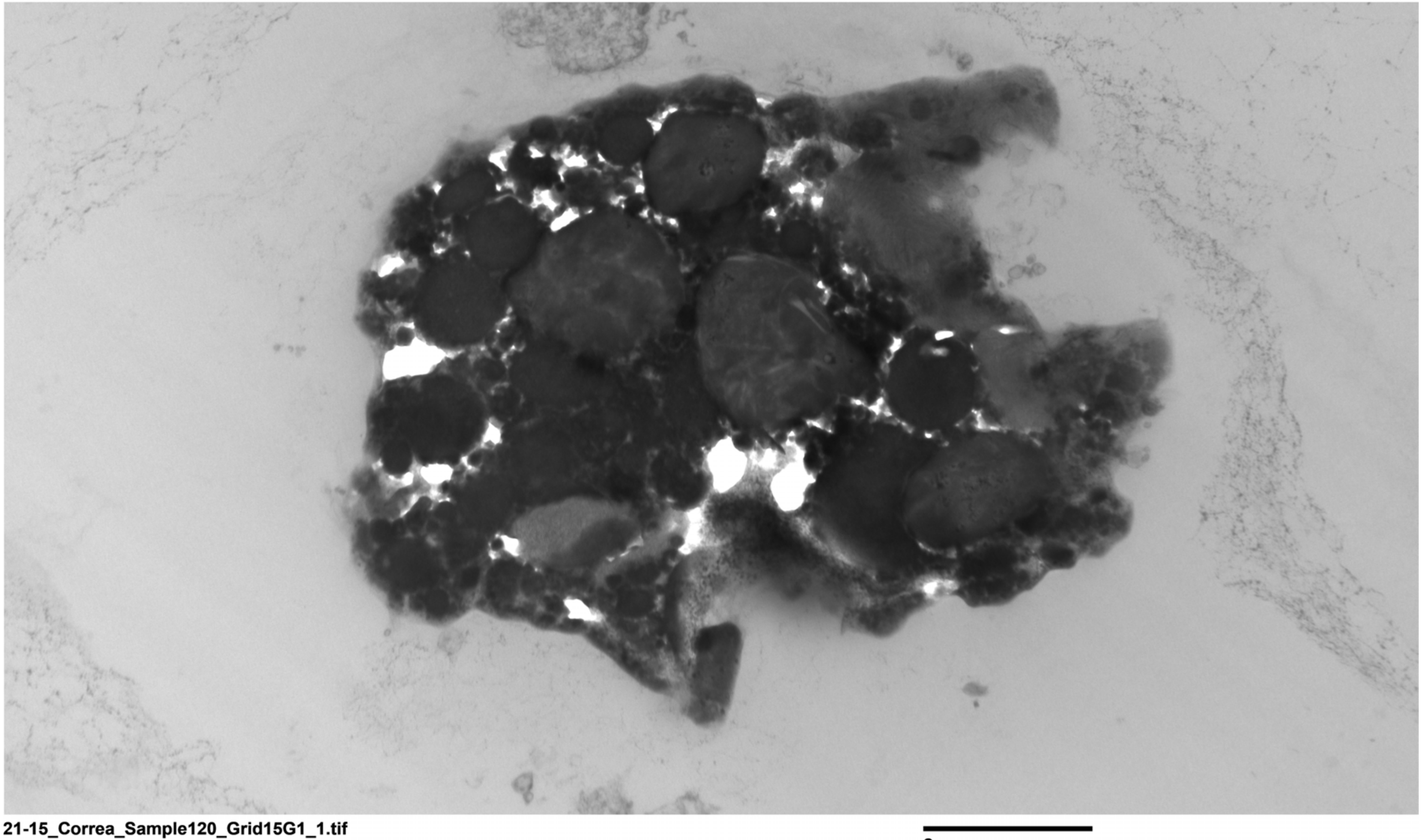

21-15_Correa_Sample120_Grid15G1_1.tif
Rice University
SEA
Biological Electron Microscopy Lab
Microscopist: Meyer

2 µm
HV=80kV
Direct Mag: 2000 x

Cell 7

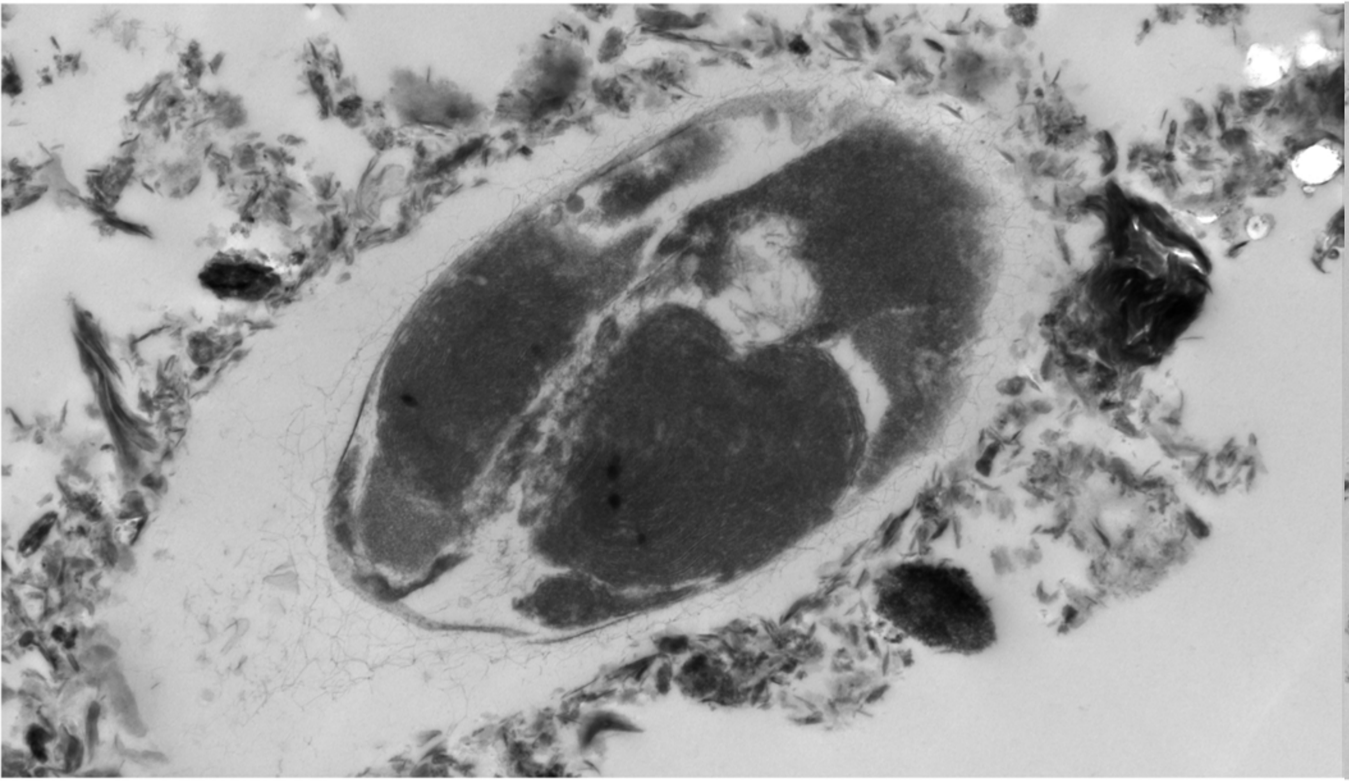

21-15_Correa_Sample120_Grid15F4_3.tif
Rice University
SEA
Biological Electron Microscopy Lab
Microscopist: Meyer

1 µm
HV=80kV
Direct Mag: 4000 x

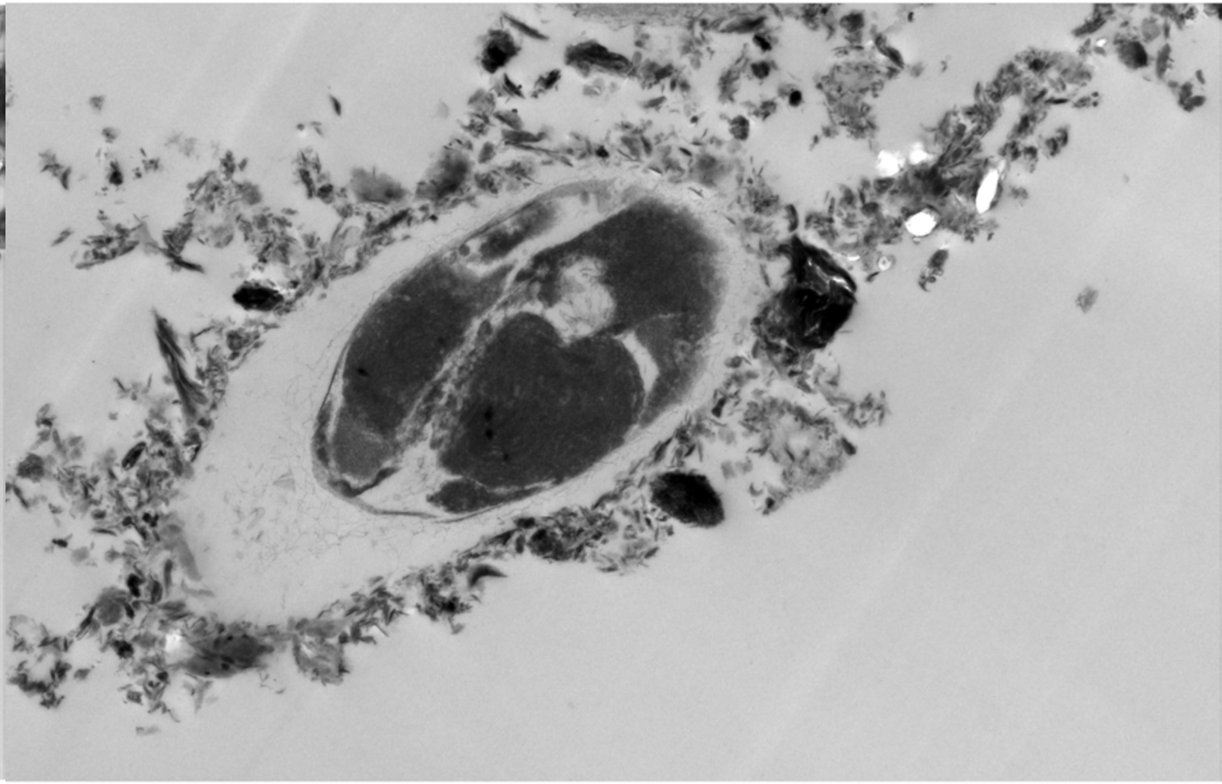

ample120_Grid15F4_2.tif
ron Microscopy Lab
eyer

1 µm
HV=80kV
Direct Mag: 2500 x

Cell 8

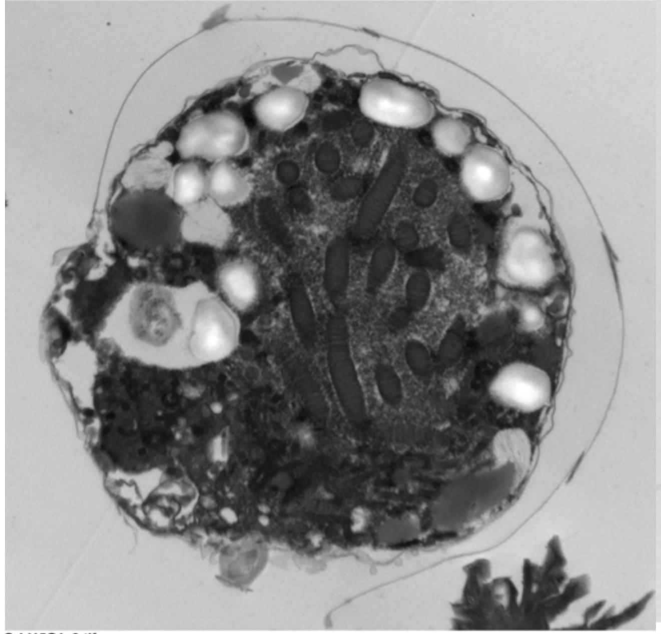

Grid15G1_3.tif

copy Lab

2 µm
HV=80kV
Direct Mag: 1500 x

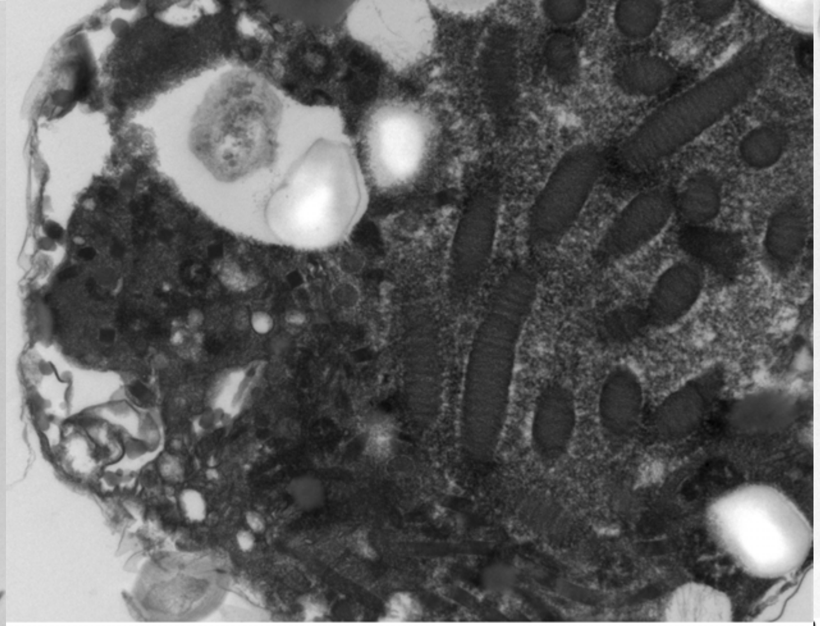

1 µm
HV=80kV
Direct Mag: 3000 x

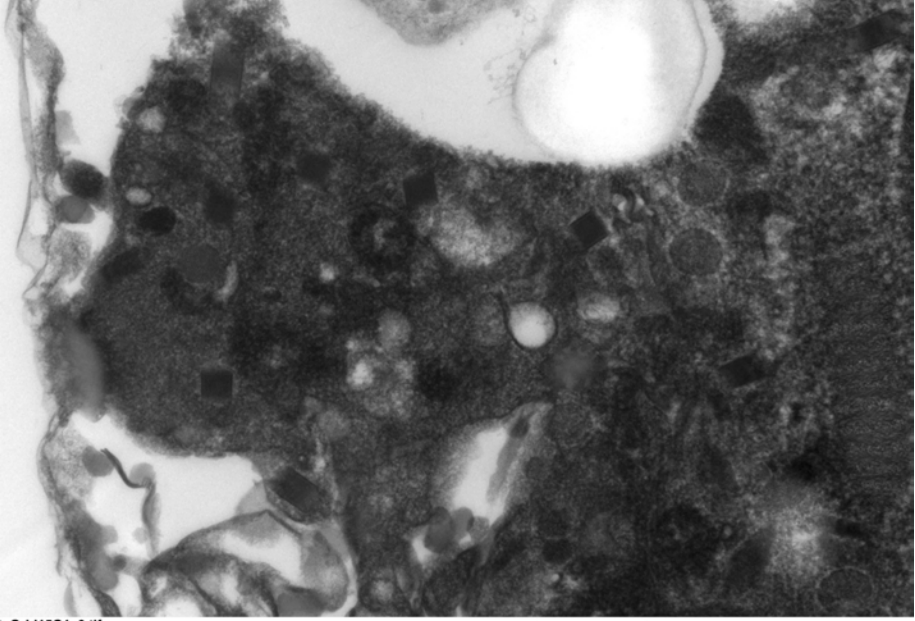

_Grid15G1_6.tif

scopy Lab

600 nm
HV=80kV
Direct Mag: 6000 x

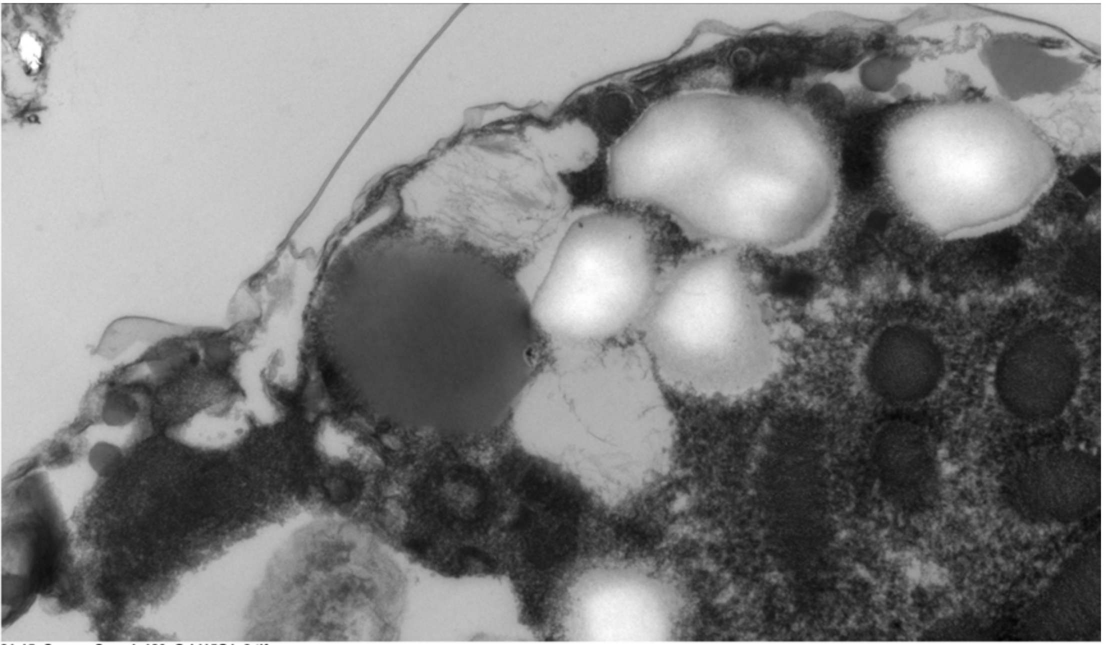

21-15_Correa_Sample120_Grid15G1_8.tif
Rice University
SEA
Biological Electron Microscopy Lab
Microscopist: Meyer

800 nm
HV=80kV
Direct Mag: 5000 x

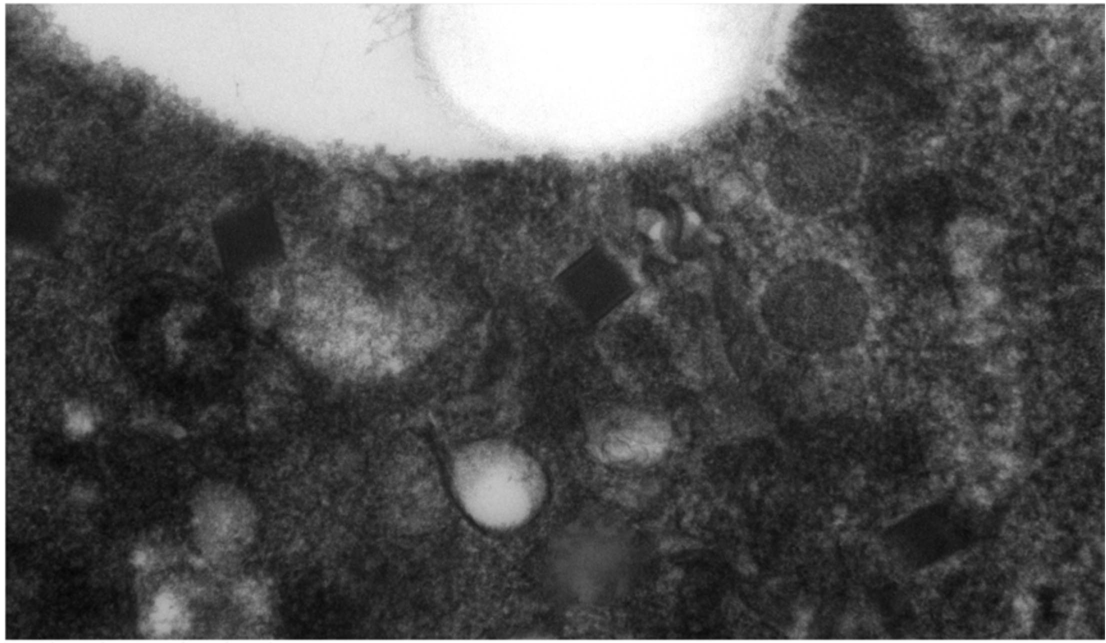

21-15_Correa_Sample120_Grid15G1_7.tif
Rice University
SEA
Biological Electron Microscopy Lab
Microscopist: Meyer

200 nm
HV=80kV
Direct Mag: 12000 x

Cell 9

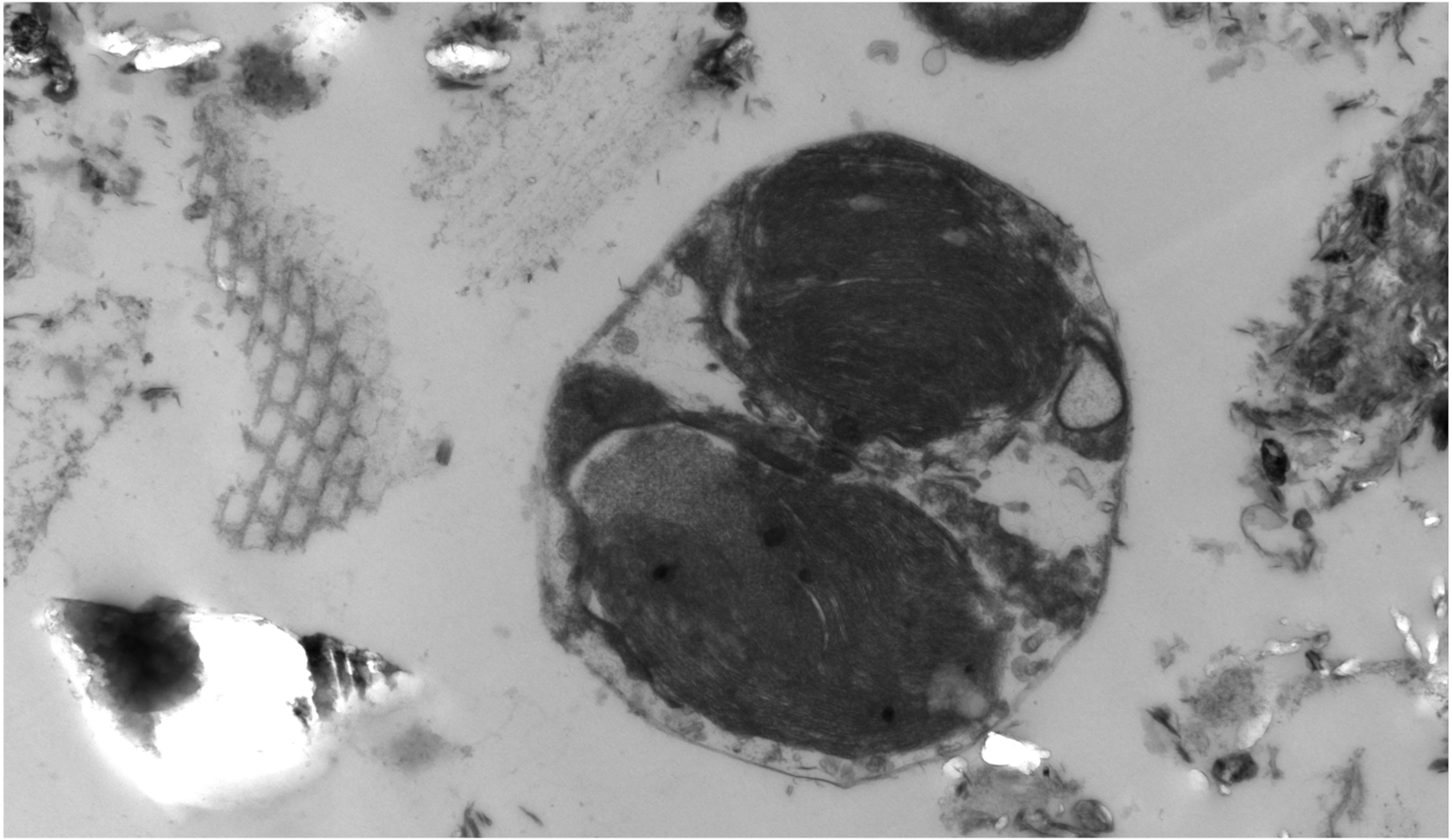

21-15_Correa_Sample120_Grid15G1_11.tif
Rice University
SEA
Biological Electron Microscopy Lab
Microscopist: Meyer

1 µm
HV=80kV
Direct Mag: 4000 x

Other images

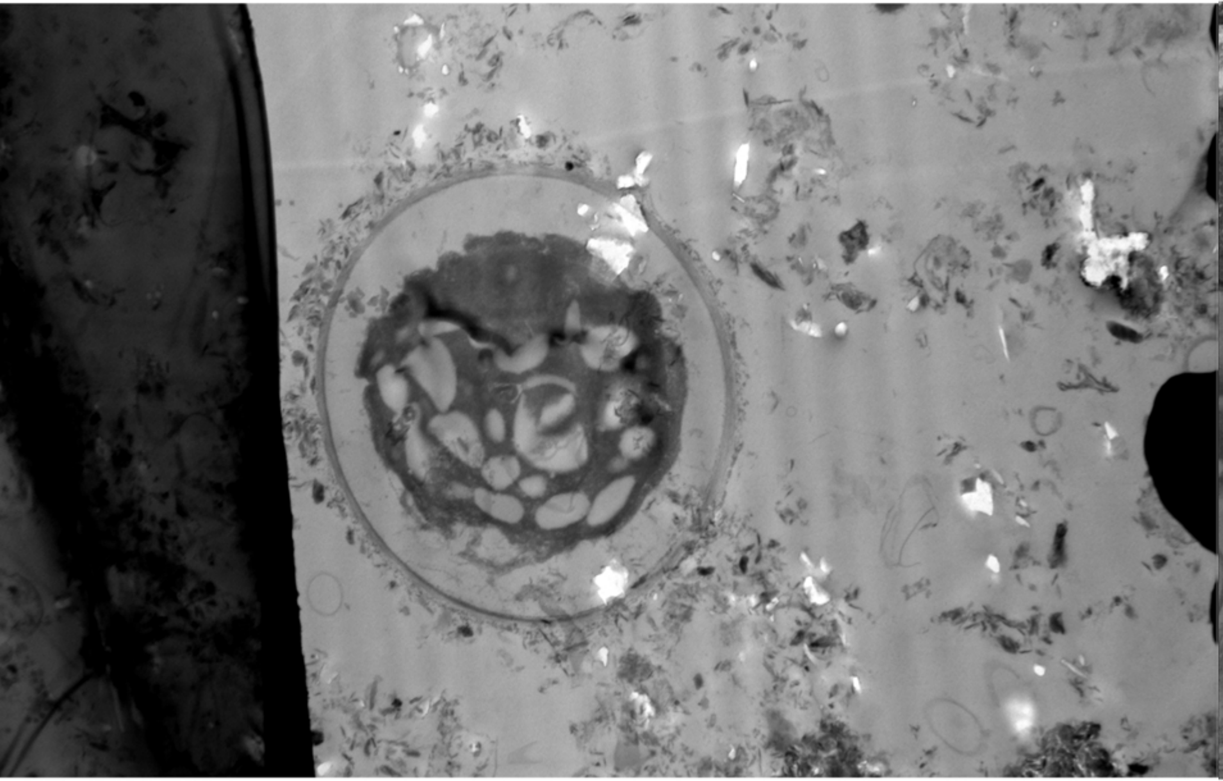

ample120_Grid15E2_007.tif
d 15E2

m/pix
leyer

1 µm
HV=80kV
Direct Mag: 2500 x

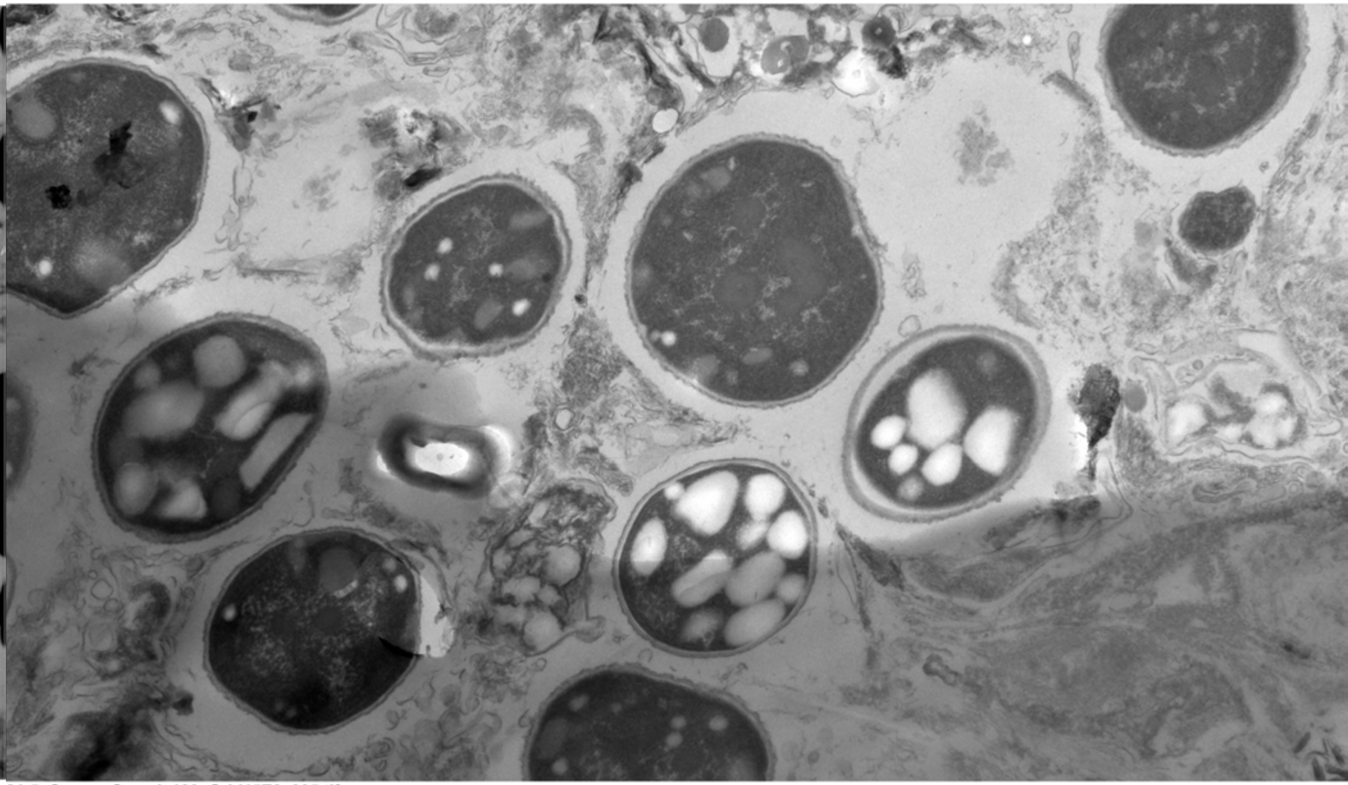

21-5_Correa_Sample120_Grid15E2_005.tif
Sample 120, grid 15E2

Cal: 0.001785 µm/pix
Microscopist: Meyer

1 µm
HV=80kV
Direct Mag: 4000 x

Other Images

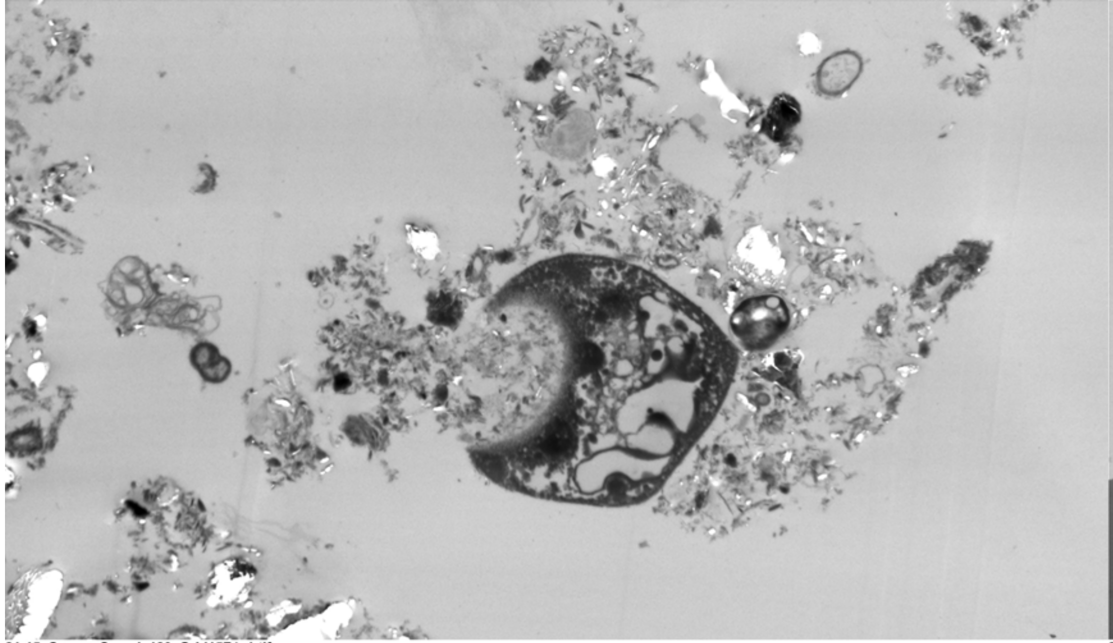

21-15_Correa_Sample120_Grid15F4_1.tif
Rice University
SEA
Biological Electron Microscopy Lab
Microscopist: Meyer

2 µm
HV=80kV
Direct Mag: 1200 x

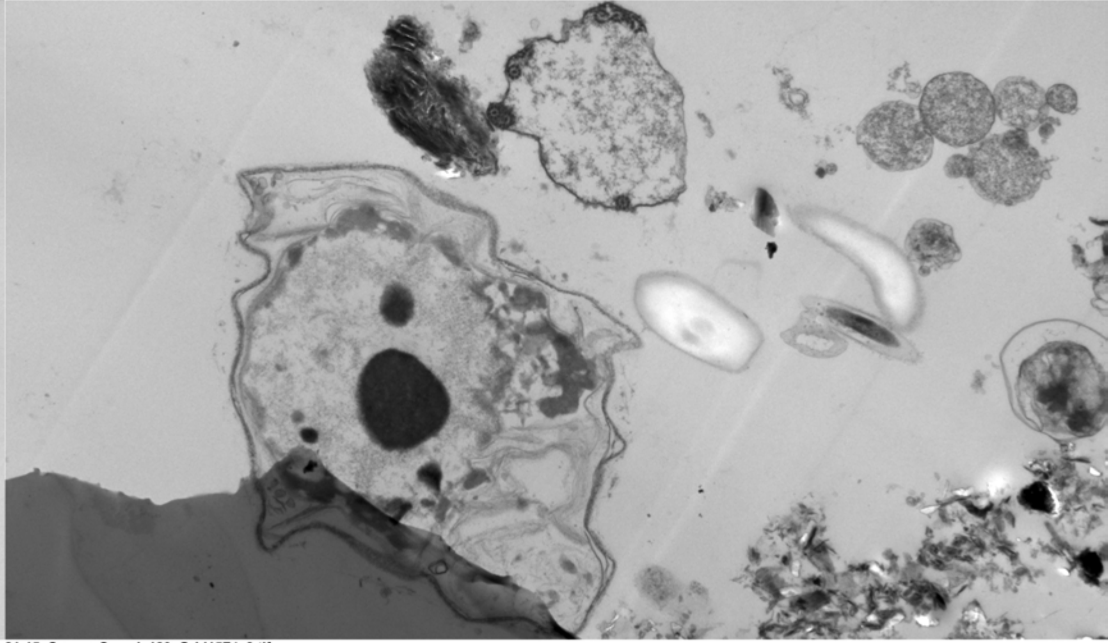

21-15_Correa_Sample120_Grid15F4_8.tif
Rice University
SEA
Biological Electron Microscopy Lab

1 µm
HV=80kV
Direct Mag: 2500 x

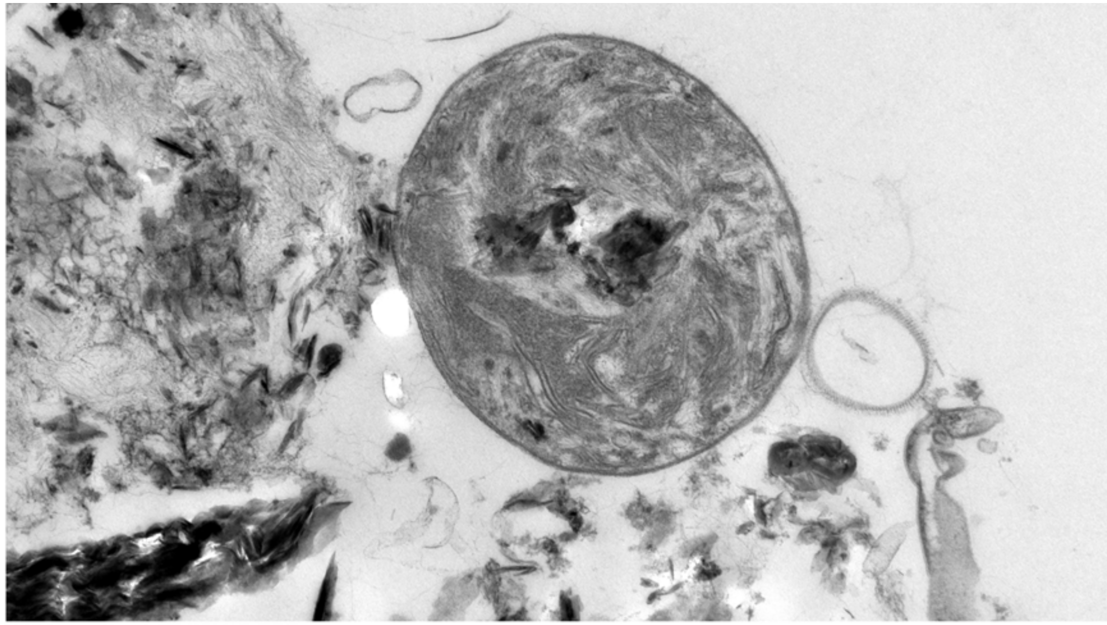

21-15_Correa_Sample120_Grid15F2_004.tif

Cal: 0.000921 µm/pix
Microscopist: Meyer

500 nm
HV=80kV
Direct Mag: 8000 x

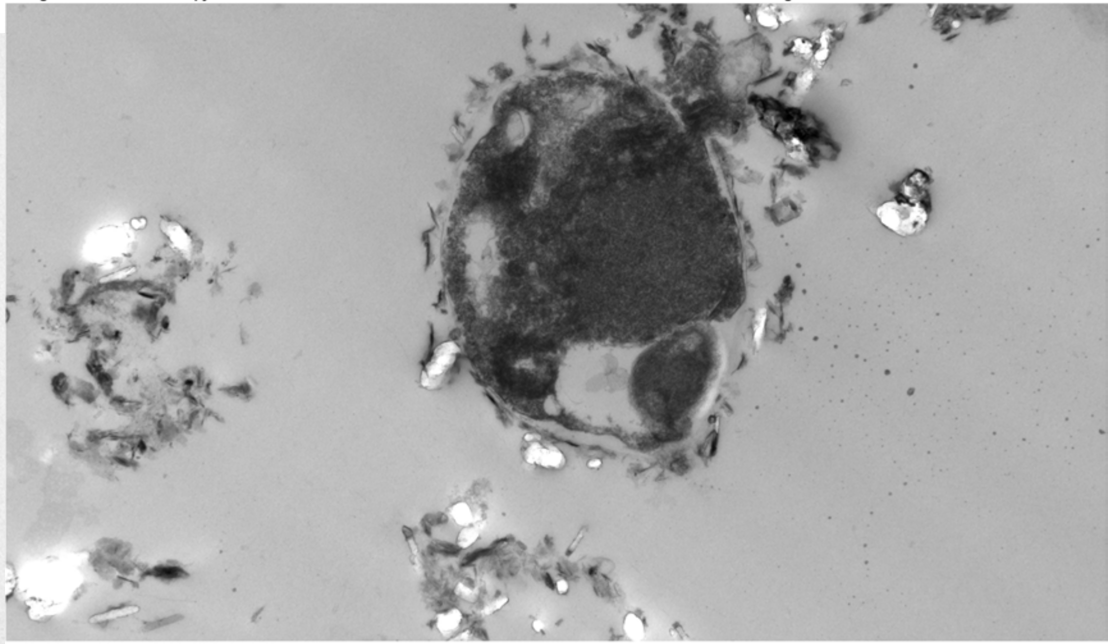

21-15_Correa_Sample120_Grid15F5_1.tif
Rice University
SEA
Biological Electron Microscopy Lab
Microscopist: Meyer

1 µm
HV=80kV
Direct Mag: 4000 x

Other images

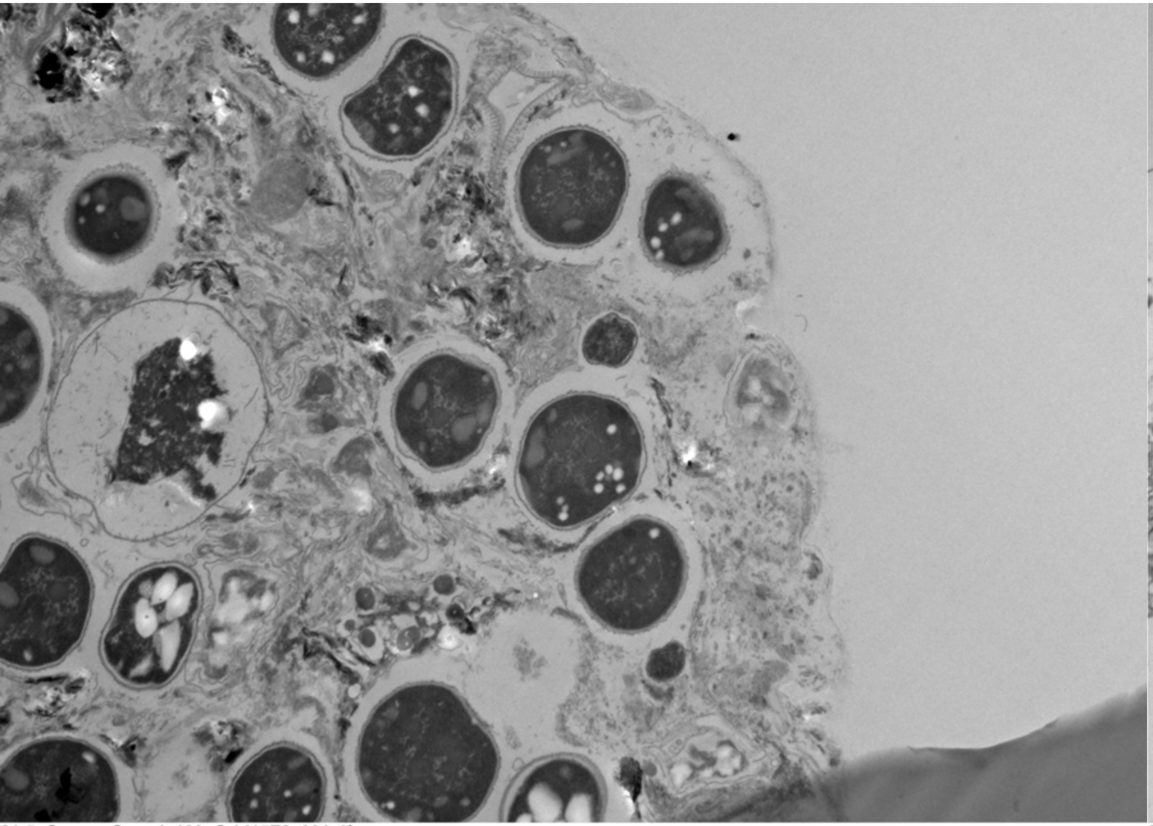

21-5_Correa_Sample120_Grid15E2_001.tif
Sample 120, grid 15E2

Cal: 0.003307 µm/pix
Microscopist: Meyer

2 µm
HV=80kV
Direct Mag: 2000 x

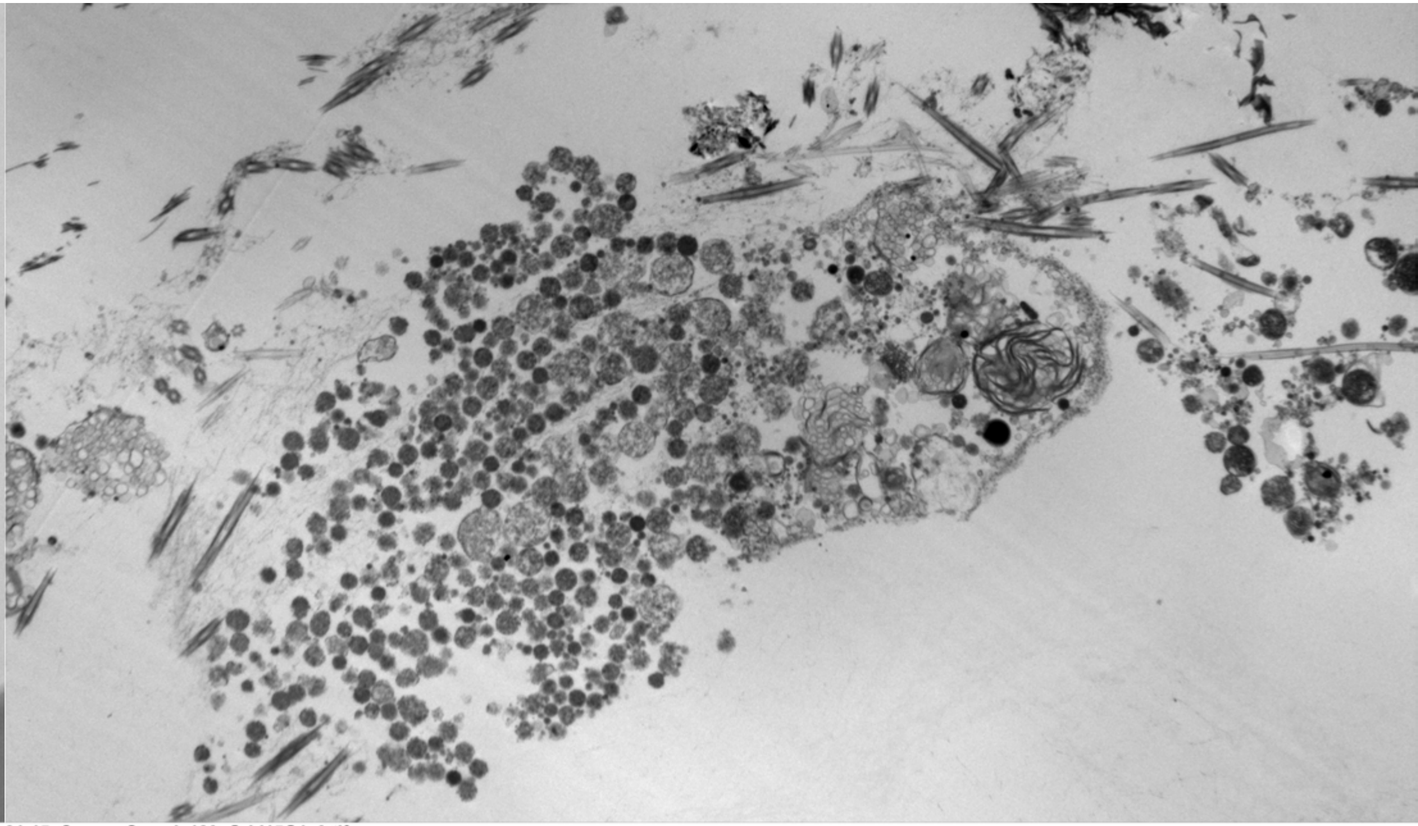

21-15_Correa_Sample120_Grid15G1_9.tif
Rice University
SEA
Biological Electron Microscopy Lab
Microscopist: Meyer

4 µm
HV=80kV
Direct Mag: 1000 x

Expelled- ACR Colony I

Cell 1

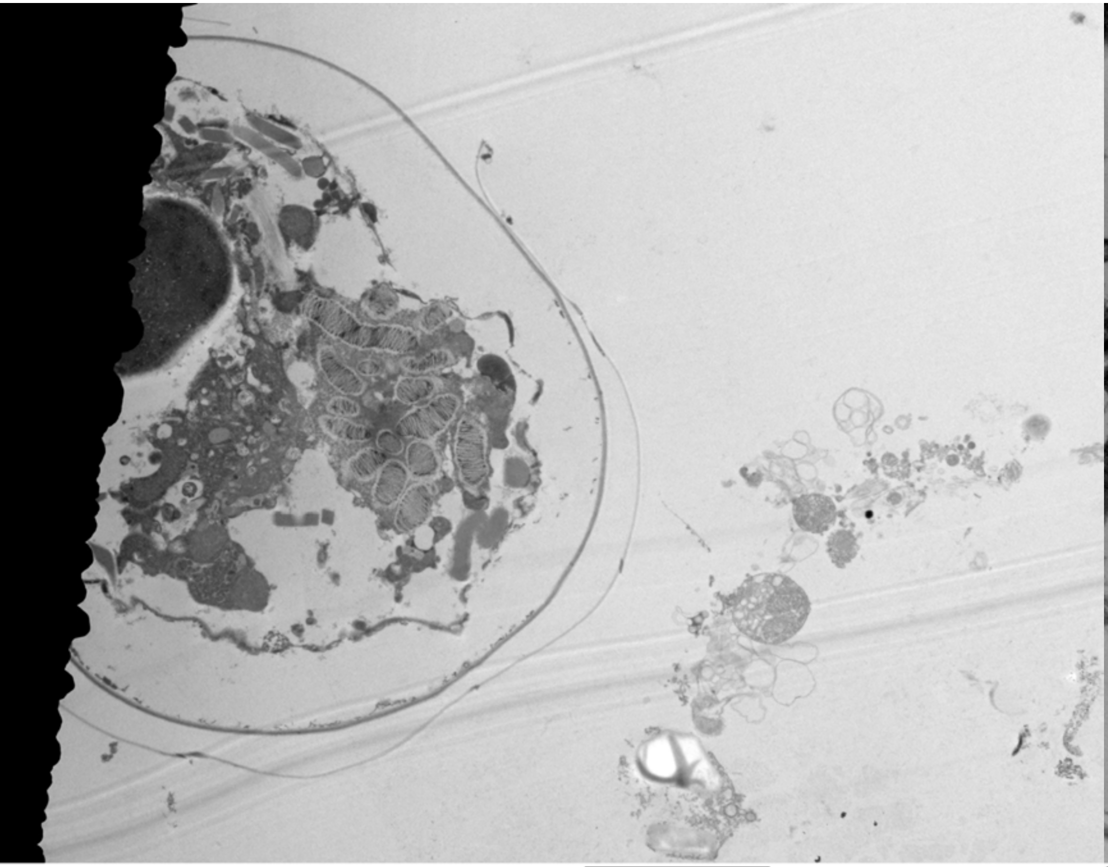

4 µm
HV=120kV
Direct Mag: 1000 x

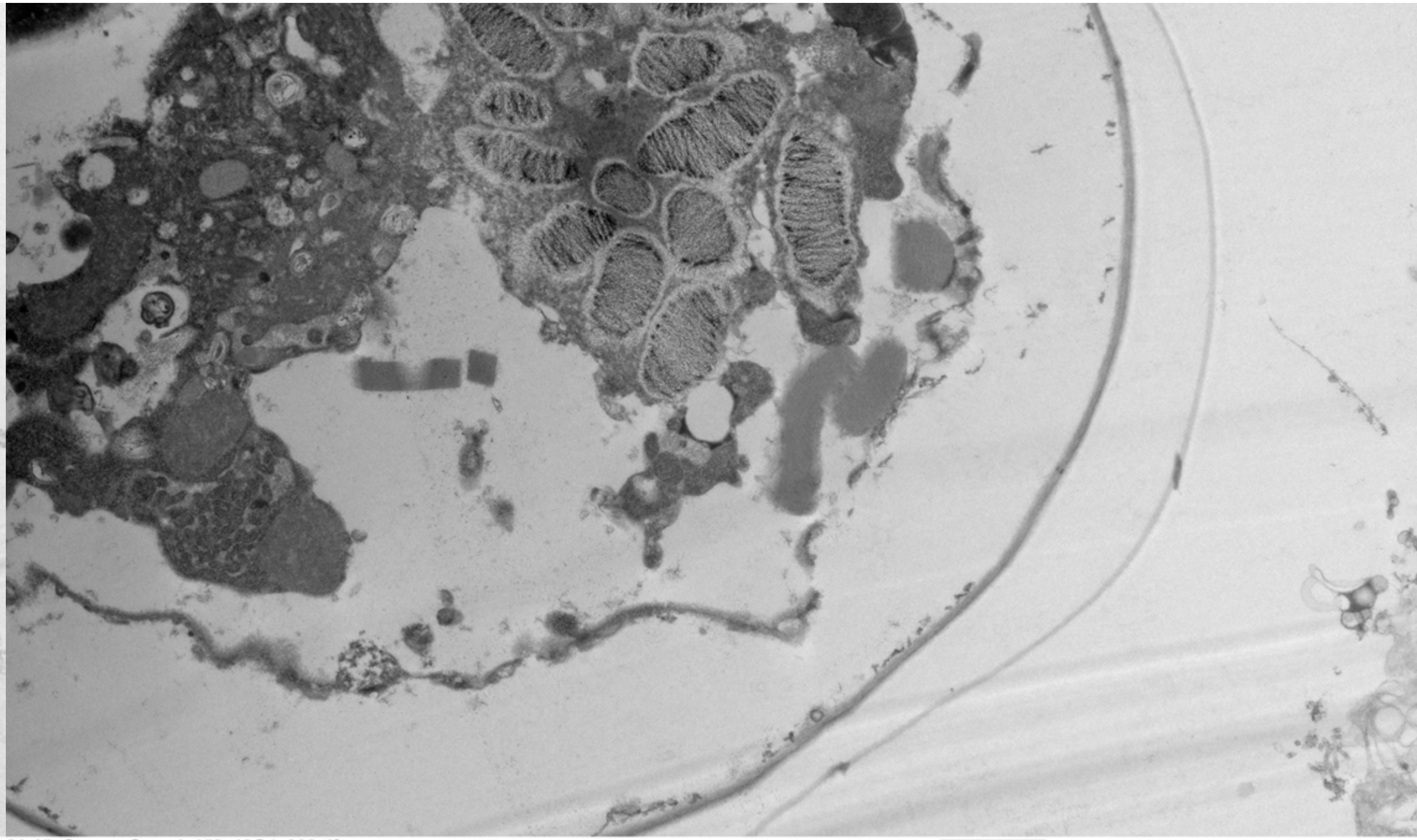

21-15_Correa_Sample158_19Q4_002.tif
Sample 158 expelled
Biological Electron Microscopy Lab
Rice University - SEA
Microscopist: MD Meyer

1 µm
HV=120kV
Direct Mag: 2500 x

Cell 1 cont.

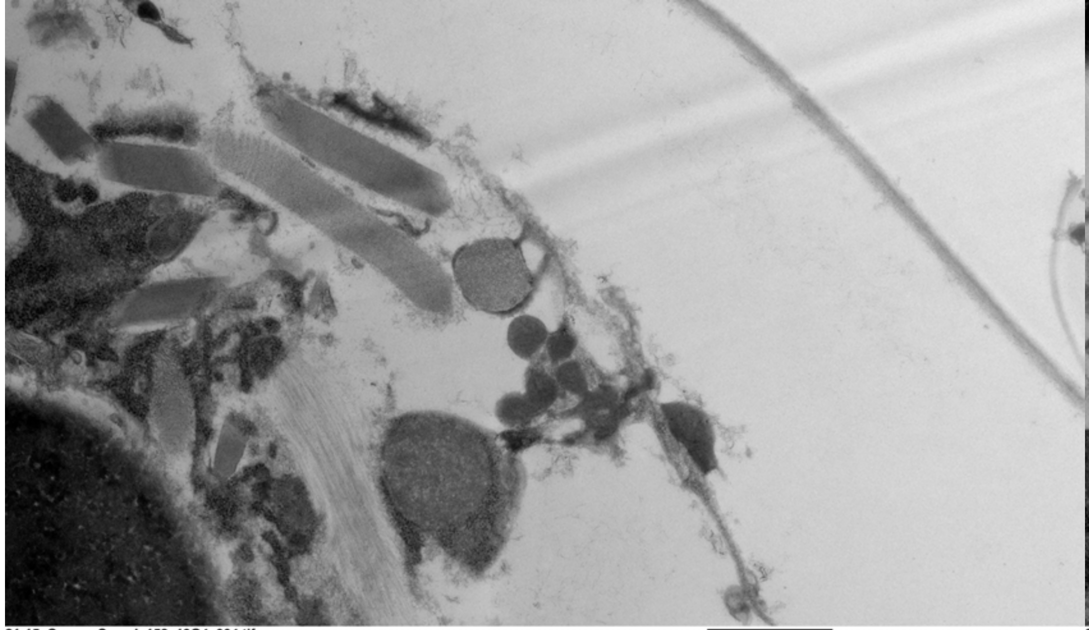

21-15_Correa_Sample158_19Q4_004.tif
Sample 158 expelled
Biological Electron Microscopy Lab
Rice University - SEA
Microscopist: MD Meyer

800 nm
HV=120kV
Direct Mag: 5000 x

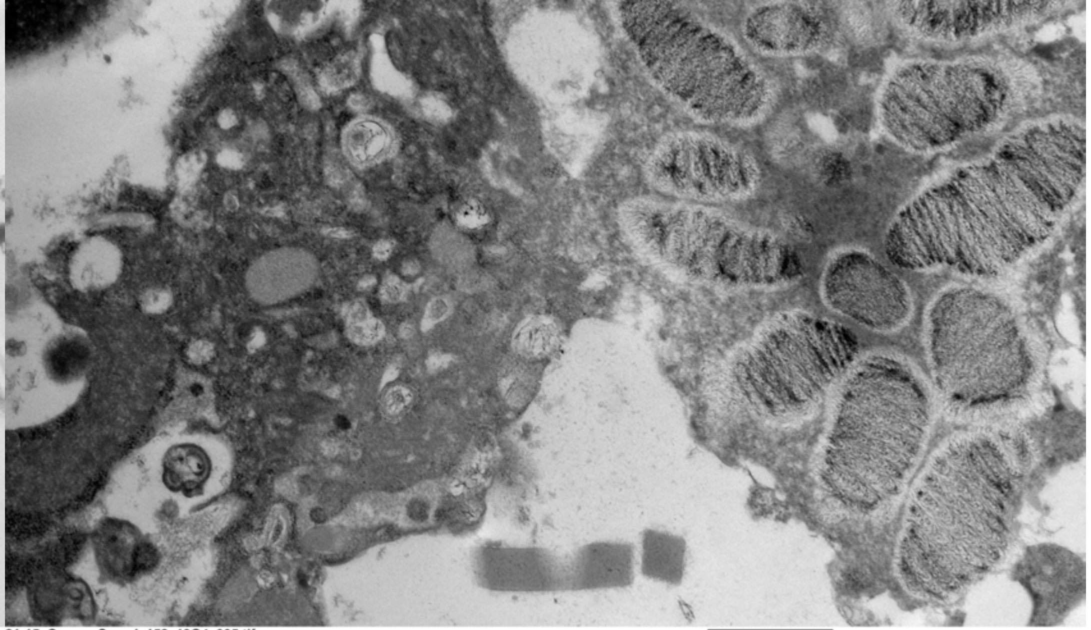

21-15_Correa_Sample158_19Q4_005.tif
Sample 158 expelled
Biological Electron Microscopy Lab
Rice University - SEA
Microscopist: MD Meyer

800 nm
HV=120kV
Direct Mag: 5000 x

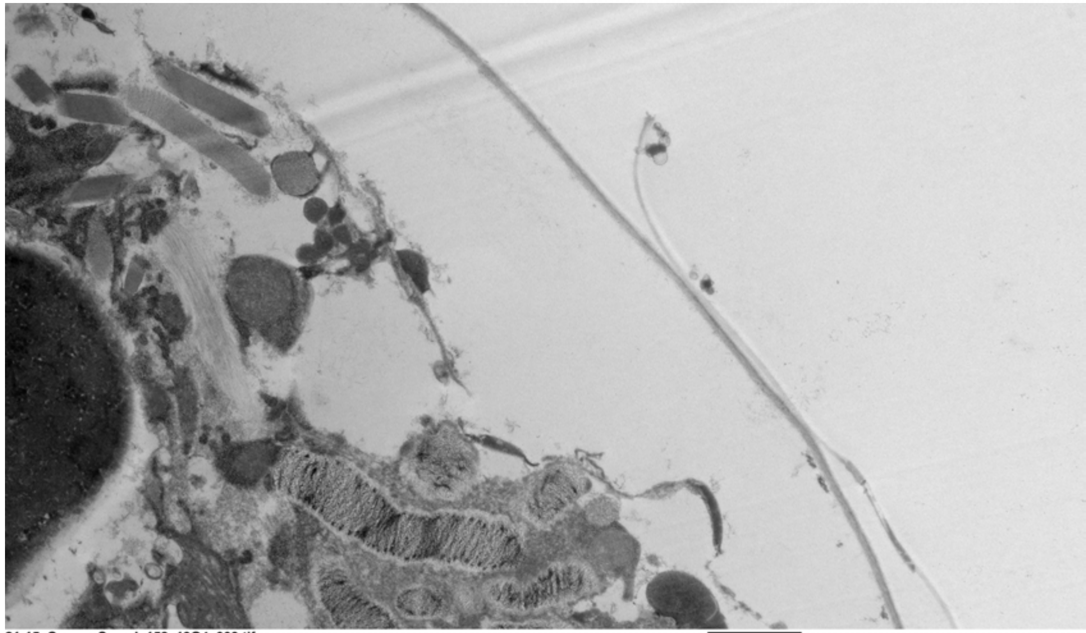

21-15_Correa_Sample158_19Q4_003.tif
Sample 158 expelled
Biological Electron Microscopy Lab
Rice University - SEA
Microscopist: MD Meyer

1 µm
HV=120kV
Direct Mag: 3000 x

Cell 2

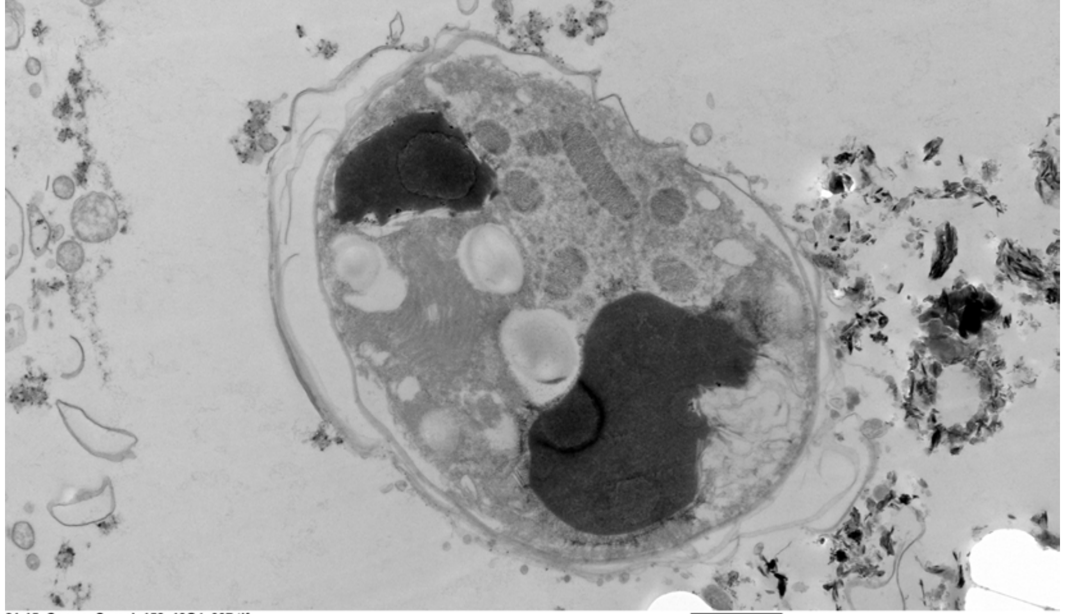

21-15_Correa_Sample158_19Q4_007.tif
Sample 158 expelled
Biological Electron Microscopy Lab
Rice University - SEA
Microscopist: MD Meyer

1 µm
HV=120kV
Direct Mag: 3000 x

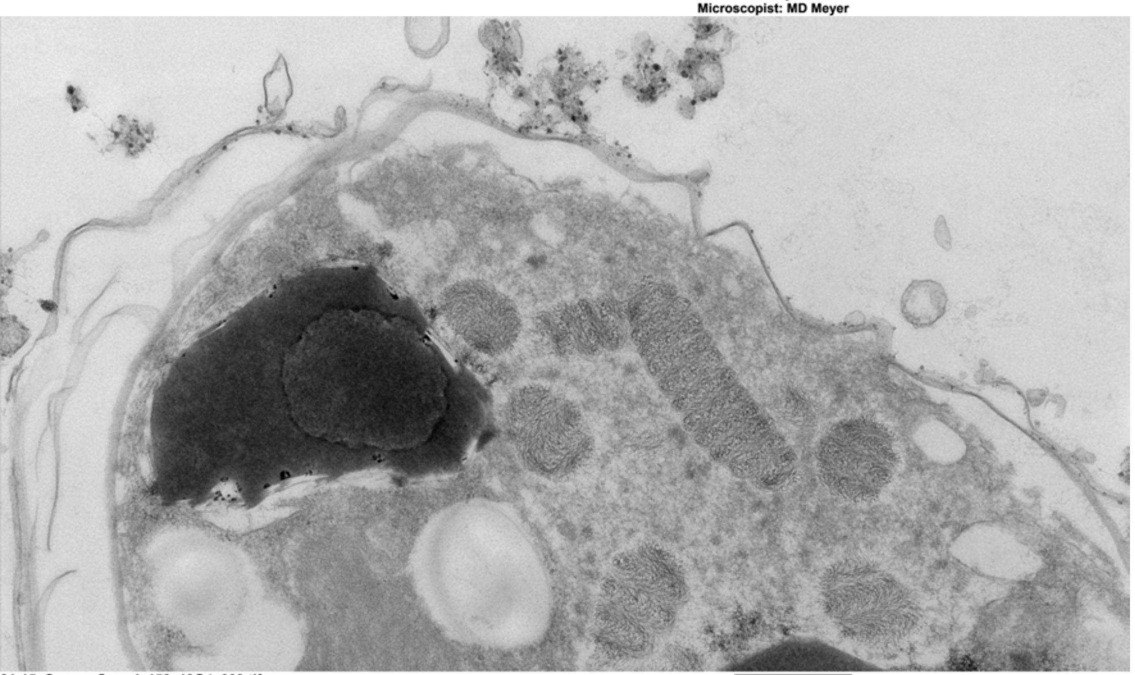

21-15_Correa_Sample158_19Q4_009.tif
Sample 158 expelled
Biological Electron Microscopy Lab
Rice University - SEA
Microscopist: MD Meyer

600 nm
HV=120kV
Direct Mag: 6000 x

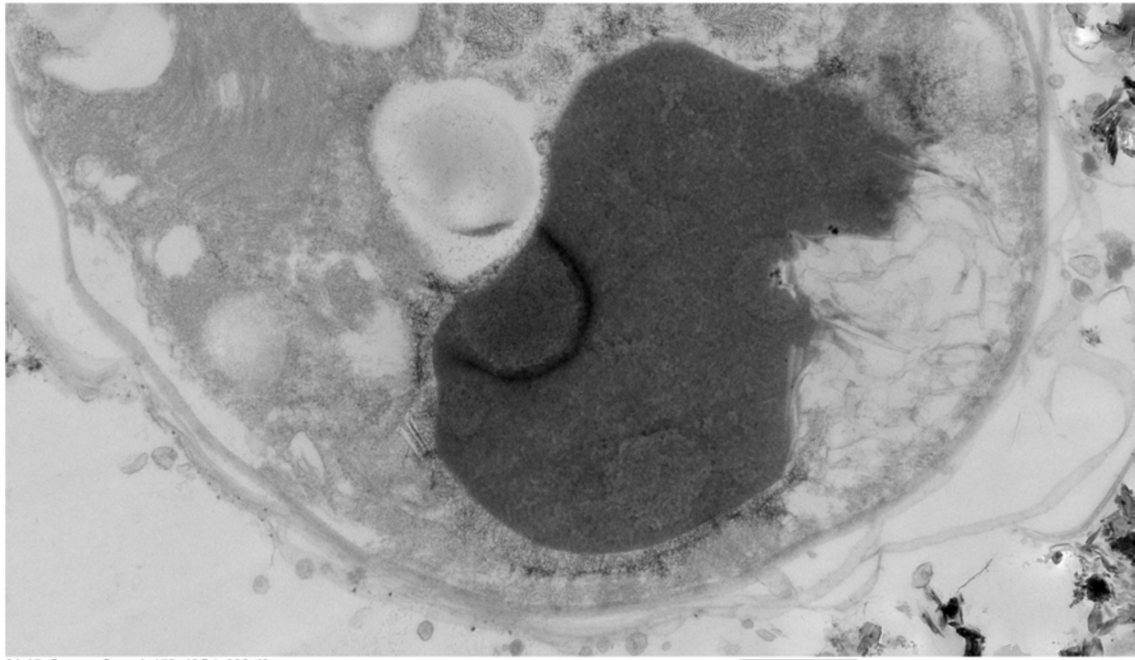

21-15_Correa_Sample158_19Q4_008.tif
Sample 158 expelled
Biological Electron Microscopy Lab
Rice University - SEA
Microscopist: MD Meyer

600 nm
HV=120kV
Direct Mag: 6000 x

Cell 3

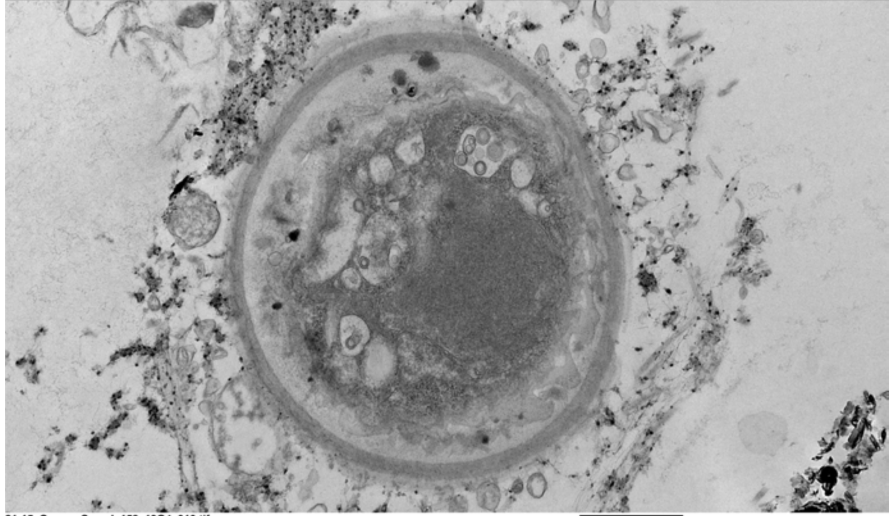

21-15_Correa_Sample158_19Q4_010.tif
Sample 158 expelled
Biological Electron Microscopy Lab
Rice University - SEA
Microscopist: MD Meyer

1 µm
HV=120kV
Direct Mag: 4000 x

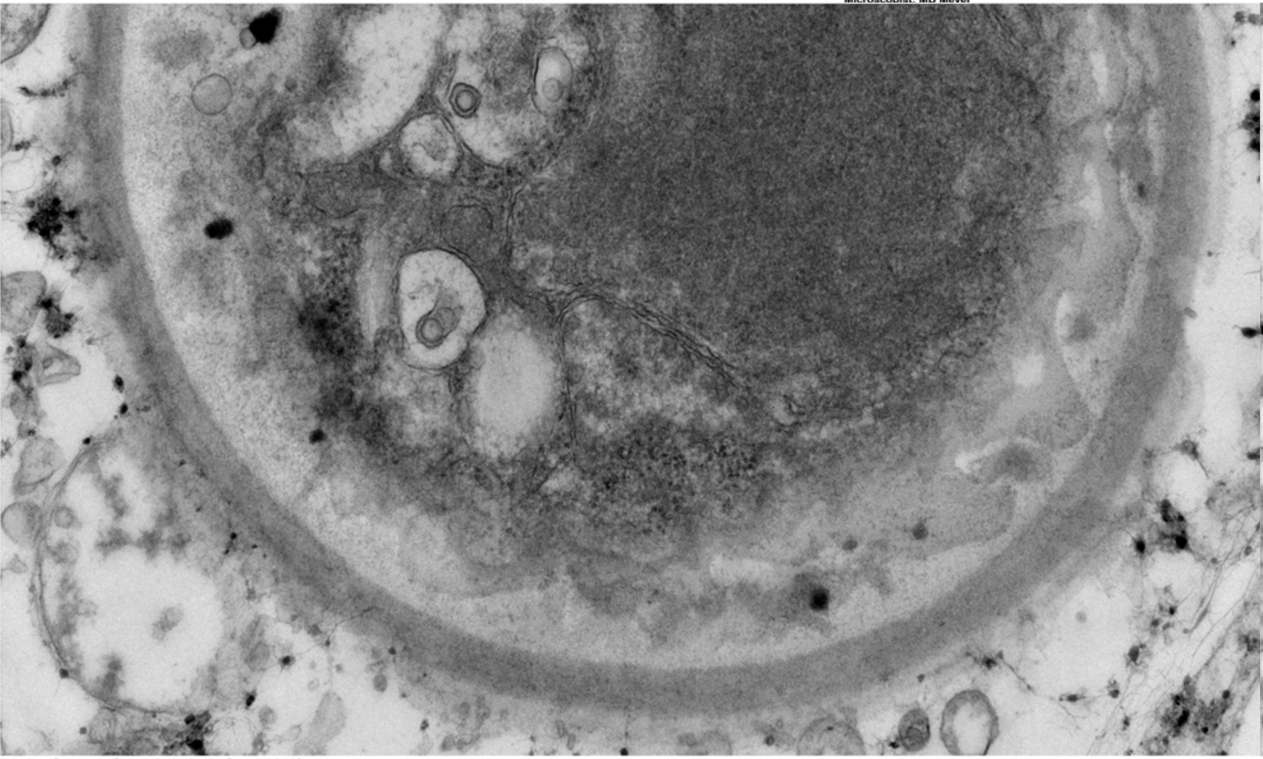

21-15_Correa_Sample158_19Q4_011.tif
Sample 158 expelled
Biological Electron Microscopy Lab
Rice University - SEA
Microscopist: MD Meyer

500 nm
HV=120kV
Direct Mag: 8000 x

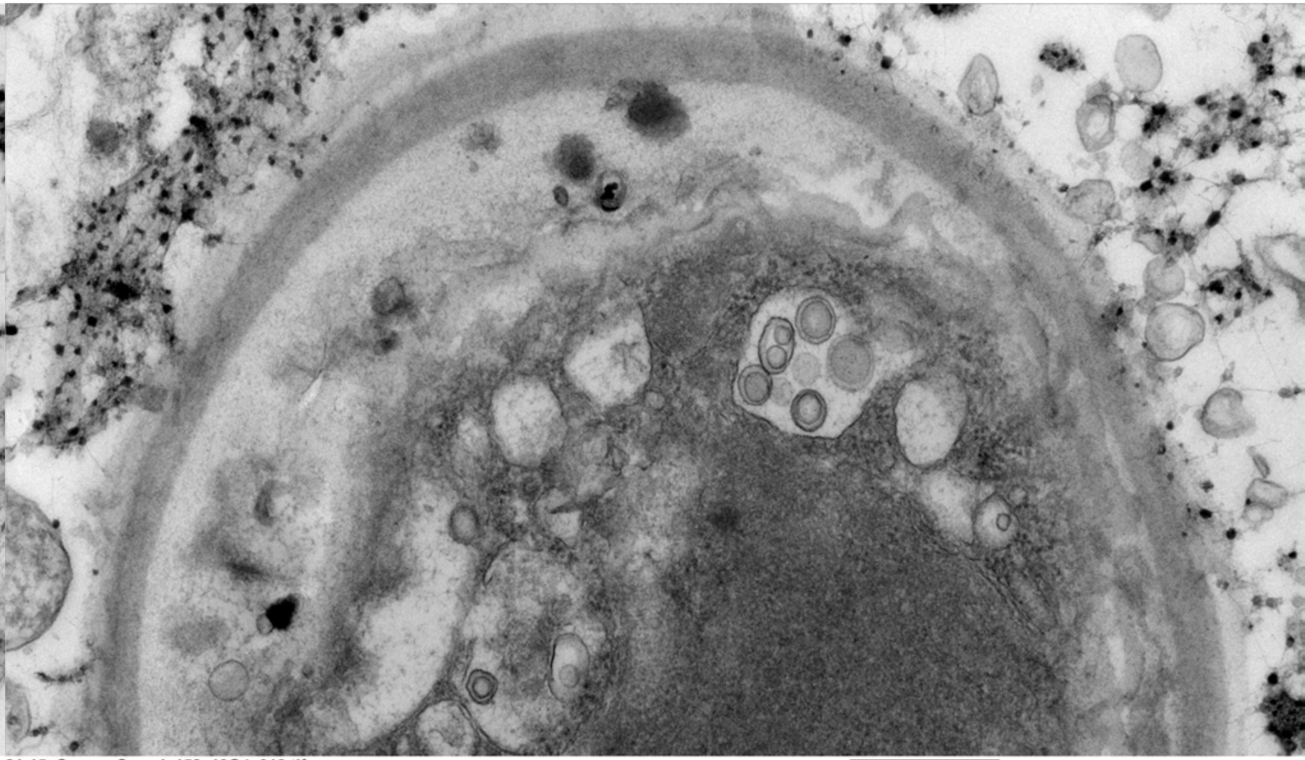

21-15_Correa_Sample158_19Q4_012.tif
Sample 158 expelled
Biological Electron Microscopy Lab
Rice University - SEA
Microscopist: MD Meyer

500 nm
HV=120kV
Direct Mag: 8000 x

Cell 4

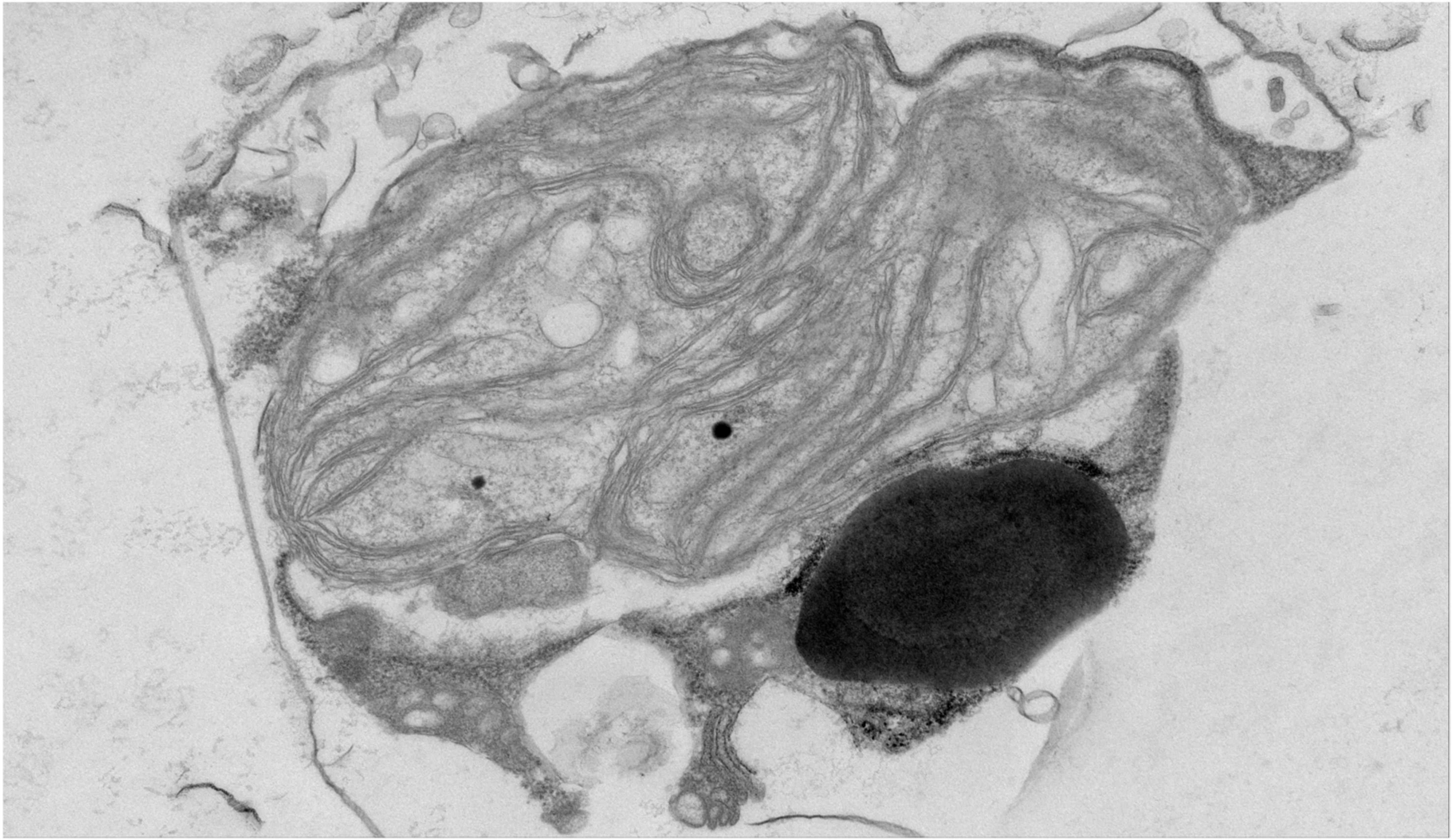

21-15_Correa_Sample158_19Q4_016.tif
Sample 158 expelled
Biological Electron Microscopy Lab
Rice University - SEA
Microscopist: MD Meyer

600 nm
HV=120kV
Direct Mag: 6000 x

Cell 5

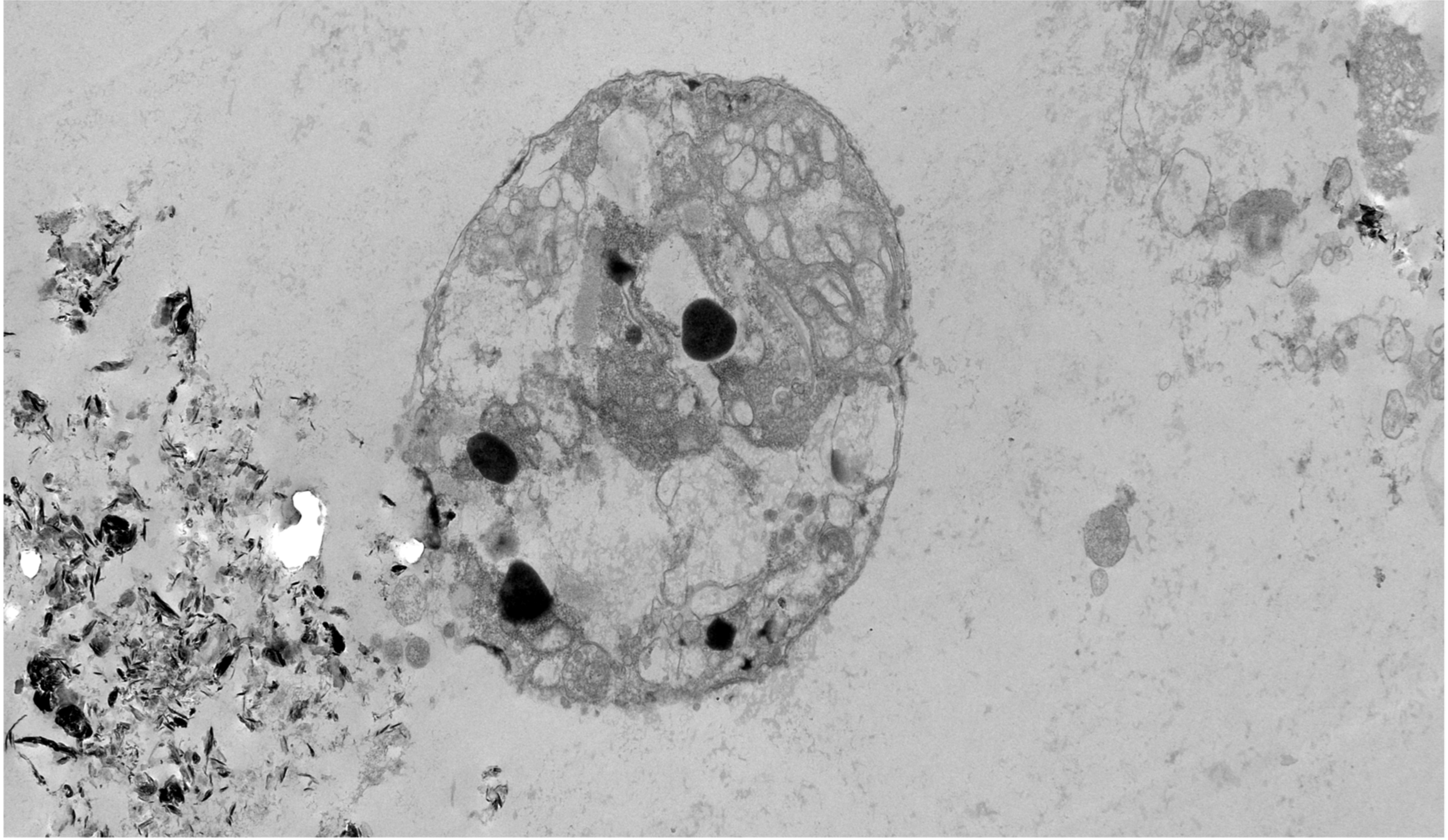

21-15_Correa_Sample158_19Q4_018.tif
Sample 158 expelled
Biological Electron Microscopy Lab
Rice University - SEA
Microscopist: MD Meyer

1 µm
HV=120kV
Direct Mag: 3000 x

Cell 6

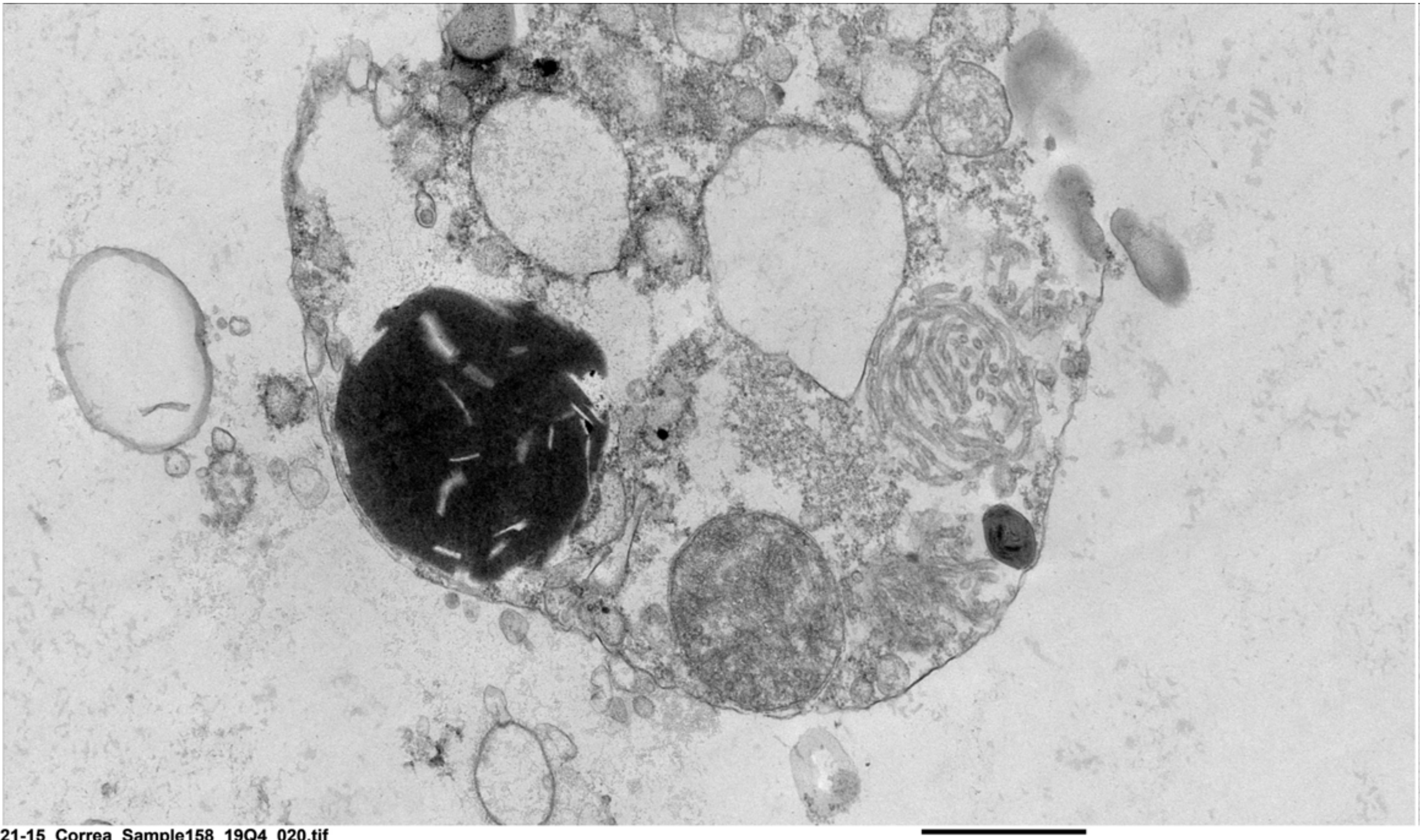

21-15_Correa_Sample158_19Q4_020.tif
Sample 158 expelled
Biological Electron Microscopy Lab
Rice University - SEA
Microscopist: MD Meyer

800 nm
HV=120kV
Direct Mag: 5000 x

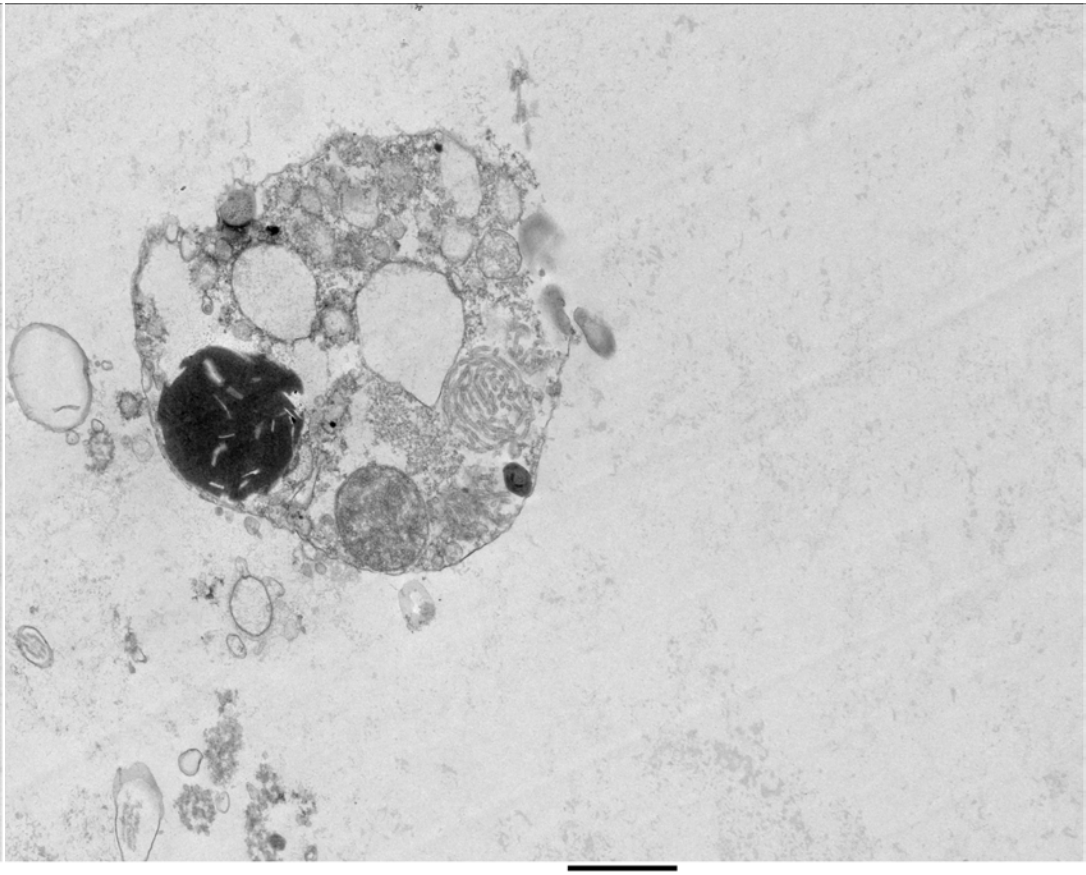

1 µm
HV=120kV
Direct Mag: 2500 x

Cell 7

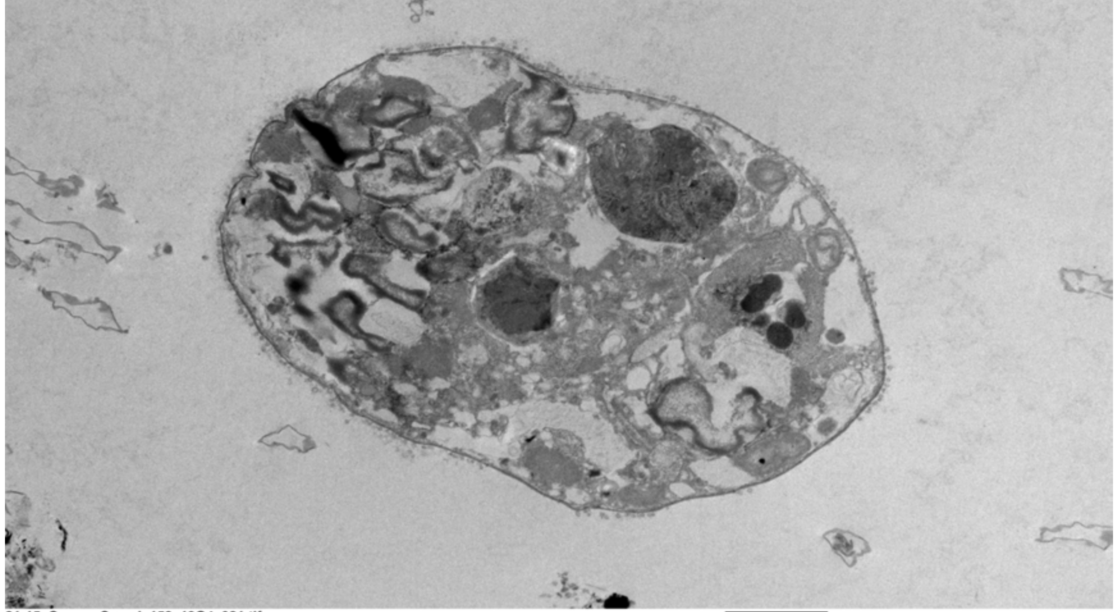

21-15_Correa_Sample158_19Q4_021.tif
Sample 158 expelled
Biological Electron Microscopy Lab
Rice University - SEA
Microscopist: MD Meyer

2 µm
HV=120kV
Direct Mag: 1500 x

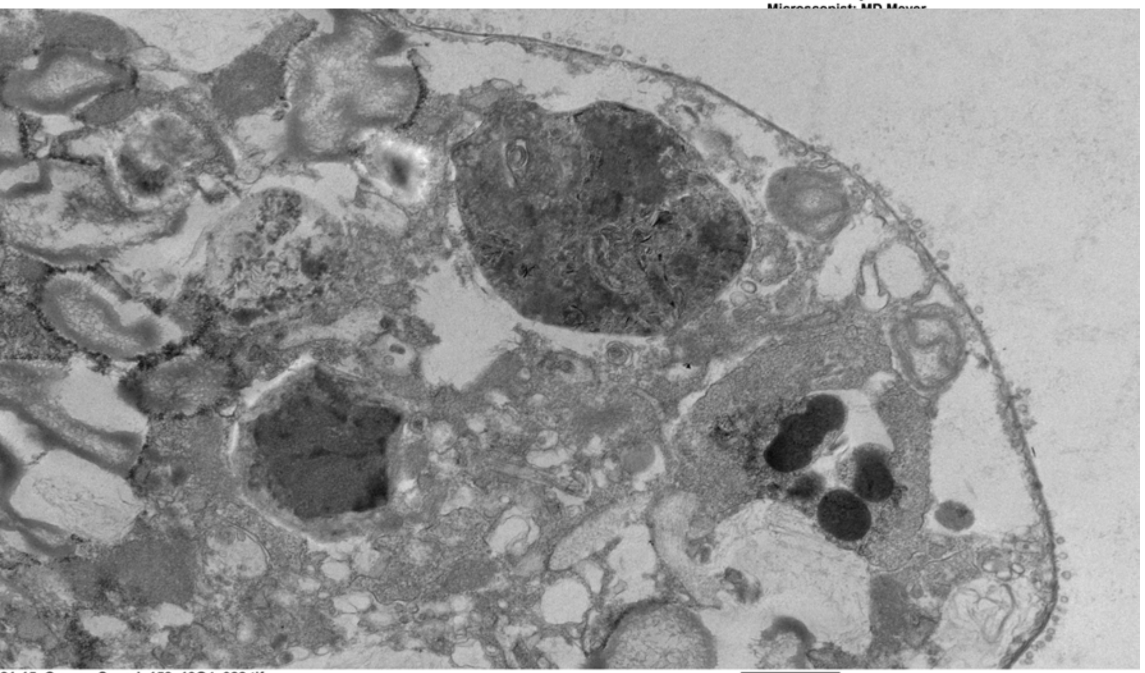

21-15_Correa_Sample158_19Q4_022.tif
Sample 158 expelled
Biological Electron Microscopy Lab
Rice University - SEA
Microscopist: MD Meyer

1 µm
HV=120kV
Direct Mag: 3000 x

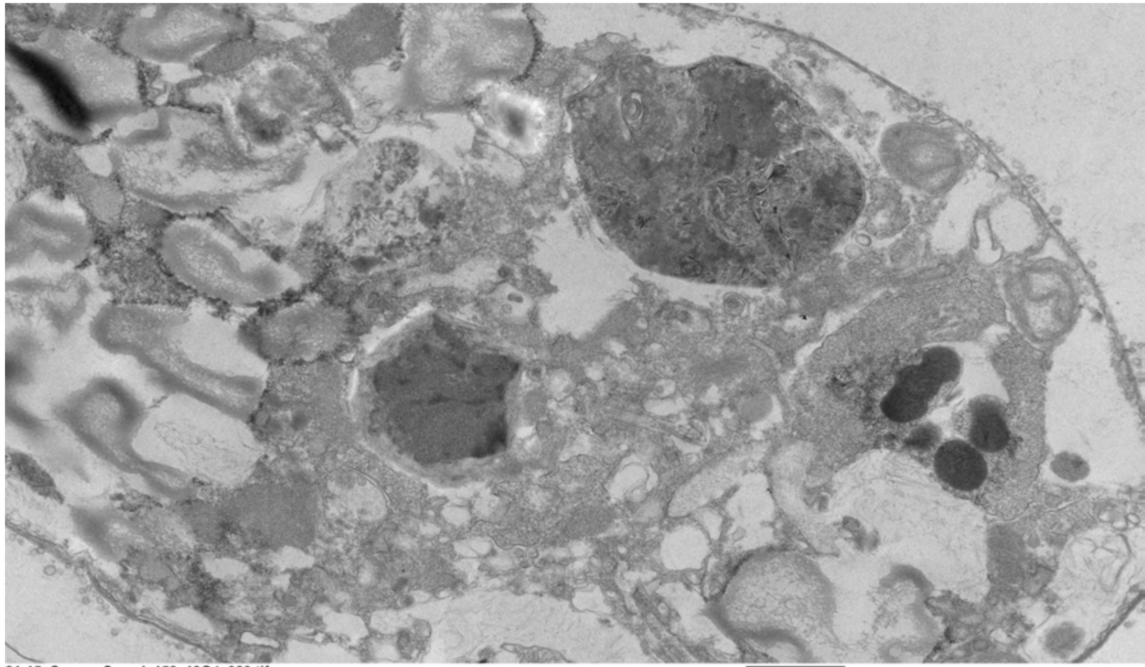

21-15_Correa_Sample158_19Q4_023.tif
Sample 158 expelled
Biological Electron Microscopy Lab
Rice University - SEA
Microscopist: MD Meyer

1 µm
HV=120kV
Direct Mag: 3000 x

Cell 8

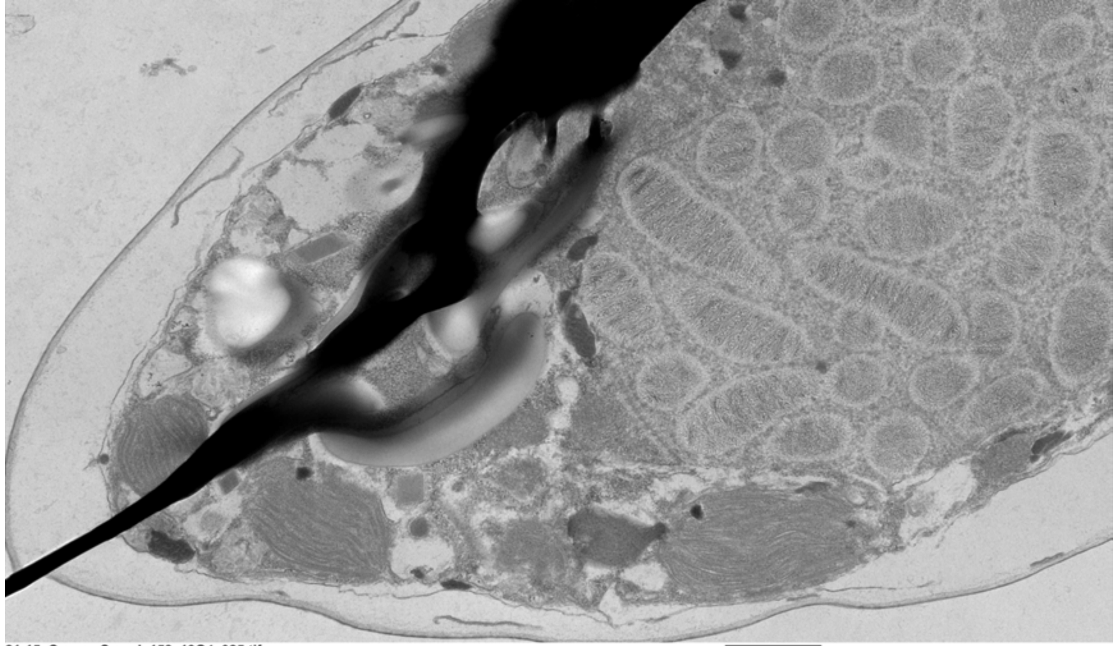

21-15_Correa_Sample158_19Q4_025.tif
Sample 158 expelled
Biological Electron Microscopy Lab
Rice University - SEA

1 µm
HV=120kV
Direct Mag: 3000 x

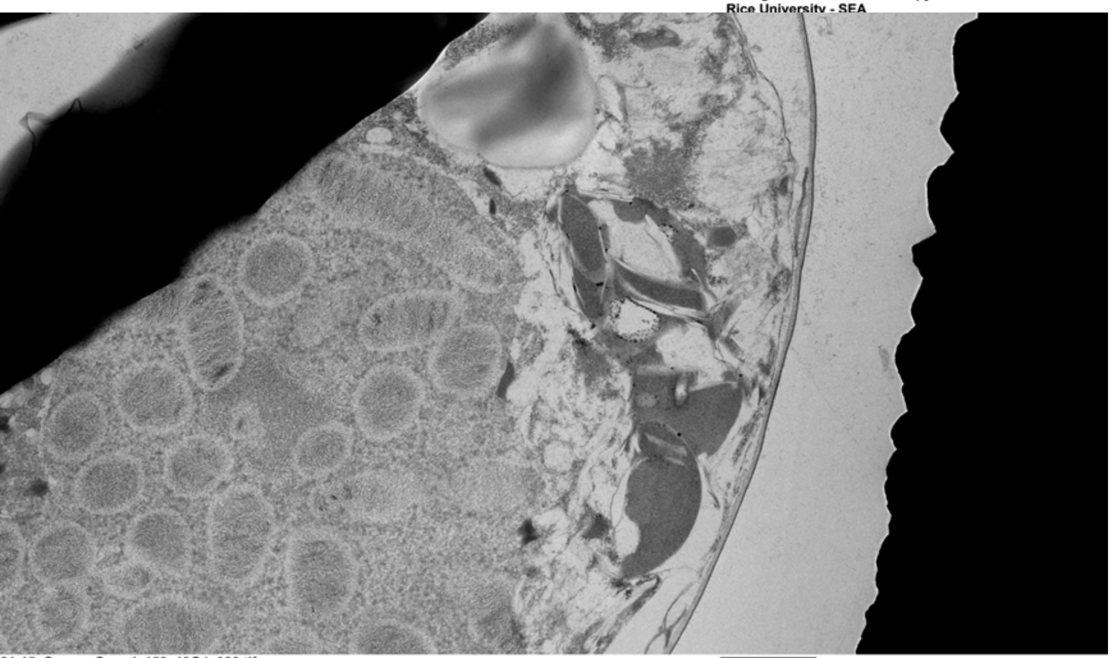

21-15_Correa_Sample158_19Q4_026.tif
Sample 158 expelled
Biological Electron Microscopy Lab
Rice University - SEA
Microscopist: MD Meyer

1 µm
HV=120kV
Direct Mag: 3000 x

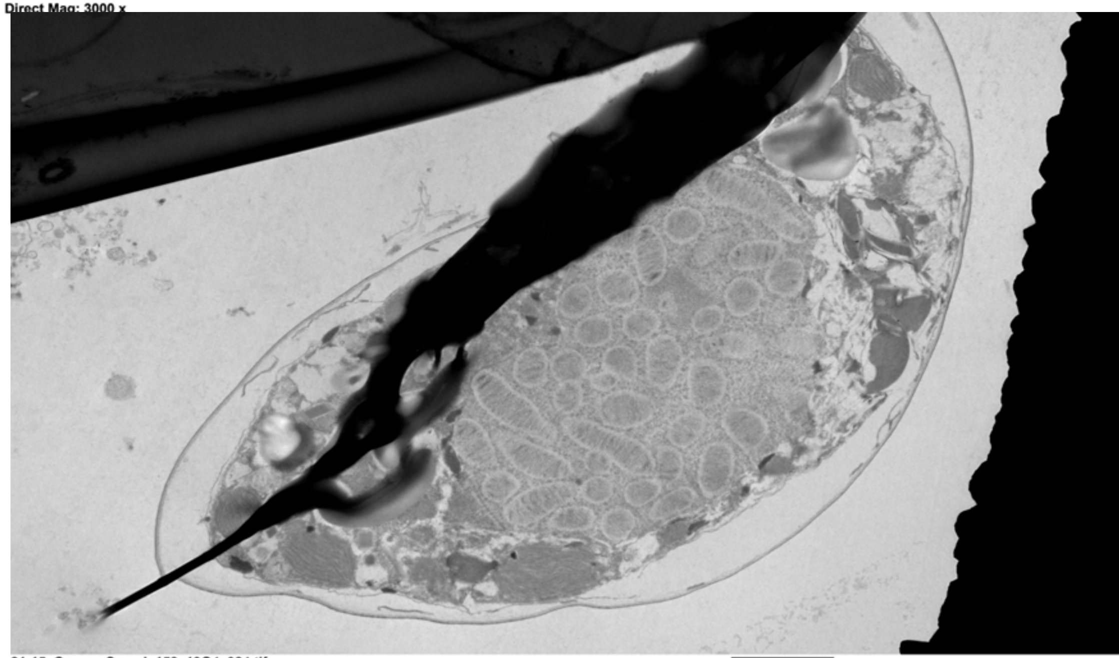

21-15_Correa_Sample158_19Q4_024.tif
Sample 158 expelled
Biological Electron Microscopy Lab
Rice University - SEA
Microscopist: MD Meyer

2 µm
HV=120kV
Direct Mag: 1500 x

Cell 9

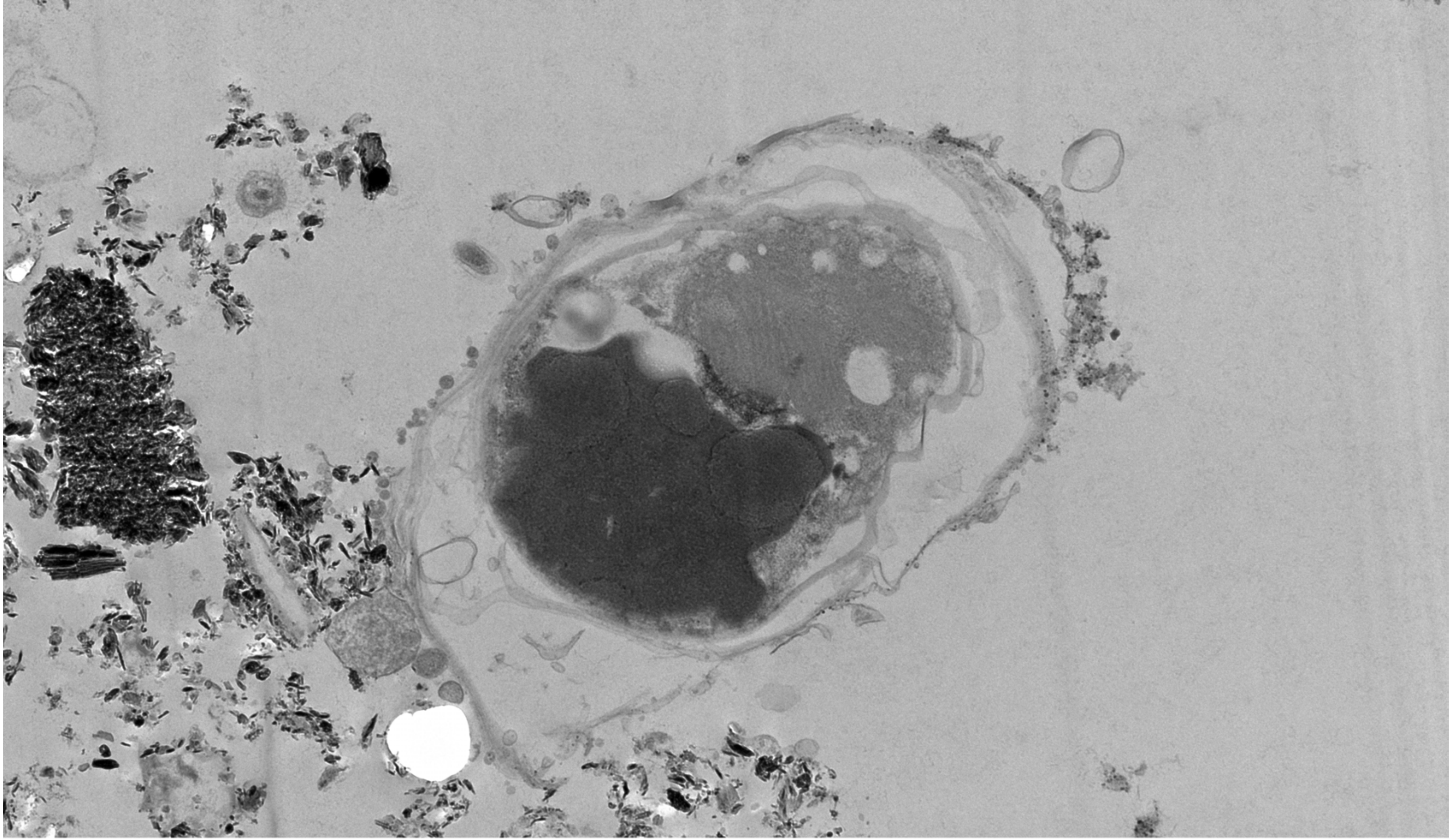

21-15_Correa_Sample158_19Q4_028.tif
Sample 158 expelled
Biological Electron Microscopy Lab
Rice University - SEA
Microscopist: MD Meyer

1 µm
HV=120kV
Direct Mag: 2500 x

Cell 10

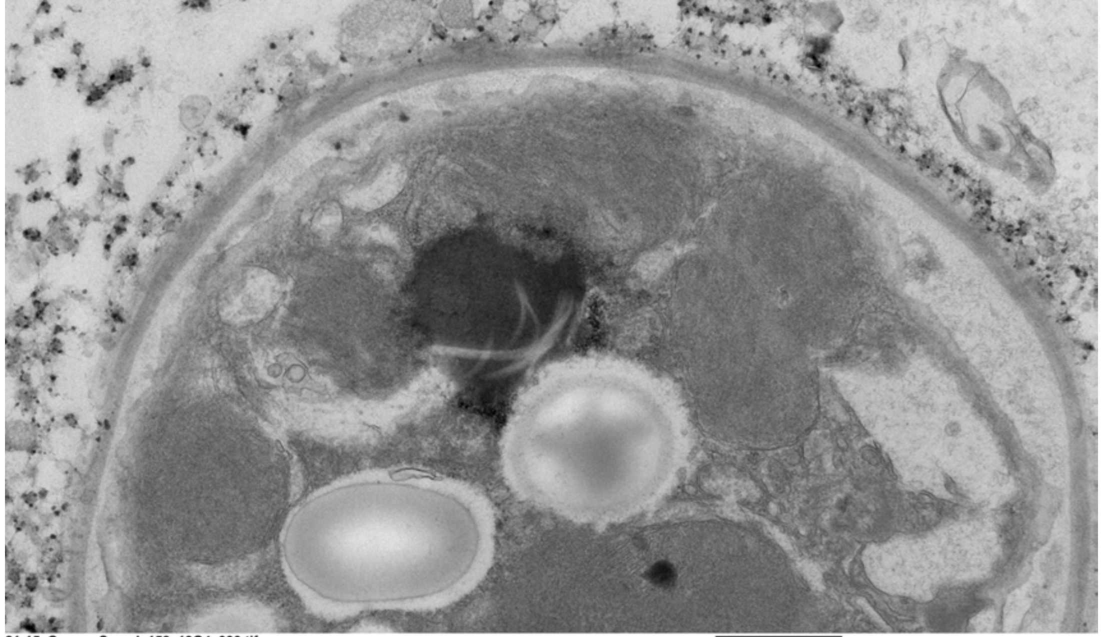

21-15_Correa_Sample158_19Q4_030.tif
Sample 158 expelled
Biological Electron Microscopy Lab
Rice University - SEA
Microscopist: MD Meyer

800 nm
HV=120kV
Direct Mag: 5000 x

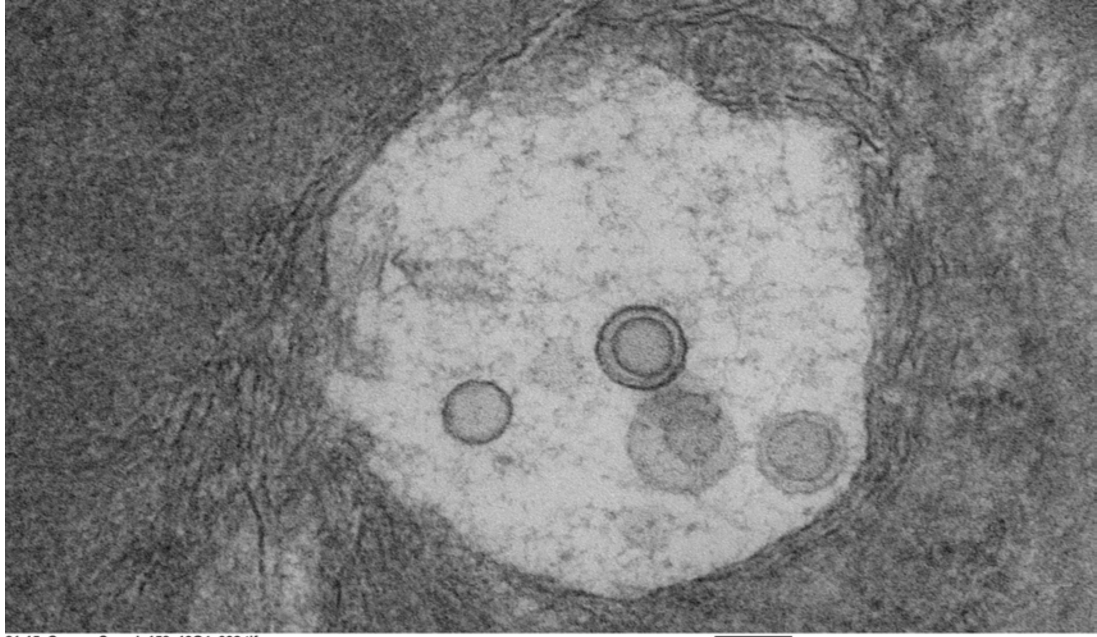

21-15_Correa_Sample158_19Q4_032.tif
Sample 158 expelled
Biological Electron Microscopy Lab
Rice University - SEA
Microscopist: MD Meyer

100 nm
HV=120kV
Direct Mag: 25000 x

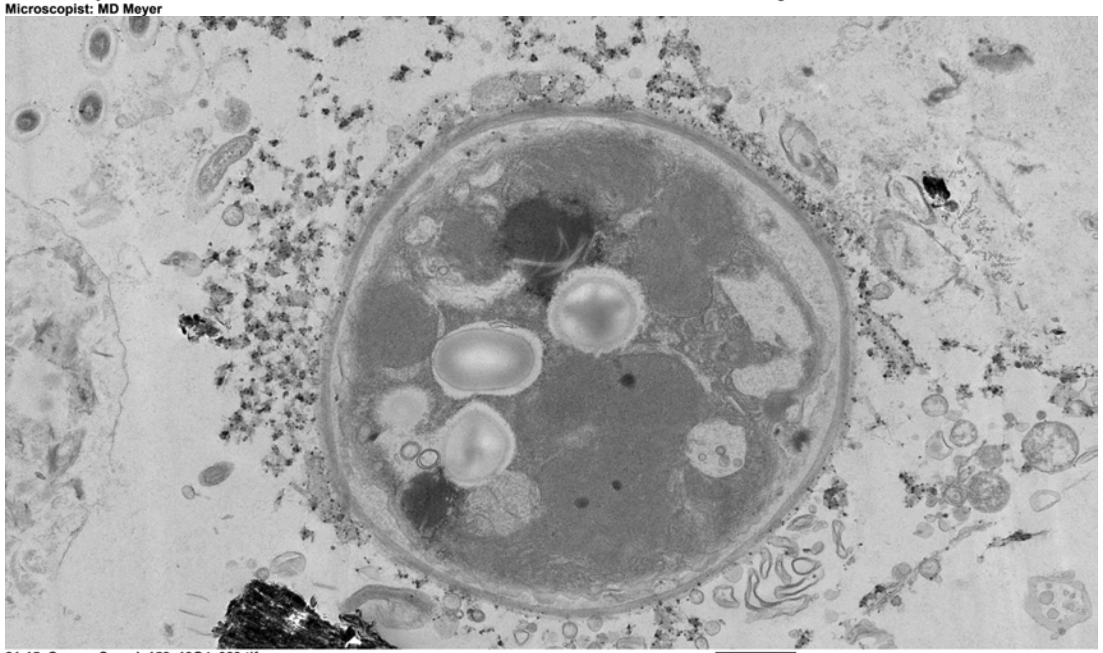

21-15_Correa_Sample158_19Q4_029.tif
Sample 158 expelled
Biological Electron Microscopy Lab
Rice University - SEA
Microscopist: MD Meyer

1 µm
HV=120kV
Direct Mag: 2500 x

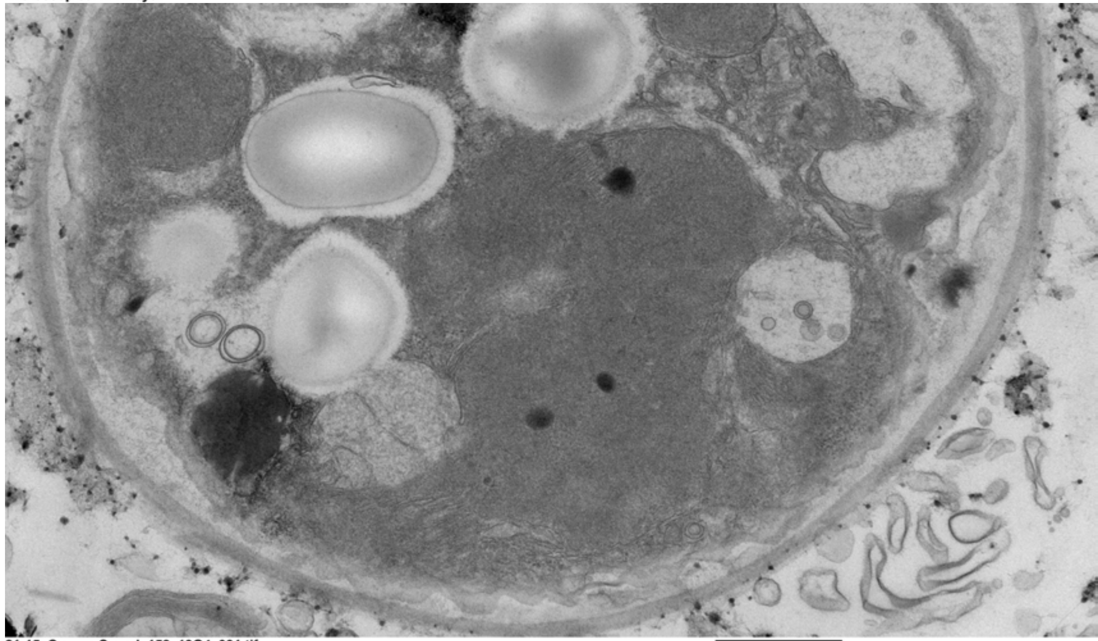

21-15_Correa_Sample158_19Q4_031.tif
Sample 158 expelled
Biological Electron Microscopy Lab
Rice University - SEA
Microscopist: MD Meyer

800 nm
HV=120kV
Direct Mag: 5000 x

Cell 11

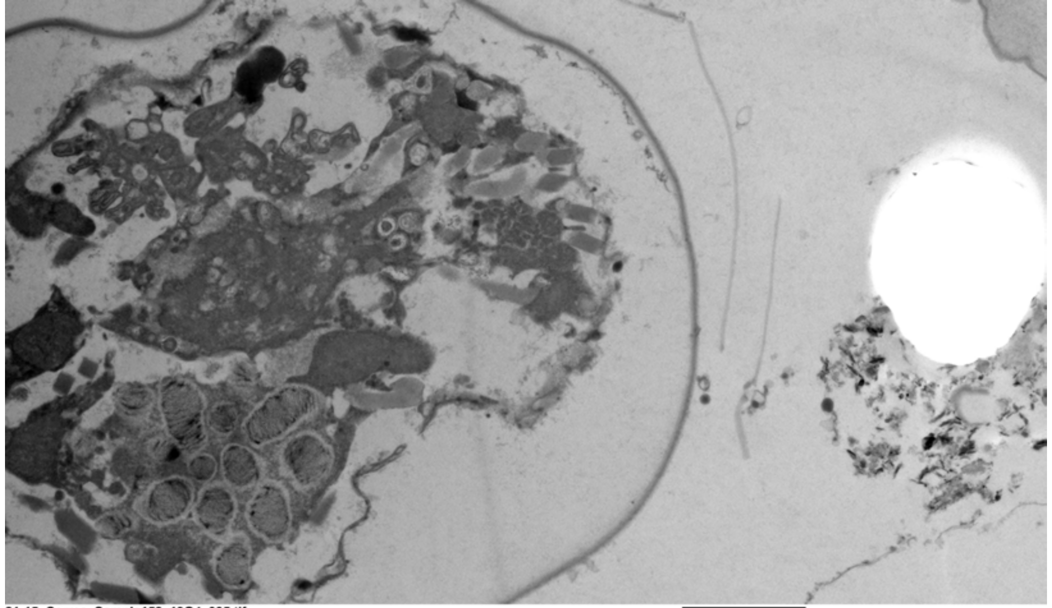

21-15_Correa_Sample158_19Q4_035.tif
Sample 158 expelled
Biological Electron Microscopy Lab
Rice University - SEA

2 µm
HV=120kV
Direct Mag: 2000 x

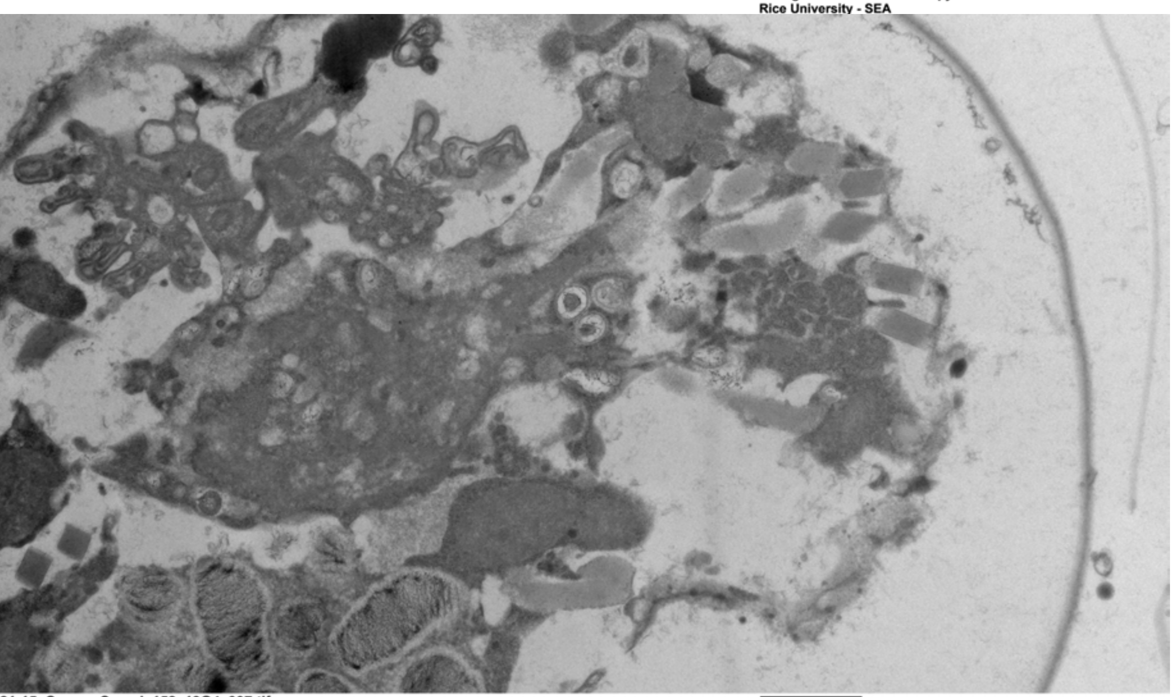

21-15_Correa_Sample158_19Q4_037.tif
Sample 158 expelled
Biological Electron Microscopy Lab
Rice University - SEA
Microscopist: MD Meyer

1 µm
HV=120kV
Direct Mag: 3000 x

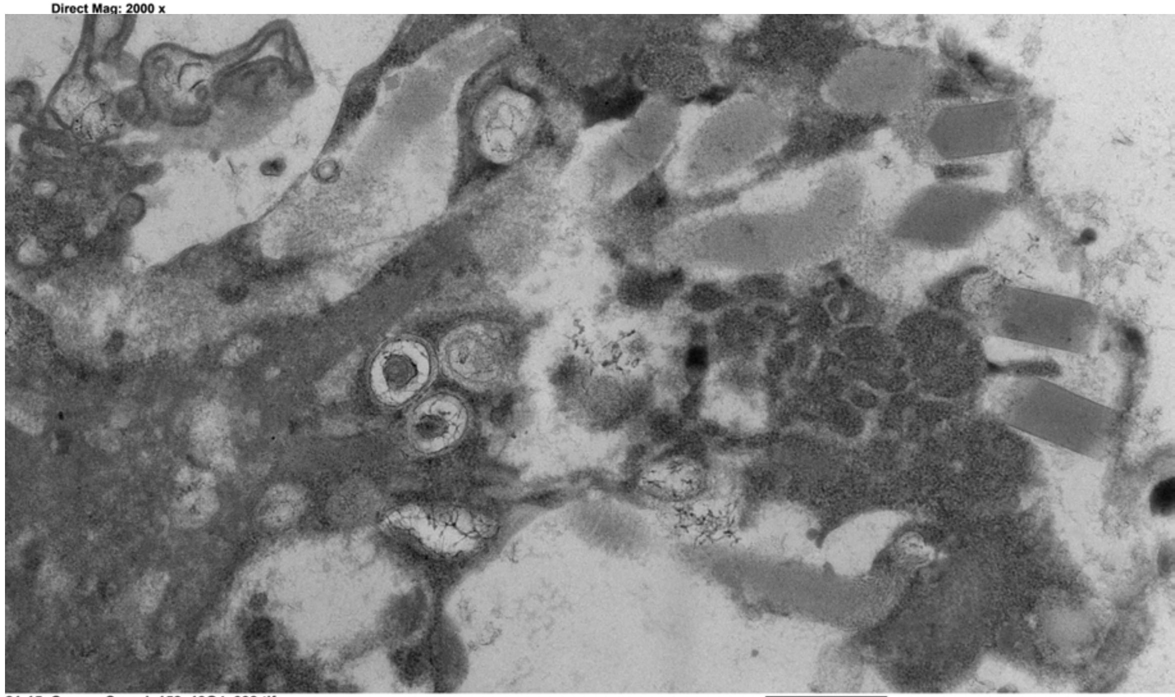

21-15_Correa_Sample158_19Q4_038.tif
Sample 158 expelled
Biological Electron Microscopy Lab
Rice University - SEA
Microscopist: MD Meyer

600 nm
HV=120kV
Direct Mag: 6000 x

Cell 12

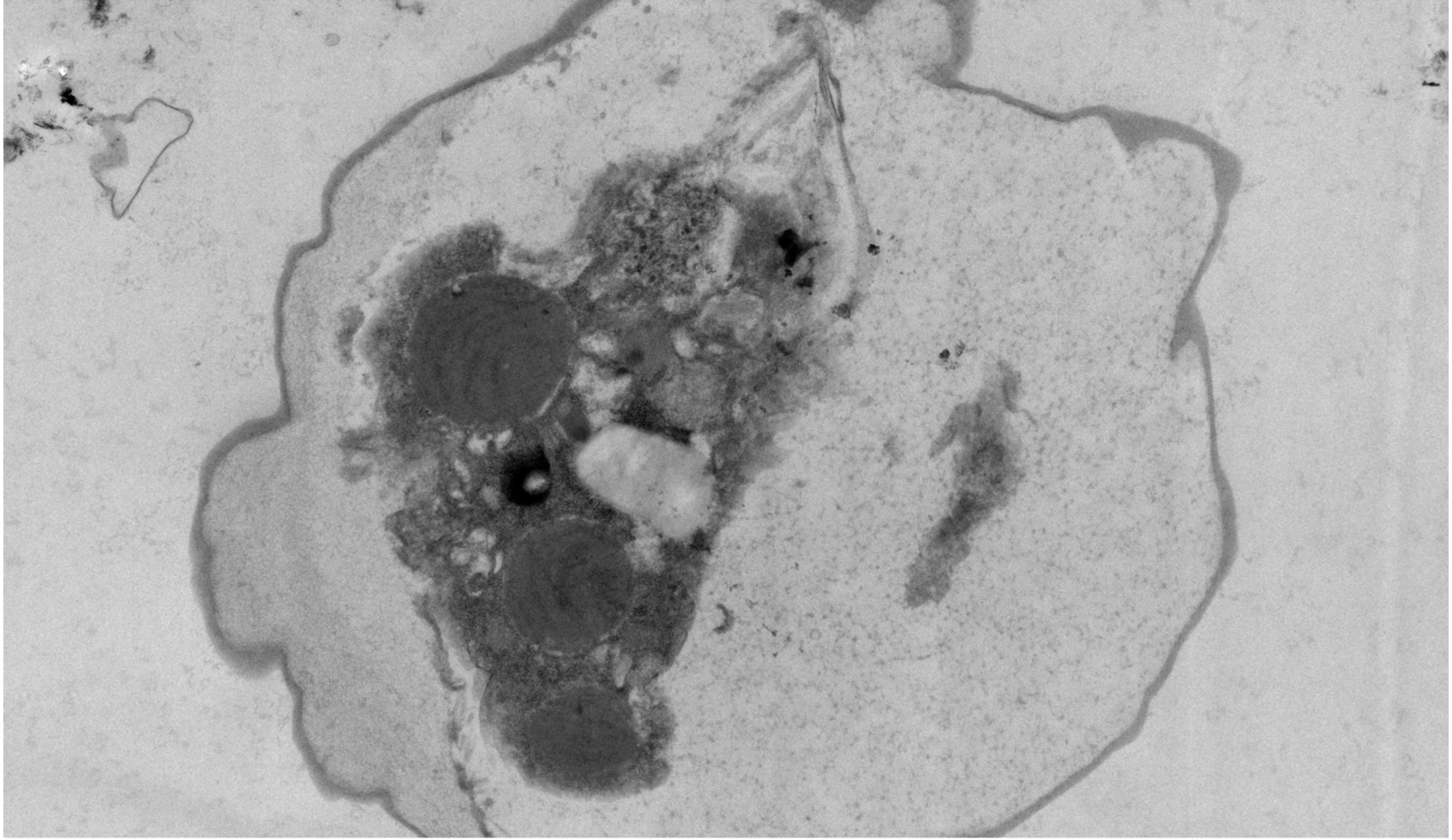

21-15_Correa_Sample158_19Q4_036.tif
Sample 158 expelled
Biological Electron Microscopy Lab
Rice University - SEA
Microscopist: MD Meyer

1 µm
HV=120kV
Direct Mag: 3000 x

Cell 13

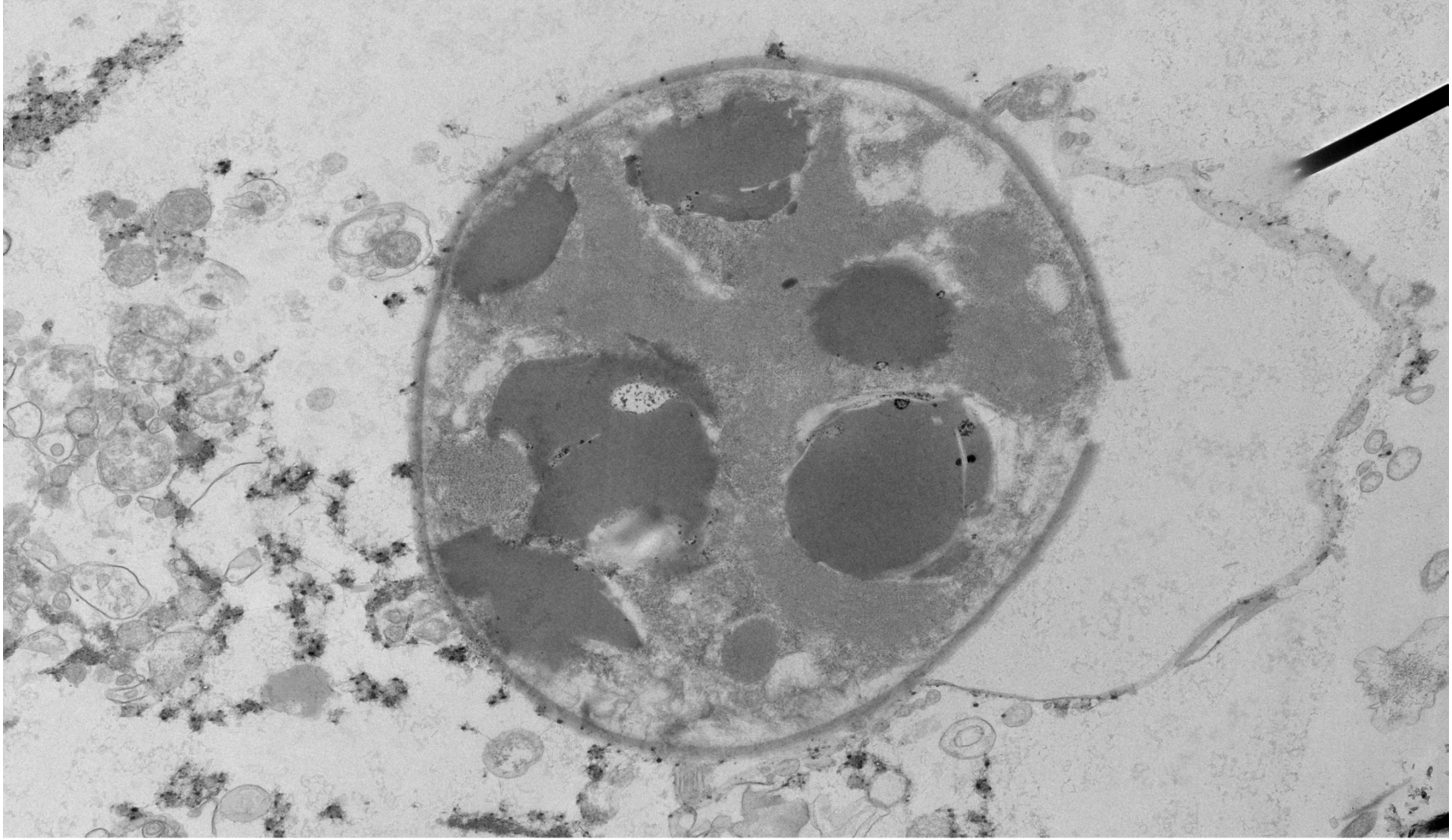

21-15_Correa_Sample158_19Q4_040.tif
Sample 158 expelled
Biological Electron Microscopy Lab
Rice University - SEA
Microscopist: MD Meyer

1 µm
HV=120kV
Direct Mag: 3000 x

Cell 14

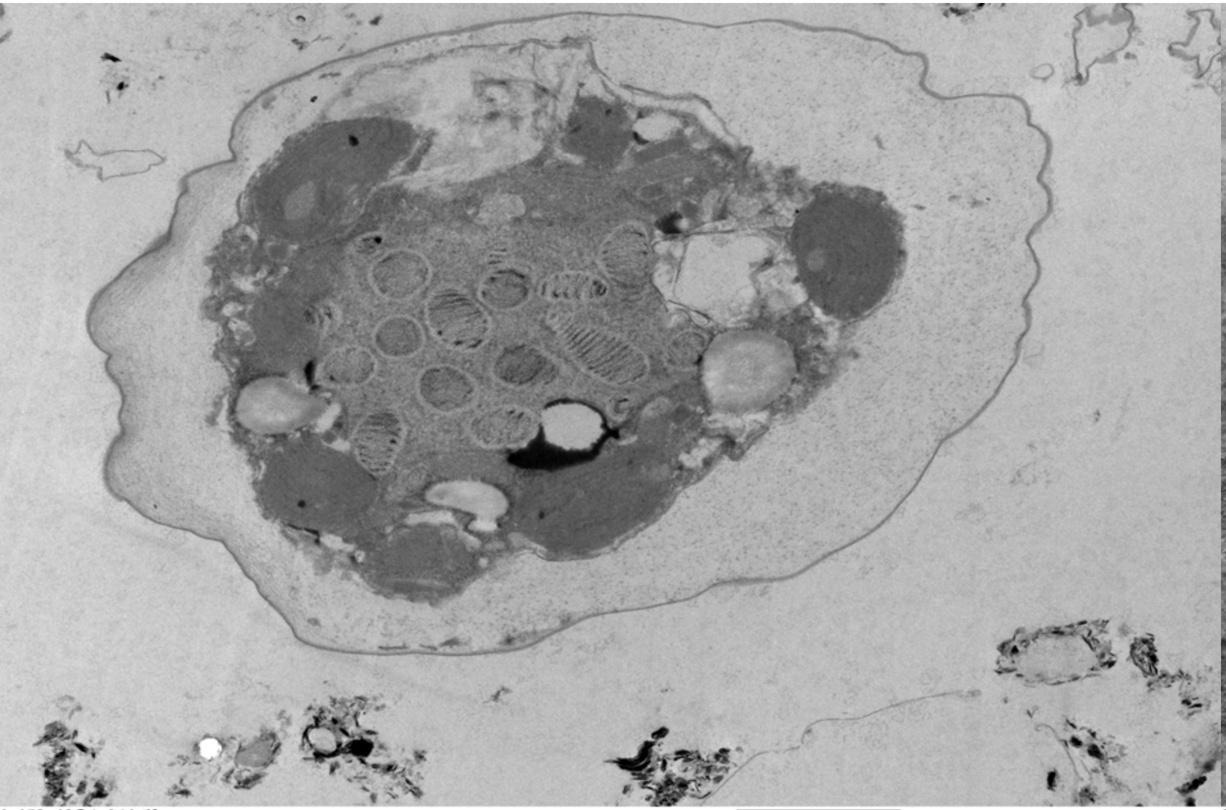

ple158_19Q4_041.tif
d
Microscopy Lab
A
eyer

2 µm
HV=120kV
Direct Mag: 2000 x

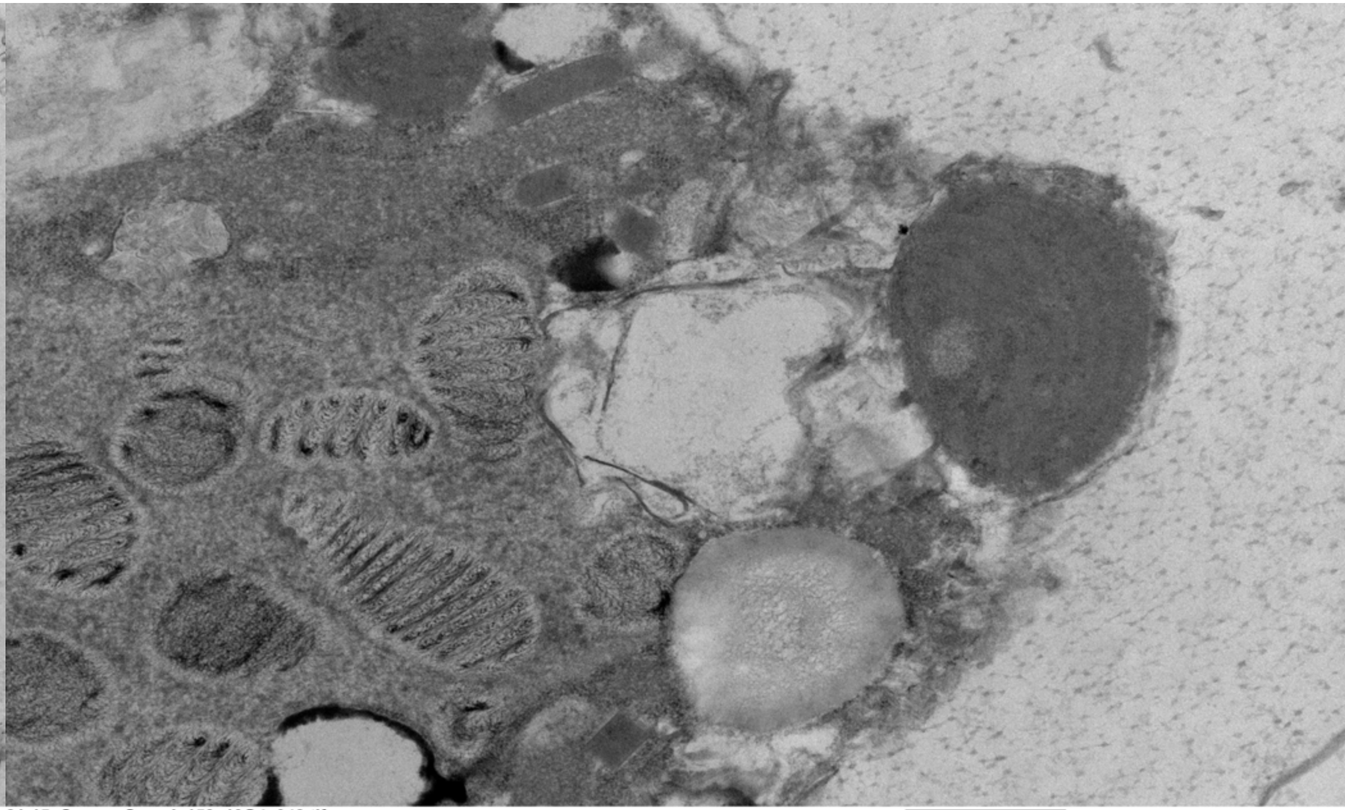

21-15_Correa_Sample158_19Q4_042.tif
Sample 158 expelled
Biological Electron Microscopy Lab
Rice University - SEA
Microscopist: MD Meyer

800 nm
HV=120kV
Direct Mag: 5000 x

Cell 15

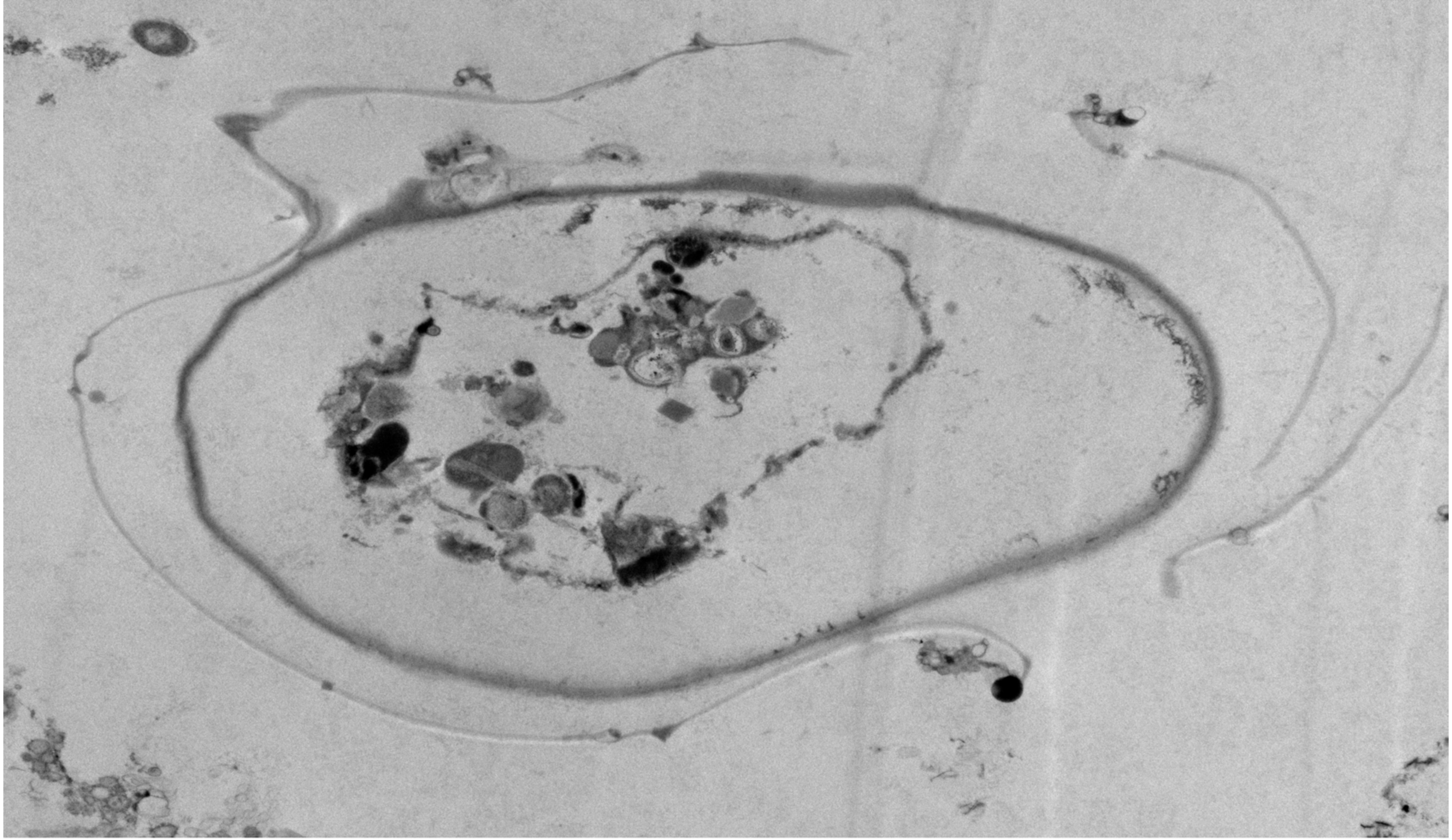

21-15_Correa_Sample158_19Q4_043.tif
Sample 158 expelled
Biological Electron Microscopy Lab
Rice University - SEA
Microscopist: MD Meyer

2 µm
HV=120kV
Direct Mag: 2000 x

Cell 16

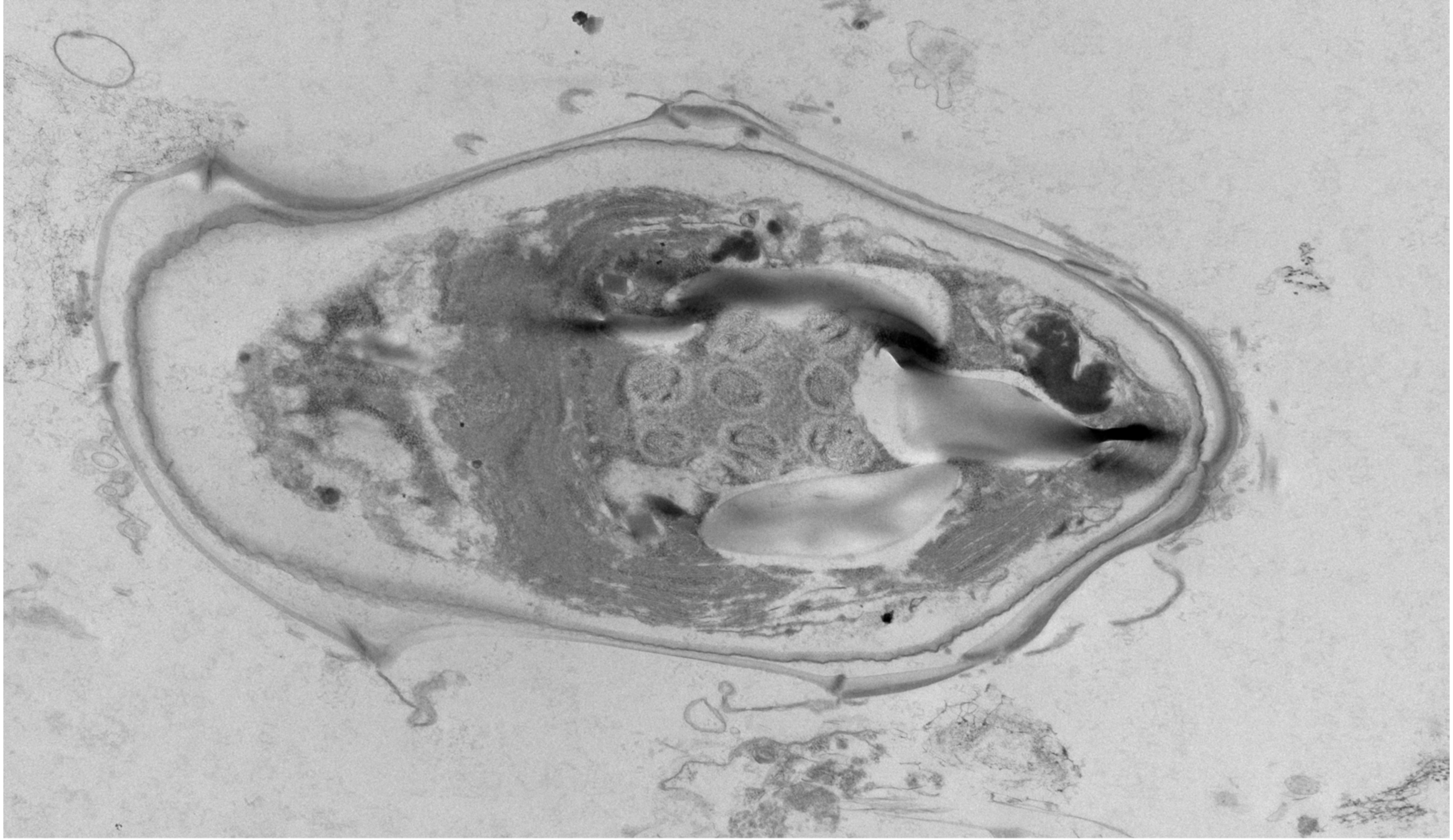

21-15_Correa_Sample158_19Q4_044.tif
Sample 158 expelled
Biological Electron Microscopy Lab
Rice University - SEA
Microscopist: MD Meyer

1 µm
HV=120kV
Direct Mag: 3000 x

Cell 17

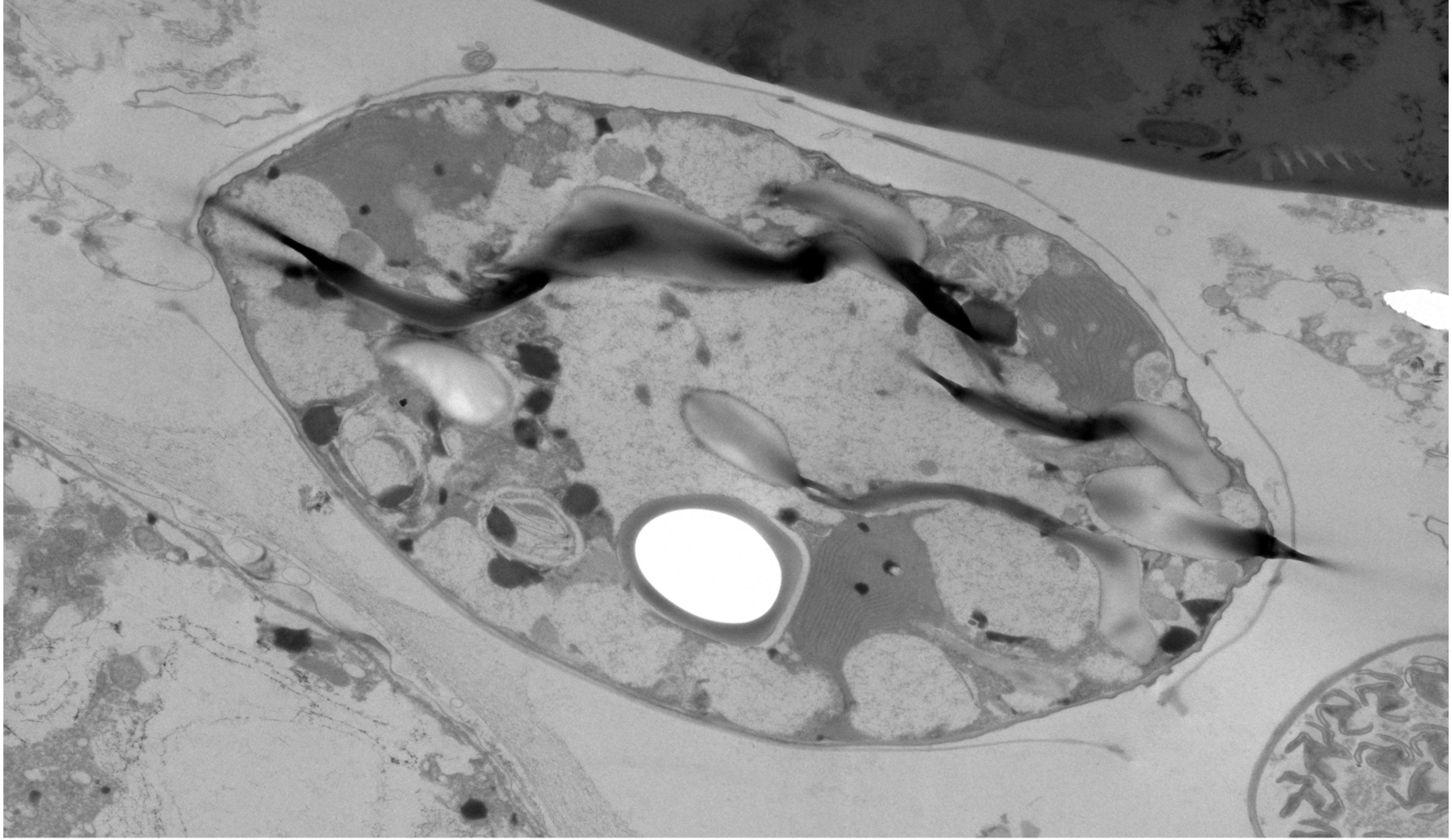

21-15_Correa_Sample158_19Q4_045.tif
Sample 158 expelled
Biological Electron Microscopy Lab
Rice University - SEA
Microscopist: MD Meyer

2 µm
HV=120kV
Direct Mag: 2000 x

Cell 18

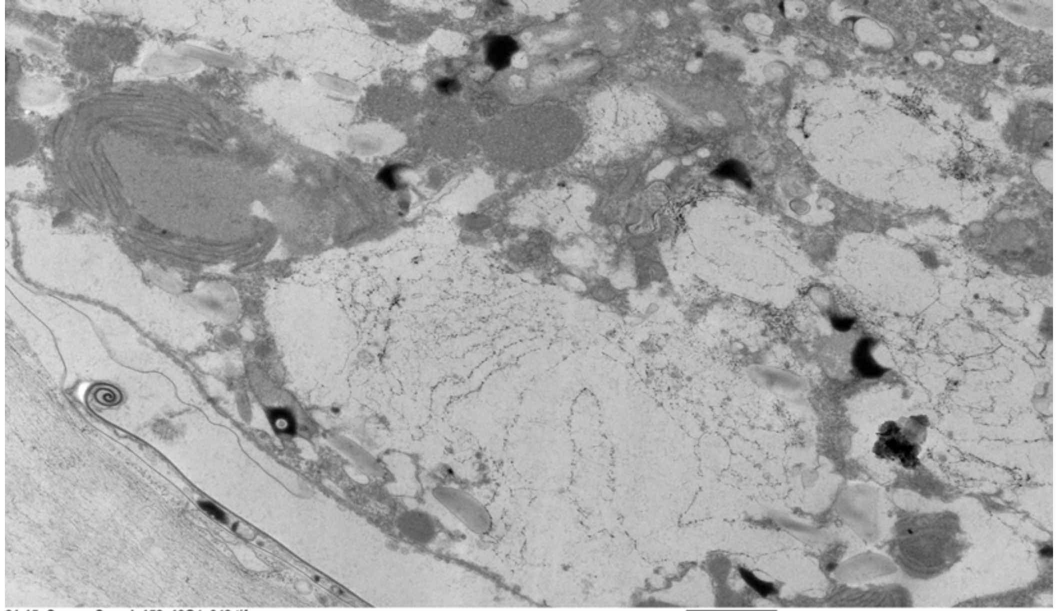

21-15_Correa_Sample158_19Q4_048.tif
Sample 158 expelled
Biological Electron Microscopy Lab
Rice University - SEA
[illegible]

1 µm
HV=120kV
Direct Mag: 3000 x

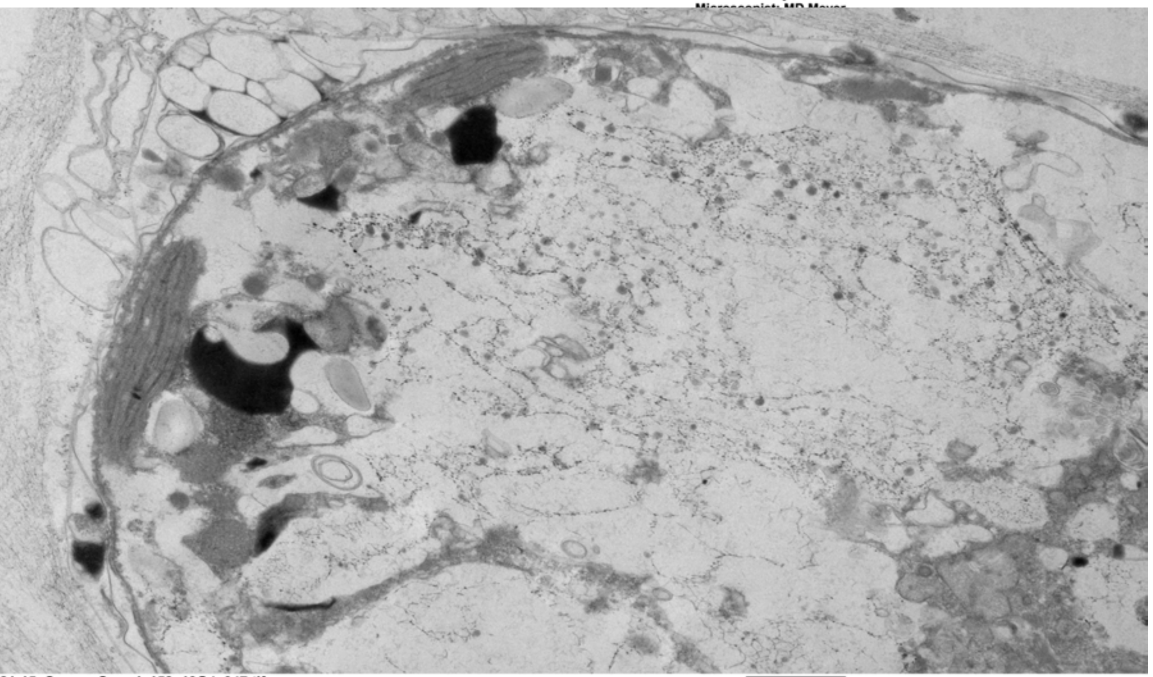

21-15_Correa_Sample158_19Q4_047.tif
Sample 158 expelled
Biological Electron Microscopy Lab
Rice University - SEA
Microscopist: MD Meyer

1 µm
HV=120kV
Direct Mag: 3000 x

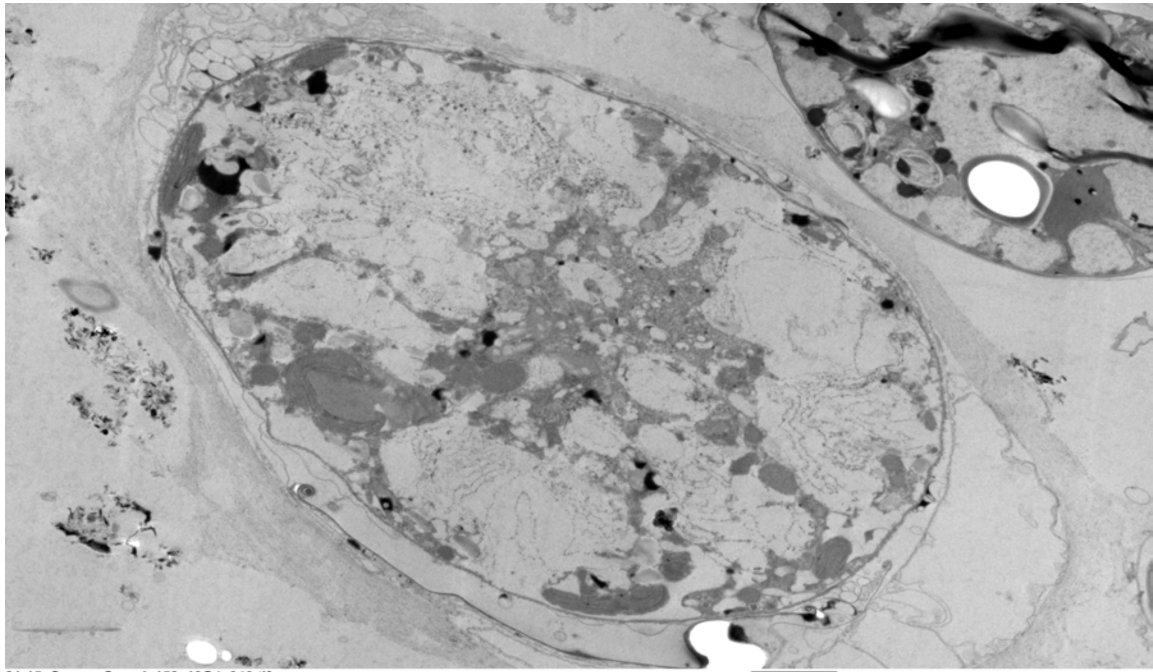

21-15_Correa_Sample158_19Q4_046.tif
Sample 158 expelled
Biological Electron Microscopy Lab
Rice University - SEA
Microscopist: MD Meyer

2 µm
HV=120kV
Direct Mag: 1200 x

Other images

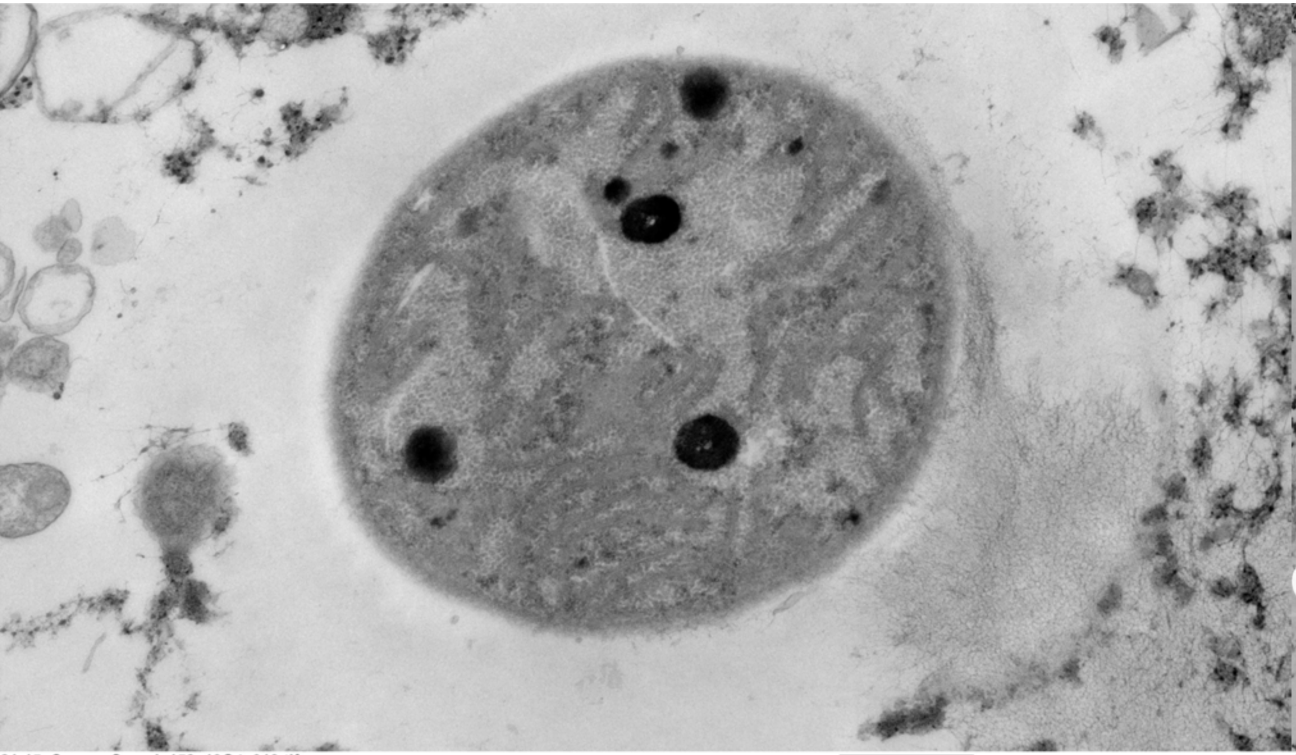

21-15_Correa_Sample158_19Q4_013.tif
Sample 158 expelled
Biological Electron Microscopy Lab
Rice University - SEA
Microscopist: MD Meyer

600 nm
HV=120kV
Direct Mag: 6000 x

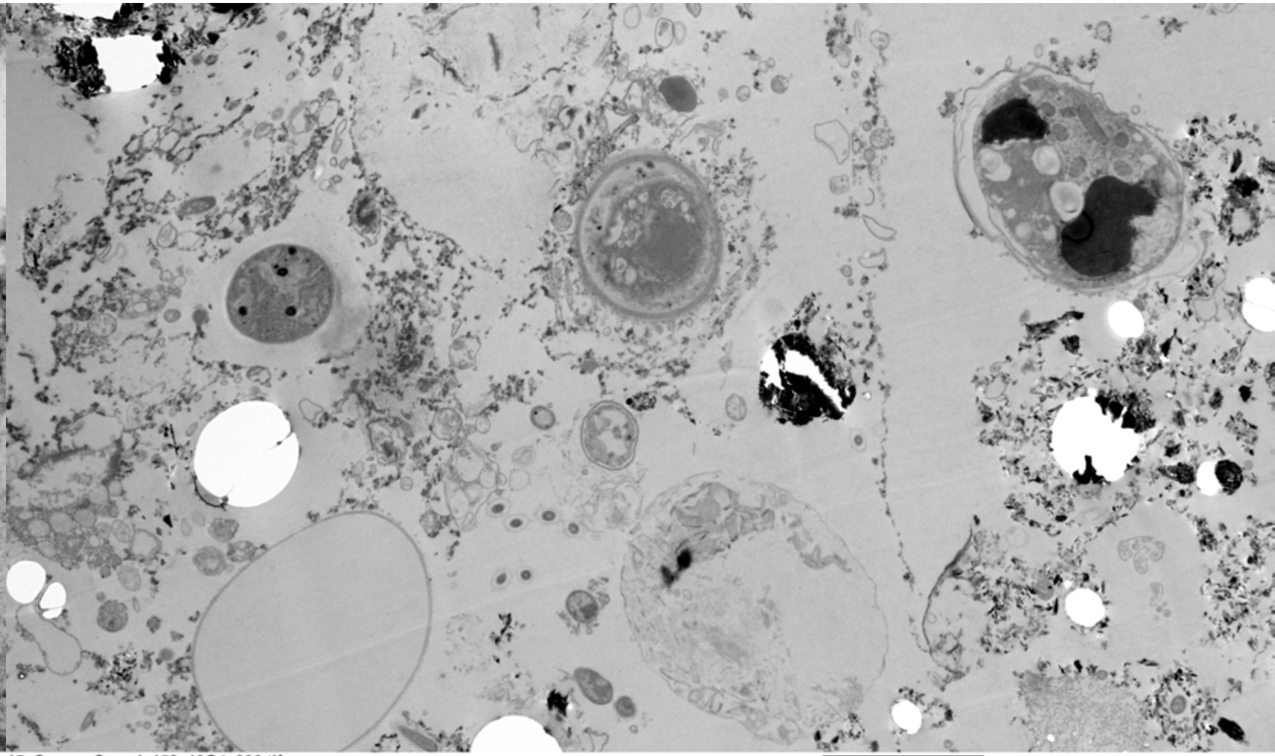

15_Correa_Sample158_19Q4_006.tif
mple 158 expelled
ological Electron Microscopy Lab
ce University - SEA
croscopist: MD Meyer

4 µm
HV=120kV
Direct Mag: 1000 x

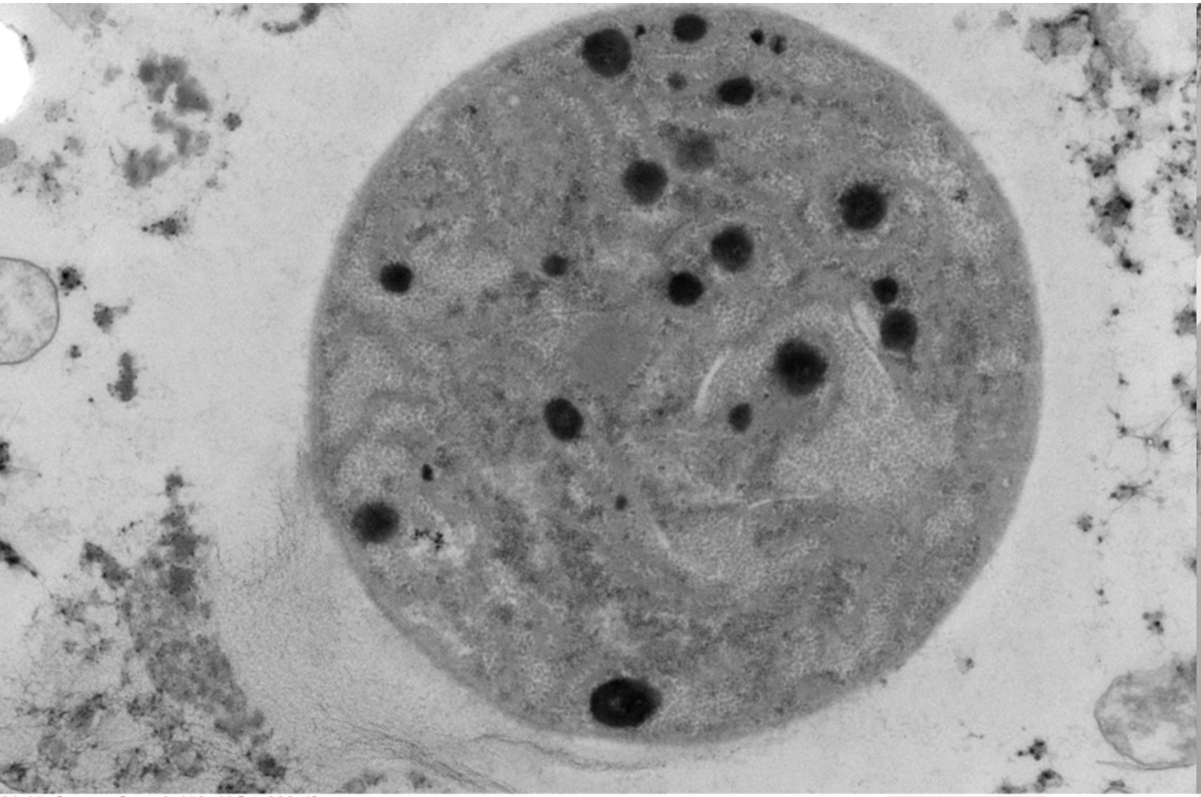

21-15_Correa_Sample158_19Q4_033.tif
Sample 158 expelled
Biological Electron Microscopy Lab
Rice University - SEA
Microscopist: MD Meyer

800 nm
HV=120kV
Direct Mag: 5000 x

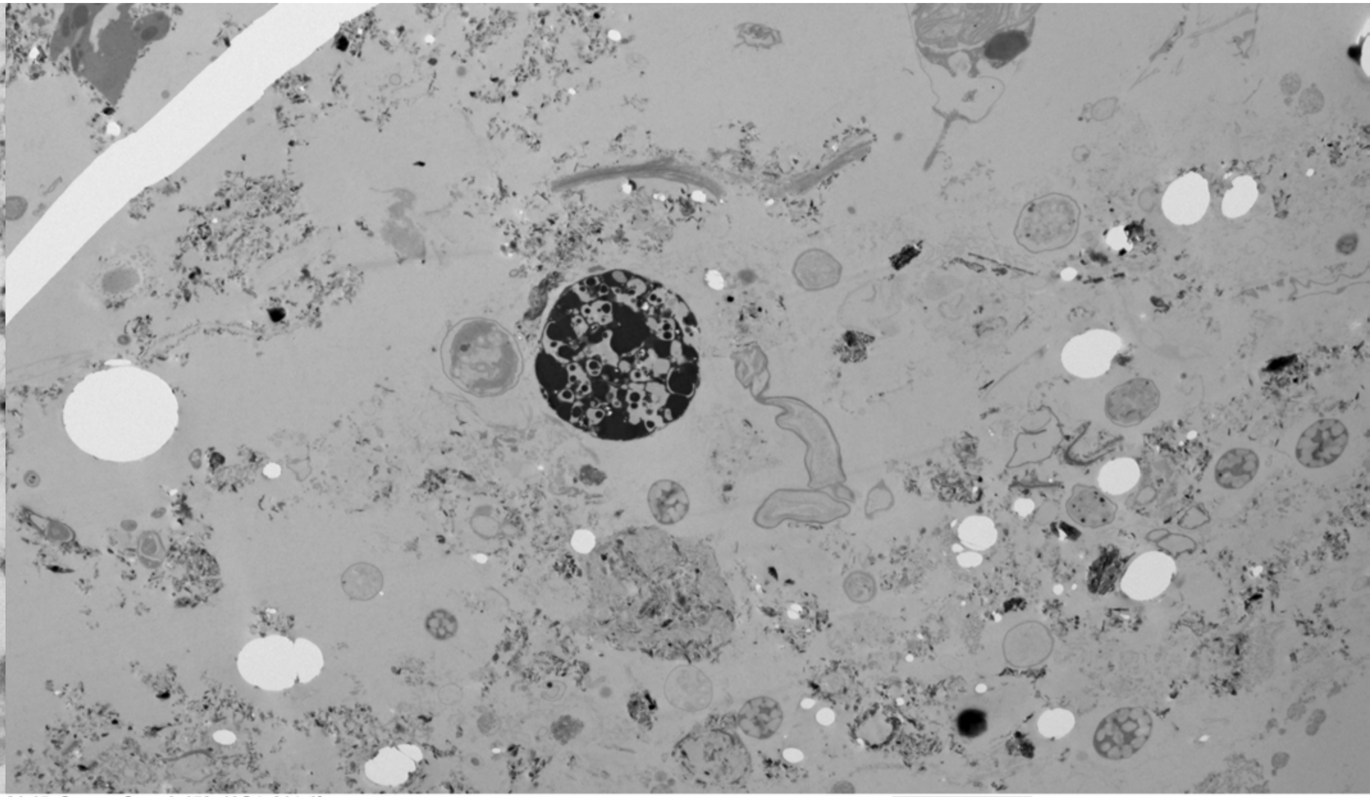

21-15_Correa_Sample158_19Q4_014.tif
Sample 158 expelled
Biological Electron Microscopy Lab
Rice University - SEA
Microscopist: MD Meyer

4 µm
HV=120kV
Direct Mag: 800 x

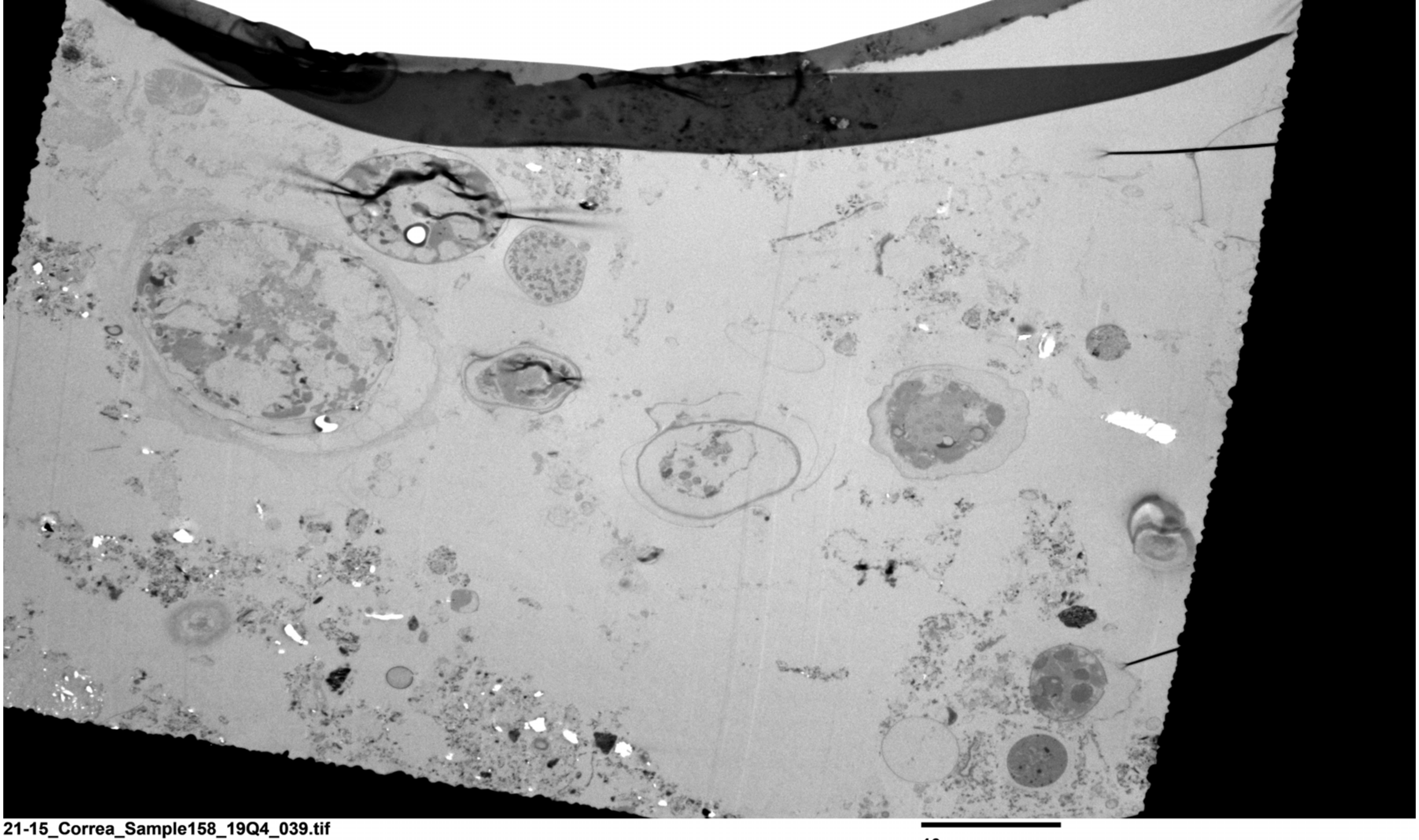

21-15_Correa_Sample158_19Q4_039.tif
Sample 158 expelled
Biological Electron Microscopy Lab
Rice University - SEA
Microscopist: MD Meyer

HV=120kV
Direct Mag: 300 x
