# Supplementary material for: Filamentous virus-like particles are present in coral dinoflagellates across genera and ocean basins
Source: ISME J. 2023 Nov 1;17(12):2389–402. doi: 10.1038/s41396-023-01526-6 (PMC10689786; doi:10.1038/s41396-023-01526-6)
Supplement: Supplementary file 2 — Supplementary TEM Images POR [file 41396_2023_1526_MOESM2_ESM.pdf]

Filamentous virus-like particles are present in coral dinoflagellates across genera and ocean basins

Supplementary Data- *Porites lobata* Symbiodiniaceae TEM images

- POR Colony L
  - Ambient (March 2018)
  - Heat (March 2019)
- POR Colony K
  - Ambient (March 2018)
  - Heat (March 2019)
- POR Colony J
  - Ambient (March 2018)
  - Heat (March 2019)
- POR Colony O
  - Ambient (March 2018)
  - Heat (March 2019)
- POR Colony M
  - Ambient (March 2018)
  - Heat (March 2019)
- POR Colony N
  - Ambient (March 2018)
  - Heat (March 2019)

Ambient (March 2018) POR Colony L

Cell 1

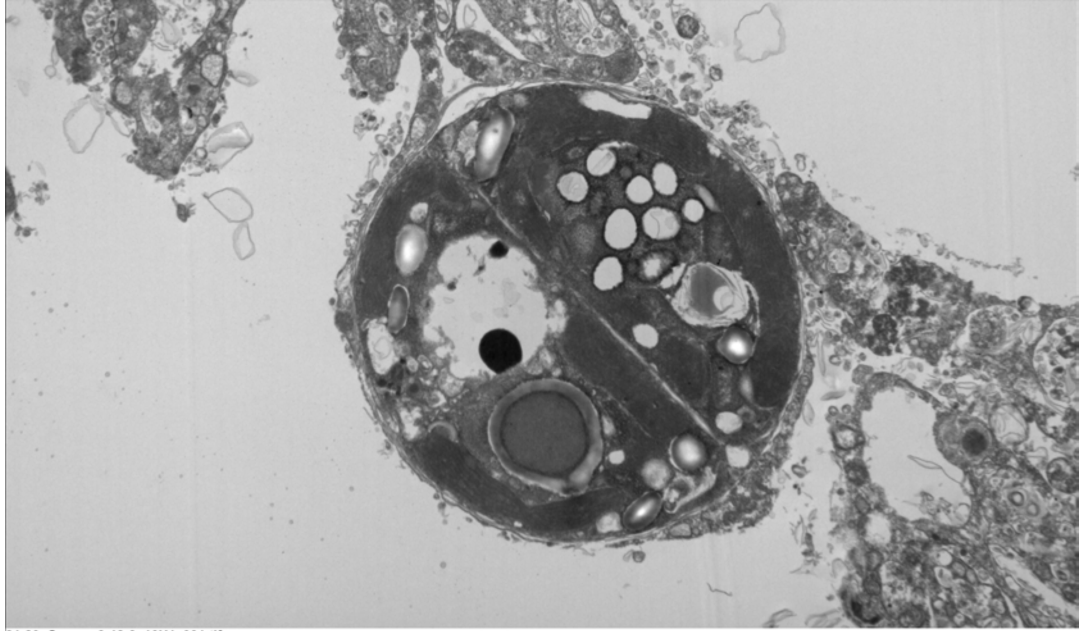

21-20_Correa_3-18-3_16H1_001.tif
Sample: 3/18, 3
Biological Electron Microscopy Lab
Rice University - SEA
Microscopist: MD Meyer

2 µm
HV=80kV
Direct Mag: 1500 x

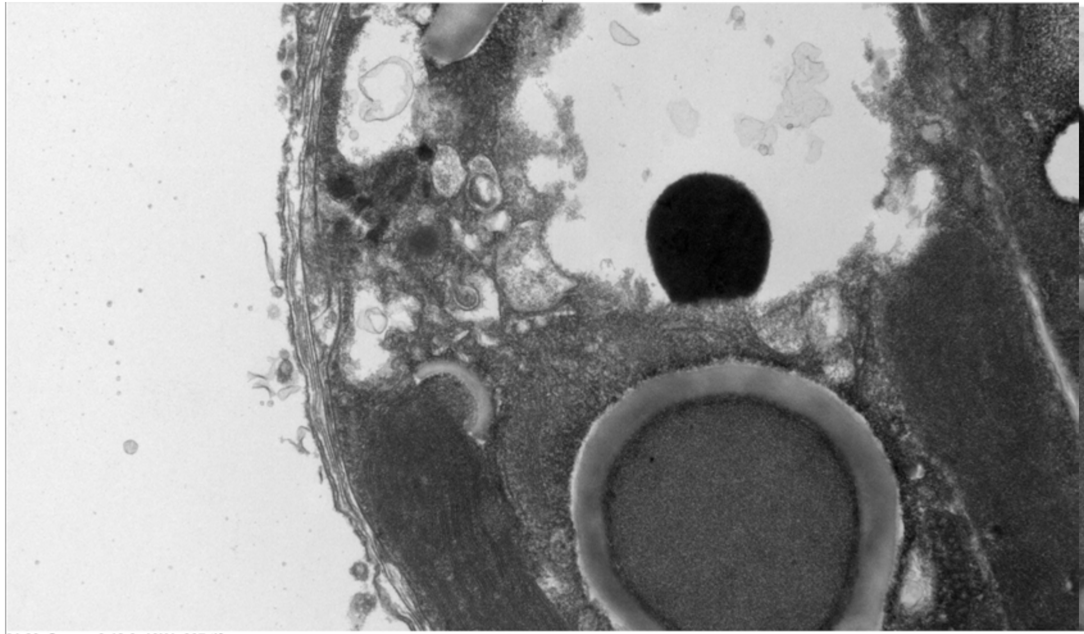

21-20_Correa_3-18-3_16H1_007.tif
Sample: 3/18, 3
Biological Electron Microscopy Lab
Rice University - SEA
Microscopist: MD Meyer

800 nm
HV=80kV
Direct Mag: 5000 x

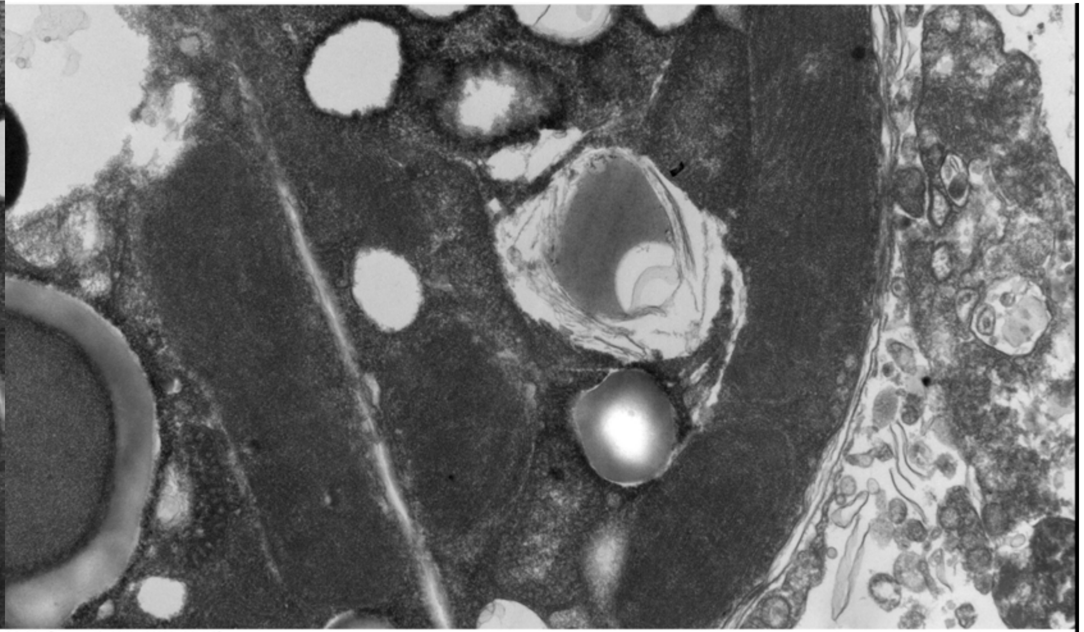

21-20_Correa_3-18-3_16H1_005.tif
Sample: 3/18, 3
Biological Electron Microscopy Lab
Rice University - SEA
Microscopist: MD Meyer

800 nm
HV=80kV
Direct Mag: 5000 x

Cell 1

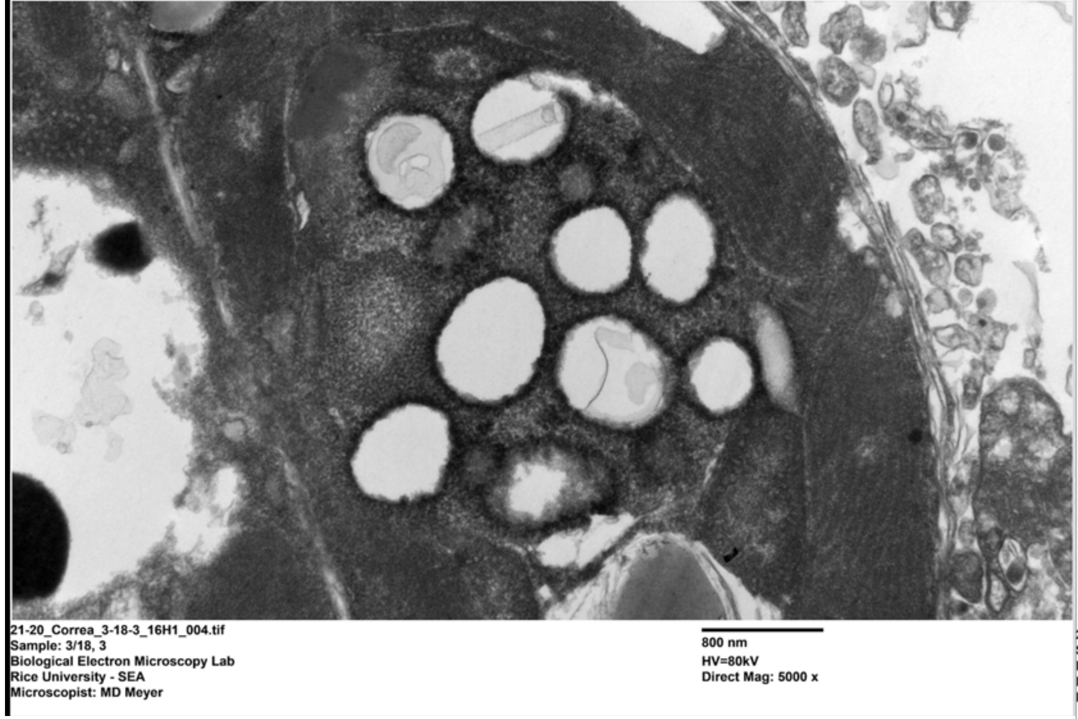
21-20_Correa_3-18-3_16H1_004.tif
Sample: 3/18, 3
Biological Electron Microscopy Lab
Rice University - SEA
Microscopist: MD Meyer
800 nm
HV=80kV
Direct Mag: 5000 x

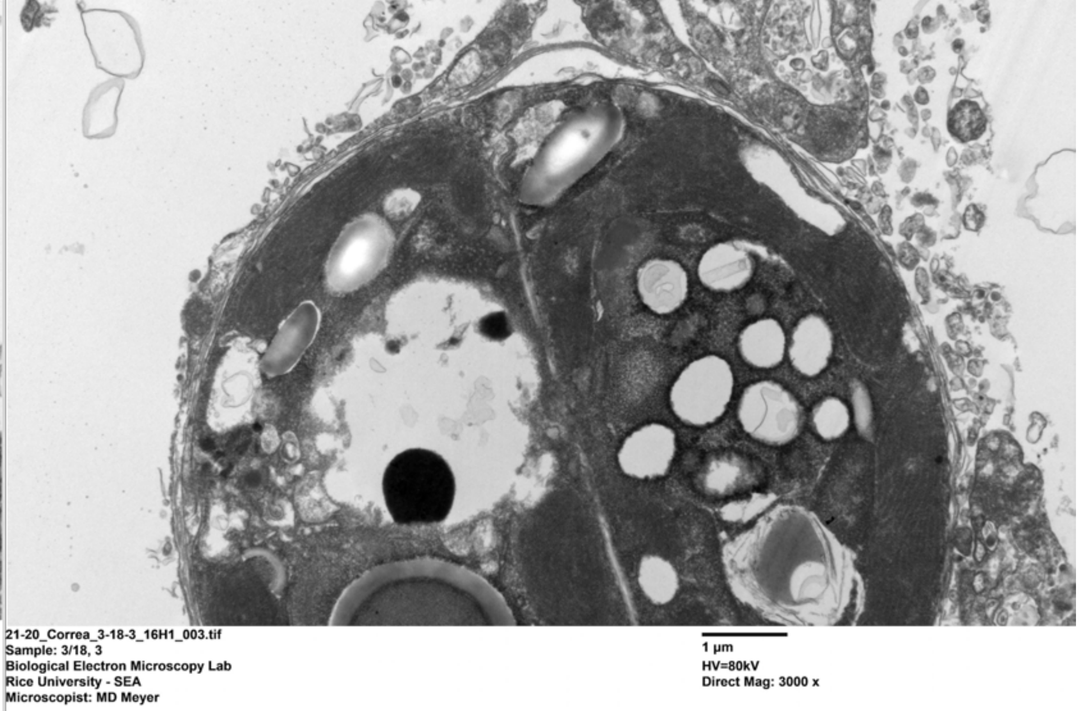
21-20_Correa_3-18-3_16H1_003.tif
Sample: 3/18, 3
Biological Electron Microscopy Lab
Rice University - SEA
Microscopist: MD Meyer
1 μm
HV=80kV
Direct Mag: 3000 x

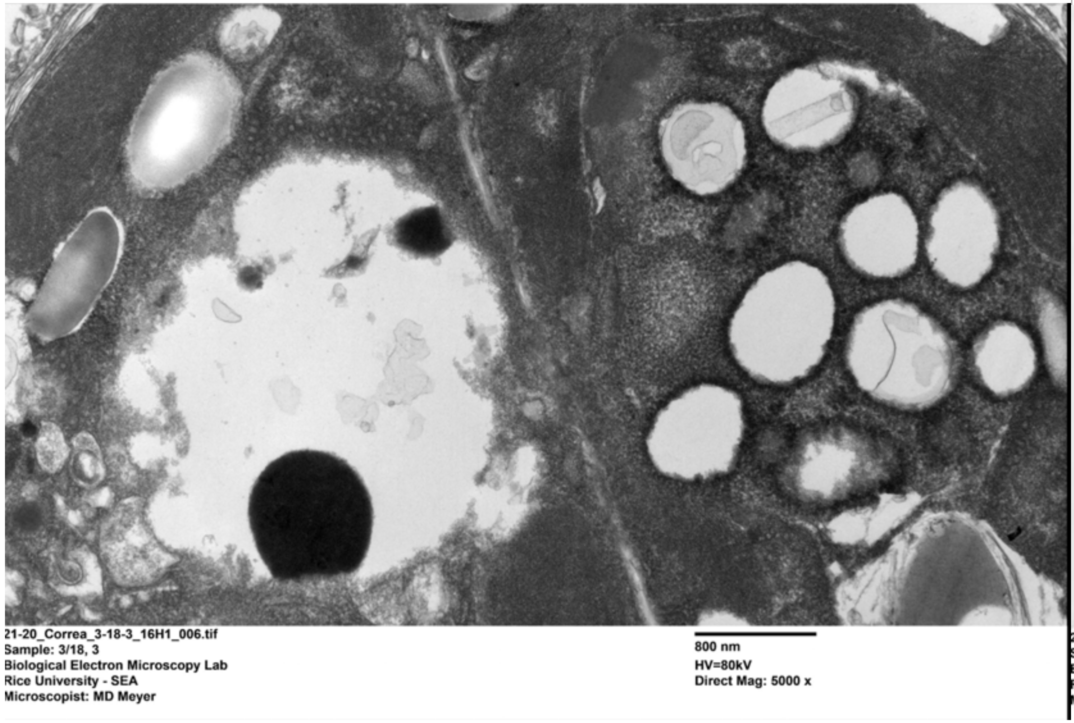
21-20_Correa_3-18-3_16H1_006.tif
Sample: 3/18, 3
Biological Electron Microscopy Lab
Rice University - SEA
Microscopist: MD Meyer
800 nm
HV=80kV
Direct Mag: 5000 x

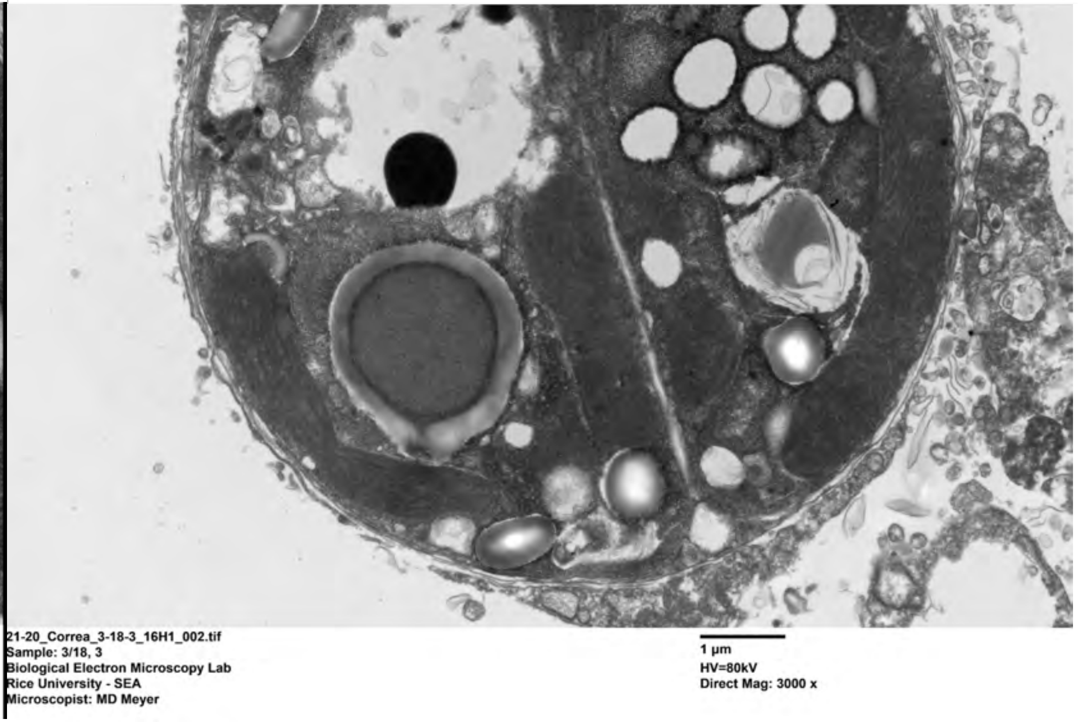
21-20_Correa_3-18-3_16H1_002.tif
Sample: 3/18, 3
Biological Electron Microscopy Lab
Rice University - SEA
Microscopist: MD Meyer
1 μm
HV=80kV
Direct Mag: 3000 x

Cell 2

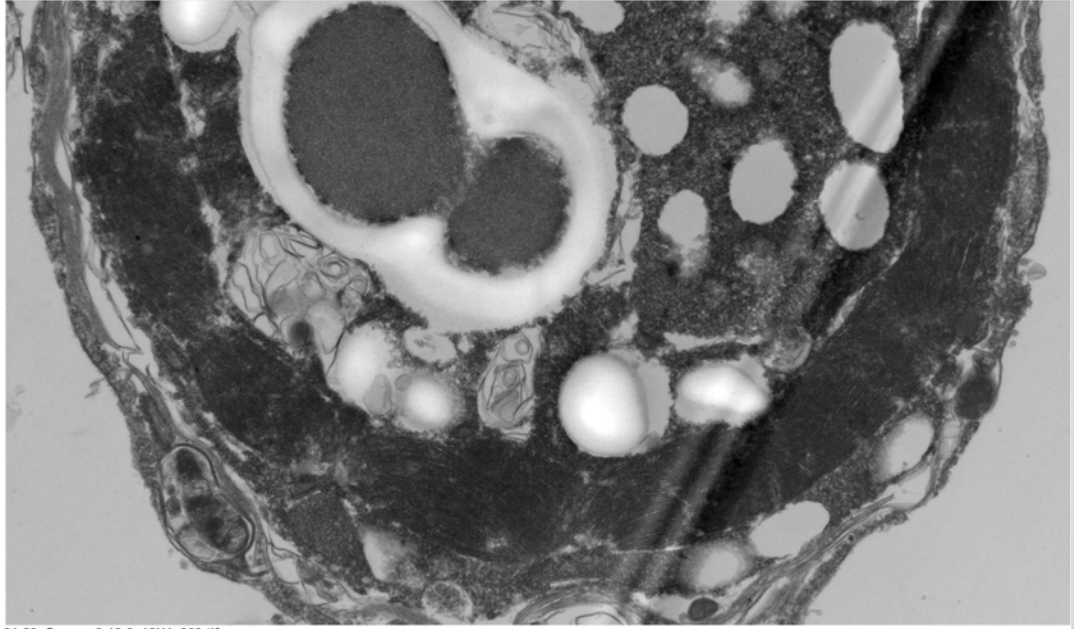

21-20_Correa_3-18-3_16H1_009.tif
Sample: 3/18, 3
Biological Electron Microscopy Lab
Rice University - SEA
Microscopist: MD Meyer

1 µm
HV=80kV
Direct Mag: 4000 x

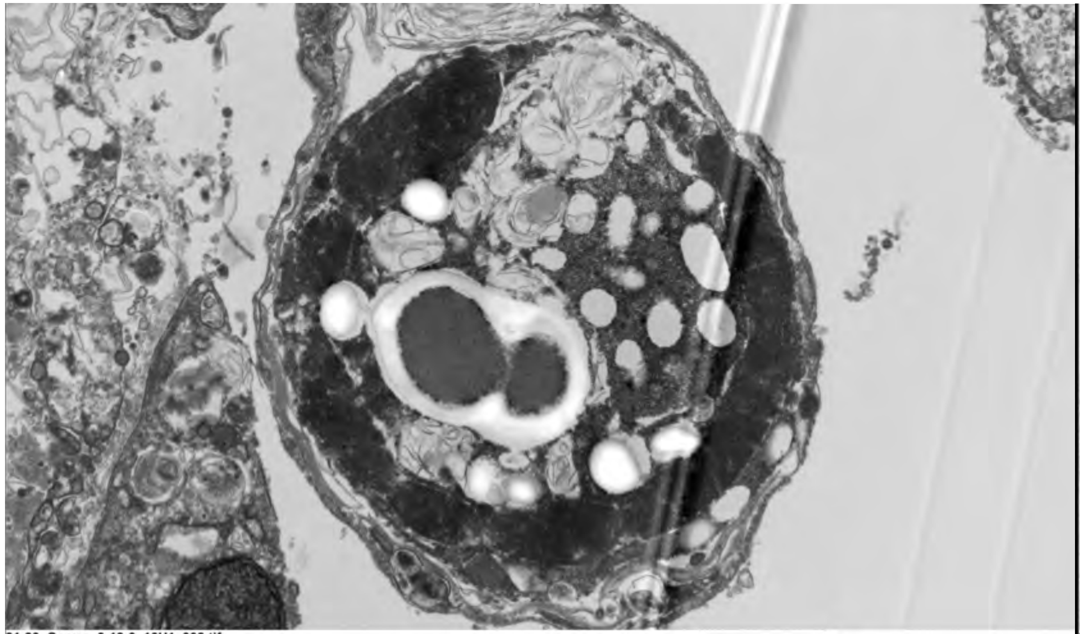

21-20_Correa_3-18-3_16H1_008.tif
Sample: 3/18, 3
Biological Electron Microscopy Lab
Rice University - SEA
Microscopist: MD Meyer

2 µm
HV=80kV
Direct Mag: 2000 x

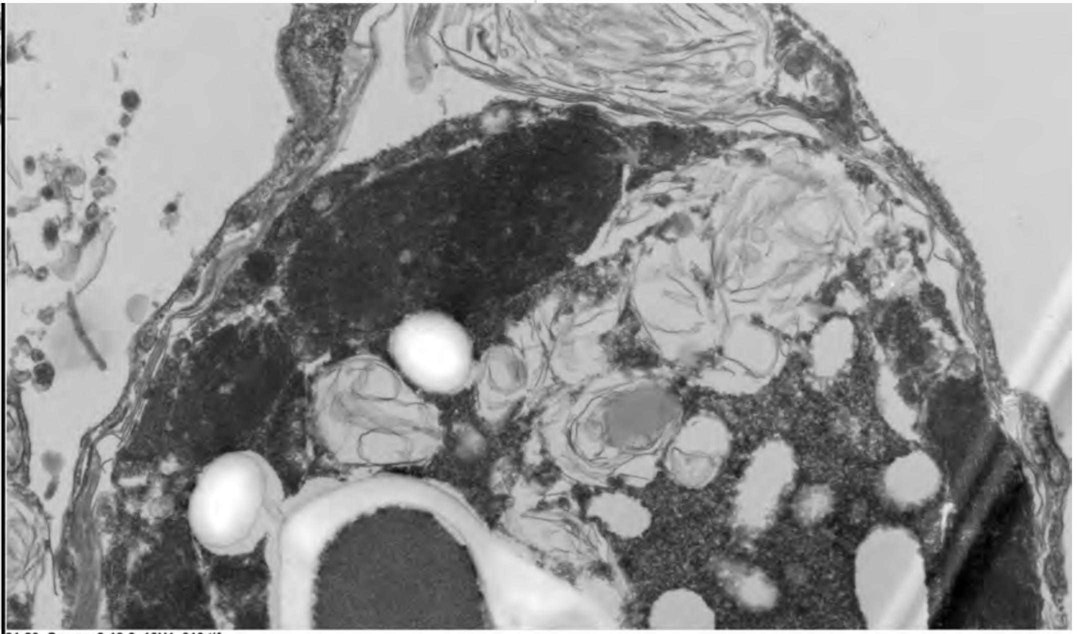

21-20_Correa_3-18-3_16H1_010.tif
Sample: 3/18, 3
Biological Electron Microscopy Lab
Rice University - SEA
Microscopist: MD Meyer

1 µm
HV=80kV
Direct Mag: 4000 x

Cell 3

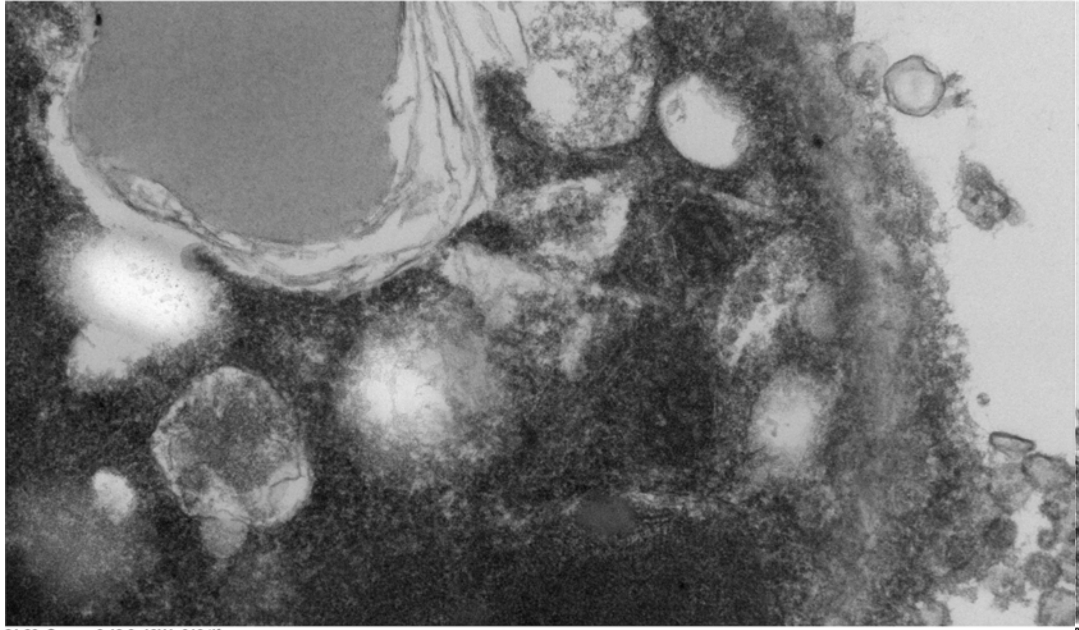

21-20_Correa_3-18-3_16H1_016.tif
Sample: 3/18, 3
Biological Electron Microscopy Lab
Rice University - SEA
Microscopist: MD Meyer

400 nm
HV=80kV
Direct Mag: 10000 x

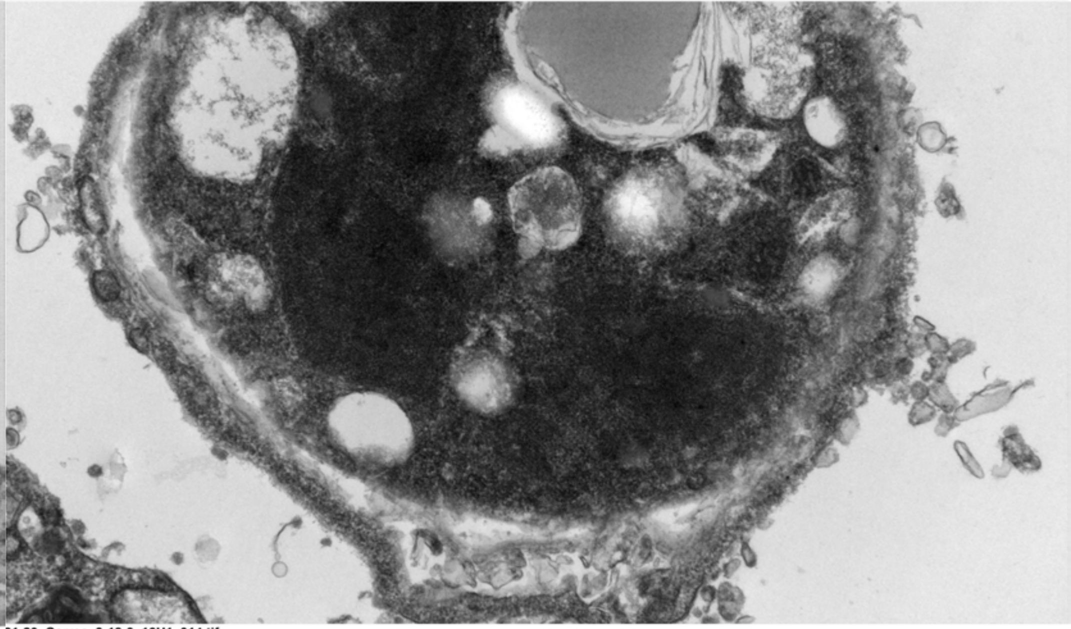

21-20_Correa_3-18-3_16H1_014.tif
Sample: 3/18, 3
Biological Electron Microscopy Lab
Rice University - SEA
Microscopist: MD Meyer

800 nm
HV=80kV
Direct Mag: 5000 x

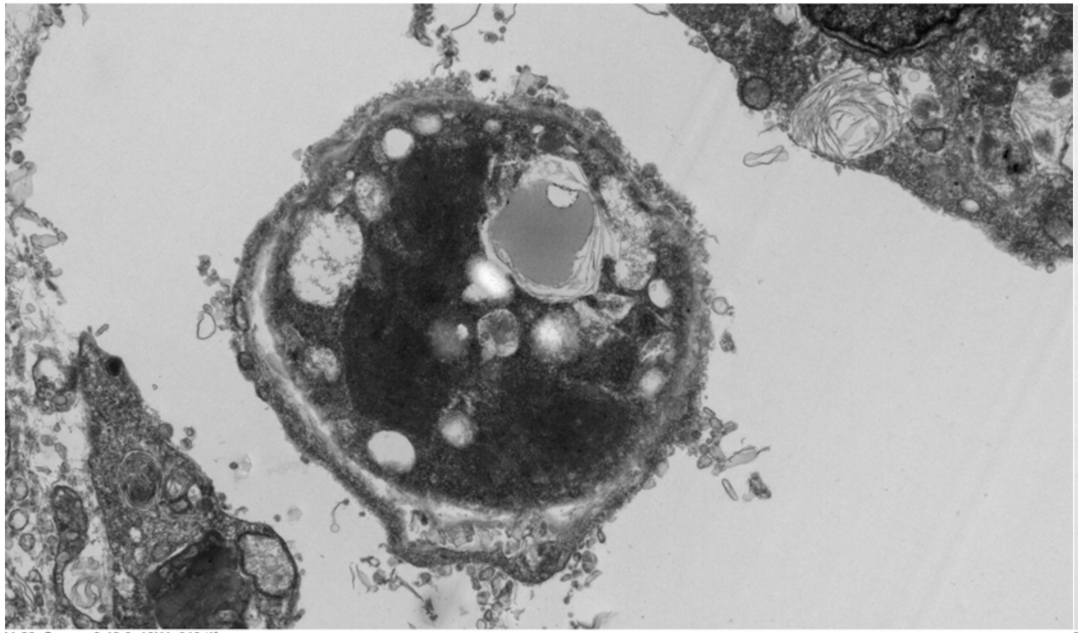

21-20_Correa_3-18-3_16H1_013.tif
ample: 3/18, 3
iological Electron Microscopy Lab
ice University - SEA
icroscopist: MD Meyer

1 µm
HV=80kV
Direct Mag: 3000 x

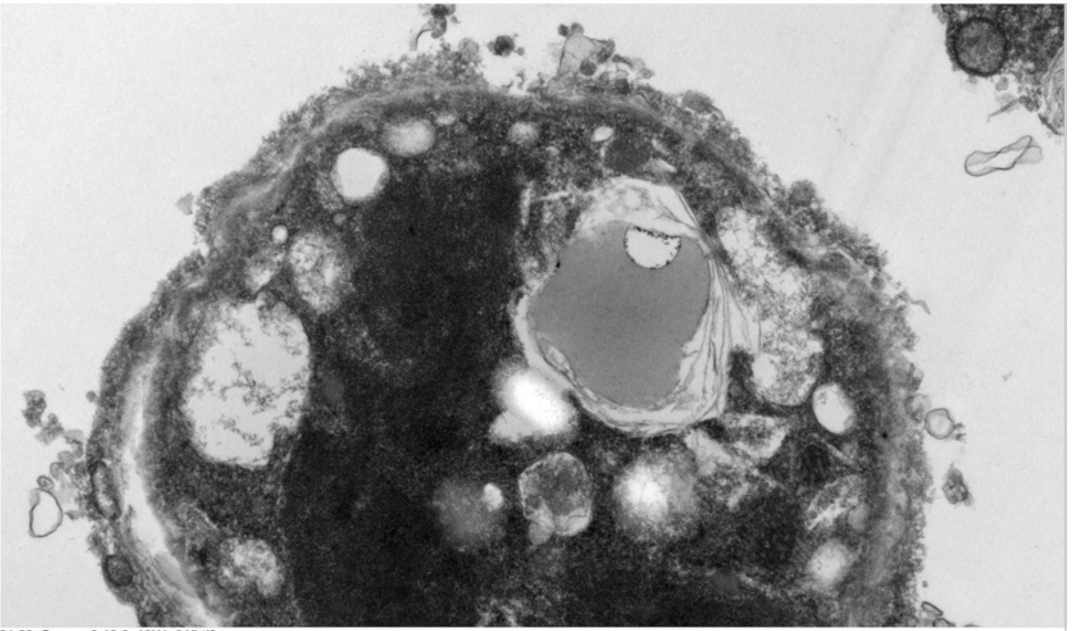

21-20_Correa_3-18-3_16H1_015.tif
Sample: 3/18, 3
Biological Electron Microscopy Lab
Rice University - SEA
Microscopist: MD Meyer

800 nm
HV=80kV
Direct Mag: 5000 x

Cell 4

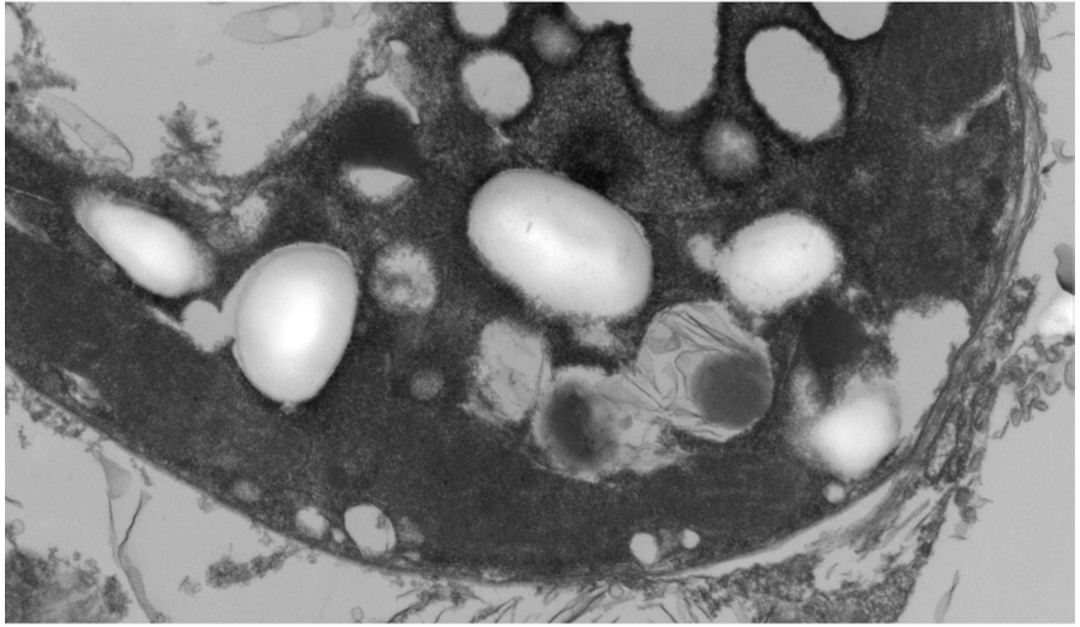

21-20_Correa_3-18-3_16H1_018.tif
Sample: 3/18, 3
Biological Electron Microscopy Lab
Rice University - SEA
Microscopist: MD Meyer

800 nm
HV=80kV
Direct Mag: 5000 x

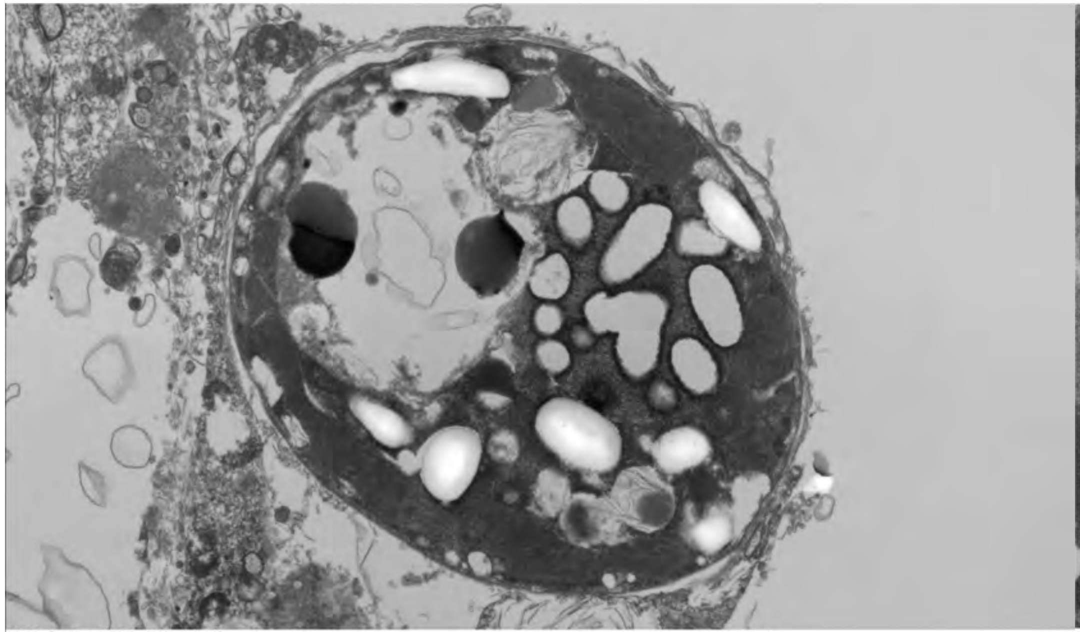

21-20_Correa_3-18-3_16H1_017.tif
Sample: 3/18, 3
Biological Electron Microscopy Lab
Rice University - SEA
Microscopist: MD Meyer

1 µm
HV=80kV
Direct Mag: 2500 x

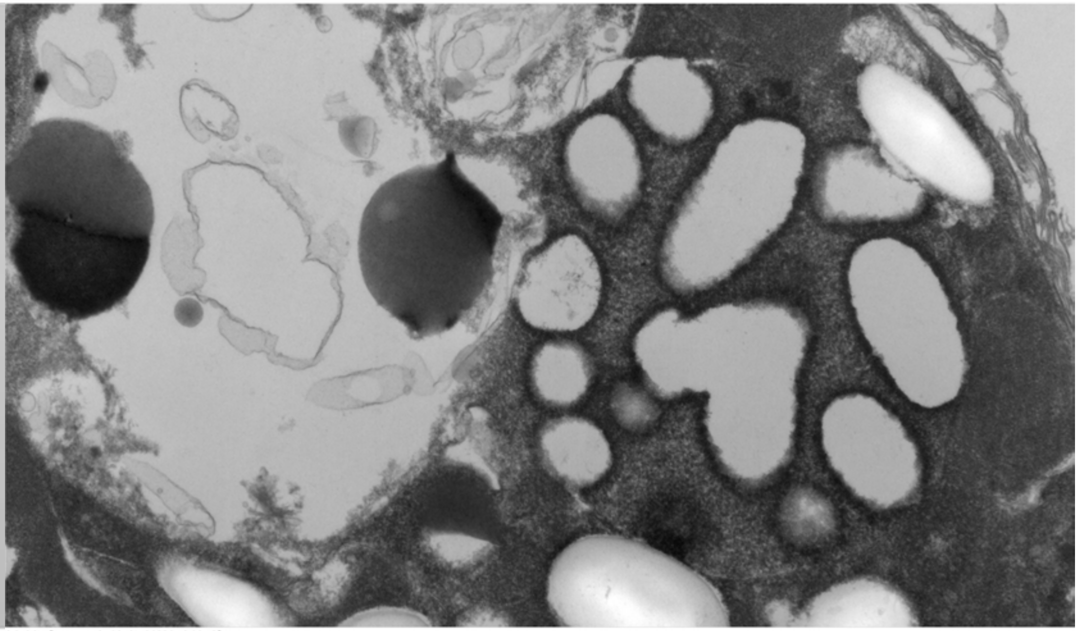

21-20_Correa_3-18-3_16H1_019.tif
Sample: 3/18, 3
Biological Electron Microscopy Lab
Rice University - SEA
Microscopist: MD Meyer

800 nm
HV=80kV
Direct Mag: 5000 x

Cell 5

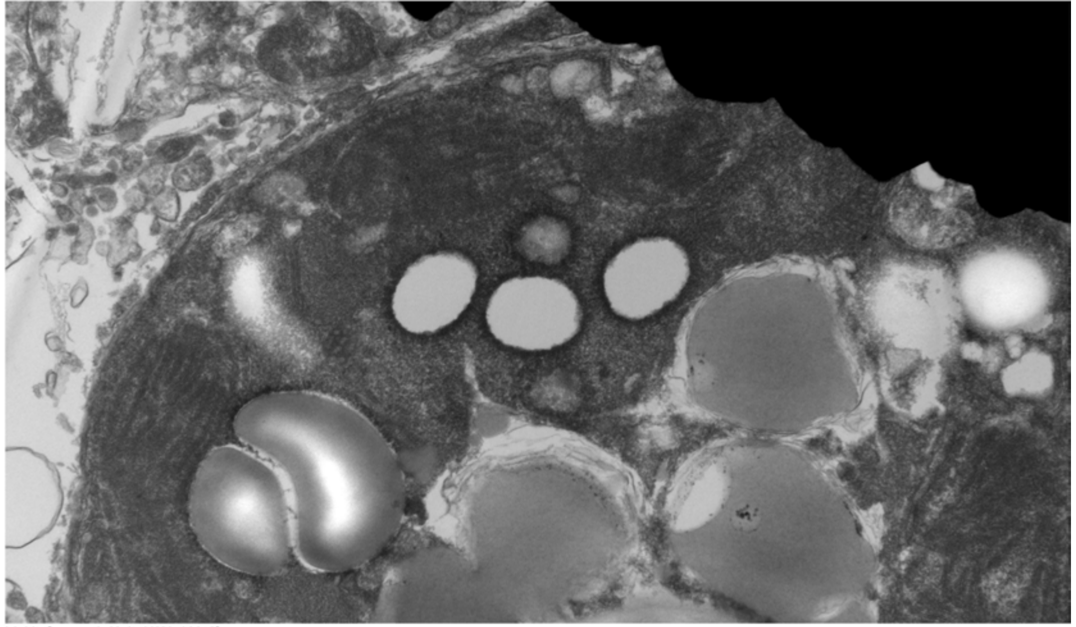

21-20_Correa_3-18-3_16H1_022.tif
Sample: 3/18, 3
Biological Electron Microscopy Lab
Rice University - SEA
Microscopist: MD Meyer

800 nm
HV=80kV
Direct Mag: 5000 x

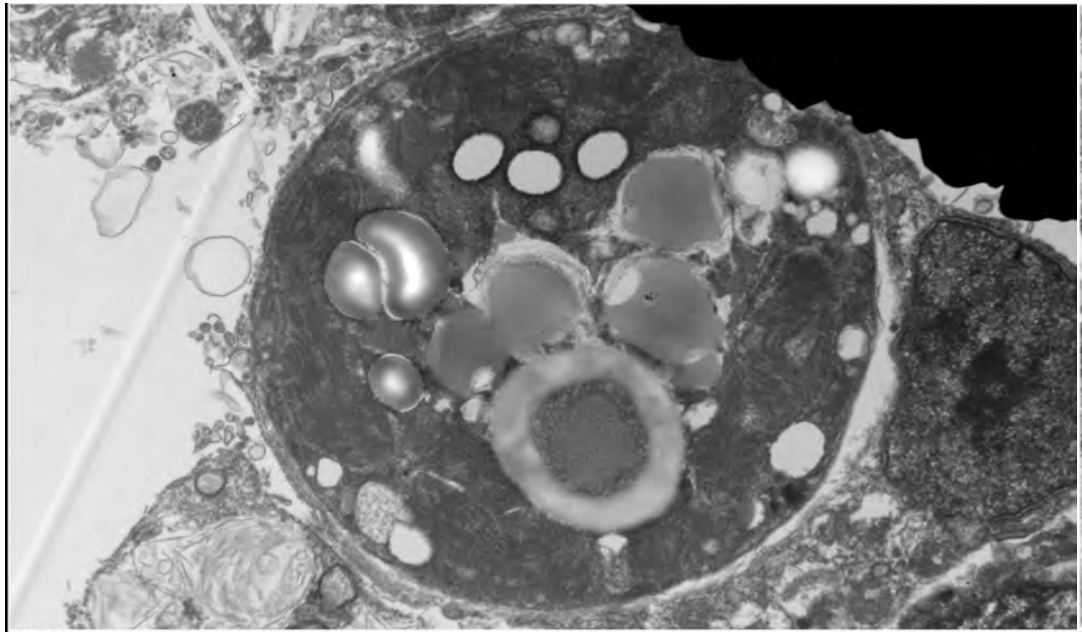

21-20_Correa_3-18-3_16H1_020.tif
Sample: 3/18, 3
Biological Electron Microscopy Lab
Rice University - SEA
Microscopist: MD Meyer

1 µm
HV=80kV
Direct Mag: 3000 x

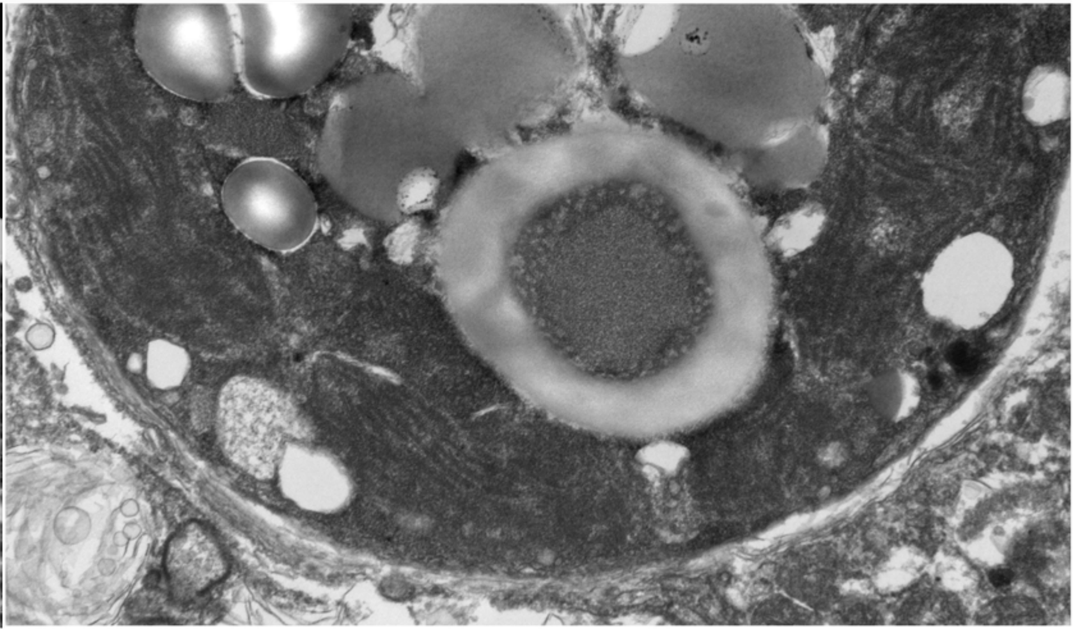

21-20_Correa_3-18-3_16H1_021.tif
Sample: 3/18, 3
Biological Electron Microscopy Lab
Rice University - SEA
Microscopist: MD Meyer

800 nm
HV=80kV
Direct Mag: 5000 x

Cell 6

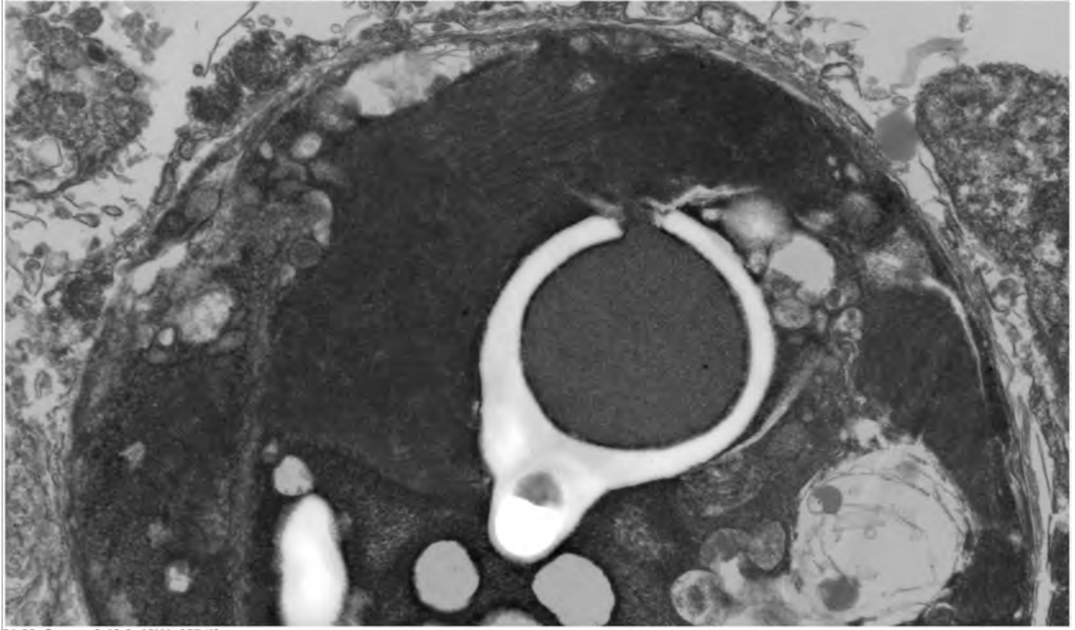

21-20_Correa_3-18-3_16H1_027.tif
Sample: 3/18, 3
Biological Electron Microscopy Lab
Rice University - SEA
Microscopist: MD Meyer

1 µm
HV=80kV
Direct Mag: 4000 x

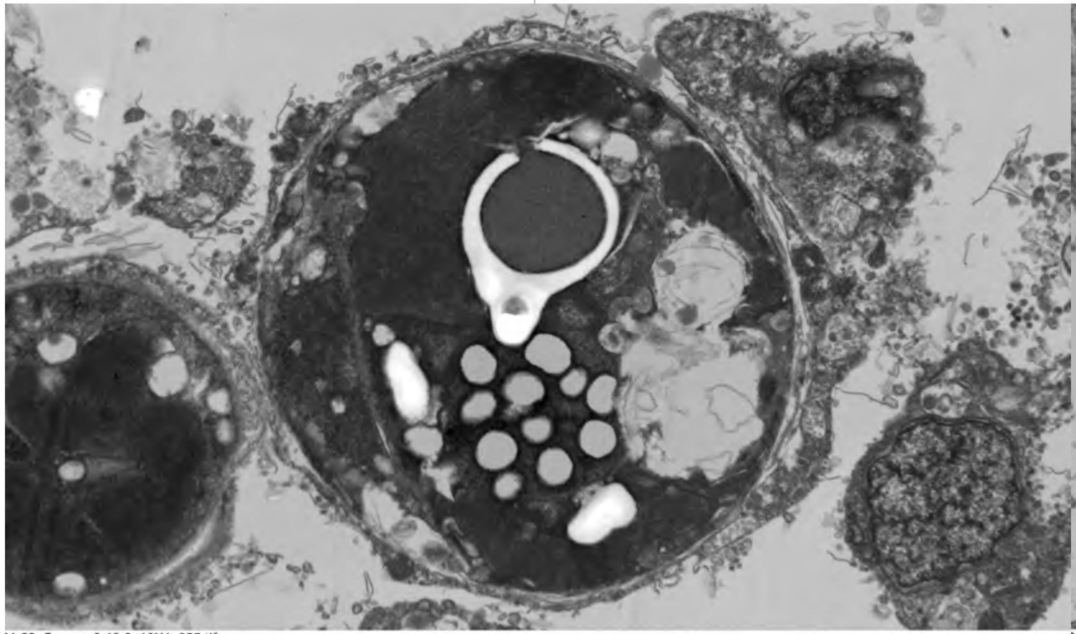

21-20_Correa_3-18-3_16H1_025.tif
Sample: 3/18, 3
Biological Electron Microscopy Lab
Rice University - SEA
Microscopist: MD Meyer

2 µm
HV=80kV
Direct Mag: 2000 x

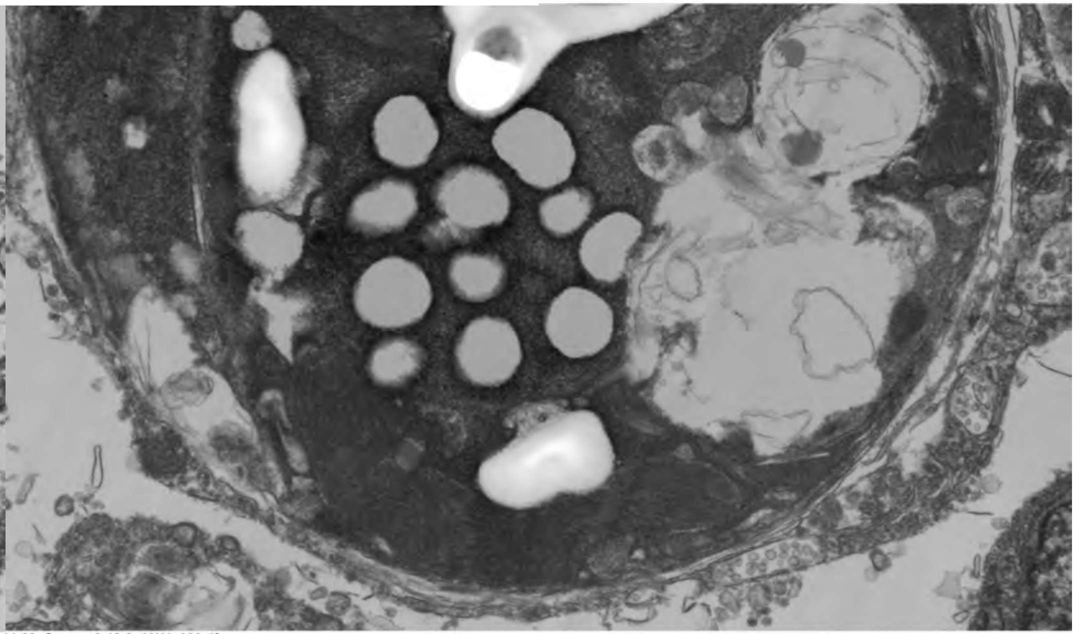

21-20_Correa_3-18-3_16H1_026.tif
Sample: 3/18, 3
Biological Electron Microscopy Lab
Rice University - SEA
Microscopist: MD Meyer

1 µm
HV=80kV
Direct Mag: 4000 x

Cell 7

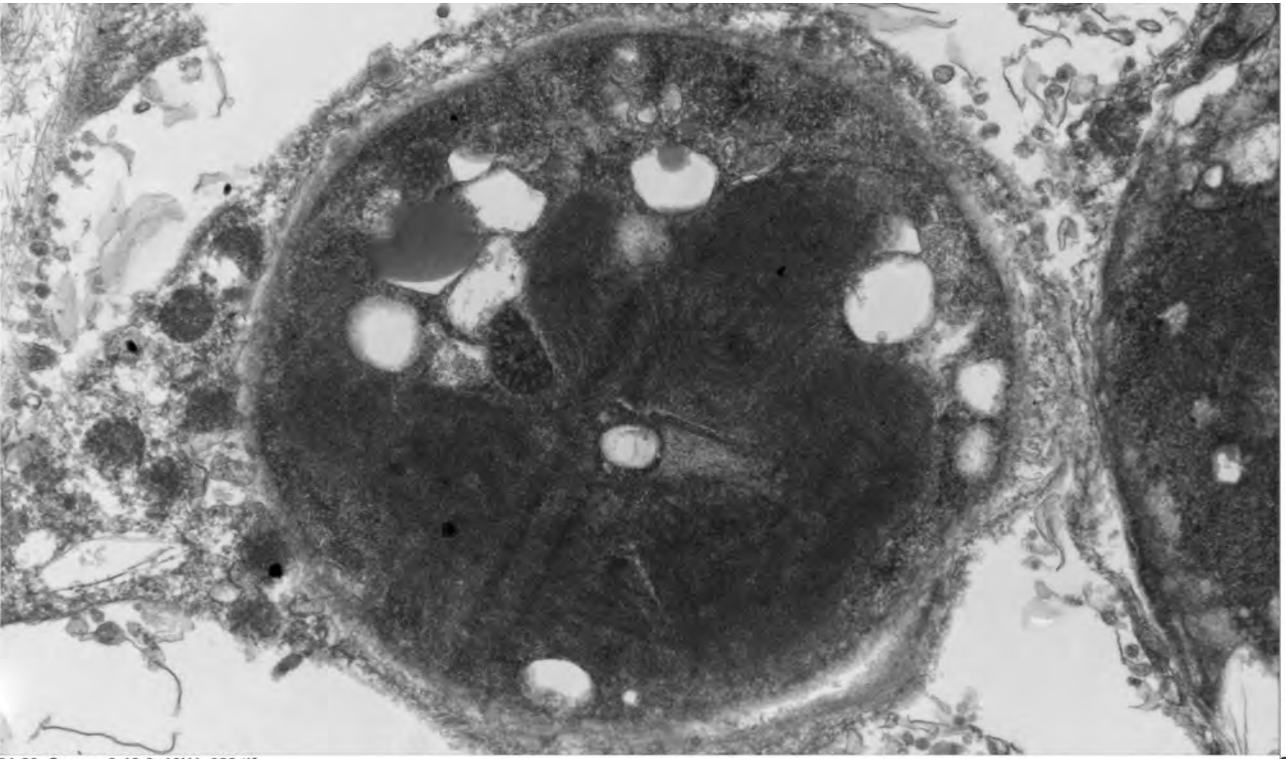

21-20_Correa_3-18-3_16H1_028.tif
Sample: 3/18, 3
Biological Electron Microscopy Lab
Rice University - SEA
Microscopist: MD Meyer

1 µm
HV=80kV
Direct Mag: 4000 x

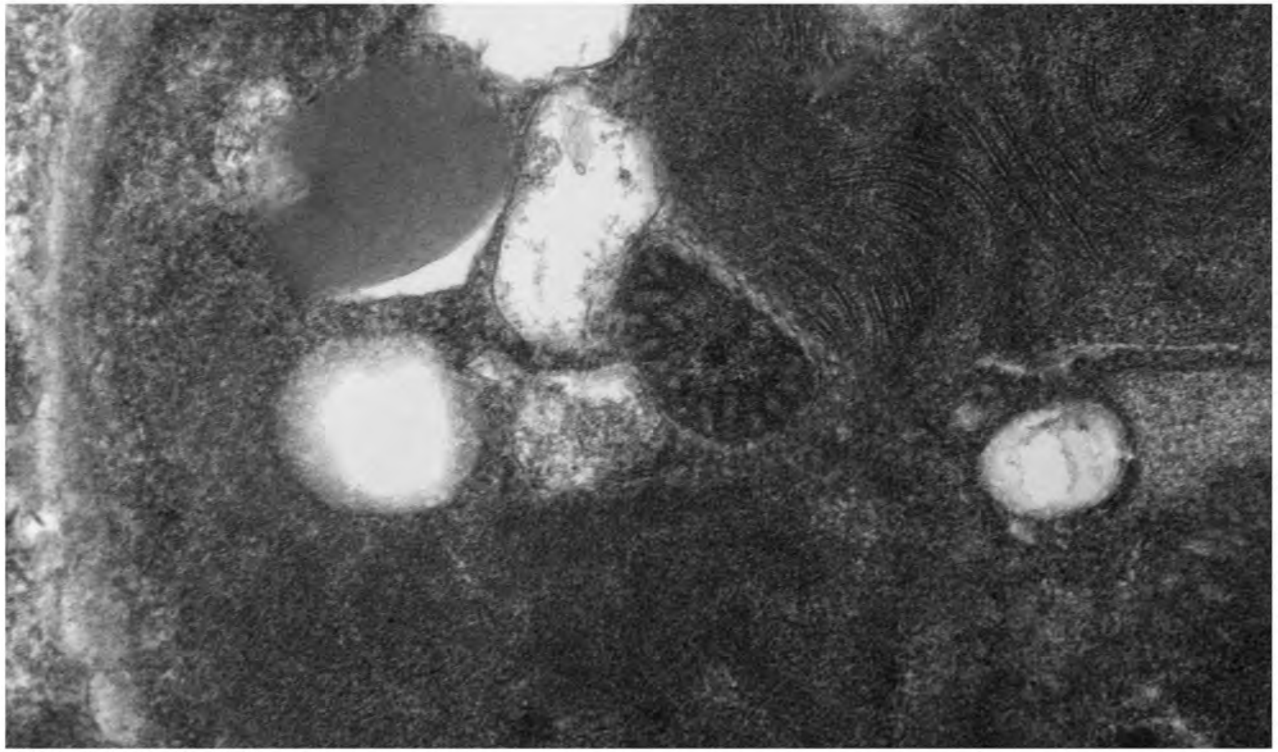

21-20_Correa_3-18-3_16H1_029.tif
Sample: 3/18, 3
Biological Electron Microscopy Lab
Rice University - SEA
Microscopist: MD Meyer

400 nm
HV=80kV
Direct Mag: 10000 x

Cell 8

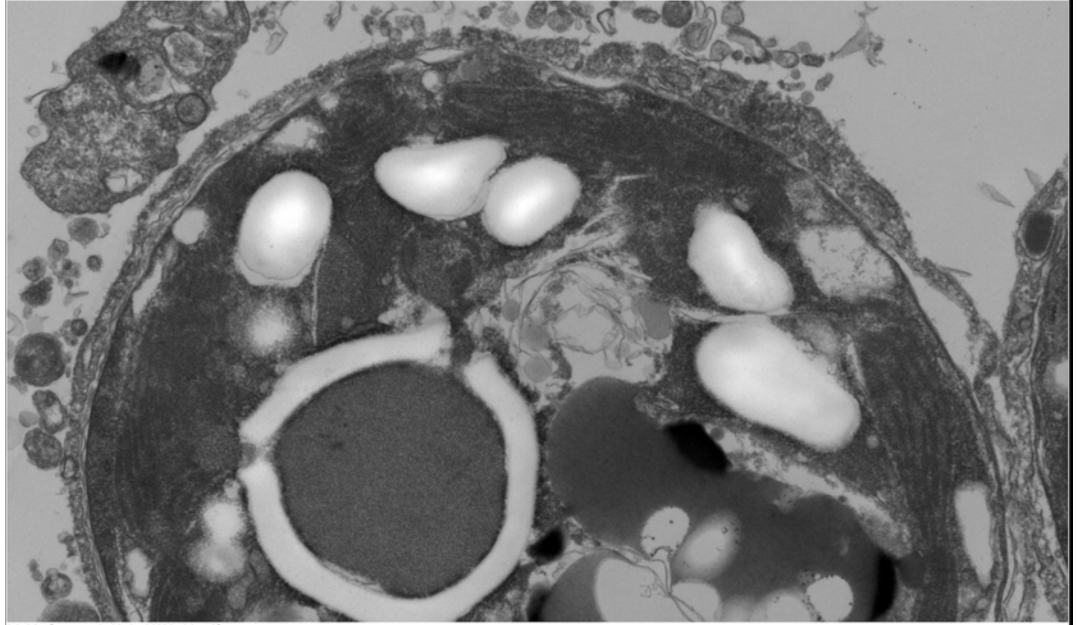

21-20_Correa_3-18-3_16H1_034.tif
Sample: 3/18, 3
Biological Electron Microscopy Lab
Rice University - SEA
Microscopist: MD Meyer

1 µm
HV=80kV
Direct Mag: 4000 x

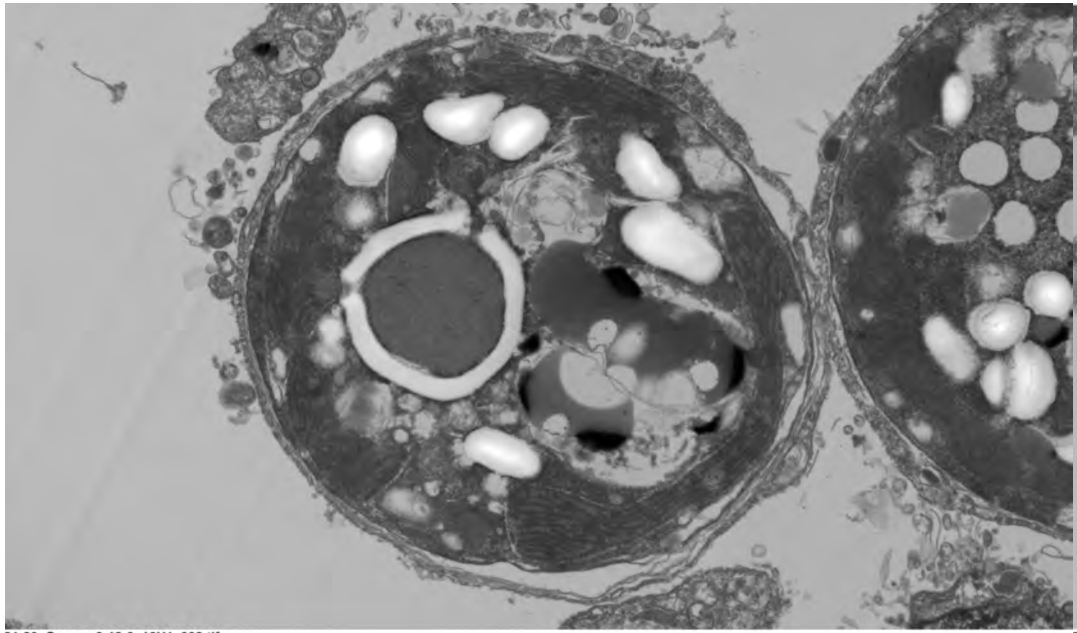

21-20_Correa_3-18-3_16H1_032.tif
Sample: 3/18, 3
Biological Electron Microscopy Lab
Rice University - SEA
Microscopist: MD Meyer

1 µm
HV=80kV
Direct Mag: 2500 x

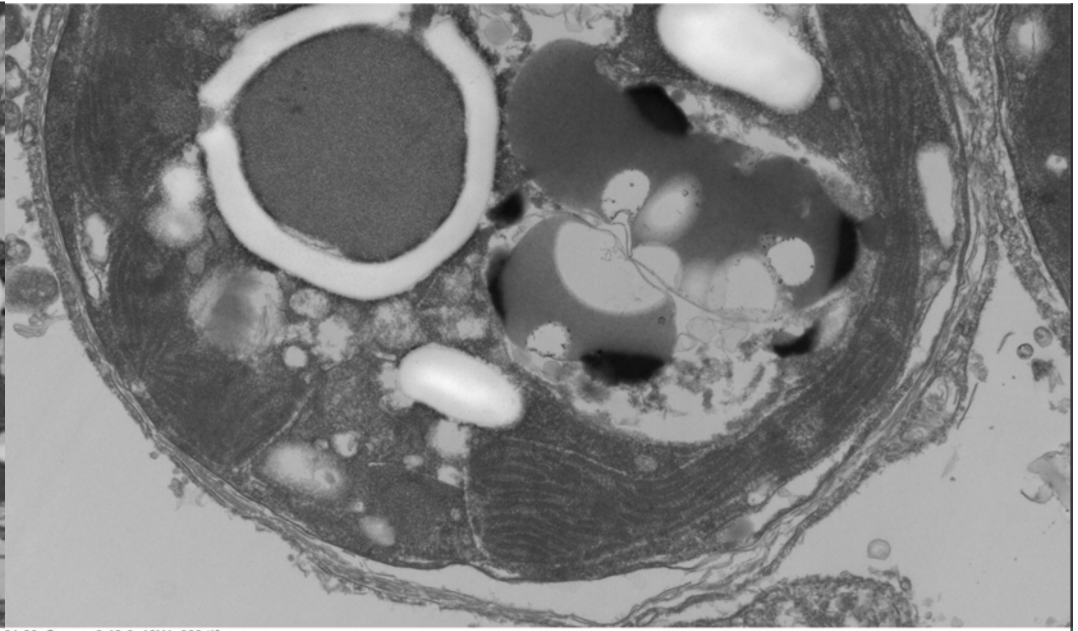

21-20_Correa_3-18-3_16H1_033.tif
Sample: 3/18, 3
Biological Electron Microscopy Lab
Rice University - SEA
Microscopist: MD Meyer

1 µm
HV=80kV
Direct Mag: 4000 x

Cell 9

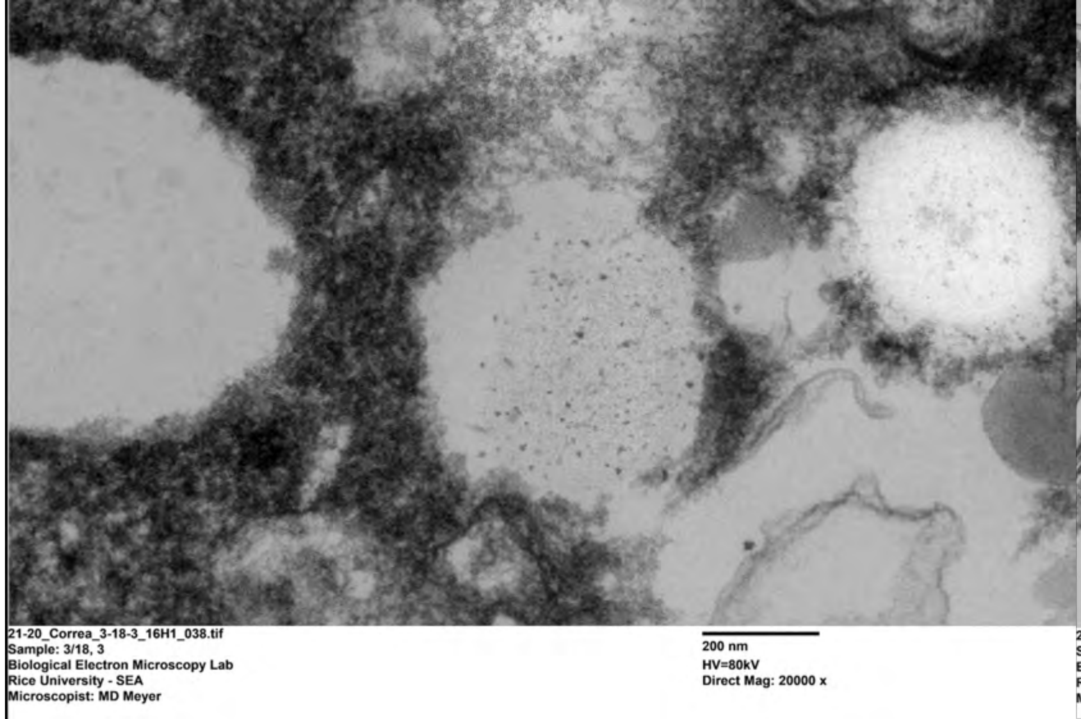
21-20_Correa_3-18-3_16H1_038.tif
Sample: 3/18, 3
Biological Electron Microscopy Lab
Rice University - SEA
Microscopist: MD Meyer
200 nm
HV=80kV
Direct Mag: 20000 x

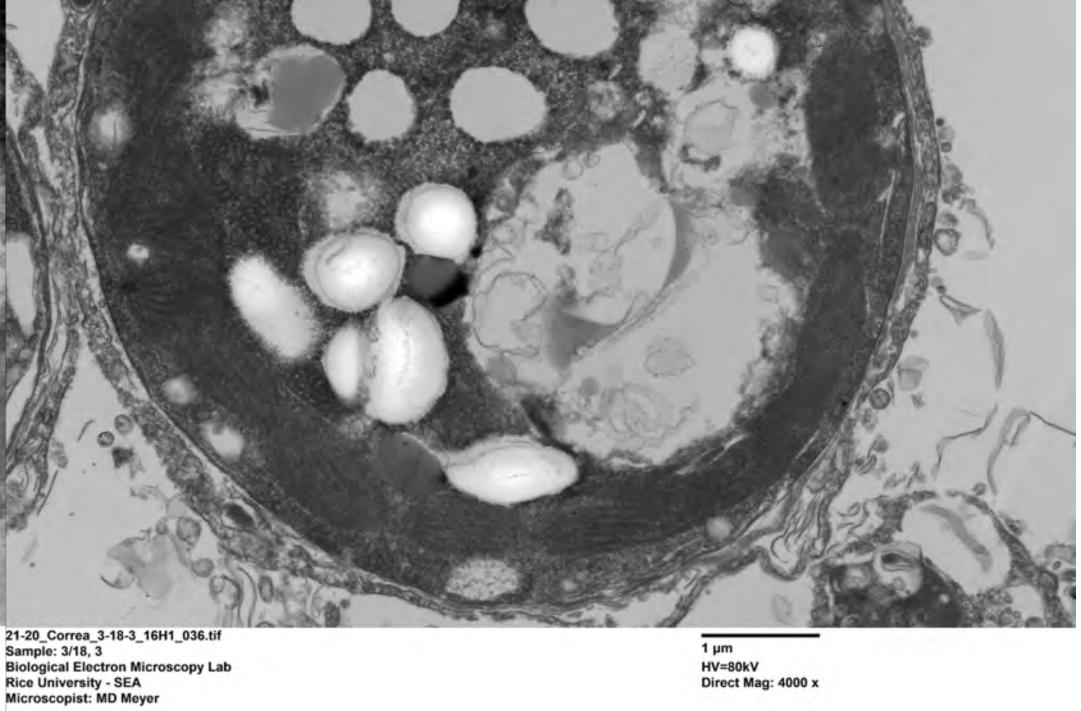
21-20_Correa_3-18-3_16H1_036.tif
Sample: 3/18, 3
Biological Electron Microscopy Lab
Rice University - SEA
Microscopist: MD Meyer
1 µm
HV=80kV
Direct Mag: 4000 x

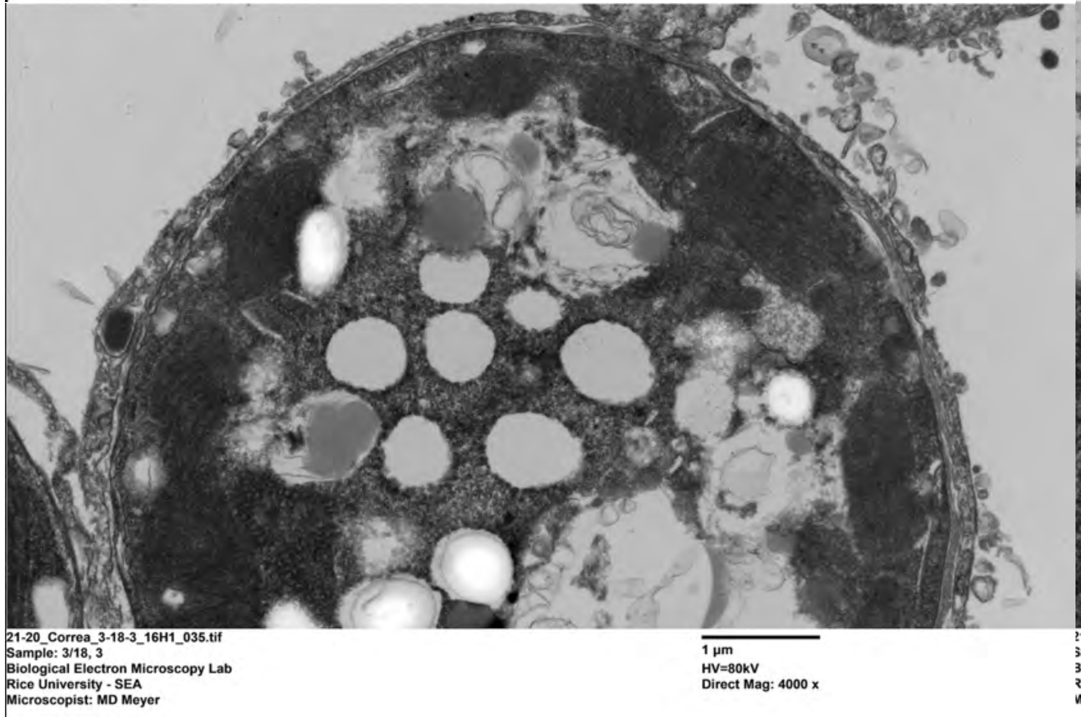
21-20_Correa_3-18-3_16H1_035.tif
Sample: 3/18, 3
Biological Electron Microscopy Lab
Rice University - SEA
Microscopist: MD Meyer
1 µm
HV=80kV
Direct Mag: 4000 x

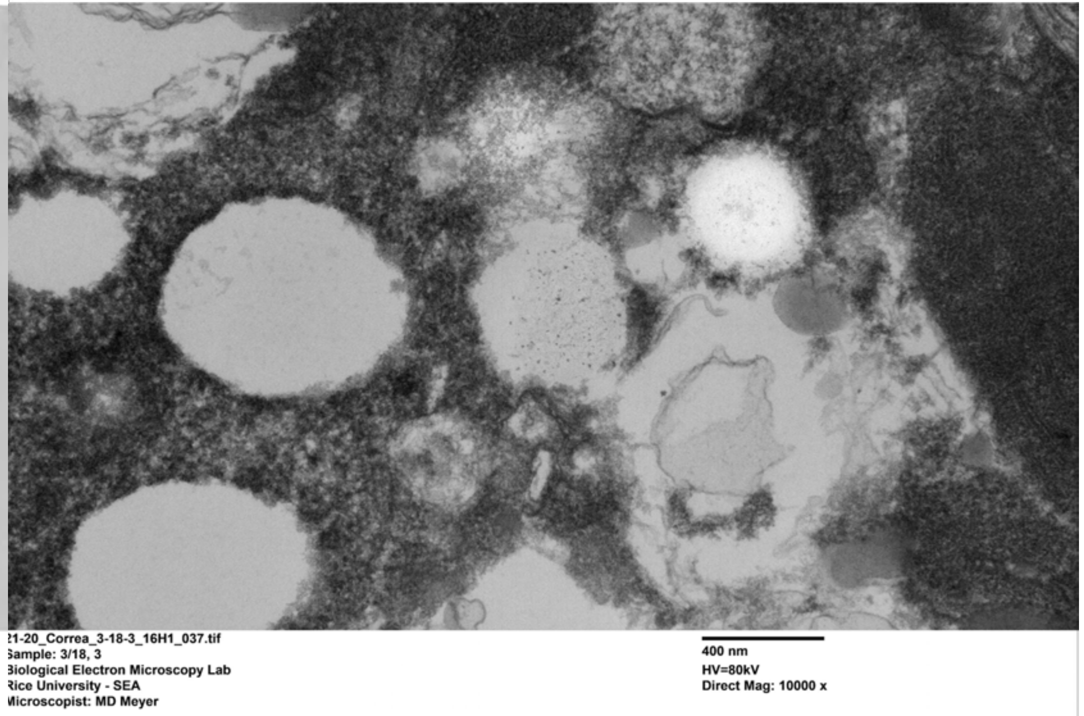
21-20_Correa_3-18-3_16H1_037.tif
Sample: 3/18, 3
Biological Electron Microscopy Lab
Rice University - SEA
Microscopist: MD Meyer
400 nm
HV=80kV
Direct Mag: 10000 x

Cell 10

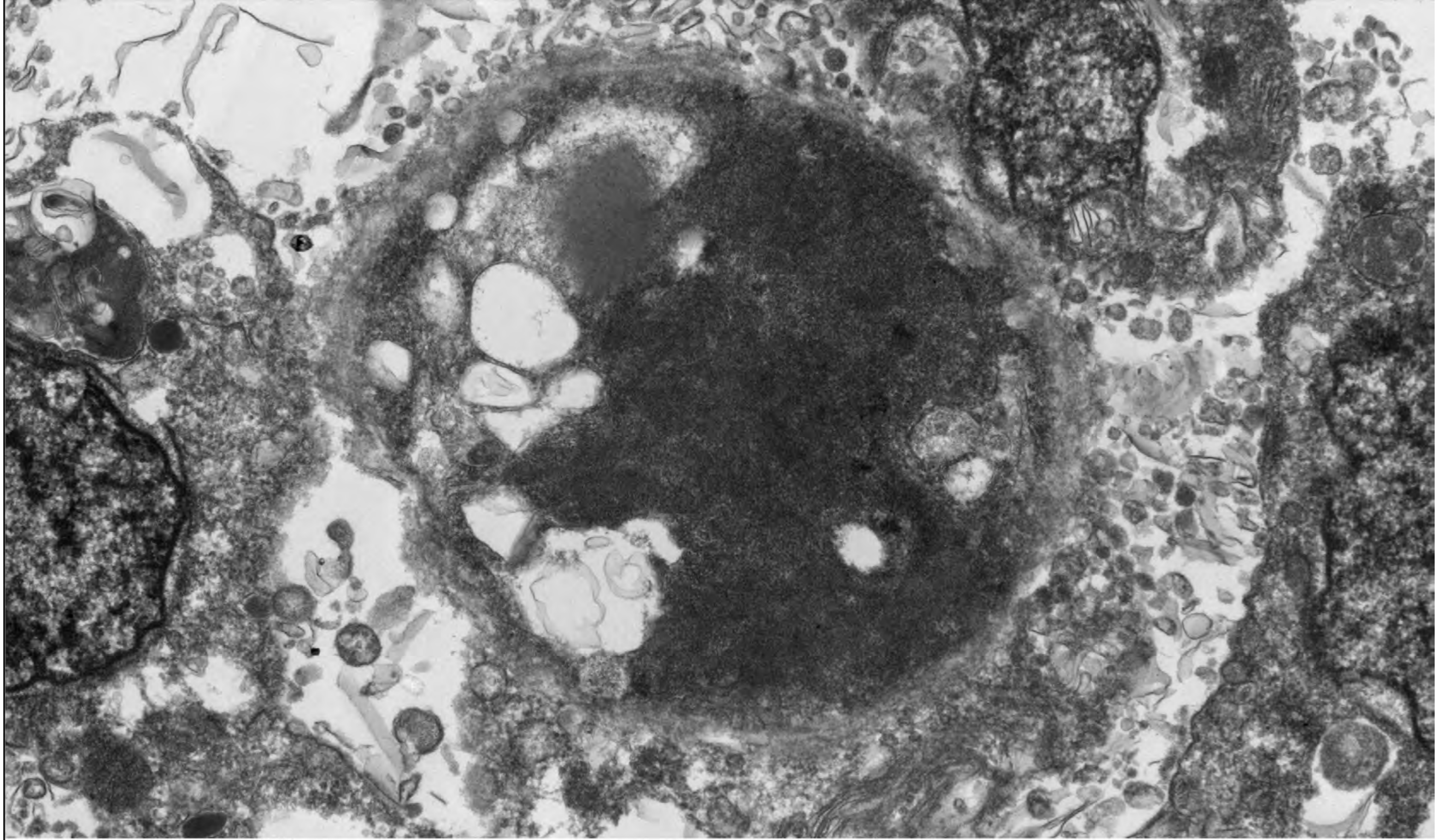

21-20_Correa_3-18-3_16H1_039.tif
Sample: 3/18, 3
Biological Electron Microscopy Lab
Rice University - SEA
Microscopist: MD Meyer

1 µm
HV=80kV
Direct Mag: 4000 x

Cell 11

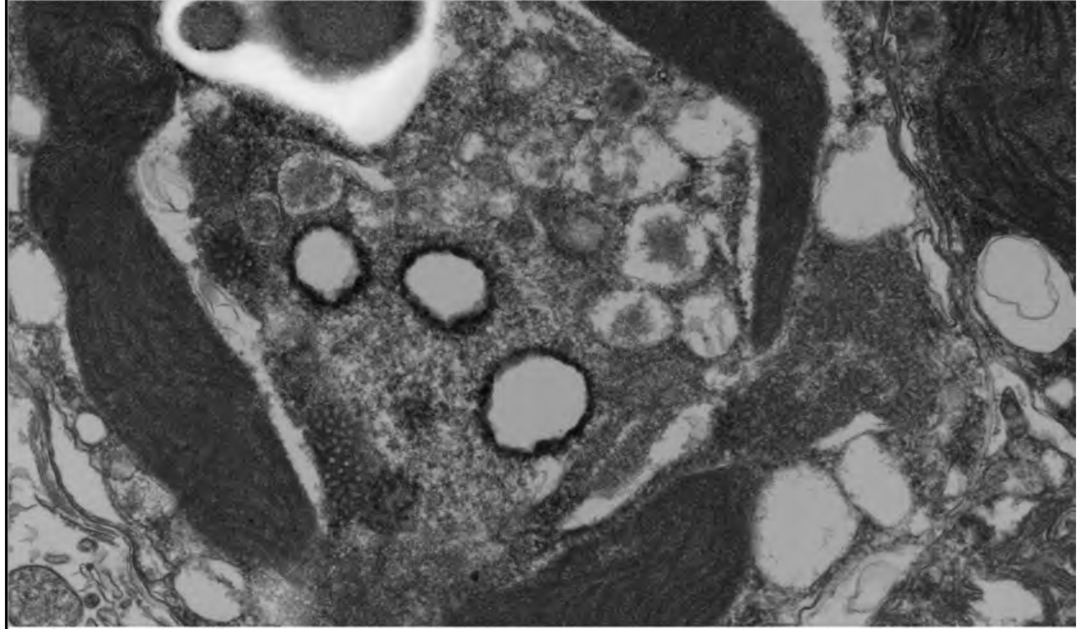

21-20_Correa_3-18-3_16H1_046.tif
Sample: 3/18, 3
Biological Electron Microscopy Lab
Rice University - SEA
Microscopist: MD Meyer

800 nm
HV=80kV
Direct Mag: 5000 x

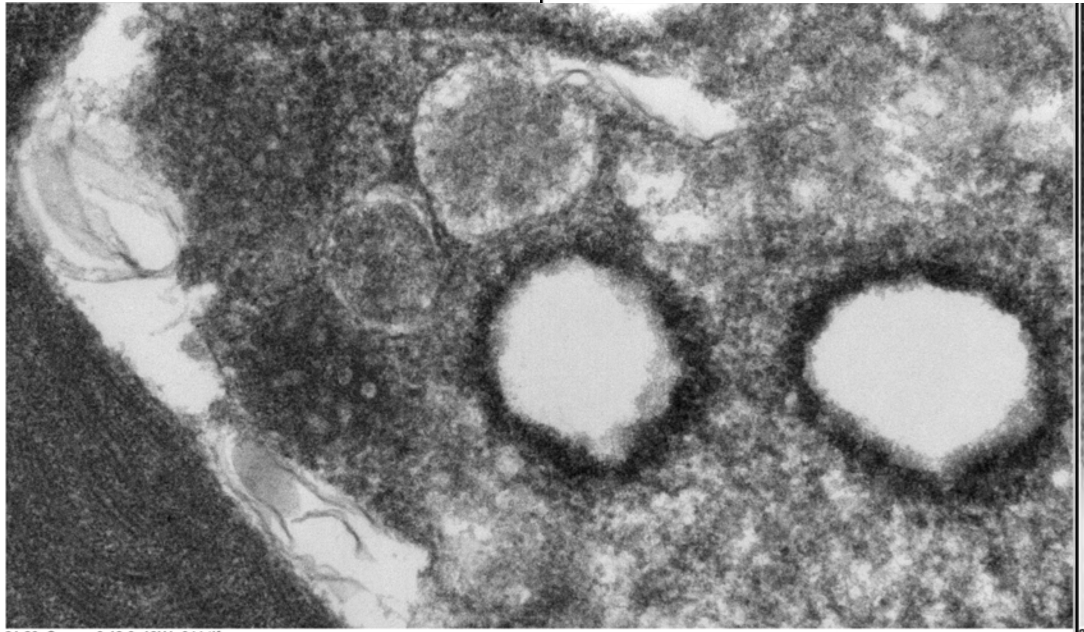

21-20_Correa_3-18-3_16H1_044.tif
Sample: 3/18, 3
Biological Electron Microscopy Lab
Rice University - SEA
Microscopist: MD Meyer

200 nm
HV=80kV
Direct Mag: 15000 x

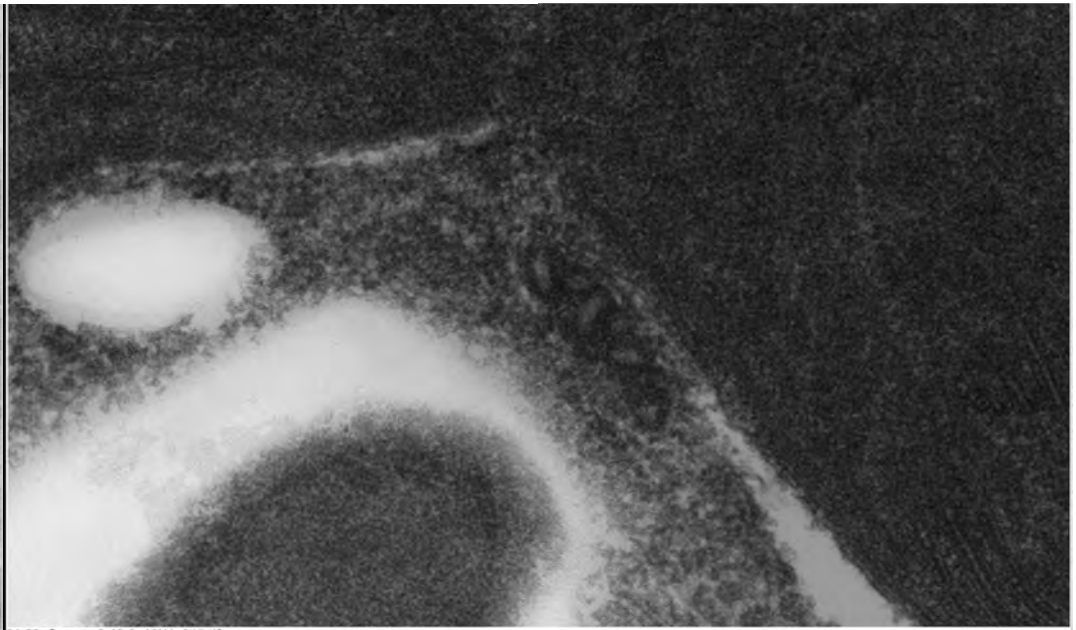

21-20_Correa_3-18-3_16H1_045.tif
Sample: 3/18, 3
Biological Electron Microscopy Lab
Rice University - SEA
Microscopist: MD Meyer

200 nm
HV=80kV
Direct Mag: 15000 x

Cell 11

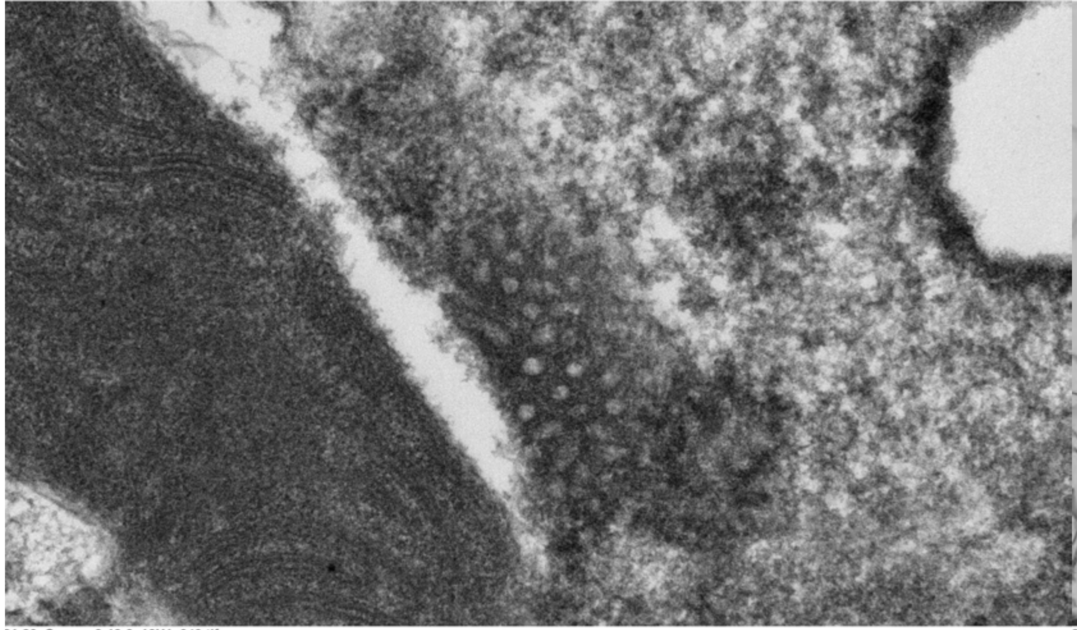

21-20_Correa_3-18-3_16H1_043.tif
Sample: 3/18, 3
Biological Electron Microscopy Lab
Rice University - SEA
Microscopist: MD Meyer

200 nm
HV=80kV
Direct Mag: 15000 x

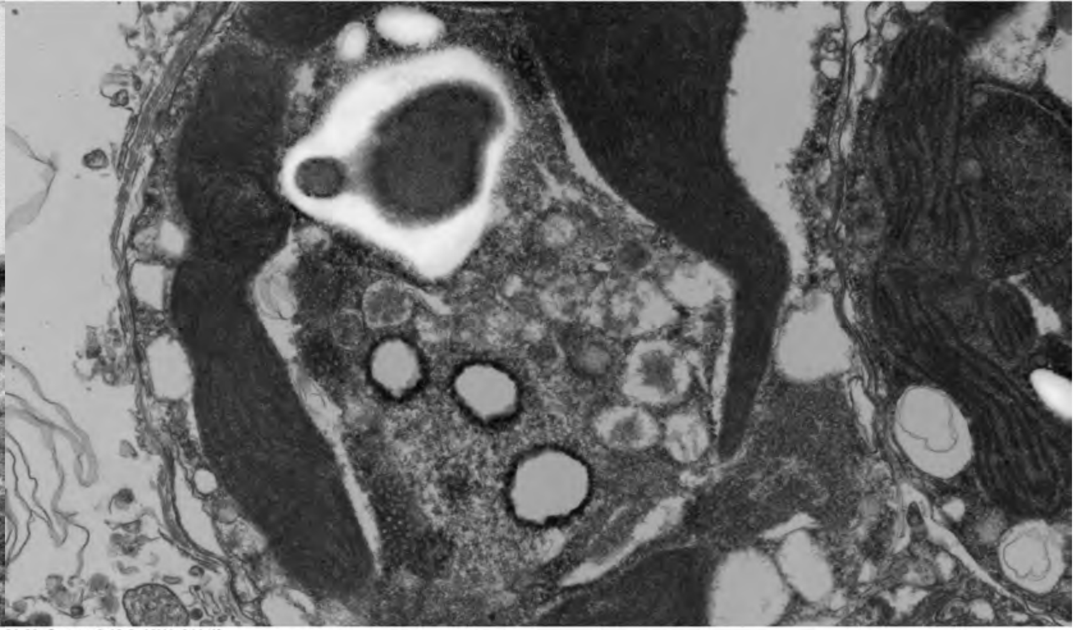

21-20_Correa_3-18-3_16H1_041.tif
Sample: 3/18, 3
Biological Electron Microscopy Lab
Rice University - SEA
Microscopist: MD Meyer

1 µm
HV=80kV
Direct Mag: 4000 x

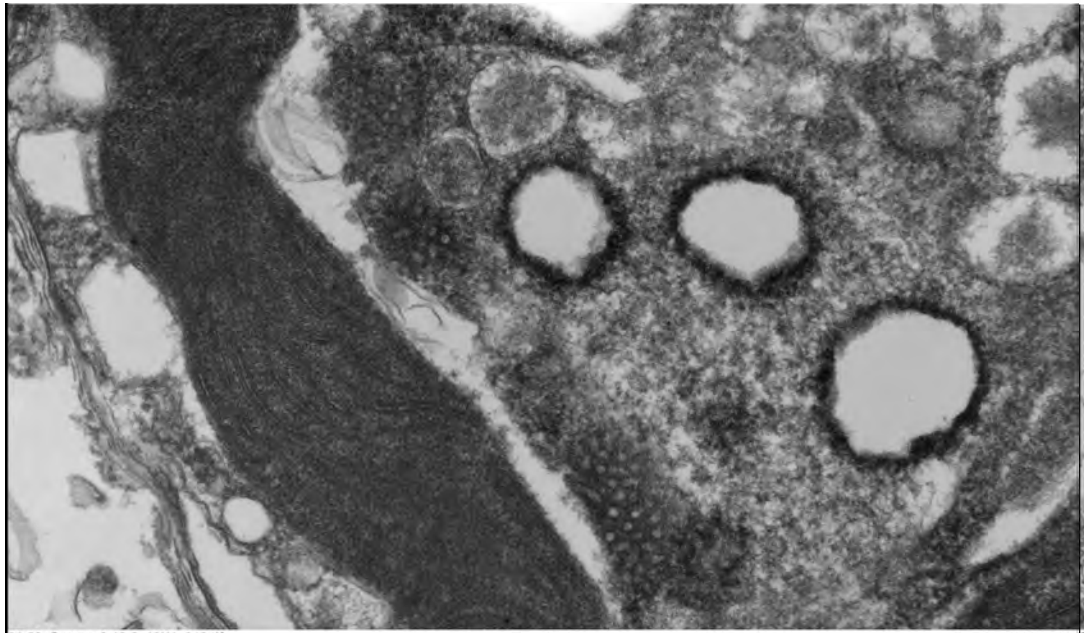

21-20_Correa_3-18-3_16H1_042.tif
Sample: 3/18, 3
Biological Electron Microscopy Lab
Rice University - SEA
Microscopist: MD Meyer

500 nm
HV=80kV
Direct Mag: 8000 x

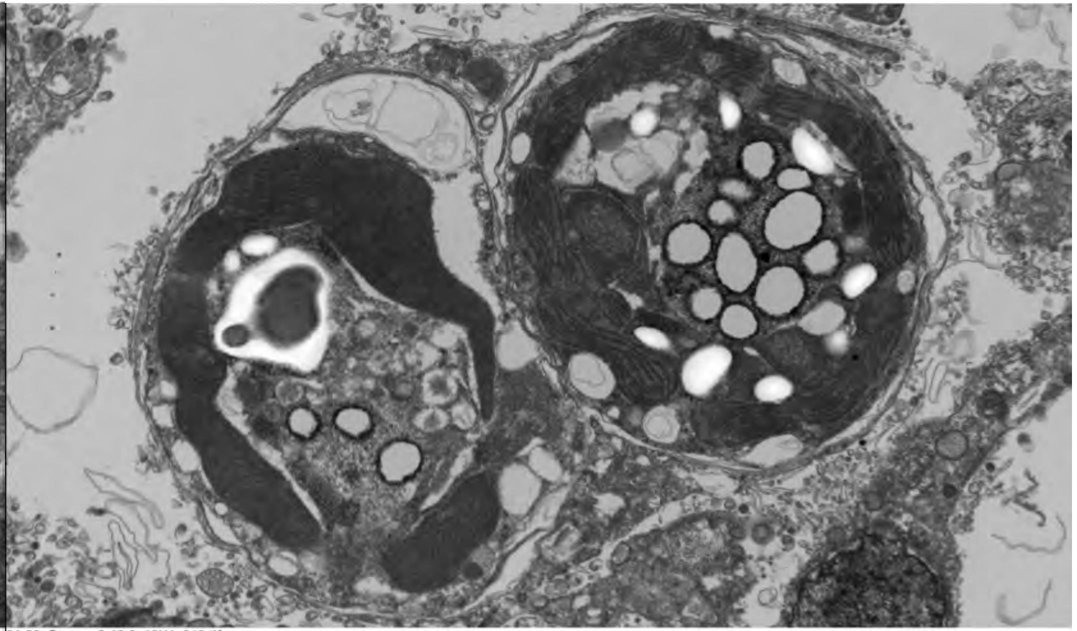

21-20_Correa_3-18-3_16H1_040.tif
Sample: 3/18, 3
Biological Electron Microscopy Lab
Rice University - SEA
Microscopist: MD Meyer

2 µm
HV=80kV
Direct Mag: 2000 x

Cell 12

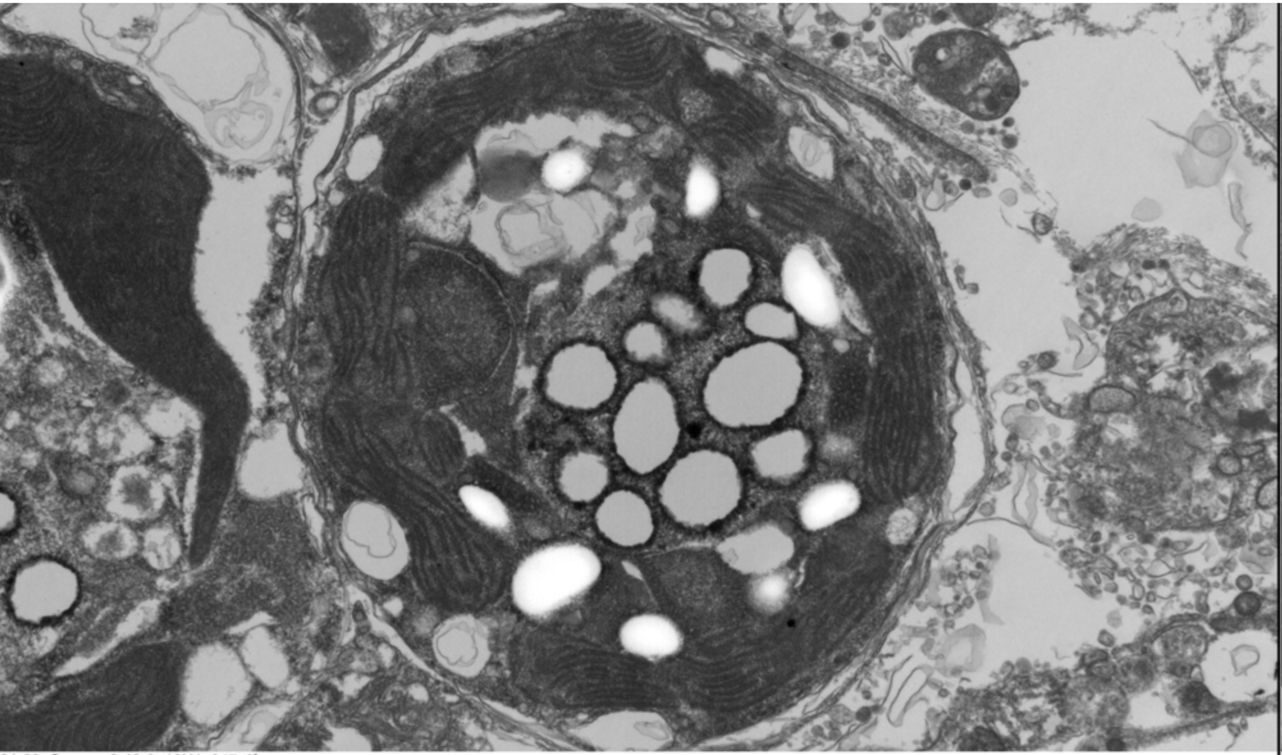

21-20_Correa_3-18-3_16H1_047.tif
Sample: 3/18, 3
Biological Electron Microscopy Lab
Rice University - SEA
Microscopist: MD Meyer

1 µm
HV=80kV
Direct Mag: 3000 x

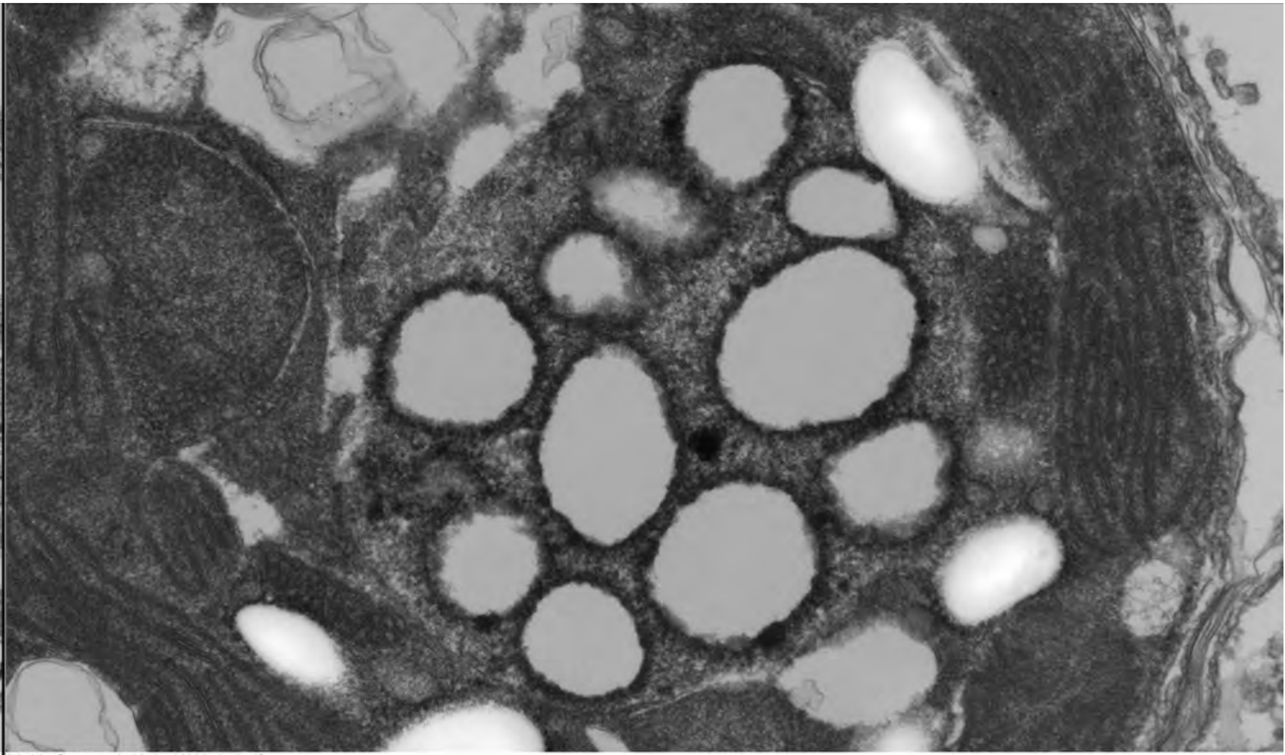

21-20_Correa_3-18-3_16H1_049.tif
Sample: 3/18, 3
Biological Electron Microscopy Lab
Rice University - SEA
Microscopist: MD Meyer

600 nm
HV=80kV
Direct Mag: 6000 x

Cell 13

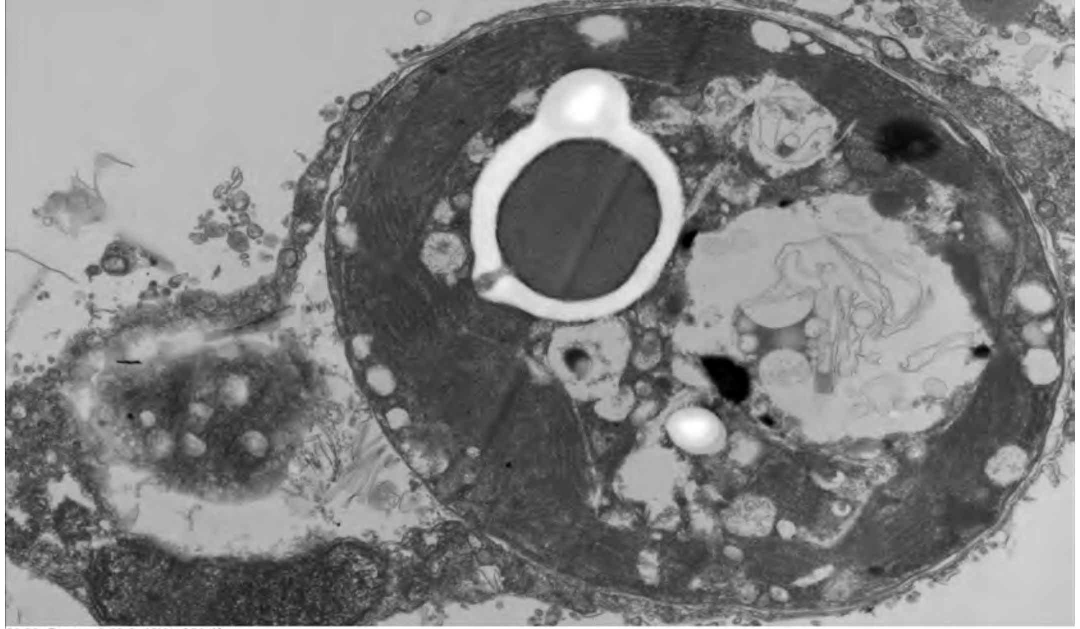

21-20_Correa_3-18-3_16H1_051.tif
Sample: 3/18, 3
Biological Electron Microscopy Lab
Rice University - SEA
Microscopist: MD Meyer

1 μm
HV=80kV
Direct Mag: 3000 x

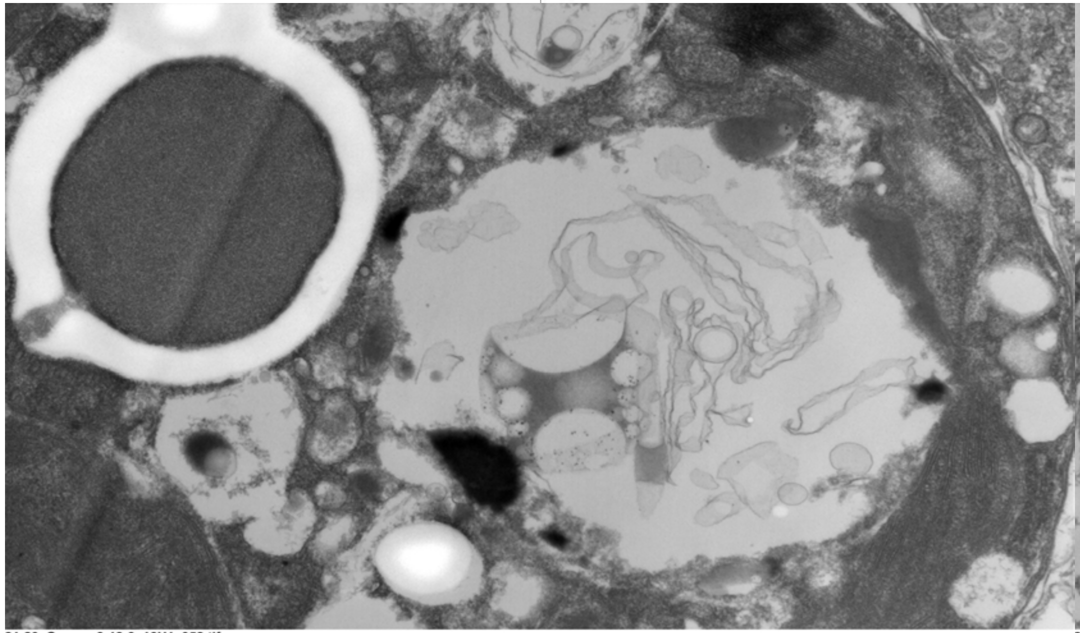

21-20_Correa_3-18-3_16H1_052.tif
Sample: 3/18, 3
Biological Electron Microscopy Lab
Rice University - SEA
Microscopist: MD Meyer

800 nm
HV=80kV
Direct Mag: 5000 x

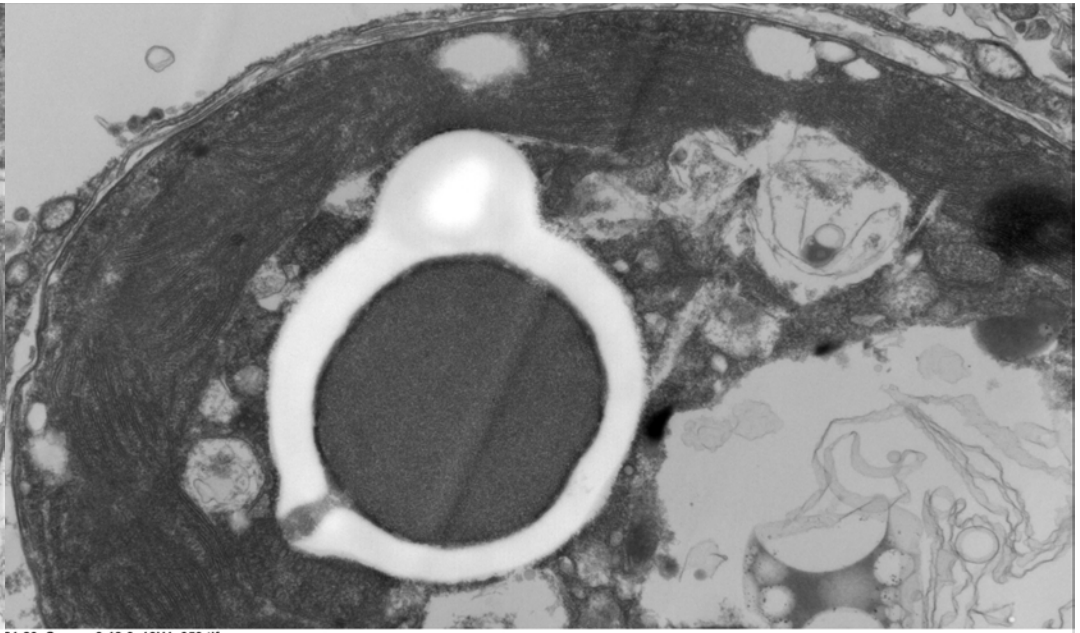

21-20_Correa_3-18-3_16H1_053.tif
Sample: 3/18, 3
Biological Electron Microscopy Lab
Rice University - SEA
Microscopist: MD Meyer

800 nm
HV=80kV
Direct Mag: 5000 x

Cell 14

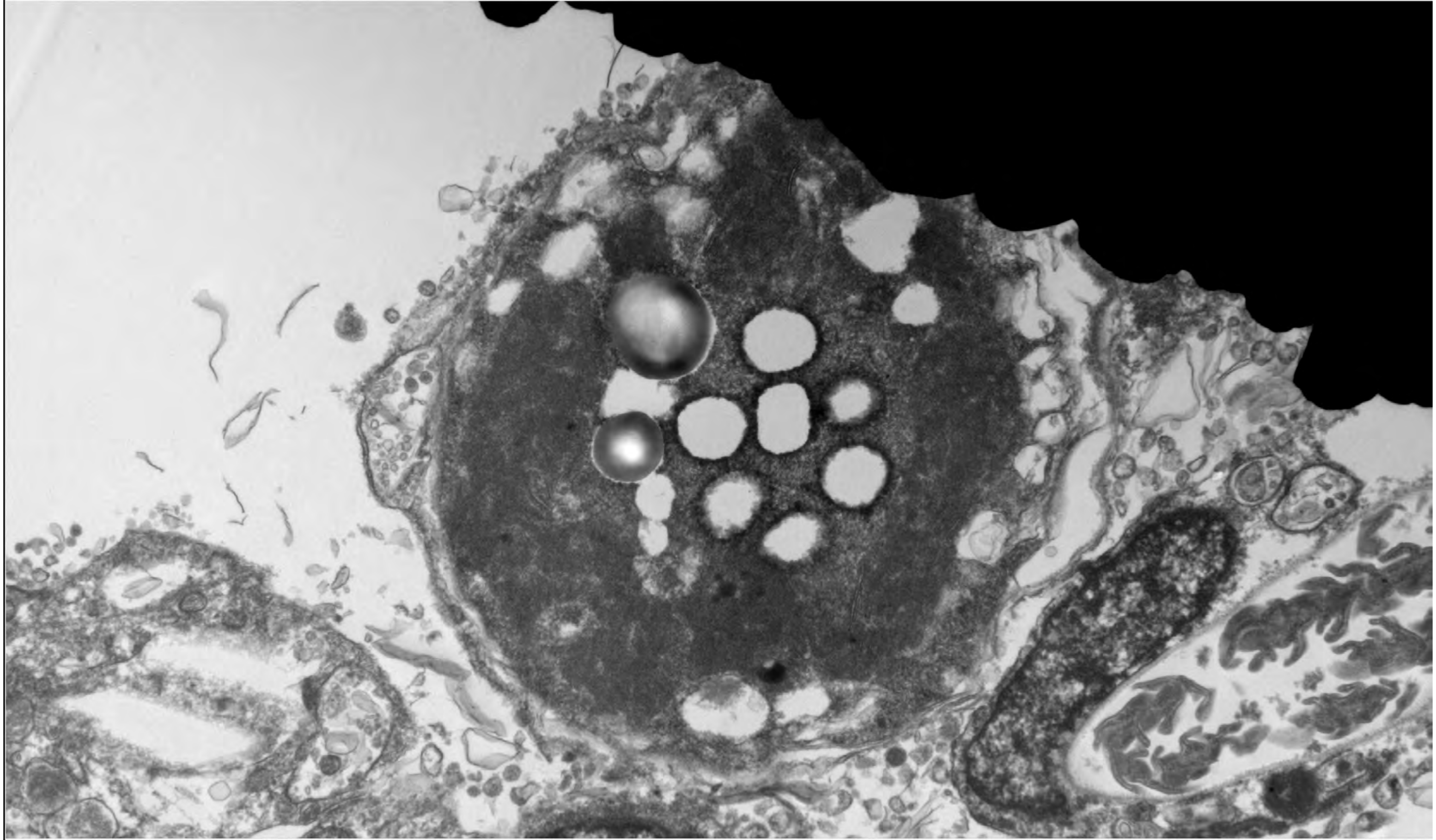

21-20_Correa_3-18-3_16H1_054.tif
Sample: 3/18, 3
Biological Electron Microscopy Lab
Rice University - SEA
Microscopist: MD Meyer

1 µm
HV=80kV
Direct Mag: 3000 x

Cell 15

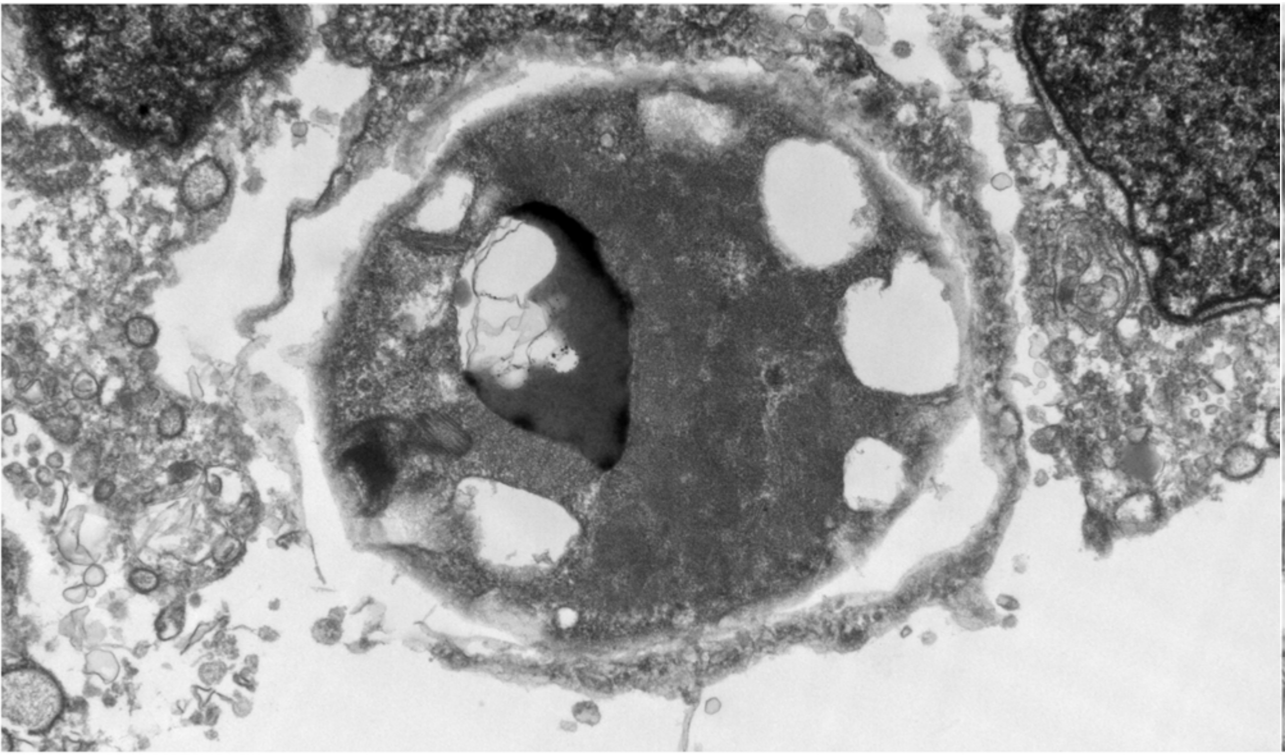

21-20_Correa_3-18-3_16H1_055.tif
Sample: 3/18, 3
Biological Electron Microscopy Lab
Rice University - SEA
Microscopist: MD Meyer

1 µm
HV=80kV
Direct Mag: 4000 x

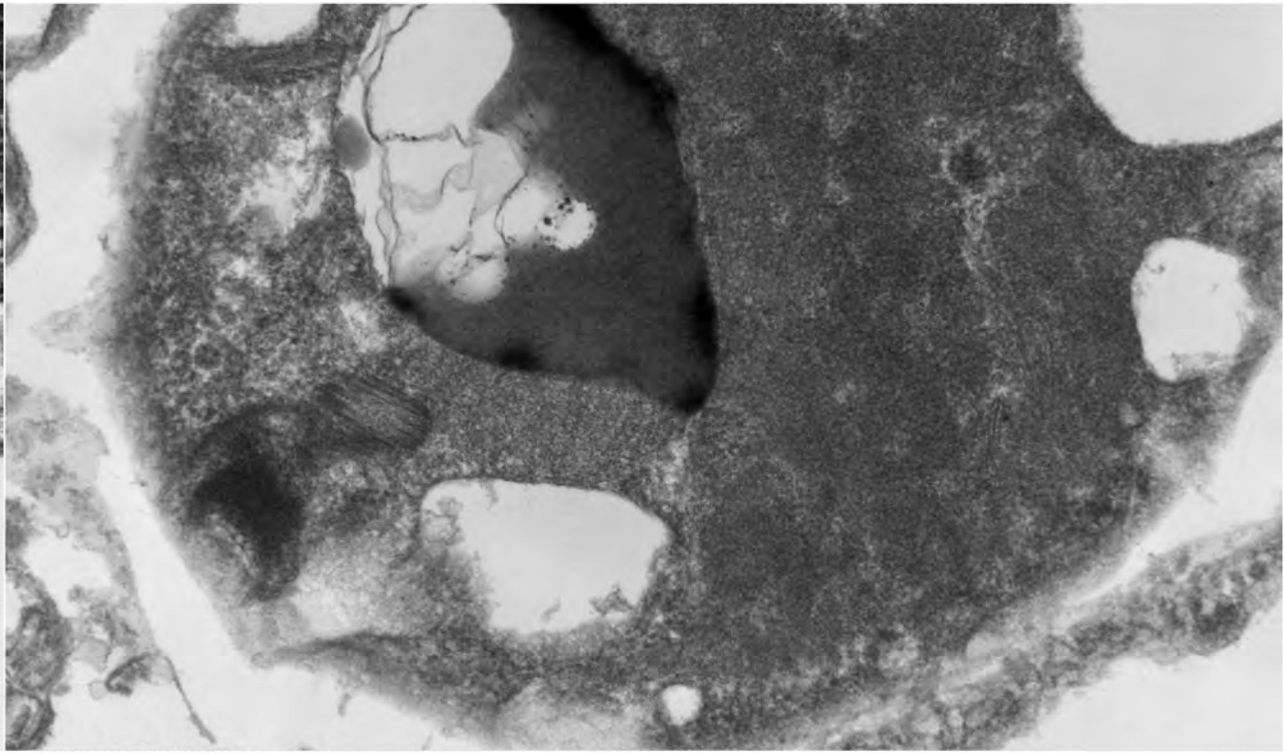

21-20_Correa_3-18-3_16H1_056.tif
Sample: 3/18, 3
Biological Electron Microscopy Lab
Rice University - SEA
Microscopist: MD Meyer

500 nm
HV=80kV
Direct Mag: 8000 x

Cell 16

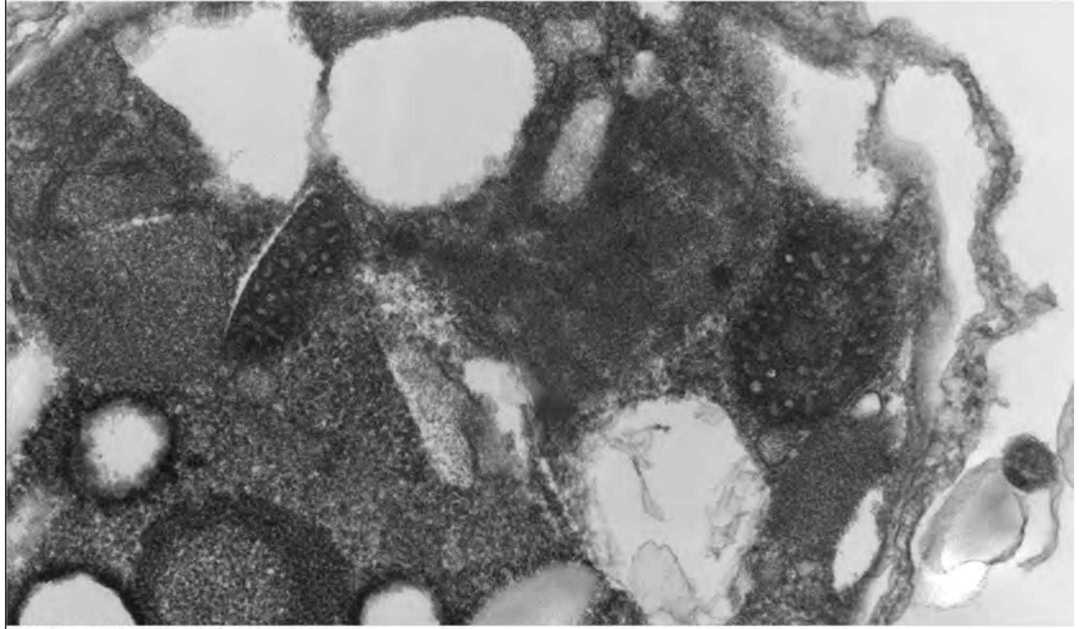

21-20_Correa_3-18-3_16H1_059.tif
Sample: 3/18, 3
Biological Electron Microscopy Lab
Rice University - SEA
Microscopist: MD Meyer

500 nm
HV=80kV
Direct Mag: 8000 x

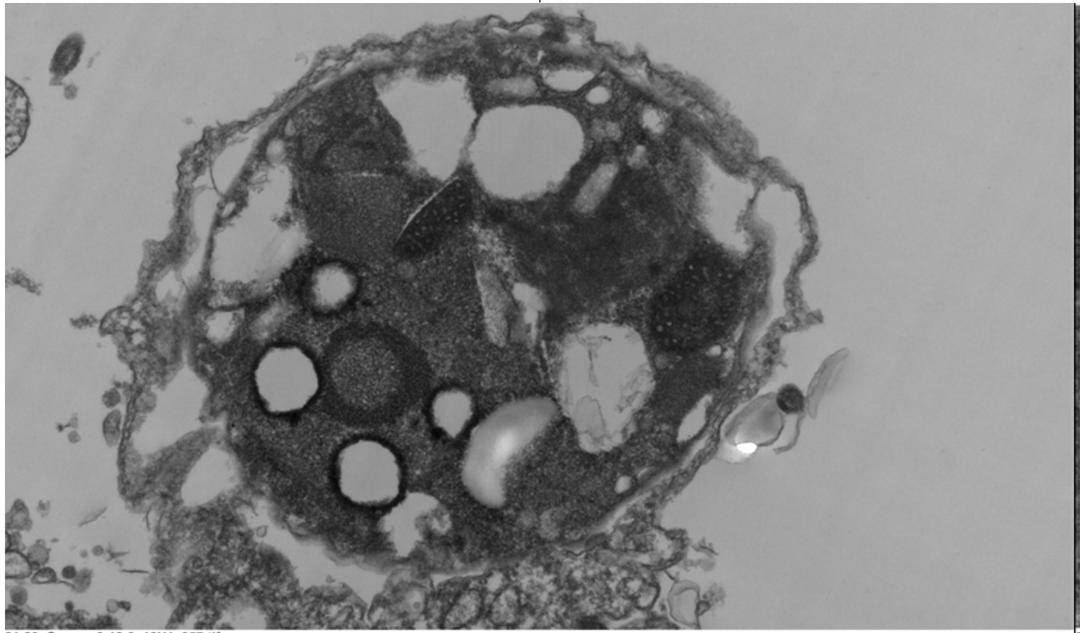

21-20_Correa_3-18-3_16H1_057.tif
Sample: 3/18, 3
Biological Electron Microscopy Lab
Rice University - SEA
Microscopist: MD Meyer

1 µm
HV=80kV
Direct Mag: 4000 x

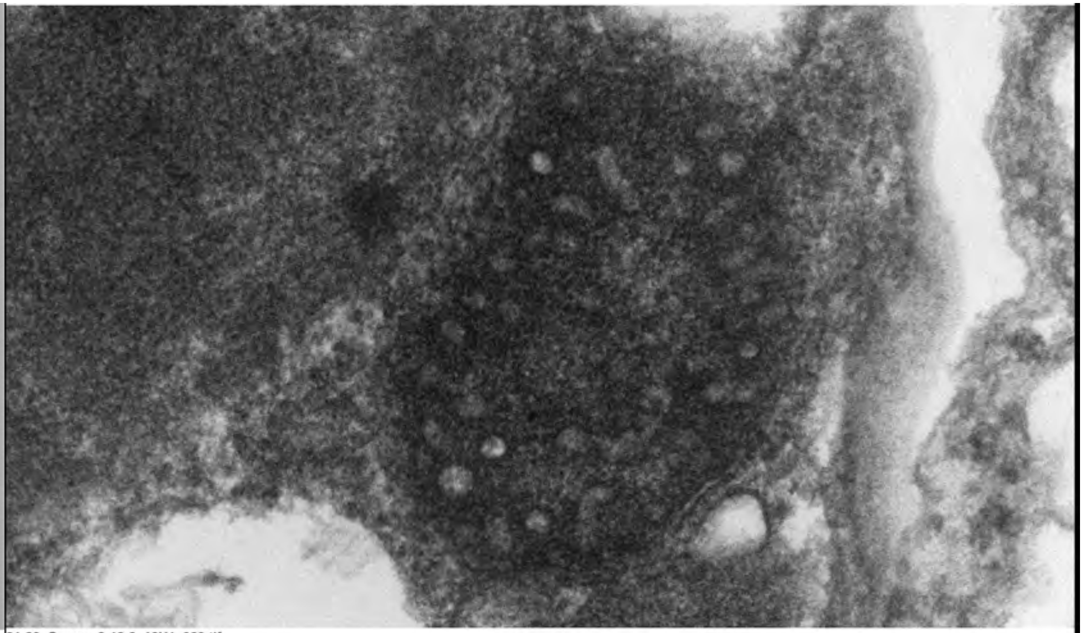

21-20_Correa_3-18-3_16H1_060.tif
Sample: 3/18, 3
Biological Electron Microscopy Lab
Rice University - SEA
Microscopist: MD Meyer

200 nm
HV=80kV
Direct Mag: 20000 x

Cell 17

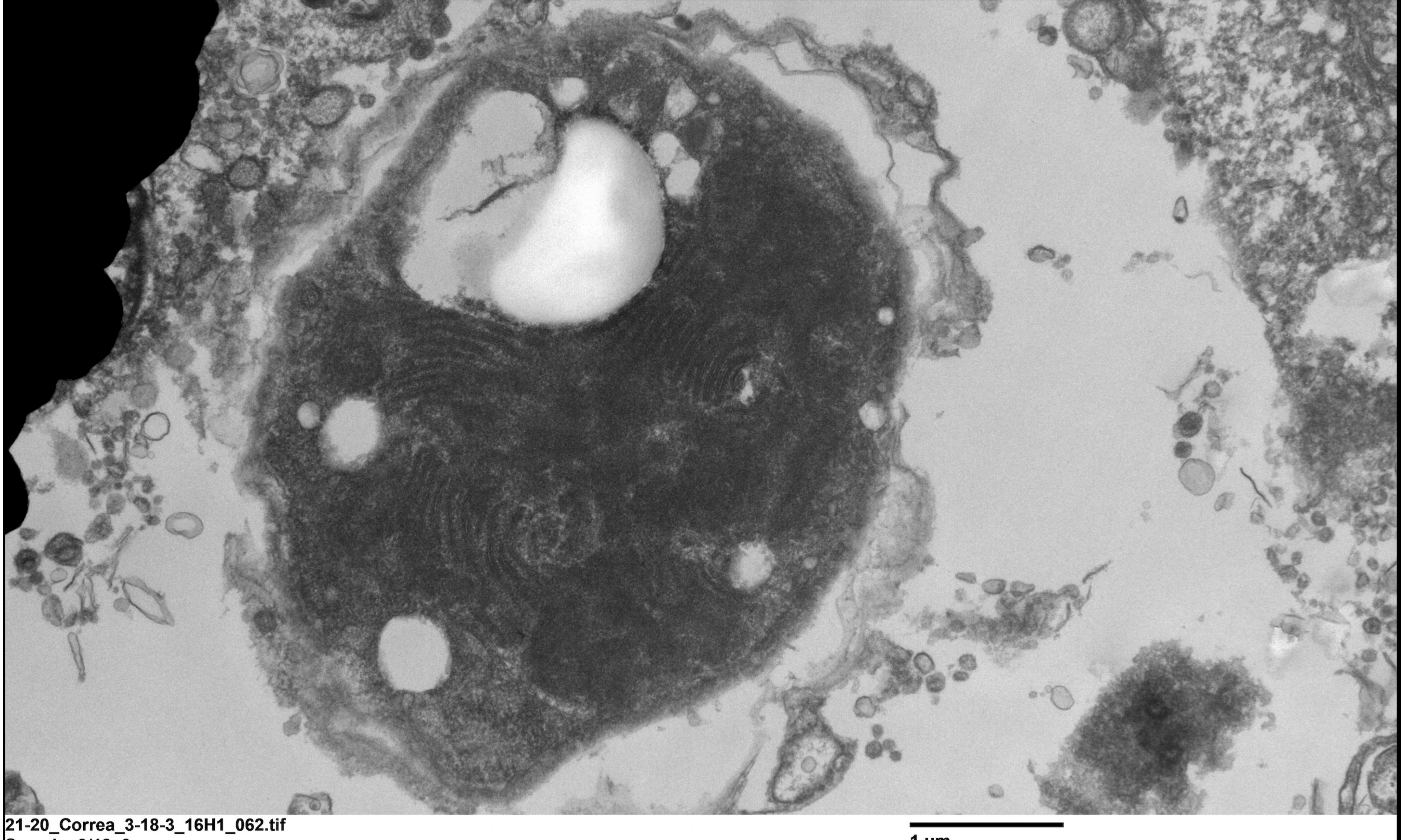

21-20_Correa_3-18-3_16H1_062.tif
Sample: 3/18, 3
Biological Electron Microscopy Lab
Rice University - SEA
Microscopist: MD Meyer

1 µm
HV=80kV
Direct Mag: 4000 x

Cell 18

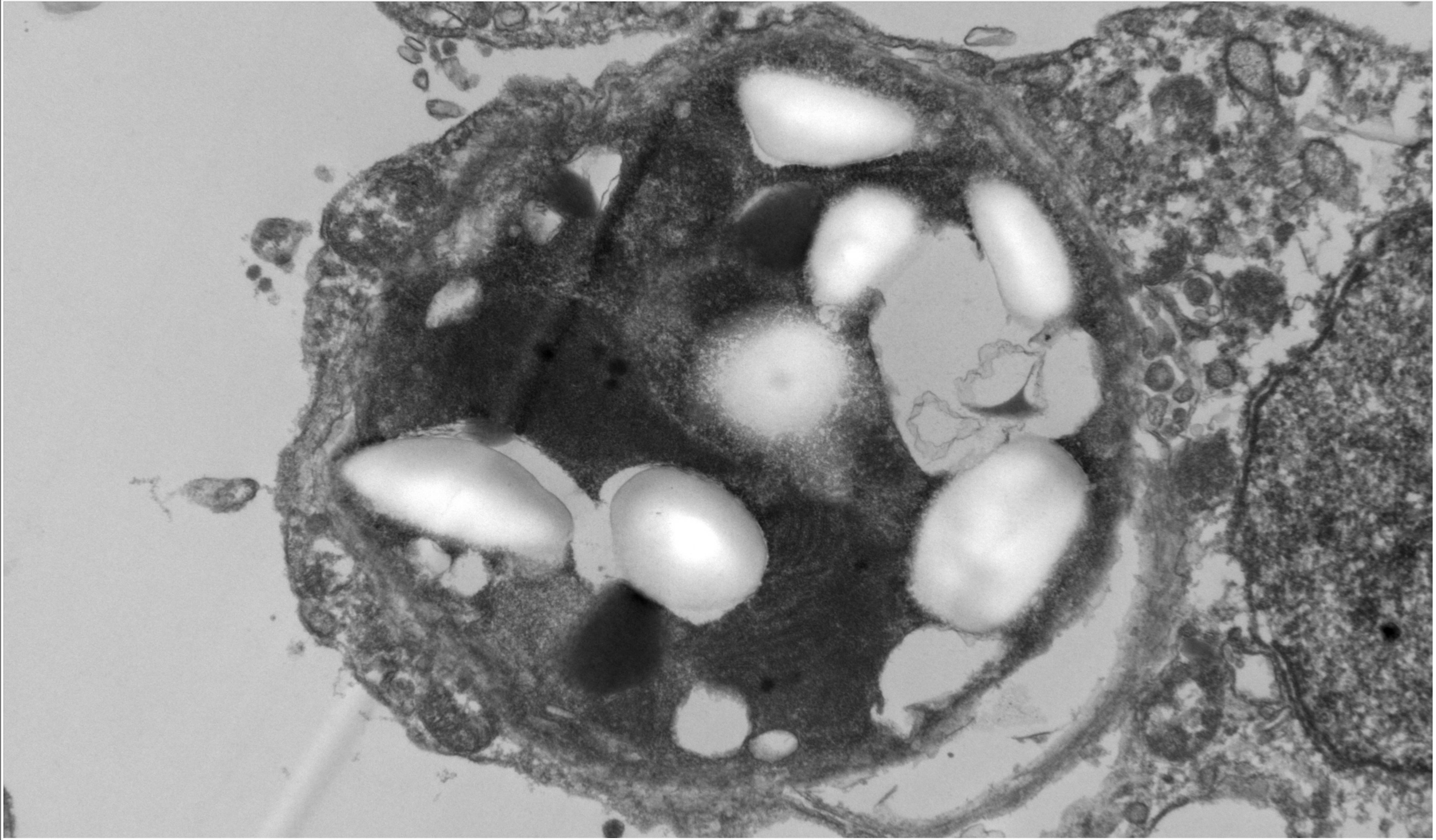

21-20_Correa_3-18-3_16H1_063.tif
Sample: 3/18, 3
Biological Electron Microscopy Lab
Rice University - SEA
Microscopist: MD Meyer

1 µm
HV=80kV
Direct Mag: 4000 x

Cell 19

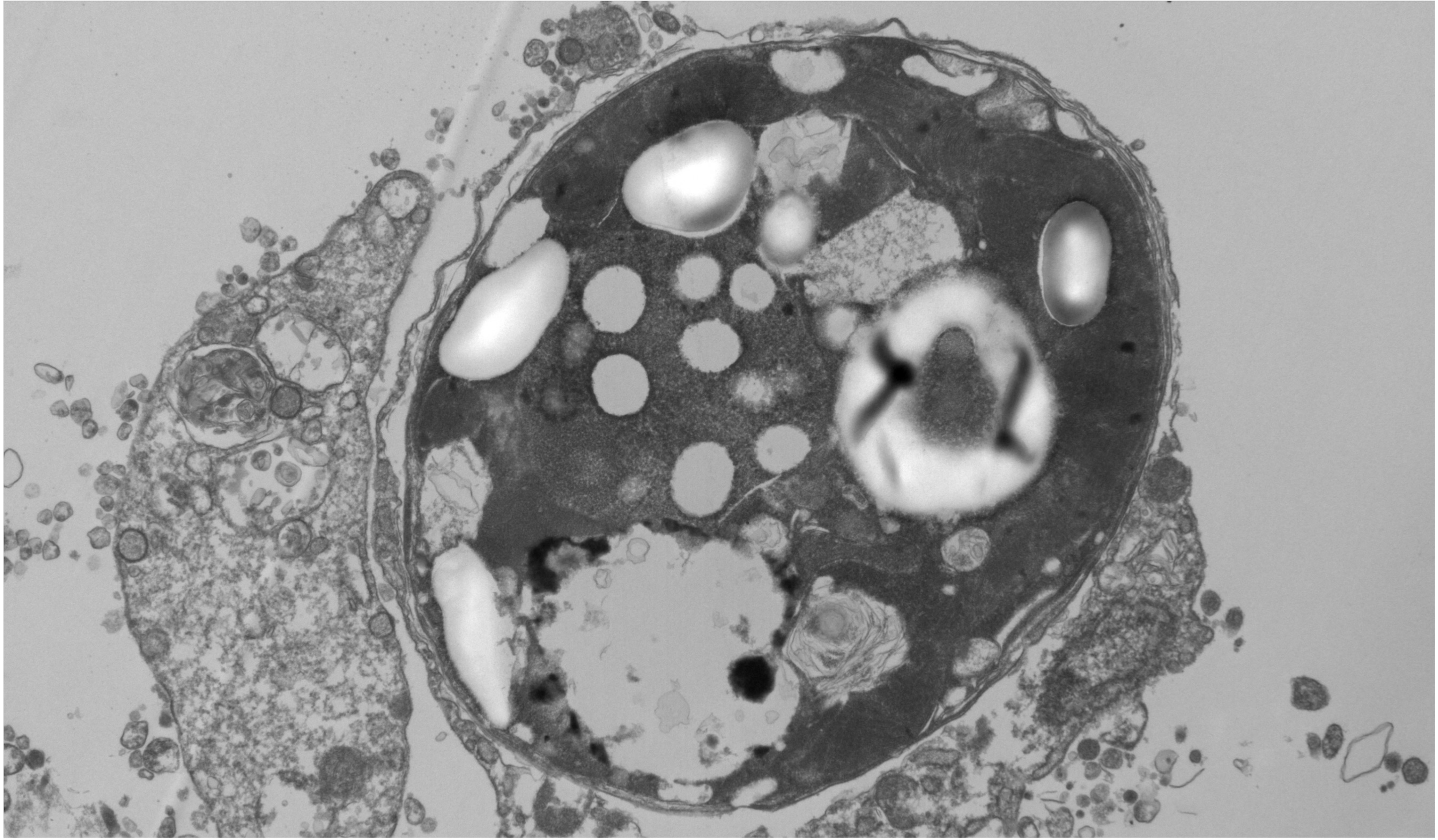

21-20_Correa_3-18-3_16H1_064.tif
Sample: 3/18, 3
Biological Electron Microscopy Lab
Rice University - SEA
Microscopist: MD Meyer

1 µm
HV=80kV
Direct Mag: 2500 x

Cell 20

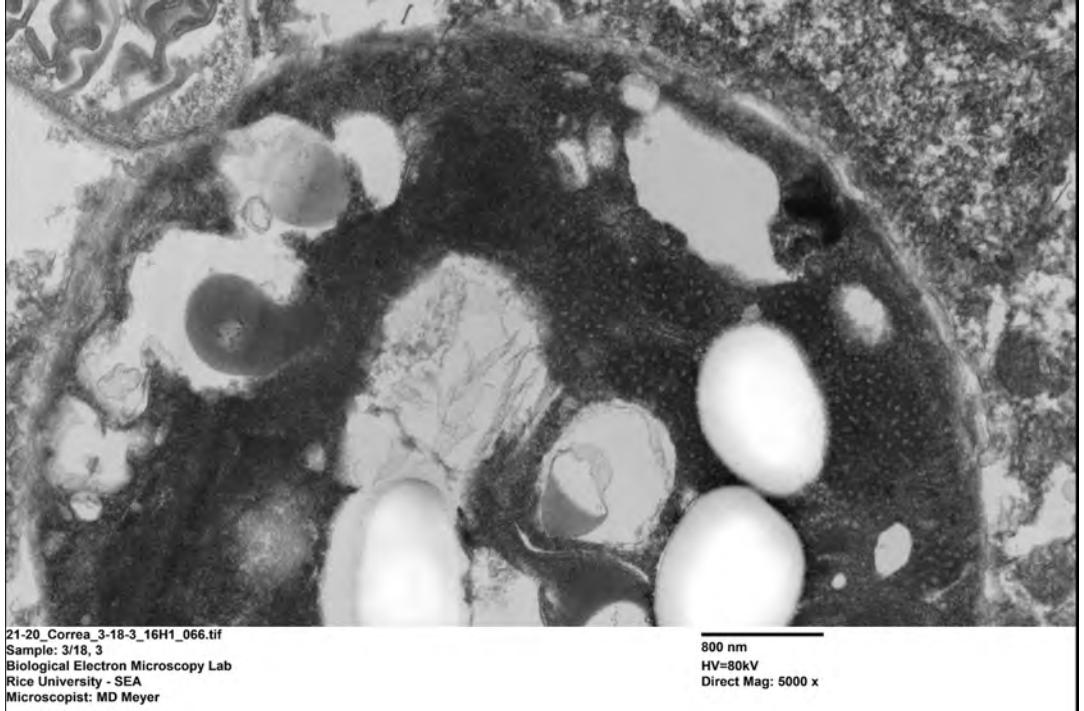
21-20_Correa_3-18-3_16H1_066.tif
Sample: 3/18, 3
Biological Electron Microscopy Lab
Rice University - SEA
Microscopist: MD Meyer
800 nm
HV=80kV
Direct Mag: 5000 x

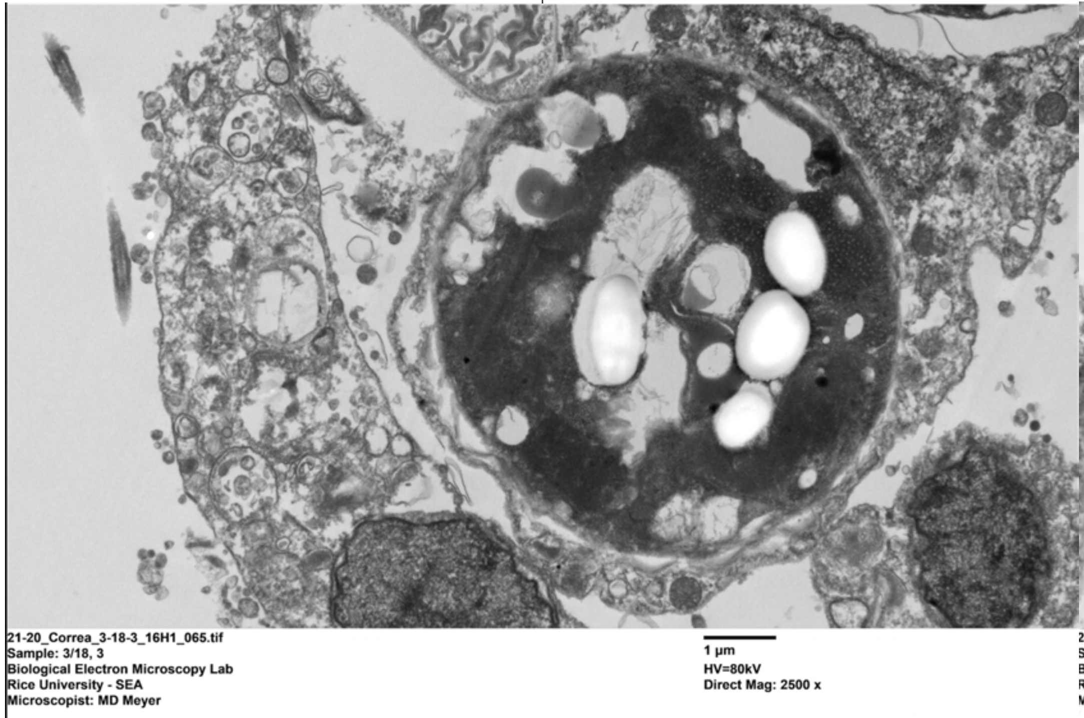
21-20_Correa_3-18-3_16H1_065.tif
Sample: 3/18, 3
Biological Electron Microscopy Lab
Rice University - SEA
Microscopist: MD Meyer
1 µm
HV=80kV
Direct Mag: 2500 x

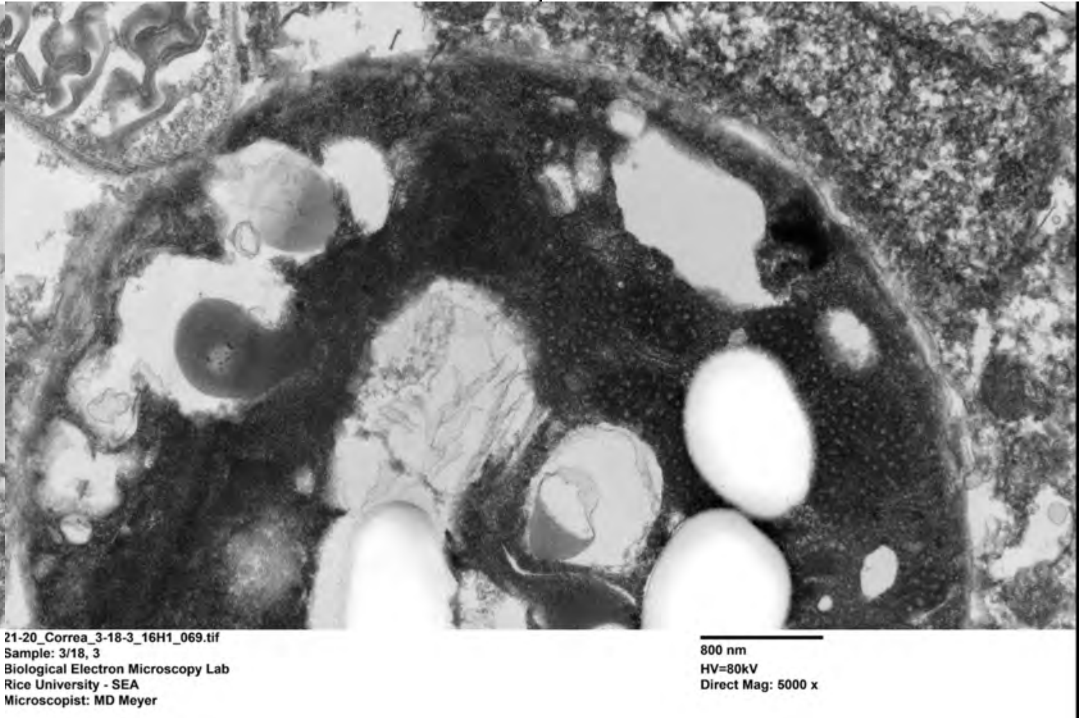
21-20_Correa_3-18-3_16H1_069.tif
Sample: 3/18, 3
Biological Electron Microscopy Lab
Rice University - SEA
Microscopist: MD Meyer
800 nm
HV=80kV
Direct Mag: 5000 x

Cell 21

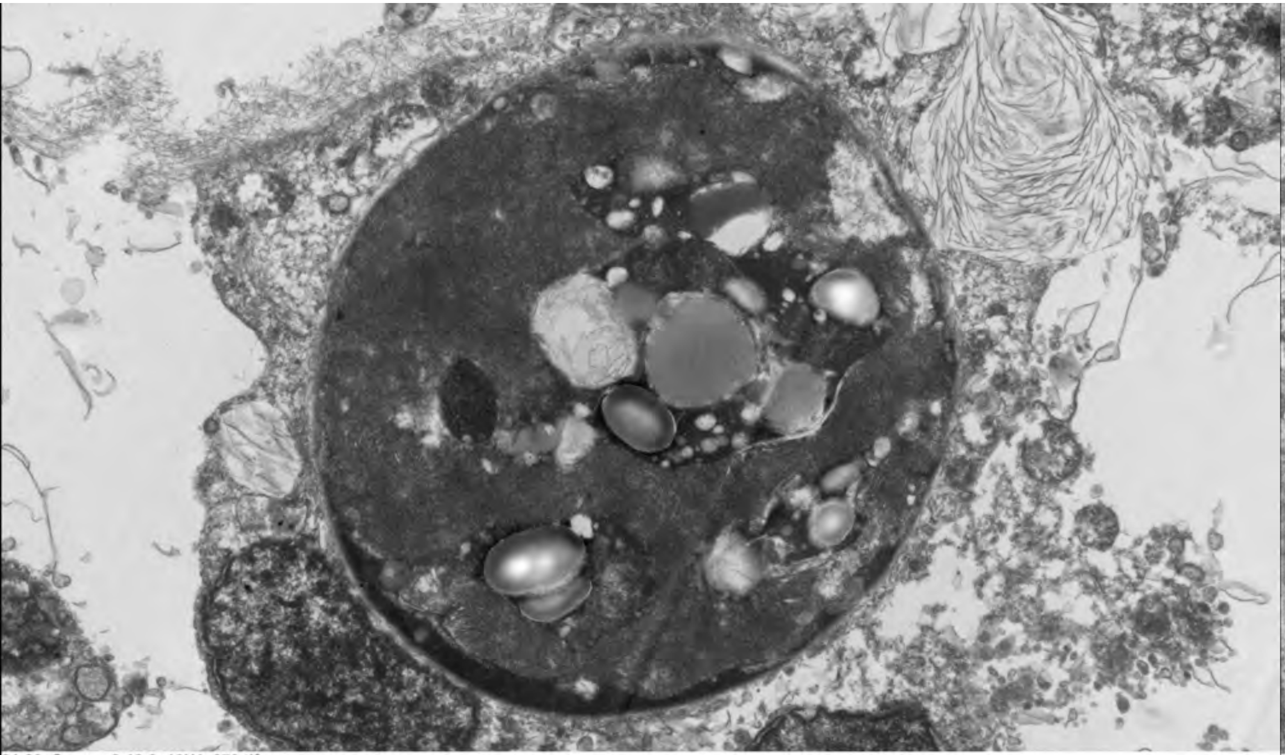

21-20_Correa_3-18-3_16H1_070.tif
Sample: 3/18, 3
Biological Electron Microscopy Lab
Rice University - SEA
Microscopist: MD Meyer

1 µm
HV=80kV
Direct Mag: 2500 x

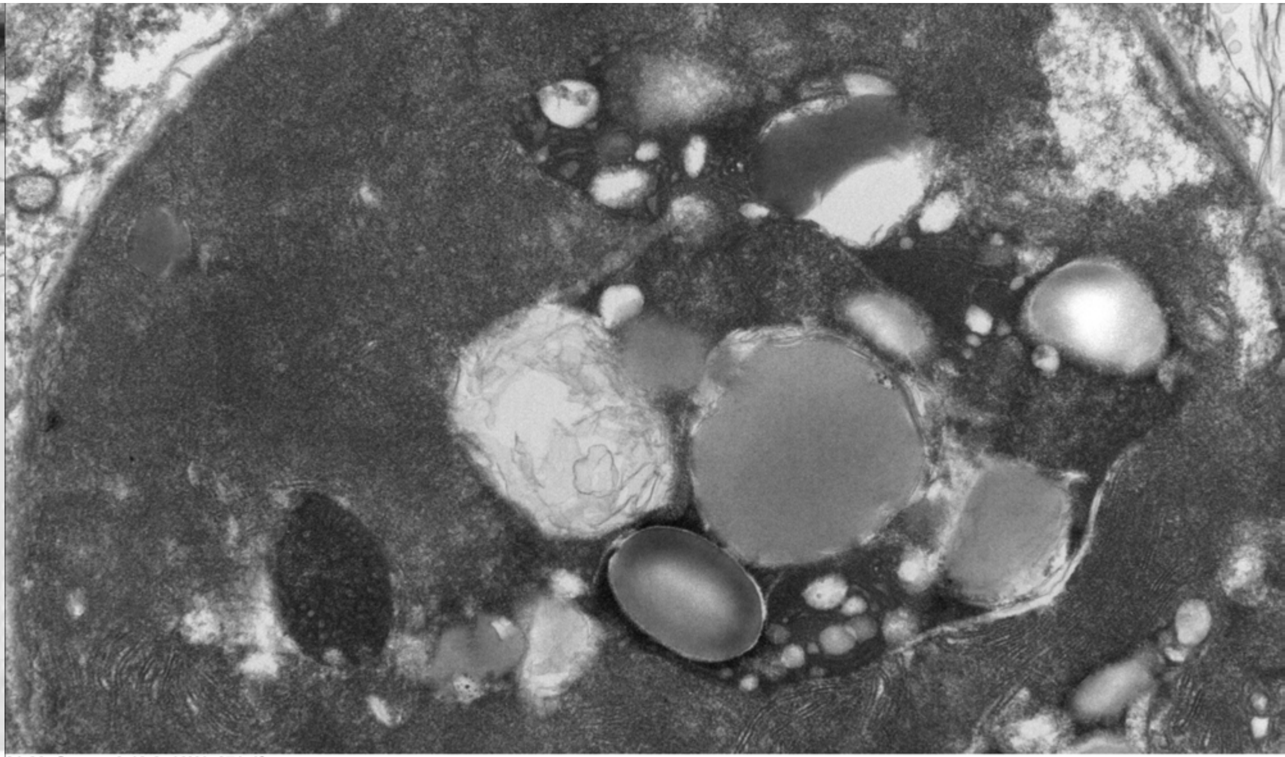

21-20_Correa_3-18-3_16H1_071.tif
Sample: 3/18, 3
Biological Electron Microscopy Lab
Rice University - SEA
Microscopist: MD Meyer

800 nm
HV=80kV
Direct Mag: 5000 x

Cell 22

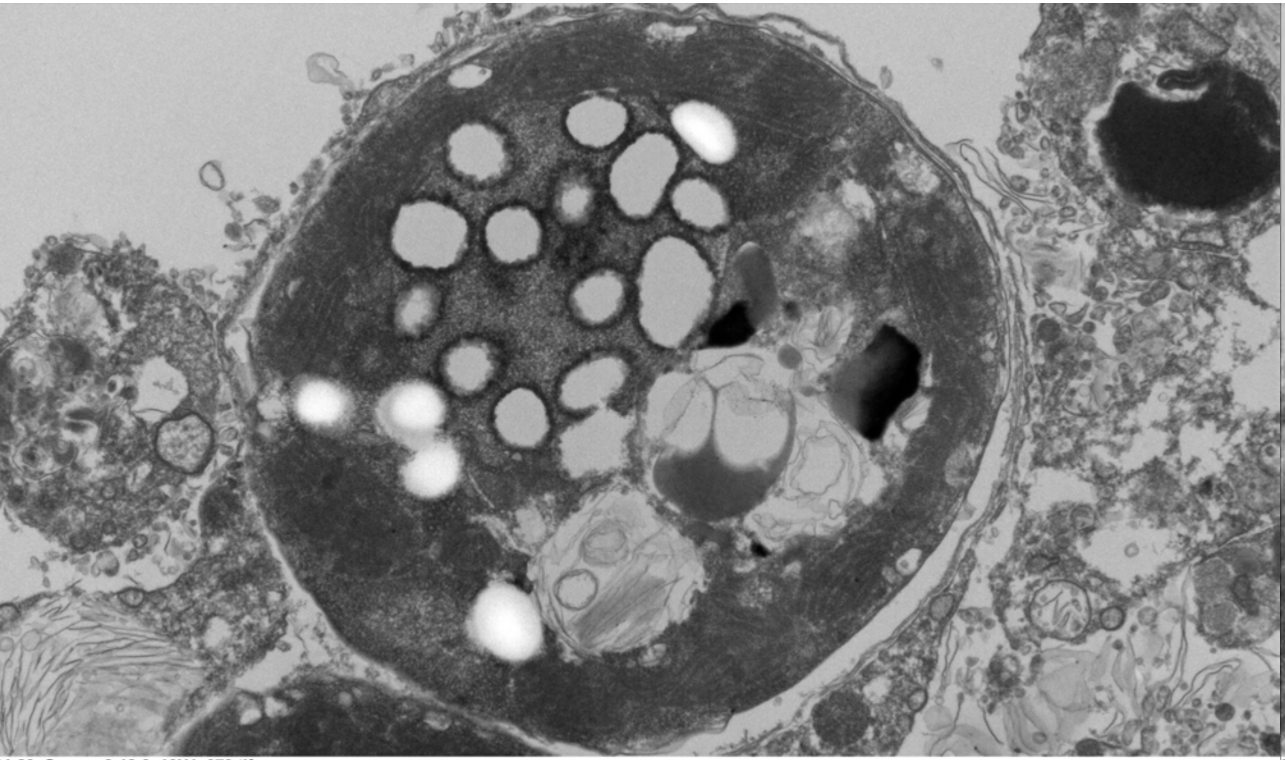

21-20_Correa_3-18-3_16H1_073.tif
Sample: 3/18, 3
Biological Electron Microscopy Lab
Rice University - SEA
Microscopist: MD Meyer

1 µm
HV=80kV
Direct Mag: 3000 x

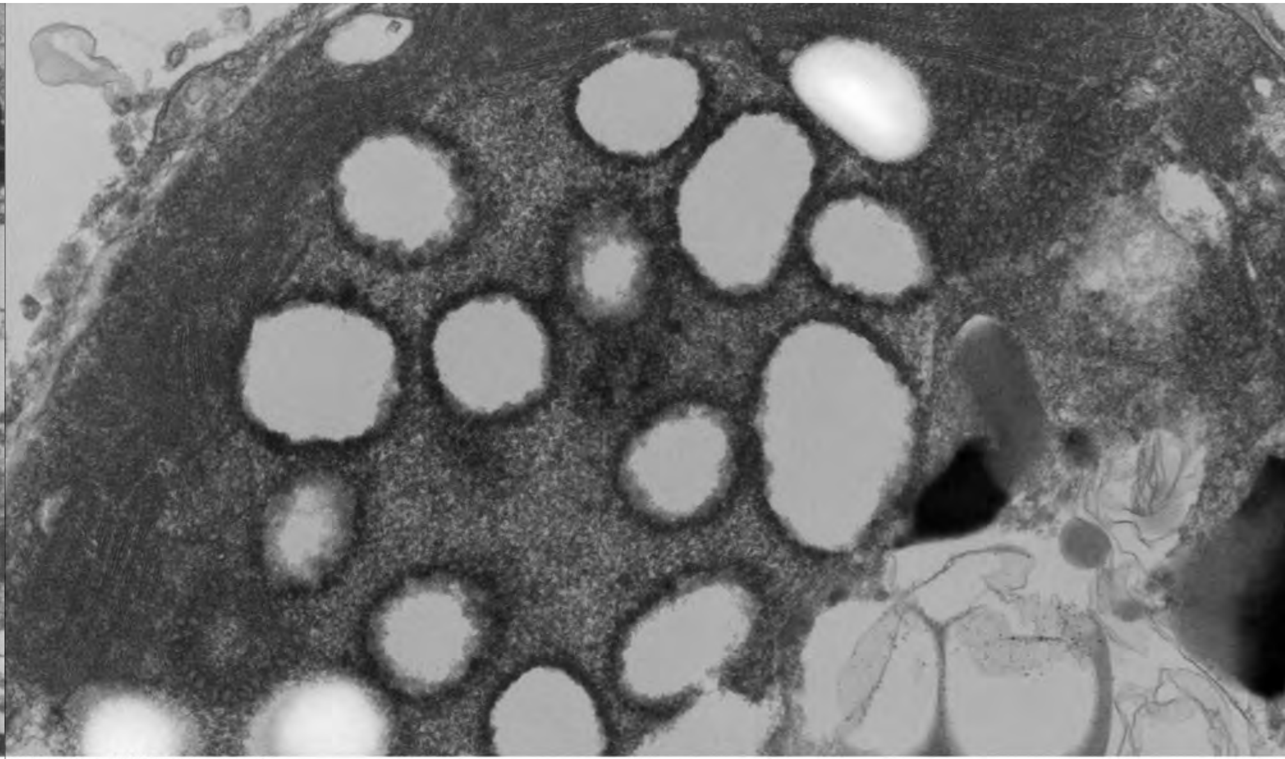

21-20_Correa_3-18-3_16H1_075.tif
Sample: 3/18, 3
Biological Electron Microscopy Lab
Rice University - SEA
Microscopist: MD Meyer

600 nm
HV=80kV
Direct Mag: 6000 x

Cell 23

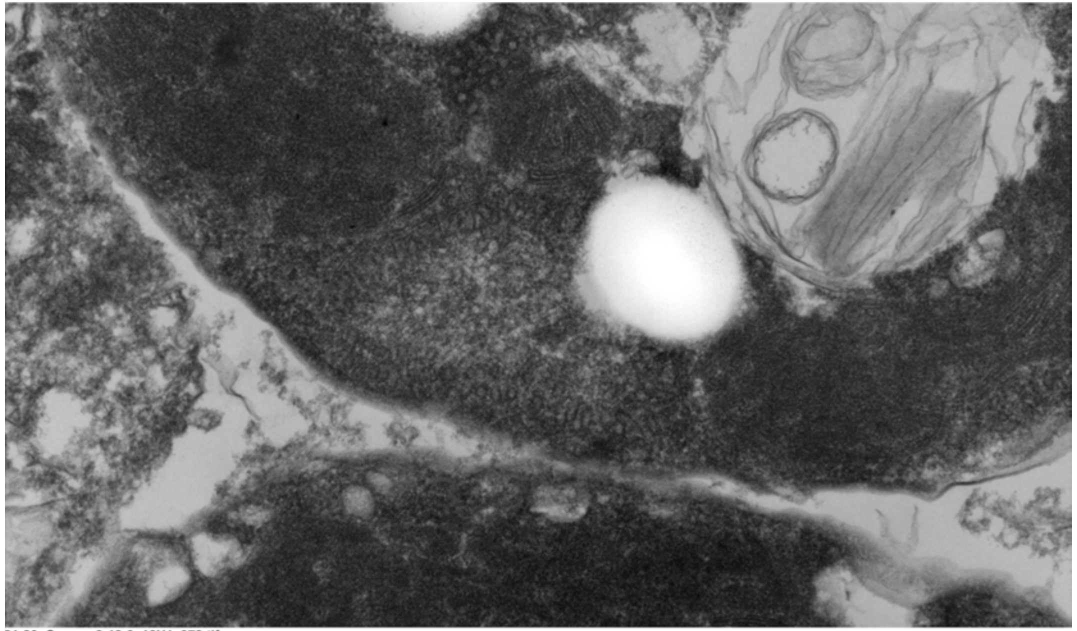

21-20_Correa_3-18-3_16H1_078.tif
Sample: 3/18, 3
Biological Electron Microscopy Lab
Rice University - SEA
Microscopist: MD Meyer

500 nm
HV=80kV
Direct Mag: 8000 x

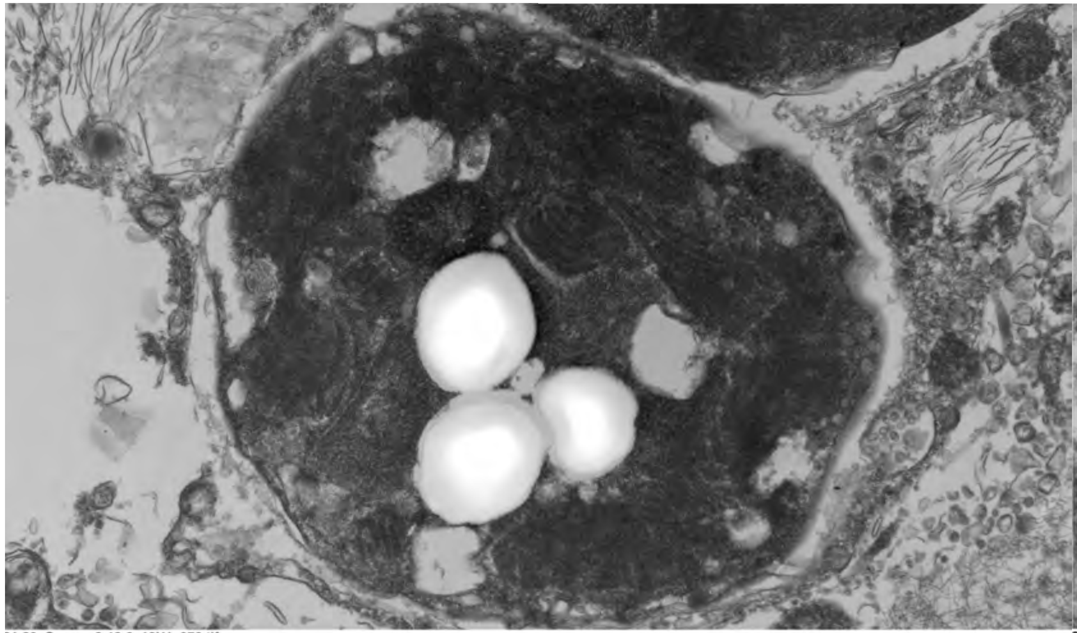

21-20_Correa_3-18-3_16H1_076.tif
Sample: 3/18, 3
Biological Electron Microscopy Lab
Rice University - SEA
Microscopist: MD Meyer

1 µm
HV=80kV
Direct Mag: 4000 x

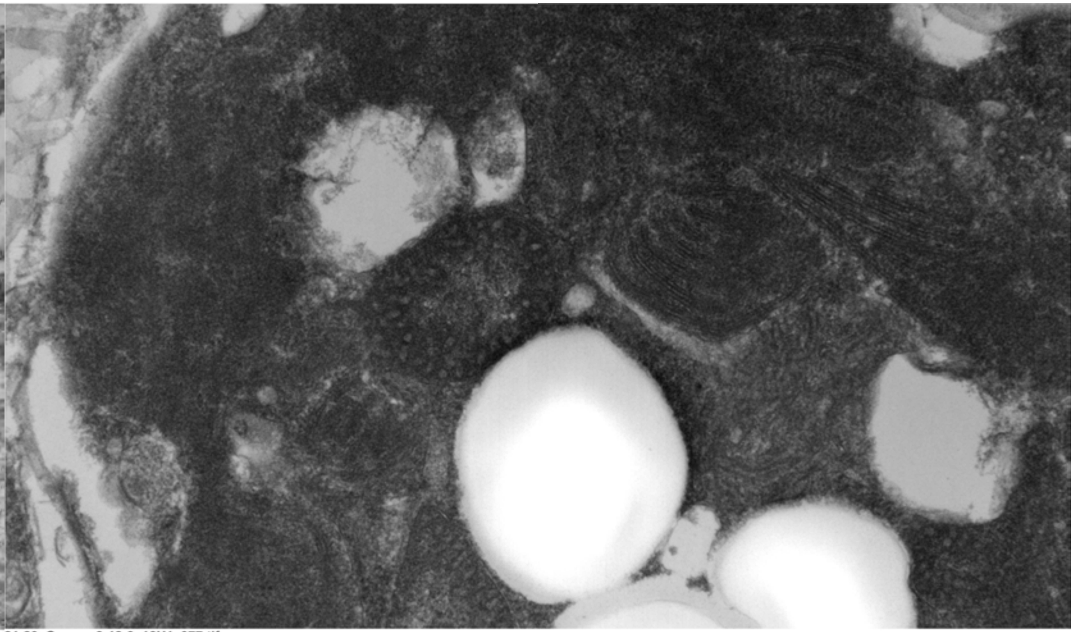

21-20_Correa_3-18-3_16H1_077.tif
Sample: 3/18, 3
Biological Electron Microscopy Lab
Rice University - SEA
Microscopist: MD Meyer

500 nm
HV=80kV
Direct Mag: 8000 x

Cell 24

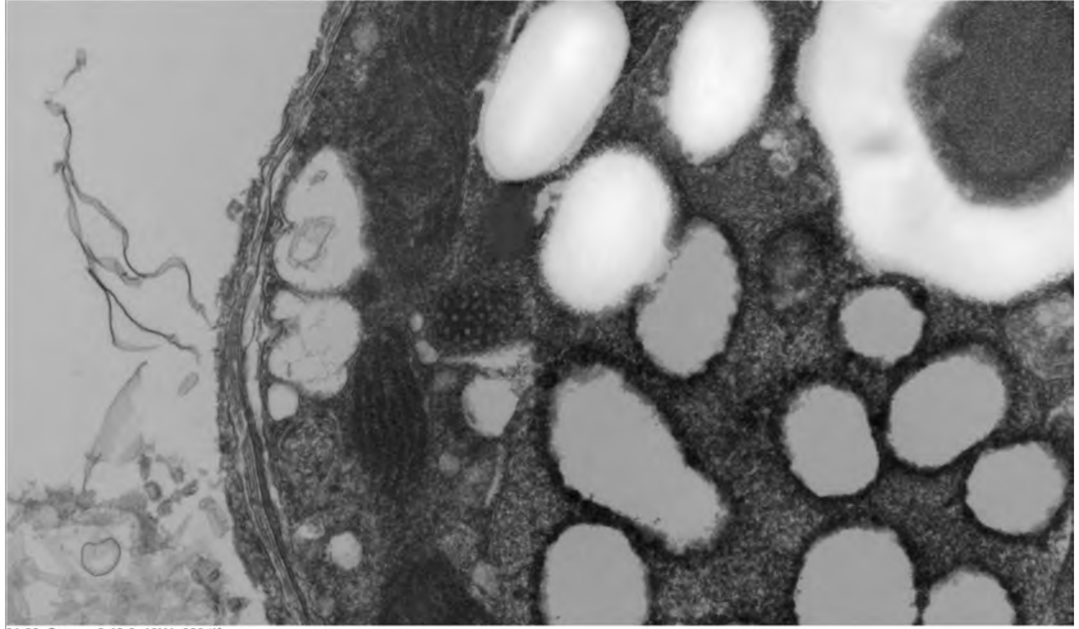

21-20_Correa_3-18-3_16H1_080.tif
Sample: 3/18, 3
Biological Electron Microscopy Lab
Rice University - SEA
Microscopist: MD Meyer

600 nm
HV=80kV
Direct Mag: 6000 x

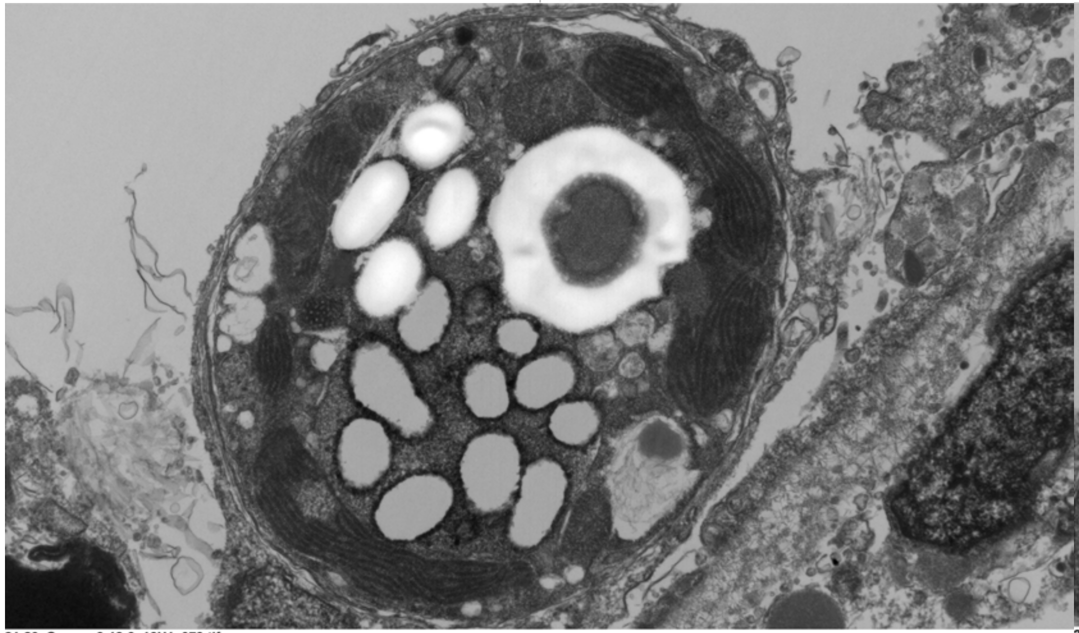

21-20_Correa_3-18-3_16H1_079.tif
Sample: 3/18, 3
Biological Electron Microscopy Lab
Rice University - SEA
Microscopist: MD Meyer

1 µm
HV=80kV
Direct Mag: 3000 x

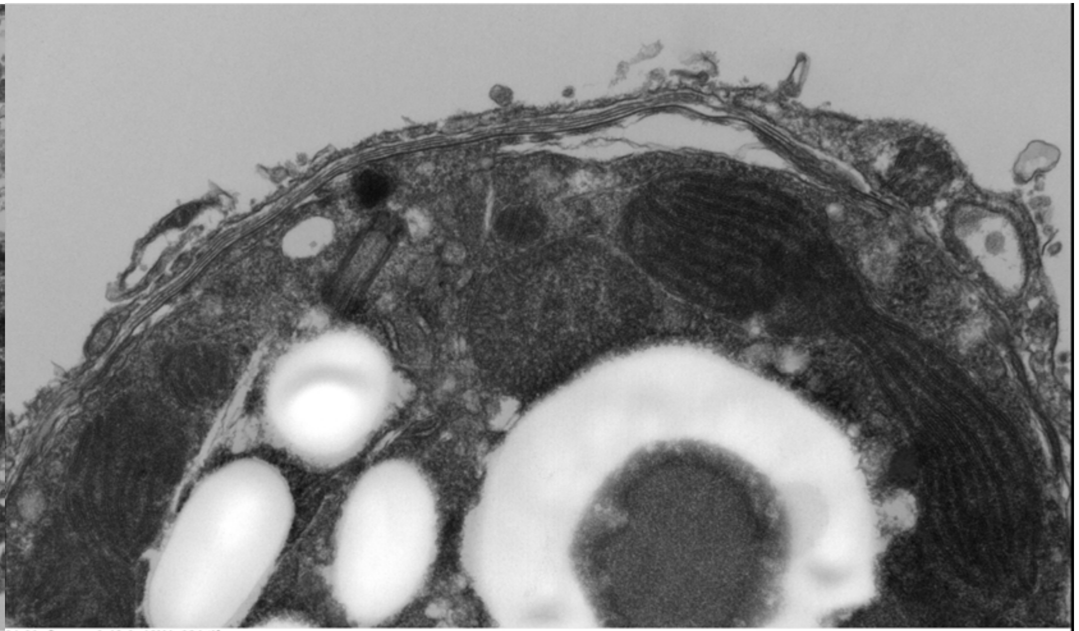

21-20_Correa_3-18-3_16H1_081.tif
Sample: 3/18, 3
Biological Electron Microscopy Lab
Rice University - SEA
Microscopist: MD Meyer

600 nm
HV=80kV
Direct Mag: 6000 x

Cell 25

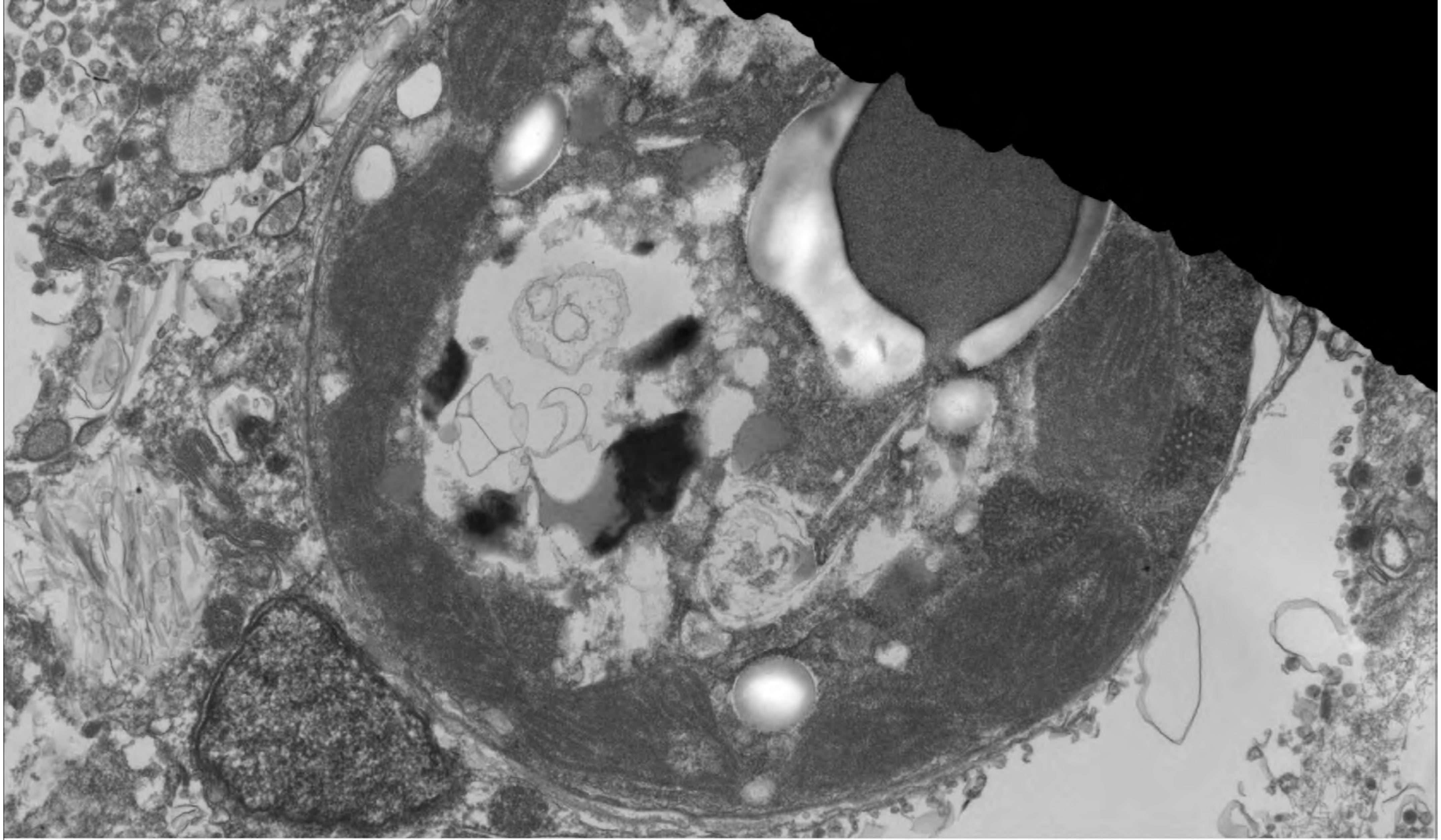

21-20_Correa_3-18-3_16H1_082.tif
Sample: 3/18, 3
Biological Electron Microscopy Lab
Rice University - SEA
Microscopist: MD Meyer

1 µm
HV=80kV
Direct Mag: 3000 x

Cell 26

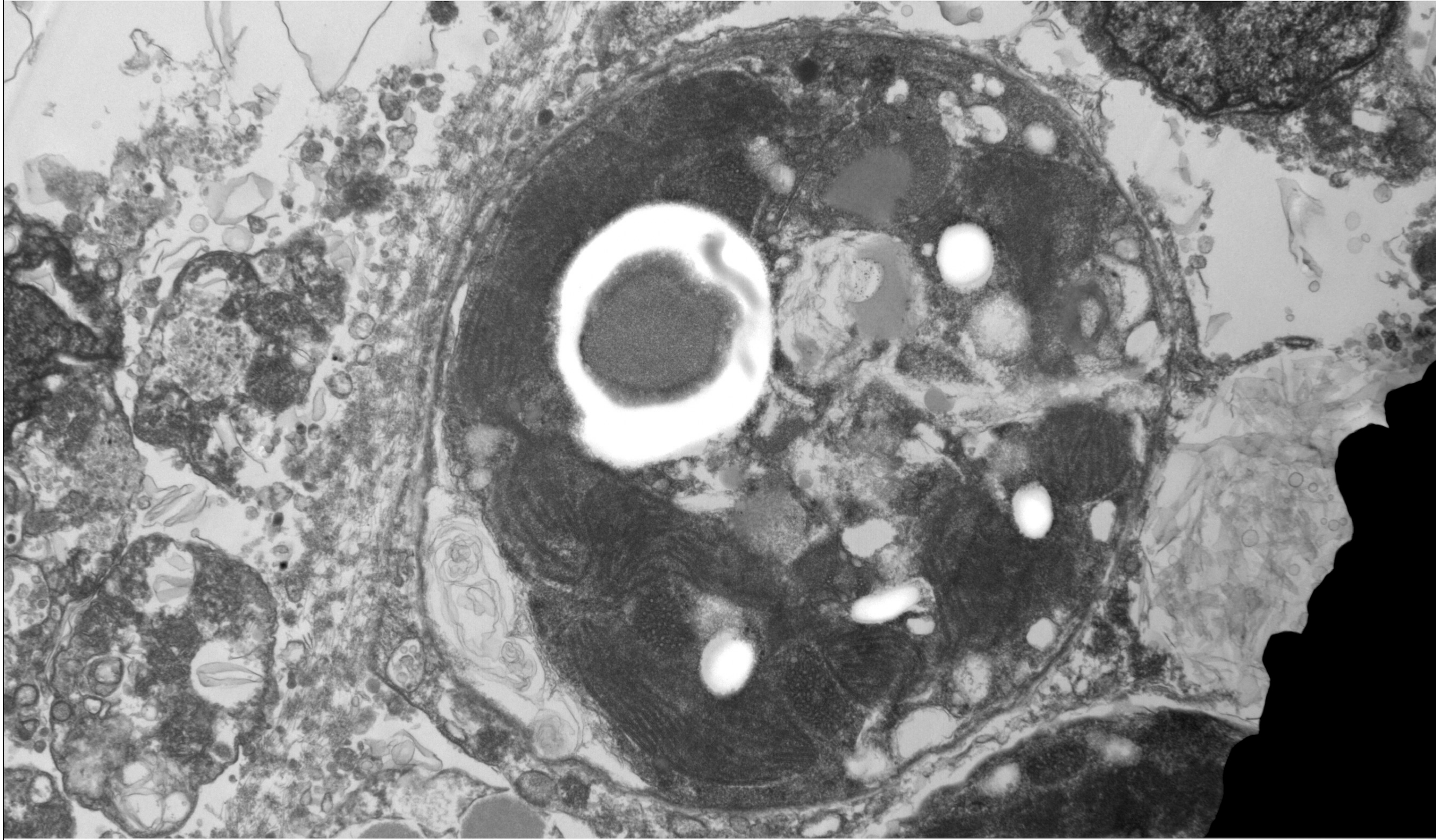

21-20_Correa_3-18-3_16H1_083.tif
Sample: 3/18, 3
Biological Electron Microscopy Lab
Rice University - SEA
Microscopist: MD Meyer

1 µm
HV=80kV
Direct Mag: 2500 x

Cell 27

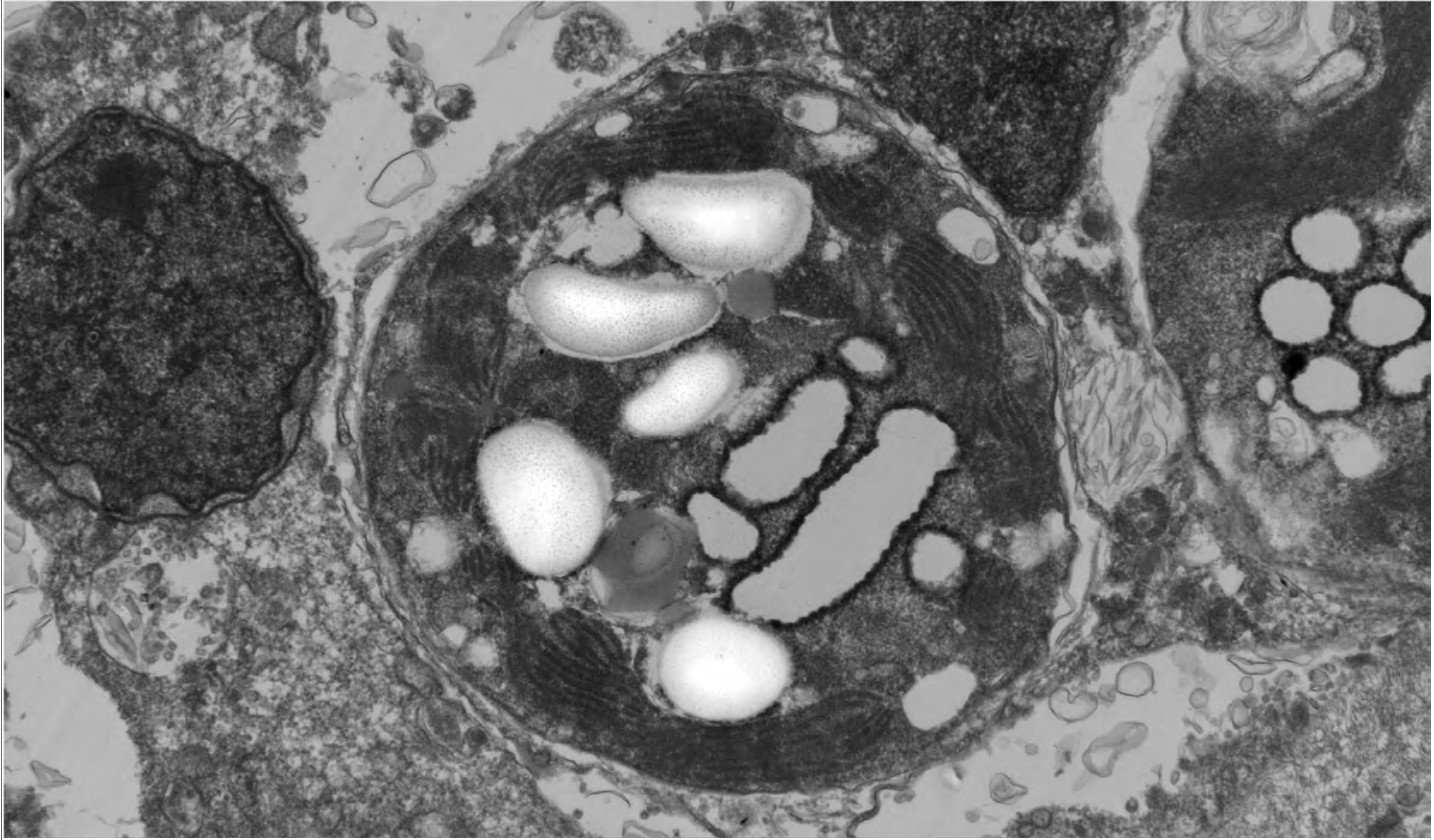

21-20_Correa_3-18-3_16H1_084.tif
Sample: 3/18, 3
Biological Electron Microscopy Lab
Rice University - SEA
Microscopist: MD Meyer

1 µm
HV=80kV
Direct Mag: 3000 x

Cell 28

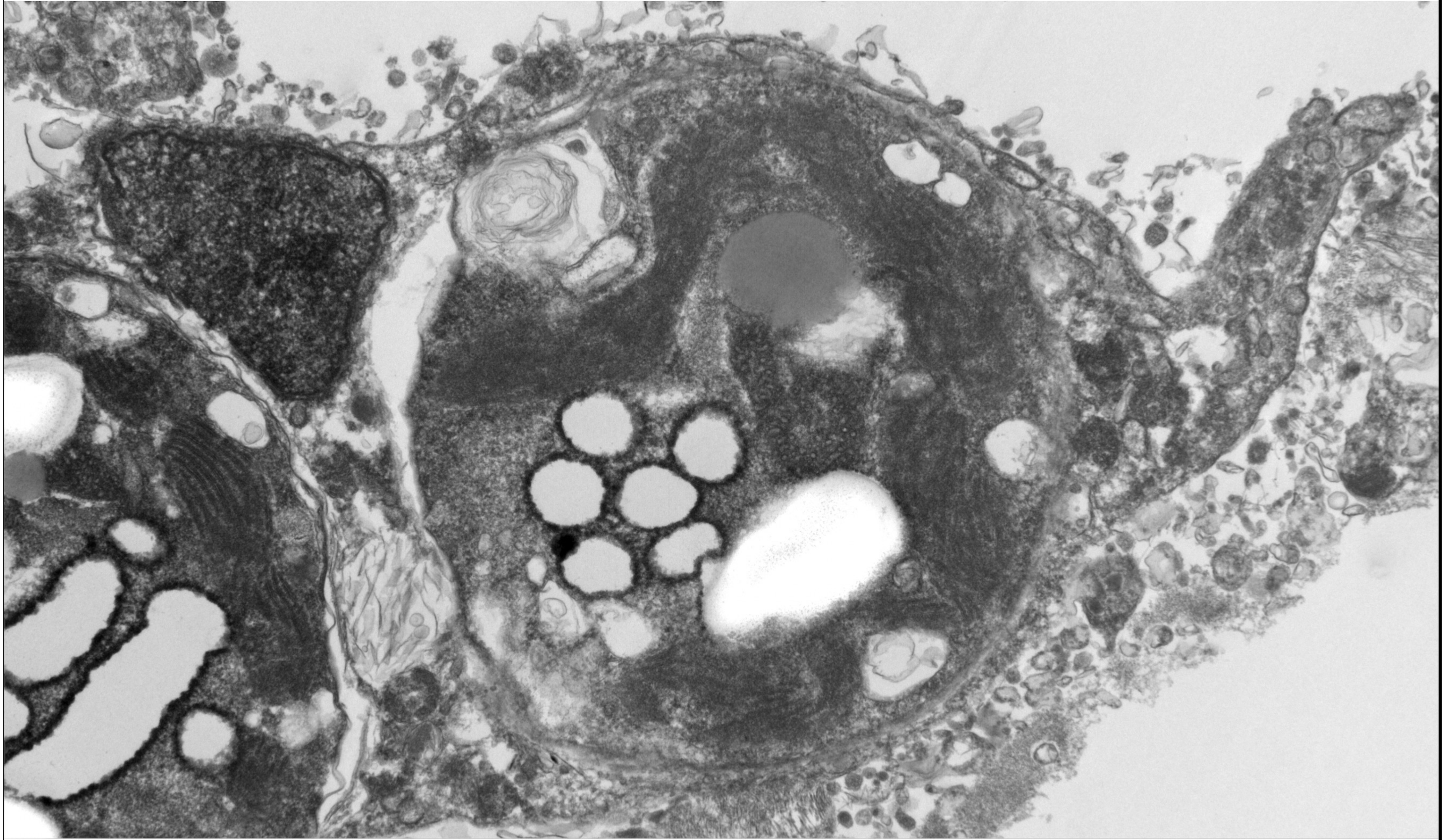

21-20_Correa_3-18-3_16H1_085.tif
Sample: 3/18, 3
Biological Electron Microscopy Lab
Rice University - SEA
Microscopist: MD Meyer

1 µm
HV=80kV
Direct Mag: 3000 x

Heat (March 2019) POR Colony L

Cell 1

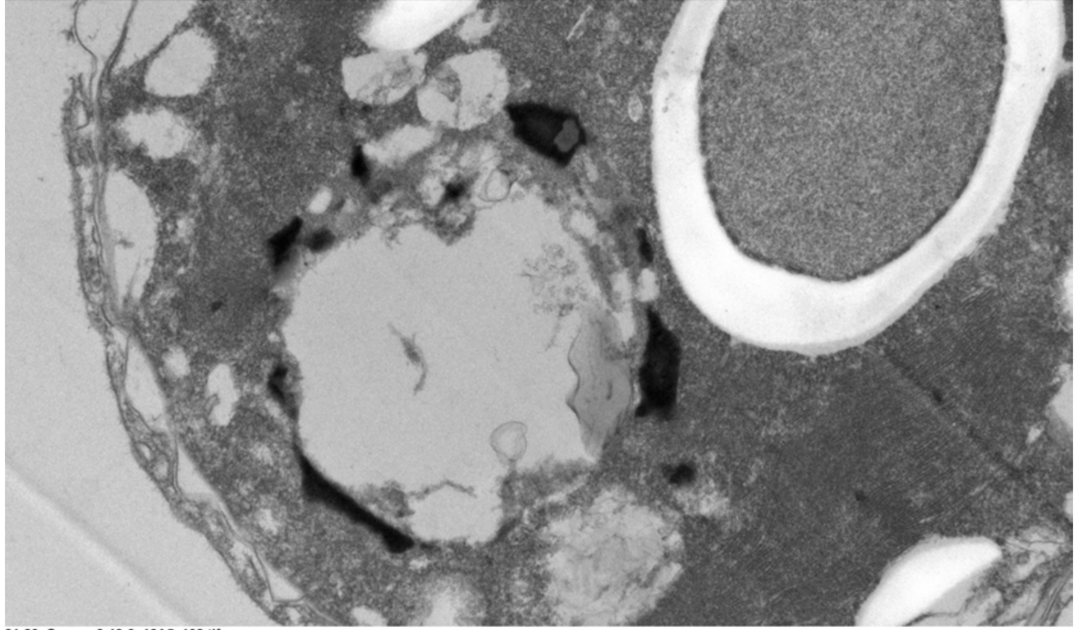

21-20_Correa_3-19-3_16A5_102.tif
Sample: 3/19, 3
Biological Electron Microscopy Lab
Rice University - SEA
Microscopist: MD Meyer

800 nm
HV=80kV
Direct Mag: 5000 x

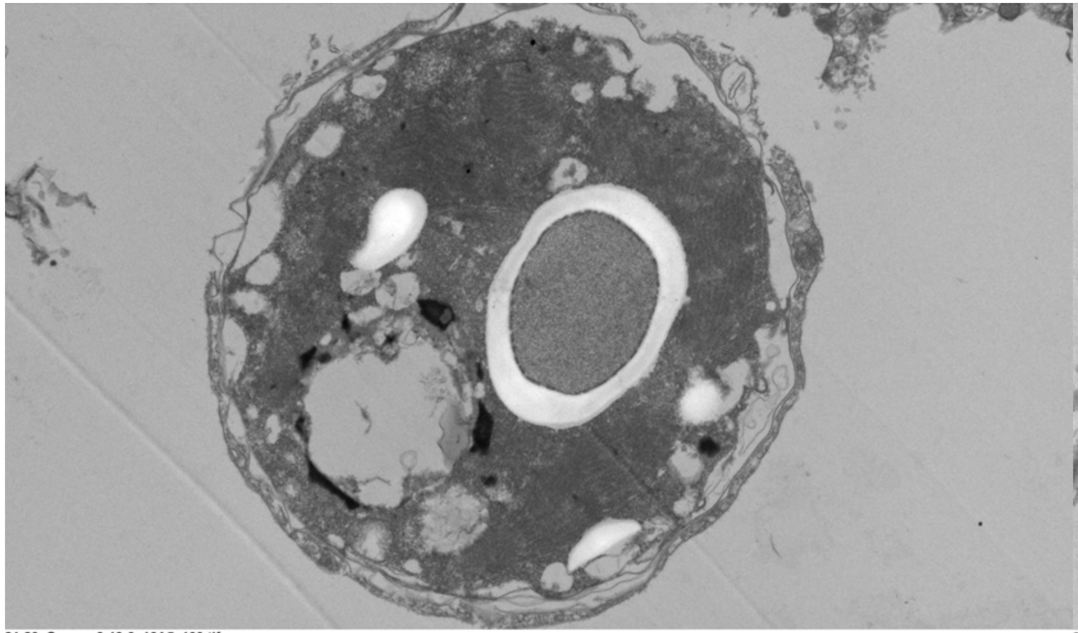

21-20_Correa_3-19-3_16A5_100.tif
Sample: 3/19, 3
Biological Electron Microscopy Lab
Rice University - SEA
Microscopist: MD Meyer

1 µm
HV=80kV
Direct Mag: 2500 x

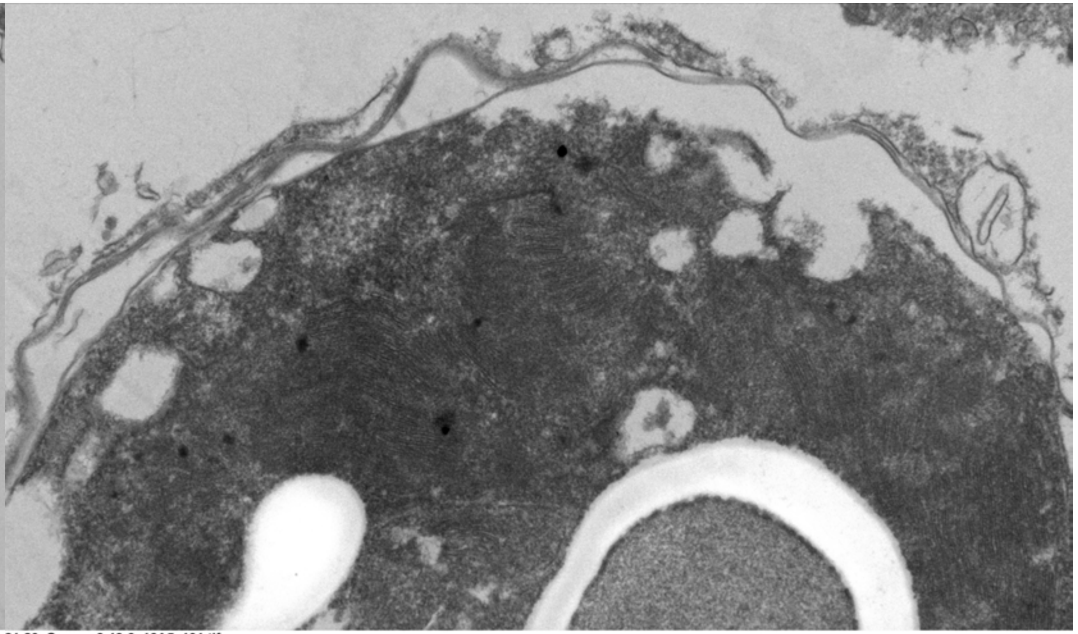

21-20_Correa_3-19-3_16A5_101.tif
Sample: 3/19, 3
Biological Electron Microscopy Lab
Rice University - SEA
Microscopist: MD Meyer

800 nm
HV=80kV
Direct Mag: 5000 x

Cell 2

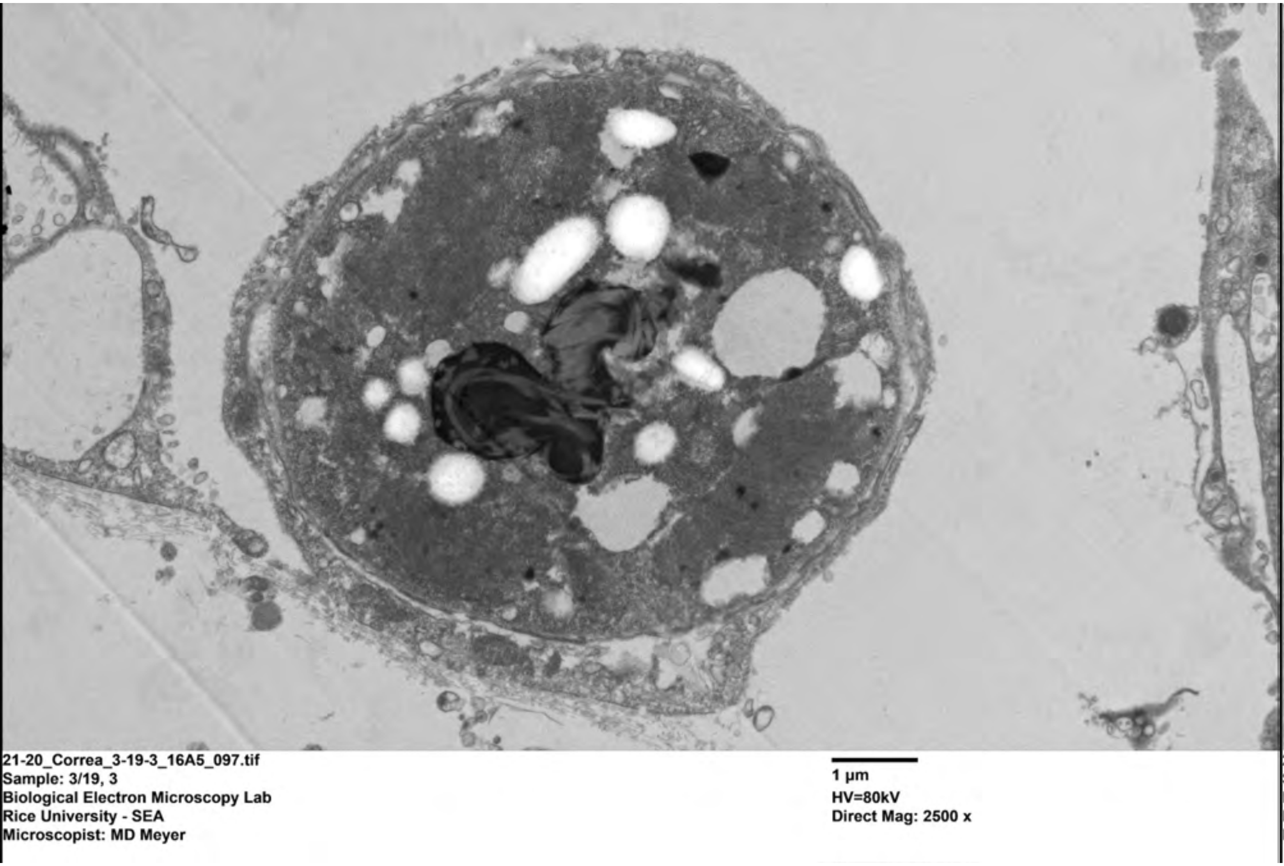
21-20_Correa_3-19-3_16A5_097.tif
Sample: 3/19, 3
Biological Electron Microscopy Lab
Rice University - SEA
Microscopist: MD Meyer
1 μm
HV=80kV
Direct Mag: 2500 x

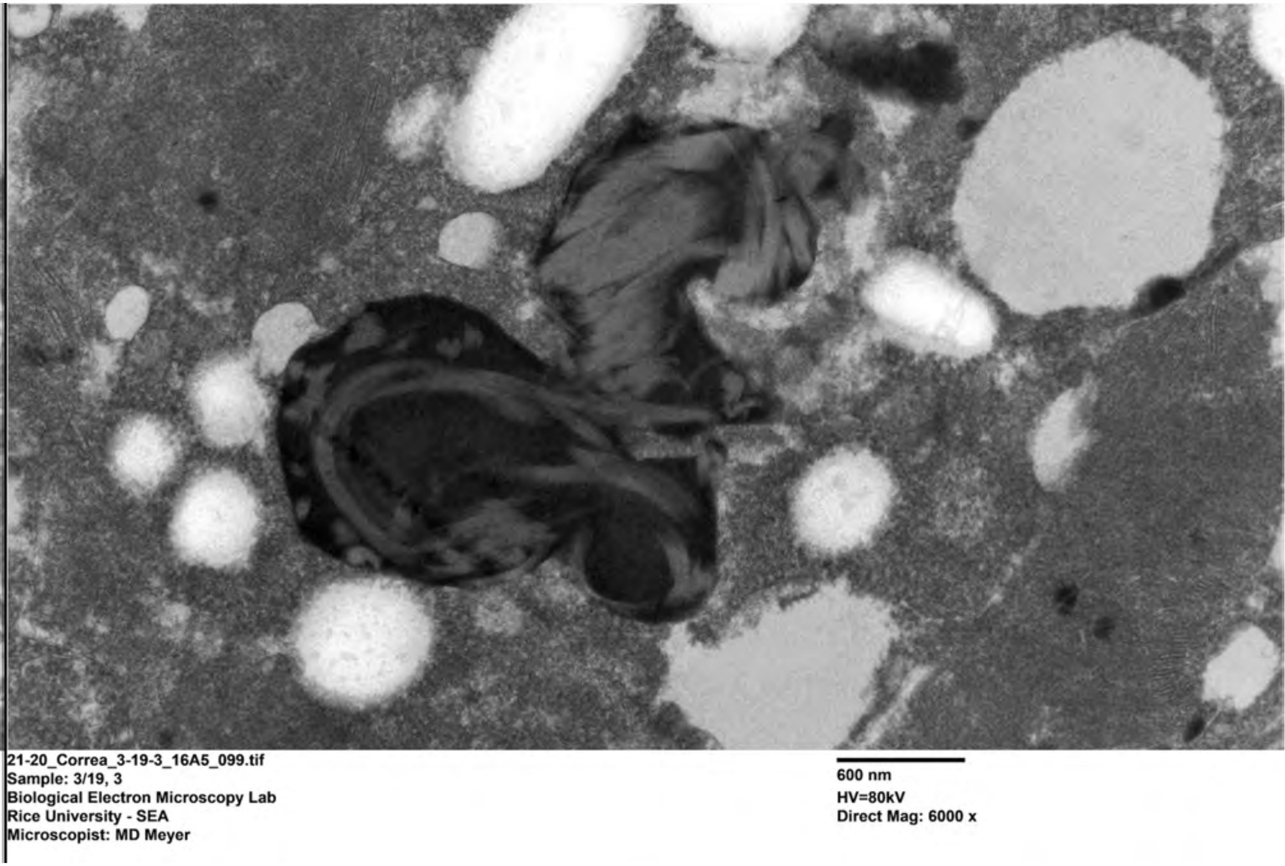
21-20_Correa_3-19-3_16A5_099.tif
Sample: 3/19, 3
Biological Electron Microscopy Lab
Rice University - SEA
Microscopist: MD Meyer
600 nm
HV=80kV
Direct Mag: 6000 x

Cell 3

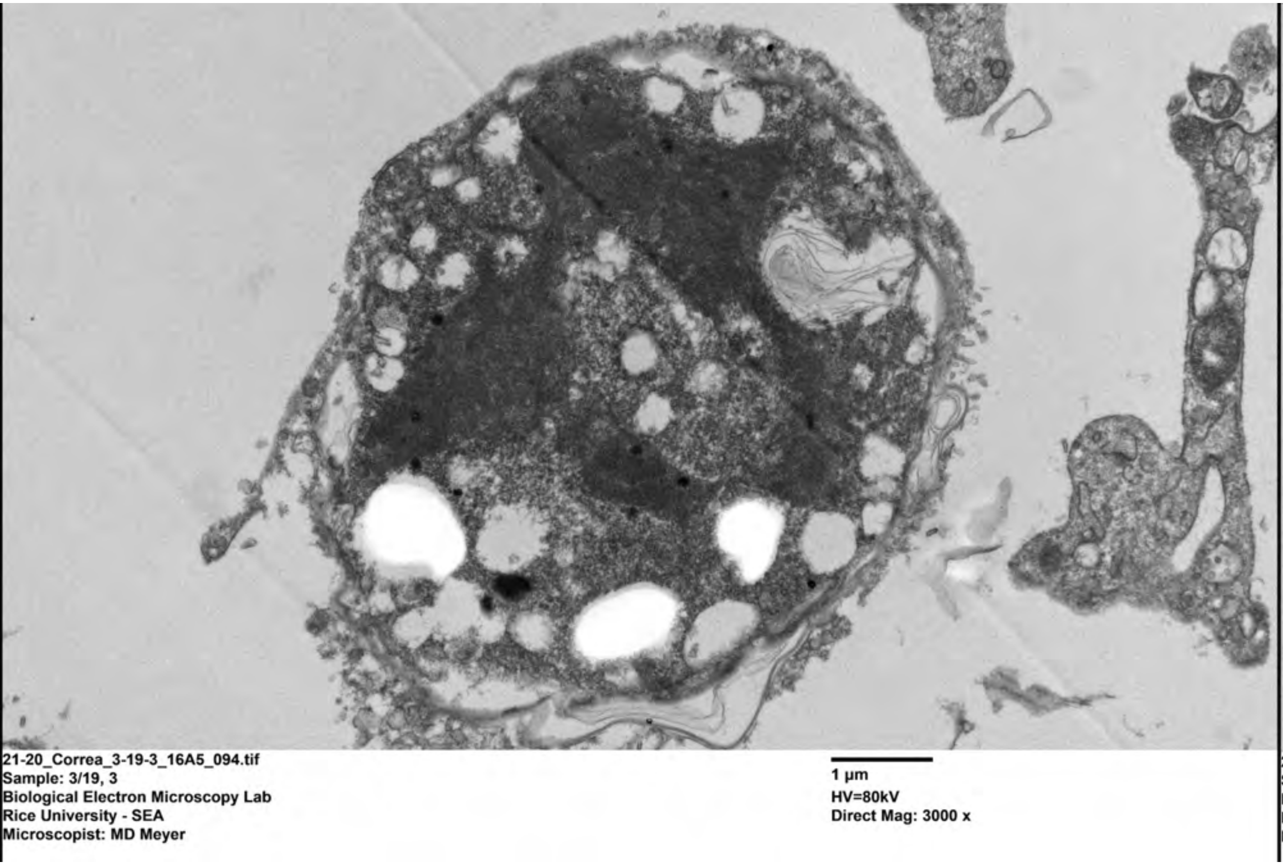
21-20_Correa_3-19-3_16A5_094.tif
Sample: 3/19, 3
Biological Electron Microscopy Lab
Rice University - SEA
Microscopist: MD Meyer
1 µm
HV=80kV
Direct Mag: 3000 x

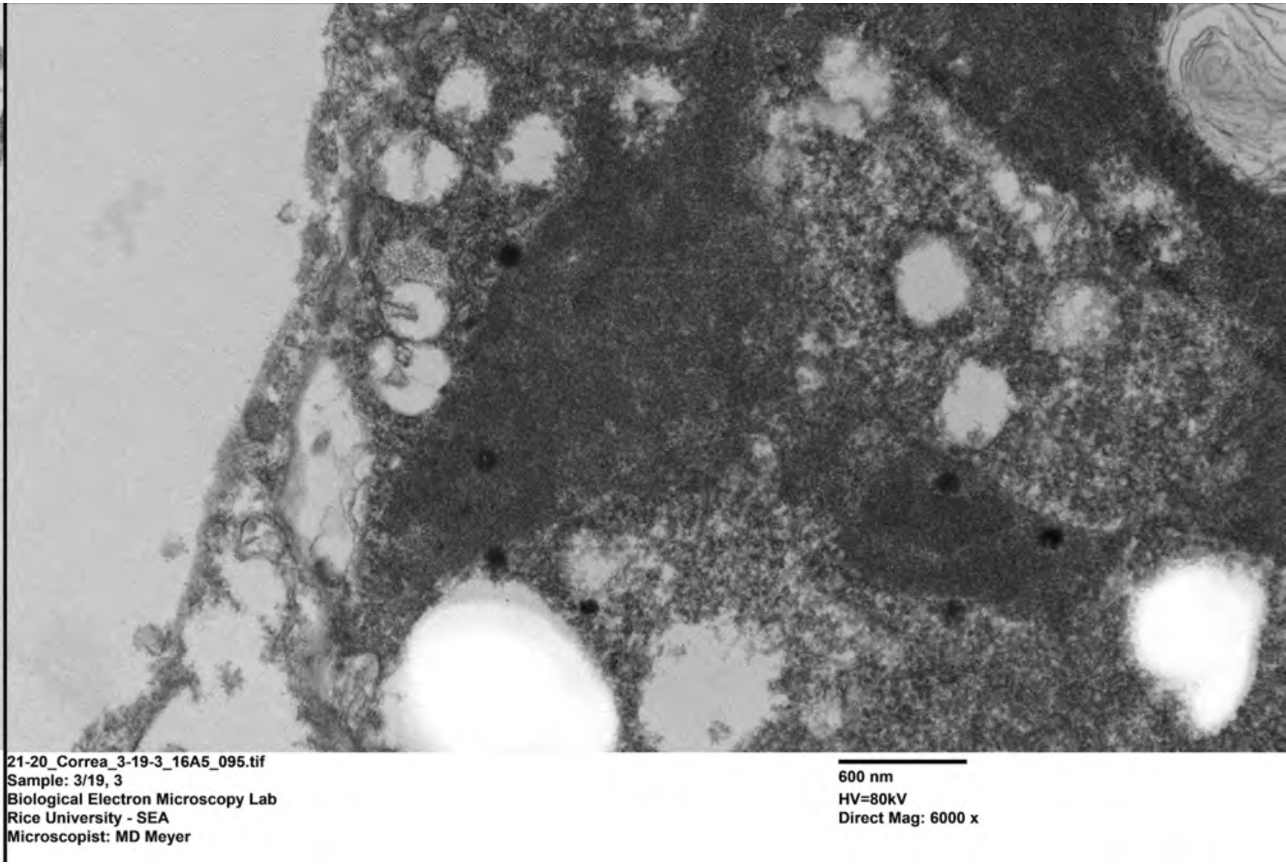
21-20_Correa_3-19-3_16A5_095.tif
Sample: 3/19, 3
Biological Electron Microscopy Lab
Rice University - SEA
Microscopist: MD Meyer
600 nm
HV=80kV
Direct Mag: 6000 x

Cell 4

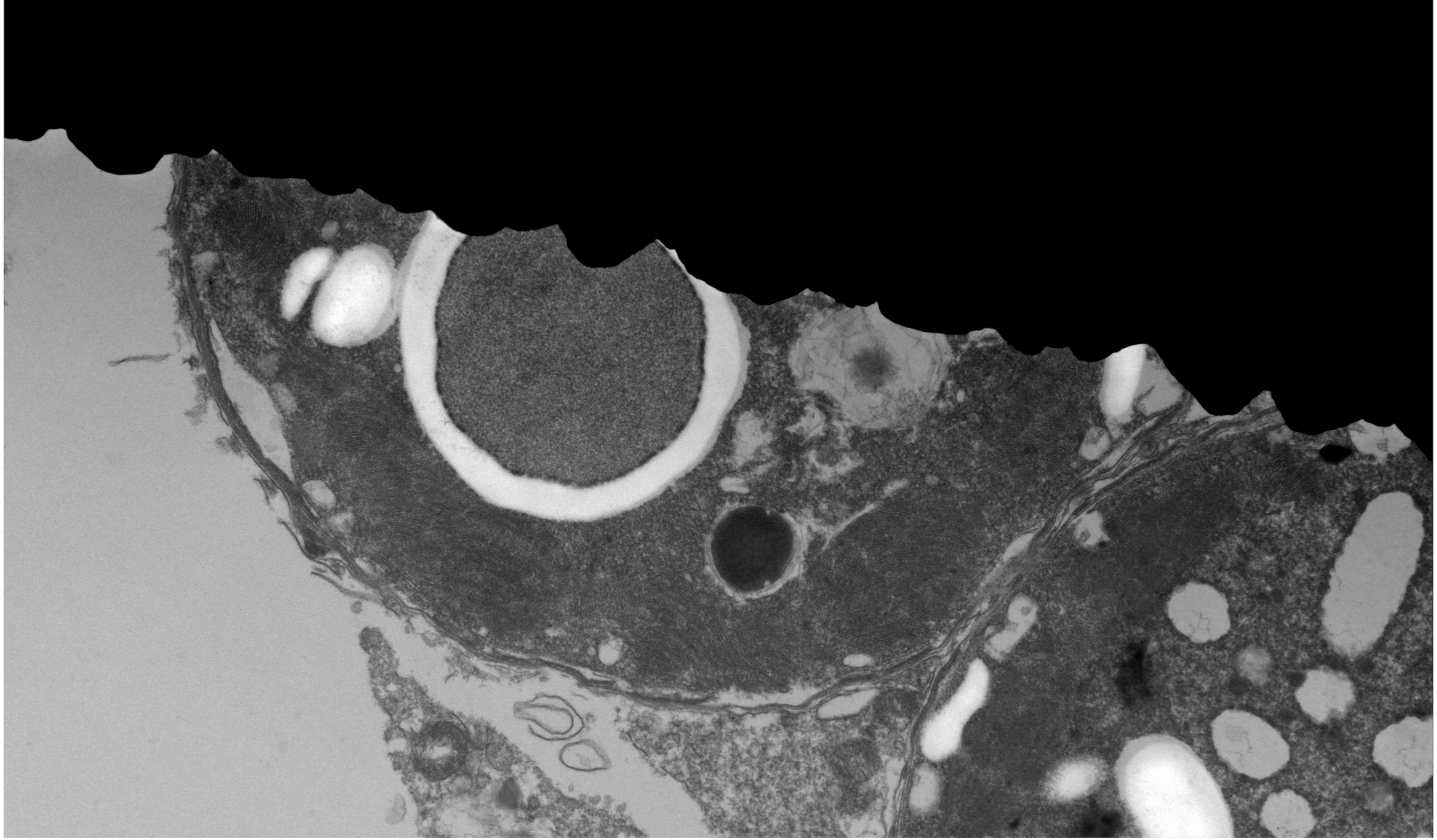

21-20_Correa_3-19-3_16A5_093.tif
Sample: 3/19, 3
Biological Electron Microscopy Lab
Rice University - SEA
Microscopist: MD Meyer

1 µm
HV=80kV
Direct Mag: 3000 x

Cell 5

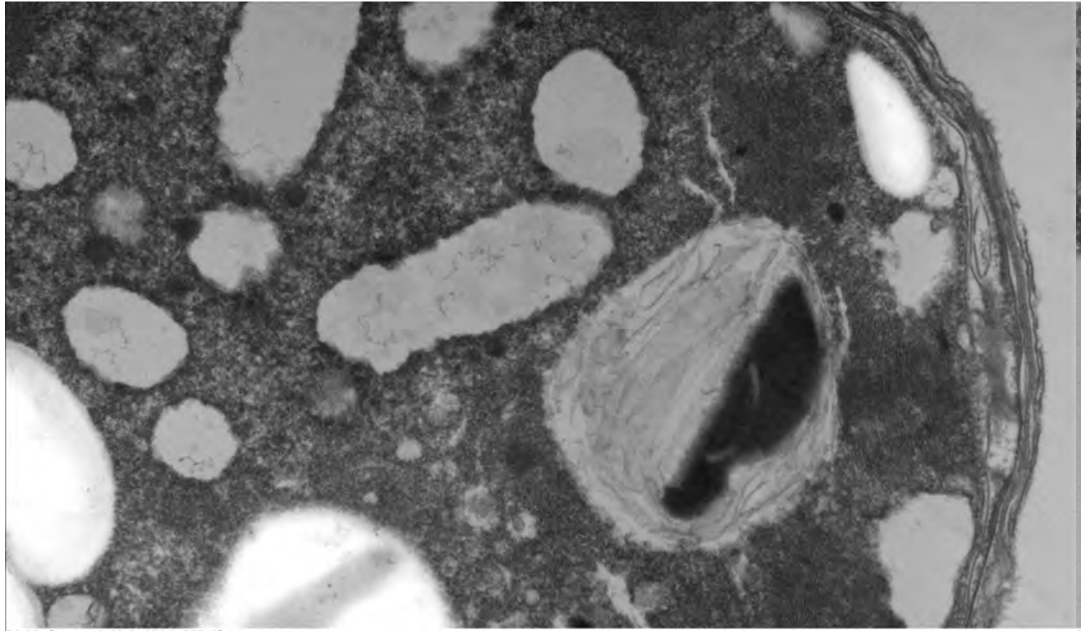

21-20_Correa_3-19-3_16A5_092.tif
Sample: 3/19, 3
Biological Electron Microscopy Lab
Rice University - SEA
Microscopist: MD Meyer

600 nm
HV=80kV
Direct Mag: 6000 x

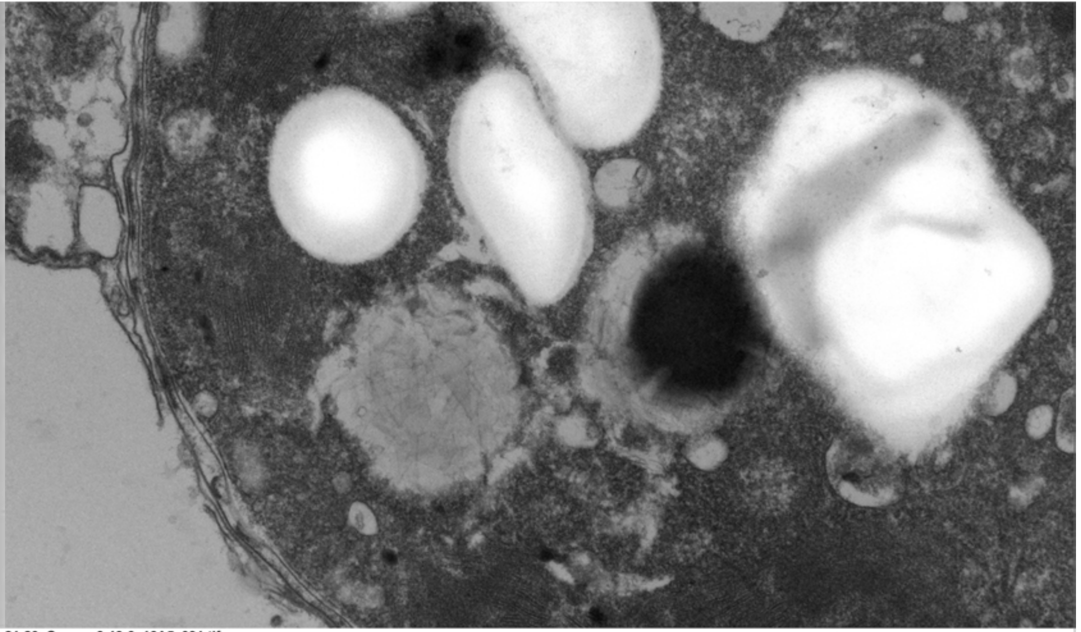

21-20_Correa_3-19-3_16A5_091.tif
Sample: 3/19, 3
Biological Electron Microscopy Lab
Rice University - SEA
Microscopist: MD Meyer

600 nm
HV=80kV
Direct Mag: 6000 x

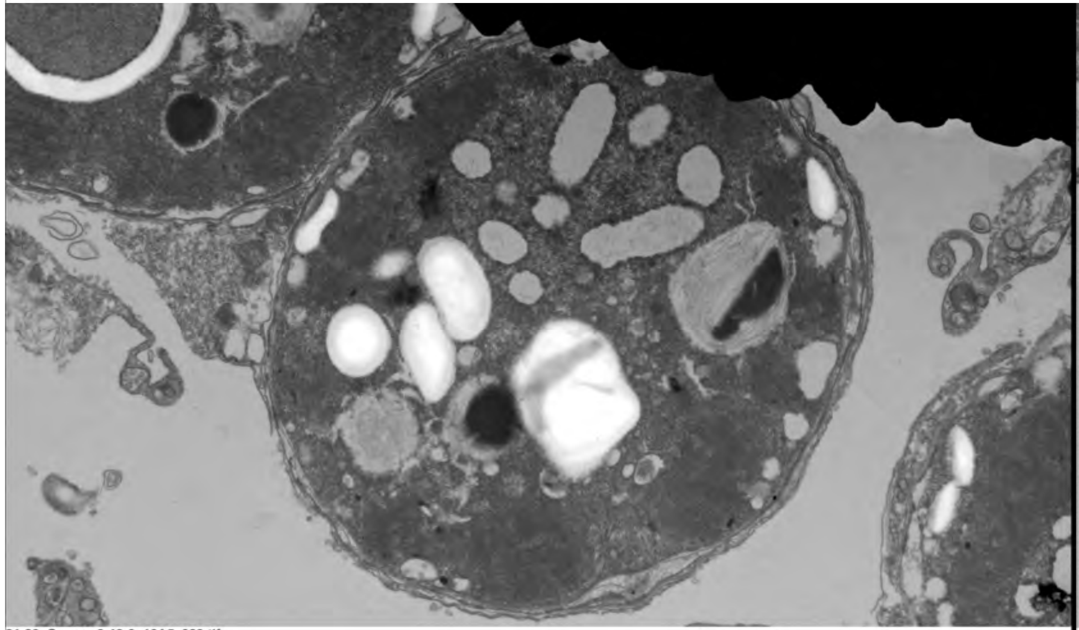

21-20_Correa_3-19-3_16A5_089.tif
Sample: 3/19, 3
Biological Electron Microscopy Lab
Rice University - SEA
Microscopist: MD Meyer

1 µm
HV=80kV
Direct Mag: 2500 x

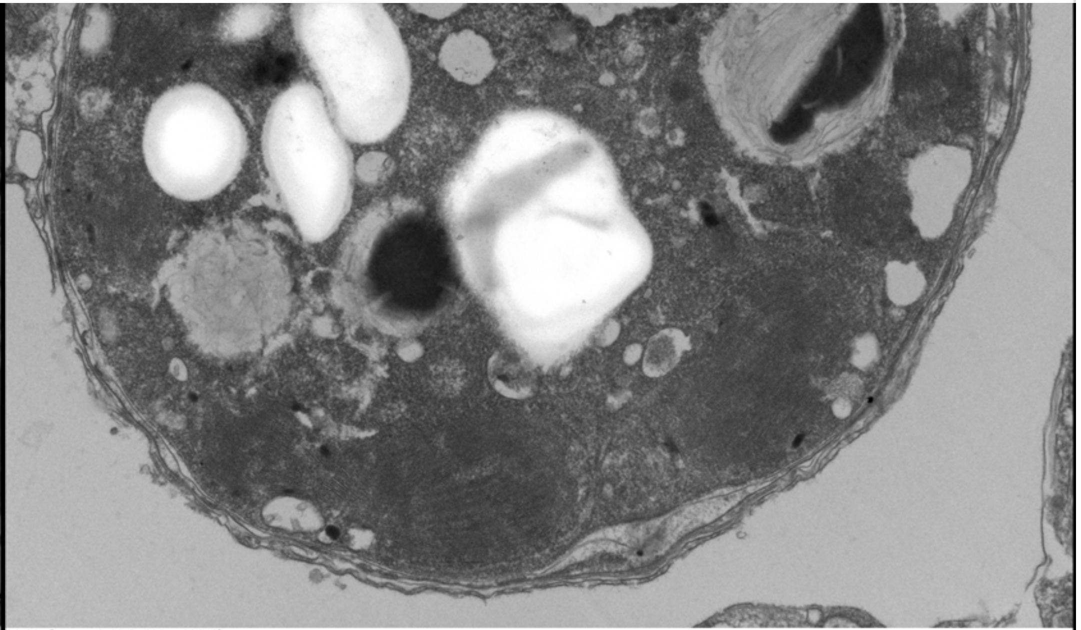

21-20_Correa_3-19-3_16A5_090.tif
Sample: 3/19, 3
Biological Electron Microscopy Lab
Rice University - SEA
Microscopist: MD Meyer

1 µm
HV=80kV
Direct Mag: 4000 x

Cell 6

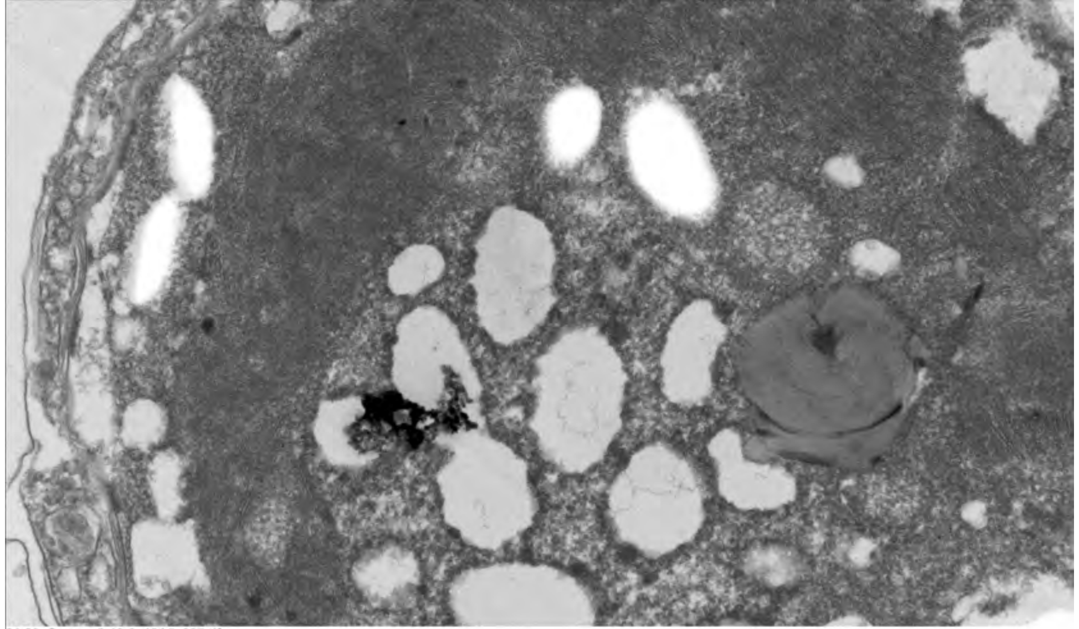

21-20_Correa_3-19-3_16A5_087.tif
Sample: 3/19, 3
Biological Electron Microscopy Lab
Rice University - SEA
Microscopist: MD Meyer

800 nm
HV=80kV
Direct Mag: 5000 x

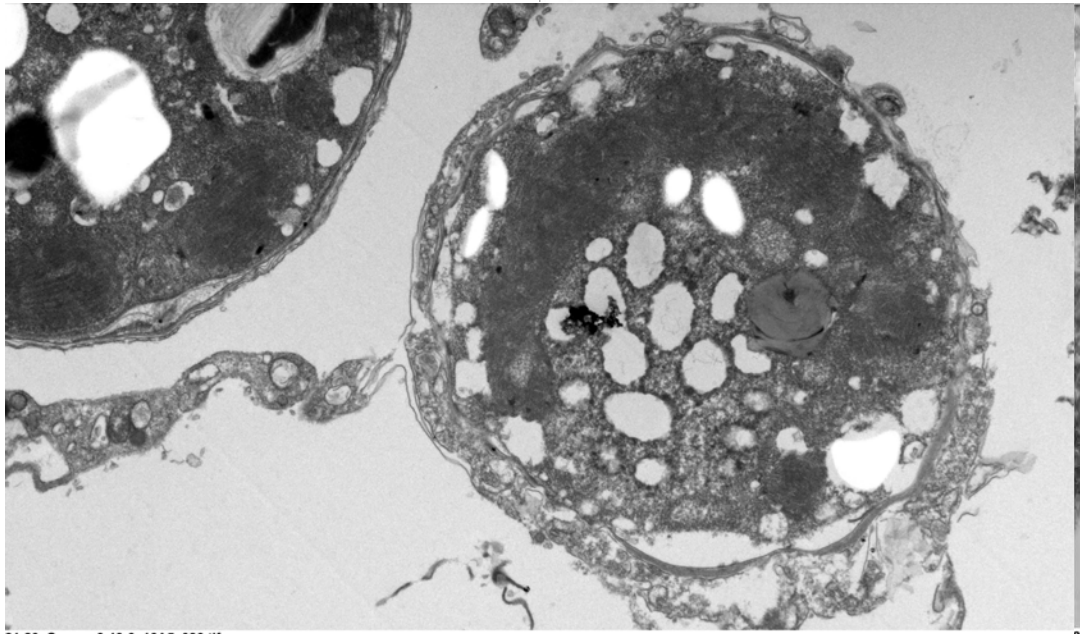

21-20_Correa_3-19-3_16A5_086.tif
Sample: 3/19, 3
Biological Electron Microscopy Lab
Rice University - SEA
Microscopist: MD Meyer

1 µm
HV=80kV
Direct Mag: 2500 x

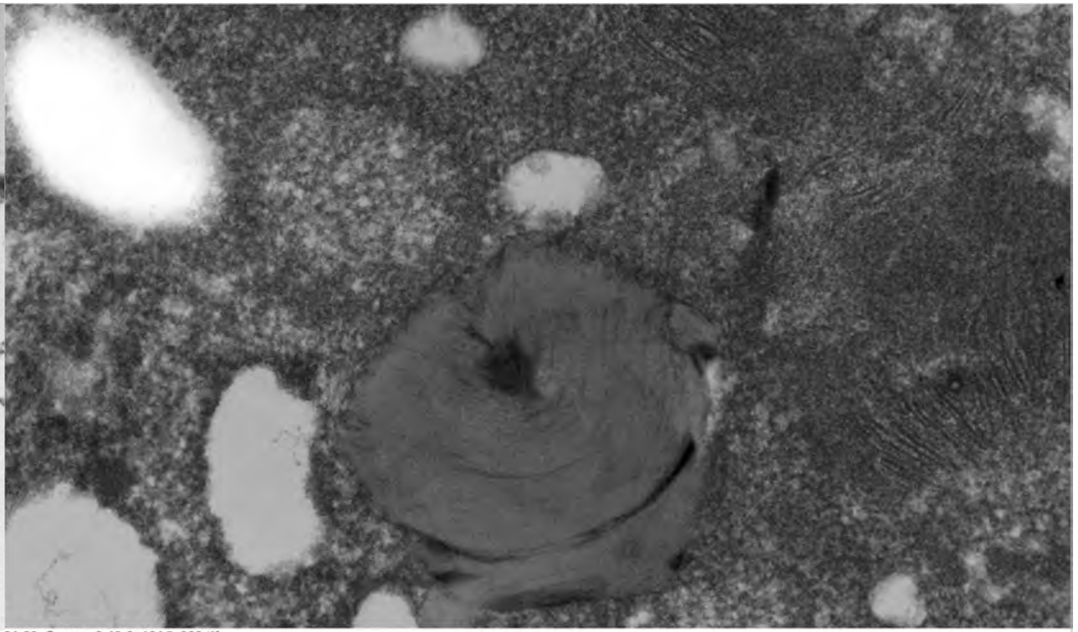

21-20_Correa_3-19-3_16A5_088.tif
Sample: 3/19, 3
Biological Electron Microscopy Lab
Rice University - SEA
Microscopist: MD Meyer

400 nm
HV=80kV
Direct Mag: 10000 x

Cell 7

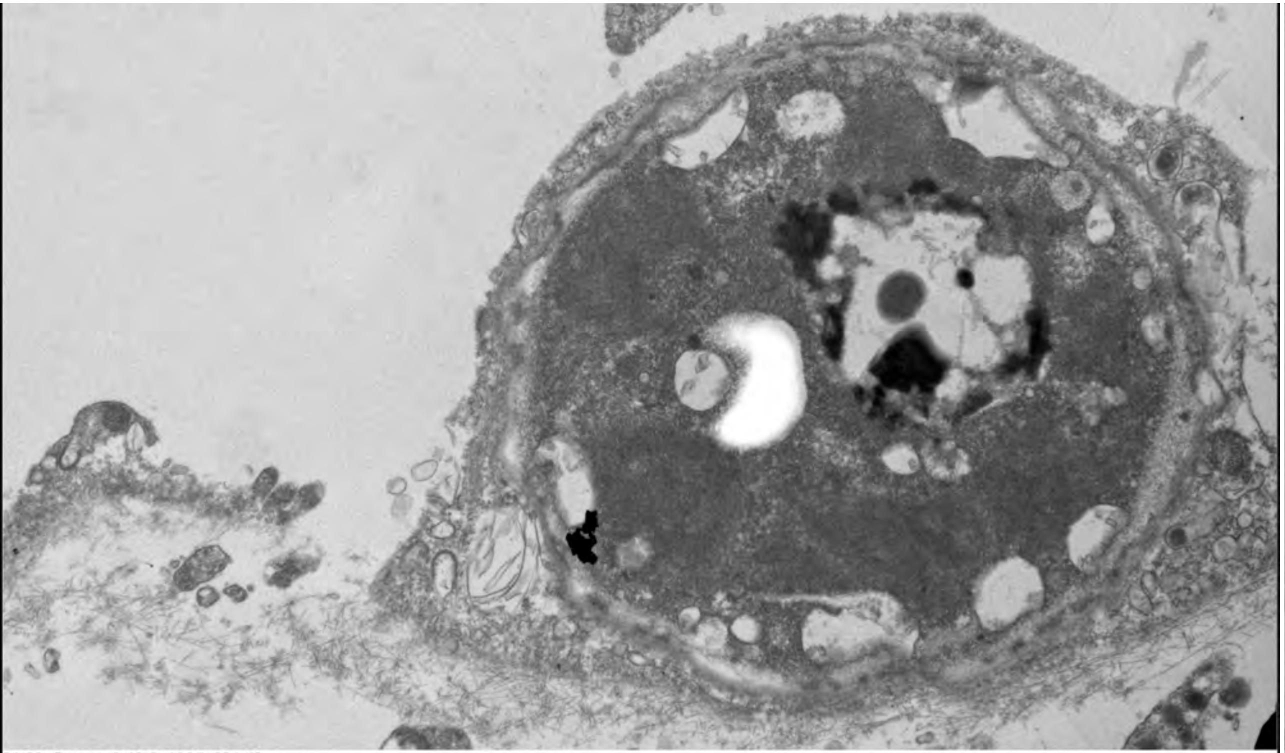

21-20_Correa_3-19-3_16A5_084.tif
Sample: 3/19, 3
Biological Electron Microscopy Lab
Rice University - SEA
Microscopist: MD Meyer

1 µm
HV=80kV
Direct Mag: 3000 x

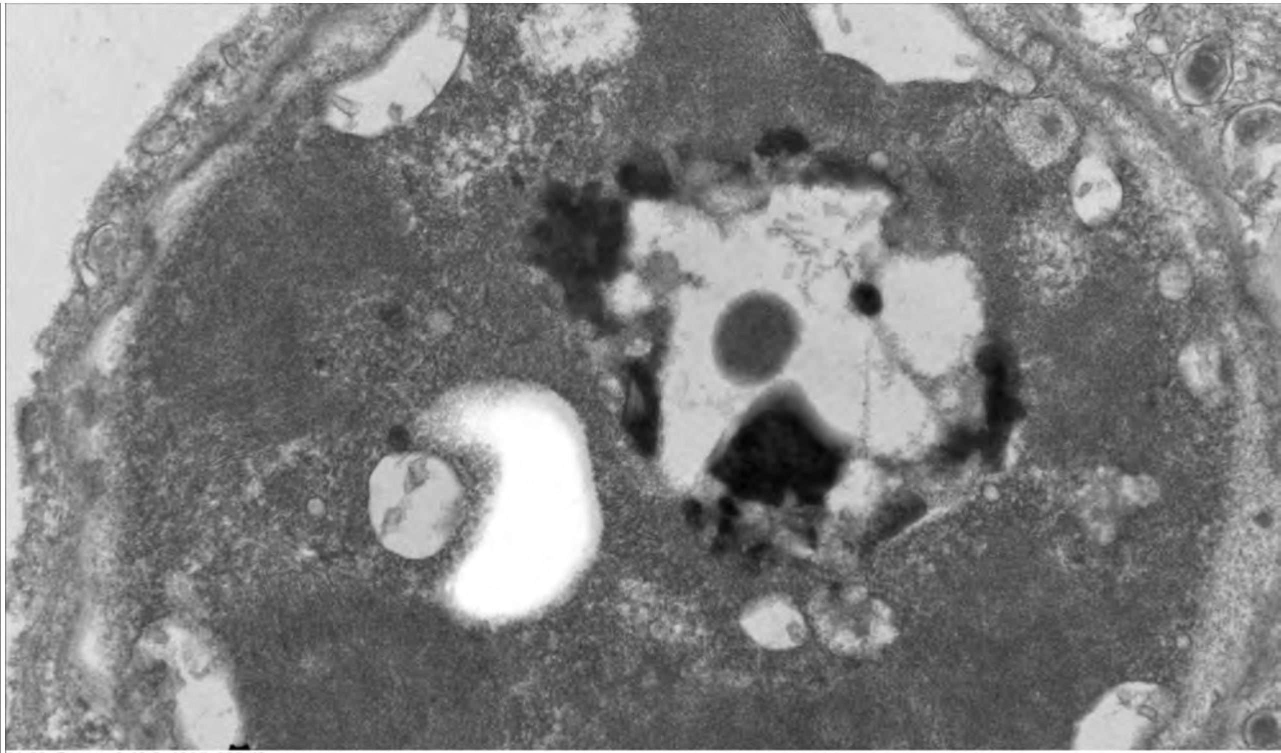

21-20_Correa_3-19-3_16A5_085.tif
Sample: 3/19, 3
Biological Electron Microscopy Lab
Rice University - SEA
Microscopist: MD Meyer

800 nm
HV=80kV
Direct Mag: 5000 x

Cell 8

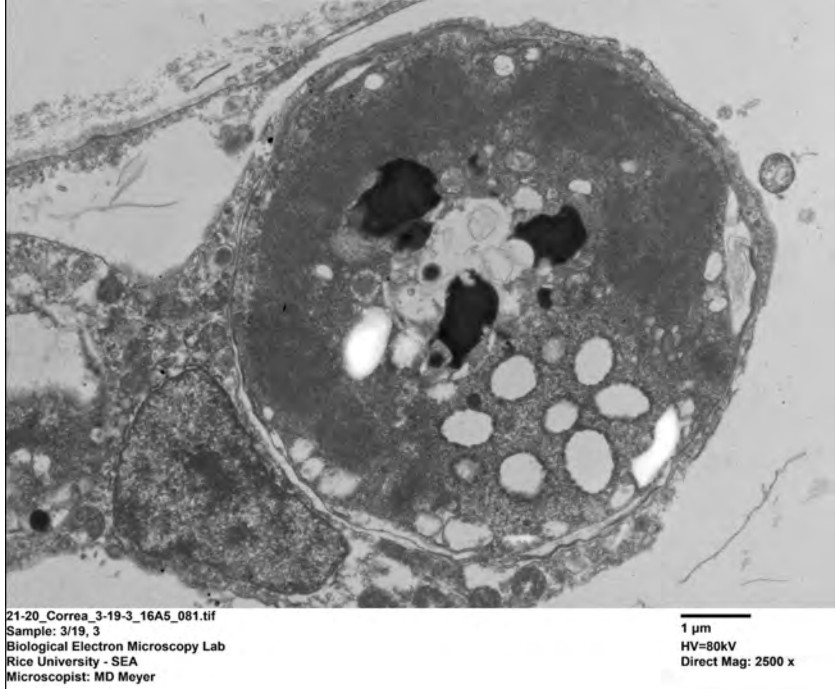
21-20_Correa_3-19-3_16A5_081.tif
Sample: 3/19, 3
Biological Electron Microscopy Lab
Rice University - SEA
Microscopist: MD Meyer
1 µm
HV=80kV
Direct Mag: 2500 x

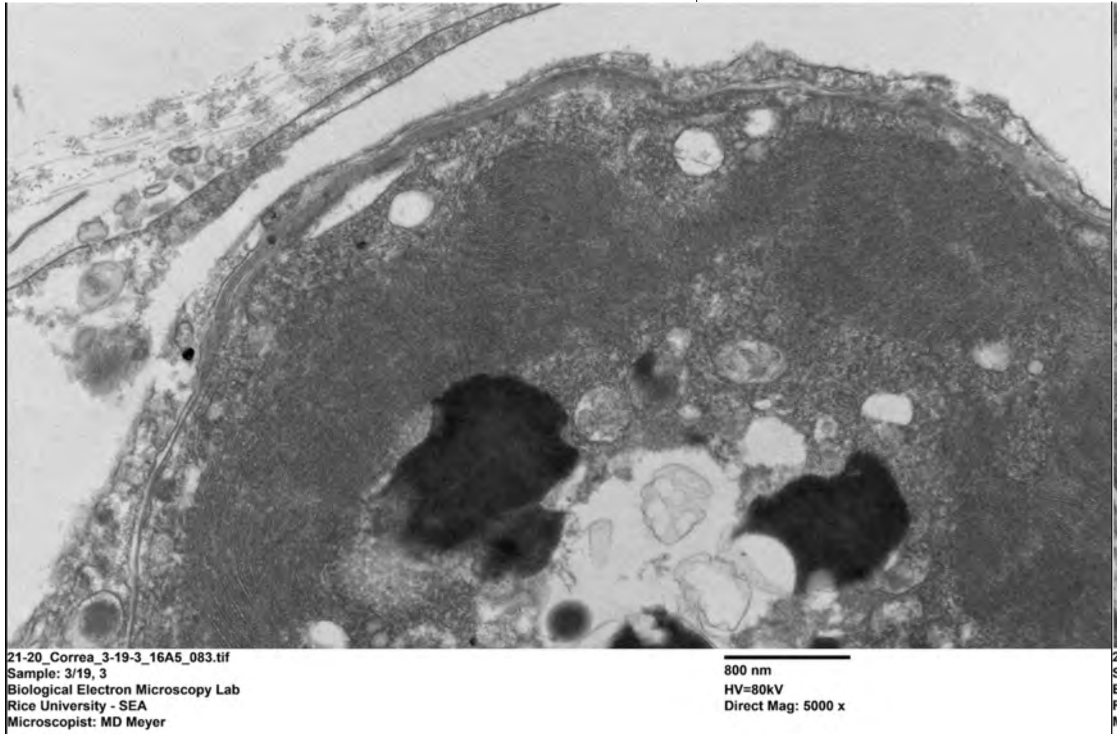
21-20_Correa_3-19-3_16A5_083.tif
Sample: 3/19, 3
Biological Electron Microscopy Lab
Rice University - SEA
Microscopist: MD Meyer
800 nm
HV=80kV
Direct Mag: 5000 x

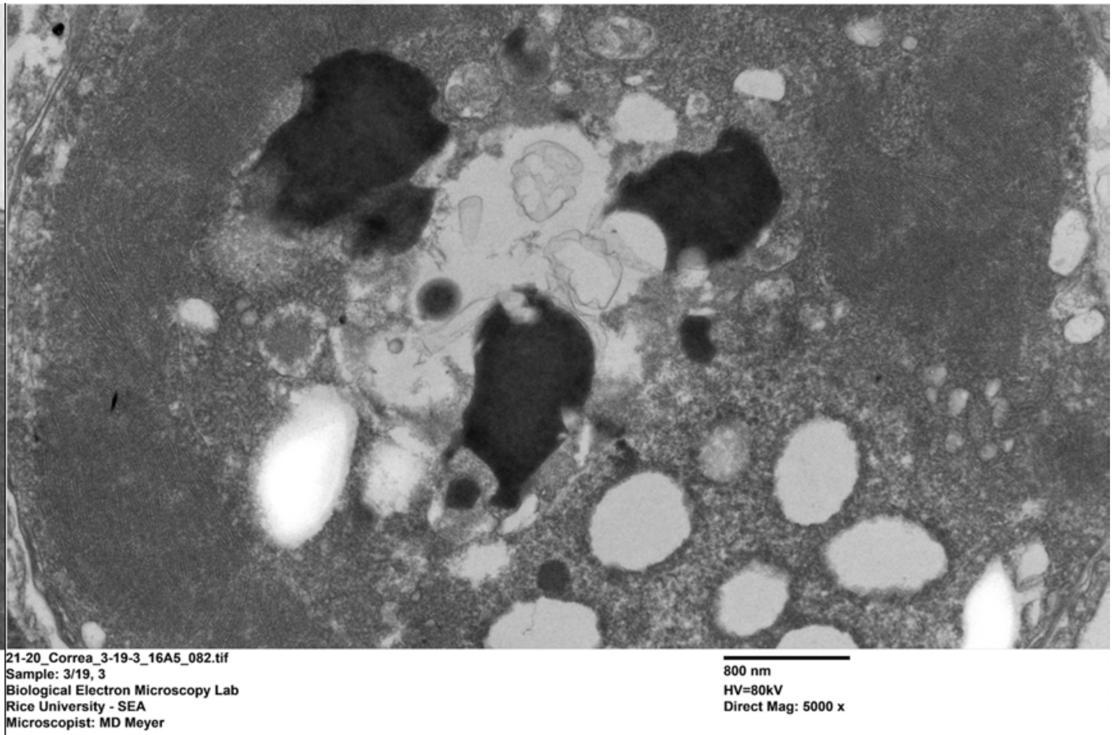
21-20_Correa_3-19-3_16A5_082.tif
Sample: 3/19, 3
Biological Electron Microscopy Lab
Rice University - SEA
Microscopist: MD Meyer
800 nm
HV=80kV
Direct Mag: 5000 x

Cell 9

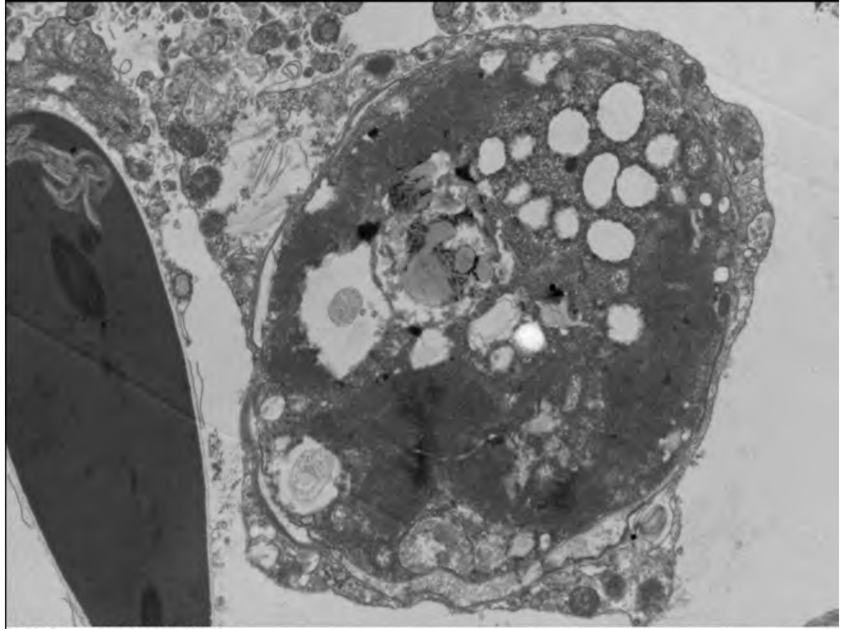

21-20_Correa_3-19-3_16A5_066.tif
Sample: 3/19, 3
Biological Electron Microscopy Lab
Rice University - SEA
Microscopist: MD Meyer

2 µm
HV=80kV
Direct Mag: 2000 x

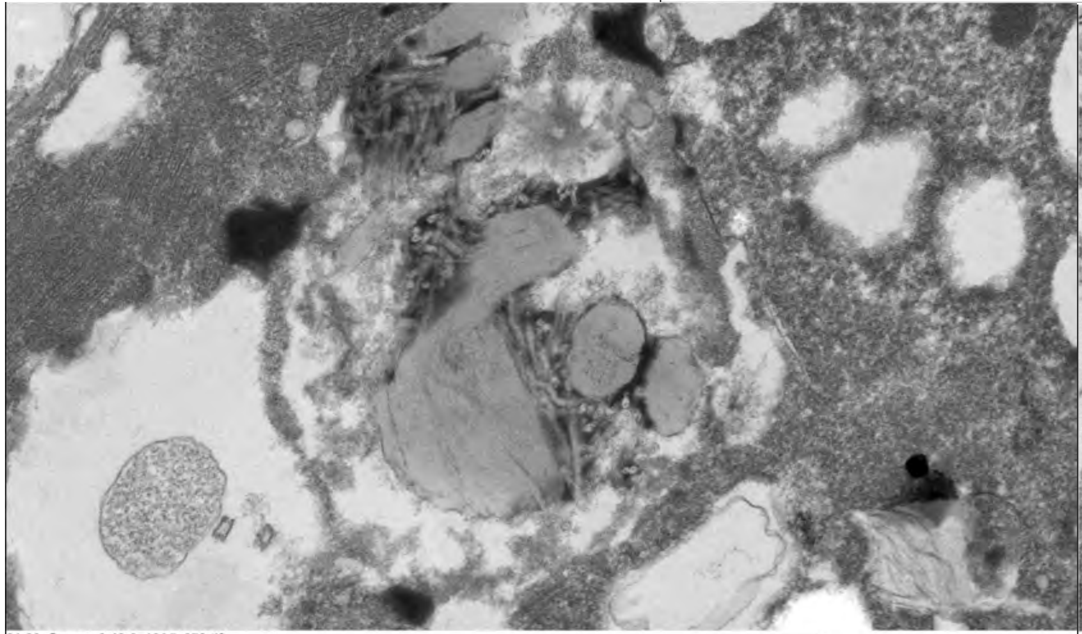

21-20_Correa_3-19-3_16A5_078.tif
Sample: 3/19, 3
Biological Electron Microscopy Lab
Rice University - SEA
Microscopist: MD Meyer

500 nm
HV=80kV
Direct Mag: 8000 x

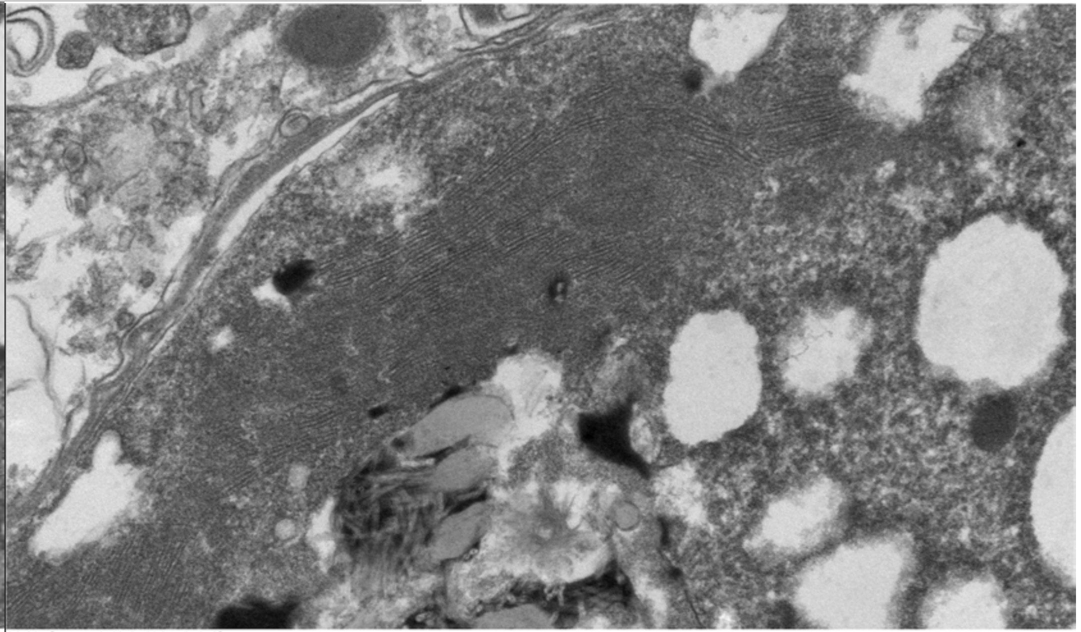

21-20_Correa_3-19-3_16A5_079.tif
Sample: 3/19, 3
Biological Electron Microscopy Lab
Rice University - SEA
Microscopist: MD Meyer

500 nm
HV=80kV
Direct Mag: 8000 x

Cell 9

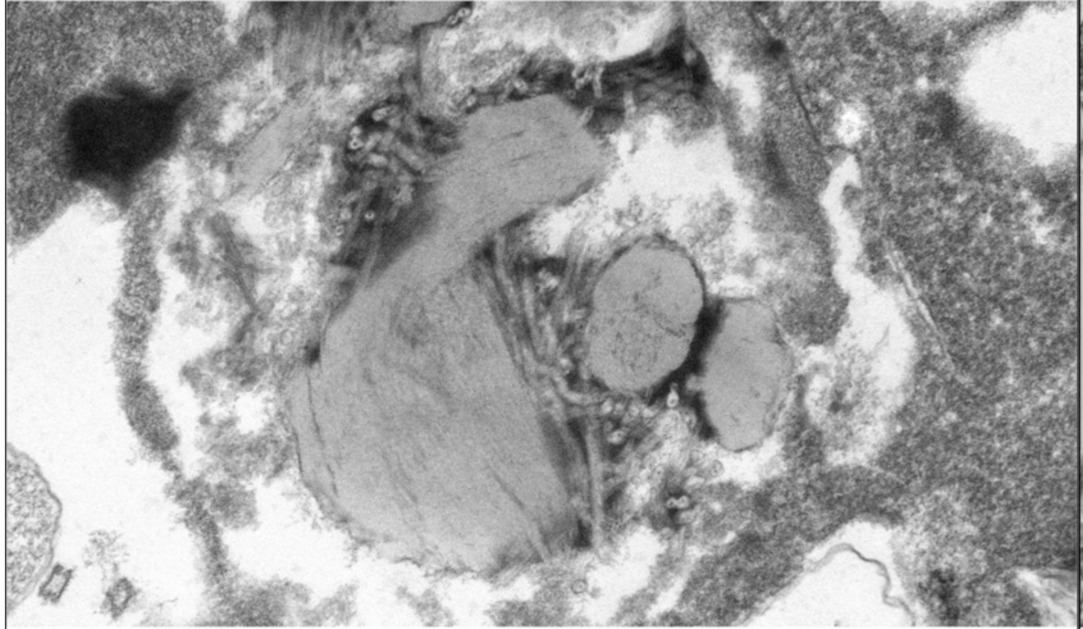

21-20_Correa_3-19-3_16A5_076.tif
Sample: 3/19, 3
Biological Electron Microscopy Lab
Rice University - SEA
Microscopist: MD Meyer

200 nm
HV=80kV
Direct Mag: 12000 x

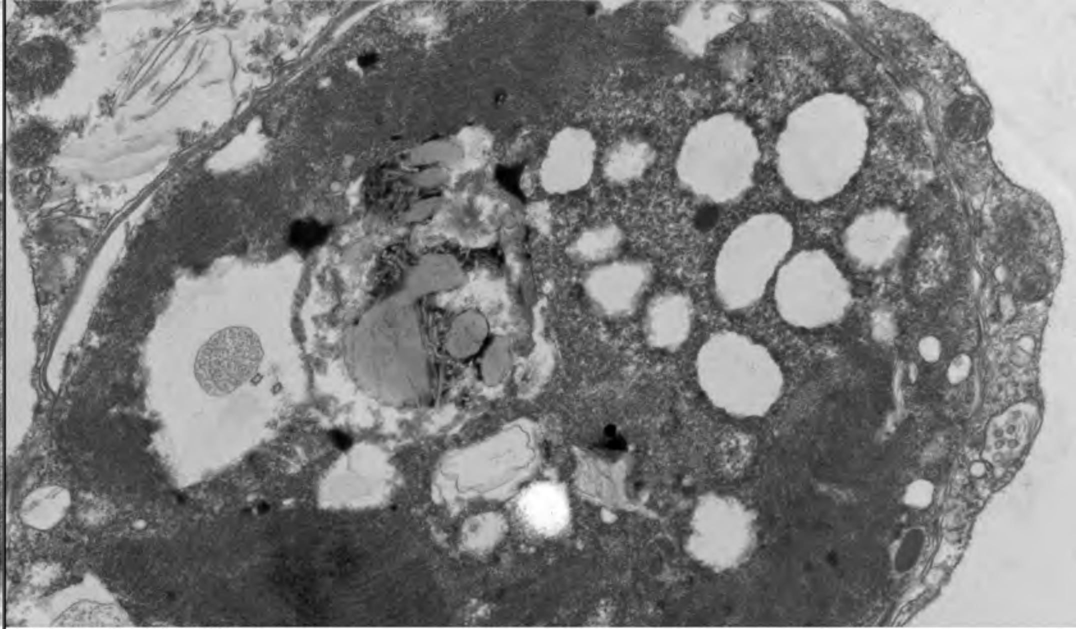

21-20_Correa_3-19-3_16A5_068.tif
Sample: 3/19, 3
Biological Electron Microscopy Lab
Rice University - SEA
Microscopist: MD Meyer

1 µm
HV=80kV
Direct Mag: 4000 x

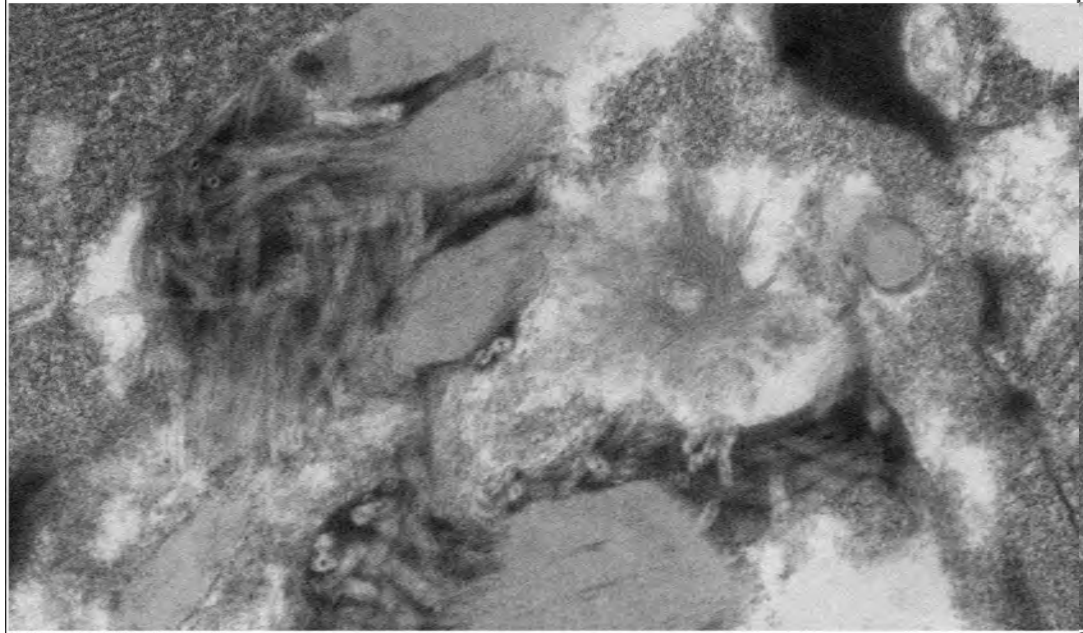

21-20_Correa_3-19-3_16A5_072.tif
Sample: 3/19, 3
Biological Electron Microscopy Lab
Rice University - SEA
Microscopist: MD Meyer

200 nm
HV=80kV
Direct Mag: 20000 x

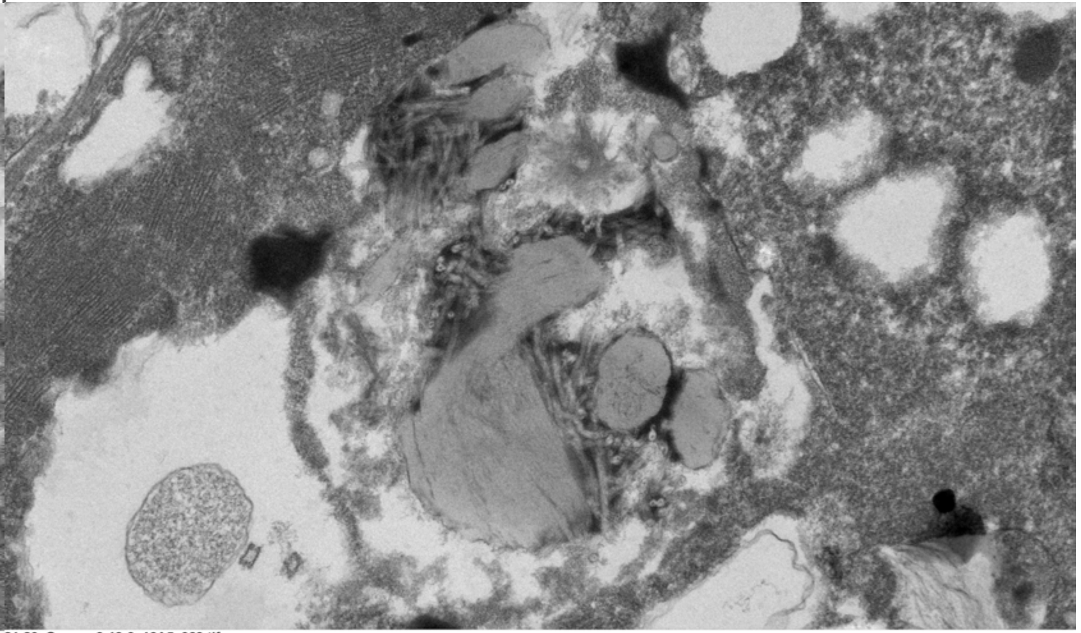

21-20_Correa_3-19-3_16A5_069.tif
Sample: 3/19, 3
Biological Electron Microscopy Lab
Rice University - SEA
Microscopist: MD Meyer

500 nm
HV=80kV
Direct Mag: 8000 x

Cell 10

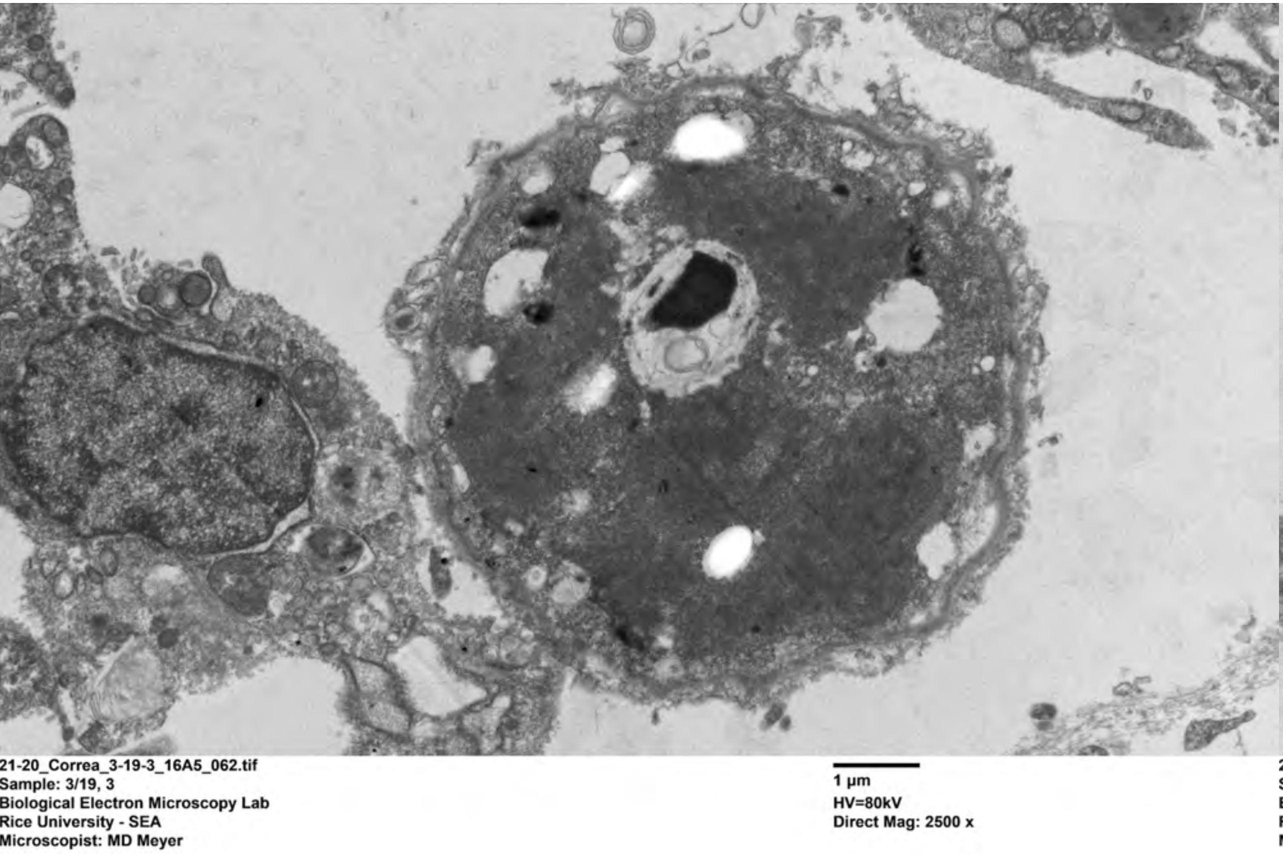
21-20_Correa_3-19-3_16A5_062.tif
Sample: 3/19, 3
Biological Electron Microscopy Lab
Rice University - SEA
Microscopist: MD Meyer
1 µm
HV=80kV
Direct Mag: 2500 x

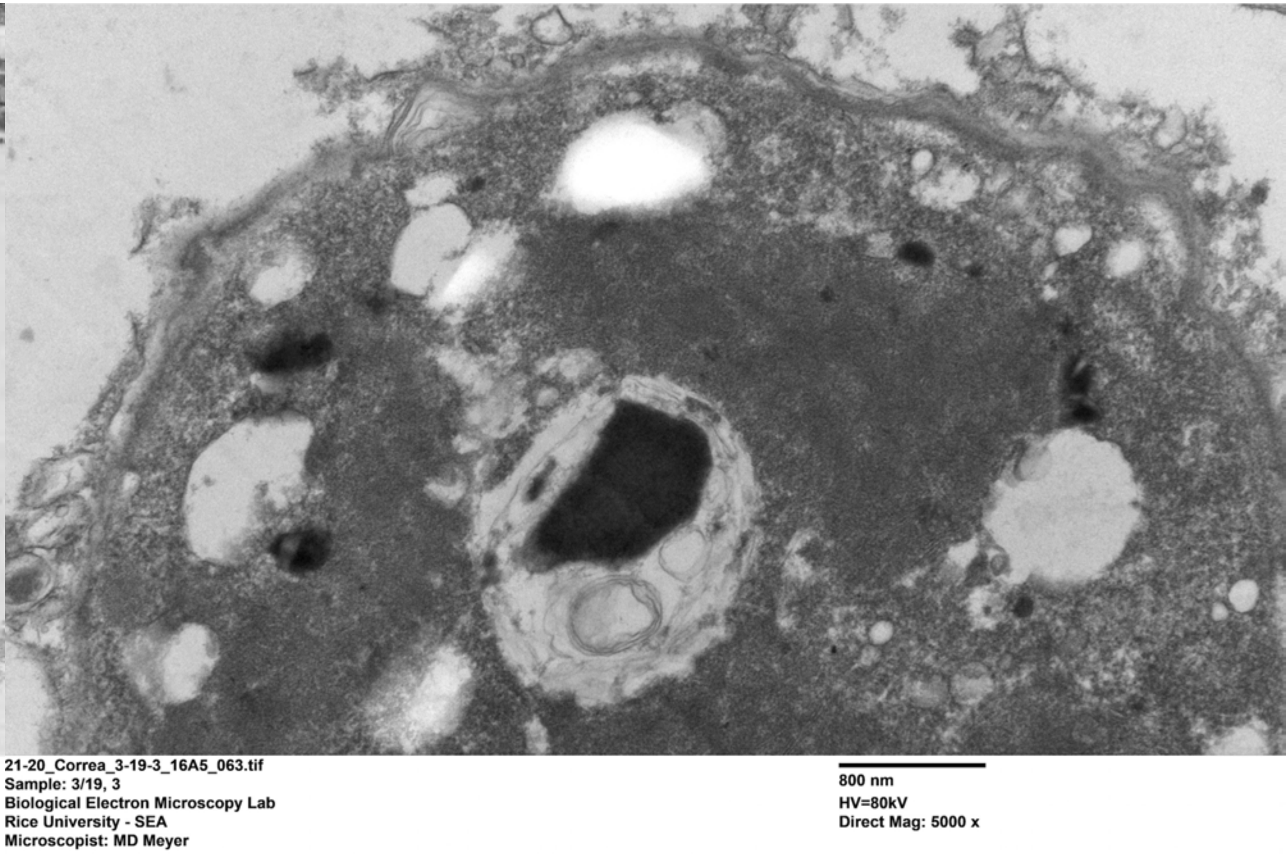
21-20_Correa_3-19-3_16A5_063.tif
Sample: 3/19, 3
Biological Electron Microscopy Lab
Rice University - SEA
Microscopist: MD Meyer
800 nm
HV=80kV
Direct Mag: 5000 x

Cell 11

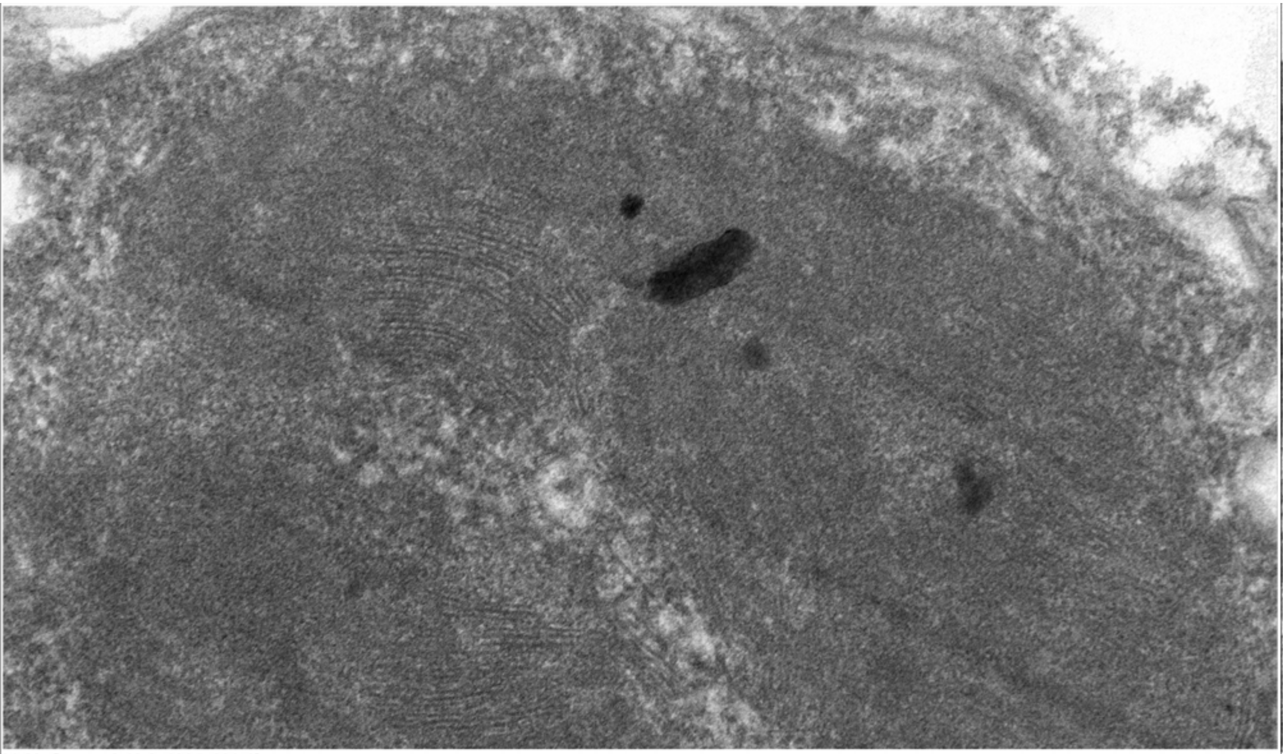

21-20_Correa_3-19-3_16A5_061.tif
Sample: 3/19, 3
Biological Electron Microscopy Lab
Rice University - SEA
Microscopist: MD Meyer

400 nm
HV=80kV
Direct Mag: 10000 x

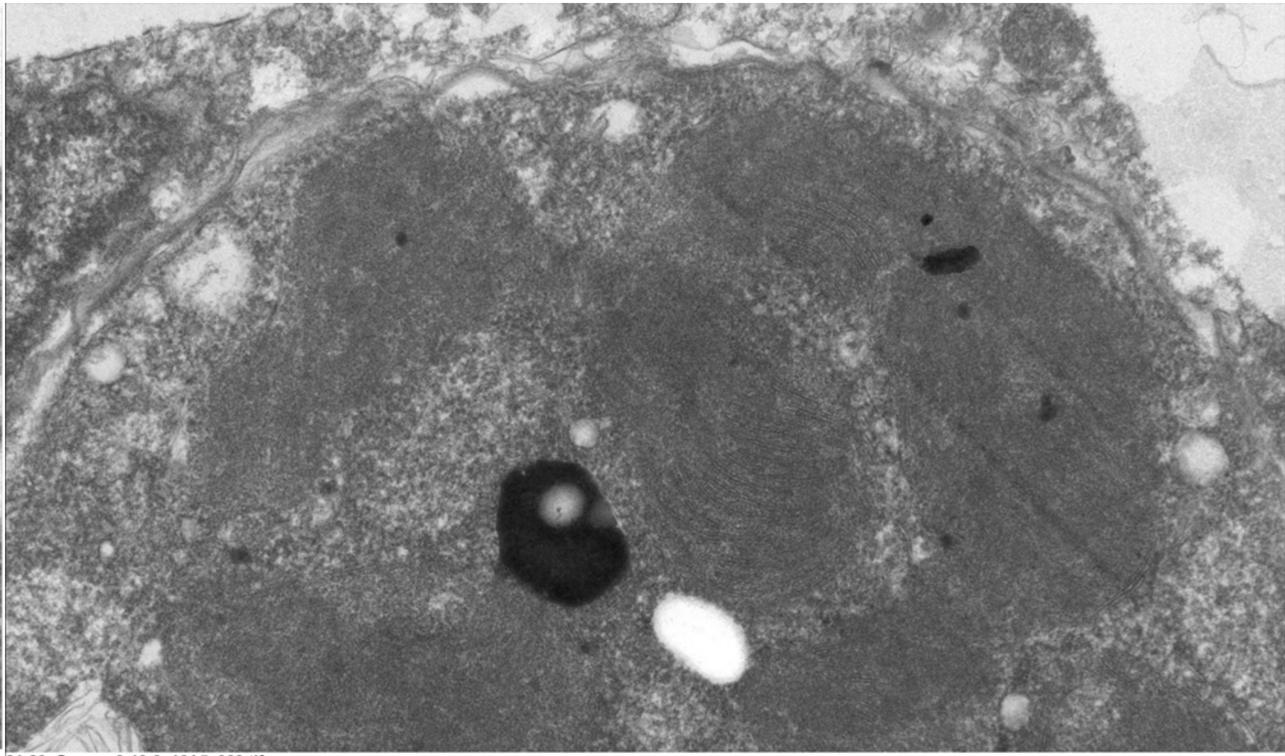

21-20_Correa_3-19-3_16A5_060.tif
Sample: 3/19, 3
Biological Electron Microscopy Lab
Rice University - SEA
Microscopist: MD Meyer

800 nm
HV=80kV
Direct Mag: 5000 x

Cell 12

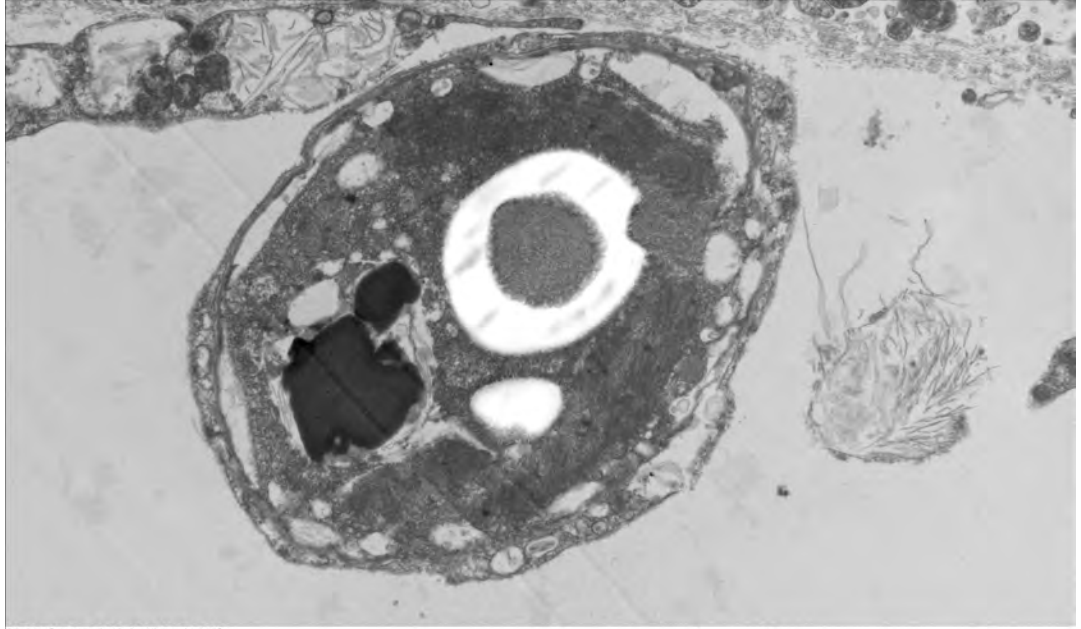

21-20_Correa_3-19-3_16A5_056.tif
Sample: 3/19, 3
Biological Electron Microscopy Lab
Rice University - SEA
Microscopist: MD Meyer

1 µm
HV=80kV
Direct Mag: 2500 x

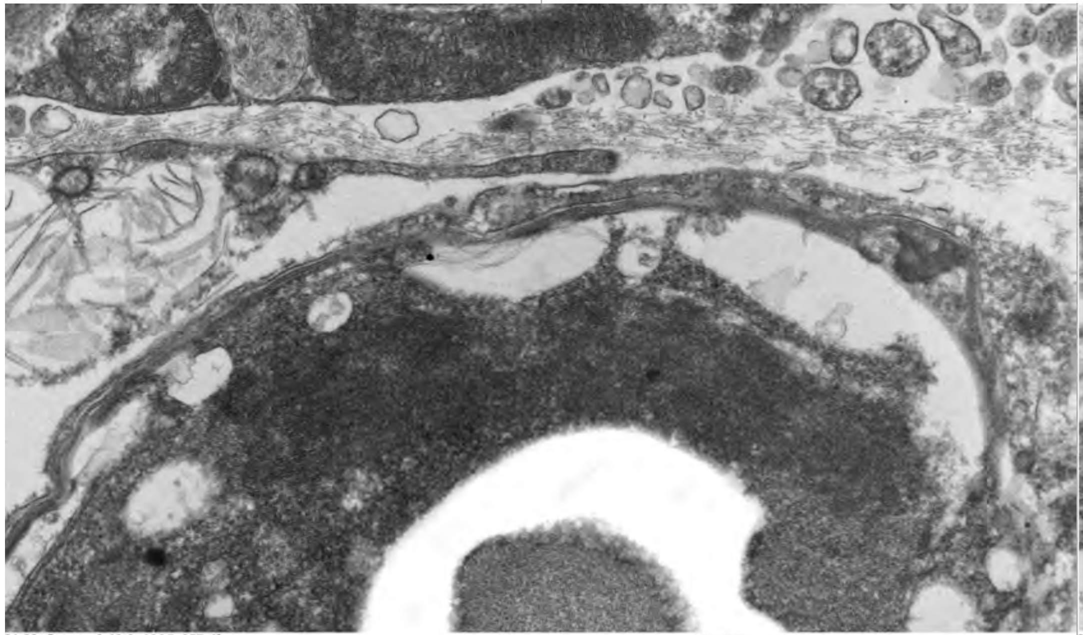

21-20_Correa_3-19-3_16A5_057.tif
Sample: 3/19, 3
Biological Electron Microscopy Lab
Rice University - SEA
Microscopist: MD Meyer

800 nm
HV=80kV
Direct Mag: 5000 x

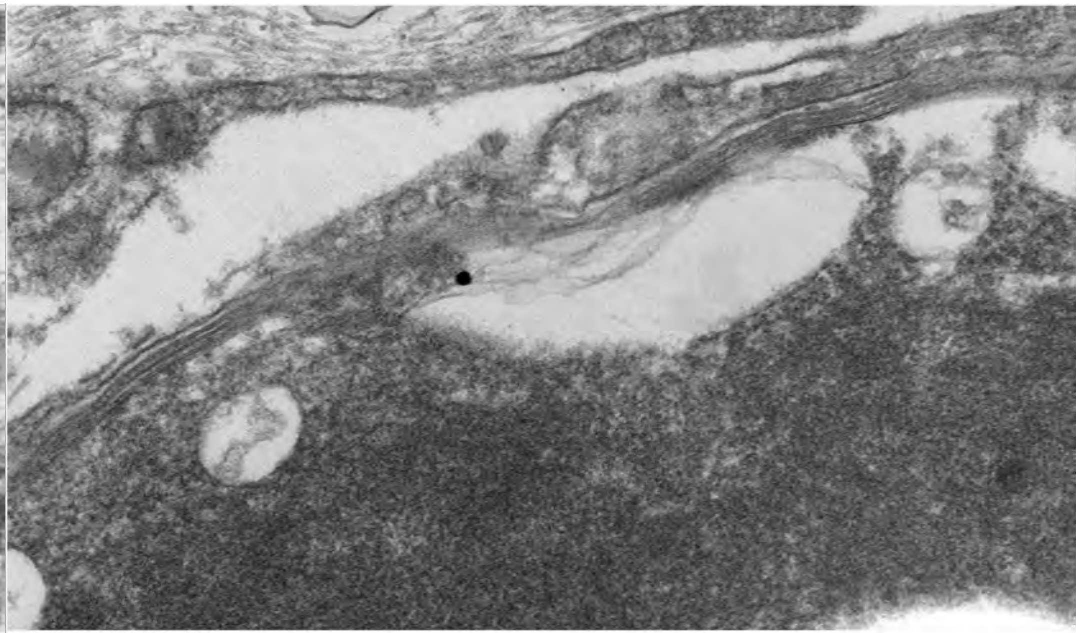

21-20_Correa_3-19-3_16A5_058.tif
Sample: 3/19, 3
Biological Electron Microscopy Lab
Rice University - SEA
Microscopist: MD Meyer

200 nm
HV=80kV
Direct Mag: 12000 x

Cell 13

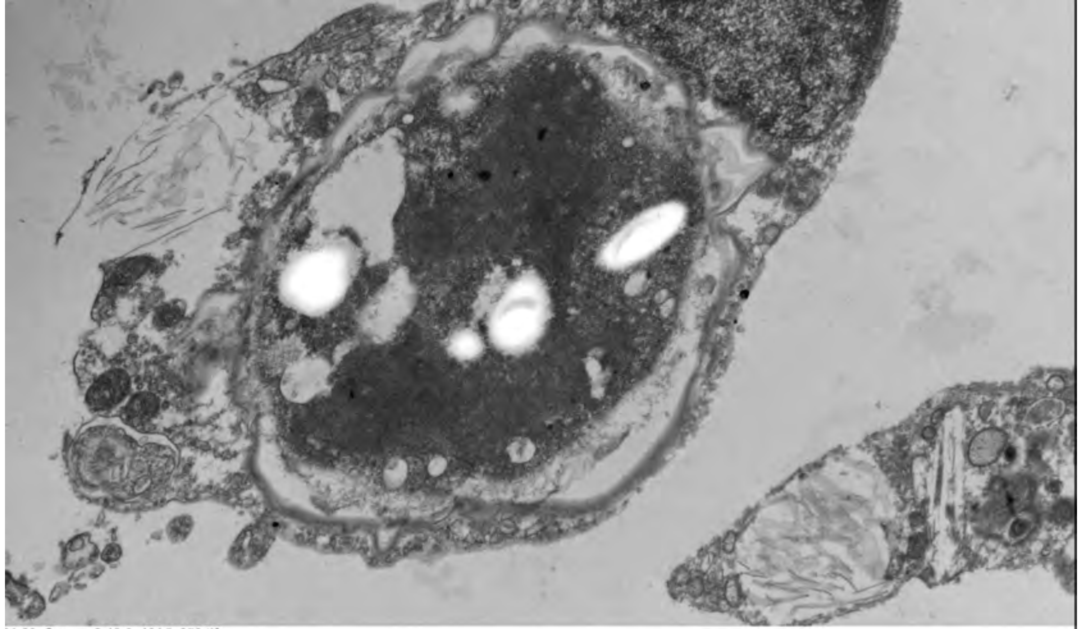

21-20_Correa_3-19-3_16A5_053.tif
Sample: 3/19, 3
Biological Electron Microscopy Lab
Rice University - SEA
Microscopist: MD Meyer

1 µm
HV=80kV
Direct Mag: 3000 x

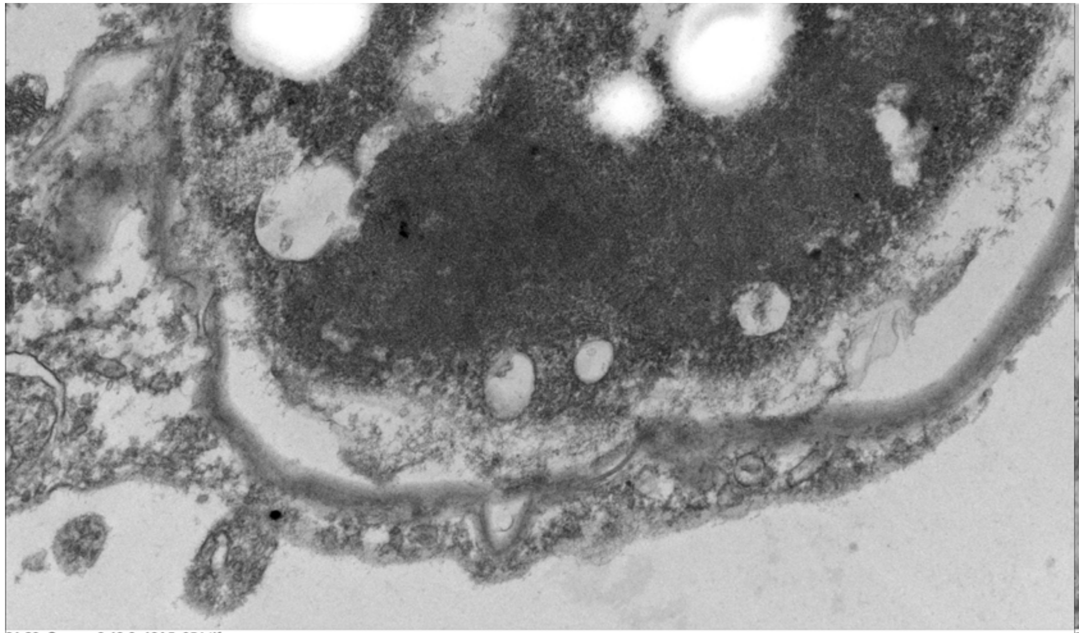

21-20_Correa_3-19-3_16A5_054.tif
Sample: 3/19, 3
Biological Electron Microscopy Lab
Rice University - SEA
Microscopist: MD Meyer

600 nm
HV=80kV
Direct Mag: 6000 x

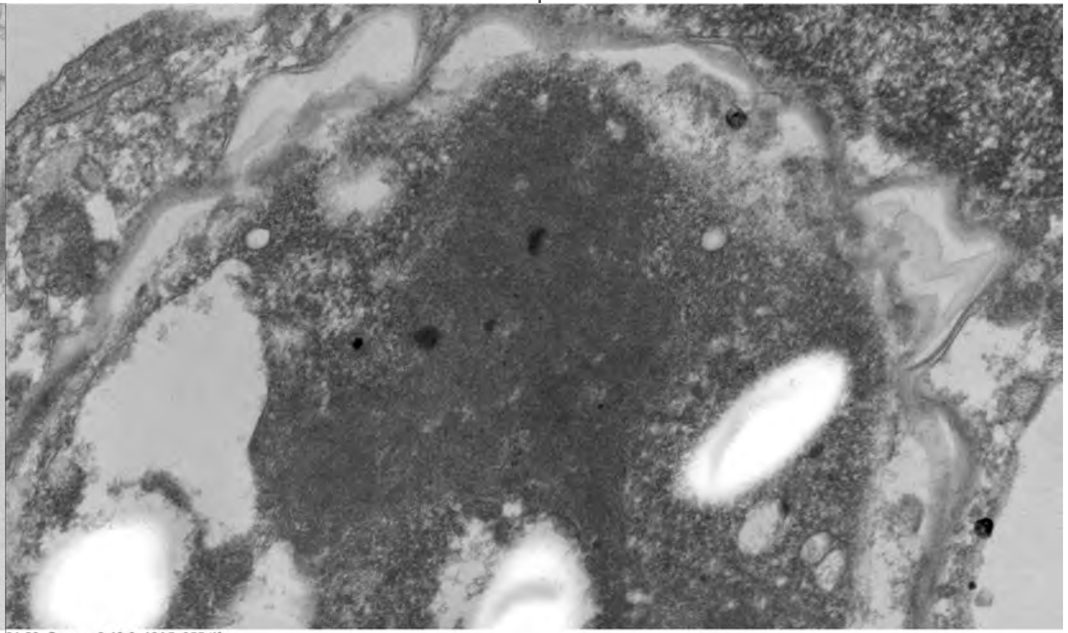

21-20_Correa_3-19-3_16A5_055.tif
Sample: 3/19, 3
Biological Electron Microscopy Lab
Rice University - SEA
Microscopist: MD Meyer

600 nm
HV=80kV
Direct Mag: 6000 x

Cell 15

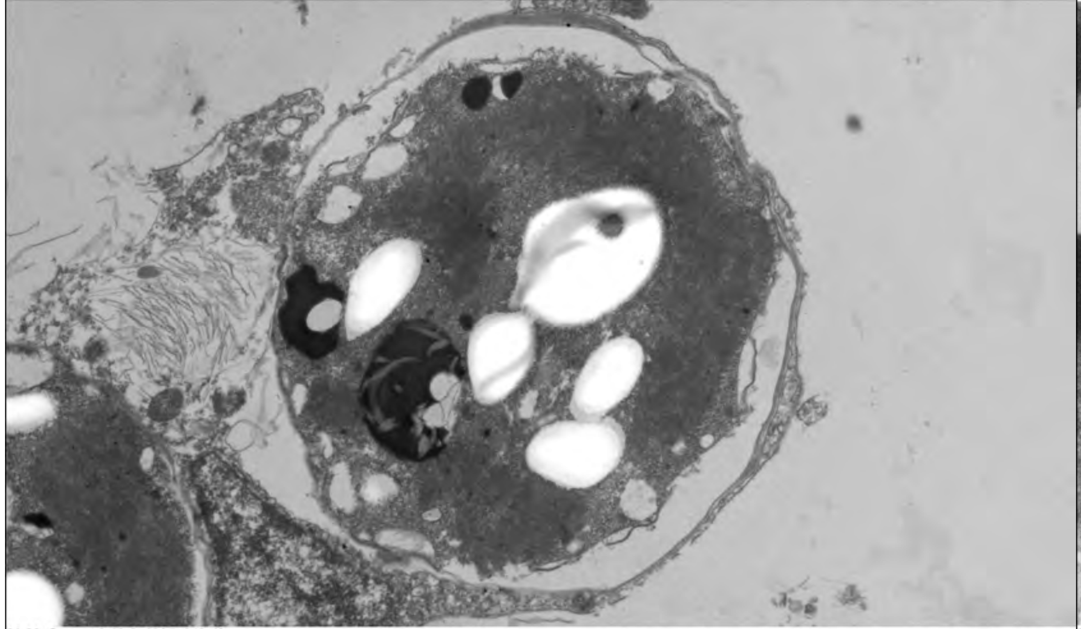

21-20_Correa_3-19-3_16A5_048.tif
Sample: 3/19, 3
Biological Electron Microscopy Lab
Rice University - SEA
Microscopist: MD Meyer

1 µm
HV=80kV
Direct Mag: 2500 x

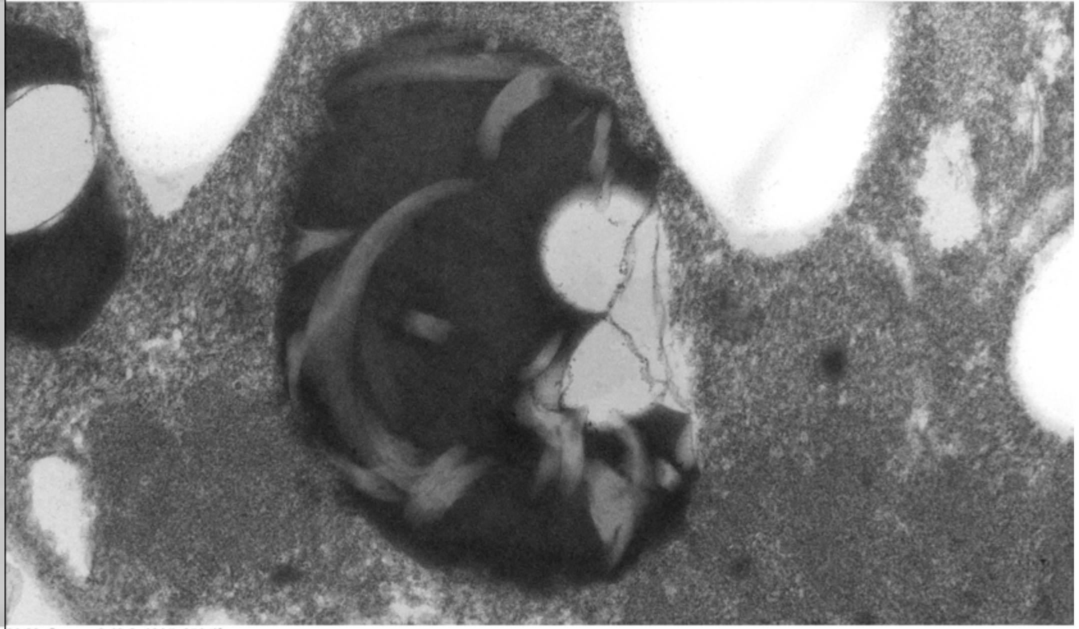

21-20_Correa_3-19-3_16A5_051.tif
Sample: 3/19, 3
Biological Electron Microscopy Lab
Rice University - SEA
Microscopist: MD Meyer

400 nm
HV=80kV
Direct Mag: 10000 x

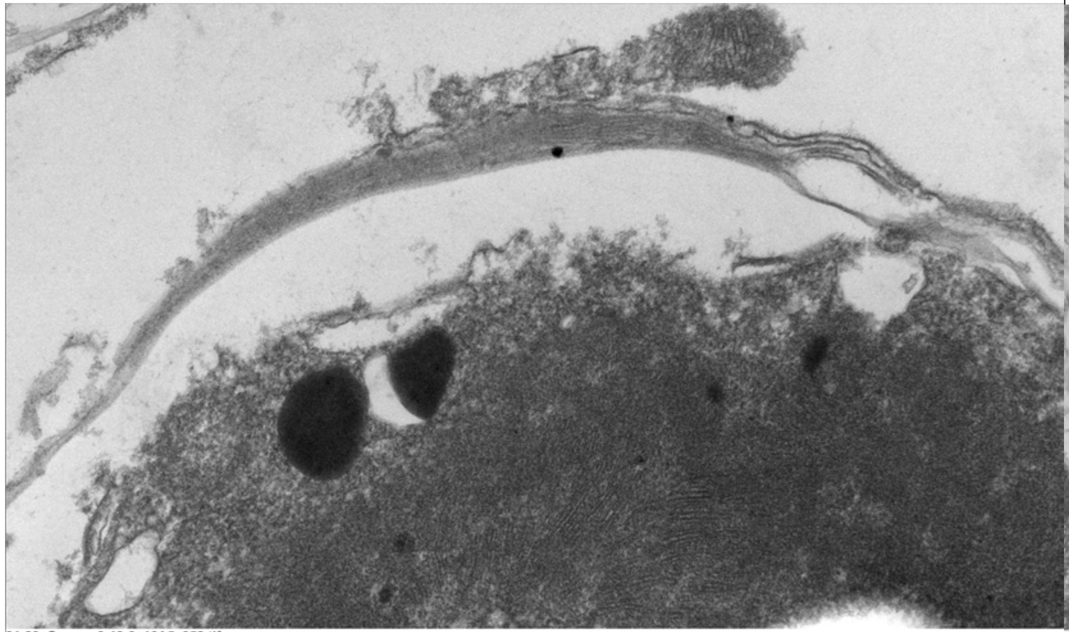

21-20_Correa_3-19-3_16A5_052.tif
Sample: 3/19, 3
Biological Electron Microscopy Lab
Rice University - SEA
Microscopist: MD Meyer

500 nm
HV=80kV
Direct Mag: 8000 x

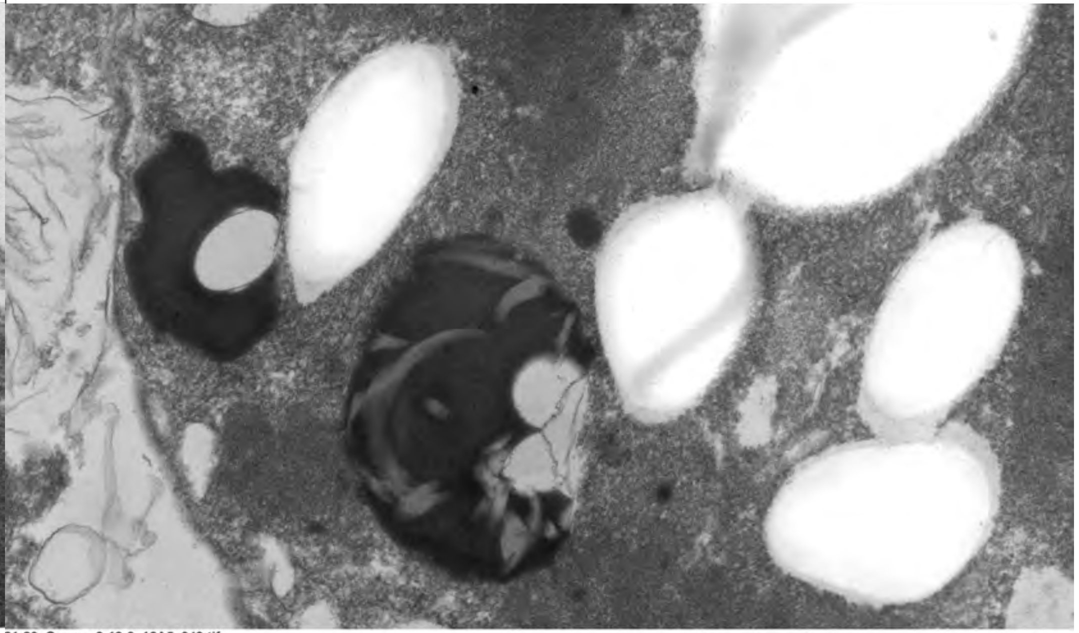

21-20_Correa_3-19-3_16A5_049.tif
Sample: 3/19, 3
Biological Electron Microscopy Lab
Rice University - SEA
Microscopist: MD Meyer

600 nm
HV=80kV
Direct Mag: 6000 x

Cell 16

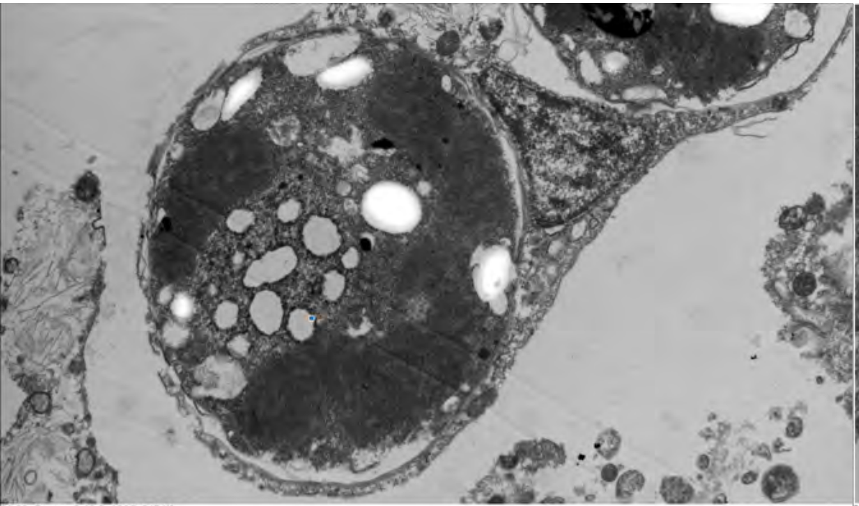

21-20_Correa_3-19-3_16A5_042.tif
Sample: 3/19, 3
Biological Electron Microscopy Lab
Rice University - SEA
Microscopist: MD Meyer

2 µm
HV=80kV
Direct Mag: 2000 x

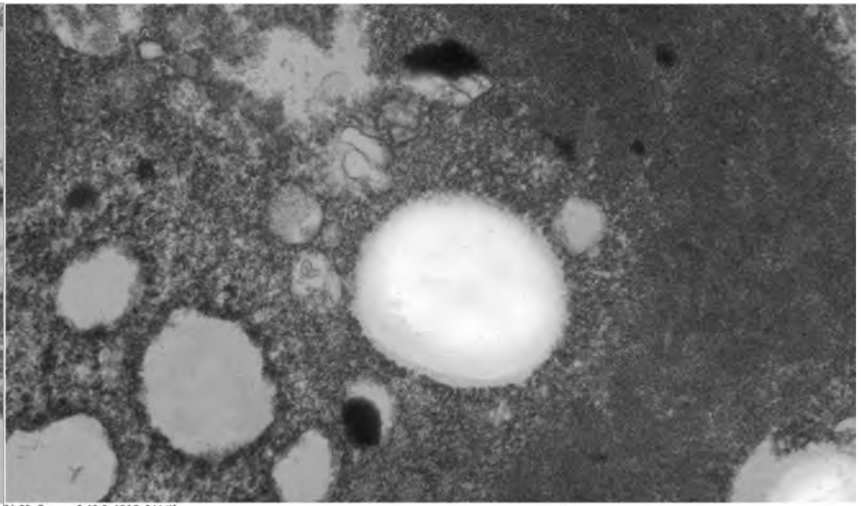

21-20_Correa_3-19-3_16A5_044.tif
Sample: 3/19, 3
Biological Electron Microscopy Lab
Rice University - SEA
Microscopist: MD Meyer

500 nm
HV=80kV
Direct Mag: 8000 x

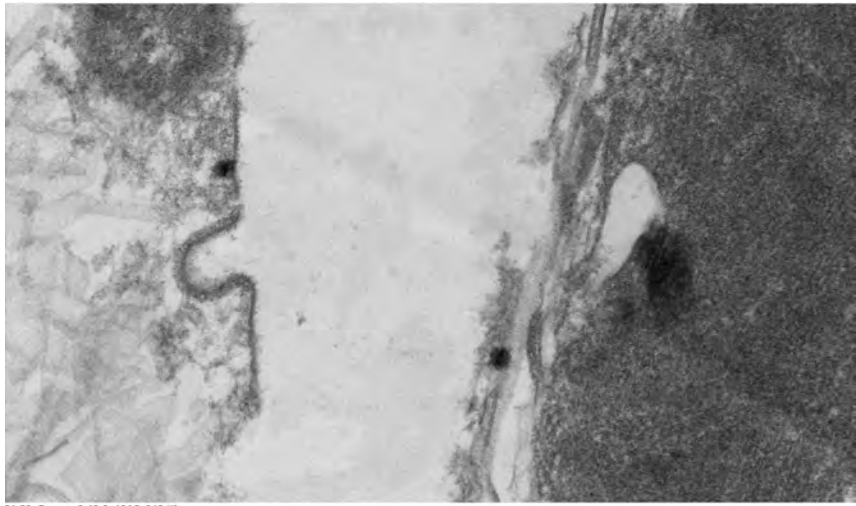

21-20_Correa_3-19-3_16A5_046.tif
Sample: 3/19, 3
Biological Electron Microscopy Lab
Rice University - SEA
Microscopist: MD Meyer

200 nm
HV=80kV
Direct Mag: 15000 x

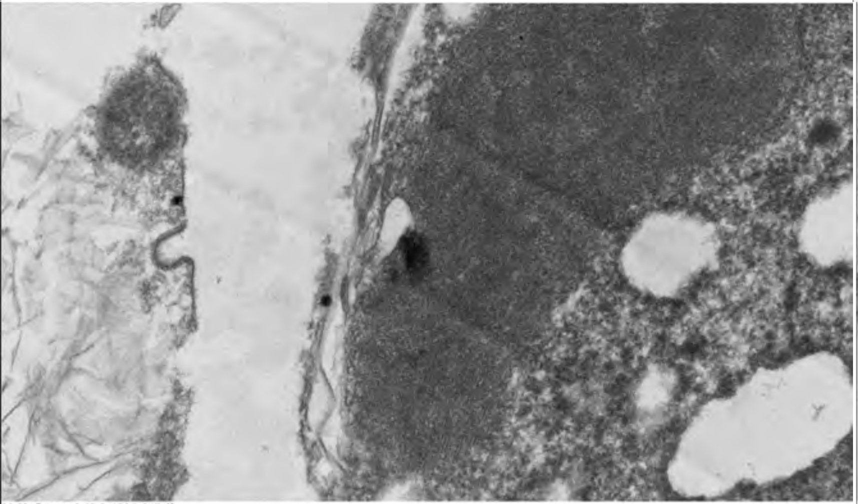

21-20_Correa_3-19-3_16A5_045.tif
Sample: 3/19, 3
Biological Electron Microscopy Lab
Rice University - SEA
Microscopist: MD Meyer

500 nm
HV=80kV
Direct Mag: 8000 x

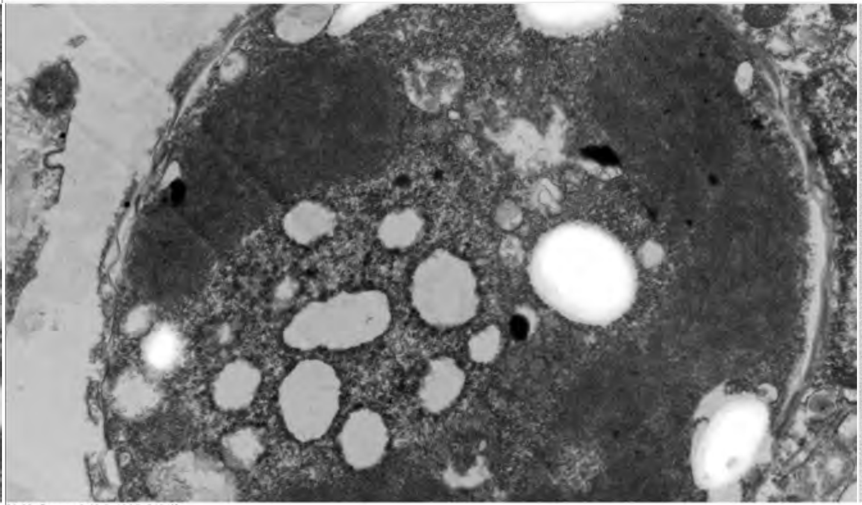

21-20_Correa_3-19-3_16A5_043.tif
Sample: 3/19, 3
Biological Electron Microscopy Lab
Rice University - SEA
Microscopist: MD Meyer

1 µm
HV=80kV
Direct Mag: 4000 x

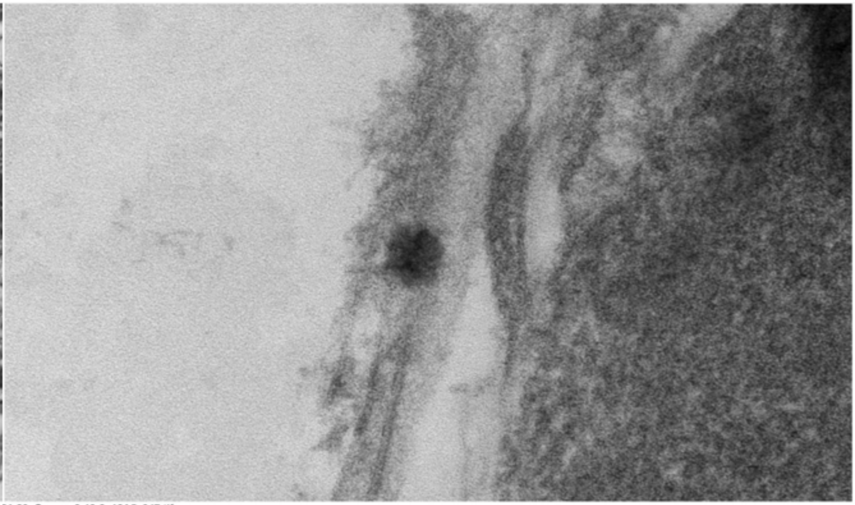

21-20_Correa_3-19-3_16A5_047.tif
Sample: 3/19, 3
Biological Electron Microscopy Lab
Rice University - SEA
Microscopist: MD Meyer

100 nm
HV=80kV
Direct Mag: 40000 x

Cell 17

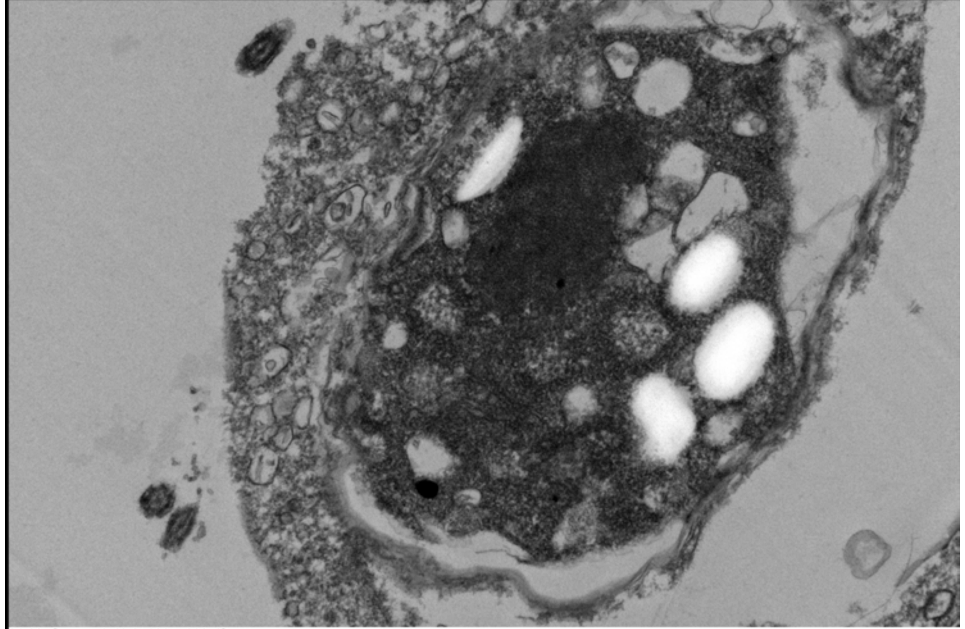

21-20_Correa_3-19-3_16A5_038.tif
Sample: 3/19, 3
Biological Electron Microscopy Lab
Rice University - SEA
Microscopist: MD Meyer

1 µm
HV=80kV
Direct Mag: 4000 x

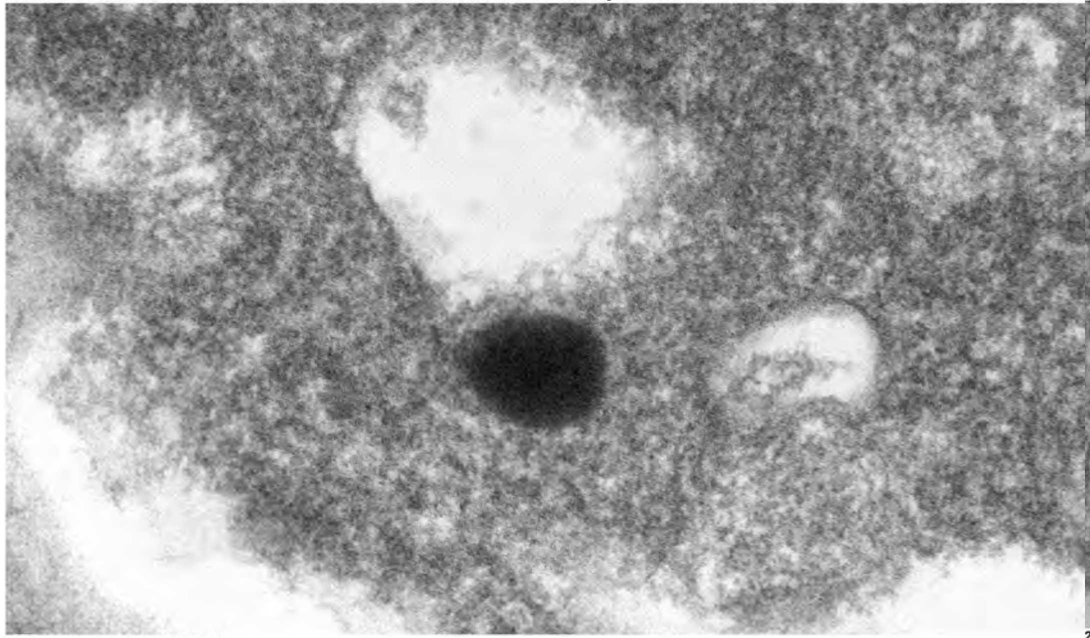

21-20_Correa_3-19-3_16A5_040.tif
Sample: 3/19, 3
Biological Electron Microscopy Lab
Rice University - SEA
Microscopist: MD Meyer

100 nm
HV=80kV
Direct Mag: 25000 x

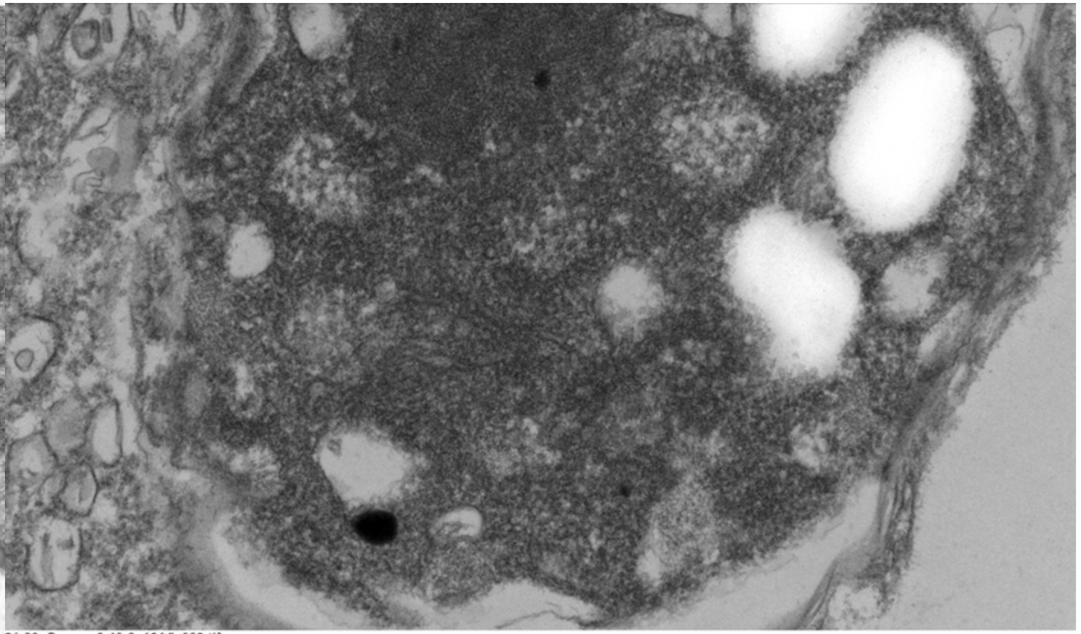

21-20_Correa_3-19-3_16A5_039.tif
Sample: 3/19, 3
Biological Electron Microscopy Lab
Rice University - SEA
Microscopist: MD Meyer

500 nm
HV=80kV
Direct Mag: 8000 x

Cell 18

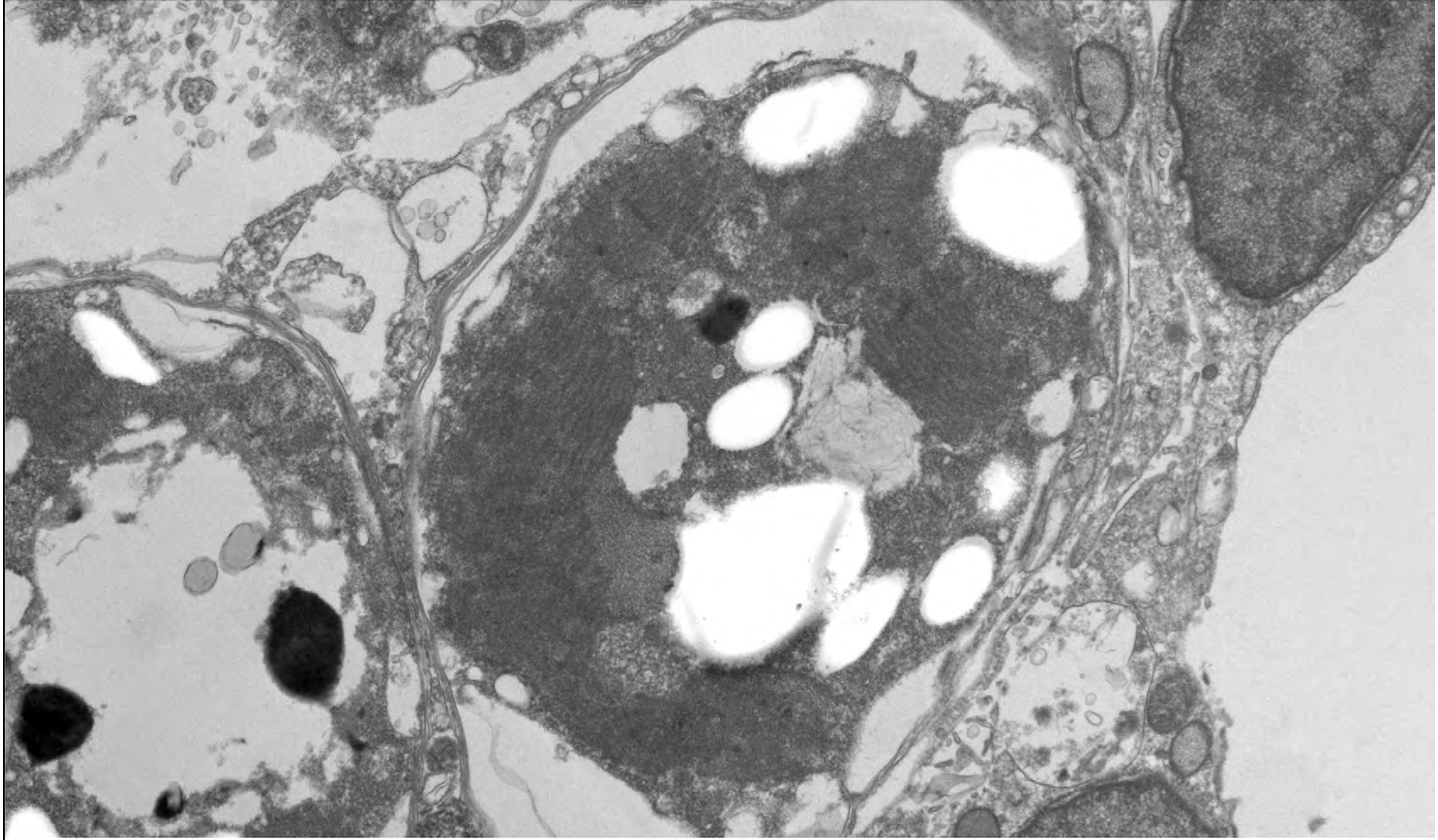

21-20_Correa_3-19-3_16A5_037.tif
Sample: 3/19, 3
Biological Electron Microscopy Lab
Rice University - SEA
Microscopist: MD Meyer

1 µm
HV=80kV
Direct Mag: 3000 x

Cell 19

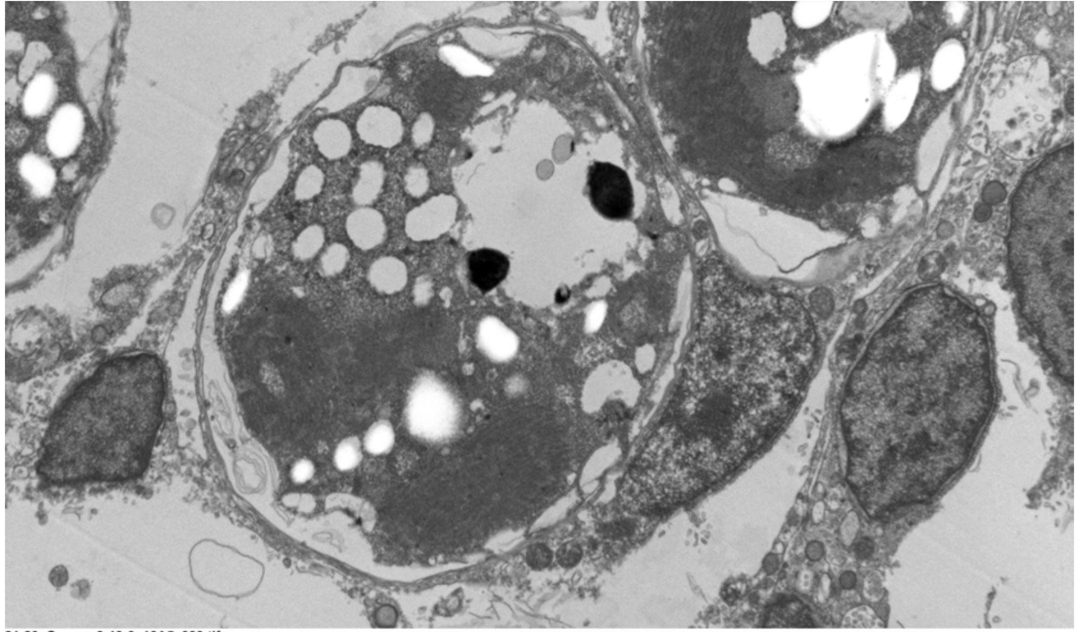

21-20_Correa_3-19-3_16A5_033.tif
Sample: 3/19, 3
Biological Electron Microscopy Lab
Rice University - SEA
Microscopist: MD Meyer

2 µm
HV=80kV
Direct Mag: 2000 x

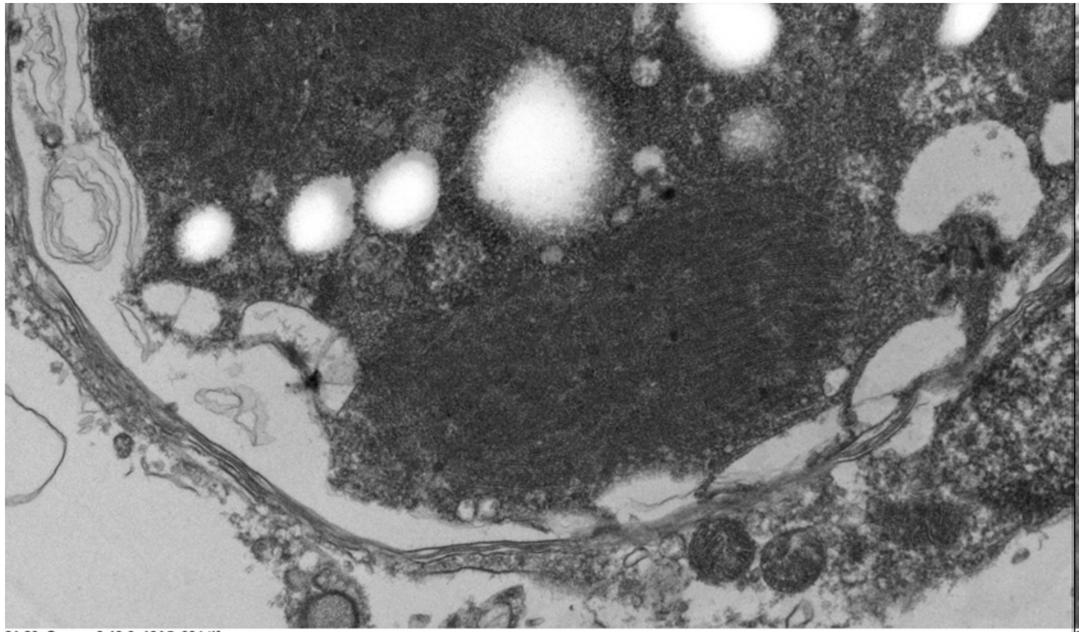

21-20_Correa_3-19-3_16A5_034.tif
Sample: 3/19, 3
Biological Electron Microscopy Lab
Rice University - SEA
Microscopist: MD Meyer

800 nm
HV=80kV
Direct Mag: 5000 x

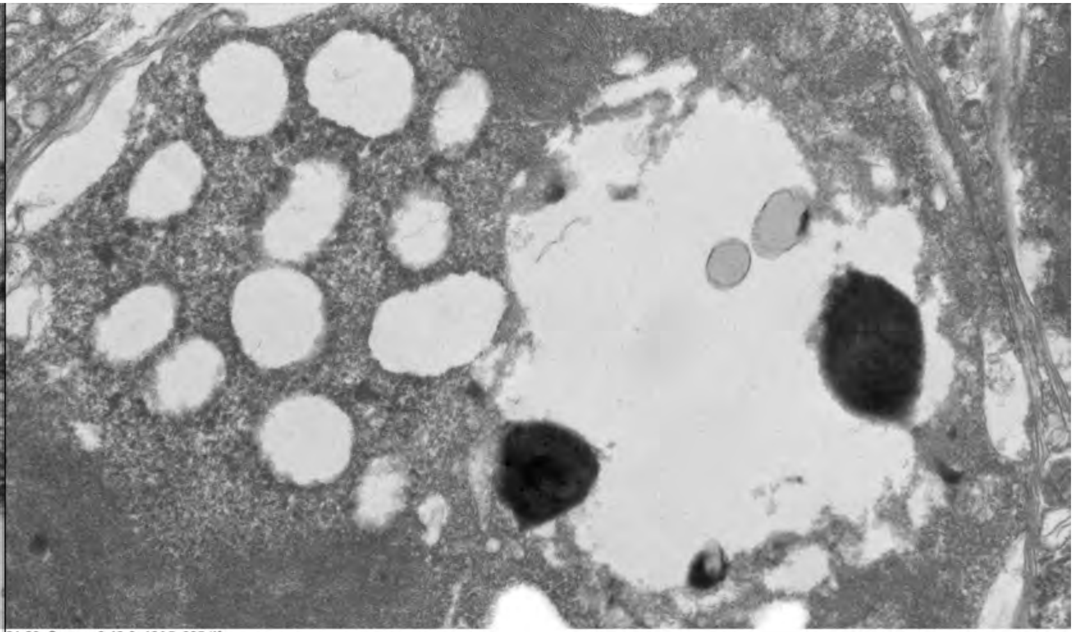

21-20_Correa_3-19-3_16A5_035.tif
Sample: 3/19, 3
Biological Electron Microscopy Lab
Rice University - SEA
Microscopist: MD Meyer

800 nm
HV=80kV
Direct Mag: 5000 x

Cell 20

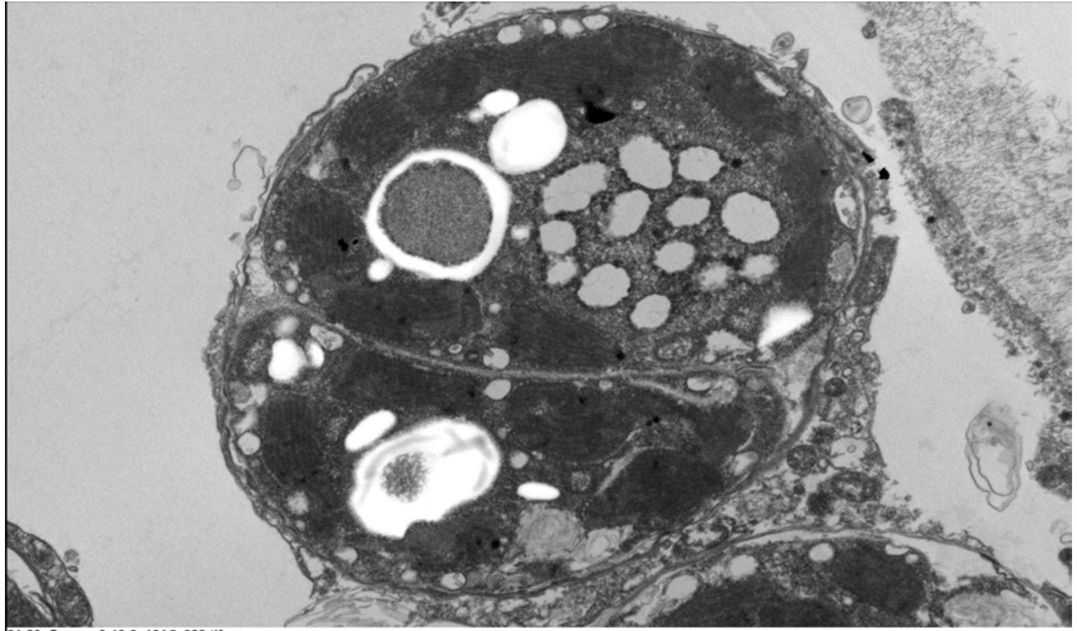

21-20_Correa_3-19-3_16A5_028.tif
Sample: 3/19, 3
Biological Electron Microscopy Lab
Rice University - SEA
Microscopist: MD Meyer

1 µm
HV=80kV
Direct Mag: 2500 x

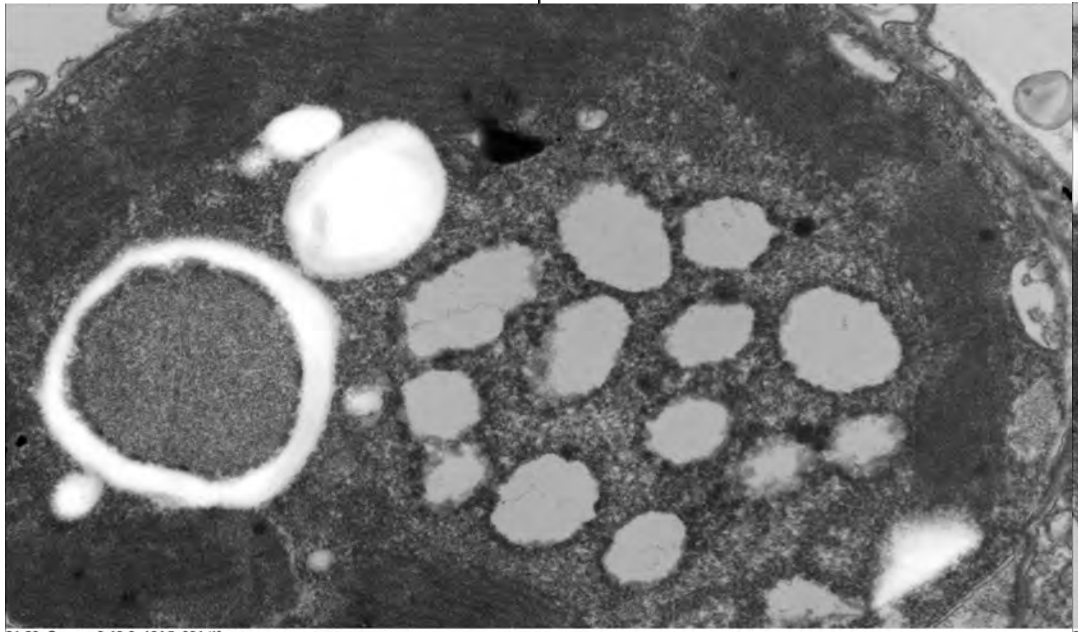

21-20_Correa_3-19-3_16A5_031.tif
Sample: 3/19, 3
Biological Electron Microscopy Lab
Rice University - SEA
Microscopist: MD Meyer

800 nm
HV=80kV
Direct Mag: 5000 x

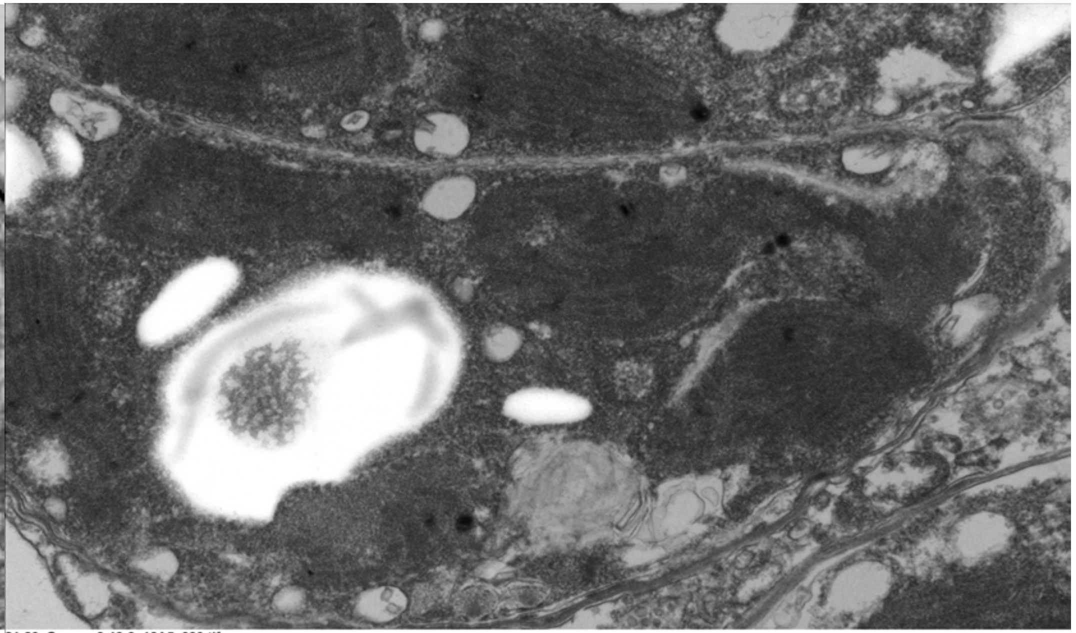

21-20_Correa_3-19-3_16A5_030.tif
Sample: 3/19, 3
Biological Electron Microscopy Lab
Rice University - SEA
Microscopist: MD Meyer

800 nm
HV=80kV
Direct Mag: 5000 x

Cell 21

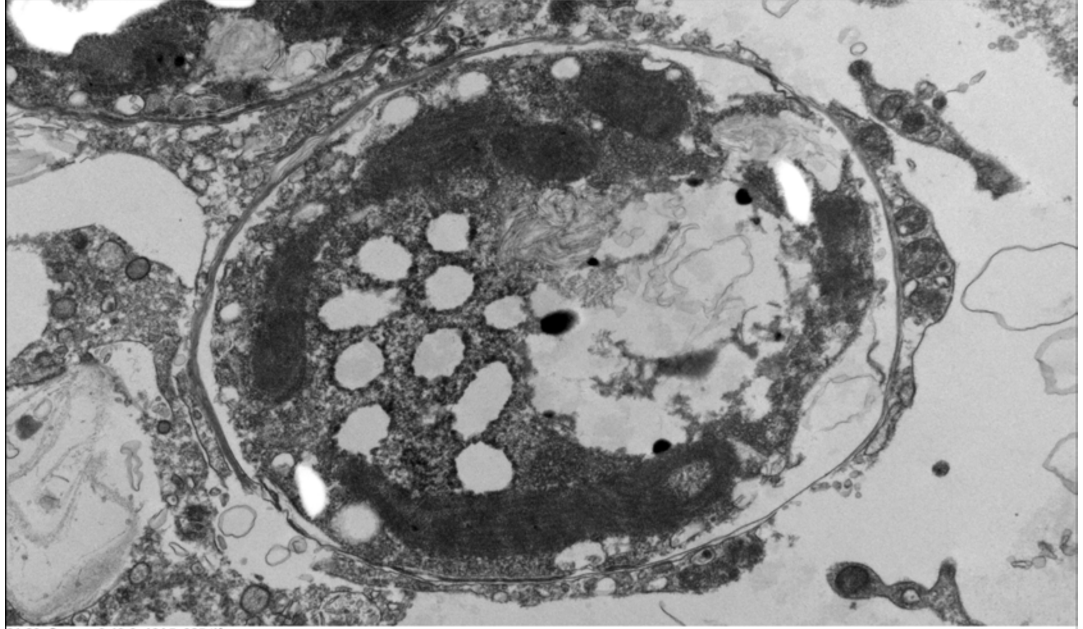

21-20_Correa_3-19-3_16A5_025.tif
Sample: 3/19, 3
Biological Electron Microscopy Lab
Rice University - SEA
Microscopist: MD Meyer

1 µm
HV=80kV
Direct Mag: 3000 x

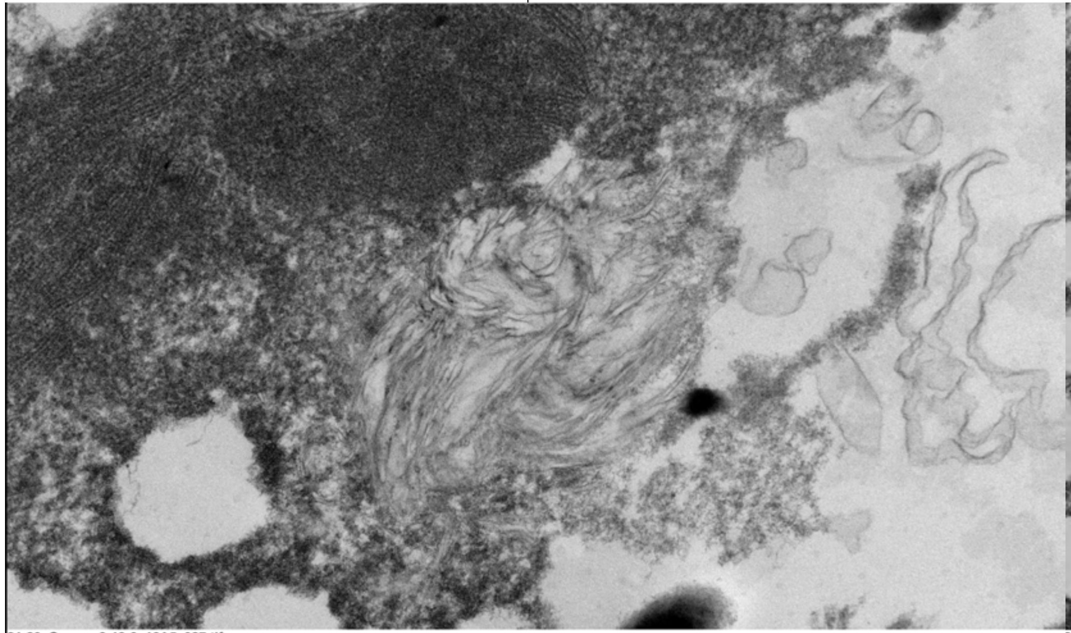

21-20_Correa_3-19-3_16A5_027.tif
Sample: 3/19, 3
Biological Electron Microscopy Lab
Rice University - SEA
Microscopist: MD Meyer

400 nm
HV=80kV
Direct Mag: 10000 x

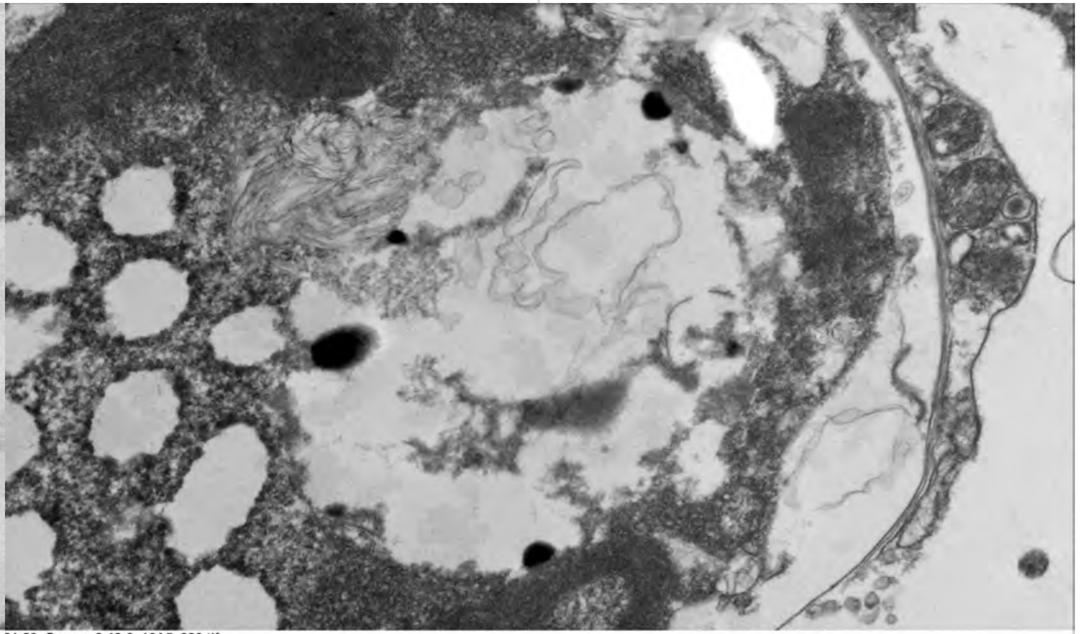

21-20_Correa_3-19-3_16A5_026.tif
Sample: 3/19, 3
Biological Electron Microscopy Lab
Rice University - SEA
Microscopist: MD Meyer

800 nm
HV=80kV
Direct Mag: 5000 x

Cell 22

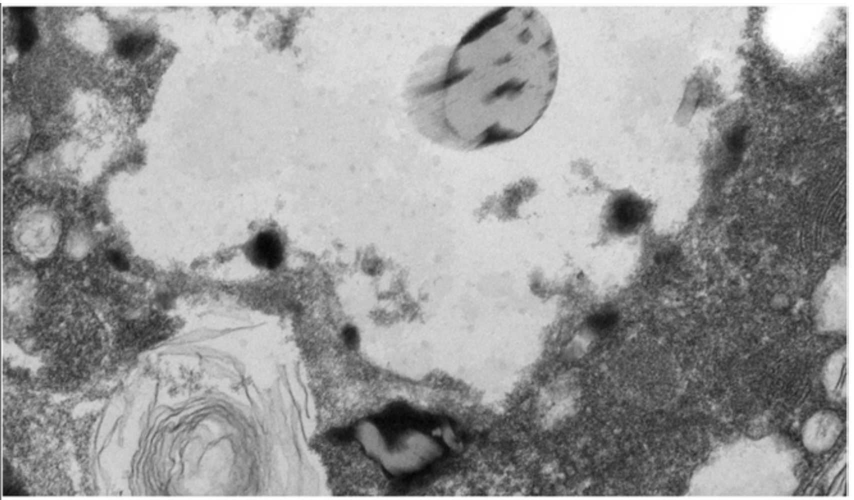

21-20_Correa_3-19-3_16A5_024.tif
Sample: 3/19, 3
Biological Electron Microscopy Lab
Rice University - SEA
Microscopist: MD Meyer
400 nm
HV=80kV
Direct Mag: 10000 x

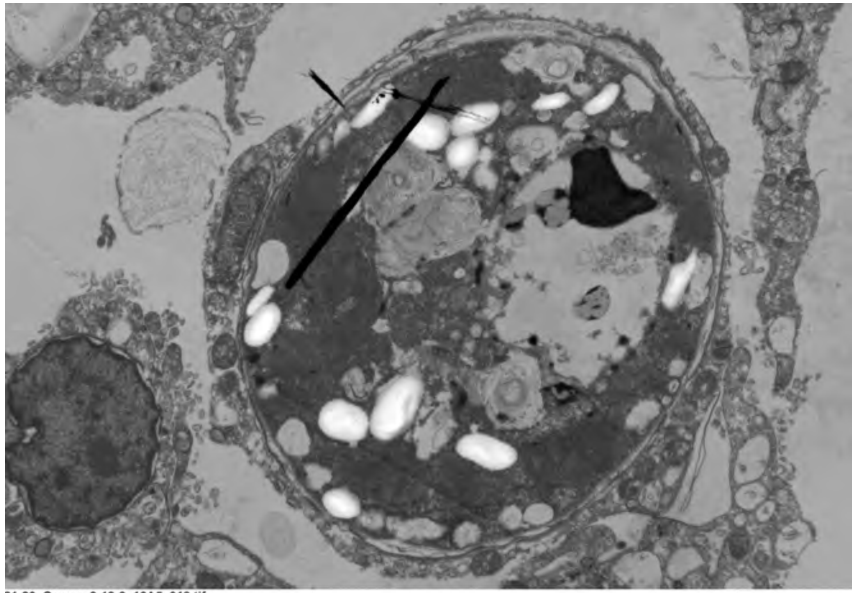

21-20_Correa_3-19-3_16A5_018.tif
Sample: 3/19, 3
Biological Electron Microscopy Lab
Rice University - SEA
Microscopist: MD Meyer
2 µm
HV=80kV
Direct Mag: 2000 x

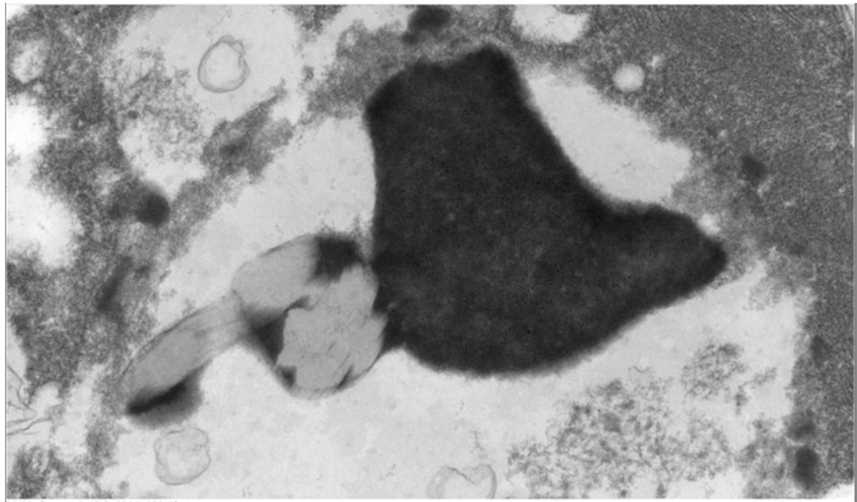

21-20_Correa_3-19-3_16A5_023.tif
Sample: 3/19, 3
Biological Electron Microscopy Lab
Rice University - SEA
Microscopist: MD Meyer
400 nm
HV=80kV
Direct Mag: 10000 x

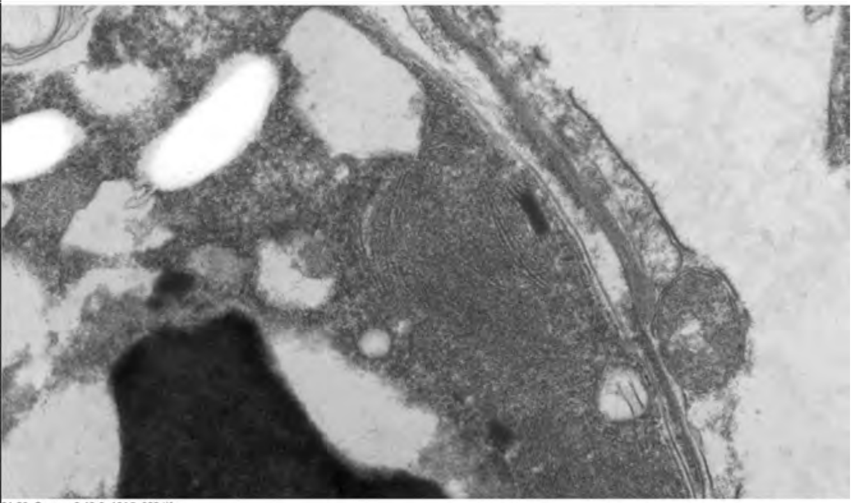

21-20_Correa_3-19-3_16A5_022.tif
Sample: 3/19, 3
Biological Electron Microscopy Lab
Rice University - SEA
Microscopist: MD Meyer
400 nm
HV=80kV
Direct Mag: 10000 x

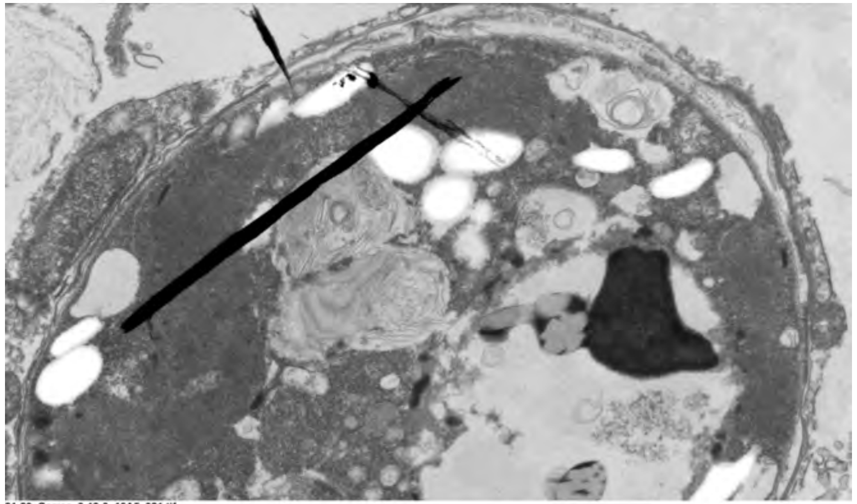

21-20_Correa_3-19-3_16A5_021.tif
Sample: 3/19, 3
Biological Electron Microscopy Lab
Rice University - SEA
Microscopist: MD Meyer
1 µm
HV=80kV
Direct Mag: 4000 x

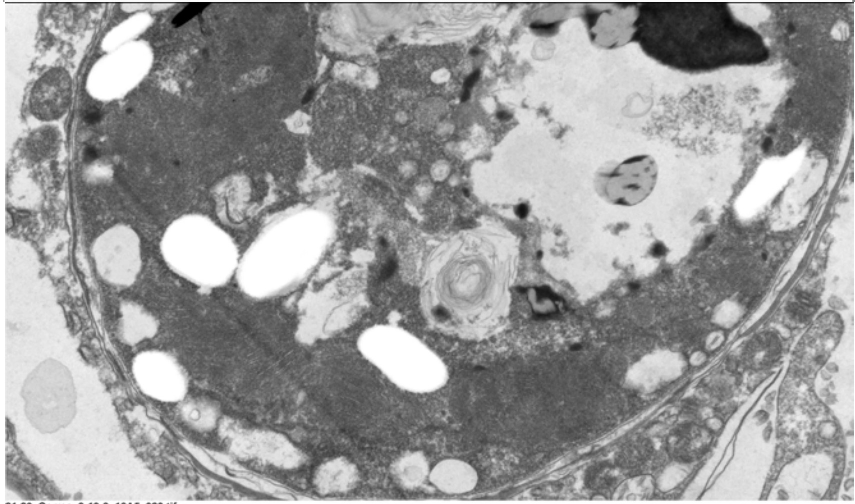

21-20_Correa_3-19-3_16A5_020.tif
Sample: 3/19, 3
Biological Electron Microscopy Lab
Rice University - SEA
Microscopist: MD Meyer
1 µm
HV=80kV
Direct Mag: 4000 x

Cell 23

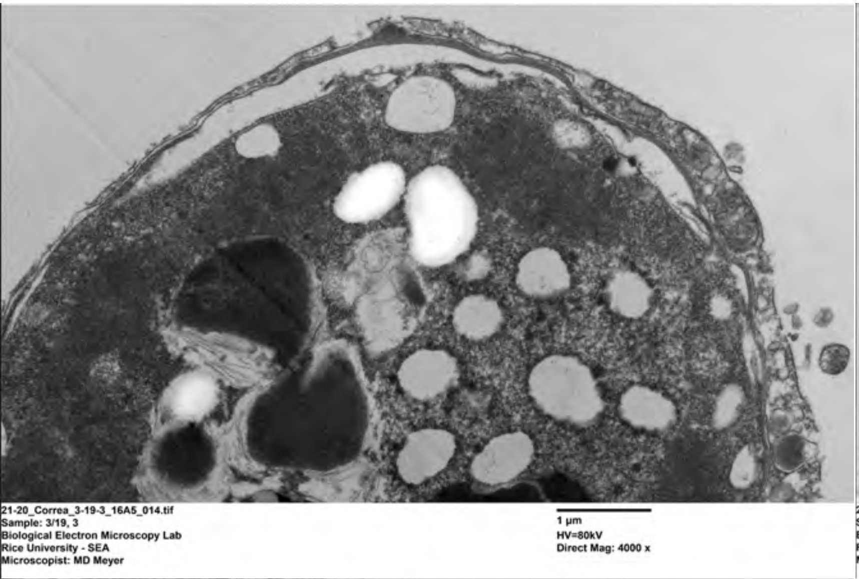
21-20_Correa_3-19-3_16A5_014.tif
Sample: 3/19, 3
Biological Electron Microscopy Lab
Rice University - SEA
Microscopist: MD Meyer
1 μm
HV=80kV
Direct Mag: 4000 x

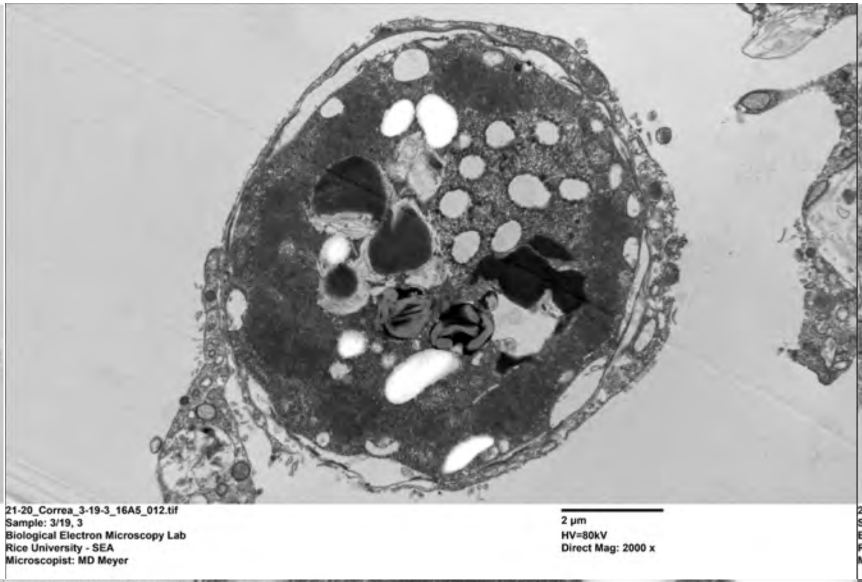
21-20_Correa_3-19-3_16A5_012.tif
Sample: 3/19, 3
Biological Electron Microscopy Lab
Rice University - SEA
Microscopist: MD Meyer
2 μm
HV=80kV
Direct Mag: 2000 x

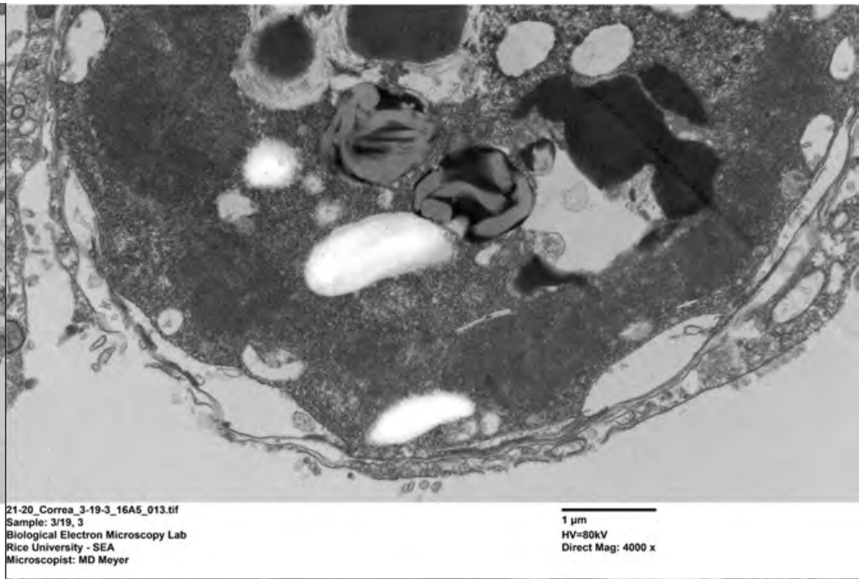
21-20_Correa_3-19-3_16A5_013.tif
Sample: 3/19, 3
Biological Electron Microscopy Lab
Rice University - SEA
Microscopist: MD Meyer
1 μm
HV=80kV
Direct Mag: 4000 x

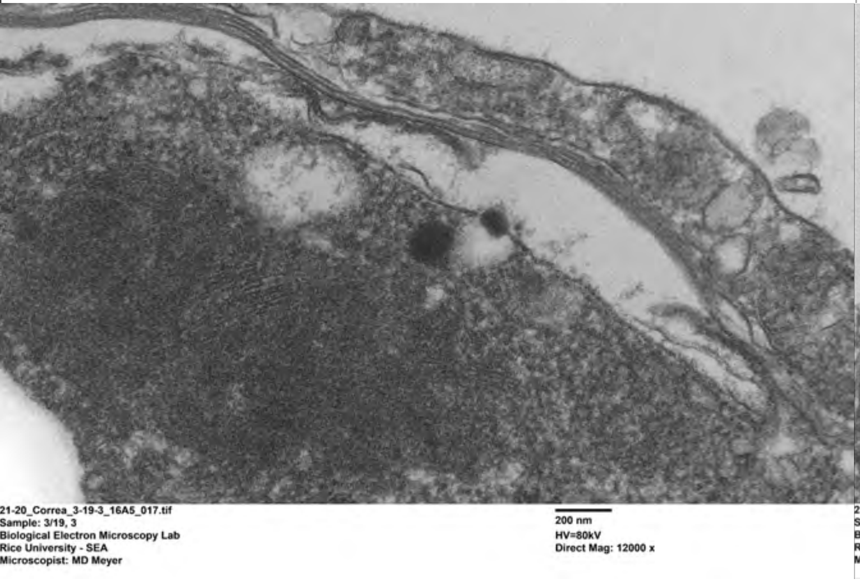
21-20_Correa_3-19-3_16A5_017.tif
Sample: 3/19, 3
Biological Electron Microscopy Lab
Rice University - SEA
Microscopist: MD Meyer
200 nm
HV=80kV
Direct Mag: 12000 x

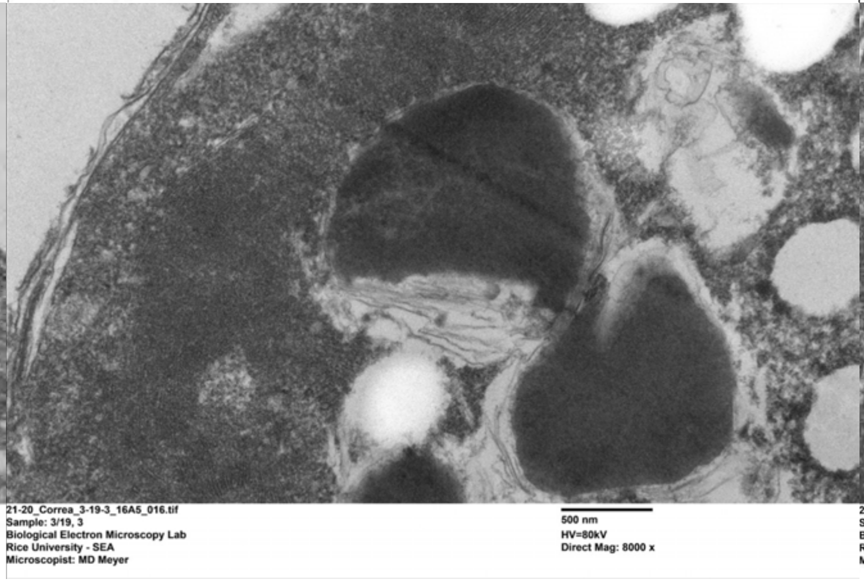
21-20_Correa_3-19-3_16A5_016.tif
Sample: 3/19, 3
Biological Electron Microscopy Lab
Rice University - SEA
Microscopist: MD Meyer
500 nm
HV=80kV
Direct Mag: 8000 x

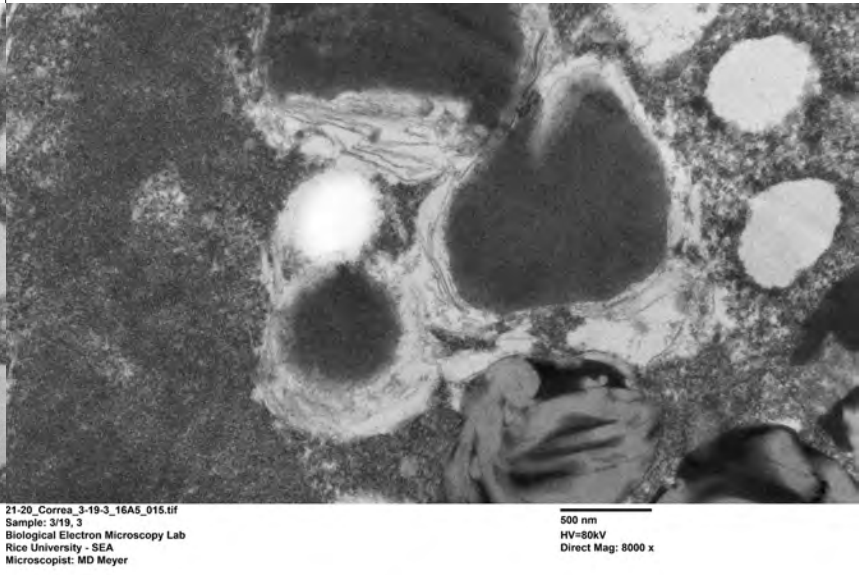
21-20_Correa_3-19-3_16A5_015.tif
Sample: 3/19, 3
Biological Electron Microscopy Lab
Rice University - SEA
Microscopist: MD Meyer
500 nm
HV=80kV
Direct Mag: 8000 x

Cell 24

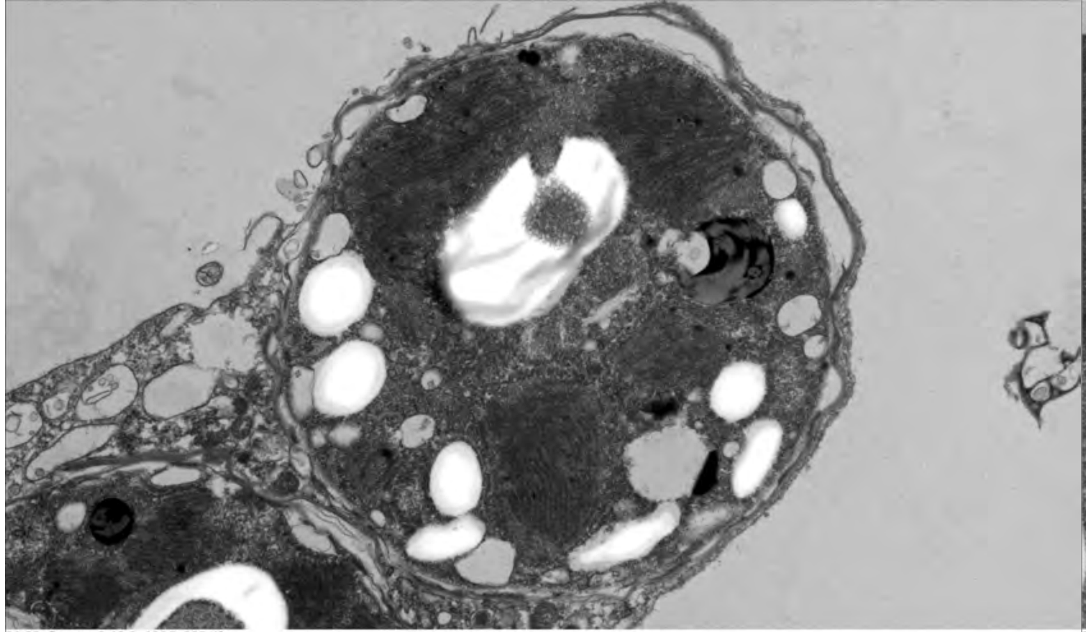

21-20_Correa_3-19-3_16A5_005.tif
Sample: 3/19, 3
Biological Electron Microscopy Lab
Rice University - SEA
Microscopist: MD Meyer

1 μm
HV=80kV
Direct Mag: 2500 x

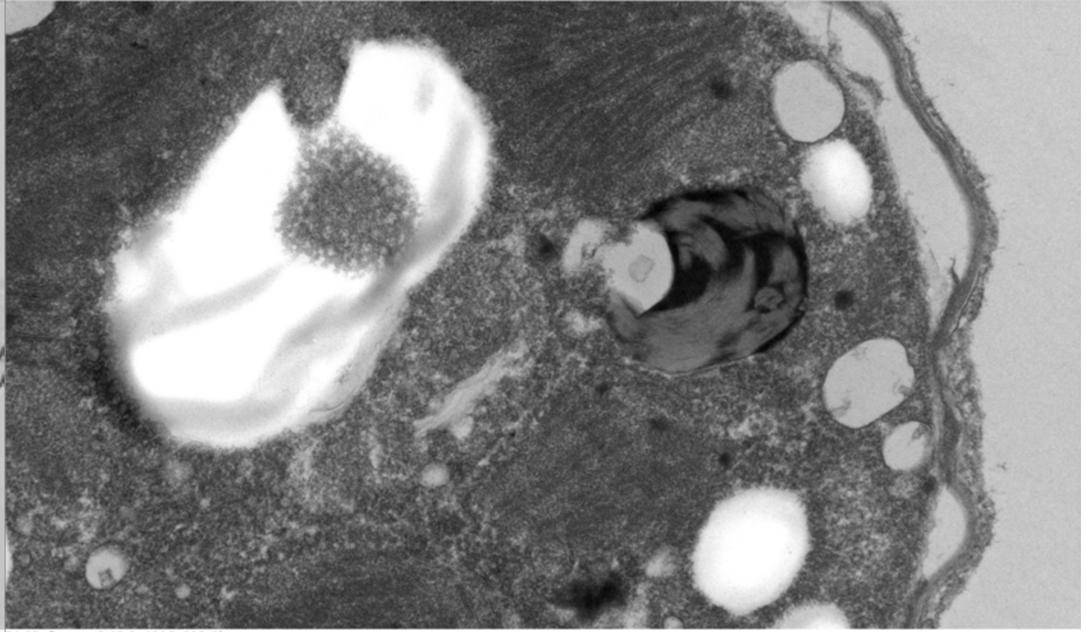

21-20_Correa_3-19-3_16A5_006.tif
Sample: 3/19, 3
Biological Electron Microscopy Lab
Rice University - SEA
Microscopist: MD Meyer

800 nm
HV=80kV
Direct Mag: 5000 x

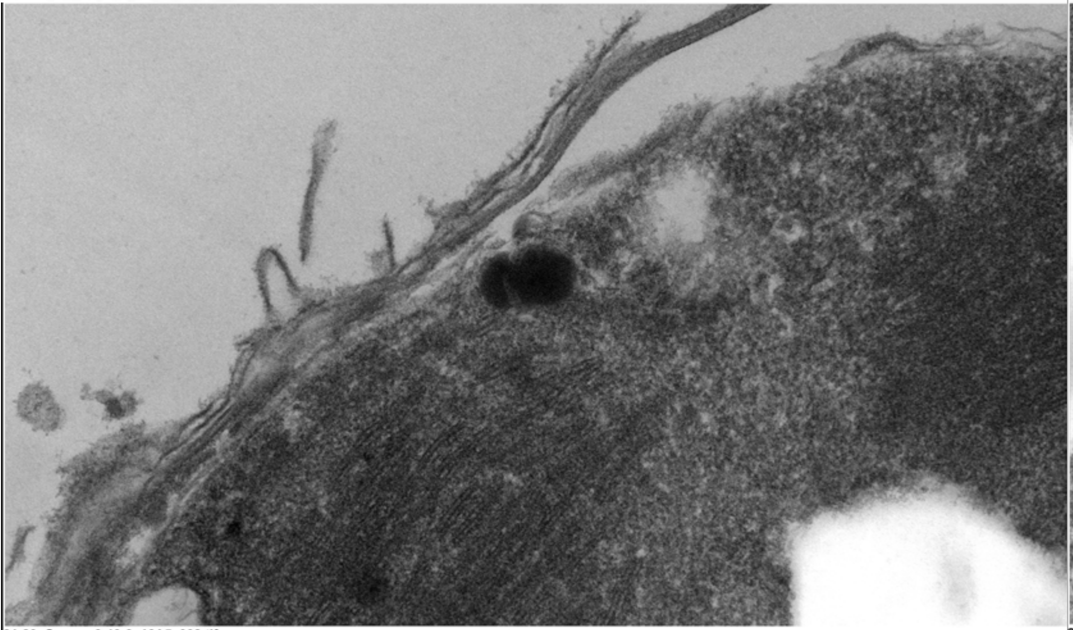

21-20_Correa_3-19-3_16A5_008.tif
Sample: 3/19, 3
Biological Electron Microscopy Lab
Rice University - SEA
Microscopist: MD Meyer

400 nm
HV=80kV
Direct Mag: 10000 x

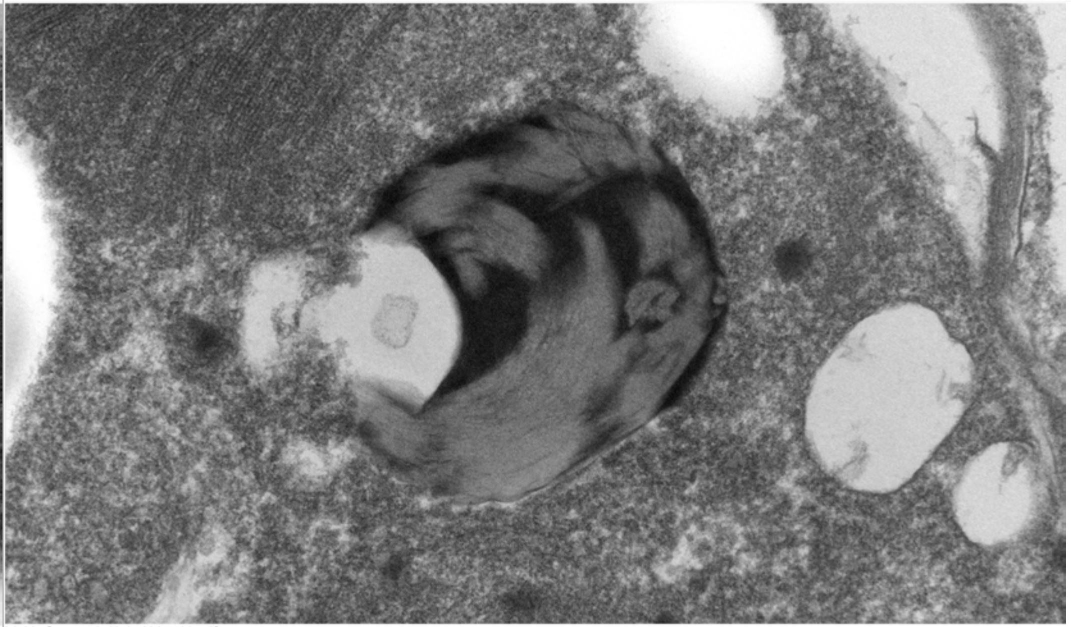

21-20_Correa_3-19-3_16A5_007.tif
Sample: 3/19, 3
Biological Electron Microscopy Lab
Rice University - SEA
Microscopist: MD Meyer

400 nm
HV=80kV
Direct Mag: 10000 x

Cell 25

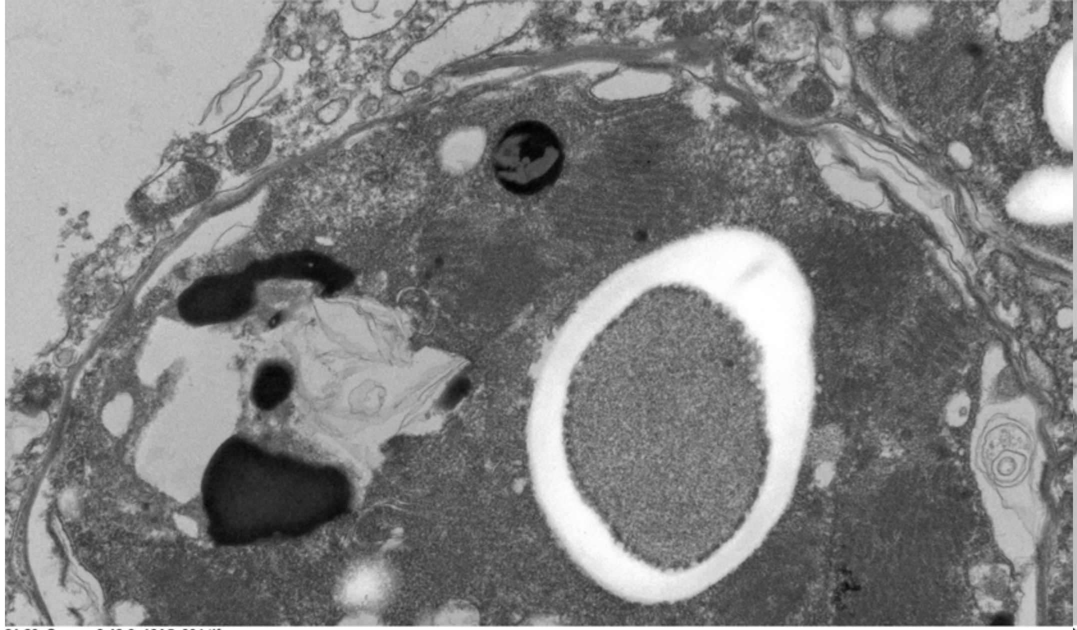

21-20_Correa_3-19-3_16A5_004.tif
Sample: 3/19, 3
Biological Electron Microscopy Lab
Rice University - SEA
Microscopist: MD Meyer

1 µm
HV=80kV
Direct Mag: 4000 x

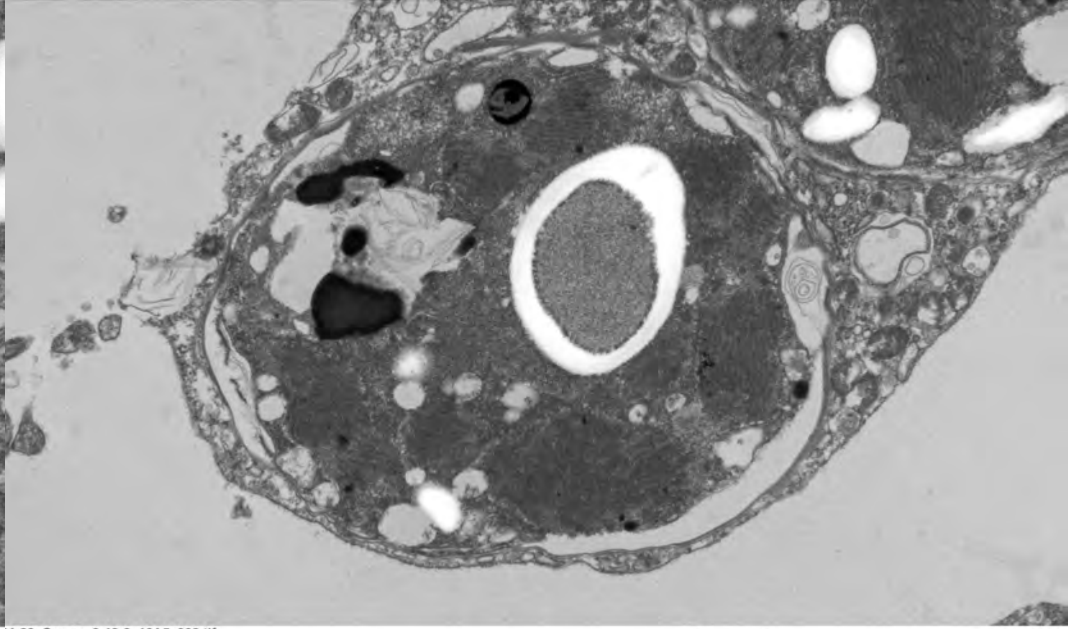

21-20_Correa_3-19-3_16A5_002.tif
Sample: 3/19, 3
Biological Electron Microscopy Lab
Rice University - SEA
Microscopist: MD Meyer

1 µm
HV=80kV
Direct Mag: 2500 x

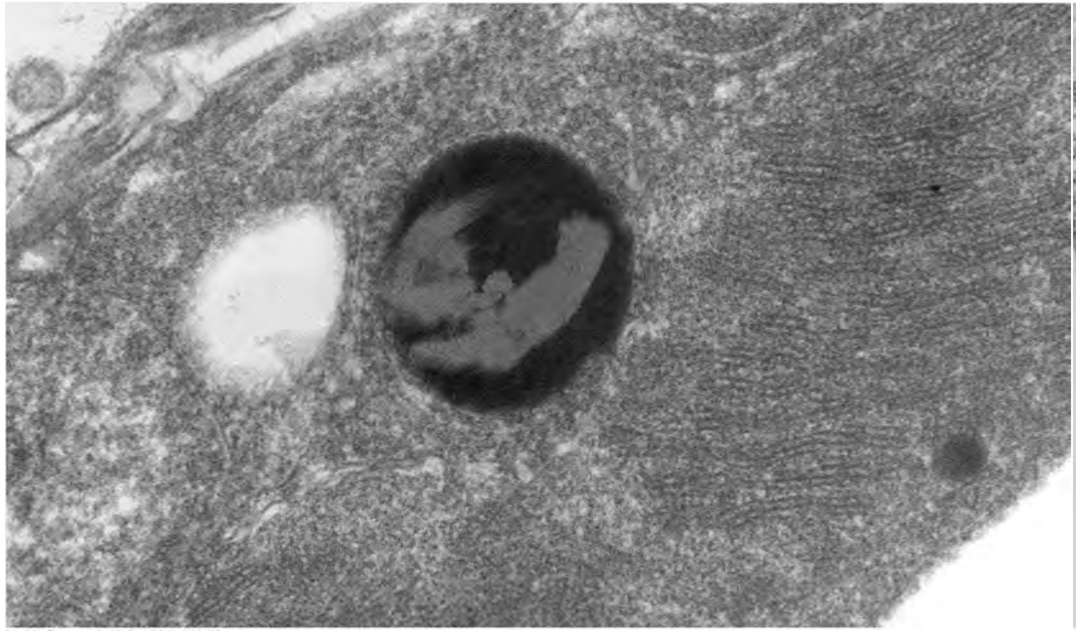

21-20_Correa_3-19-3_16A5_010.tif
Sample: 3/19, 3
Biological Electron Microscopy Lab
Rice University - SEA
Microscopist: MD Meyer

200 nm
HV=80kV
Direct Mag: 15000 x

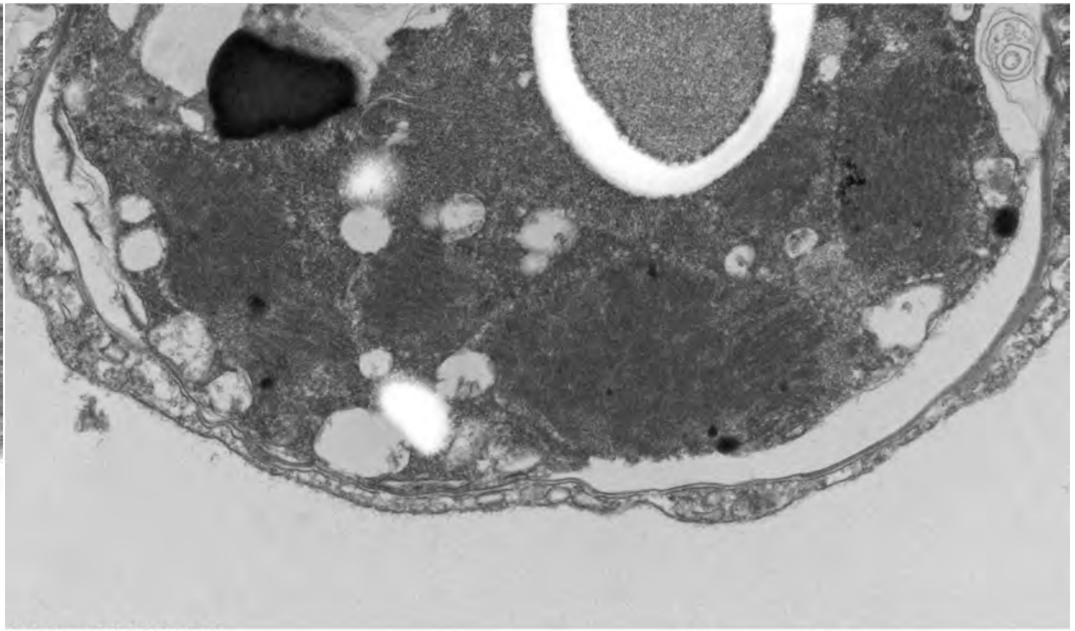

21-20_Correa_3-19-3_16A5_003.tif
Sample: 3/19, 3
Biological Electron Microscopy Lab
Rice University - SEA
Microscopist: MD Meyer

1 µm
HV=80kV
Direct Mag: 4000 x

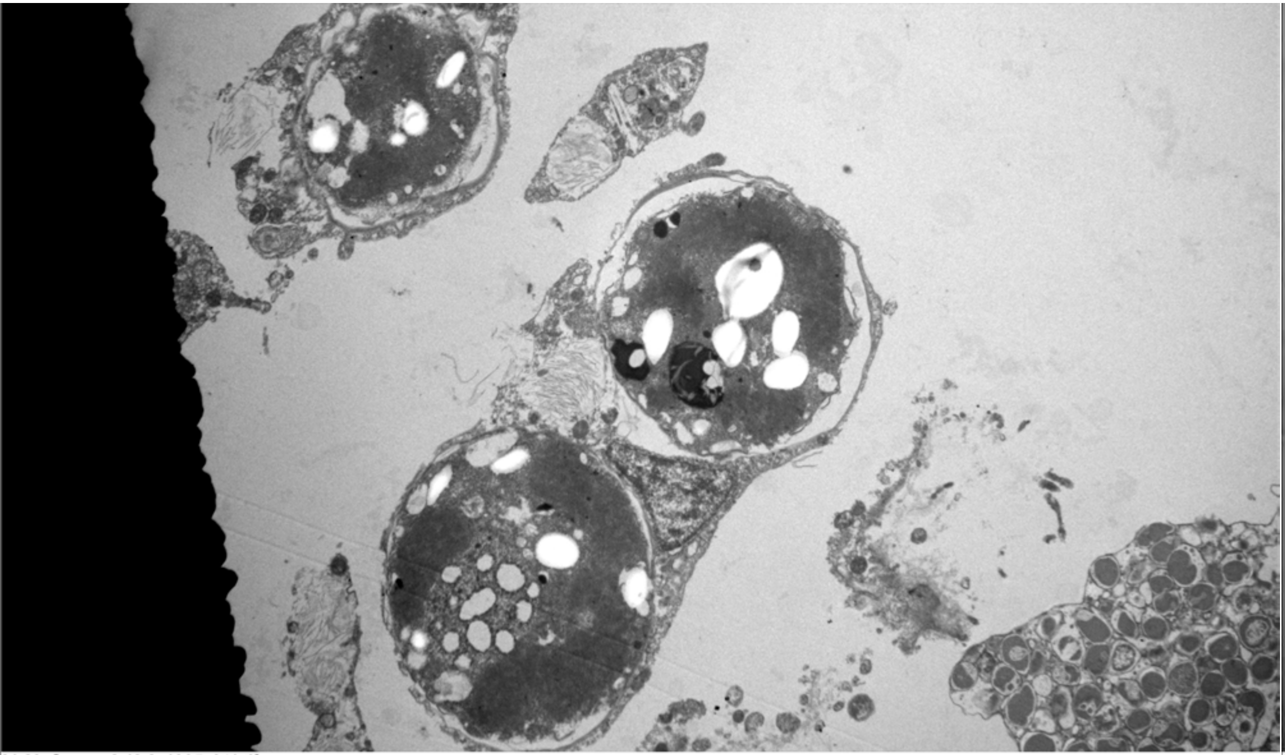

21-20_Correa_3-19-3_16A5_041.tif
Sample: 3/19, 3
Biological Electron Microscopy Lab
Rice University - SEA
Microscopist: MD Meyer

4 µm
HV=80kV
Direct Mag: 1000 x

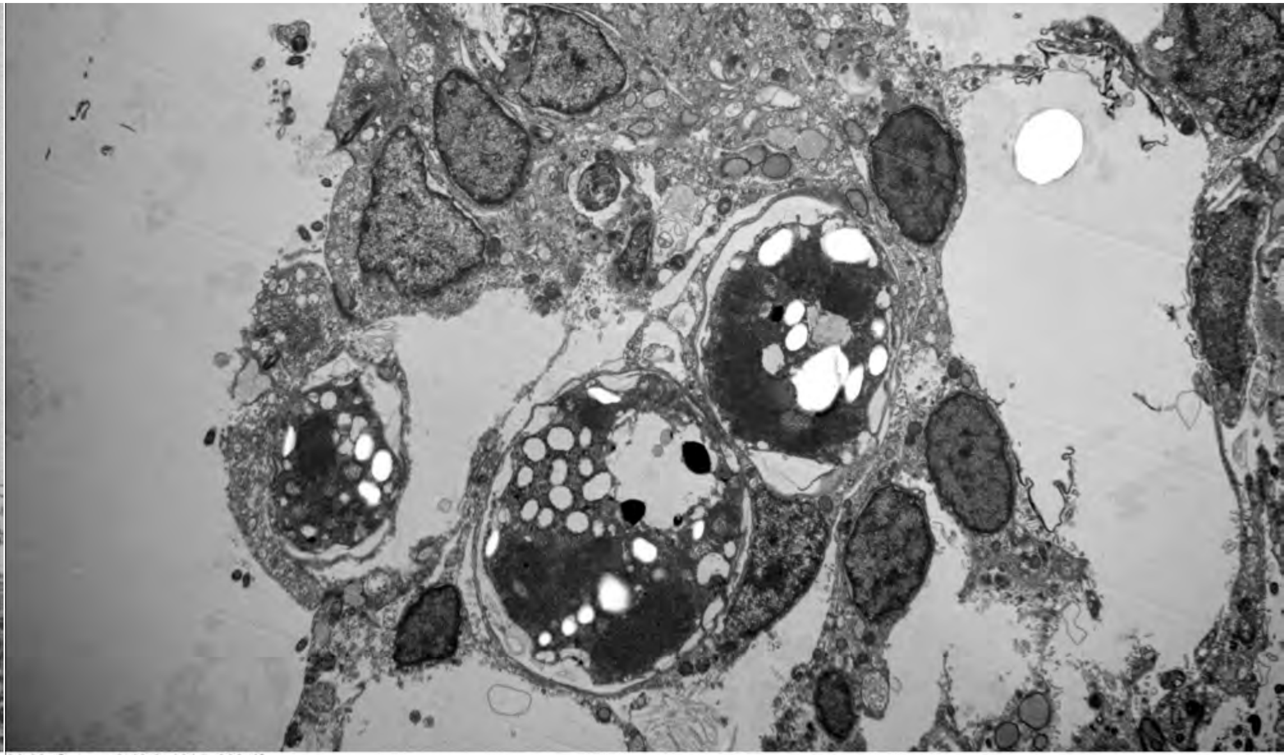

21-20_Correa_3-19-3_16A5_032.tif
Sample: 3/19, 3
Biological Electron Microscopy Lab
Rice University - SEA
Microscopist: MD Meyer

4 µm
HV=80kV
Direct Mag: 1000 x

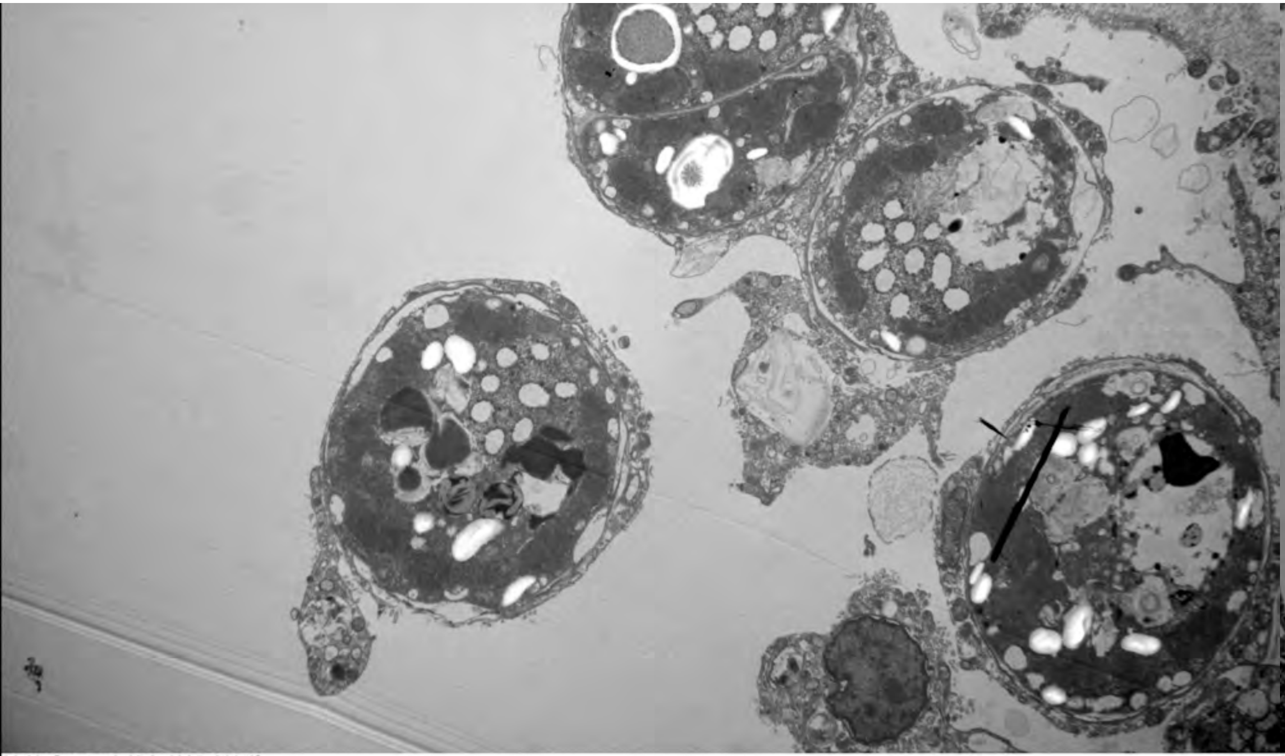

21-20_Correa_3-19-3_16A5_011.tif
Sample: 3/19, 3
Biological Electron Microscopy Lab
Rice University - SEA
Microscopist: MD Meyer

4 µm
HV=80kV
Direct Mag: 1000 x

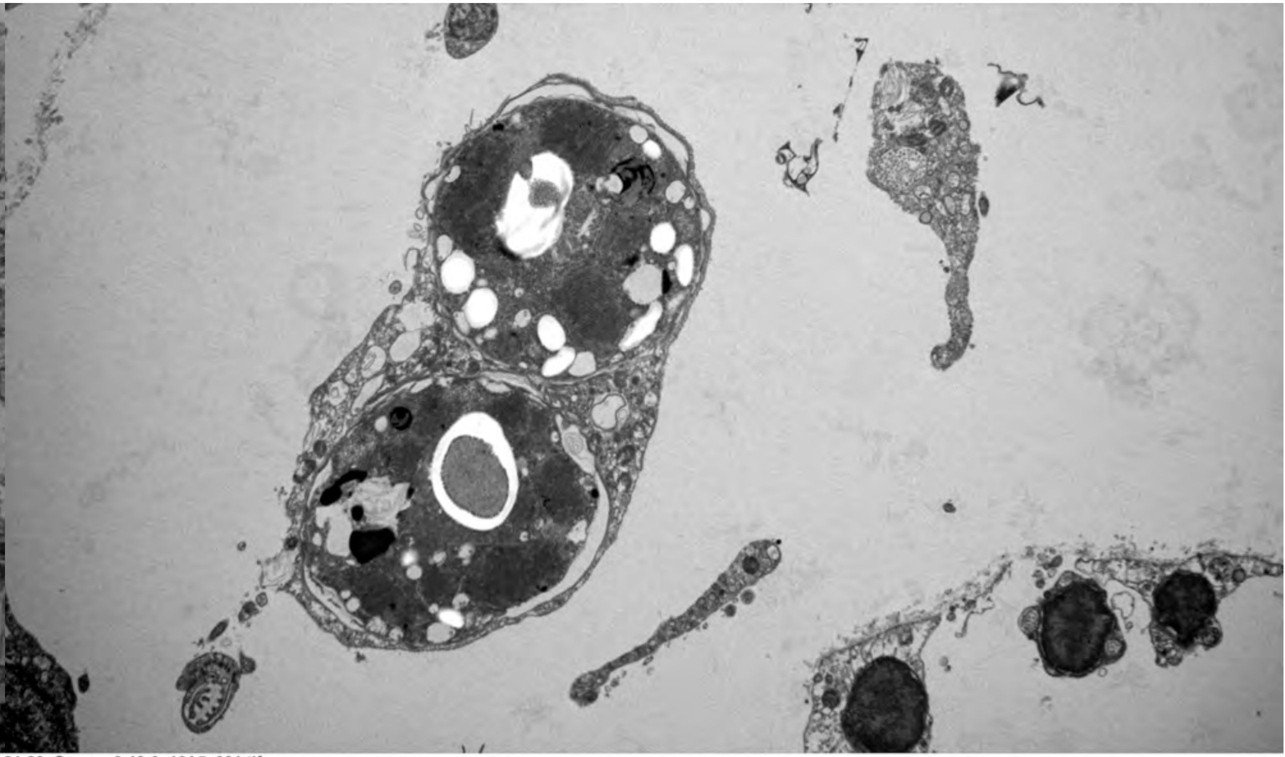

21-20_Correa_3-19-3_16A5_001.tif
Sample: 3/19, 3
Biological Electron Microscopy Lab
Rice University - SEA
Microscopist: MD Meyer

4 µm
HV=80kV
Direct Mag: 1000 x

Ambient (March 2018) POR Colony K

Cell 1

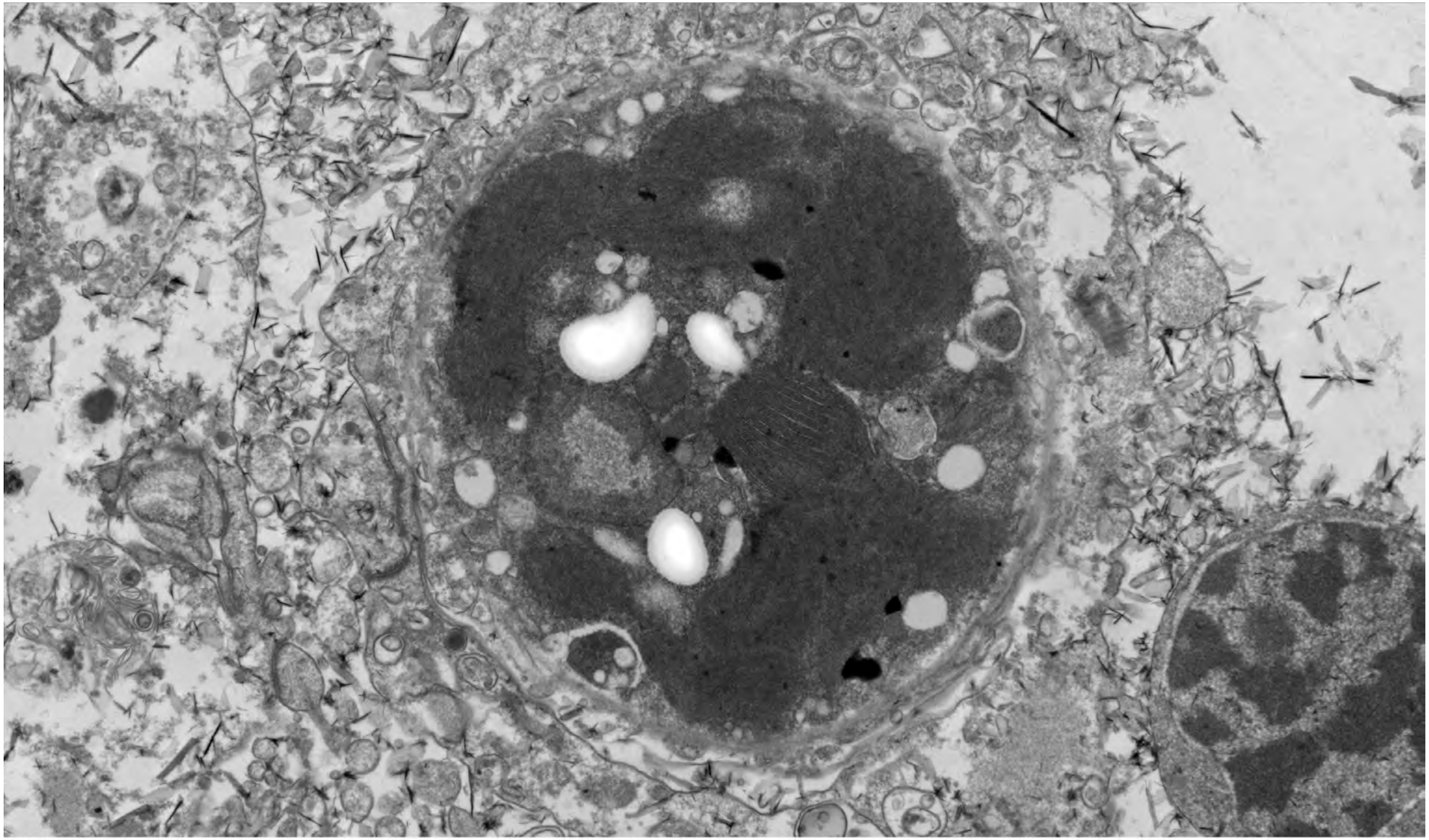

21-20_Correa_3-18-25_16I3_060.tif
Sample: 3/18, 25
Biological Electron Microscopy Lab
Rice University - SEA
Microscopist: MD Meyer

1 µm
HV=80kV
Direct Mag: 3000 x

Cell 2

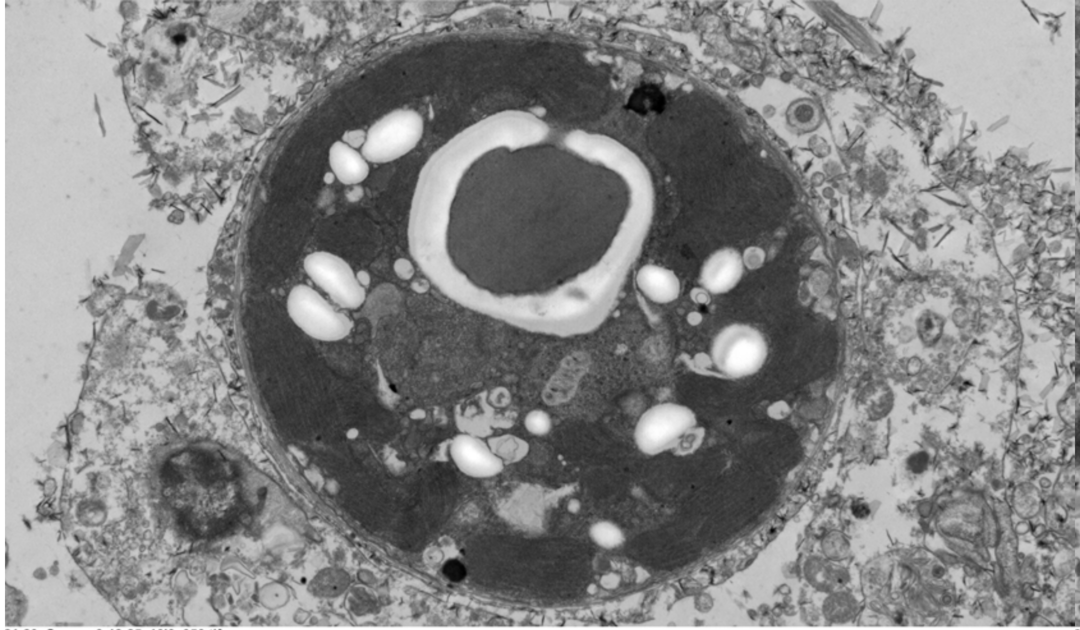

21-20_Correa_3-18-25_16I3_056.tif
Sample: 3/18, 25
Biological Electron Microscopy Lab
Rice University - SEA
Microscopist: MD Meyer

1 µm
HV=80kV
Direct Mag: 2500 x

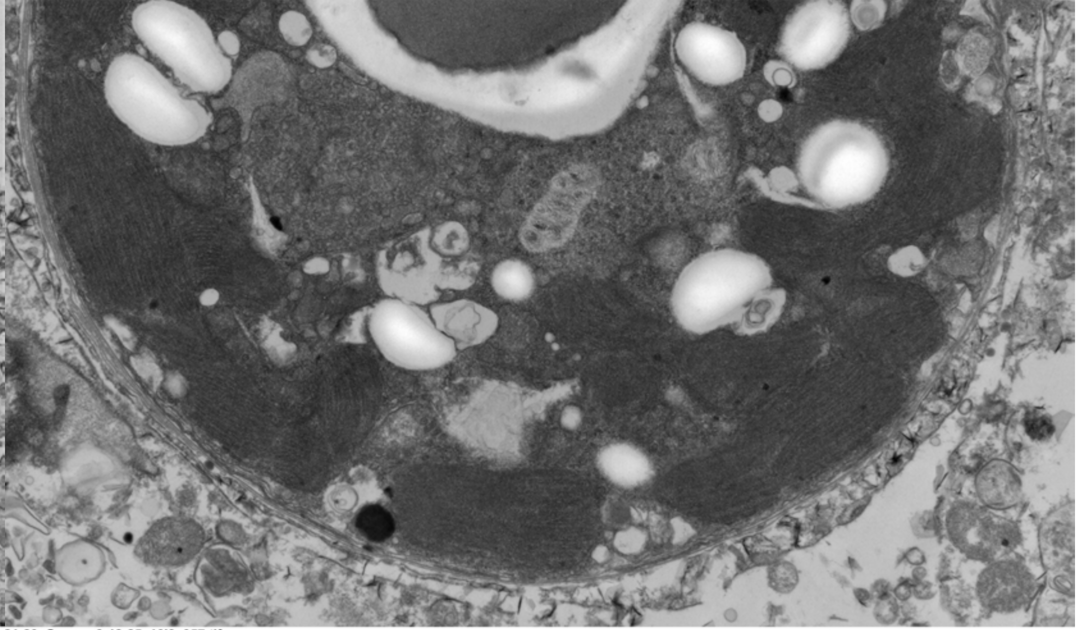

21-20_Correa_3-18-25_16I3_057.tif
Sample: 3/18, 25
Biological Electron Microscopy Lab
Rice University - SEA
Microscopist: MD Meyer

1 µm
HV=80kV
Direct Mag: 4000 x

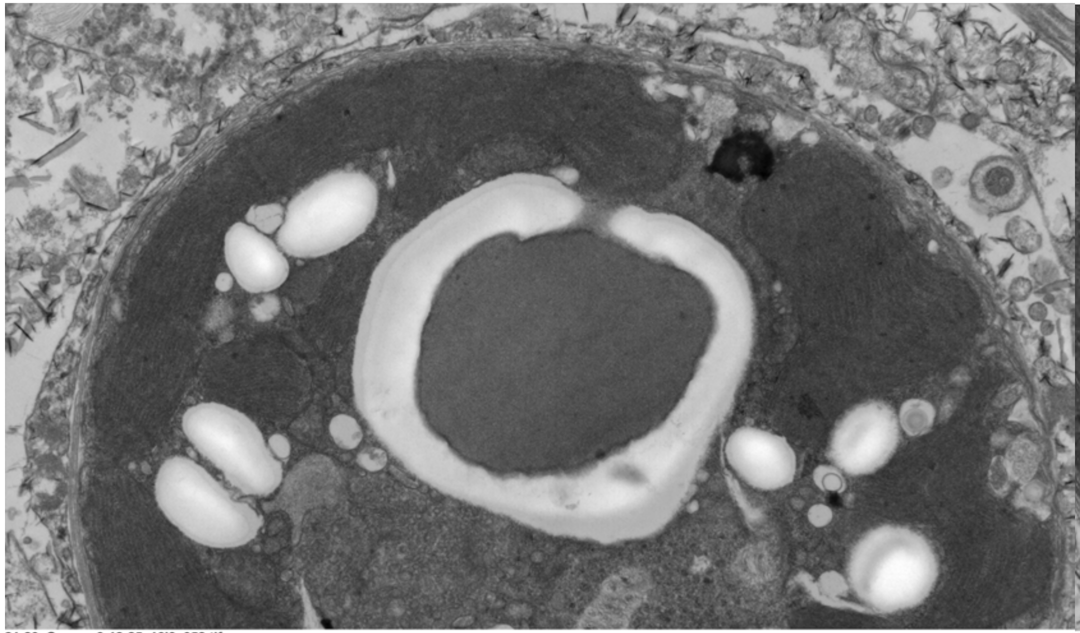

21-20_Correa_3-18-25_16I3_058.tif
Sample: 3/18, 25
Biological Electron Microscopy Lab
Rice University - SEA
Microscopist: MD Meyer

1 µm
HV=80kV
Direct Mag: 4000 x

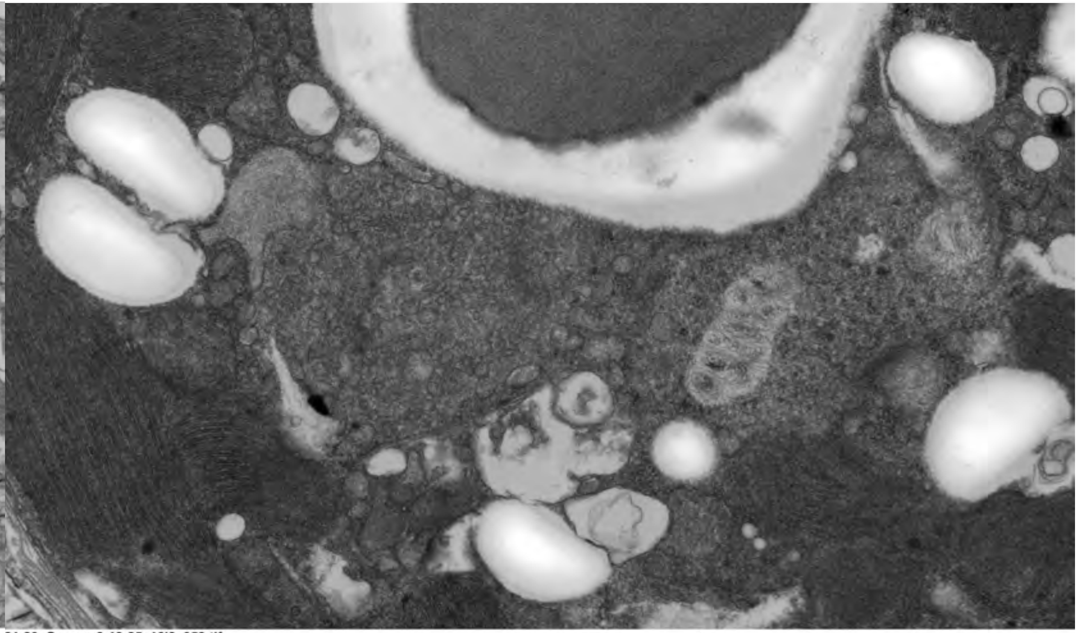

21-20_Correa_3-18-25_16I3_059.tif
Sample: 3/18, 25
Biological Electron Microscopy Lab
Rice University - SEA
Microscopist: MD Meyer

600 nm
HV=80kV
Direct Mag: 6000 x

Cell 3

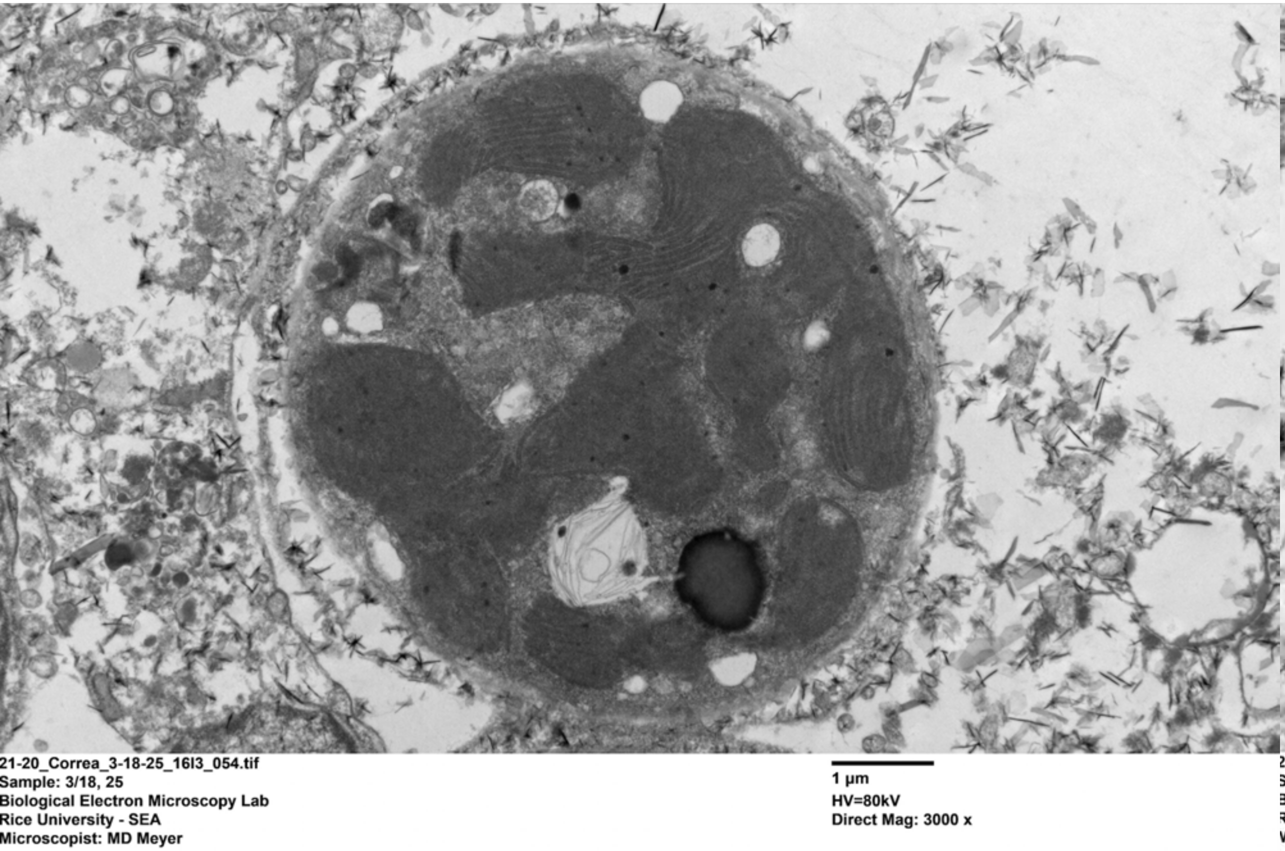
21-20_Correa_3-18-25_16l3_054.tif
Sample: 3/18, 25
Biological Electron Microscopy Lab
Rice University - SEA
Microscopist: MD Meyer
1 µm
HV=80kV
Direct Mag: 3000 x

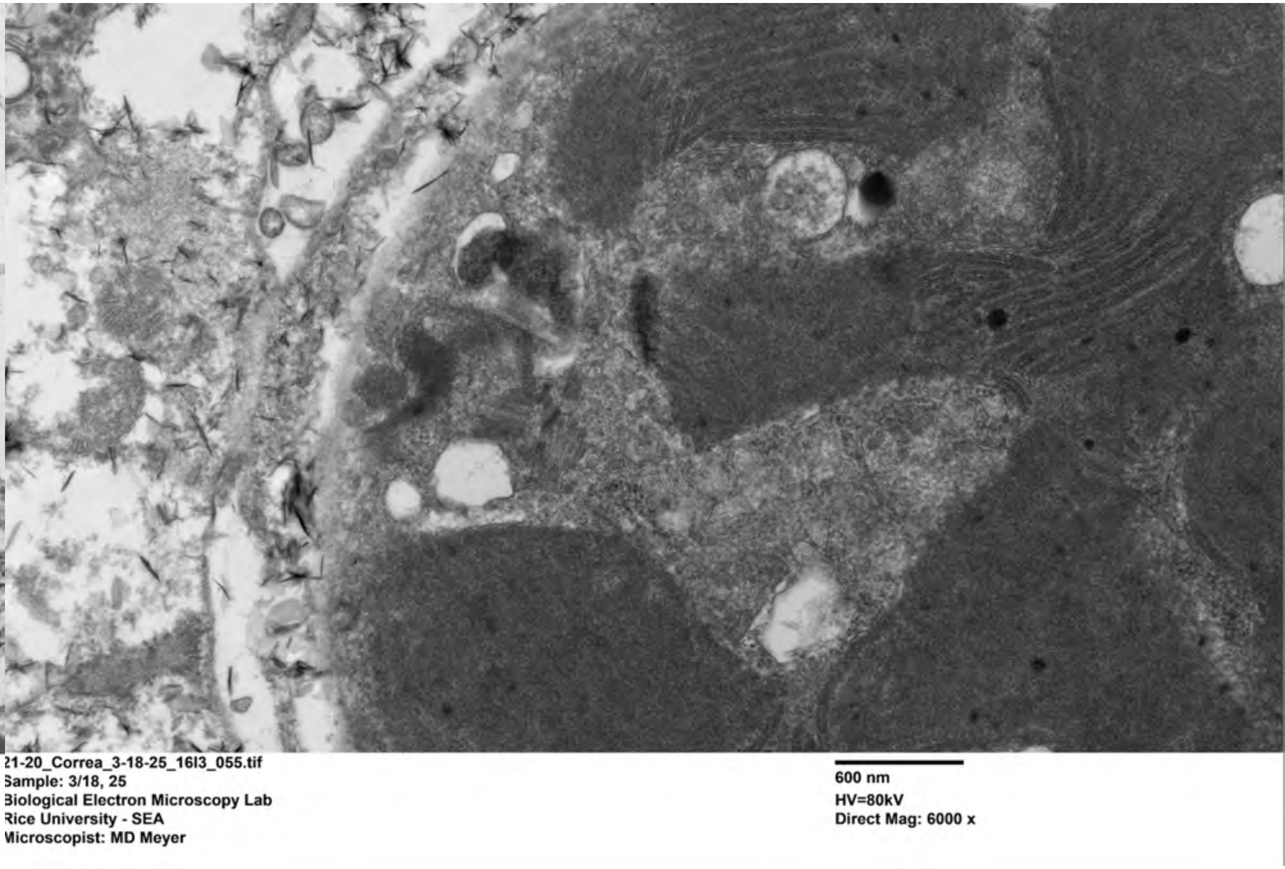
21-20_Correa_3-18-25_16l3_055.tif
Sample: 3/18, 25
Biological Electron Microscopy Lab
Rice University - SEA
Microscopist: MD Meyer
600 nm
HV=80kV
Direct Mag: 6000 x

Cell 4

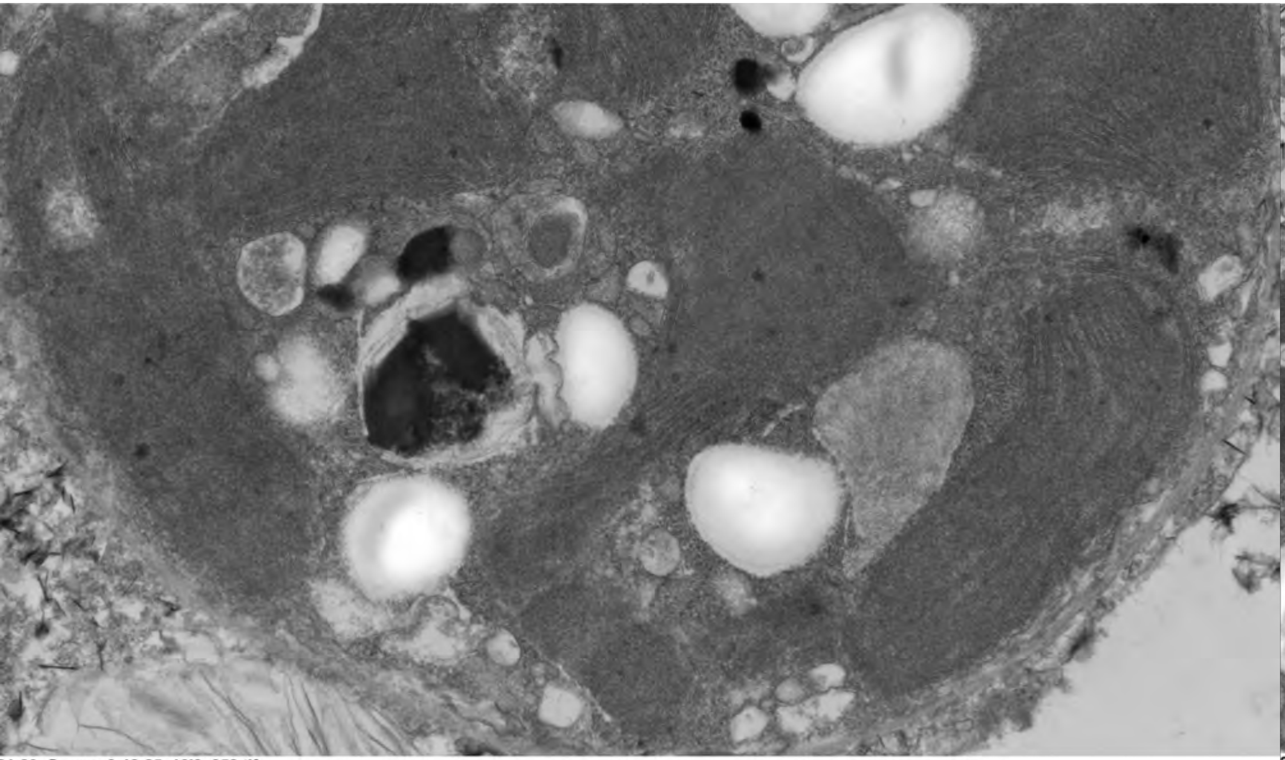

21-20_Correa_3-18-25_16l3_052.tif
Sample: 3/18, 25
Biological Electron Microscopy Lab
Rice University - SEA
Microscopist: MD Meyer

800 nm
HV=80kV
Direct Mag: 5000 x

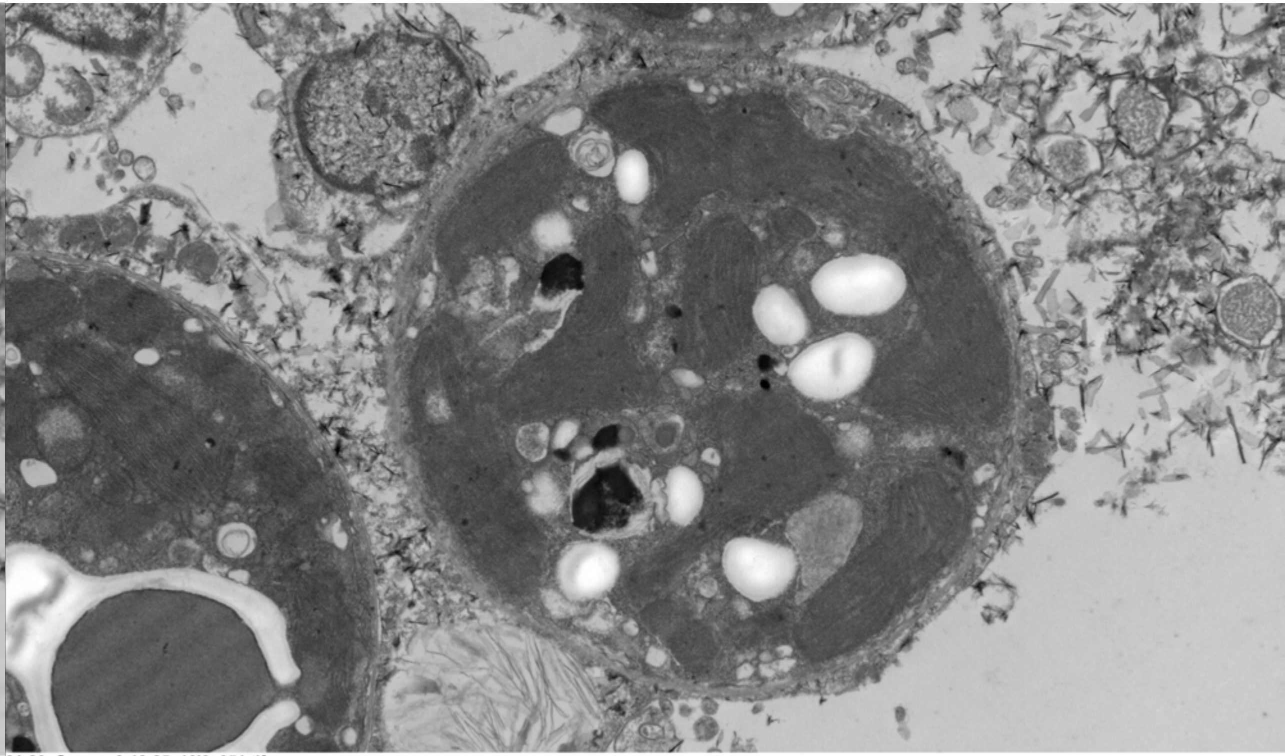

21-20_Correa_3-18-25_16l3_051.tif
Sample: 3/18, 25
Biological Electron Microscopy Lab
Rice University - SEA
Microscopist: MD Meyer

1 μm
HV=80kV
Direct Mag: 2500 x

Cell 5

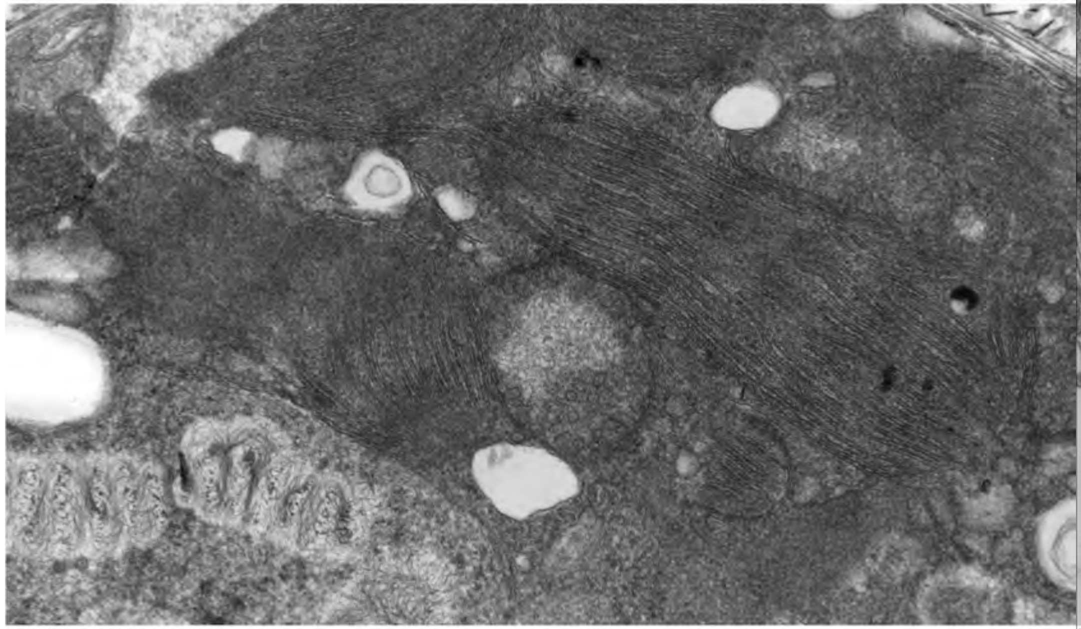

21-20_Correa_3-18-25_16I3_048.tif
Sample: 3/18, 25
Biological Electron Microscopy Lab
Rice University - SEA
Microscopist: MD Meyer

500 nm
HV=80kV
Direct Mag: 8000 x

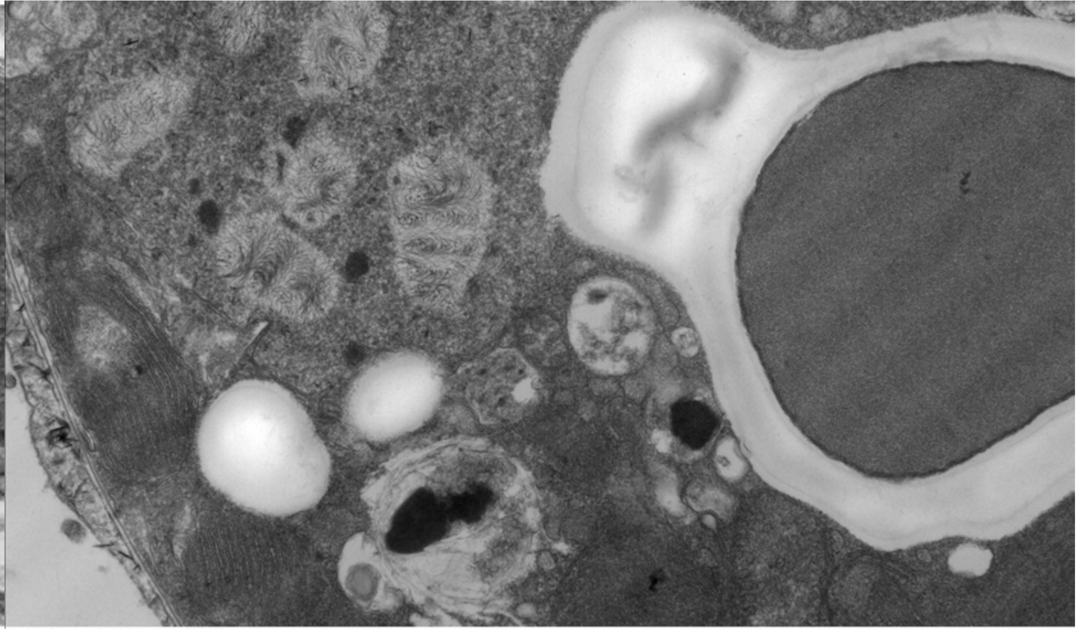

21-20_Correa_3-18-25_16I3_049.tif
Sample: 3/18, 25
Biological Electron Microscopy Lab
Rice University - SEA
Microscopist: MD Meyer

600 nm
HV=80kV
Direct Mag: 6000 x

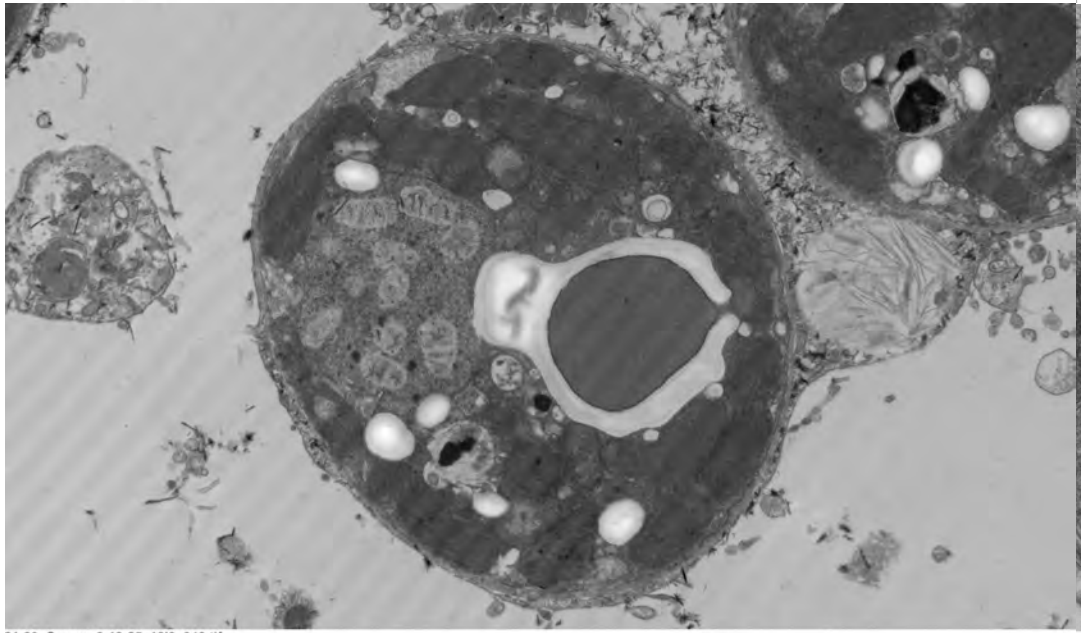

21-20_Correa_3-18-25_16I3_046.tif
Sample: 3/18, 25
Biological Electron Microscopy Lab
Rice University - SEA
Microscopist: MD Meyer

2 µm
HV=80kV
Direct Mag: 2000 x

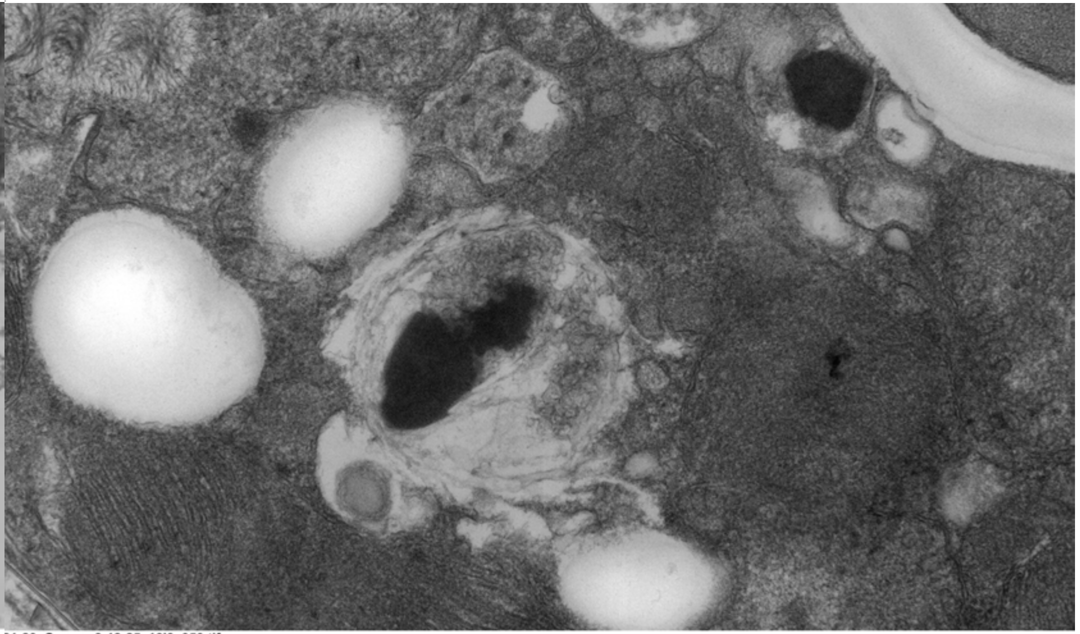

21-20_Correa_3-18-25_16I3_050.tif
Sample: 3/18, 25
Biological Electron Microscopy Lab
Rice University - SEA
Microscopist: MD Meyer

400 nm
HV=80kV
Direct Mag: 10000 x

Cell 6

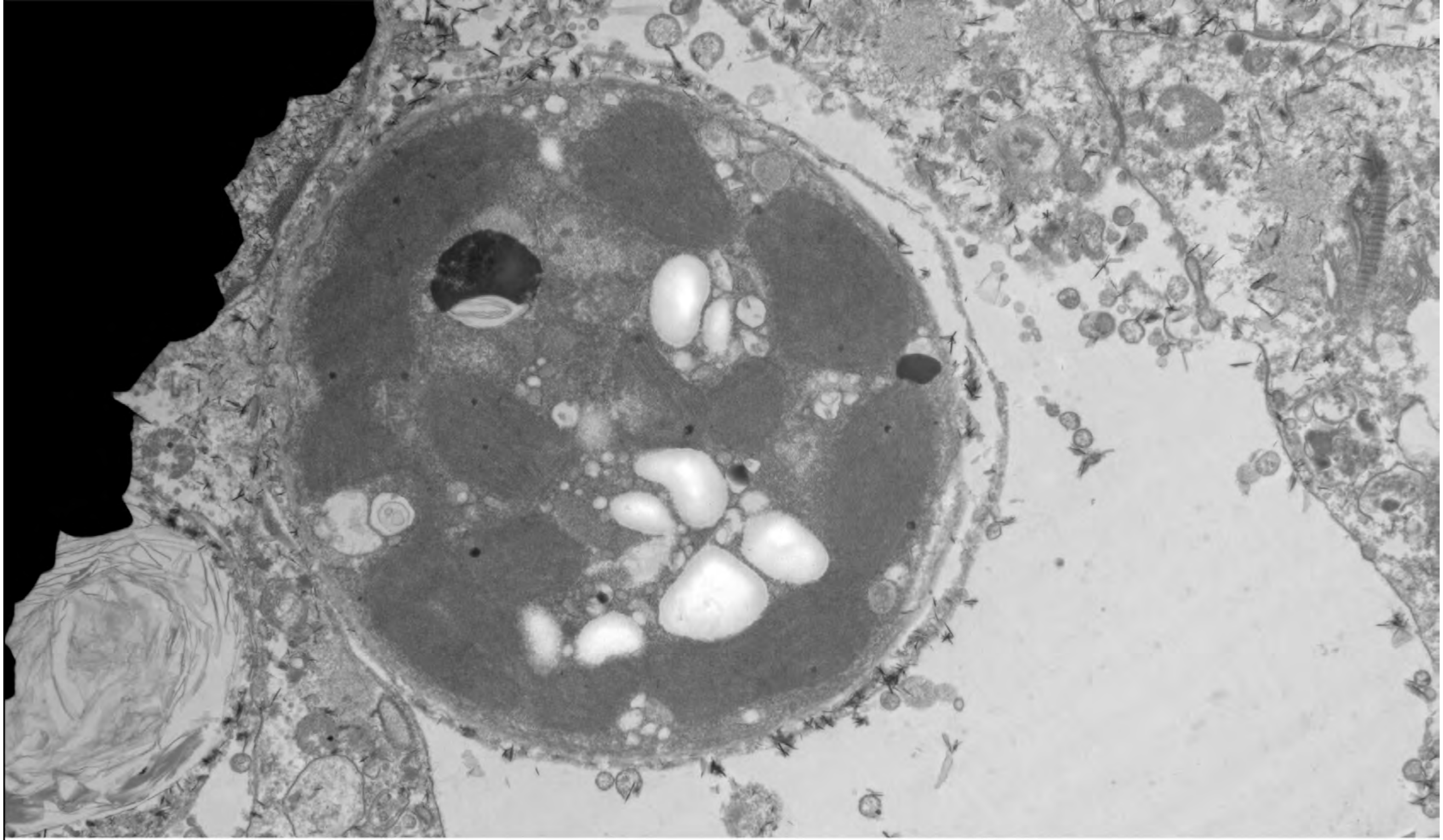

21-20_Correa_3-18-25_16I3_045.tif
Sample: 3/18, 25
Biological Electron Microscopy Lab
Rice University - SEA
Microscopist: MD Meyer

1 µm
HV=80kV
Direct Mag: 2500 x

Cell 7

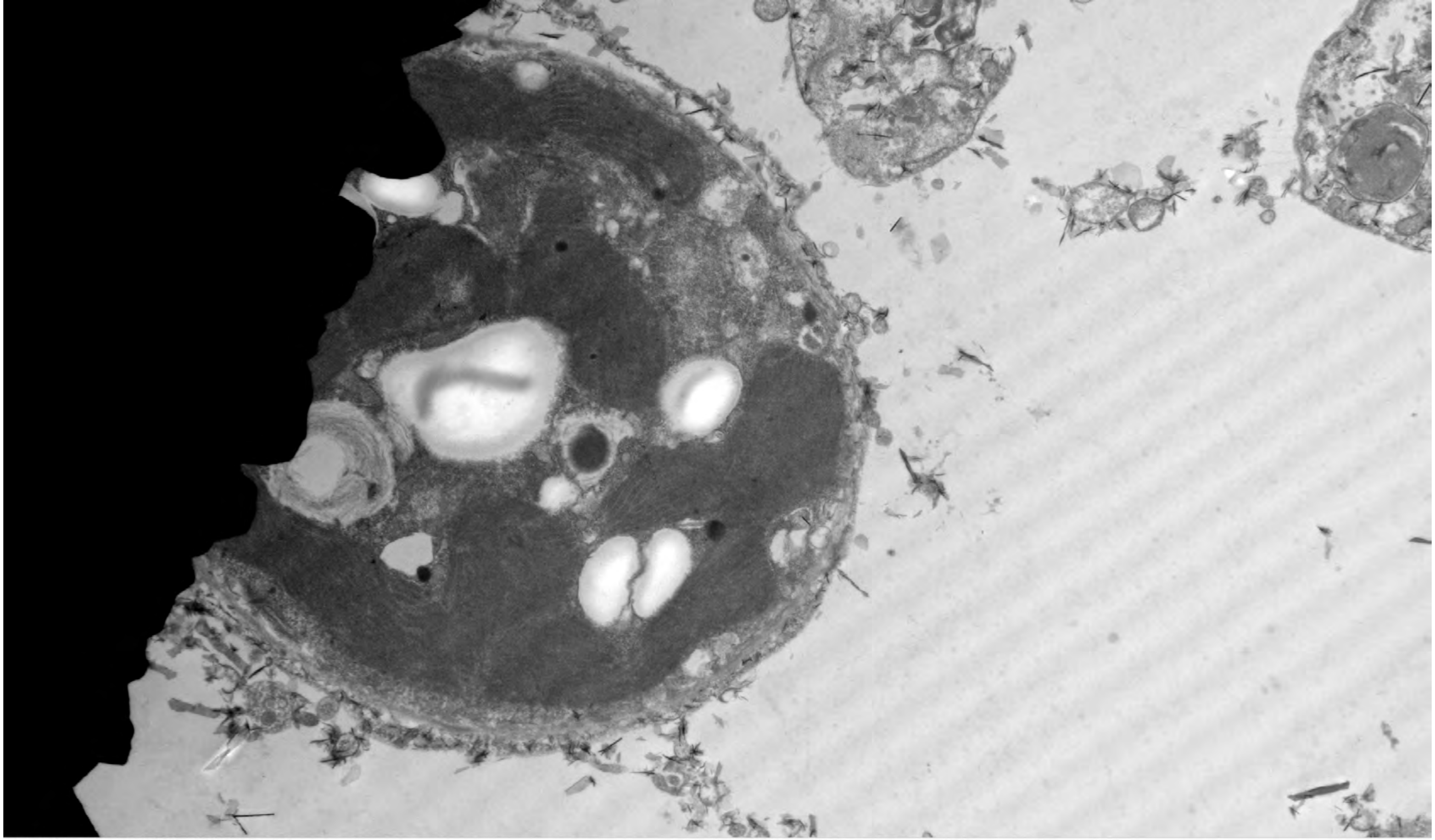

21-20_Correa_3-18-25_16l3_044.tif
Sample: 3/18, 25
Biological Electron Microscopy Lab
Rice University - SEA
Microscopist: MD Meyer

1 µm
HV=80kV
Direct Mag: 2500 x

Cell 8

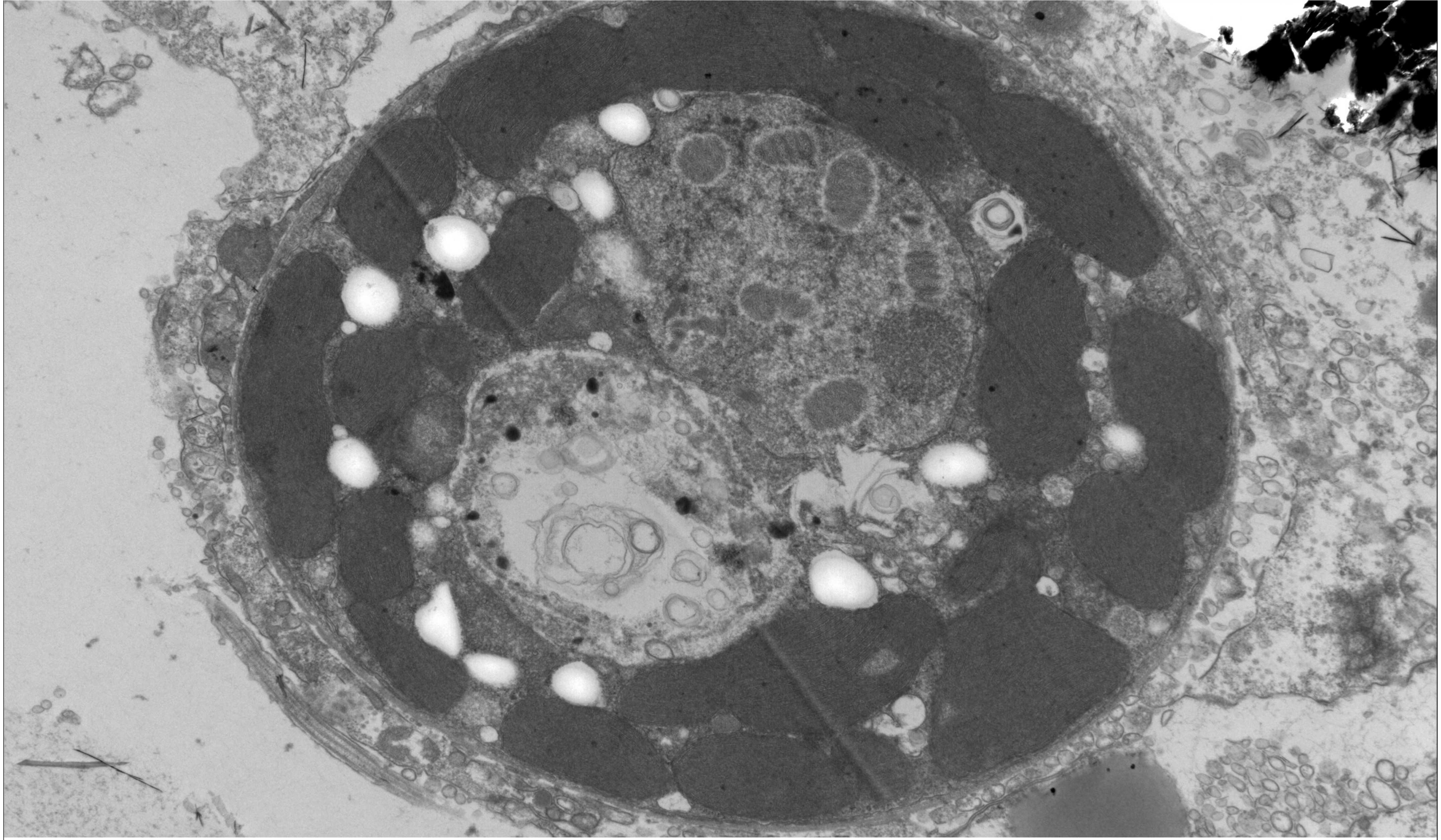

21-20_Correa_3-18-25_16I3_042.tif
Sample: 3/18, 25
Biological Electron Microscopy Lab
Rice University - SEA
Microscopist: MD Meyer

1 µm
HV=80kV
Direct Mag: 2500 x

Cell 9

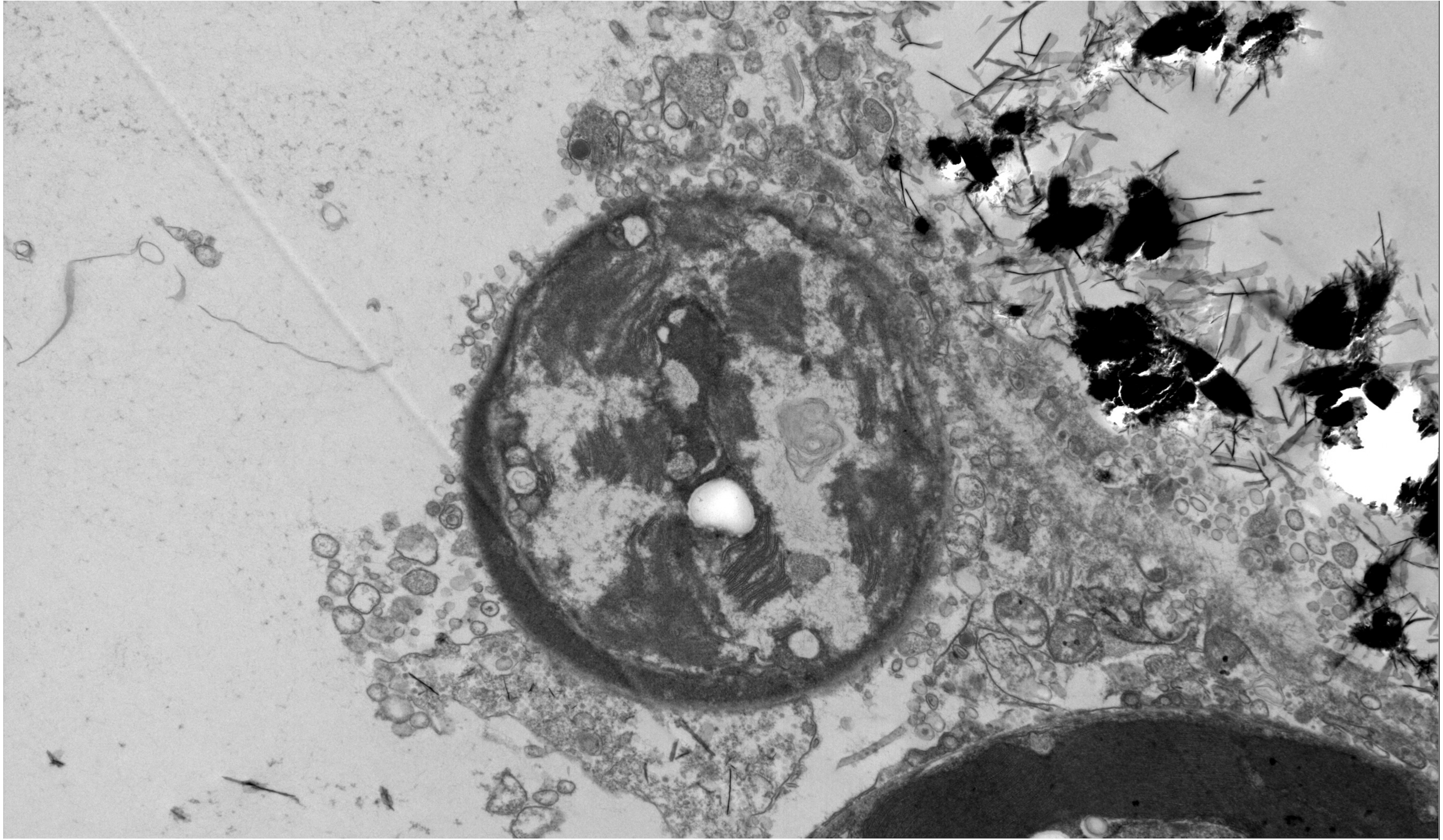

21-20_Correa_3-18-25_16I3_041.tif
Sample: 3/18, 25
Biological Electron Microscopy Lab
Rice University - SEA
Microscopist: MD Meyer

1 µm
HV=80kV
Direct Mag: 2500 x

Cell 10

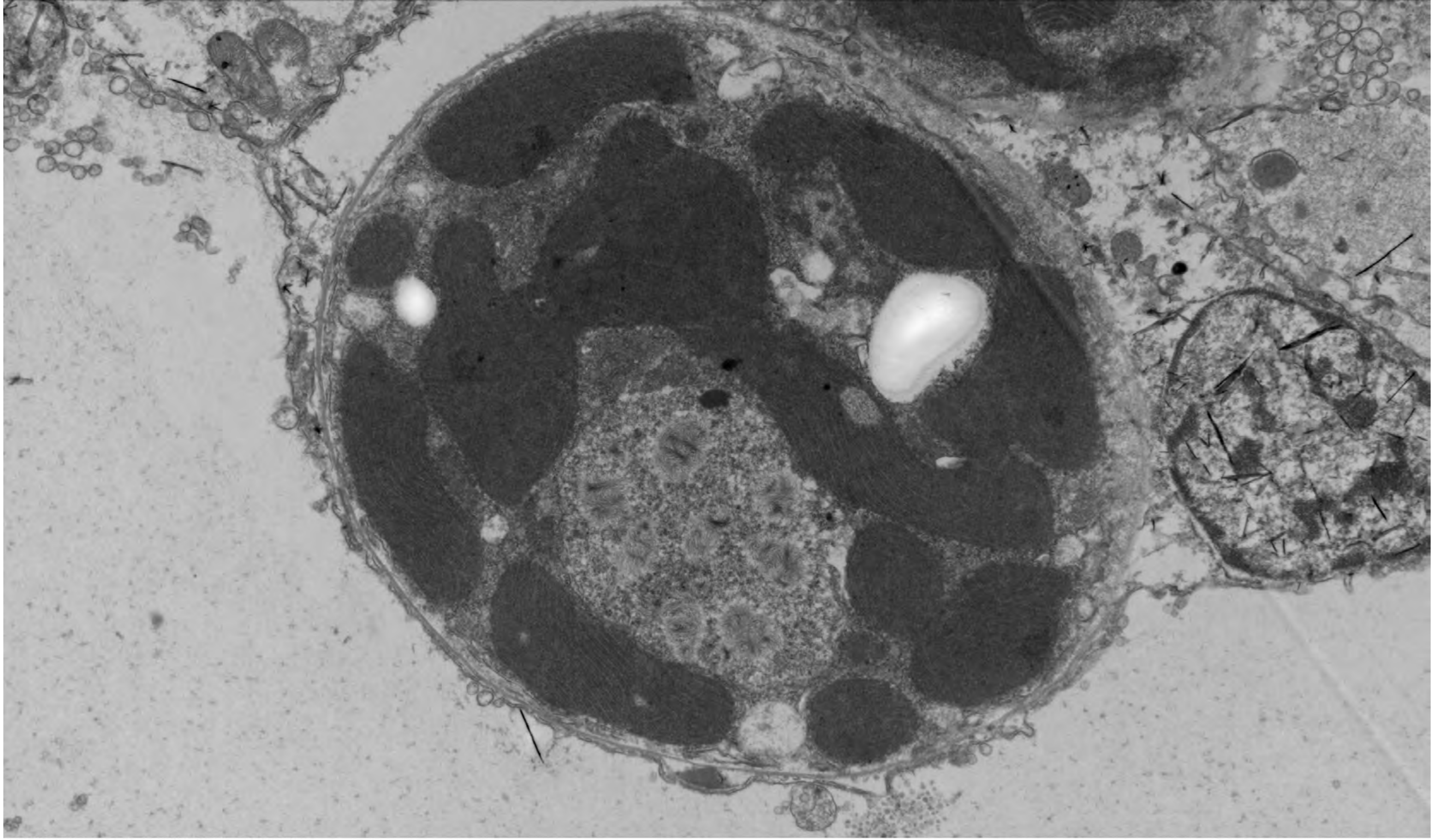

21-20_Correa_3-18-25_16l3_040.tif
Sample: 3/18, 25
Biological Electron Microscopy Lab
Rice University - SEA
Microscopist: MD Meyer

1 µm
HV=80kV
Direct Mag: 2500 x

Cell 11

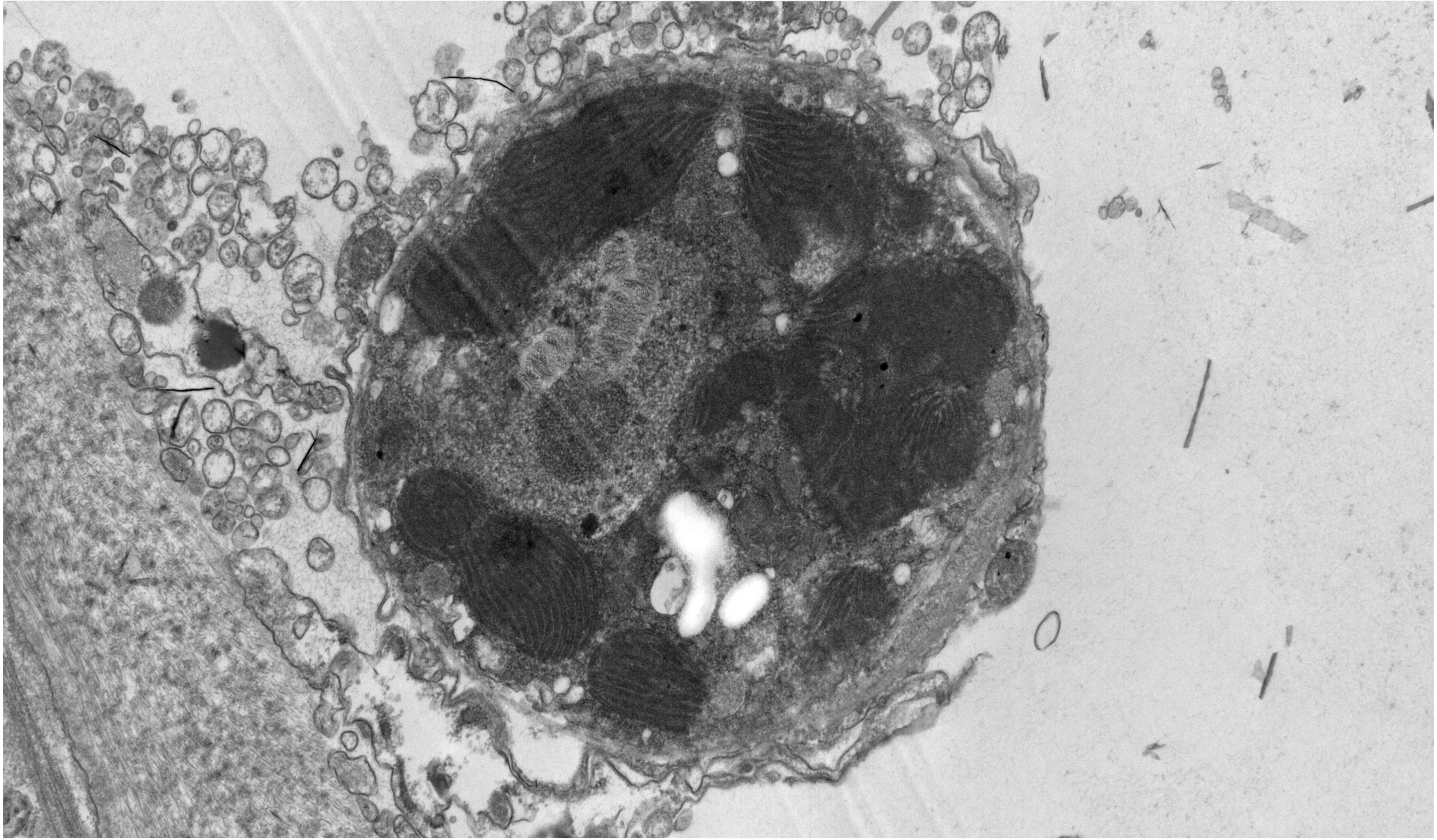

21-20_Correa_3-18-25_16l3_039.tif
Sample: 3/18, 25
Biological Electron Microscopy Lab
Rice University - SEA
Microscopist: MD Meyer

1 µm
HV=80kV
Direct Mag: 3000 x

Cell 12

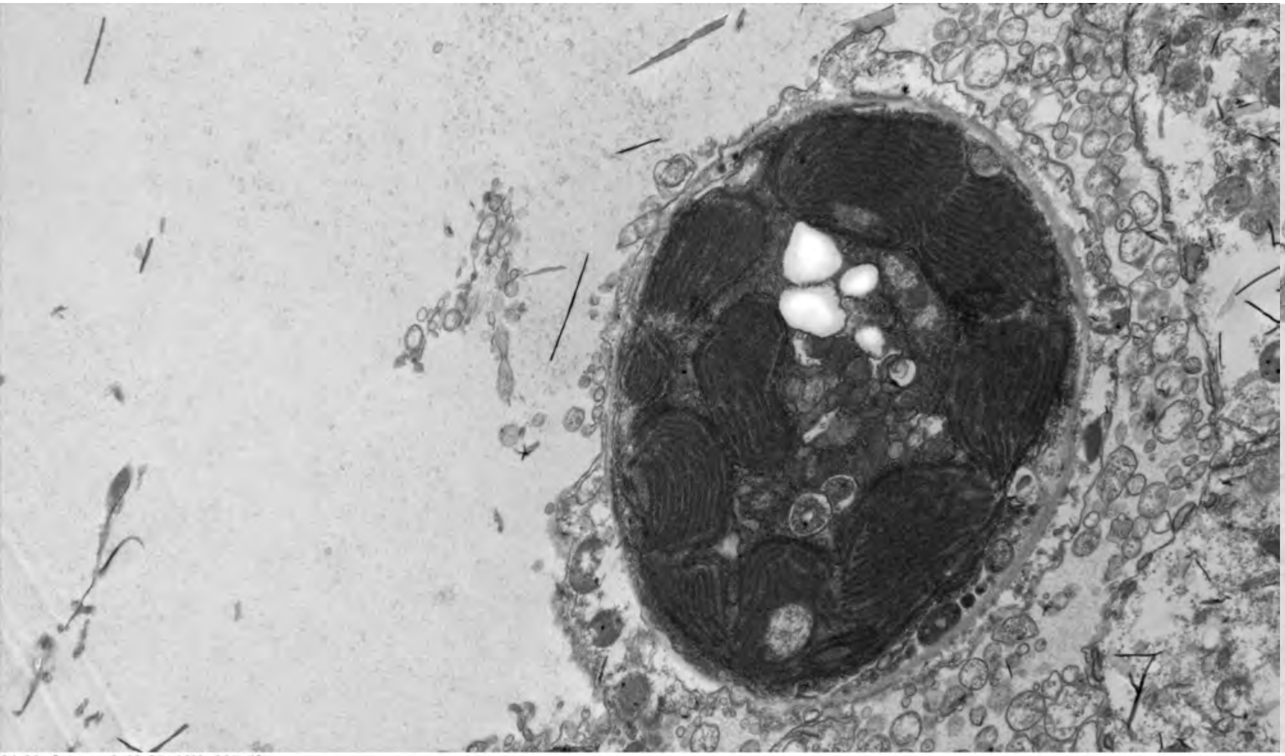

21-20_Correa_3-18-25_16l3_037.tif
Sample: 3/18, 25
Biological Electron Microscopy Lab
Rice University - SEA
Microscopist: MD Meyer

1 µm
HV=80kV
Direct Mag: 2500 x

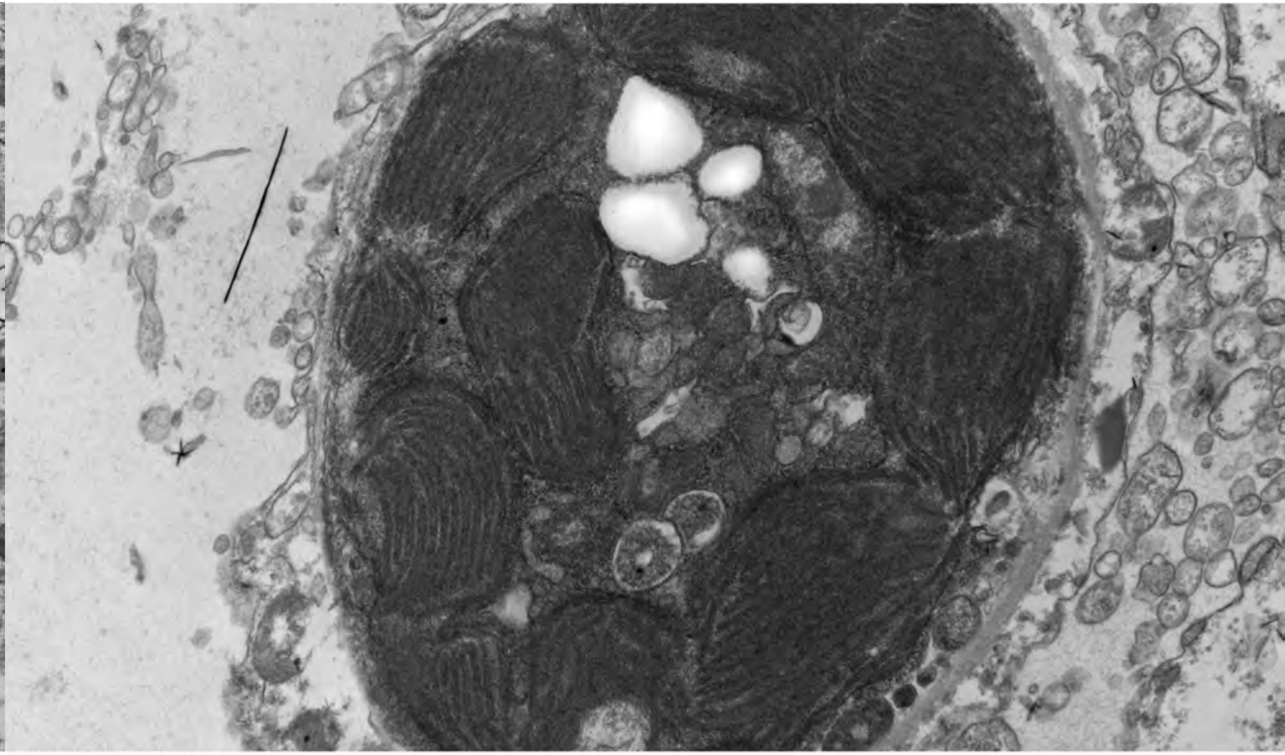

21-20_Correa_3-18-25_16l3_038.tif
Sample: 3/18, 25
Biological Electron Microscopy Lab
Rice University - SEA
Microscopist: MD Meyer

1 µm
HV=80kV
Direct Mag: 4000 x

Cell 13

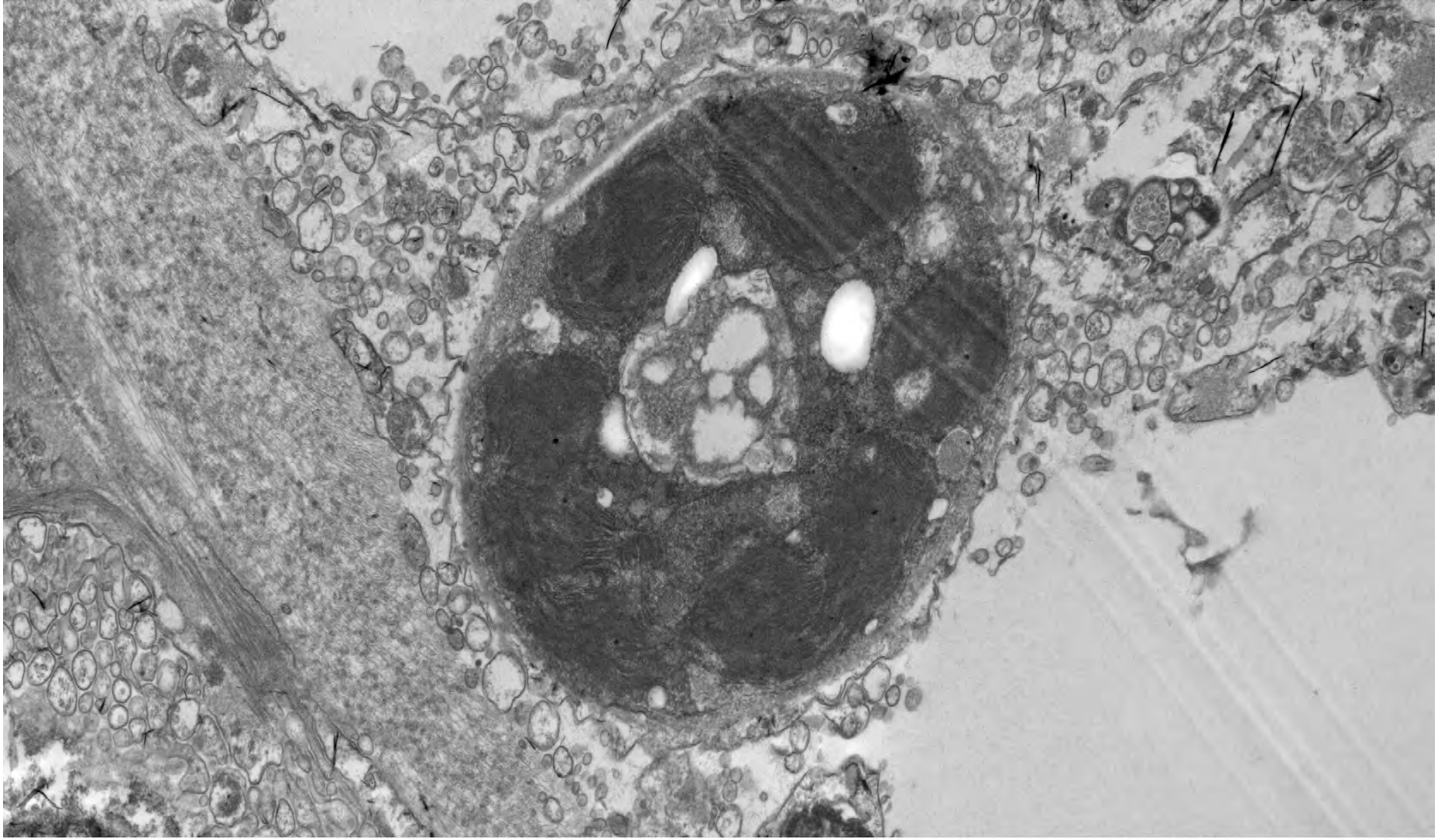

21-20_Correa_3-18-25_16I3_036.tif
Sample: 3/18, 25
Biological Electron Microscopy Lab
Rice University - SEA
Microscopist: MD Meyer

1 µm
HV=80kV
Direct Mag: 2500 x

Cell 14

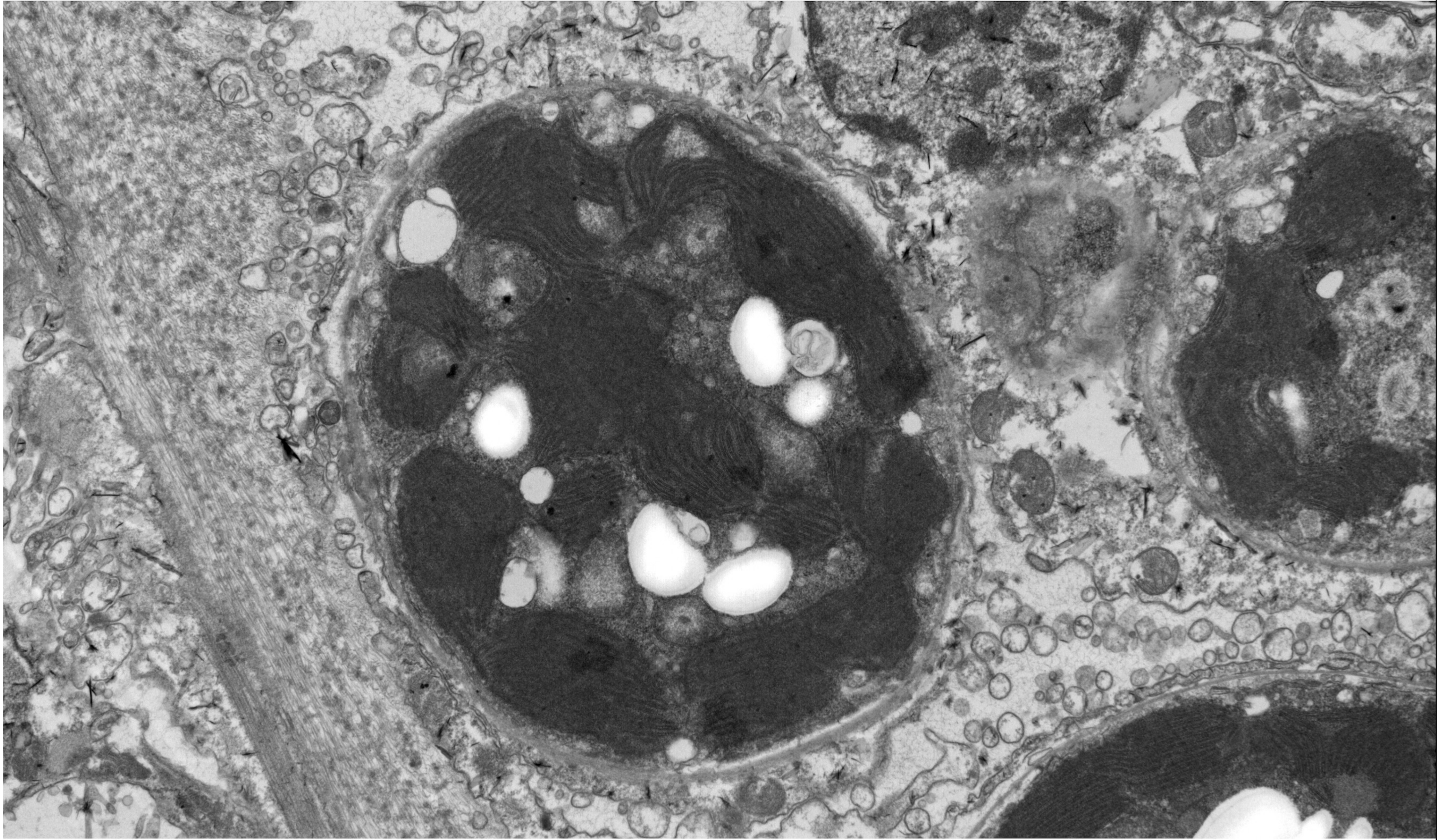

21-20_Correa_3-18-25_16I3_034.tif
Sample: 3/18, 25
Biological Electron Microscopy Lab
Rice University - SEA
Microscopist: MD Meyer

1 µm
HV=80kV
Direct Mag: 2500 x

Cell 15

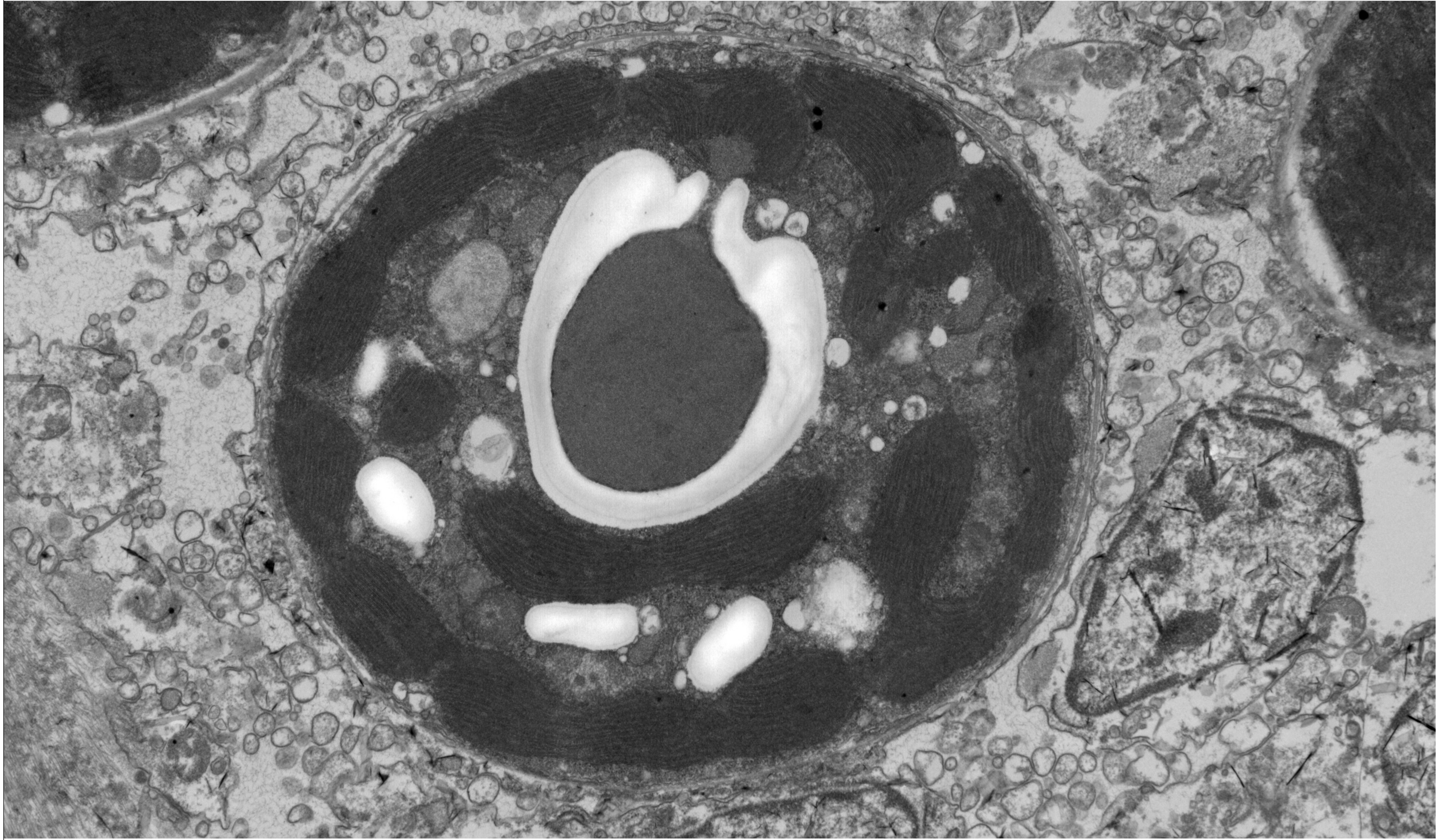

21-20_Correa_3-18-25_16l3_032.tif
Sample: 3/18, 25
Biological Electron Microscopy Lab
Rice University - SEA
Microscopist: MD Meyer

1 µm
HV=80kV
Direct Mag: 2500 x

Cell 16

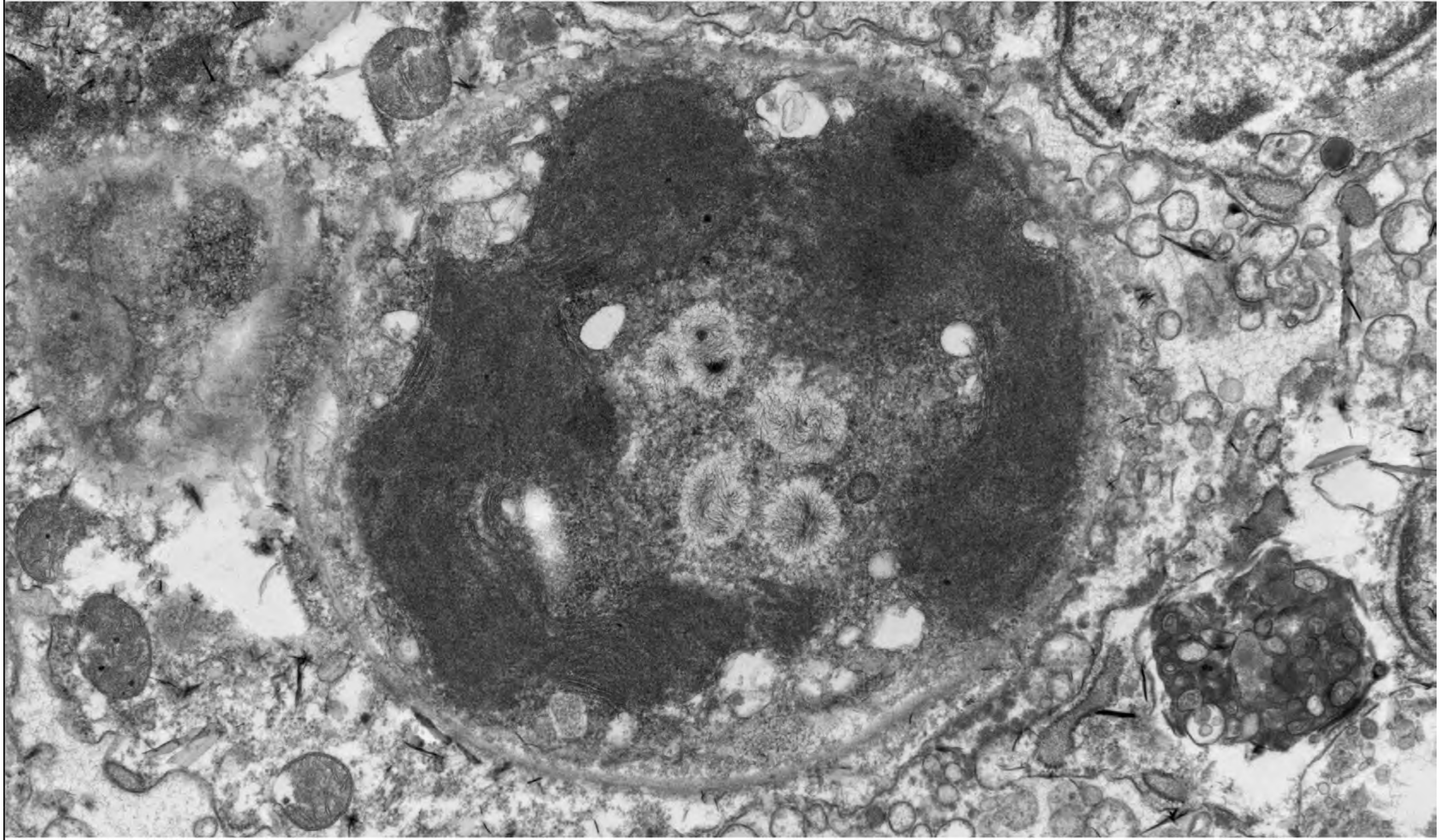

21-20_Correa_3-18-25_16l3_031.tif
Sample: 3/18, 25
Biological Electron Microscopy Lab
Rice University - SEA
Microscopist: MD Meyer

1 µm
HV=80kV
Direct Mag: 4000 x

Cell 17

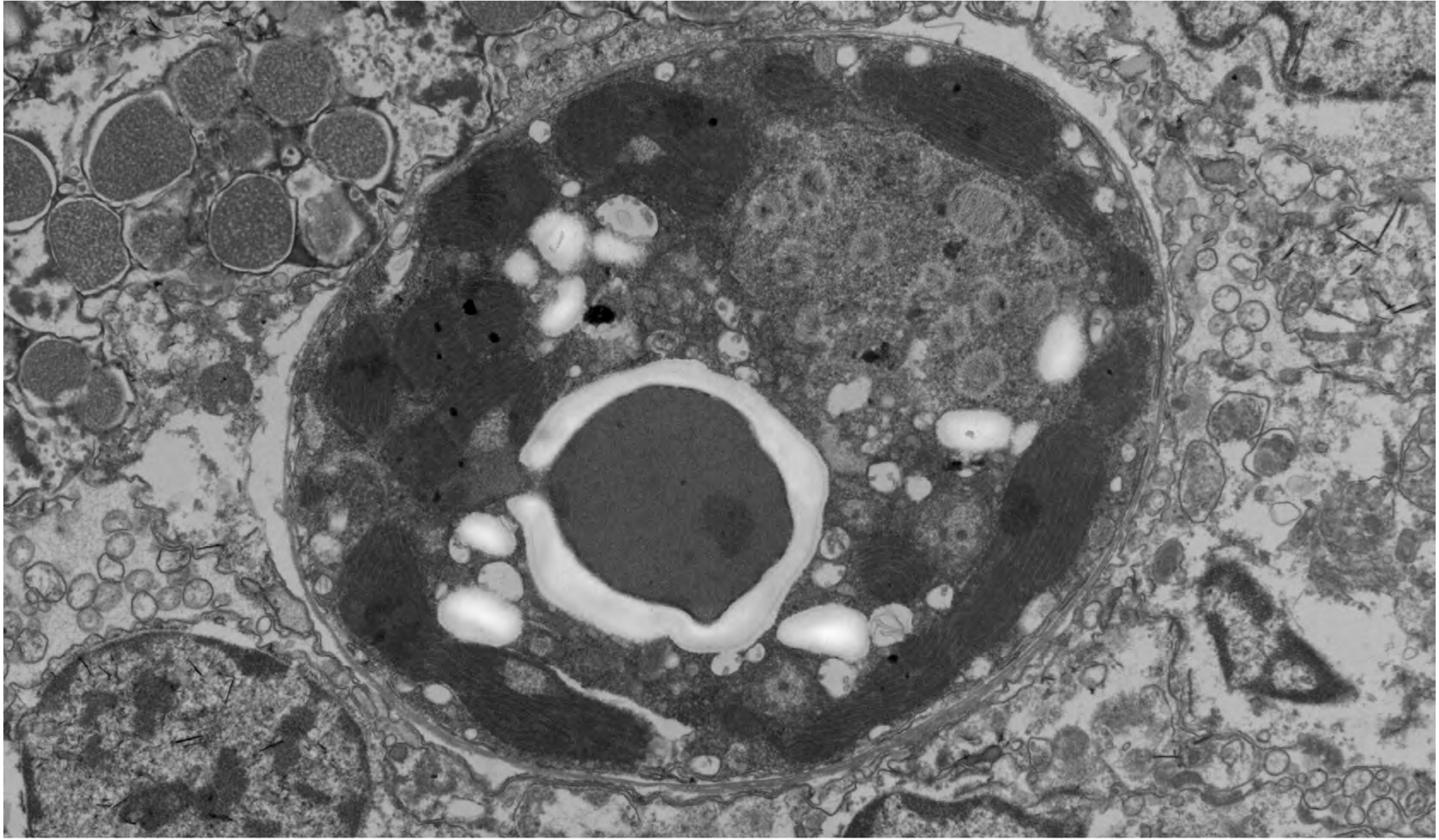

21-20_Correa_3-18-25_16I3_029.tif
Sample: 3/18, 25
Biological Electron Microscopy Lab
Rice University - SEA
Microscopist: MD Meyer

1 µm
HV=80kV
Direct Mag: 2500 x

Cell 18

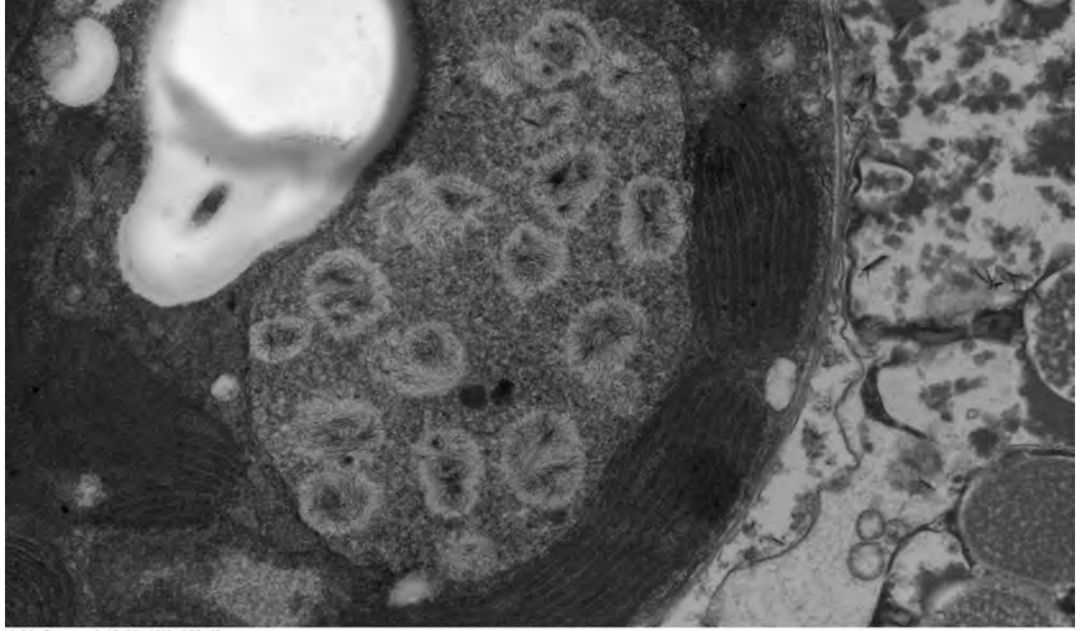

21-20_Correa_3-18-25_16I3_028.tif
Sample: 3/18, 25
Biological Electron Microscopy Lab
Rice University - SEA
Microscopist: MD Meyer

800 nm
HV=80kV
Direct Mag: 5000 x

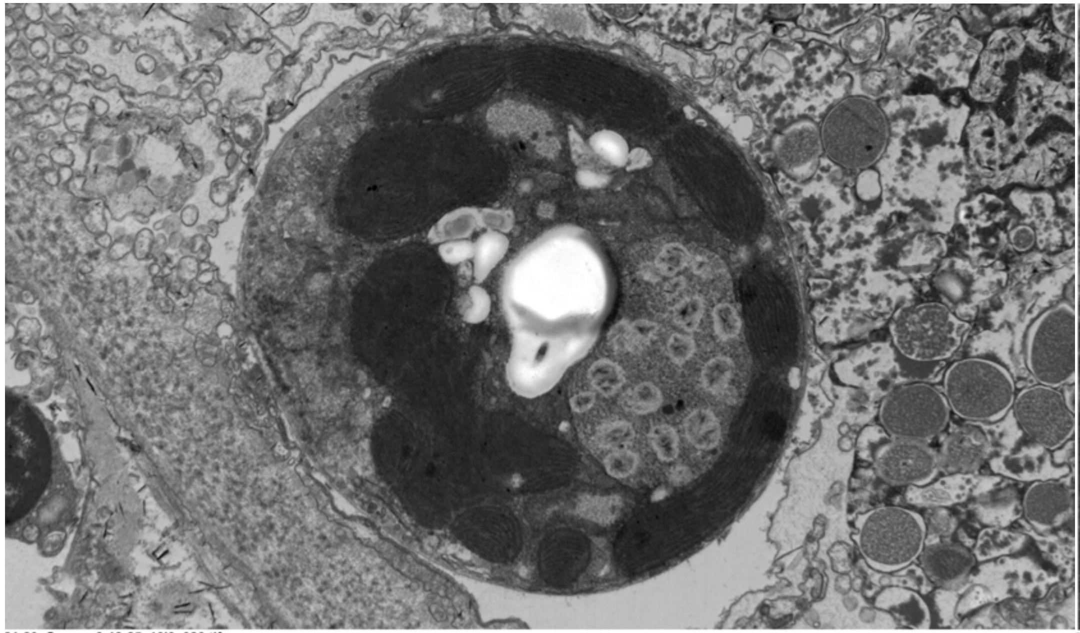

21-20_Correa_3-18-25_16I3_026.tif
Sample: 3/18, 25
Biological Electron Microscopy Lab
Rice University - SEA
Microscopist: MD Meyer

2 µm
HV=80kV
Direct Mag: 2000 x

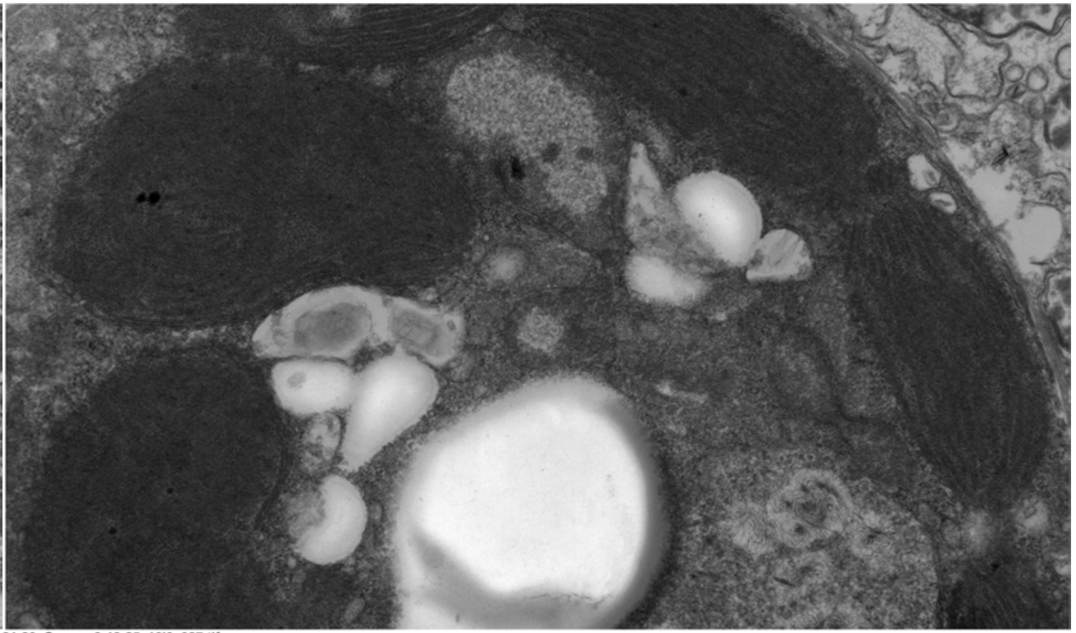

21-20_Correa_3-18-25_16I3_027.tif
Sample: 3/18, 25
Biological Electron Microscopy Lab
Rice University - SEA
Microscopist: MD Meyer

800 nm
HV=80kV
Direct Mag: 5000 x

Cell 19

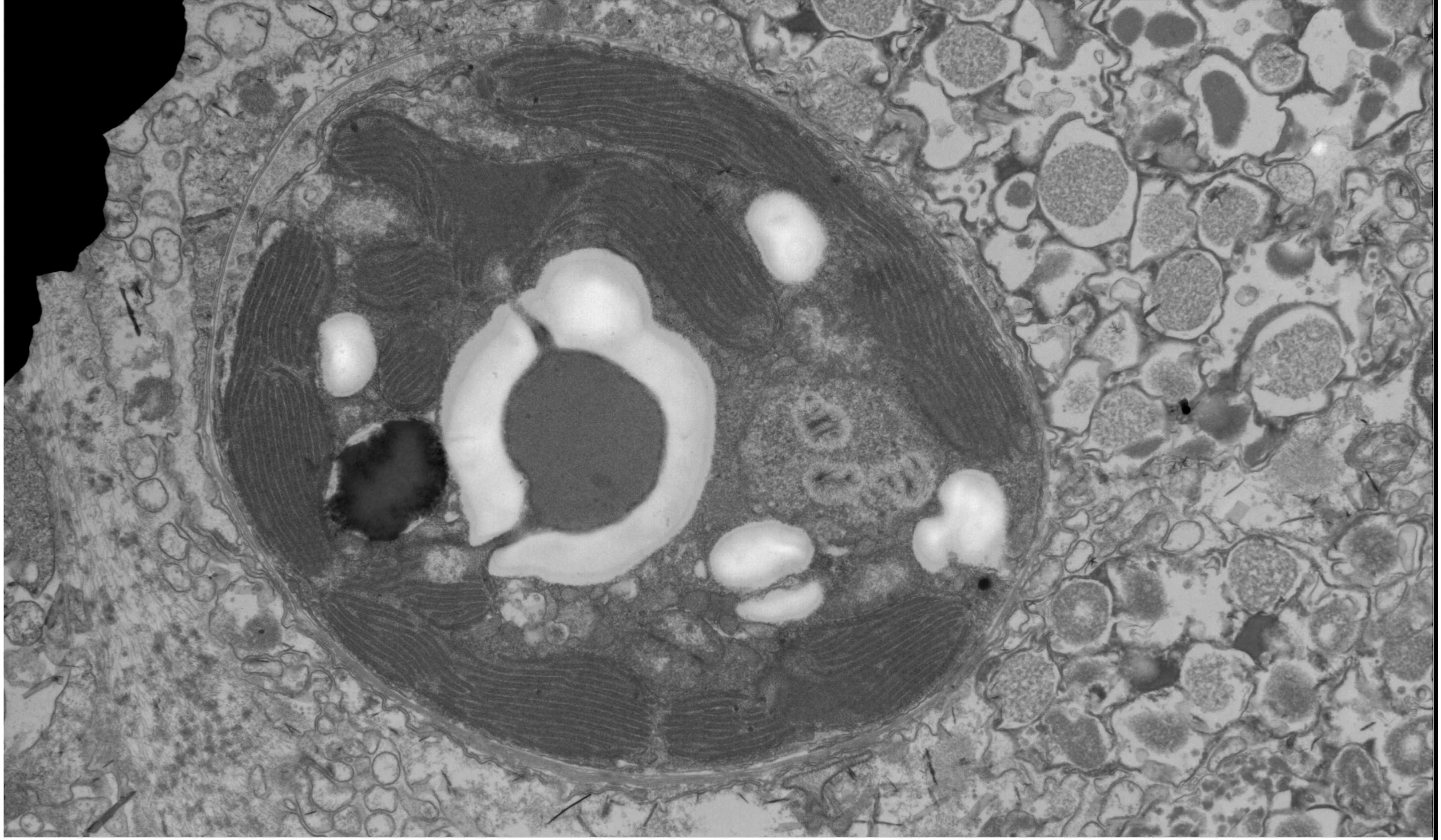

21-20_Correa_3-18-25_16I3_025.tif
Sample: 3/18, 25
Biological Electron Microscopy Lab
Rice University - SEA
Microscopist: MD Meyer

1 µm
HV=80kV
Direct Mag: 2500 x

Cell 20

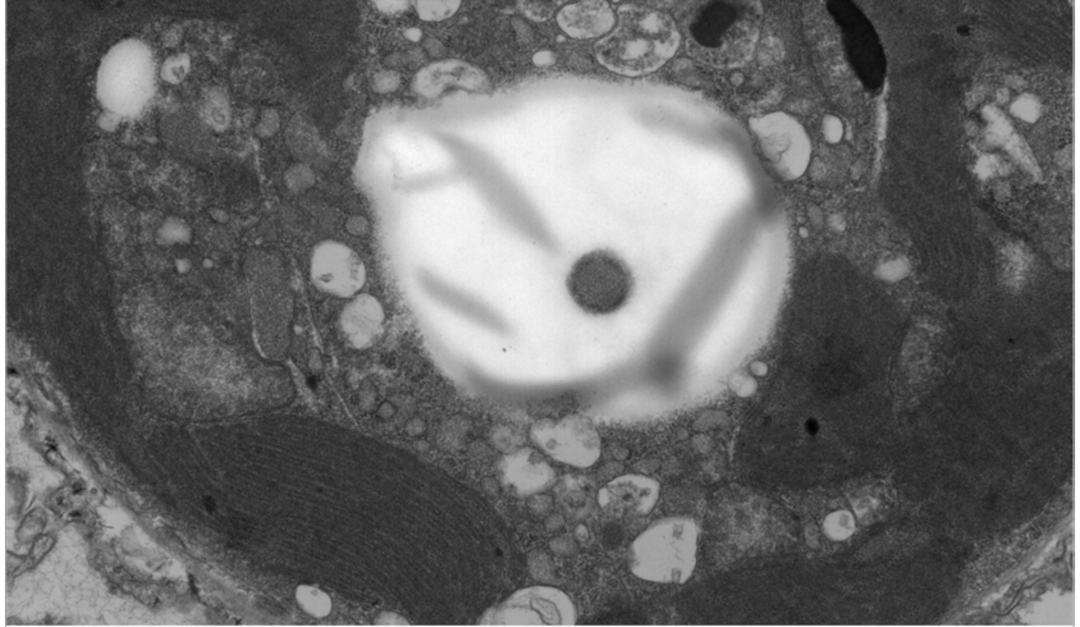

21-20_Correa_3-18-25_16I3_023.tif
Sample: 3/18, 25
Biological Electron Microscopy Lab
Rice University - SEA
Microscopist: MD Meyer

800 nm
HV=80kV
Direct Mag: 5000 x

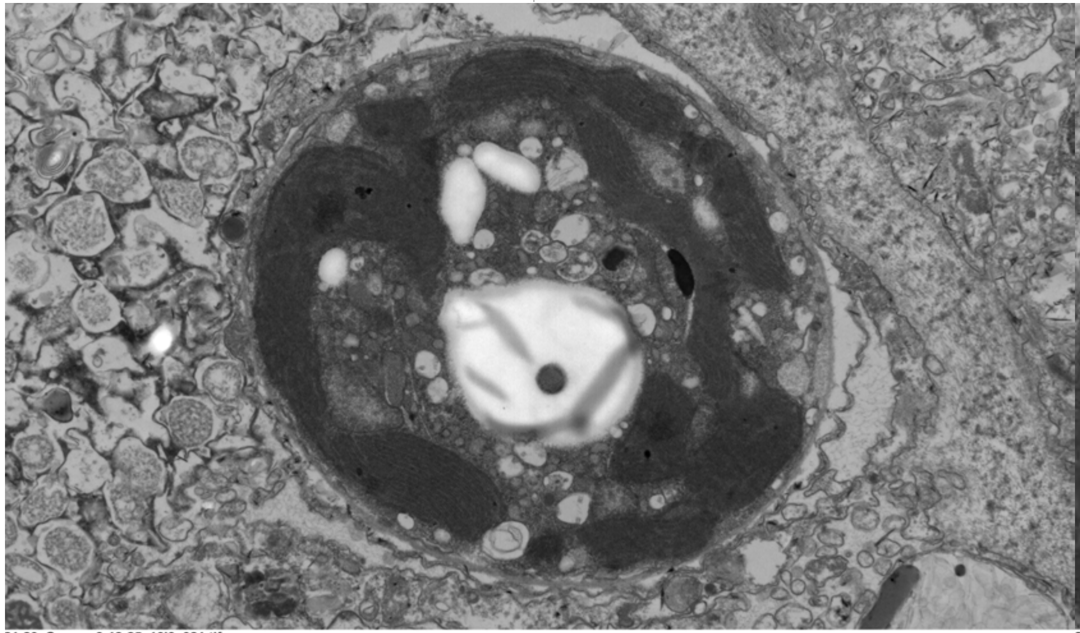

21-20_Correa_3-18-25_16I3_021.tif
Sample: 3/18, 25
Biological Electron Microscopy Lab
Rice University - SEA
Microscopist: MD Meyer

1 µm
HV=80kV
Direct Mag: 2500 x

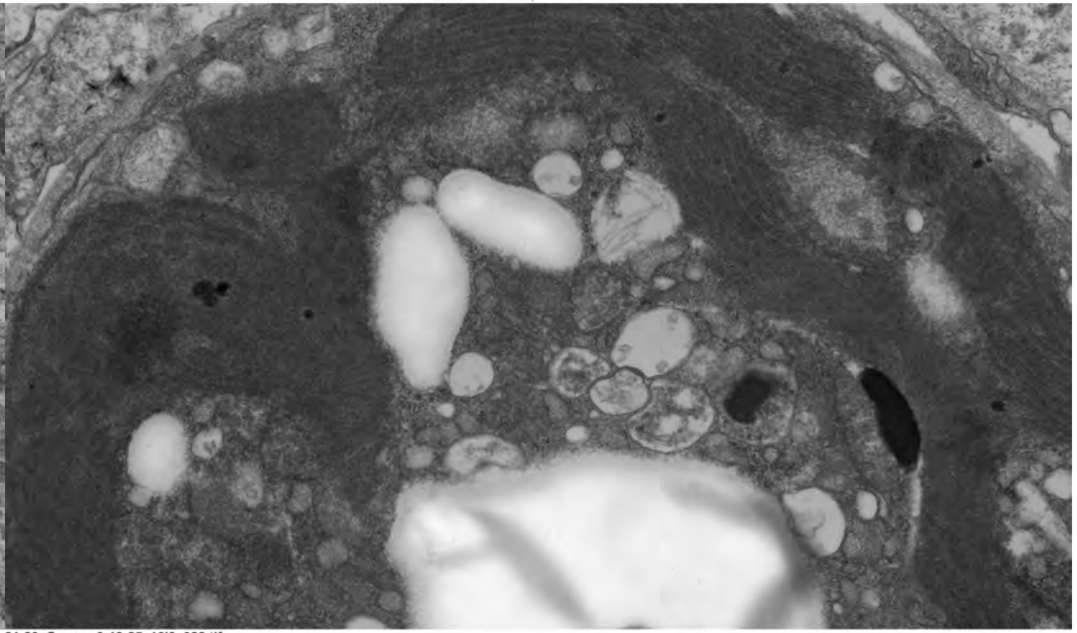

21-20_Correa_3-18-25_16I3_022.tif
Sample: 3/18, 25
Biological Electron Microscopy Lab
Rice University - SEA
Microscopist: MD Meyer

800 nm
HV=80kV
Direct Mag: 5000 x

Cell 21

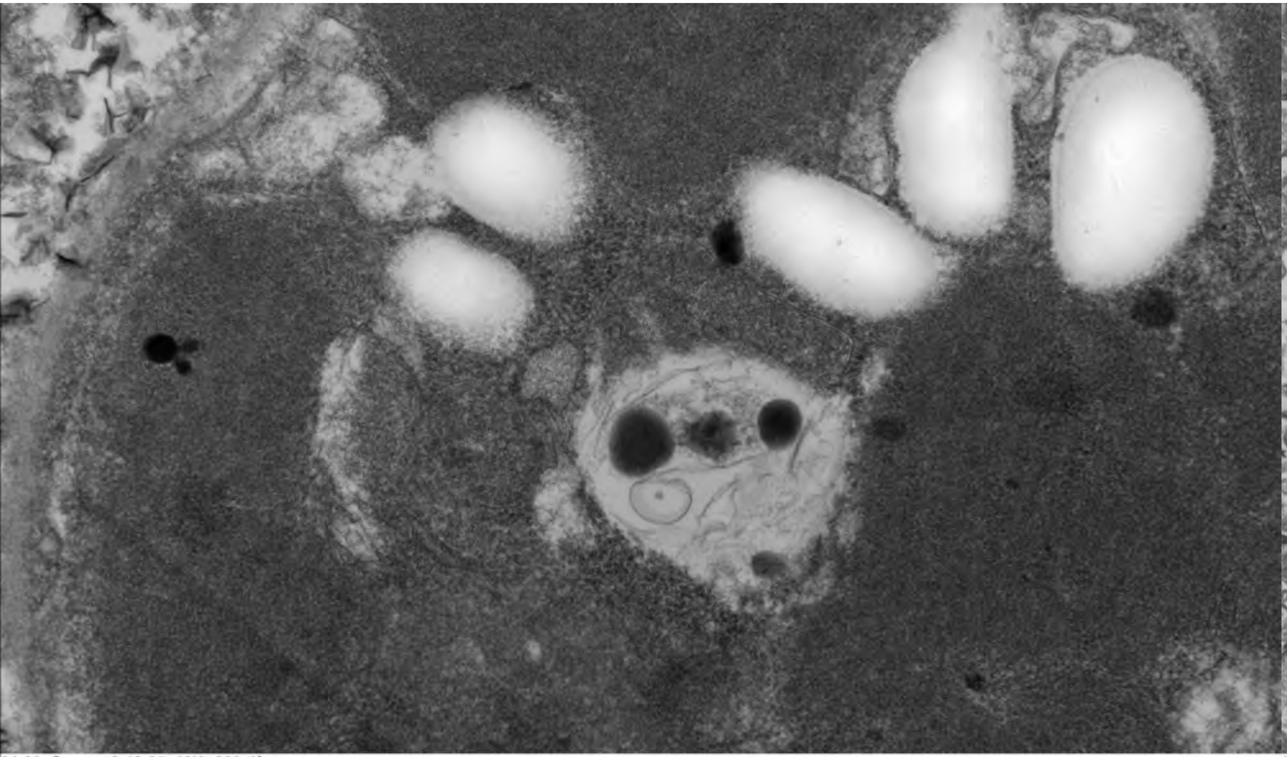

21-20_Correa_3-18-25_16l3_020.tif
Sample: 3/18, 25
Biological Electron Microscopy Lab
Rice University - SEA
Microscopist: MD Meyer

500 nm
HV=80kV
Direct Mag: 8000 x

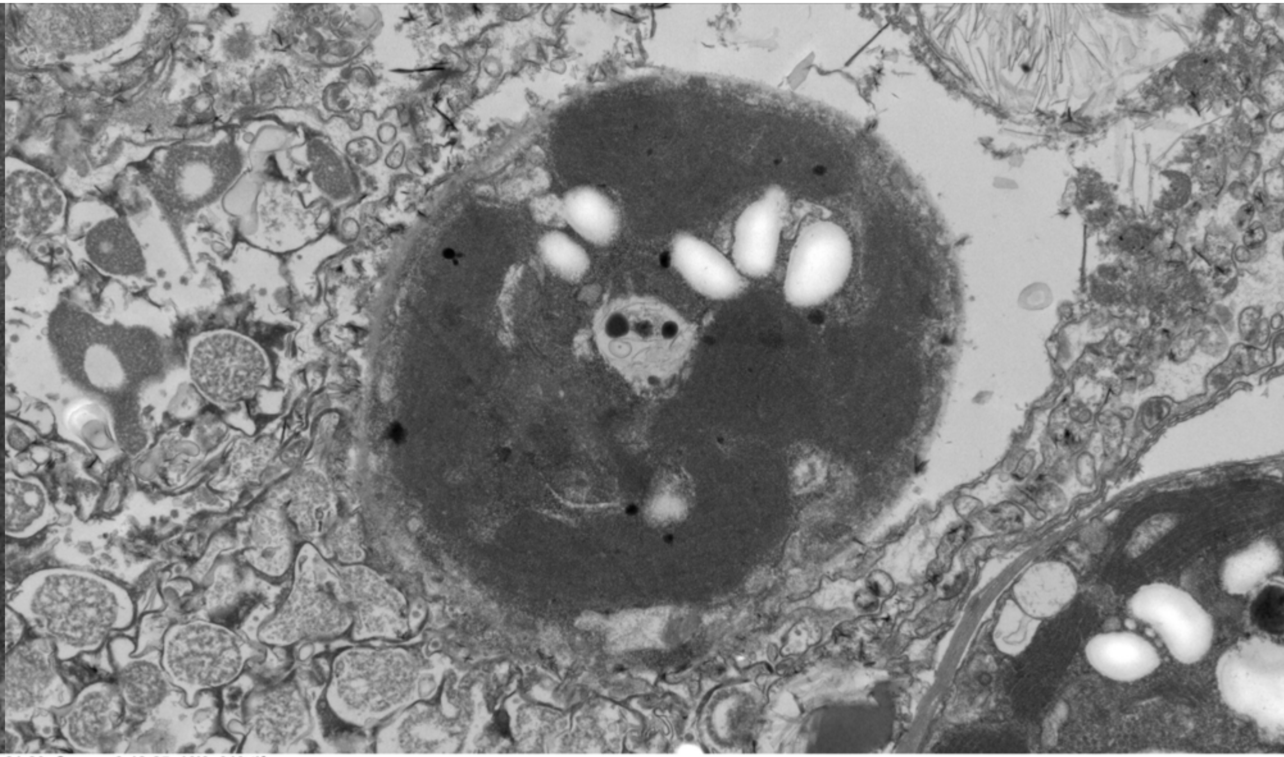

21-20_Correa_3-18-25_16l3_019.tif
Sample: 3/18, 25
Biological Electron Microscopy Lab
Rice University - SEA
Microscopist: MD Meyer

1 µm
HV=80kV
Direct Mag: 3000 x

Cell 22

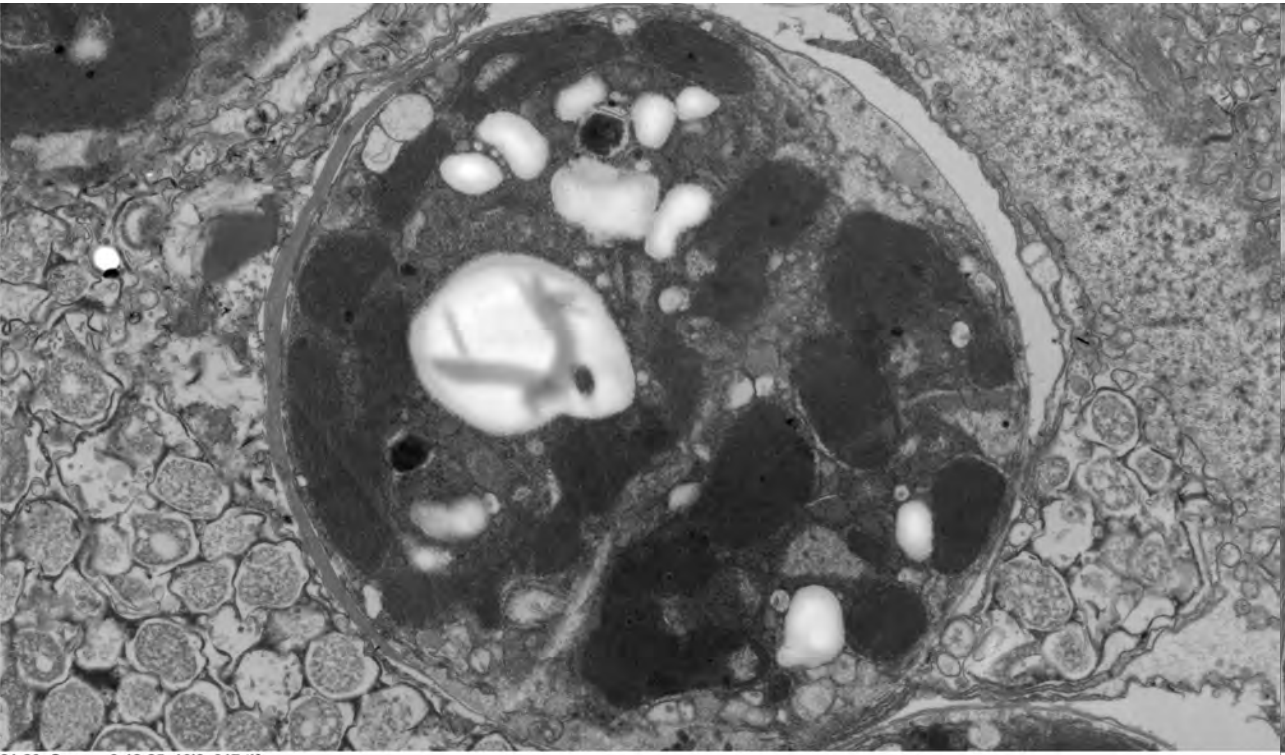

21-20_Correa_3-18-25_16I3_017.tif
Sample: 3/18, 25
Biological Electron Microscopy Lab
Rice University - SEA
Microscopist: MD Meyer

1 µm
HV=80kV
Direct Mag: 2500 x

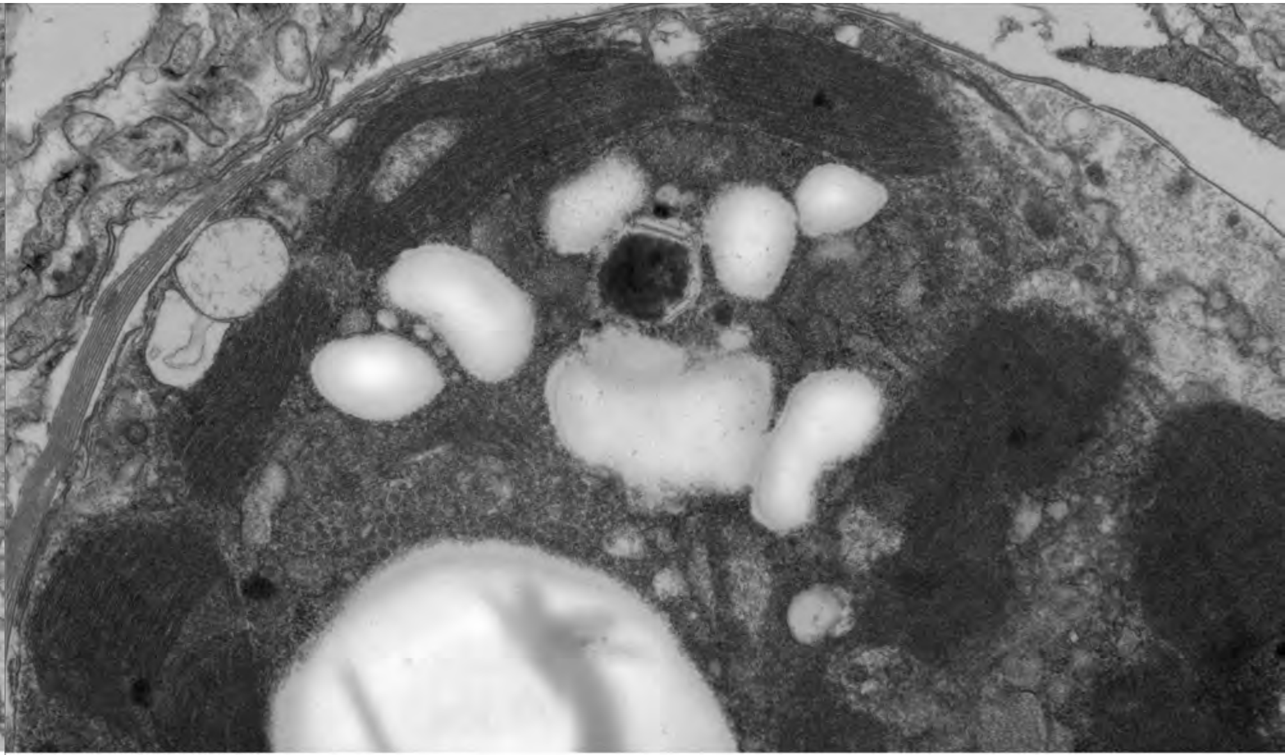

21-20_Correa_3-18-25_16I3_018.tif
Sample: 3/18, 25
Biological Electron Microscopy Lab
Rice University - SEA
Microscopist: MD Meyer

800 nm
HV=80kV
Direct Mag: 5000 x

Cell 23

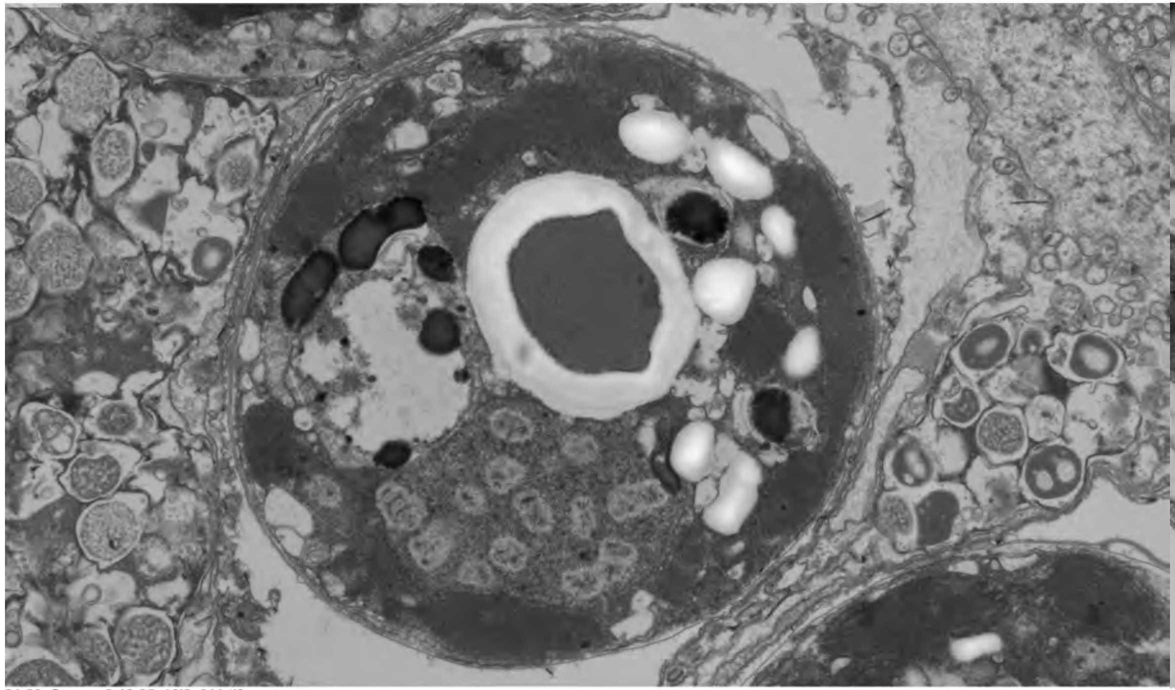

21-20_Correa_3-18-25_16I3_011.tif
Sample: 3/18, 25
Biological Electron Microscopy Lab
Rice University - SEA
Microscopist: MD Meyer

1 µm
HV=80kV
Direct Mag: 2500 x

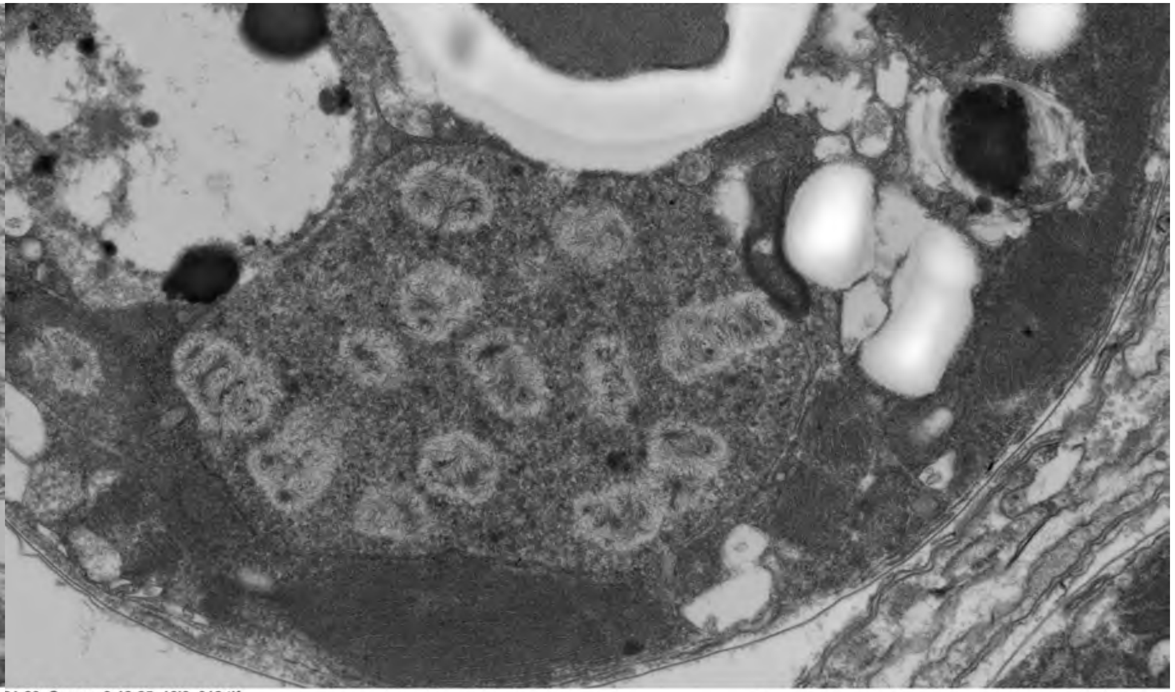

21-20_Correa_3-18-25_16I3_012.tif
Sample: 3/18, 25
Biological Electron Microscopy Lab
Rice University - SEA
Microscopist: MD Meyer

800 nm
HV=80kV
Direct Mag: 5000 x

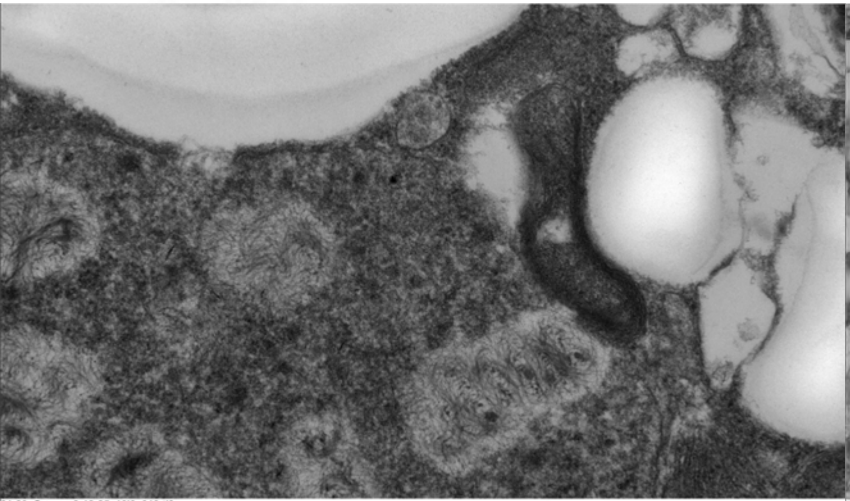

21-20_Correa_3-18-25_16I3_013.tif
Sample: 3/18, 25
Biological Electron Microscopy Lab
Rice University - SEA
Microscopist: MD Meyer

200 nm
HV=80kV
Direct Mag: 12000 x

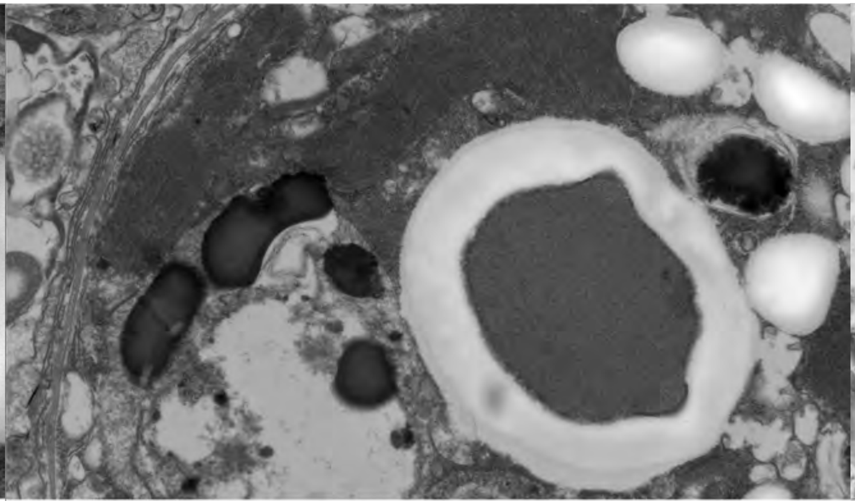

21-20_Correa_3-18-25_16I3_015.tif
Sample: 3/18, 25
Biological Electron Microscopy Lab
Rice University - SEA
Microscopist: MD Meyer

800 nm
HV=80kV
Direct Mag: 5000 x

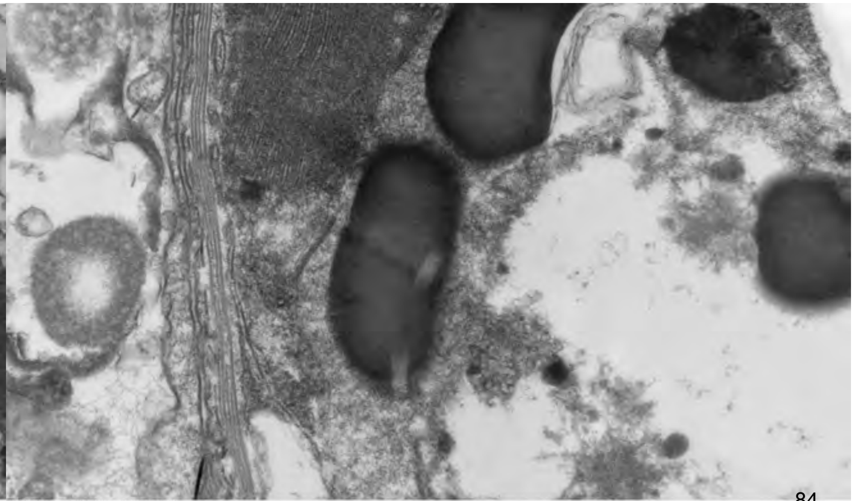

21-20_Correa_3-18-25_16I3_016.tif
Sample: 3/18, 25
Biological Electron Microscopy Lab
Rice University - SEA
Microscopist: MD Meyer

400 nm
HV=80kV
Direct Mag: 10000 x

Cell 24

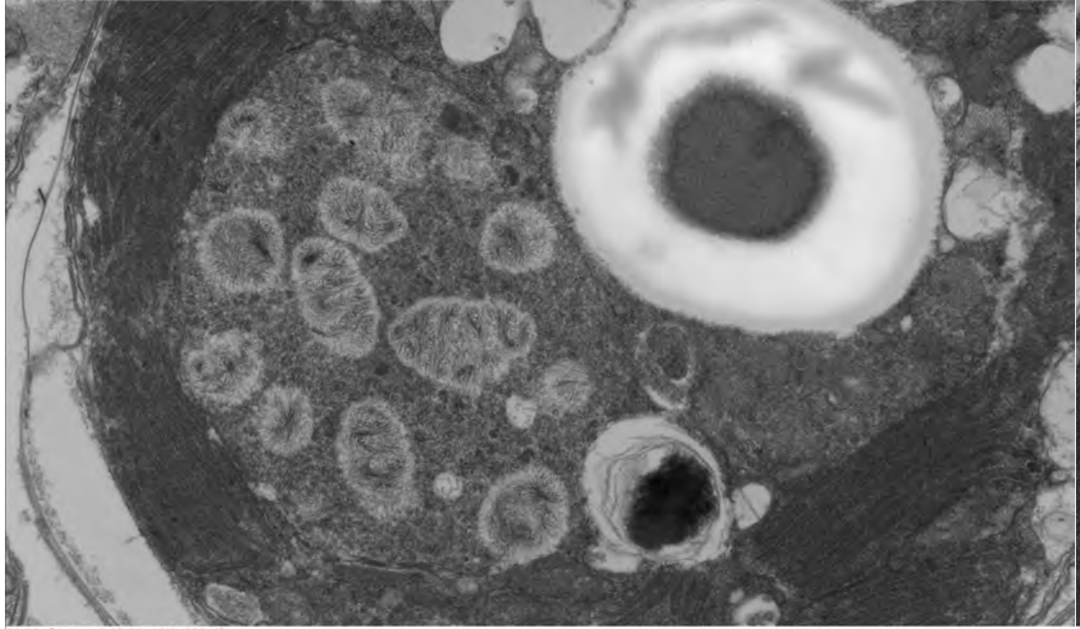

21-20_Correa_3-18-25_16I3_008.tif
Sample: 3/18, 25
Biological Electron Microscopy Lab
Rice University - SEA
Microscopist: MD Meyer

800 nm
HV=80kV
Direct Mag: 5000 x

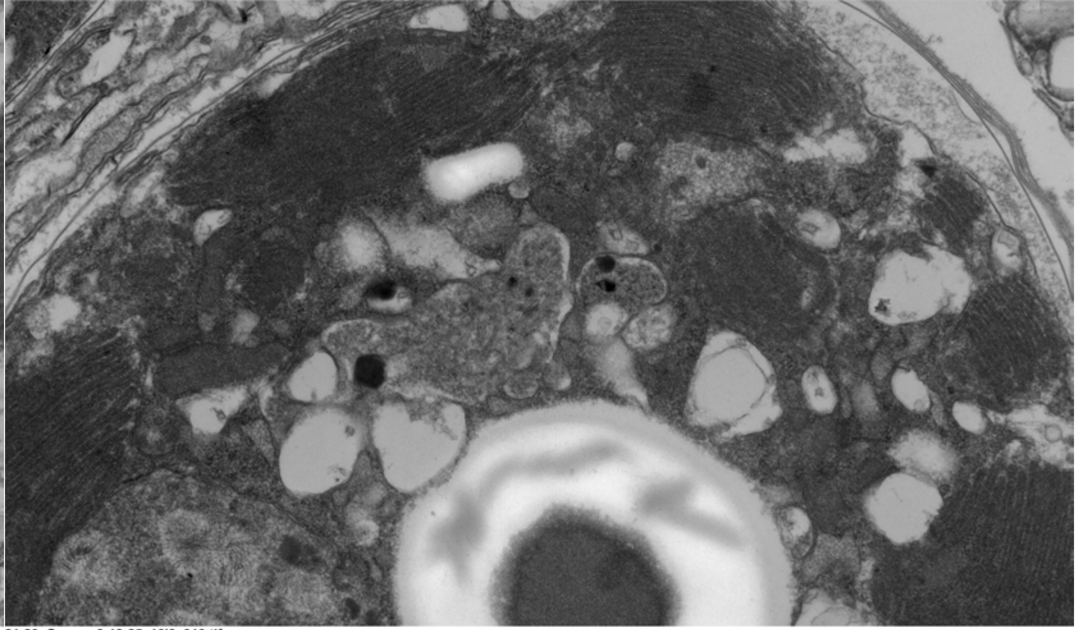

21-20_Correa_3-18-25_16I3_010.tif
Sample: 3/18, 25
Biological Electron Microscopy Lab
Rice University - SEA
Microscopist: MD Meyer

800 nm
HV=80kV
Direct Mag: 5000 x

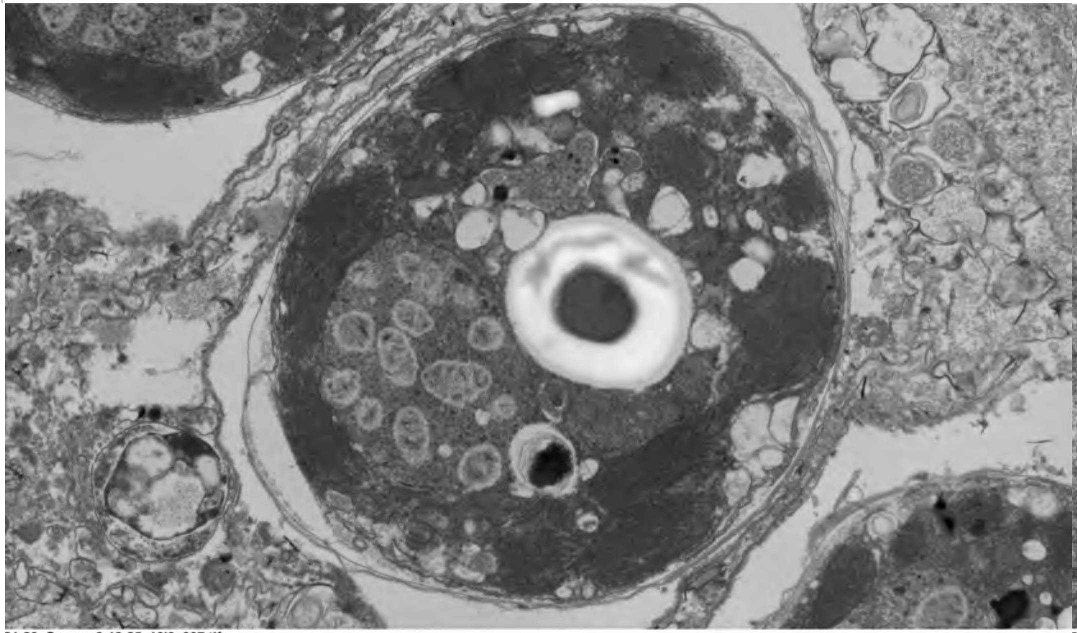

21-20_Correa_3-18-25_16I3_007.tif
Sample: 3/18, 25
Biological Electron Microscopy Lab
Rice University - SEA
Microscopist: MD Meyer

1 µm
HV=80kV
Direct Mag: 2500 x

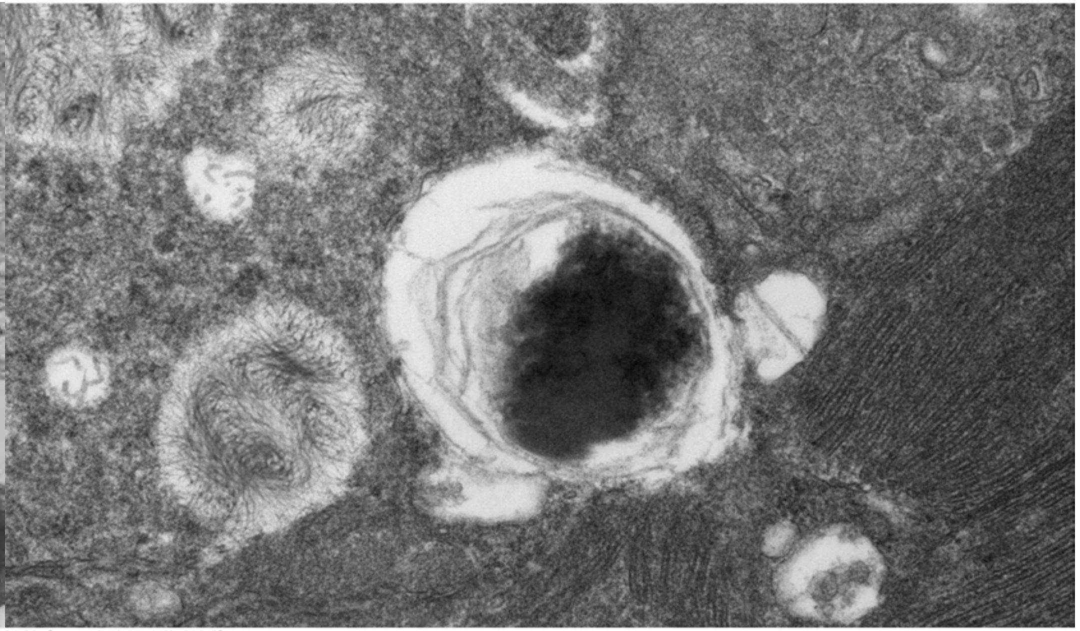

21-20_Correa_3-18-25_16I3_009.tif
Sample: 3/18, 25
Biological Electron Microscopy Lab
Rice University - SEA
Microscopist: MD Meyer

200 nm
HV=80kV
Direct Mag: 12000 x

Cell 25

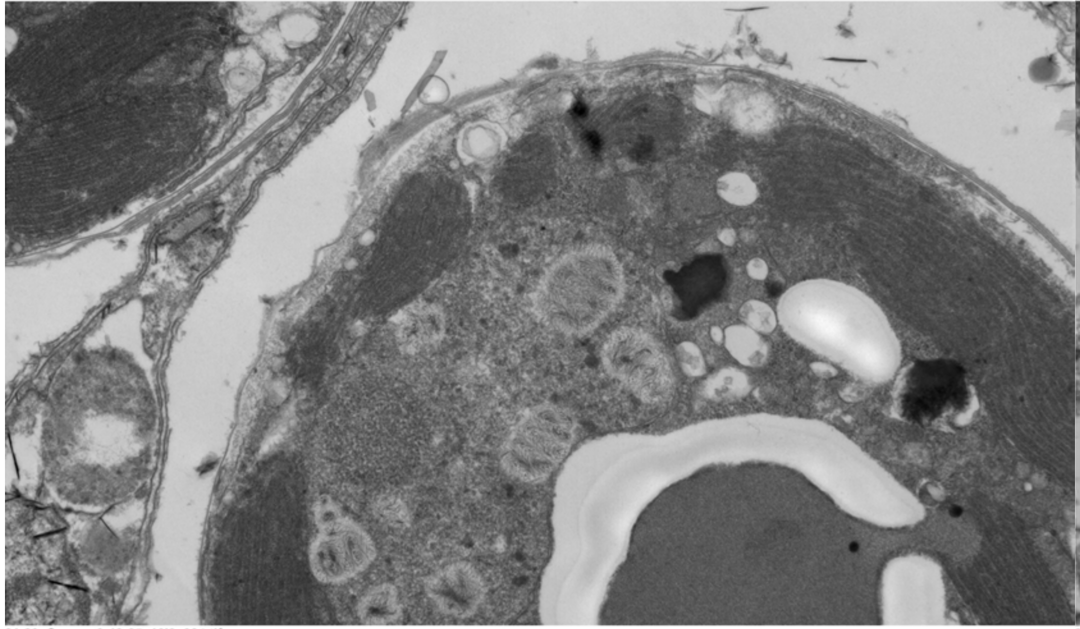

21-20_Correa_3-18-25_16I3_004.tif
Sample: 3/18, 25
Biological Electron Microscopy Lab
Rice University - SEA
Microscopist: MD Meyer

1 µm
HV=80kV
Direct Mag: 4000 x

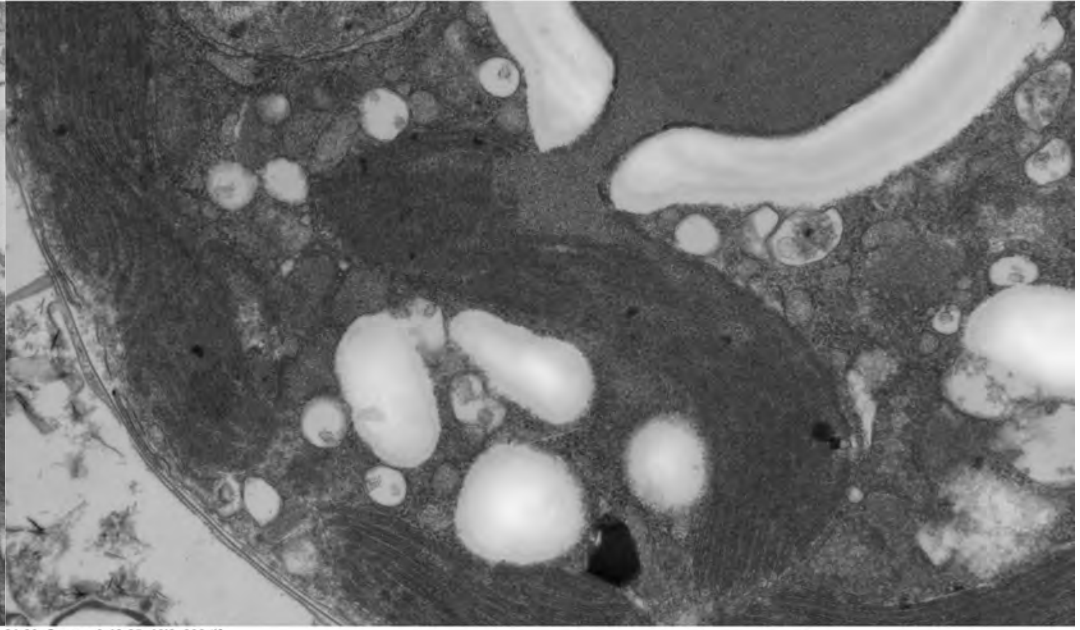

21-20_Correa_3-18-25_16I3_006.tif
Sample: 3/18, 25
Biological Electron Microscopy Lab
Rice University - SEA
Microscopist: MD Meyer

600 nm
HV=80kV
Direct Mag: 6000 x

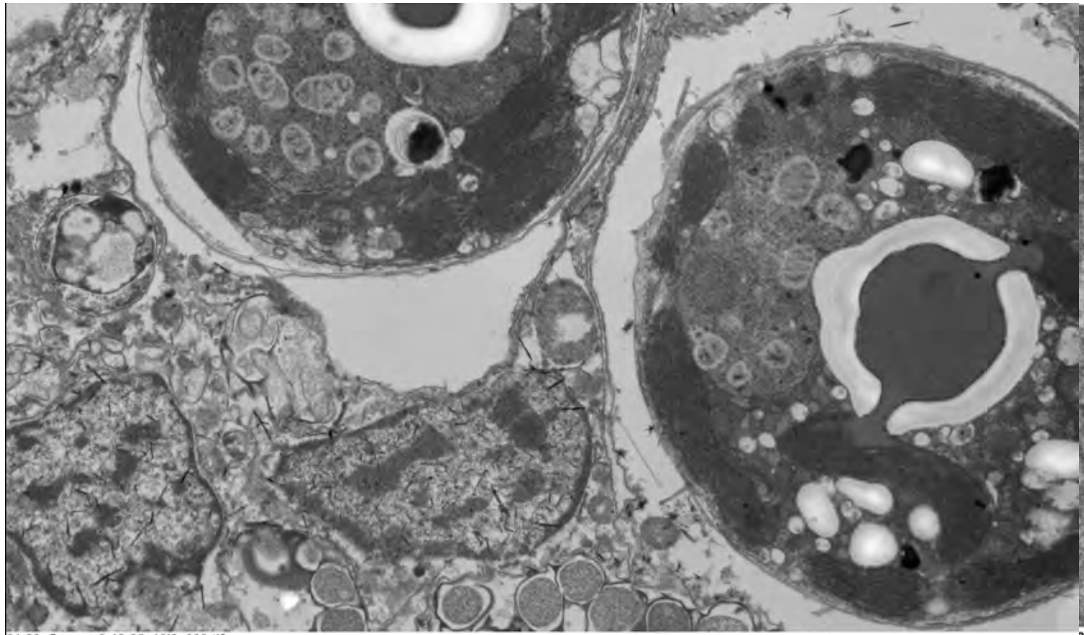

21-20_Correa_3-18-25_16I3_003.tif
Sample: 3/18, 25
Biological Electron Microscopy Lab
Rice University - SEA
Microscopist: MD Meyer

2 µm
HV=80kV
Direct Mag: 2000 x

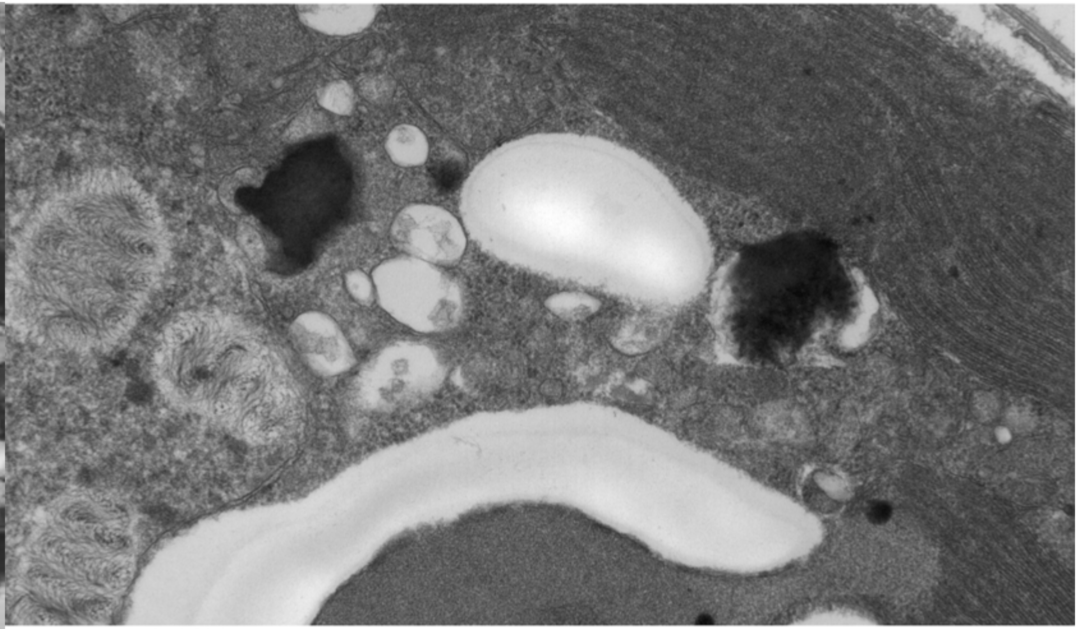

21-20_Correa_3-18-25_16I3_005.tif
Sample: 3/18, 25
Biological Electron Microscopy Lab
Rice University - SEA
Microscopist: MD Meyer

500 nm
HV=80kV
Direct Mag: 8000 x

Heat (March 2019) POR Colony K

Cell 29

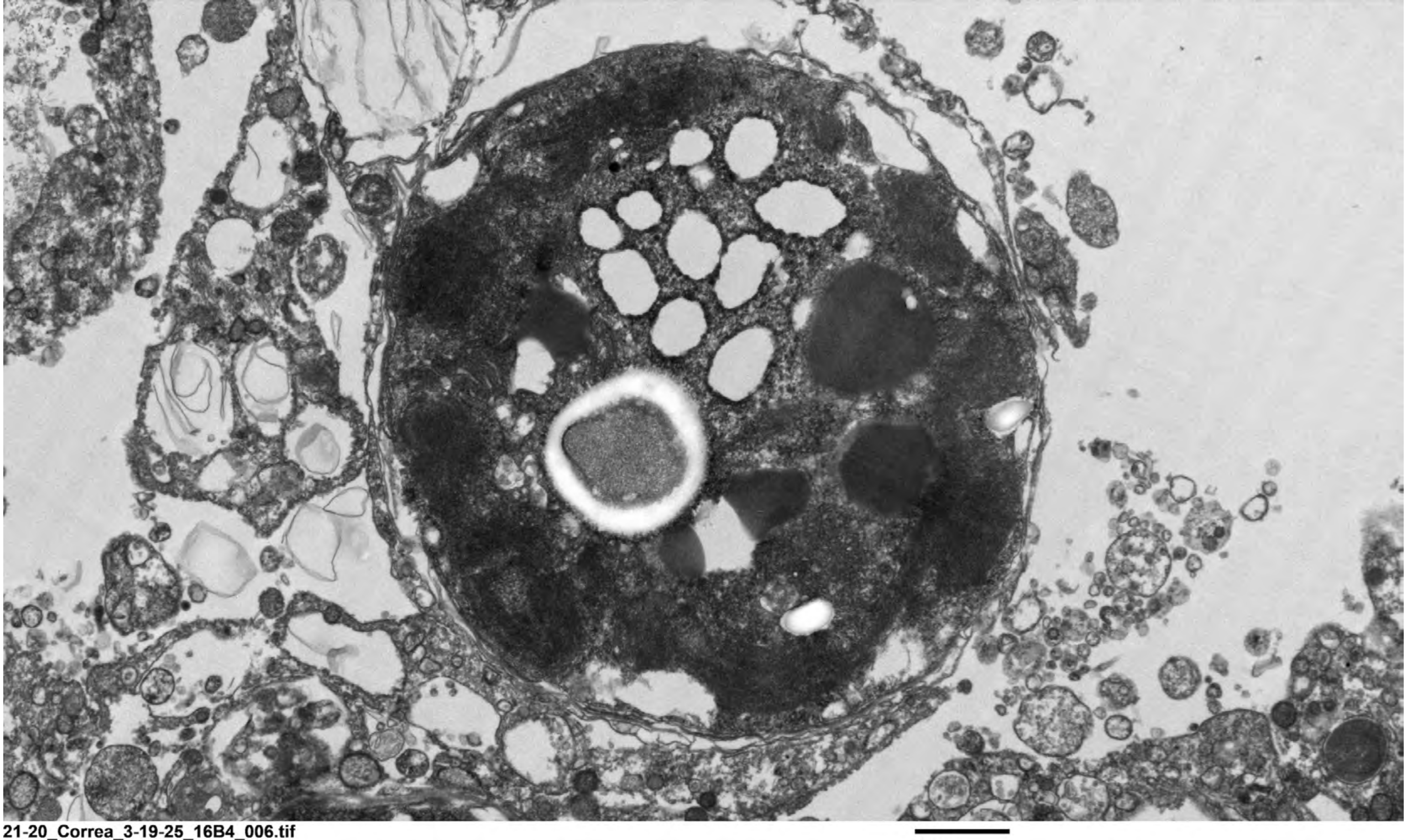

21-20_Correa_3-19-25_16B4_006.tif
Sample: 3/19, 25
Biological Electron Microscopy Lab
Rice University - SEA
Microscopist: MD Meyer

1 μm
HV=80kV
Direct Mag: 2500 x

Cell 1

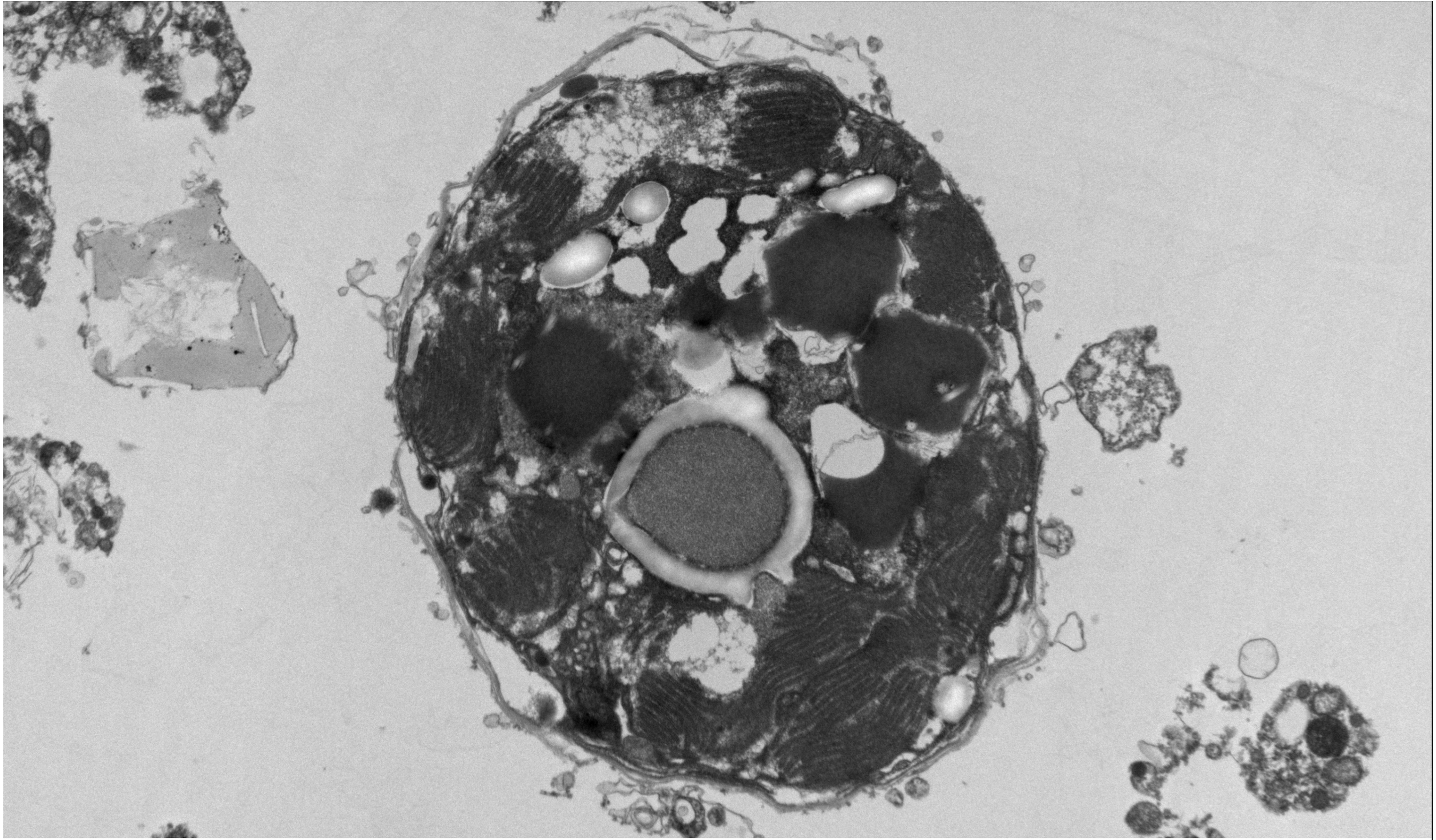

21-20_Correa_3-19-25_16B4_123.tif
Sample: 3/19, 25
Biological Electron Microscopy Lab
Rice University - SEA
Microscopist: MD Meyer

2 µm
HV=80kV
Direct Mag: 2000 x

Cell 2

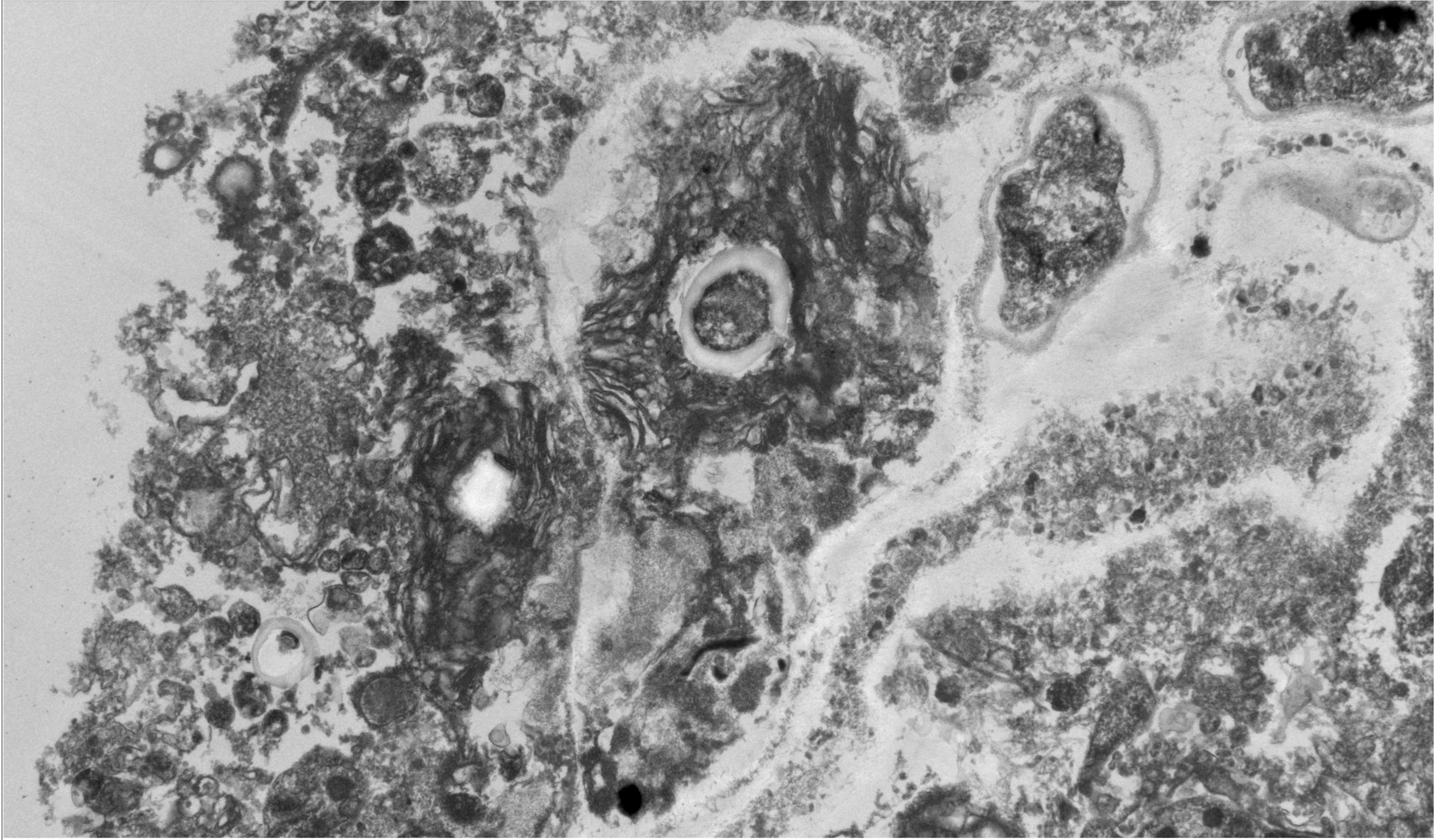

21-20_Correa_3-19-25_16B4_122.tif
Sample: 3/19, 25
Biological Electron Microscopy Lab
Rice University - SEA
Microscopist: MD Meyer

1 µm
HV=80kV
Direct Mag: 3000 x

Cell 3

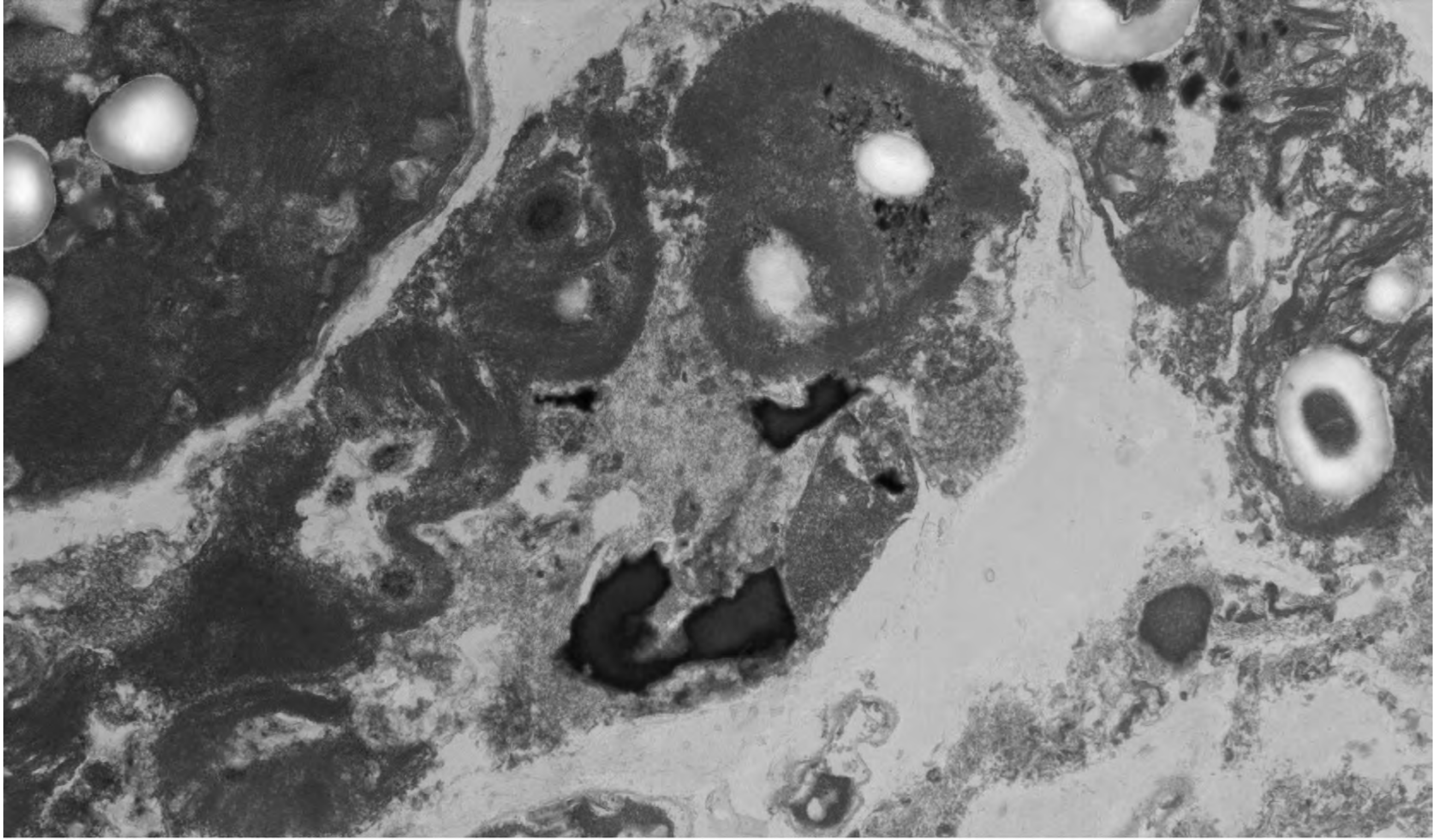

21-20_Correa_3-19-25_16B4_121.tif
Sample: 3/19, 25
Biological Electron Microscopy Lab
Rice University - SEA
Microscopist: MD Meyer

1 µm
HV=80kV
Direct Mag: 3000 x

Cell 4

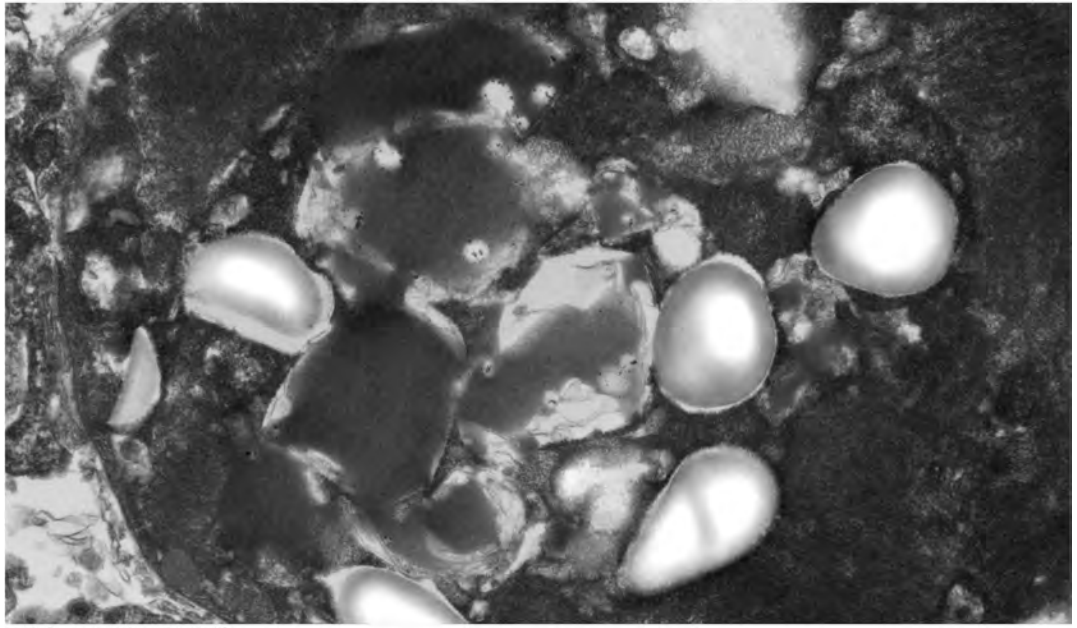

21-20_Correa_3-19-25_16B4_120.tif
Sample: 3/19, 25
Biological Electron Microscopy Lab
Rice University - SEA
Microscopist: MD Meyer

800 nm
HV=80kV
Direct Mag: 5000 x

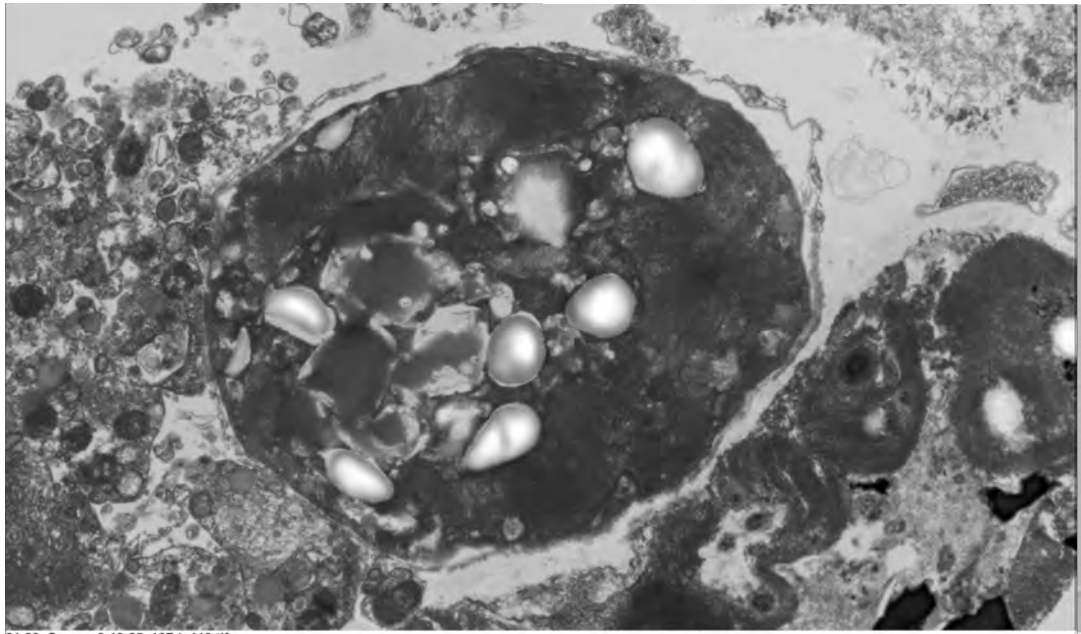

21-20_Correa_3-19-25_16B4_119.tif
Sample: 3/19, 25
Biological Electron Microscopy Lab
Rice University - SEA
Microscopist: MD Meyer

1 µm
HV=80kV
Direct Mag: 2500 x

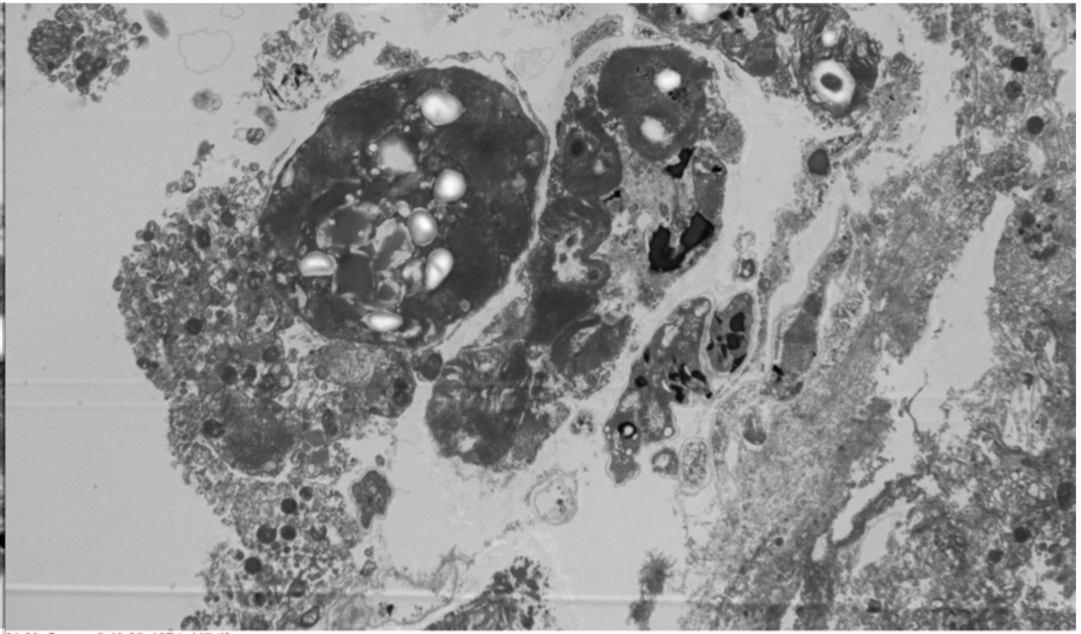

21-20_Correa_3-19-25_16B4_117.tif
Sample: 3/19, 25
Biological Electron Microscopy Lab
Rice University - SEA
Microscopist: MD Meyer

2 µm
HV=80kV
Direct Mag: 1200 x

Cell 5

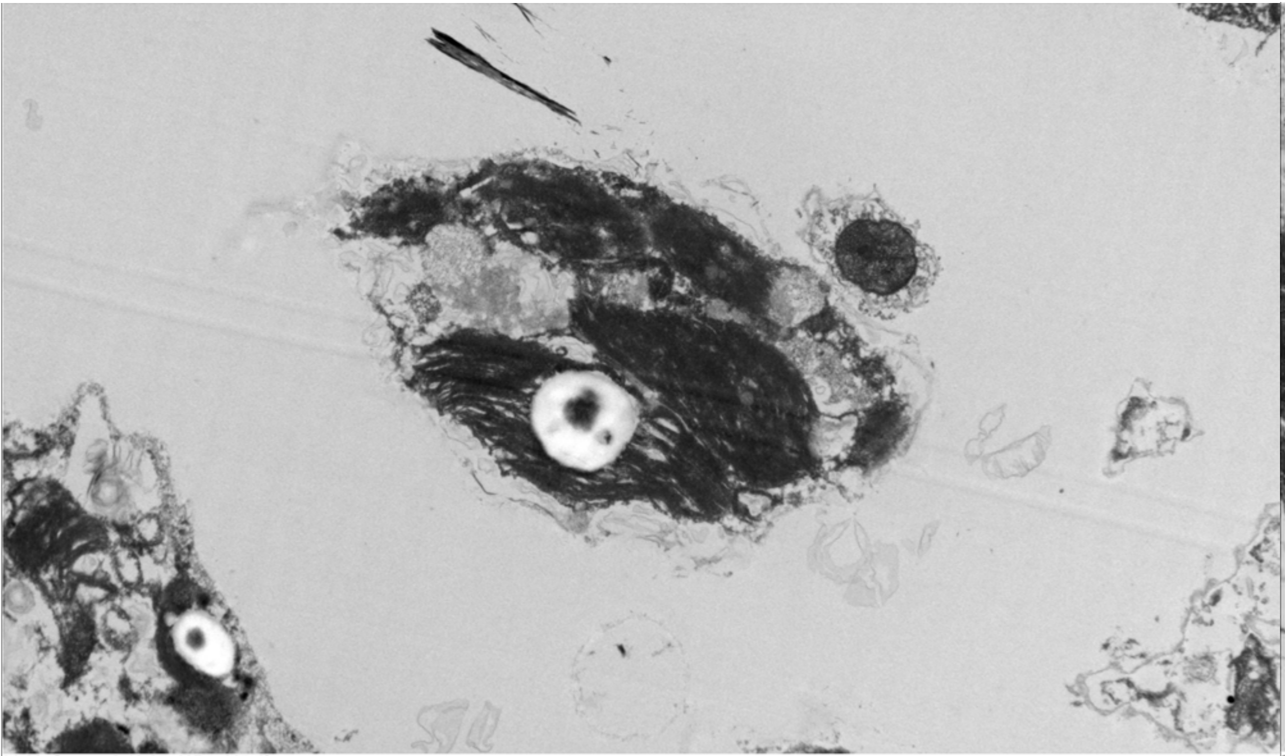

21-20_Correa_3-19-25_16B4_115.tif
Sample: 3/19, 25
Biological Electron Microscopy Lab
Rice University - SEA
Microscopist: MD Meyer

2 µm
HV=80kV
Direct Mag: 2000 x

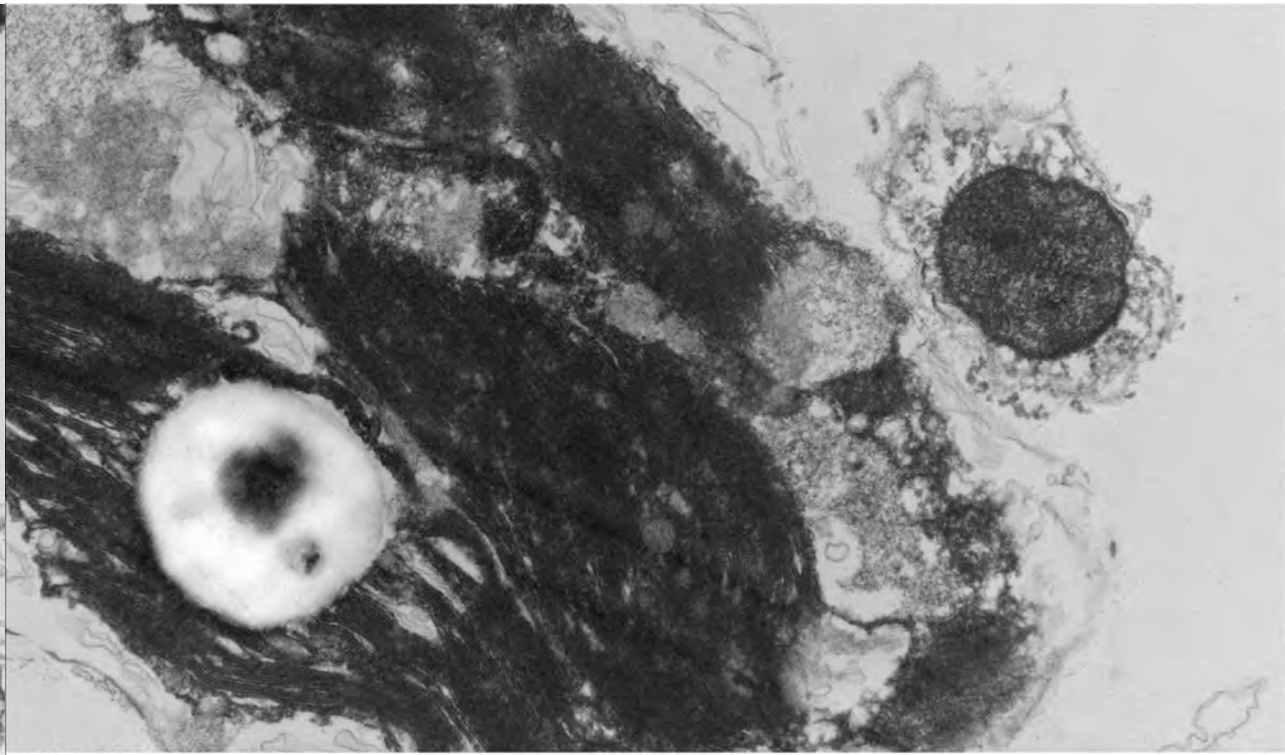

21-20_Correa_3-19-25_16B4_116.tif
Sample: 3/19, 25
Biological Electron Microscopy Lab
Rice University - SEA
Microscopist: MD Meyer

800 nm
HV=80kV
Direct Mag: 5000 x

Cells 6 + 7

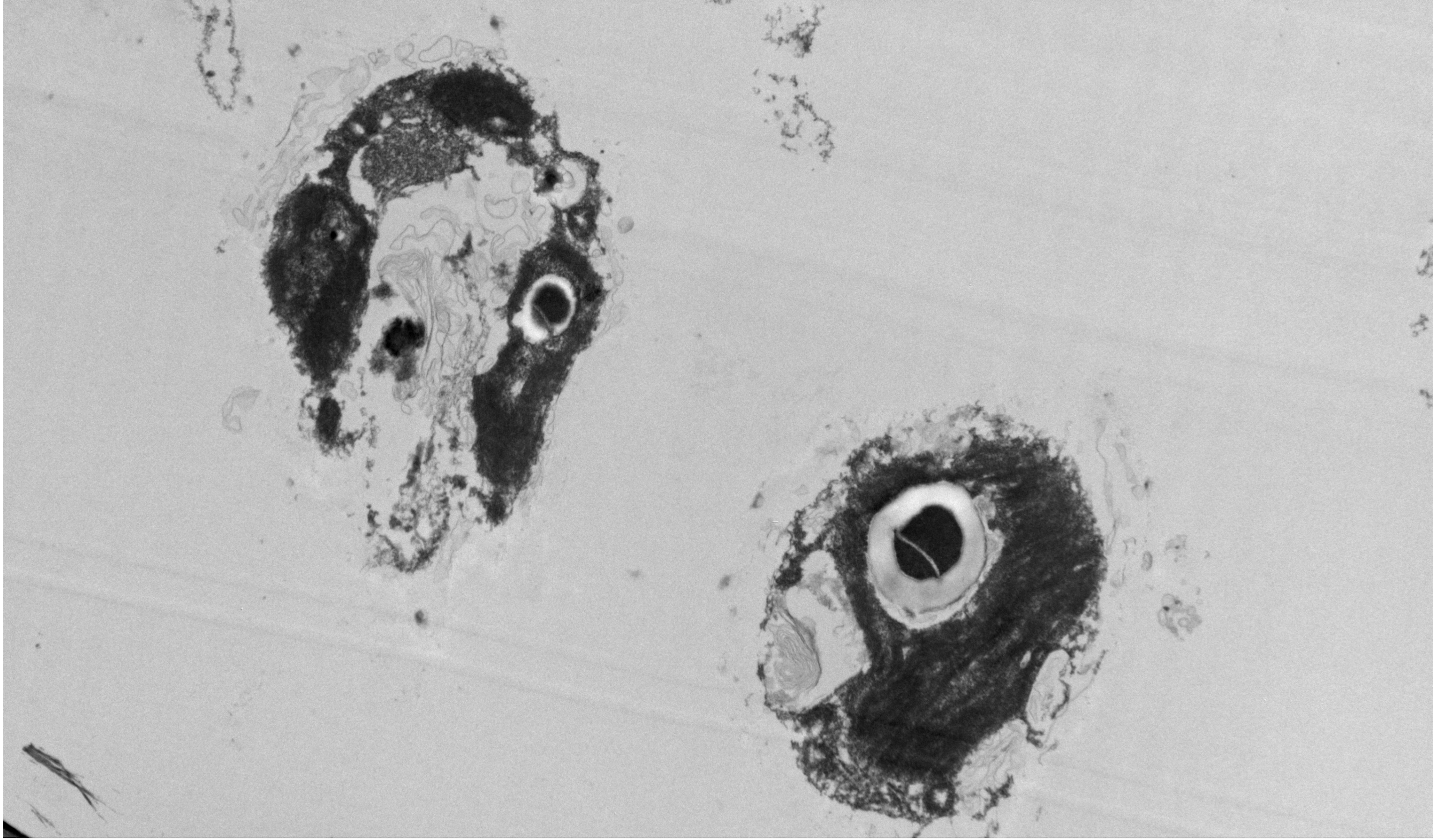

21-20_Correa_3-19-25_16B4_114.tif
Sample: 3/19, 25
Biological Electron Microscopy Lab
Rice University - SEA
Microscopist: MD Meyer

2 µm
HV=80kV
Direct Mag: 2000 x

Cell 8

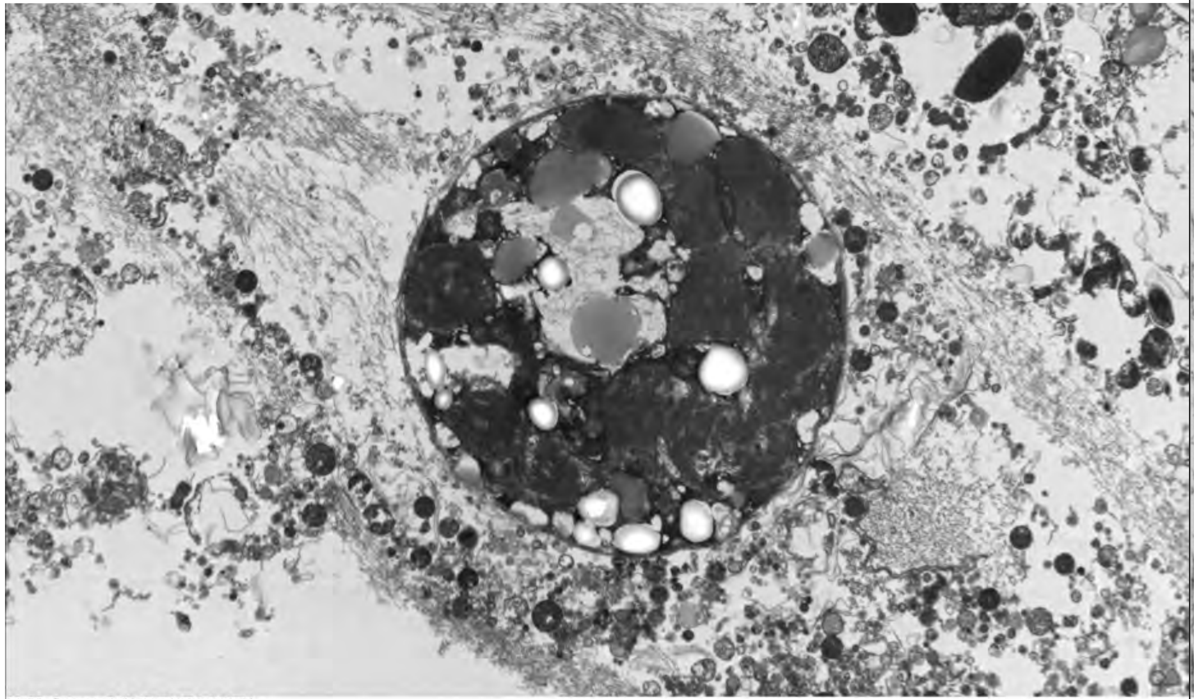

21-20_Correa_3-19-25_16B4_111.tif
Sample: 3/19, 25
Biological Electron Microscopy Lab
Rice University - SEA
Microscopist: MD Meyer

2 µm
HV=80kV
Direct Mag: 1500 x

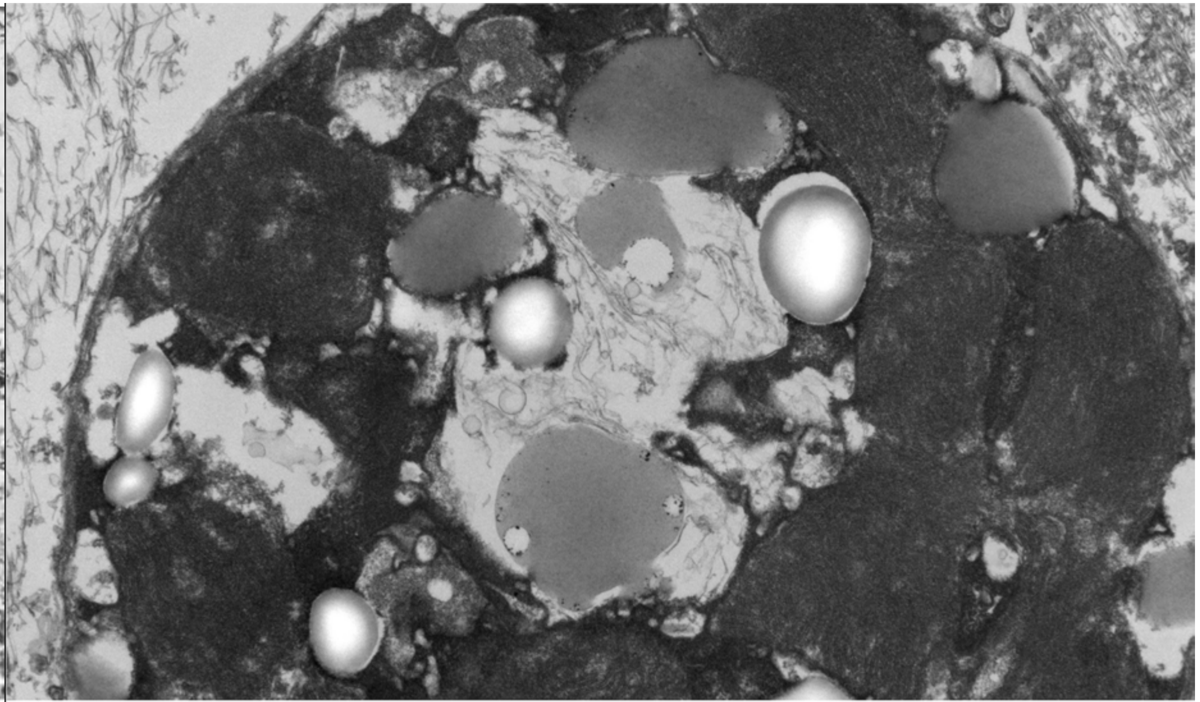

21-20_Correa_3-19-25_16B4_049.tif
Sample: 3/19, 25
Biological Electron Microscopy Lab
Rice University - SEA
Microscopist: MD Meyer

1 µm
HV=80kV
Direct Mag: 4000 x

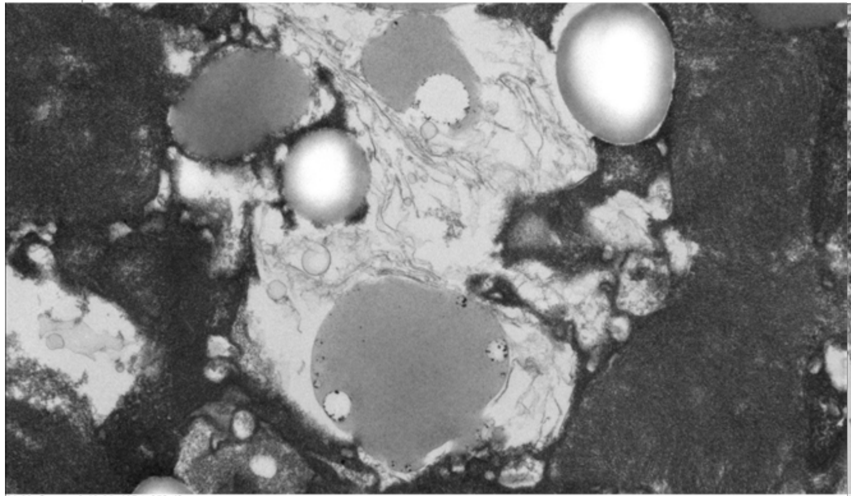

21-20_Correa_3-19-25_16B4_052.tif
Sample: 3/19, 25
Biological Electron Microscopy Lab
Rice University - SEA
Microscopist: MD Meyer

600 nm
HV=80kV
Direct Mag: 6000 x

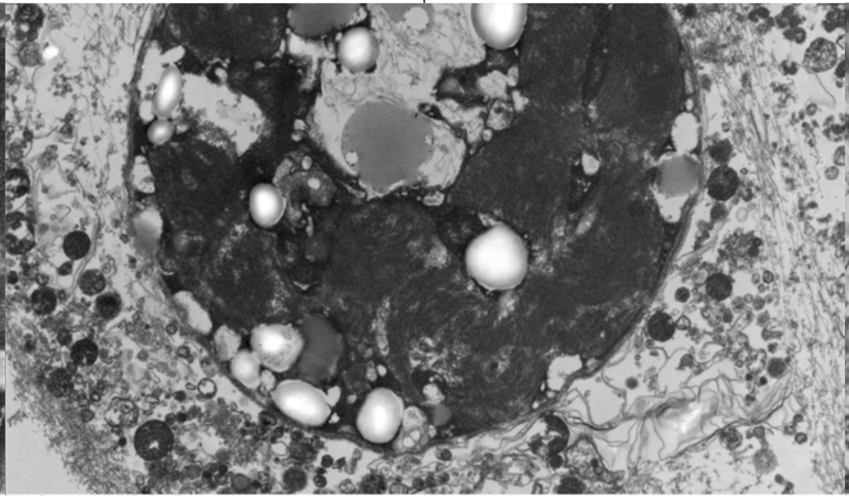

21-20_Correa_3-19-25_16B4_113.tif
Sample: 3/19, 25
Biological Electron Microscopy Lab
Rice University - SEA
Microscopist: MD Meyer

1 µm
HV=80kV
Direct Mag: 3000 x

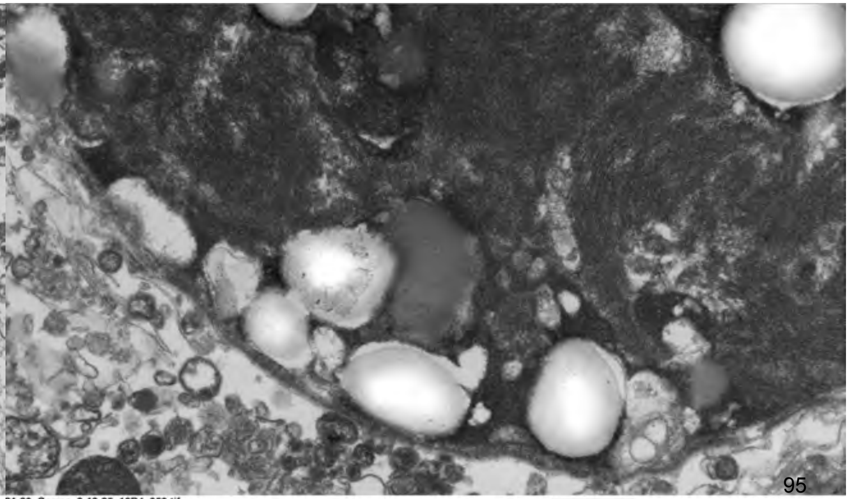

21-20_Correa_3-19-25_16B4_050.tif
Sample: 3/19, 25
Biological Electron Microscopy Lab
Rice University - SEA
Microscopist: MD Meyer

600 nm
HV=80kV
Direct Mag: 6000 x

Cell 9

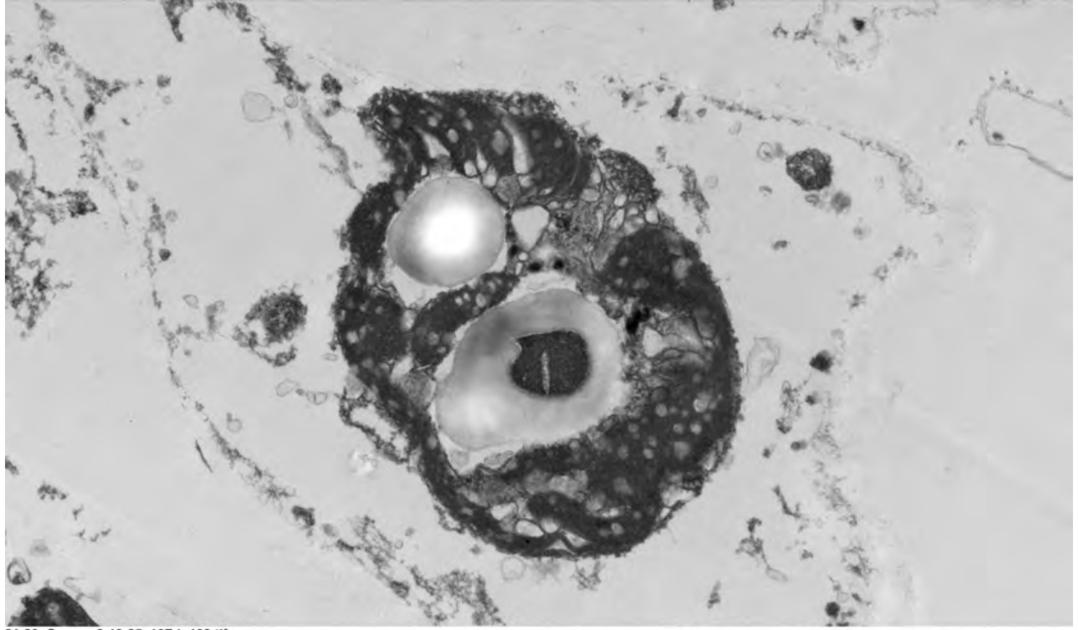

21-20_Correa_3-19-25_16B4_108.tif
Sample: 3/19, 25
Biological Electron Microscopy Lab
Rice University - SEA
Microscopist: MD Meyer

1 μm
HV=80kV
Direct Mag: 4000 x

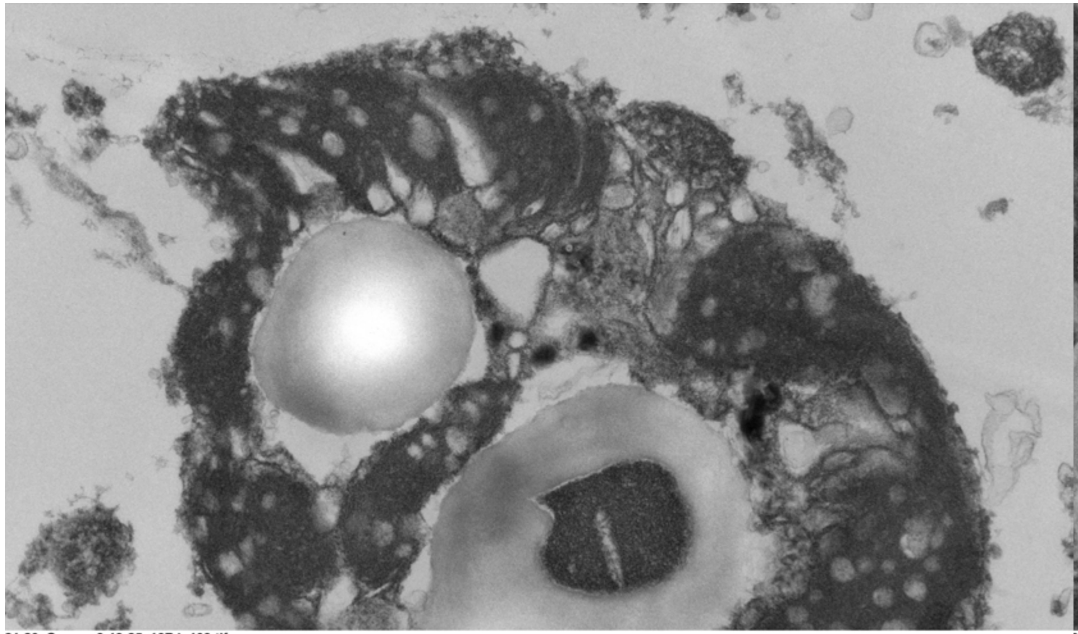

21-20_Correa_3-19-25_16B4_109.tif
Sample: 3/19, 25
Biological Electron Microscopy Lab
Rice University - SEA
Microscopist: MD Meyer

500 nm
HV=80kV
Direct Mag: 8000 x

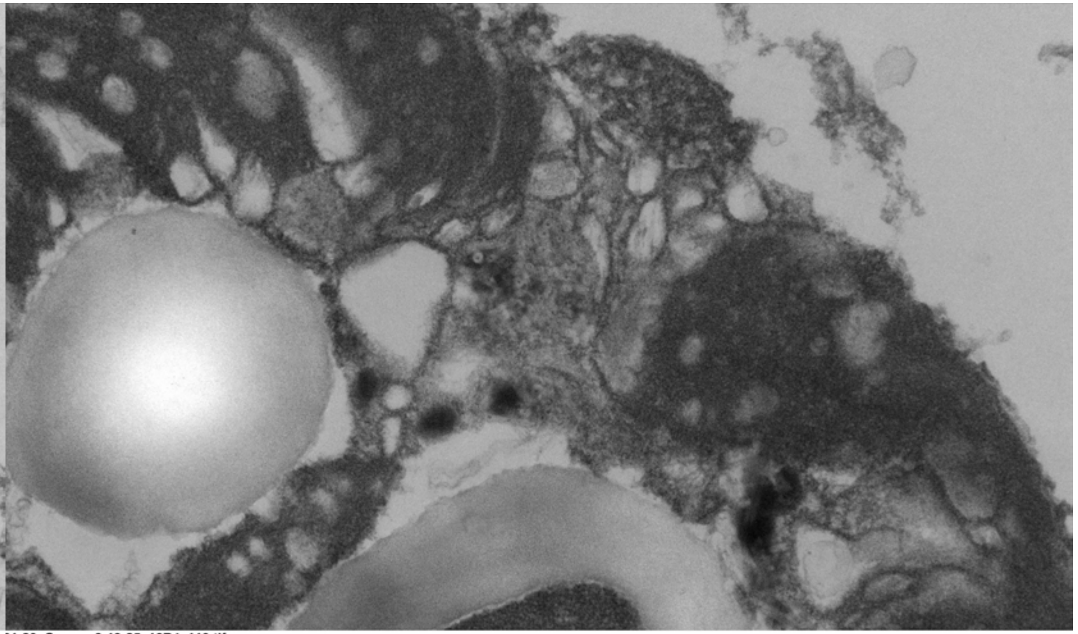

21-20_Correa_3-19-25_16B4_110.tif
Sample: 3/19, 25
Biological Electron Microscopy Lab
Rice University - SEA
Microscopist: MD Meyer

200 nm
HV=80kV
Direct Mag: 12000 x

Cell 10

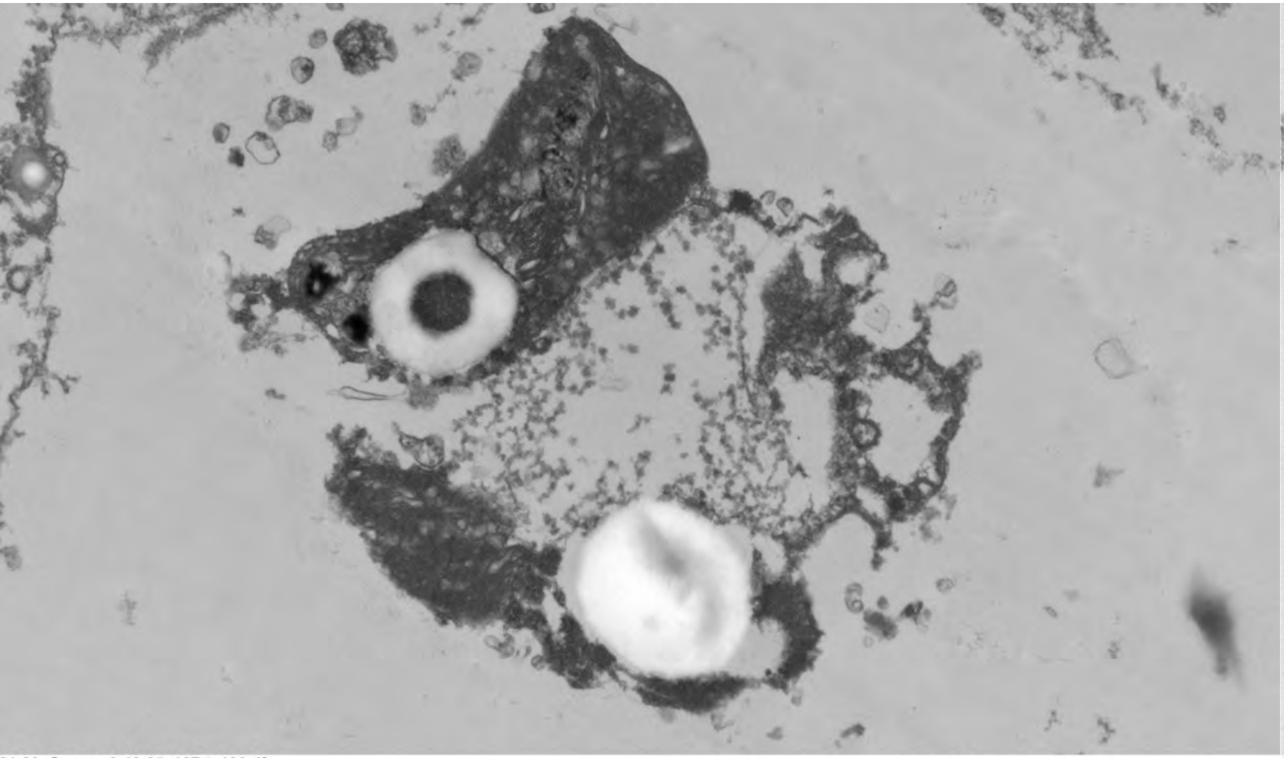

21-20_Correa_3-19-25_16B4_106.tif
Sample: 3/19, 25
Biological Electron Microscopy Lab
Rice University - SEA
Microscopist: MD Meyer

1 µm
HV=80kV
Direct Mag: 4000 x

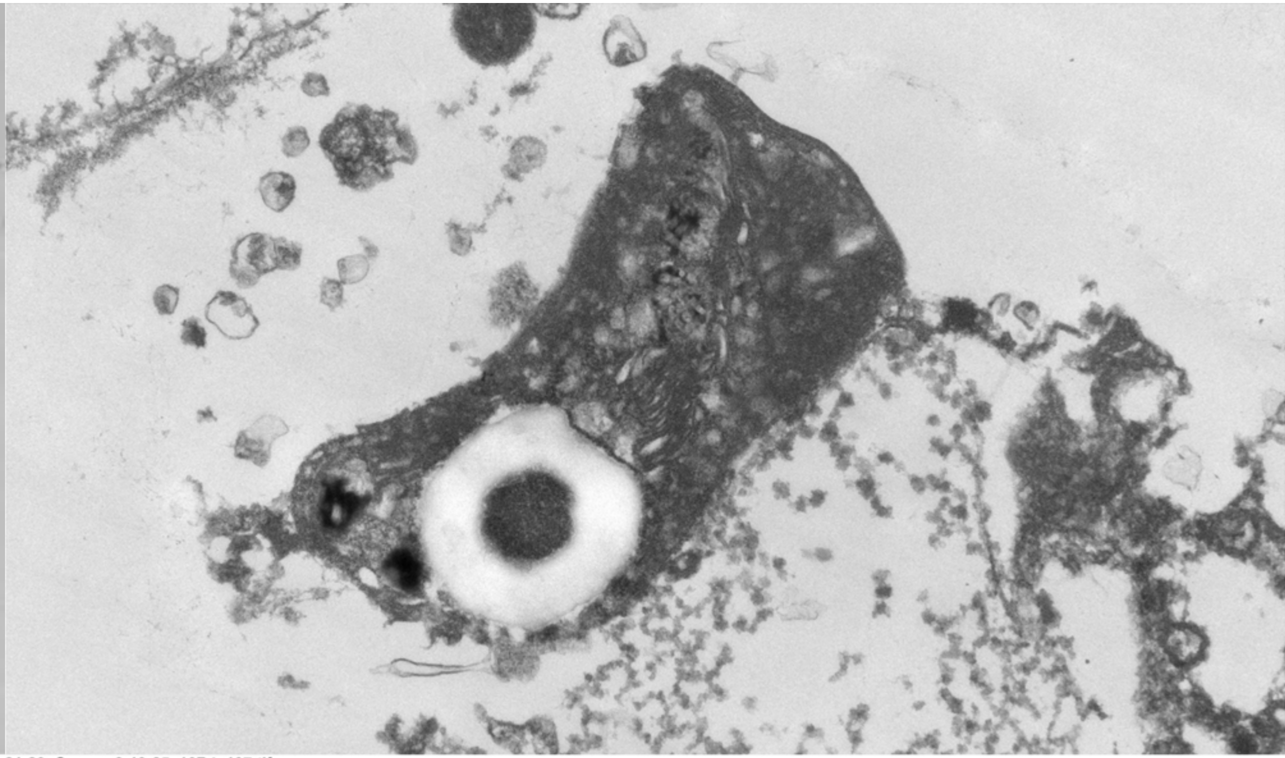

21-20_Correa_3-19-25_16B4_107.tif
Sample: 3/19, 25
Biological Electron Microscopy Lab
Rice University - SEA
Microscopist: MD Meyer

600 nm
HV=80kV
Direct Mag: 6000 x

Cell 11

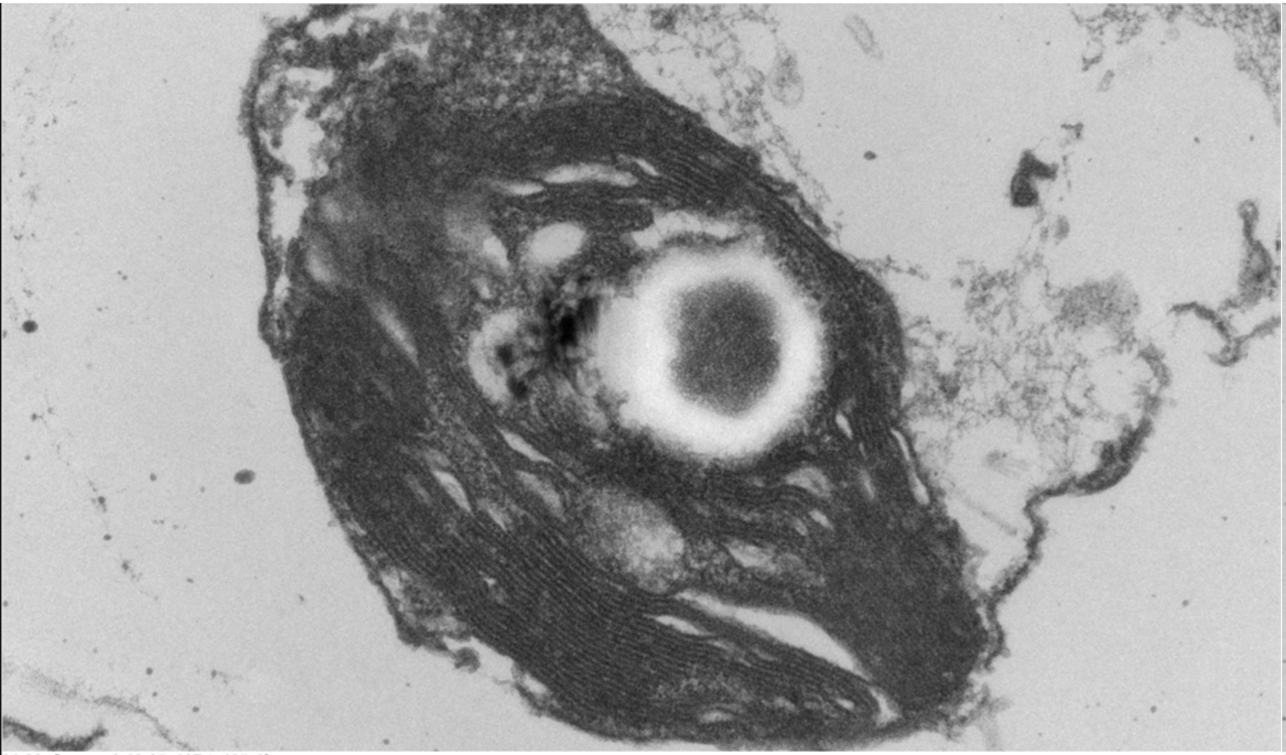

21-20_Correa_3-19-25_16B4_105.tif
Sample: 3/19, 25
Biological Electron Microscopy Lab
Rice University - SEA
Microscopist: MD Meyer

400 nm
HV=80kV
Direct Mag: 10000 x

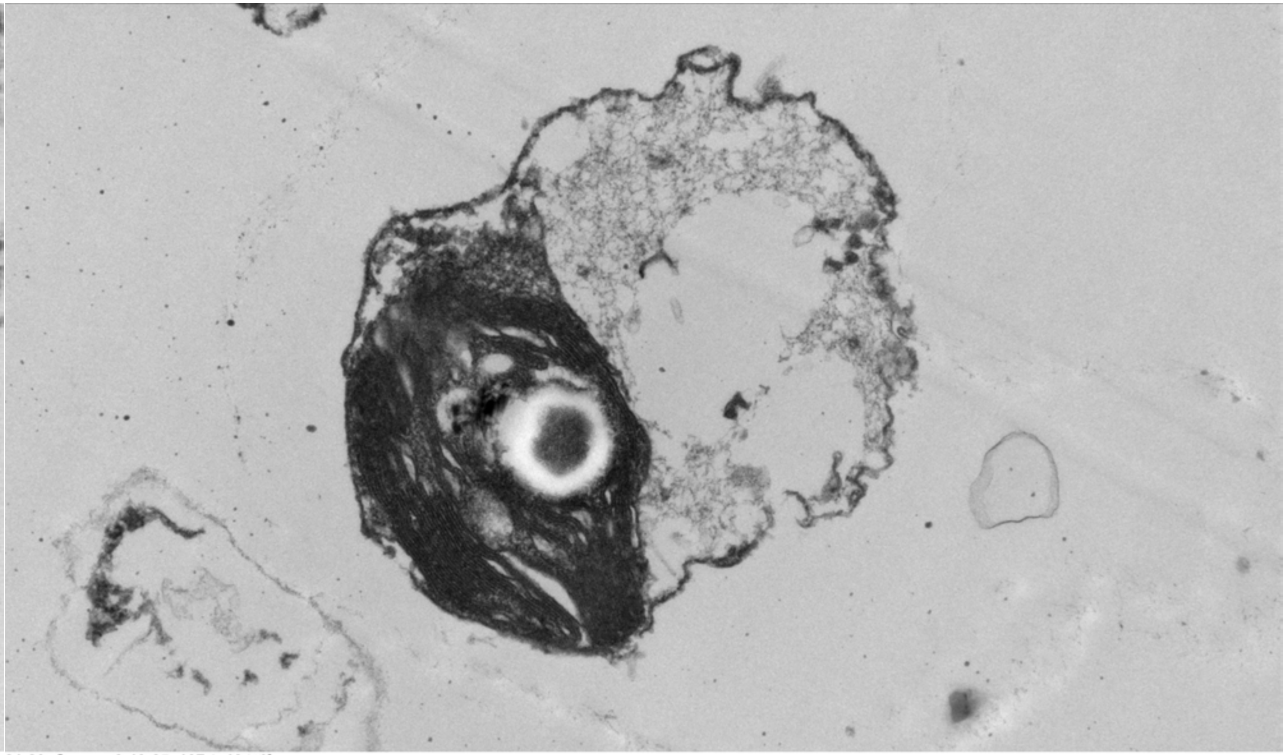

21-20_Correa_3-19-25_16B4_104.tif
Sample: 3/19, 25
Biological Electron Microscopy Lab
Rice University - SEA
Microscopist: MD Meyer

800 nm
HV=80kV
Direct Mag: 5000 x

Cell 12

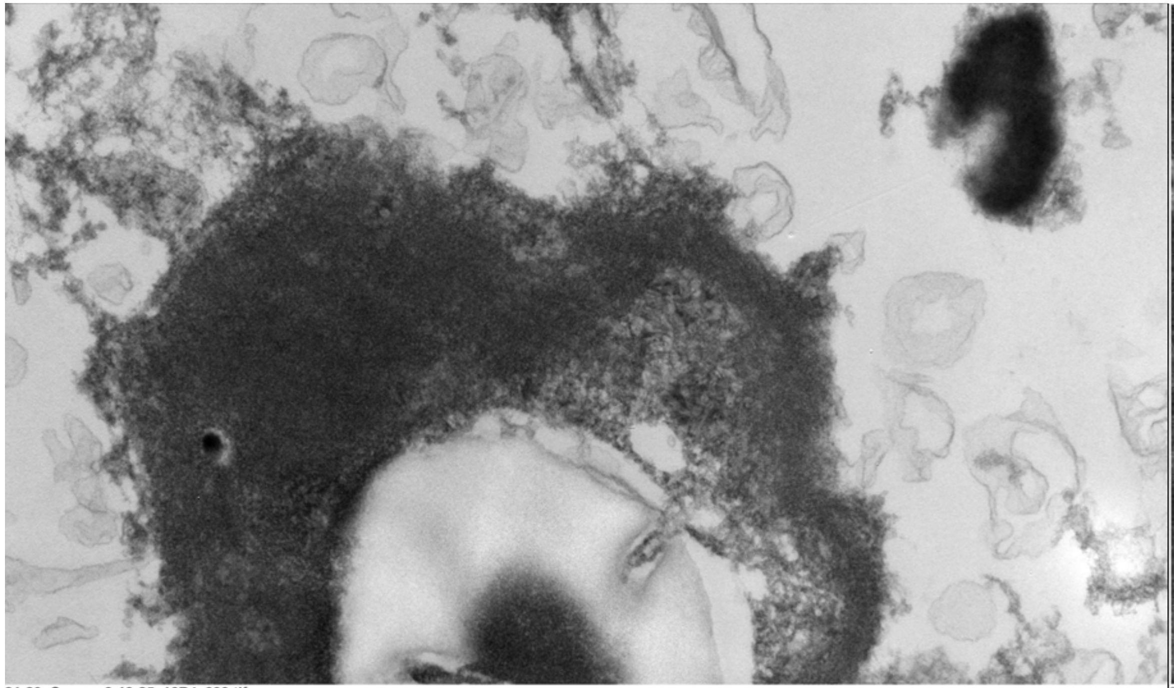

21-20_Correa_3-19-25_16B4_099.tif
Sample: 3/19, 25
Biological Electron Microscopy Lab
Rice University - SEA
Microscopist: MD Meyer

400 nm
HV=80kV
Direct Mag: 10000 x

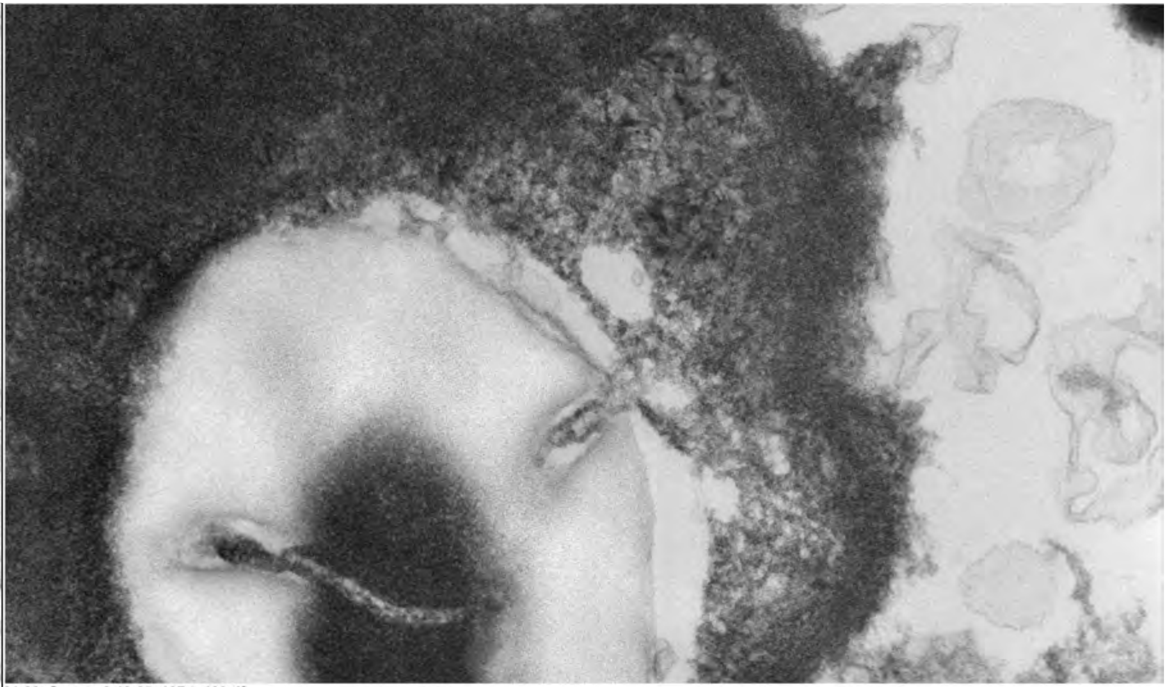

21-20_Correa_3-19-25_16B4_103.tif
Sample: 3/19, 25
Biological Electron Microscopy Lab
Rice University - SEA
Microscopist: MD Meyer

200 nm
HV=80kV
Direct Mag: 15000 x

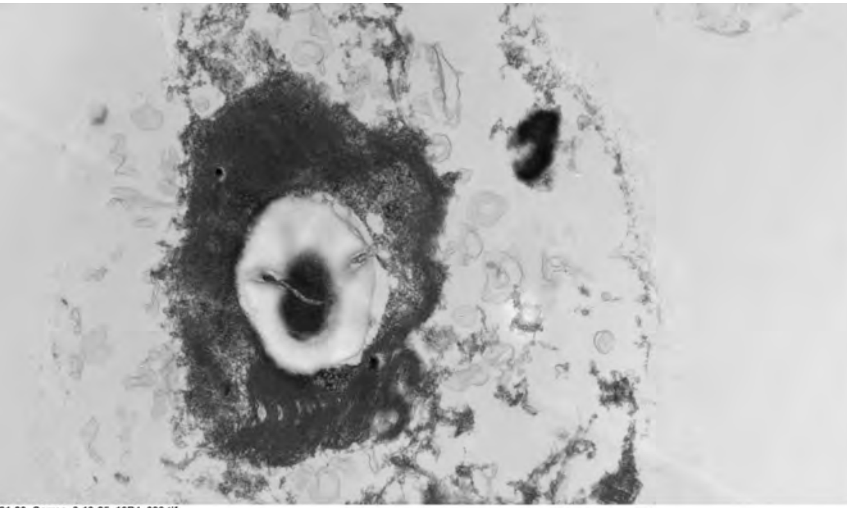

21-20_Correa_3-19-25_16B4_098.tif
Sample: 3/19, 25
Biological Electron Microscopy Lab
Rice University - SEA
Microscopist: MD Meyer

800 nm
HV=80kV
Direct Mag: 5000 x

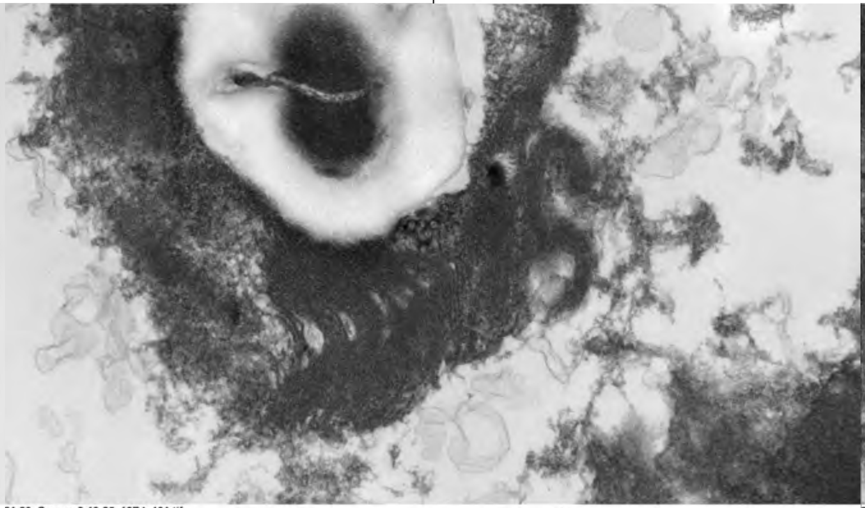

21-20_Correa_3-19-25_16B4_101.tif
Sample: 3/19, 25
Biological Electron Microscopy Lab
Rice University - SEA
Microscopist: MD Meyer

400 nm
HV=80kV
Direct Mag: 10000 x

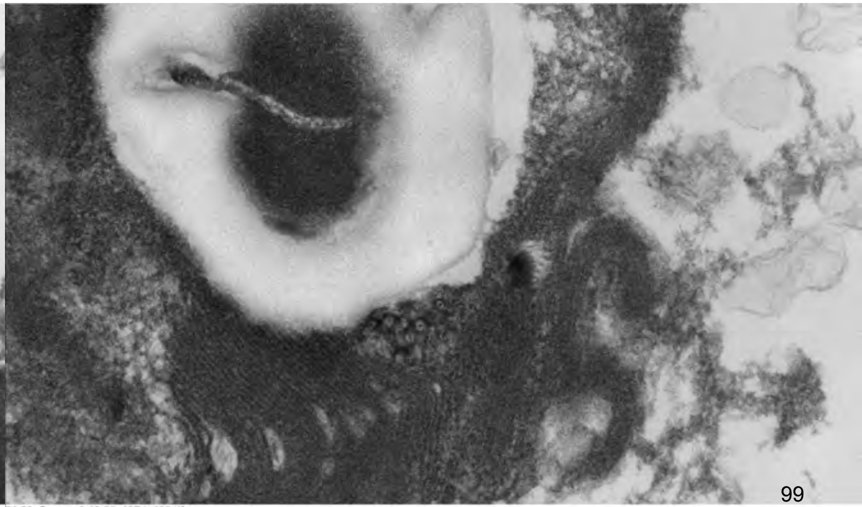

21-20_Correa_3-19-25_16B4_102.tif
Sample: 3/19, 25
Biological Electron Microscopy Lab
Rice University - SEA
Microscopist: MD Meyer

200 nm
HV=80kV
Direct Mag: 15000 x

Cell 13 – 13_2

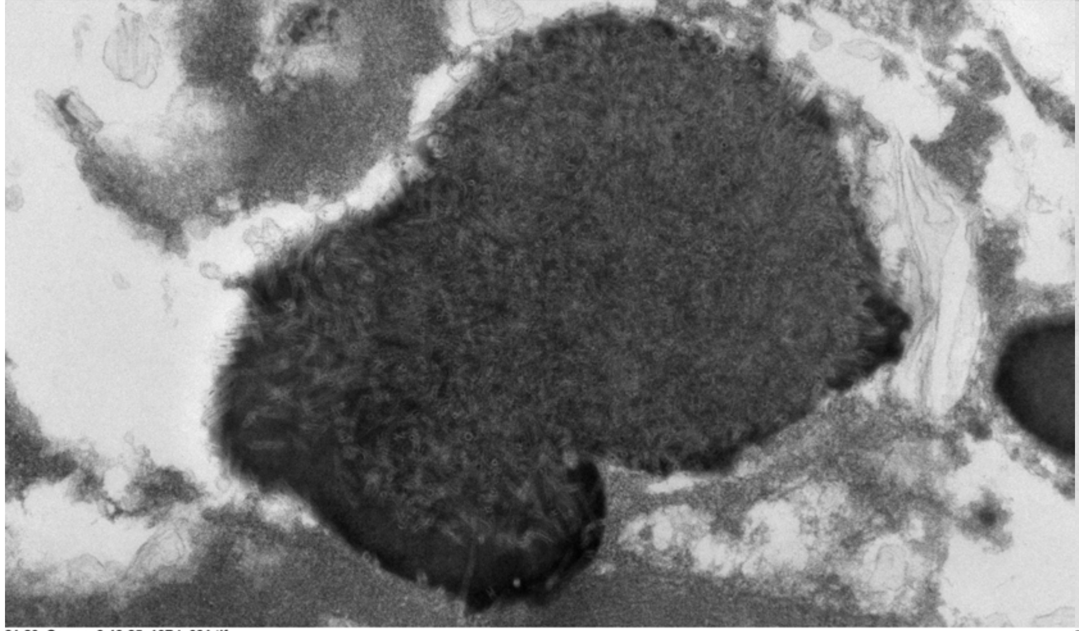

21-20_Correa_3-19-25_16B4_091.tif
Sample: 3/19, 25
Biological Electron Microscopy Lab
Rice University - SEA
Microscopist: MD Meyer

200 nm
HV=80kV
Direct Mag: 12000 x

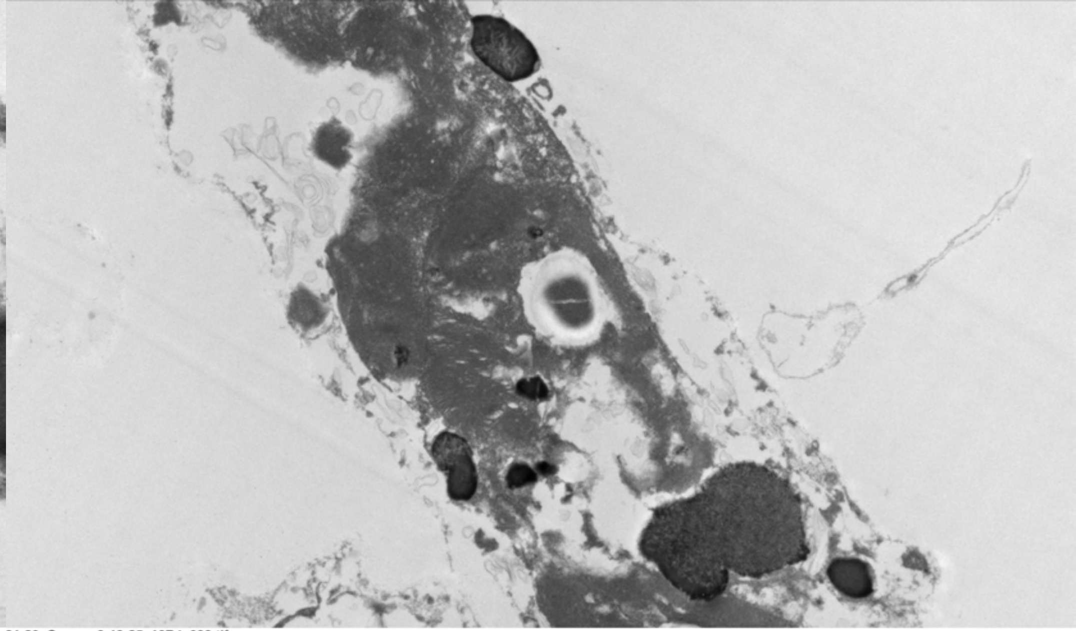

21-20_Correa_3-19-25_16B4_093.tif
Sample: 3/19, 25
Biological Electron Microscopy Lab
Rice University - SEA
Microscopist: MD Meyer

1 µm
HV=80kV
Direct Mag: 3000 x

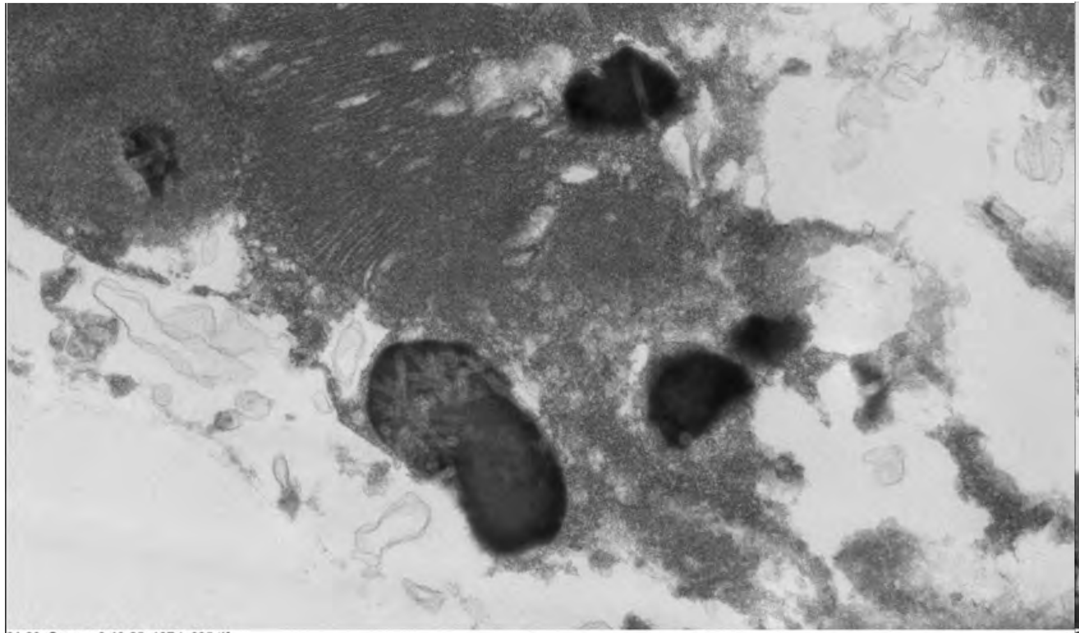

21-20_Correa_3-19-25_16B4_095.tif
Sample: 3/19, 25
Biological Electron Microscopy Lab
Rice University - SEA
Microscopist: MD Meyer

400 nm
HV=80kV
Direct Mag: 10000 x

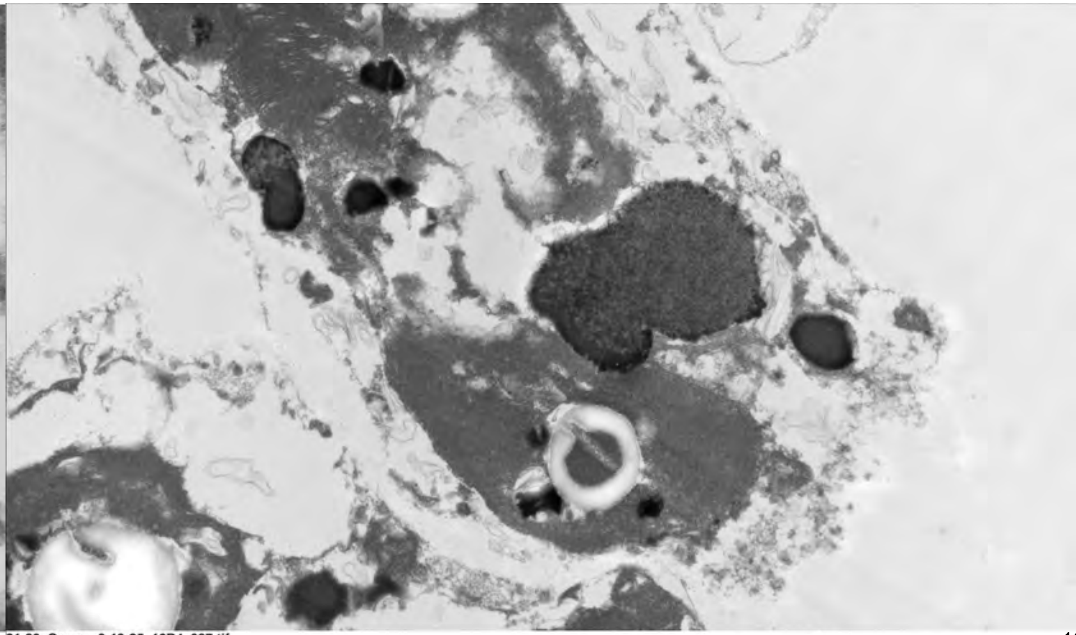

21-20_Correa_3-19-25_16B4_087.tif
Sample: 3/19, 25
Biological Electron Microscopy Lab
Rice University - SEA
Microscopist: MD Meyer

1 µm
HV=80kV
Direct Mag: 4000 x

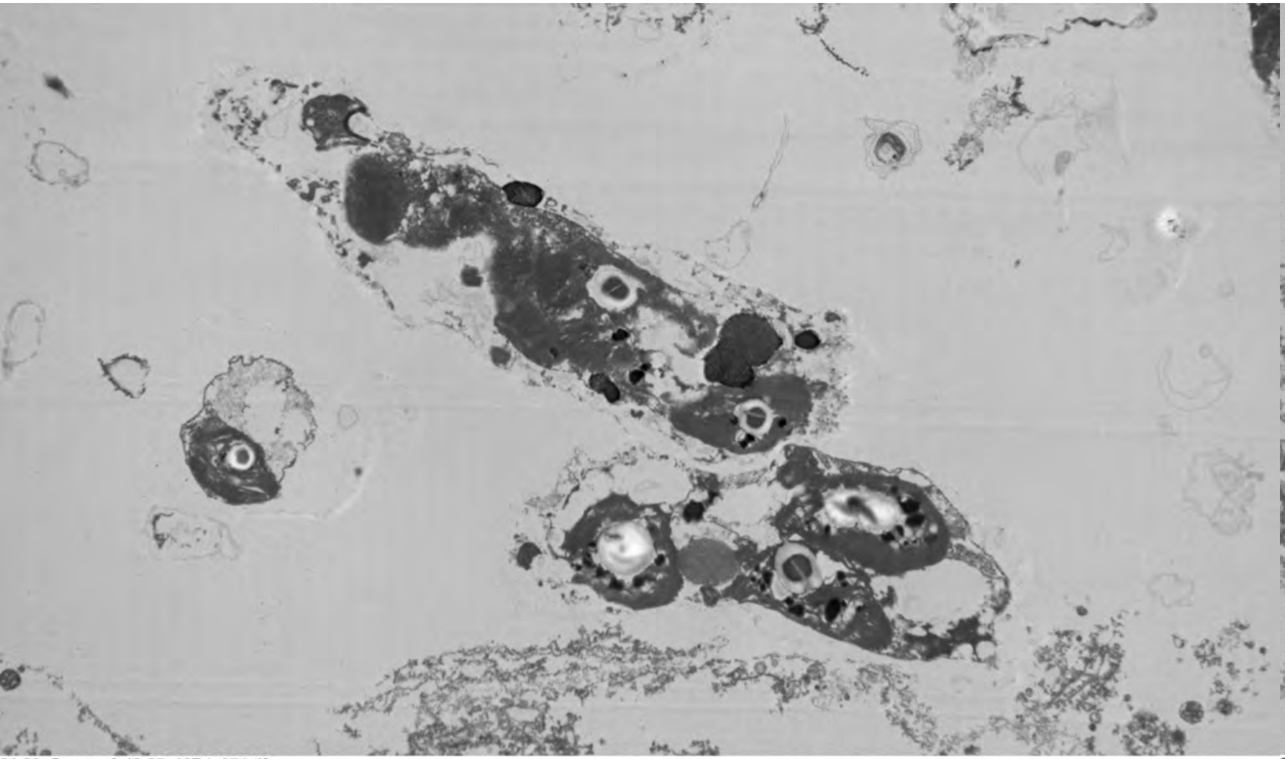

21-20_Correa_3-19-25_16B4_074.tif
Sample: 3/19, 25
Biological Electron Microscopy Lab
Rice University - SEA
Microscopist: MD Meyer

2 µm
HV=80kV
Direct Mag: 1200 x

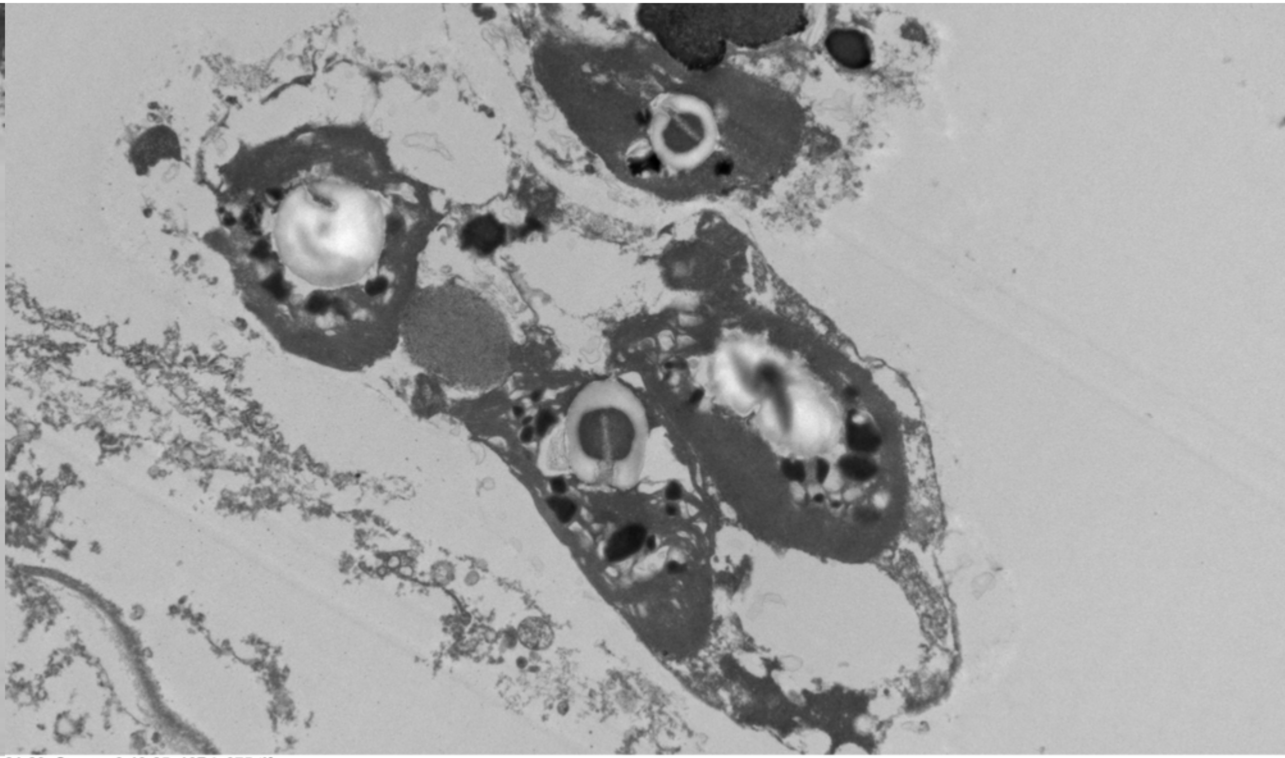

21-20_Correa_3-19-25_16B4_075.tif
Sample: 3/19, 25
Biological Electron Microscopy Lab
Rice University - SEA
Microscopist: MD Meyer

1 µm
HV=80kV
Direct Mag: 2500 x

Cell 14

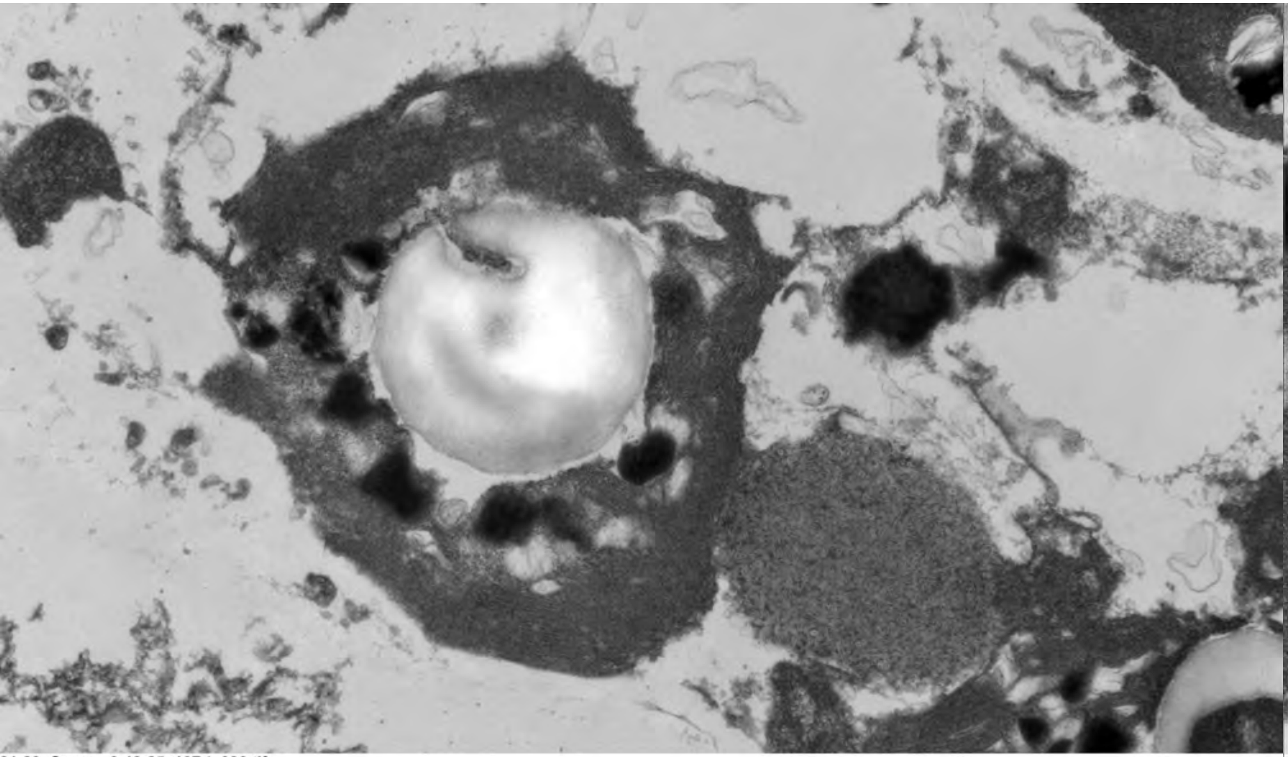

21-20_Correa_3-19-25_16B4_086.tif
Sample: 3/19, 25
Biological Electron Microscopy Lab
Rice University - SEA
Microscopist: MD Meyer

600 nm
HV=80kV
Direct Mag: 6000 x

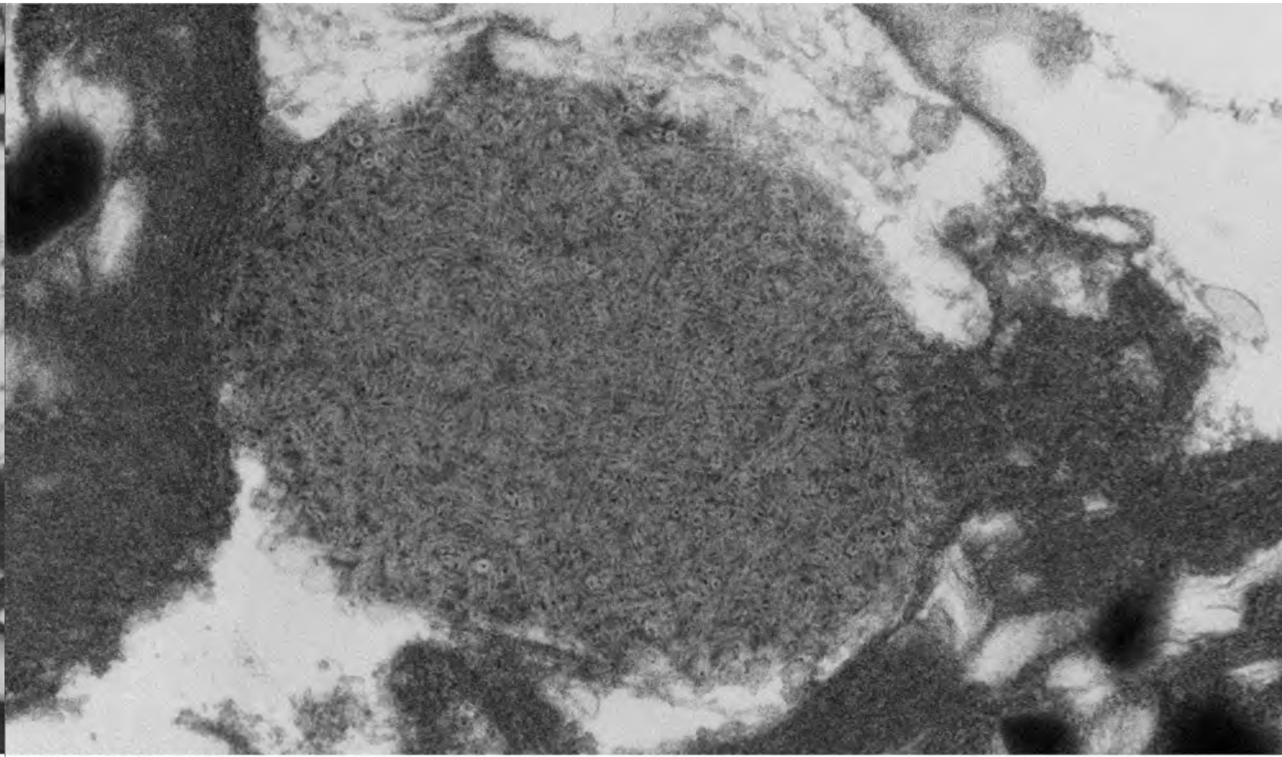

21-20_Correa_3-19-25_16B4_083.tif
Sample: 3/19, 25
Biological Electron Microscopy Lab
Rice University - SEA
Microscopist: MD Meyer

200 nm
HV=80kV
Direct Mag: 15000 x

Cell 15

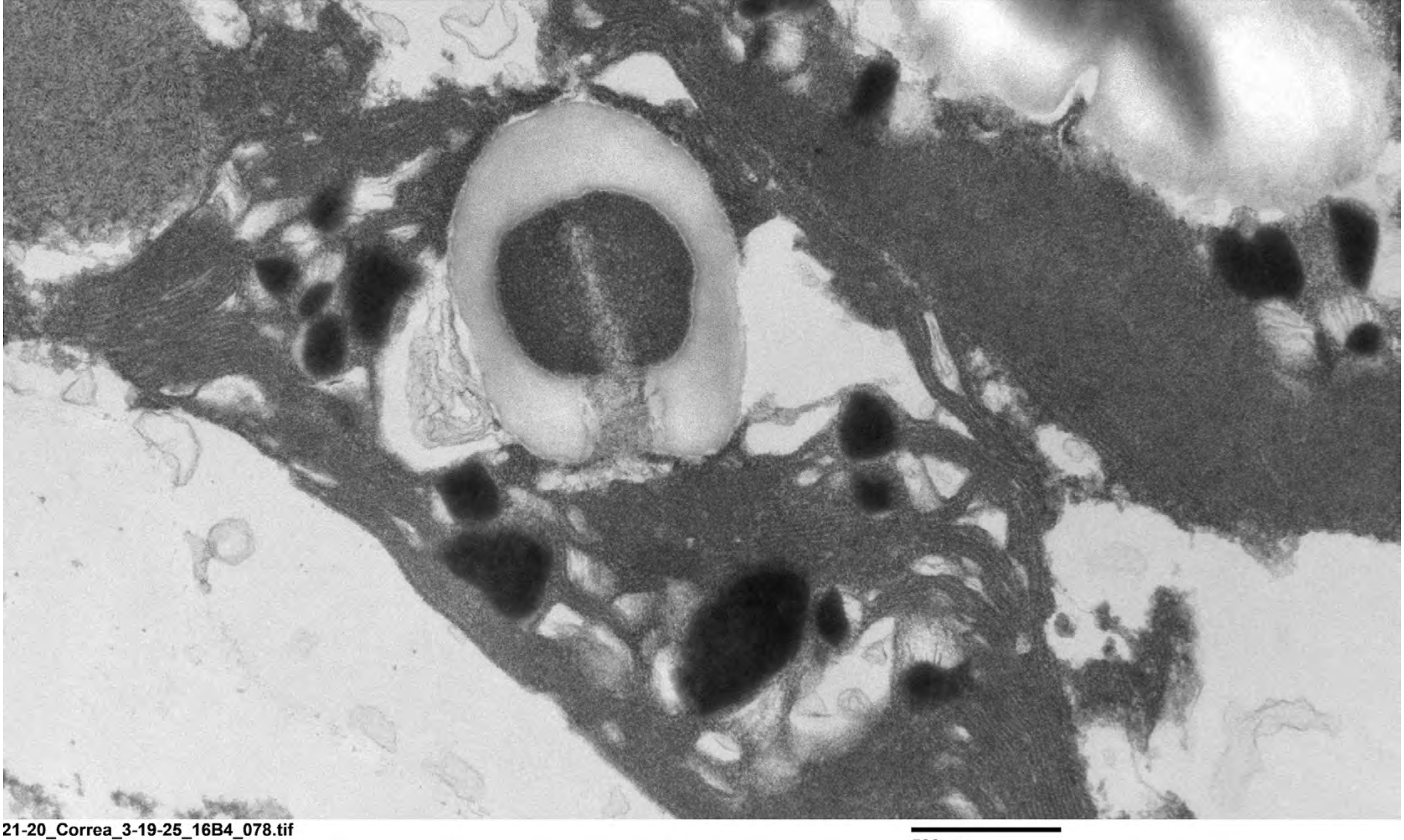

21-20_Correa_3-19-25_16B4_078.tif
Sample: 3/19, 25
Biological Electron Microscopy Lab
Rice University - SEA
Microscopist: MD Meyer

500 nm
HV=80kV
Direct Mag: 8000 x

Cell 16

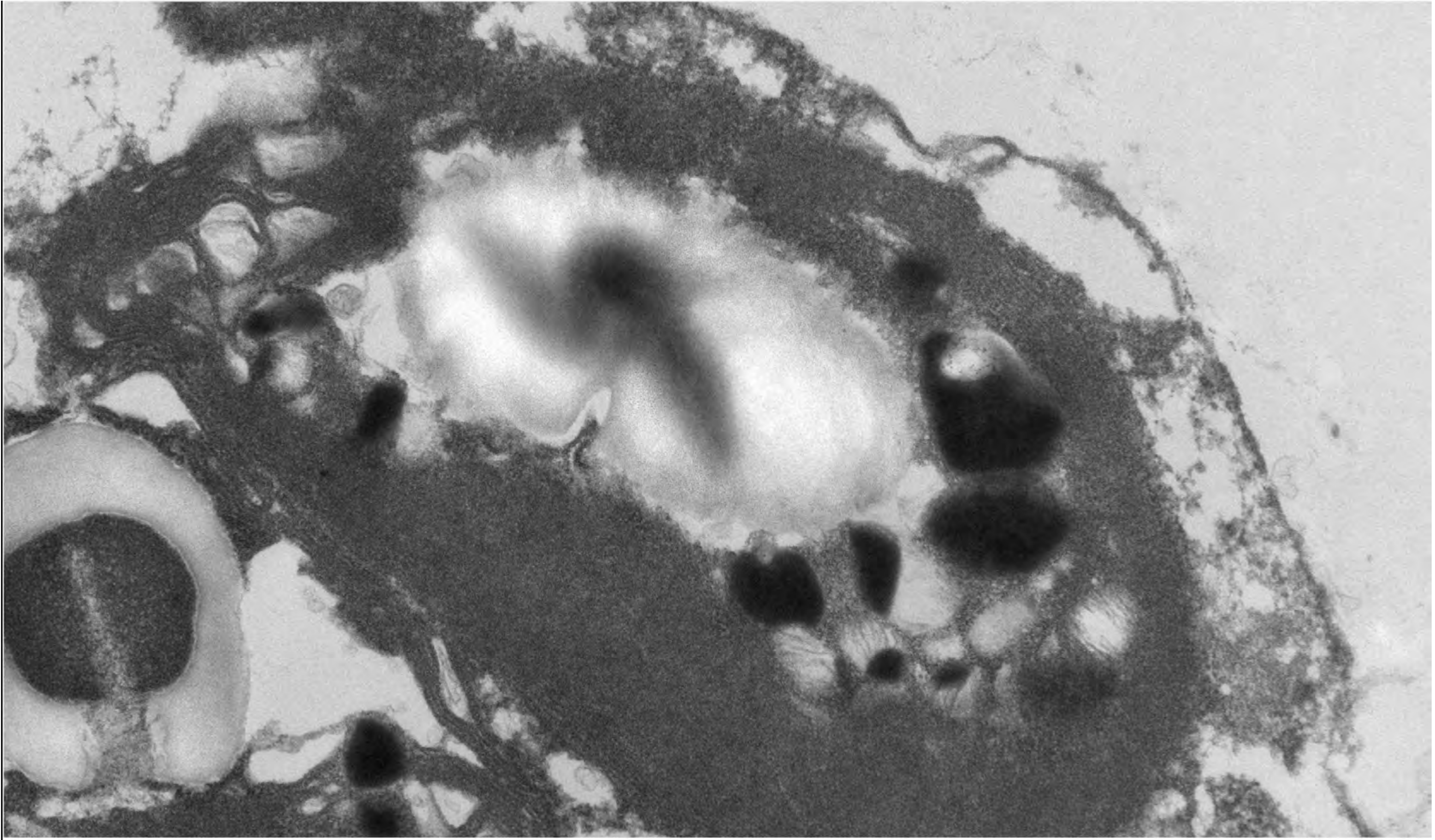

21-20_Correa_3-19-25_16B4_077.tif
Sample: 3/19, 25
Biological Electron Microscopy Lab
Rice University - SEA
Microscopist: MD Meyer

500 nm
HV=80kV
Direct Mag: 8000 x

Cell 17

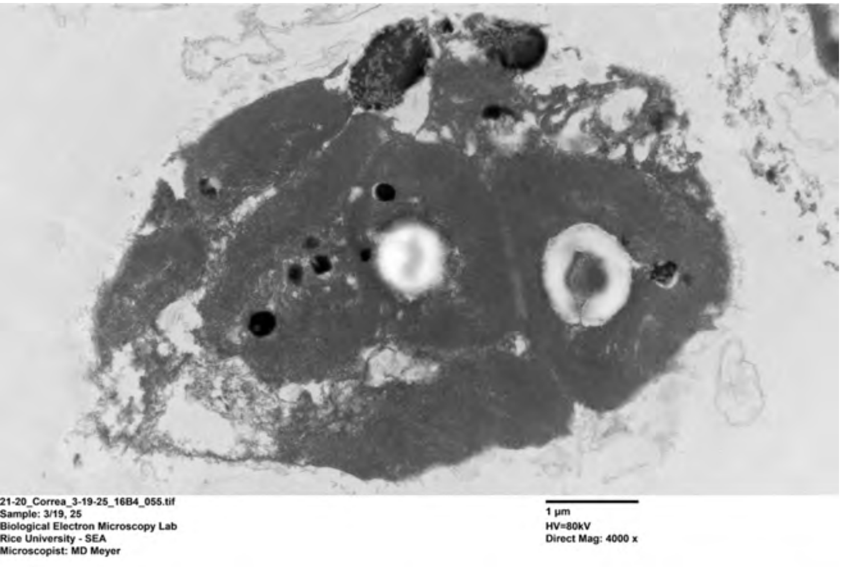
21-20_Correa_3-19-25_16B4_055.tif
Sample: 3/19, 25
Biological Electron Microscopy Lab
Rice University - SEA
Microscopist: MD Meyer
1 µm
HV=80kV
Direct Mag: 4000 x

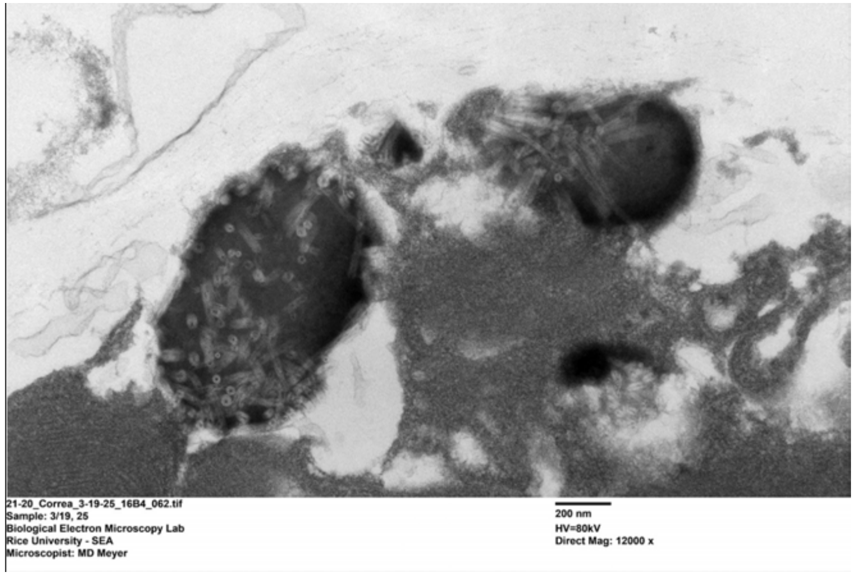
21-20_Correa_3-19-25_16B4_062.tif
Sample: 3/19, 25
Biological Electron Microscopy Lab
Rice University - SEA
Microscopist: MD Meyer
200 nm
HV=80kV
Direct Mag: 12000 x

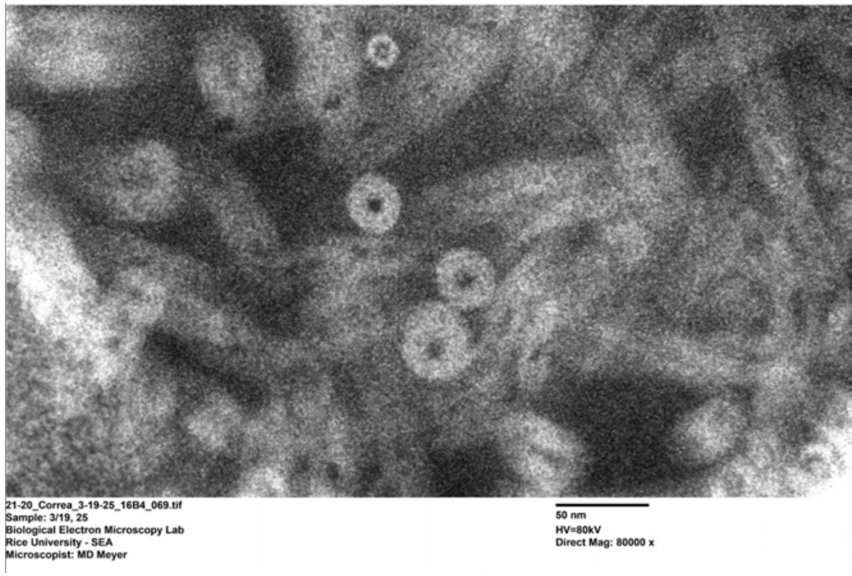
21-20_Correa_3-19-25_16B4_069.tif
Sample: 3/19, 25
Biological Electron Microscopy Lab
Rice University - SEA
Microscopist: MD Meyer
50 nm
HV=80kV
Direct Mag: 80000 x

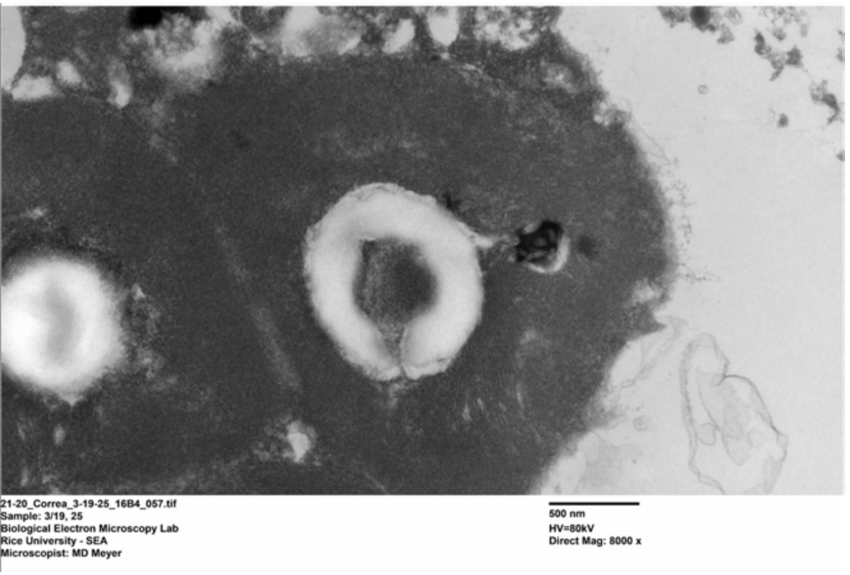
21-20_Correa_3-19-25_16B4_057.tif
Sample: 3/19, 25
Biological Electron Microscopy Lab
Rice University - SEA
Microscopist: MD Meyer
500 nm
HV=80kV
Direct Mag: 8000 x

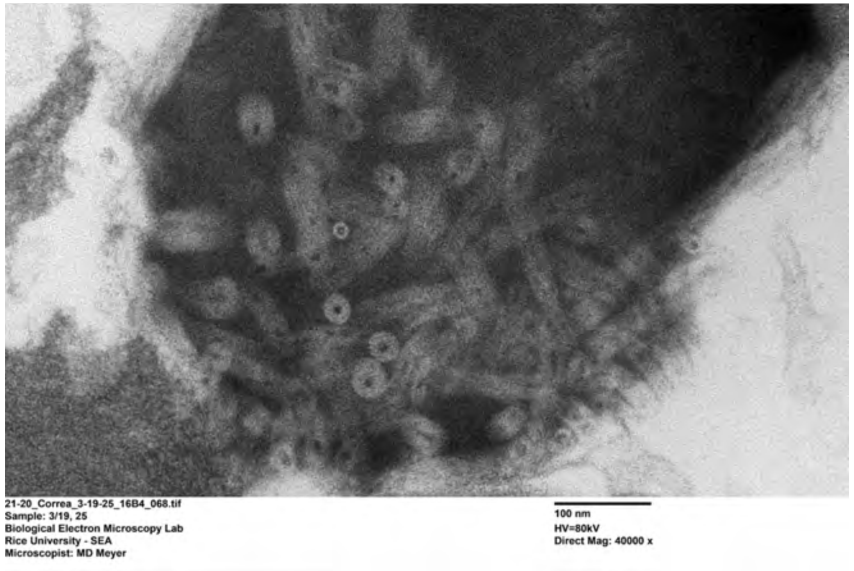
21-20_Correa_3-19-25_16B4_068.tif
Sample: 3/19, 25
Biological Electron Microscopy Lab
Rice University - SEA
Microscopist: MD Meyer
100 nm
HV=80kV
Direct Mag: 40000 x

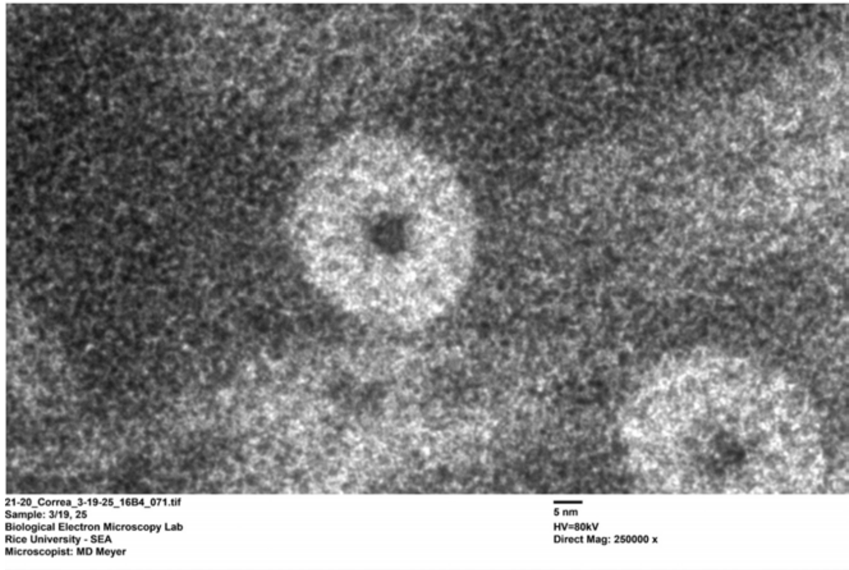
21-20_Correa_3-19-25_16B4_071.tif
Sample: 3/19, 25
Biological Electron Microscopy Lab
Rice University - SEA
Microscopist: MD Meyer
5 nm
HV=80kV
Direct Mag: 250000 x

Cell 18

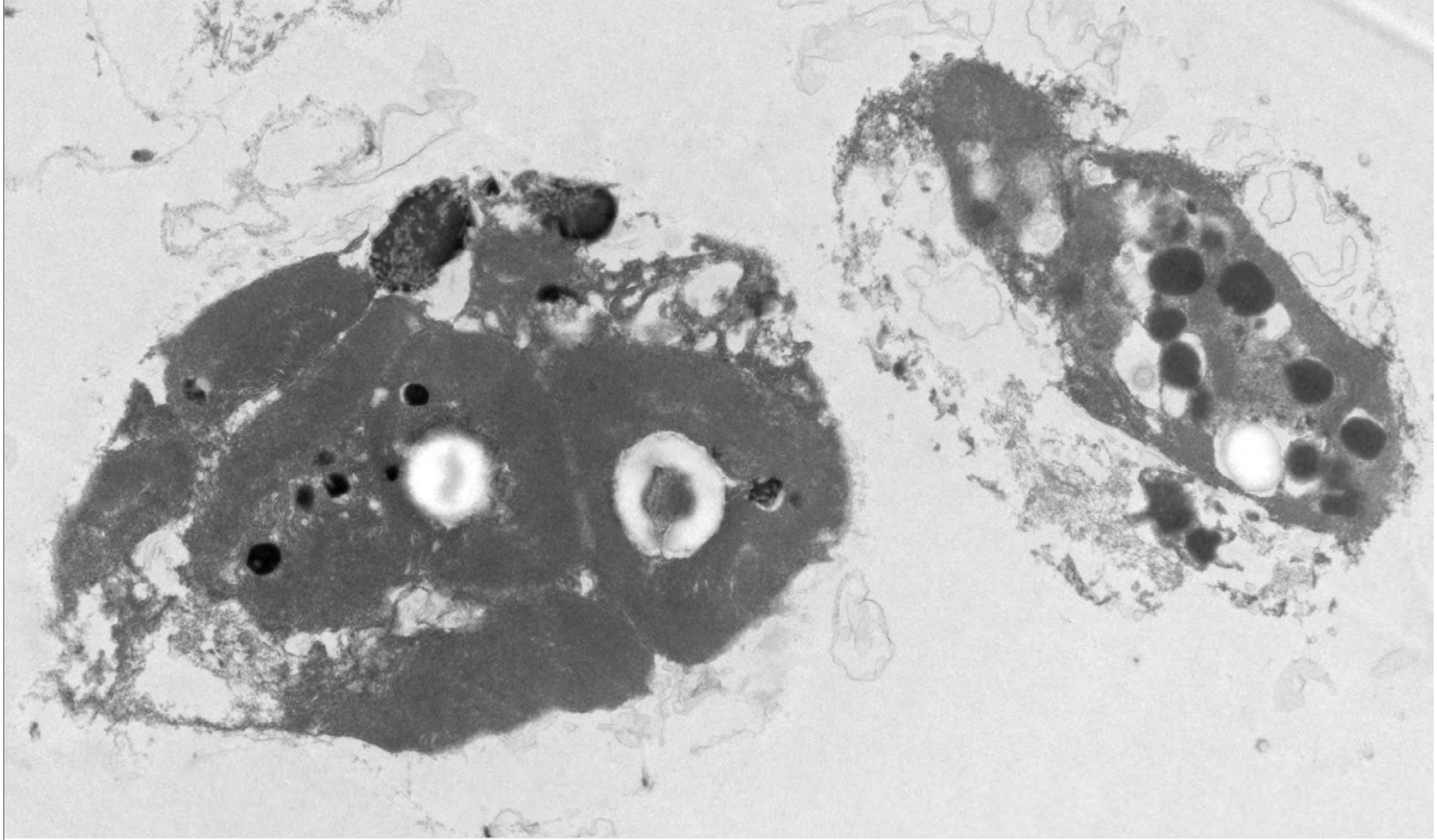

21-20_Correa_3-19-25_16B4_053.tif
Sample: 3/19, 25
Biological Electron Microscopy Lab
Rice University - SEA
Microscopist: MD Meyer

1 µm
HV=80kV
Direct Mag: 3000 x

Cell 20

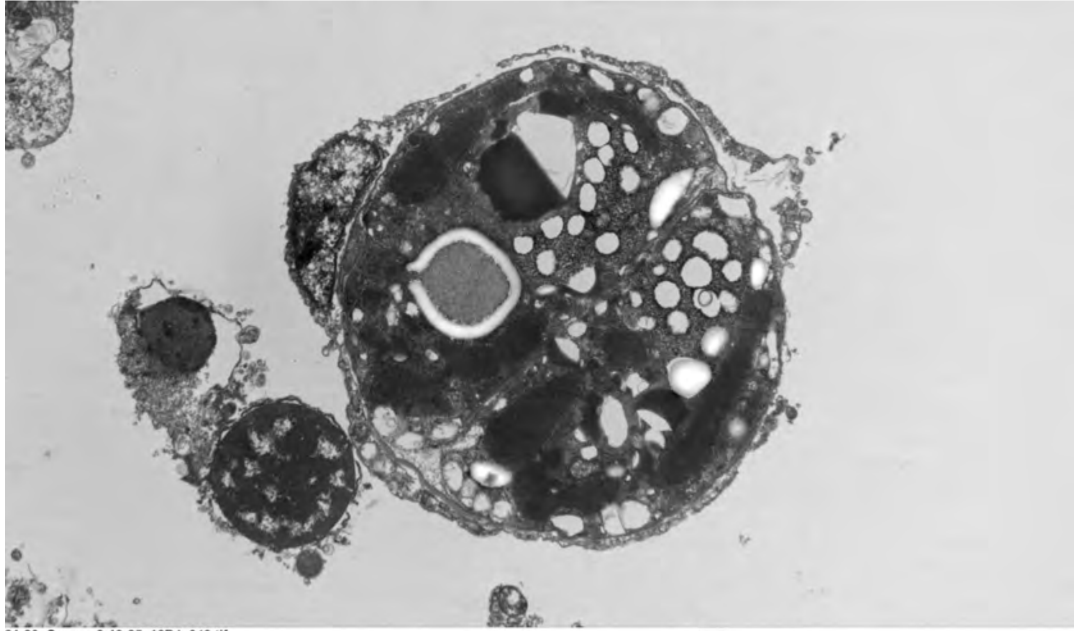

21-20_Correa_3-19-25_16B4_043.tif
Sample: 3/19, 25
Biological Electron Microscopy Lab
Rice University - SEA
Microscopist: MD Meyer

2 µm
HV=80kV
Direct Mag: 1500 x

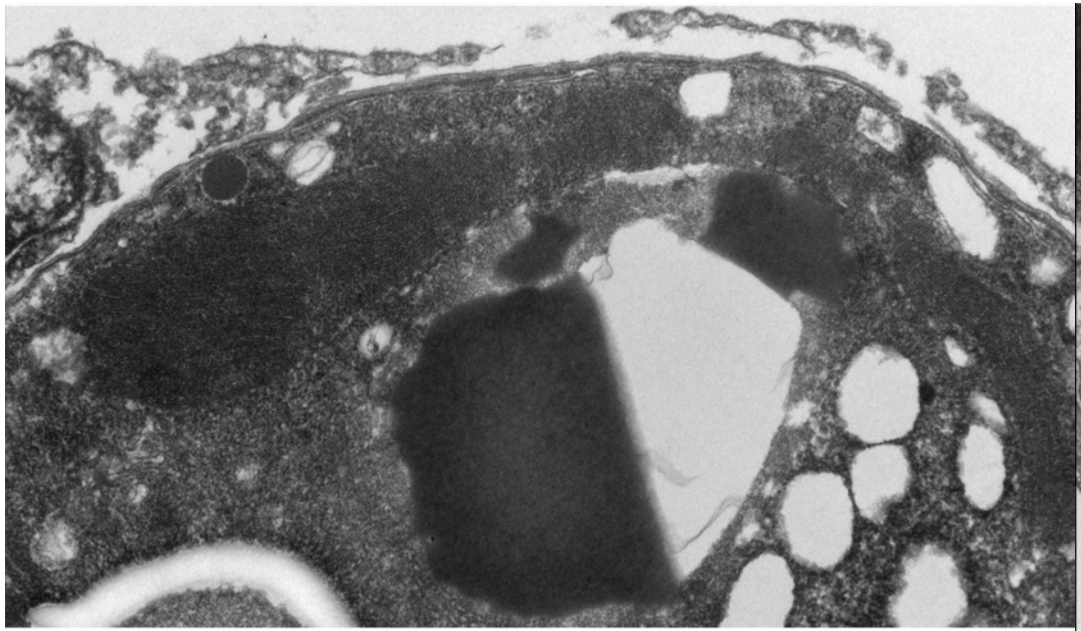

21-20_Correa_3-19-25_16B4_045.tif
Sample: 3/19, 25
Biological Electron Microscopy Lab
Rice University - SEA
Microscopist: MD Meyer

600 nm
HV=80kV
Direct Mag: 6000 x

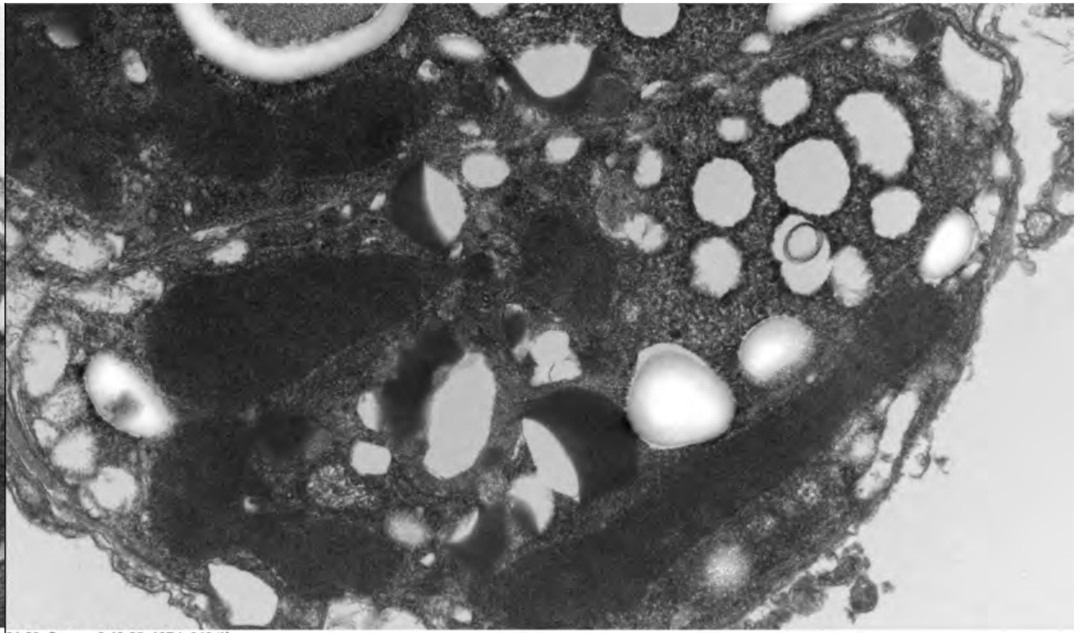

21-20_Correa_3-19-25_16B4_046.tif
Sample: 3/19, 25
Biological Electron Microscopy Lab
Rice University - SEA
Microscopist: MD Meyer

1 µm
HV=80kV
Direct Mag: 4000 x

Cell 21

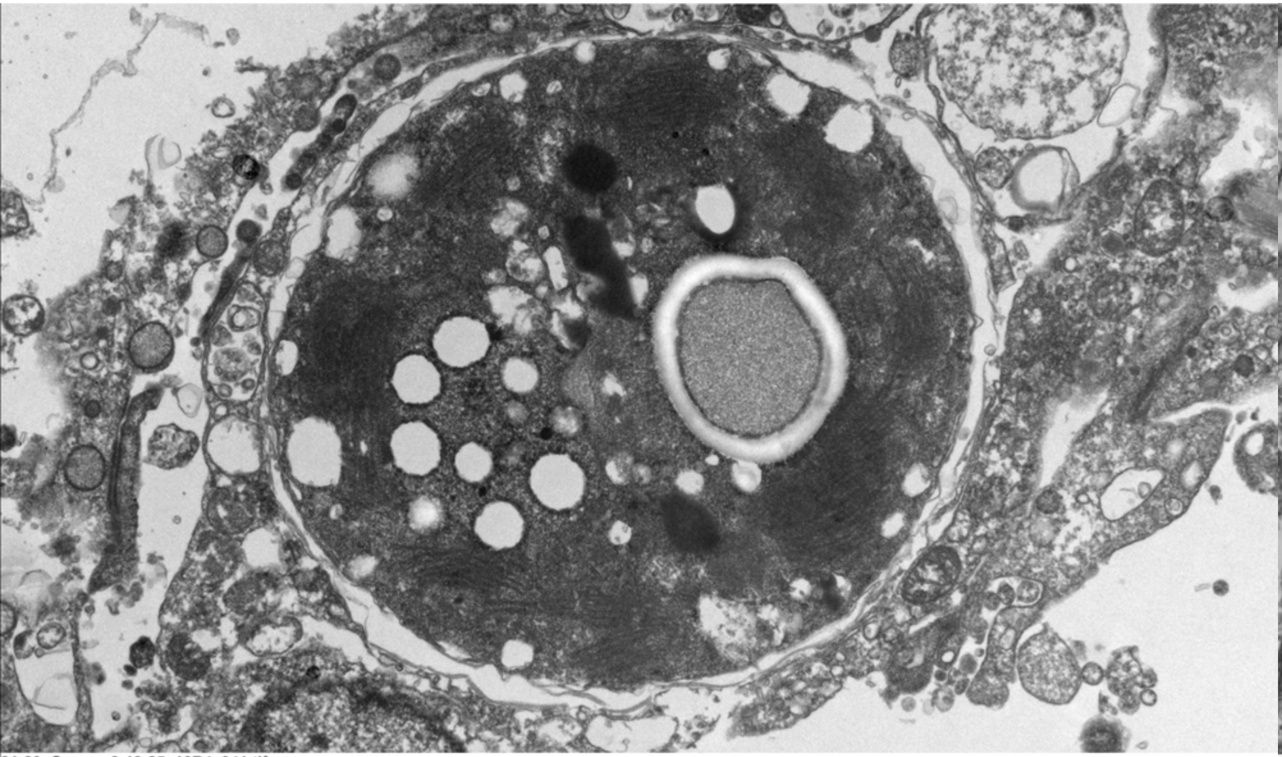

21-20_Correa_3-19-25_16B4_041.tif
Sample: 3/19, 25
Biological Electron Microscopy Lab
Rice University - SEA
Microscopist: MD Meyer

1 µm
HV=80kV
Direct Mag: 2500 x

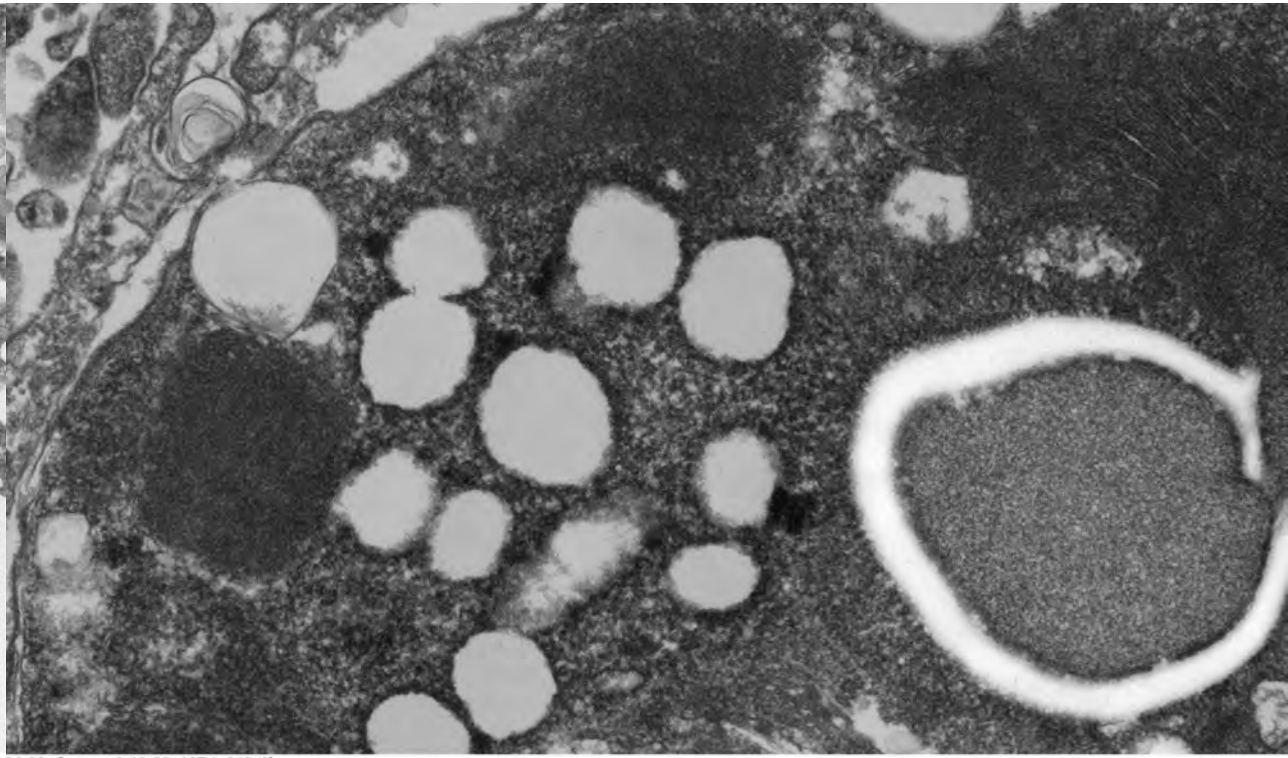

21-20_Correa_3-19-25_16B4_040.tif
Sample: 3/19, 25
Biological Electron Microscopy Lab
Rice University - SEA
Microscopist: MD Meyer

600 nm
HV=80kV
Direct Mag: 6000 x

Cell 22

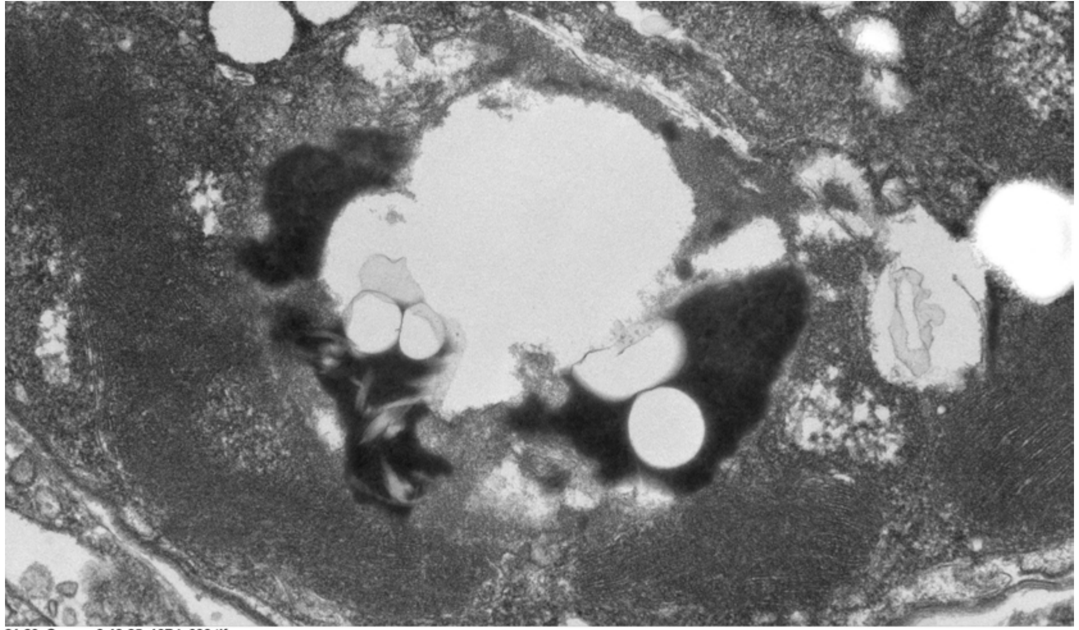

21-20_Correa_3-19-25_16B4_036.tif
Sample: 3/19, 25
Biological Electron Microscopy Lab
Rice University - SEA
Microscopist: MD Meyer

600 nm
HV=80kV
Direct Mag: 6000 x

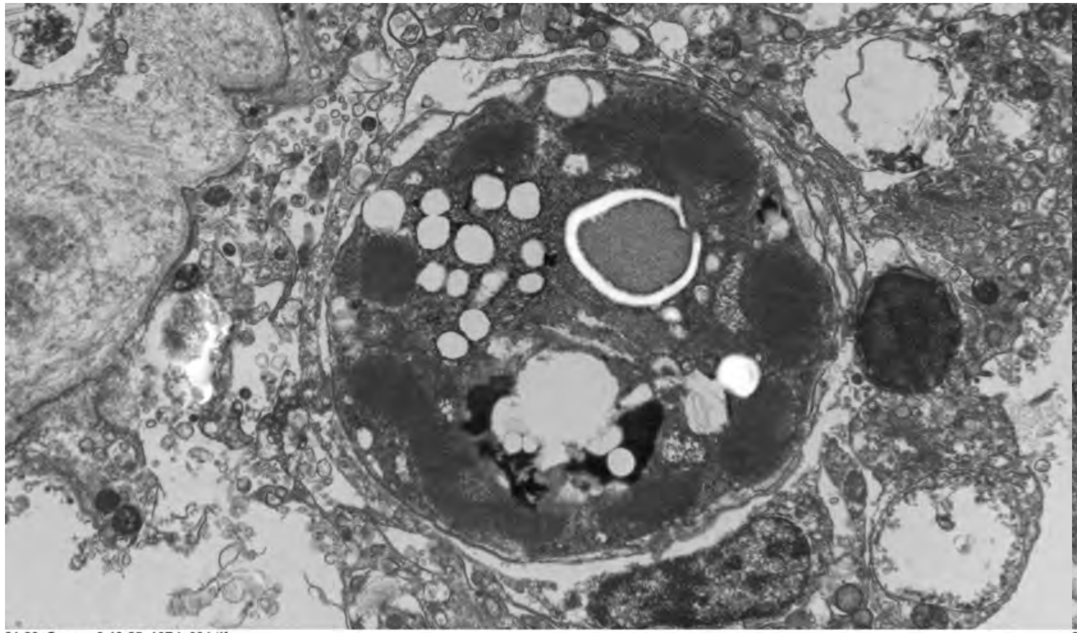

21-20_Correa_3-19-25_16B4_034.tif
Sample: 3/19, 25
Biological Electron Microscopy Lab
Rice University - SEA
Microscopist: MD Meyer

2 µm
HV=80kV
Direct Mag: 2000 x

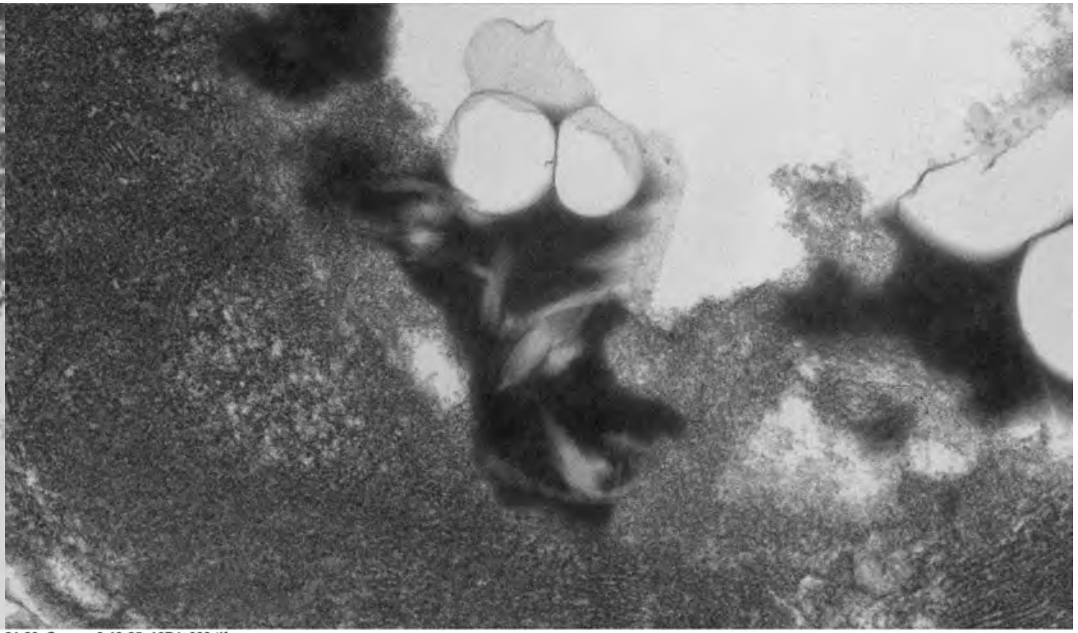

21-20_Correa_3-19-25_16B4_039.tif
Sample: 3/19, 25
Biological Electron Microscopy Lab
Rice University - SEA
Microscopist: MD Meyer

200 nm
HV=80kV
Direct Mag: 12000 x

Cell 23

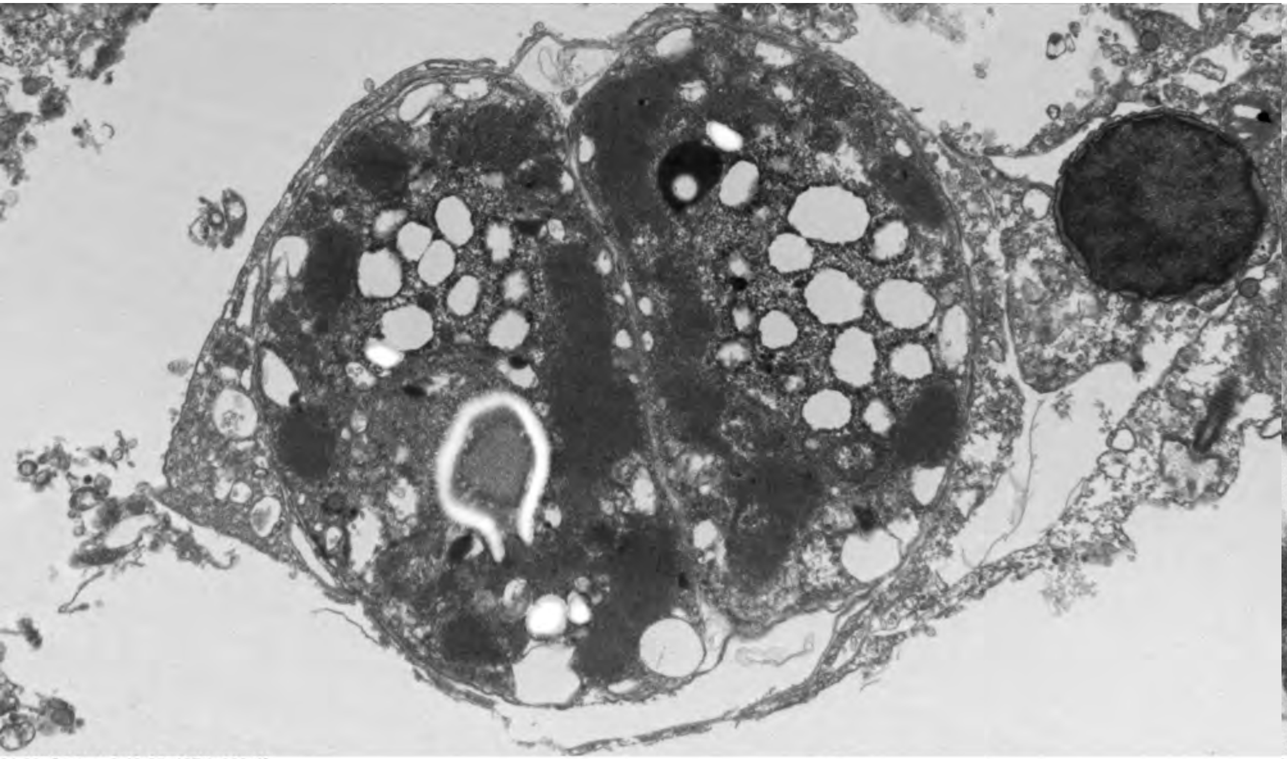

21-20_Correa_3-19-25_16B4_028.tif
Sample: 3/19, 25
Biological Electron Microscopy Lab
Rice University - SEA
Microscopist: MD Meyer

2 µm
HV=80kV
Direct Mag: 2000 x

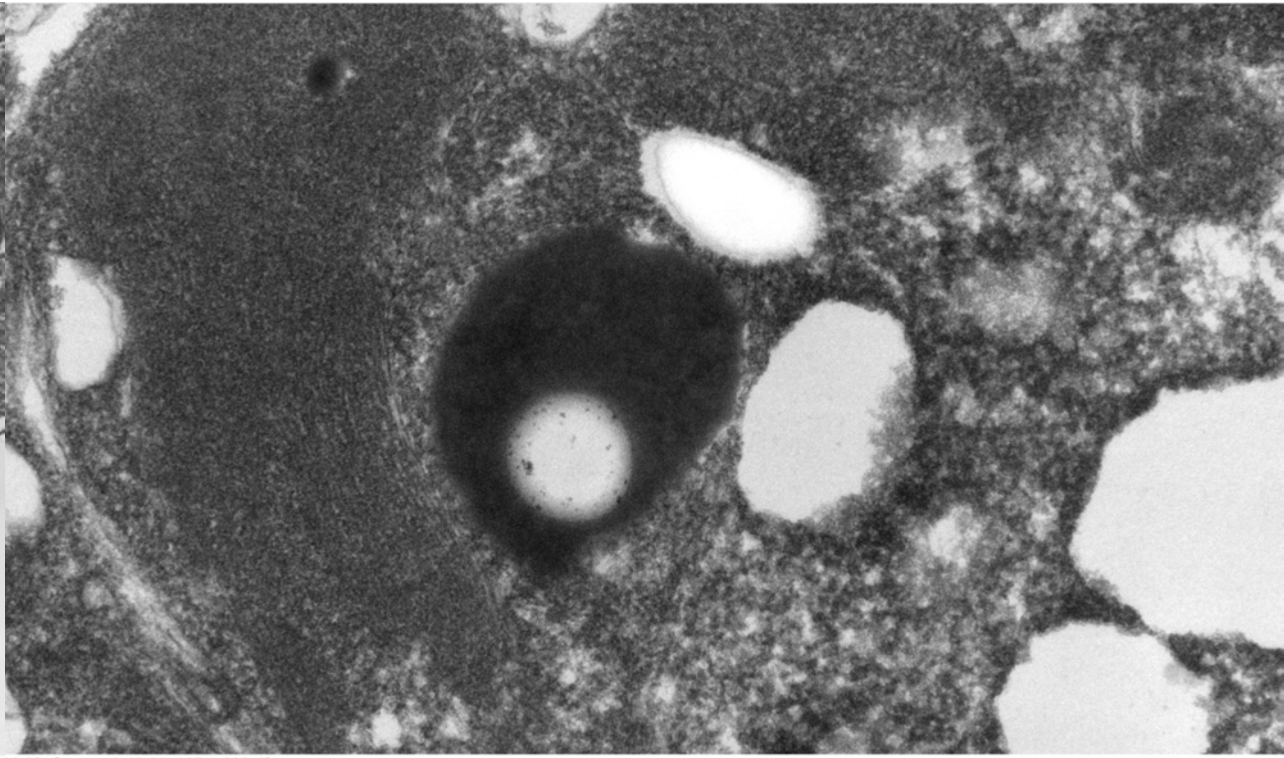

21-20_Correa_3-19-25_16B4_032.tif
Sample: 3/19, 25
Biological Electron Microscopy Lab
Rice University - SEA
Microscopist: MD Meyer

400 nm
HV=80kV
Direct Mag: 10000 x

Cell 24

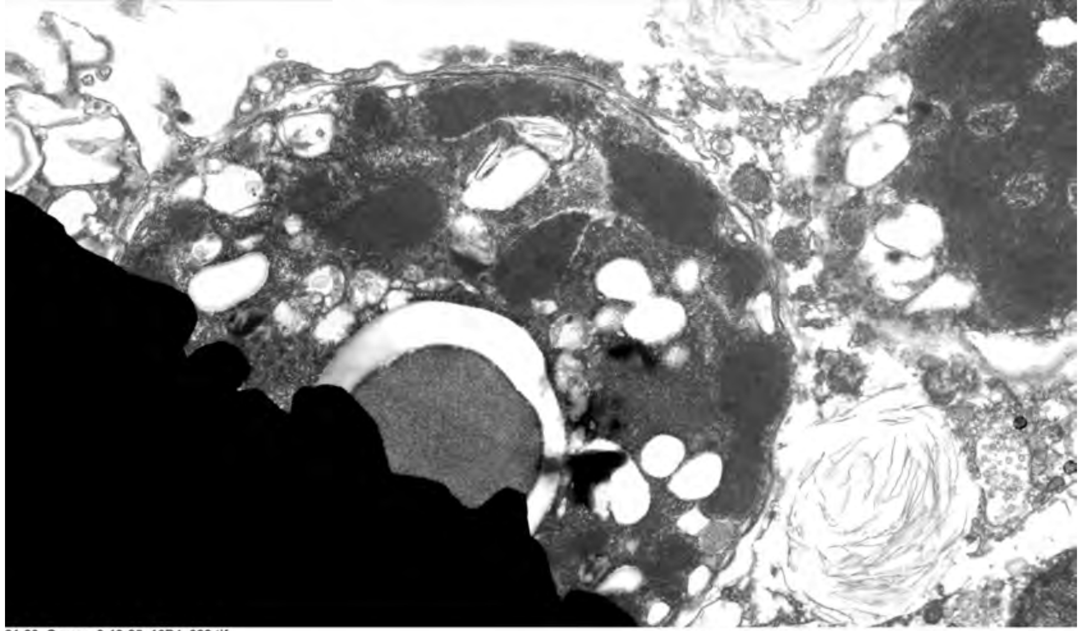

21-20_Correa_3-19-25_16B4_022.tif
Sample: 3/19, 25
Biological Electron Microscopy Lab
Rice University - SEA
Microscopist: MD Meyer

1 µm
HV=80kV
Direct Mag: 3000 x

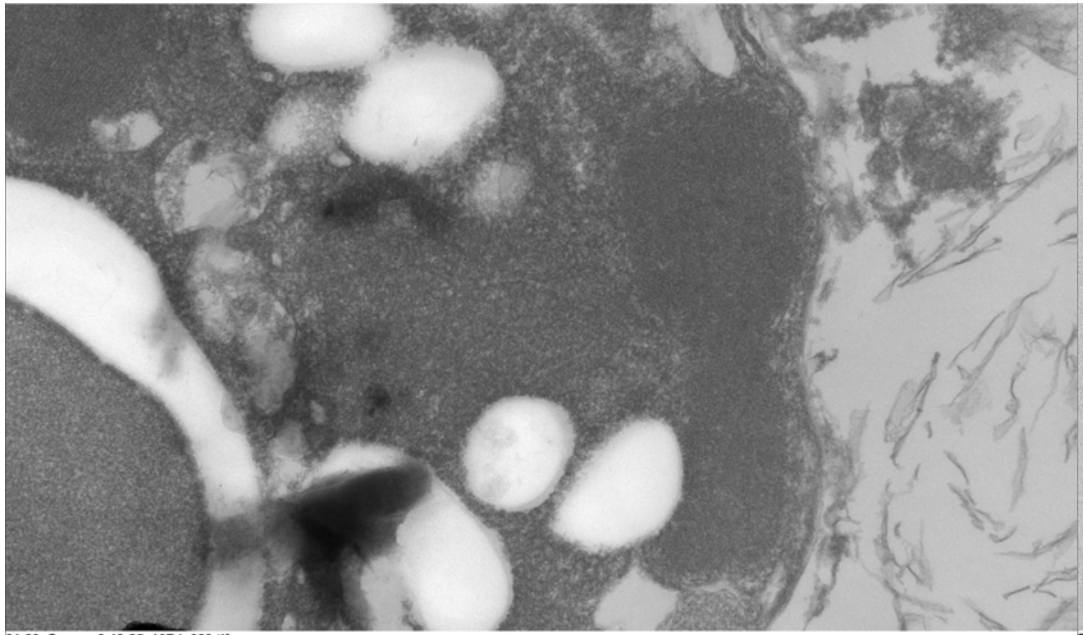

21-20_Correa_3-19-25_16B4_023.tif
Sample: 3/19, 25
Biological Electron Microscopy Lab
Rice University - SEA
Microscopist: MD Meyer

500 nm
HV=80kV
Direct Mag: 8000 x

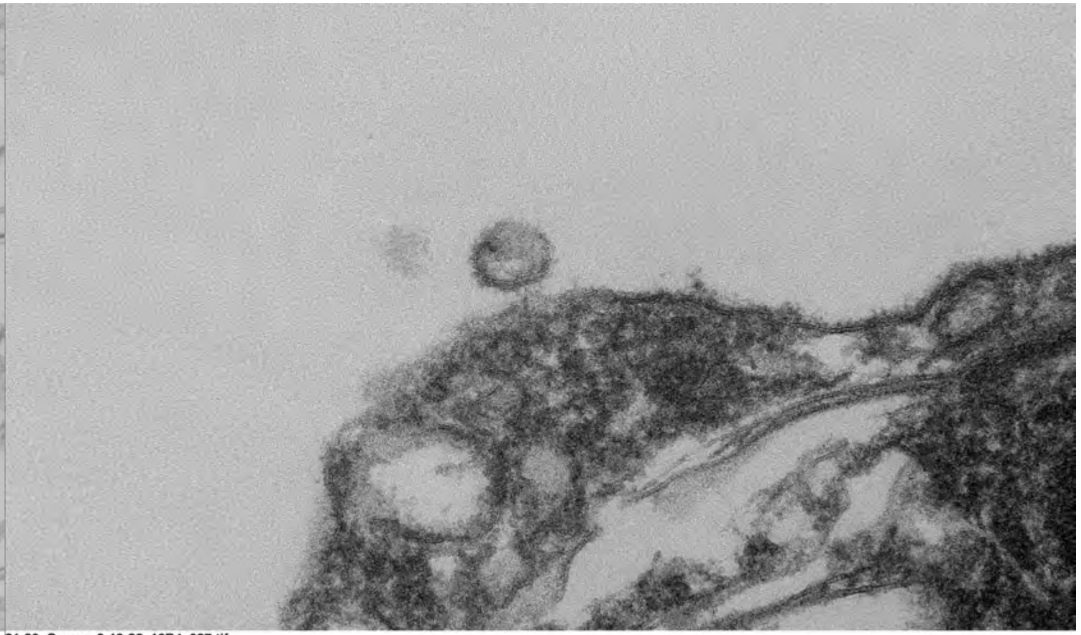

21-20_Correa_3-19-25_16B4_027.tif
Sample: 3/19, 25
Biological Electron Microscopy Lab
Rice University - SEA
Microscopist: MD Meyer

200 nm
HV=80kV
Direct Mag: 20000 x

Cell 25

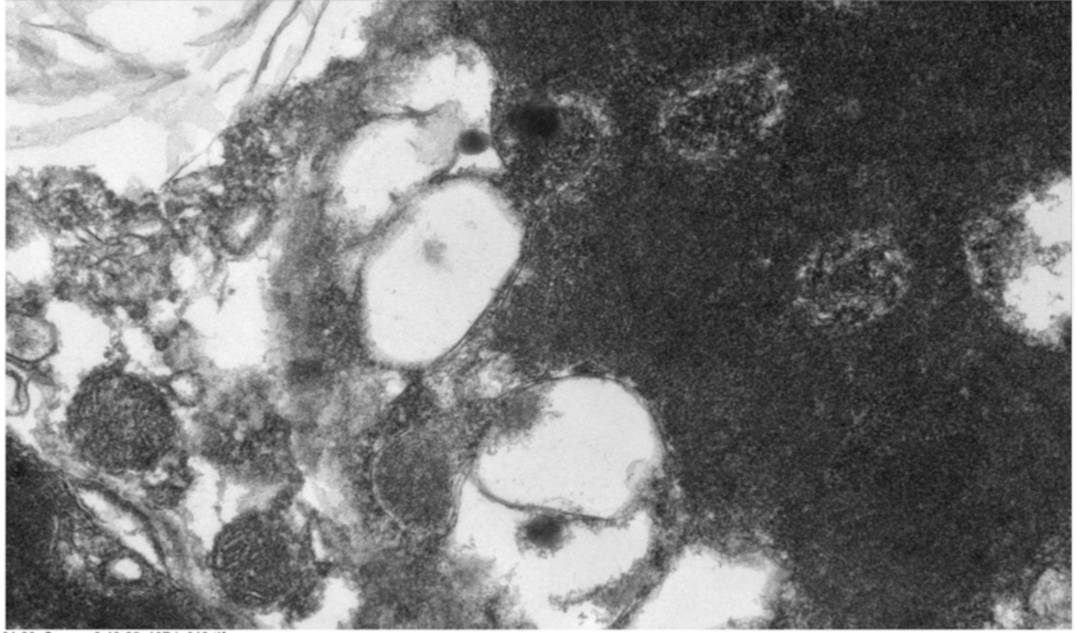

21-20_Correa_3-19-25_16B4_018.tif
Sample: 3/19, 25
Biological Electron Microscopy Lab
Rice University - SEA
Microscopist: MD Meyer

500 nm
HV=80kV
Direct Mag: 8000 x

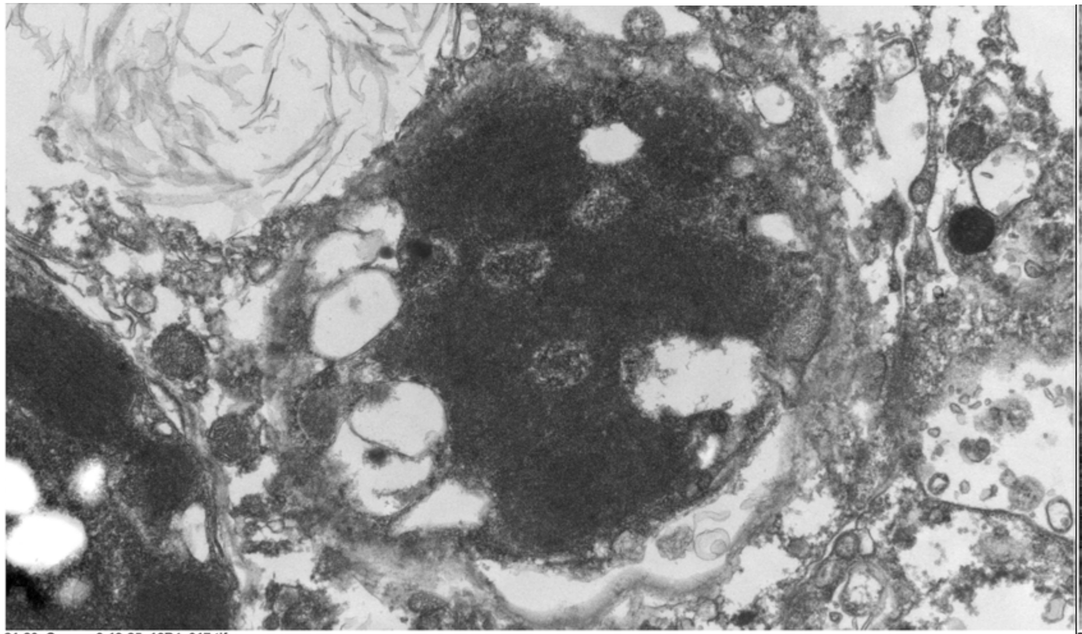

21-20_Correa_3-19-25_16B4_017.tif
Sample: 3/19, 25
Biological Electron Microscopy Lab
Rice University - SEA
Microscopist: MD Meyer

1 µm
HV=80kV
Direct Mag: 4000 x

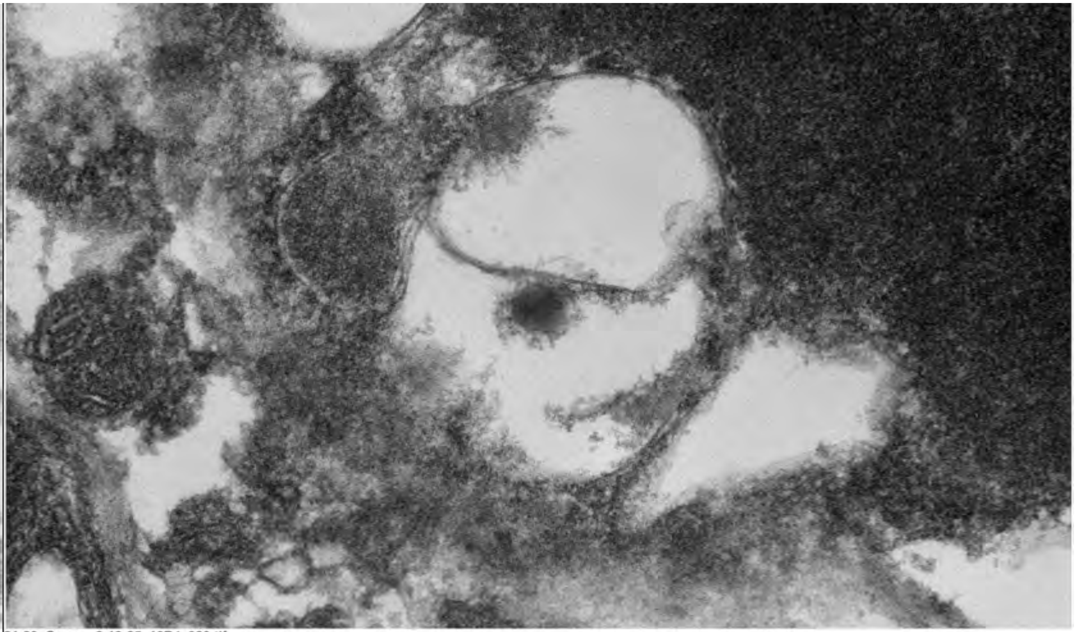

21-20_Correa_3-19-25_16B4_020.tif
Sample: 3/19, 25
Biological Electron Microscopy Lab
Rice University - SEA
Microscopist: MD Meyer

200 nm
HV=80kV
Direct Mag: 12000 x

Cell 26

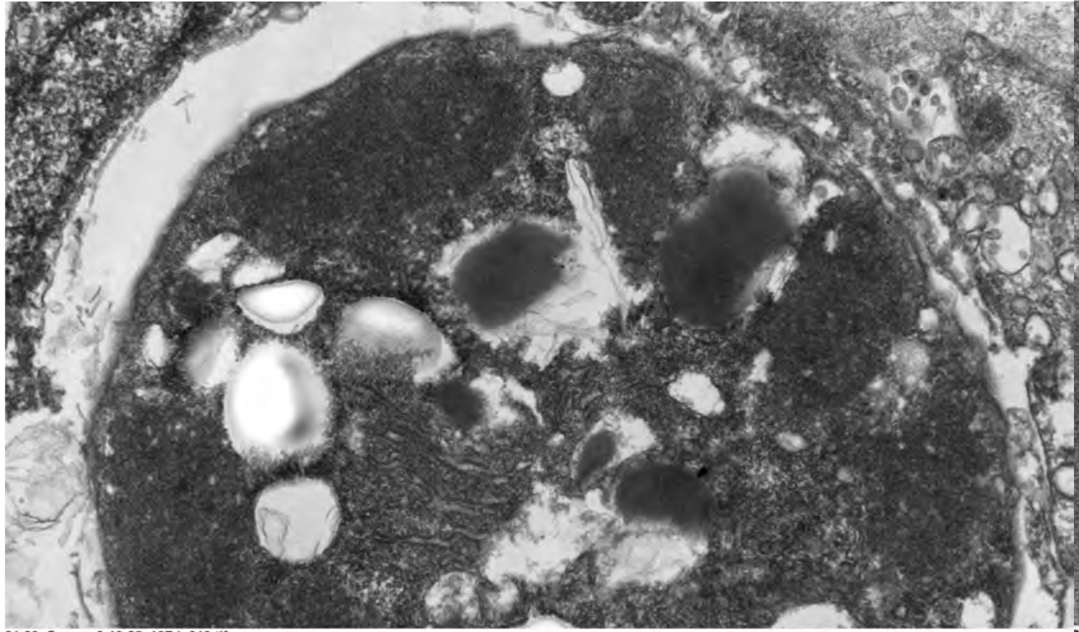

21-20_Correa_3-19-25_16B4_013.tif
Sample: 3/19, 25
Biological Electron Microscopy Lab
Rice University - SEA
Microscopist: MD Meyer

800 nm
HV=80kV
Direct Mag: 5000 x

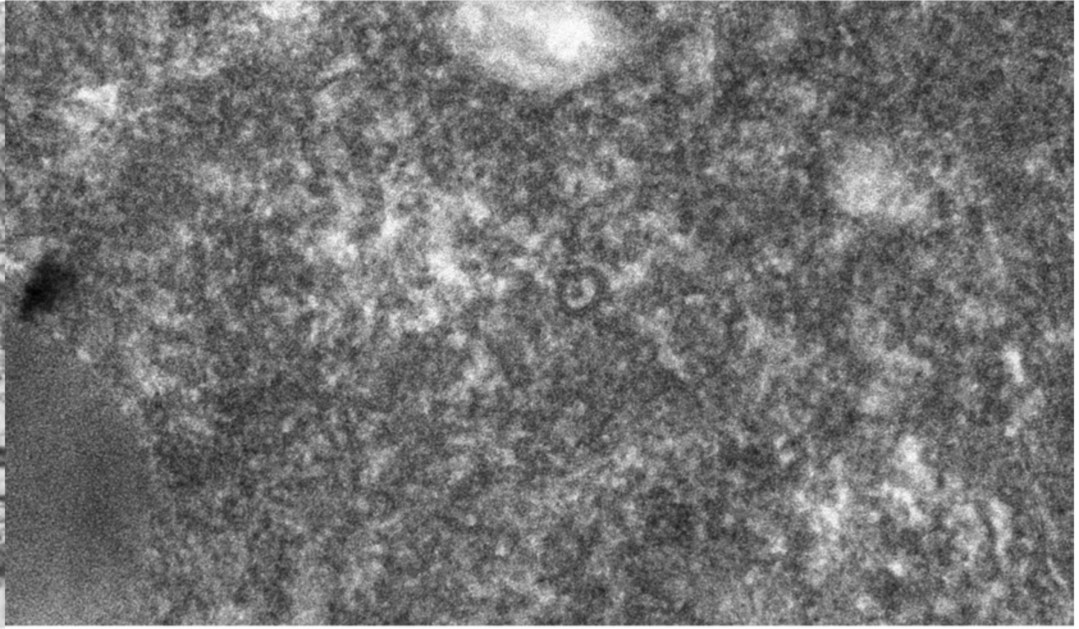

21-20_Correa_3-19-25_16B4_015.tif
Sample: 3/19, 25
Biological Electron Microscopy Lab
Rice University - SEA
Microscopist: MD Meyer

100 nm
HV=80kV
Direct Mag: 30000 x

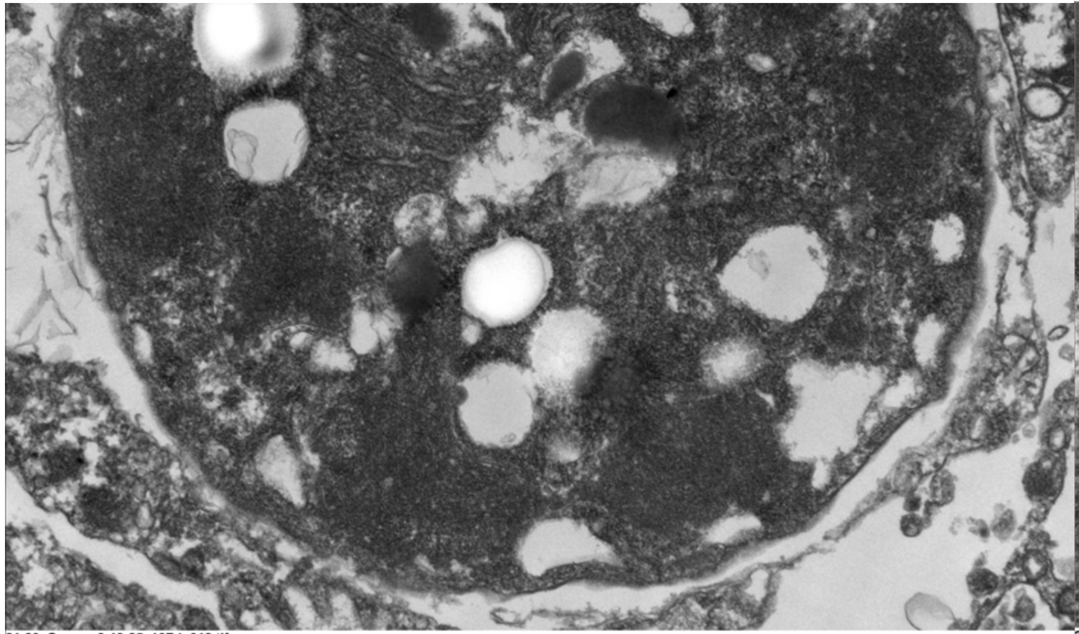

21-20_Correa_3-19-25_16B4_012.tif
Sample: 3/19, 25
Biological Electron Microscopy Lab
Rice University - SEA
Microscopist: MD Meyer

800 nm
HV=80kV
Direct Mag: 5000 x

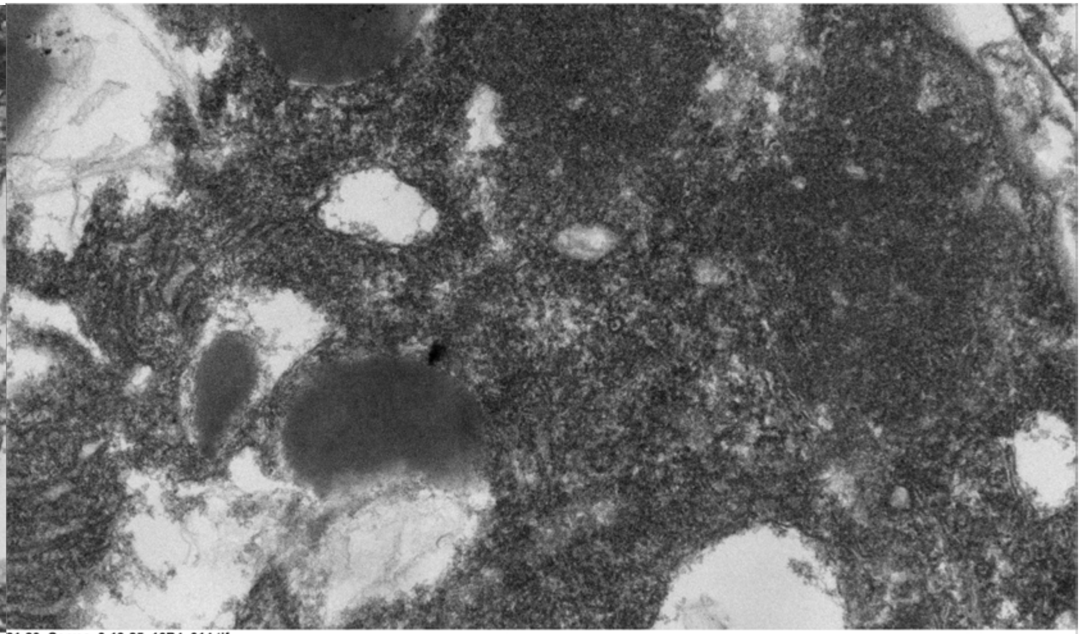

21-20_Correa_3-19-25_16B4_014.tif
Sample: 3/19, 25
Biological Electron Microscopy Lab
Rice University - SEA
Microscopist: MD Meyer

400 nm
HV=80kV
Direct Mag: 10000 x

Cell 27

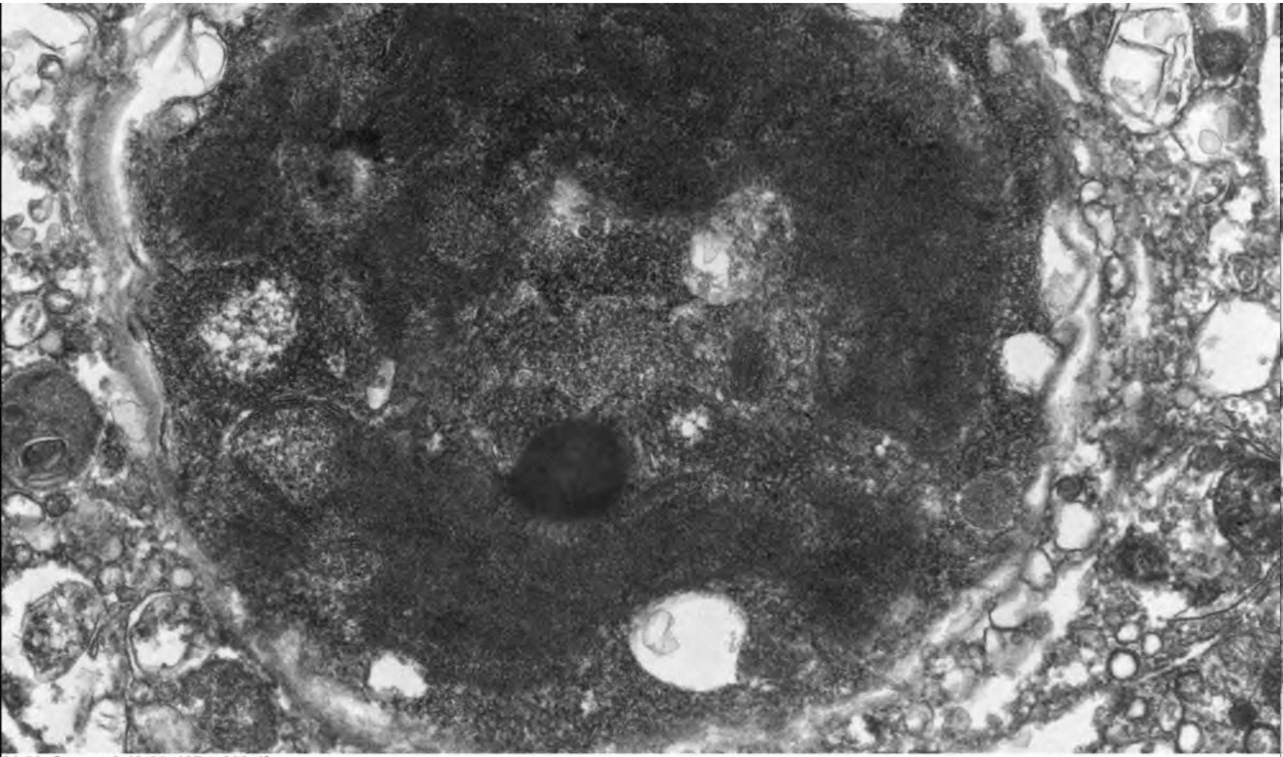

21-20_Correa_3-19-25_16B4_008.tif
Sample: 3/19, 25
Biological Electron Microscopy Lab
Rice University - SEA
Microscopist: MD Meyer

800 nm
HV=80kV
Direct Mag: 5000 x

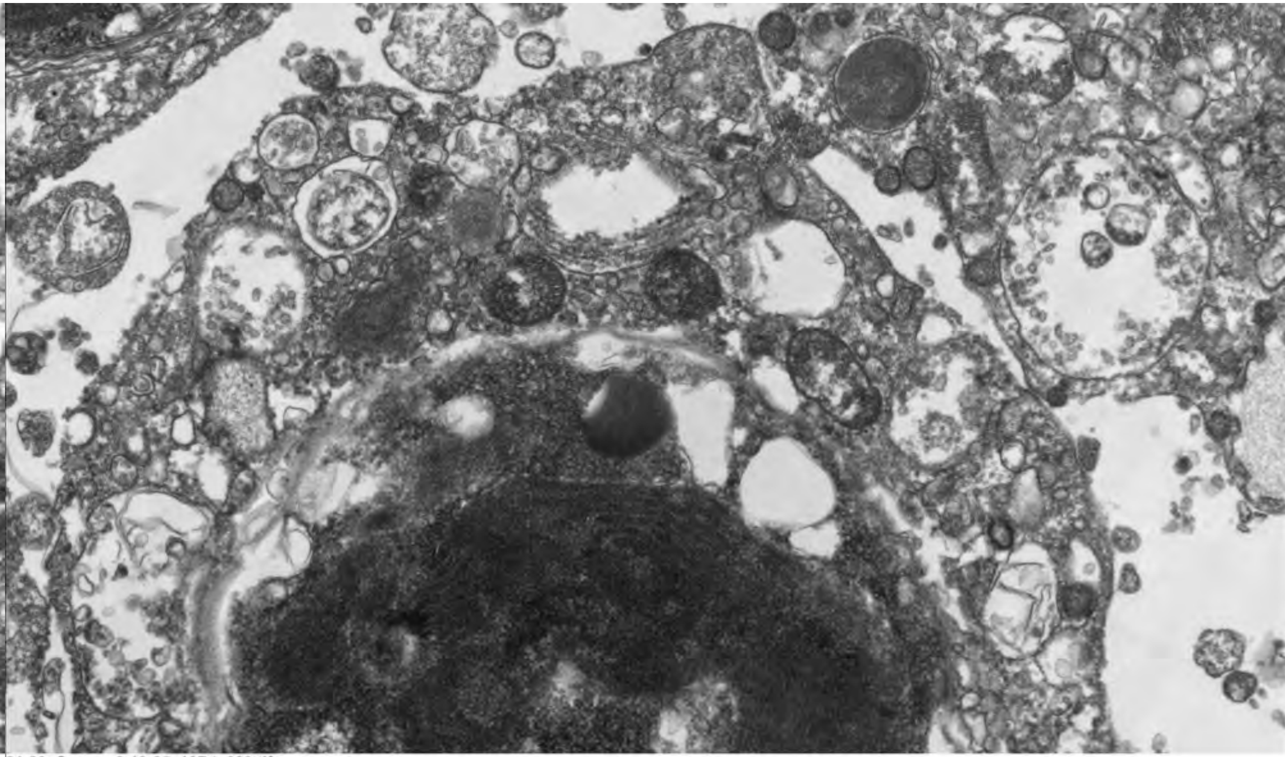

21-20_Correa_3-19-25_16B4_009.tif
Sample: 3/19, 25
Biological Electron Microscopy Lab
Rice University - SEA
Microscopist: MD Meyer

1 µm
HV=80kV
Direct Mag: 4000 x

Cell 28

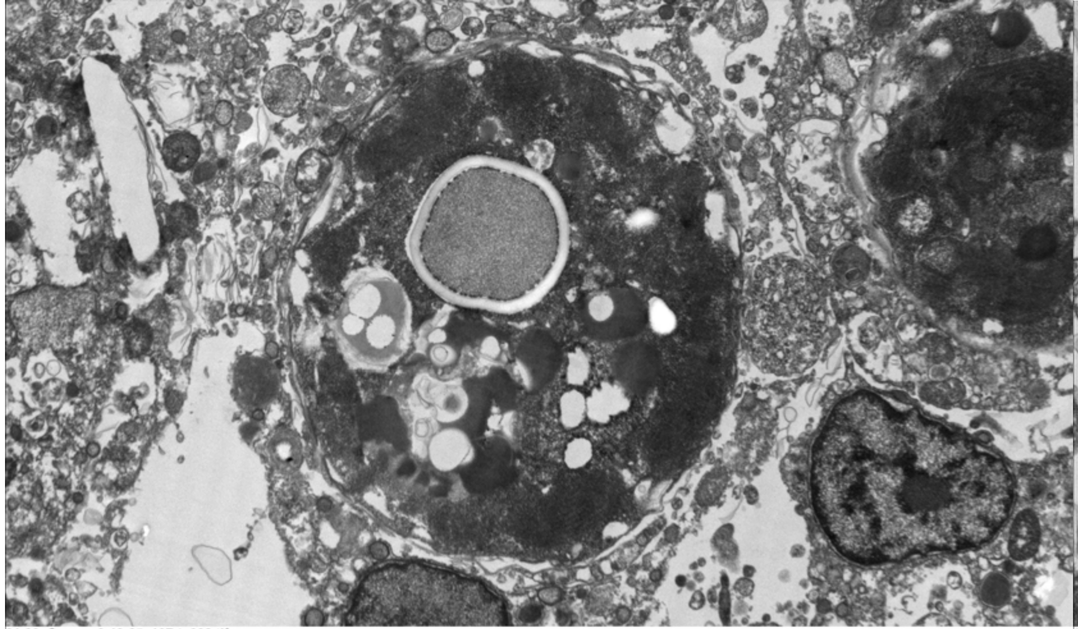

21-20_Correa_3-19-25_16B4_002.tif
Sample: 3/19, 25
Biological Electron Microscopy Lab
Rice University - SEA
Microscopist: MD Meyer

2 µm
HV=80kV
Direct Mag: 2000 x

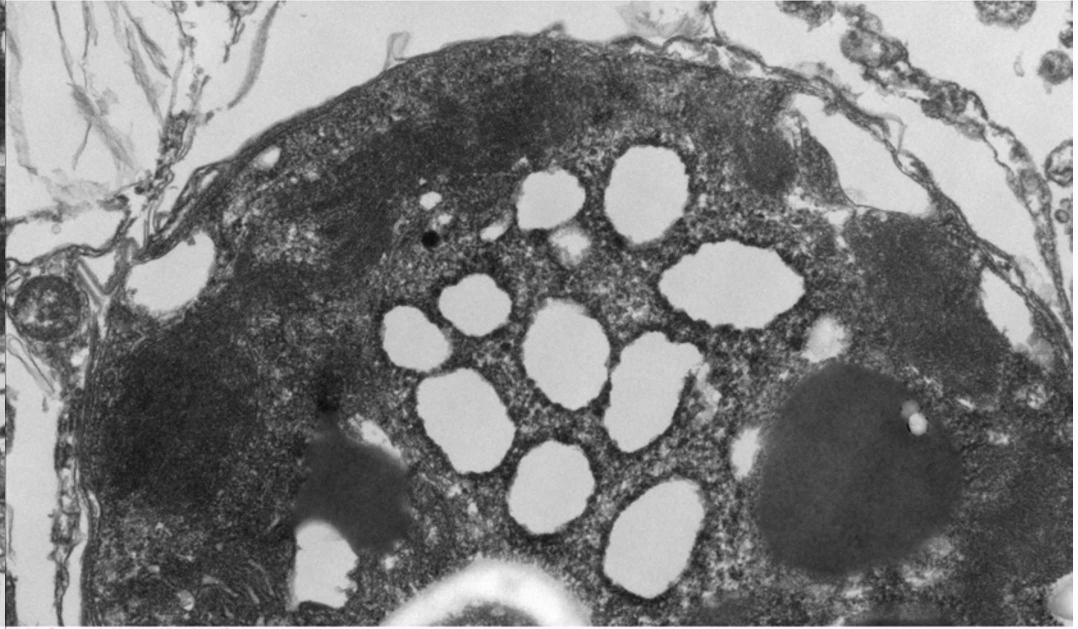

21-20_Correa_3-19-25_16B4_007.tif
Sample: 3/19, 25
Biological Electron Microscopy Lab
Rice University - SEA
Microscopist: MD Meyer

800 nm
HV=80kV
Direct Mag: 5000 x

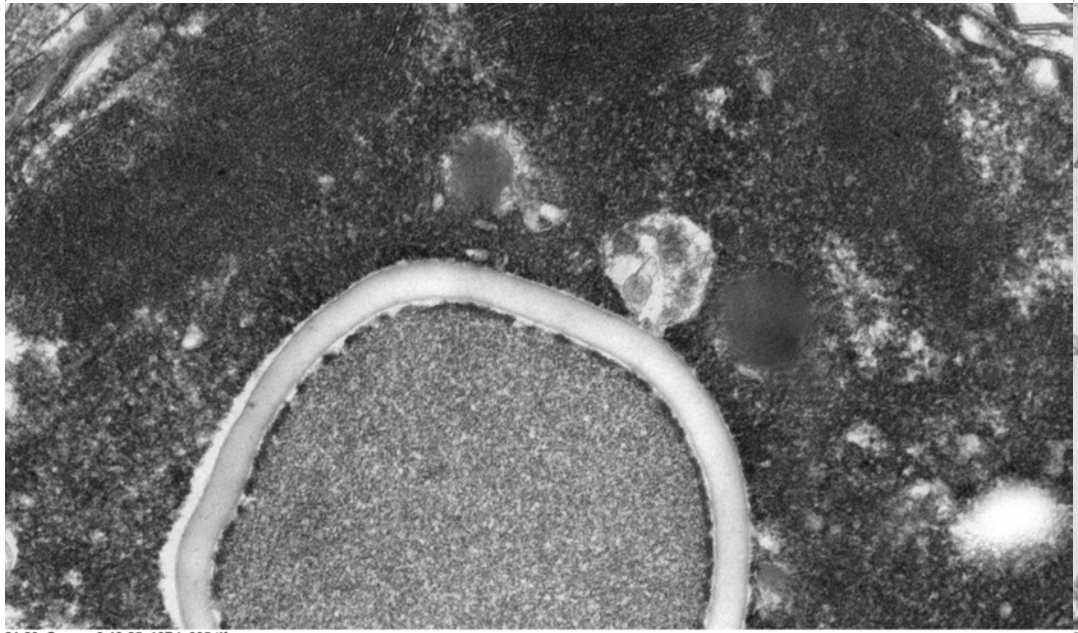

21-20_Correa_3-19-25_16B4_005.tif
Sample: 3/19, 25
Biological Electron Microscopy Lab
Rice University - SEA
Microscopist: MD Meyer

500 nm
HV=80kV
Direct Mag: 8000 x

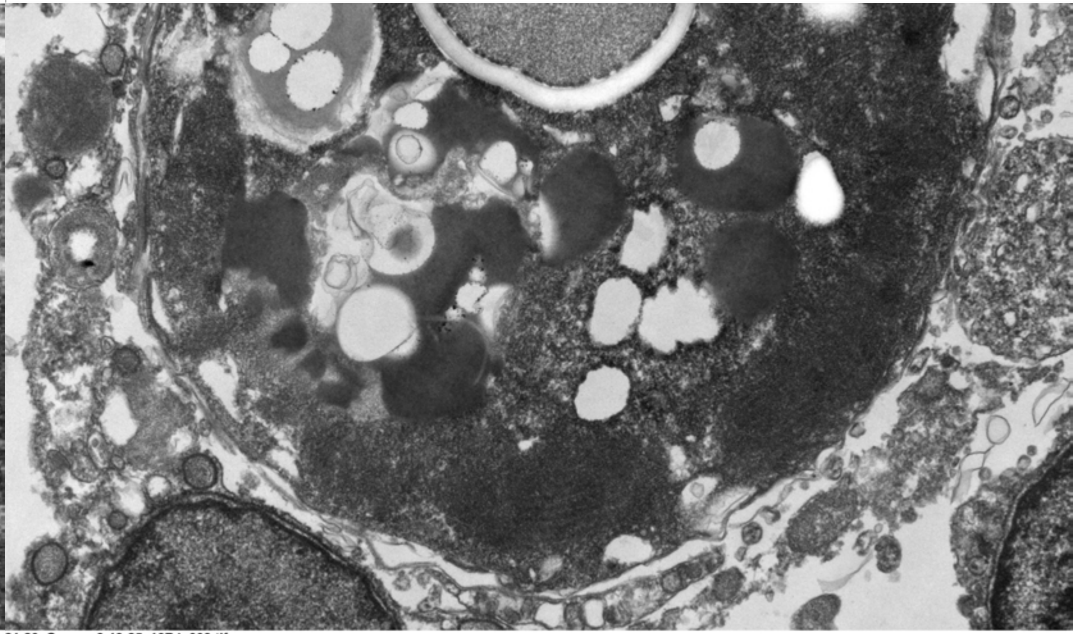

21-20_Correa_3-19-25_16B4_003.tif
Sample: 3/19, 25
Biological Electron Microscopy Lab
Rice University - SEA
Microscopist: MD Meyer

1 µm
HV=80kV
Direct Mag: 4000 x

Ambient (March 2018) POR Colony J

Cell 1

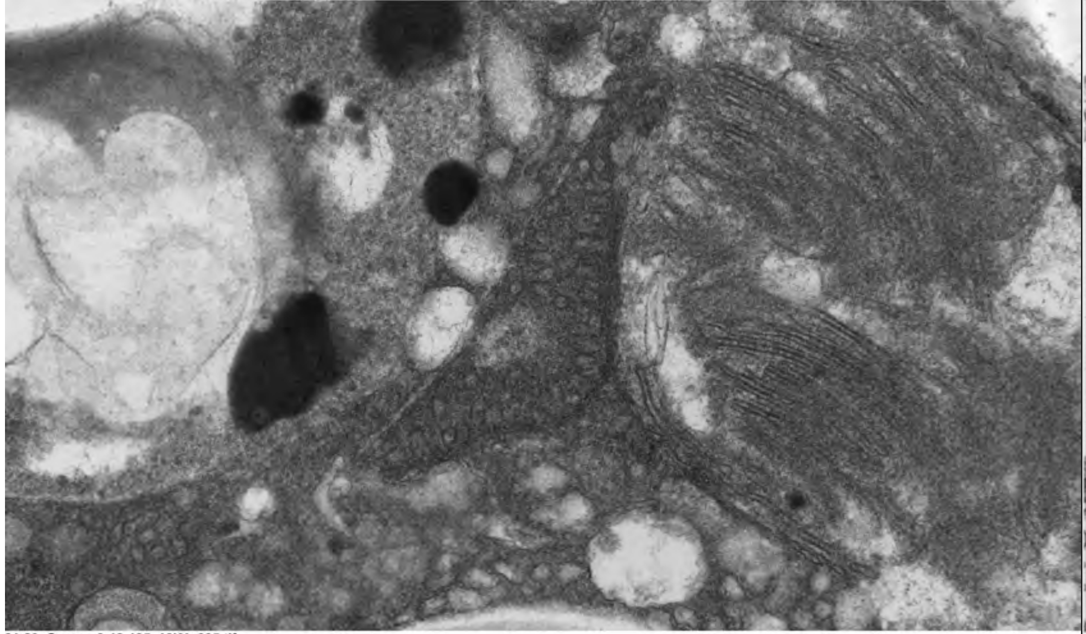

21-20_Correa_3-18-135_16K1_005.tif
Sample: 3/18, 135
Biological Electron Microscopy Lab
Rice University - SEA
Microscopist: MD Meyer

400 nm
HV=80kV
Direct Mag: 10000 x

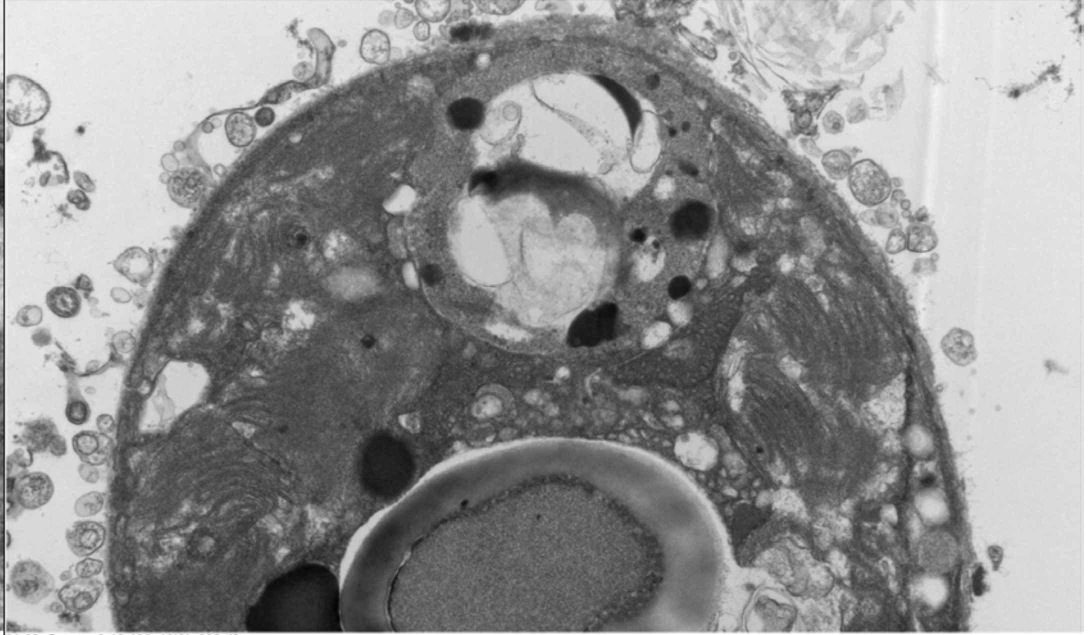

21-20_Correa_3-18-135_16K1_003.tif
Sample: 3/18, 135
Biological Electron Microscopy Lab
Rice University - SEA
Microscopist: MD Meyer

1 µm
HV=80kV
Direct Mag: 4000 x

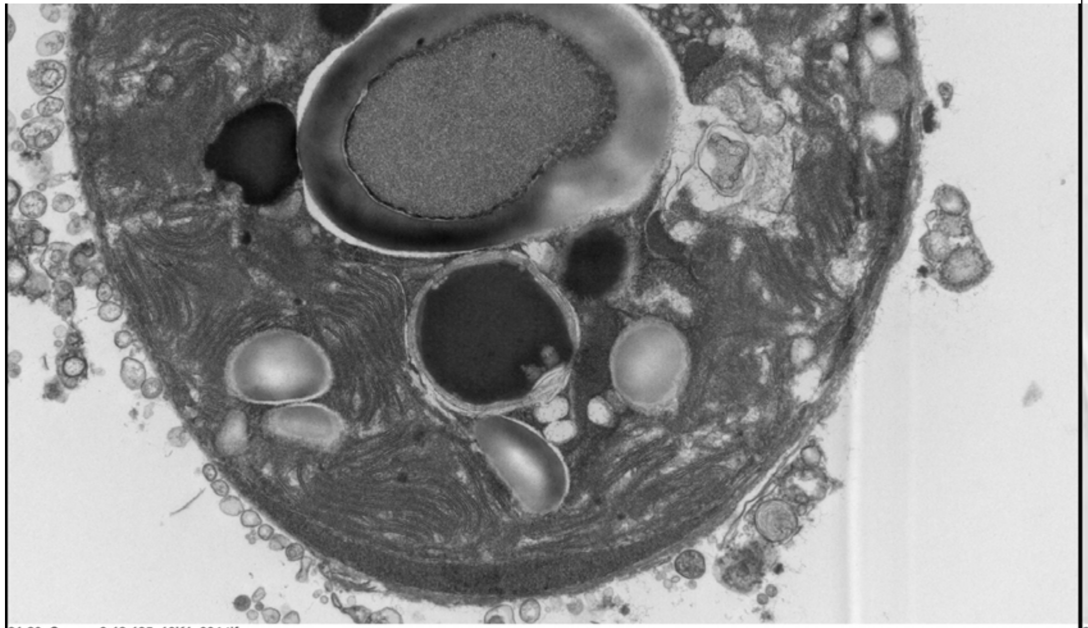

21-20_Correa_3-18-135_16K1_004.tif
Sample: 3/18, 135
Biological Electron Microscopy Lab
Rice University - SEA
Microscopist: MD Meyer

1 µm
HV=80kV
Direct Mag: 4000 x

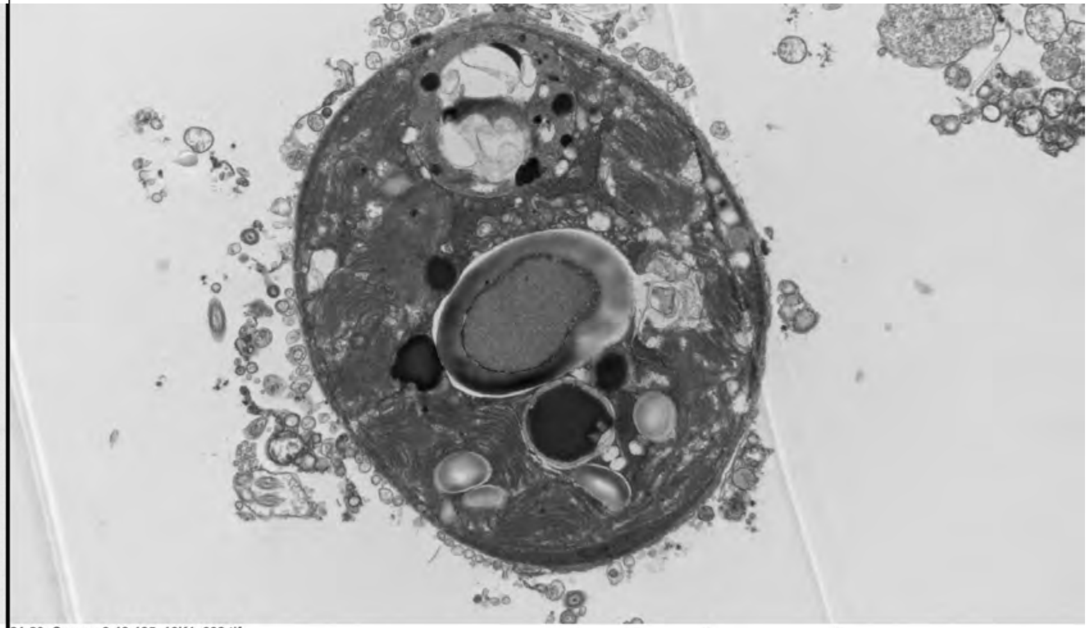

21-20_Correa_3-18-135_16K1_002.tif
Sample: 3/18, 135
Biological Electron Microscopy Lab
Rice University - SEA
Microscopist: MD Meyer

2 µm
HV=80kV
Direct Mag: 2000 x

Cell 2

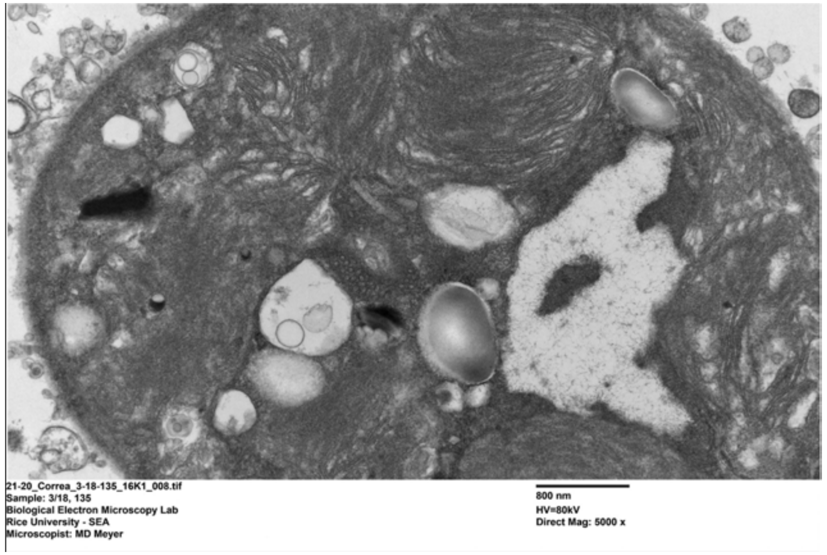
21-20_Correa_3-18-135_16K1_008.tif
Sample: 3/18, 135
Biological Electron Microscopy Lab
Rice University - SEA
Microscopist: MD Meyer
800 nm
HV=80kV
Direct Mag: 5000 x

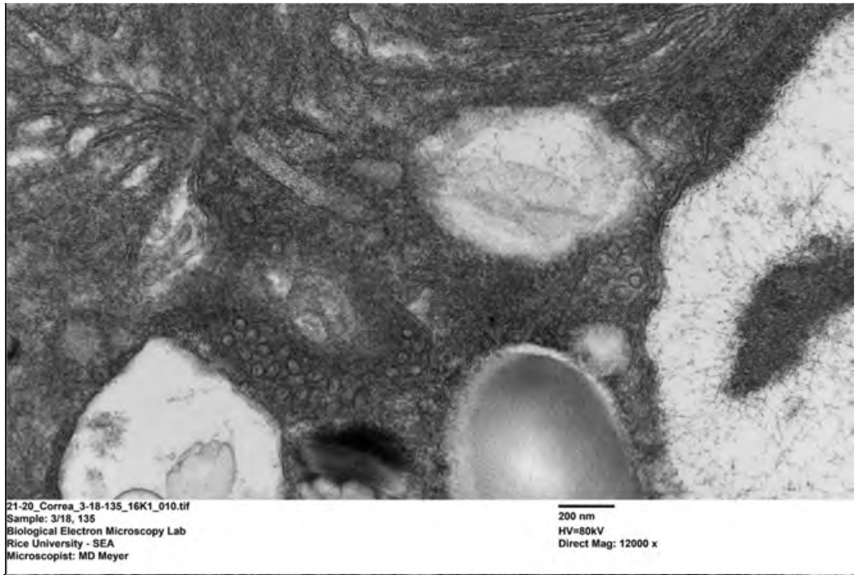
21-20_Correa_3-18-135_16K1_010.tif
Sample: 3/18, 135
Biological Electron Microscopy Lab
Rice University - SEA
Microscopist: MD Meyer
200 nm
HV=80kV
Direct Mag: 12000 x

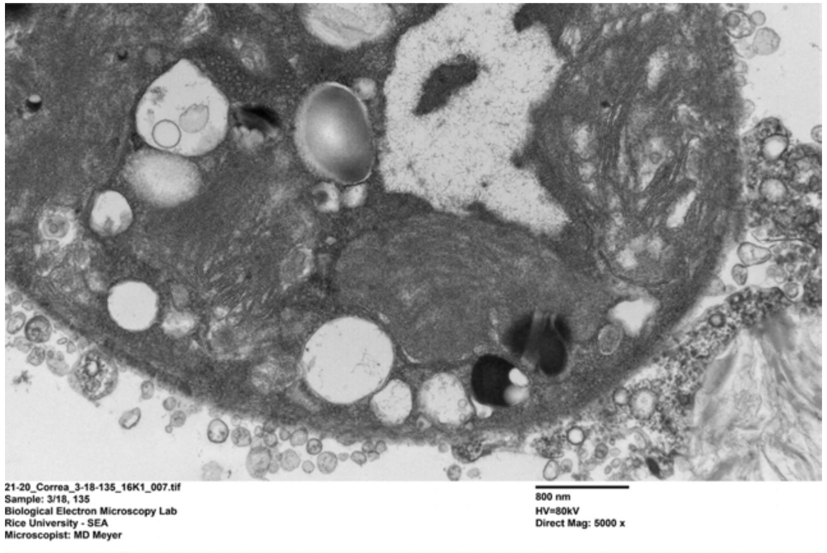
21-20_Correa_3-18-135_16K1_007.tif
Sample: 3/18, 135
Biological Electron Microscopy Lab
Rice University - SEA
Microscopist: MD Meyer
800 nm
HV=80kV
Direct Mag: 5000 x

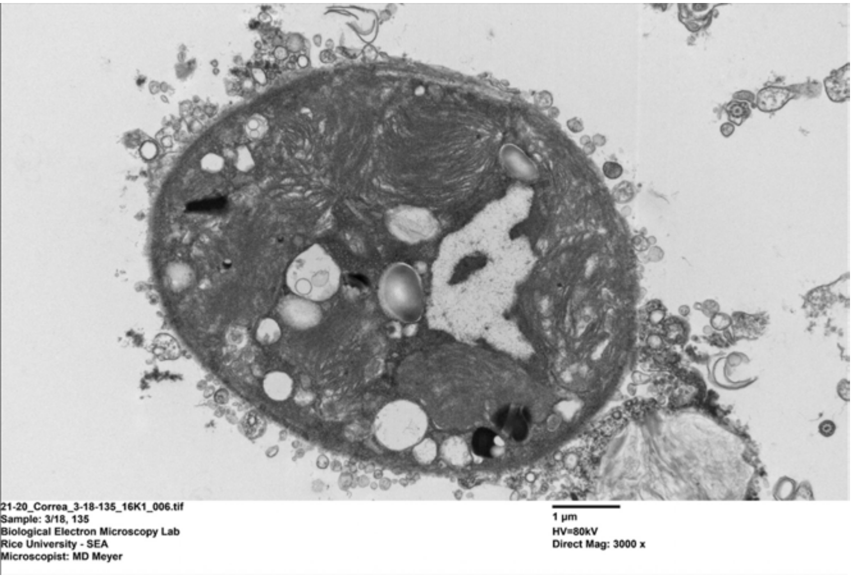
21-20_Correa_3-18-135_16K1_006.tif
Sample: 3/18, 135
Biological Electron Microscopy Lab
Rice University - SEA
Microscopist: MD Meyer
1 µm
HV=80kV
Direct Mag: 3000 x

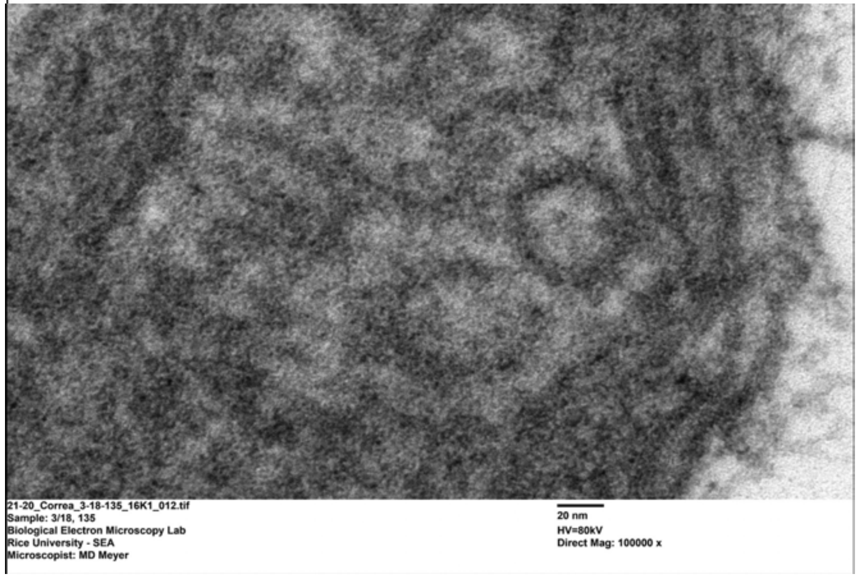
21-20_Correa_3-18-135_16K1_012.tif
Sample: 3/18, 135
Biological Electron Microscopy Lab
Rice University - SEA
Microscopist: MD Meyer
20 nm
HV=80kV
Direct Mag: 100000 x

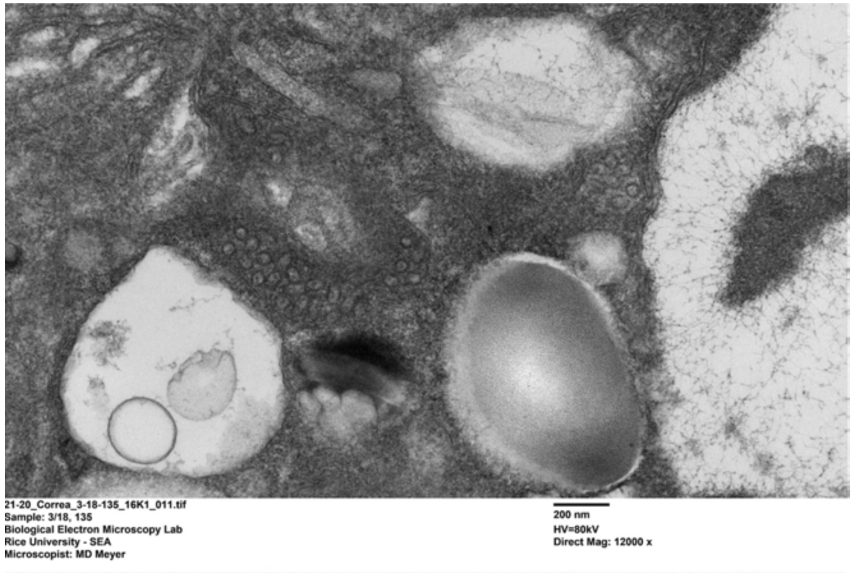
21-20_Correa_3-18-135_16K1_011.tif
Sample: 3/18, 135
Biological Electron Microscopy Lab
Rice University - SEA
Microscopist: MD Meyer
200 nm
HV=80kV
Direct Mag: 12000 x

Cell 3

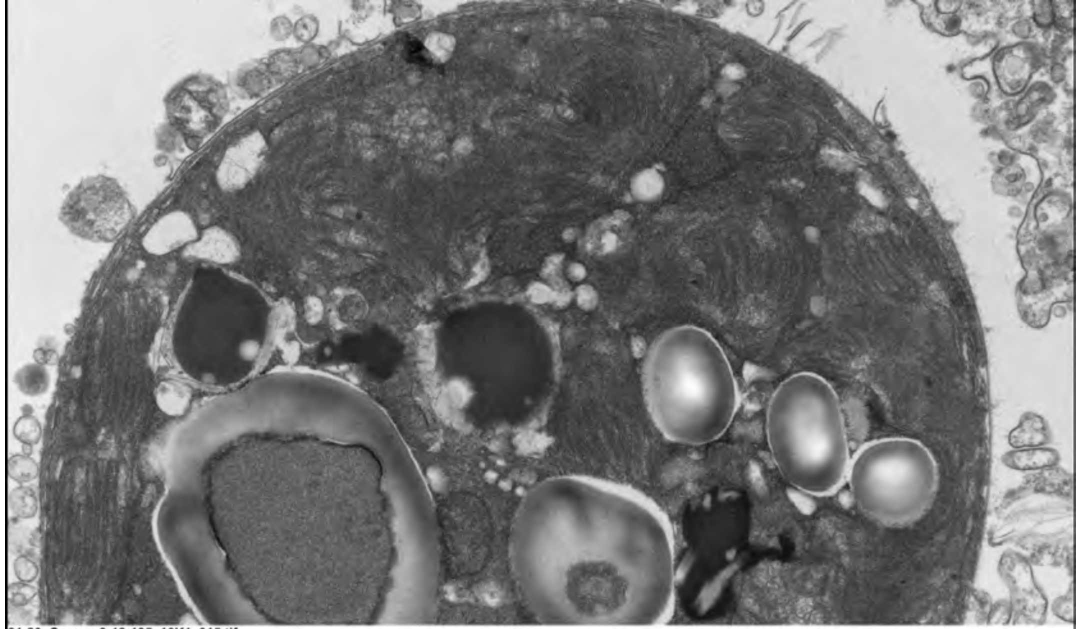

21-20_Correa_3-18-135_16K1_015.tif
Sample: 3/18, 135
Biological Electron Microscopy Lab
Rice University - SEA
Microscopist: MD Meyer

1 µm
HV=80kV
Direct Mag: 4000 x

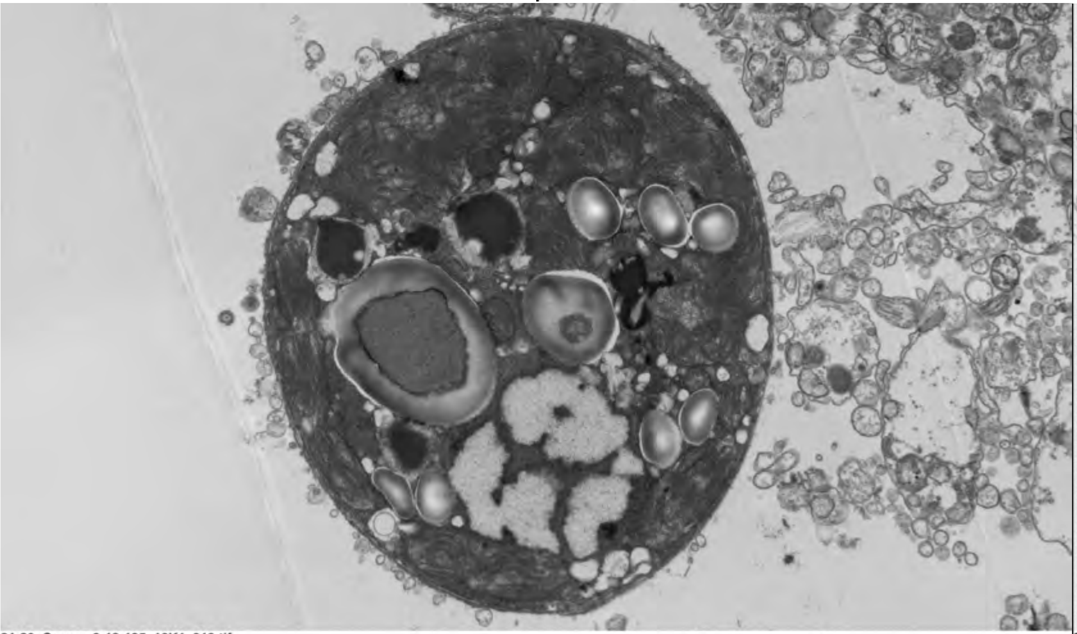

21-20_Correa_3-18-135_16K1_013.tif
Sample: 3/18, 135
Biological Electron Microscopy Lab
Rice University - SEA
Microscopist: MD Meyer

2 µm
HV=80kV
Direct Mag: 2000 x

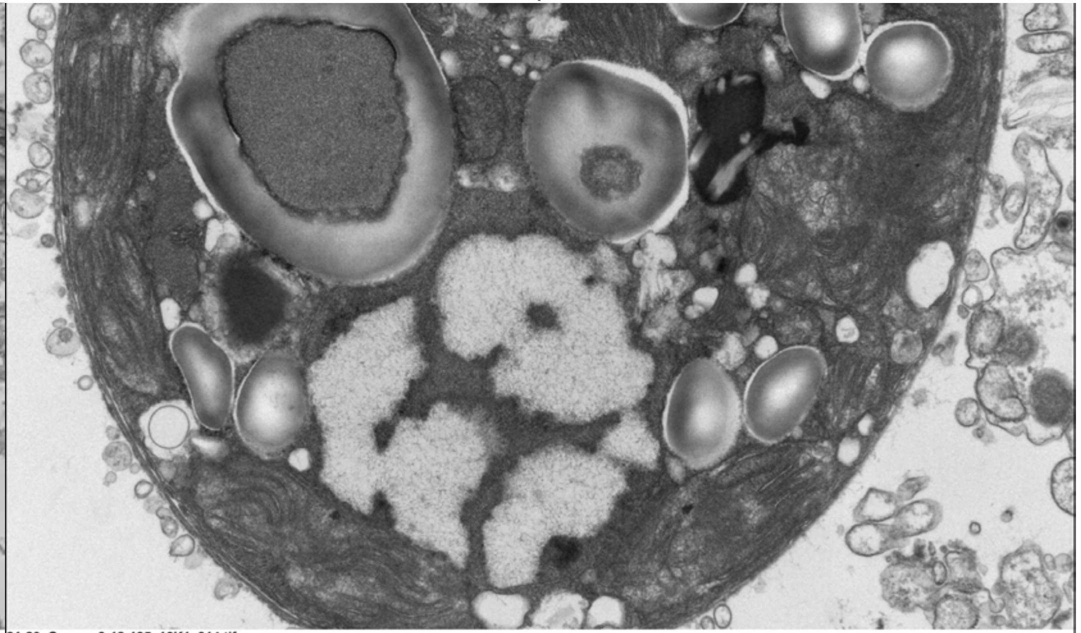

21-20_Correa_3-18-135_16K1_014.tif
Sample: 3/18, 135
Biological Electron Microscopy Lab
Rice University - SEA
Microscopist: MD Meyer

1 µm
HV=80kV
Direct Mag: 4000 x

Cell 4

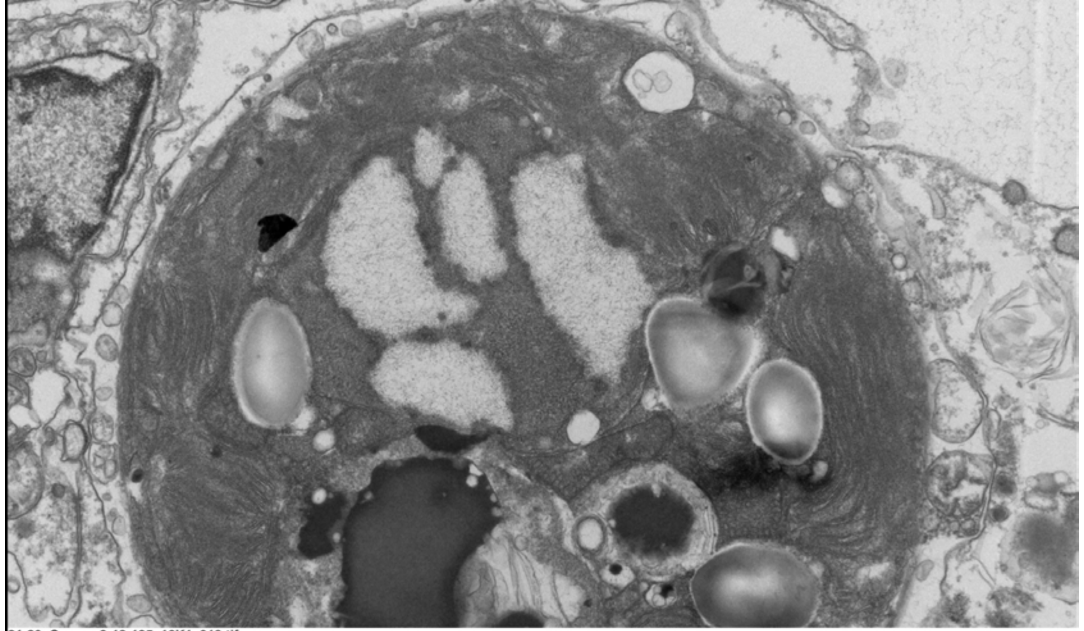

21-20_Correa_3-18-135_16K1_018.tif
Sample: 3/18, 135
Biological Electron Microscopy Lab
Rice University - SEA
Microscopist: MD Meyer

1 µm
HV=80kV
Direct Mag: 4000 x

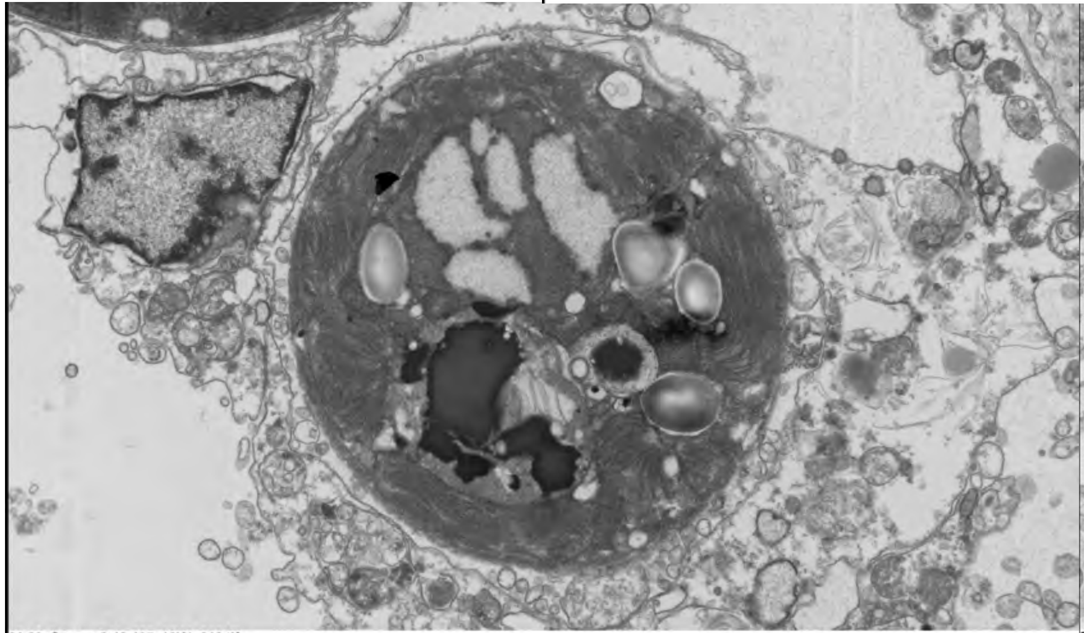

21-20_Correa_3-18-135_16K1_016.tif
Sample: 3/18, 135
Biological Electron Microscopy Lab
Rice University - SEA
Microscopist: MD Meyer

1 µm
HV=80kV
Direct Mag: 2500 x

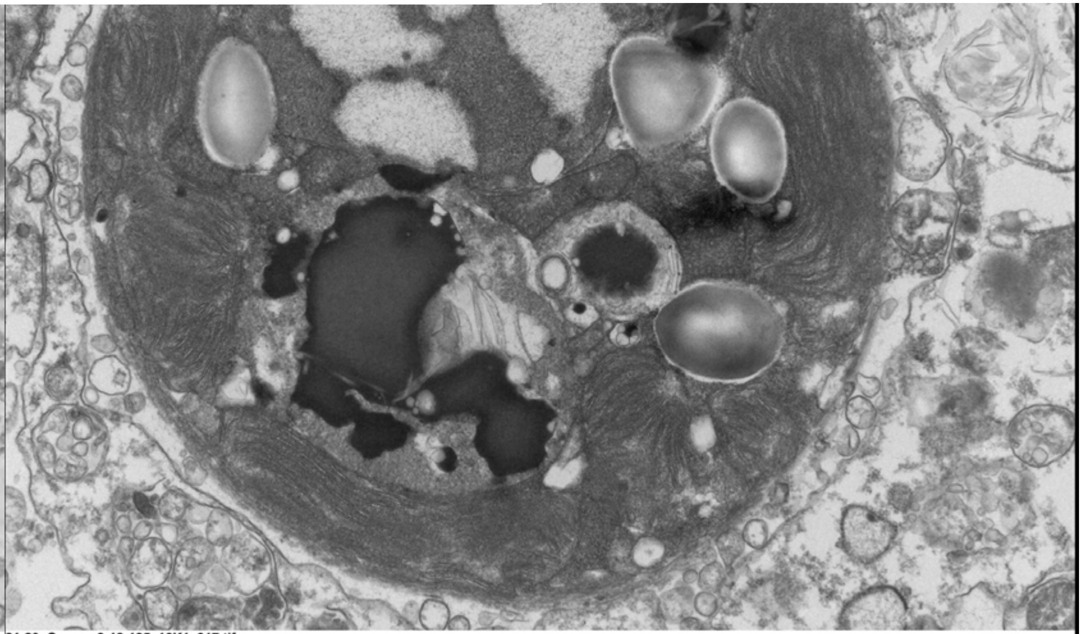

21-20_Correa_3-18-135_16K1_017.tif
Sample: 3/18, 135
Biological Electron Microscopy Lab
Rice University - SEA
Microscopist: MD Meyer

1 µm
HV=80kV
Direct Mag: 4000 x

Cell 5

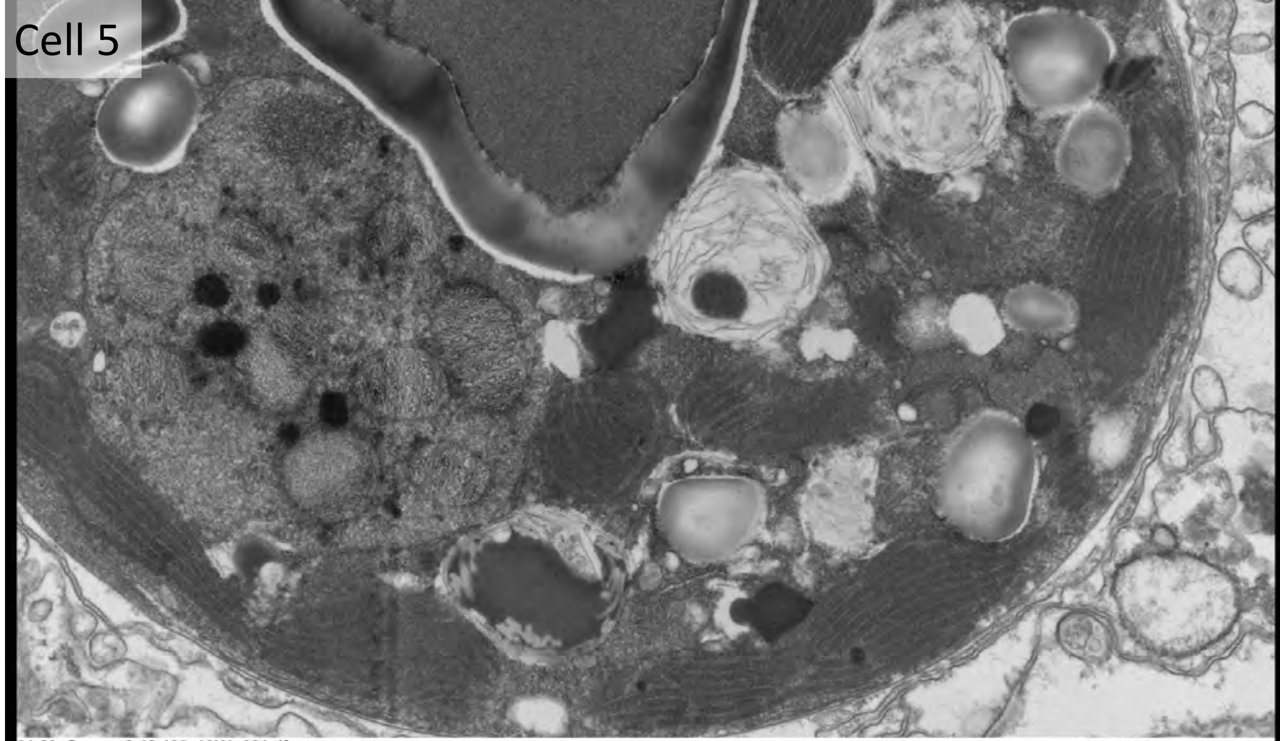

21-20_Correa_3-18-135_16K1_021.tif
Sample: 3/18, 135
Biological Electron Microscopy Lab
Rice University - SEA
Microscopist: MD Meyer

1 µm
HV=80kV
Direct Mag: 4000 x

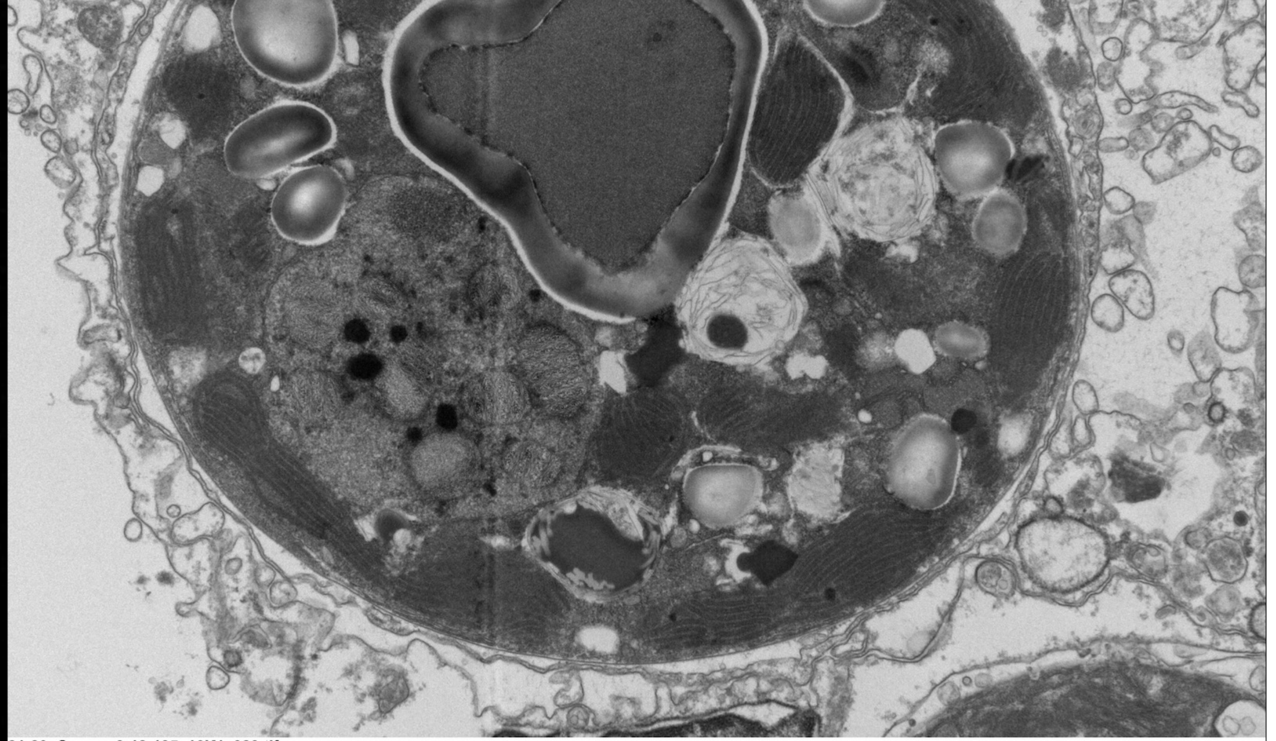

21-20_Correa_3-18-135_16K1_020.tif
Sample: 3/18, 135
Biological Electron Microscopy Lab
Rice University - SEA
Microscopist: MD Meyer

1 µm
HV=80kV
Direct Mag: 3000 x

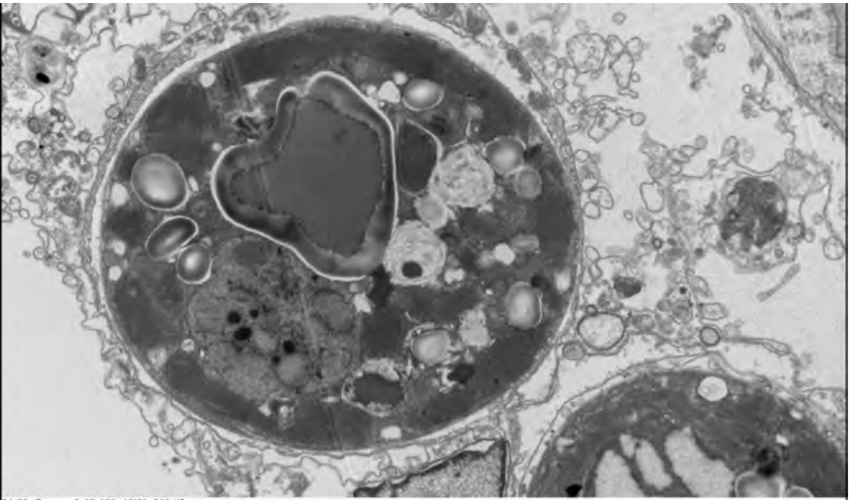

21-20_Correa_3-18-135_16K1_019.tif
Sample: 3/18, 135
Biological Electron Microscopy Lab
Rice University - SEA
Microscopist: MD Meyer

2 µm
HV=80kV
Direct Mag: 2000 x

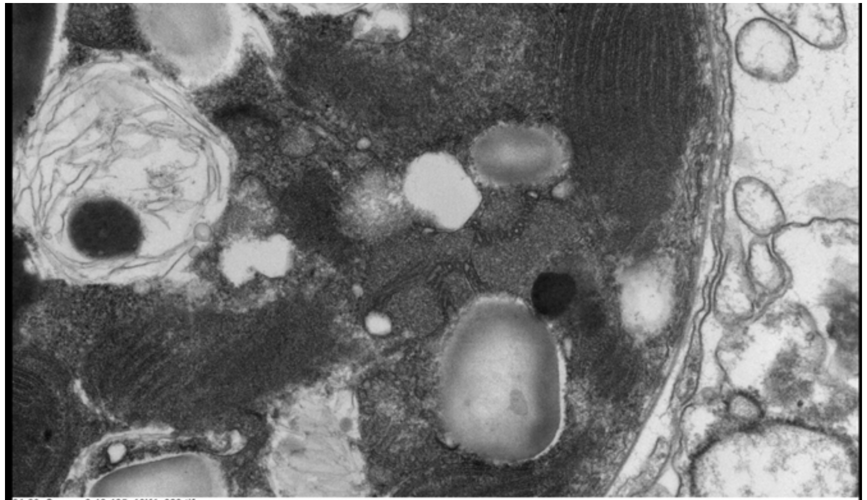

21-20_Correa_3-18-135_16K1_023.tif
Sample: 3/18, 135
Biological Electron Microscopy Lab
Rice University - SEA
Microscopist: MD Meyer

500 nm
HV=80kV
Direct Mag: 8000 x

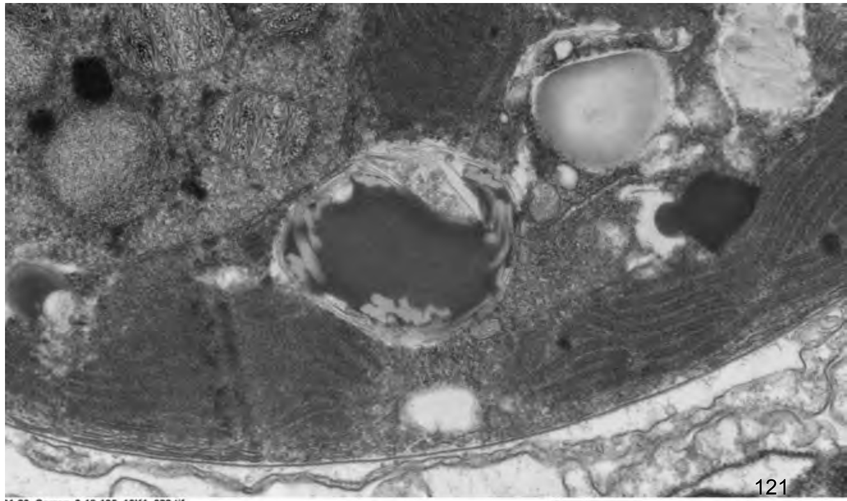

21-20_Correa_3-18-135_16K1_022.tif
Sample: 3/18, 135
Biological Electron Microscopy Lab
Rice University - SEA
Microscopist: MD Meyer

500 nm
HV=80kV
Direct Mag: 8000 x

Cell 6

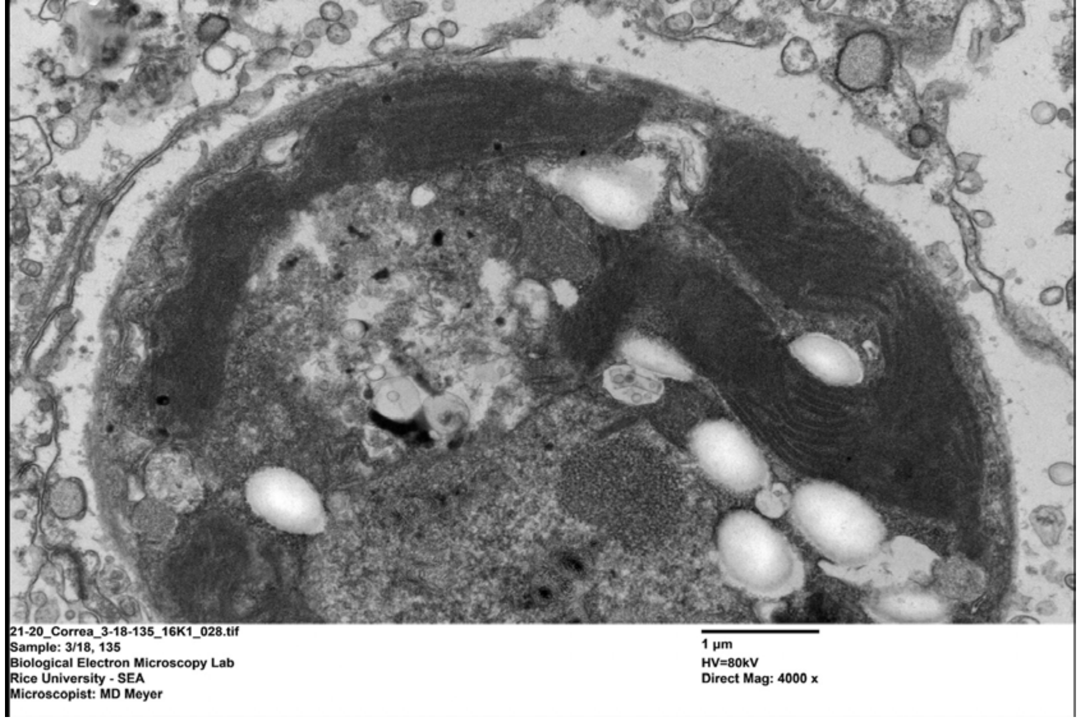
21-20_Correa_3-18-135_16K1_028.tif
Sample: 3/18, 135
Biological Electron Microscopy Lab
Rice University - SEA
Microscopist: MD Meyer
1 µm
HV=80kV
Direct Mag: 4000 x

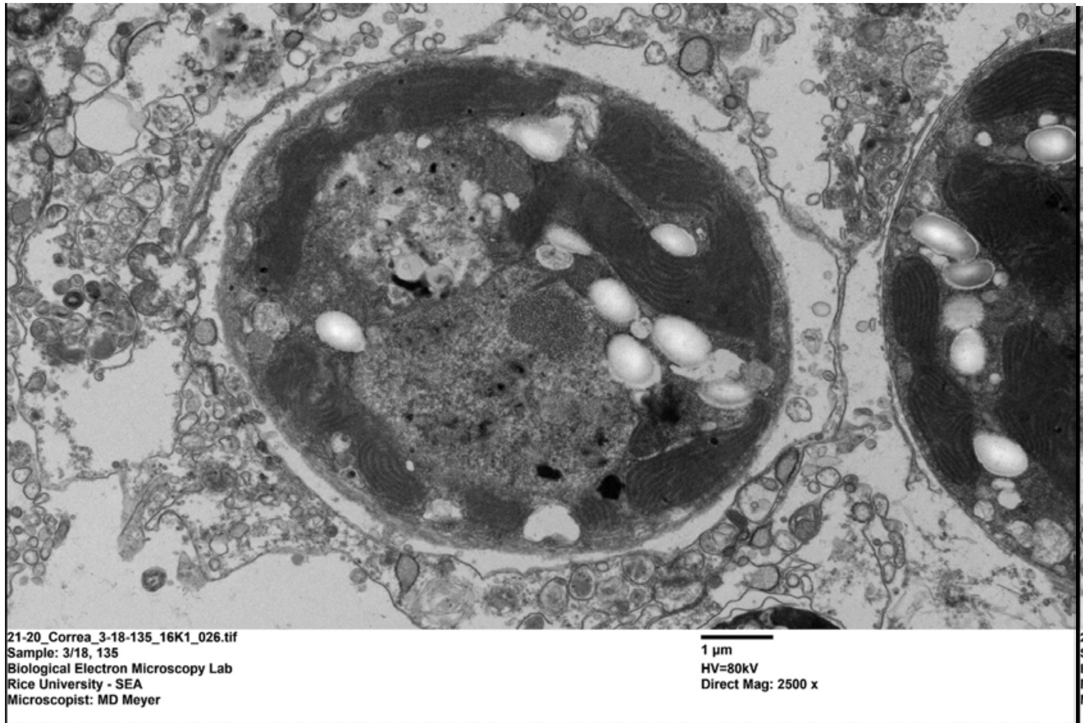
21-20_Correa_3-18-135_16K1_026.tif
Sample: 3/18, 135
Biological Electron Microscopy Lab
Rice University - SEA
Microscopist: MD Meyer
1 µm
HV=80kV
Direct Mag: 2500 x

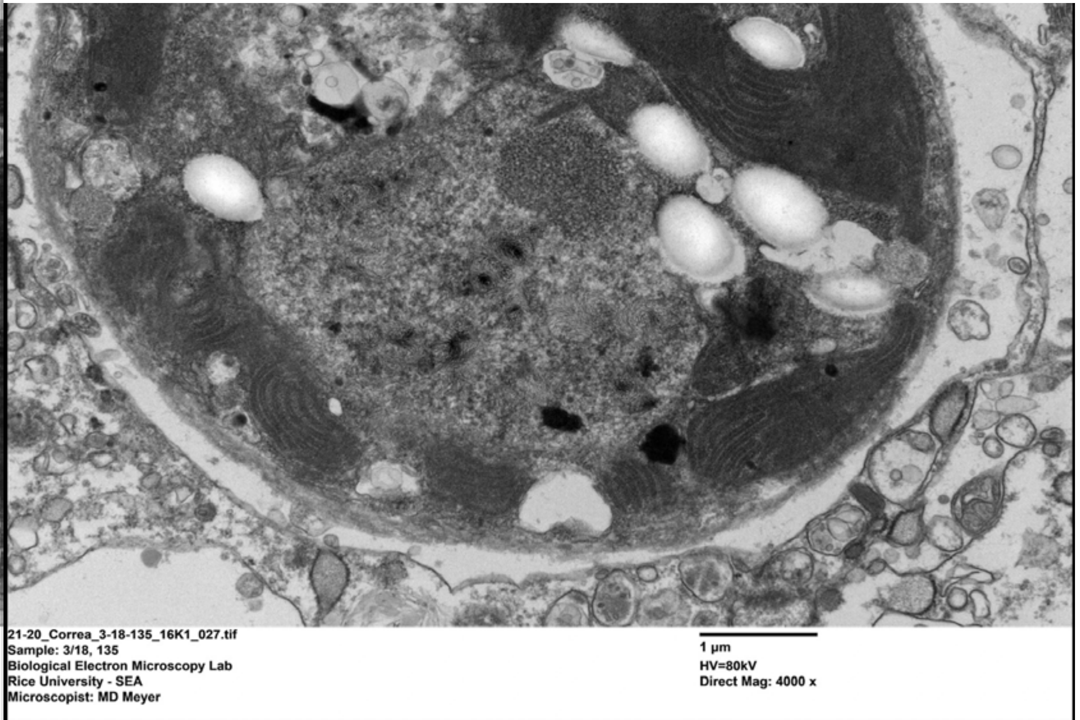
21-20_Correa_3-18-135_16K1_027.tif
Sample: 3/18, 135
Biological Electron Microscopy Lab
Rice University - SEA
Microscopist: MD Meyer
1 µm
HV=80kV
Direct Mag: 4000 x

Cell 7

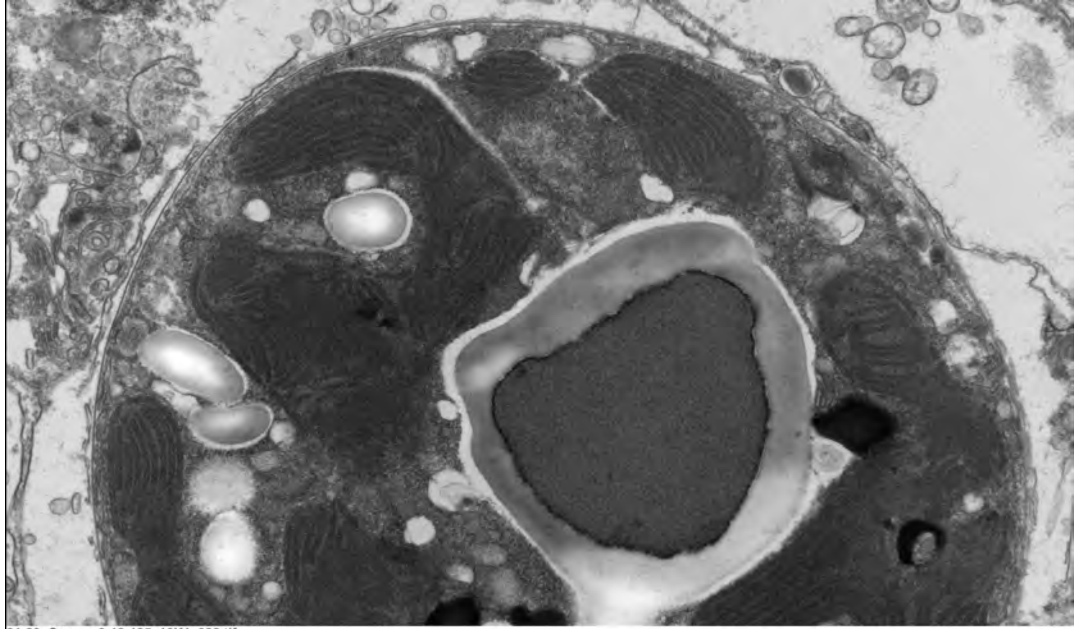

21-20_Correa_3-18-135_16K1_032.tif
Sample: 3/18, 135
Biological Electron Microscopy Lab
Rice University - SEA
Microscopist: MD Meyer

1 µm
HV=80kV
Direct Mag: 4000 x

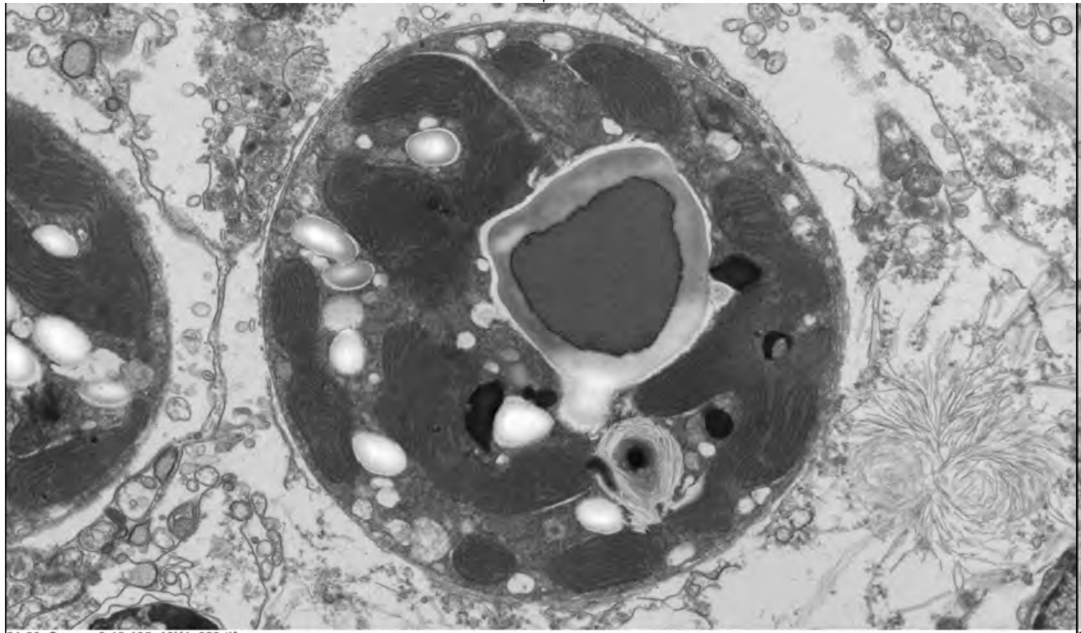

21-20_Correa_3-18-135_16K1_029.tif
Sample: 3/18, 135
Biological Electron Microscopy Lab
Rice University - SEA
Microscopist: MD Meyer

1 µm
HV=80kV
Direct Mag: 2500 x

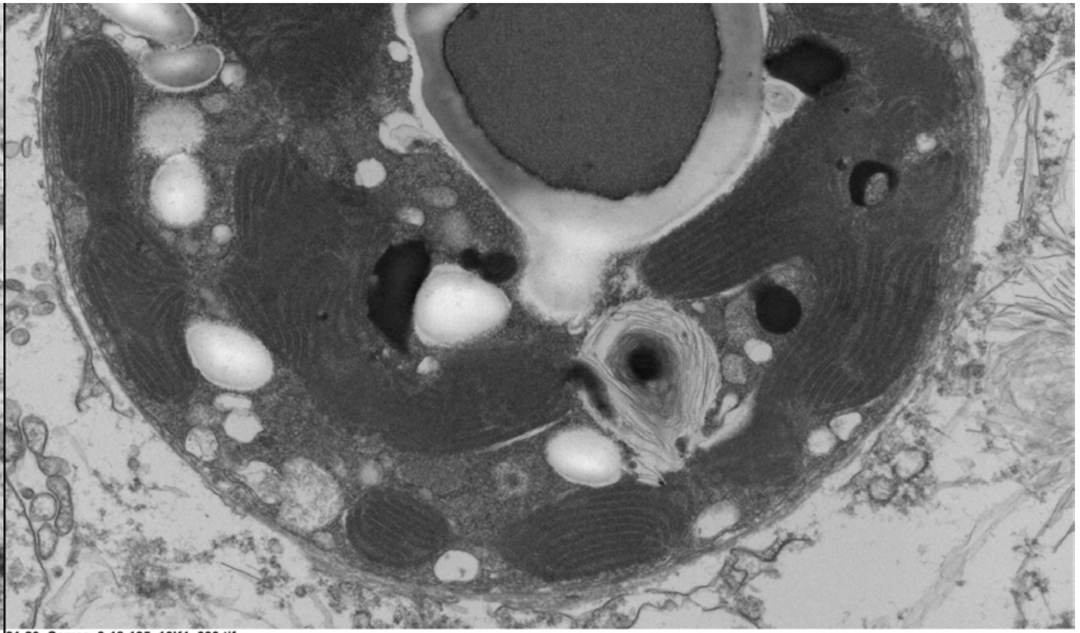

21-20_Correa_3-18-135_16K1_030.tif
Sample: 3/18, 135
Biological Electron Microscopy Lab
Rice University - SEA
Microscopist: MD Meyer

1 µm
HV=80kV
Direct Mag: 4000 x

Cell 8

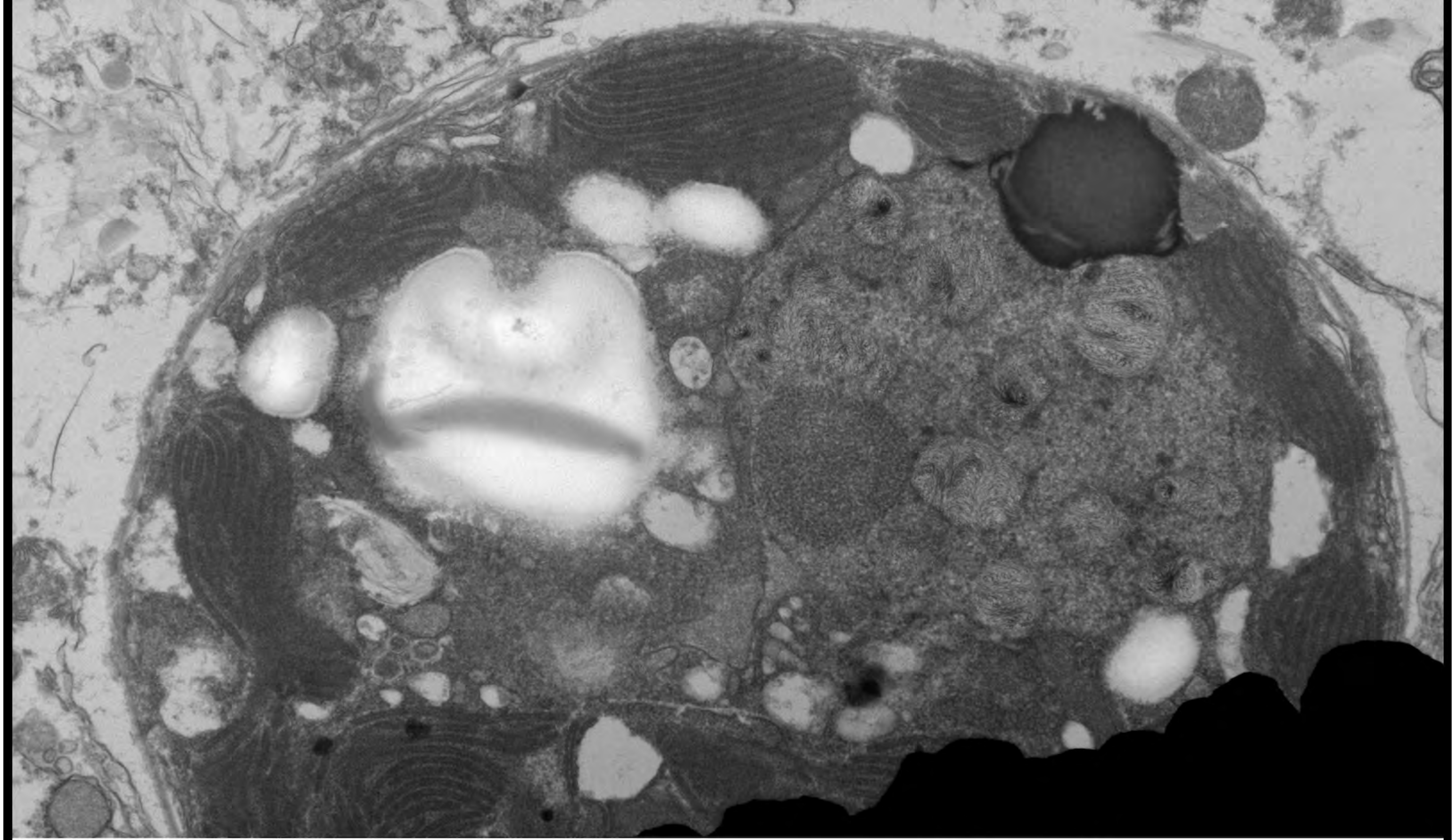

21-20_Correa_3-18-135_16K1_033.tif
Sample: 3/18, 135
Biological Electron Microscopy Lab
Rice University - SEA
Microscopist: MD Meyer

1 µm
HV=80kV
Direct Mag: 4000 x

Cell 9

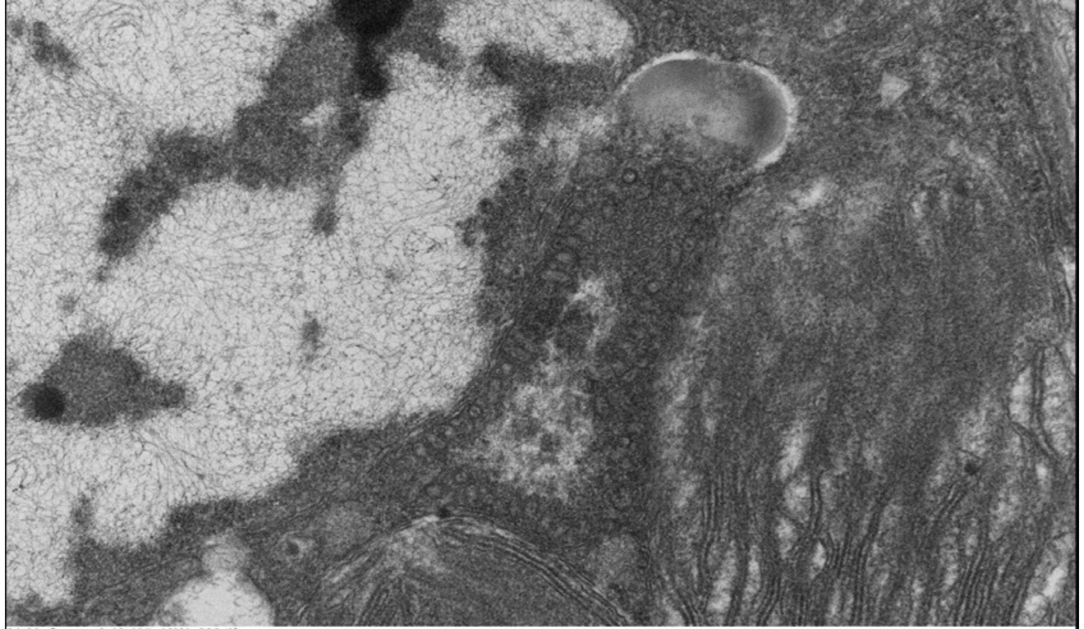

21-20_Correa_3-18-135_16K1_036.tif
Sample: 3/18, 135
Biological Electron Microscopy Lab
Rice University - SEA
Microscopist: MD Meyer

400 nm
HV=80kV
Direct Mag: 10000 x

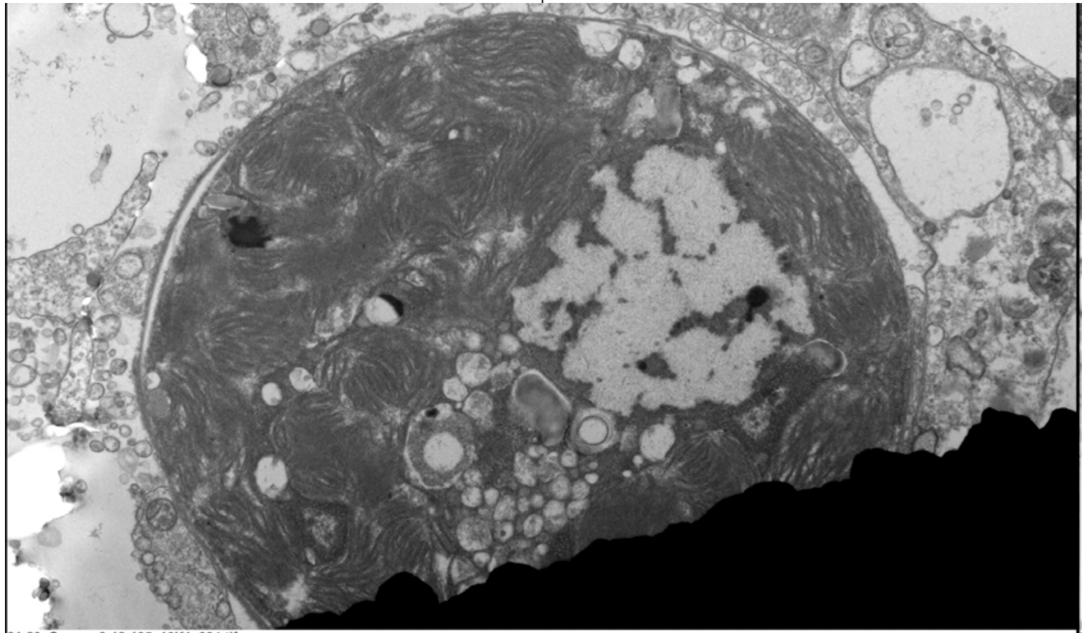

21-20_Correa_3-18-135_16K1_034.tif
Sample: 3/18, 135
Biological Electron Microscopy Lab
Rice University - SEA
Microscopist: MD Meyer

1 µm
HV=80kV
Direct Mag: 2500 x

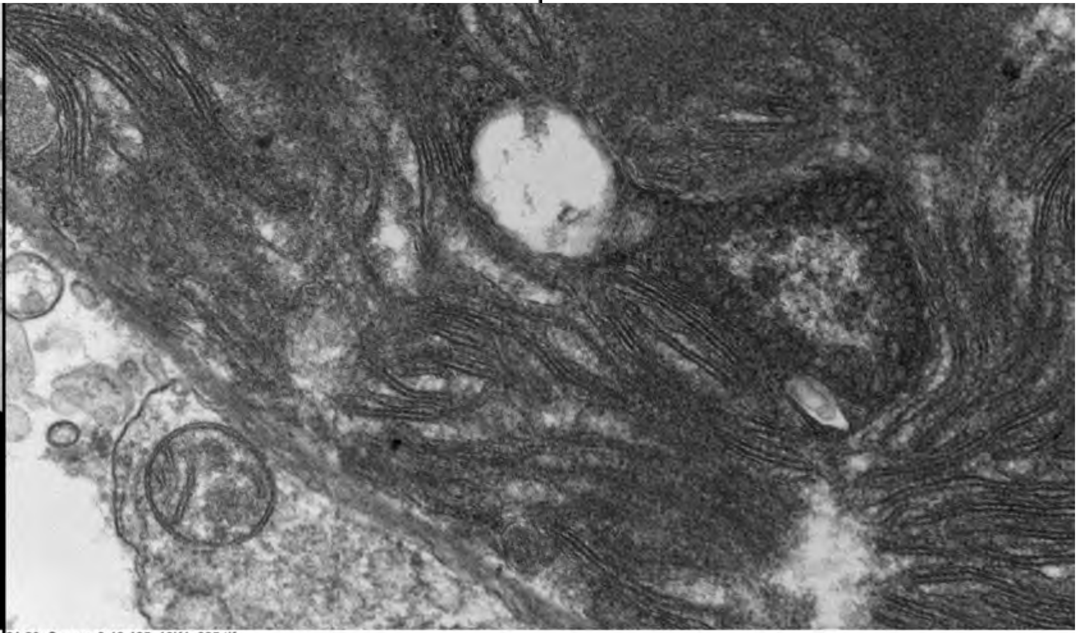

21-20_Correa_3-18-135_16K1_035.tif
Sample: 3/18, 135
Biological Electron Microscopy Lab
Rice University - SEA
Microscopist: MD Meyer

400 nm
HV=80kV
Direct Mag: 10000 x

Cell 10

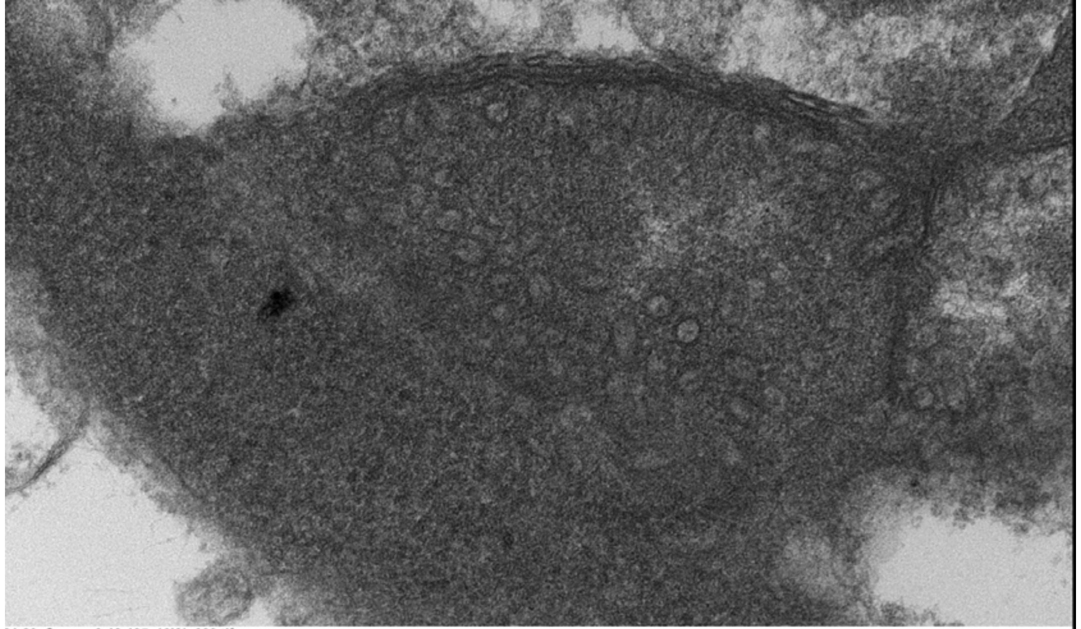

21-20_Correa_3-18-135_16K1_039.tif
Sample: 3/18, 135
Biological Electron Microscopy Lab
Rice University - SEA
Microscopist: MD Meyer

200 nm
HV=80kV
Direct Mag: 20000 x

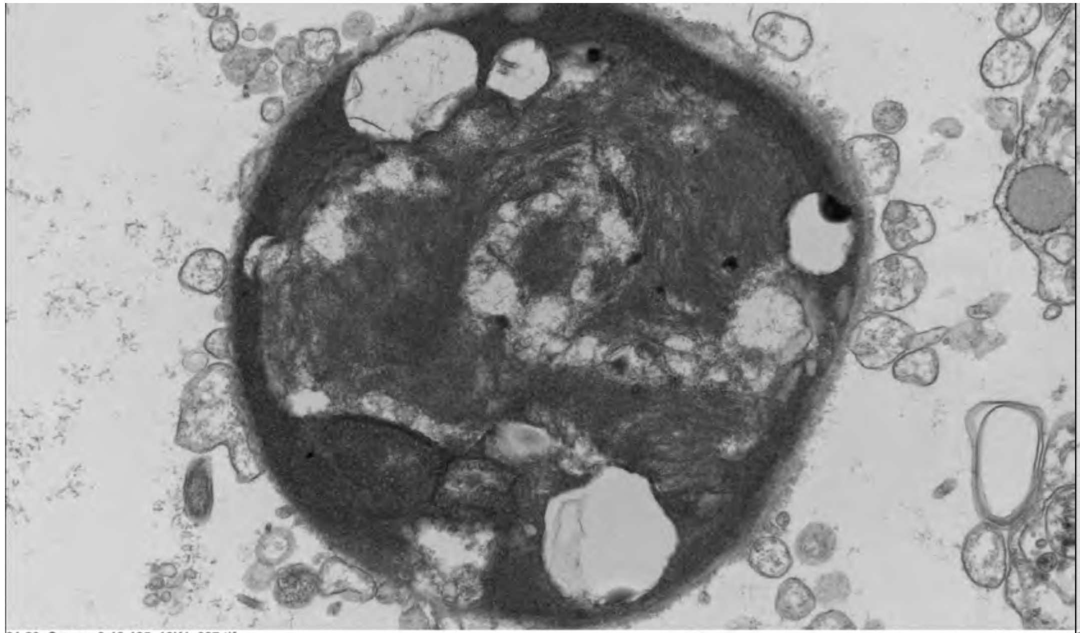

21-20_Correa_3-18-135_16K1_037.tif
Sample: 3/18, 135
Biological Electron Microscopy Lab
Rice University - SEA
Microscopist: MD Meyer

1 µm
HV=80kV
Direct Mag: 4000 x

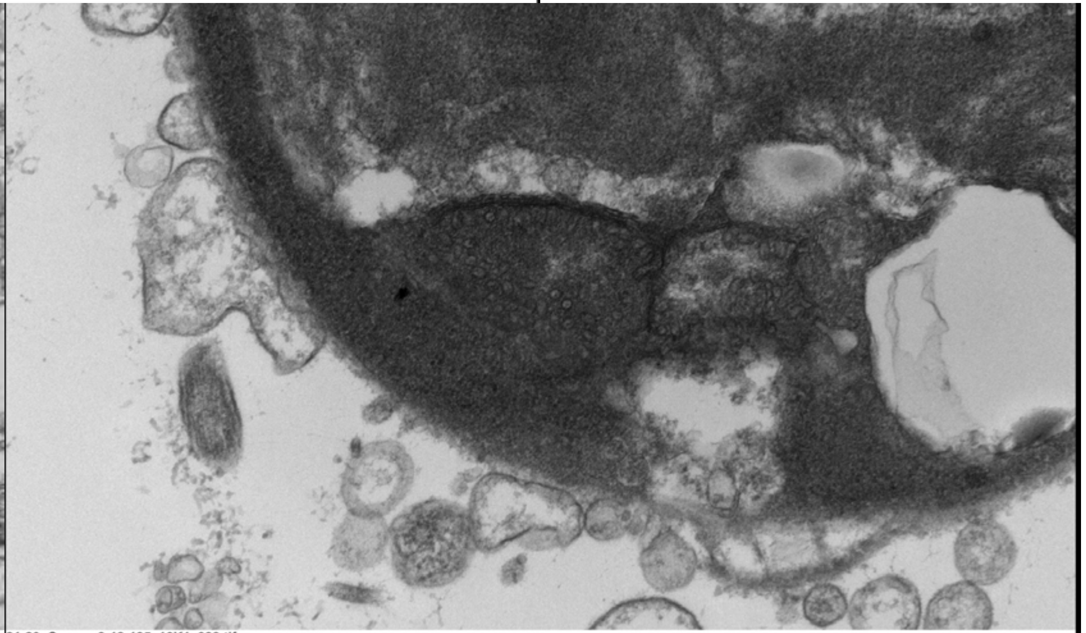

21-20_Correa_3-18-135_16K1_038.tif
Sample: 3/18, 135
Biological Electron Microscopy Lab
Rice University - SEA
Microscopist: MD Meyer

500 nm
HV=80kV
Direct Mag: 8000 x

Cell 11

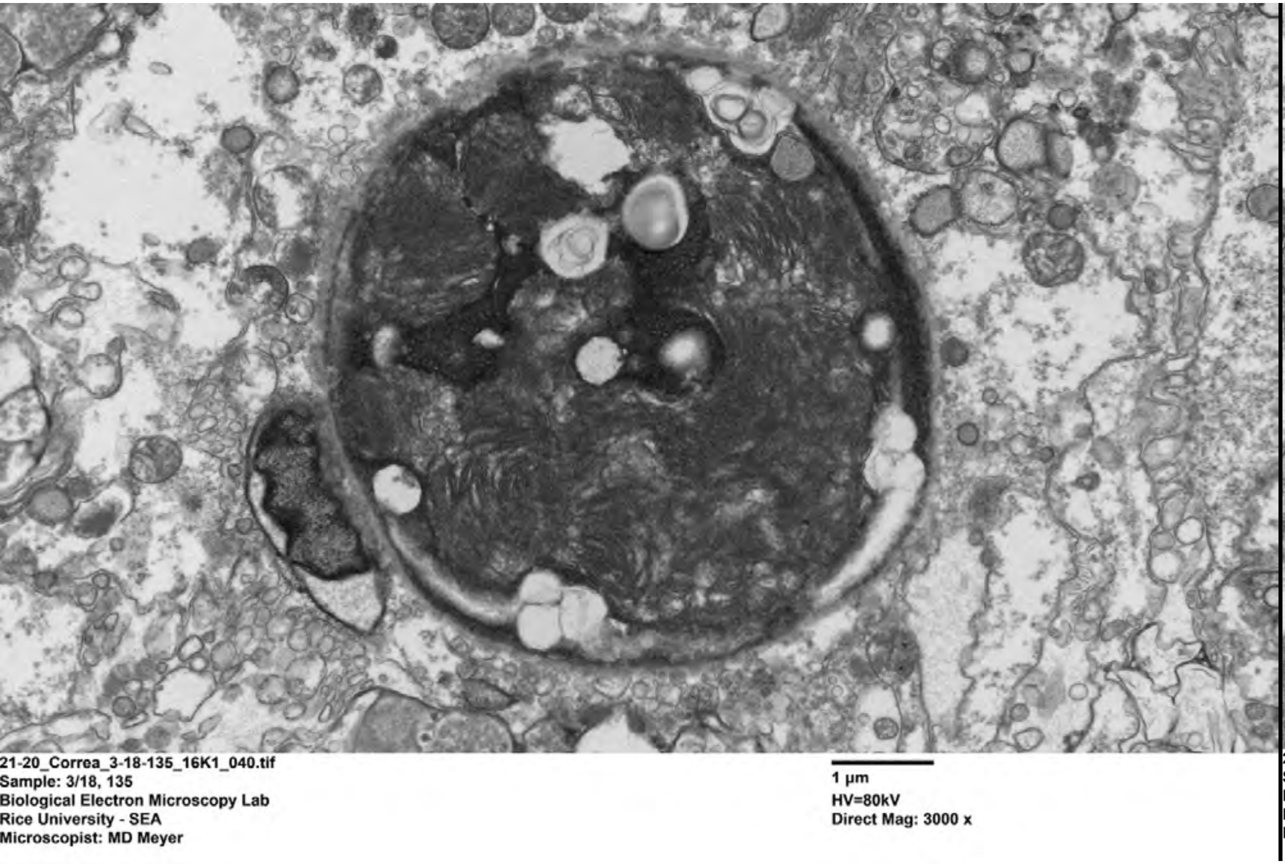
21-20_Correa_3-18-135_16K1_040.tif
Sample: 3/18, 135
Biological Electron Microscopy Lab
Rice University - SEA
Microscopist: MD Meyer
1 µm
HV=80kV
Direct Mag: 3000 x

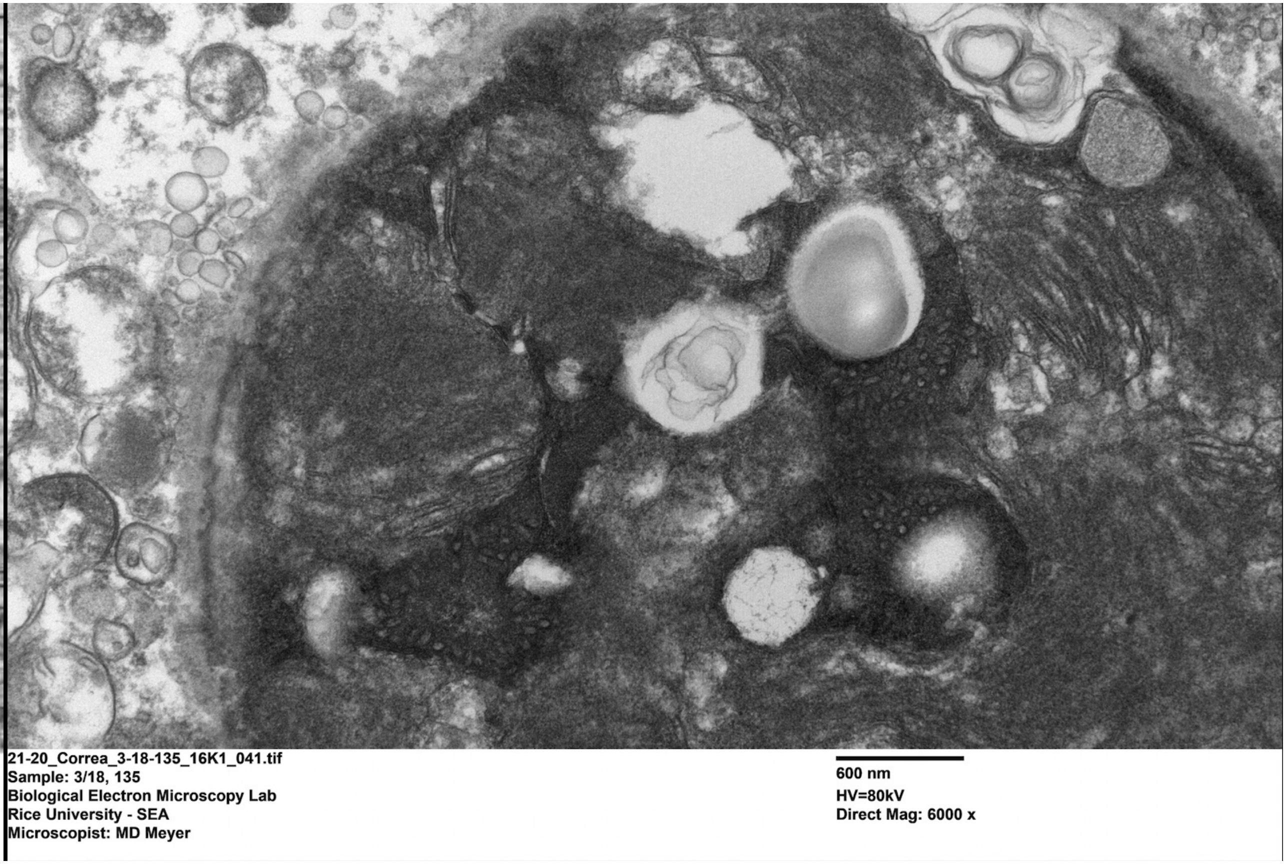
21-20_Correa_3-18-135_16K1_041.tif
Sample: 3/18, 135
Biological Electron Microscopy Lab
Rice University - SEA
Microscopist: MD Meyer
600 nm
HV=80kV
Direct Mag: 6000 x

Cell 12

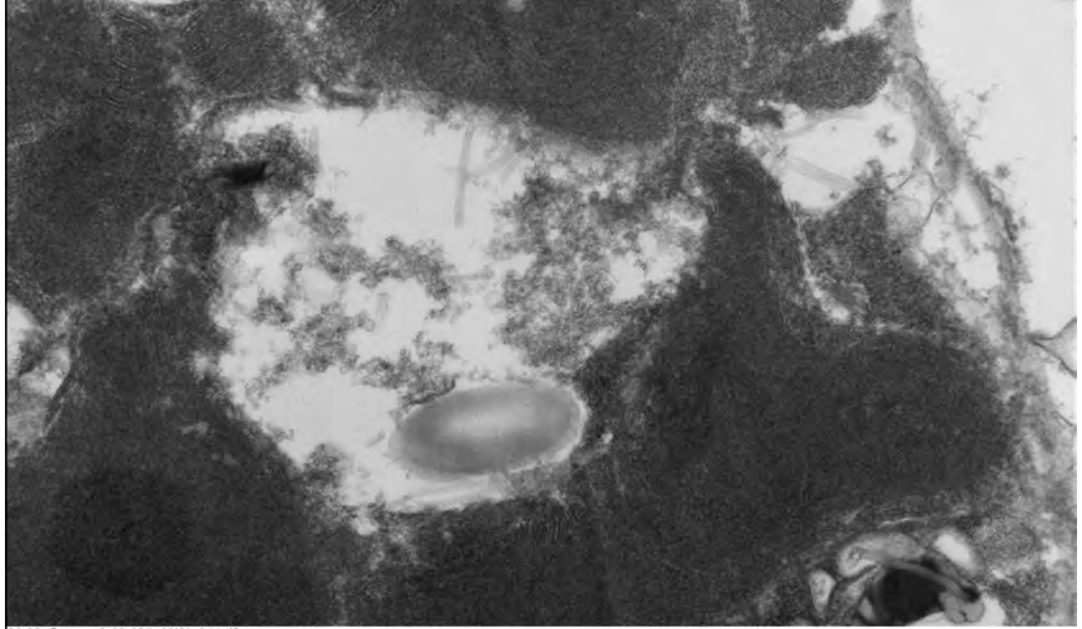

21-20_Correa_3-18-135_16K1_044.tif
Sample: 3/18, 135
Biological Electron Microscopy Lab
Rice University - SEA
Microscopist: MD Meyer

500 nm
HV=80kV
Direct Mag: 8000 x

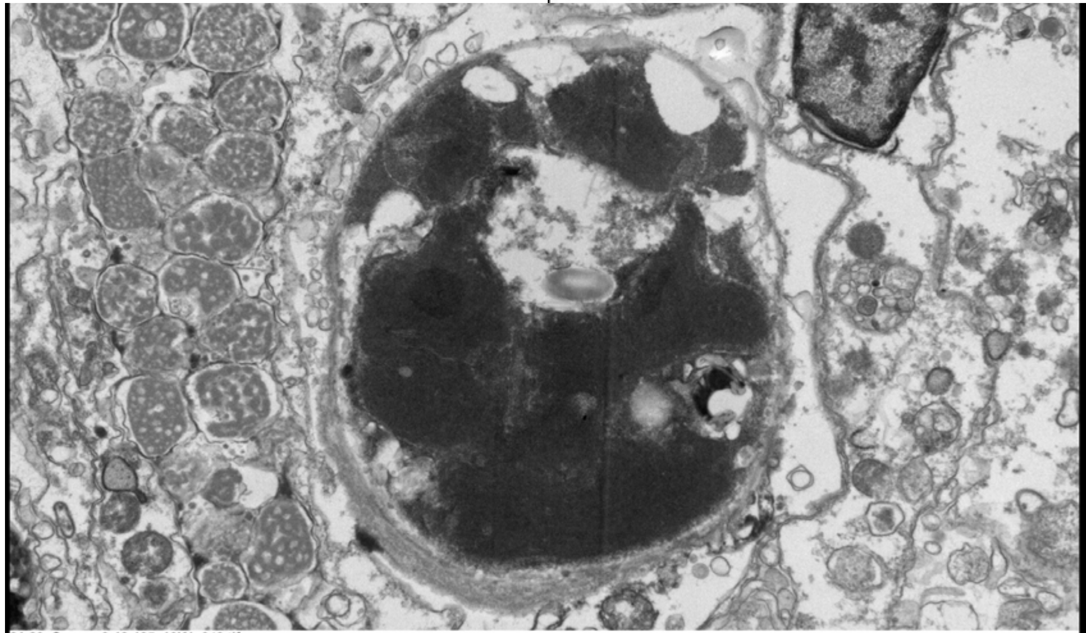

21-20_Correa_3-18-135_16K1_042.tif
Sample: 3/18, 135
Biological Electron Microscopy Lab
Rice University - SEA
Microscopist: MD Meyer

1 µm
HV=80kV
Direct Mag: 3000 x

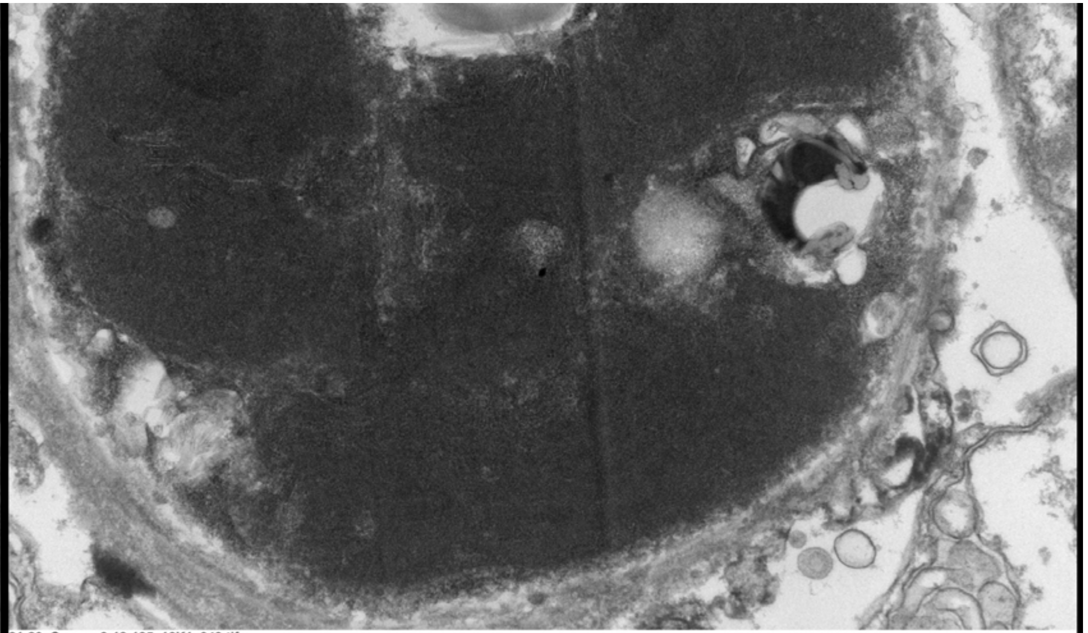

21-20_Correa_3-18-135_16K1_043.tif
Sample: 3/18, 135
Biological Electron Microscopy Lab
Rice University - SEA
Microscopist: MD Meyer

600 nm
HV=80kV
Direct Mag: 6000 x

Cell 13

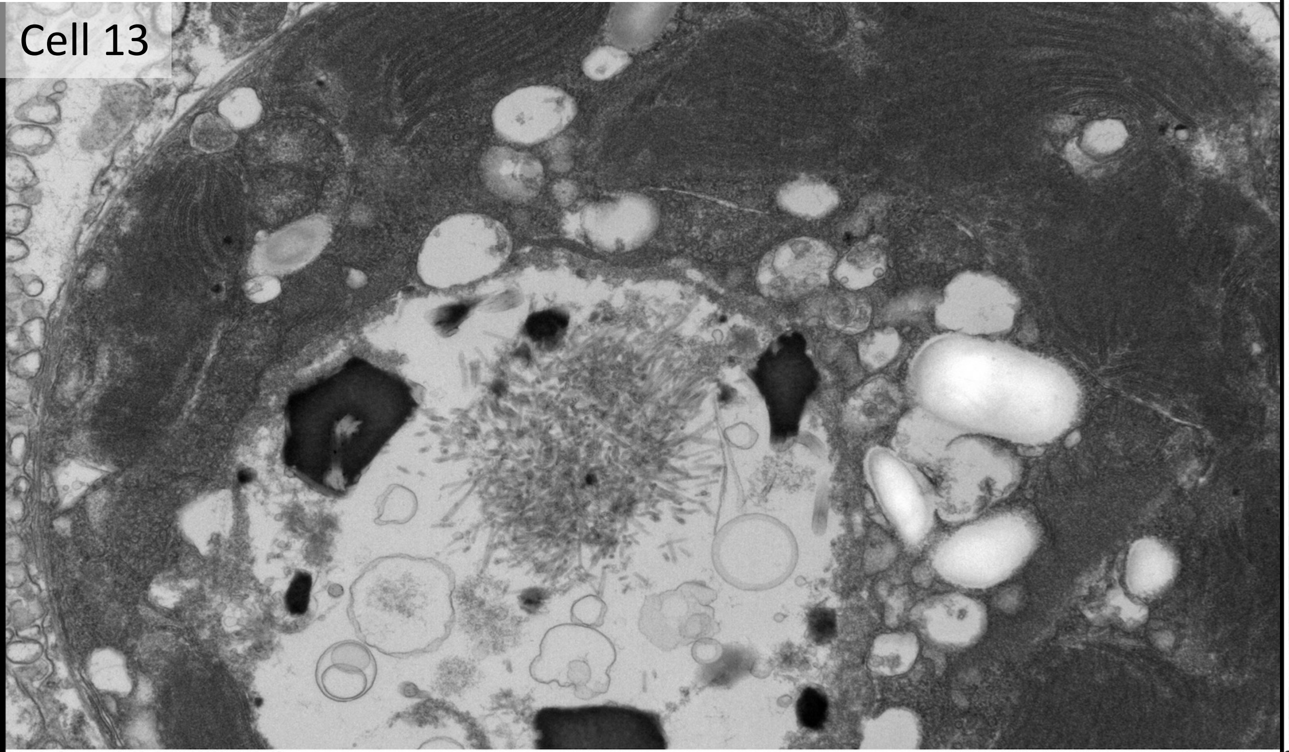

21-20_Correa_3-18-135_16K1_046.tif
Sample: 3/18, 135
Biological Electron Microscopy Lab
Rice University - SEA
Microscopist: MD Meyer

1 µm
HV=80kV
Direct Mag: 4000 x

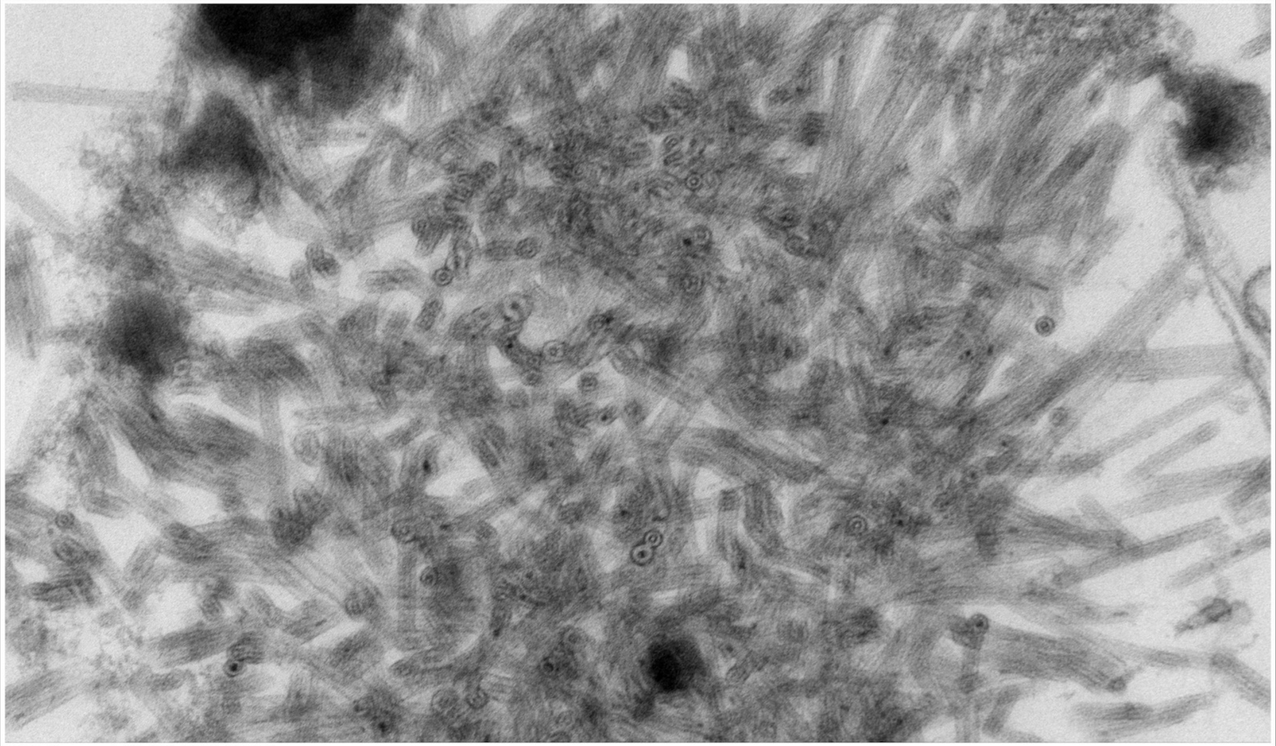

21-20_Correa_3-18-135_16K1_048.tif
Sample: 3/18, 135
Biological Electron Microscopy Lab
Rice University - SEA
Microscopist: MD Meyer

200 nm
HV=80kV
Direct Mag: 20000 x

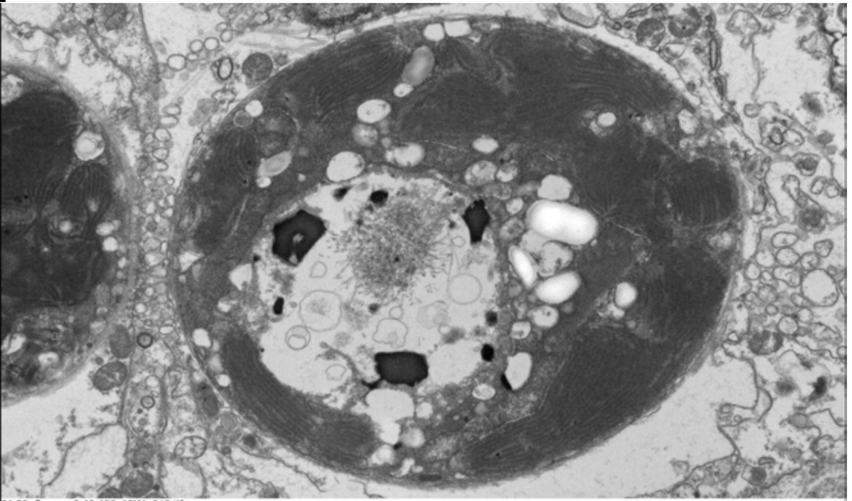

21-20_Correa_3-18-135_16K1_045.tif
Sample: 3/18, 135
Biological Electron Microscopy Lab
Rice University - SEA
Microscopist: MD Meyer

1 µm
HV=80kV
Direct Mag: 2500 x

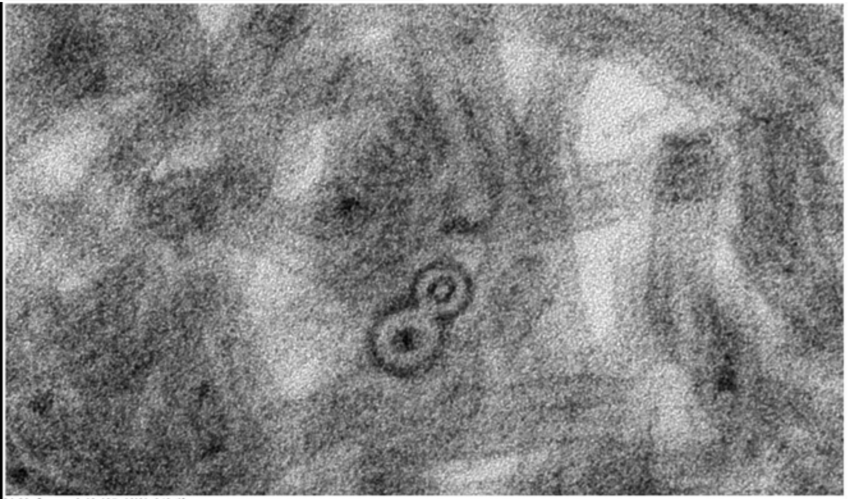

21-20_Correa_3-18-135_16K1_049.tif
Sample: 3/18, 135
Biological Electron Microscopy Lab
Rice University - SEA
Microscopist: MD Meyer

20 nm
HV=80kV
Direct Mag: 100000 x

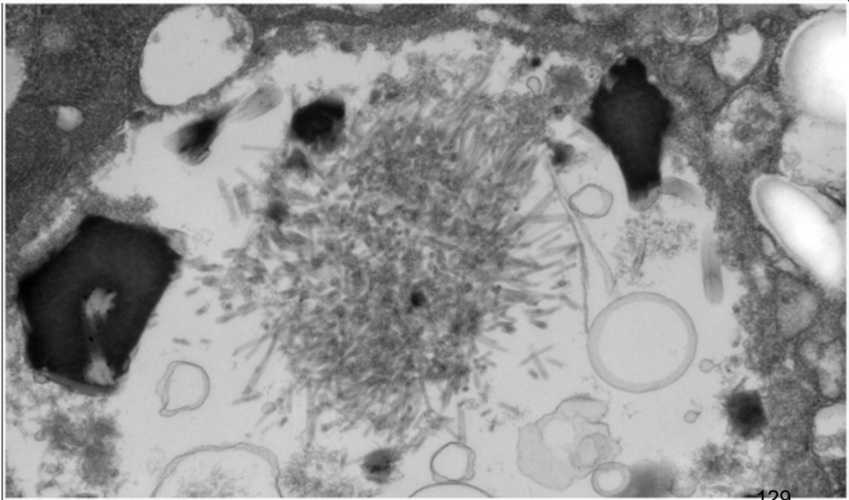

21-20_Correa_3-18-135_16K1_047.tif
Sample: 3/18, 135
Biological Electron Microscopy Lab
Rice University - SEA
Microscopist: MD Meyer

500 nm
HV=80kV
Direct Mag: 8000 x

Cell 14

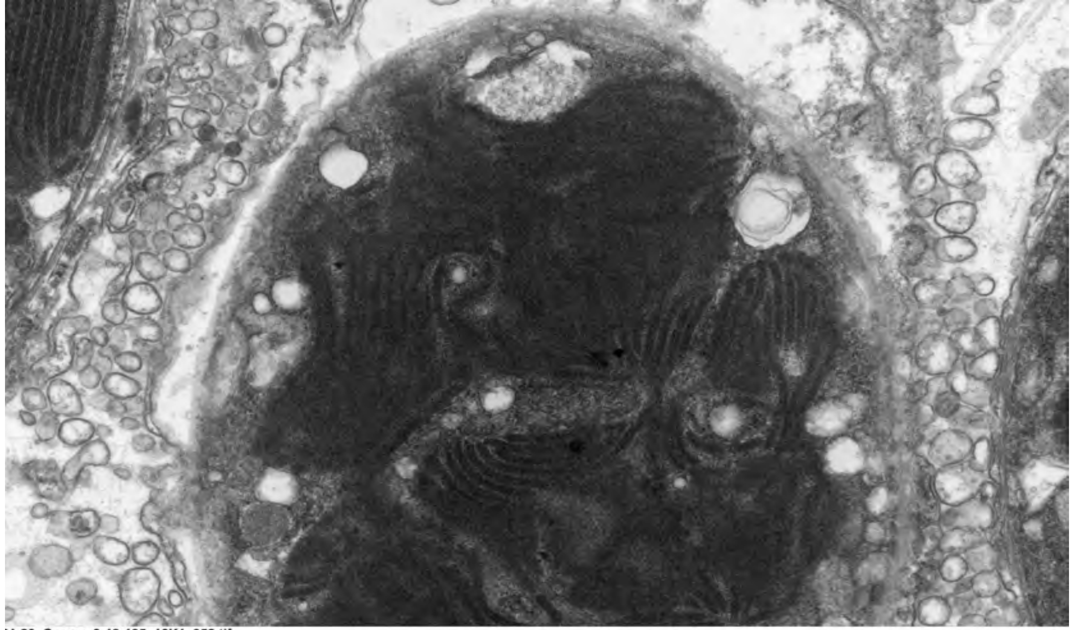

21-20_Correa_3-18-135_16K1_052.tif
Sample: 3/18, 135
Biological Electron Microscopy Lab
Rice University - SEA
Microscopist: MD Meyer

800 nm
HV=80kV
Direct Mag: 5000 x

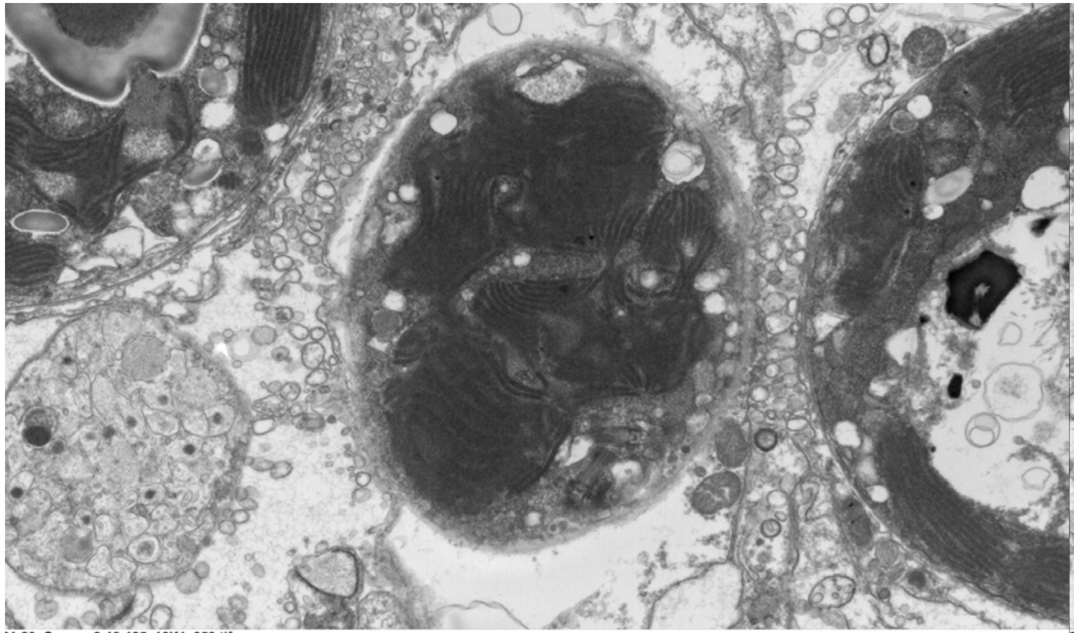

21-20_Correa_3-18-135_16K1_050.tif
Sample: 3/18, 135
Biological Electron Microscopy Lab
Rice University - SEA
Microscopist: MD Meyer

1 µm
HV=80kV
Direct Mag: 3000 x

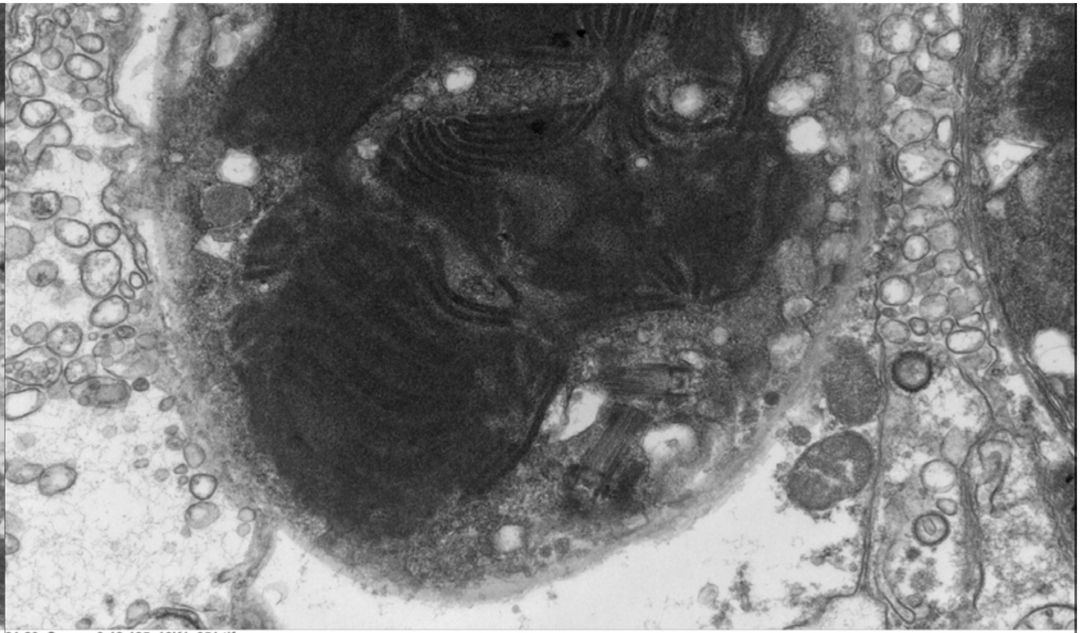

21-20_Correa_3-18-135_16K1_051.tif
Sample: 3/18, 135
Biological Electron Microscopy Lab
Rice University - SEA
Microscopist: MD Meyer

800 nm
HV=80kV
Direct Mag: 5000 x

Cell 15

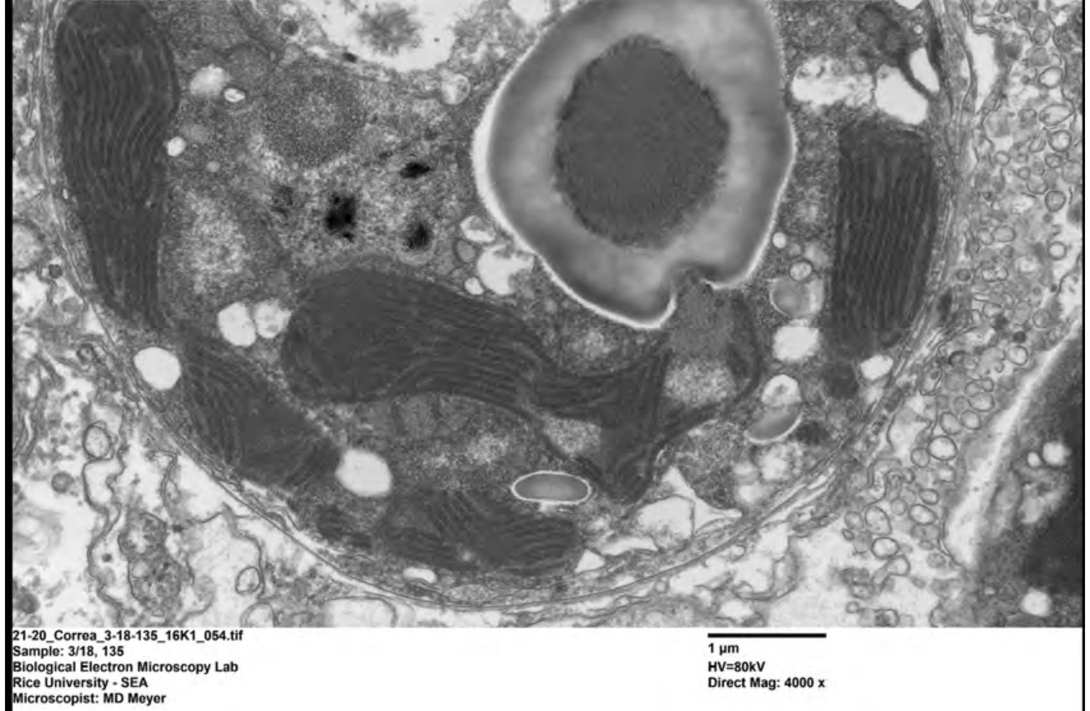
21-20_Correa_3-18-135_16K1_054.tif
Sample: 3/18, 135
Biological Electron Microscopy Lab
Rice University - SEA
Microscopist: MD Meyer
1 µm
HV=80kV
Direct Mag: 4000 x

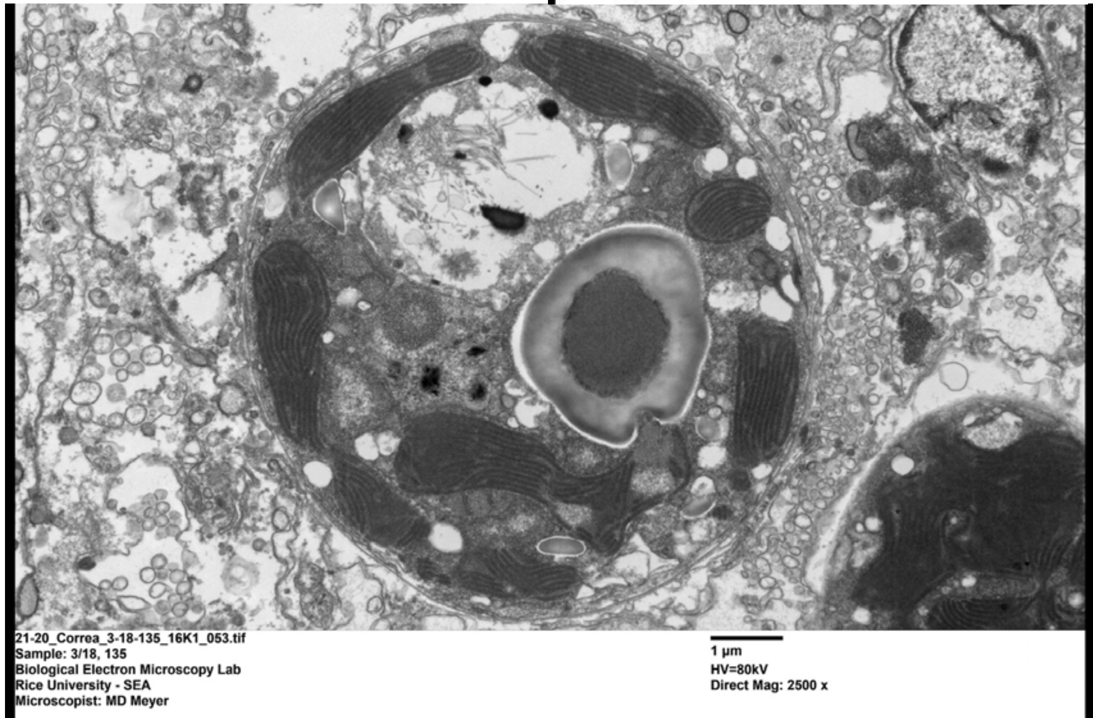
21-20_Correa_3-18-135_16K1_053.tif
Sample: 3/18, 135
Biological Electron Microscopy Lab
Rice University - SEA
Microscopist: MD Meyer
1 µm
HV=80kV
Direct Mag: 2500 x

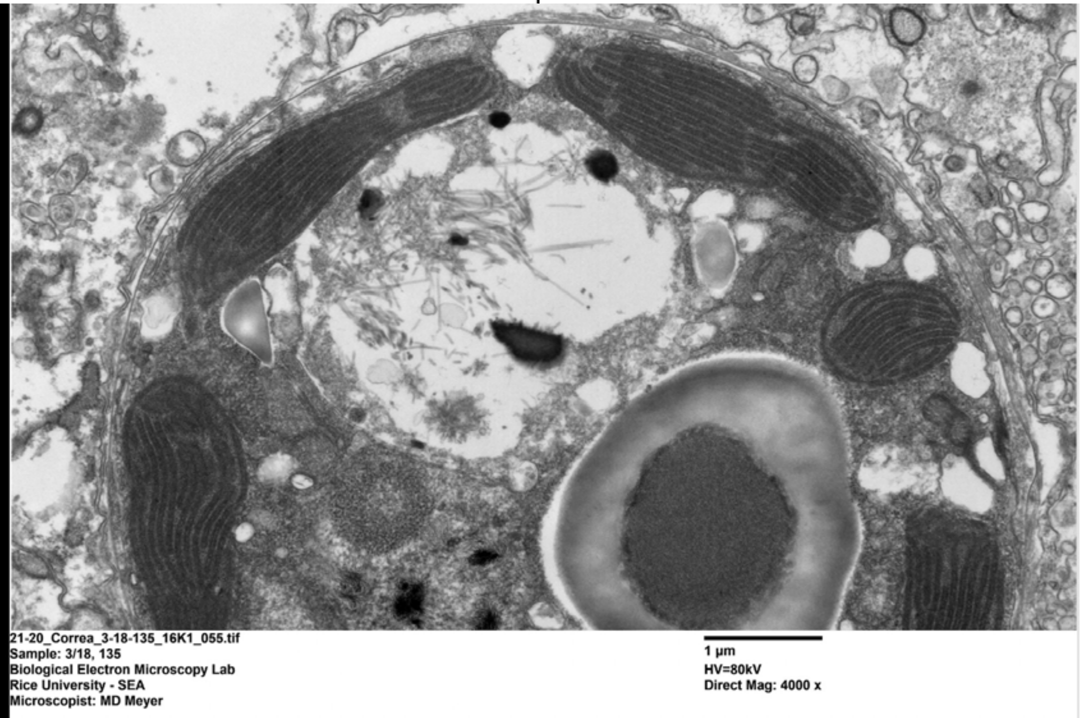
21-20_Correa_3-18-135_16K1_055.tif
Sample: 3/18, 135
Biological Electron Microscopy Lab
Rice University - SEA
Microscopist: MD Meyer
1 µm
HV=80kV
Direct Mag: 4000 x

Cell 16

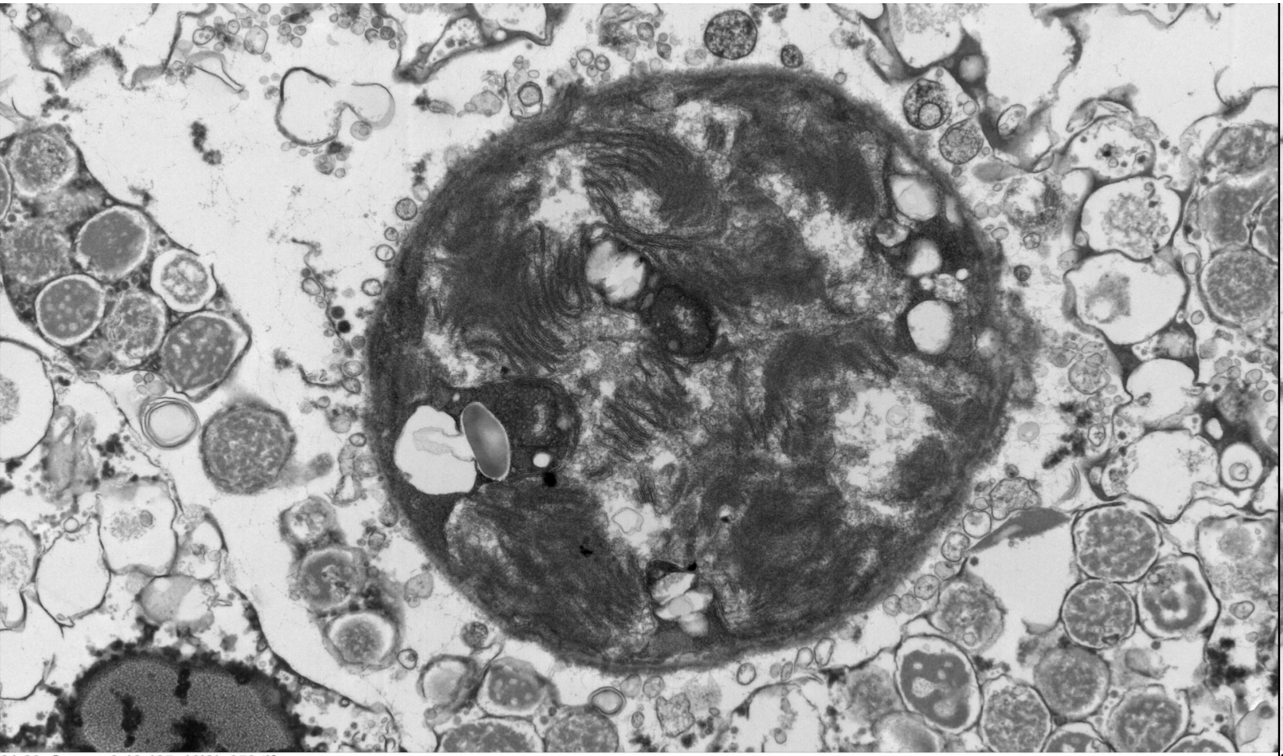

21-20_Correa_3-18-135_16K1_056.tif
Sample: 3/18, 135
Biological Electron Microscopy Lab
Rice University - SEA
Microscopist: MD Meyer

1 µm
HV=80kV
Direct Mag: 3000 x

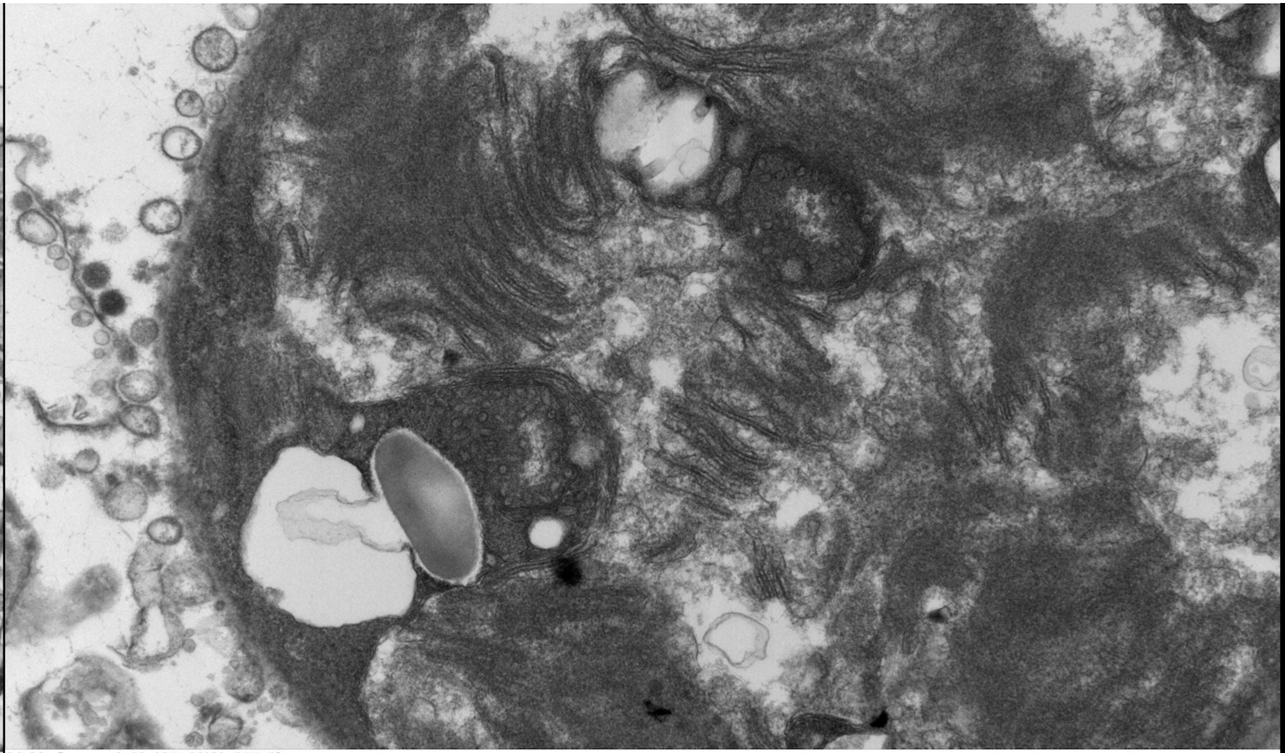

21-20_Correa_3-18-135_16K1_057.tif
Sample: 3/18, 135
Biological Electron Microscopy Lab
Rice University - SEA
Microscopist: MD Meyer

600 nm
HV=80kV
Direct Mag: 6000 x

Cell 17

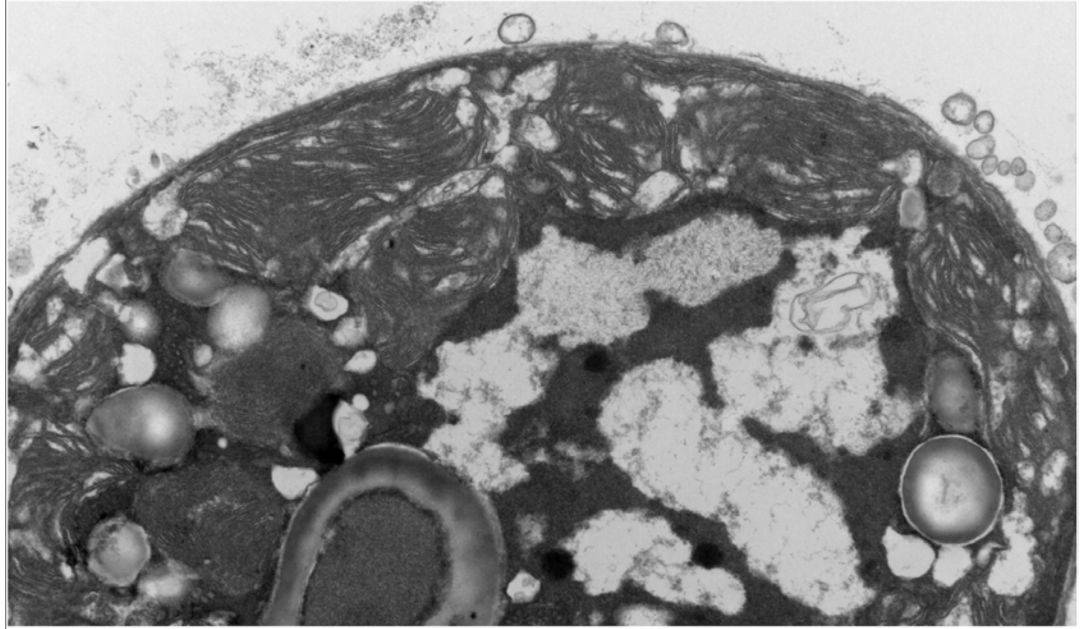

21-20_Correa_3-18-135_16K1_060.tif
Sample: 3/18, 135
Biological Electron Microscopy Lab
Rice University - SEA
Microscopist: MD Meyer

1 µm
HV=80kV
Direct Mag: 4000 x

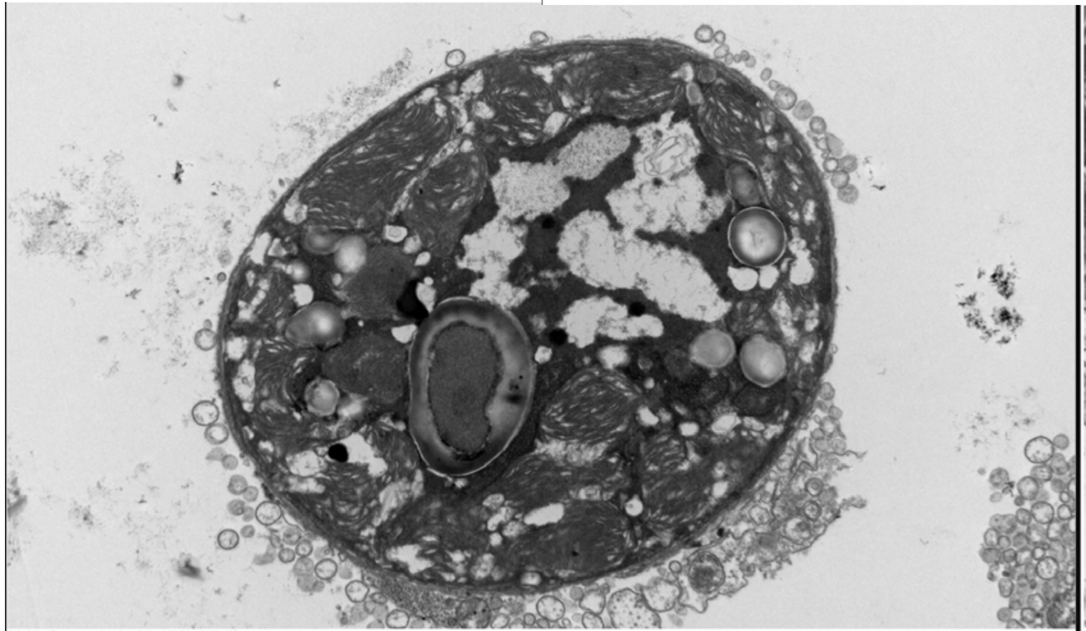

21-20_Correa_3-18-135_16K1_058.tif
Sample: 3/18, 135
Biological Electron Microscopy Lab
Rice University - SEA
Microscopist: MD Meyer

2 µm
HV=80kV
Direct Mag: 2000 x

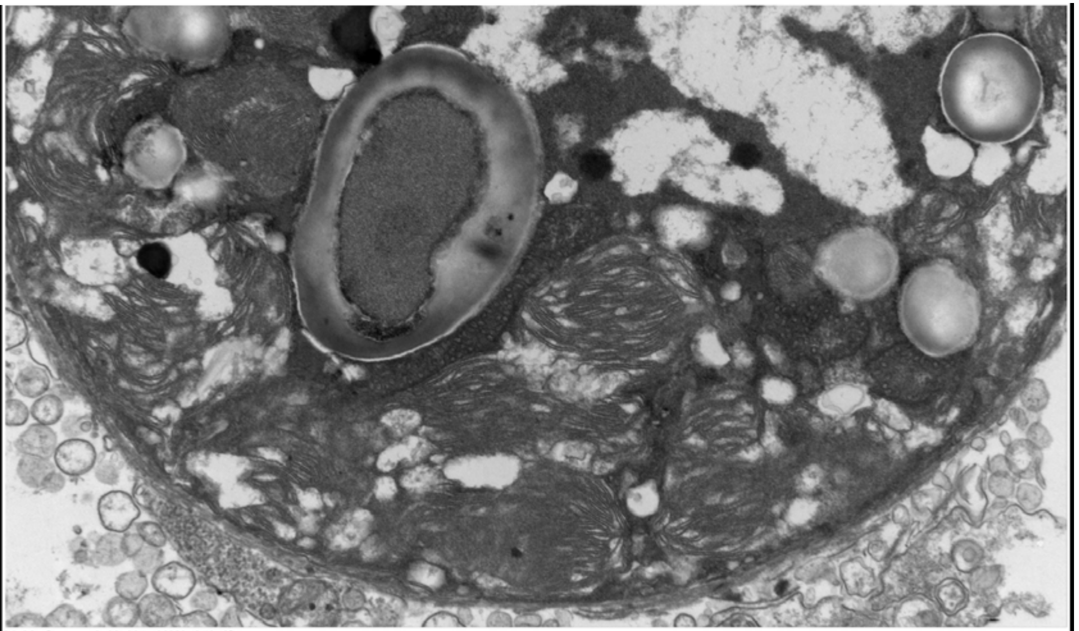

21-20_Correa_3-18-135_16K1_059.tif
Sample: 3/18, 135
Biological Electron Microscopy Lab
Rice University - SEA
Microscopist: MD Meyer

1 µm
HV=80kV
Direct Mag: 4000 x

Cell 18

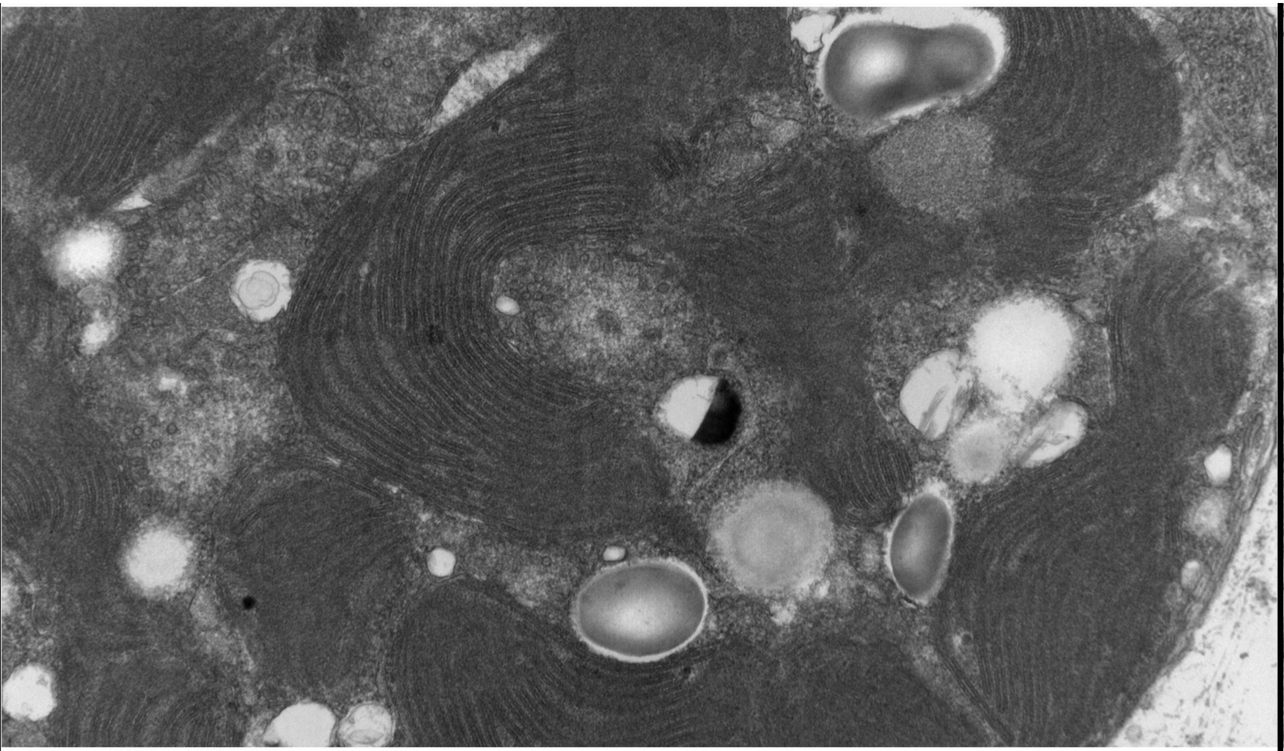

21-20_Correa_3-18-135_16K1_062.tif
Sample: 3/18, 135
Biological Electron Microscopy Lab
Rice University - SEA
Microscopist: MD Meyer

800 nm
HV=80kV
Direct Mag: 5000 x

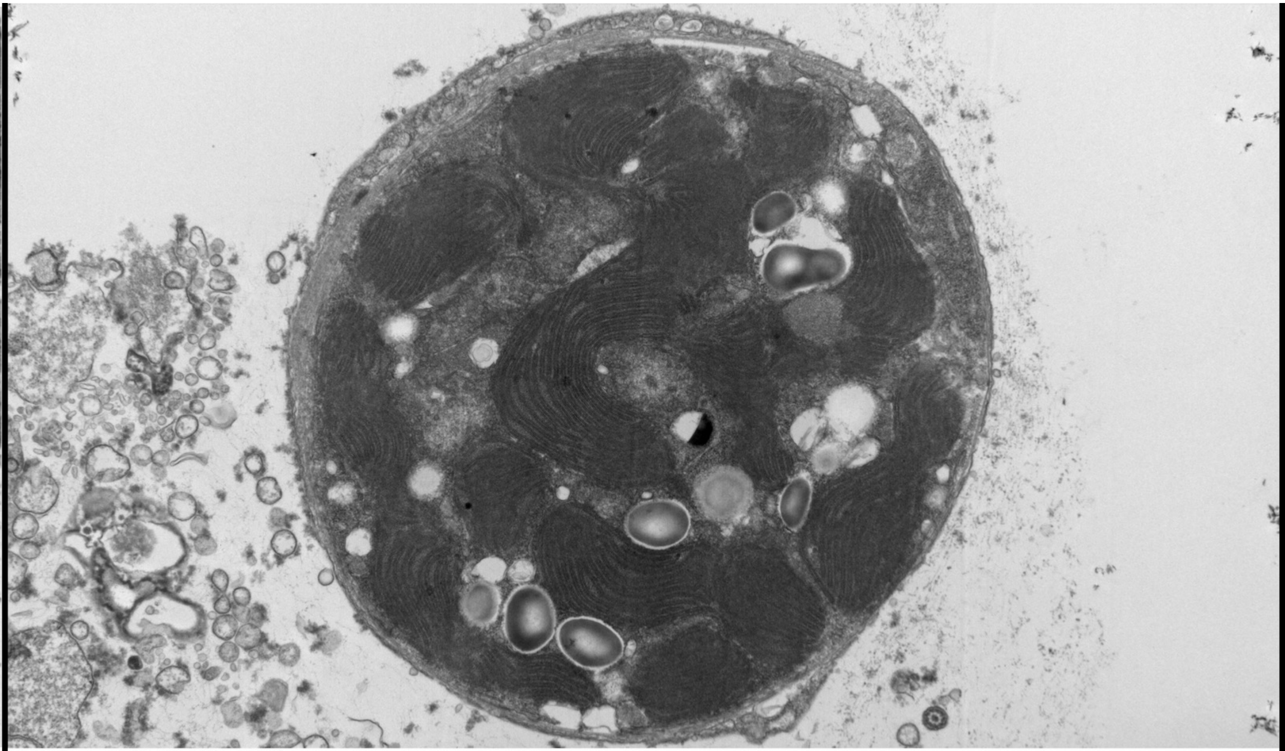

21-20_Correa_3-18-135_16K1_061.tif
Sample: 3/18, 135
Biological Electron Microscopy Lab
Rice University - SEA
Microscopist: MD Meyer

1 µm
HV=80kV
Direct Mag: 2500 x

Cell 19

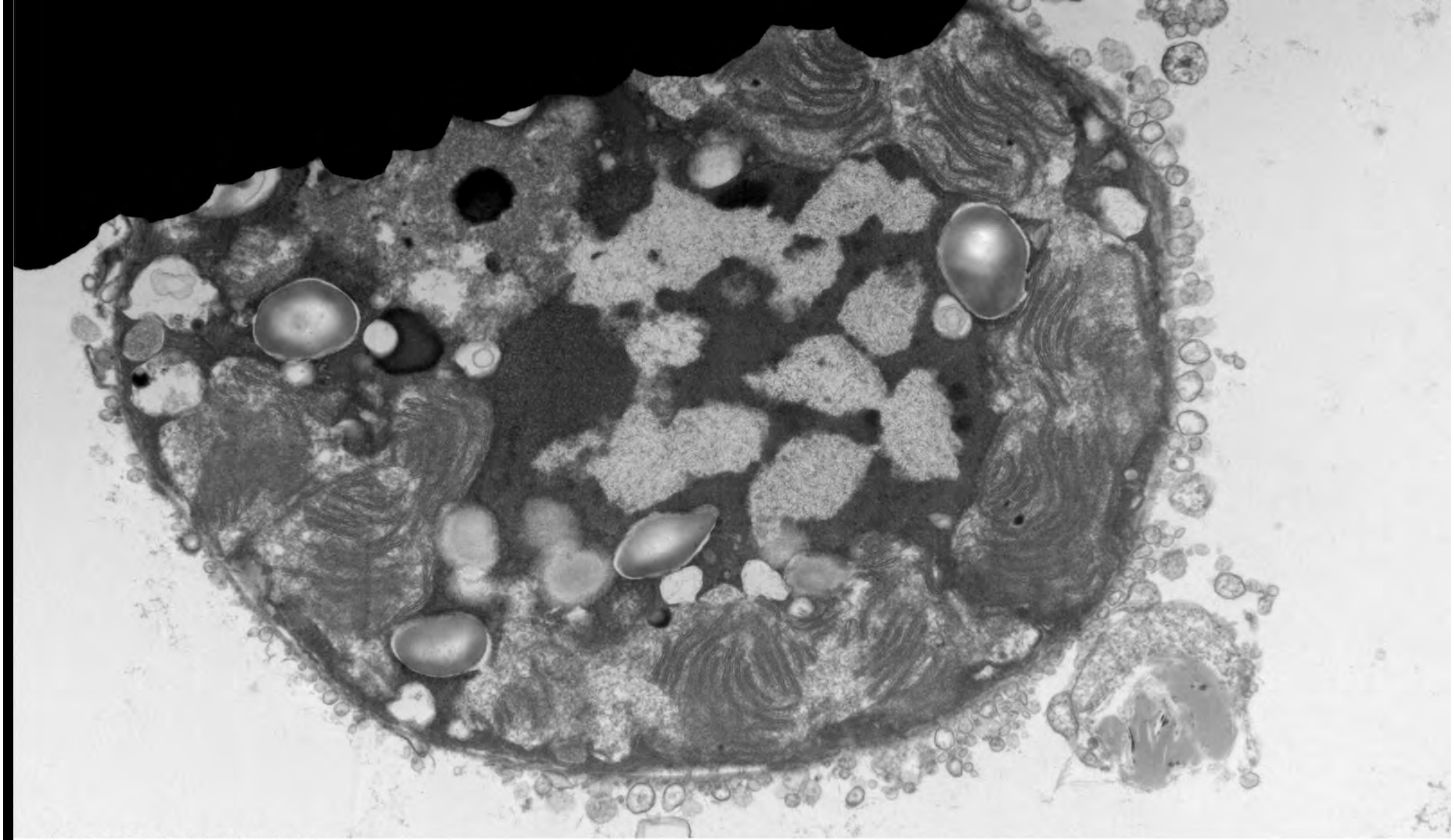

21-20_Correa_3-18-135_16K1_063.tif
Sample: 3/18, 135
Biological Electron Microscopy Lab
Rice University - SEA
Microscopist: MD Meyer

1 µm
HV=80kV
Direct Mag: 3000 x

Cell 20

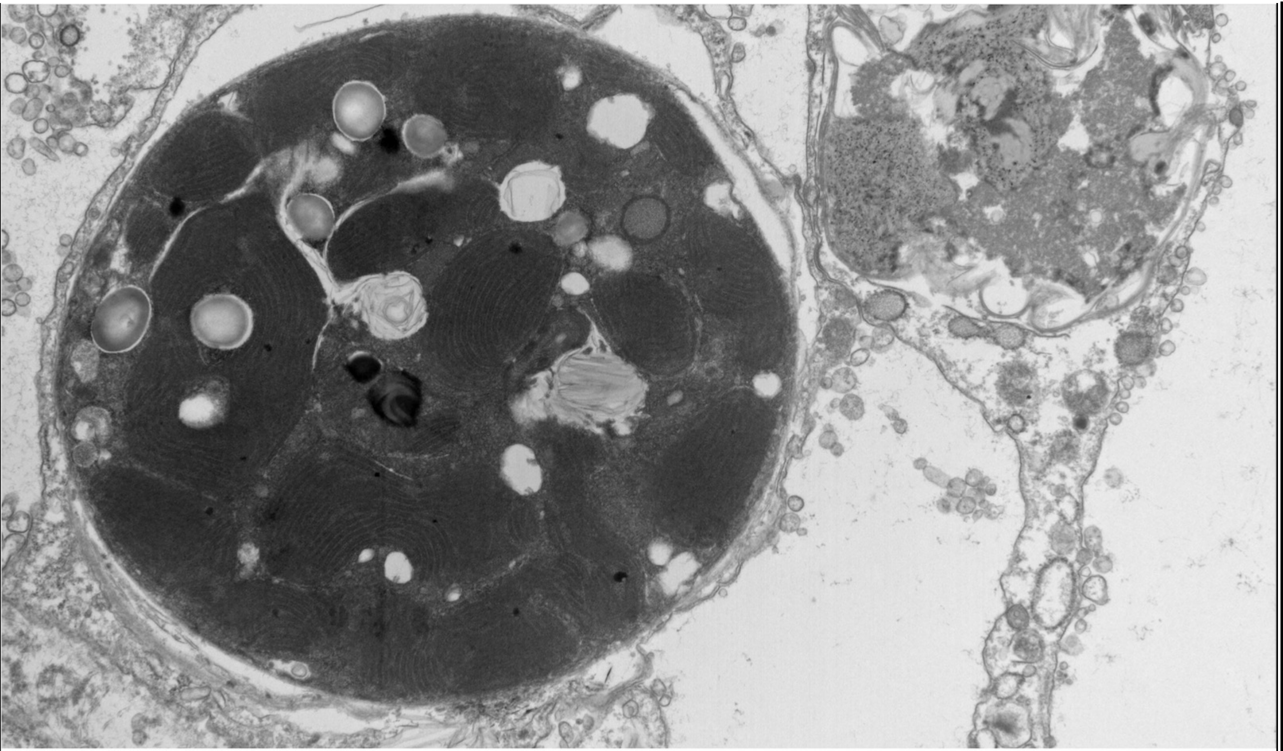

21-20_Correa_3-18-135_16K1_064.tif
Sample: 3/18, 135
Biological Electron Microscopy Lab
Rice University - SEA
Microscopist: MD Meyer

1 µm
HV=80kV
Direct Mag: 2500 x

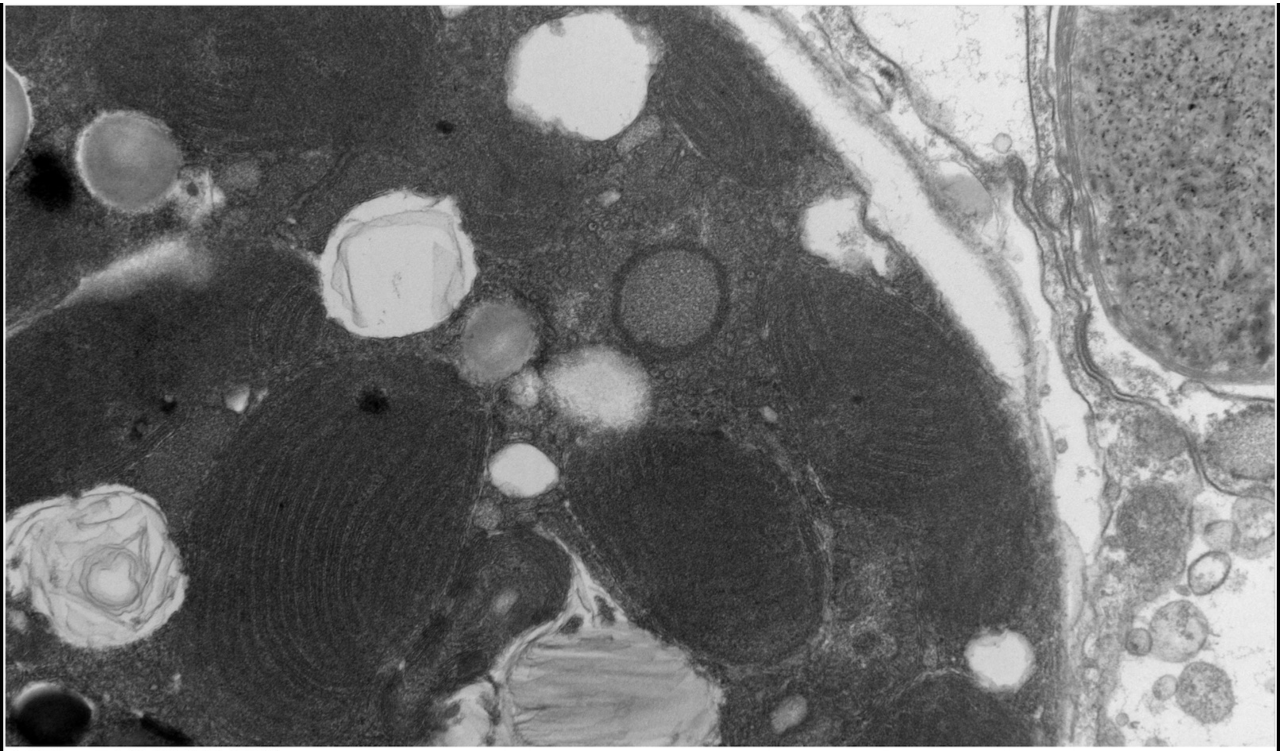

21-20_Correa_3-18-135_16K1_065.tif
Sample: 3/18, 135
Biological Electron Microscopy Lab
Rice University - SEA
Microscopist: MD Meyer

600 nm
HV=80kV
Direct Mag: 6000 x

Cell 21

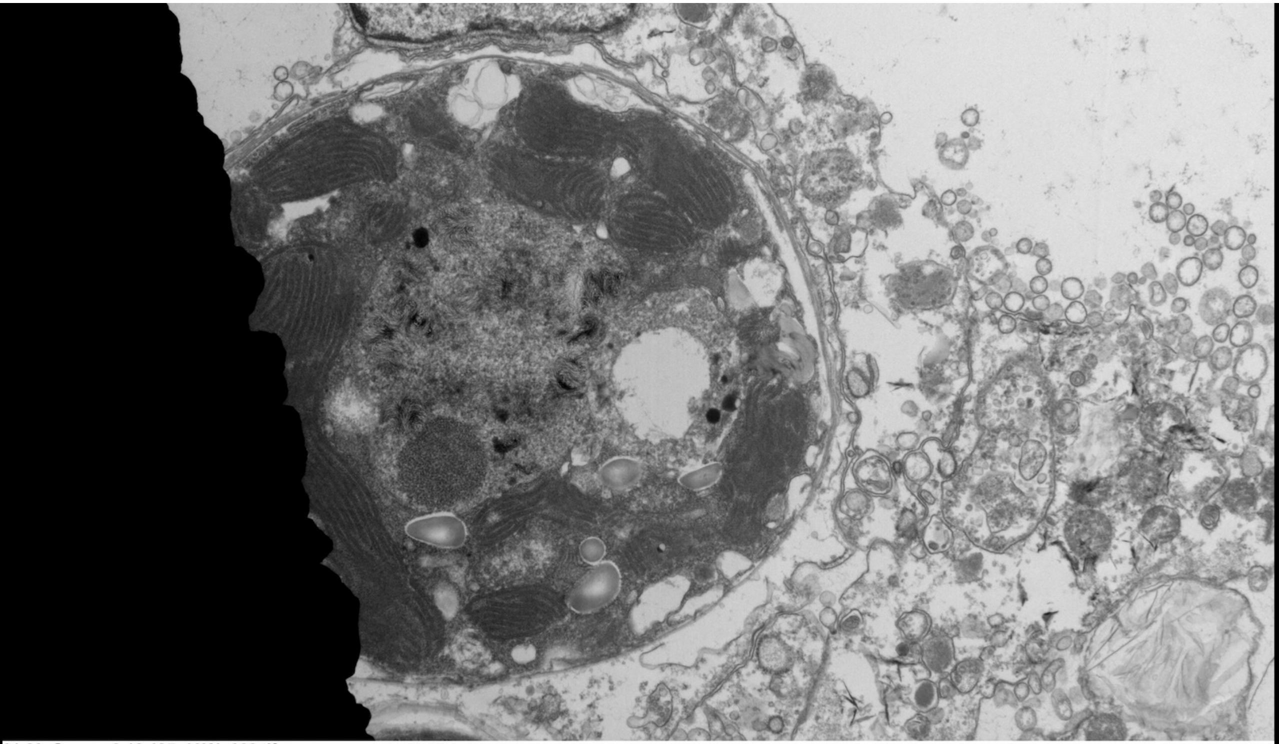

21-20_Correa_3-18-135_16K1_066.tif
Sample: 3/18, 135
Biological Electron Microscopy Lab
Rice University - SEA
Microscopist: MD Meyer

1 µm
HV=80kV
Direct Mag: 2500 x

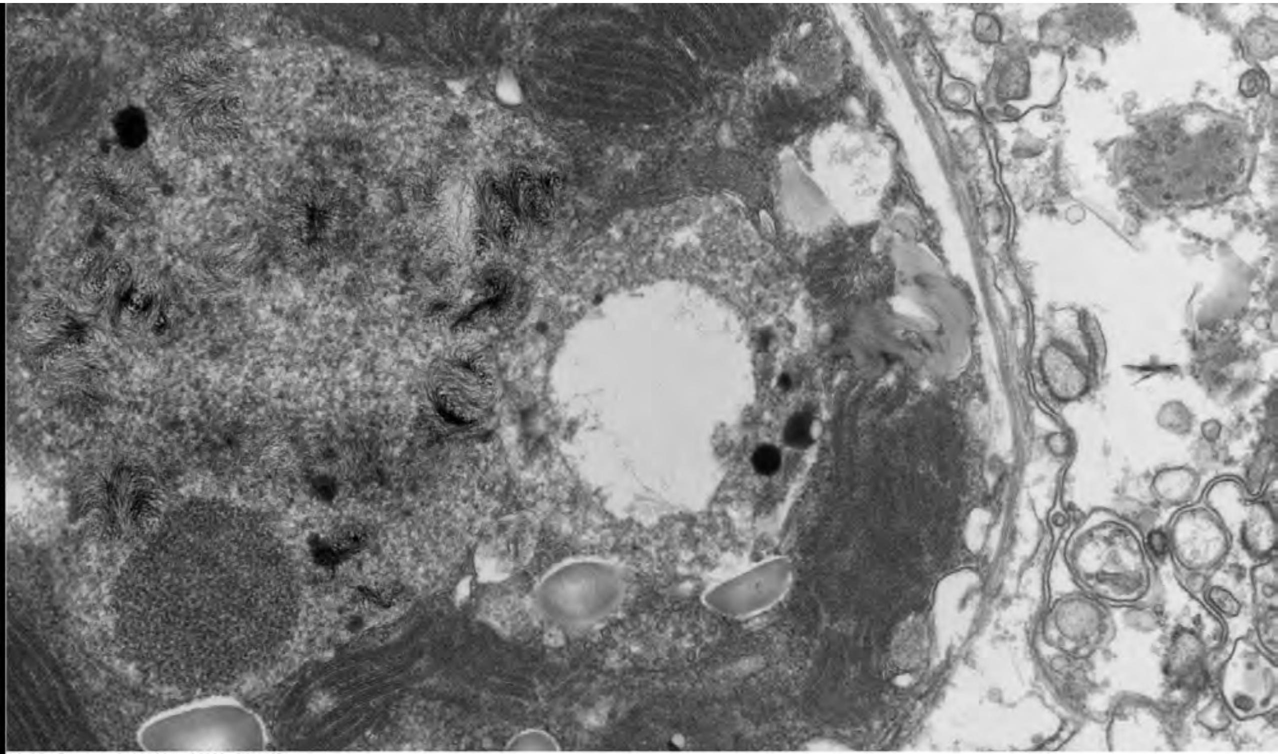

21-20_Correa_3-18-135_16K1_067.tif
Sample: 3/18, 135
Biological Electron Microscopy Lab
Rice University - SEA
Microscopist: MD Meyer

800 nm
HV=80kV
Direct Mag: 5000 x

Cell 22

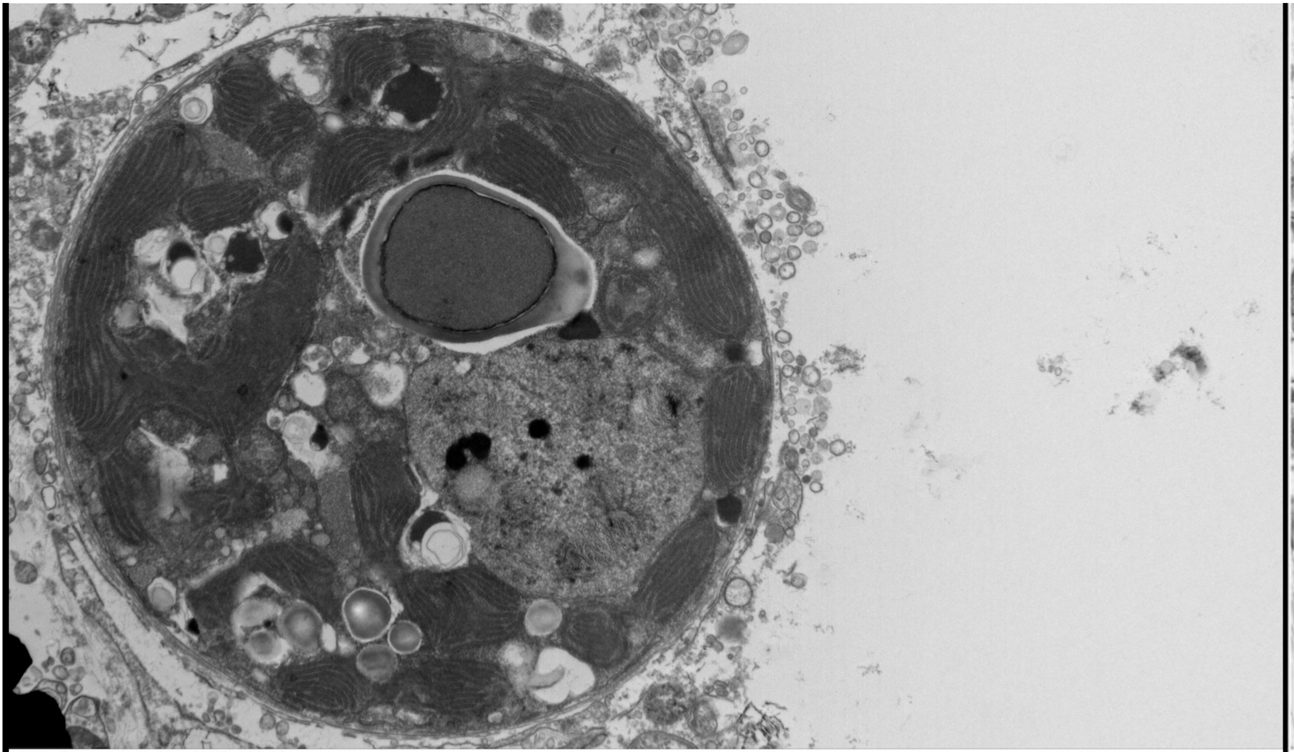

21-20_Correa_3-18-135_16K1_068.tif
Sample: 3/18, 135
Biological Electron Microscopy Lab
Rice University - SEA
Microscopist: MD Meyer

2 µm
HV=80kV
Direct Mag: 2000 x

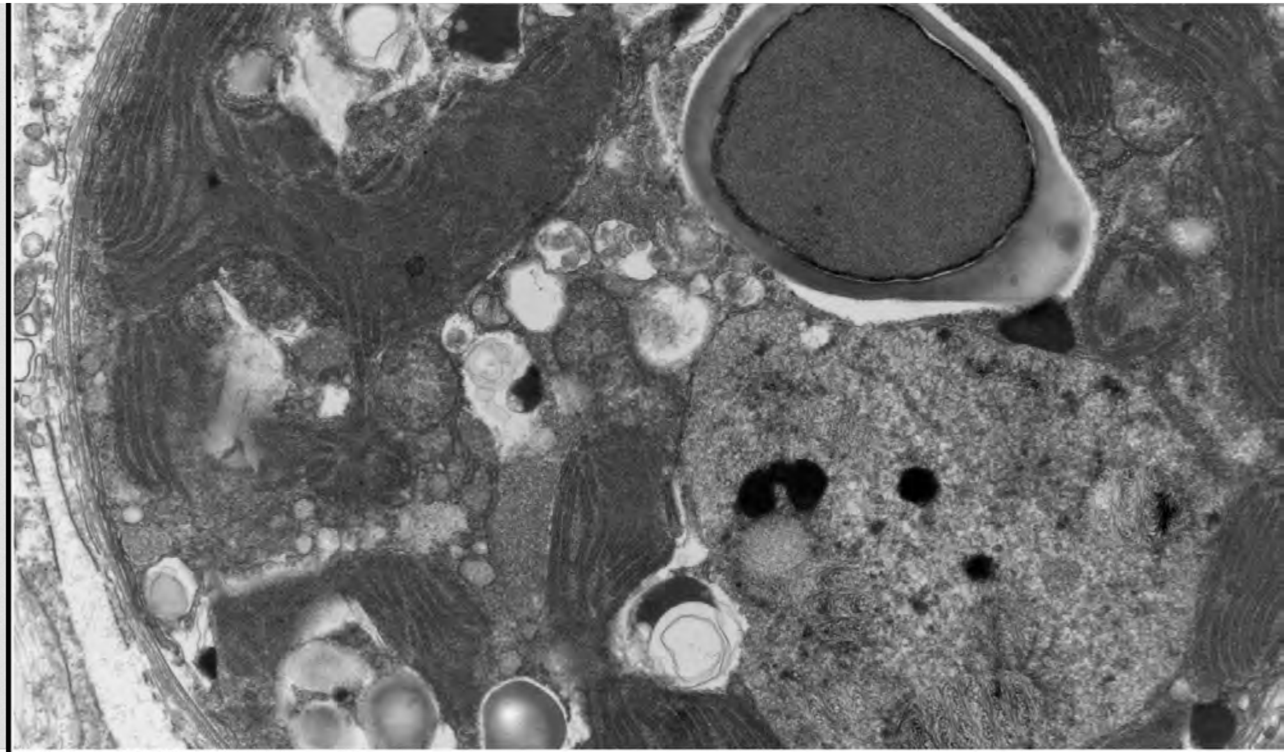

21-20_Correa_3-18-135_16K1_069.tif
Sample: 3/18, 135
Biological Electron Microscopy Lab
Rice University - SEA
Microscopist: MD Meyer

1 µm
HV=80kV
Direct Mag: 4000 x

Cell 23

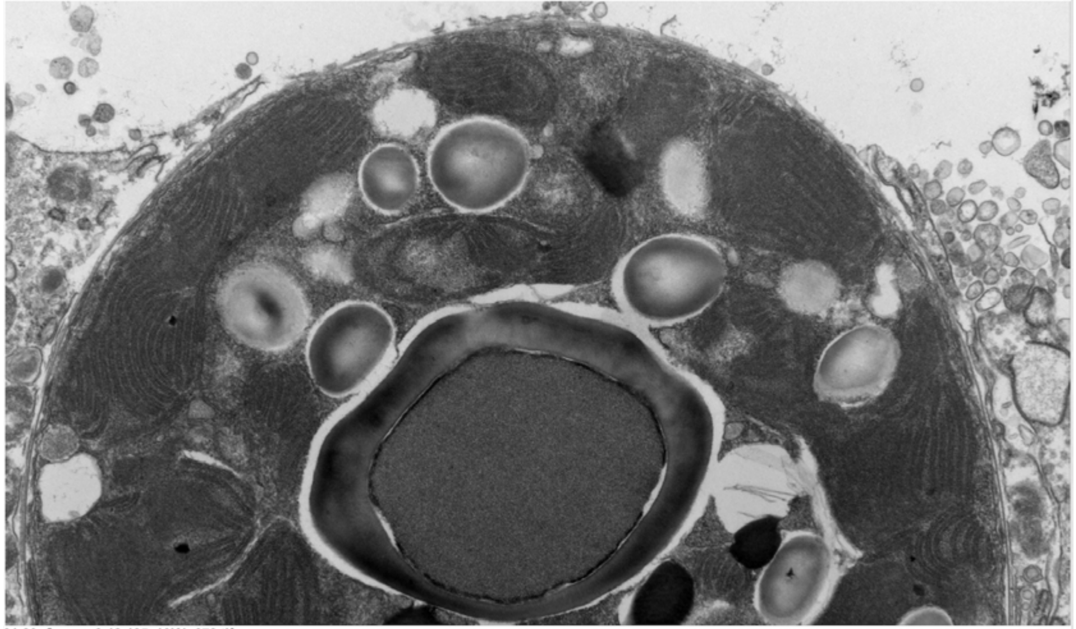

21-20_Correa_3-18-135_16K1_072.tif
Sample: 3/18, 135
Biological Electron Microscopy Lab
Rice University - SEA
Microscopist: MD Meyer

1 µm
HV=80kV
Direct Mag: 4000 x

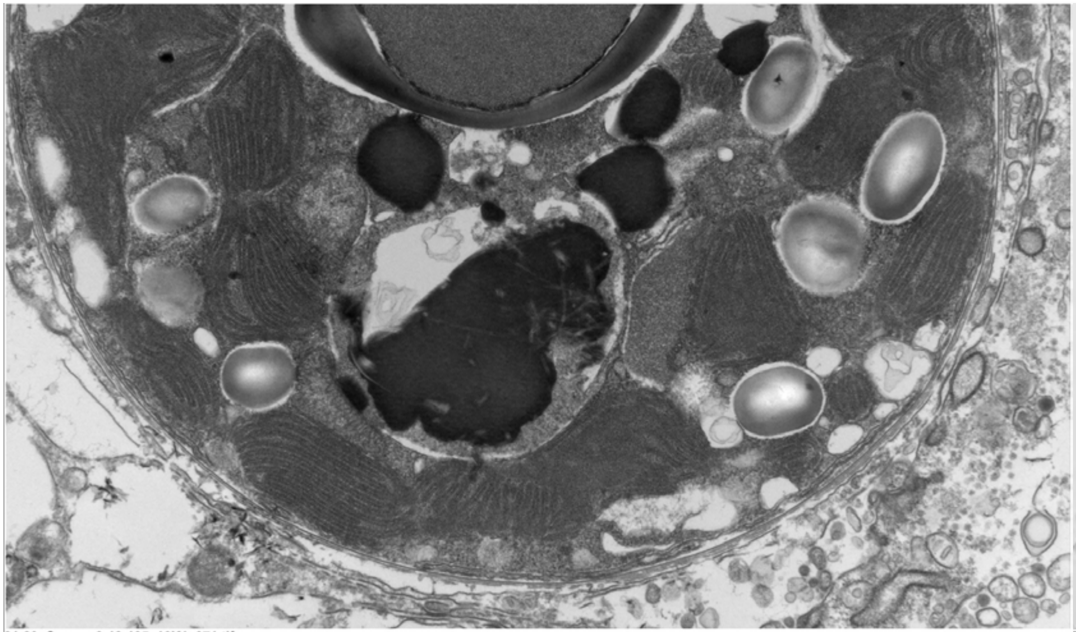

21-20_Correa_3-18-135_16K1_071.tif
Sample: 3/18, 135
Biological Electron Microscopy Lab
Rice University - SEA
Microscopist: MD Meyer

1 µm
HV=80kV
Direct Mag: 4000 x

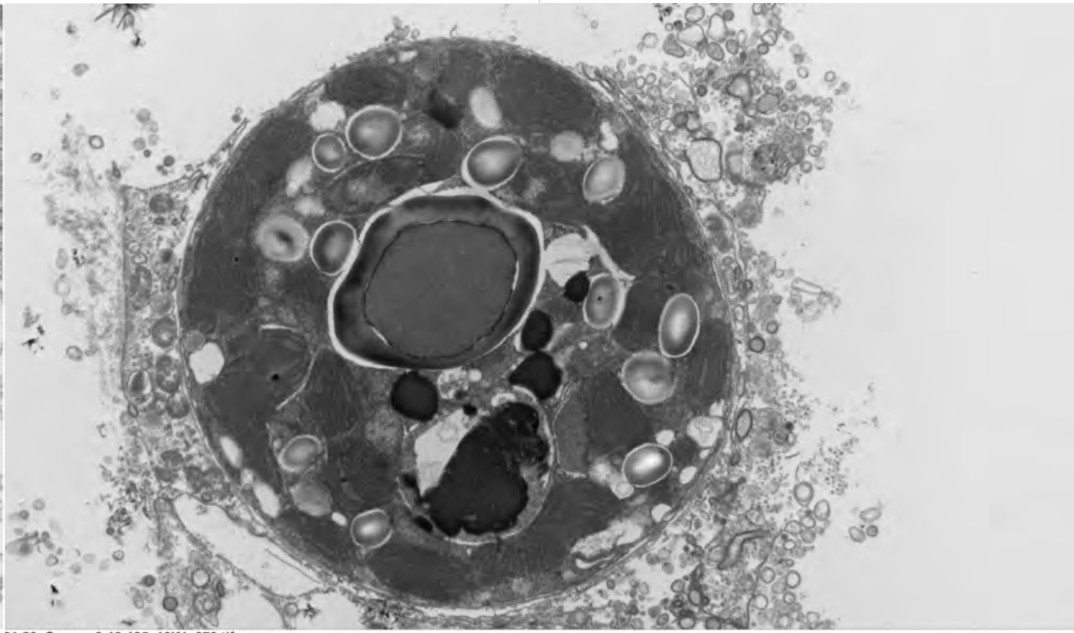

21-20_Correa_3-18-135_16K1_070.tif
Sample: 3/18, 135
Biological Electron Microscopy Lab
Rice University - SEA
Microscopist: MD Meyer

2 µm
HV=80kV
Direct Mag: 2000 x

Cell 24

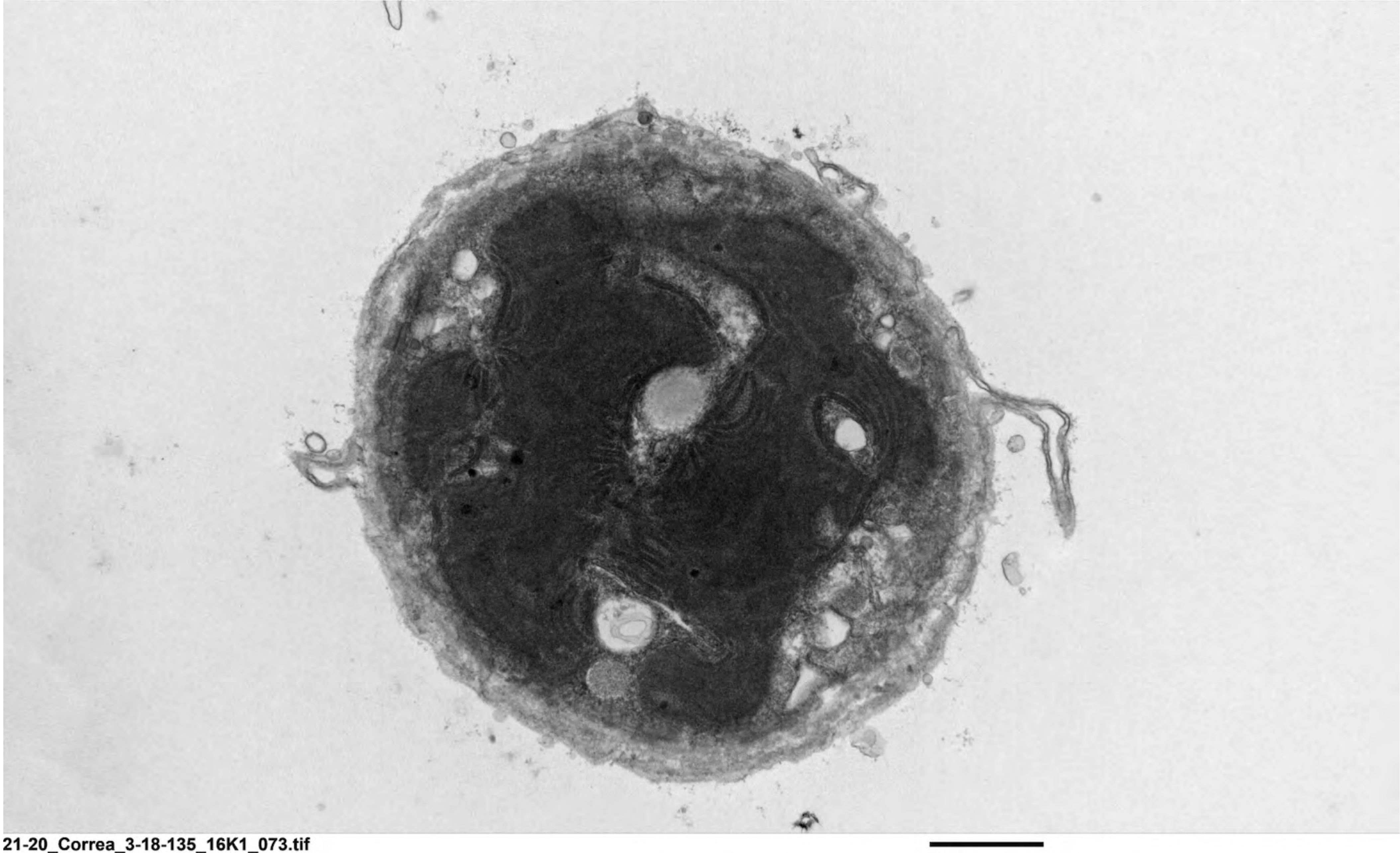

21-20_Correa_3-18-135_16K1_073.tif
Sample: 3/18, 135
Biological Electron Microscopy Lab
Rice University - SEA
Microscopist: MD Meyer

1 µm
HV=80kV
Direct Mag: 3000 x

Cell 25

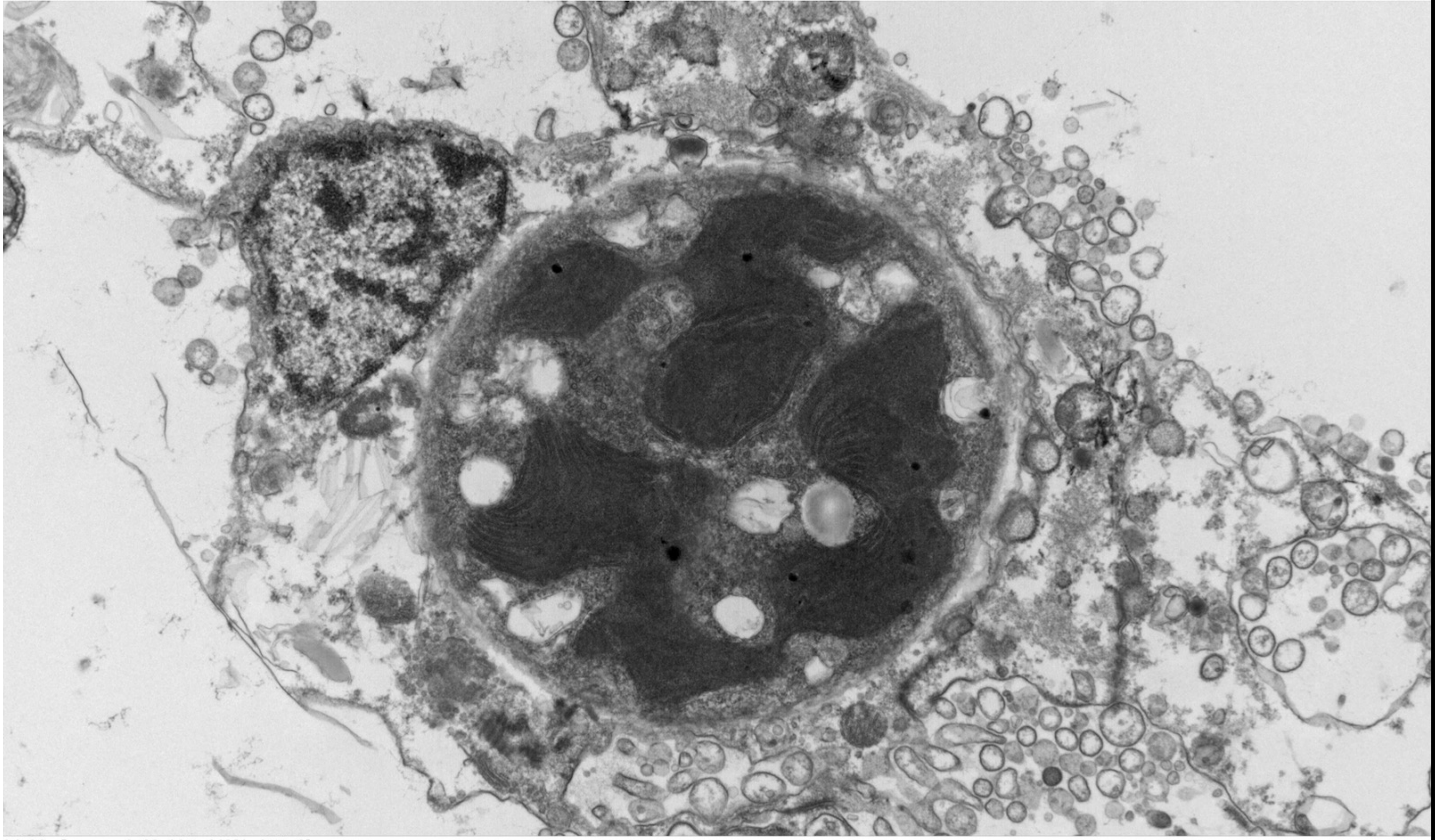

21-20_Correa_3-18-135_16K1_074.tif
Sample: 3/18, 135
Biological Electron Microscopy Lab
Rice University - SEA
Microscopist: MD Meyer

1 µm
HV=80kV
Direct Mag: 3000 x

Cell 26

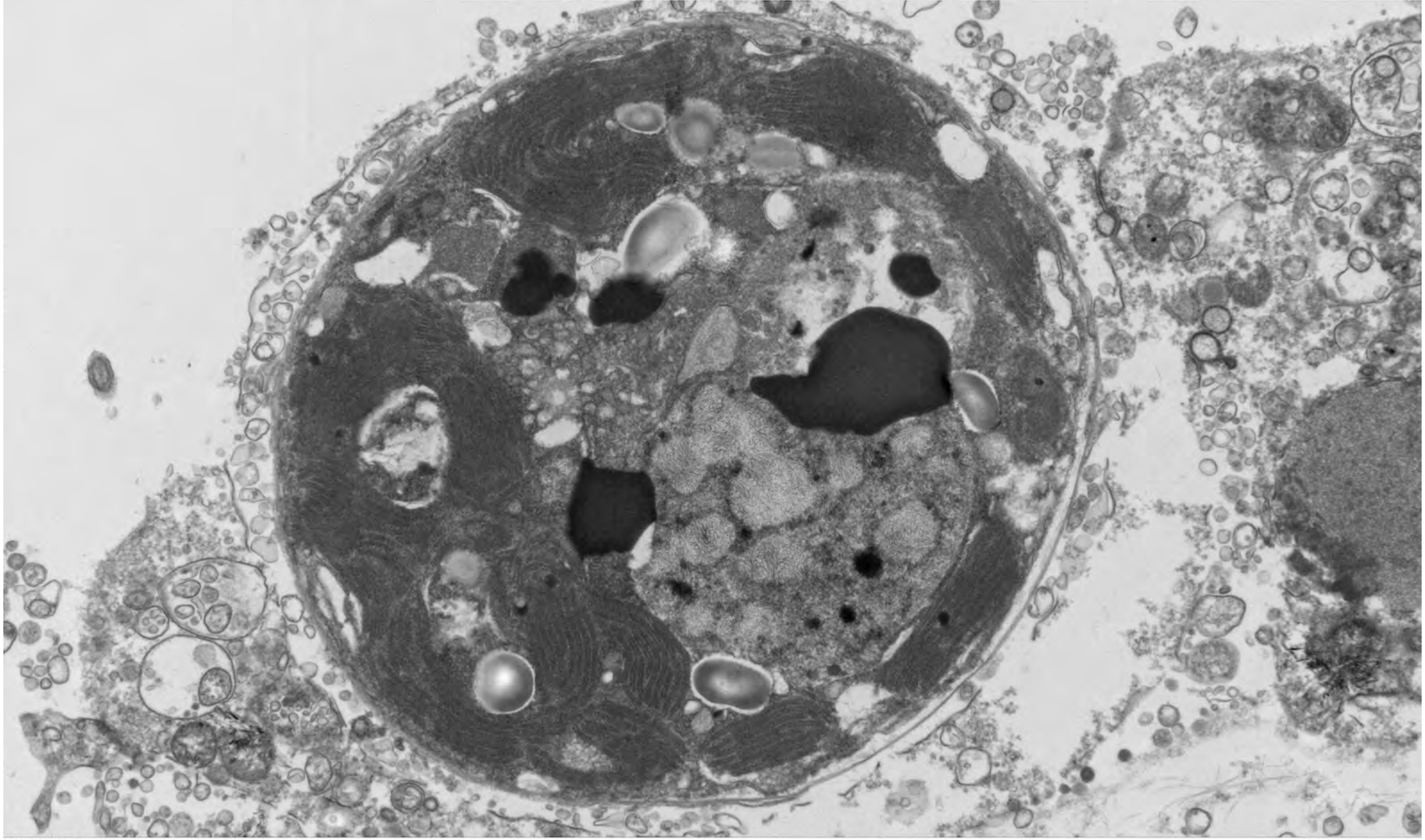

21-20_Correa_3-18-135_16K1_075.tif
Sample: 3/18, 135
Biological Electron Microscopy Lab
Rice University - SEA
Microscopist: MD Meyer

1 µm
HV=80kV
Direct Mag: 2500 x

Cell 27

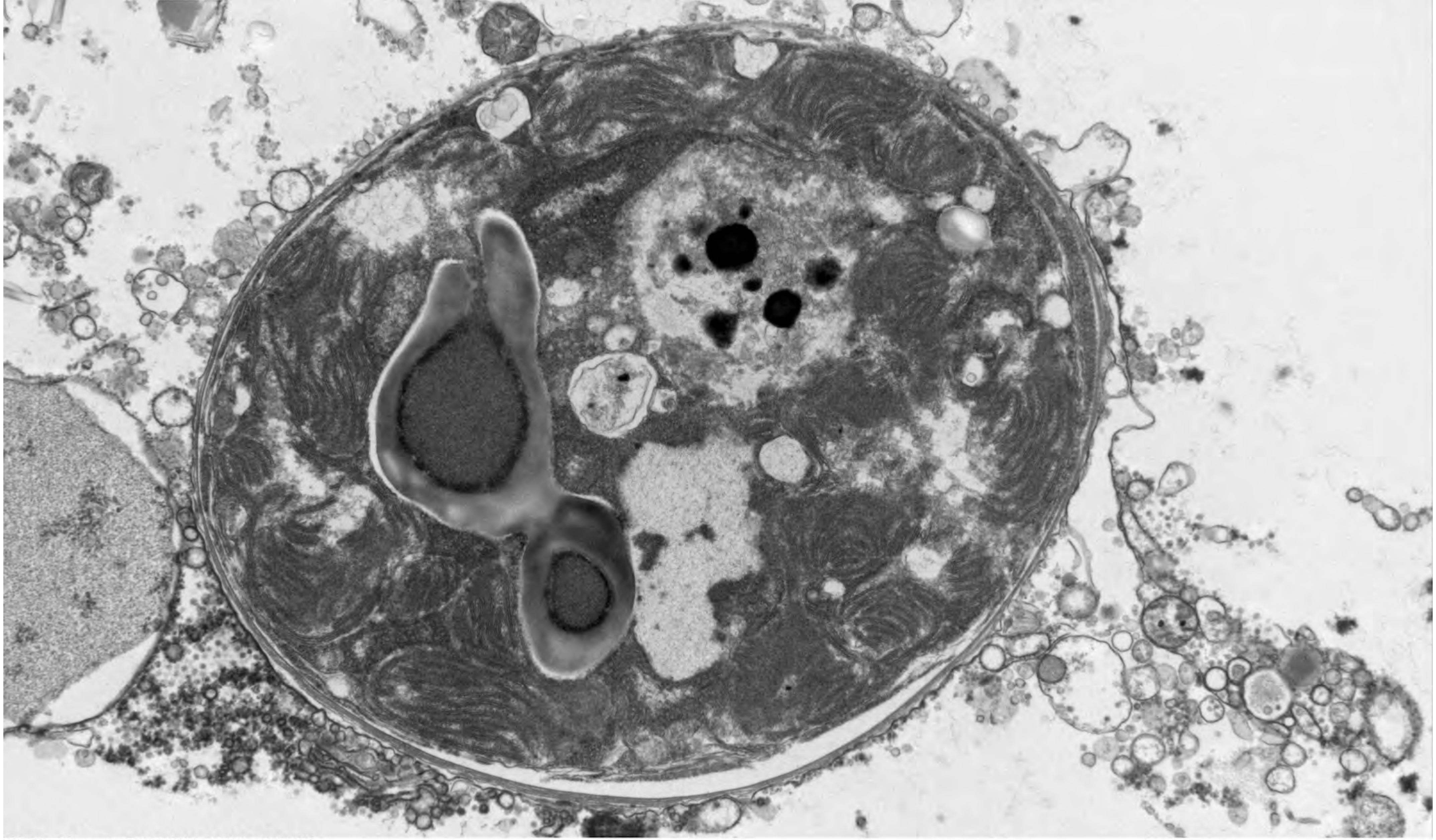

21-20_Correa_3-18-135_16K1_077.tif
Sample: 3/18, 135
Biological Electron Microscopy Lab
Rice University - SEA
Microscopist: MD Meyer

1 µm
HV=80kV
Direct Mag: 2500 x

Cell 28

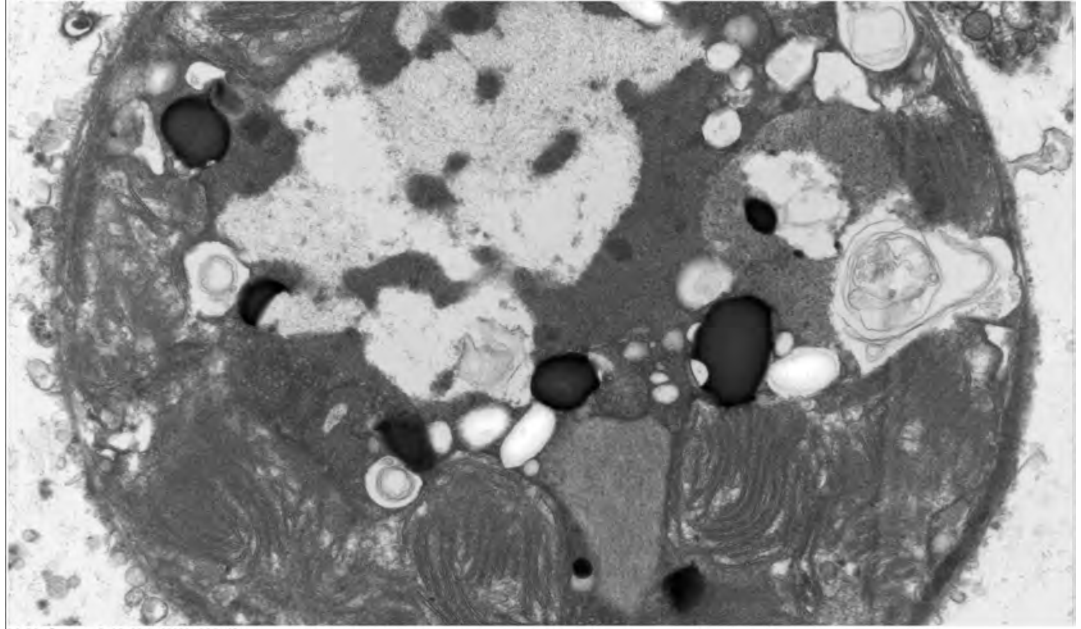

21-20_Correa_3-18-135_16K1_080.tif
Sample: 3/18, 135
Biological Electron Microscopy Lab
Rice University - SEA
Microscopist: MD Meyer

1 µm
HV=80kV
Direct Mag: 4000 x

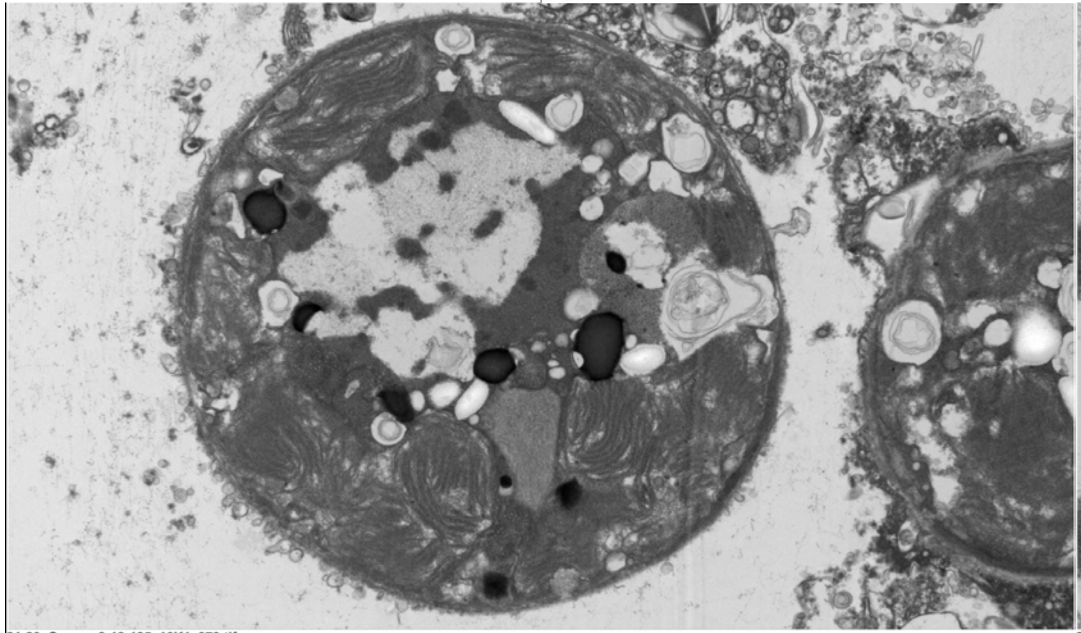

21-20_Correa_3-18-135_16K1_079.tif
Sample: 3/18, 135
Biological Electron Microscopy Lab
Rice University - SEA
Microscopist: MD Meyer

1 µm
HV=80kV
Direct Mag: 2500 x

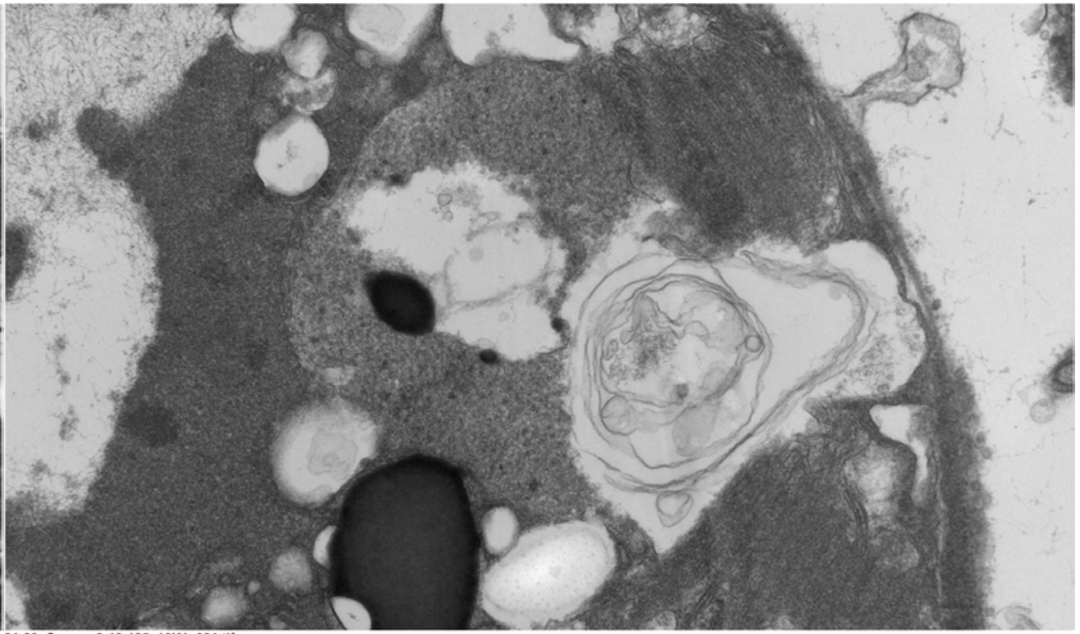

21-20_Correa_3-18-135_16K1_081.tif
Sample: 3/18, 135
Biological Electron Microscopy Lab
Rice University - SEA
Microscopist: MD Meyer

500 nm
HV=80kV
Direct Mag: 8000 x

Cell 29

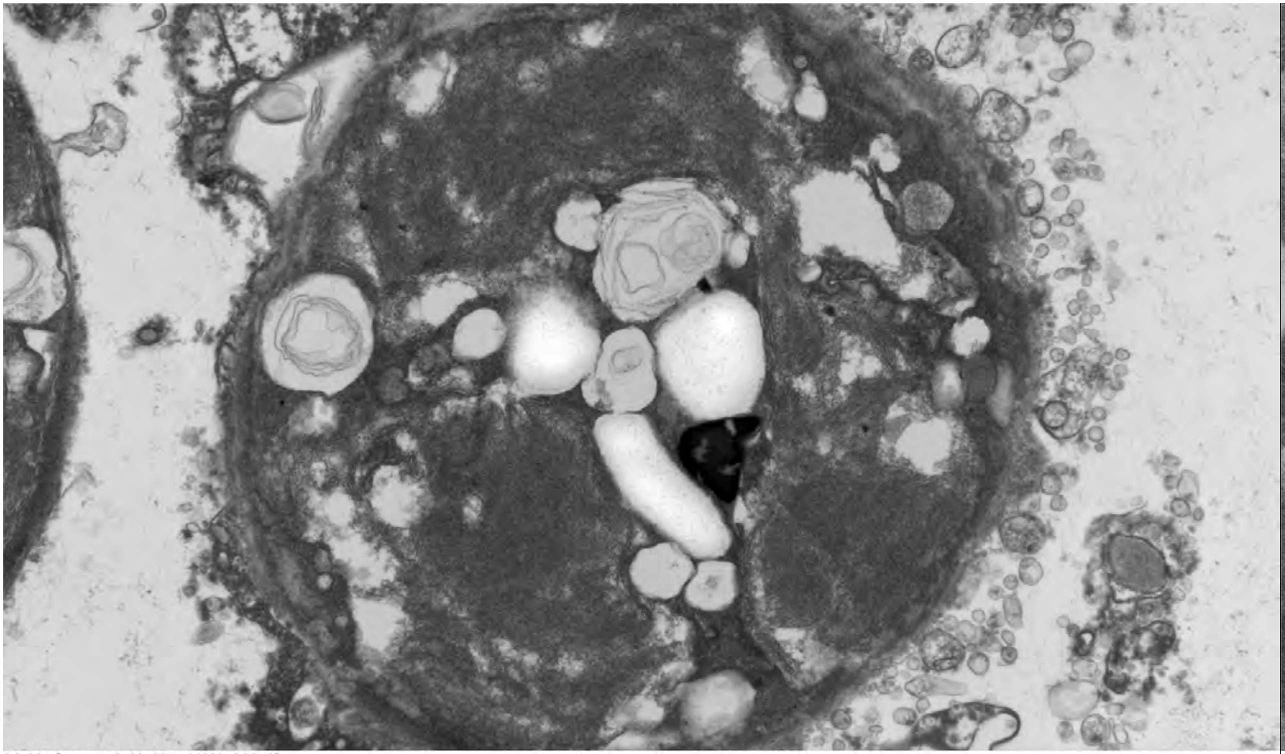

21-20_Correa_3-18-135_16K1_082.tif
Sample: 3/18, 135
Biological Electron Microscopy Lab
Rice University - SEA
Microscopist: MD Meyer

1 µm
HV=80kV
Direct Mag: 4000 x

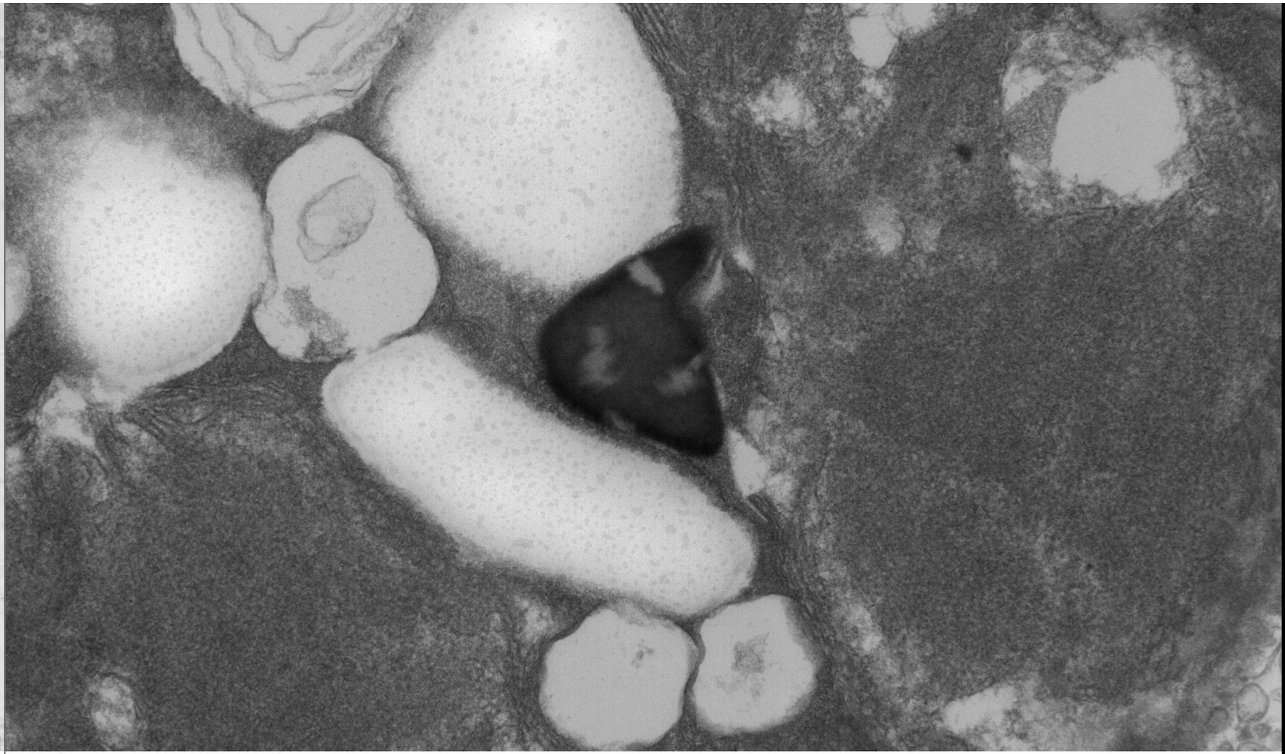

21-20_Correa_3-18-135_16K1_083.tif
Sample: 3/18, 135
Biological Electron Microscopy Lab
Rice University - SEA
Microscopist: MD Meyer

400 nm
HV=80kV
Direct Mag: 10000 x

Cell 30

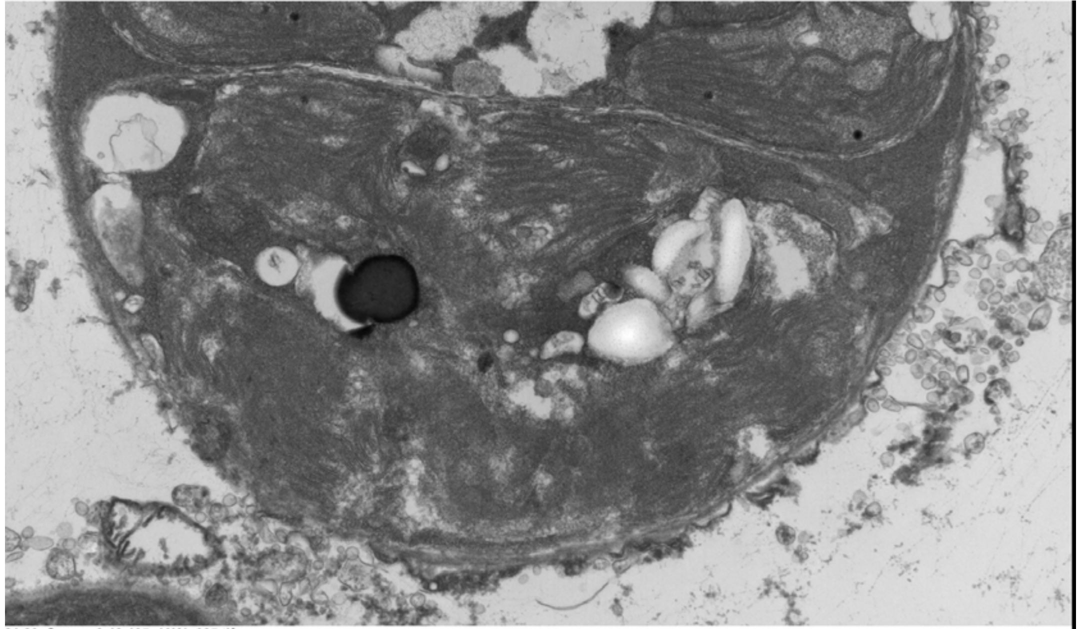

21-20_Correa_3-18-135_16K1_085.tif
Sample: 3/18, 135
Biological Electron Microscopy Lab
Rice University - SEA
Microscopist: MD Meyer

1 µm
HV=80kV
Direct Mag: 4000 x

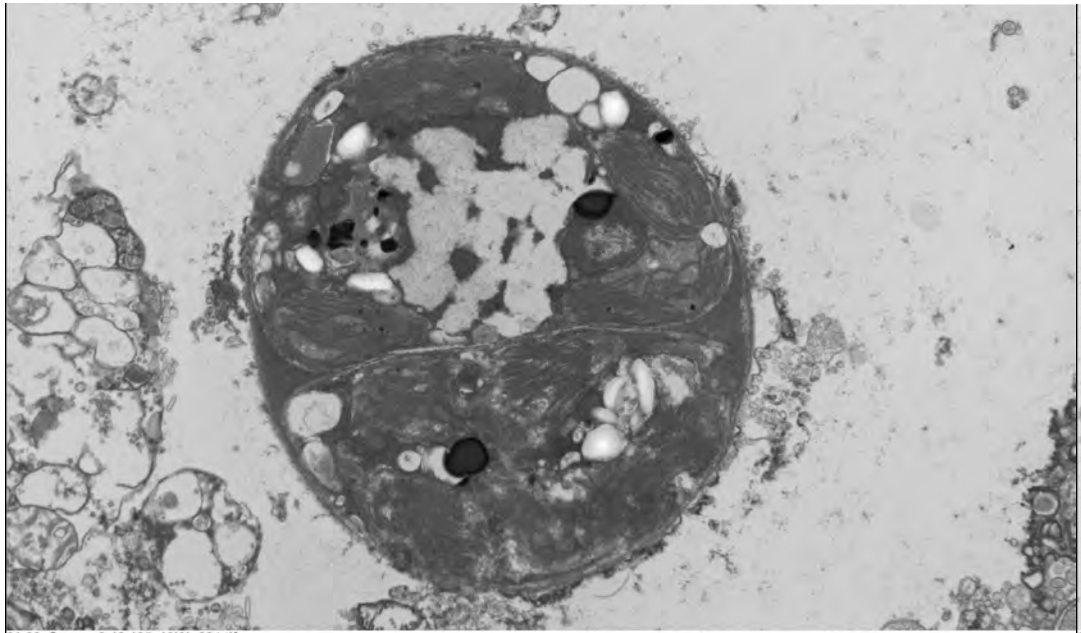

21-20_Correa_3-18-135_16K1_084.tif
Sample: 3/18, 135
Biological Electron Microscopy Lab
Rice University - SEA
Microscopist: MD Meyer

2 µm
HV=80kV
Direct Mag: 2000 x

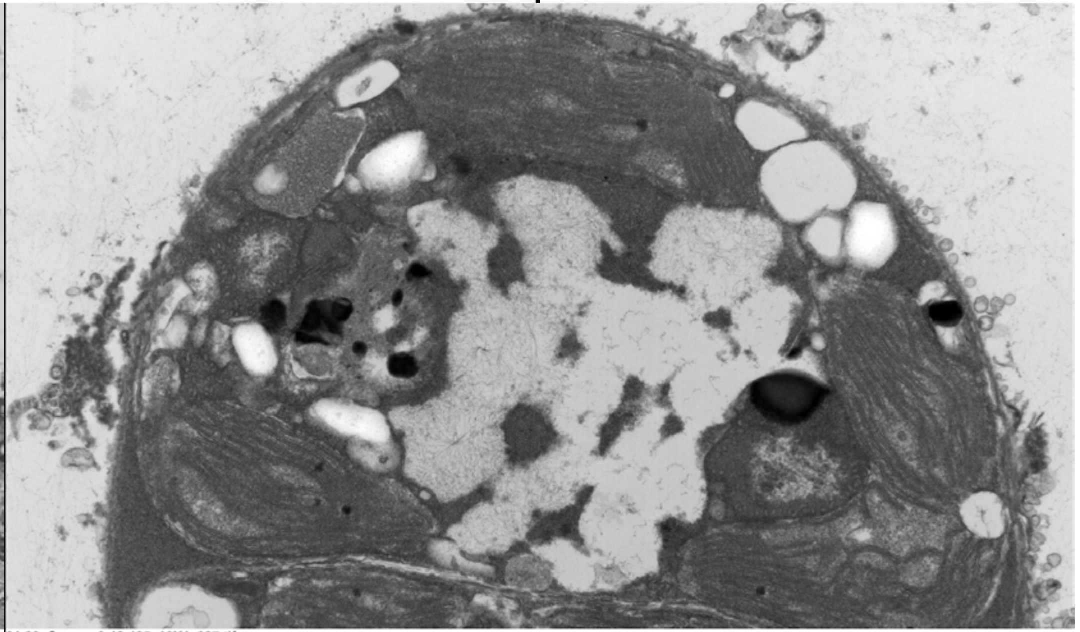

21-20_Correa_3-18-135_16K1_087.tif
Sample: 3/18, 135
Biological Electron Microscopy Lab
Rice University - SEA
Microscopist: MD Meyer

1 µm
HV=80kV
Direct Mag: 4000 x

Cell 31

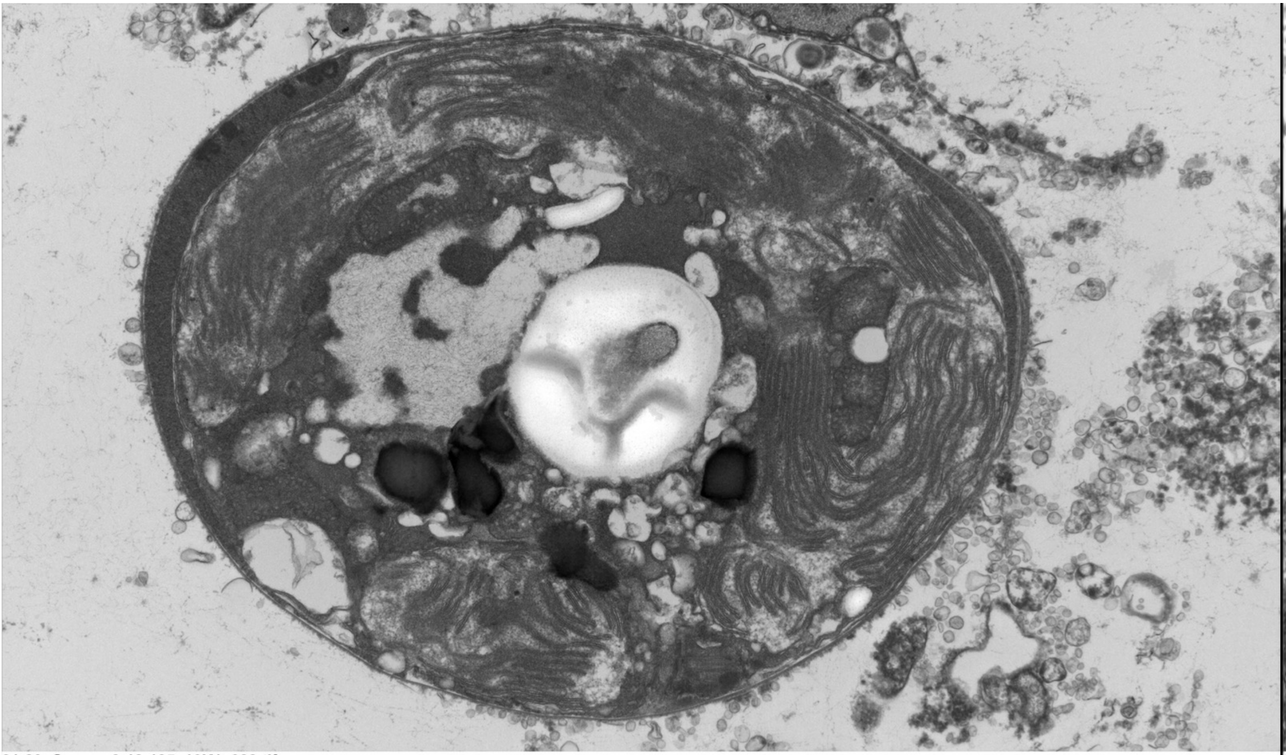

21-20_Correa_3-18-135_16K1_088.tif
Sample: 3/18, 135
Biological Electron Microscopy Lab
Rice University - SEA
Microscopist: MD Meyer

1 µm
HV=80kV
Direct Mag: 3000 x

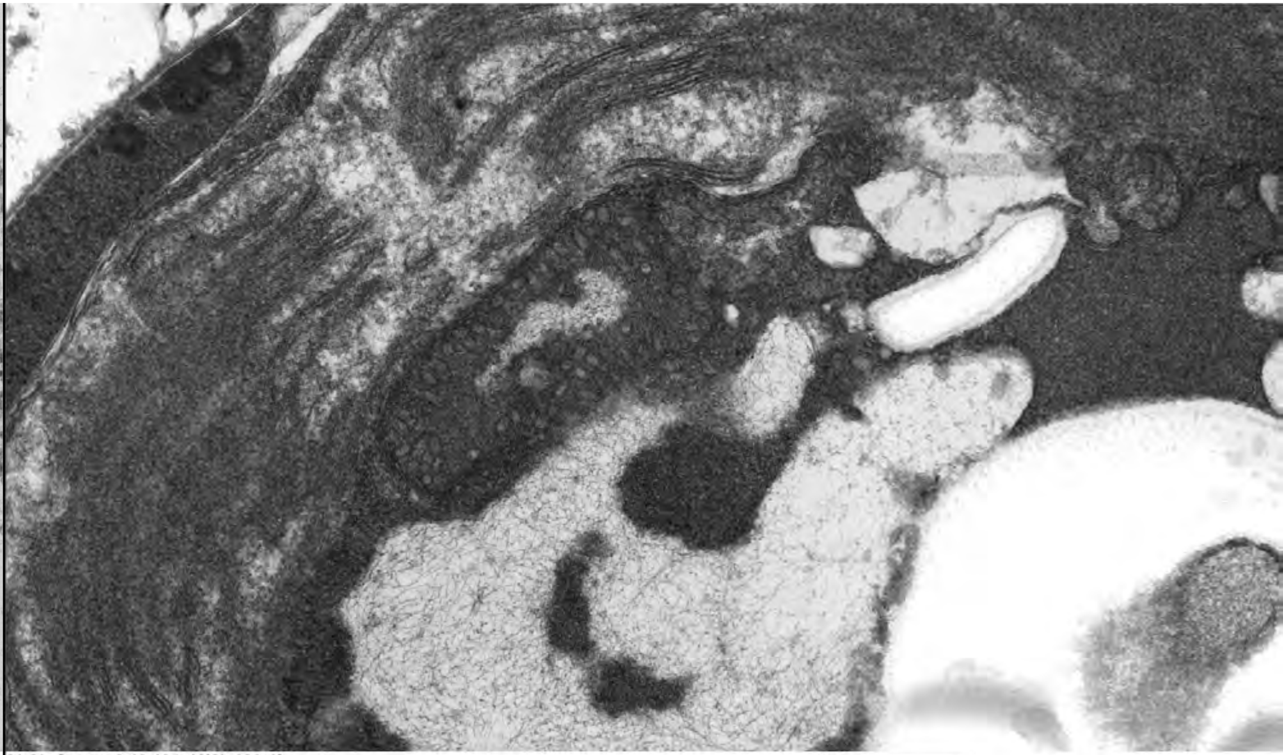

21-20_Correa_3-18-135_16K1_090.tif
Sample: 3/18, 135
Biological Electron Microscopy Lab
Rice University - SEA
Microscopist: MD Meyer

500 nm
HV=80kV
Direct Mag: 8000 x

Heat (March 2019) POR Colony J

Cell 1

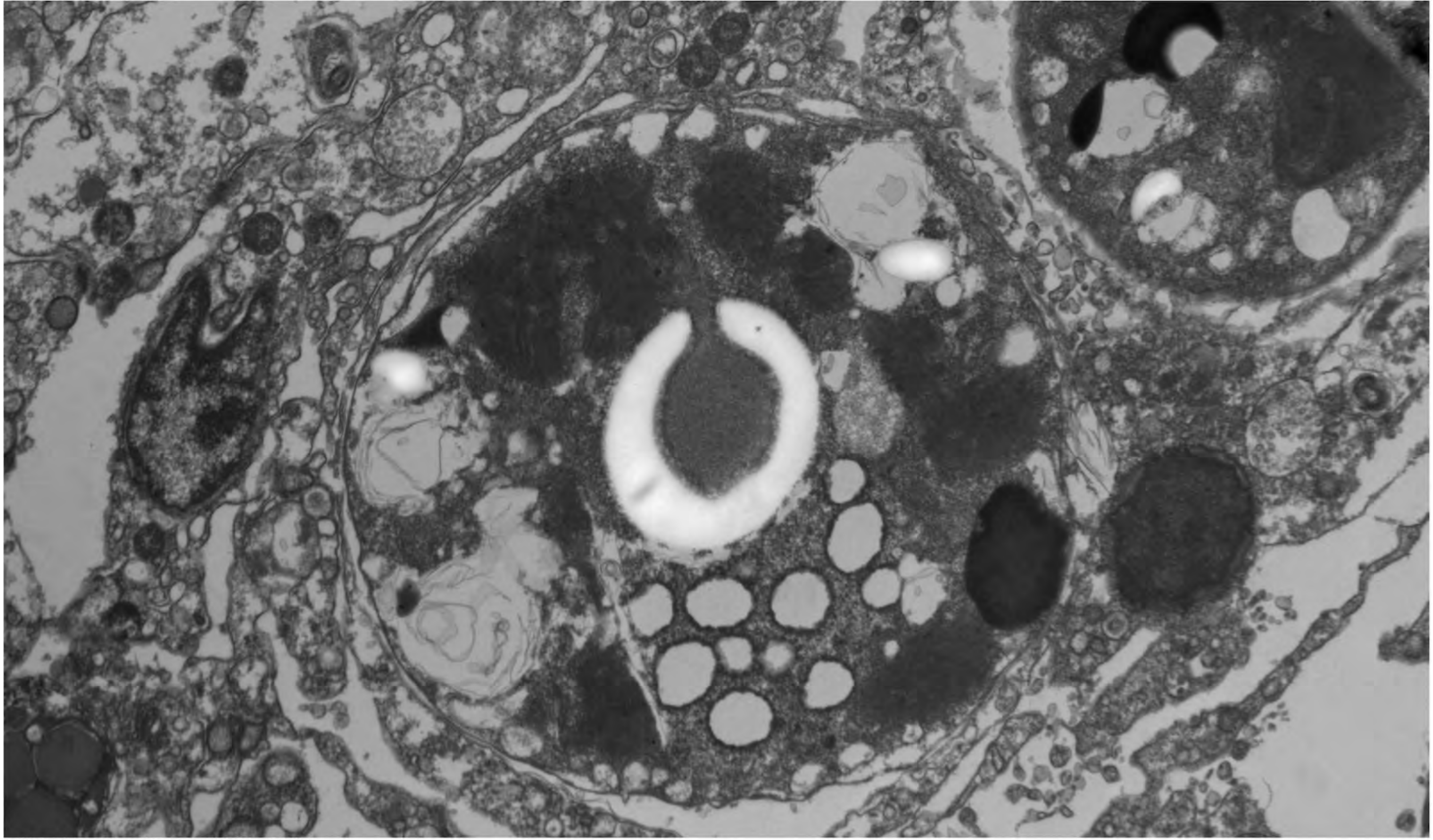

21-20_Correa_3-19-135_16D2_092.tif
Sample: 3/19, 135
Biological Electron Microscopy Lab
Rice University - SEA
Microscopist: MD Meyer

2 µm
HV=80kV
Direct Mag: 2000 x

Cell 2

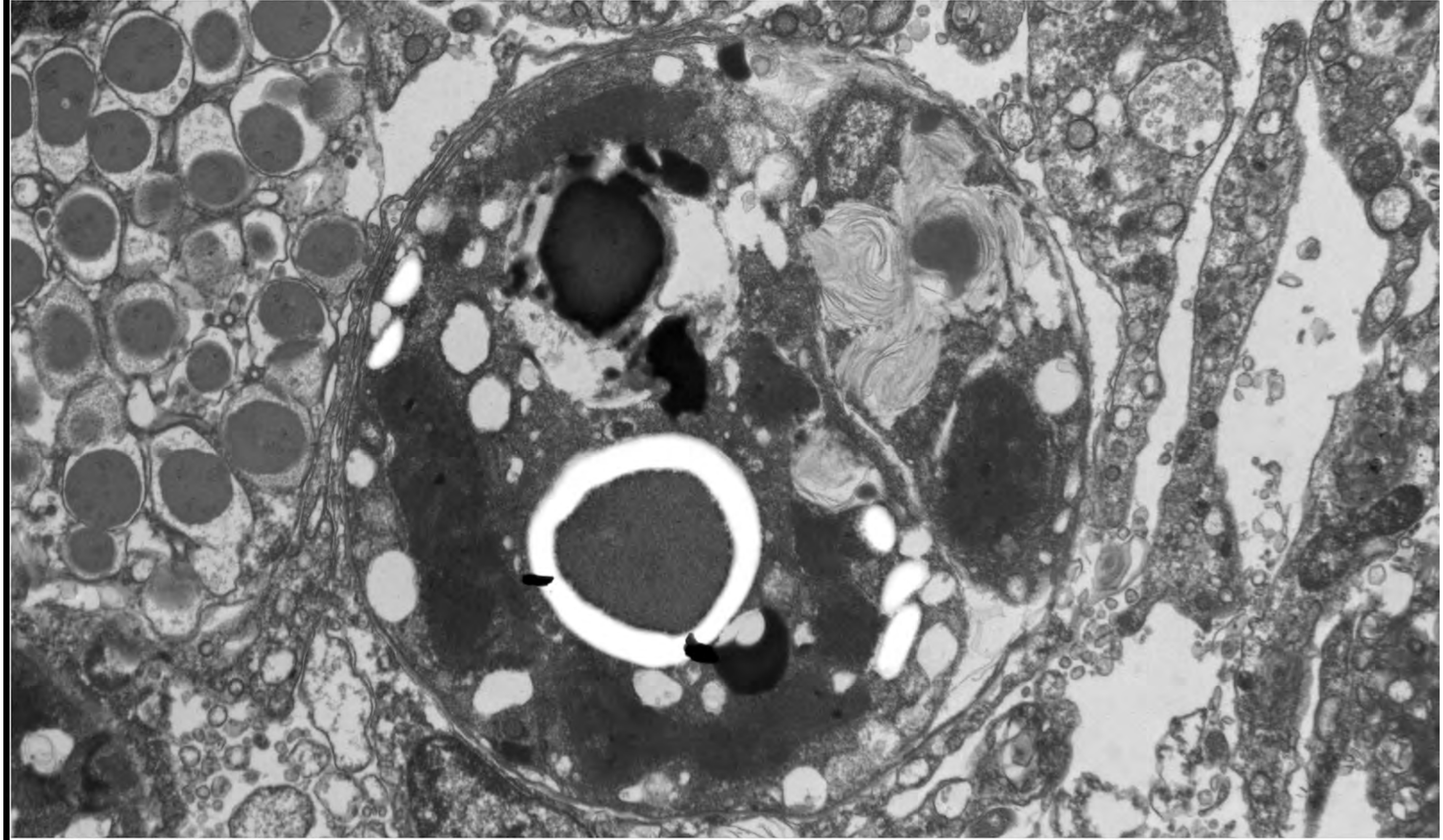

21-20_Correa_3-19-135_16D2_091.tif
Sample: 3/19, 135
Biological Electron Microscopy Lab
Rice University - SEA
Microscopist: MD Meyer

2 µm
HV=80kV
Direct Mag: 2000 x

Cell 3

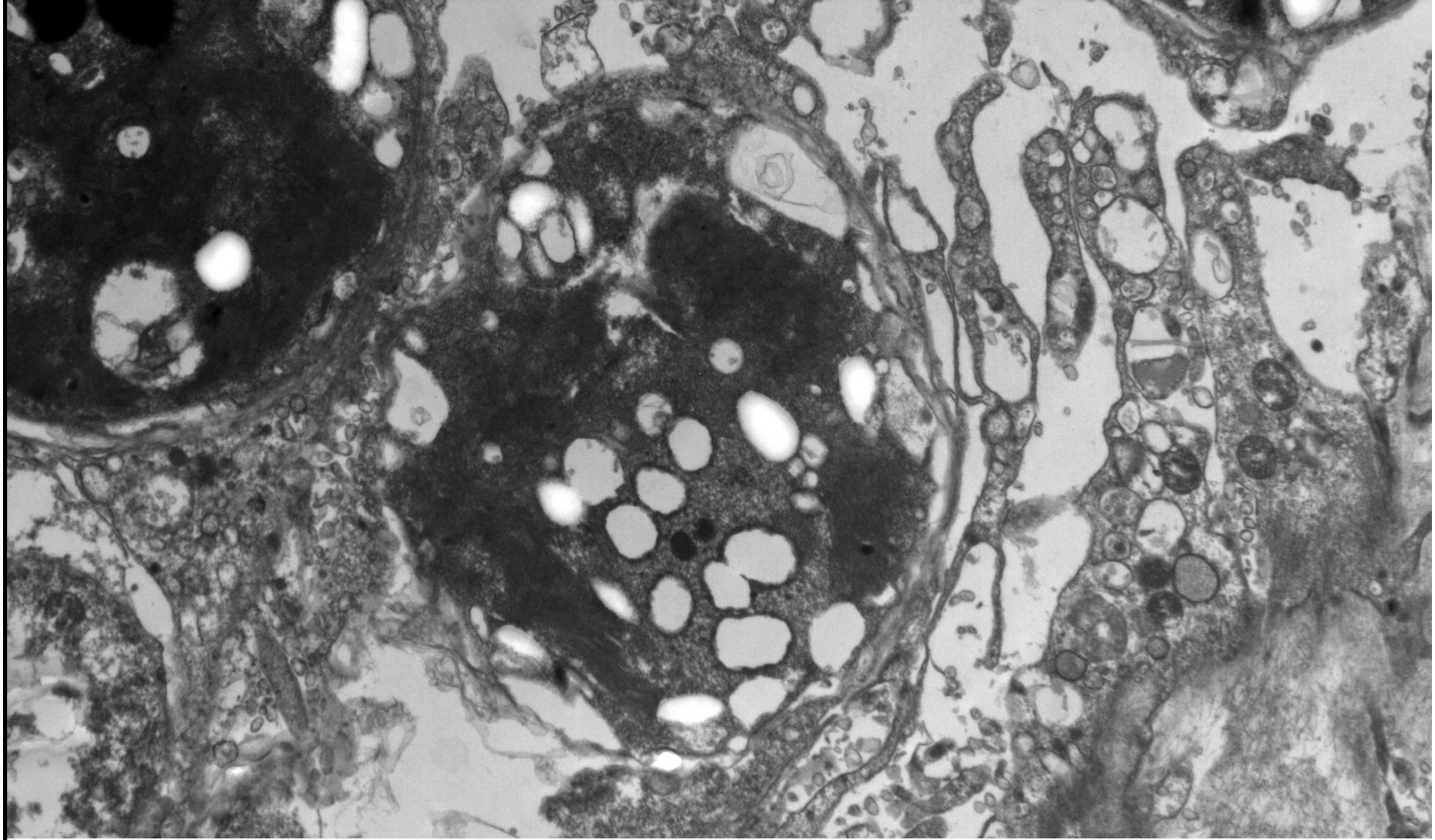

21-20_Correa_3-19-135_16D2_090.tif
Sample: 3/19, 135
Biological Electron Microscopy Lab
Rice University - SEA
Microscopist: MD Meyer

2 µm
HV=80kV
Direct Mag: 2000 x

Cell 4

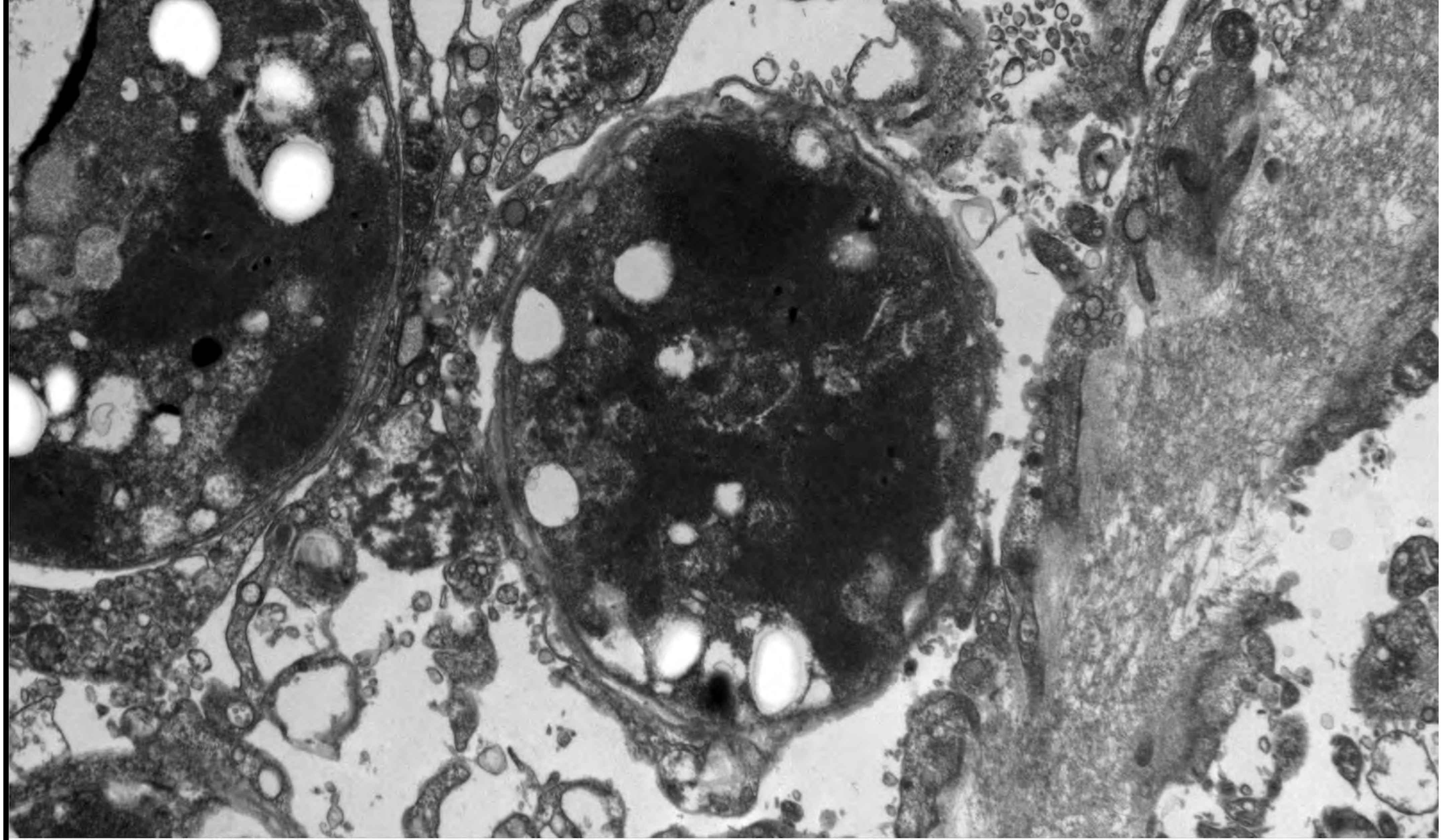

21-20_Correa_3-19-135_16D2_089.tif
Sample: 3/19, 135
Biological Electron Microscopy Lab
Rice University - SEA
Microscopist: MD Meyer

2 µm
HV=80kV
Direct Mag: 2000 x

Cell 5

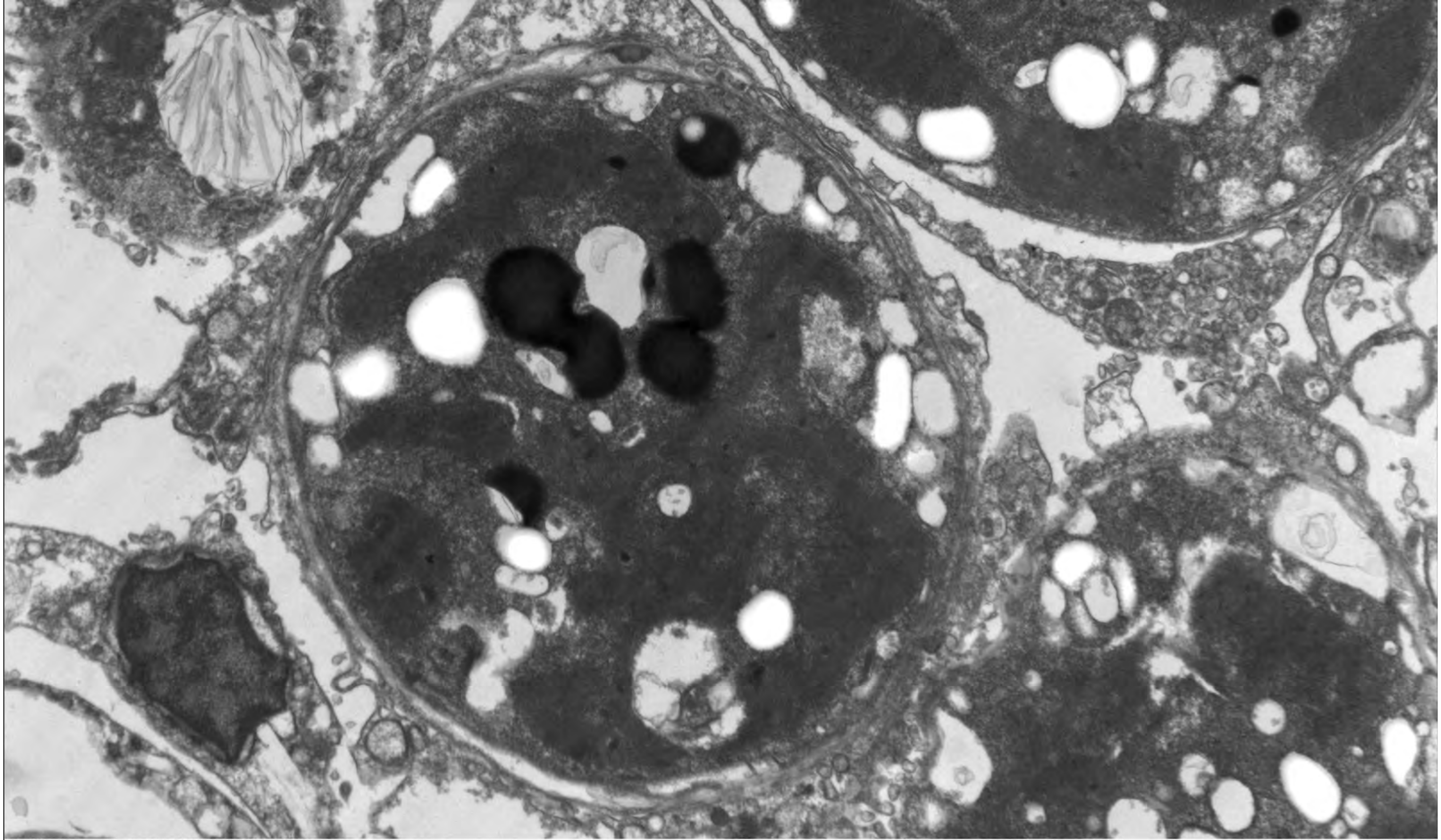

21-20_Correa_3-19-135_16D2_088.tif
Sample: 3/19, 135
Biological Electron Microscopy Lab
Rice University - SEA
Microscopist: MD Meyer

2 µm
HV=80kV
Direct Mag: 2000 x

Cell 6

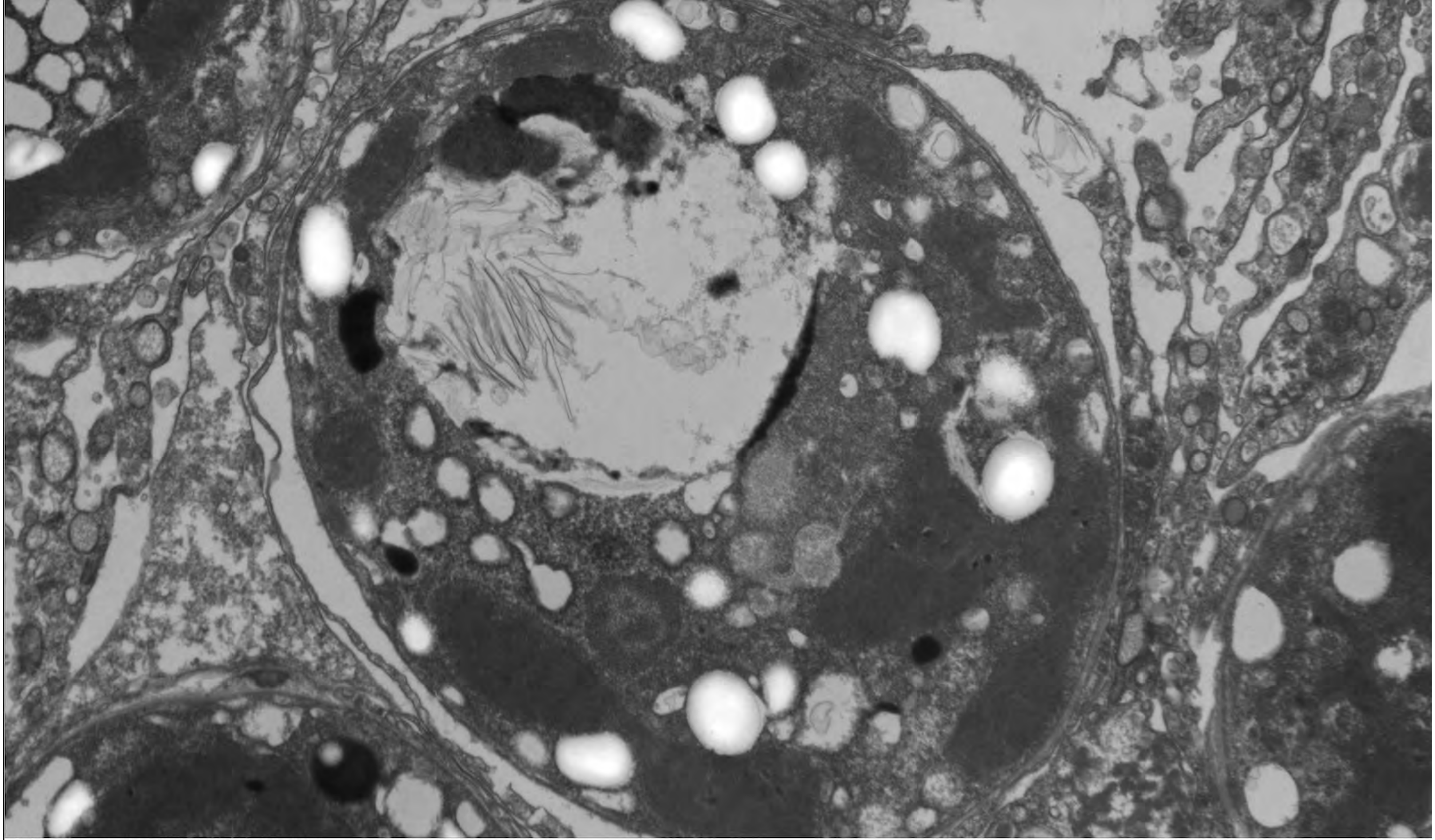

21-20_Correa_3-19-135_16D2_087.tif
Sample: 3/19, 135
Biological Electron Microscopy Lab
Rice University - SEA
Microscopist: MD Meyer

2 µm
HV=80kV
Direct Mag: 2000 x

Cell 7

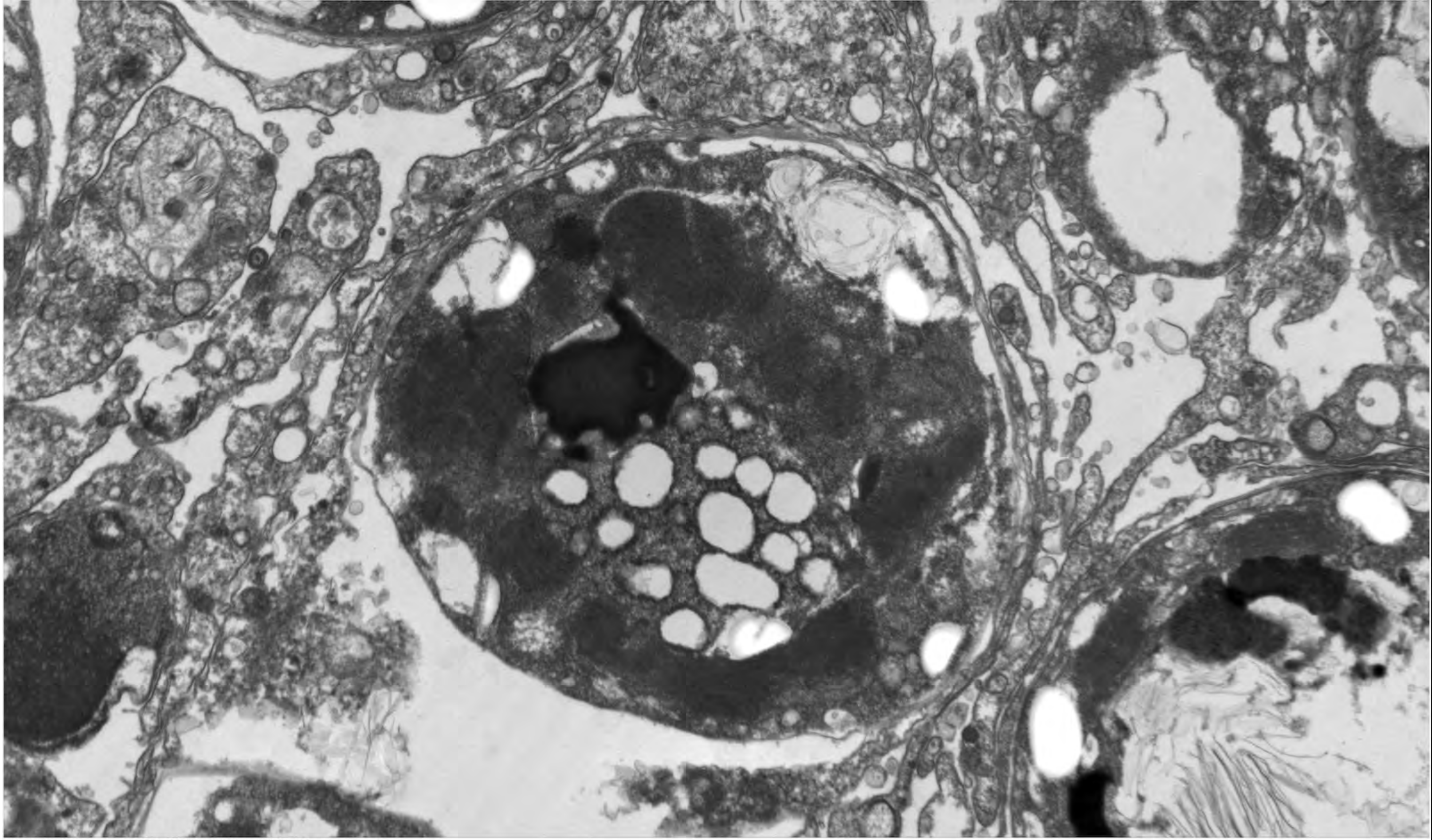

21-20_Correa_3-19-135_16D2_086.tif
Sample: 3/19, 135
Biological Electron Microscopy Lab
Rice University - SEA
Microscopist: MD Meyer

2 µm
HV=80kV
Direct Mag: 2000 x

Cell 8

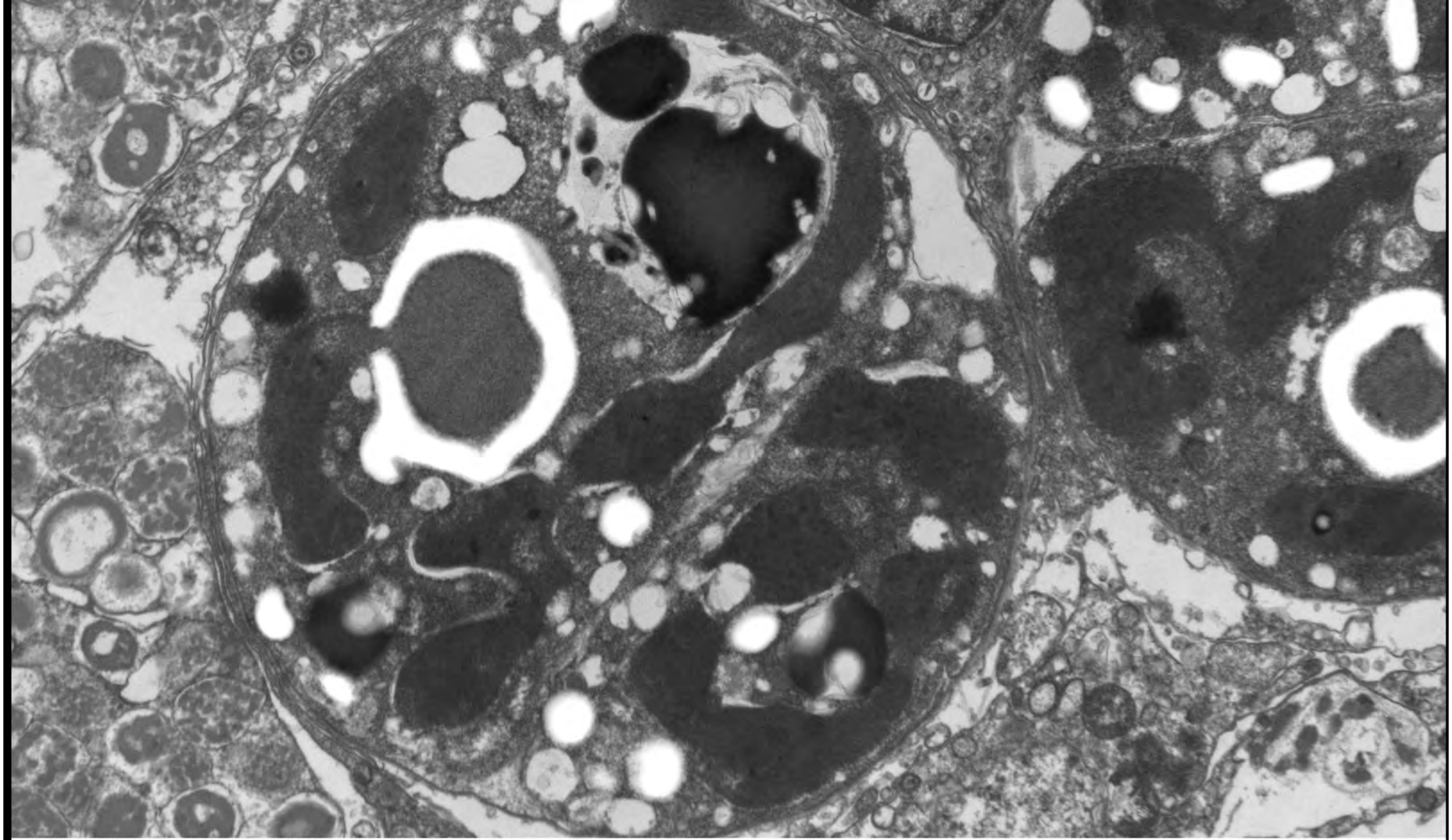

21-20_Correa_3-19-135_16D2_085.tif
Sample: 3/19, 135
Biological Electron Microscopy Lab
Rice University - SEA
Microscopist: MD Meyer

2 µm
HV=80kV
Direct Mag: 2000 x

Cell 9

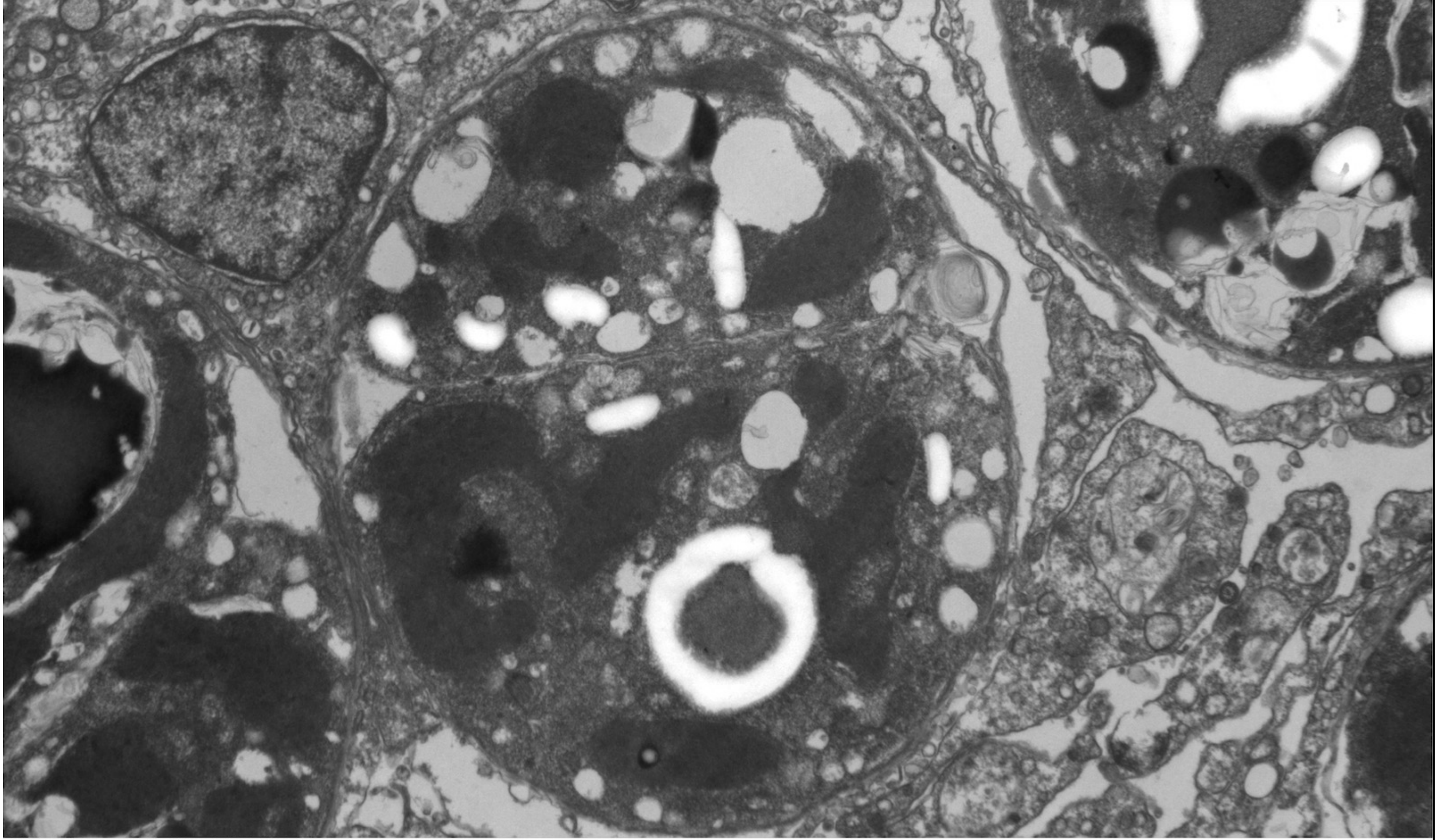

21-20_Correa_3-19-135_16D2_084.tif
Sample: 3/19, 135
Biological Electron Microscopy Lab
Rice University - SEA
Microscopist: MD Meyer

2 µm
HV=80kV
Direct Mag: 2000 x

Cell 10

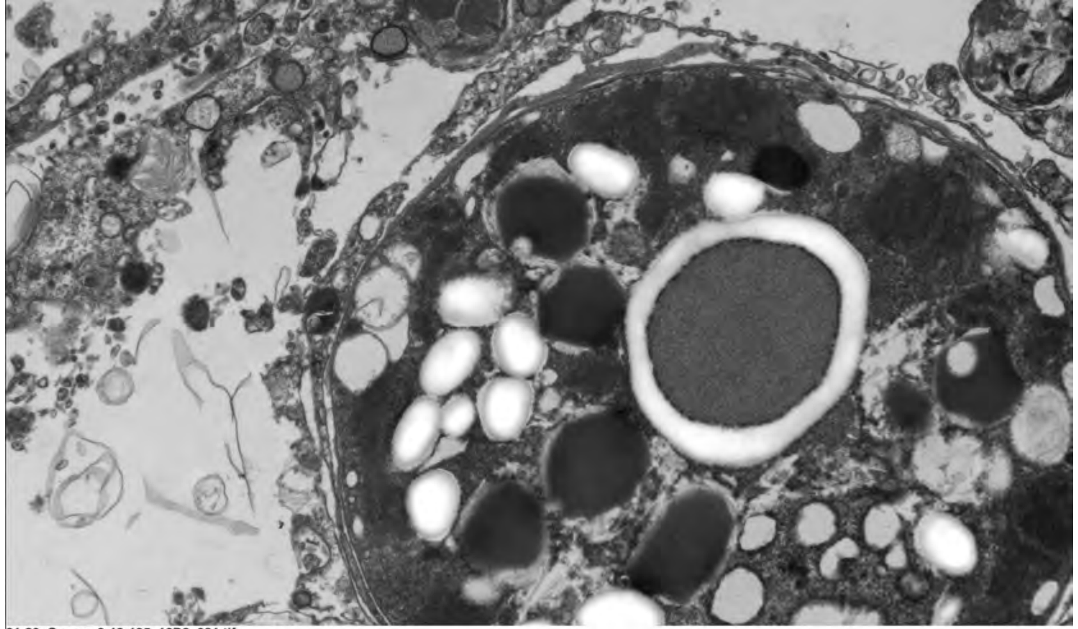

21-20_Correa_3-19-135_16D2_081.tif
Sample: 3/19, 135
Biological Electron Microscopy Lab
Rice University - SEA
Microscopist: MD Meyer

1 µm
HV=80kV
Direct Mag: 2500 x

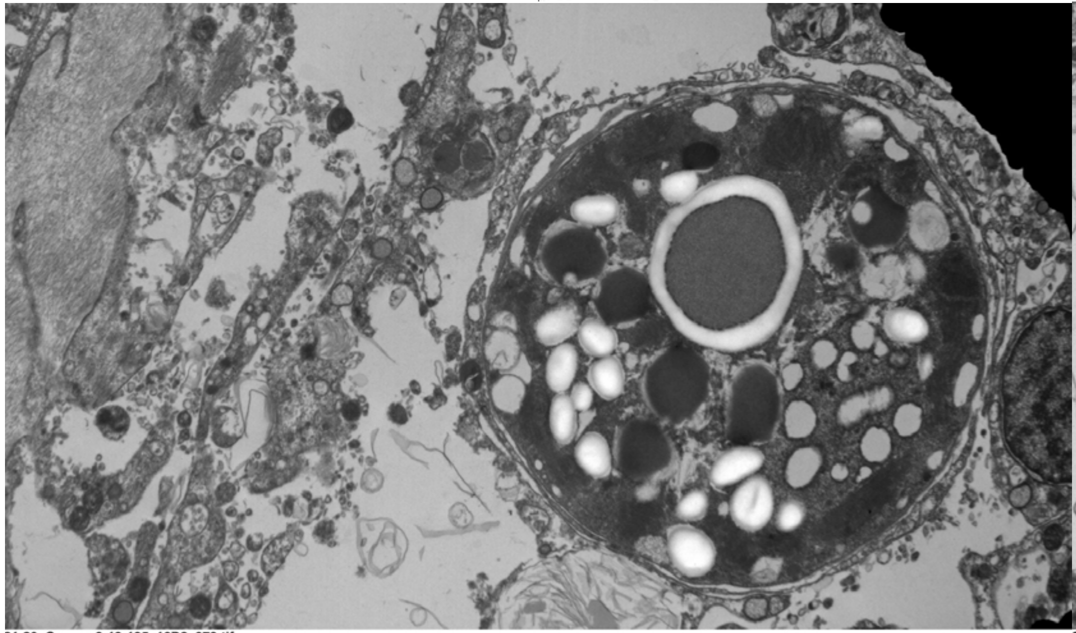

21-20_Correa_3-19-135_16D2_079.tif
Sample: 3/19, 135
Biological Electron Microscopy Lab
Rice University - SEA
Microscopist: MD Meyer

2 µm
HV=80kV
Direct Mag: 1500 x

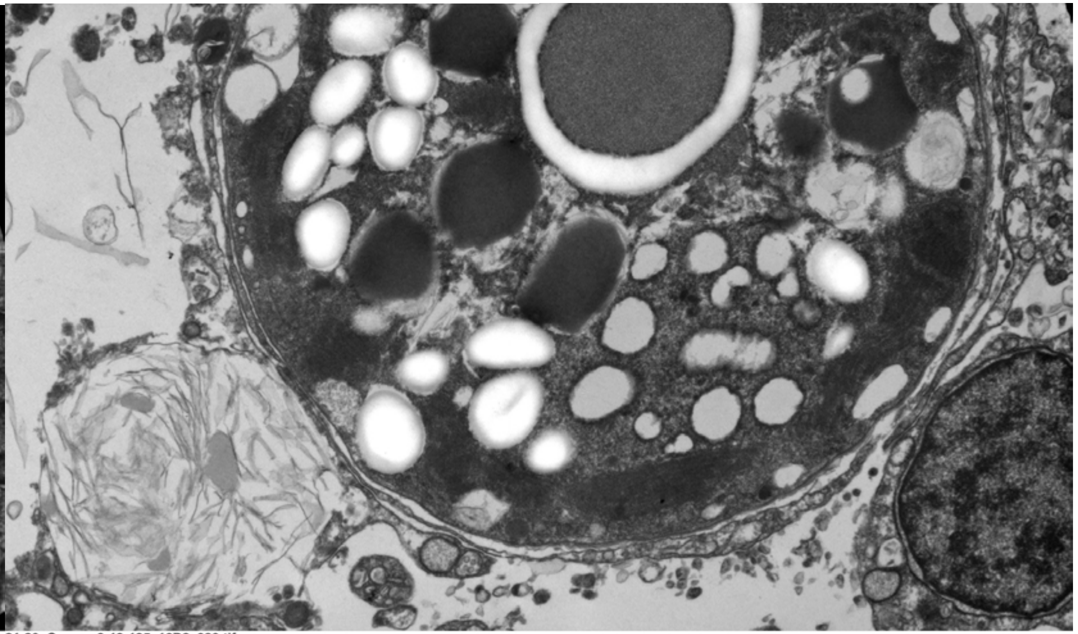

21-20_Correa_3-19-135_16D2_080.tif
Sample: 3/19, 135
Biological Electron Microscopy Lab
Rice University - SEA
Microscopist: MD Meyer

1 µm
HV=80kV
Direct Mag: 2500 x

Cell 11

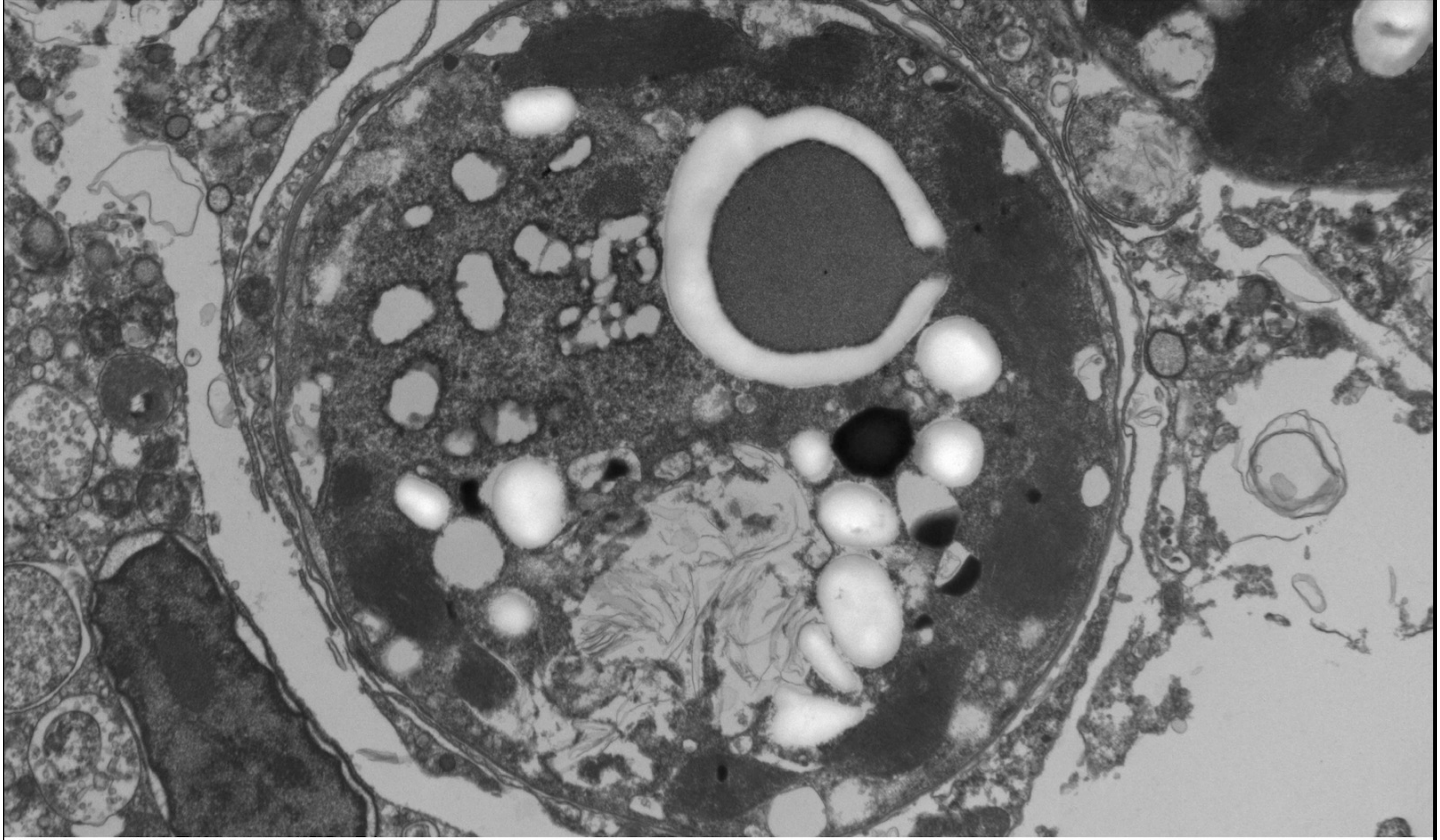

21-20_Correa_3-19-135_16D2_078.tif
Sample: 3/19, 135
Biological Electron Microscopy Lab
Rice University - SEA
Microscopist: MD Meyer

2 µm
HV=80kV
Direct Mag: 2000 x

Cell 12

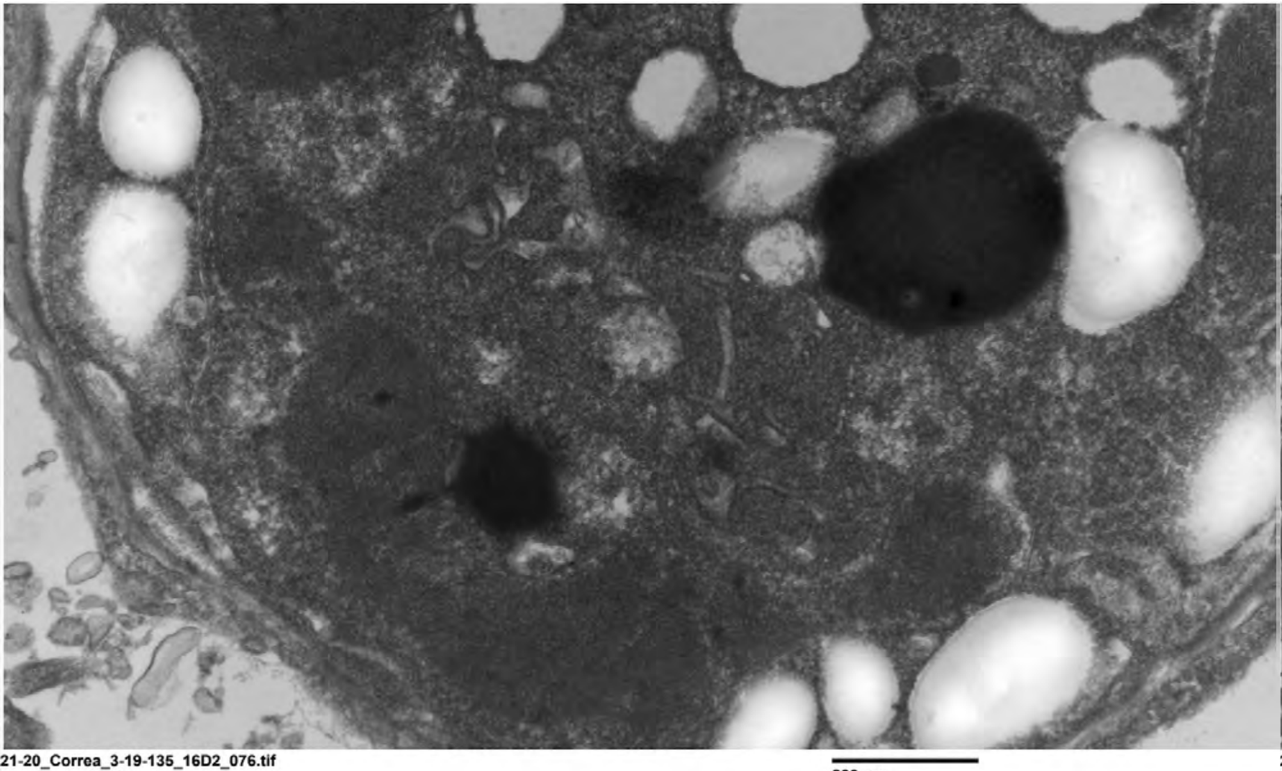

21-20_Correa_3-19-135_16D2_076.tif
Sample: 3/19, 135
Biological Electron Microscopy Lab
Rice University - SEA
Microscopist: MD Meyer

HV=80kV
Direct Mag: 5000 x

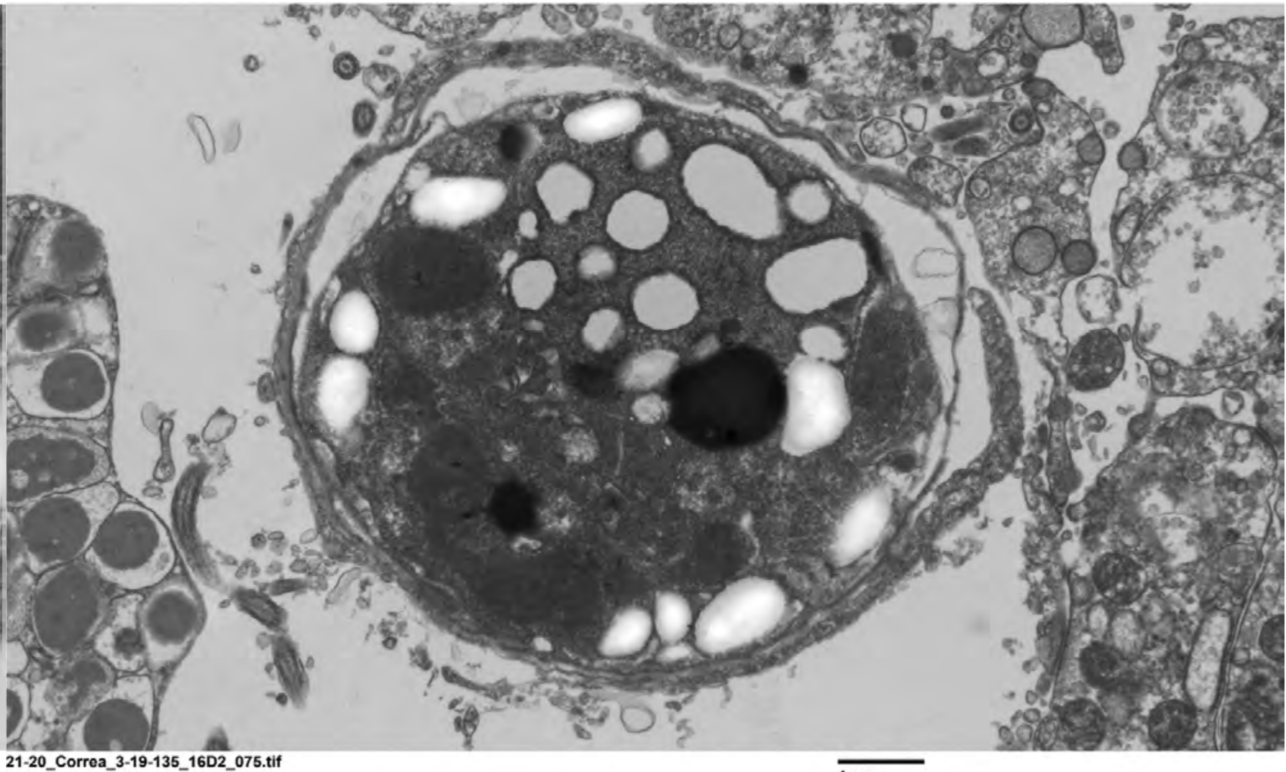

21-20_Correa_3-19-135_16D2_075.tif
Sample: 3/19, 135
Biological Electron Microscopy Lab
Rice University - SEA
Microscopist: MD Meyer

HV=80kV
Direct Mag: 2500 x

Cell 13

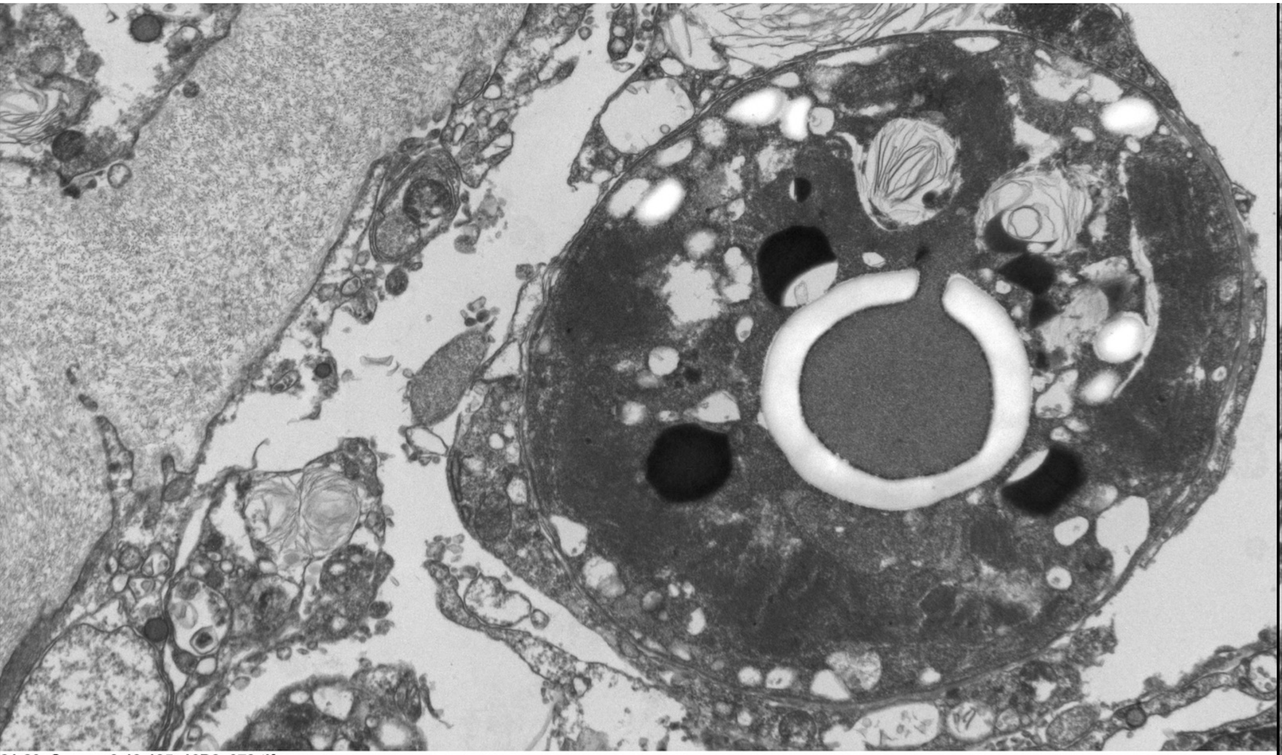

21-20_Correa_3-19-135_16D2_073.tif
Sample: 3/19, 135
Biological Electron Microscopy Lab
Rice University - SEA
Microscopist: MD Meyer

2 µm
HV=80kV
Direct Mag: 2000 x

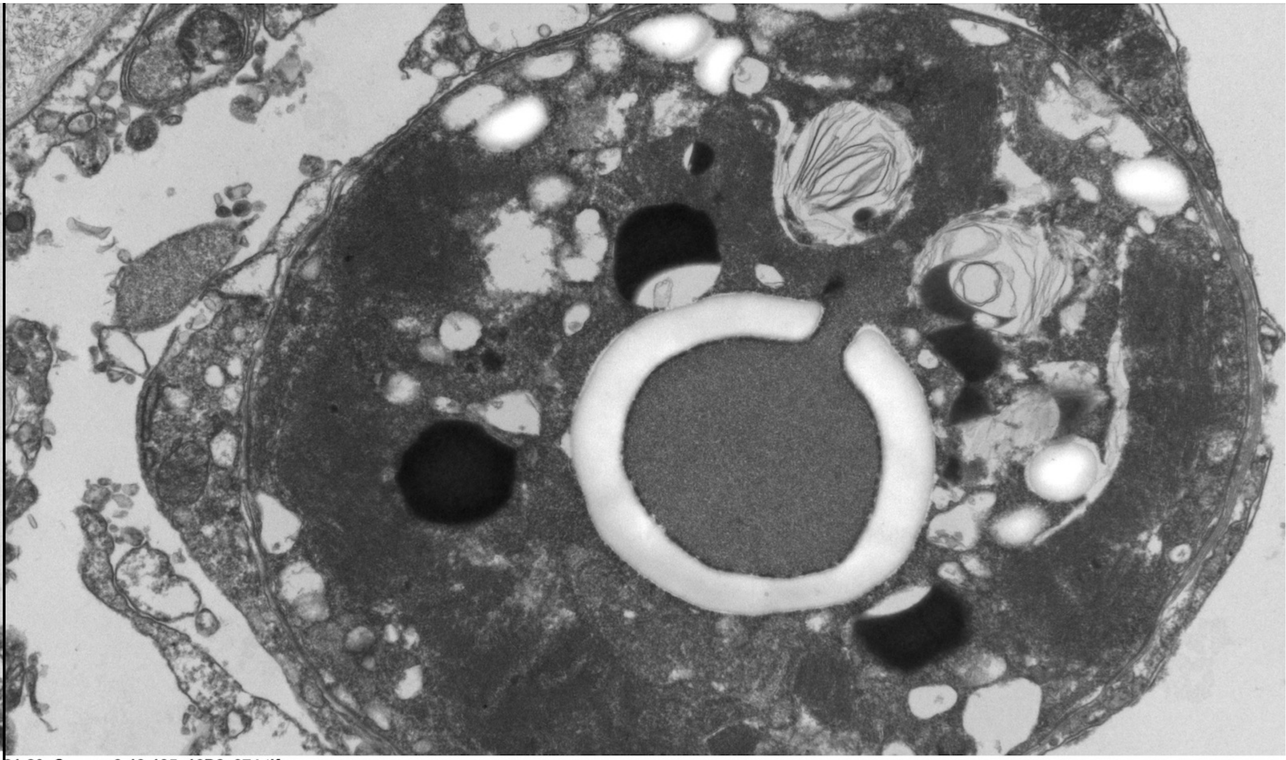

21-20_Correa_3-19-135_16D2_074.tif
Sample: 3/19, 135
Biological Electron Microscopy Lab
Rice University - SEA
Microscopist: MD Meyer

1 µm
HV=80kV
Direct Mag: 3000 x

Cell 14

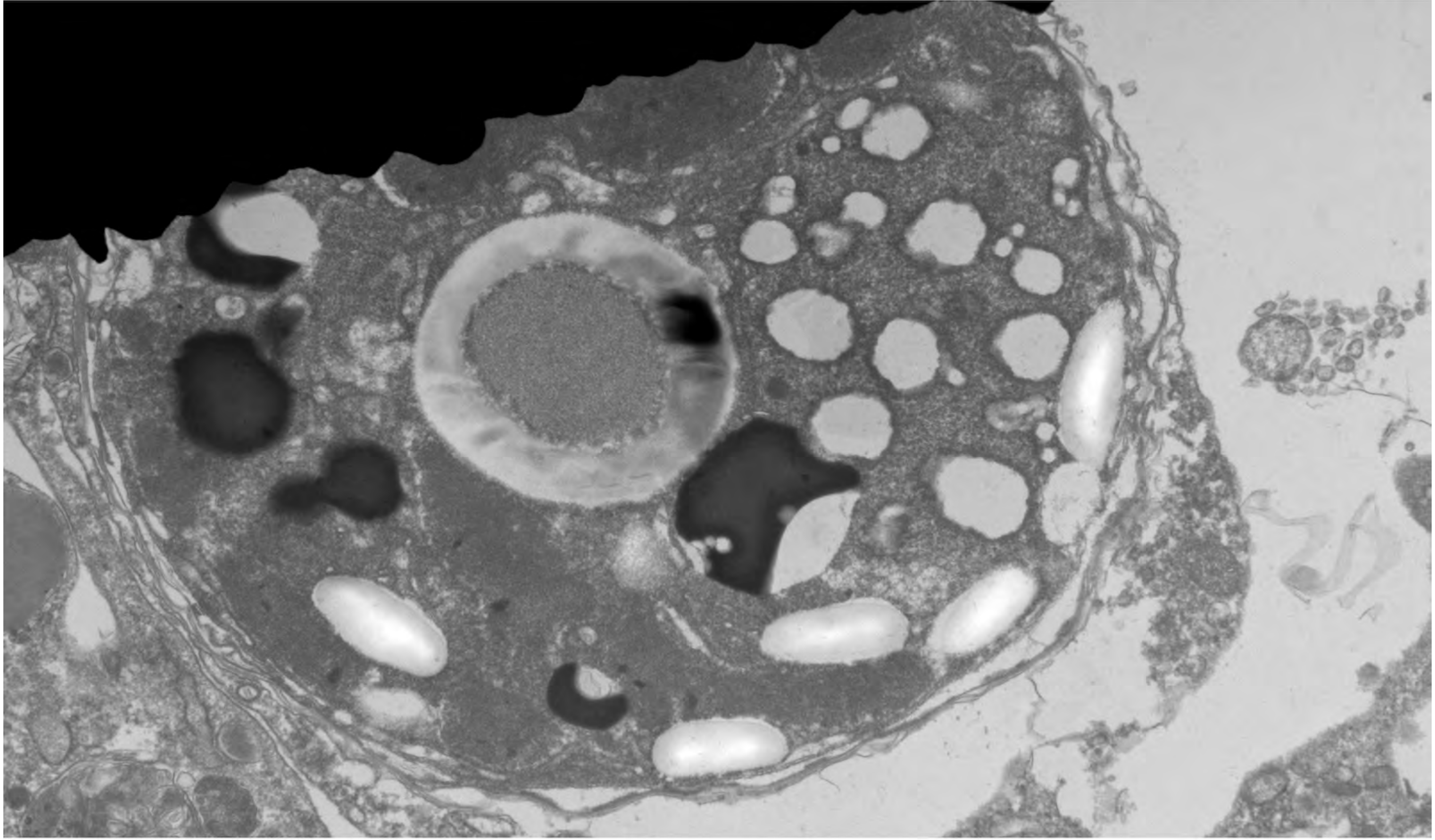

21-20_Correa_3-19-135_16D2_072.tif
Sample: 3/19, 135
Biological Electron Microscopy Lab
Rice University - SEA
Microscopist: MD Meyer

1 µm
HV=80kV
Direct Mag: 3000 x

Cell 15

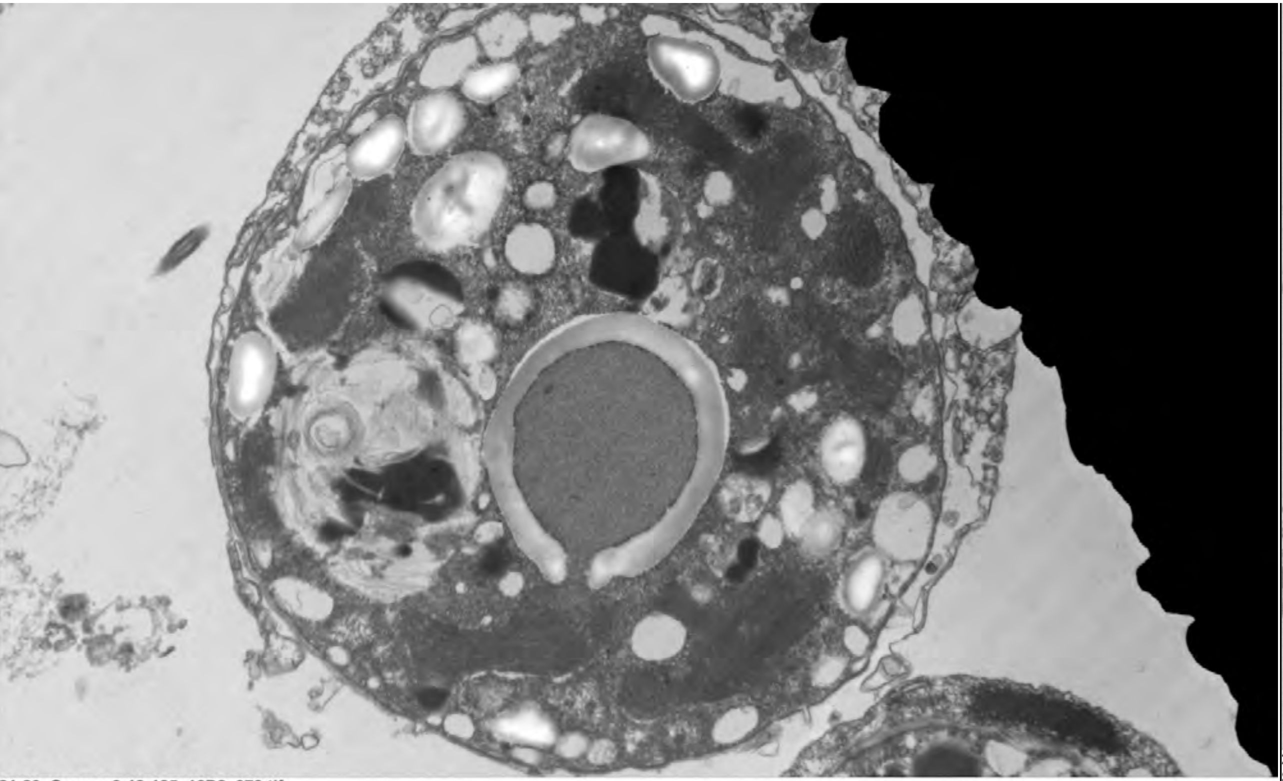

21-20_Correa_3-19-135_16D2_070.tif
Sample: 3/19, 135
Biological Electron Microscopy Lab
Rice University - SEA
Microscopist: MD Meyer

2 µm
HV=80kV
Direct Mag: 2000 x

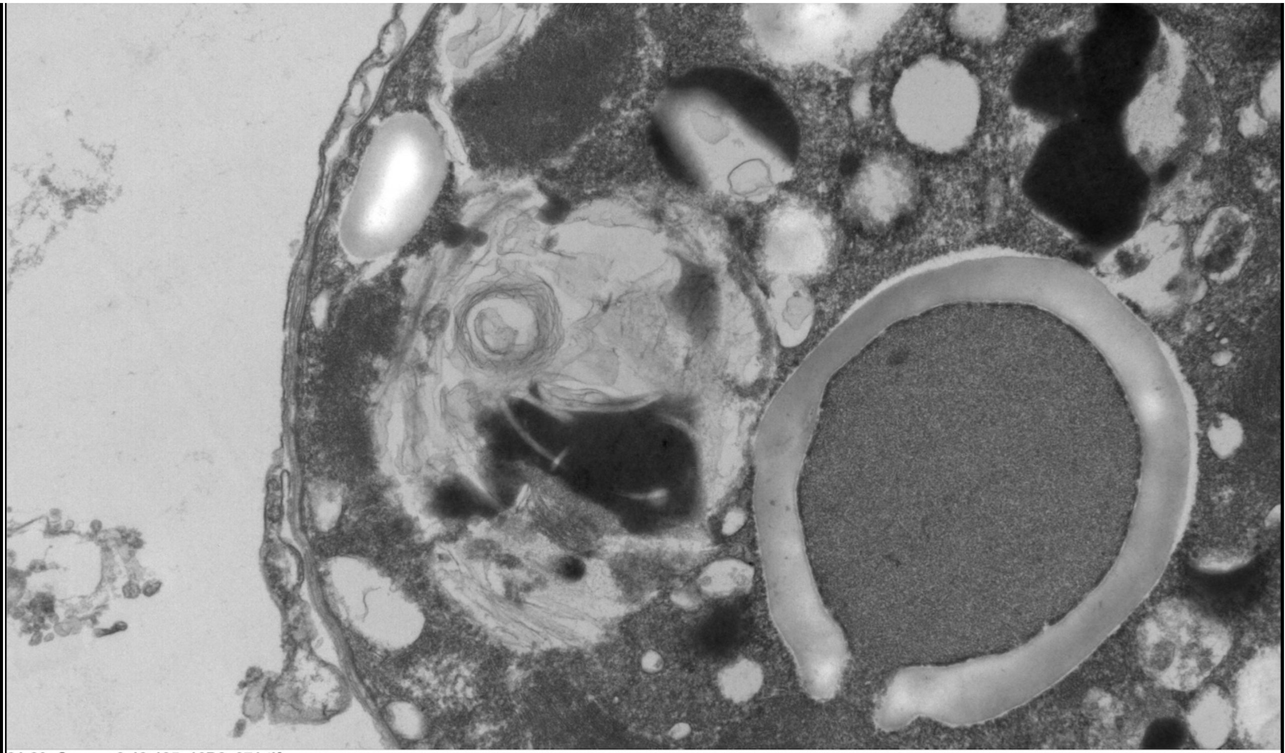

21-20_Correa_3-19-135_16D2_071.tif
Sample: 3/19, 135
Biological Electron Microscopy Lab
Rice University - SEA
Microscopist: MD Meyer

1 µm
HV=80kV
Direct Mag: 4000 x

Cell 16

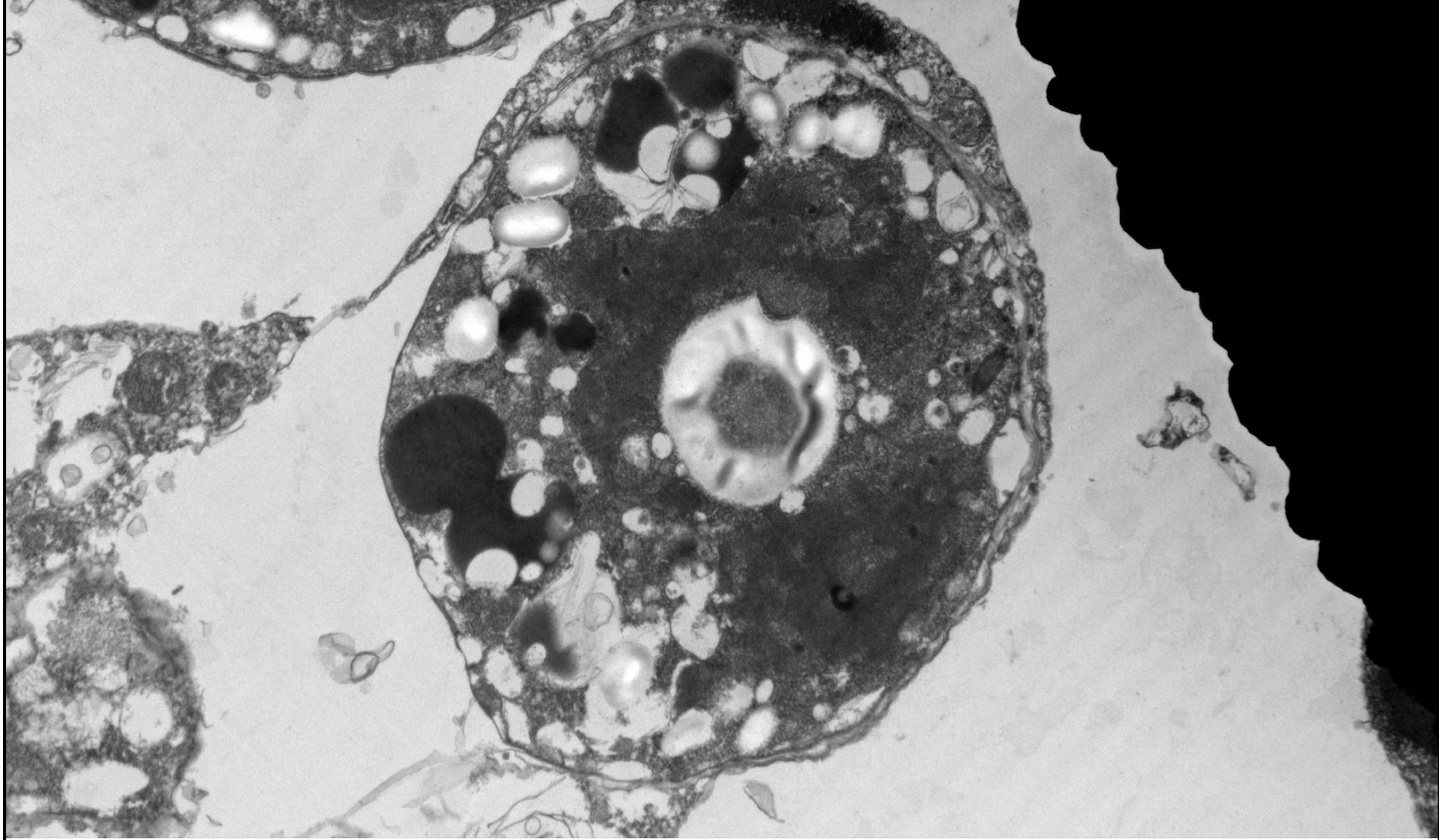

21-20_Correa_3-19-135_16D2_069.tif
Sample: 3/19, 135
Biological Electron Microscopy Lab
Rice University - SEA
Microscopist: MD Meyer

2 µm
HV=80kV
Direct Mag: 2000 x

Cell 17

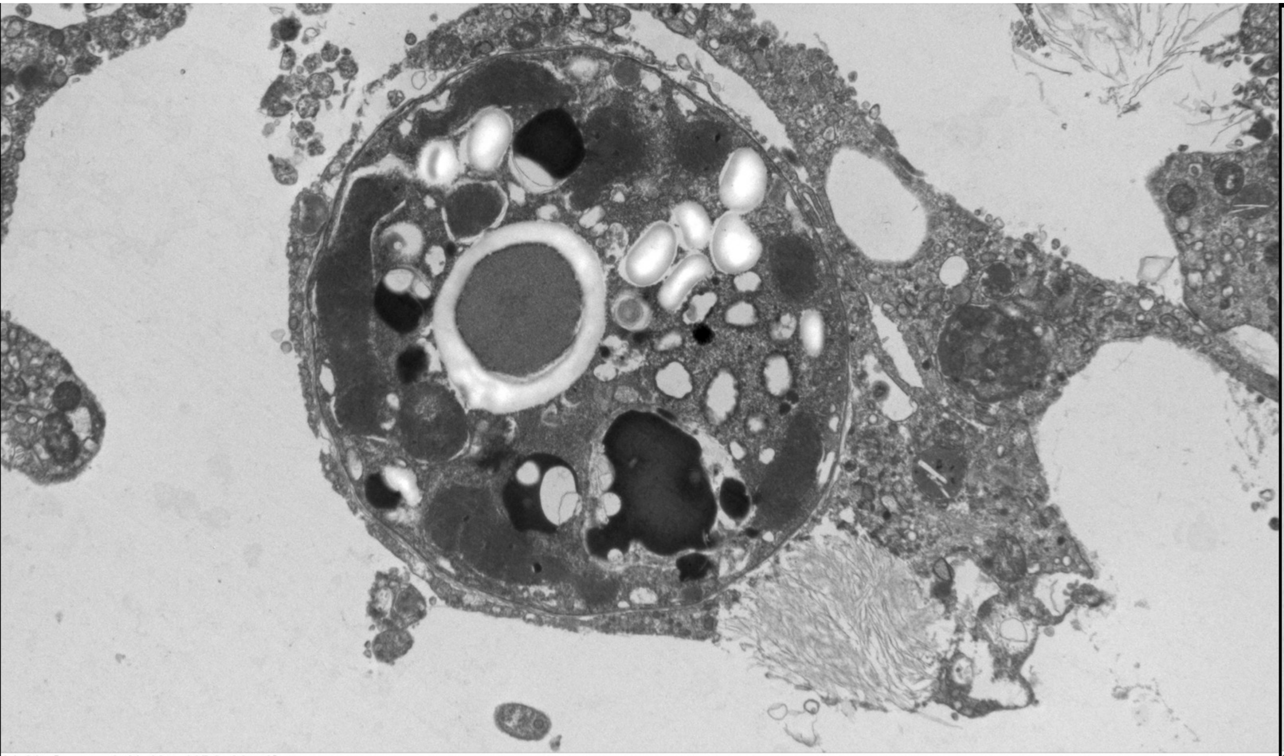

21-20_Correa_3-19-135_16D2_065.tif
Sample: 3/19, 135
Biological Electron Microscopy Lab
Rice University - SEA
Microscopist: MD Meyer

2 µm
HV=80kV
Direct Mag: 1500 x

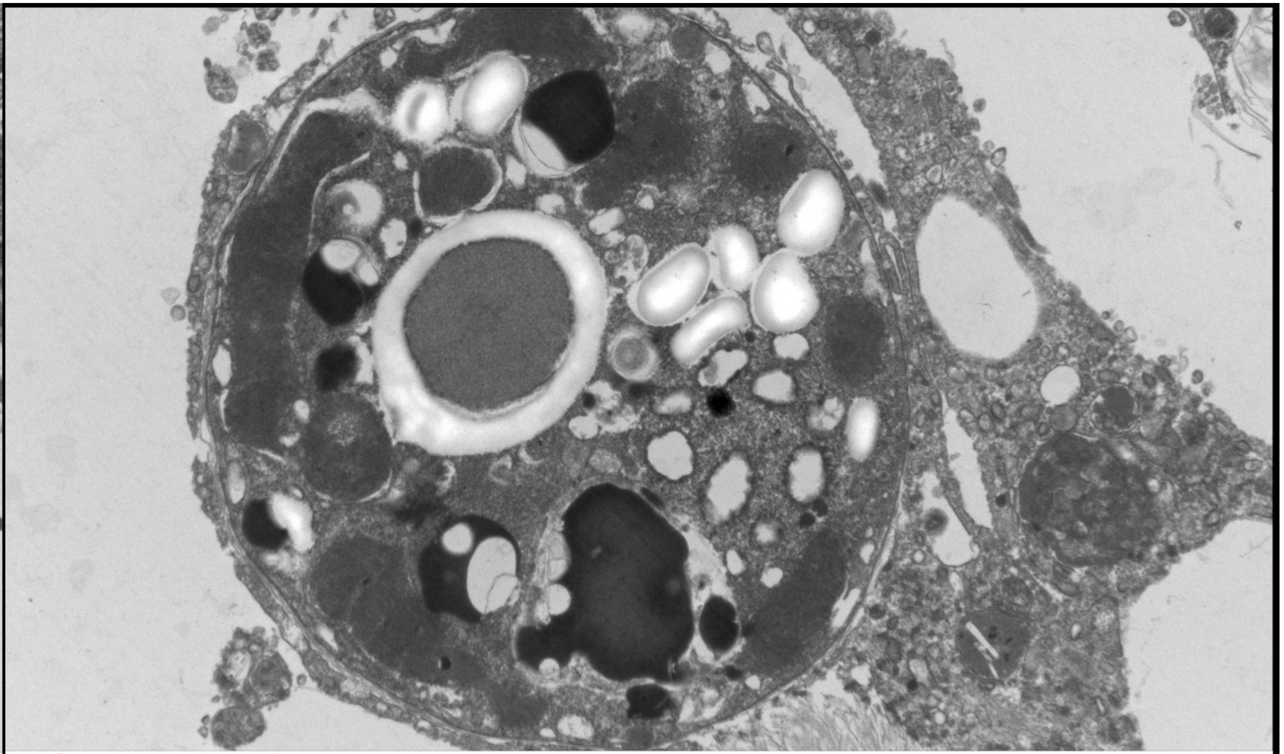

21-20_Correa_3-19-135_16D2_066.tif
Sample: 3/19, 135
Biological Electron Microscopy Lab
Rice University - SEA
Microscopist: MD Meyer

2 µm
HV=80kV
Direct Mag: 2000 x

Cell 18

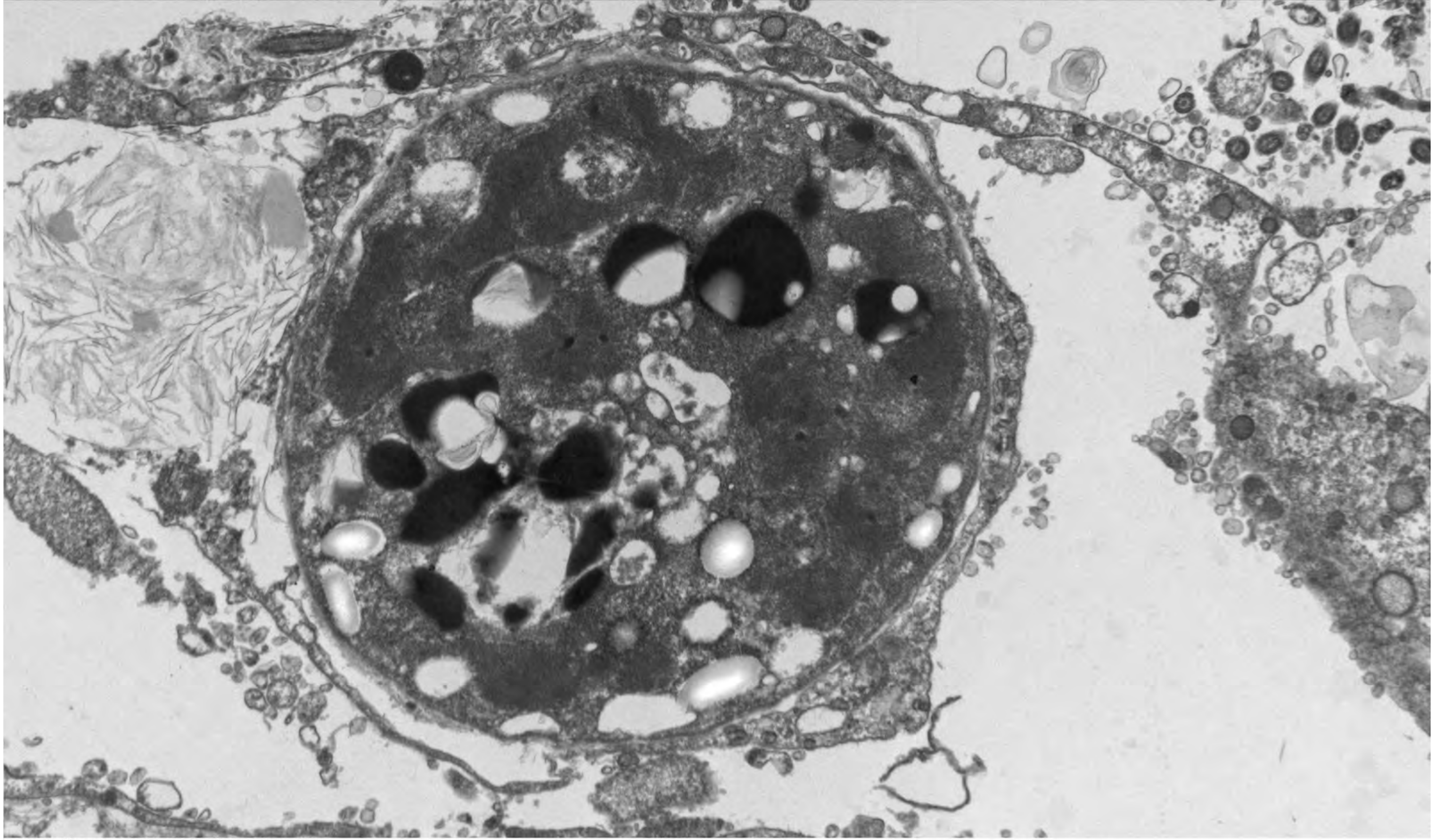

21-20_Correa_3-19-135_16D2_064.tif
Sample: 3/19, 135
Biological Electron Microscopy Lab
Rice University - SEA
Microscopist: MD Meyer

2 µm
HV=80kV
Direct Mag: 2000 x

Cell 19

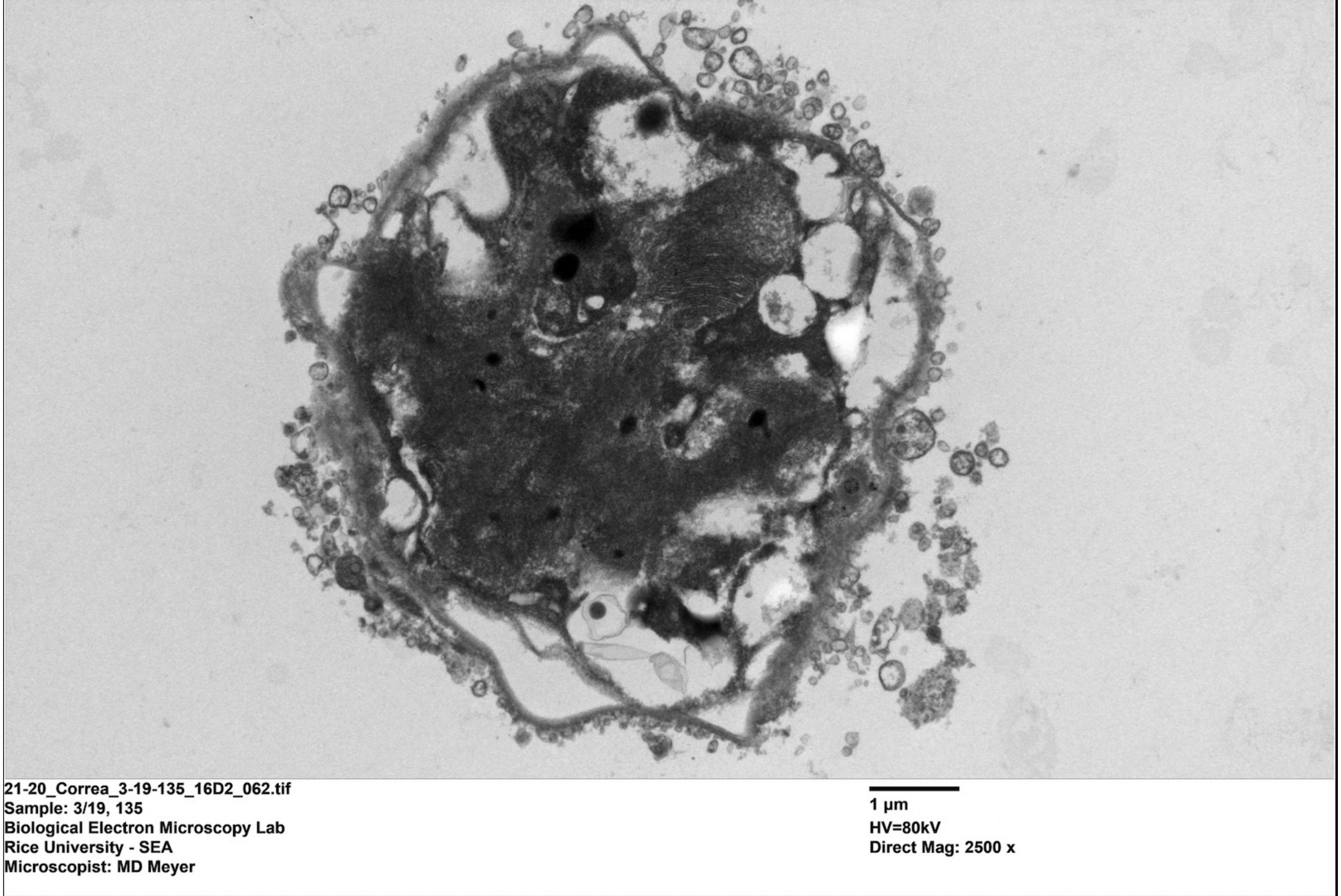
21-20_Correa_3-19-135_16D2_062.tif
Sample: 3/19, 135
Biological Electron Microscopy Lab
Rice University - SEA
Microscopist: MD Meyer
1 µm
HV=80kV
Direct Mag: 2500 x

Cell 20

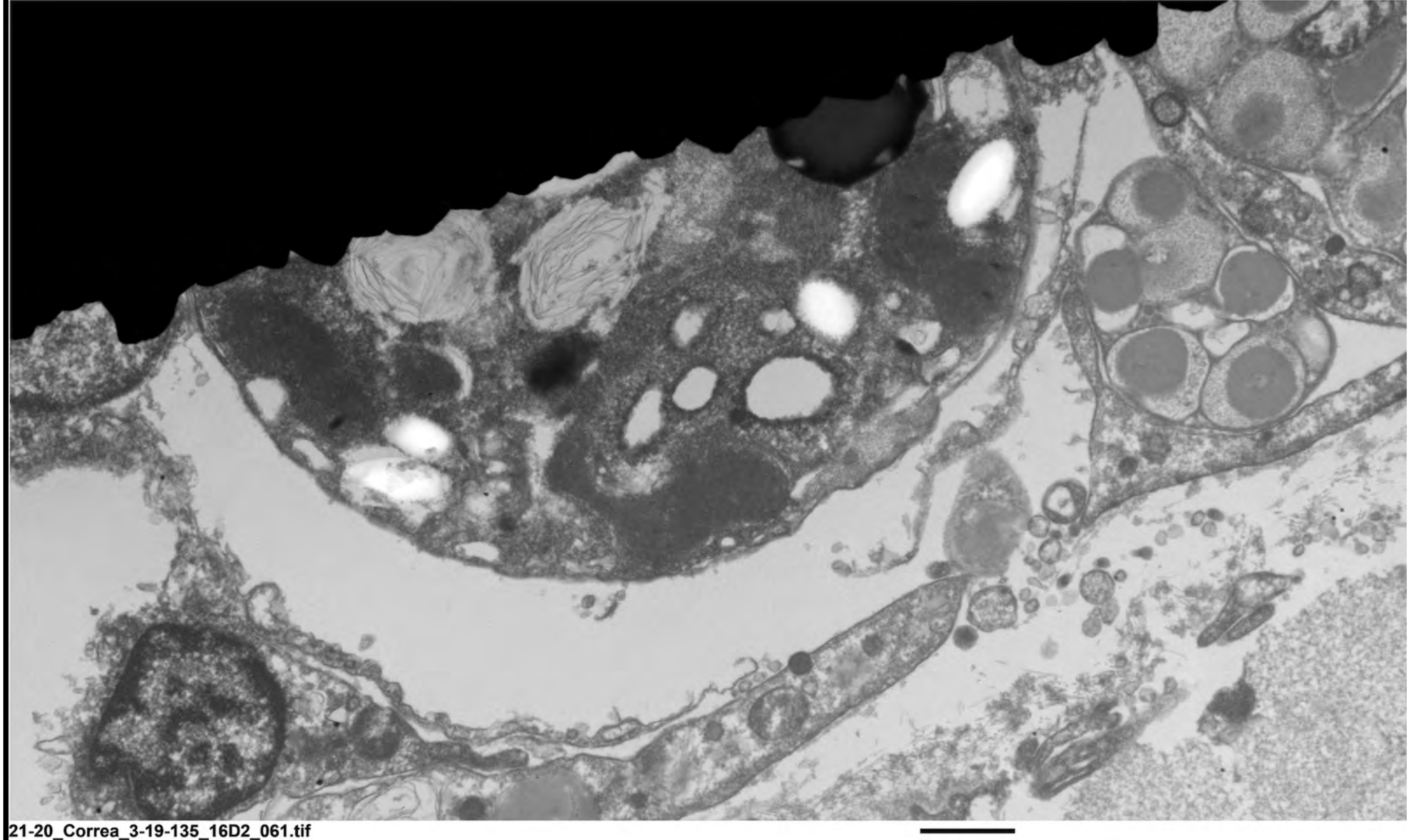

21-20_Correa_3-19-135_16D2_061.tif
Sample: 3/19, 135
Biological Electron Microscopy Lab
Rice University - SEA
Microscopist: MD Meyer

HV=80kV
Direct Mag: 2500 x

Cell 21

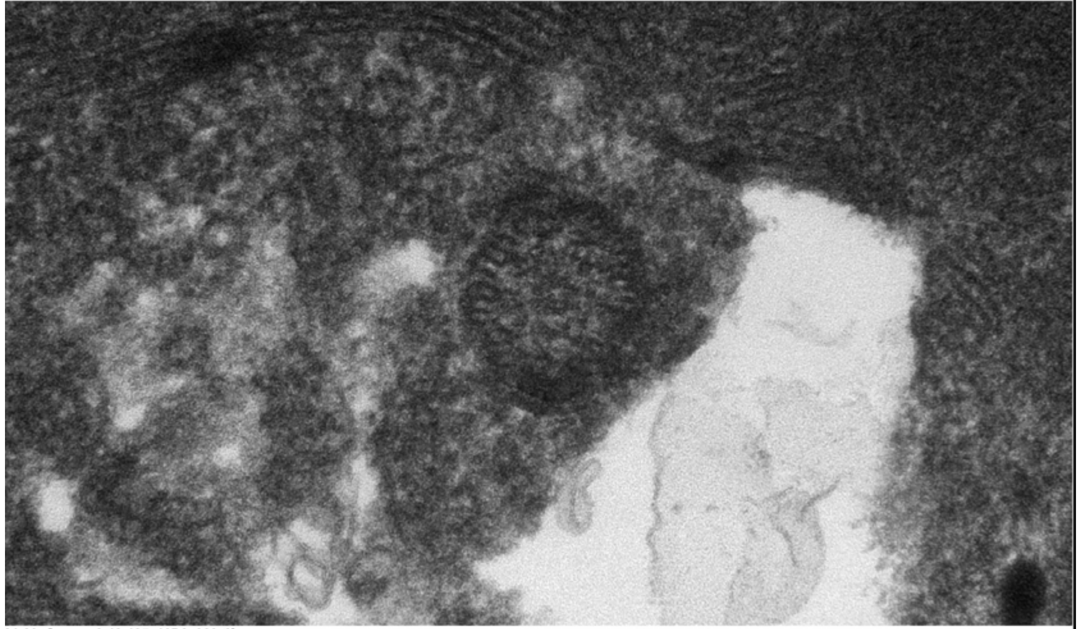

21-20_Correa_3-19-135_16D2_060.tif
Sample: 3/19, 135
Biological Electron Microscopy Lab
Rice University - SEA
Microscopist: MD Meyer

100 nm
HV=80kV
Direct Mag: 25000 x

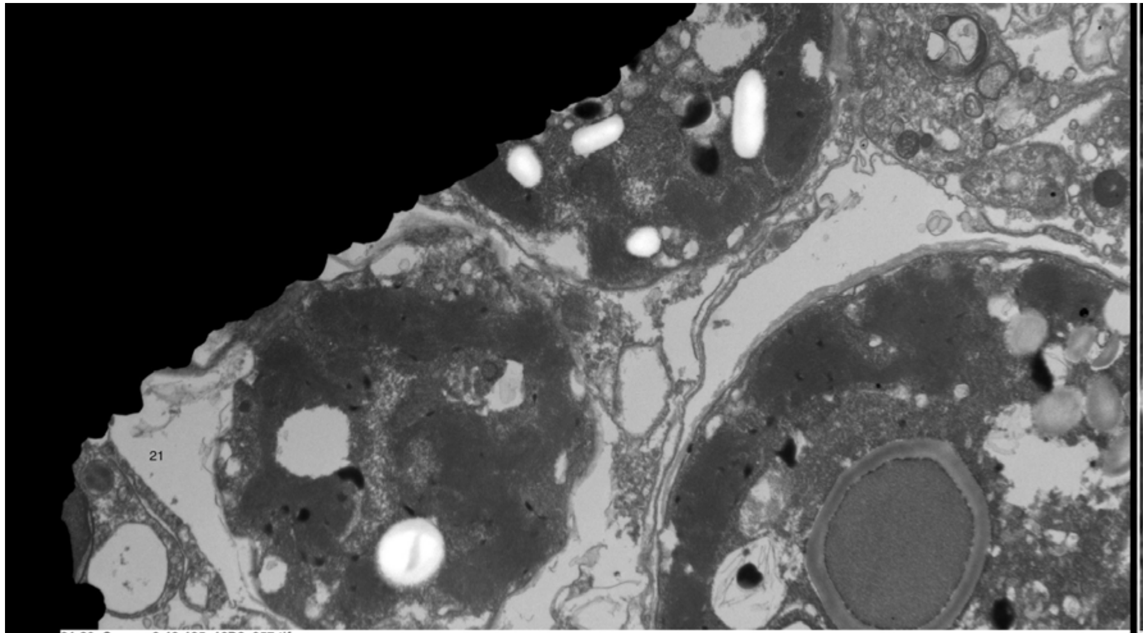

21-20_Correa_3-19-135_16D2_057.tif
Sample: 3/19, 135
Biological Electron Microscopy Lab
Rice University - SEA
Microscopist: MD Meyer

2 µm
HV=80kV
Direct Mag: 2000 x

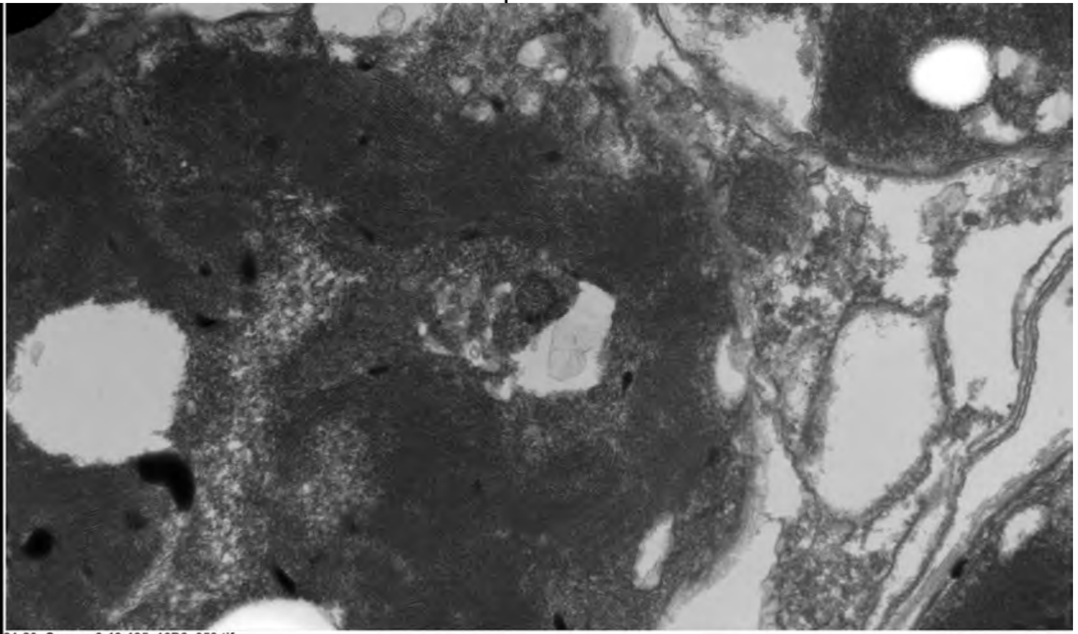

21-20_Correa_3-19-135_16D2_058.tif
Sample: 3/19, 135
Biological Electron Microscopy Lab
Rice University - SEA
Microscopist: MD Meyer

800 nm
HV=80kV
Direct Mag: 5000 x

Cell 21_2

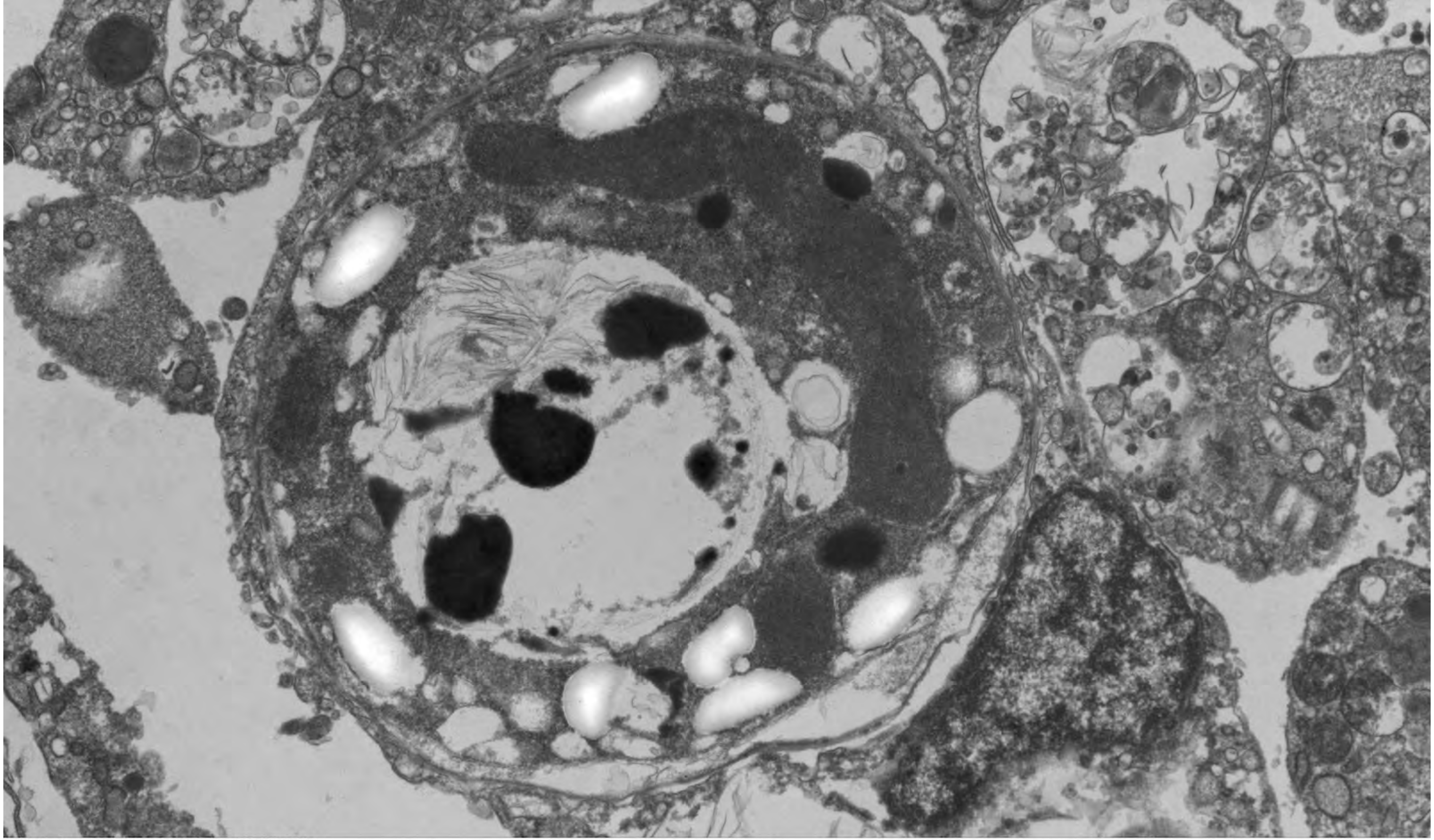

21-20_Correa_3-19-135_16D2_067.tif
Sample: 3/19, 135
Biological Electron Microscopy Lab
Rice University - SEA
Microscopist: MD Meyer

1 µm
HV=80kV
Direct Mag: 2500 x

Cell 22

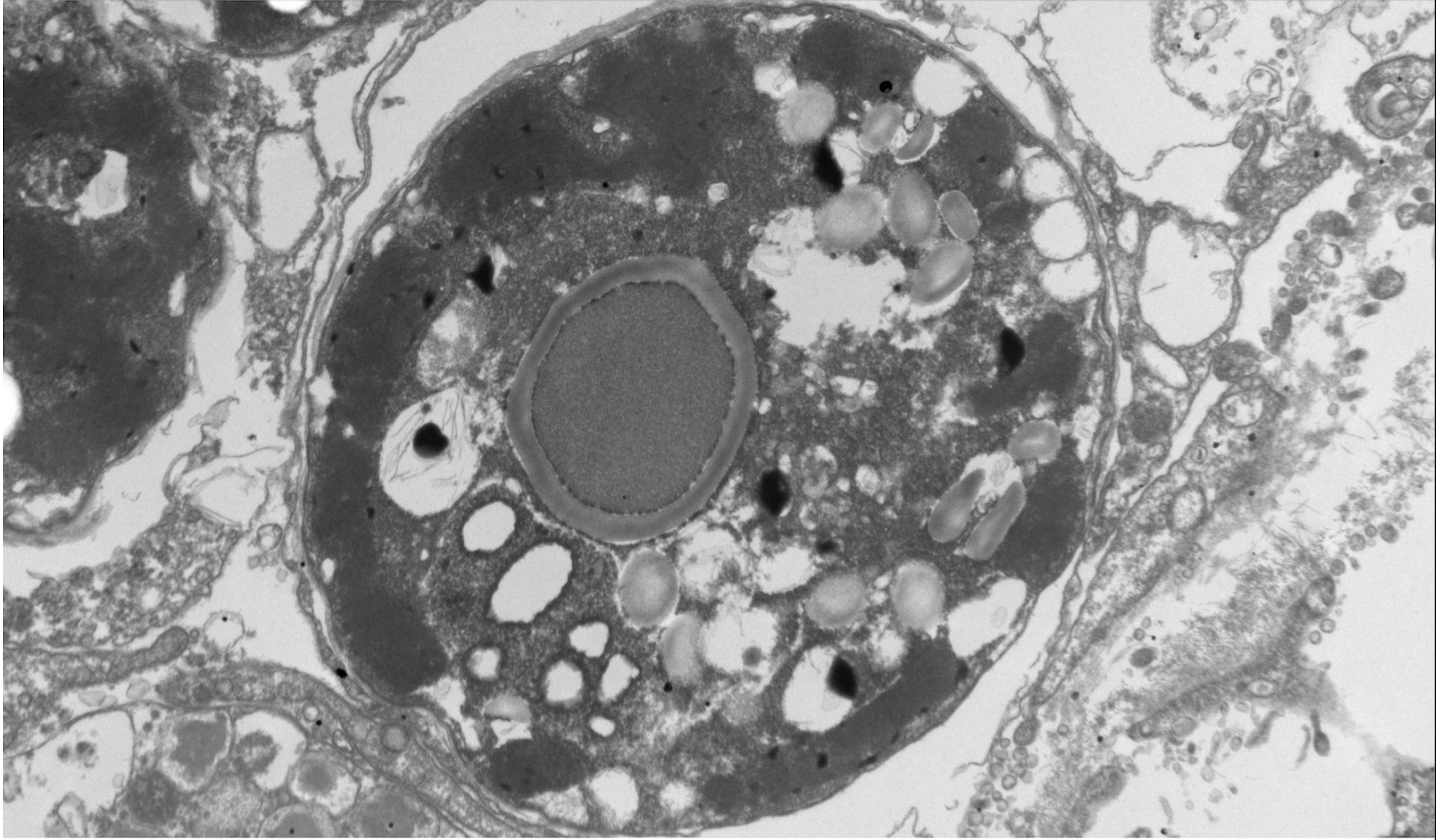

21-20_Correa_3-19-135_16D2_056.tif
Sample: 3/19, 135
Biological Electron Microscopy Lab
Rice University - SEA
Microscopist: MD Meyer

2 µm
HV=80kV
Direct Mag: 2000 x

Cell 23

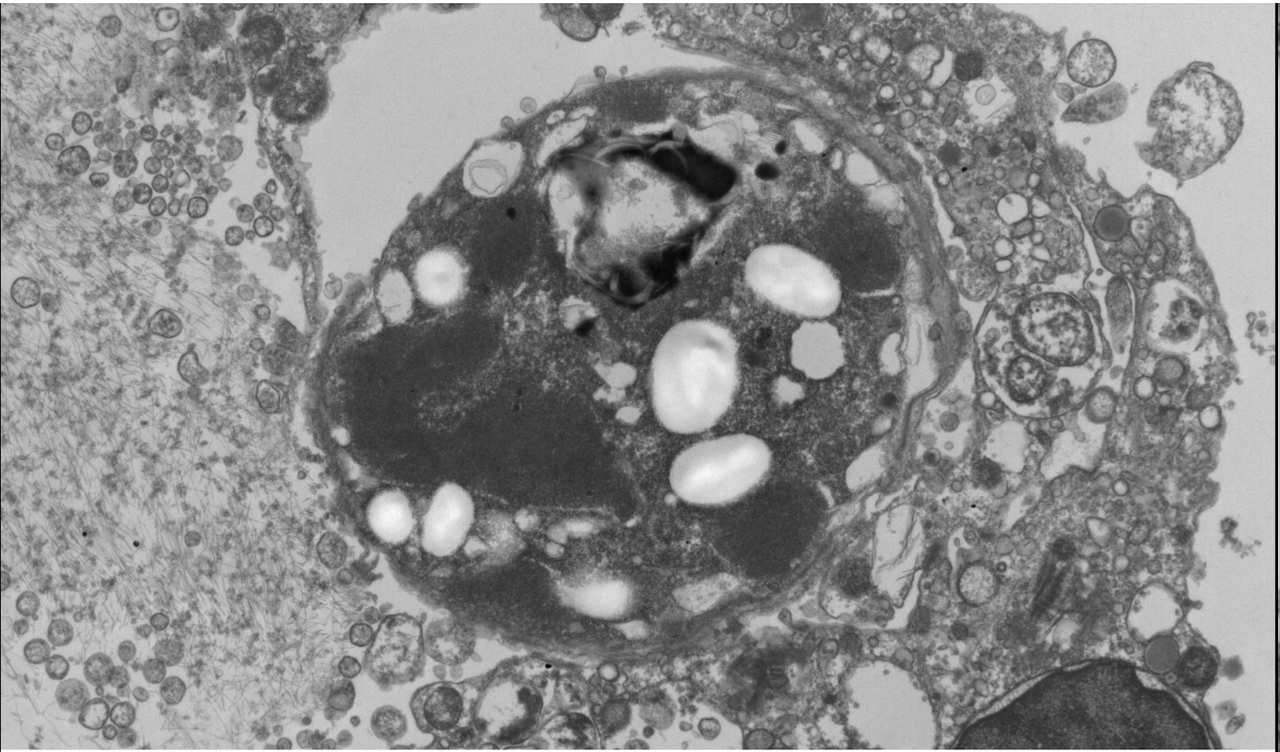

21-20_Correa_3-19-135_16D2_054.tif
Sample: 3/19, 135
Biological Electron Microscopy Lab
Rice University - SEA
Microscopist: MD Meyer

1 µm
HV=80kV
Direct Mag: 2500 x

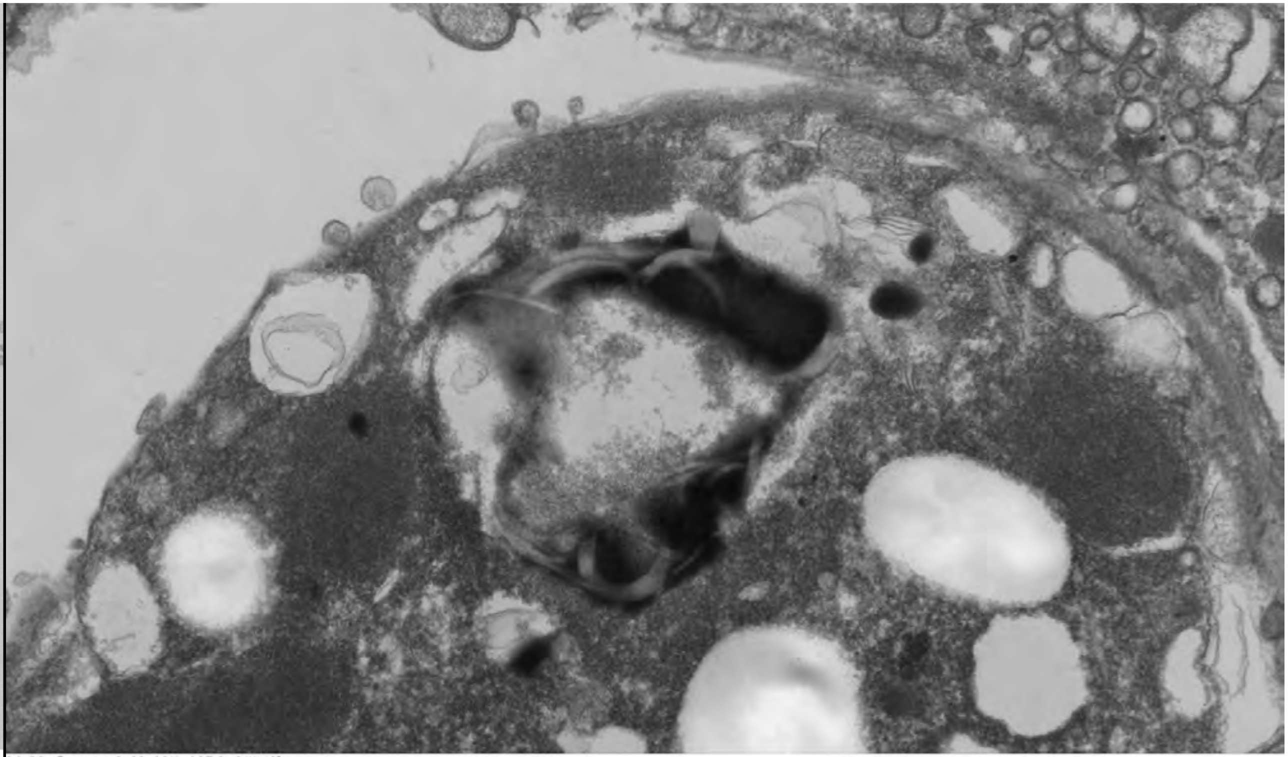

21-20_Correa_3-19-135_16D2_055.tif
Sample: 3/19, 135
Biological Electron Microscopy Lab
Rice University - SEA
Microscopist: MD Meyer

800 nm
HV=80kV
Direct Mag: 5000 x

Cell 24

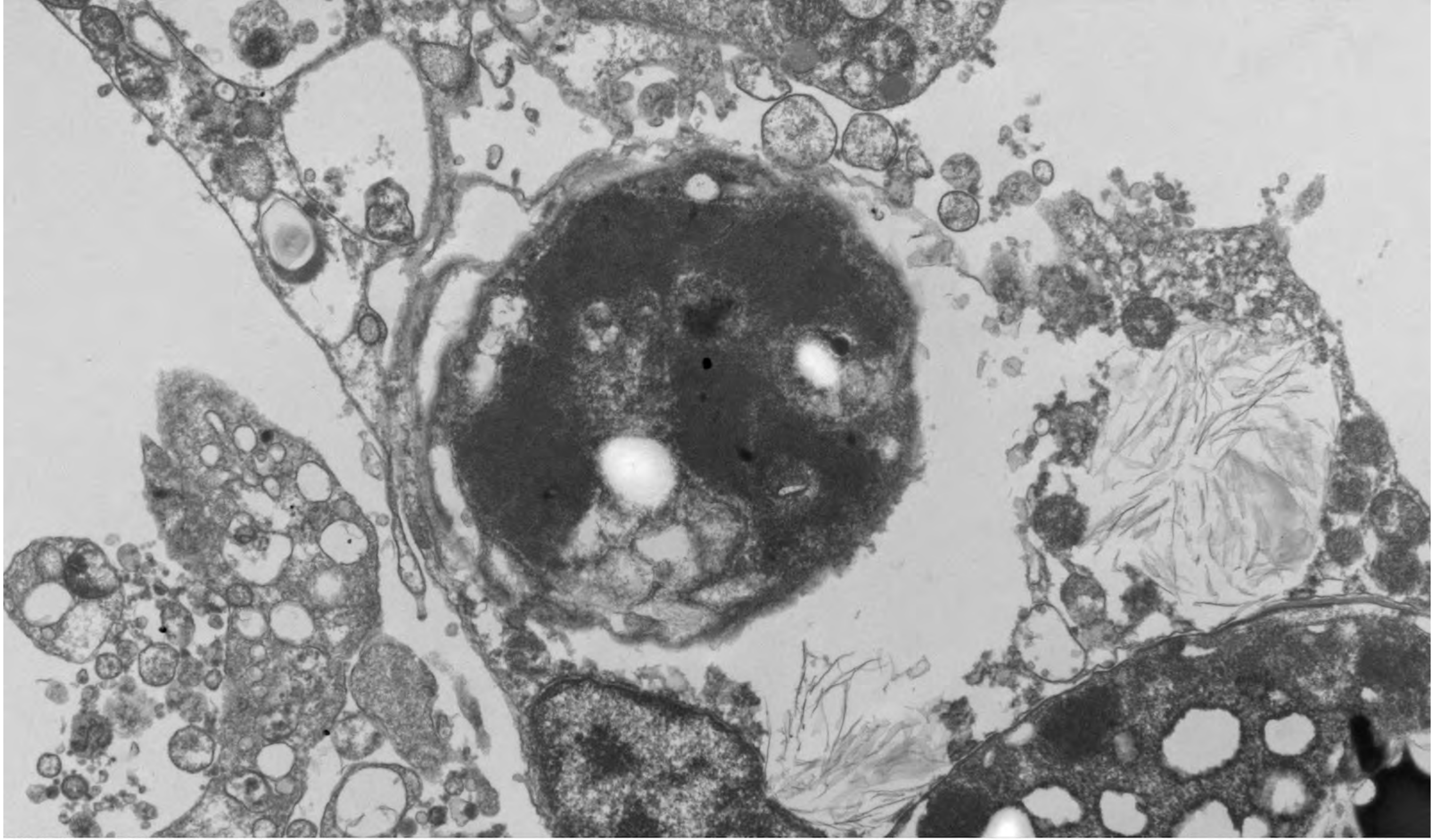

21-20_Correa_3-19-135_16D2_053.tif
Sample: 3/19, 135
Biological Electron Microscopy Lab
Rice University - SEA
Microscopist: MD Meyer

1 µm
HV=80kV
Direct Mag: 2500 x

Cell 25

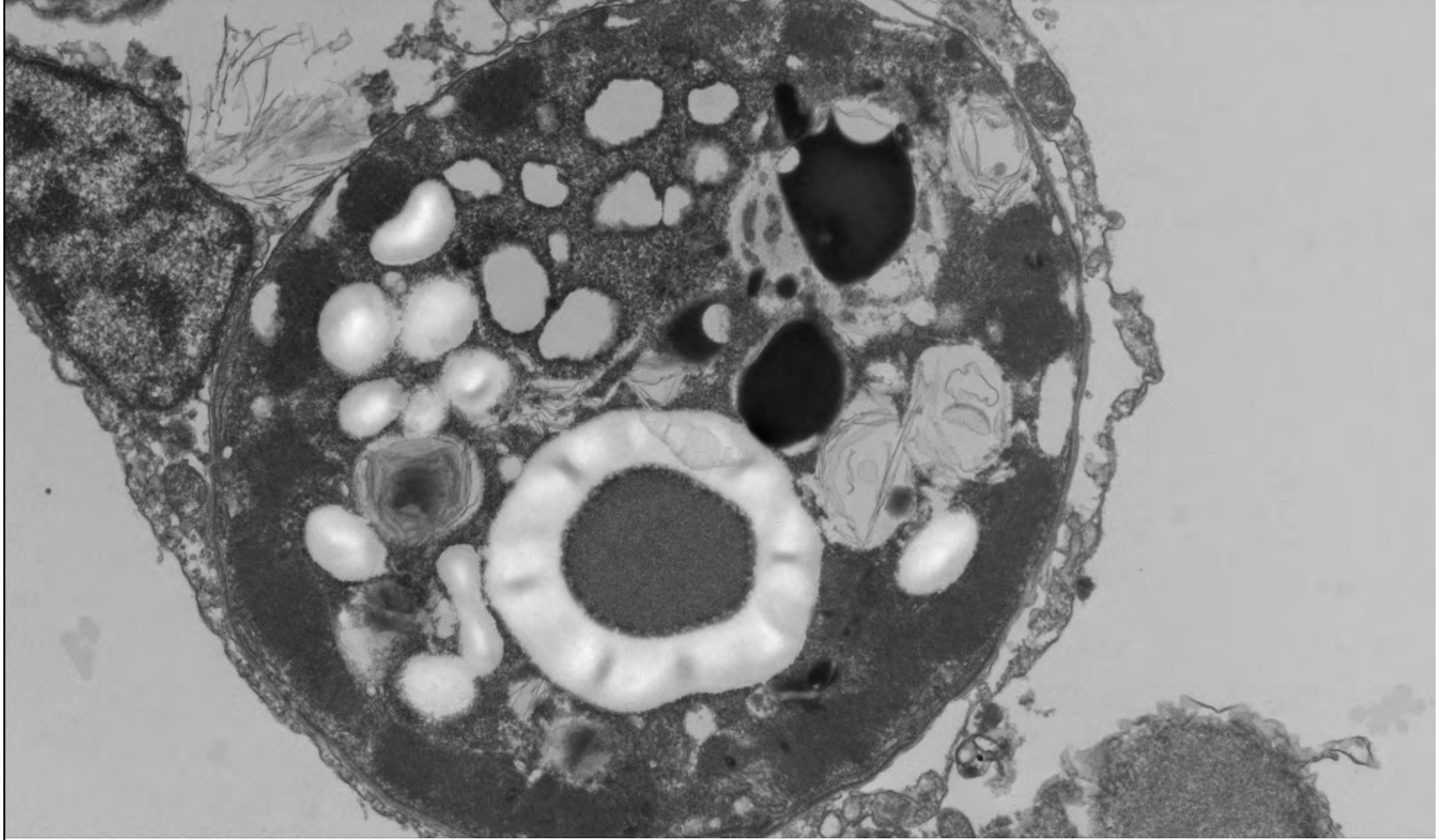

21-20_Correa_3-19-135_16D2_051.tif
Sample: 3/19, 135
Biological Electron Microscopy Lab
Rice University - SEA
Microscopist: MD Meyer

1 µm
HV=80kV
Direct Mag: 2500 x

Cell 26

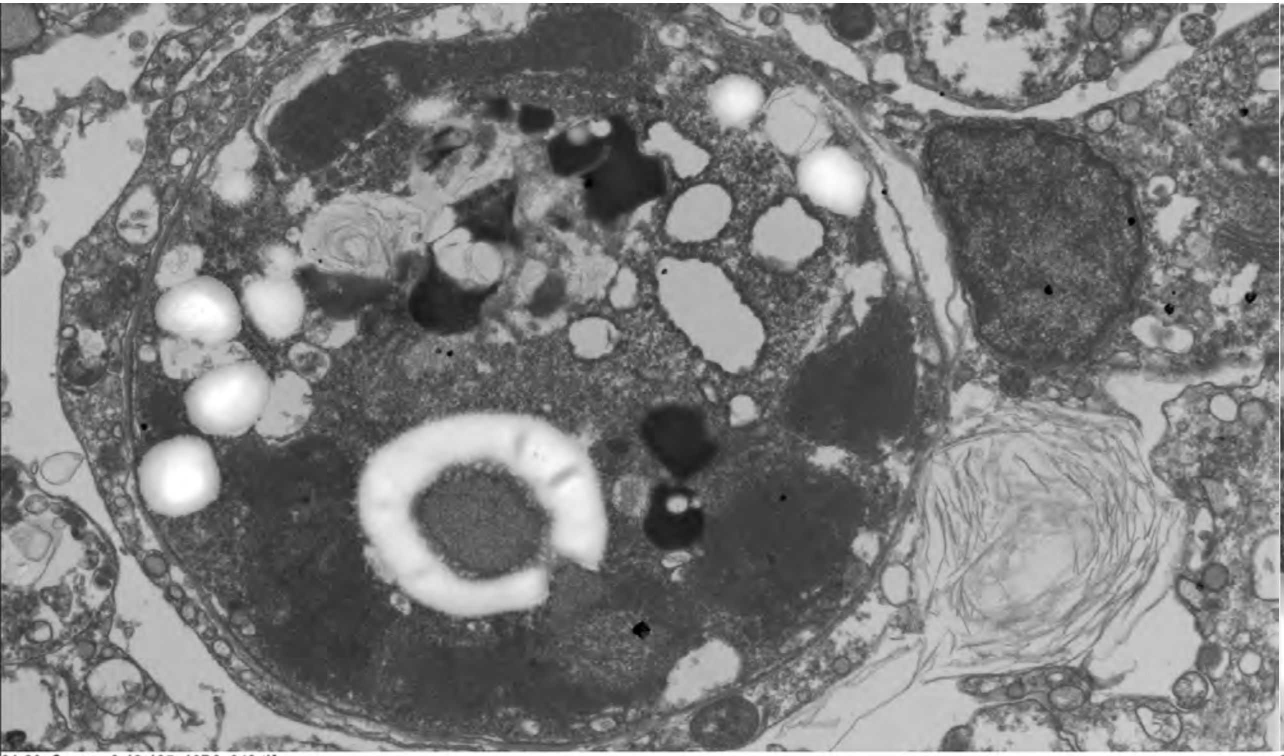

21-20_Correa_3-19-135_16D2_048.tif
Sample: 3/19, 135
Biological Electron Microscopy Lab
Rice University - SEA
Microscopist: MD Meyer

1 µm
HV=80kV
Direct Mag: 2500 x

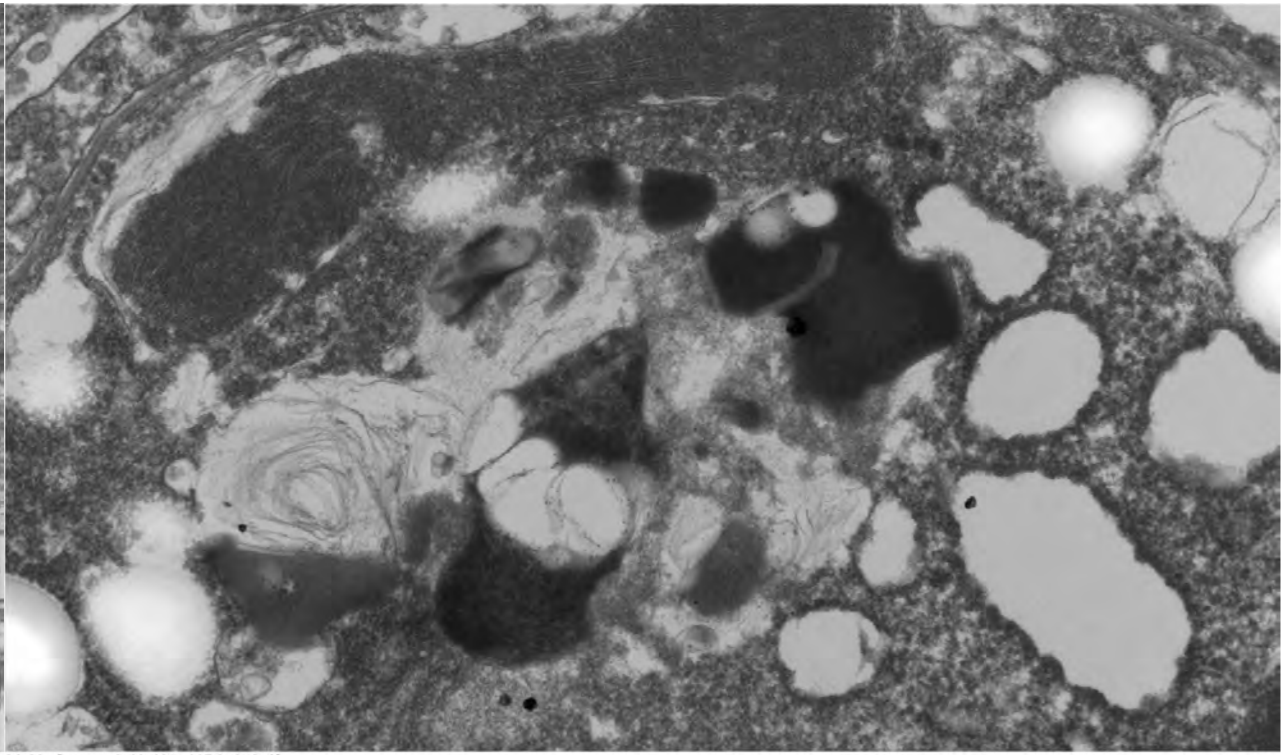

21-20_Correa_3-19-135_16D2_049.tif
Sample: 3/19, 135
Biological Electron Microscopy Lab
Rice University - SEA
Microscopist: MD Meyer

800 nm
HV=80kV
Direct Mag: 5000 x

Cell 27

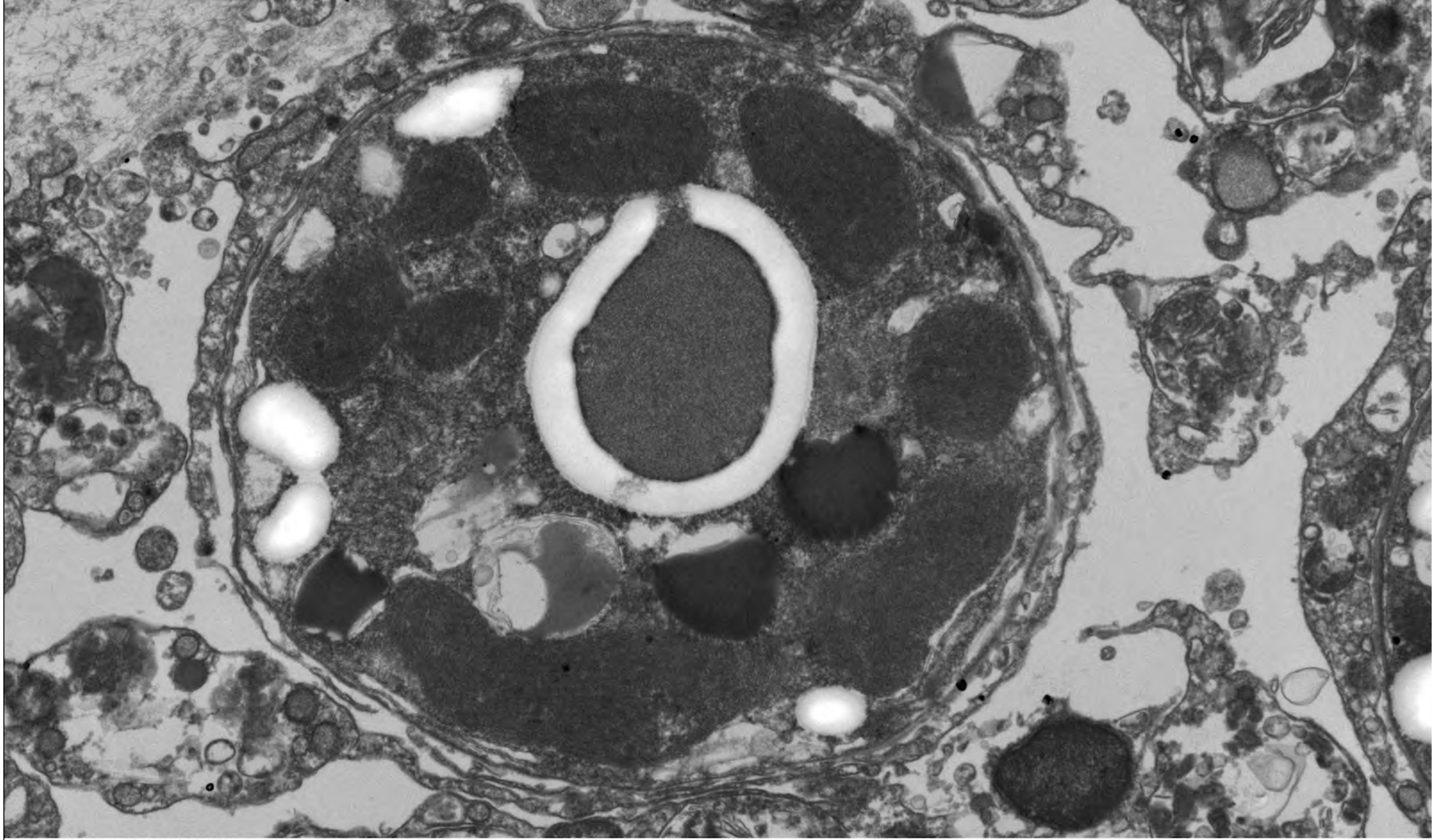

21-20_Correa_3-19-135_16D2_046.tif
Sample: 3/19, 135
Biological Electron Microscopy Lab
Rice University - SEA
Microscopist: MD Meyer

1 µm
HV=80kV
Direct Mag: 2500 x

Cell 28

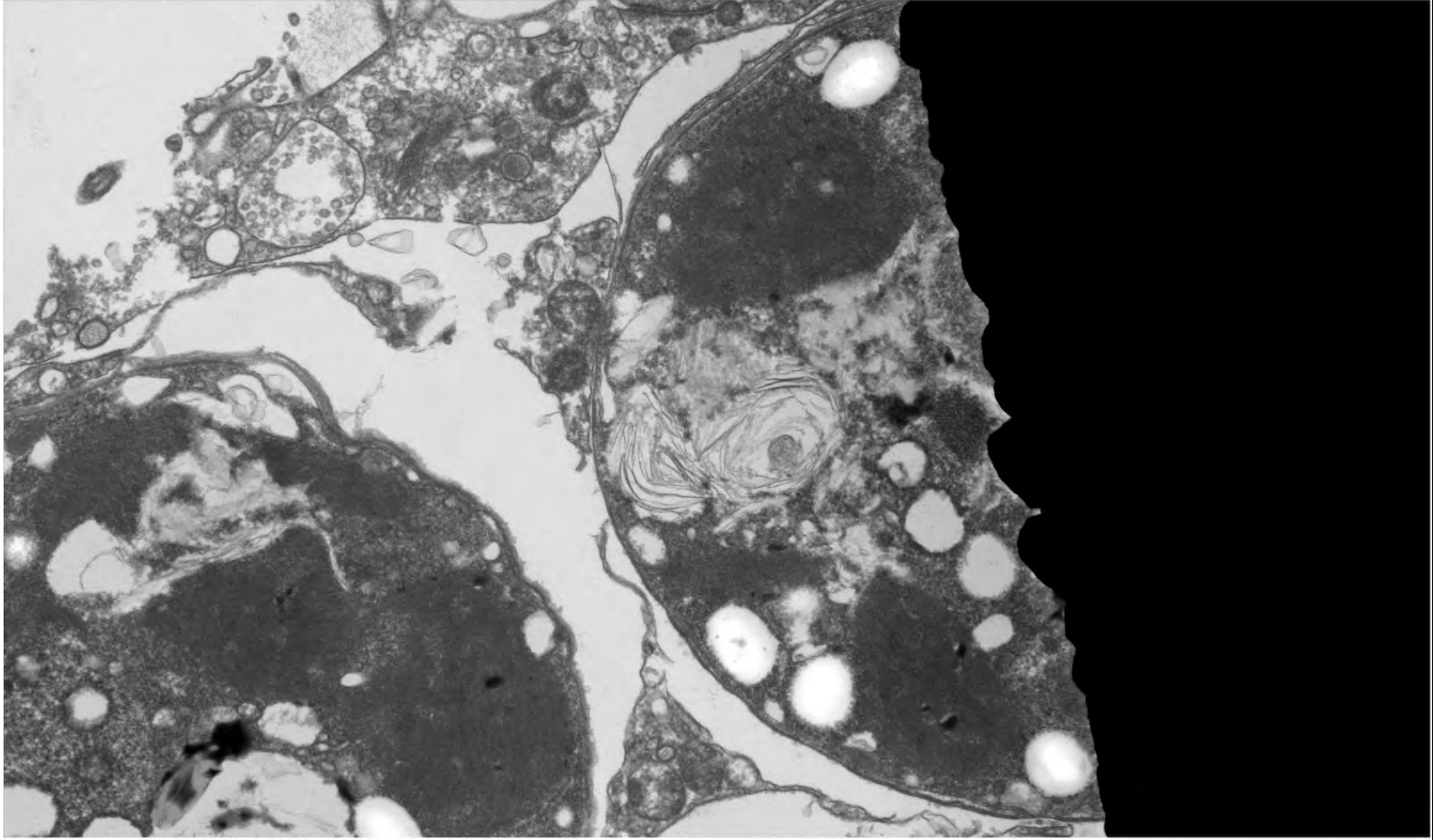

21-20_Correa_3-19-135_16D2_044.tif
Sample: 3/19, 135
Biological Electron Microscopy Lab
Rice University - SEA
Microscopist: MD Meyer

1 µm
HV=80kV
Direct Mag: 2500 x

Cell 29

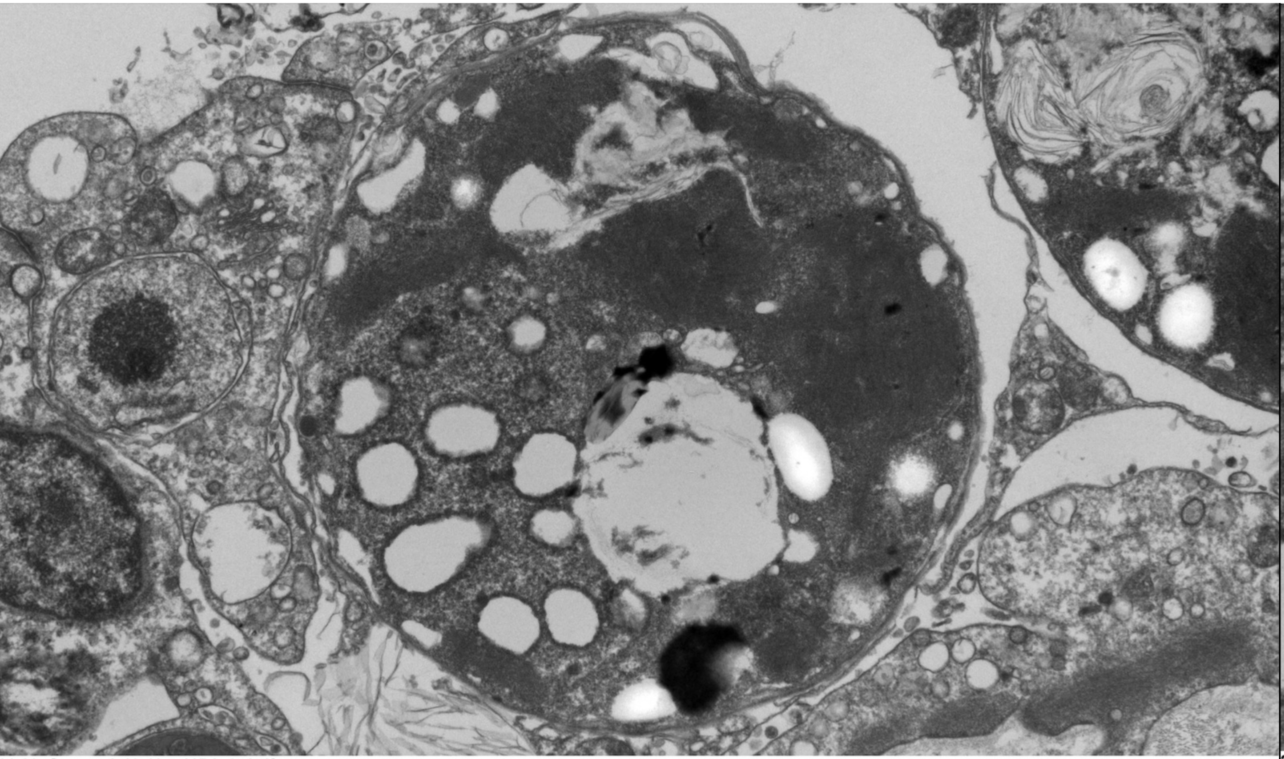

21-20_Correa_3-19-135_16D2_042.tif
Sample: 3/19, 135
Biological Electron Microscopy Lab
Rice University - SEA
Microscopist: MD Meyer

1 µm
HV=80kV
Direct Mag: 2500 x

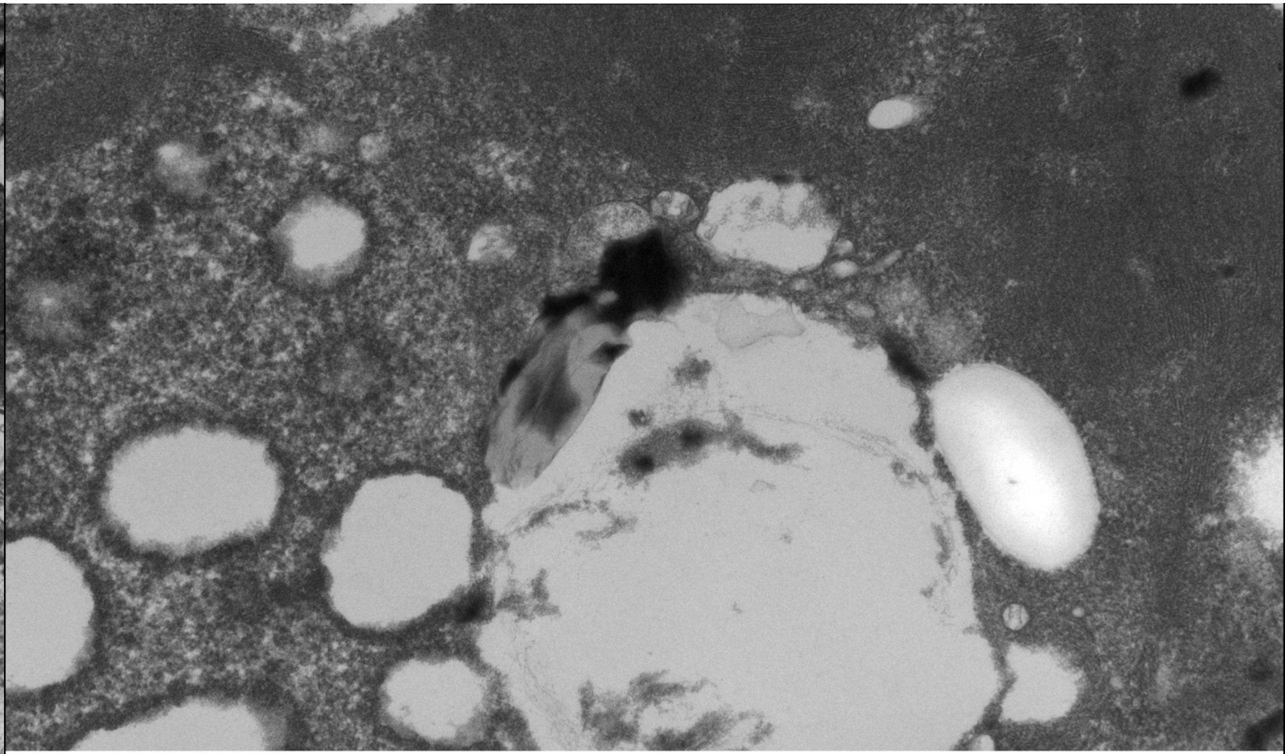

21-20_Correa_3-19-135_16D2_043.tif
Sample: 3/19, 135
Biological Electron Microscopy Lab
Rice University - SEA
Microscopist: MD Meyer

600 nm
HV=80kV
Direct Mag: 6000 x

Cell 30

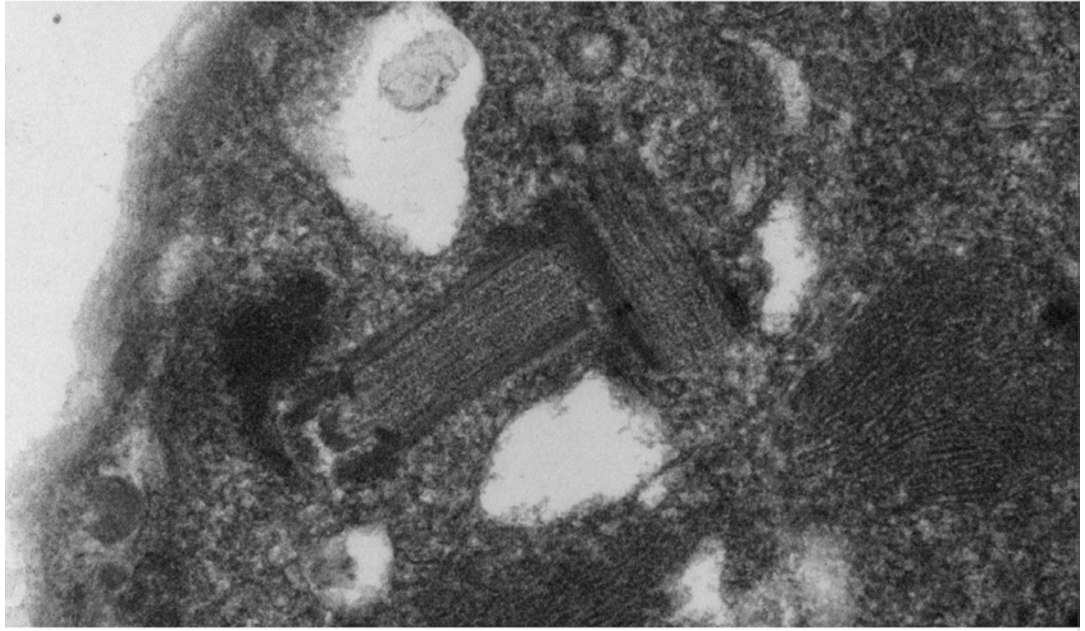

21-20_Correa_3-19-135_16D2_039.tif
Sample: 3/19, 135
Biological Electron Microscopy Lab
Rice University - SEA
Microscopist: MD Meyer

200 nm
HV=80kV
Direct Mag: 15000 x

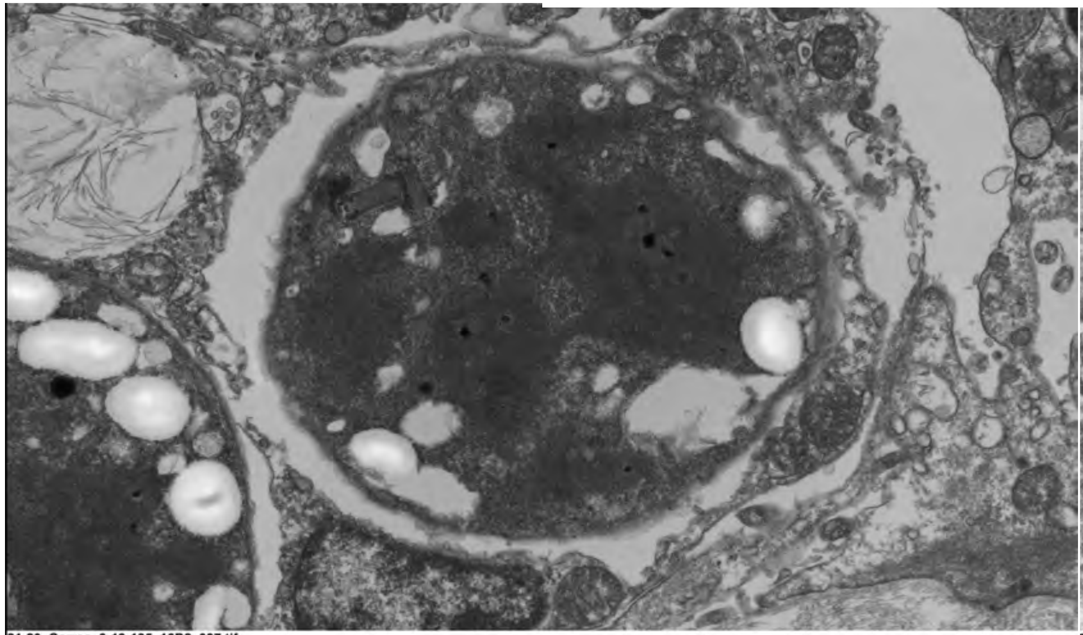

21-20_Correa_3-19-135_16D2_037.tif
Sample: 3/19, 135
Biological Electron Microscopy Lab
Rice University - SEA
Microscopist: MD Meyer

1 µm
HV=80kV
Direct Mag: 3000 x

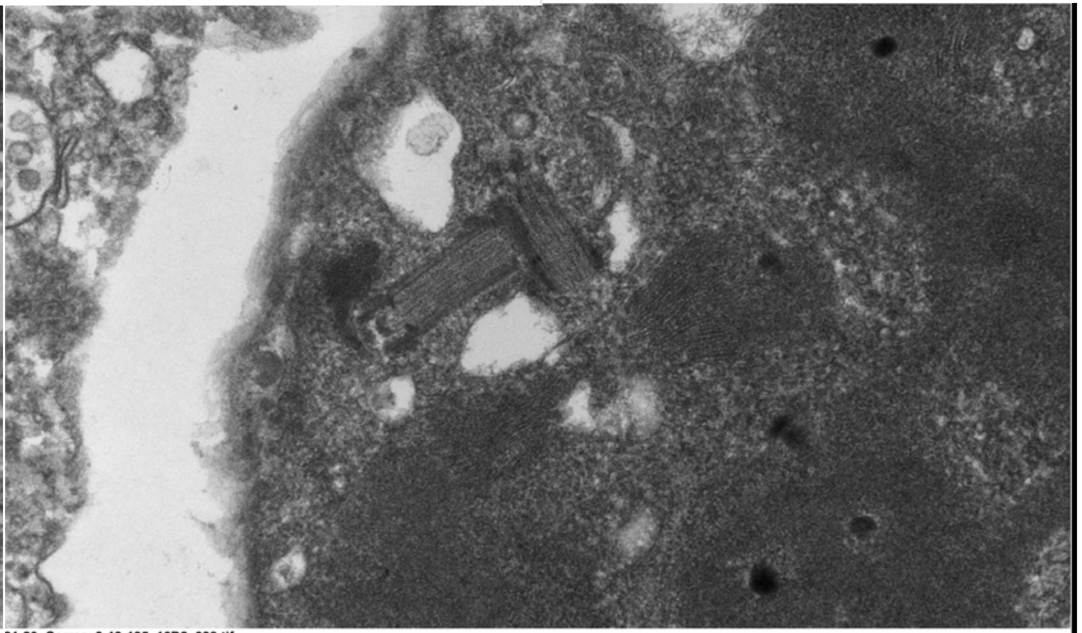

21-20_Correa_3-19-135_16D2_038.tif
Sample: 3/19, 135
Biological Electron Microscopy Lab
Rice University - SEA
Microscopist: MD Meyer

500 nm
HV=80kV
Direct Mag: 8000 x

Cell 31

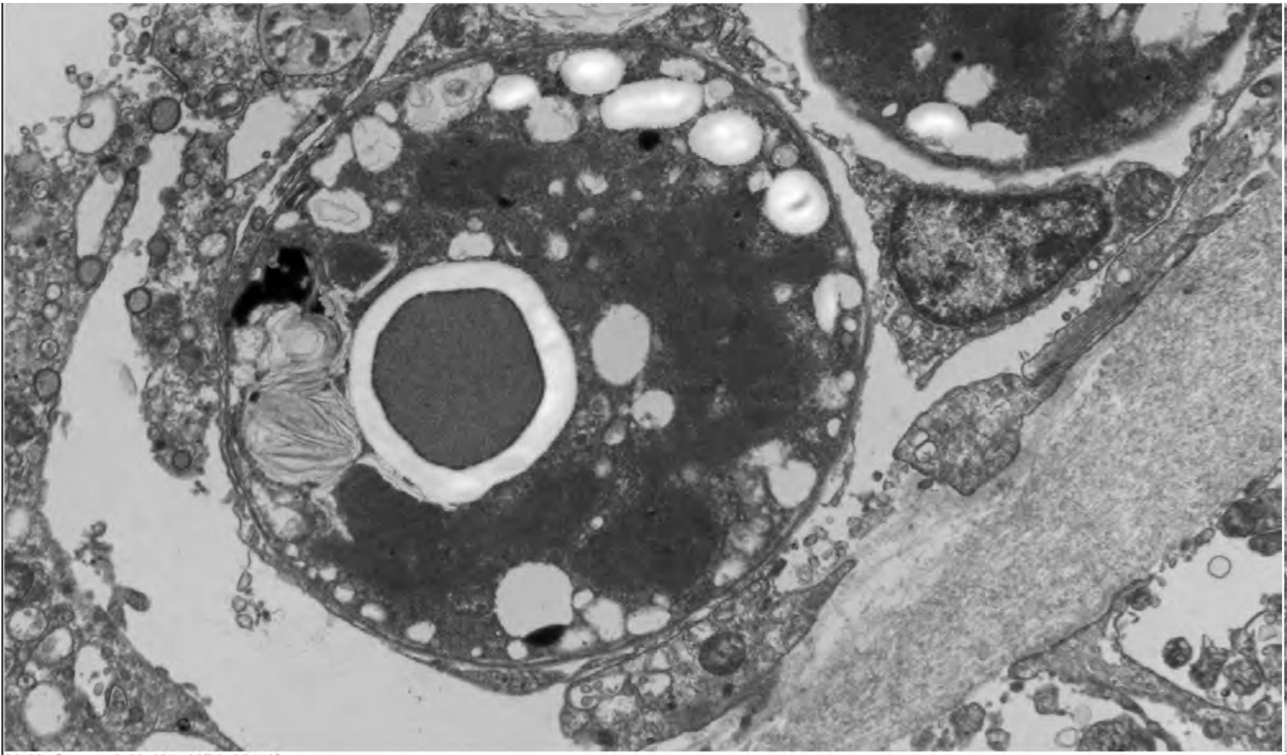

21-20_Correa_3-19-135_16D2_035.tif
Sample: 3/19, 135
Biological Electron Microscopy Lab
Rice University - SEA
Microscopist: MD Meyer

2 µm
HV=80kV
Direct Mag: 2000 x

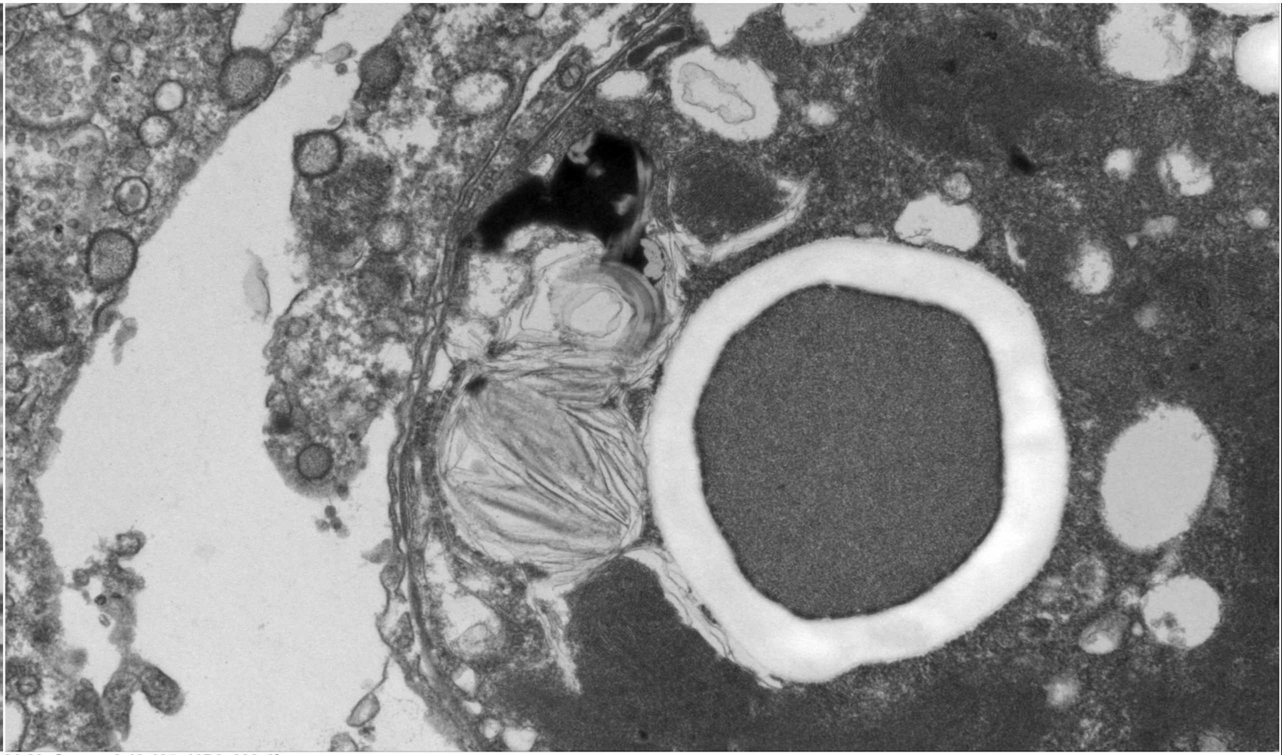

21-20_Correa_3-19-135_16D2_036.tif
Sample: 3/19, 135
Biological Electron Microscopy Lab
Rice University - SEA
Microscopist: MD Meyer

1 µm
HV=80kV
Direct Mag: 4000 x

Cell 32

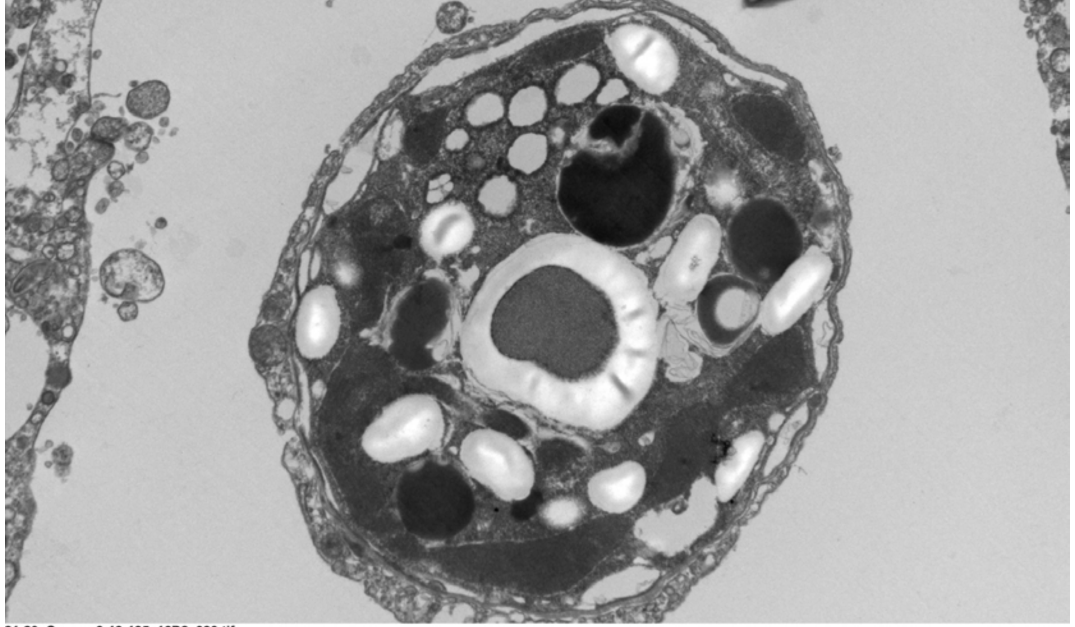

21-20_Correa_3-19-135_16D2_030.tif
Sample: 3/19, 135
Biological Electron Microscopy Lab
Rice University - SEA
Microscopist: MD Meyer

2 µm
HV=80kV
Direct Mag: 2000 x

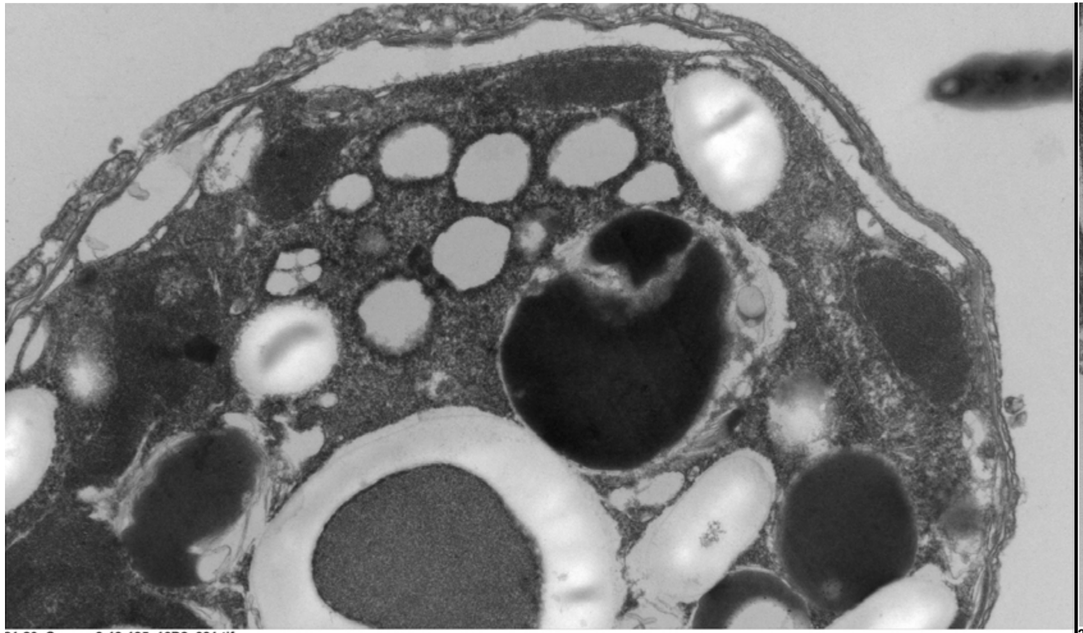

21-20_Correa_3-19-135_16D2_031.tif
Sample: 3/19, 135
Biological Electron Microscopy Lab
Rice University - SEA
Microscopist: MD Meyer

1 µm
HV=80kV
Direct Mag: 4000 x

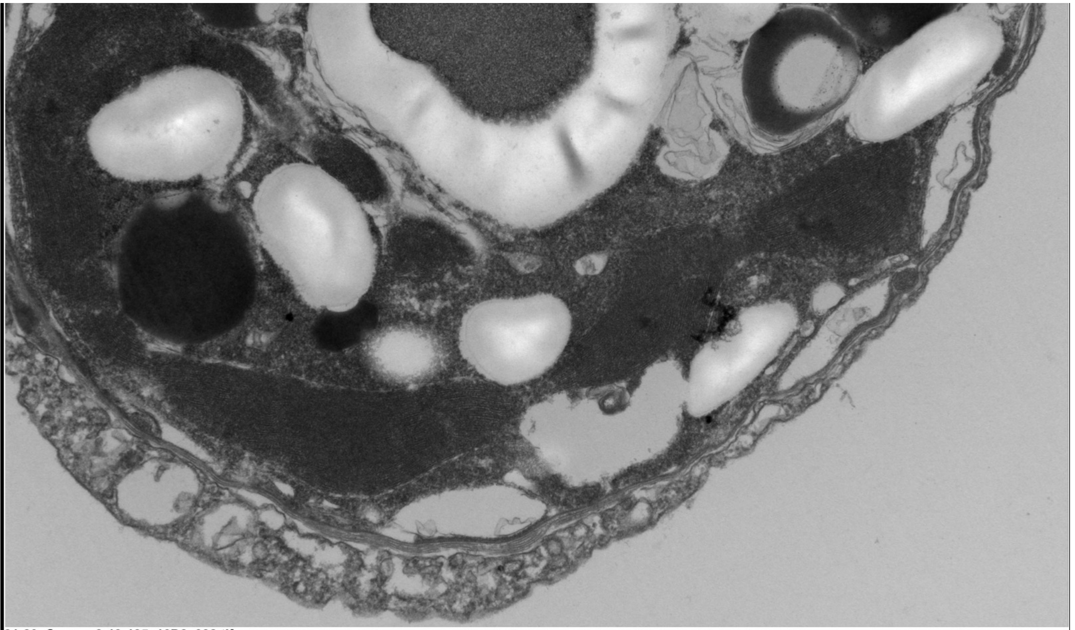

21-20_Correa_3-19-135_16D2_032.tif
Sample: 3/19, 135
Biological Electron Microscopy Lab
Rice University - SEA
Microscopist: MD Meyer

1 µm
HV=80kV
Direct Mag: 4000 x

Cell 33

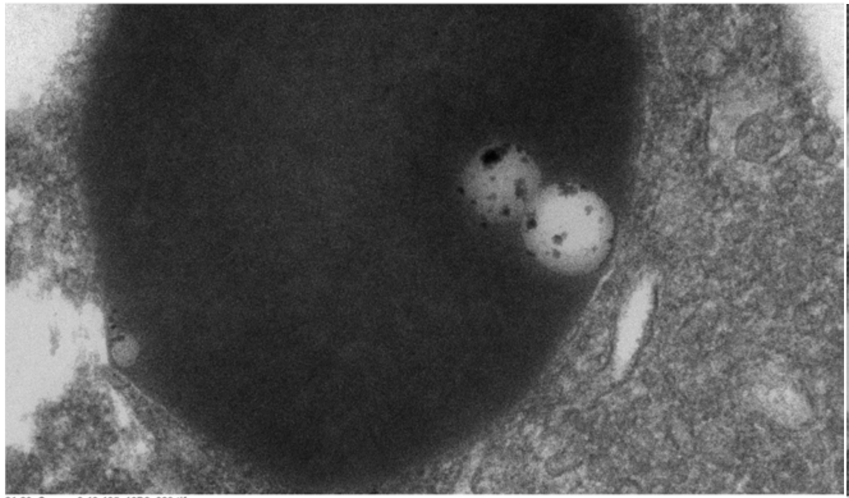

21-20_Correa_3-19-135_16D2_028.tif
Sample: 3/19, 135
Biological Electron Microscopy Lab
Rice University - SEA
Microscopist: MD Meyer
200 nm
HV=80kV
Direct Mag: 20000 x

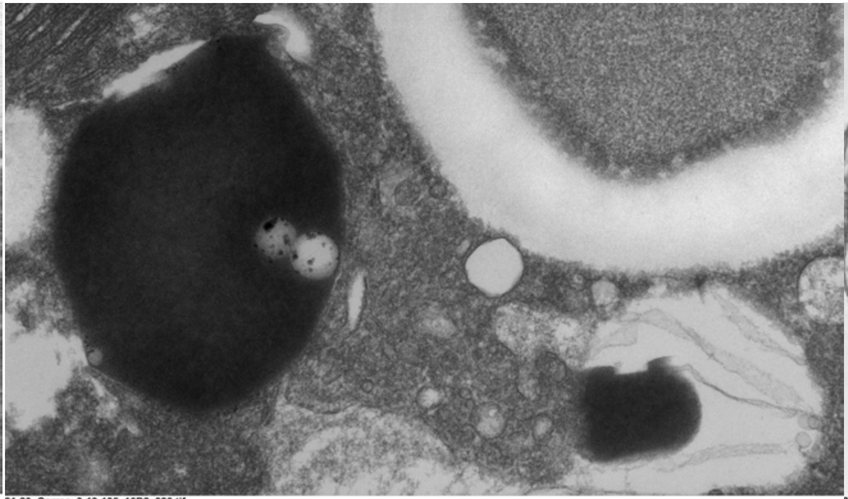

21-20_Correa_3-19-135_16D2_026.tif
Sample: 3/19, 135
Biological Electron Microscopy Lab
Rice University - SEA
Microscopist: MD Meyer
400 nm
HV=80kV
Direct Mag: 10000 x

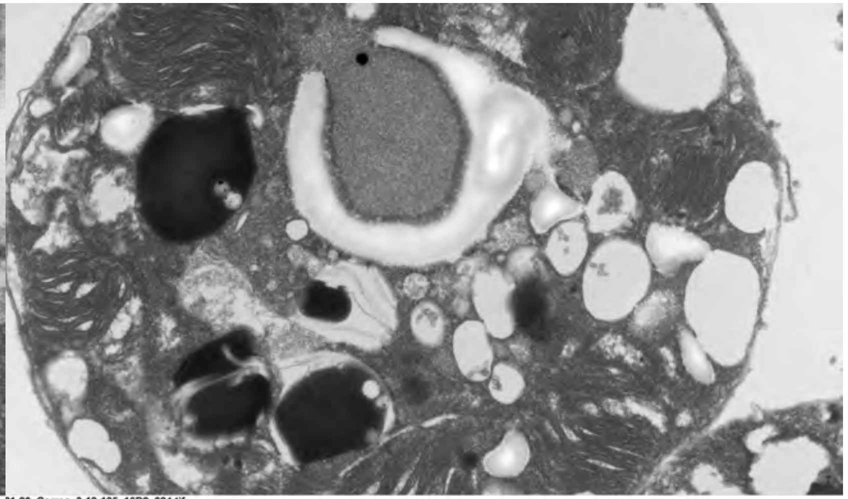

21-20_Correa_3-19-135_16D2_024.tif
Sample: 3/19, 135
Biological Electron Microscopy Lab
Rice University - SEA
Microscopist: MD Meyer
1 μm
HV=80kV
Direct Mag: 4000 x

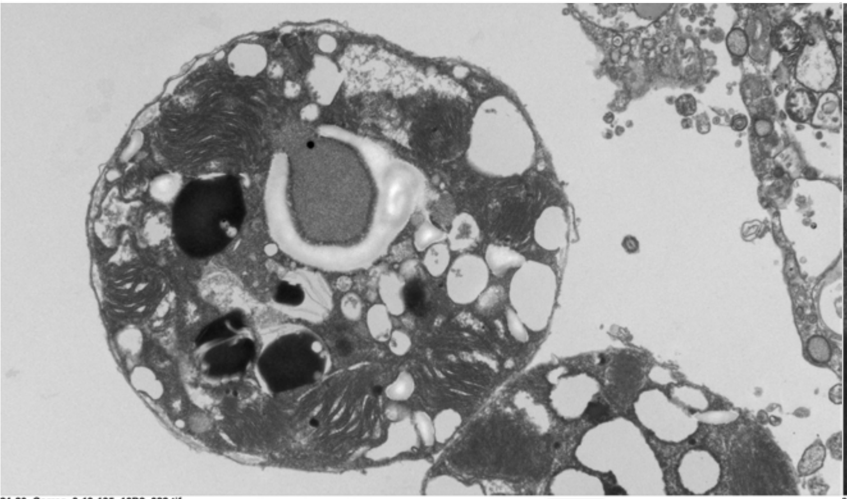

21-20_Correa_3-19-135_16D2_022.tif
Sample: 3/19, 135
Biological Electron Microscopy Lab
Rice University - SEA
Microscopist: MD Meyer
1 μm
HV=80kV
Direct Mag: 2500 x

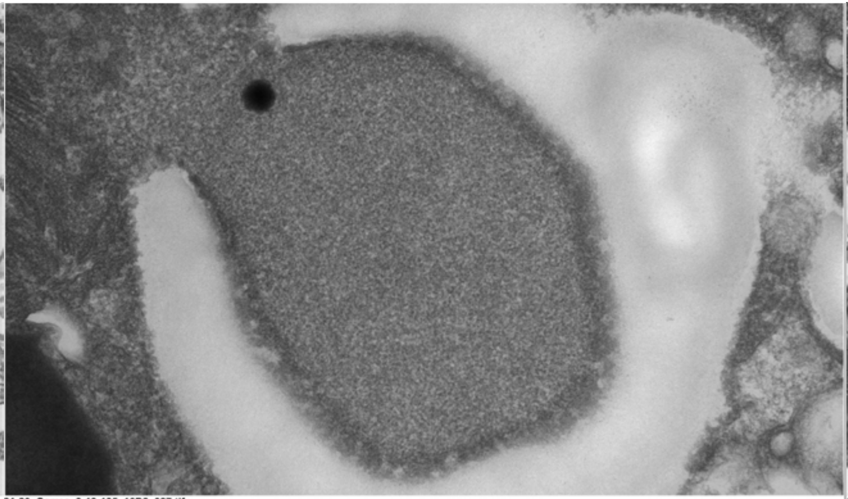

21-20_Correa_3-19-135_16D2_027.tif
Sample: 3/19, 135
Biological Electron Microscopy Lab
Rice University - SEA
Microscopist: MD Meyer
400 nm
HV=80kV
Direct Mag: 10000 x

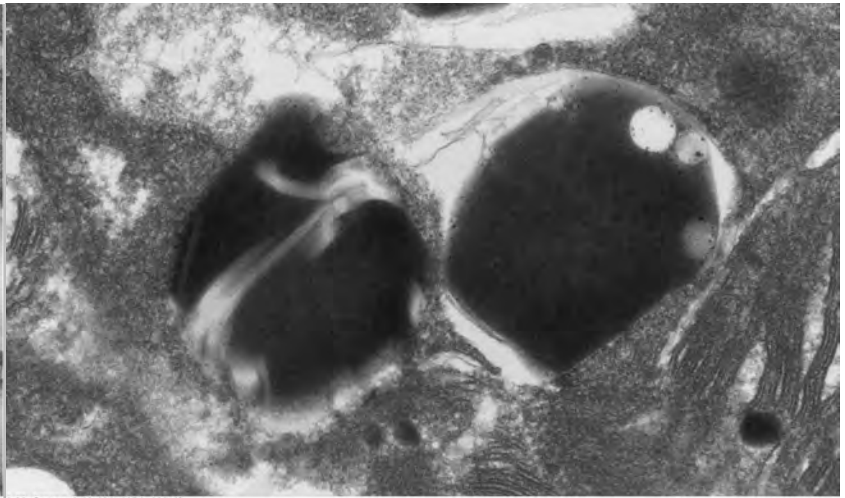

1-20_Correa_3-19-135_16D2_025.tif
ample: 3/19, 135
iological Electron Microscopy Lab
ice University - SEA
licroscopist: MD Meyer
400 nm
HV=80kV
Direct Mag: 10000 x

Cell 34

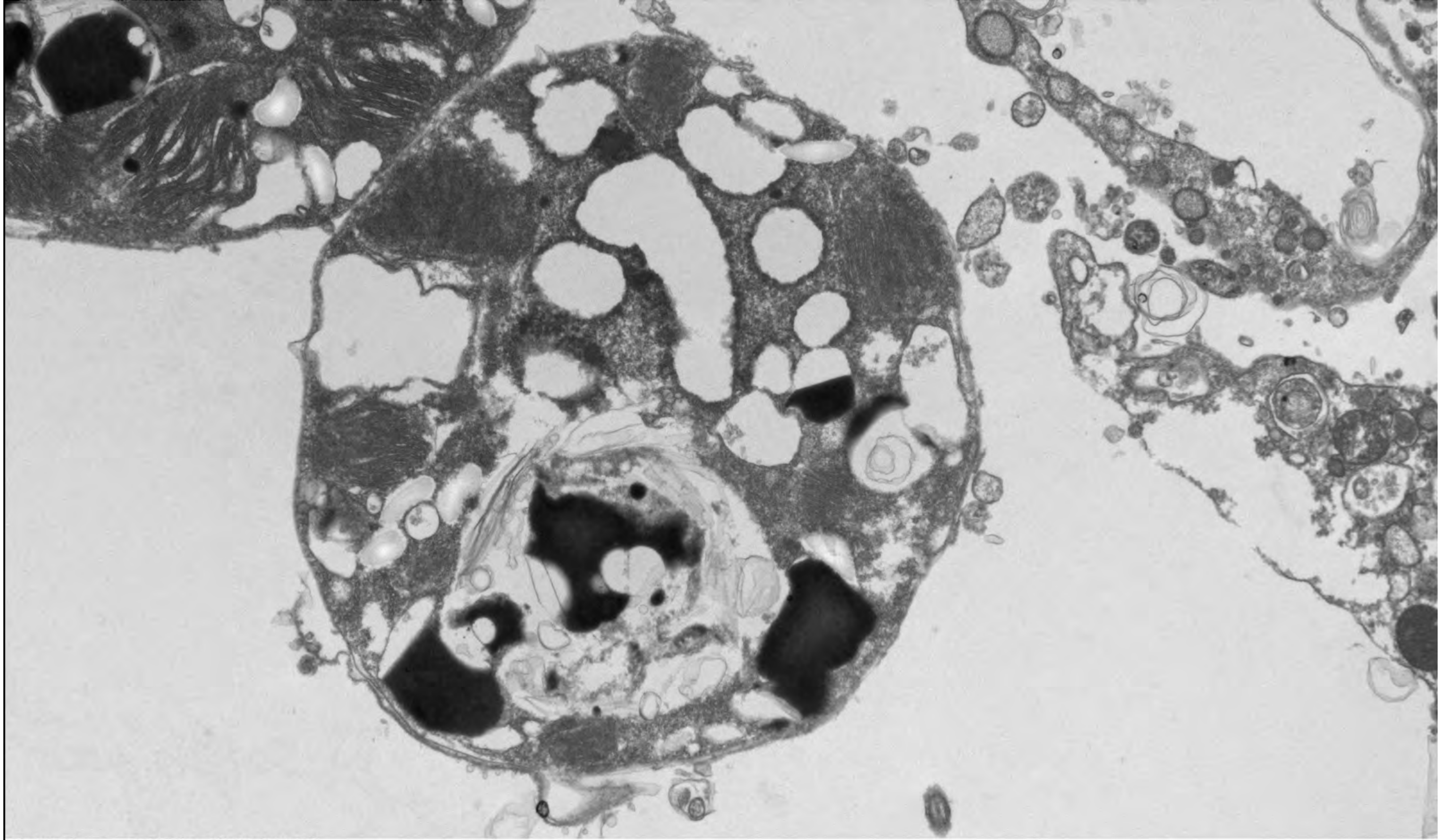

21-20_Correa_3-19-135_16D2_021.tif
Sample: 3/19, 135
Biological Electron Microscopy Lab
Rice University - SEA
Microscopist: MD Meyer

1 µm
HV=80kV
Direct Mag: 2500 x

Cell 35

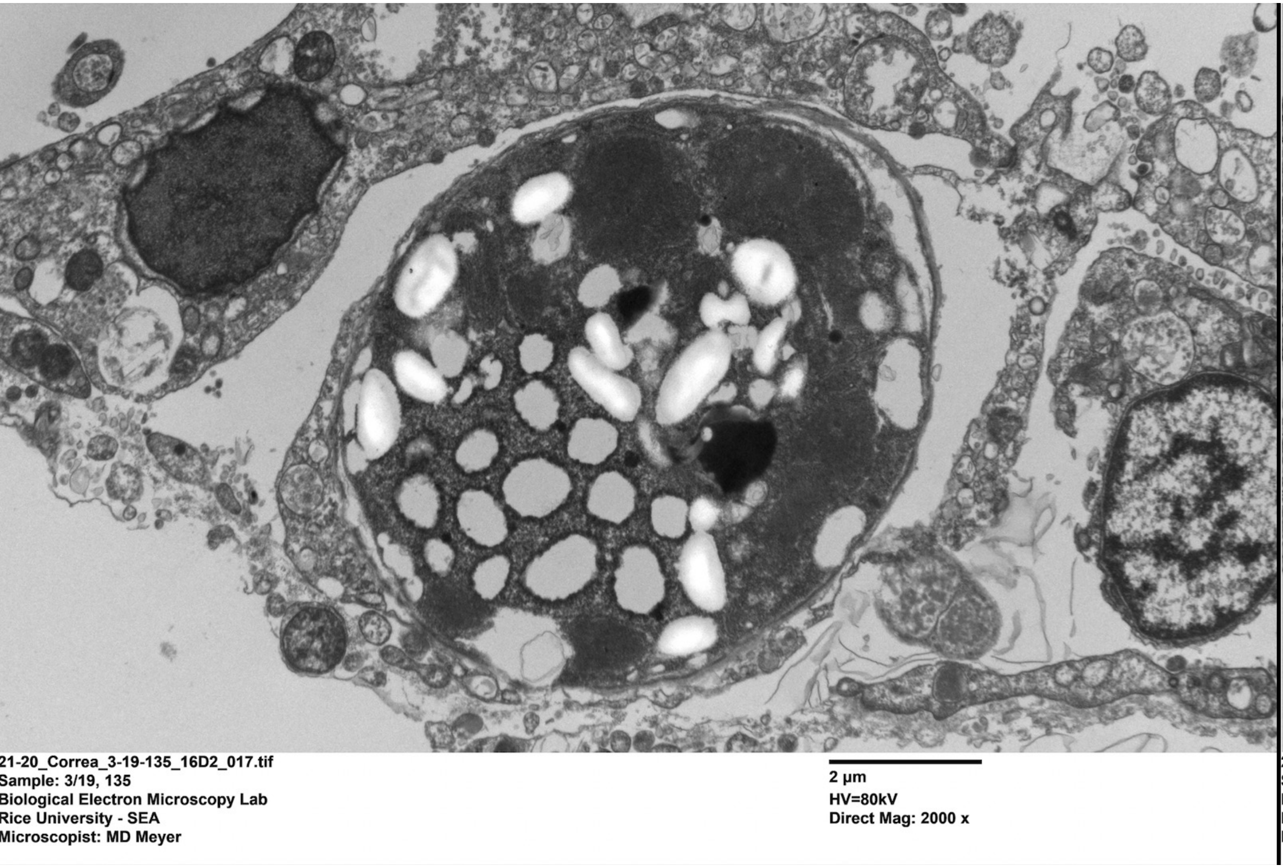
21-20_Correa_3-19-135_16D2_017.tif
Sample: 3/19, 135
Biological Electron Microscopy Lab
Rice University - SEA
Microscopist: MD Meyer
2 µm
HV=80kV
Direct Mag: 2000 x

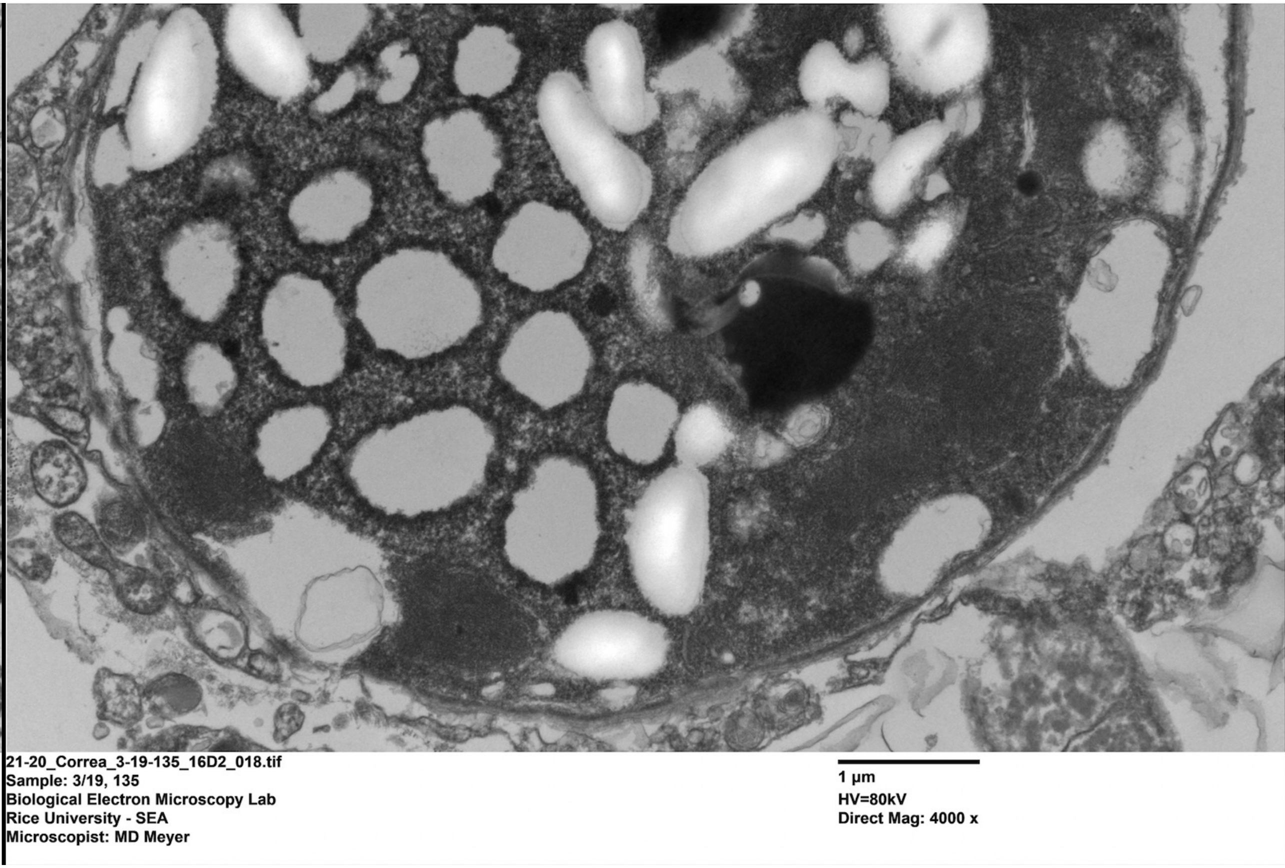
21-20_Correa_3-19-135_16D2_018.tif
Sample: 3/19, 135
Biological Electron Microscopy Lab
Rice University - SEA
Microscopist: MD Meyer
1 µm
HV=80kV
Direct Mag: 4000 x

Cell 36

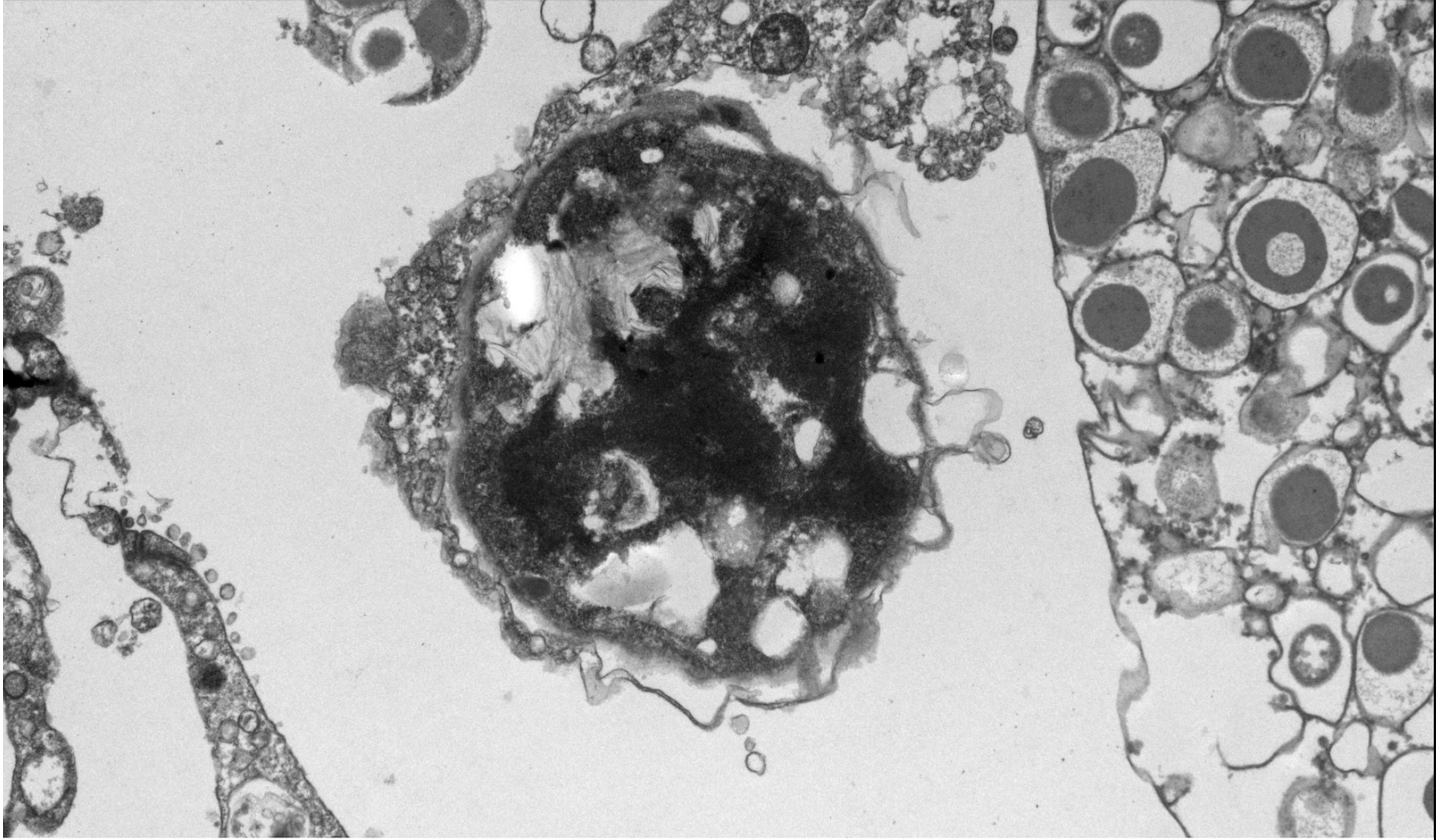

21-20_Correa_3-19-135_16D2_016.tif
Sample: 3/19, 135
Biological Electron Microscopy Lab
Rice University - SEA
Microscopist: MD Meyer

1 µm
HV=80kV
Direct Mag: 2500 x

Cell 37

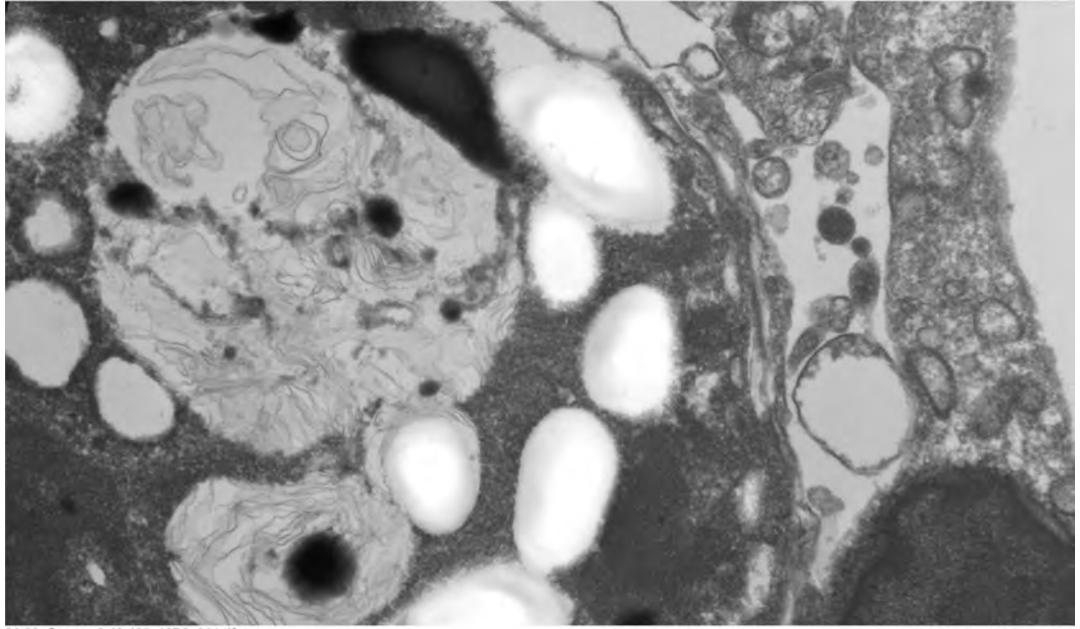

21-20_Correa_3-19-135_16D2_014.tif
Sample: 3/19, 135
Biological Electron Microscopy Lab
Rice University - SEA
Microscopist: MD Meyer

800 nm
HV=80kV
Direct Mag: 5000 x

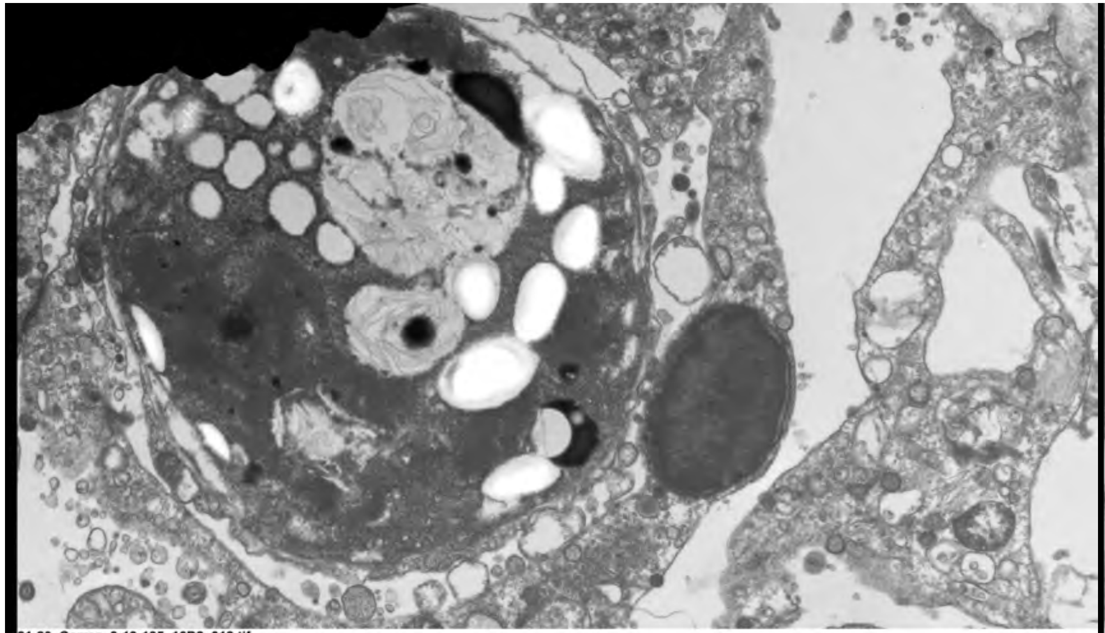

21-20_Correa_3-19-135_16D2_012.tif
Sample: 3/19, 135
Biological Electron Microscopy Lab
Rice University - SEA
Microscopist: MD Meyer

1 µm
HV=80kV
Direct Mag: 2500 x

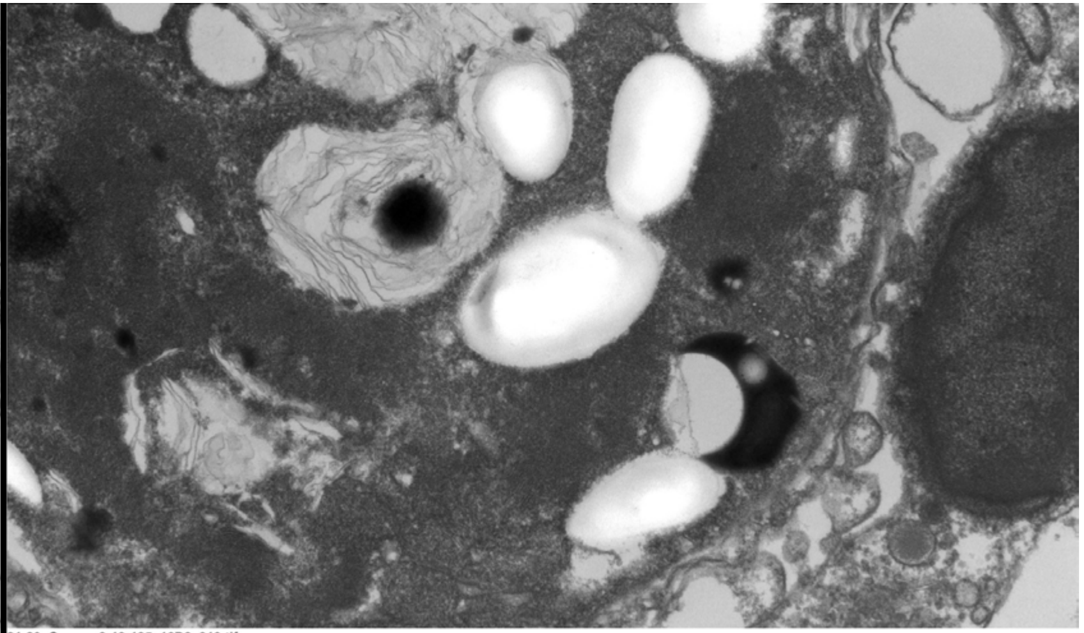

21-20_Correa_3-19-135_16D2_013.tif
Sample: 3/19, 135
Biological Electron Microscopy Lab
Rice University - SEA
Microscopist: MD Meyer

800 nm
HV=80kV
Direct Mag: 5000 x

Cell 38

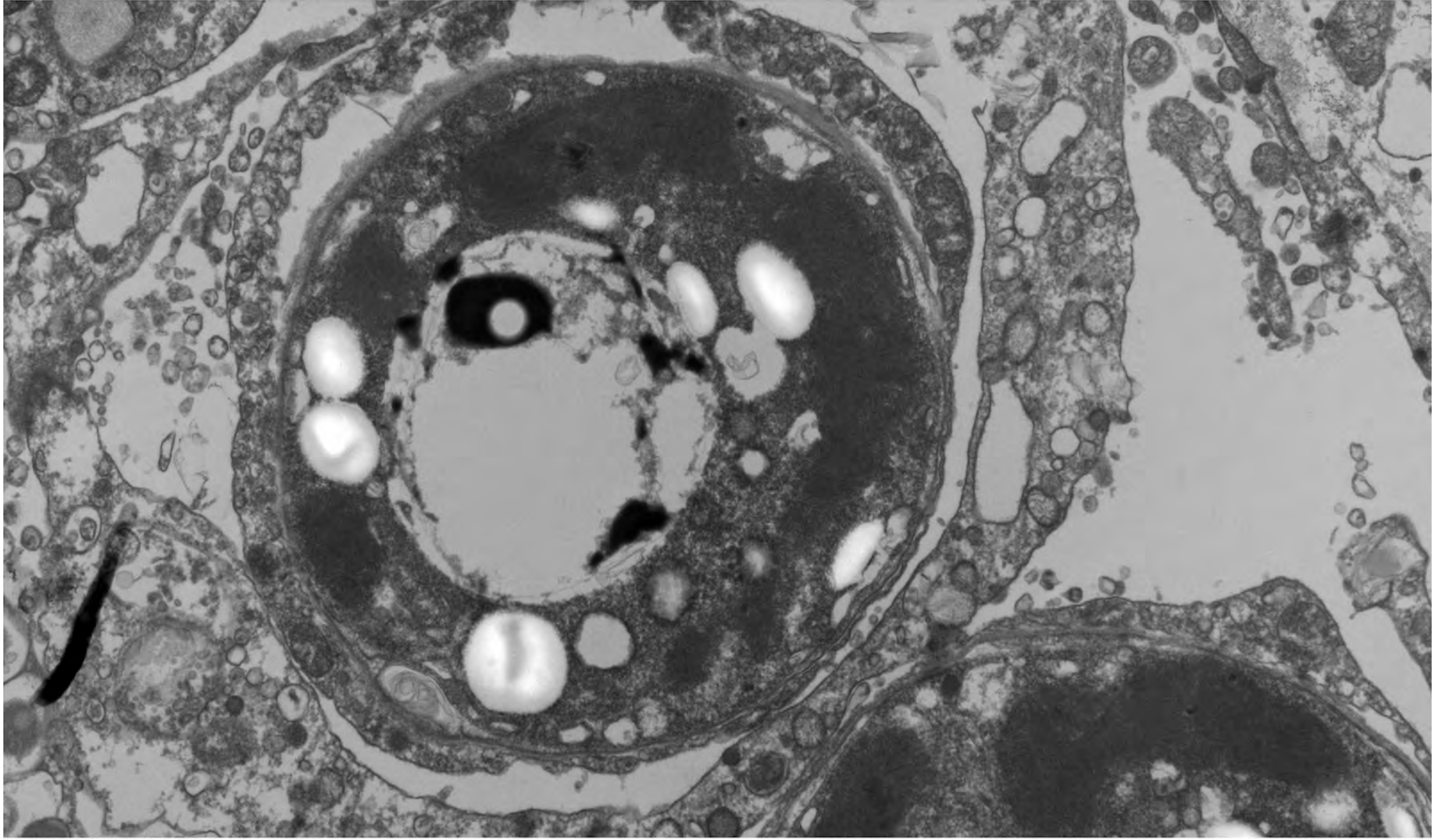

21-20_Correa_3-19-135_16D2_011.tif
Sample: 3/19, 135
Biological Electron Microscopy Lab
Rice University - SEA
Microscopist: MD Meyer

1 µm
HV=80kV
Direct Mag: 2500 x

Cell 39

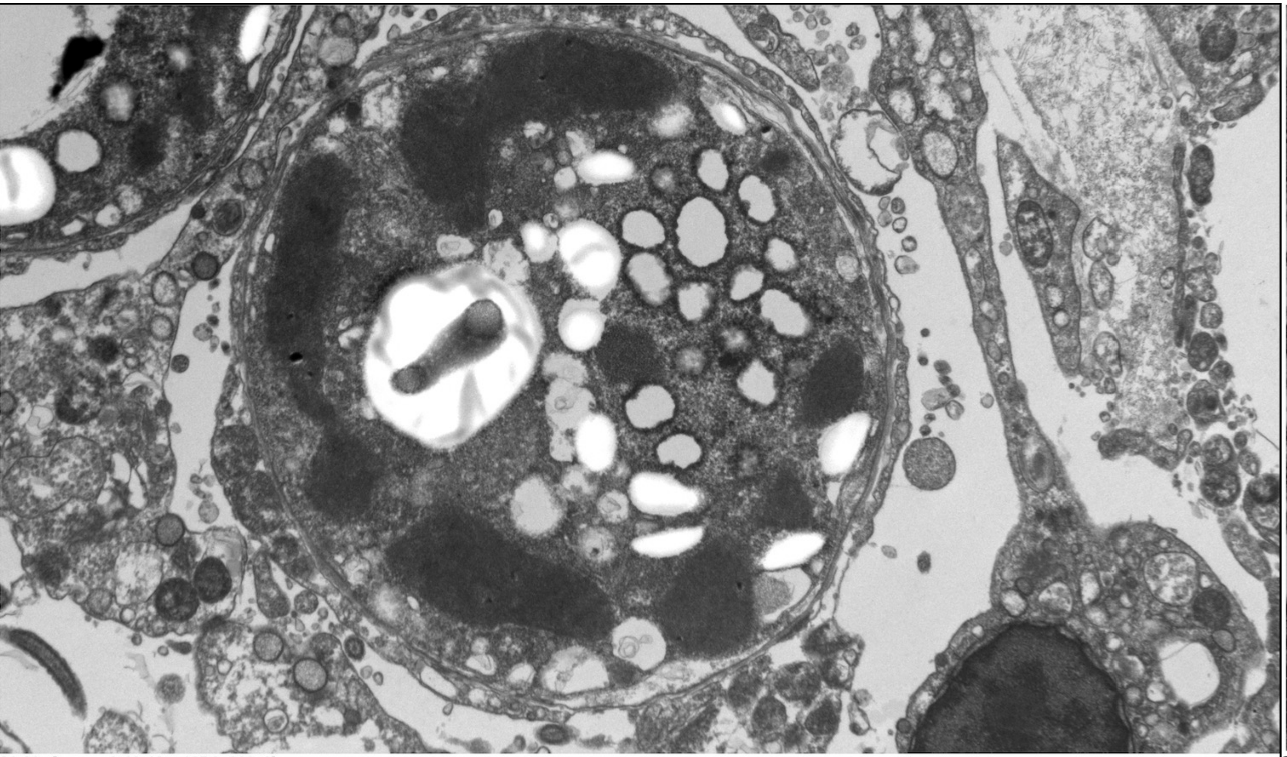

21-20_Correa_3-19-135_16D2_009.tif
Sample: 3/19, 135
Biological Electron Microscopy Lab
Rice University - SEA
Microscopist: MD Meyer

2 µm
HV=80kV
Direct Mag: 2000 x

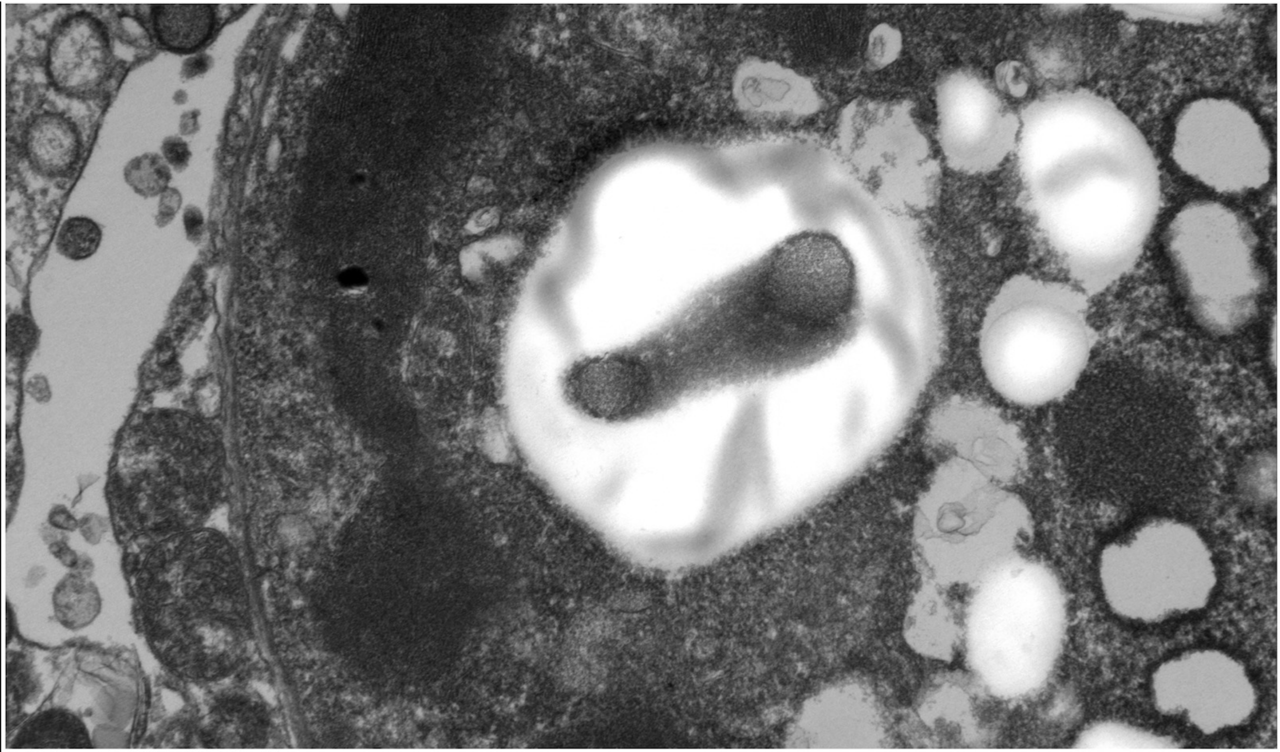

21-20_Correa_3-19-135_16D2_010.tif
Sample: 3/19, 135
Biological Electron Microscopy Lab
Rice University - SEA
Microscopist: MD Meyer

800 nm
HV=80kV
Direct Mag: 5000 x

Cell 40

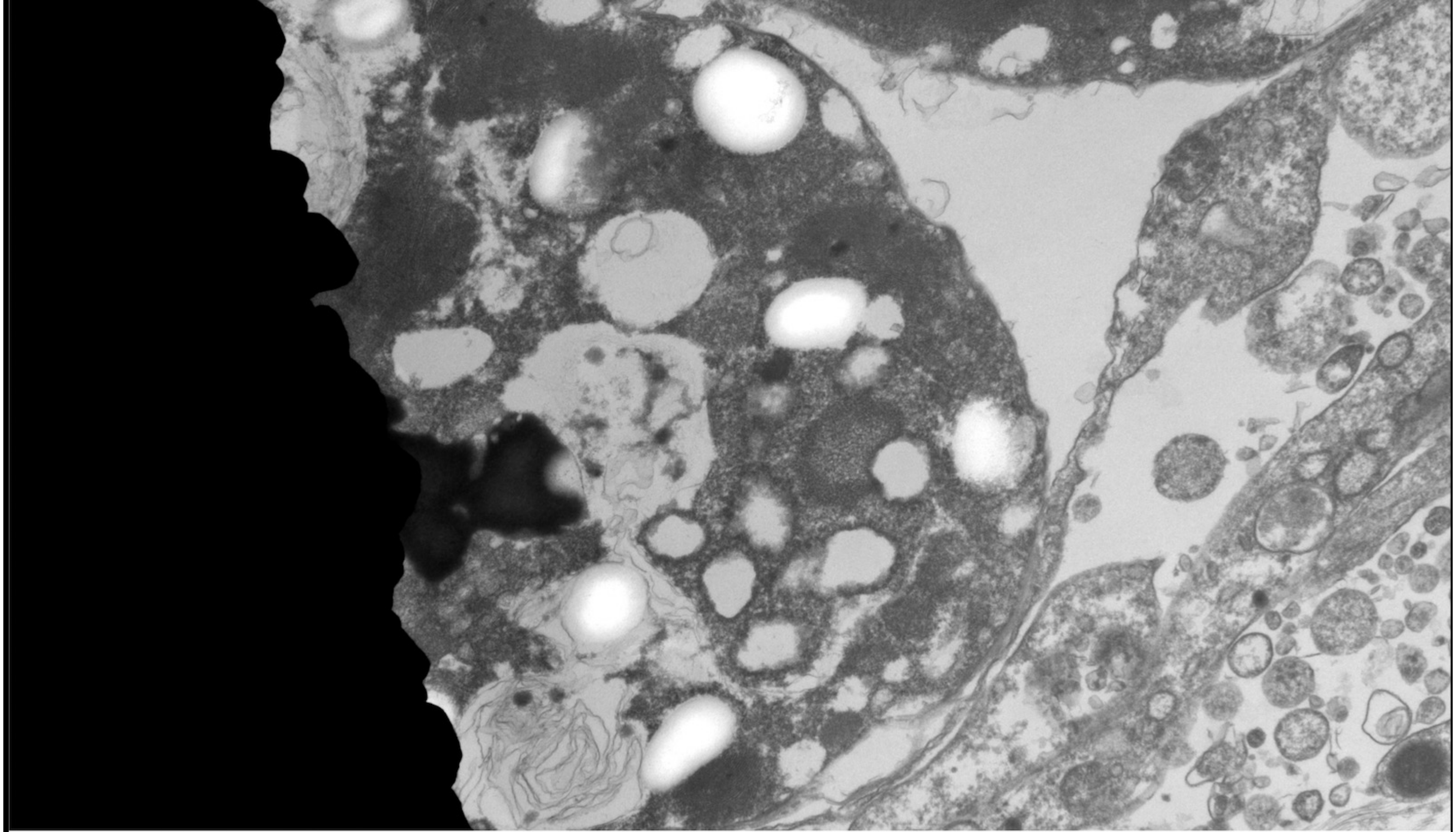

21-20_Correa_3-19-135_16D2_007.tif
Sample: 3/19, 135
Biological Electron Microscopy Lab
Rice University - SEA
Microscopist: MD Meyer

1 µm
HV=80kV
Direct Mag: 3000 x

Cell 41

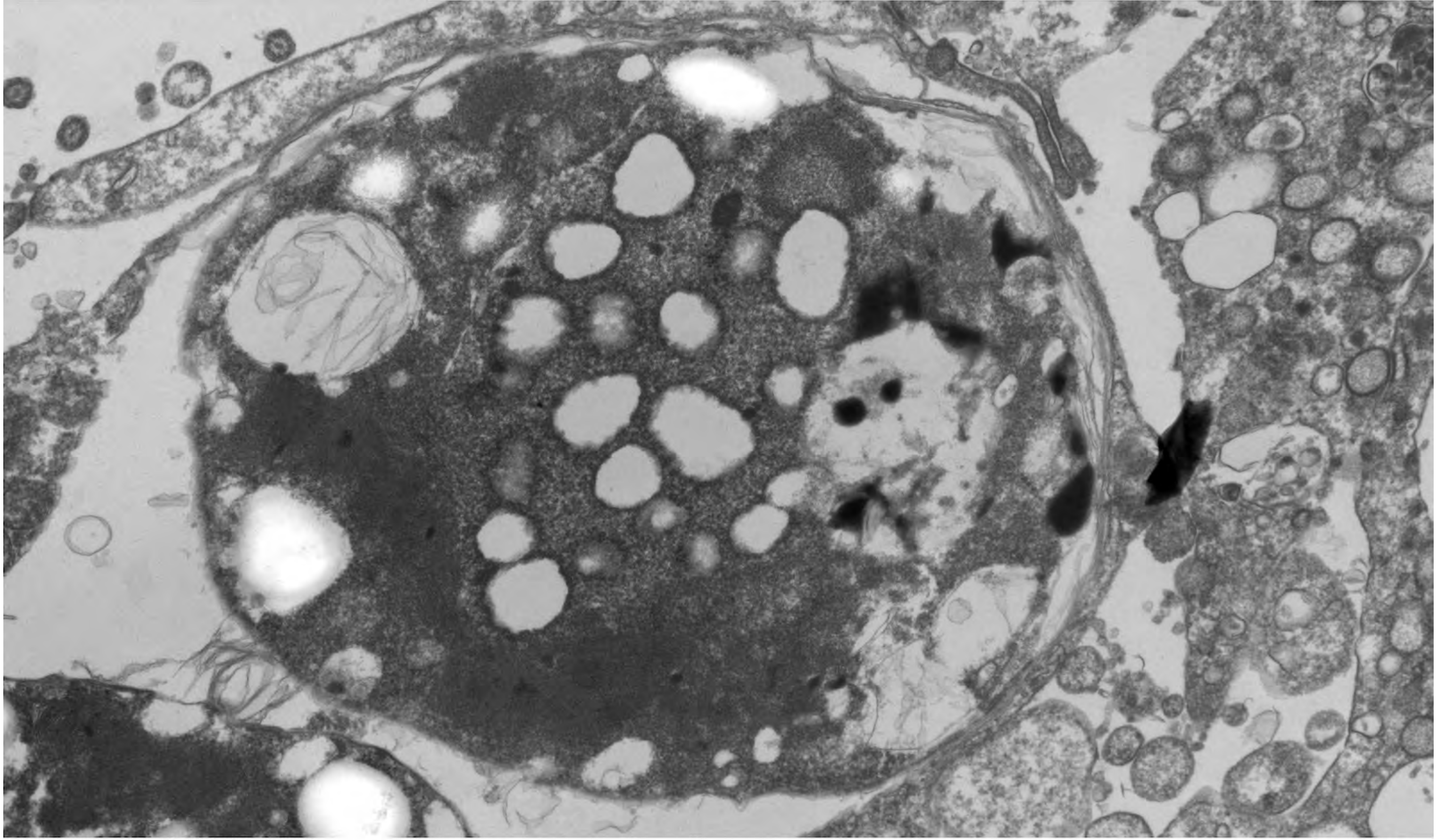

21-20_Correa_3-19-135_16D2_006.tif
Sample: 3/19, 135
Biological Electron Microscopy Lab
Rice University - SEA
Microscopist: MD Meyer

1 µm
HV=80kV
Direct Mag: 3000 x

Cell 42

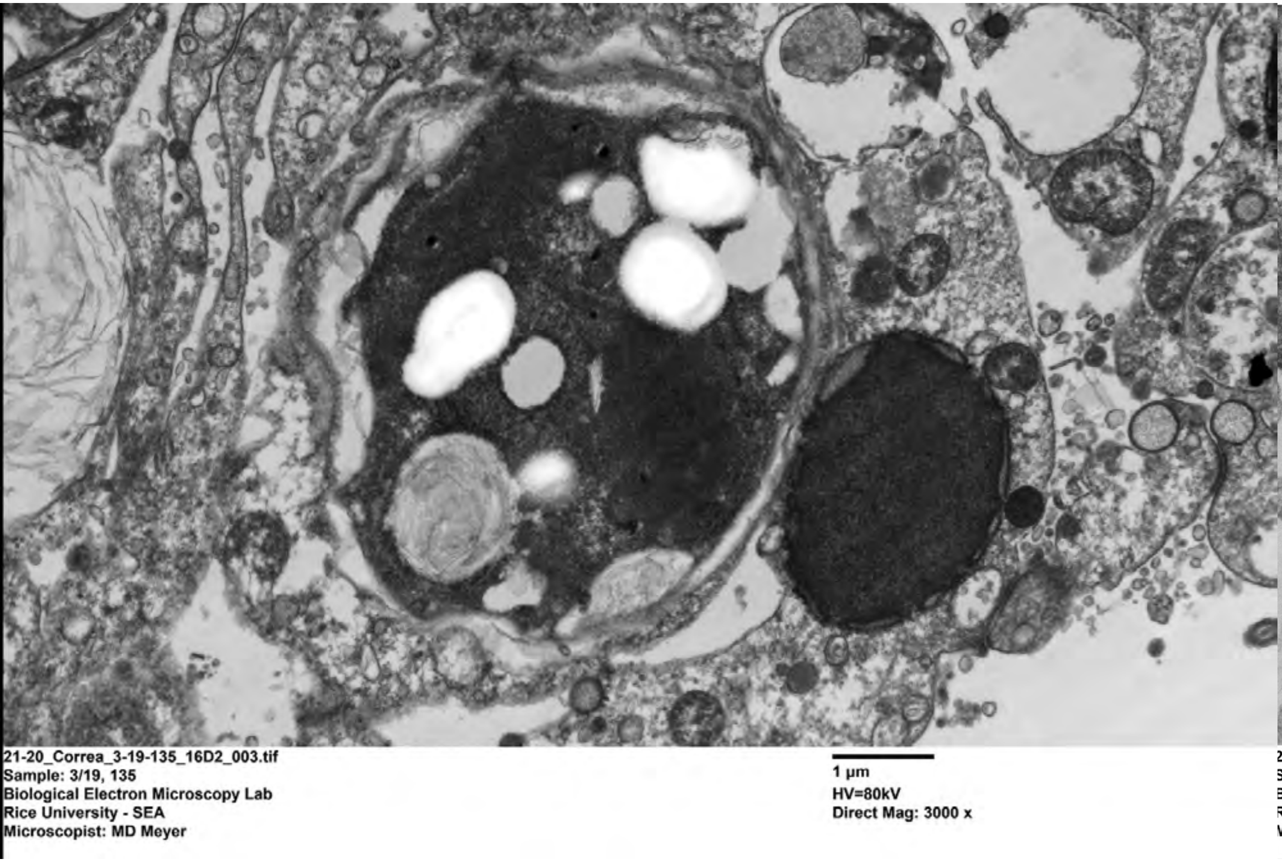
21-20_Correa_3-19-135_16D2_003.tif
Sample: 3/19, 135
Biological Electron Microscopy Lab
Rice University - SEA
Microscopist: MD Meyer
1 µm
HV=80kV
Direct Mag: 3000 x

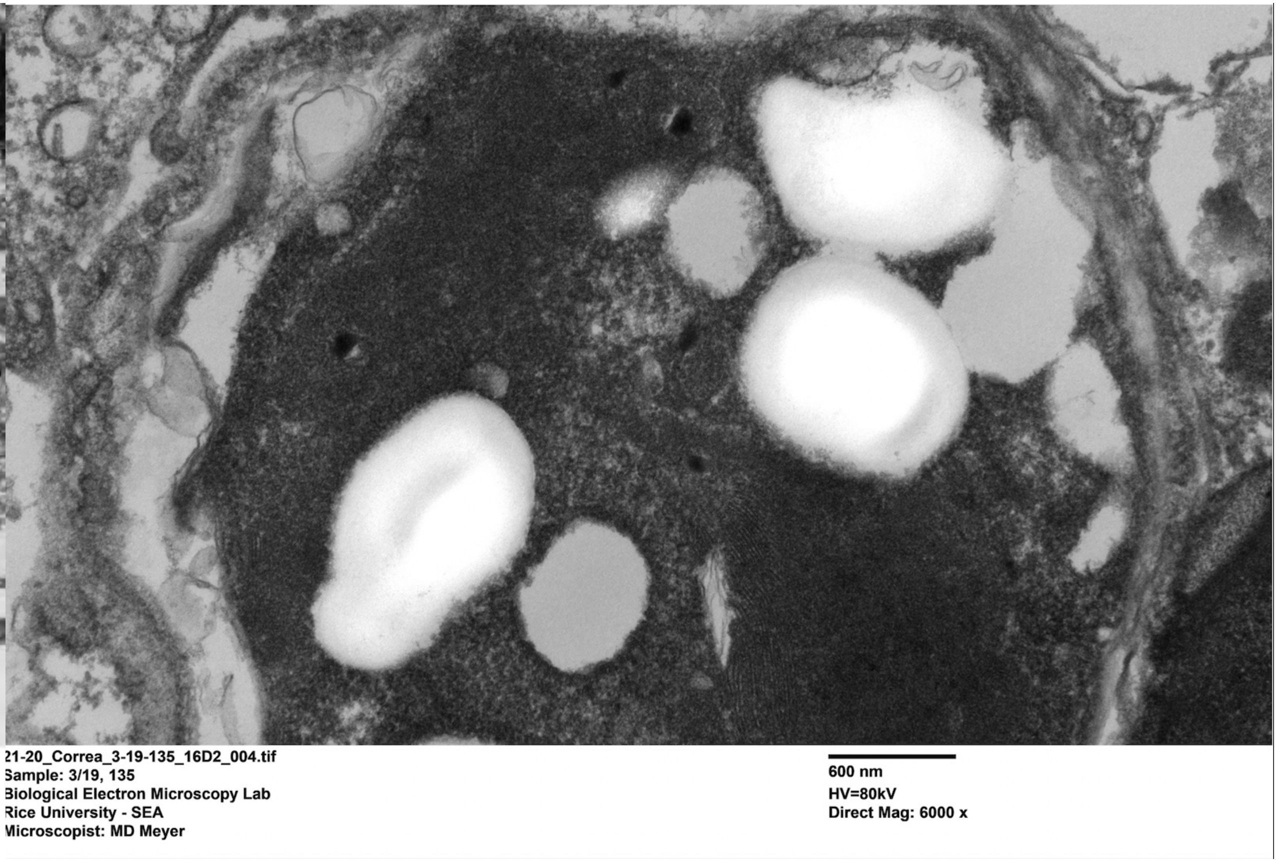
21-20_Correa_3-19-135_16D2_004.tif
Sample: 3/19, 135
Biological Electron Microscopy Lab
Rice University - SEA
Microscopist: MD Meyer
600 nm
HV=80kV
Direct Mag: 6000 x

Cell 43

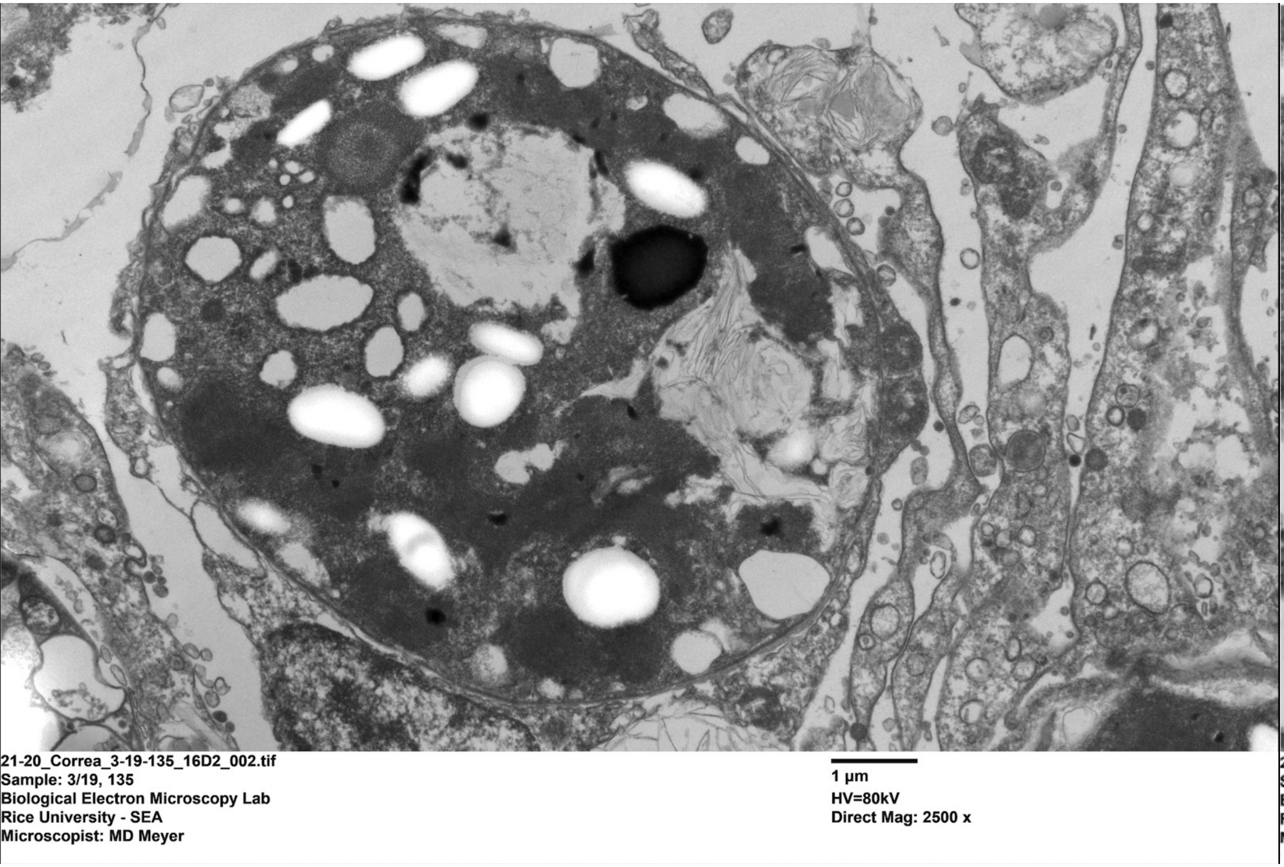
21-20_Correa_3-19-135_16D2_002.tif
Sample: 3/19, 135
Biological Electron Microscopy Lab
Rice University - SEA
Microscopist: MD Meyer
1 µm
HV=80kV
Direct Mag: 2500 x

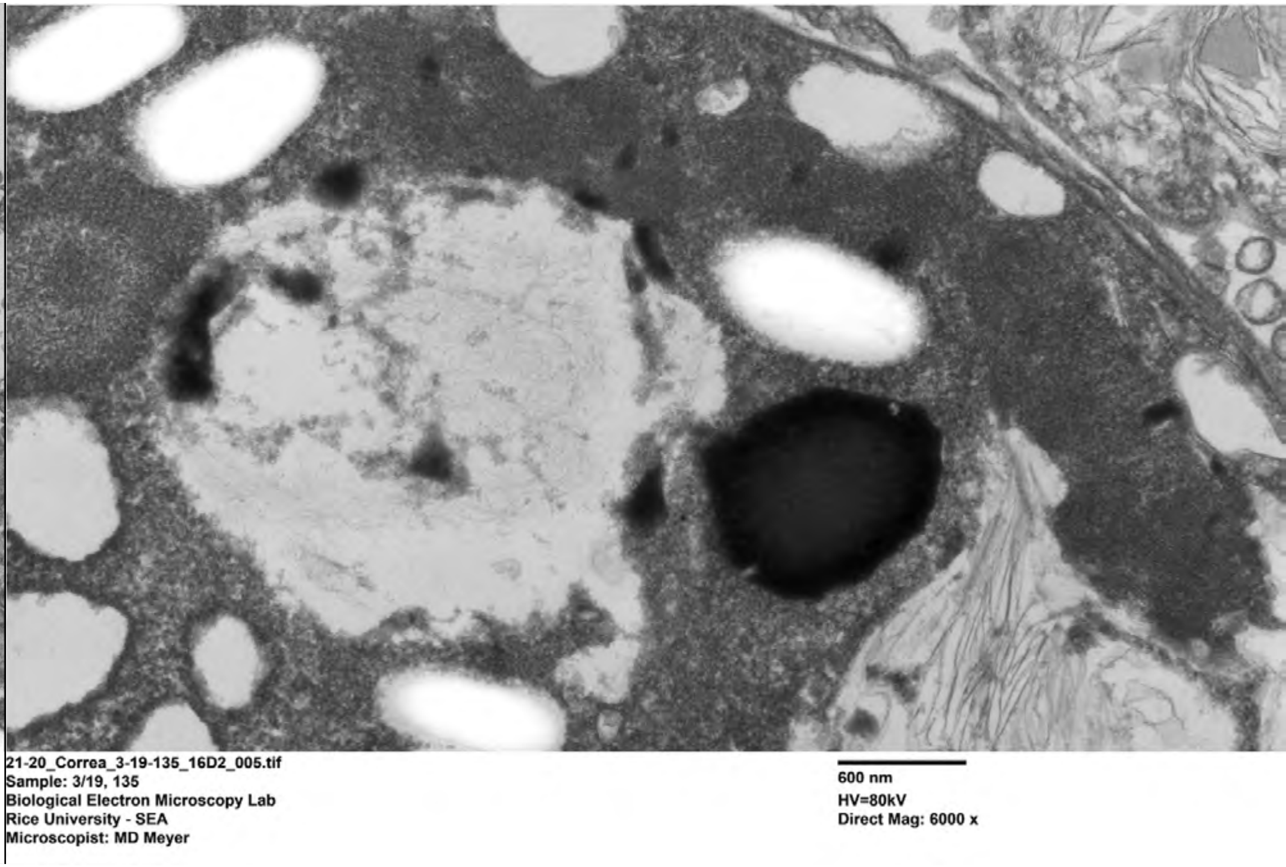
21-20_Correa_3-19-135_16D2_005.tif
Sample: 3/19, 135
Biological Electron Microscopy Lab
Rice University - SEA
Microscopist: MD Meyer
600 nm
HV=80kV
Direct Mag: 6000 x

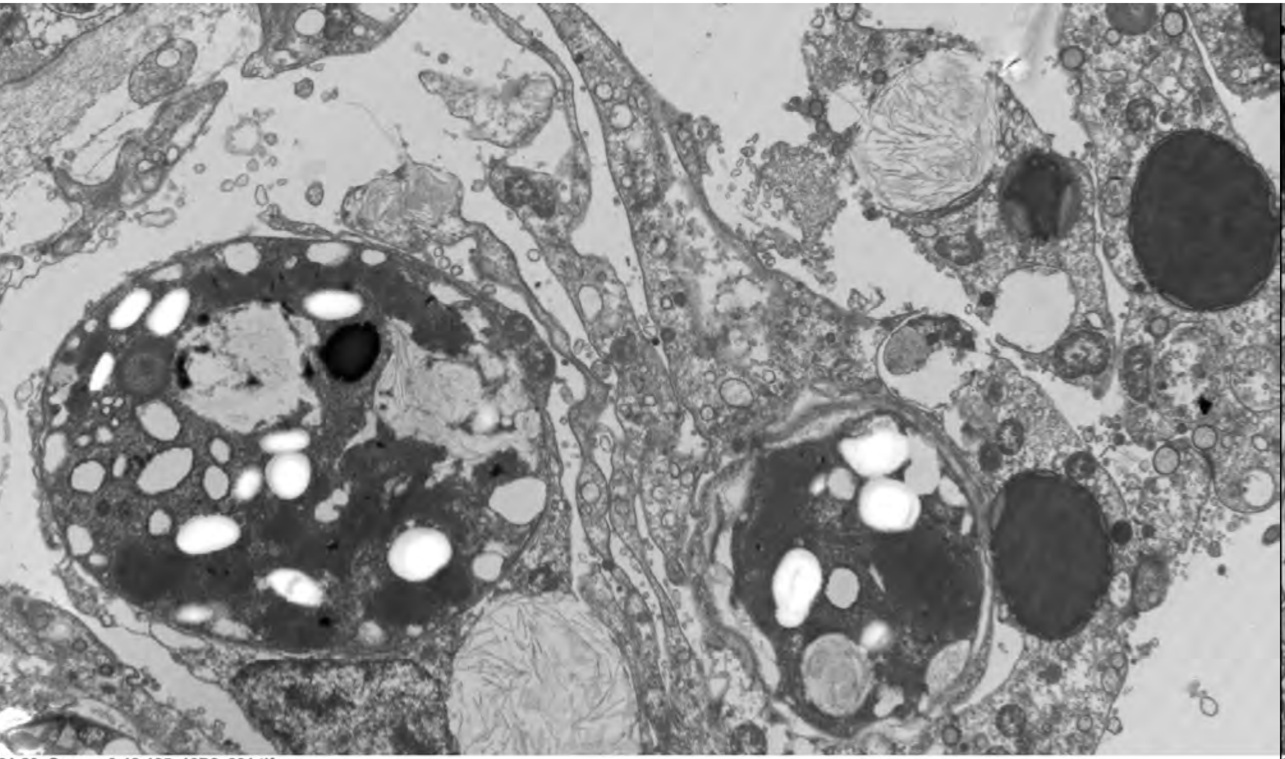

21-20_Correa_3-19-135_16D2_001.tif
Sample: 3/19, 135
Biological Electron Microscopy Lab
Rice University - SEA
Microscopist: MD Meyer

2 μm
HV=80kV
Direct Mag: 1500 x

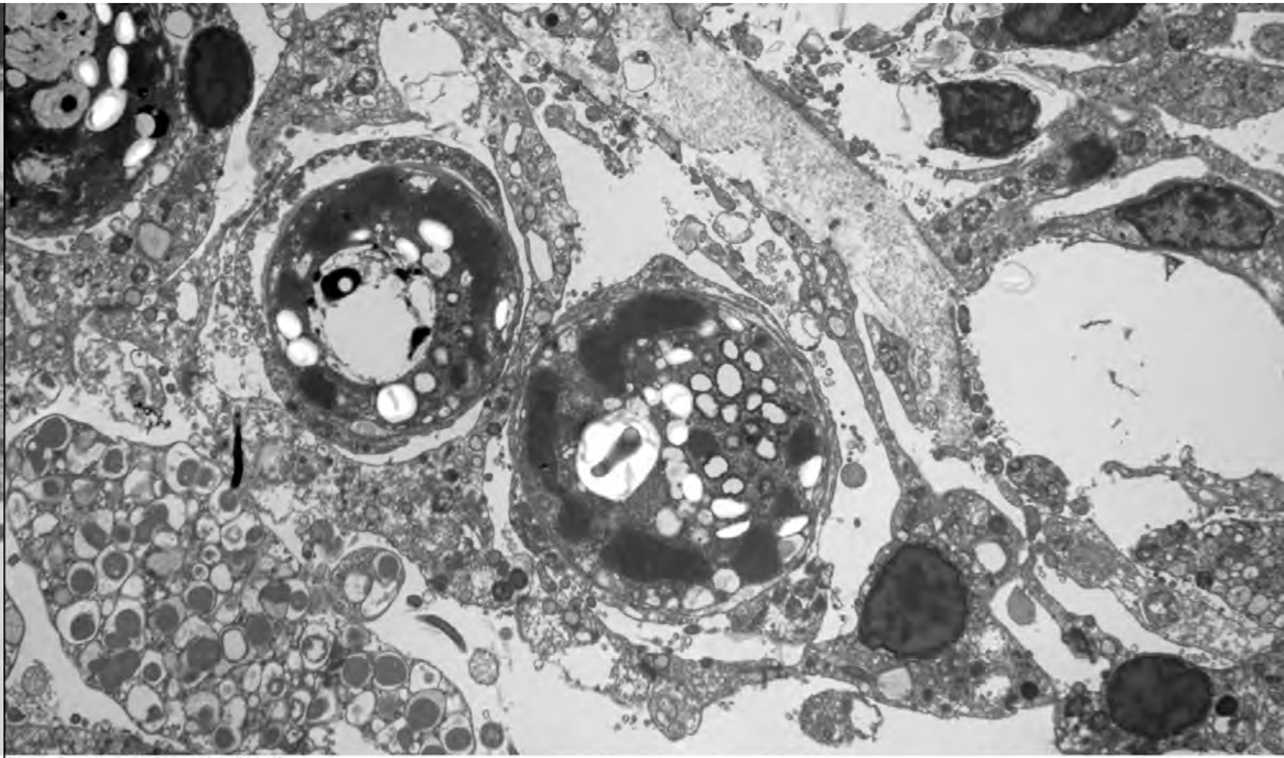

21-20_Correa_3-19-135_16D2_008.tif
Sample: 3/19, 135
Biological Electron Microscopy Lab
Rice University - SEA
Microscopist: MD Meyer

4 μm
HV=80kV
Direct Mag: 1000 x

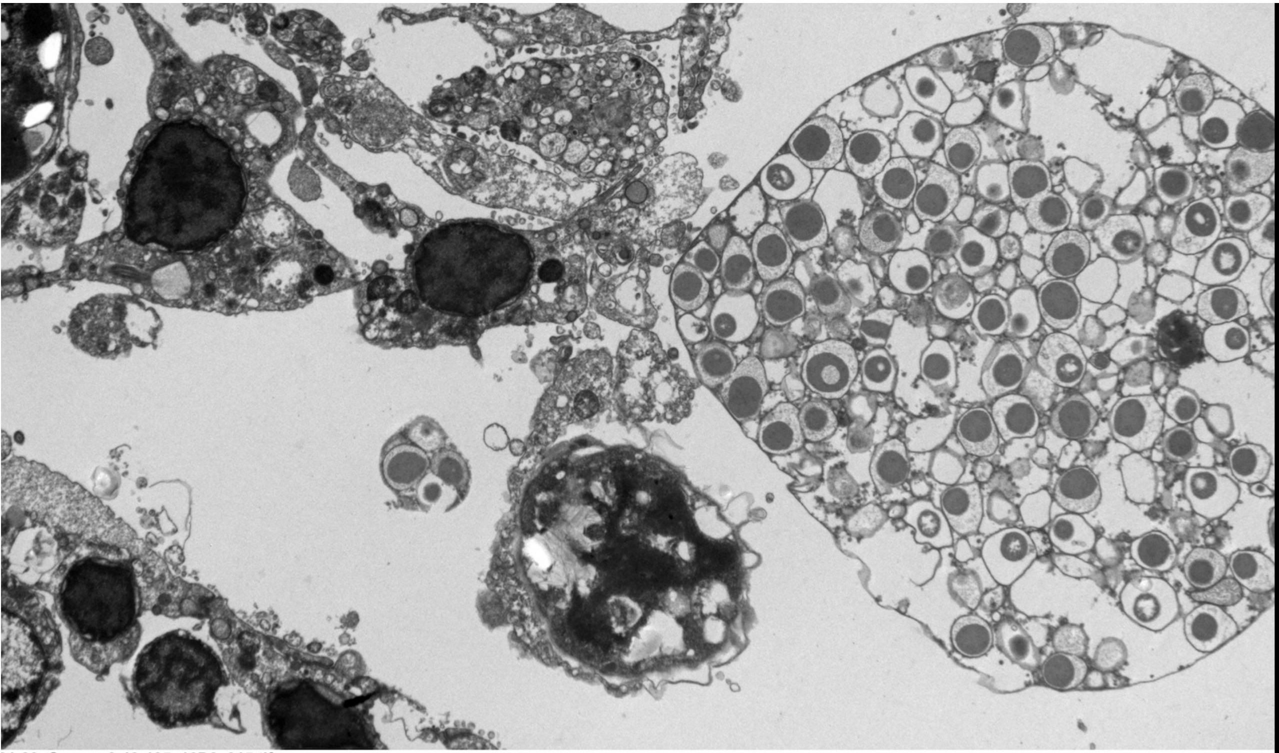

21-20_Correa_3-19-135_16D2_015.tif
Sample: 3/19, 135
Biological Electron Microscopy Lab
Rice University - SEA
Microscopist: MD Meyer

2 µm
HV=80kV
Direct Mag: 1200 x

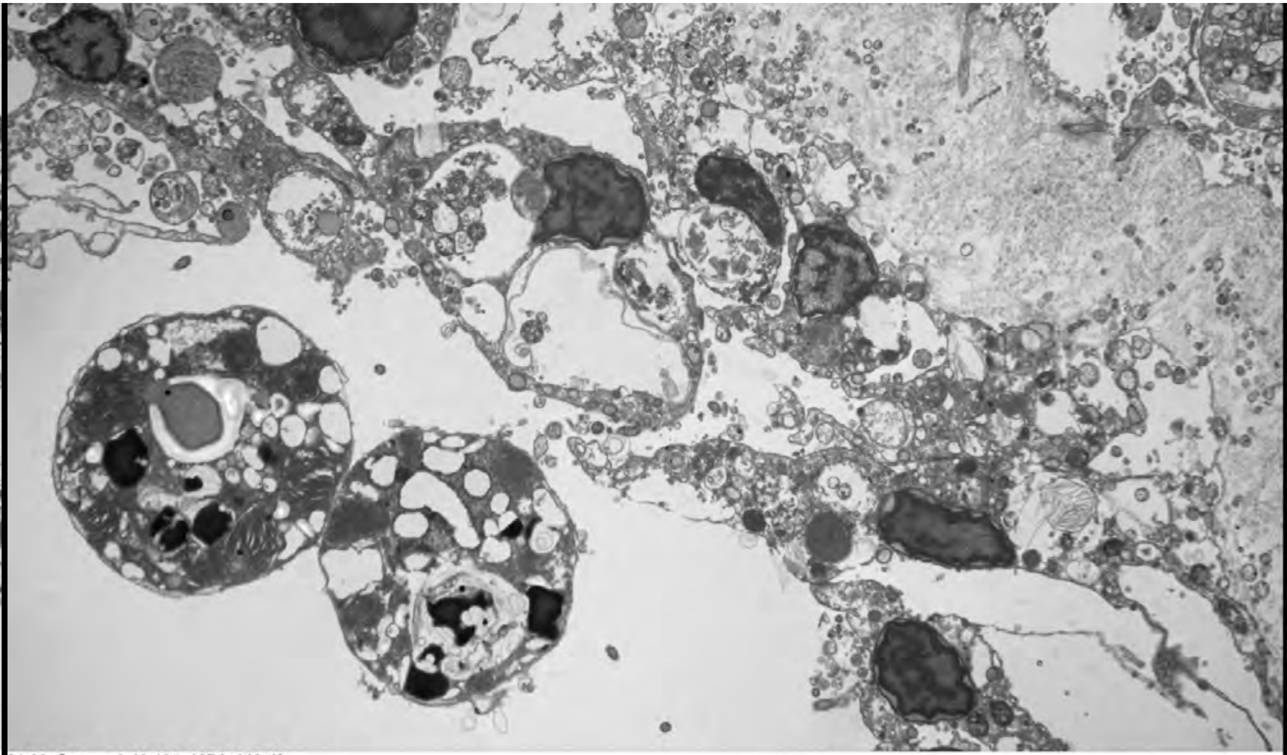

21-20_Correa_3-19-135_16D2_019.tif
Sample: 3/19, 135
Biological Electron Microscopy Lab
Rice University - SEA
Microscopist: MD Meyer

4 µm
HV=80kV
Direct Mag: 1000 x

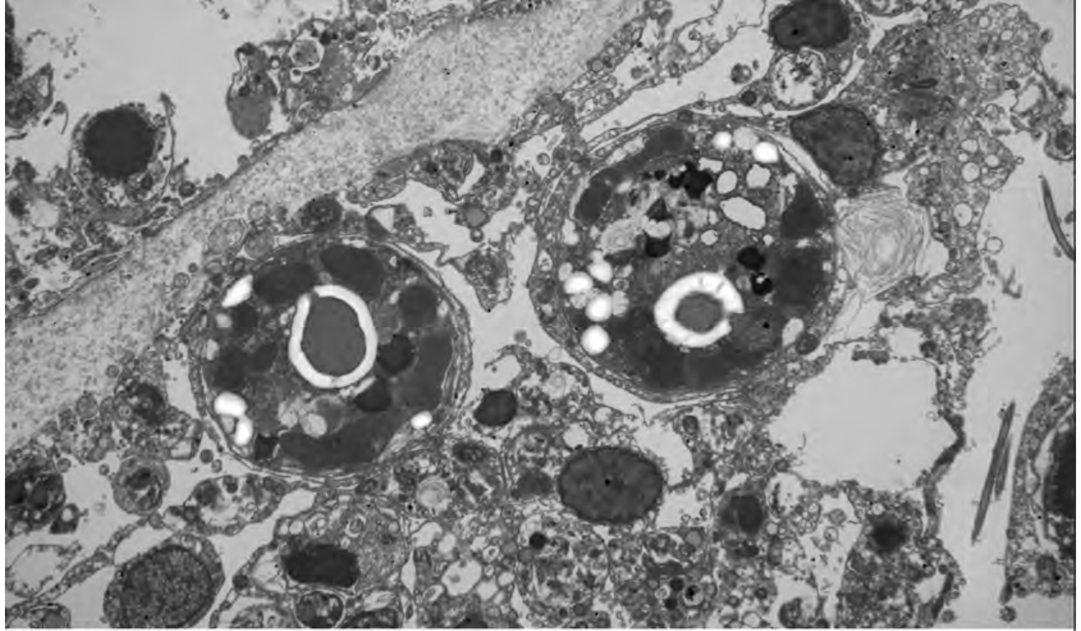

21-20_Correa_3-19-135_16D2_045.tif
Sample: 3/19, 135
Biological Electron Microscopy Lab
Rice University - SEA
Microscopist: MD Meyer

4 µm
HV=80kV
Direct Mag: 1000 x

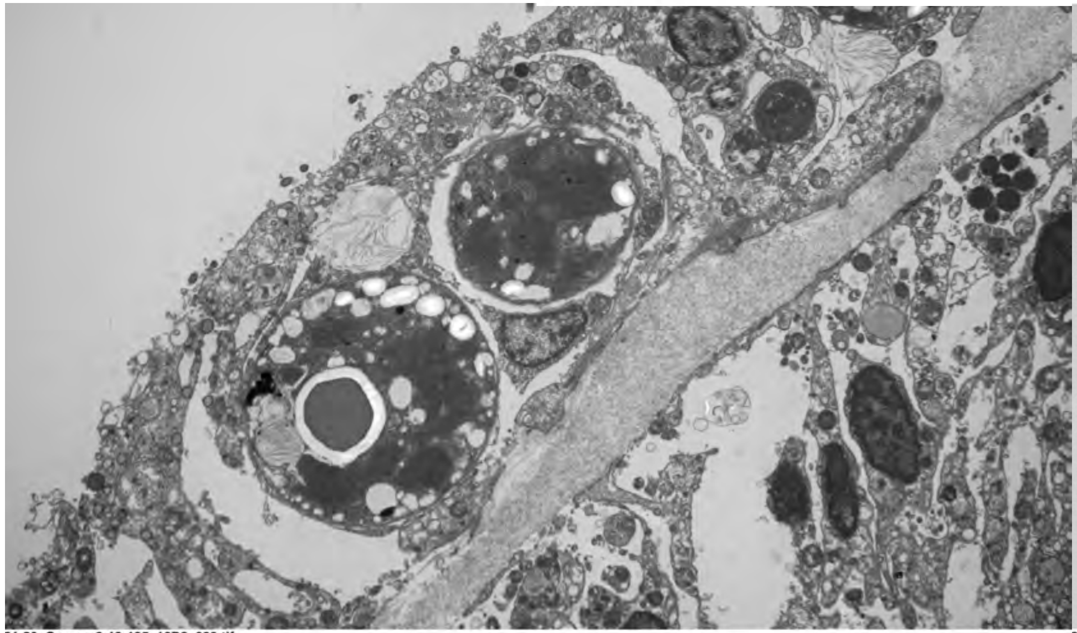

21-20_Correa_3-19-135_16D2_033.tif
Sample: 3/19, 135
Biological Electron Microscopy Lab
Rice University - SEA
Microscopist: MD Meyer

4 µm
HV=80kV
Direct Mag: 1000 x

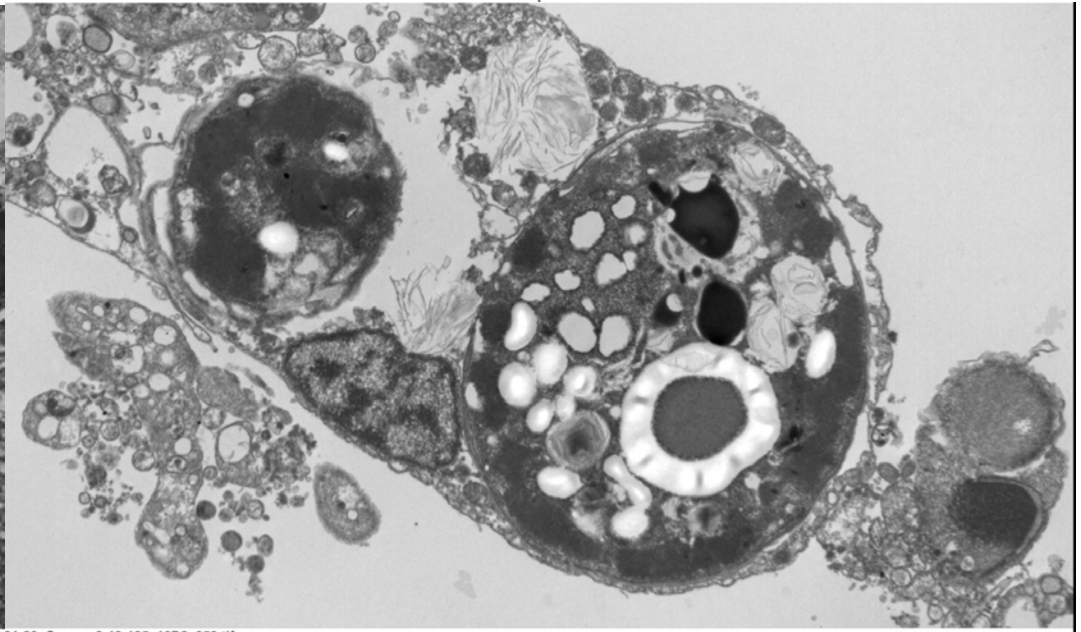

21-20_Correa_3-19-135_16D2_050.tif
Sample: 3/19, 135
Biological Electron Microscopy Lab
Rice University - SEA
Microscopist: MD Meyer

2 µm
HV=80kV
Direct Mag: 1500 x

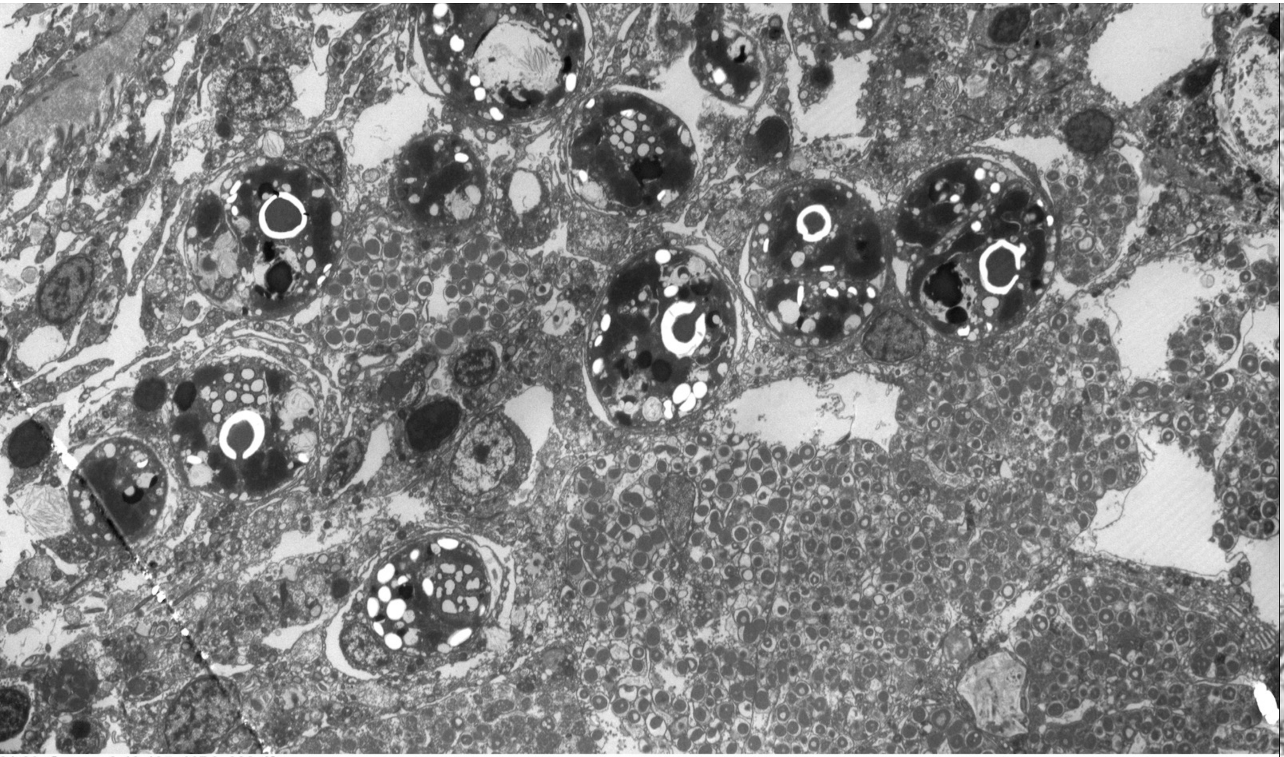

21-20_Correa_3-19-135_16D2_082.tif
Sample: 3/19, 135
Biological Electron Microscopy Lab
Rice University - SEA
Microscopist: MD Meyer

8 µm
HV=80kV
Direct Mag: 500 x

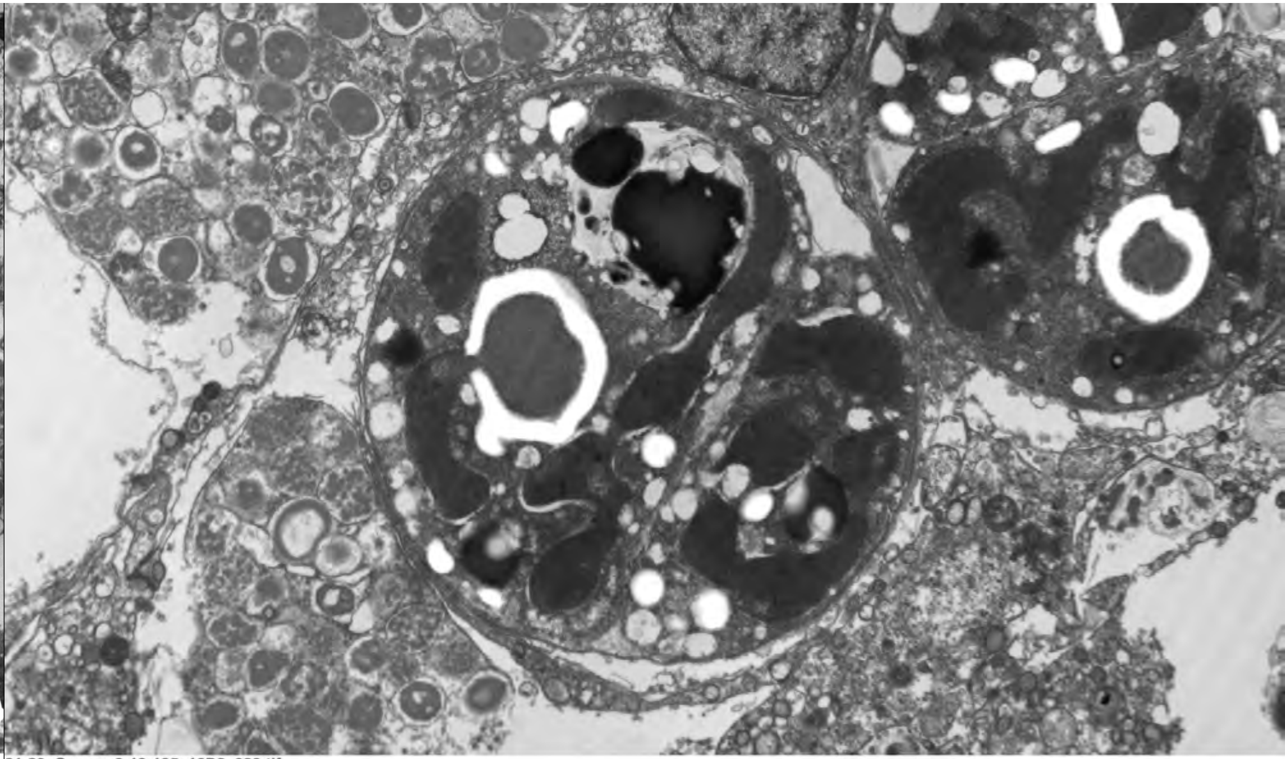

21-20_Correa_3-19-135_16D2_083.tif
Sample: 3/19, 135
Biological Electron Microscopy Lab
Rice University - SEA
Microscopist: MD Meyer

2 µm
HV=80kV
Direct Mag: 1500 x

Ambient (March 2018) POR Colony O

Cell 1

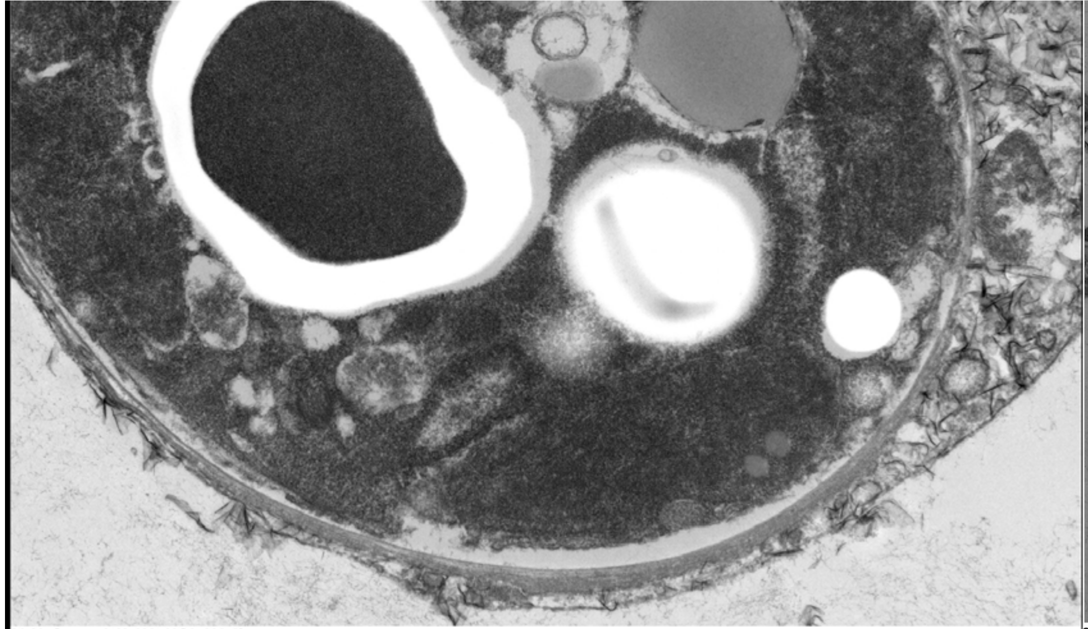

21-20_Correa_3-18-138_16G2_006.tif
Sample: 3/18, 138
Biological Electron Microscopy Lab
Rice University - SEA
Microscopist: MD Meyer

800 nm
HV=80kV
Direct Mag: 5000 x

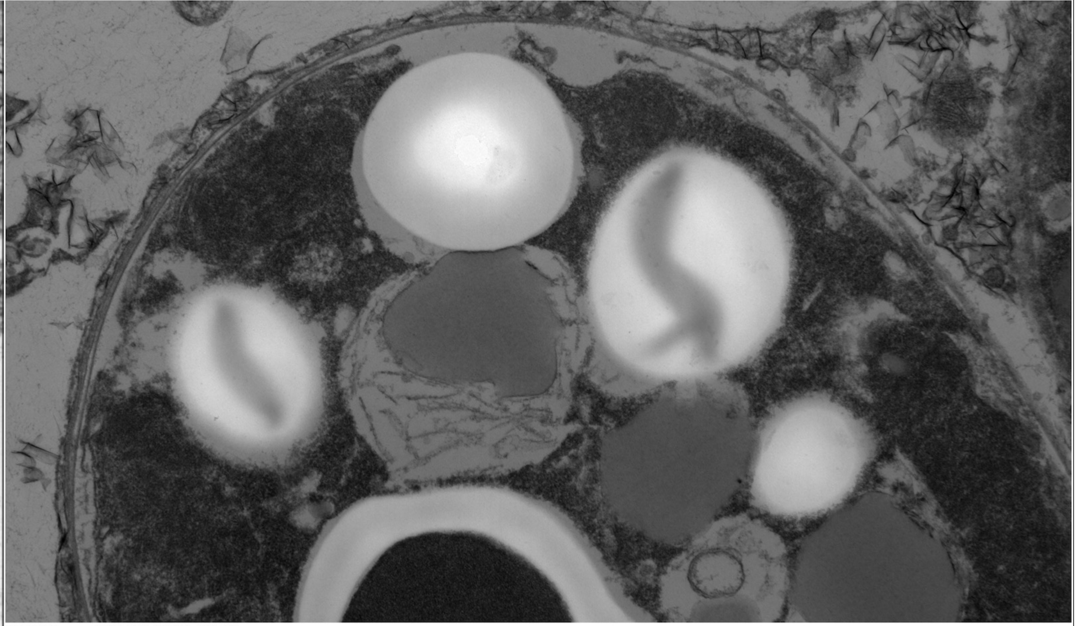

21-20_Correa_3-18-138_16G2_003.tif
Sample: 3/18, 138
Biological Electron Microscopy Lab
Rice University - SEA
Microscopist: MD Meyer

800 nm
HV=80kV
Direct Mag: 5000 x

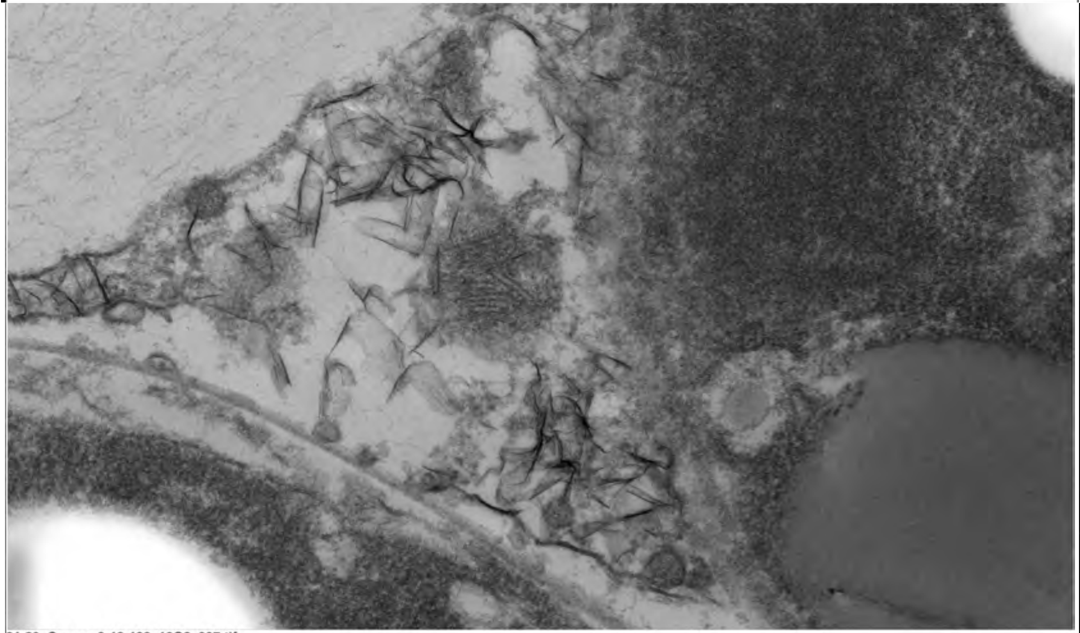

21-20_Correa_3-18-138_16G2_007.tif
Sample: 3/18, 138
Biological Electron Microscopy Lab
Rice University - SEA
Microscopist: MD Meyer

400 nm
HV=80kV
Direct Mag: 10000 x

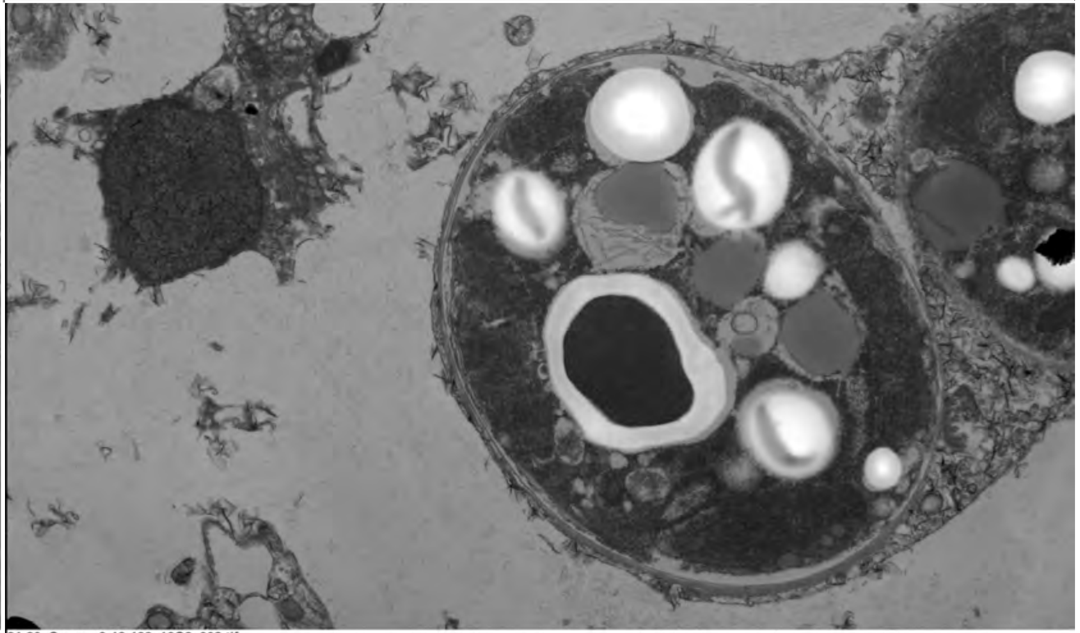

21-20_Correa_3-18-138_16G2_002.tif
Sample: 3/18, 138
Biological Electron Microscopy Lab
Rice University - SEA
Microscopist: MD Meyer

1 µm
HV=80kV
Direct Mag: 2500 x

Cell 2

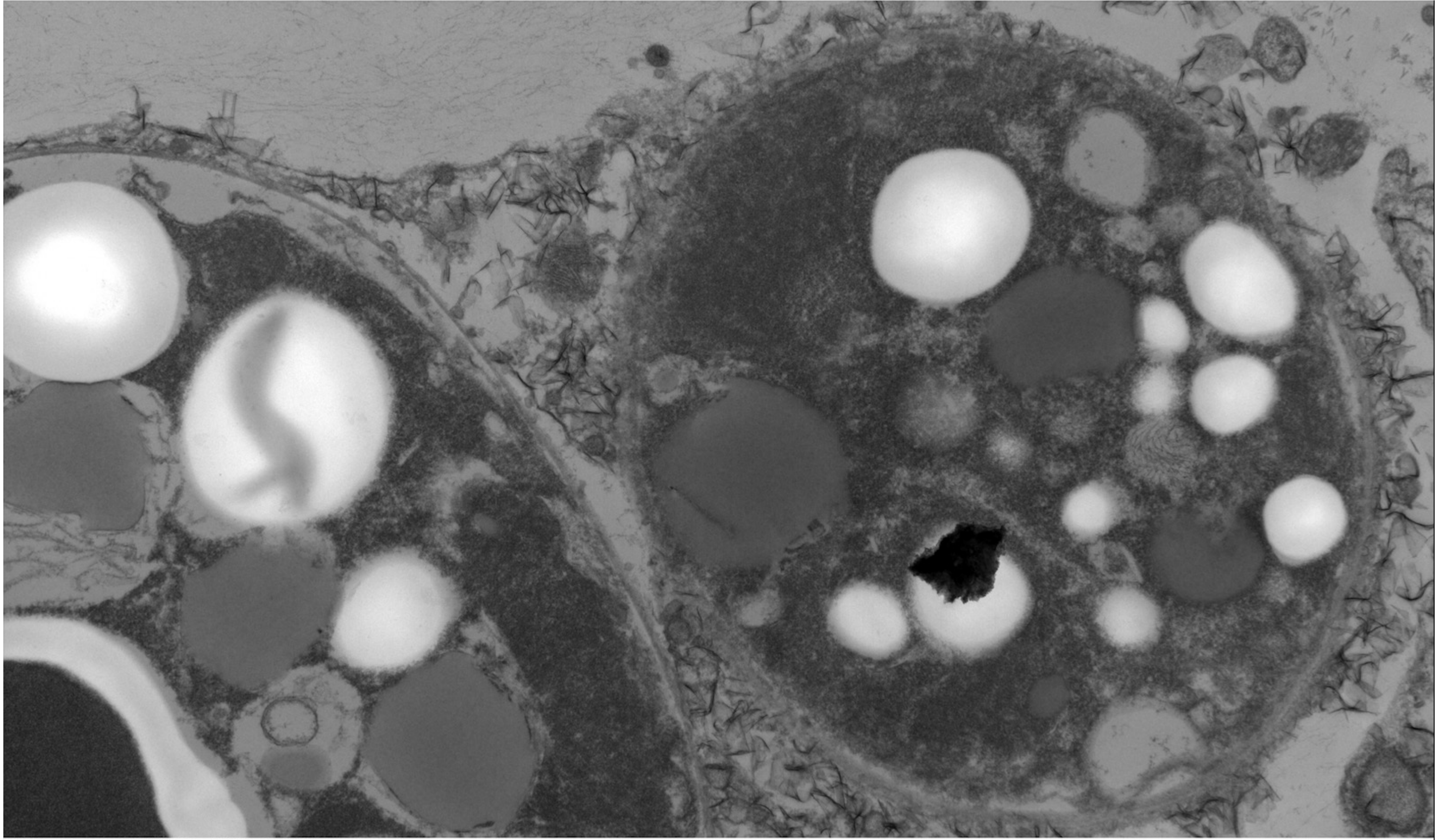

21-20_Correa_3-18-138_16G2_008.tif
Sample: 3/18, 138
Biological Electron Microscopy Lab
Rice University - SEA
Microscopist: MD Meyer

1 µm
HV=80kV
Direct Mag: 4000 x

Cell 3

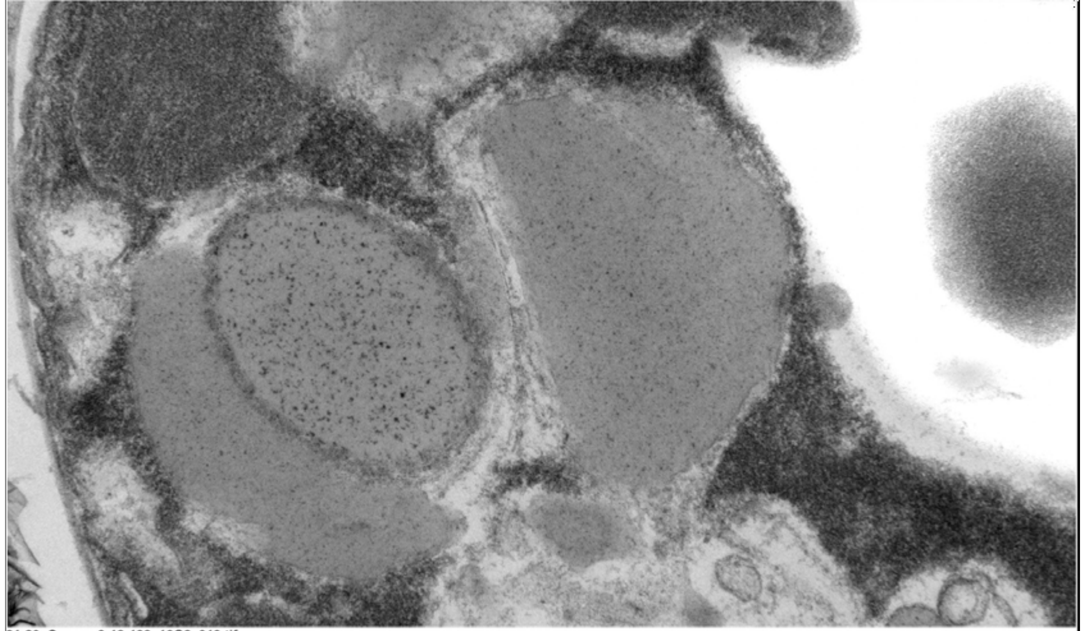

21-20_Correa_3-18-138_16G2_013.tif
Sample: 3/18, 138
Biological Electron Microscopy Lab
Rice University - SEA
Microscopist: MD Meyer

400 nm
HV=80kV
Direct Mag: 10000 x

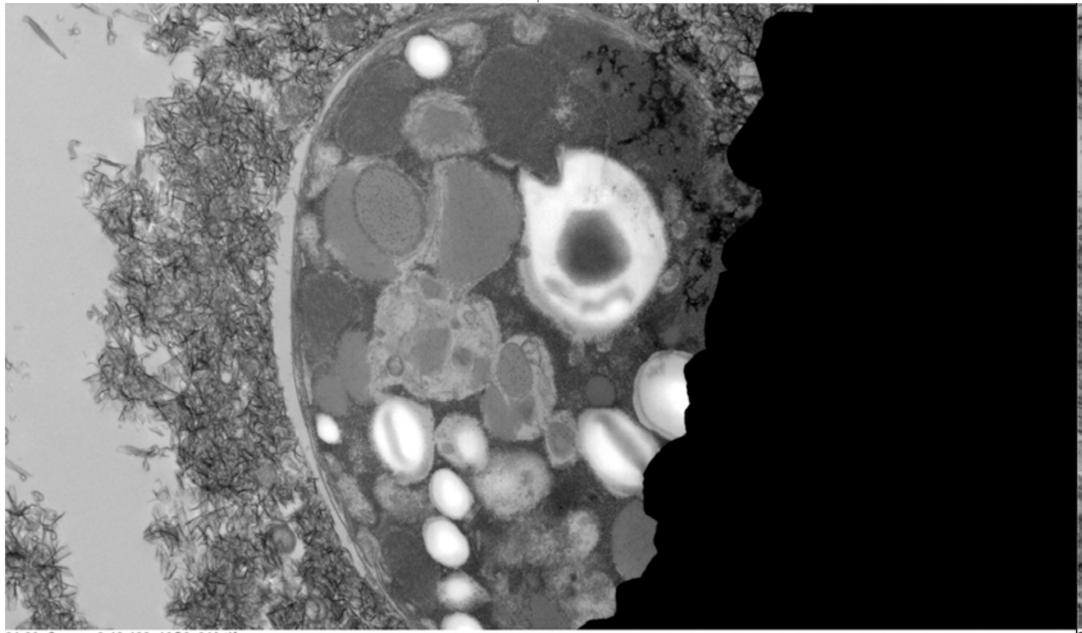

21-20_Correa_3-18-138_16G2_010.tif
Sample: 3/18, 138
Biological Electron Microscopy Lab
Rice University - SEA
Microscopist: MD Meyer

1 µm
HV=80kV
Direct Mag: 3000 x

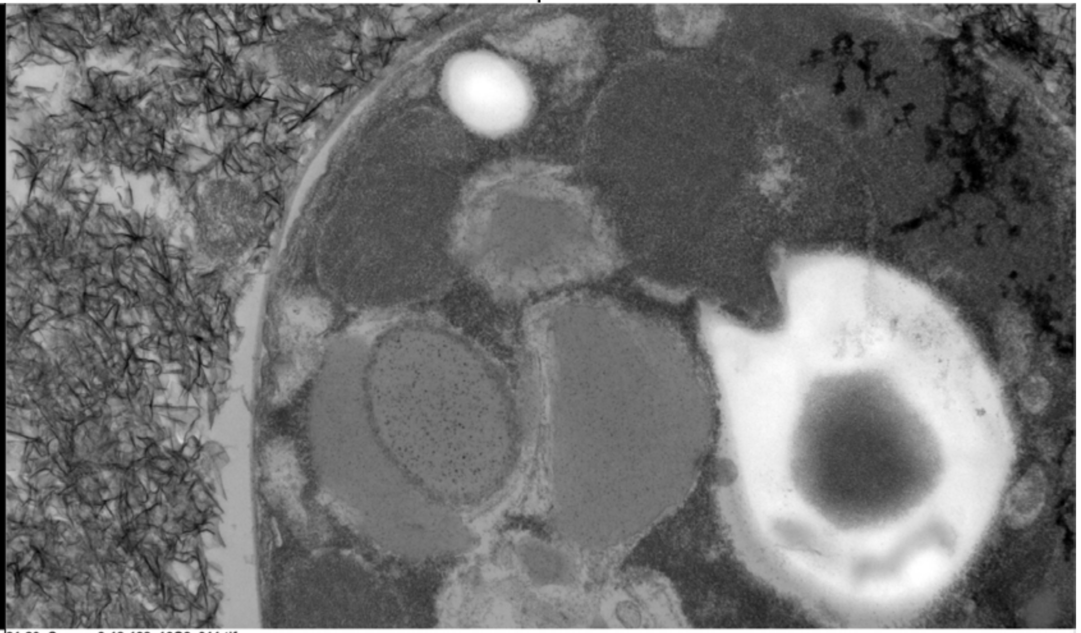

21-20_Correa_3-18-138_16G2_011.tif
Sample: 3/18, 138
Biological Electron Microscopy Lab
Rice University - SEA
Microscopist: MD Meyer

600 nm
HV=80kV
Direct Mag: 6000 x

Cell 4

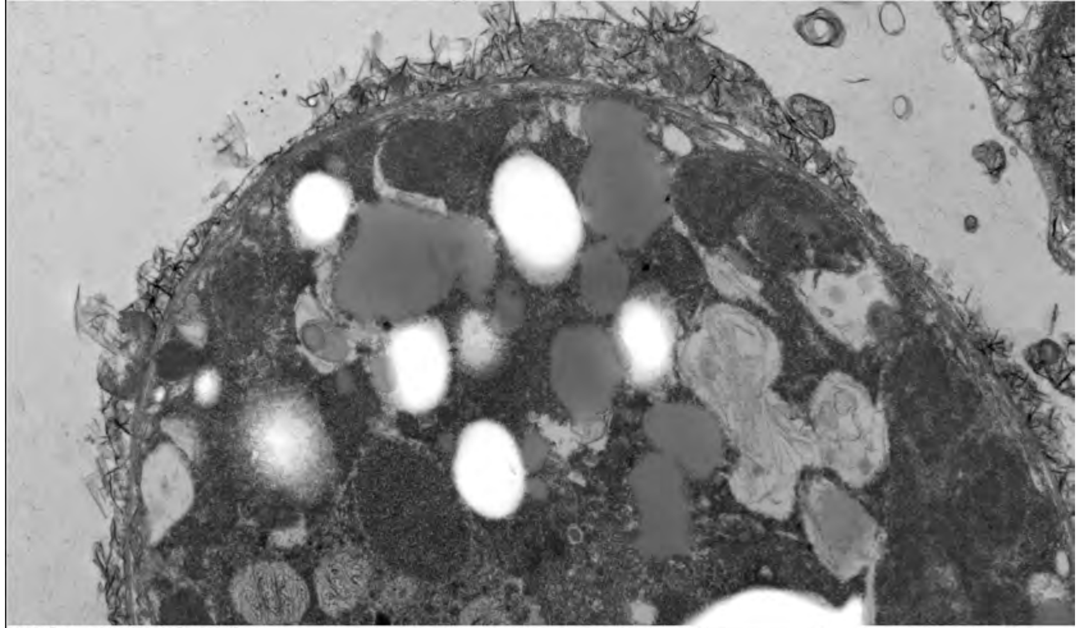

21-20_Correa_3-18-138_16G2_018.tif
Sample: 3/18, 138
Biological Electron Microscopy Lab
Rice University - SEA
Microscopist: MD Meyer

1 µm
HV=80kV
Direct Mag: 4000 x

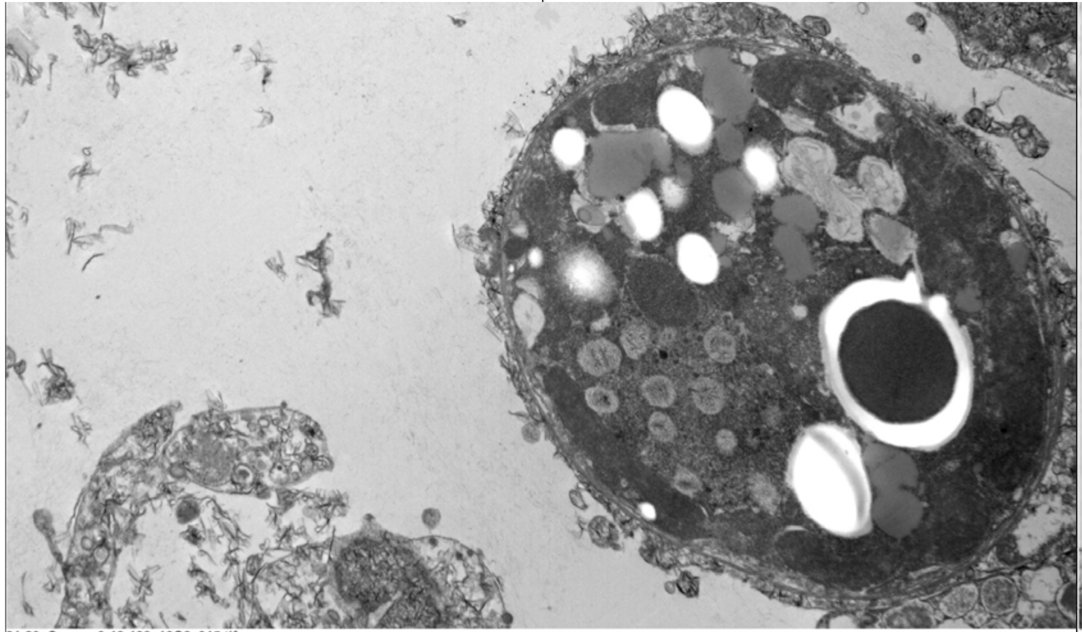

21-20_Correa_3-18-138_16G2_015.tif
Sample: 3/18, 138
Biological Electron Microscopy Lab
Rice University - SEA
Microscopist: MD Meyer

2 µm
HV=80kV
Direct Mag: 2000 x

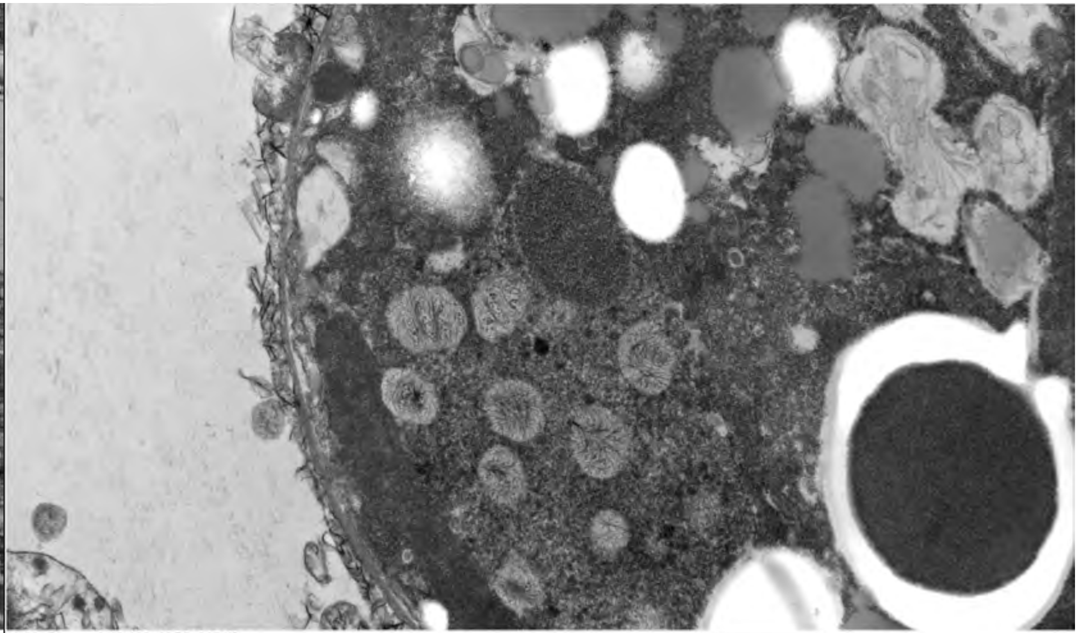

21-20_Correa_3-18-138_16G2_017.tif
Sample: 3/18, 138
Biological Electron Microscopy Lab
Rice University - SEA
Microscopist: MD Meyer

1 µm
HV=80kV
Direct Mag: 4000 x

Cell 5

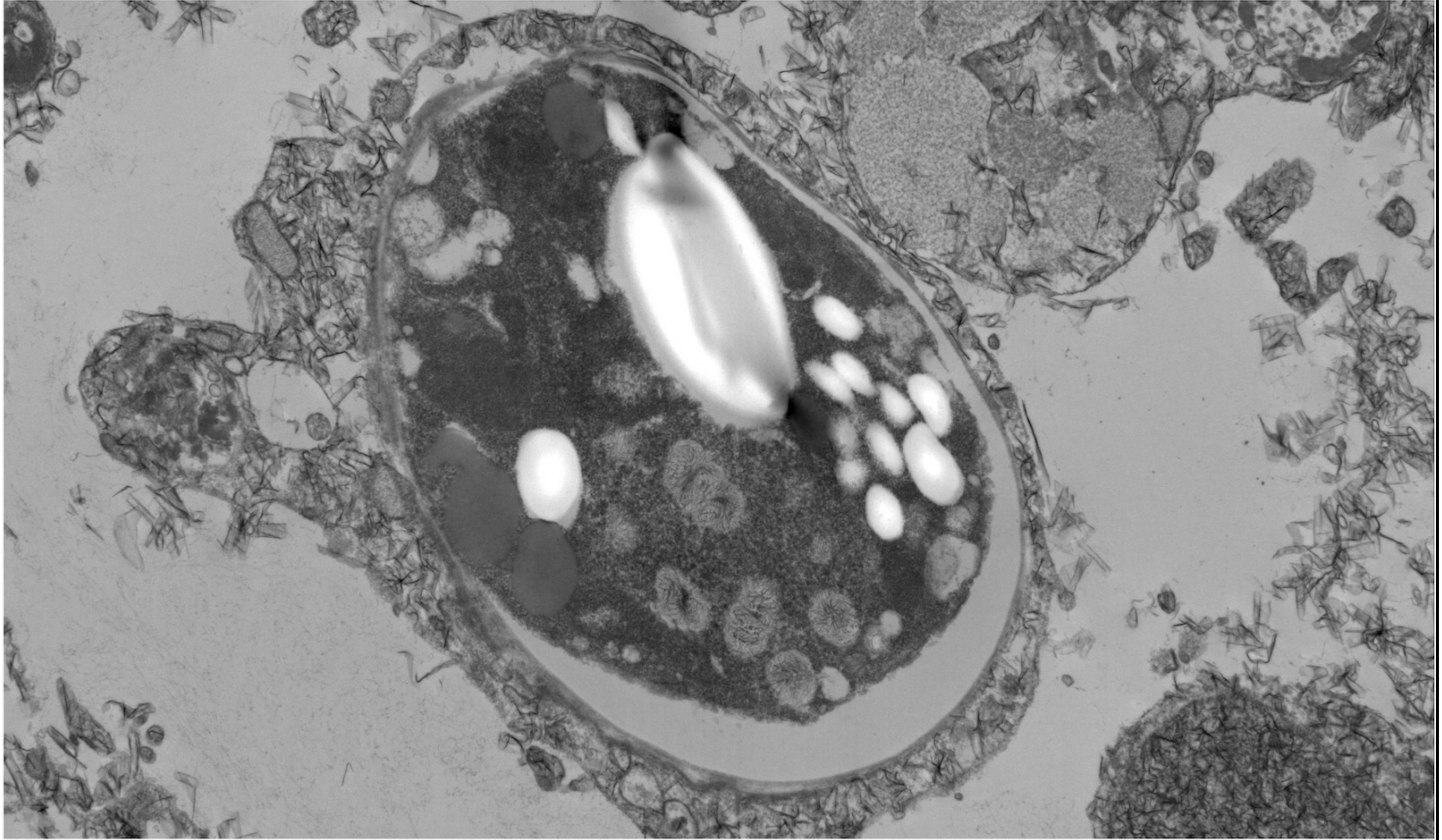

21-20_Correa_3-18-138_16G2_019.tif
Sample: 3/18, 138
Biological Electron Microscopy Lab
Rice University - SEA
Microscopist: MD Meyer

1 µm
HV=80kV
Direct Mag: 3000 x

Cell 6

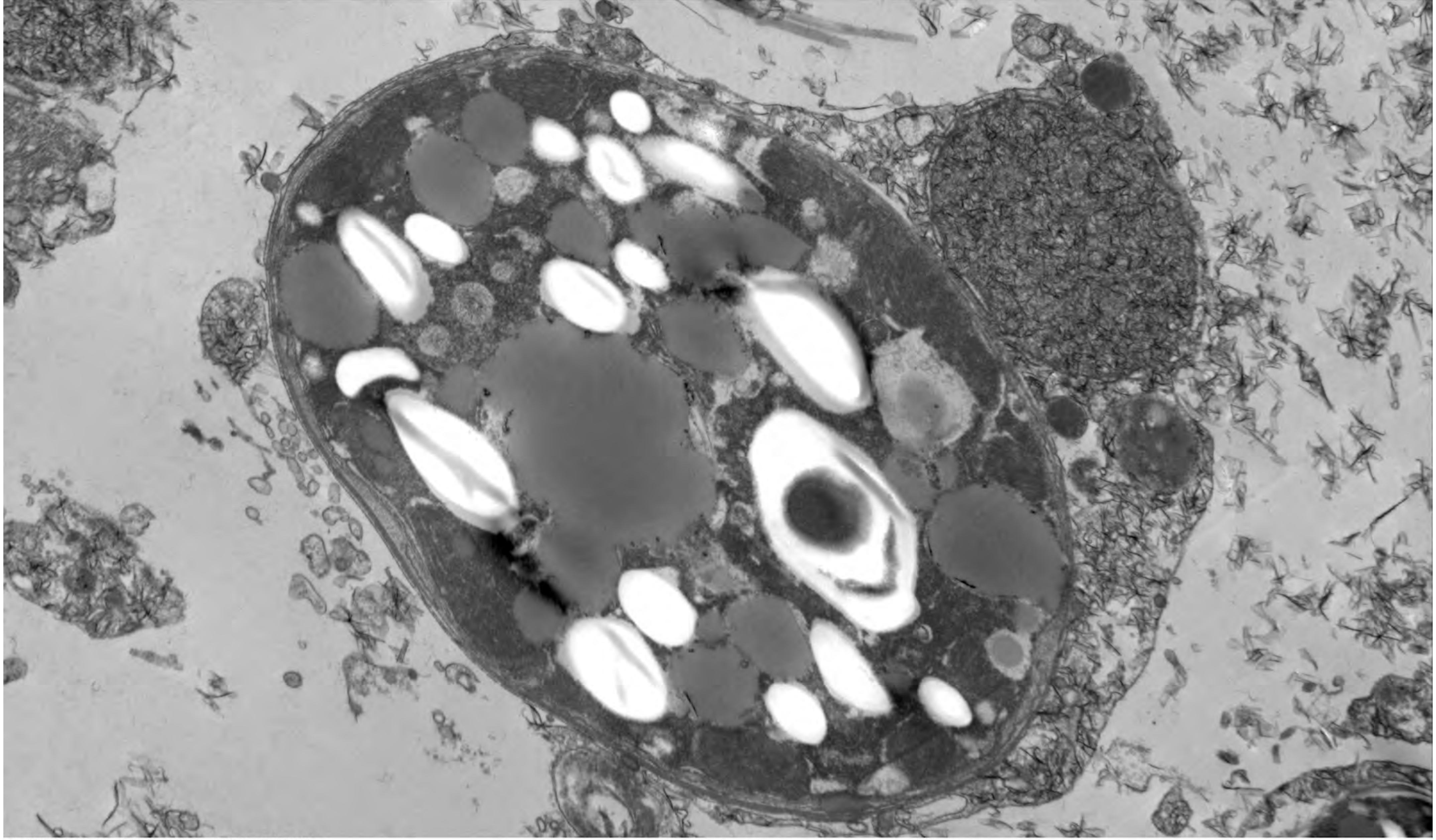

21-20_Correa_3-18-138_16G2_022.tif
Sample: 3/18, 138
Biological Electron Microscopy Lab
Rice University - SEA
Microscopist: MD Meyer

2 μm
HV=80kV
Direct Mag: 2000 x

Cell 7

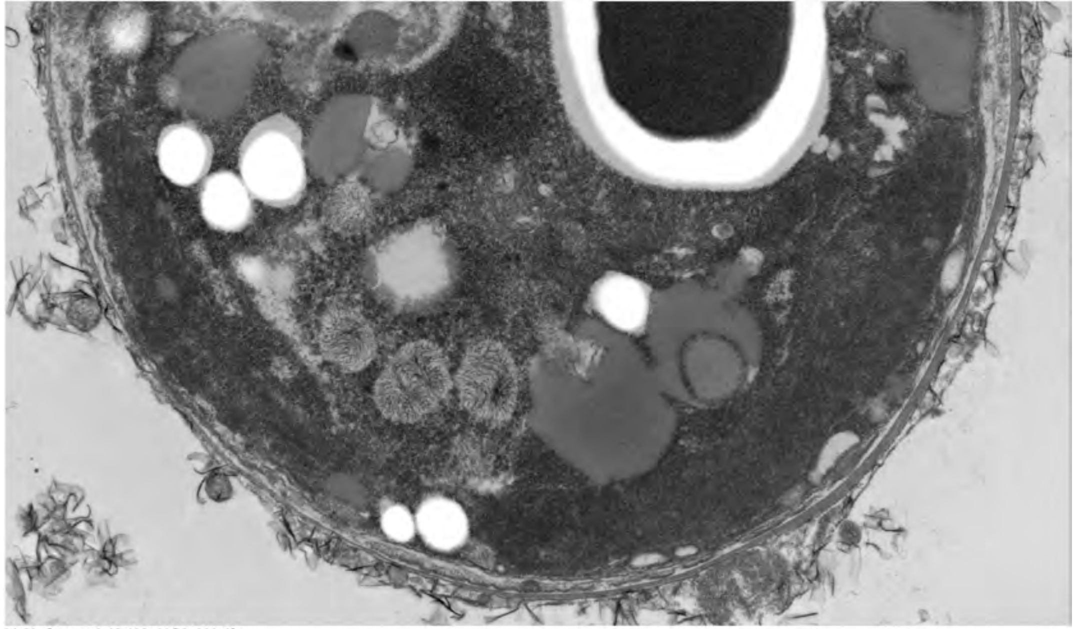
21-20_Correa_3-18-138_16G2_026.tif
Sample: 3/18, 138
Biological Electron Microscopy Lab
Rice University - SEA
Microscopist: MD Meyer
1 µm
HV=80kV
Direct Mag: 4000 x

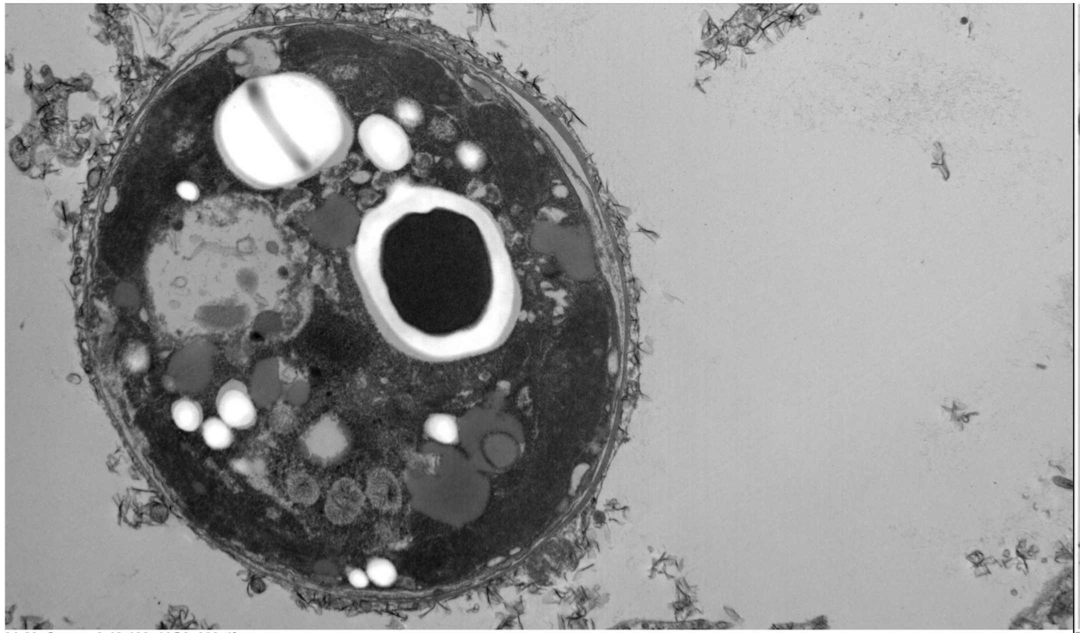
21-20_Correa_3-18-138_16G2_023.tif
Sample: 3/18, 138
Biological Electron Microscopy Lab
Rice University - SEA
Microscopist: MD Meyer
2 µm
HV=80kV
Direct Mag: 2000 x

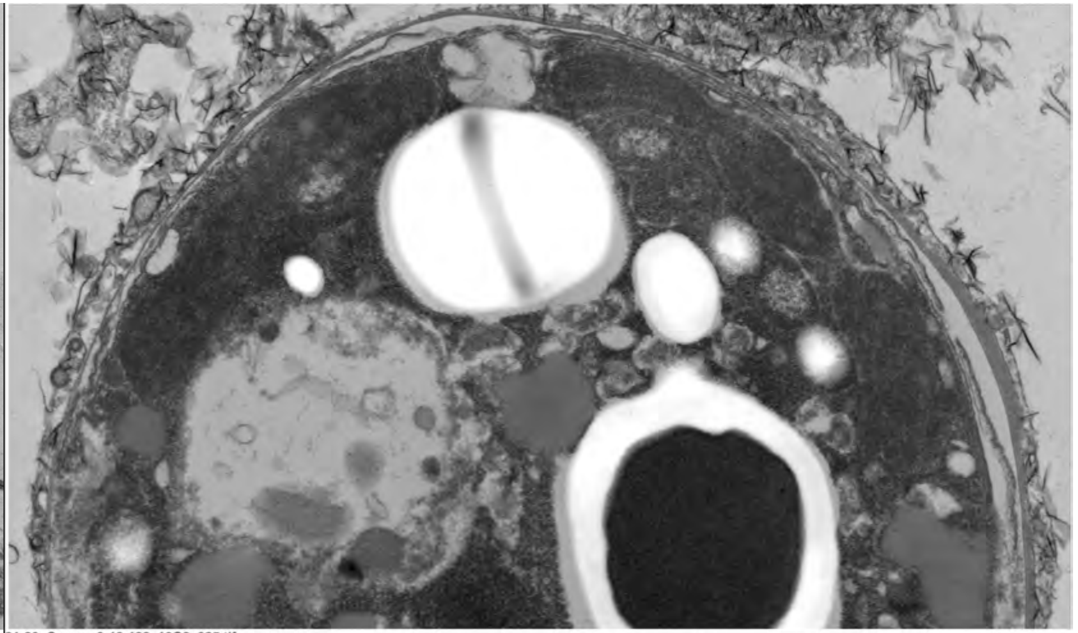
21-20_Correa_3-18-138_16G2_025.tif
Sample: 3/18, 138
Biological Electron Microscopy Lab
Rice University - SEA
Microscopist: MD Meyer
1 µm
HV=80kV
Direct Mag: 4000 x

Cell 8

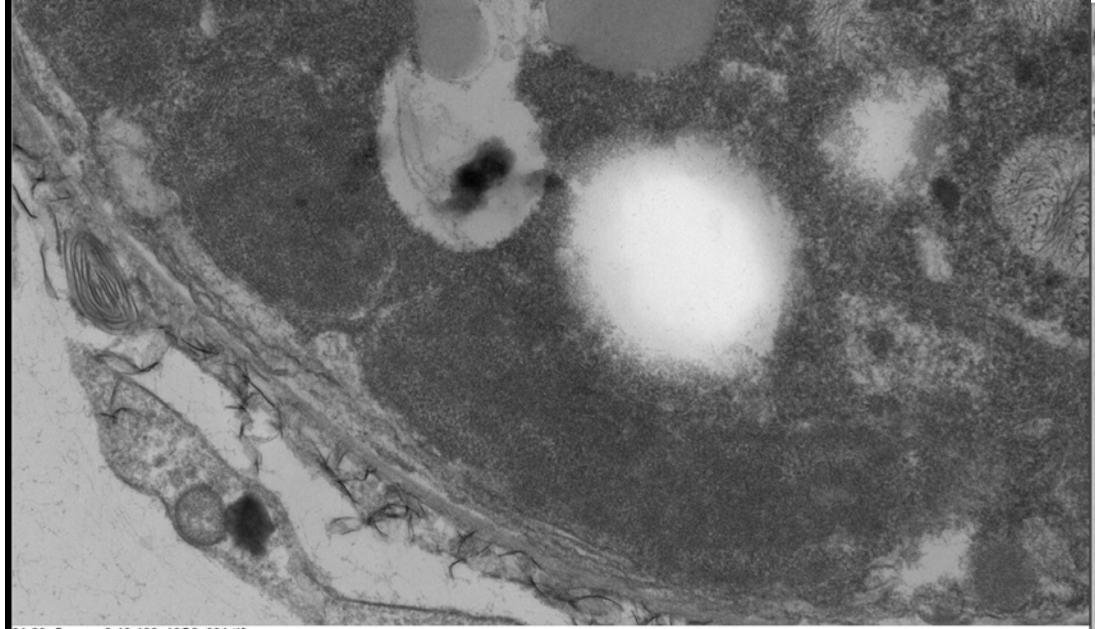

21-20_Correa_3-18-138_16G2_031.tif
Sample: 3/18, 138
Biological Electron Microscopy Lab
Rice University - SEA
Microscopist: MD Meyer

500 nm
HV=80kV
Direct Mag: 8000 x

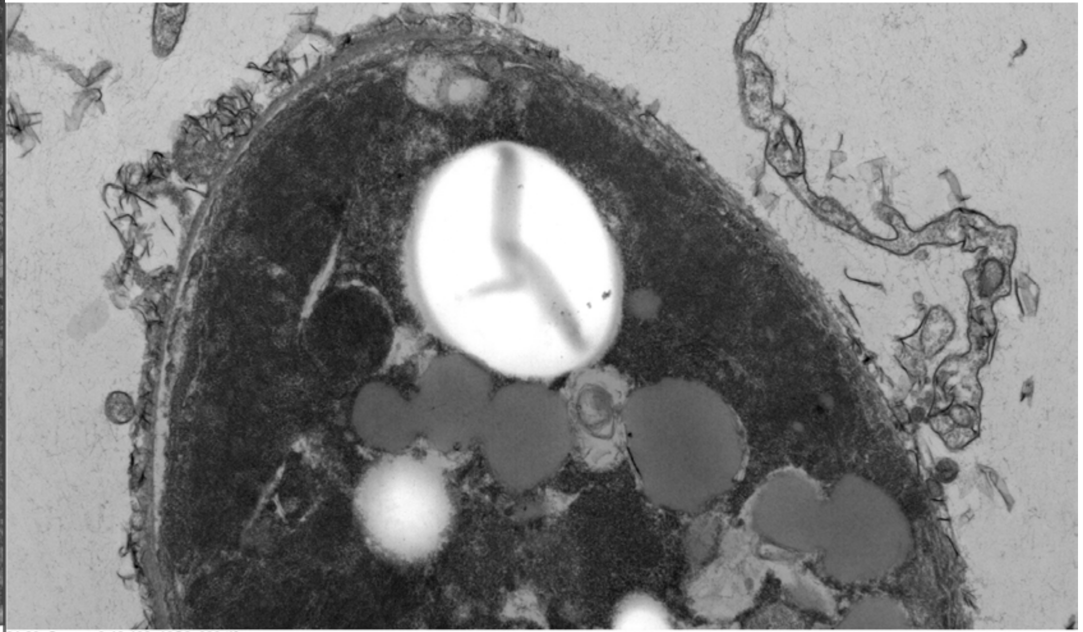

21-20_Correa_3-18-138_16G2_030.tif
Sample: 3/18, 138
Biological Electron Microscopy Lab
Rice University - SEA
Microscopist: MD Meyer

1 µm
HV=80kV
Direct Mag: 4000 x

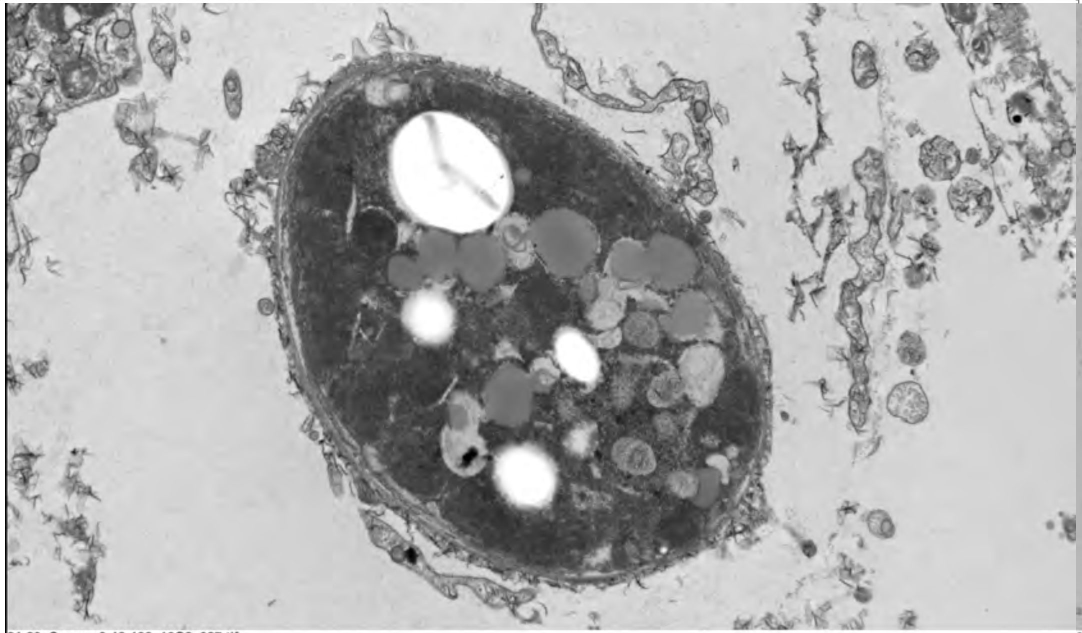

21-20_Correa_3-18-138_16G2_027.tif
Sample: 3/18, 138
Biological Electron Microscopy Lab
Rice University - SEA
Microscopist: MD Meyer

2 µm
HV=80kV
Direct Mag: 2000 x

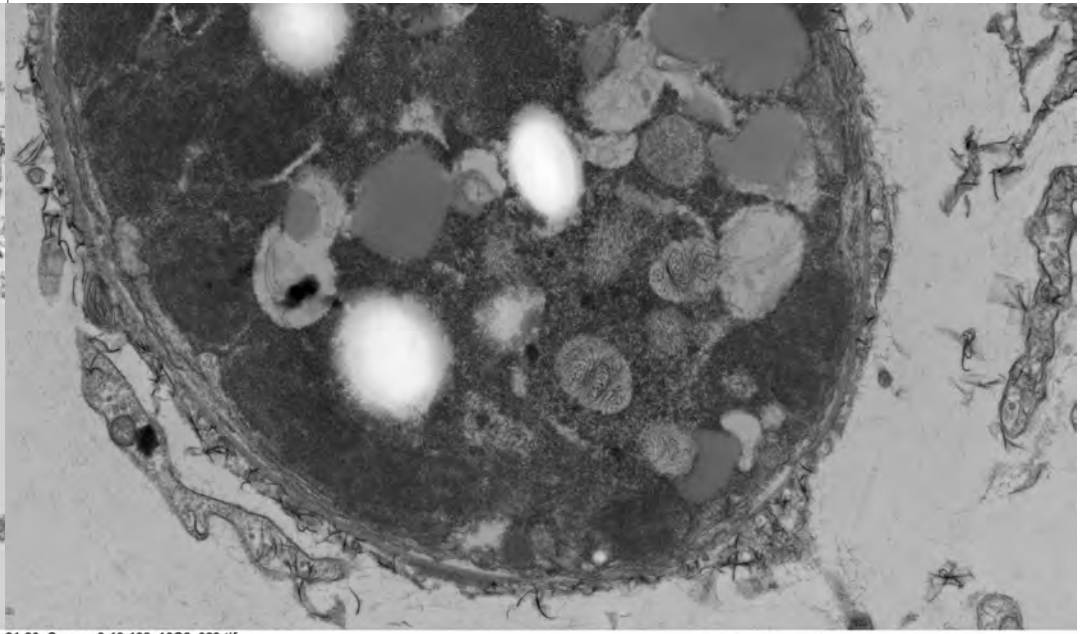

21-20_Correa_3-18-138_16G2_029.tif
Sample: 3/18, 138
Biological Electron Microscopy Lab
Rice University - SEA
Microscopist: MD Meyer

1 µm
HV=80kV
Direct Mag: 4000 x

Cell 9

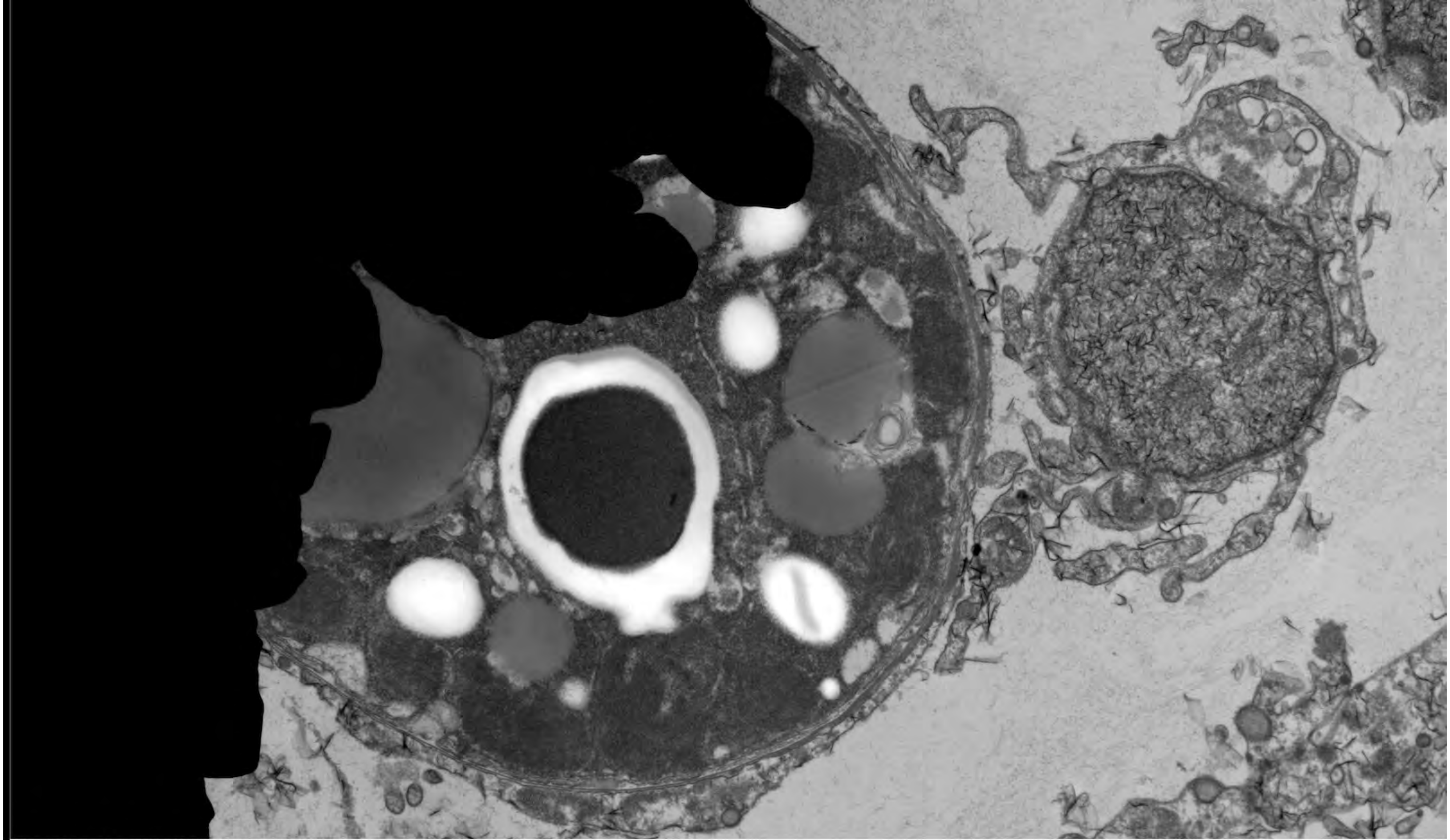

21-20_Correa_3-18-138_16G2_032.tif
Sample: 3/18, 138
Biological Electron Microscopy Lab
Rice University - SEA
Microscopist: MD Meyer

1 µm
HV=80kV
Direct Mag: 2500 x

Cell 10

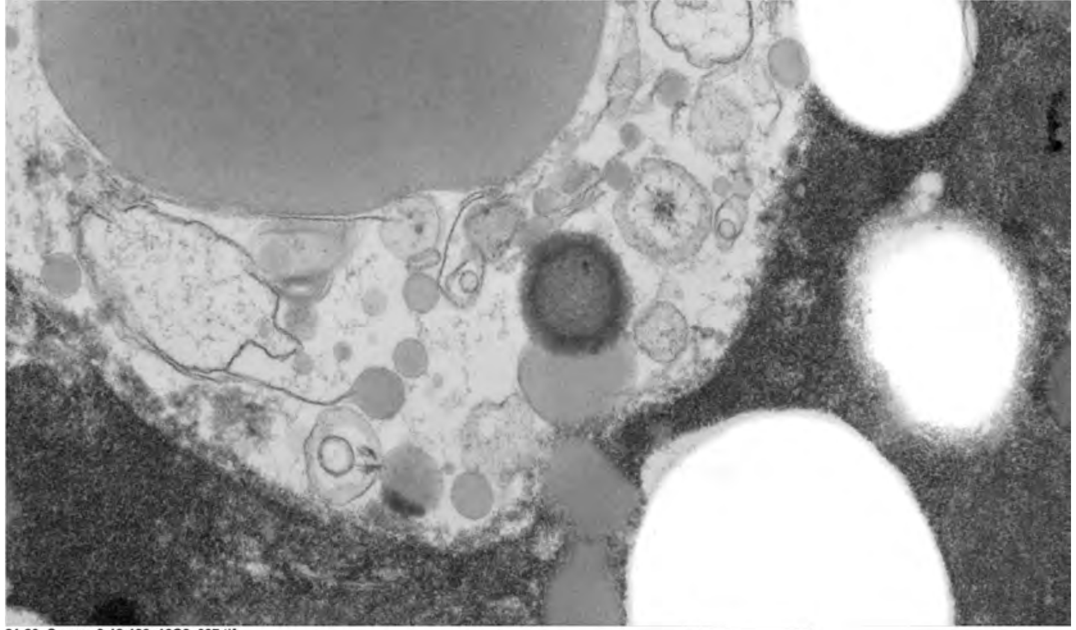

21-20_Correa_3-18-138_16G2_037.tif
Sample: 3/18, 138
Biological Electron Microscopy Lab
Rice University - SEA
Microscopist: MD Meyer

500 nm
HV=80kV
Direct Mag: 8000 x

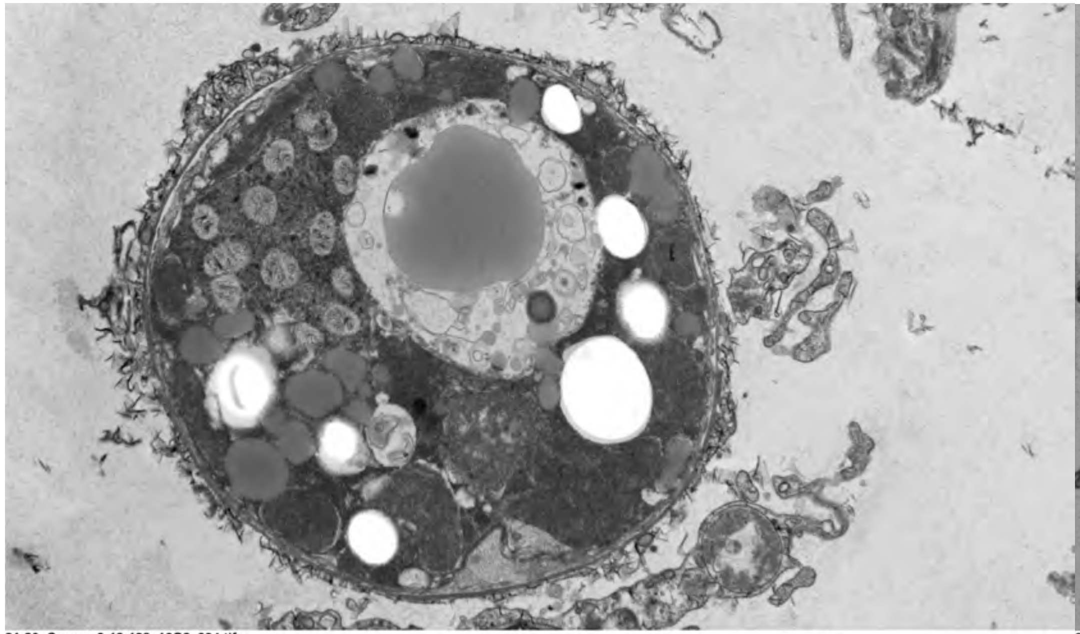

21-20_Correa_3-18-138_16G2_034.tif
Sample: 3/18, 138
Biological Electron Microscopy Lab
Rice University - SEA
Microscopist: MD Meyer

2 µm
HV=80kV
Direct Mag: 2000 x

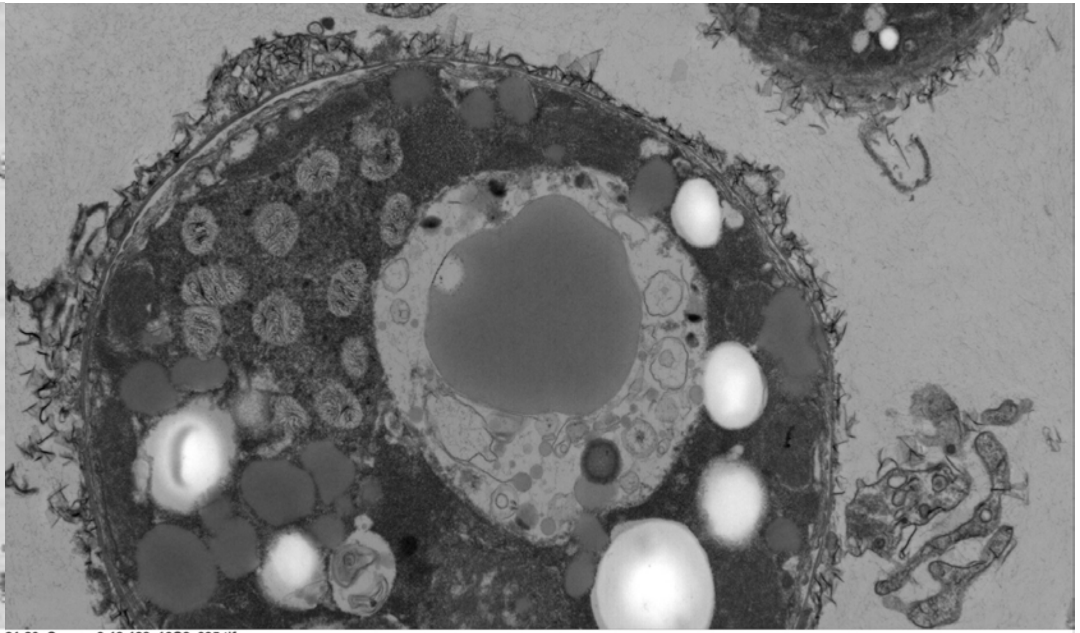

21-20_Correa_3-18-138_16G2_035.tif
Sample: 3/18, 138
Biological Electron Microscopy Lab
Rice University - SEA
Microscopist: MD Meyer

1 µm
HV=80kV
Direct Mag: 3000 x

Cell 11

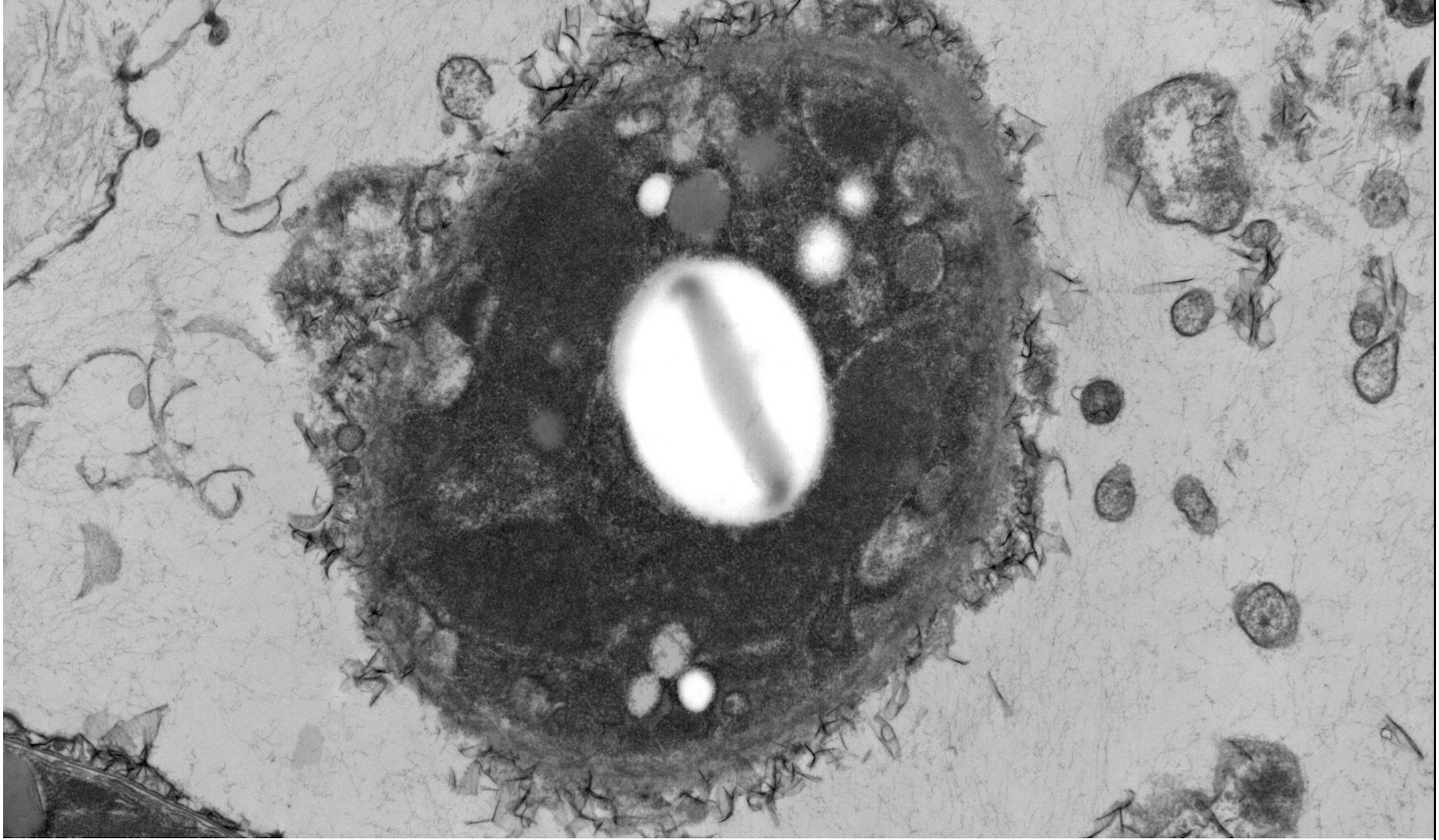

21-20_Correa_3-18-138_16G2_038.tif
Sample: 3/18, 138
Biological Electron Microscopy Lab
Rice University - SEA
Microscopist: MD Meyer

1 µm
HV=80kV
Direct Mag: 4000 x

Cell 12

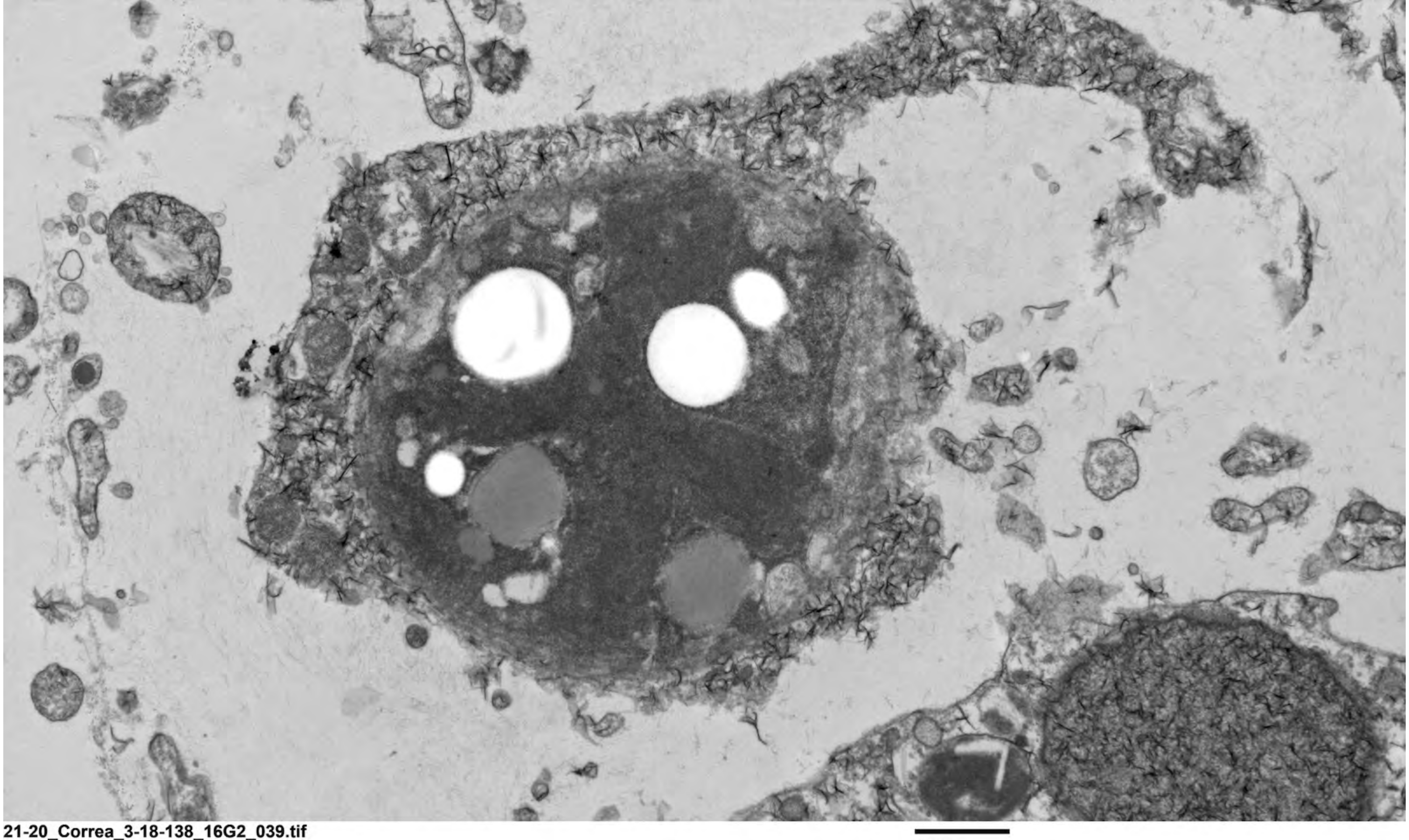

21-20_Correa_3-18-138_16G2_039.tif
Sample: 3/18, 138
Biological Electron Microscopy Lab
Rice University - SEA
Microscopist: MD Meyer

HV=80kV
Direct Mag: 2500 x

Cell 13

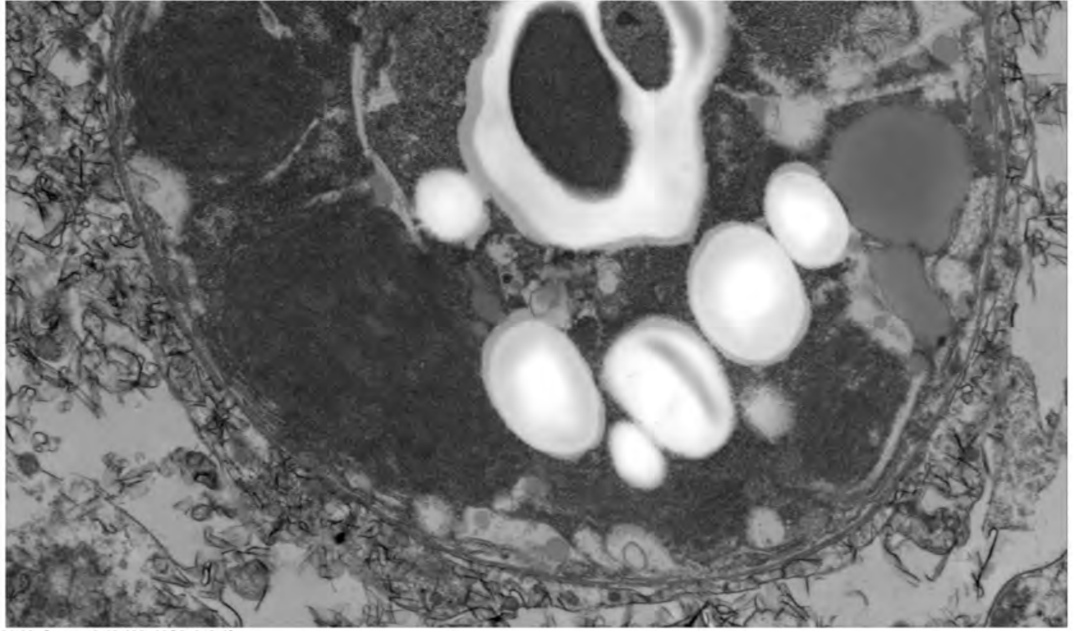

21-20_Correa_3-18-138_16G2_042.tif
Sample: 3/18, 138
Biological Electron Microscopy Lab
Rice University - SEA
Microscopist: MD Meyer

1 µm
HV=80kV
Direct Mag: 4000 x

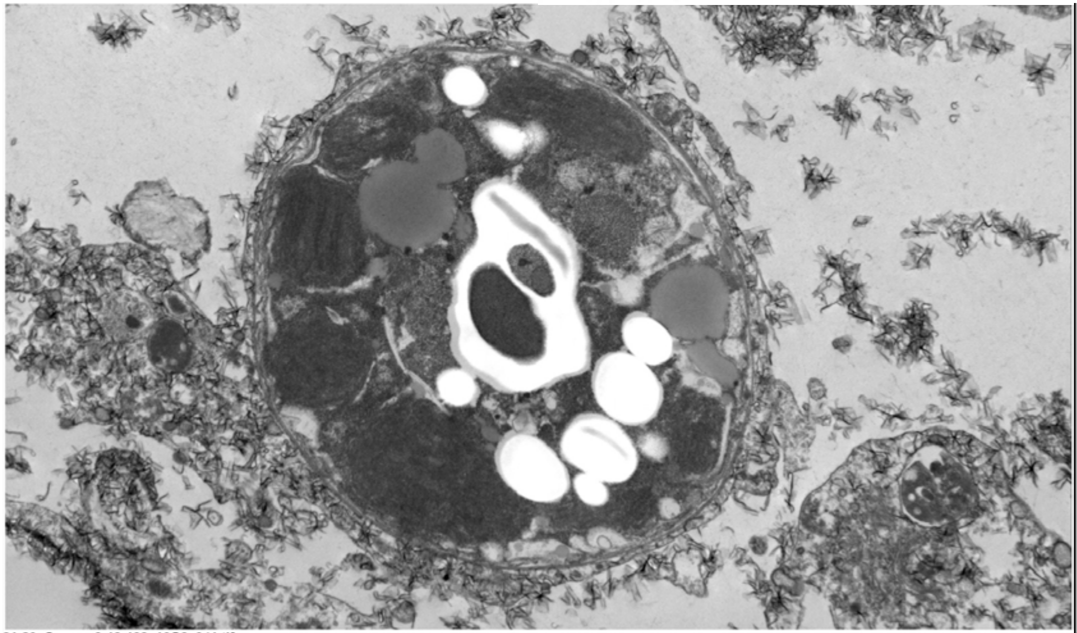

21-20_Correa_3-18-138_16G2_041.tif
Sample: 3/18, 138
Biological Electron Microscopy Lab
Rice University - SEA
Microscopist: MD Meyer

2 µm
HV=80kV
Direct Mag: 2000 x

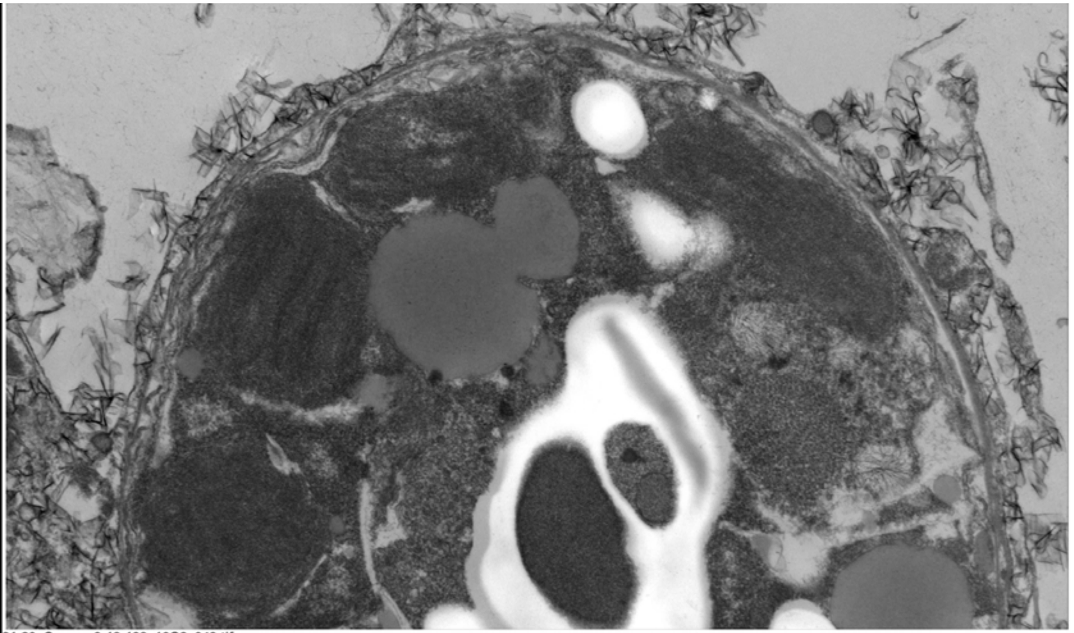

21-20_Correa_3-18-138_16G2_043.tif
Sample: 3/18, 138
Biological Electron Microscopy Lab
Rice University - SEA
Microscopist: MD Meyer

1 µm
HV=80kV
Direct Mag: 4000 x

Cell 14

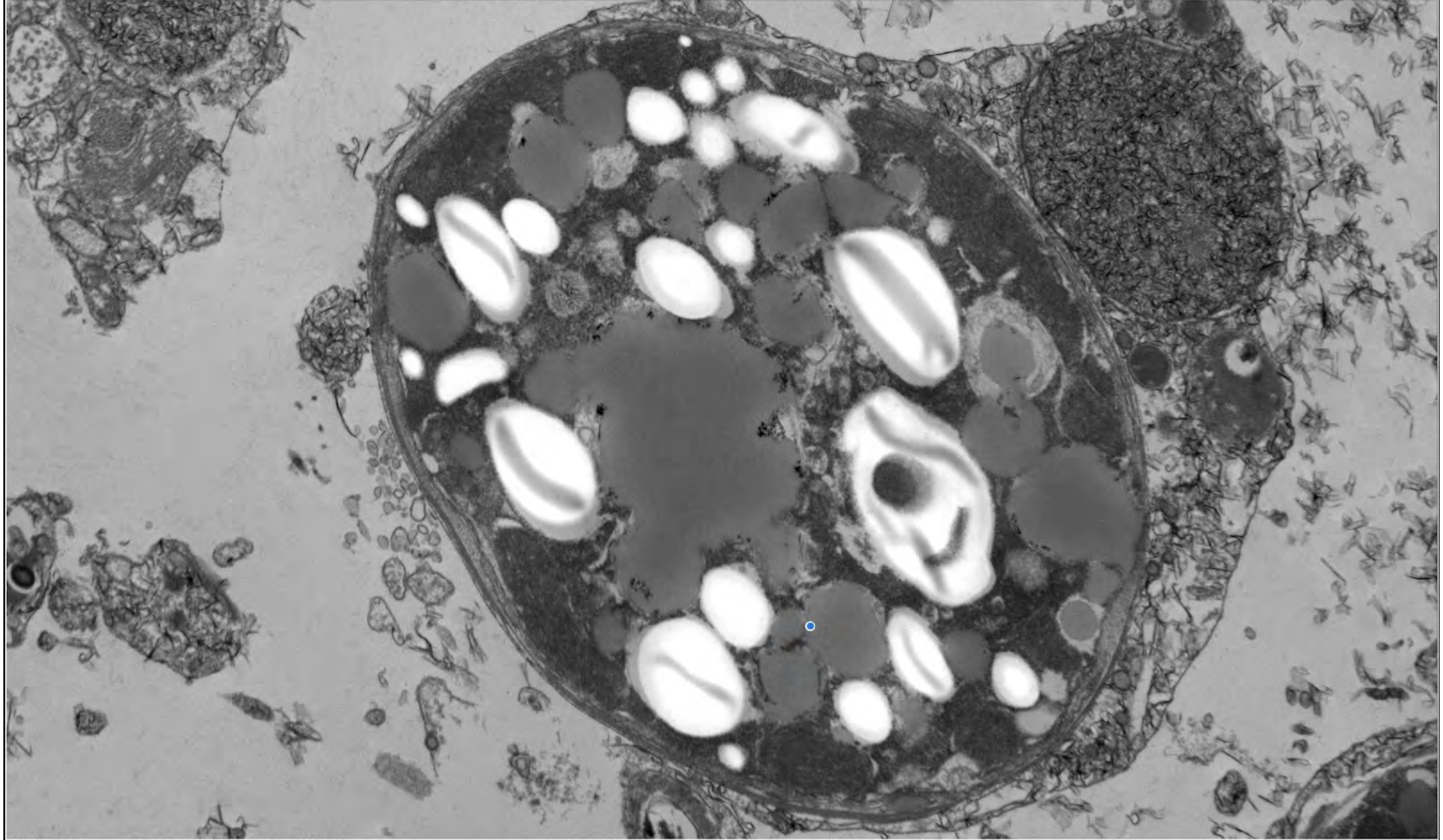

21-20_Correa_3-18-138_16G2_044.tif
Sample: 3/18, 138
Biological Electron Microscopy Lab
Rice University - SEA
Microscopist: MD Meyer

2 µm
HV=80kV
Direct Mag: 2000 x

Cell 15

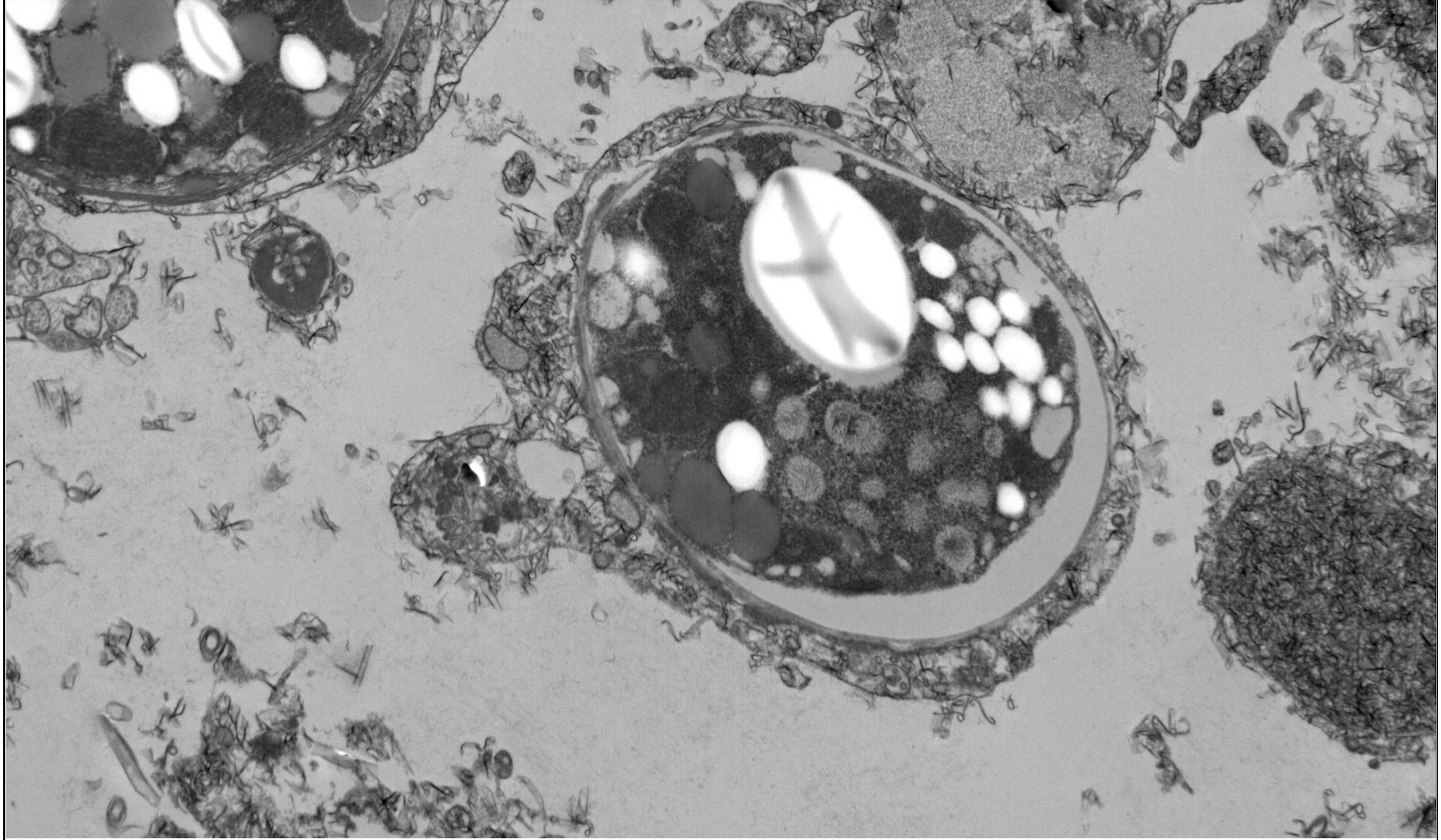

21-20_Correa_3-18-138_16G2_046.tif
Sample: 3/18, 138
Biological Electron Microscopy Lab
Rice University - SEA
Microscopist: MD Meyer

2 µm
HV=80kV
Direct Mag: 2000 x

Cell 16

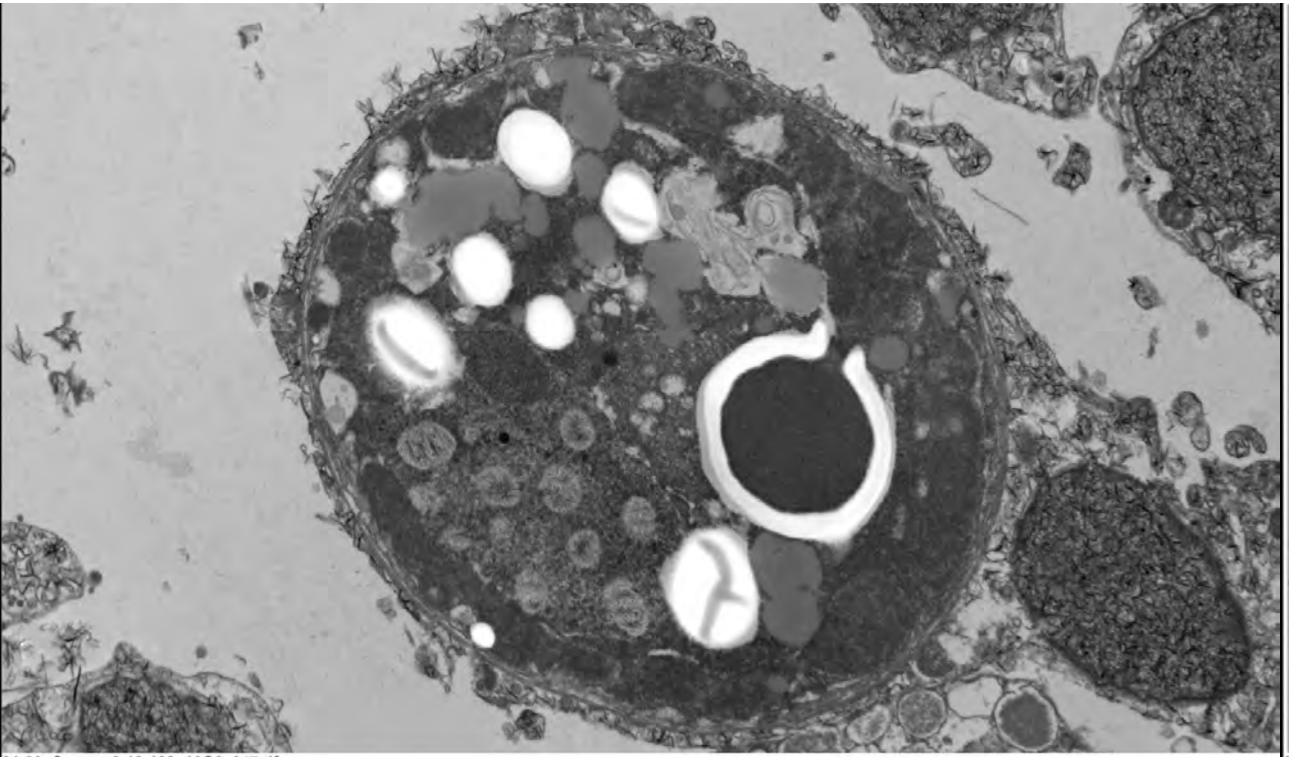

21-20_Correa_3-18-138_16G2_047.tif
Sample: 3/18, 138
Biological Electron Microscopy Lab
Rice University - SEA
Microscopist: MD Meyer

2 µm
HV=80kV
Direct Mag: 2000 x

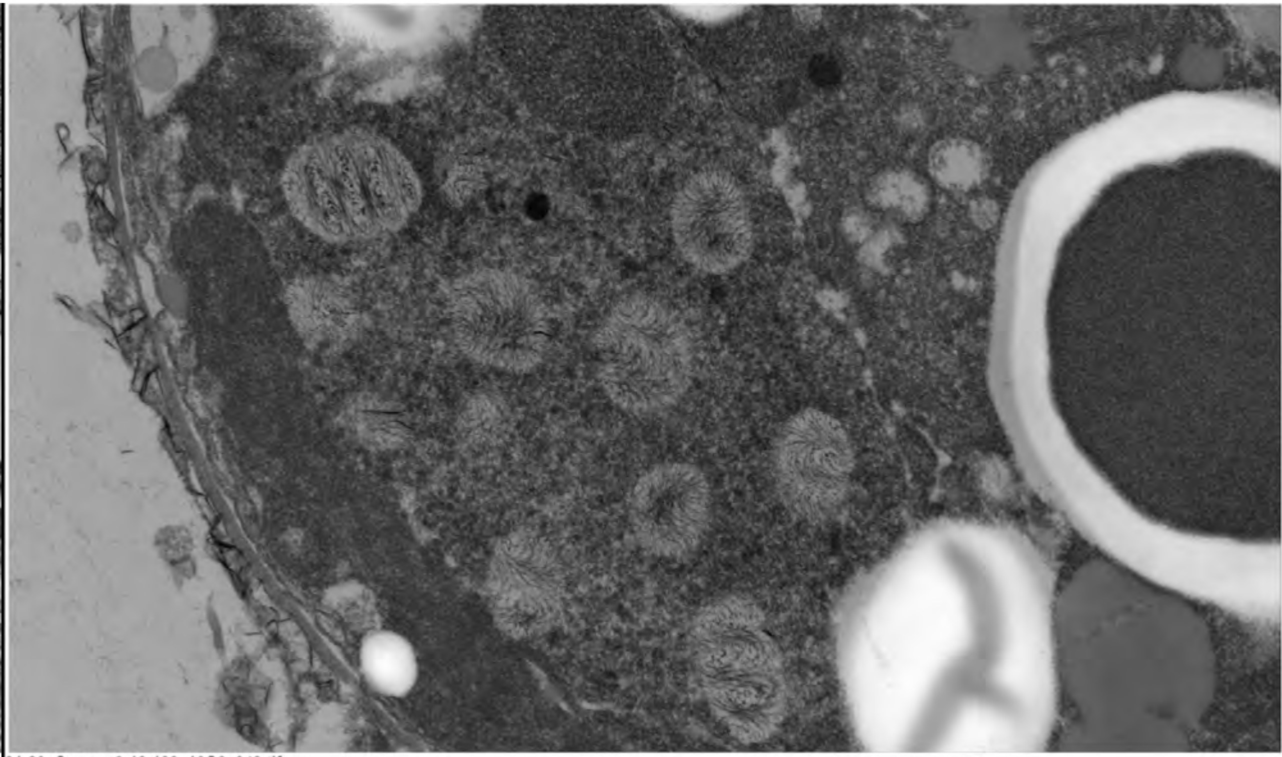

21-20_Correa_3-18-138_16G2_048.tif
Sample: 3/18, 138
Biological Electron Microscopy Lab
Rice University - SEA
Microscopist: MD Meyer

800 nm
HV=80kV
Direct Mag: 5000 x

Cell 17 + 18

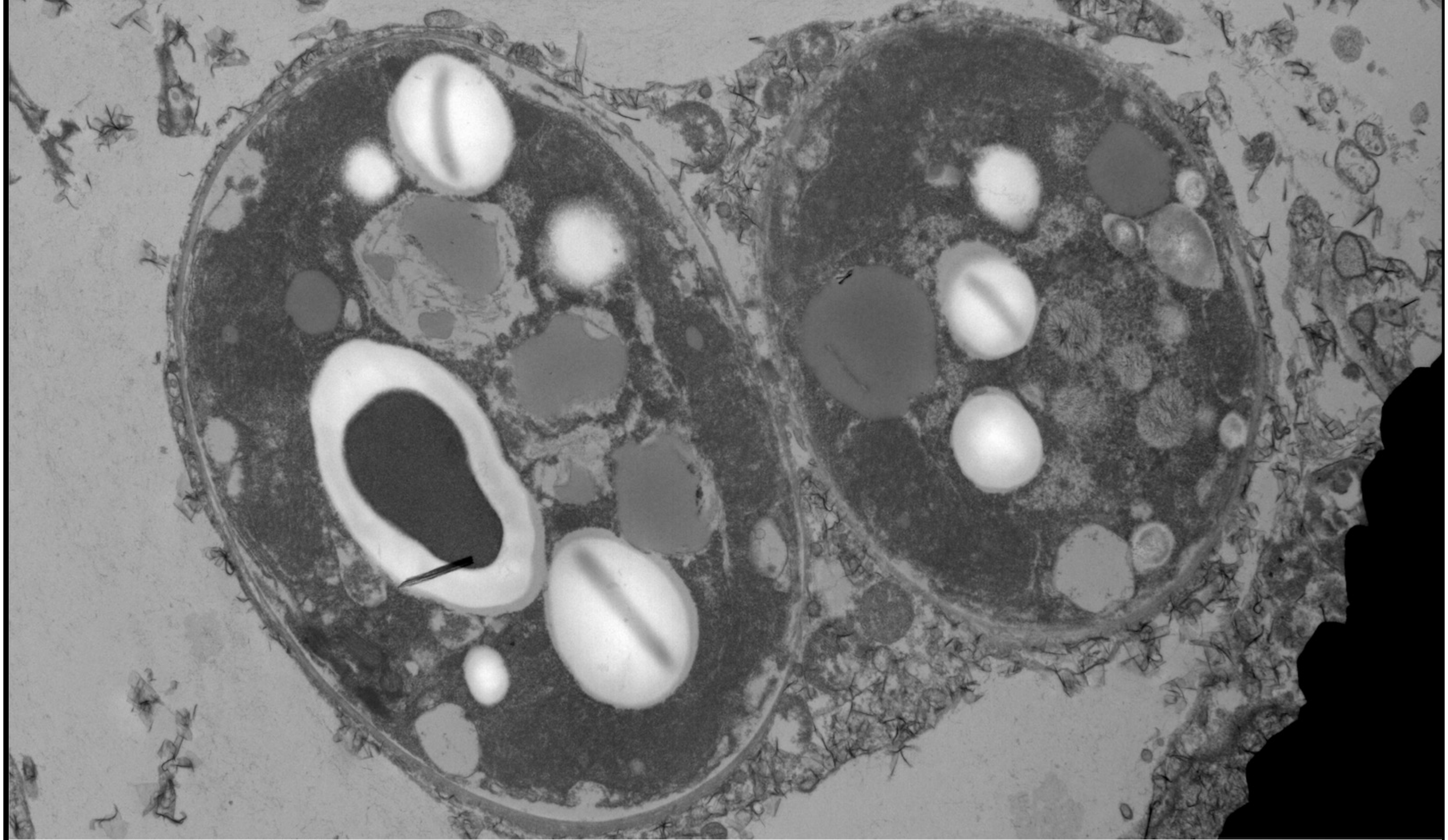

21-20_Correa_3-18-138_16G2_050.tif
Sample: 3/18, 138
Biological Electron Microscopy Lab
Rice University - SEA
Microscopist: MD Meyer

1 µm
HV=80kV
Direct Mag: 2500 x

Heat (March 2019) POR Colony 138

Cell 1

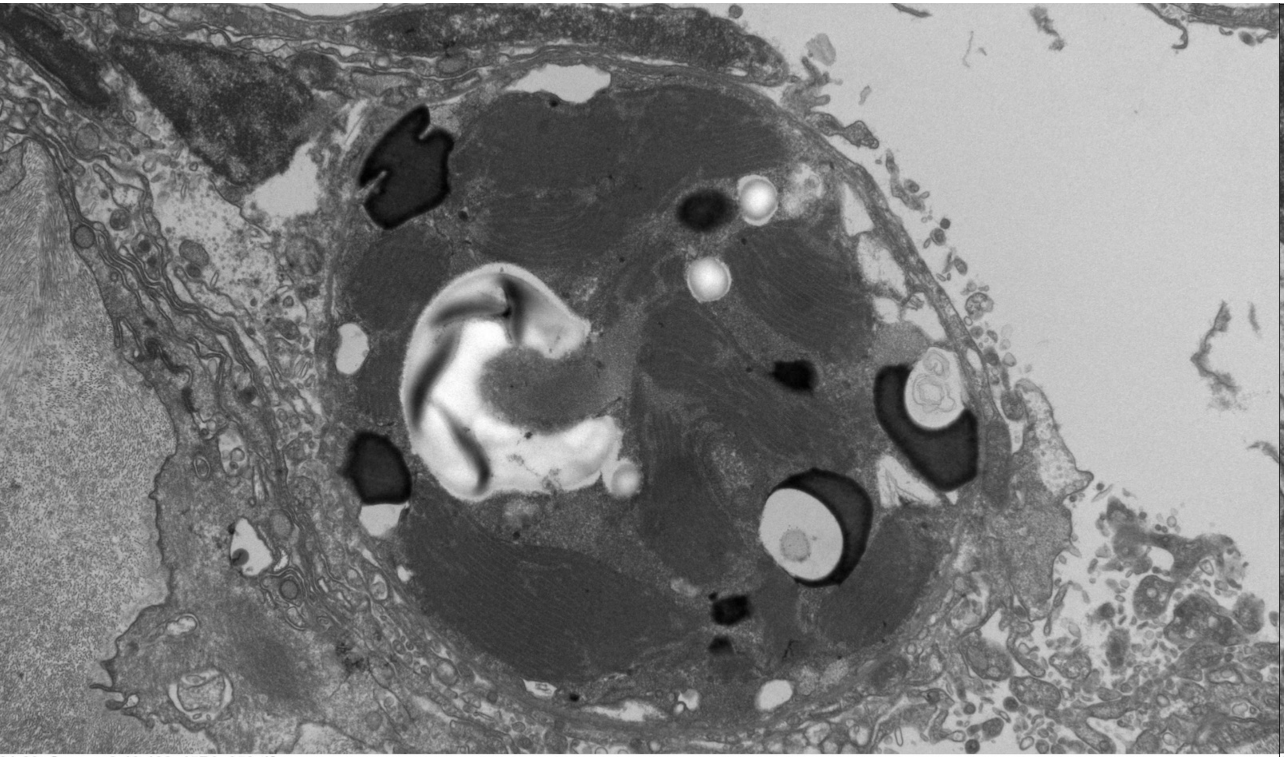

21-20_Correa_3-19-138_15R3_053.tif
Sample: 3/19, 138
Biological Electron Microscopy Lab
Rice University - SEA
Microscopist: MD Meyer

1 µm
HV=80kV
Direct Mag: 2500 x

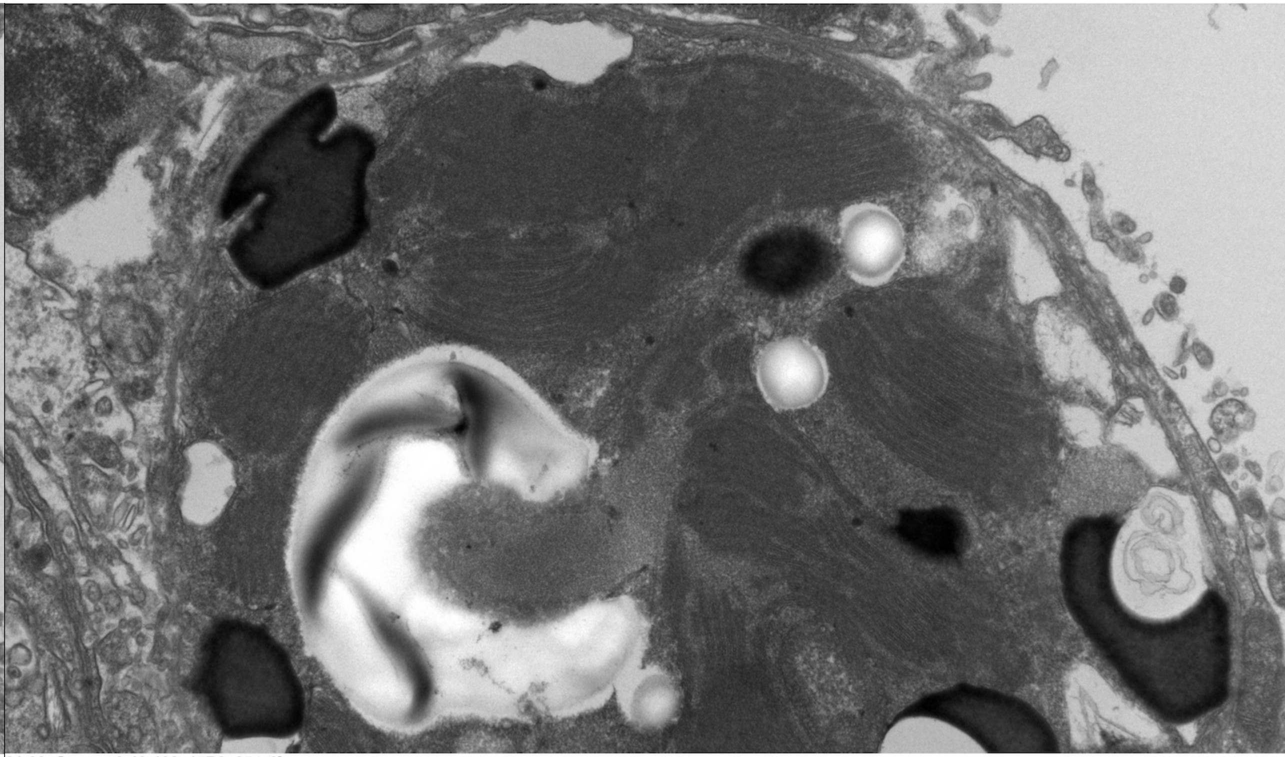

21-20_Correa_3-19-138_15R3_054.tif
Sample: 3/19, 138
Biological Electron Microscopy Lab
Rice University - SEA
Microscopist: MD Meyer

1 µm
HV=80kV
Direct Mag: 4000 x

Cell 2 + 3

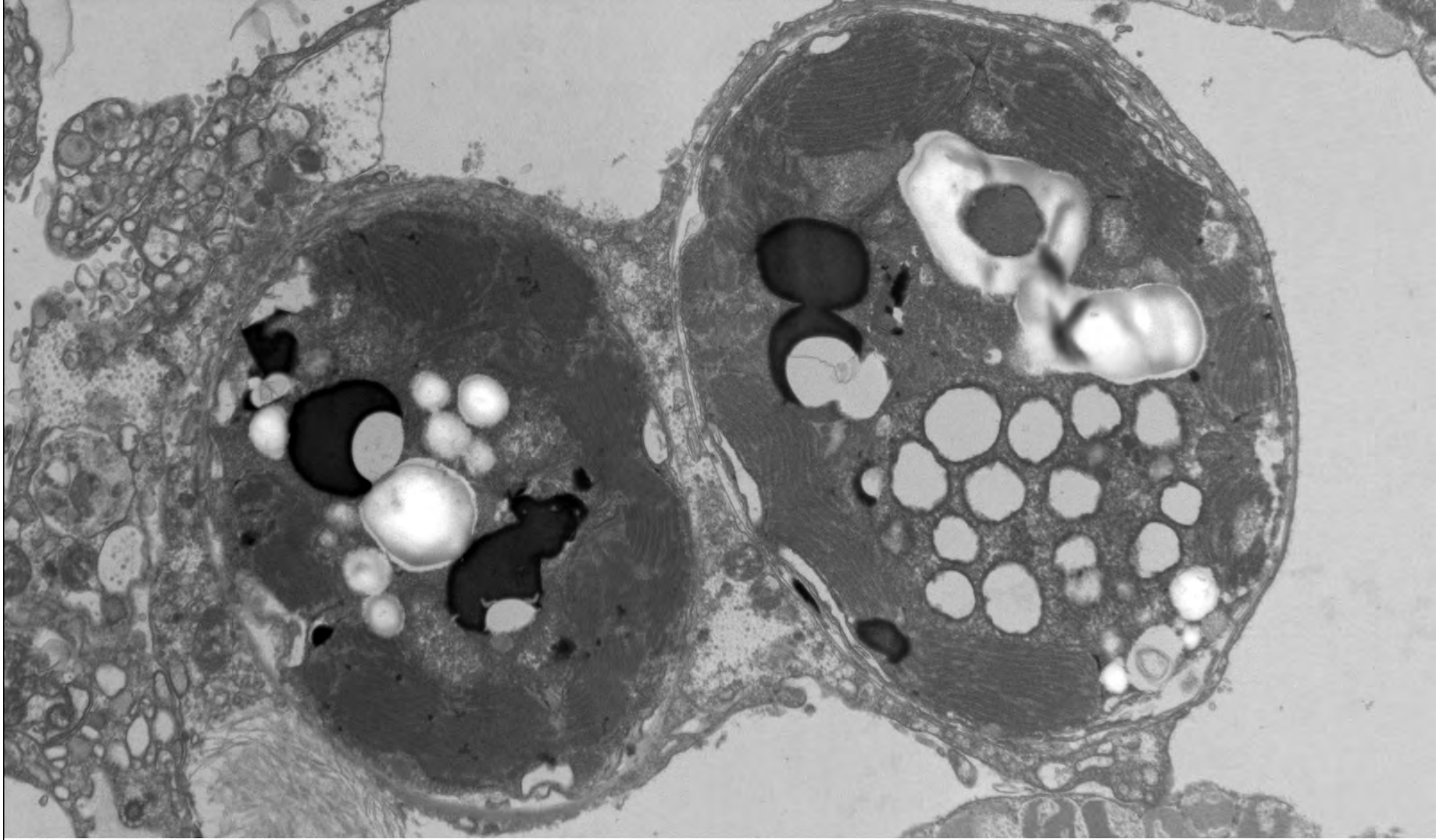

21-20_Correa_3-19-138_15R3_051.tif
Sample: 3/19, 138
Biological Electron Microscopy Lab
Rice University - SEA
Microscopist: MD Meyer

2 µm
HV=80kV
Direct Mag: 2000 x

Cell 4

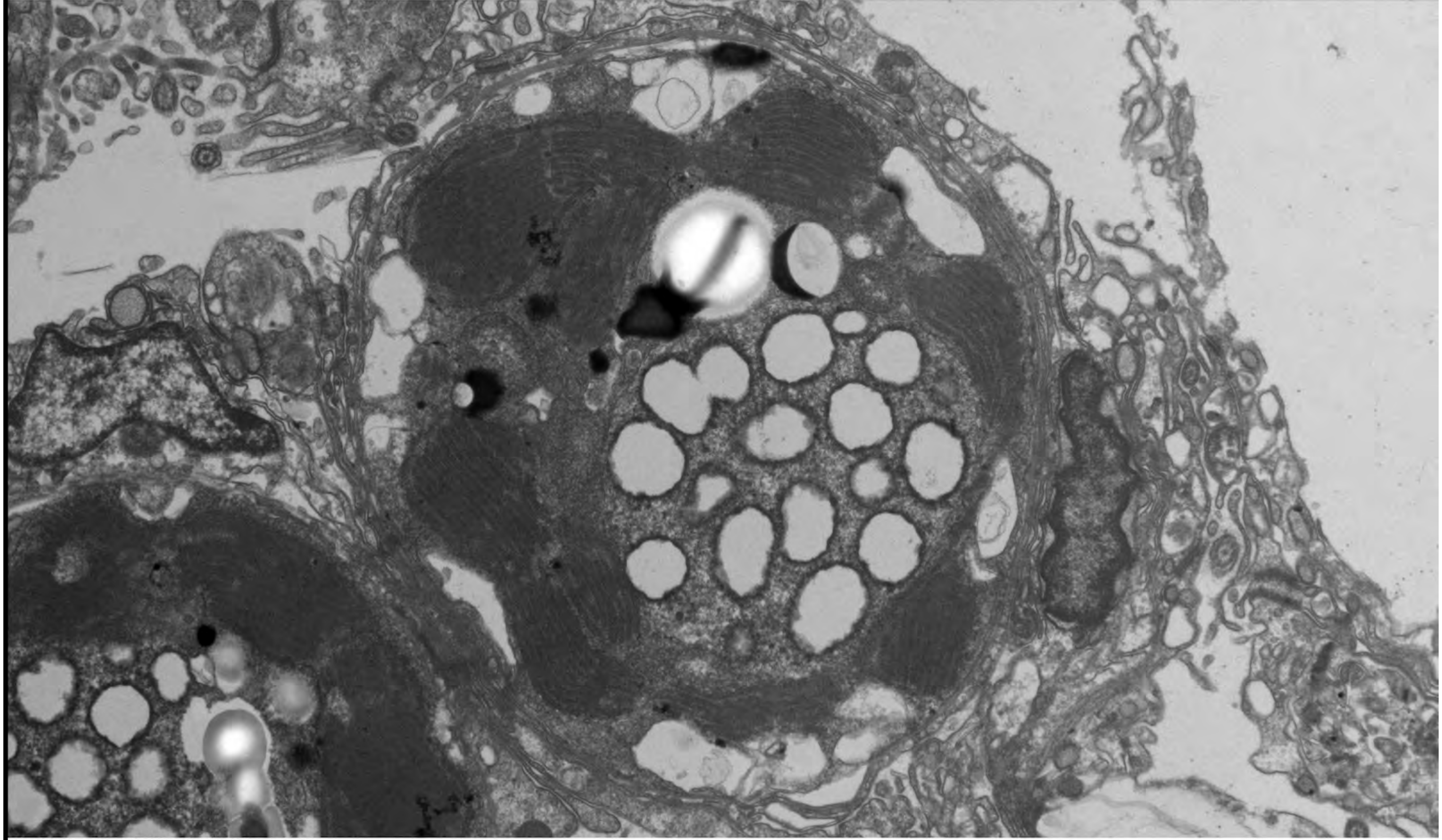

21-20_Correa_3-19-138_15R3_050.tif
Sample: 3/19, 138
Biological Electron Microscopy Lab
Rice University - SEA
Microscopist: MD Meyer

1 µm
HV=80kV
Direct Mag: 2500 x

Cell 5

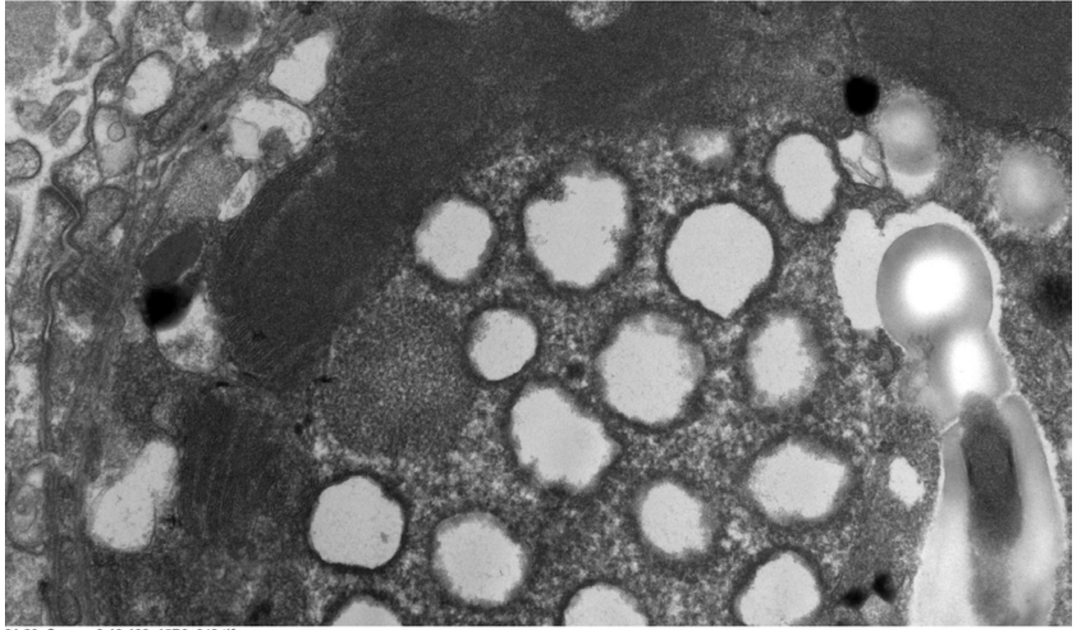

21-20_Correa_3-19-138_15R3_049.tif
Sample: 3/19, 138
Biological Electron Microscopy Lab
Rice University - SEA
Microscopist: MD Meyer

600 nm
HV=80kV
Direct Mag: 6000 x

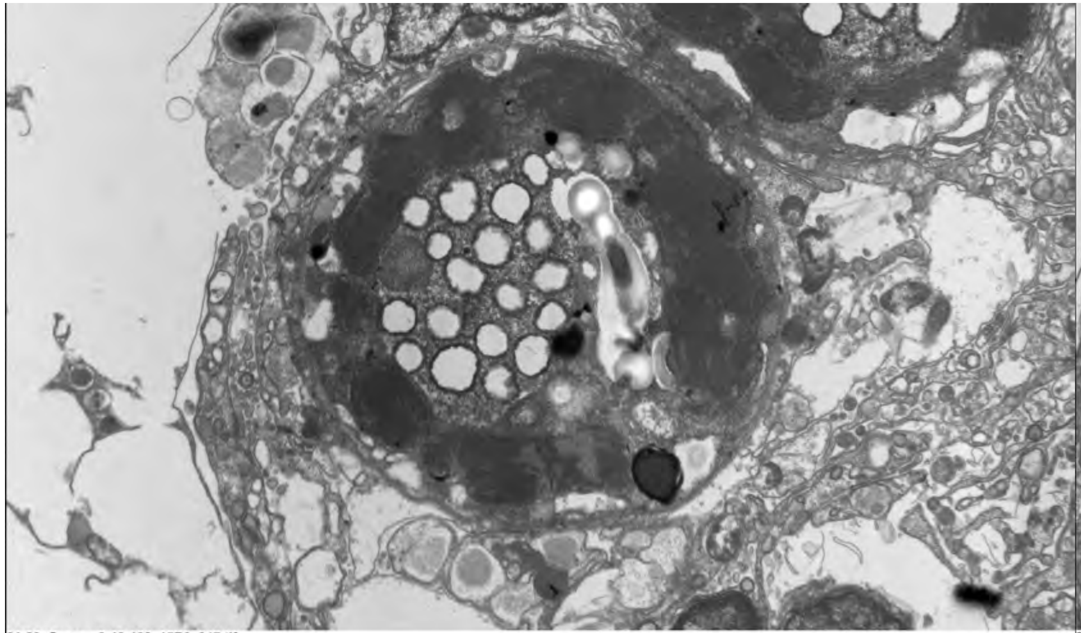

21-20_Correa_3-19-138_15R3_047.tif
Sample: 3/19, 138
Biological Electron Microscopy Lab
Rice University - SEA
Microscopist: MD Meyer

2 µm
HV=80kV
Direct Mag: 2000 x

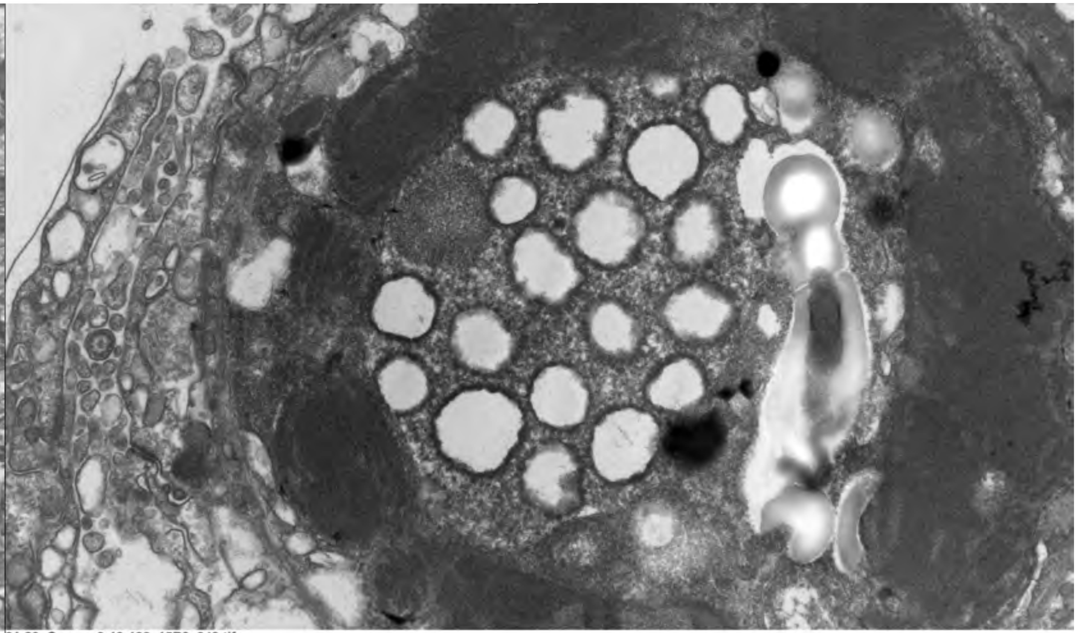

21-20_Correa_3-19-138_15R3_048.tif
Sample: 3/19, 138
Biological Electron Microscopy Lab
Rice University - SEA
Microscopist: MD Meyer

1 µm
HV=80kV
Direct Mag: 4000 x

Cell 6

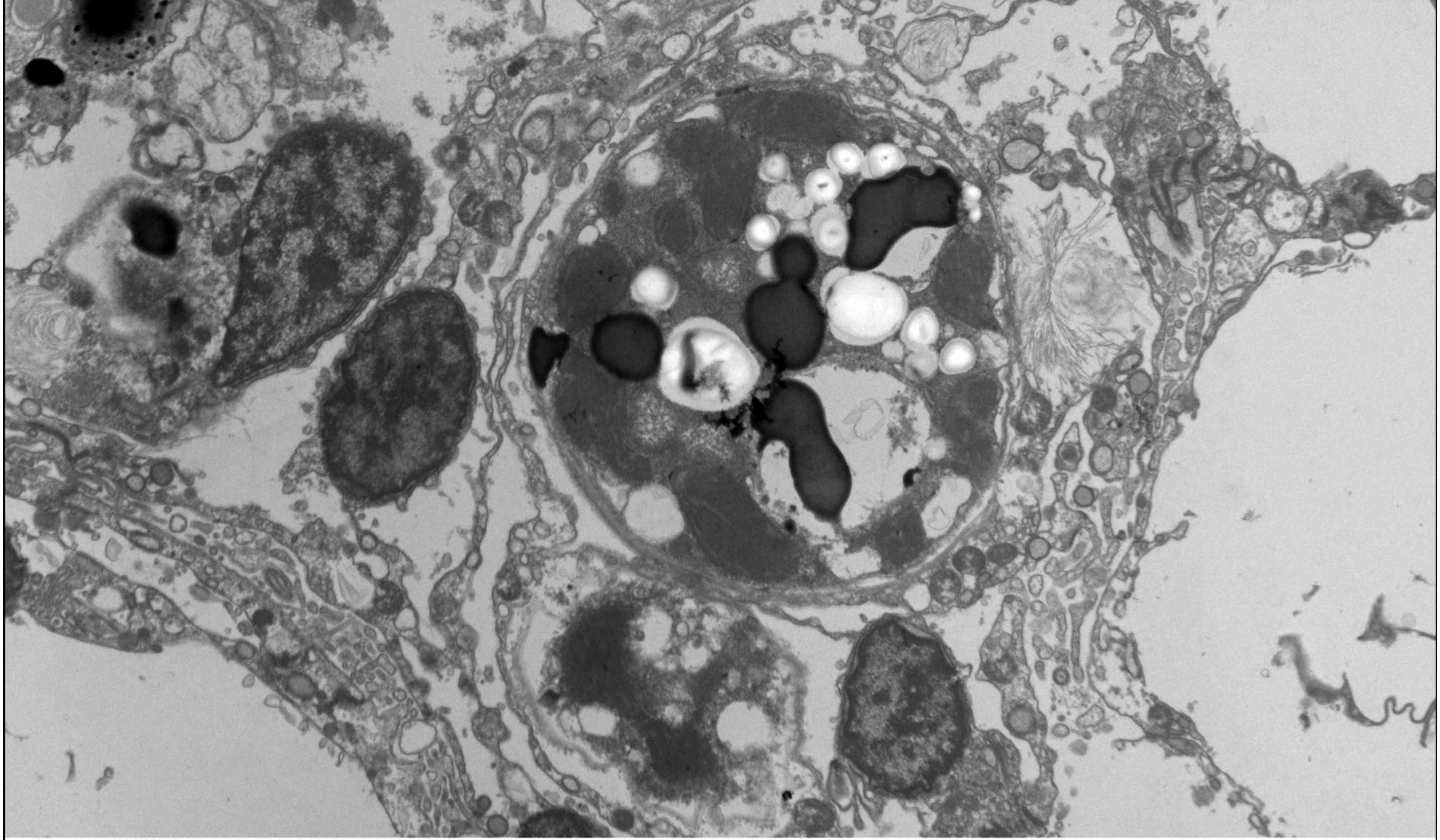

21-20_Correa_3-19-138_15R3_044.tif
Sample: 3/19, 138
Biological Electron Microscopy Lab
Rice University - SEA
Microscopist: MD Meyer

2 µm
HV=80kV
Direct Mag: 1500 x

Cell 7

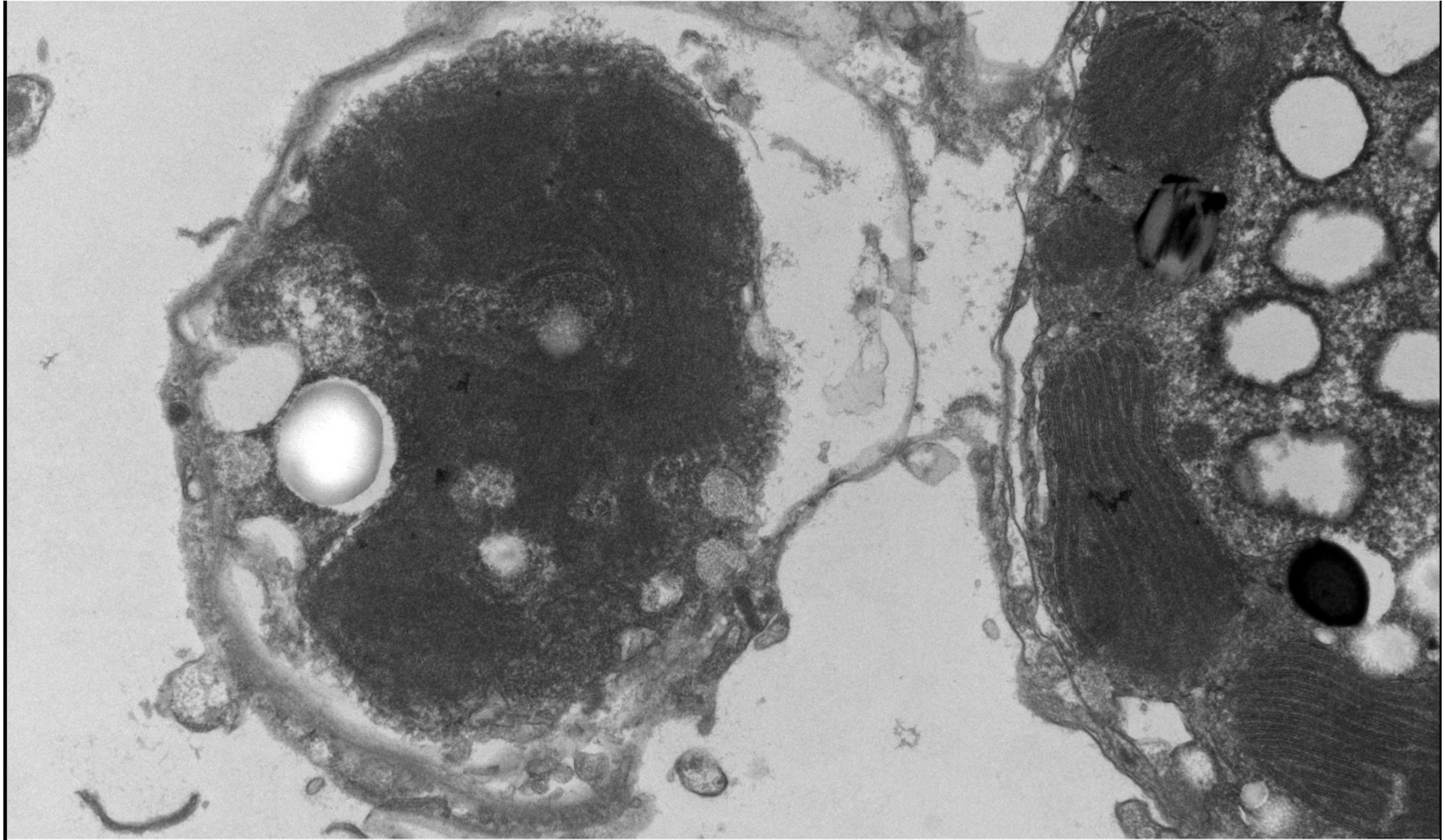

21-20_Correa_3-19-138_15R3_043.tif
Sample: 3/19, 138
Biological Electron Microscopy Lab
Rice University - SEA
Microscopist: MD Meyer

1 µm
HV=80kV
Direct Mag: 4000 x

Cell 8

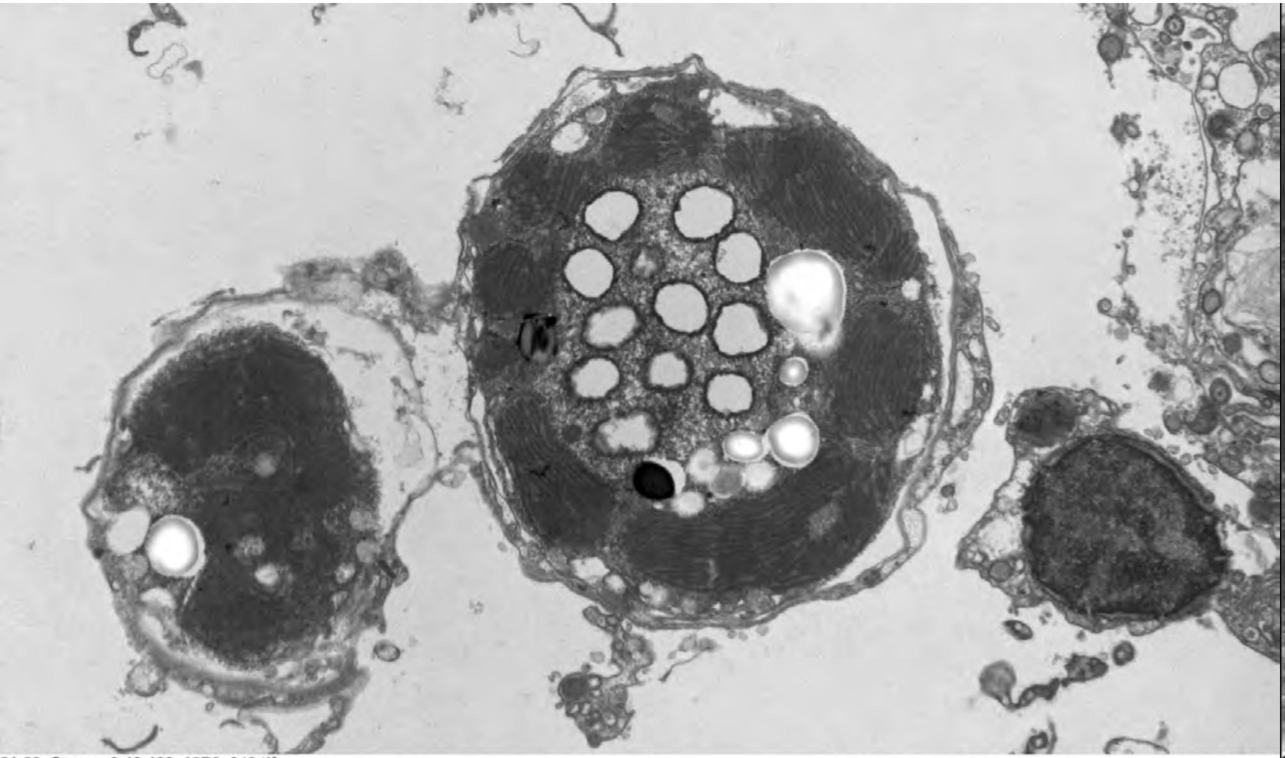

21-20_Correa_3-19-138_15R3_040.tif
Sample: 3/19, 138
Biological Electron Microscopy Lab
Rice University - SEA
Microscopist: MD Meyer

2 µm
HV=80kV
Direct Mag: 2000 x

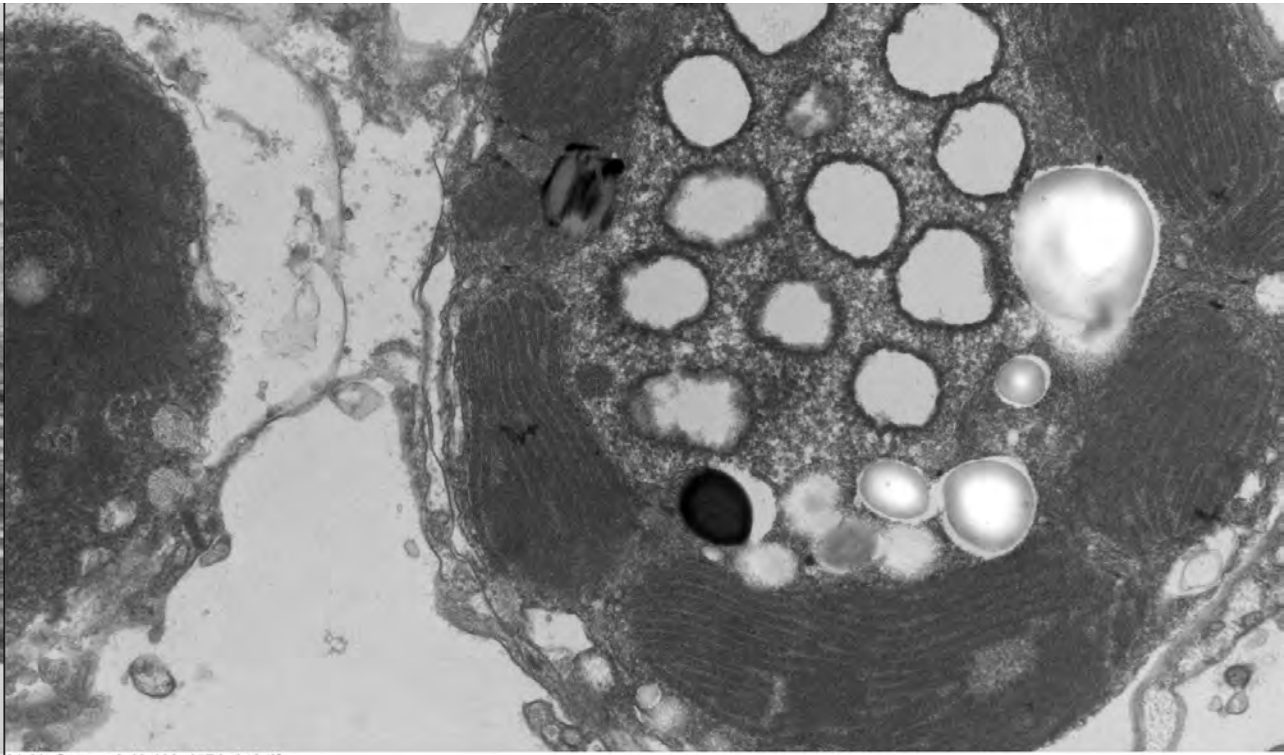

21-20_Correa_3-19-138_15R3_042.tif
Sample: 3/19, 138
Biological Electron Microscopy Lab
Rice University - SEA
Microscopist: MD Meyer

1 µm
HV=80kV
Direct Mag: 4000 x

Cell 9

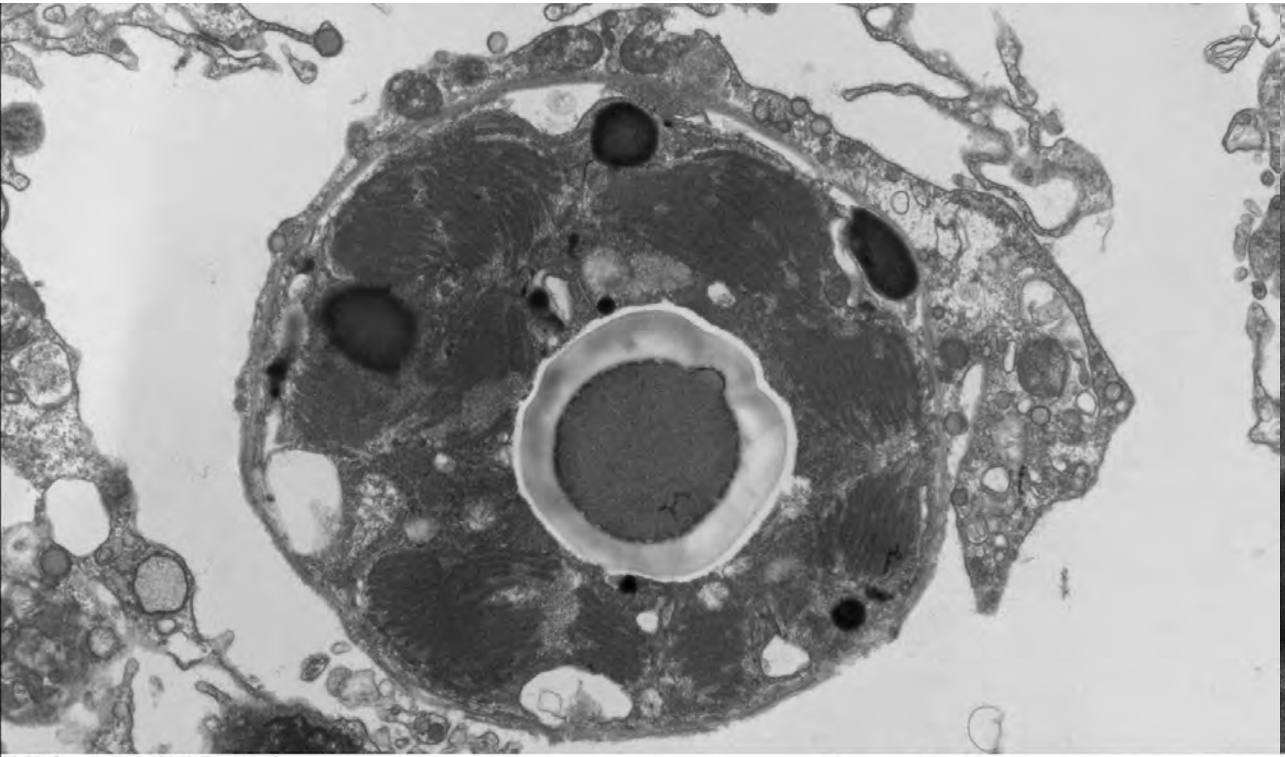

21-20_Correa_3-19-138_15R3_037.tif
Sample: 3/19, 138
Biological Electron Microscopy Lab
Rice University - SEA
Microscopist: MD Meyer

1 µm
HV=80kV
Direct Mag: 3000 x

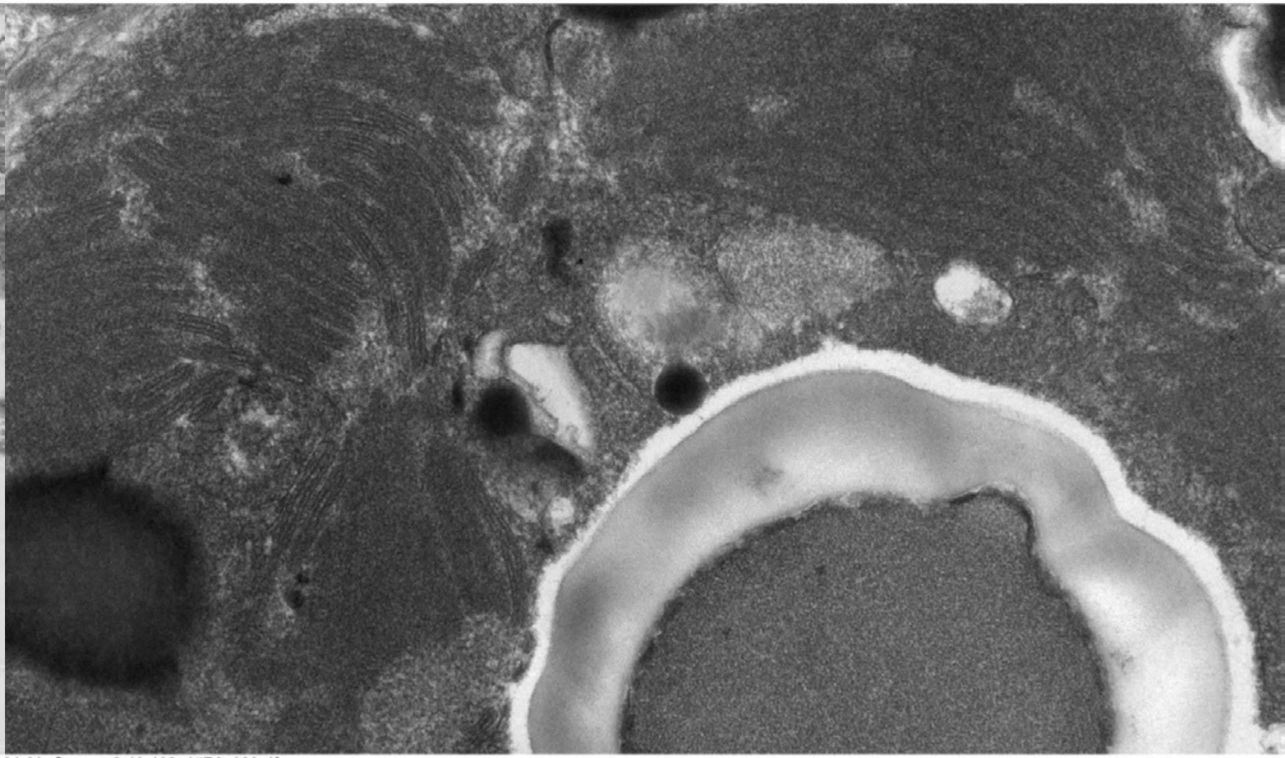

21-20_Correa_3-19-138_15R3_038.tif
Sample: 3/19, 138
Biological Electron Microscopy Lab
Rice University - SEA
Microscopist: MD Meyer

500 nm
HV=80kV
Direct Mag: 8000 x

Cell 10

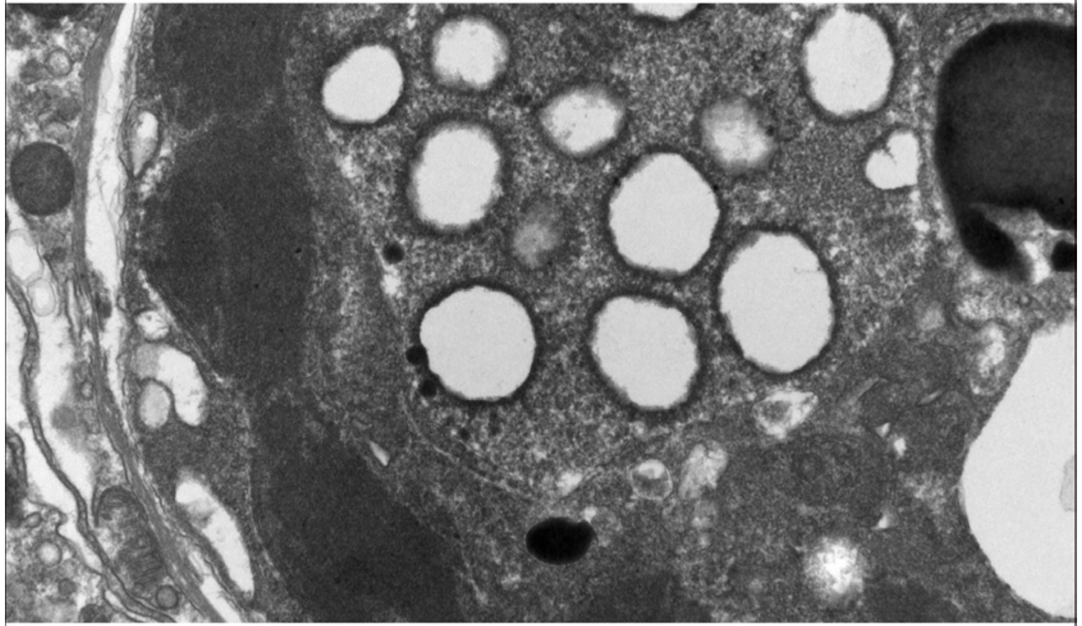

21-20_Correa_3-19-138_15R3_034.tif
Sample: 3/19, 138
Biological Electron Microscopy Lab
Rice University - SEA
Microscopist: MD Meyer

800 nm
HV=80kV
Direct Mag: 5000 x

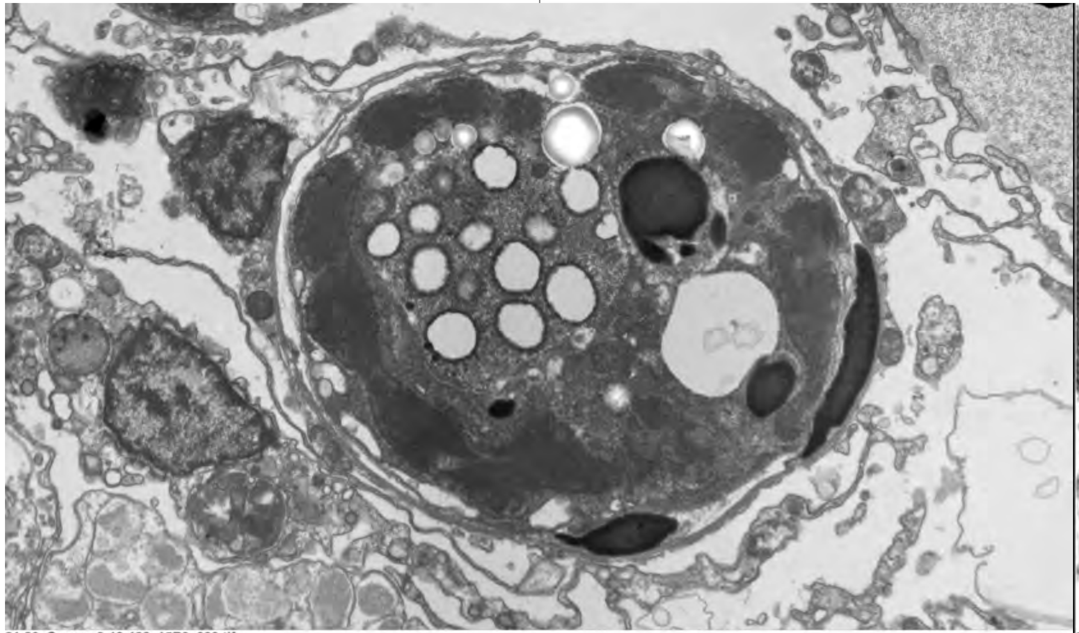

21-20_Correa_3-19-138_15R3_033.tif
Sample: 3/19, 138
Biological Electron Microscopy Lab
Rice University - SEA
Microscopist: MD Meyer

2 µm
HV=80kV
Direct Mag: 2000 x

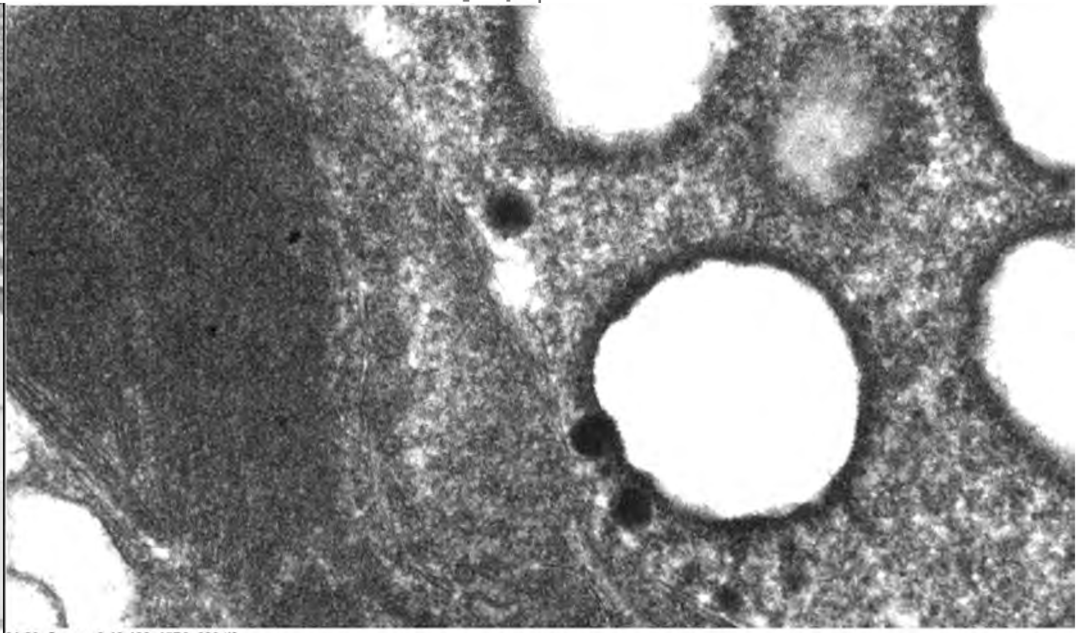

21-20_Correa_3-19-138_15R3_036.tif
Sample: 3/19, 138
Biological Electron Microscopy Lab
Rice University - SEA
Microscopist: MD Meyer

200 nm
HV=80kV
Direct Mag: 12000 x

Cell 11

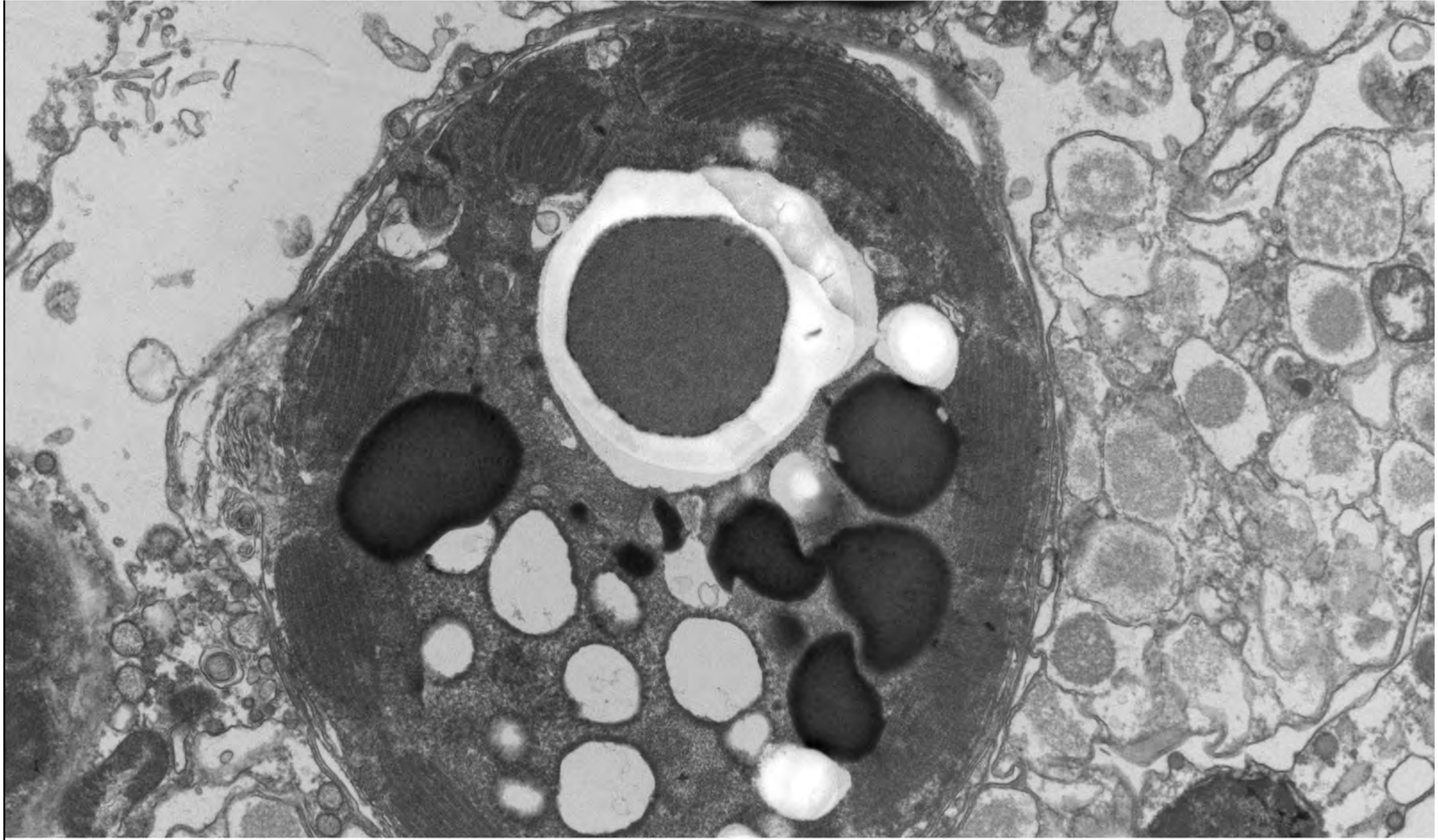

21-20_Correa_3-19-138_15R3_030.tif
Sample: 3/19, 138
Biological Electron Microscopy Lab
Rice University - SEA
Microscopist: MD Meyer

1 µm
HV=80kV
Direct Mag: 3000 x

Cell 12

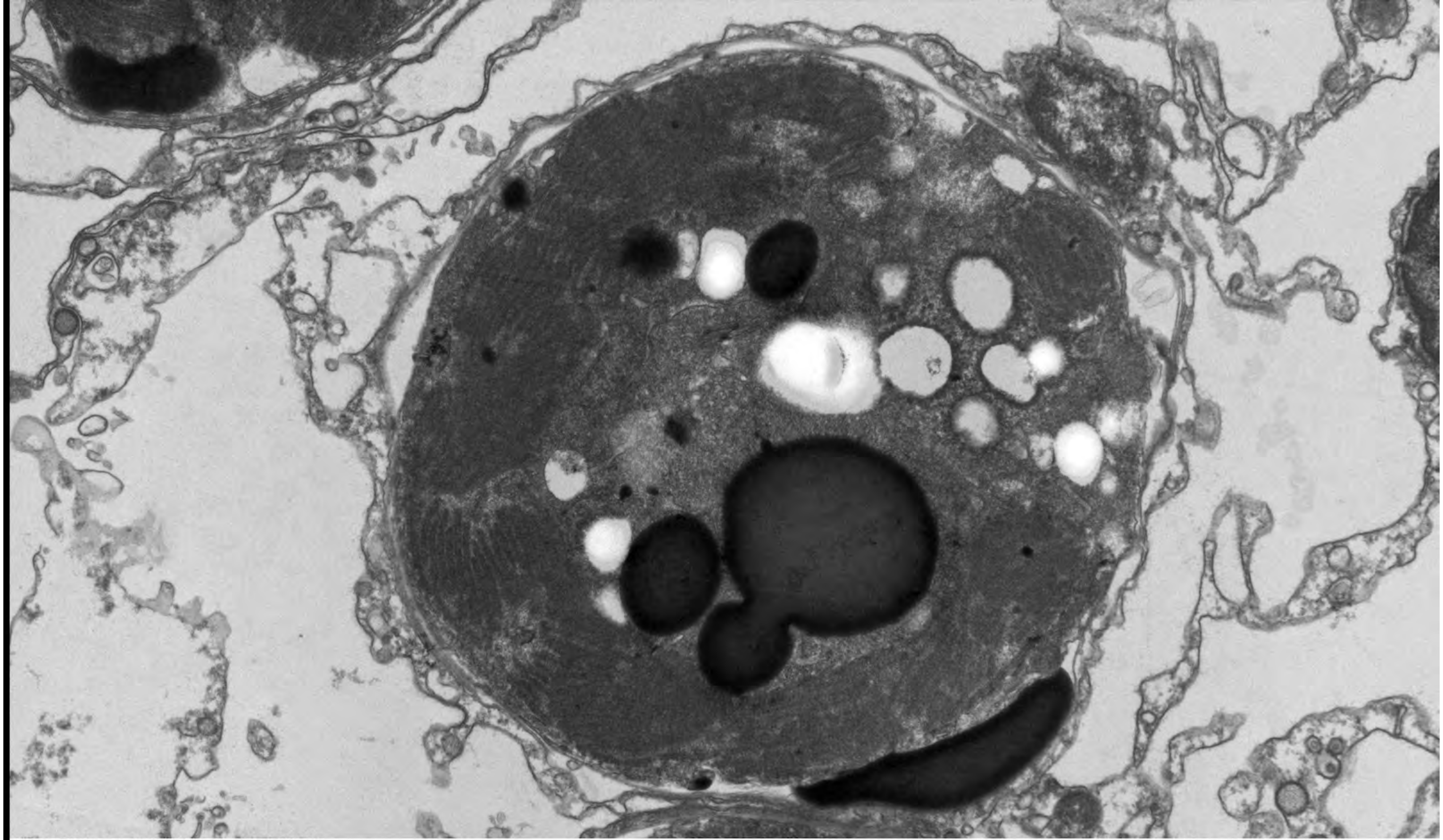

21-20_Correa_3-19-138_15R3_029.tif
Sample: 3/19, 138
Biological Electron Microscopy Lab
Rice University - SEA
Microscopist: MD Meyer

1 µm
HV=80kV
Direct Mag: 3000 x

Cell 13

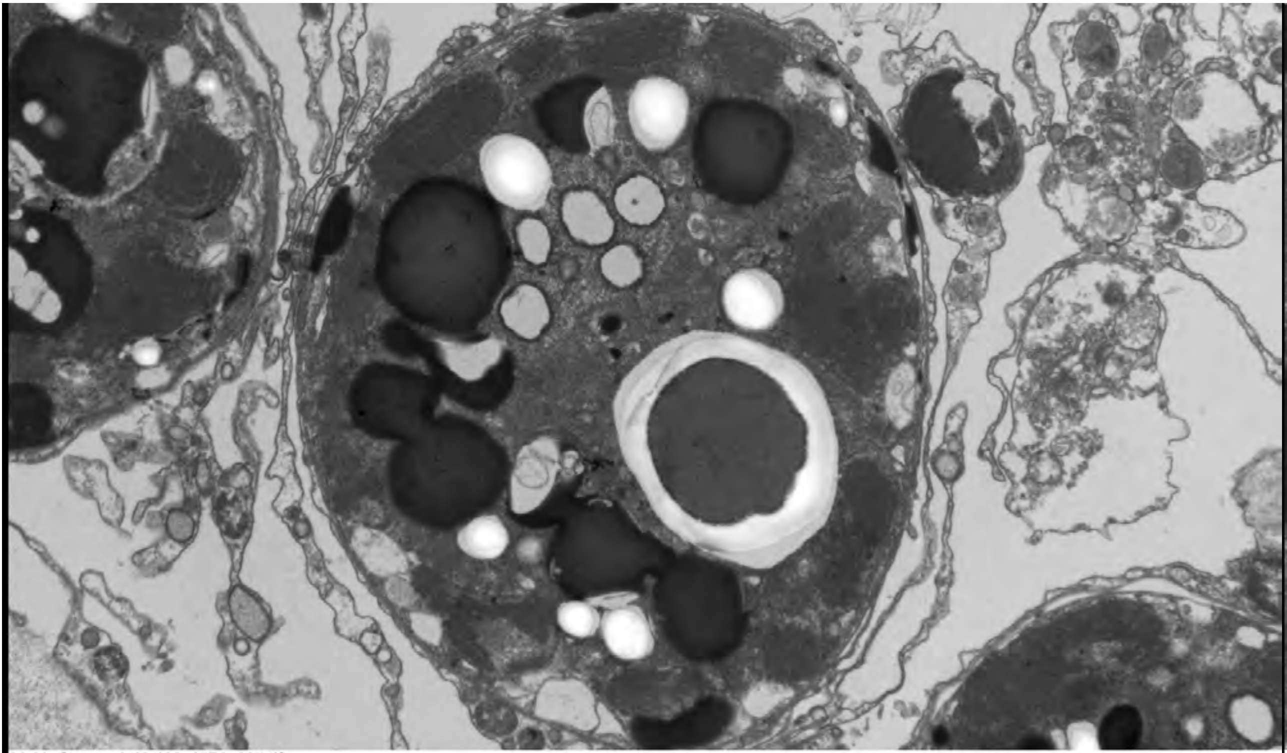

21-20_Correa_3-19-138_15R3_027.tif
Sample: 3/19, 138
Biological Electron Microscopy Lab
Rice University - SEA
Microscopist: MD Meyer

2 µm
HV=80kV
Direct Mag: 2000 x

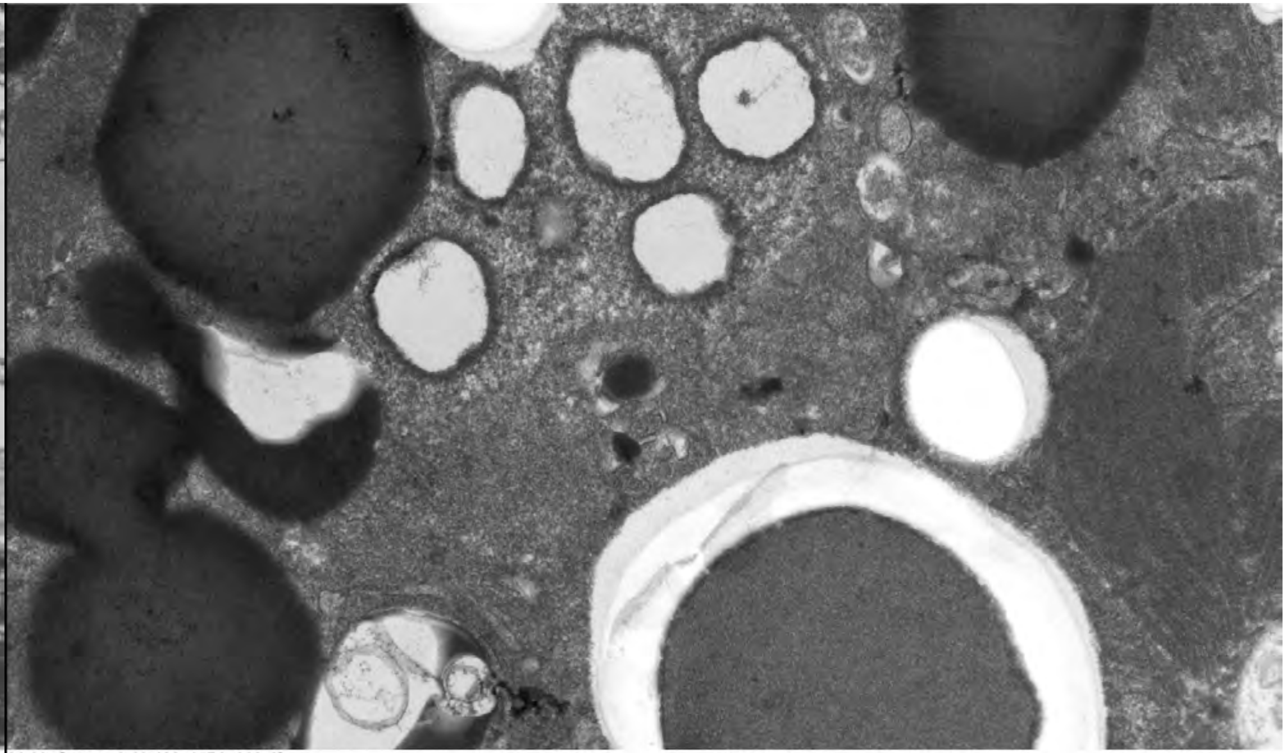

21-20_Correa_3-19-138_15R3_028.tif
Sample: 3/19, 138
Biological Electron Microscopy Lab
Rice University - SEA
Microscopist: MD Meyer

800 nm
HV=80kV
Direct Mag: 5000 x

Cell 14

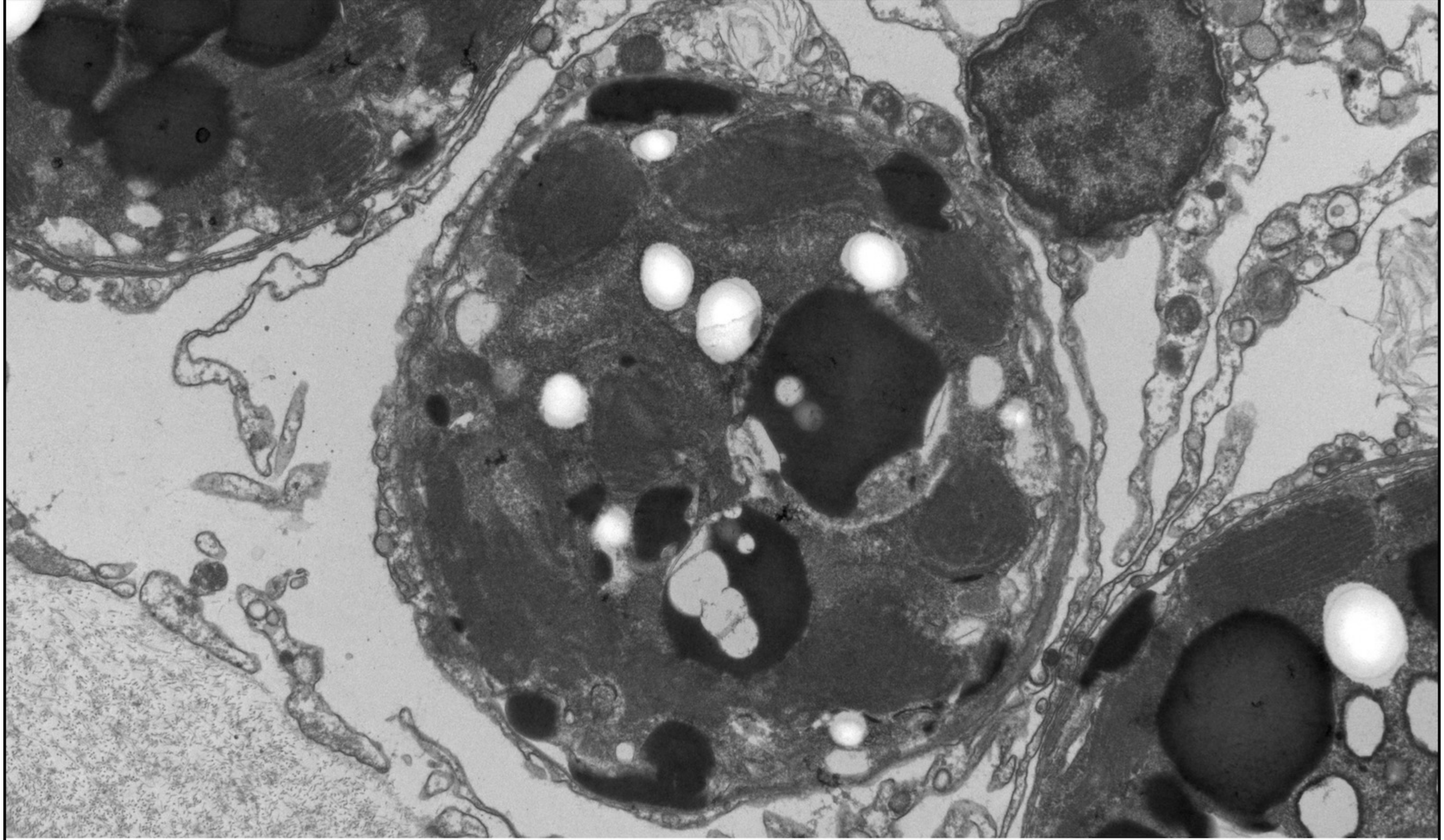

21-20_Correa_3-19-138_15R3_026.tif
Sample: 3/19, 138
Biological Electron Microscopy Lab
Rice University - SEA
Microscopist: MD Meyer

1 µm
HV=80kV
Direct Mag: 2500 x

Cell 15

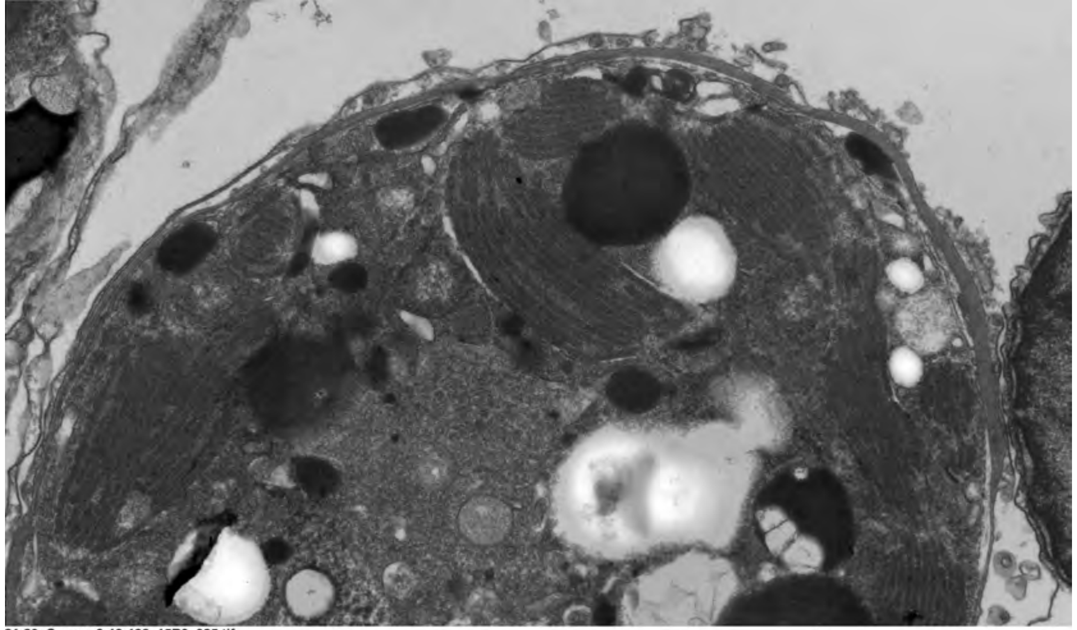

21-20_Correa_3-19-138_15R3_025.tif
Sample: 3/19, 138
Biological Electron Microscopy Lab
Rice University - SEA
Microscopist: MD Meyer

1 µm
HV=80kV
Direct Mag: 4000 x

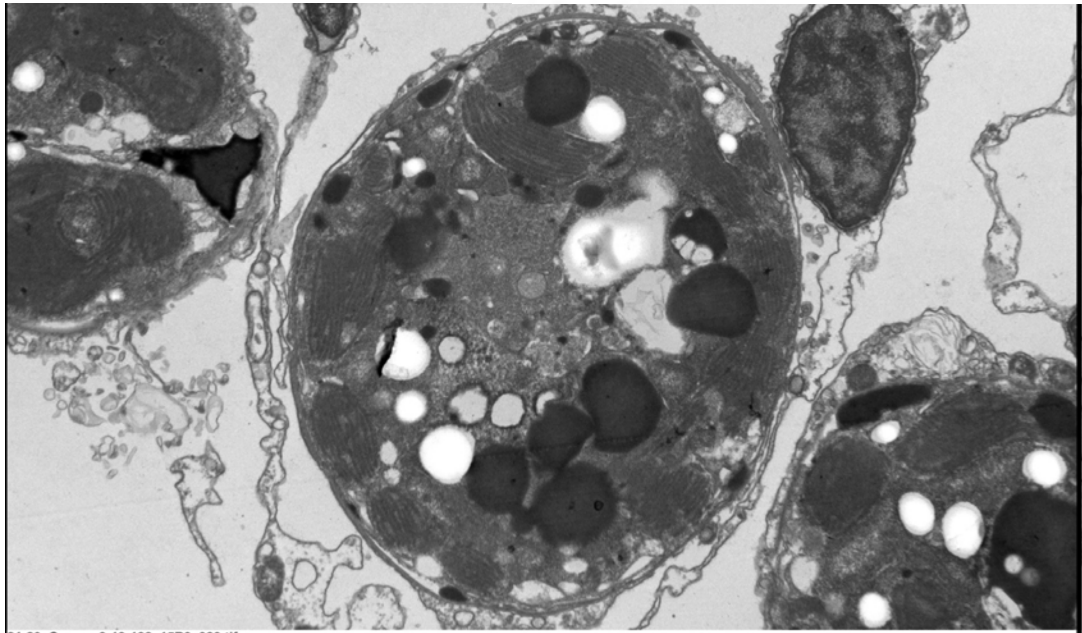

21-20_Correa_3-19-138_15R3_023.tif
Sample: 3/19, 138
Biological Electron Microscopy Lab
Rice University - SEA
Microscopist: MD Meyer

2 µm
HV=80kV
Direct Mag: 2000 x

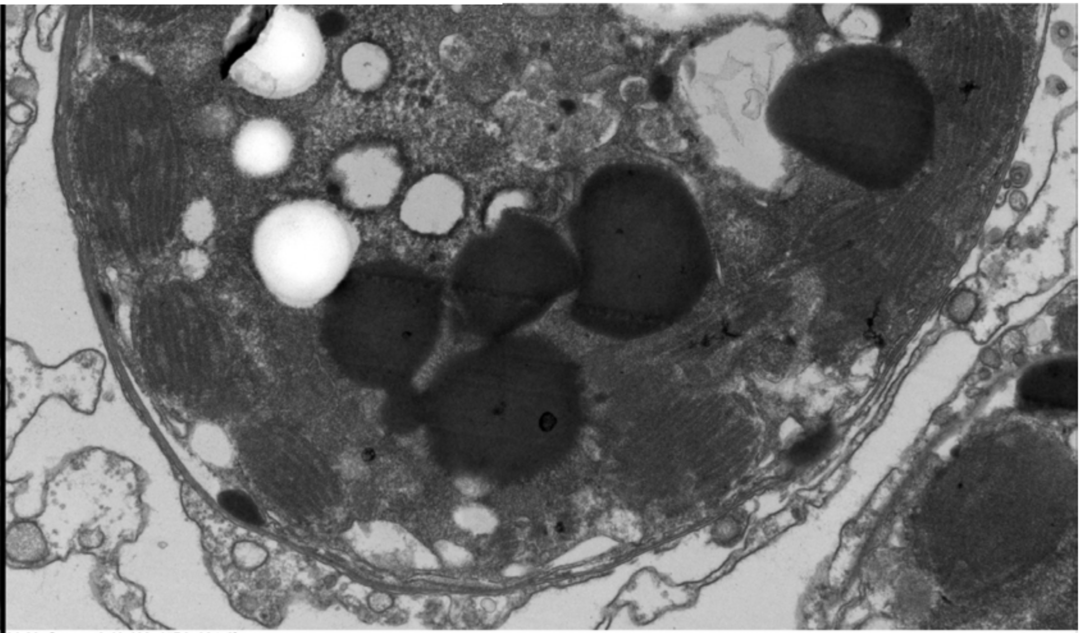

21-20_Correa_3-19-138_15R3_024.tif
Sample: 3/19, 138
Biological Electron Microscopy Lab
Rice University - SEA
Microscopist: MD Meyer

1 µm
HV=80kV
Direct Mag: 4000 x

Cell 16

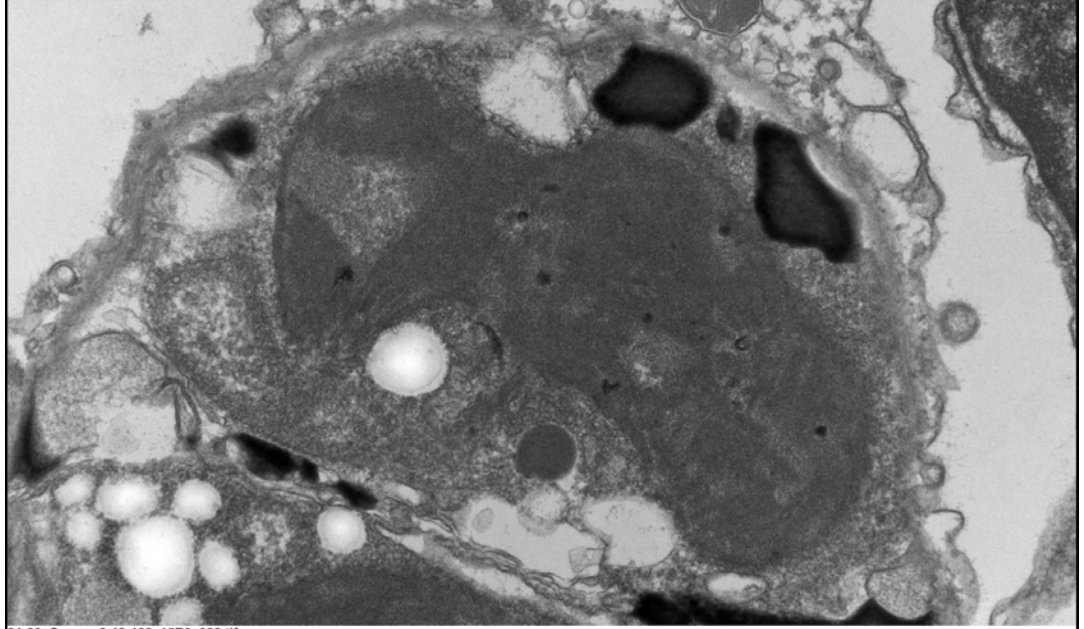
21-20_Correa_3-19-138_15R3_022.tif
Sample: 3/19, 138
Biological Electron Microscopy Lab
Rice University - SEA
Microscopist: MD Meyer
800 nm
HV=80kV
Direct Mag: 5000 x

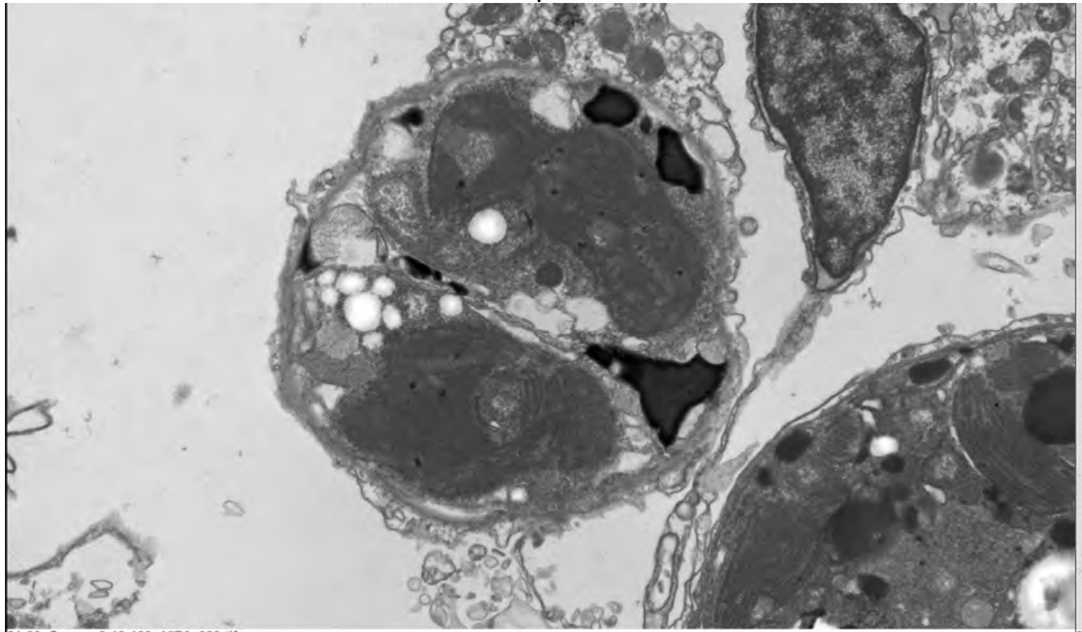
21-20_Correa_3-19-138_15R3_020.tif
Sample: 3/19, 138
Biological Electron Microscopy Lab
Rice University - SEA
Microscopist: MD Meyer
1 µm
HV=80kV
Direct Mag: 2500 x

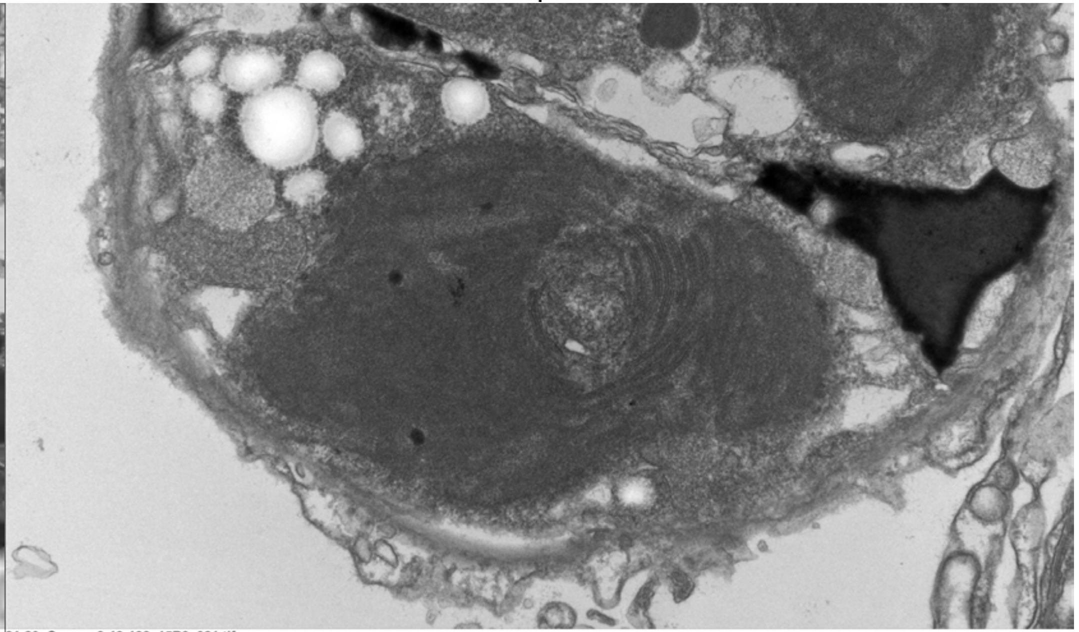
21-20_Correa_3-19-138_15R3_021.tif
Sample: 3/19, 138
Biological Electron Microscopy Lab
Rice University - SEA
Microscopist: MD Meyer
800 nm
HV=80kV
Direct Mag: 5000 x

Cell 17

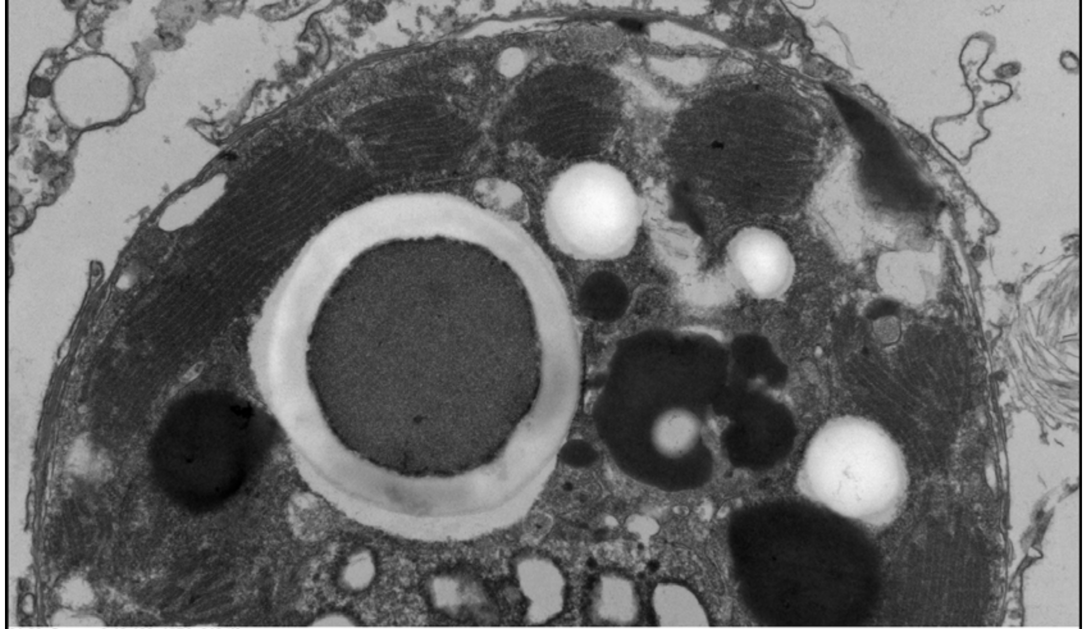

21-20_Correa_3-19-138_15R3_018.tif
Sample: 3/19, 138
Biological Electron Microscopy Lab
Rice University - SEA
Microscopist: MD Meyer

1 µm
HV=80kV
Direct Mag: 4000 x

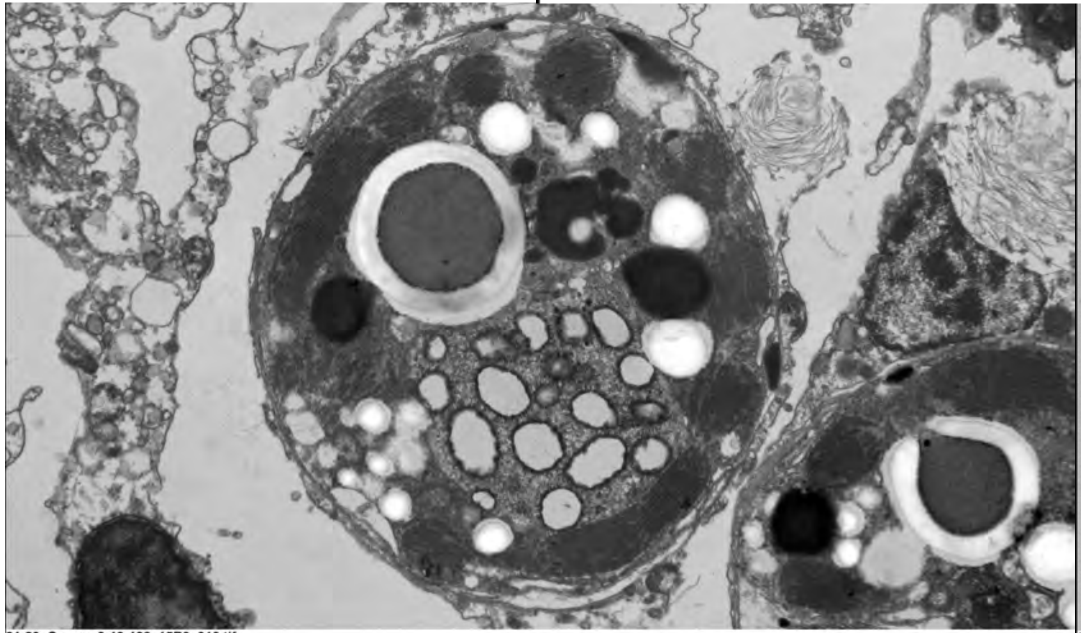

21-20_Correa_3-19-138_15R3_016.tif
Sample: 3/19, 138
Biological Electron Microscopy Lab
Rice University - SEA
Microscopist: MD Meyer

2 µm
HV=80kV
Direct Mag: 2000 x

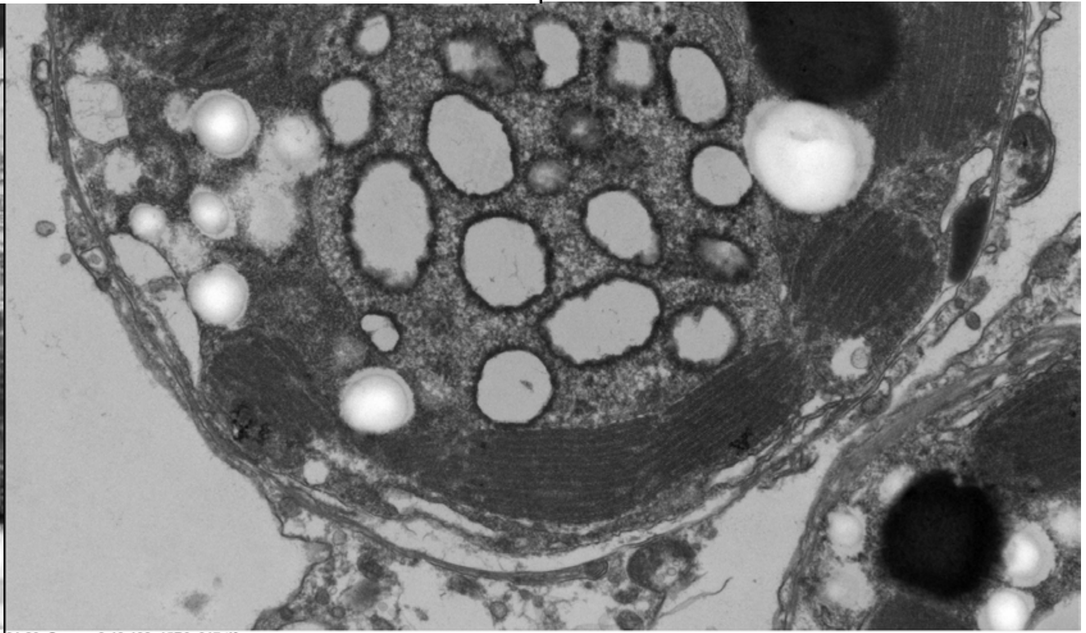

21-20_Correa_3-19-138_15R3_017.tif
Sample: 3/19, 138
Biological Electron Microscopy Lab
Rice University - SEA
Microscopist: MD Meyer

1 µm
HV=80kV
Direct Mag: 4000 x

Cell 18

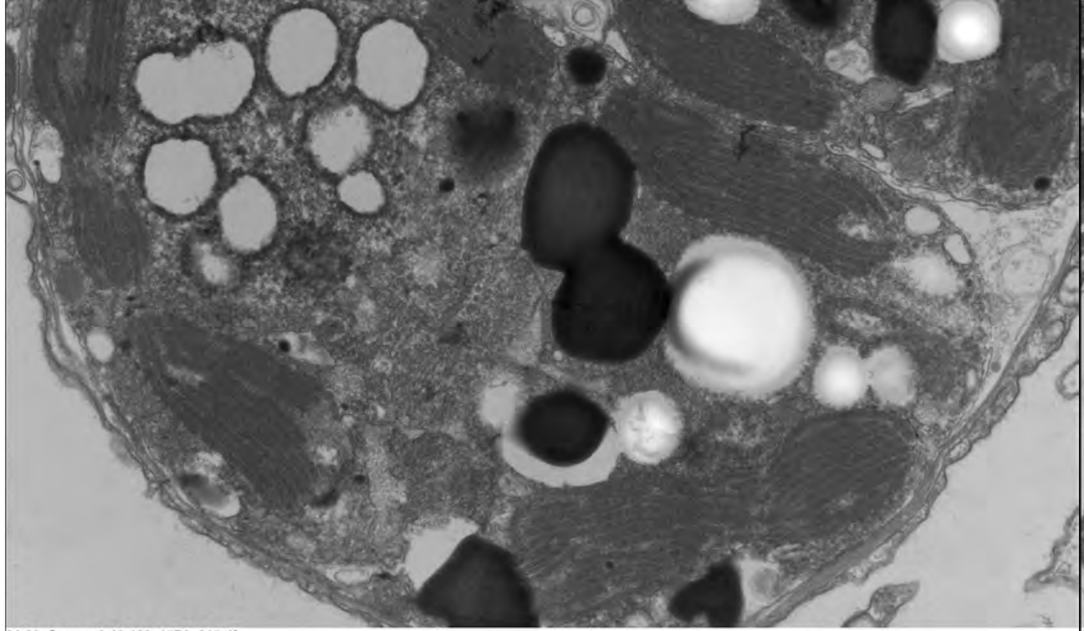

21-20_Correa_3-19-138_15R3_015.tif
Sample: 3/19, 138
Biological Electron Microscopy Lab
Rice University - SEA
Microscopist: MD Meyer

1 µm
HV=80kV
Direct Mag: 4000 x

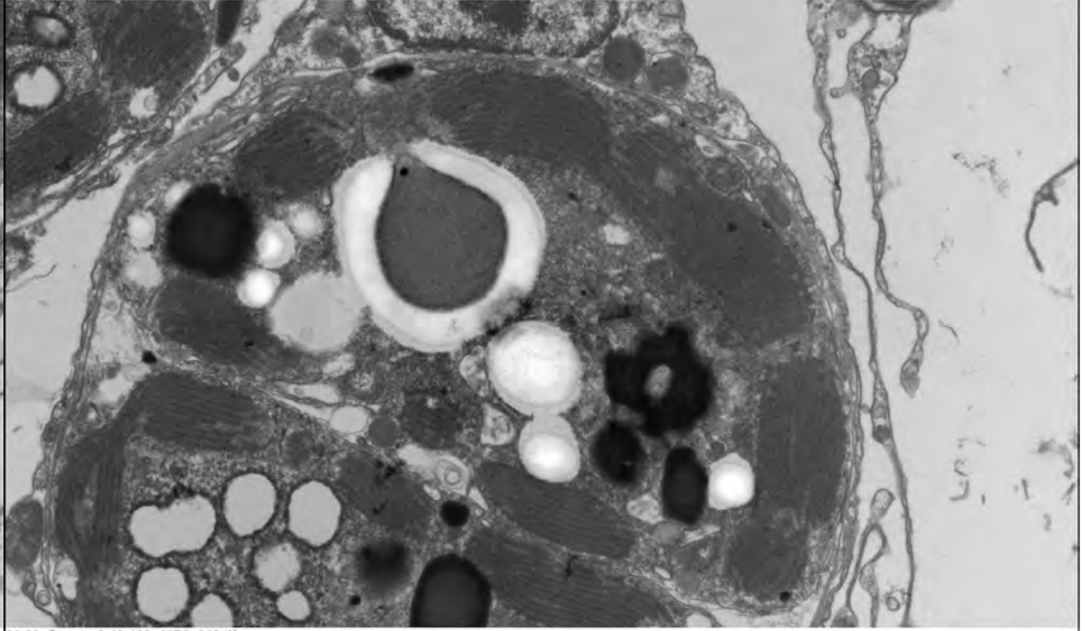

21-20_Correa_3-19-138_15R3_012.tif
Sample: 3/19, 138
Biological Electron Microscopy Lab
Rice University - SEA
Microscopist: MD Meyer

1 µm
HV=80kV
Direct Mag: 3000 x

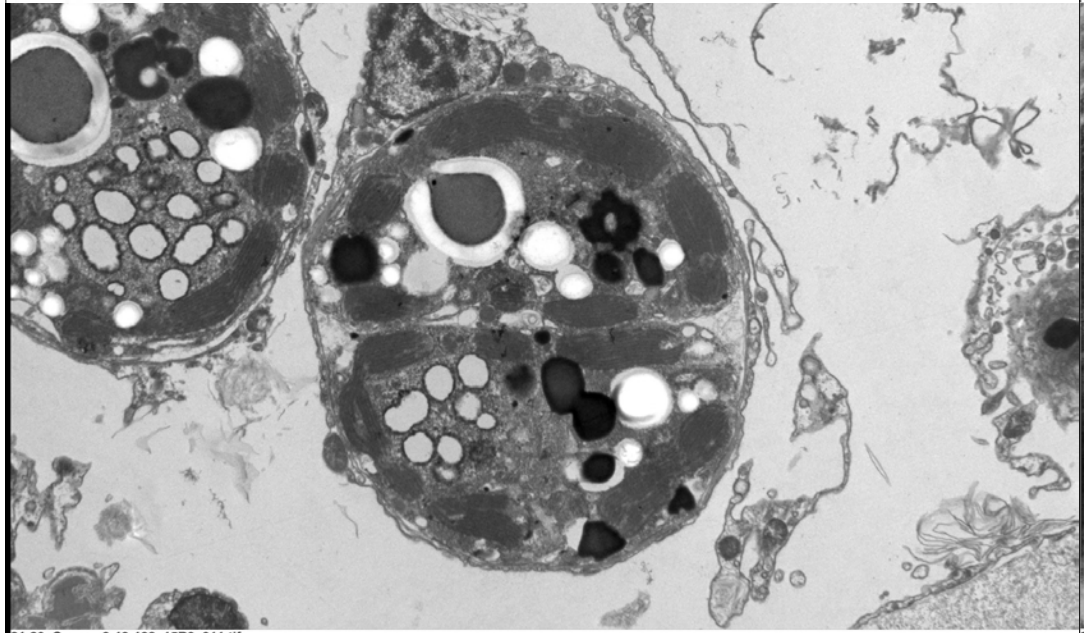

21-20_Correa_3-19-138_15R3_011.tif
Sample: 3/19, 138
Biological Electron Microscopy Lab
Rice University - SEA
Microscopist: MD Meyer

2 µm
HV=80kV
Direct Mag: 1500 x

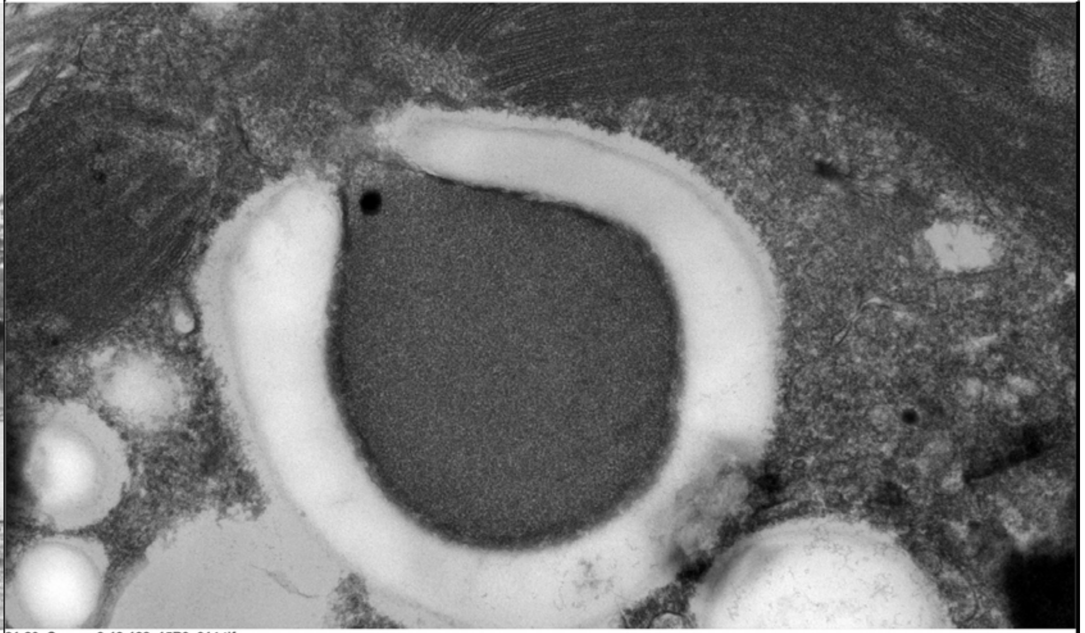

21-20_Correa_3-19-138_15R3_014.tif
Sample: 3/19, 138
Biological Electron Microscopy Lab
Rice University - SEA
Microscopist: MD Meyer

500 nm
HV=80kV
Direct Mag: 8000 x

Cell 19

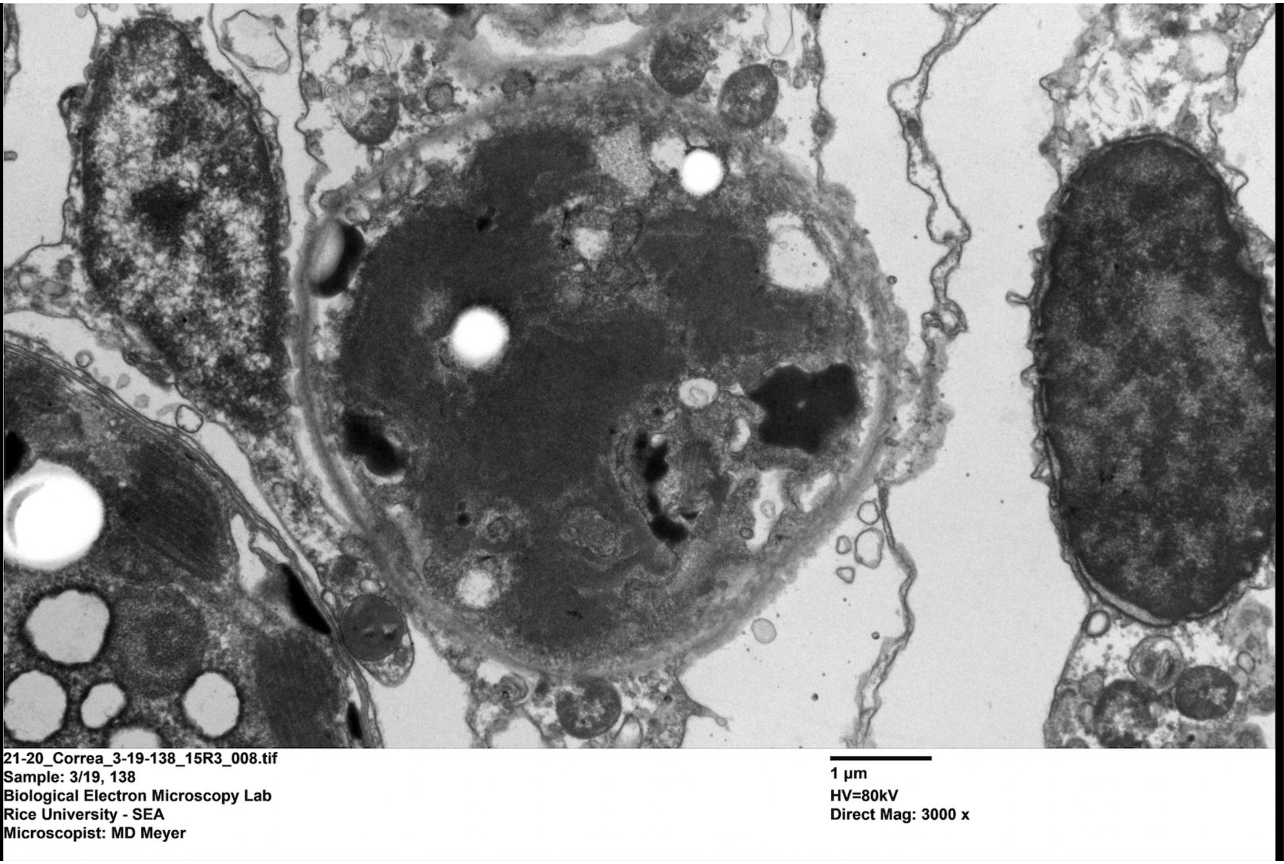
21-20_Correa_3-19-138_15R3_008.tif
Sample: 3/19, 138
Biological Electron Microscopy Lab
Rice University - SEA
Microscopist: MD Meyer
1 µm
HV=80kV
Direct Mag: 3000 x

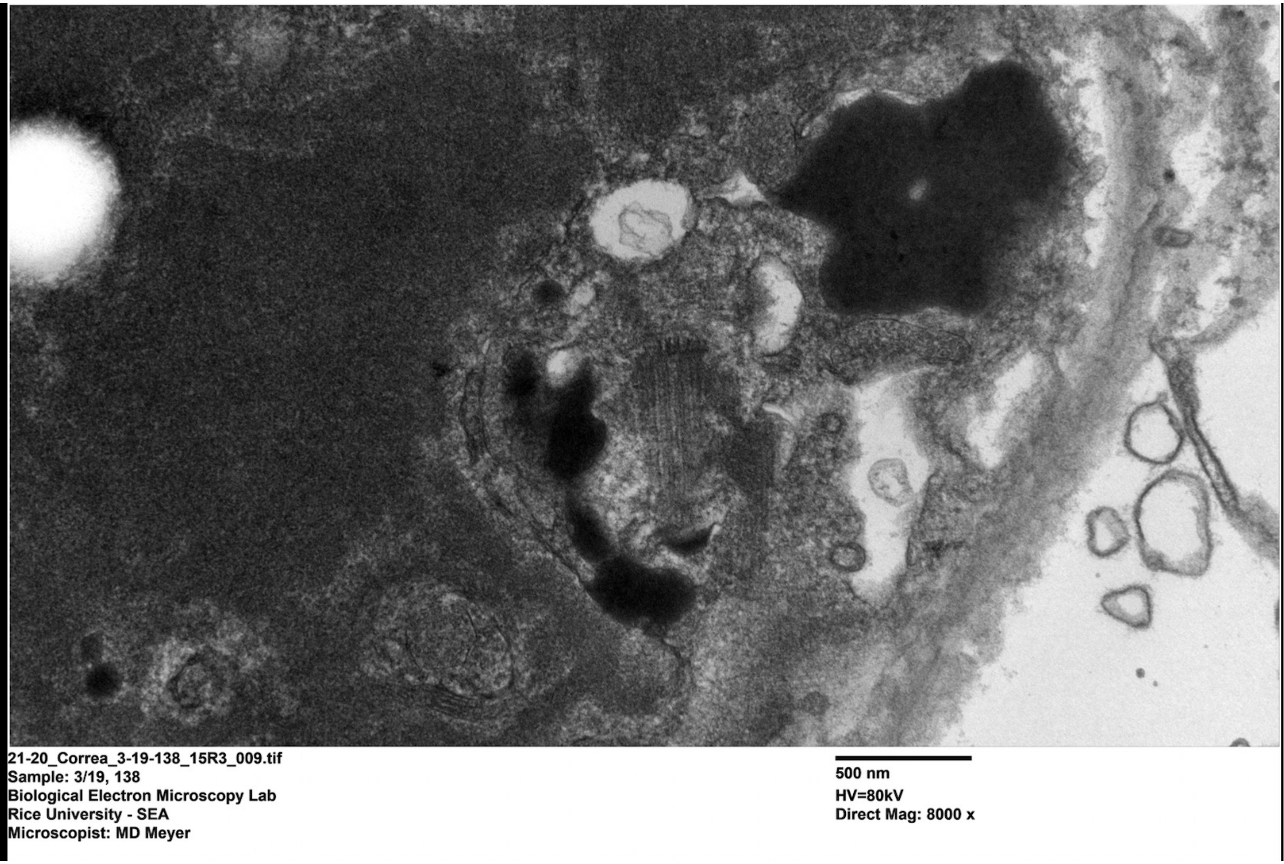
21-20_Correa_3-19-138_15R3_009.tif
Sample: 3/19, 138
Biological Electron Microscopy Lab
Rice University - SEA
Microscopist: MD Meyer
500 nm
HV=80kV
Direct Mag: 8000 x

Cell 20

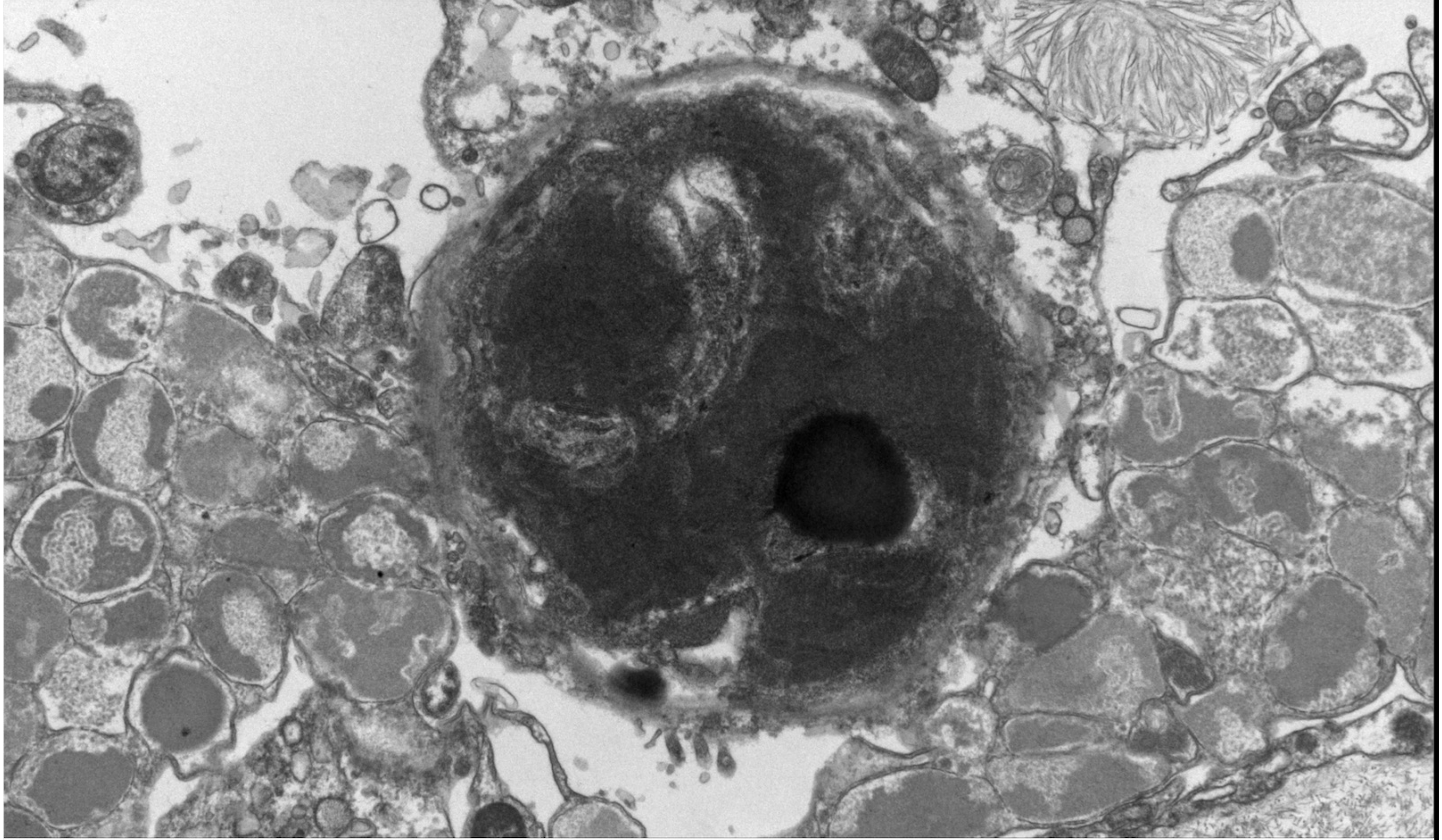

21-20_Correa_3-19-138_15R3_007.tif
Sample: 3/19, 138
Biological Electron Microscopy Lab
Rice University - SEA
Microscopist: MD Meyer

1 µm
HV=80kV
Direct Mag: 3000 x

Cell 21

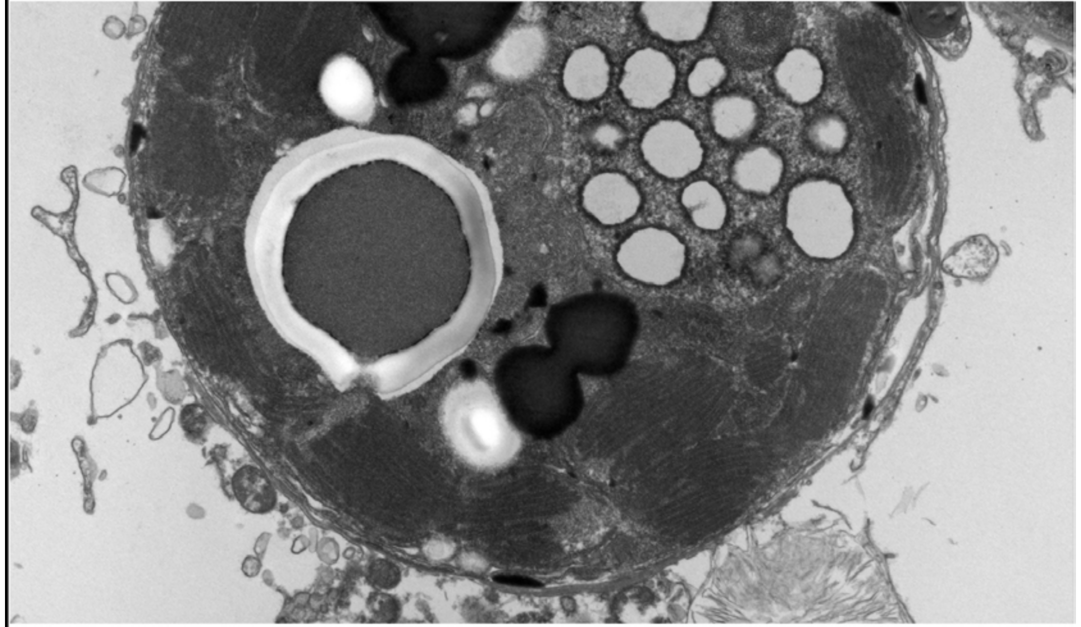

21-20_Correa_3-19-138_15R3_004.tif
Sample: 3/19, 138
Biological Electron Microscopy Lab
Rice University - SEA
Microscopist: MD Meyer

1 µm
HV=80kV
Direct Mag: 3000 x

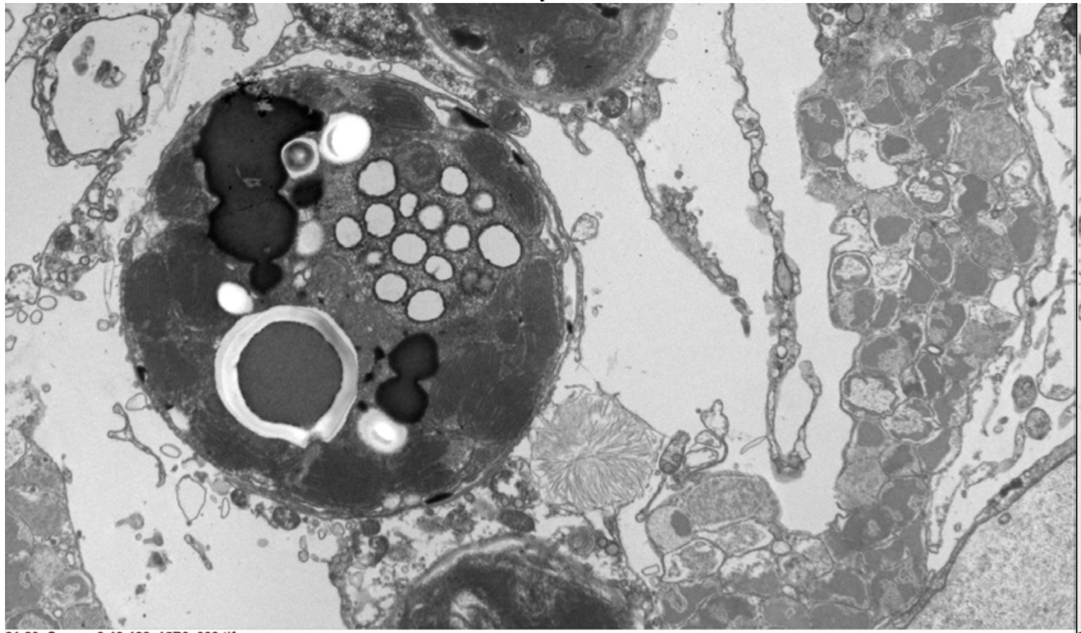

21-20_Correa_3-19-138_15R3_003.tif
Sample: 3/19, 138
Biological Electron Microscopy Lab
Rice University - SEA
Microscopist: MD Meyer

2 µm
HV=80kV
Direct Mag: 1500 x

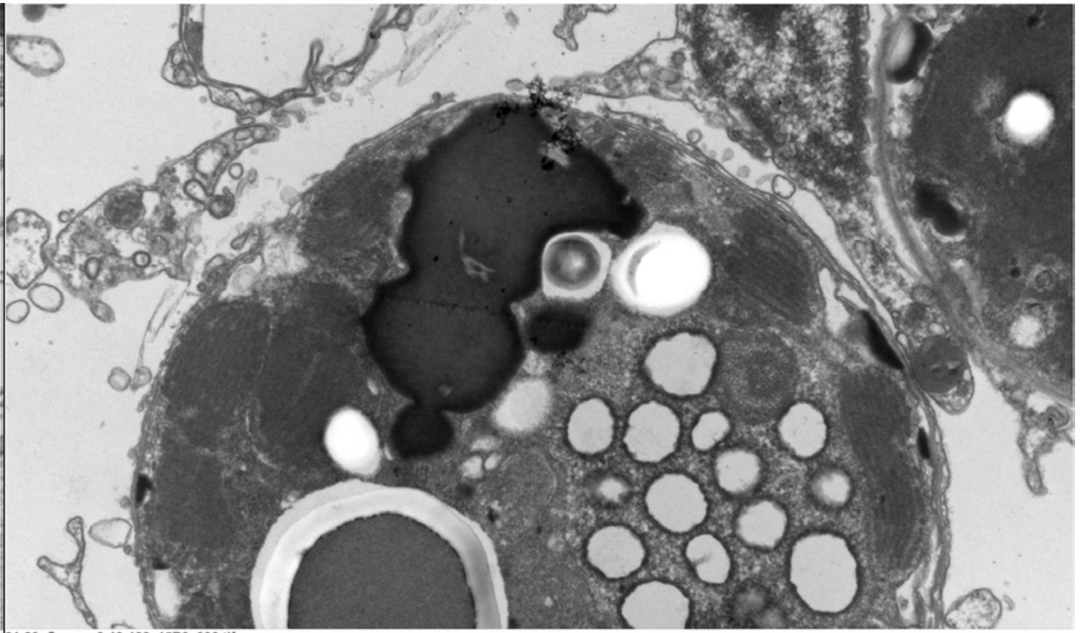

21-20_Correa_3-19-138_15R3_006.tif
Sample: 3/19, 138
Biological Electron Microscopy Lab
Rice University - SEA
Microscopist: MD Meyer

1 µm
HV=80kV
Direct Mag: 3000 x

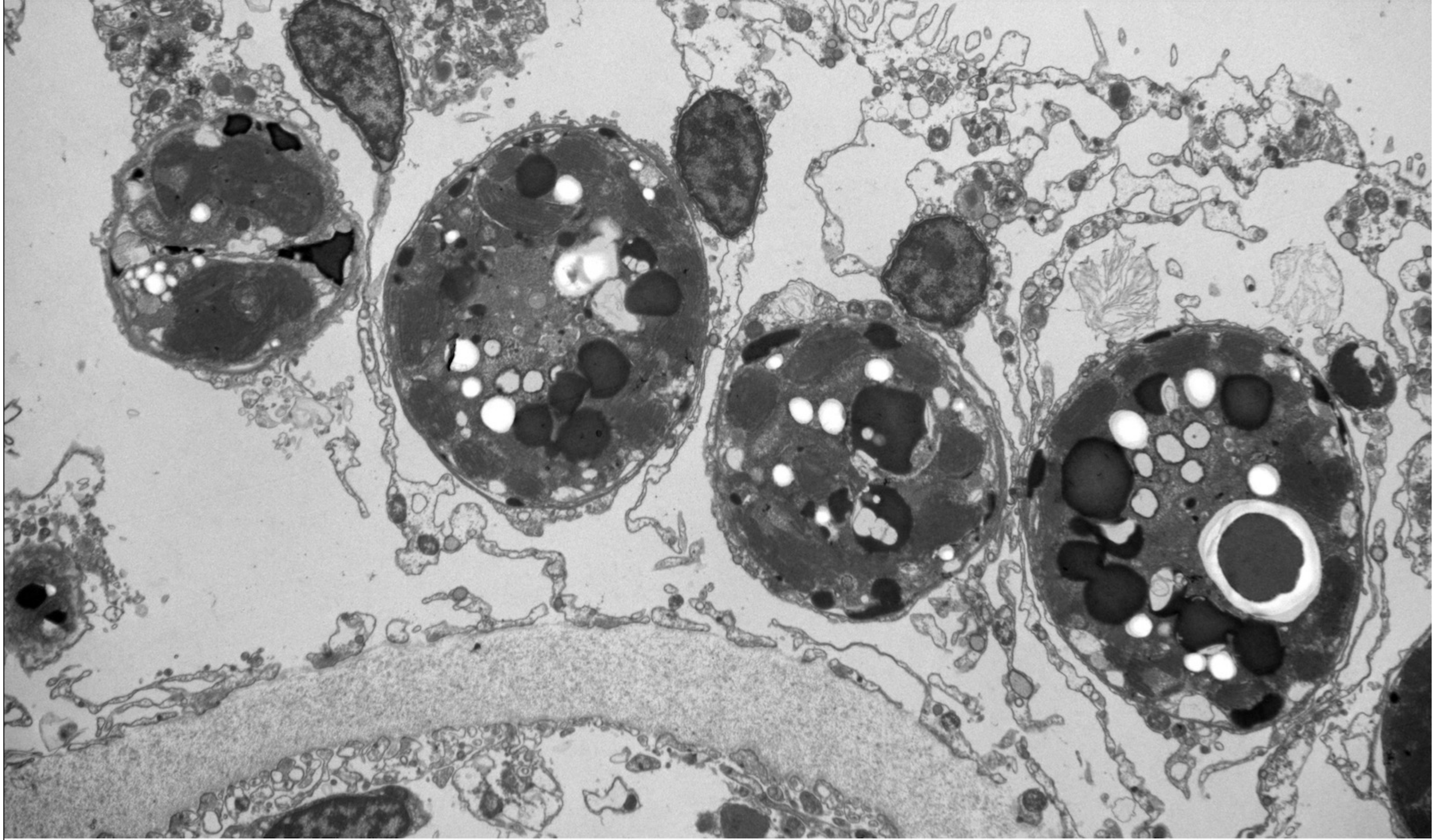

21-20_Correa_3-19-138_15R3_019.tif
Sample: 3/19, 138
Biological Electron Microscopy Lab
Rice University - SEA
Microscopist: MD Meyer

4 µm
HV=80kV
Direct Mag: 1000 x

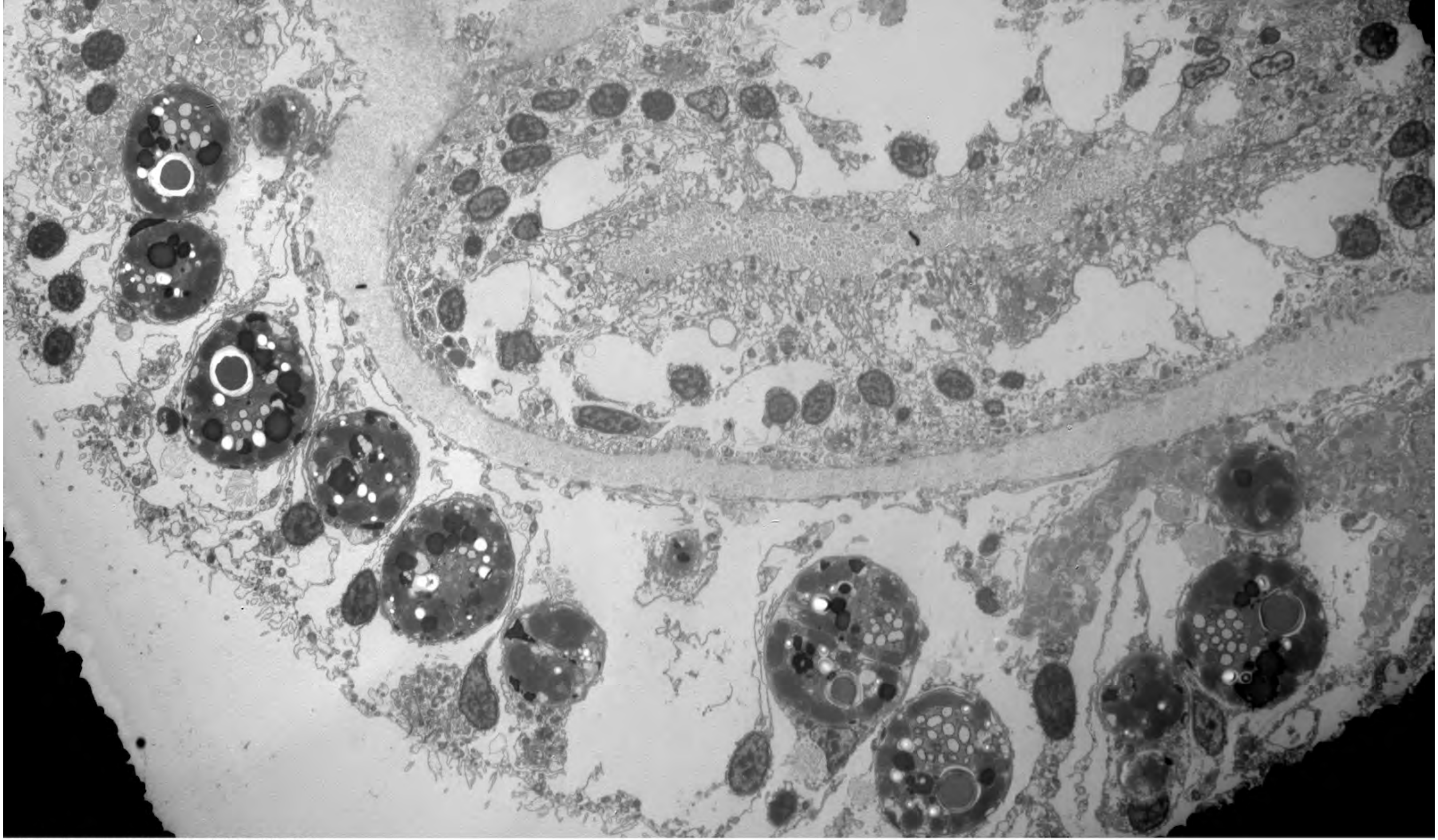

21-20_Correa_3-19-138_15R3_001.tif
Sample: 3/19, 138
Biological Electron Microscopy Lab
Rice University - SEA
Microscopist: MD Meyer

10 µm
HV=80kV
Direct Mag: 400 x

Ambient (March 2018) POR Colony M

Cell 1

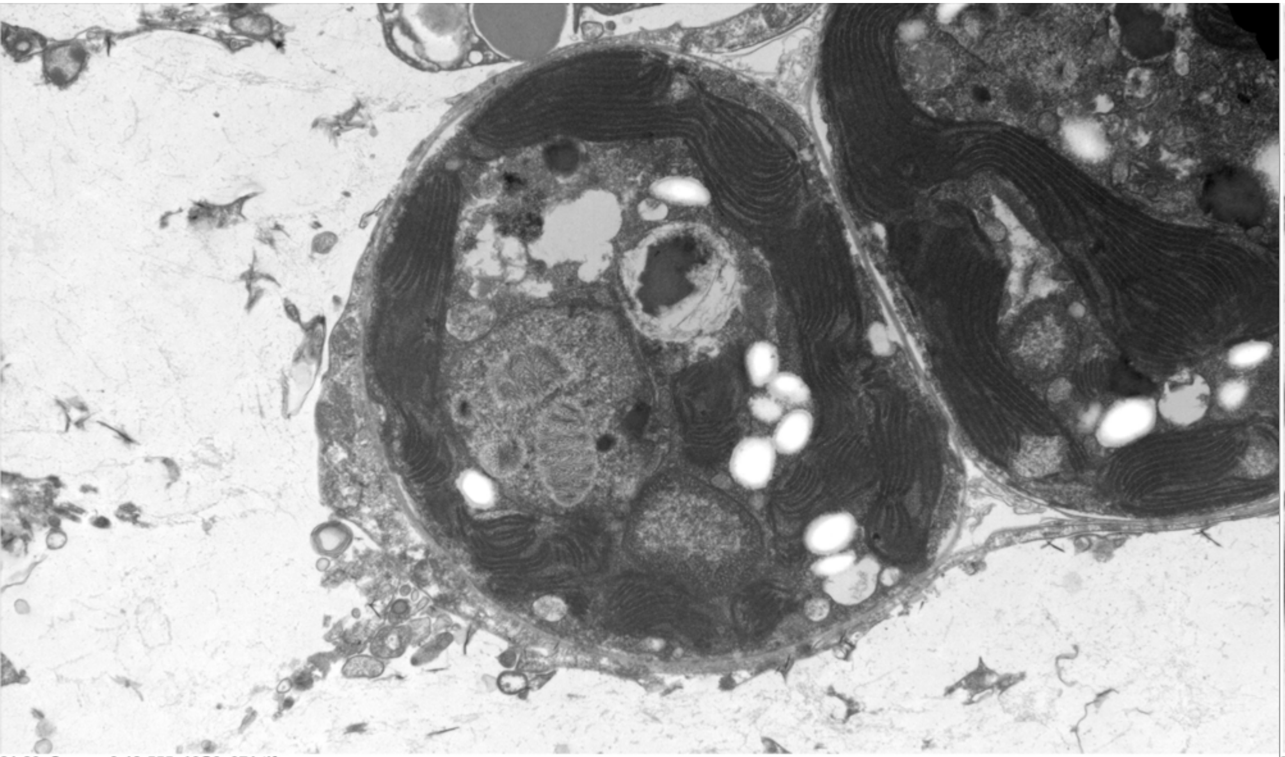

21-20_Correa_3-18-555_16G3_071.tif
Sample: 3/18, 555
Biological Electron Microscopy Lab
Rice University - SEA
Microscopist: MD Meyer

1 µm
HV=80kV
Direct Mag: 2500 x

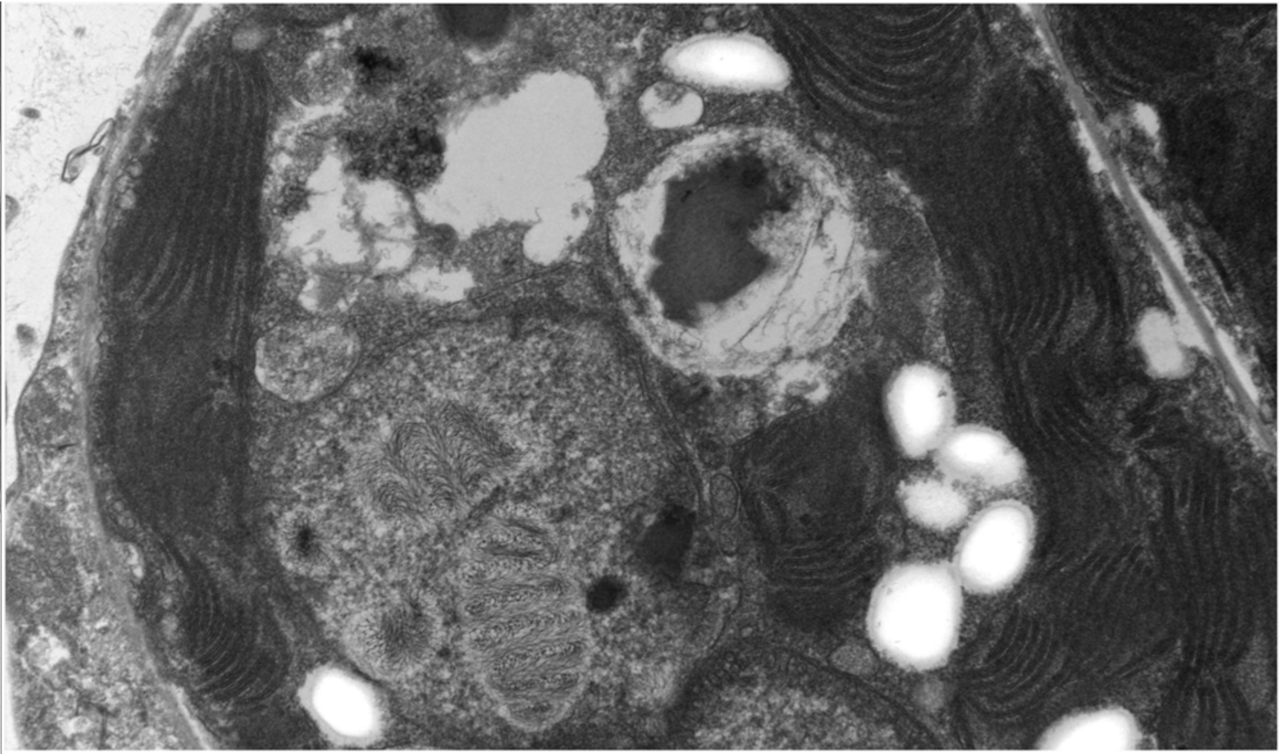

21-20_Correa_3-18-555_16G3_073.tif
Sample: 3/18, 555
Biological Electron Microscopy Lab
Rice University - SEA
Microscopist: MD Meyer

800 nm
HV=80kV
Direct Mag: 5000 x

Cell 2

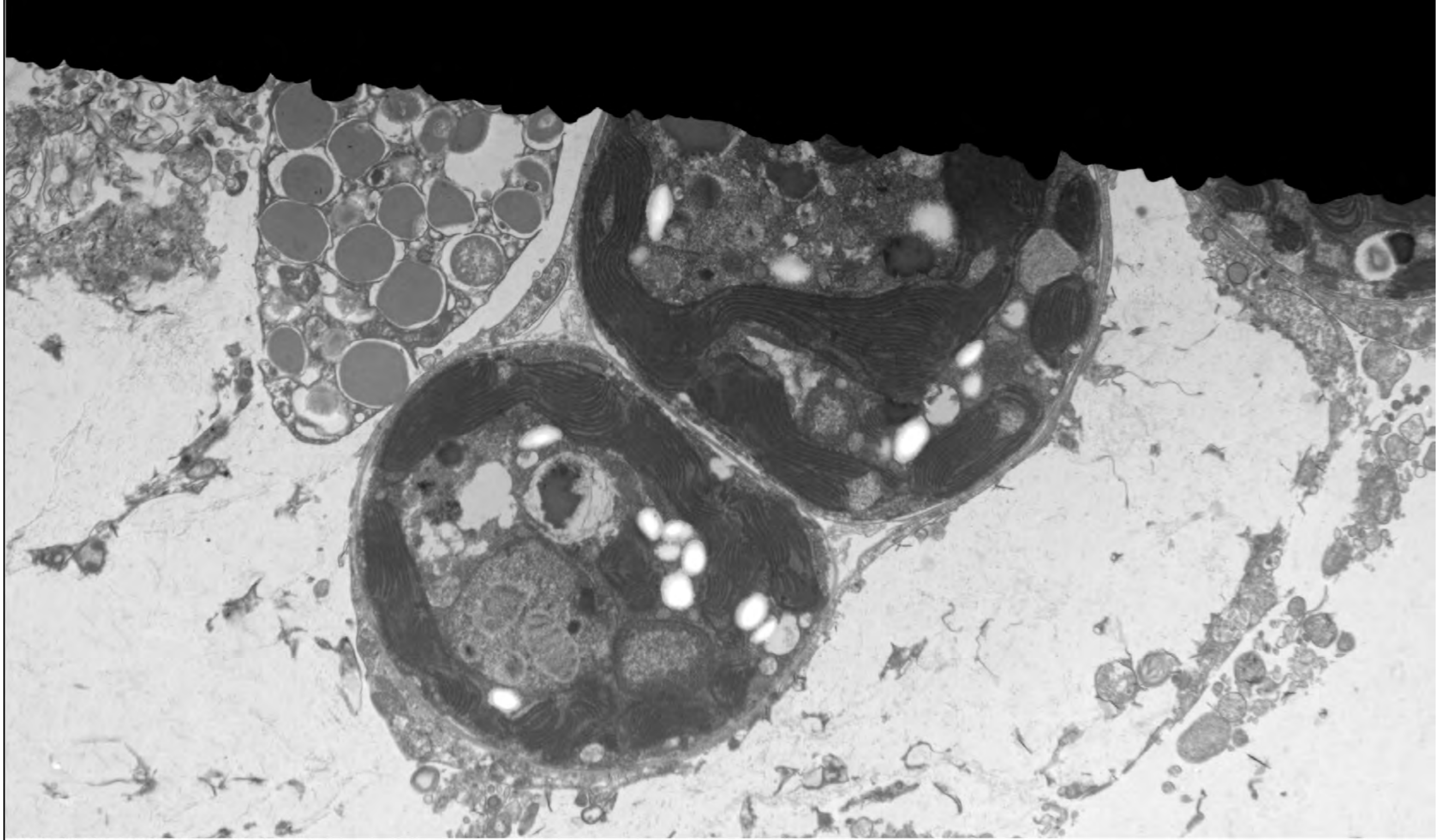

21-20_Correa_3-18-555_16G3_070.tif
Sample: 3/18, 555
Biological Electron Microscopy Lab
Rice University - SEA
Microscopist: MD Meyer

2 µm
HV=80kV
Direct Mag: 1500 x

Cell 3

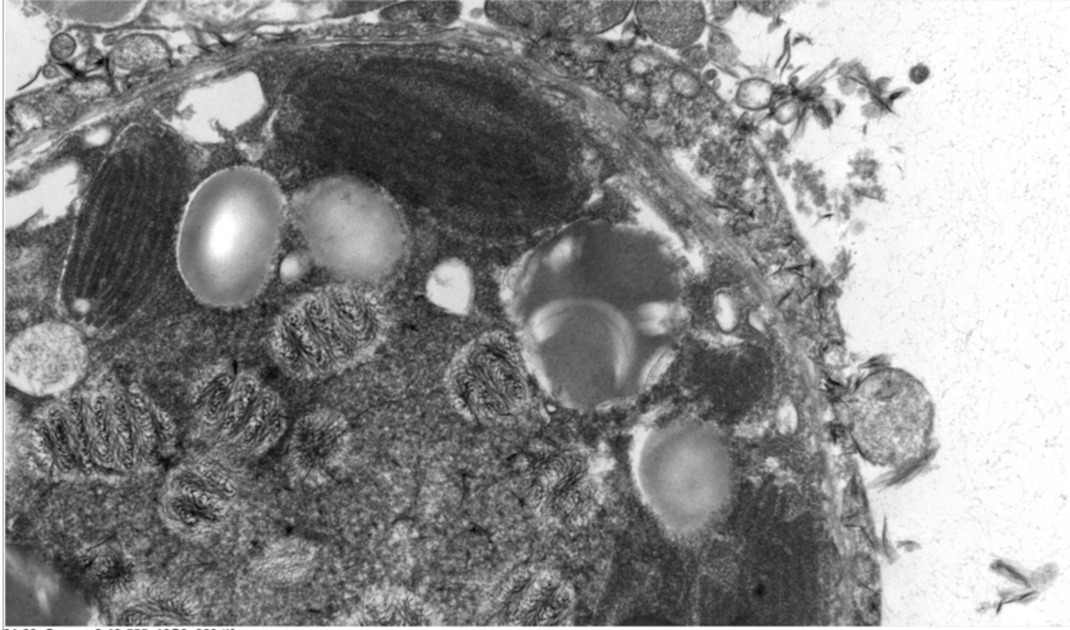

21-20_Correa_3-18-555_16G3_069.tif
Sample: 3/18, 555
Biological Electron Microscopy Lab
Rice University - SEA
Microscopist: MD Meyer

600 nm
HV=80kV
Direct Mag: 6000 x

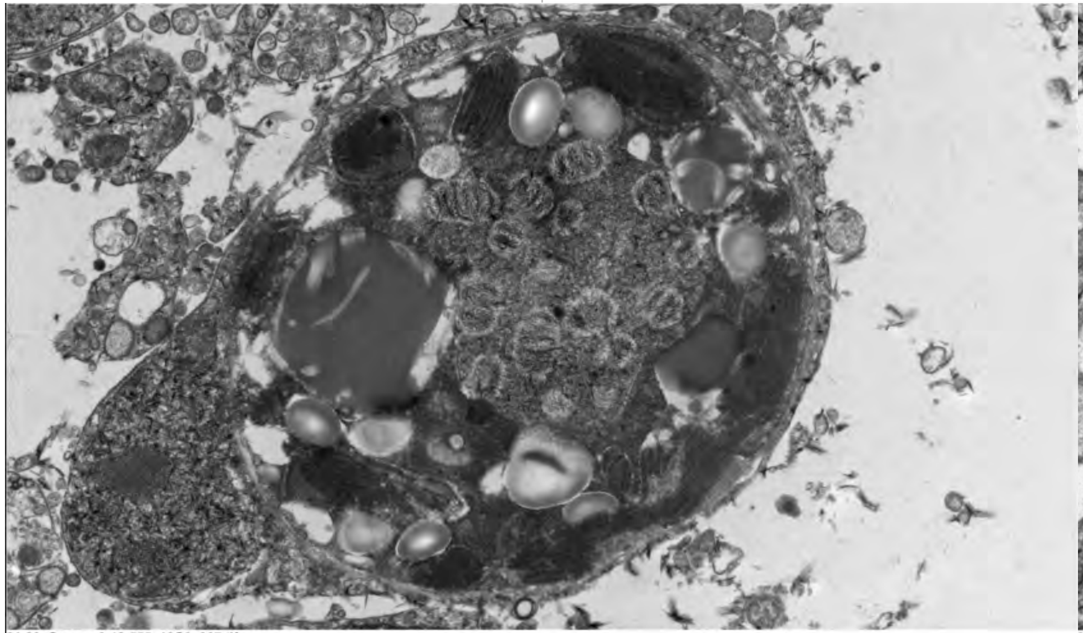

21-20_Correa_3-18-555_16G3_067.tif
Sample: 3/18, 555
Biological Electron Microscopy Lab
Rice University - SEA
Microscopist: MD Meyer

1 µm
HV=80kV
Direct Mag: 3000 x

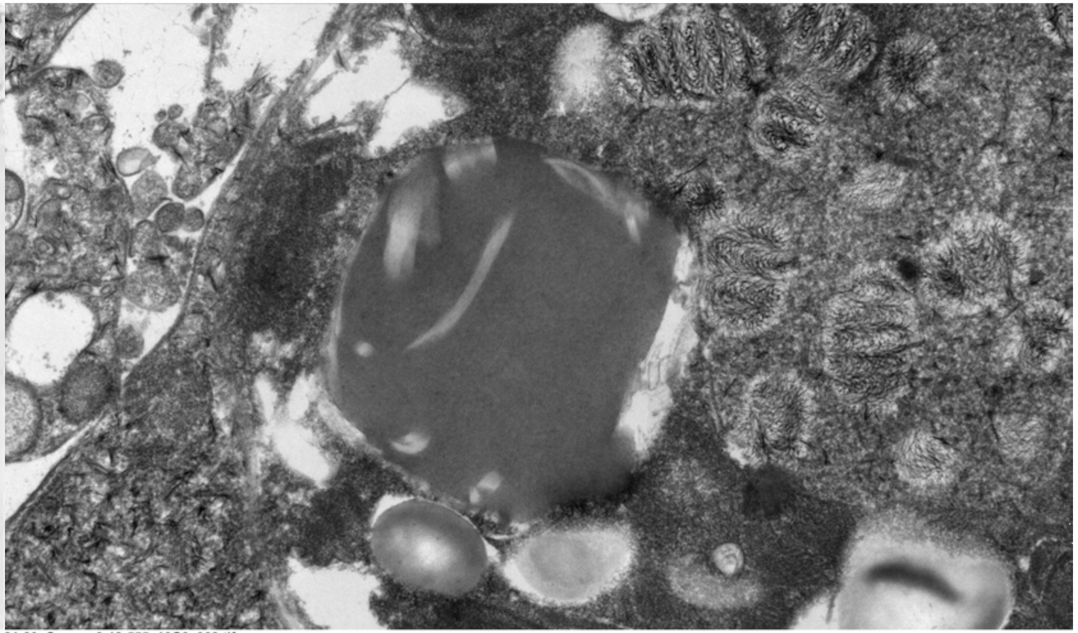

21-20_Correa_3-18-555_16G3_068.tif
Sample: 3/18, 555
Biological Electron Microscopy Lab
Rice University - SEA
Microscopist: MD Meyer

600 nm
HV=80kV
Direct Mag: 6000 x

Cell 4

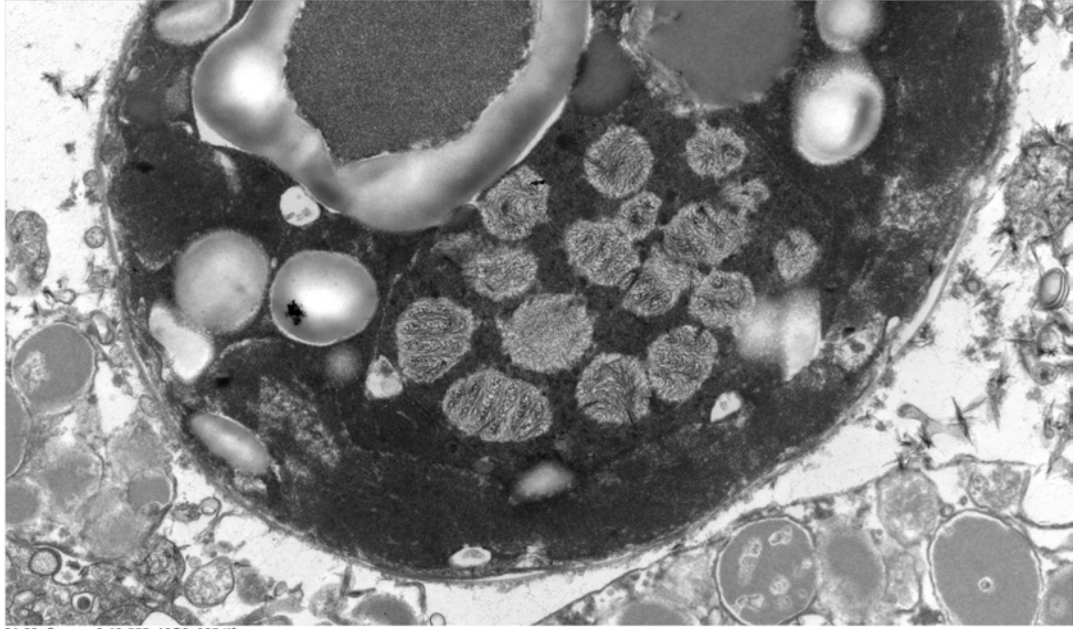

21-20_Correa_3-18-555_16G3_065.tif
Sample: 3/18, 555
Biological Electron Microscopy Lab
Rice University - SEA
Microscopist: MD Meyer

1 µm
HV=80kV
Direct Mag: 4000 x

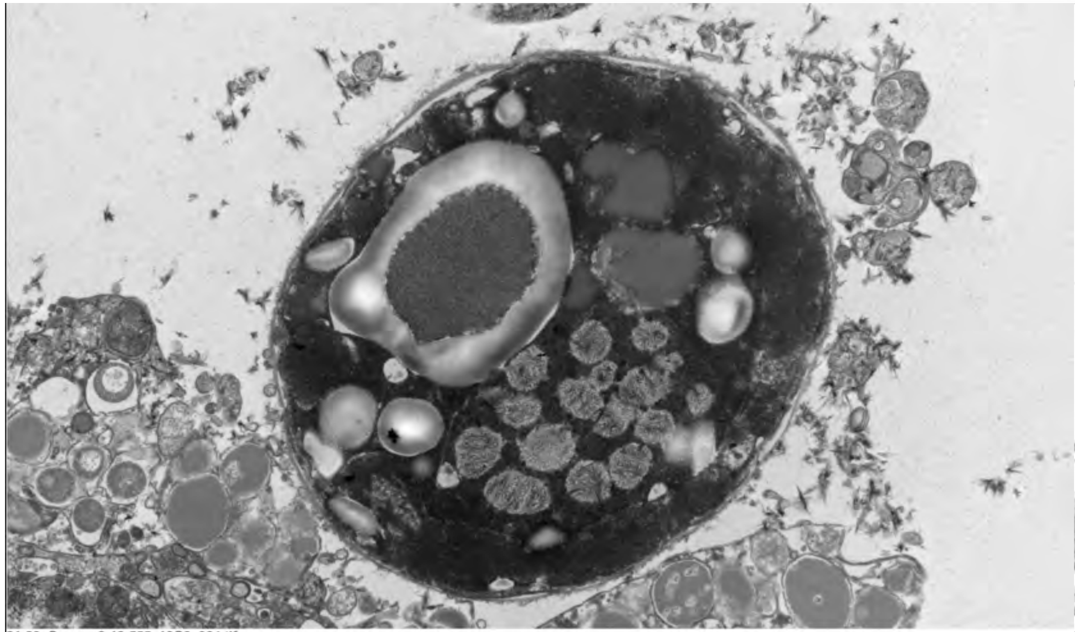

21-20_Correa_3-18-555_16G3_064.tif
Sample: 3/18, 555
Biological Electron Microscopy Lab
Rice University - SEA
Microscopist: MD Meyer

1 µm
HV=80kV
Direct Mag: 2500 x

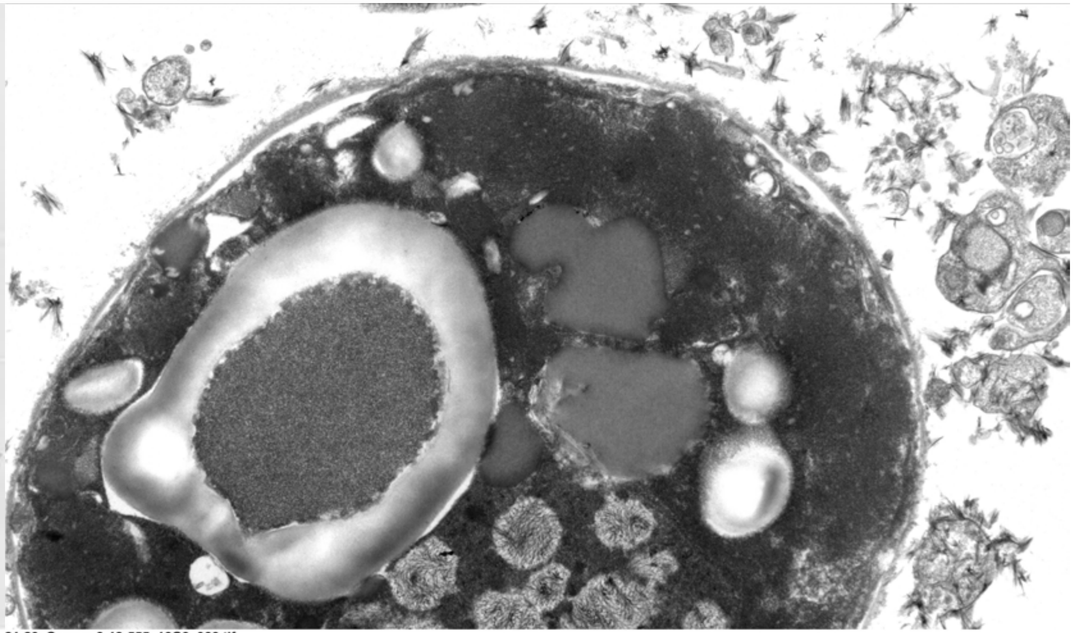

21-20_Correa_3-18-555_16G3_066.tif
Sample: 3/18, 555
Biological Electron Microscopy Lab
Rice University - SEA
Microscopist: MD Meyer

1 µm
HV=80kV
Direct Mag: 4000 x

Cell 5

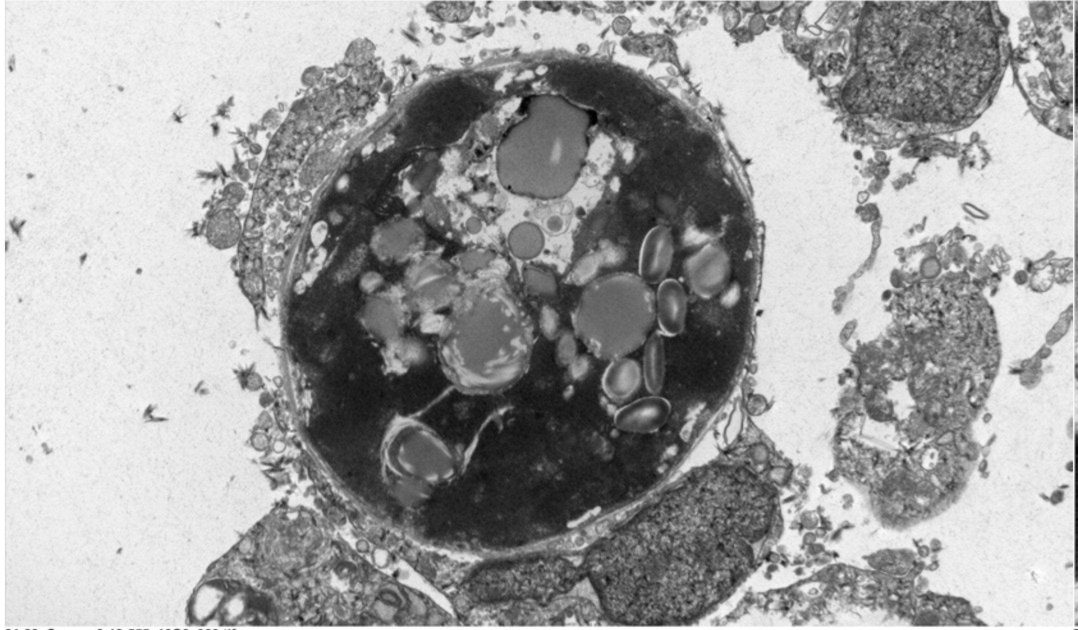

21-20_Correa_3-18-555_16G3_060.tif
Sample: 3/18, 555
Biological Electron Microscopy Lab
Rice University - SEA
Microscopist: MD Meyer

2 µm
HV=80kV
Direct Mag: 2000 x

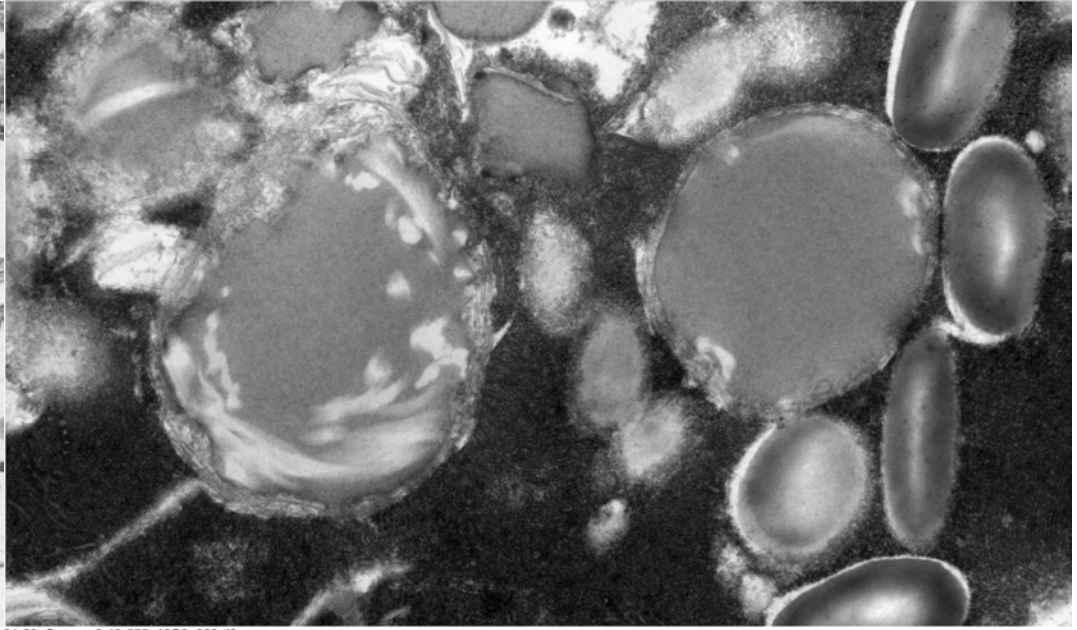

21-20_Correa_3-18-555_16G3_063.tif
Sample: 3/18, 555
Biological Electron Microscopy Lab
Rice University - SEA
Microscopist: MD Meyer

500 nm
HV=80kV
Direct Mag: 8000 x

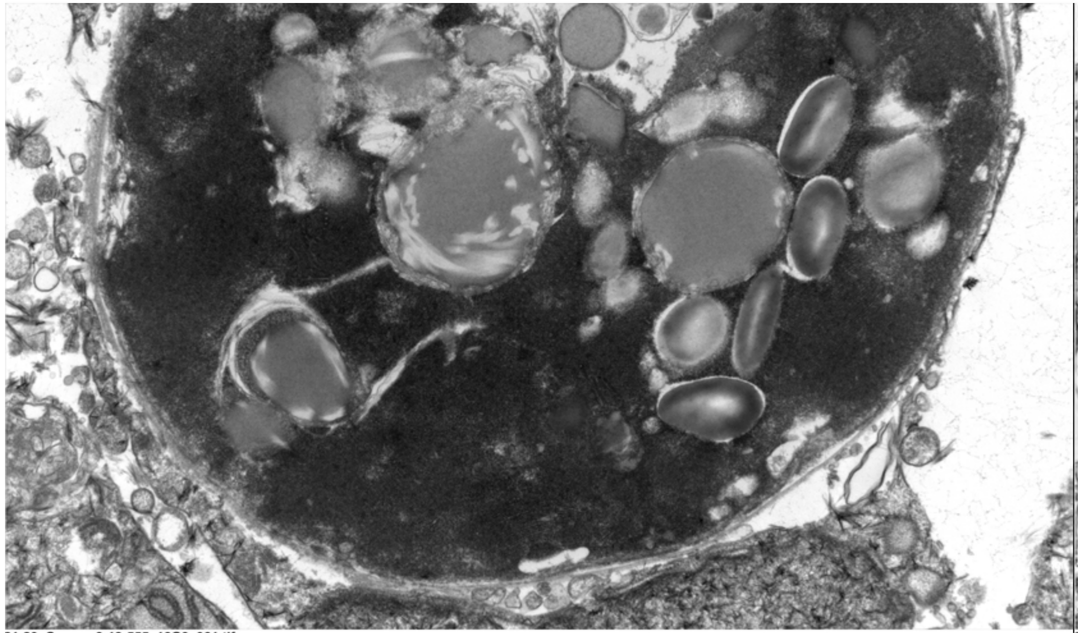

21-20_Correa_3-18-555_16G3_061.tif
Sample: 3/18, 555
Biological Electron Microscopy Lab
Rice University - SEA
Microscopist: MD Meyer

1 µm
HV=80kV
Direct Mag: 4000 x

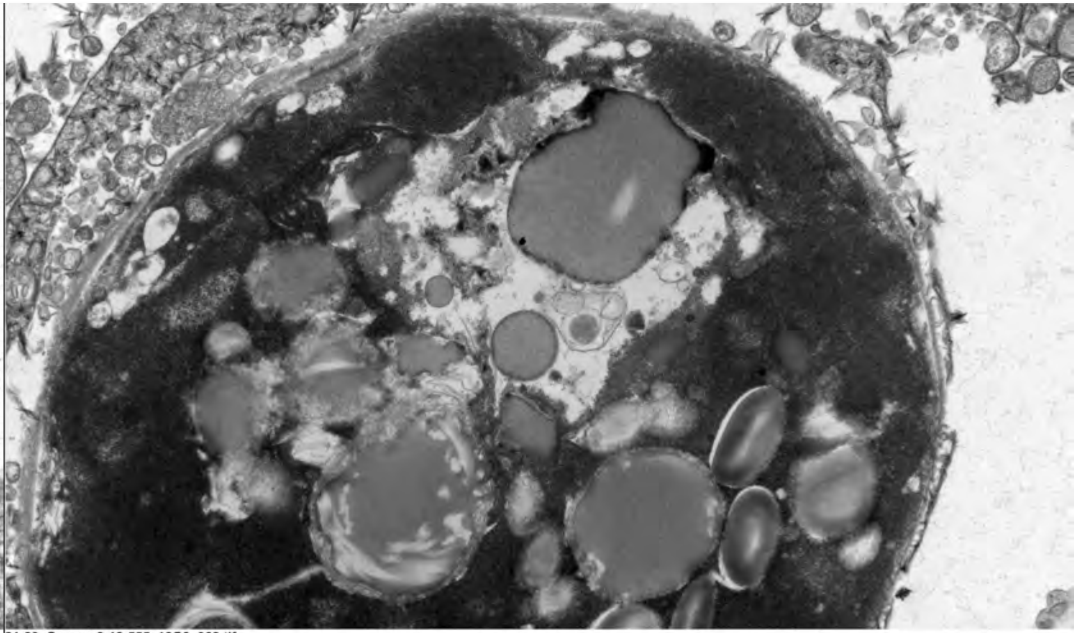

21-20_Correa_3-18-555_16G3_062.tif
Sample: 3/18, 555
Biological Electron Microscopy Lab
Rice University - SEA
Microscopist: MD Meyer

1 µm
HV=80kV
Direct Mag: 4000 x

Cell 6

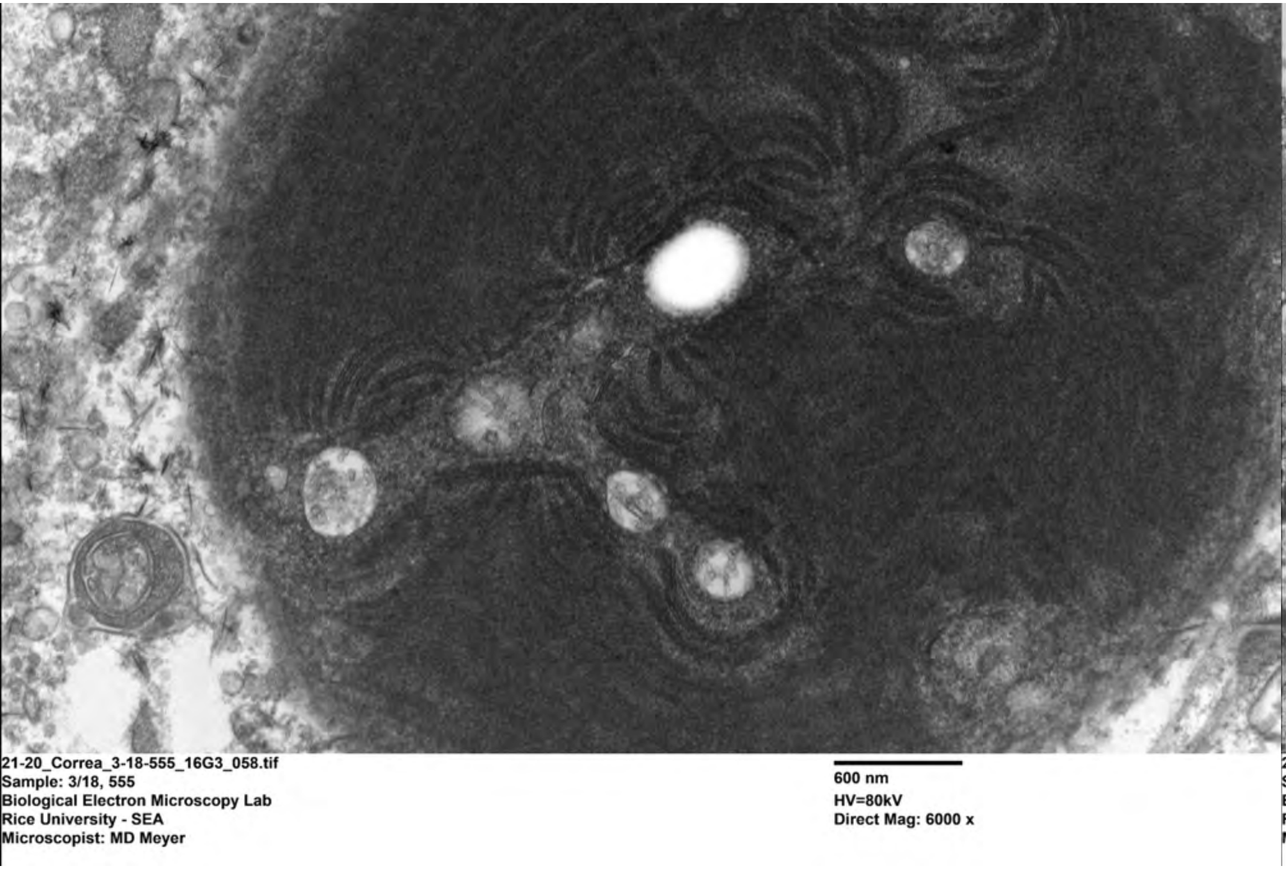
21-20_Correa_3-18-555_16G3_058.tif
Sample: 3/18, 555
Biological Electron Microscopy Lab
Rice University - SEA
Microscopist: MD Meyer
600 nm
HV=80kV
Direct Mag: 6000 x

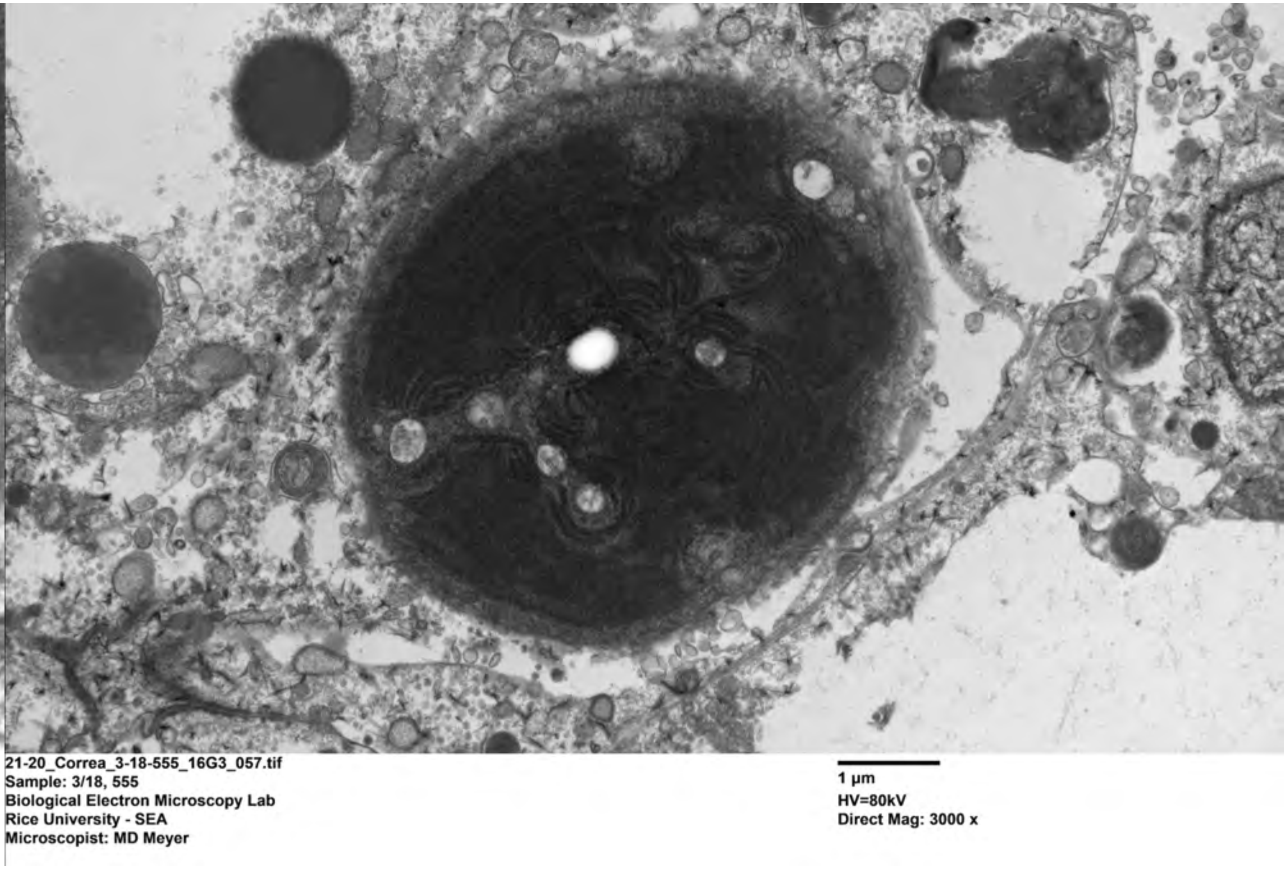
21-20_Correa_3-18-555_16G3_057.tif
Sample: 3/18, 555
Biological Electron Microscopy Lab
Rice University - SEA
Microscopist: MD Meyer
1 µm
HV=80kV
Direct Mag: 3000 x

Cell 7

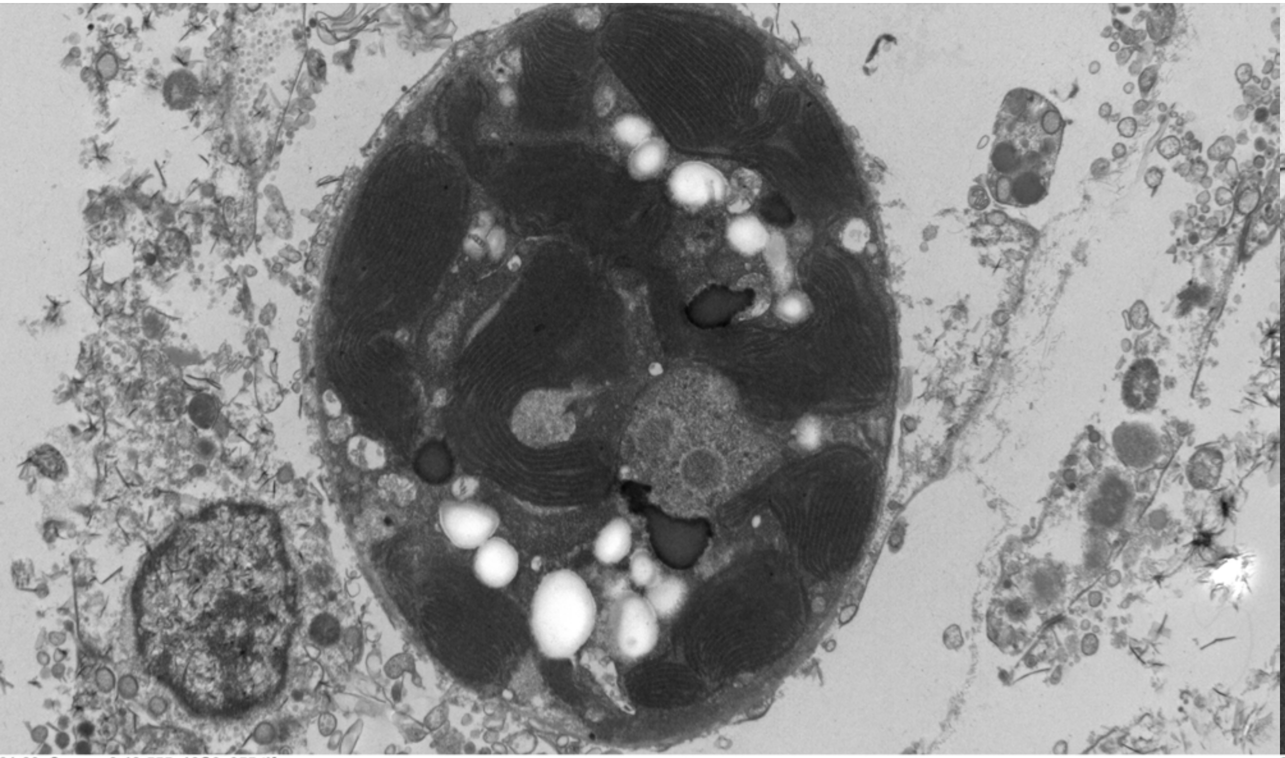

21-20_Correa_3-18-555_16G3_055.tif
Sample: 3/18, 555
Biological Electron Microscopy Lab
Rice University - SEA
Microscopist: MD Meyer

2 µm
HV=80kV
Direct Mag: 2000 x

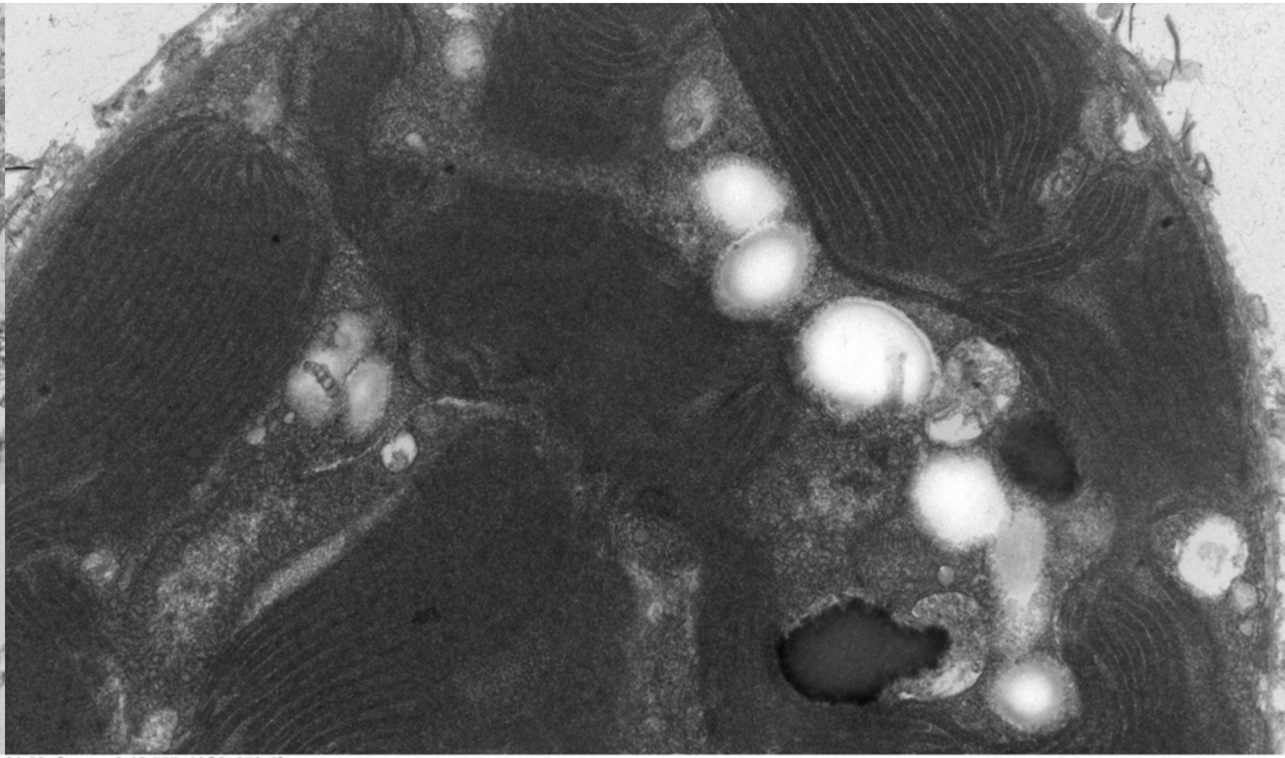

21-20_Correa_3-18-555_16G3_056.tif
Sample: 3/18, 555
Biological Electron Microscopy Lab
Rice University - SEA
Microscopist: MD Meyer

800 nm
HV=80kV
Direct Mag: 5000 x

Cell 8

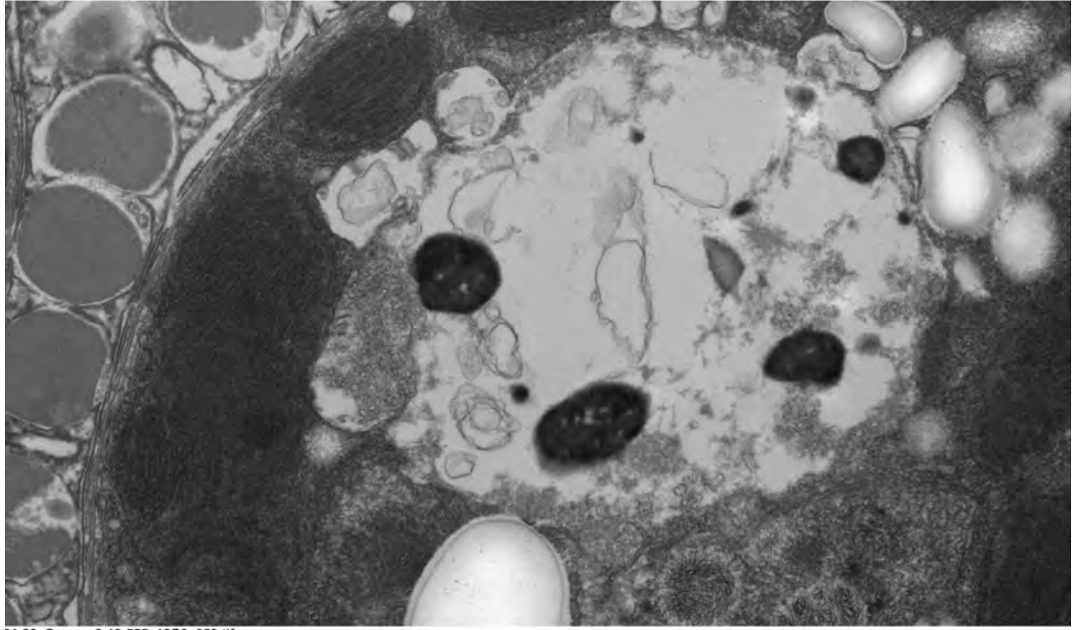

21-20_Correa_3-18-555_16G3_053.tif
Sample: 3/18, 555
Biological Electron Microscopy Lab
Rice University - SEA
Microscopist: MD Meyer

800 nm
HV=80kV
Direct Mag: 5000 x

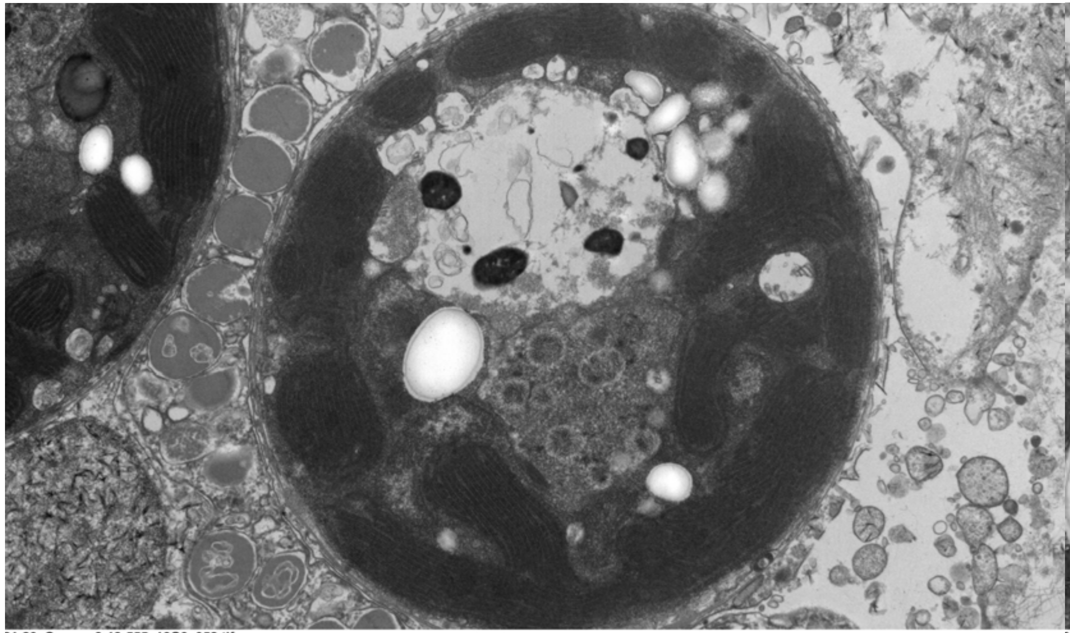

21-20_Correa_3-18-555_16G3_052.tif
Sample: 3/18, 555
Biological Electron Microscopy Lab
Rice University - SEA
Microscopist: MD Meyer

1 µm
HV=80kV
Direct Mag: 2500 x

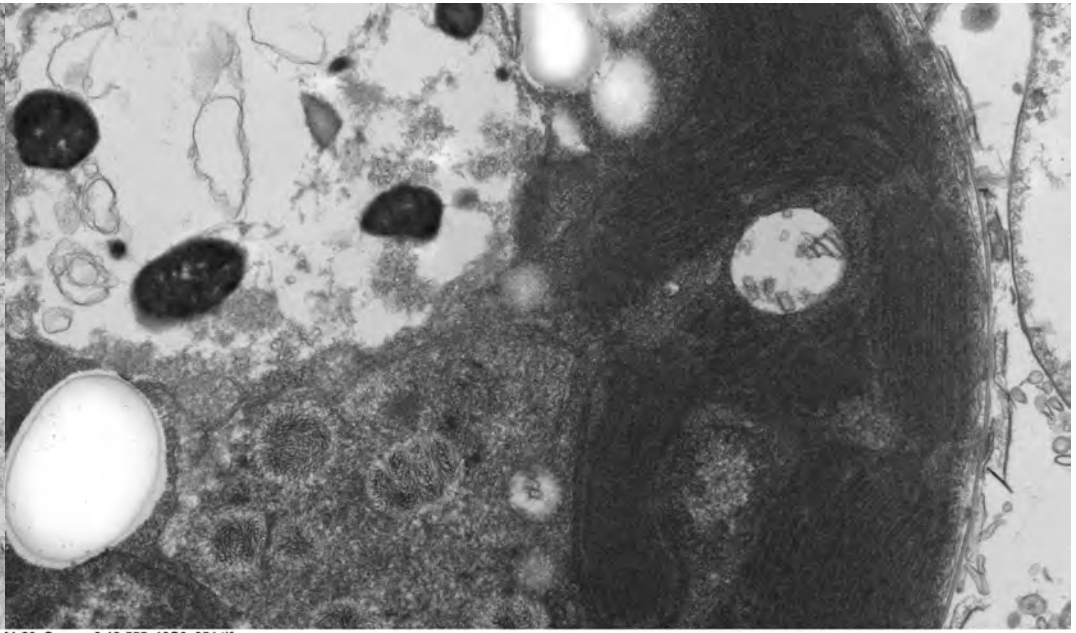

21-20_Correa_3-18-555_16G3_054.tif
Sample: 3/18, 555
Biological Electron Microscopy Lab
Rice University - SEA
Microscopist: MD Meyer

800 nm
HV=80kV
Direct Mag: 5000 x

Cell 9

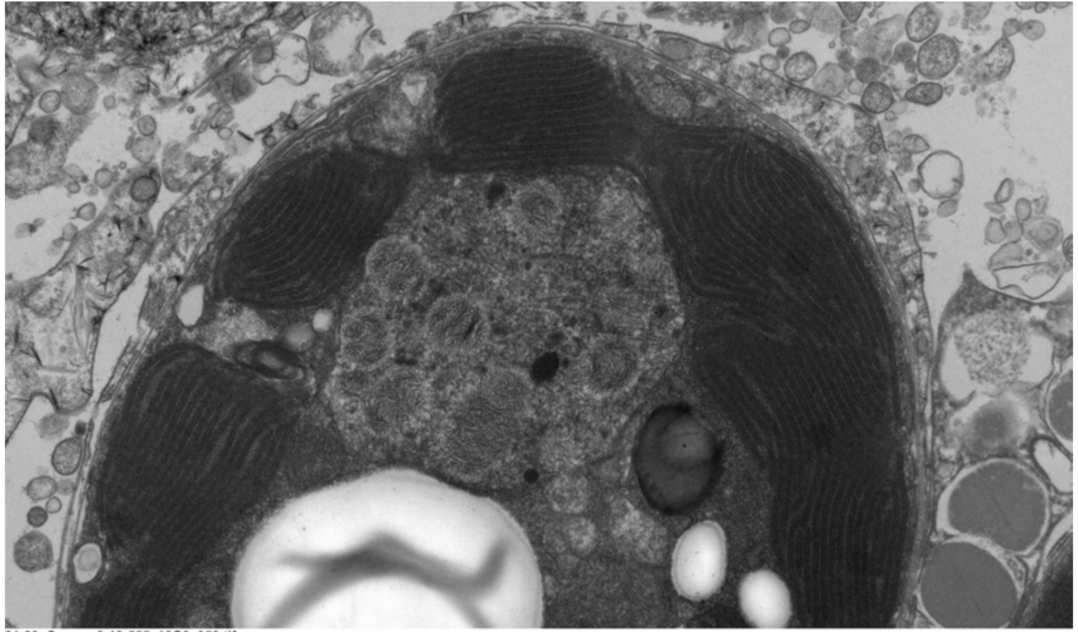

21-20_Correa_3-18-555_16G3_050.tif
Sample: 3/18, 555
Biological Electron Microscopy Lab
Rice University - SEA
Microscopist: MD Meyer

1 µm
HV=80kV
Direct Mag: 4000 x

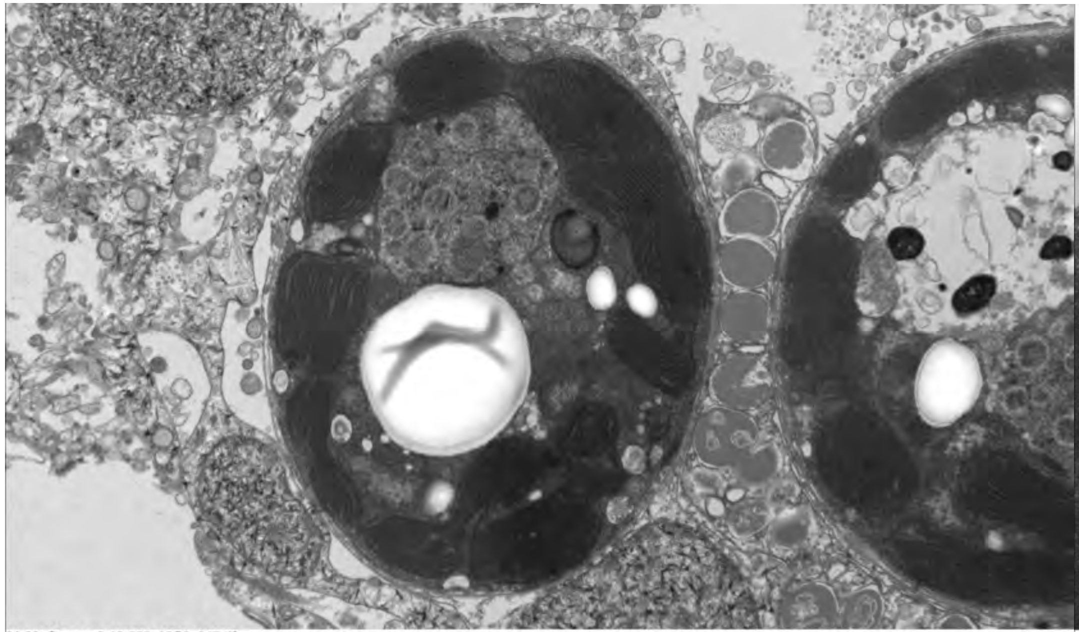

21-20_Correa_3-18-555_16G3_047.tif
Sample: 3/18, 555
Biological Electron Microscopy Lab
Rice University - SEA
Microscopist: MD Meyer

2 µm
HV=80kV
Direct Mag: 2000 x

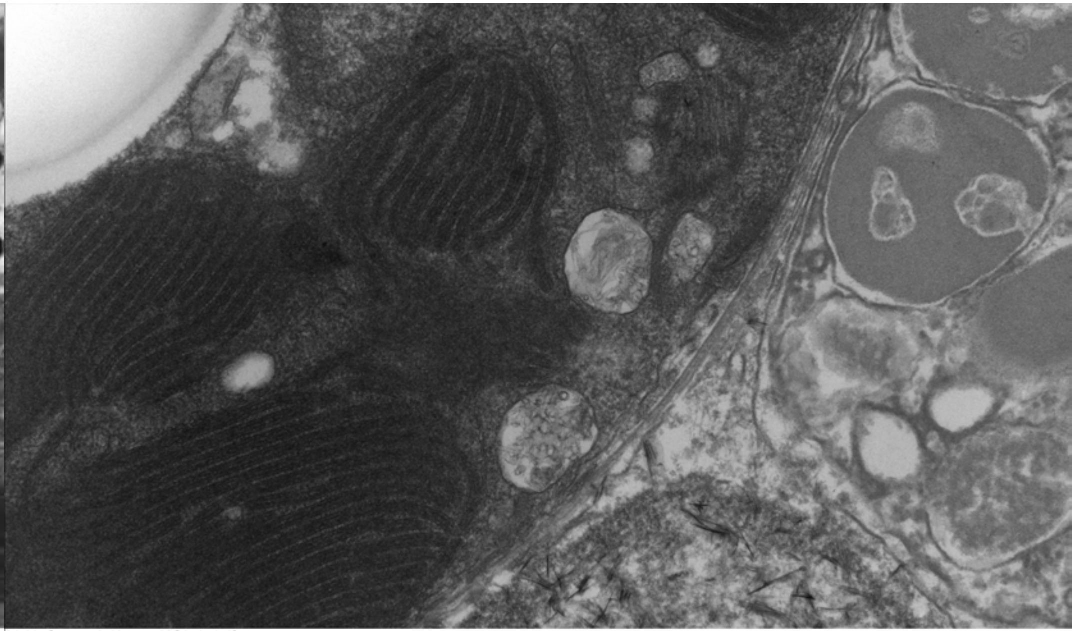

21-20_Correa_3-18-555_16G3_051.tif
Sample: 3/18, 555
Biological Electron Microscopy Lab
Rice University - SEA
Microscopist: MD Meyer

500 nm
HV=80kV
Direct Mag: 8000 x

Cell 10

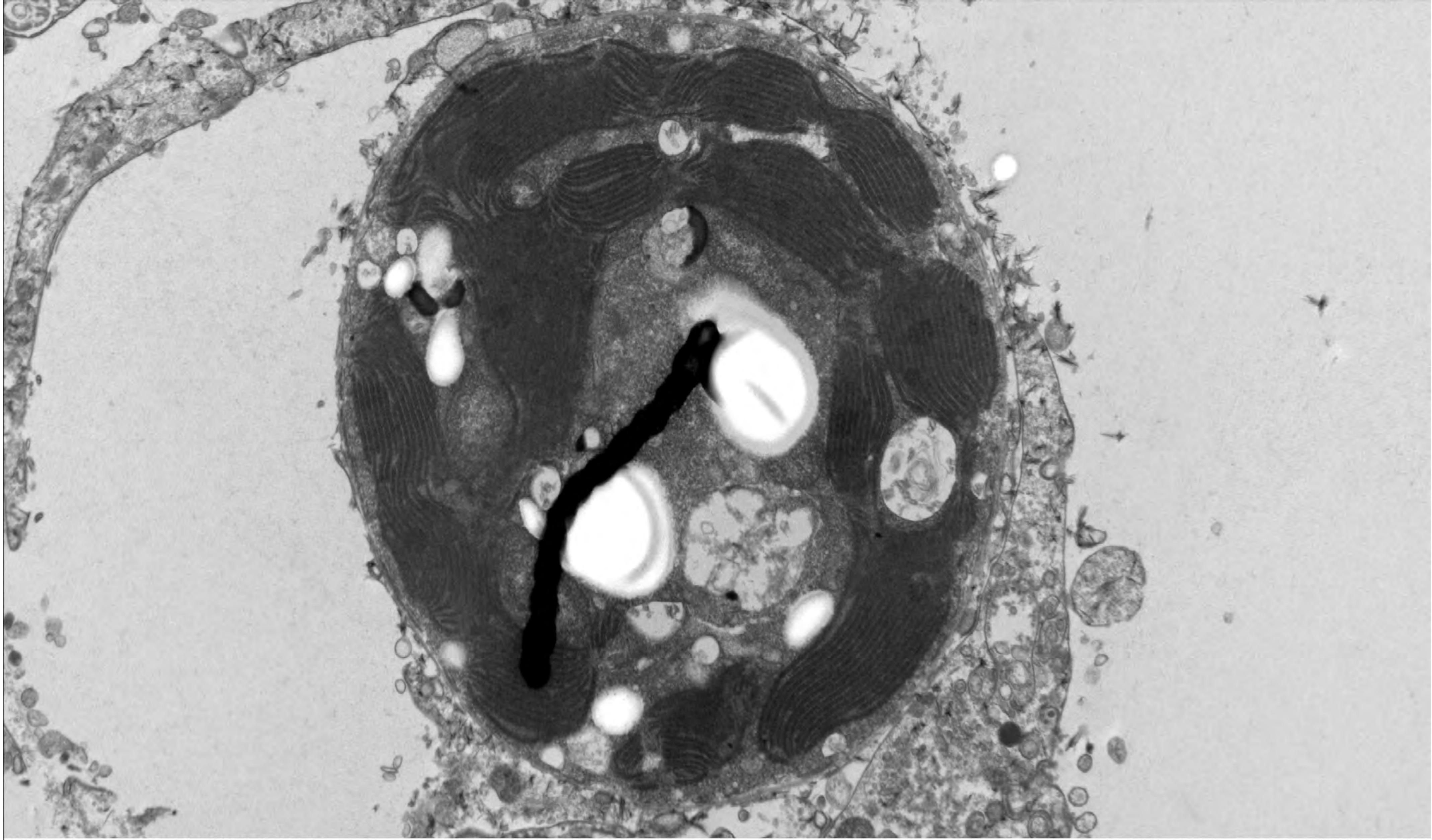

21-20_Correa_3-18-555_16G3_045.tif
Sample: 3/18, 555
Biological Electron Microscopy Lab
Rice University - SEA
Microscopist: MD Meyer

2 µm
HV=80kV
Direct Mag: 2000 x

Cell 11

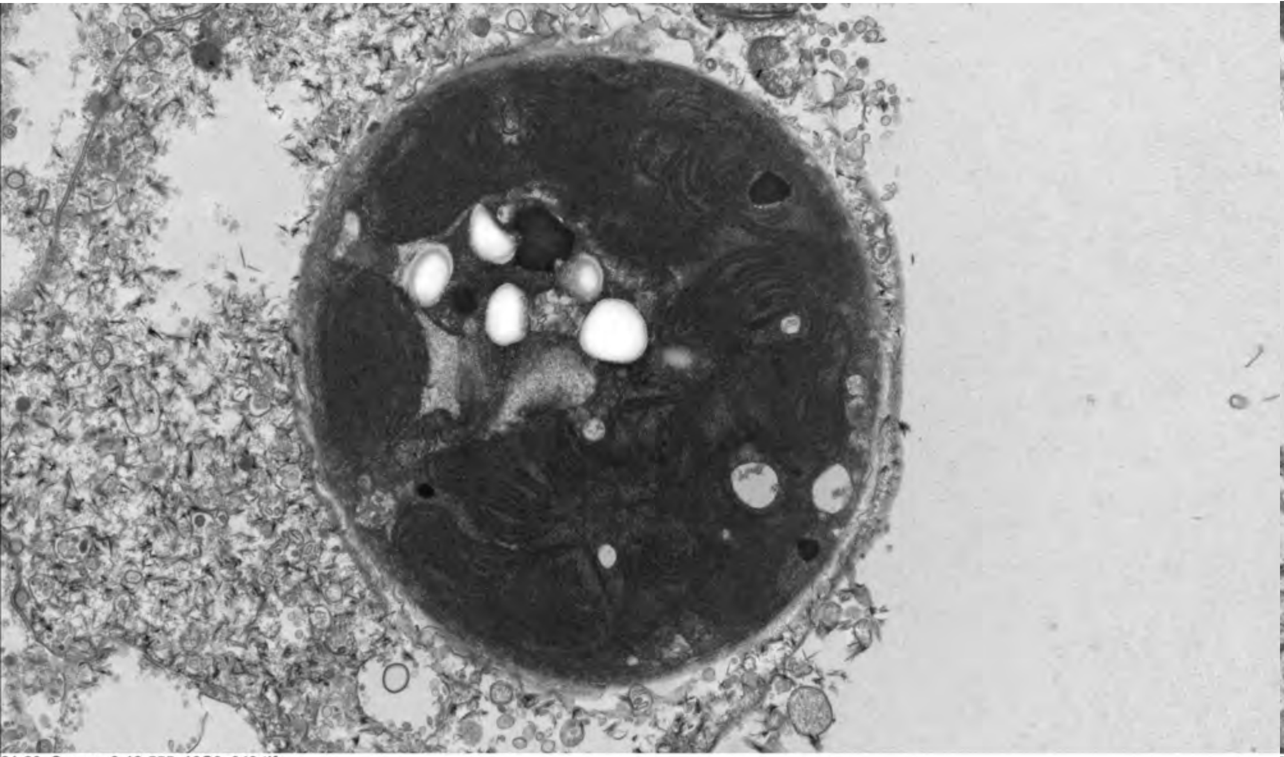

21-20_Correa_3-18-555_16G3_043.tif
Sample: 3/18, 555
Biological Electron Microscopy Lab
Rice University - SEA
Microscopist: MD Meyer

1 μm
HV=80kV
Direct Mag: 2500 x

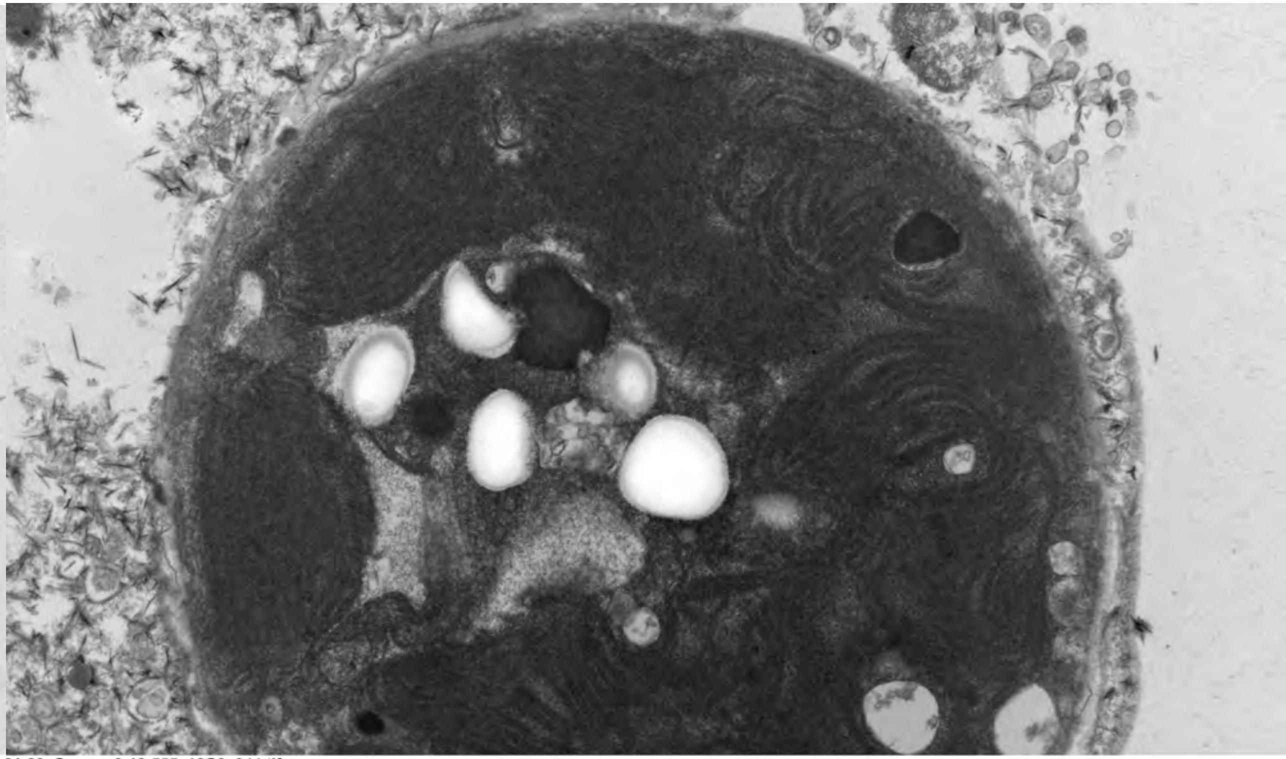

21-20_Correa_3-18-555_16G3_044.tif
Sample: 3/18, 555
Biological Electron Microscopy Lab
Rice University - SEA
Microscopist: MD Meyer

1 μm
HV=80kV
Direct Mag: 4000 x

Cell 12

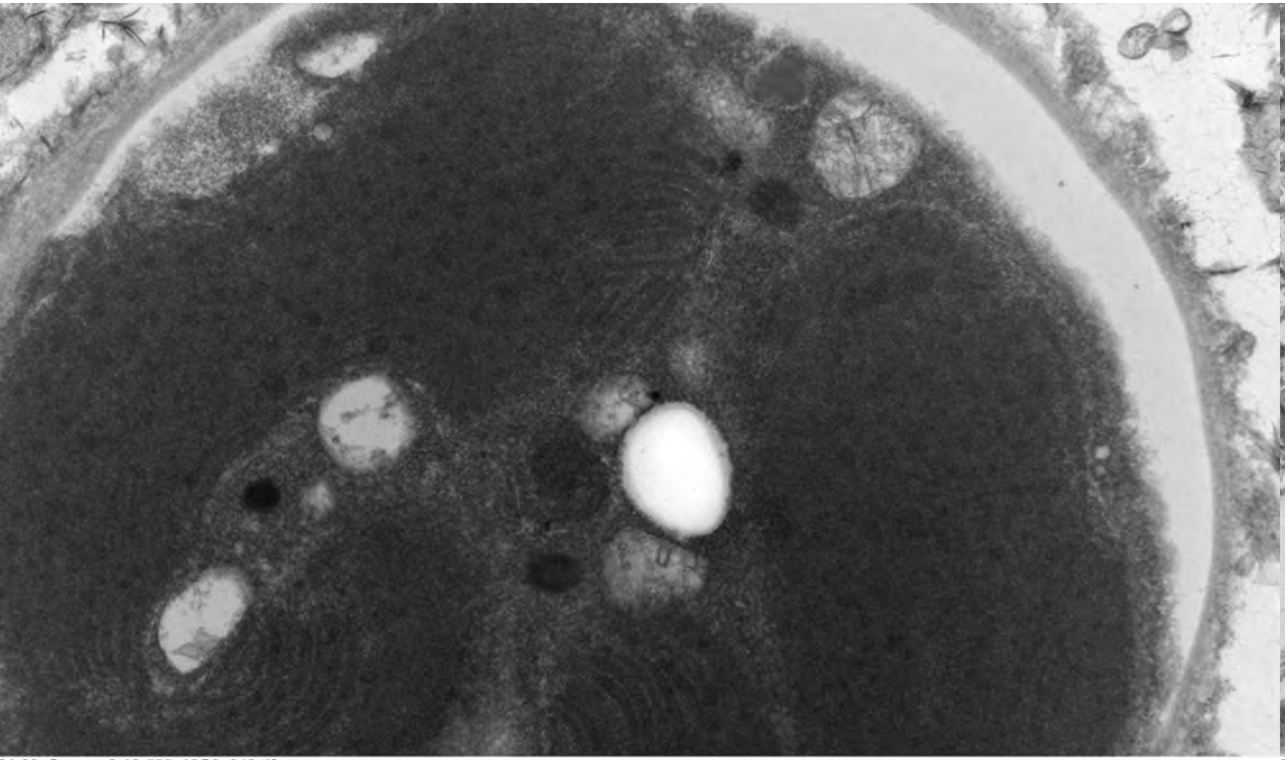

21-20_Correa_3-18-555_16G3_042.tif
Sample: 3/18, 555
Biological Electron Microscopy Lab
Rice University - SEA
Microscopist: MD Meyer

600 nm
HV=80kV
Direct Mag: 6000 x

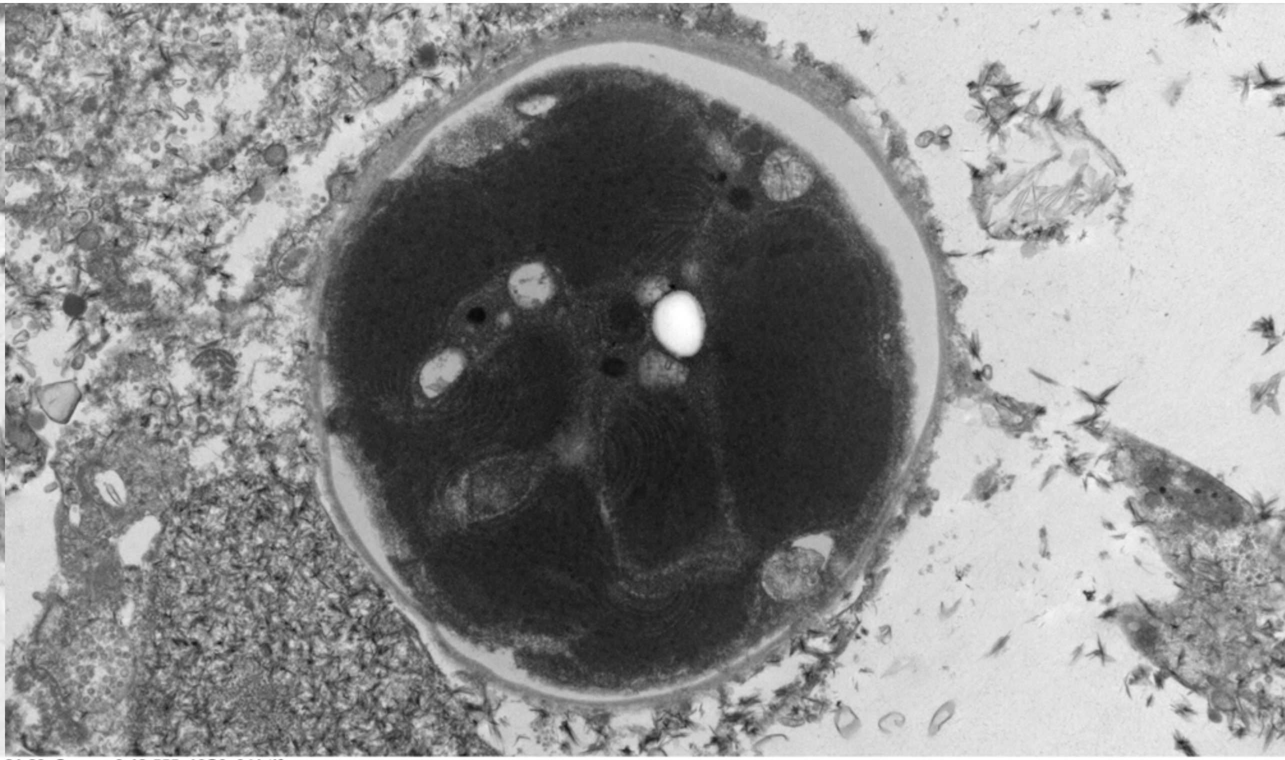

21-20_Correa_3-18-555_16G3_041.tif
Sample: 3/18, 555
Biological Electron Microscopy Lab
Rice University - SEA
Microscopist: MD Meyer

1 μm
HV=80kV
Direct Mag: 3000 x

Cell 13

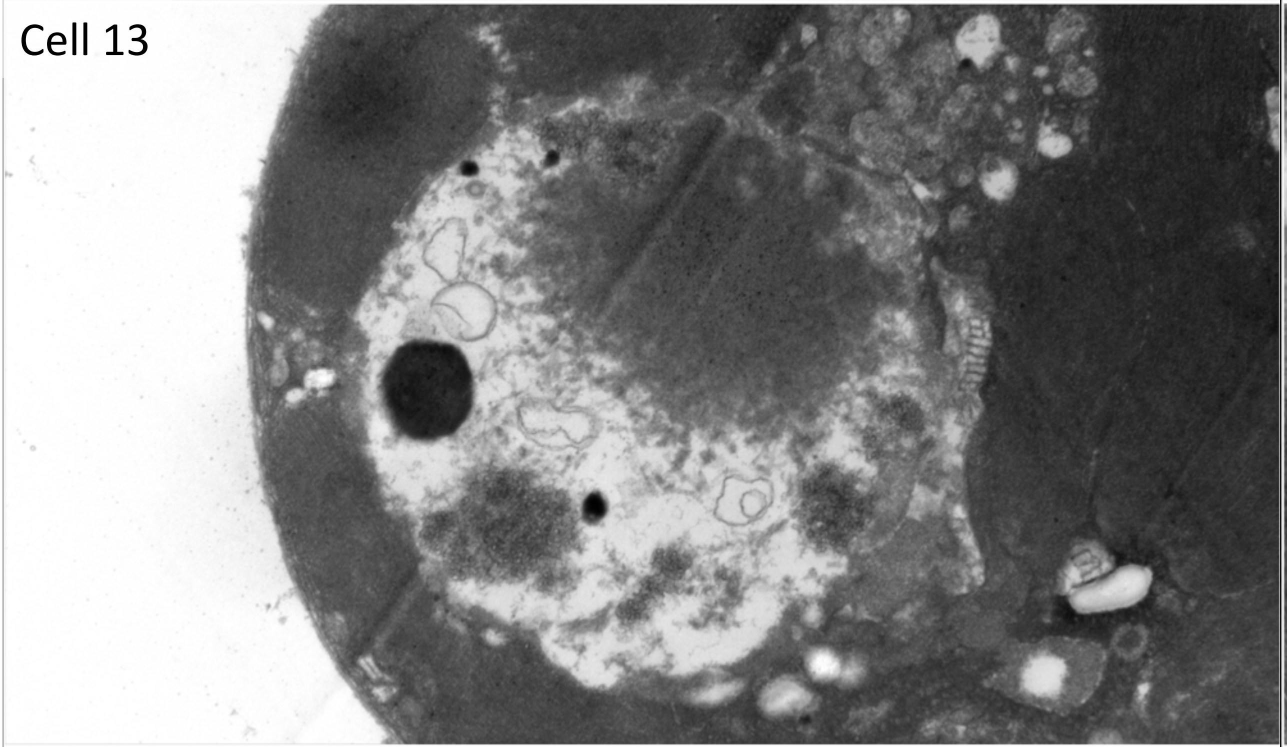

21-20_Correa_3-18-555_16G3_035.tif
Sample: 3/18, 555
Biological Electron Microscopy Lab
Rice University - SEA
Microscopist: MD Meyer

1 µm
HV=80kV
Direct Mag: 4000 x

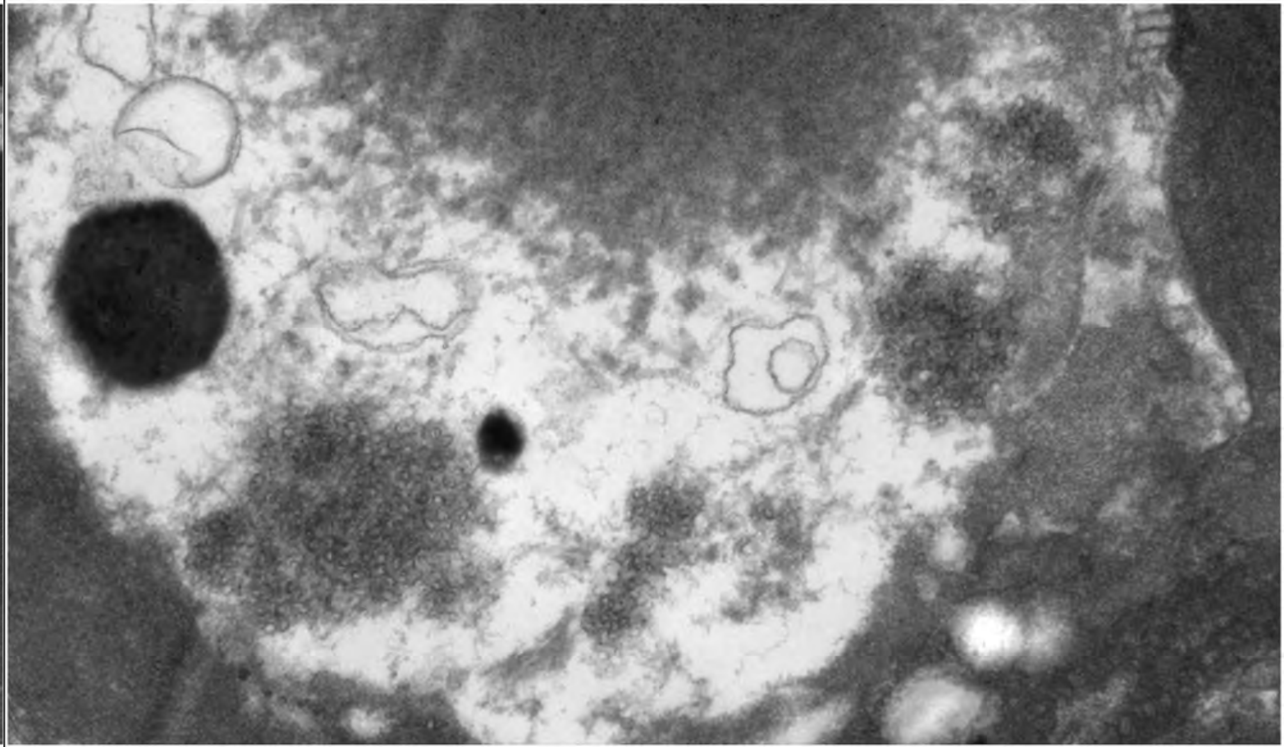

21-20_Correa_3-18-555_16G3_037.tif
Sample: 3/18, 555
Biological Electron Microscopy Lab
Rice University - SEA
Microscopist: MD Meyer

500 nm
HV=80kV
Direct Mag: 8000 x

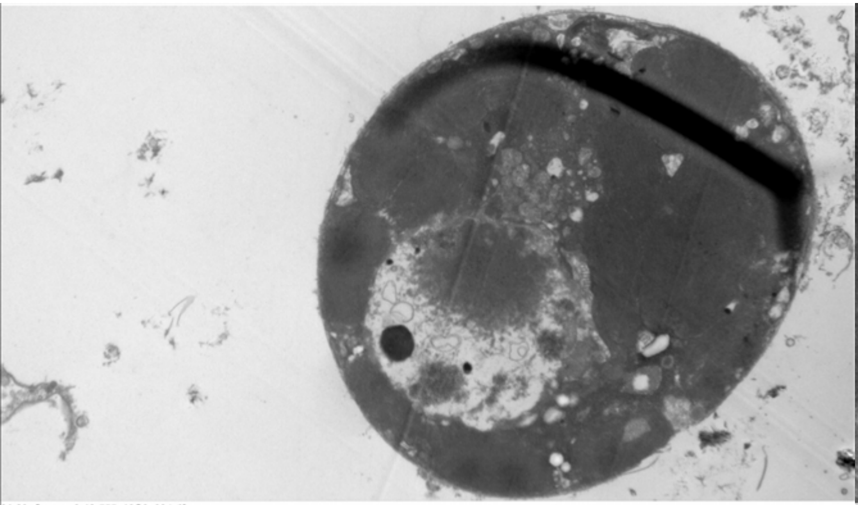

21-20_Correa_3-18-555_16G3_034.tif
Sample: 3/18, 555
Biological Electron Microscopy Lab
Rice University - SEA
Microscopist: MD Meyer

2 µm
HV=80kV
Direct Mag: 2000 x

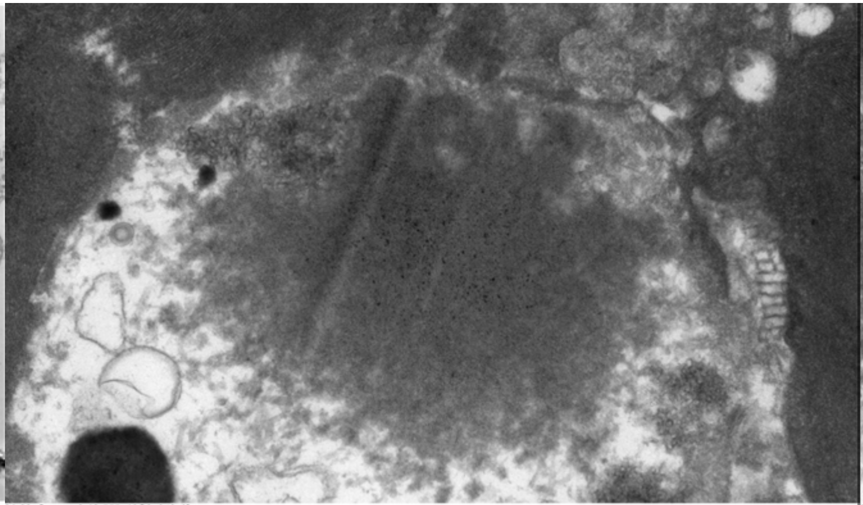

21-20_Correa_3-18-555_16G3_040.tif
Sample: 3/18, 555
Biological Electron Microscopy Lab
Rice University - SEA
Microscopist: MD Meyer

500 nm
HV=80kV
Direct Mag: 8000 x

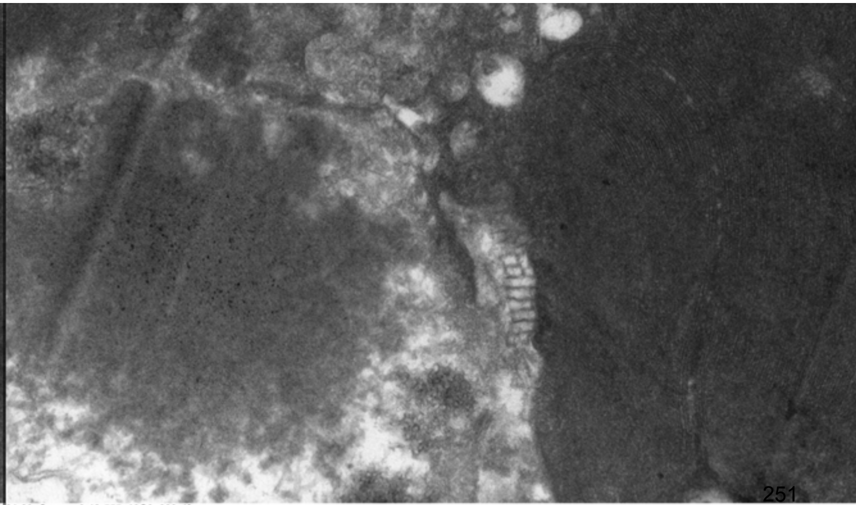

21-20_Correa_3-18-555_16G3_039.tif
Sample: 3/18, 555
Biological Electron Microscopy Lab
Rice University - SEA
Microscopist: MD Meyer

500 nm
HV=80kV
Direct Mag: 8000 x

Cell 14

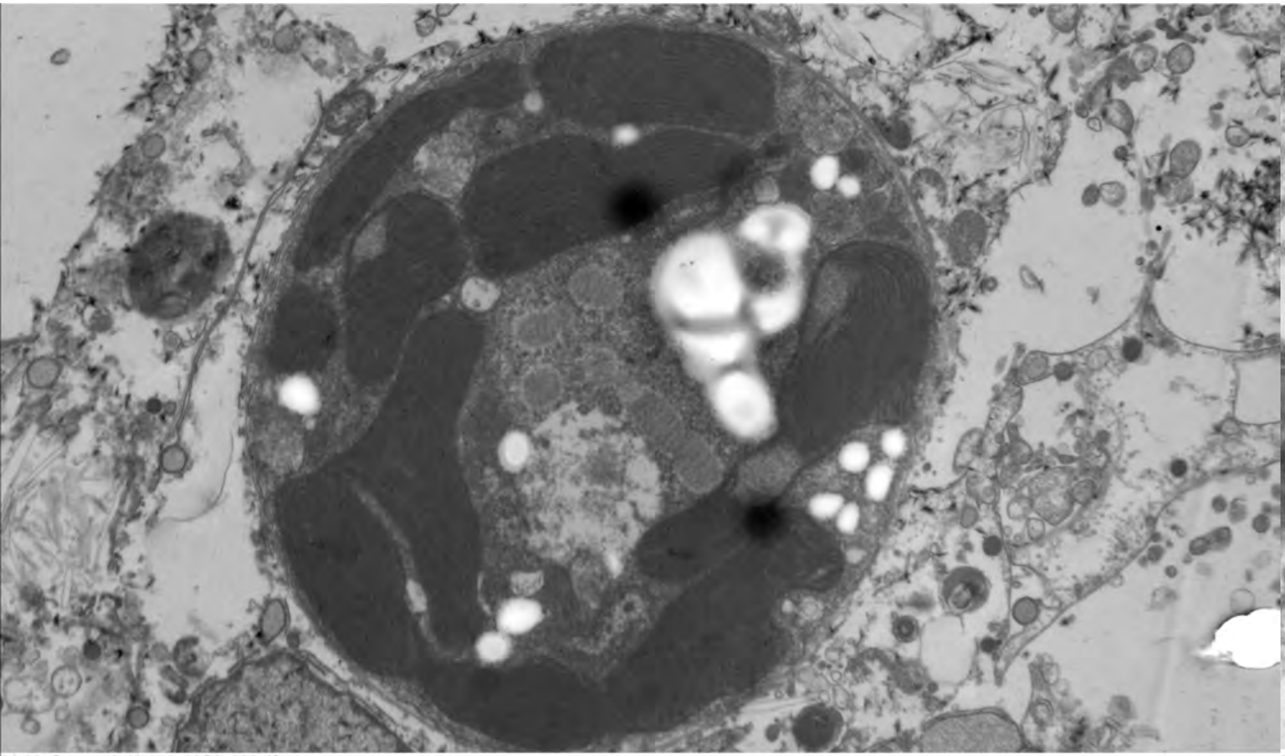

21-20_Correa_3-18-555_16G3_032.tif
Sample: 3/18, 555
Biological Electron Microscopy Lab
Rice University - SEA
Microscopist: MD Meyer

2 µm
HV=80kV
Direct Mag: 2000 x

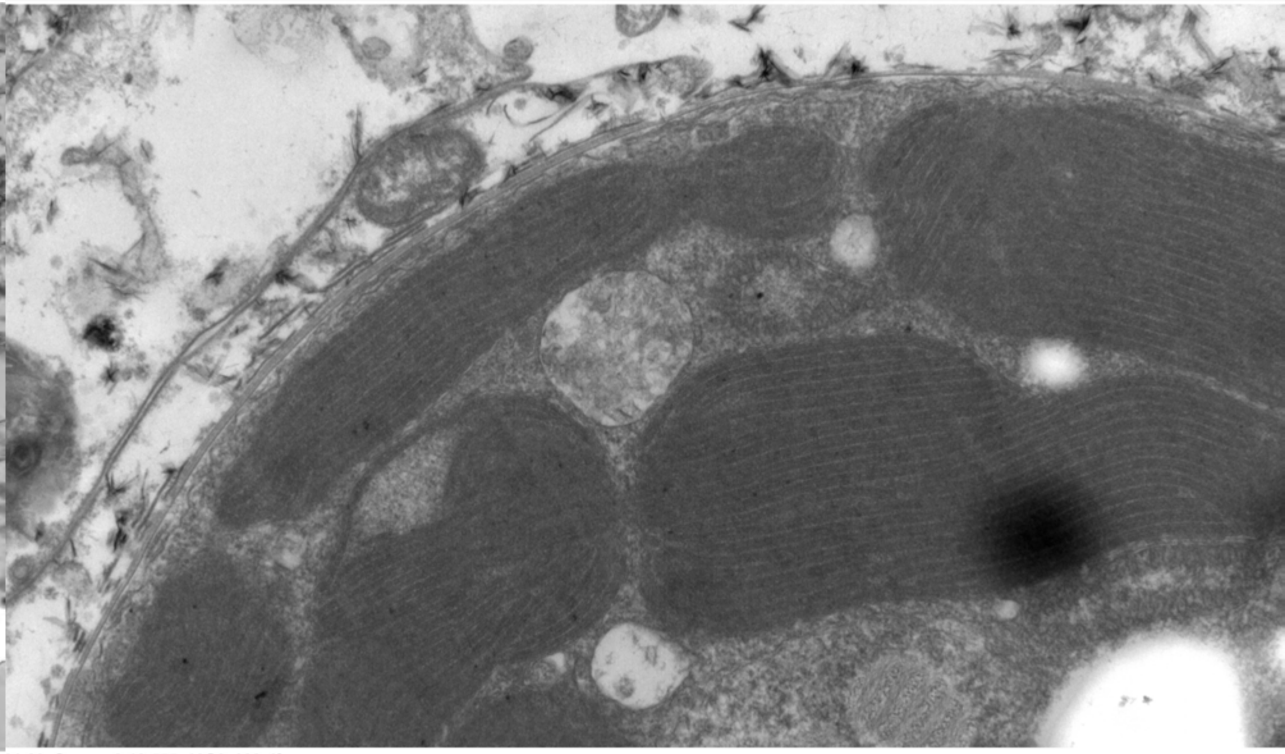

21-20_Correa_3-18-555_16G3_033.tif
Sample: 3/18, 555
Biological Electron Microscopy Lab
Rice University - SEA
Microscopist: MD Meyer

800 nm
HV=80kV
Direct Mag: 5000 x

Cell 15

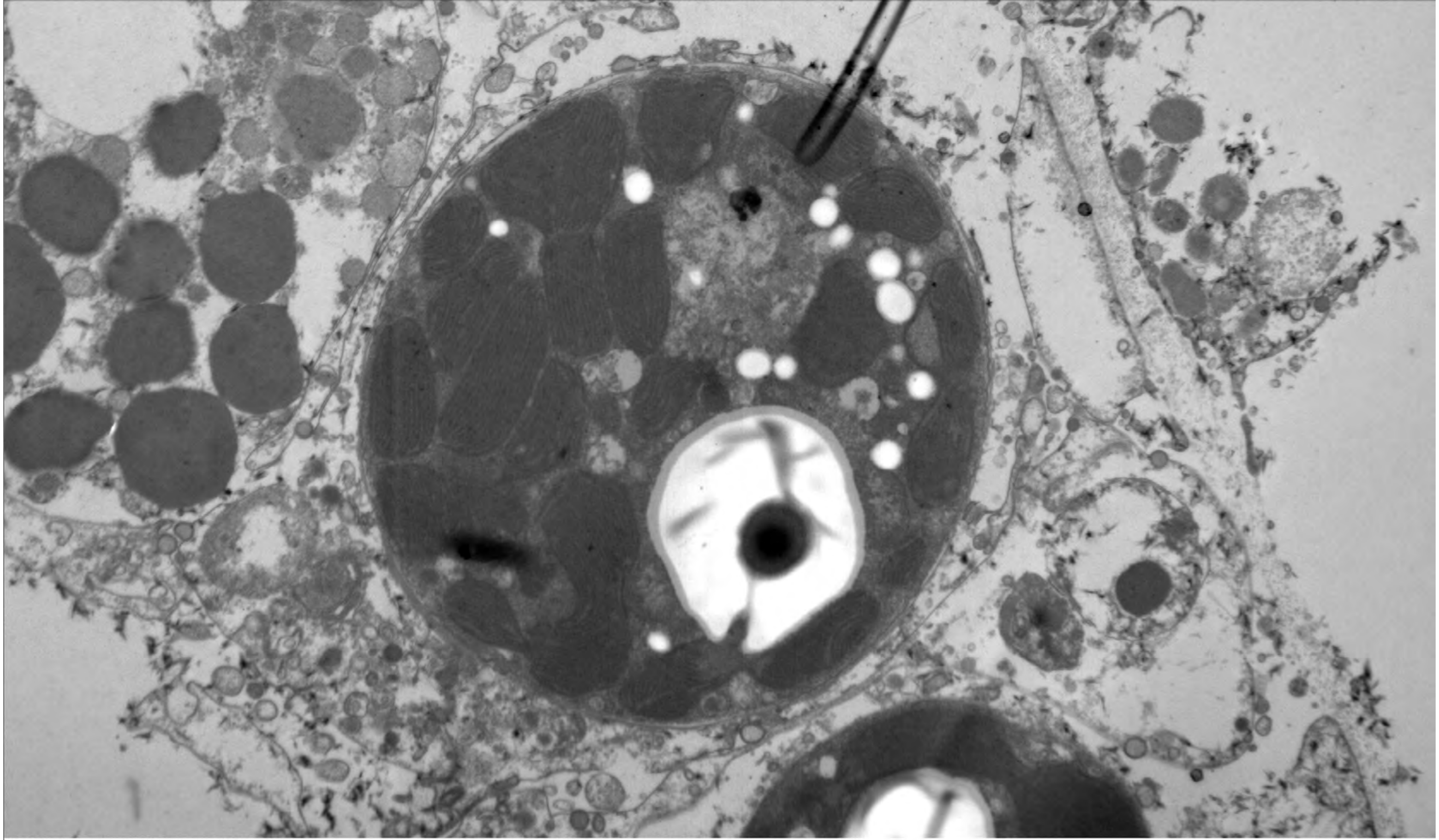

21-20_Correa_3-18-555_16G3_029.tif
Sample: 3/18, 555
Biological Electron Microscopy Lab
Rice University - SEA
Microscopist: MD Meyer

2 µm
HV=80kV
Direct Mag: 1500 x

Cell 16

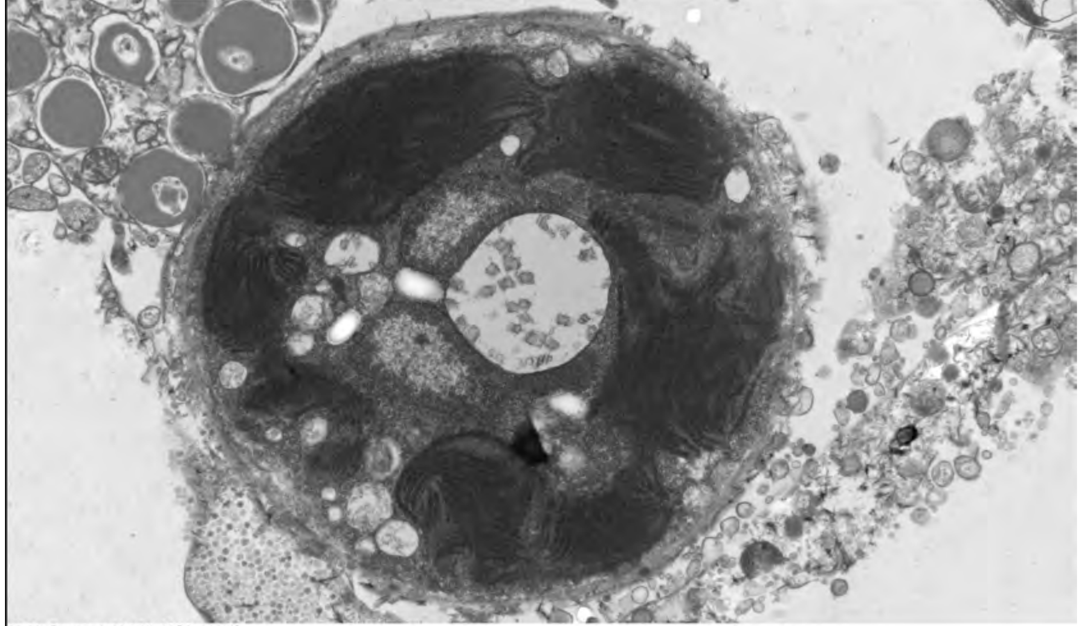

21-20_Correa_3-18-555_16G3_026.tif
Sample: 3/18, 555
Biological Electron Microscopy Lab
Rice University - SEA
Microscopist: MD Meyer

1 µm
HV=80kV
Direct Mag: 3000 x

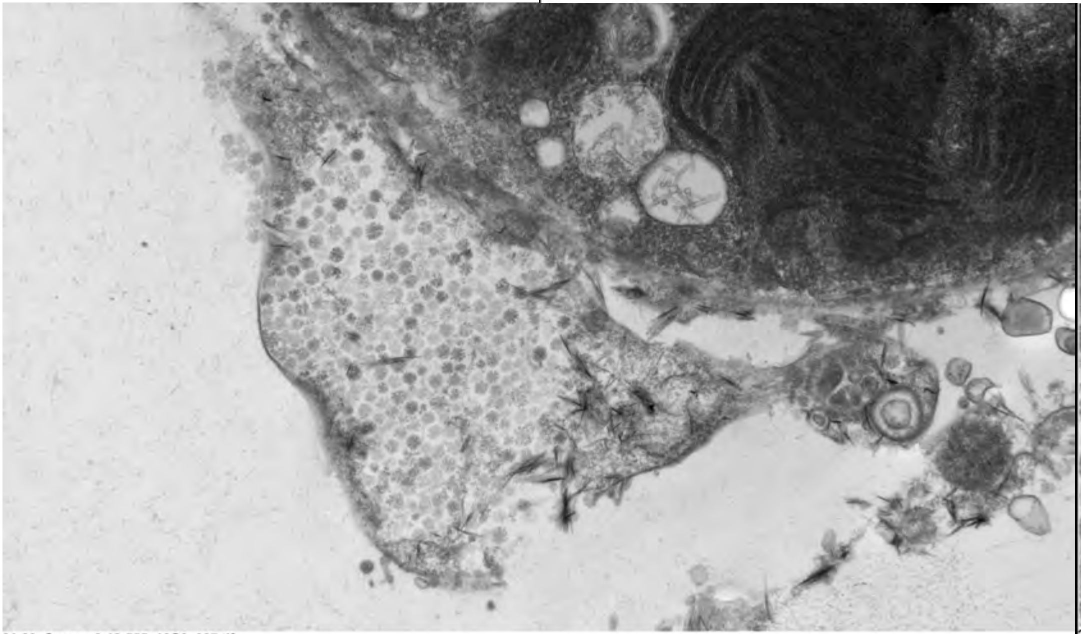

21-20_Correa_3-18-555_16G3_027.tif
Sample: 3/18, 555
Biological Electron Microscopy Lab
Rice University - SEA
Microscopist: MD Meyer

600 nm
HV=80kV
Direct Mag: 6000 x

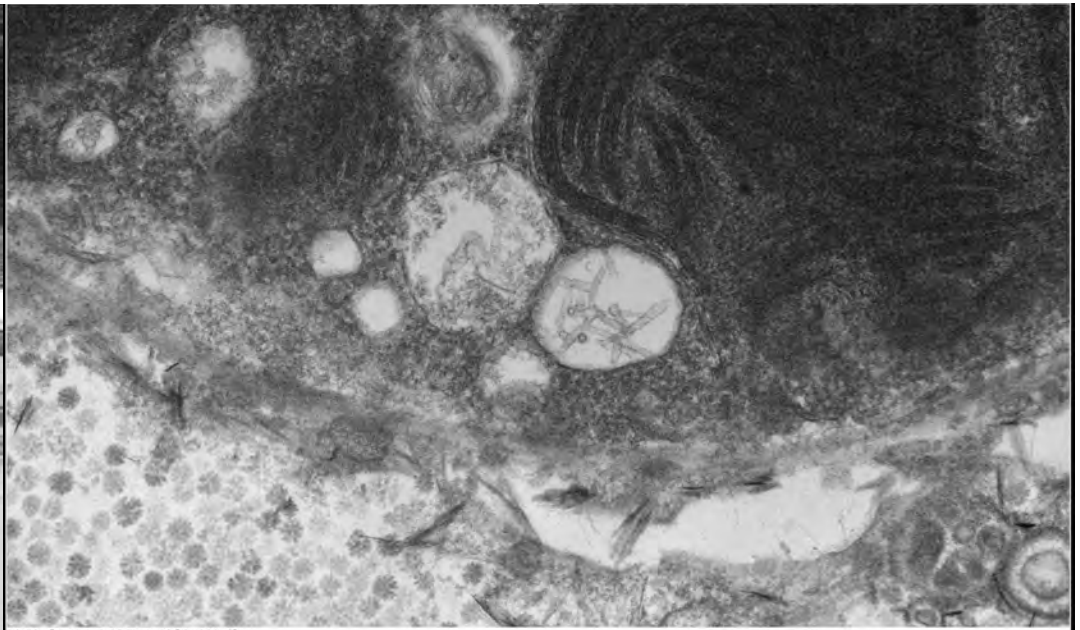

21-20_Correa_3-18-555_16G3_028.tif
Sample: 3/18, 555
Biological Electron Microscopy Lab
Rice University - SEA
Microscopist: MD Meyer

400 nm
HV=80kV
Direct Mag: 10000 x

Cell 17

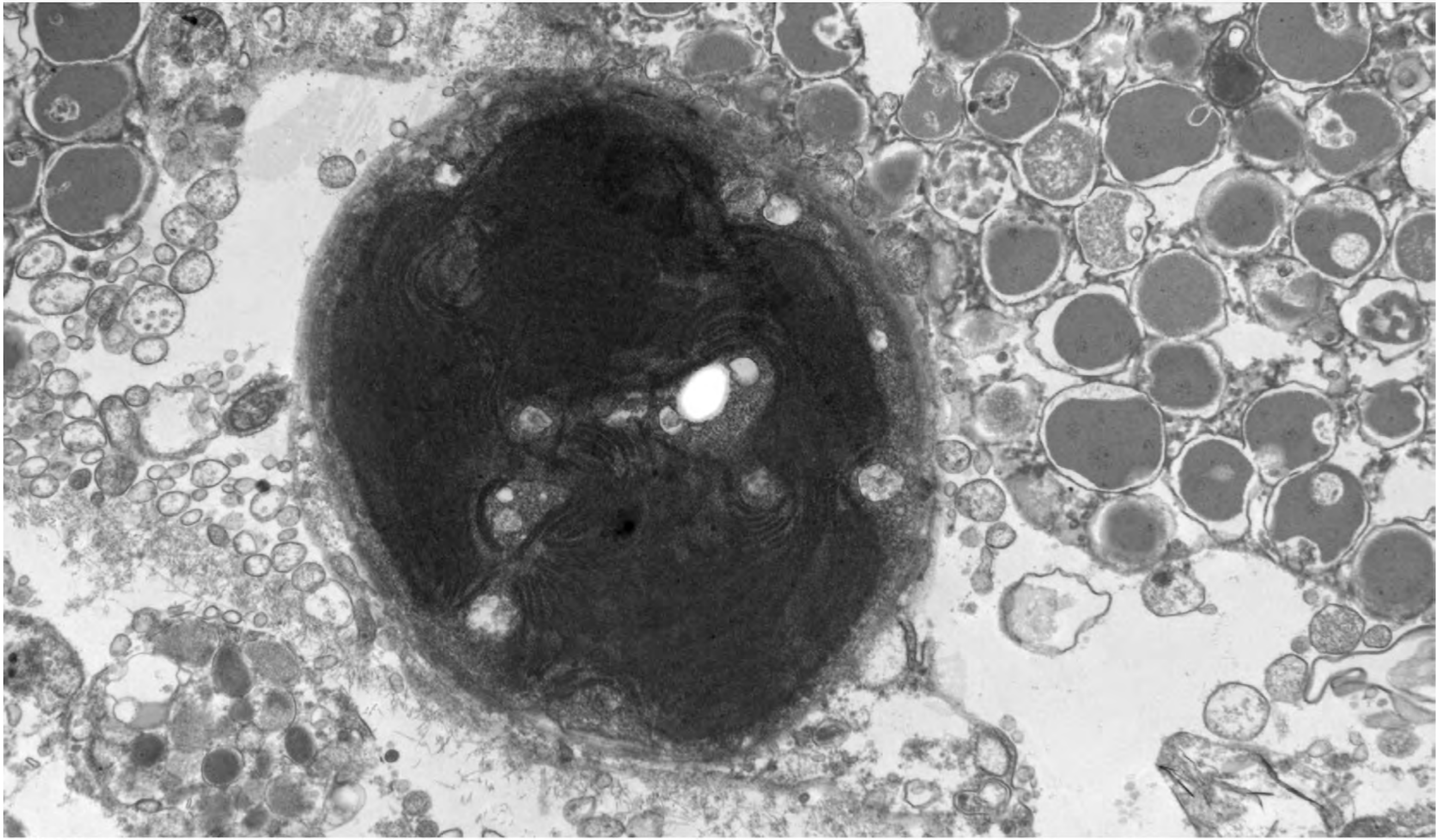

21-20_Correa_3-18-555_16G3_025.tif
Sample: 3/18, 555
Biological Electron Microscopy Lab
Rice University - SEA
Microscopist: MD Meyer

1 µm
HV=80kV
Direct Mag: 3000 x

Cell 18

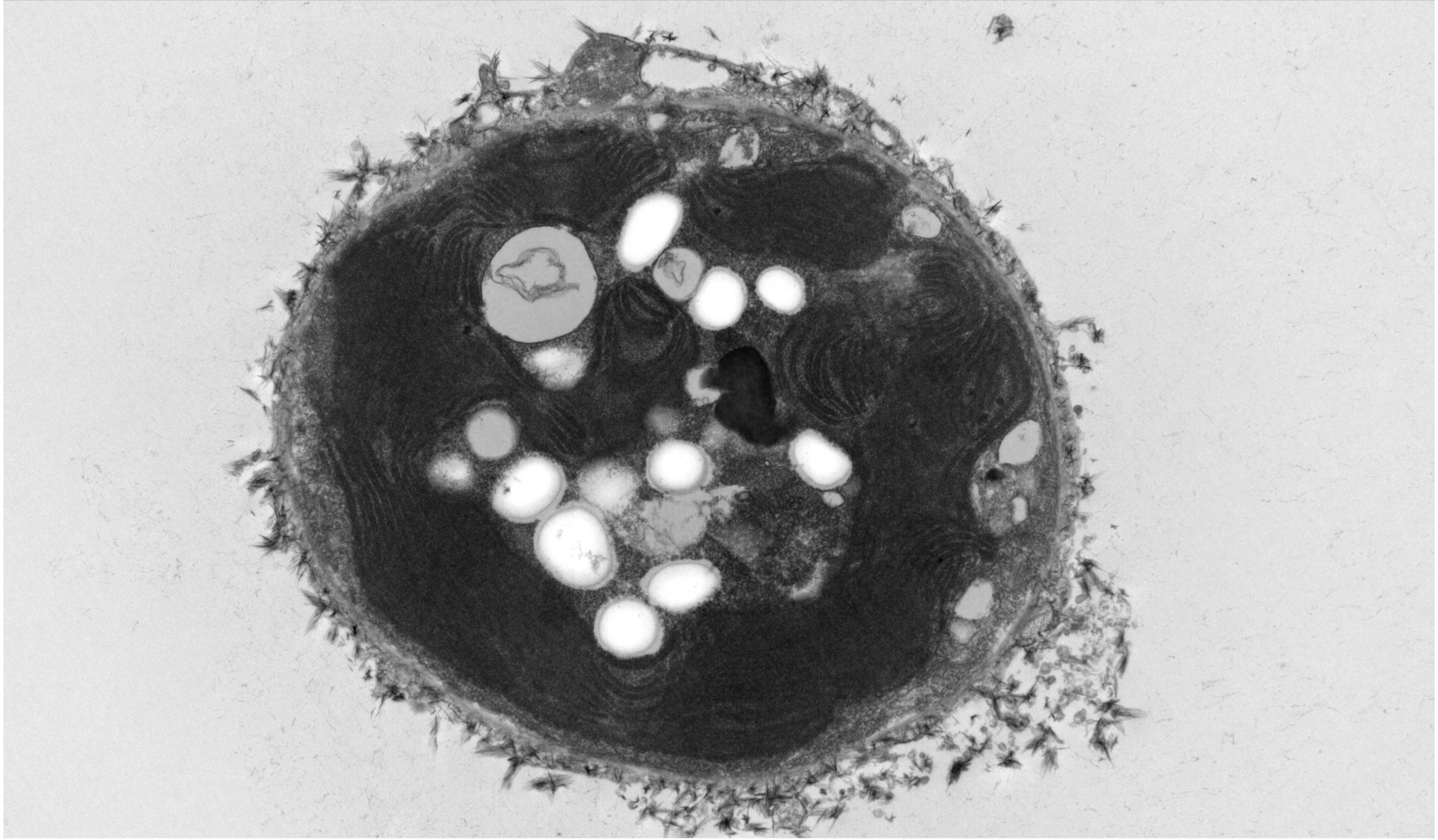

21-20_Correa_3-18-555_16G3_024.tif
Sample: 3/18, 555
Biological Electron Microscopy Lab
Rice University - SEA
Microscopist: MD Meyer

1 µm
HV=80kV
Direct Mag: 3000 x

Cell 19

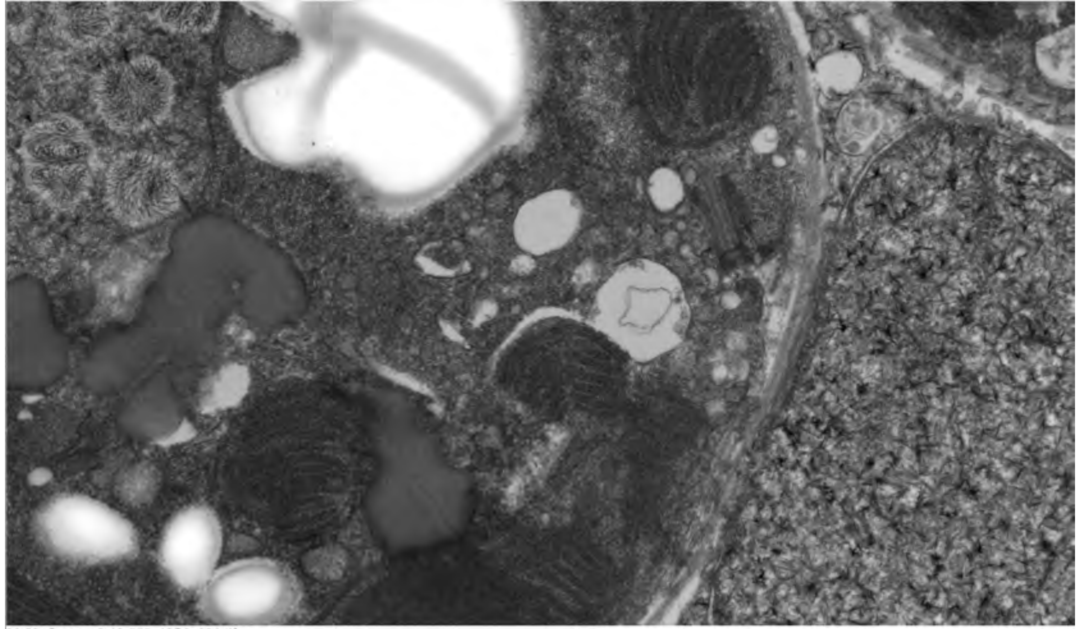

21-20_Correa_3-18-555_16G3_021.tif
Sample: 3/18, 555
Biological Electron Microscopy Lab
Rice University - SEA
Microscopist: MD Meyer

800 nm
HV=80kV
Direct Mag: 5000 x

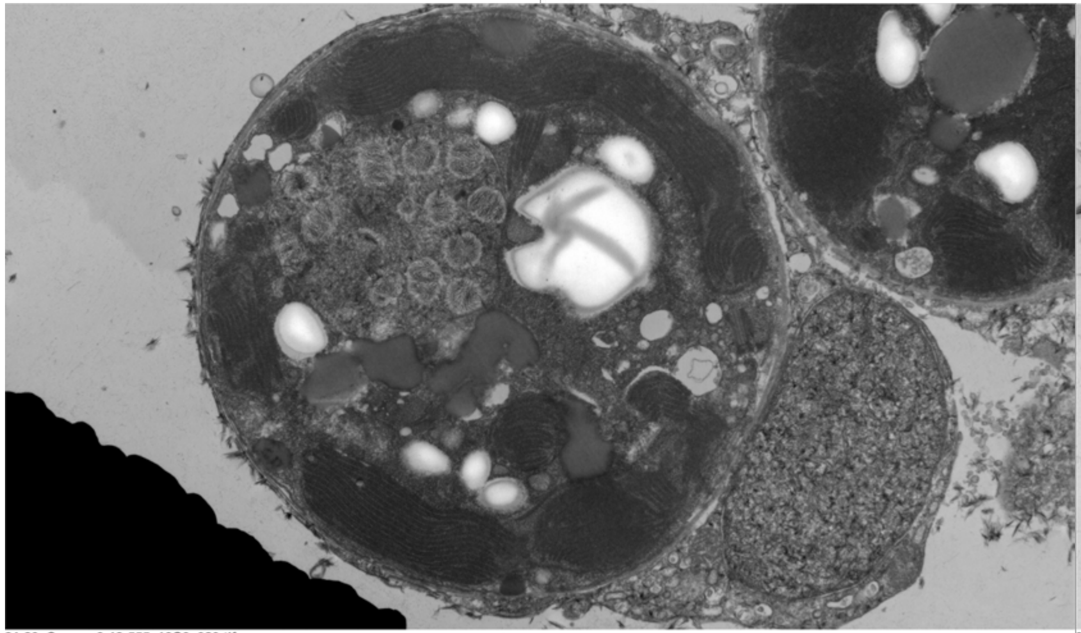

21-20_Correa_3-18-555_16G3_020.tif
Sample: 3/18, 555
Biological Electron Microscopy Lab
Rice University - SEA
Microscopist: MD Meyer

1 µm
HV=80kV
Direct Mag: 2500 x

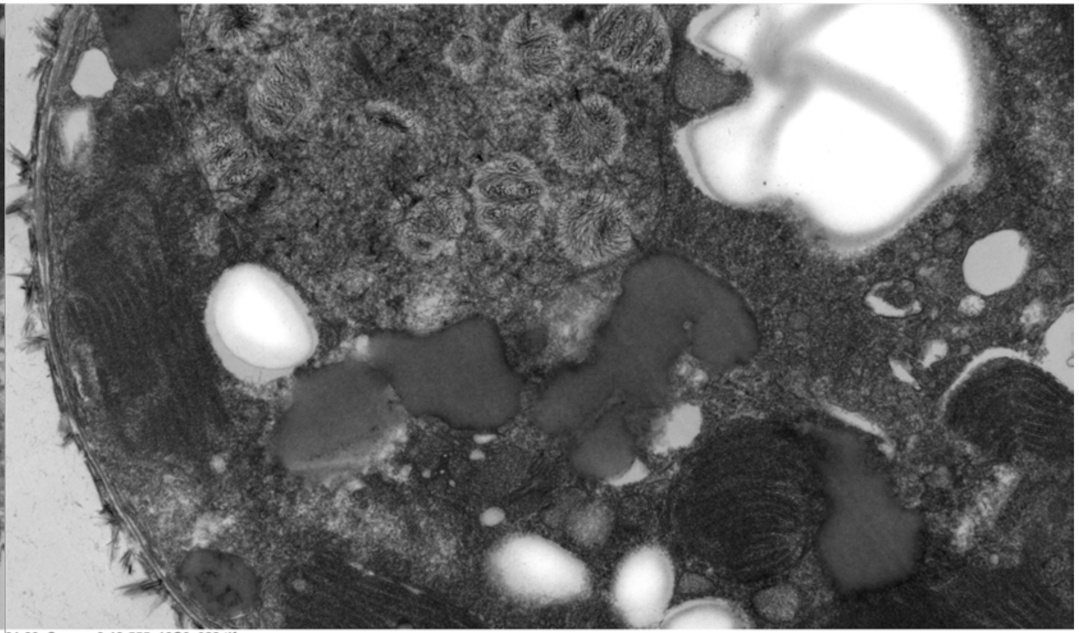

21-20_Correa_3-18-555_16G3_022.tif
Sample: 3/18, 555
Biological Electron Microscopy Lab
Rice University - SEA
Microscopist: MD Meyer

800 nm
HV=80kV
Direct Mag: 5000 x

Cell 20

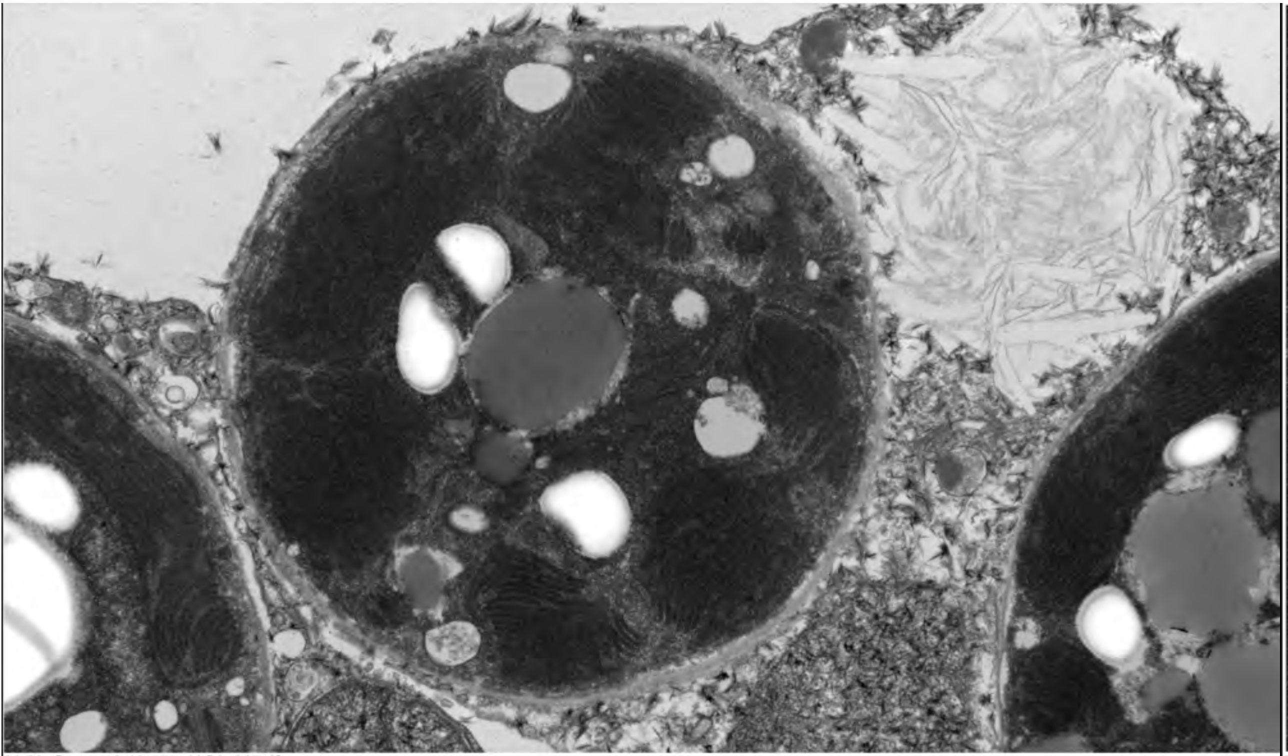

21-20_Correa_3-18-555_16G3_017.tif
Sample: 3/18, 555
Biological Electron Microscopy Lab
Rice University - SEA
Microscopist: MD Meyer

1 µm
HV=80kV
Direct Mag: 3000 x

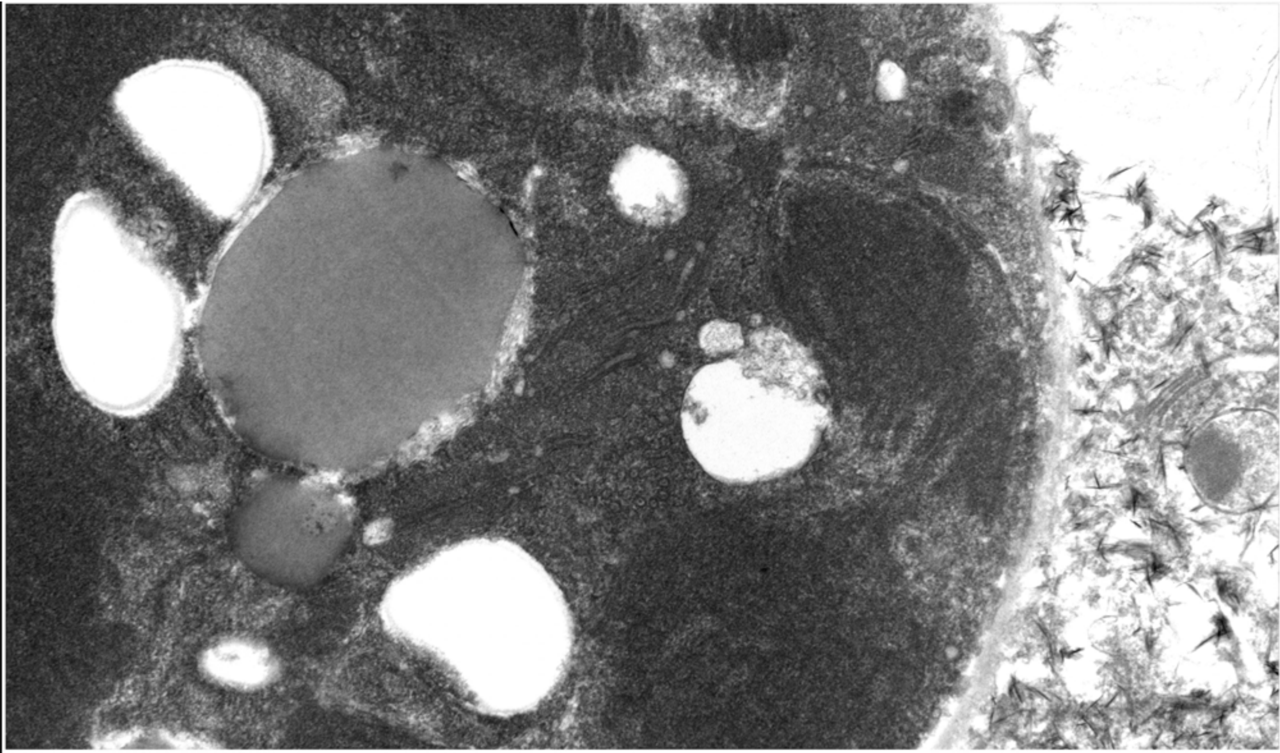

21-20_Correa_3-18-555_16G3_019.tif
Sample: 3/18, 555
Biological Electron Microscopy Lab
Rice University - SEA
Microscopist: MD Meyer

600 nm
HV=80kV
Direct Mag: 6000 x

Cell 21

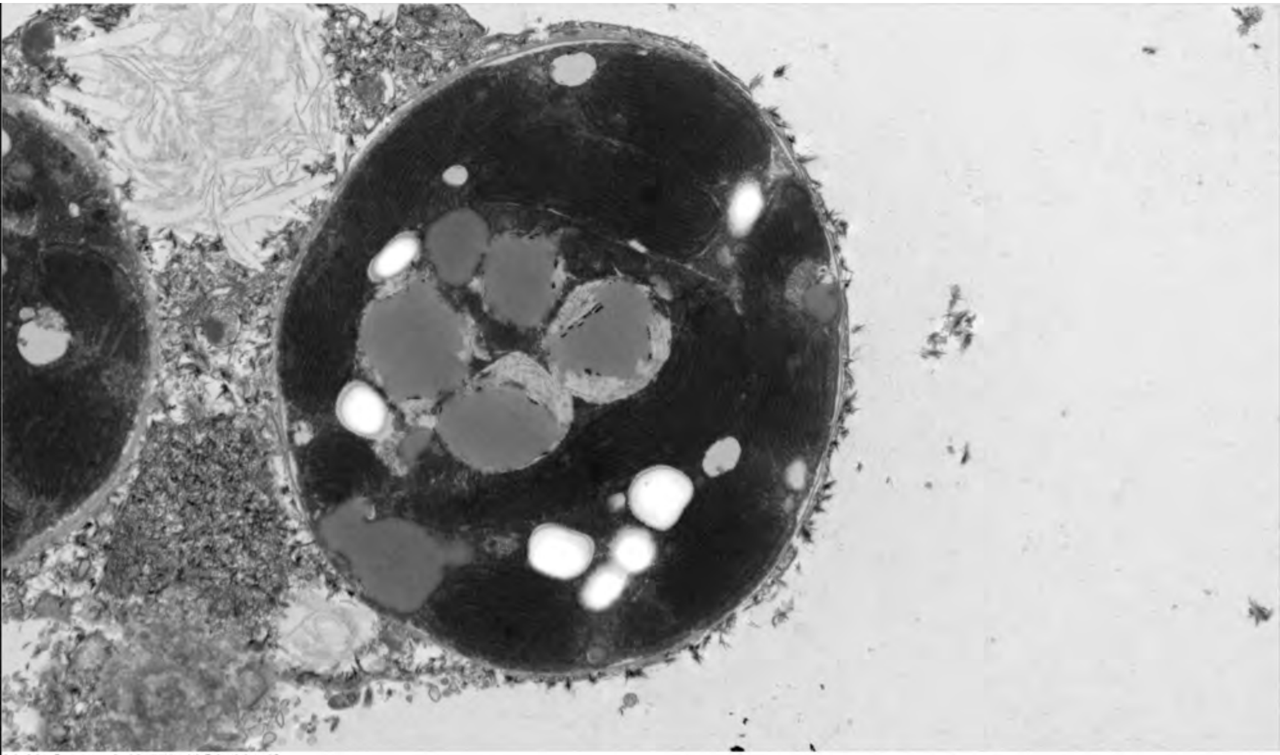

21-20_Correa_3-18-555_16G3_014.tif
Sample: 3/18, 555
Biological Electron Microscopy Lab
Rice University - SEA
Microscopist: MD Meyer

2 µm
HV=80kV
Direct Mag: 2000 x

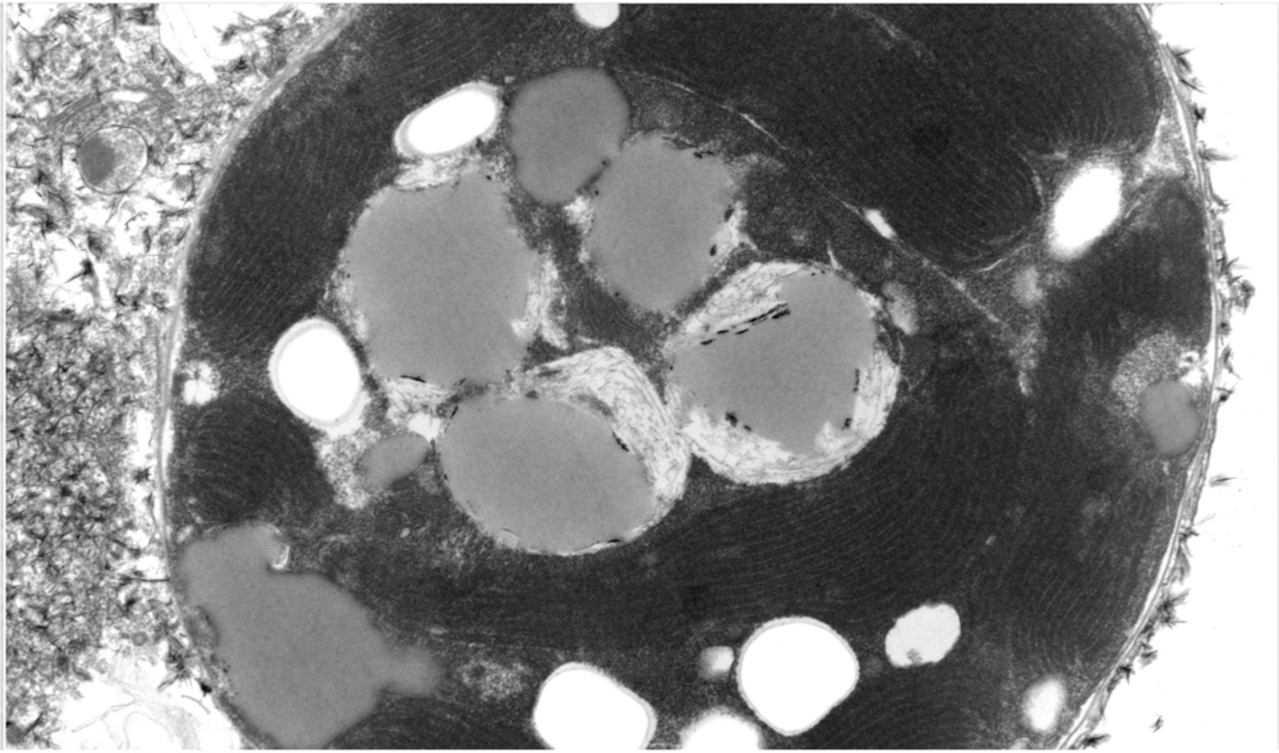

21-20_Correa_3-18-555_16G3_016.tif
Sample: 3/18, 555
Biological Electron Microscopy Lab
Rice University - SEA
Microscopist: MD Meyer

1 µm
HV=80kV
Direct Mag: 4000 x

Cell 22

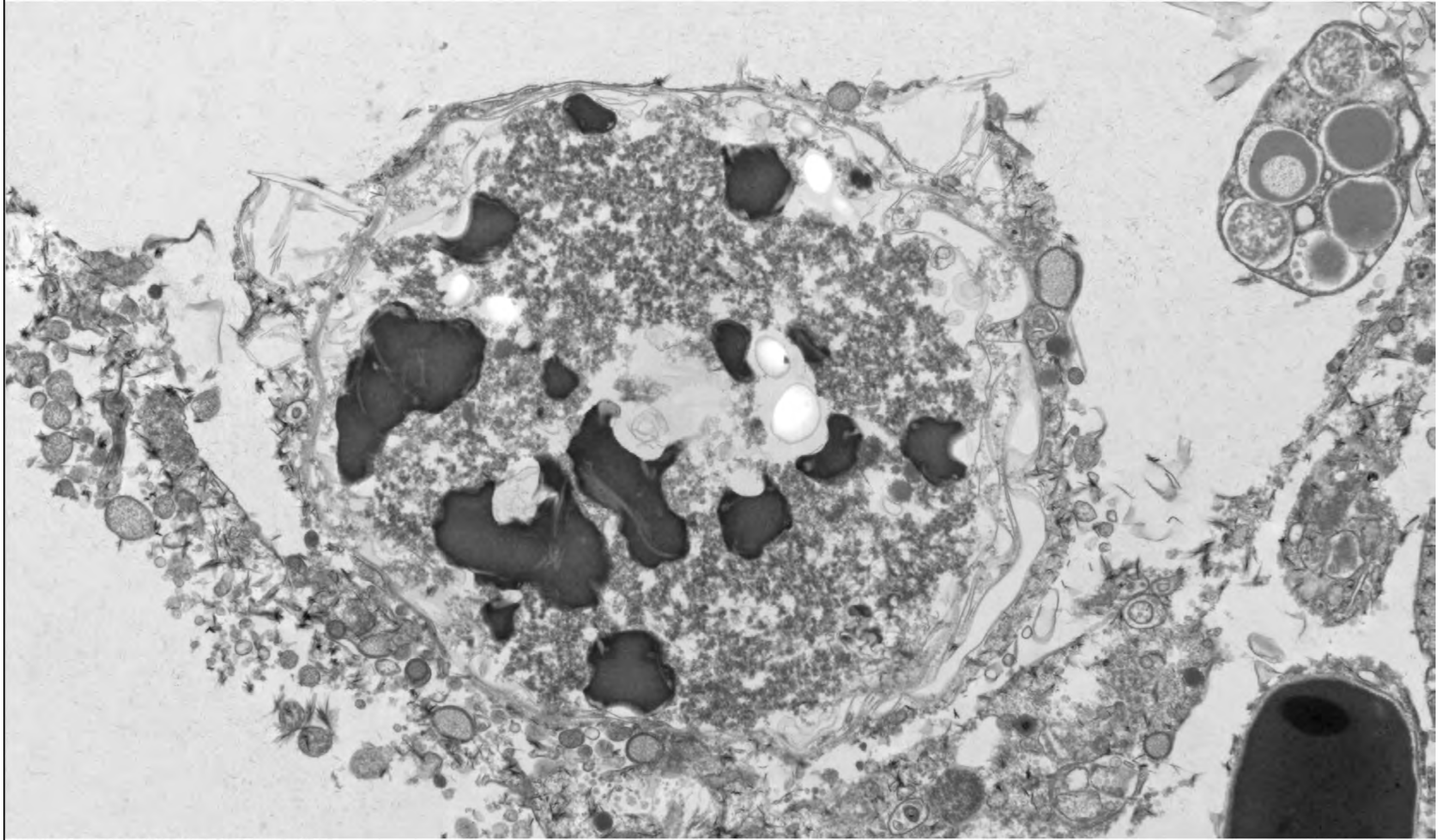

21-20_Correa_3-18-555_16G3_012.tif
Sample: 3/18, 555
Biological Electron Microscopy Lab
Rice University - SEA
Microscopist: MD Meyer

1 µm
HV=80kV
Direct Mag: 2500 x

Cell 23

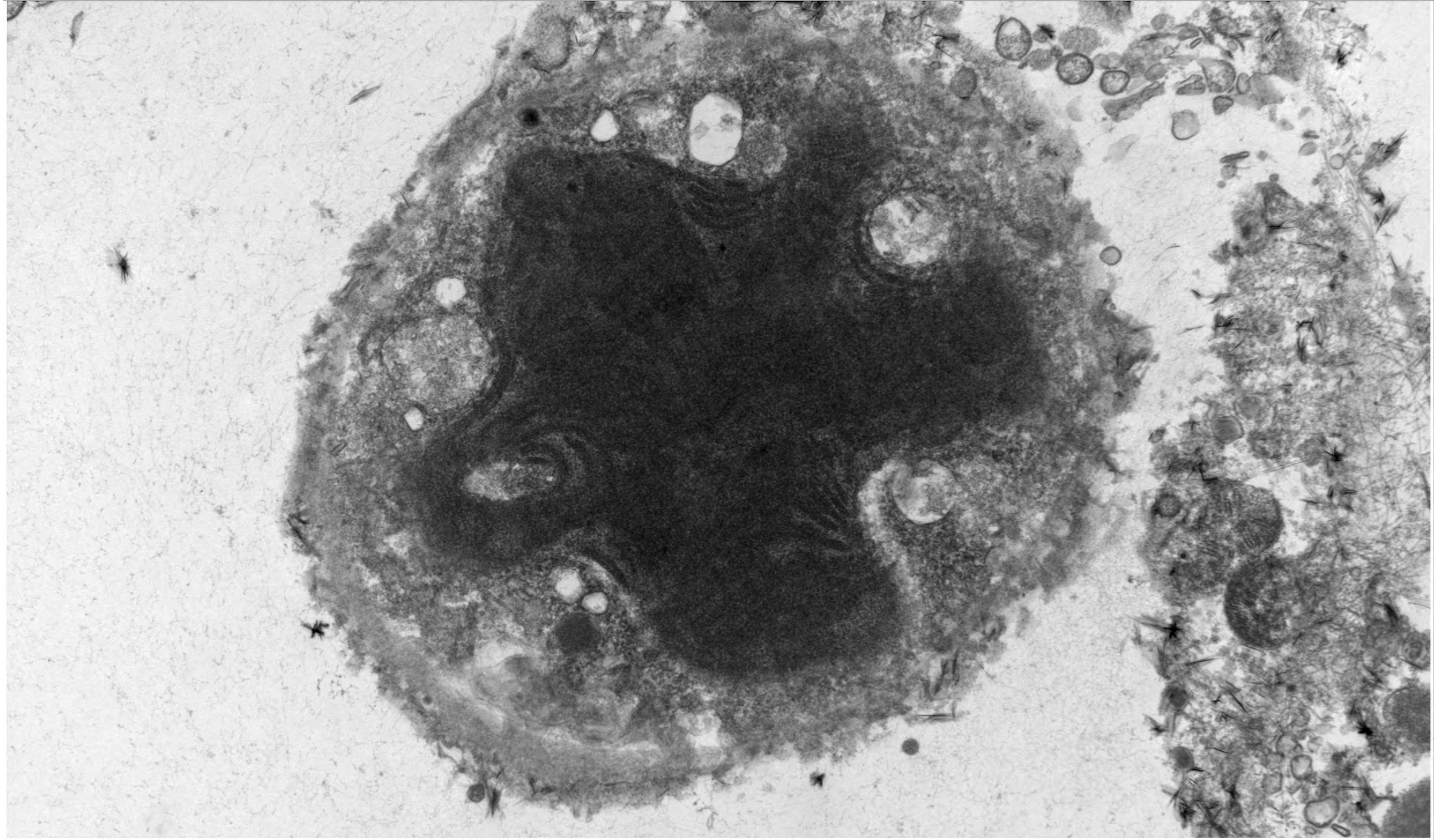

21-20_Correa_3-18-555_16G3_011.tif
Sample: 3/18, 555
Biological Electron Microscopy Lab
Rice University - SEA
Microscopist: MD Meyer

1 µm
HV=80kV
Direct Mag: 4000 x

Cell 24

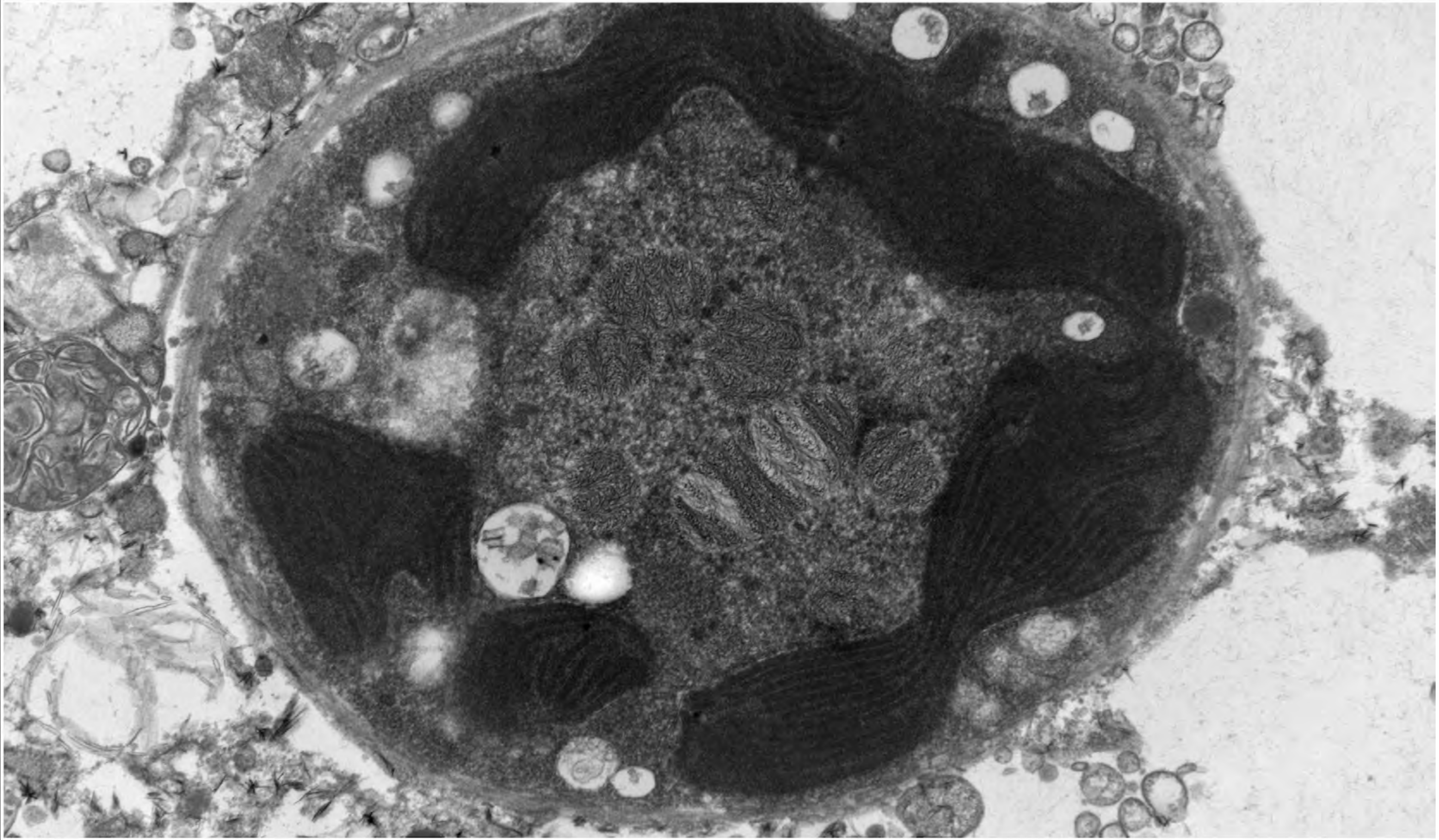

21-20_Correa_3-18-555_16G3_010.tif
Sample: 3/18, 555
Biological Electron Microscopy Lab
Rice University - SEA
Microscopist: MD Meyer

1 µm
HV=80kV
Direct Mag: 4000 x

Cell 25

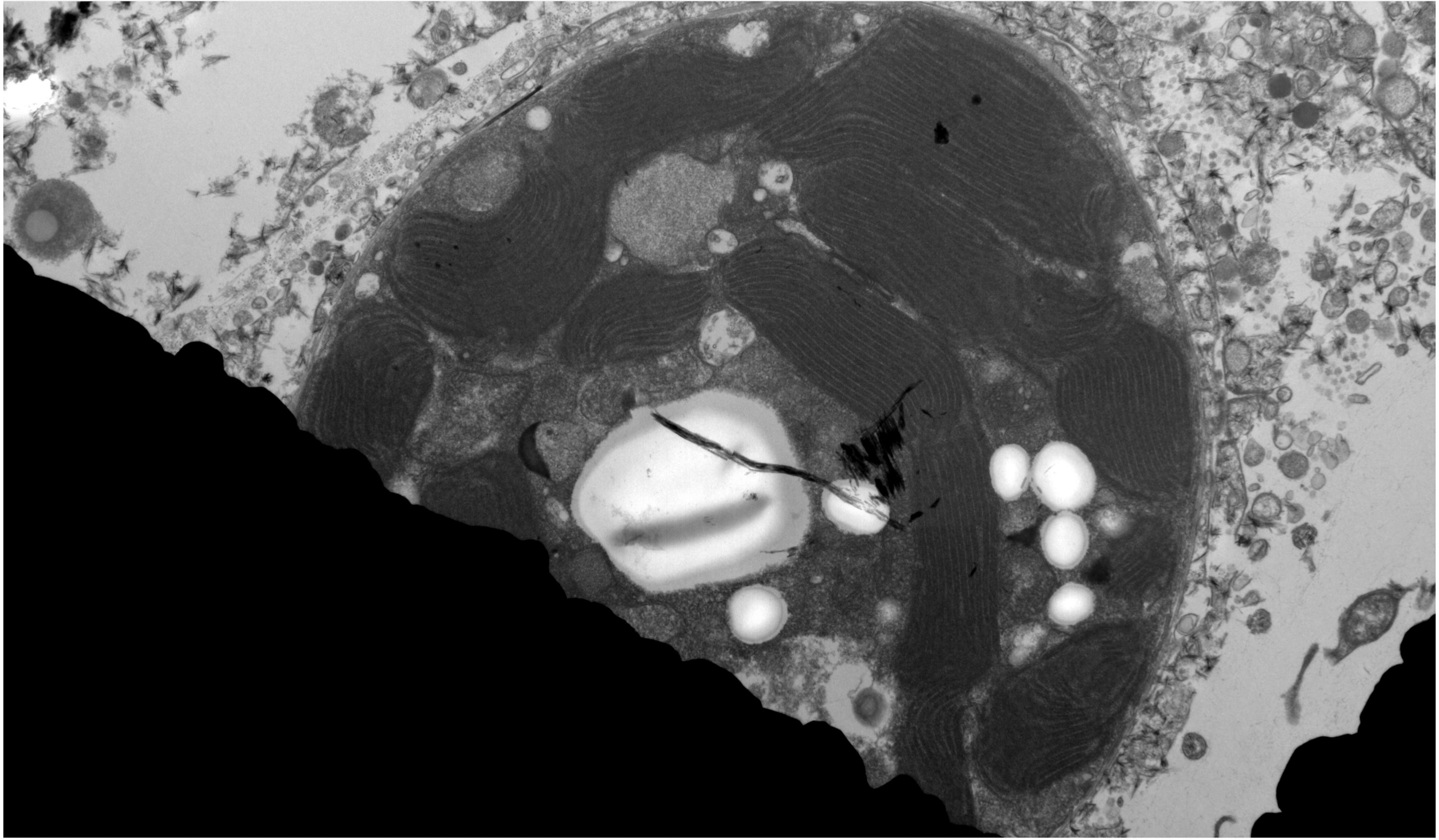

21-20_Correa_3-18-555_16G3_008.tif
Sample: 3/18, 555
Biological Electron Microscopy Lab
Rice University - SEA
Microscopist: MD Meyer

1 µm
HV=80kV
Direct Mag: 2500 x

Cell 26

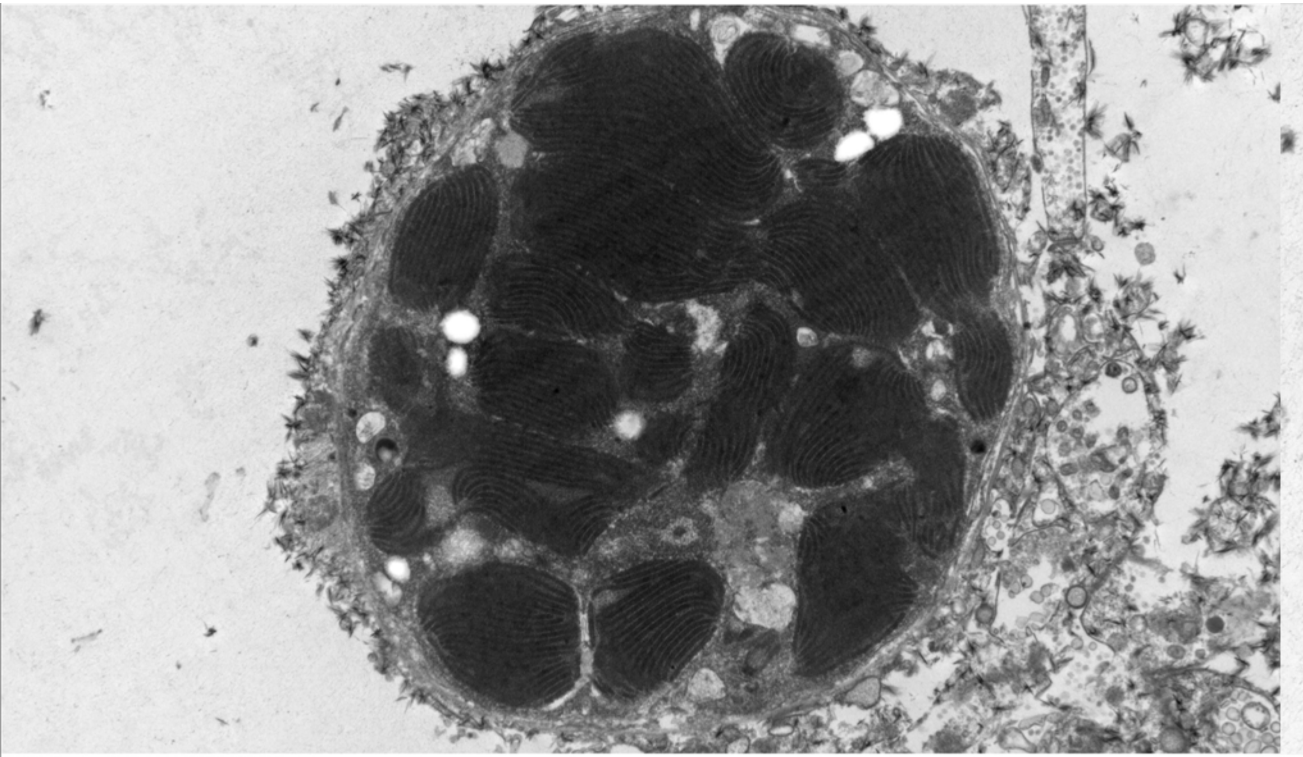

21-20_Correa_3-18-555_16G3_006.tif
Sample: 3/18, 555
Biological Electron Microscopy Lab
Rice University - SEA
Microscopist: MD Meyer

1 µm
HV=80kV
Direct Mag: 2500 x

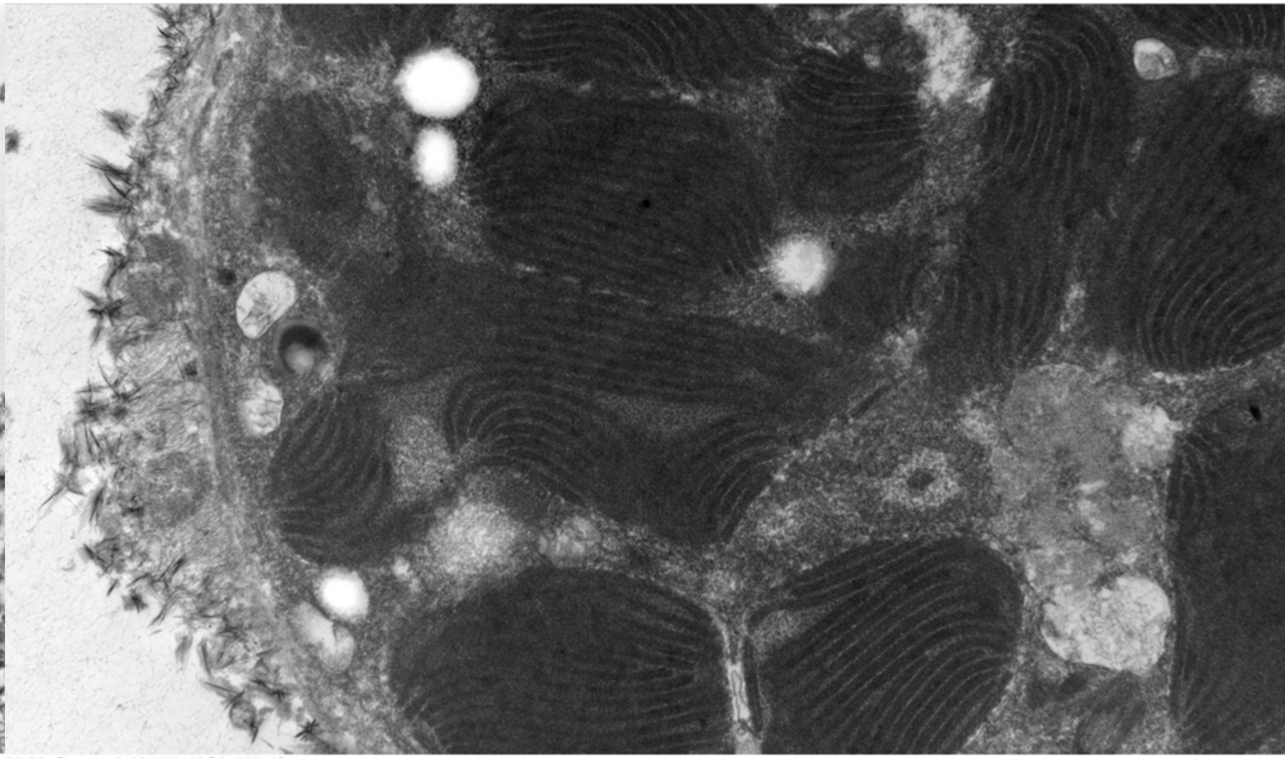

21-20_Correa_3-18-555_16G3_007.tif
Sample: 3/18, 555
Biological Electron Microscopy Lab
Rice University - SEA
Microscopist: MD Meyer

800 nm
HV=80kV
Direct Mag: 5000 x

Cell 27

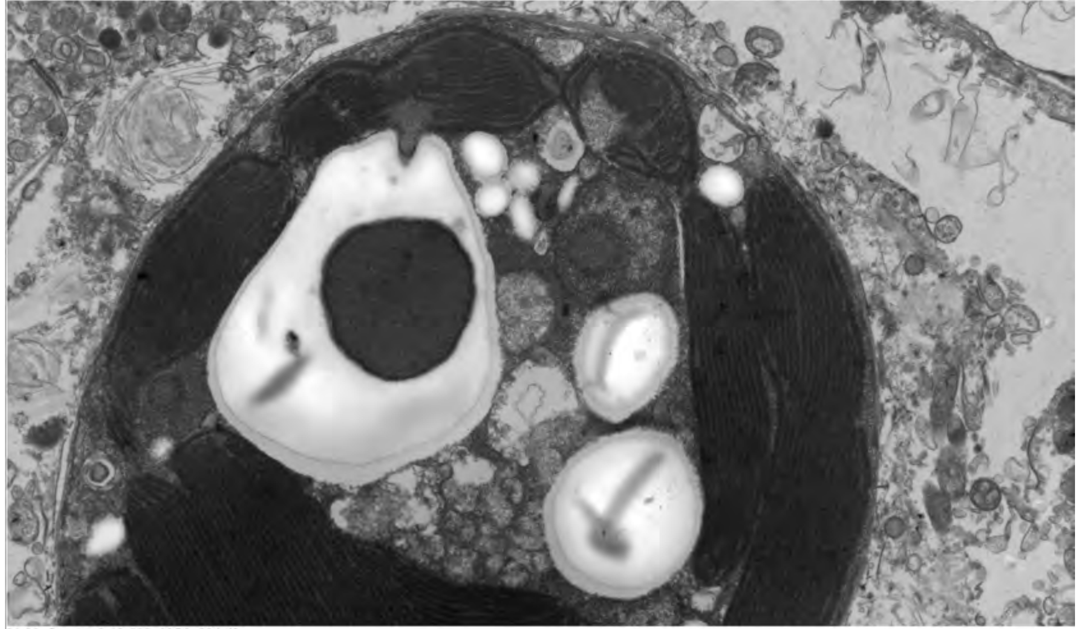
21-20_Correa_3-18-555_16G3_004.tif
Sample: 3/18, 555
Biological Electron Microscopy Lab
Rice University - SEA
Microscopist: MD Meyer
1 µm
HV=80kV
Direct Mag: 3000 x

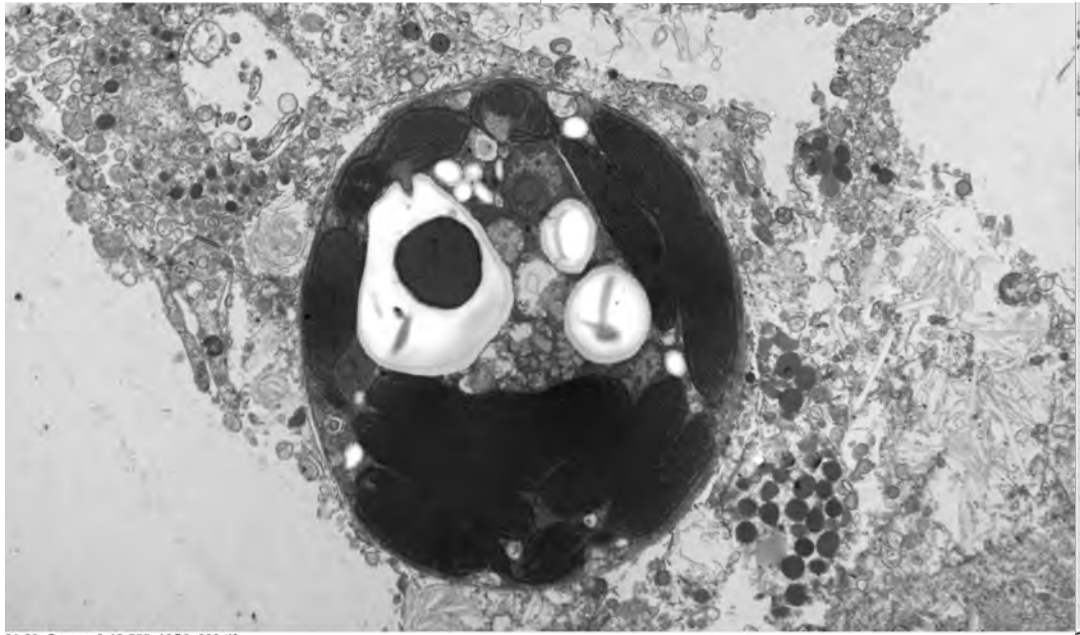
21-20_Correa_3-18-555_16G3_003.tif
Sample: 3/18, 555
Biological Electron Microscopy Lab
Rice University - SEA
Microscopist: MD Meyer
2 µm
HV=80kV
Direct Mag: 1500 x

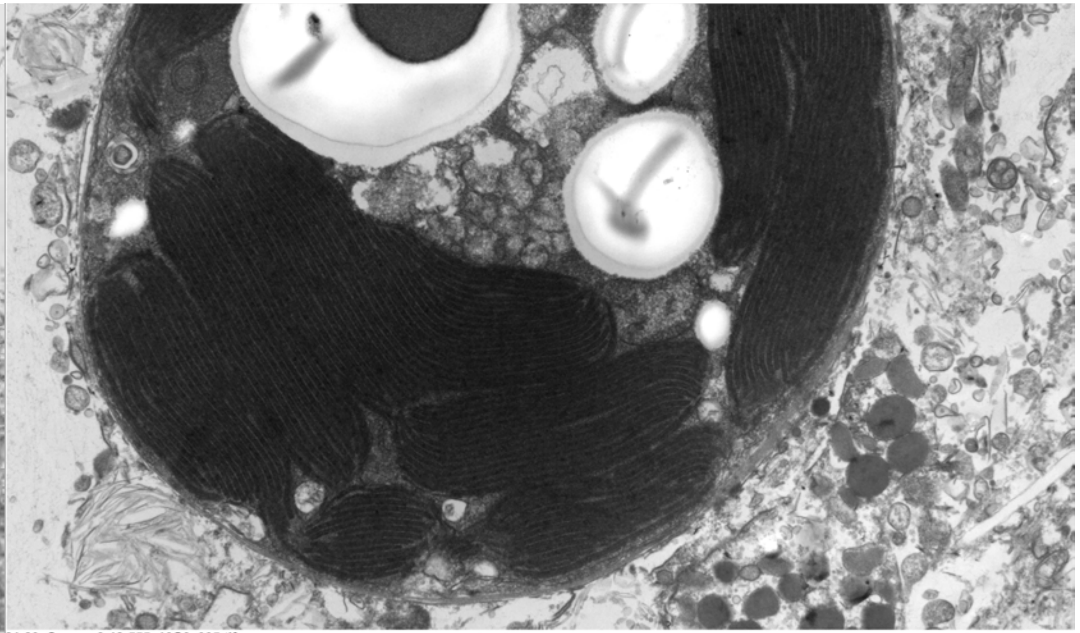
21-20_Correa_3-18-555_16G3_005.tif
Sample: 3/18, 555
Biological Electron Microscopy Lab
Rice University - SEA
Microscopist: MD Meyer
1 µm
HV=80kV
Direct Mag: 3000 x

Cell 28

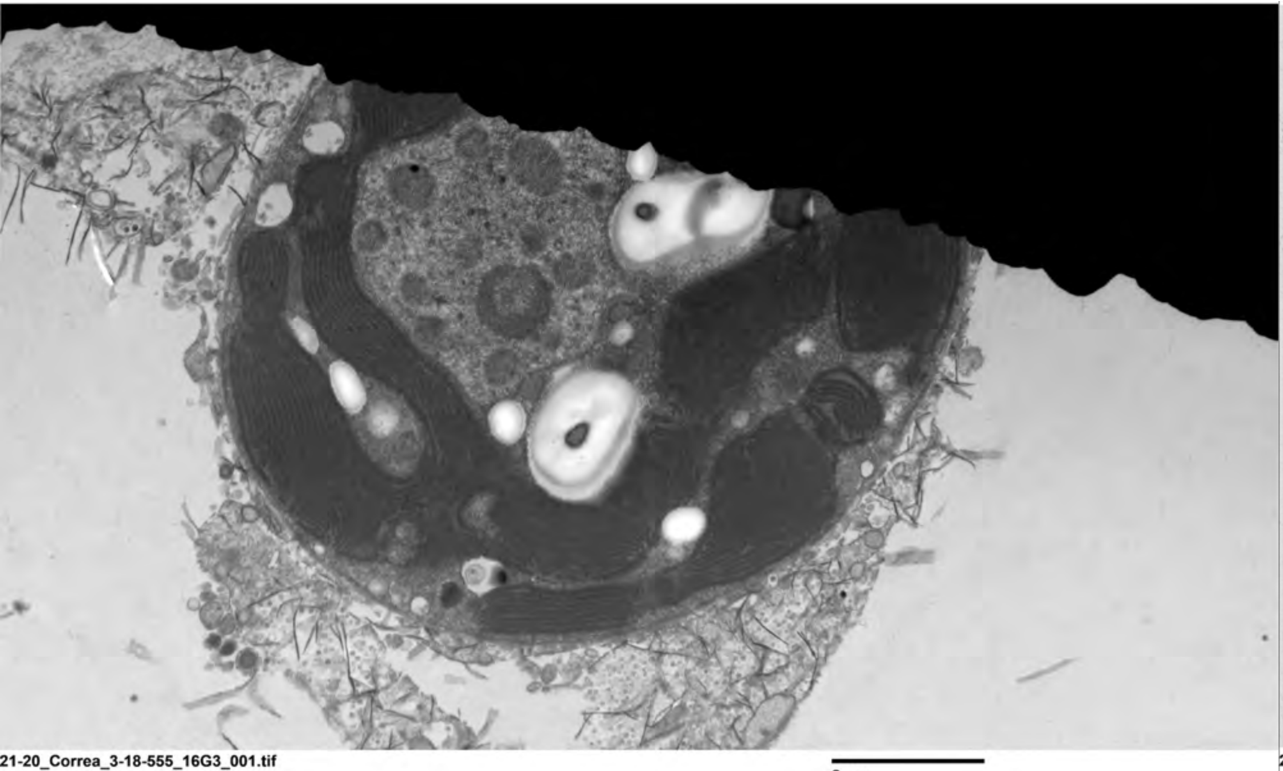

21-20_Correa_3-18-555_16G3_001.tif
Sample: 3/18, 555
Biological Electron Microscopy Lab
Rice University - SEA
Microscopist: MD Meyer

HV=80kV
Direct Mag: 2000 x

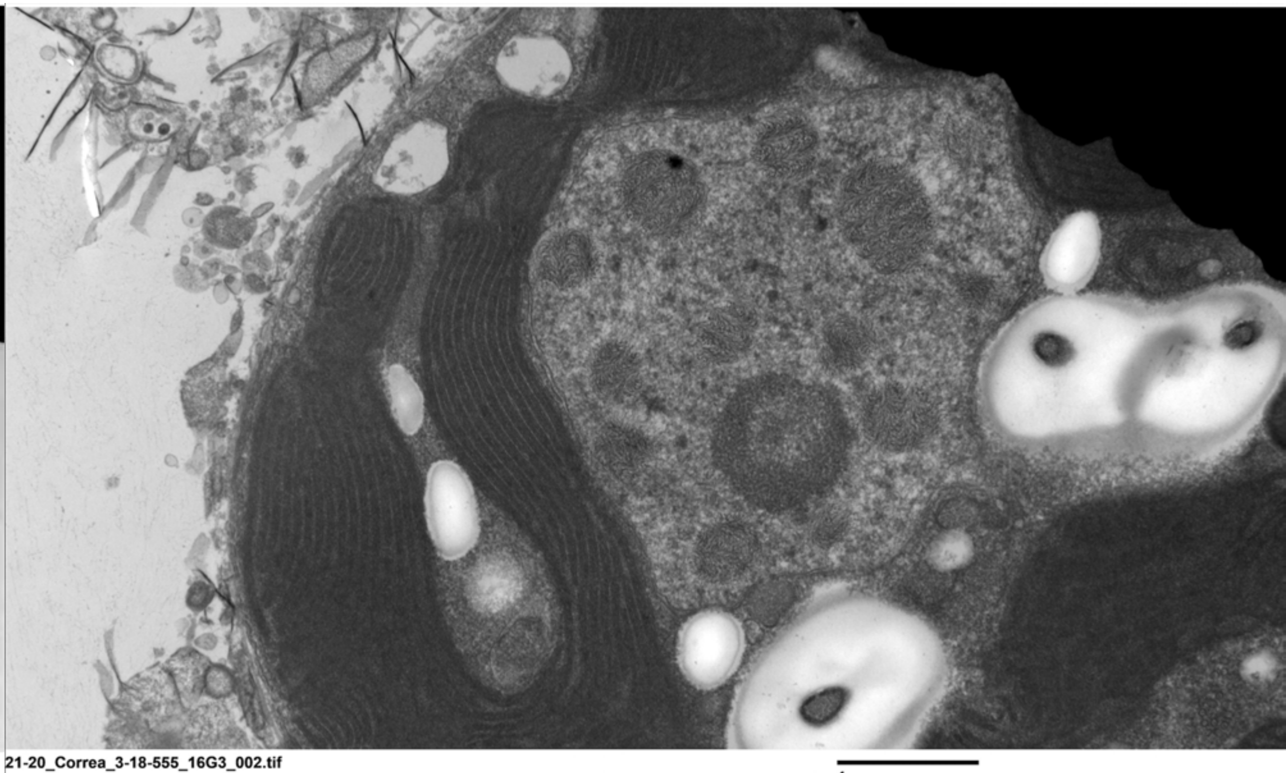

21-20_Correa_3-18-555_16G3_002.tif
Sample: 3/18, 555
Biological Electron Microscopy Lab
Rice University - SEA
Microscopist: MD Meyer

HV=80kV
Direct Mag: 4000 x

Heat (March 2019) POR Colony M

Cell 1

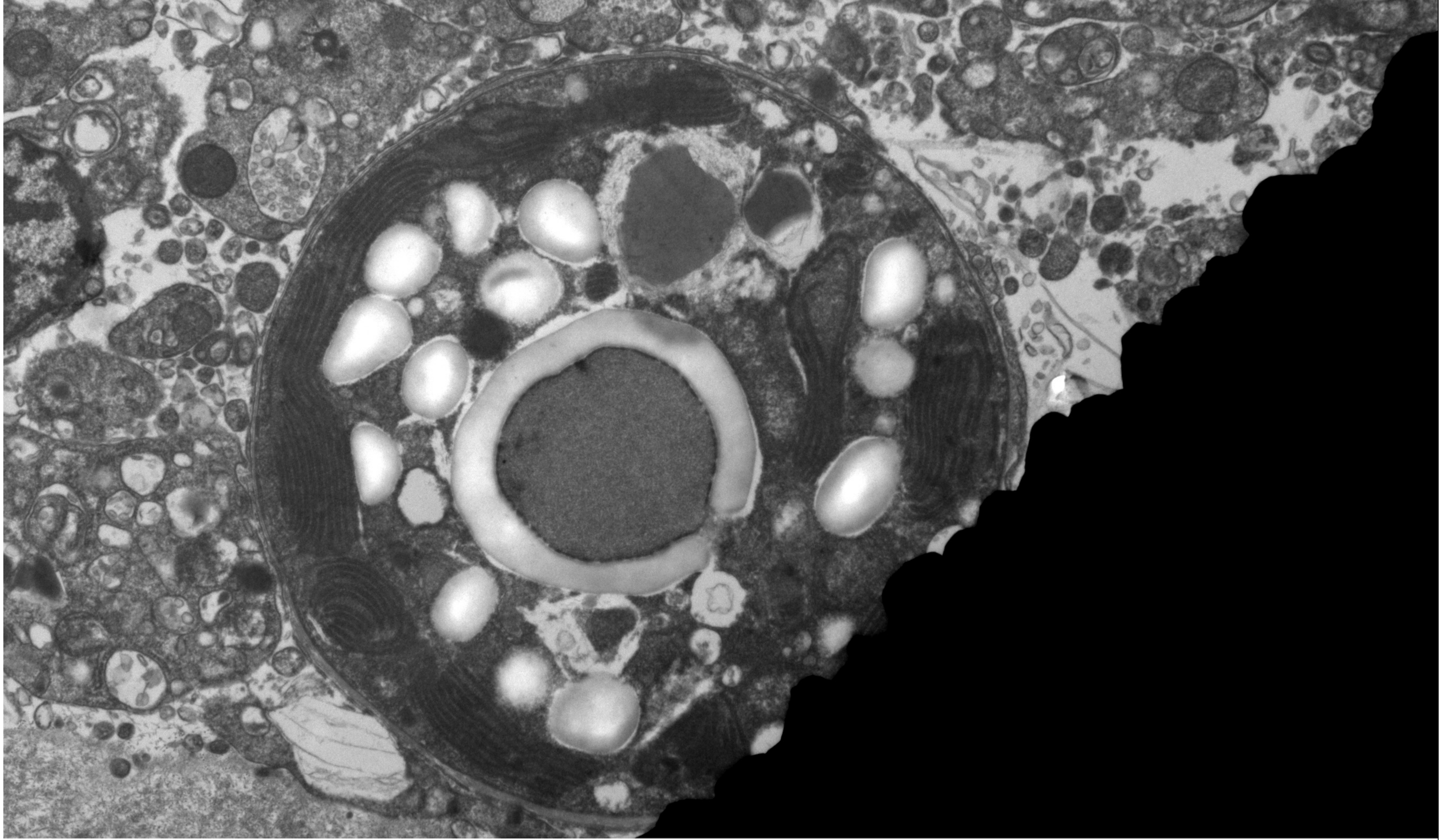

21-20_Correa_3-19-555_16A1_062.tif
Sample: 3/19, 555
Biological Electron Microscopy Lab
Rice University - SEA
Microscopist: MD Meyer

1 µm
HV=80kV
Direct Mag: 2500 x

Cell 2

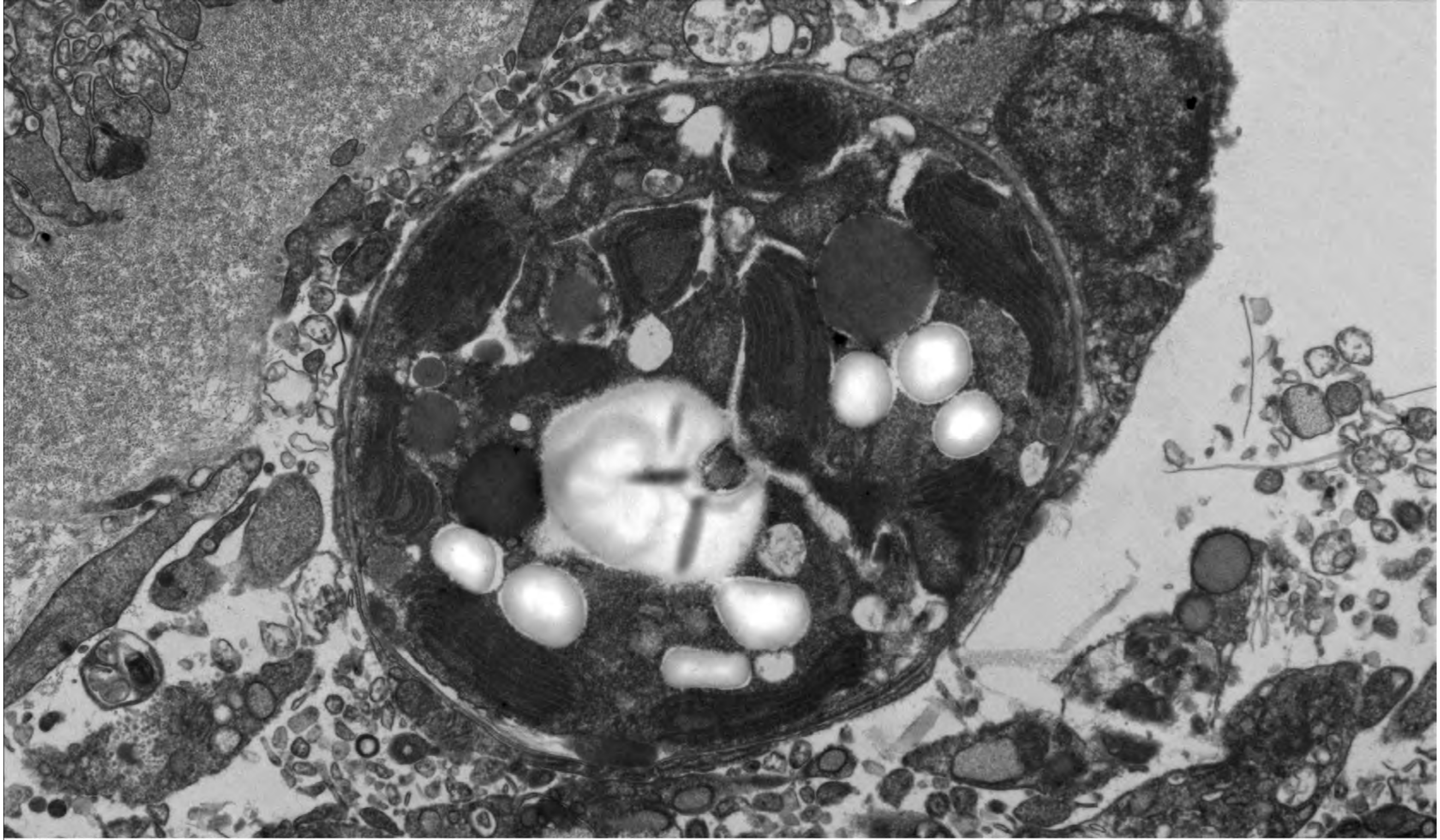

21-20_Correa_3-19-555_16A1_061.tif
Sample: 3/19, 555
Biological Electron Microscopy Lab
Rice University - SEA
Microscopist: MD Meyer

1 µm
HV=80kV
Direct Mag: 2500 x

Cell 3

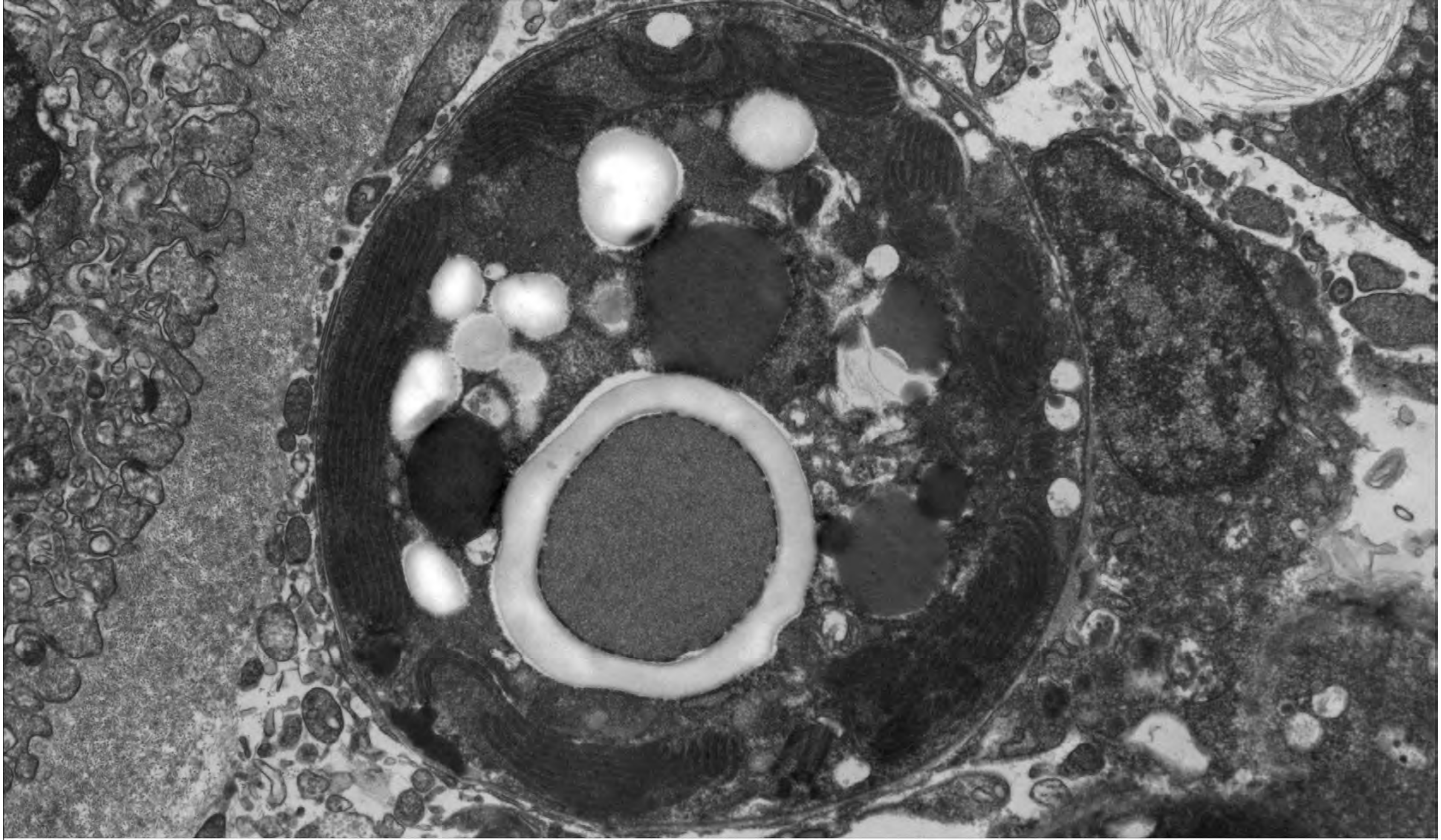

21-20_Correa_3-19-555_16A1_060.tif
Sample: 3/19, 555
Biological Electron Microscopy Lab
Rice University - SEA
Microscopist: MD Meyer

1 µm
HV=80kV
Direct Mag: 2500 x

Cell 4

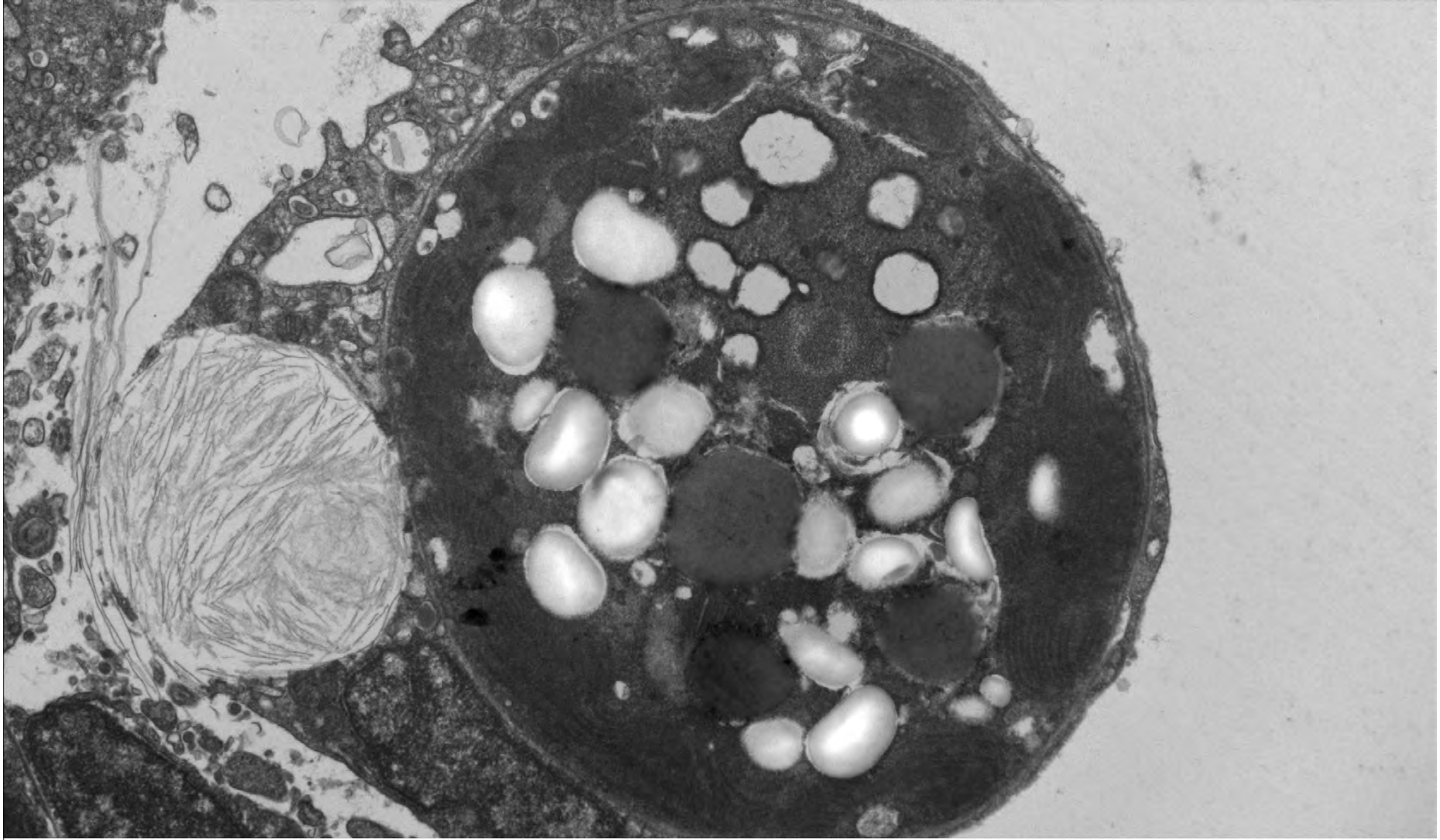

21-20_Correa_3-19-555_16A1_059.tif
Sample: 3/19, 555
Biological Electron Microscopy Lab
Rice University - SEA
Microscopist: MD Meyer

1 µm
HV=80kV
Direct Mag: 2500 x

Cell 5

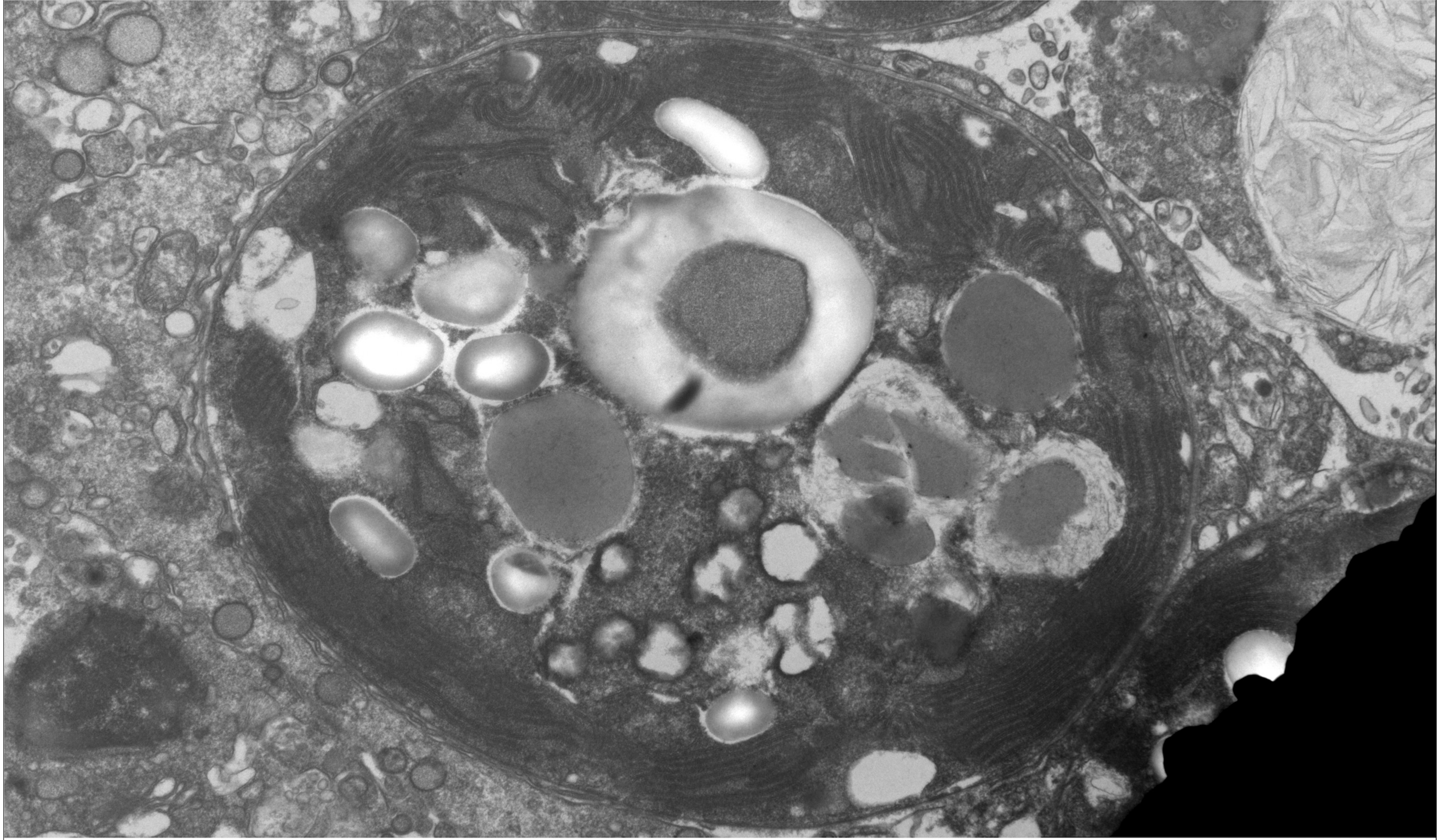

21-20_Correa_3-19-555_16A1_058.tif
Sample: 3/19, 555
Biological Electron Microscopy Lab
Rice University - SEA
Microscopist: MD Meyer

1 µm
HV=80kV
Direct Mag: 3000 x

Cell 6

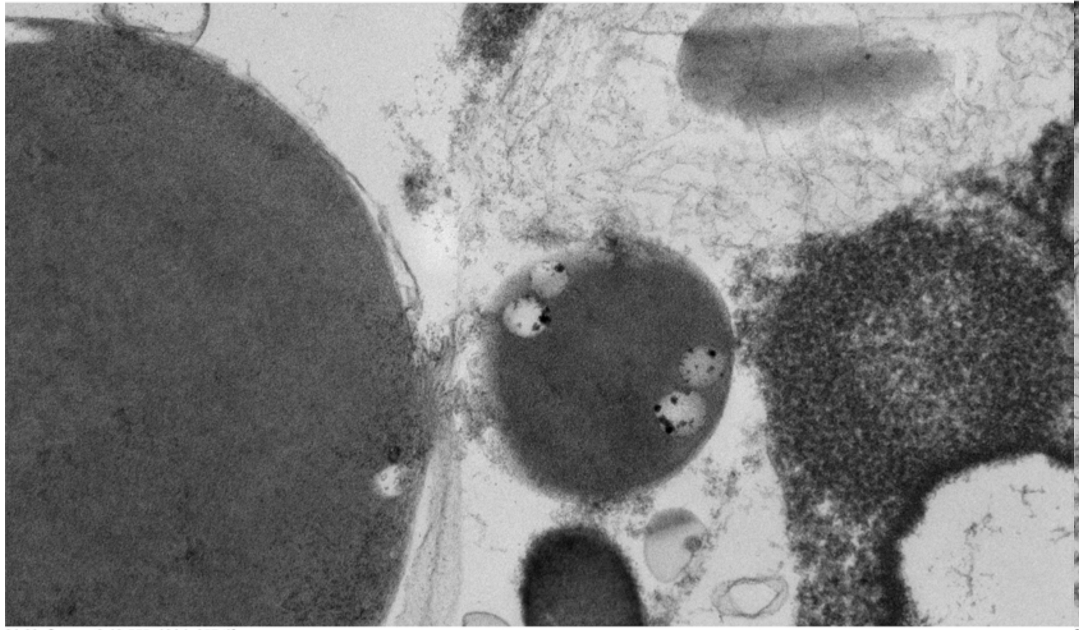

21-20_Correa_3-19-555_16A1_057.tif
Sample: 3/19, 555
Biological Electron Microscopy Lab
Rice University - SEA
Microscopist: MD Meyer

200 nm
HV=80kV
Direct Mag: 12000 x

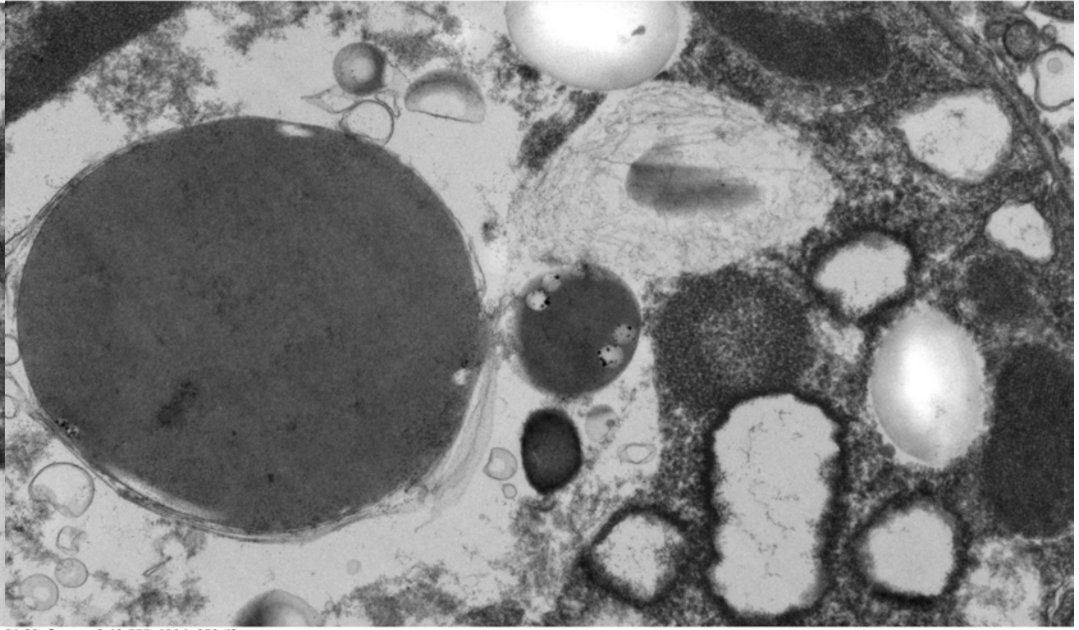

21-20_Correa_3-19-555_16A1_056.tif
Sample: 3/19, 555
Biological Electron Microscopy Lab
Rice University - SEA
Microscopist: MD Meyer

600 nm
HV=80kV
Direct Mag: 6000 x

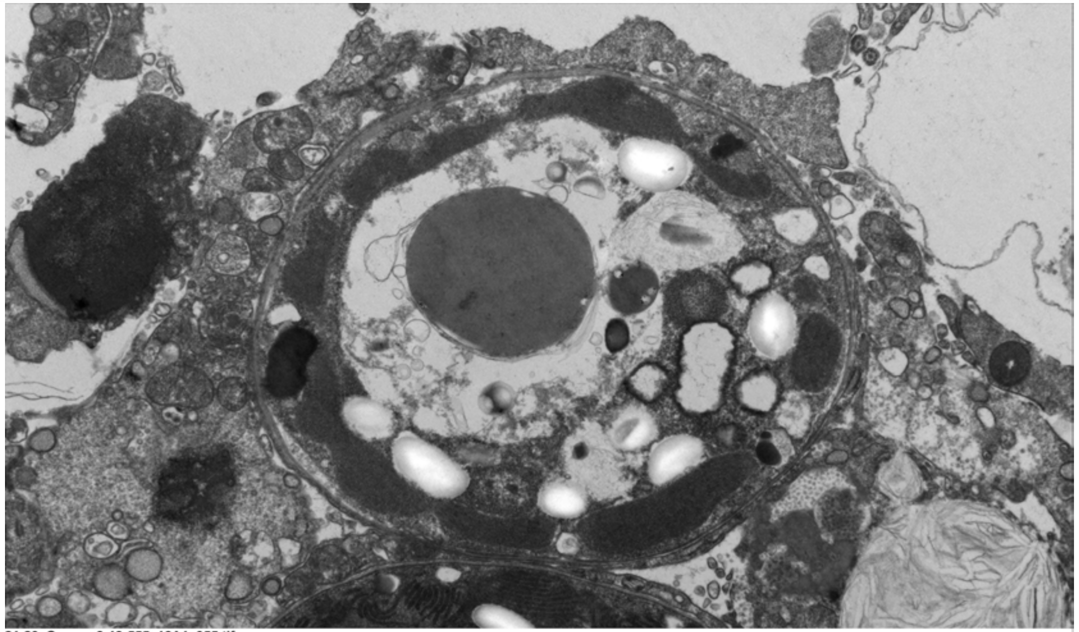

21-20_Correa_3-19-555_16A1_055.tif
Sample: 3/19, 555
Biological Electron Microscopy Lab
Rice University - SEA
Microscopist: MD Meyer

1 µm
HV=80kV
Direct Mag: 2500 x

Cell 7

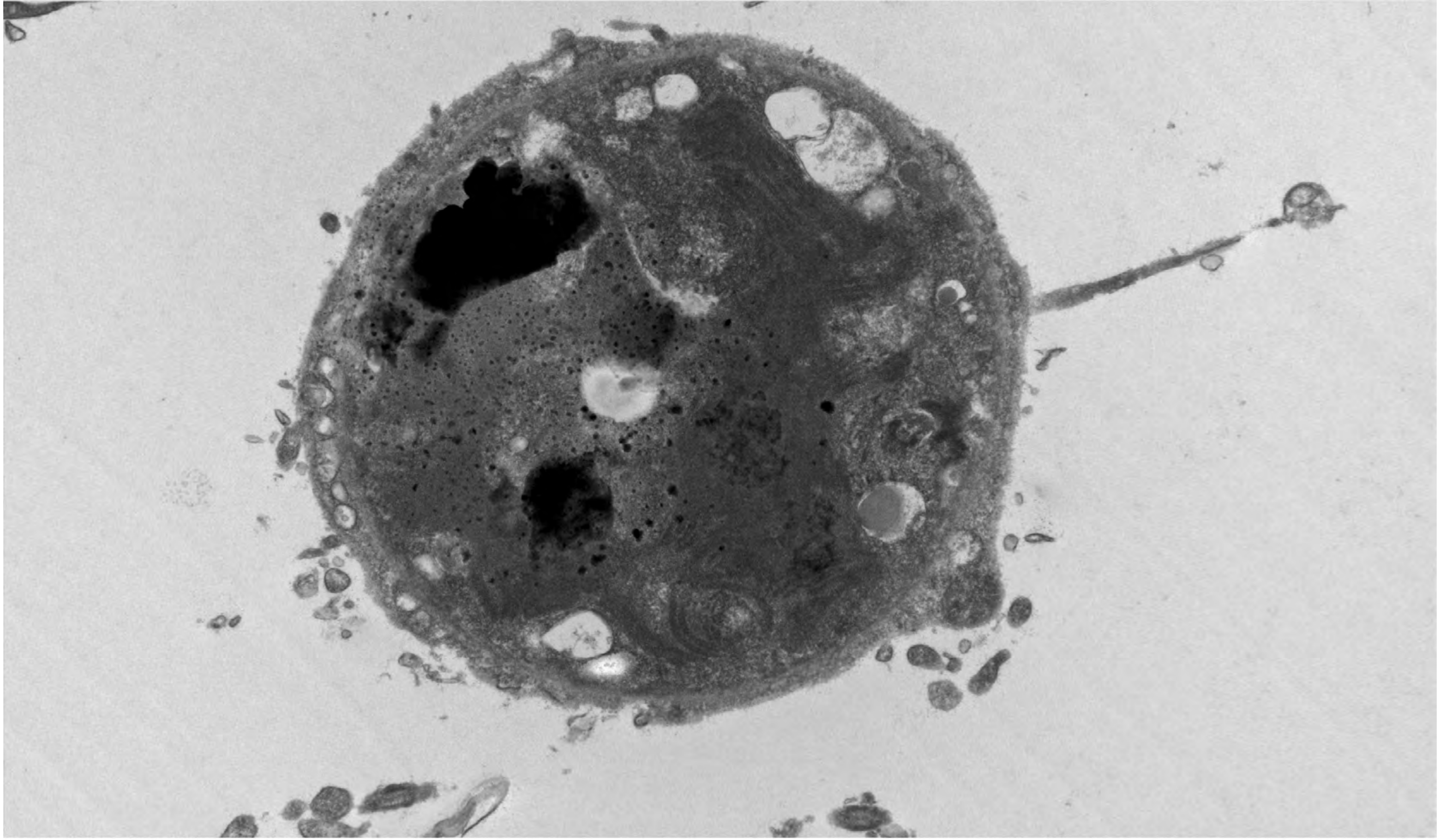

21-20_Correa_3-19-555_16A1_054.tif
Sample: 3/19, 555
Biological Electron Microscopy Lab
Rice University - SEA
Microscopist: MD Meyer

1 µm
HV=80kV
Direct Mag: 3000 x

Cell 8

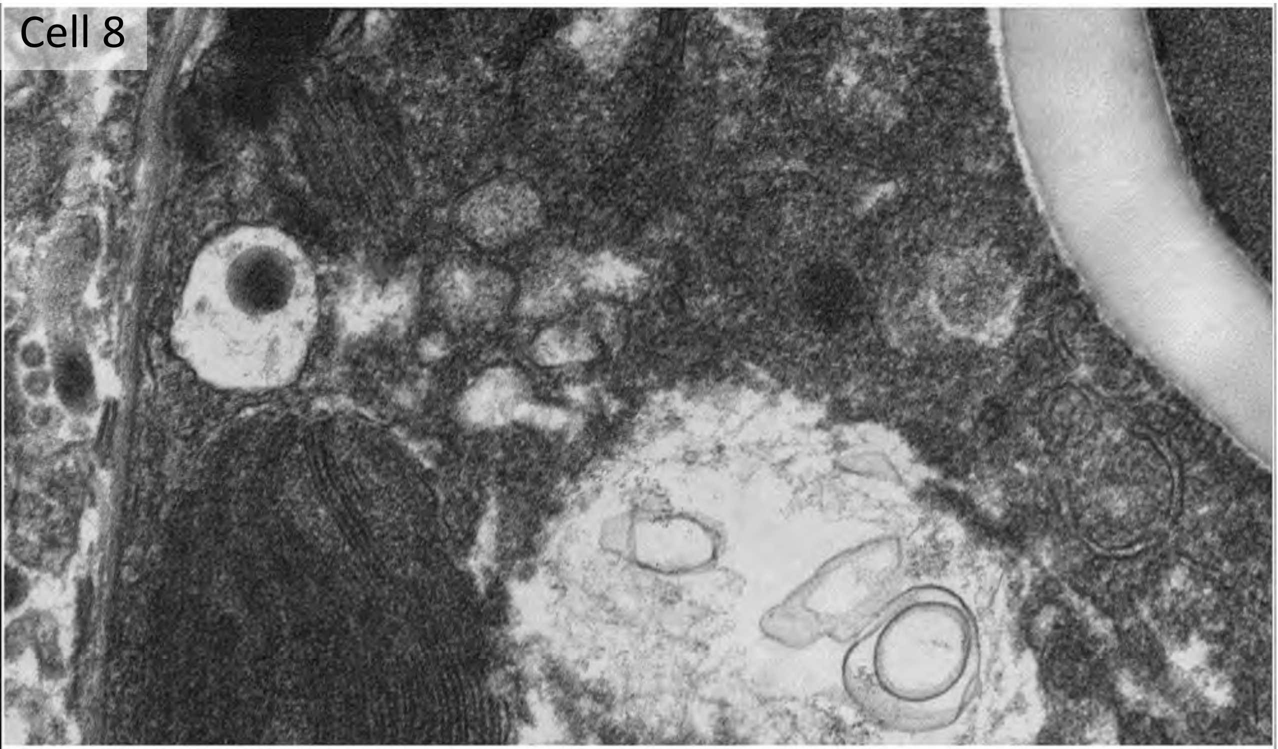

21-20_Correa_3-19-555_16A1_053.tif
Sample: 3/19, 555
Biological Electron Microscopy Lab
Rice University - SEA
Microscopist: MD Meyer

200 nm
HV=80kV
Direct Mag: 12000 x

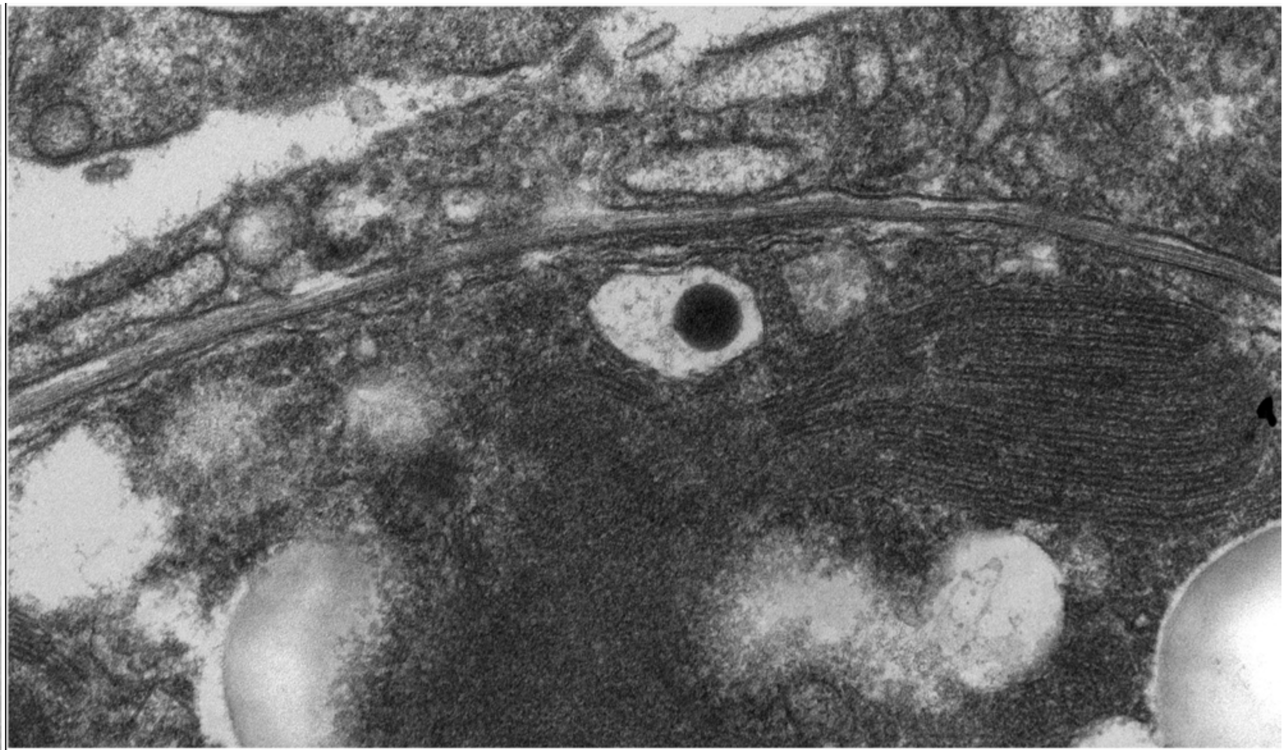

21-20_Correa_3-19-555_16A1_052.tif
Sample: 3/19, 555
Biological Electron Microscopy Lab
Rice University - SEA
Microscopist: MD Meyer

200 nm
HV=80kV
Direct Mag: 12000 x

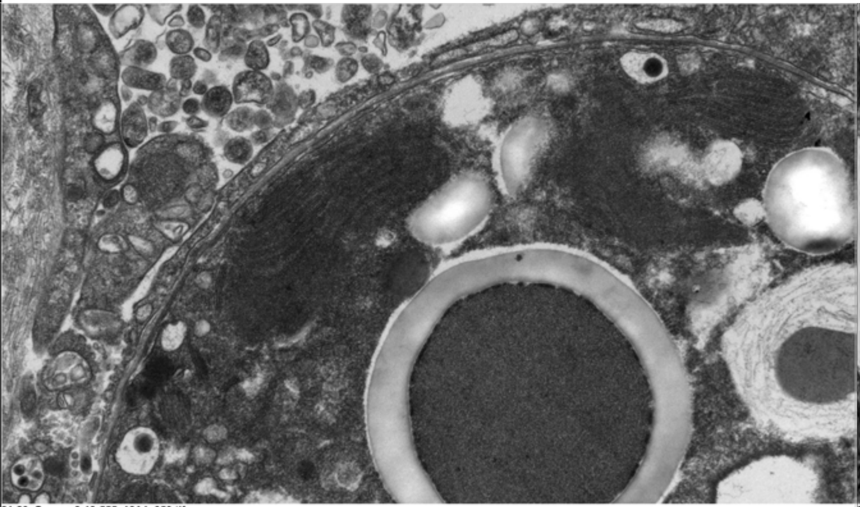

21-20_Correa_3-19-555_16A1_050.tif
Sample: 3/19, 555
Biological Electron Microscopy Lab
Rice University - SEA
Microscopist: MD Meyer

800 nm
HV=80kV
Direct Mag: 5000 x

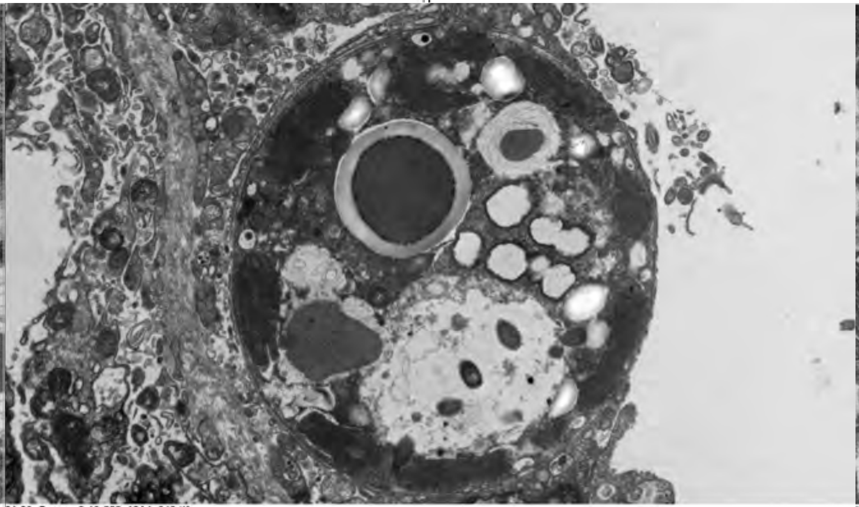

21-20_Correa_3-19-555_16A1_048.tif
Sample: 3/19, 555
Biological Electron Microscopy Lab
Rice University - SEA
Microscopist: MD Meyer

2 µm
HV=80kV
Direct Mag: 2000 x

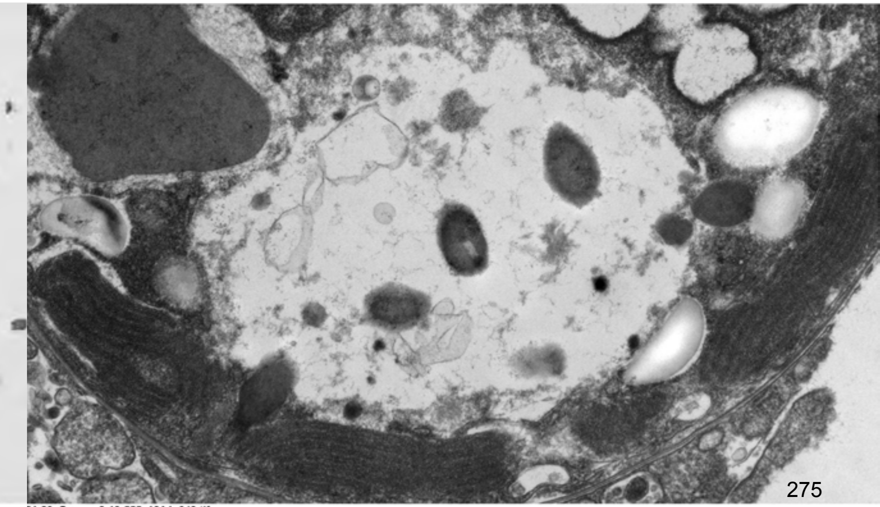

21-20_Correa_3-19-555_16A1_049.tif
Sample: 3/19, 555
Biological Electron Microscopy Lab
Rice University - SEA
Microscopist: MD Meyer

800 nm
HV=80kV
Direct Mag: 5000 x

Cell 9

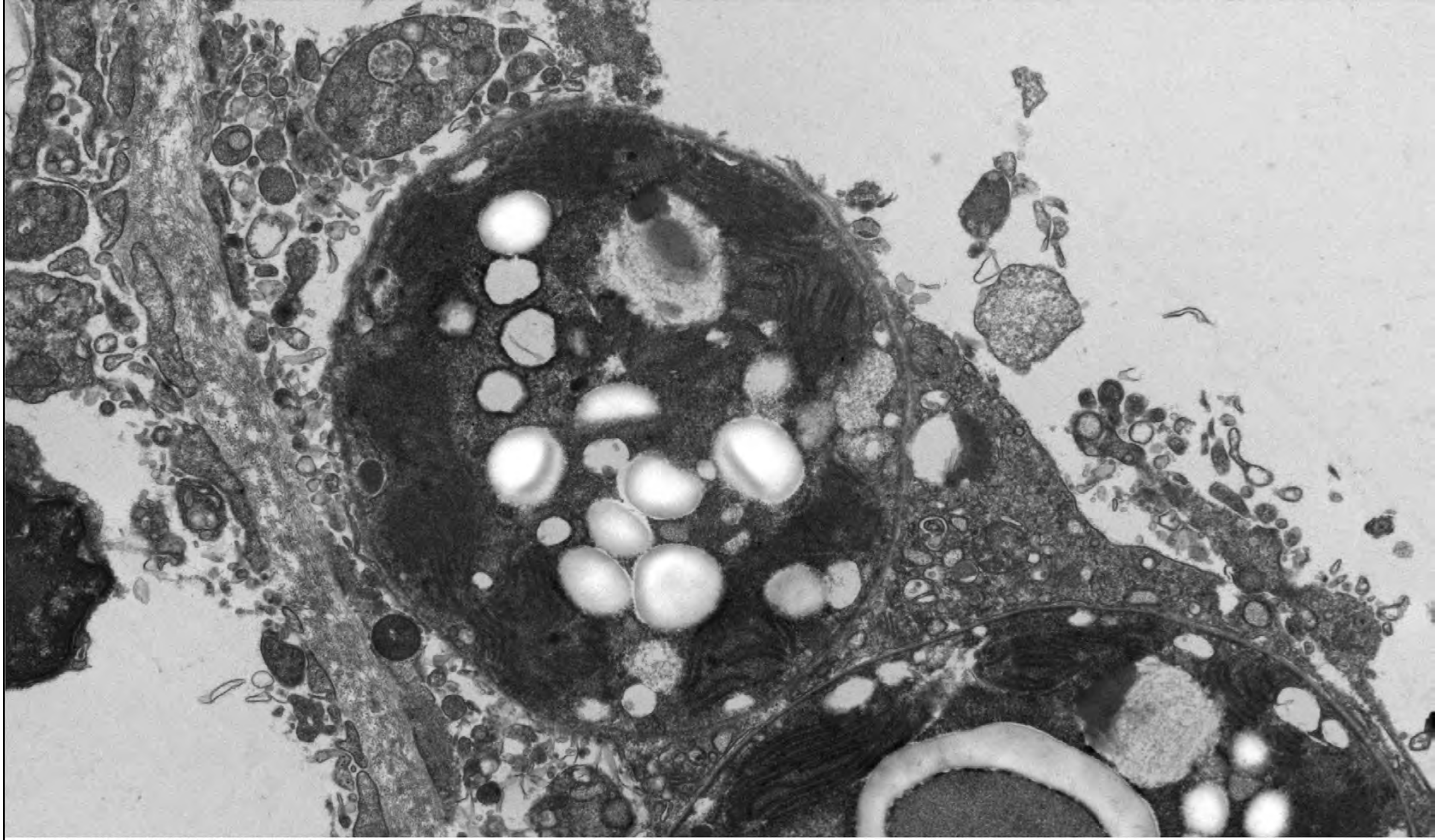

21-20_Correa_3-19-555_16A1_047.tif
Sample: 3/19, 555
Biological Electron Microscopy Lab
Rice University - SEA
Microscopist: MD Meyer

1 µm
HV=80kV
Direct Mag: 2500 x

Cell 10

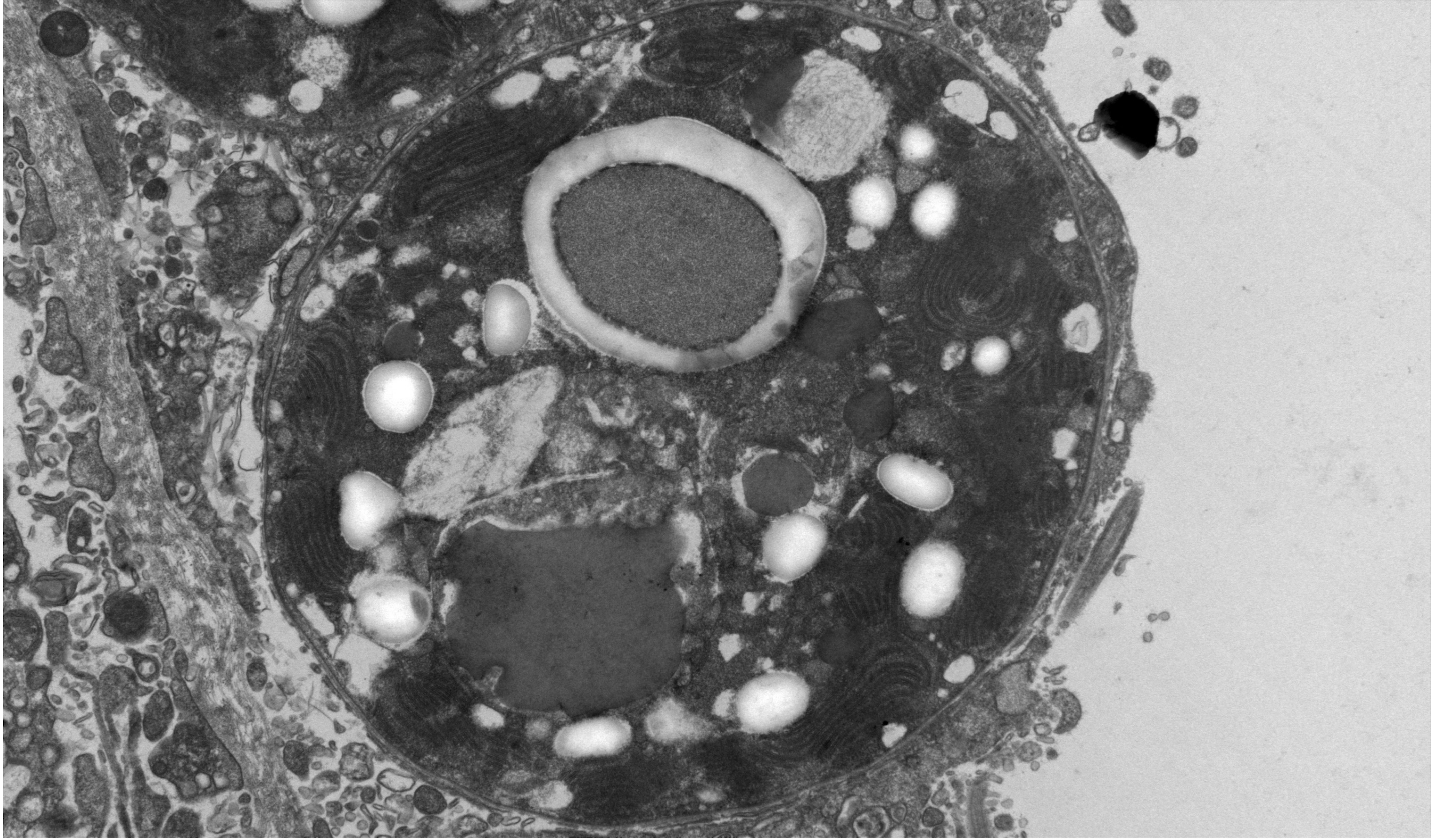

21-20_Correa_3-19-555_16A1_046.tif
Sample: 3/19, 555
Biological Electron Microscopy Lab
Rice University - SEA
Microscopist: MD Meyer

1 µm
HV=80kV
Direct Mag: 2500 x

Cell 11

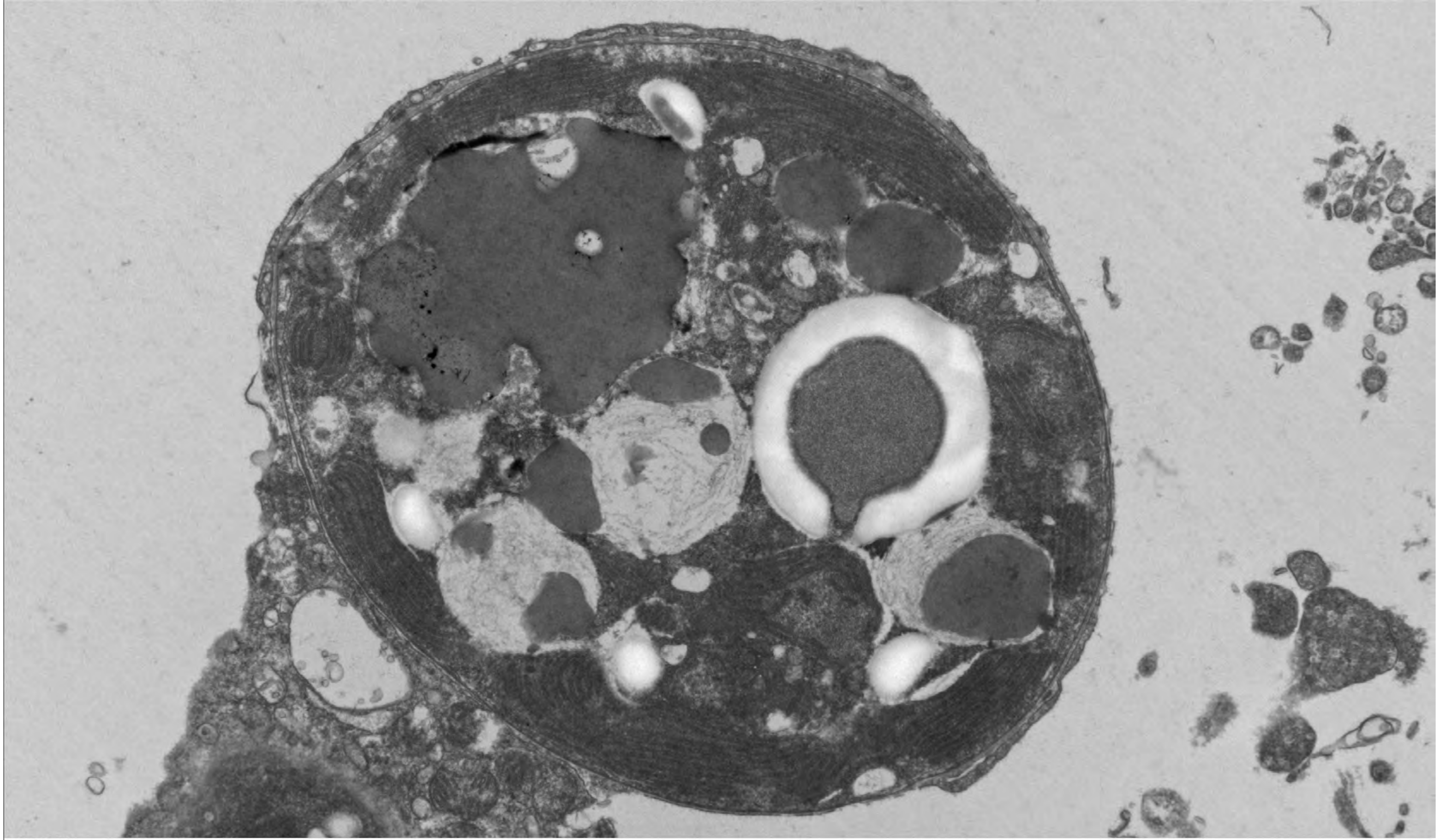

21-20_Correa_3-19-555_16A1_044.tif
Sample: 3/19, 555
Biological Electron Microscopy Lab
Rice University - SEA
Microscopist: MD Meyer

1 µm
HV=80kV
Direct Mag: 2500 x

Cell 12

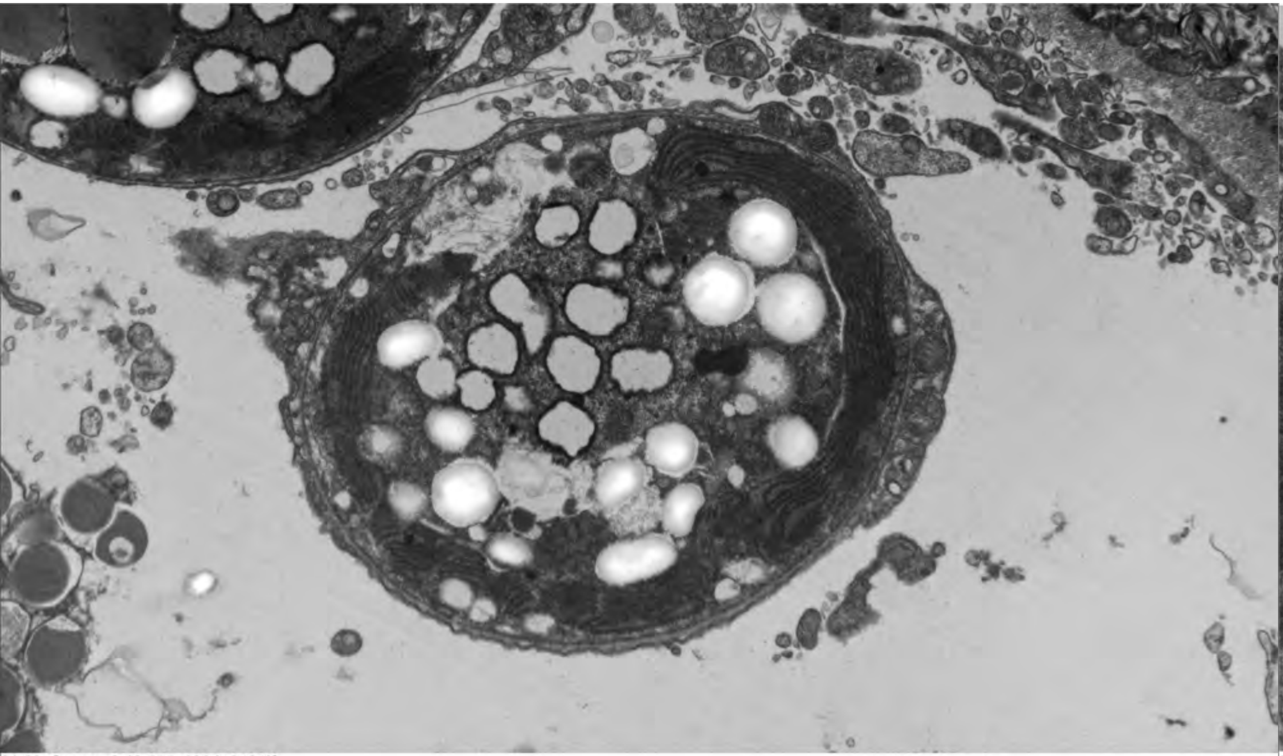

21-20_Correa_3-19-555_16A1_042.tif
Sample: 3/19, 555
Biological Electron Microscopy Lab
Rice University - SEA
Microscopist: MD Meyer

2 μm
HV=80kV
Direct Mag: 2000 x

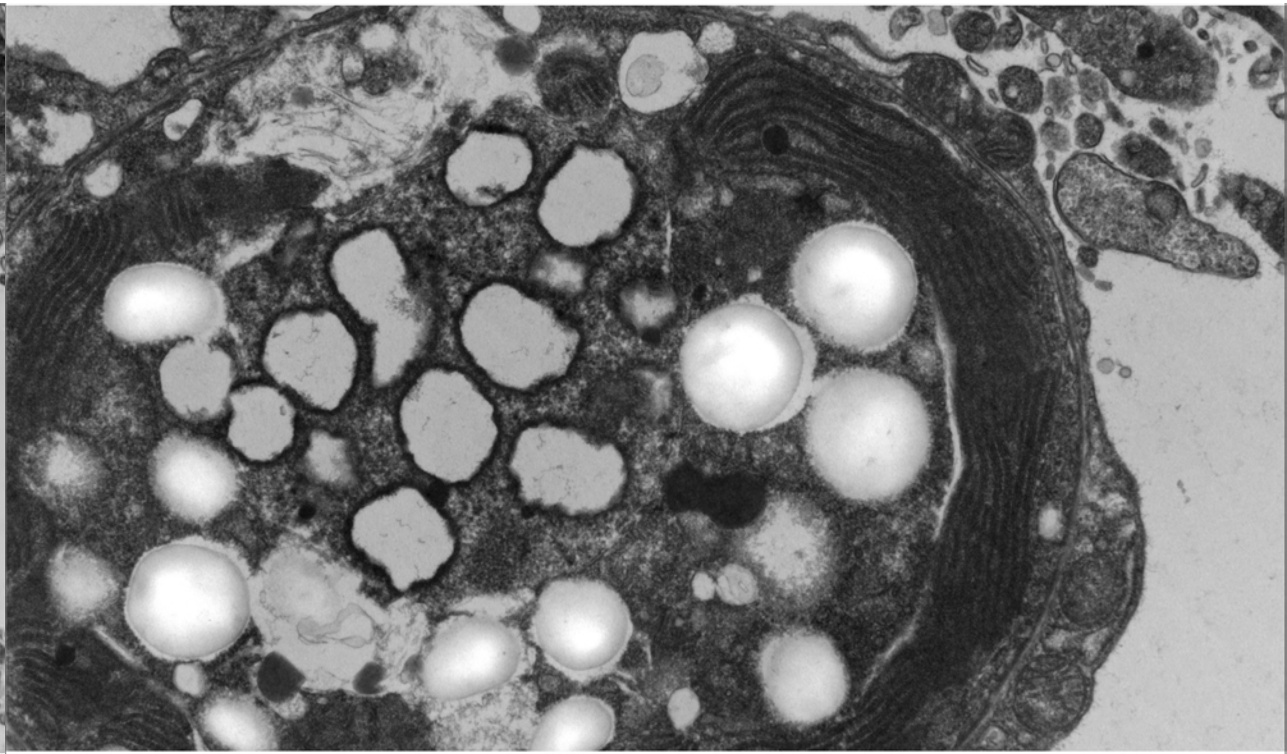

21-20_Correa_3-19-555_16A1_043.tif
Sample: 3/19, 555
Biological Electron Microscopy Lab
Rice University - SEA
Microscopist: MD Meyer

1 μm
HV=80kV
Direct Mag: 4000 x

Cell 13

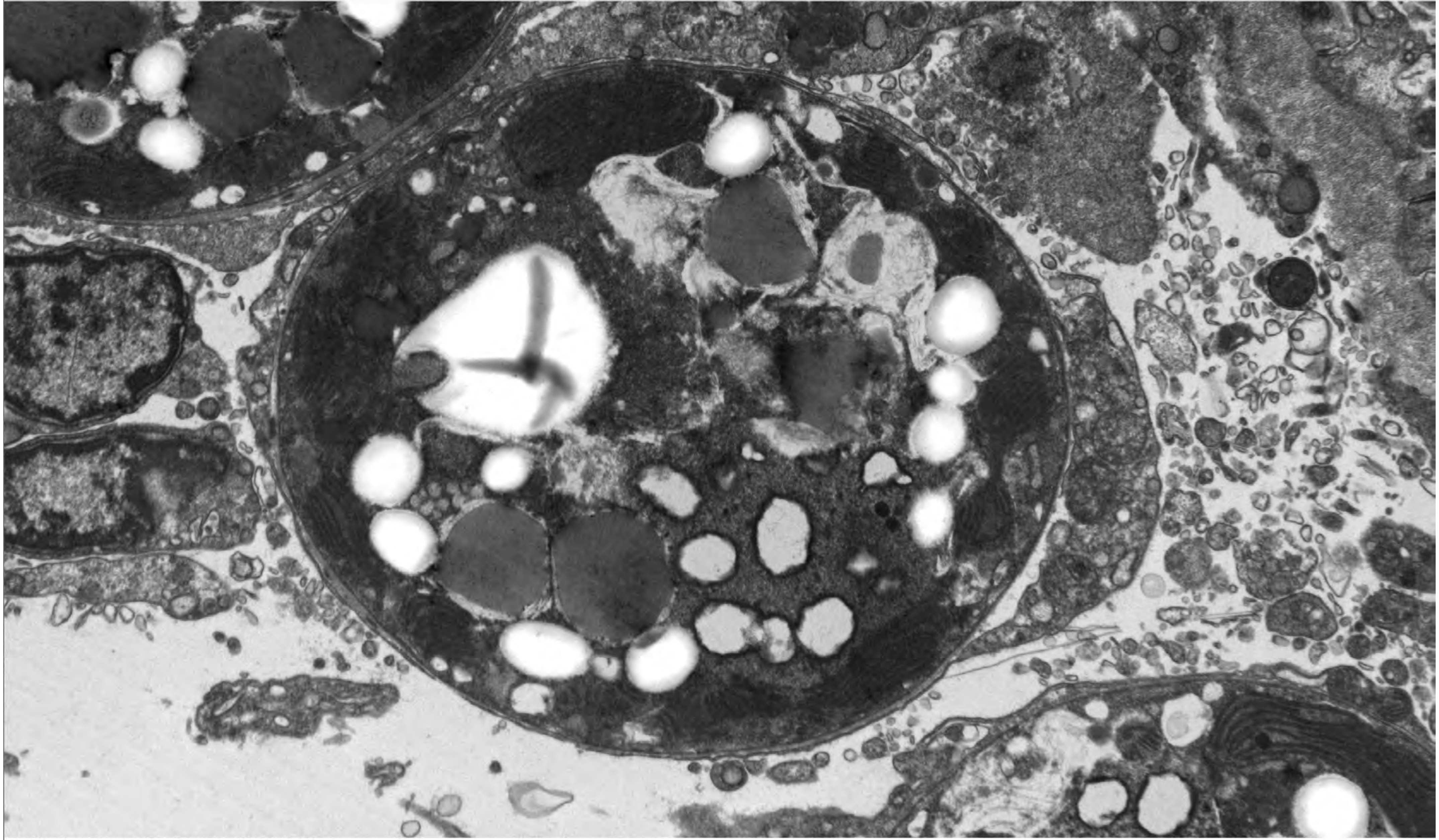

21-20_Correa_3-19-555_16A1_041.tif
Sample: 3/19, 555
Biological Electron Microscopy Lab
Rice University - SEA
Microscopist: MD Meyer

2 µm
HV=80kV
Direct Mag: 2000 x

Cell 14

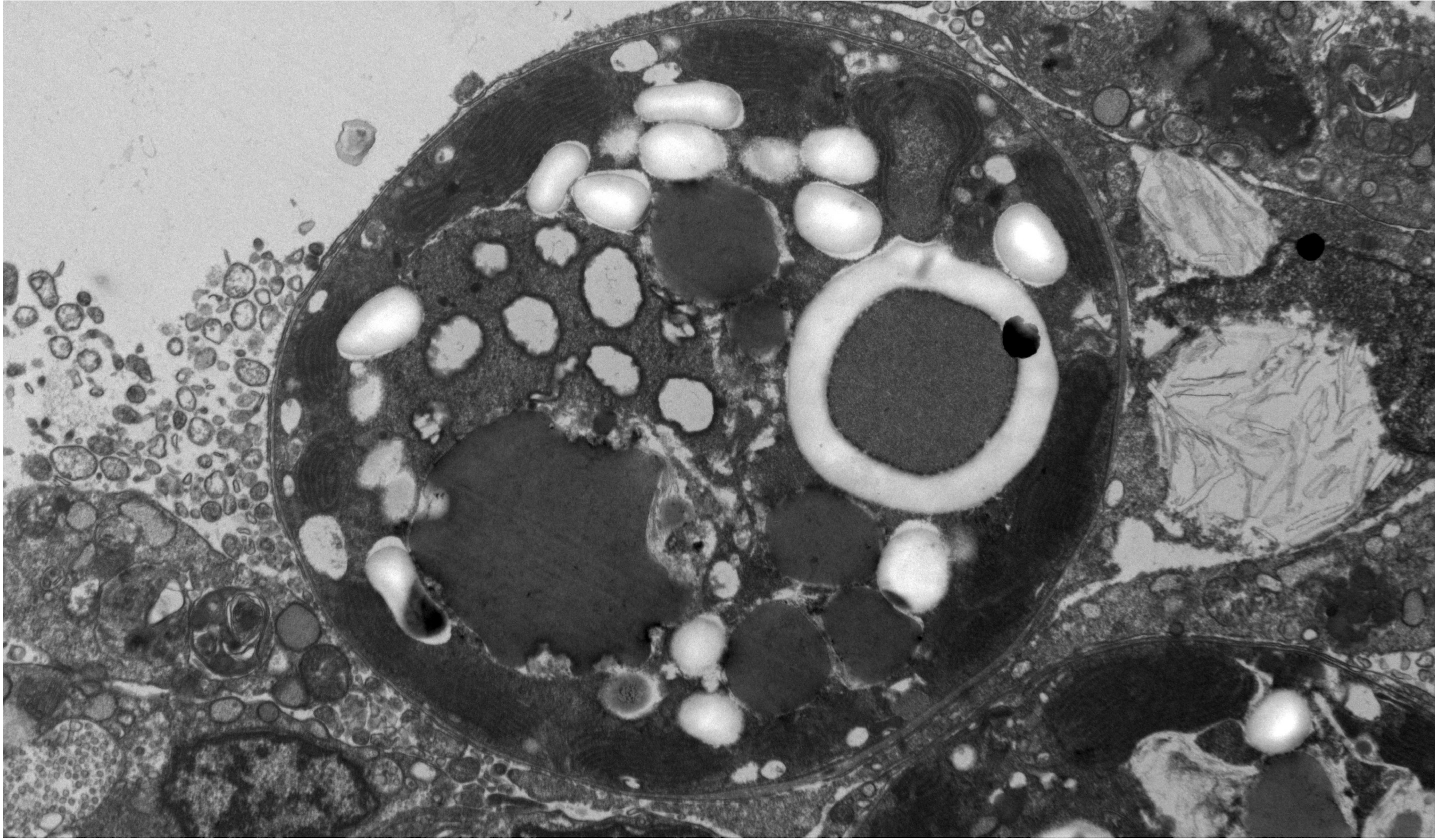

21-20_Correa_3-19-555_16A1_040.tif
Sample: 3/19, 555
Biological Electron Microscopy Lab
Rice University - SEA
Microscopist: MD Meyer

2 µm
HV=80kV
Direct Mag: 2000 x

Cell 15

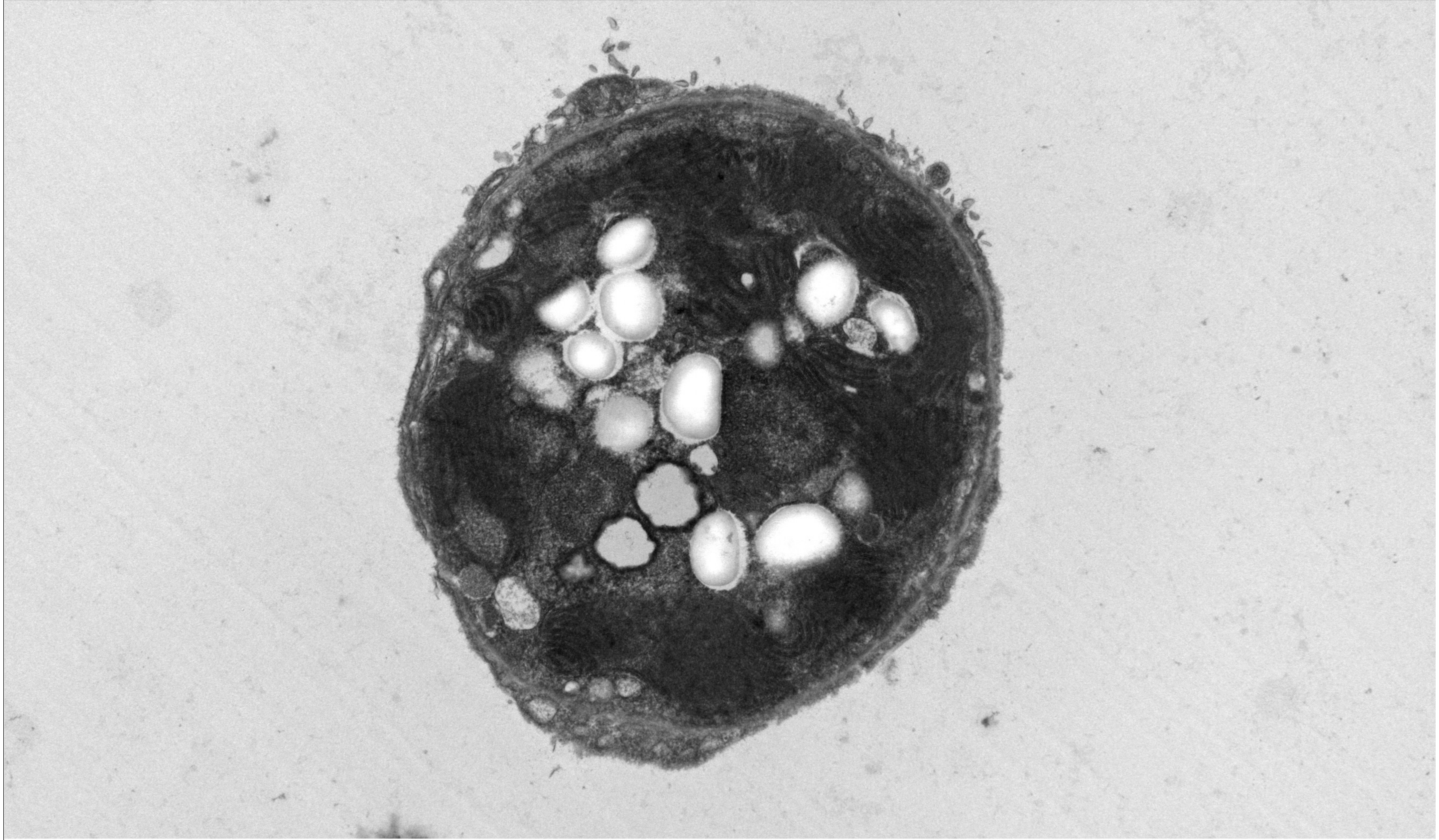

21-20_Correa_3-19-555_16A1_038.tif
Sample: 3/19, 555
Biological Electron Microscopy Lab
Rice University - SEA
Microscopist: MD Meyer

1 µm
HV=80kV
Direct Mag: 2500 x

Cell 16

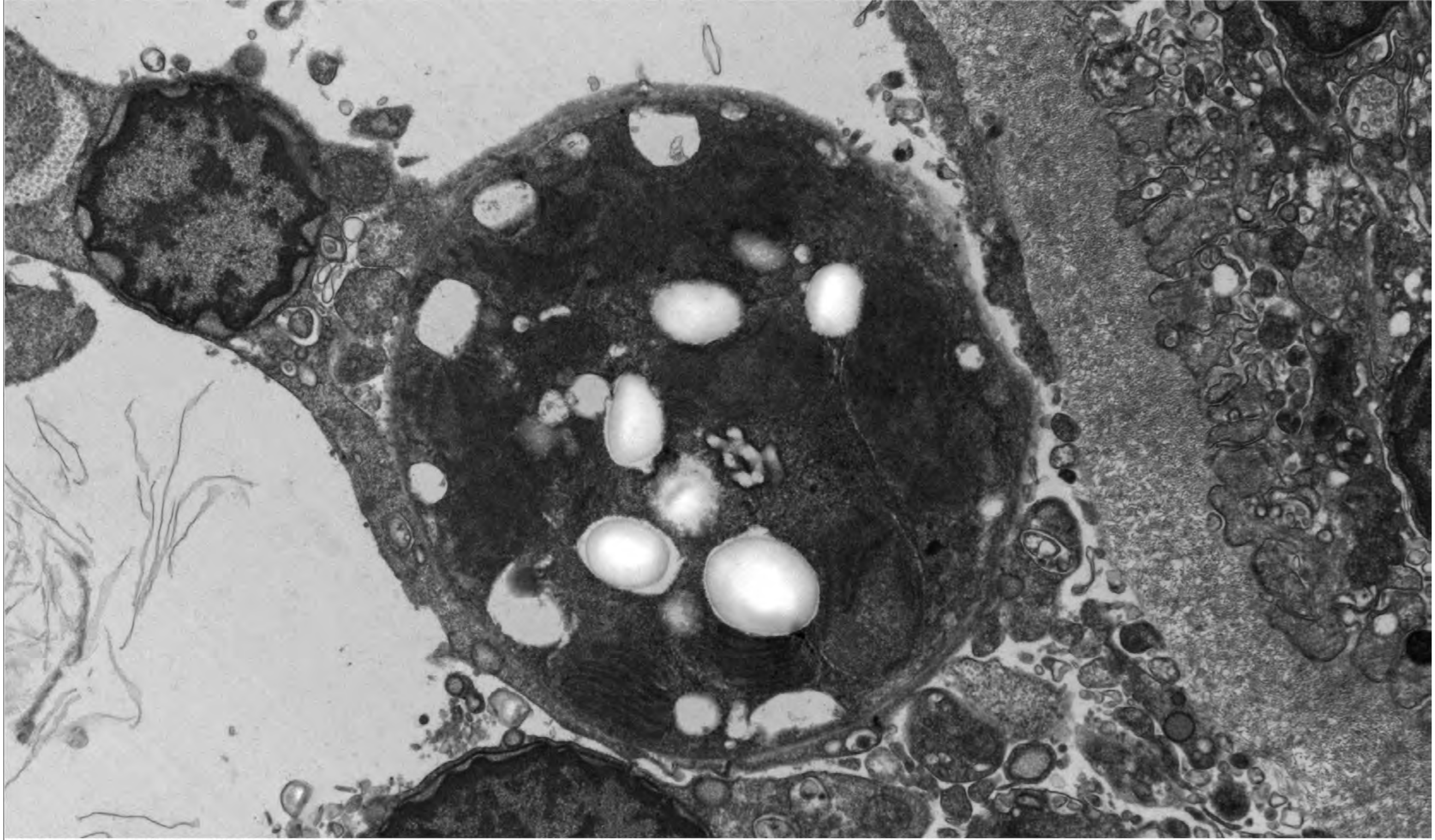

21-20_Correa_3-19-555_16A1_037.tif
Sample: 3/19, 555
Biological Electron Microscopy Lab
Rice University - SEA
Microscopist: MD Meyer

1 µm
HV=80kV
Direct Mag: 2500 x

Cell 17

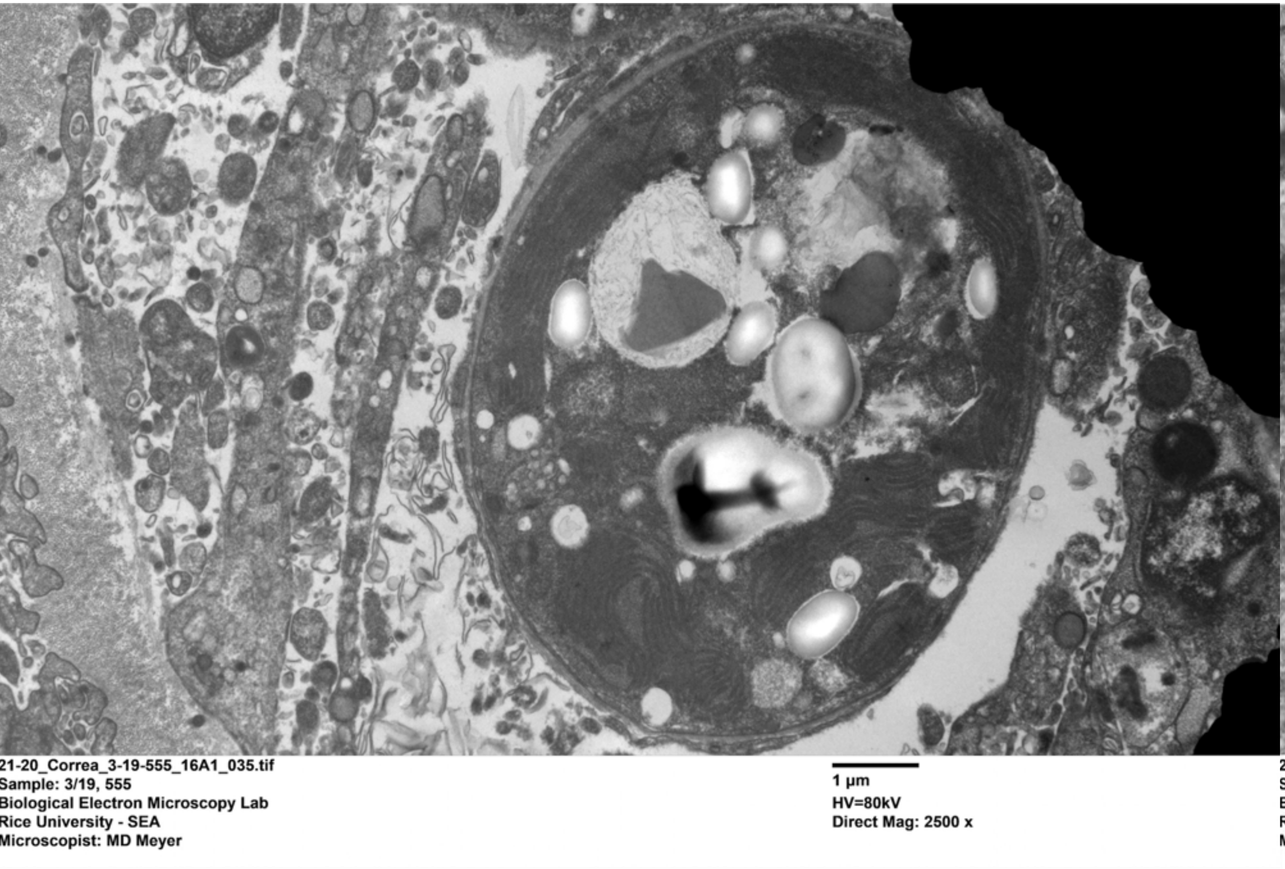
21-20_Correa_3-19-555_16A1_035.tif
Sample: 3/19, 555
Biological Electron Microscopy Lab
Rice University - SEA
Microscopist: MD Meyer
1 µm
HV=80kV
Direct Mag: 2500 x

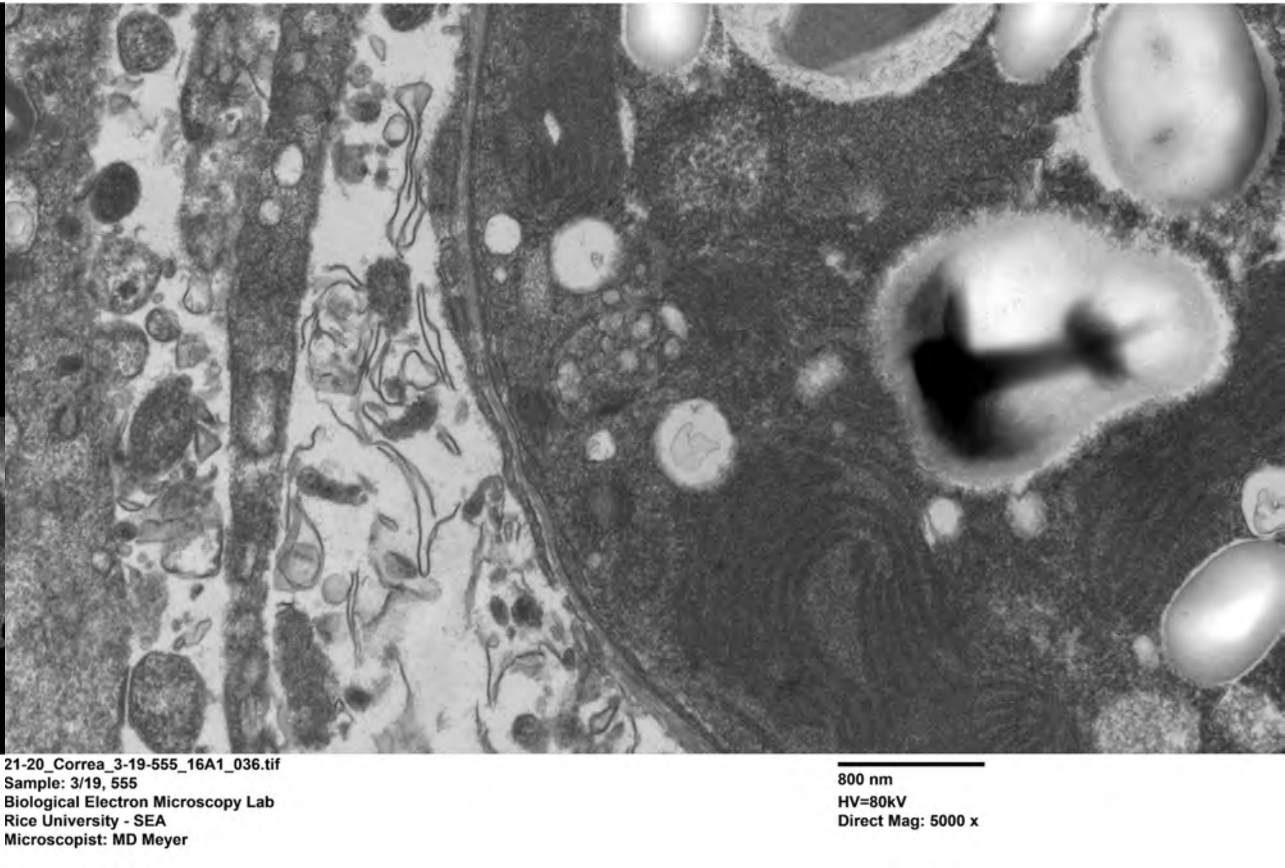
21-20_Correa_3-19-555_16A1_036.tif
Sample: 3/19, 555
Biological Electron Microscopy Lab
Rice University - SEA
Microscopist: MD Meyer
800 nm
HV=80kV
Direct Mag: 5000 x

Cell 18

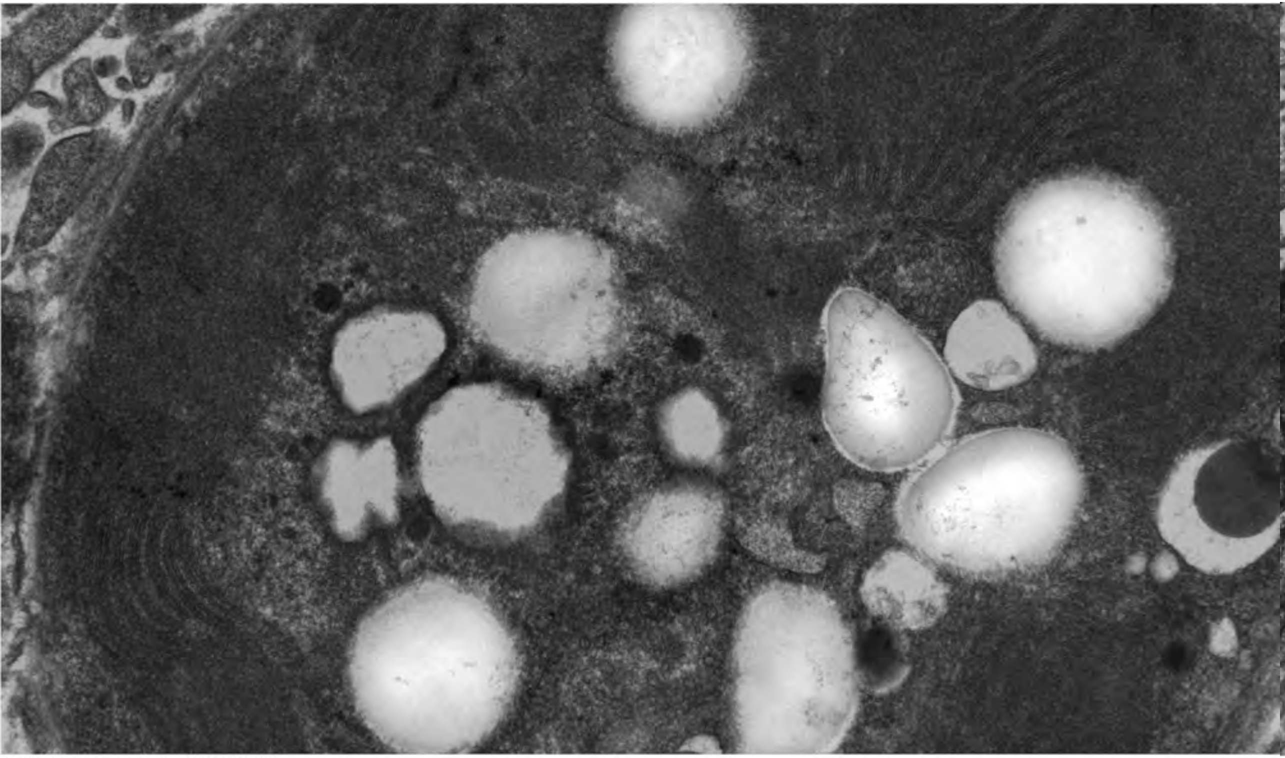

21-20_Correa_3-19-555_16A1_034.tif
Sample: 3/19, 555
Biological Electron Microscopy Lab
Rice University - SEA
Microscopist: MD Meyer

600 nm
HV=80kV
Direct Mag: 6000 x

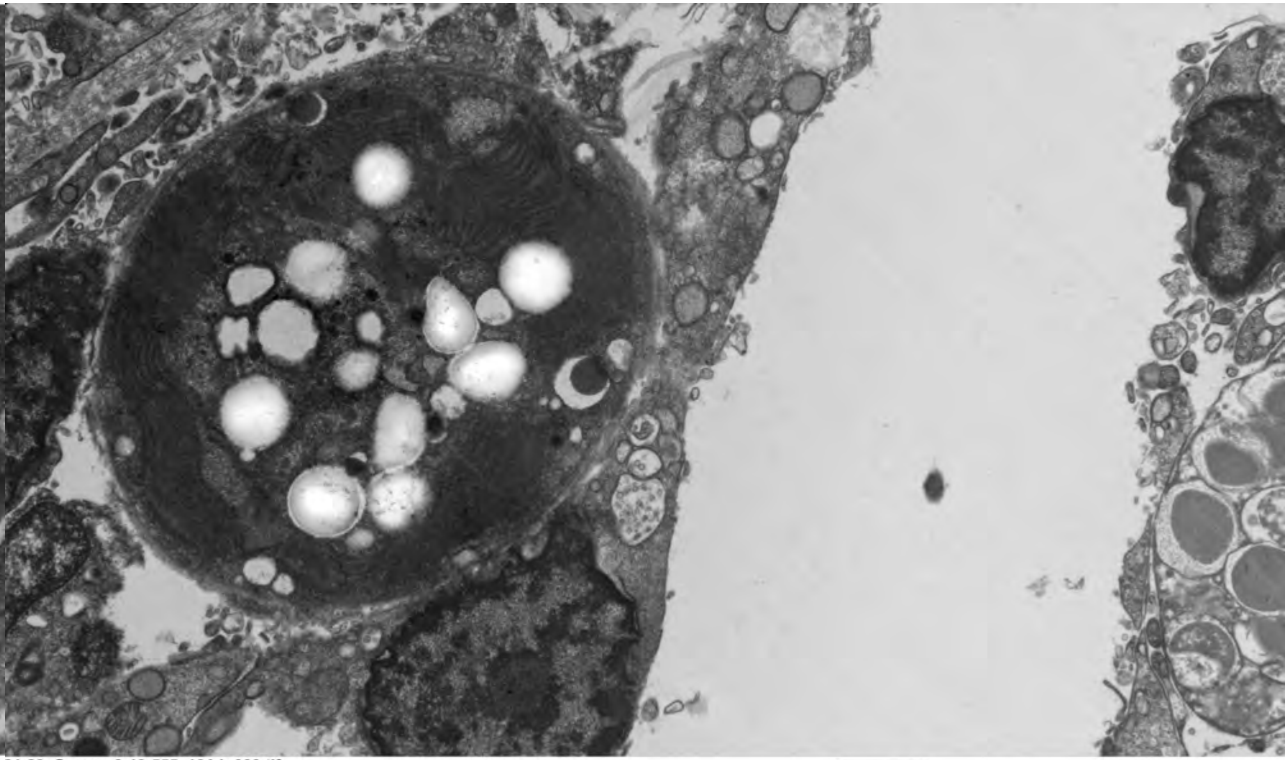

21-20_Correa_3-19-555_16A1_033.tif
Sample: 3/19, 555
Biological Electron Microscopy Lab
Rice University - SEA
Microscopist: MD Meyer

1 µm
HV=80kV
Direct Mag: 2500 x

Cell 19

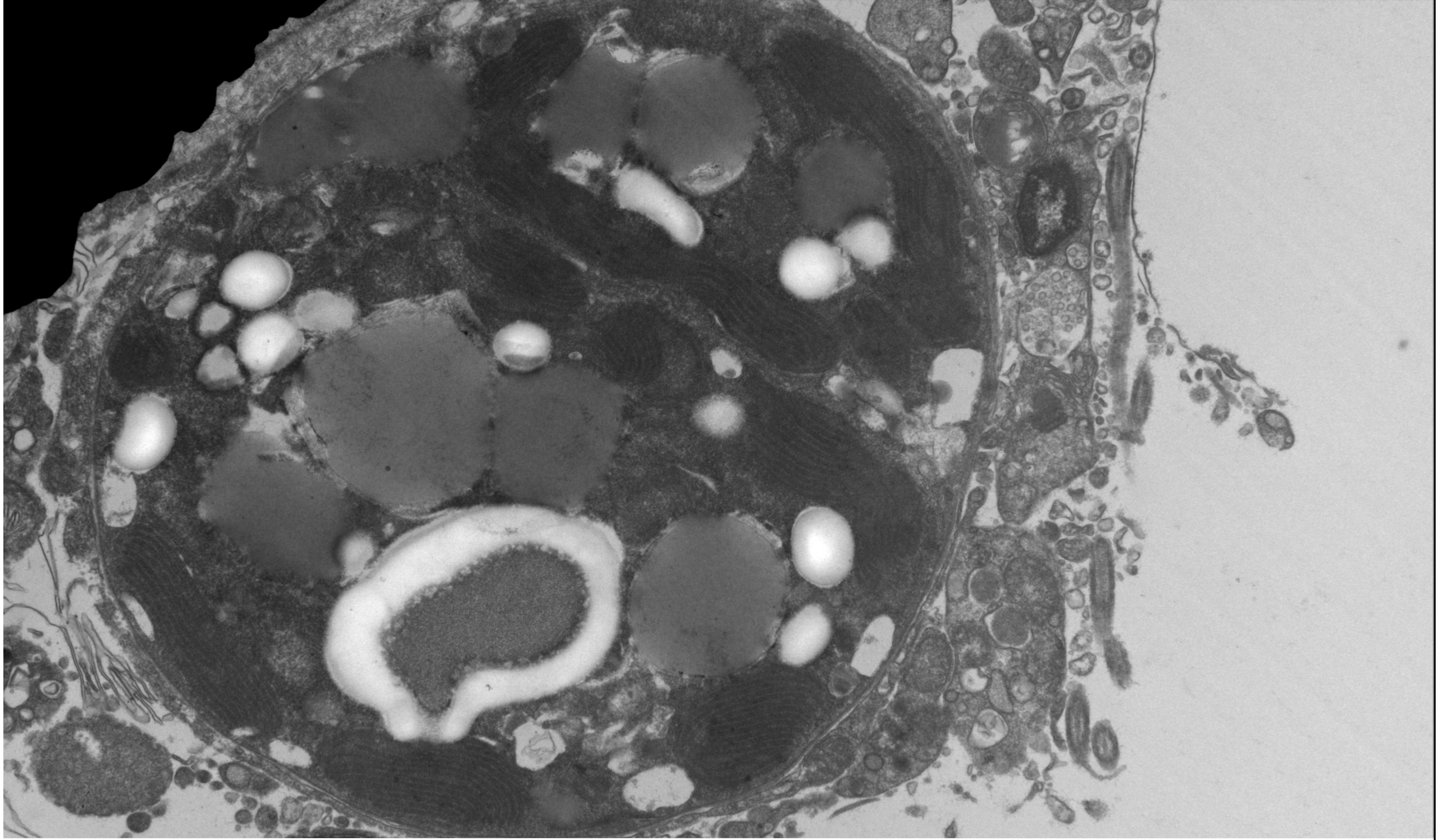

21-20_Correa_3-19-555_16A1_031.tif
Sample: 3/19, 555
Biological Electron Microscopy Lab
Rice University - SEA
Microscopist: MD Meyer

1 µm
HV=80kV
Direct Mag: 2500 x

Cell 20

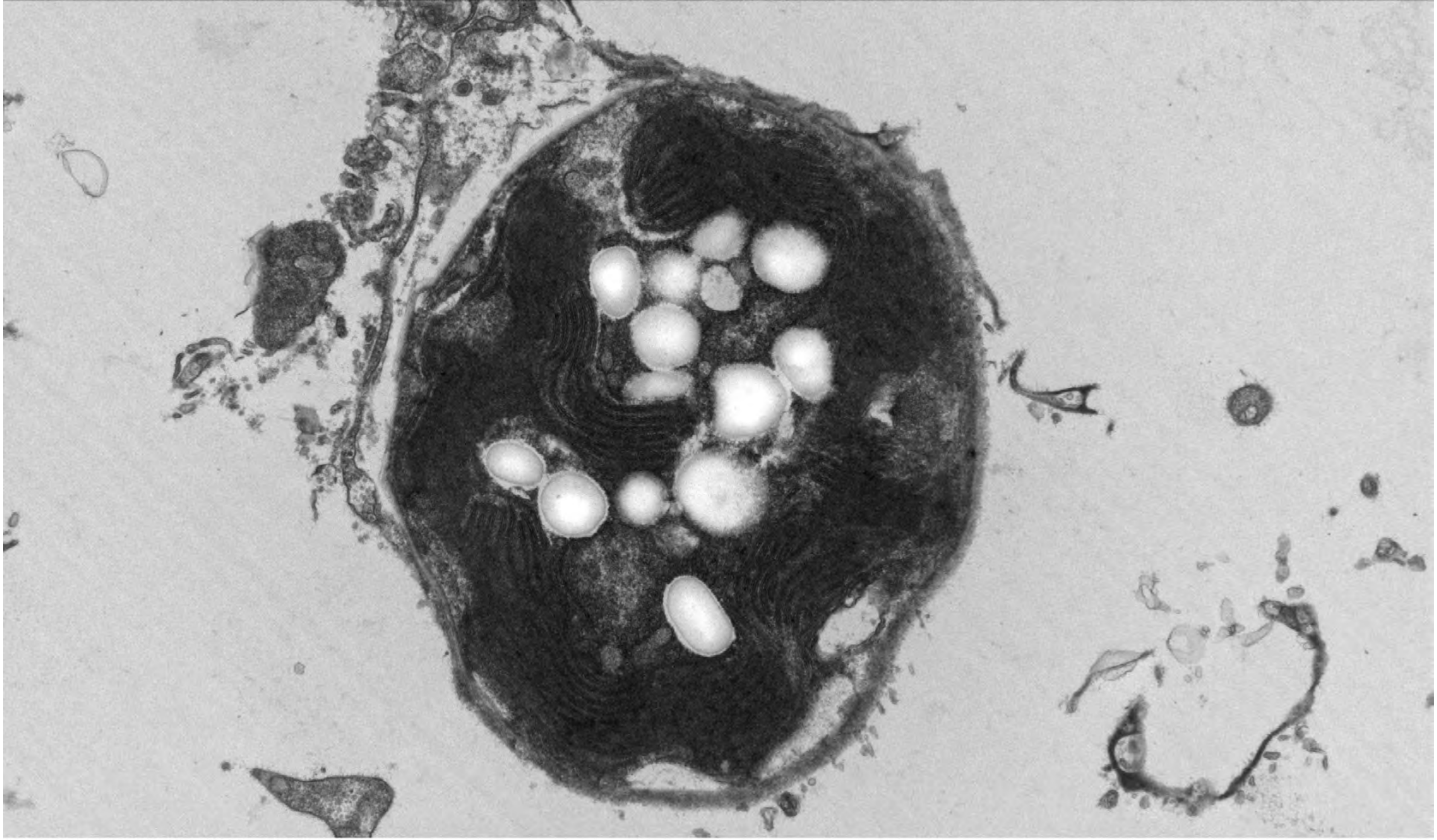

21-20_Correa_3-19-555_16A1_030.tif
Sample: 3/19, 555
Biological Electron Microscopy Lab
Rice University - SEA
Microscopist: MD Meyer

1 µm
HV=80kV
Direct Mag: 3000 x

Cell 21

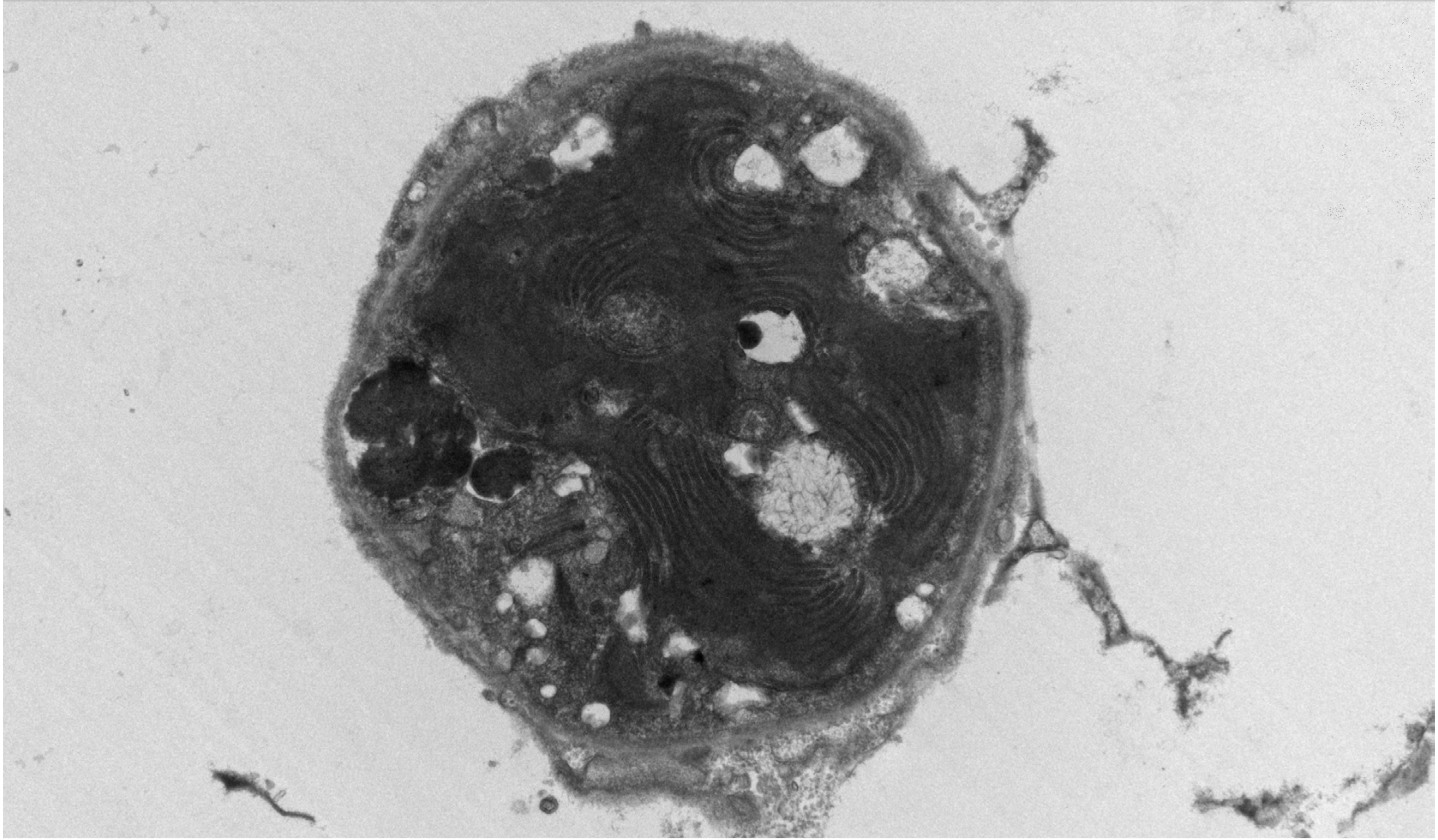

21-20_Correa_3-19-555_16A1_029.tif
Sample: 3/19, 555
Biological Electron Microscopy Lab
Rice University - SEA
Microscopist: MD Meyer

1 µm
HV=80kV
Direct Mag: 3000 x

Cell 22

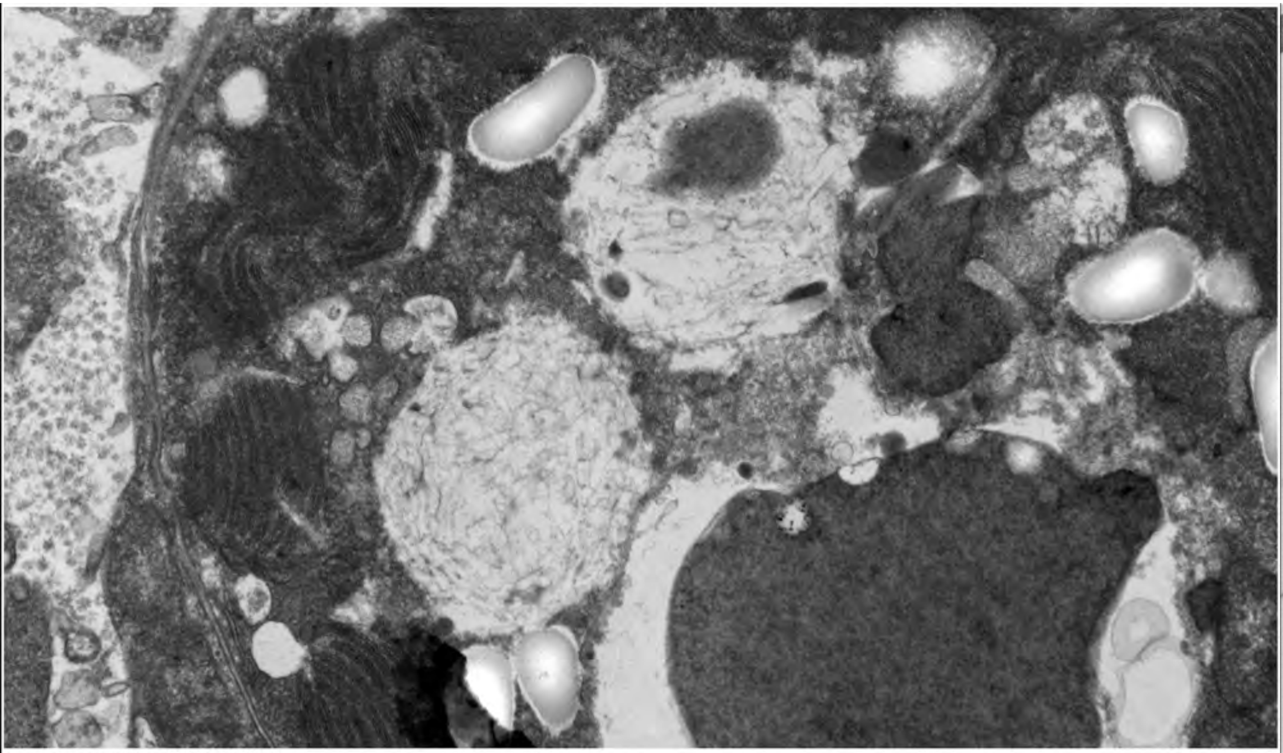

21-20_Correa_3-19-555_16A1_028.tif
Sample: 3/19, 555
Biological Electron Microscopy Lab
Rice University - SEA
Microscopist: MD Meyer

800 nm
HV=80kV
Direct Mag: 5000 x

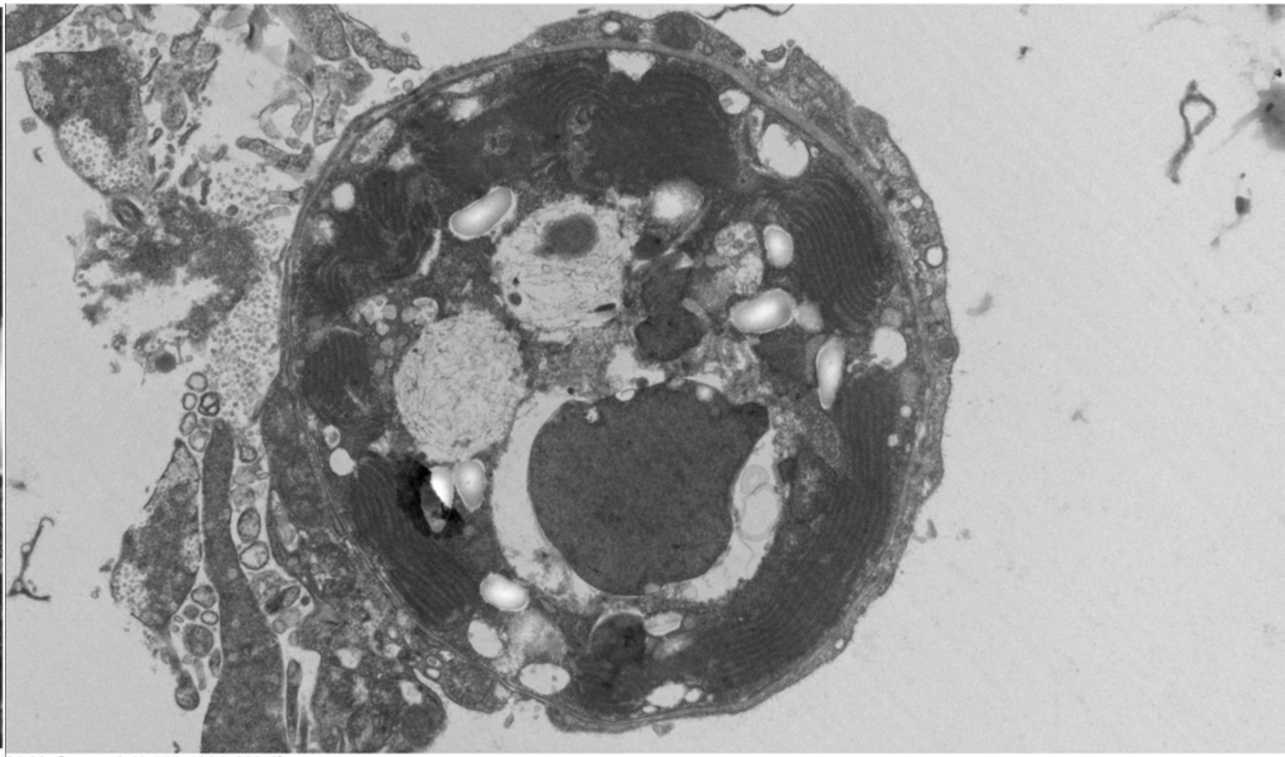

21-20_Correa_3-19-555_16A1_026.tif
Sample: 3/19, 555
Biological Electron Microscopy Lab
Rice University - SEA
Microscopist: MD Meyer

1 μm
HV=80kV
Direct Mag: 2500 x

Cell 23

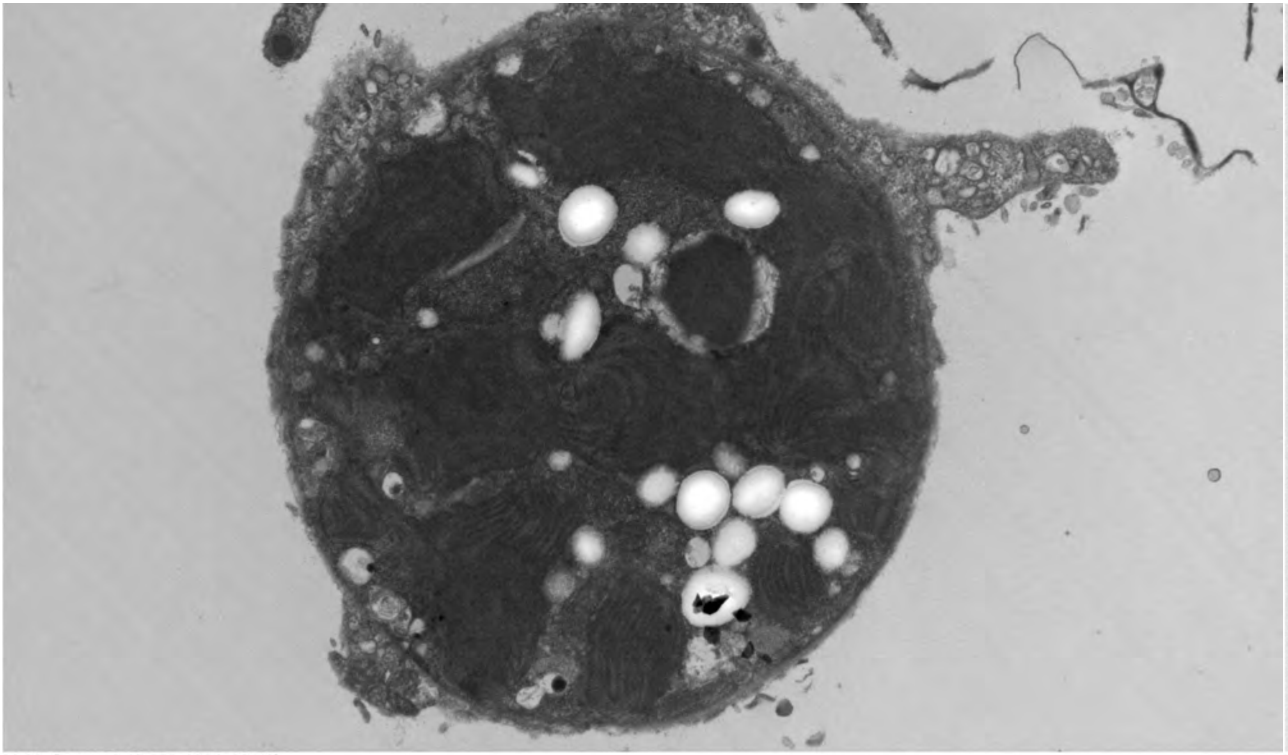

21-20_Correa_3-19-555_16A1_024.tif
Sample: 3/19, 555
Biological Electron Microscopy Lab
Rice University - SEA
Microscopist: MD Meyer

1 μm
HV=80kV
Direct Mag: 2500 x

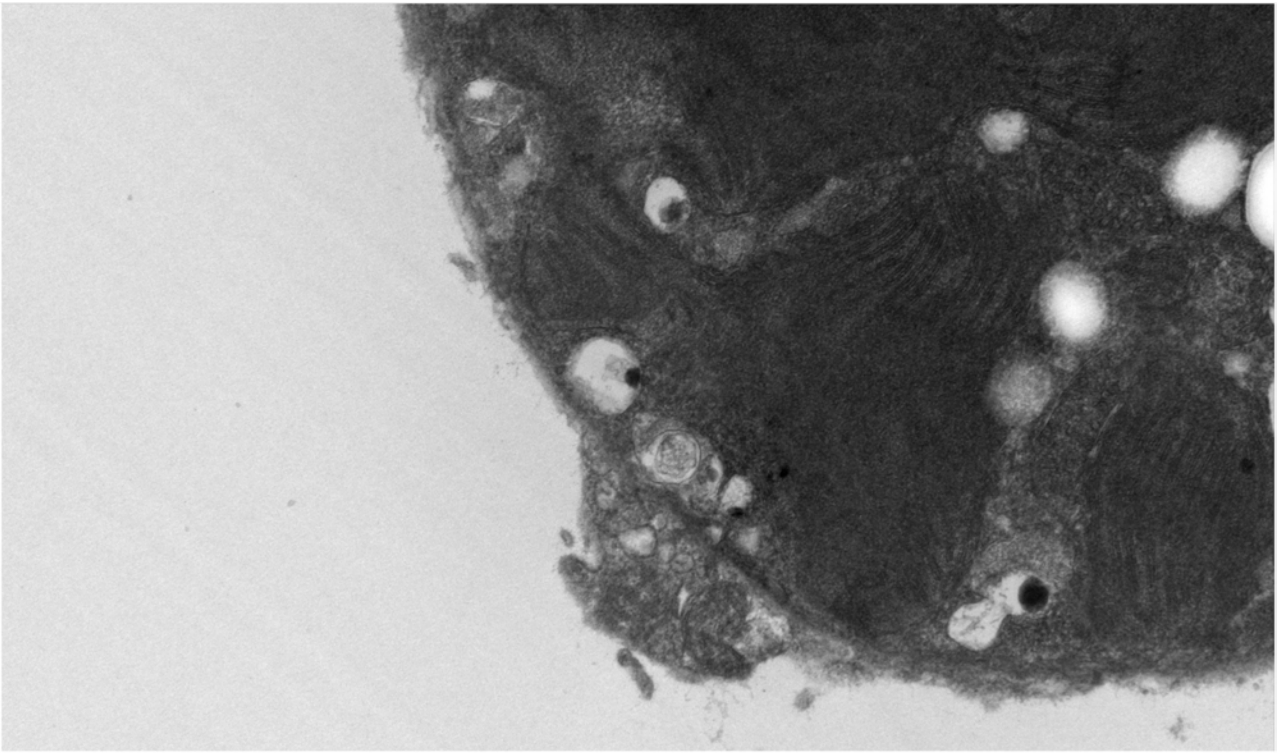

21-20_Correa_3-19-555_16A1_025.tif
Sample: 3/19, 555
Biological Electron Microscopy Lab
Rice University - SEA
Microscopist: MD Meyer

800 nm
HV=80kV
Direct Mag: 5000 x

Cell 24

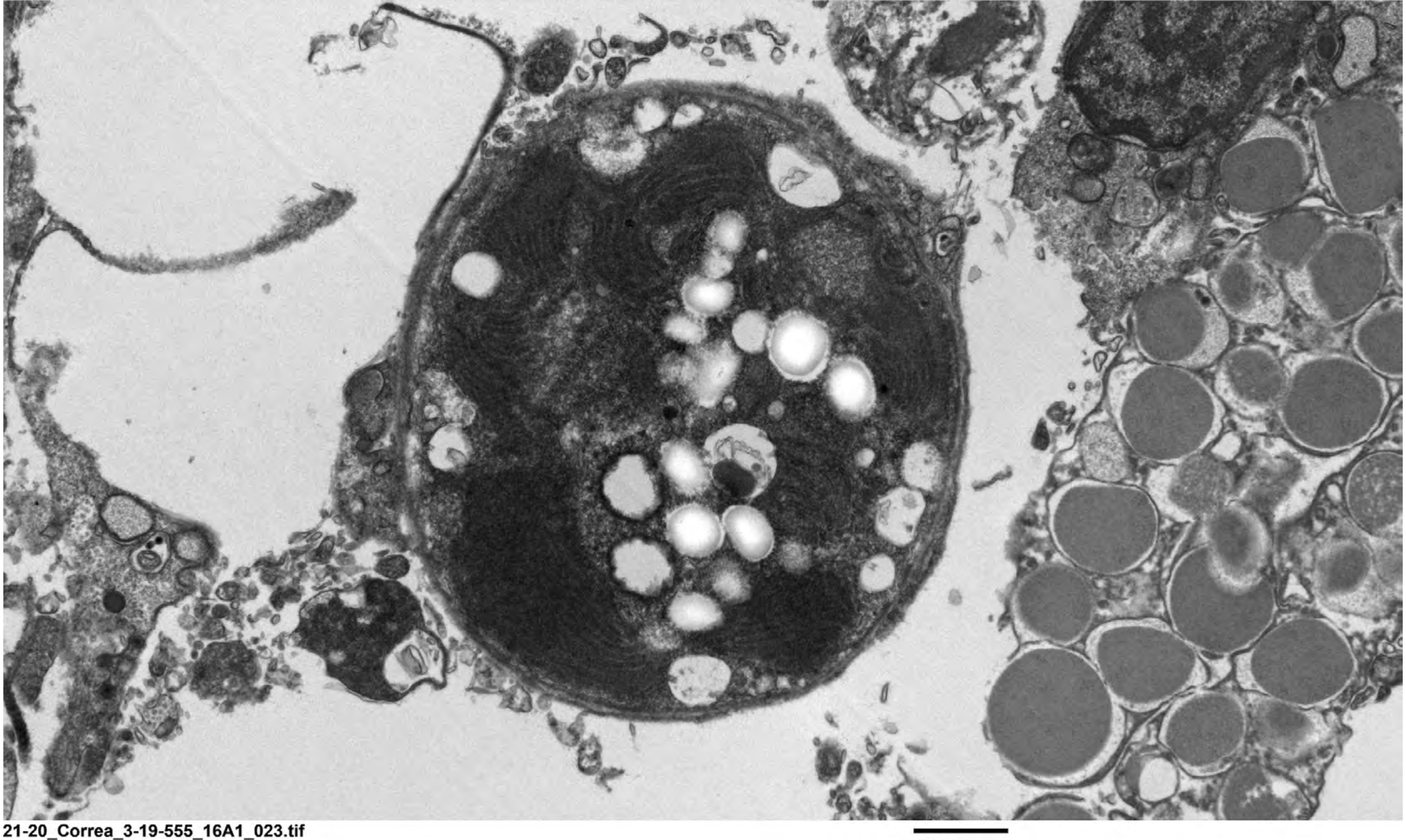

21-20_Correa_3-19-555_16A1_023.tif
Sample: 3/19, 555
Biological Electron Microscopy Lab
Rice University - SEA
Microscopist: MD Meyer

1 µm
HV=80kV
Direct Mag: 2500 x

Cell 25

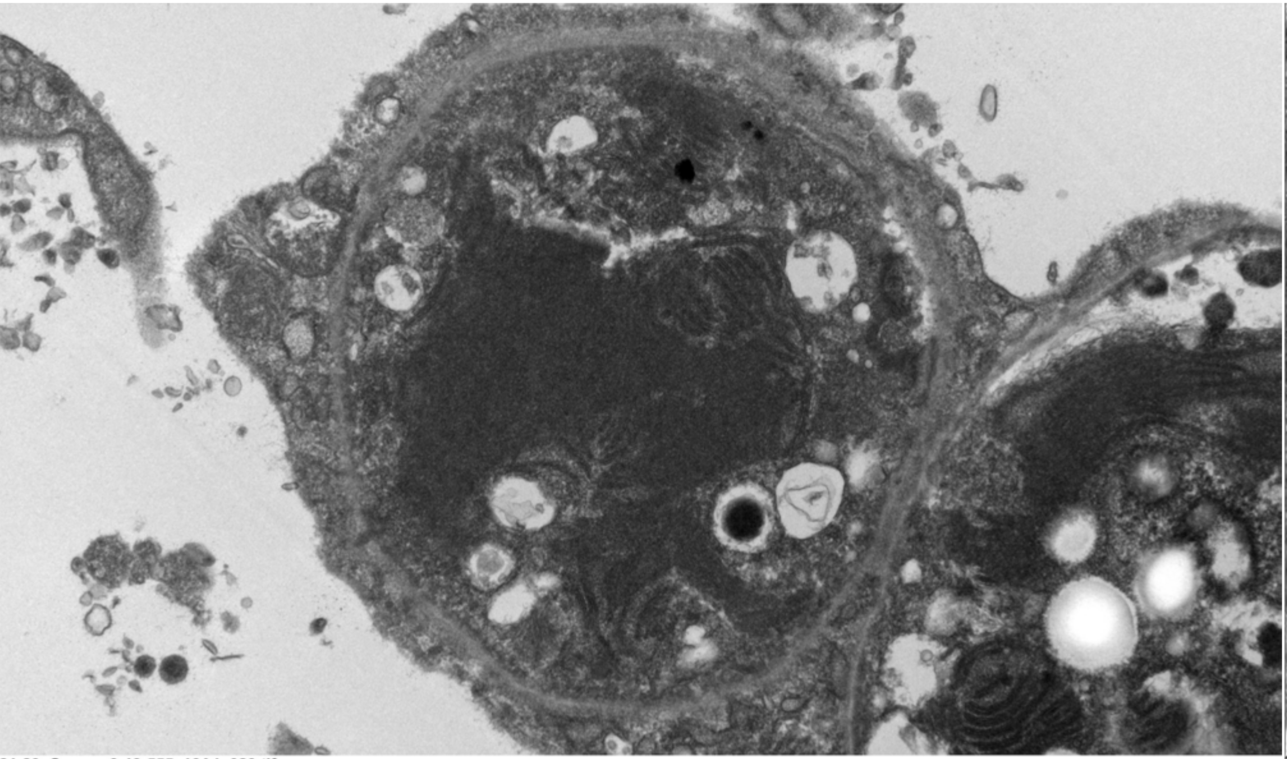

21-20_Correa_3-19-555_16A1_020.tif
Sample: 3/19, 555
Biological Electron Microscopy Lab
Rice University - SEA
Microscopist: MD Meyer

1 µm
HV=80kV
Direct Mag: 4000 x

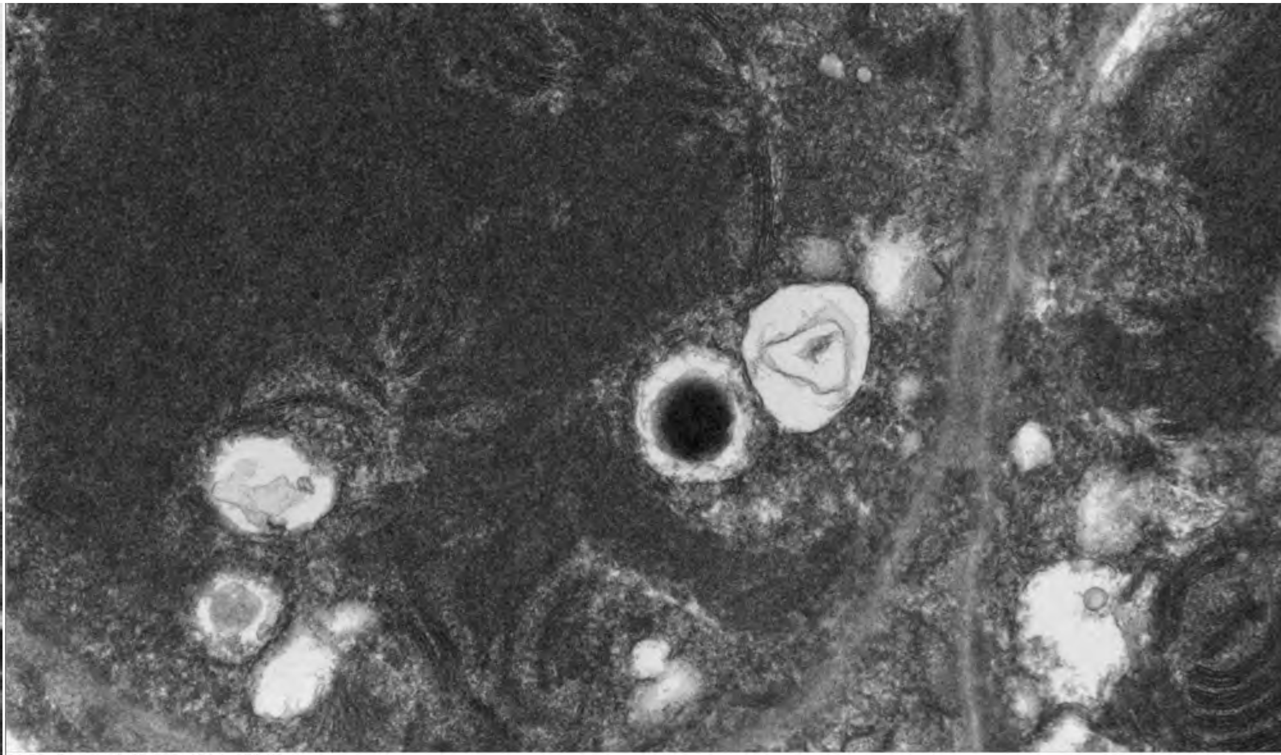

21-20_Correa_3-19-555_16A1_021.tif
Sample: 3/19, 555
Biological Electron Microscopy Lab
Rice University - SEA
Microscopist: MD Meyer

500 nm
HV=80kV
Direct Mag: 8000 x

Cell 26

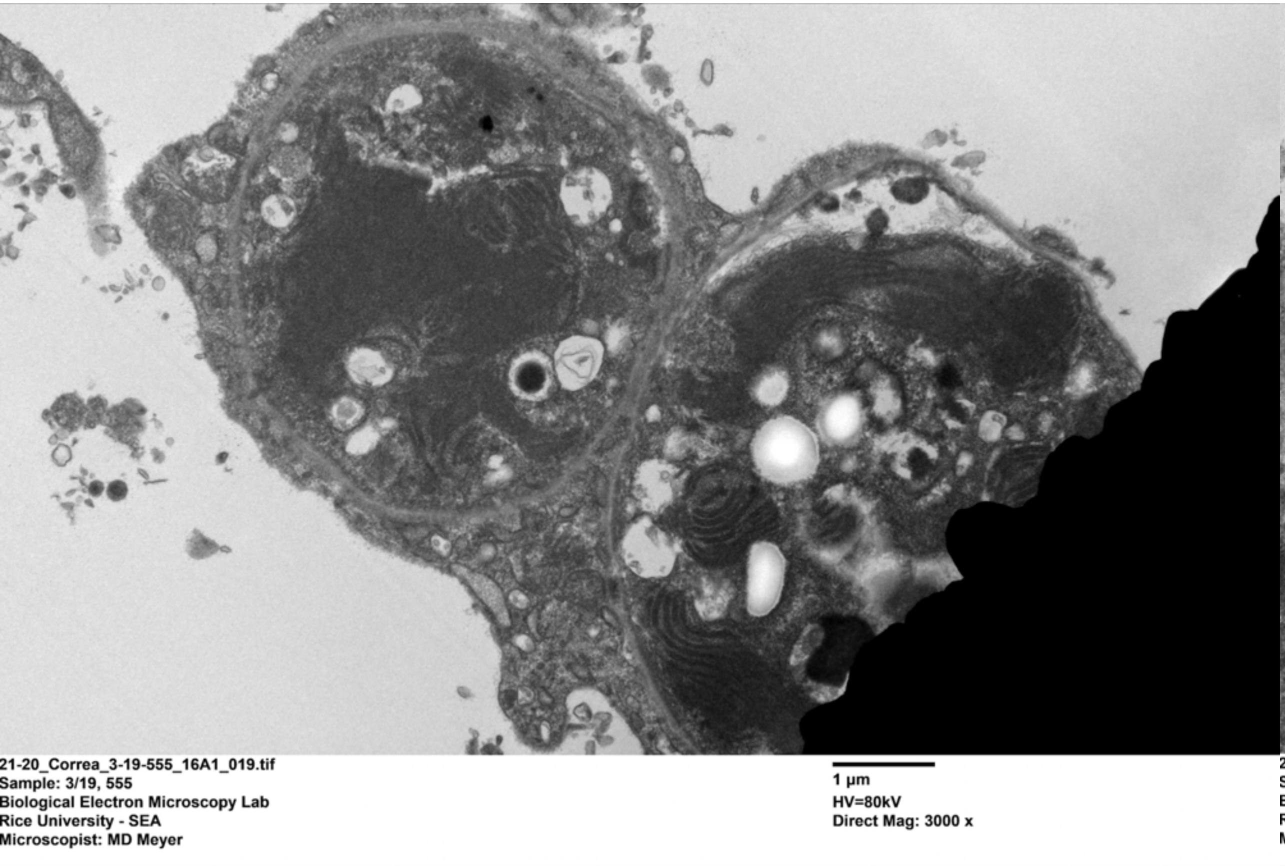
21-20_Correa_3-19-555_16A1_019.tif
Sample: 3/19, 555
Biological Electron Microscopy Lab
Rice University - SEA
Microscopist: MD Meyer
1 µm
HV=80kV
Direct Mag: 3000 x

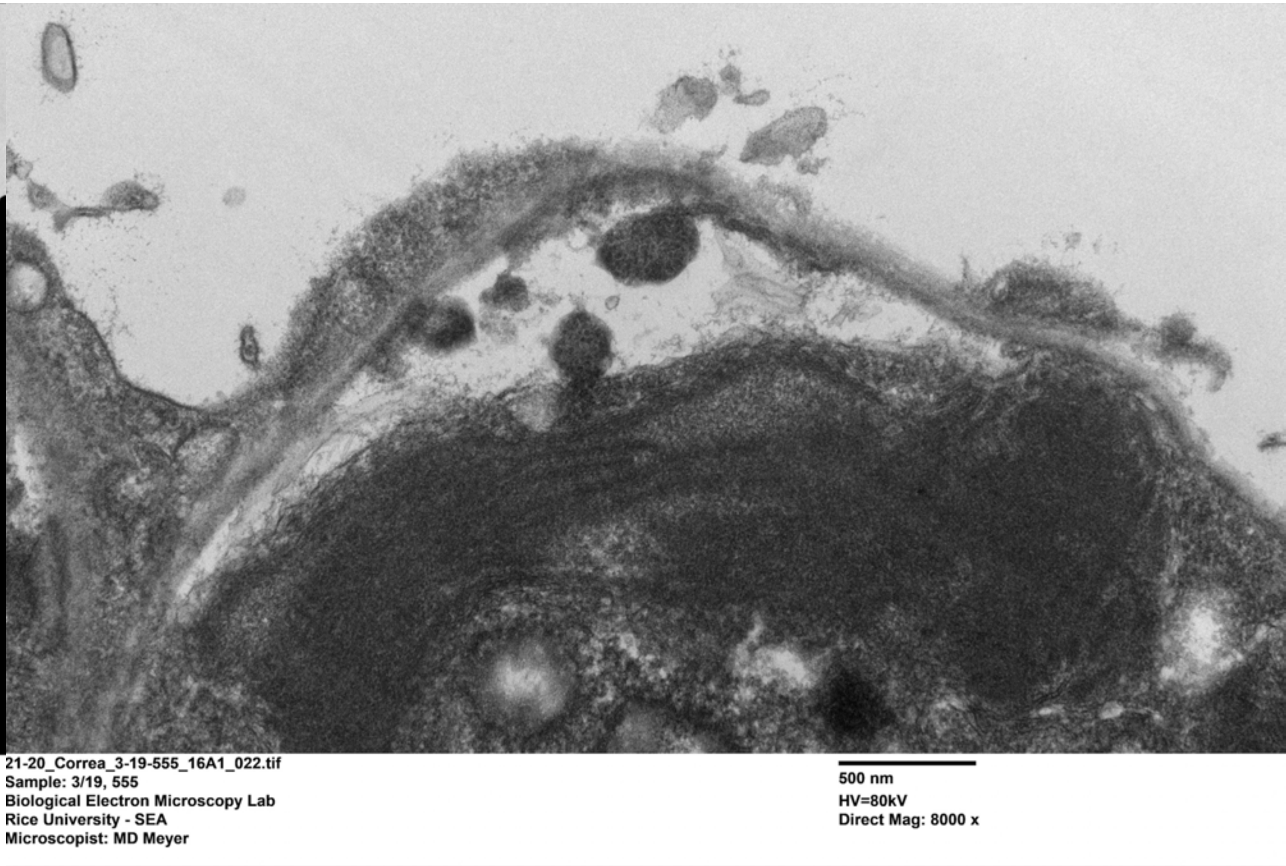
21-20_Correa_3-19-555_16A1_022.tif
Sample: 3/19, 555
Biological Electron Microscopy Lab
Rice University - SEA
Microscopist: MD Meyer
500 nm
HV=80kV
Direct Mag: 8000 x

Cell 27

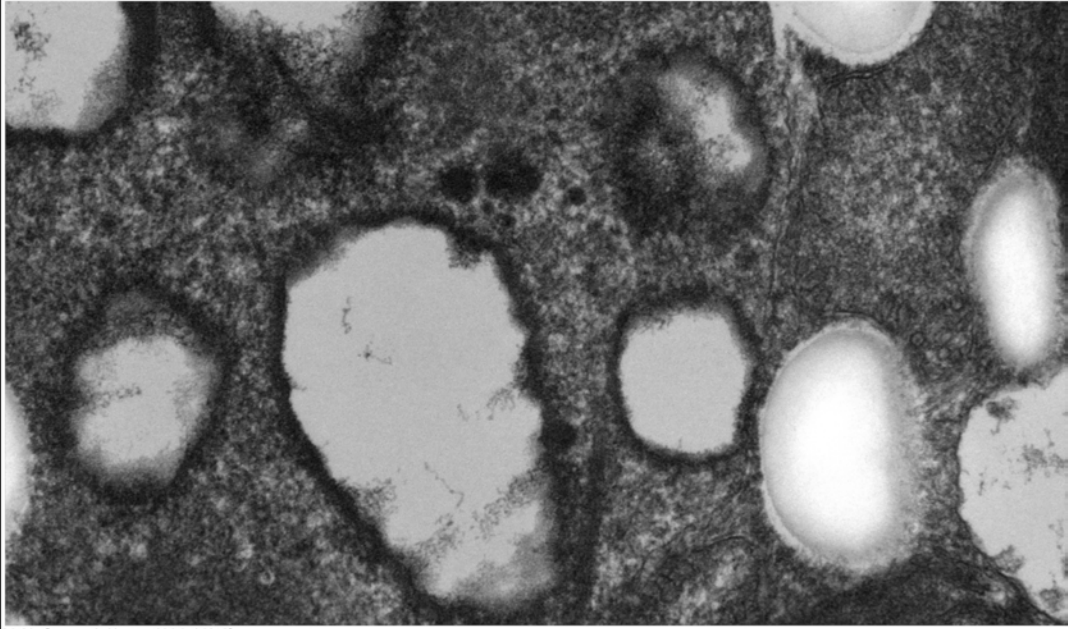

21-20_Correa_3-19-555_16A1_018.tif
Sample: 3/19, 555
Biological Electron Microscopy Lab
Rice University - SEA
Microscopist: MD Meyer

400 nm
HV=80kV
Direct Mag: 10000 x

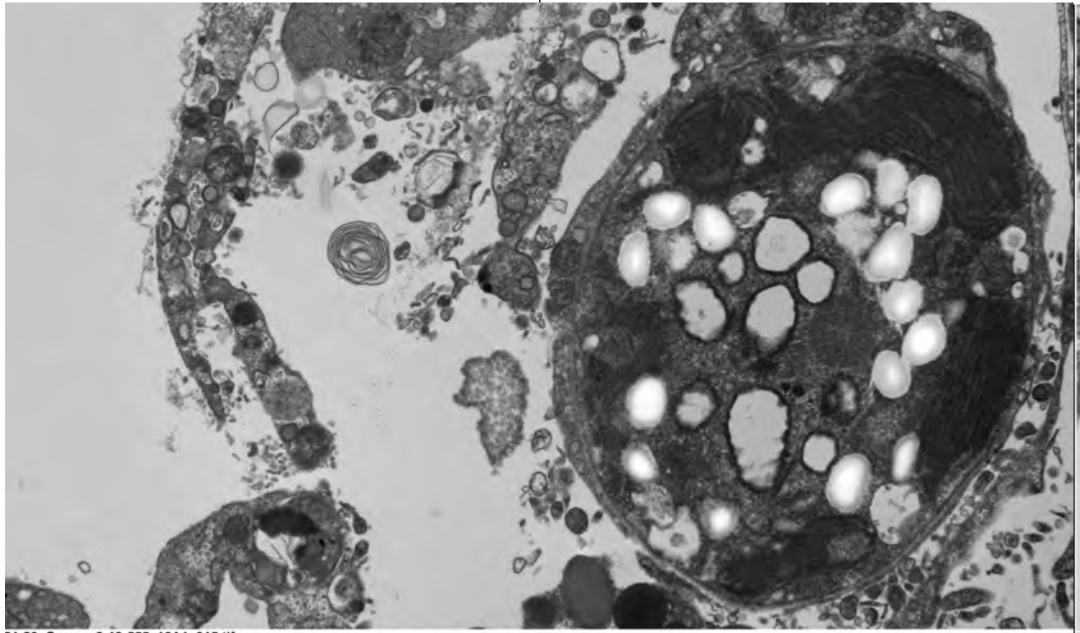

21-20_Correa_3-19-555_16A1_015.tif
Sample: 3/19, 555
Biological Electron Microscopy Lab
Rice University - SEA
Microscopist: MD Meyer

1 µm
HV=80kV
Direct Mag: 2500 x

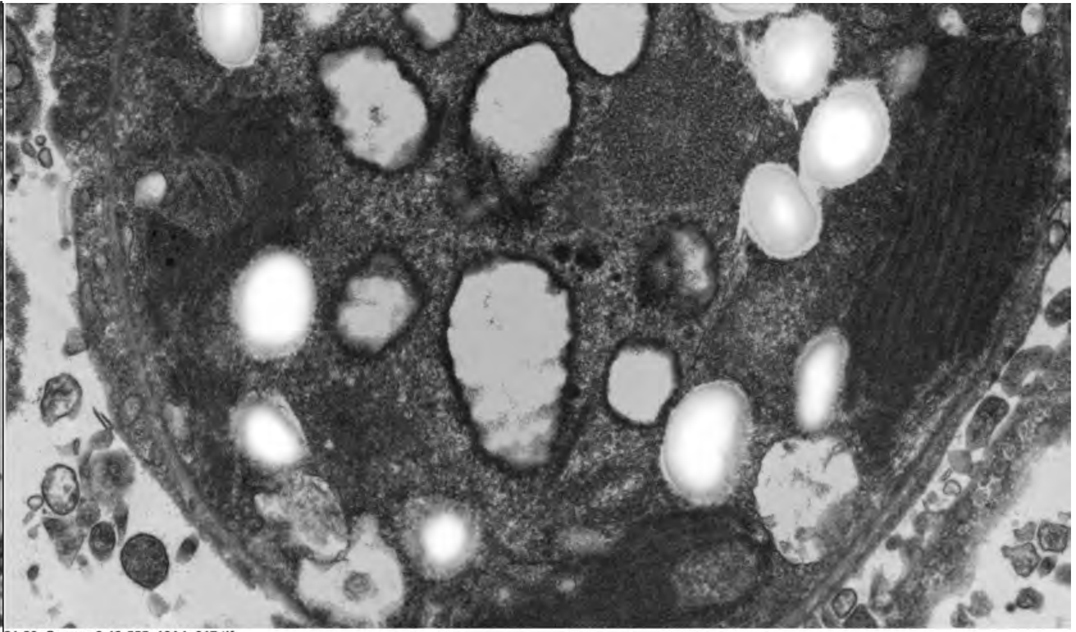

21-20_Correa_3-19-555_16A1_017.tif
Sample: 3/19, 555
Biological Electron Microscopy Lab
Rice University - SEA
Microscopist: MD Meyer

800 nm
HV=80kV
Direct Mag: 5000 x

Cell 28

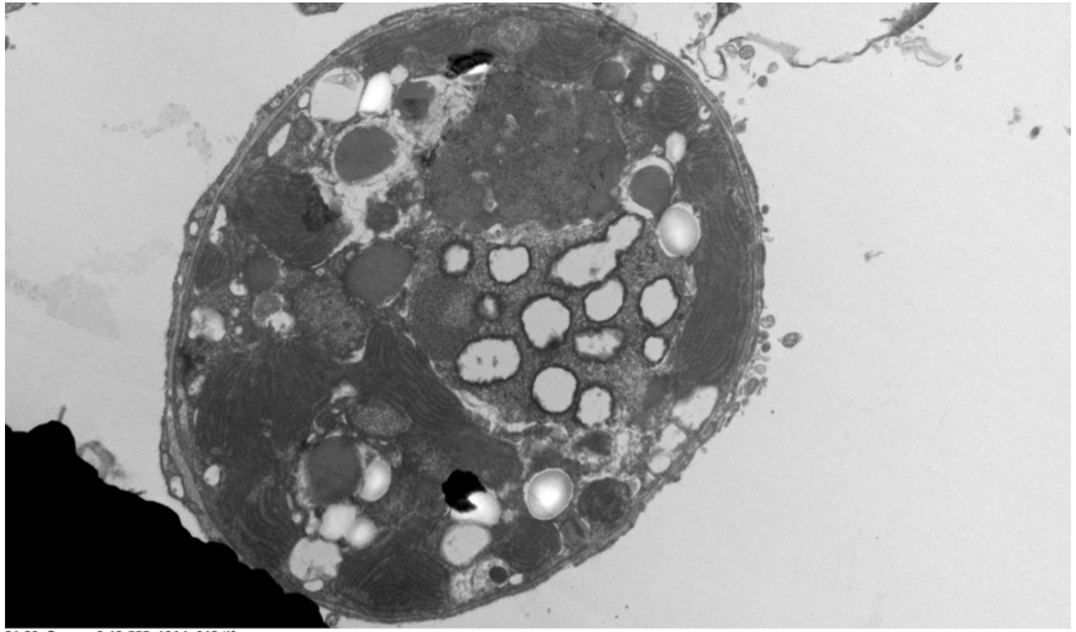

21-20_Correa_3-19-555_16A1_012.tif
Sample: 3/19, 555
Biological Electron Microscopy Lab
Rice University - SEA
Microscopist: MD Meyer

2 µm
HV=80kV
Direct Mag: 2000 x

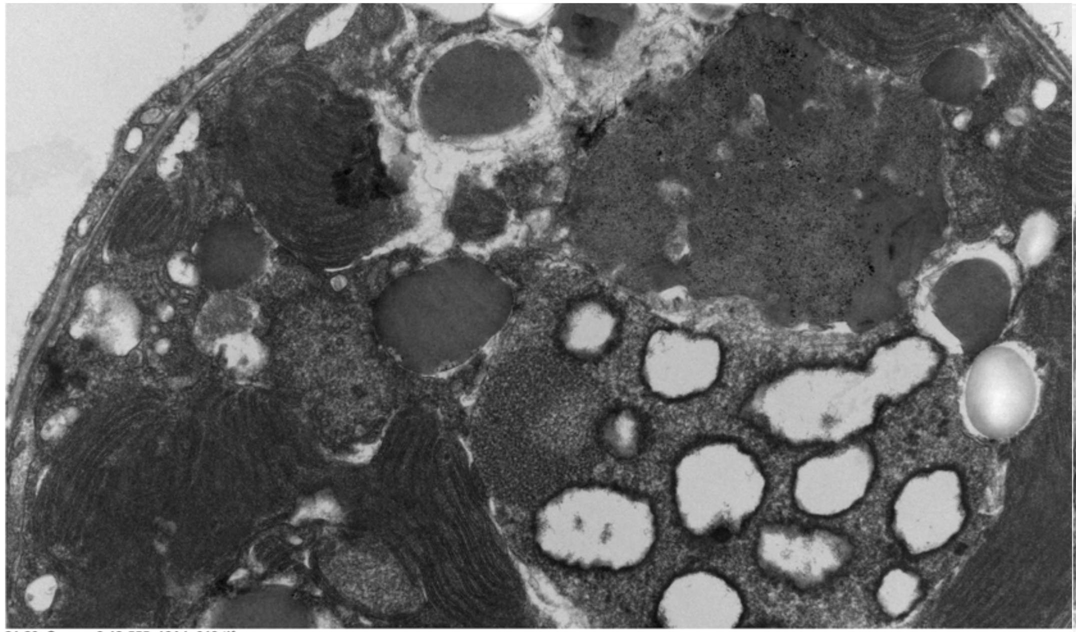

21-20_Correa_3-19-555_16A1_013.tif
Sample: 3/19, 555
Biological Electron Microscopy Lab
Rice University - SEA
Microscopist: MD Meyer

1 µm
HV=80kV
Direct Mag: 4000 x

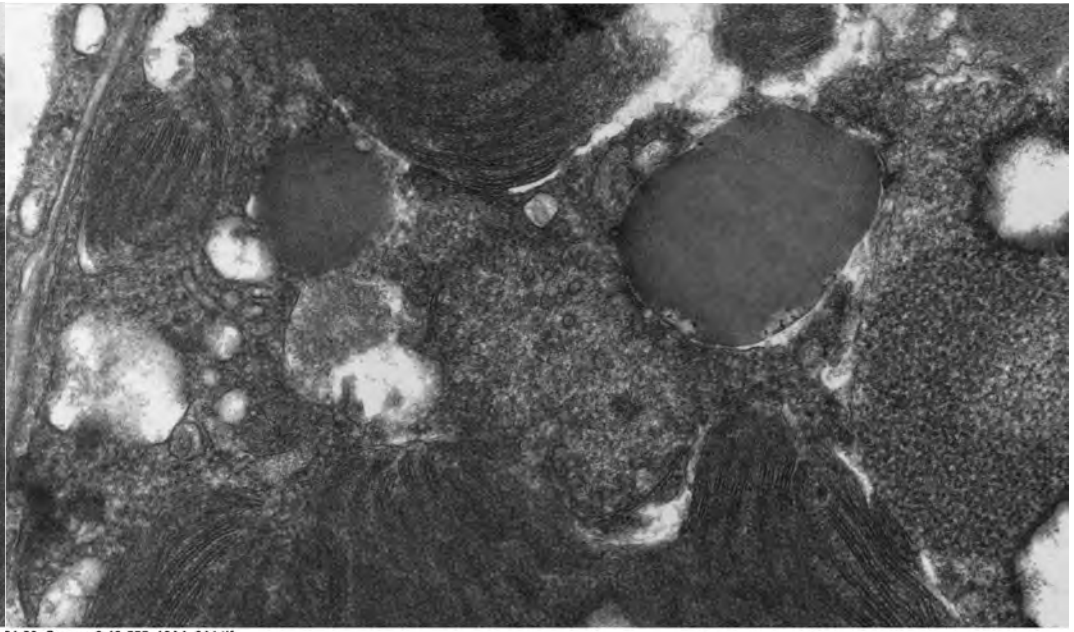

21-20_Correa_3-19-555_16A1_014.tif
Sample: 3/19, 555
Biological Electron Microscopy Lab
Rice University - SEA
Microscopist: MD Meyer

500 nm
HV=80kV
Direct Mag: 8000 x

Cell 29

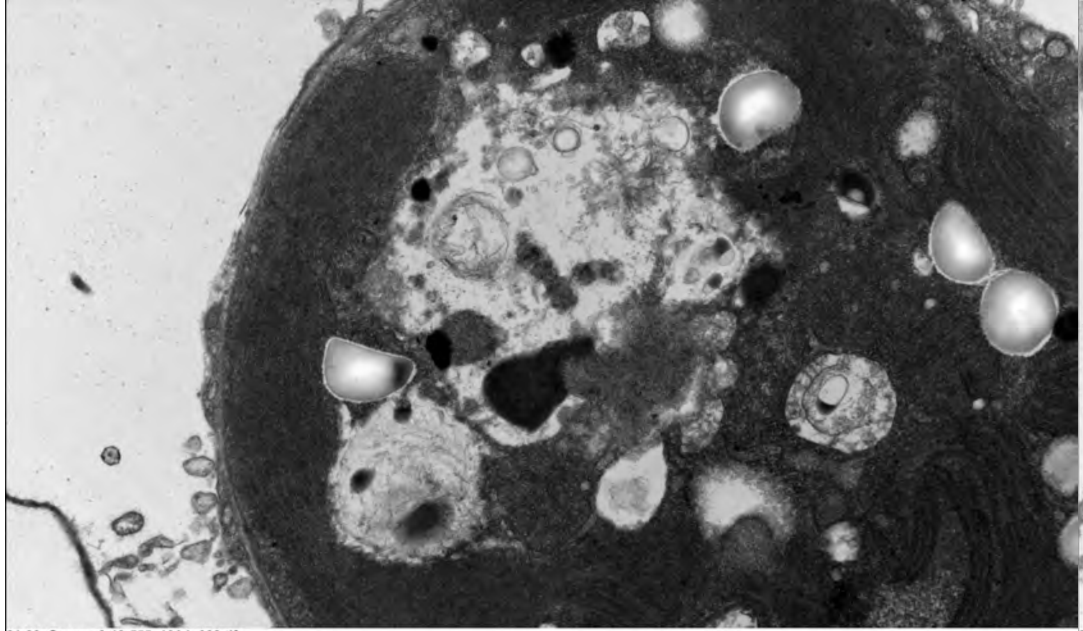

21-20_Correa_3-19-555_16A1_008.tif
Sample: 3/19, 555
Biological Electron Microscopy Lab
Rice University - SEA
Microscopist: MD Meyer

1 µm
HV=80kV
Direct Mag: 4000 x

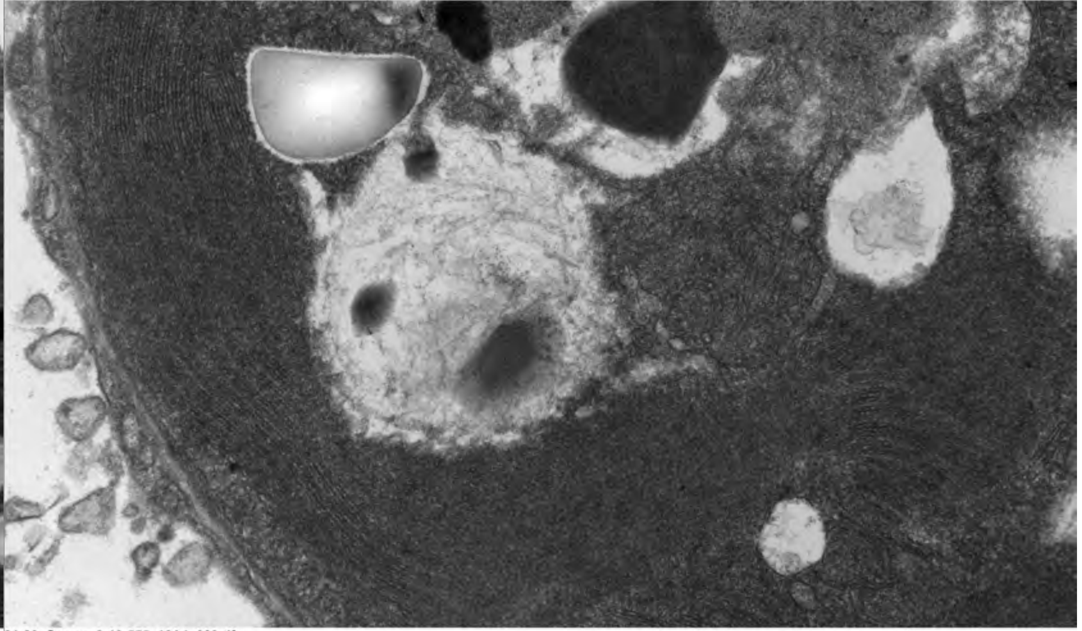

21-20_Correa_3-19-555_16A1_009.tif
Sample: 3/19, 555
Biological Electron Microscopy Lab
Rice University - SEA
Microscopist: MD Meyer

500 nm
HV=80kV
Direct Mag: 8000 x

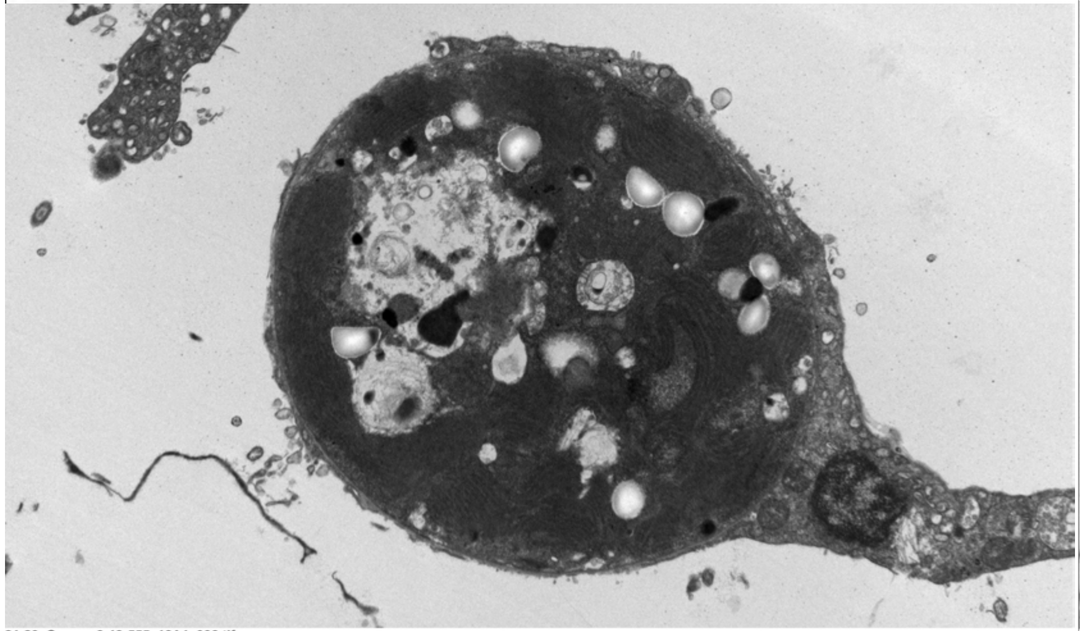

21-20_Correa_3-19-555_16A1_006.tif
Sample: 3/19, 555
Biological Electron Microscopy Lab
Rice University - SEA
Microscopist: MD Meyer

2 µm
HV=80kV
Direct Mag: 2000 x

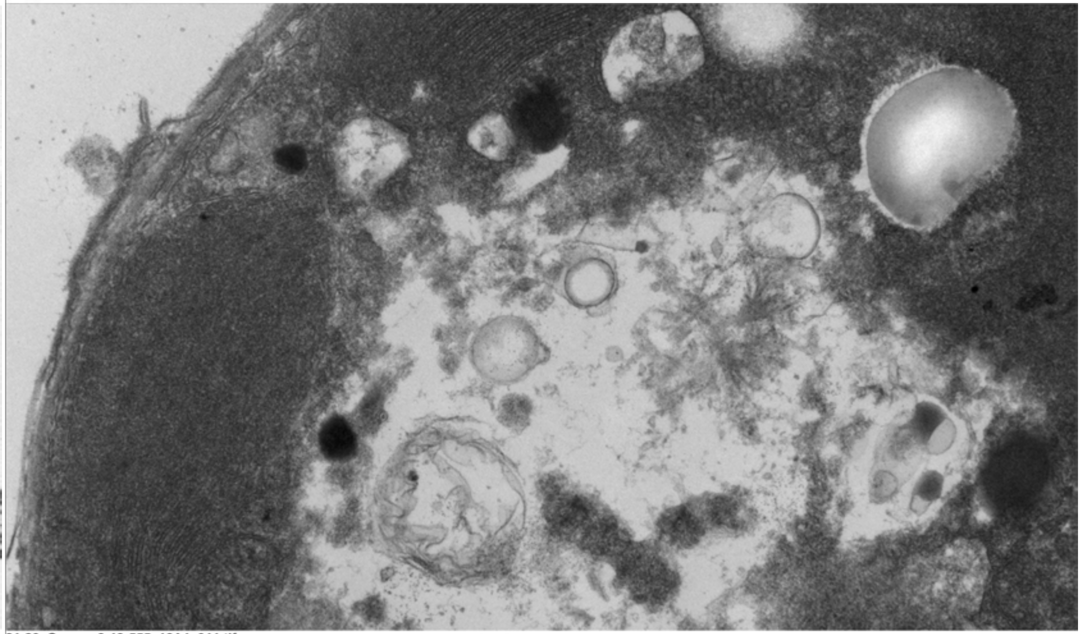

21-20_Correa_3-19-555_16A1_011.tif
Sample: 3/19, 555
Biological Electron Microscopy Lab
Rice University - SEA
Microscopist: MD Meyer

500 nm
HV=80kV
Direct Mag: 8000 x

Cell 30

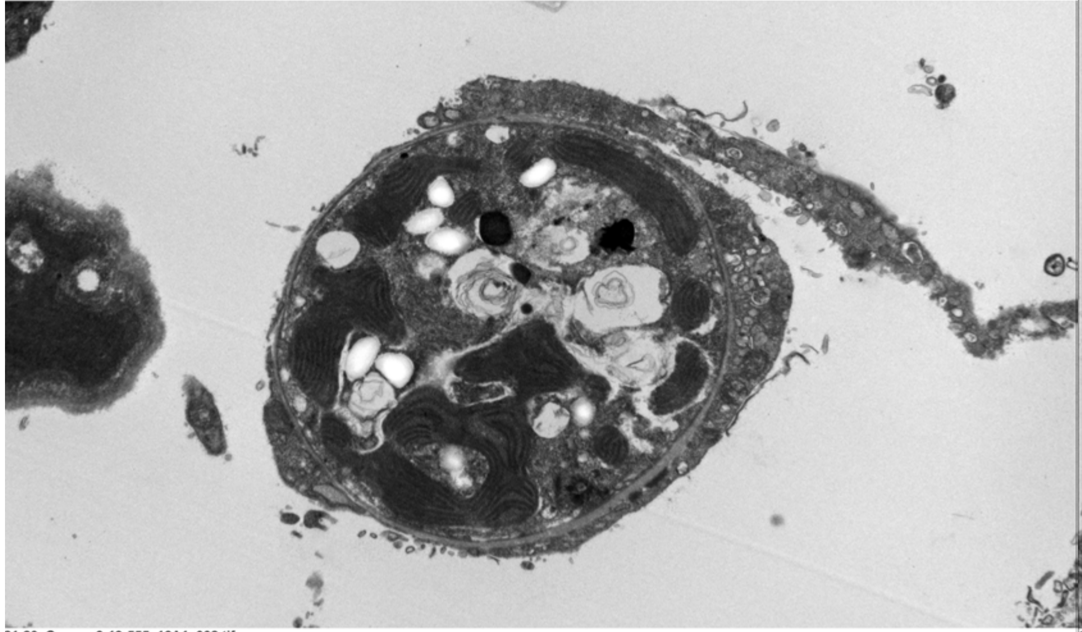

21-20_Correa_3-19-555_16A1_002.tif
Sample: 3/19, 555
Biological Electron Microscopy Lab
Rice University - SEA
Microscopist: MD Meyer

2 µm
HV=80kV
Direct Mag: 2000 x

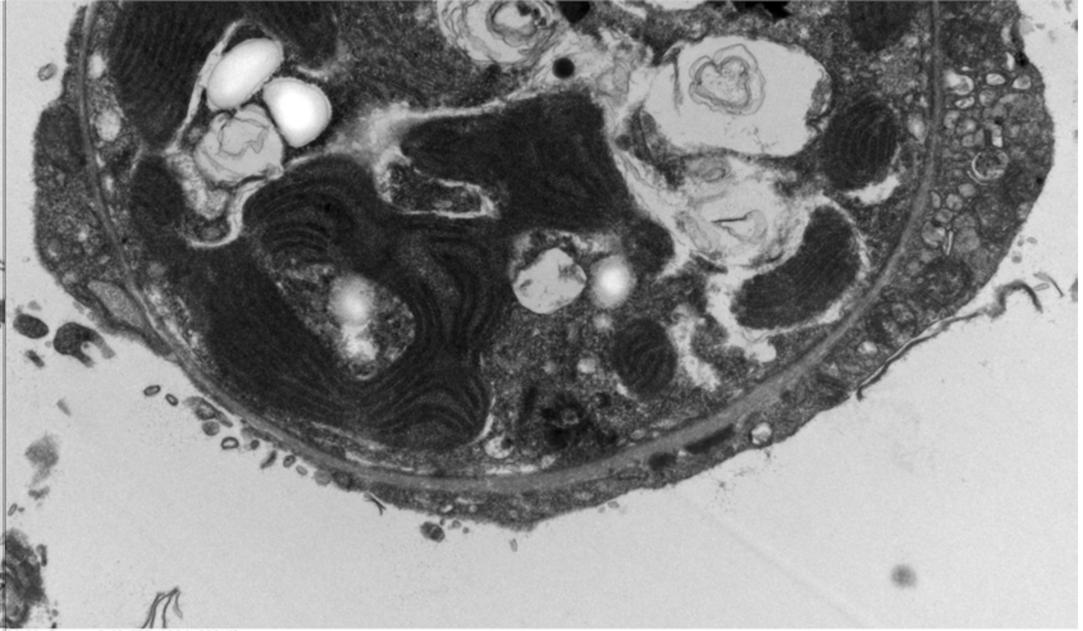

21-20_Correa_3-19-555_16A1_003.tif
Sample: 3/19, 555
Biological Electron Microscopy Lab
Rice University - SEA
Microscopist: MD Meyer

1 µm
HV=80kV
Direct Mag: 4000 x

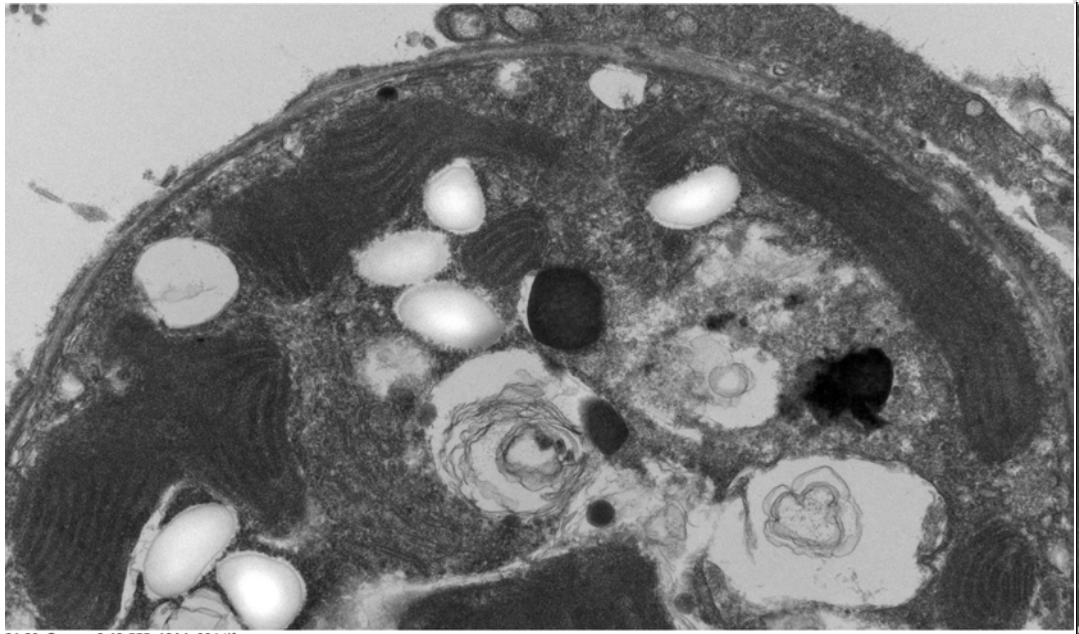

21-20_Correa_3-19-555_16A1_004.tif
Sample: 3/19, 555
Biological Electron Microscopy Lab
Rice University - SEA
Microscopist: MD Meyer

800 nm
HV=80kV
Direct Mag: 5000 x

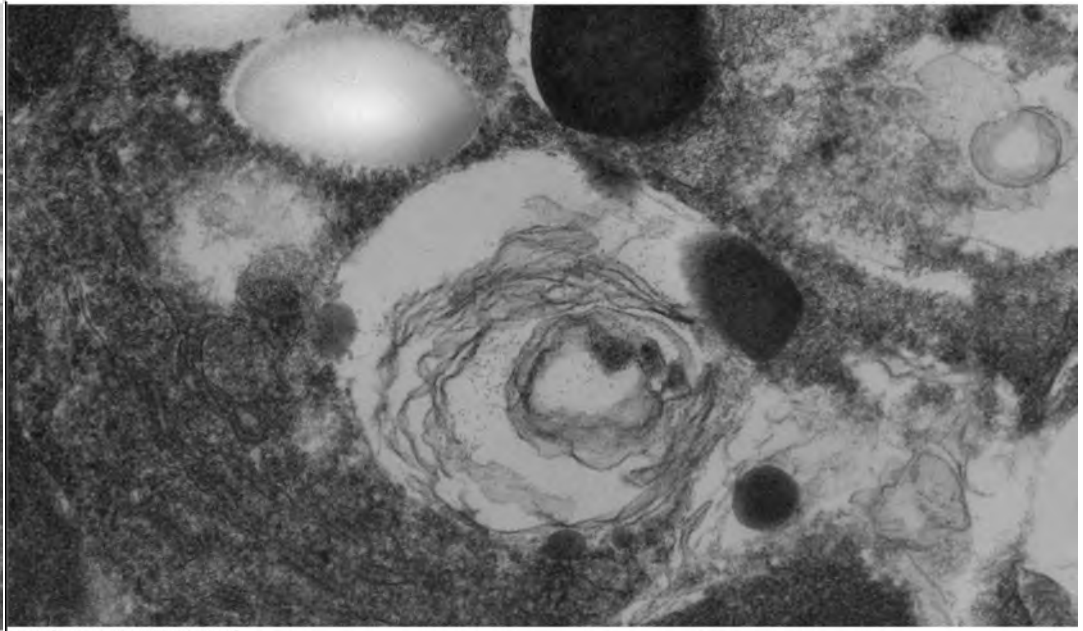

21-20_Correa_3-19-555_16A1_005.tif
Sample: 3/19, 555
Biological Electron Microscopy Lab
Rice University - SEA
Microscopist: MD Meyer

200 nm
HV=80kV
Direct Mag: 12000 x

Ambient (March 2018) POR Colony N

Cell 1

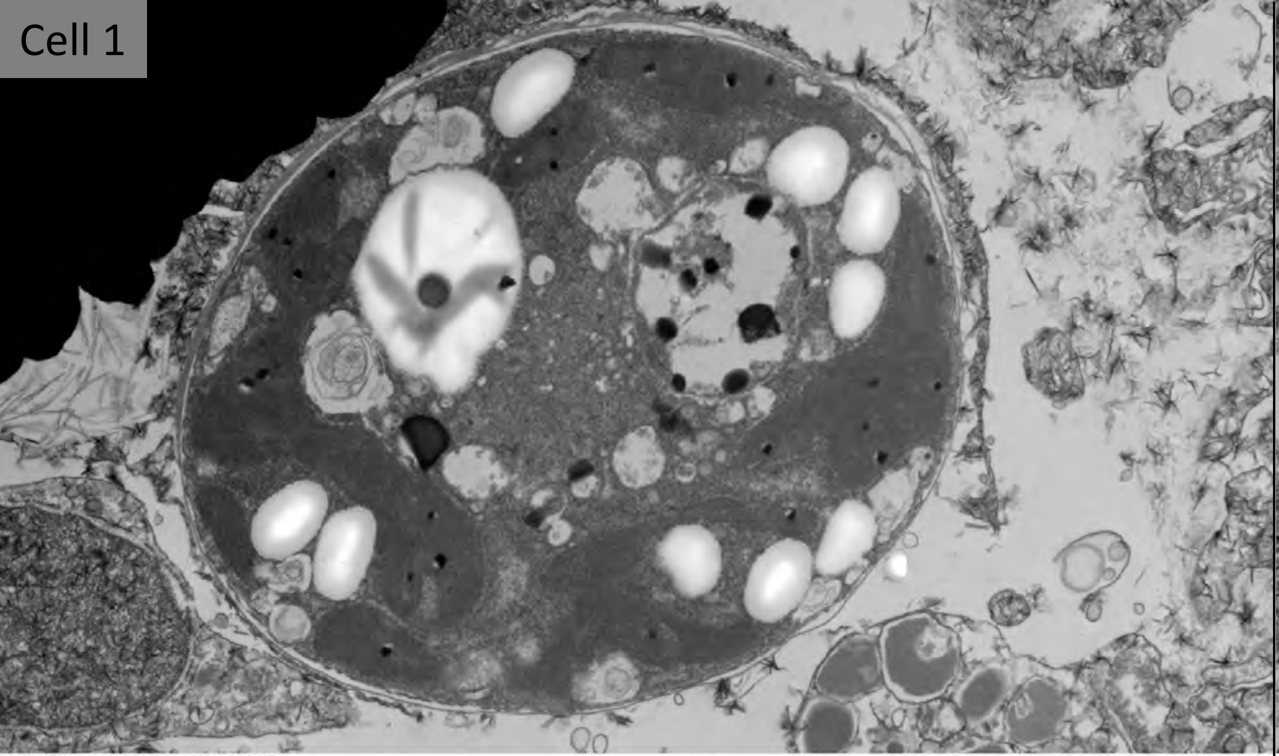

21-20_Correa_3-18-590_16K4_039.tif
Sample: 3/18, 590
Biological Electron Microscopy Lab
Rice University - SEA
Microscopist: MD Meyer

1 μm
HV=80kV
Direct Mag: 2500 x

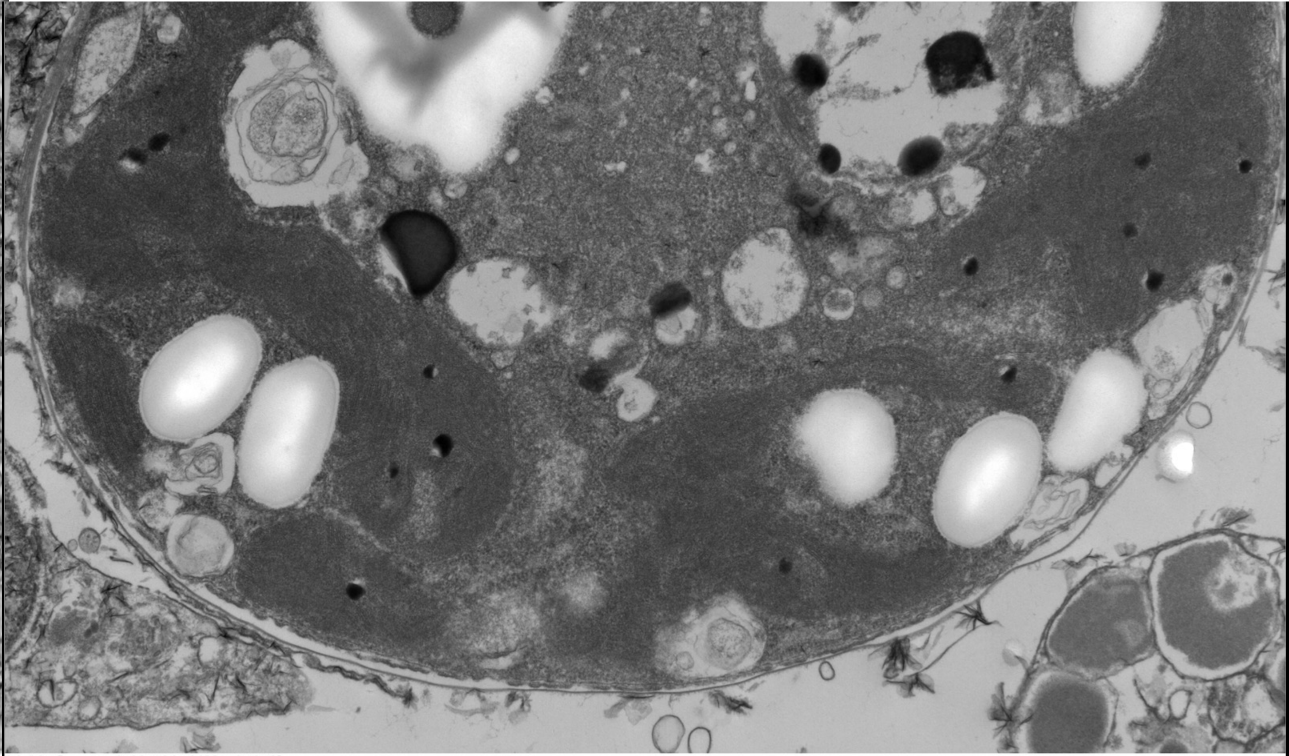

21-20_Correa_3-18-590_16K4_040.tif
Sample: 3/18, 590
Biological Electron Microscopy Lab
Rice University - SEA
Microscopist: MD Meyer

1 μm
HV=80kV
Direct Mag: 4000 x

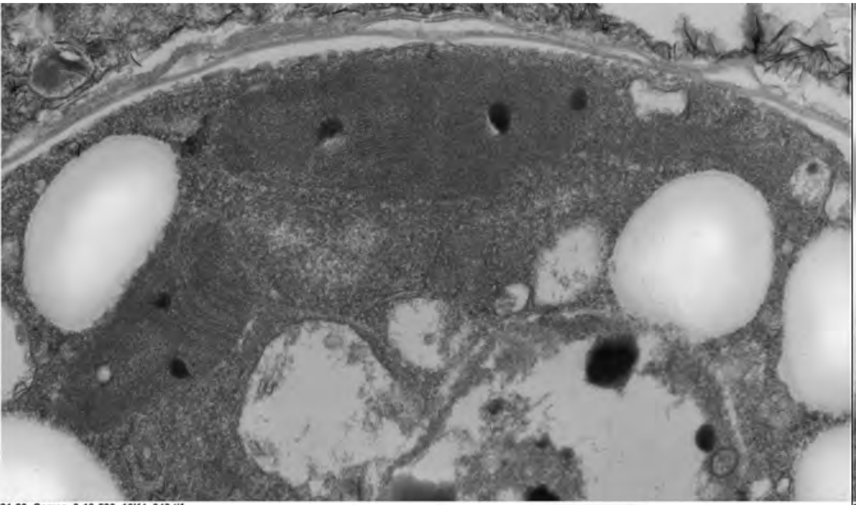

21-20_Correa_3-18-590_16K4_043.tif
Sample: 3/18, 590
Biological Electron Microscopy Lab
Rice University - SEA
Microscopist: MD Meyer

500 nm
HV=80kV
Direct Mag: 8000 x

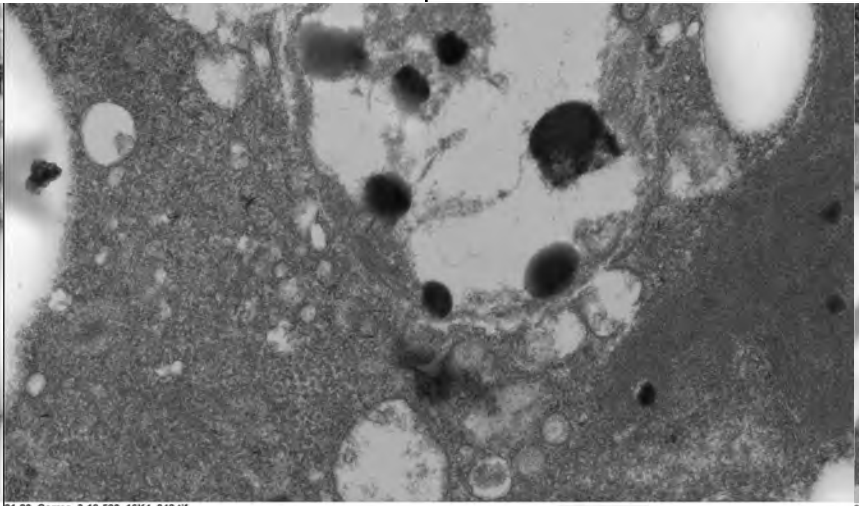

21-20_Correa_3-18-590_16K4_042.tif
Sample: 3/18, 590
Biological Electron Microscopy Lab
Rice University - SEA
Microscopist: MD Meyer

500 nm
HV=80kV
Direct Mag: 8000 x

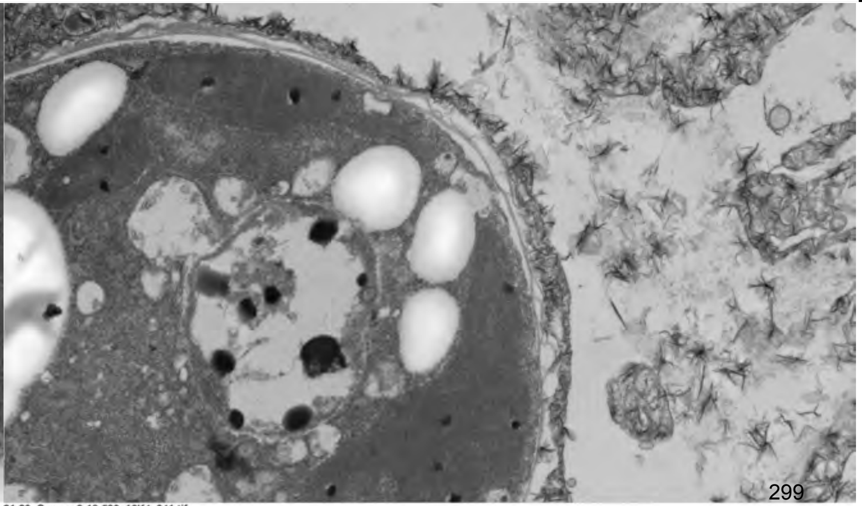

21-20_Correa_3-18-590_16K4_041.tif
Sample: 3/18, 590
Biological Electron Microscopy Lab
Rice University - SEA
Microscopist: MD Meyer

1 μm
HV=80kV
Direct Mag: 4000 x

Cell 2

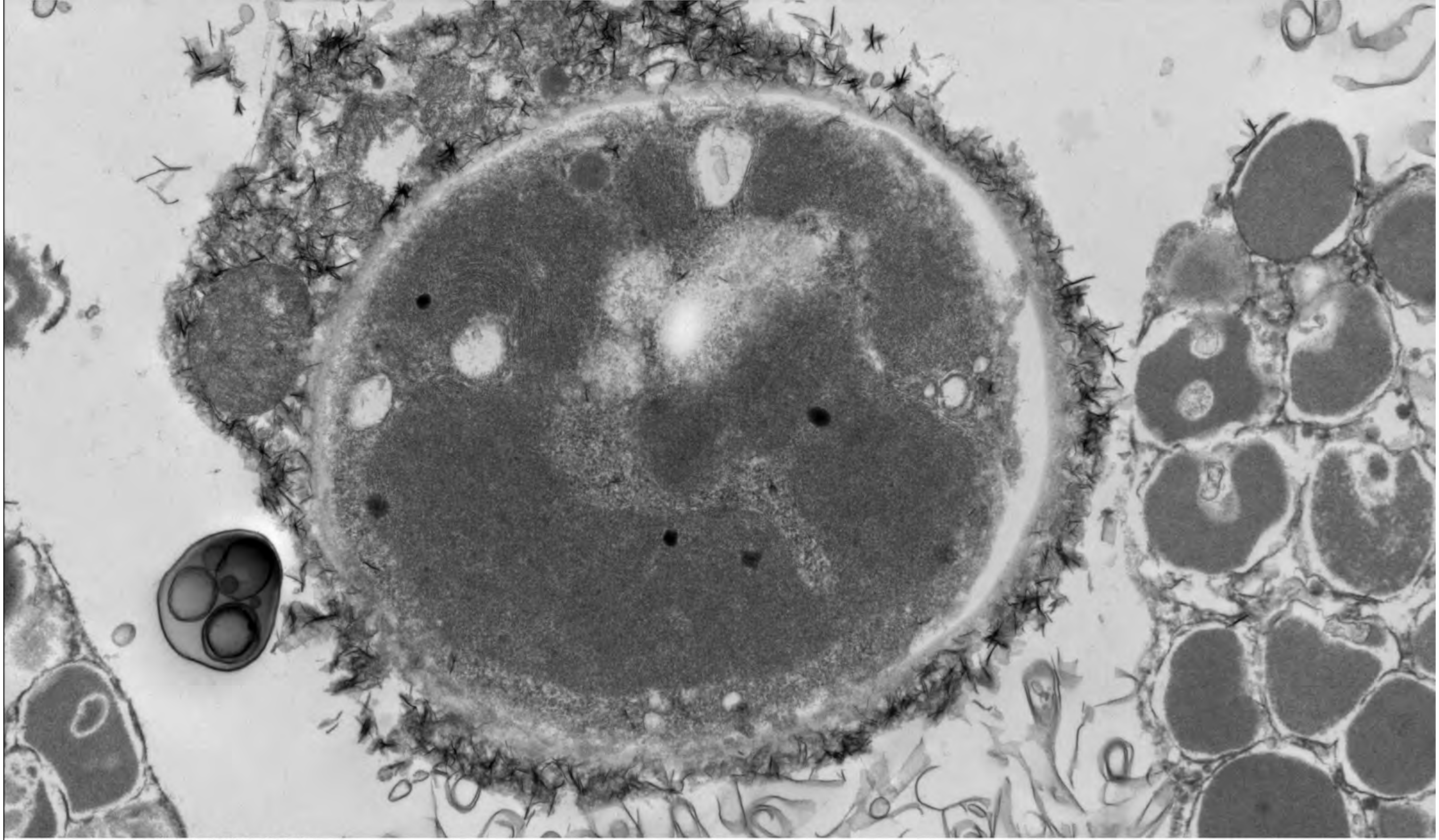

21-20_Correa_3-18-590_16K4_038.tif
Sample: 3/18, 590
Biological Electron Microscopy Lab
Rice University - SEA
Microscopist: MD Meyer

1 µm
HV=80kV
Direct Mag: 4000 x

Cell 3

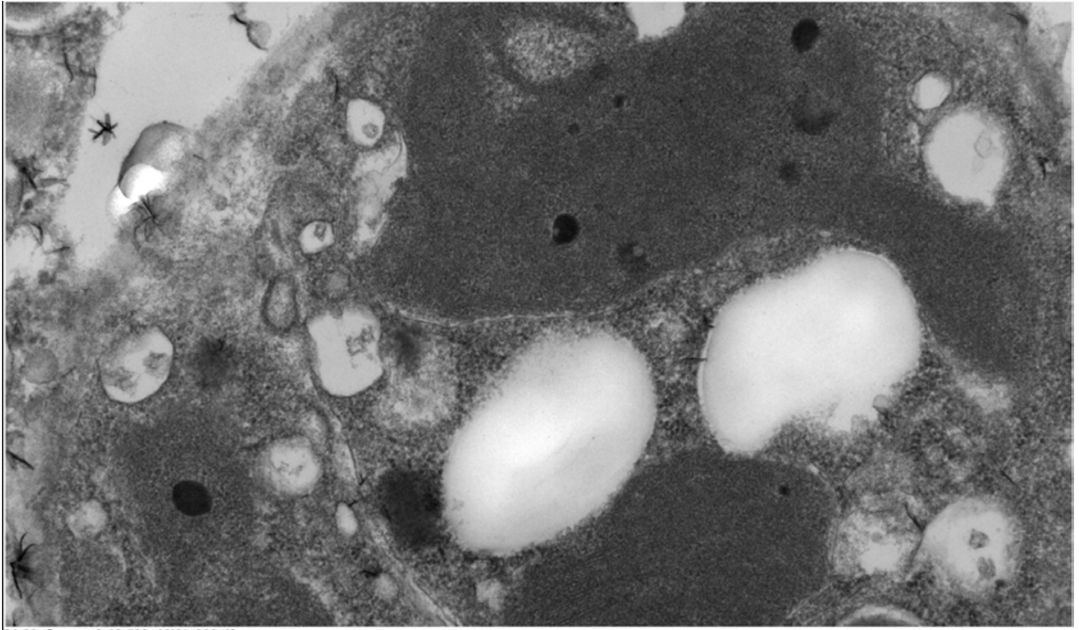

21-20_Correa_3-18-590_16K4_036.tif
Sample: 3/18, 590
Biological Electron Microscopy Lab
Rice University - SEA
Microscopist: MD Meyer

500 nm
HV=80kV
Direct Mag: 8000 x

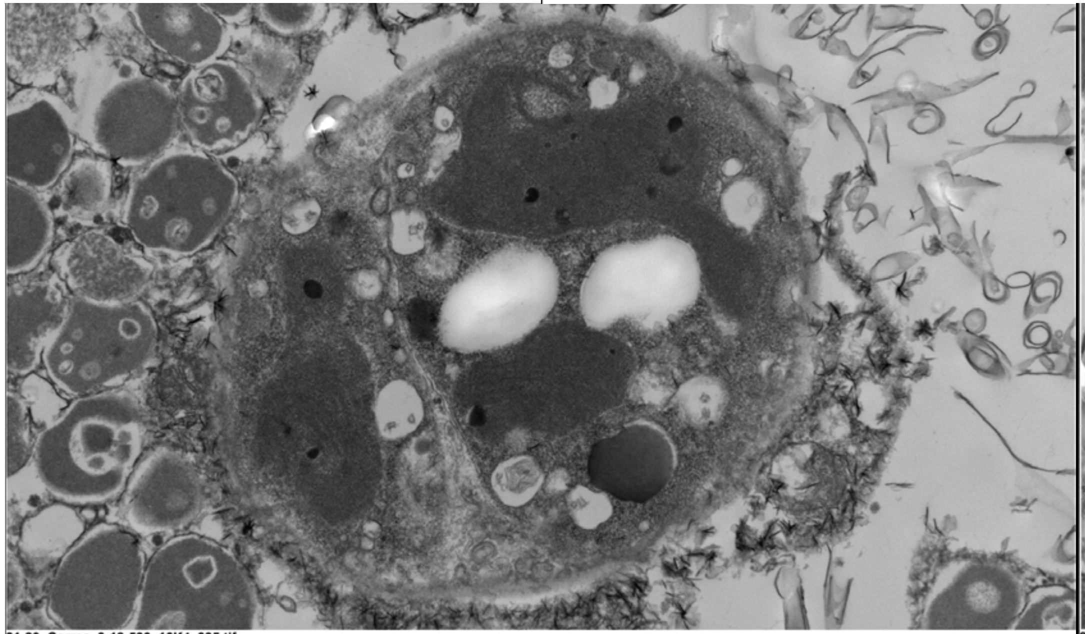

21-20_Correa_3-18-590_16K4_035.tif
Sample: 3/18, 590
Biological Electron Microscopy Lab
Rice University - SEA
Microscopist: MD Meyer

1 µm
HV=80kV
Direct Mag: 4000 x

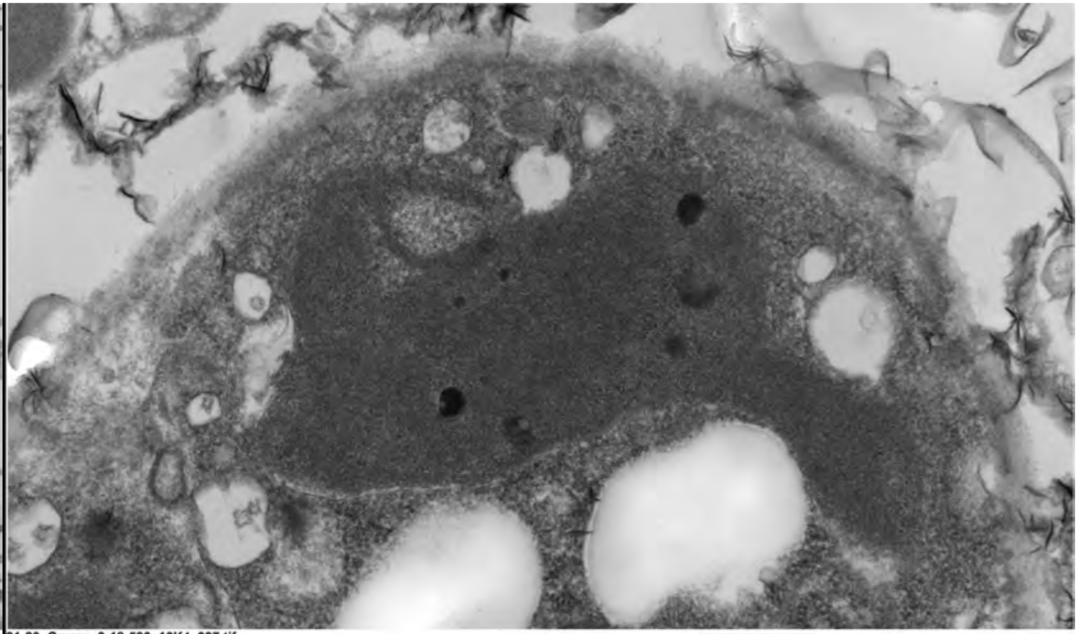

21-20_Correa_3-18-590_16K4_037.tif
Sample: 3/18, 590
Biological Electron Microscopy Lab
Rice University - SEA
Microscopist: MD Meyer

500 nm
HV=80kV
Direct Mag: 8000 x

Cell 4

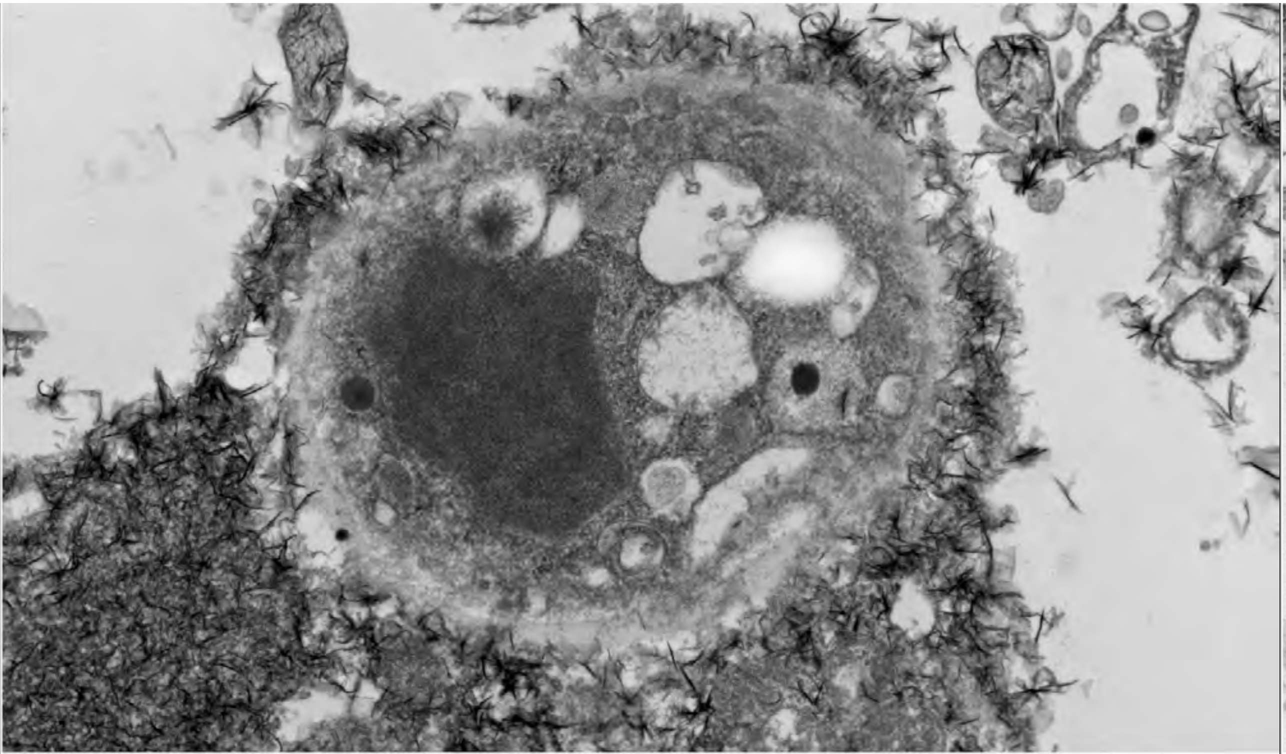

21-20_Correa_3-18-590_16K4_032.tif
Sample: 3/18, 590
Biological Electron Microscopy Lab
Rice University - SEA
Microscopist: MD Meyer

800 nm
HV=80kV
Direct Mag: 5000 x

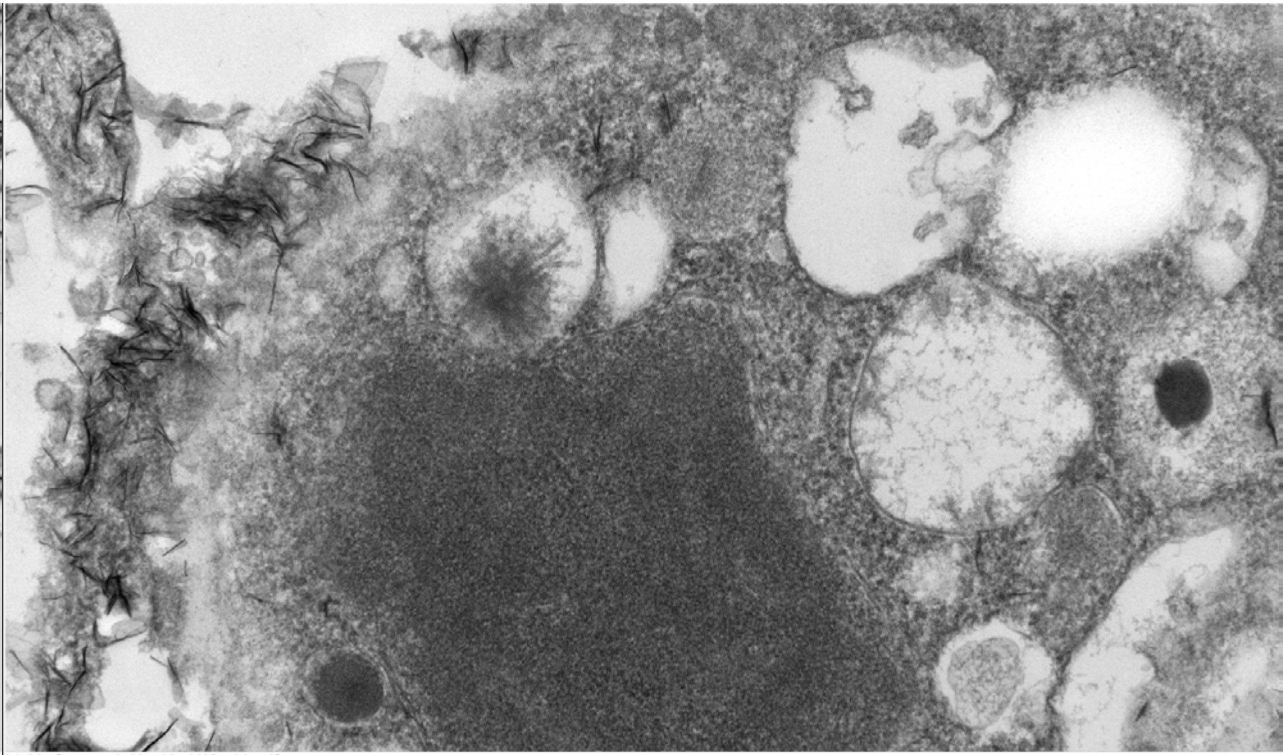

21-20_Correa_3-18-590_16K4_034.tif
Sample: 3/18, 590
Biological Electron Microscopy Lab
Rice University - SEA
Microscopist: MD Meyer

400 nm
HV=80kV
Direct Mag: 10000 x

Cell 7

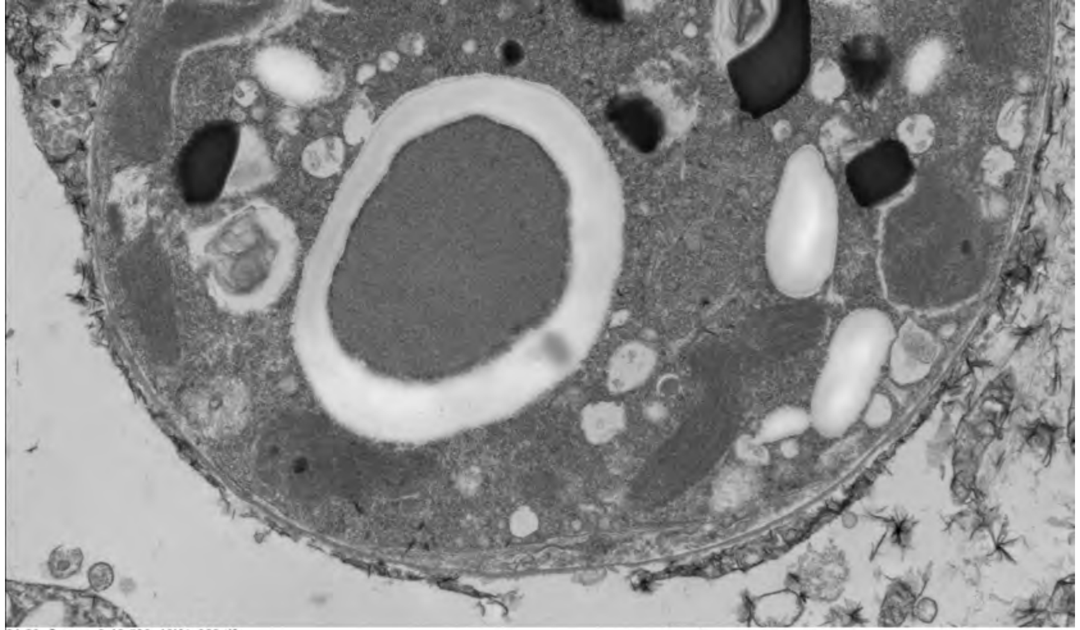

21-20_Correa_3-18-590_16K4_029.tif
Sample: 3/18, 590
Biological Electron Microscopy Lab
Rice University - SEA
Microscopist: MD Meyer

1 µm
HV=80kV
Direct Mag: 4000 x

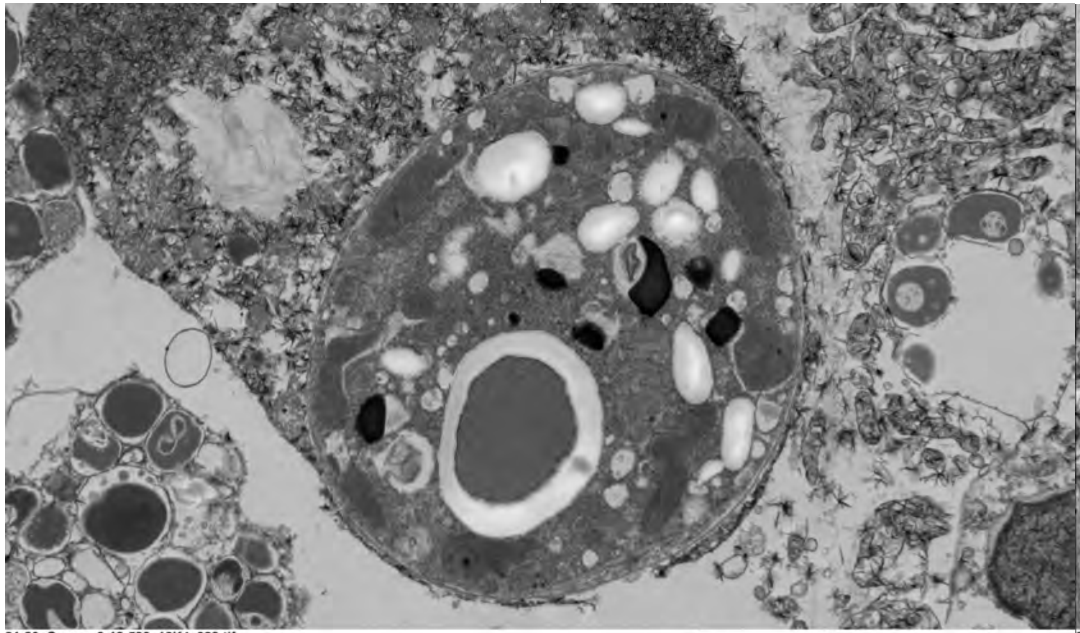

21-20_Correa_3-18-590_16K4_028.tif
Sample: 3/18, 590
Biological Electron Microscopy Lab
Rice University - SEA
Microscopist: MD Meyer

2 µm
HV=80kV
Direct Mag: 2000 x

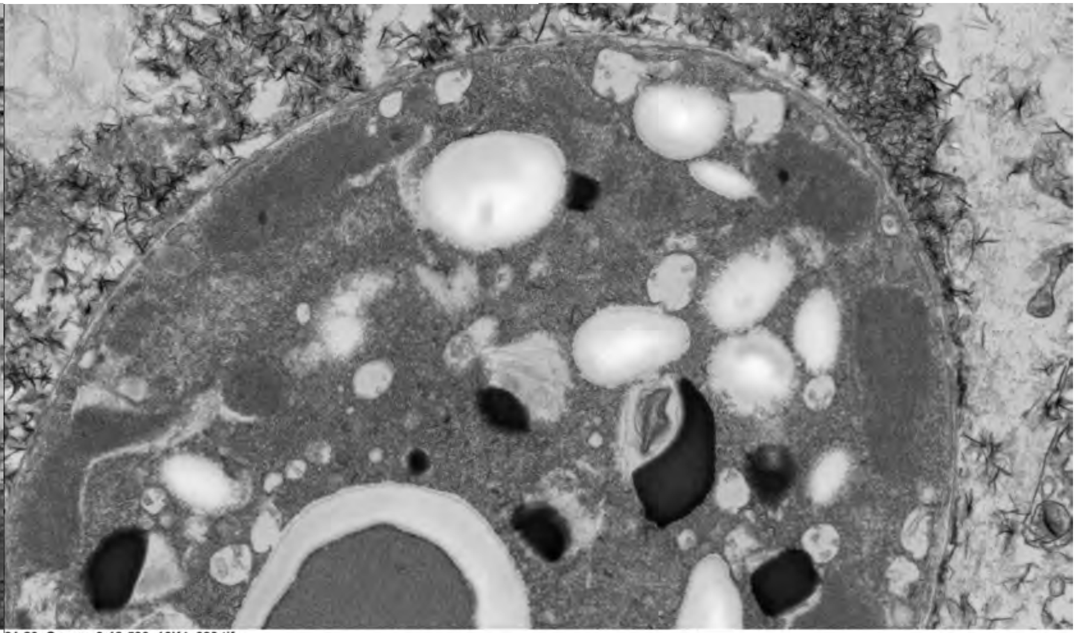

21-20_Correa_3-18-590_16K4_030.tif
Sample: 3/18, 590
Biological Electron Microscopy Lab
Rice University - SEA
Microscopist: MD Meyer

1 µm
HV=80kV
Direct Mag: 4000 x

Cell 6

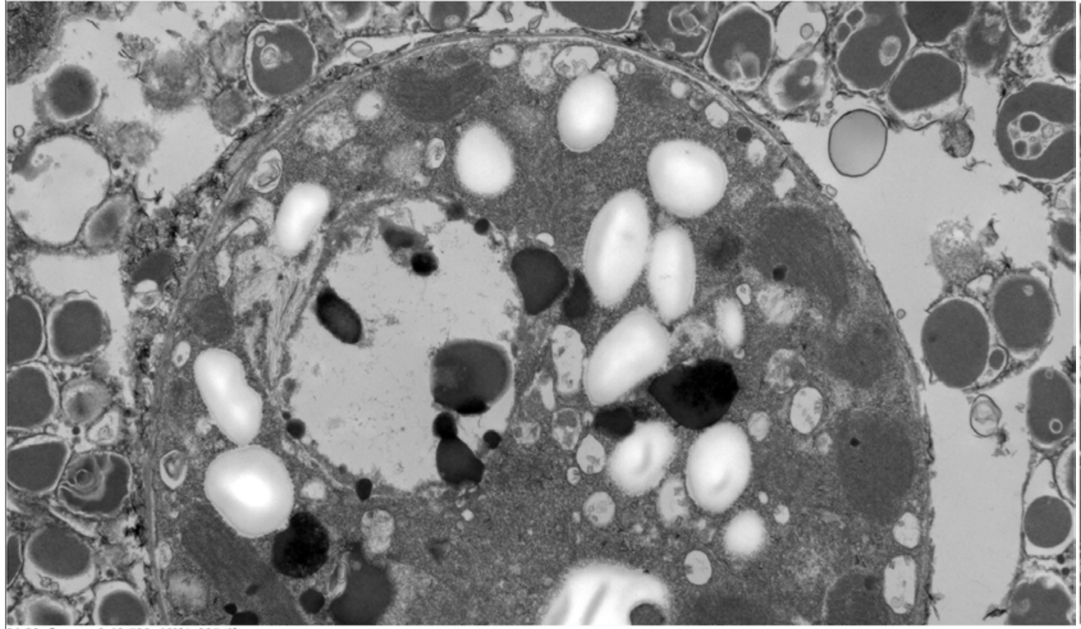

21-20_Correa_3-18-590_16K4_025.tif
Sample: 3/18, 590
Biological Electron Microscopy Lab
Rice University - SEA
Microscopist: MD Meyer

1 µm
HV=80kV
Direct Mag: 3000 x

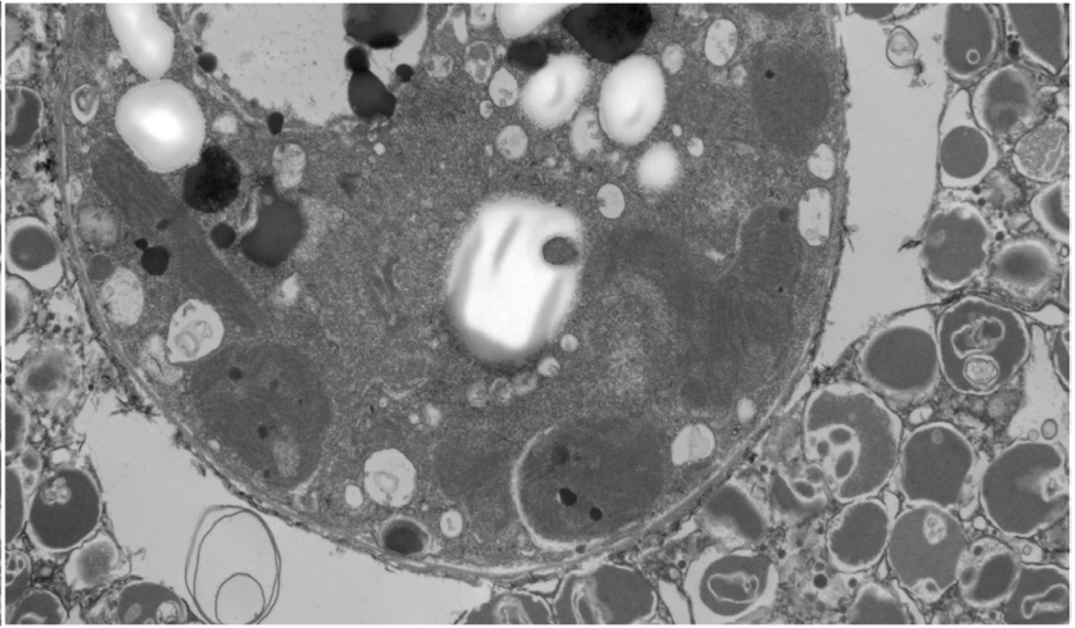

21-20_Correa_3-18-590_16K4_024.tif
Sample: 3/18, 590
Biological Electron Microscopy Lab
Rice University - SEA
Microscopist: MD Meyer

1 µm
HV=80kV
Direct Mag: 3000 x

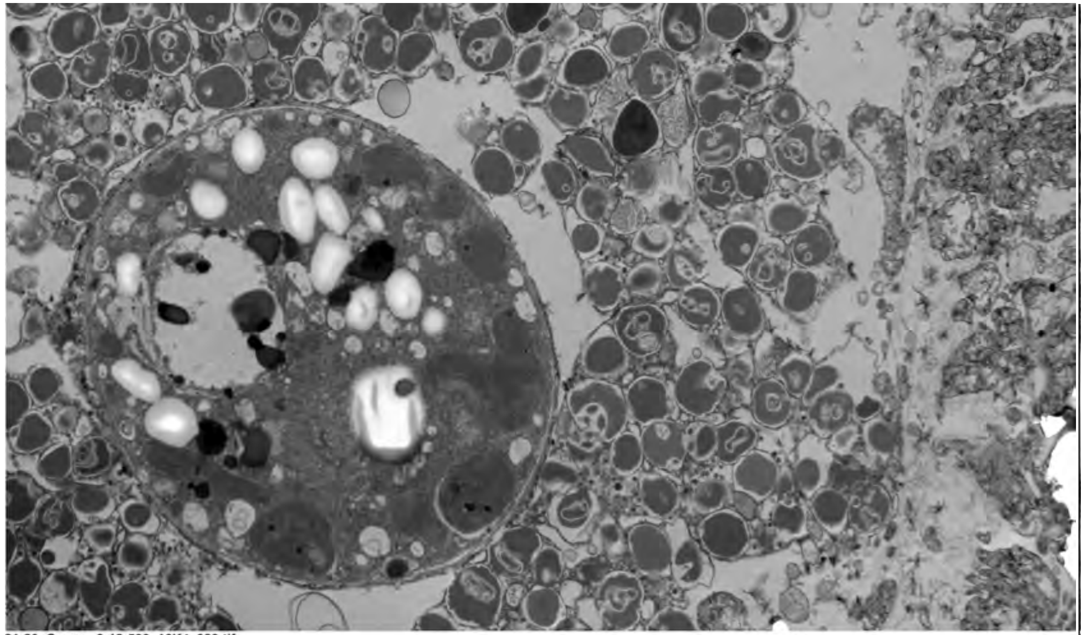

21-20_Correa_3-18-590_16K4_023.tif
Sample: 3/18, 590
Biological Electron Microscopy Lab
Rice University - SEA
Microscopist: MD Meyer

2 µm
HV=80kV
Direct Mag: 1500 x

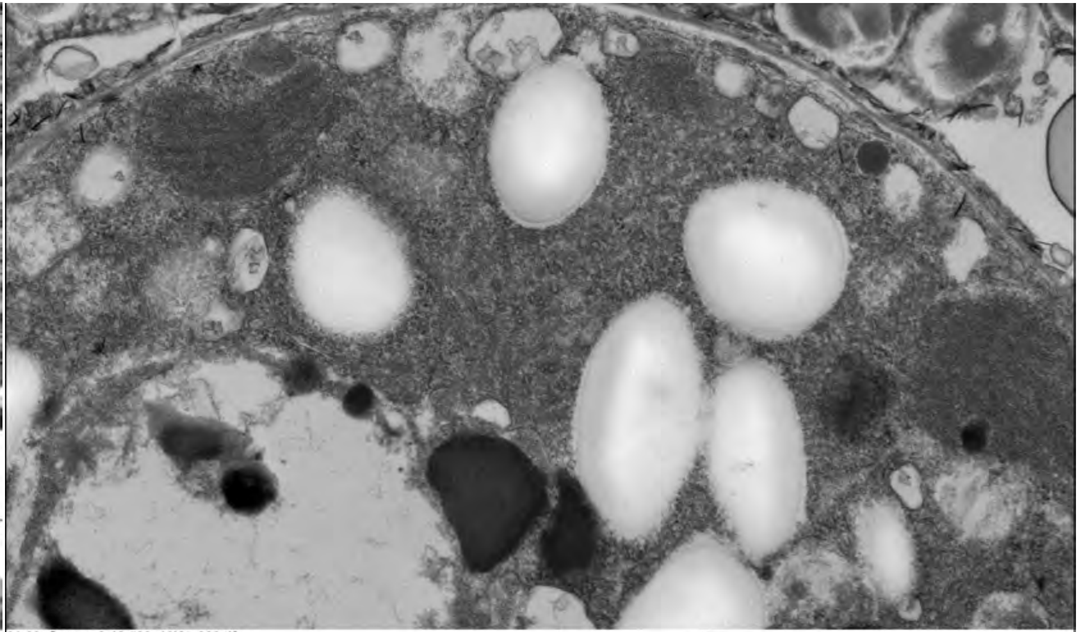

21-20_Correa_3-18-590_16K4_026.tif
Sample: 3/18, 590
Biological Electron Microscopy Lab
Rice University - SEA
Microscopist: MD Meyer

600 nm
HV=80kV
Direct Mag: 6000 x

Cell 7

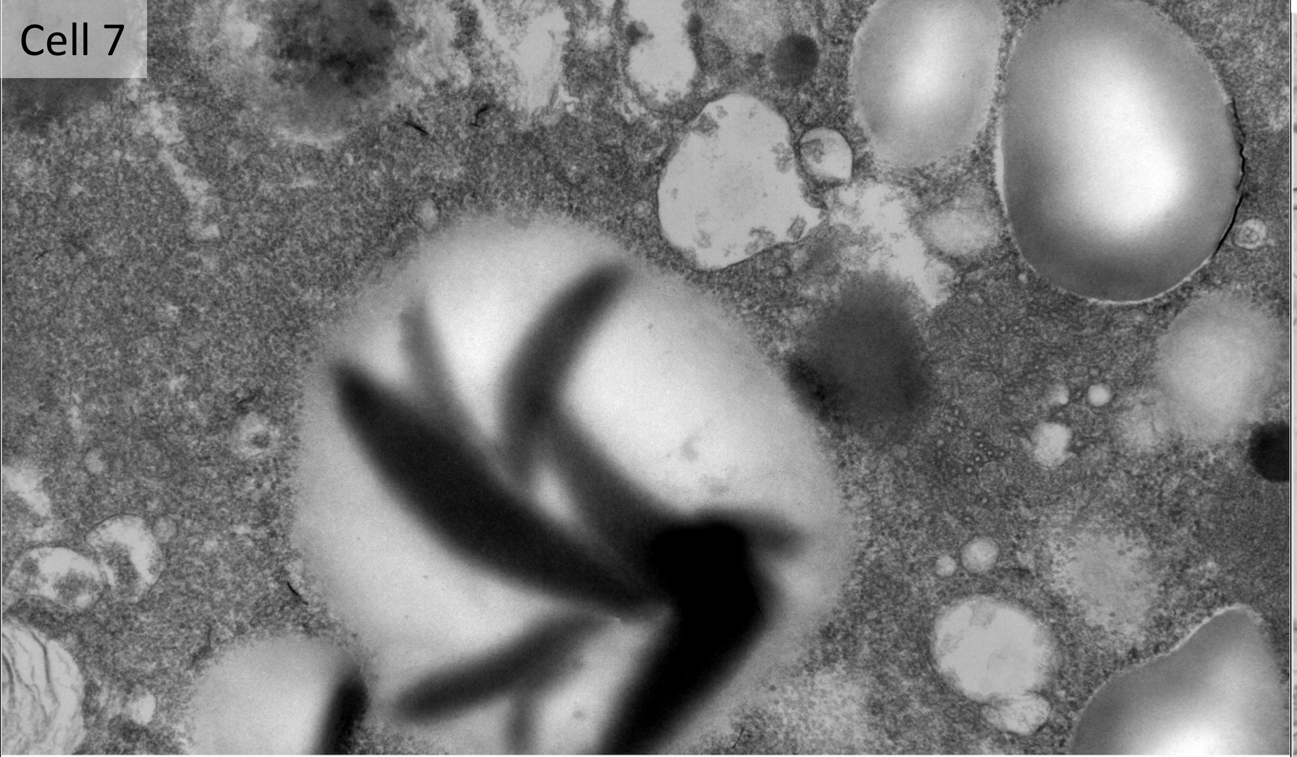

21-20_Correa_3-18-590_16K4_020.tif
Sample: 3/18, 590
Biological Electron Microscopy Lab
Rice University - SEA
Microscopist: MD Meyer

500 nm
HV=80kV
Direct Mag: 8000 x

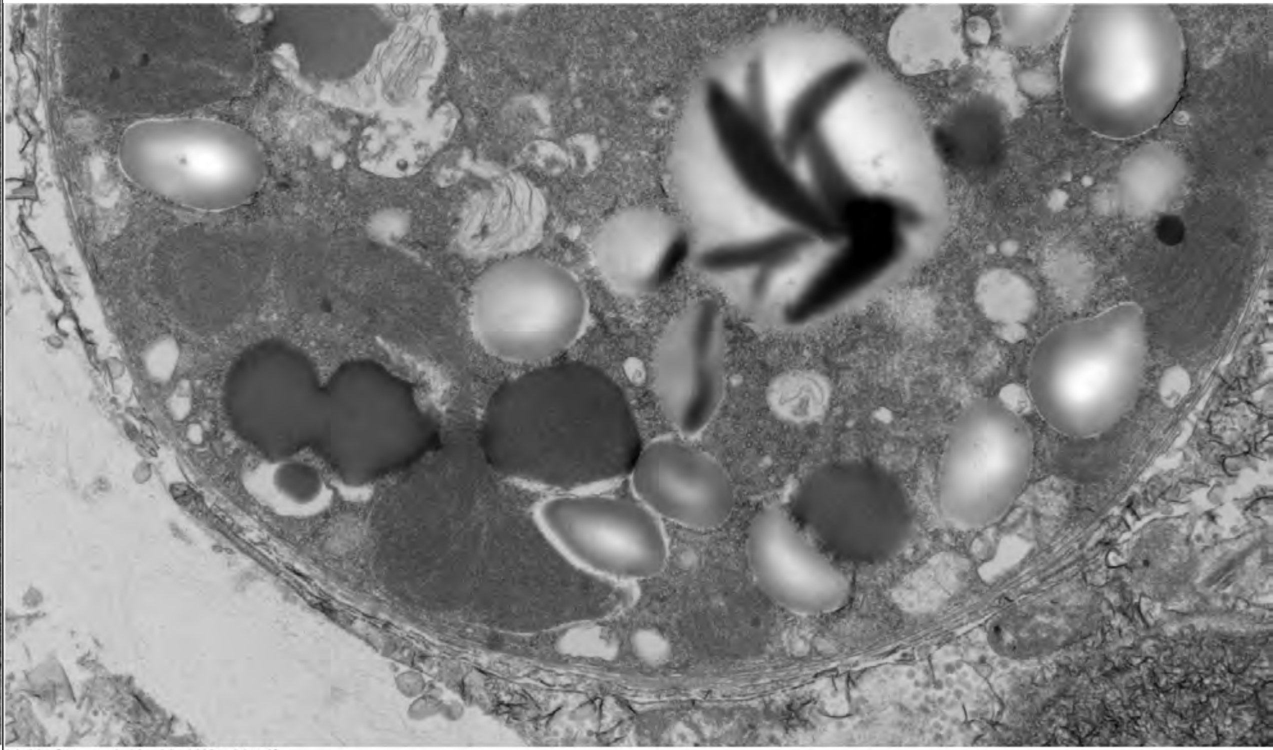

21-20_Correa_3-18-590_16K4_017.tif
Sample: 3/18, 590
Biological Electron Microscopy Lab
Rice University - SEA
Microscopist: MD Meyer

1 µm
HV=80kV
Direct Mag: 4000 x

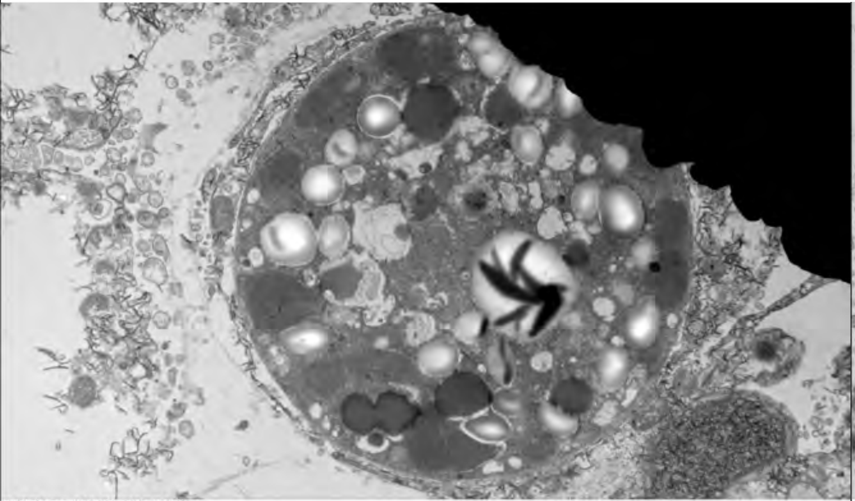

21-20_Correa_3-18-590_16K4_016.tif
Sample: 3/18, 590
Biological Electron Microscopy Lab
Rice University - SEA
Microscopist: MD Meyer

2 µm
HV=80kV
Direct Mag: 2000 x

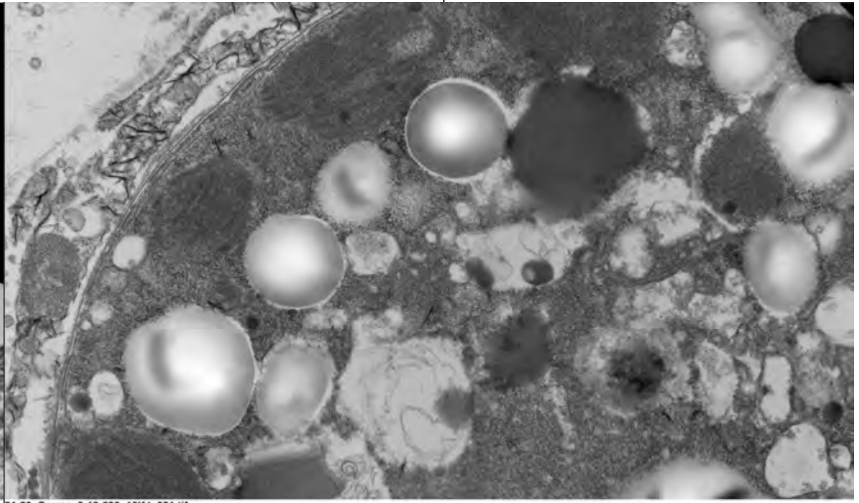

21-20_Correa_3-18-590_16K4_021.tif
Sample: 3/18, 590
Biological Electron Microscopy Lab
Rice University - SEA
Microscopist: MD Meyer

800 nm
HV=80kV
Direct Mag: 5000 x

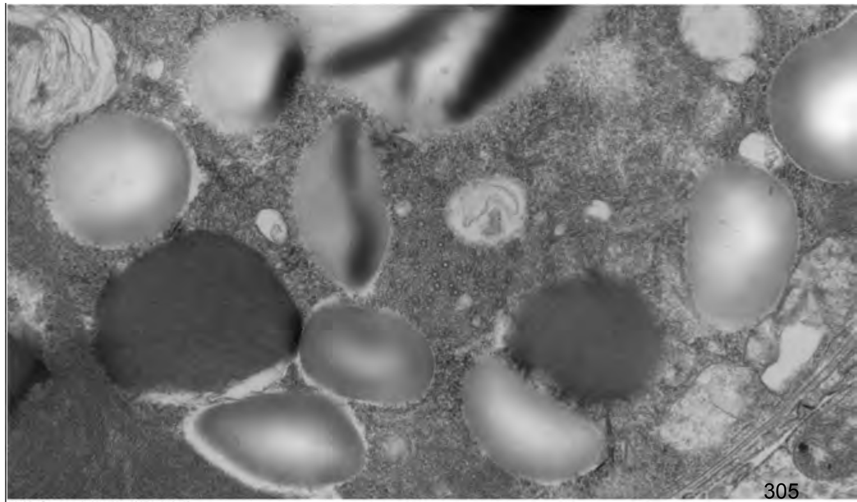

21-20_Correa_3-18-590_16K4_018.tif
Sample: 3/18, 590
Biological Electron Microscopy Lab
Rice University - SEA
Microscopist: MD Meyer

500 nm
HV=80kV
Direct Mag: 8000 x

Cell 8

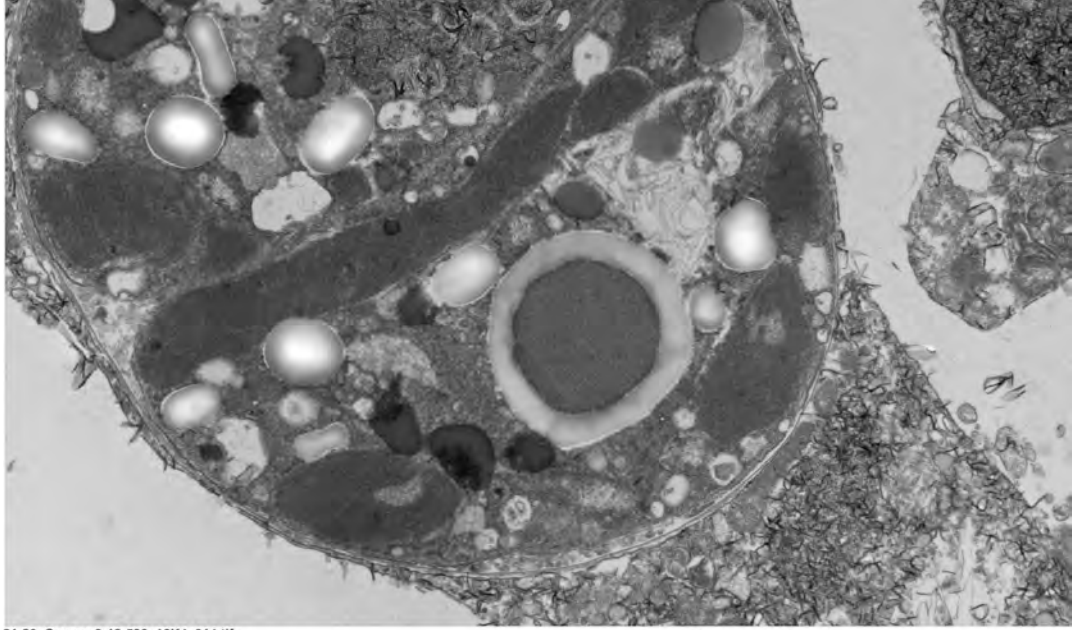

21-20_Correa_3-18-590_16K4_014.tif
Sample: 3/18, 590
Biological Electron Microscopy Lab
Rice University - SEA
Microscopist: MD Meyer

1 µm
HV=80kV
Direct Mag: 3000 x

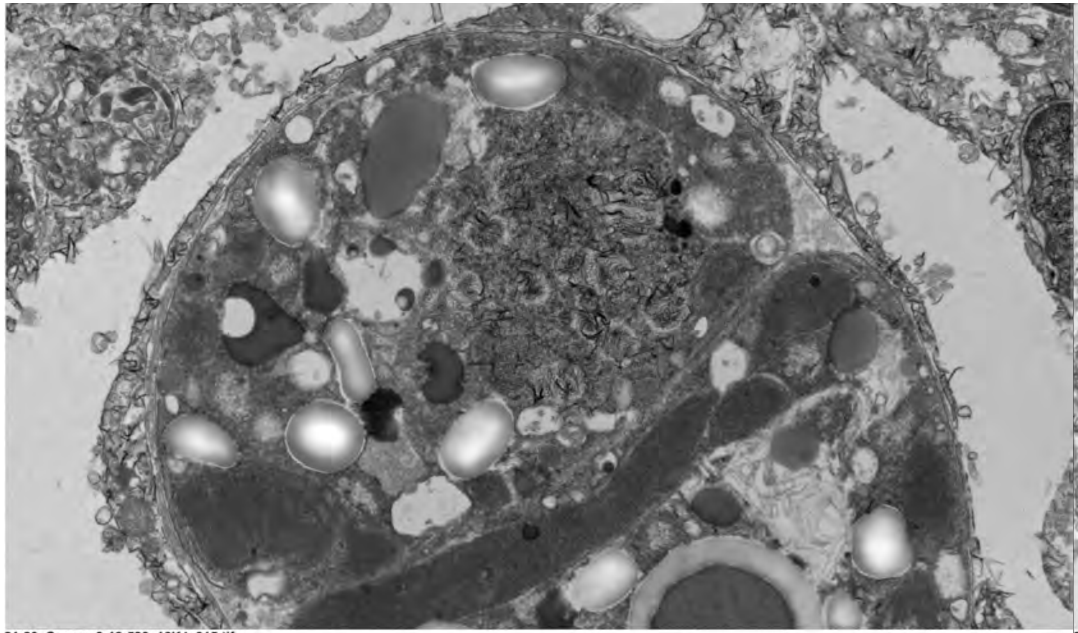

21-20_Correa_3-18-590_16K4_015.tif
Sample: 3/18, 590
Biological Electron Microscopy Lab
Rice University - SEA
Microscopist: MD Meyer

1 µm
HV=80kV
Direct Mag: 3000 x

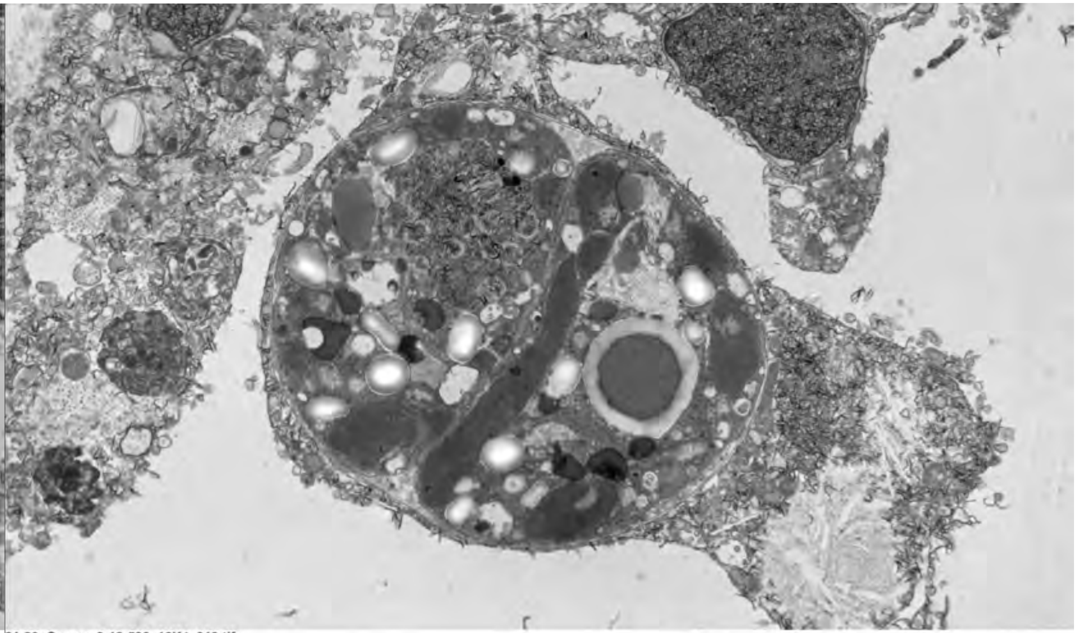

21-20_Correa_3-18-590_16K4_013.tif
Sample: 3/18, 590
Biological Electron Microscopy Lab
Rice University - SEA
Microscopist: MD Meyer

2 µm
HV=80kV
Direct Mag: 1500 x

Cell 9

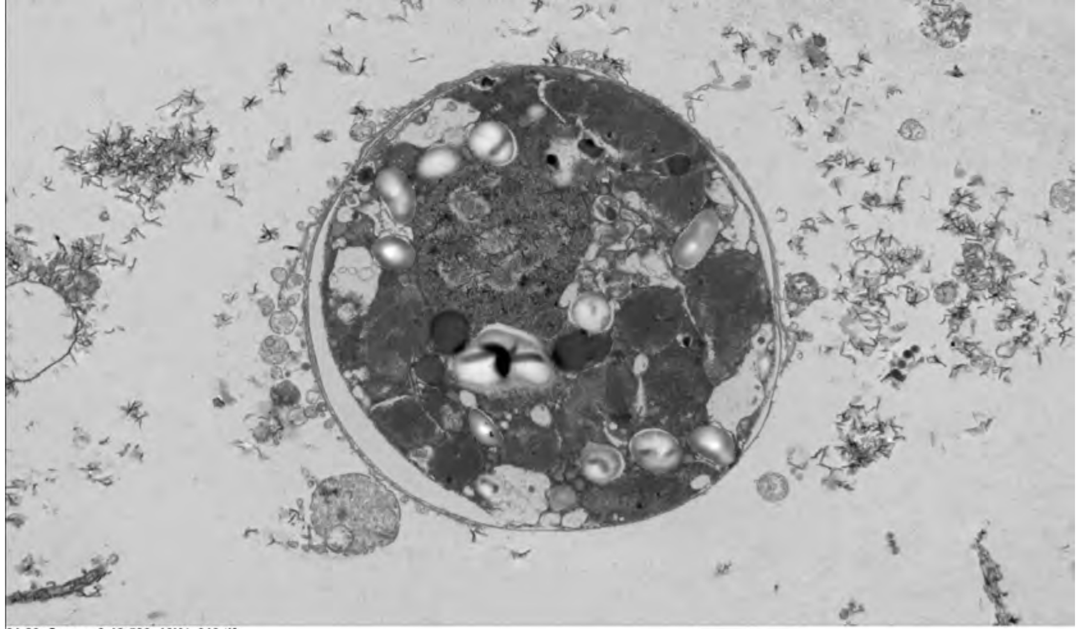

21-20_Correa_3-18-590_16K4_010.tif
Sample: 3/18, 590
Biological Electron Microscopy Lab
Rice University - SEA
Microscopist: MD Meyer

2 µm
HV=80kV
Direct Mag: 1500 x

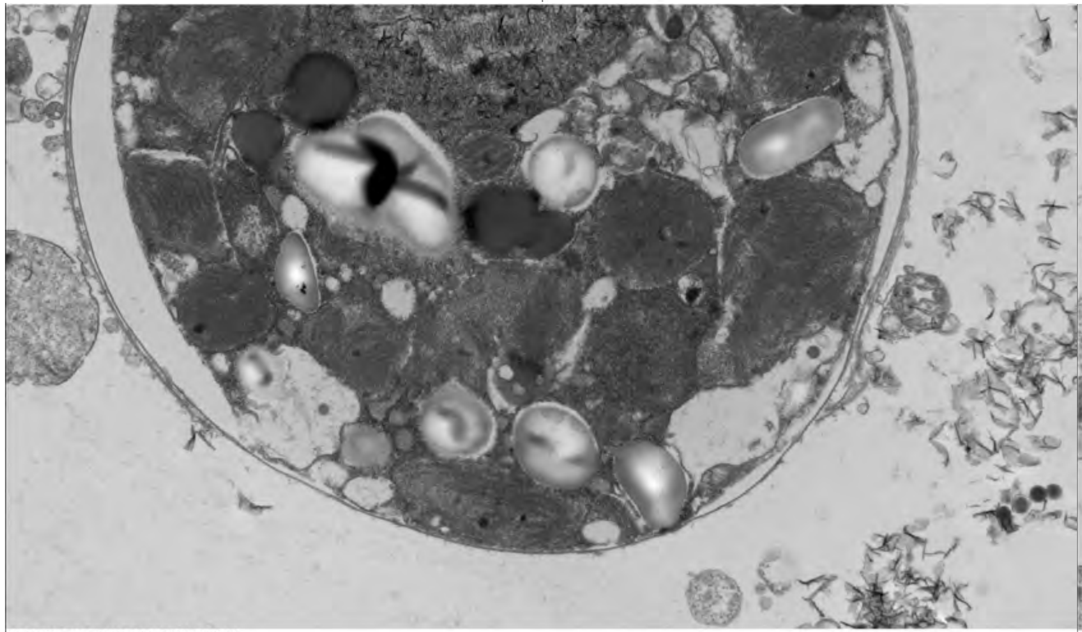

21-20_Correa_3-18-590_16K4_011.tif
Sample: 3/18, 590
Biological Electron Microscopy Lab
Rice University - SEA
Microscopist: MD Meyer

1 µm
HV=80kV
Direct Mag: 3000 x

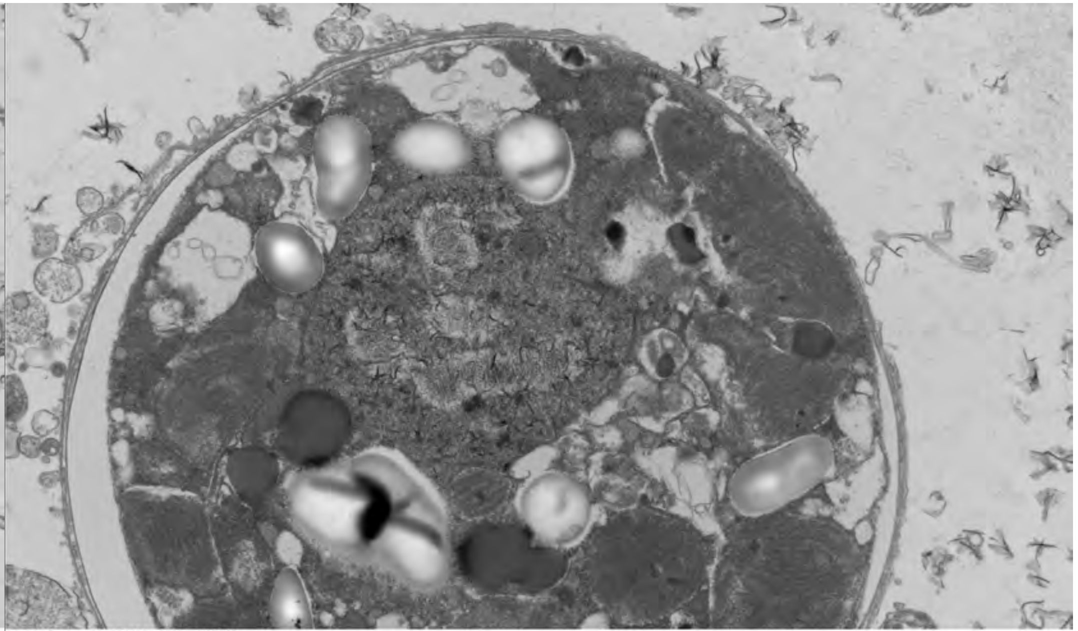

21-20_Correa_3-18-590_16K4_012.tif
Sample: 3/18, 590
Biological Electron Microscopy Lab
Rice University - SEA
Microscopist: MD Meyer

1 µm
HV=80kV
Direct Mag: 3000 x

Cell 10

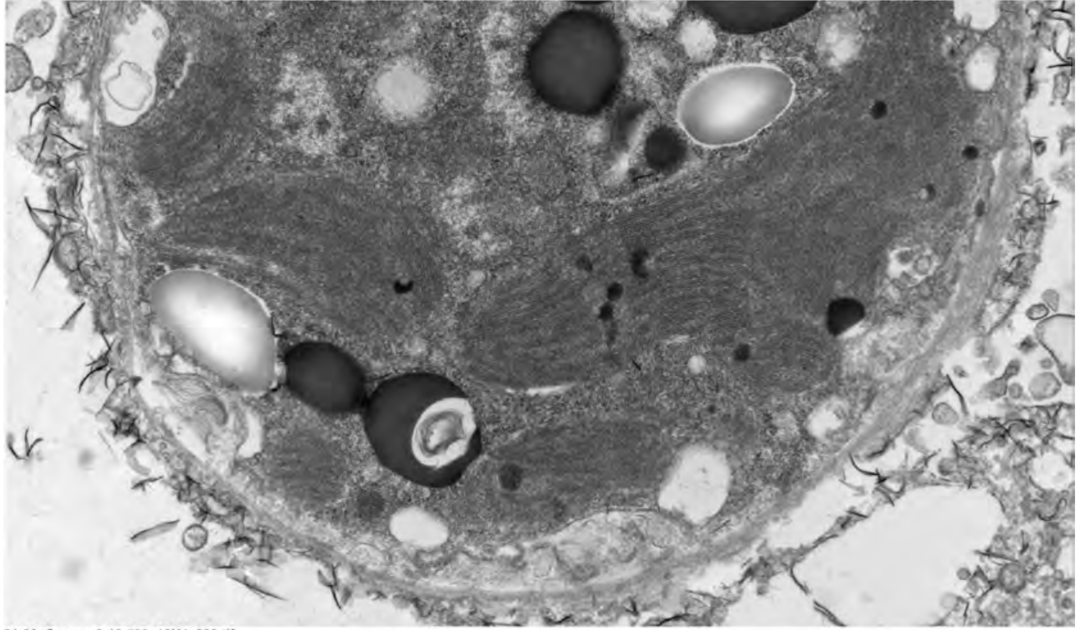

21-20_Correa_3-18-590_16K4_008.tif
Sample: 3/18, 590
Biological Electron Microscopy Lab
Rice University - SEA
Microscopist: MD Meyer

800 nm
HV=80kV
Direct Mag: 5000 x

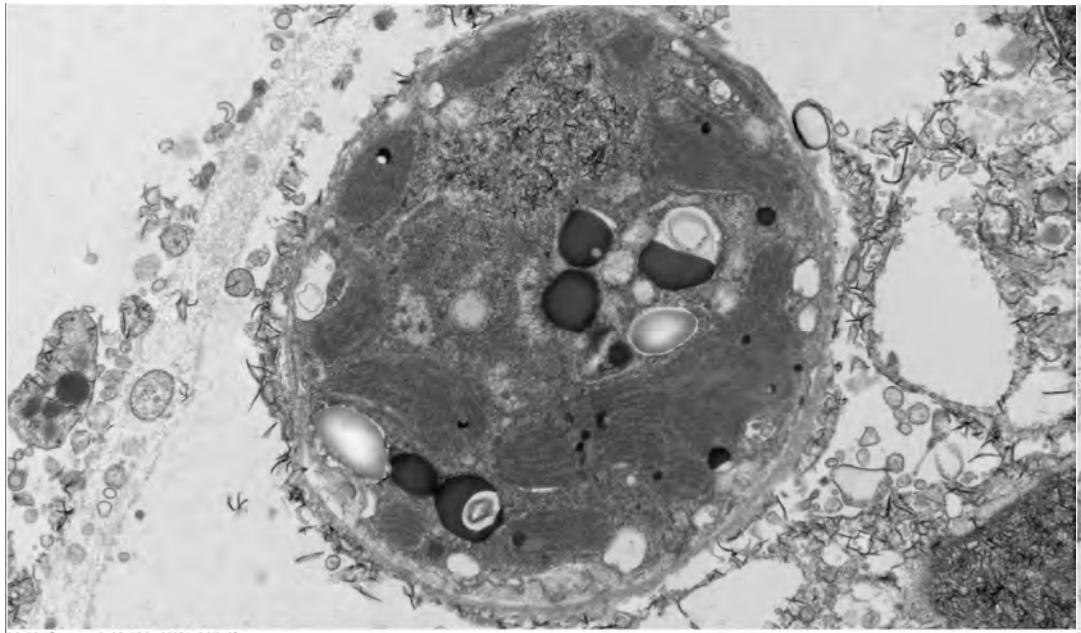

21-20_Correa_3-18-590_16K4_007.tif
Sample: 3/18, 590
Biological Electron Microscopy Lab
Rice University - SEA
Microscopist: MD Meyer

1 µm
HV=80kV
Direct Mag: 3000 x

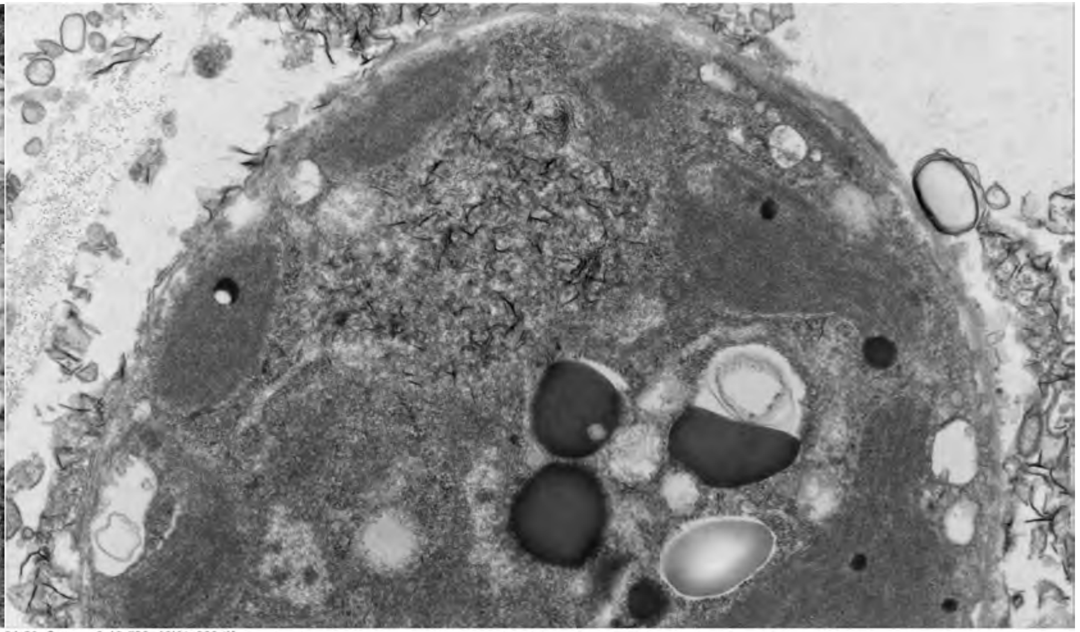

21-20_Correa_3-18-590_16K4_009.tif
Sample: 3/18, 590
Biological Electron Microscopy Lab
Rice University - SEA
Microscopist: MD Meyer

800 nm
HV=80kV
Direct Mag: 5000 x

Cell 11

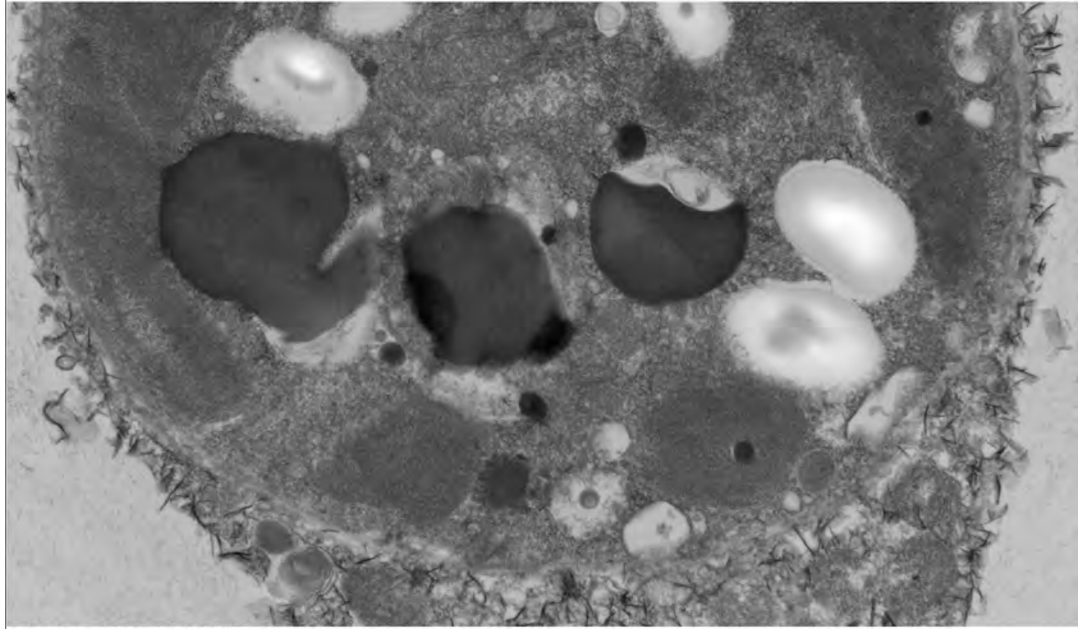

21-20_Correa_3-18-590_16K4_005.tif
Sample: 3/18, 590
Biological Electron Microscopy Lab
Rice University - SEA
Microscopist: MD Meyer

800 nm
HV=80kV
Direct Mag: 5000 x

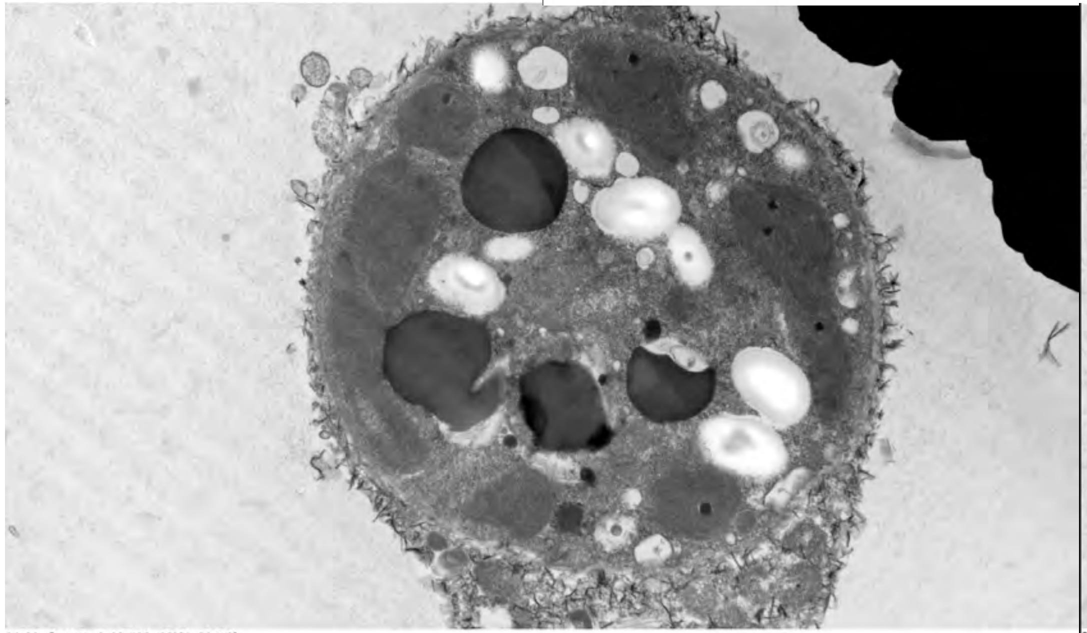

21-20_Correa_3-18-590_16K4_004.tif
Sample: 3/18, 590
Biological Electron Microscopy Lab
Rice University - SEA
Microscopist: MD Meyer

1 µm
HV=80kV
Direct Mag: 3000 x

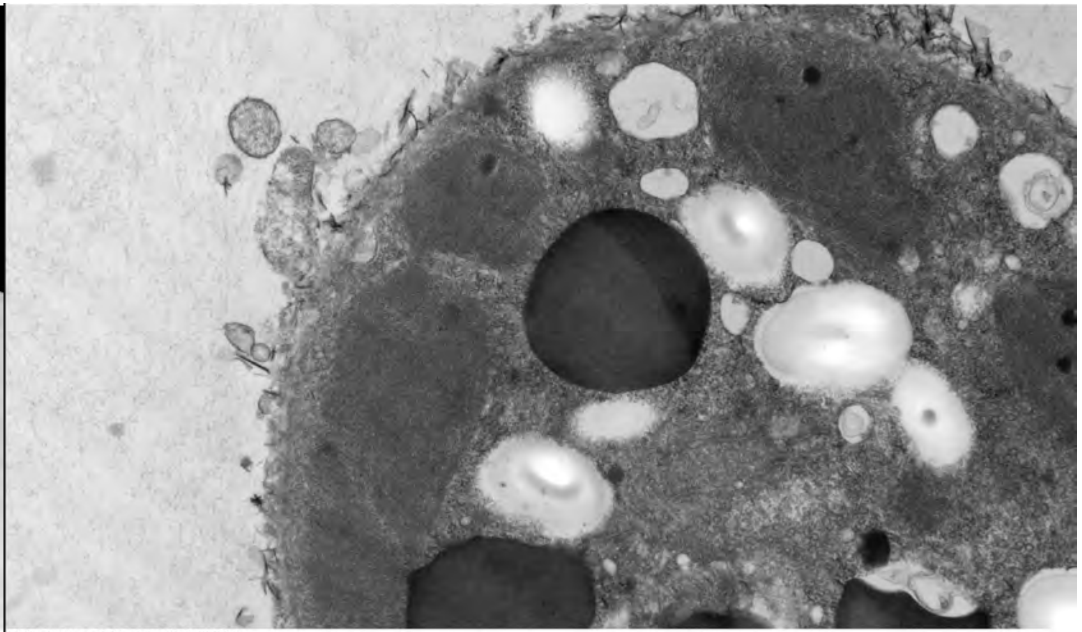

21-20_Correa_3-18-590_16K4_006.tif
Sample: 3/18, 590
Biological Electron Microscopy Lab
Rice University - SEA
Microscopist: MD Meyer

800 nm
HV=80kV
Direct Mag: 5000 x

Cell 12

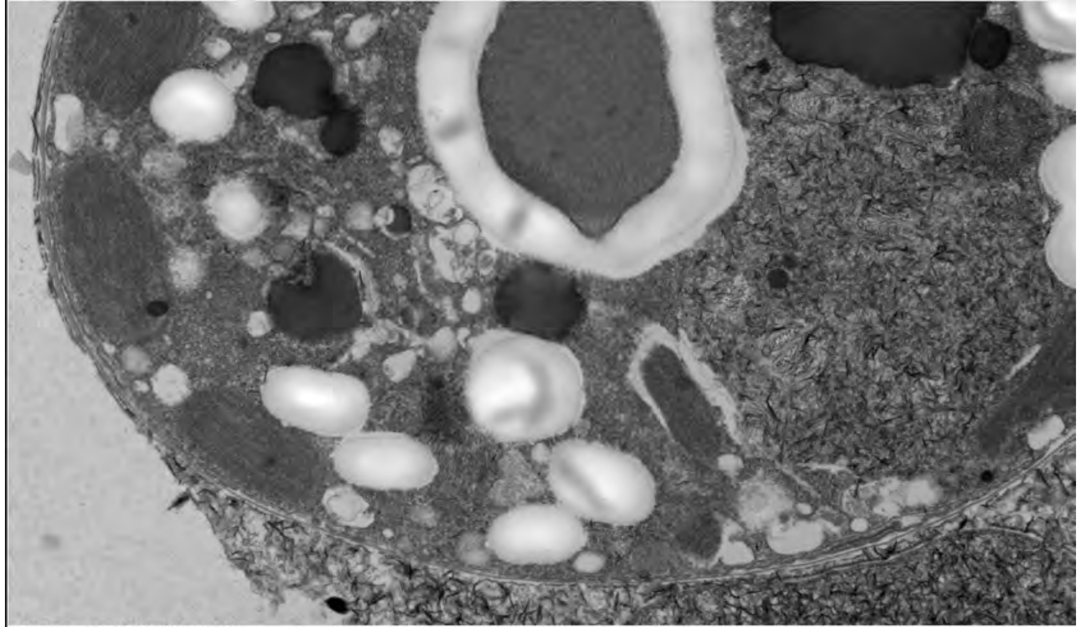

21-20_Correa_3-18-590_16K4_002.tif
Sample: 3/18, 590
Biological Electron Microscopy Lab
Rice University - SEA
Microscopist: MD Meyer

1 µm
HV=80kV
Direct Mag: 4000 x

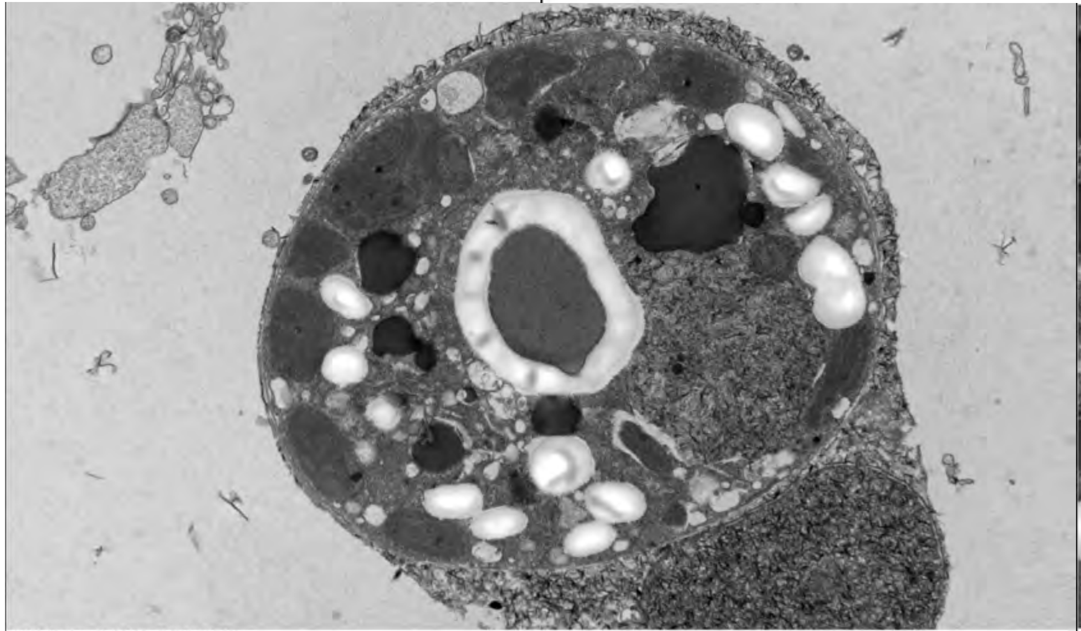

21-20_Correa_3-18-590_16K4_001.tif
Sample: 3/18, 590
Biological Electron Microscopy Lab
Rice University - SEA
Microscopist: MD Meyer

2 µm
HV=80kV
Direct Mag: 2000 x

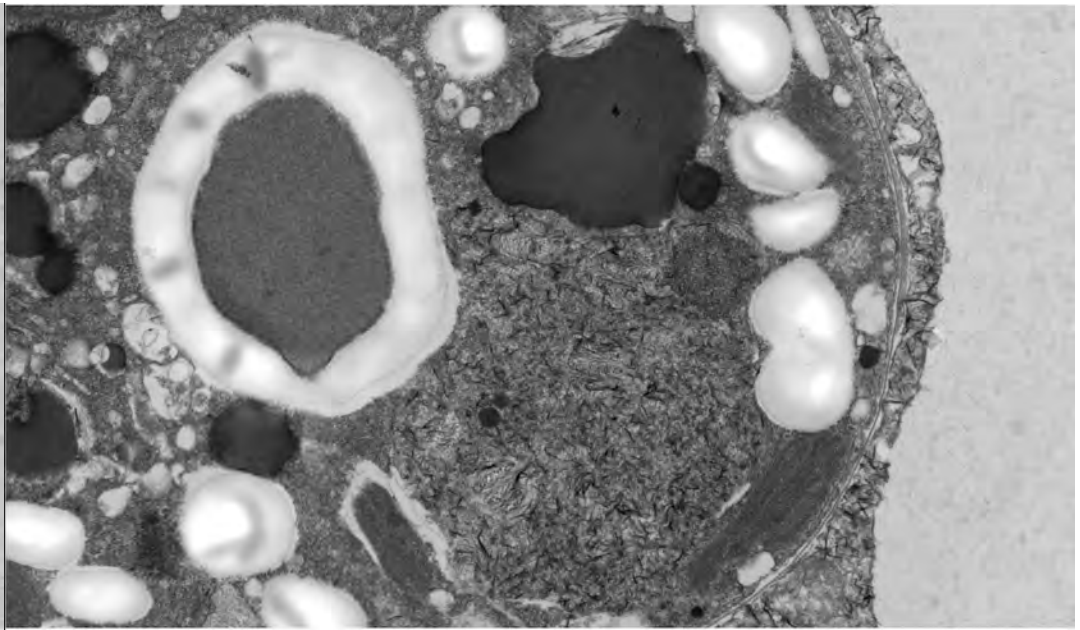

21-20_Correa_3-18-590_16K4_003.tif
Sample: 3/18, 590
Biological Electron Microscopy Lab
Rice University - SEA
Microscopist: MD Meyer

1 µm
HV=80kV
Direct Mag: 4000 x

Heat (March 2019) POR Colony N

Cell 1

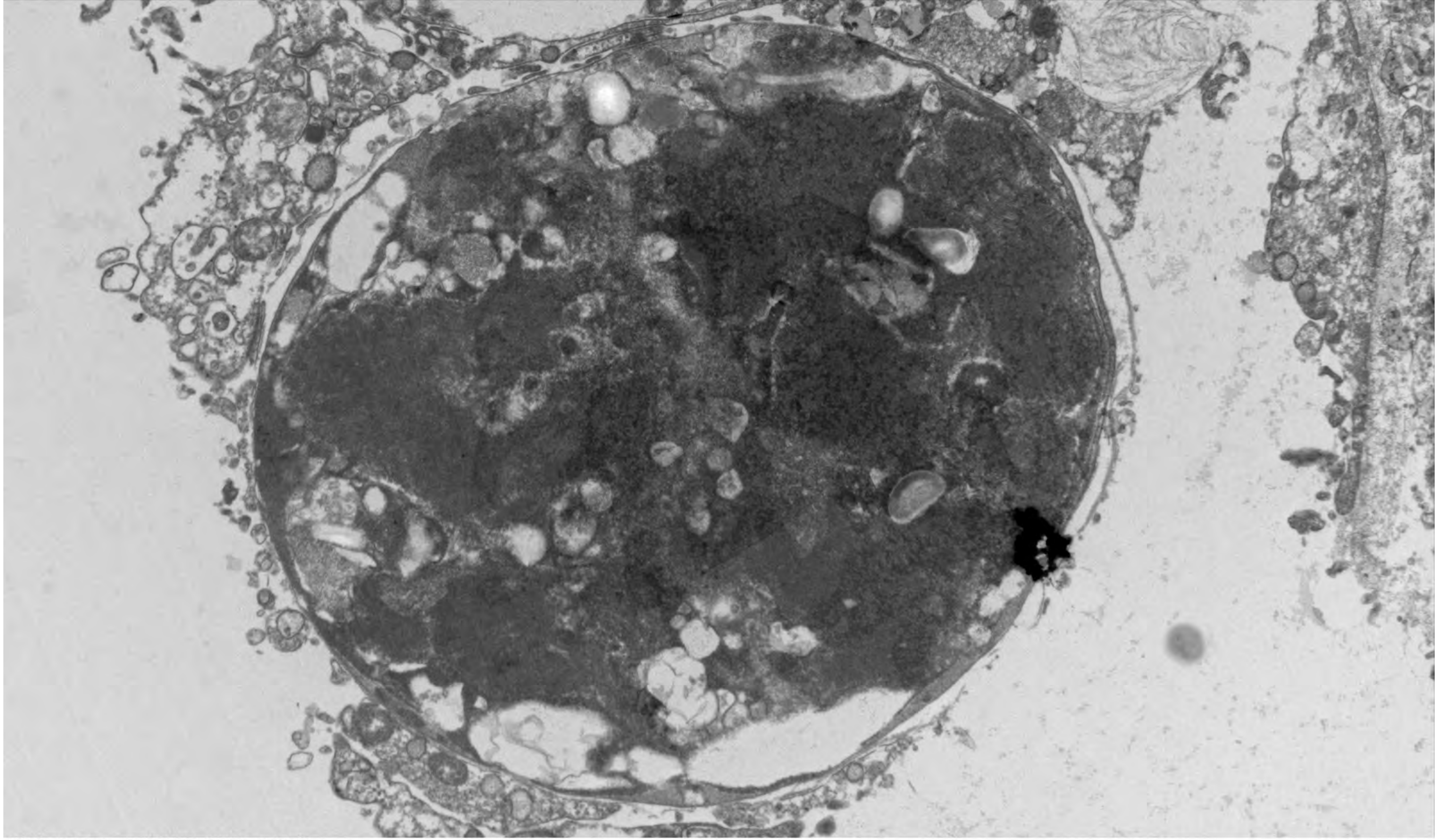

21-20_Correa_3-19-590_16C3_095.tif
Sample: 3/19, 590
Biological Electron Microscopy Lab
Rice University - SEA
Microscopist: MD Meyer

2 µm
HV=80kV
Direct Mag: 2000 x

Cell 2

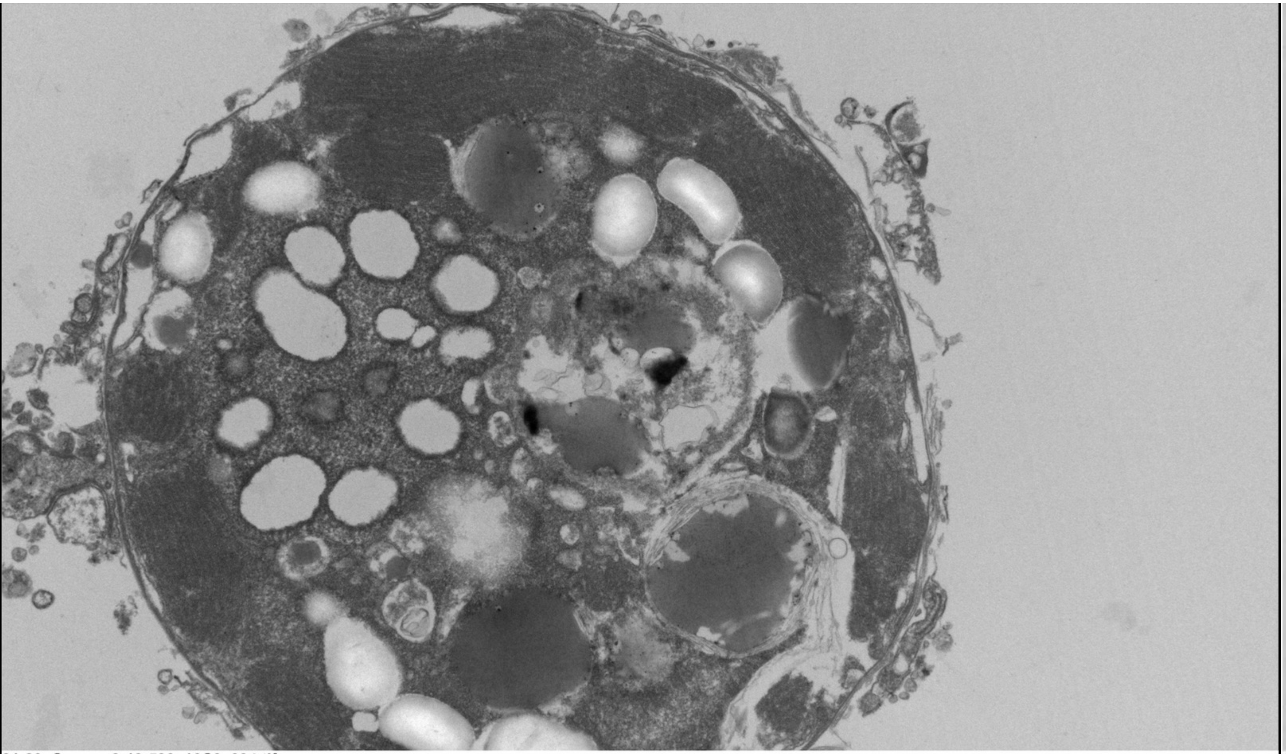

21-20_Correa_3-19-590_16C3_094.tif
Sample: 3/19, 590
Biological Electron Microscopy Lab
Rice University - SEA
Microscopist: MD Meyer
1 μm
HV=80kV
Direct Mag: 3000 x

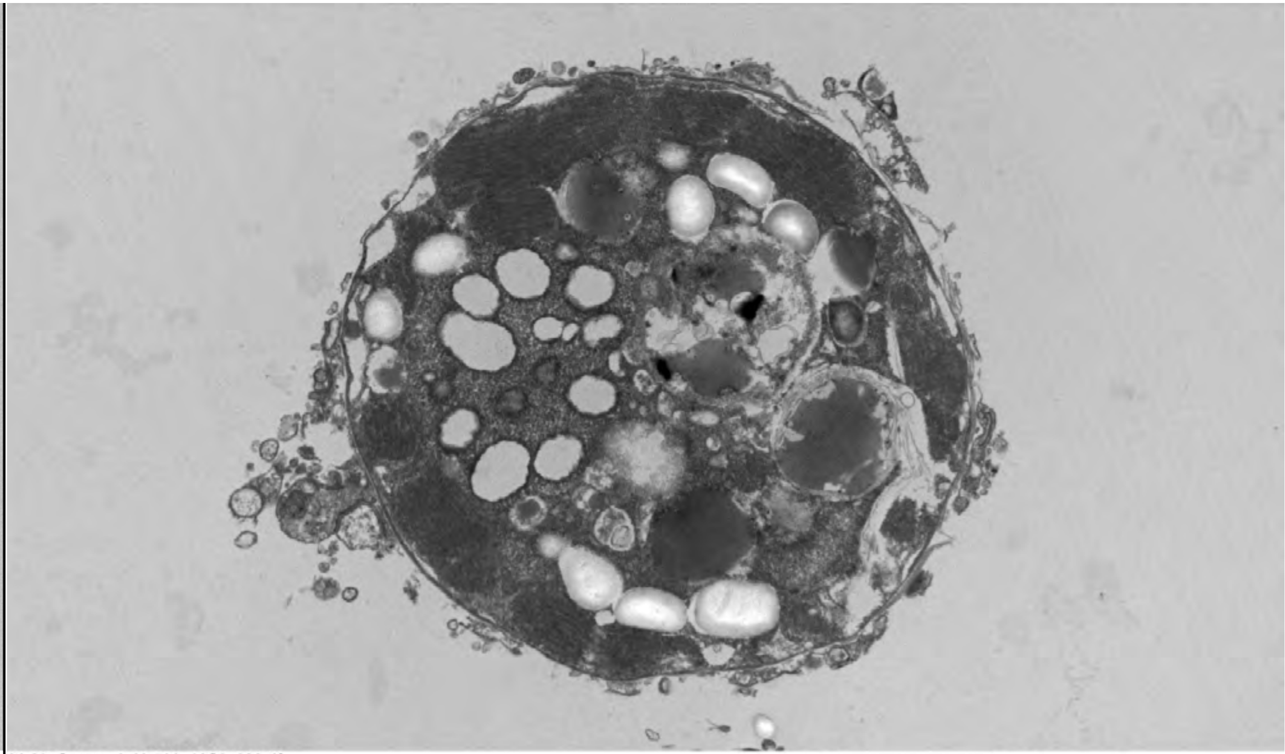

21-20_Correa_3-19-590_16C3_093.tif
Sample: 3/19, 590
Biological Electron Microscopy Lab
Rice University - SEA
Microscopist: MD Meyer
2 μm
HV=80kV
Direct Mag: 2000 x

Cell 3

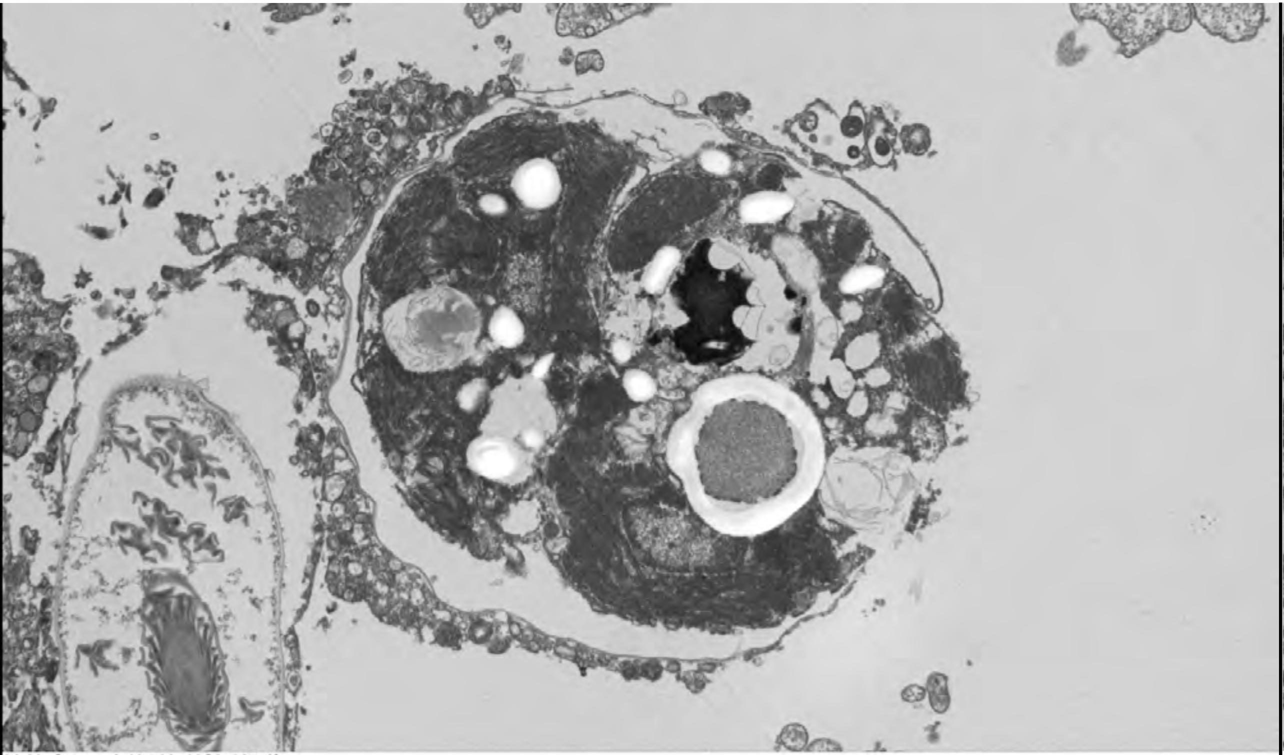

21-20_Correa_3-19-590_16C3_087.tif
Sample: 3/19, 590
Biological Electron Microscopy Lab
Rice University - SEA
Microscopist: MD Meyer

2 µm
HV=80kV
Direct Mag: 1500 x

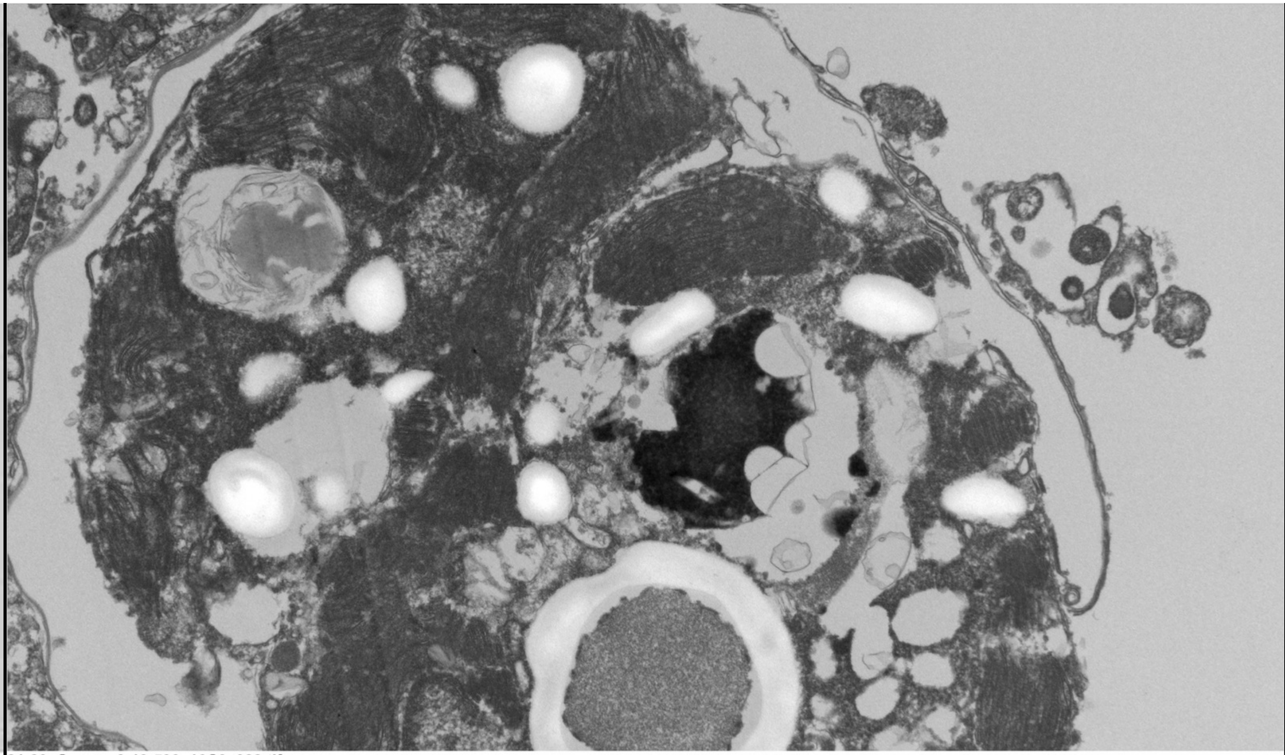

21-20_Correa_3-19-590_16C3_088.tif
Sample: 3/19, 590
Biological Electron Microscopy Lab
Rice University - SEA
Microscopist: MD Meyer

1 µm
HV=80kV
Direct Mag: 3000 x

Cell 4

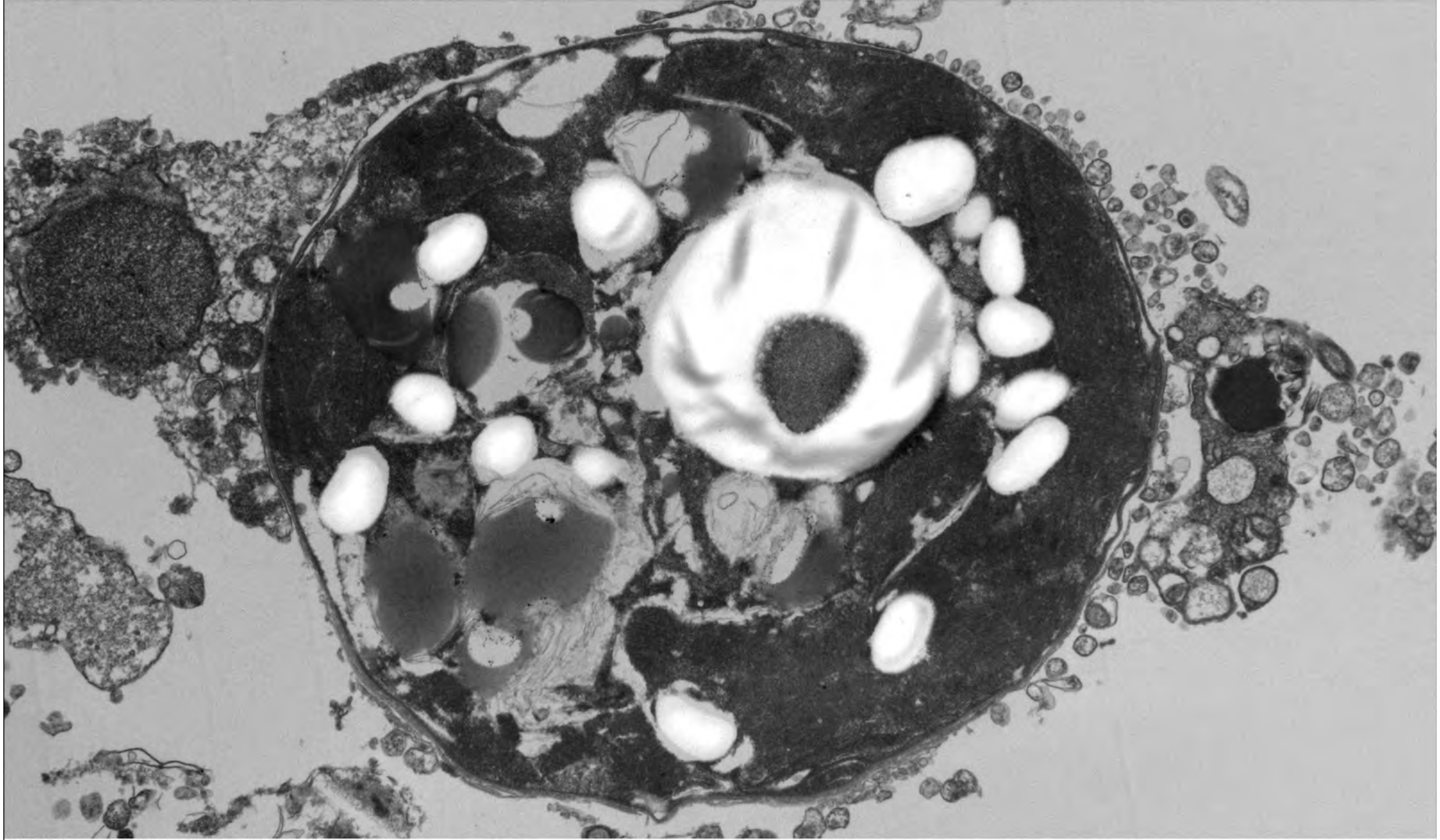

21-20_Correa_3-19-590_16C3_085.tif
Sample: 3/19, 590
Biological Electron Microscopy Lab
Rice University - SEA
Microscopist: MD Meyer

1 μm
HV=80kV
Direct Mag: 2500 x

Cell 5

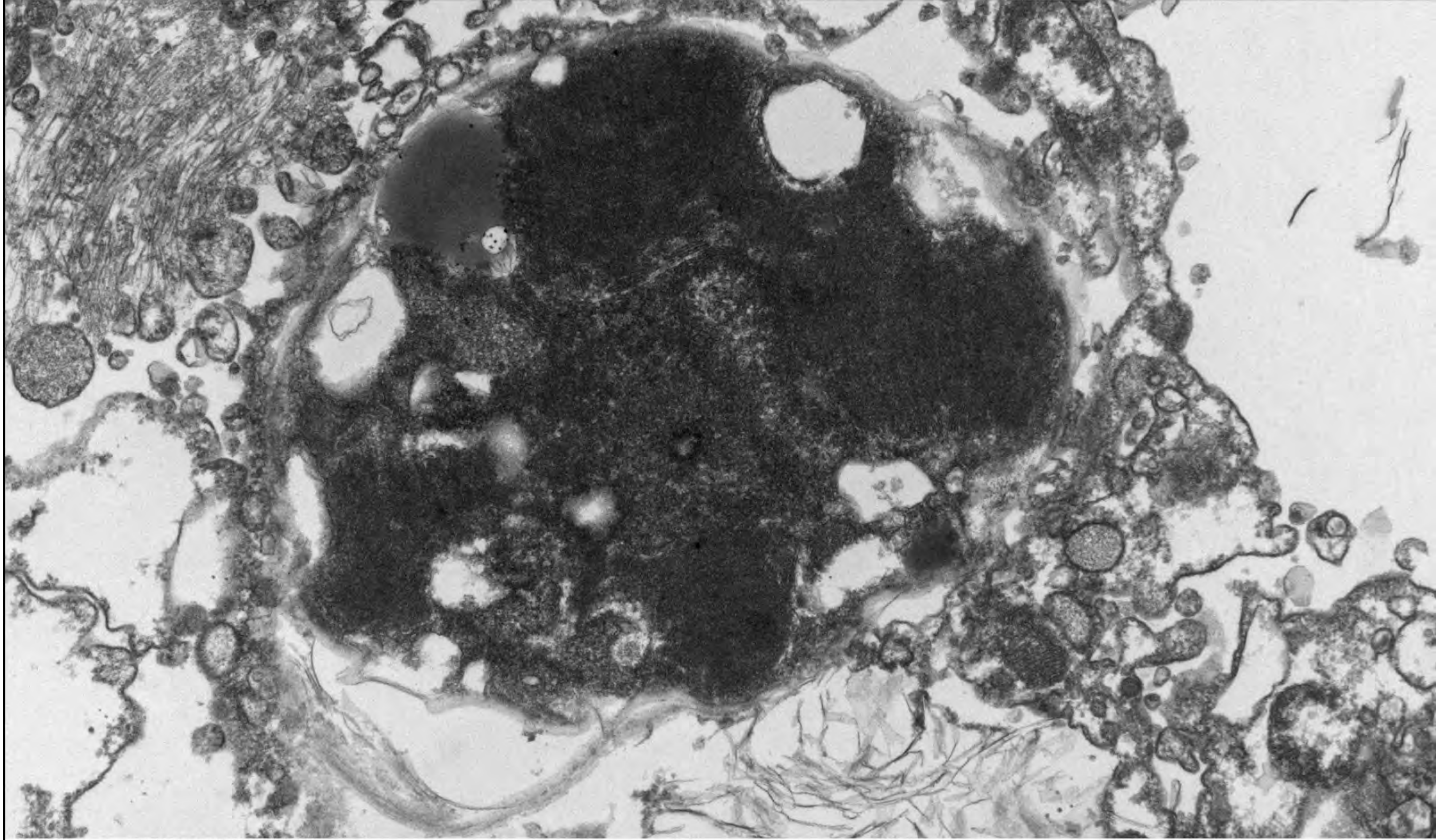

21-20_Correa_3-19-590_16C3_084.tif
Sample: 3/19, 590
Biological Electron Microscopy Lab
Rice University - SEA
Microscopist: MD Meyer

1 µm
HV=80kV
Direct Mag: 4000 x

Cell 6

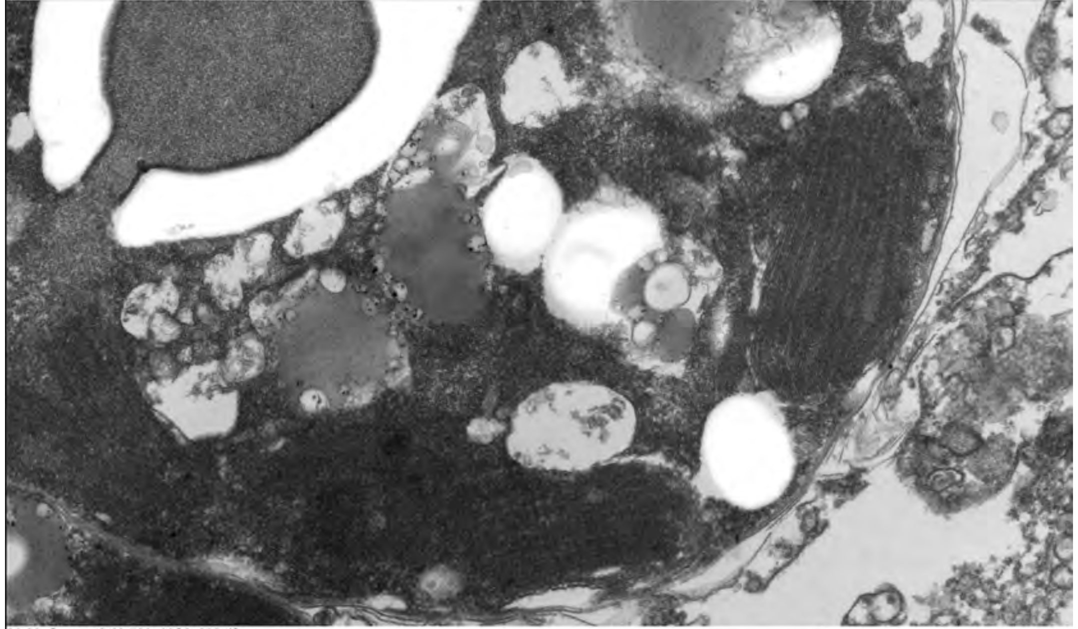

21-20_Correa_3-19-590_16C3_082.tif
Sample: 3/19, 590
Biological Electron Microscopy Lab
Rice University - SEA
Microscopist: MD Meyer

800 nm
HV=80kV
Direct Mag: 5000 x

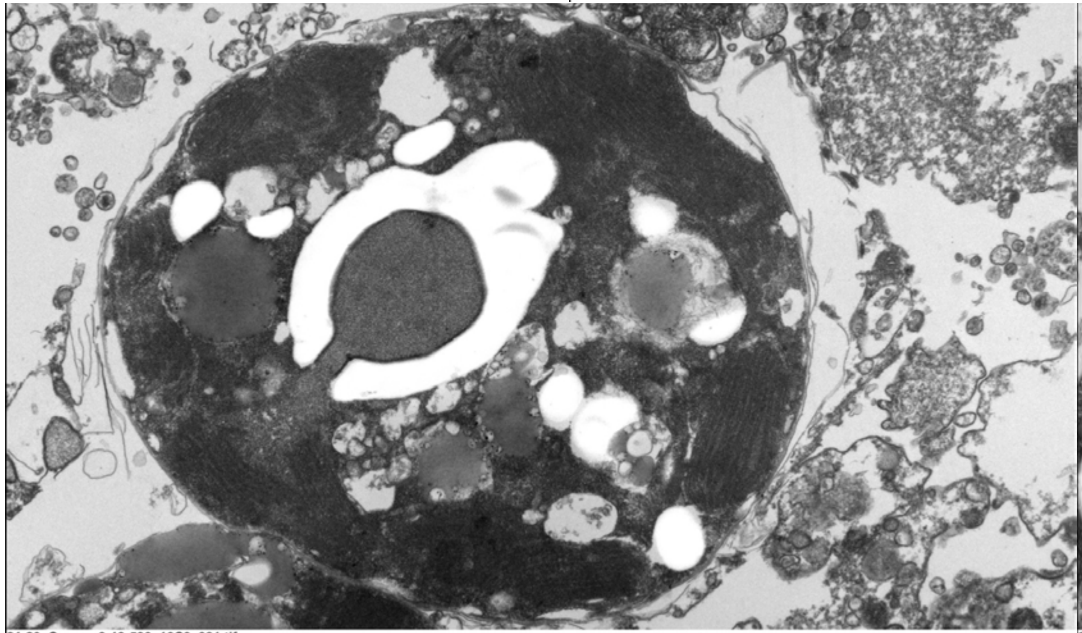

21-20_Correa_3-19-590_16C3_081.tif
Sample: 3/19, 590
Biological Electron Microscopy Lab
Rice University - SEA
Microscopist: MD Meyer

1 µm
HV=80kV
Direct Mag: 3000 x

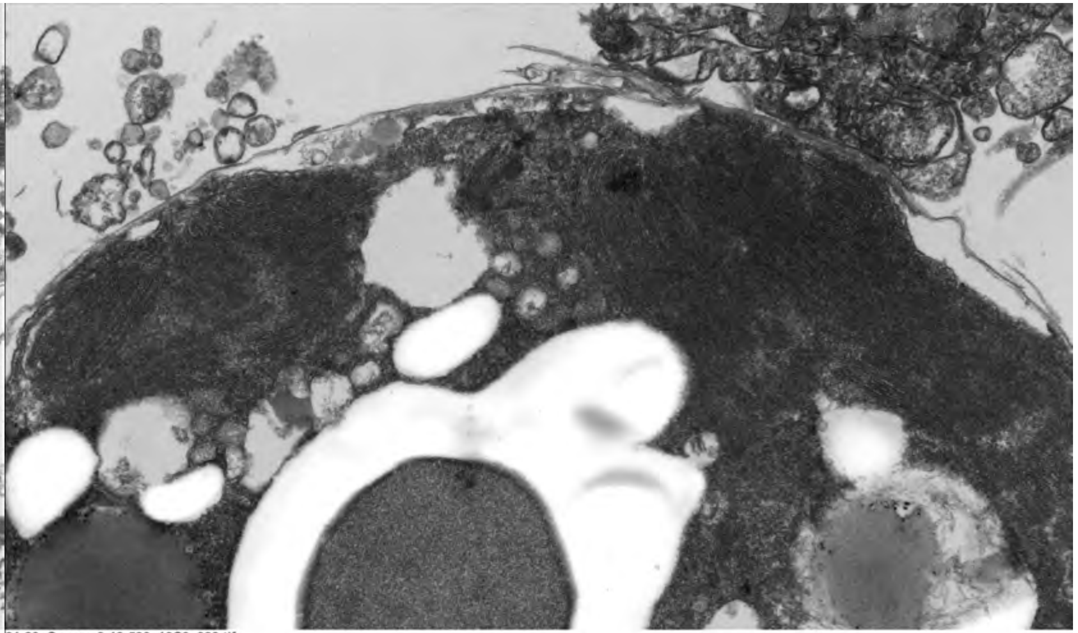

21-20_Correa_3-19-590_16C3_083.tif
Sample: 3/19, 590
Biological Electron Microscopy Lab
Rice University - SEA
Microscopist: MD Meyer

800 nm
HV=80kV
Direct Mag: 5000 x

Cell 7

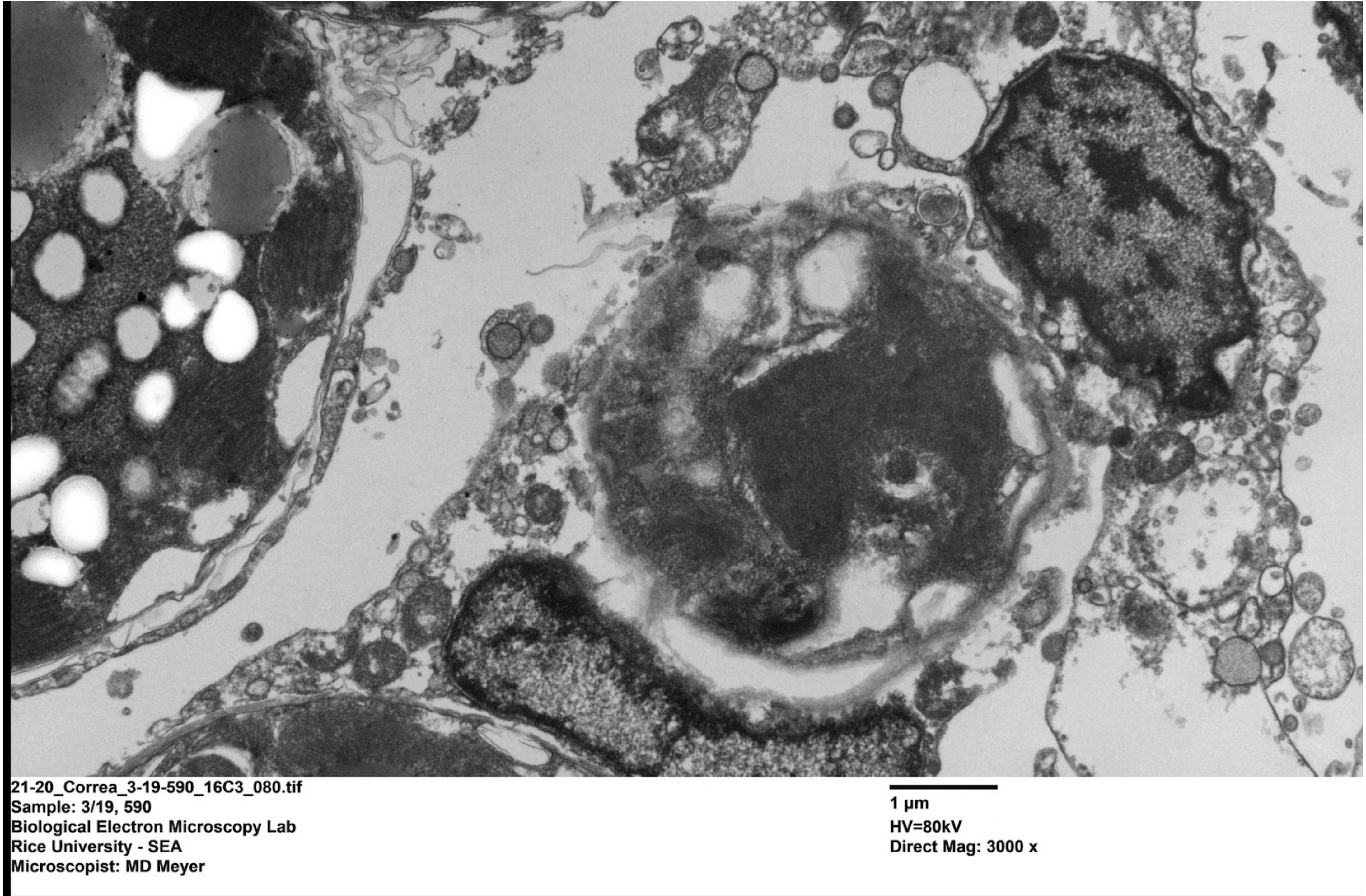
21-20_Correa_3-19-590_16C3_080.tif
Sample: 3/19, 590
Biological Electron Microscopy Lab
Rice University - SEA
Microscopist: MD Meyer
1 µm
HV=80kV
Direct Mag: 3000 x

Cell 8

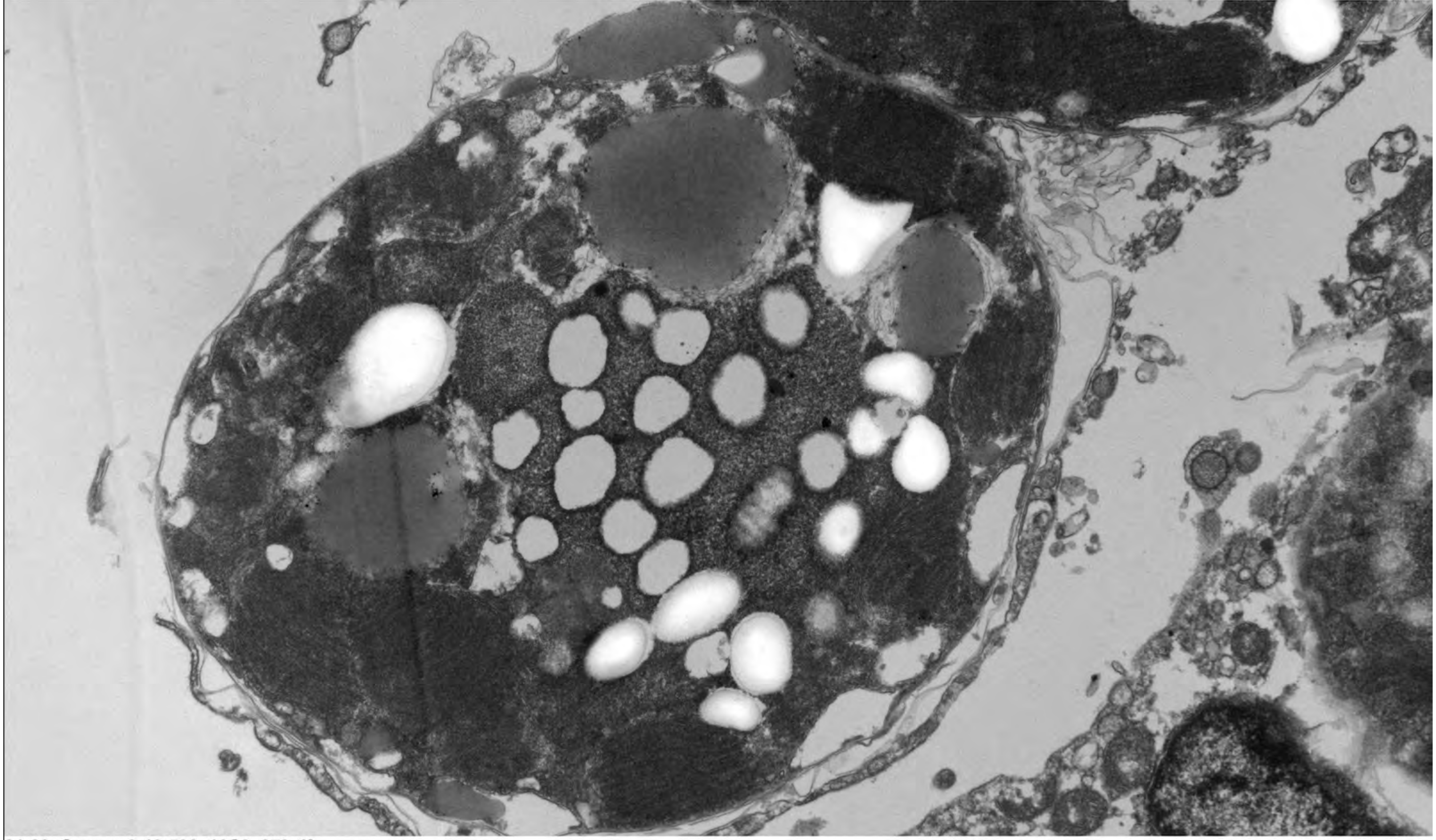

21-20_Correa_3-19-590_16C3_079.tif
Sample: 3/19, 590
Biological Electron Microscopy Lab
Rice University - SEA
Microscopist: MD Meyer

1 µm
HV=80kV
Direct Mag: 3000 x

Cell 9

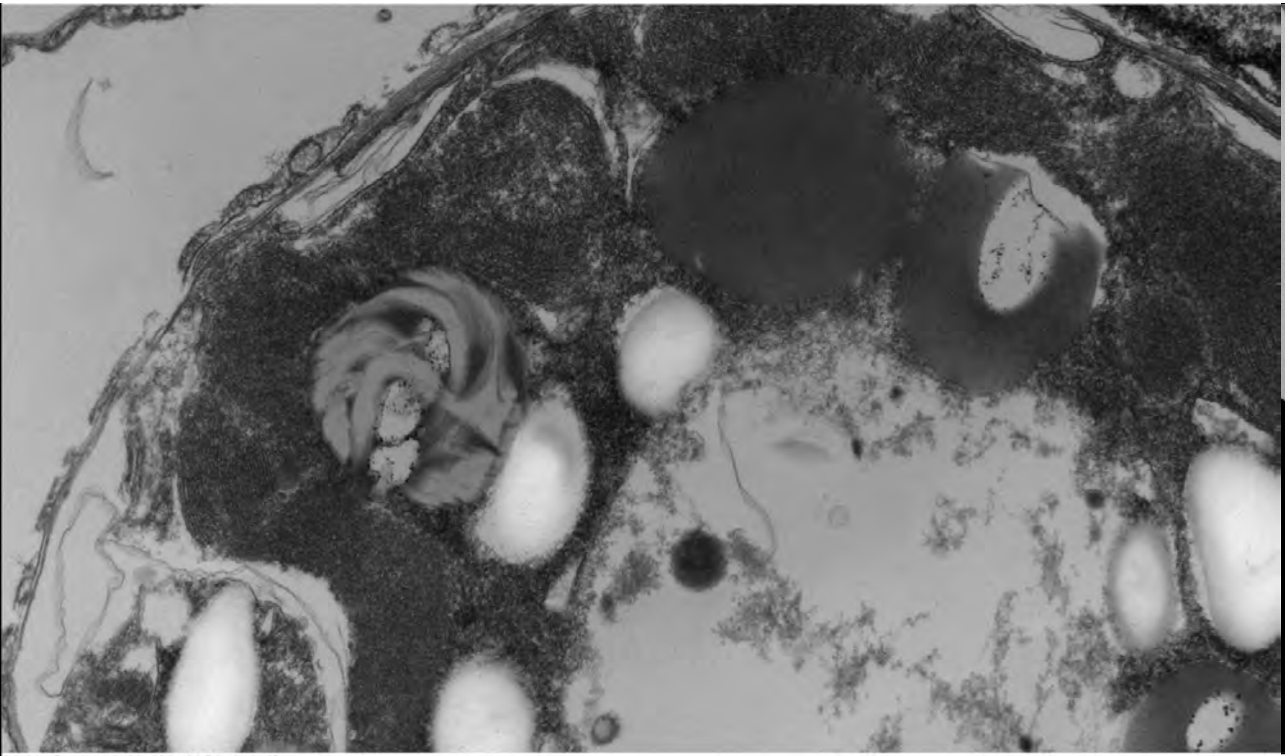

21-20_Correa_3-19-590_16C3_078.tif
Sample: 3/19, 590
Biological Electron Microscopy Lab
Rice University - SEA
Microscopist: MD Meyer

800 nm
HV=80kV
Direct Mag: 5000 x

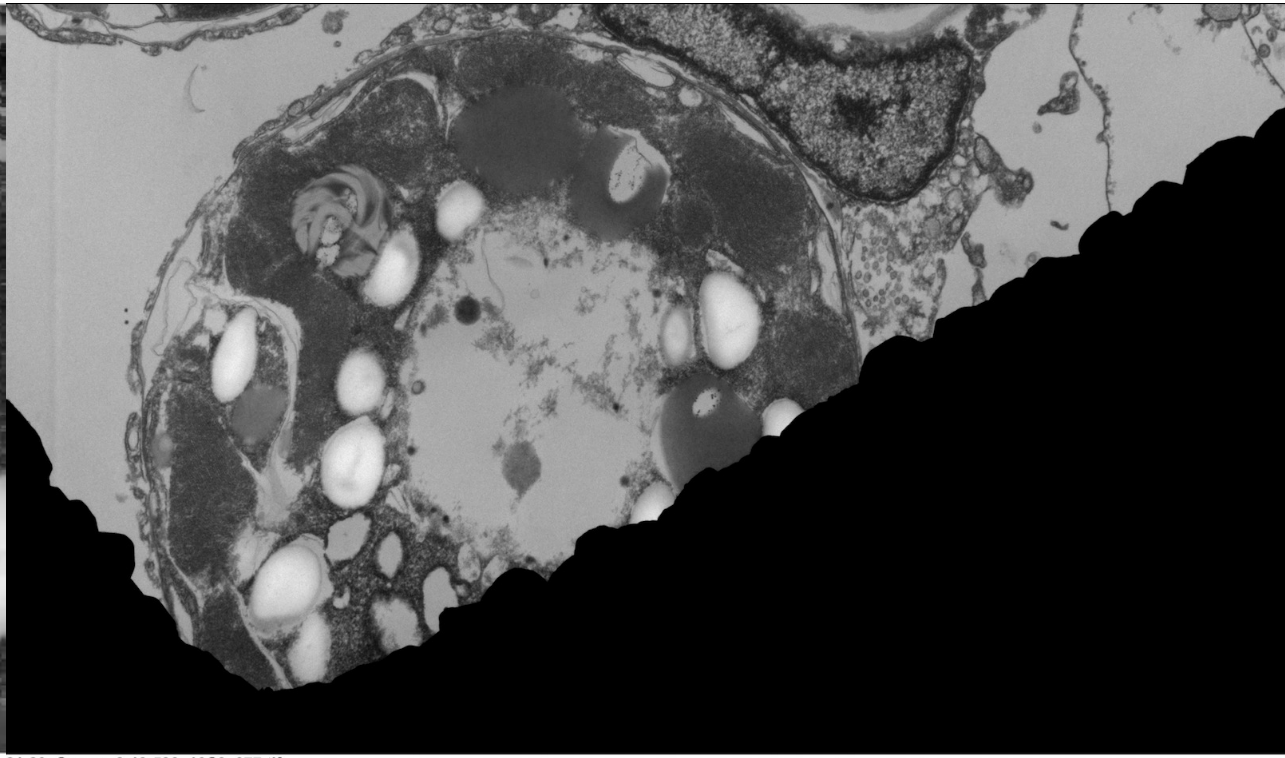

21-20_Correa_3-19-590_16C3_077.tif
Sample: 3/19, 590
Biological Electron Microscopy Lab
Rice University - SEA
Microscopist: MD Meyer

1 μm
HV=80kV
Direct Mag: 2500 x

Cell 10

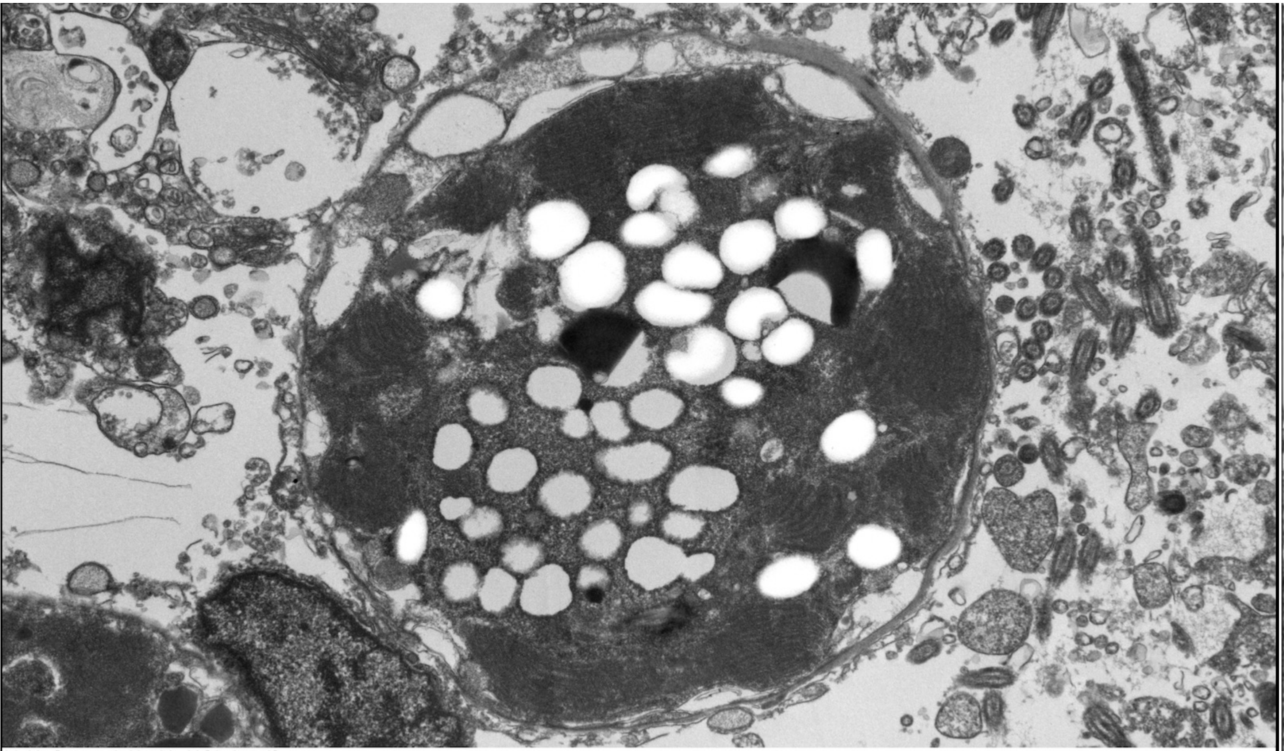

21-20_Correa_3-19-590_16C3_074.tif
Sample: 3/19, 590
Biological Electron Microscopy Lab
Rice University - SEA
Microscopist: MD Meyer

1 µm
HV=80kV
Direct Mag: 2500 x

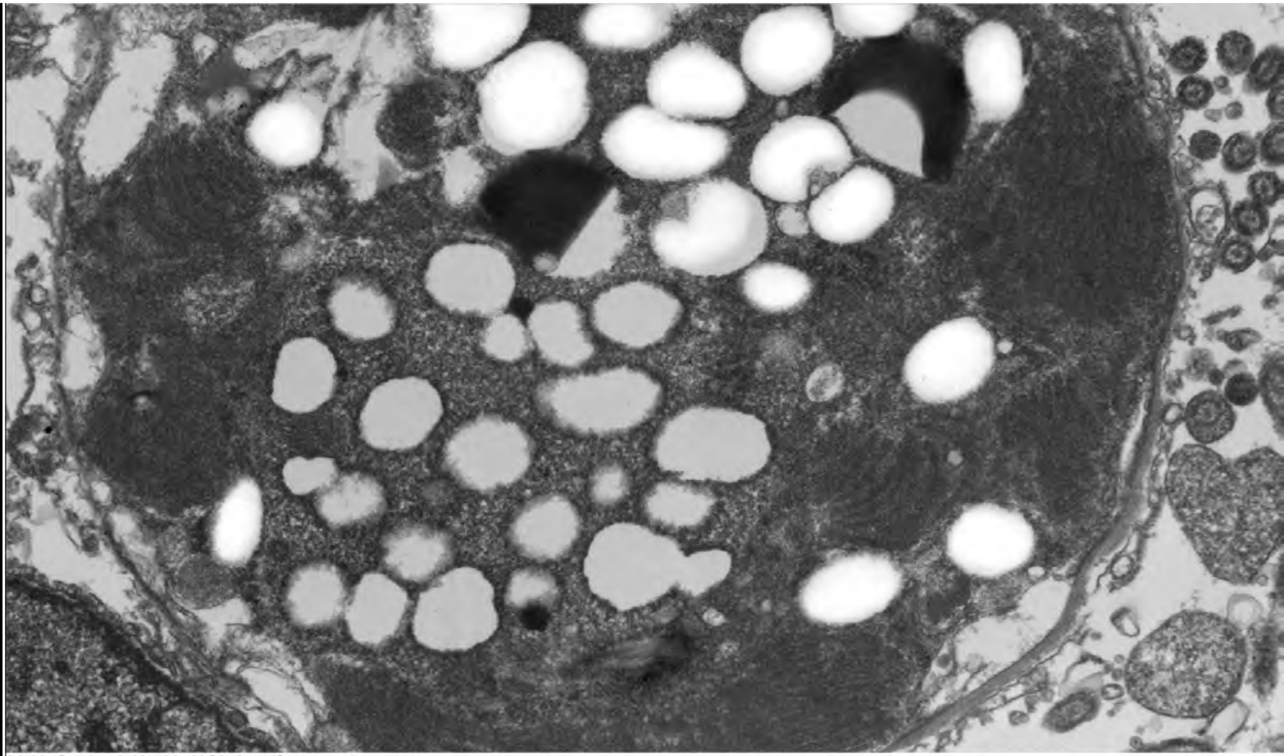

21-20_Correa_3-19-590_16C3_075.tif
Sample: 3/19, 590
Biological Electron Microscopy Lab
Rice University - SEA
Microscopist: MD Meyer

1 µm
HV=80kV
Direct Mag: 4000 x

Cell 11

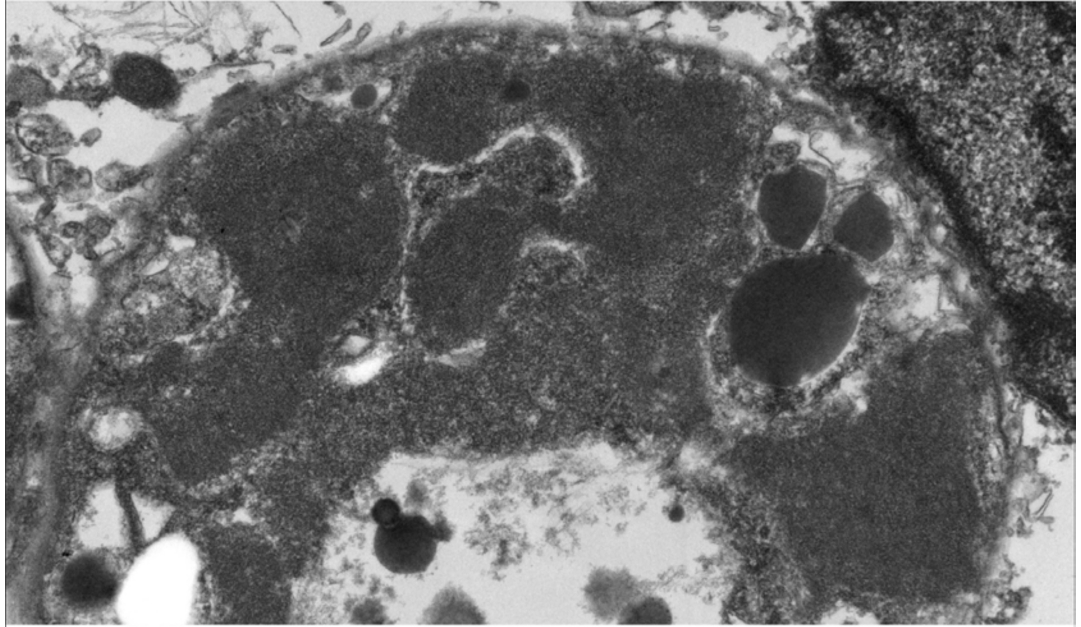

21-20_Correa_3-19-590_16C3_072.tif
Sample: 3/19, 590
Biological Electron Microscopy Lab
Rice University - SEA
Microscopist: MD Meyer

800 nm
HV=80kV
Direct Mag: 5000 x

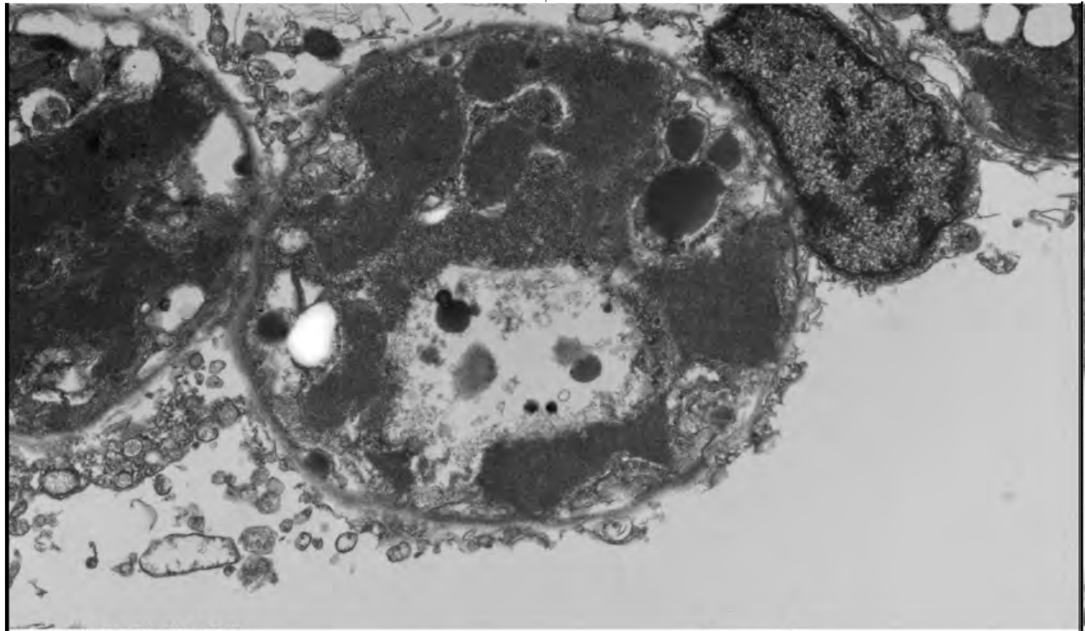

21-20_Correa_3-19-590_16C3_070.tif
Sample: 3/19, 590
Biological Electron Microscopy Lab
Rice University - SEA
Microscopist: MD Meyer

1 µm
HV=80kV
Direct Mag: 3000 x

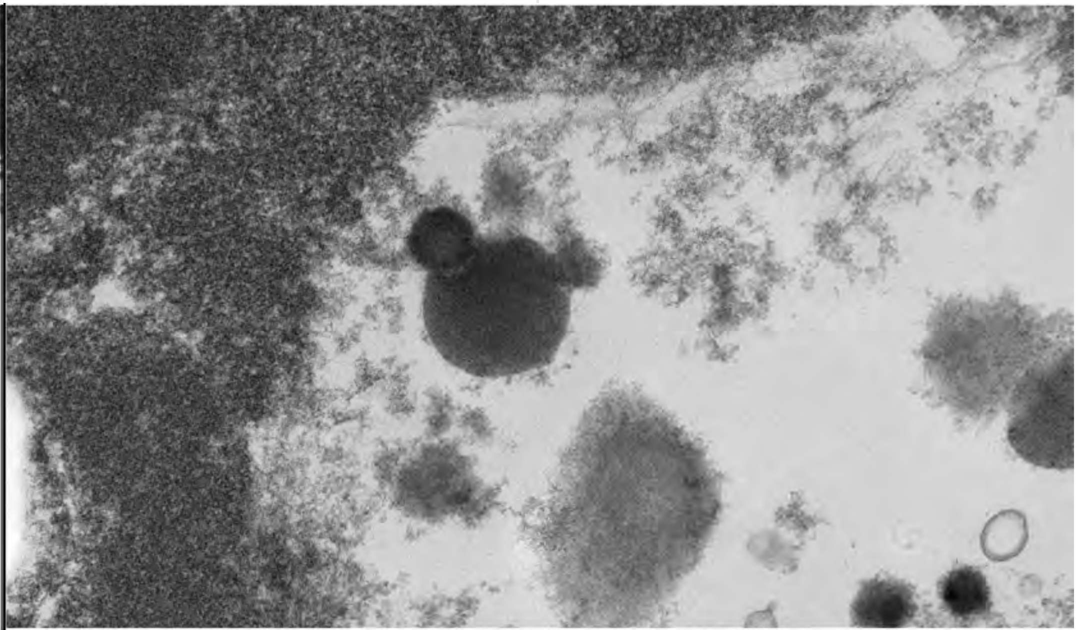

21-20_Correa_3-19-590_16C3_073.tif
Sample: 3/19, 590
Biological Electron Microscopy Lab
Rice University - SEA
Microscopist: MD Meyer

200 nm
HV=80kV
Direct Mag: 12000 x

Cell 12

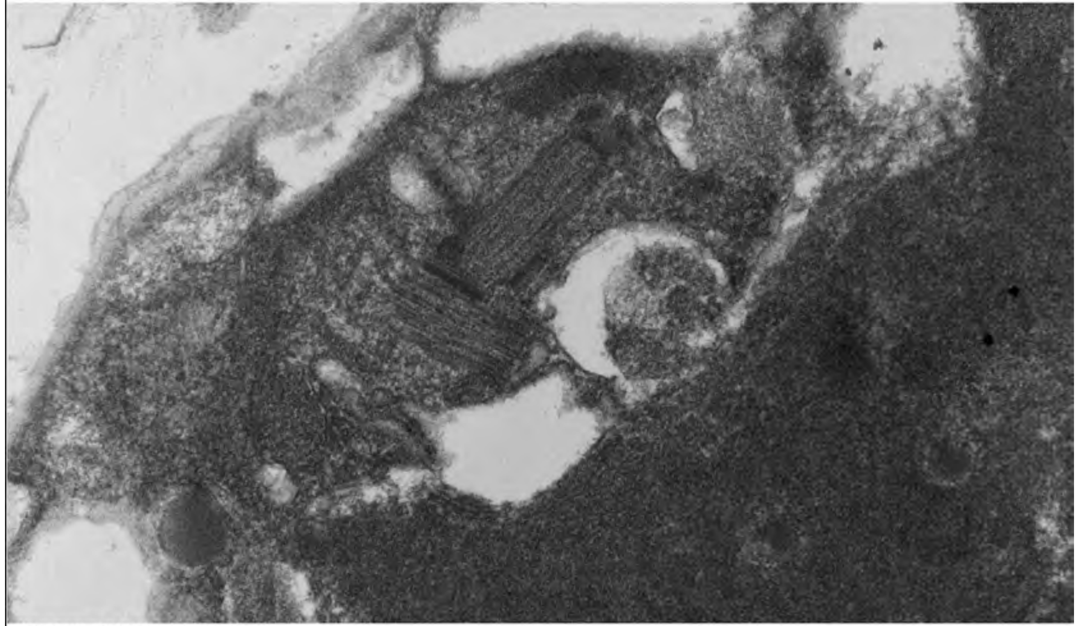

21-20_Correa_3-19-590_16C3_068.tif
Sample: 3/19, 590
Biological Electron Microscopy Lab
Rice University - SEA
Microscopist: MD Meyer

400 nm
HV=80kV
Direct Mag: 10000 x

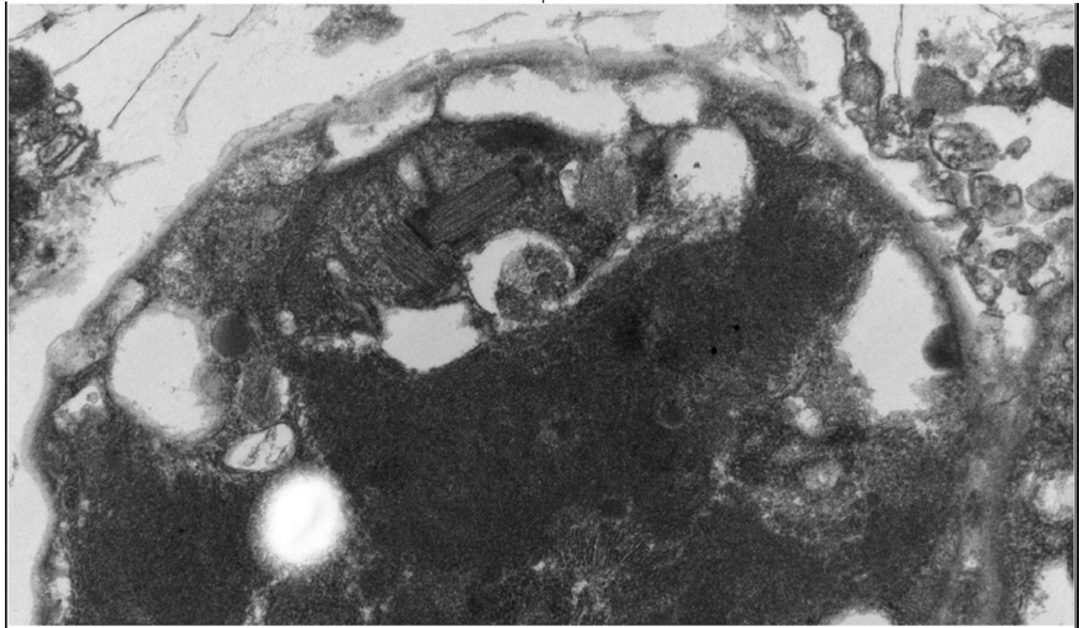

21-20_Correa_3-19-590_16C3_067.tif
Sample: 3/19, 590
Biological Electron Microscopy Lab
Rice University - SEA
Microscopist: MD Meyer

600 nm
HV=80kV
Direct Mag: 6000 x

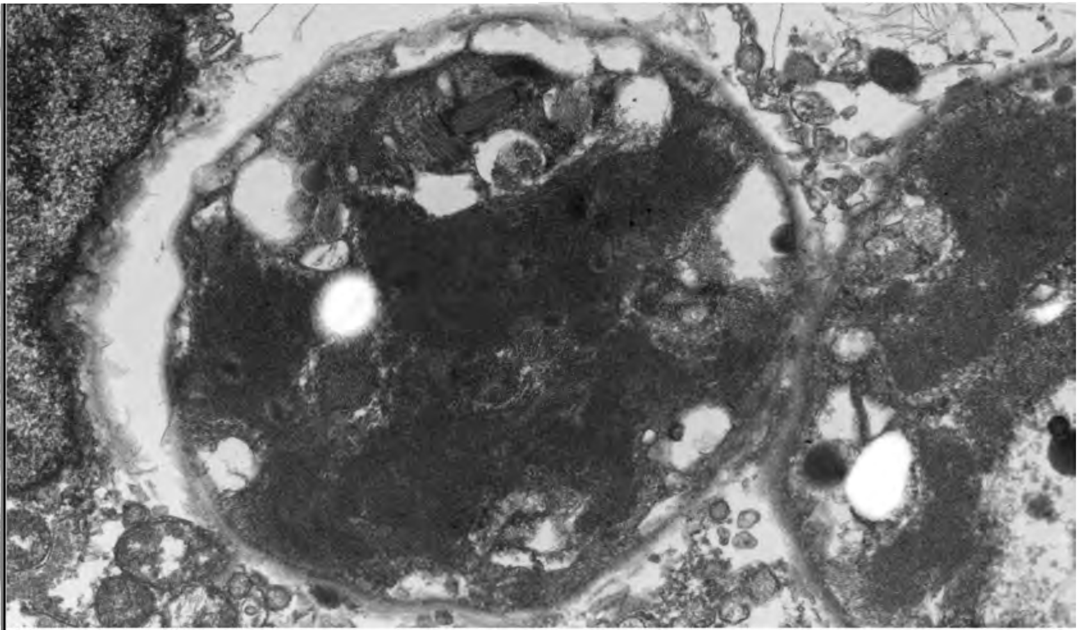

21-20_Correa_3-19-590_16C3_066.tif
Sample: 3/19, 590
Biological Electron Microscopy Lab
Rice University - SEA
Microscopist: MD Meyer

1 µm
HV=80kV
Direct Mag: 4000 x

Cell 13

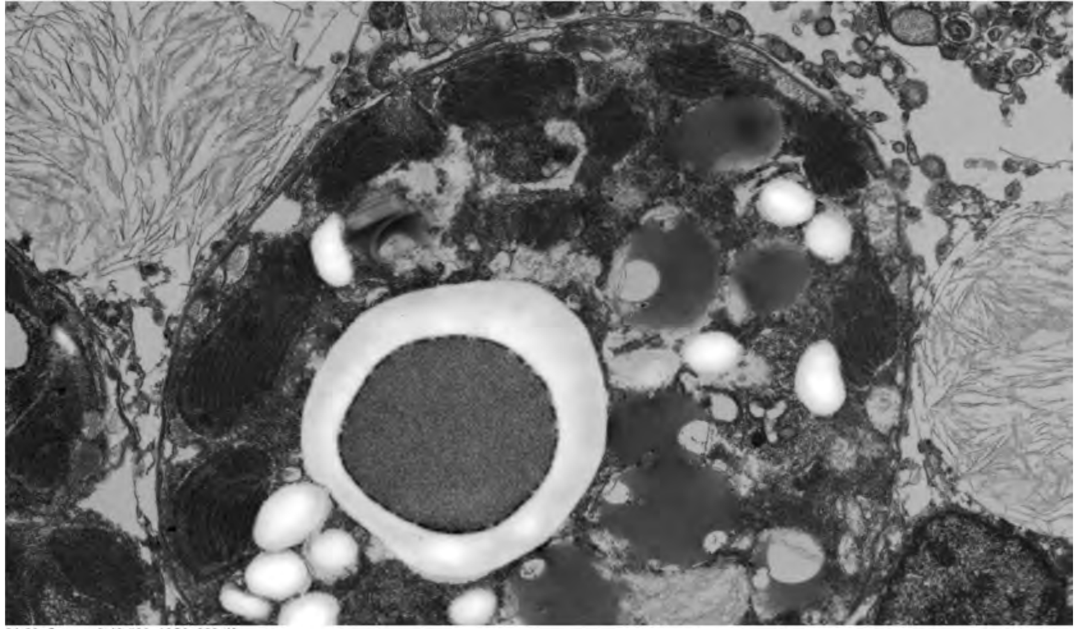

21-20_Correa_3-19-590_16C3_062.tif
Sample: 3/19, 590
Biological Electron Microscopy Lab
Rice University - SEA
Microscopist: MD Meyer

1 µm
HV=80kV
Direct Mag: 3000 x

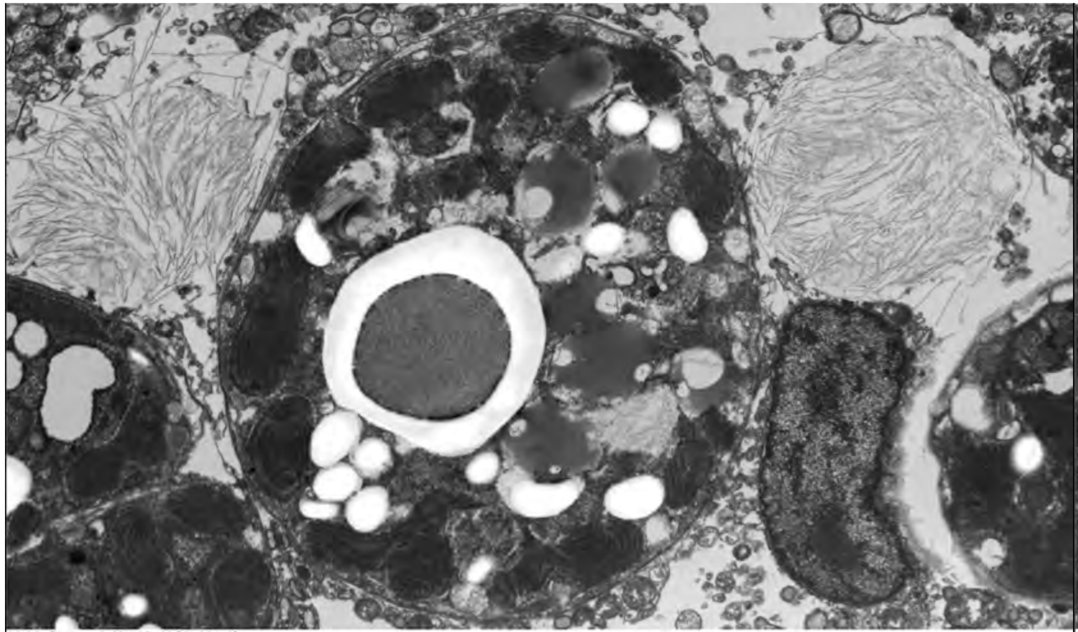

21-20_Correa_3-19-590_16C3_064.tif
Sample: 3/19, 590
Biological Electron Microscopy Lab
Rice University - SEA
Microscopist: MD Meyer

2 µm
HV=80kV
Direct Mag: 2000 x

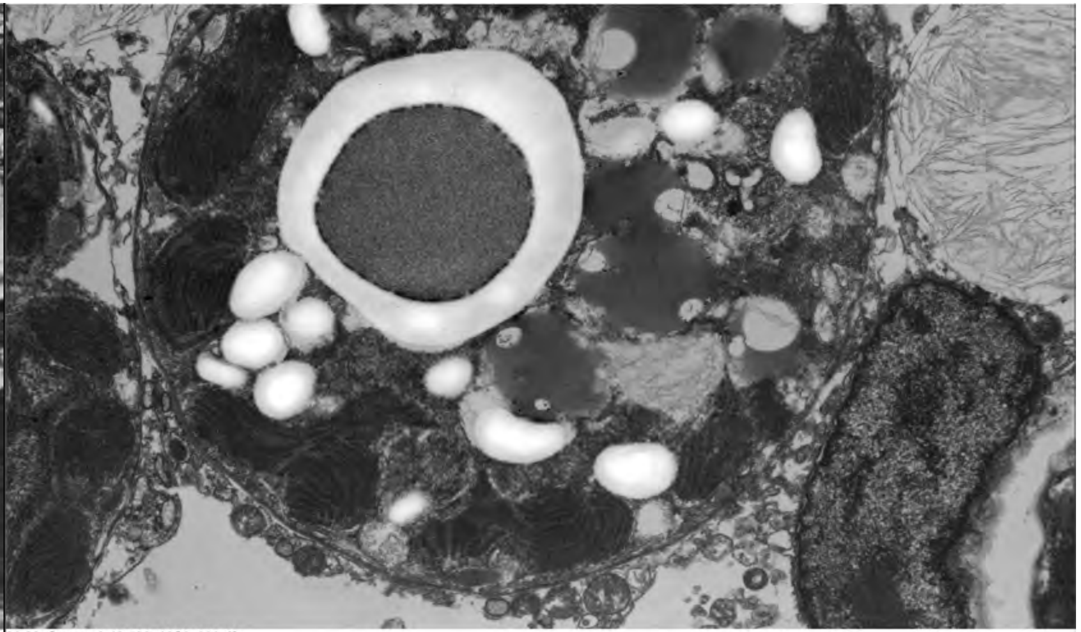

21-20_Correa_3-19-590_16C3_063.tif
Sample: 3/19, 590
Biological Electron Microscopy Lab
Rice University - SEA
Microscopist: MD Meyer

1 µm
HV=80kV
Direct Mag: 3000 x

Cell 14

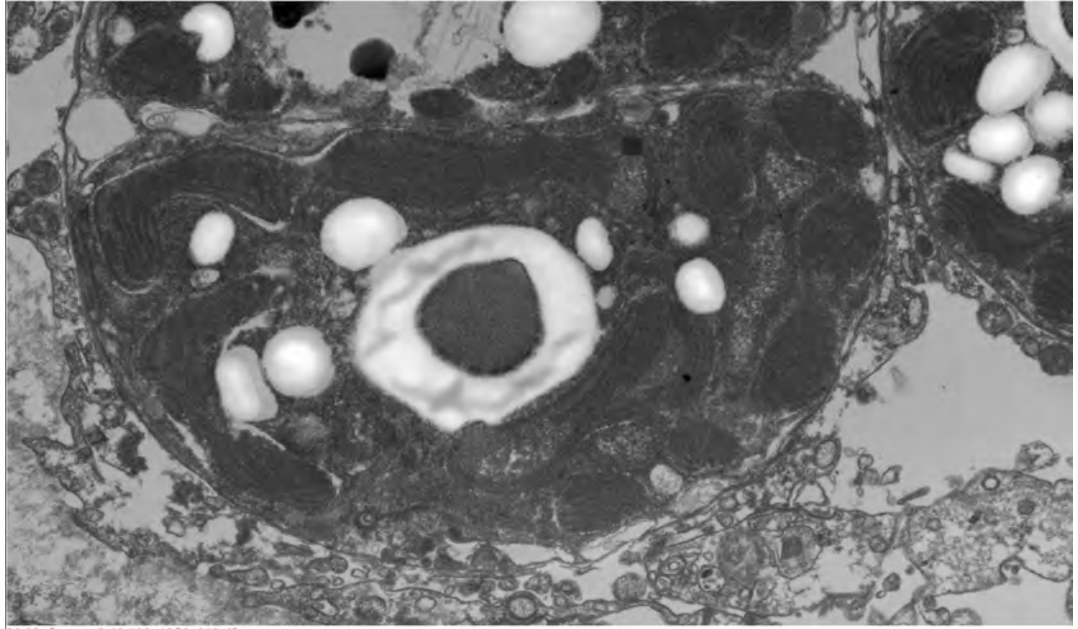

21-20_Correa_3-19-590_16C3_059.tif
Sample: 3/19, 590
Biological Electron Microscopy Lab
Rice University - SEA
Microscopist: MD Meyer

1 µm
HV=80kV
Direct Mag: 3000 x

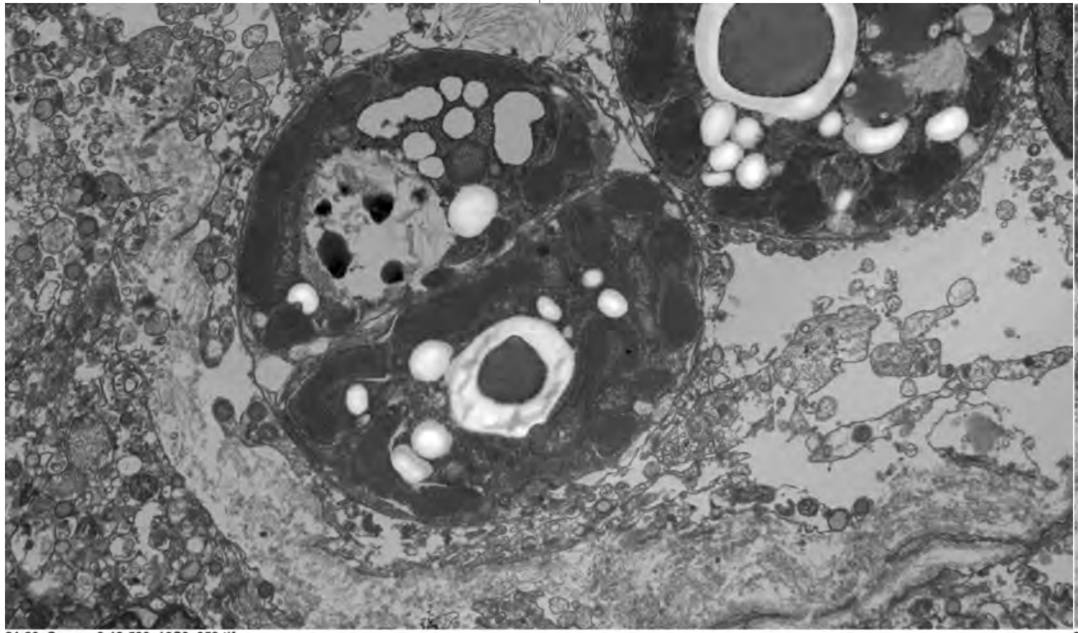

21-20_Correa_3-19-590_16C3_058.tif
Sample: 3/19, 590
Biological Electron Microscopy Lab
Rice University - SEA
Microscopist: MD Meyer

2 µm
HV=80kV
Direct Mag: 1500 x

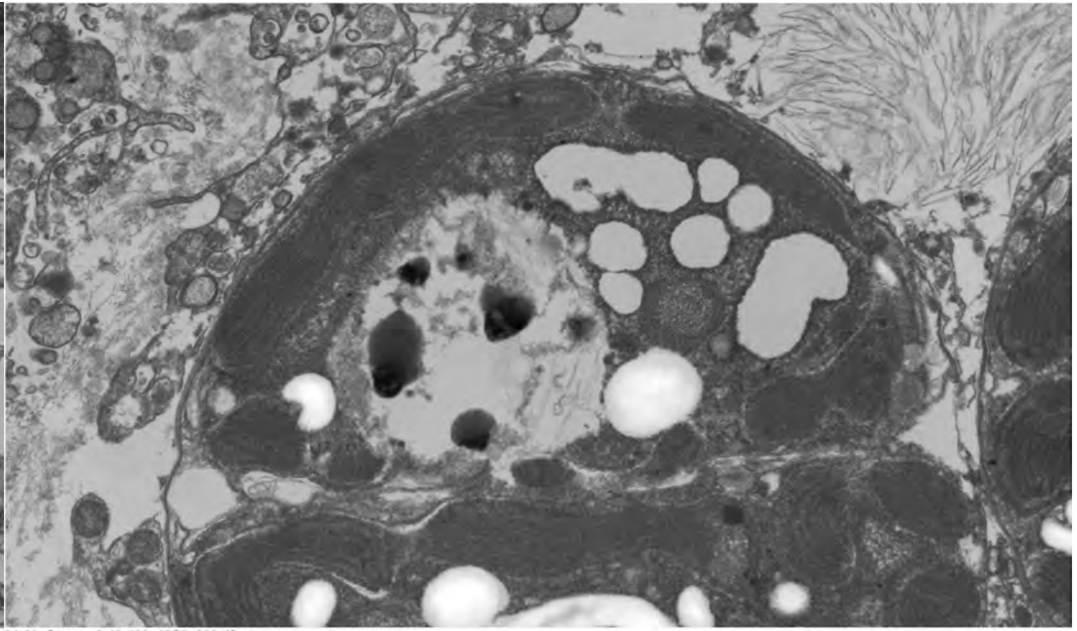

21-20_Correa_3-19-590_16C3_060.tif
Sample: 3/19, 590
Biological Electron Microscopy Lab
Rice University - SEA
Microscopist: MD Meyer

1 µm
HV=80kV
Direct Mag: 3000 x

Cell 15

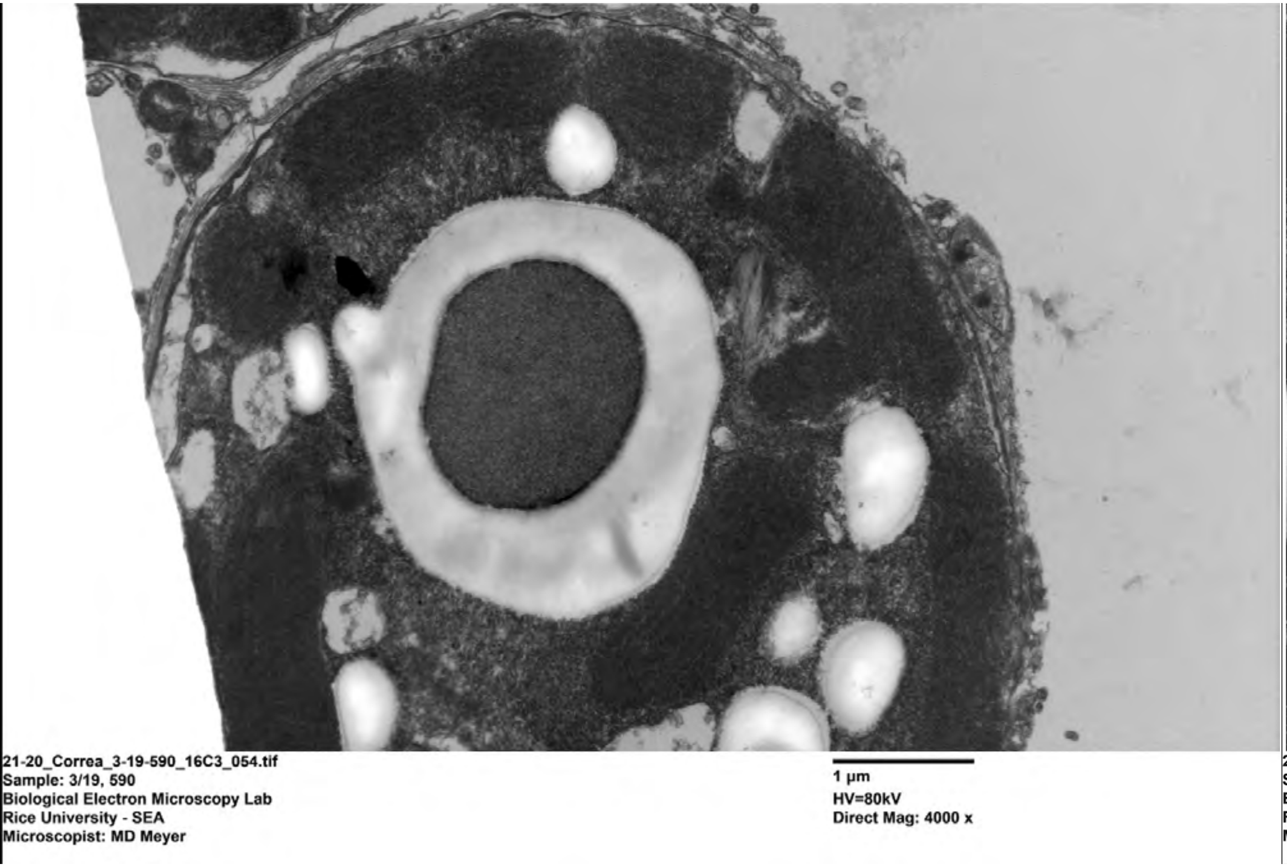
21-20_Correa_3-19-590_16C3_054.tif
Sample: 3/19, 590
Biological Electron Microscopy Lab
Rice University - SEA
Microscopist: MD Meyer
1 μm
HV=80kV
Direct Mag: 4000 x

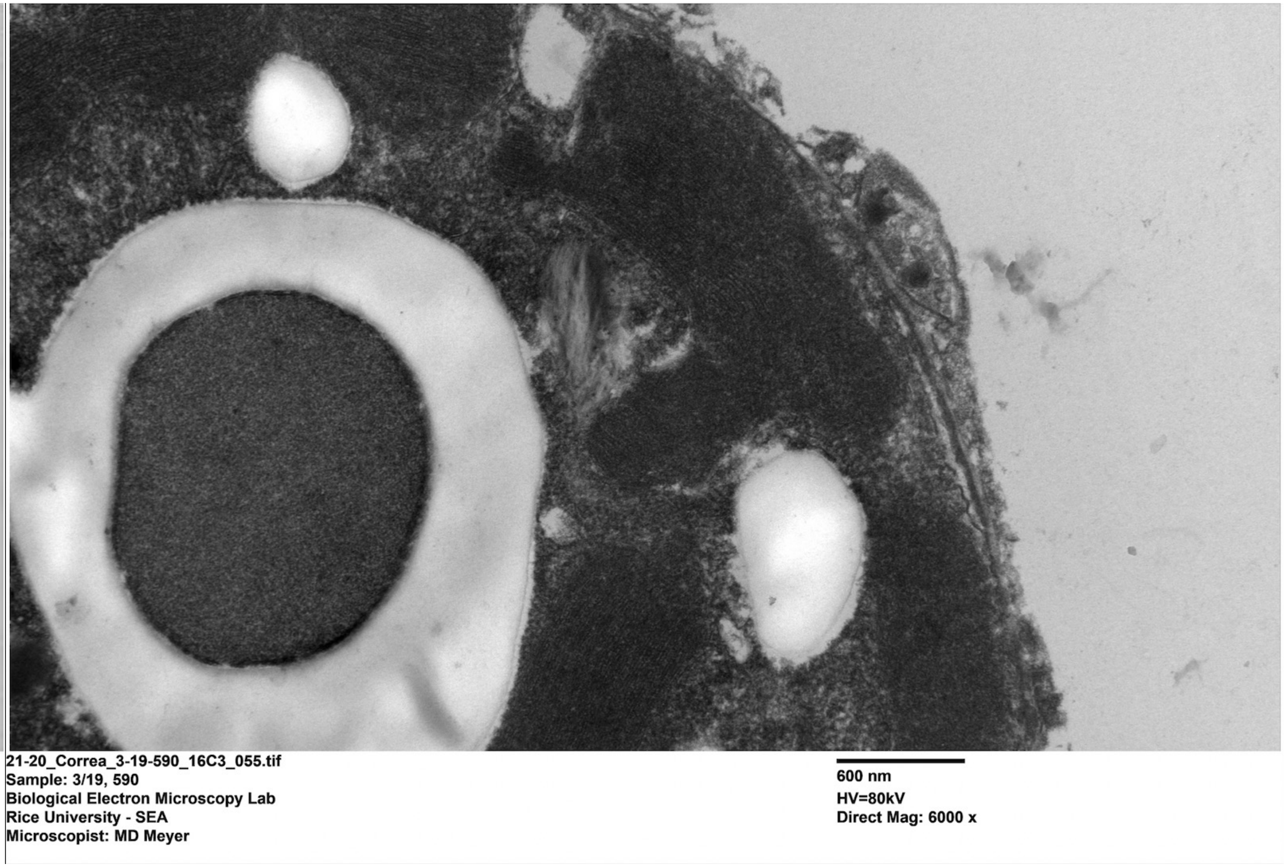
21-20_Correa_3-19-590_16C3_055.tif
Sample: 3/19, 590
Biological Electron Microscopy Lab
Rice University - SEA
Microscopist: MD Meyer
600 nm
HV=80kV
Direct Mag: 6000 x

Cell 16

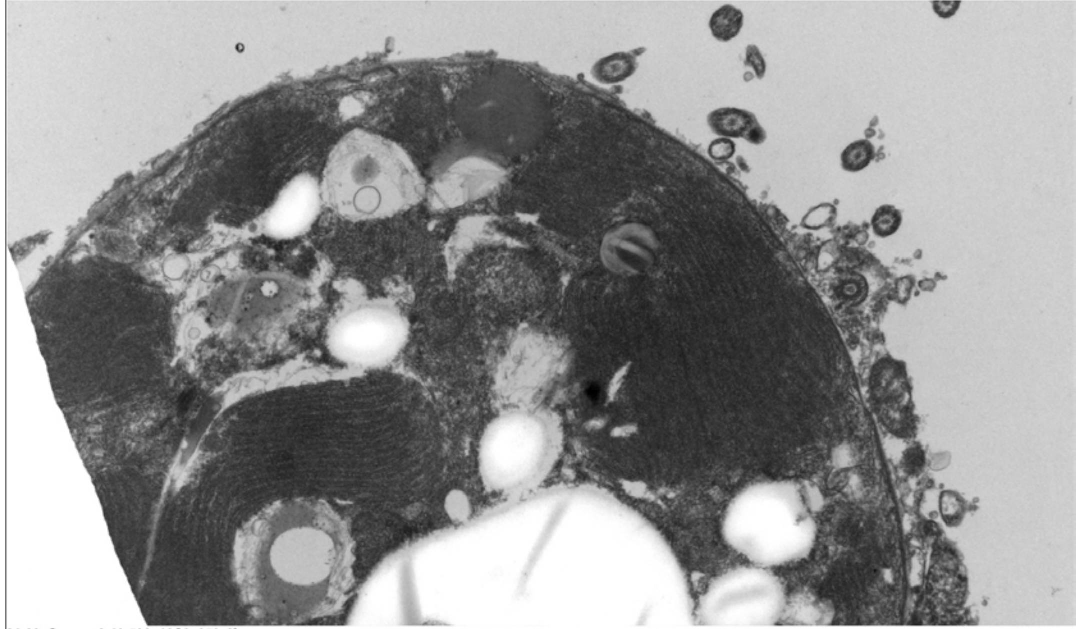

21-20_Correa_3-19-590_16C3_050.tif
Sample: 3/19, 590
Biological Electron Microscopy Lab
Rice University - SEA
Microscopist: MD Meyer

1 µm
HV=80kV
Direct Mag: 4000 x

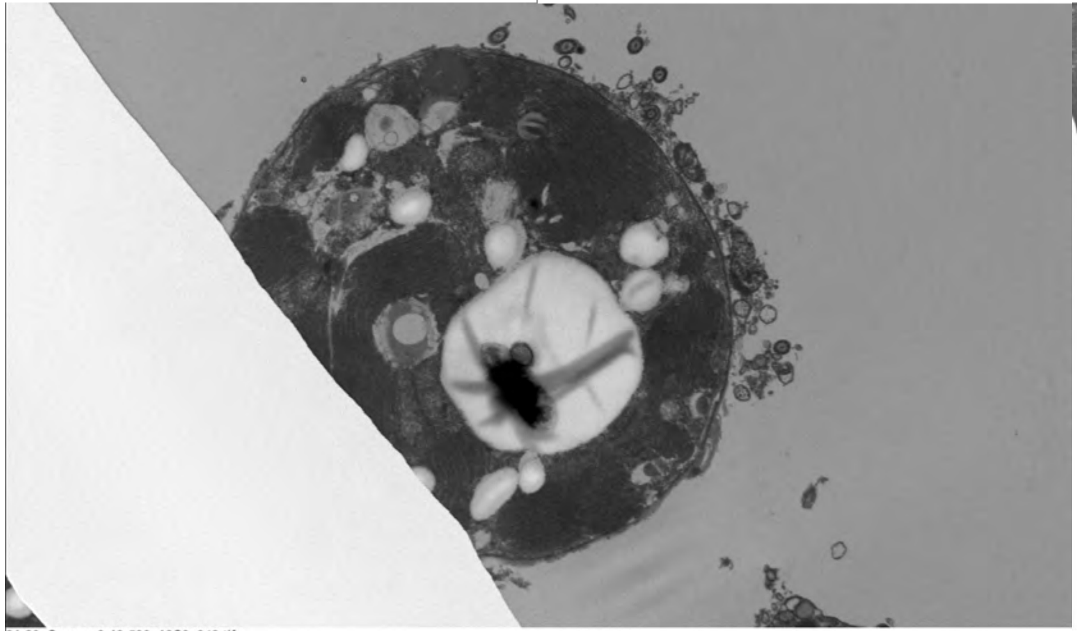

21-20_Correa_3-19-590_16C3_048.tif
Sample: 3/19, 590
Biological Electron Microscopy Lab
Rice University - SEA
Microscopist: MD Meyer

2 µm
HV=80kV
Direct Mag: 2000 x

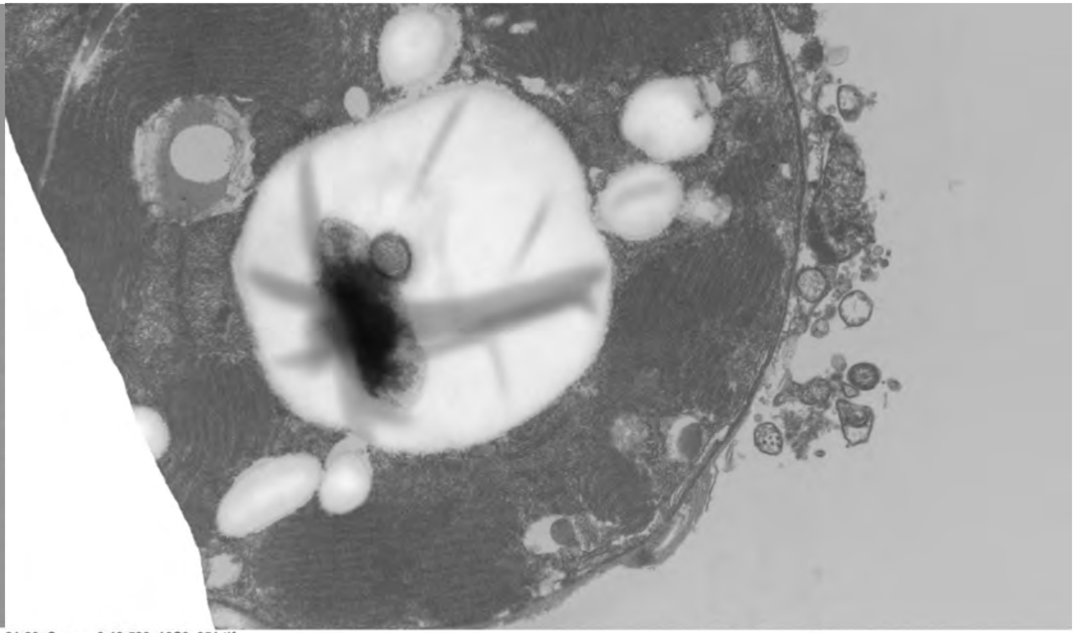

21-20_Correa_3-19-590_16C3_051.tif
Sample: 3/19, 590
Biological Electron Microscopy Lab
Rice University - SEA
Microscopist: MD Meyer

1 µm
HV=80kV
Direct Mag: 4000 x

Cell 17

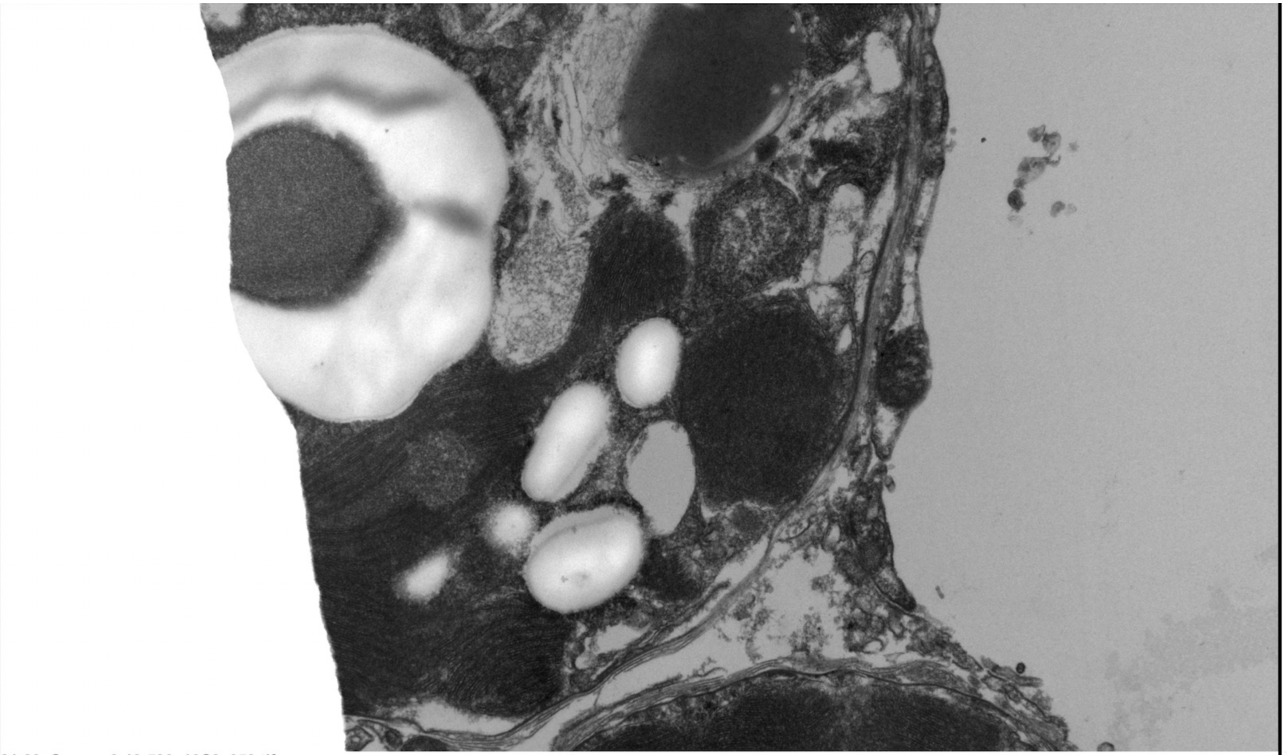

21-20_Correa_3-19-590_16C3_053.tif
Sample: 3/19, 590
Biological Electron Microscopy Lab
Rice University - SEA
Microscopist: MD Meyer

1 µm
HV=80kV
Direct Mag: 4000 x

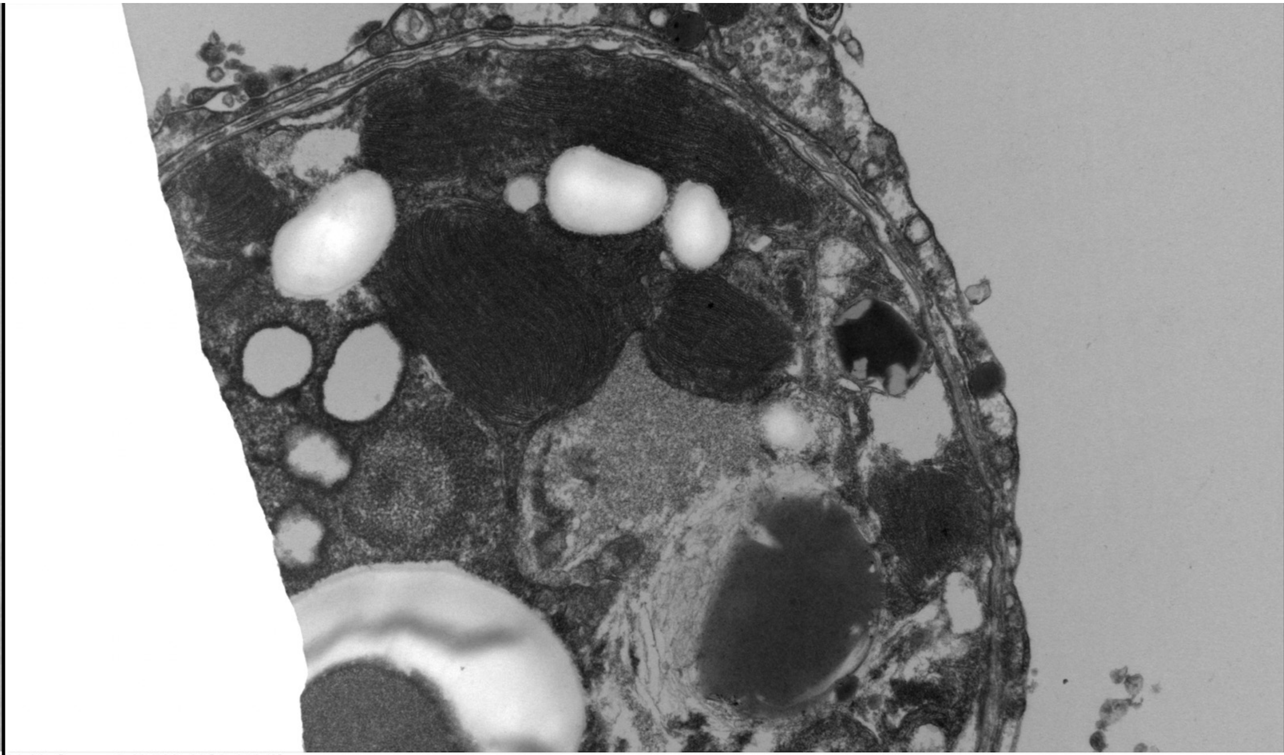

21-20_Correa_3-19-590_16C3_052.tif
Sample: 3/19, 590
Biological Electron Microscopy Lab
Rice University - SEA
Microscopist: MD Meyer

1 µm
HV=80kV
Direct Mag: 4000 x

Cell 18

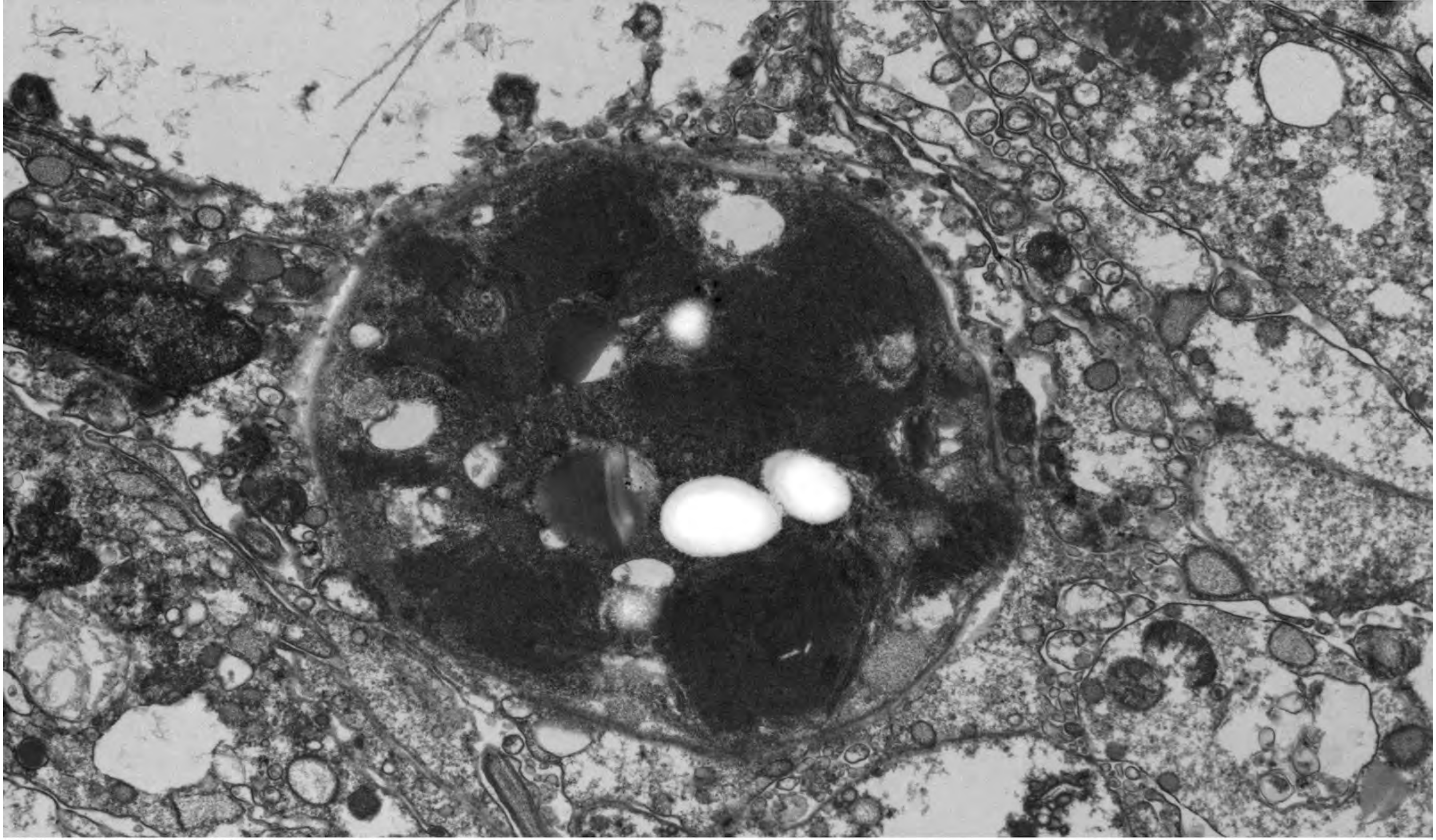

21-20_Correa_3-19-590_16C3_045.tif
Sample: 3/19, 590
Biological Electron Microscopy Lab
Rice University - SEA
Microscopist: MD Meyer

1 µm
HV=80kV
Direct Mag: 3000 x

Cell 19

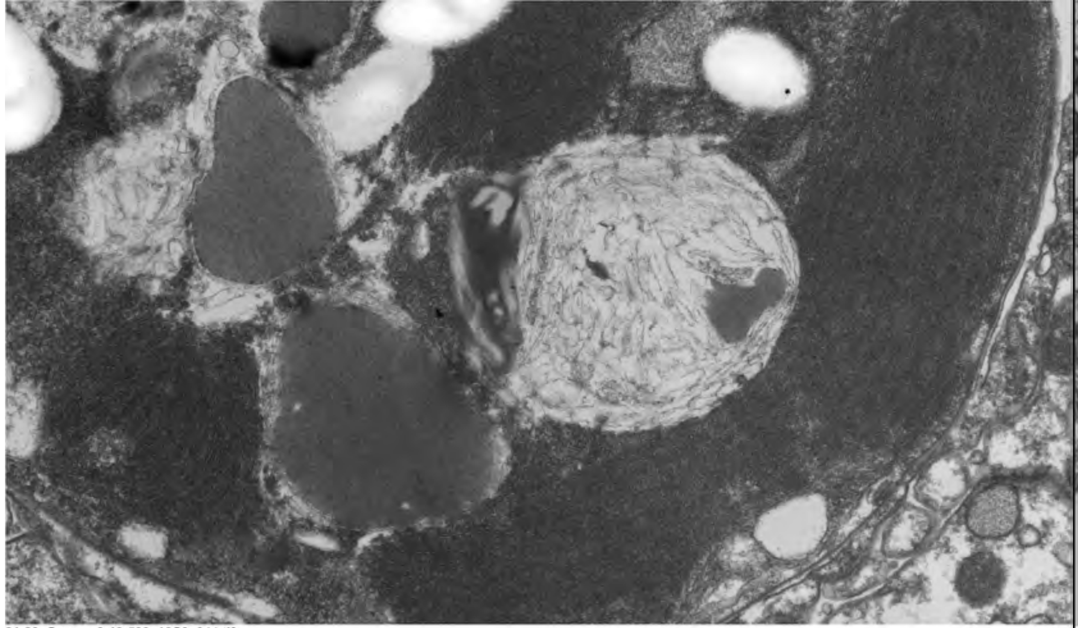

21-20_Correa_3-19-590_16C3_044.tif
Sample: 3/19, 590
Biological Electron Microscopy Lab
Rice University - SEA
Microscopist: MD Meyer

600 nm
HV=80kV
Direct Mag: 6000 x

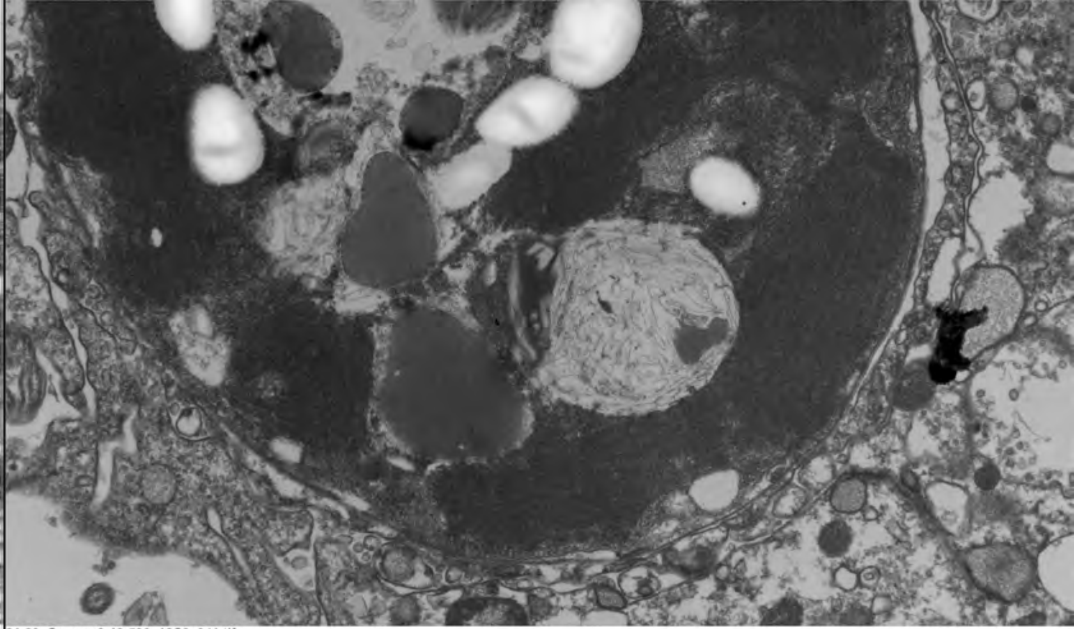

21-20_Correa_3-19-590_16C3_041.tif
Sample: 3/19, 590
Biological Electron Microscopy Lab
Rice University - SEA
Microscopist: MD Meyer

1 µm
HV=80kV
Direct Mag: 4000 x

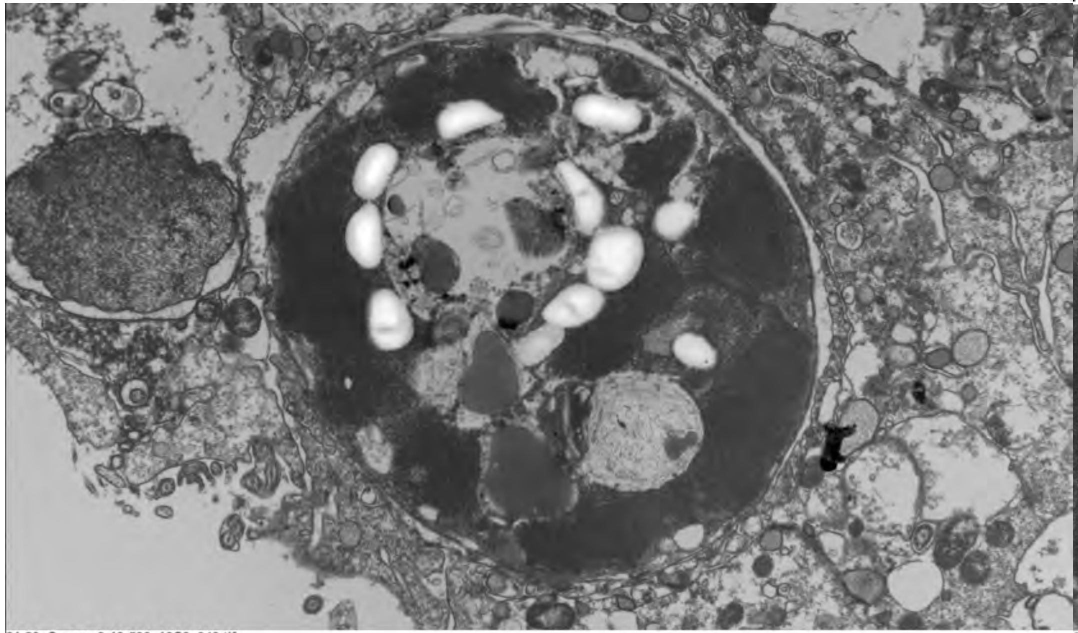

21-20_Correa_3-19-590_16C3_040.tif
Sample: 3/19, 590
Biological Electron Microscopy Lab
Rice University - SEA
Microscopist: MD Meyer

1 µm
HV=80kV
Direct Mag: 2500 x

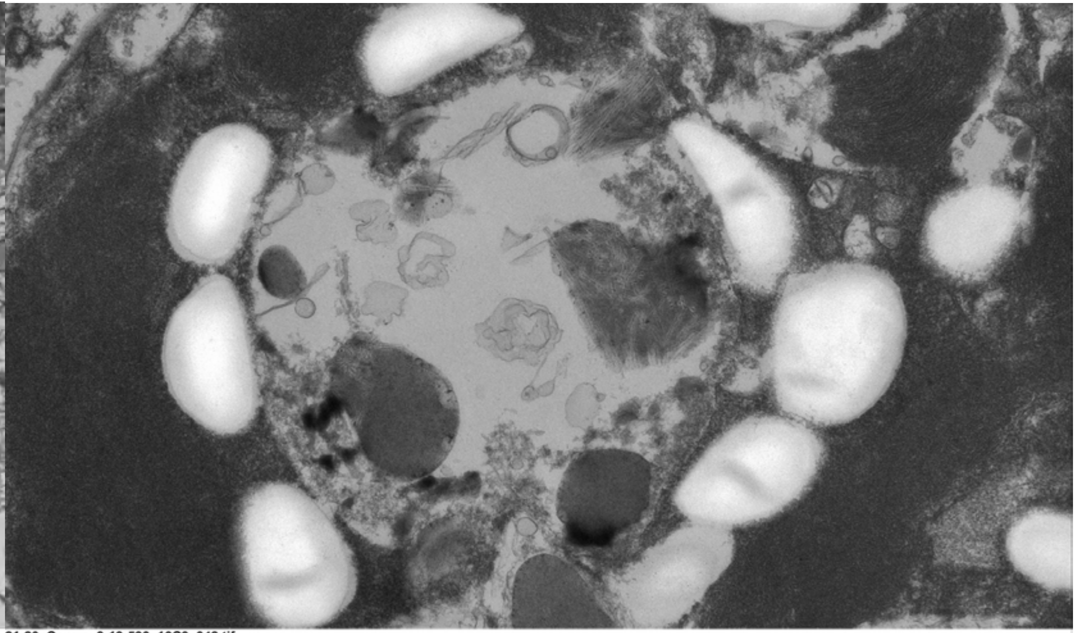

21-20_Correa_3-19-590_16C3_042.tif
Sample: 3/19, 590
Biological Electron Microscopy Lab
Rice University - SEA
Microscopist: MD Meyer

600 nm
HV=80kV
Direct Mag: 6000 x

Cell 20

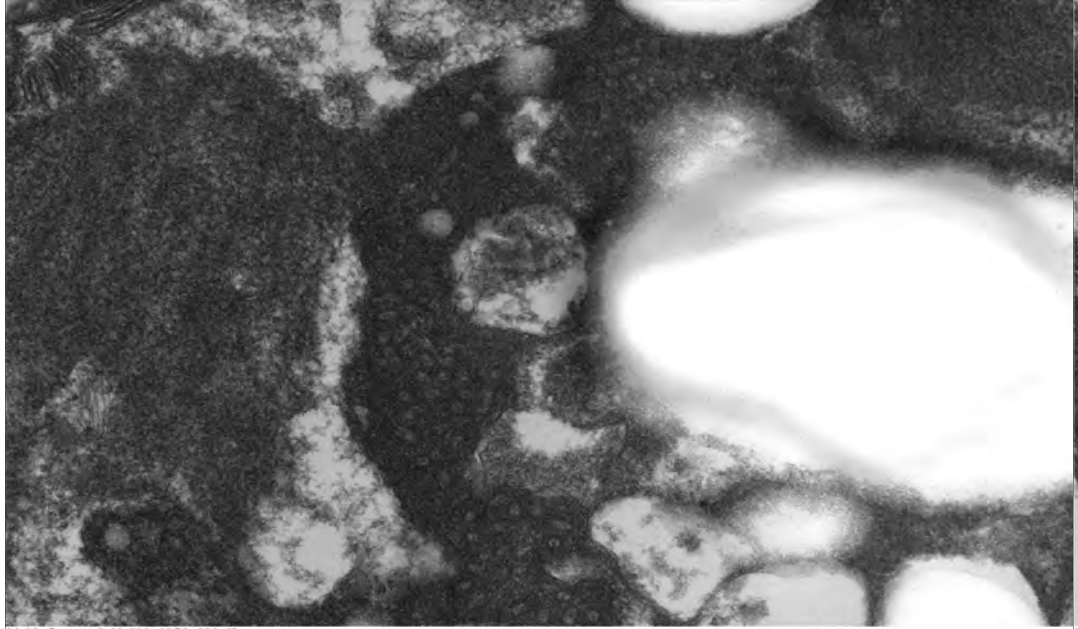

21-20_Correa_3-19-590_16C3_036.tif
Sample: 3/19, 590
Biological Electron Microscopy Lab
Rice University - SEA
Microscopist: MD Meyer

400 nm
HV=80kV
Direct Mag: 10000 x

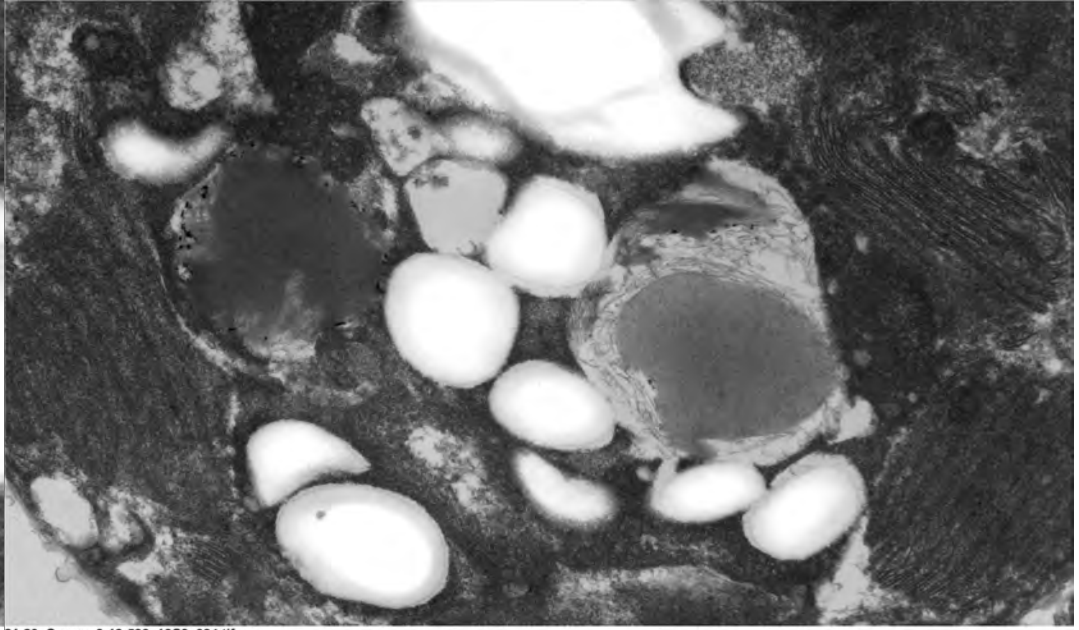

21-20_Correa_3-19-590_16C3_034.tif
Sample: 3/19, 590
Biological Electron Microscopy Lab
Rice University - SEA
Microscopist: MD Meyer

600 nm
HV=80kV
Direct Mag: 6000 x

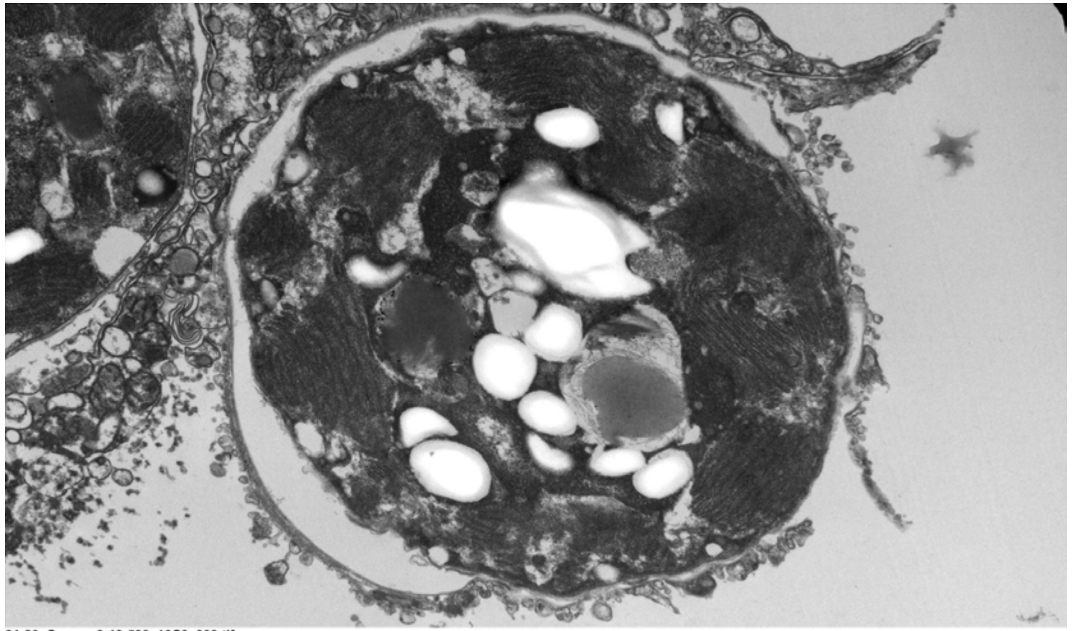

21-20_Correa_3-19-590_16C3_033.tif
Sample: 3/19, 590
Biological Electron Microscopy Lab
Rice University - SEA
Microscopist: MD Meyer

1 µm
HV=80kV
Direct Mag: 3000 x

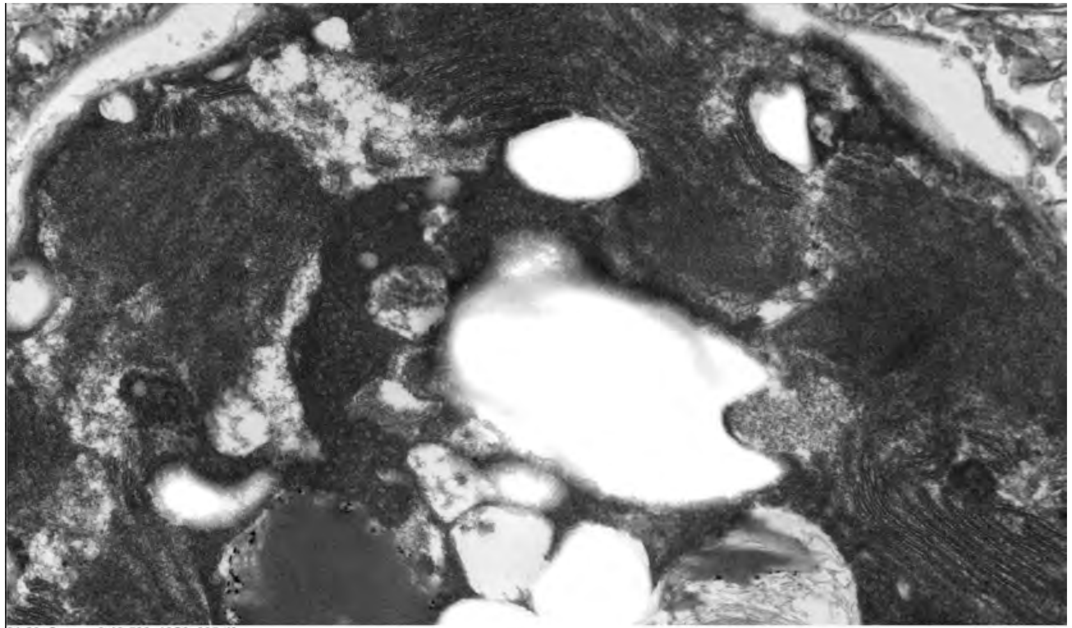

21-20_Correa_3-19-590_16C3_035.tif
Sample: 3/19, 590
Biological Electron Microscopy Lab
Rice University - SEA
Microscopist: MD Meyer

600 nm
HV=80kV
Direct Mag: 6000 x

Cell 21

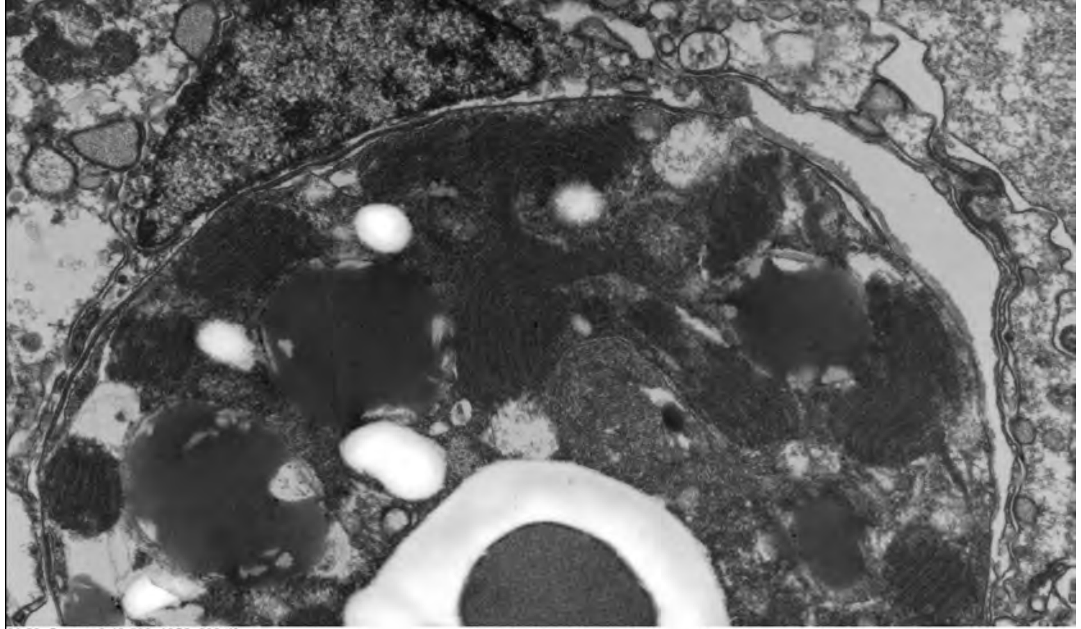

21-20_Correa_3-19-590_16C3_032.tif
Sample: 3/19, 590
Biological Electron Microscopy Lab
Rice University - SEA
Microscopist: MD Meyer

1 µm
HV=80kV
Direct Mag: 4000 x

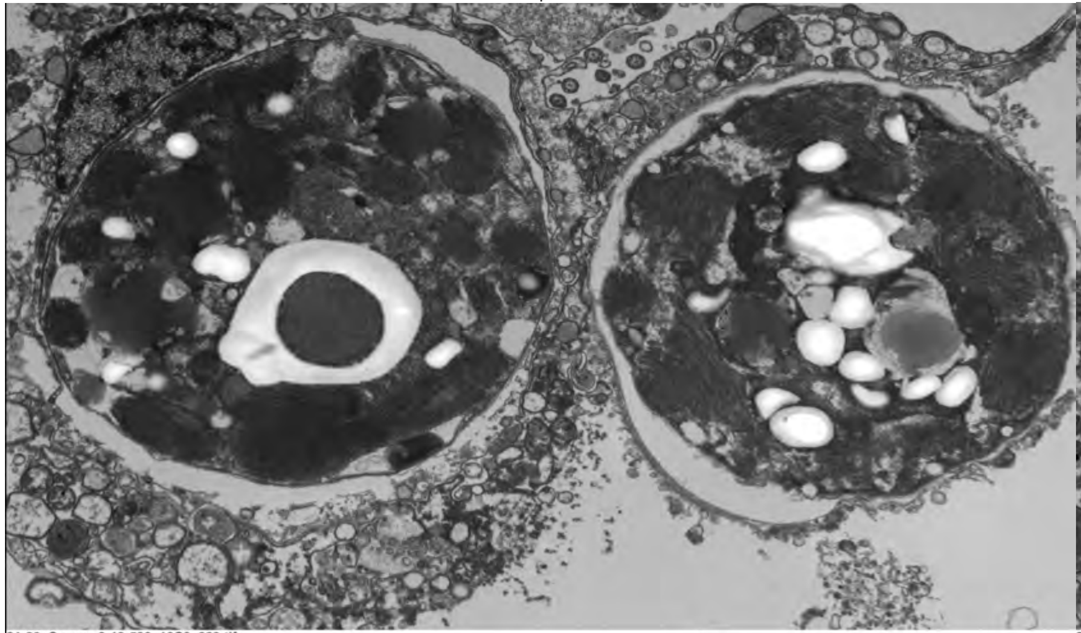

21-20_Correa_3-19-590_16C3_029.tif
Sample: 3/19, 590
Biological Electron Microscopy Lab
Rice University - SEA
Microscopist: MD Meyer

2 µm
HV=80kV
Direct Mag: 2000 x

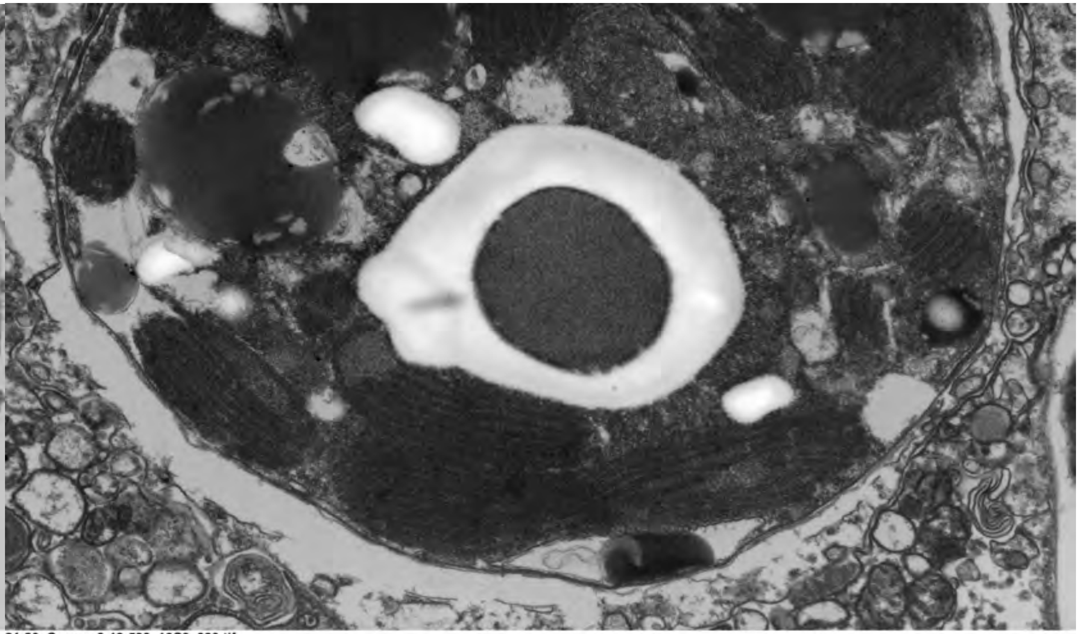

21-20_Correa_3-19-590_16C3_030.tif
Sample: 3/19, 590
Biological Electron Microscopy Lab
Rice University - SEA
Microscopist: MD Meyer

1 µm
HV=80kV
Direct Mag: 4000 x

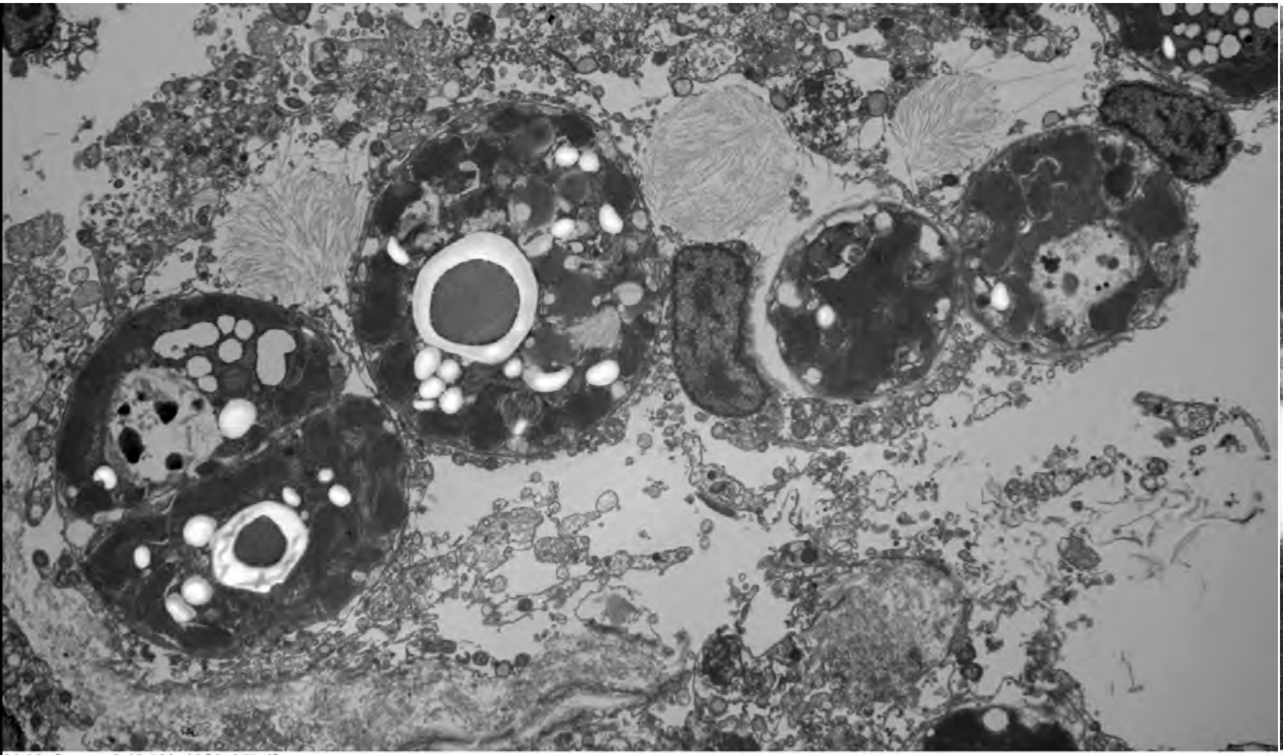

21-20_Correa_3-19-590_16C3_057.tif
Sample: 3/19, 590
Biological Electron Microscopy Lab
Rice University - SEA
Microscopist: MD Meyer

4 µm
HV=80kV
Direct Mag: 1000 x

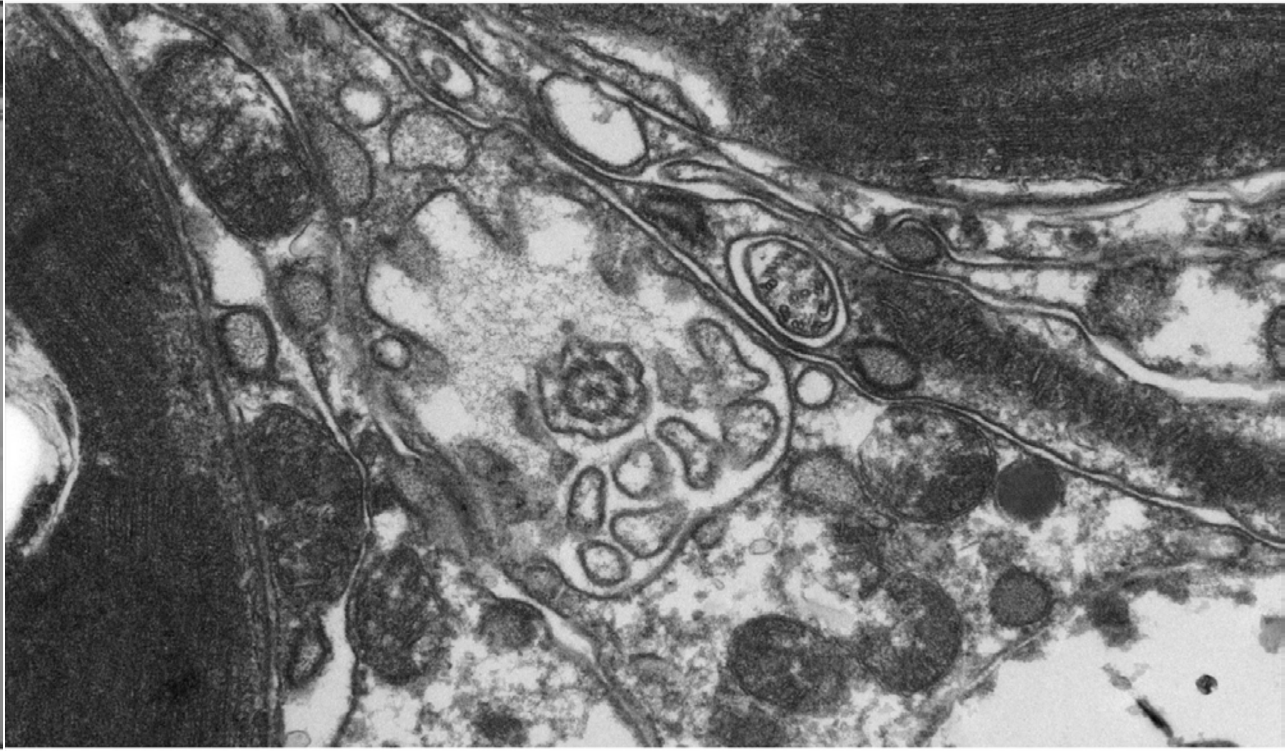

21-20_Correa_3-19-590_16C3_010.tif
Sample: 3/19, 590
Biological Electron Microscopy Lab
Rice University - SEA
Microscopist: MD Meyer

500 nm
HV=80kV
Direct Mag: 8000 x

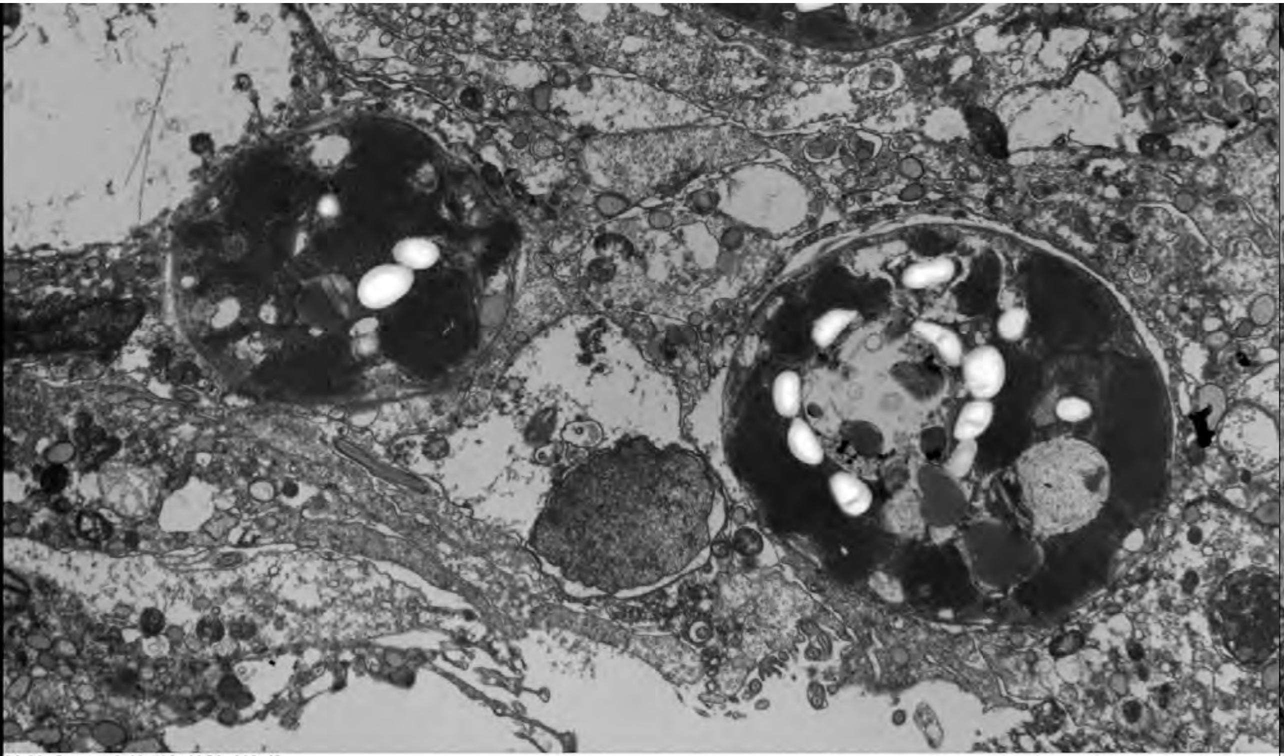

21-20_Correa_3-19-590_16C3_039.tif
Sample: 3/19, 590
Biological Electron Microscopy Lab
Rice University - SEA
Microscopist: MD Meyer

2 µm
HV=80kV
Direct Mag: 1500 x

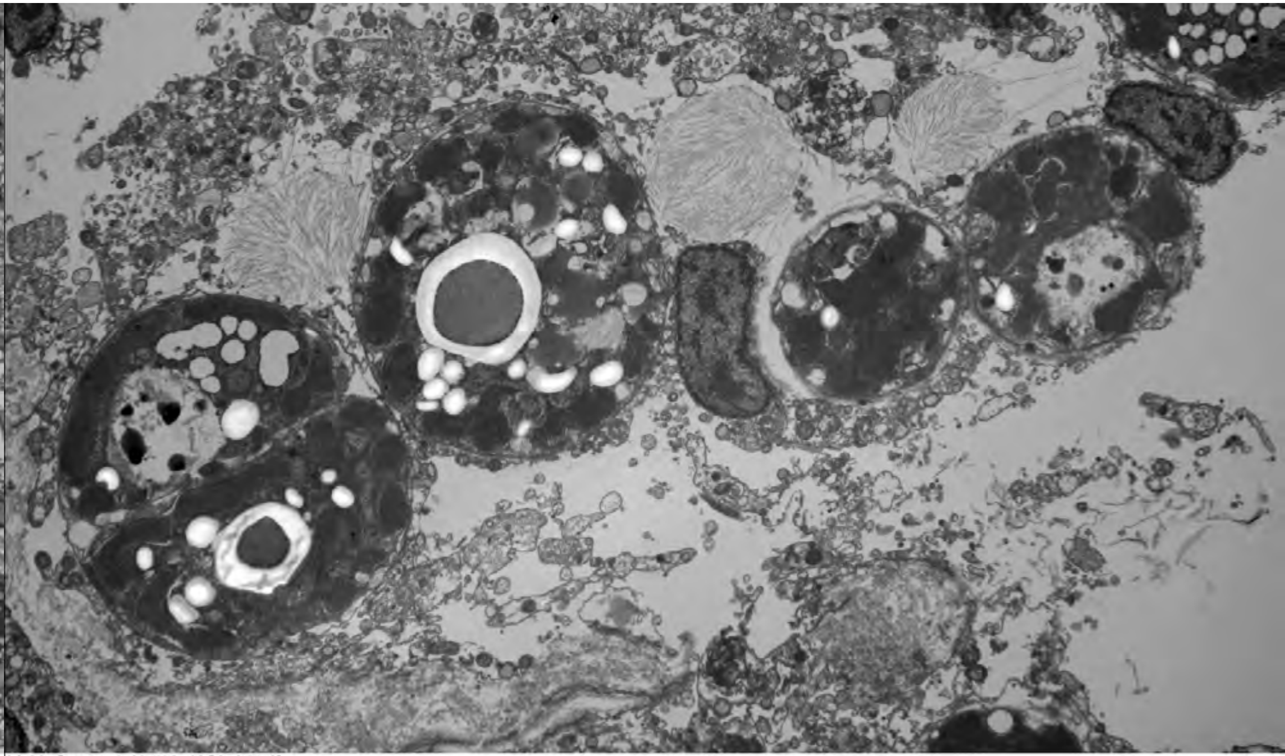

21-20_Correa_3-19-590_16C3_057.tif
Sample: 3/19, 590
Biological Electron Microscopy Lab
Rice University - SEA
Microscopist: MD Meyer

4 µm
HV=80kV
Direct Mag: 1000 x

Cell 22

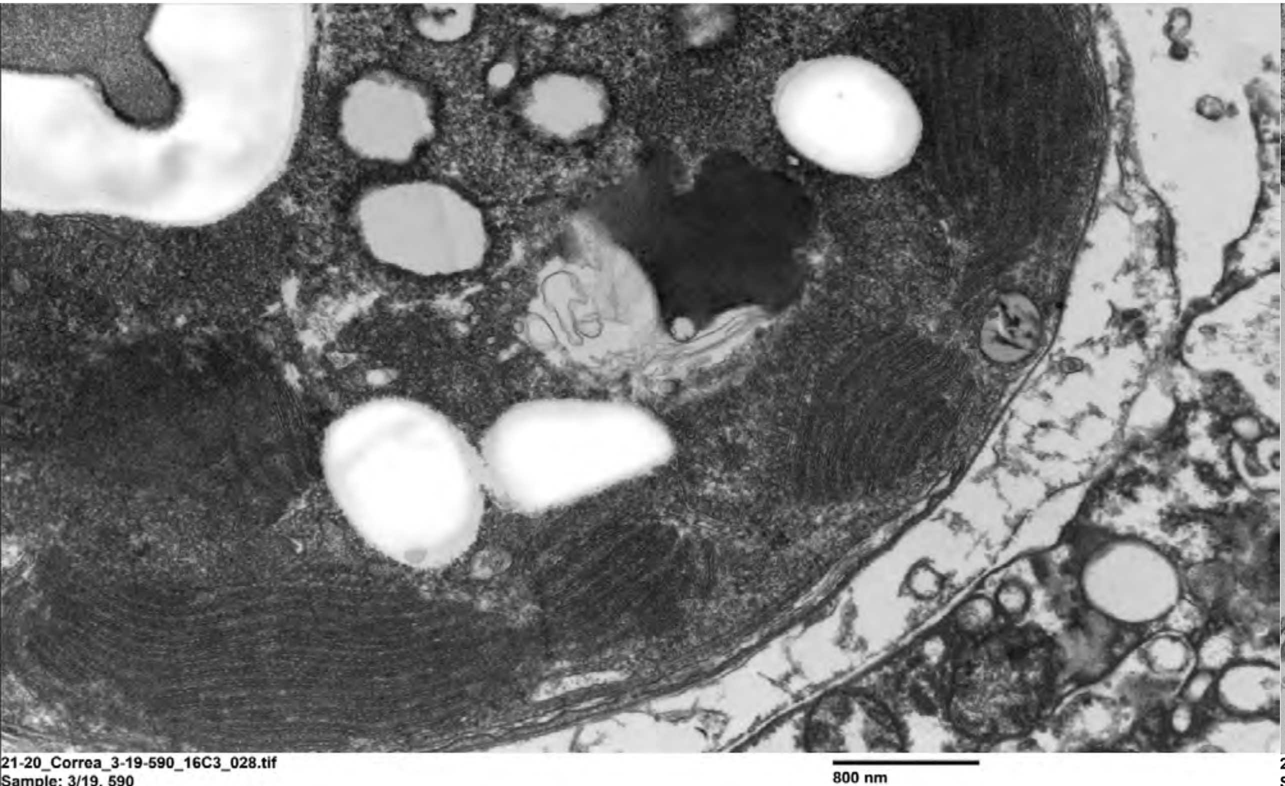

21-20_Correa_3-19-590_16C3_028.tif
Sample: 3/19, 590
Biological Electron Microscopy Lab
Rice University - SEA
Microscopist: MD Meyer

HV=80kV
Direct Mag: 5000 x

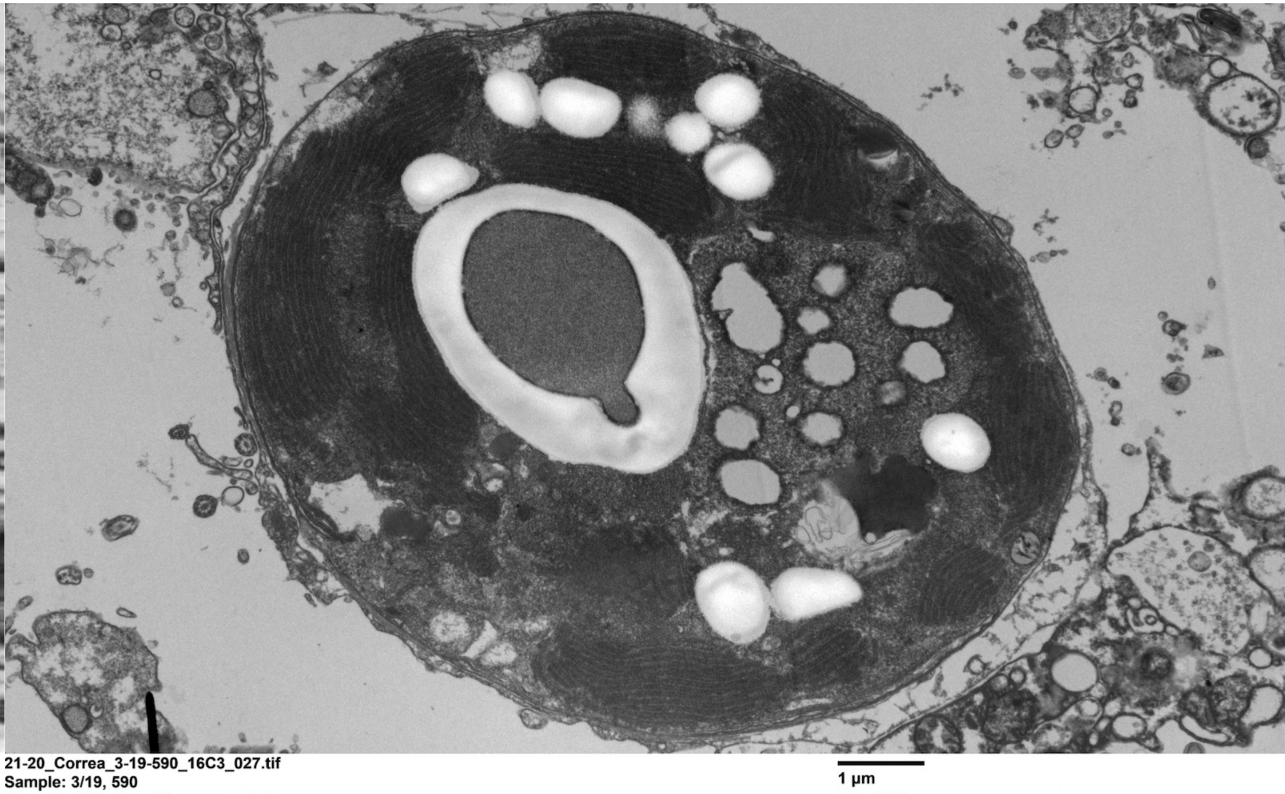

21-20_Correa_3-19-590_16C3_027.tif
Sample: 3/19, 590
Biological Electron Microscopy Lab
Rice University - SEA
Microscopist: MD Meyer

HV=80kV
Direct Mag: 2500 x

Cell 23

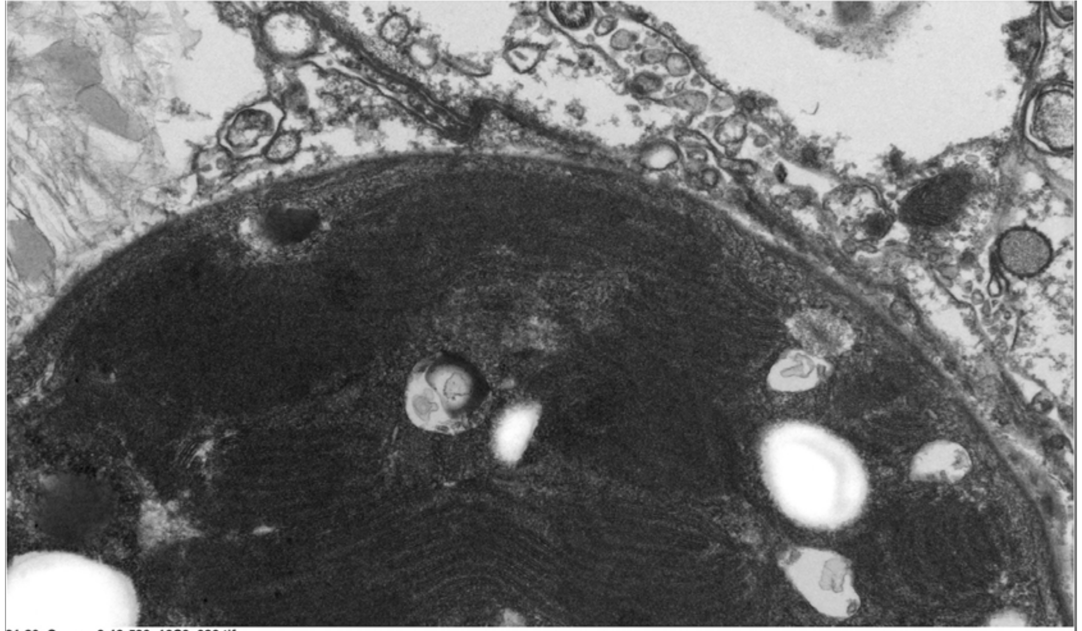

21-20_Correa_3-19-590_16C3_026.tif
Sample: 3/19, 590
Biological Electron Microscopy Lab
Rice University - SEA
Microscopist: MD Meyer

800 nm
HV=80kV
Direct Mag: 5000 x

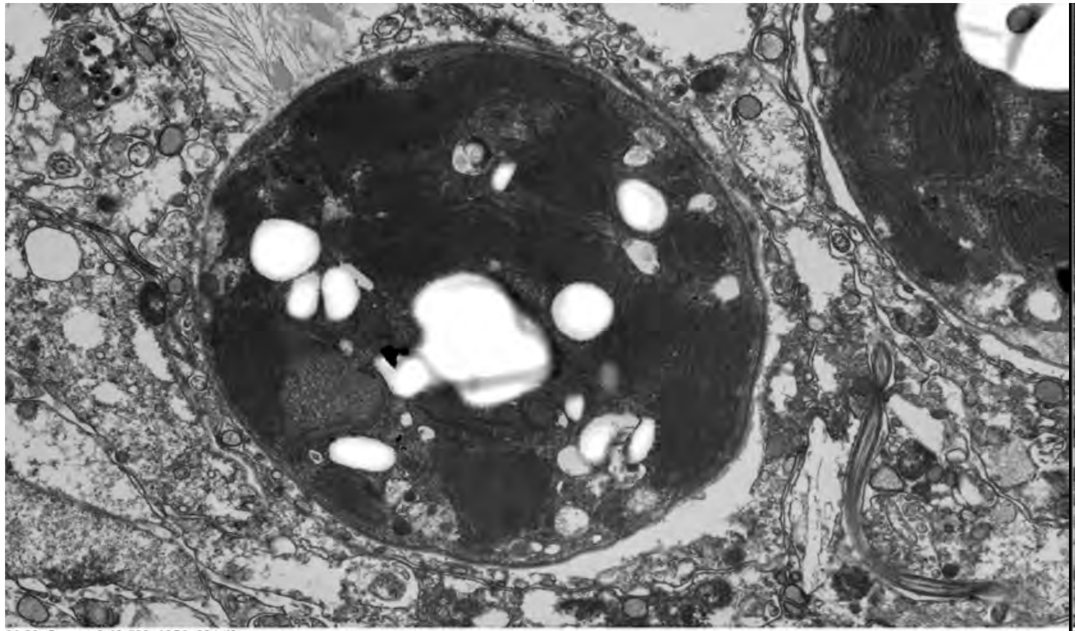

21-20_Correa_3-19-590_16C3_024.tif
Sample: 3/19, 590
Biological Electron Microscopy Lab
Rice University - SEA
Microscopist: MD Meyer

1 µm
HV=80kV
Direct Mag: 2500 x

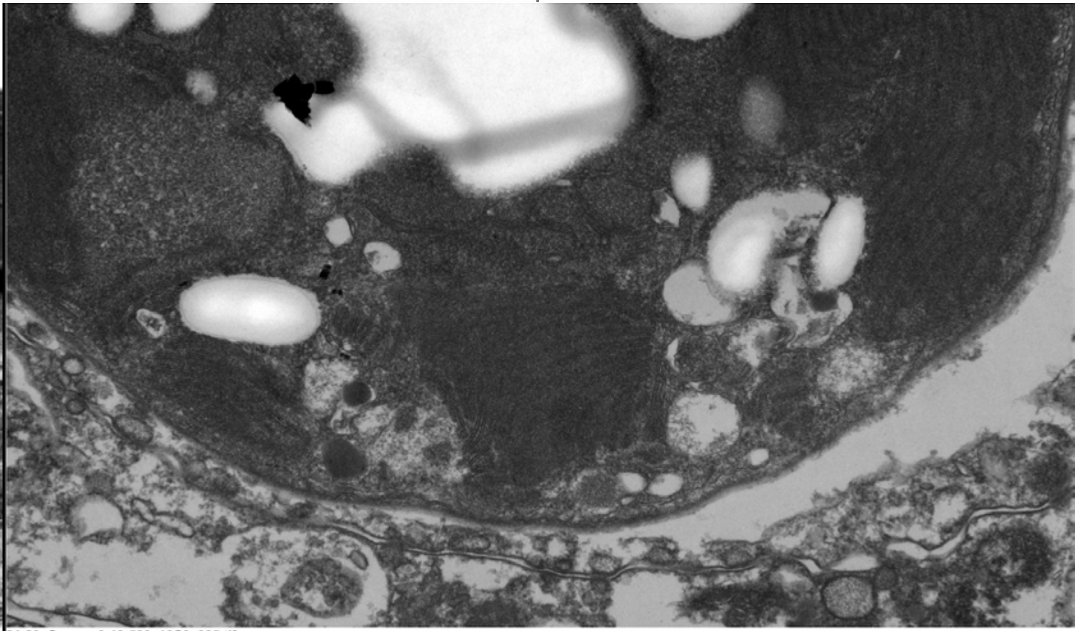

21-20_Correa_3-19-590_16C3_025.tif
Sample: 3/19, 590
Biological Electron Microscopy Lab
Rice University - SEA
Microscopist: MD Meyer

800 nm
HV=80kV
Direct Mag: 5000 x

Cell 24

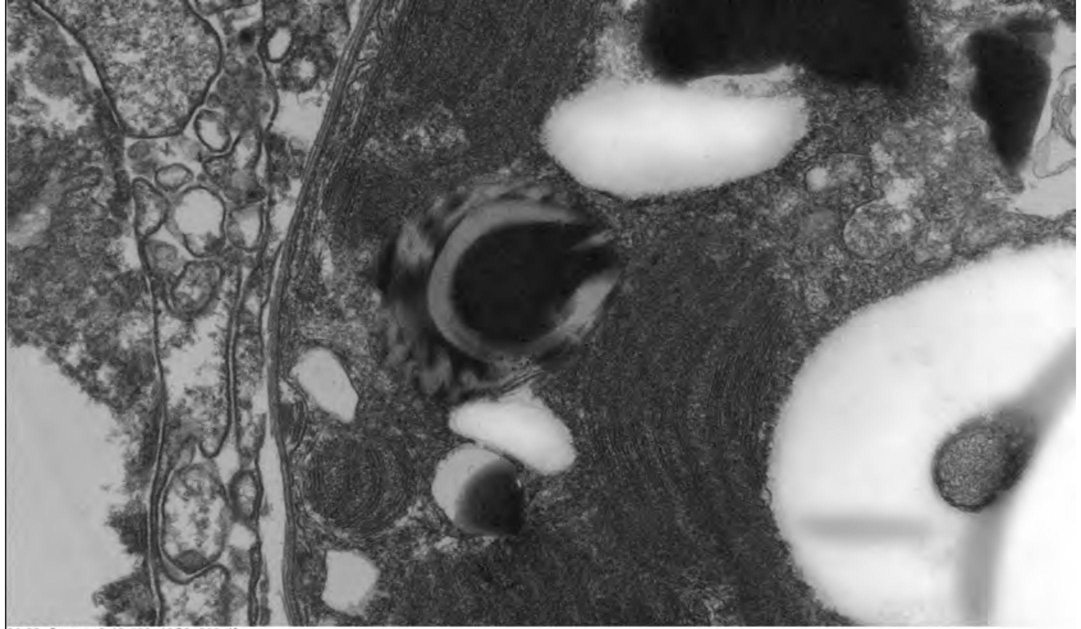

21-20_Correa_3-19-590_16C3_023.tif
Sample: 3/19, 590
Biological Electron Microscopy Lab
Rice University - SEA
Microscopist: MD Meyer

500 nm
HV=80kV
Direct Mag: 8000 x

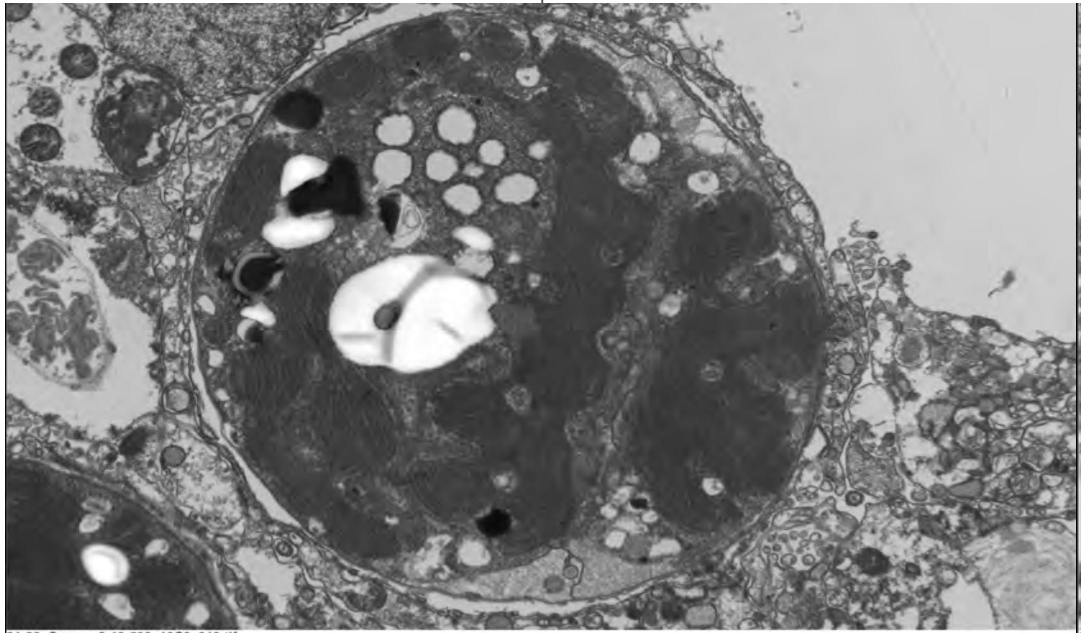

21-20_Correa_3-19-590_16C3_019.tif
Sample: 3/19, 590
Biological Electron Microscopy Lab
Rice University - SEA
Microscopist: MD Meyer

2 µm
HV=80kV
Direct Mag: 2000 x

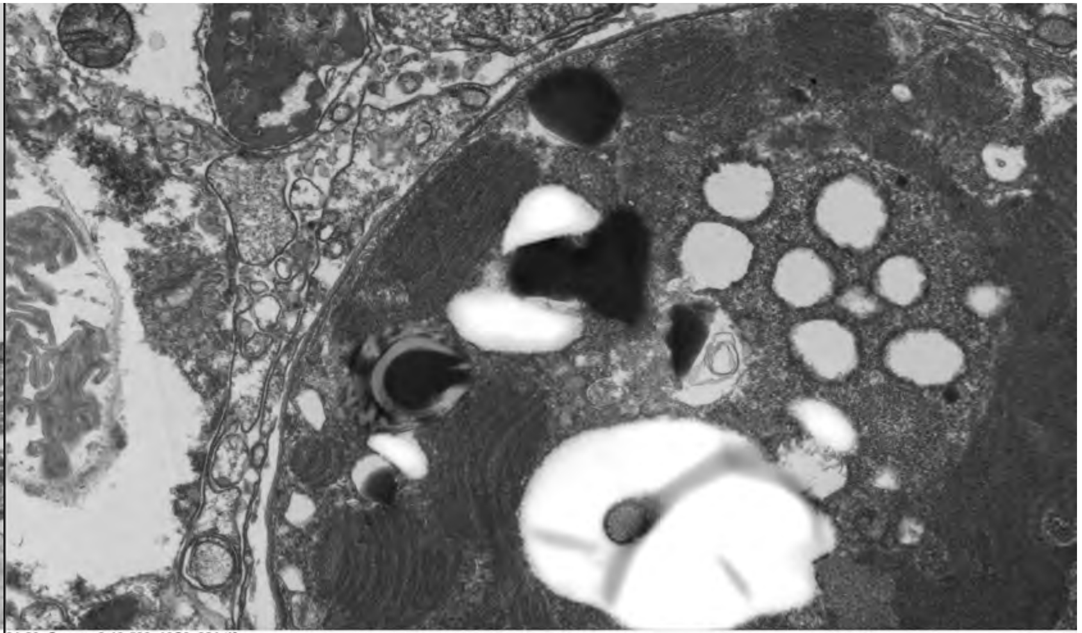

21-20_Correa_3-19-590_16C3_021.tif
Sample: 3/19, 590
Biological Electron Microscopy Lab
Rice University - SEA
Microscopist: MD Meyer

1 µm
HV=80kV
Direct Mag: 4000 x

Cell 25

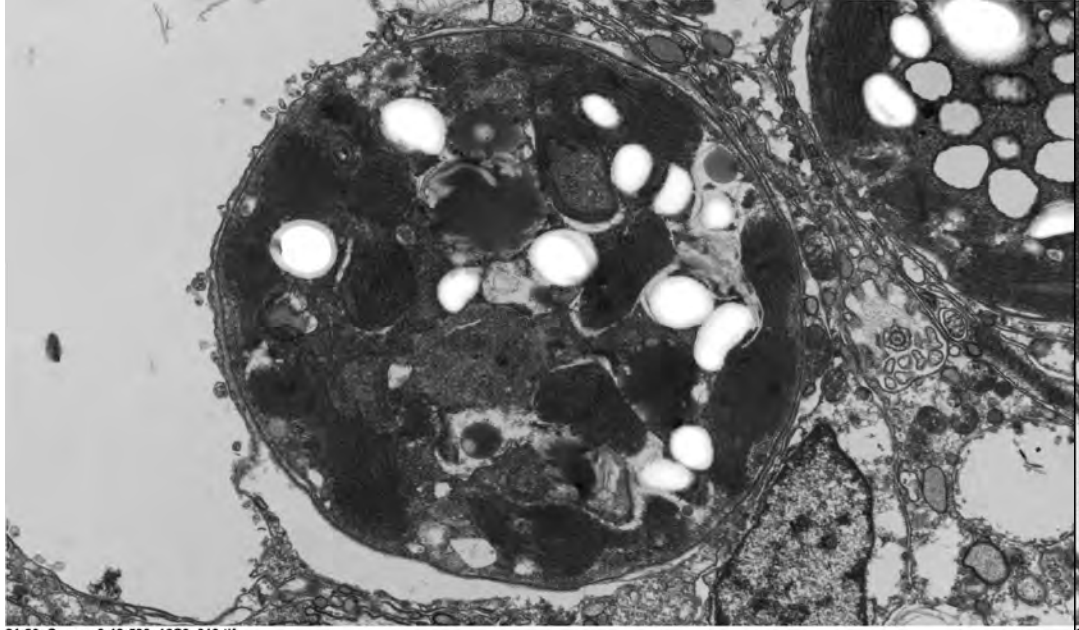

21-20_Correa_3-19-590_16C3_013.tif
Sample: 3/19, 590
Biological Electron Microscopy Lab
Rice University - SEA
Microscopist: MD Meyer

1 µm
HV=80kV
Direct Mag: 2500 x

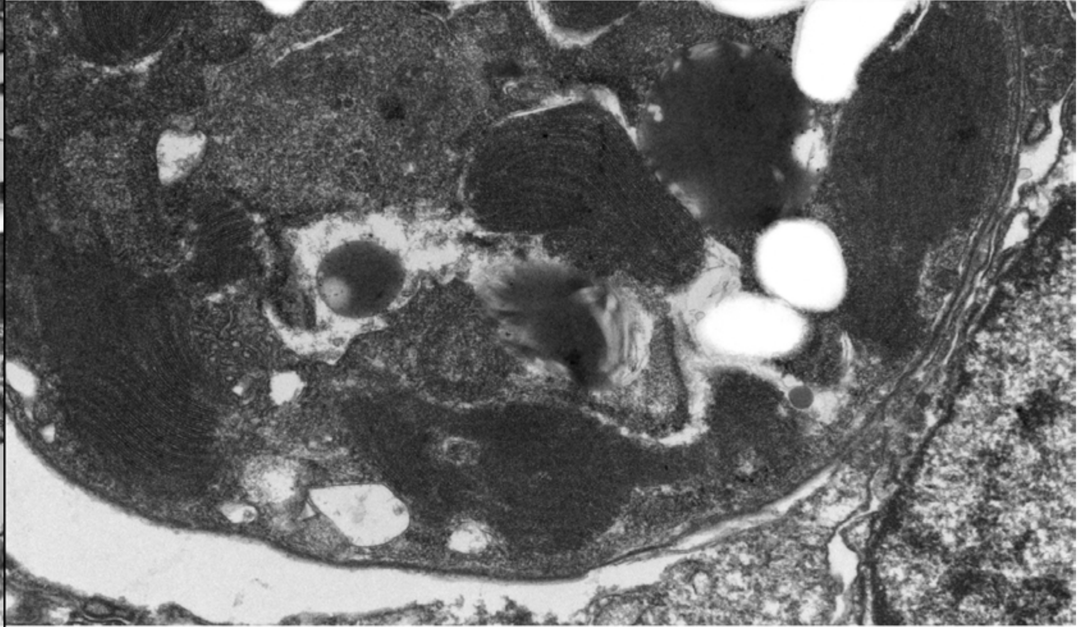

21-20_Correa_3-19-590_16C3_014.tif
Sample: 3/19, 590
Biological Electron Microscopy Lab
Rice University - SEA
Microscopist: MD Meyer

800 nm
HV=80kV
Direct Mag: 5000 x

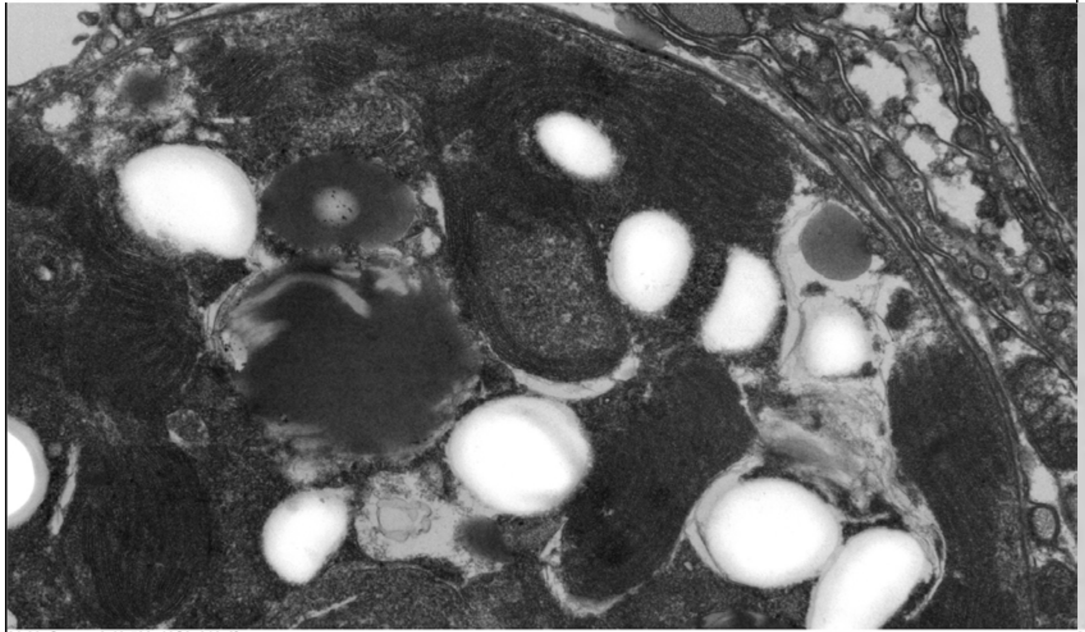

21-20_Correa_3-19-590_16C3_016.tif
Sample: 3/19, 590
Biological Electron Microscopy Lab
Rice University - SEA
Microscopist: MD Meyer

800 nm
HV=80kV
Direct Mag: 5000 x

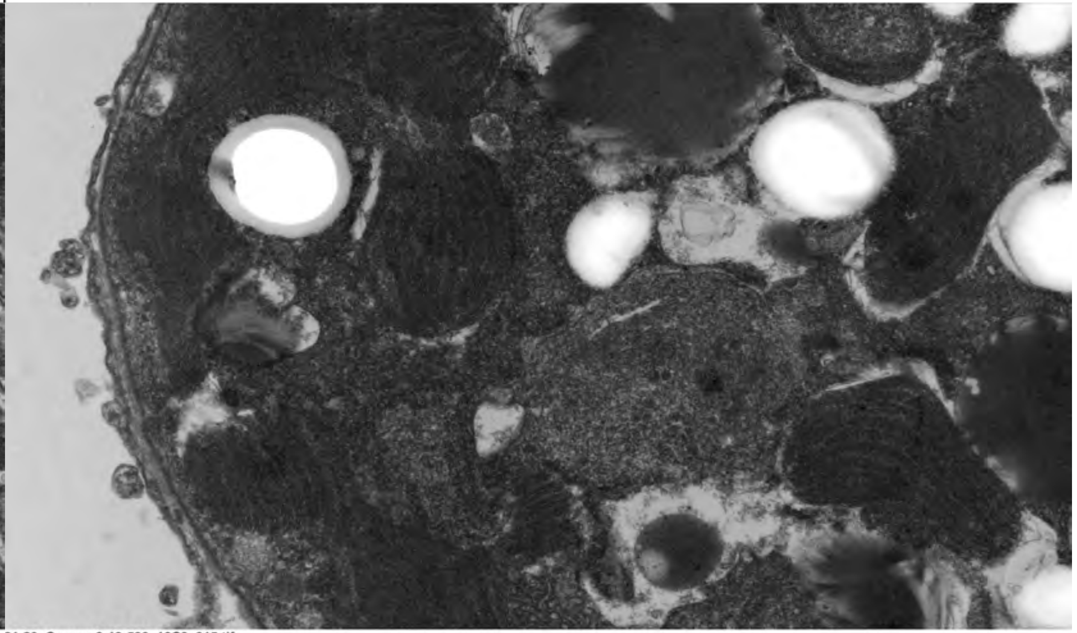

21-20_Correa_3-19-590_16C3_015.tif
Sample: 3/19, 590
Biological Electron Microscopy Lab
Rice University - SEA
Microscopist: MD Meyer

800 nm
HV=80kV
Direct Mag: 5000 x

Cell 26

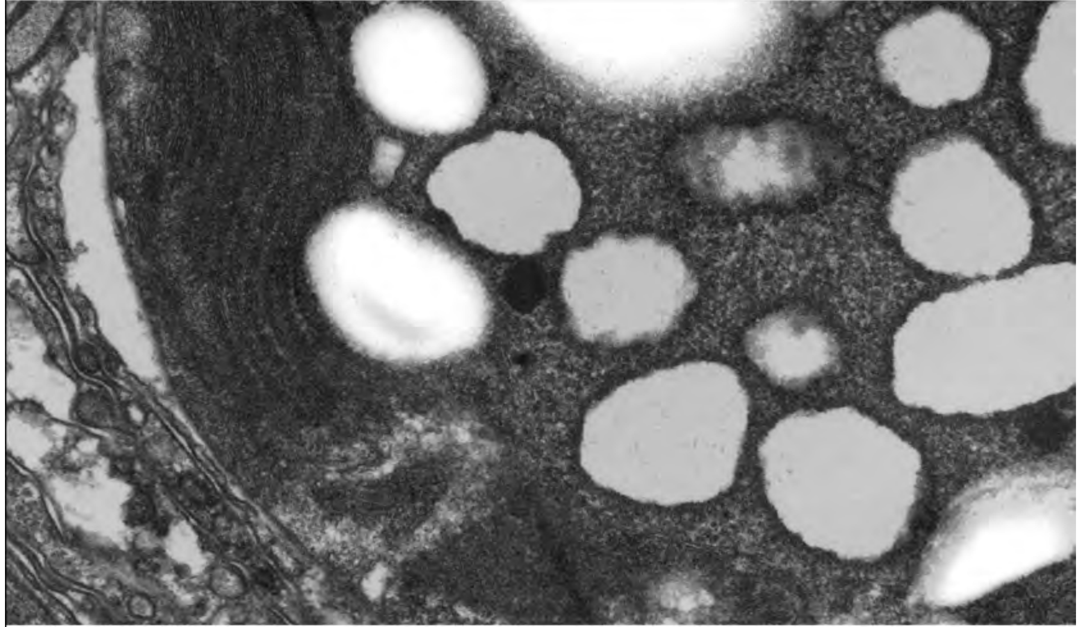

21-20_Correa_3-19-590_16C3_012.tif
Sample: 3/19, 590
Biological Electron Microscopy Lab
Rice University - SEA
Microscopist: MD Meyer

500 nm
HV=80kV
Direct Mag: 8000 x

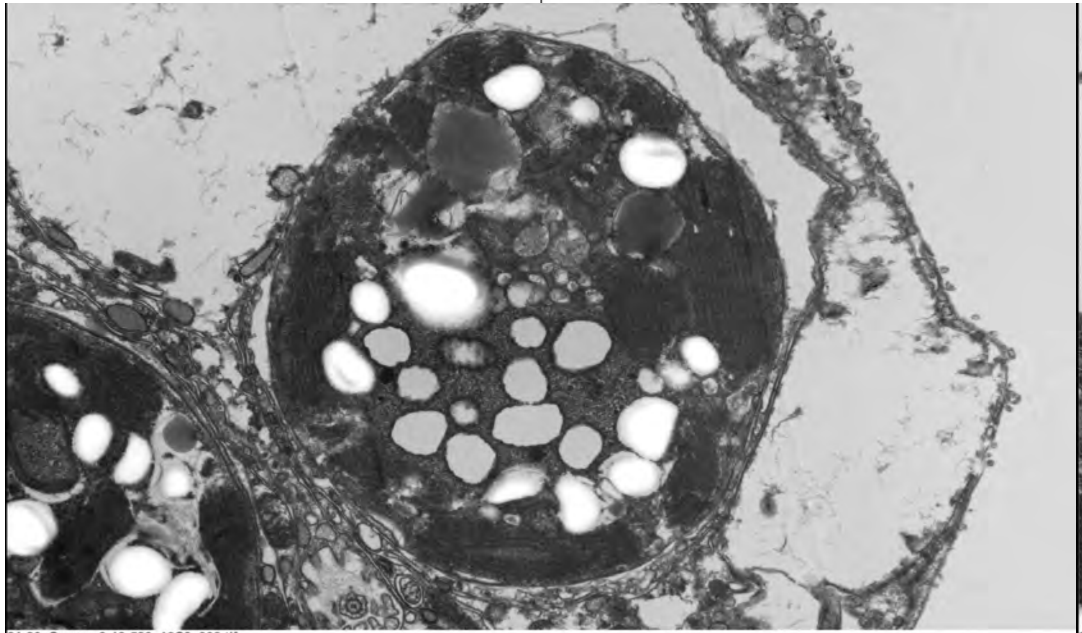

21-20_Correa_3-19-590_16C3_008.tif
Sample: 3/19, 590
Biological Electron Microscopy Lab
Rice University - SEA
Microscopist: MD Meyer

1 µm
HV=80kV
Direct Mag: 2500 x

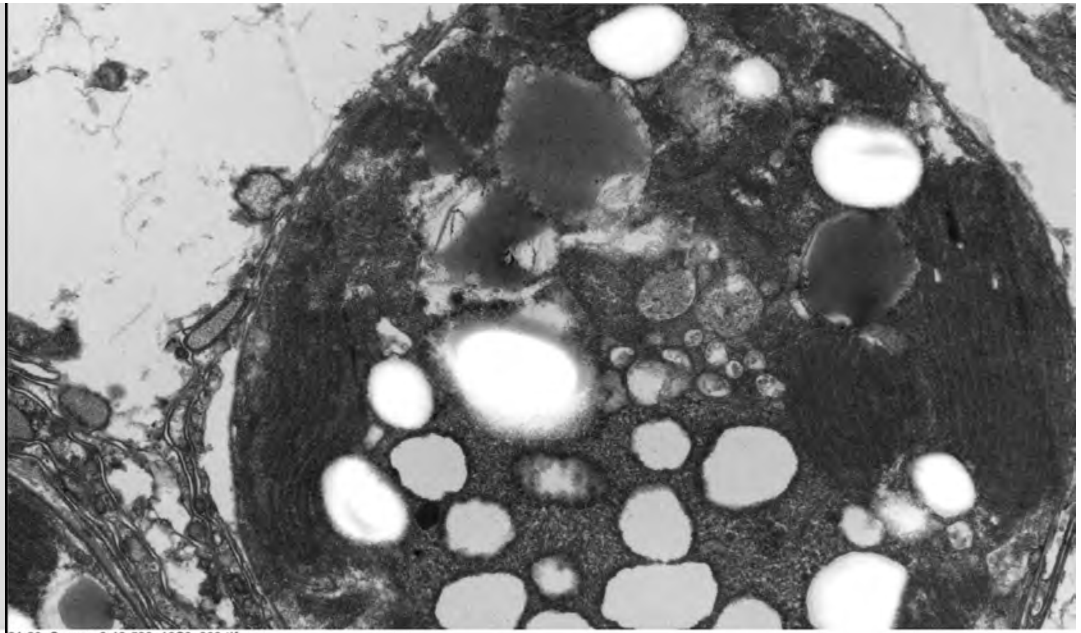

21-20_Correa_3-19-590_16C3_009.tif
Sample: 3/19, 590
Biological Electron Microscopy Lab
Rice University - SEA
Microscopist: MD Meyer

1 µm
HV=80kV
Direct Mag: 4000 x

Cell 27

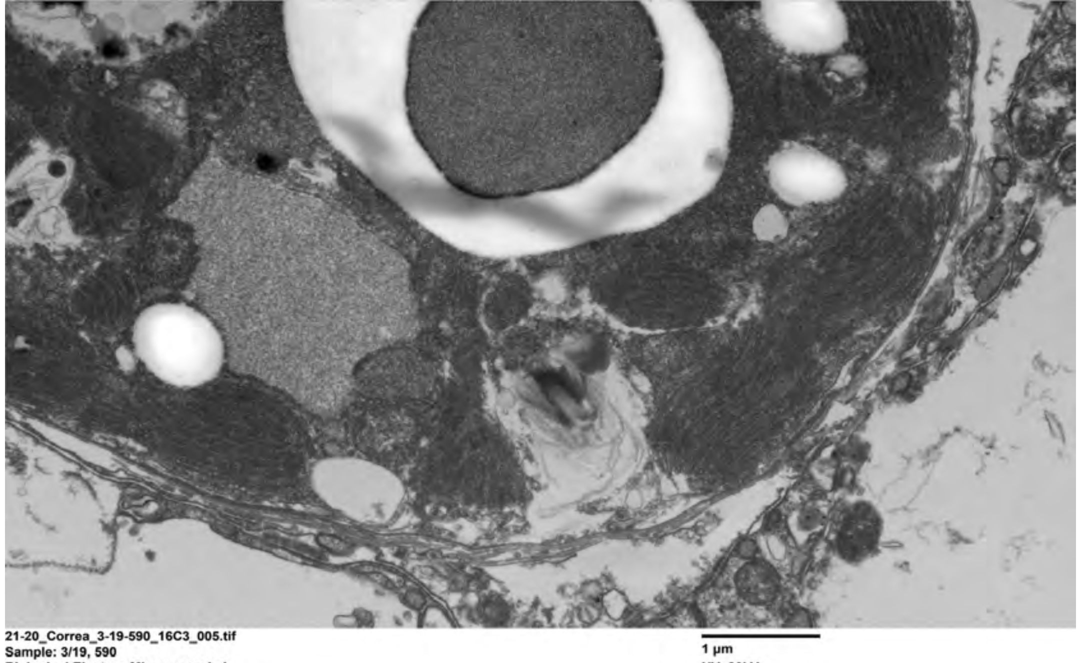

21-20_Correa_3-19-590_16C3_005.tif
Sample: 3/19, 590
Biological Electron Microscopy Lab
Rice University - SEA
Microscopist: MD Meyer

HV=80kV
Direct Mag: 4000 x

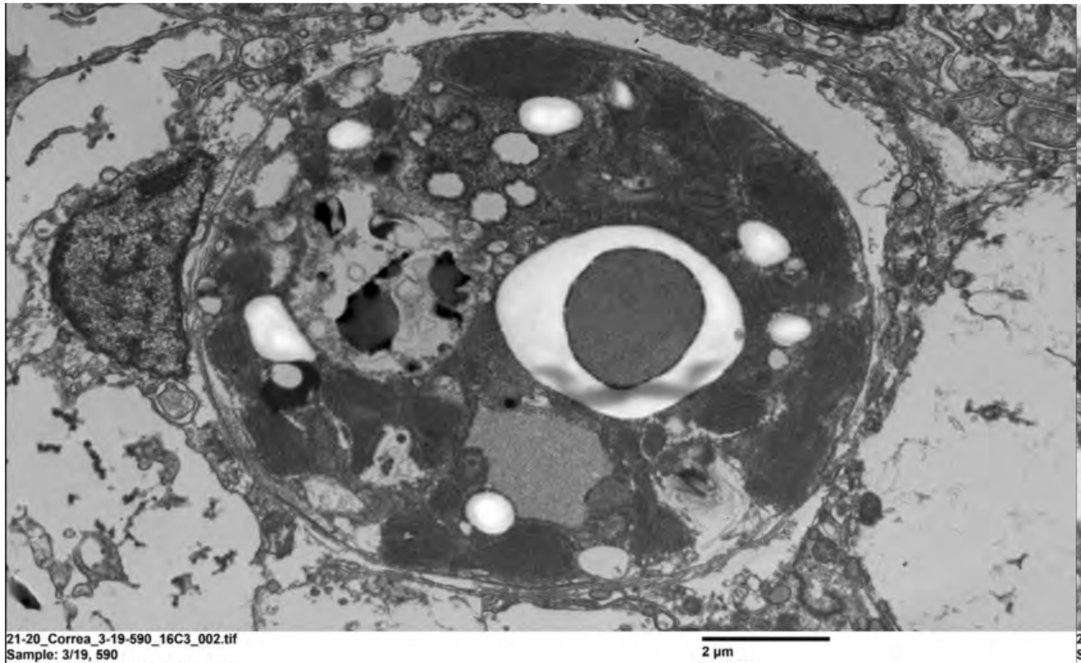

21-20_Correa_3-19-590_16C3_002.tif
Sample: 3/19, 590
Biological Electron Microscopy Lab
Rice University - SEA
Microscopist: MD Meyer

HV=80kV
Direct Mag: 2000 x

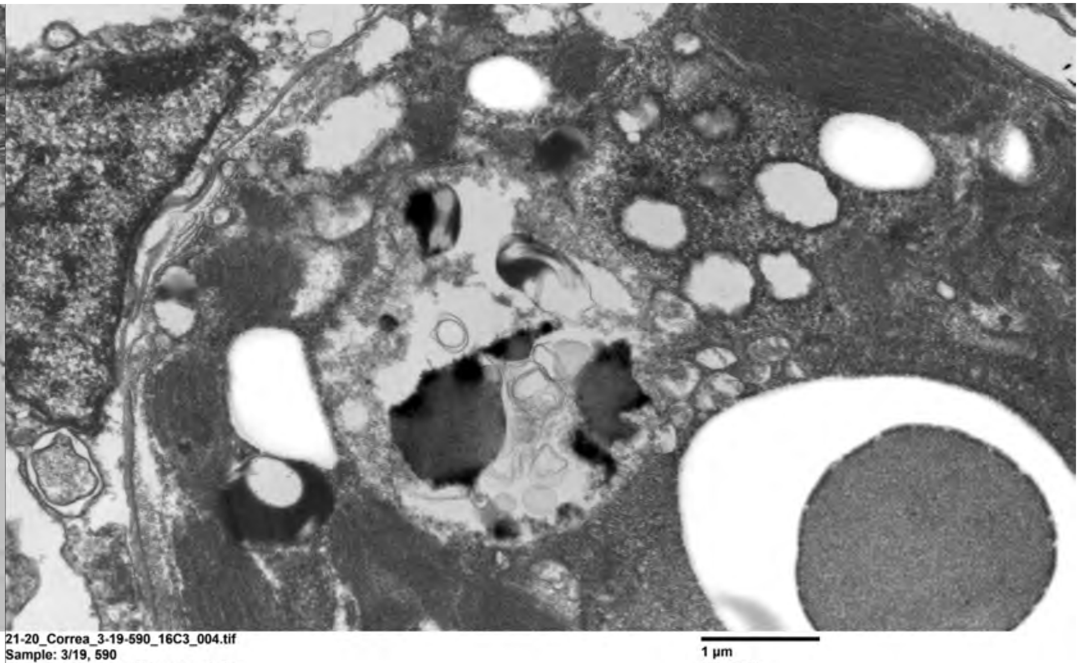

21-20_Correa_3-19-590_16C3_004.tif
Sample: 3/19, 590
Biological Electron Microscopy Lab
Rice University - SEA
Microscopist: MD Meyer

HV=80kV
Direct Mag: 4000 x
