# Supplementary material for: Filamentous virus-like particles are present in coral dinoflagellates across genera and ocean basins
Source: ISME J. 2023 Nov 1;17(12):2389–402. doi: 10.1038/s41396-023-01526-6 (PMC10689786; doi:10.1038/s41396-023-01526-6)
Supplement: Supplementary file 4 — Supplementary TEM Images ACR aquaria experiment [file 41396_2023_1526_MOESM4_ESM.pdf]

Filamentous virus-like particles are present in coral dinoflagellates across genera and ocean basins

Supplementary Data- Aquaria Experiment *Acropora hyacinthus* Symbiodiniaceae TEM images

ACR Colony A
- Ambient
- Heat

ACR Colony B
- Ambient
- Heat

ACR Colony C
- Ambient
- Heat

ACR Colony D
- Ambient
- Heat

Ambient - ACR Colony A

Cell 1

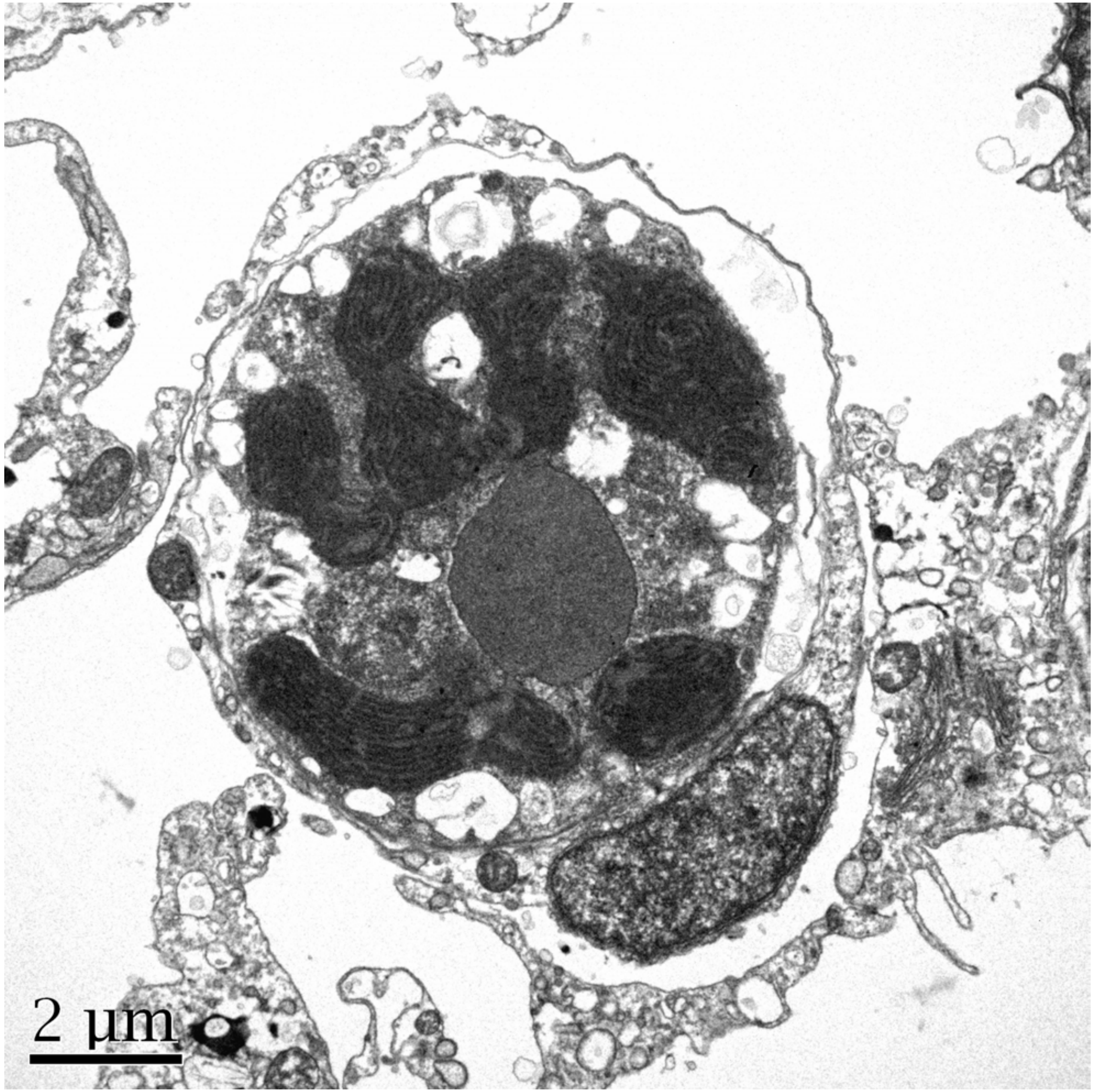

18-14_Correa_JC4_4E8_3.tif

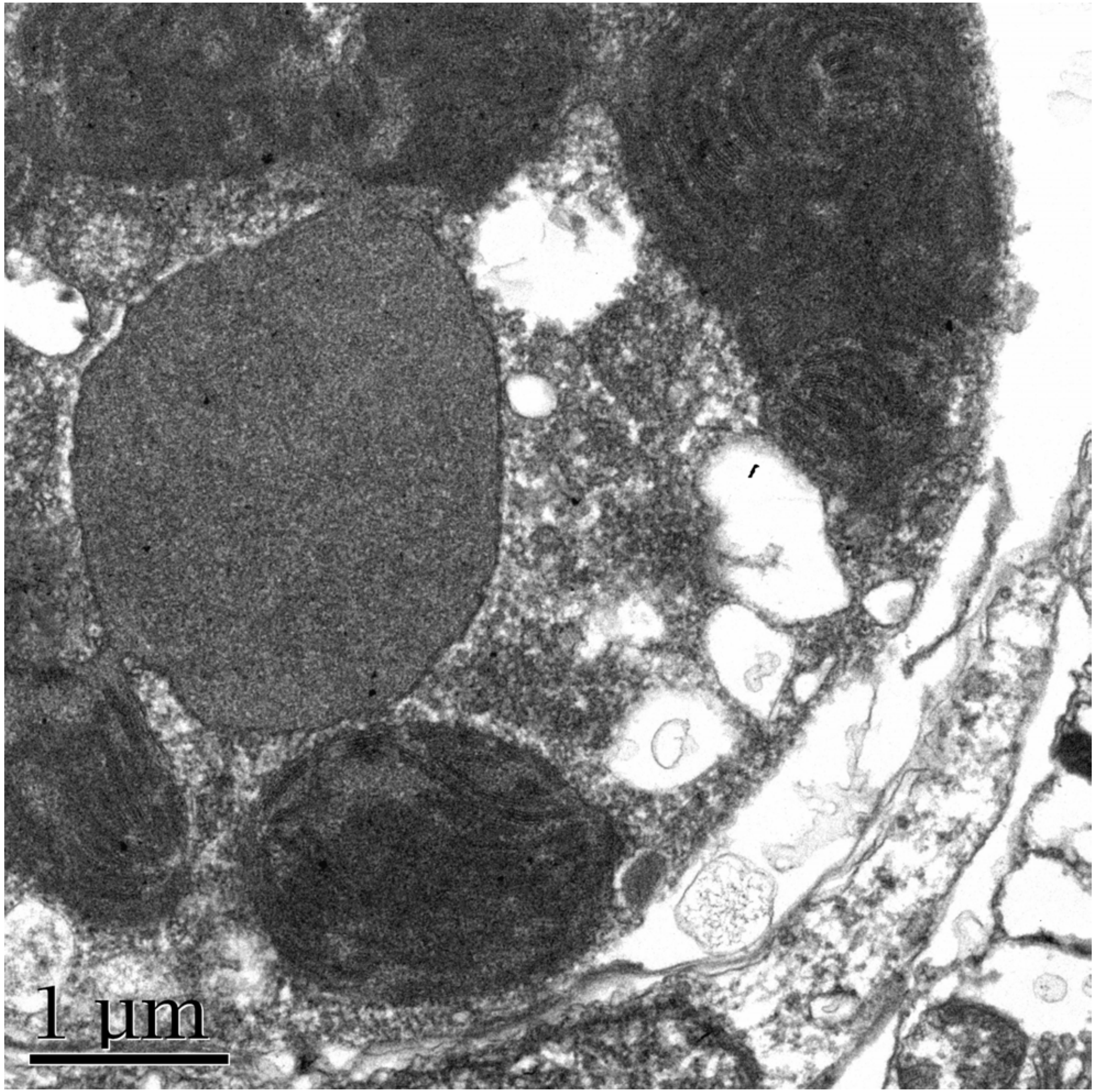

18-14_Correa_JC4_4E8_5.tif

Cell 2

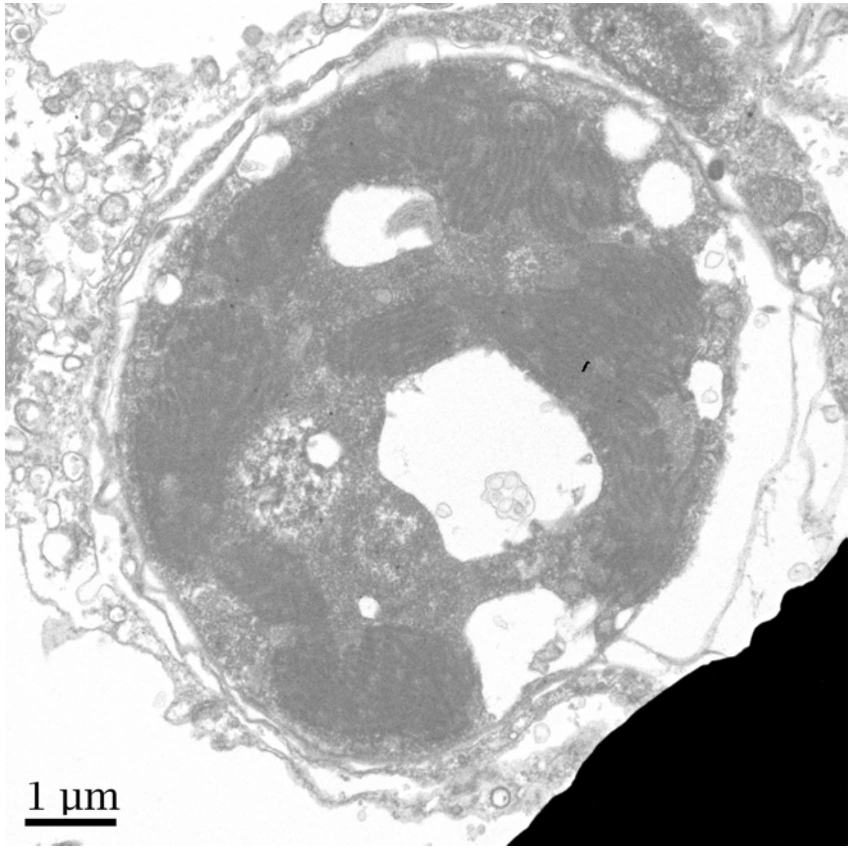

18-14_Correa_JC4_4E8_14.tif

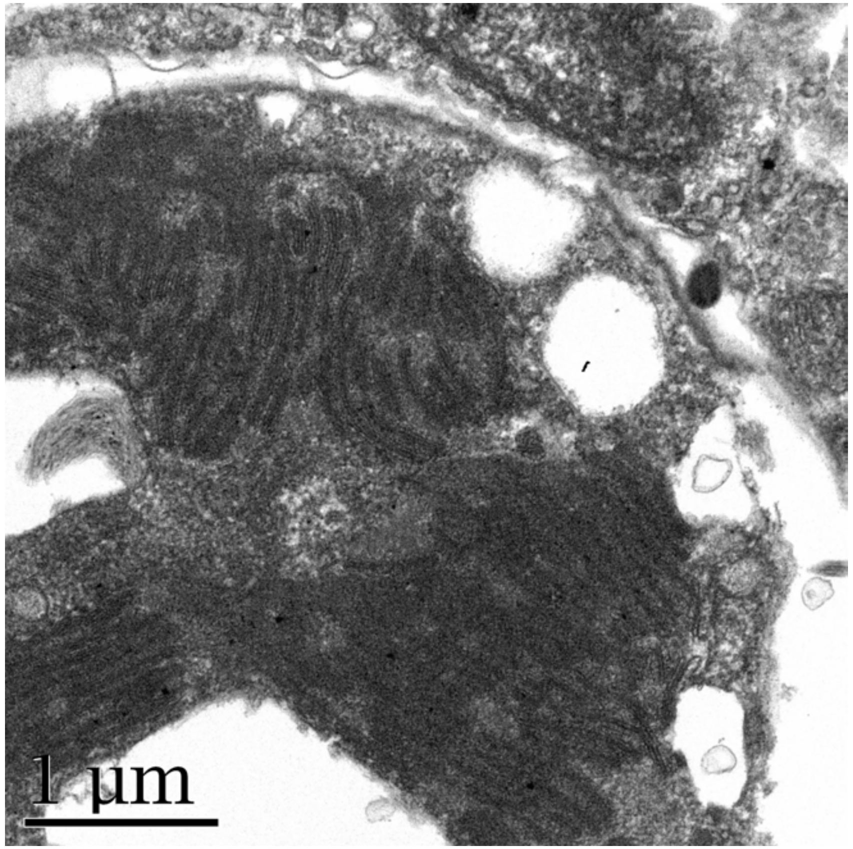

18-14_Correa_JC4_4E8_19.tif

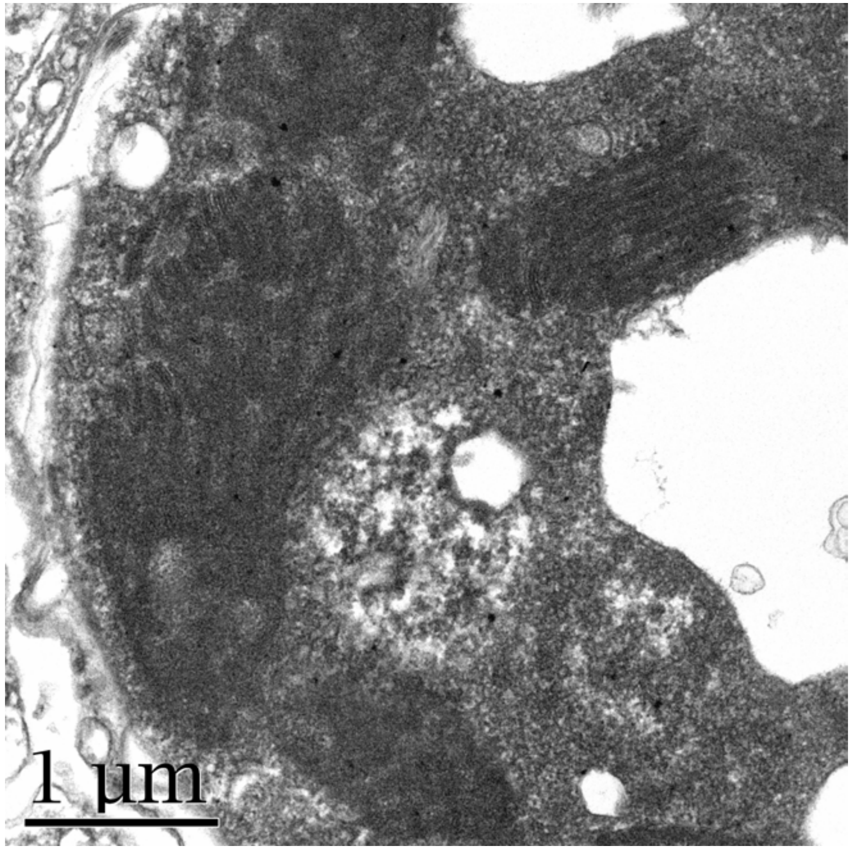

18-14_Correa_JC4_4E8_16.tif

Cell 3

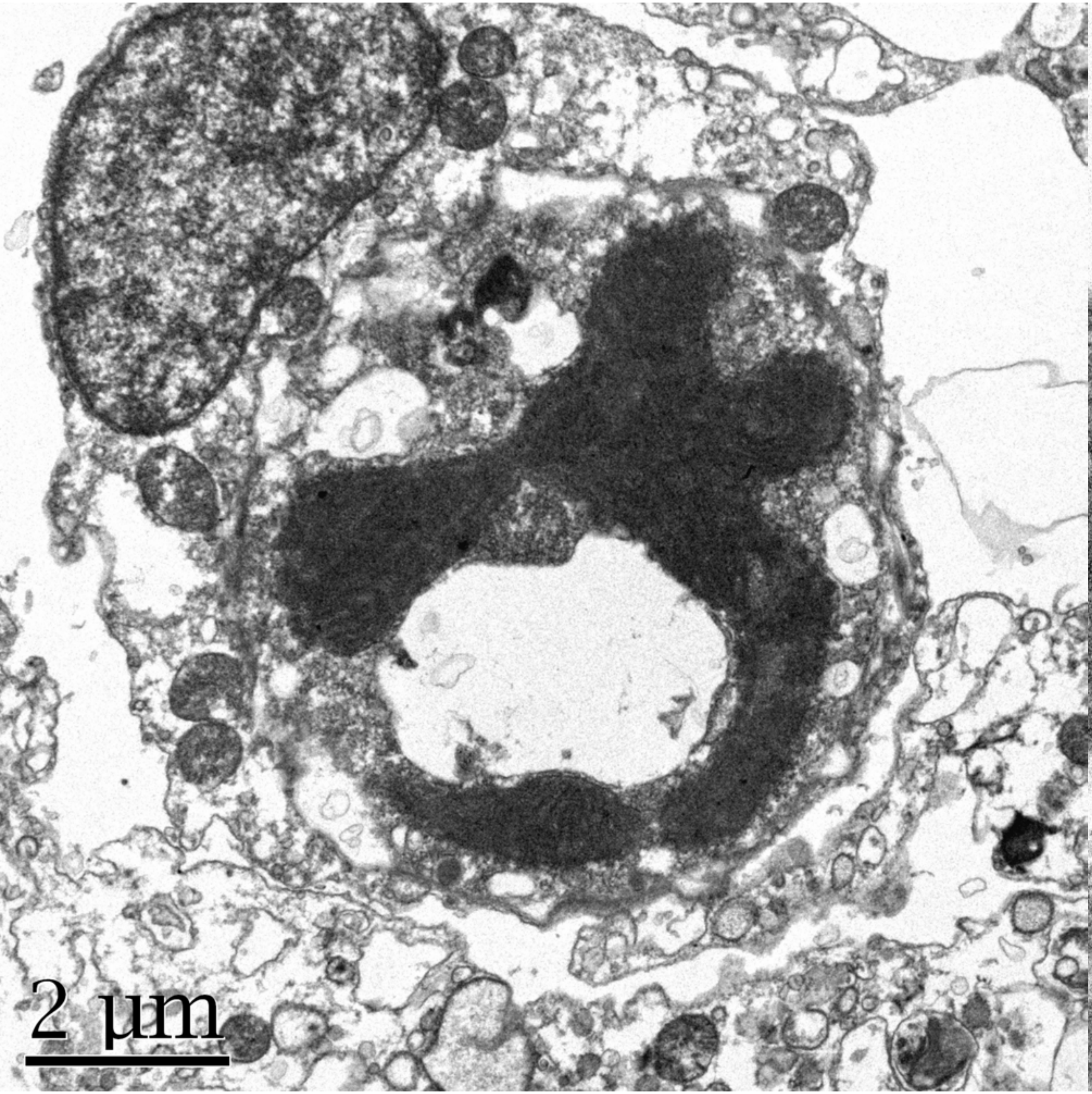

18-14_Correa_JC4_4E8_21.tif

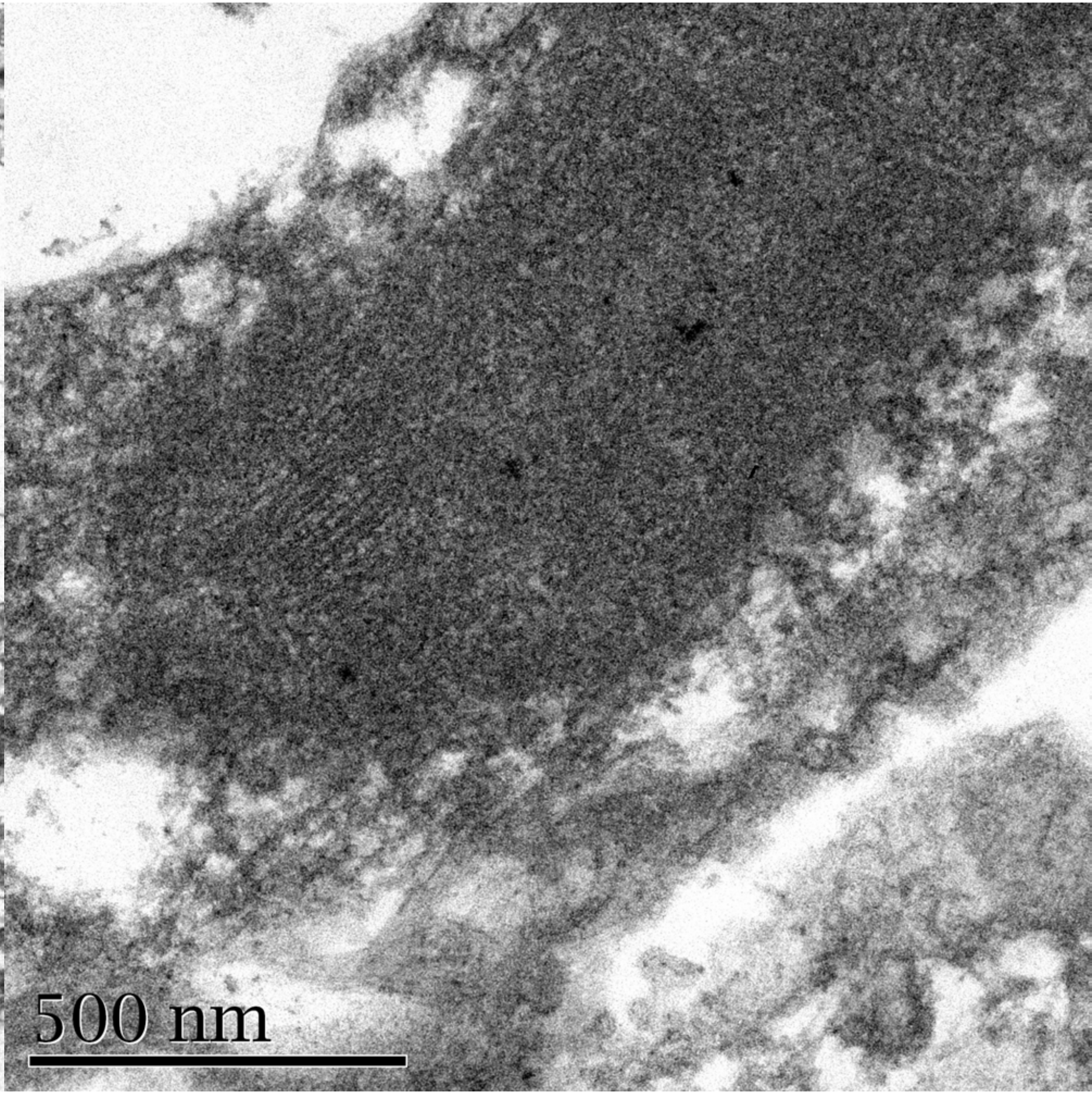

18-14_Correa_JC4_4E8_27.tif

Cell 4

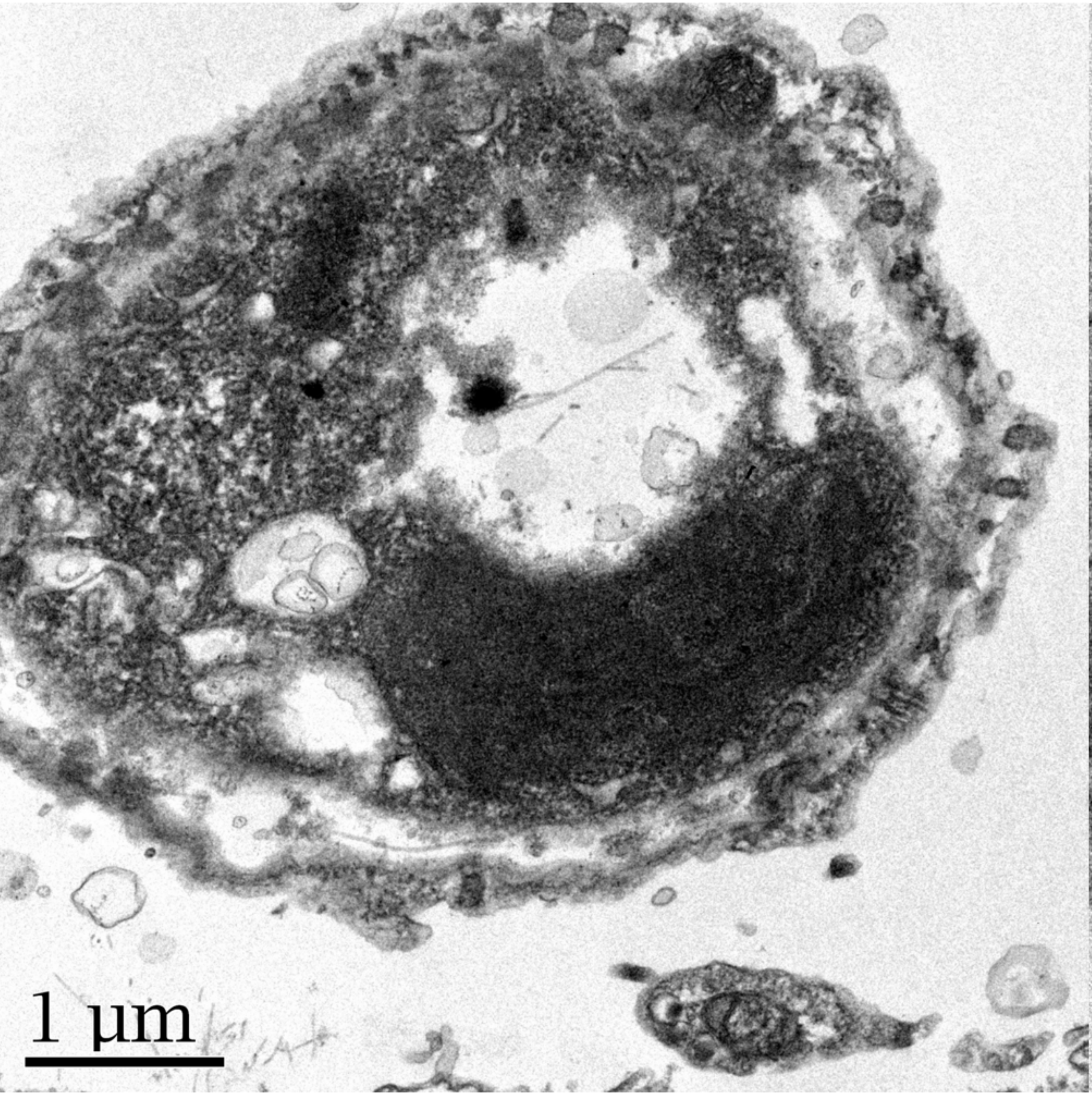

18-14_Correa_JC4_4E8_29.tif

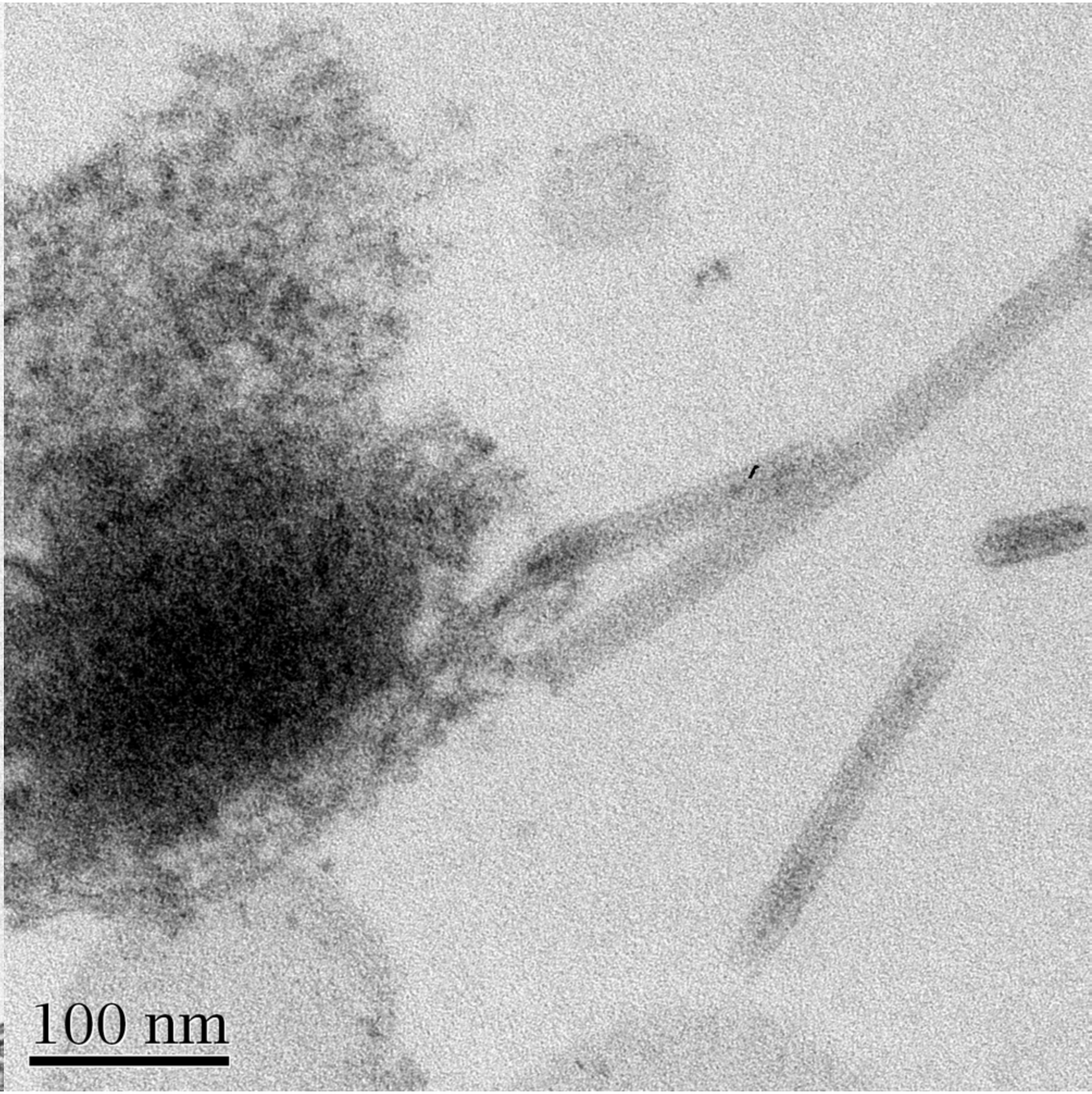

18-14_Correa_JC4_4E8_34.tif

Cell 5

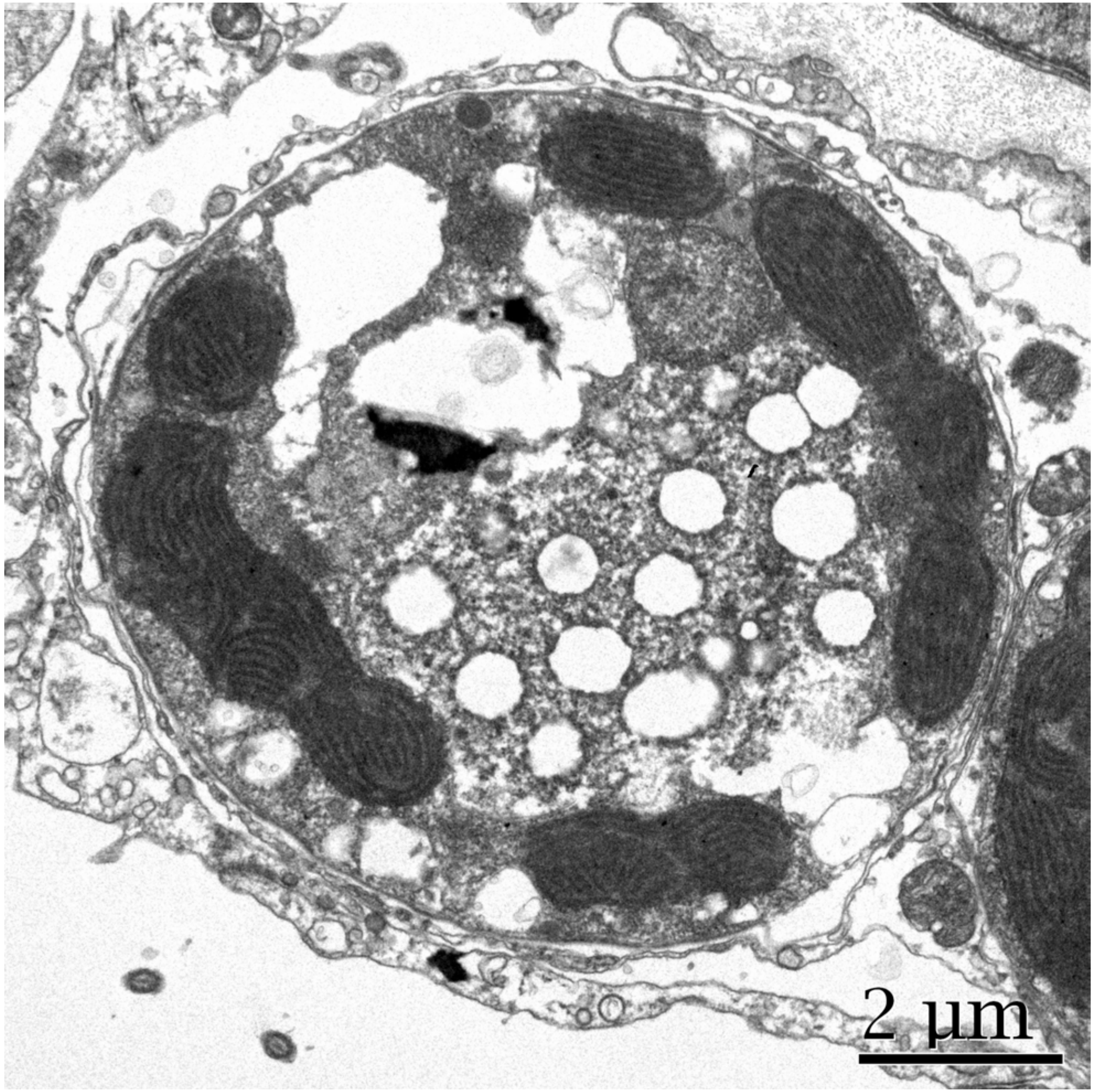

18-14_Correa_JC4_4E8_38.tif

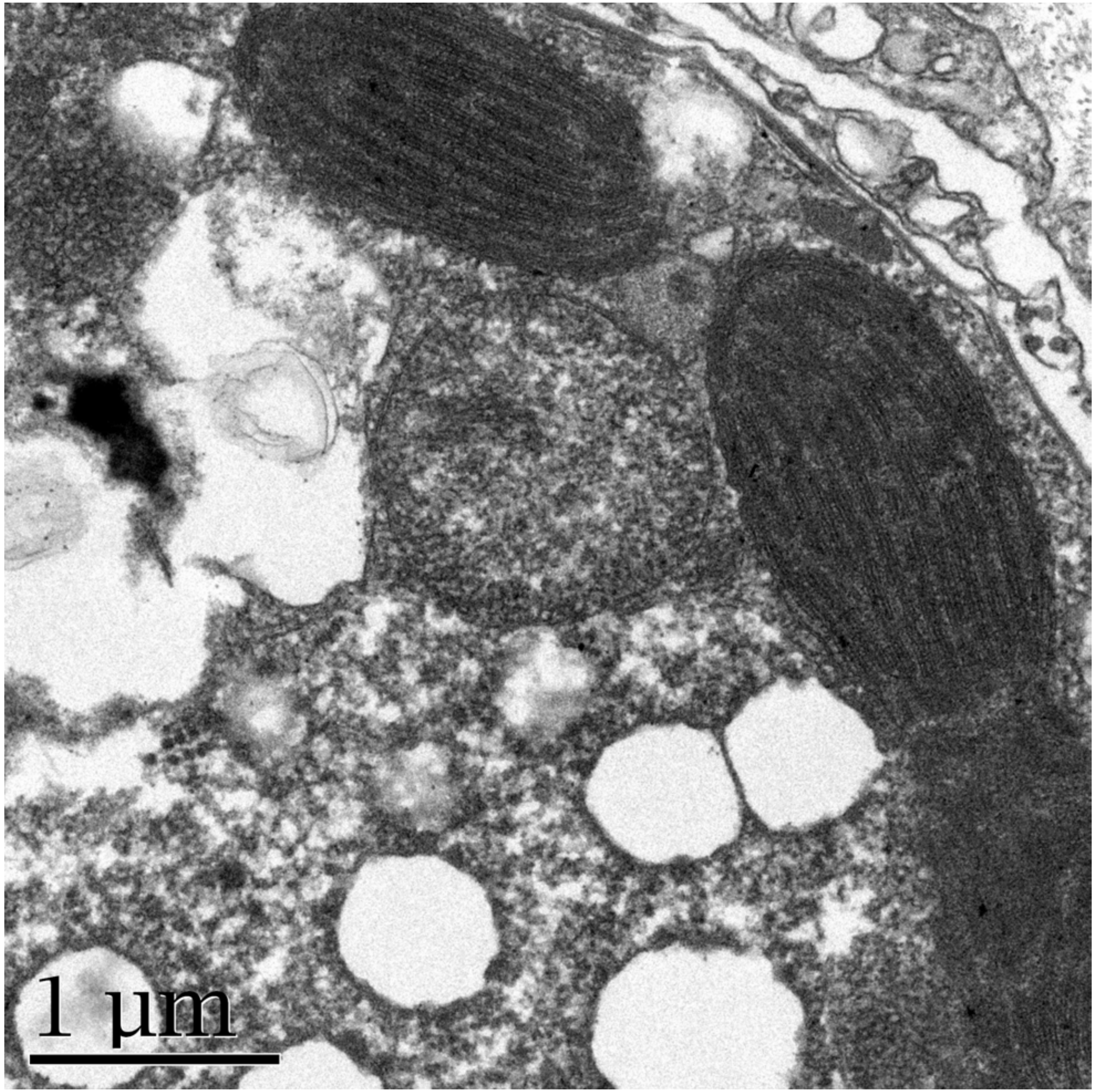

18-14_Correa_JC4_4E8_44.tif

Cell 6

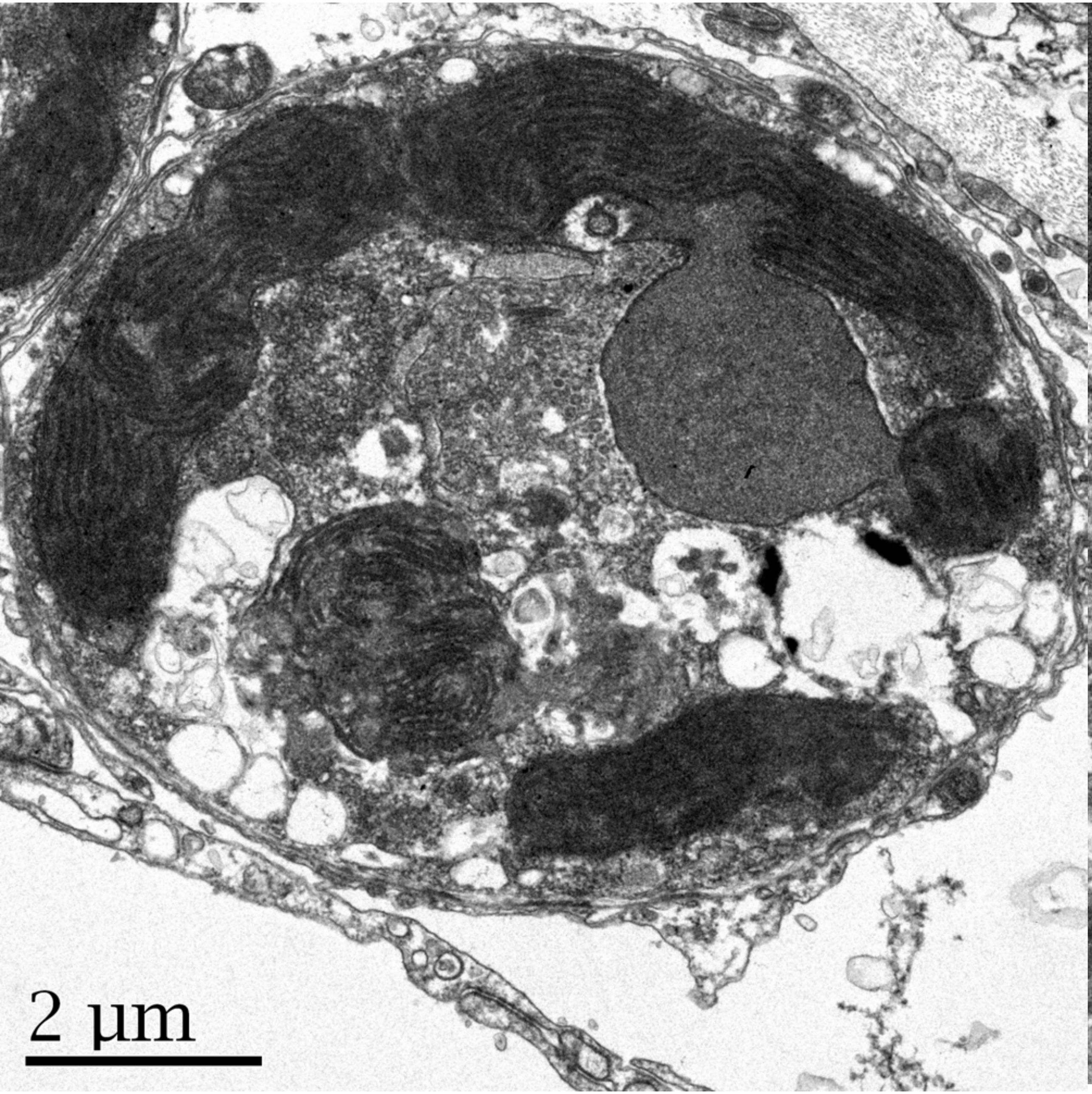

18-14_Correa_JC4_4E8_47.tif

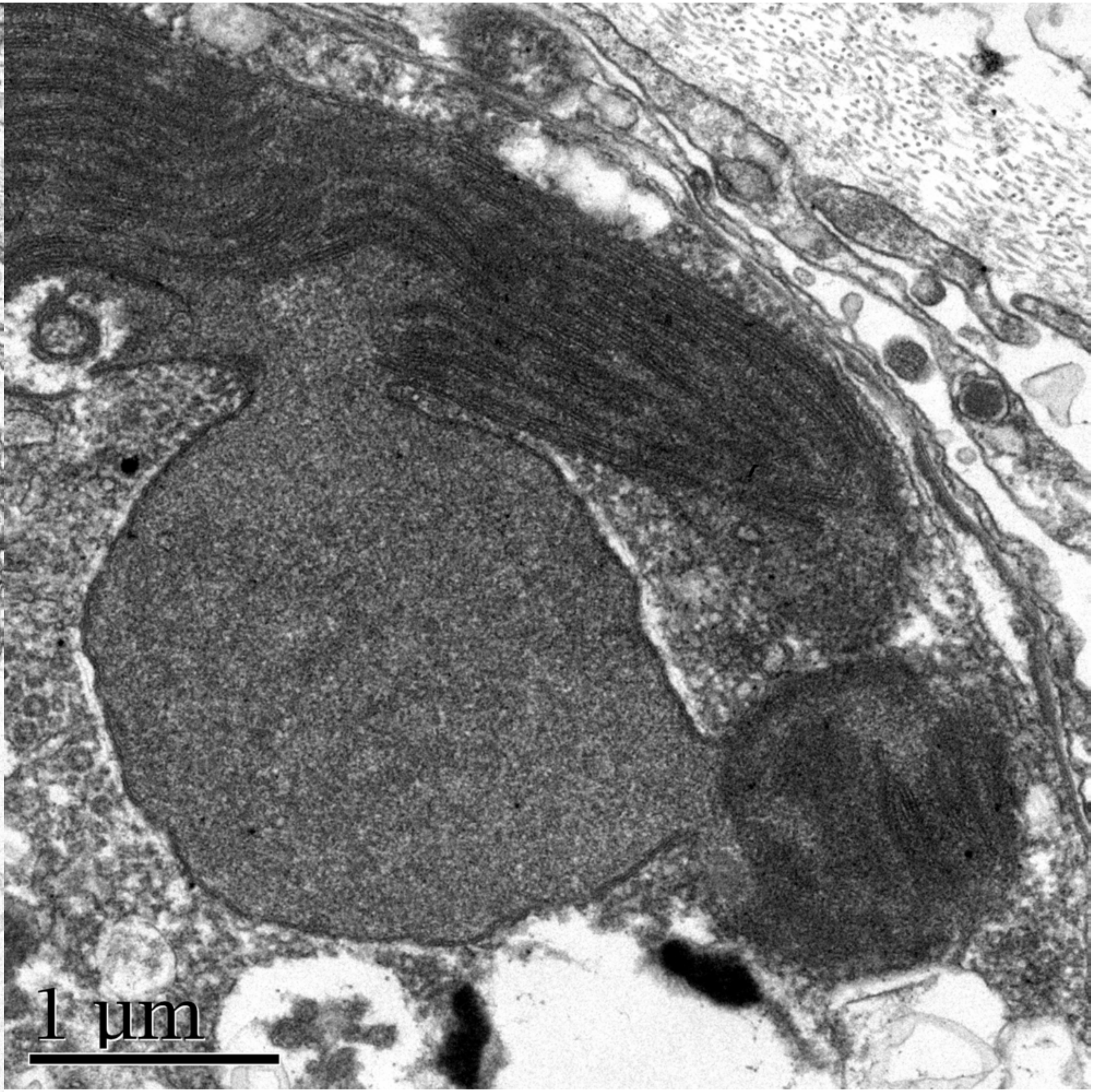

18-14_Correa_JC4_4E8_49.tif

Cell 7

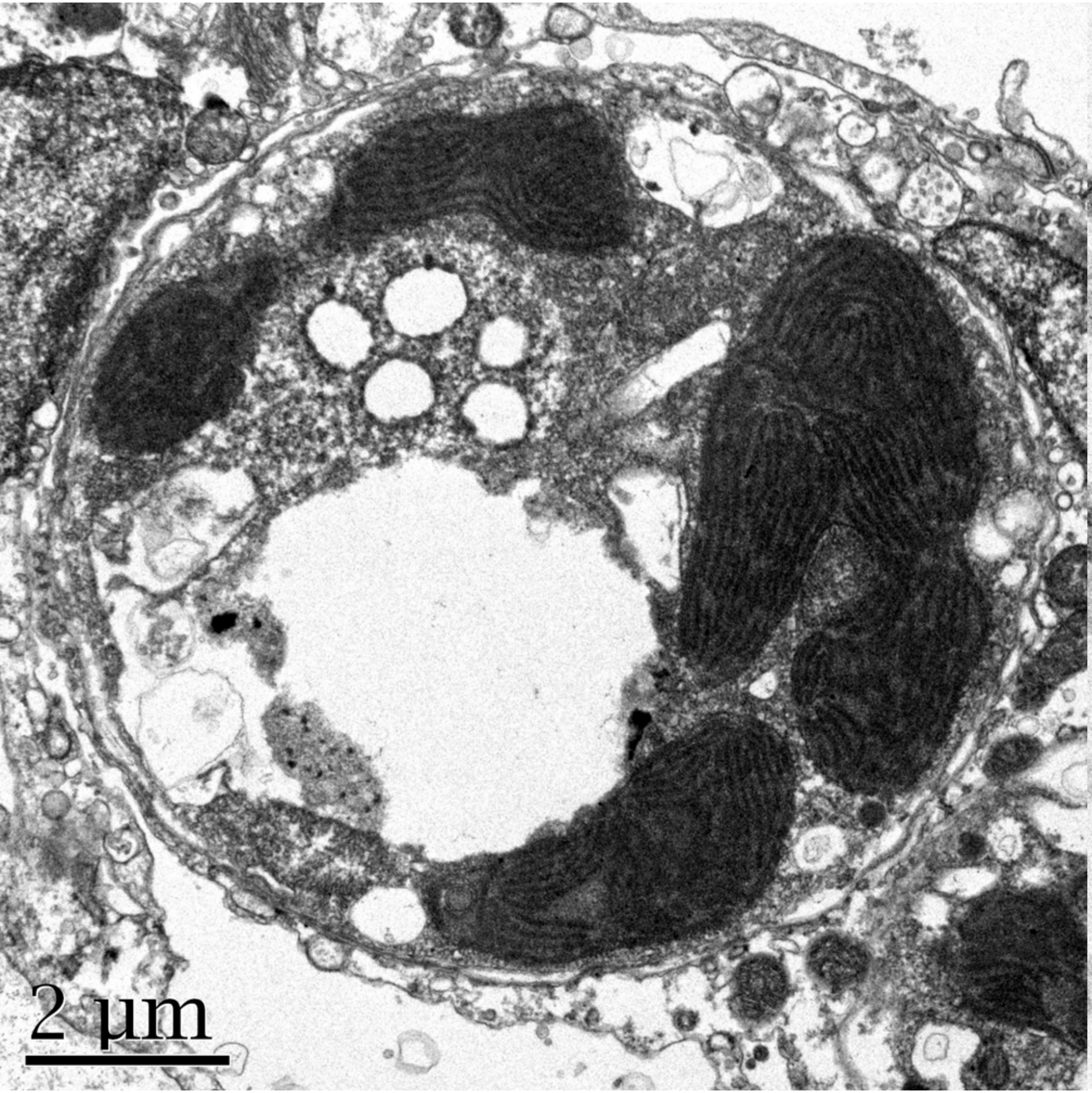

18-14_Correa_JC4_4E8_52.tif

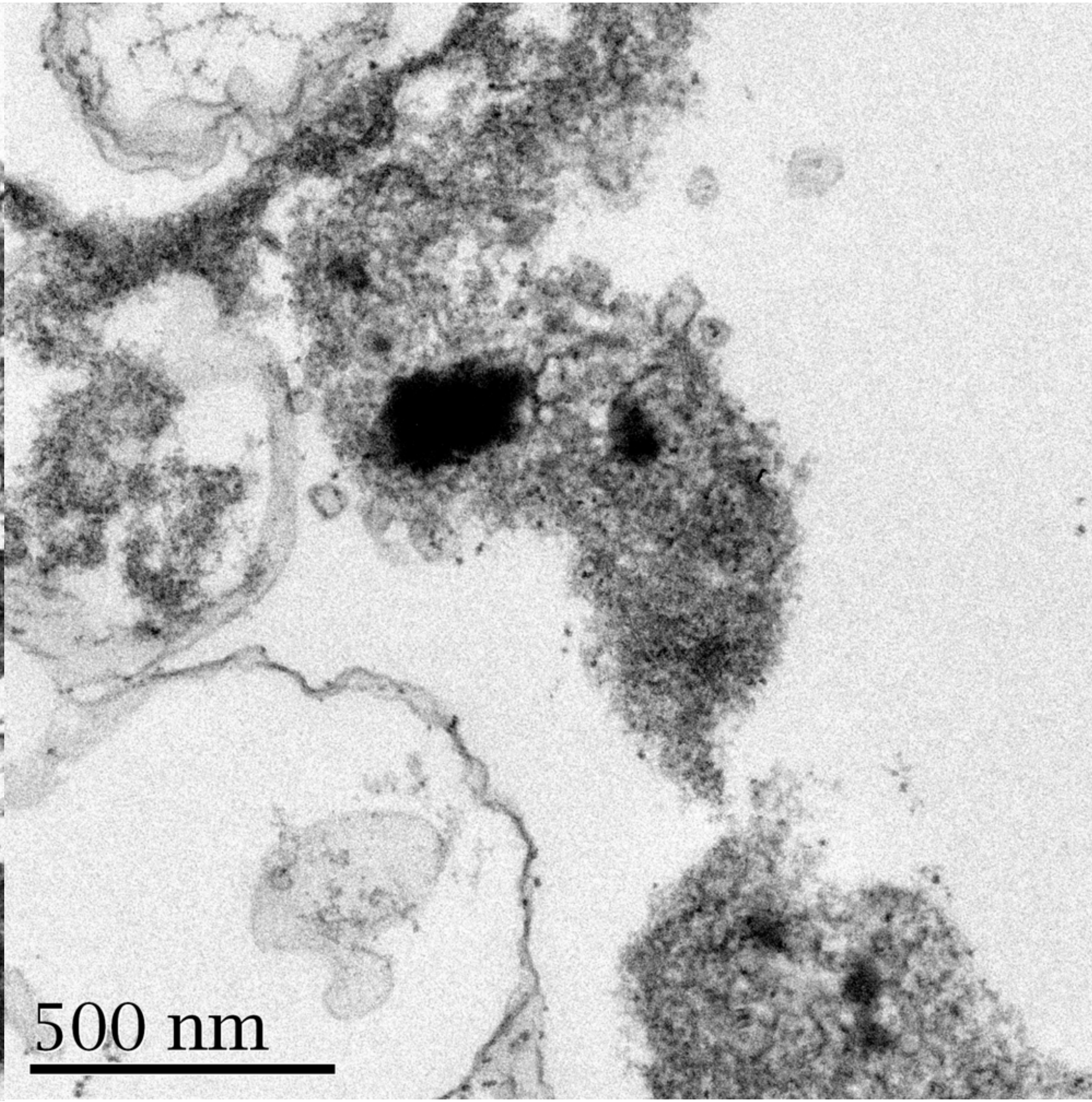

18-14_Correa_JC4_4E8_56.tif

Cell 8

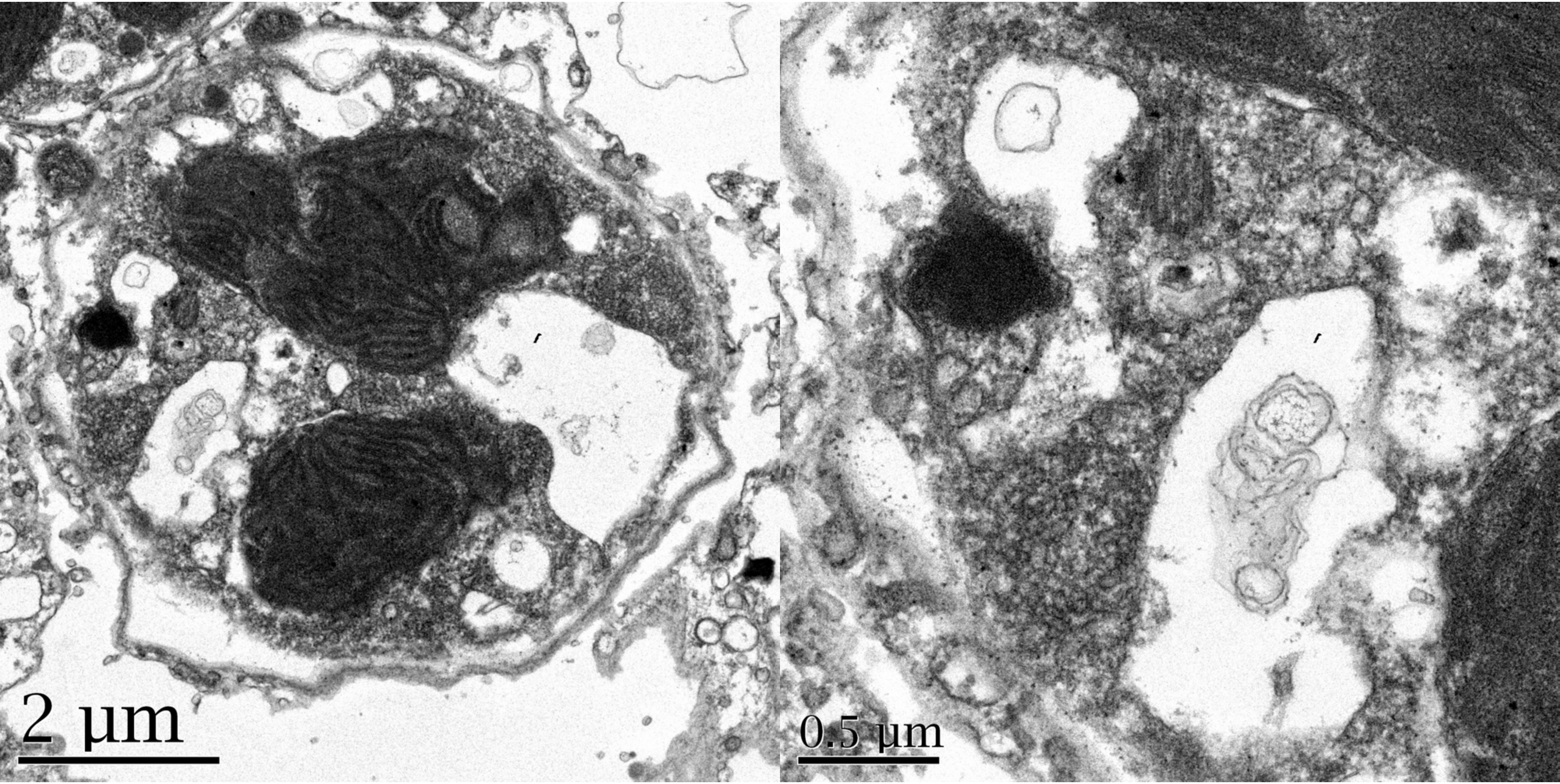

18-14_Correa_JC4_4E8_59.tif

18-14_Correa_JC4_4E8_60.tif

Cell 9

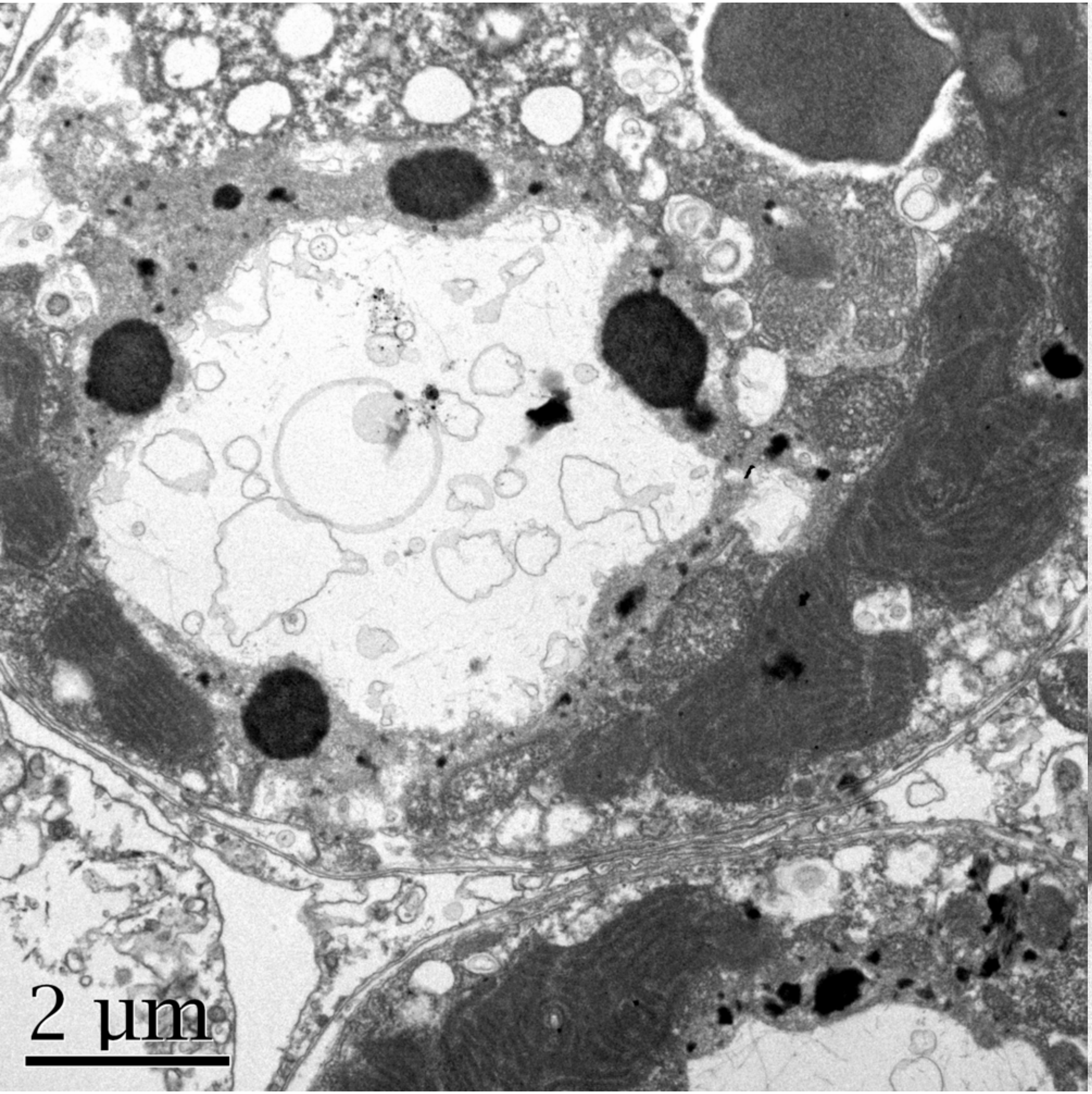

18-14_Correa_JC4_4E8_64.tif

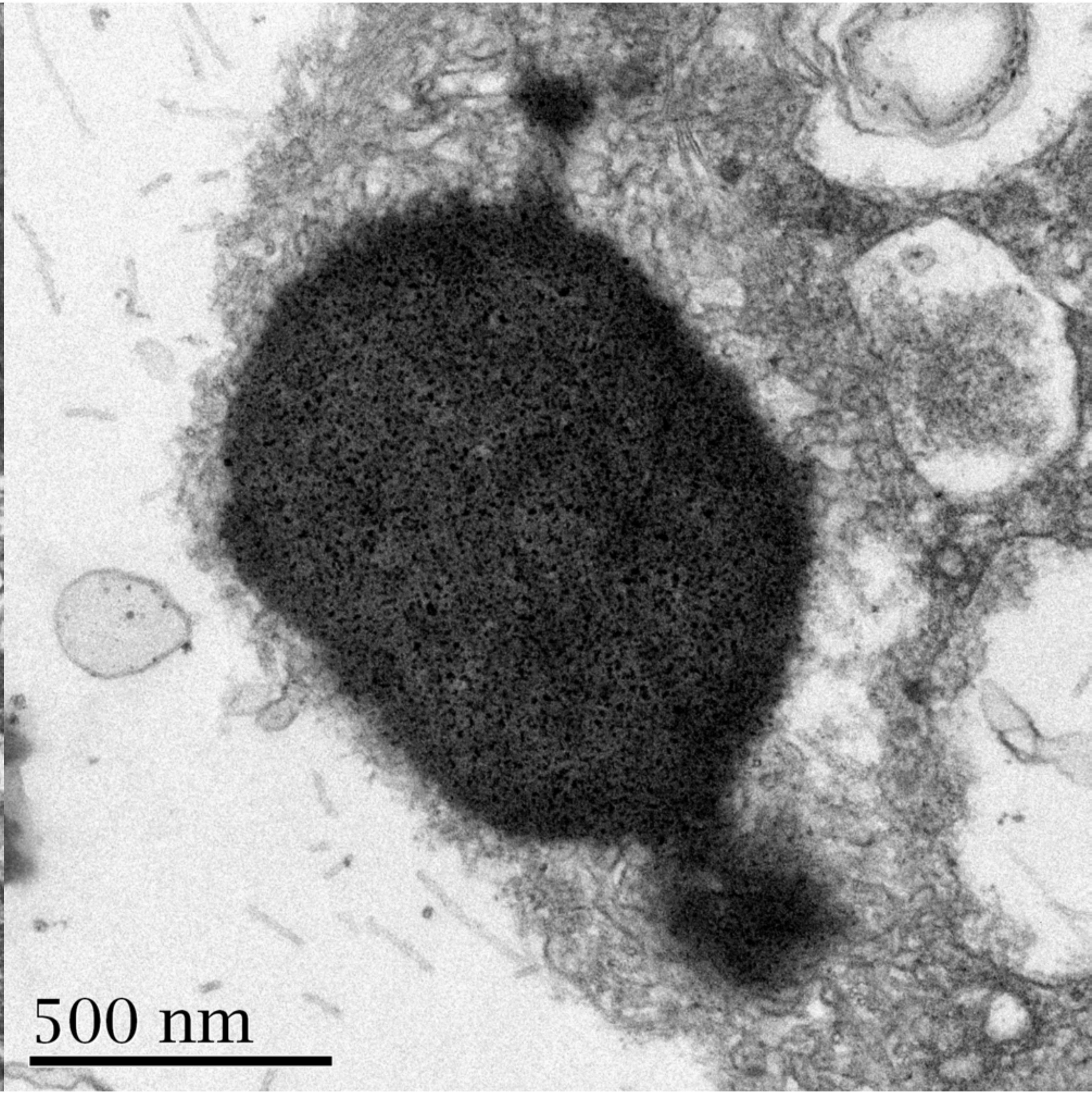

18-14_Correa_JC4_4E8_66.tif

Cell 10

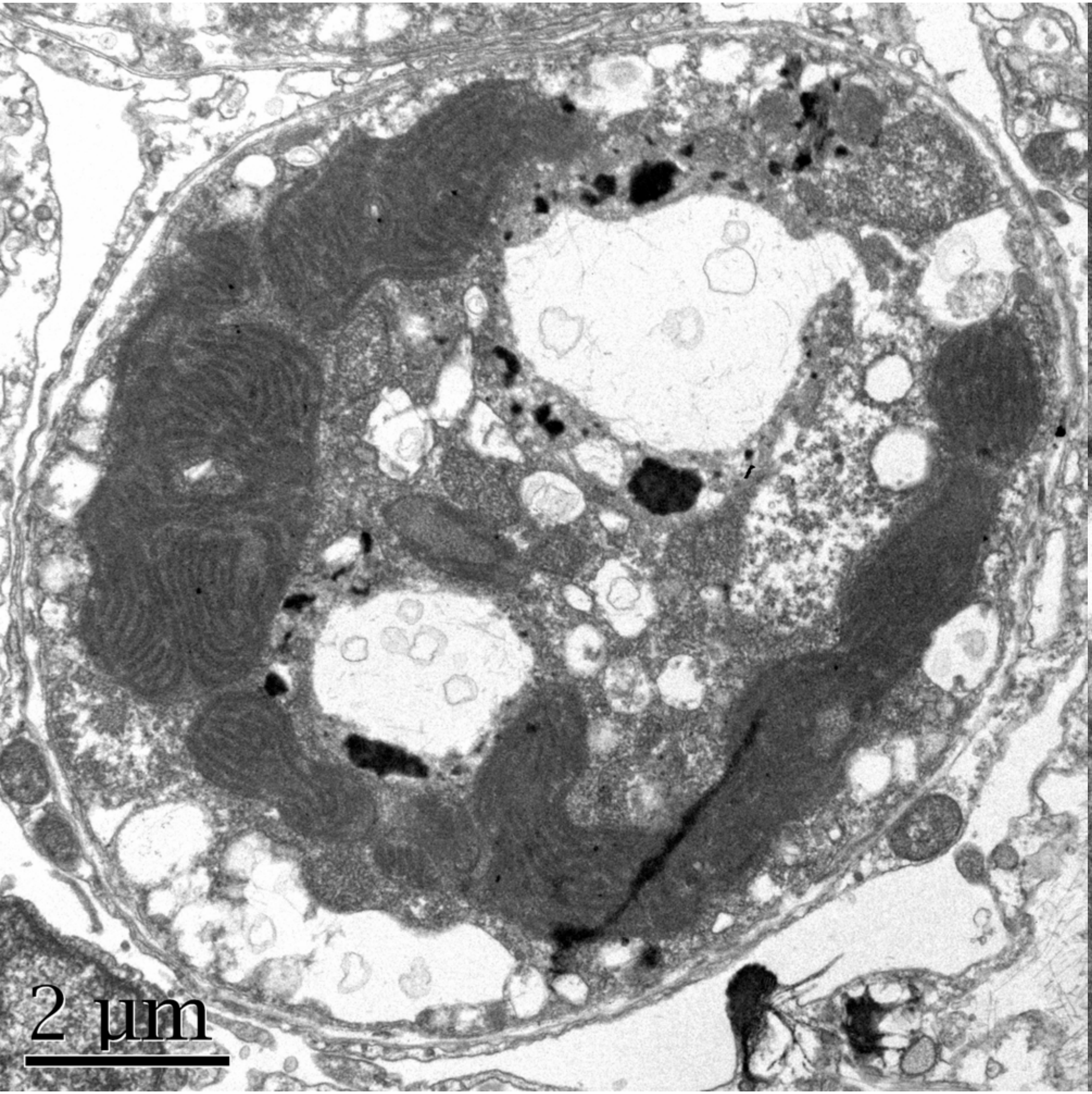

18-14_Correa_JC4_4E8_69.tif

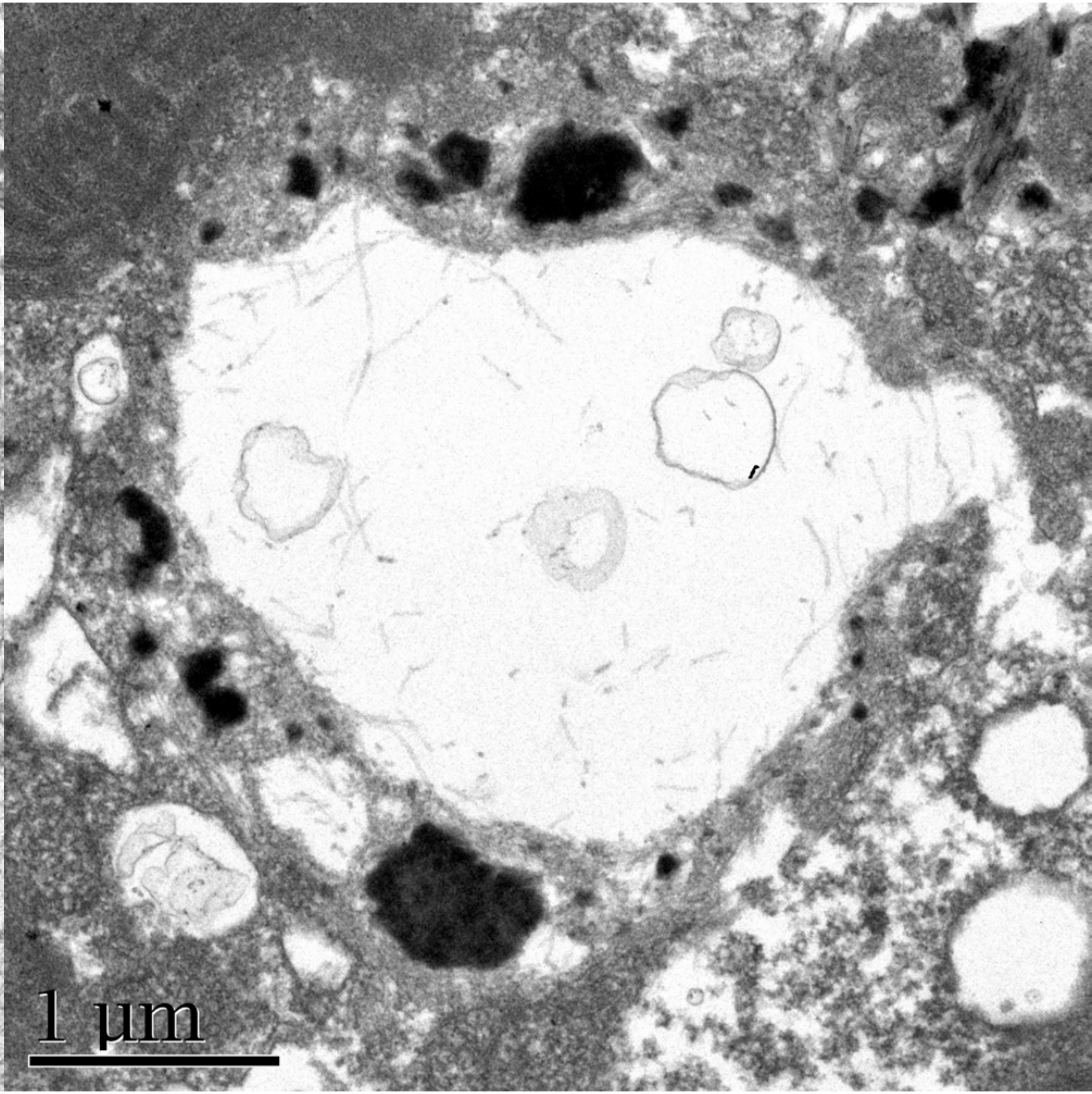

18-14_Correa_JC4_4E8_70.tif

Cell 11

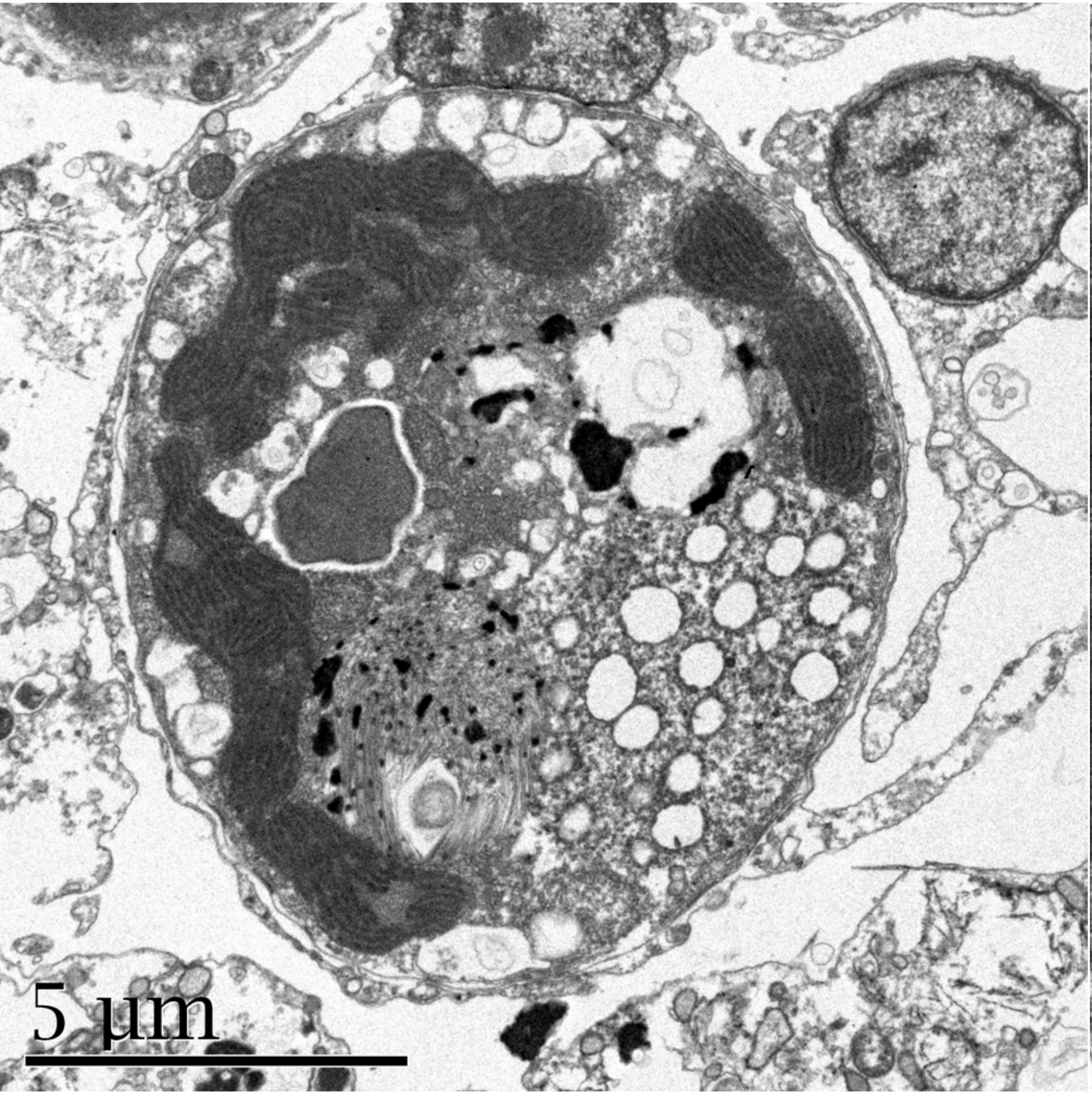

18-14_Correa_JC4_4E8_71.tif

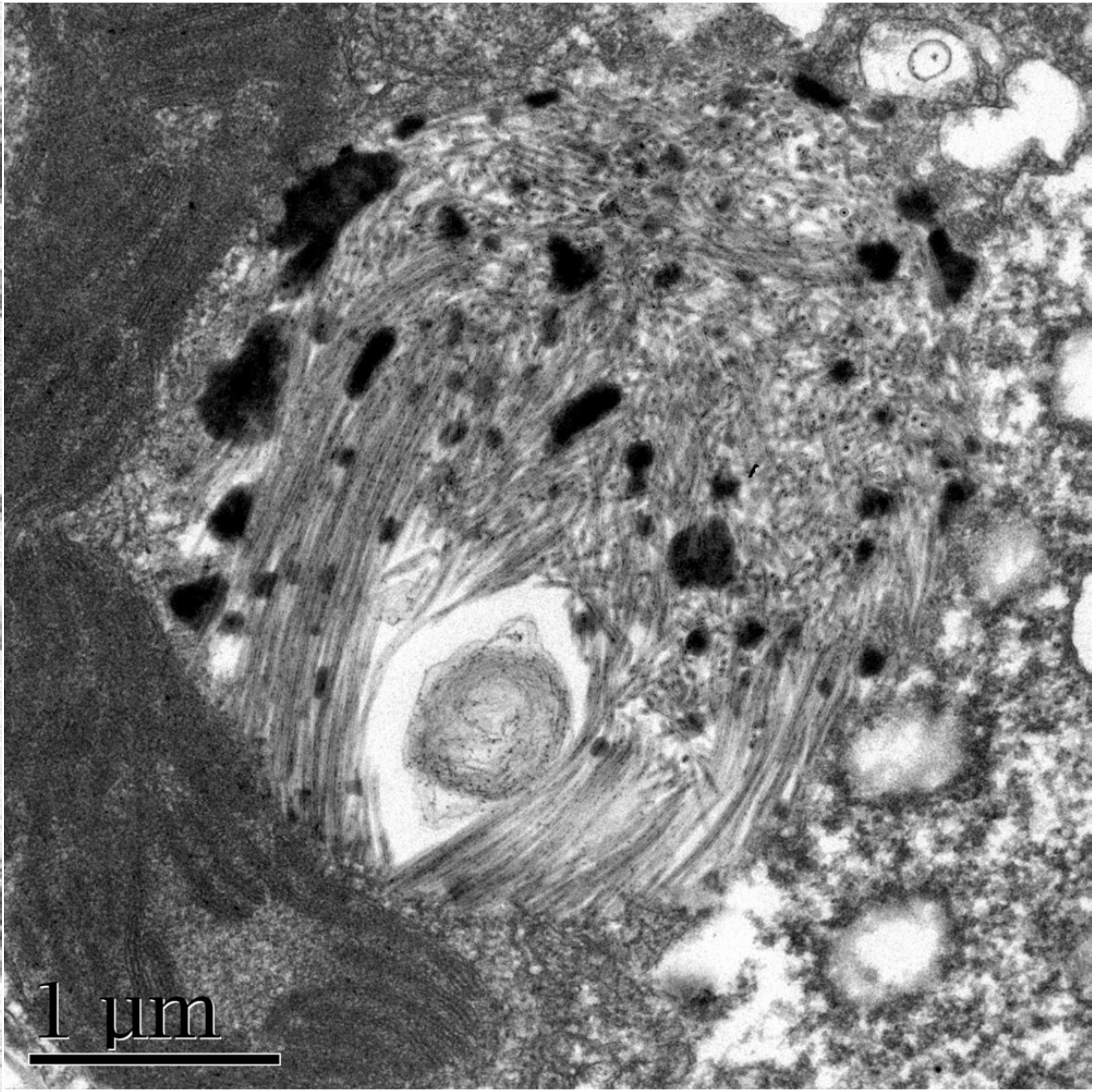

18-14_Correa_JC4_4E8_72.tif

Cell 12

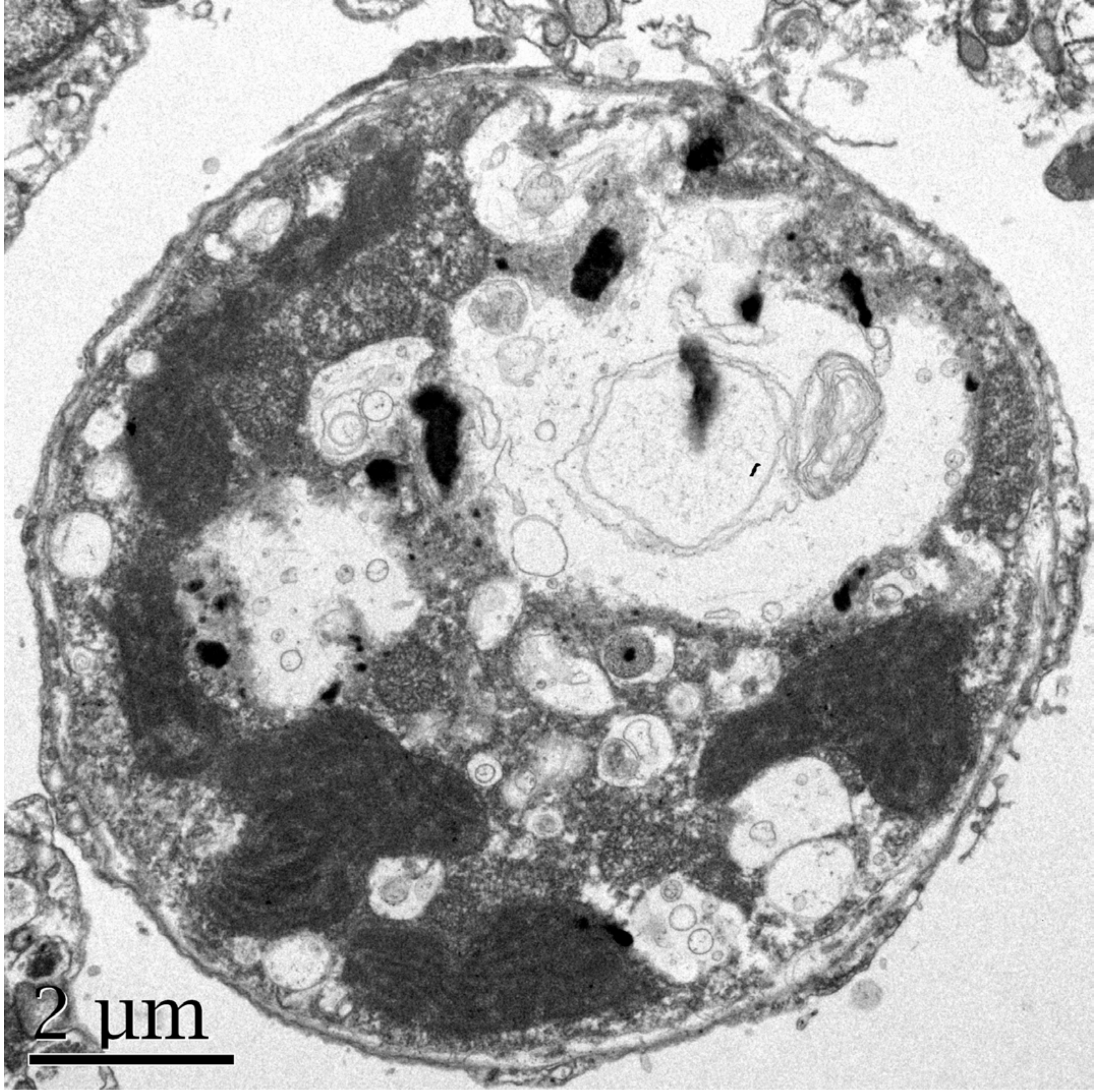

18-14_Correa_JC4_4E8_73.tif

Heat Treatment - ACR Colony A

Cell 1

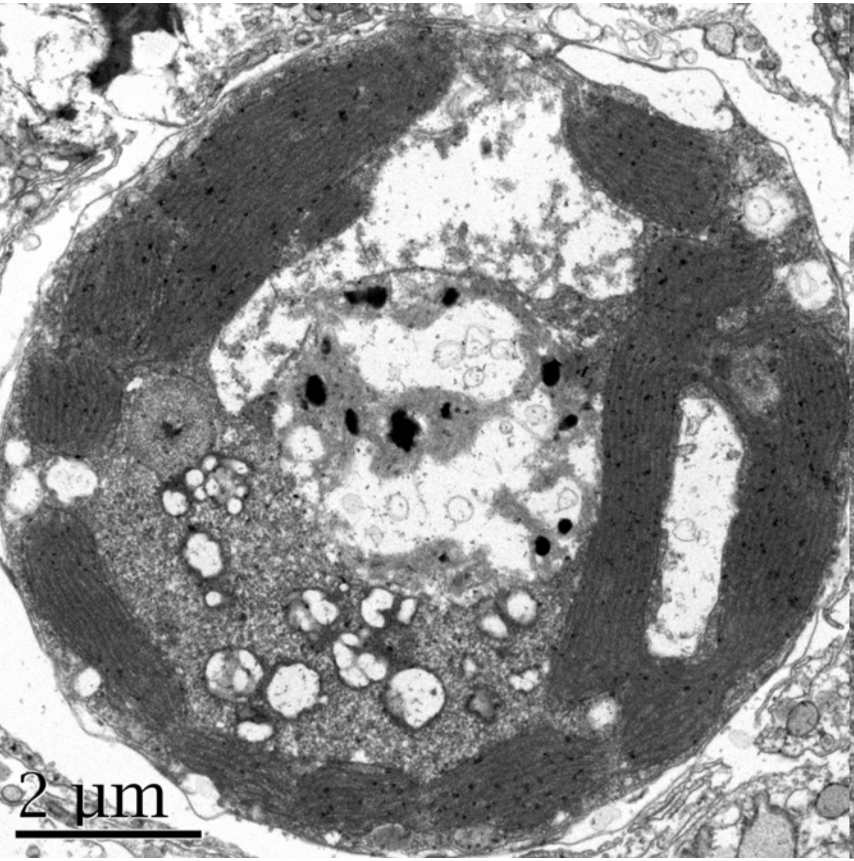

18-14_Correa_JH4_5A2_3.tif

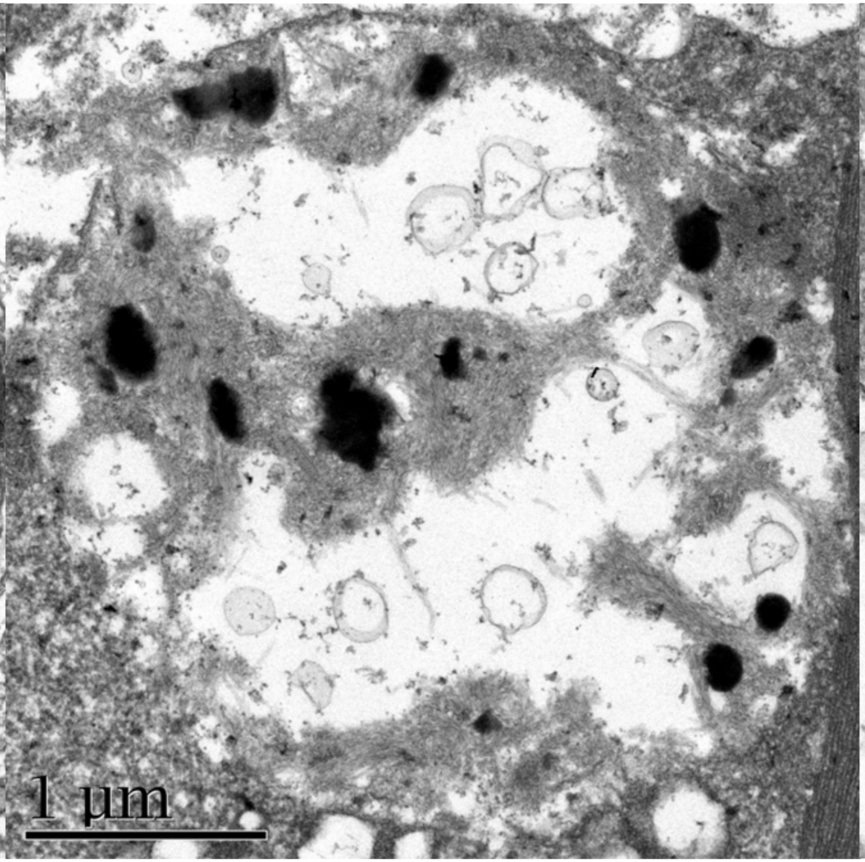

18-14_Correa_JH4_5A2_10.tif

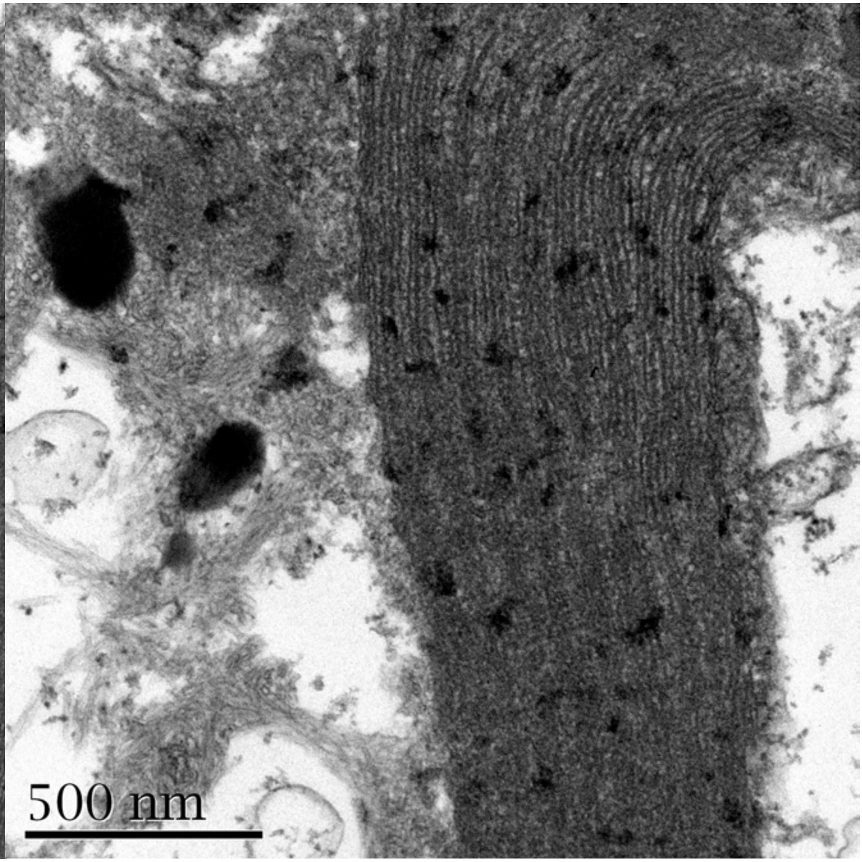

18-14_Correa_JH4_5A2_8.tif

Cell 2

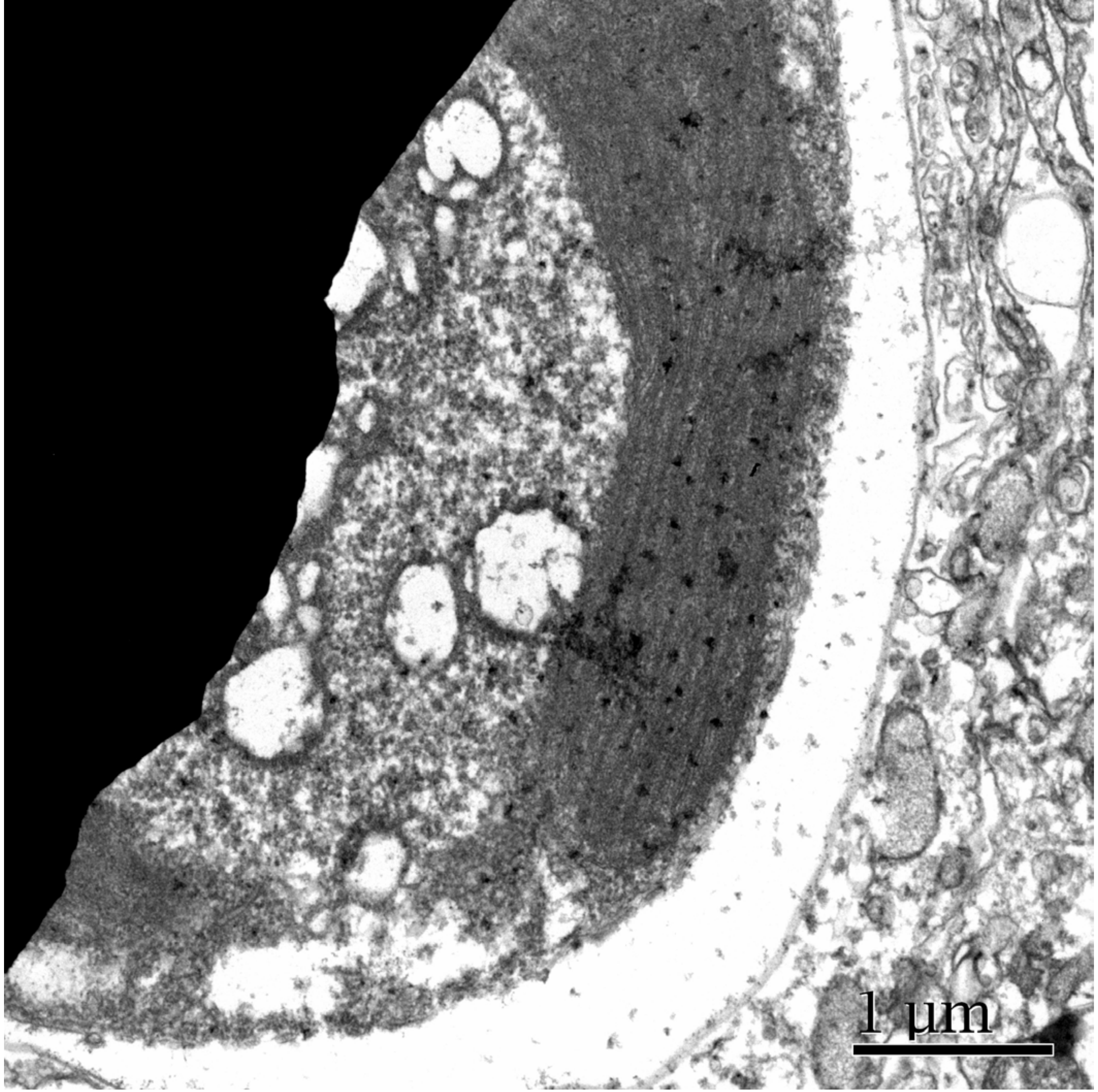

18-14_Correa_JH4_5A2_14.tif

Cell 3

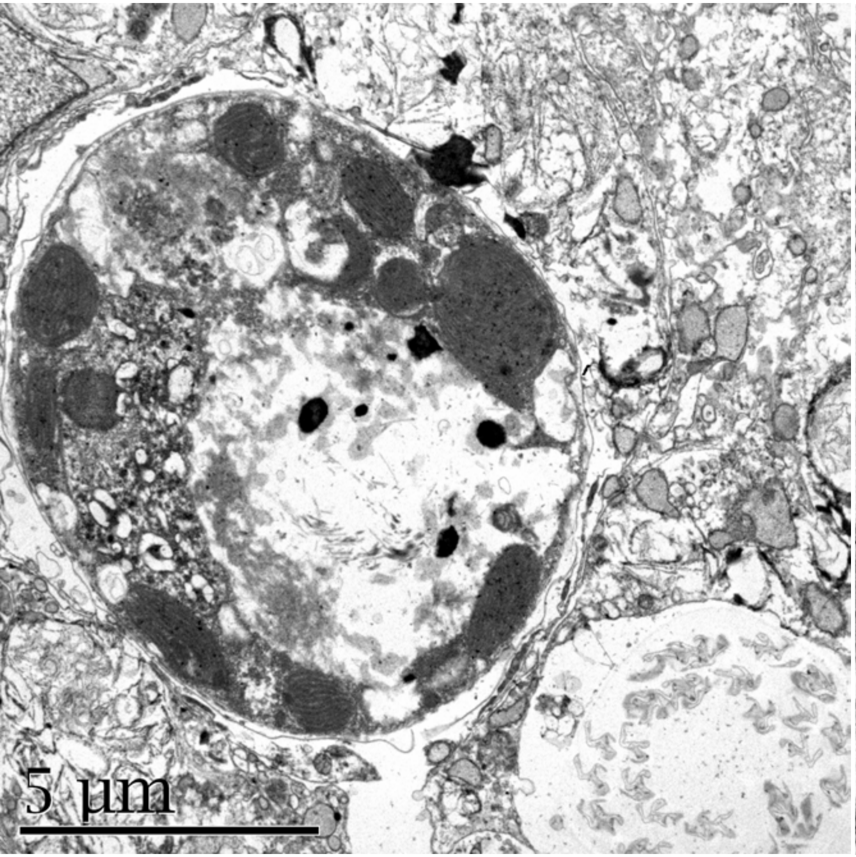

18-14_Correa_JH4_5A2_15.tif

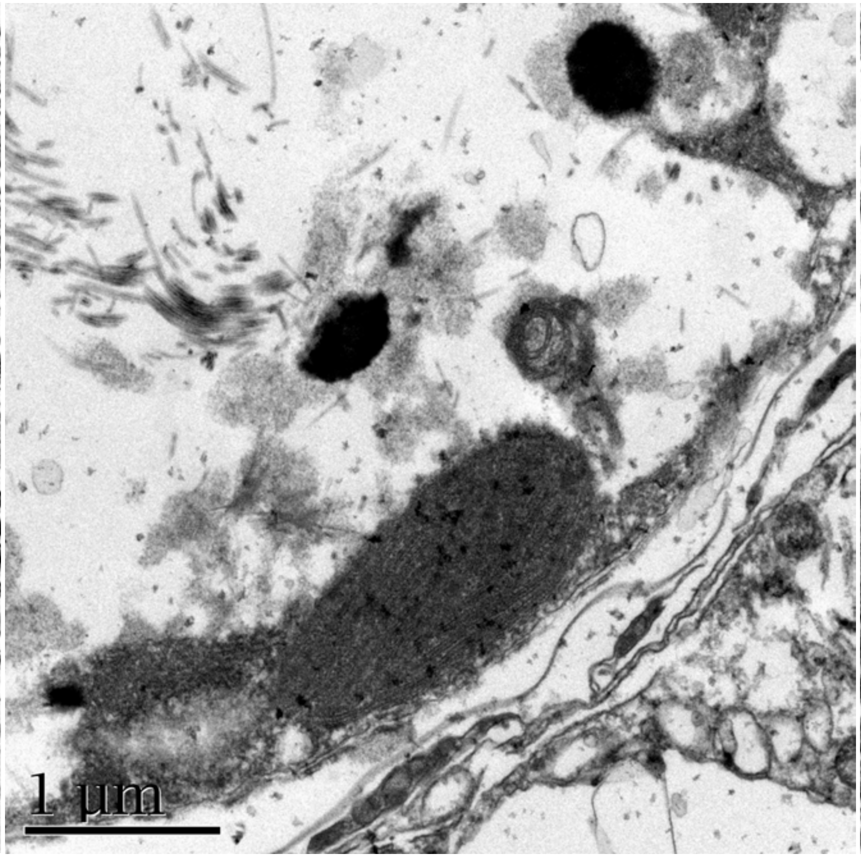

18-14_Correa_JH4_5A2_17.tif

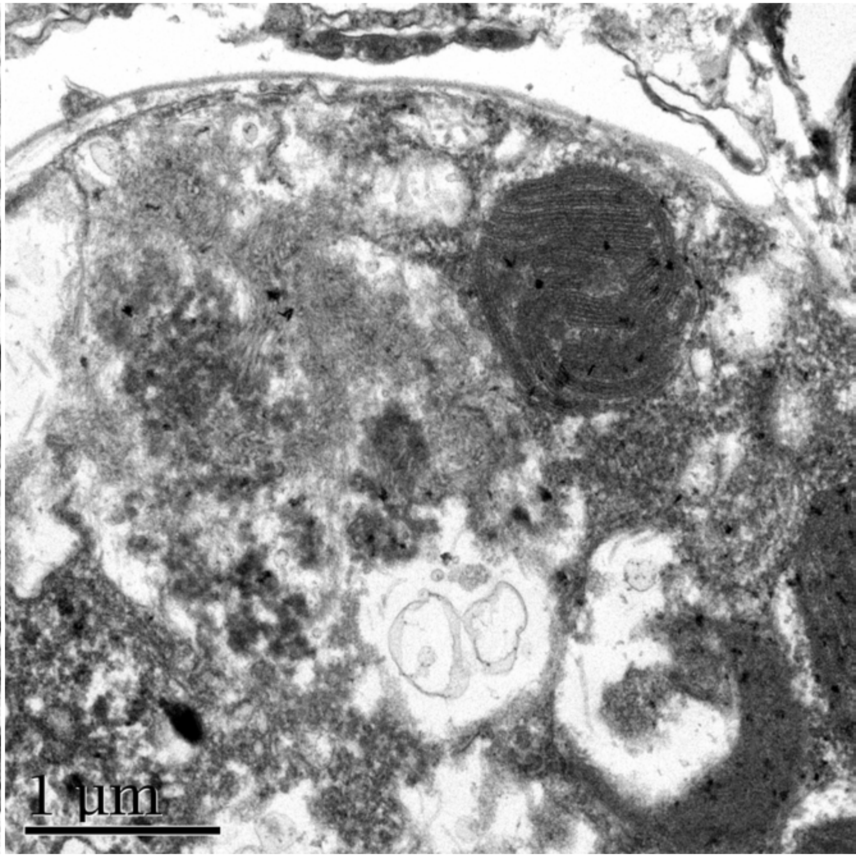

18-14_Correa_JH4_5A2_20.tif

Cell 4

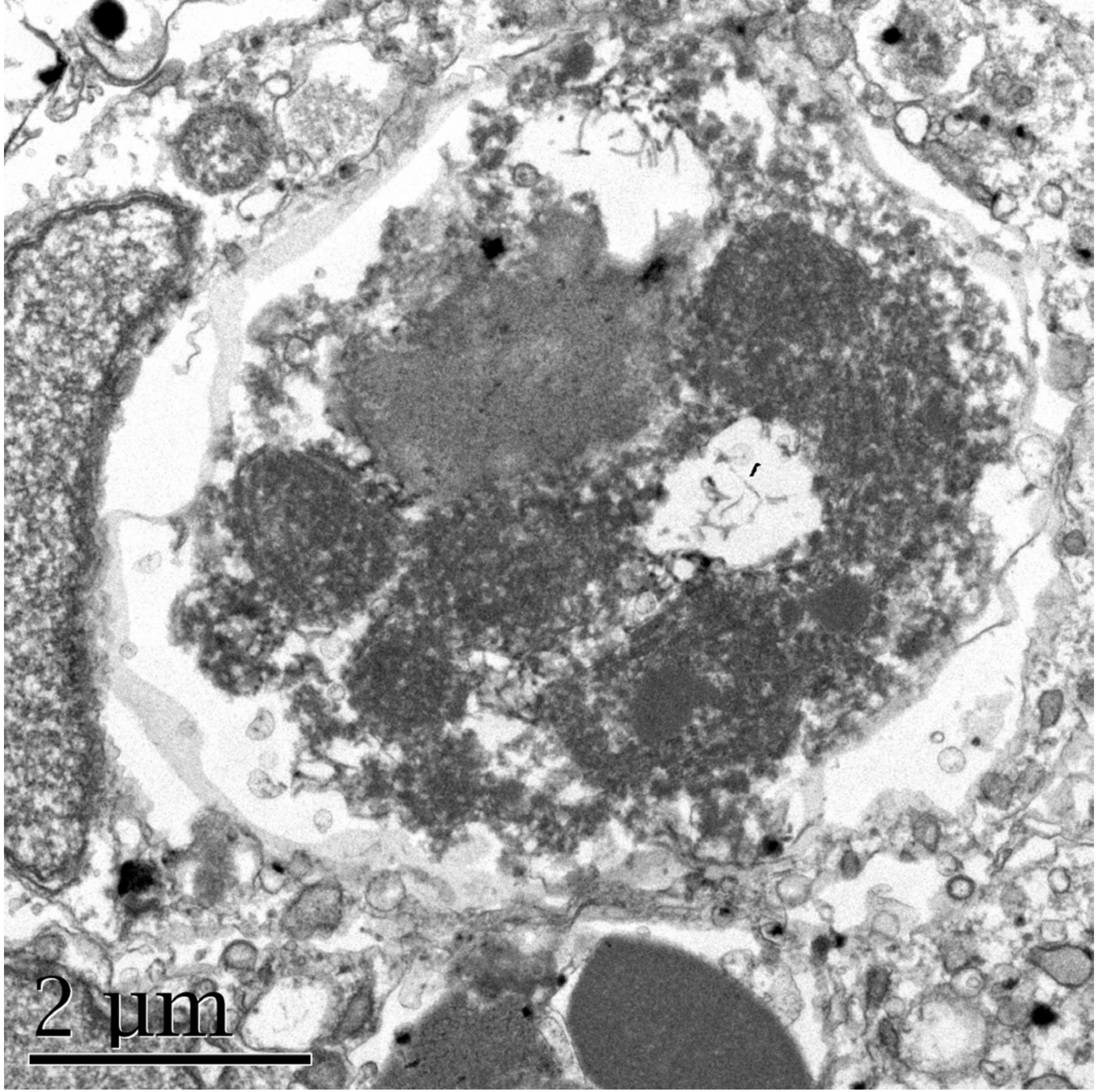

18-14_Correa_JH4_5B1_3.tif

Cell 5

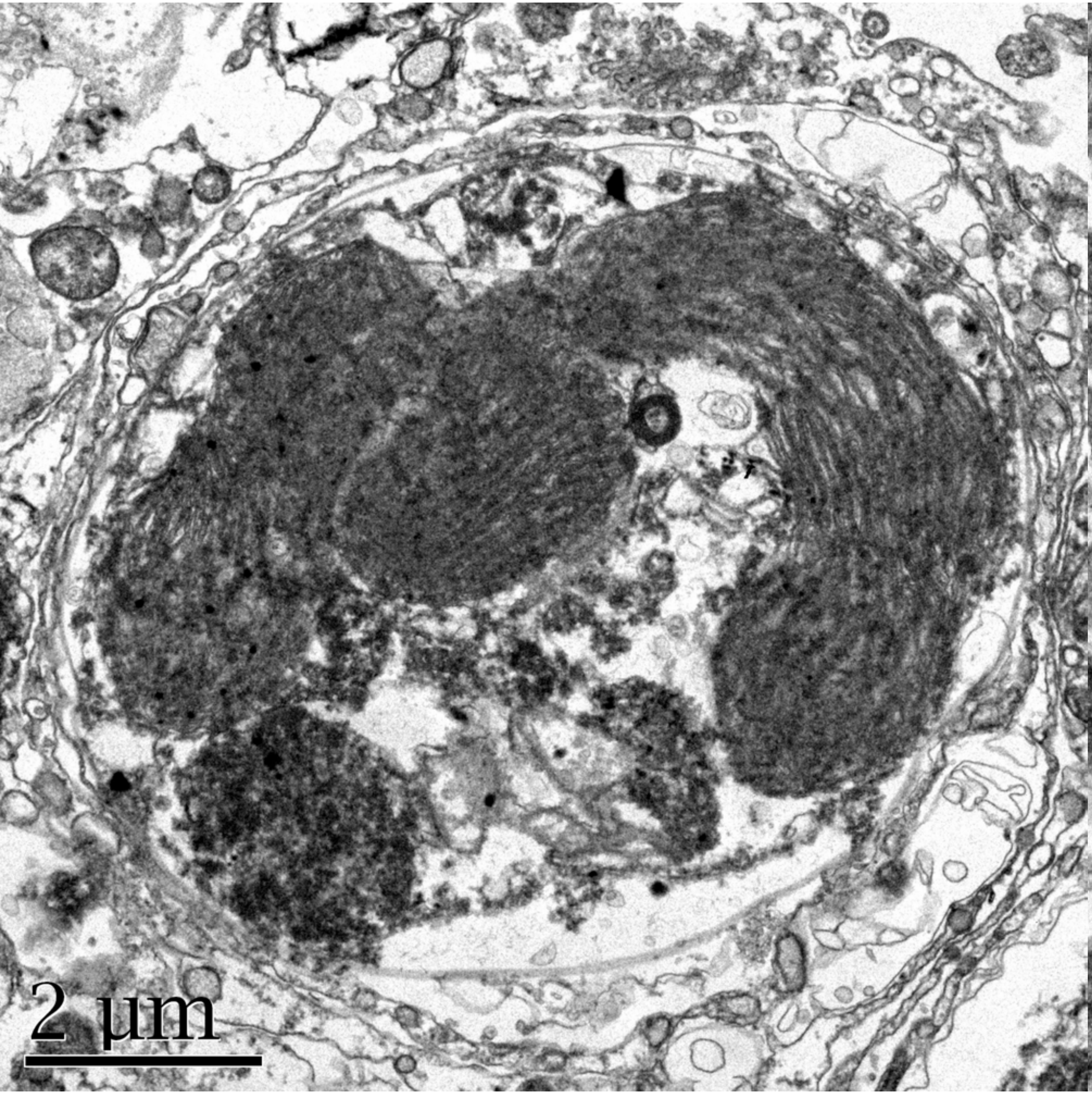

18-14_Correa_JH4_5B1_6.tif

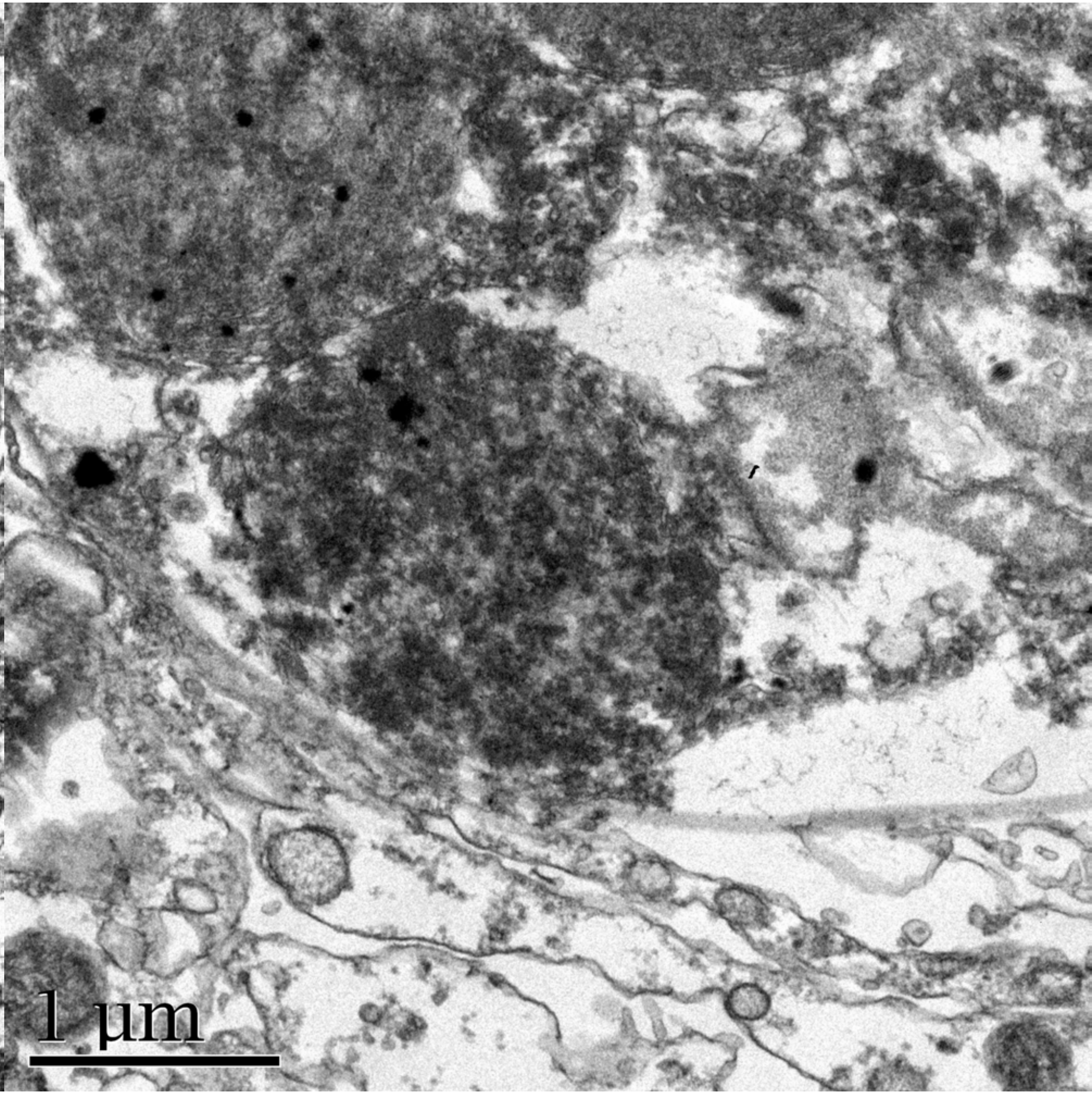

18-14_Correa_JH4_5B1_7.tif

Cell 6

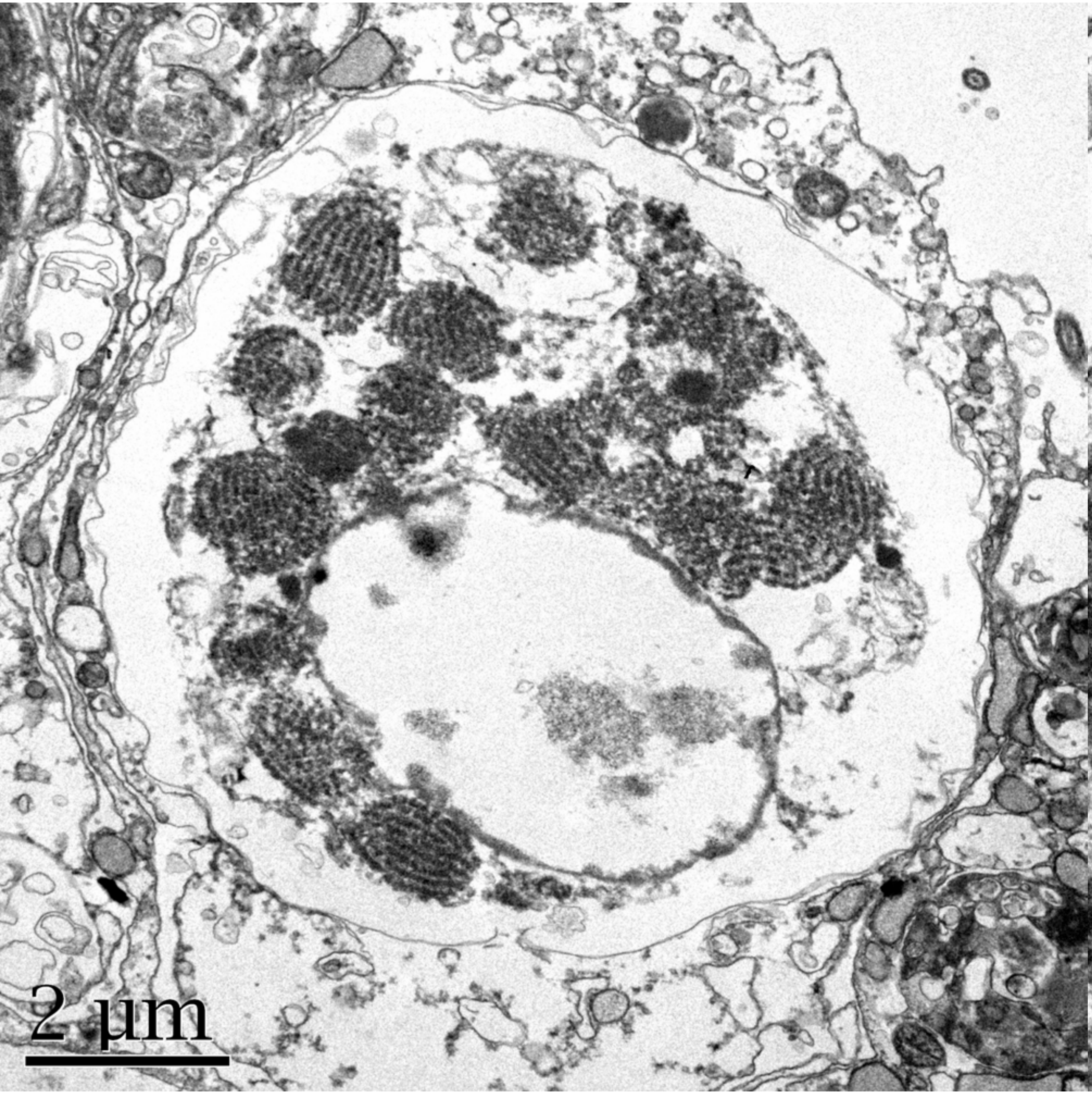

18-14_Correa_JH4_5B1_11.tif

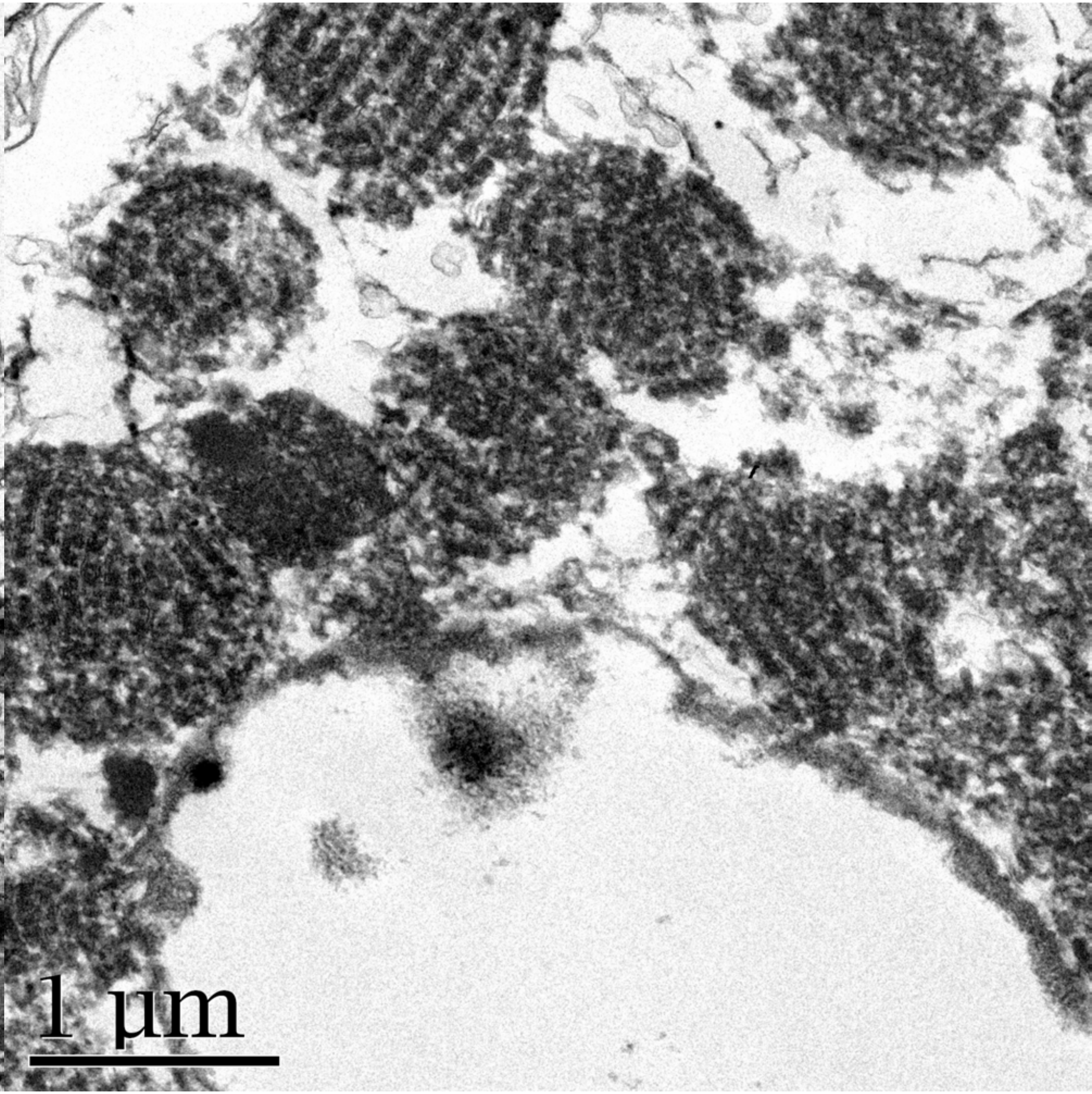

18-14_Correa_JH4_5B1_12.tif

Cell 7

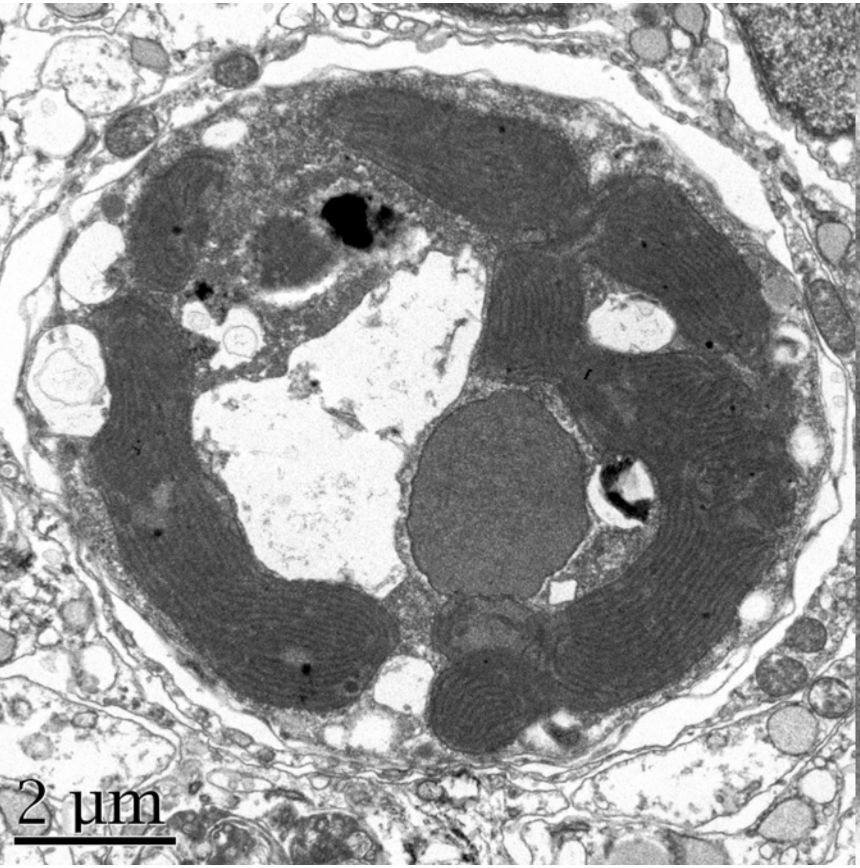

18-14_Correa_JH4_5B1_14.tif

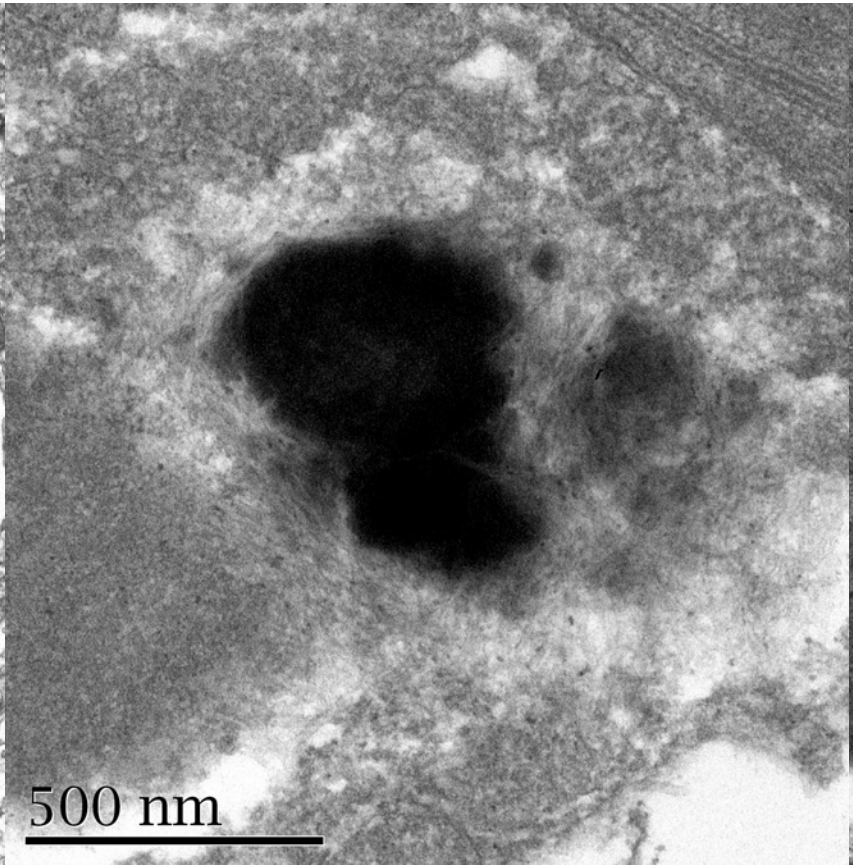

18-14_Correa_JH4_5B1_19.tif

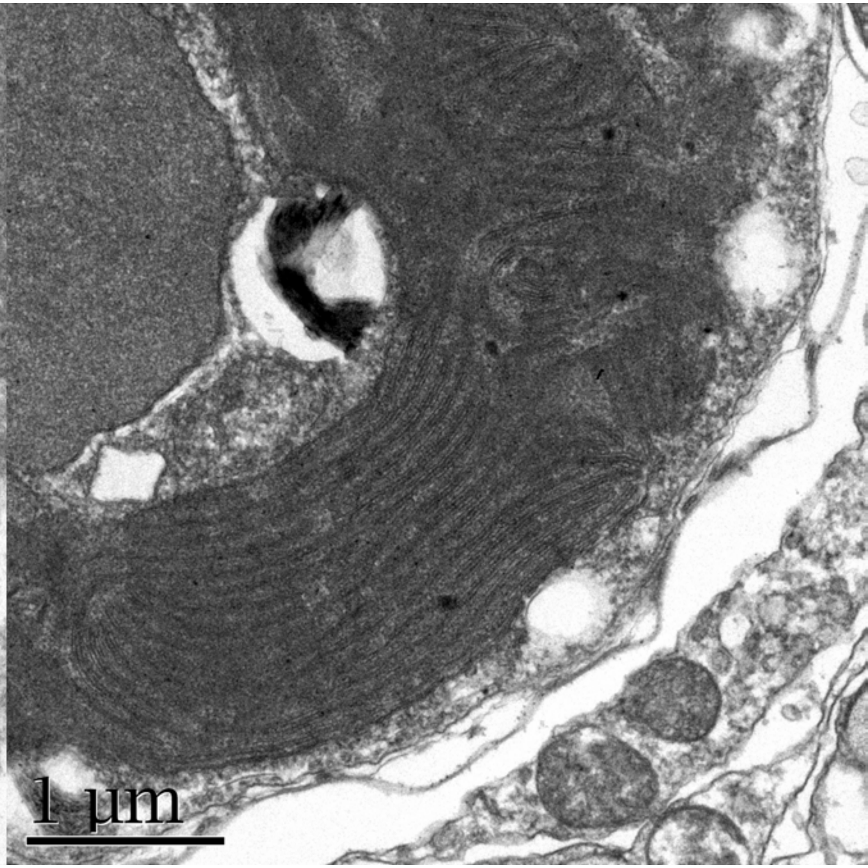

18-14_Correa_JH4_5B1_16.tif

Cell 8

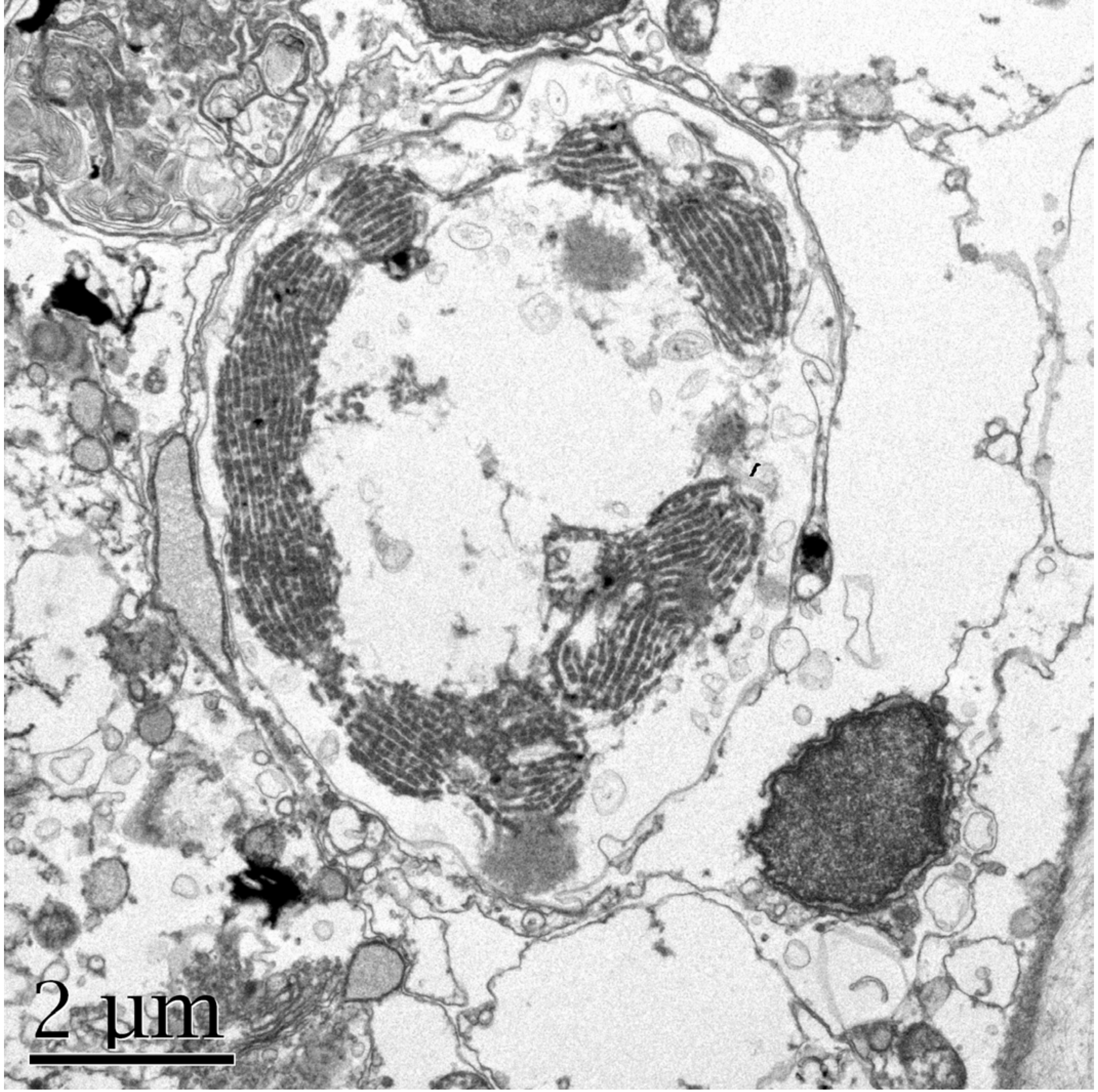

18-14_Correa_JH4_5B1_23.tif

Cell 9

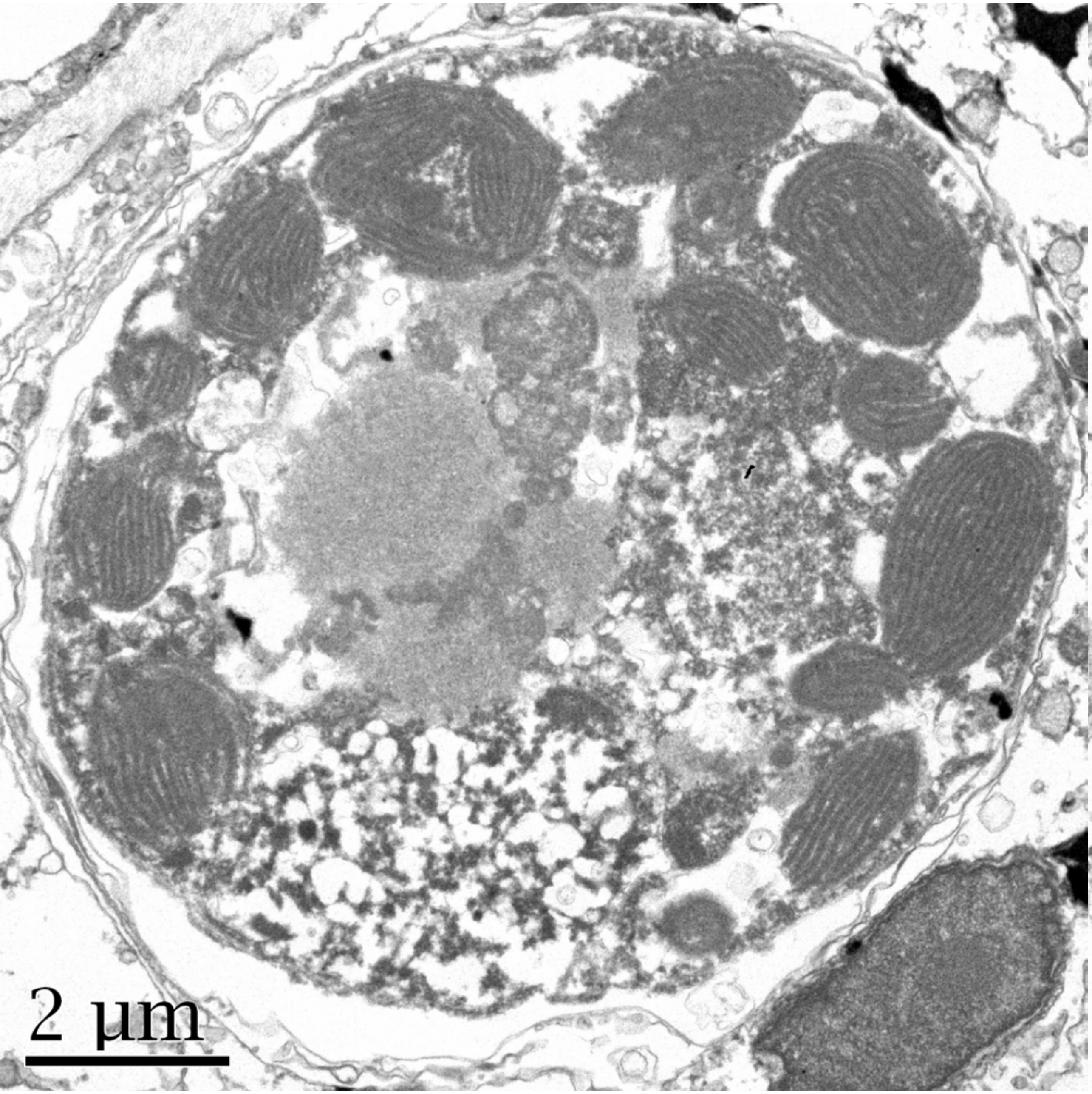

18-14_Correa_JH4_5B1_24.tif

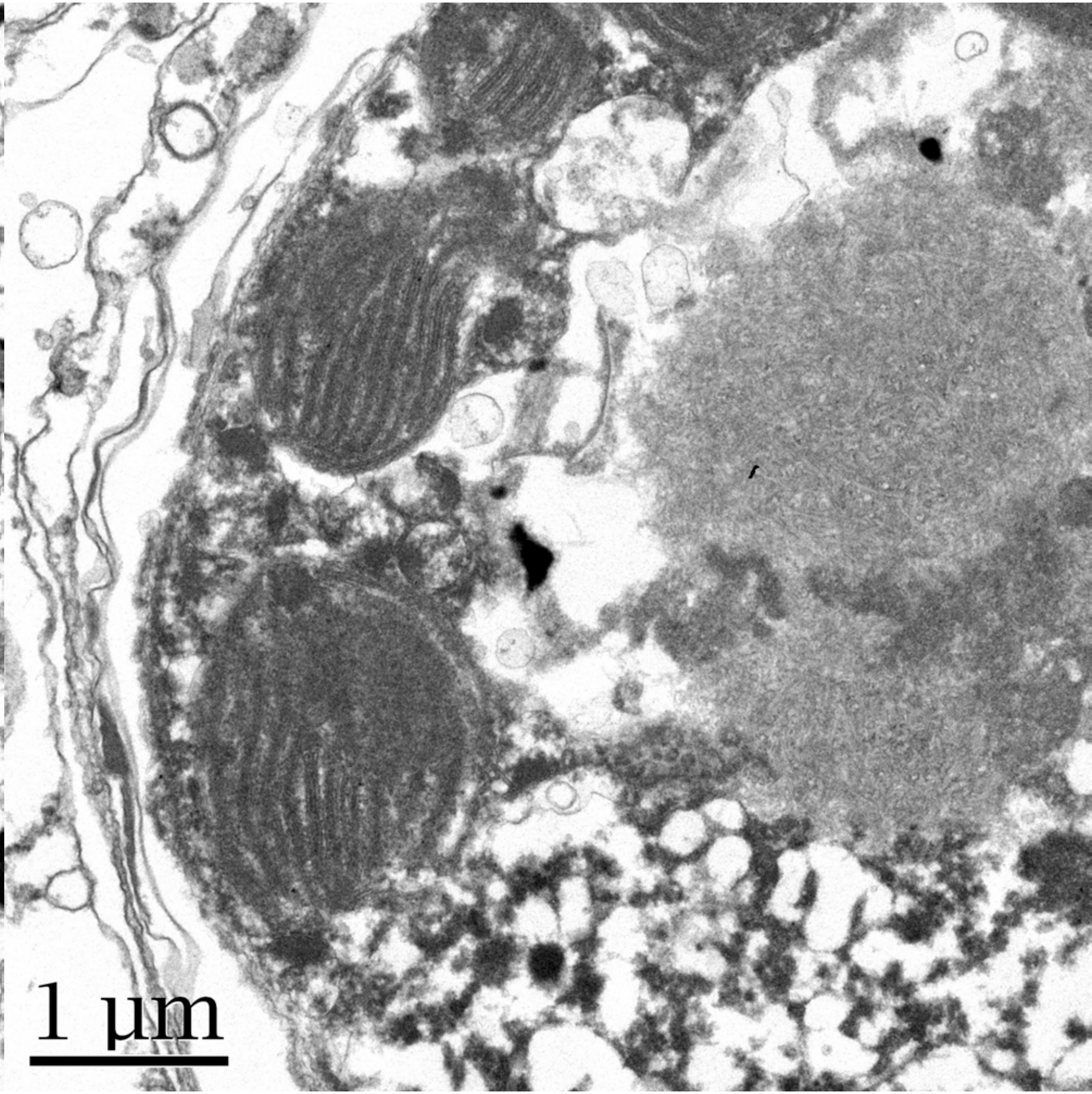

18-14_Correa_JH4_5B1_27.tif

Cell 10

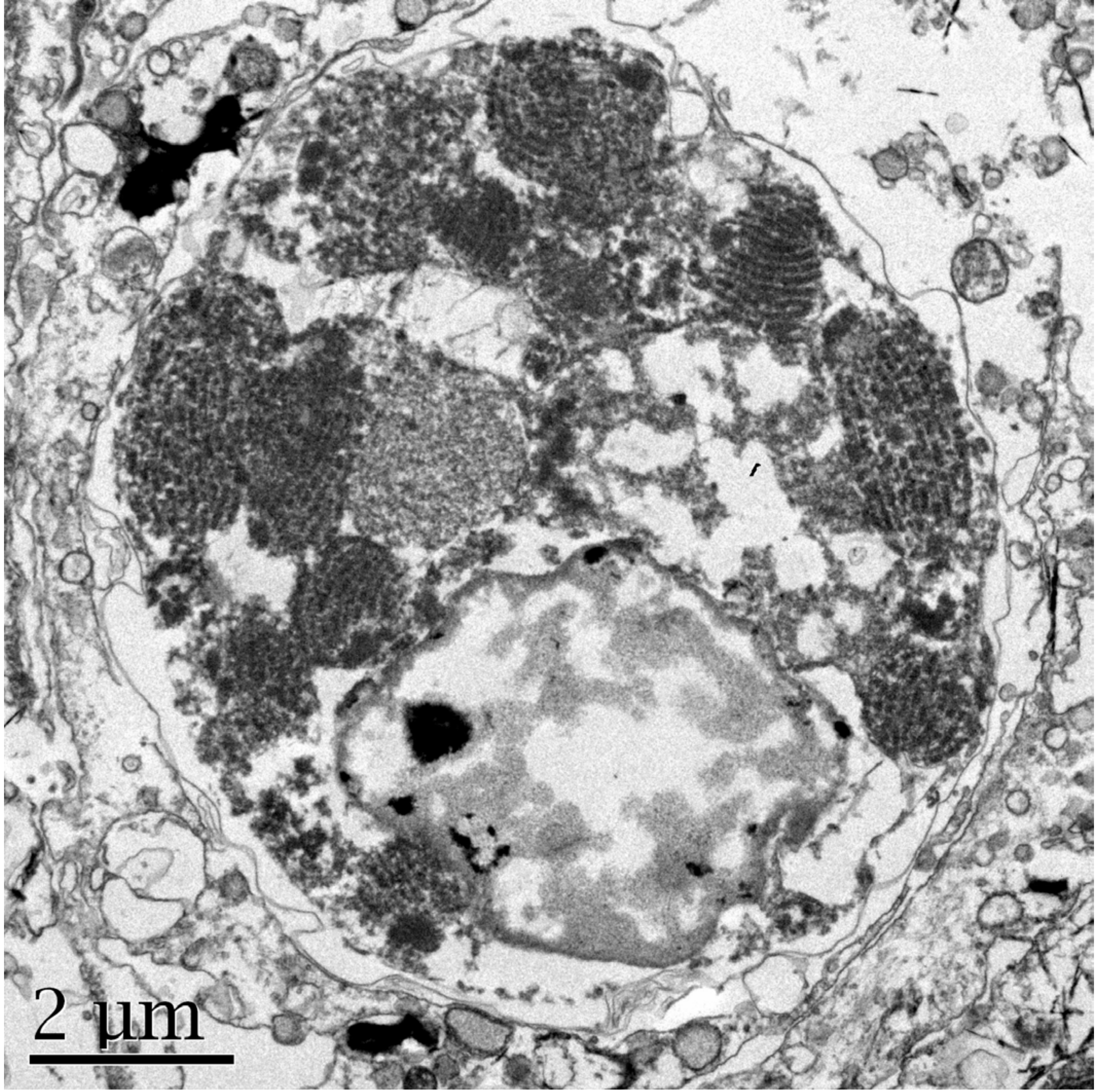

18-14_Correa_JH4_5B1_31.tif

Cell 11

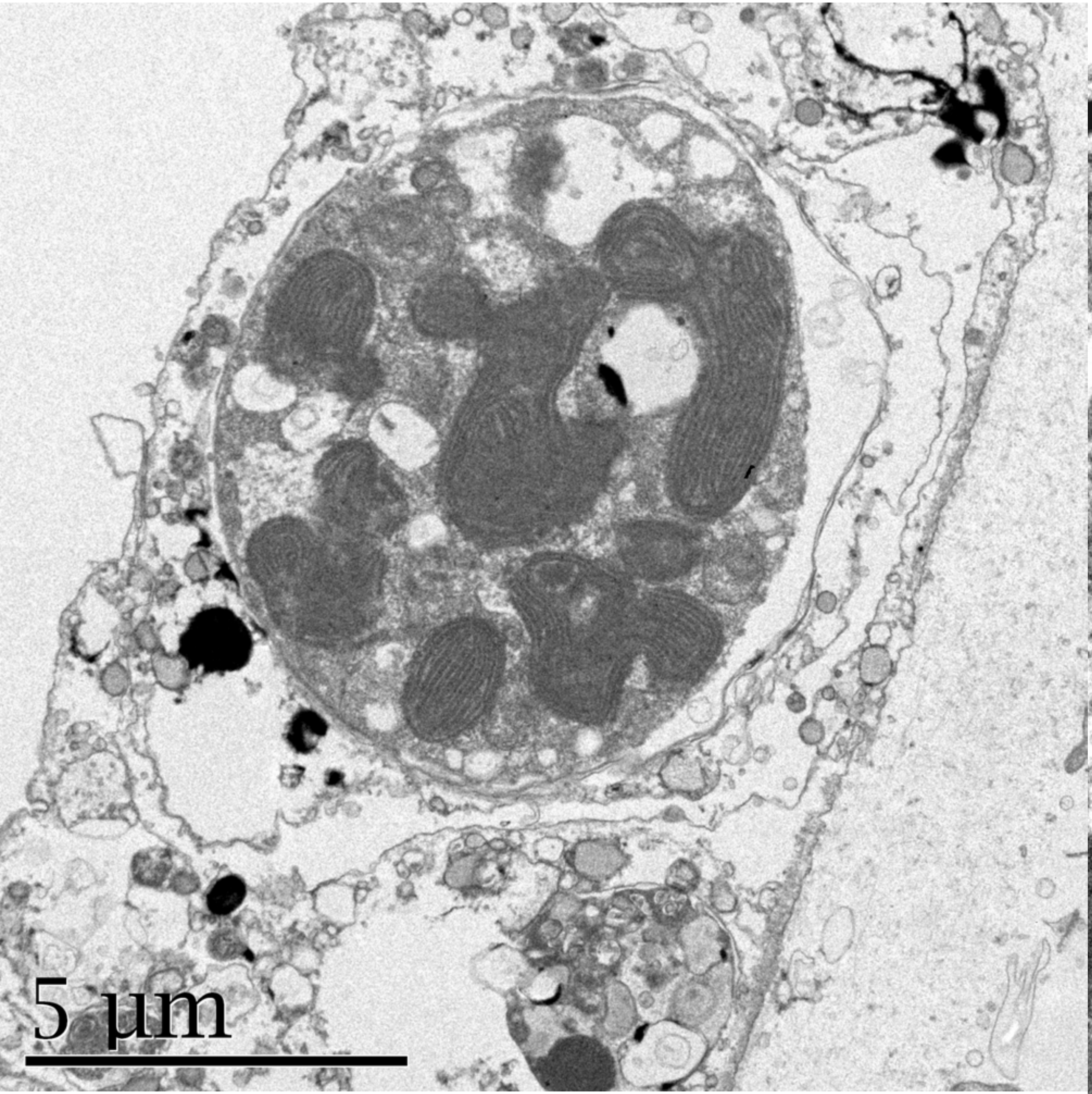

18-14_Correa_JH4_5B1_32.tif

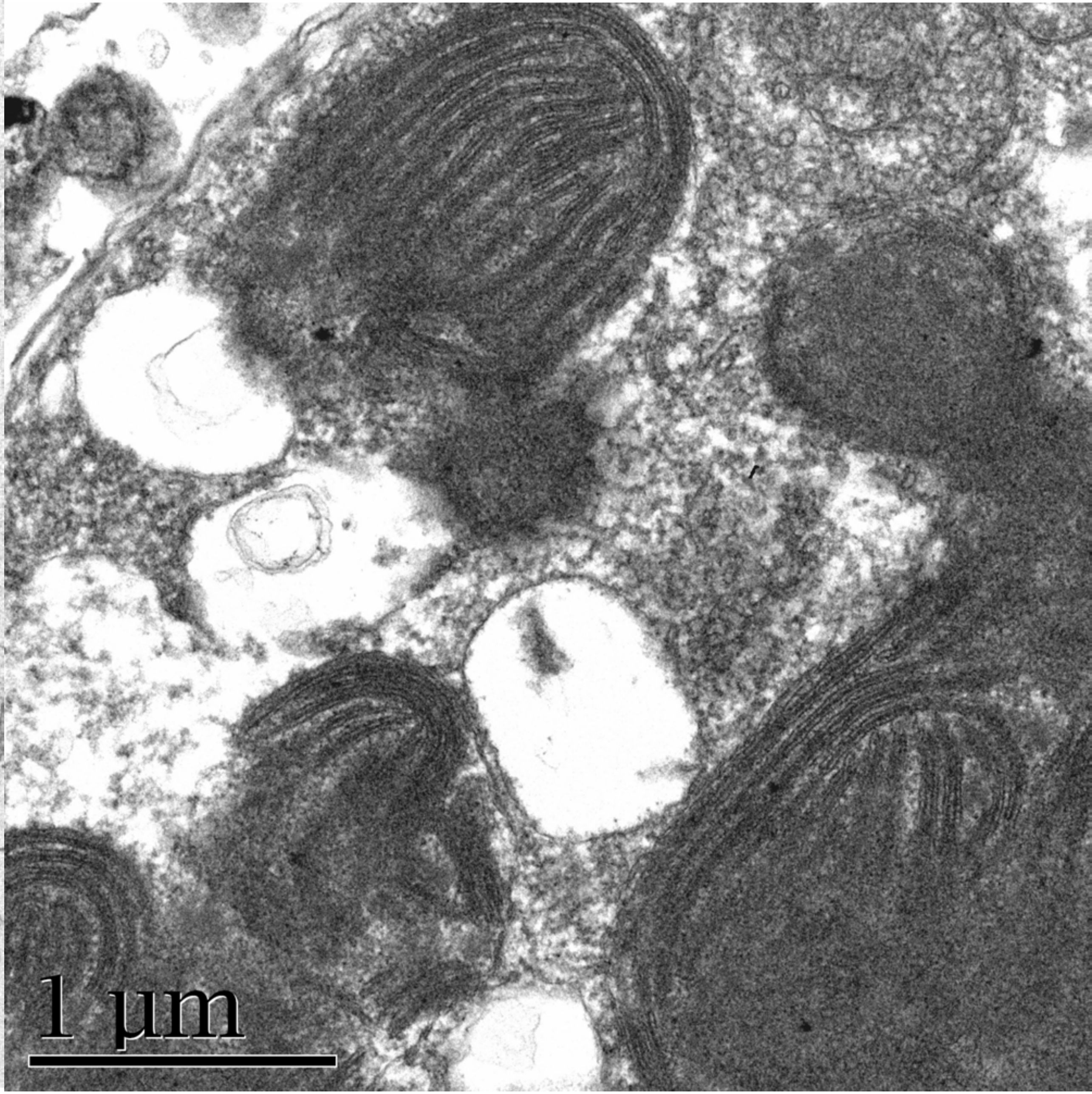

18-14_Correa_JH4_5B1_39.tif

Cell 12

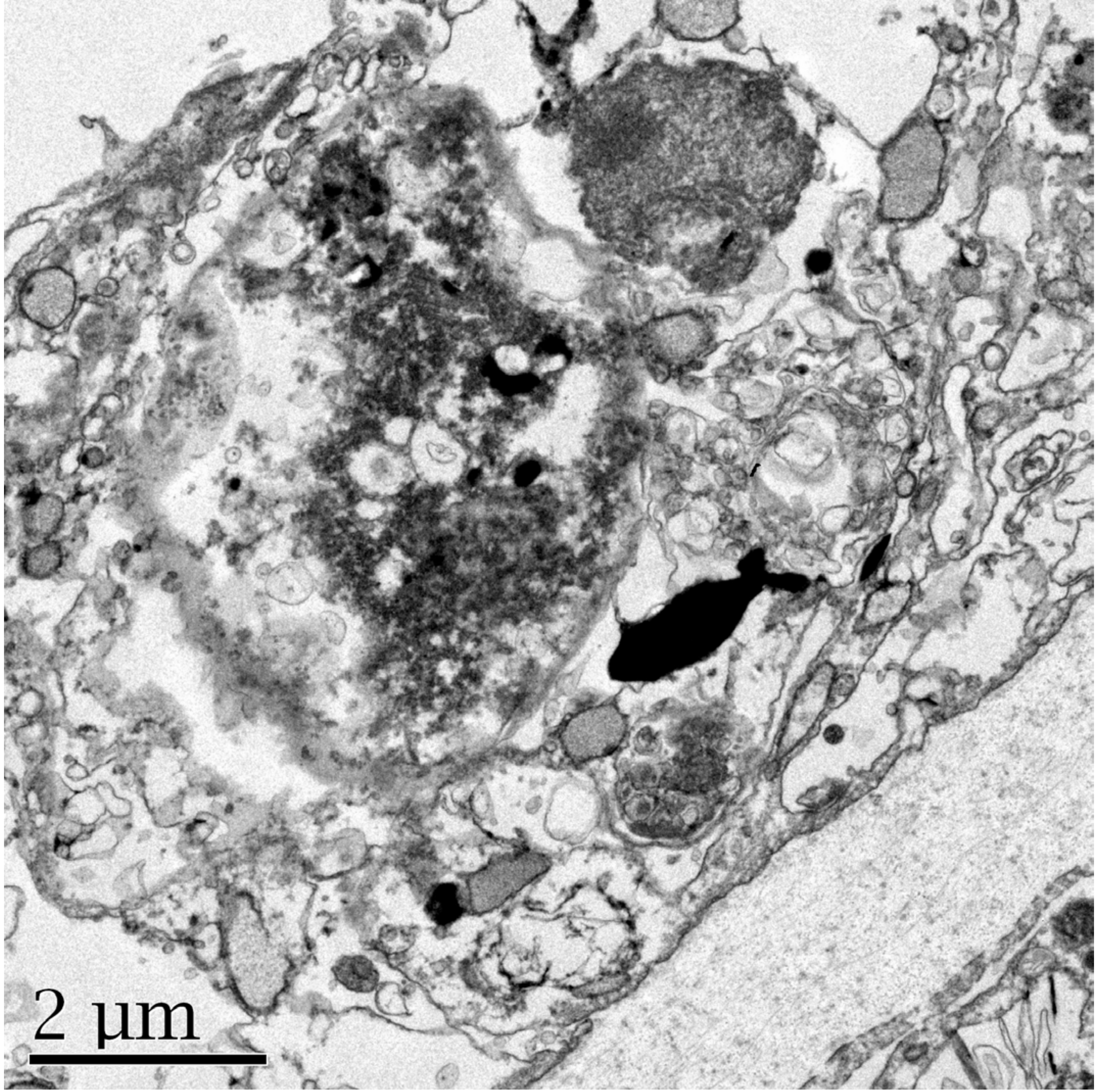

18-14_Correa_JH4_5B1_40.tif

Cell 13

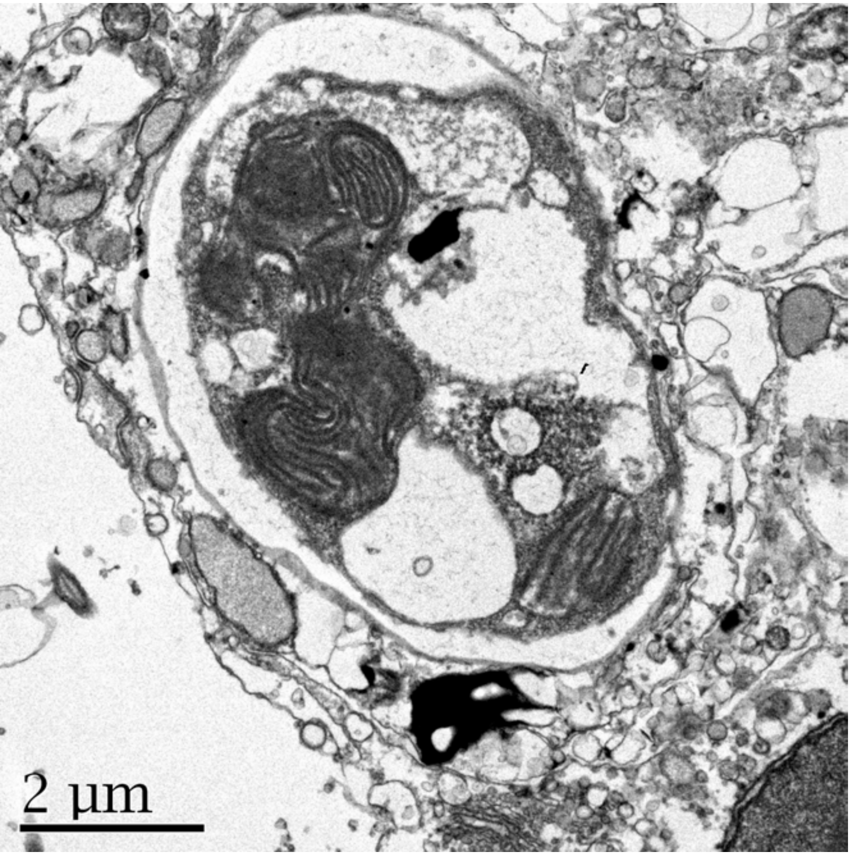

18-14_Correa_JH4_5B1_44.tif

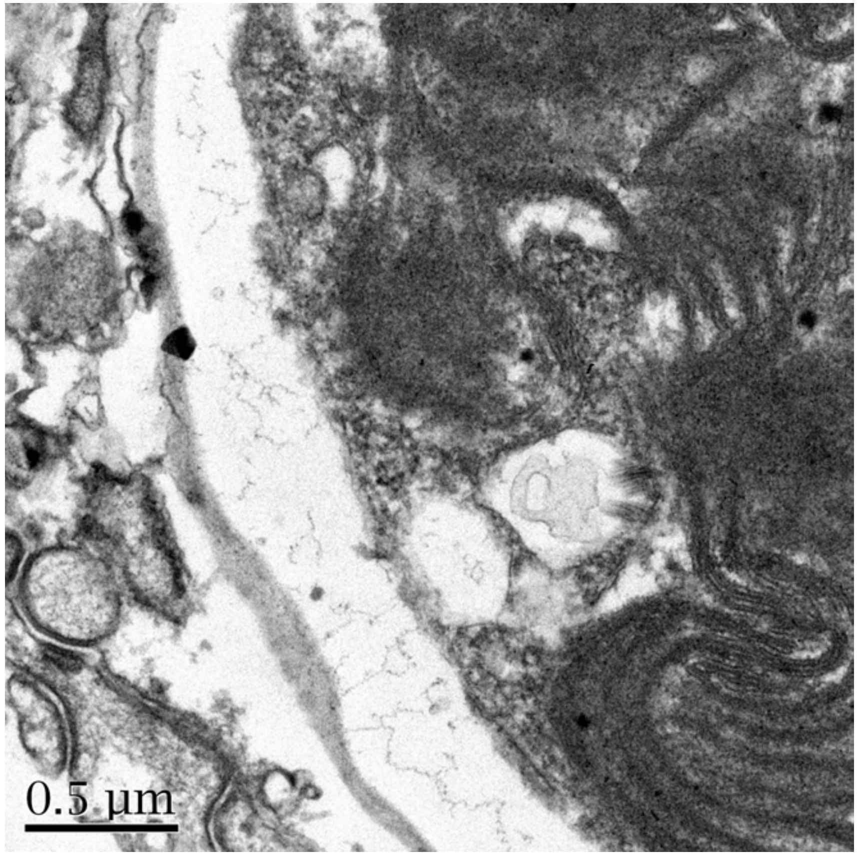

18-14_Correa_JH4_5B1_52.tif

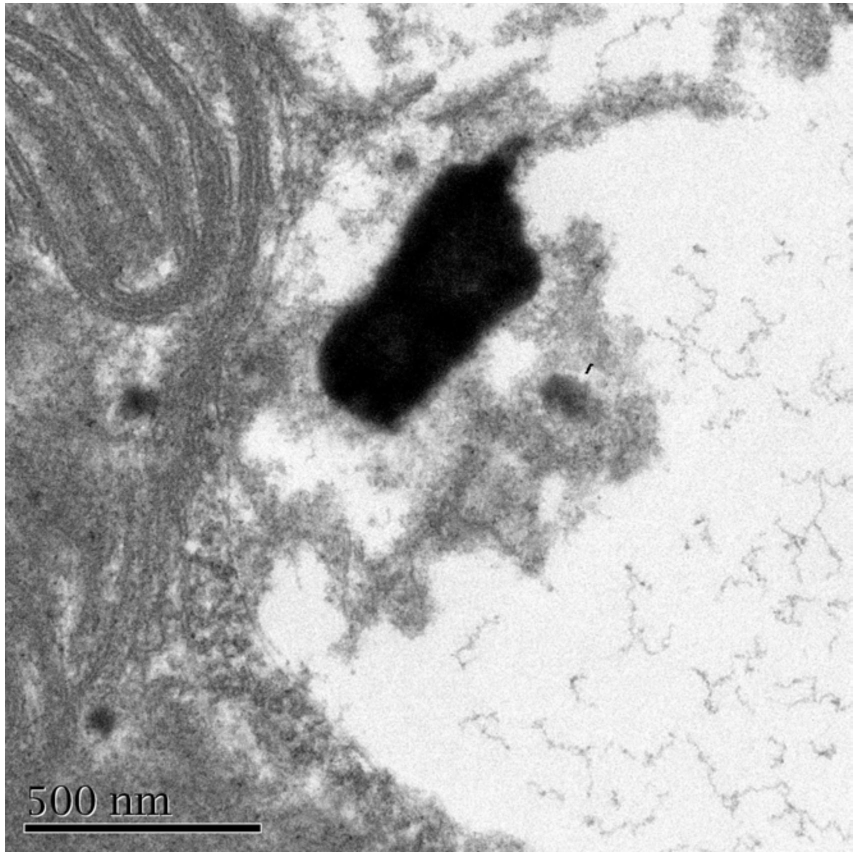

18-14_Correa_JH4_5B1_50.tif

Cell 14

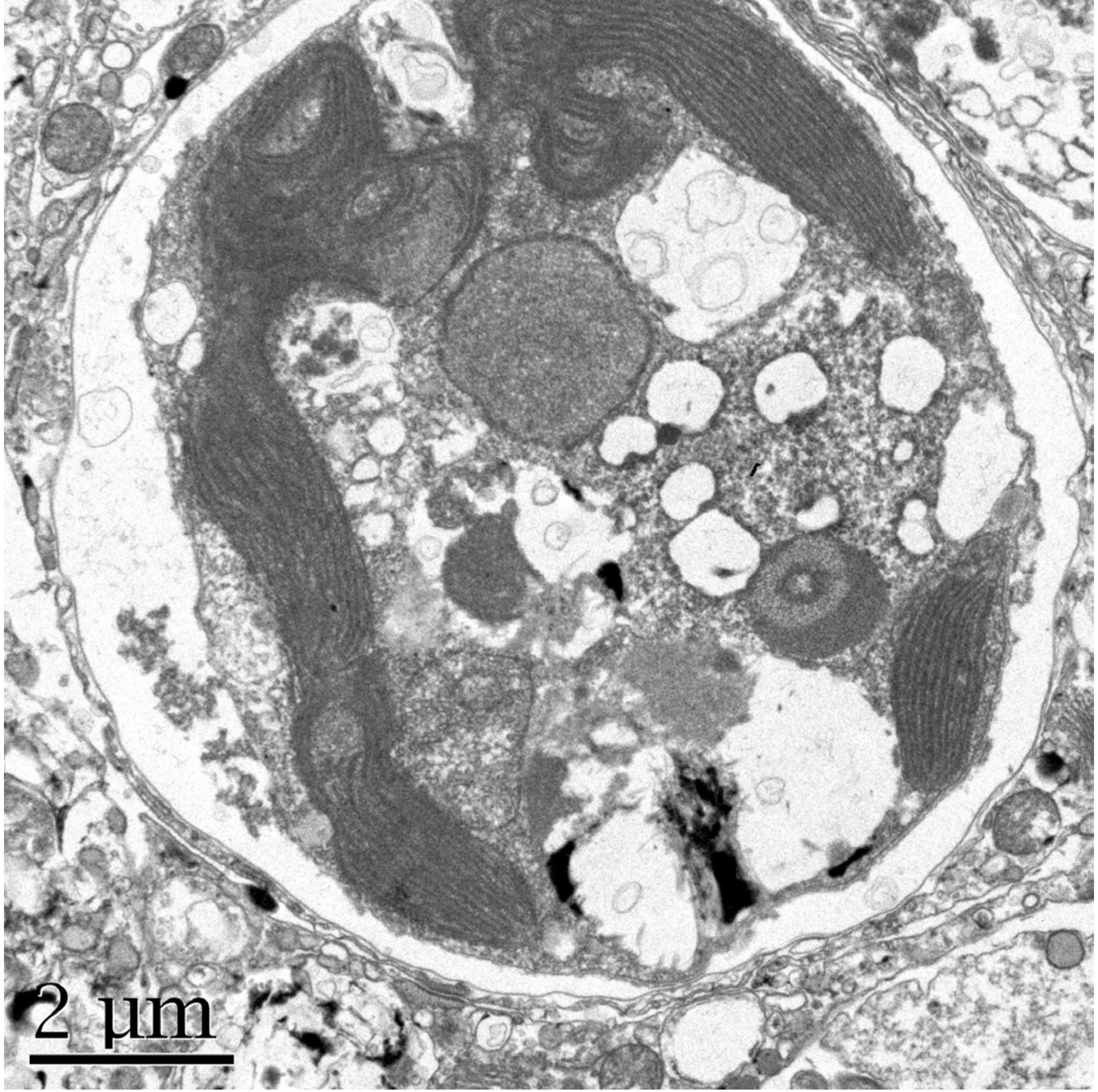

18-14_Correa_JH4_5B1_57.tif

Cell 15

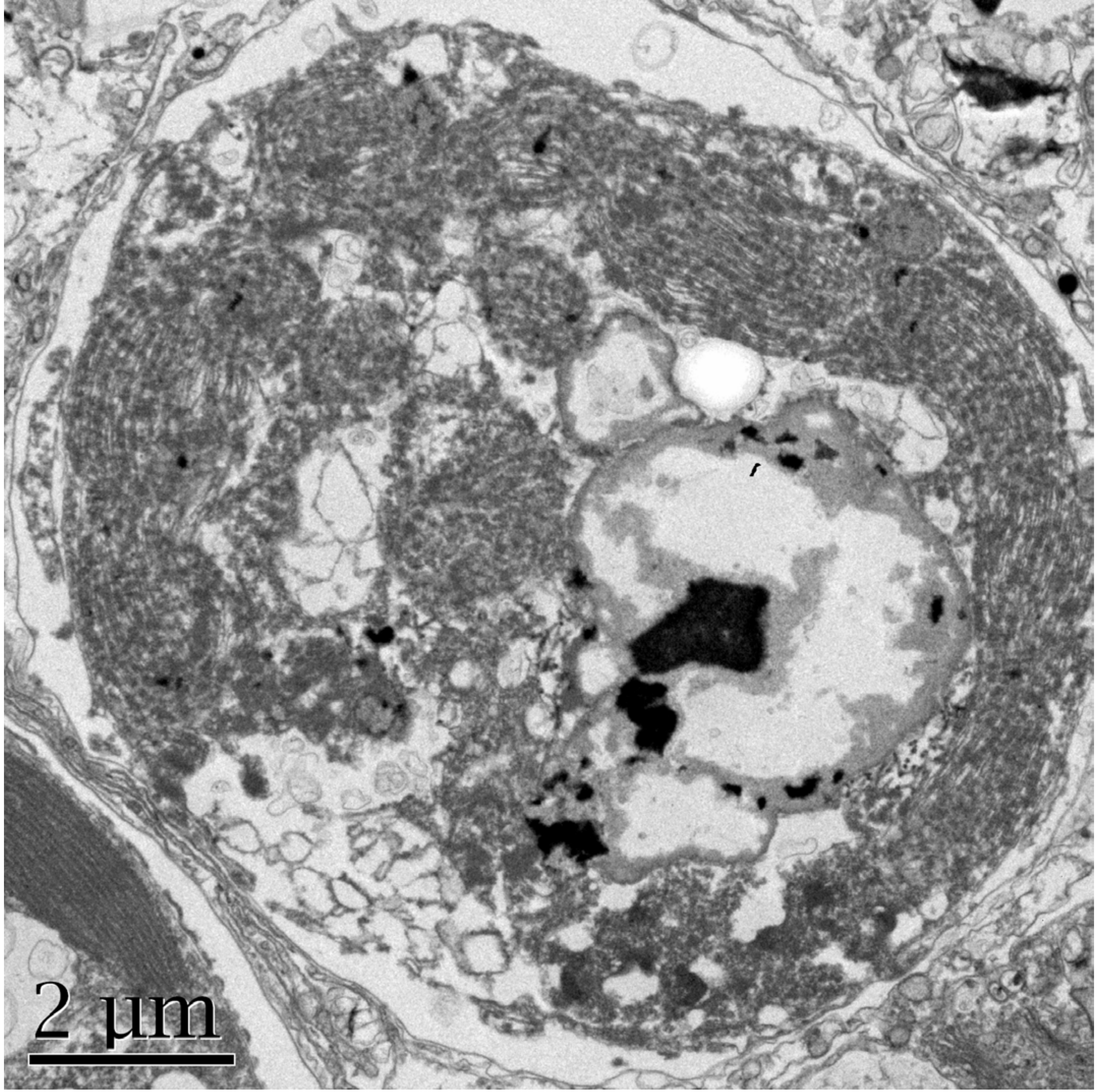

18-14_Correa_JH4_5B1_58.tif

Cell 16

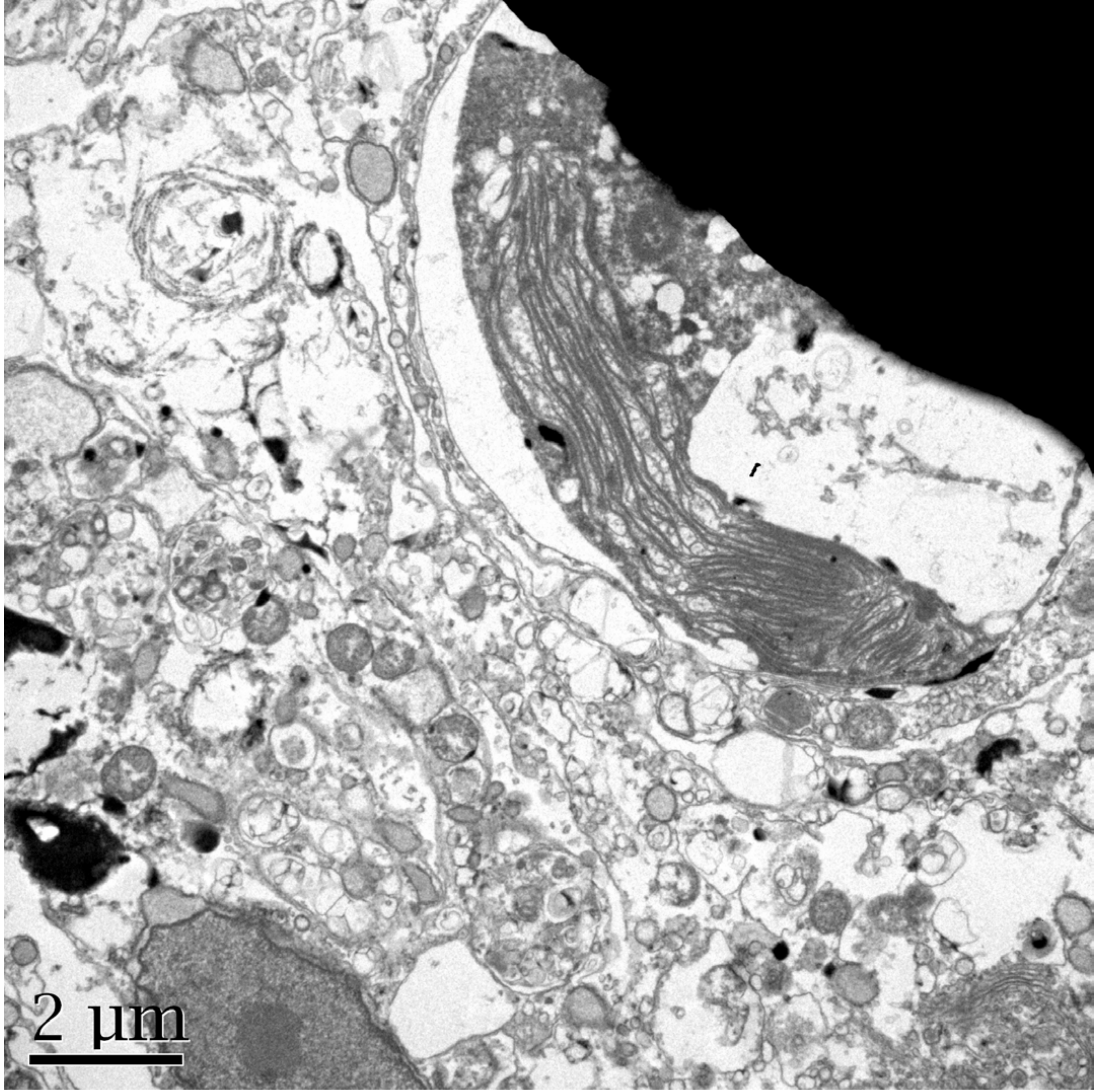

18-14_Correa_JH4_5B1_59.tif

Cell 17

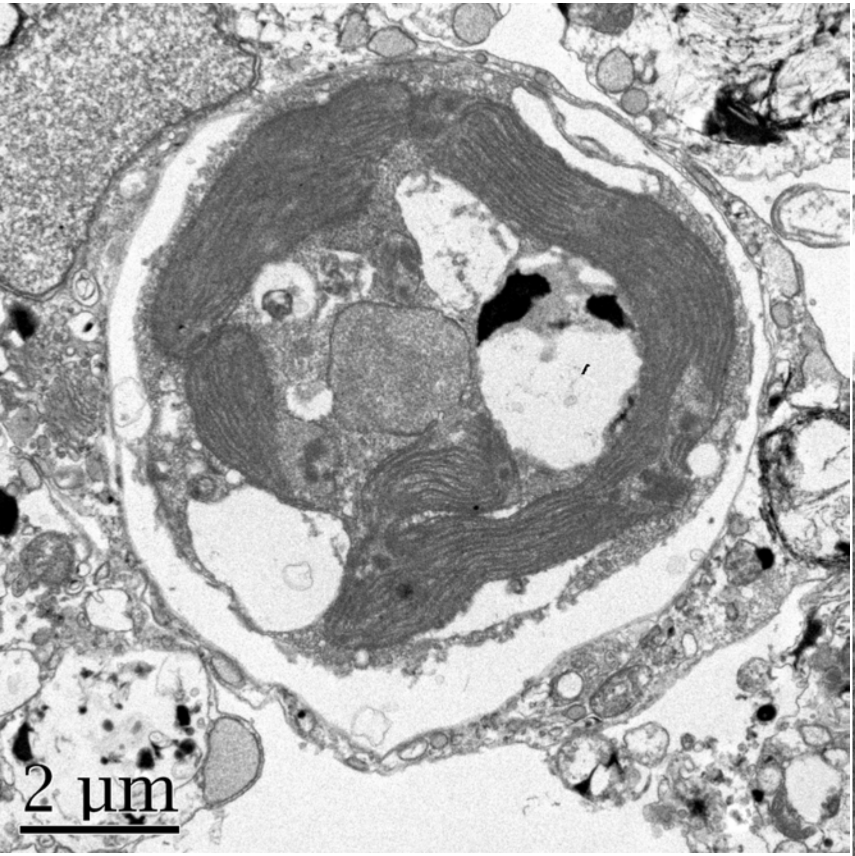

18-14_Correa_JH4_5B1_61.tif

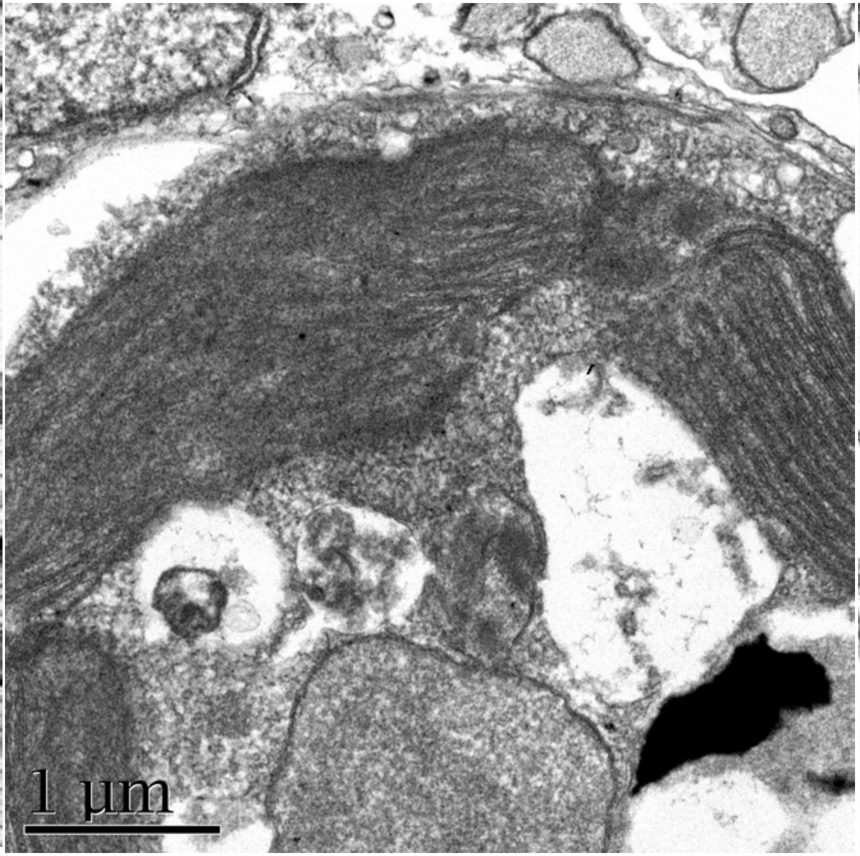

18-14_Correa_JH4_5B1_66.tif

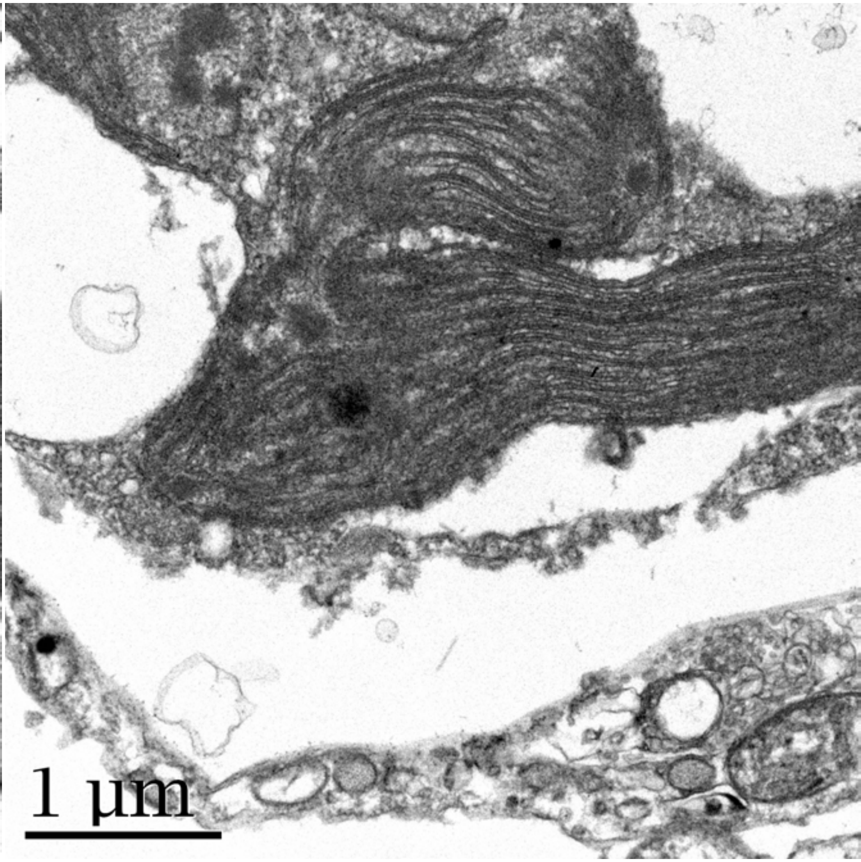

18-14_Correa_JH4_5B1_62.tif

Ambient - ACR Colony B

Cell 1

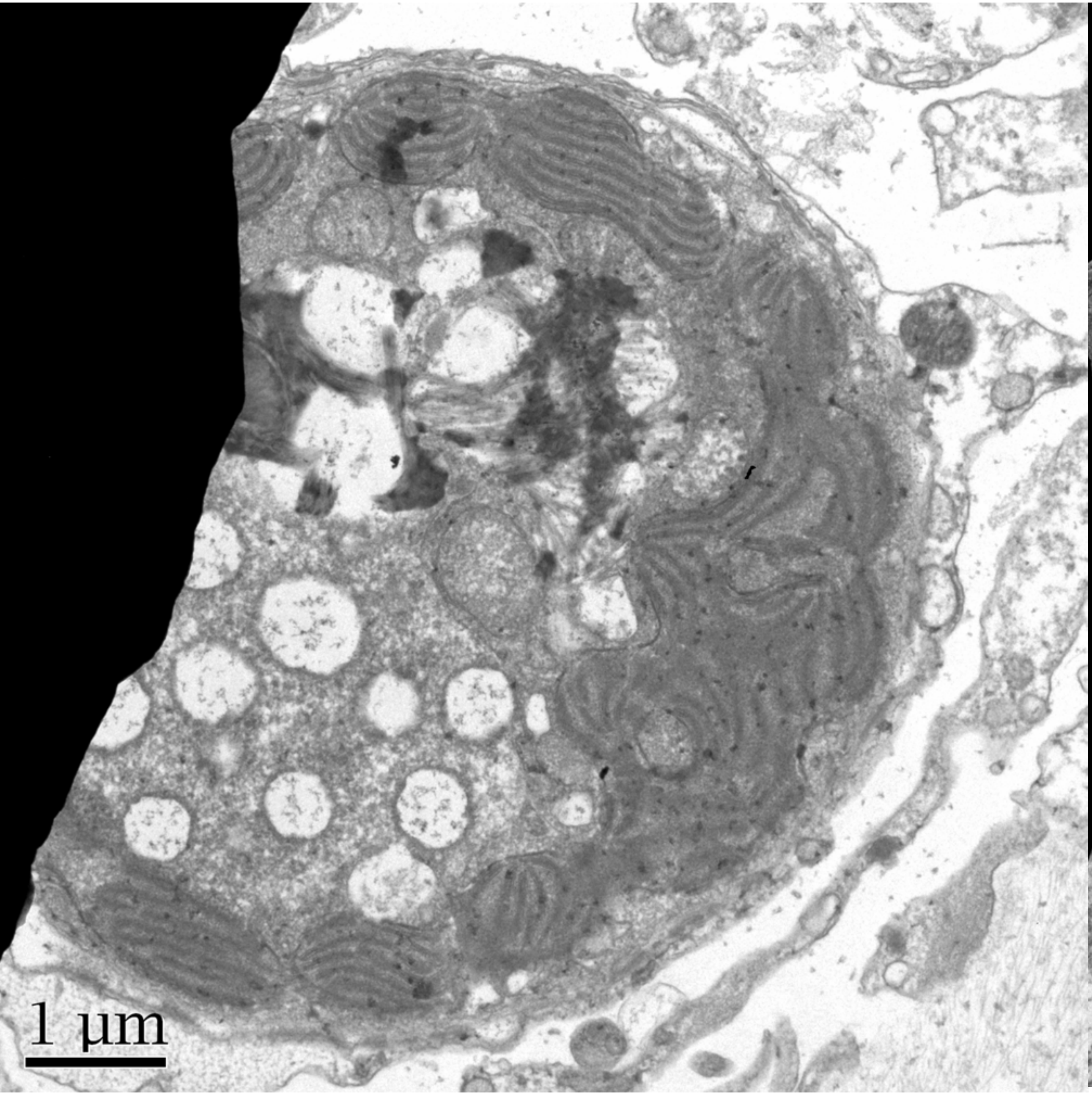

18-14_Correa_KC4_5A5_2.tif

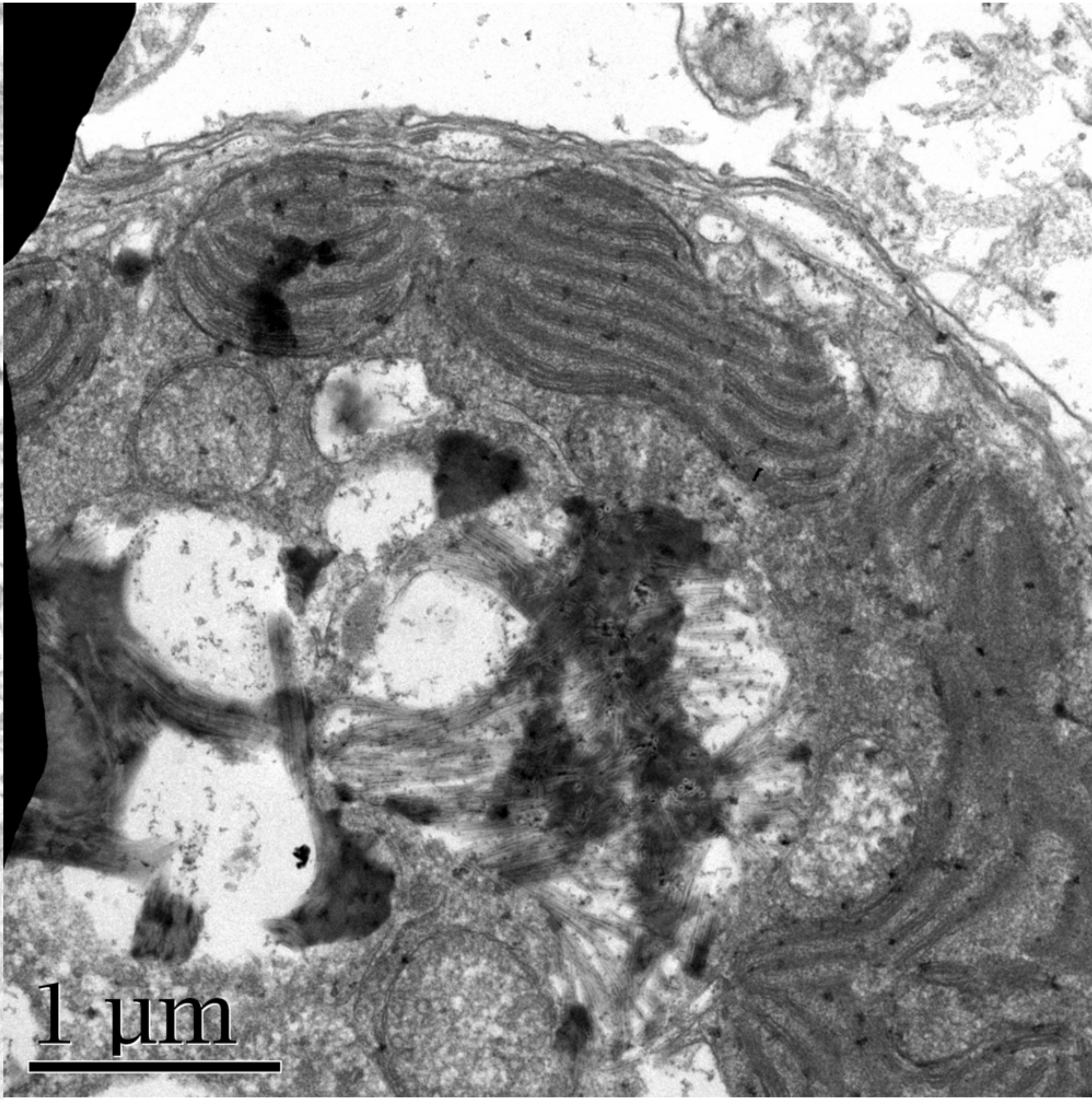

18-14_Correa_KC4_5A5_6.tif

Cell 2

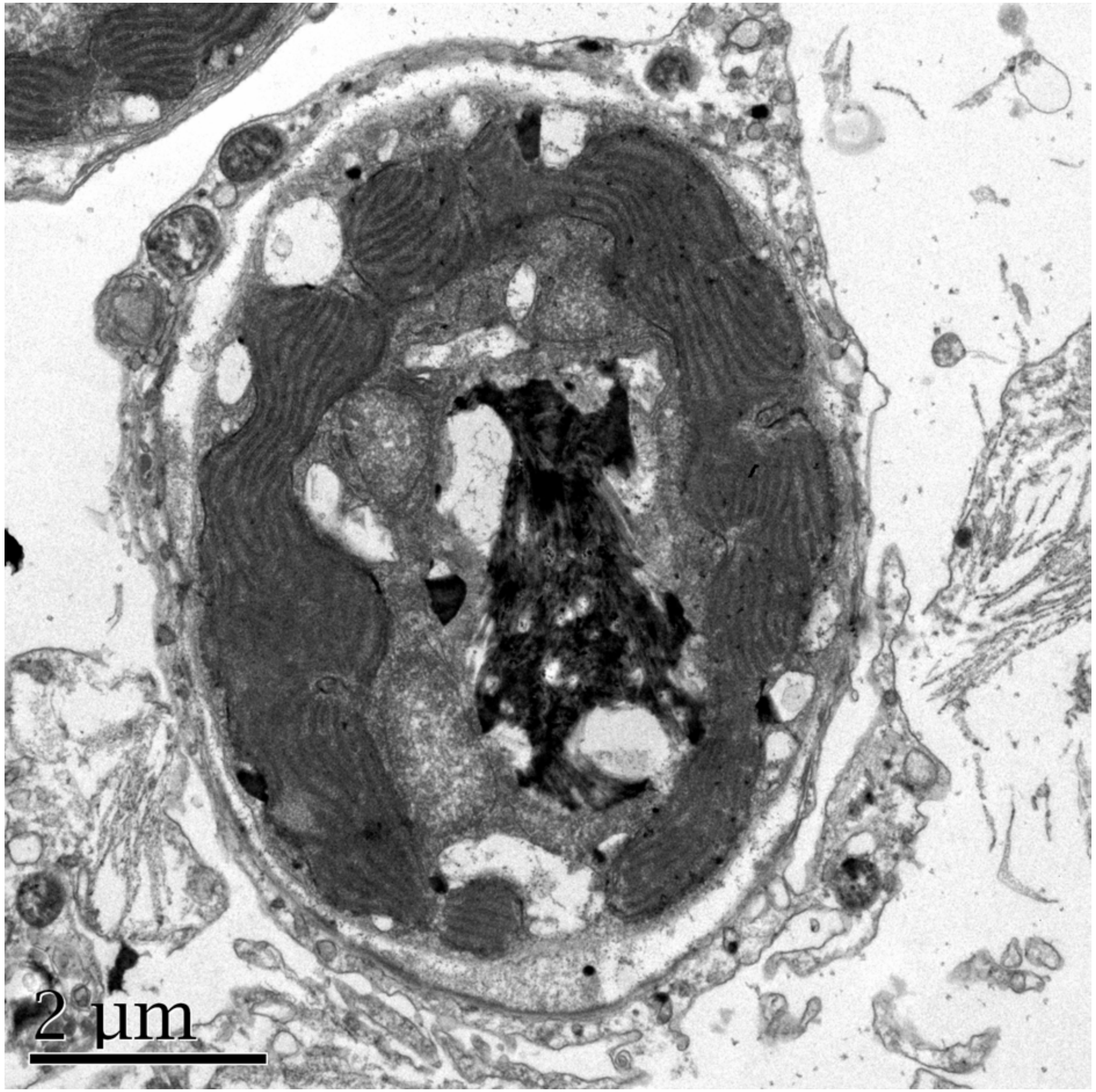

18-14_Correa_KC4_5A5_9.tif

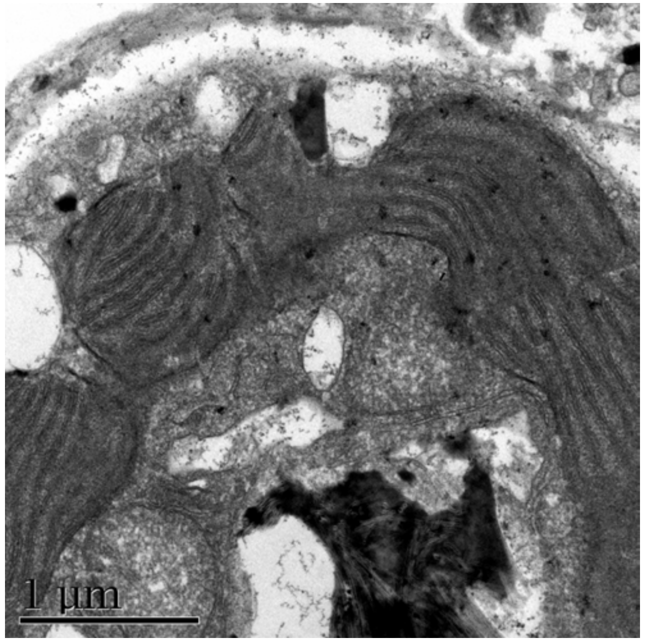

18-14_Correa_KC4_5A5_14.tif

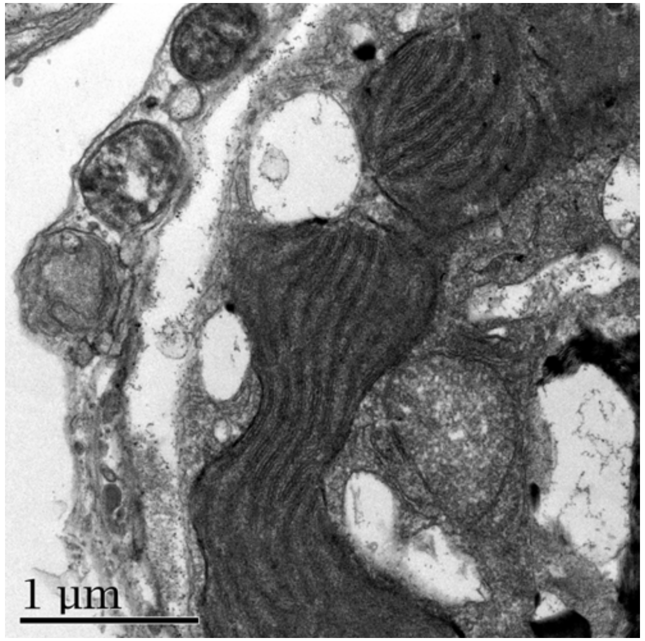

18-14_Correa_KC4_5A5_12.tif

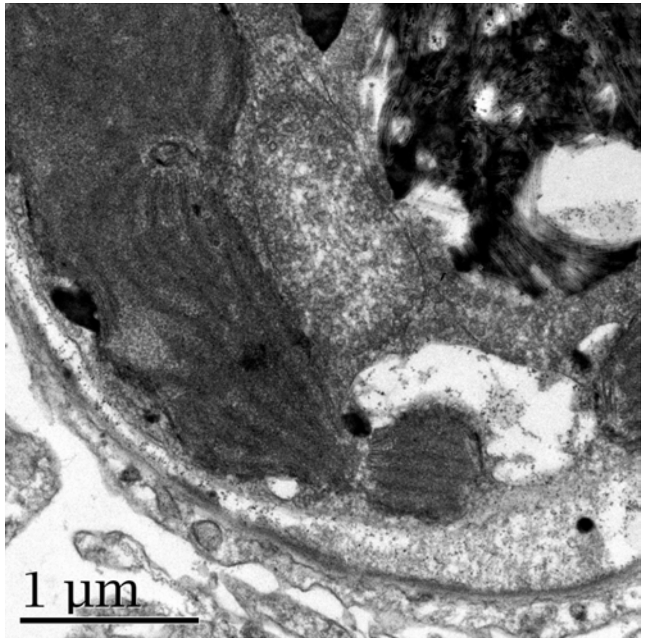

18-14_Correa_KC4_5A5_16.tif

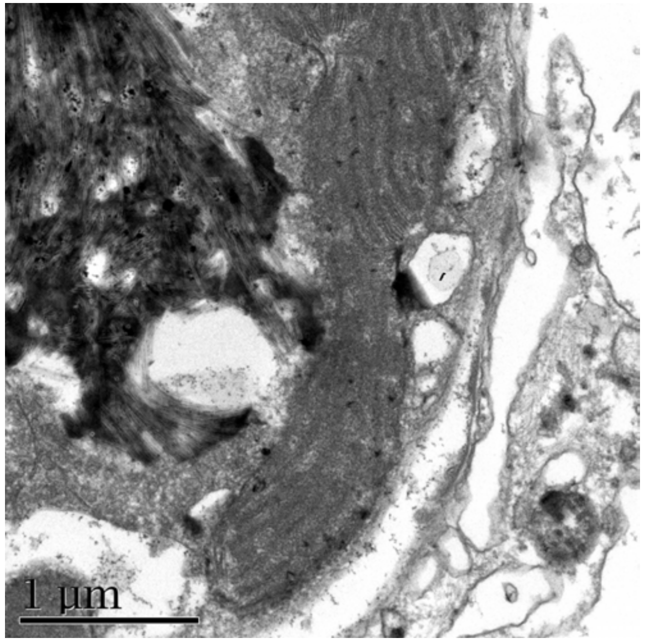

18-14_Correa_KC4_5A5_15.tif

Cell 3

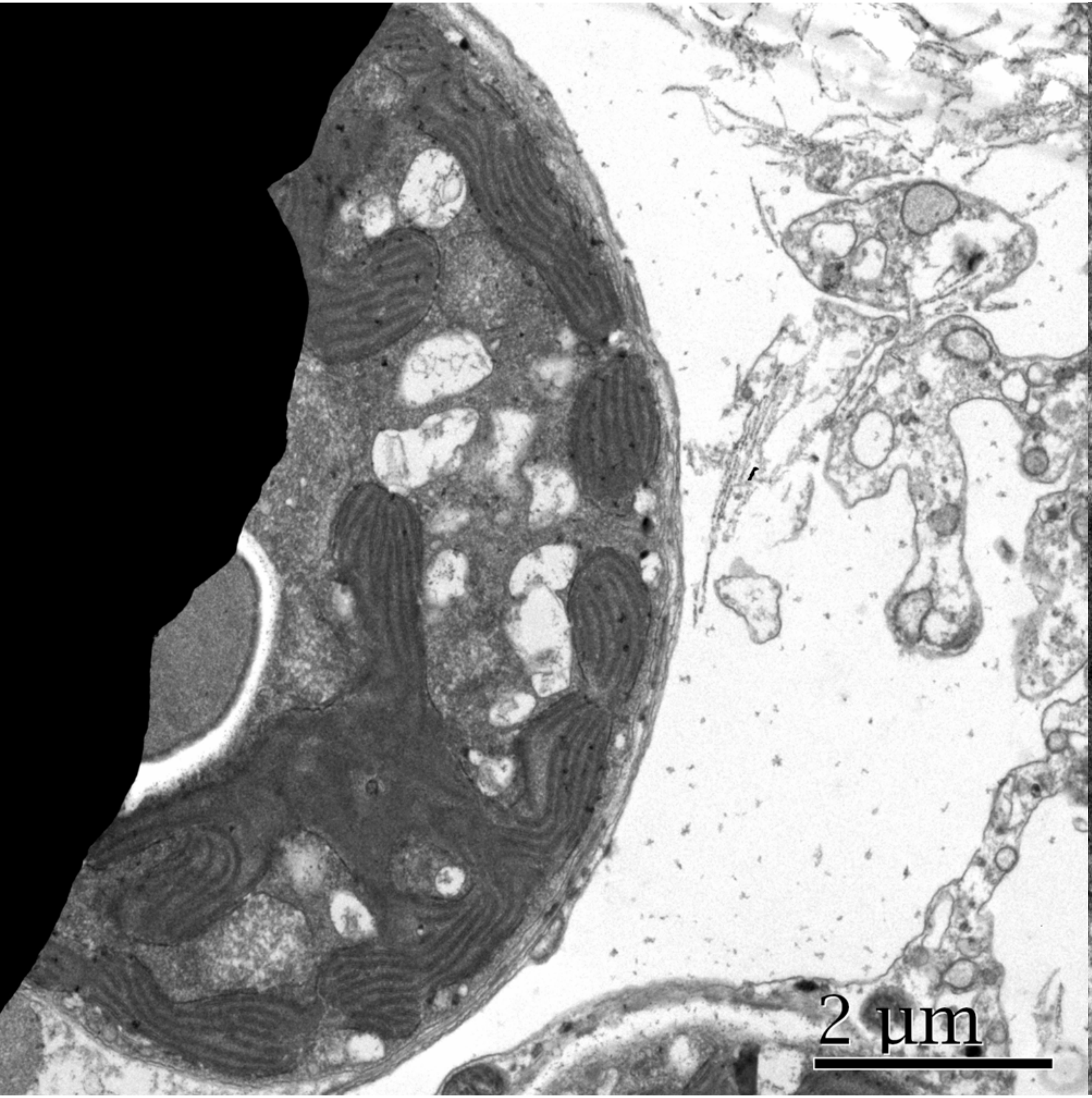

18-14_Correa_KC4_5A5_17.tif

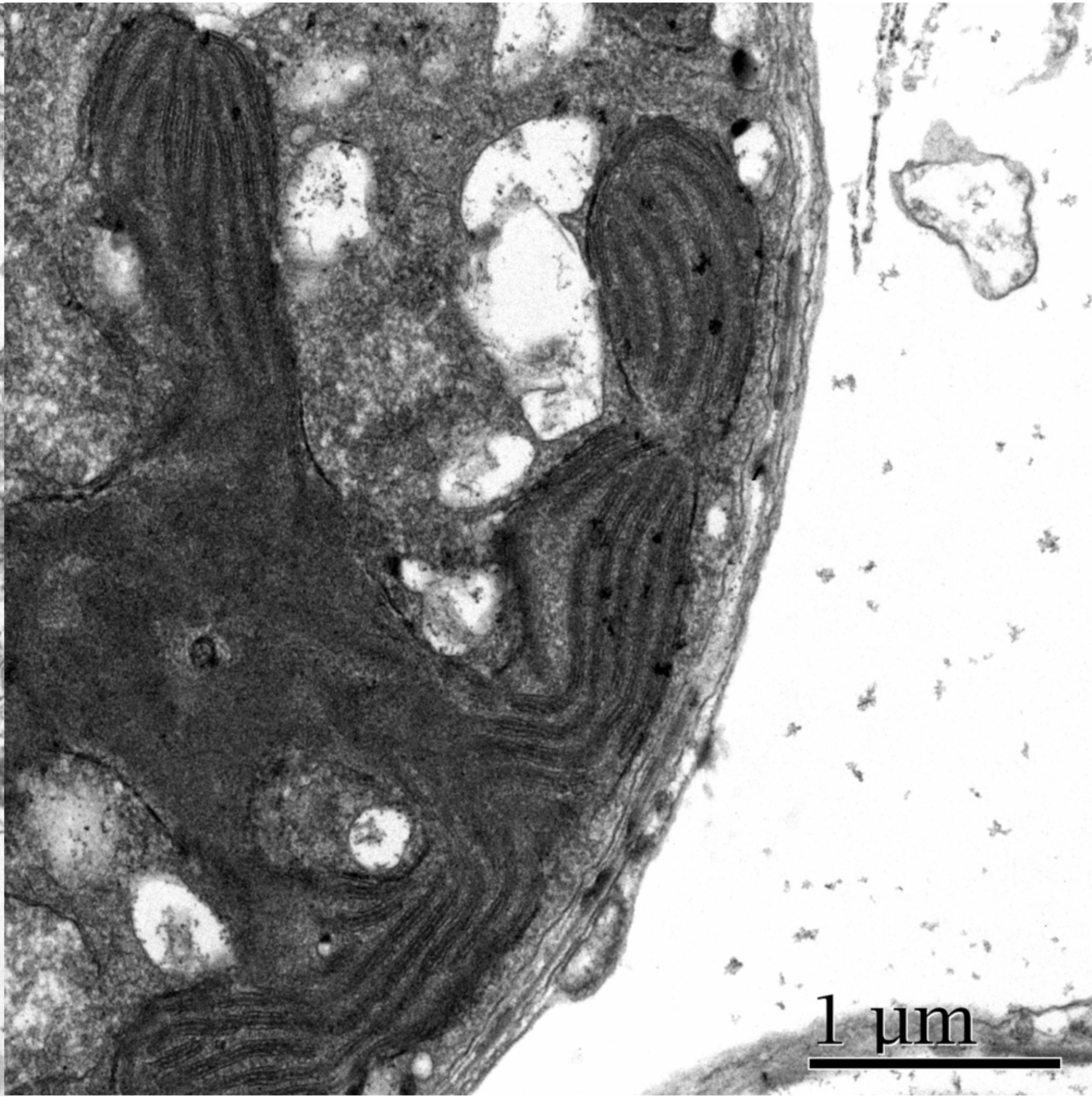

18-14_Correa_KC4_5A5_19.tif

Cell 4

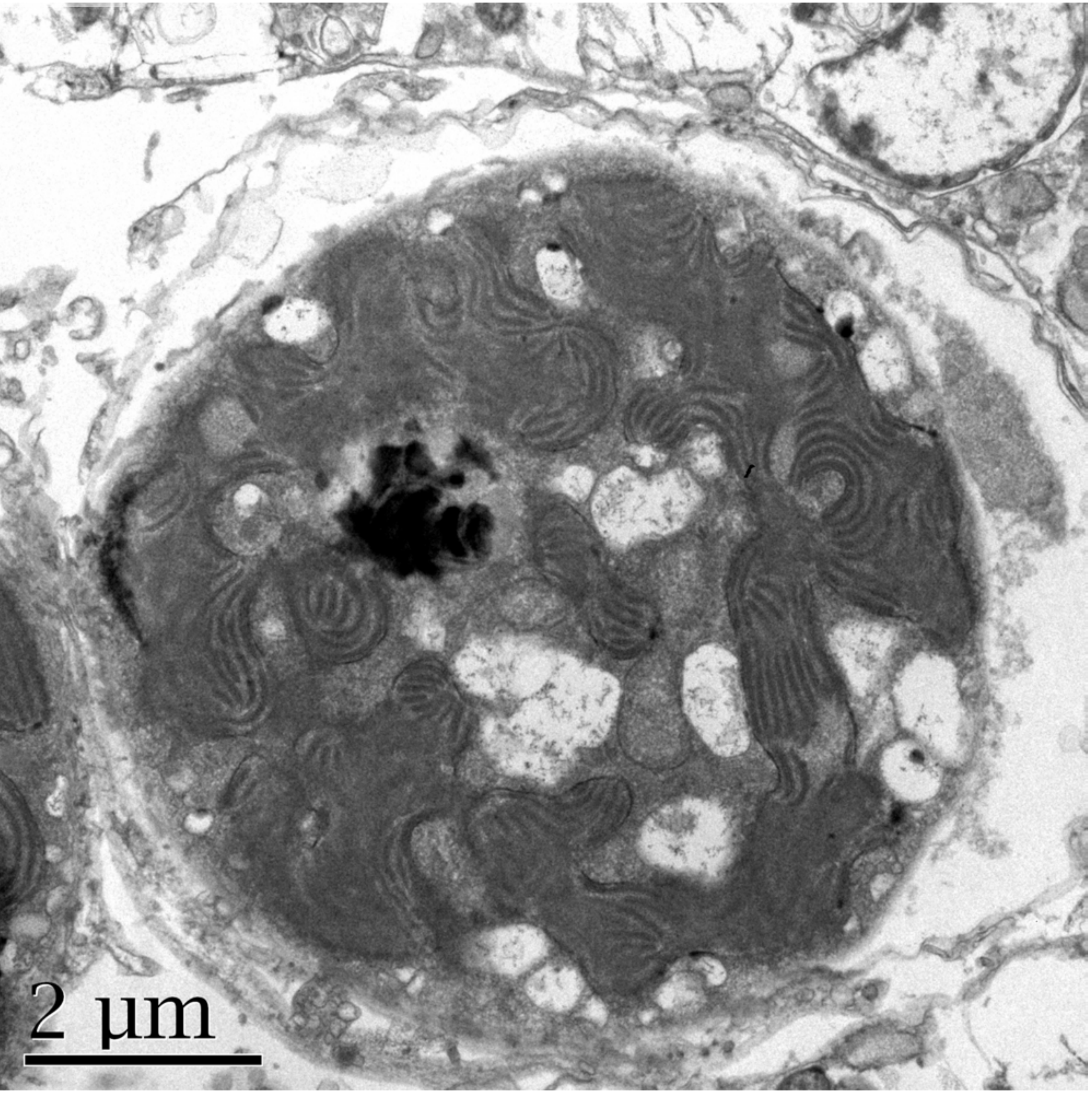

18-14_Correa_KC4_5A6_2.tif

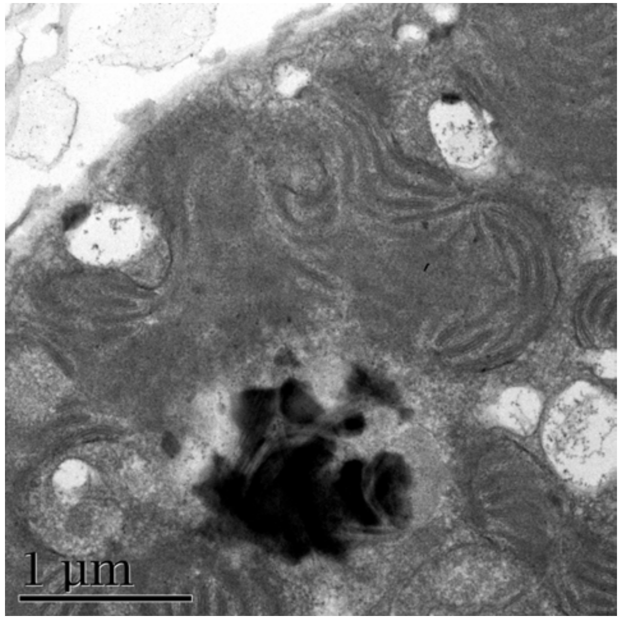

18-14_Correa_KC4_5A6_5.tif

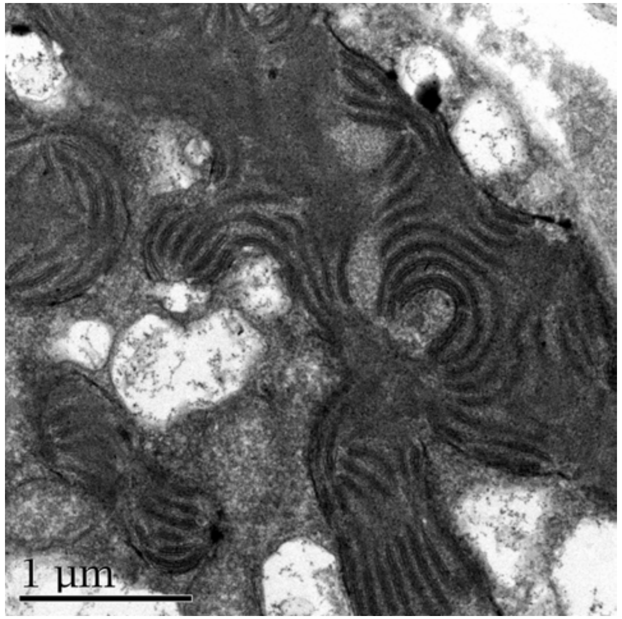

18-14_Correa_KC4_5A6_4.tif

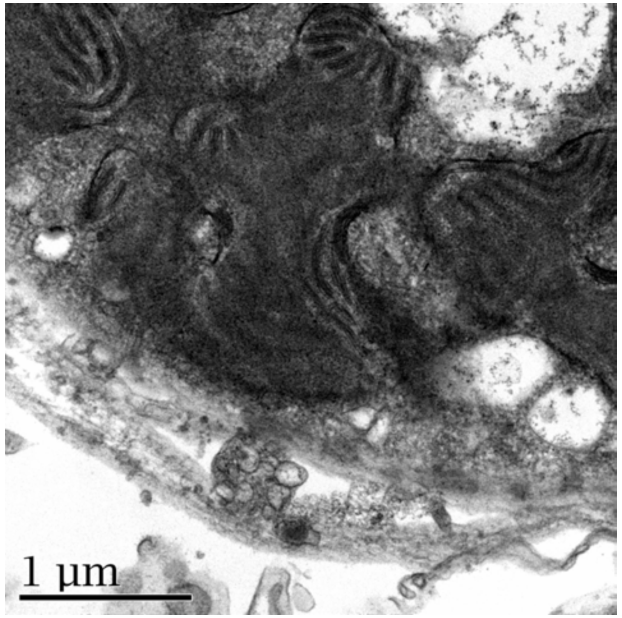

18-14_Correa_KC4_5A6_6.tif

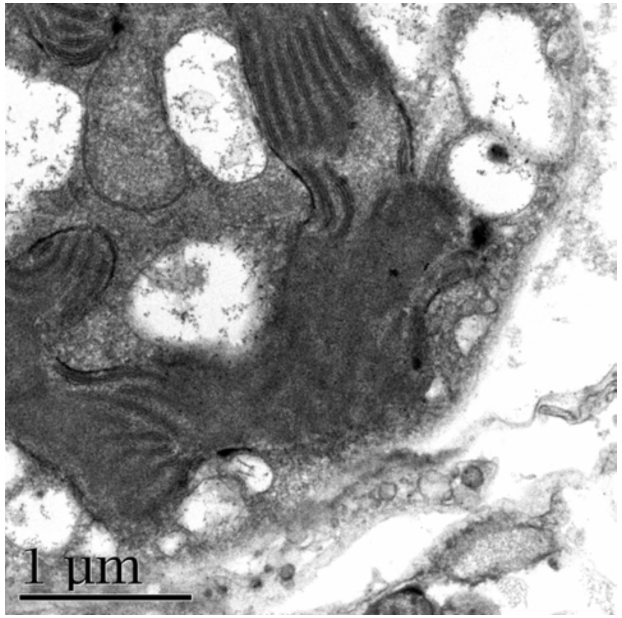

18-14_Correa_KC4_5A6_3.tif

Cell 5

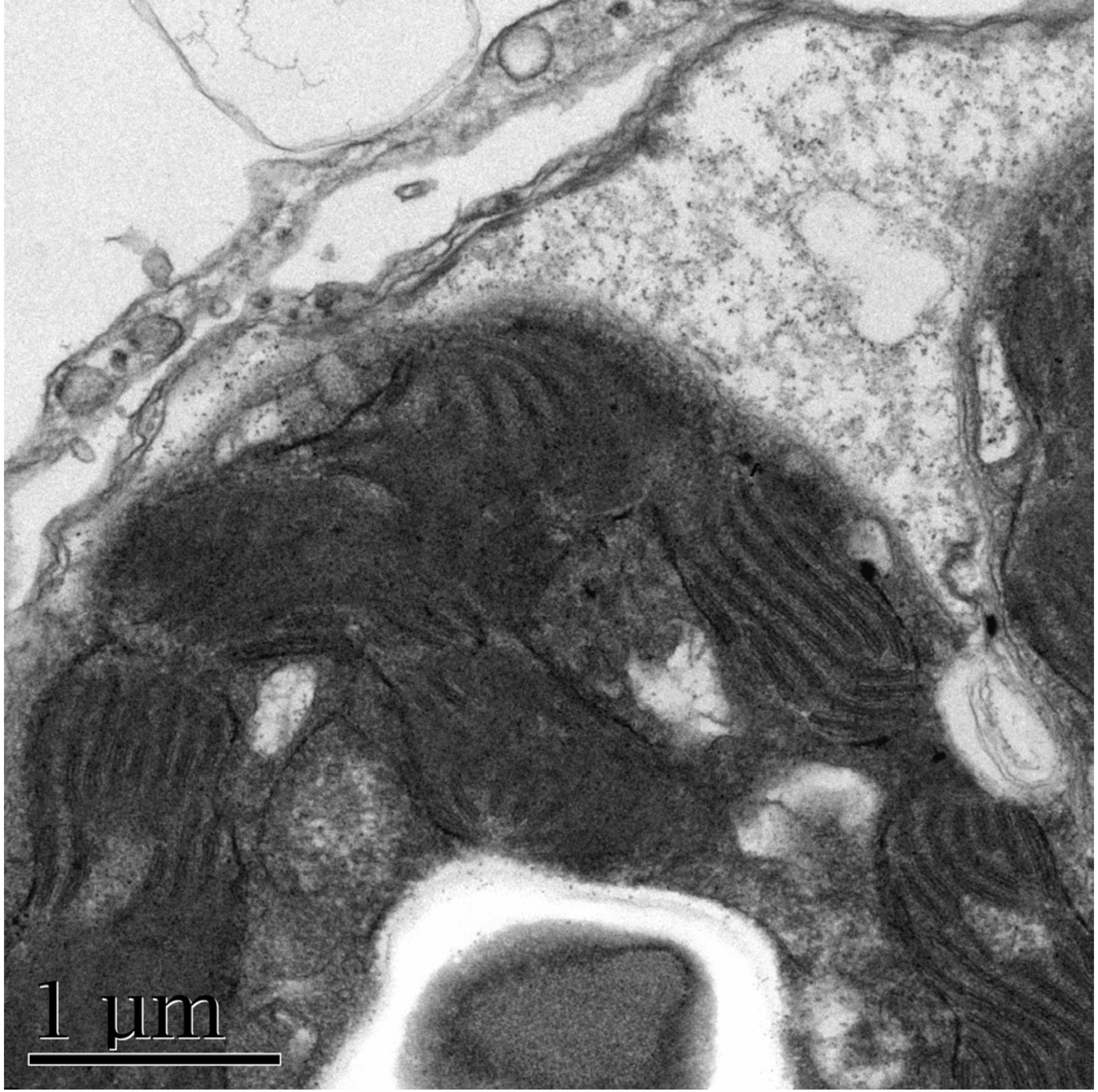

18-14_Correa_KC4_5A6_8.tif

Cell 6

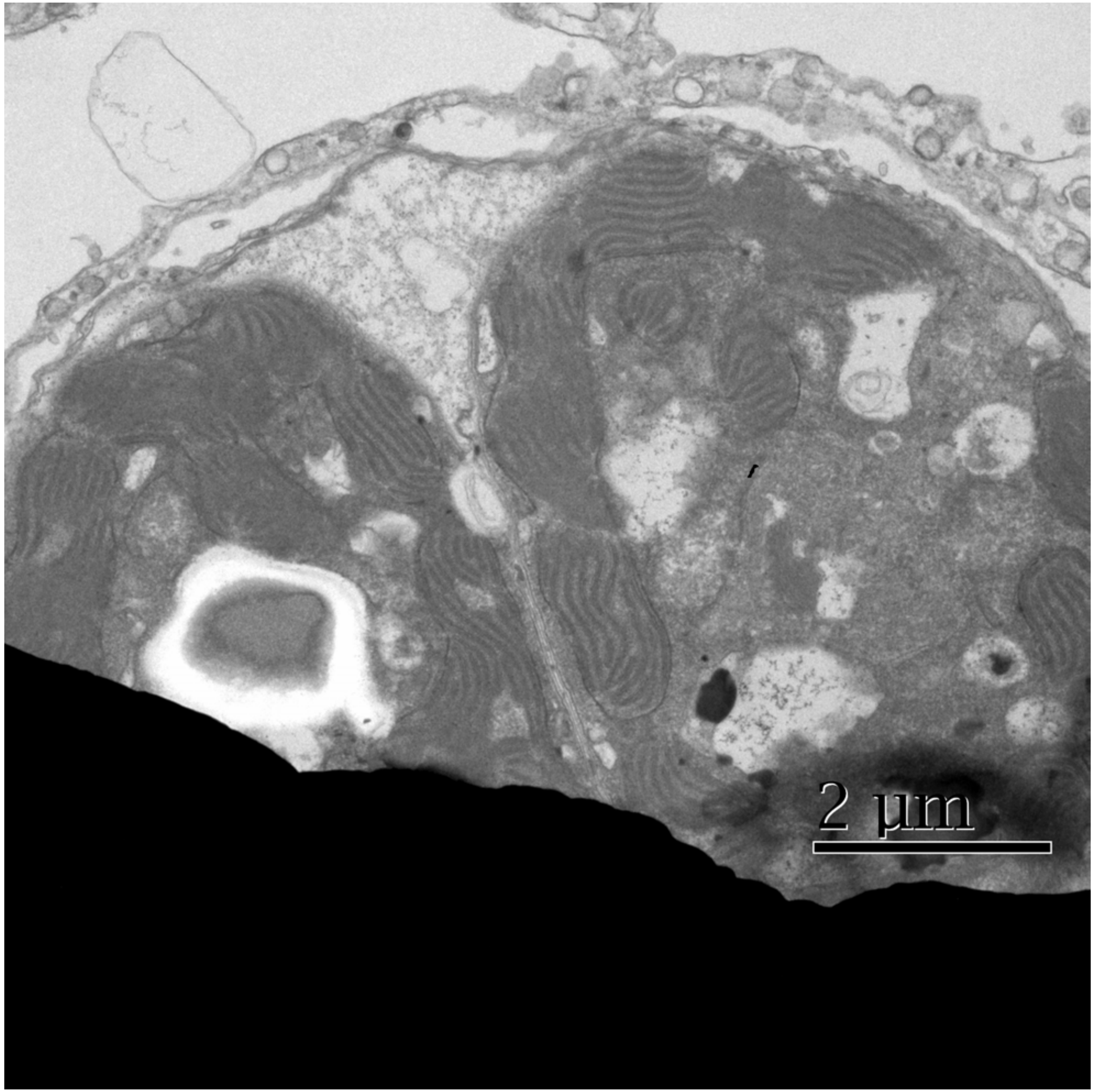

18-14_Correa_KC4_5A6_9.tif

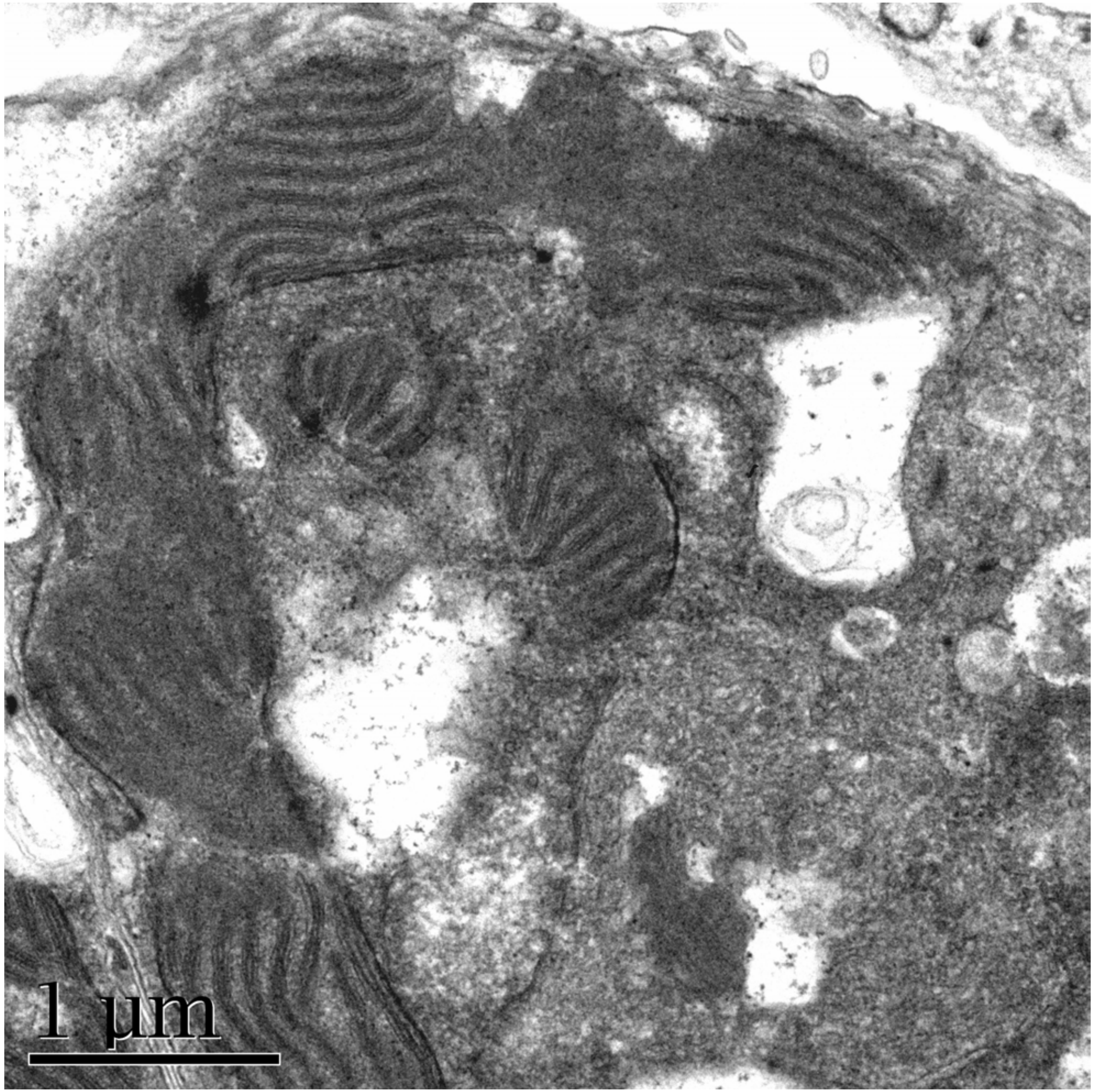

18-14_Correa_KC4_5A6_9.tif

Cell 7

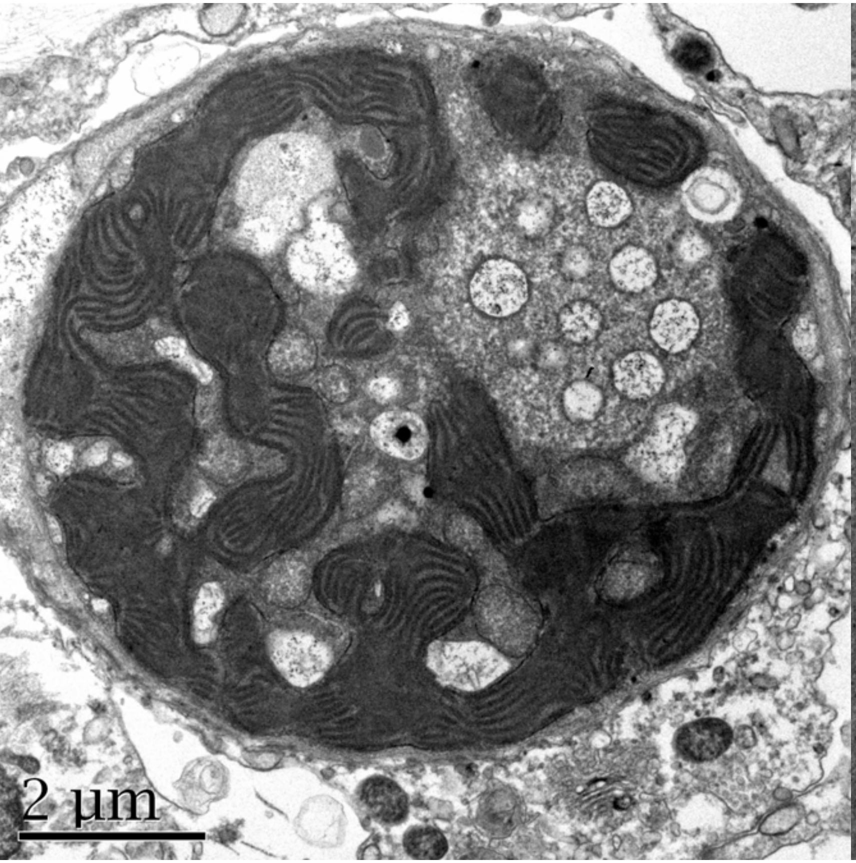

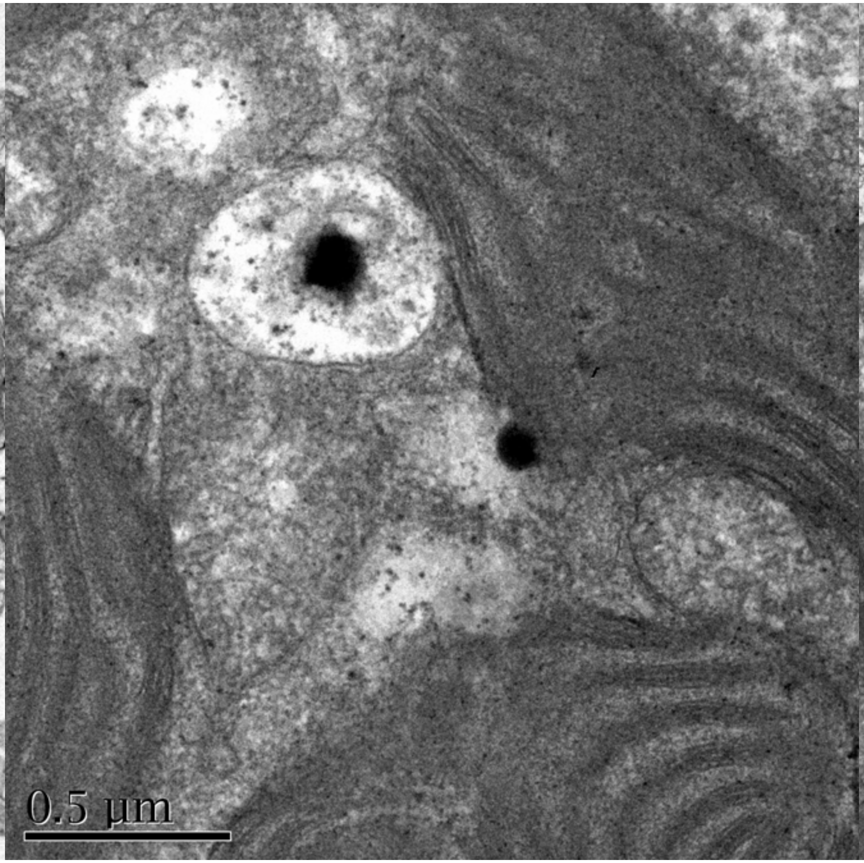

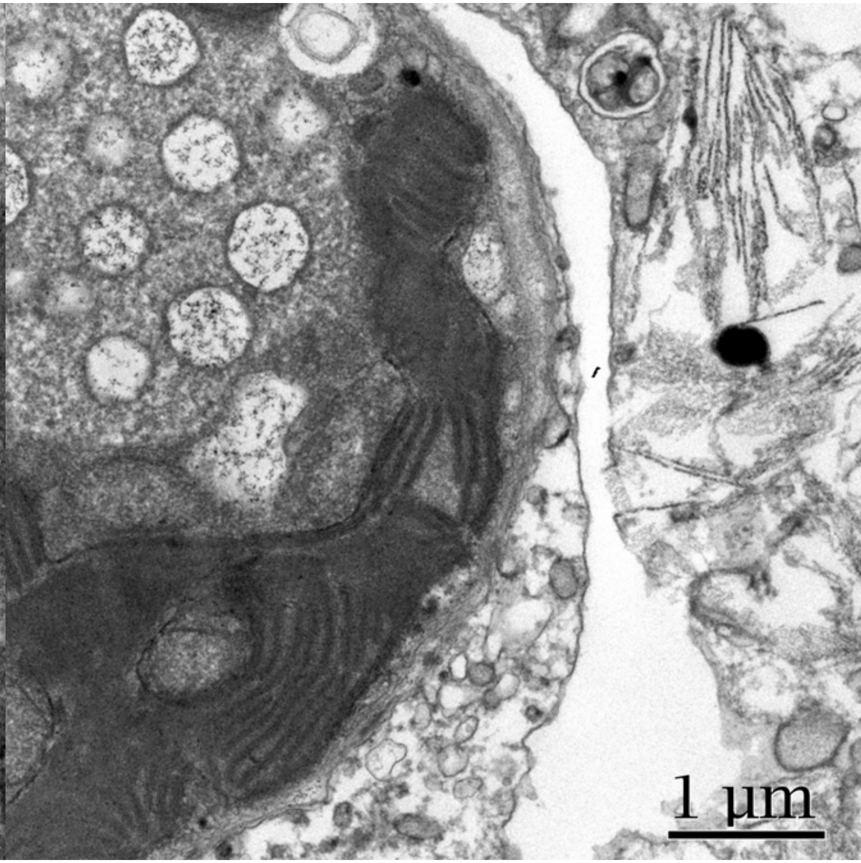

18-14_Correa_KC4_5A6_13.tif

18-14_Correa_KC4_5A6_13.tif

18-14_Correa_KC4_5A6_14.tif

Cell 8

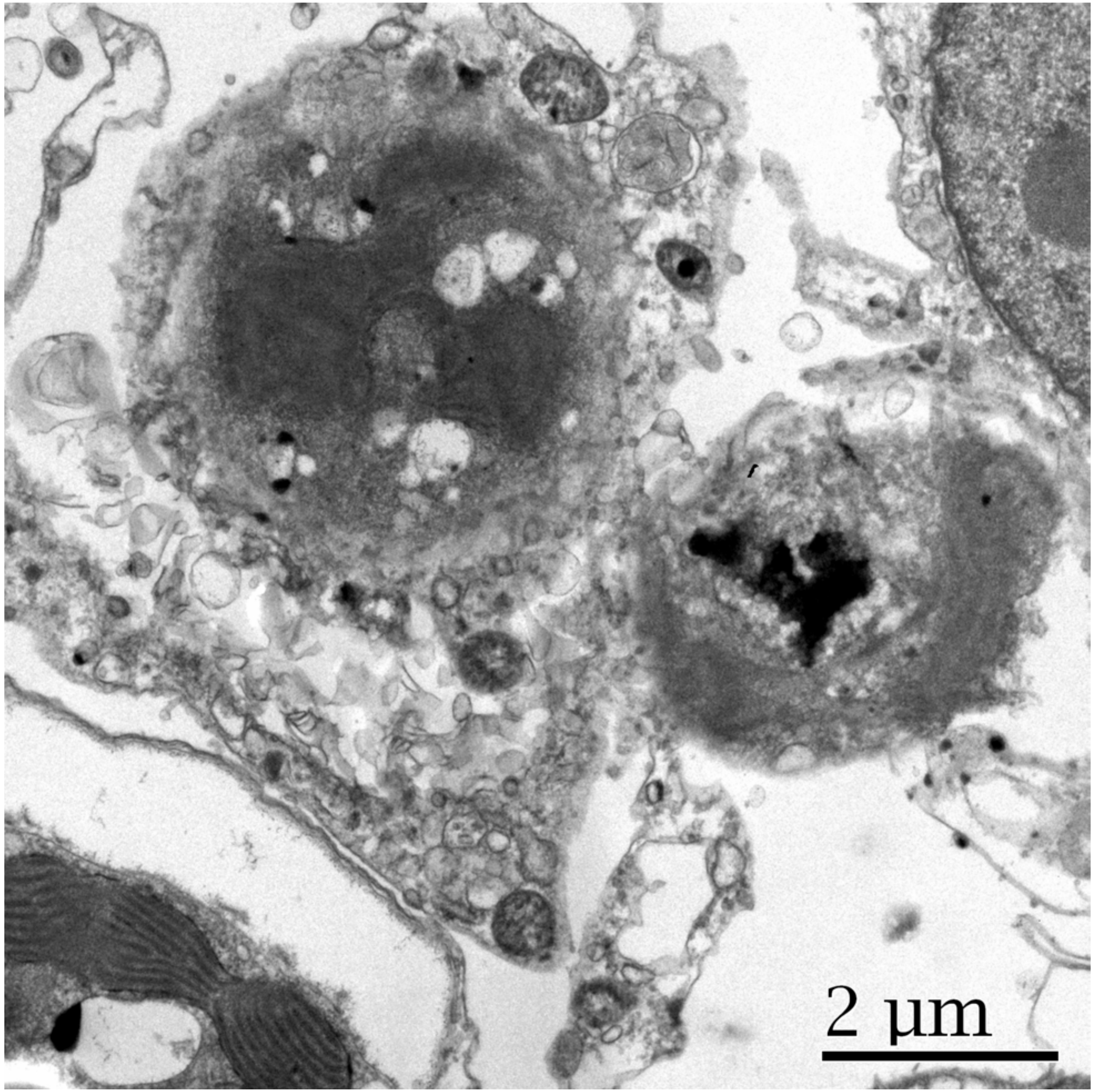

18-14_Correa_KC4_5A6_17.tif

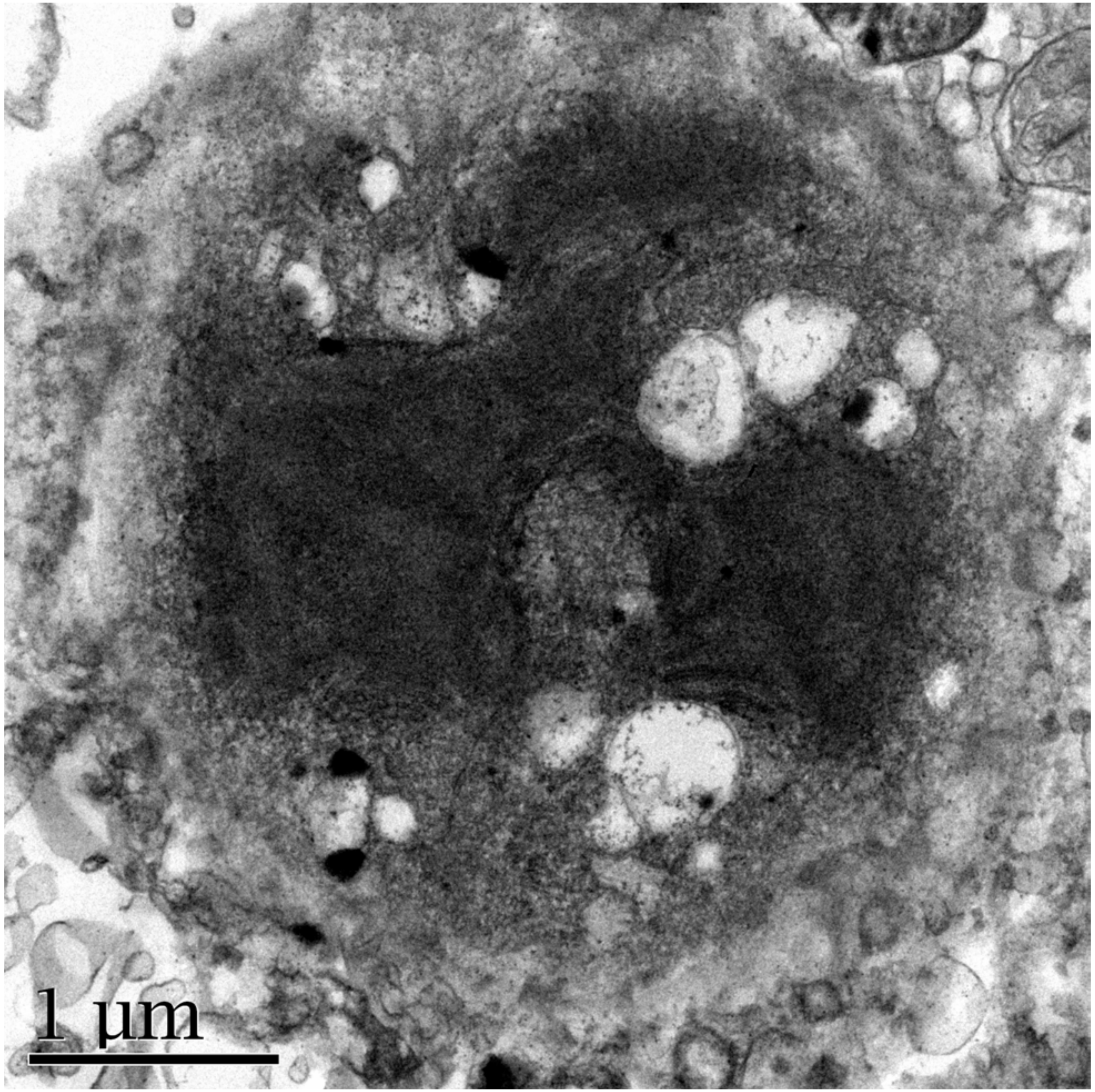

18-14_Correa_KC4_5A6_18.tif

Cell 9

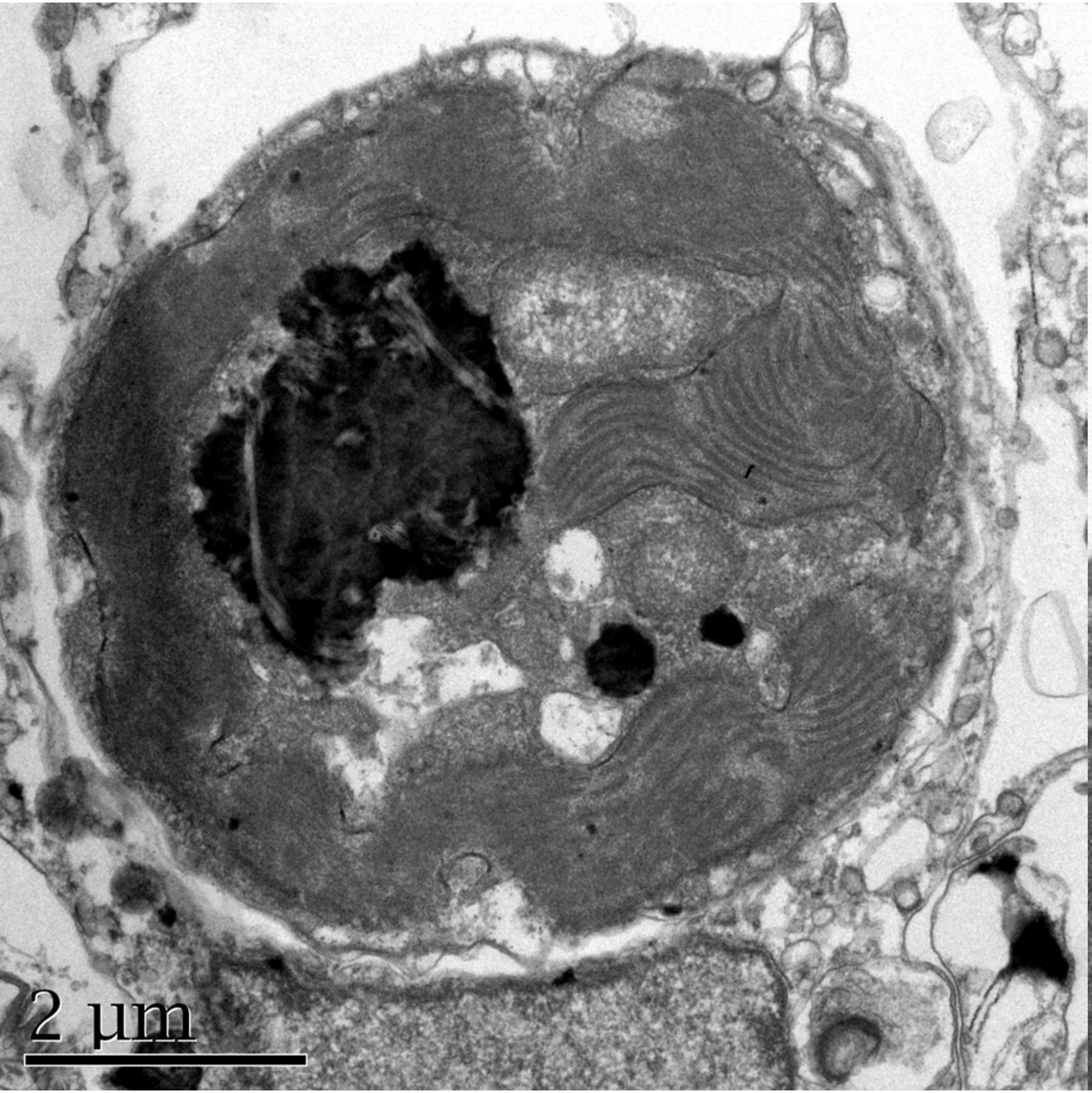

18-14_Correa_KC4_5A6_20.tif

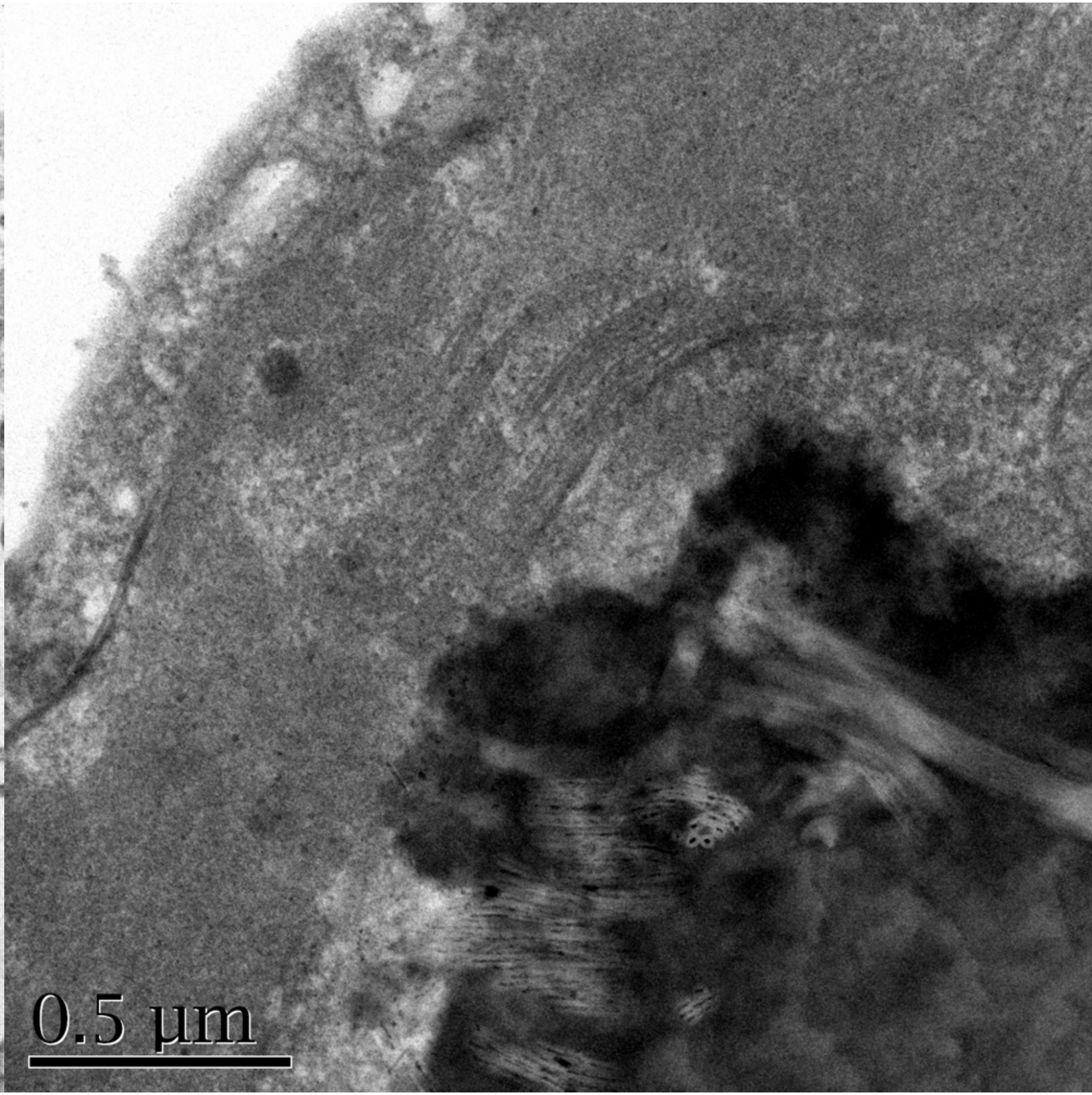

18-14_Correa_KC4_5A6_24.tif

Cell 10

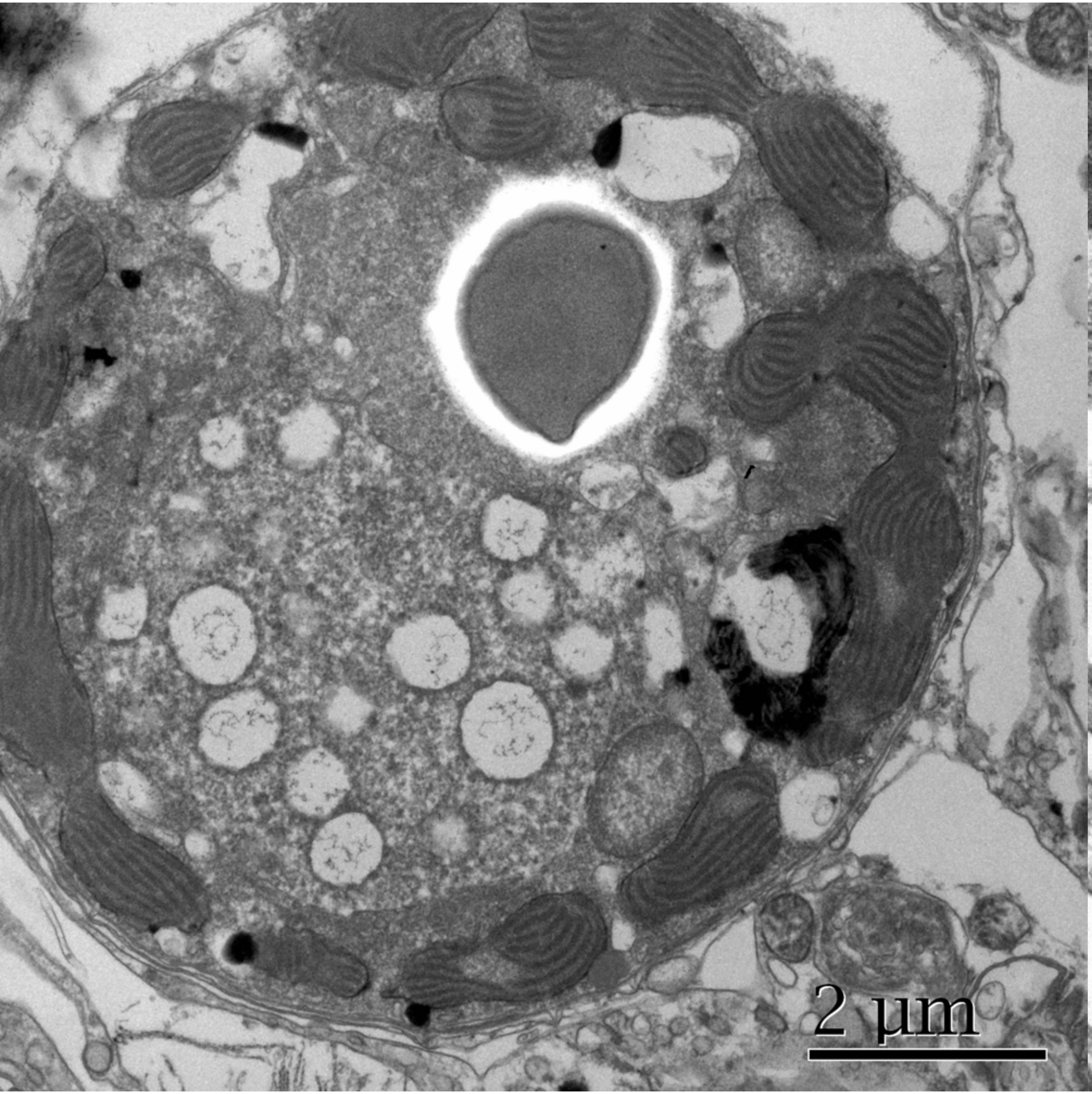

18-14_Correa_KC4_5A6_27.tif

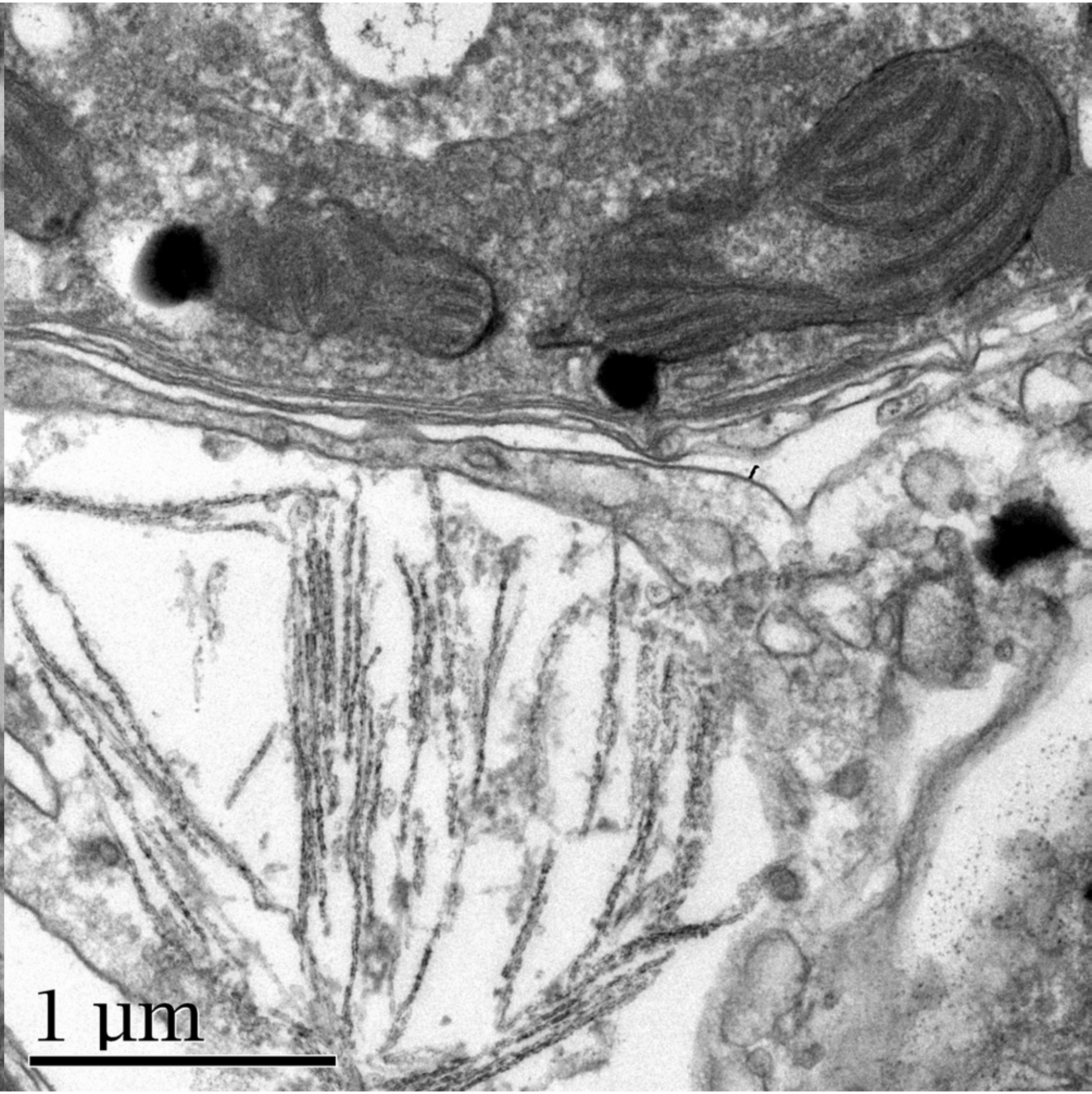

18-14_Correa_KC4_5A6_28.tif

Cell 11

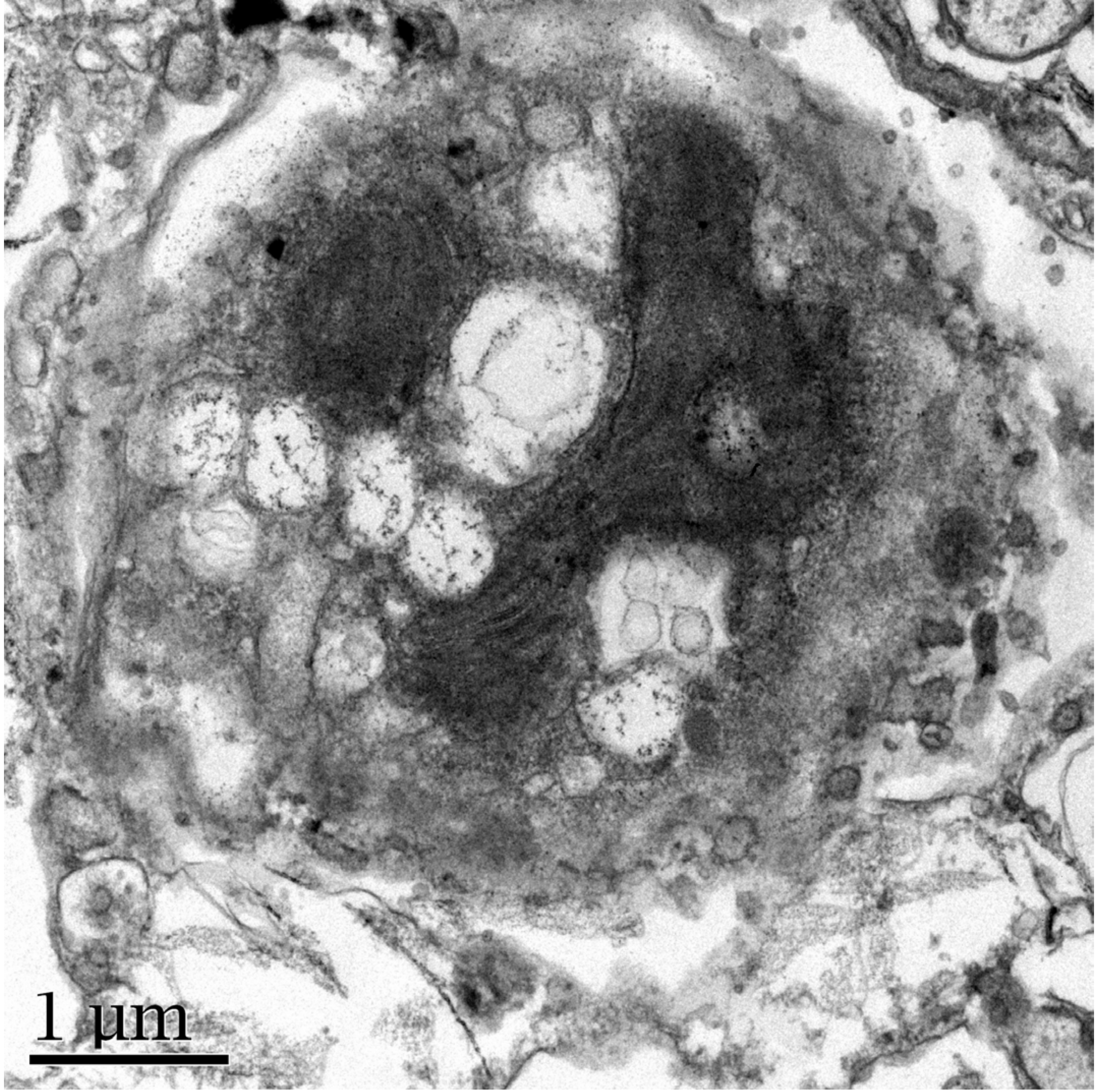

18-14_Correa_KC4_5A6_33.tif

Cell 12

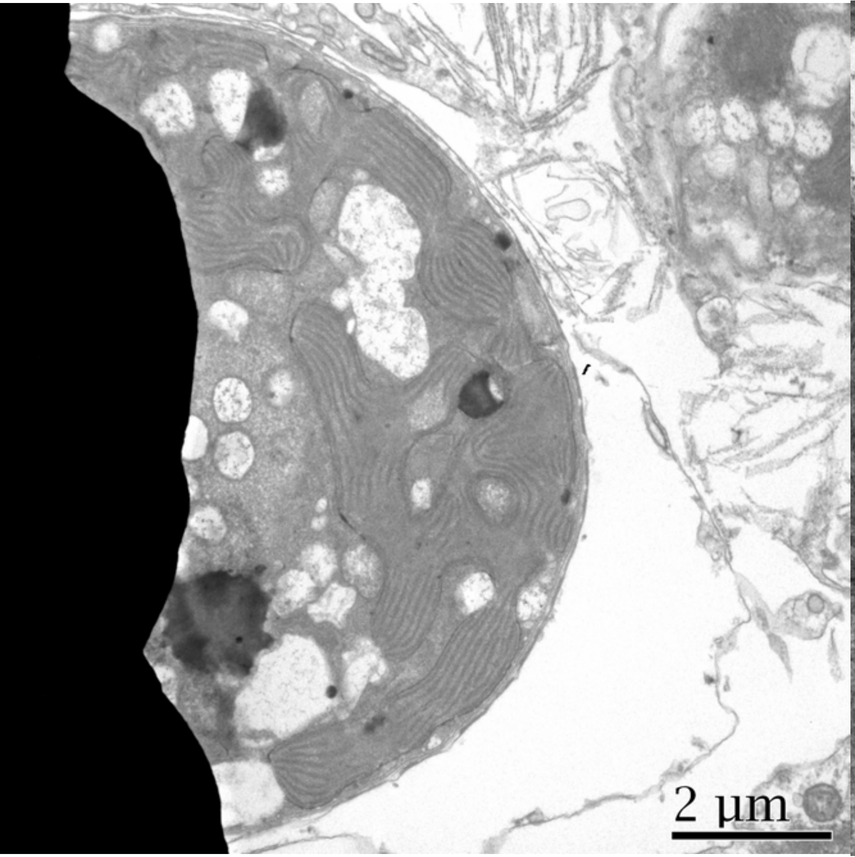

18-14_Correa_KC4_5A6_38.tif

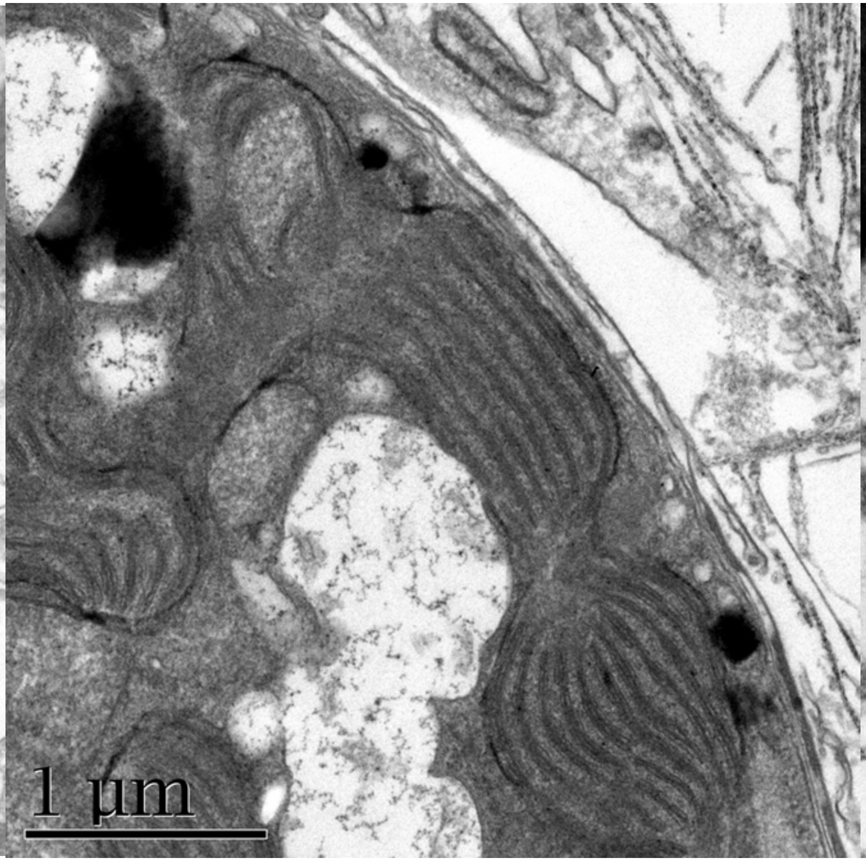

18-14_Correa_KC4_5A6_38.tif

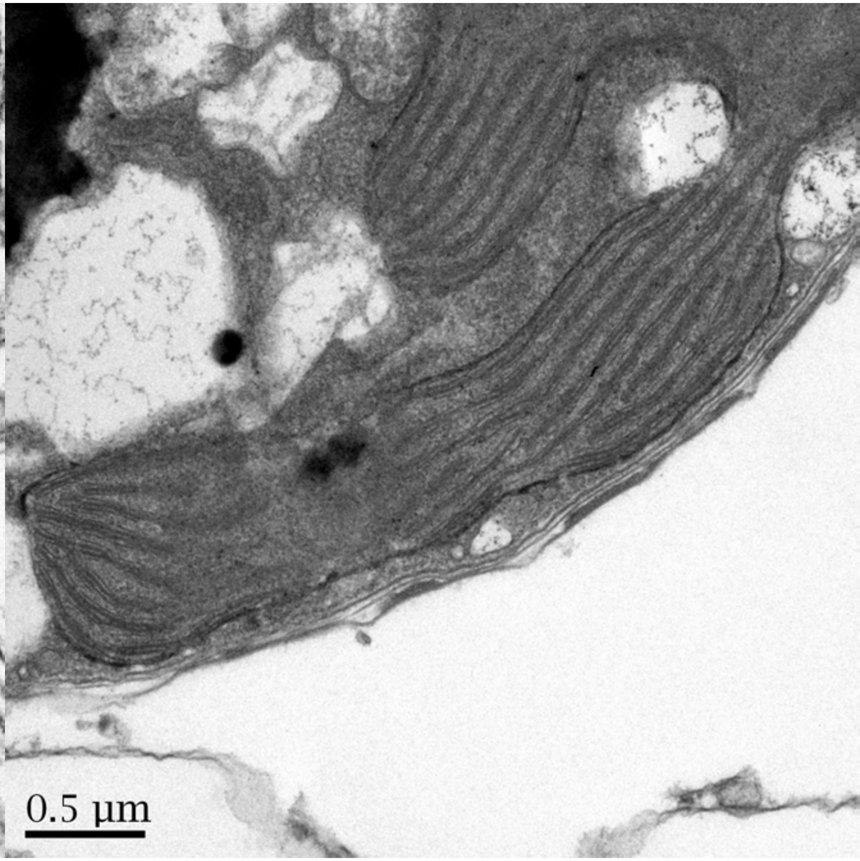

18-14_Correa_KC4_5A6_35.tif

Cell 13

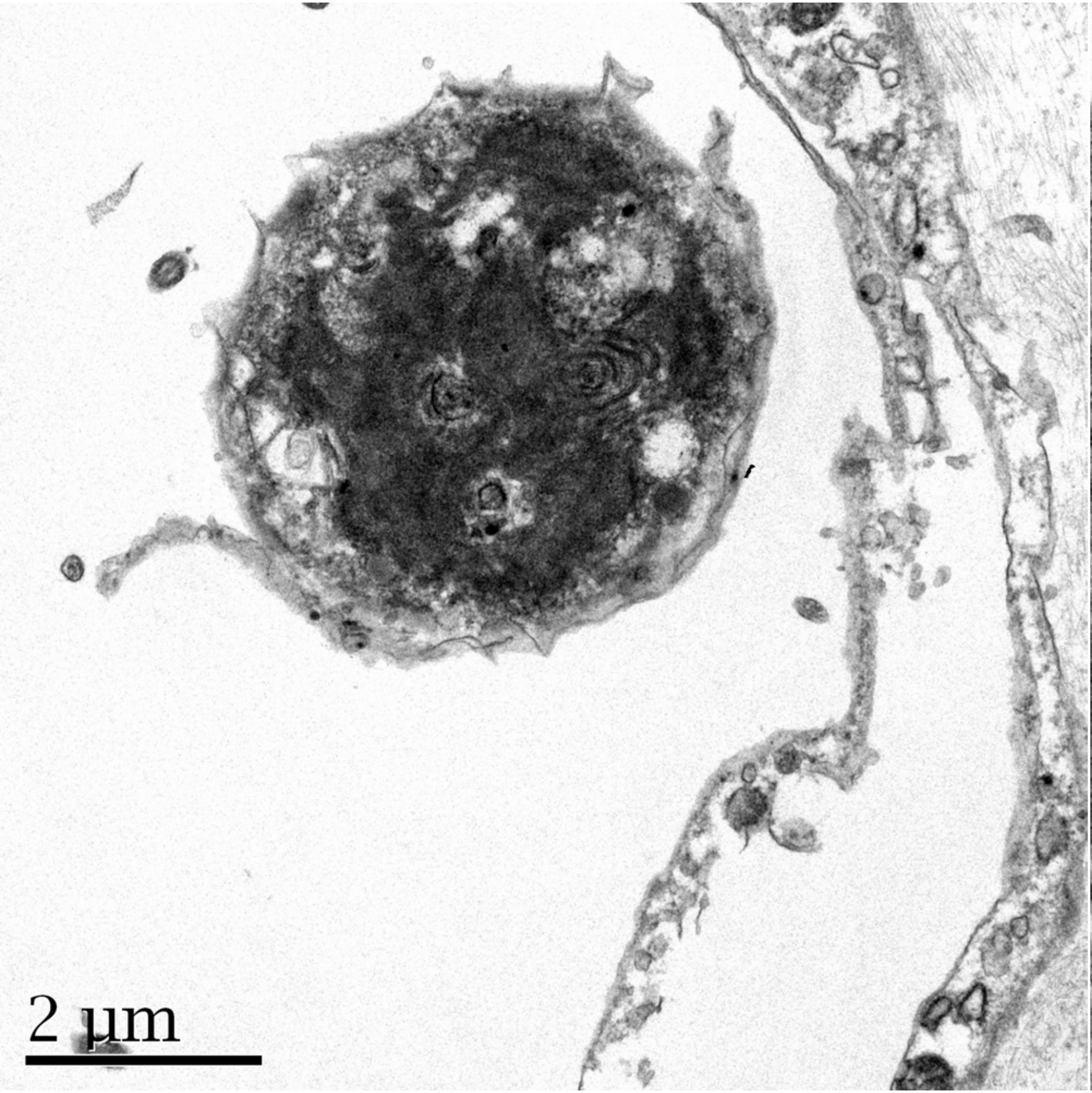

18-14_Correa_KC4_5A6_39.tif

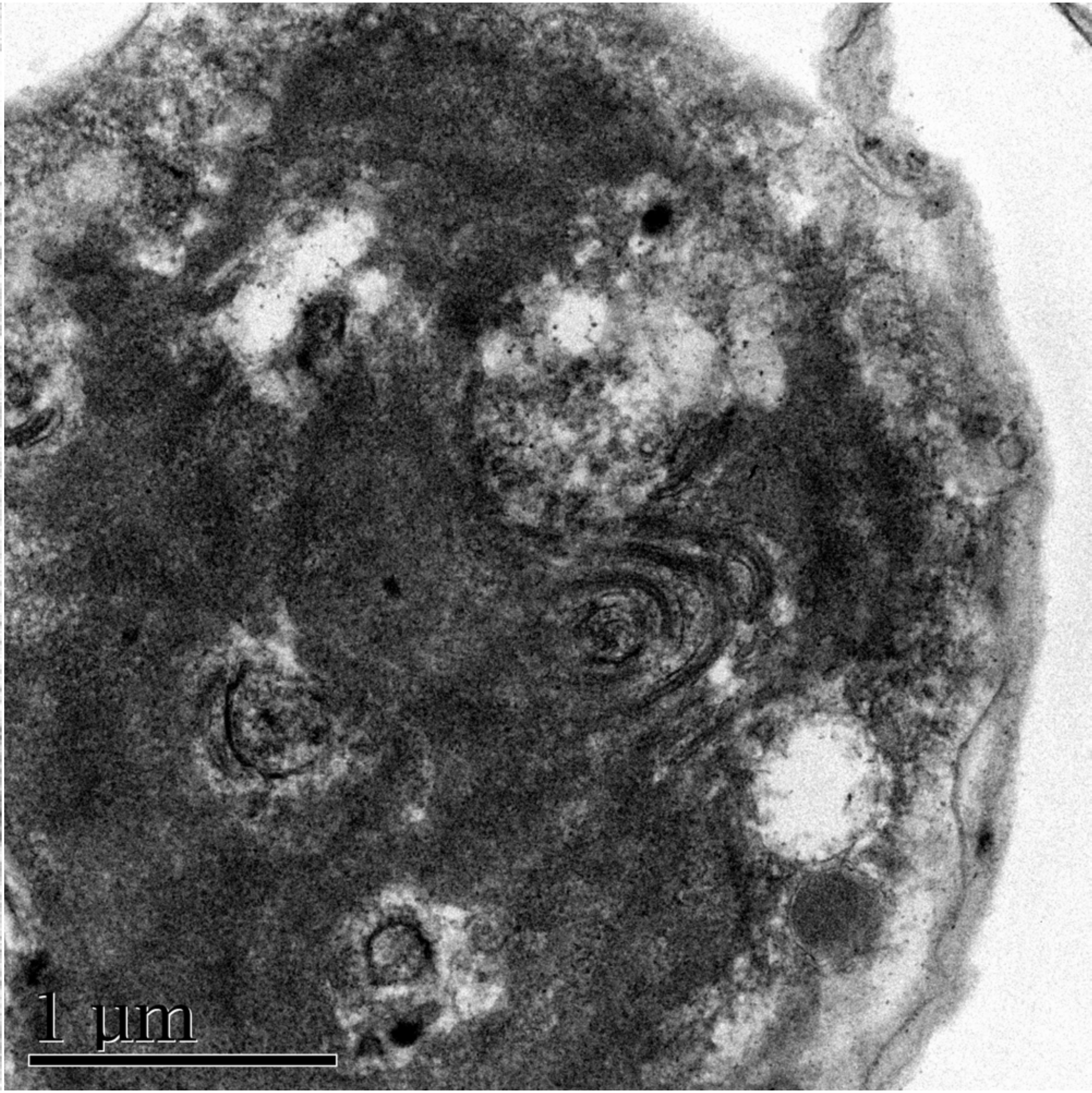

18-14_Correa_KC4_5A6_40.tif

Cell 14

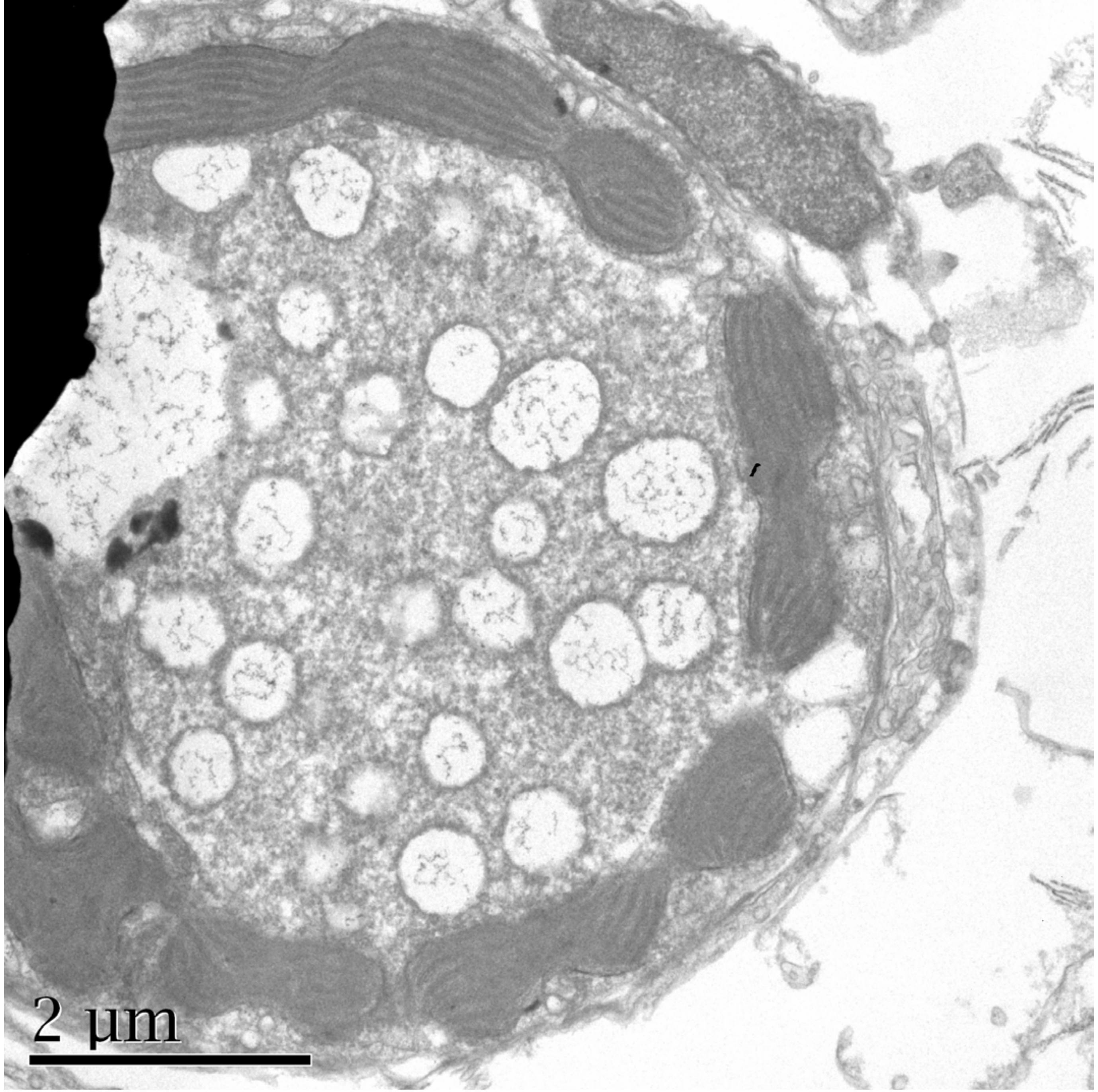

18-14_Correa_KC4_5A6_41.tif

Cell 15

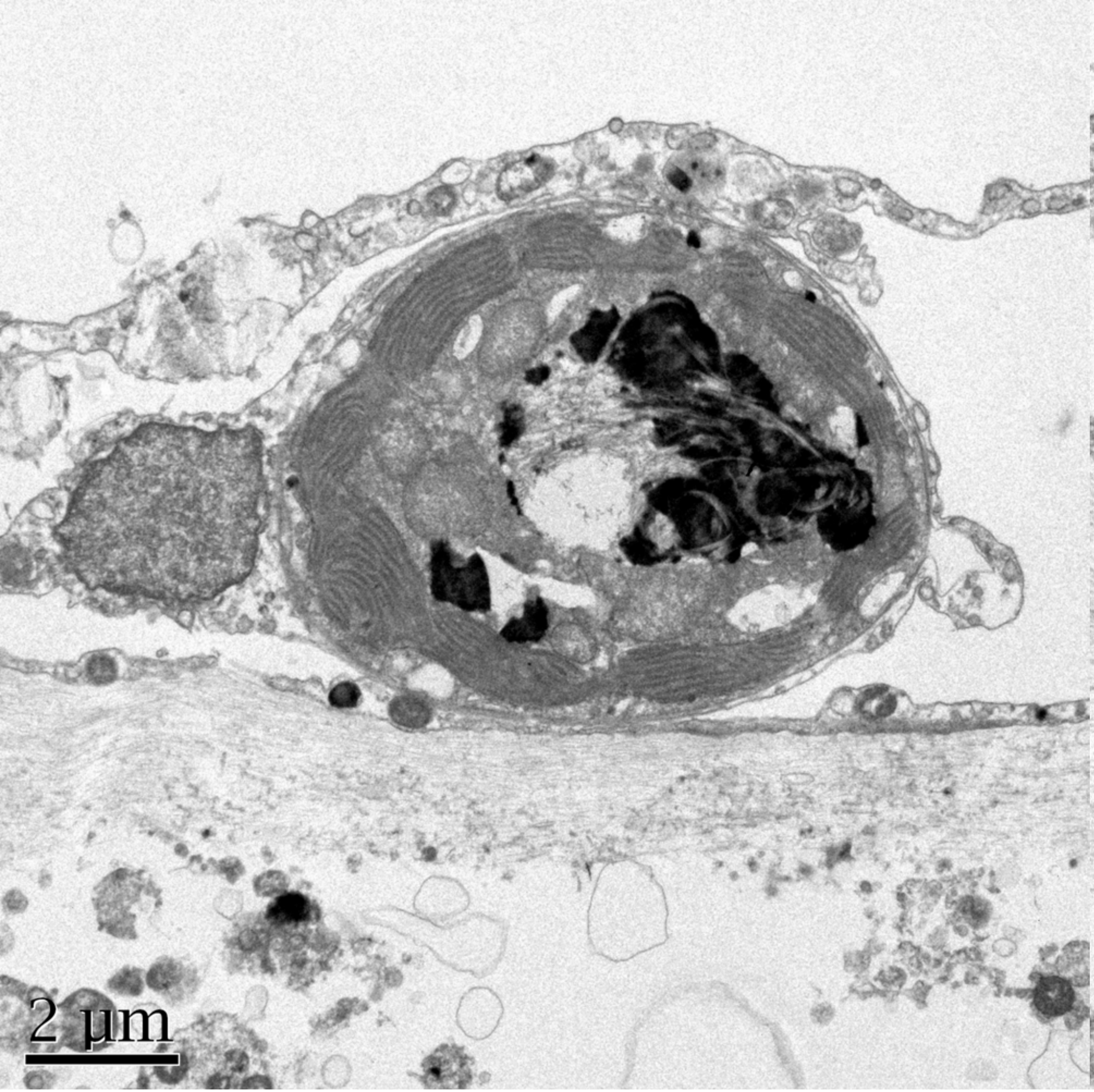

18-14_Correa_KC4_5A6_42.tif

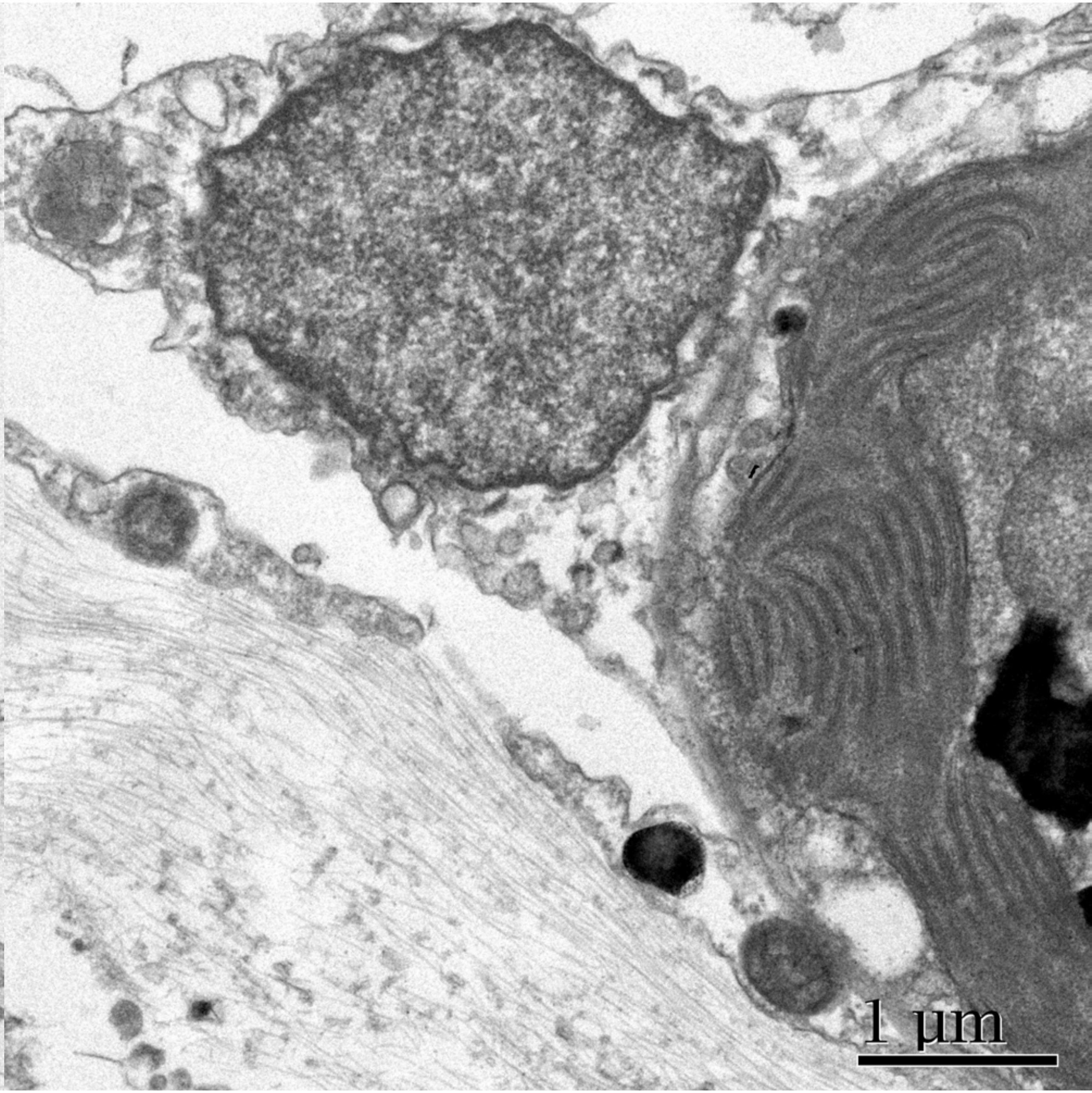

18-14_Correa_KC4_5A6_43.tif

Cell 16

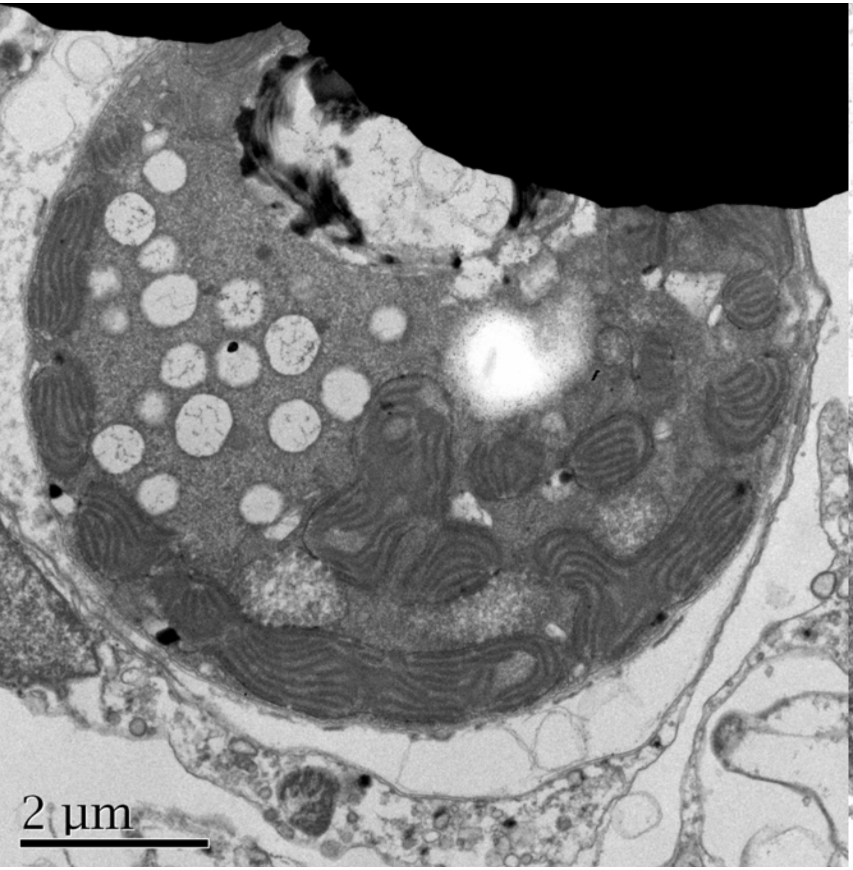

18-14_Correa_KC4_5A6_45.tif

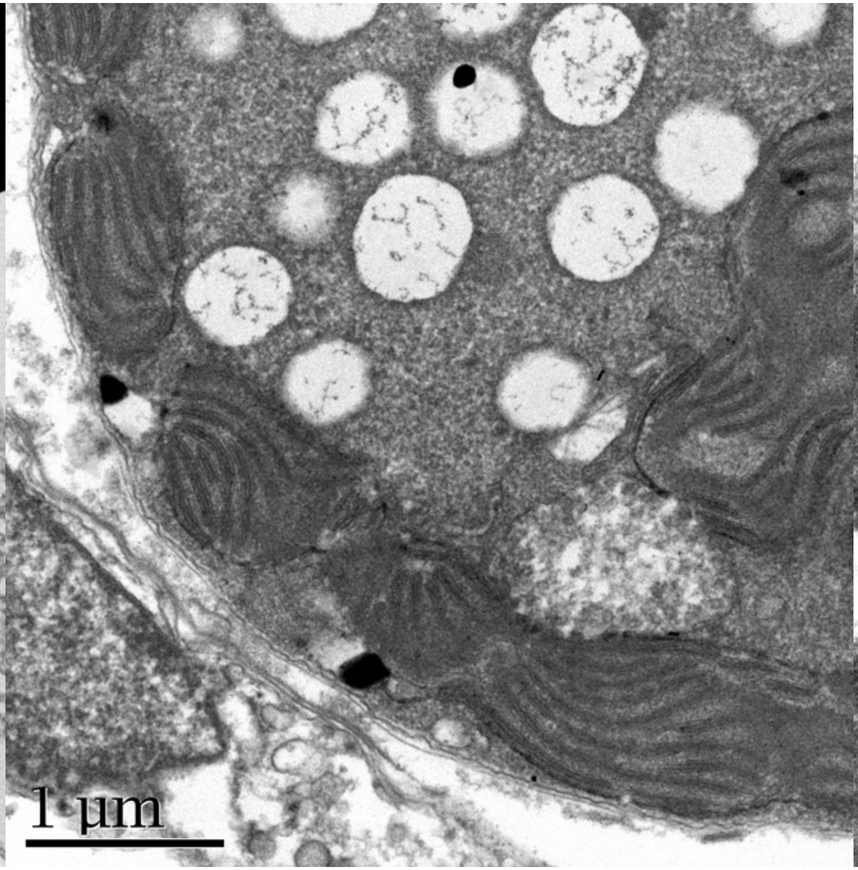

18-14_Correa_KC4_5A6_47.tif

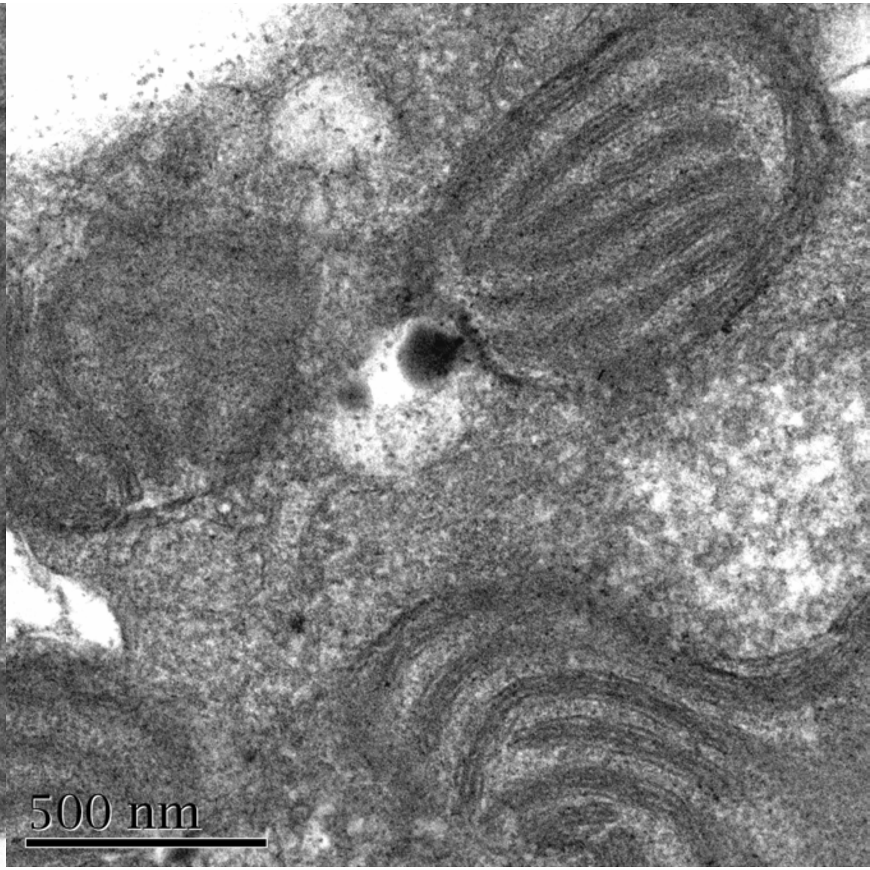

18-14_Correa_KC4_5A6_50.tif

Cell 17

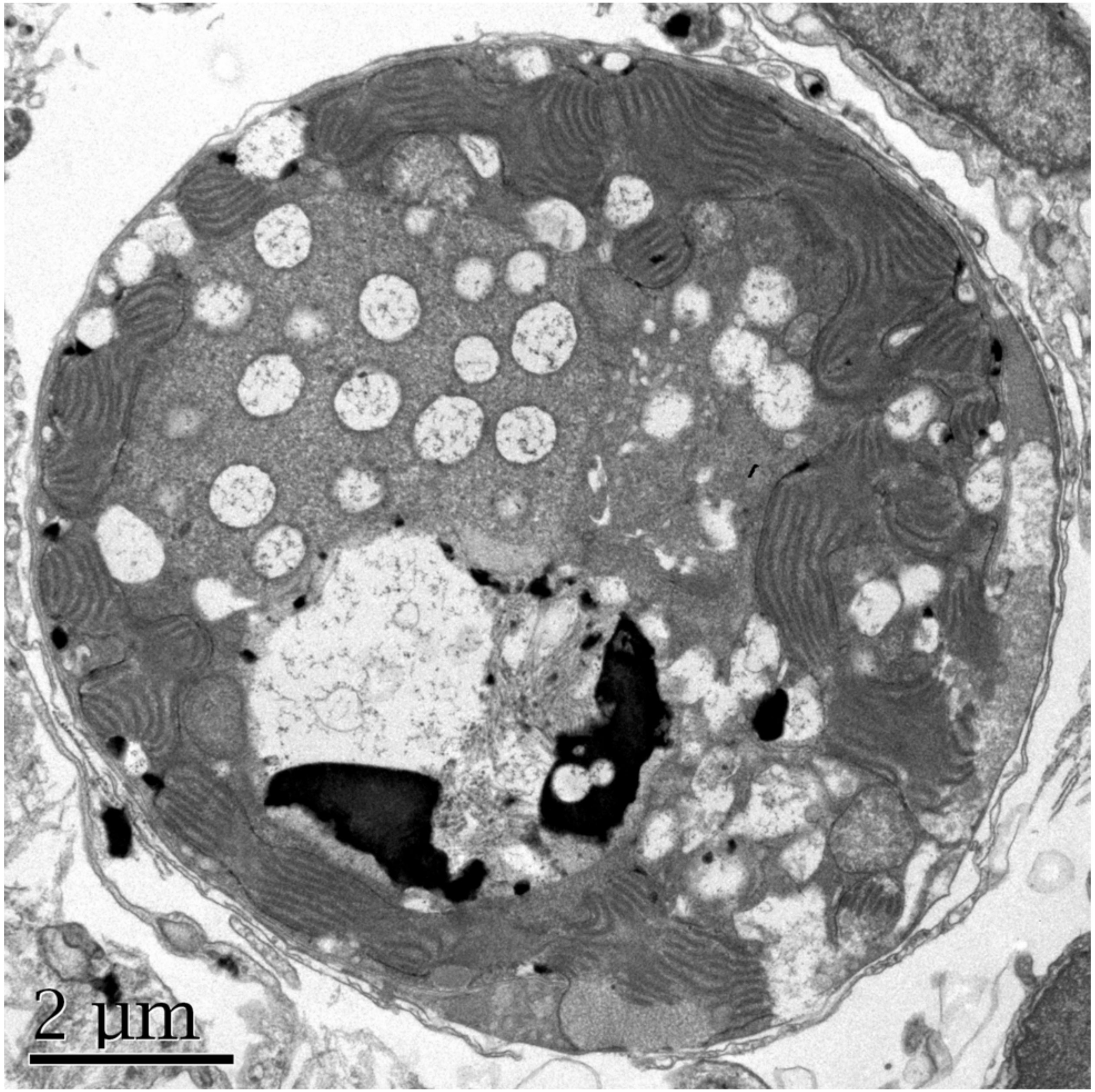

18-14_Correa_KC4_5A6_51.tif

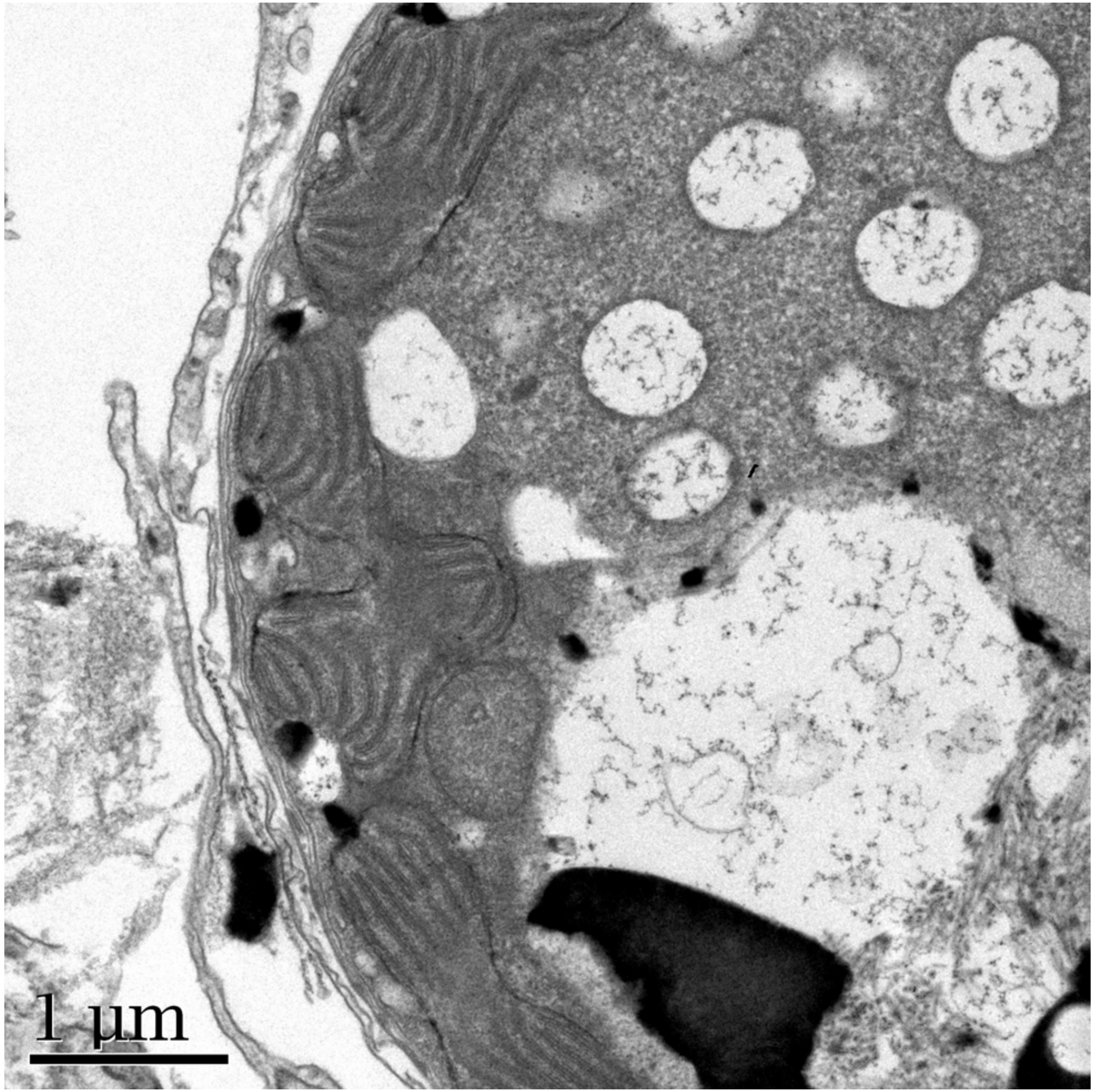

18-14_Correa_KC4_5A6_52.tif

Cell 18

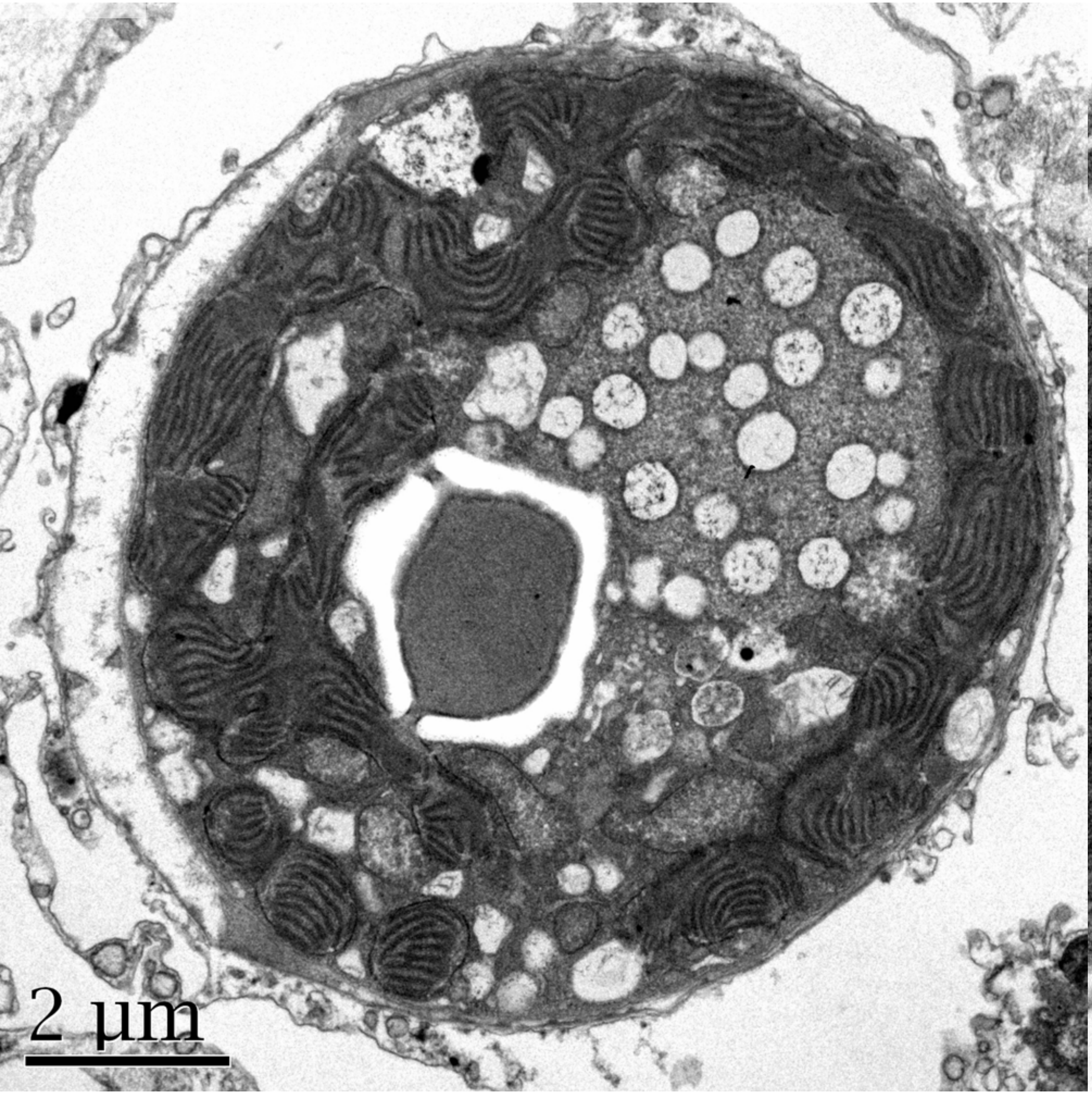

18-14_Correa_KC4_5A6_54.tif

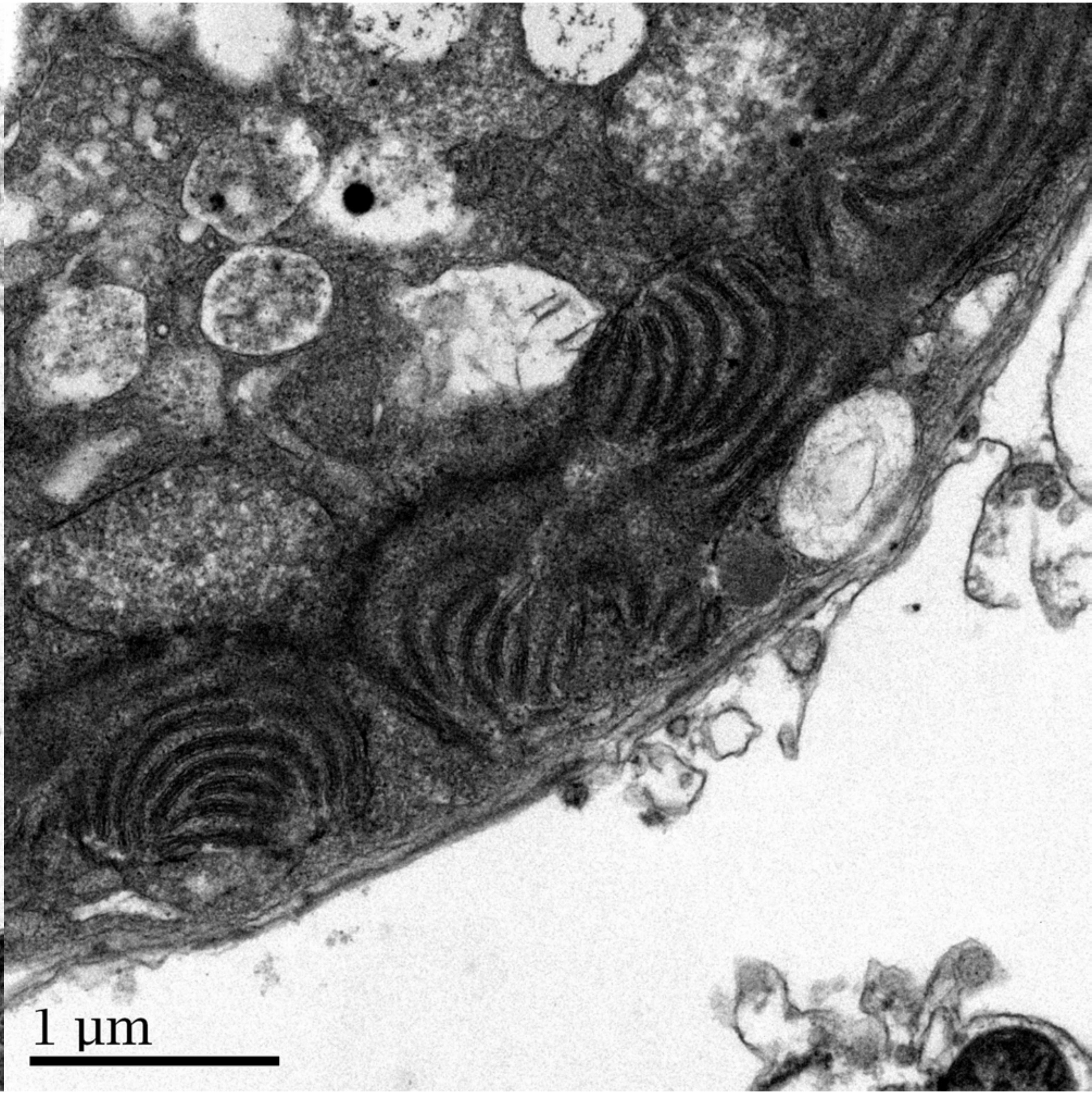

18-14_Correa_KC4_5A6_55.tif

Cell 19

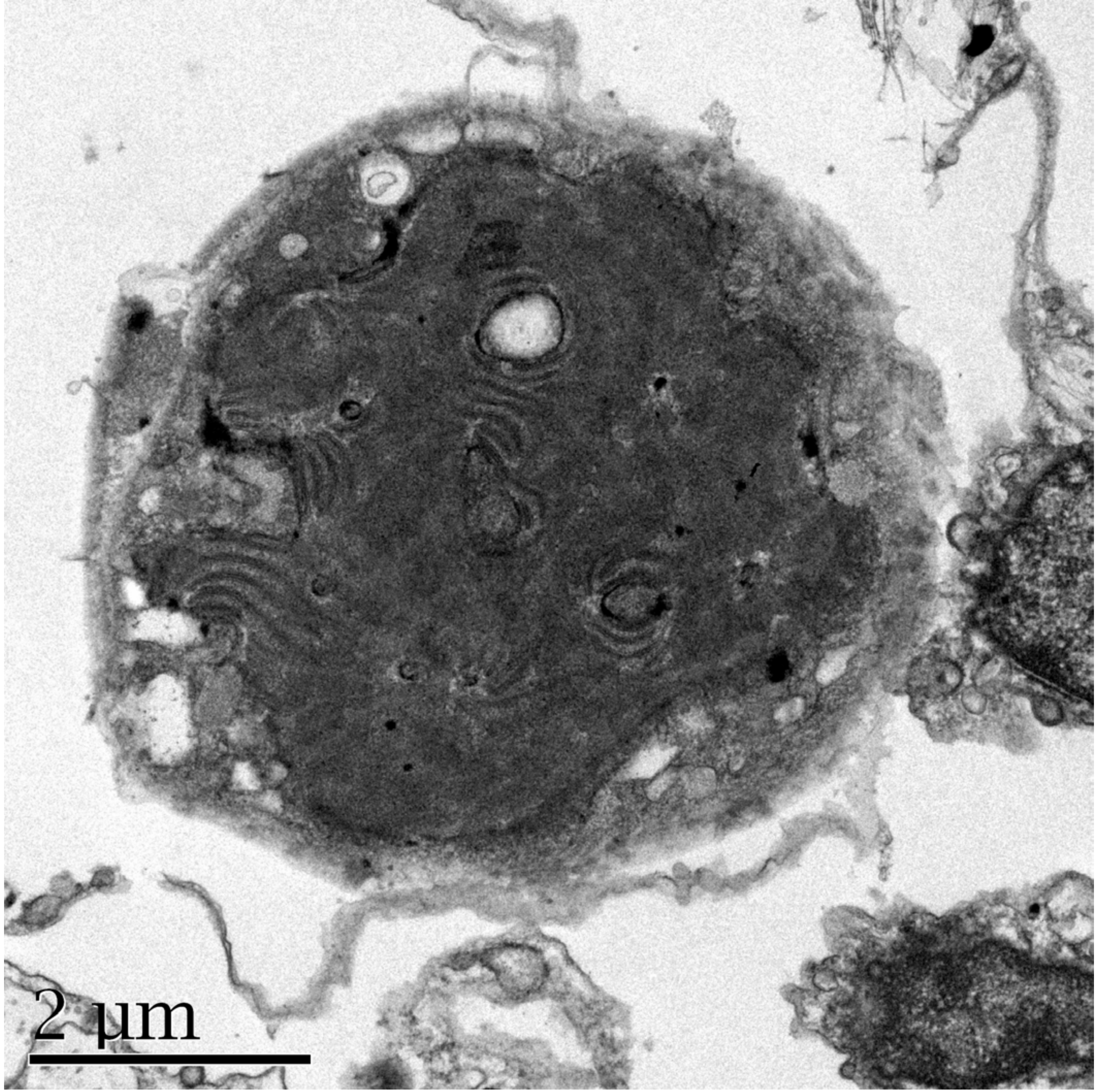

18-14_Correa_KC4_5A6_56.tif

Cell 20

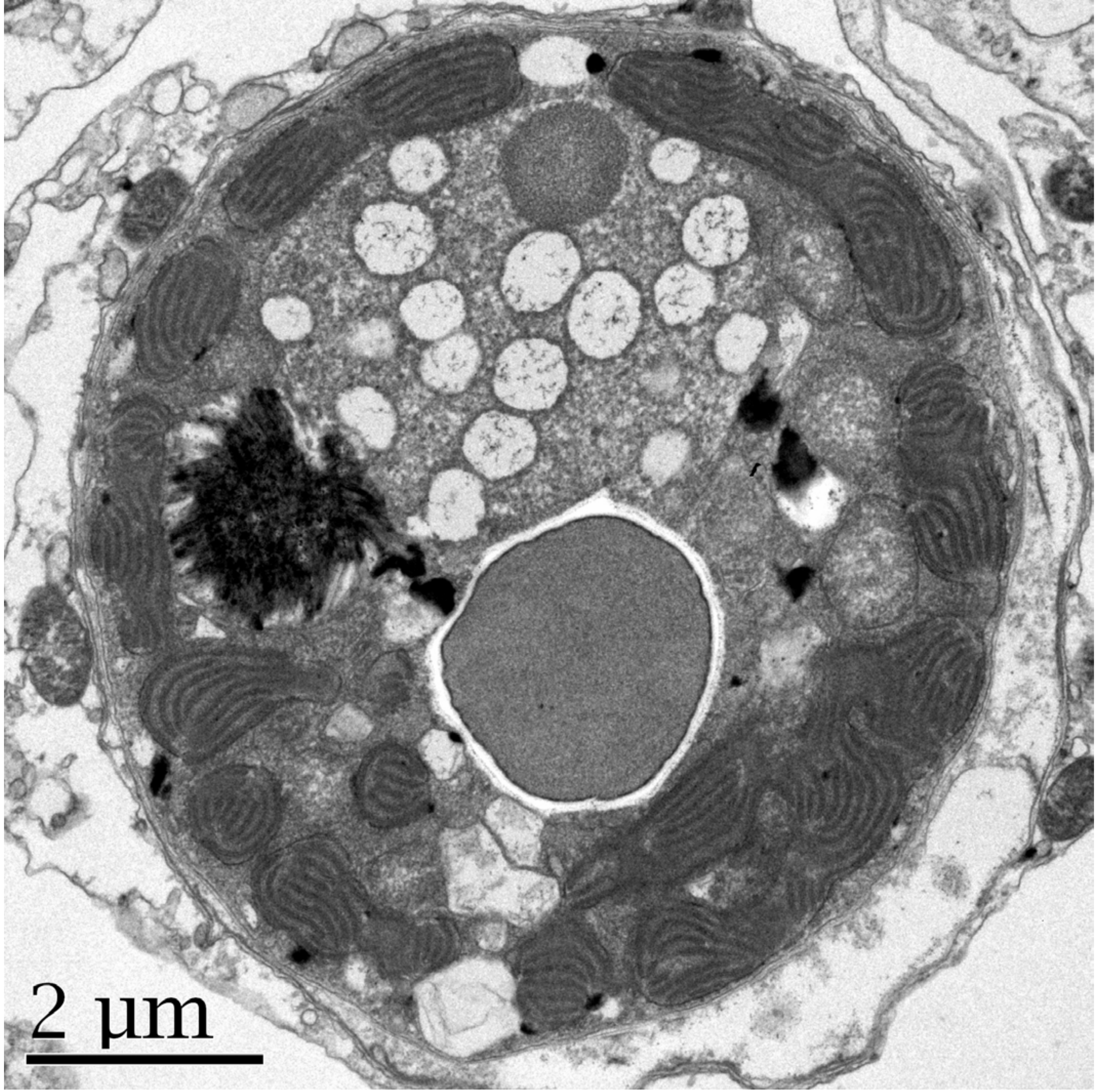

18-14_Correa_KC4_5A6_59.tif

Cell 21

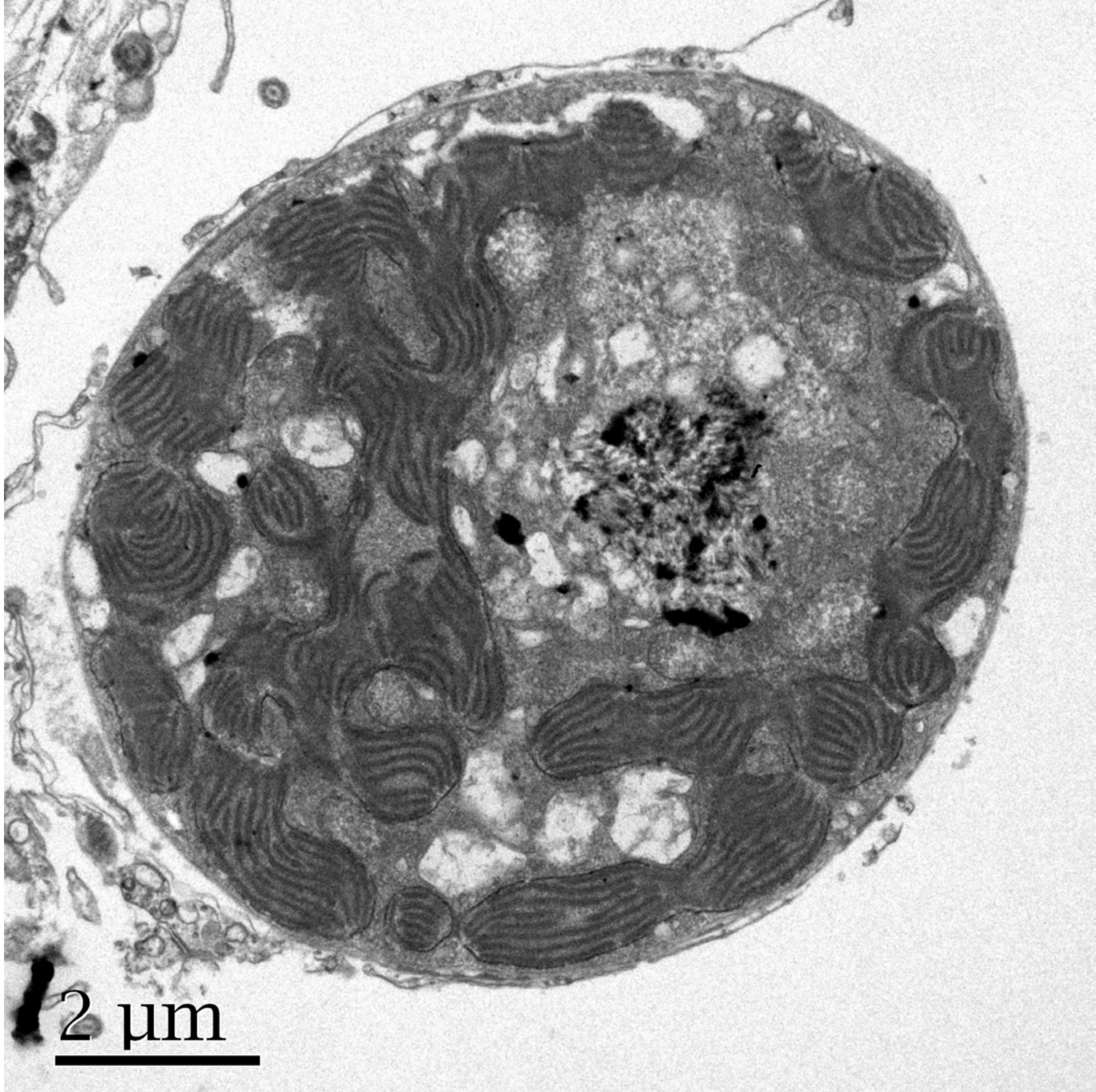

18-14_Correa_KC4_5A6_61.tif

Cell 22

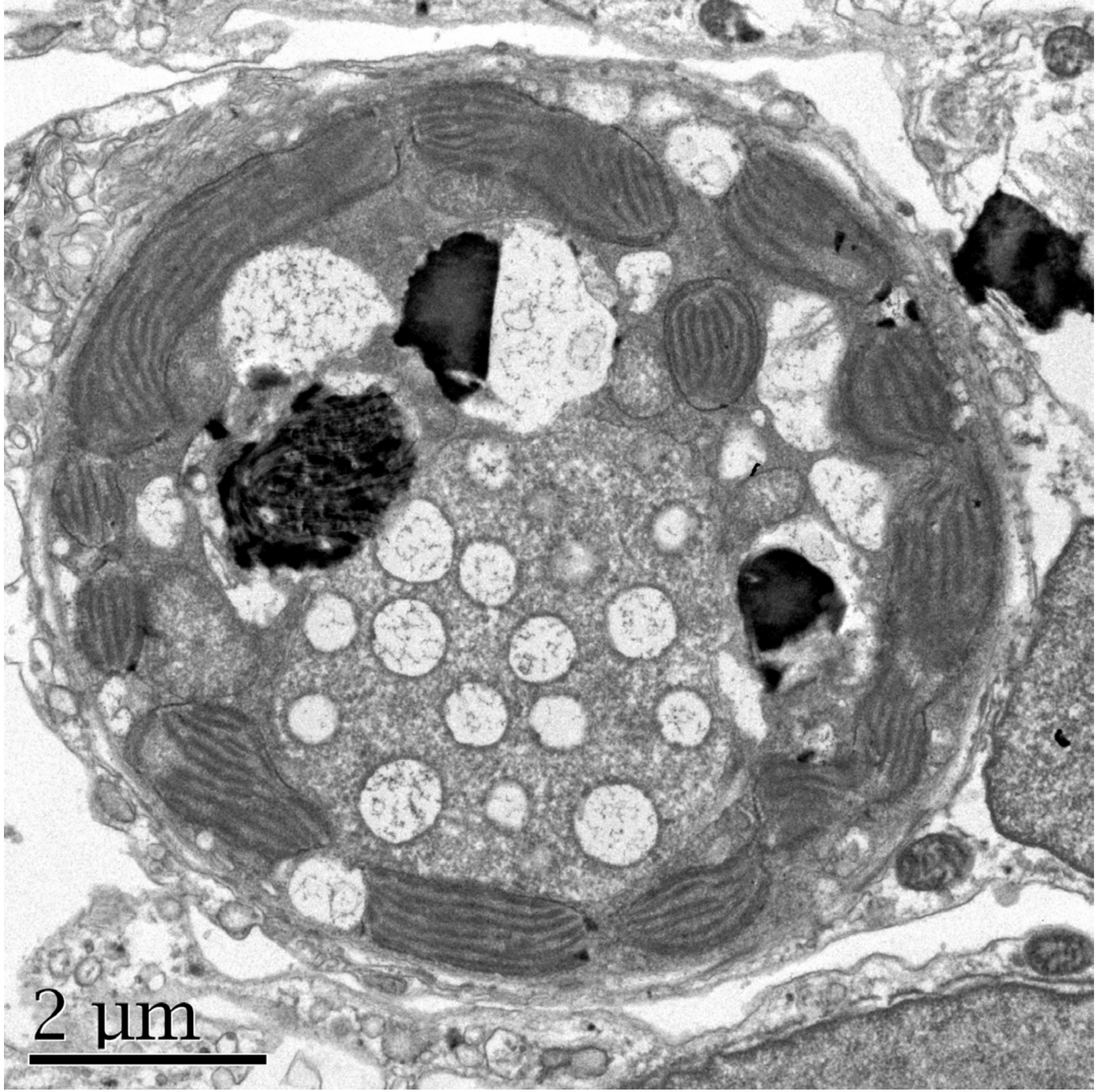

18-14_Correa_KC4_5A6_63.tif

Cell 23

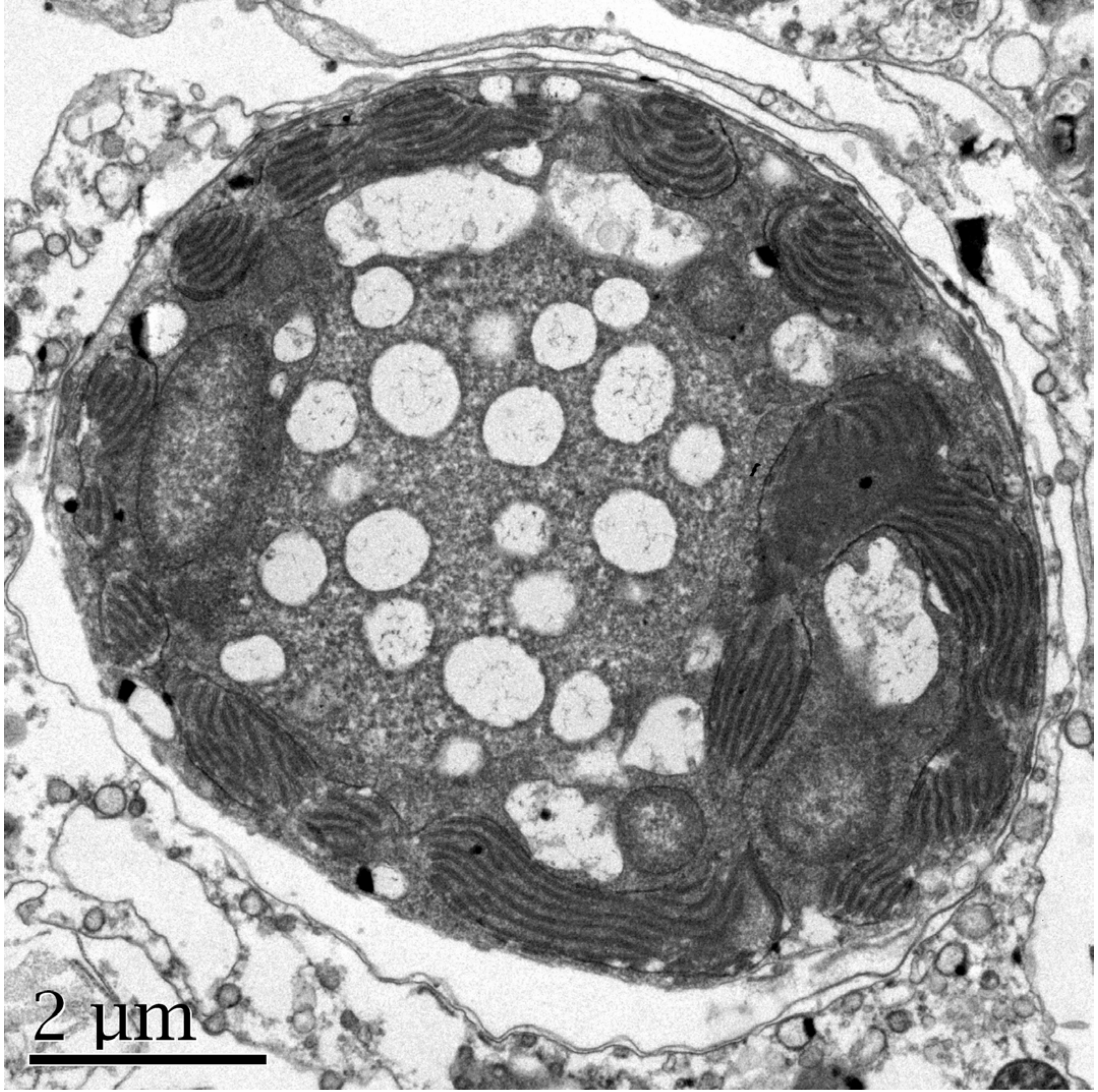

18-14_Correa_KC4_5A6_64.tif

Cell 24

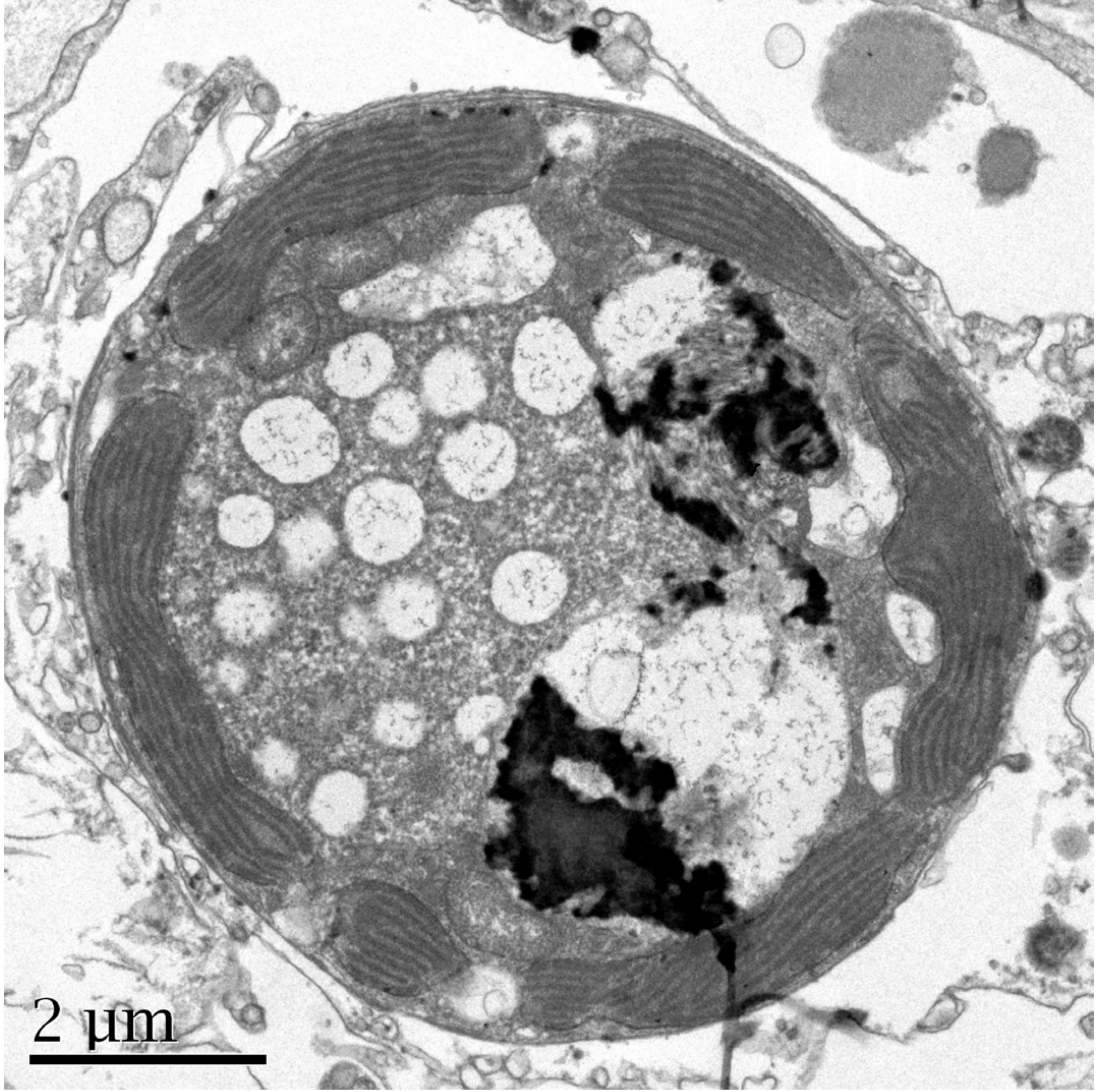

18-14_Correa_KC4_5A6_65.tif

Heat Treatment - ACR Colony B

Cell 1

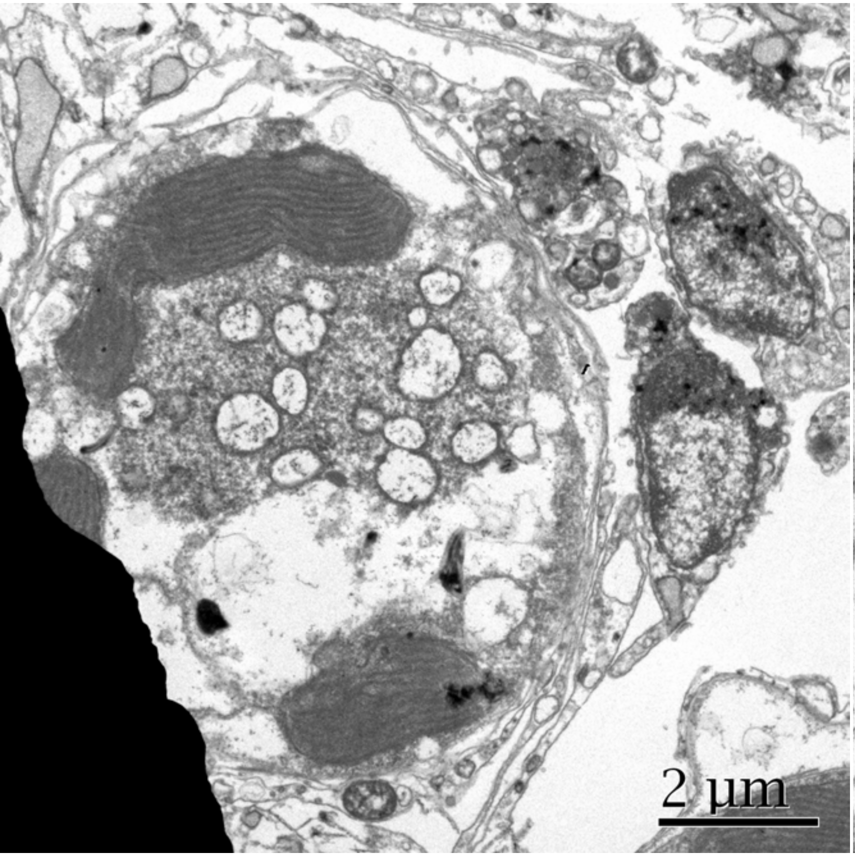

18-14_Correa_KH4_5A1_2.tif

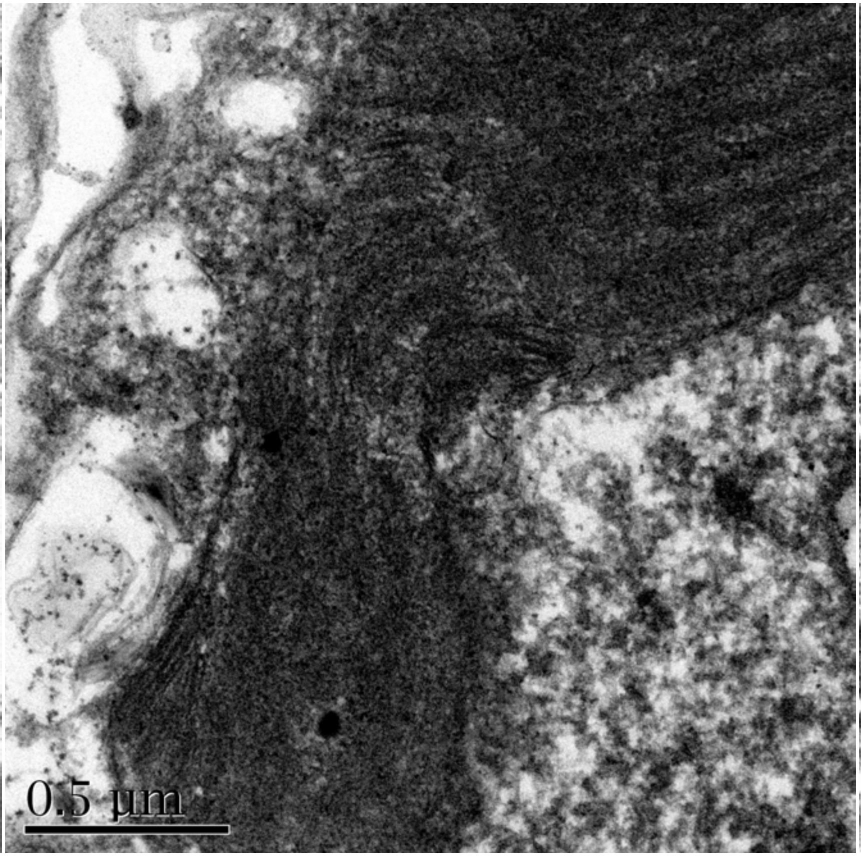

18-14_Correa_KH4_5A1_5.tif

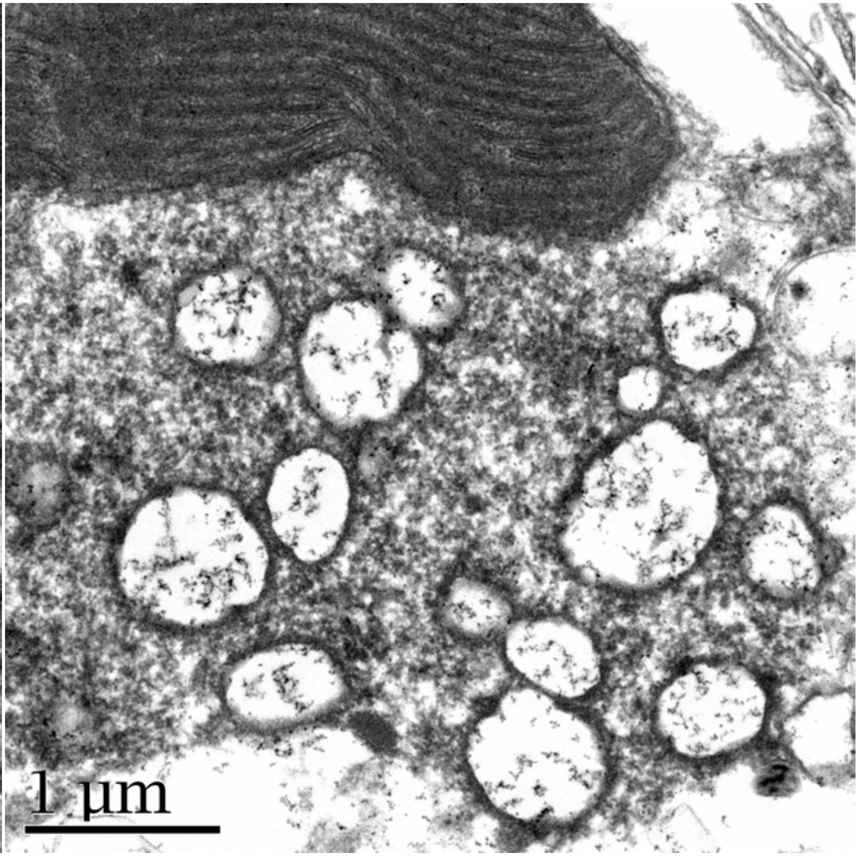

18-14_Correa_KH4_5A1_3.tif

Cell 2

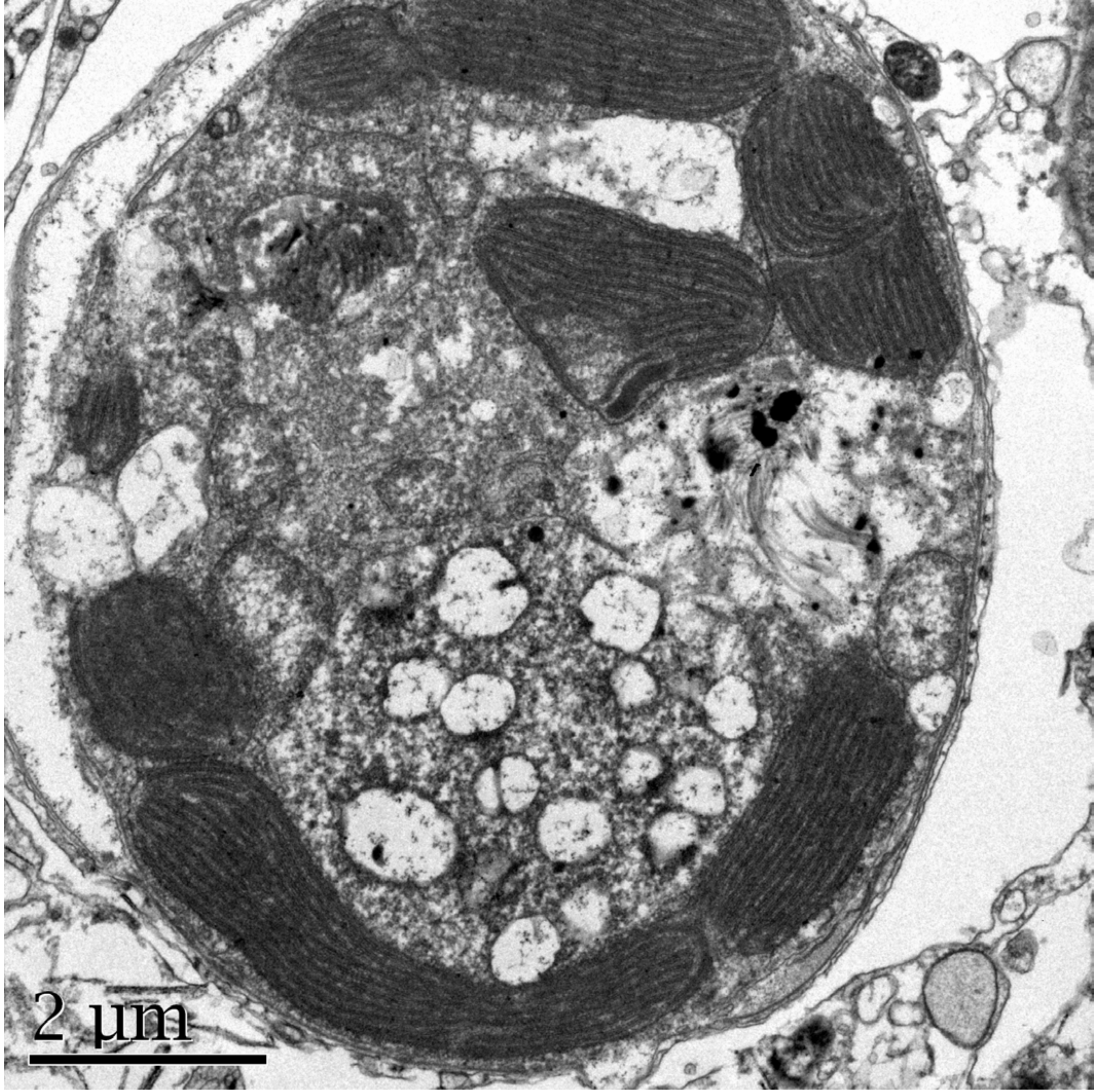

18-14_Correa_KH4_5A1_7.tif

Cell 3

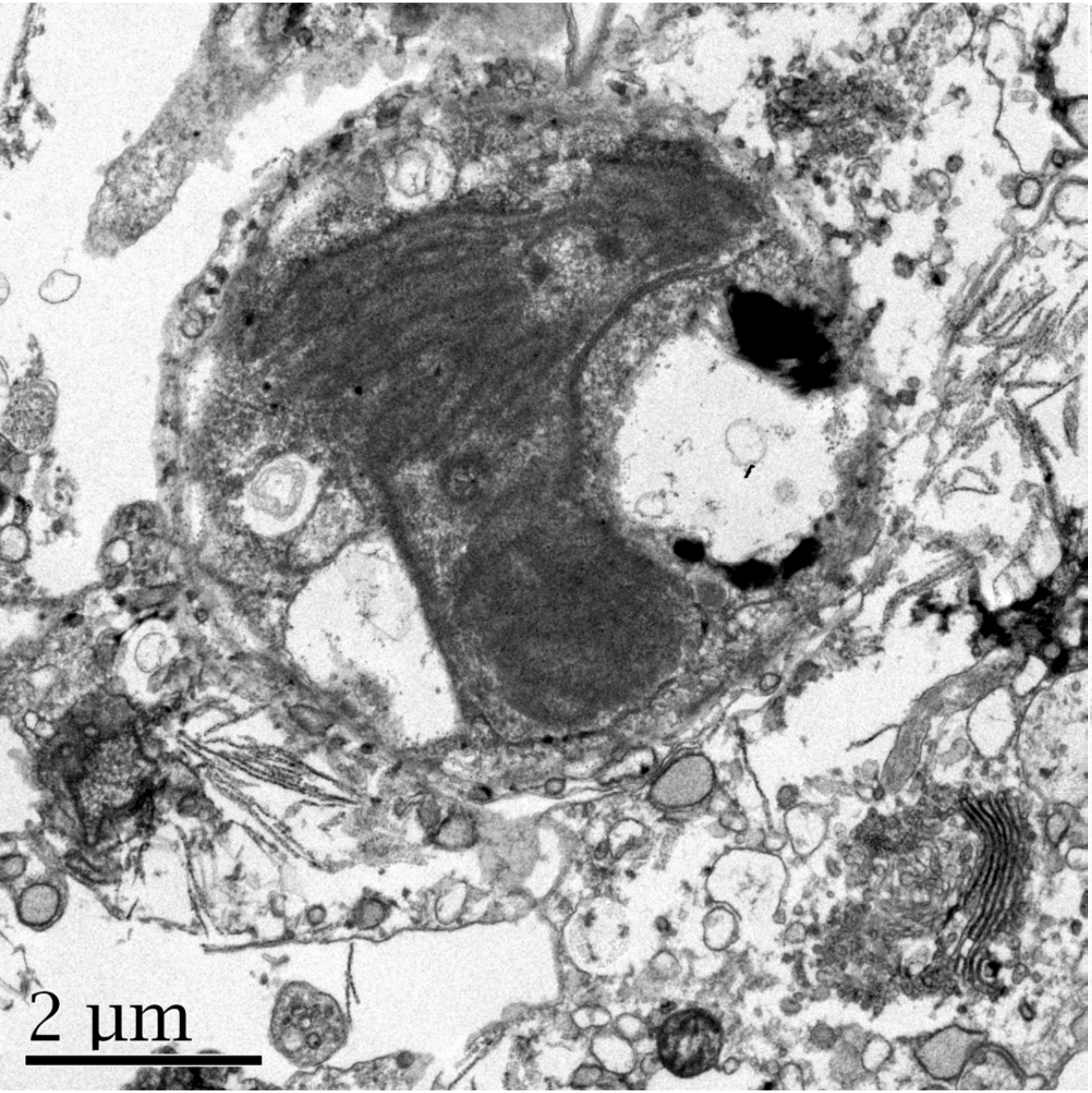

18-14_Correa_KH4_5A1_9.tif

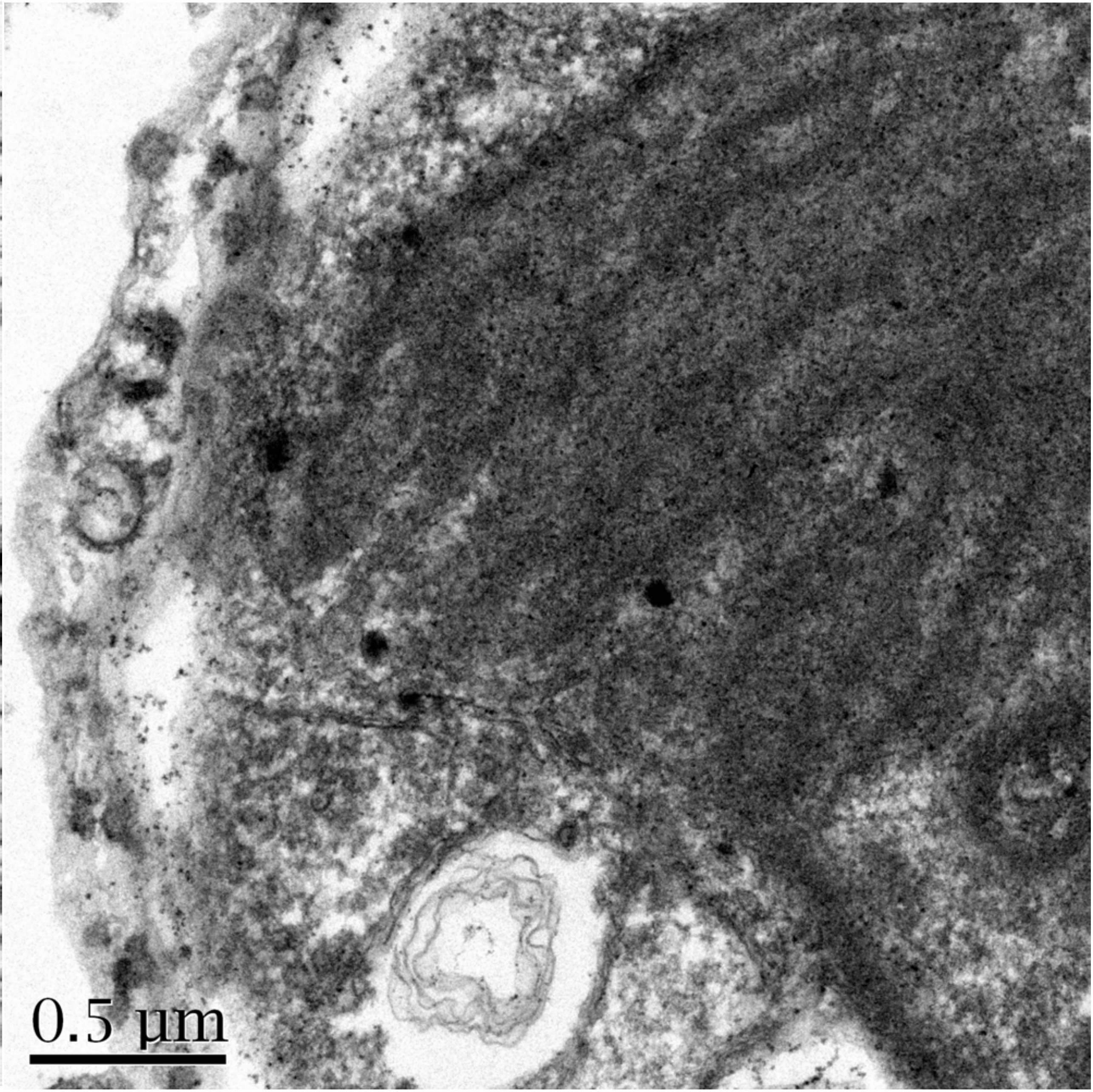

18-14_Correa_KH4_5A1_11.tif

Cell 4

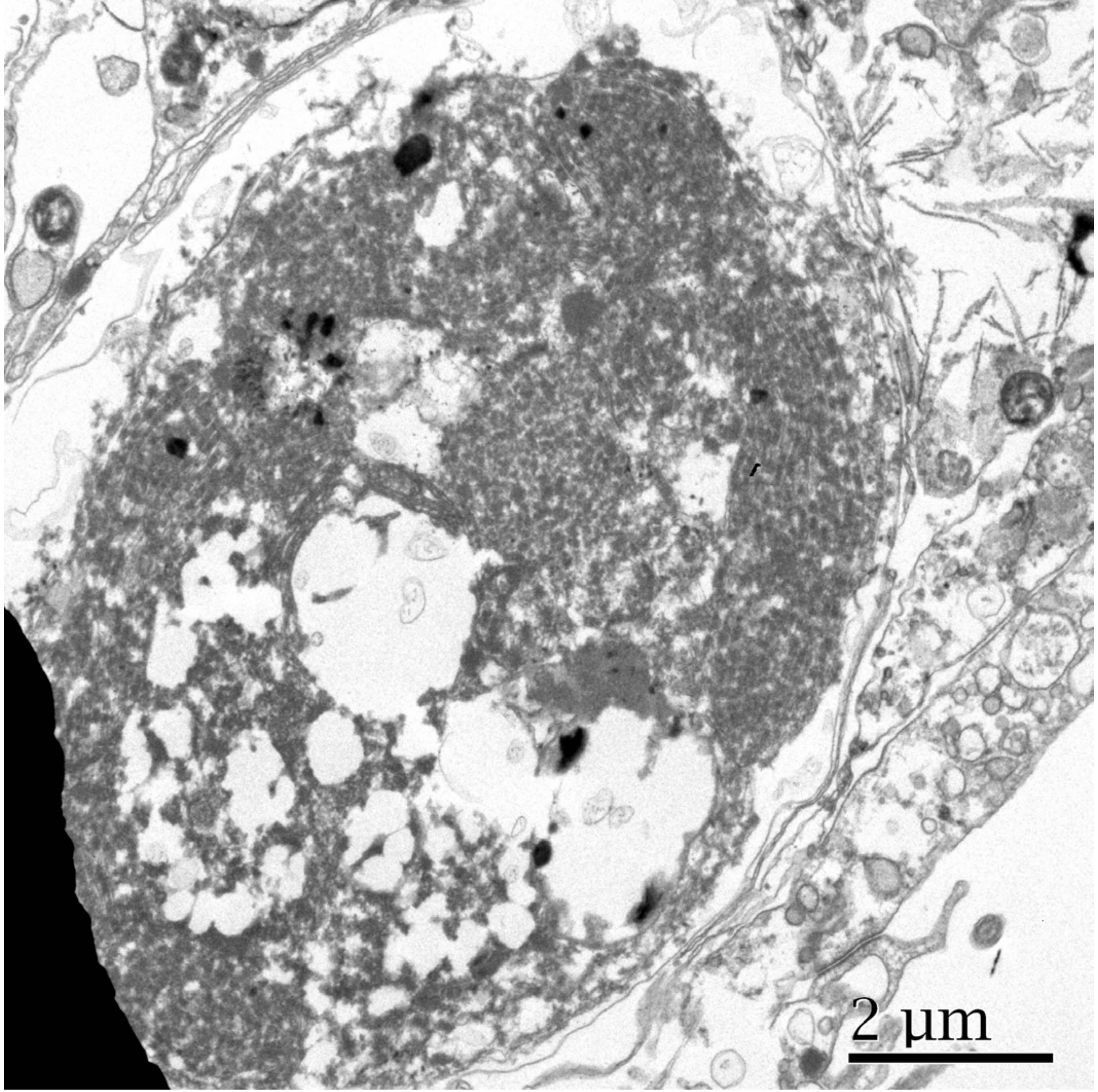

18-14_Correa_KH4_5A1_12.tif

Cell 5

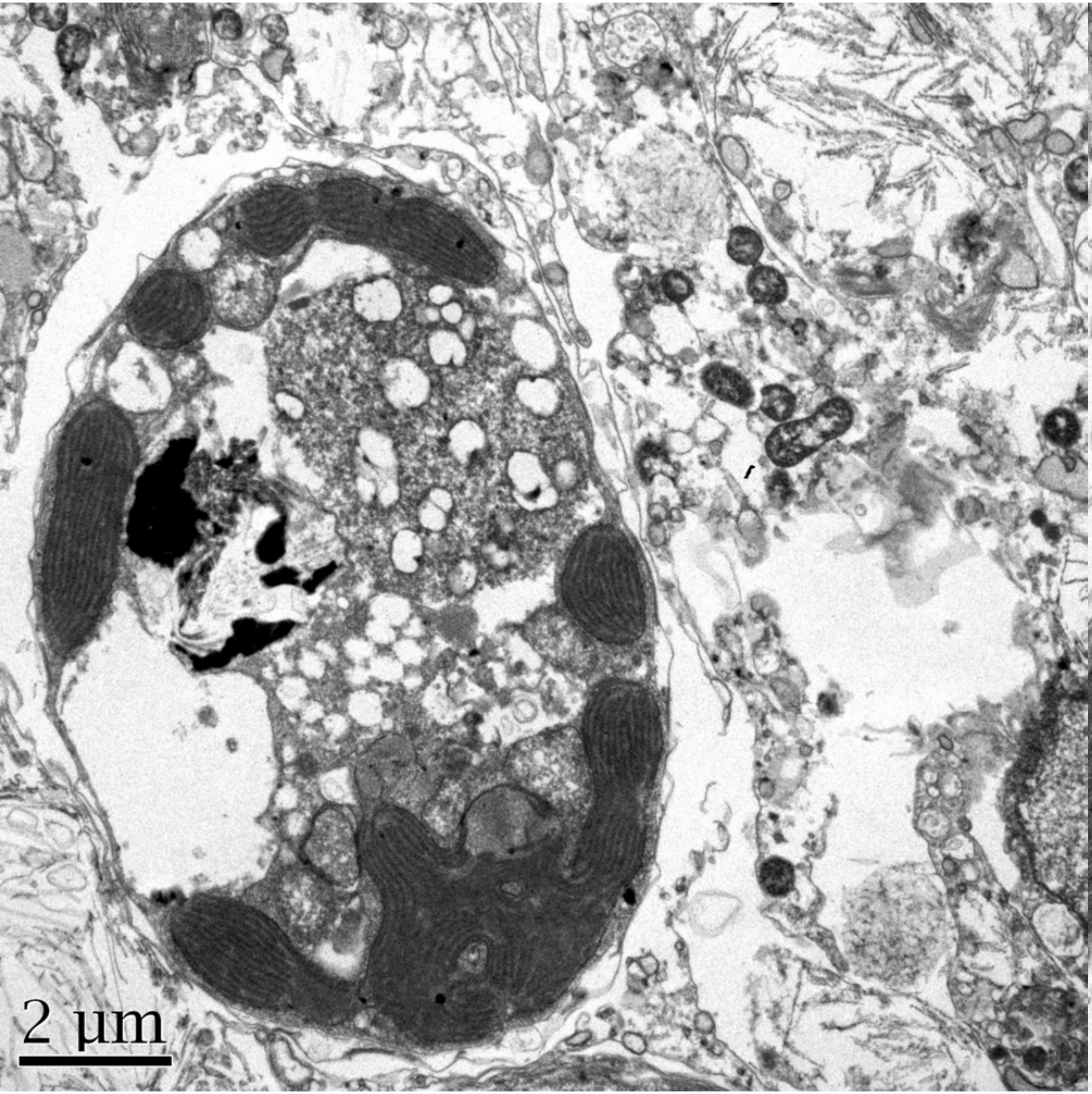

18-14_Correa_KH4_5A1_14.tif

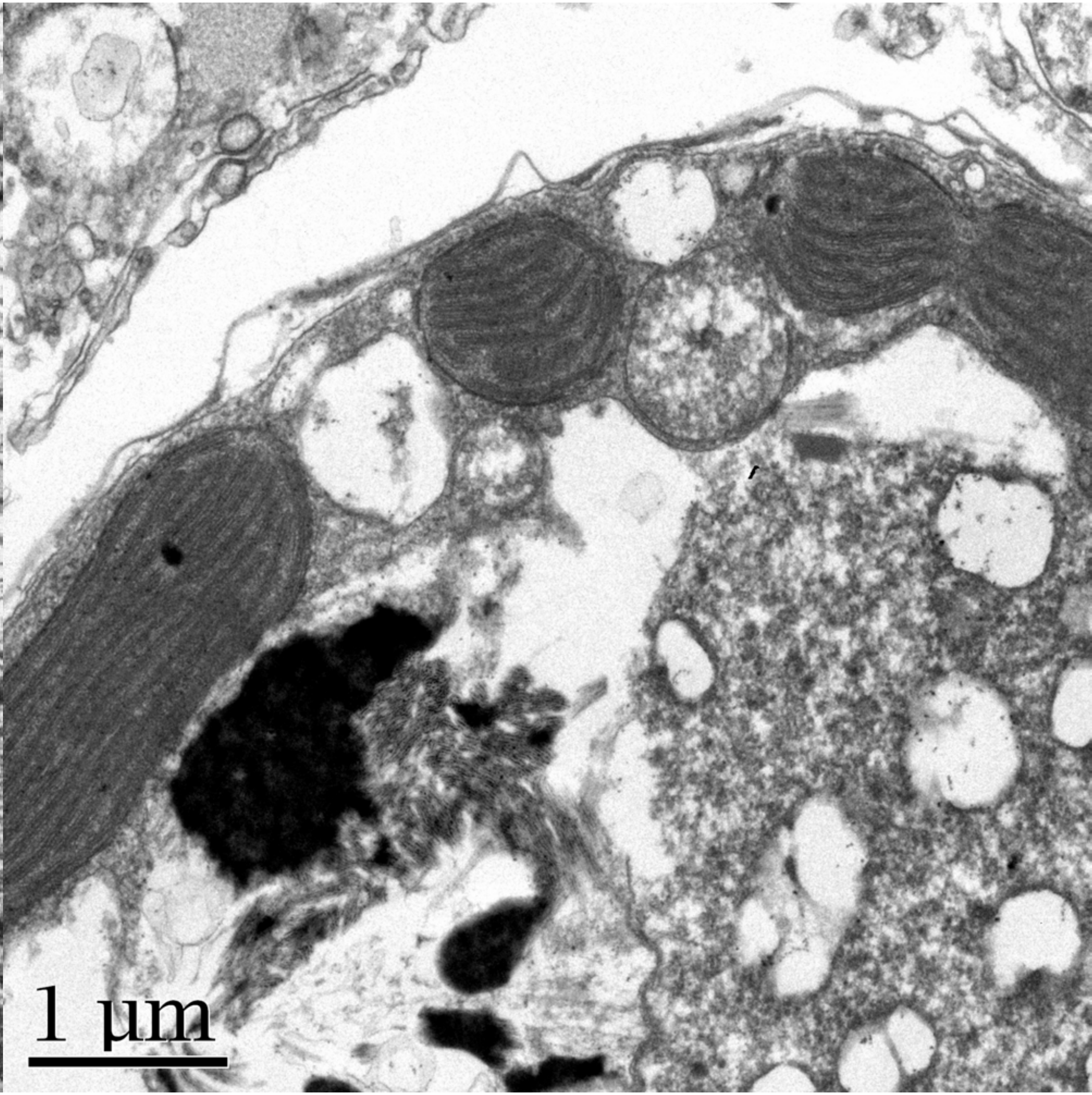

18-14_Correa_KH4_5A1_16.tif

Cell 6

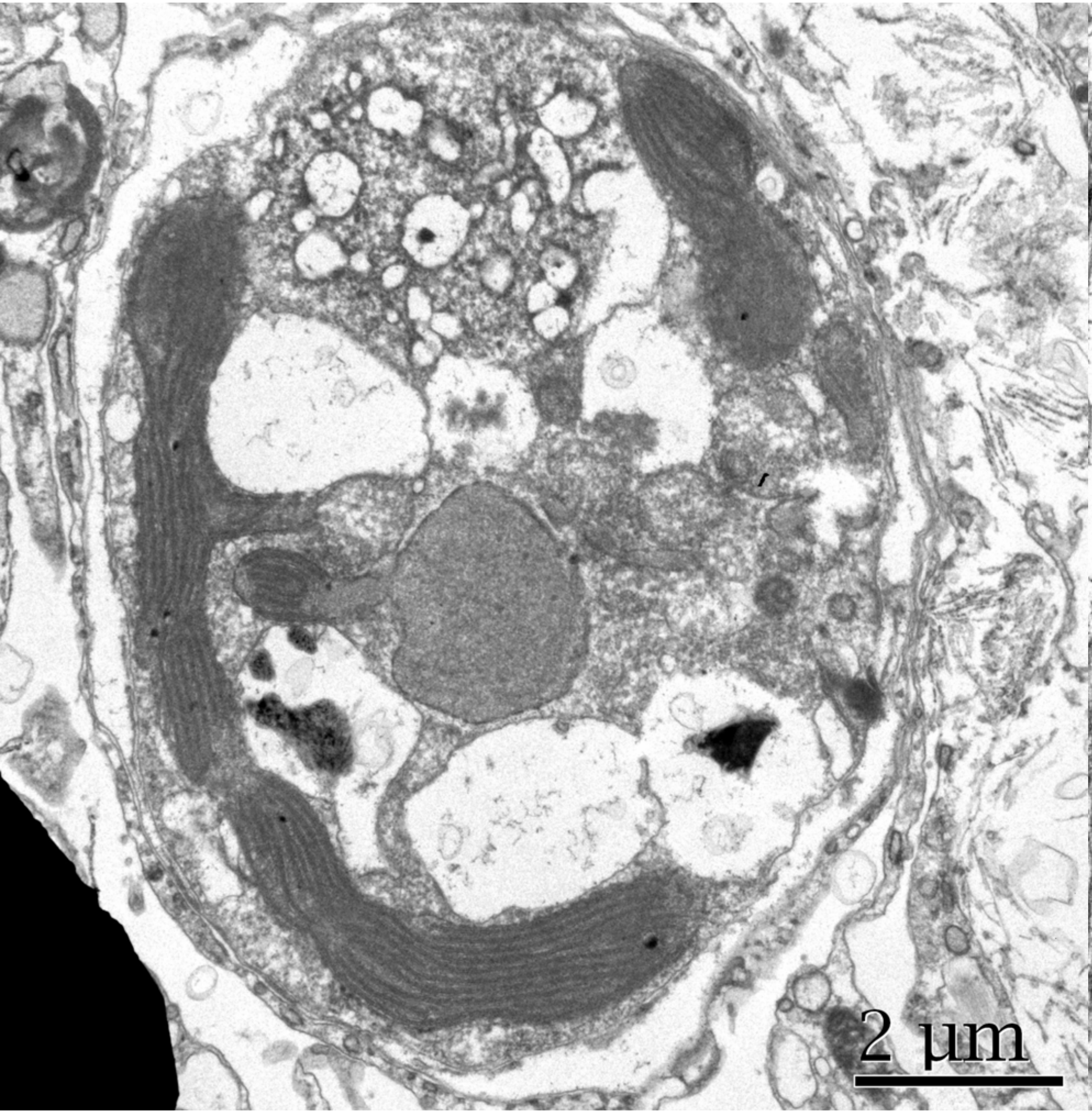

18-14_Correa_KH4_5A1_18.tif

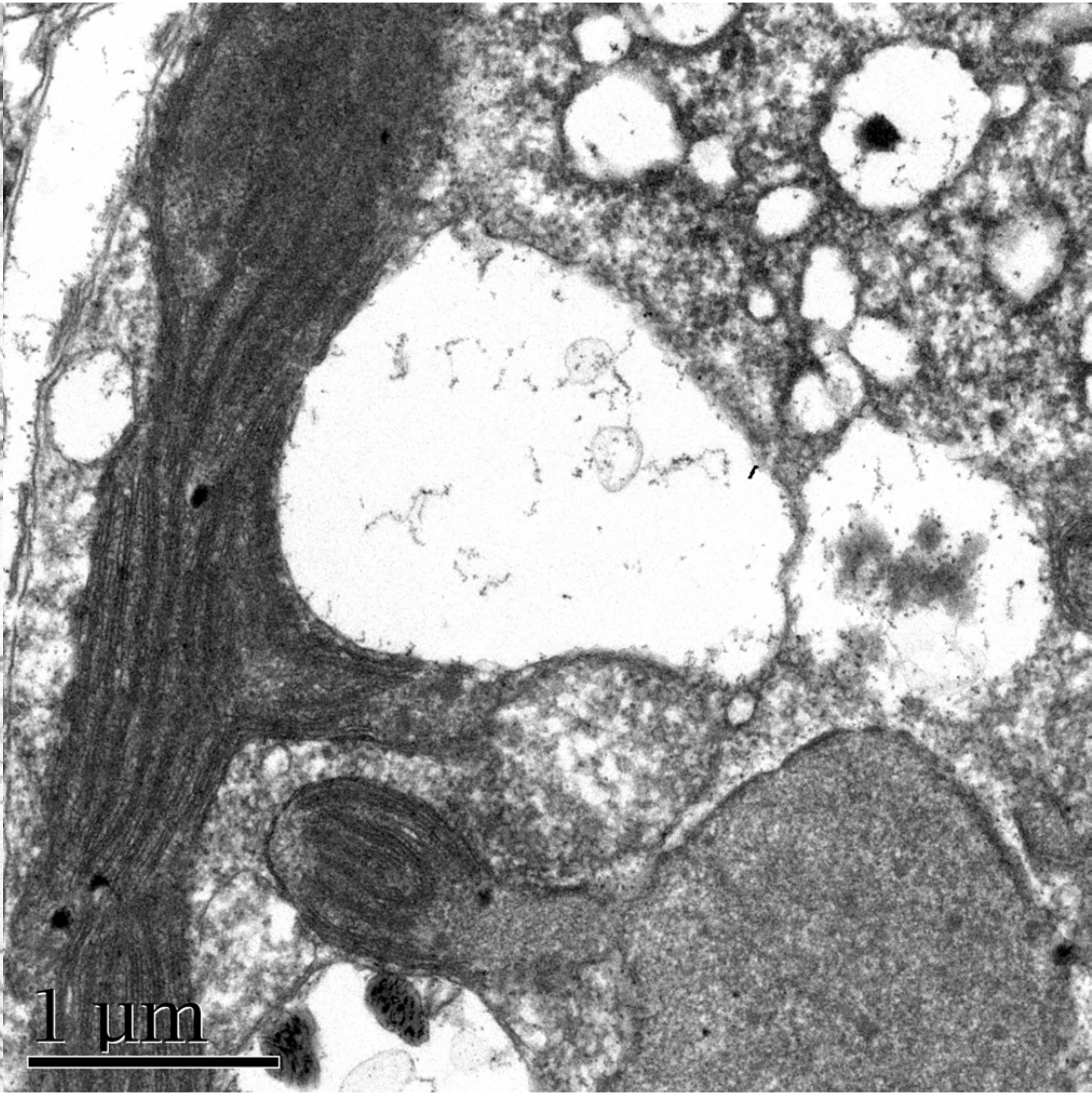

18-14_Correa_KH4_5A1_21.tif

Cell 7

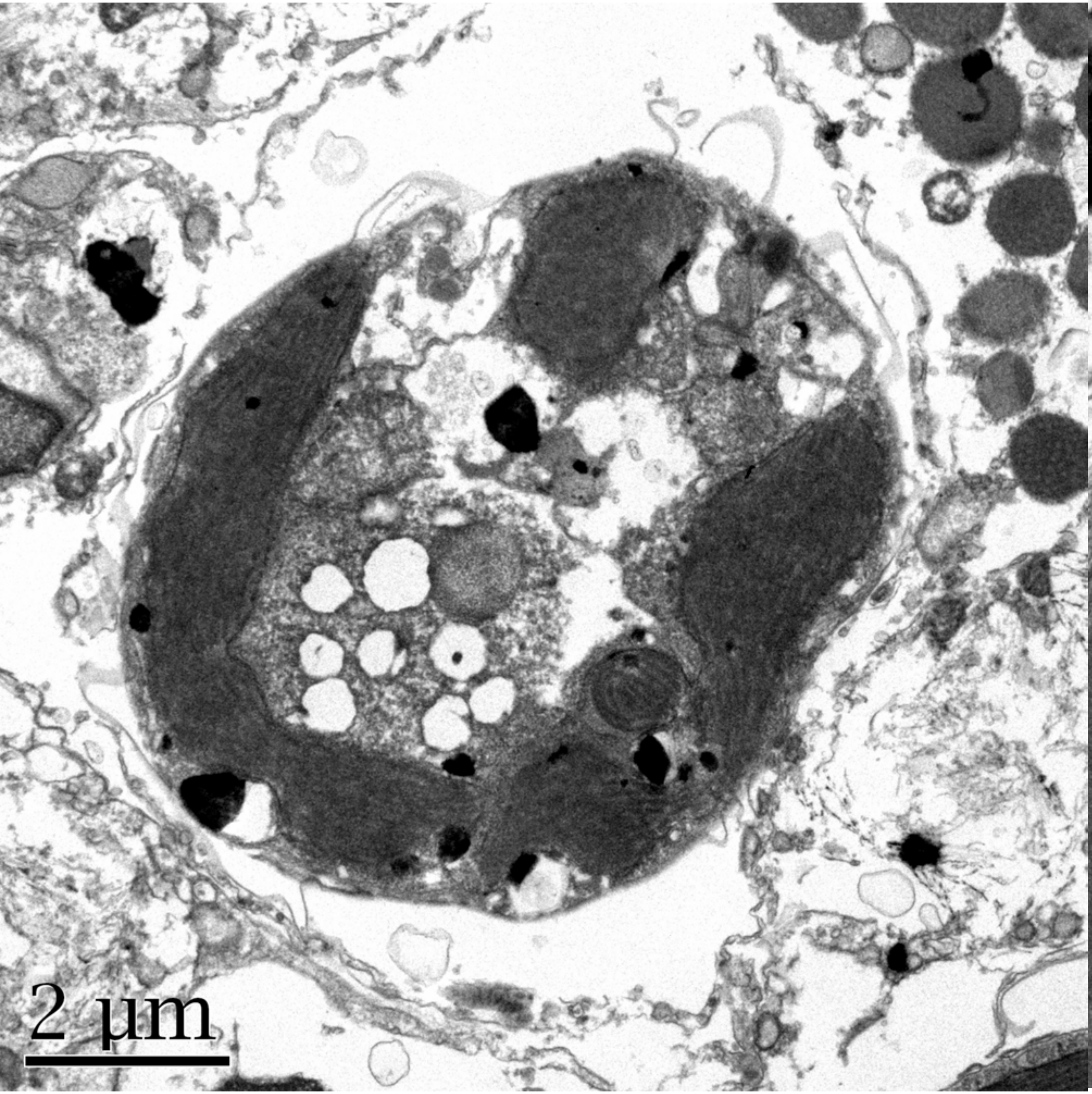

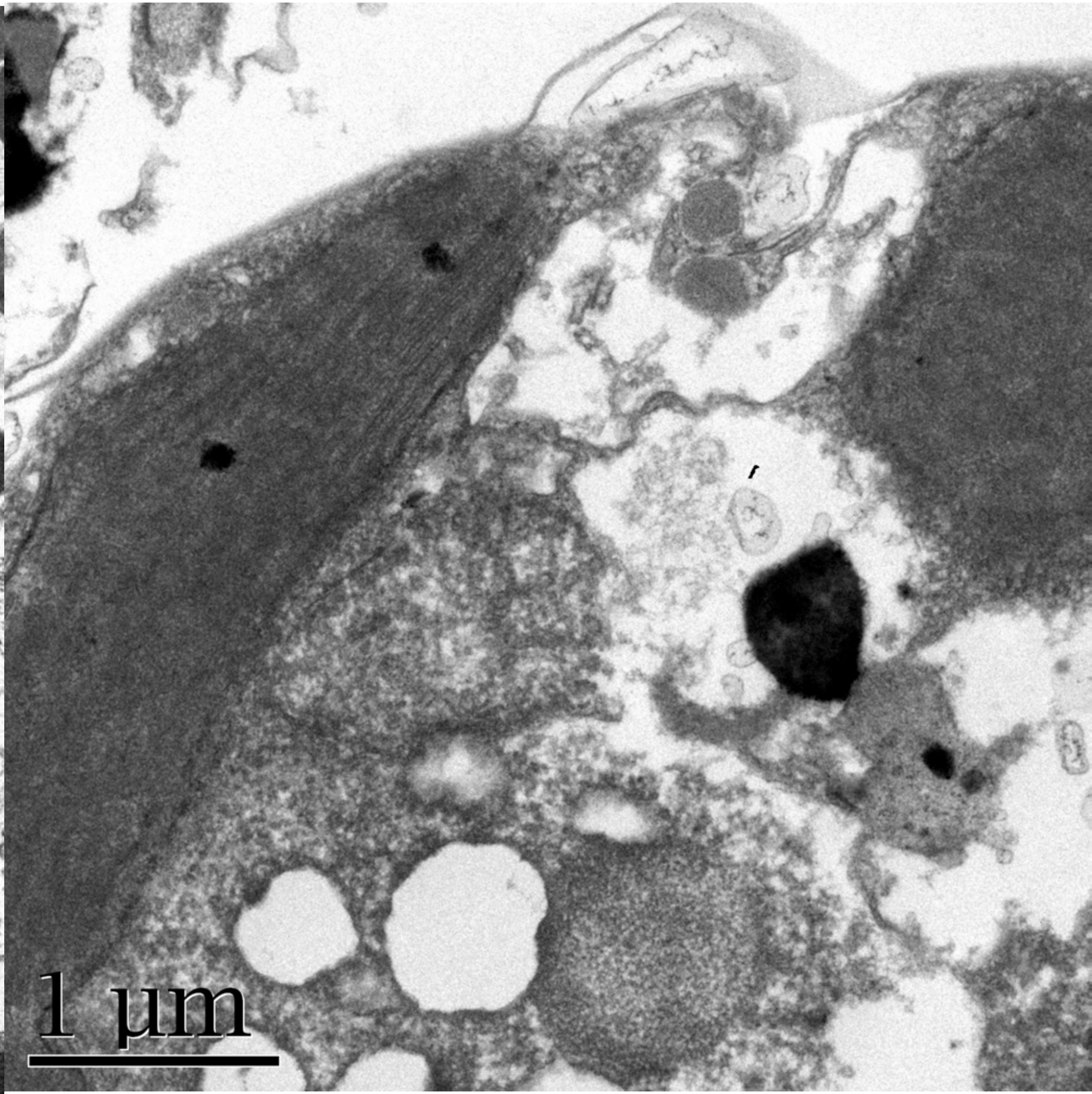

18-14_Correa_KH4_5A1_25.tif

18-14_Correa_KH4_5A1_27.tif

Cell 8

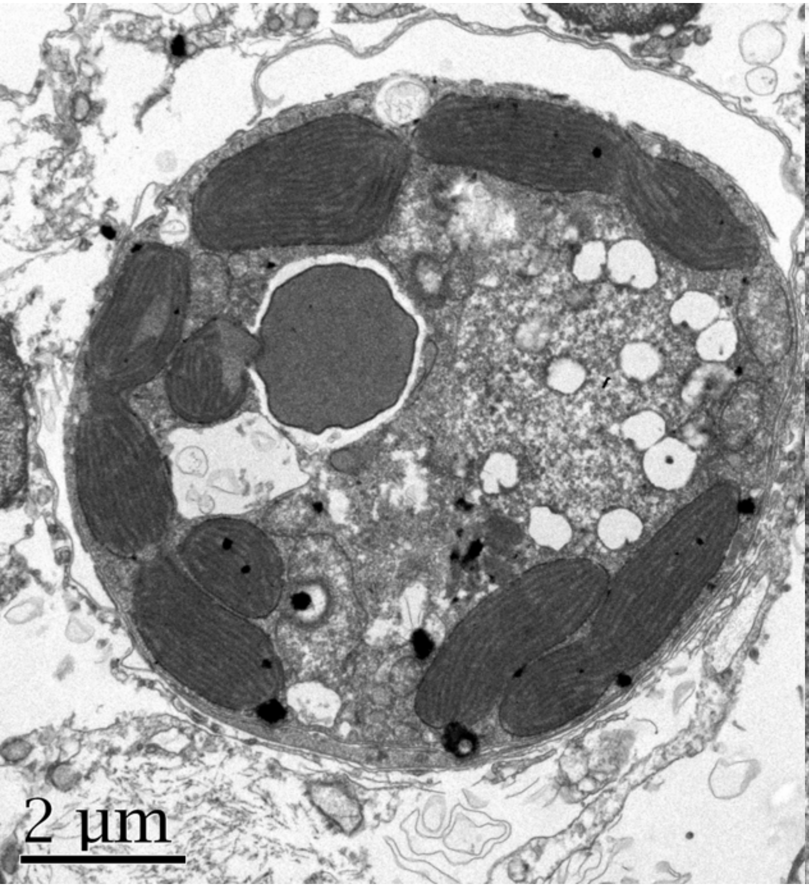

18-14_Correa_KH4_5A1_31.tif

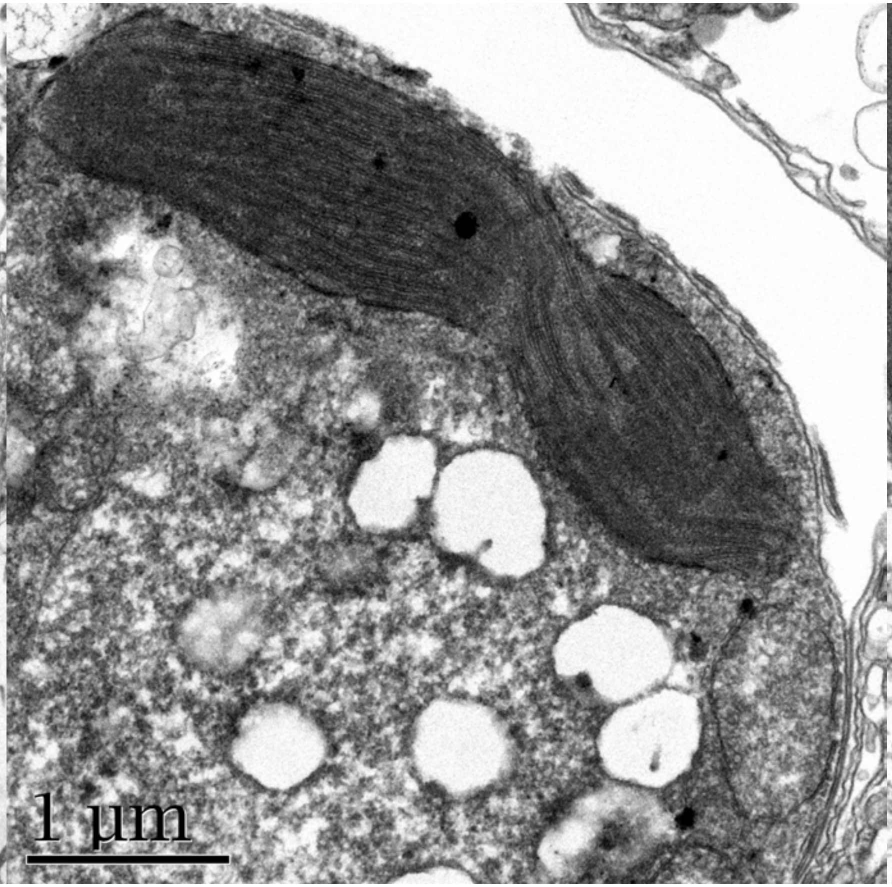

18-14_Correa_KH4_5A1_36.tif

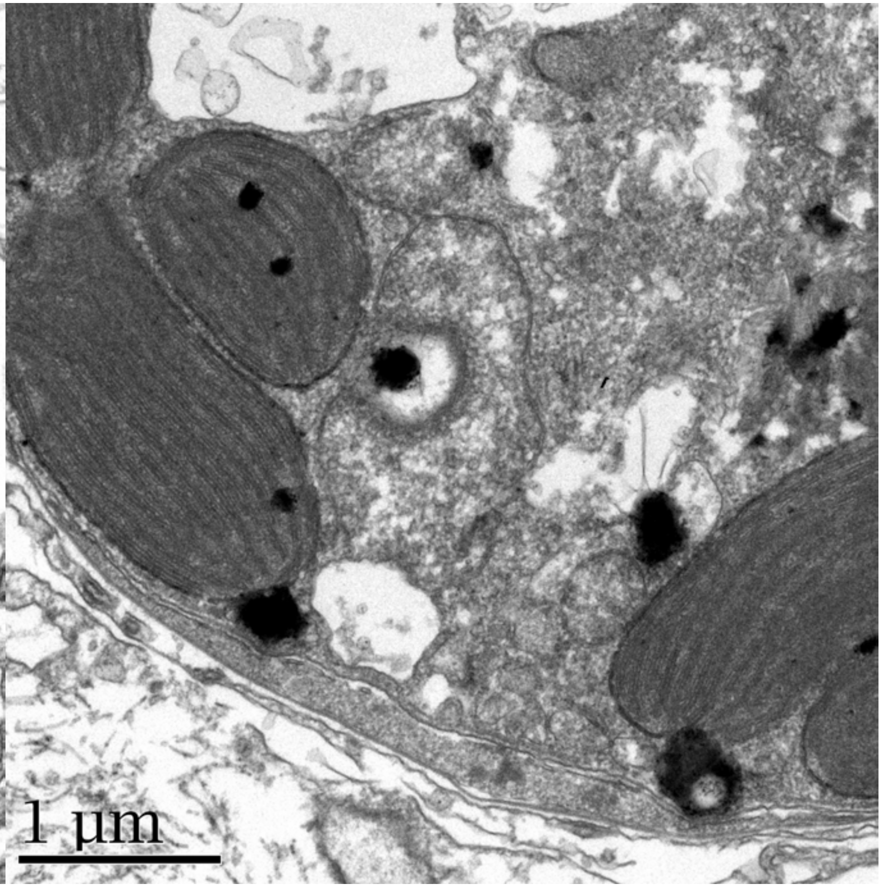

18-14_Correa_KH4_5A1_32.tif

Cell 9

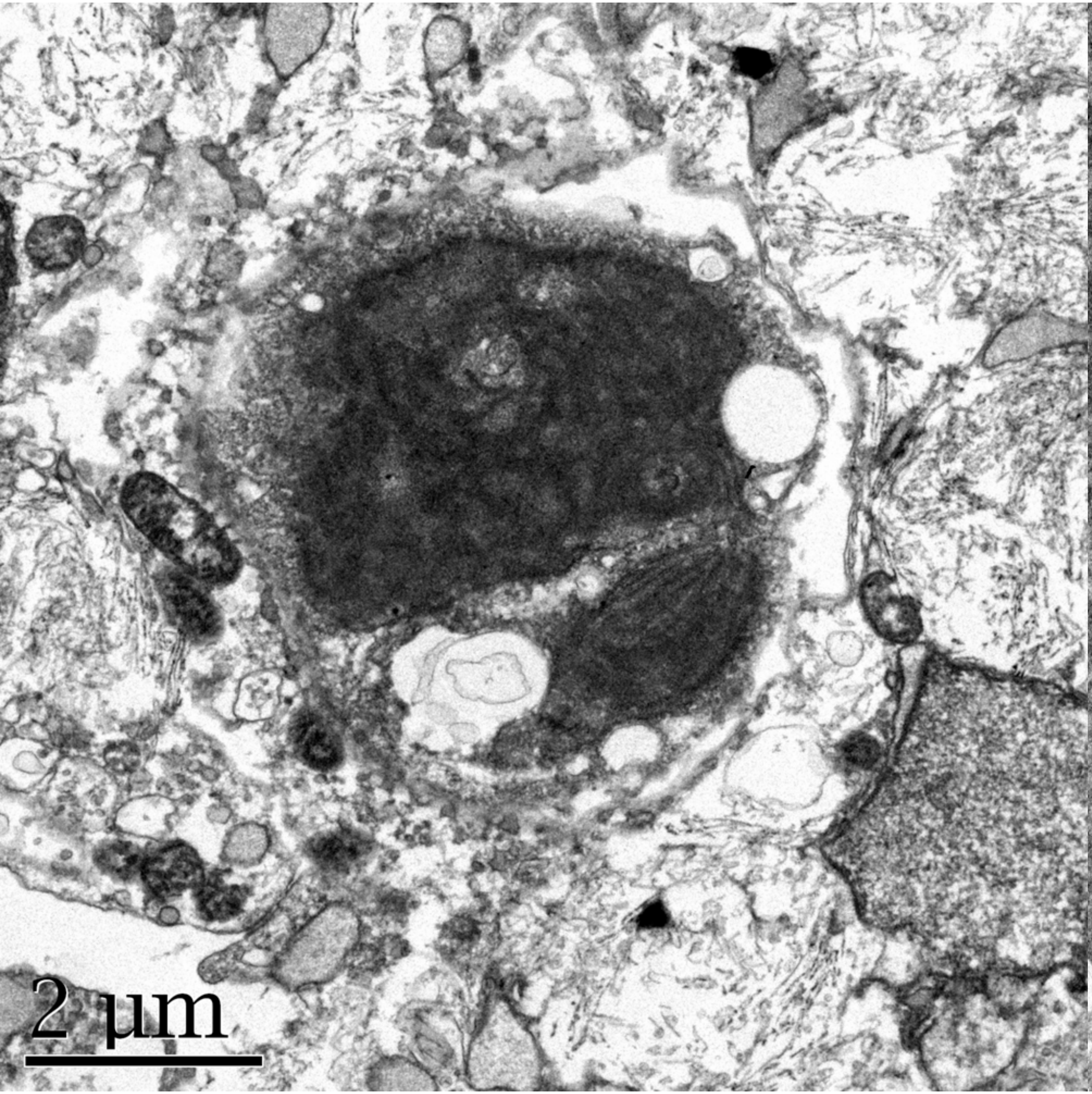

18-14_Correa_KH4_5A1_40.tif

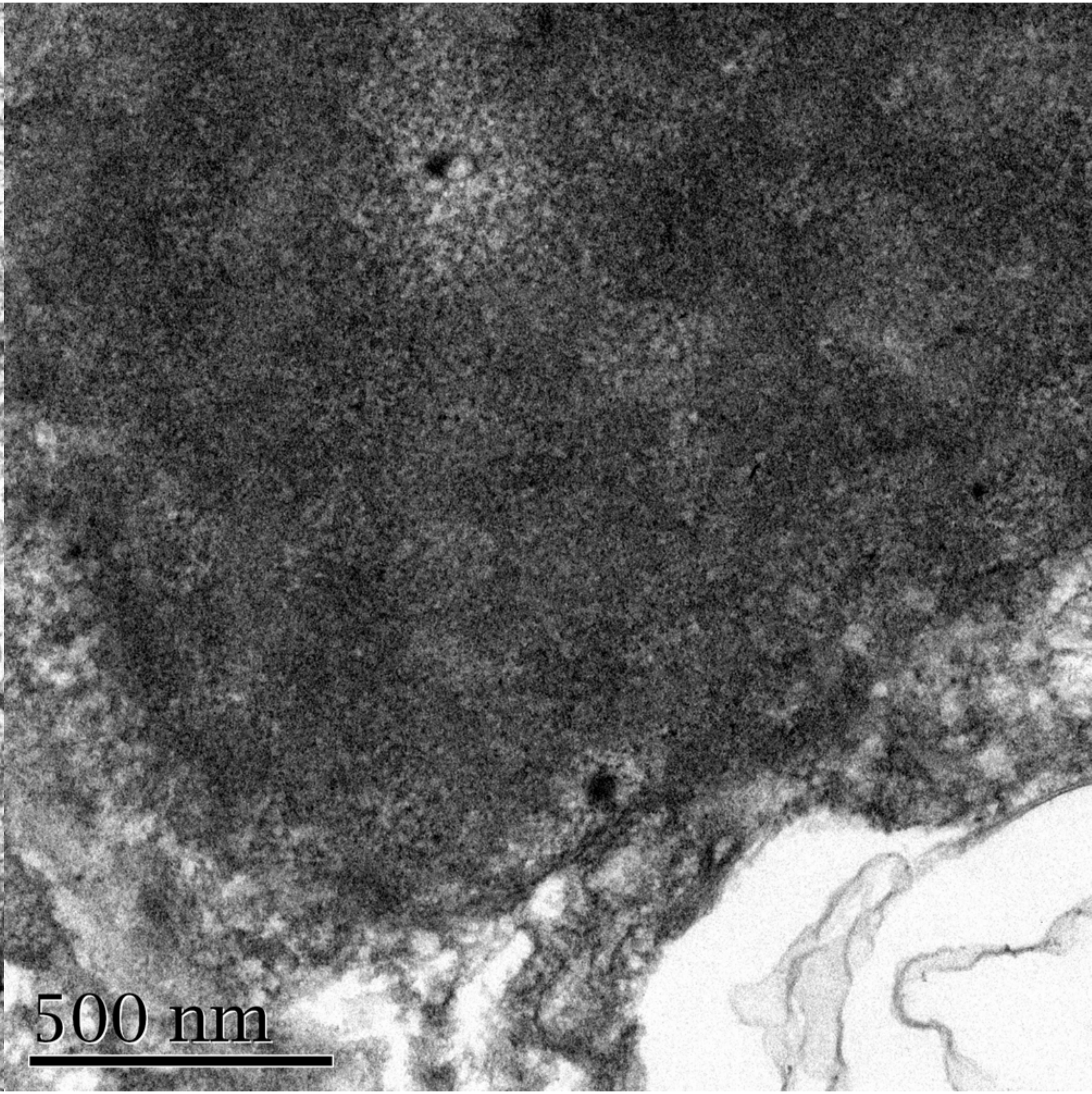

18-14_Correa_KH4_5A1_42.tif

Cell 10

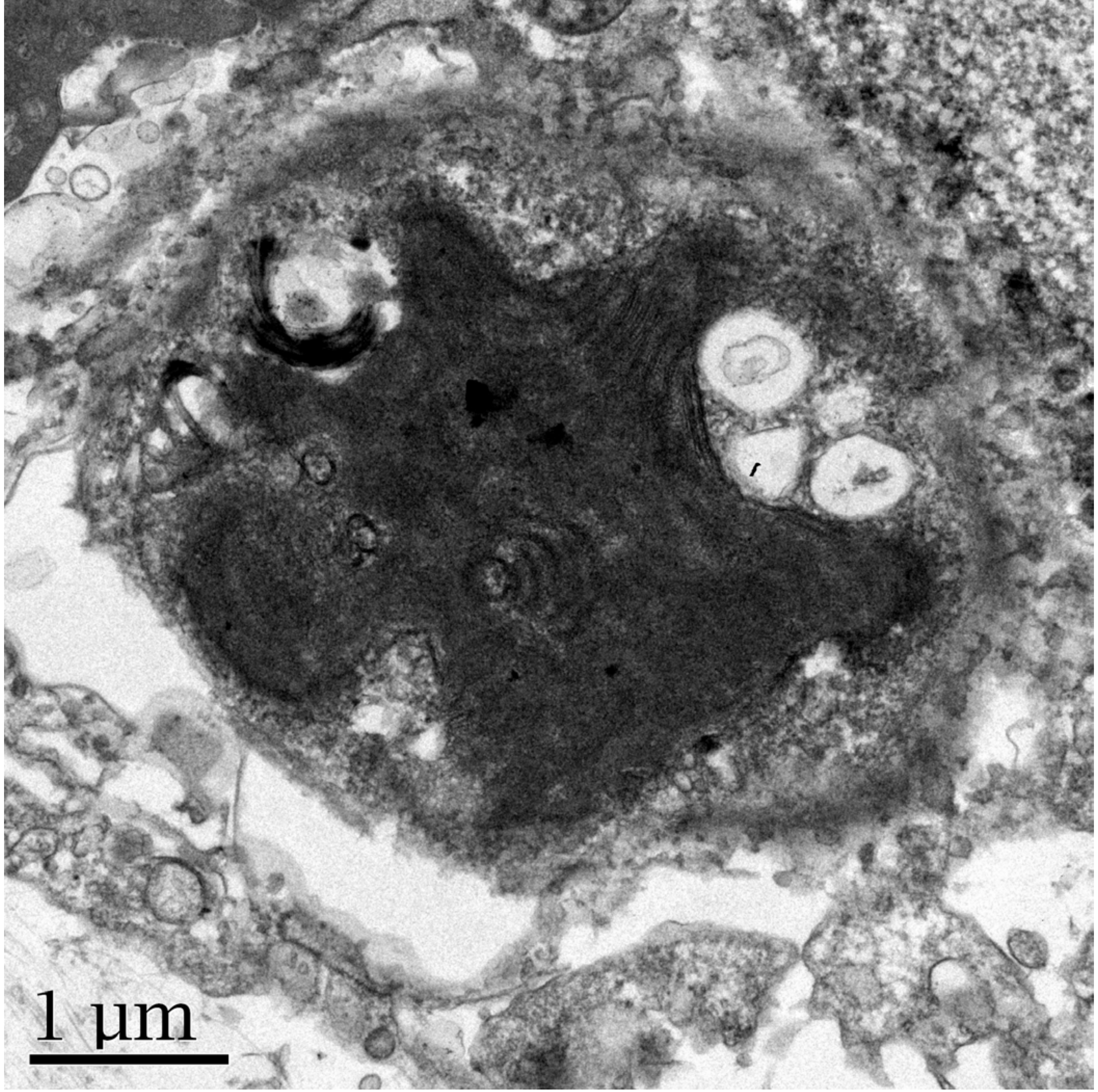

18-14_Correa_KH4_5A1_43.tif

Cell 11

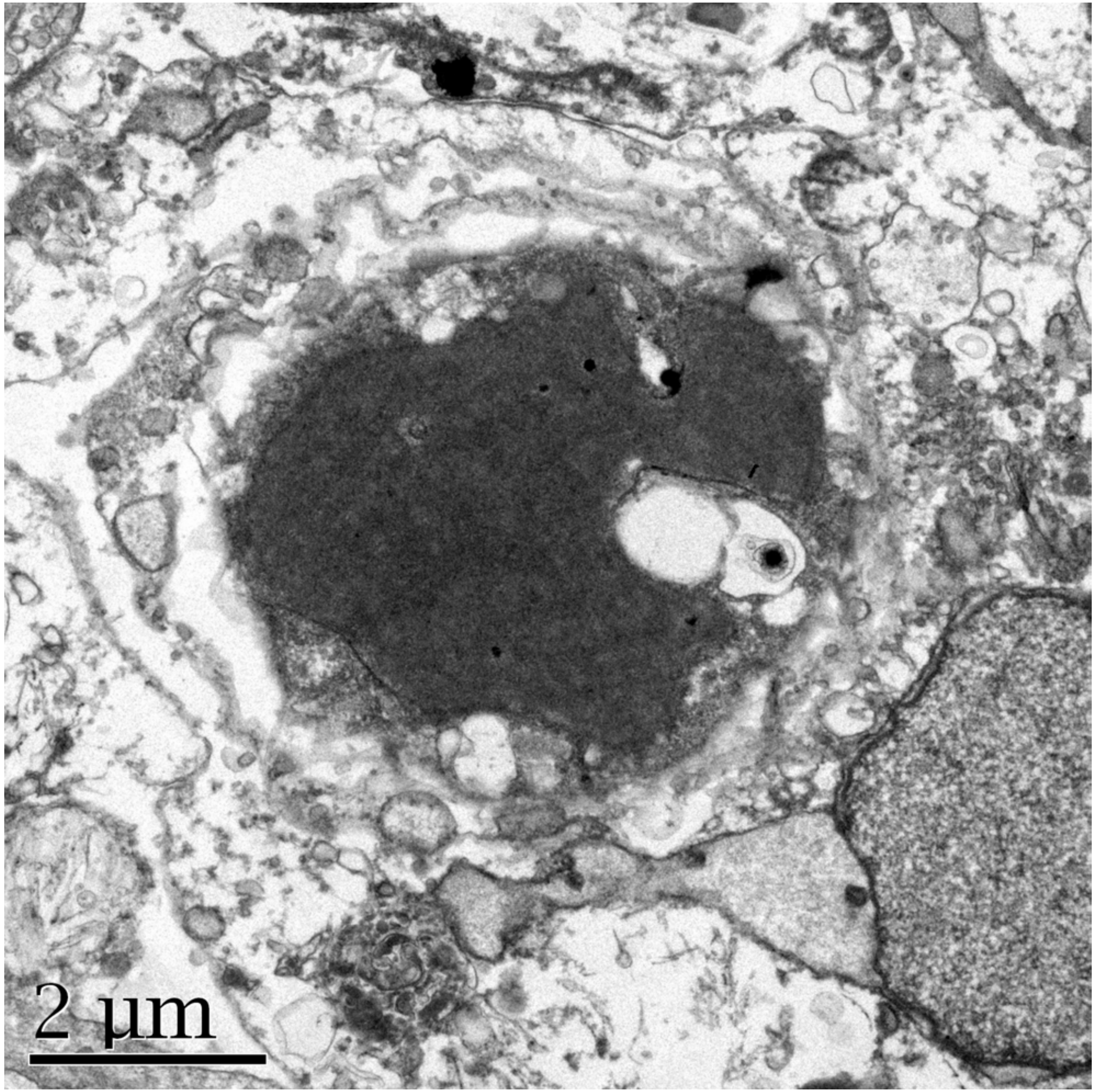

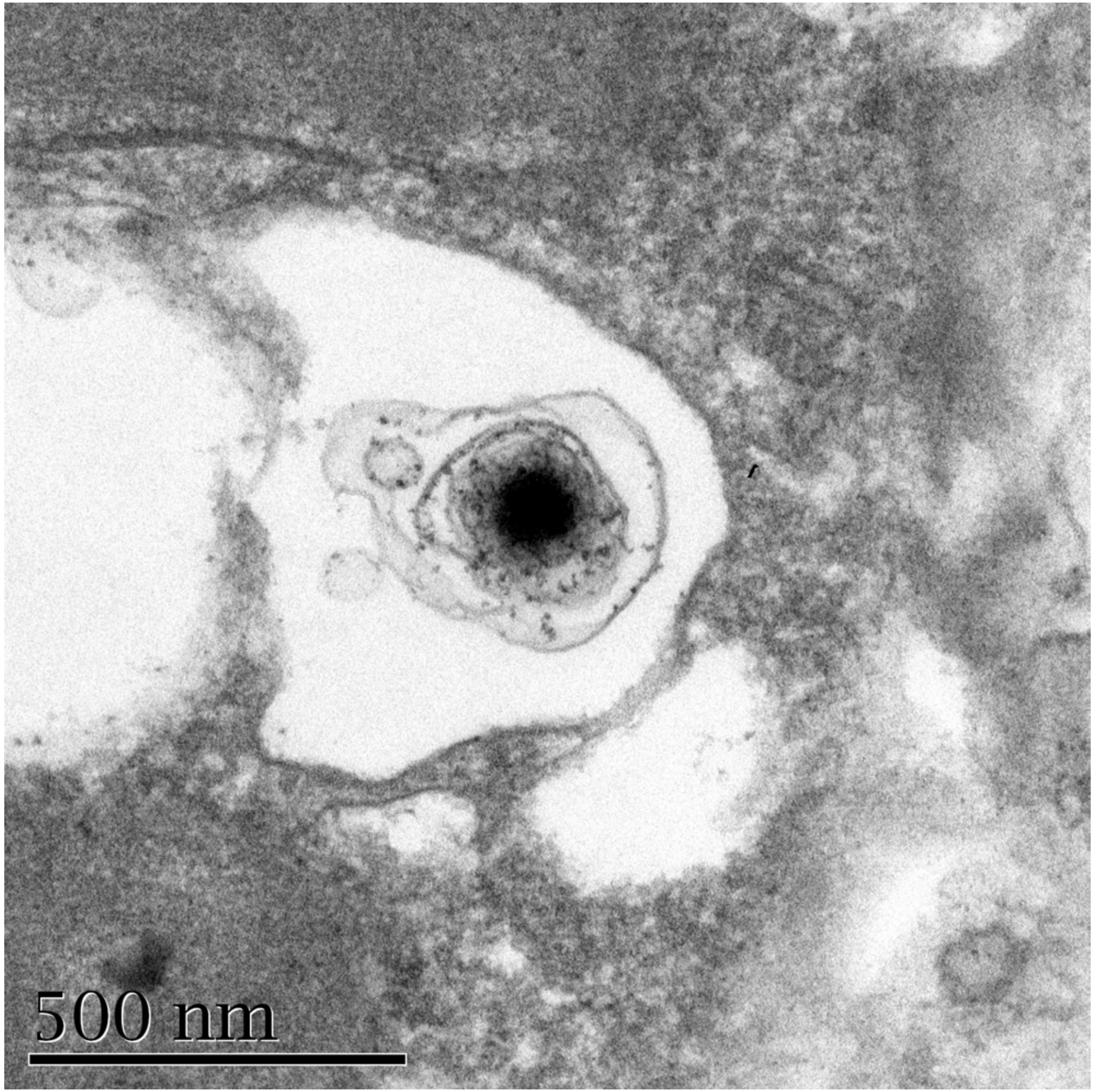

18-14_Correa_KH4_5A1_45.tif

18-14_Correa_KH4_5A1_48.tif

Cell 12

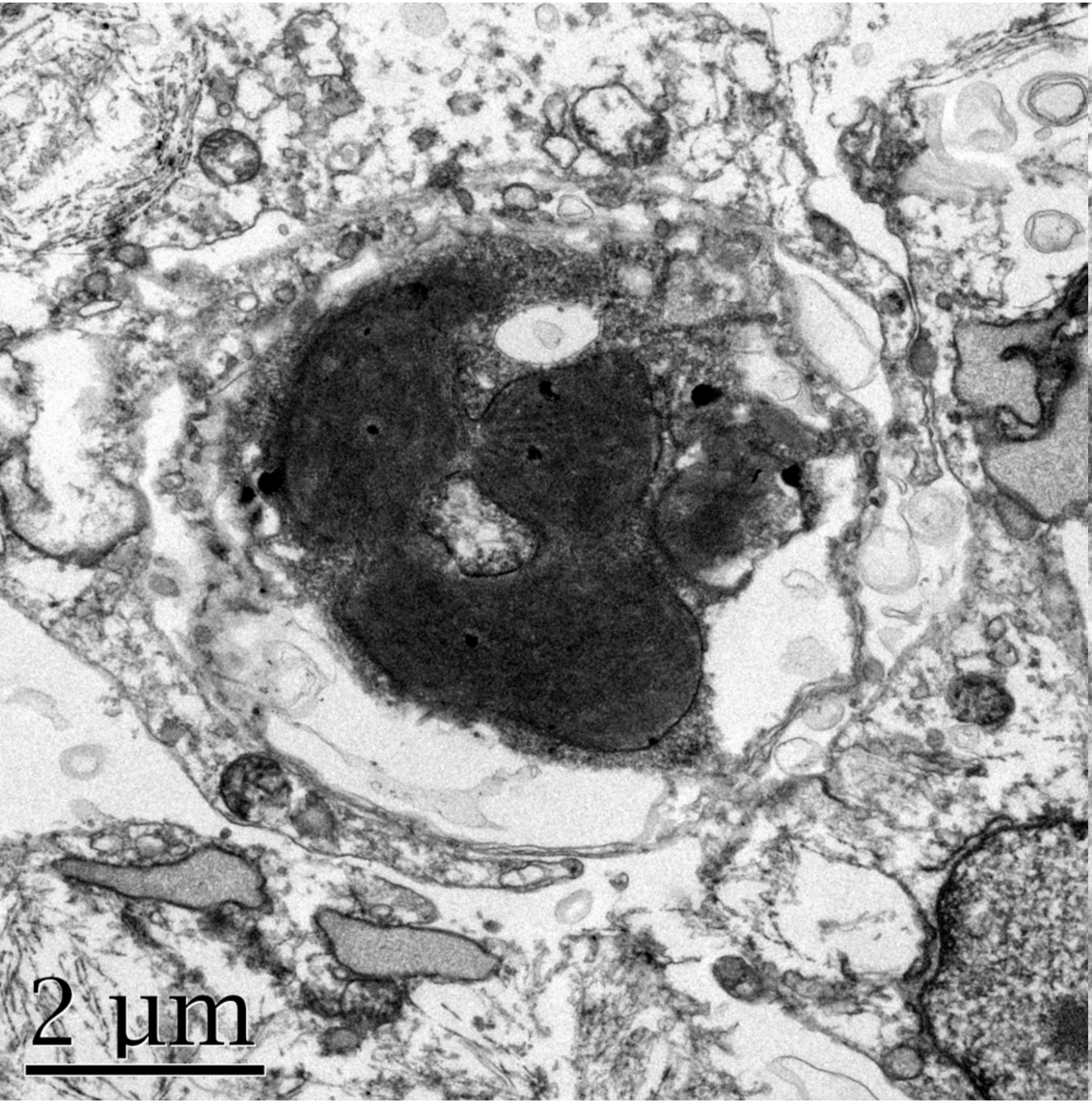

18-14_Correa_KH4_5A1_50.tif

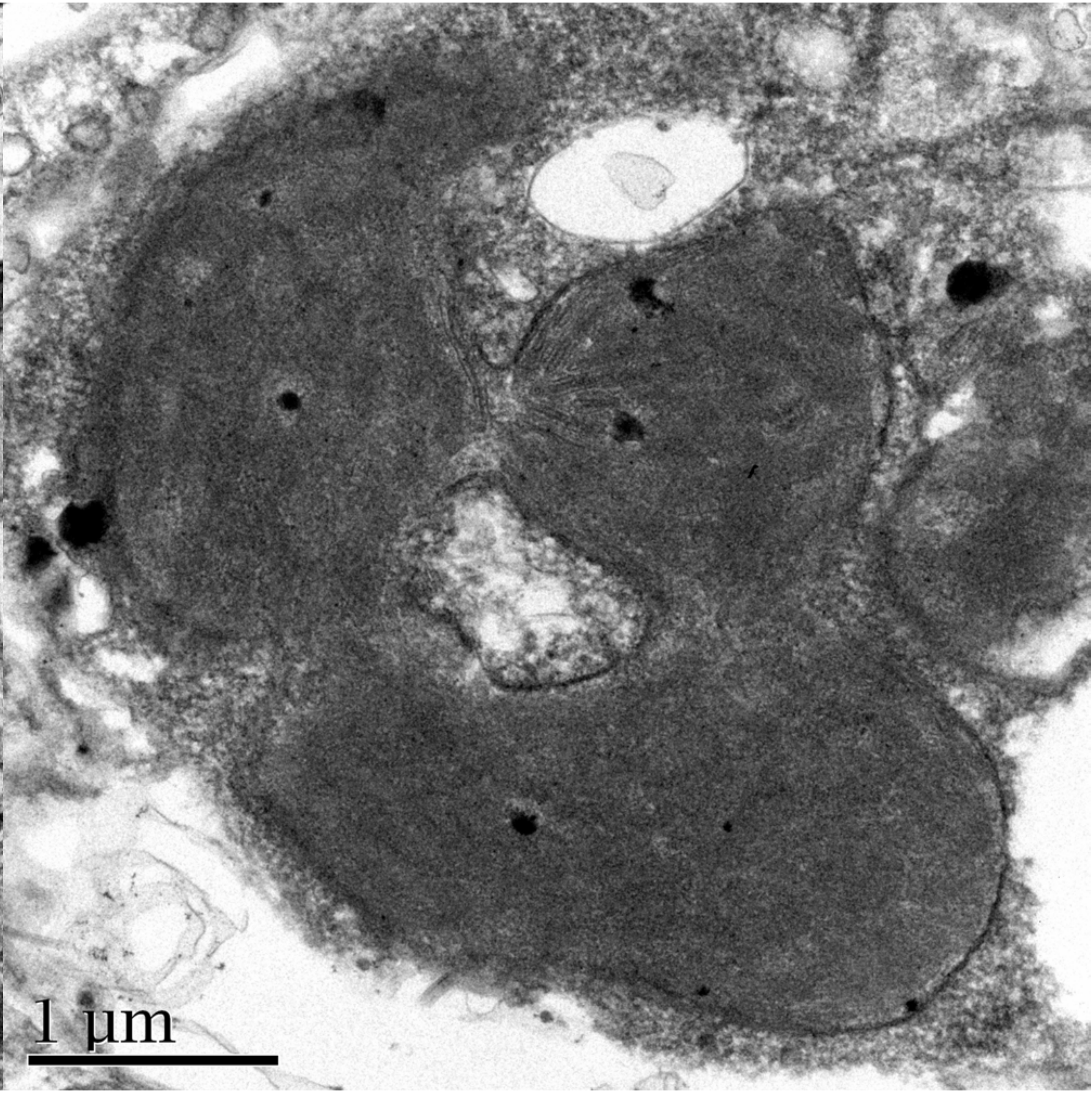

18-14_Correa_KH4_5A1_51.tif

Cell 13

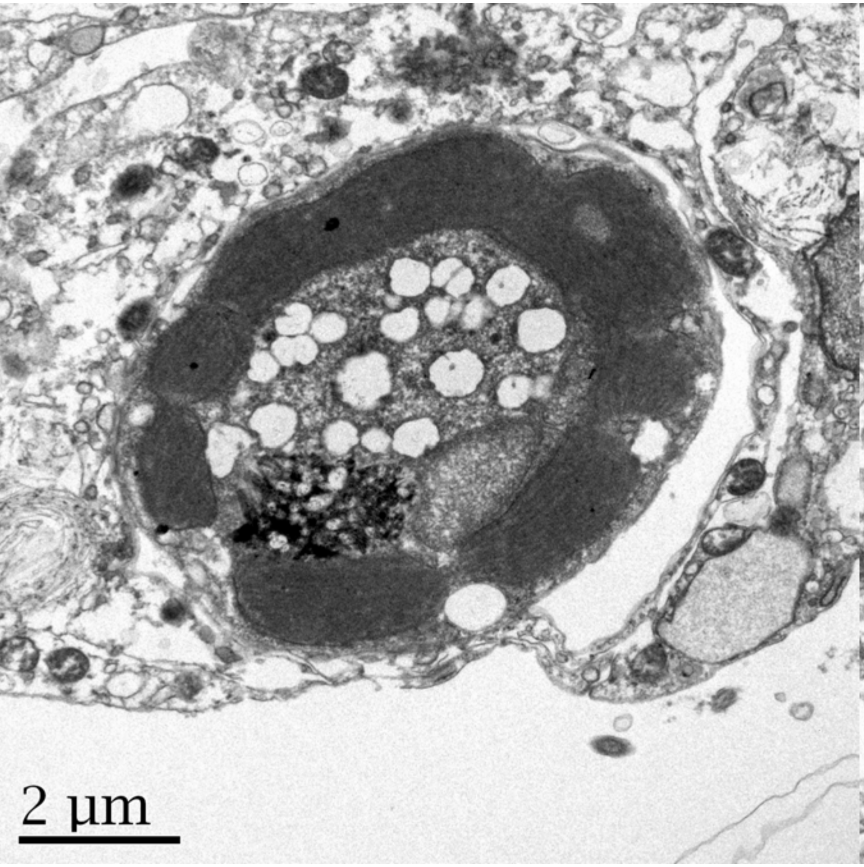

18-14_Correa_KH4_5A1_54.tif

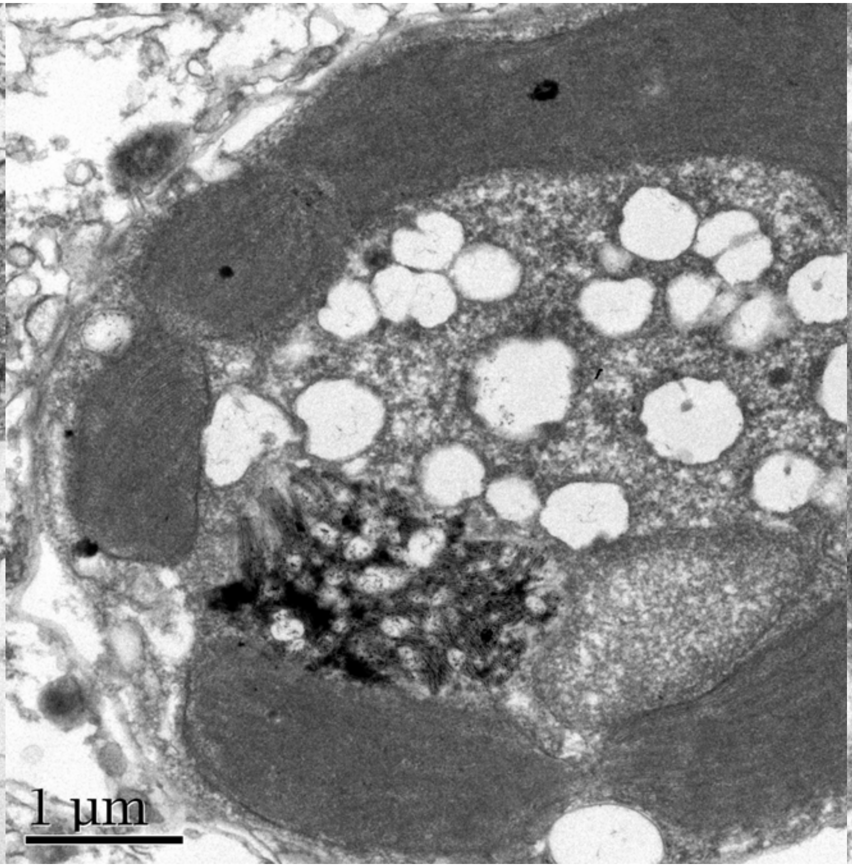

18-14_Correa_KH4_5A1_57.tif

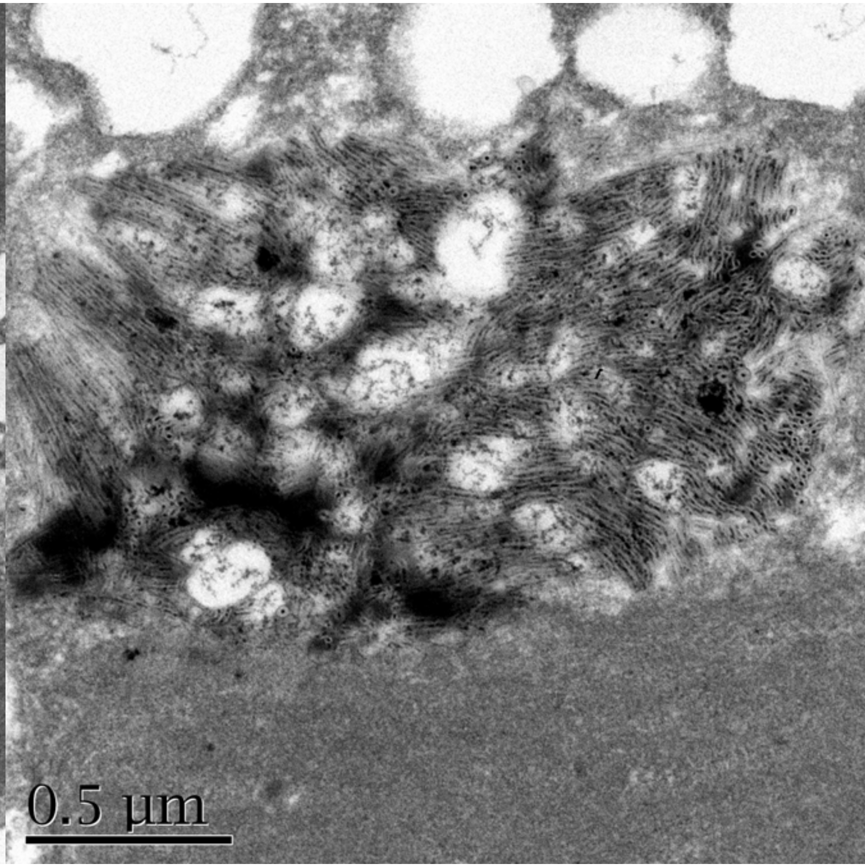

18-14_Correa_KH4_5A1_58.tif

Cell 14

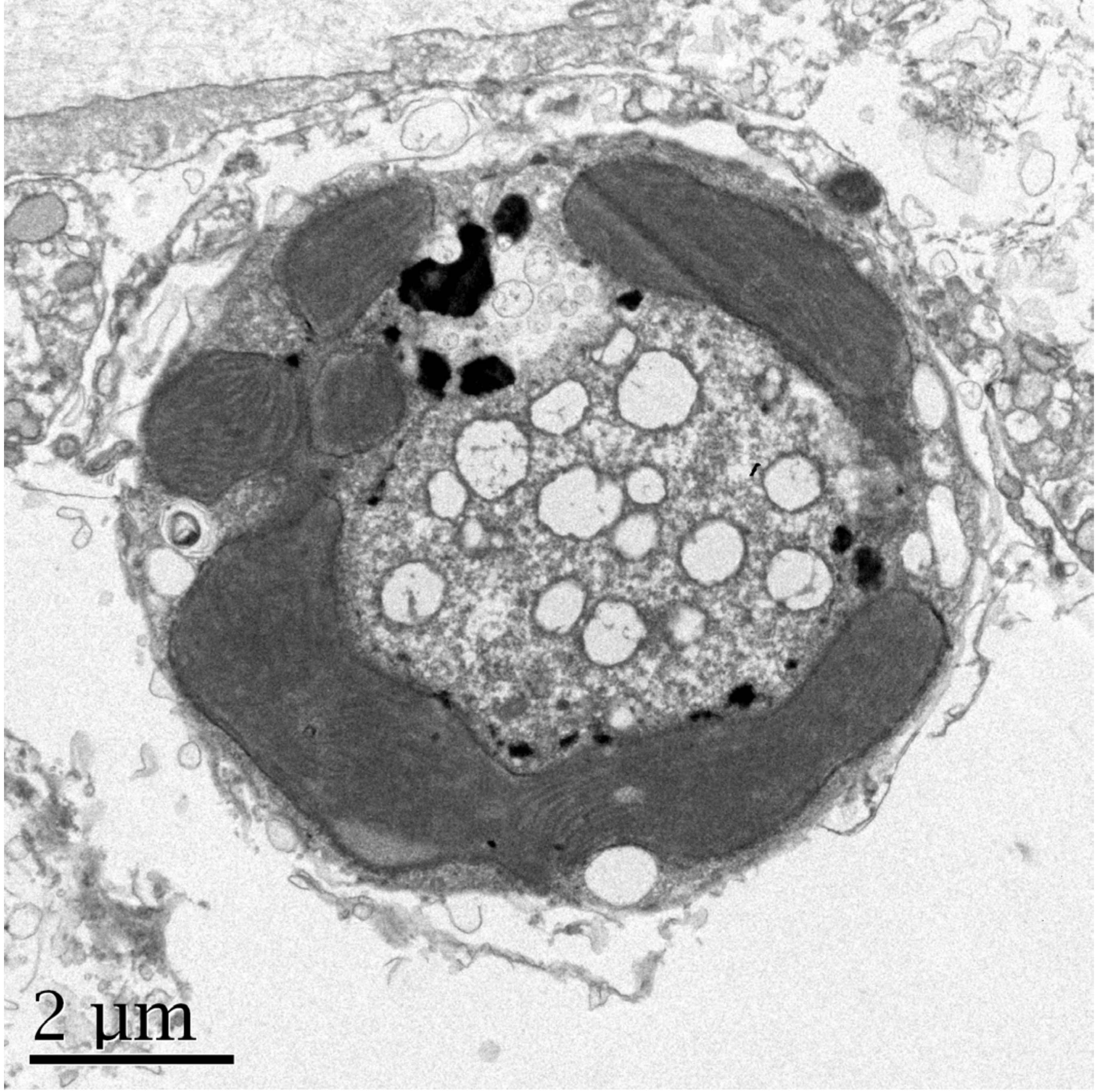

18-14_Correa_KH4_5A1_61.tif

Cell 15

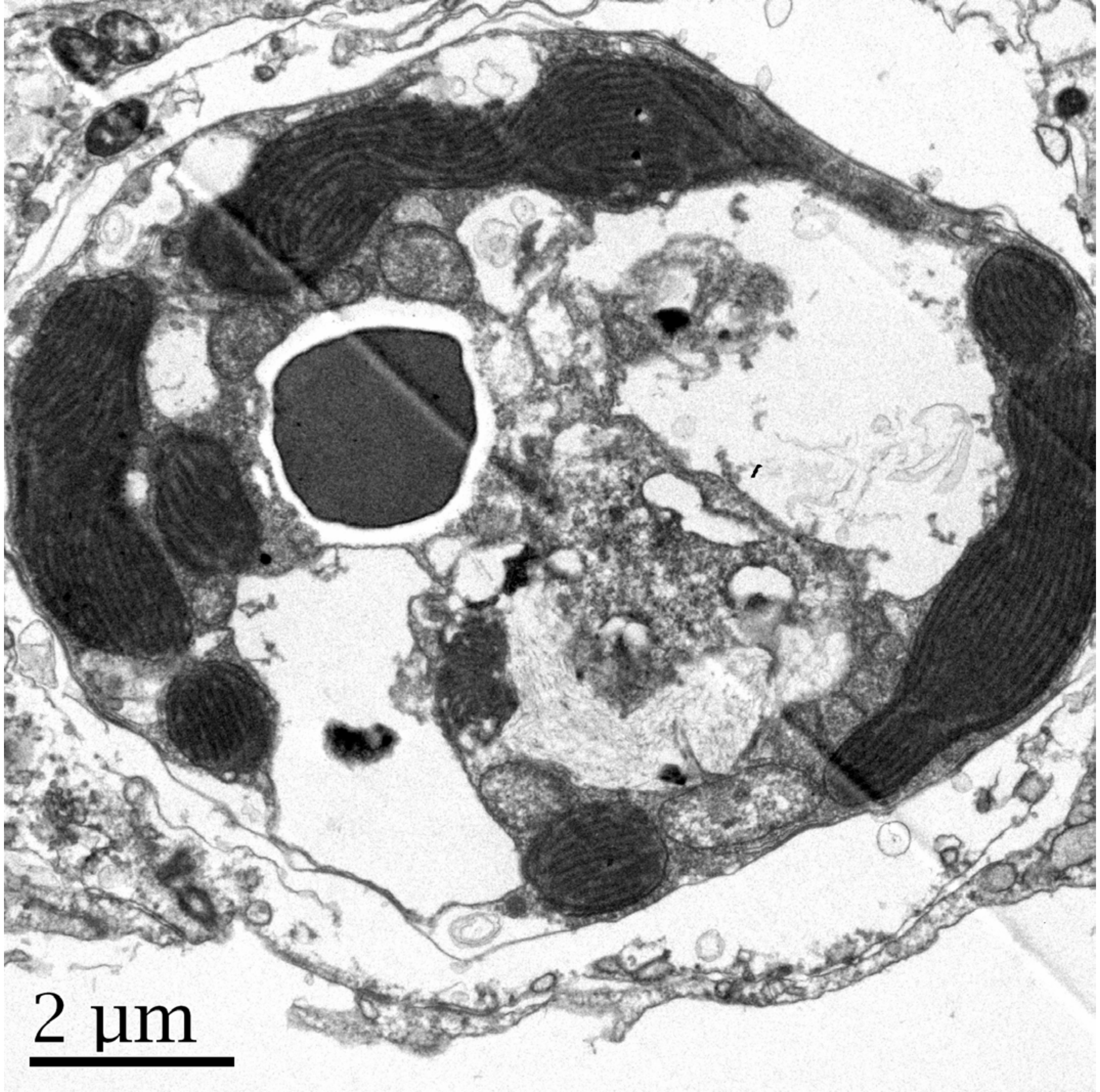

18-14_Correa_KH4_5A1_64.tif

Cell 16

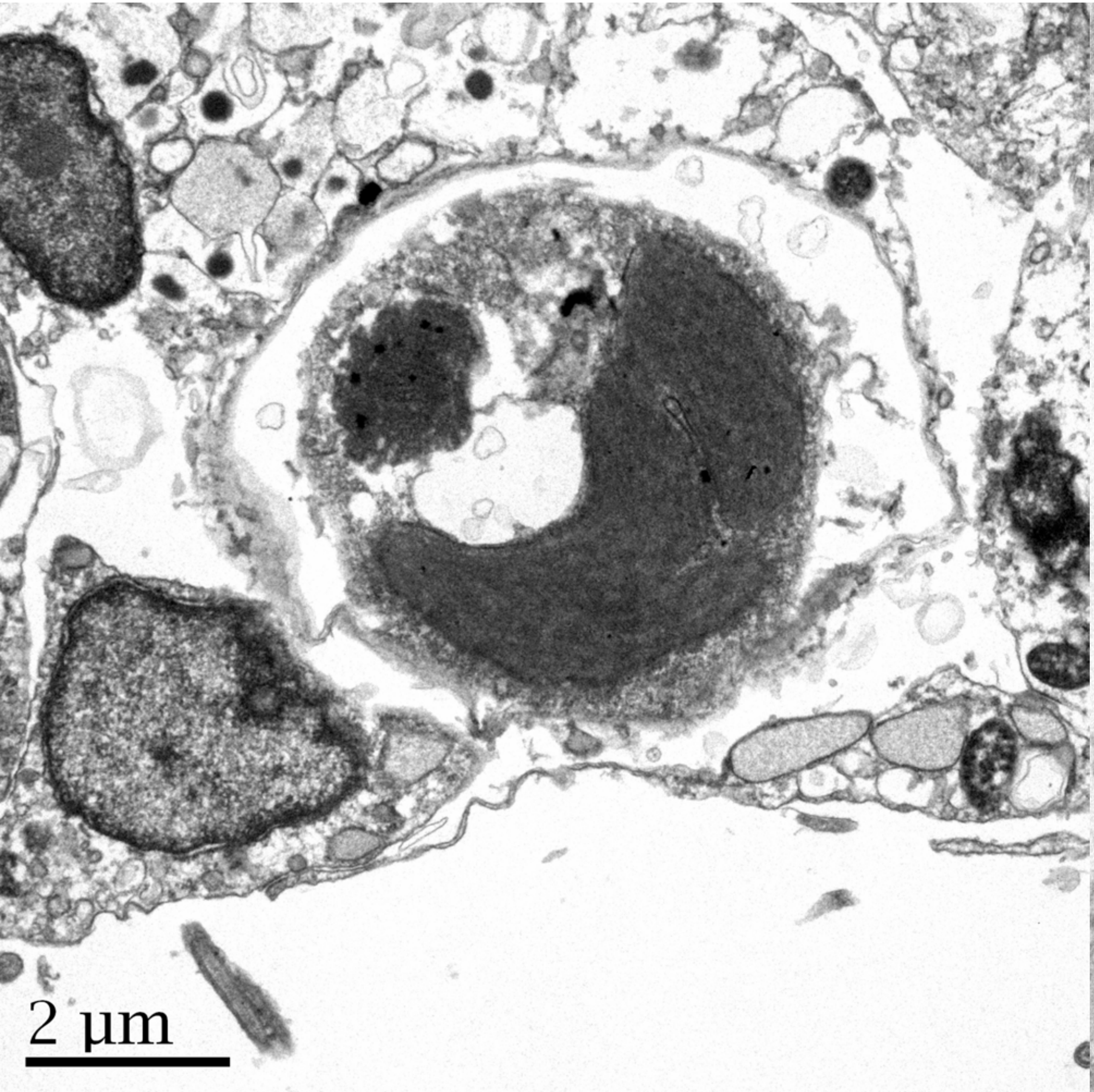

18-14_Correa_KH4_5A1_65.tif

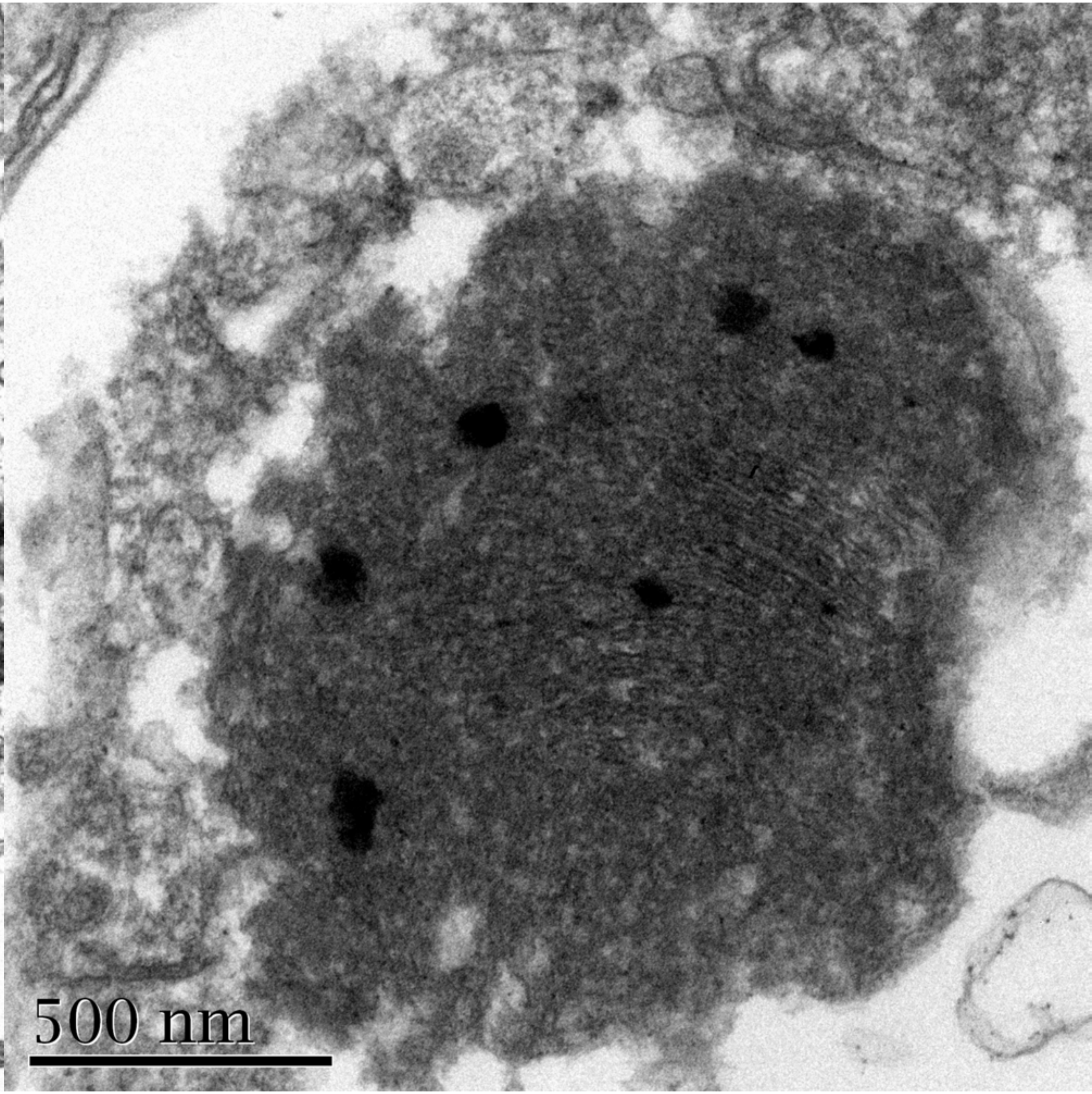

18-14_Correa_KH4_5A1_67.tif

Cell 17

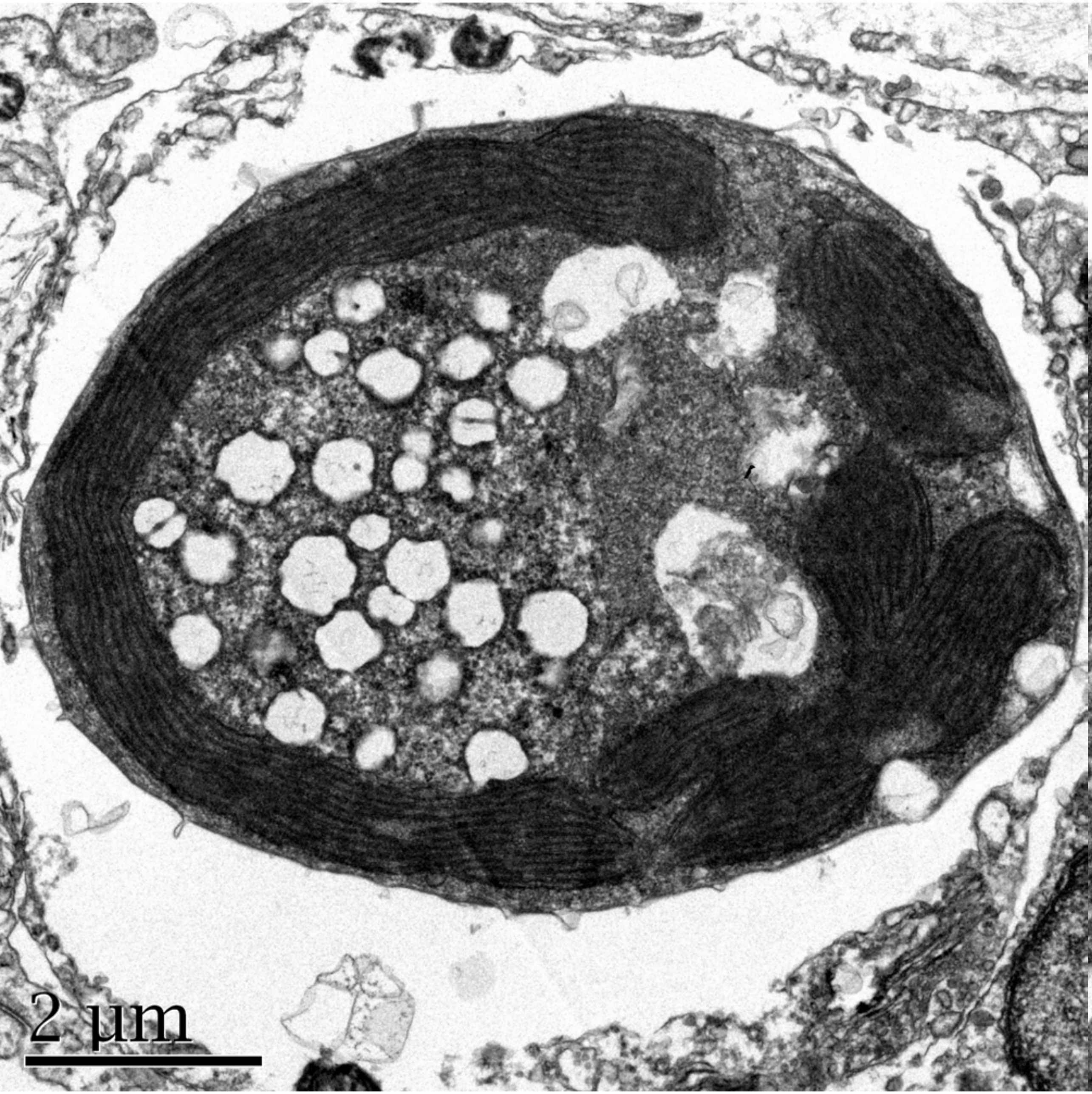

18-14_Correa_KH4_5A1_68.tif

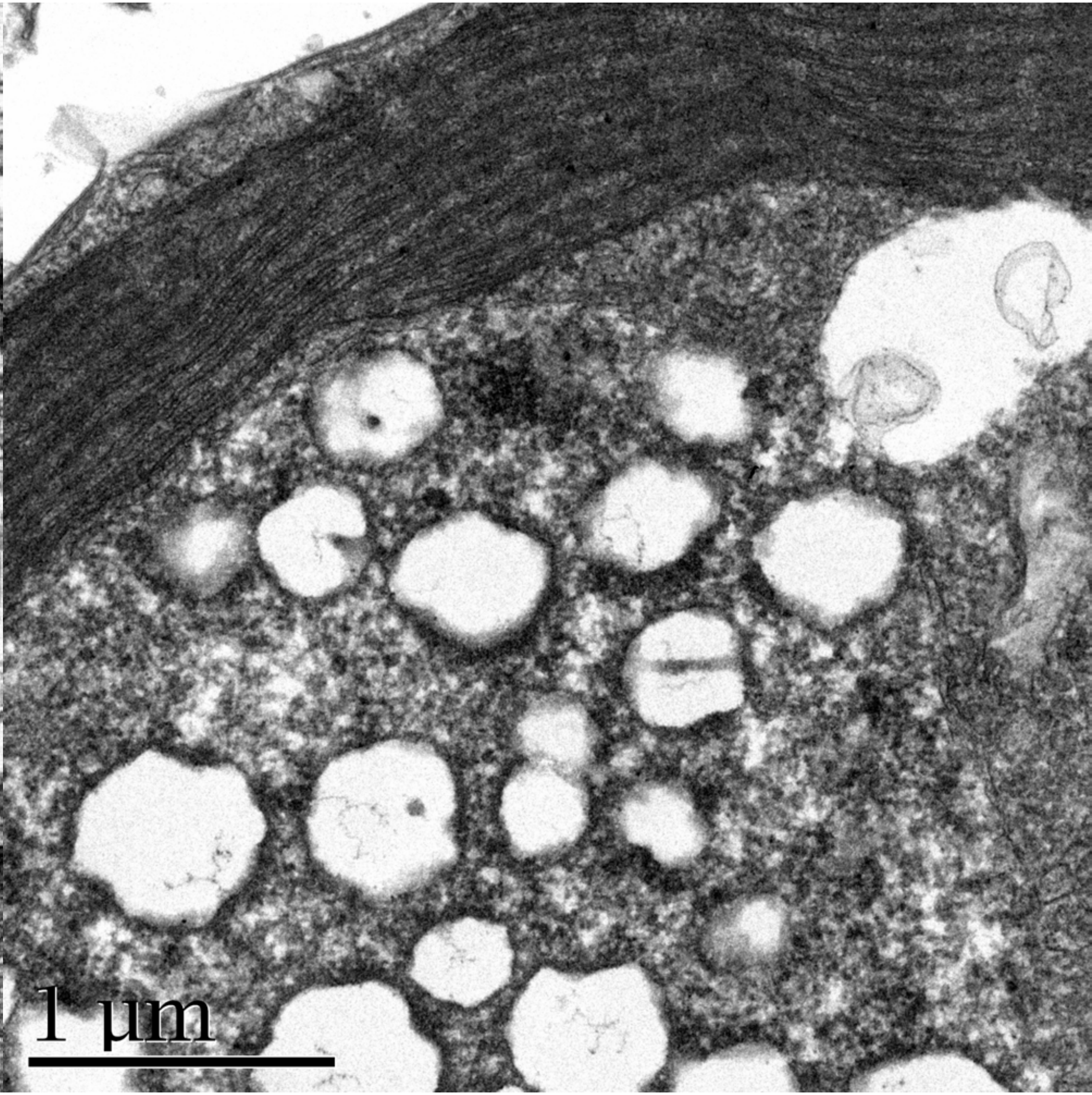

18-14_Correa_KH4_5A1_69.tif

Cell 18

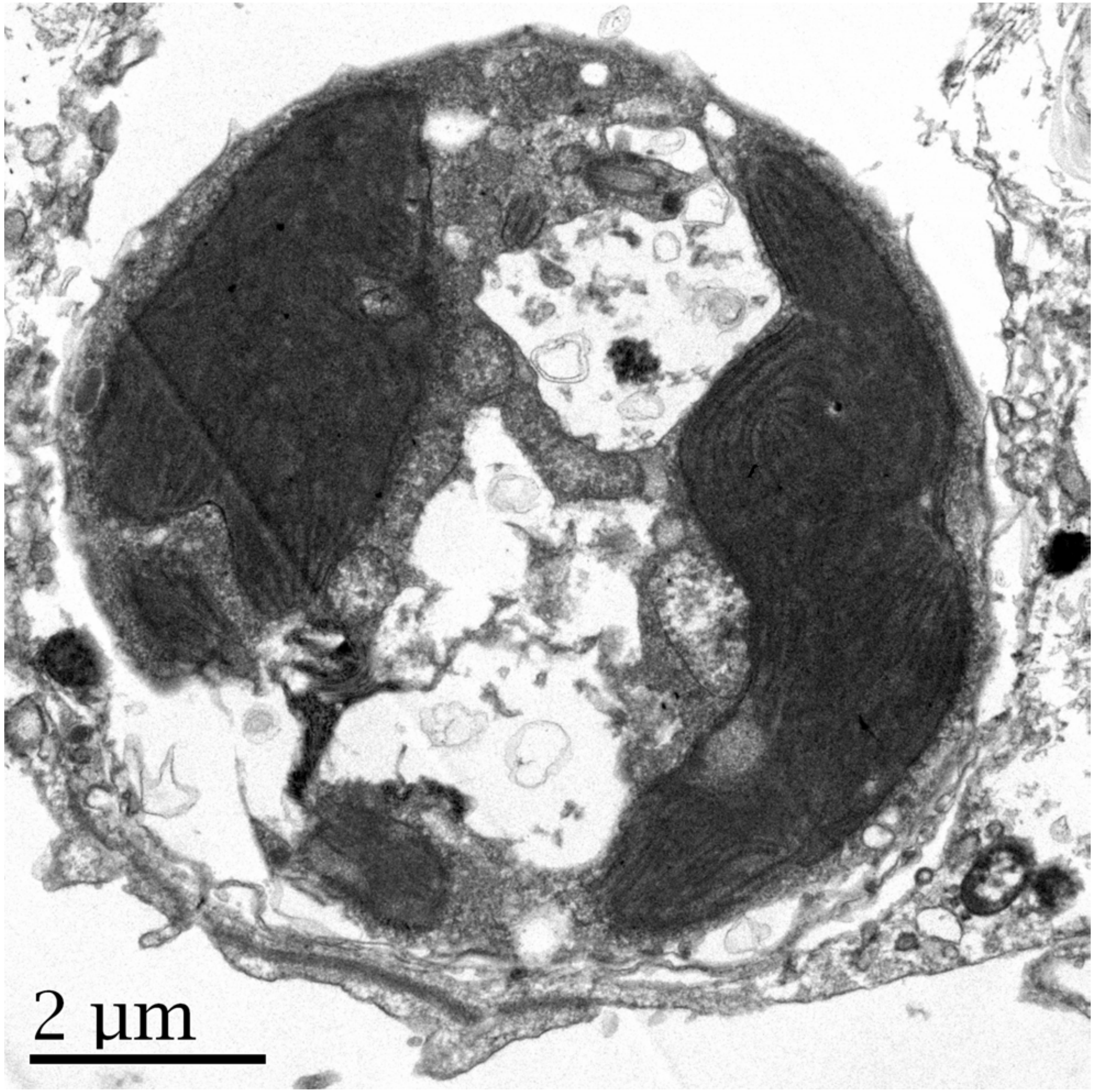

18-14_Correa_KH4_5A1_70.tif

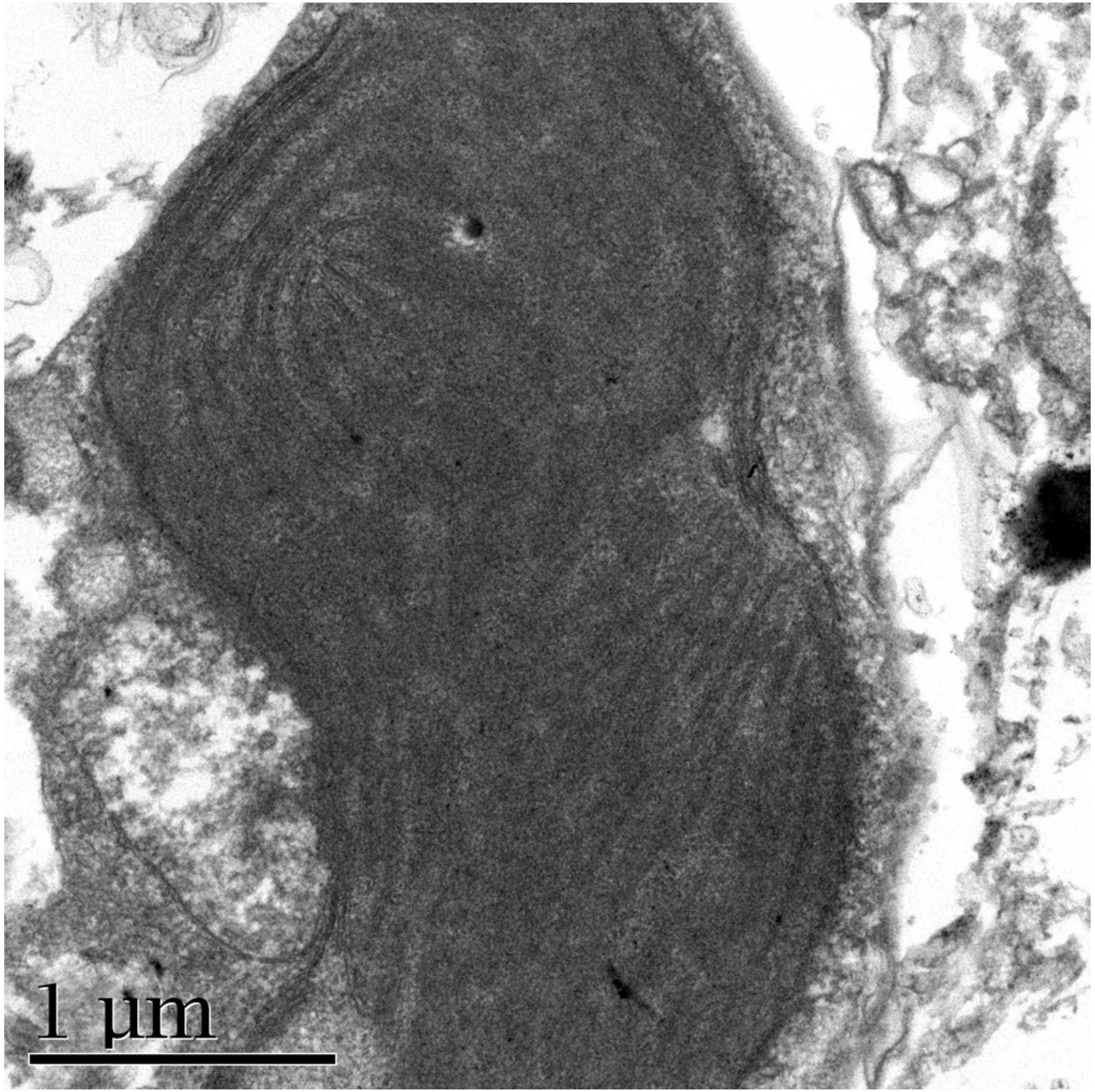

18-14_Correa_KH4_5A1_71.tif

Ambient - ACR Colony C

Cell 1

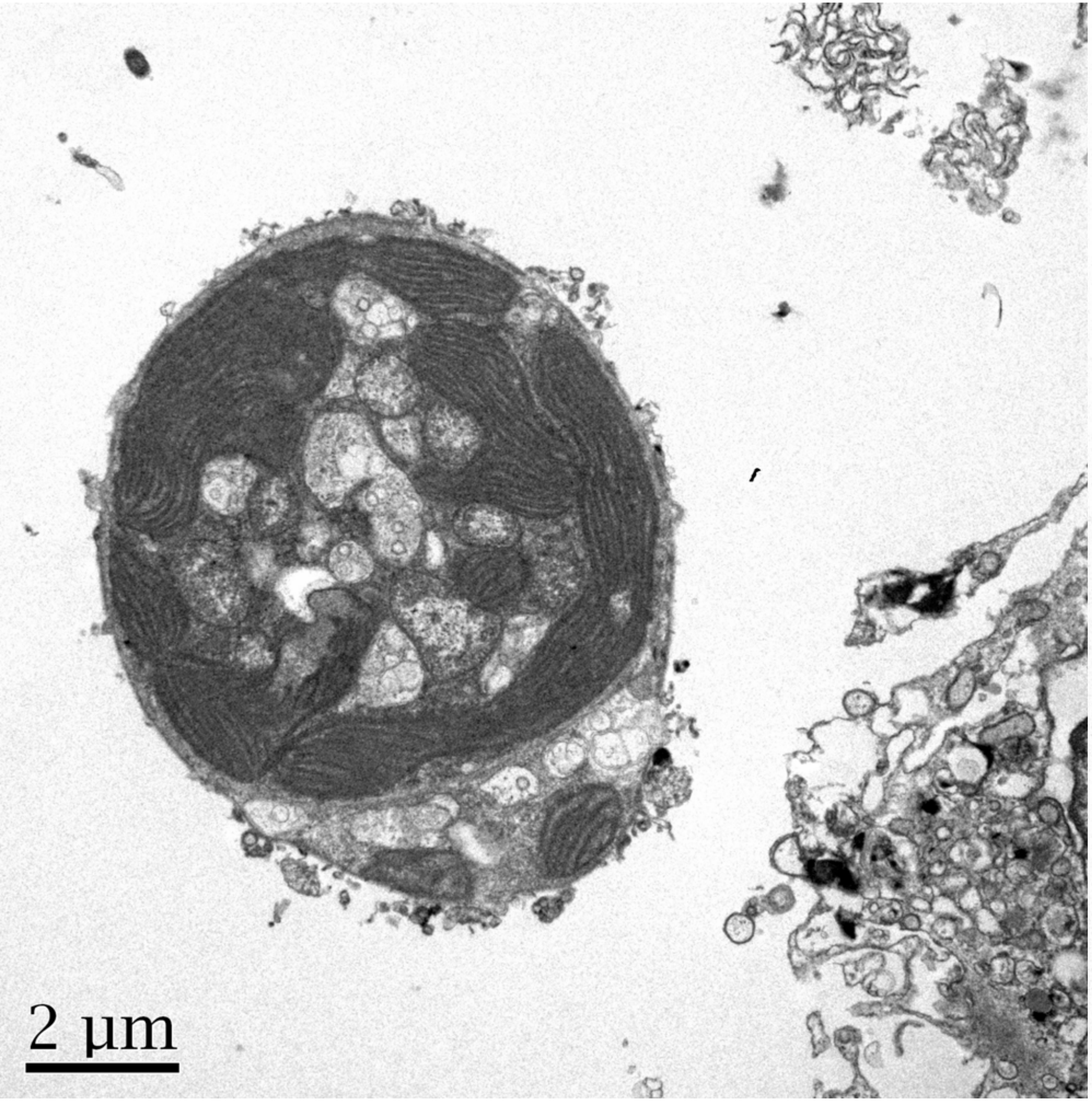

18-14_Correa_XC4_5C3_1.tif

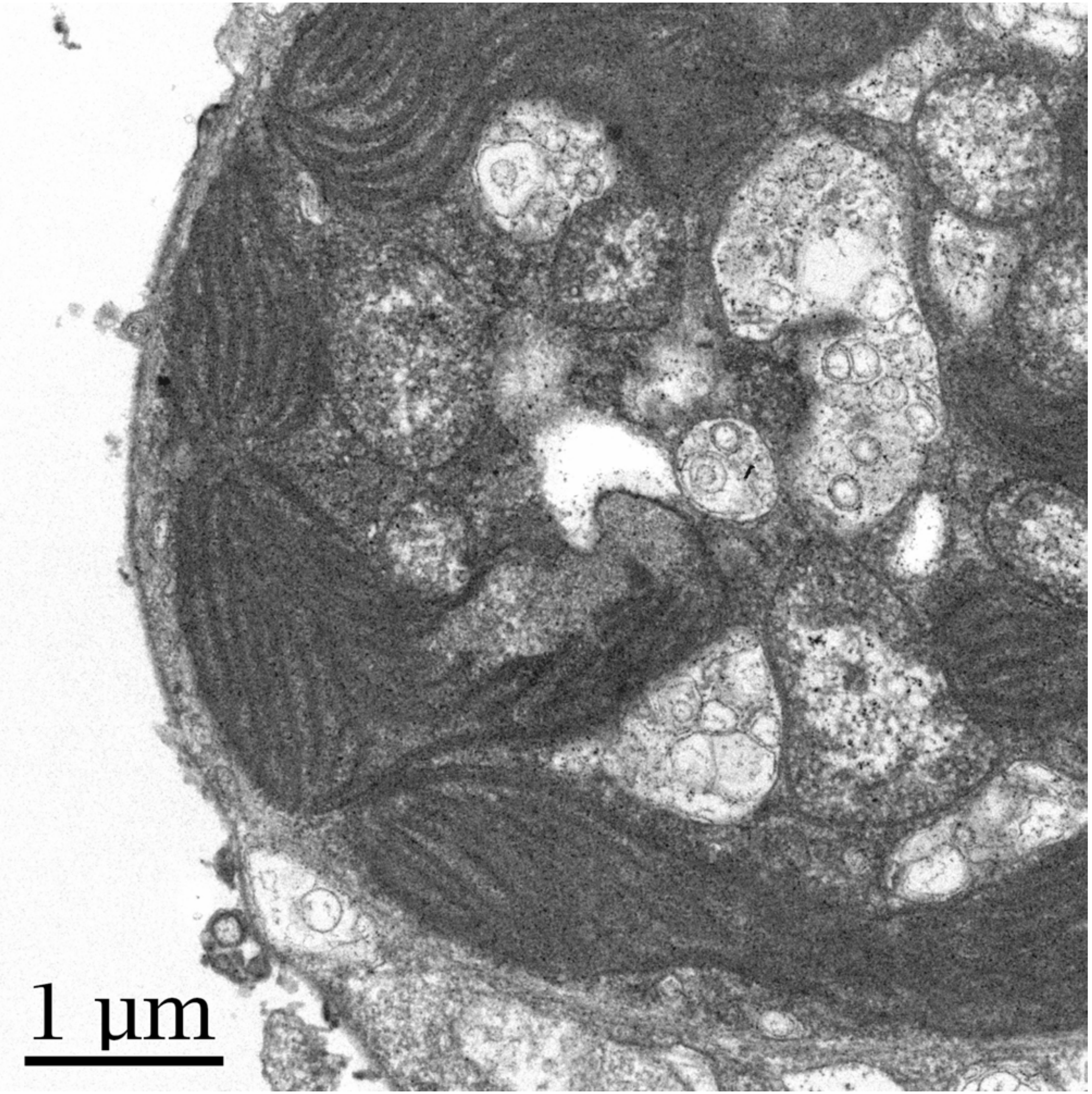

18-14_Correa_XC4_5C3_7.tif

Cell 2

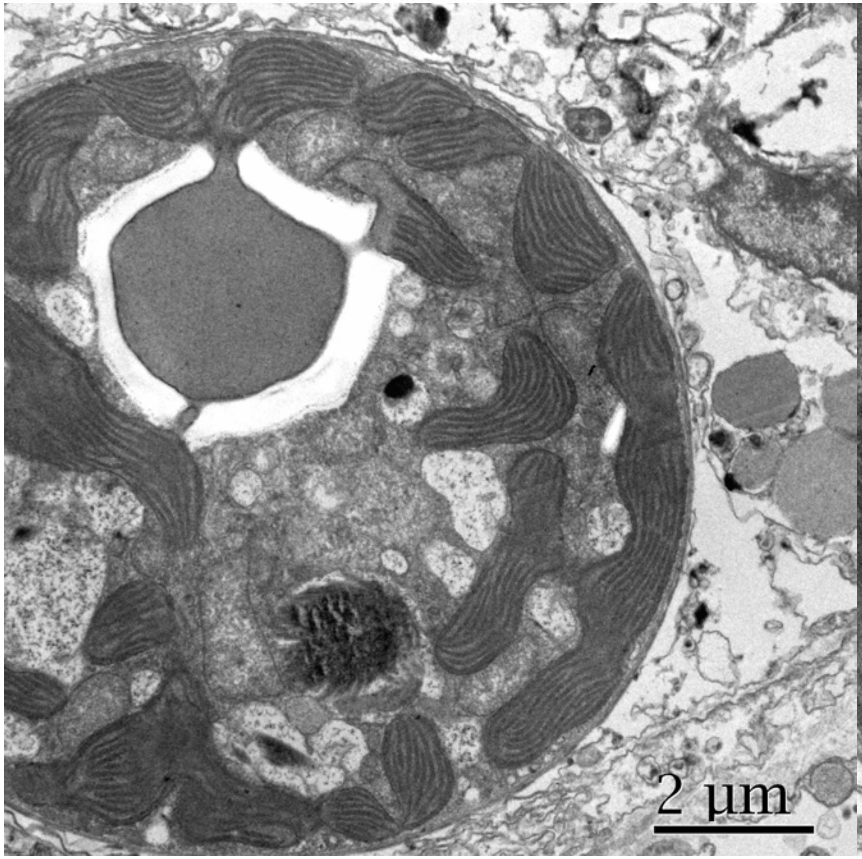

18-14_Correa_XC4_5C3_12.tif

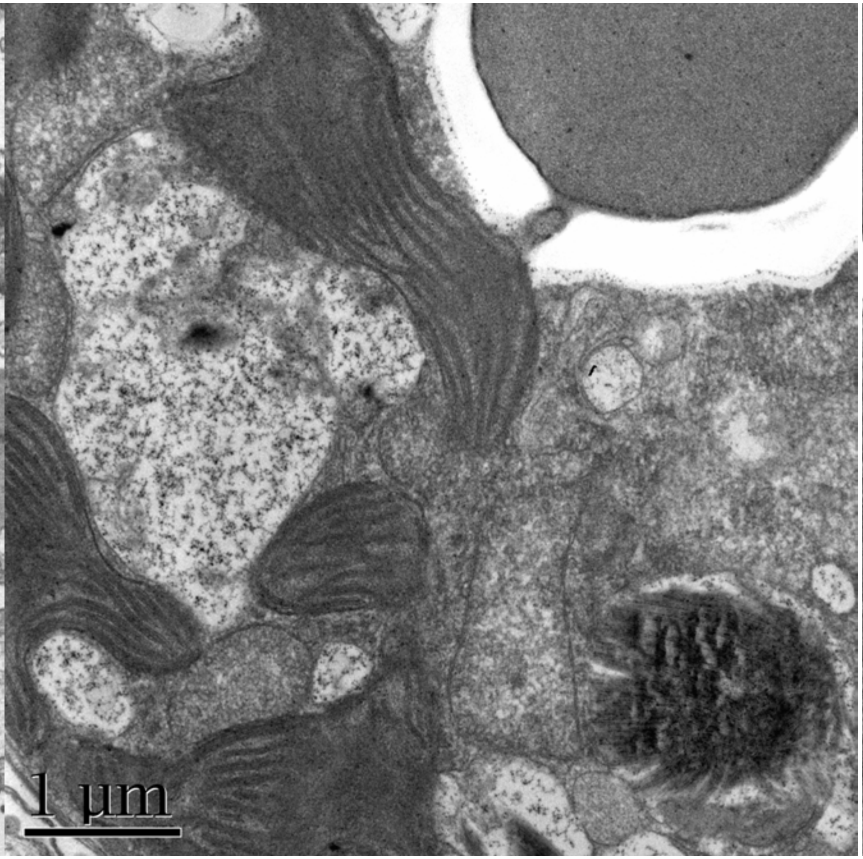

18-14_Correa_XC4_5C3_13.tif

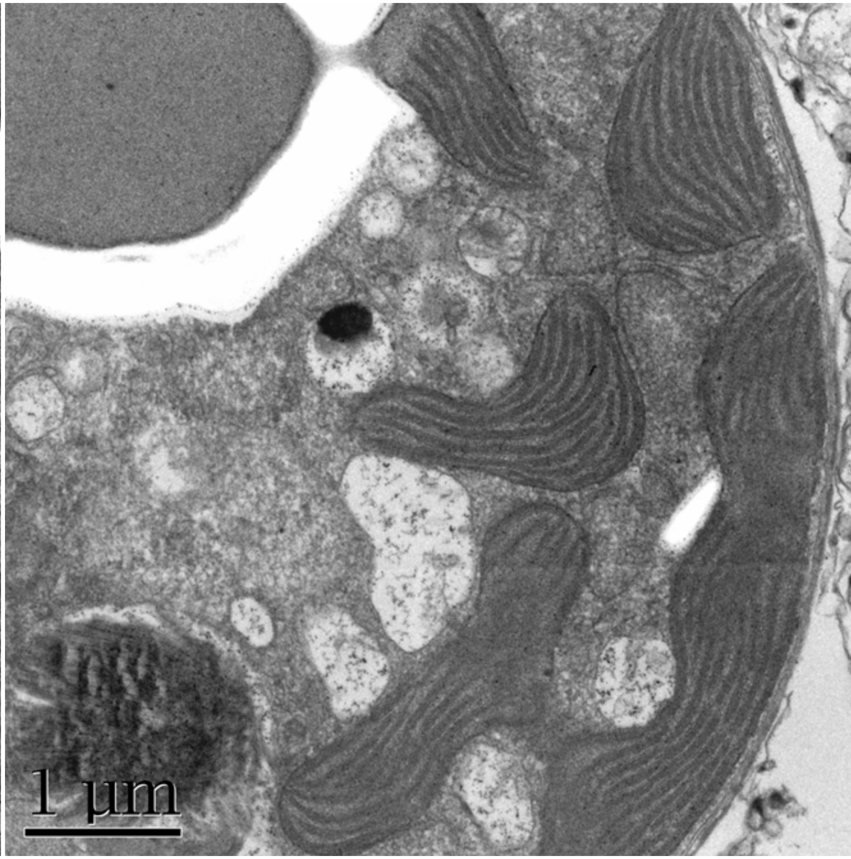

18-14_Correa_XC4_5C3_18.tif

Cell 3

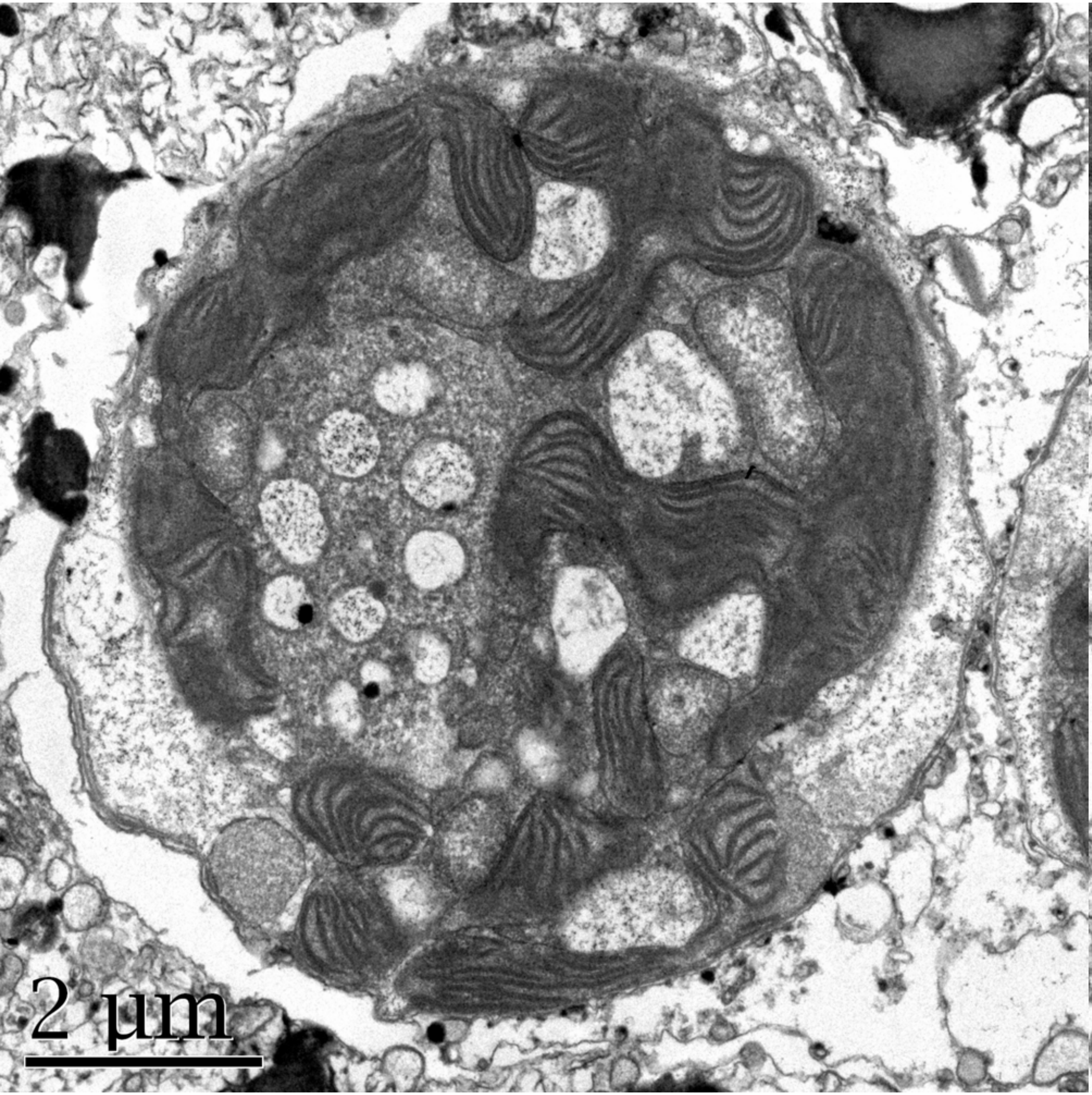

18-14_Correa_XC4_5C3_22.tif

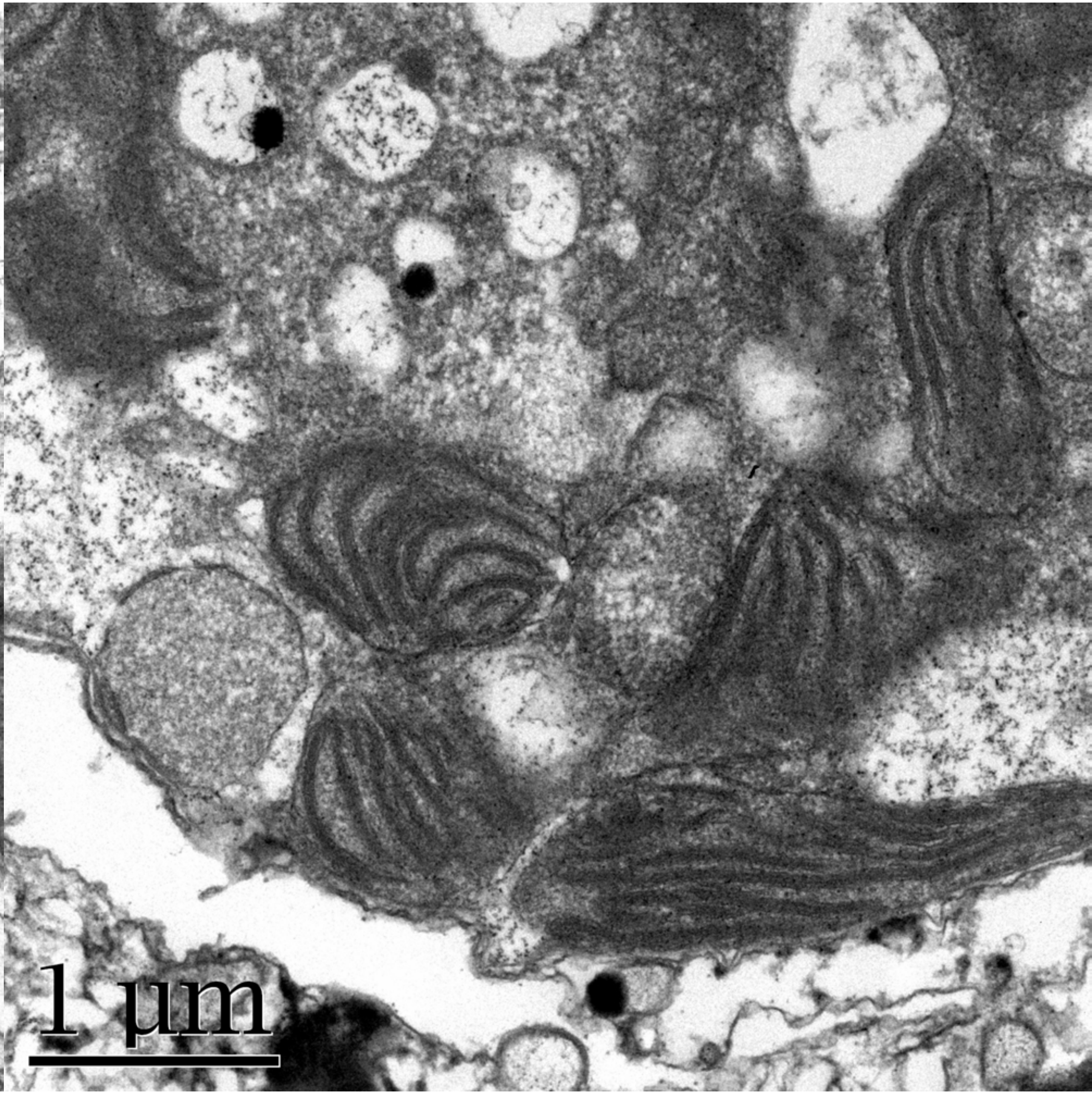

18-14_Correa_XC4_5C3_23.tif

Cell 4

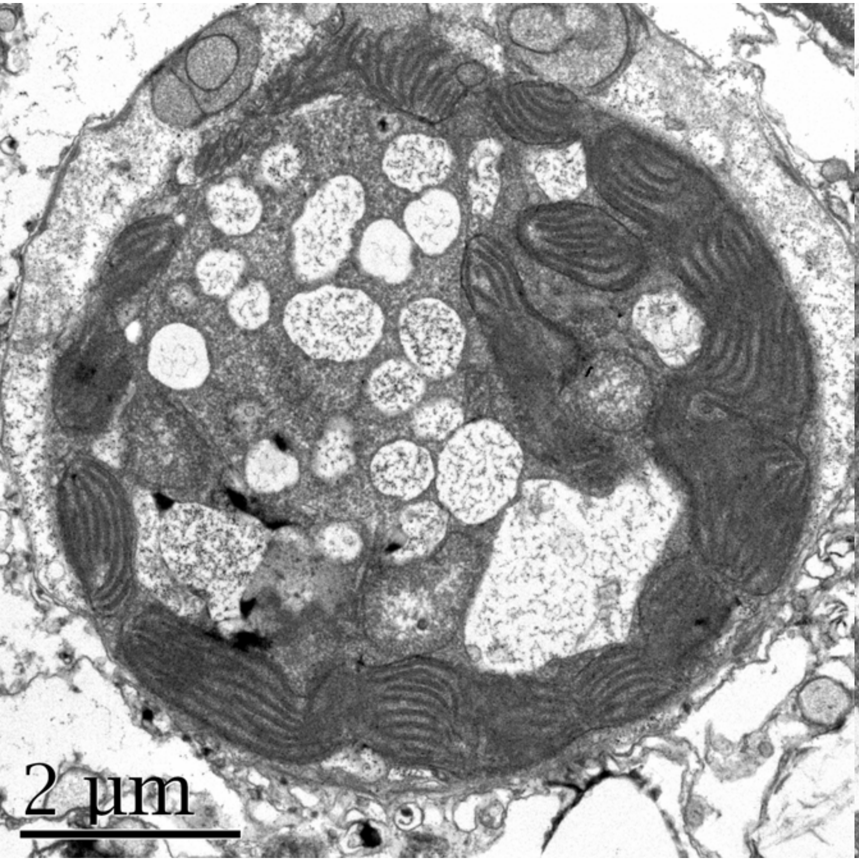

18-14_Correa_XC4_5C3_31.tif

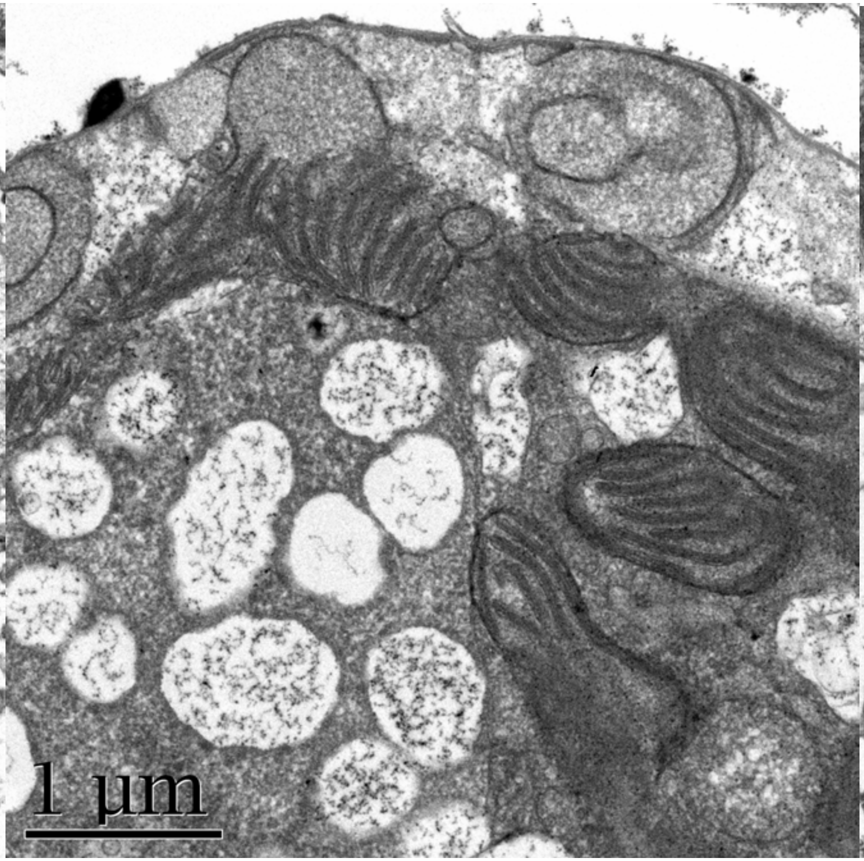

18-14_Correa_XC4_5C3_34.tif

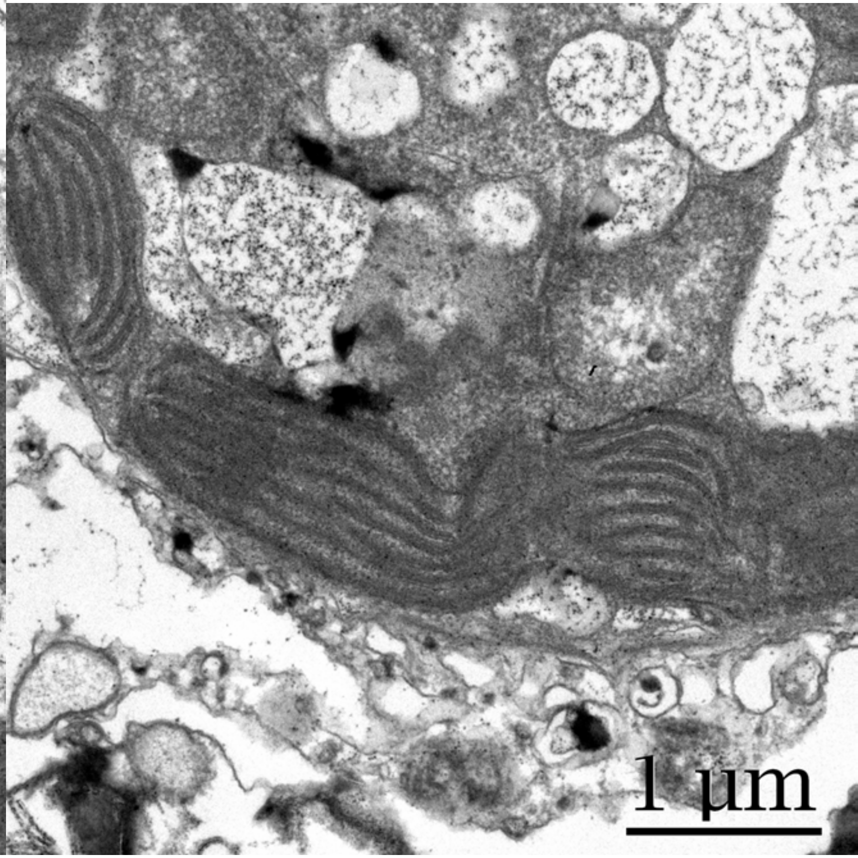

18-14_Correa_XC4_5C3_32.tif

Cell 5

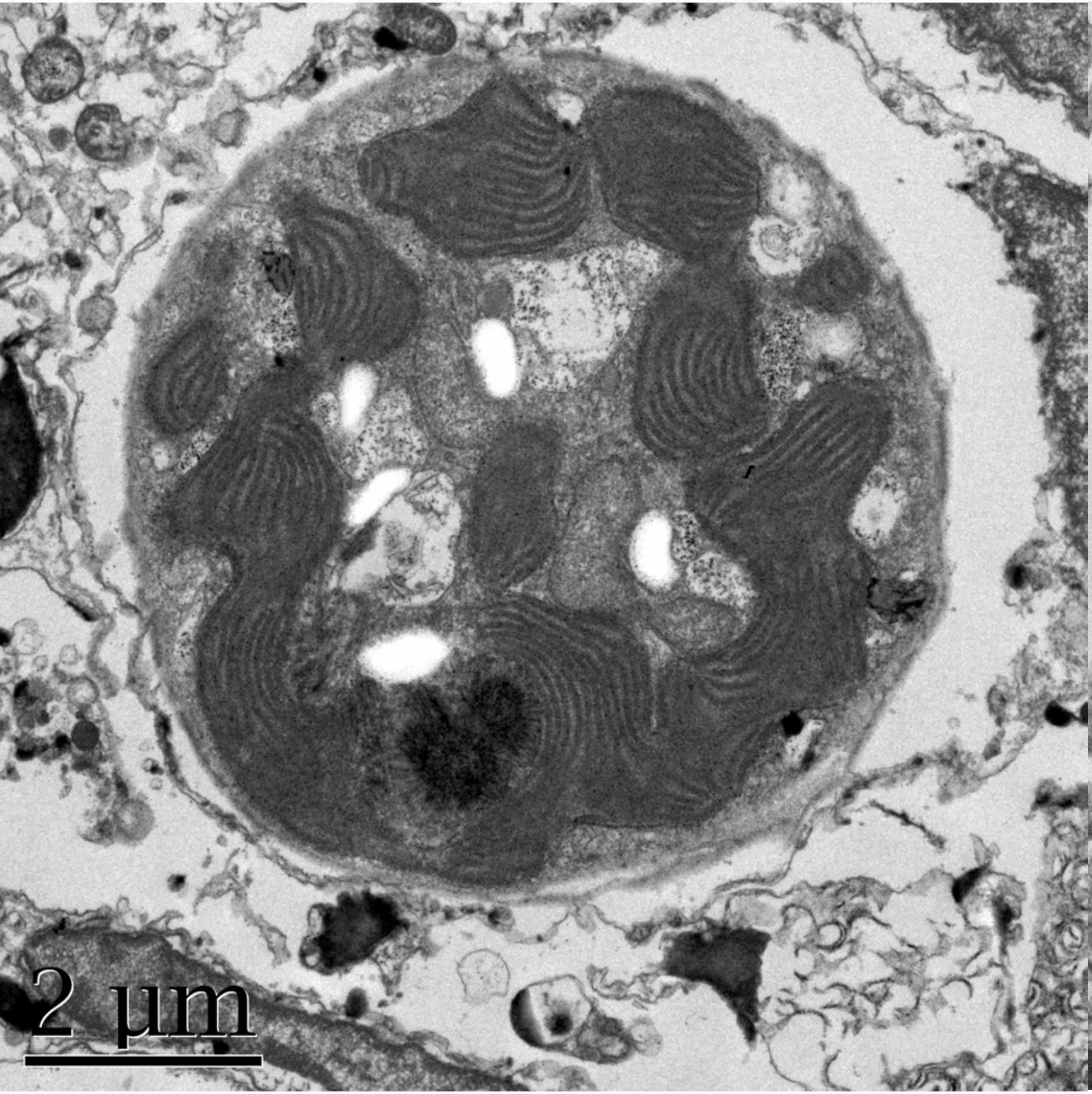

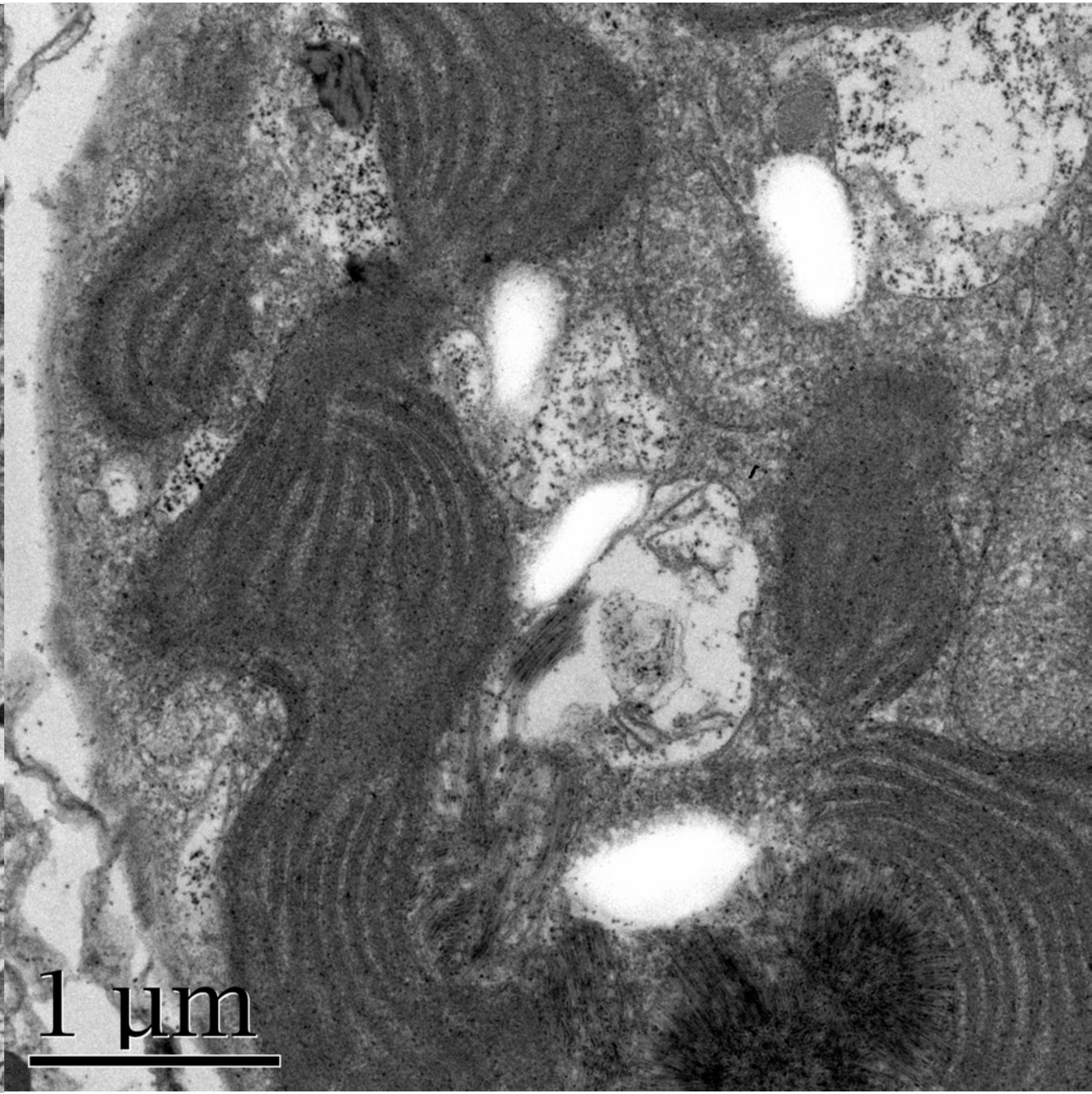

18-14_Correa_XC4_5C3_35.tif

18-14_Correa_XC4_5C3_36.tif

Cell 6

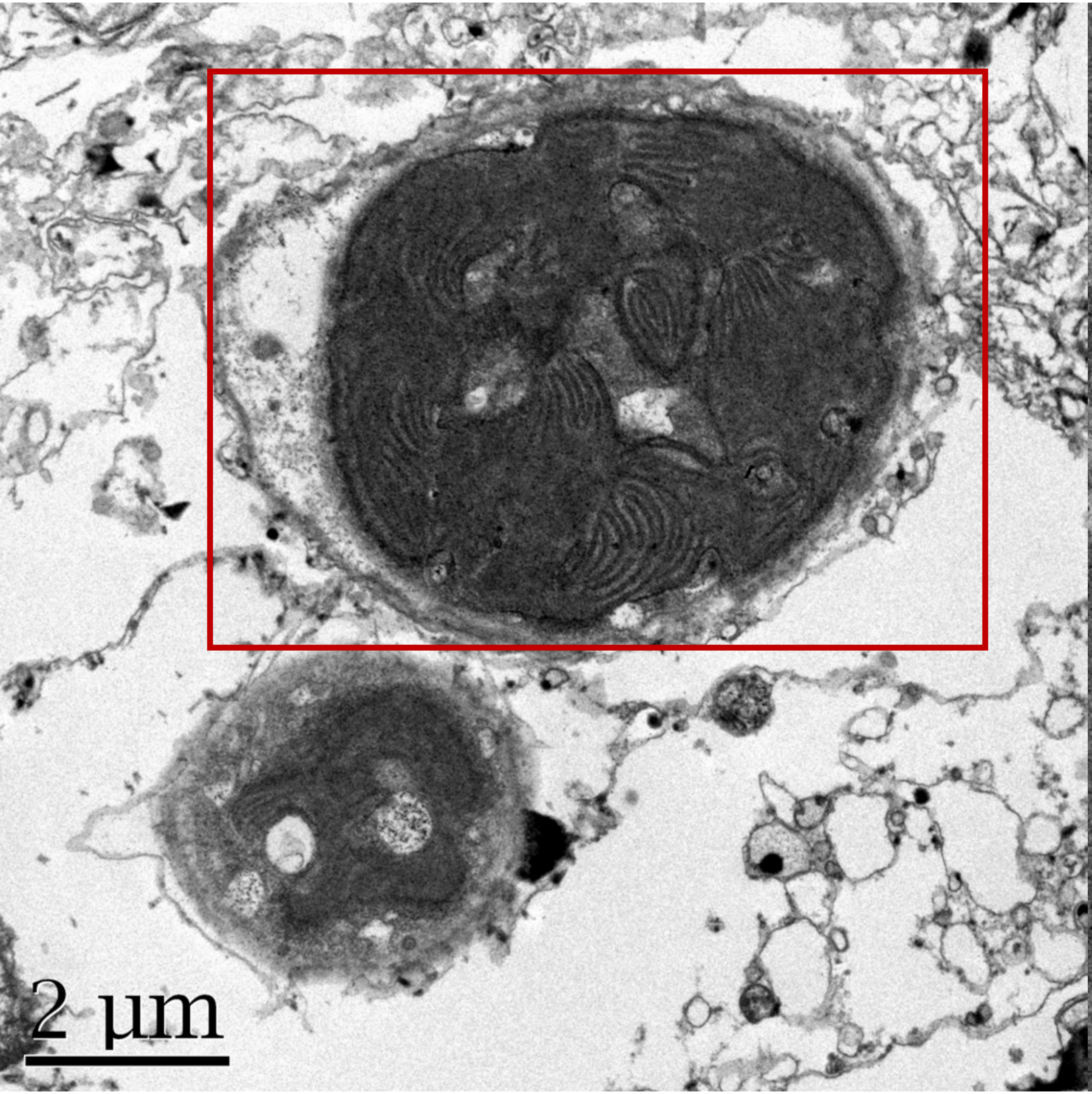

18-14_Correa_XC4_5C3_38.tif

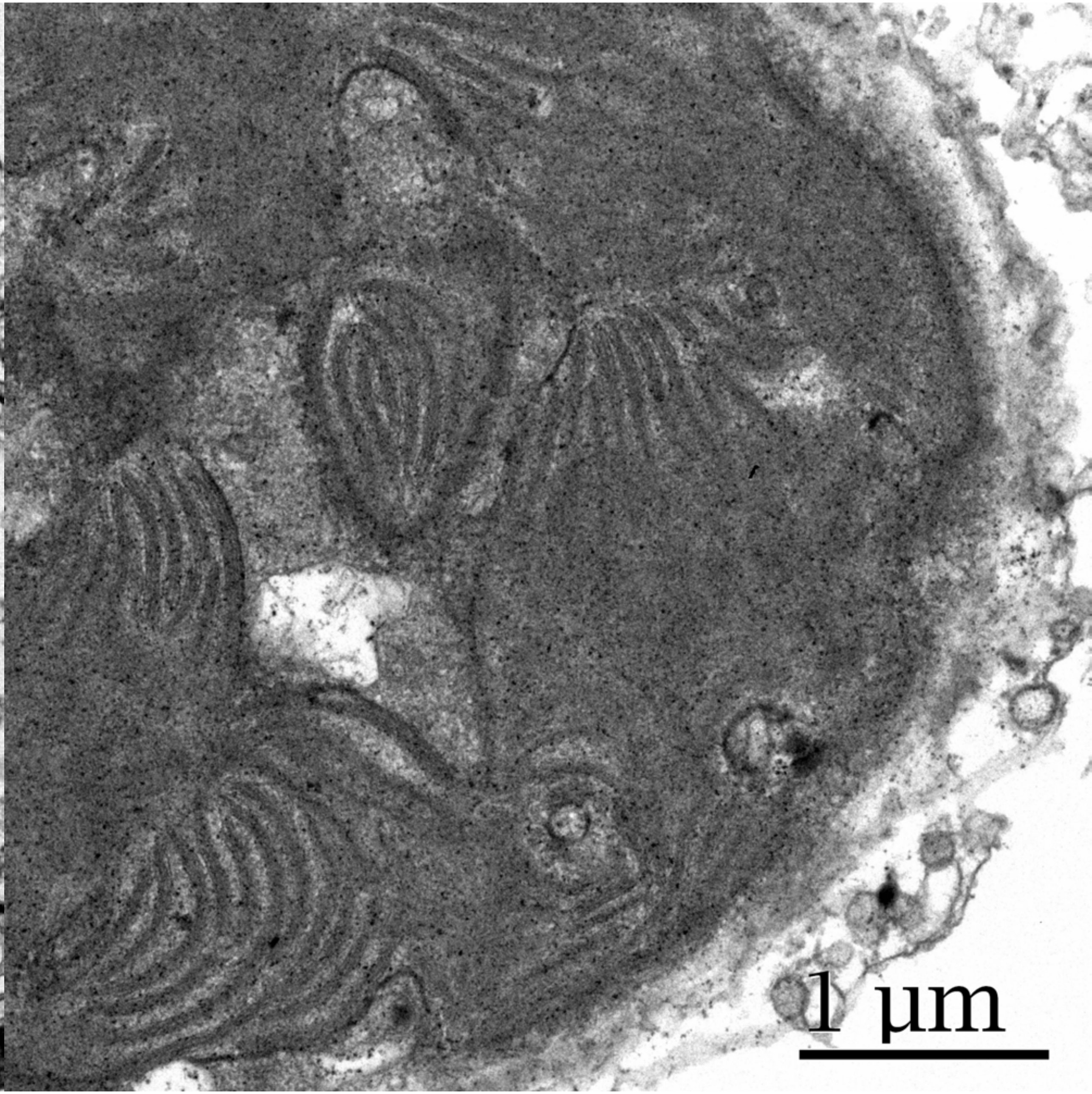

18-14_Correa_XC4_5C3_41.tif

Cell 7

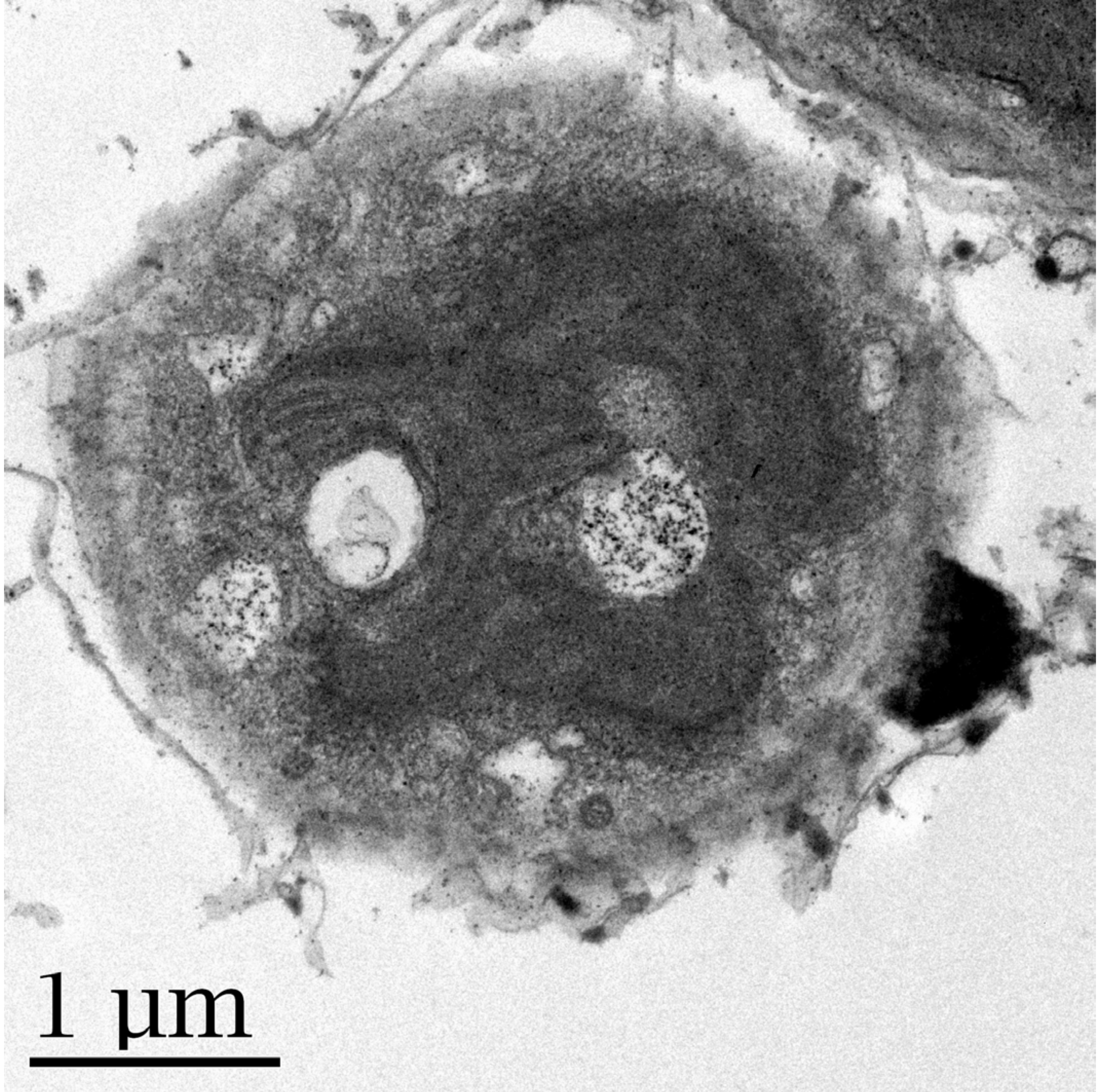

18-14_Correa_XC4_5C3_39.tif

Cell 8

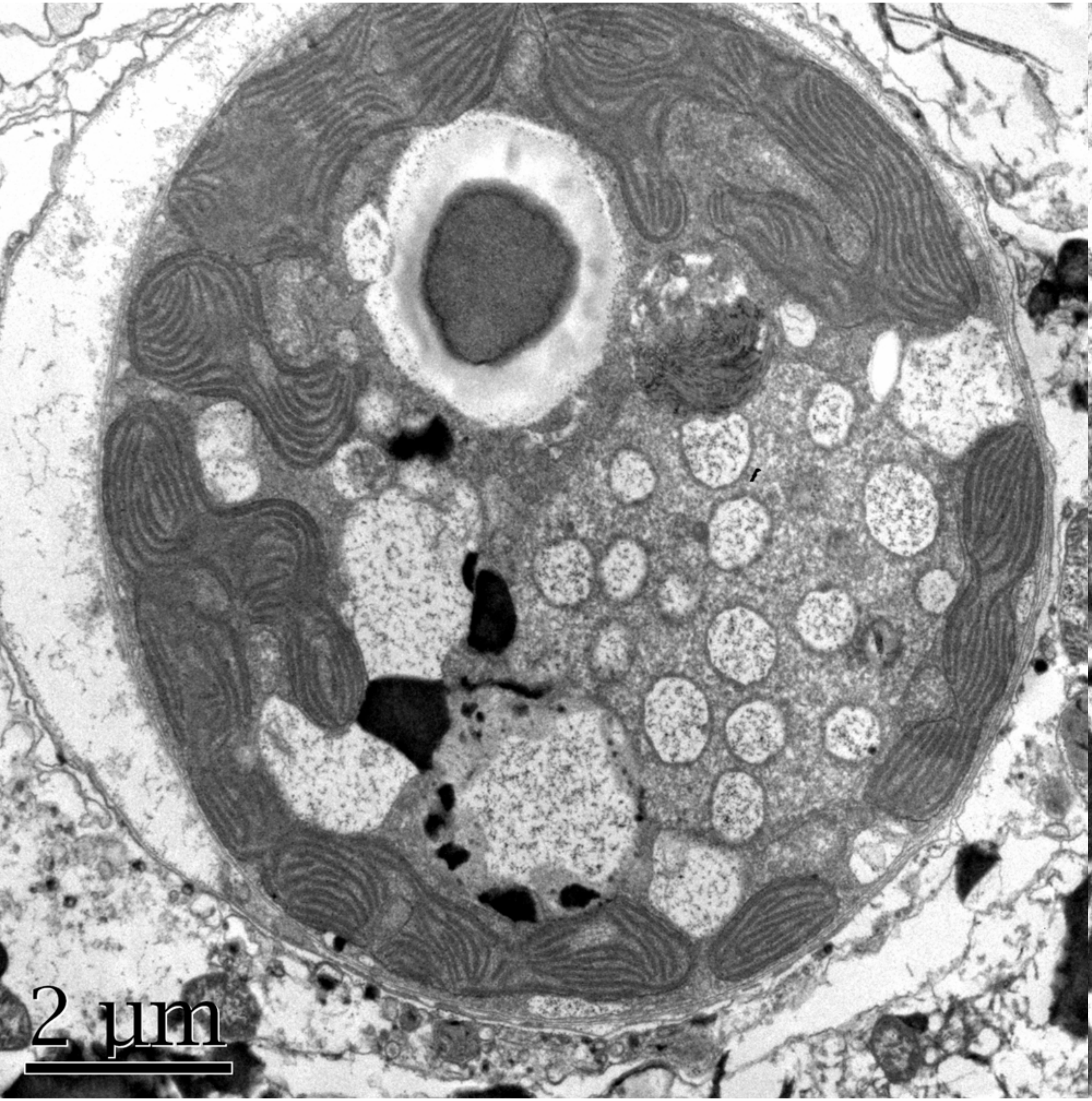

18-14_Correa_XC4_5C3_43.tif

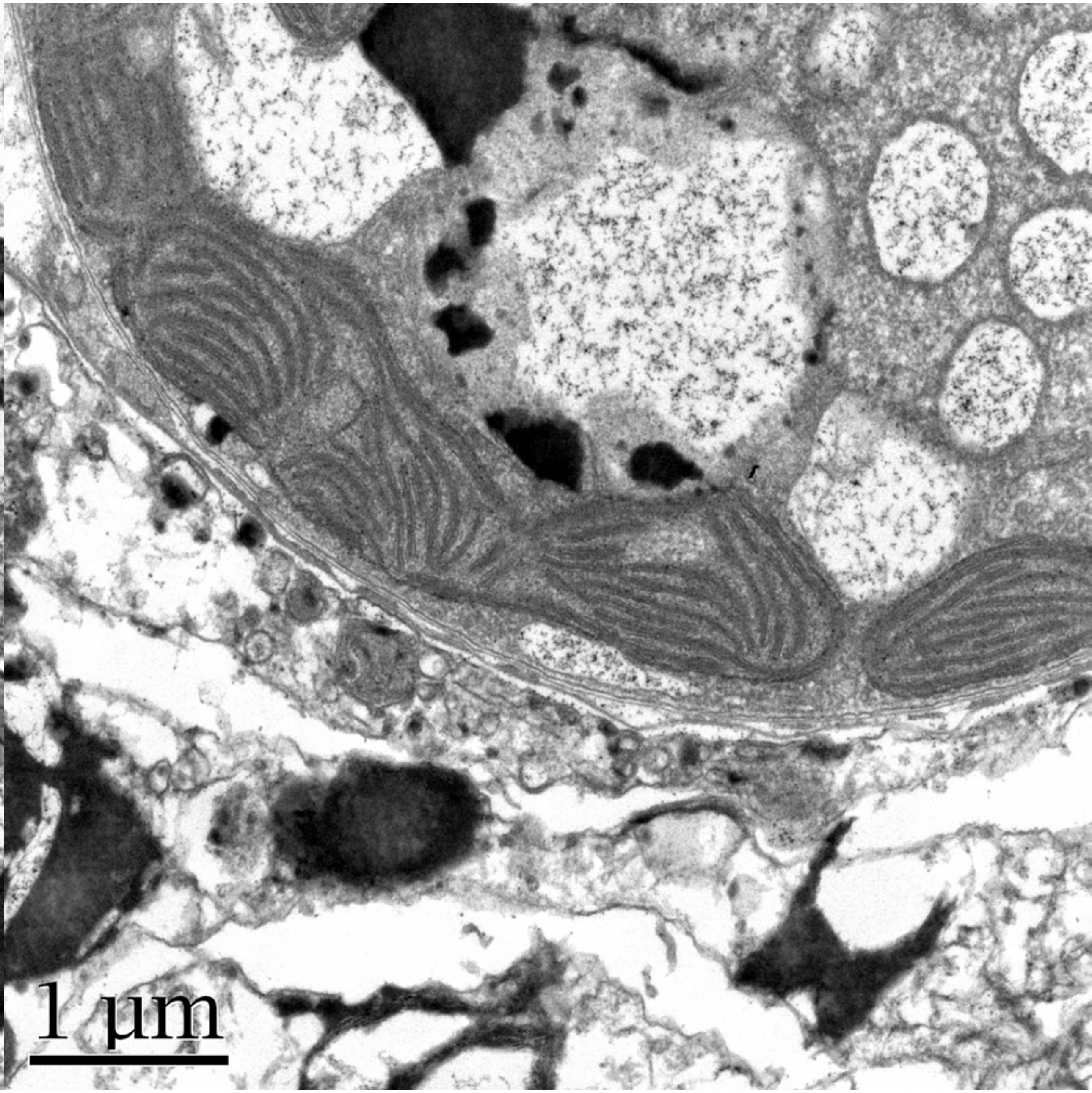

18-14_Correa_XC4_5C3_44.tif

Cell 9

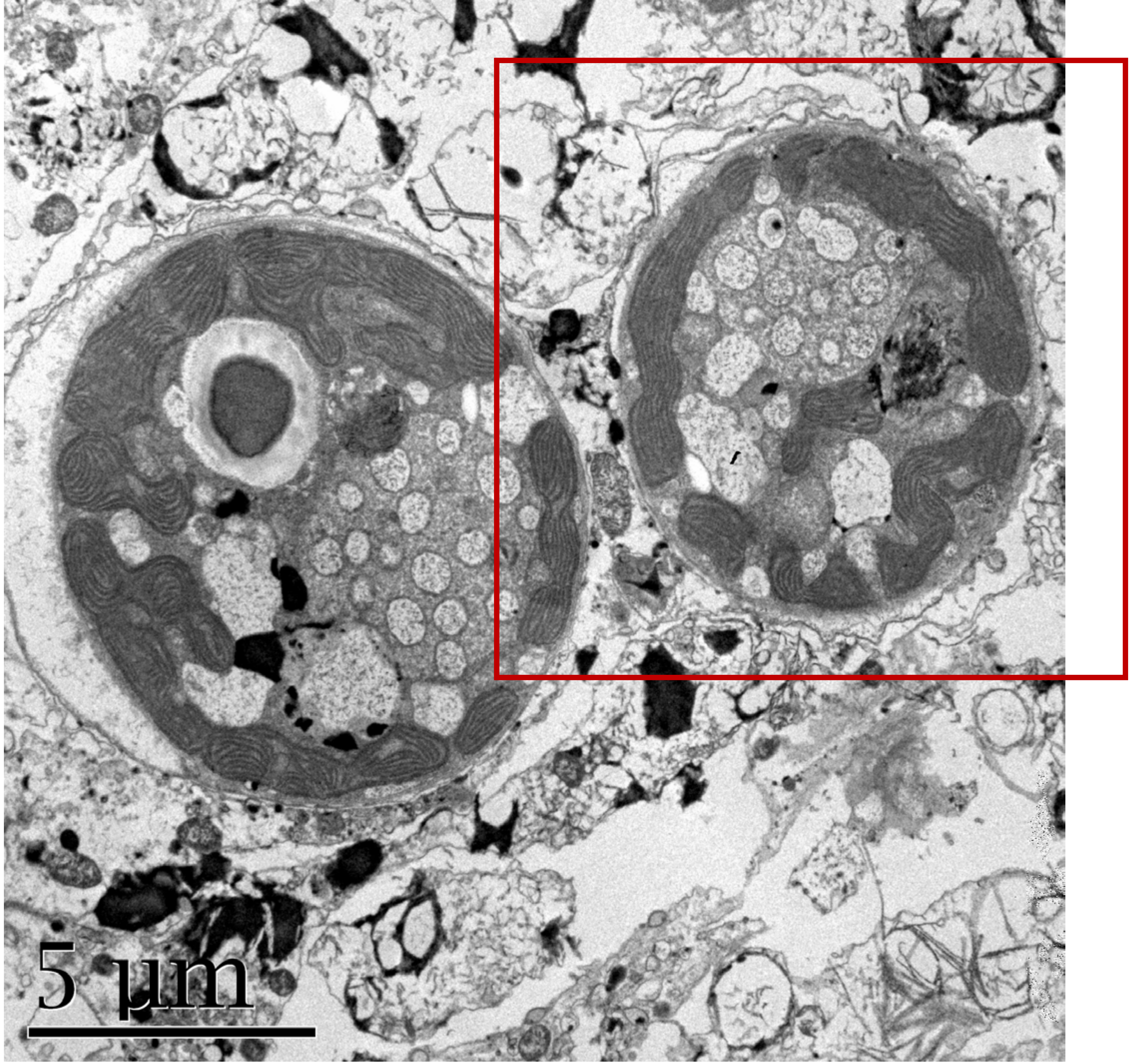

18-14_Correa_XC4_5C3_42.tif

Cell 10

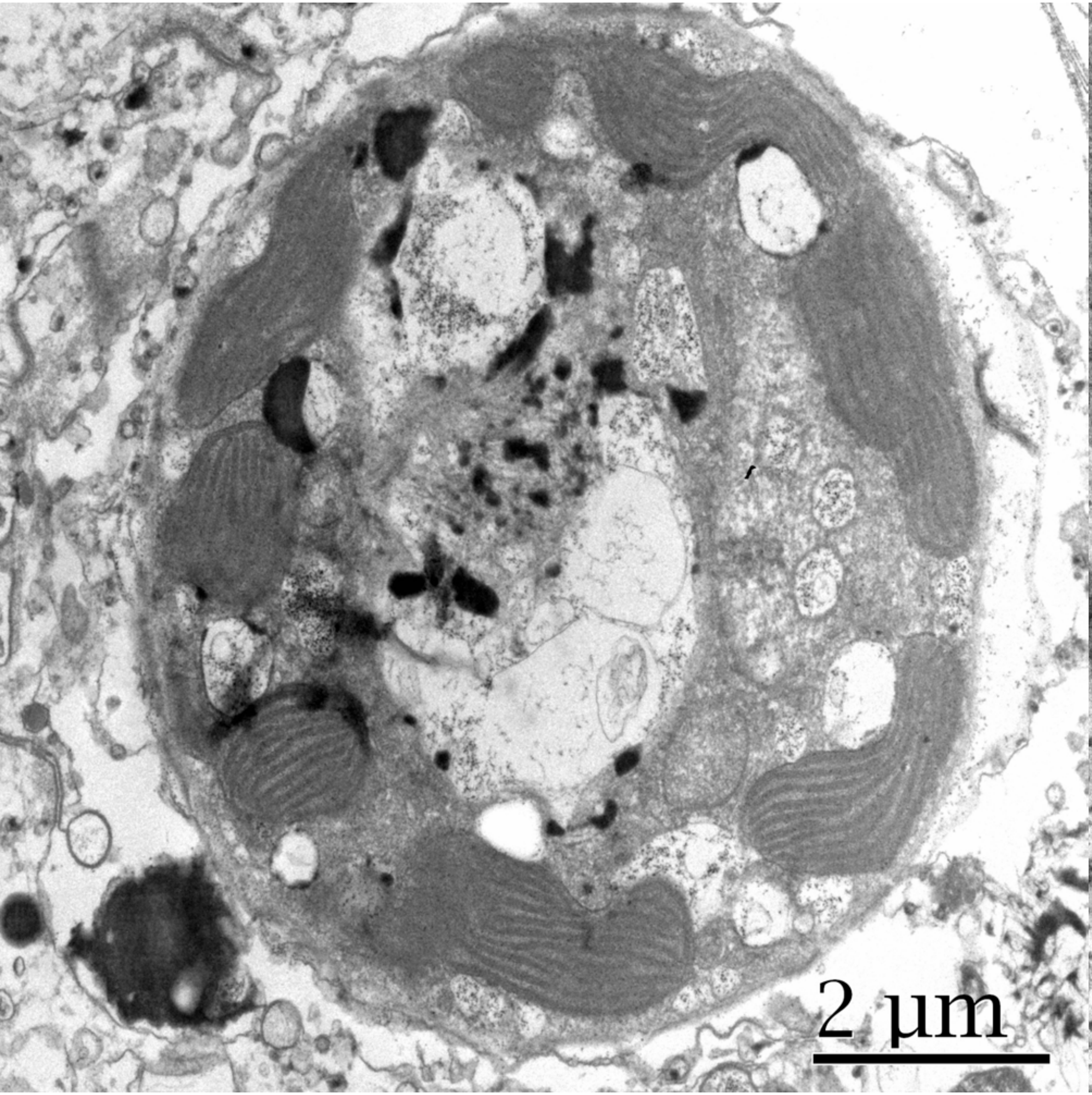

18-14_Correa_XC4_5C3_55.tif

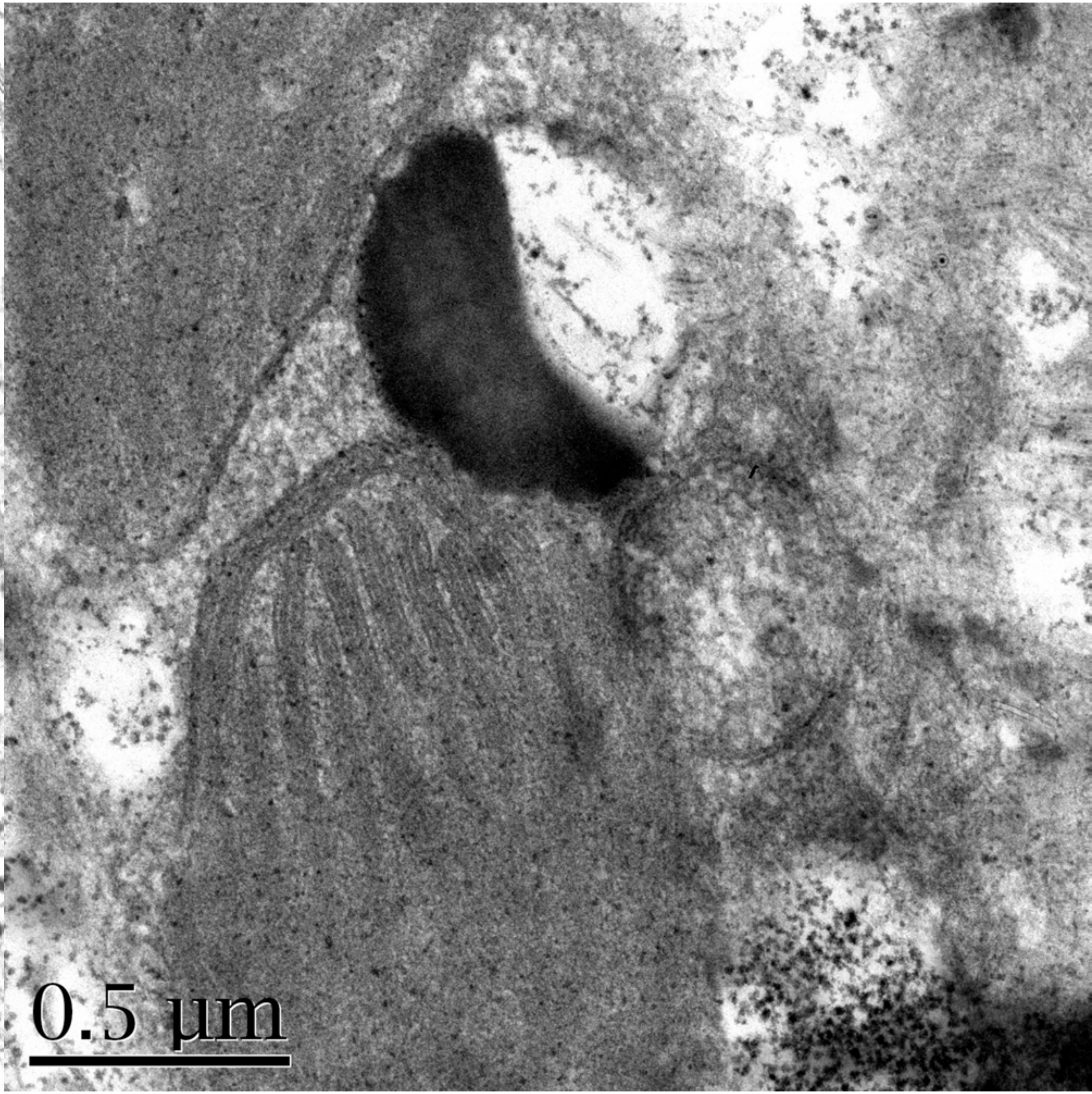

18-14_Correa_XC4_5C3_61.tif

Cell 11

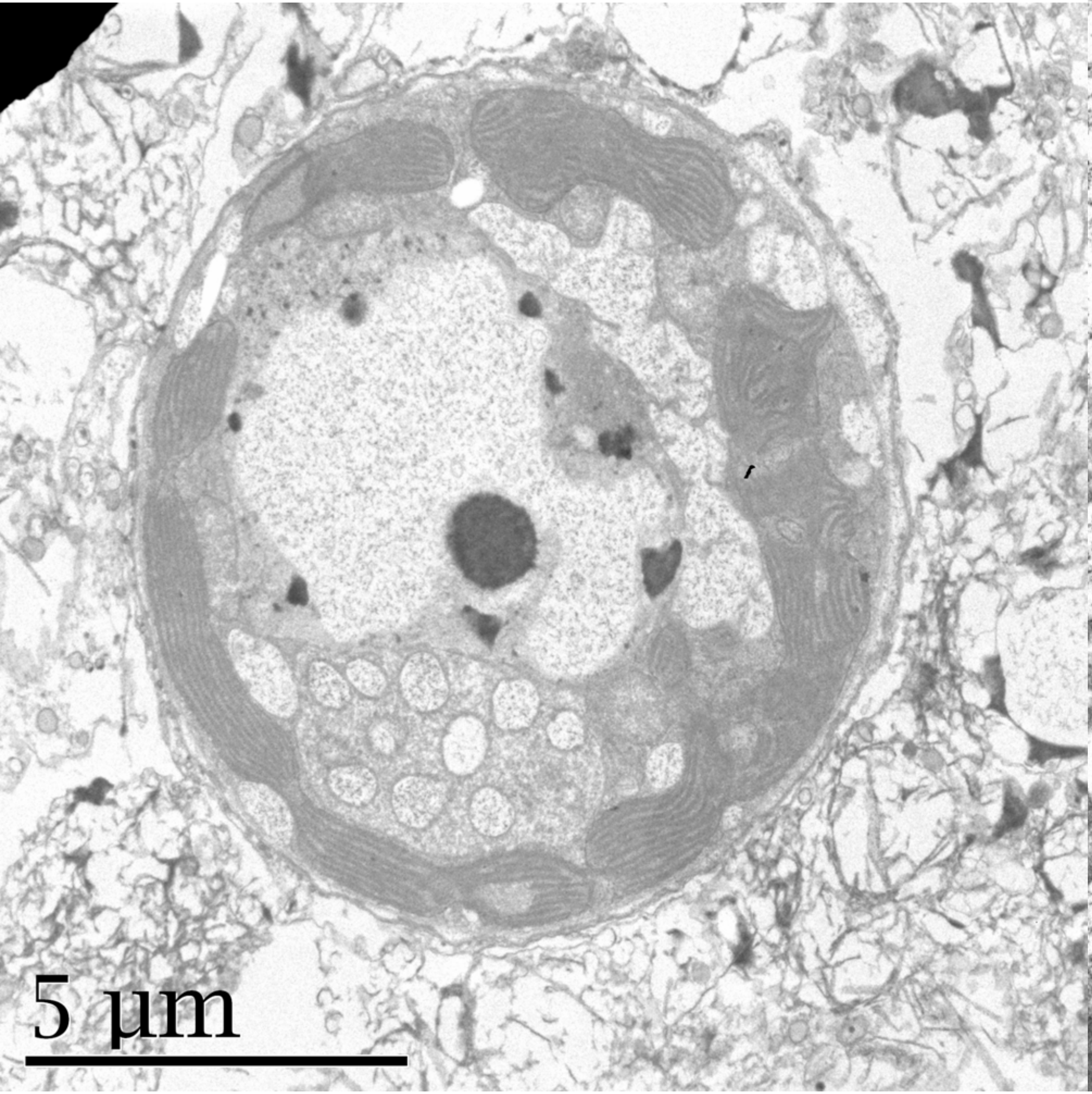

18-14_Correa_XC4_5C3_62.tif

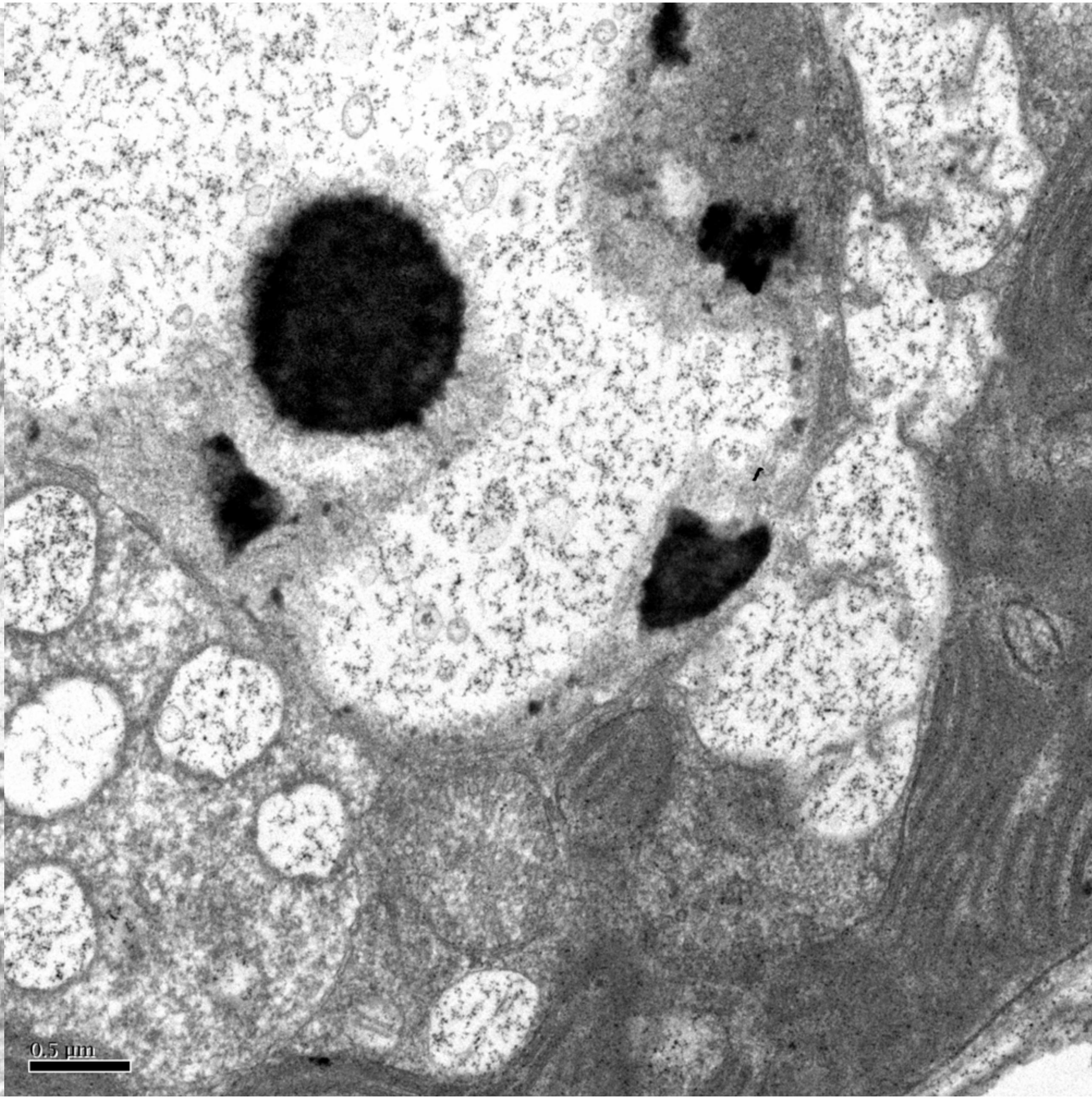

18-14_Correa_XC4_5C3_63.tif

Cell 12

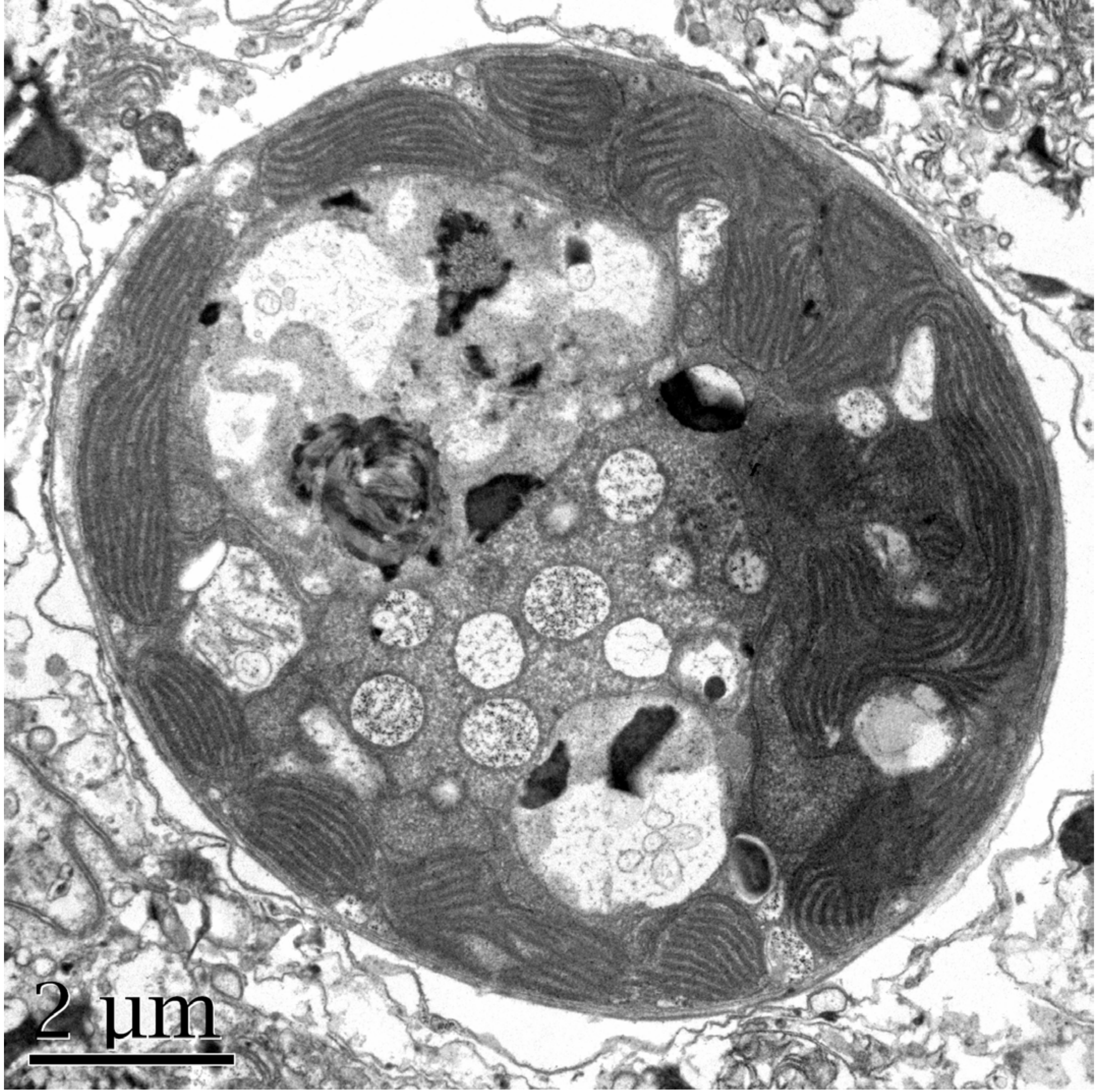

18-14_Correa_XC4_5C3_70.tif

Cell 13

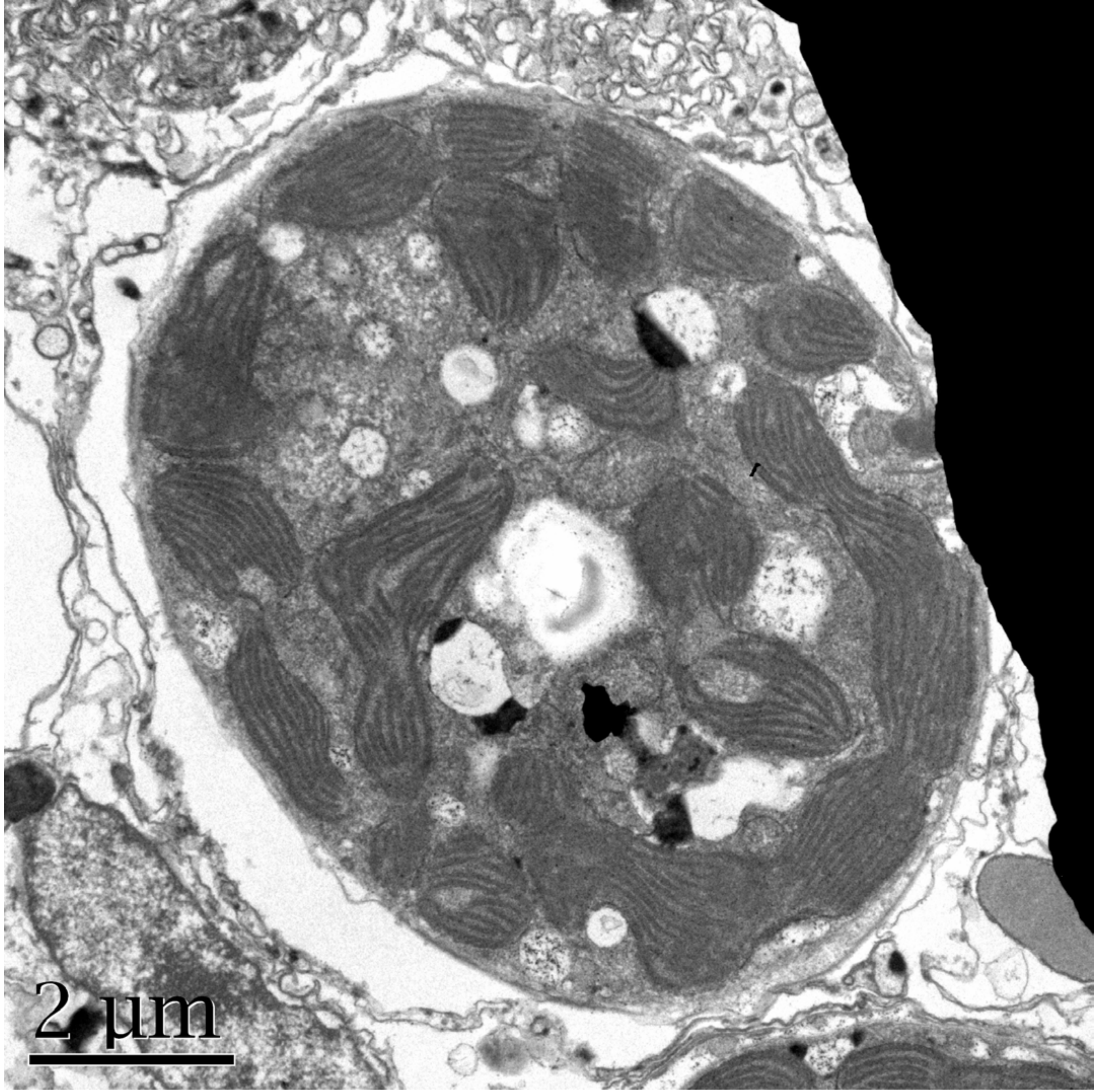

18-14_Correa_XC4_5C3_71.tif

Cell 14

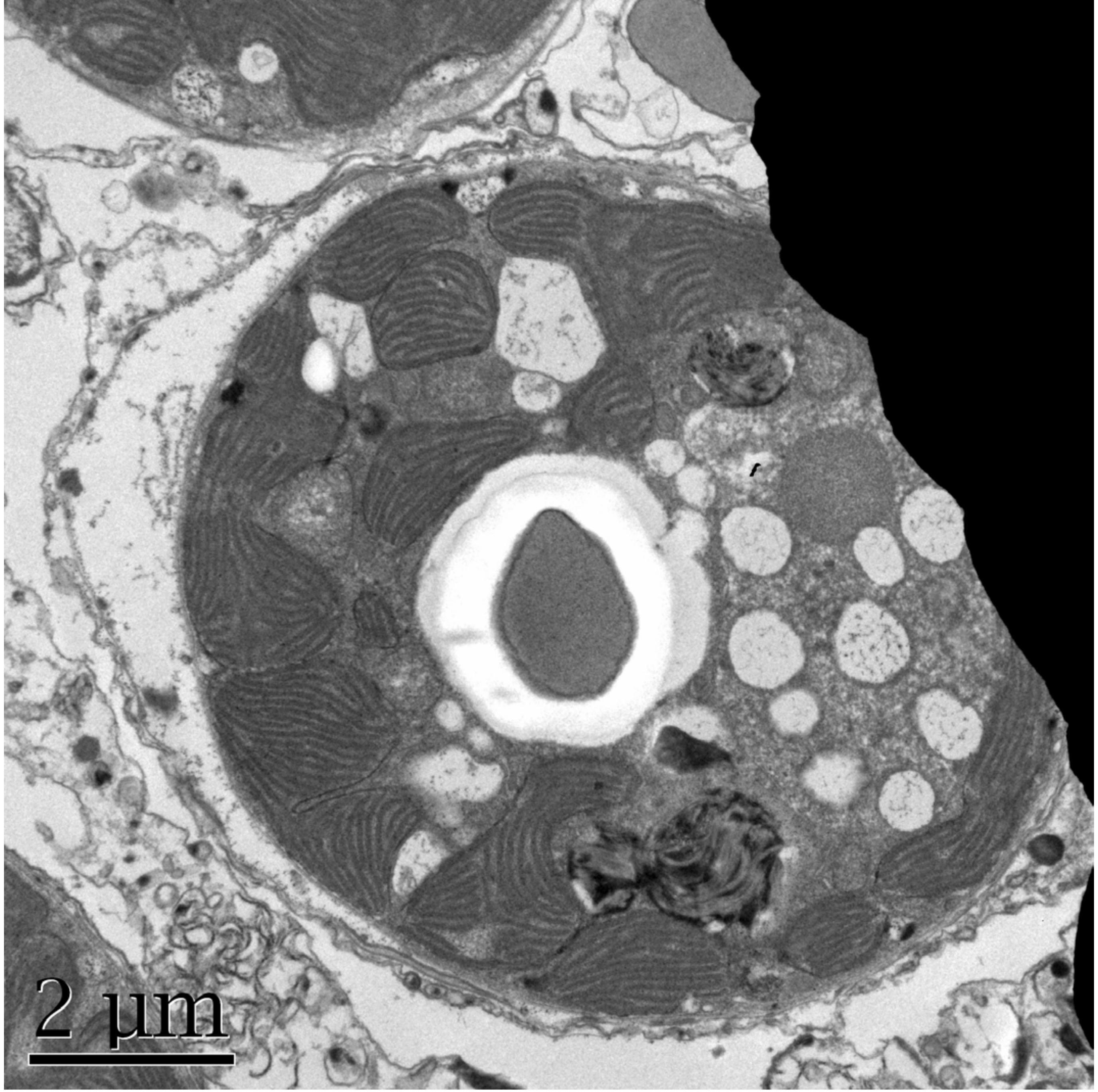

18-14_Correa_XC4_5C3_72.tif

Cell 15

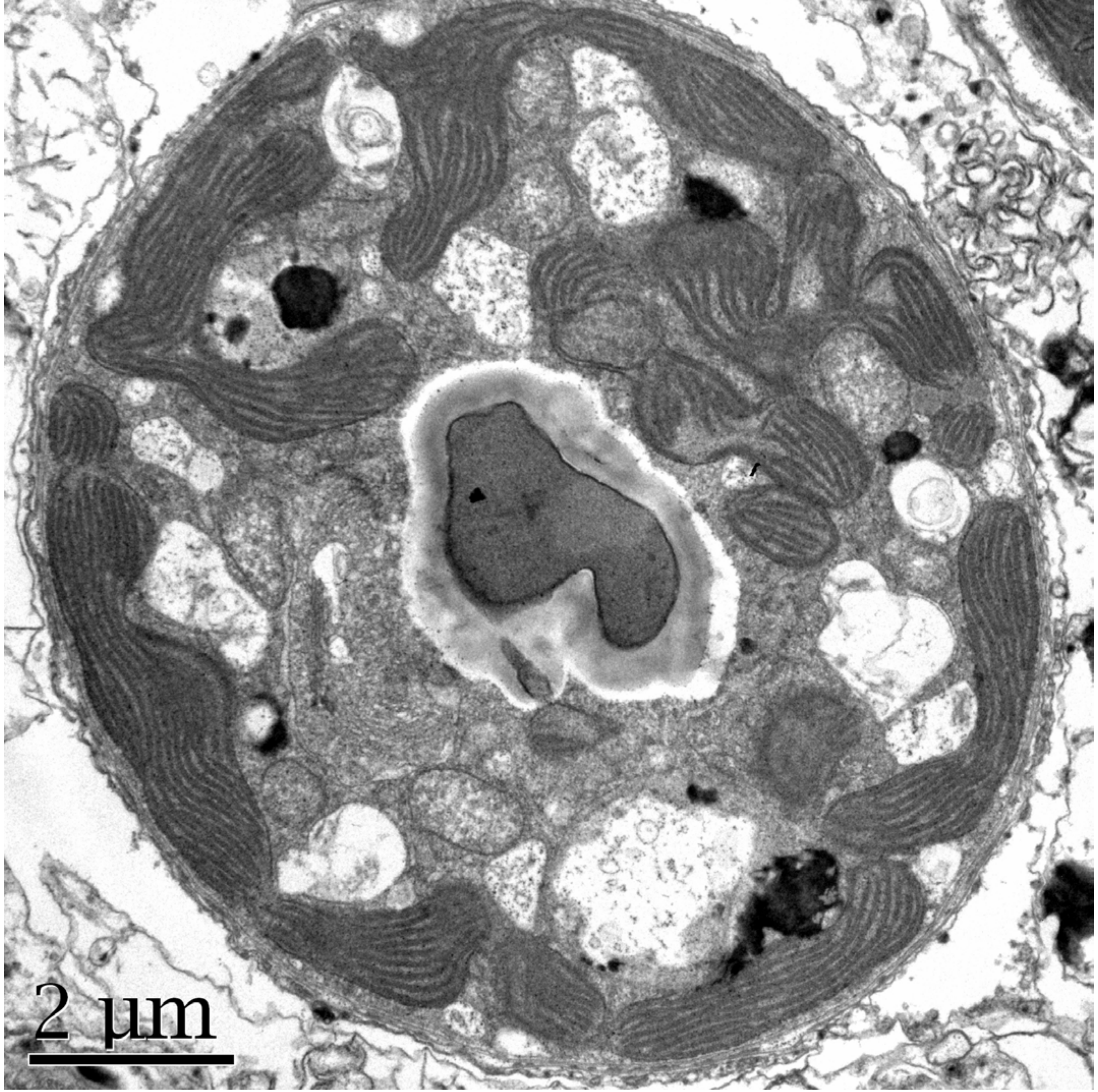

18-14_Correa_XC4_5C3_73.tif

Cell 16

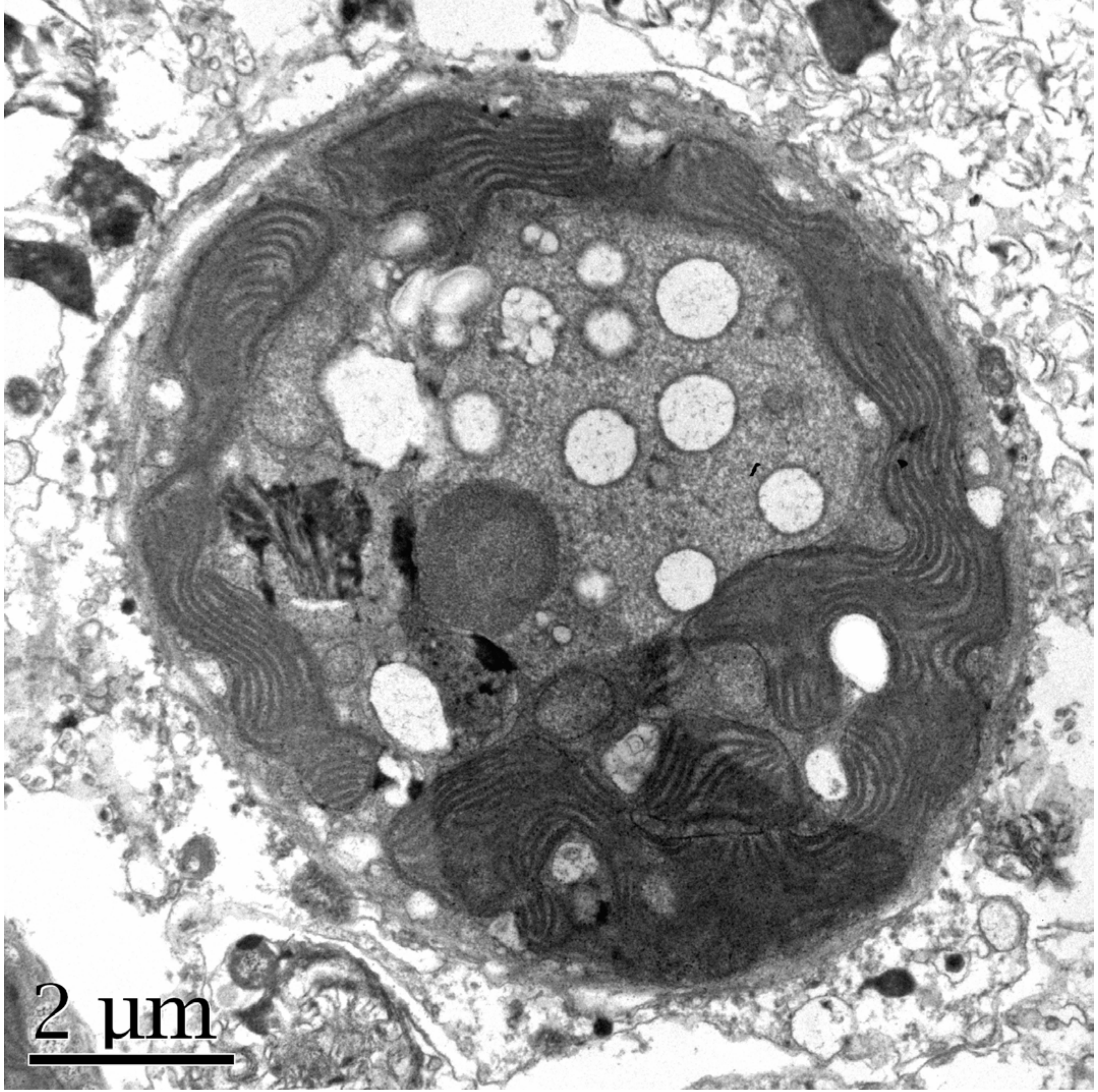

18-14_Correa_XC4_5C3_74.tif

Cell 17

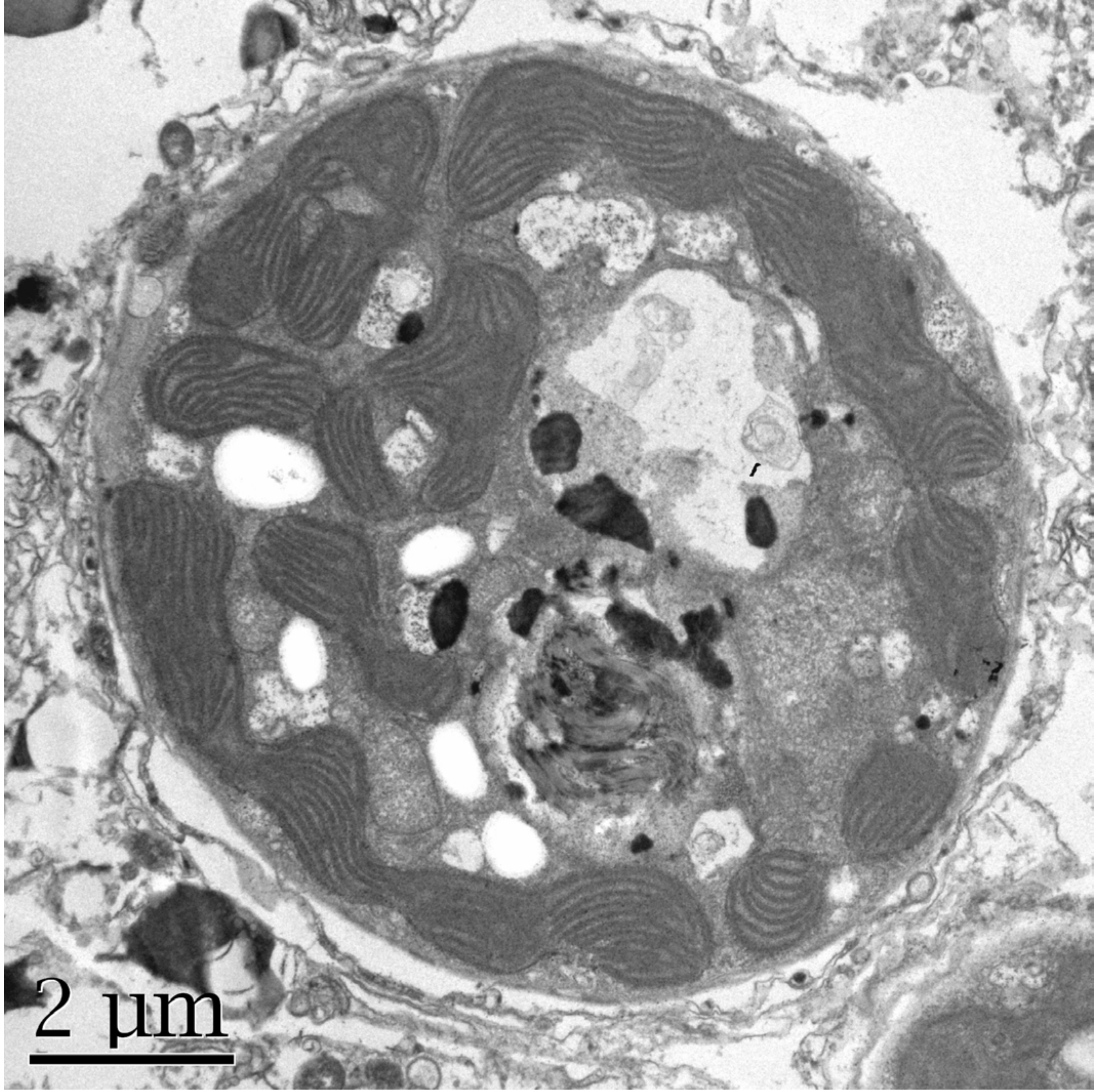

18-14_Correa_XC4_5C3_75.tif

Cell 18

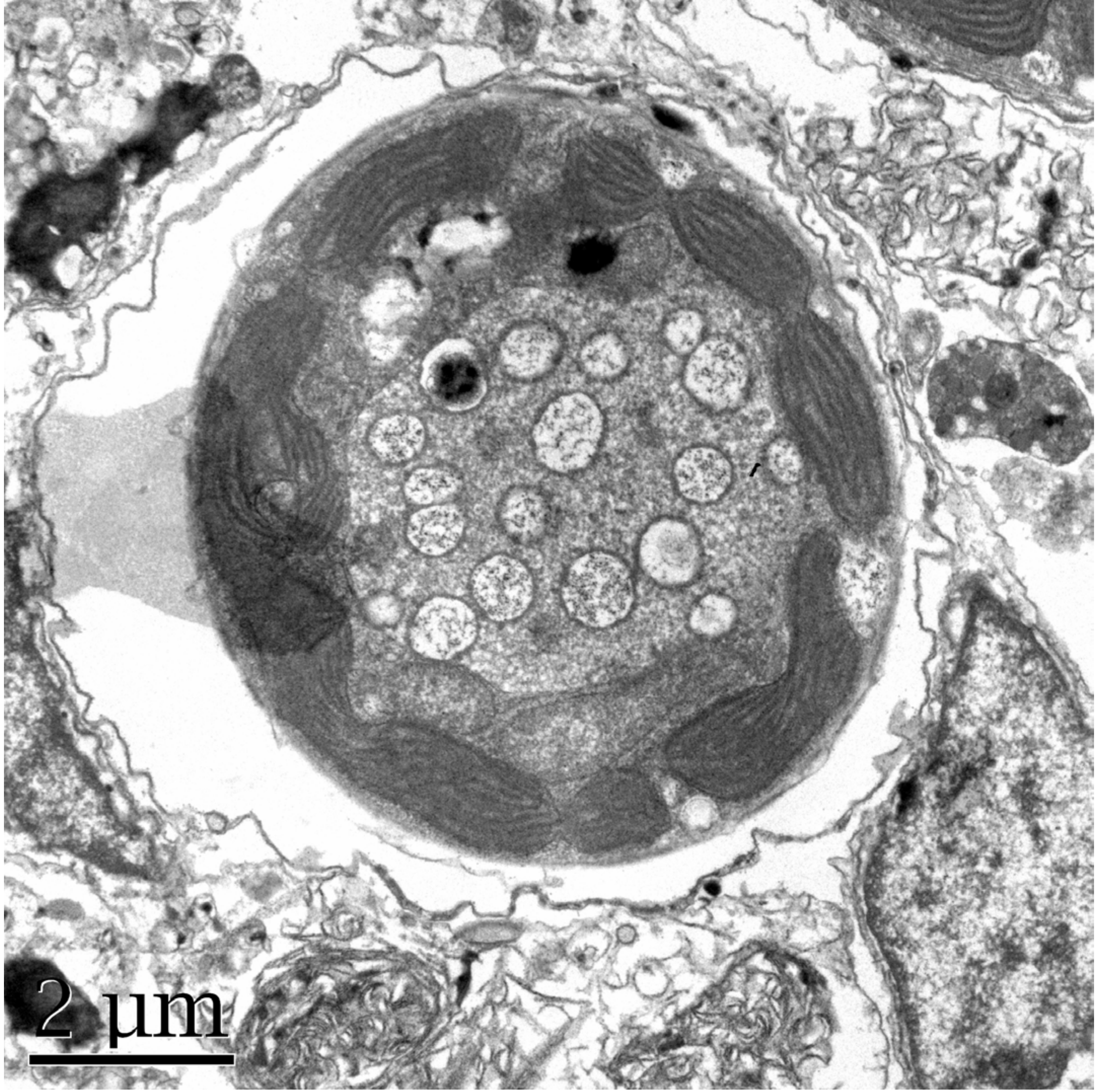

18-14_Correa_XC4_5C3_76.tif

Cell 19

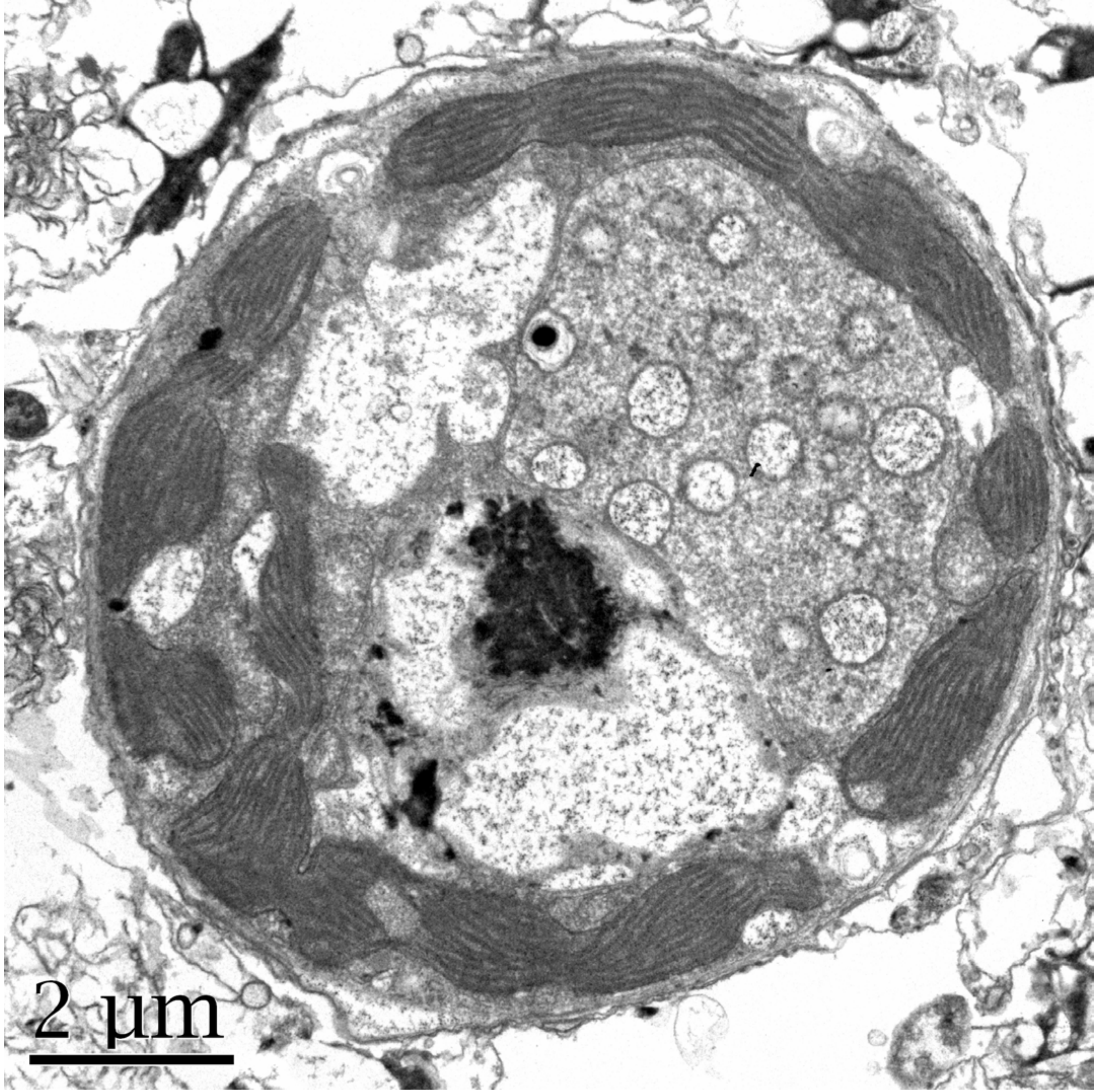

18-14_Correa_XC4_5C3_77.tif

Cell 20

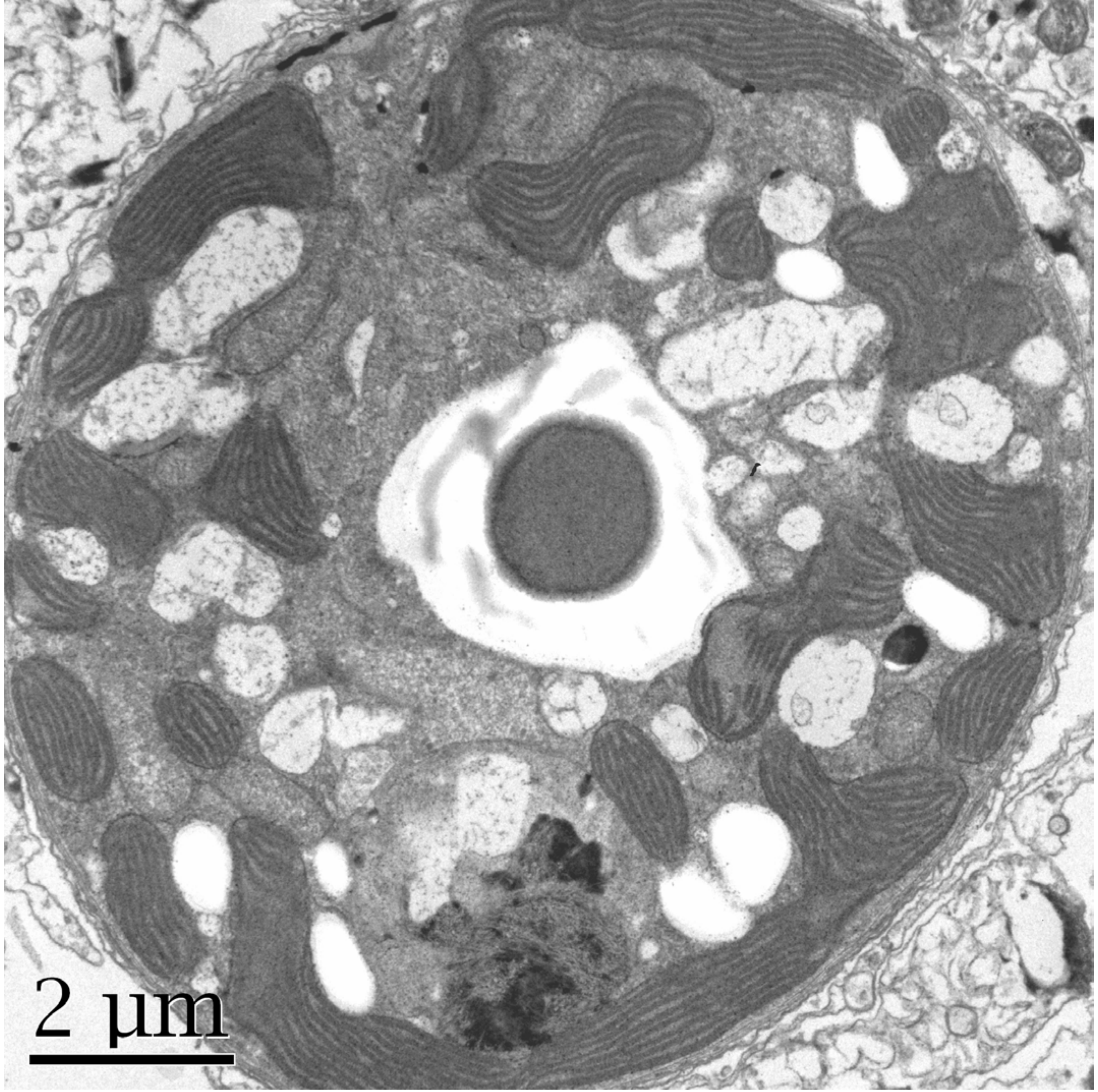

18-14_Correa_XC4_5C3_79.tif

Heat Treatment - ACR Colony C

Cell 1

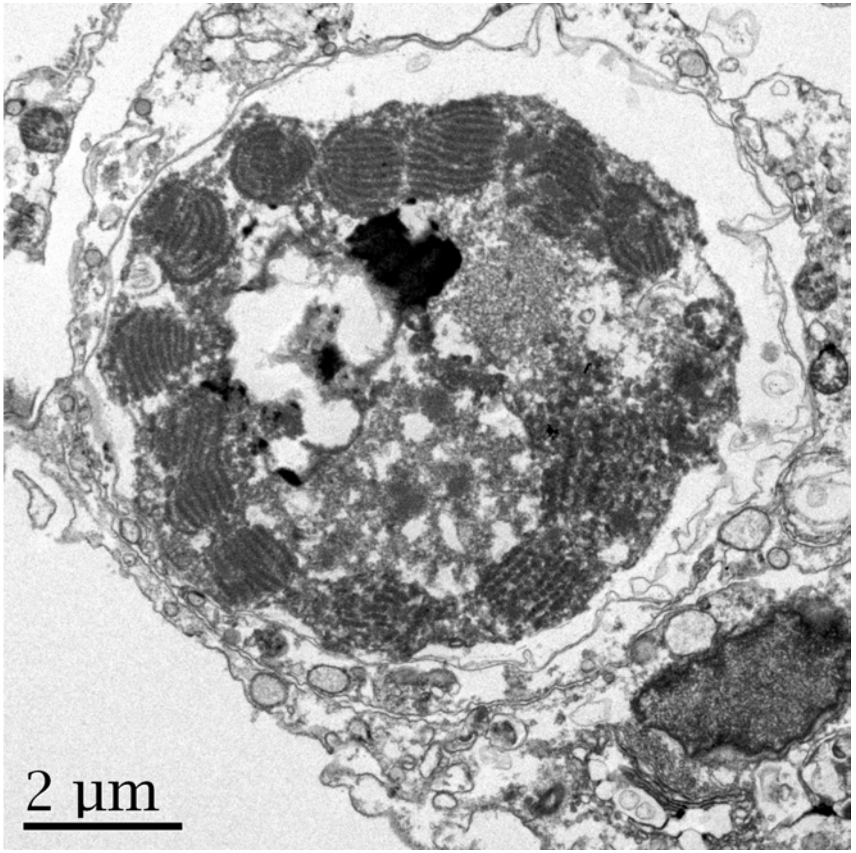

18-14_Correa_XH4_5C5_2.tif

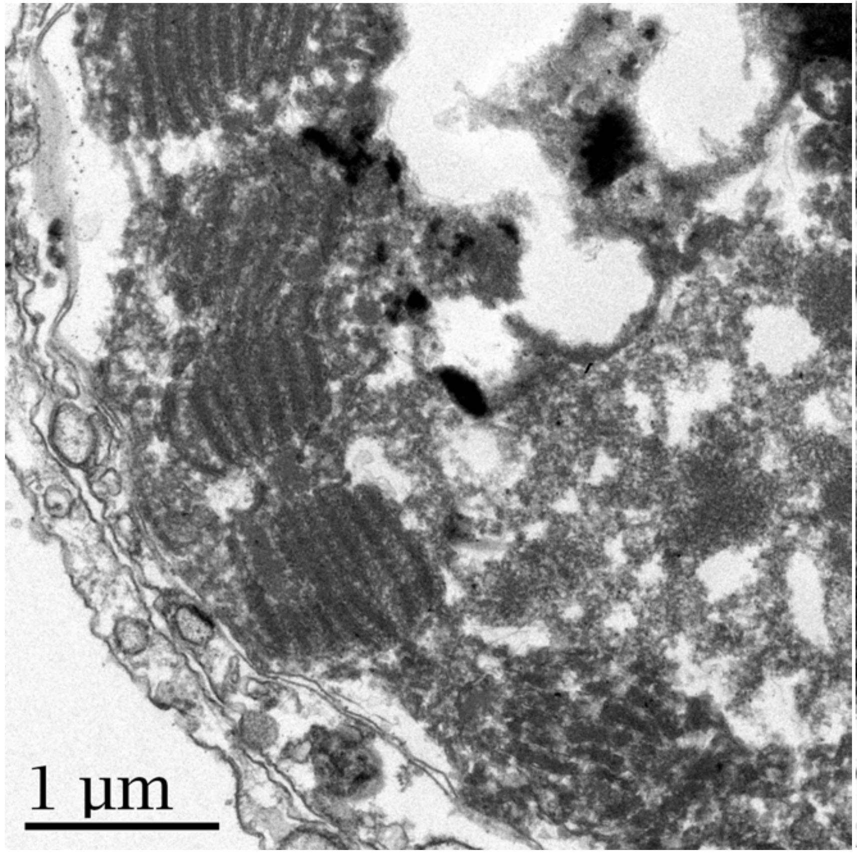

18-14_Correa_XH4_5C5_3.tif

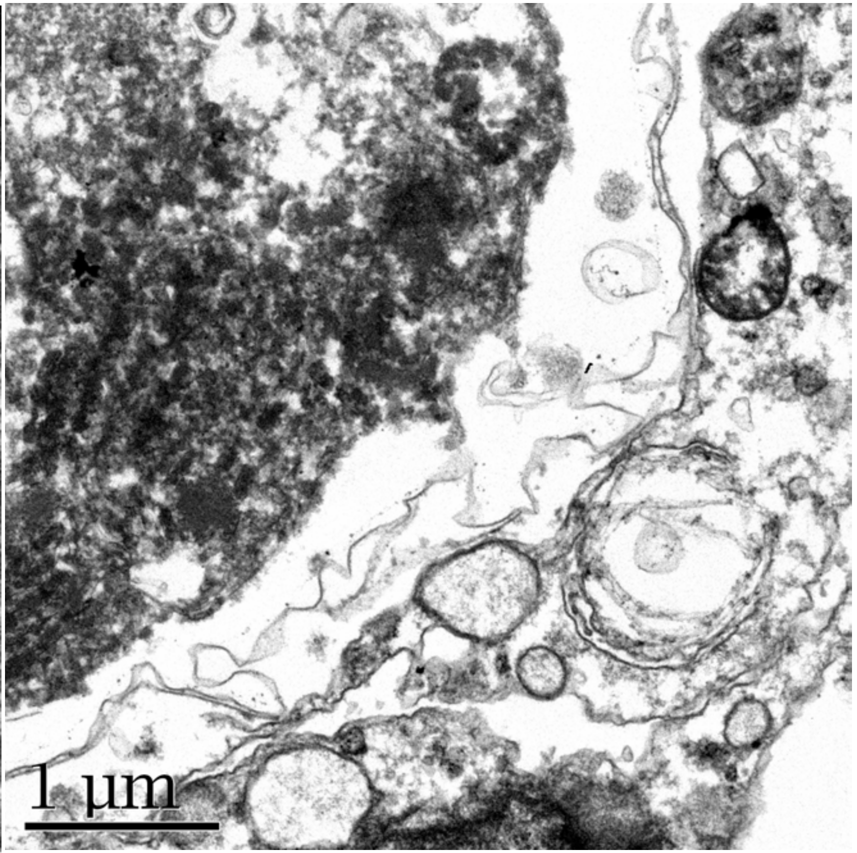

18-14_Correa_XH4_5C5_6.tif

Cell 2

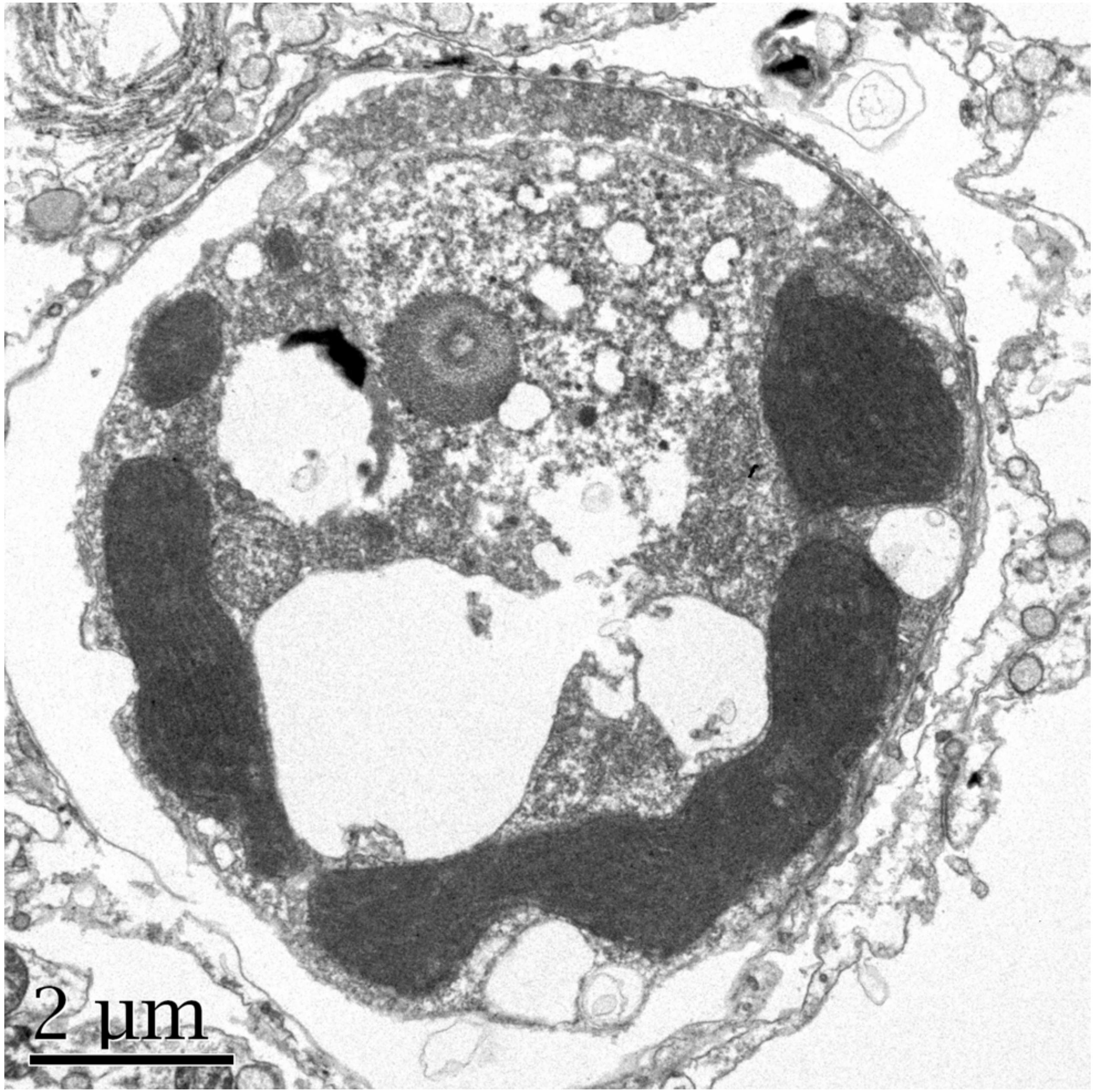

18-14_Correa_XH4_5C5_8.tif

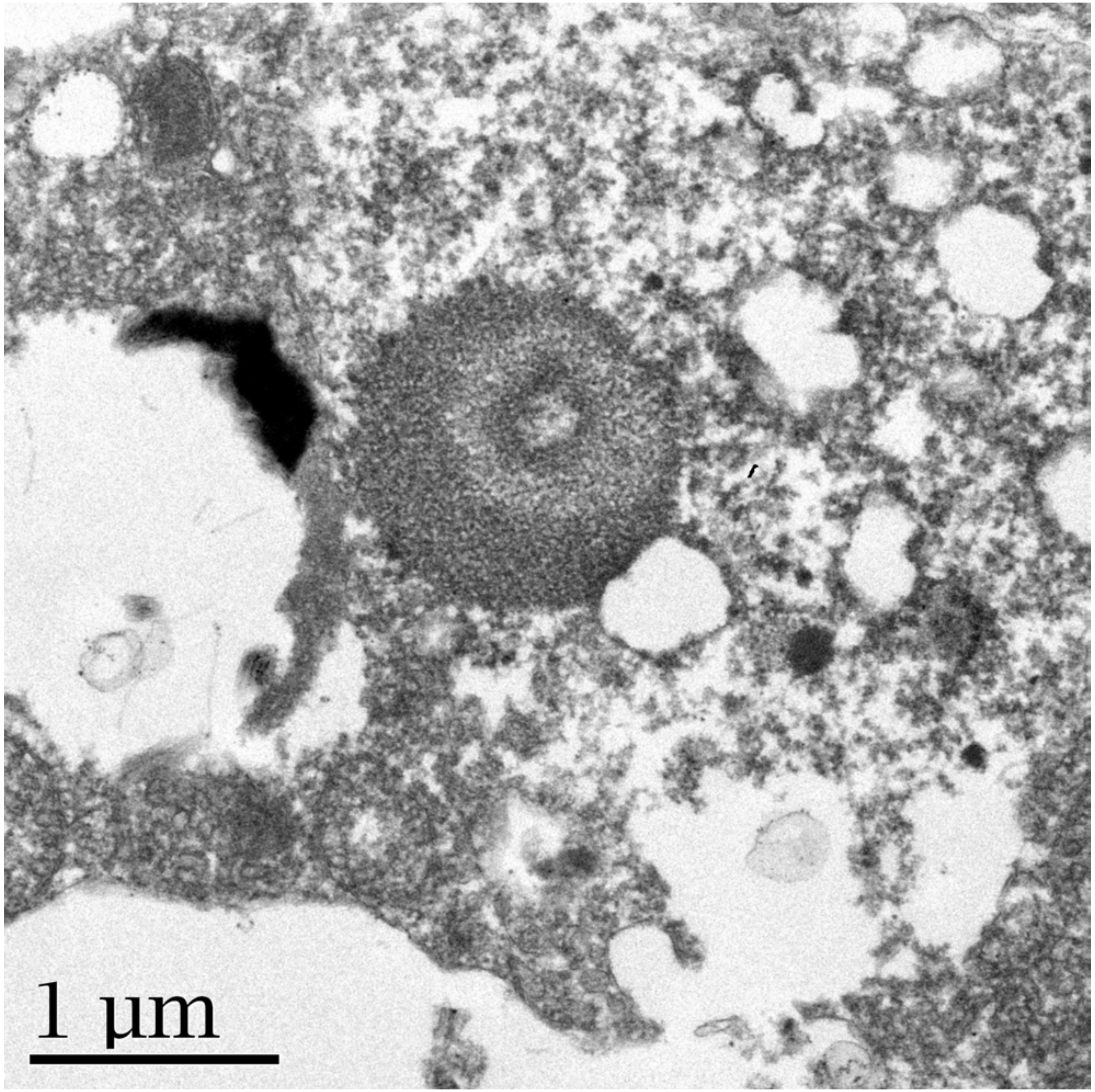

18-14_Correa_XH4_5C5_9.tif

Cell 3

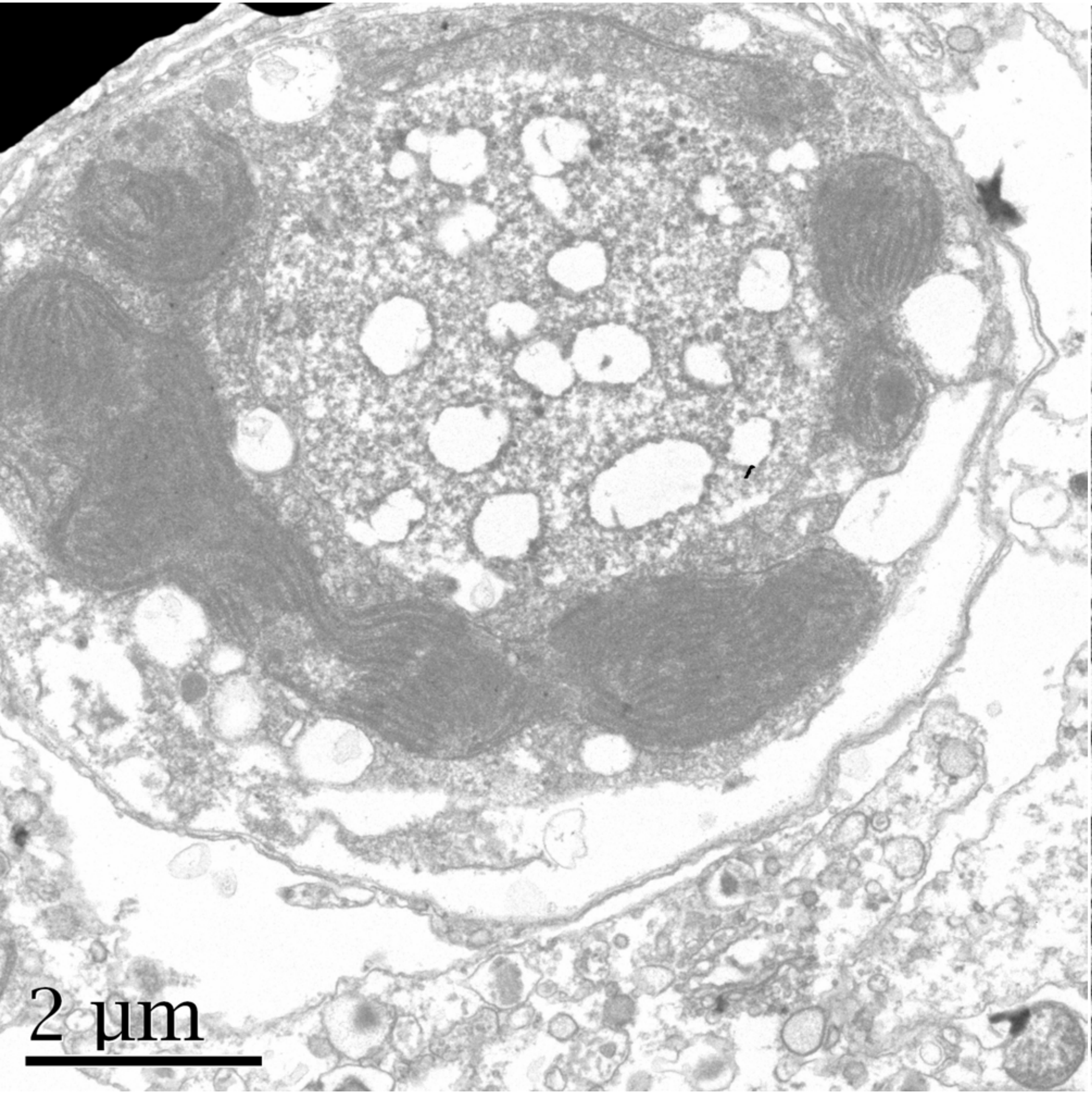

18-14_Correa_XH4_5C5_12.tif

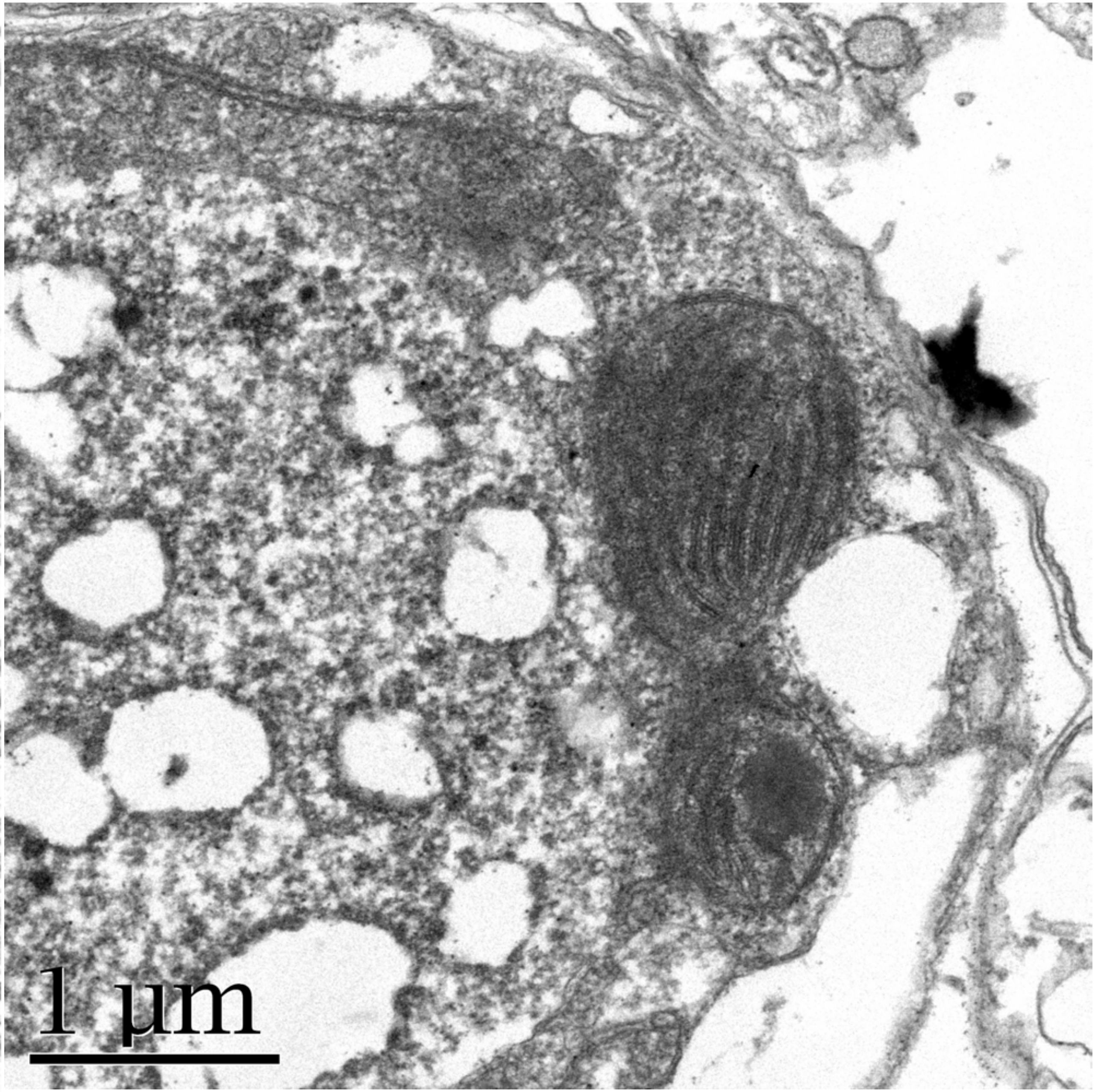

18-14_Correa_XH4_5C5_15.tif

Cell 4

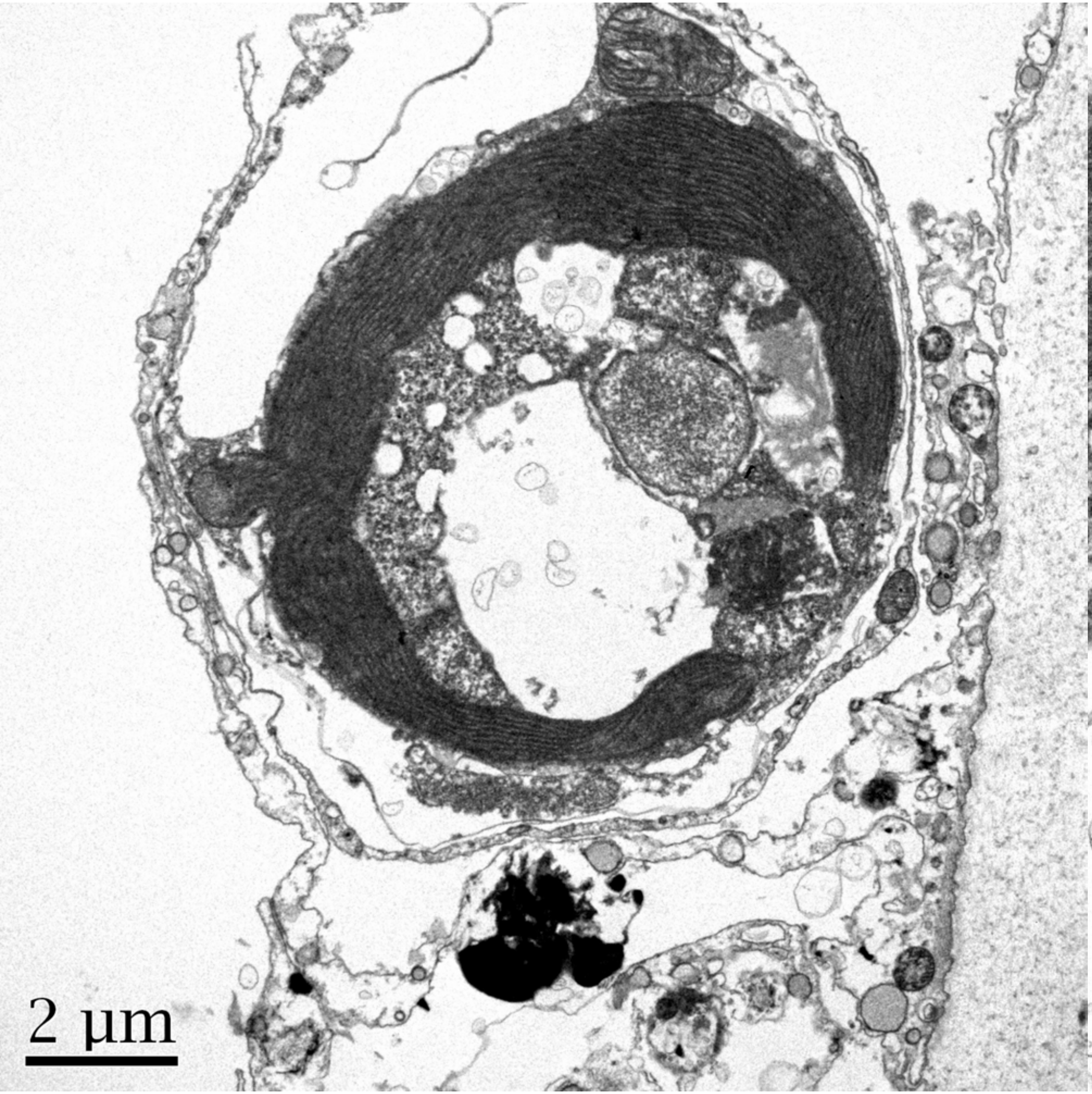

18-14_Correa_XH4_5C5_20.tif

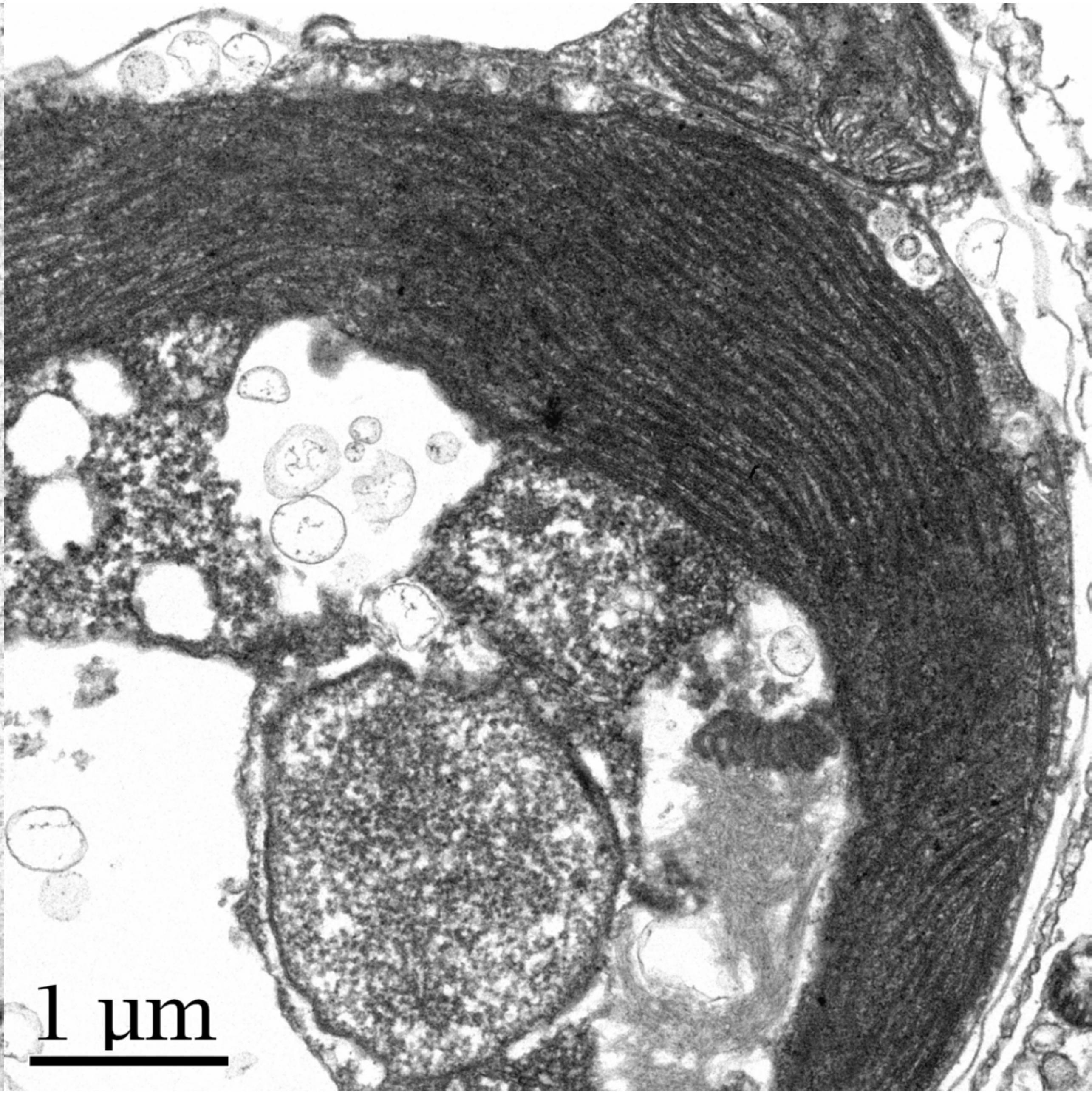

18-14_Correa_XH4_5C5_25.tif

Cell 5

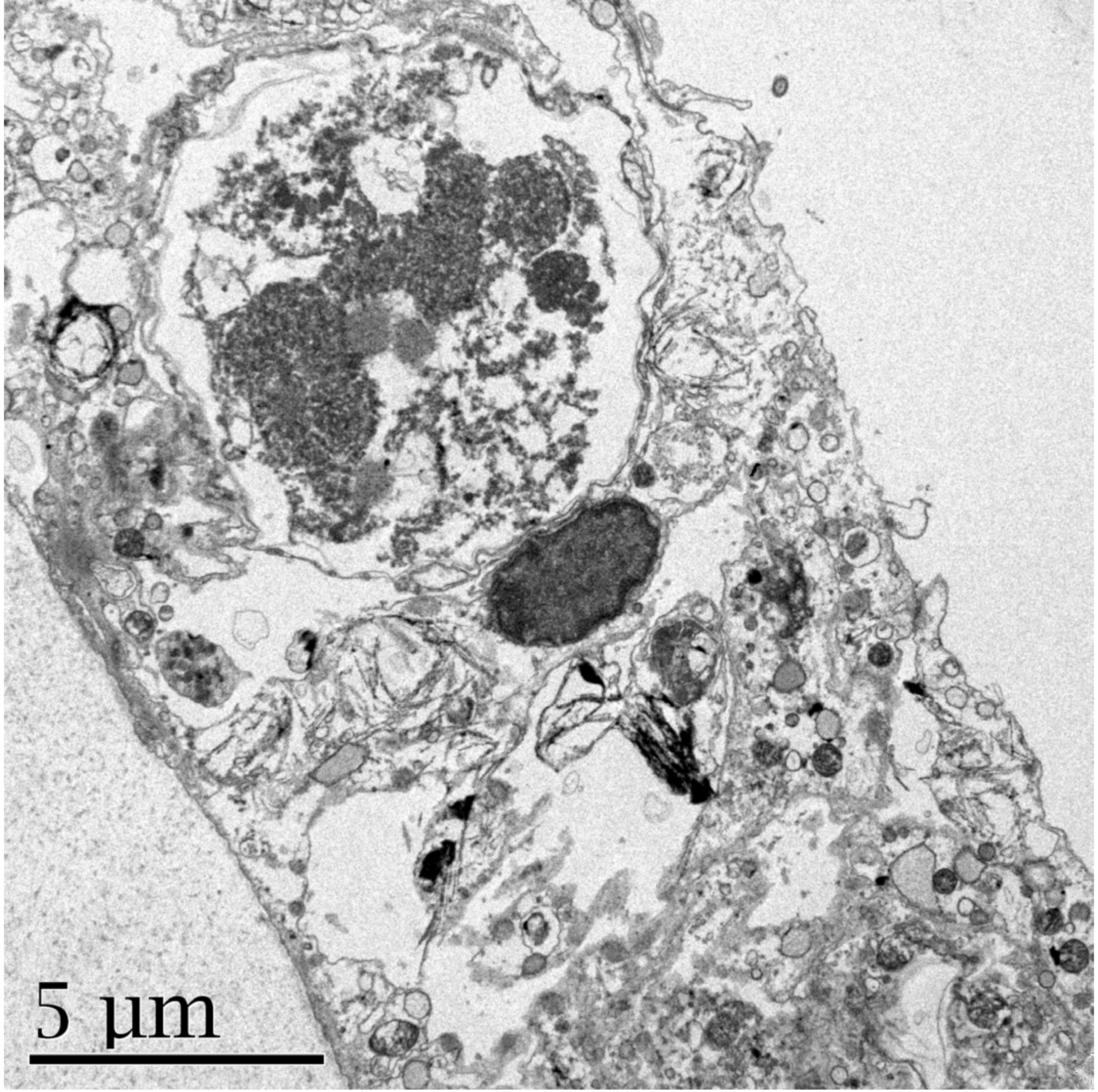

18-14_Correa_XH4_5C5_28.tif

Cell 6

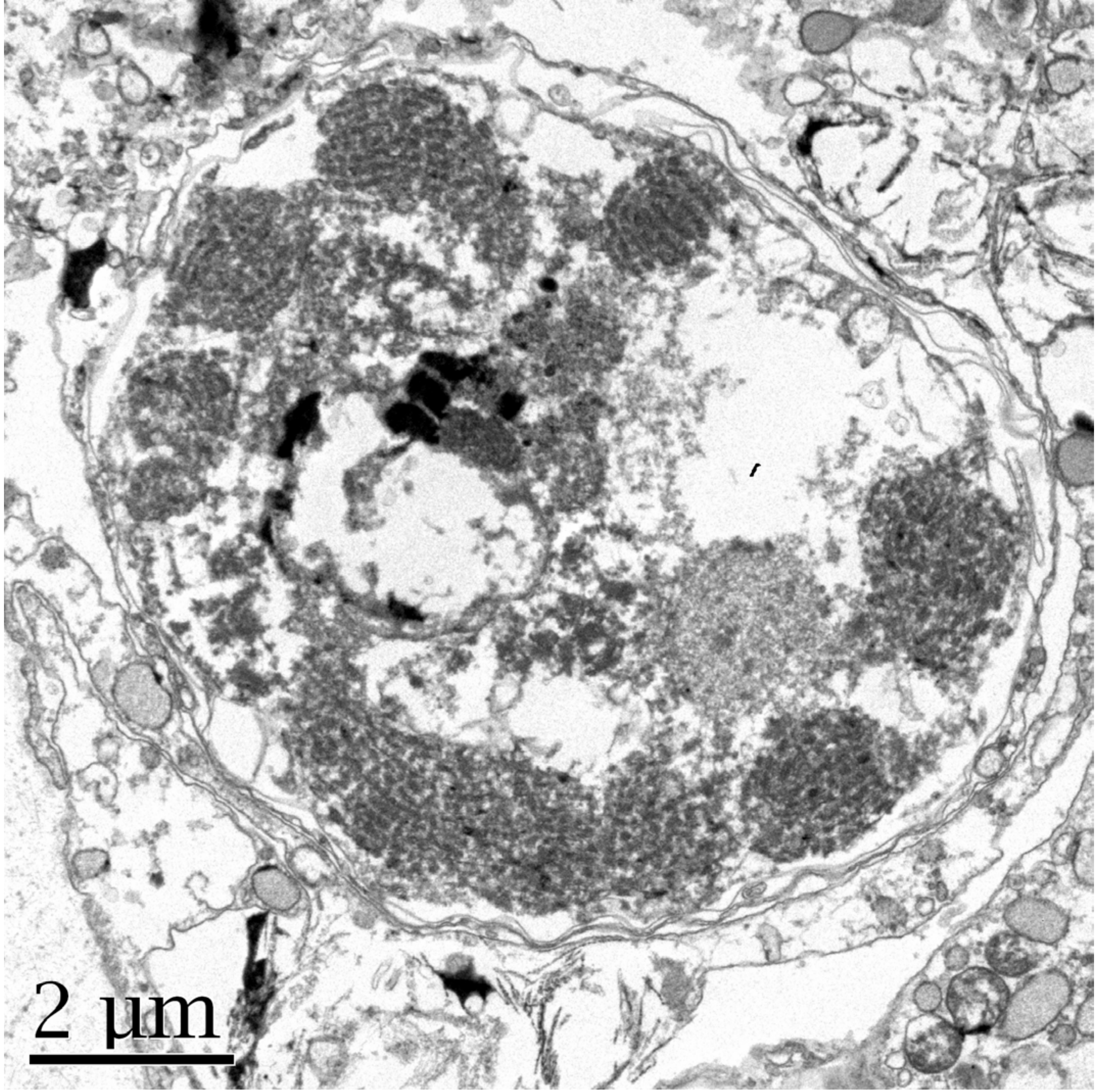

18-14_Correa_XH4_5C5_30.tif

Cell 7

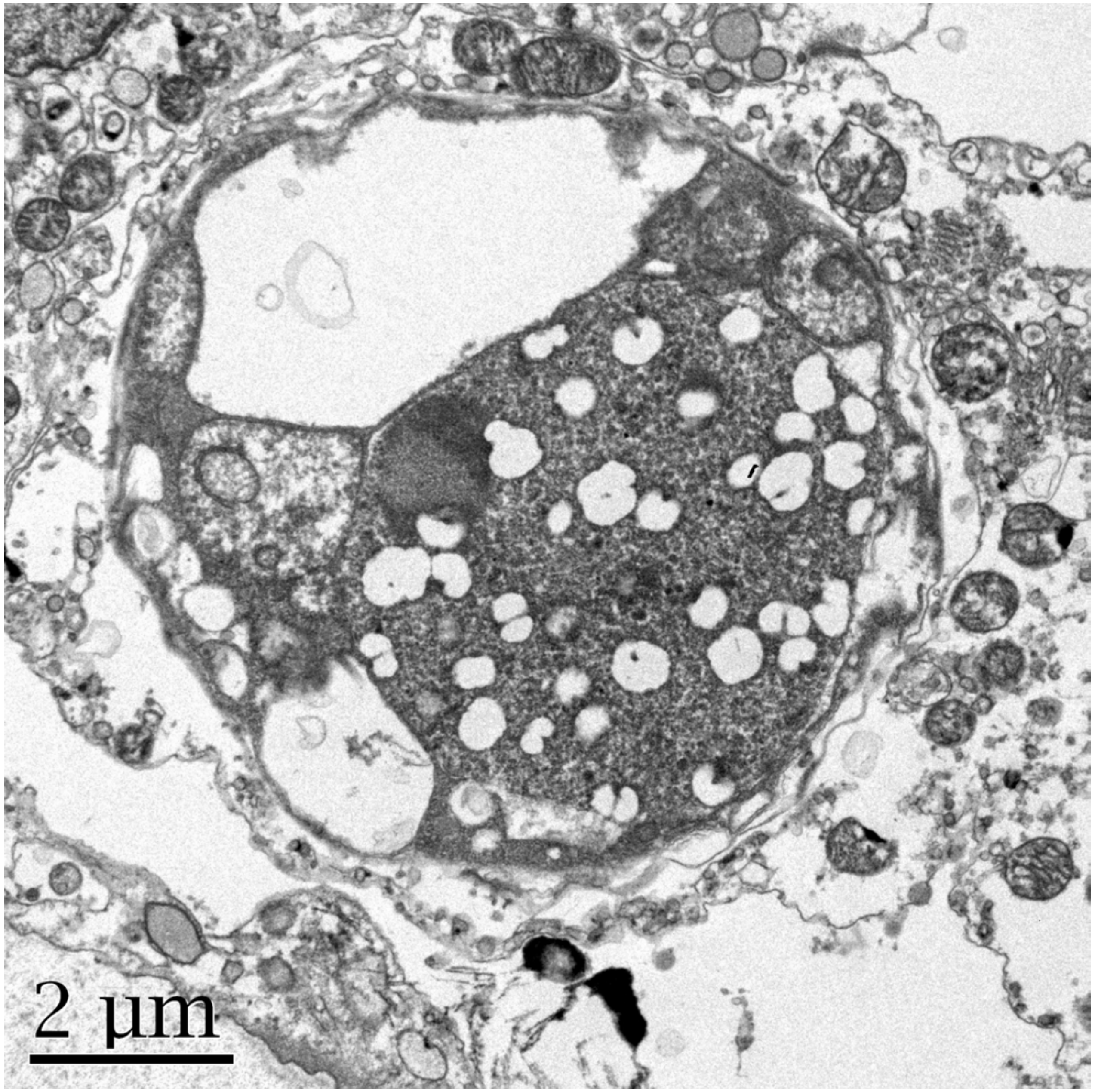

18-14_Correa_XH4_5C5_31.tif

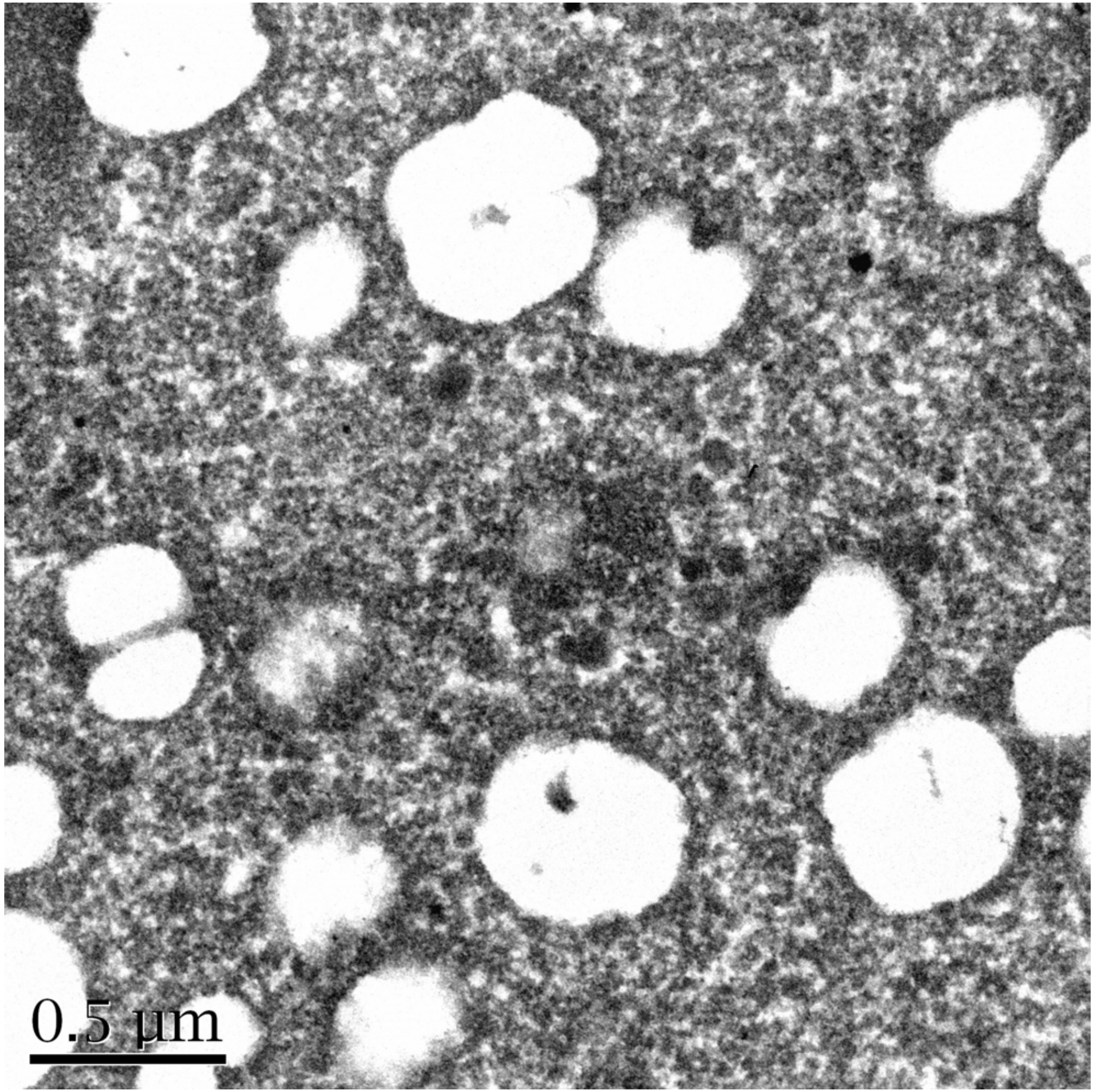

18-14_Correa_XH4_5C5_33.tif

Cell 8

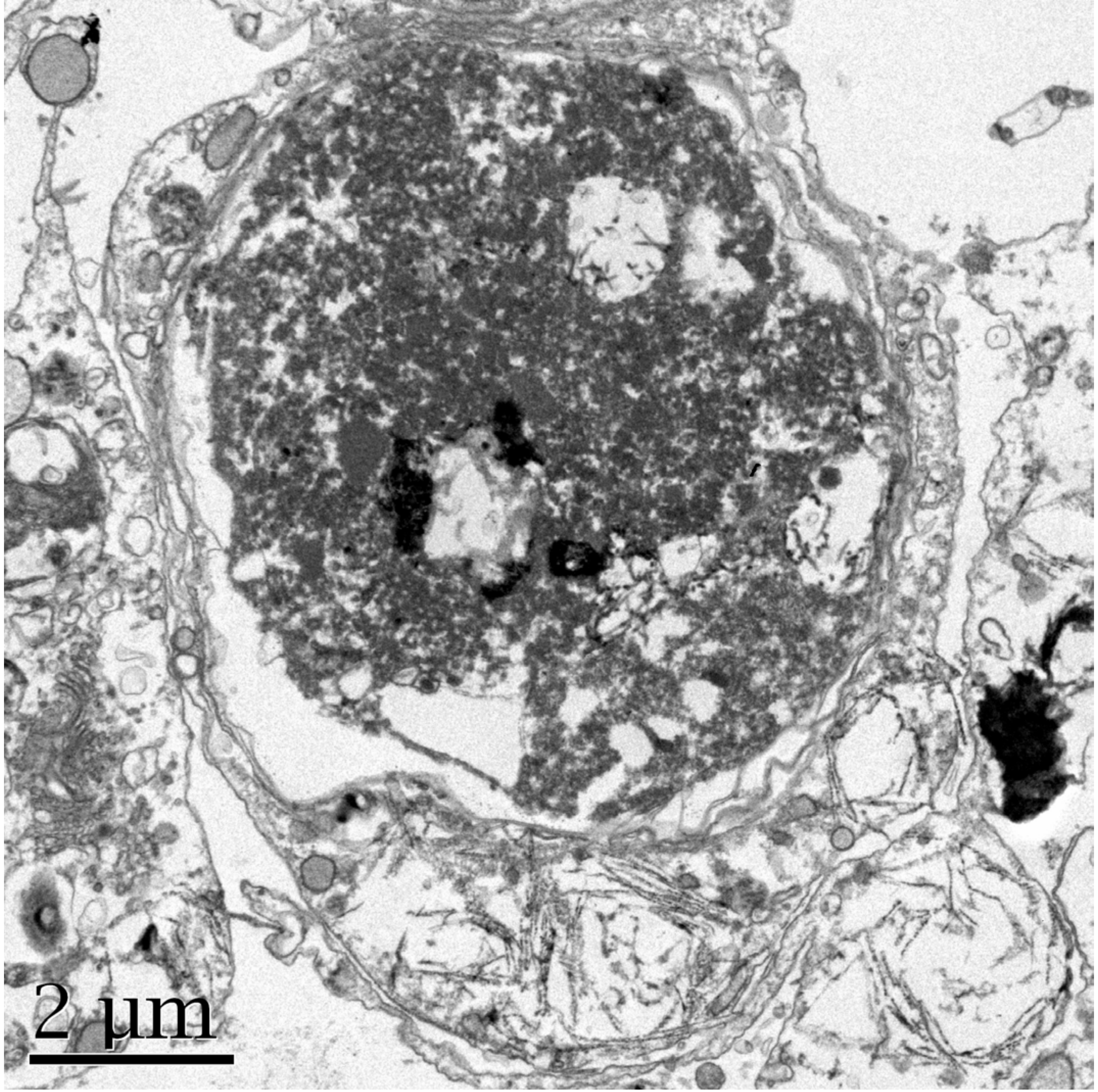

18-14_Correa_XH4_5C5_35.tif

Cell 9

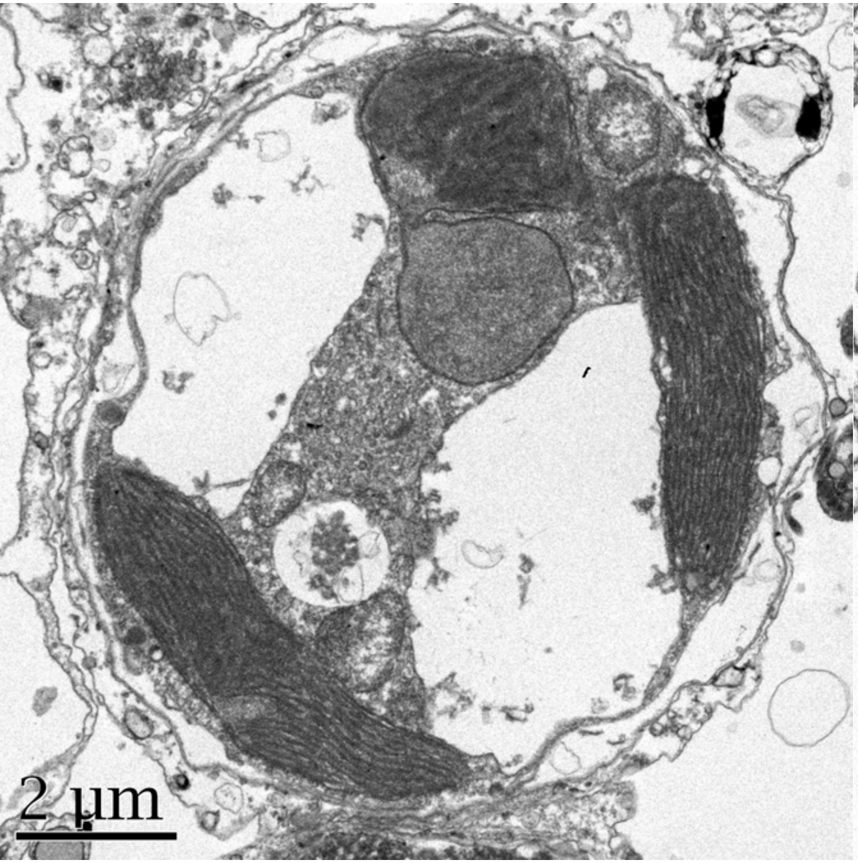

18-14_Correa_XH4_5C5_36.tif

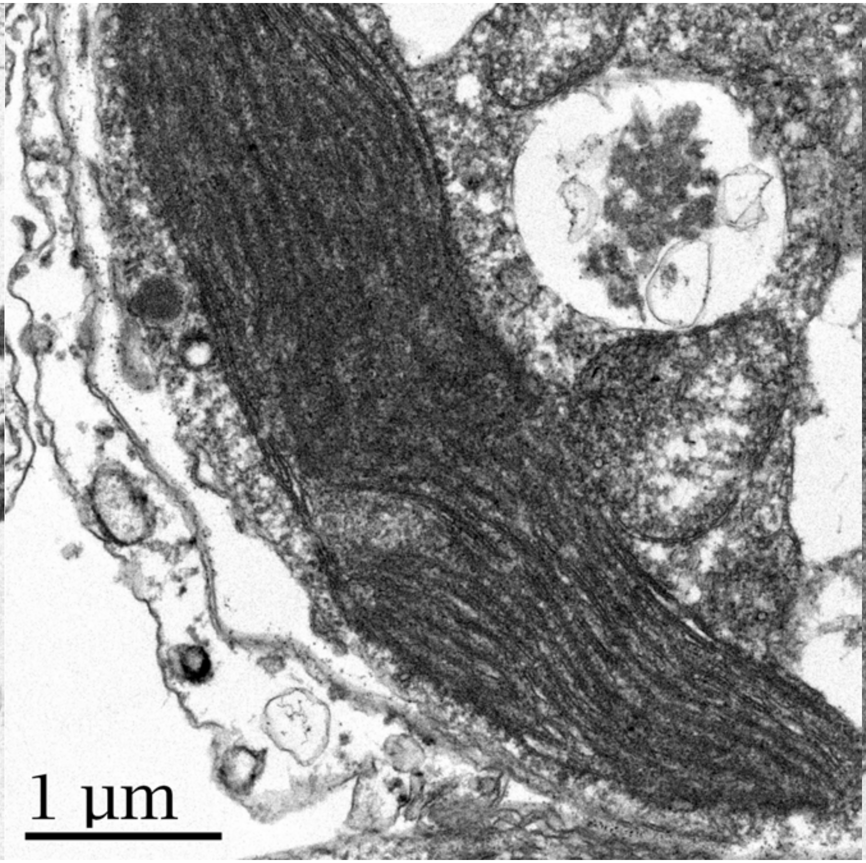

18-14_Correa_XH4_5C5_37.tif

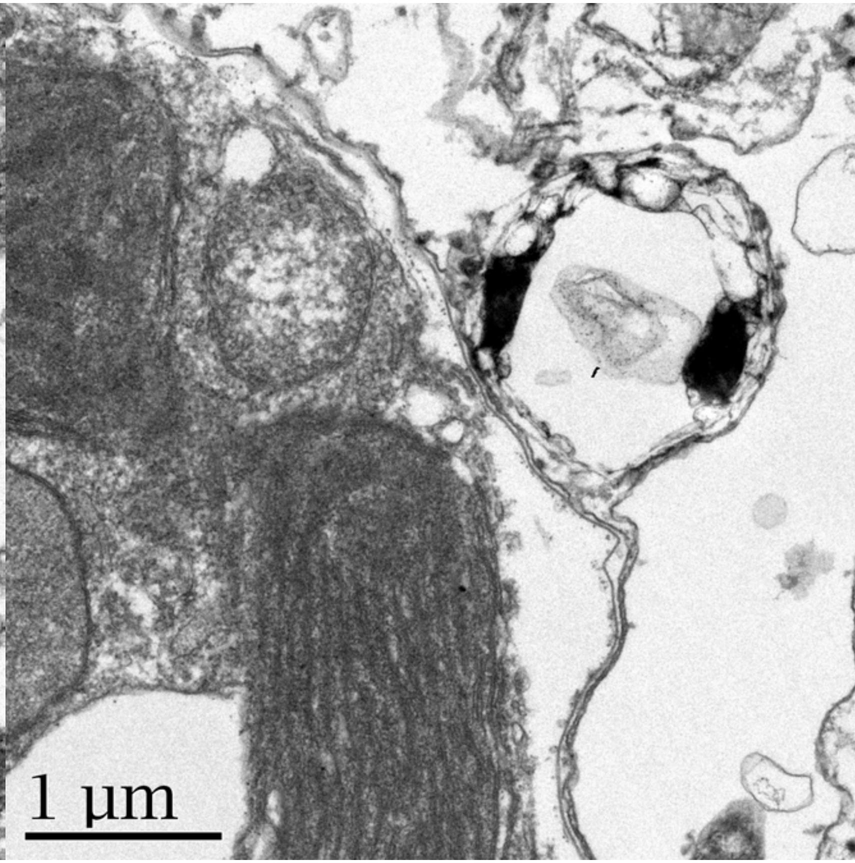

18-14_Correa_XH4_5C5_39.tif

Cell 10

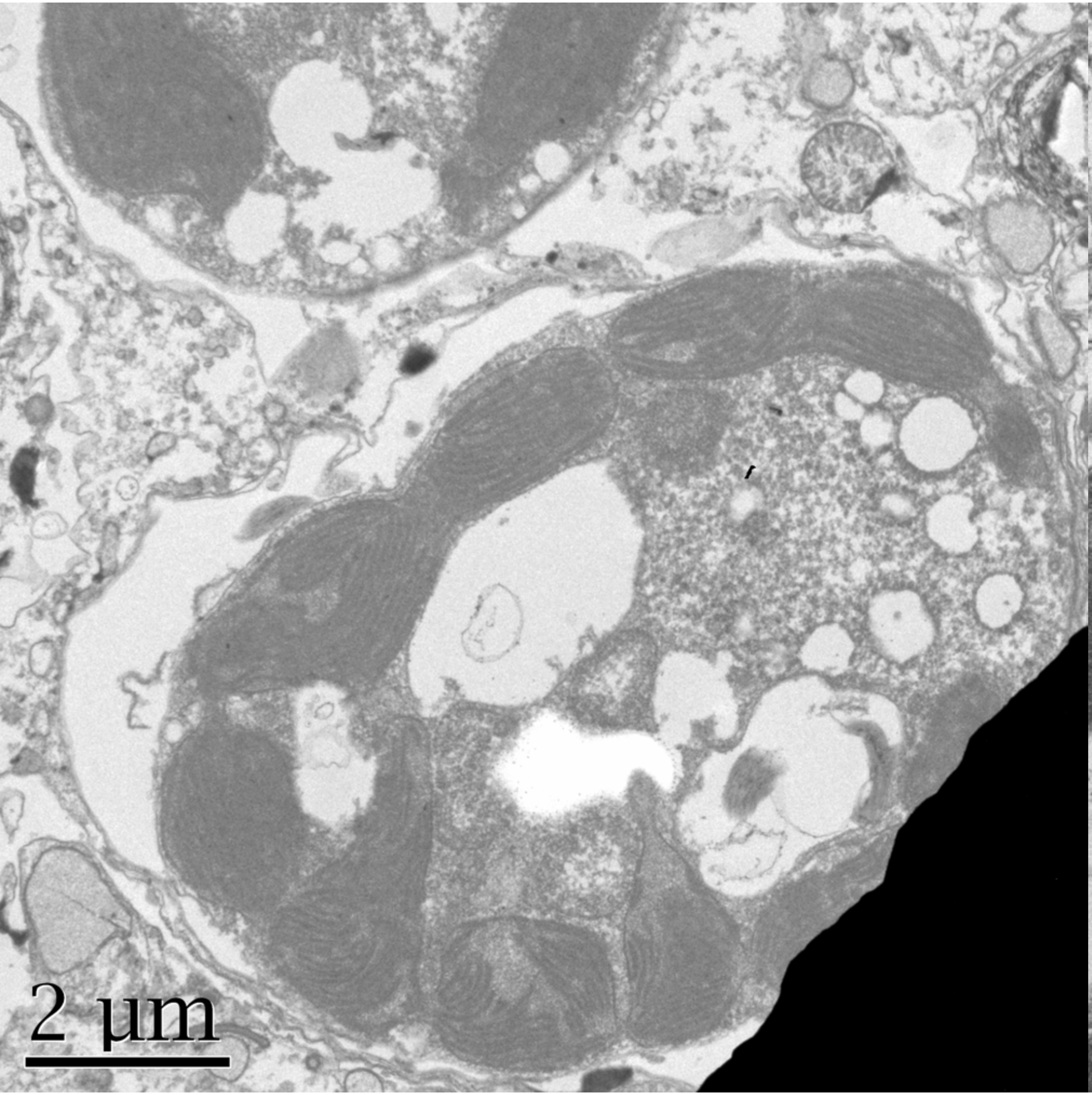

18-14_Correa_XH4_5C5_43.tif

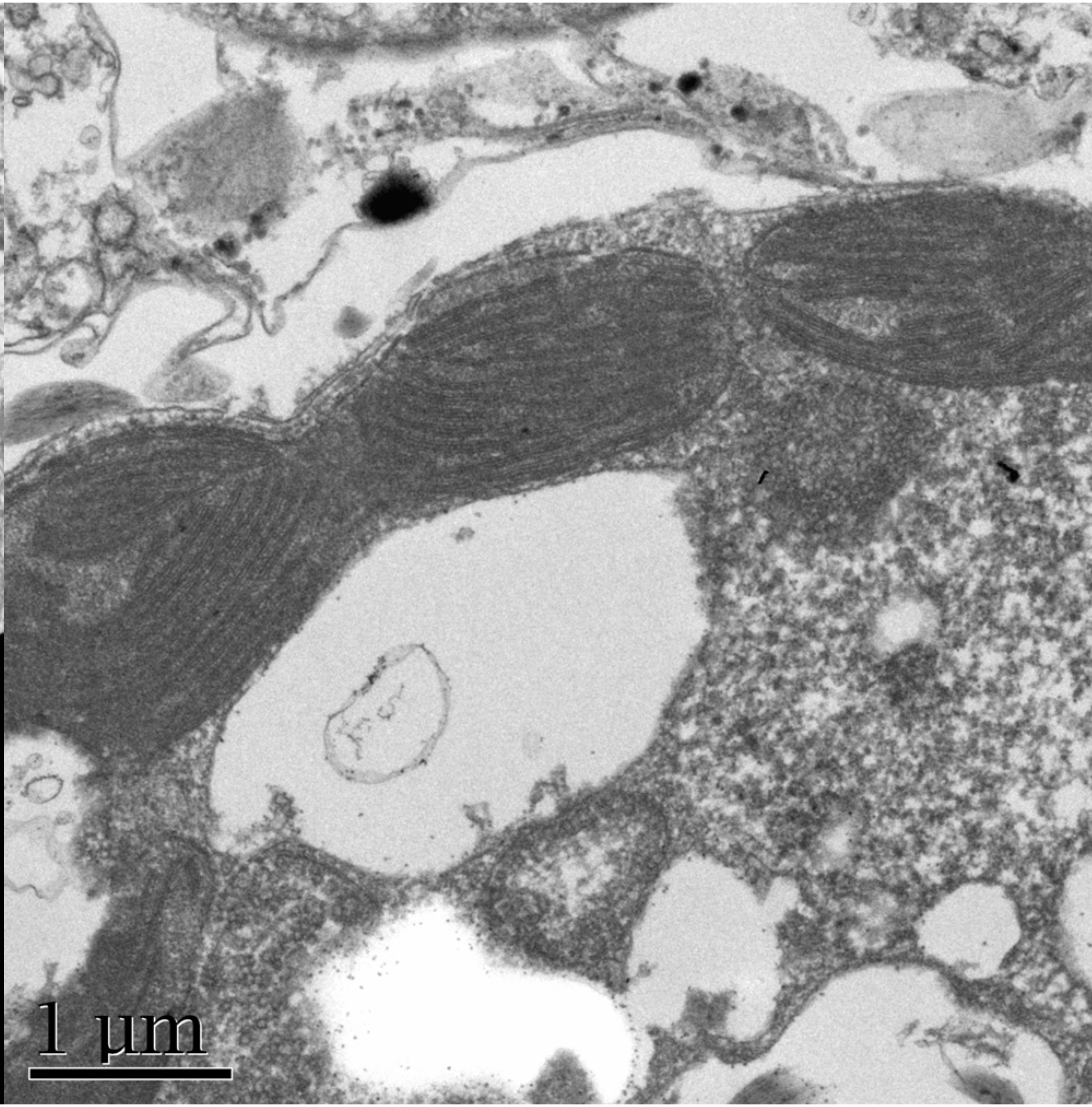

18-14_Correa_XH4_5C5_44.tif

Cell 11

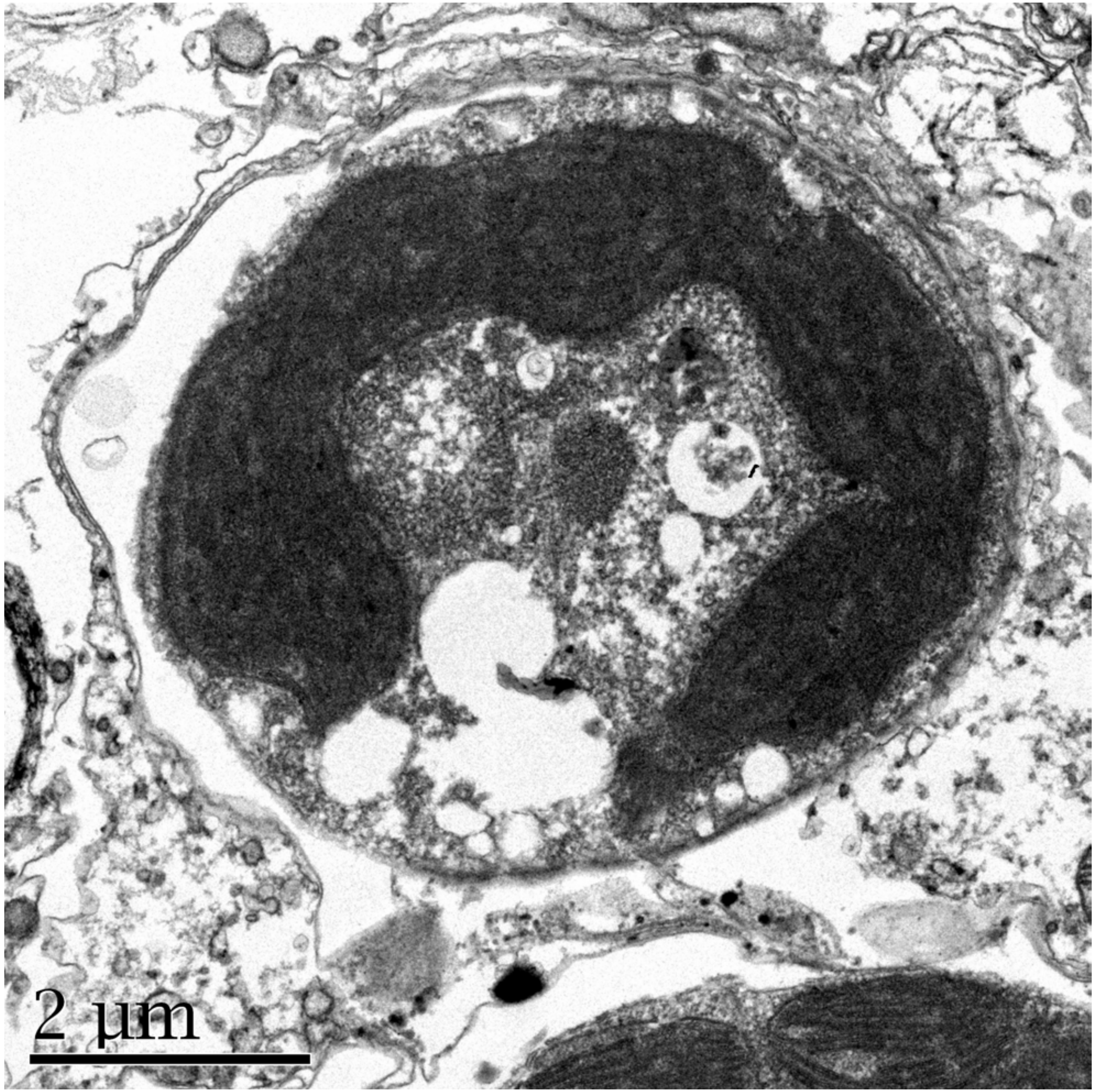

18-14_Correa_XH4_5C5_45.tif

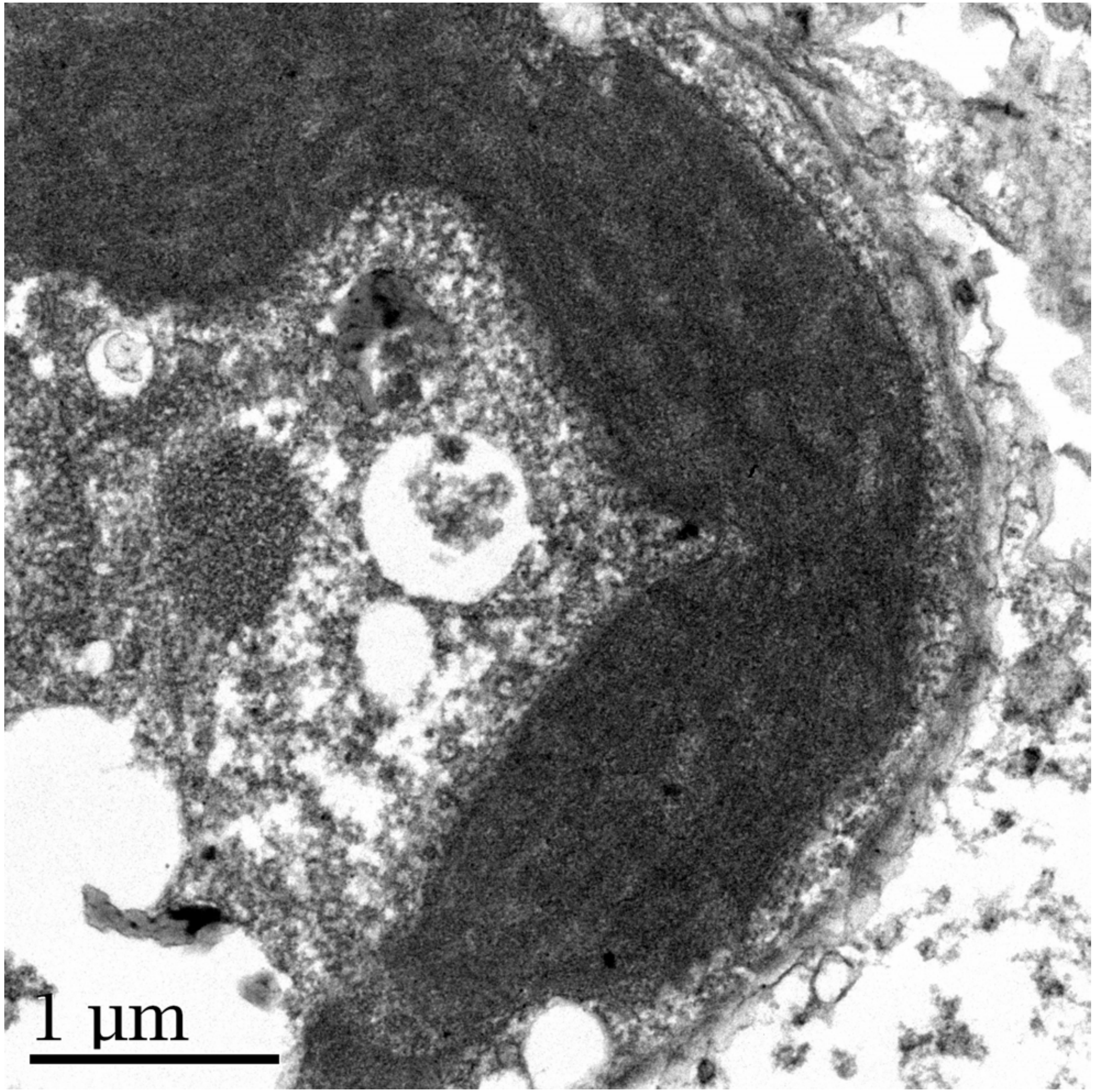

18-14_Correa_XH4_5C5_46.tif

Cell 12

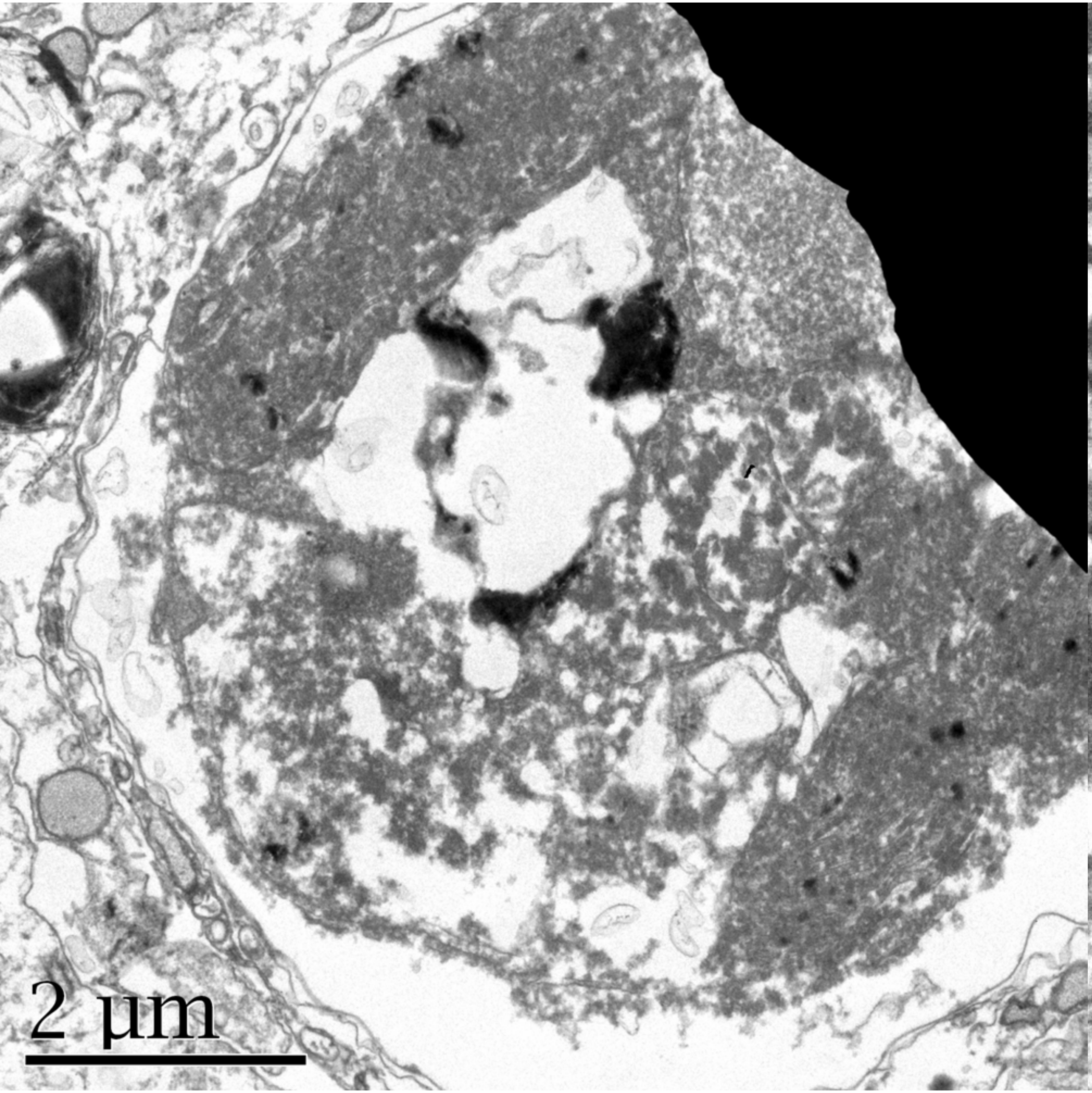

18-14_Correa_XH4_5C5_50.tif

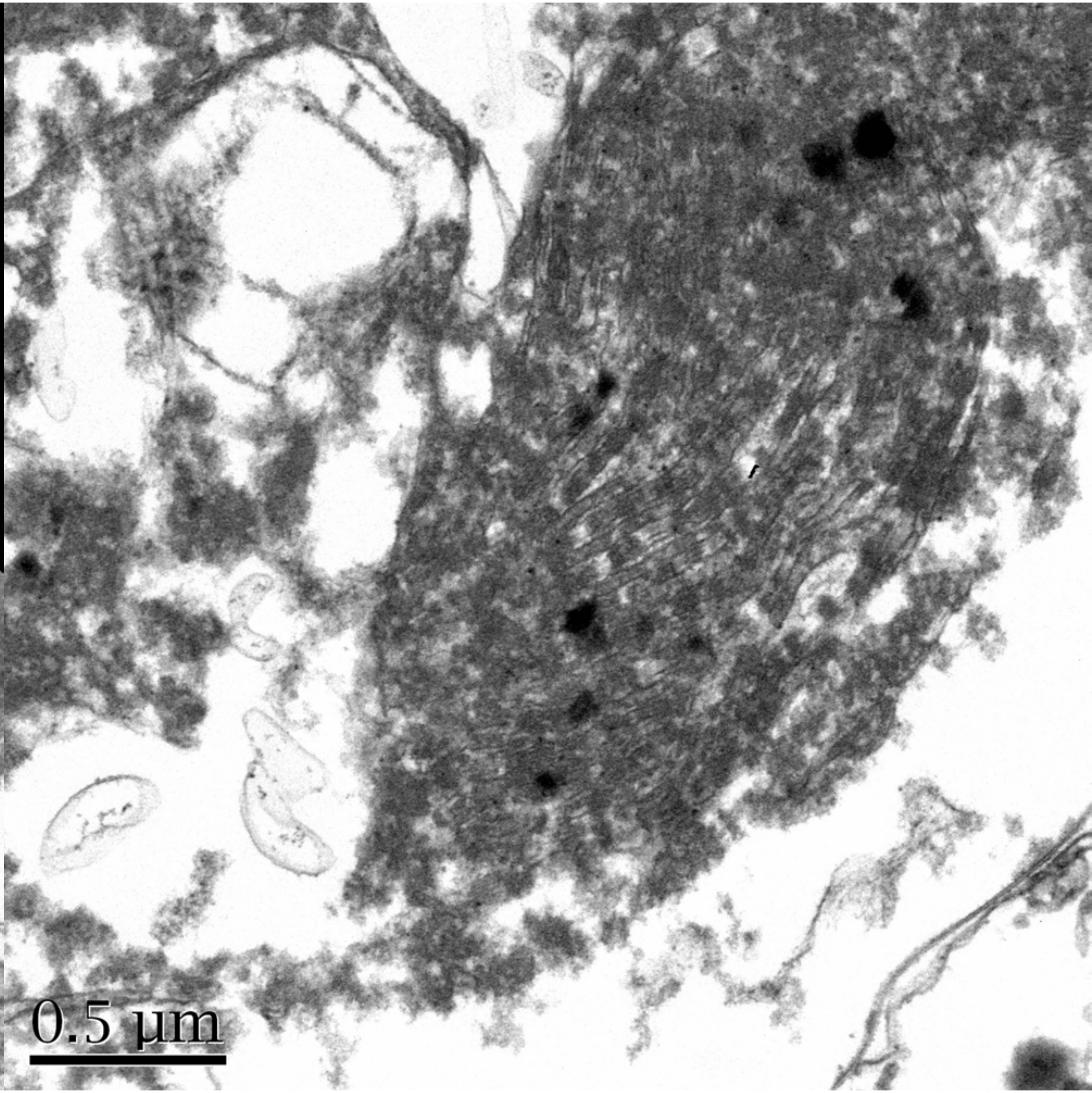

18-14_Correa_XH4_5C5_51.tif

Cell 13

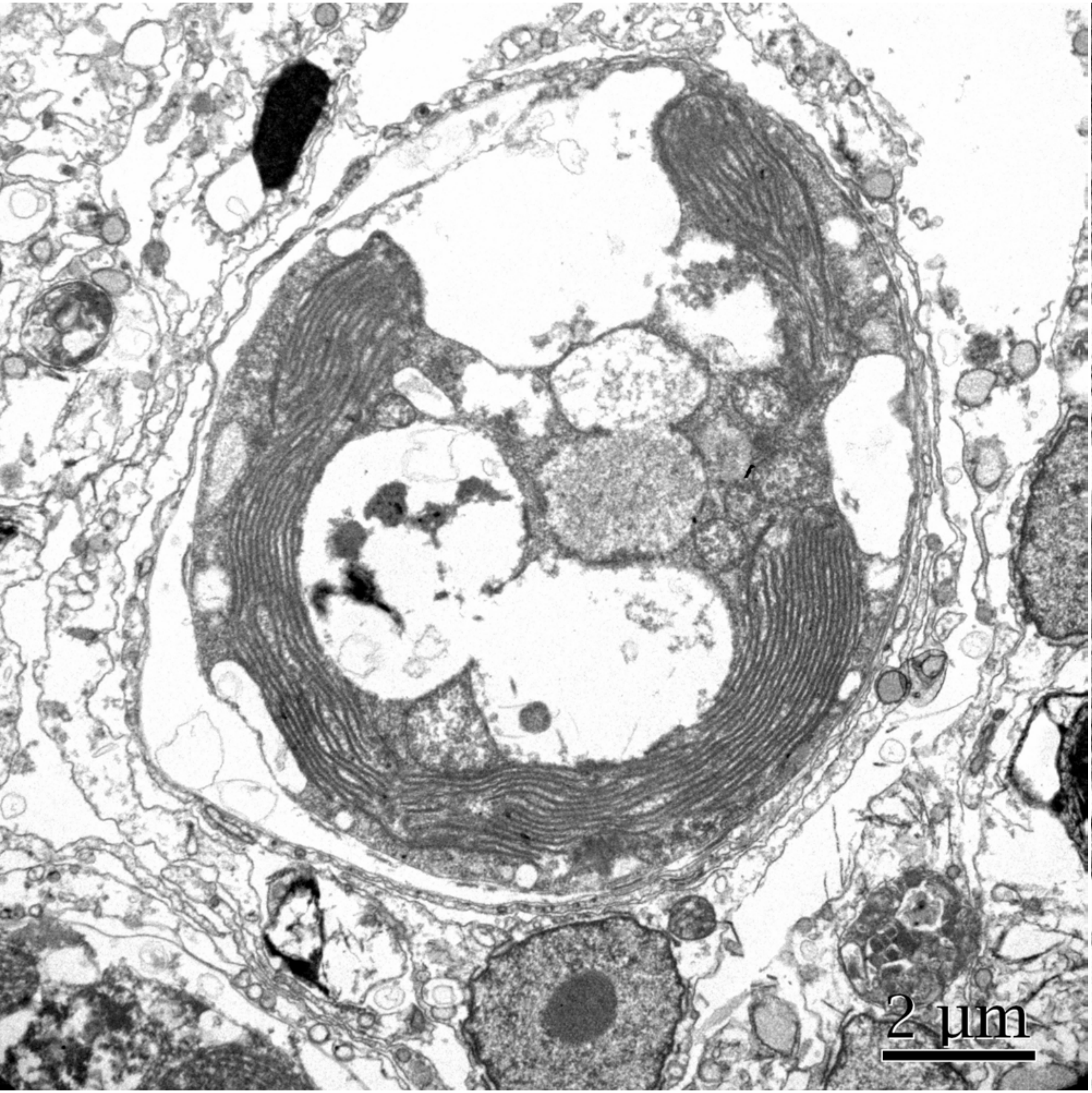

18-14_Correa_XH4_5C5_55.tif

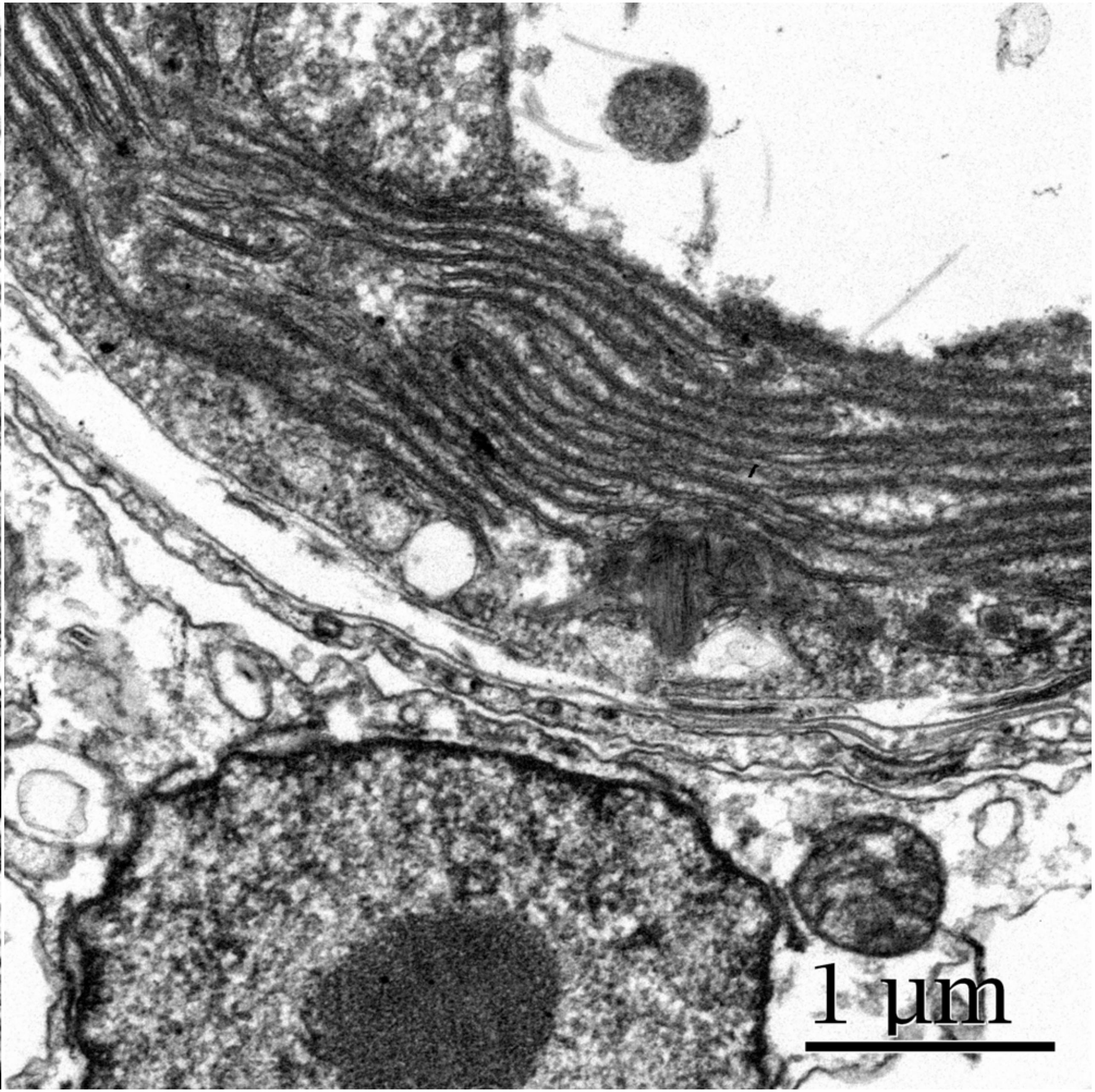

18-14_Correa_XH4_5C5_56.tif

Ambient - ACR Colony D

Cell 1

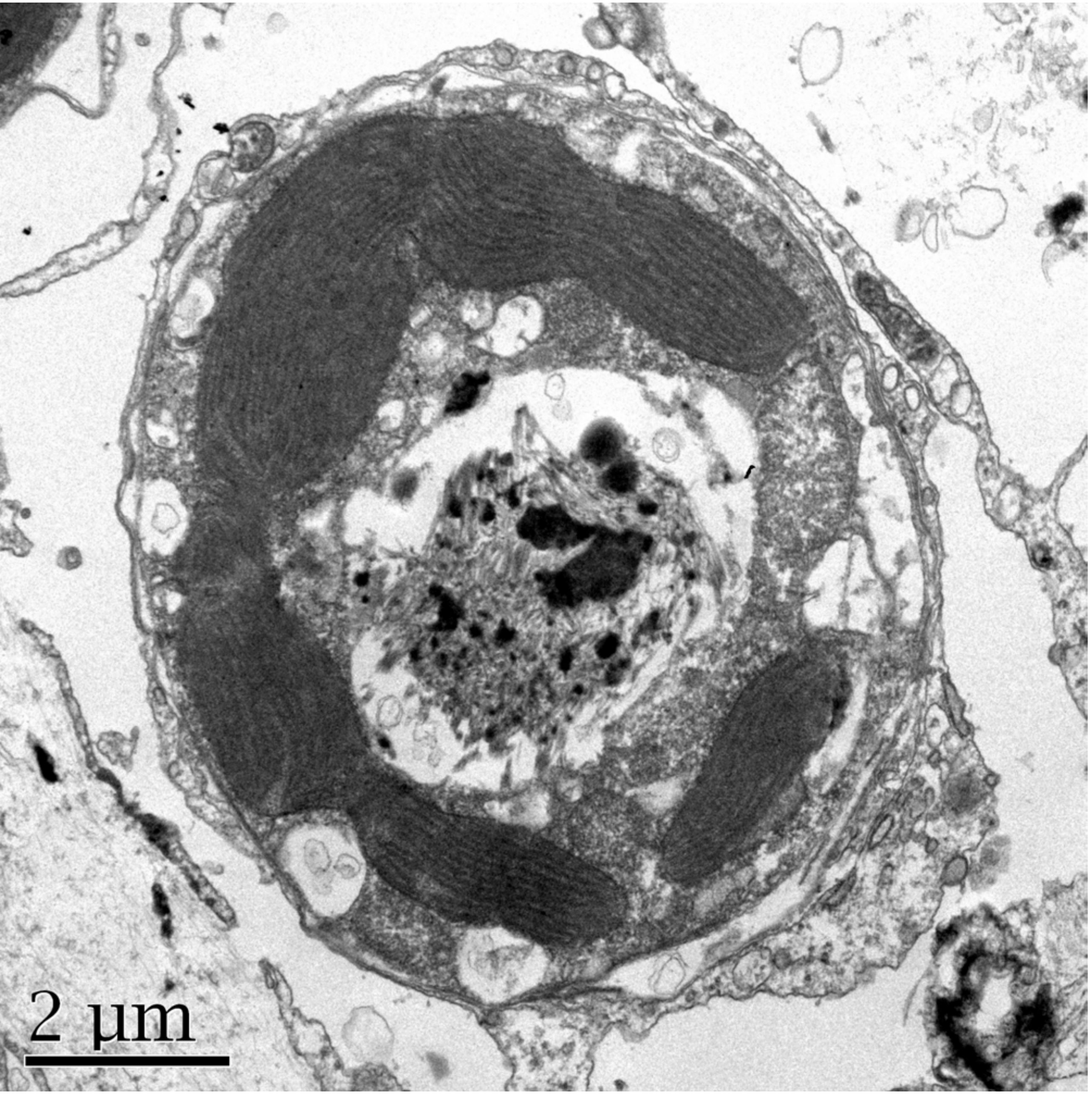

18-14_Correa_YC4_5C9_2.tif

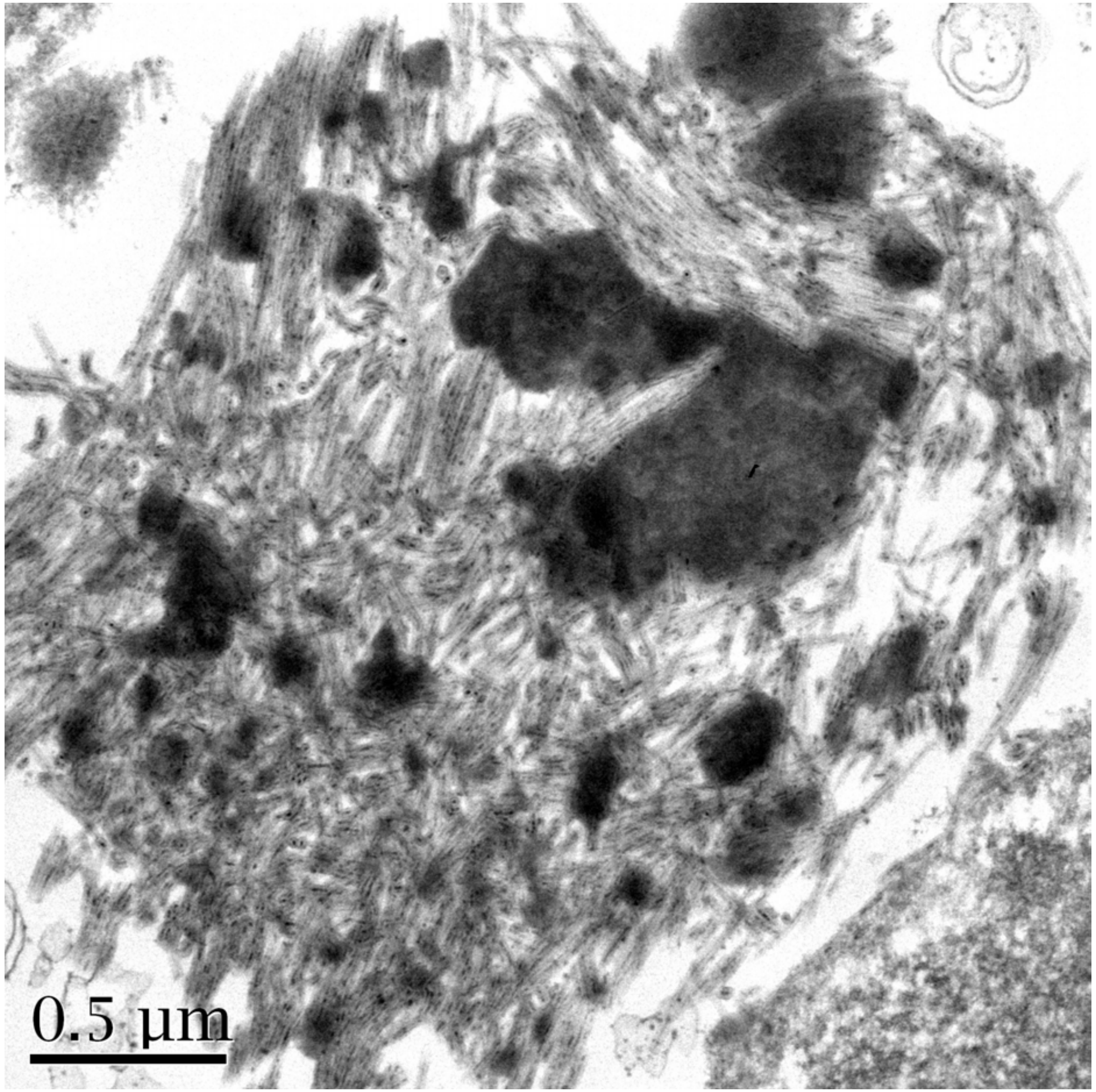

18-14_Correa_YC4_5C9_4.tif

Cell 2

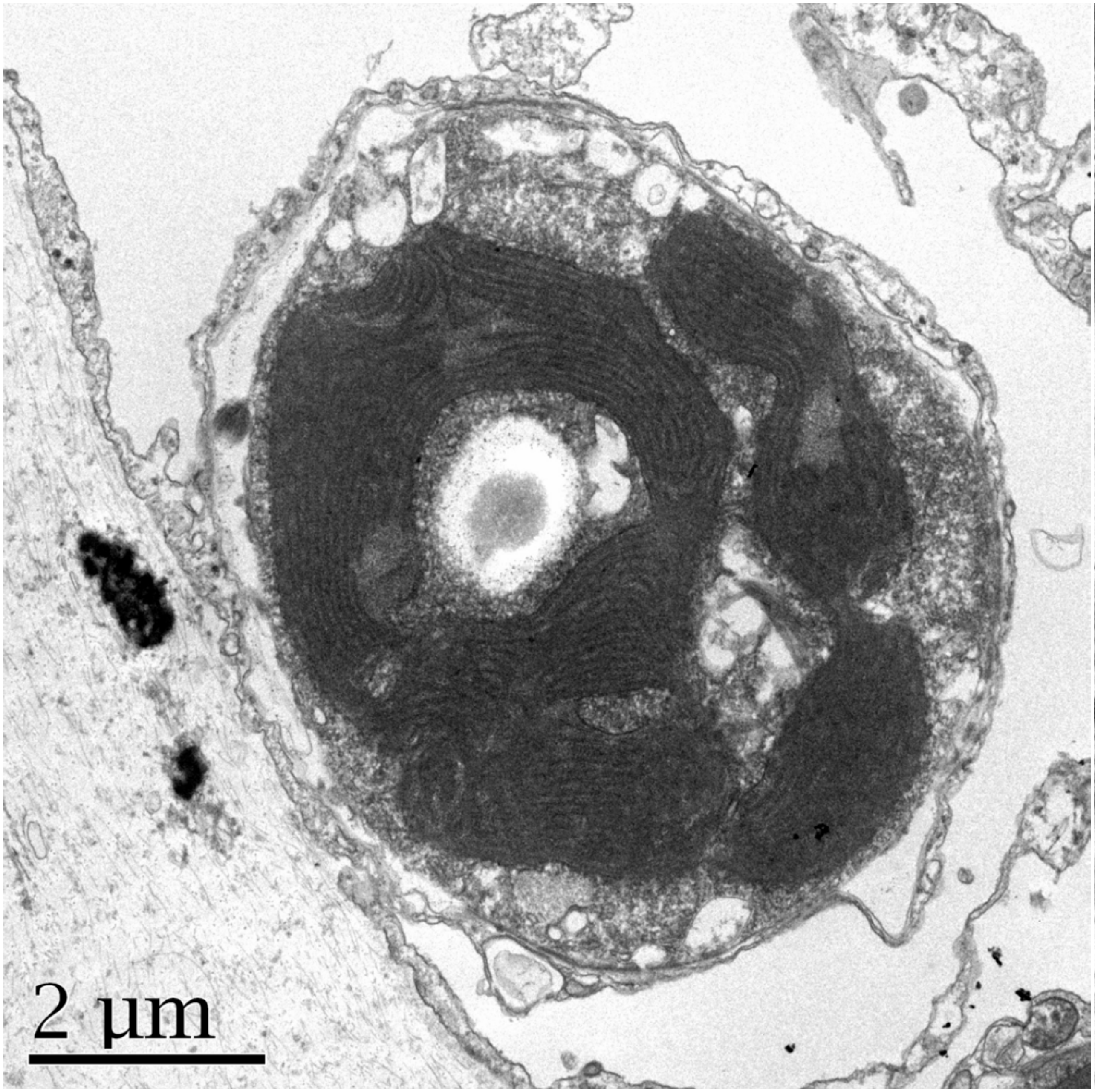

18-14_Correa_YC4_5C9_6.tif

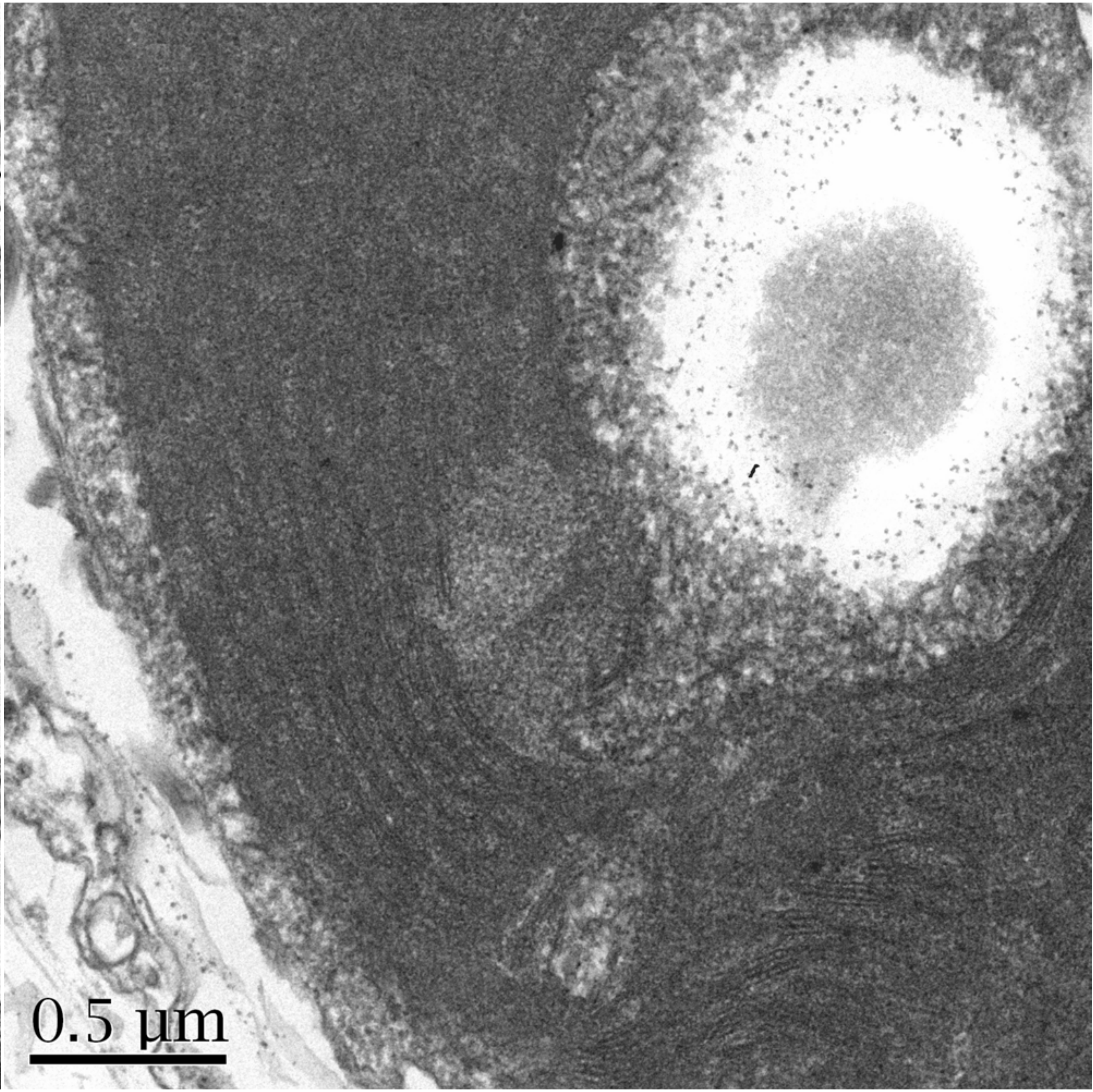

18-14_Correa_YC4_5C9_10.tif

Cell 3

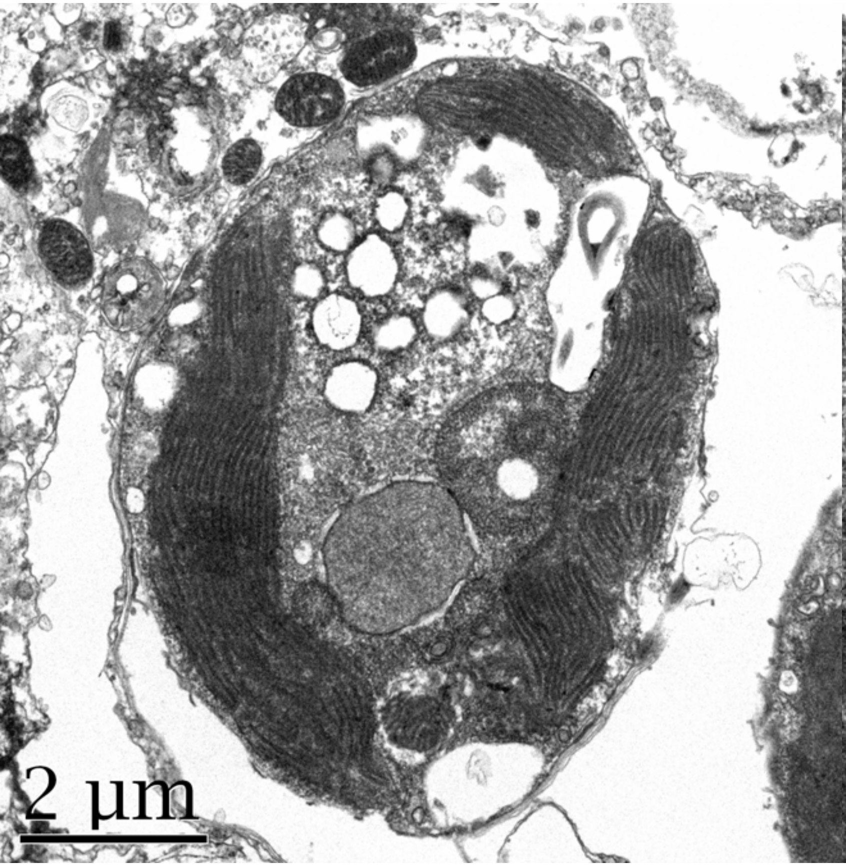

18-14_Correa_YC4_5C9_12.tif

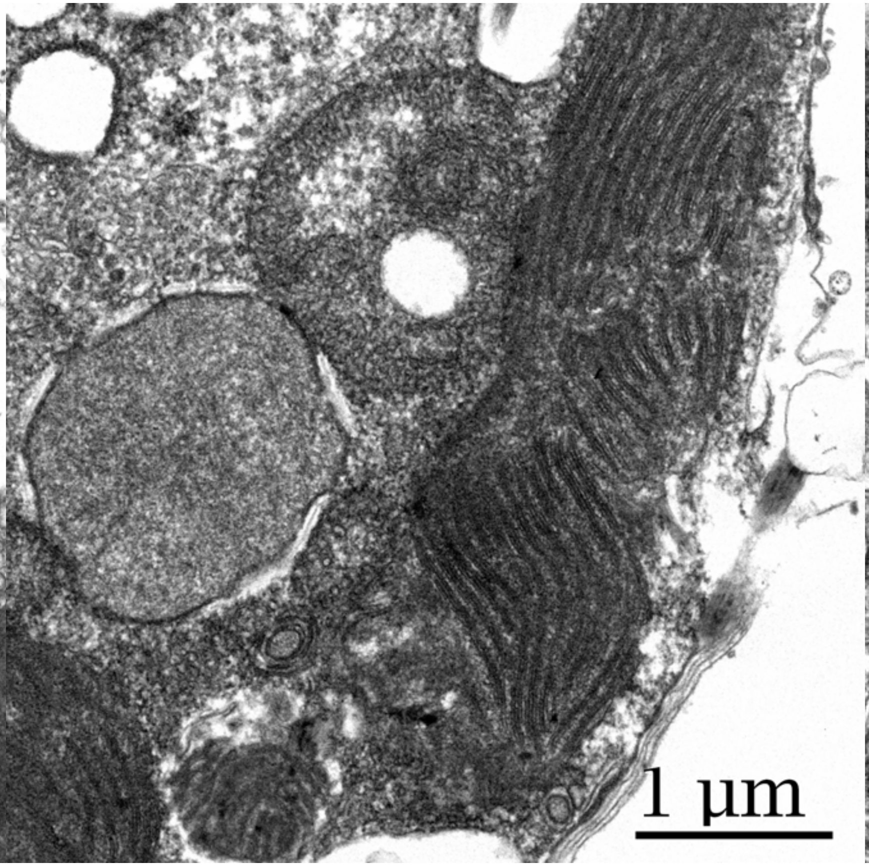

18-14_Correa_YC4_5C9_13.tif

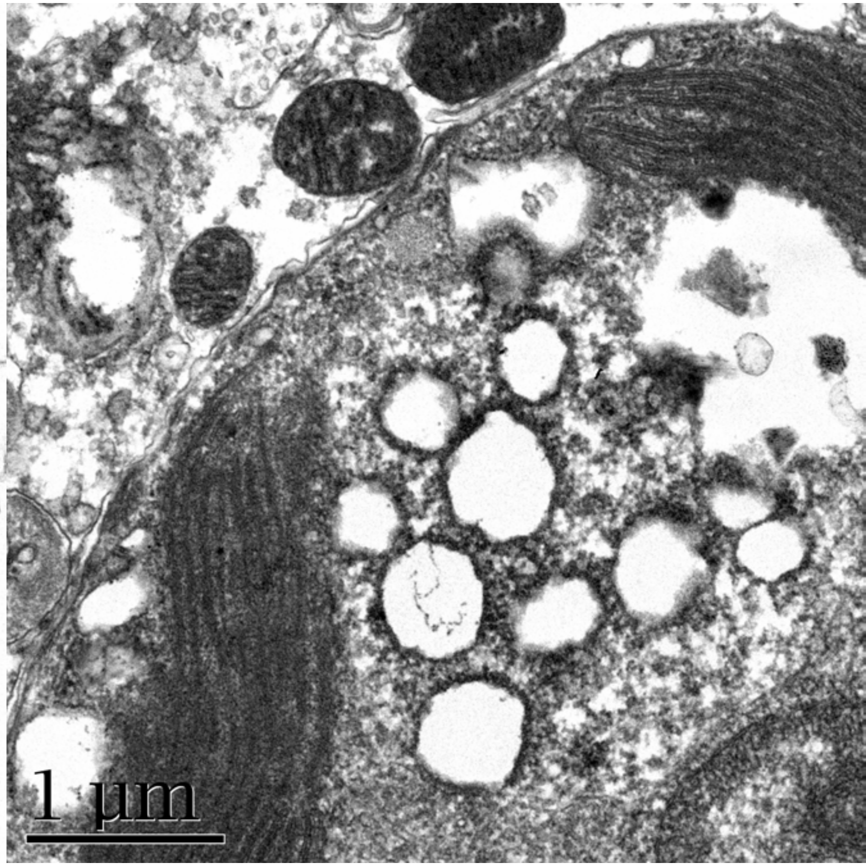

18-14_Correa_YC4_5C9_14.tif

Cell 4

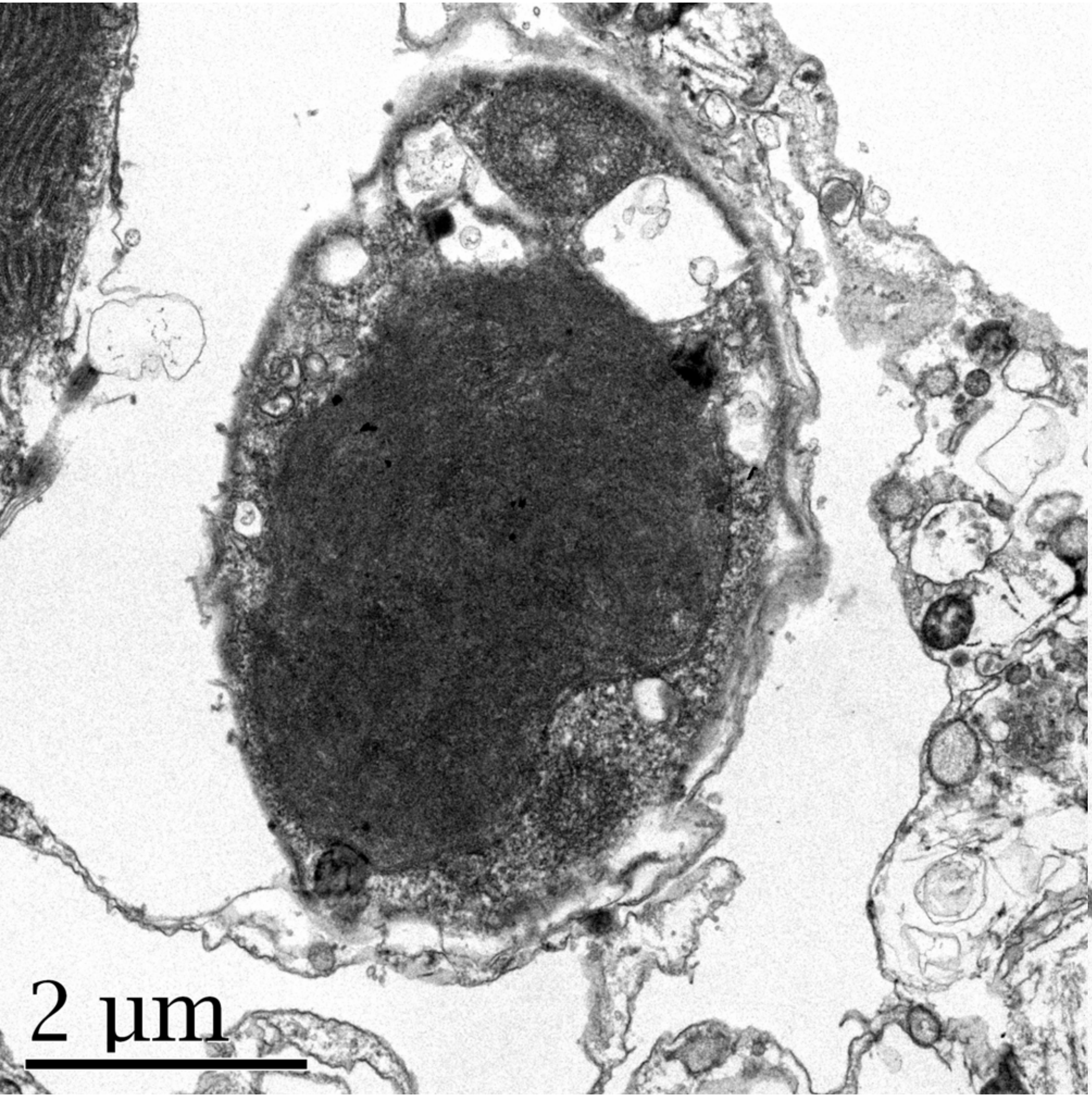

18-14_Correa_YC4_5C9_16.tif

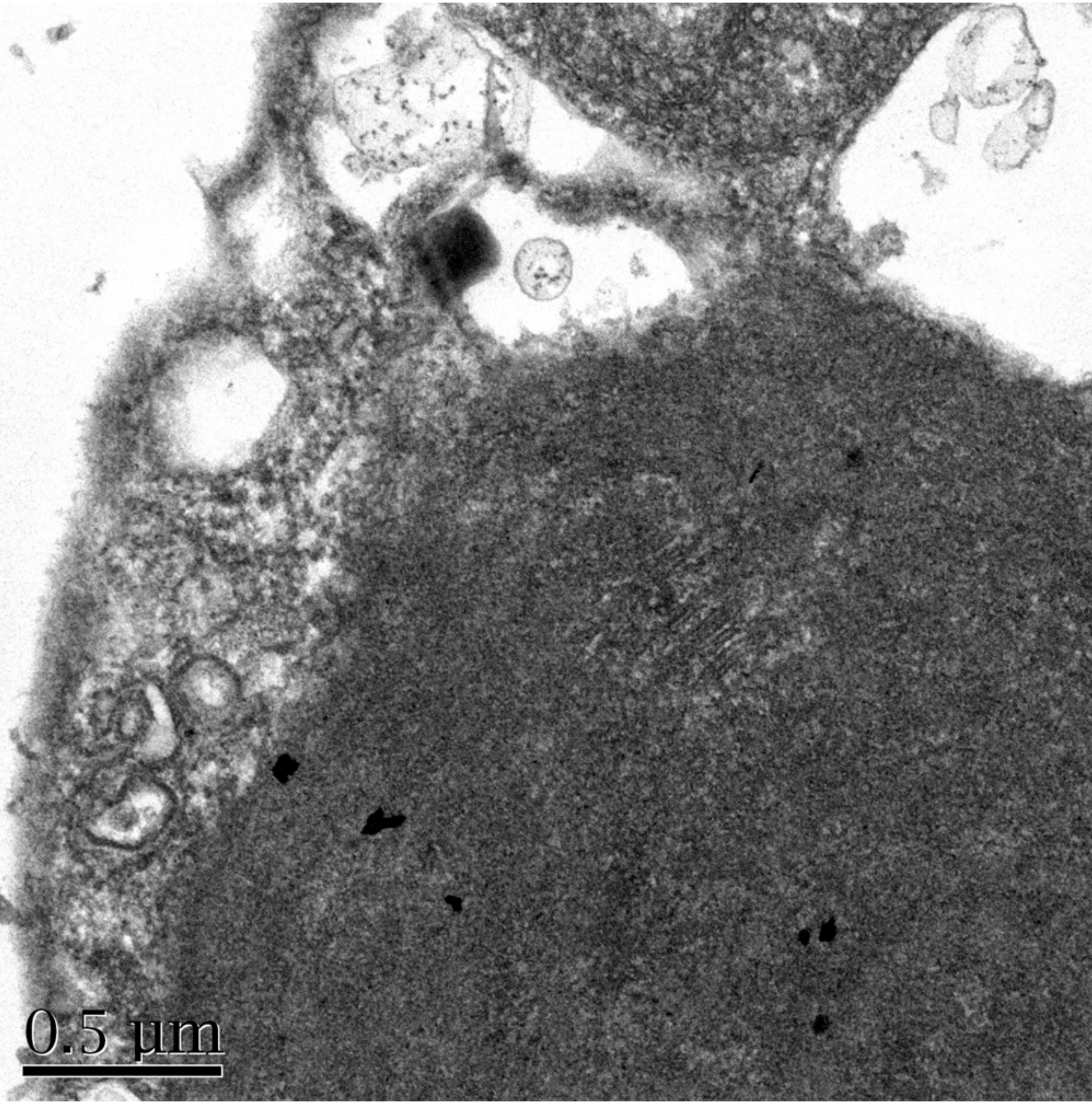

18-14_Correa_YC4_5C9_17.tif

Cell 5

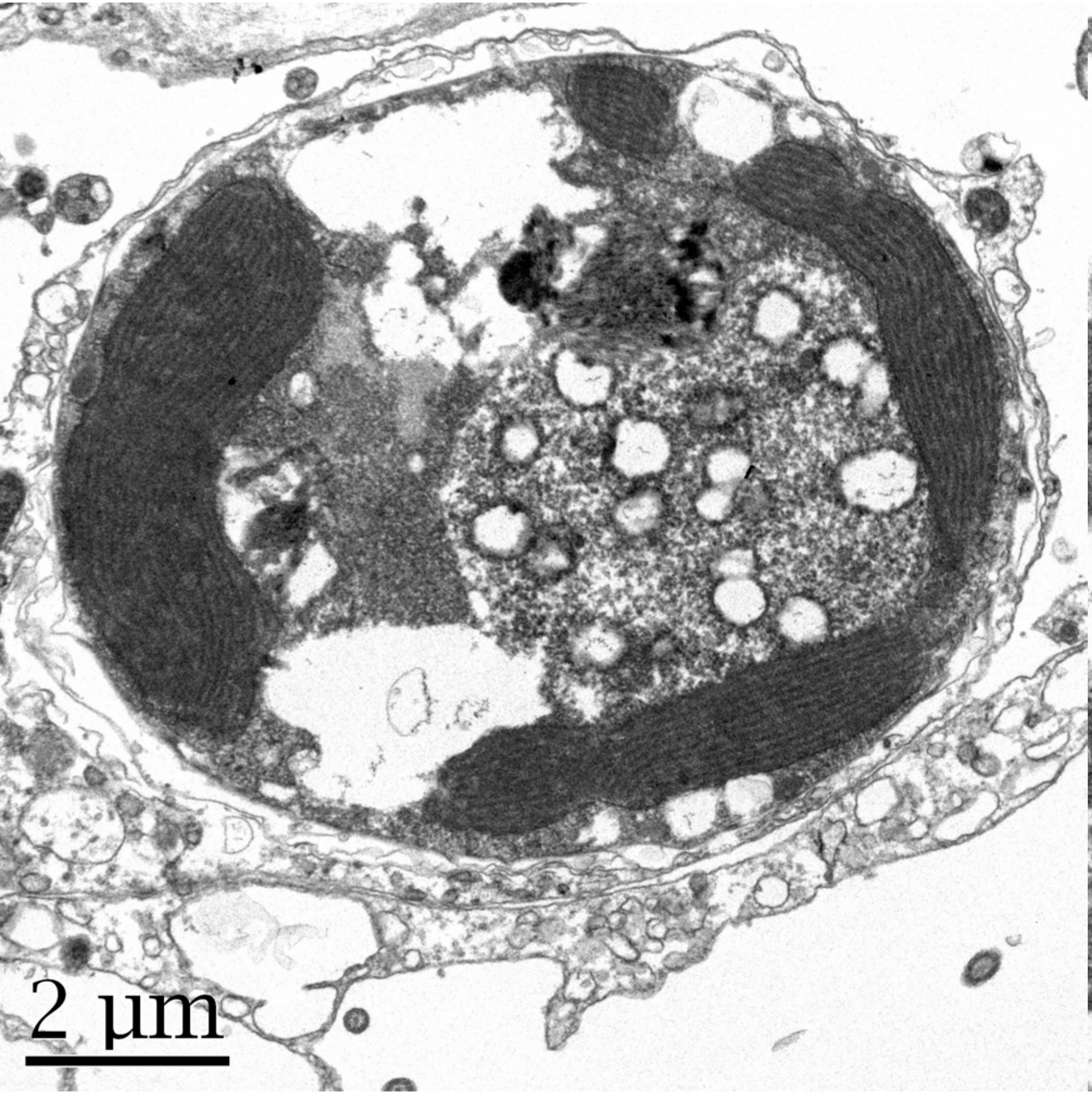

18-14_Correa_YC4_5C9_20.tif

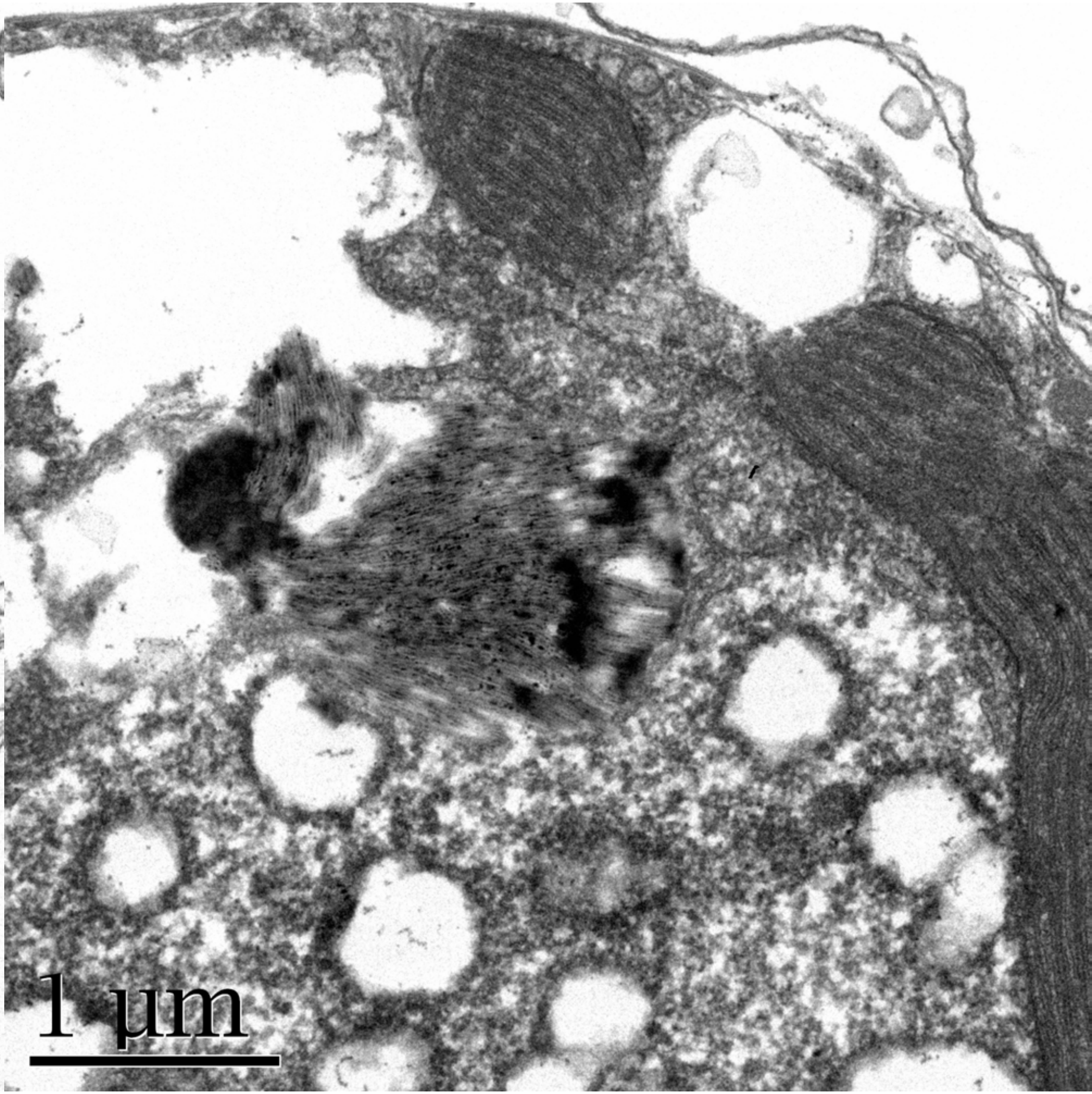

18-14_Correa_YC4_5C9_22.tif

Cell 6

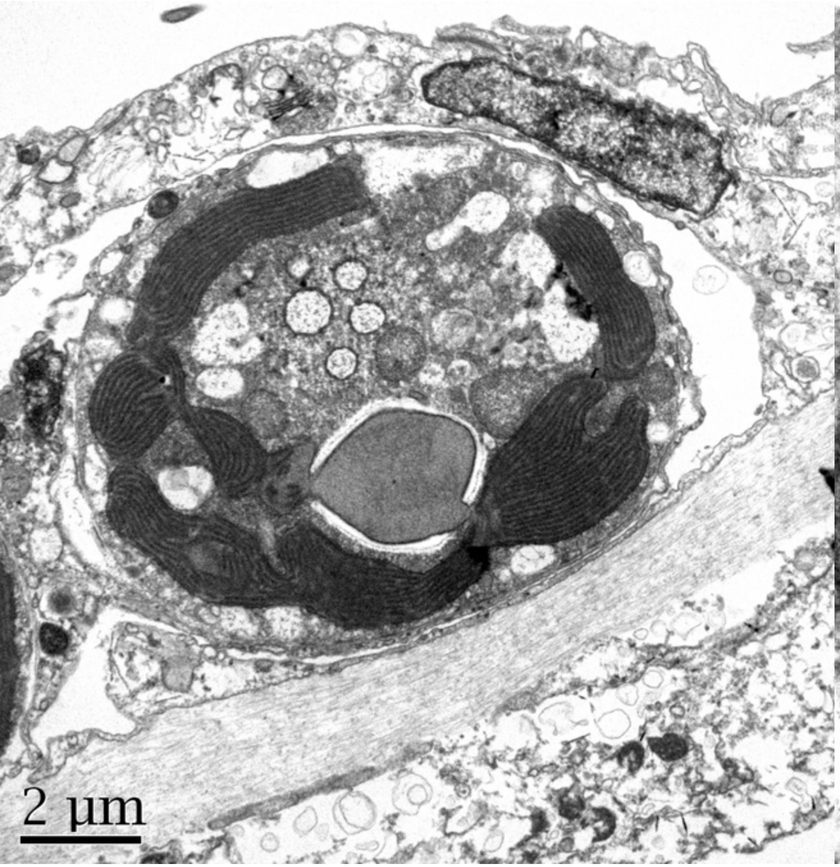

18-14_Correa_YC4_5C9_23.tif

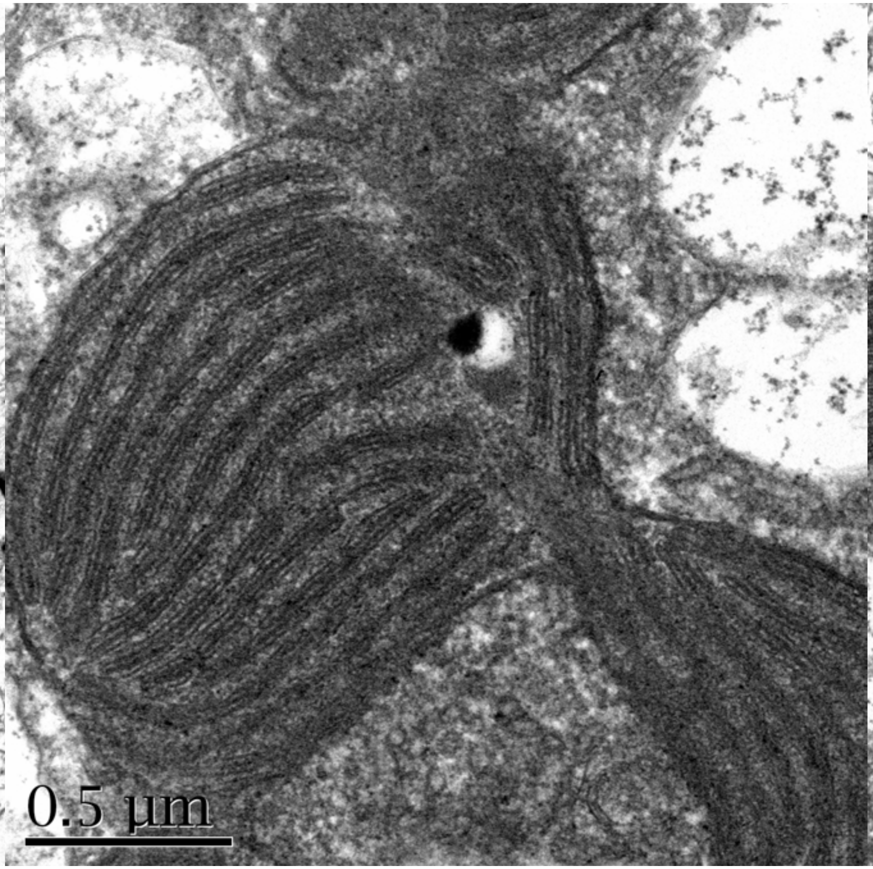

18-14_Correa_YC4_5C9_26.tif

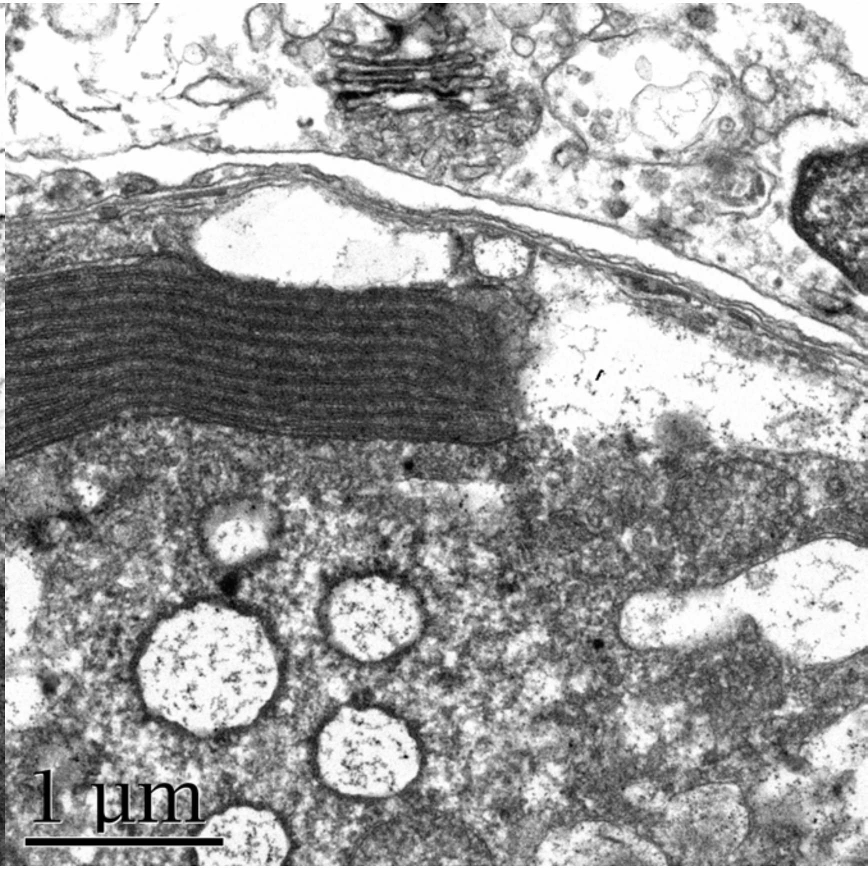

18-14_Correa_YC4_5C9_28.tif

Cell 7

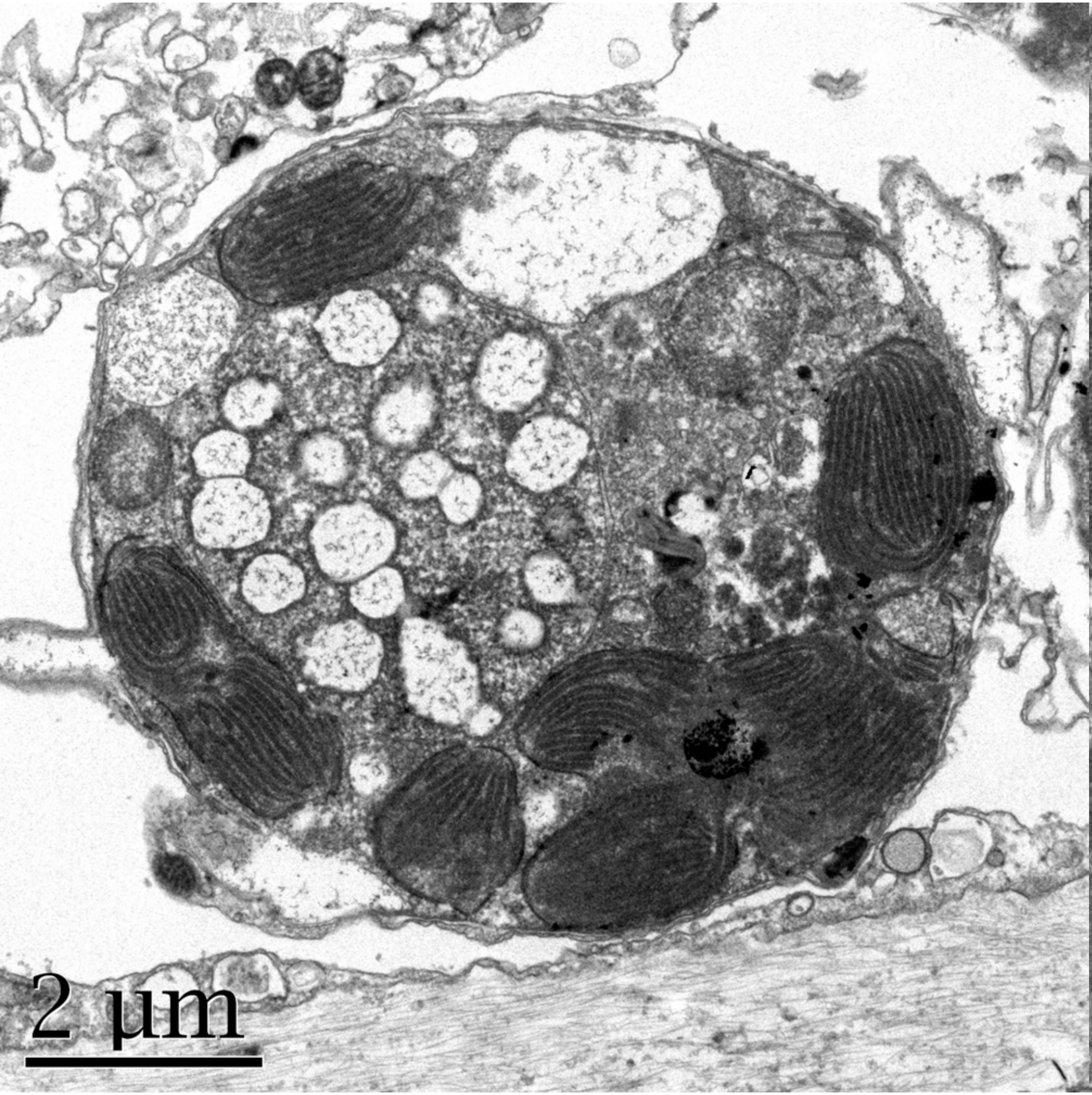

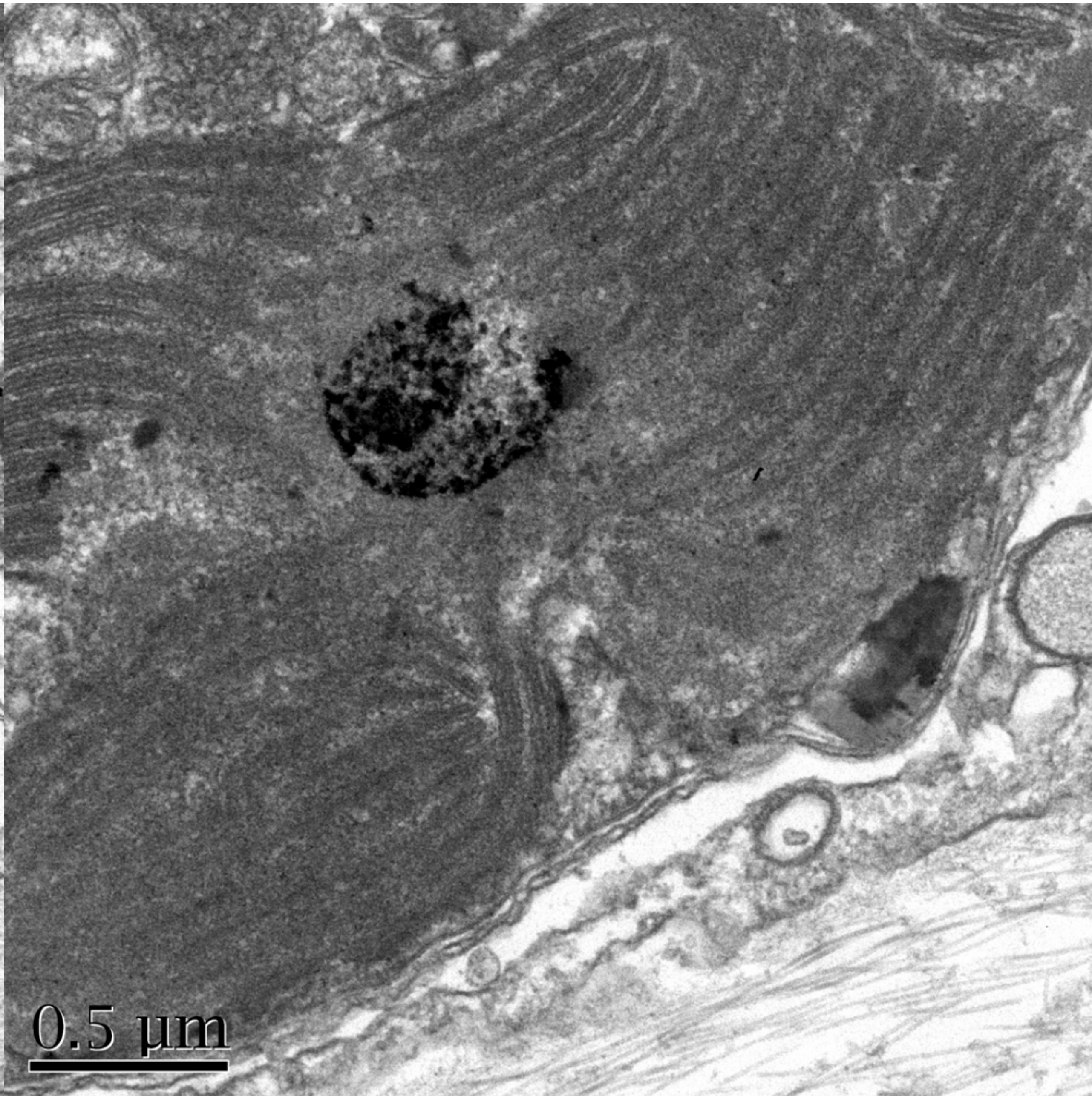

18-14_Correa_YC4_5C9_30.tif

18-14_Correa_YC4_5C9_31.tif

Cell 8

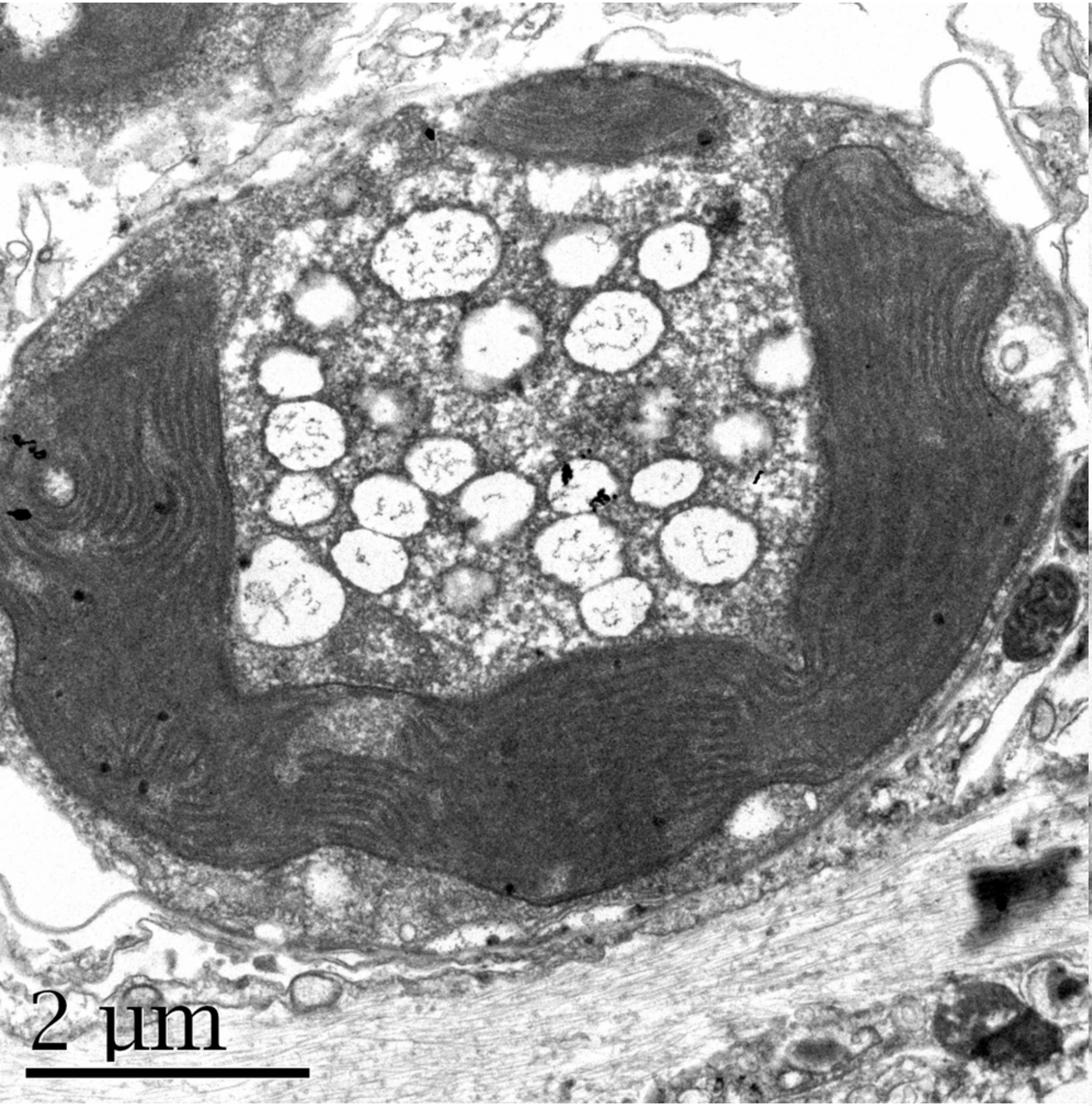

18-14_Correa_YC4_5C9_36.tif

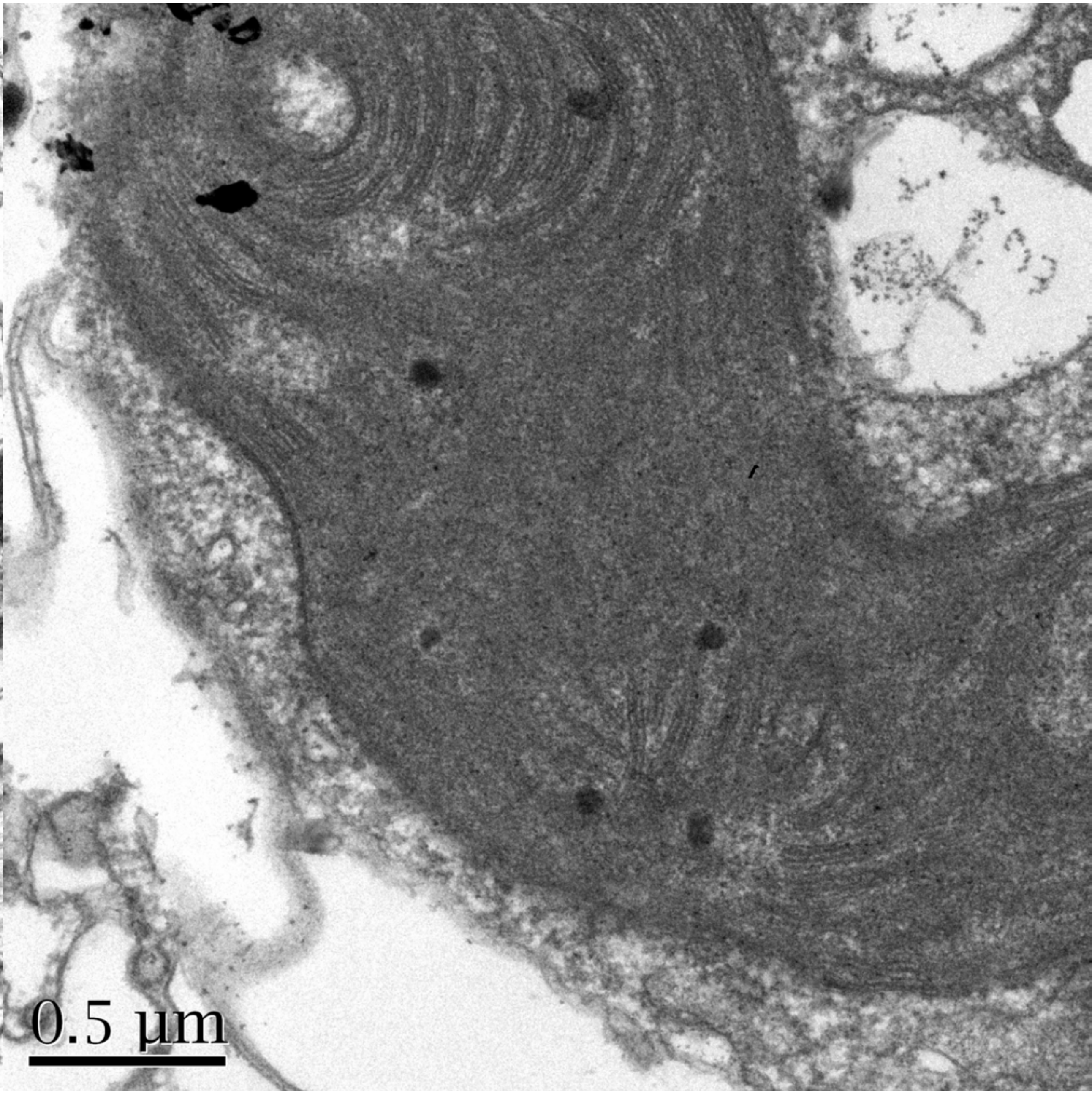

18-14_Correa_YC4_5C9_37.tif

Cell 9

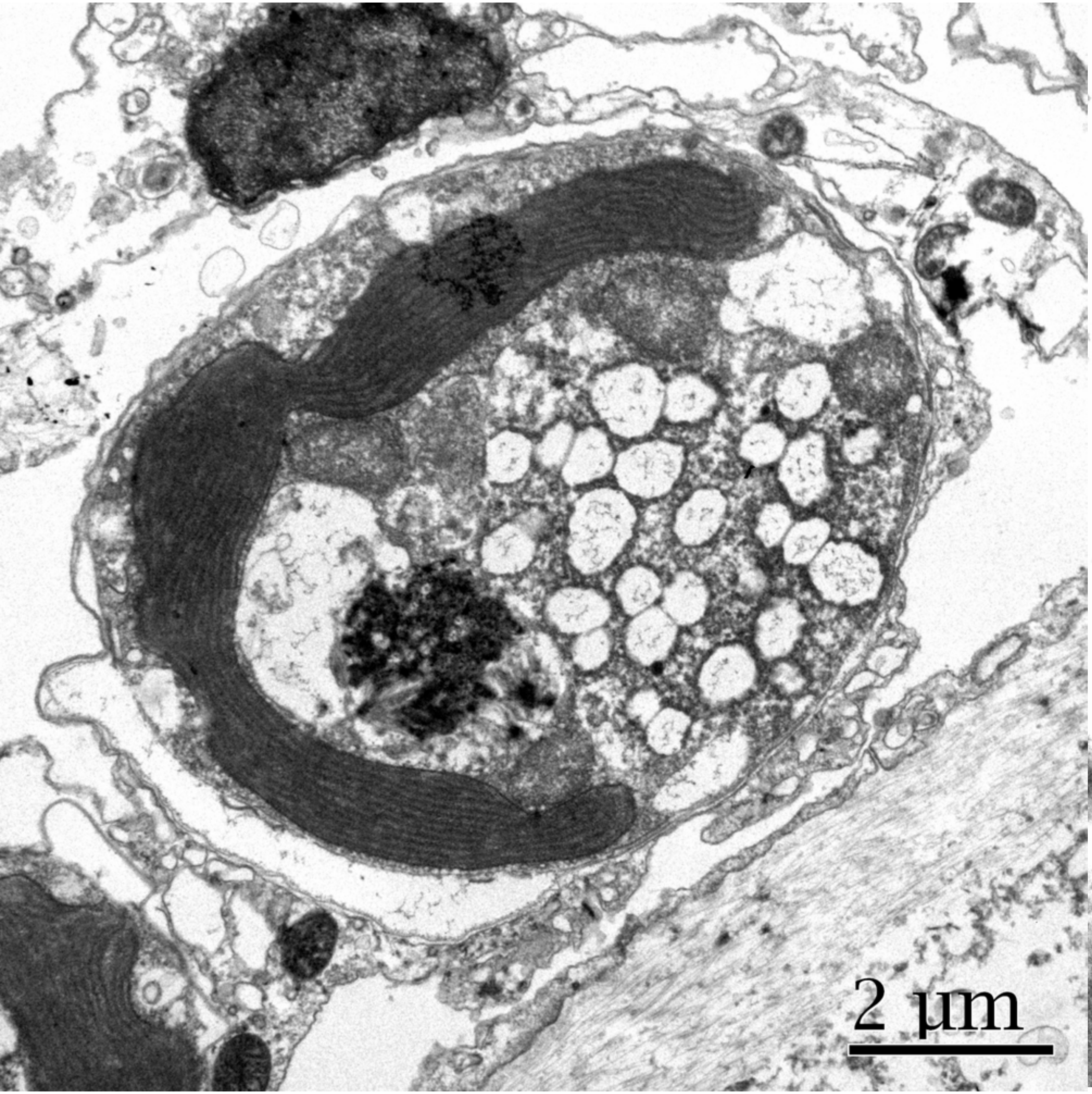

18-14_Correa_YC4_5C9_41.tif

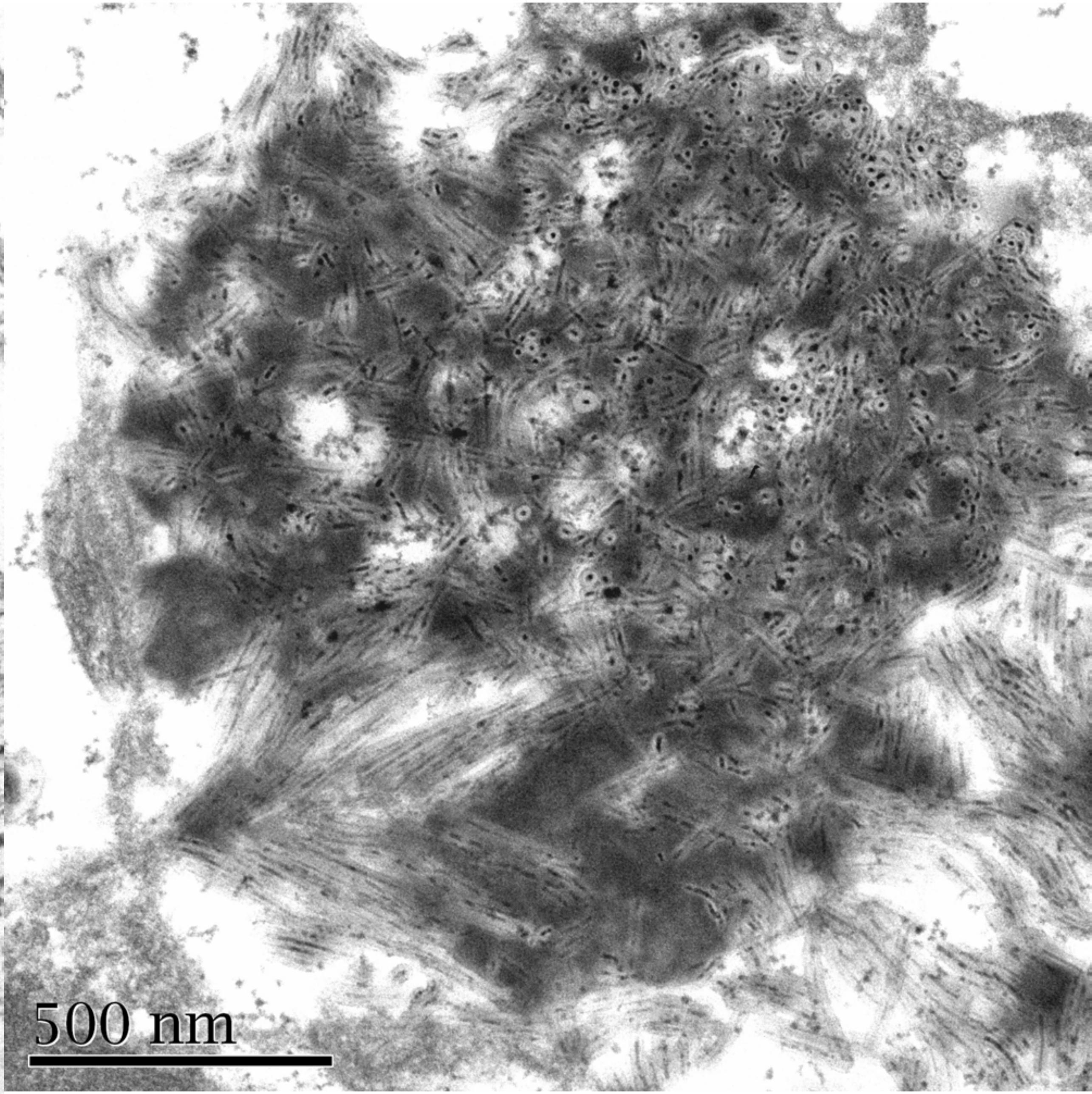

18-14_Correa_YC4_5C9_44.tif

Cell 10

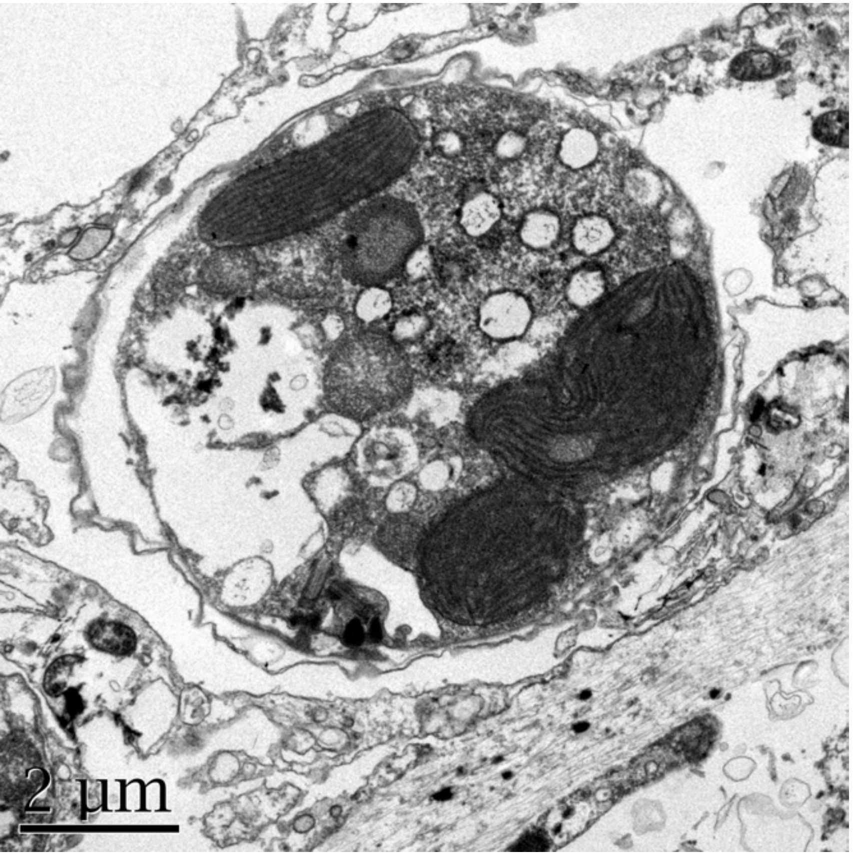

18-14_Correa_YC4_5C9_46.tif

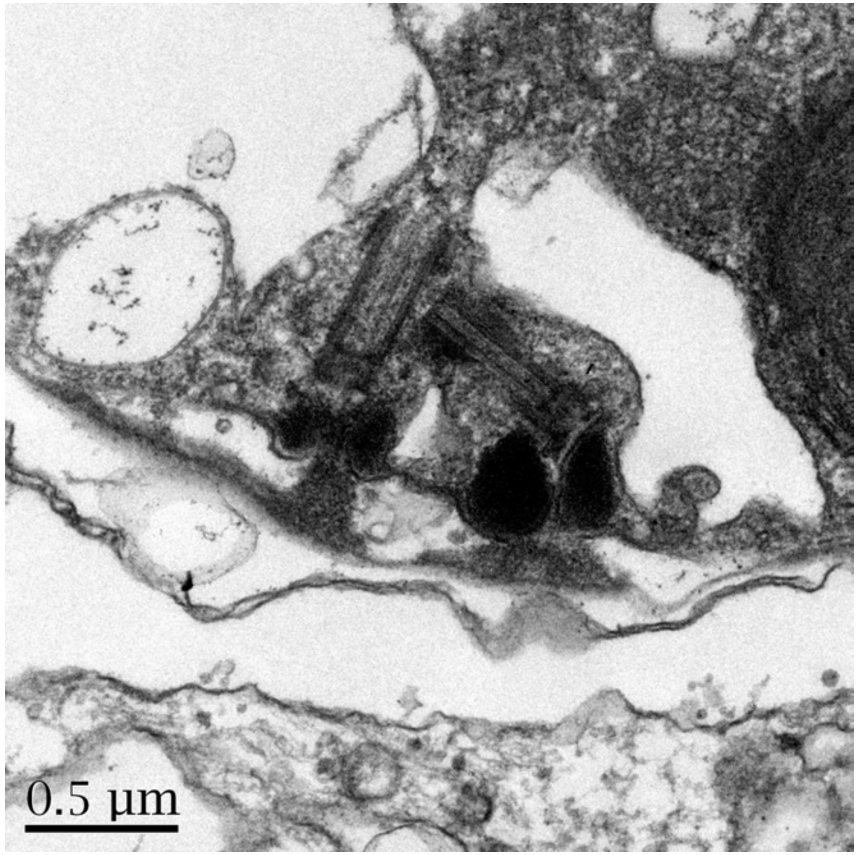

18-14_Correa_YC4_5C9_47.tif

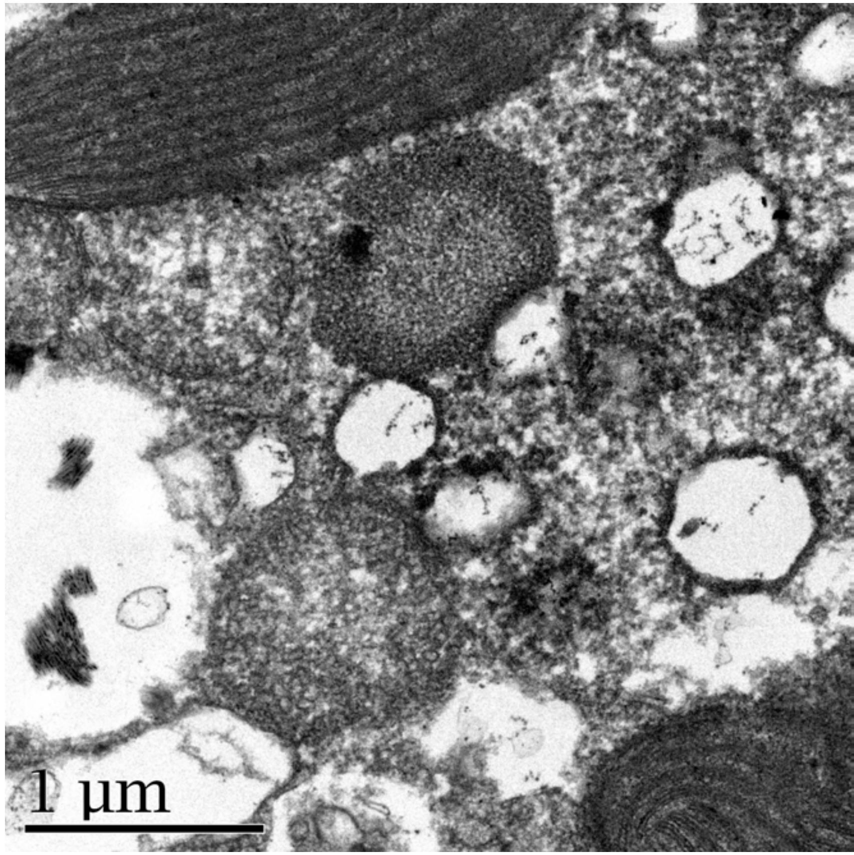

18-14_Correa_YC4_5C9_50.tif

Cell 11

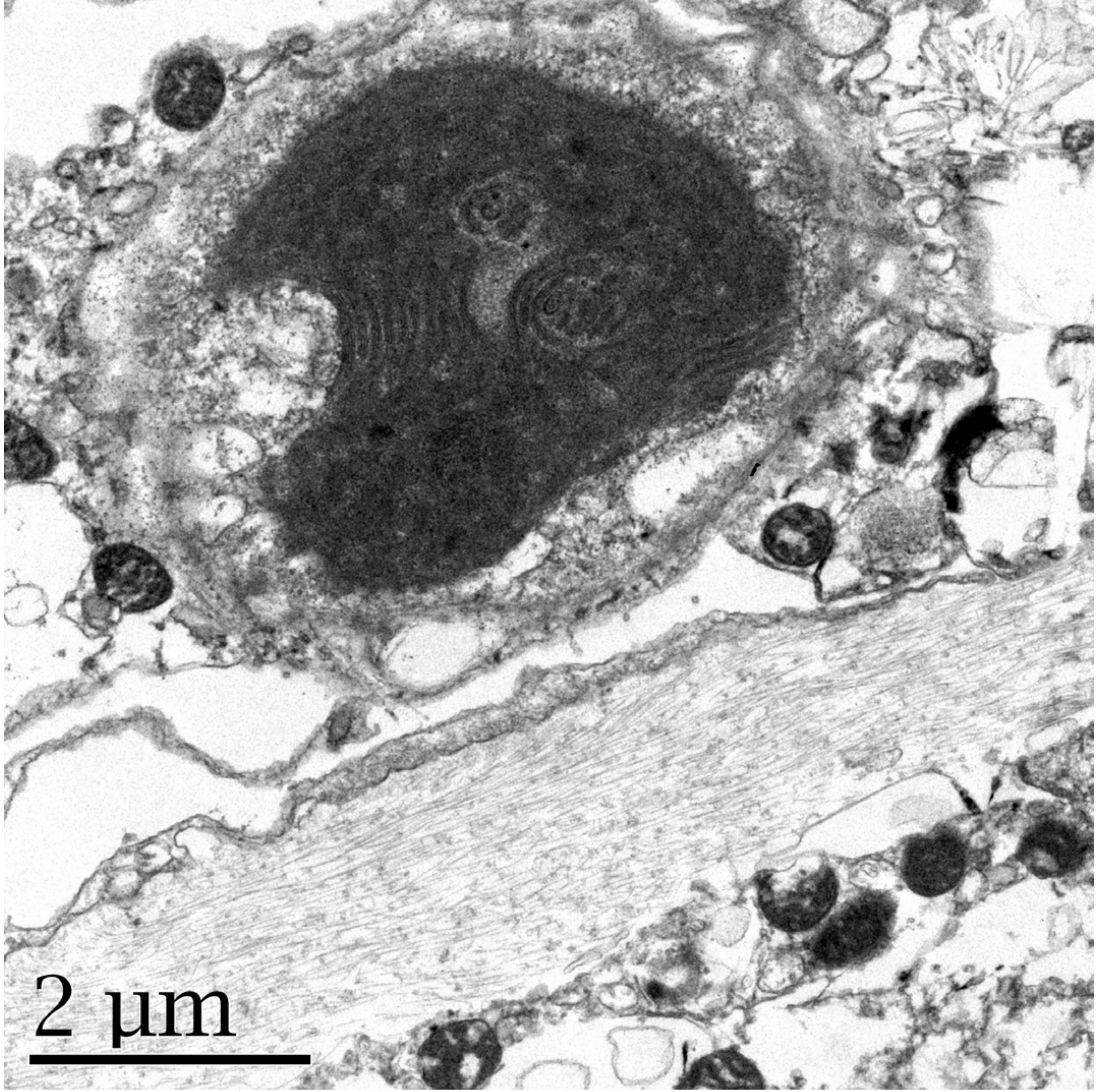

18-14_Correa_YC4_5C9_53.tif

Cell 12

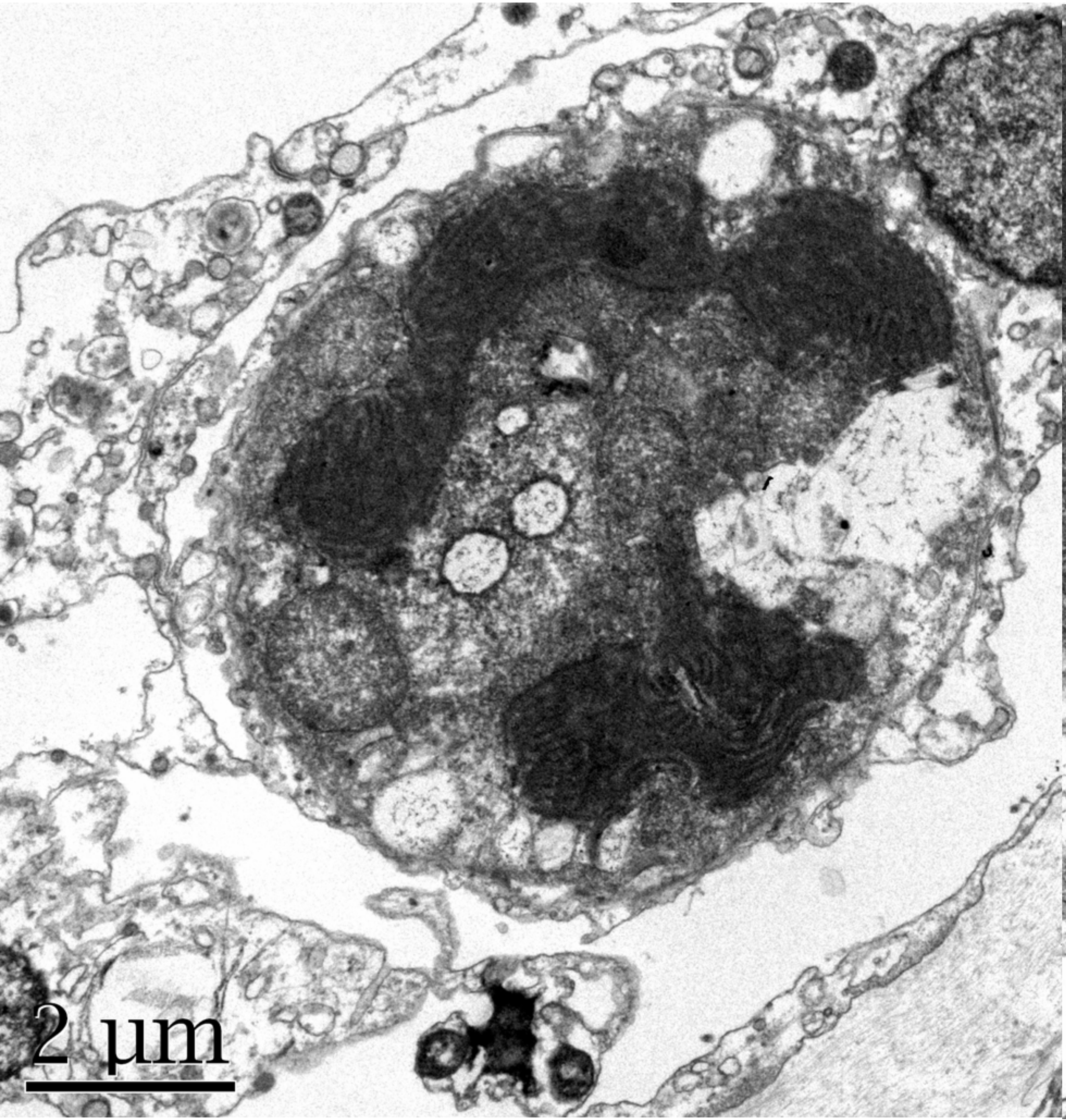

18-14_Correa_YC4_5C9_54.tif

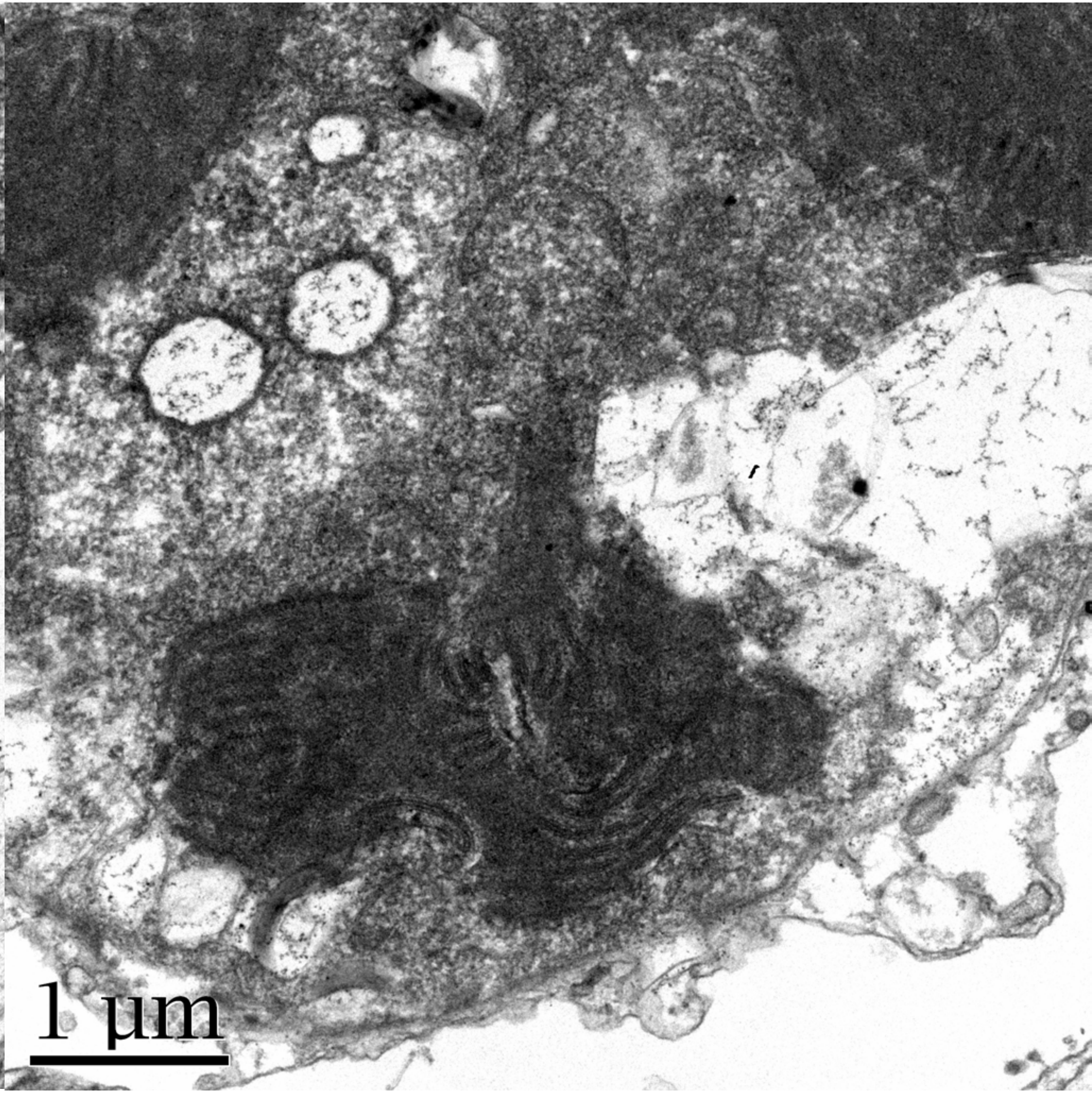

18-14_Correa_YC4_5C9_55.tif

Cell 13

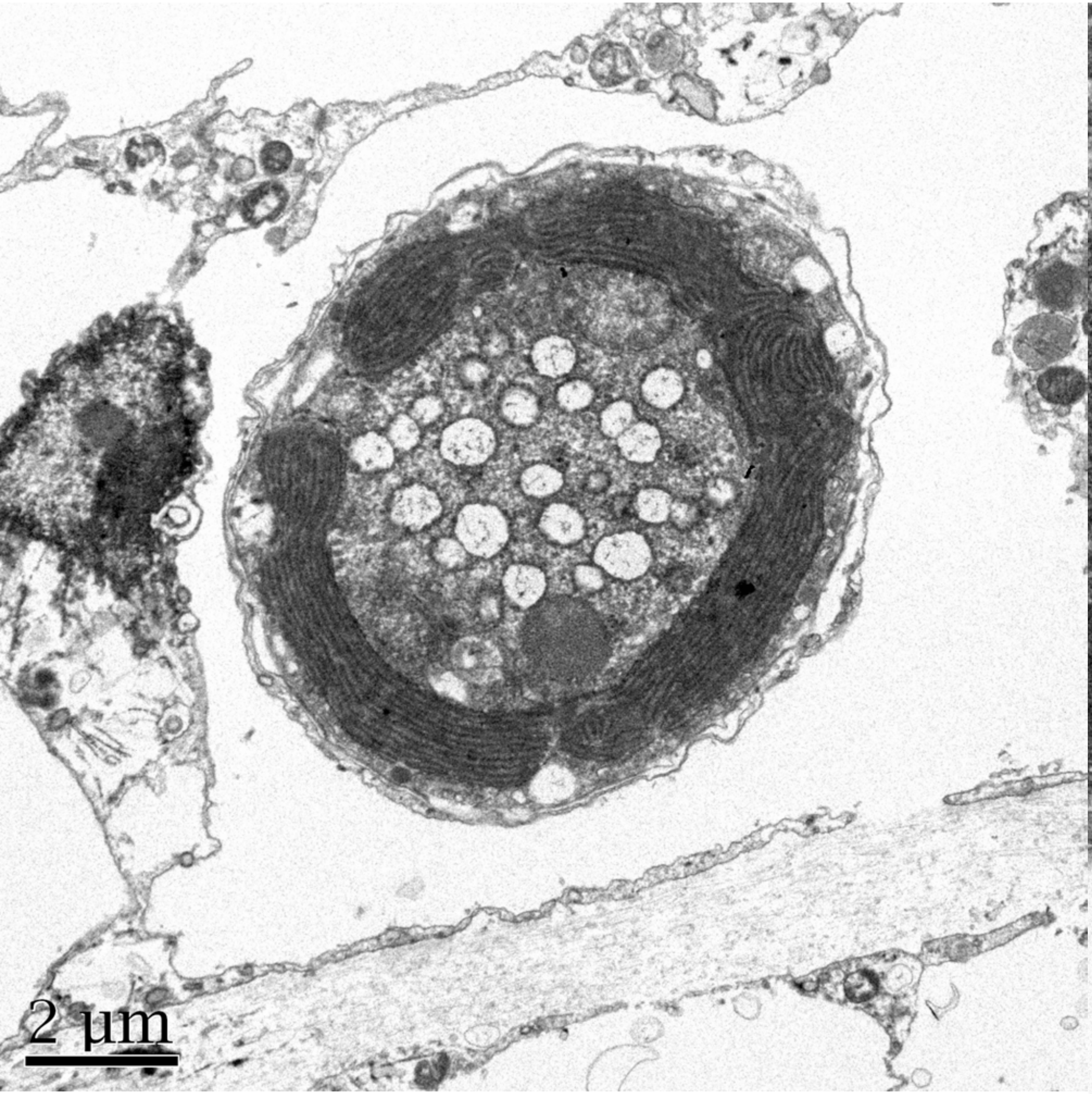

18-14_Correa_YC4_5C9_58.tif

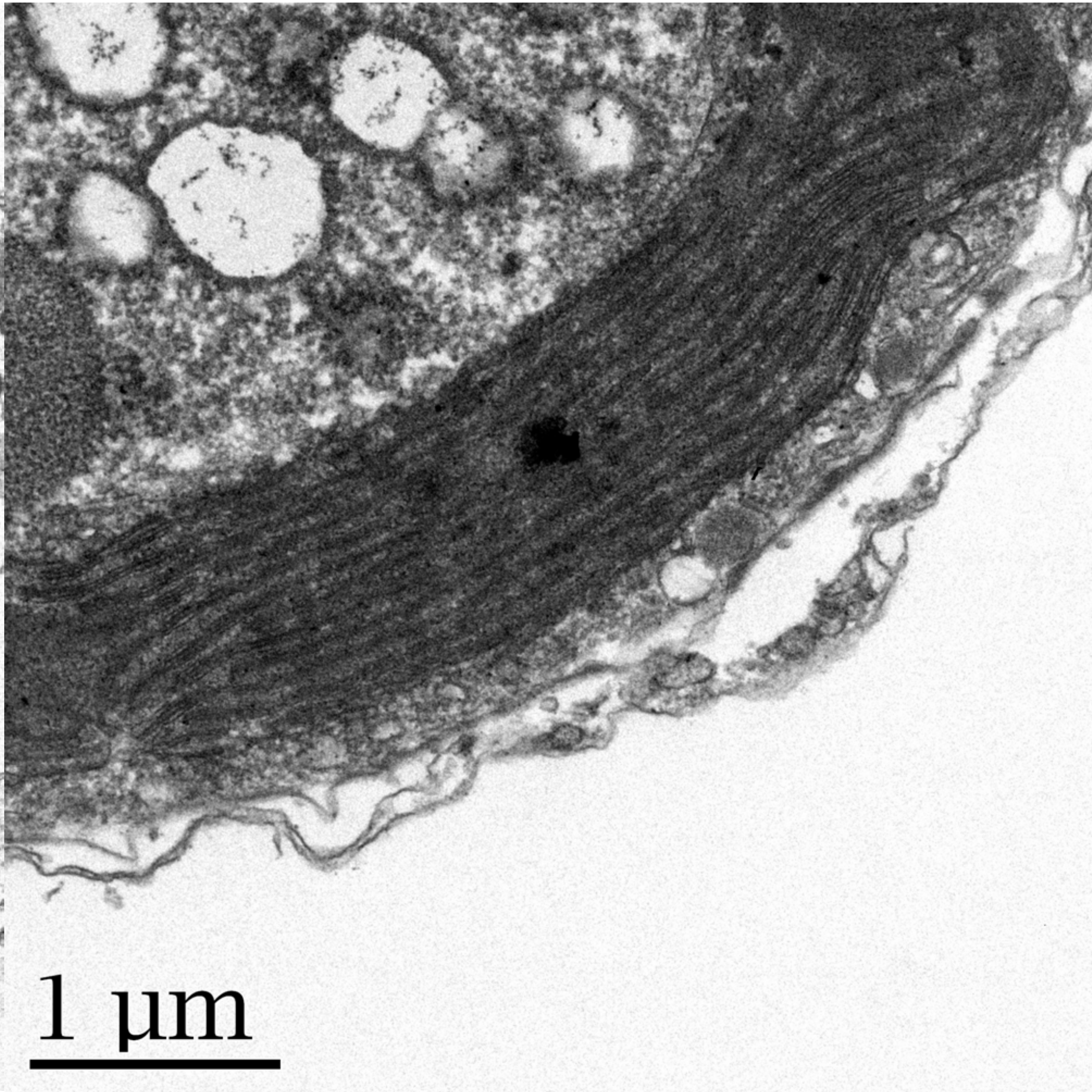

18-14_Correa_YC4_5C9_59.tif

Cell 14

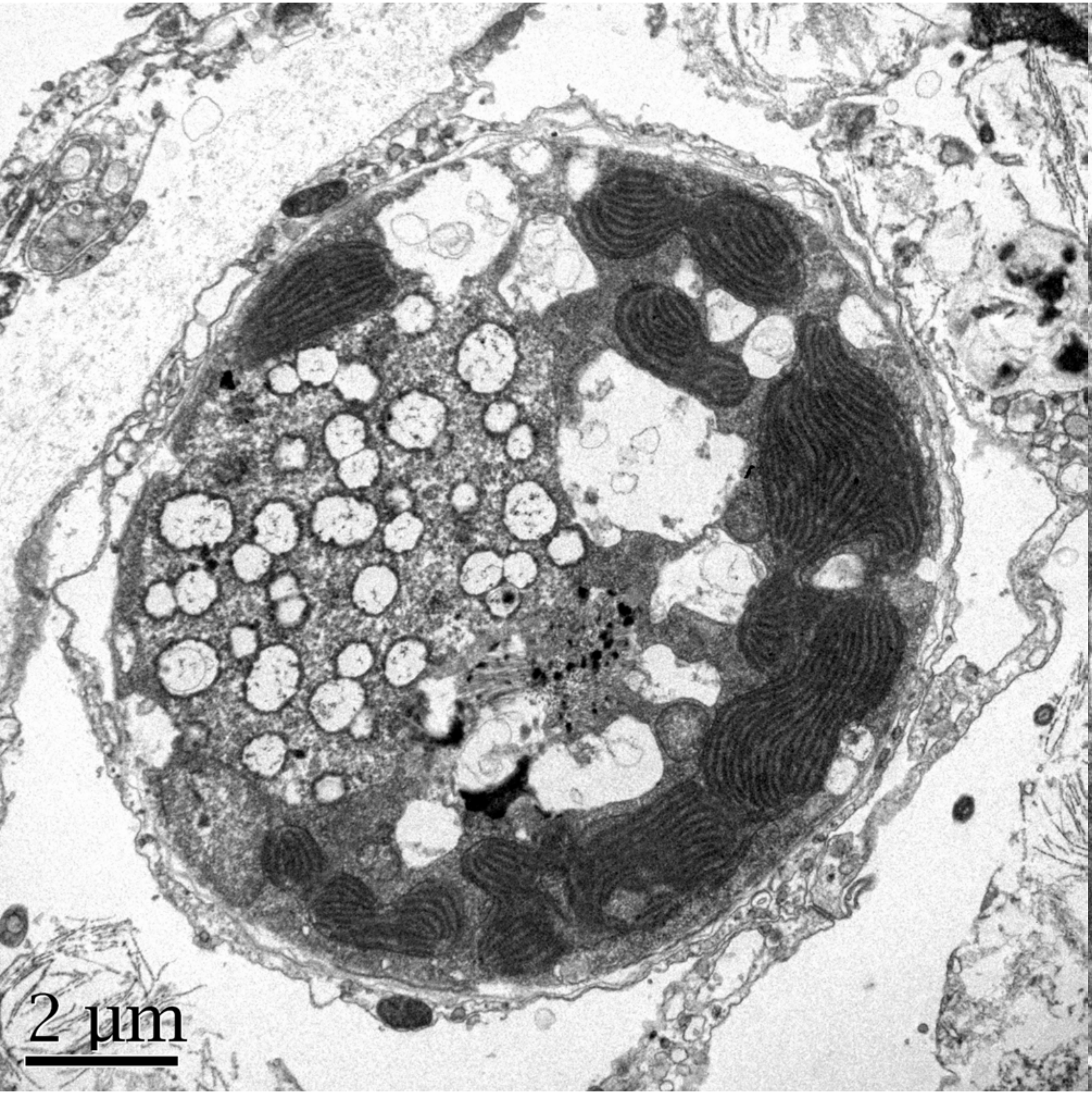

18-14_Correa_YC4_5C9_60.tif

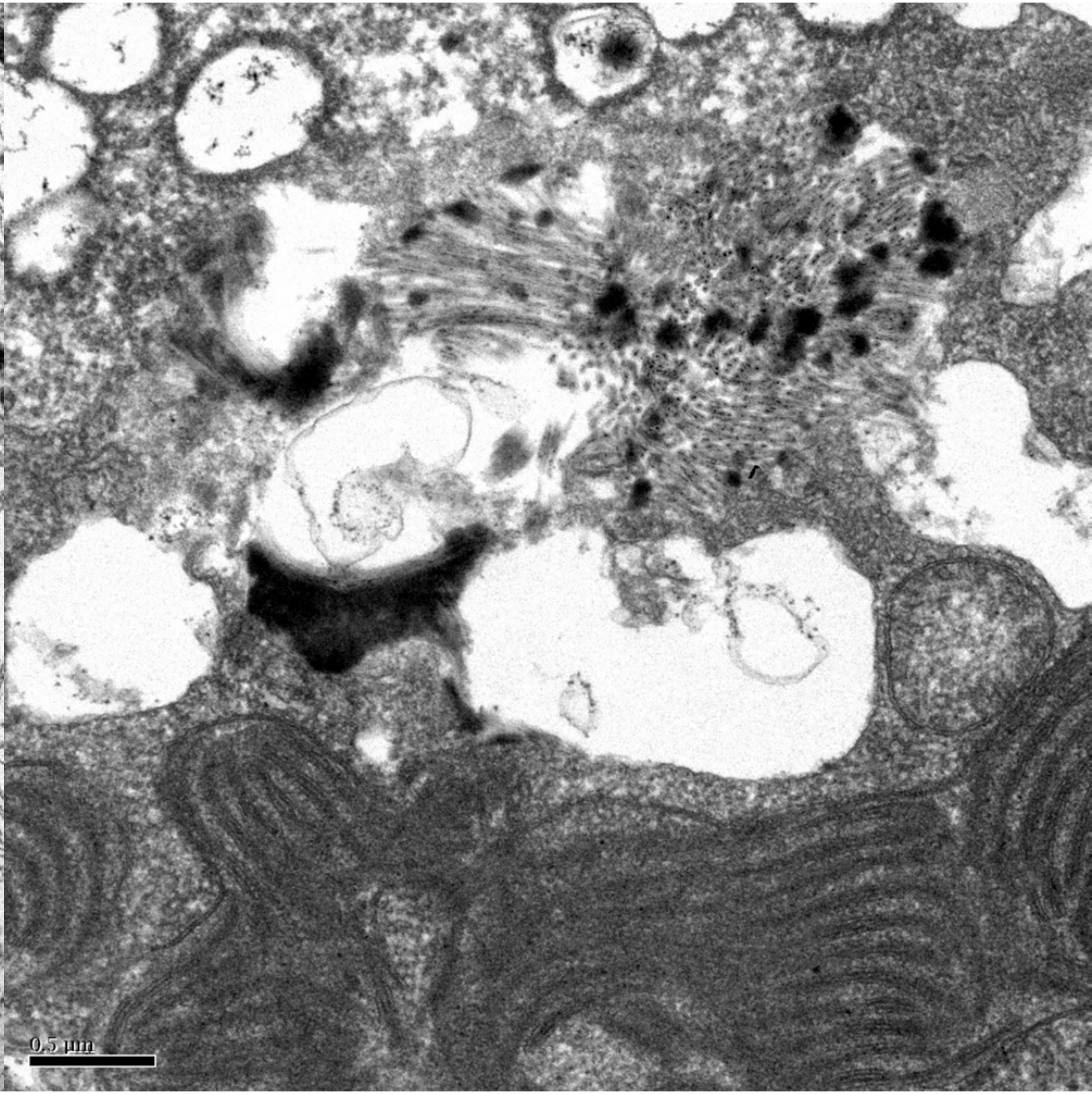

18-14_Correa_YC4_5C9_61.tif

Cell 15

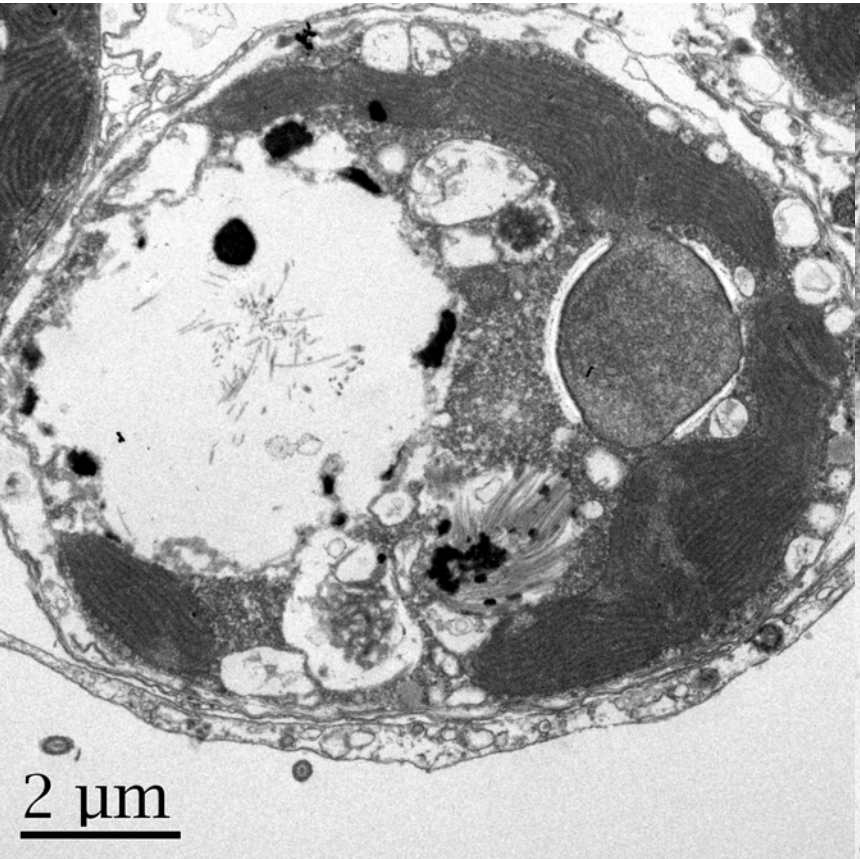

18-14_Correa_YC4_5C9_64.tif

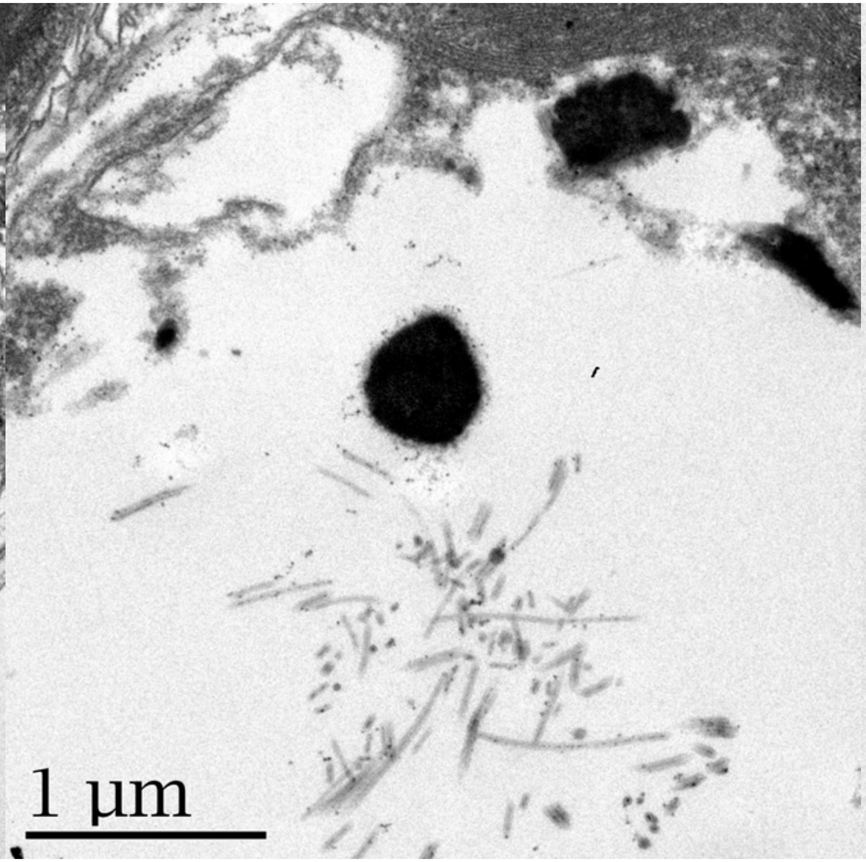

18-14_Correa_YC4_5C9_65.tif

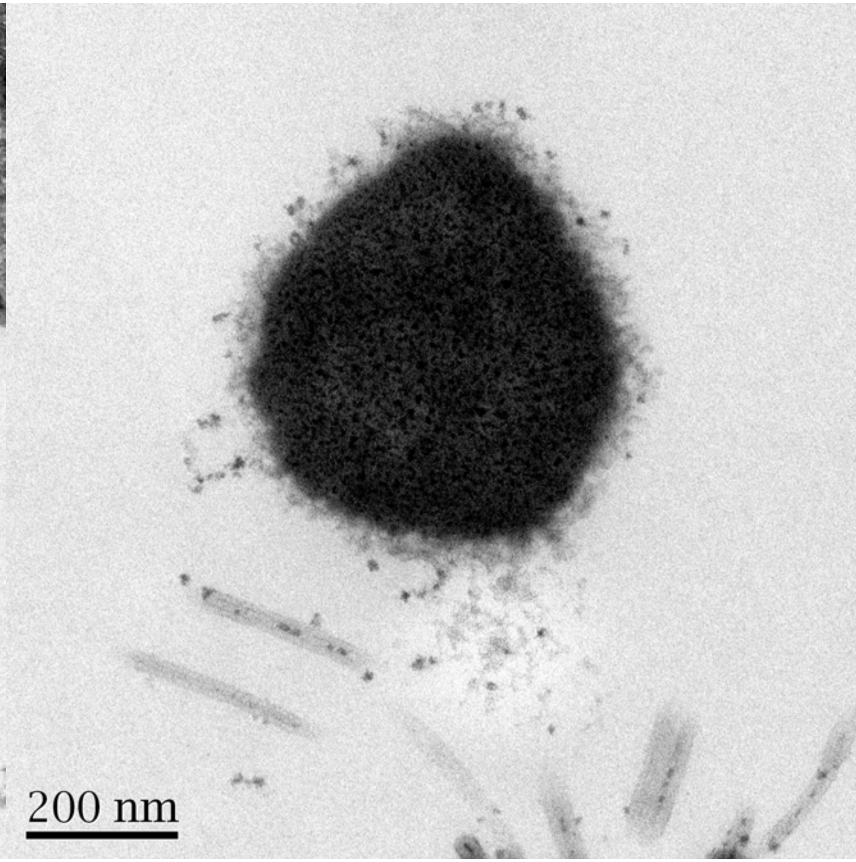

18-14_Correa_YC4_5C9_66.tif

Cell 16

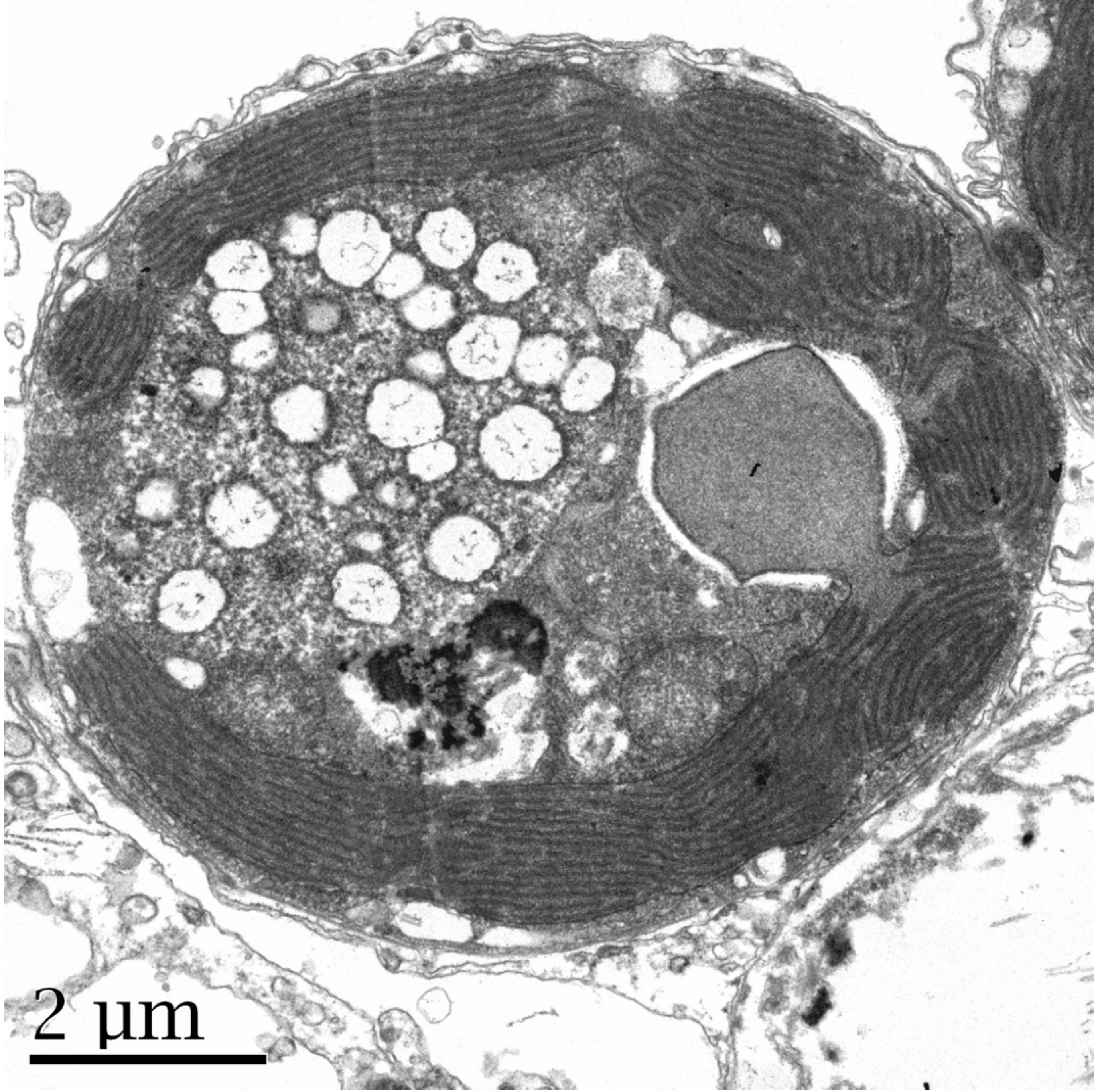

18-14_Correa_YC4_5C9_70.tif

Cell 17

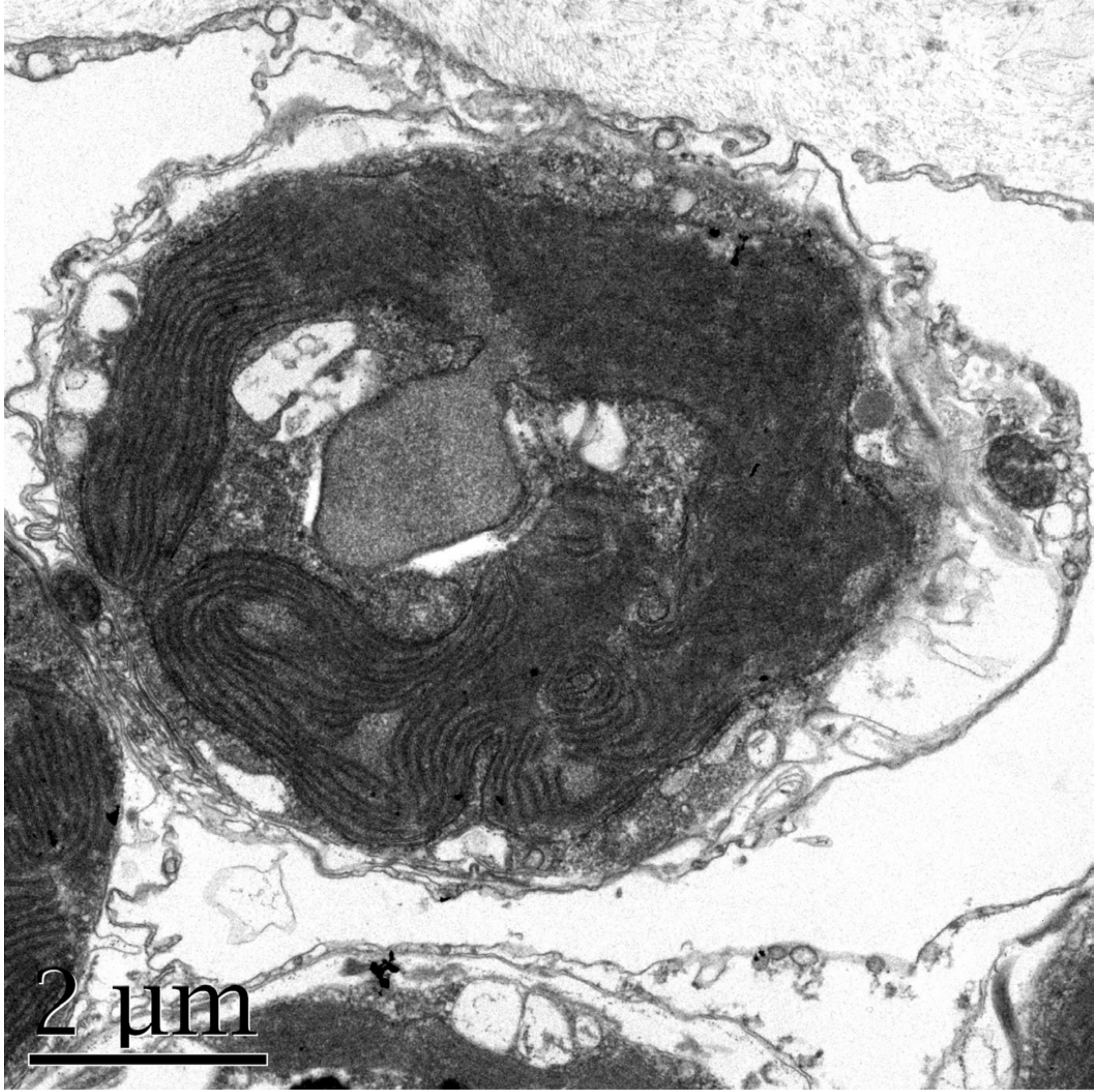

18-14_Correa_YC4_5C9_71.tif

Cell 18

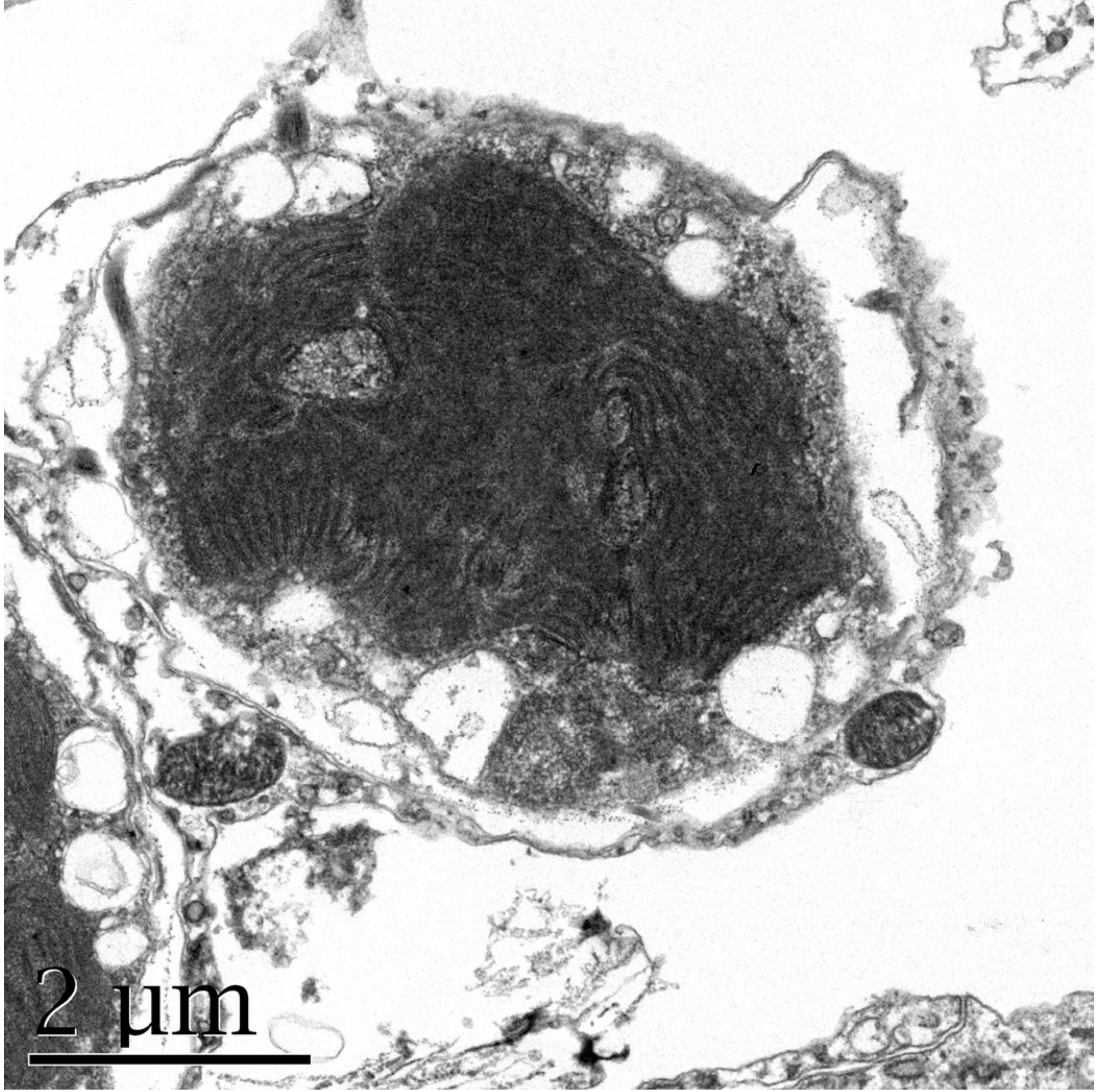

18-14_Correa_YC4_5C9_73.tif

Cell 19

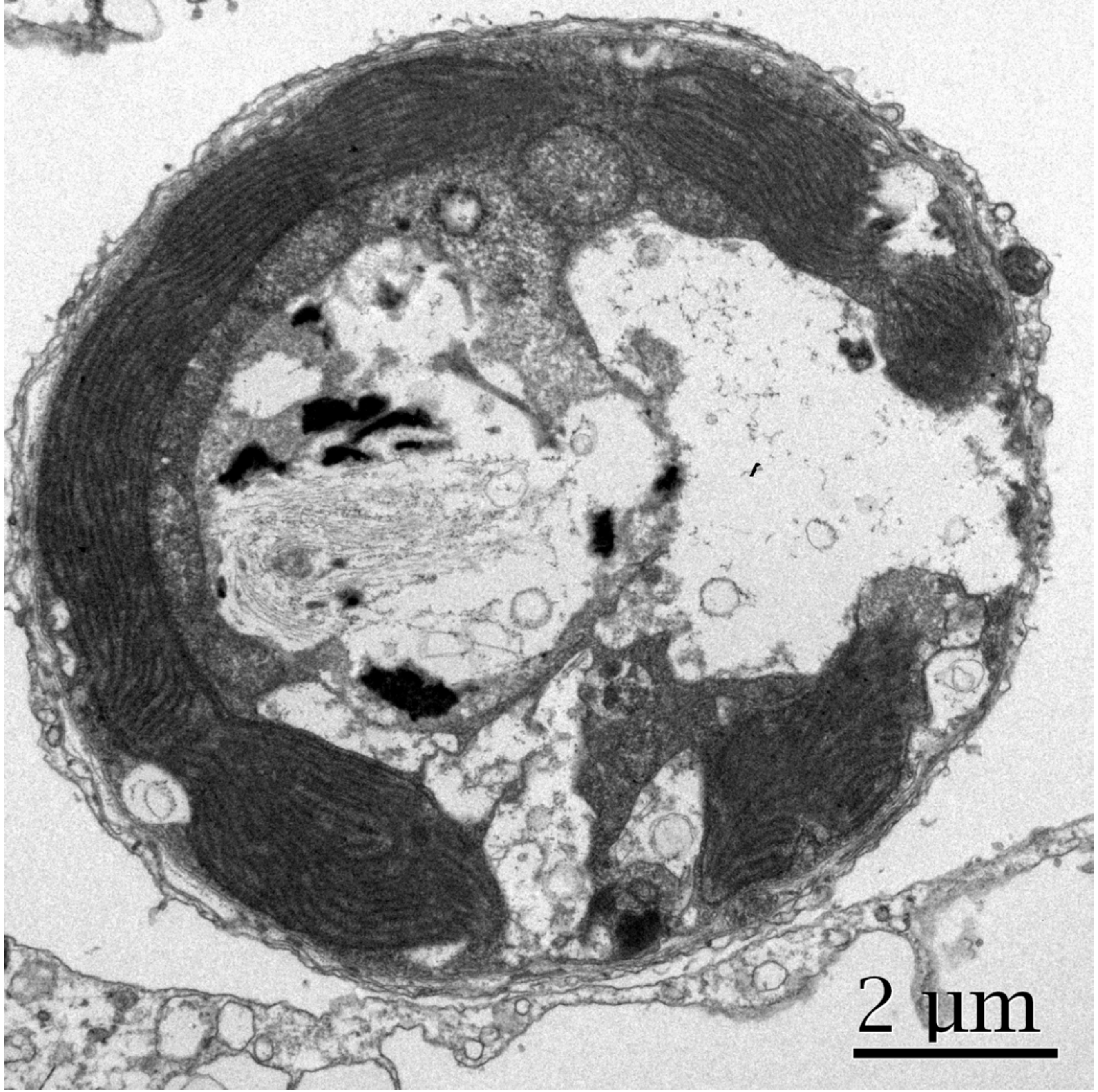

18-14_Correa_YC4_5C9_75.tif

Heat Treatment - ACR Colony D

Cell 1

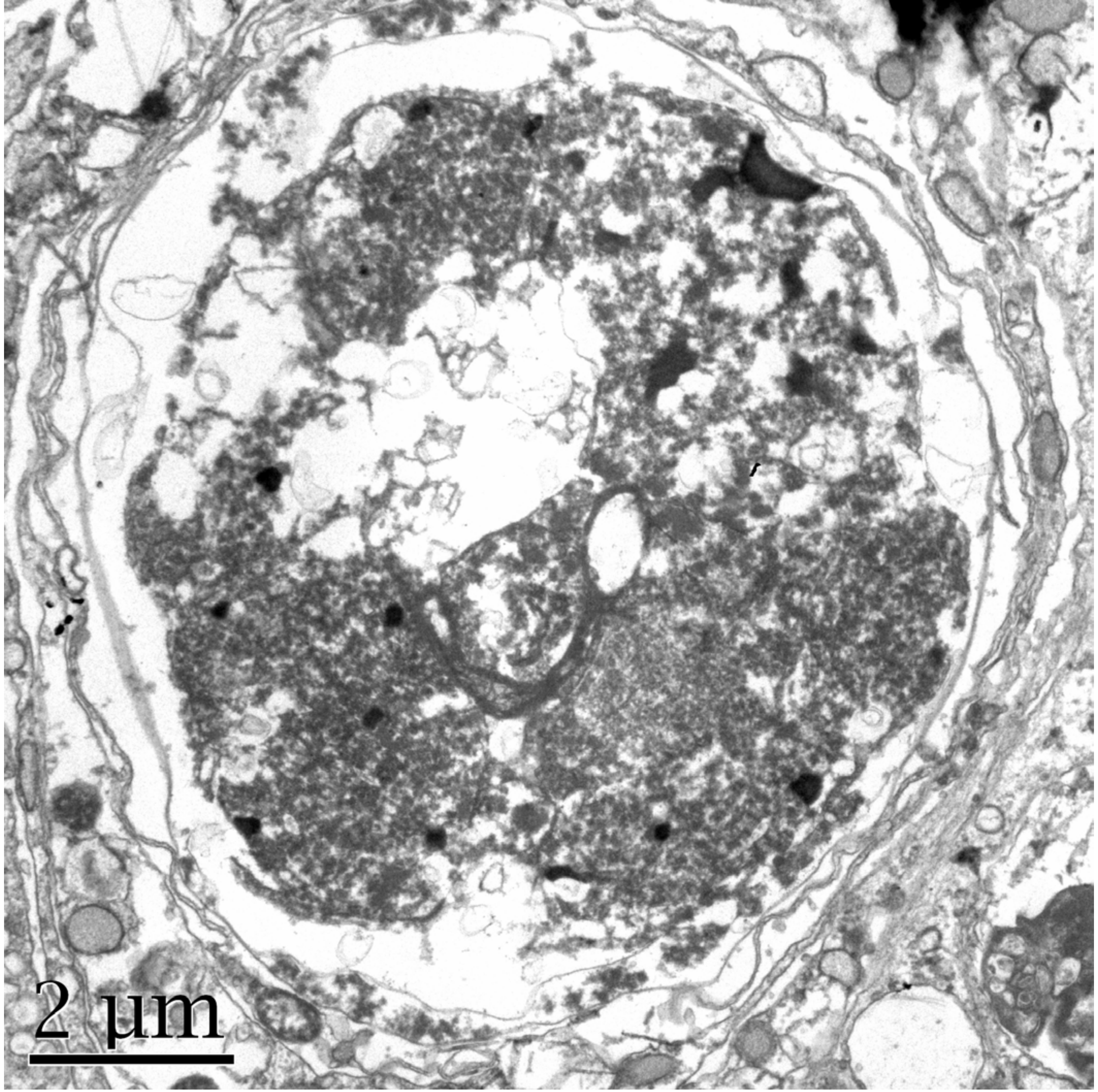

18-14_Correa_YH4_5D3_2.tif

Cell 2

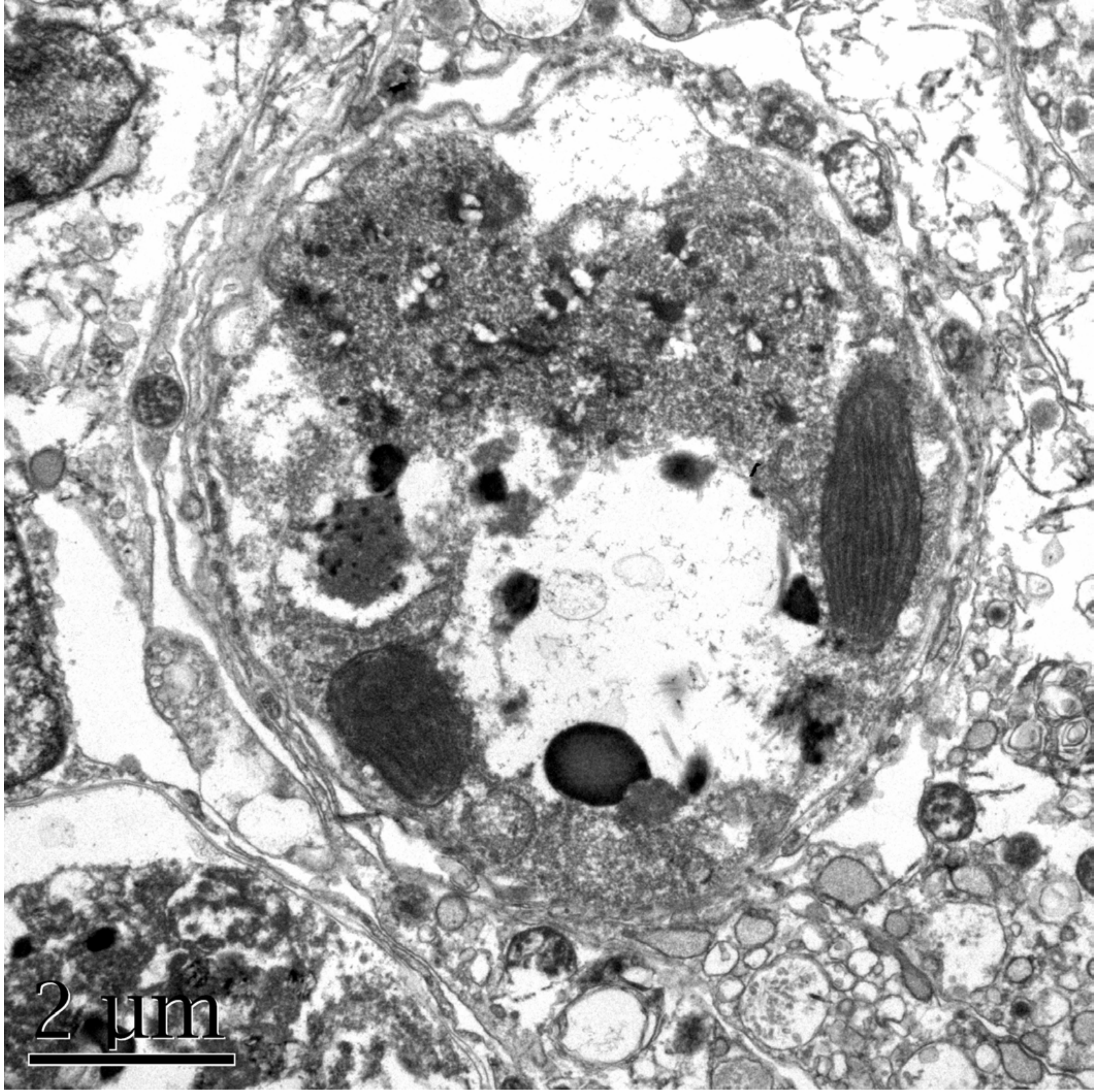

18-14_Correa_YH4_5D3_3.tif

Cell 3

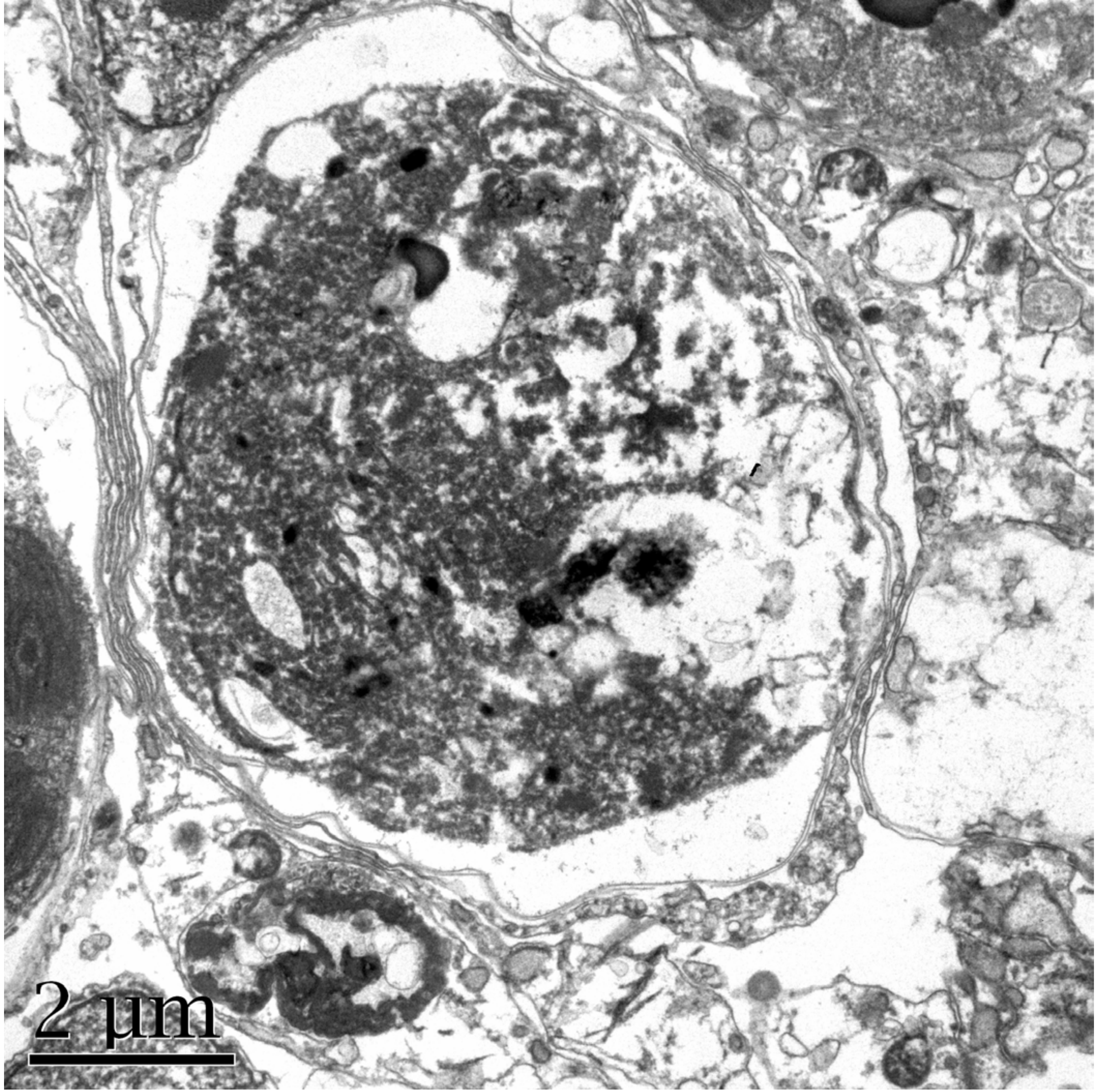

18-14_Correa_YH4_5D3_5.tif

Cell 4

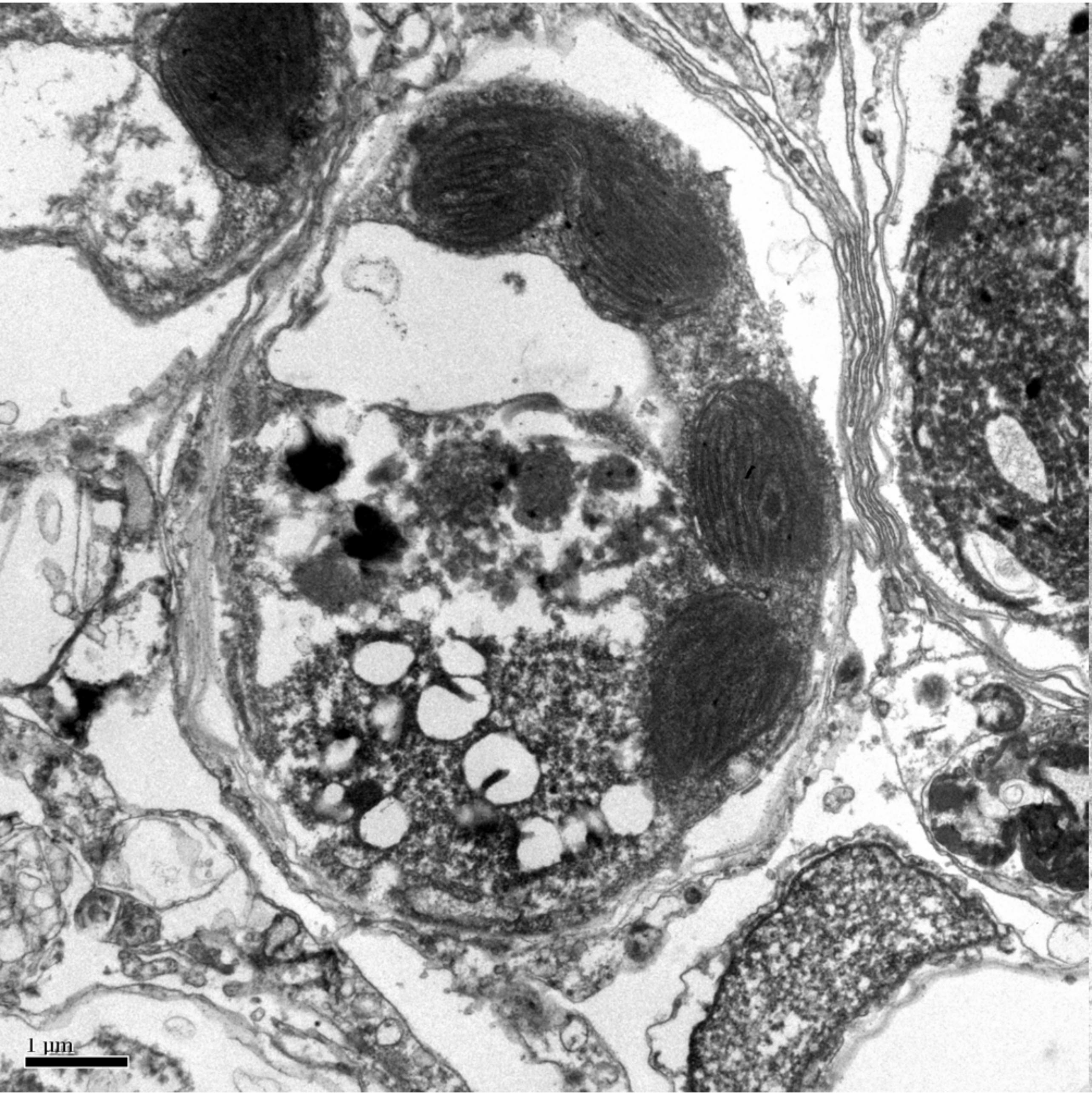

18-14_Correa_YH4_5D3_6.tif

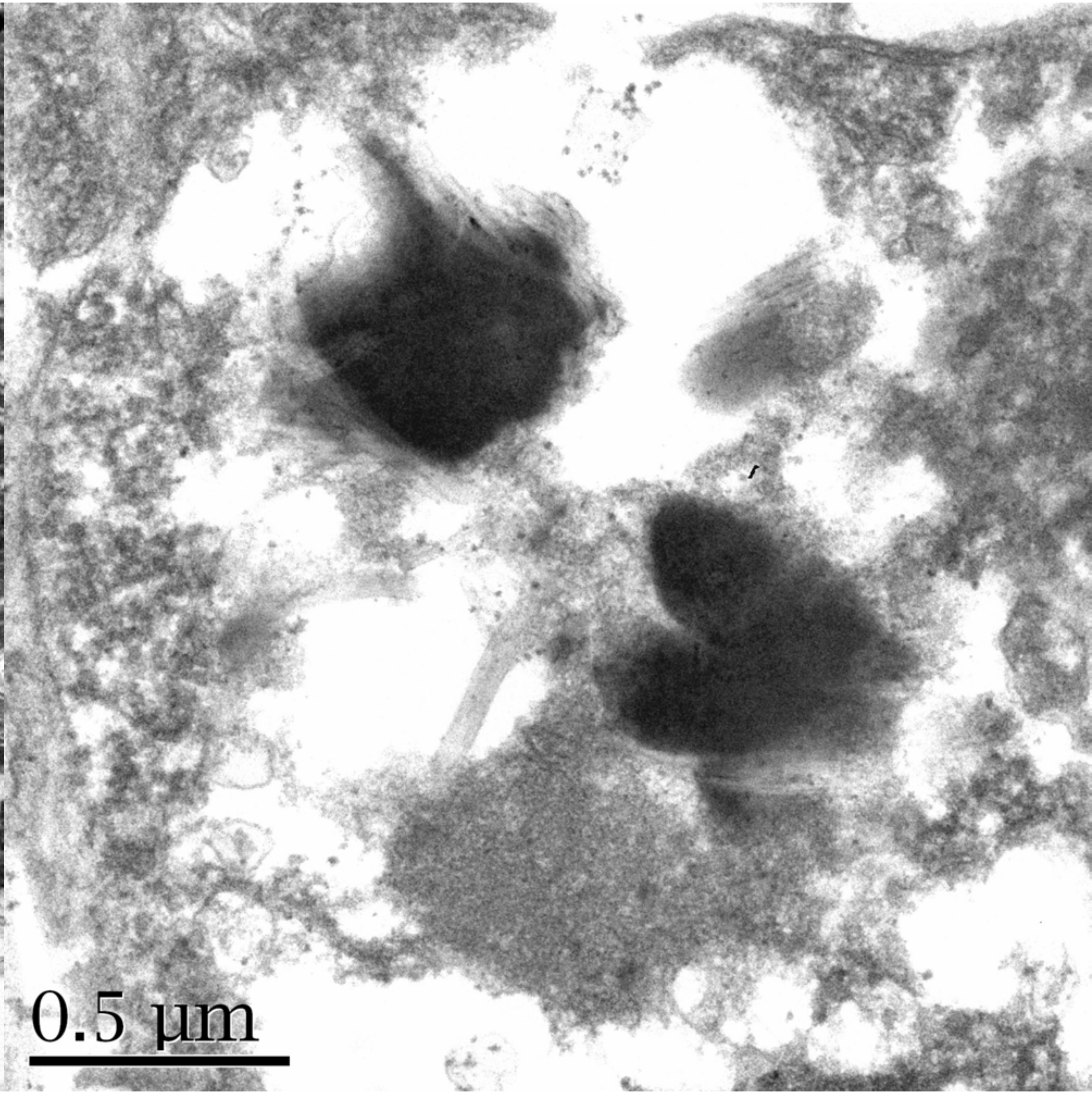

18-14_Correa_YH4_5D3_9.tif

Cell 5

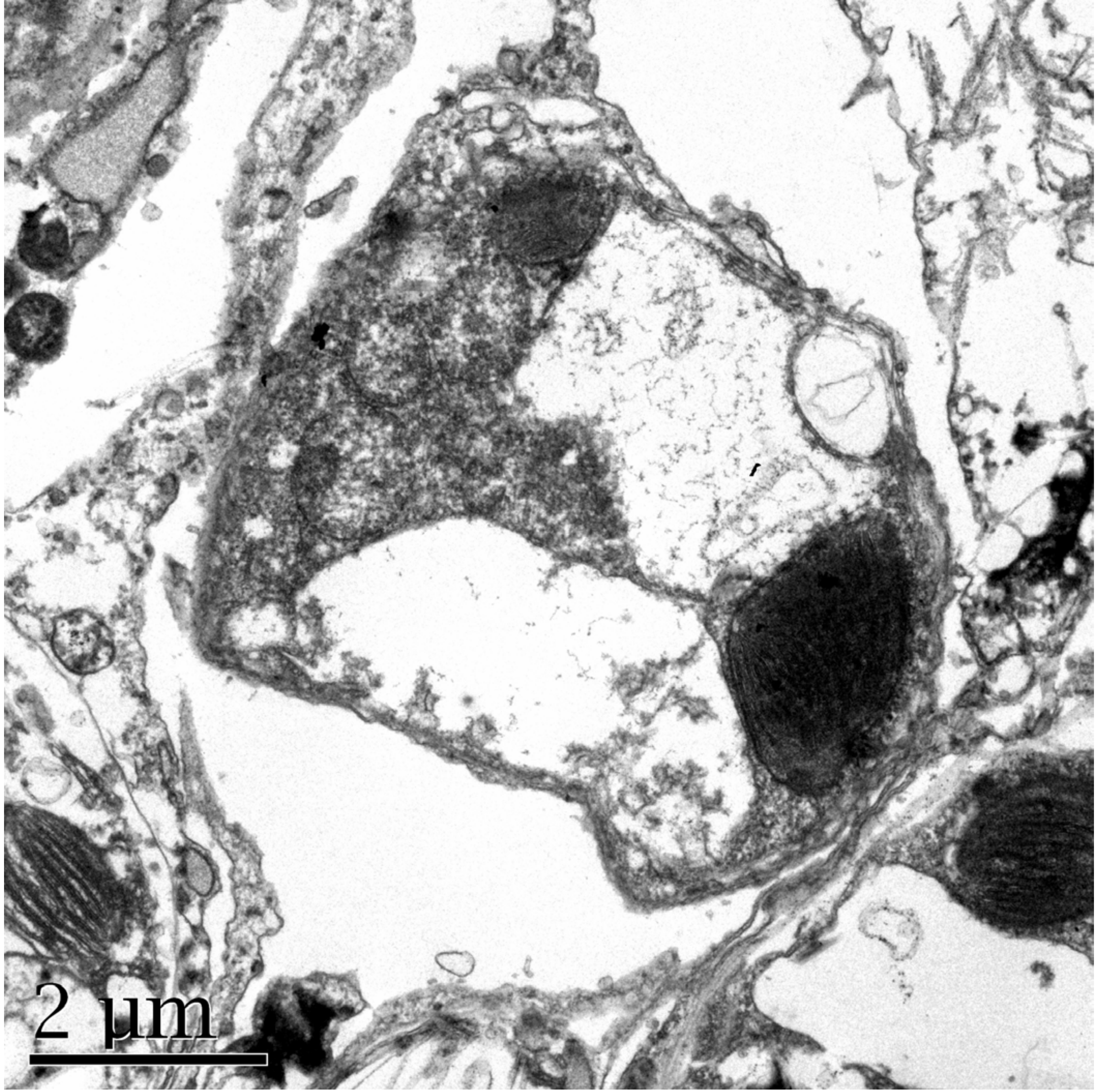

18-14_Correa_YH4_5D3_12.tif

Cell 6

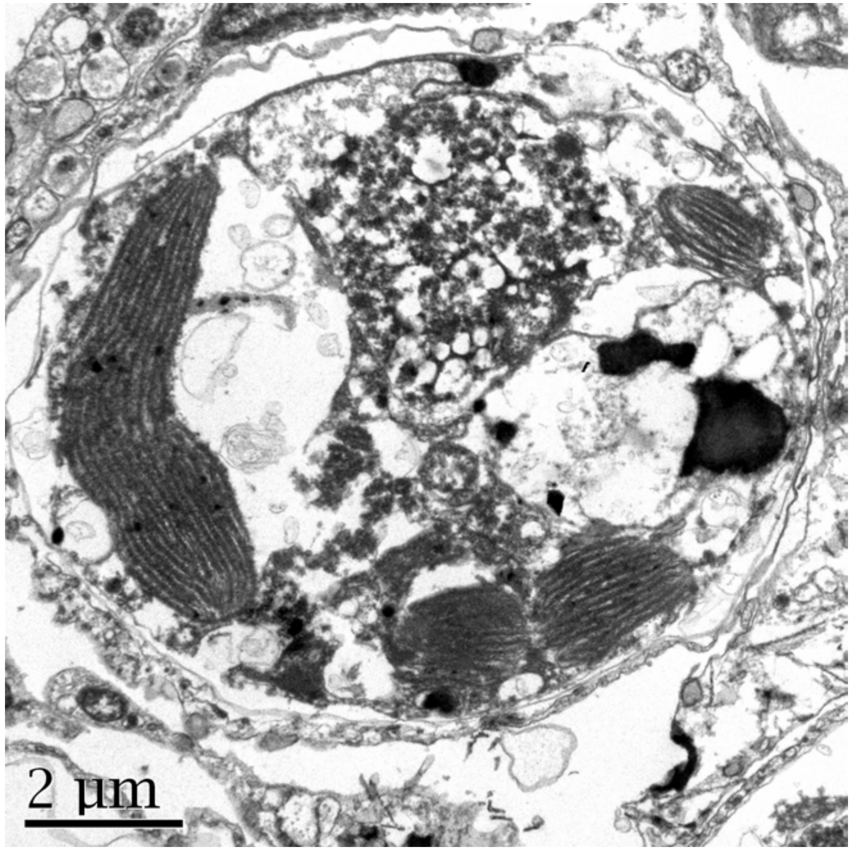

18-14_Correa_YH4_5D3_13.tif

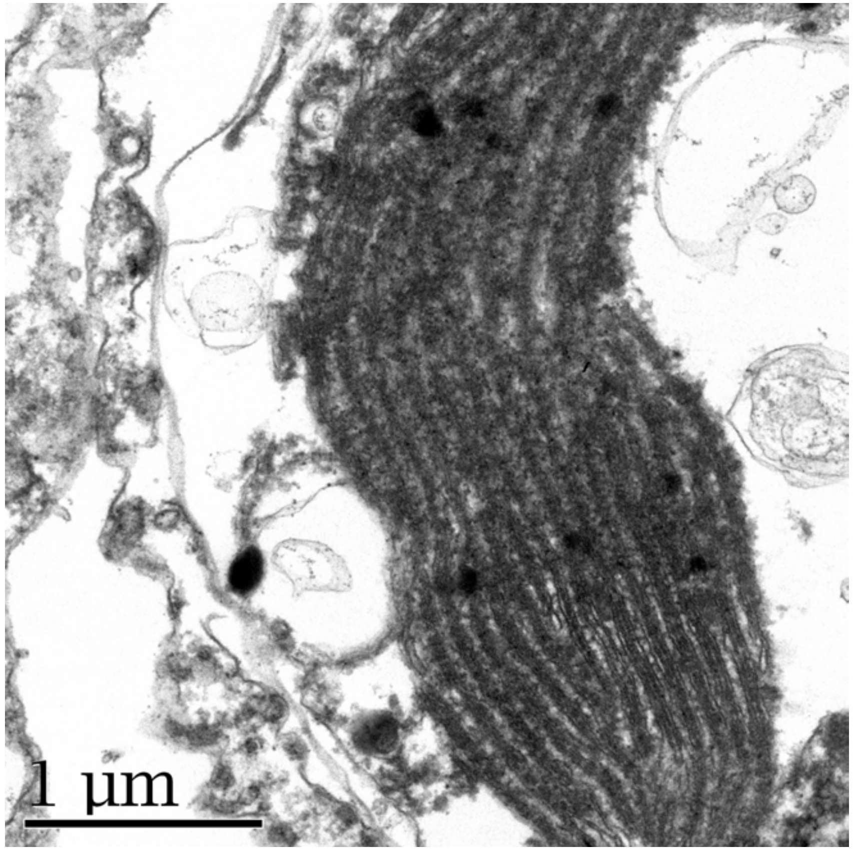

18-14_Correa_YH4_5D3_16.tif

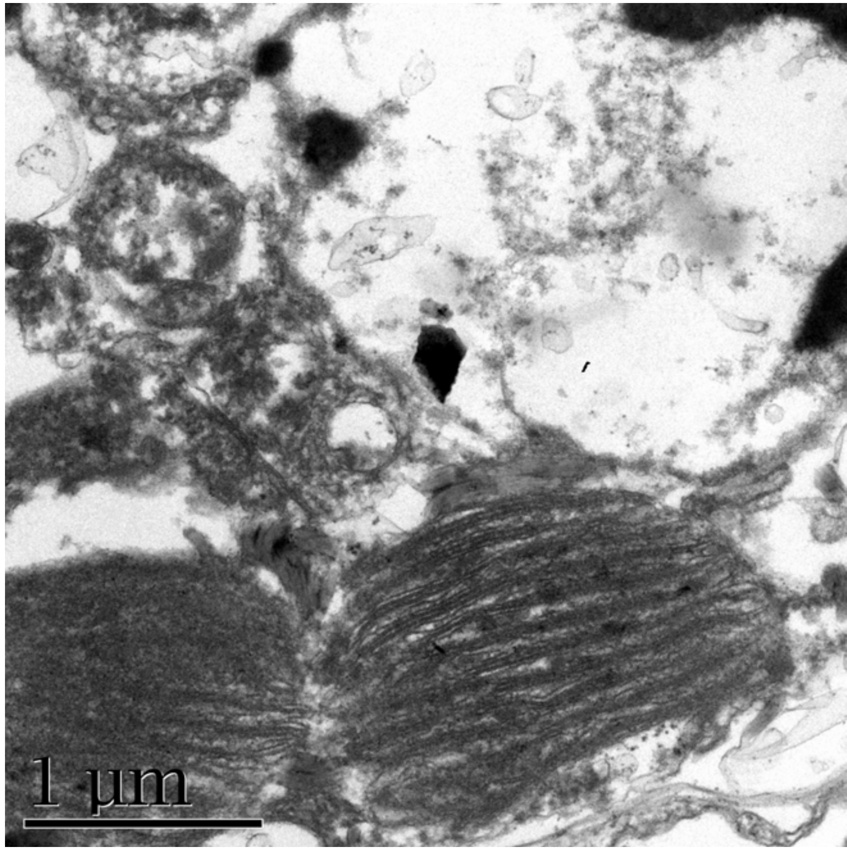

18-14_Correa_YH4_5D3_14.tif

Cell 7

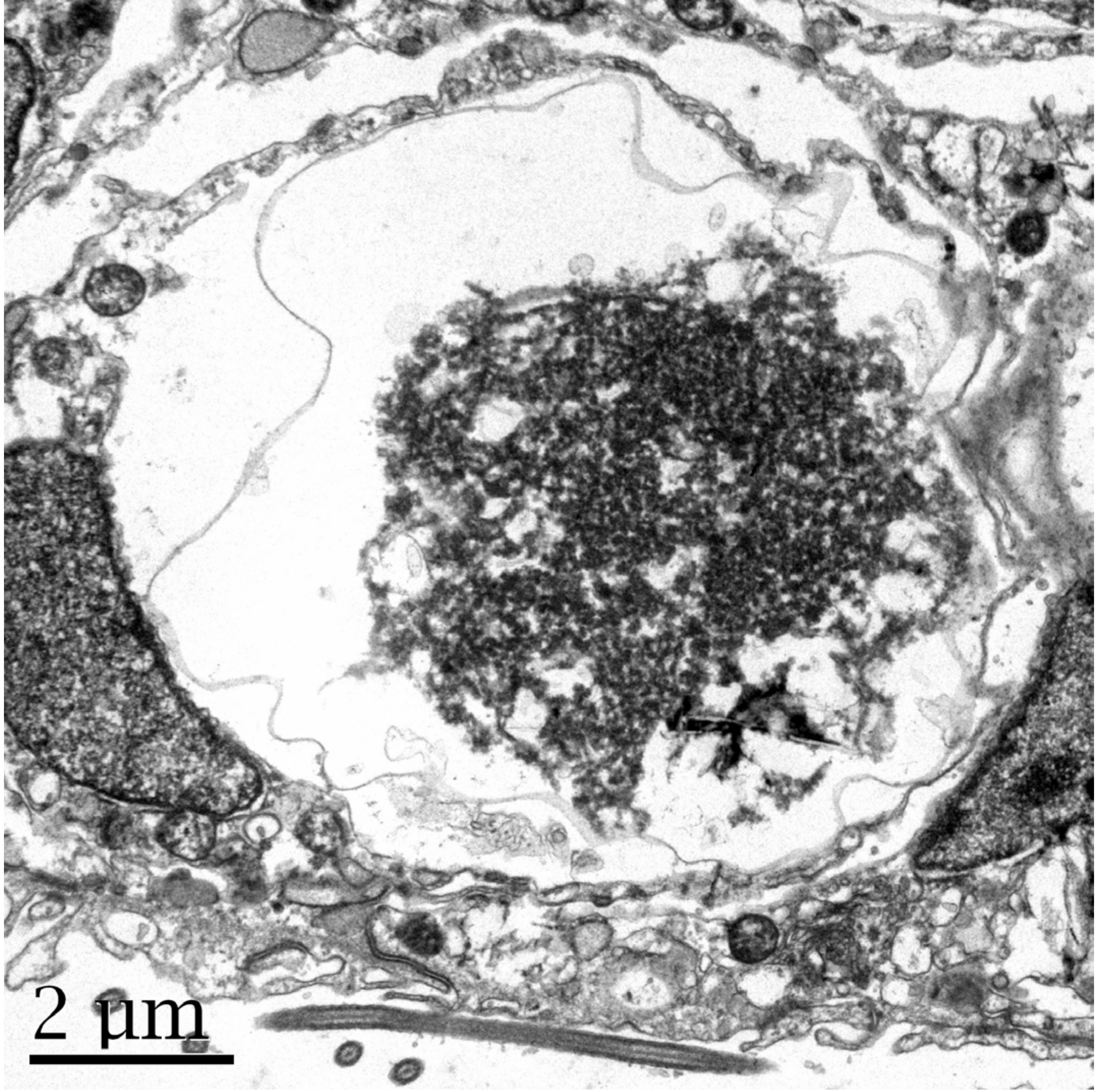

18-14_Correa_YH4_5D3_17.tif

Cell 8

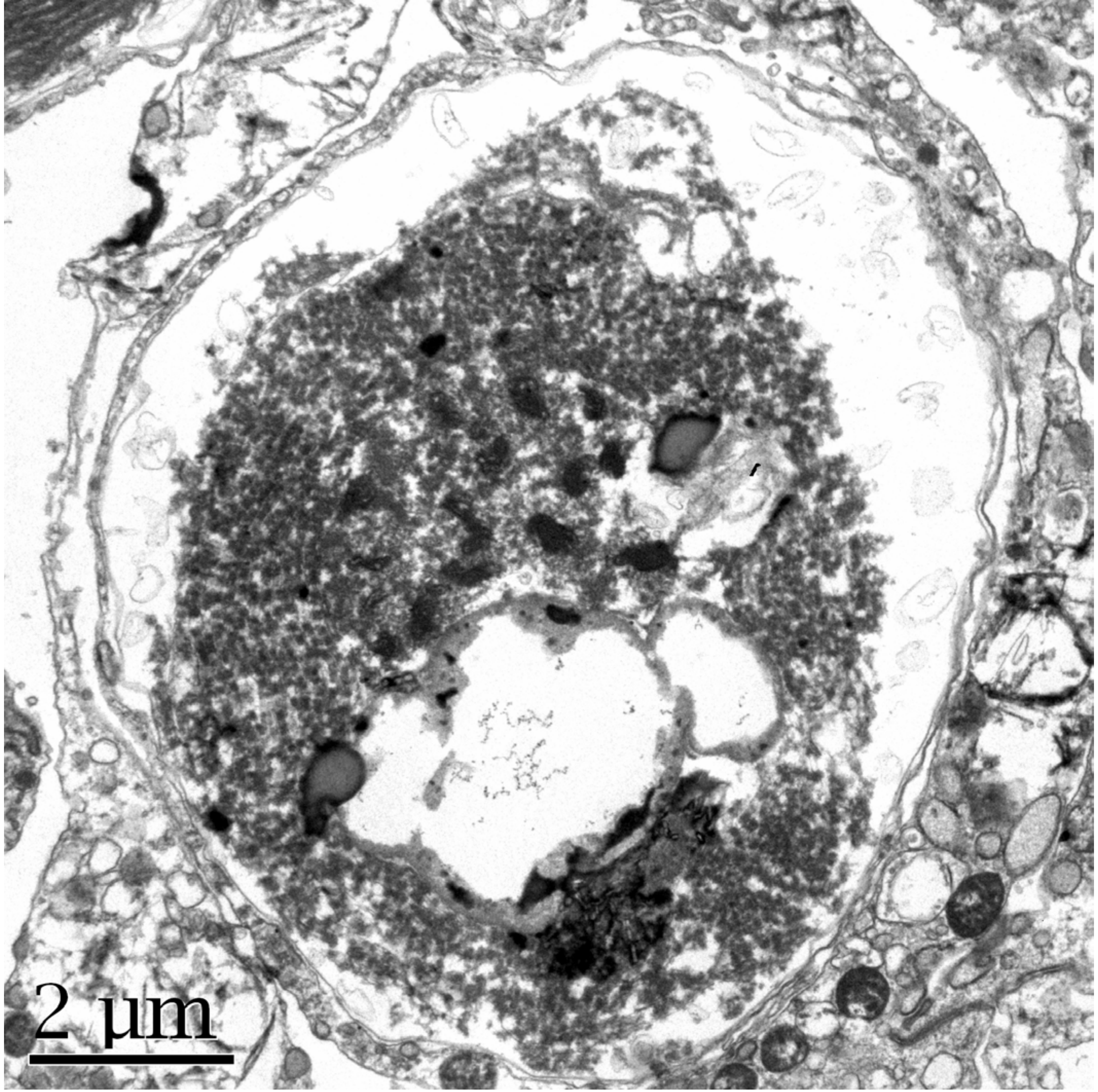

18-14_Correa_YH4_5D3_18.tif

Cell 9

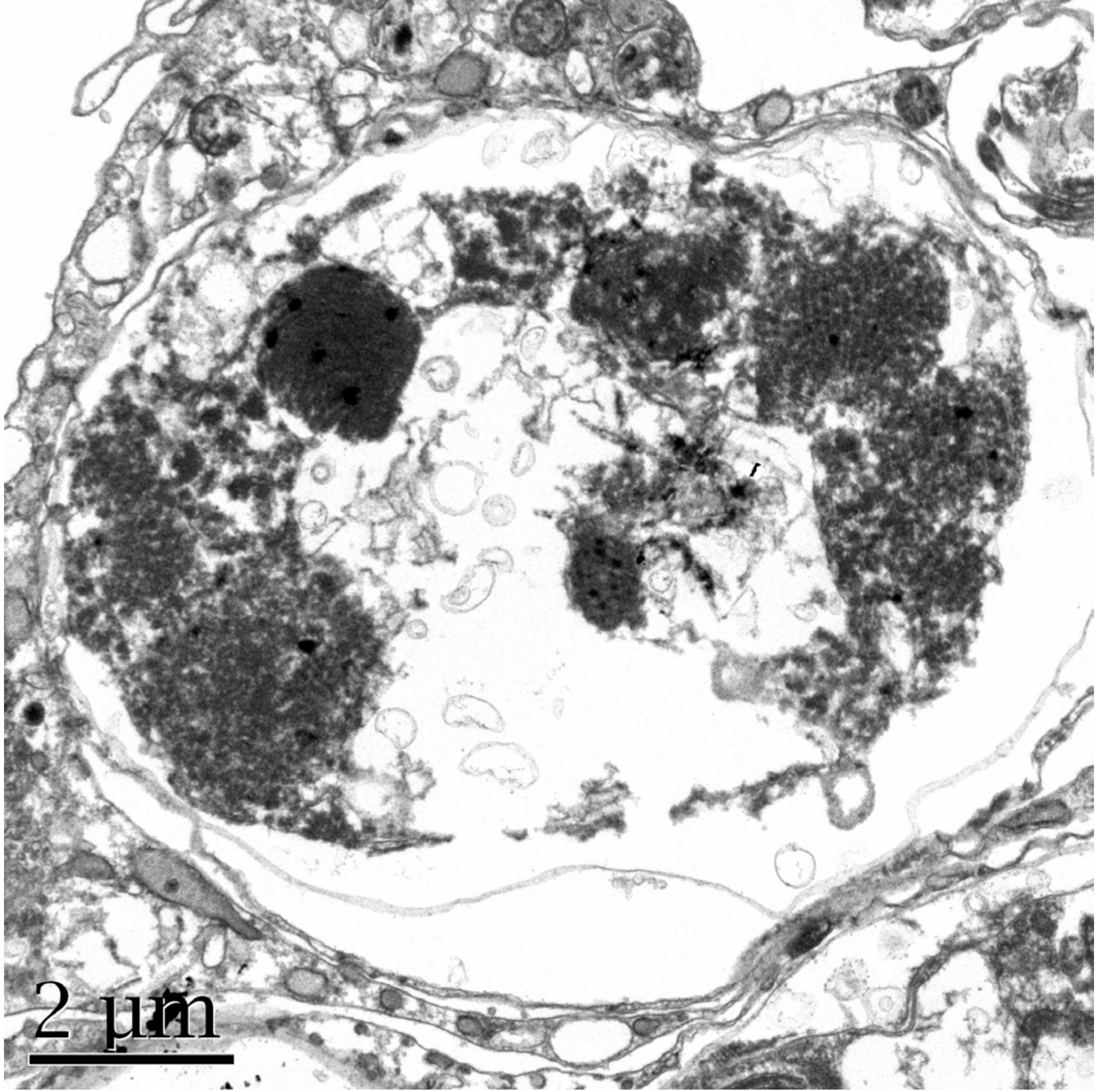

18-14_Correa_YH4_5D3_20.tif

Cell 10

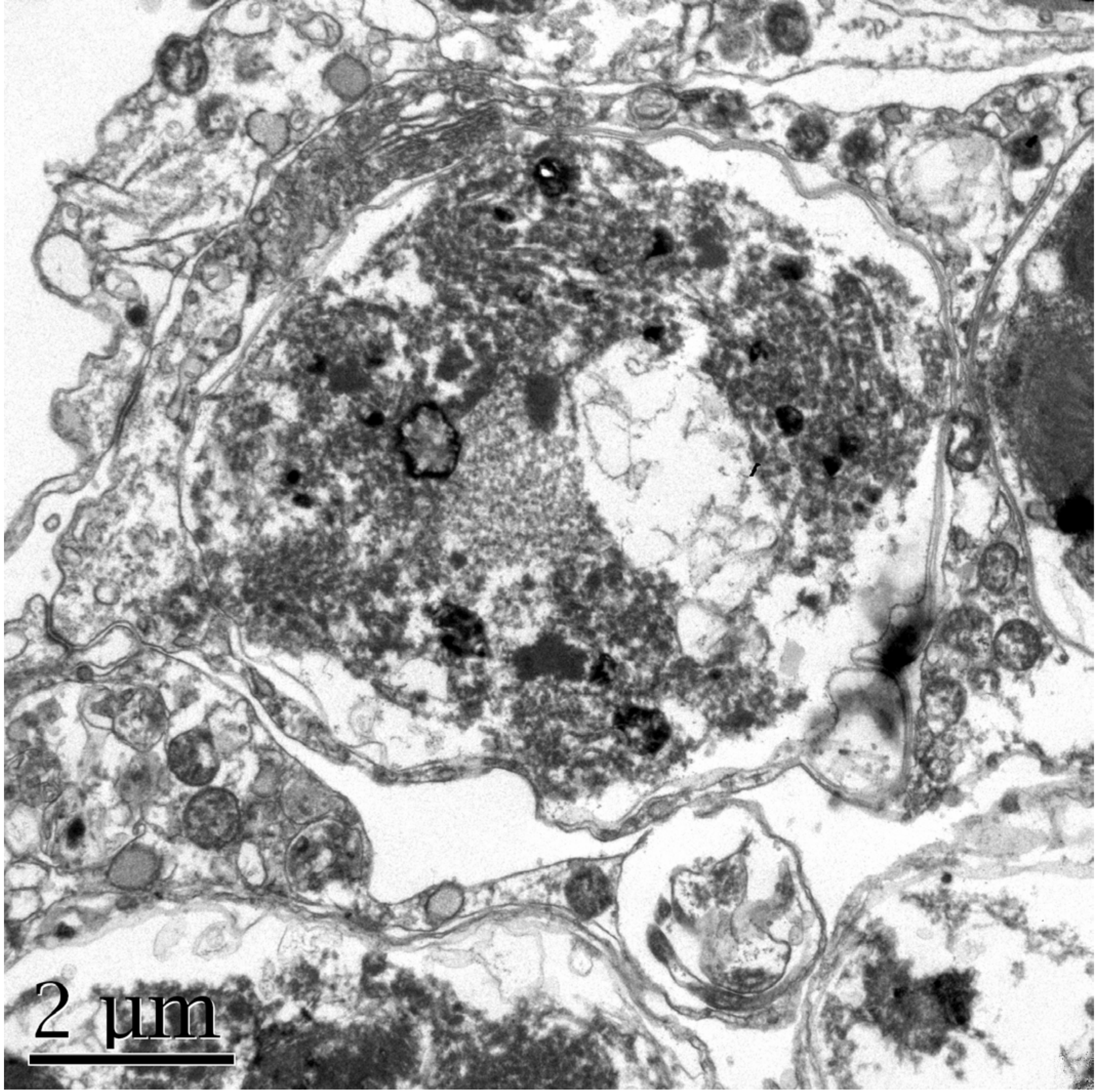

18-14_Correa_YH4_5D3_21.tif

Cell 11

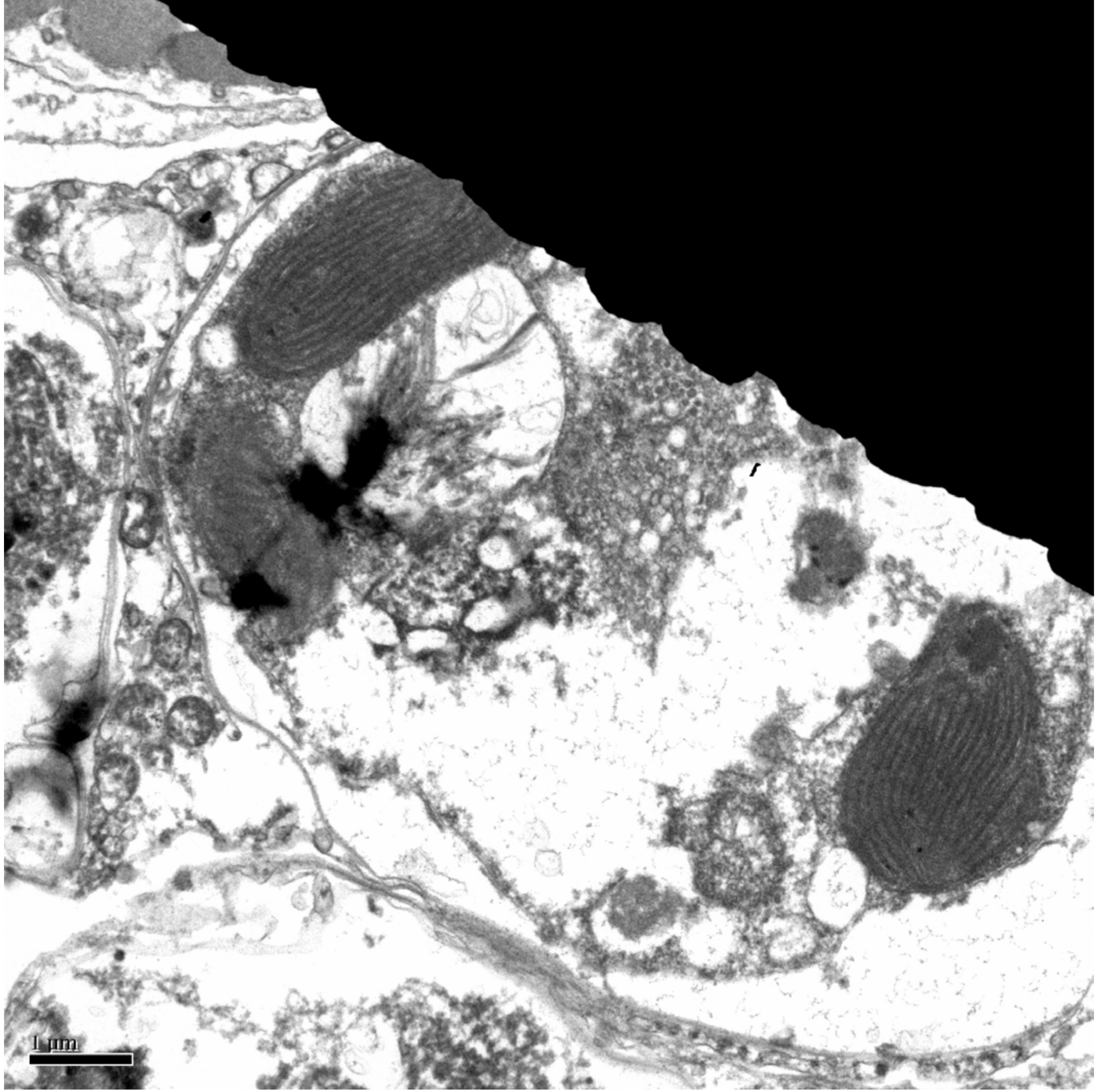

18-14_Correa_YH4_5D3_22.tif

Cell 12

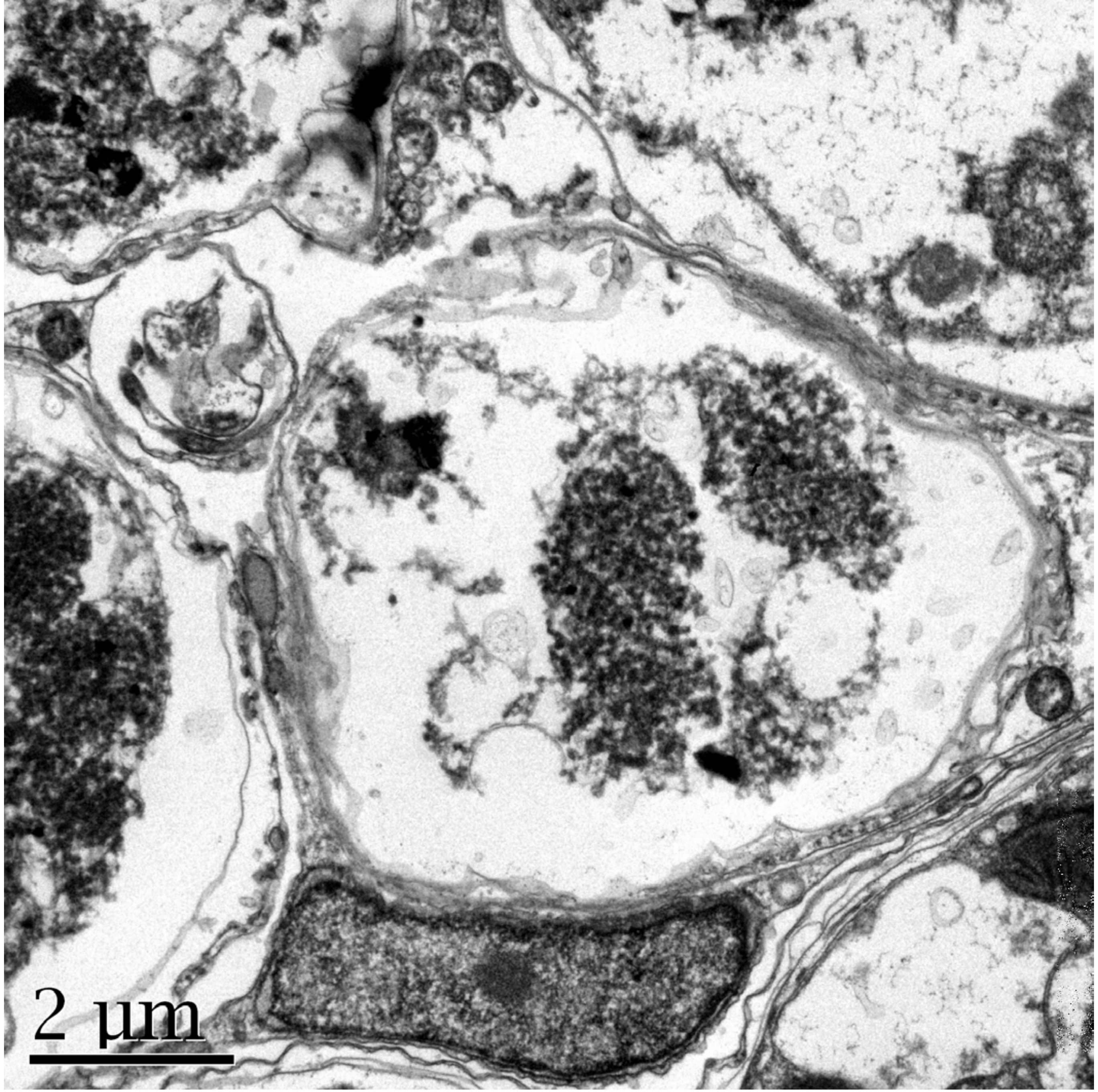

18-14_Correa_YH4_5D3_23.tif

Cell 13

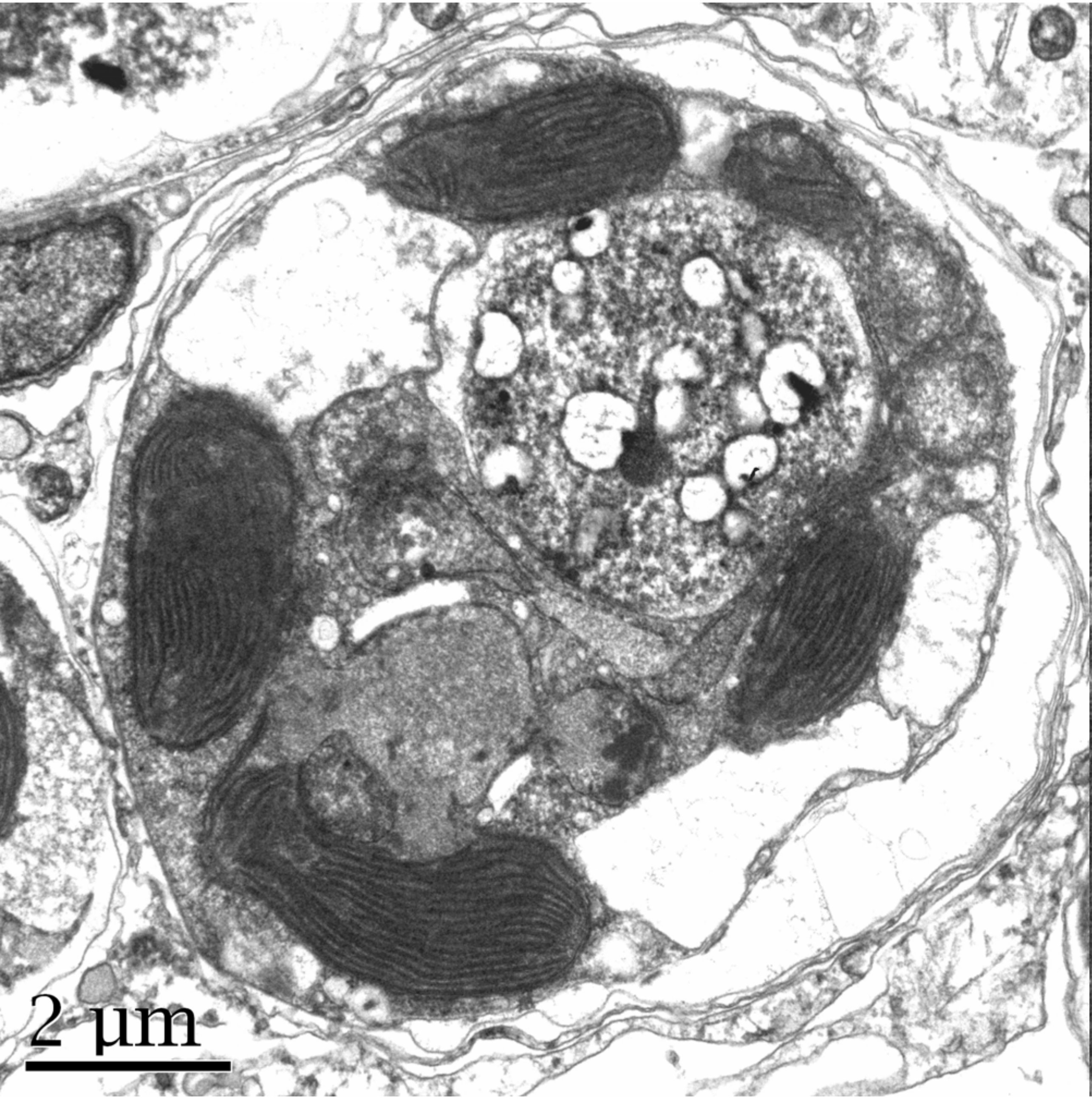

18-14_Correa_YH4_5D3_24.tif

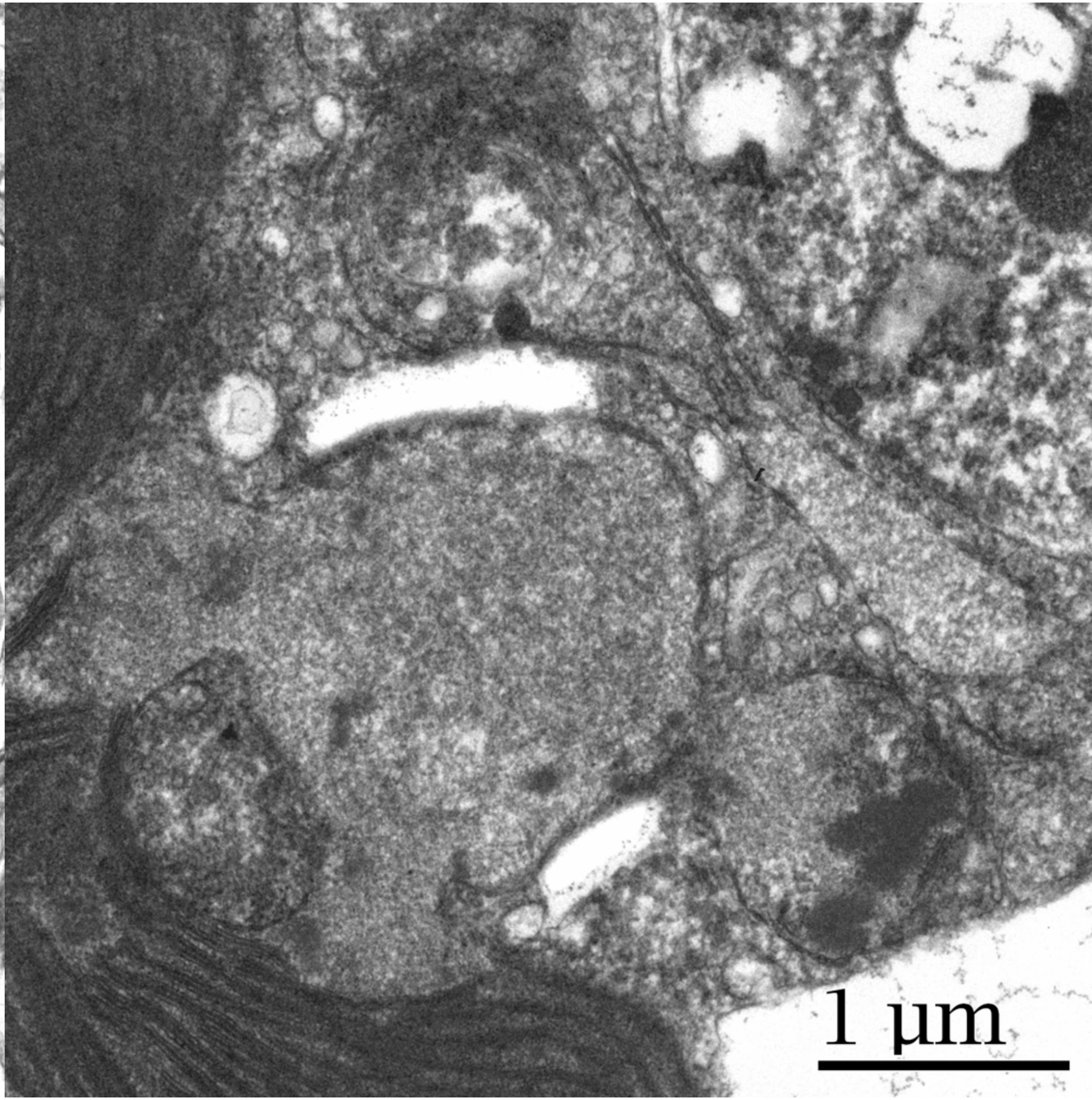

18-14_Correa_YH4_5D3_25.tif

Cell 14

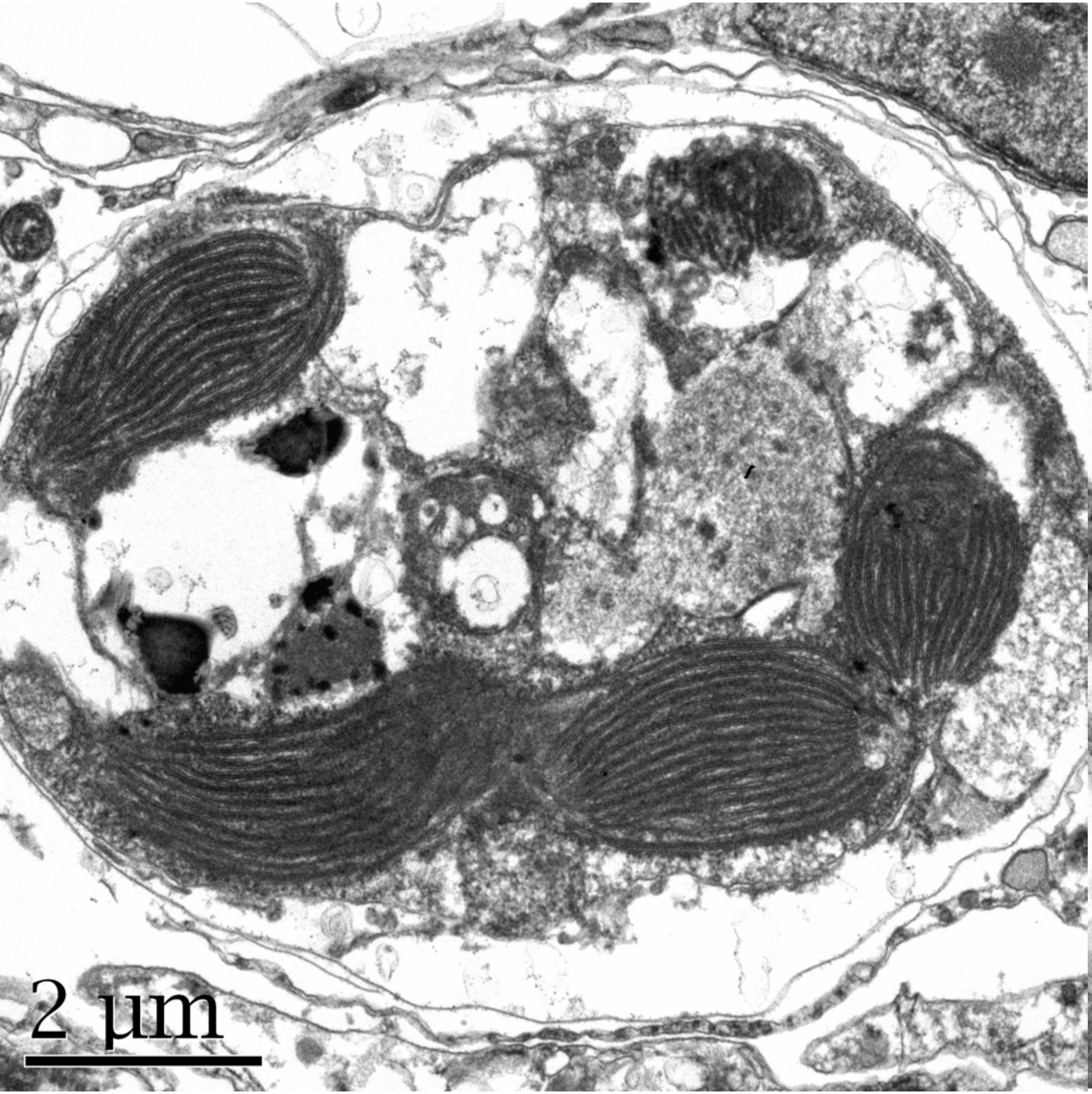

18-14_Correa_YH4_5D3_26.tif

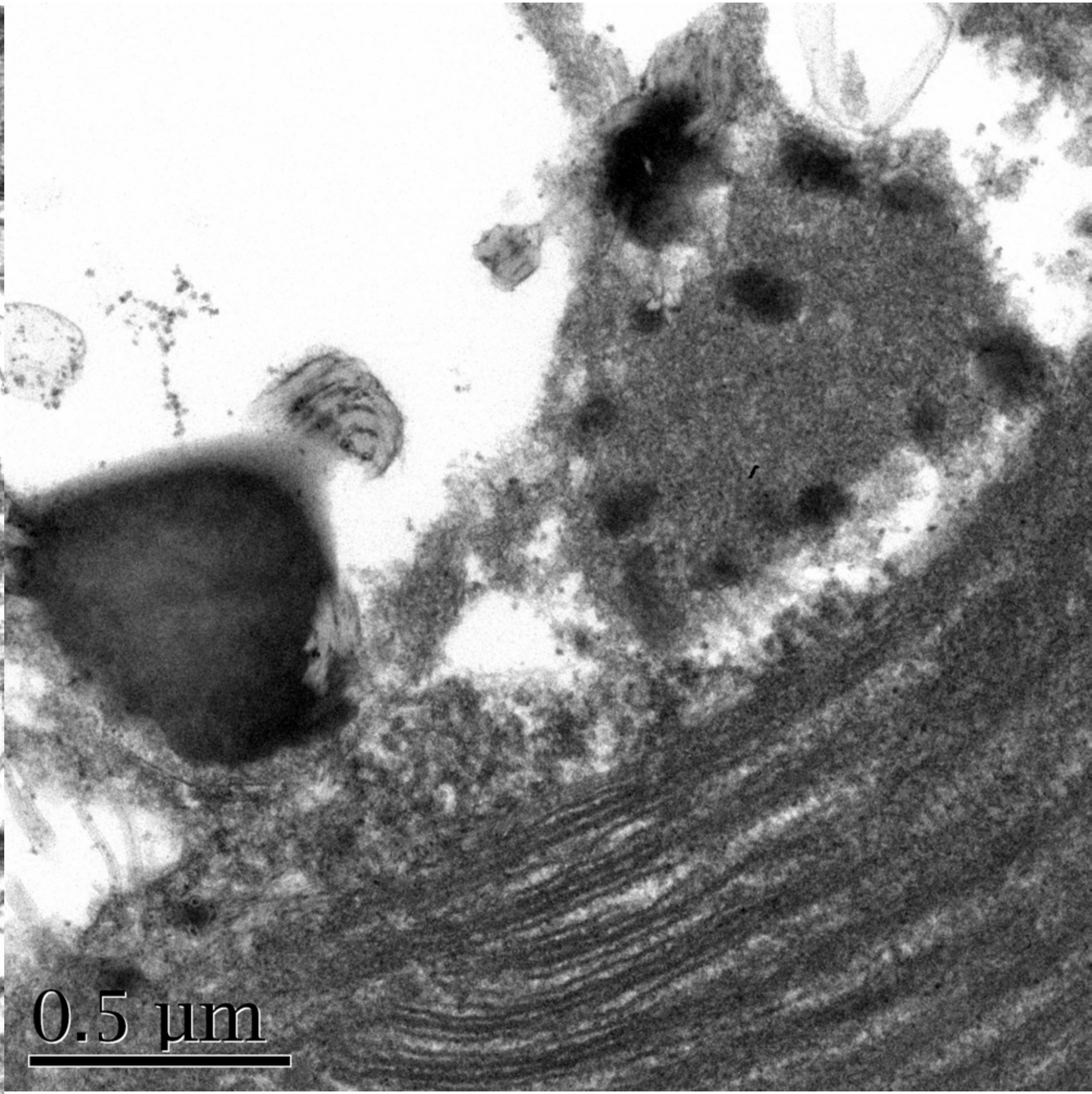

18-14_Correa_YH4_5D3_27.tif

Cell 15

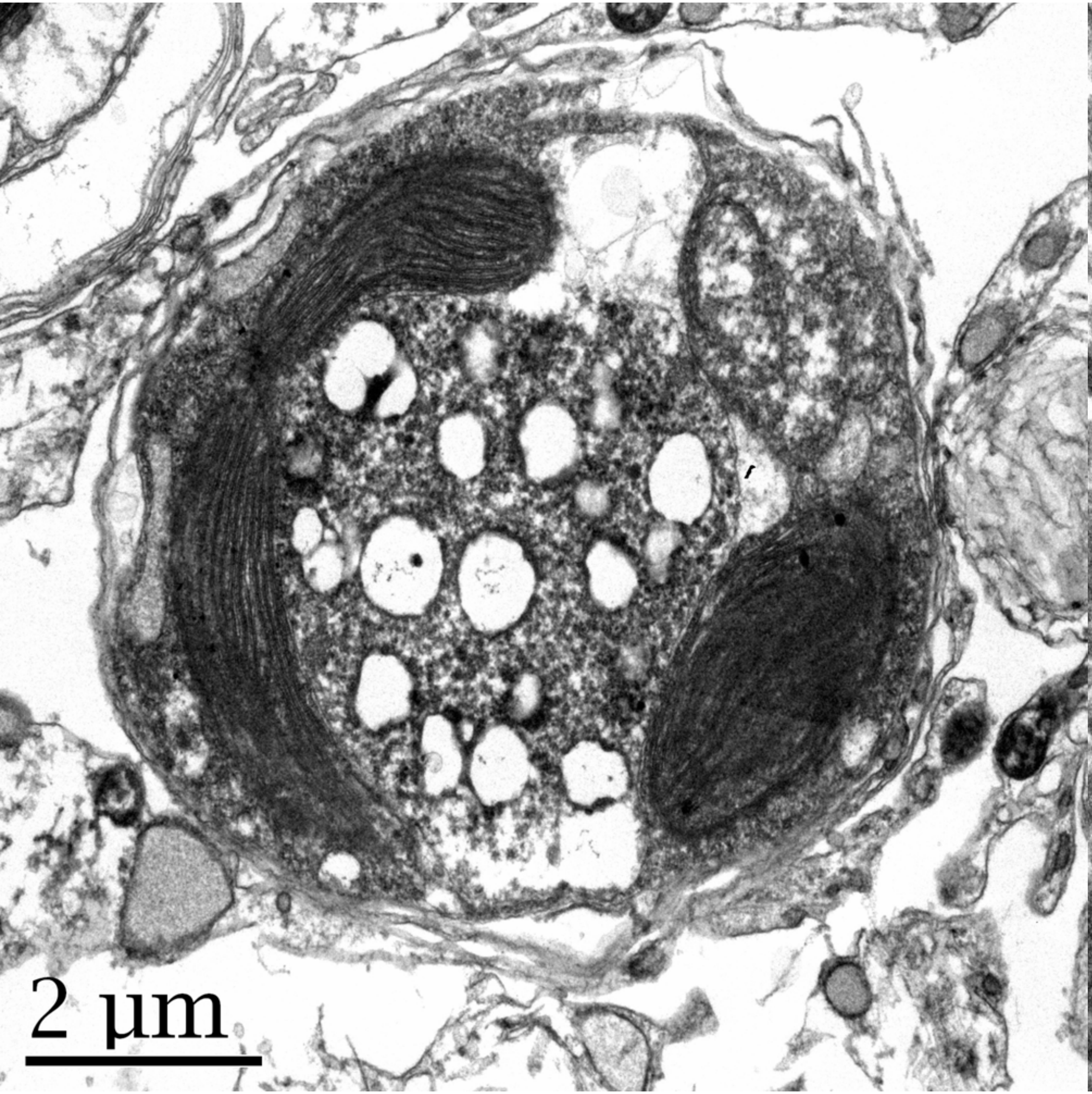

18-14_Correa_YH4_5D3_29.tif

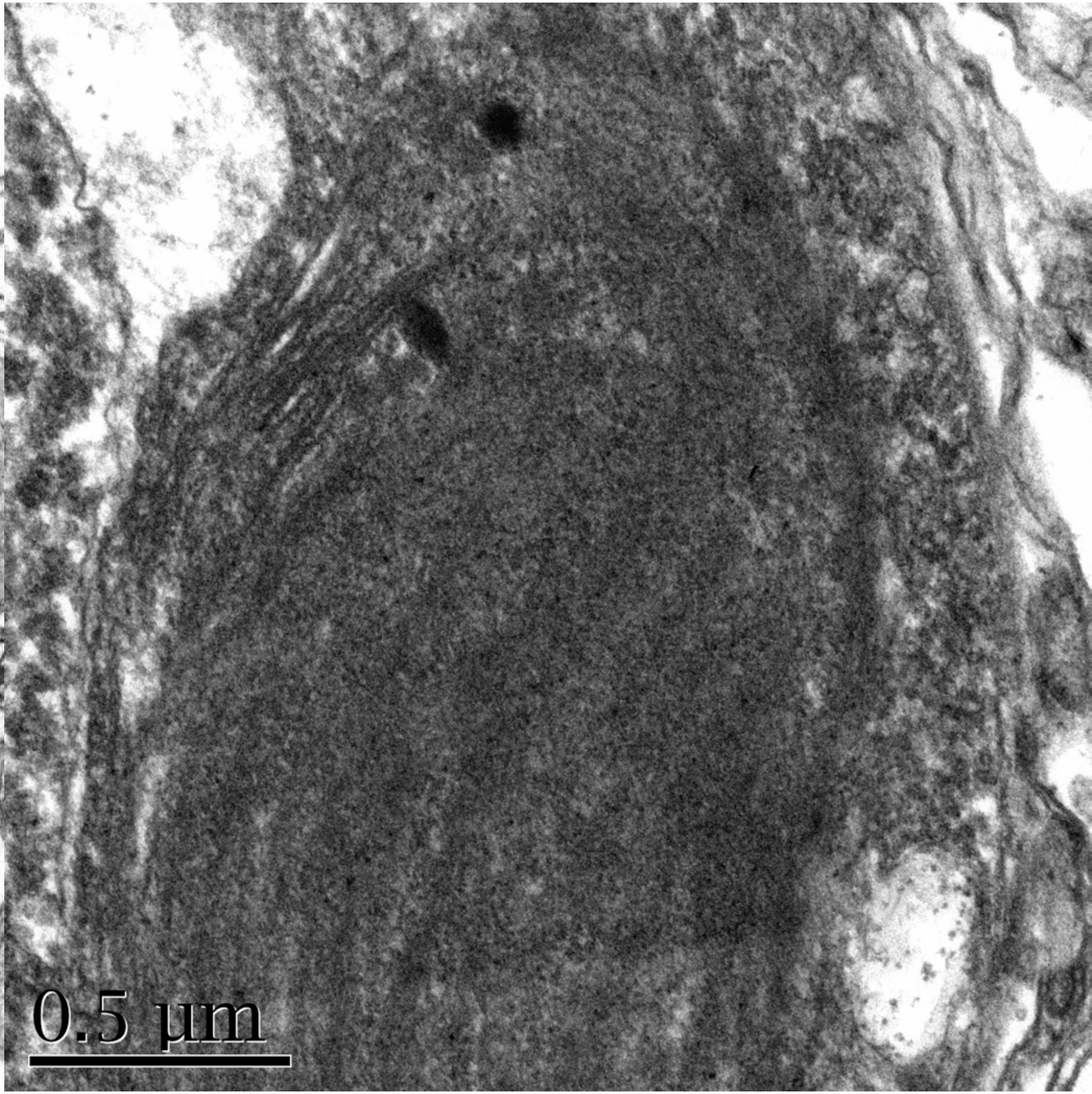

18-14_Correa_YH4_5D3_30.tif

Cell 16

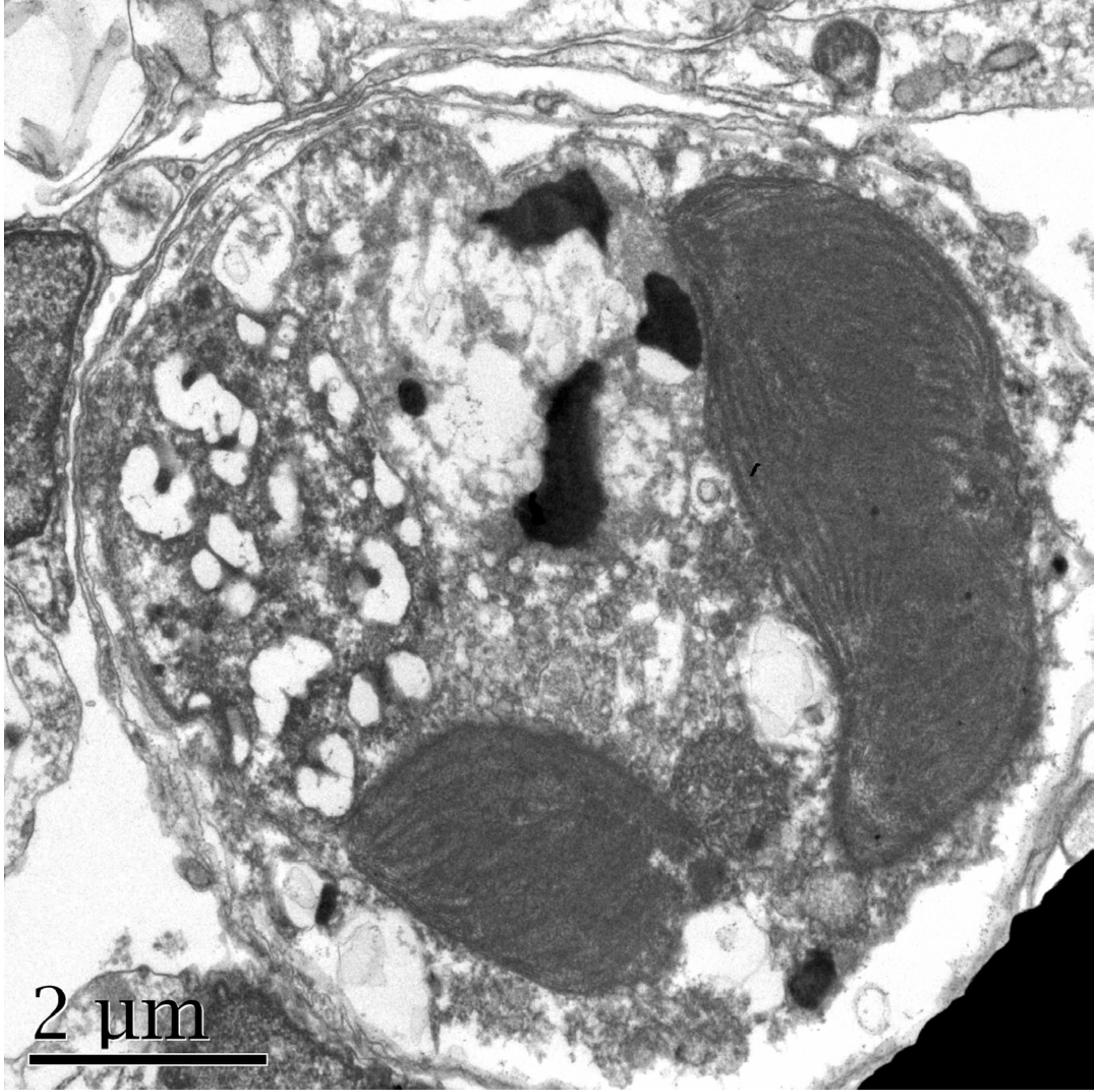

18-14_Correa_YH4_5D3_31.tif

Cell 17

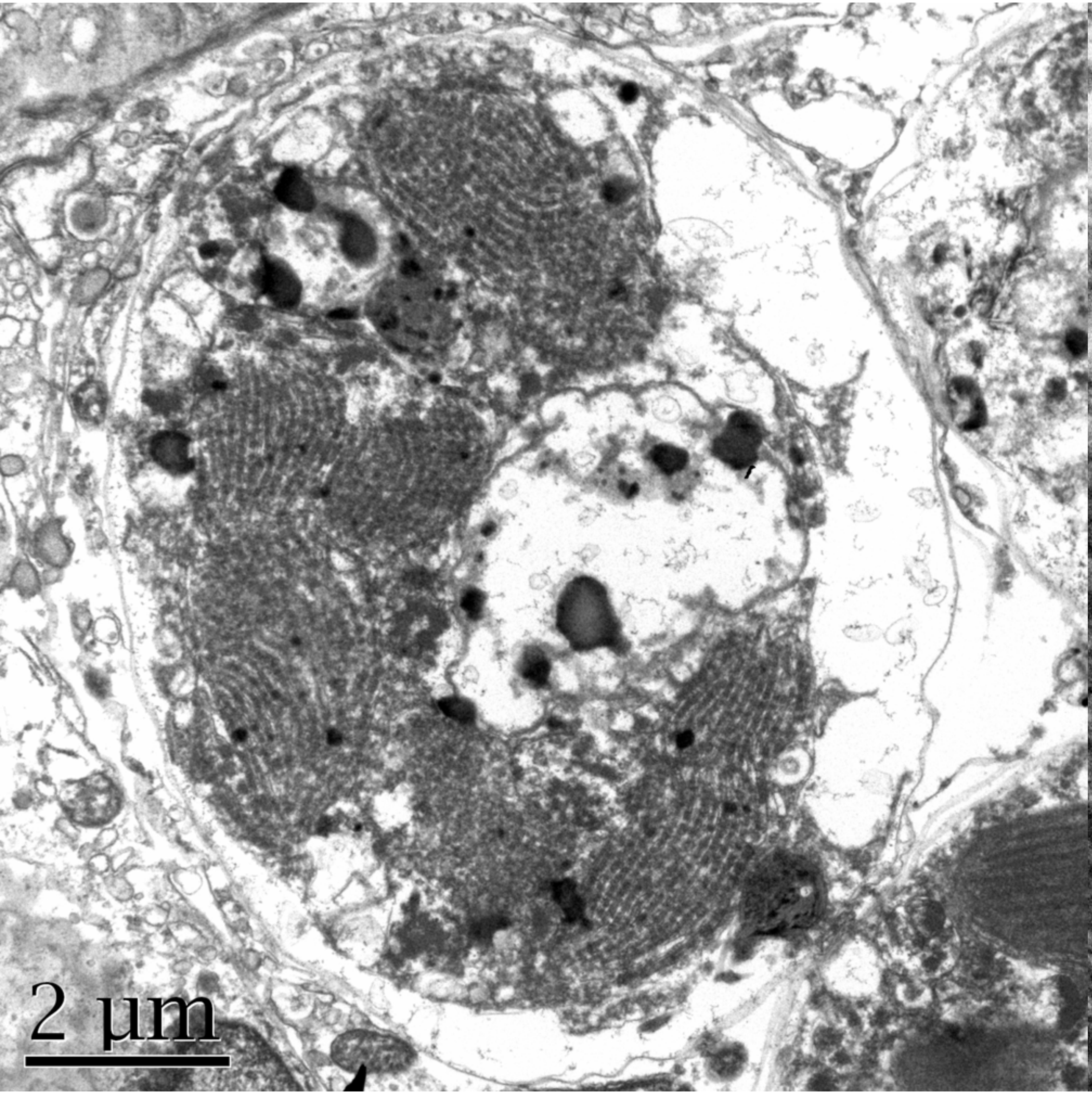

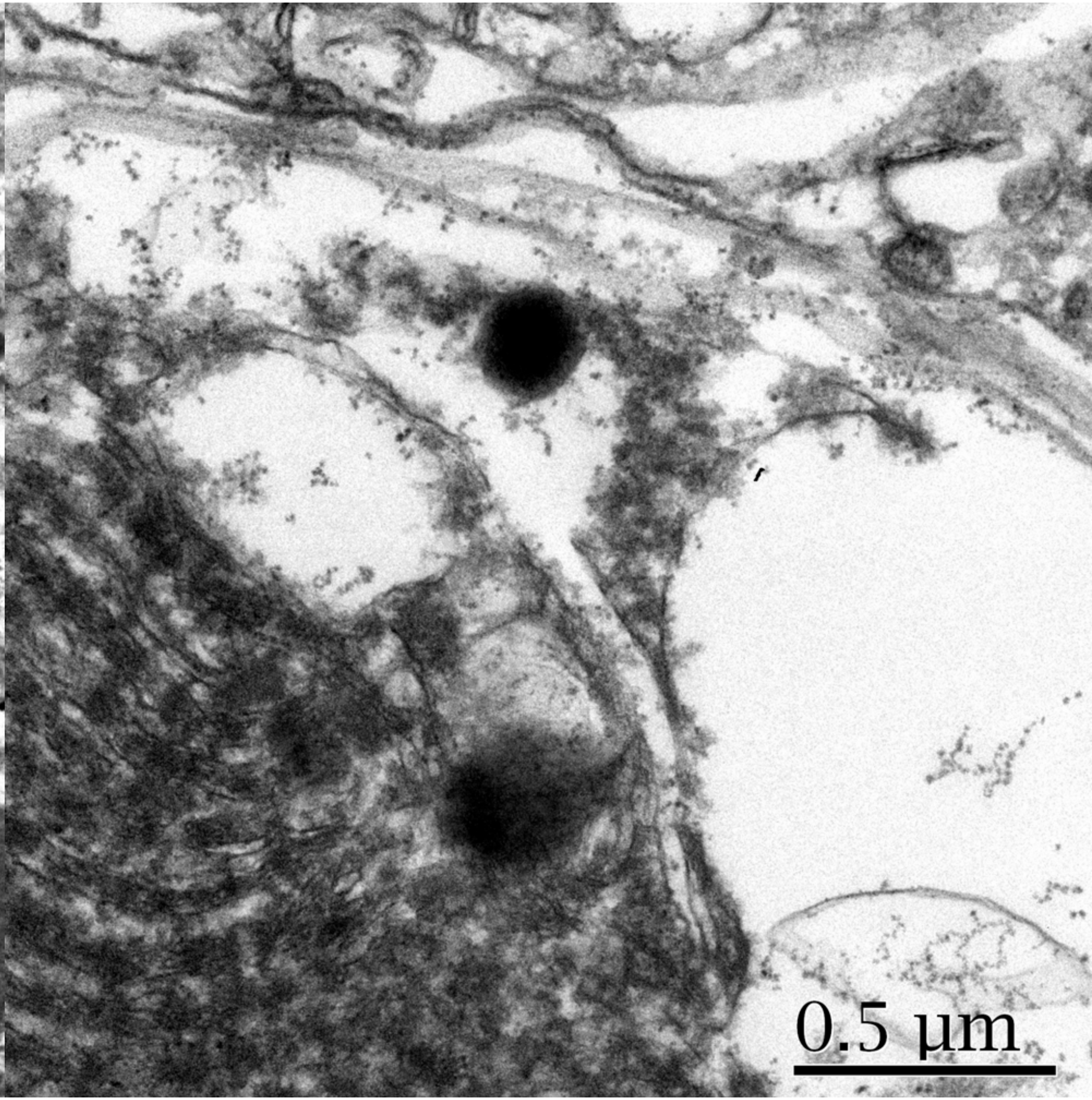

18-14_Correa_YH4_5D3_33.tif

18-14_Correa_YH4_5D3_35.tif

Cell 18

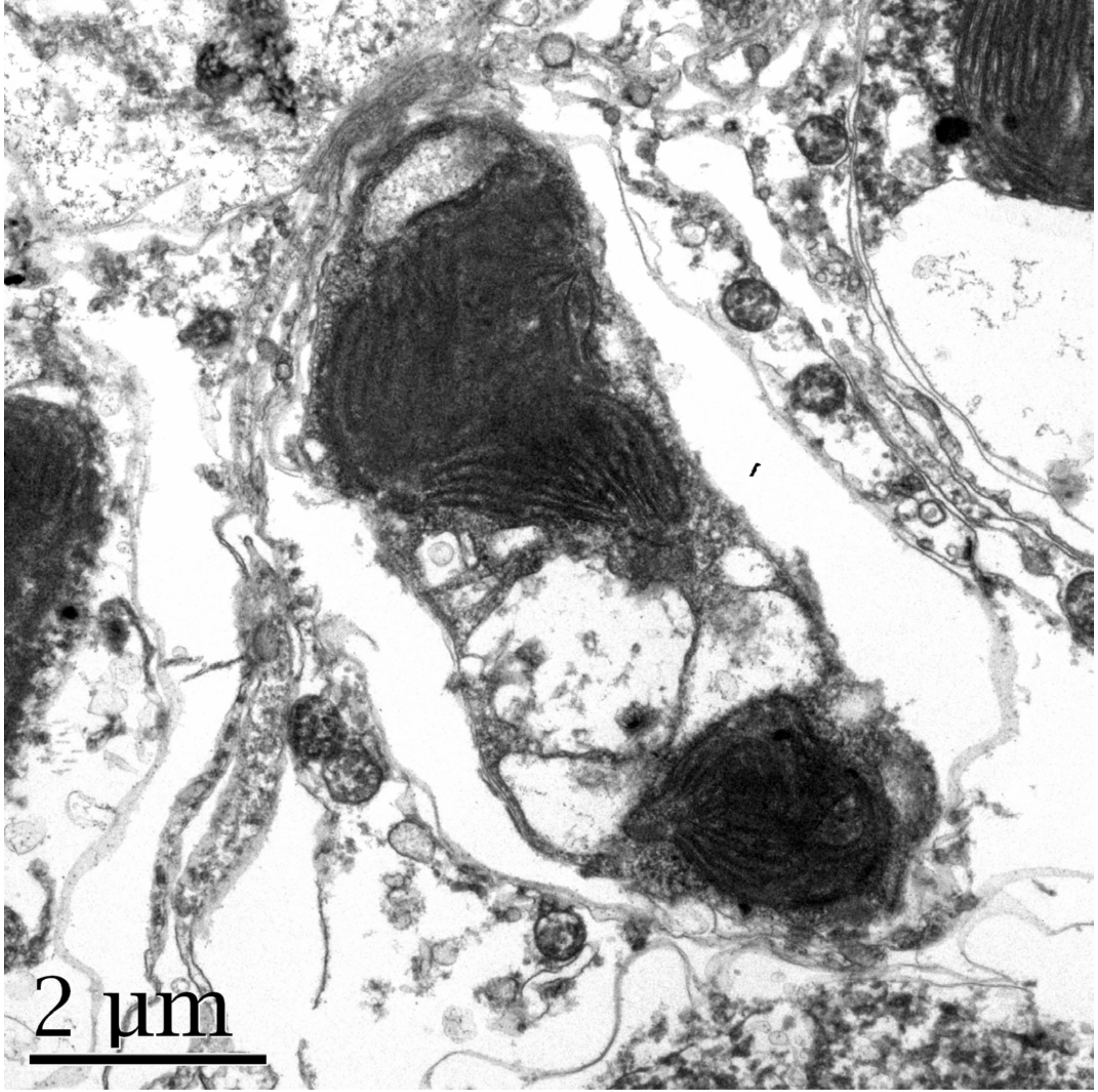

18-14_Correa_YH4_5D3_37.tif

Cell 19

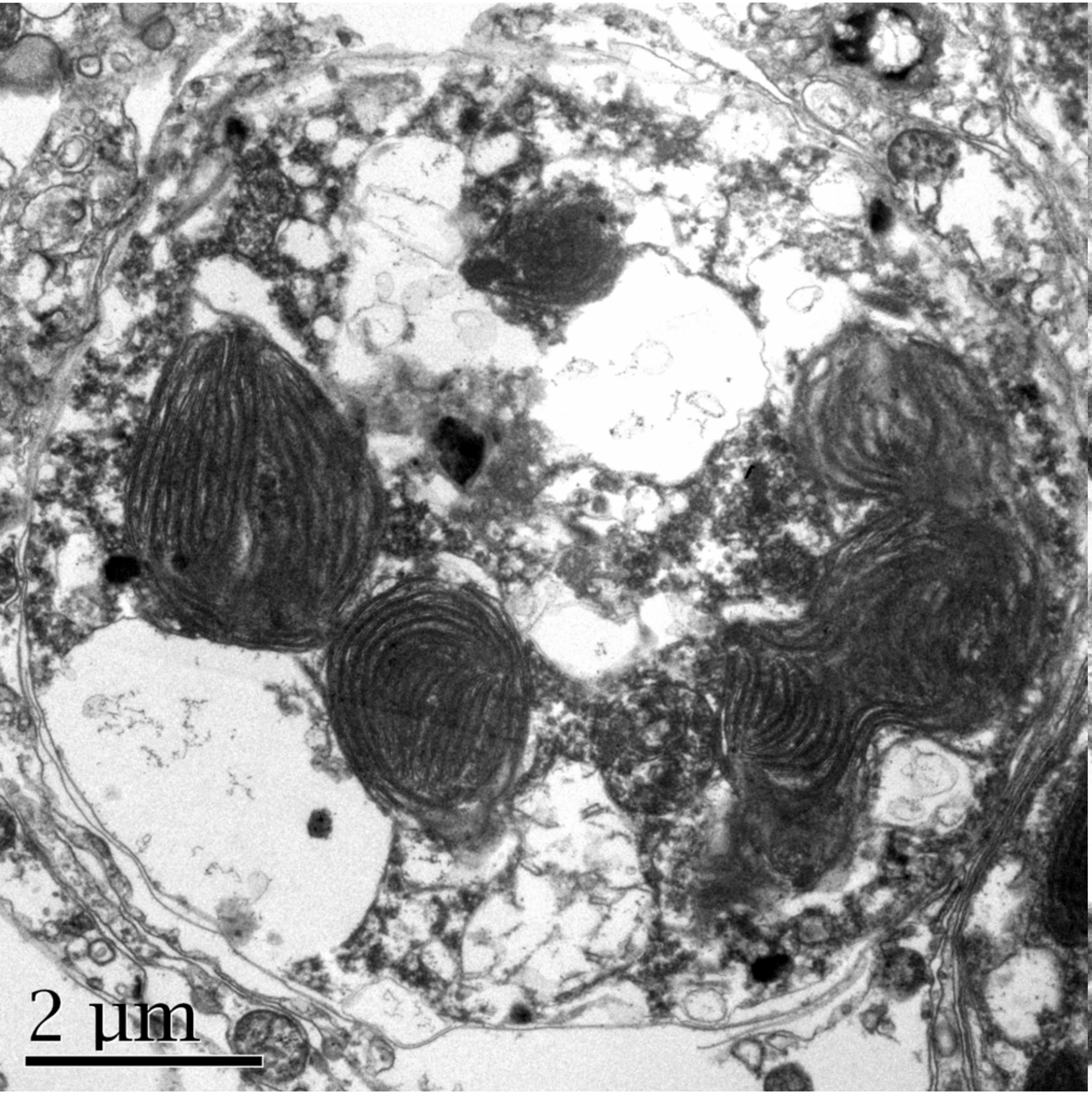

18-14_Correa_YH4_5D3_38.tif

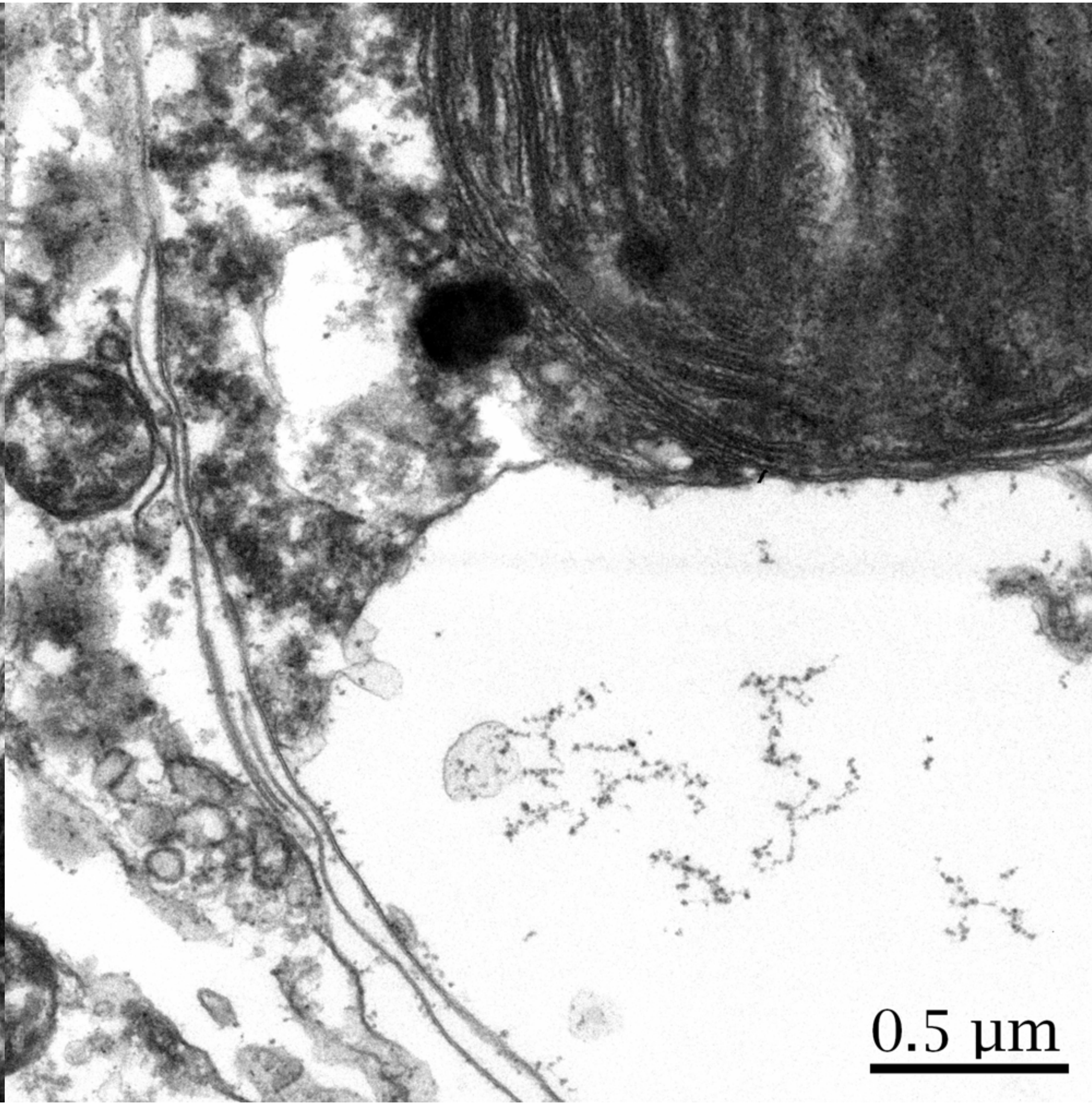

18-14_Correa_YH4_5D3_39.tif

Cell 20

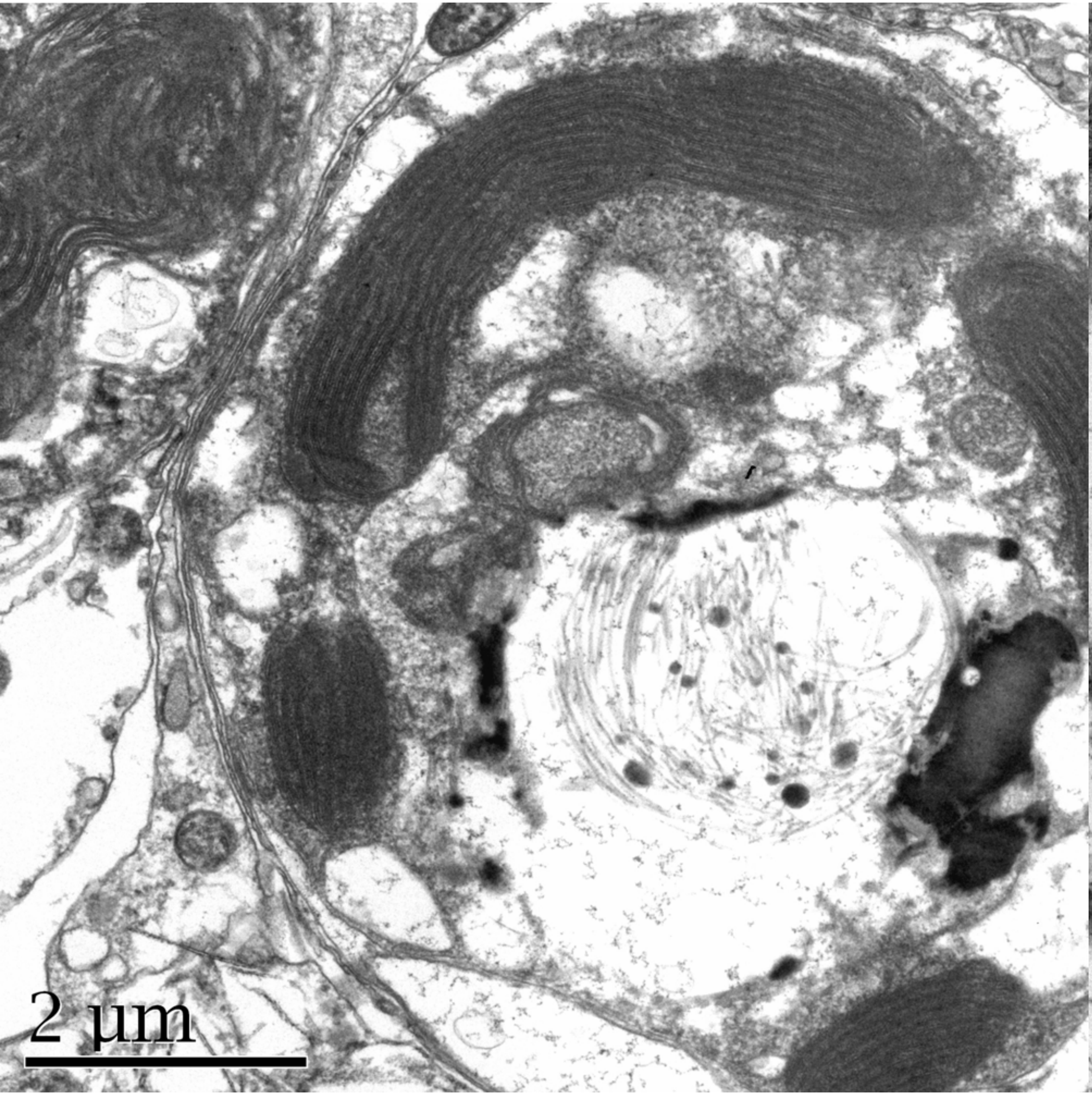

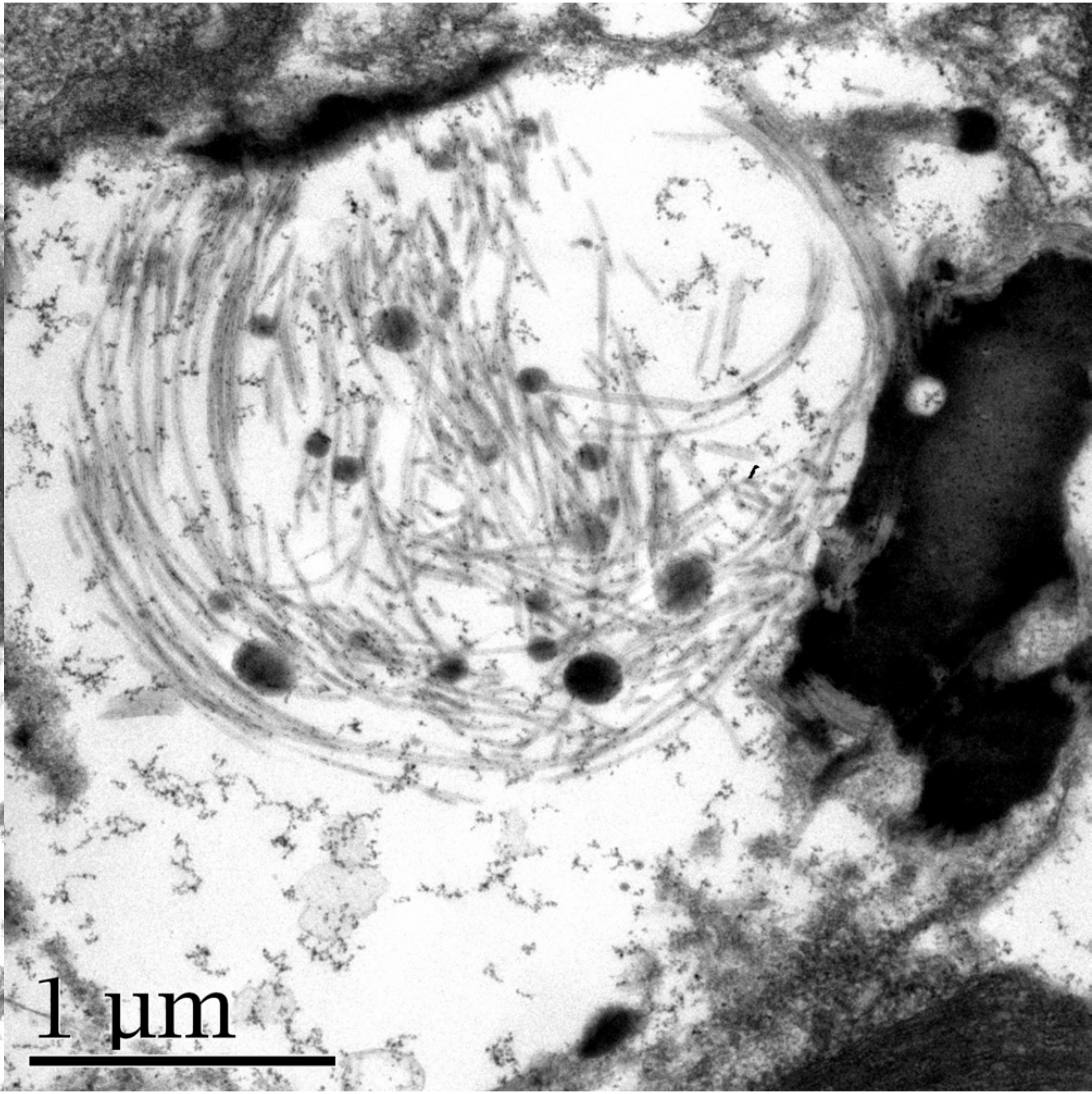

18-14_Correa_YH4_5D3_38.tif

18-14_Correa_YH4_5D3_41.tif

Cell 21

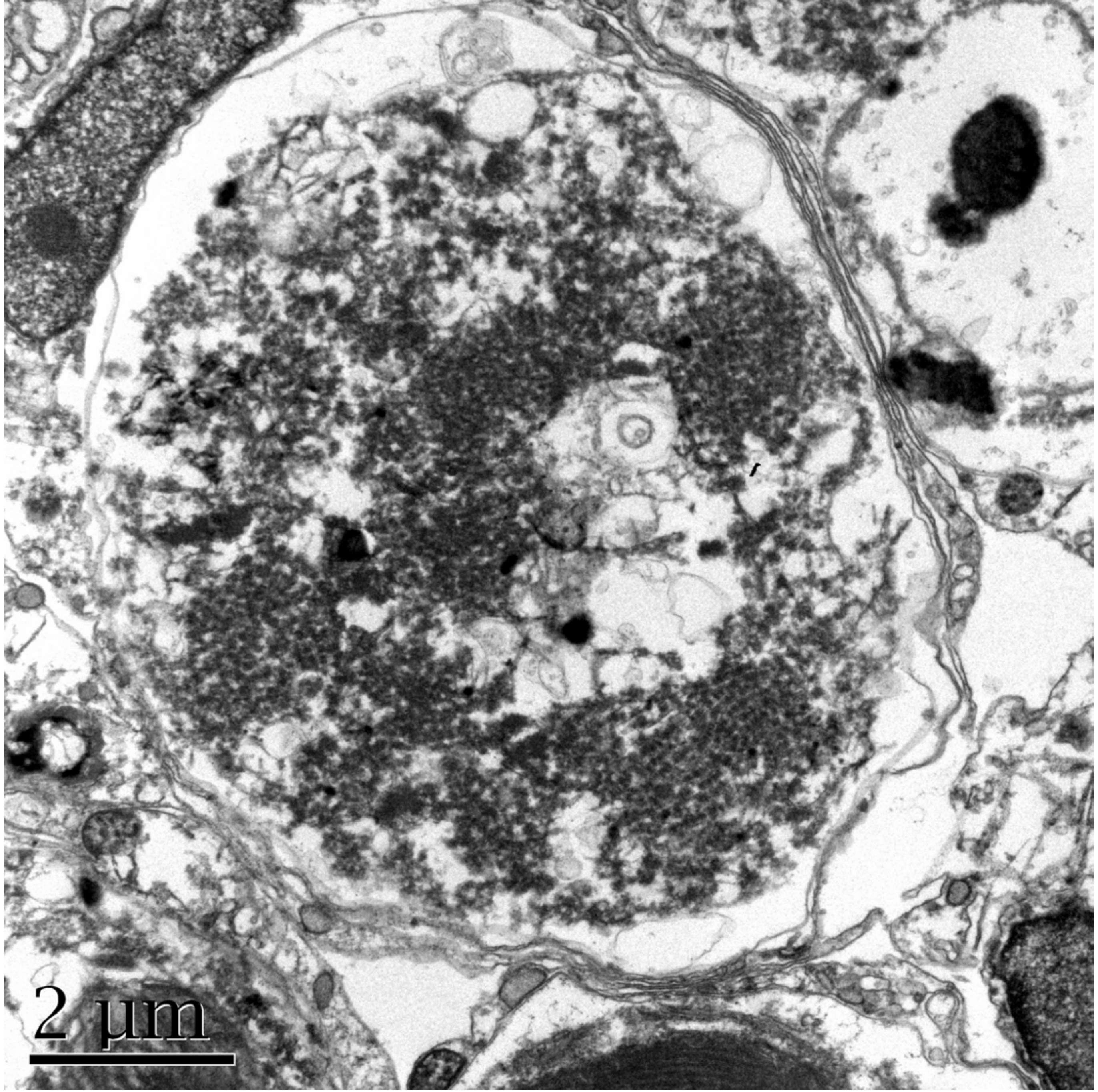

18-14_Correa_YH4_5D3_42.tif

Cell 22

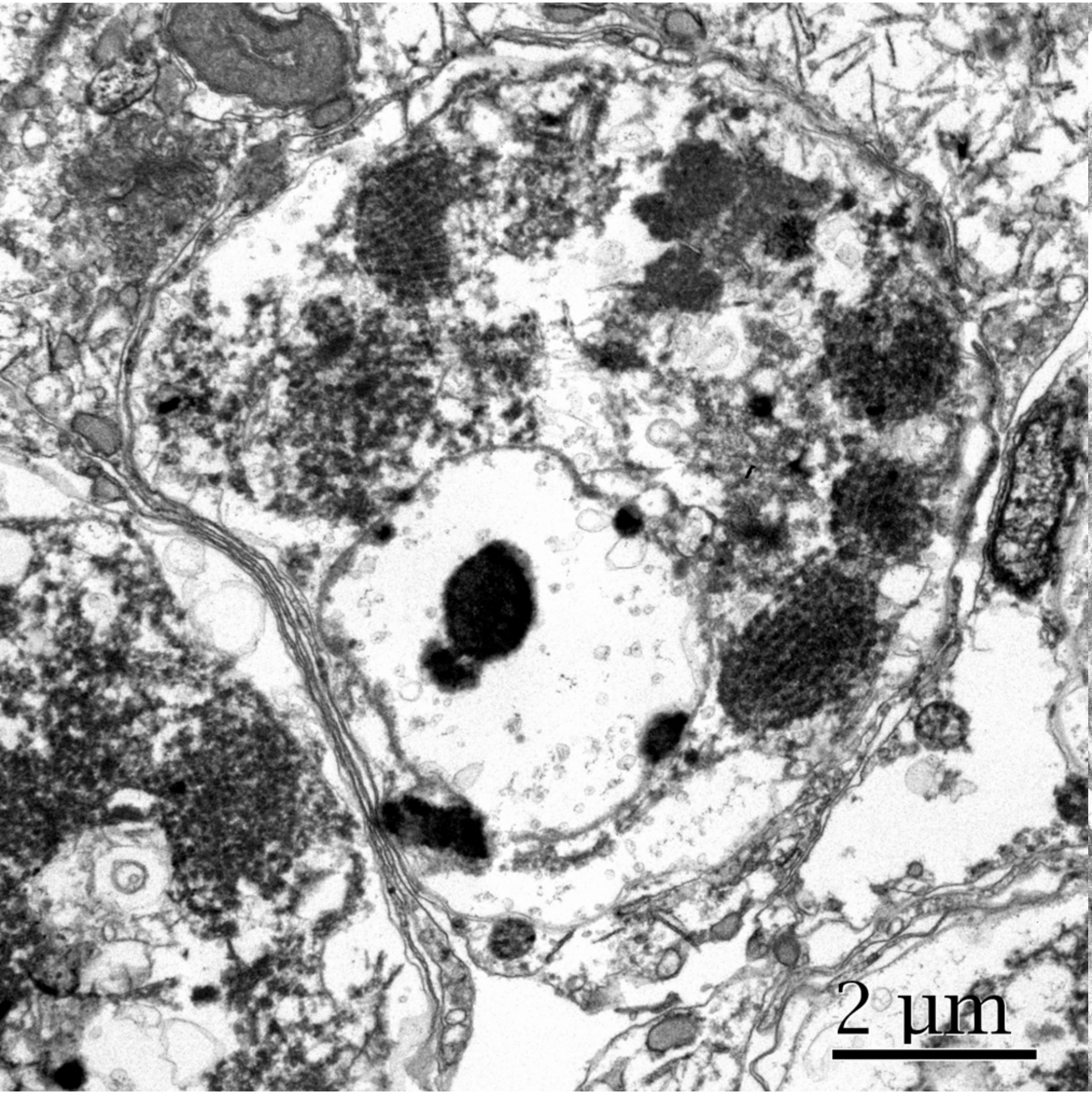

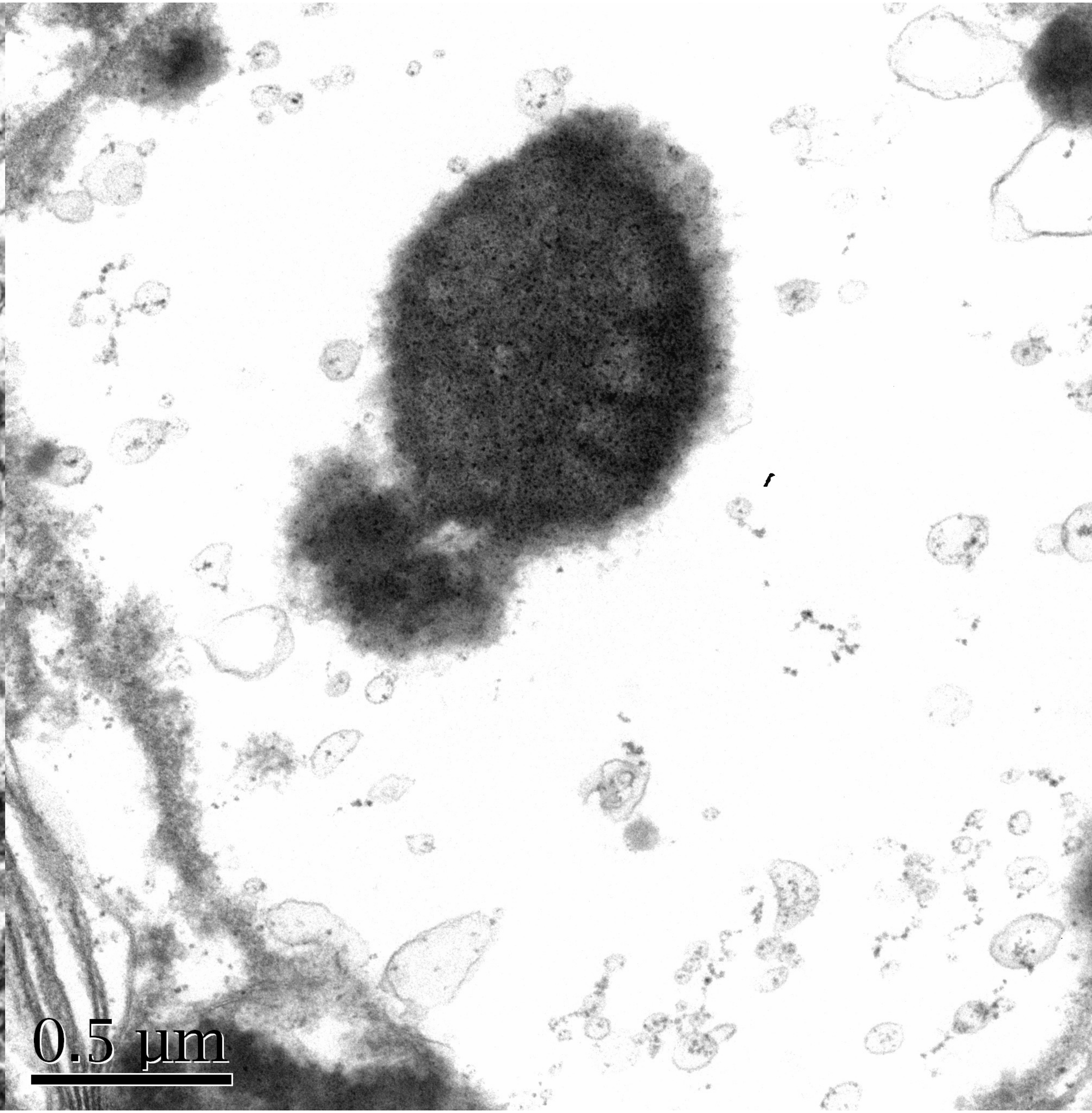

18-14_Correa_YH4_5D3_43.tif

18-14_Correa_YH4_5D3_45.tif

Cell 23

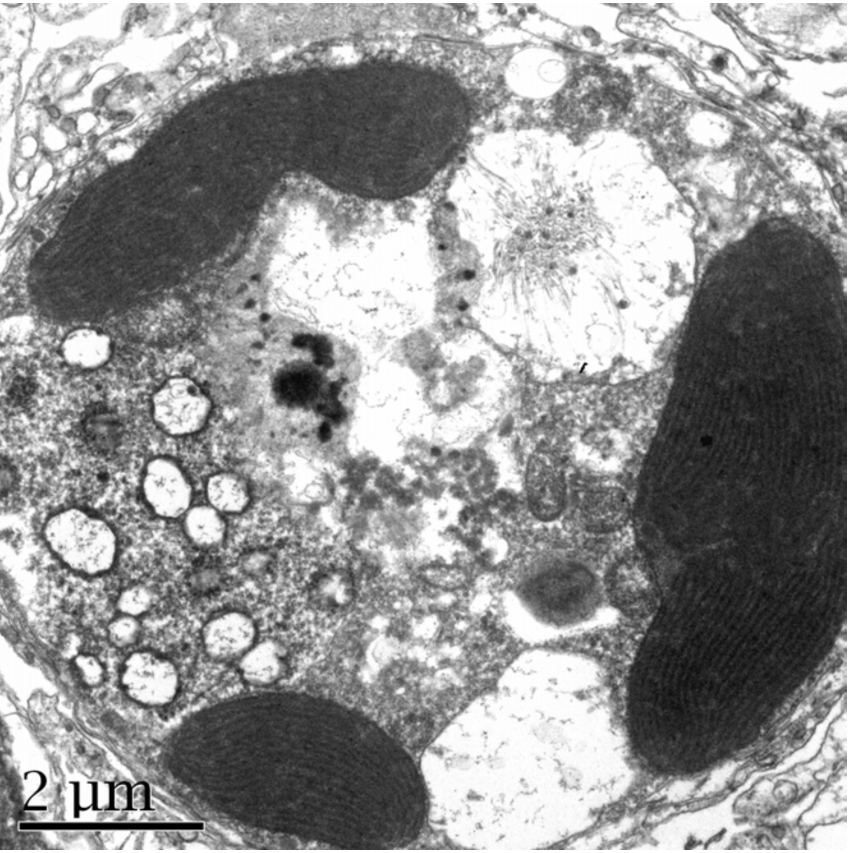

18-14_Correa_YH4_5D3_47.tif

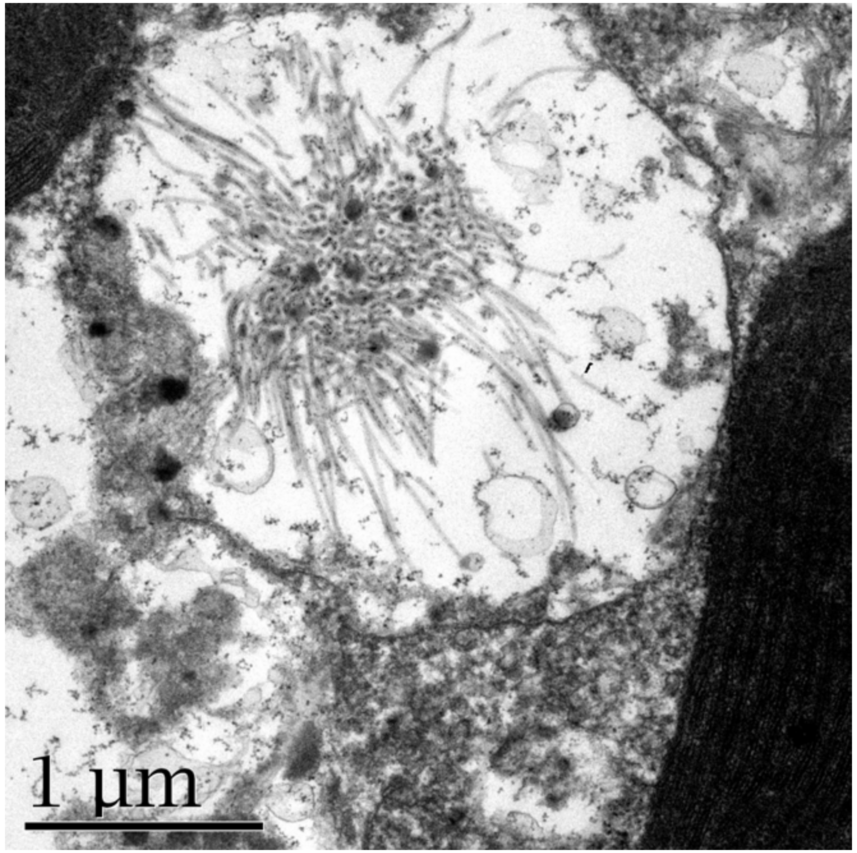

18-14_Correa_YH4_5D3_48.tif

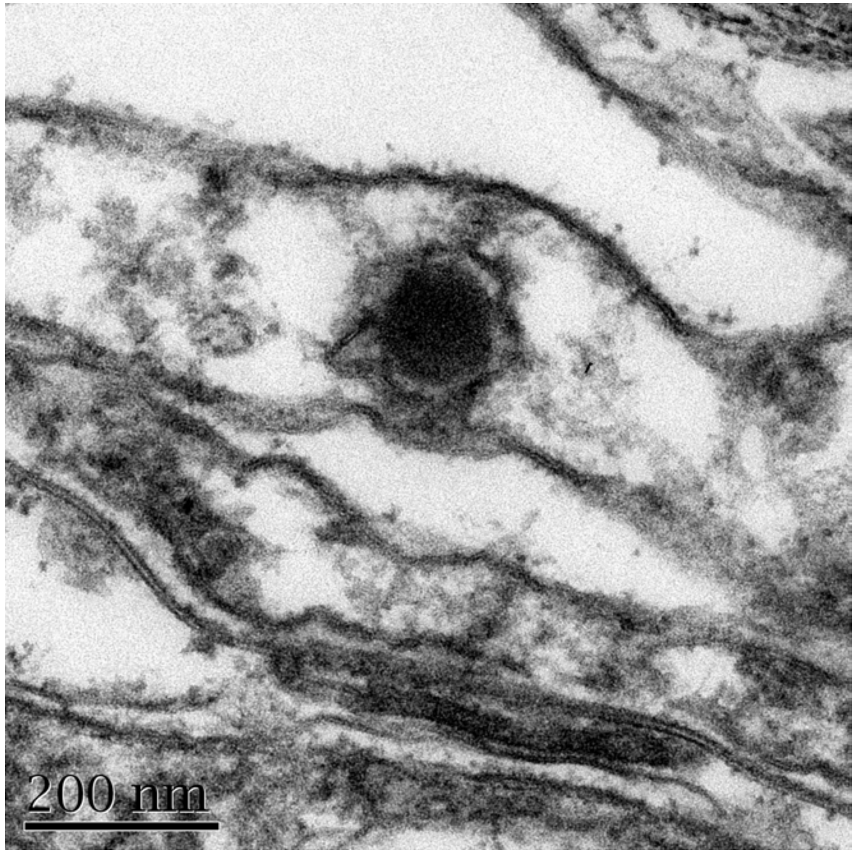

18-14_Correa_YH4_5D3_51.tif

Cell 24

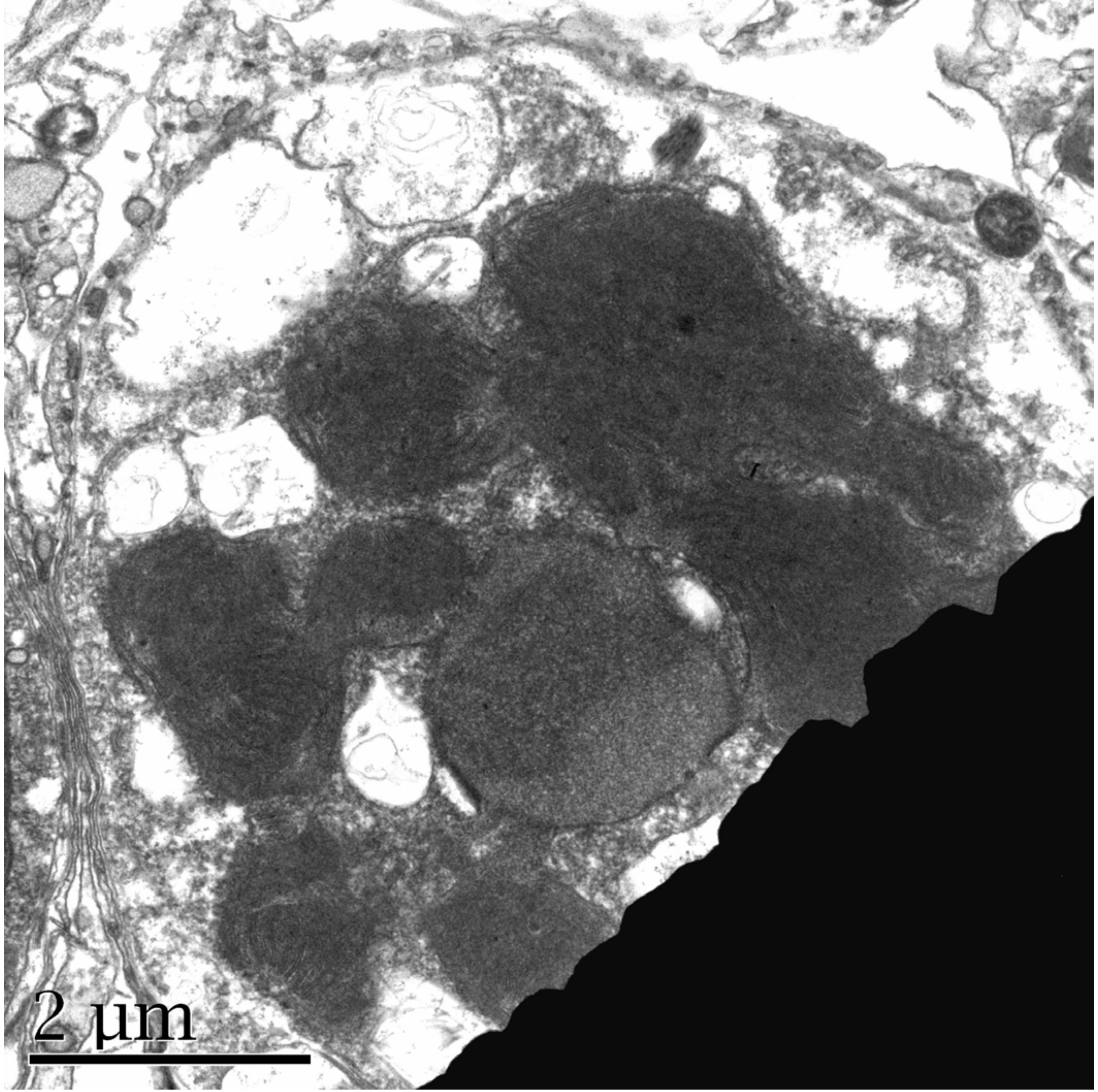

18-14_Correa_YH4_5D3_52.tif

Cell 25

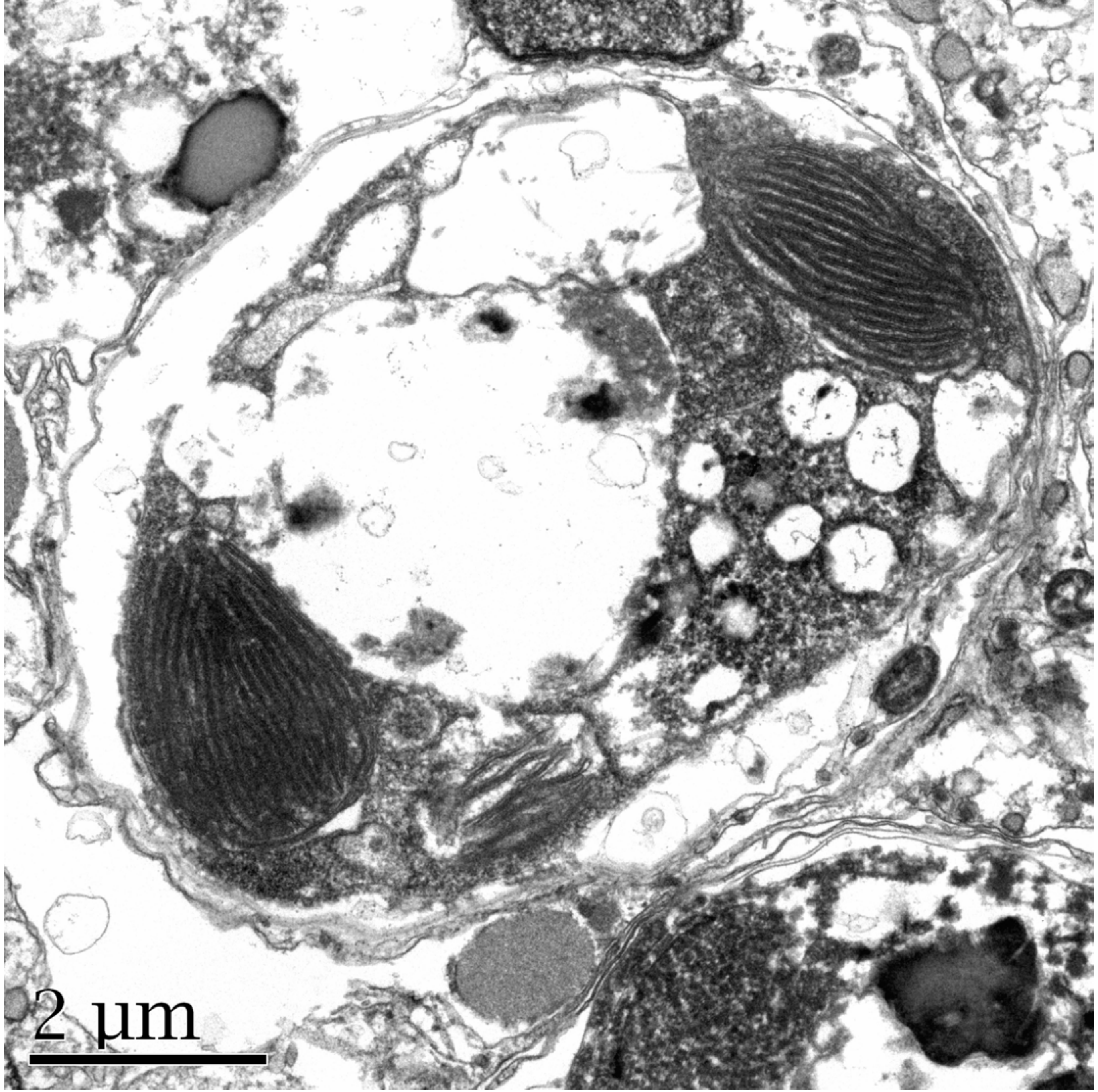

18-14_Correa_YH4_5D3_53.tif

Cell 26

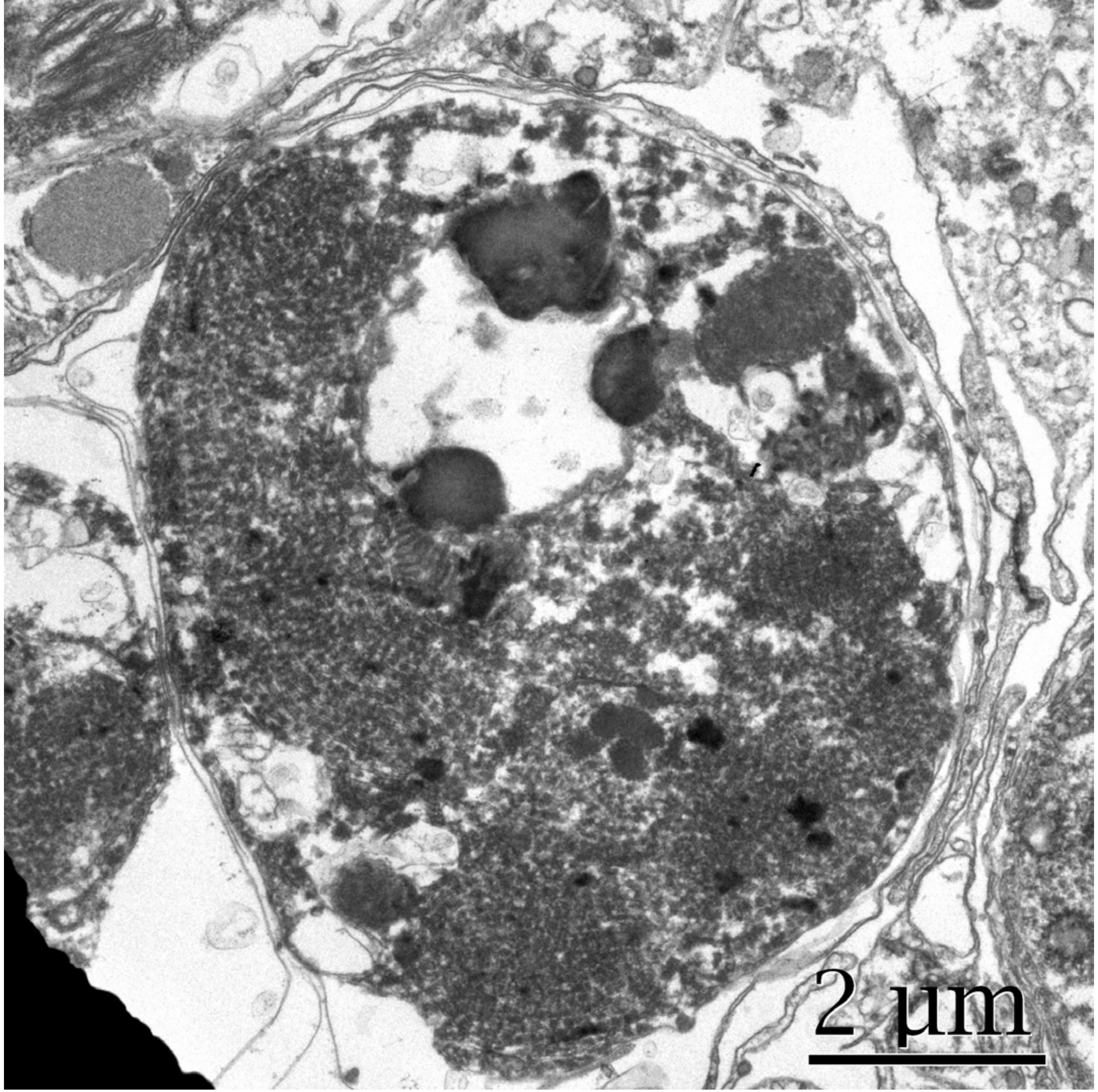

18-14_Correa_YH4_5D3_54.tif

Cell 27

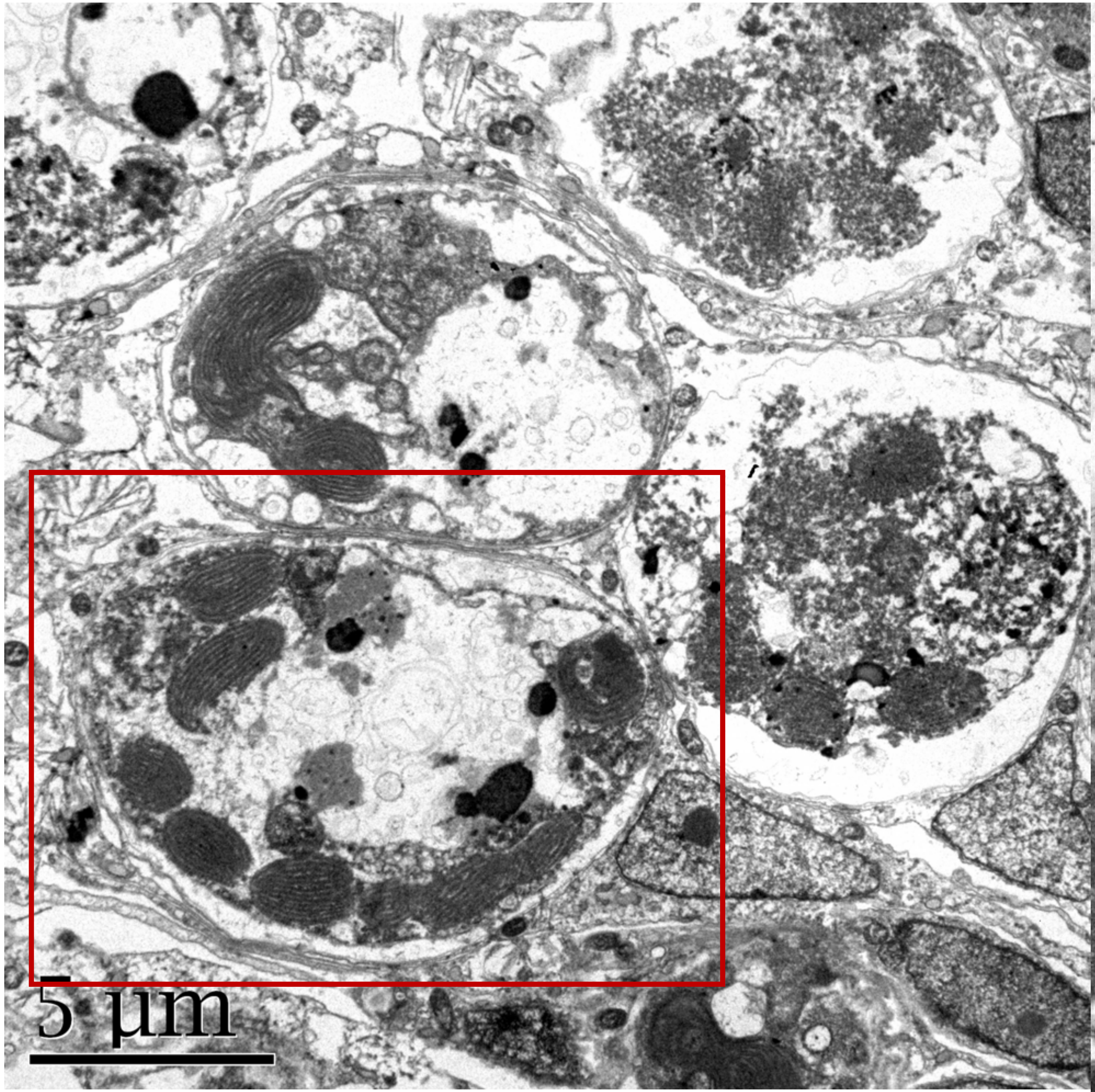

18-14_Correa_YH4_5D3_55.tif

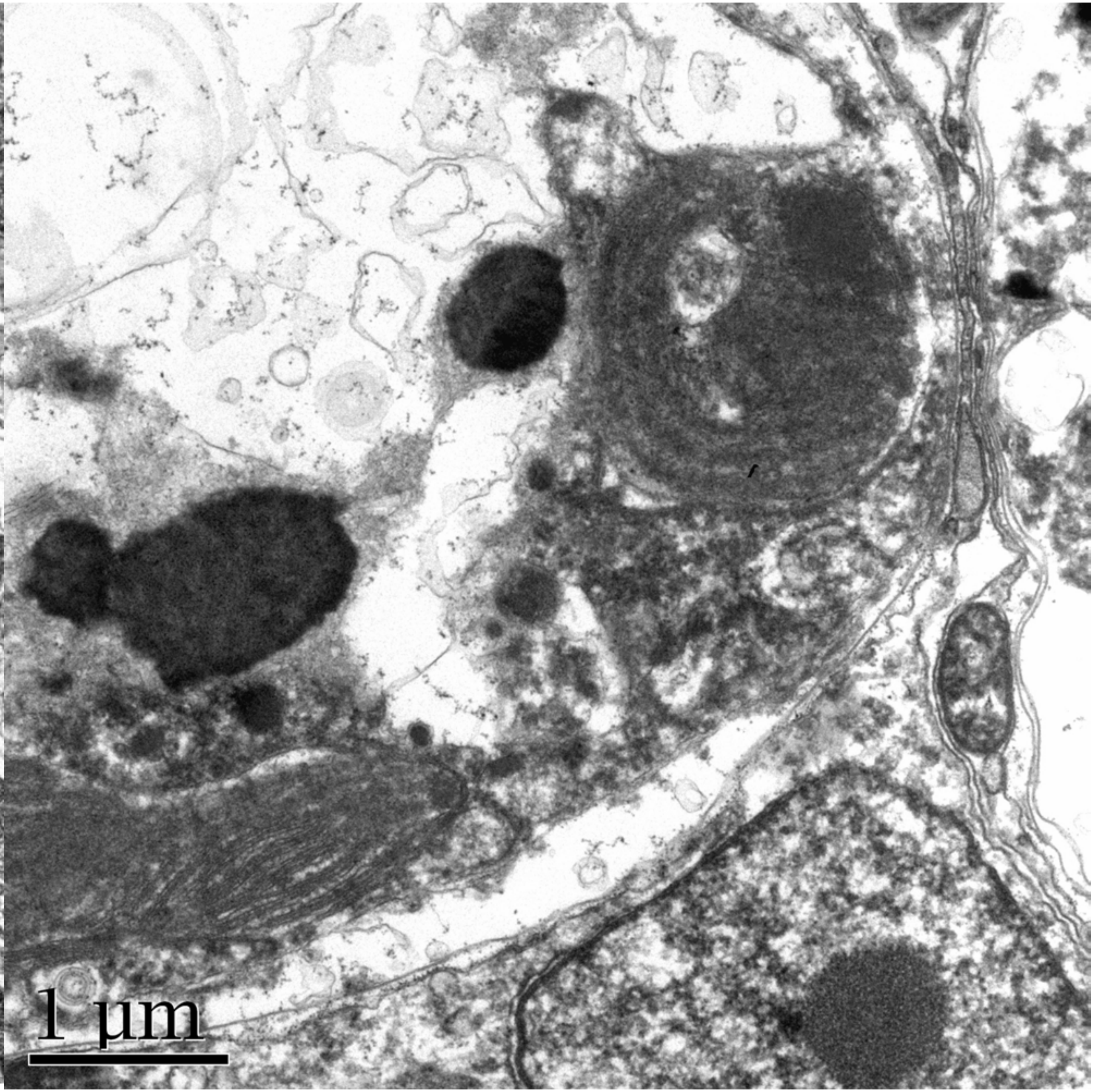

18-14_Correa_YH4_5D3_56.tif

Cell 28

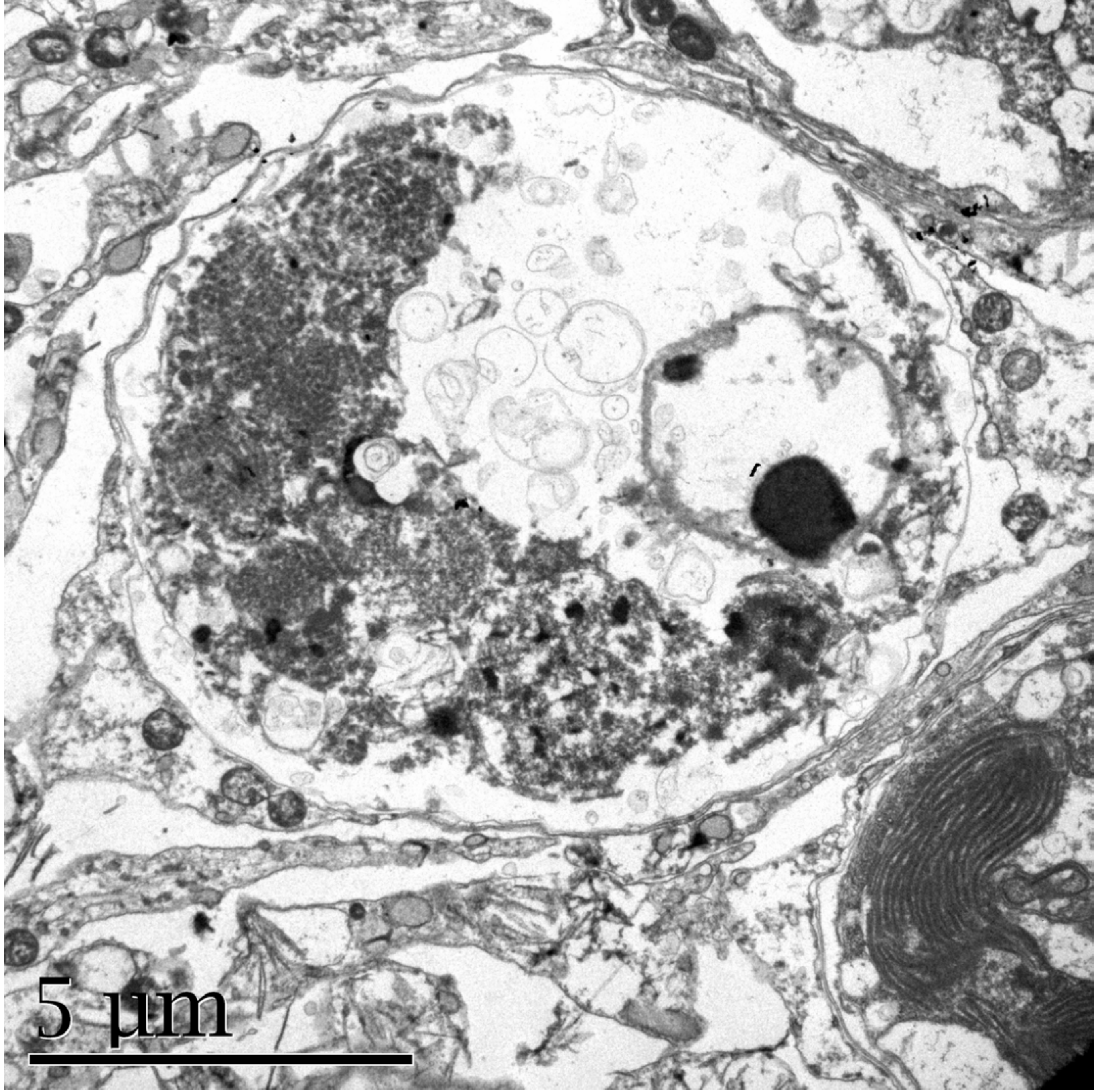

18-14_Correa_YH4_5D3_57.tif

Cell 29

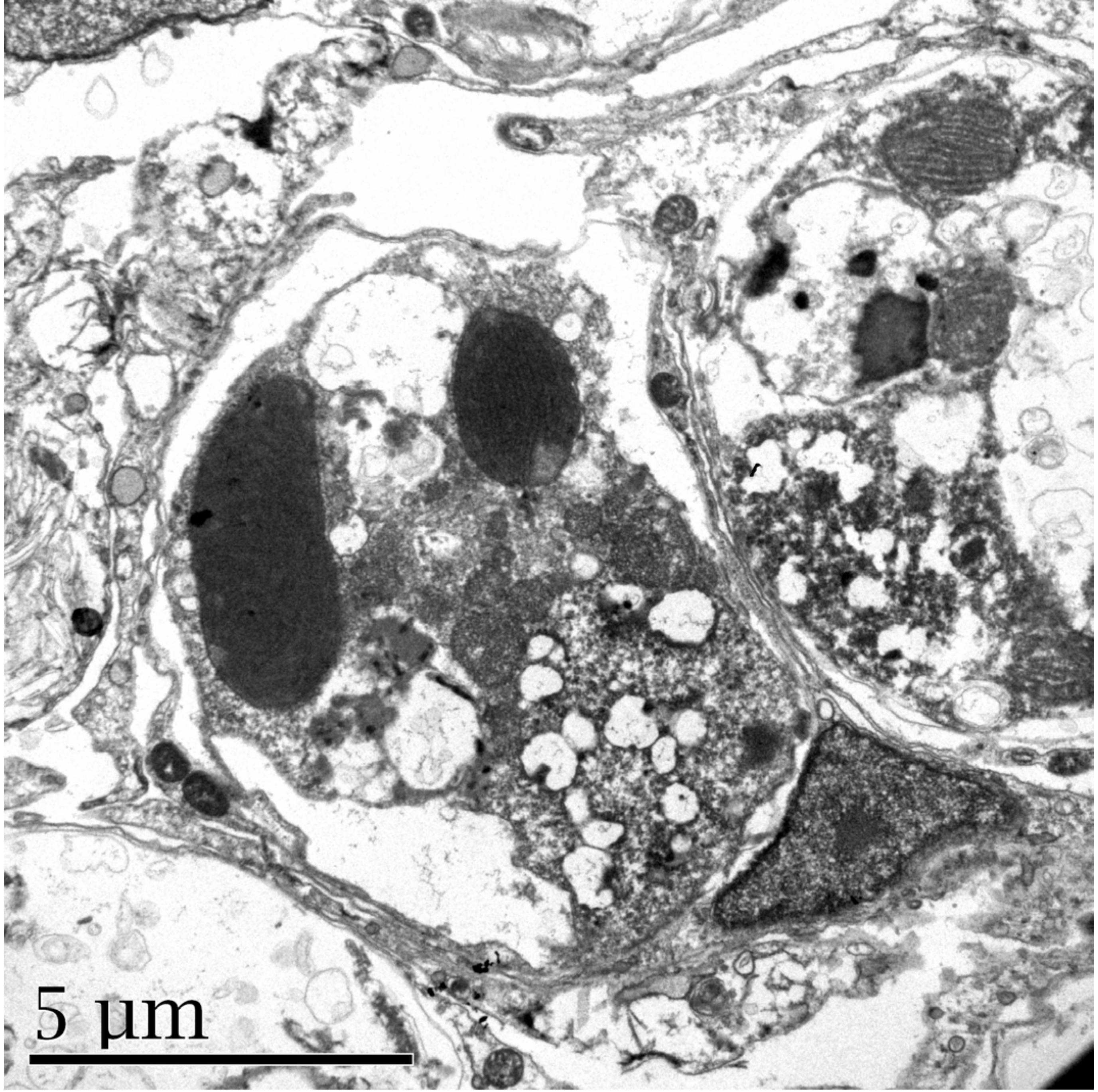

18-14_Correa_YH4_5D3_58.tif

Cell 30

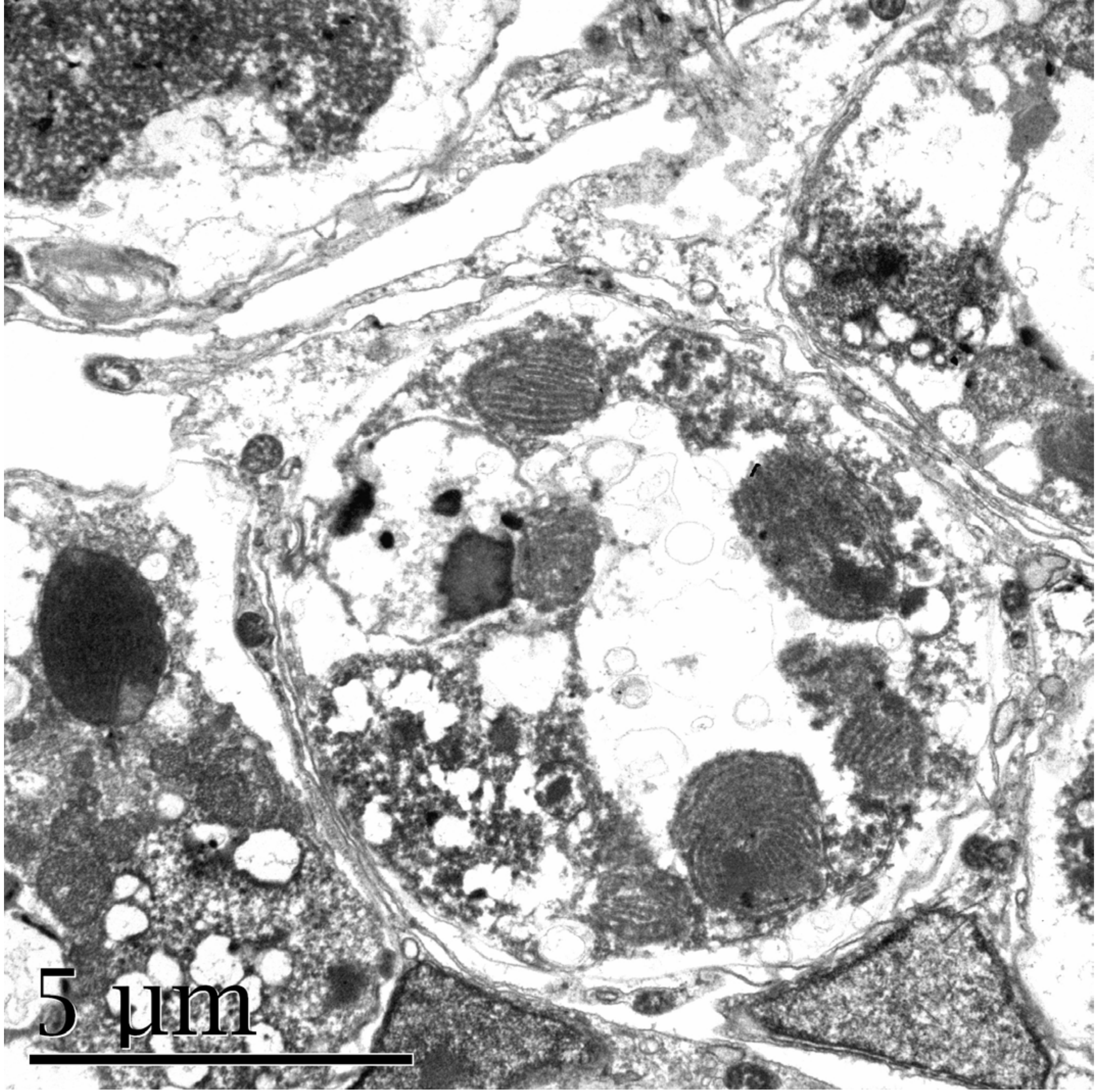

18-14_Correa_YH4_5D3_59.tif
